# Detecting adaptive changes in gene copy number distribution accompanying the human out-of-Africa expansion

**DOI:** 10.1038/s41439-024-00293-w

**Published:** 2024-09-23

**Authors:** Moritz Otto, Yichen Zheng, Paul Grablowitz, Thomas Wiehe

**Affiliations:** 1 Institue for Genetics, University of Cologne, Cologne, Germany; 2 Department of Computer Science, University of Tübingen, Tübingen, Germany

**Keywords:** Structural variation, Evolutionary biology

## Abstract

Genes with multiple copies are likely to be maintained by stabilizing selection, which puts a bound to unlimited expansion of copy number. We designed a model in which copy number variation is generated by unequal recombination, which fits well with several genes surveyed in three human populations. Based on this theoretical model and computer simulations, we were interested in determining whether the gene copy number distribution in the derived European and Asian populations can be explained by a purely demographic scenario or whether shifts in the distribution are signatures of adaptation. Although the copy number distribution in most of the analyzed gene clusters can be explained by a bottleneck, such as in the out-of-Africa expansion of Homo sapiens 60–10 kyrs ago, we identified several candidate genes, such as **AMY1A** and **PGA3**, whose copy numbers are likely to differ among African, Asian, and European populations.

## Traces of human evolutionary history in gene copy numbers

Gene copy number variation can influence evolution and adaptation. This study uses modeling and simulations to investigate these forces’ impact on CNV in human populations. The method combines real data analysis with simulations of human population history and genetic evolution, highlighting the importance of both historical events and survival pressures in shaping gene copy numbers. The results suggest that changes in gene copy numbers across different populations can not be fully explained by population history alone, implying that adaptive changes in survival pressures also play a key role. Specifically, genes related to diet and immune response show significant variations, suggesting a possible link to dietary habits and disease resistance. The study concludes that both population shifts and natural selection have influenced human genetic diversity, with survival changes contributing significantly to the observed variations in gene copy numbers. This summary was initially drafted using artificial intelligence, then revised and fact-checked by the author.

## Introduction

Gene copy number variation (CNV) refers to the presence of multiple copies of a gene family within a genome resulting from duplications, deletions, or rearrangements.

Combined with their high mutation rate, CNVs constitute a significant driver of genomic variability that allows for rapid adaptive evolution in response to environmental changes^1–5^.

A well-studied example of CNV within the human population is the salivary amylase gene, whose variation in the number of copies is hypothesized to correlate with the extent of dietary starch consumption not only in humans but also in other species^6–11^.

In general, CNV may result from different evolutionary forces acting upon them. Demographic events, such as population migrations and expansions, can lead to changes in gene frequencies and distributions over time. Simultaneously, natural selection acts on genetic variations, favoring advantageous alleles and promoting their proliferation within populations.

Both demographic effects and selection may produce similar patterns in single nucleotides as well as in structural variants, making it difficult to disentangle these forces^12,13^. For SNP or allele frequency data, there have been well-developed statistics^14,15^ that are standardized so that a genomic baseline can be established, from which loci under selection may be detected. However, such a genomic baseline is not available for gene CNV data. Therefore, we resort to a more basic approach involving modeling and computer simulations.

We have recently examined the evolutionary dynamics of multicopy gene families with respect to selective pressure and unequal recombination^16^. This study focused on analyzing the impact of stabilizing selection on gene copy numbers while considering the role of recombination as a randomizing mechanism that introduces variability within the population.

By expanding this model, we aimed to assess whether gene copy number alterations observed within human populations could be solely attributed to demographic events or whether selective pressures play a role in shaping these variations.

In this study, we conducted extensive simulations under various scenarios of human demography and selective changes. By disentangling the effects of these two forces, we sought to gain a deeper understanding of the evolutionary processes driving gene CNV in human populations. Based on empirical data of human gene copy numbers, we identified several candidate genes whose copy numbers are likely to be selected differently among African, Asian, and European populations.

## Materials and methods

### Gene CNV in humans

We started with the dataset provided by Brahmachary et al.^1^. Using NanoString technology, they estimated the gene copy numbers of 180 gene families in 165 individuals of three populations (60 African Yoruba – YRI, 60 Central Europe – CEU, and 45 Asia – CHB) based on data collected in the framework of the 1000 Genomes Project^17^.

While some of these loci presented copy numbers of >100 copies (**DUX4** even up to 600), we focused on intermediate copy numbers and removed all satellite loci, genes on sex chromosomes, genes with minimum copy numbers less than 2, and genes with mean copy numbers (in YRI) <5 or >60. For genes that have two primer sets, only one is used.

**Fig. 1:**
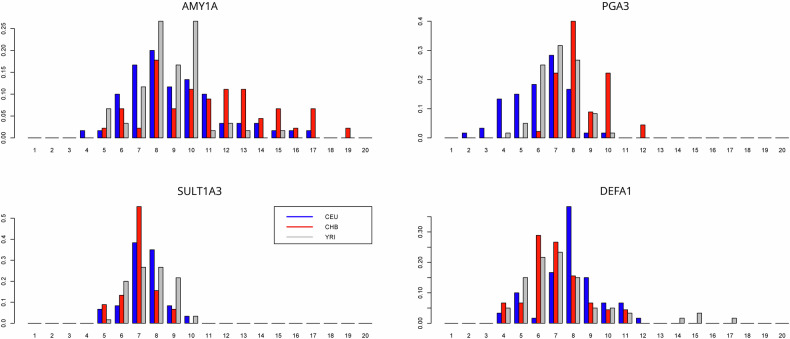
Gene copy number distribution in four exemplary gene families in three human populations: CEU, CHB, and YRI. The data were adapted from Brahmachary et al.^1^. The *y* axis indicates the frequency of individuals carrying the number of copies shown on the *x* axis.

The filtering procedure is described in the supplementary material, which resulted in 49 gene families. For these analyses, we used *t* tests and *F* tests to select gene families with significant differences in means or standard deviations between the YRI–CHB or YRI–CEU comparisons and removed those that showed no statistical evidence (*α* = 5%) in any of these comparisons. The remaining 42 gene families are shown in Table 1, and the copy number distributions of four of them are shown in Fig. 1.

**Table 1:** Differences in means and variances between populations.

Gene	YRI mean	CEU mean		CHB mean		YRI sd	CEU sd		CHB sd	
AMY1A	8.628	0	9.151	+	11.128	1.907	+	2.713	+	3.35
ANKRD20A3	26.414	+	29.148	+	29.904	1.804	0	1.718	0	1.698
BOLA2B	7.235	-	6.679	-	6.49	1.012	0	0.914	0	0.839
CBWD3	12.146	0	12.374	+	13.068	0.995	0	0.949	+	1.81
CDC37P1	14.941	+	19.977	0	16.582	4.11	+	5.619	+	5.579
CLEC18A	7.799	+	8.362	0	7.932	1.331	0	1.216	0	1.392
CSH	6.738	+	7.182	+	7.474	0.497	0	0.555	0	0.575
DEFA1	7.442	0	7.891	0	7.056	2.643	-	1.671	-	1.604
DEFB130	5.081	+	5.315	0	5.243	0.562	0	0.532	0	0.462
FAM72A	6.914	+	7.573	+	7.561	0.617	+	0.86	0	0.651
FAM75A1	11.859	0	11.972	+	13.362	1.473	0	1.391	+	2.019
FAM75A5	11.693	0	11.522	+	12.533	1.115	0	1.197	+	1.751
FCGBP	5.282	+	5.693	+	5.79	1.291	-	0.678	0	1.046
FOXD4L2	13.013	+	13.694	+	14.55	1.015	0	0.994	+	1.877
GOLGA6L9	27.683	0	28.586	+	29.181	2.615	0	2.532	0	2.59
GOLGA8G	29.209	+	31.641	+	30.37	3.065	0	2.783	0	2.35
GUSBP1	12.95	+	15.886	+	13.987	2.249	0	2.585	0	2.213
HIST2	8.436	+	8.709	+	8.894	0.528	0	0.673	0	0.644
LIMS3	5.829	-	5.408	-	5.661	0.346	0	0.354	0	0.39
LOC23117	50.194	0	50.304	-	48.639	3.685	0	2.963	0	2.789
LOC653606	6.56	0	6.403	-	5.999	0.486	0	0.621	+	0.917
MUC12	11.845	+	14.098	0	12.123	2.586	0	2.01	-	1.803
NBPF11	49.963	-	48.002	0	48.68	4.203	-	3.114	0	3.311
NBPF16	45.25	0	46.436	+	47.006	4.706	0	5.023	0	3.988
NPIP	51.171	-	49.488	-	48.938	2.16	0	2.327	0	2.224
PGA3	7.044	-	6.181	+	8.473	1.205	+	1.565	0	1.353
PPIAP21	43.141	+	48.632	+	49.493	3.765	0	4.315	0	3.881
PRAMEF14	10.516	+	11.835	+	11.888	1.295	+	2.246	+	1.937
PRAMEF20	7.253	0	7.415	+	7.576	0.566	0	0.723	+	0.924
PRAMEF5	17.844	-	16.475	-	15.804	1.721	+	2.386	+	2.578
PRAMEF8	5.919	0	5.787	0	5.842	0.652	+	1.281	0	0.819
PRR11	6.868	+	8.298	+	8.305	0.923	0	0.965	0	0.708
PRR20A	20.639	-	17.284	-	14.85	6.903	-	5.288	0	5.584
PSG3	14.943	+	15.624	0	15.087	1.314	0	1.238	+	1.843
RGPD1	13.959	0	14.037	0	14.151	0.791	+	1.309	+	1.266
SPDYE3	34.611	-	31.656	-	32.828	2.836	-	2.105	0	2.617
SULT1A3	7.627	0	7.406	-	7.017	1.197	0	1.087	0	0.904
TBC1D3	45.515	-	33.191	-	39.306	6.337	0	6.888	+	8.381
TCEB3C	33.02	-	28.574	-	25.895	7	0	7.383	0	6.299
TP53TG3	9.172	0	8.904	-	6.735	1.825	0	2.08	0	1.666
TRIM49L1	12.353	+	14.078	+	14.112	1.664	0	2.06	0	1.874
ZNF658B	5.544	+	6.273	+	6.647	0.727	0	0.827	+	1.01

Zero indicates no significant change, ‘+’ indicates a significant increase, and ‘−’ indicates a significant decrease (*t* test for the mean and F test for the standard deviation; *α* = 0.05). The four candidate genes shown in Fig. 1 are highlighted with a light gray background.

### Unequal recombination model

In a recently developed model, we considered unequal recombination and selection to describe the evolution of tandem gene arrays^16^.

We briefly summarize the main findings. Consider two chromosomes with gene arrays of size *y*_*1*_ and *y*_*2*_. A recombination event occurs at rate *r* and may produce a gamete of gene array size according to the trapezoidal distribution, such that

$${Prob}\left({y|}{y}_{1},{y}_{2}\right)=\frac{1}{{y}_{1}{y}_{2}}\left\{\begin{array}{ll}0, & y\, <\, 1\\ y, & 1\le y \,< \,\min ({y}_{1},{y}_{2})\\ \min ({y}_{1},{y}_{2}), & \min ({y}_{1},{y}_{2})\le y\, < \,\max ({y}_{1},{y}_{2})\\ {y}_{1}+{y}_{2}-y, & \max ({y}_{1},{y}_{2})\le y \,< \,{y}_{1}+{y}_{2}-1\\ 0, & y\ge {y}_{1}+{y}_{2}\end{array}\right.$$

**Fig. 2:**
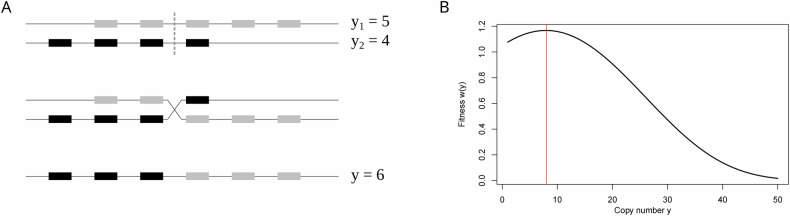
Recombination process and fitness function. **A** Sketch of the unequal recombination process. Starting with two chromosomes with *y*_*1*_ = *5* and *y*_*2*_ = *4* gene copies, two break points are chosen. One of the recombinants is then propagated. Its copy number (here *y* = 6) is trapezoidal, as shown in ref. ^16^. **B** Example of the fitness function *w*(*y*) (Eq. (1)) with *ε* = 0.05, *s*_x_ = 0.05, and *s*_y_ = 0.0025, which leads to an optimal copy number, *y*_opt_, of ~8 copies.

See Fig. 2A for an illustration. We apply a fitness function, where each newly arising copy has a positive yet decreasing benefit *s*_*x*_. This is motivated by the assumption of a beneficial effect, yet with diminishing returns, either of increased gene dosage or of increased allelic diversity within an individual^16^. At the same time, we assume that additional copies are selected with increasing selective disadvantage *s*_*y*_. This is motivated by the increasing cost of replication, gene processing and maintaining genome integrity. Both effects are cast in a double-epistatic fitness function with two selection coefficients (*s*_*x*_, *s*_*y*_), governed by a single epistasis parameter (*ε*). To avoid trivial long-term evolution equilibrium of one copy, we assume that *s*_*x*_ *>* *s*_*y*_. Furthermore, we assume that *ε* = 0.05 is constant. In summary, the fitness of a diploid individual with total gene copy number *y* is given by

1 $$\omega \left(y\right)=\exp \left\{\frac{1}{\varepsilon }\left(\left({s}_{x}+{s}_{y}\right)-{s}_{x}\cdot {e}^{-\varepsilon y}-{s}_{y}\cdot {e}^{\varepsilon \left(y-2\right)}\right)\right\}$$

This leads to an optimal copy number *y*_*opt*_ of

2 $${y}_{{opt}}=1+\frac{\log \left({s}_{x}/{s}_{y}\right)}{2\cdot \varepsilon }$$

which is determined by the ratio *s*_*x*_*/s*_*y*_ when *ε* is fixed. See Fig. 2B for an example. The population is then simulated according to a Wright‒Fisher model with nonoverlapping generations and with selection and recombination described above. In the deterministic model, the equilibrium copy number distribution is centered on *y*_opt_ and is well approximated by a gamma distribution^16^. Furthermore, the coefficient of variation *C*_*V*_ = *σ/y* is correlated with the logarithm of the recombination-selection ratio *log(r/s*_*x*_*)*. With strong selection and low recombination, the distribution is tightly distributed around the optimal value, whereas higher *r* and lower *s*_*x*_ lead to a widespread distribution. For convenience, we introduce two new parameters:

- *q*_*S*_ *= s*_*x*_*/s*_*y*_, the *‘selection ratio’*, which determines the optimal copy number such that for *ε* = 0.05, we find
  $${y}_{{opt}}={y}_{{opt}}\left({q}_{S}\right)=1+10\cdot \log \left({q}_{S}\right)$$
- *q*_*R*_ = *r/s*_*x*_, the *‘recombination/selection ratio’*, which measures the impact of unequal recombination compared with the selective pressure of the fitness function and therefore determines the coefficient of variation *C*_*V*_ = *σ/y* of the equilibrium distribution.

Note that this selection model affects solely the copy number of a gene family, not its sequence. We calculated Tajima’s *D* in a 1 Mb interval around each copy of the 42 gene families in sliding windows of 10 kb via data from the 1000 Genomes Project [http://ftp.1000genomes.ebi.ac.uk/vol1/ftp/release/20130502/]^18^ to test whether selective sweeps may also affect the copy number distribution. Furthermore, we also tested whether these regions are affected by the introgression of archaic hominins. With the data of Browning et al.^19^, we counted the number of introgressed SNPs of Neanderthals or Denisovans in an interval of 10 Mb around the candidate genes to test whether admixture affected the copy number distribution in humans.

### Regression

We aim to quantify the effect of *(r, s*_*x*_*, s*_*y*_*)* on the resulting equilibrium copy number distribution and, vice versa, to estimate the underlying parameter triple for given empirical data. We simulated the population evolution under different parameter settings to analyze the equilibrium distribution of the unequal recombination process under drift. The codes for all the following simulations are available on GitHub https://github.com/y-zheng/gCNV-human. The population size is kept at *N* = 5000 and assumed to be at an initial state of 5 copies on each chromosome. The different input parameters are given in Table 2.

**Table 2:** Parameters for regression simulations.

4 recombination rates *r*	0.1%, 0.2%, 0.5%, and 1%
9 recombination/selection ratios *q*_R_ = r/s_x_	0.01, 0.02, 0.05, 0.1, 0.5, 1.0, 2.0, 5.0
9 optimal copy number values *y*_opt_	10, 15, 20, 25, 30, 35, 40, 45, 50

Together, they define 324 triples *r, s*_*x*_*, s*_*y*_. Additionally, we generated 160 random pairs such that *q*_*R*_ is between 0.01 and 5 and *y*_*opt*_ is between 4 and 60 and combined them with the four recombination rates, leading to a total parameter set of 964 combinations, where we disregarded those triples with selective strengths *s*_*x*_ > *0.1* to maintain a realistic parameter range.

For each of these parameter combinations, we evolve the population under the given selection scheme for 5 million generations. The first 200,000 generations were discarded as burn-in, and the population statistics (mean copy number *y* and standard deviation *σ*) were recorded every 20,000 generations. Note that in contrast to the deterministic model with an infinitely large population size, the population does not reach a stationary distribution but rather fluctuates around the equilibrium distribution. However, since we included an extensive burn-in phase, we are confident that the population is close to equilibrium.

In total, this results in ≈185,000 data points, which we used to determine the relationships between the input parameters *(r, s*_*x*_*, s*_*y*_*)* and the output population statistics *(y, σ)*.

As indicated in Otto et al.^16^, we suggest a mean copy number *y* close to its optimal value *y*_*opt*_ and a correlation of the *C*_*V*_ to *log(q*_*R*_*)*. Indeed, with coefficients of determination (*r*^*2*^) of 0.9842 and 0.9088, we find

3 $$\begin{array}{l}\bar{y}=0.0379+0.983\cdot {y}_{{opt}}\\ {C}_{V}=\displaystyle{\frac{\sigma}{\bar{y}}}=0.323+0.0566\cdot \log \left({q}_{R}\right)-0.00152\cdot {y}_{{opt}}-0.000036\cdot \log \left({q}_{R}\right)\cdot {y}_{{opt}}\end{array}$$

We calculated the *q*_*S*_ and *q*_*R*_ ratios based on *y* and *C*_*V*_ from gene copy numbers (see Table 1) via regression Formula (3), with four recombination rates, *r* = 0.001, 0.002, 0.005, and 0.01.

The results for the four candidate genes shown in Fig. 1 are given in Table 3.

**Table 3:** Estimates of selection coefficients *s*_*x*_, *s*_*y*_ under four recombination rates *r* = 0.001…,0.01 based on regression Eq. (3).

					*r* = 0.001		*r* = 0.002		*r* = 0.005		*r* = 0.01	
Gene	Pop	Mean	SD	y_opt_	*s*_*x*_	*s*_*y*_	*s*_*x*_	*s*_*y*_	*s*_*x*_	*s*_*y*_	*s*_*x*_	*s*_*y*_
AMY1A	CEU	9.1511	2.7133	9.2708	0.0012	0.0005	0.0025	0.0011	0.0062	0.0027	0.0125	0.0055
	CHB	11.128	3.3503	11.282	0.0011	0.0004	0.0022	0.0008	0.0054	0.0019	0.0109	0.0039
	YRI	8.6279	1.9074	8.7386	0.0048	0.0022	0.0097	0.0045	0.0242	0.0111	0.0483	0.0223
PGA3	CEU	6.1808	1.5646	6.2491	0.0029	0.0017	0.0058	0.0035	0.0146	0.0086	(0.0292)	(0.0173)
	CHB	8.4731	1.3526	8.5811	0.0144	0.0068	0.0289	0.0135	0.0722	0.0338	(0.1445)	(0.0677)
	YRI	7.0444	1.2053	7.1277	0.0122	0.0066	0.0245	0.0133	0.0611	0.0331	(0.1223)	(0.0663)
SULT1A3	CEU	7.4058	1.0872	7.4953	0.0186	0.0097	0.0373	0.0195	0.0932	0.0487	(0.1865)	(0.0974)
	CHB	7.0165	0.9041	7.0993	0.0259	0.0141	0.0518	0.0282	0.1295	0.0704	(0.2591)	(0.1408)
	YRI	7.6269	1.1971	7.7202	0.0155	0.0079	0.0311	0.0158	0.0774	0.0395	(0.1548)	(0.0791)
DEFA1	CEU	7.8911	1.6708	7.9889	(0.0058)	(0.0029)	(0.0116)	(0.0058)	0.0291	0.0145	0.0581	0.0289
	CHB	7.0561	1.6041	7.1396	(0.0045)	(0.0024)	(0.0091)	(0.0049)	0.0225	0.0122	0.0451	0.0244
	YRI	7.4421	2.6428	7.5321	(0.0005)	(0.0002)	(0.0009)	(0.0005)	0.0023	0.0012	0.0046	0.0024

The displayed gene families are the ones in Fig. 1 for all three populations. The values in parentheses are outside the range of 0.001 < *s*_*x*_ < 0.1 in YRI and hence are not used in simulations.

### Demography simulations

**Fig. 3:**
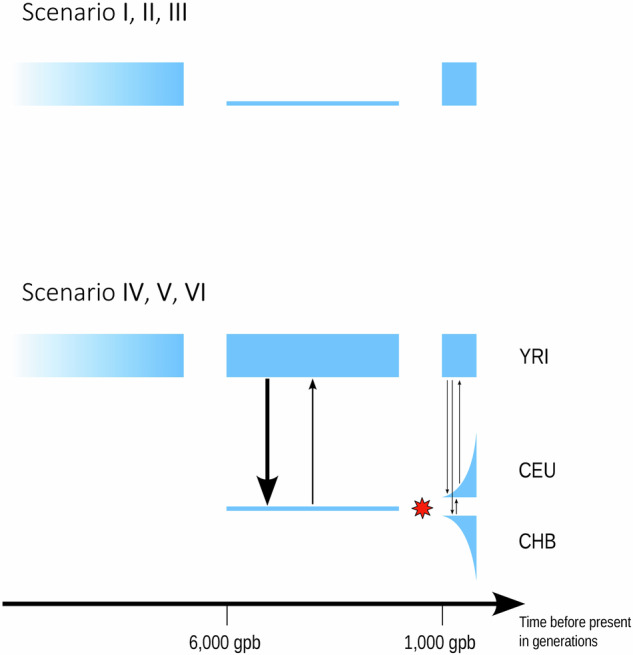
Illustration of the six different scenarios investigated. Scenarios I–III: simple bottleneck lasting 5000 generations. Reduction to 1% (scenario I), 5% (II), and 10% (III) of its original size (*N* = 10,000). Scenarios IV–VI: GADMA model of human demographic history. There was no change in selection intensity (scenario IV), with a change in selection intensity (represented by the red star) 896 generations ago (scenario V) and 500 generations ago (VI). The black arrows indicate the direction and frequency of migration between subpopulations.

To determine whether significant changes in the mean and variance of the copy number distribution (Table 1) can be explained by the demographic history of human populations, we examined a total of 6 different scenarios (enumerated as I–VI), as shown in Fig. 3.

**Simulation of the bottleneck model** First, we ran a simple bottleneck model of three different population reductions. Each is divided into three phases:

1. Burn-in phase. For each gene, we used the estimated *(r, s*_*x*_*, s*_*y*_*)*-triple based on the dataset from YRI. These parameters were chosen as inputs to produce an equilibrium population of *N* = 10,000 via a burn-in process of 200,000 generations. Independent equilibrium populations are produced by recording the population state every 20,000 generations.
2. Bottleneck. From equilibrium, we reduced the population size to *N* = 100, 500, or 1000, denoted scenarios I, II, and III, and maintained it for 5000 generations.
3. Recovery phase. At the end of the bottleneck, the population is reset to *N* = 10,000, and the copy number distribution is recorded every 50 generations until generation 1000 after the bottleneck.

We ran the bottleneck simulations I–III on all gene families given in Table 1, with recombination rates *r* = 0.001, 0.002, 0.005 and 0.01, and discarded parameter combinations with *s*_*x*_ outside the interval [0.001, 0.1] in YRI. This resulted in a total of 42 gene families and 95 gene-*r* combinations. For each gene, recombination rate and bottleneck population size combination, 10,000 replicates are produced (from 100 ‘independent’ starting equilibria). We then traced the means and *C*_*V*_ along the recovery phase and compared them with the empirical data from the CHB and CEU populations.

**Simulation of human population history** A more realistic population history of humans is given by the genetic algorithm for demographic model analysis (GADMA)^20^, which also includes migration between subpopulations. We ran simulations on four candidate genes (**AMY1A, PGA3, SULT1A3, DEFA1**) with the following modification of the GADMA-demography: As an ancestral population (*N* = 9900 in GADMA), we used the equilibrium populations (*N* = 10,000) from the previous section. Therefore, we started the simulation 5992 generations before the present, roughly corresponding to the onset of the out-of-Africa expansion, when the Eurasian population split from the ancestral population and experienced a sharp bottleneck. To reduce computation time, we did not simulate the continued evolution of the African (YRI) population, since we assumed it to be in equilibrium; for migration from YRI to Eurasian populations, we drew samples from the ancestral population. At 896 generations before present, CEU and CHB split from each other and started to evolve, including reciprocal migration and an exponentially increasing population size. In the following, we refer to this simulation as scenario IV. At present, copy number distributions (mean and variance) were recorded. For each gene and recombination rate combination, 10,000 replicates were produced.

We also ran the same population model with a change in the selection parameter either at 500 generations or 896 generations before present (the latter being the CEU/CHB split time). The new selection parameters *s*_*x*_ and *s*_*y*_) are different for the CEU and CHB populations and are estimated from the present CEU/CHB distributions (see Table 3). These simulations are hereafter called scenario V (896 generations of selection change before present) and VI (500 gpb).

### Rejecting a purely demographic model

By observing the copy number distribution for a gene family in the ancestral (YRI) population, we seek to answer the question of whether the observed distribution in the derived population (CEU or CHB) can be explained by a purely demographic model (various bottlenecks but keeping selection pressure constant as modeled in scenarios I to IV) or not (demography plus change in selection pressure as modeled in scenarios V or VI). We use the following strategy to decide this.

For each scenario I–VI and each parameter triple estimated from the YRI population, 10,000 replicates were produced. From each resulting equilibrium distribution, we record the mean *y* and standard deviation *σ*. This results in a parameter distribution for each scenario. If a chosen empirical dataset has a mean *y* or standard deviation *σ*, which are not in the 95% quantile of the 10,000 simulated values, we conclude that this scenario is rejected as a possible explanation of the data. We reject a purely demographic explanation if scenarios I–IV are rejected.

## Results and discussion

In this study, we conducted an analysis of multicopy gene family evolution via a model that incorporates unequal recombination and selection. Our investigation aimed to examine the copy number changes observed in subpopulations of Europe, Asia, and Africa and to determine whether these changes could be attributed to either constant selective pressure and demographic factors or an adaptive change in selection together with demography. Our findings reveal that the observed CNVs in several genes cannot adequately be explained by demographic processes alone, suggesting a possibly adaptive change in selective pressure in the derived populations.

Based on the data of Brahmachary et al.^1^, we chose 42 gene families with intermediate copy numbers that presented significant differences in distribution among different populations (Table 1).

Although the raw data rely on phase I of the 1000 Genomes project, they proved to be most suitable for our analyses. More recent data, for example, from the human pangenome project^21^, still lack sufficient coverage of the different subpopulations.

When we compared the copy number distributions of the 42 candidate genes in the Asian and European populations with those in the African population (assumed to be in equilibrium), we observed 61 significant changes in the mean copy number and 29 significant changes in the variance (Table 1), of which only seven showed a decrease in variance (one example was **DEFA1**). Within our model, assuming a constant recombination rate among subpopulations and no demographic changes, a decreased variance (or standard deviation) can be achieved only by an increase in positive selection (*s*_*x*_), since the *C*_*V*_ is determined by *q*_*R*_ = *r/s*_*x*_; see Eq. (3). However, the most common case is that of a consistent significant shift in the mean in both derived populations without affecting the variance, i.e., either **(++| 0 0)** or **(−−| 0 0)**, which occurs in 12 of the 42 analyzed genes. Only one gene (**PGA3**) showed opposite significant changes in the mean (increasing in Asia but decreasing in Europe).

In this model from Otto et al.^16^, selection does not act on allele sequences, but it acts on the number of gene copies such that an individual with *y*_*opt*_ (2) many copies has the highest fitness. Several methods exist to detect sequence traces of recent selective sweeps, ranging from classical statistics, such as Tajima’s D^15^ or Fu and Way’s *H*^22^, to recent machine learning methods—see, for example, Lauterbur et al.^23^. It is conceivable that selective sweeps in regions close to CNV loci may, by hitchhiking, also affect the gene copy number distribution. A prominent example is the selective sweep in the Asian population in the **EDAR** gene^24^, which is located at ~300 kb to **LIMS1** and ~500 kb to the **LIMS3** gene family from our set. To determine how generally this phenomenon might be and whether selection on allele sequences may correlate, or coincide, with selection on gene copy number, we calculated Tajima’s *D* in an area of 1 Mb approximately 33 MANE-selected genes^25^. Our results suggest that these two forces are independent of each other. For example, **AMY1A** shows a negative Tajima’s *D* in CHB, positive in CEU and approximately zero in YRI (see Supplementary material), but the estimated selection coefficients *s*_*x*_ for a recombination rate of *r* = 0.005 (see Table 3) are 0.6% in CHB, 0.5% in CEU and 2.4% in YRI. **LIMS3** showed only small differences in estimates of *s*_*x*_ (8.3% in CEU, 7.7% in CHB and 9.1% in YRI for *r* = 0.001), and the strong selective sweep of **EDAR** in CHB seems to have no effect.

However, sequence-based summary statistics (such as Tajima’s *D*) calculated across CNV loci need to be interpreted with caution: correct alignment and correct variant calling remain challenging problems, especially when gene copies are highly similar. Biased test statistics are imminently dangerous in such cases^26^.

Another force that may affect the copy number distribution is introgression. Derived populations are known to have interbred with archaic hominins (i.e., Neanderthals and Denisovans). If the archaic hominins had a greater copy number than did *Homo sapiens*, this might have had a significant effect on the distribution of the derived population. Hence, we counted the introgressed SNPs of Neanderthals and Denisovans^19^ in an area of 10 Mb around the candidate gene families. We find the analyzed genes to seem to be in *introgression deserts*^27^, i.e., regions that lack introgression (see Supplementary). However, as a Markovian process, the equilibrium distribution of gene copy number is independent of its initial distribution and hence robust against outliers. Therefore, even if an archaic hominins would have introduced a higher (or lower) copy number, the effect would vanish over time as the distribution would return to its derived equilibrium. In addition, the same note of caution toward technical shortcomings, as mentioned above, applies here as well: marker allele mapping to a gene family may be counted only once rather than multiple times, thereby leading to an underestimation of the amount of introgression.

**Fig. 4:**
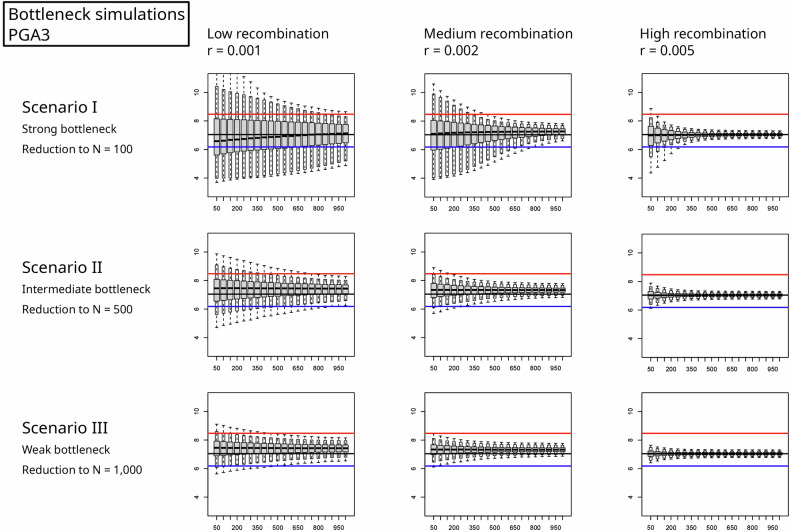
Mean copy number over time. After population reduction to *N* = 100, 500, or 1000 (top to bottom), we traced the mean value (*y* axis) of 10,000 replicates over time (*x* axis in generations). The input parameters s_x_ and s_y_ were estimated for *r* = 0.001, 0.002, and 0.005 (from left to right) from the YRI dataset for the candidate gene **PGA3** (see Table 3) and kept constant over time to determine the effects of the bottleneck and recovery. Whiskers indicate the 99% quantile, and white boxes indicate the 95% quantile. The horizontal lines mark the values from the original dataset of Brahmachary et al.^1^ (black: YRI; red: CHB; blue: CEU).

To test whether a change in population size is sufficient to explain these significant differences in copy number statistics (shown in Table 1), we ran simple bottleneck scenarios I–III with 95 parameter combinations *(r, s*_*x*_*, s*_*y*_*)* on the basis of the ancestral copy number distribution of YRI and the regression Eq. (3). As an example, for **PGA3** and *r* = 0.001, we ran simulations with selection coefficients of *s*_*x*_ = 0.0122 and *s*_*y*_ = 0.0066; see Table 3. Fig. 4 shows the mean gene copy number of 10,000 simulated bottleneck populations over time for each recombination strength (*r* = 0.001, 0.002, and 0.005). Note that for this gene, the value of *r* = 0.01 was neglected since the selection strength *s*_*x*_ would exceed the threshold of 0.1. The gray boxes indicate the centered 50% quantile, the white boxes indicate the 95% quantile, and the whiskers indicate the 99% quantile. With a strong bottleneck (reduction to *N* = 100 for 5000 generations) and under low recombination and hence weak selection (*r* = 0.001, and *q*_*R*_ = *r/s*_*x*_*, q*_*S*_ *=* *s*_*x*_*/s*_*y*_ constant), we find the widest variation among the 10,000 replicates. A higher *r* and stronger selection result in a mean value close to that of the YRI population, i.e., the value we would expect from the initial parameter estimation.

In this example, the empirical data show a significantly greater mean copy number of **PGA3** in CHB (red line) and a lower mean value in CEU (blue line) than in YRI (black horizontal line). It is the only gene in our set that shows significant shifts in the mean copy number in opposite directions. These changes could be explained only under a strong bottleneck and with low recombination without invoking a change in selection intensity.

The results for all 95 parameter combinations obtained for scenario I (strong bottleneck) are summarized in Table 4. To test whether the observed means or standard deviations can be explained by scenario I, we considered the time point after 1000 generations of recovery (first row of Fig. 4, last boxplot in each panel). If the mean or *C*_*V*_ lies within the 95% quantile, we indicate nonsignificant differences with a value of 0. Significant changes are marked with a single * (*α* = 5%) or double asterisk ** (*α* = 1%). Taking again **PGA3** as an example, we find a mean value that is significantly smaller in CEU than in YRI (marked with –). When *r* = 0.001, this might be explained by a bottleneck (denoted by 0), whereas when *r* = 0.002 and *r* = 0.005, we find a significant difference (**) and that the bottleneck explanation is highly unlikely. Higher recombination (*r* = 0.01) led to *s*_*x*_ values >0.1 in CHB and YRI (see Table 3) and hence were omitted.

**Table 4:** Results of bottleneck simulations.

	CEU mean					CEU sd					CHB mean					CHB sd				
Gene	*t-test*	r1	r2	r5	r10	*F-test*	r1	r2	r5	r10	*t-test*	r1	r2	r5	r10	*F-test*	r1	r2	r5	r10
AMY1A	0	0	0	0	*	+	0	*	*	**	+	*	**	**	**	+	*	**	**	**
ANKRD20A3	+	**	**	.	.	0	0	0	.	.	+	**	**	.	.	0	0	0	.	.
BOLA2B	–	0	**	.	.	0	*	**	.	.	–	0	**	.	.	0	*	**	.	.
CBWD3	0	0	.	.	.	0	0	.	.	.	+	0	.	.	.	+	*	.	.	.
CDC37P1	+	**	**	**	**	+	*	**	**	**	0	0	0	*	**	+	*	**	**	**
CLEC18A	+	0	0	*	.	0	0	*	*	.	0	0	0	0	.	0	0	0	0	.
CSH	+	0	.	.	.	0	**	.	.	.	+	**	.	.	.	0	**	0	0	0
DEFA1	0	.	.	0	0	–	.	.	0	**	0	.	.	0	0	–	.	.	0	**
DEFB130	+	0	*	.	.	0	**	**	.	.	0	0	0	.	.	0	**	**	.	.
FAM72A	+	0	.	.	.	+	0	.	.	.	+	0	.	.	.	0	**	.	.	.
FAM75A1	0	0	0	.	.	0	0	0	.	.	+	*	**	.	.	+	0	**	.	.
FAM75A5	0	0	0	.	.	0	0	0	.	.	+	0	**	.	.	+	0	**	.	.
FCGBP	+	0	0	0	0	–	*	**	**	**	+	0	0	0	0	0	0	0	**	**
FOXD4L2	+	0	.	.	.	0	0	.	.	.	+	**	.	.	.	+	*	.	.	.
GOLGA6L9	0	0	0	.	.	0	0	0	.	.	+	0	*	.	.	0	0	0	.	.
GOLGA8G	+	*	**	.	.	0	0	0	.	.	+	0	0	.	.	0	0	0	.	.
GSUBP1	+	**	**	**	.	0	0	0	0	.	+	0	0	**	.	0	0	0	0	.
HIST2	+	0	.	.	.	0	0	.	.	.	+	*	.	.	.	0	0	.	.	.
LIMS3	–	*	.	.	.	0	**	.	.	.	–	0	.	.	.	0	**	.	.	.
LOC23117	0	0	0	.	.	0	*	*	.	.	–	*	**	.	.	0	*	**	.	.
LOC653606	0	0	.	.	.	0	0	.	.	.	–	**	.	.	.	+	0	.	.	.
MUC12	+	*	**	**	**	0	0	*	**	**	0	0	0	0	0	–	0	**	**	**
NBPF11	–	*	**	**	.	–	*	**	**	.	0	0	**	**	.	0	*	*	*	.
NBPF16	0	0	0	0	.	0	0	0	0	.	+	0	0	0	.	0	0	0	0	.
NPIP	–	**	**	.	.	0	*	*	.	.	–	**	**	.	.	0	*	*	.	.
PGA3	–	0	**	**	.	+	0	0	0	.	+	*	**	**	.	0	0	0	0	.
PPIAP21	+	**	**	.	.	0	0	0	.	.	+	**	**	.	.	0	0	0	.	.
PRAMEF14	+	*	**	.	.	+	**	**	.	.	+	**	**	.	.	+	0	**	.	.
PRAMEF20	0	0	.	.	.	0	0	.	.	.	+	0	.	.	.	+	0	.	.	.
PRAMEF5	–	**	**	.	.	+	*	*	.	.	–	**	**	.	.	+	*	**	.	.
PRAMEF8	0	0	0	.	.	+	0	**	.	.	0	0	0	.	.	0	0	0	.	.
PRR11	+	**	**	.	.	0	0	*	.	.	+	**	**	.	.	0	**	**	.	.
PRR20A	–	.	.	0	**	–	.	.	0	*	–	.	.	0	**	0	.	.	0	0
PSG3	+	0	*	.	.	0	0	0	.	.	0	0	0	.	.	+	0	*	.	.
RGPD1	0	0	.	.	.	+	0	.	.	.	0	0	.	.	.	+	0	.	.	.
SPYDE3	–	**	**	.	.	–	0	0	.	.	–	**	**	.	.	0	0	0	.	.
SULT1A3	0	0	0	0	.	0	0	*	*	.	–	0	**	**	.	0	**	**	**	.
TBC1D3	–	**	**	**	**	0	0	0	0	0	–	**	**	**	**	+	0	*	**	**
TCEB3C	–	0	**	**	**	0	0	0	0	0	–	0	**	**	**	0	0	0	0	0
TP53TG3	0	0	0	0	0	0	0	0	0	0	–	0	**	**	**	0	0	0	*	*
TRIM49L1	+	**	**	.	.	0	0	0	.	.	+	**	**	.	.	0	0	0	.	.
ZNF658B	+	0	**	.	.	0	0	0	.	.	+	**	**	.	.	+	0	0	.	.

We ran simulations of scenario I (the strongest bottleneck with a reduction to *N* = 100) with parameters (*r*, *s*_*x*_, *s*_*y*_) estimated from the YRI data and tested whether, after 1000 generations of recovery, the mean and standard deviation *σ* of the CEU and CHB data could be explained by a bottleneck. The blank space indicates that this parameter combination led to an *s*_*x*_ value out of the range of 0.001–0.1; hence, no simulation was run. The columns with 0, + and – indicate whether there is a significant difference from the empirical dataset (see Table 1). The column names r1–r10 indicate recombination rates ranging from 0.001 to 0.01, and a value of 0 in that column indicates that the data can be explained by a bottleneck. * and ** represent significant differences (5% and 1%, respectively) between the simulated and empirical data. The four candidate genes that were used for further simulations are highlighted with a light gray background.

If we consider a significant difference in the mean of CEU compared with YRI (28 genes; the first column in Table 4 is nonzero), we find that only 17 out of 65 simulated parameter combinations in scenario I can explain these differences. For significant mean changes in CHB (33 genes; Table 4), 22 out of 72 parameter combinations are compatible with the observations, whereas the remaining 50 cannot explain the significant difference. For other examples, **AMY1A** and **PGA3** both presented increased mean values in CHB. In neither case nor for either parameter combination is scenario I sufficient to explain the observation.

From the candidates with a significant difference in mean or variance, we selected well-studied genes with known functions and annotations and chose three genes coding for digestive enzymes, **AMY1A,** **SULT1A3,** **PGA3**, and the defense gene **DEFA1**, for a more detailed analysis and tested the GADMA model^20^ without and with selection changes according to the estimates from regression (scenarios IV–VI).

**Fig. 5:**
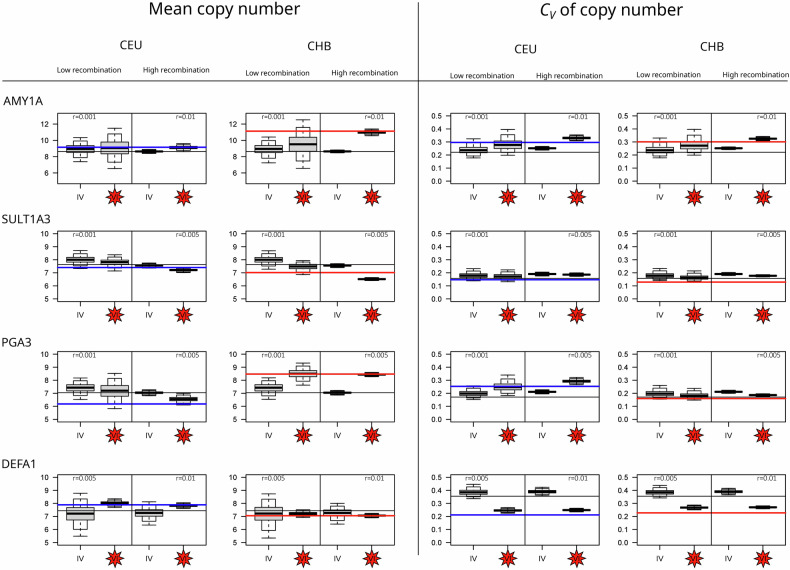
Mean copy number *y* and coefficient of variation *C*_*V*_ = *σ/y* for four candidate genes (**AMY1A,** **SULT1A3,** **PGA3**, and **DEFA1**). Boxplots: simulation results for the GADMA demographic model without (scenario IV) and with (scenario VI) a change in the selection and for two settings of the recombination rate (low and high; see Table 3). Horizontal lines: means and *C*_*V*_ of the experimental data in YRI (black), CEU (blue), and CHB (red).

Figure 5 shows the mean copy number and coefficient of variation (*C*_*V*_) at present, which are simulated according to scenarios IV and VI for 10,000 replicates each.

As in scenarios I–III, the terminal values generated in scenario IV are close to the initial values of the ancestral YRI dataset (black line). Therefore, even the more realistic GADMA migration model often fails to explain the data found in CEU and CHB when constant selection parameters derived from the ancestral YRI population are considered.

However, when selection strength is allowed to change, as in scenario VI, a different picture emerges: Consider a change in *s*_*x*_ and *s*_*y*_ at 500 generations before being present with respect to the values estimated from Eq. (3), given in Table 3. Then, the simulations return the mean and *C*_*V*_, which are closer to the values found in the CEU and CHB data. Indeed, the empirical data often lie within the 95%- or 99%-quantiles of the simulated data distributions. We observe no strong difference between the results of scenarios V and VI, suggesting that even 500 generations represents a sufficiently large time span to reach a new equilibrium.

Hence, one possible explanation for the shift in the copy number distribution of the four candidate genes is a change in selection pressure and adaptation.

The **AMY1A** gene, which encodes amylase, an enzyme that breaks down starch, has strongly increased mean and *σ* values in the Asian population, which is likely linked to adaptations to high grain intake. In the European population, while the variation increased, the change in the mean copy number was small.

These findings agree with the results of several studies that indicate that individuals from populations with high-starch diets have more gene copies on average than those with traditional low-starch diets^6,9,10^. Our model selection strength is relaxed in CEU and CHB with a factor of 4, such that a higher copy number is not selected, and a more widespread distribution of CNVs can evolve. A recent study^28^ suggested a more complicated model of Amylase evolution involving two steps: expansion from one to several copies after the human–Neanderthal split but before the separation of modern human populations and a subsequent shift in the optimal gene copy number, independently in different populations. This study also suggested that the increase in **AMY1** copy number occurred in South America even more dramatically than in East Asia, a hypothesis that should be tested in the framework of our model as soon as suitable data become available.

**SULT1A3** is a gene in the SULT (sulfotransferase) family that catalyzes the sulfation of a variety of substrates, especially catecholamines, including dopamine and epinephrine^29,30^. Polymorphisms in **SULT1A3** and **SULT1A4** have been shown to affect the metabolism of therapeutic drugs^31,32^, and these genes have therefore been studied extensively in the framework of medico- and pharmacogenetics^33,34^. In the dataset analyzed, there was a reduced mean copy number in Asia but not in Europe. The reduced mean (from 7.6 in YRI to 7.0 in CHB) is a significant difference, which cannot be explained by a simple bottleneck scenario with a recombination rate higher than 0.002 (see Table 4). If one considers a change in selection, as in scenarios V and VI, we expect a stronger selective pressure (rising from *s*_*x*_ = 0.03 to *s*_*x*_ = 0.05 for *r* = 0.002) in CHB. There have been few studies on the copy numbers of **SULT1A3/4** genes. Hildebrandt et al.^33^ first noted possible duplication of **SULT1A3** and identified a duplicated copy in all four different human populations. More recently, a study of 172 human individuals revealed variable **SULT1A3/4** copy numbers ranging from 1 to 10 and associated its copy number with the risk and onset of neurodegenerative disease^35^. Note that **SULT1A3** and **SULT1A4** are closely related paralogs that are often difficult to distinguish, and studies on copy numbers usually combine them.

**PGA3** (Pepsinogen, a precursor for pepsin, an enzyme that breaks down protein into smaller peptides) is associated with prostate-specific antigen production. It is the only gene in our list that has opposite changes in two derived populations: its mean copy number increases in Asia and decreases in Europe. As Asian and European humans share most of the same bottleneck period, the diverging copy number distribution is highly unlikely to be a demographic effect, and complex selection patterns are needed to explain the data. Indeed, the bottleneck simulations shown in Table 4 and simulations V and VI with a change in selection parameters, as shown in Fig. 5, support this hypothesis. When considering the estimates of Table 3, we observe a small increase in *s*_*x*_ in Asia compared with Africa but a strong decrease in both *s*_*x*_ and *s*_*y*_ in Europe to cope with the increased variance in copy number in CEU.

CNV in the pepsinogen (PGA) locus was originally discovered via electrophoresis, and three individual genes (**PGA** 3, 4, 5) were initially identified^36^. Pepsinogen genes have been shown to duplicate and become recurrently lost in vertebrates^37^. The pepsinogen genes were also shown to have variable expression levels in tumor cells, particularly a reduction in PGA expression in specific stomach and thyroid cancers^38^. This could be an additional source of selective pressure in addition to protein metabolism. While the simplest explanation is that dietary differences between Asian and European populations during the spread of agriculture (in the last 5000–10,000 years) are the drivers of **PGA** copy number changes, alternative hypotheses involving tumor suppression or interaction with other enzymes must be considered.

Finally, we analyzed the immune gene alpha-defensin **DEFA1**. It codes for defensins, proteins that are involved in innate (non-learned) immunity, specifically in antimicrobial defense against a broad spectrum of microorganisms, including bacteria, fungi, and viruses. **DEFA1** shows a decrease in variance in both Asia and Europe, indicating stronger selective pressures. More precisely, when considering the distribution in Fig. 1, one observes four individuals in the YRI population with high copy numbers, which indicates relaxed selective pressure in Africa. With Eq. (3), we find selection coefficients 10-fold smaller in Africa than in Europe and Asia (see Table 3). Alpha defensins are expressed in neutrophil cells and intestinal epithelial cells and act as microbiocidal agents^39–41^. The genes **DEFA1** and **DEFA3** encode some of the alpha-defensins (HNP1/2/3) and appear to be “interchangeable variant cassettes” within a tandem array of 19 kb^42^. CNV of **DEFA1** is present in all apes, including gibbons, but the version identified as **DEFA3** is human-specific; the copy number has also been demonstrated to affect the expression level^42^. A low copy number of **DEFA1/3** has been shown to be associated with hospital-acquired infection^43^ as well as kidney diseases^44^. On the other hand, and counterintuitively, a high copy number of **DEFA1/3** may lead to more severe cases of sepsis^45,46^ and is associated with Crohn’s disease^47^; thus, this gene was selected against. The trade-off between infective and autoimmune diseases could lead to a selection toward an intermediate copy number of alpha-defensins. Therefore, our results suggest that out-of-Africa expansion may be accompanied by a change in environmental pathogen diversity such that a delicately tuned dosage of defensin is needed. This can be corroborated by the fact that YRI has a few individuals with very high (outliers) copy numbers of **DEFA1**, which cannot be found in CHB or CEU.

In conclusion, while both demographic effects and shifts in selection schemes can result in changes in copy number distributions, in some of our candidate genes, the former is not sufficient to explain the observation. Adaptive processes can induce new relationships between copy number and fitness and impact the resulting copy number distribution. Importantly, changes in the strength or direction of selection may manifest not only in the mean copy number but also in the variance or compound statistics, such as the coefficient of variation.

## Supplementary information

Supplementary tables and figures

2_TajimasD_data

3_Introgression_data

## Data Availability

Data and custom codes are available at https://github.com/y-zheng/gCNV-human.
