# Supplementary material for: Detecting adaptive changes in gene copy number distribution accompanying the human out-of-Africa expansion
Source: Hum Genome Var. 2024 Sep 23;11:37. doi: 10.1038/s41439-024-00293-w (PMC11420239; doi:10.1038/s41439-024-00293-w)
Supplement: Supplementary file 1 — Supplementary tables and figures [file 41439_2024_293_MOESM1_ESM.pdf]

Supplementary tables and figures
„Detecting adaptive changes in gene copy number distribution accompanying the human out-of-Africa expansion“ by Otto et al.

Suppl. 1:

Table S1: List of gene families from Brahmachary *et al.* (2014) and description of filtering procedure. After filtering, 42 gene families were chosen for further analysis

Suppl. 2:

Table S2: Positions in genome assembly hg38 of gene copies from 33 MANE (Morales *et al.* 2022) selected gene families. Note: this is a subset of the 42 gene families which remained after filtering (Table S1).

Suppl. 3:

Tajima’s *D* profiles.
Tajima’s *D* was calculated in a 1 Mb region centered in the respective gene family (positions from Suppl. Table S2) in sliding windows of size 10 kb and based on SNPs from the 1k human genomes project phase III (http://ftp.1000genomes.ebi.ac.uk/vol1/ftp/release/20130502/)

(Raw data shown in separate file "TajD-raw.pdf")

Suppl. 4 and 5:

Introgression profiles for (a) individual gene families and (b) summed over all gene families. Introgression in gene families was calculated as the total number of introgressed SNPs in a 10Mb window centered in the respective gene family and in sliding windows of size 100 kb. Introgression data from Browning et al. (2018).

(Raw data shown in separate file "introgression-raw.pdf")

Table S1 List of all gene families from Brahmachary *et al.* (2014). We applied three levels of filtering: first (comment in gray), we excluded microsatellites and all genes which are sex-chromosome linked. Second (comment in orange), we excluded gene families which displayed either very little variation (mean copy number in YRI is less than 5 copies; one or more individuals in CEU or CHB have only one copy; coefficient of variation smaller than 0.004) or which are extremely large (mean copy number in YRI larger than 60). Third (comment in red), gene families were excluded for which neither the t-test nor the F-test between YRI and CEU or CHB turned out to be significant ('homogeneous'). The remaining families (green checkmark) were investigated further.

Gene Name	Filtering	Gene Name	Filtering	Gene Name	Filtering
AMY1A	✓	GSTM	<5 copies	NXF2	sex-linked
ANAPC11	min copies <2	GSTT2	min copies <2	OPN1MW	sex-linked
ANKRD20A3	✓	GTF2H2C	<5 copies	ORM1	<5 copies
ANXA8L1	<5 copies	GTPBP6	sex-linked	P4HB	min copies <2
AREG	min copies <2	GUSBP1	✓	PCYT2	min copies <2
ARL17B	<5 copies	H2AFB1	sex-linked	PGA3	✓
ATG4B	min copies <2	HGD	min copies <2	PGAM4	sex-linked
AURKAIP1	min copies <2	HIST2	✓	PLGLB2	low CV
BOLA2B	✓	HP	min copies <2	PPIAP21	✓
BPY2	sex-linked	HRNR	min copies <2	PRAMEF14	✓
BTNL8	min copies <2	HSFY2	sex-linked	PRAMEF20	✓
C16orf54	min copies <2	KIF22	min copies <2	PRAMEF5	✓
C4B	<5 copies	KIR2DL2	homogeneous	PRAMEF8	✓
C6orf124	min copies <2	KIR2DL4	min copies <2	PRR11	✓
CBWD3	✓	KIR2DL5A	homogeneous	PRR20A	✓
CCL3	min copies <2	KIR2DS2	homogeneous	PRR20A (probe2)	excluded (duplicate)
CCL4	min copies <2	KIR3DP1	min copies <2	PRY2	sex-linked
CCR2	min copies <2	KLRC2	<5 copies	PSG11	sex-linked
CCR5	min copies <2	KPNA2	<5 copies	PSG3	✓
CCRL2	min copies <2	KRT39	min copies <2	PTPN20A	<5 copies
CDC37P1	✓	KRT40	min copies <2	PYCR1	min copies <2
CDY1B	sex-linked	KRTAP1-1	min copies <2	RBMY1E	sex-linked
CFC1	<5 copies	KRTAP2-1	min copies <2	REXO1L1	>60 copies
CKMT1A	<5 copies	KRTAP3-1	min copies <2	REXO1L1 (probe2)	>60 copies
CLEC18A	✓	KRTAP4-1	min copies <2	RGPD1	✓
CR1	<5 copies	KRTAP5-1	min copies <2	RGPD6	low CV
CRLF2	sex-linked	KRTAP7-1	min copies <2	RHOXF2	sex-linked
CSAG2	sex-linked	KRTAP8-1	min copies <2	RPS17	<5 copies
CSH	✓	KRTAP9-1	<5 copies	RSPH10B	<5 copies
CT45	sex-linked	LCE2	<5 copies	SCRT1	min copies <2
CT47 (probe 1)	sex-linked	LIMS3	✓	SCXB	min copies <2
CT47 (probe 2)	sex-linked	LOC100506776	min copies <2	SERF1B	min copies <2
CTAG1B	sex-linked	LOC23117	✓	SFTPA1	<5 copies
D2HGDH	min copies <2	LOC389852	sex-linked	SIRT7	min copies <2
DAZ1	sex-linked	LOC653606	✓	SLX1B	homogeneous
DEFA1	✓	LOC727800	min copies <2	SMN2	min copies <2
DEFB103A	min copies <2	LOC728932	<5 copies	SNAR-A1	homogeneous
DEFB104A	<5 copies	LPA	<5 copies	SPAG11	min copies <2
DEFB105A	min copies <2	MAFG	<5 copies	SPANXA1	sex-linked
DEFB106A	min copies <2	MAGEA2	sex-linked	SPDYE3	✓
DEFB107A	min copies <2	MAGEA9	sex-linked	SSX4	sex-linked
DEFB130	✓	MGC59937	min copies <2	SULT1A1	min copies <2
DGAT1	min copies <2	MRC1	min copies <2	SULT1A3	✓
DHRS4	<5 copies	MSat1	microsatellite	TAF9B	sex-linked
DMRTC1	sex-linked	MSat10	microsatellite	TAS1R3	min copies <2
DTYMK	min copies <2	MSat11	microsatellite	TBC1D3	✓
DUX4	>60 copies	MSat12	microsatellite	TBC1D3 (probe2)	excluded (duplicate)
EIF3C	<5 copies	MSat13	microsatellite	TCEB3C	✓
ELA	<5 copies	MSat14	microsatellite	THAP4	min copies <2
F8A2	sex-linked	MSat15	microsatellite	TMEM106A	min copies <2
FAM156A	sex-linked	MSat2	microsatellite	TMEM167B	min copies <2
FAM203A	min copies <2	MSat5	microsatellite	TMPRSS11E	min copies <2
FAM21B	<5 copies	MSat6	microsatellite	TP53TG3	✓
FAM23A	min copies <2	MSat7	microsatellite	TRIM49L1	✓
FAM72A	✓	MSat8	microsatellite	TSPY4	sex-linked
FAM75A1	✓	MSat9	microsatellite	UGT2B15	min copies <2
FAM75A5	✓	MUC12	✓	USP17	>60 copies
FAM90A	>60 copies	MUC5AC	min copies <2	XAGE1	sex-linked
FCGBP	✓	NBPF10	>60 copies	XAGE2	sex-linked
FCGR	min copies <2	NBPF11	✓	XKRY2	sex-linked
FOXD4L2	✓	NBPF16	✓	ZFP62	min copies <2
FUSIP1	<5 copies	NDUFB4	min copies <2	ZG16	min copies <2
GAGE6	sex-linked	NEB	min copies <2	ZNF658B	✓
GOLGA6L9	✓	NPB	min copies <2		
GOLGA8G	✓	NPIP	✓		

Table S2: Positions of genes in hg38

Gene	Chromosome	Start	End
AMY1A	chr1	103655519	103664554
BOLA2B	chr16	29453588	29454964
BOLA2B	chr16	30192932	30193666
CBWD3	chr9	65666374	65734041
CBWD3	chr9	68241866	68300035
CLEC18A	chr16	69950232	69968478
CSH	chr7	123763708	123877481
CSH	chr16	81081945	81096395
CSH	chr17	18327873	18363560
CSH	chr17	63872012	63873729
CSH	chr17	63894918	63896574
CSH	chr17	63909602	63911280
CSH	chr19	11435290	11450968
CSH	chr19	58498333	58512028
DEFA1	chr8	6977649	6980120
DEFA1	chr8	6996766	6999198
DEFB130	chr8	12064389	12071747
DEFB130	chr8	12310962	12318316
FAM72A	chr1	206186179	206205773
FAM75A1	chr9	39355667	39361962
FAM75A5	chr9	60914372	60920653
FOXD4L2	chr9	65737146	65738396
FOXD4L2	chr9	68302867	68305084
GOLGA6L9	chr15	82429363	82439153
GOLGA8G	chr15	28519611	28533014
HIST2	chr1	143902386	143905977
HIST2	chr1	149782689	149812370
HIST2	chr1	149813225	149813693
HIST2	chr1	149832657	149833052
HIST2	chr1	149840687	149841208
HIST2	chr1	149842218	149842750
HIST2	chr1	149851061	149851594
HIST2	chr1	149852608	149853125
HIST2	chr1	149853262	149861159
HIST2	chr1	149884459	149886682
HIST2	chr1	149886918	149887411
HIST2	chr1	149887469	149887965

LIMS3	chr2	109898688	109925625
LIMS3	chr2	110386341	110473075
MUC12	chr7	100969565	101018936
NBPF11	chr1	148102151	148152281
NBPF16	chr1	144421390	144461669
NPIP	chr1	44221070	44355298
NPIP	chr16	11927263	11976643
NPIP	chr16	14703411	14726342
NPIP	chr16	14742533	14765462
NPIP	chr16	14937443	14952056
NPIP	chr16	15363613	15381467
NPIP	chr16	16379140	16393972
NPIP	chr16	18317919	18336736
NPIP	chr16	21834568	21857756
NPIP	chr16	22503599	22536535
NPIP	chr16	28342517	28364780
NPIP	chr16	28456329	28472336
NPIP	chr16	28637891	28658744
NPIP	chr16	29381310	29406711
NPIP	chr16	30222897	30248479
NPIP	chr16	74374986	74392115
PGA3	chr11	61203515	61213098
PRAMEF14	chr1	13341892	13347134
PRAMEF20	chr1	13410450	13421328
PRAMEF5	chr1	13254198	13263435
PRAMEF8	chr1	13281223	13285137
PRR11	chr17	59155355	59206709
PRR20A	chr13	57140918	57143939
PRR20A	chr13	57147488	57150509
PRR20A	chr13	57154061	57157082
PRR20A	chr13	57160632	57163653
PRR20A	chr13	57167197	57170218
PSG3	chr19	42721642	42740486
RGPD1	chr2	86913577	87013976
SPDYE3	chr7	100306798	100322196
SULT1A3	chr16	30199255	30204310
TBC1D3	chr6	121079494	121335384
TBC1D3	chr8	123041961	123165173
TBC1D3	chr12	64759484	64881033
TBC1D3	chr17	36165683	36176636

TBC1D3	chr17	36253639	36264601
TBC1D3	chr17	36322242	36334759
TBC1D3	chr17	36377531	36388452
TBC1D3	chr17	37923805	37935371
TBC1D3	chr17	37978155	37989060
TBC1D3	chr17	38003976	38014908
TBC1D3	chr17	38057693	38068634
TBC1D3	chr17	38118408	38138868
TBC1D3	chr17	38181659	38192555
TP53TG3	chr16	32251603	32255928
TP53TG3	chr16	32673528	32676165
TP53TG3	chr16	33191770	33196858
TP53TG3	chr16	33301850	33306945
TP53TG3	chr16	33360841	33363478
TP53TG3	chr16	33457156	33462249
TRIM49L1	chr11	89924064	89933063

1 CEU AMY1A

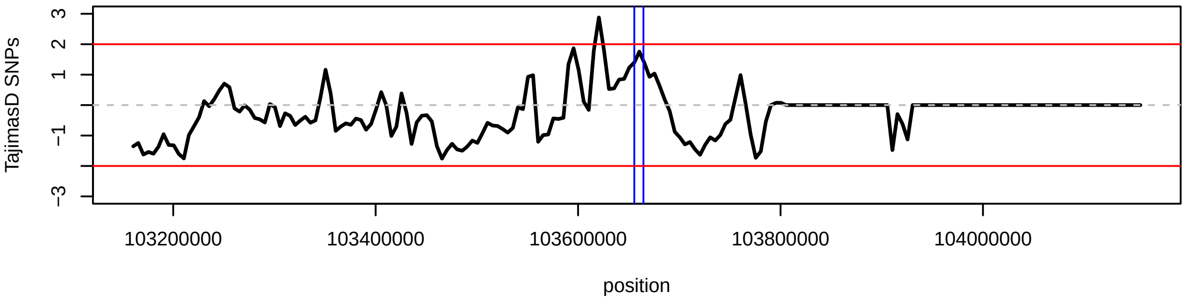

1 CHB AMY1A

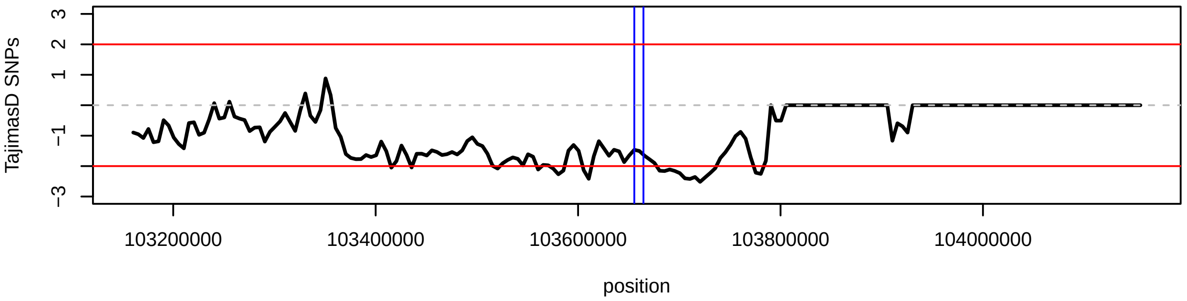

1 YRI AMY1A

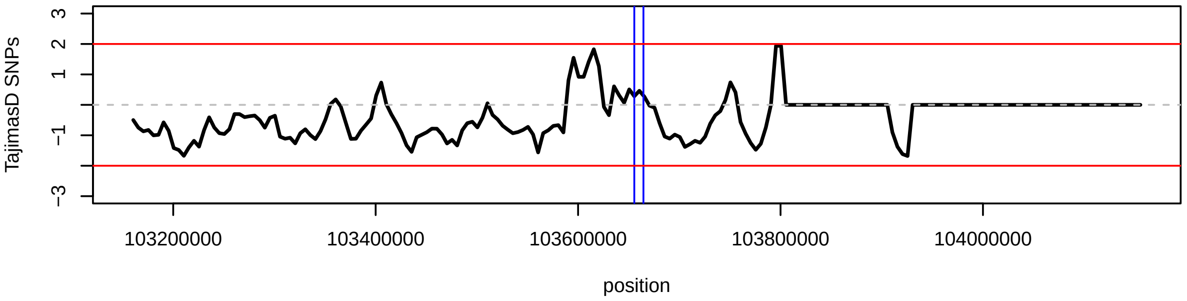

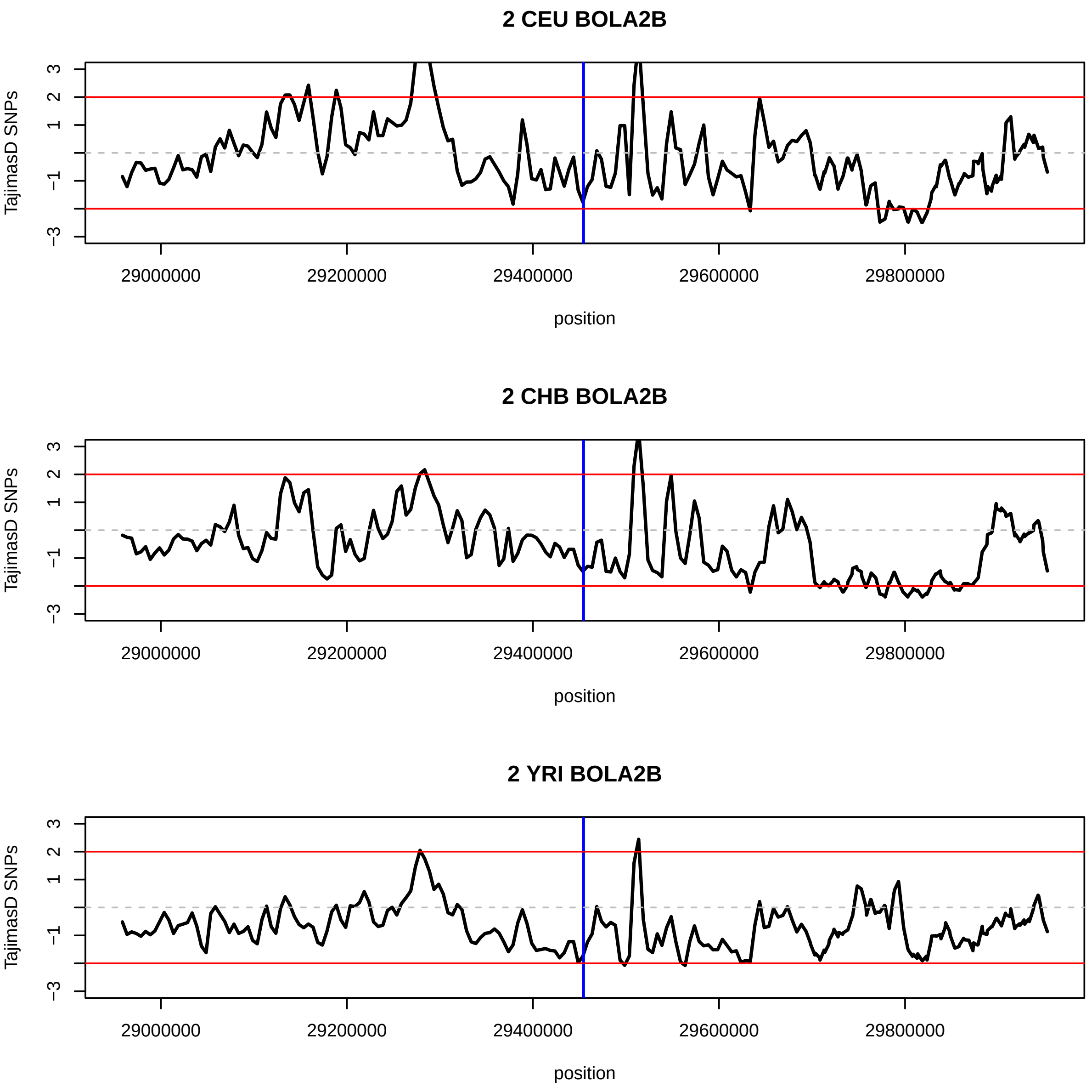
2 CEU BOLA2B
TajimasD SNPs
position
29000000
29200000
29400000
29600000
29800000
2 CHB BOLA2B
TajimasD SNPs
position
2 YRI BOLA2B
TajimasD SNPs
position

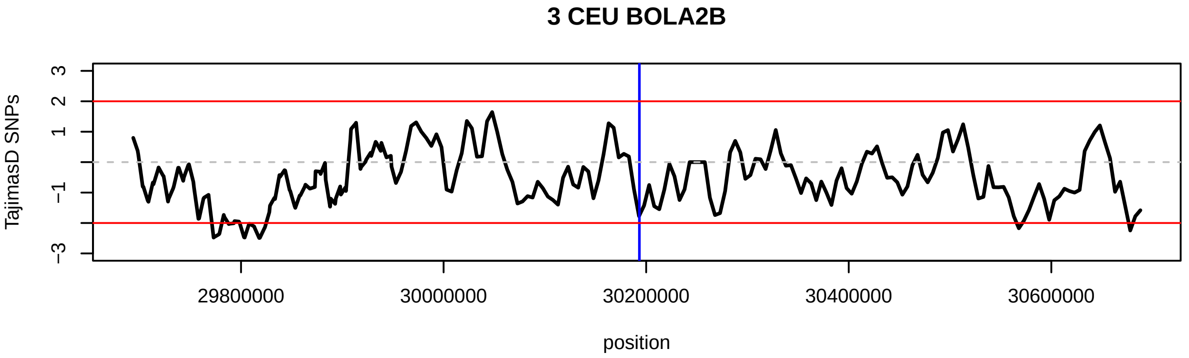
3 CEU BOLA2B
TajimasD SNPs
3
2
1
−1
−3
29800000
30000000
30200000
30400000
30600000
position

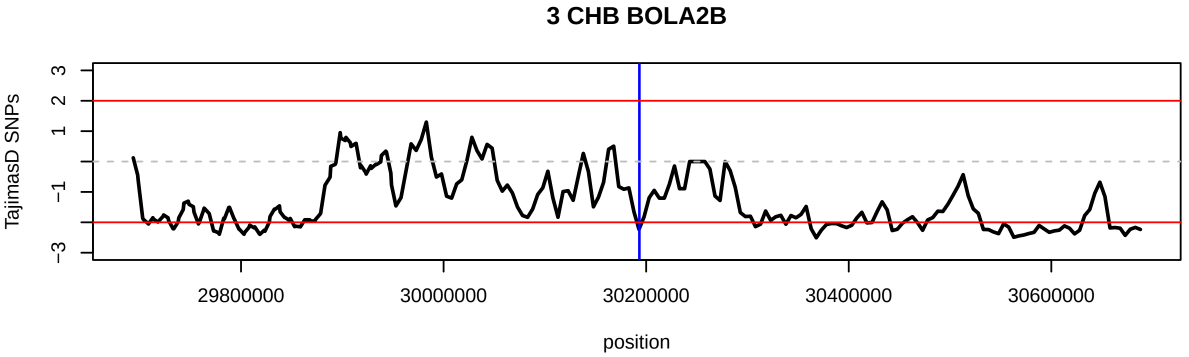
3 CHB BOLA2B
TajimasD SNPs
3
2
1
−1
−3
29800000
30000000
30200000
30400000
30600000
position

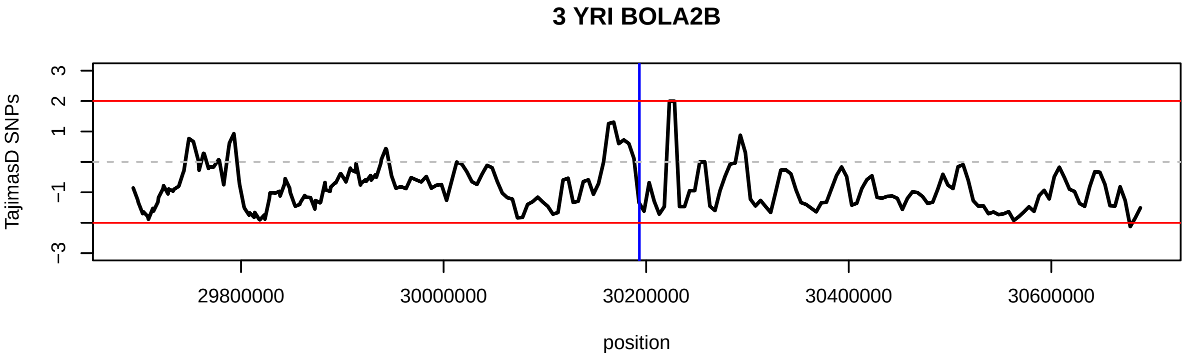
3 YRI BOLA2B
TajimasD SNPs
3
2
1
−1
−3
29800000
30000000
30200000
30400000
30600000
position

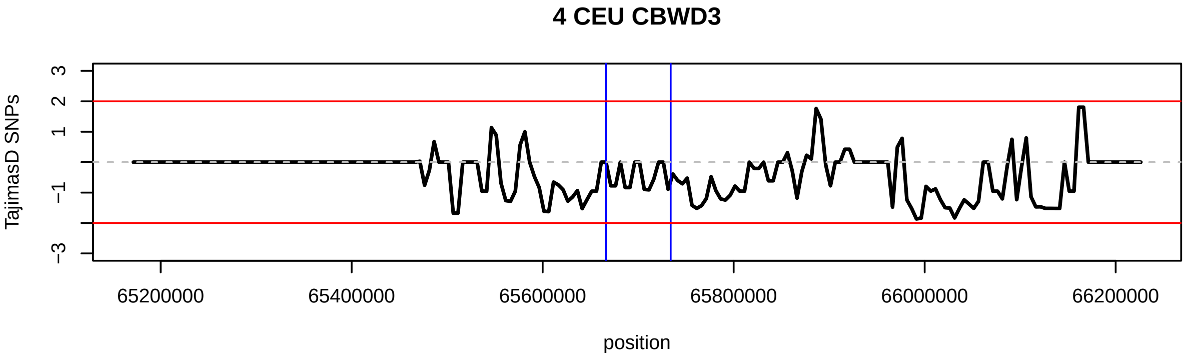
4 CEU CBWD3
TajimasD SNPs
3
2
1
−1
−3
65200000
65400000
65600000
65800000
66000000
66200000
position

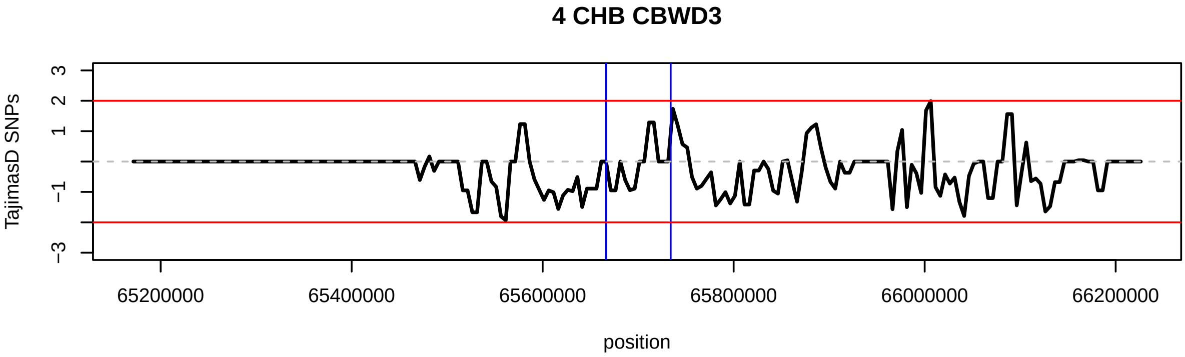
4 CHB CBWD3
TajimasD SNPs
3
2
1
−1
−3
65200000
65400000
65600000
65800000
66000000
66200000
position

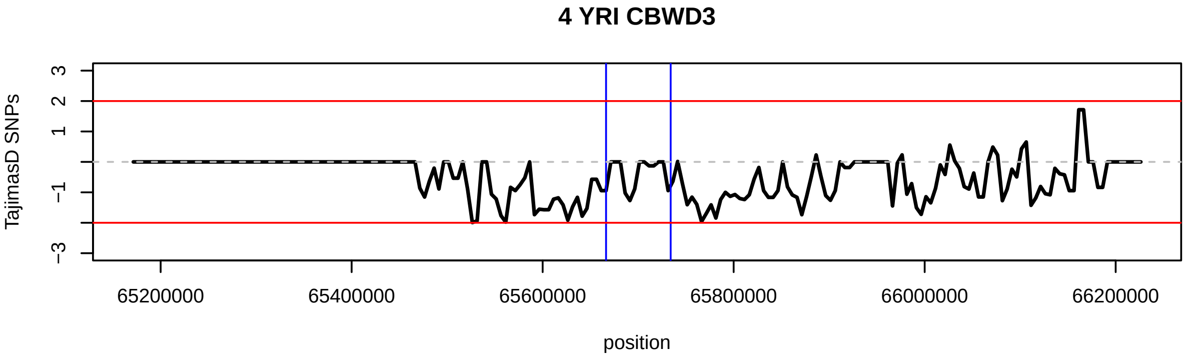
4 YRI CBWD3
TajimasD SNPs
3
2
1
−1
−3
65200000
65400000
65600000
65800000
66000000
66200000
position

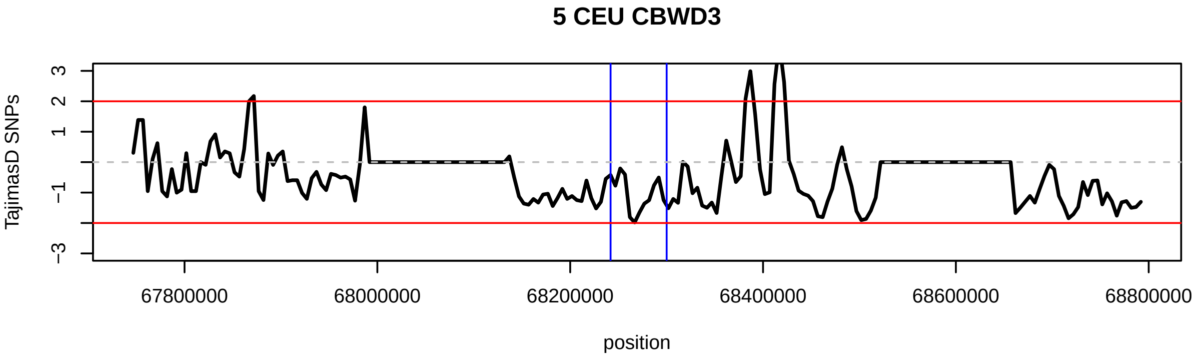
5 CEU CBWD3
TajimasD SNPs
3
2
1
−1
−3
67800000
68000000
68200000
68400000
68600000
68800000
position

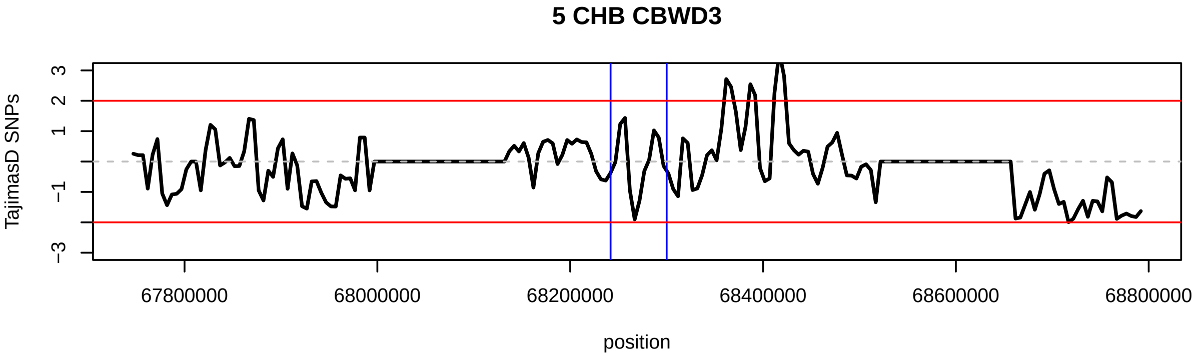
5 CHB CBWD3
TajimasD SNPs
3
2
1
−1
−3
67800000
68000000
68200000
68400000
68600000
68800000
position

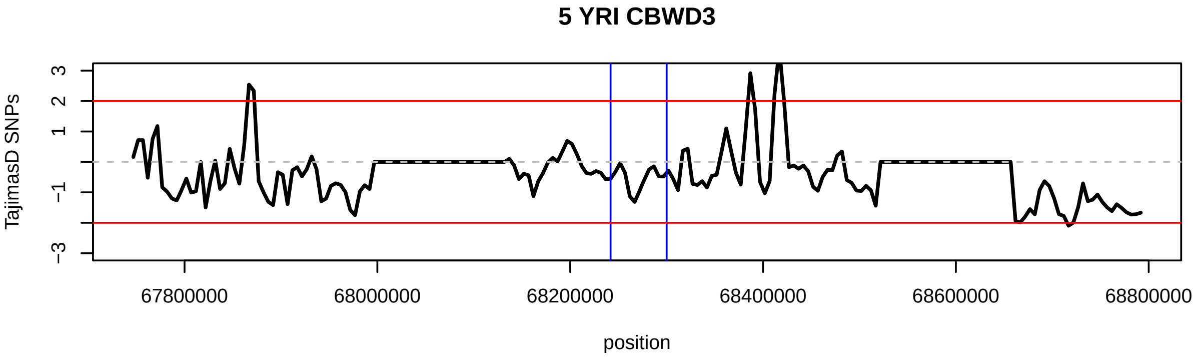
5 YRI CBWD3
TajimasD SNPs
3
2
1
−1
−3
67800000
68000000
68200000
68400000
68600000
68800000
position

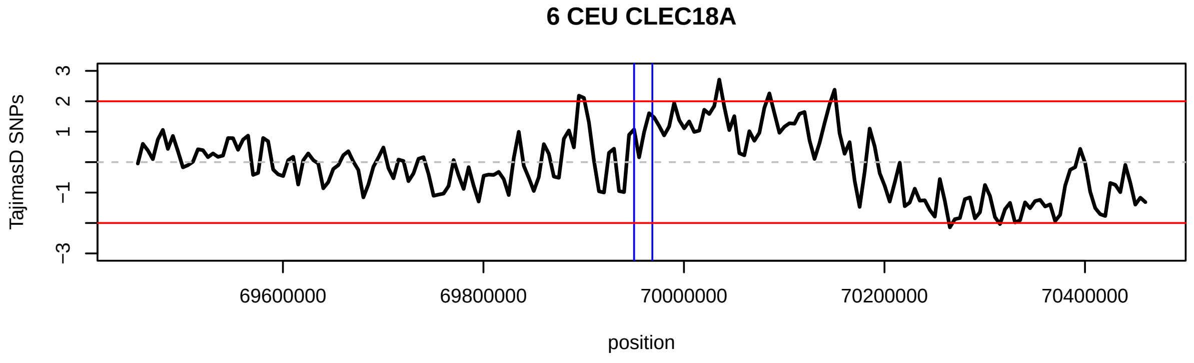
6 CEU CLEC18A
TajimasD SNPs
3
2
1
−1
−3
69600000
69800000
70000000
70200000
70400000
position

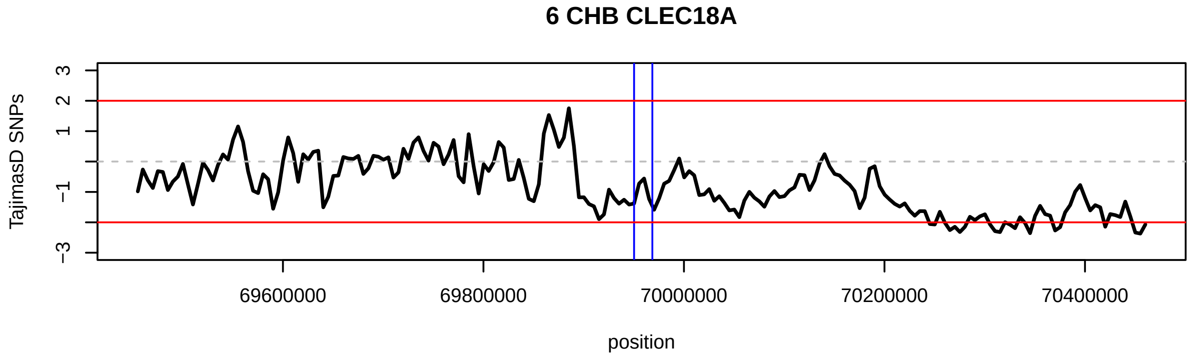
6 CHB CLEC18A
TajimasD SNPs
3
2
1
−1
−3
69600000
69800000
70000000
70200000
70400000
position

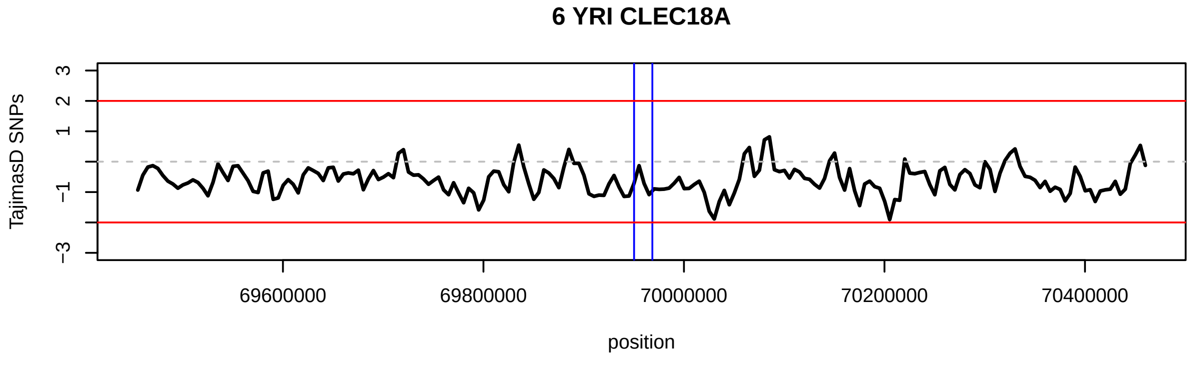
6 YRI CLEC18A
TajimasD SNPs
3
2
1
−1
−3
69600000
69800000
70000000
70200000
70400000
position

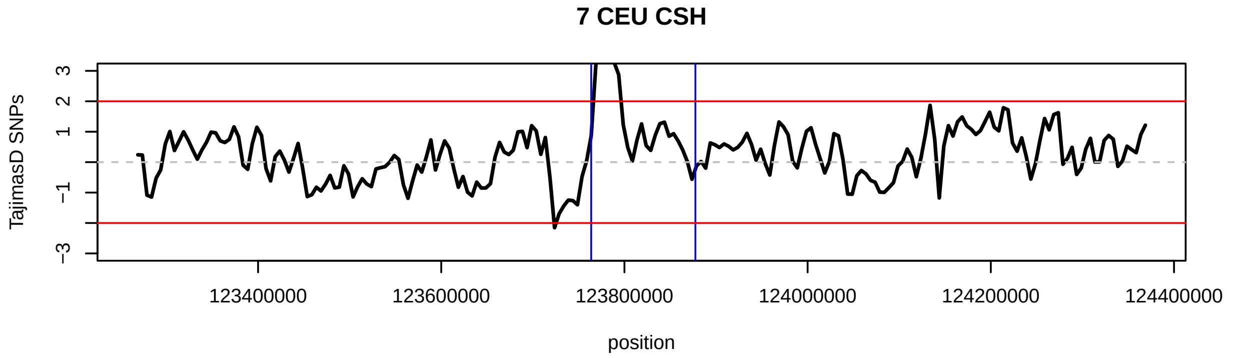
7 CEU CSH
TajimasD SNPs
−3
−1
1
2
3
123400000
123600000
123800000
124000000
124200000
124400000
position

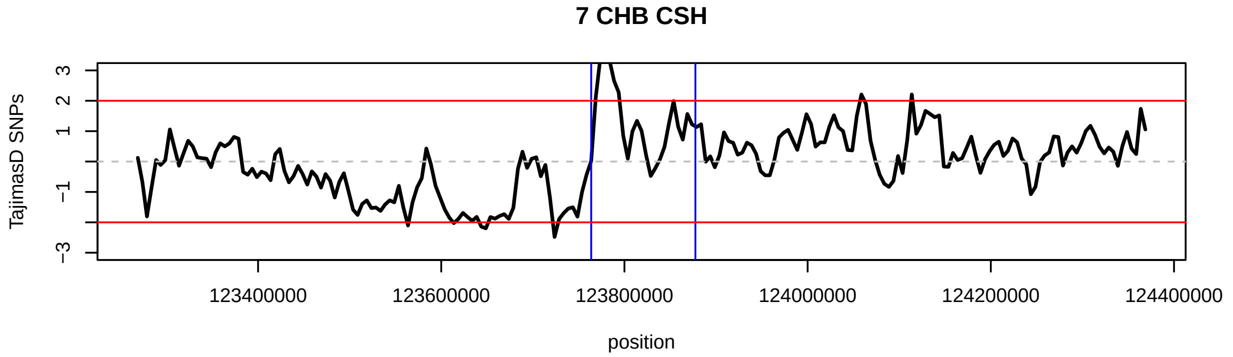
7 CHB CSH
TajimasD SNPs
−3
−1
1
2
3
123400000
123600000
123800000
124000000
124200000
124400000
position

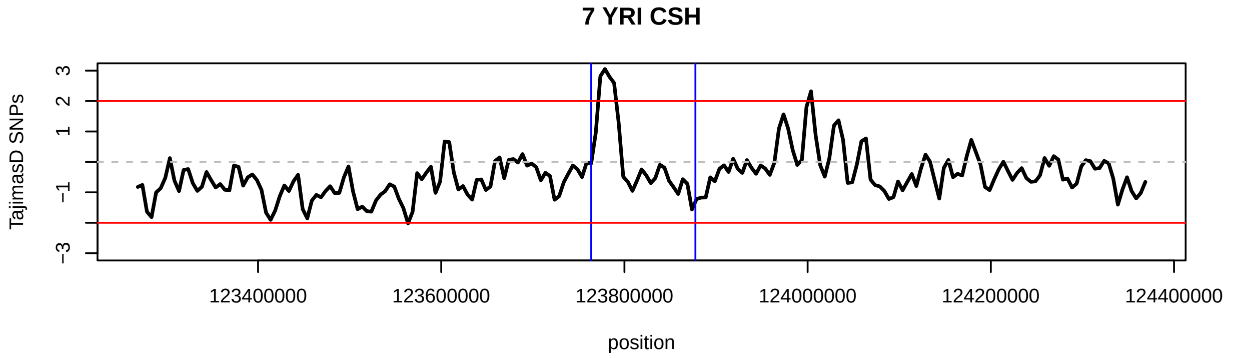
7 YRI CSH
TajimasD SNPs
−3
−1
1
2
3
123400000
123600000
123800000
124000000
124200000
124400000
position

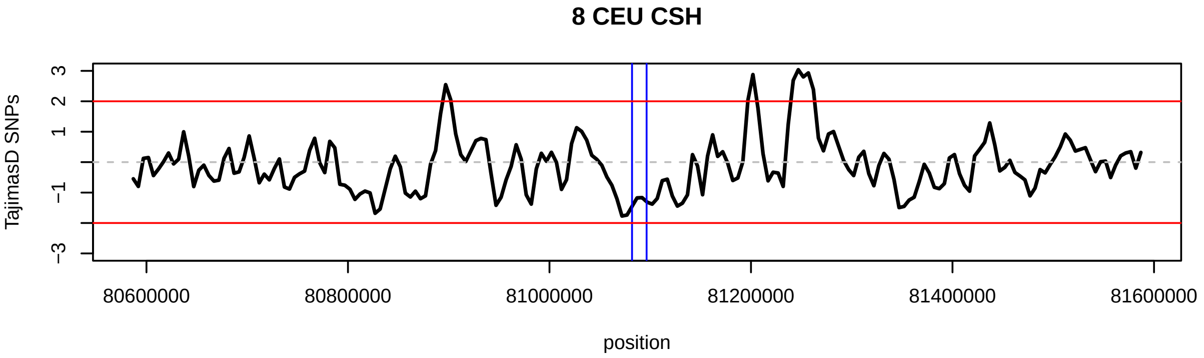
8 CEU CSH
TajimasD SNPs
3
2
1
−1
−3
80600000
80800000
81000000
81200000
81400000
81600000
position

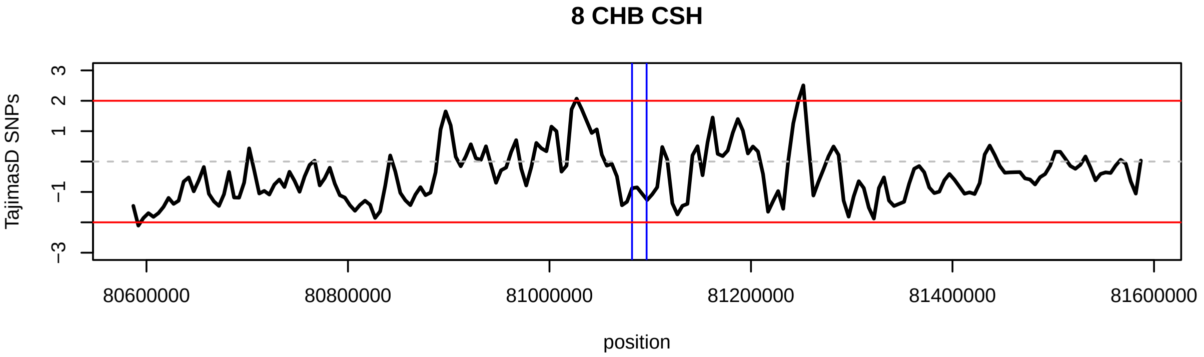
8 CHB CSH
TajimasD SNPs
3
2
1
−1
−3
80600000
80800000
81000000
81200000
81400000
81600000
position

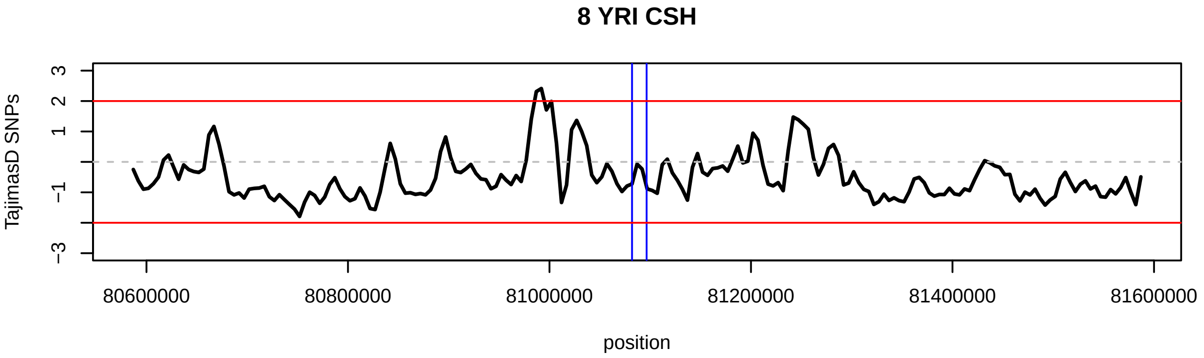
8 YRI CSH
TajimasD SNPs
3
2
1
−1
−3
80600000
80800000
81000000
81200000
81400000
81600000
position

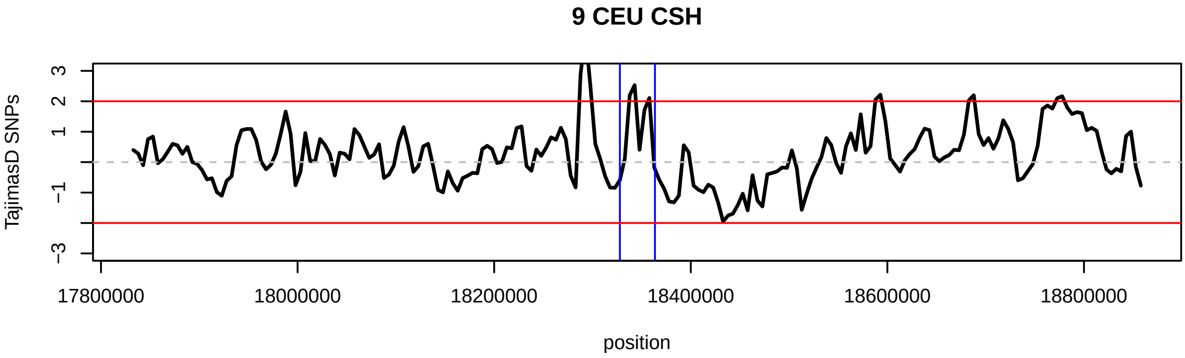
9 CEU CSH
TajimasD SNPs
3
2
1
−1
−3
17800000
18000000
18200000
18400000
18600000
18800000
position

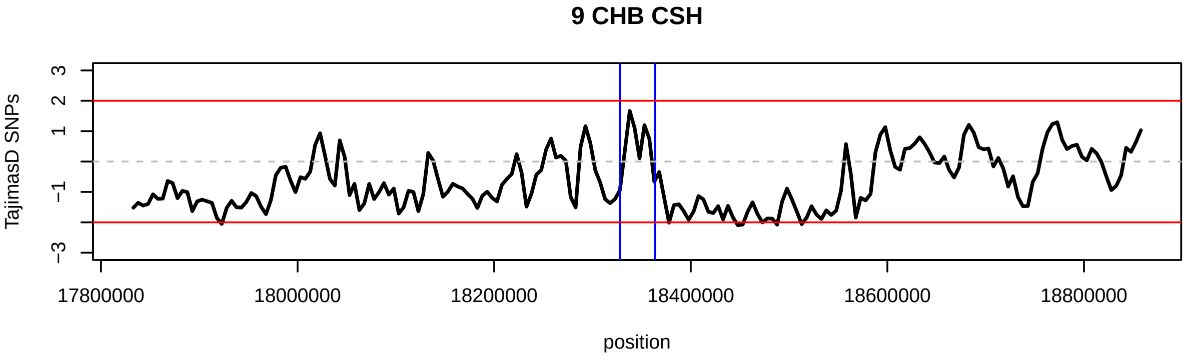
9 CHB CSH
TajimasD SNPs
3
2
1
−1
−3
17800000
18000000
18200000
18400000
18600000
18800000
position

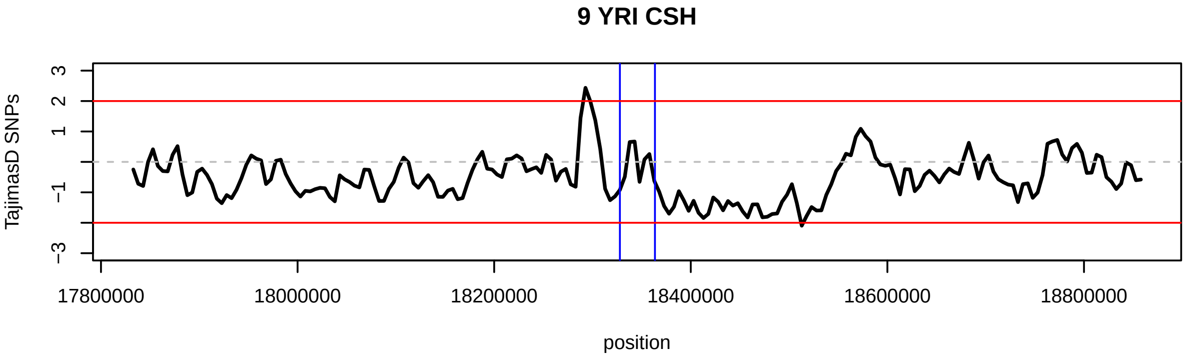
9 YRI CSH
TajimasD SNPs
3
2
1
−1
−3
17800000
18000000
18200000
18400000
18600000
18800000
position

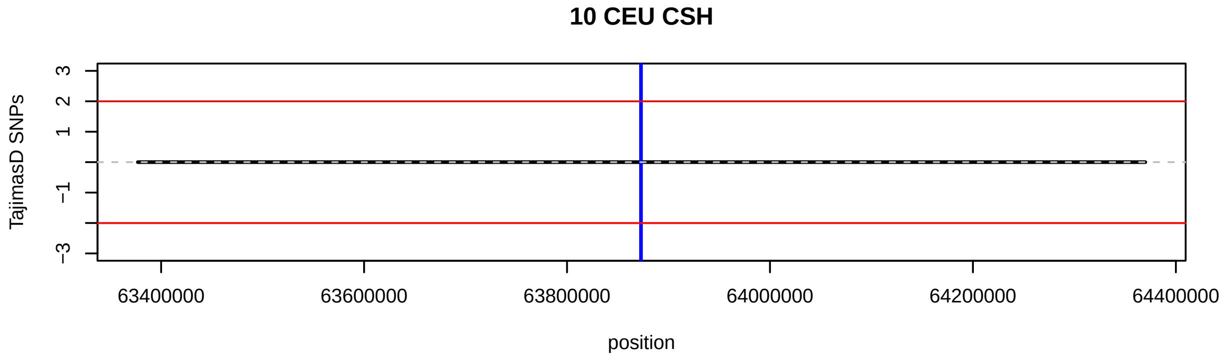
10 CEU CSH
TajimasD SNPs
3
2
1
−1
−3
63400000
63600000
63800000
64000000
64200000
64400000
position

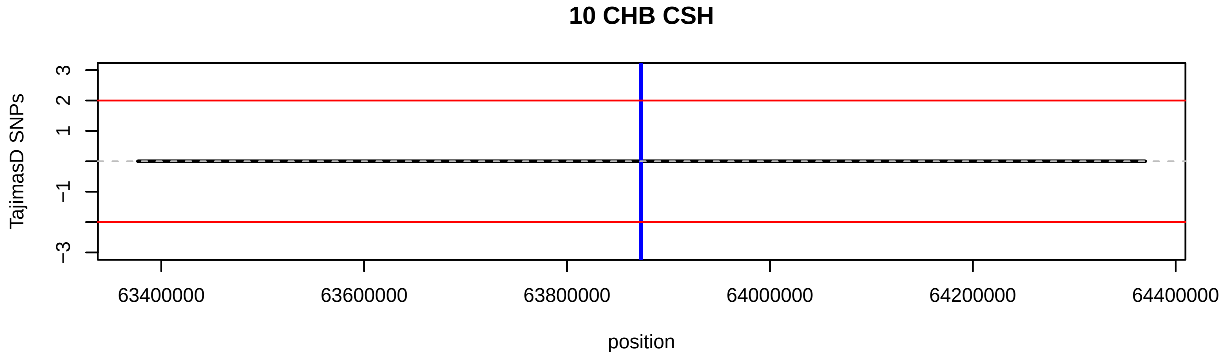
10 CHB CSH
TajimasD SNPs
3
2
1
−1
−3
63400000
63600000
63800000
64000000
64200000
64400000
position

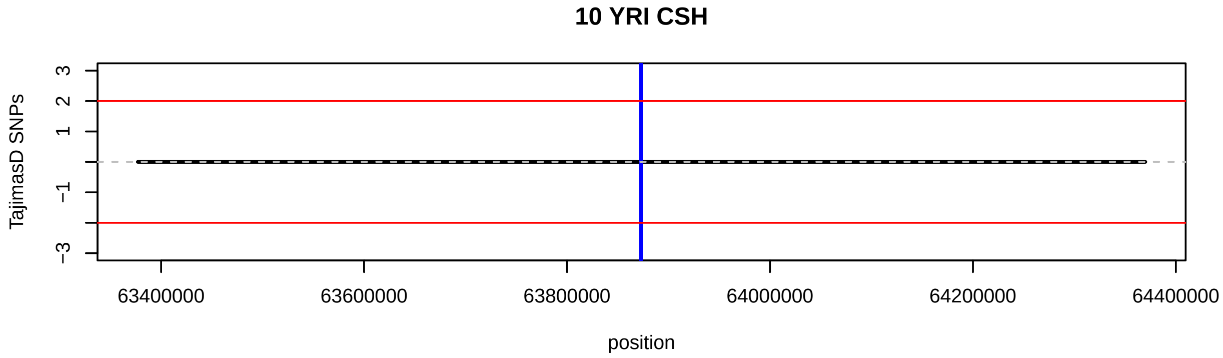
10 YRI CSH
TajimasD SNPs
3
2
1
−1
−3
63400000
63600000
63800000
64000000
64200000
64400000
position

11 CEU CSH

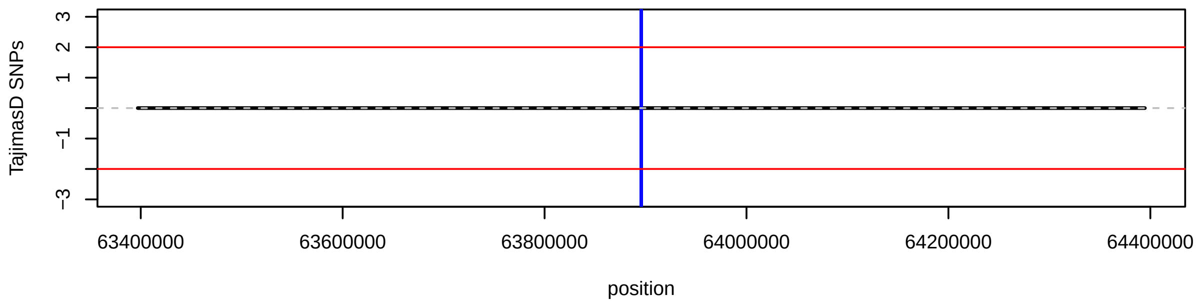

11 CHB CSH

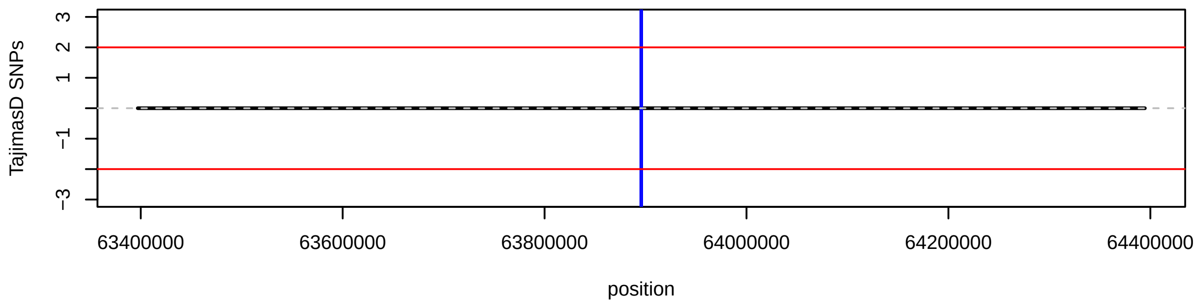

11 YRI CSH

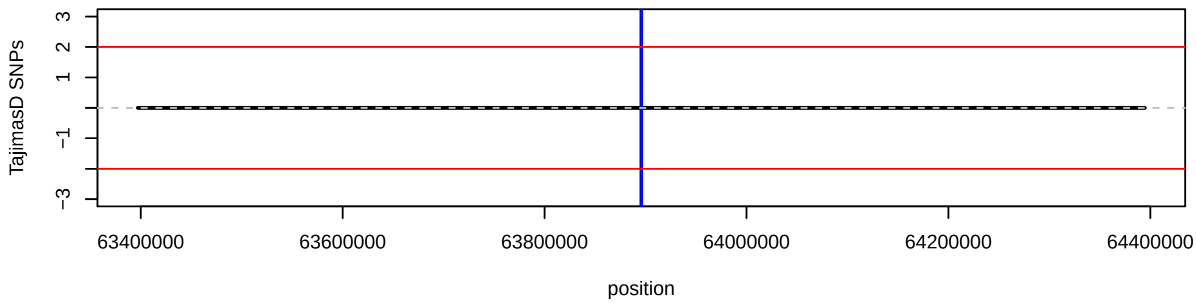

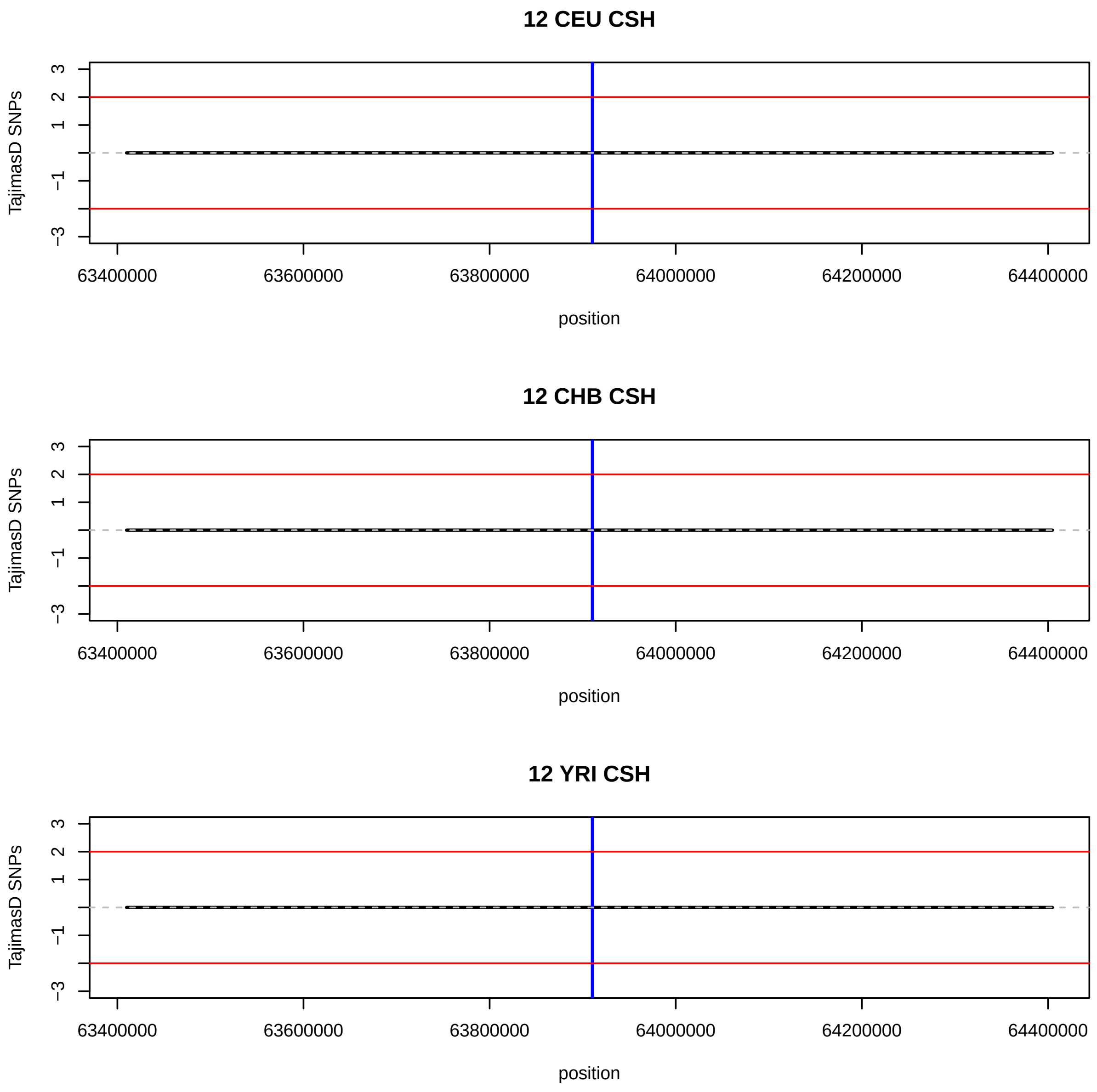
12 CEU CSH
TajimasD SNPs
3
2
1
−1
−3
63400000
63600000
63800000
64000000
64200000
64400000
position
12 CHB CSH
TajimasD SNPs
3
2
1
−1
−3
63400000
63600000
63800000
64000000
64200000
64400000
position
12 YRI CSH
TajimasD SNPs
3
2
1
−1
−3
63400000
63600000
63800000
64000000
64200000
64400000
position

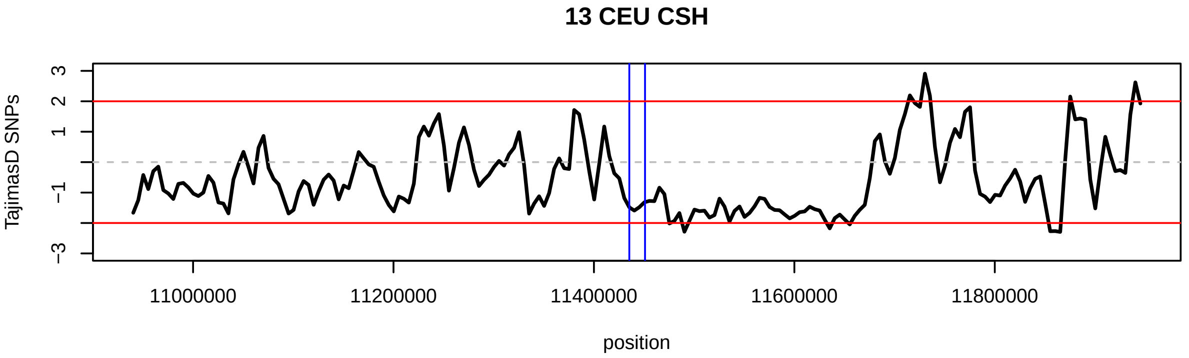
13 CEU CSH
TajimasD SNPs
3
2
1
−1
−3
11000000
11200000
11400000
11600000
11800000
position

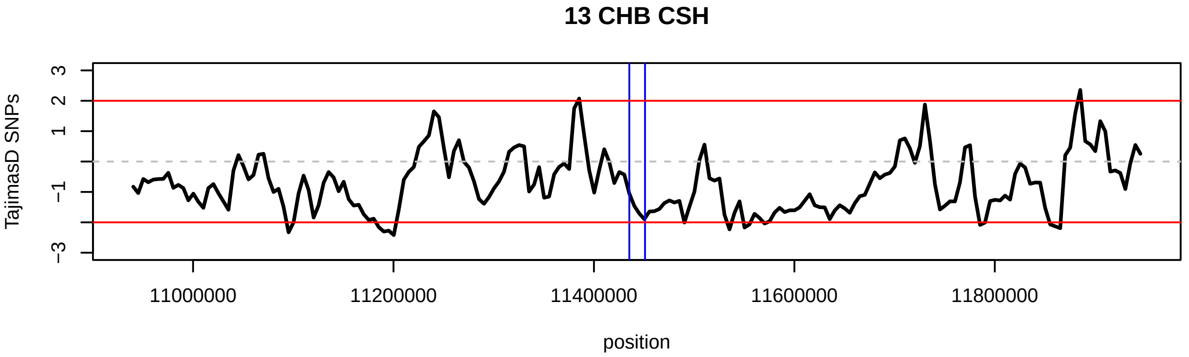
13 CHB CSH
TajimasD SNPs
3
2
1
−1
−3
11000000
11200000
11400000
11600000
11800000
position

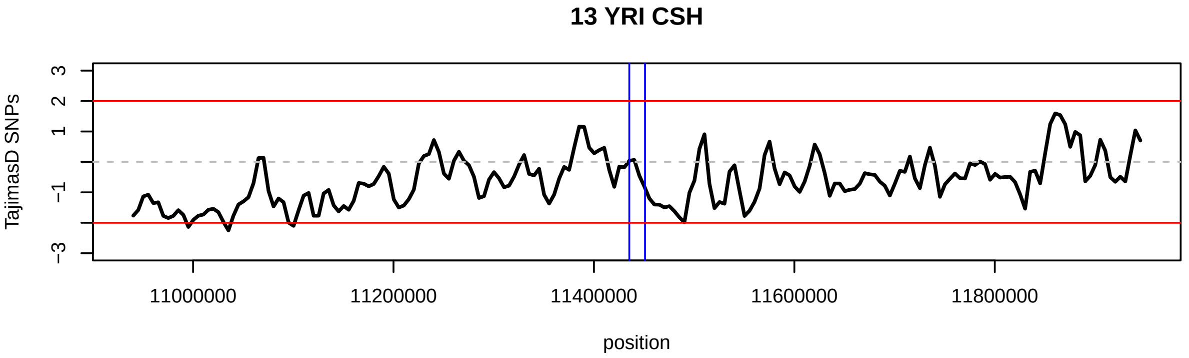
13 YRI CSH
TajimasD SNPs
3
2
1
−1
−3
11000000
11200000
11400000
11600000
11800000
position

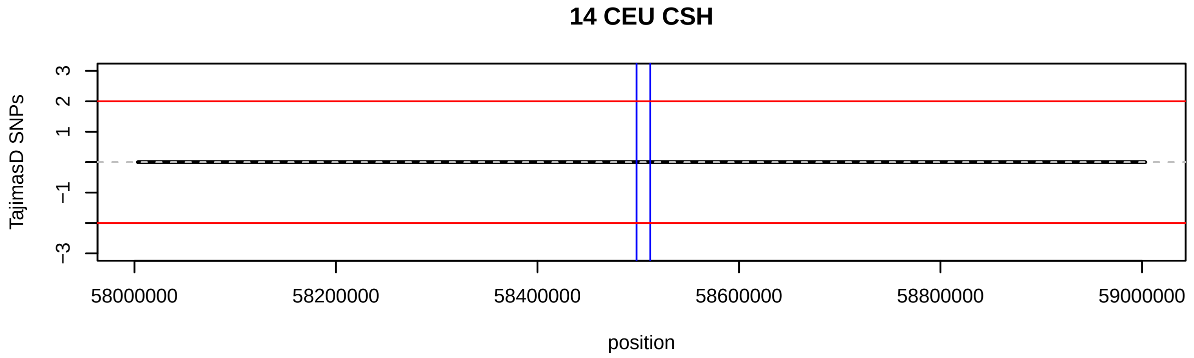
14 CEU CSH
TajimasD SNPs
3
2
1
−1
−3
58000000
58200000
58400000
58600000
58800000
59000000
position

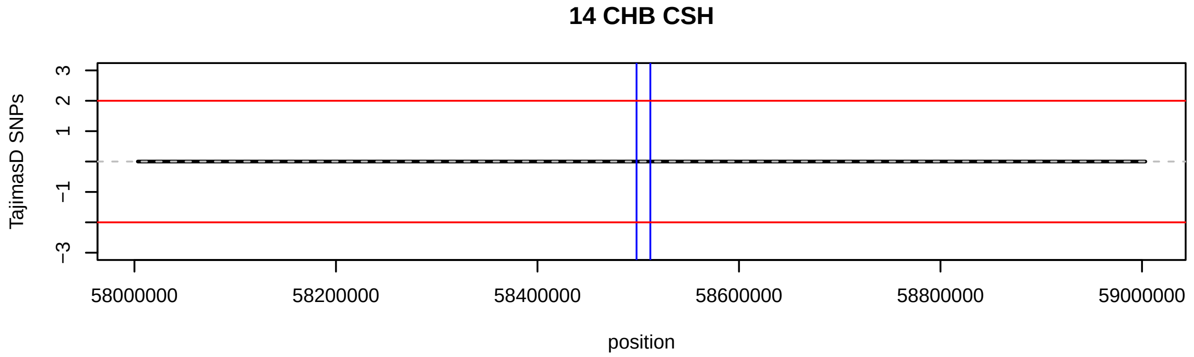
14 CHB CSH
TajimasD SNPs
3
2
1
−1
−3
58000000
58200000
58400000
58600000
58800000
59000000
position

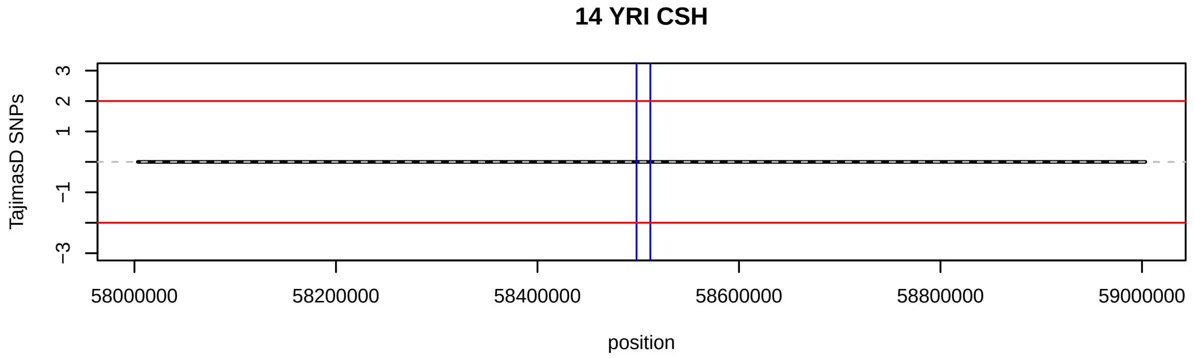
14 YRI CSH
TajimasD SNPs
3
2
1
−1
−3
58000000
58200000
58400000
58600000
58800000
59000000
position

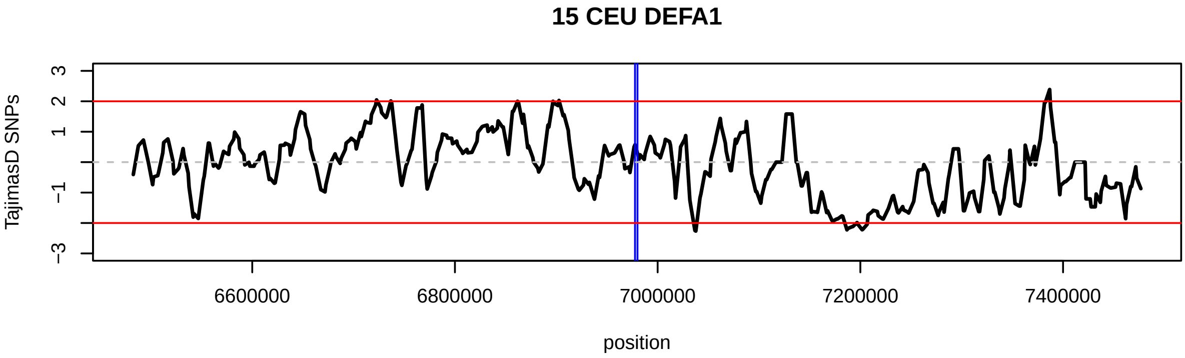

15 CEU DEFA1
TajimasD SNPs
3
2
1
−1
−3
6600000
6800000
7000000
7200000
7400000
position

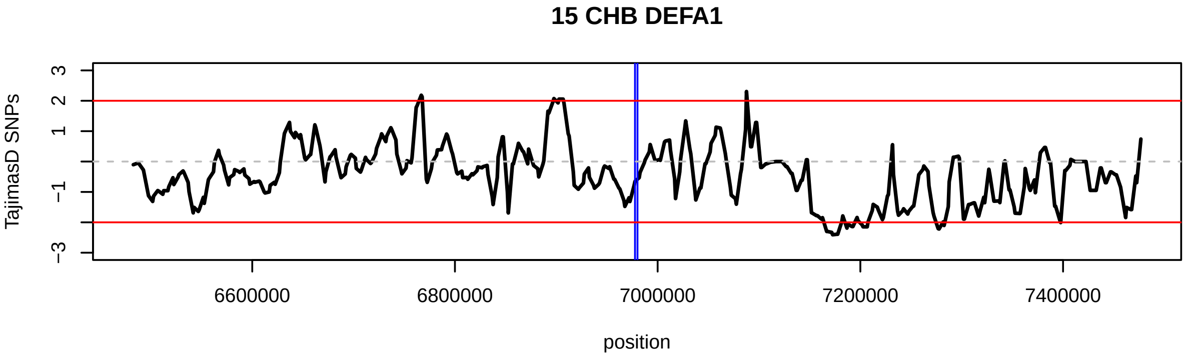

15 CHB DEFA1
TajimasD SNPs
3
2
1
−1
−3
6600000
6800000
7000000
7200000
7400000
position

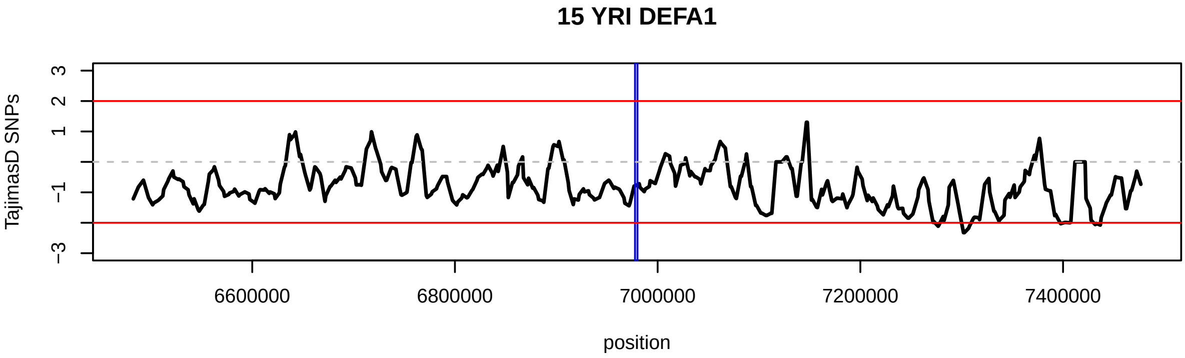

15 YRI DEFA1
TajimasD SNPs
3
2
1
−1
−3
6600000
6800000
7000000
7200000
7400000
position

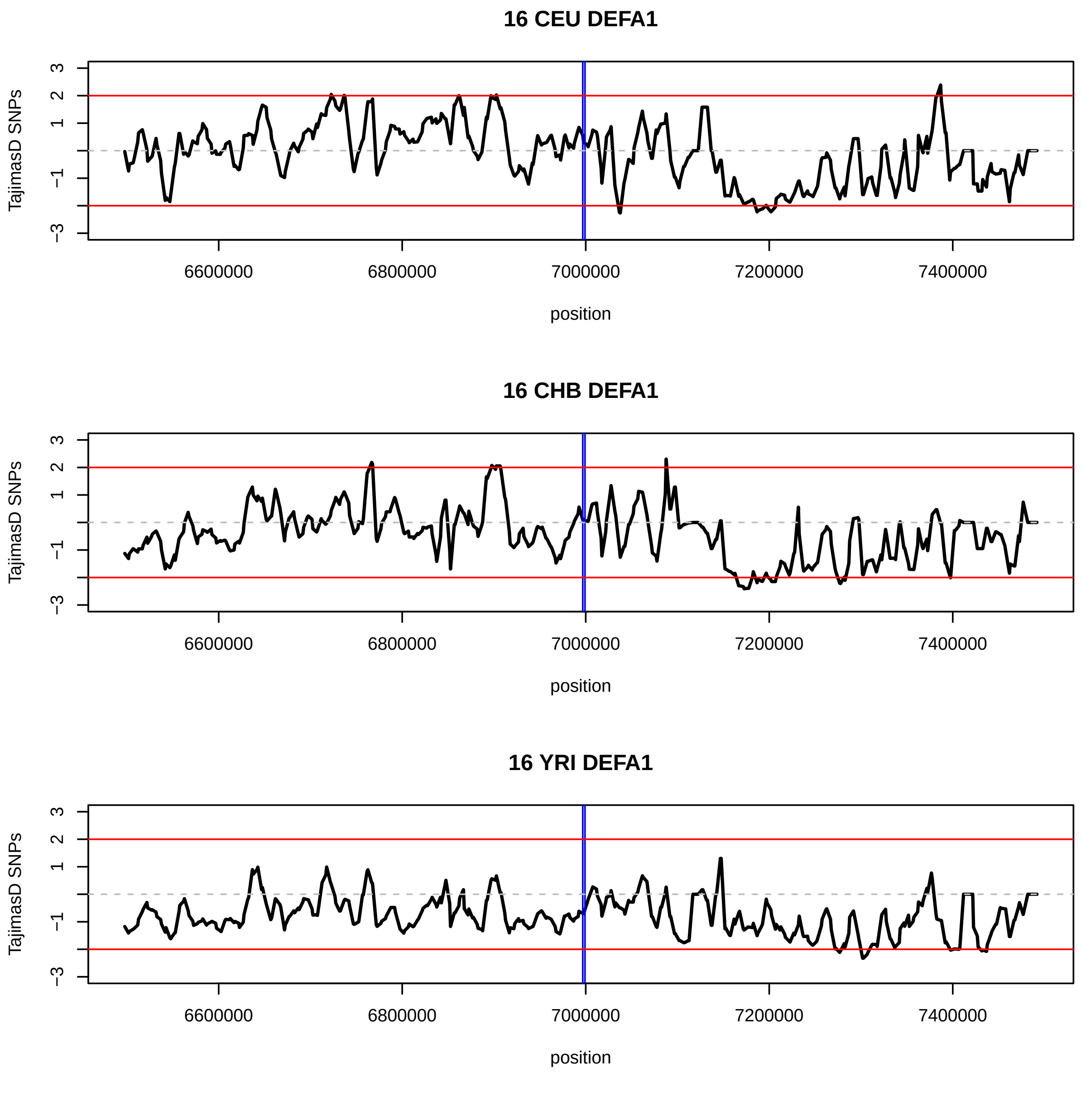
16 CEU DEFA1
TajimasD SNPs
3
2
1
−1
−3
6600000
6800000
7000000
7200000
7400000
position
16 CHB DEFA1
TajimasD SNPs
3
2
1
−1
−3
6600000
6800000
7000000
7200000
7400000
position
16 YRI DEFA1
TajimasD SNPs
3
2
1
−1
−3
6600000
6800000
7000000
7200000
7400000
position

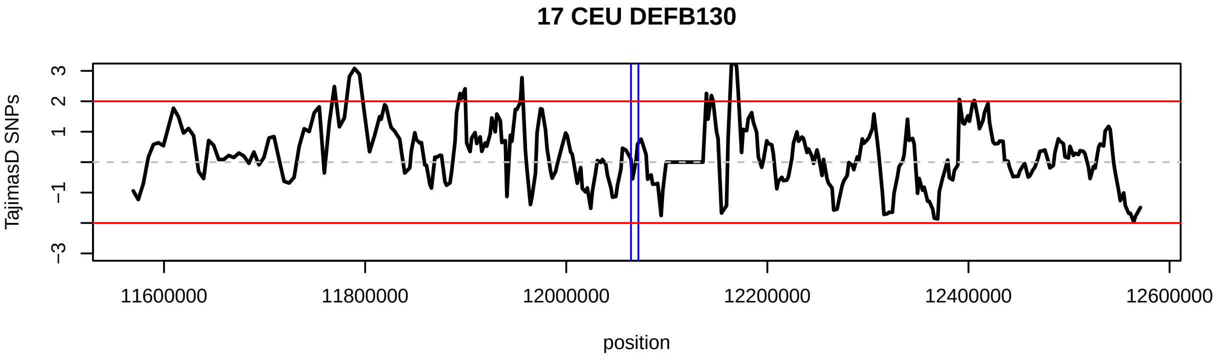
17 CEU DEFB130
TajimasD SNPs
3
2
1
−1
−3
11600000
11800000
12000000
12200000
12400000
12600000
position

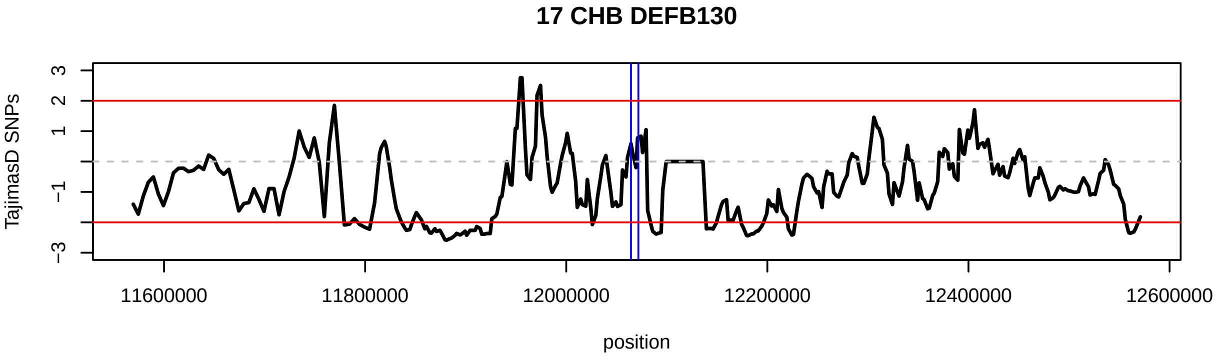
17 CHB DEFB130
TajimasD SNPs
3
2
1
−1
−3
11600000
11800000
12000000
12200000
12400000
12600000
position

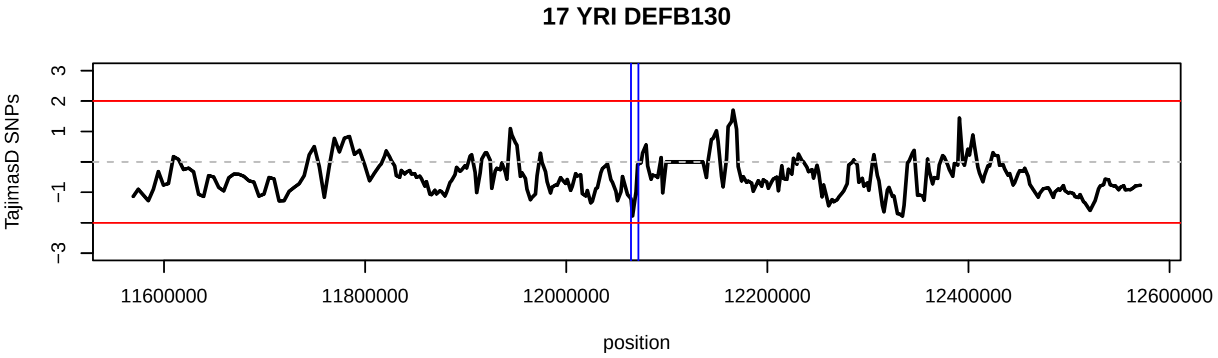
17 YRI DEFB130
TajimasD SNPs
3
2
1
−1
−3
11600000
11800000
12000000
12200000
12400000
12600000
position

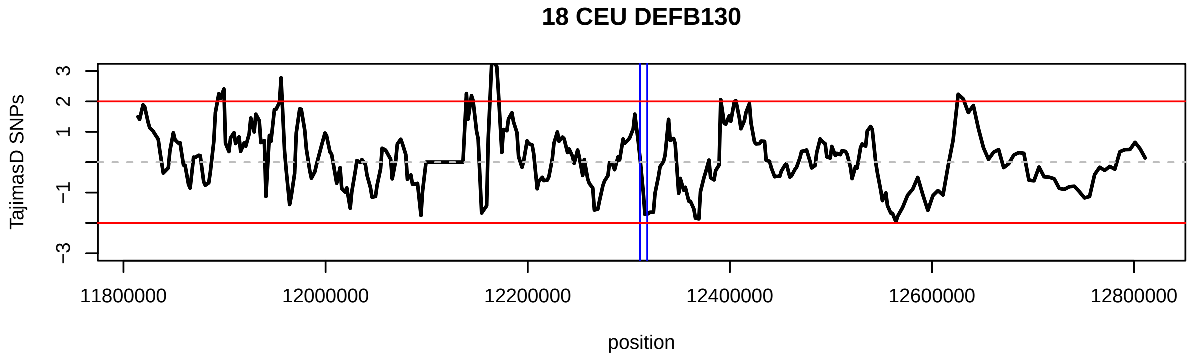
18 CEU DEFB130
TajimasD SNPs
3
2
1
−1
−3
11800000
12000000
12200000
12400000
12600000
12800000
position

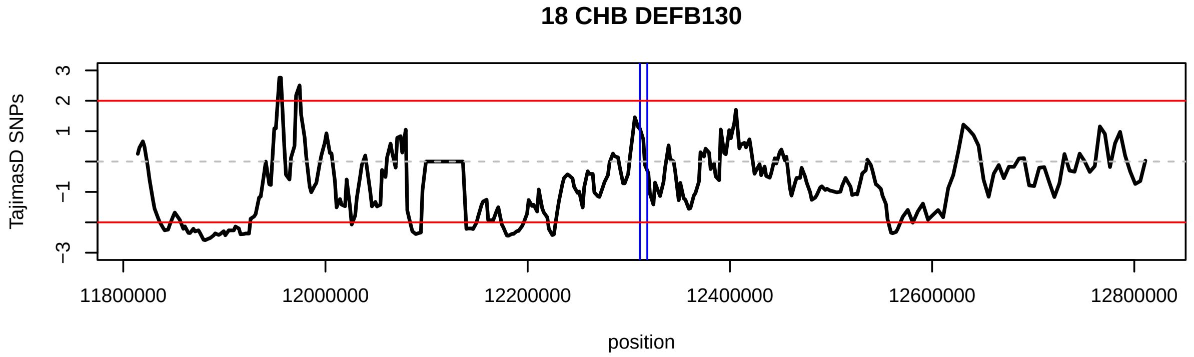
18 CHB DEFB130
TajimasD SNPs
3
2
1
−1
−3
11800000
12000000
12200000
12400000
12600000
12800000
position

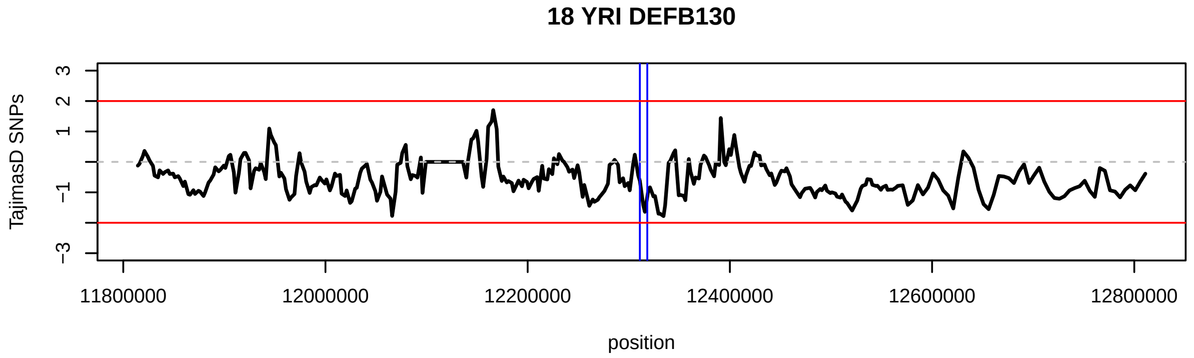
18 YRI DEFB130
TajimasD SNPs
3
2
1
−1
−3
11800000
12000000
12200000
12400000
12600000
12800000
position

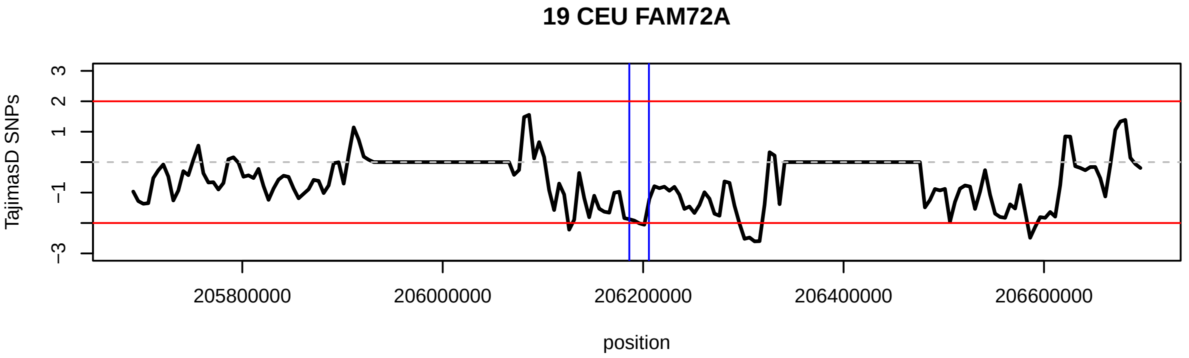
19 CEU FAM72A
TajimasD SNPs
3
2
1
−1
−3
205800000
206000000
206200000
206400000
206600000
position

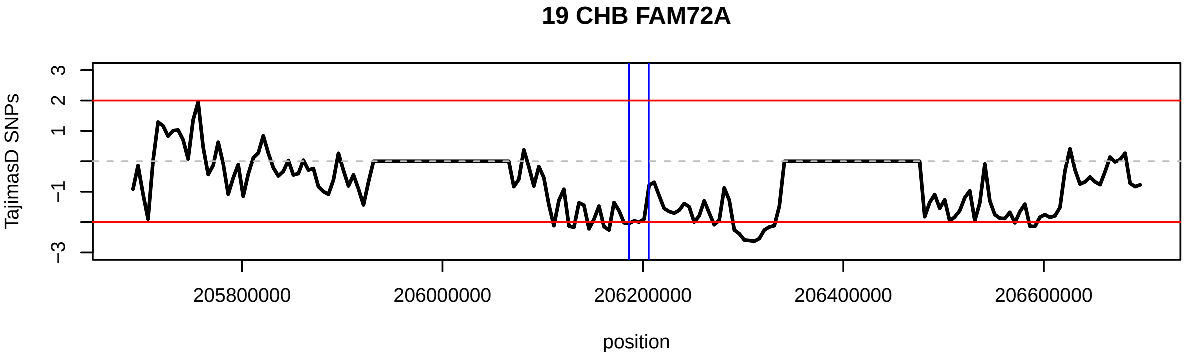
19 CHB FAM72A
TajimasD SNPs
3
2
1
−1
−3
205800000
206000000
206200000
206400000
206600000
position

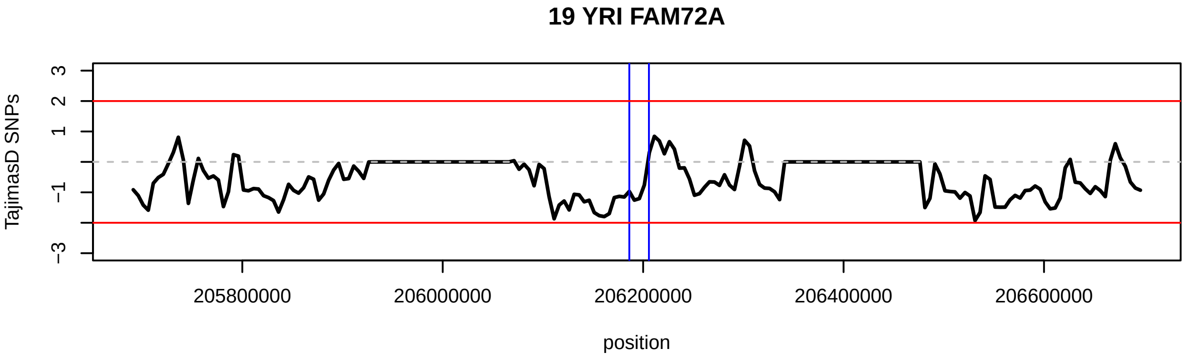
19 YRI FAM72A
TajimasD SNPs
3
2
1
−1
−3
205800000
206000000
206200000
206400000
206600000
position

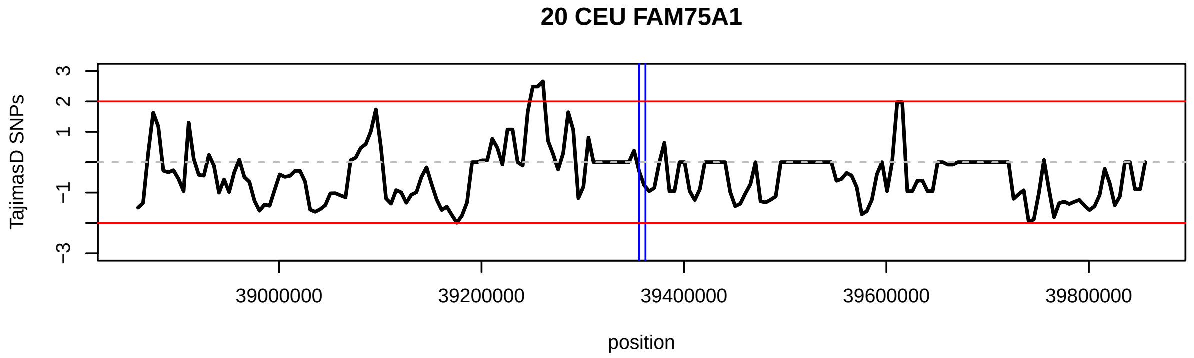
20 CEU FAM75A1
TajimasD SNPs
3
2
1
−1
−3
39000000
39200000
39400000
39600000
39800000
position

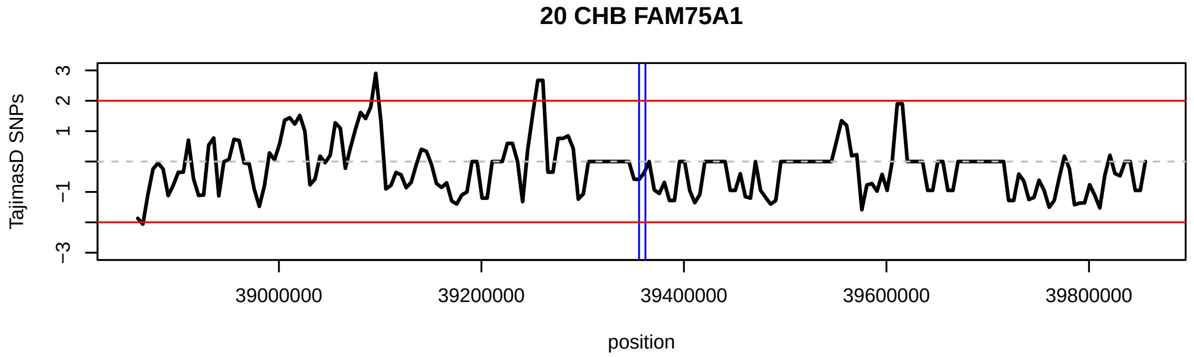
20 CHB FAM75A1
TajimasD SNPs
3
2
1
−1
−3
39000000
39200000
39400000
39600000
39800000
position

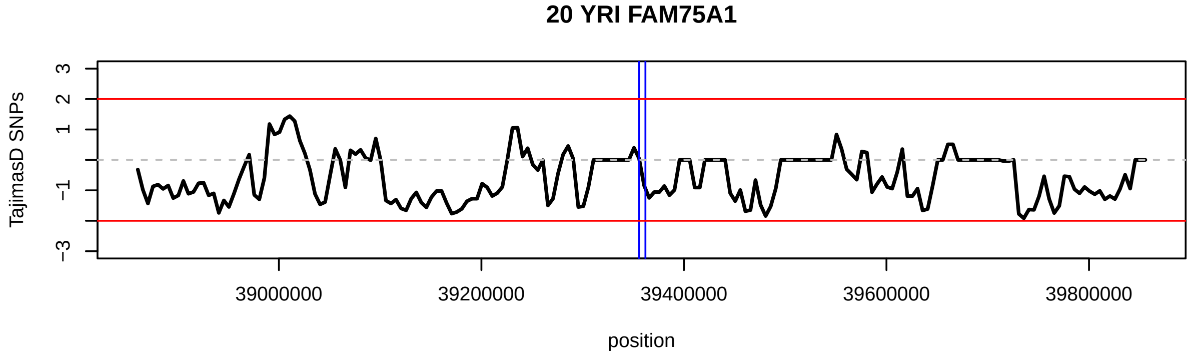
20 YRI FAM75A1
TajimasD SNPs
3
2
1
−1
−3
39000000
39200000
39400000
39600000
39800000
position

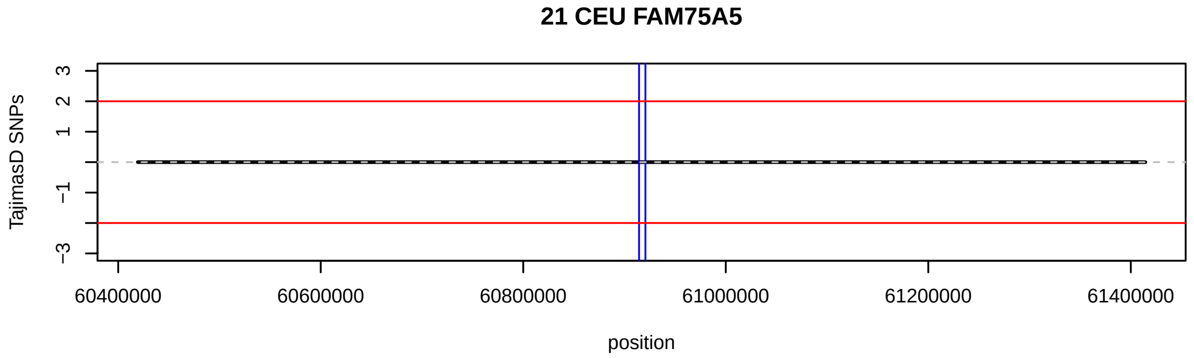
21 CEU FAM75A5
TajimasD SNPs
3
2
1
−1
−3
60400000
60600000
60800000
61000000
61200000
61400000
position

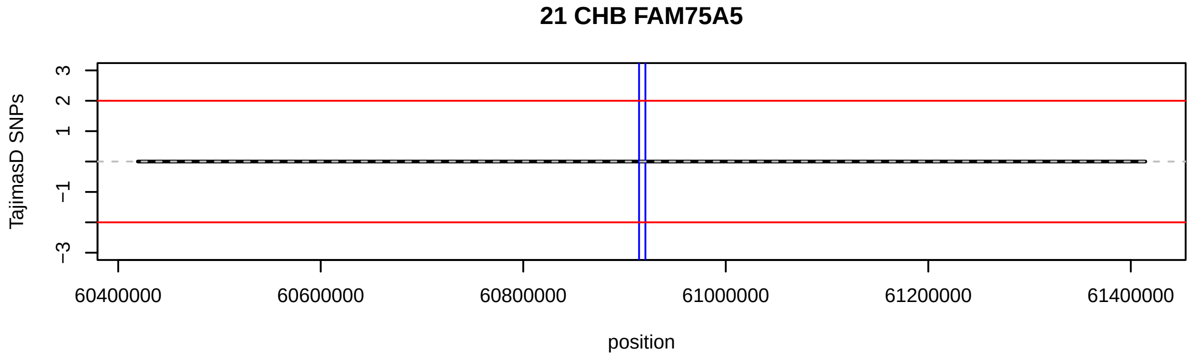
21 CHB FAM75A5
TajimasD SNPs
3
2
1
−1
−3
60400000
60600000
60800000
61000000
61200000
61400000
position

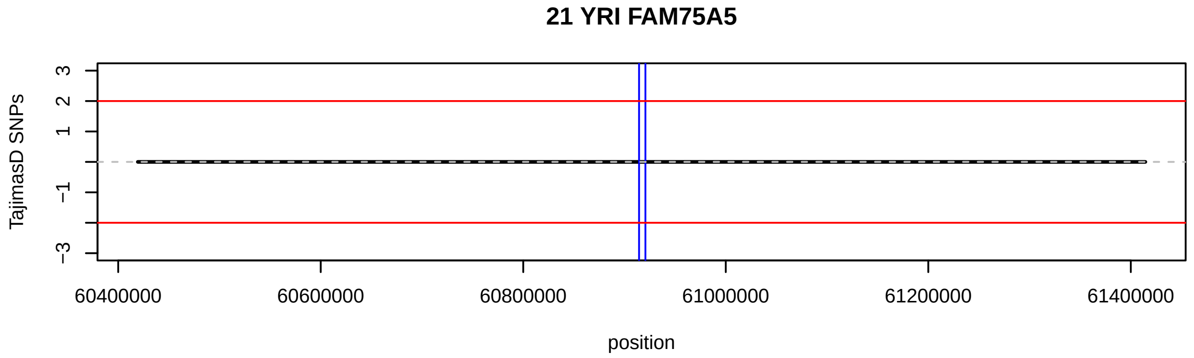
21 YRI FAM75A5
TajimasD SNPs
3
2
1
−1
−3
60400000
60600000
60800000
61000000
61200000
61400000
position

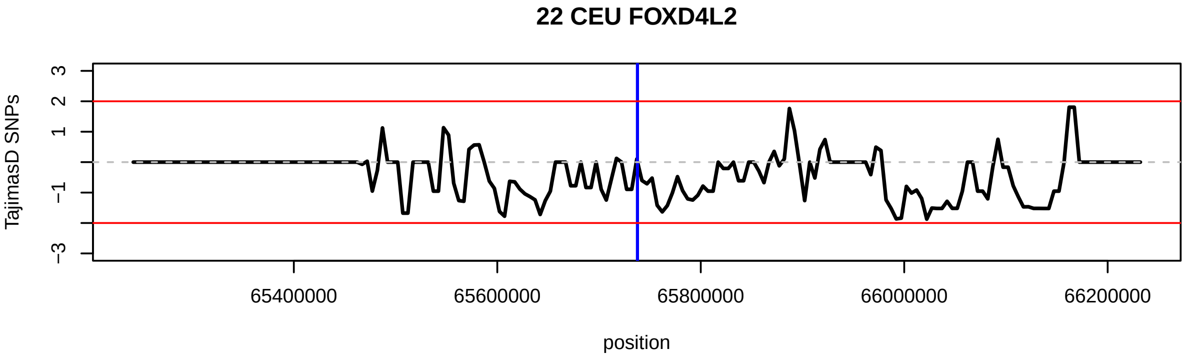
22 CEU FOXD4L2
TajimasD SNPs
3
2
1
−1
−3
65400000
65600000
65800000
66000000
66200000
position

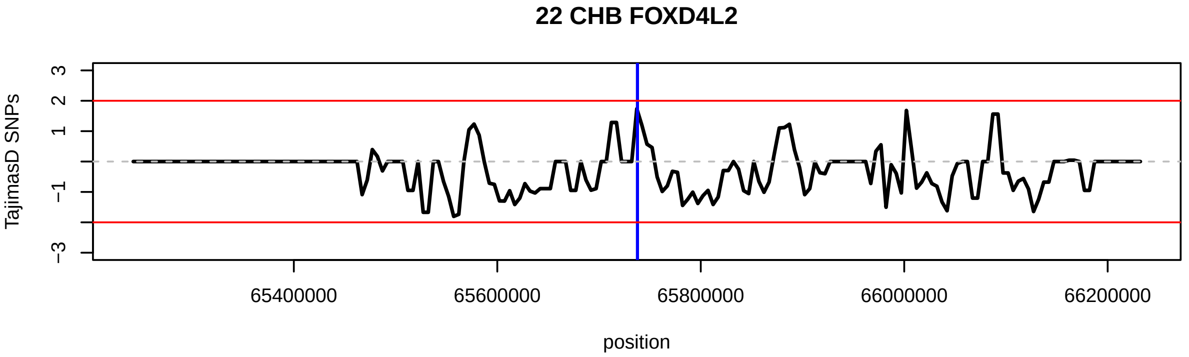
22 CHB FOXD4L2
TajimasD SNPs
3
2
1
−1
−3
65400000
65600000
65800000
66000000
66200000
position

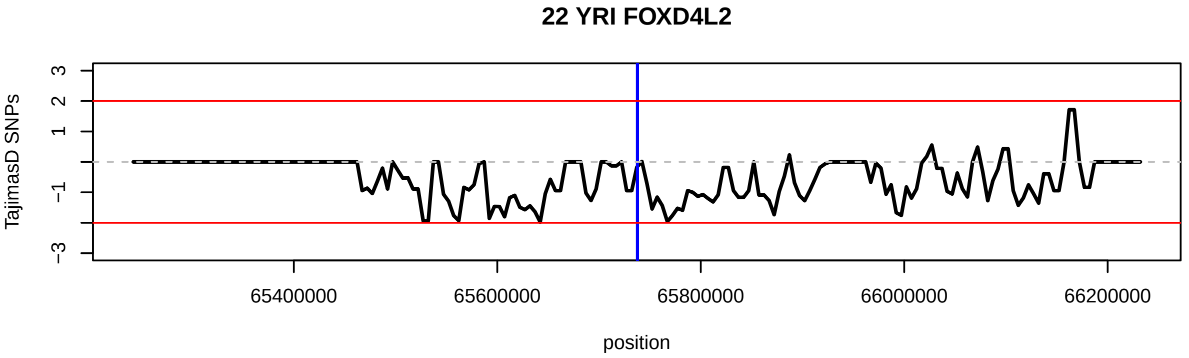
22 YRI FOXD4L2
TajimasD SNPs
3
2
1
−1
−3
65400000
65600000
65800000
66000000
66200000
position

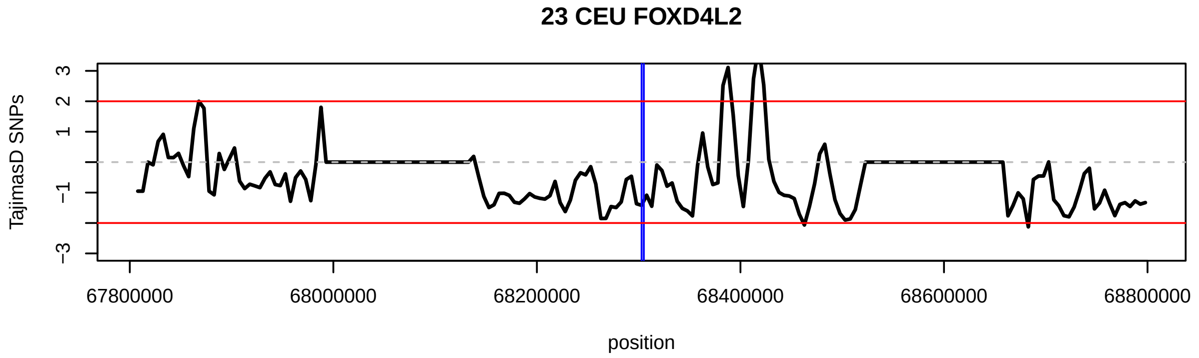

23 CEU FOXD4L2
TajimasD SNPs
position

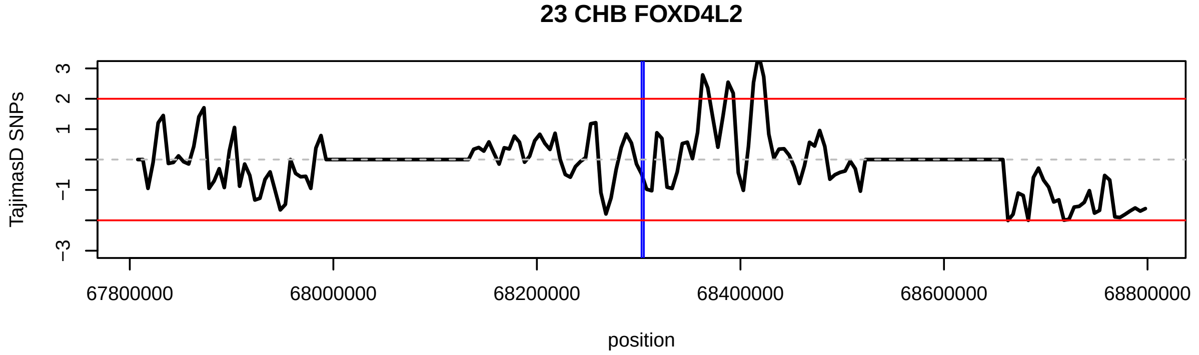

23 CHB FOXD4L2
TajimasD SNPs
position

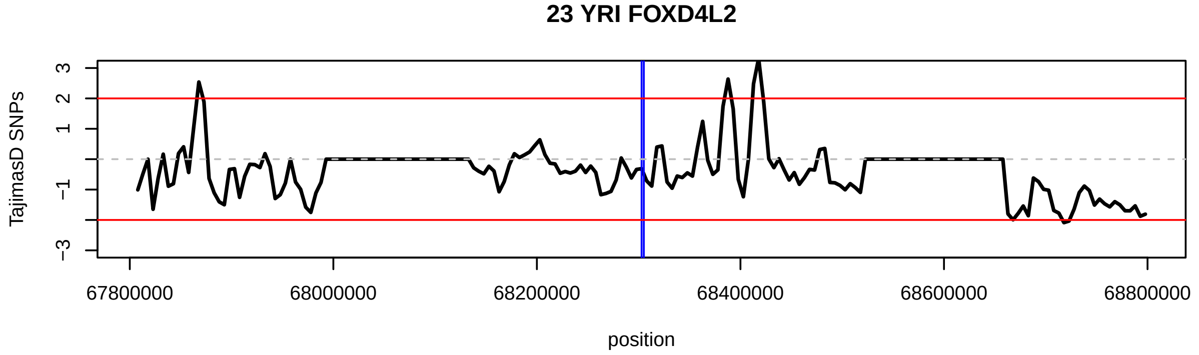

23 YRI FOXD4L2
TajimasD SNPs
position

24 CEU GOLGA6L9

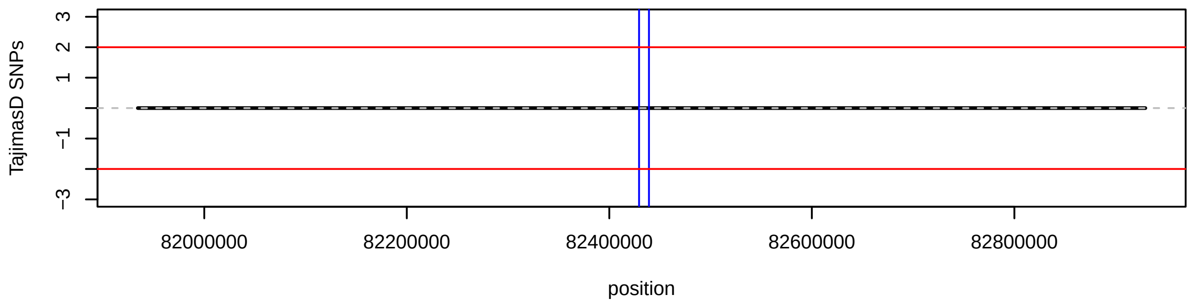

24 CHB GOLGA6L9

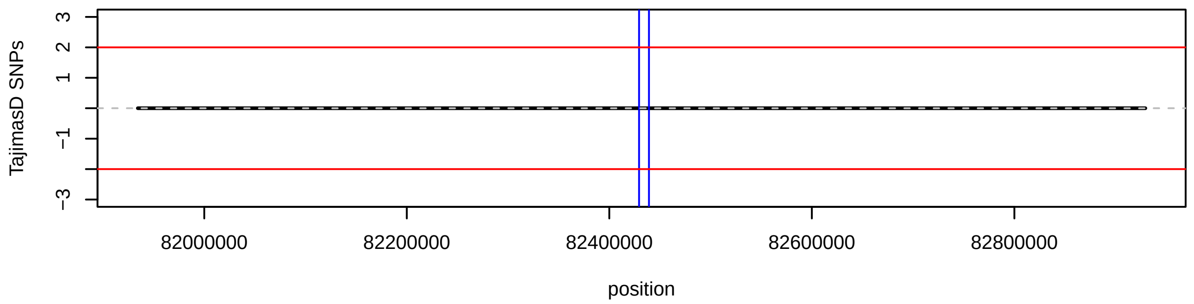

24 YRI GOLGA6L9

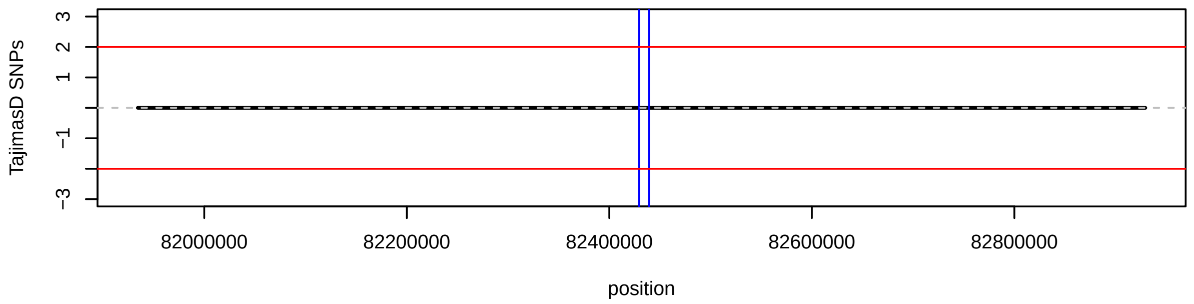

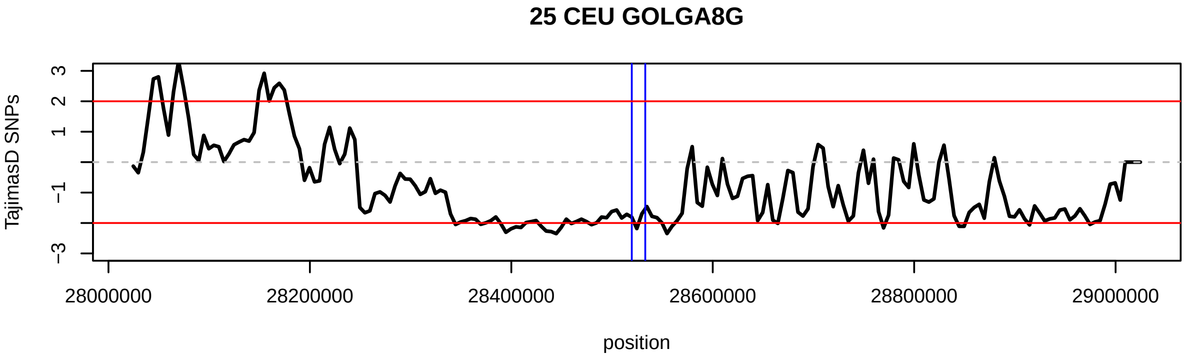
25 CEU GOLGA8G
TajimasD SNPs
3
2
1
−1
−3
28000000
28200000
28400000
28600000
28800000
29000000
position

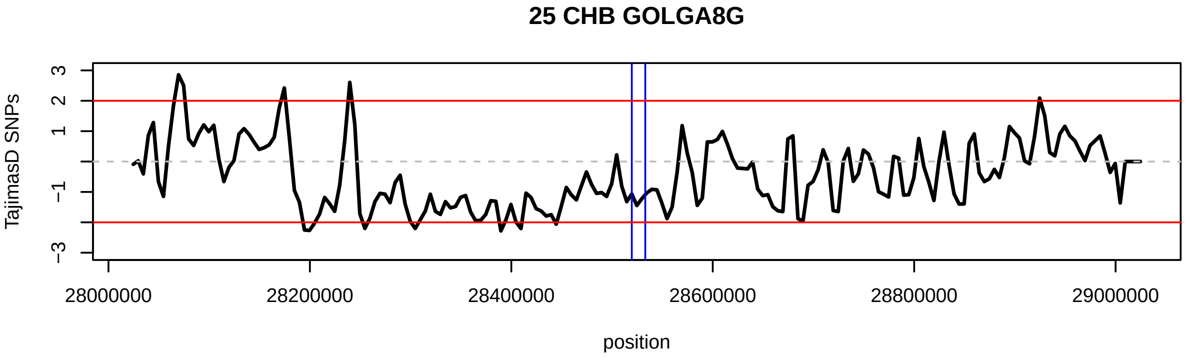
25 CHB GOLGA8G
TajimasD SNPs
3
2
1
−1
−3
28000000
28200000
28400000
28600000
28800000
29000000
position

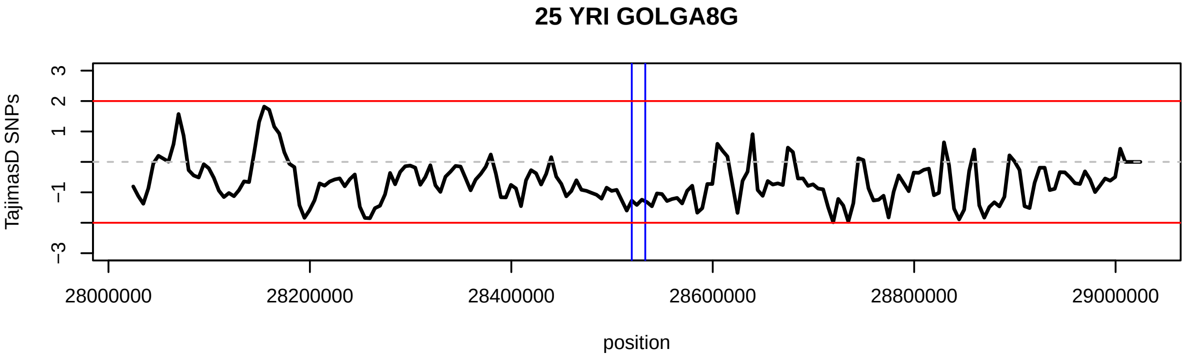
25 YRI GOLGA8G
TajimasD SNPs
3
2
1
−1
−3
28000000
28200000
28400000
28600000
28800000
29000000
position

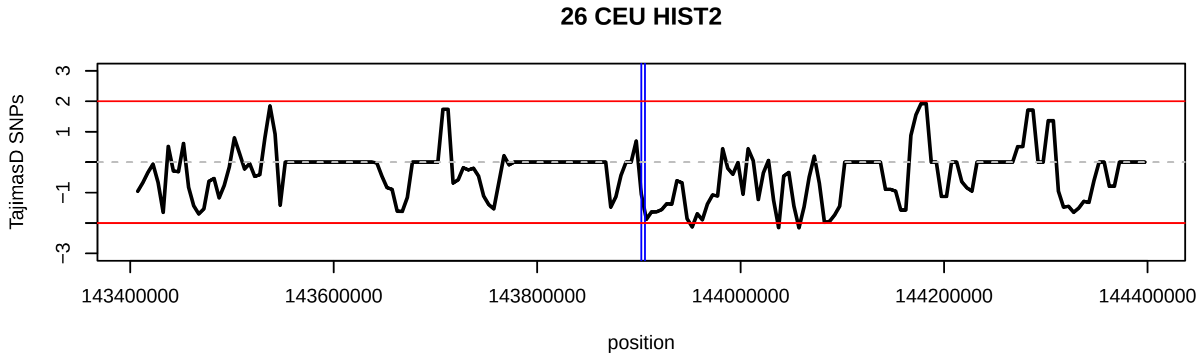
26 CEU HIST2
TajimasD SNPs
3
2
1
−1
−3
143400000
143600000
143800000
144000000
144200000
144400000
position

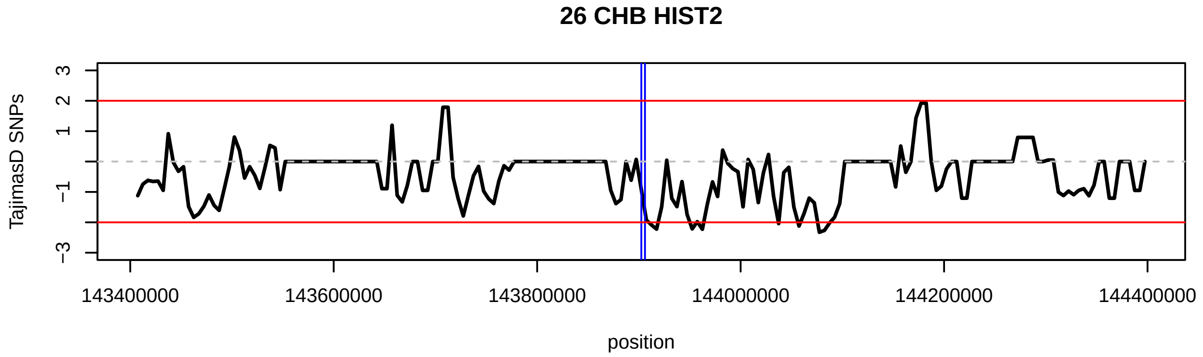
26 CHB HIST2
TajimasD SNPs
3
2
1
−1
−3
143400000
143600000
143800000
144000000
144200000
144400000
position

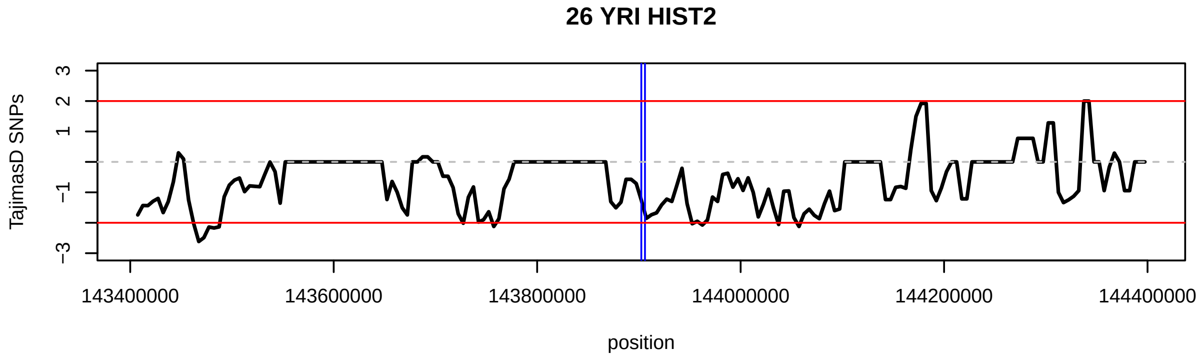
26 YRI HIST2
TajimasD SNPs
3
2
1
−1
−3
143400000
143600000
143800000
144000000
144200000
144400000
position

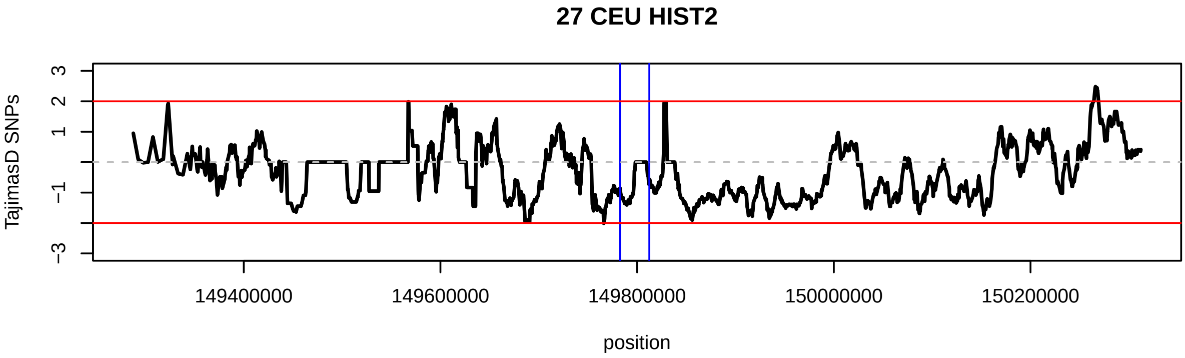
27 CEU HIST2
TajimasD SNPs
3
2
1
−1
−3
149400000
149600000
149800000
150000000
150200000
position

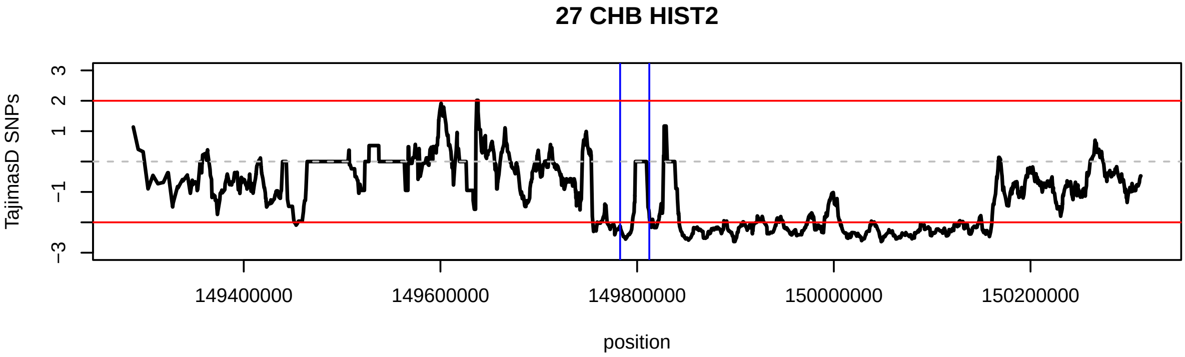
27 CHB HIST2
TajimasD SNPs
3
2
1
−1
−3
149400000
149600000
149800000
150000000
150200000
position

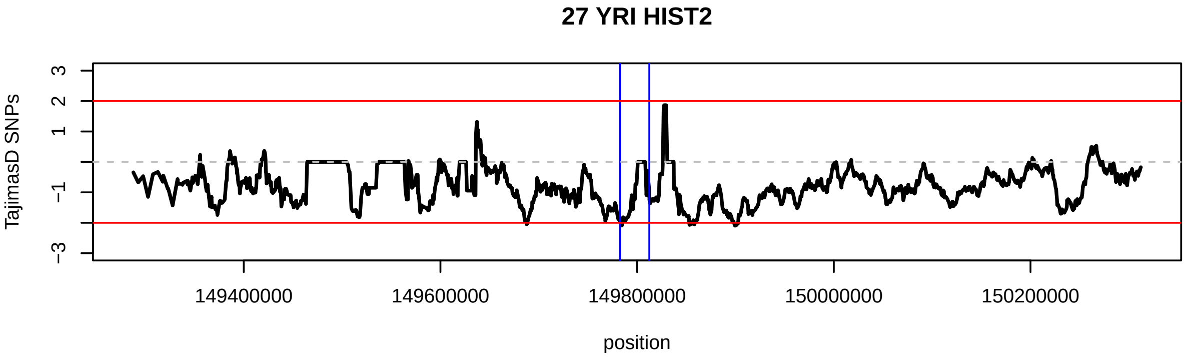
27 YRI HIST2
TajimasD SNPs
3
2
1
−1
−3
149400000
149600000
149800000
150000000
150200000
position

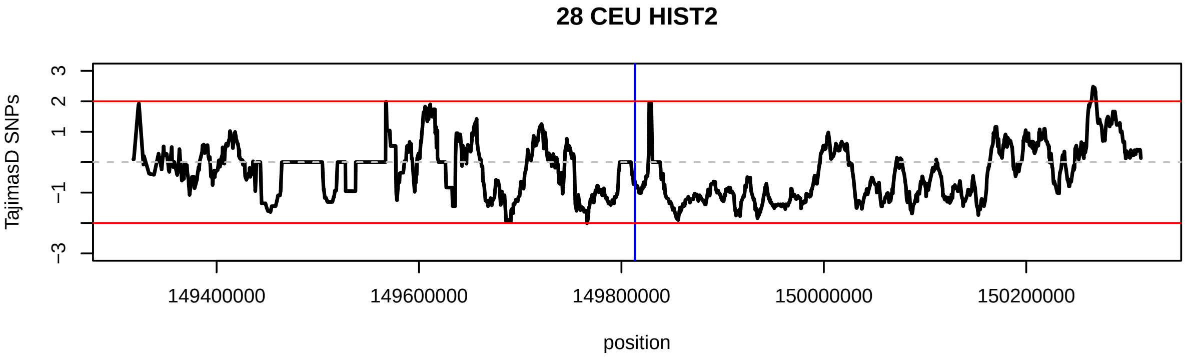
28 CEU HIST2
TajimasD SNPs
3
2
1
−1
−3
149400000
149600000
149800000
150000000
150200000
position

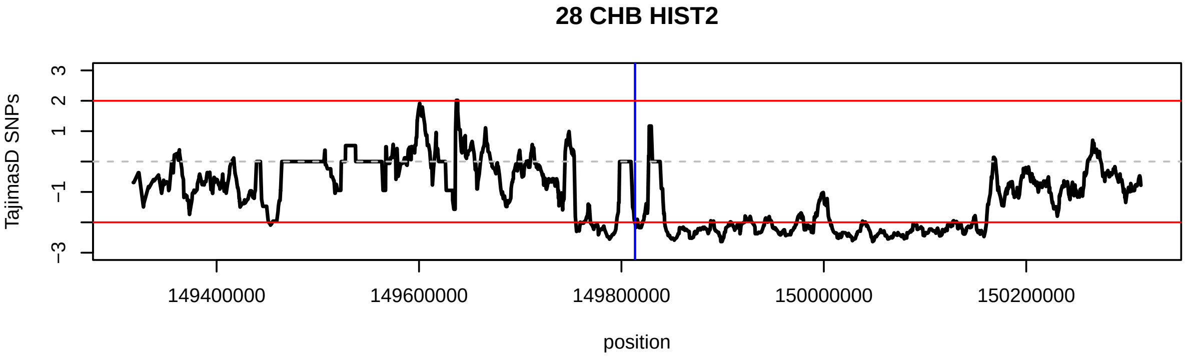
28 CHB HIST2
TajimasD SNPs
3
2
1
−1
−3
149400000
149600000
149800000
150000000
150200000
position

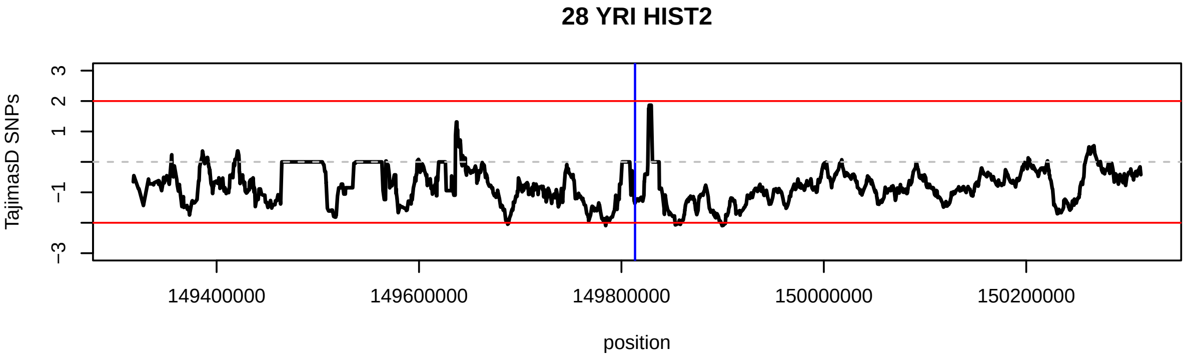
28 YRI HIST2
TajimasD SNPs
3
2
1
−1
−3
149400000
149600000
149800000
150000000
150200000
position

29 CEU HIST2

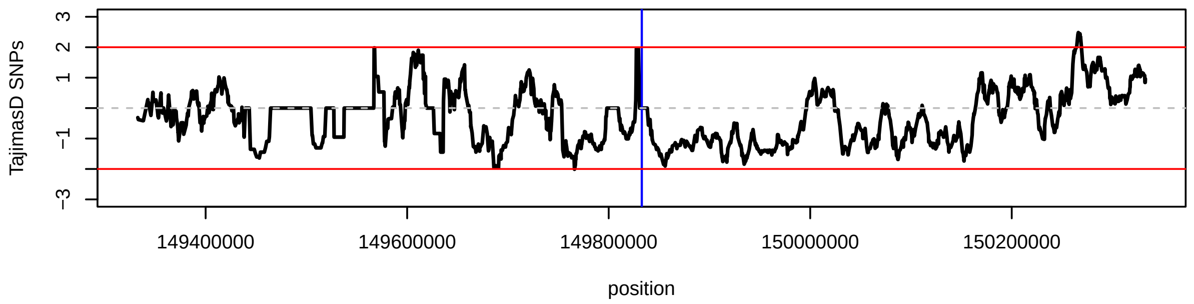

29 CHB HIST2

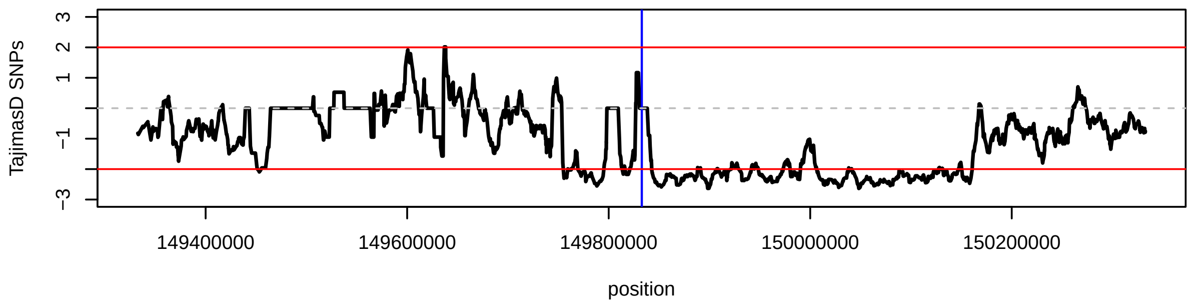

29 YRI HIST2

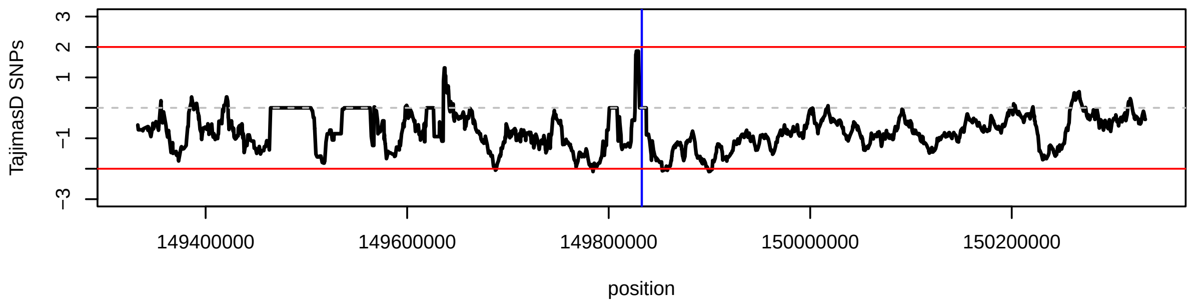

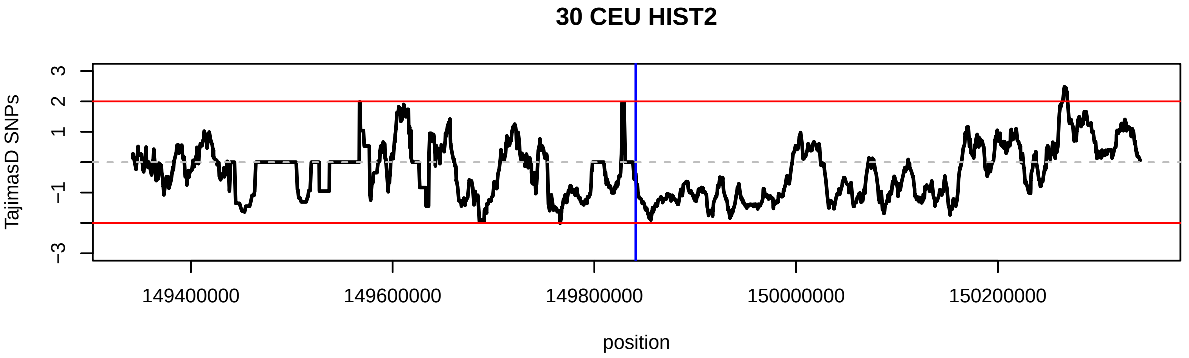
30 CEU HIST2
TajimasD SNPs
3
2
1
−1
−3
149400000
149600000
149800000
150000000
150200000
position

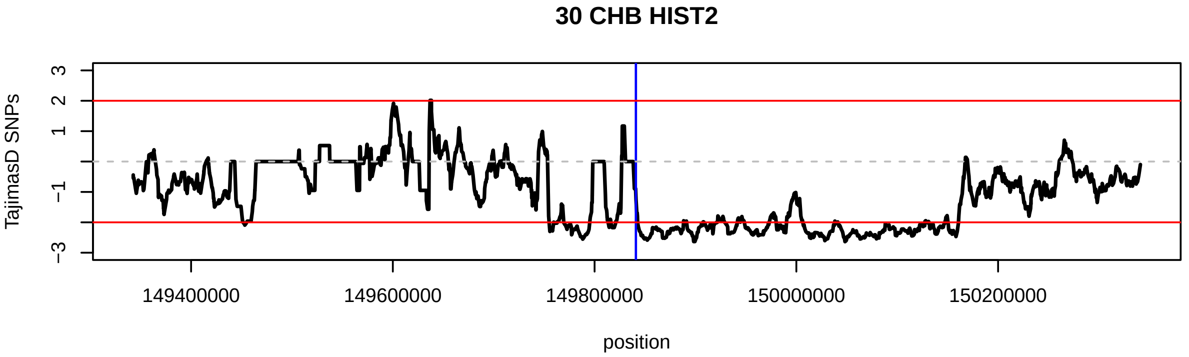
30 CHB HIST2
TajimasD SNPs
3
2
1
−1
−3
149400000
149600000
149800000
150000000
150200000
position

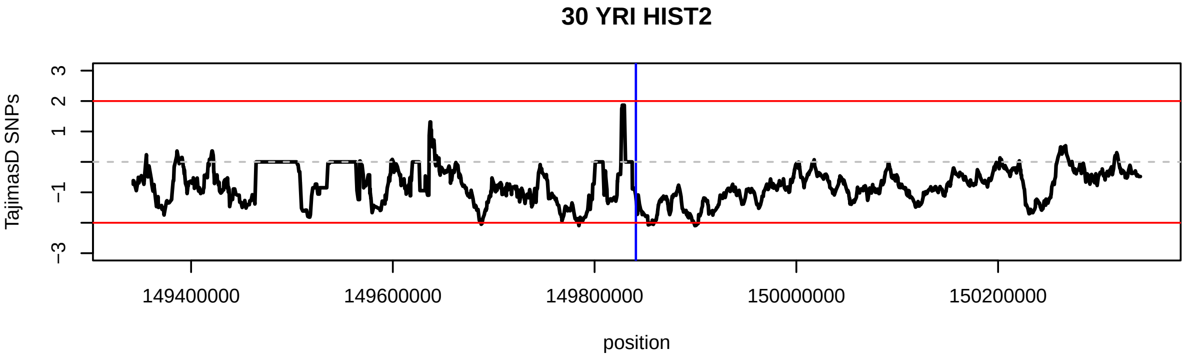
30 YRI HIST2
TajimasD SNPs
3
2
1
−1
−3
149400000
149600000
149800000
150000000
150200000
position

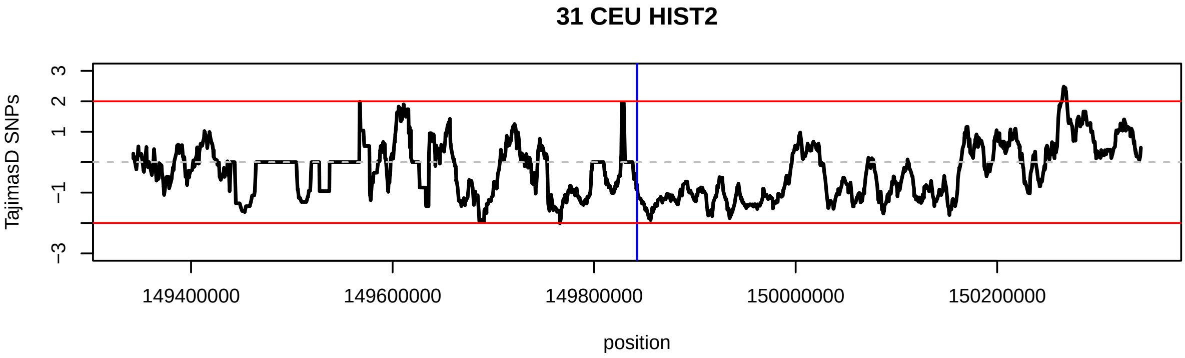

31 CEU HIST2
TajimasD SNPs
3
2
1
−1
−3
149400000
149600000
149800000
150000000
150200000
position

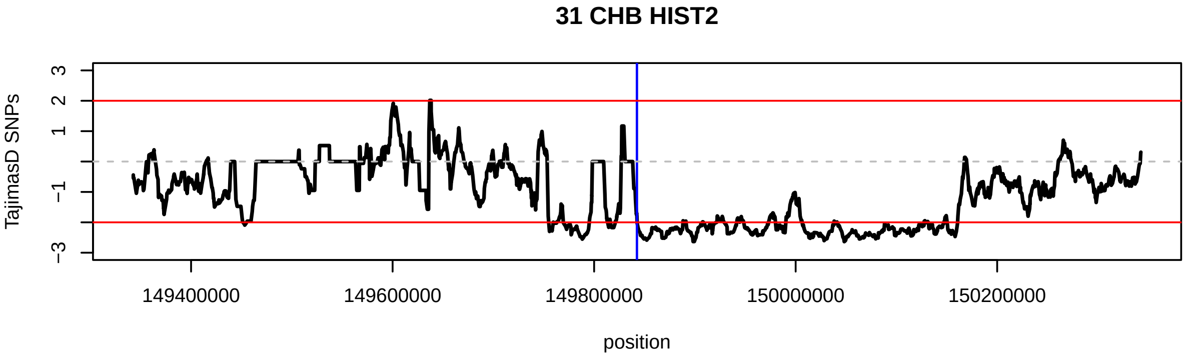

31 CHB HIST2
TajimasD SNPs
3
2
1
−1
−3
149400000
149600000
149800000
150000000
150200000
position

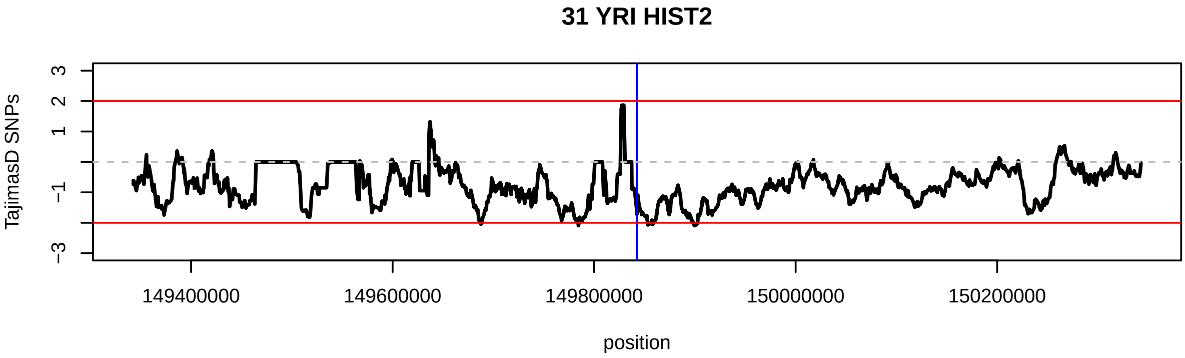

31 YRI HIST2
TajimasD SNPs
3
2
1
−1
−3
149400000
149600000
149800000
150000000
150200000
position

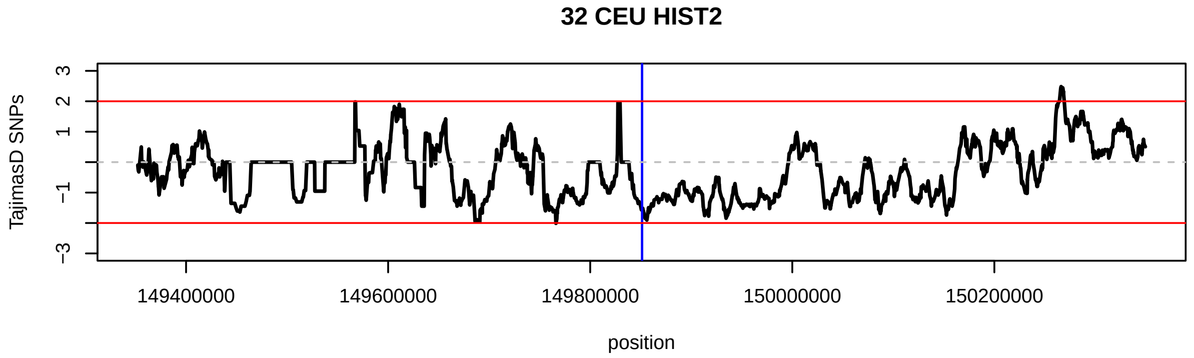
32 CEU HIST2
TajimasD SNPs
position
149400000
149600000
149800000
150000000
150200000

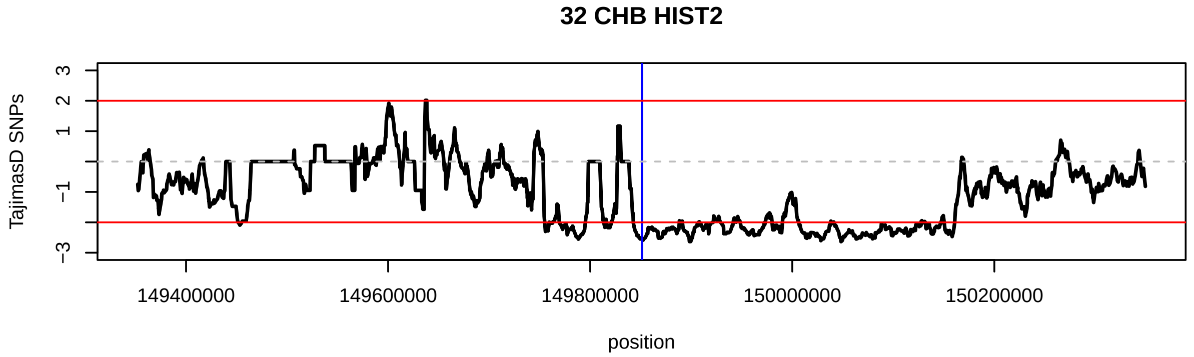
32 CHB HIST2
TajimasD SNPs
position
149400000
149600000
149800000
150000000
150200000

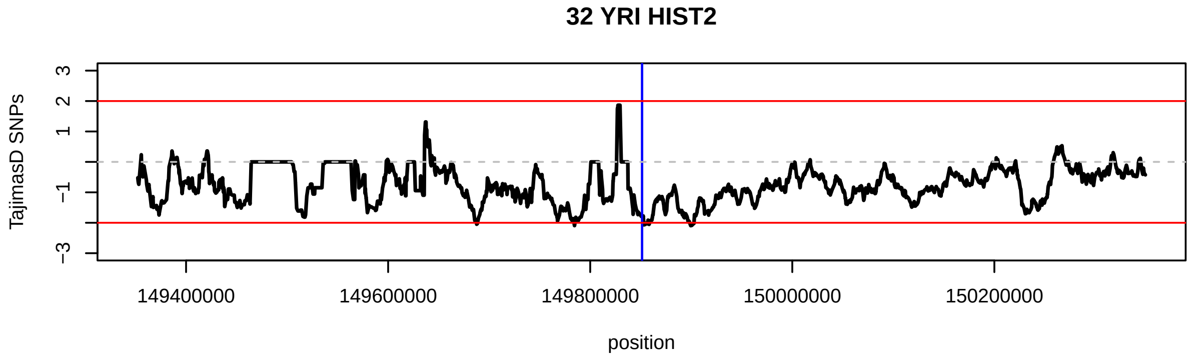
32 YRI HIST2
TajimasD SNPs
position
149400000
149600000
149800000
150000000
150200000

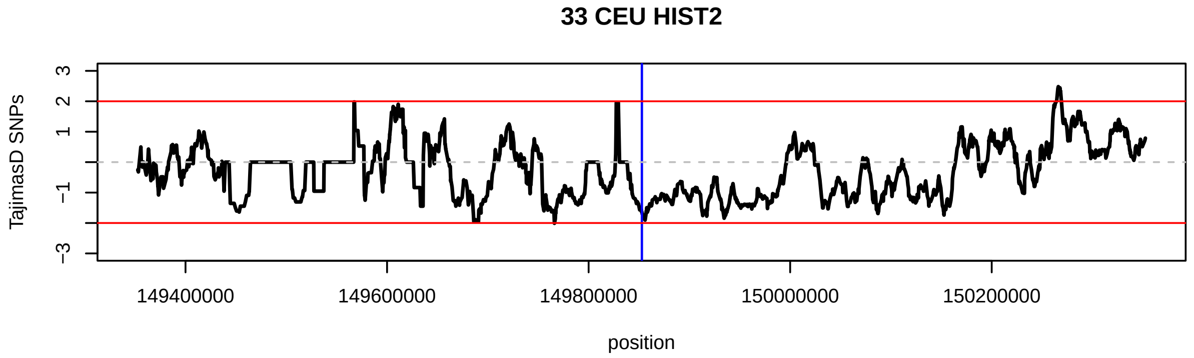
33 CEU HIST2
TajimasD SNPs
3
2
1
−1
−3
149400000
149600000
149800000
150000000
150200000
position

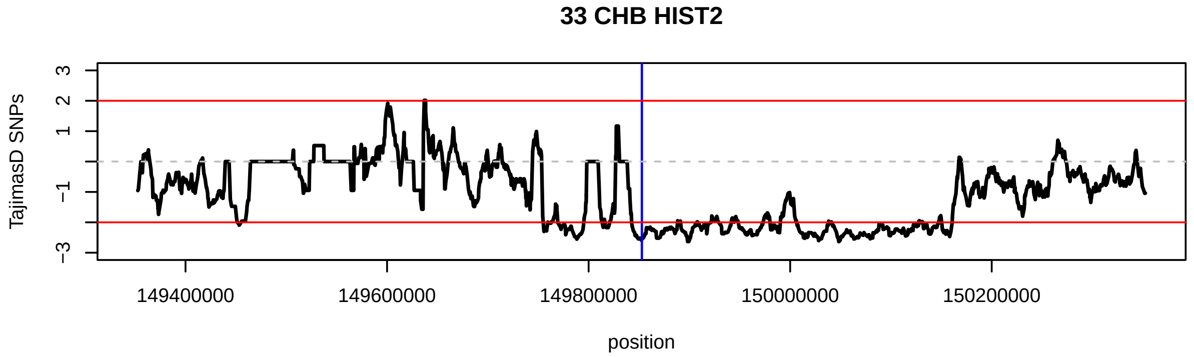
33 CHB HIST2
TajimasD SNPs
3
2
1
−1
−3
149400000
149600000
149800000
150000000
150200000
position

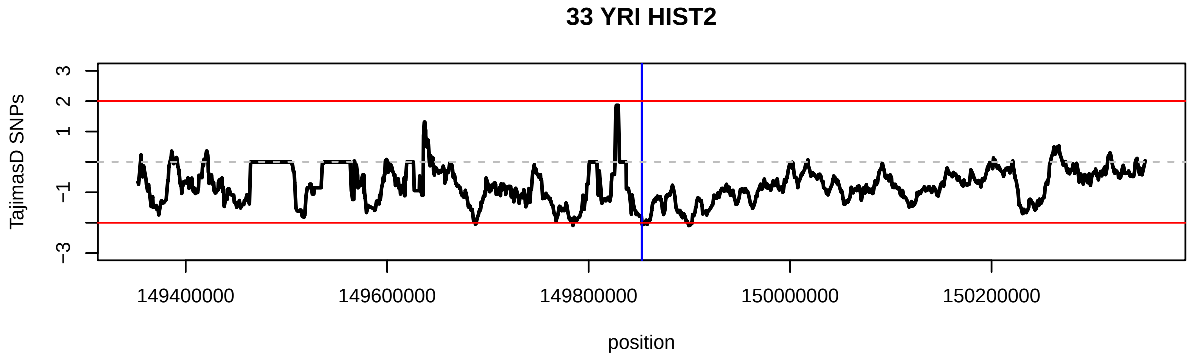
33 YRI HIST2
TajimasD SNPs
3
2
1
−1
−3
149400000
149600000
149800000
150000000
150200000
position

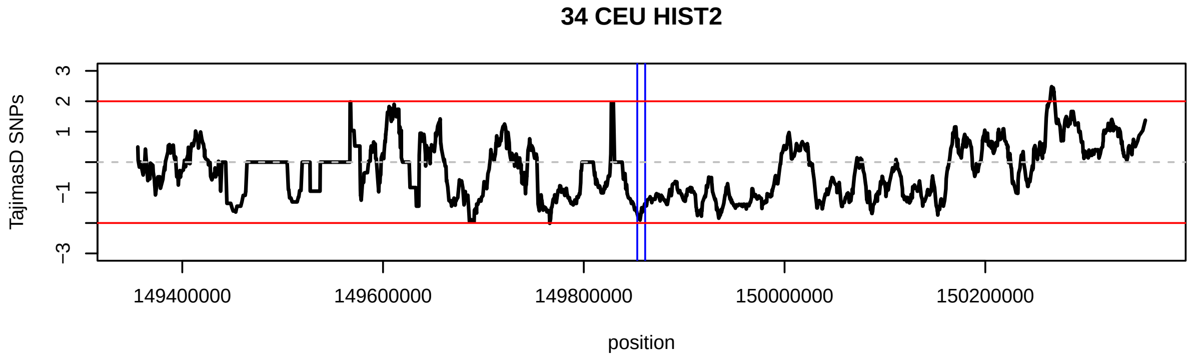
34 CEU HIST2
TajimasD SNPs
3
2
1
−1
−3
149400000
149600000
149800000
150000000
150200000
position

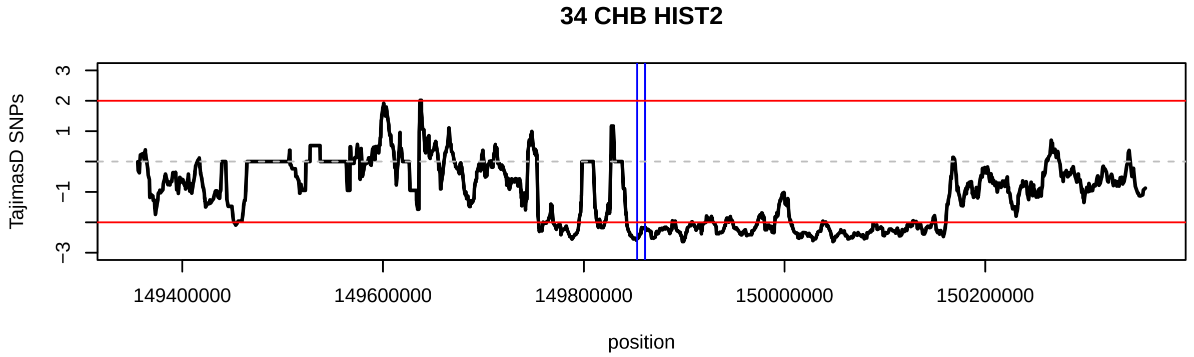
34 CHB HIST2
TajimasD SNPs
3
2
1
−1
−3
149400000
149600000
149800000
150000000
150200000
position

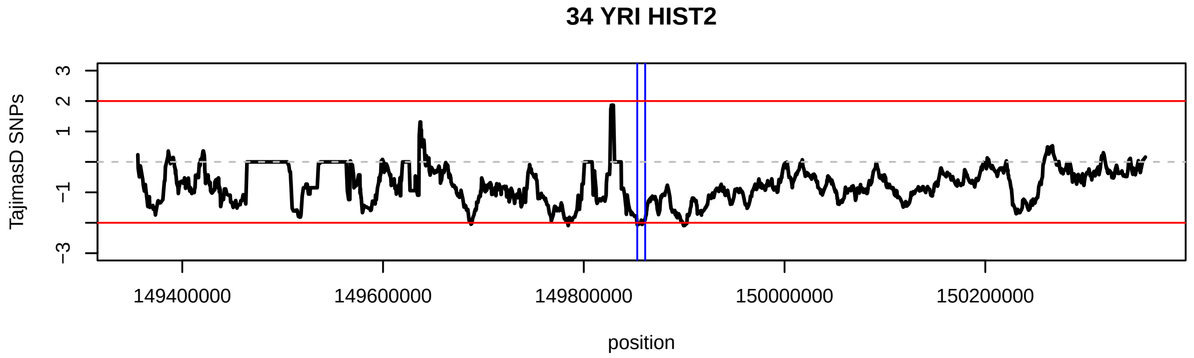
34 YRI HIST2
TajimasD SNPs
3
2
1
−1
−3
149400000
149600000
149800000
150000000
150200000
position

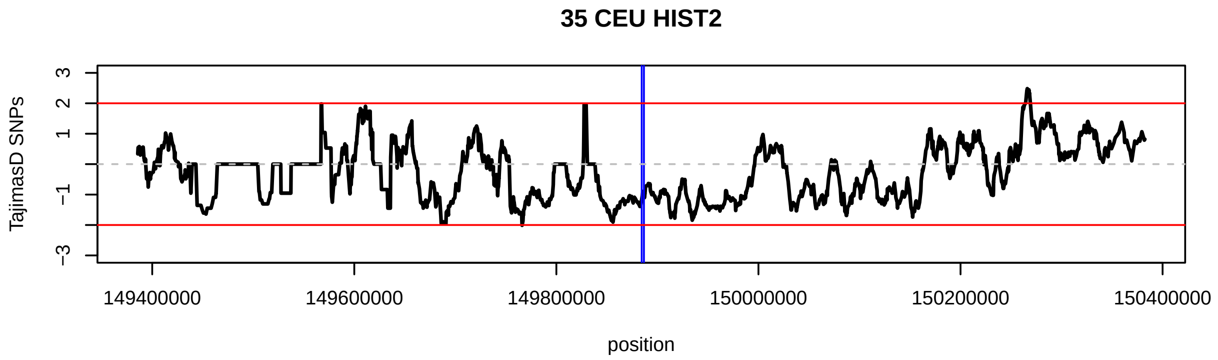
35 CEU HIST2
TajimasD SNPs
3
2
1
−1
−3
149400000
149600000
149800000
150000000
150200000
150400000
position

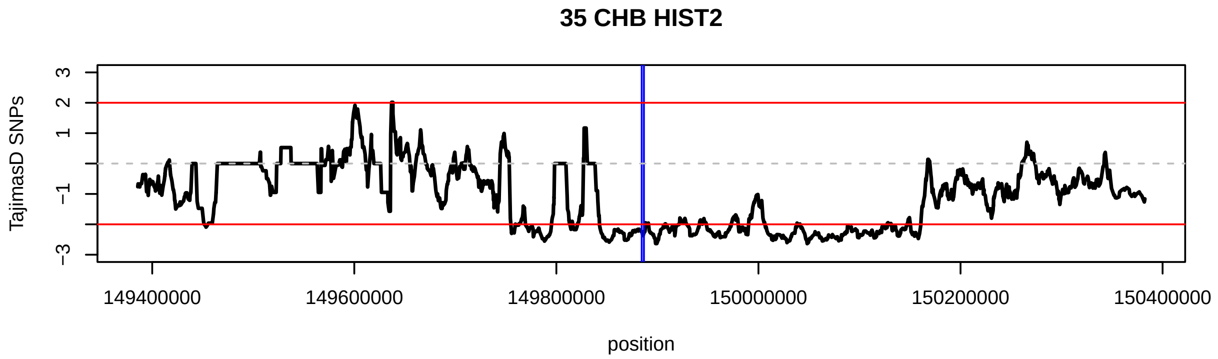
35 CHB HIST2
TajimasD SNPs
3
2
1
−1
−3
149400000
149600000
149800000
150000000
150200000
150400000
position

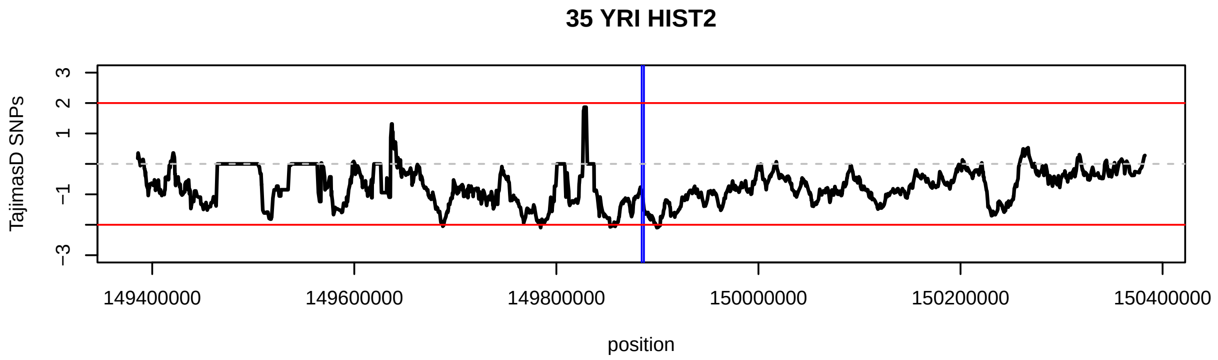
35 YRI HIST2
TajimasD SNPs
3
2
1
−1
−3
149400000
149600000
149800000
150000000
150200000
150400000
position

36 CEU HIST2

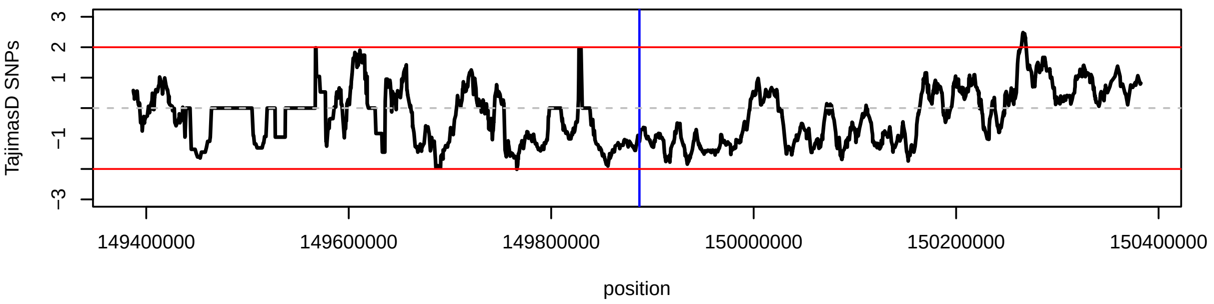

36 CHB HIST2

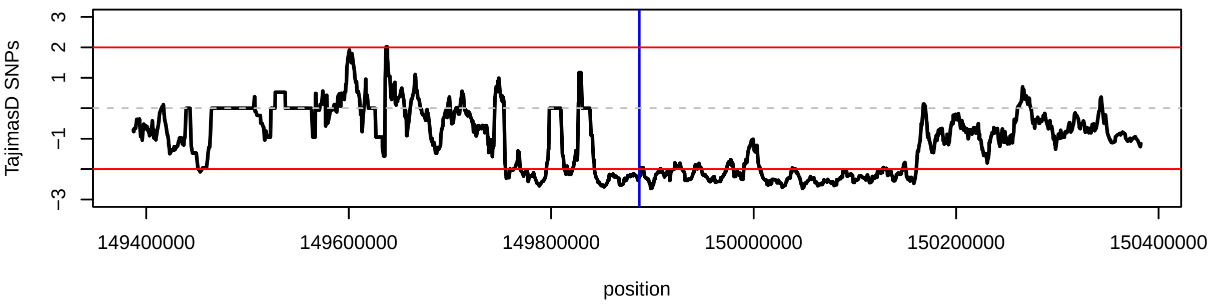

36 YRI HIST2

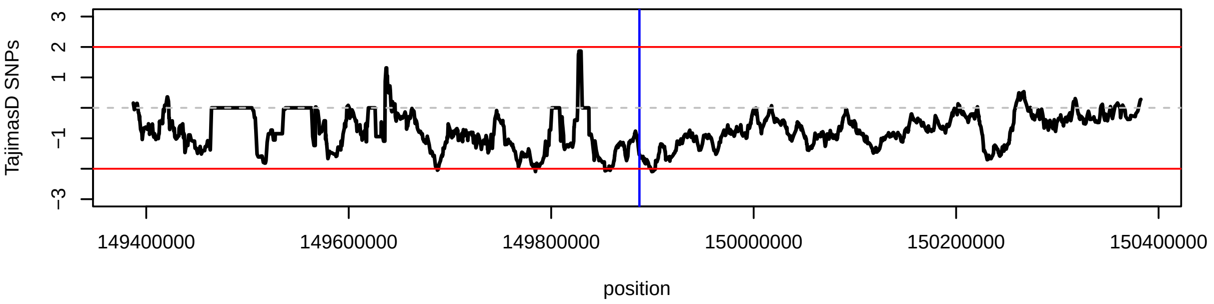

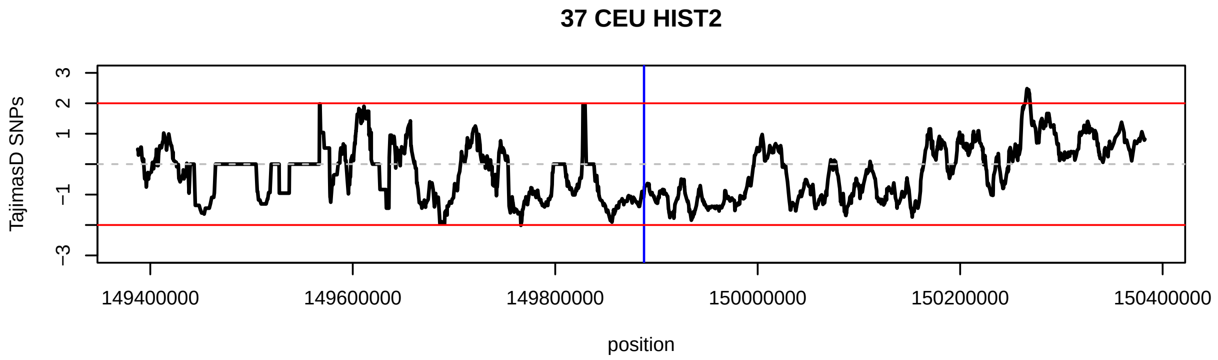
37 CEU HIST2
TajimasD SNPs
3
2
1
−1
−3
149400000
149600000
149800000
150000000
150200000
150400000
position

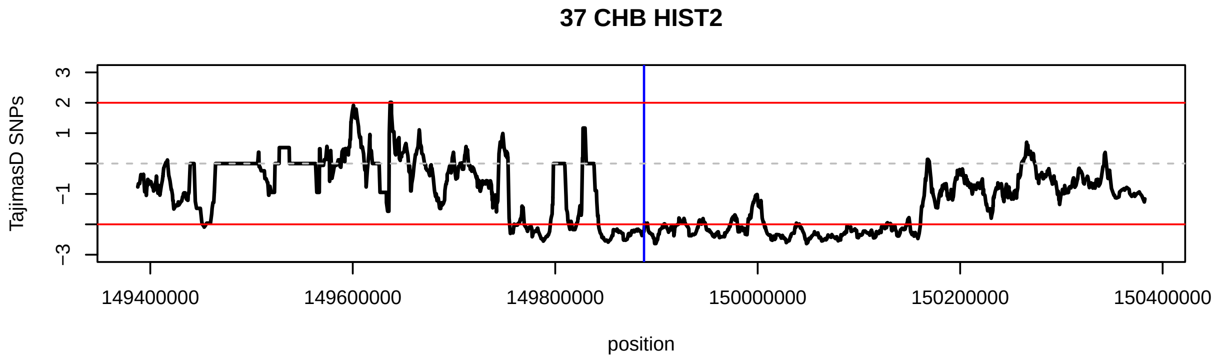
37 CHB HIST2
TajimasD SNPs
3
2
1
−1
−3
149400000
149600000
149800000
150000000
150200000
150400000
position

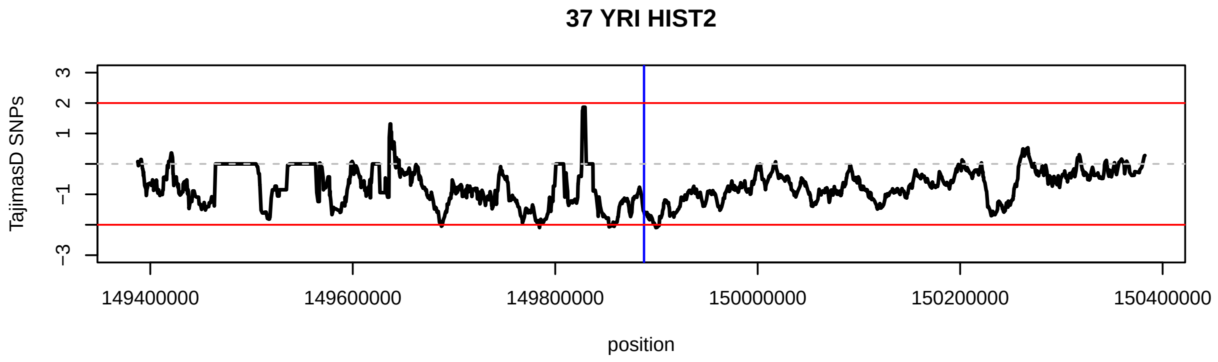
37 YRI HIST2
TajimasD SNPs
3
2
1
−1
−3
149400000
149600000
149800000
150000000
150200000
150400000
position

38 CEU LIMS3

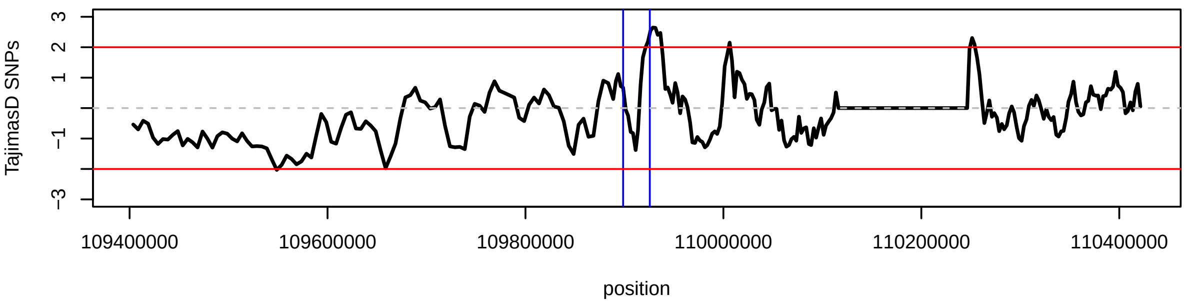

38 CHB LIMS3

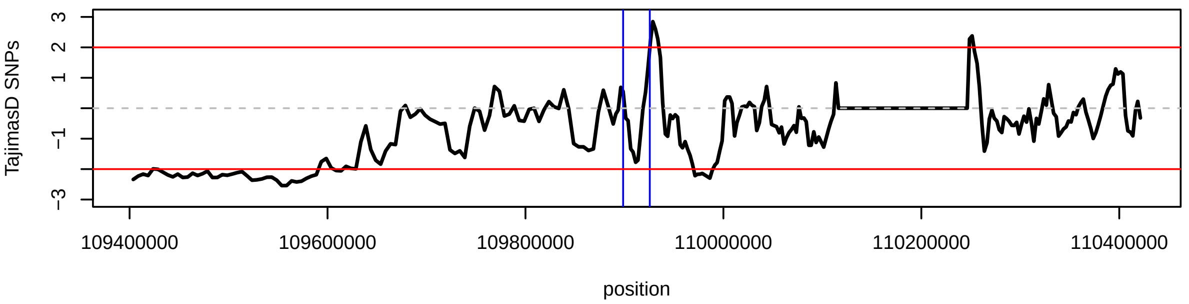

38 YRI LIMS3

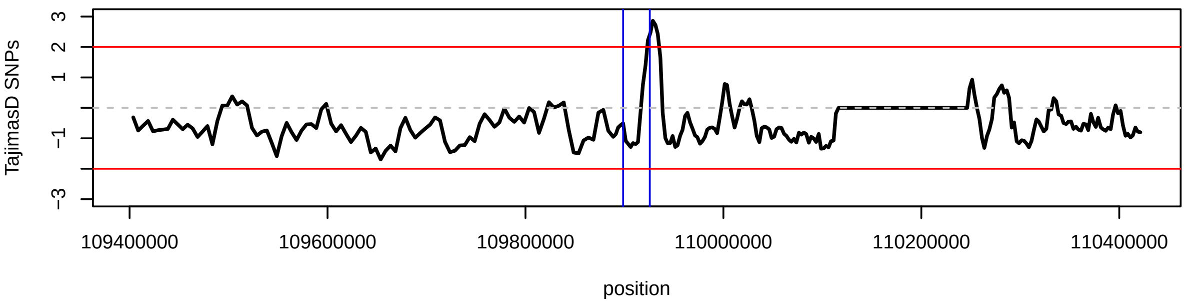

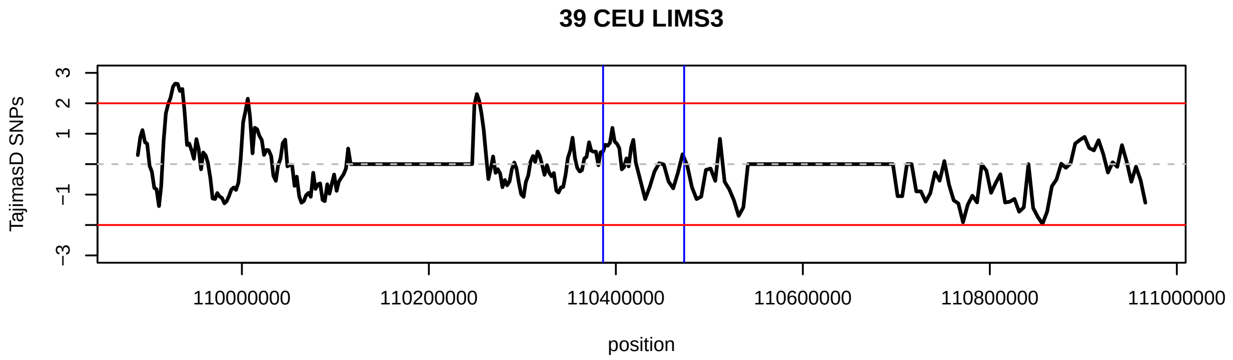
39 CEU LIMS3
TajimasD SNPs
3
2
1
−1
−3
110000000
110200000
110400000
110600000
110800000
111000000
position

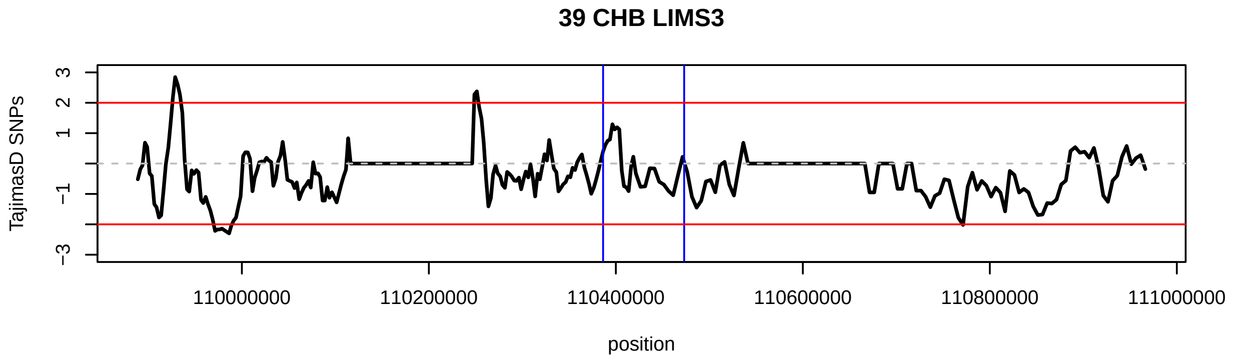
39 CHB LIMS3
TajimasD SNPs
3
2
1
−1
−3
110000000
110200000
110400000
110600000
110800000
111000000
position

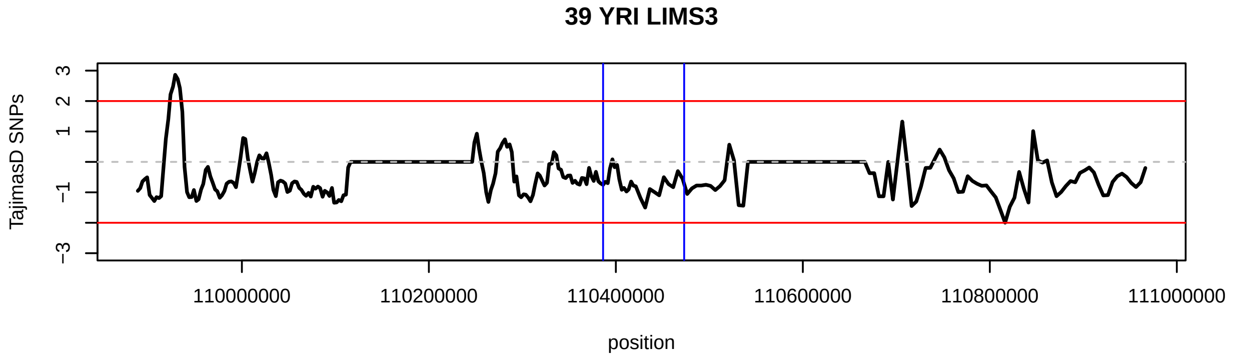
39 YRI LIMS3
TajimasD SNPs
3
2
1
−1
−3
110000000
110200000
110400000
110600000
110800000
111000000
position

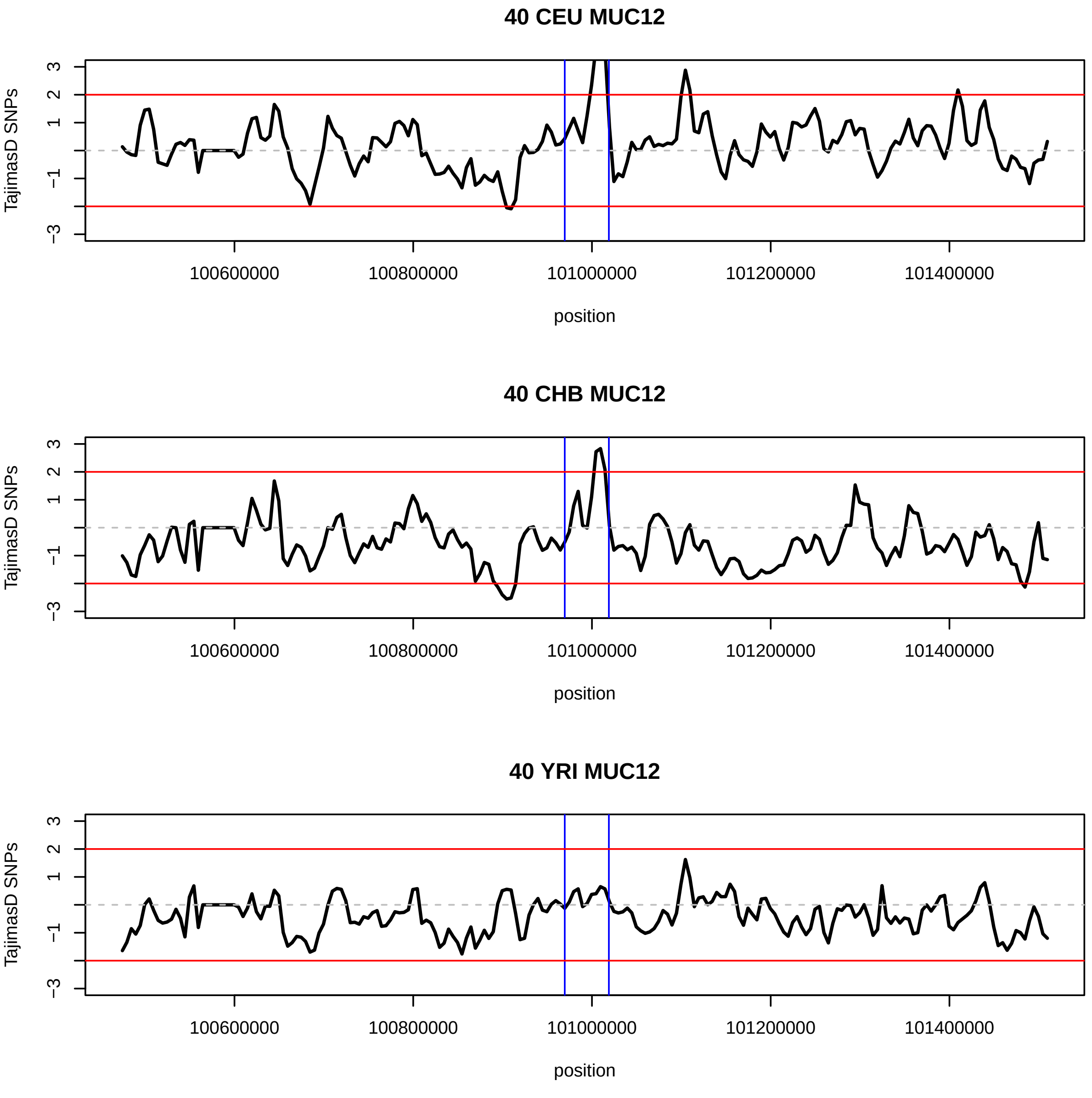
40 CEU MUC12
TajimasD SNPs
3
2
1
−1
−3
100600000
100800000
101000000
101200000
101400000
position
40 CHB MUC12
TajimasD SNPs
3
2
1
−1
−3
100600000
100800000
101000000
101200000
101400000
position
40 YRI MUC12
TajimasD SNPs
3
2
1
−1
−3
100600000
100800000
101000000
101200000
101400000
position

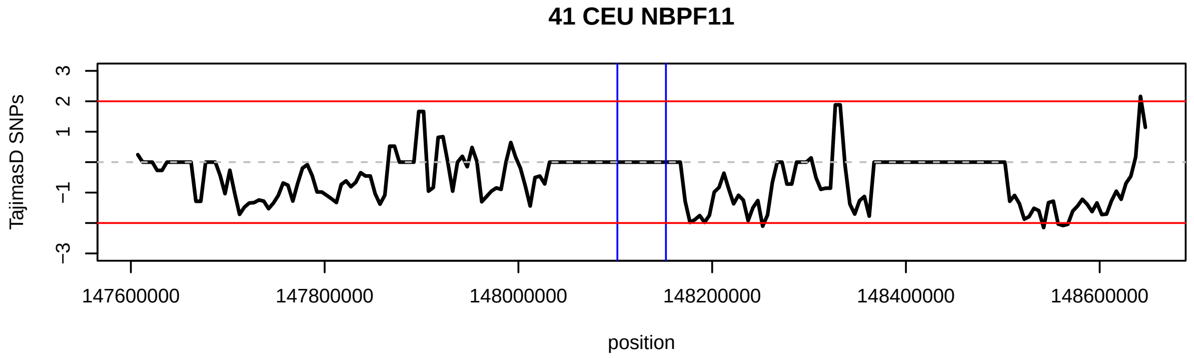
41 CEU NBPF11
TajimasD SNPs
3
2
1
−1
−3
147600000
147800000
148000000
148200000
148400000
148600000
position

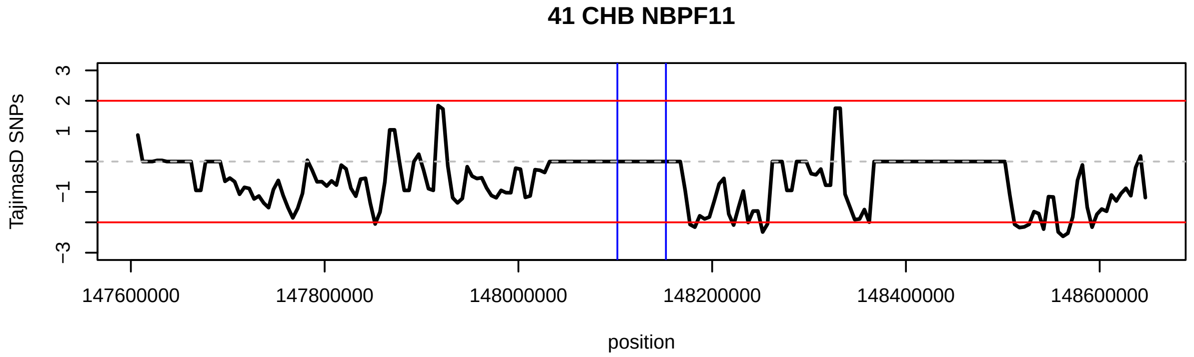
41 CHB NBPF11
TajimasD SNPs
3
2
1
−1
−3
147600000
147800000
148000000
148200000
148400000
148600000
position

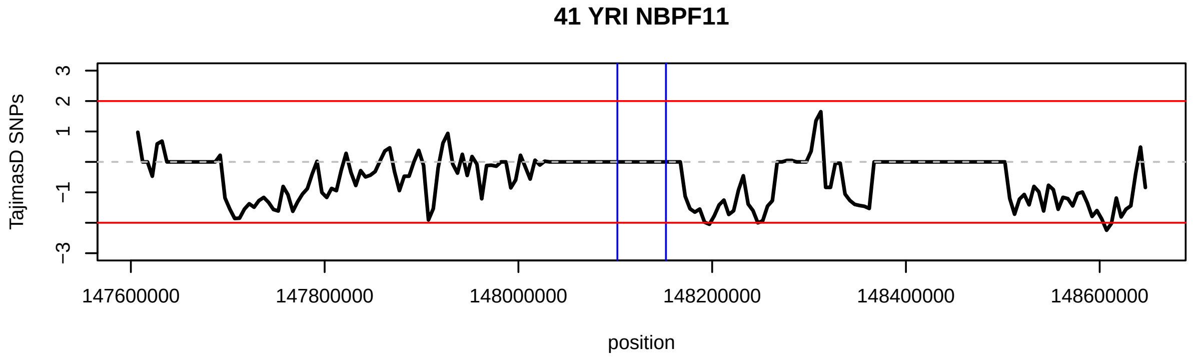
41 YRI NBPF11
TajimasD SNPs
3
2
1
−1
−3
147600000
147800000
148000000
148200000
148400000
148600000
position

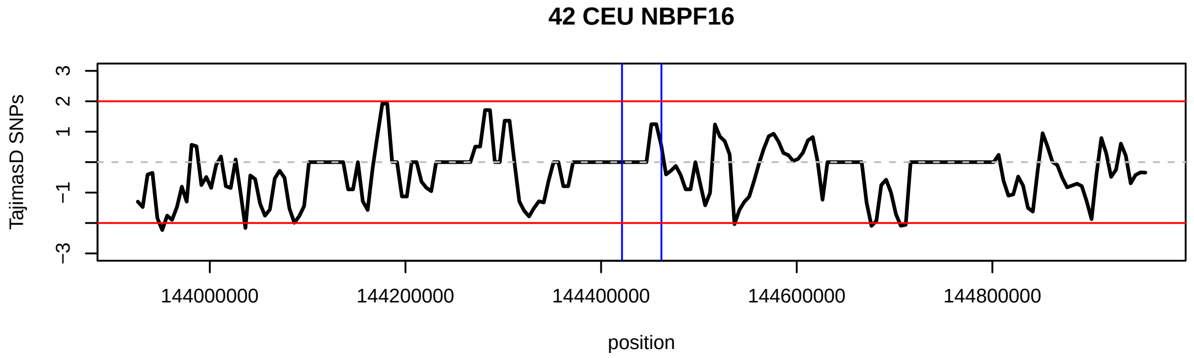
42 CEU NBPF16
TajimasD SNPs
3
2
1
−1
−3
144000000
144200000
144400000
144600000
144800000
position

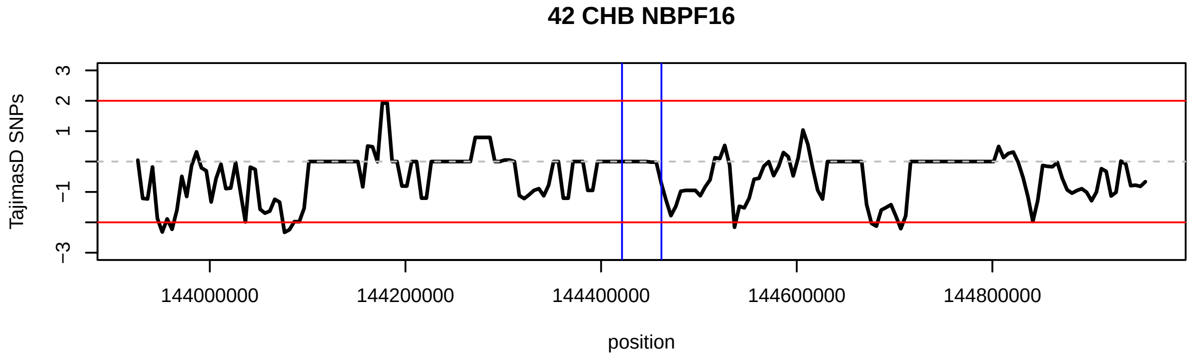
42 CHB NBPF16
TajimasD SNPs
3
2
1
−1
−3
144000000
144200000
144400000
144600000
144800000
position

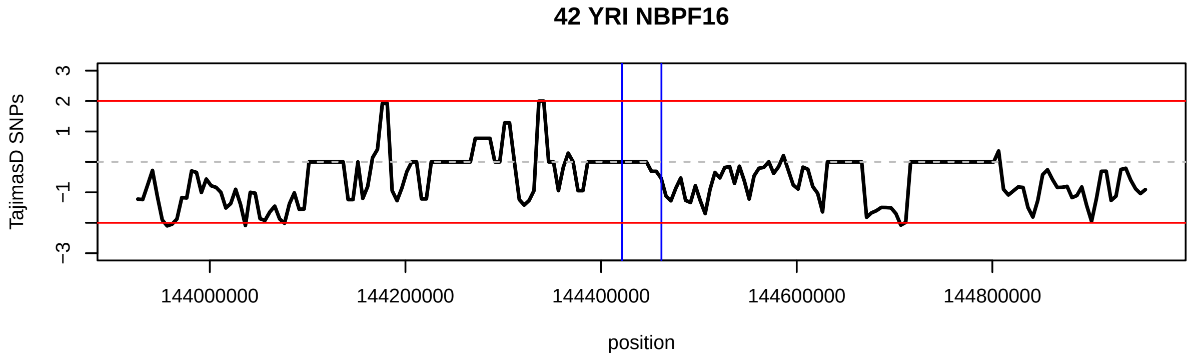
42 YRI NBPF16
TajimasD SNPs
3
2
1
−1
−3
144000000
144200000
144400000
144600000
144800000
position

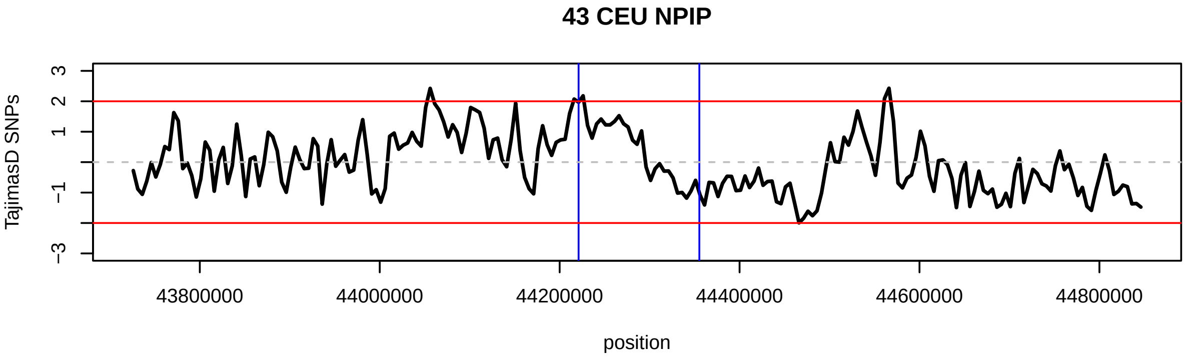
43 CEU NPIP
TajimasD SNPs
3
2
1
−1
−3
43800000
44000000
44200000
44400000
44600000
44800000
position

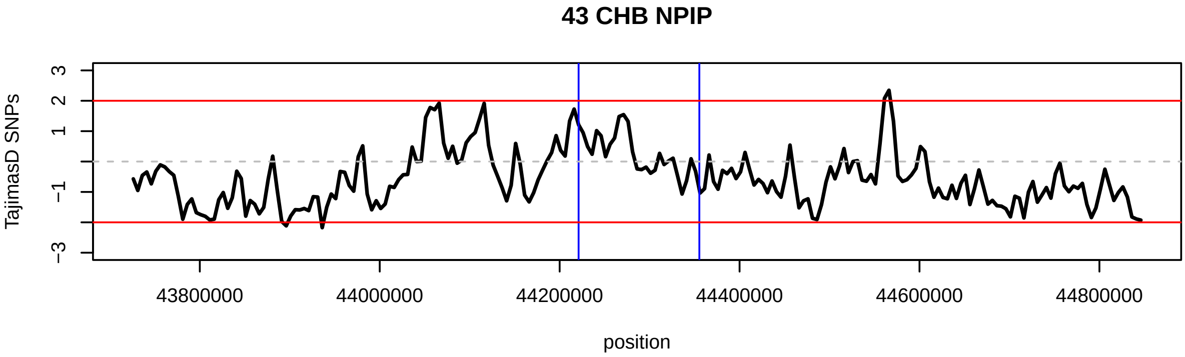
43 CHB NPIP
TajimasD SNPs
3
2
1
−1
−3
43800000
44000000
44200000
44400000
44600000
44800000
position

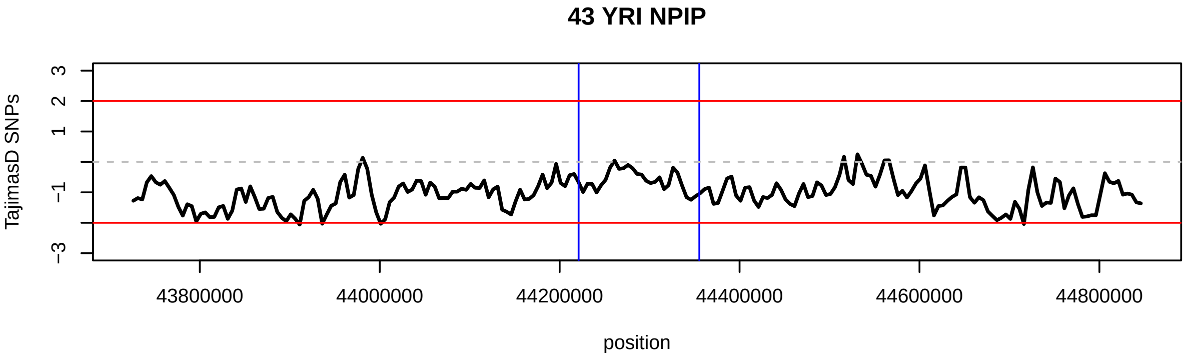
43 YRI NPIP
TajimasD SNPs
3
2
1
−1
−3
43800000
44000000
44200000
44400000
44600000
44800000
position

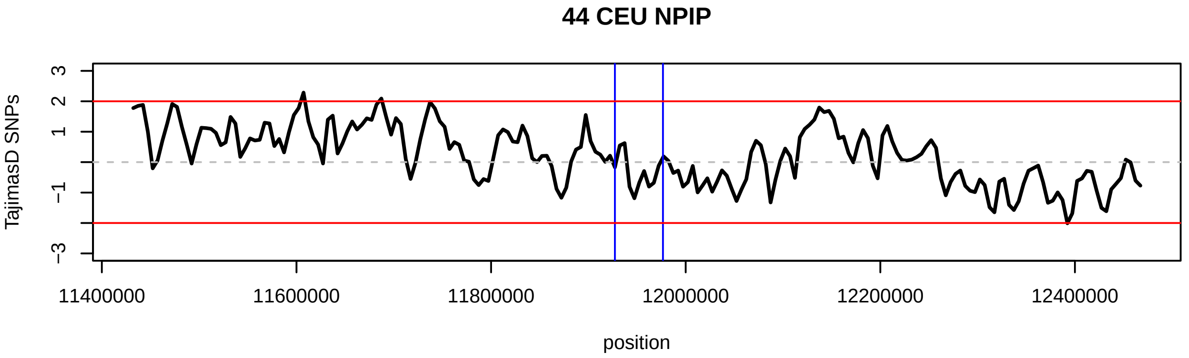
44 CEU NPIP
TajimasD SNPs
3
2
1
−1
−3
11400000
11600000
11800000
12000000
12200000
12400000
position

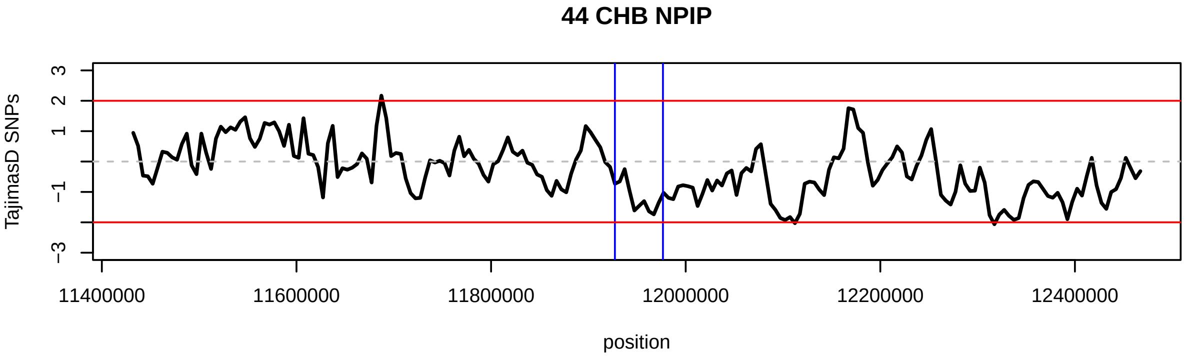
44 CHB NPIP
TajimasD SNPs
3
2
1
−1
−3
11400000
11600000
11800000
12000000
12200000
12400000
position

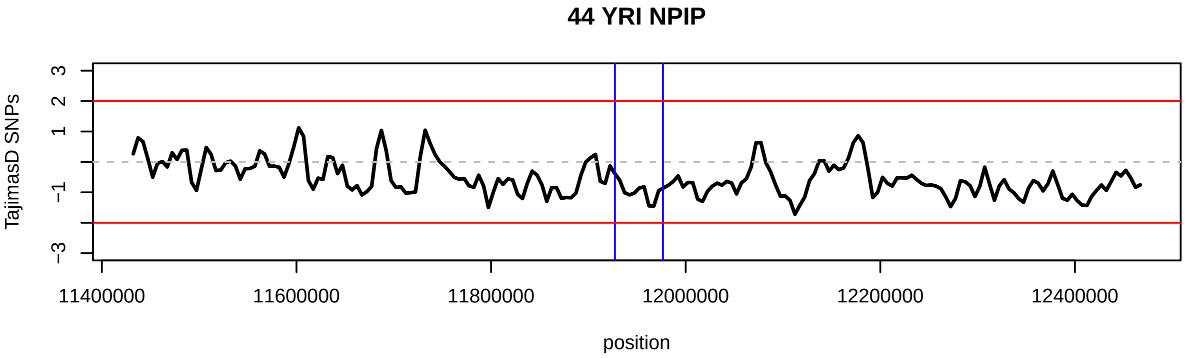
44 YRI NPIP
TajimasD SNPs
3
2
1
−1
−3
11400000
11600000
11800000
12000000
12200000
12400000
position

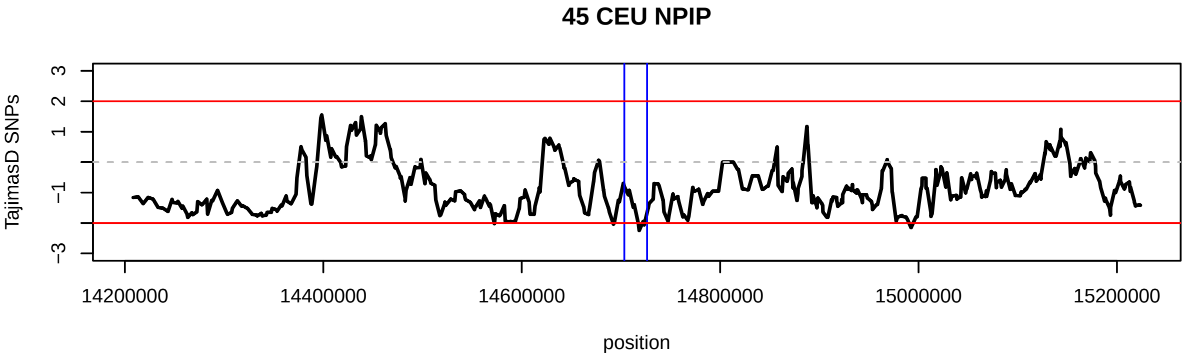
45 CEU NPIP
TajimasD SNPs
3
2
1
−1
−3
14200000
14400000
14600000
14800000
15000000
15200000
position

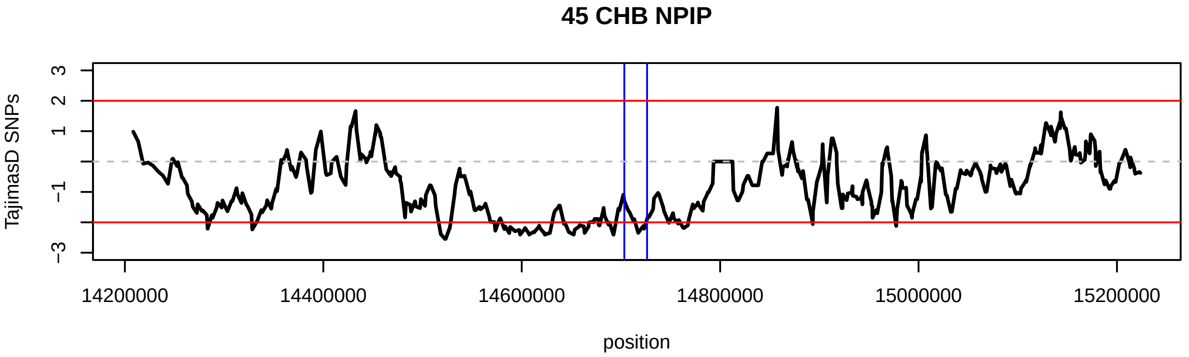
45 CHB NPIP
TajimasD SNPs
3
2
1
−1
−3
14200000
14400000
14600000
14800000
15000000
15200000
position

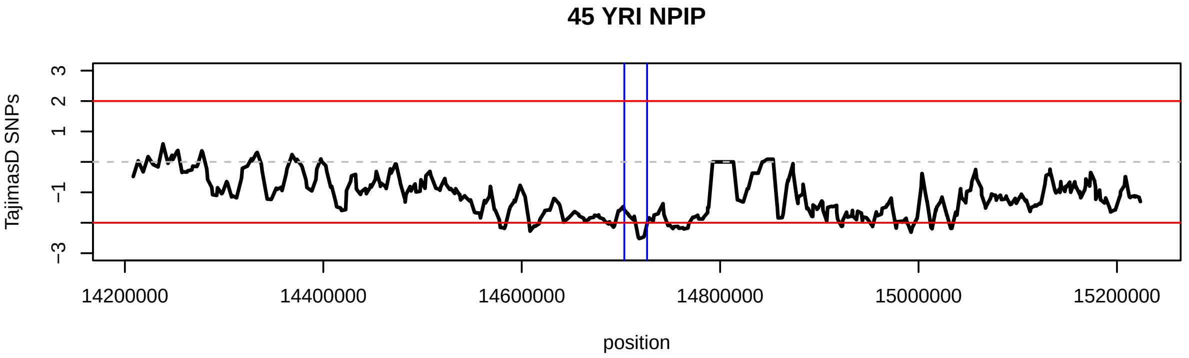
45 YRI NPIP
TajimasD SNPs
3
2
1
−1
−3
14200000
14400000
14600000
14800000
15000000
15200000
position

46 CEU NPIP

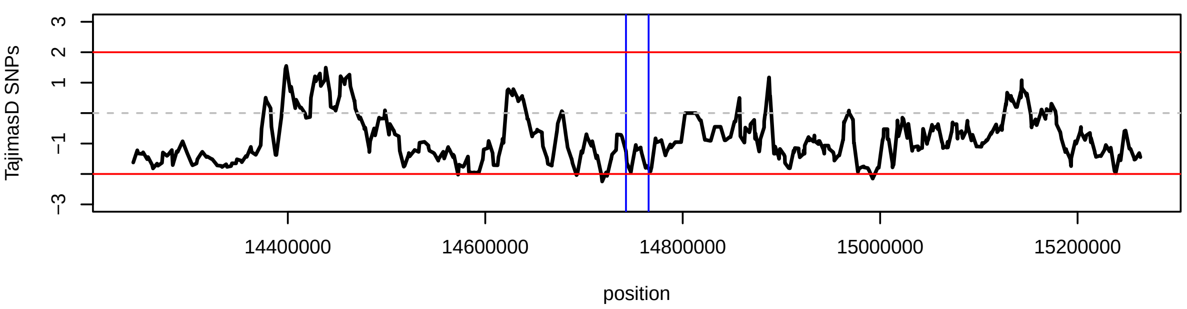

46 CHB NPIP

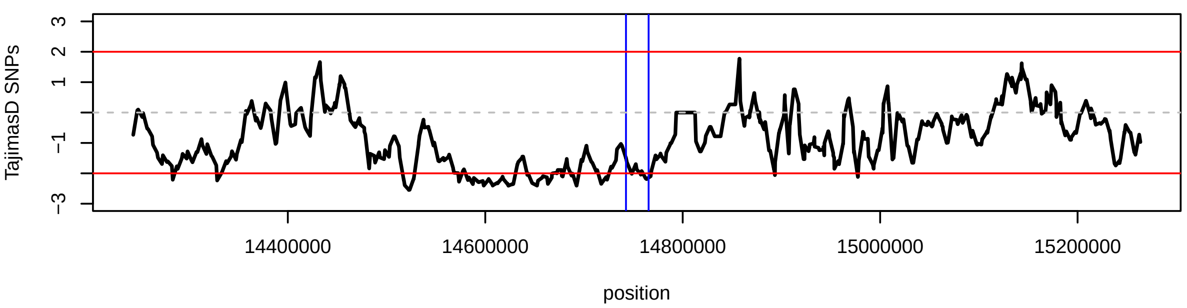

46 YRI NPIP

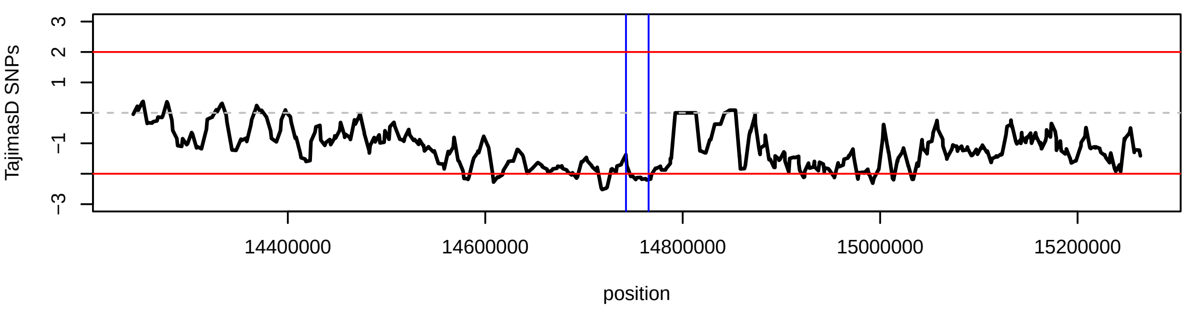

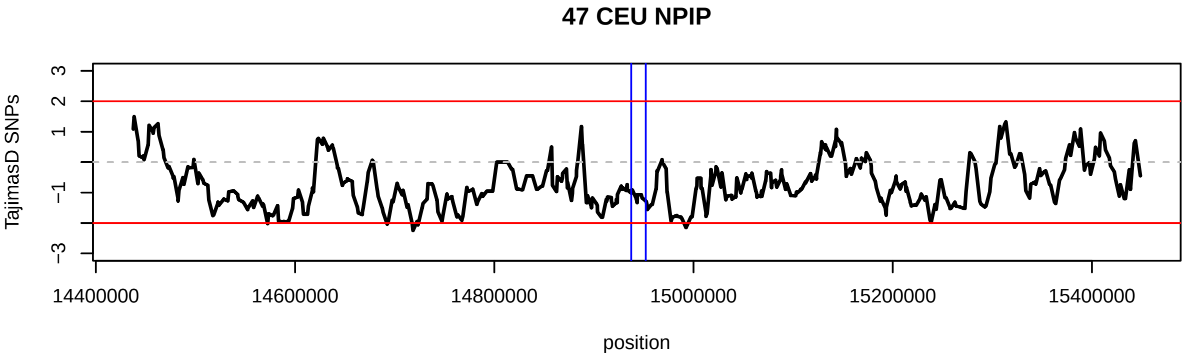
47 CEU NPIP
TajimasD SNPs
3
2
1
−1
−3
14400000
14600000
14800000
15000000
15200000
15400000
position

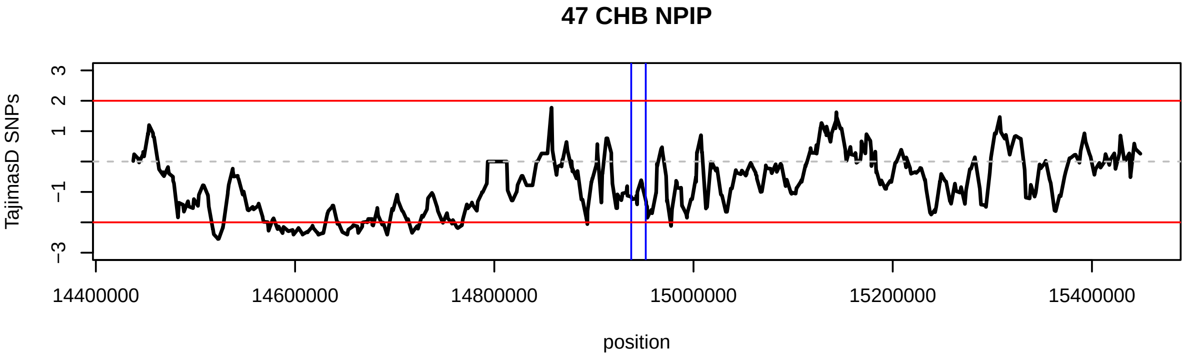
47 CHB NPIP
TajimasD SNPs
3
2
1
−1
−3
14400000
14600000
14800000
15000000
15200000
15400000
position

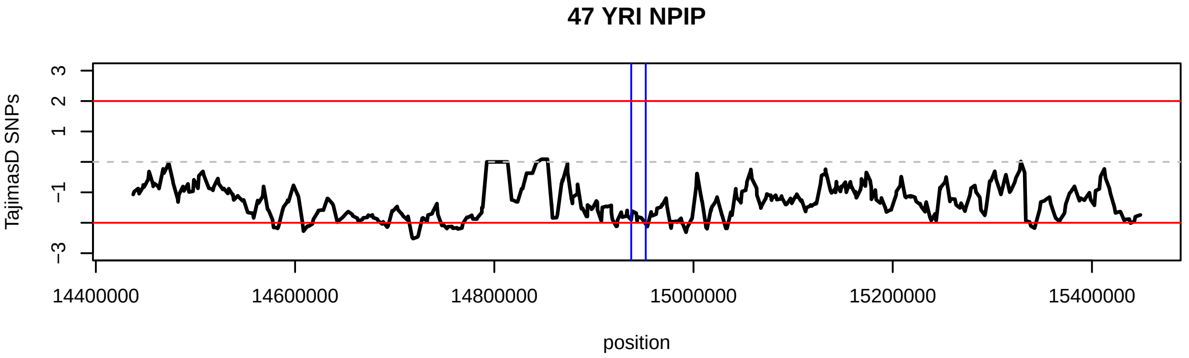
47 YRI NPIP
TajimasD SNPs
3
2
1
−1
−3
14400000
14600000
14800000
15000000
15200000
15400000
position

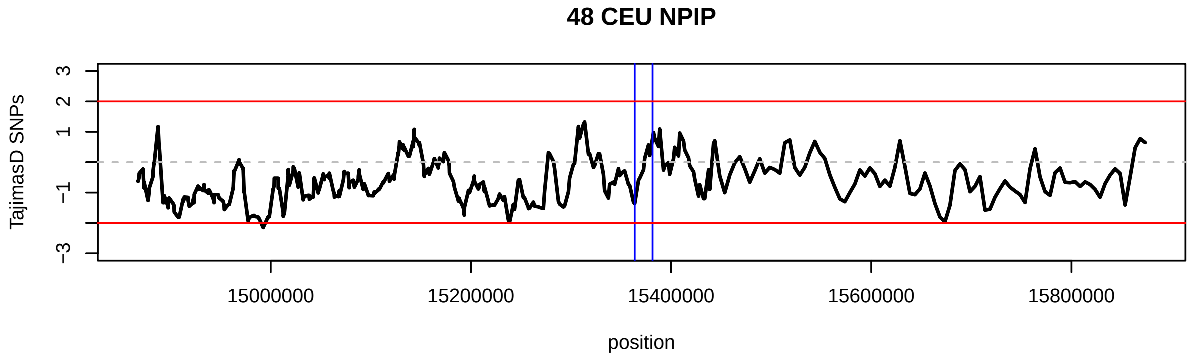
48 CEU NPIP
TajimasD SNPs
3
2
1
−1
−3
15000000
15200000
15400000
15600000
15800000
position

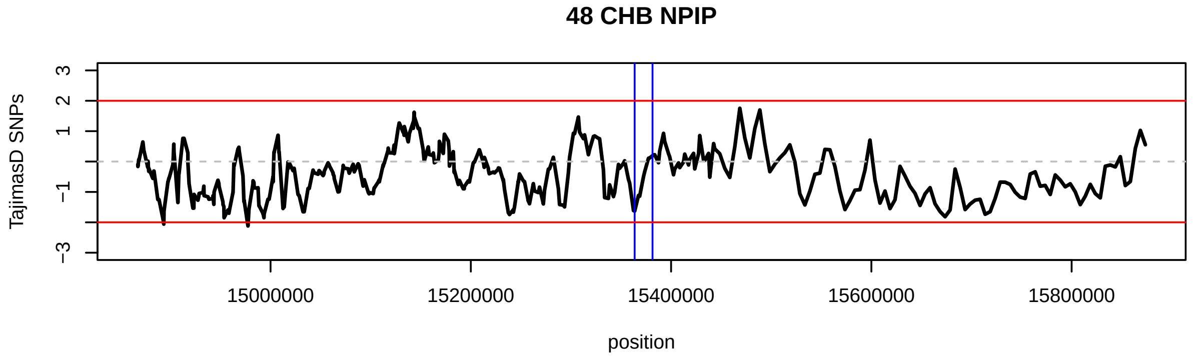
48 CHB NPIP
TajimasD SNPs
3
2
1
−1
−3
15000000
15200000
15400000
15600000
15800000
position

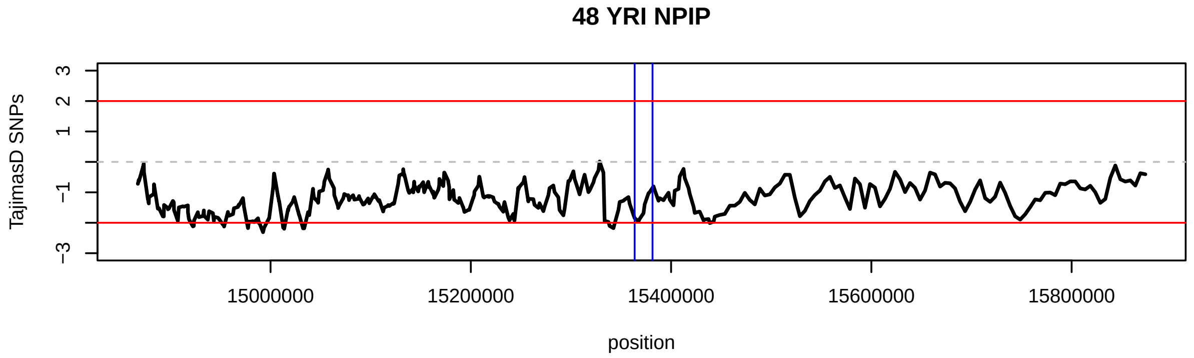
48 YRI NPIP
TajimasD SNPs
3
2
1
−1
−3
15000000
15200000
15400000
15600000
15800000
position

49 CEU NPIP

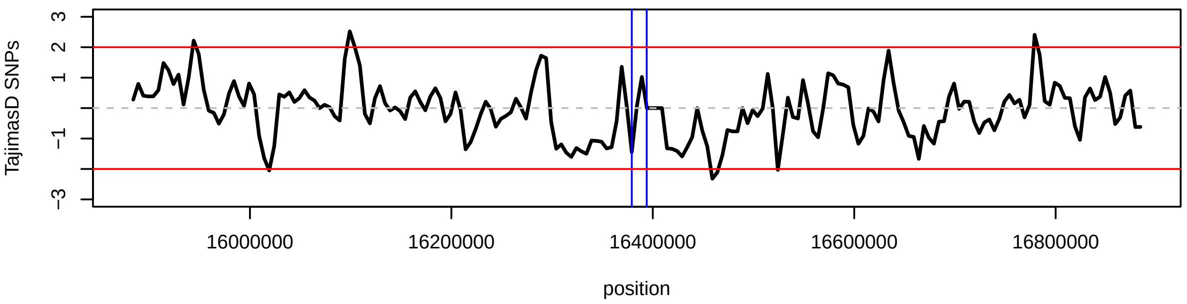

49 CHB NPIP

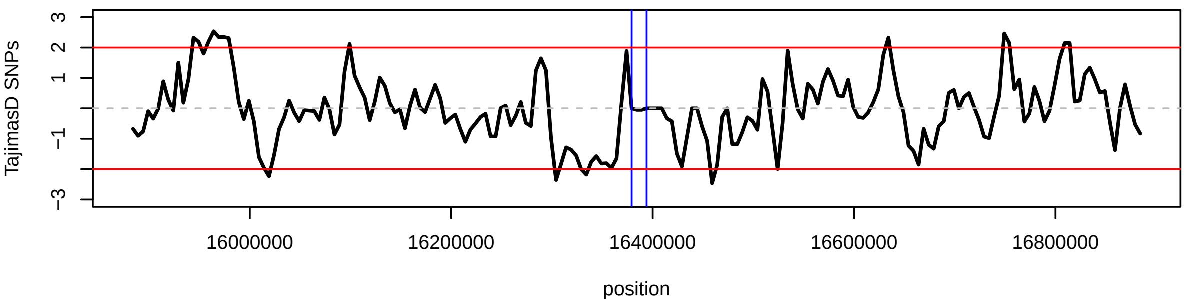

49 YRI NPIP

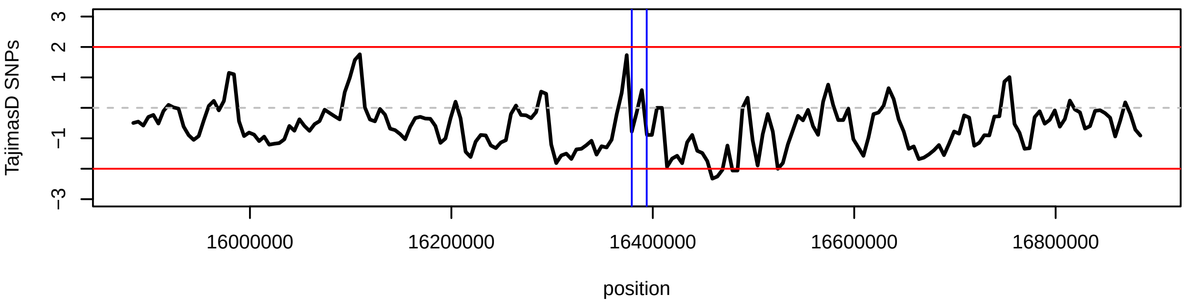

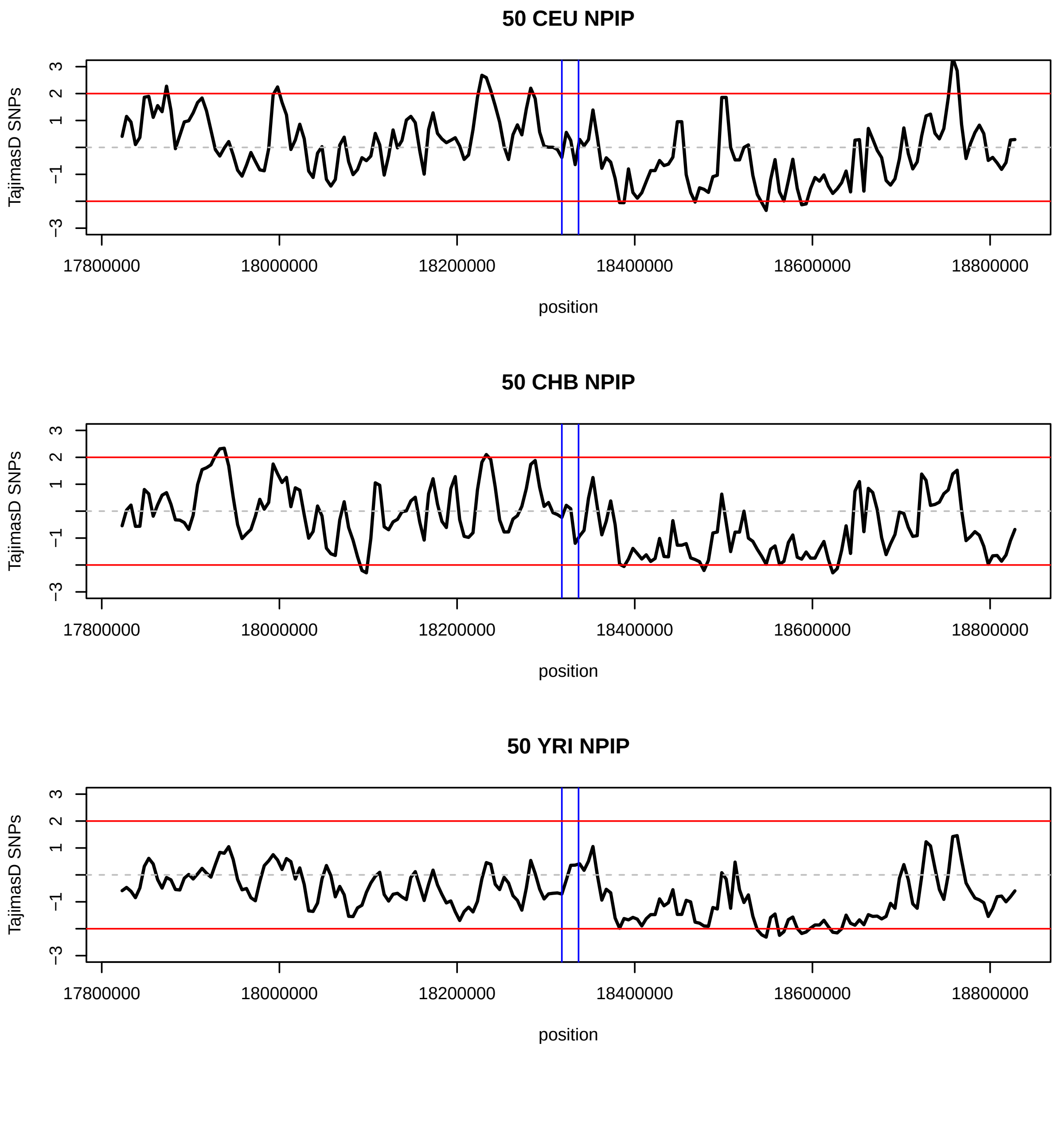
50 CEU NPIP
TajimasD SNPs
position
50 CHB NPIP
TajimasD SNPs
position
50 YRI NPIP
TajimasD SNPs
position
17800000
18000000
18200000
18400000
18600000
18800000

51 CEU NPIP

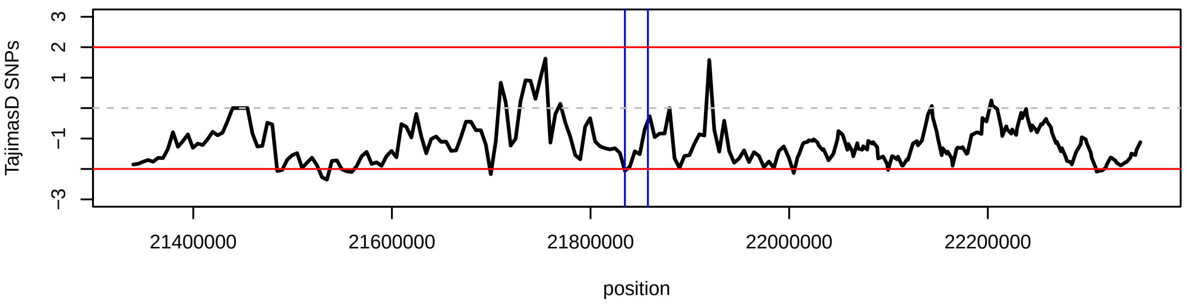

51 CHB NPIP

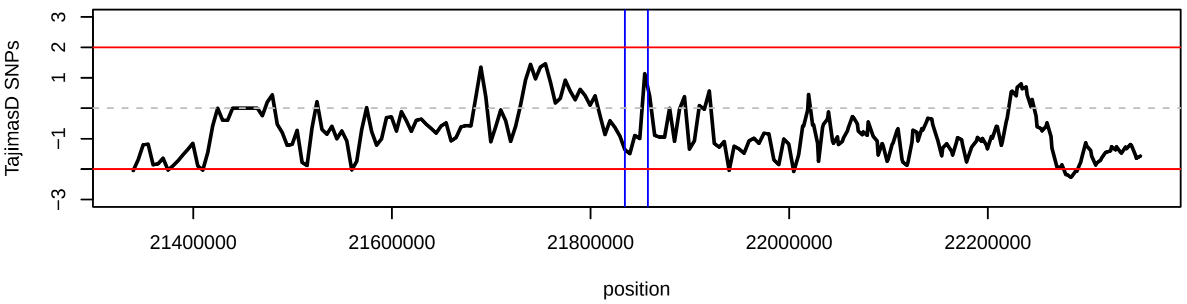

51 YRI NPIP

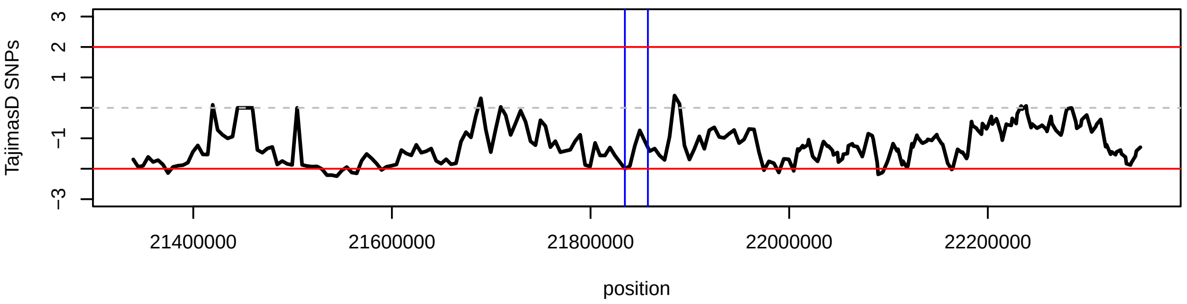

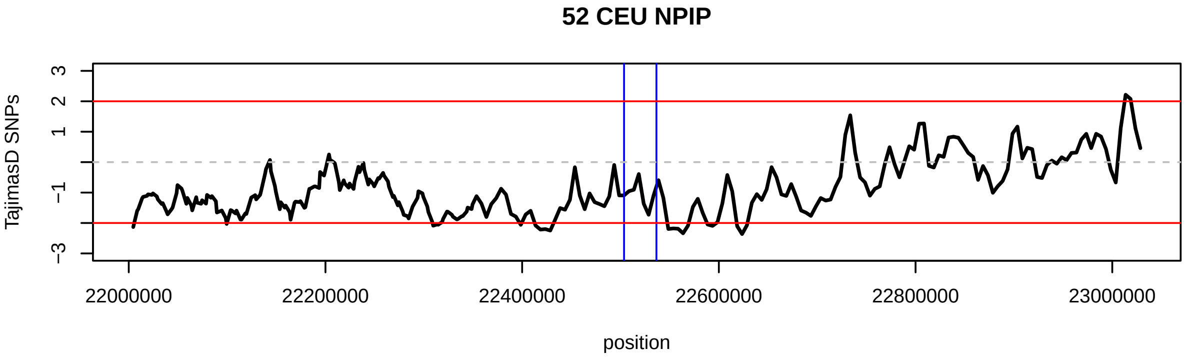
52 CEU NPIP
TajimasD SNPs
3
2
1
−1
−3
22000000
22200000
22400000
22600000
22800000
23000000
position

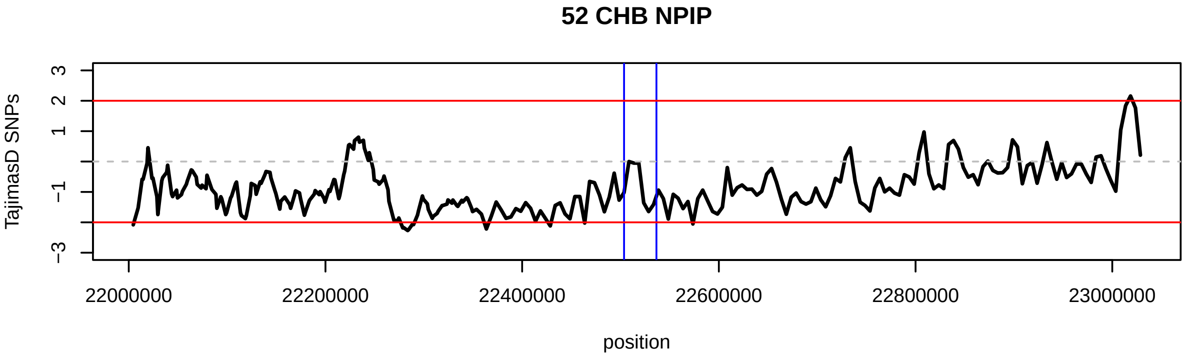
52 CHB NPIP
TajimasD SNPs
3
2
1
−1
−3
22000000
22200000
22400000
22600000
22800000
23000000
position

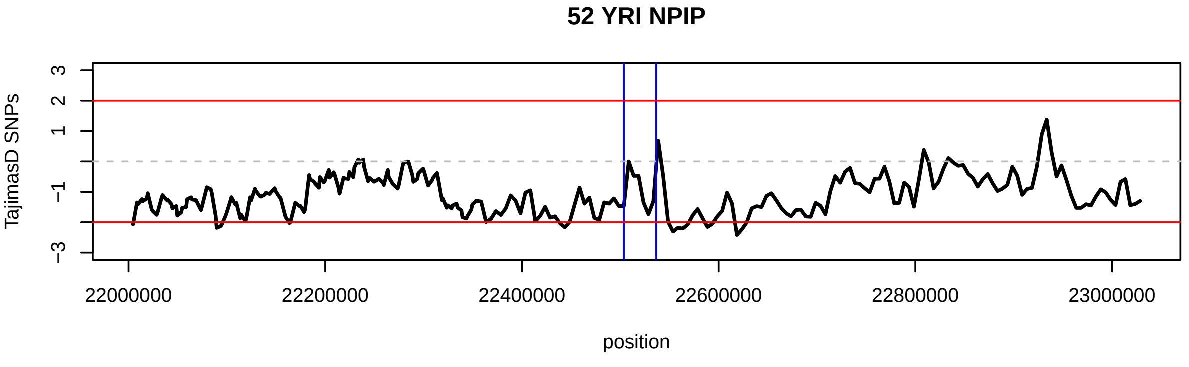
52 YRI NPIP
TajimasD SNPs
3
2
1
−1
−3
22000000
22200000
22400000
22600000
22800000
23000000
position

53 CEU NPIP

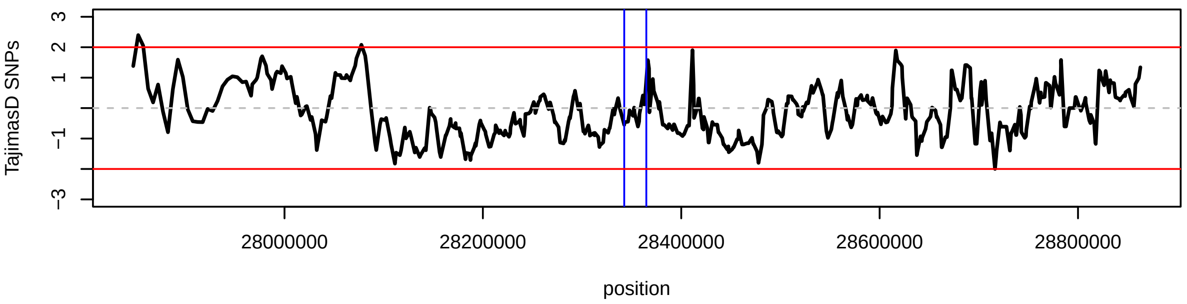

53 CHB NPIP

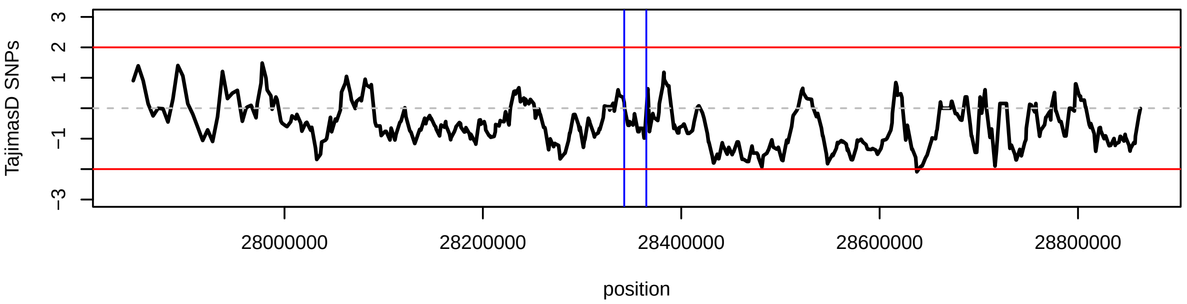

53 YRI NPIP

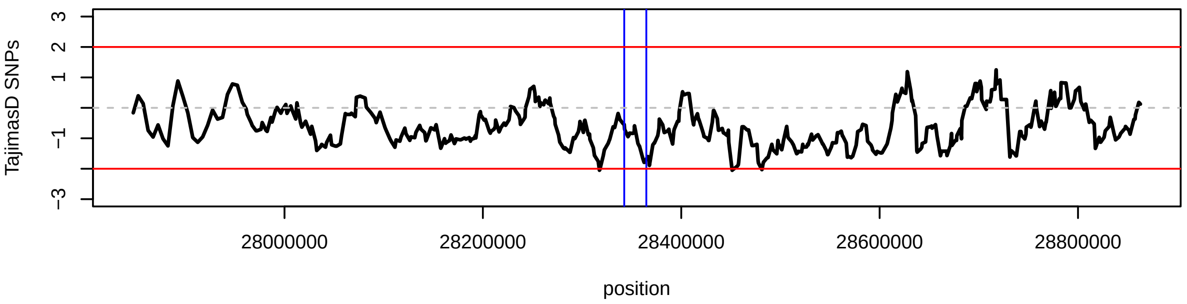

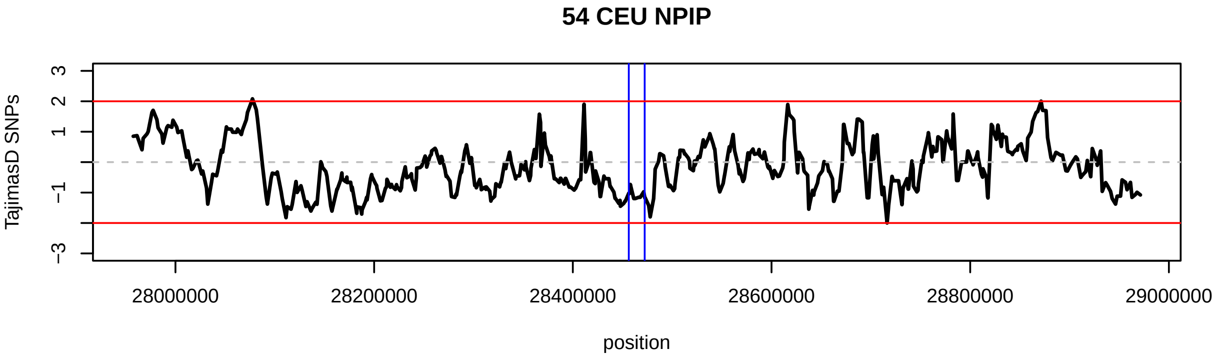
54 CEU NPIP
TajimasD SNPs
3
2
1
−1
−3
28000000
28200000
28400000
28600000
28800000
29000000
position

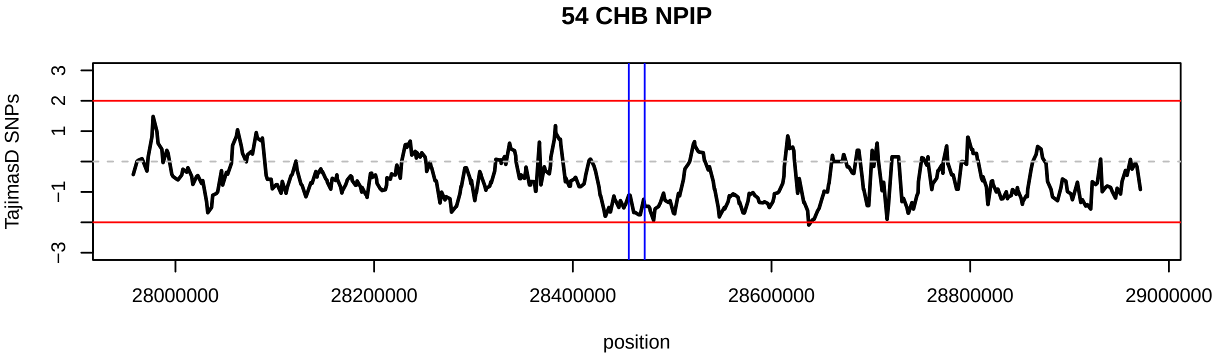
54 CHB NPIP
TajimasD SNPs
3
2
1
−1
−3
28000000
28200000
28400000
28600000
28800000
29000000
position

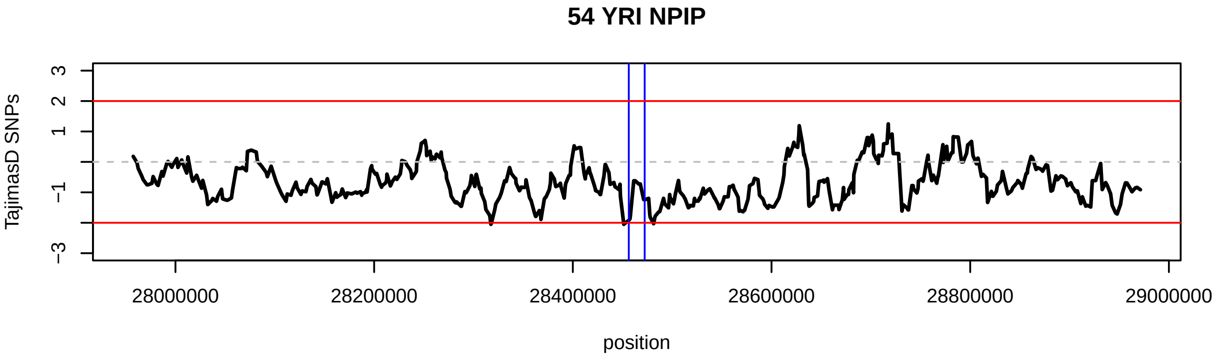
54 YRI NPIP
TajimasD SNPs
3
2
1
−1
−3
28000000
28200000
28400000
28600000
28800000
29000000
position

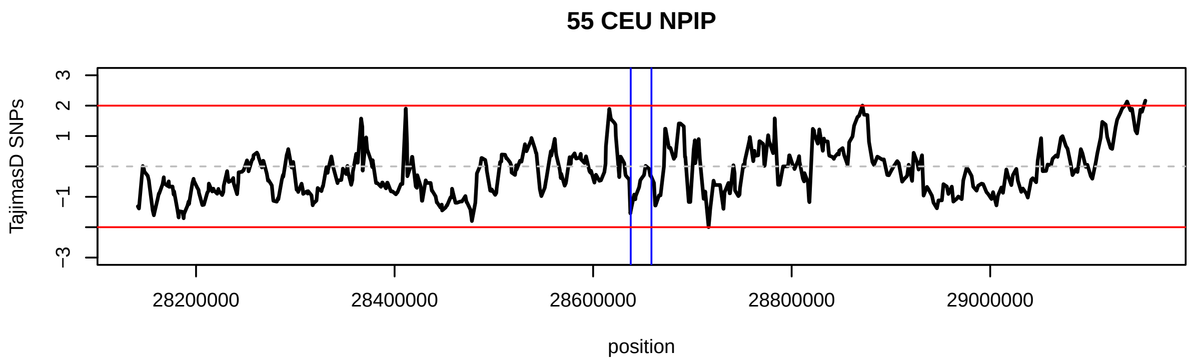
55 CEU NPIP
TajimasD SNPs
3
2
1
−1
−3
28200000
28400000
28600000
28800000
29000000
position

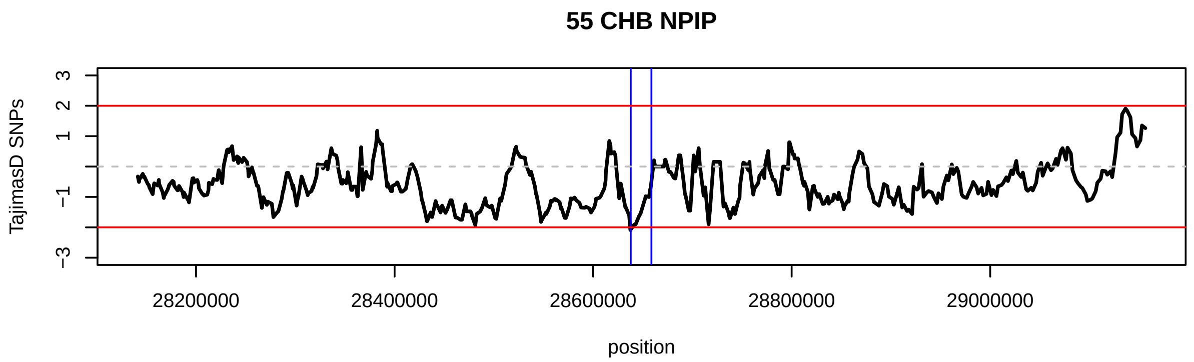
55 CHB NPIP
TajimasD SNPs
3
2
1
−1
−3
28200000
28400000
28600000
28800000
29000000
position

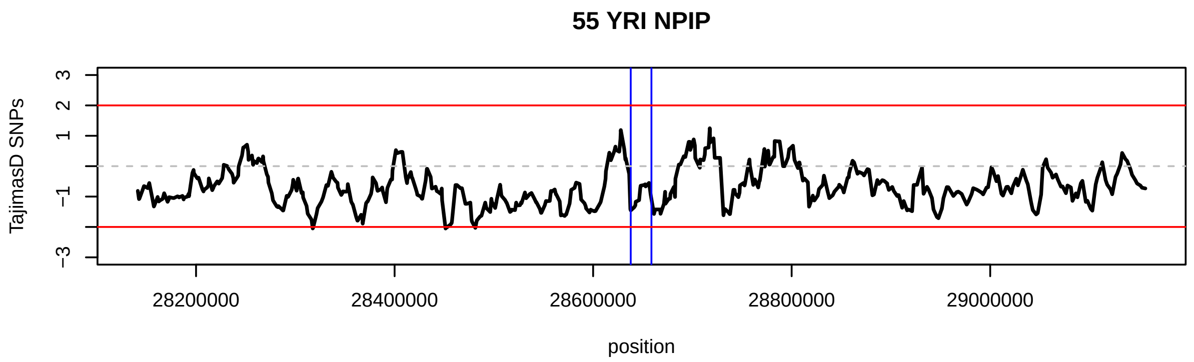
55 YRI NPIP
TajimasD SNPs
3
2
1
−1
−3
28200000
28400000
28600000
28800000
29000000
position

56 CEU NPIP

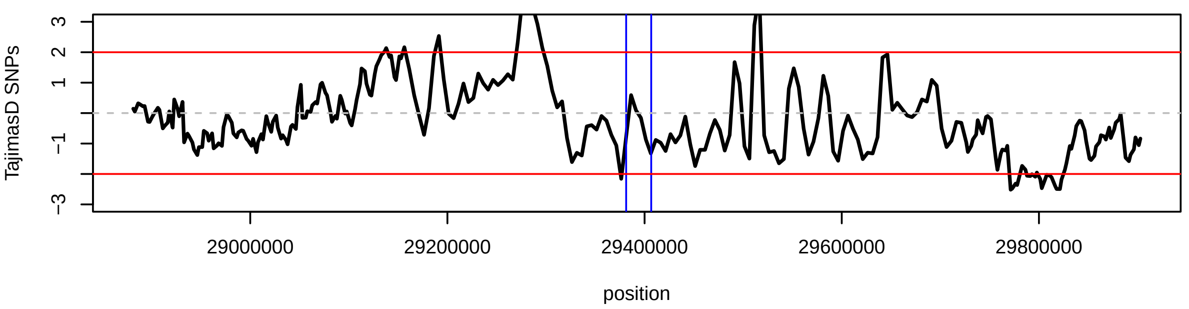

56 CHB NPIP

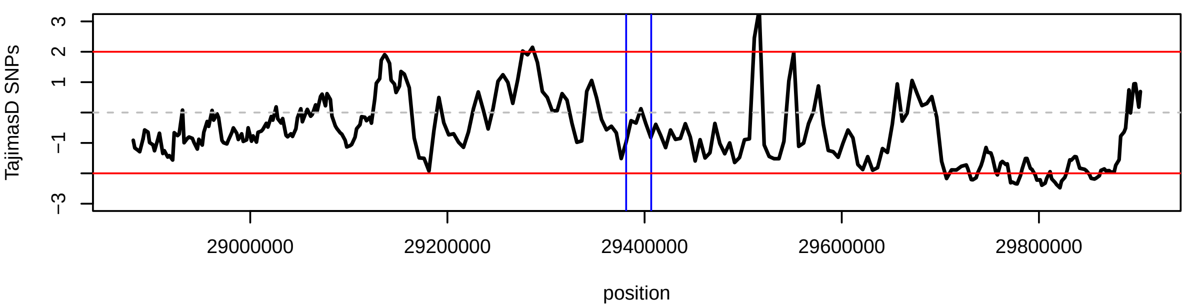

56 YRI NPIP

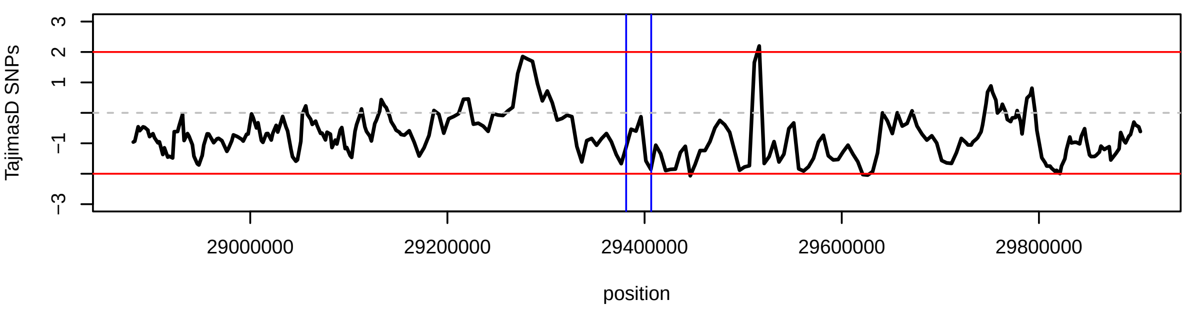

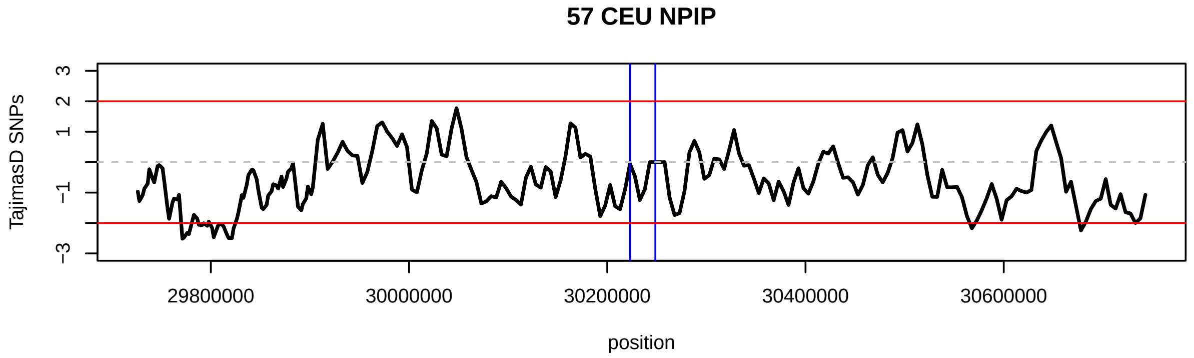
57 CEU NPIP
TajimasD SNPs
3
2
1
−1
−3
29800000
30000000
30200000
30400000
30600000
position

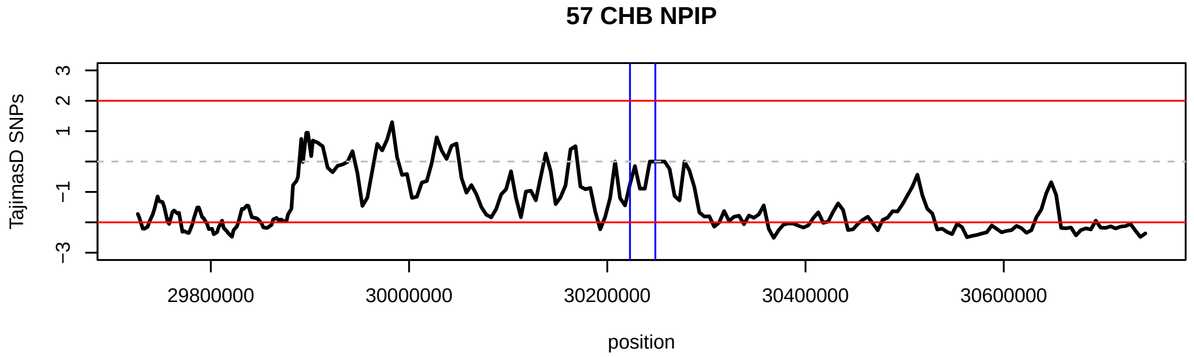
57 CHB NPIP
TajimasD SNPs
3
2
1
−1
−3
29800000
30000000
30200000
30400000
30600000
position

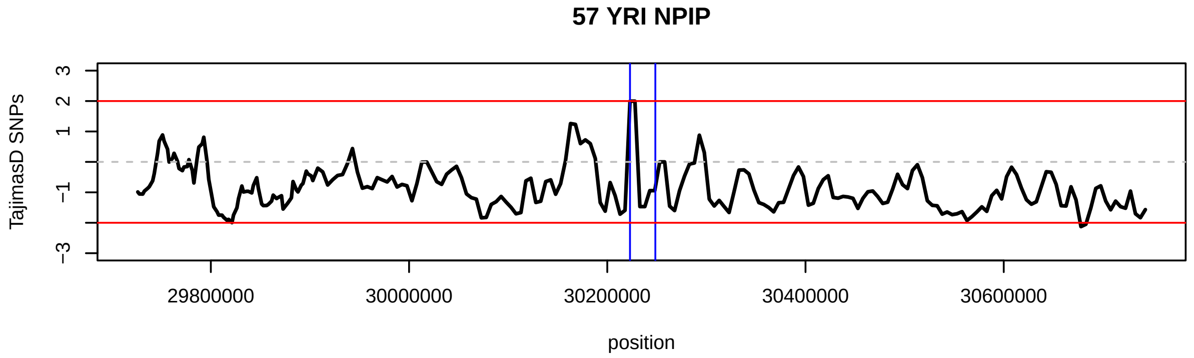
57 YRI NPIP
TajimasD SNPs
3
2
1
−1
−3
29800000
30000000
30200000
30400000
30600000
position

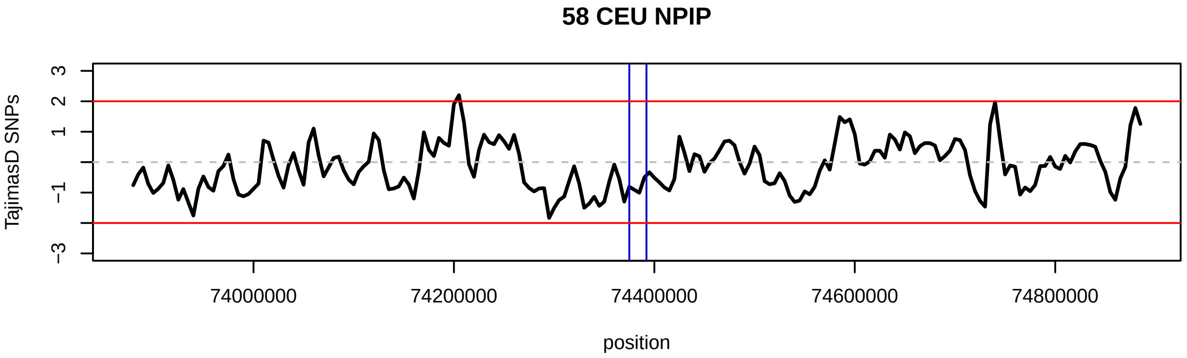
58 CEU NPIP
TajimasD SNPs
3
2
1
−1
−3
74000000
74200000
74400000
74600000
74800000
position

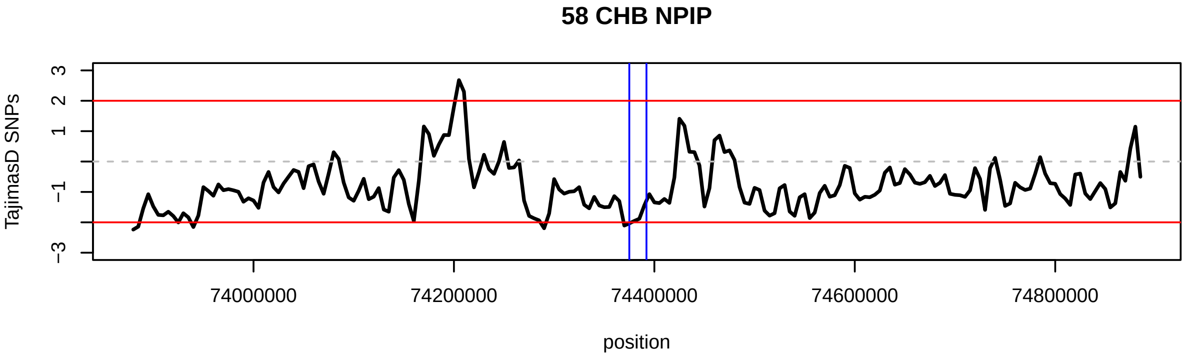
58 CHB NPIP
TajimasD SNPs
3
2
1
−1
−3
74000000
74200000
74400000
74600000
74800000
position

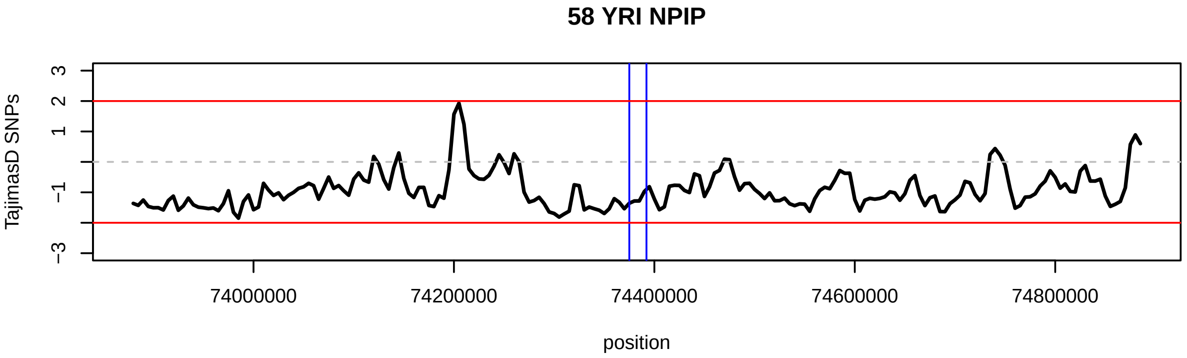
58 YRI NPIP
TajimasD SNPs
3
2
1
−1
−3
74000000
74200000
74400000
74600000
74800000
position

59 CEU PGA3

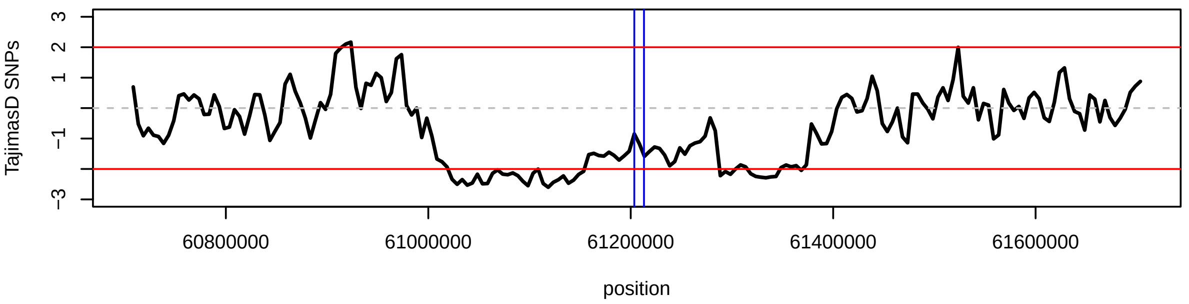

59 CHB PGA3

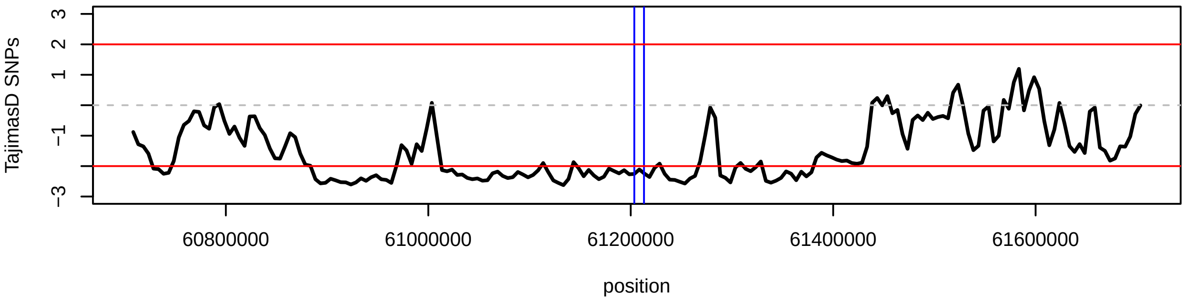

59 YRI PGA3

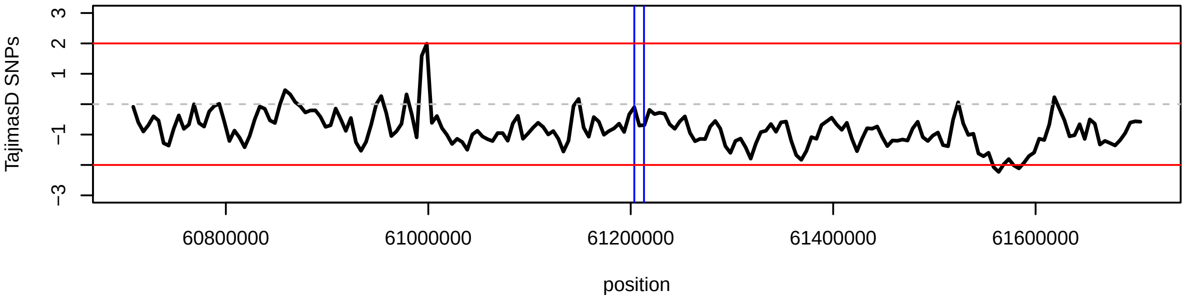

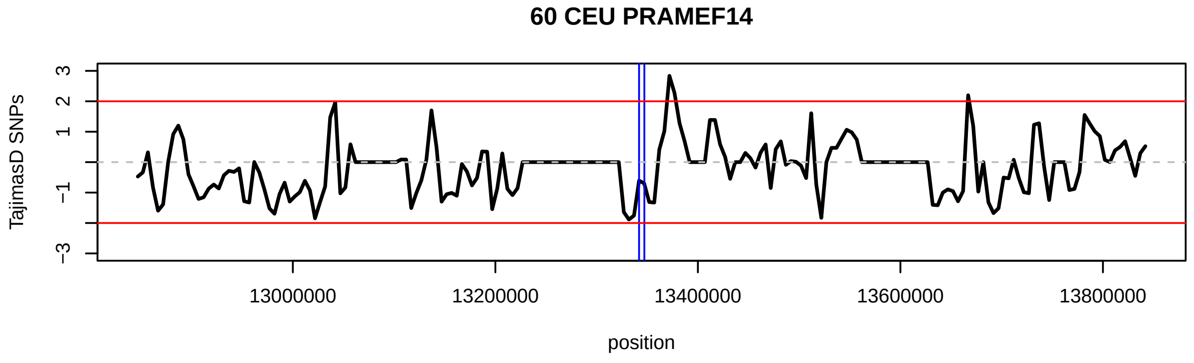
60 CEU PRAMEF14
TajimasD SNPs
3
2
1
−1
−3
13000000
13200000
13400000
13600000
13800000
position

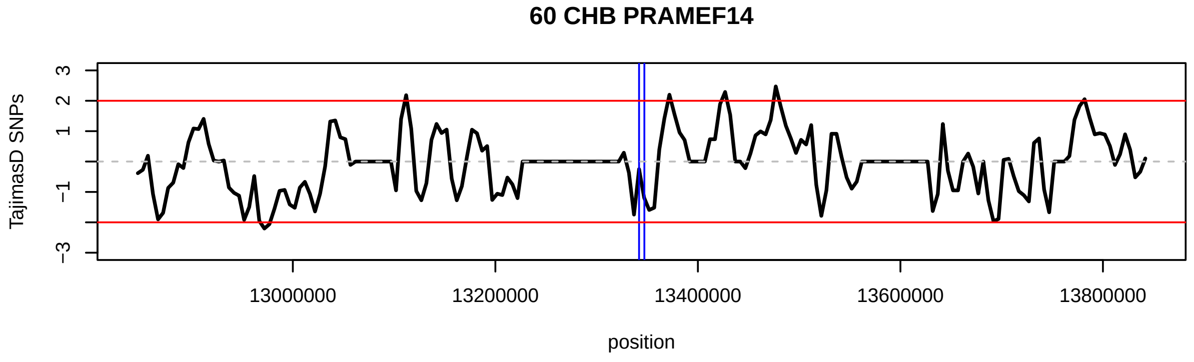
60 CHB PRAMEF14
TajimasD SNPs
3
2
1
−1
−3
13000000
13200000
13400000
13600000
13800000
position

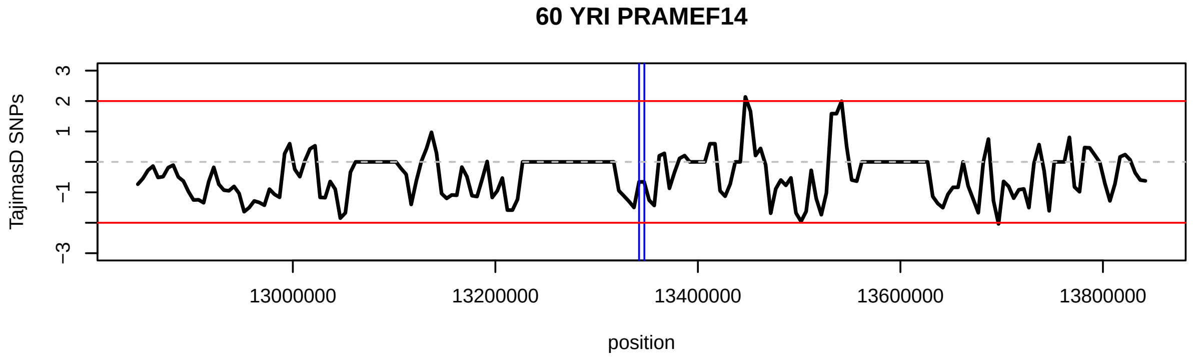
60 YRI PRAMEF14
TajimasD SNPs
3
2
1
−1
−3
13000000
13200000
13400000
13600000
13800000
position

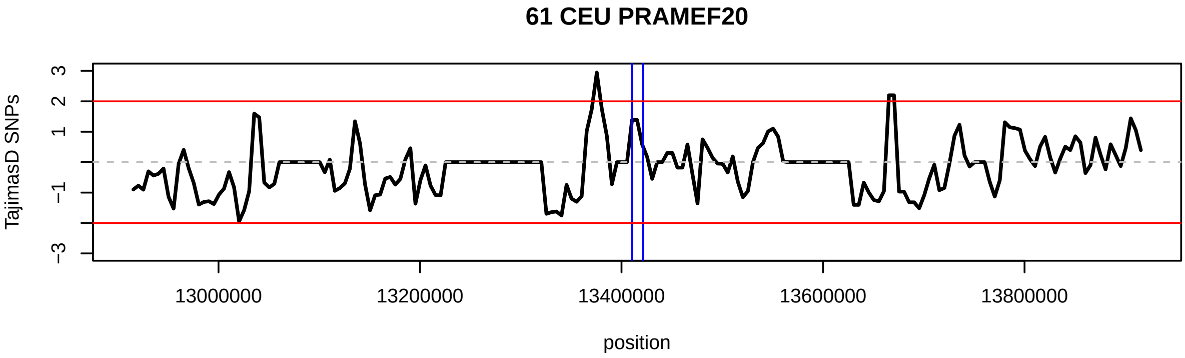
61 CEU PRAMEF20
TajimasD SNPs
3
2
1
−1
−3
13000000
13200000
13400000
13600000
13800000
position

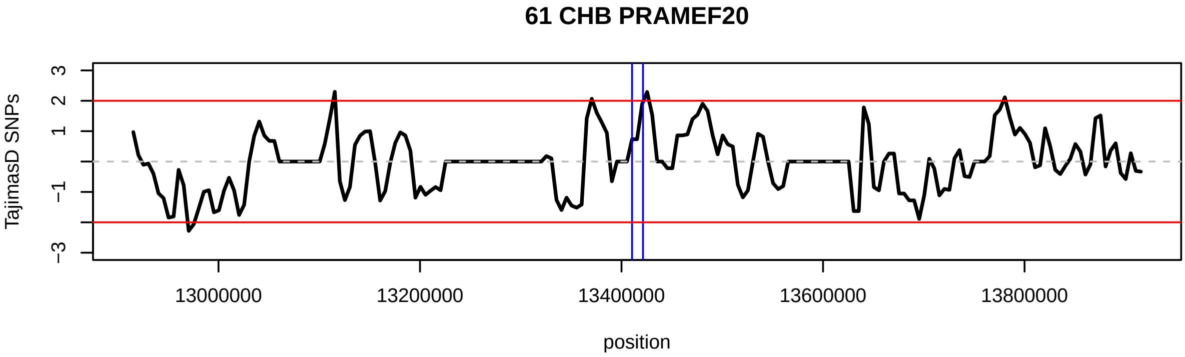
61 CHB PRAMEF20
TajimasD SNPs
3
2
1
−1
−3
13000000
13200000
13400000
13600000
13800000
position

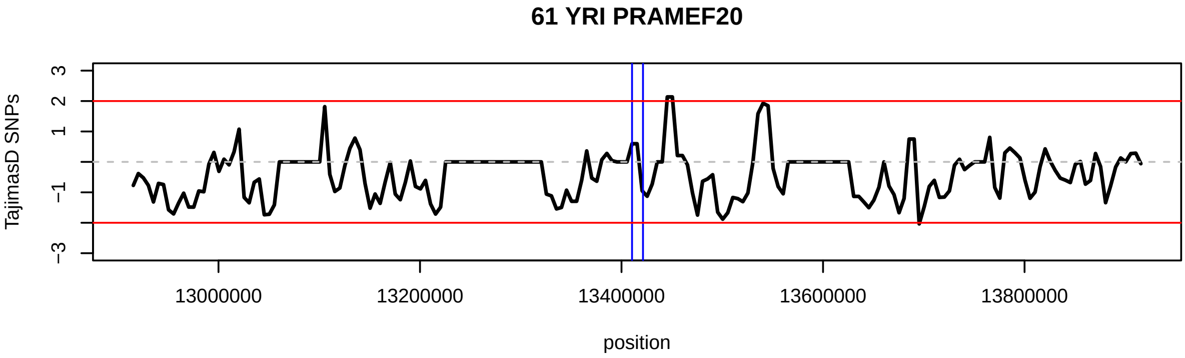
61 YRI PRAMEF20
TajimasD SNPs
3
2
1
−1
−3
13000000
13200000
13400000
13600000
13800000
position

62 CEU PRAMEF5

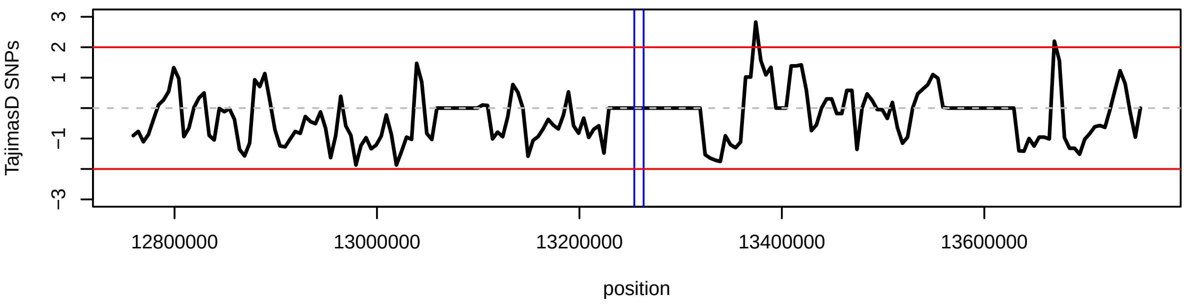

62 CHB PRAMEF5

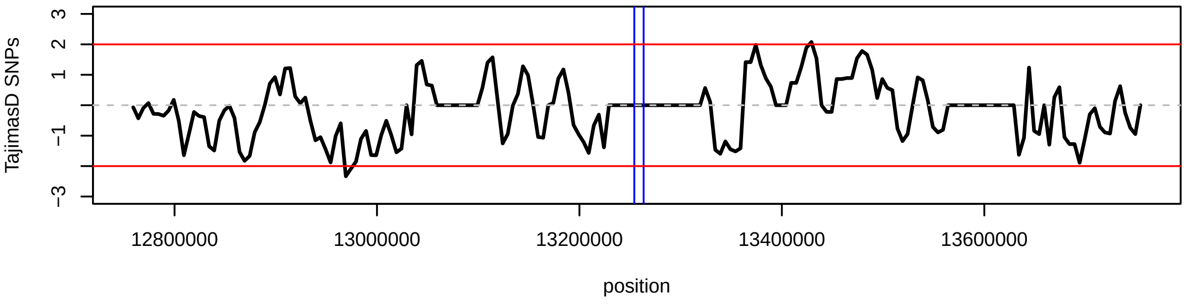

62 YRI PRAMEF5

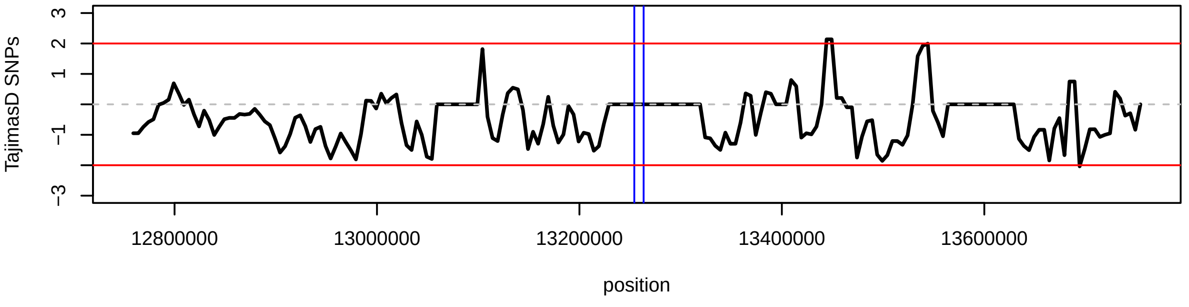

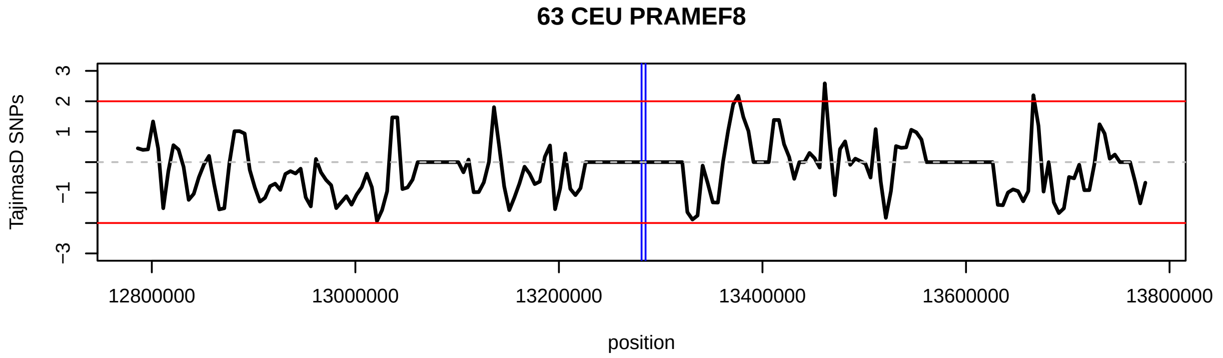
63 CEU PRAMEF8
TajimasD SNPs
−3
−1
1
2
3
12800000
13000000
13200000
13400000
13600000
13800000
position

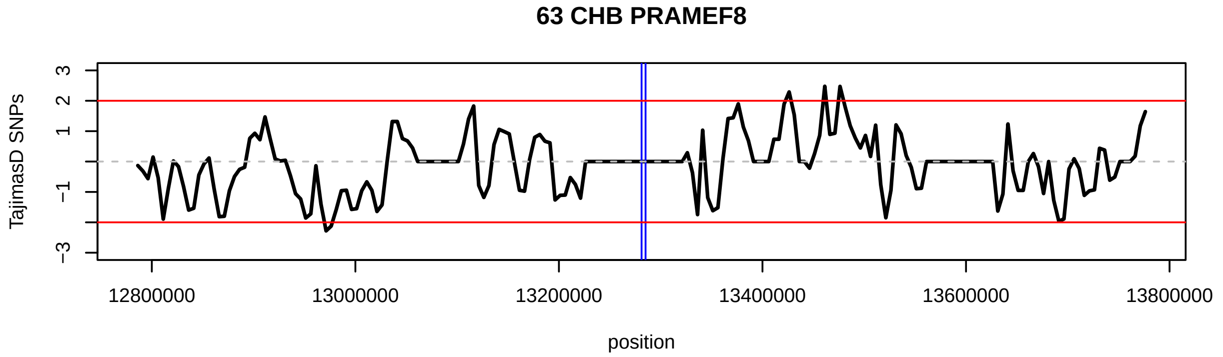
63 CHB PRAMEF8
TajimasD SNPs
−3
−1
1
2
3
12800000
13000000
13200000
13400000
13600000
13800000
position

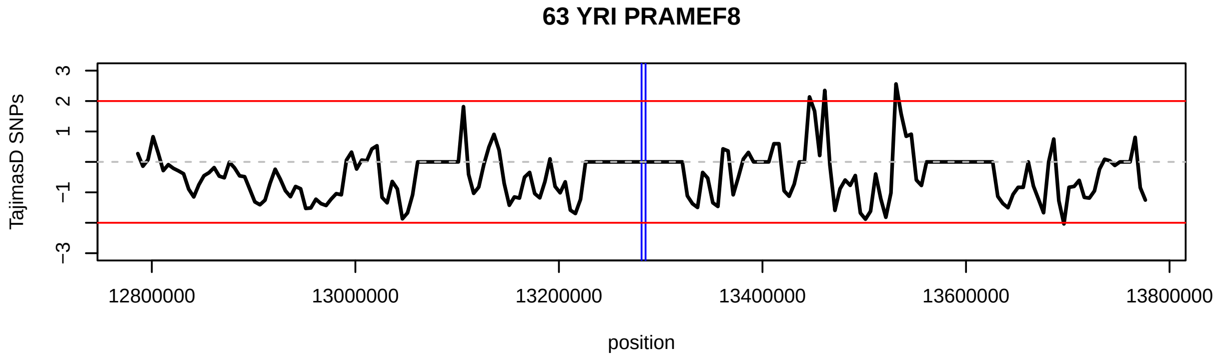
63 YRI PRAMEF8
TajimasD SNPs
−3
−1
1
2
3
12800000
13000000
13200000
13400000
13600000
13800000
position

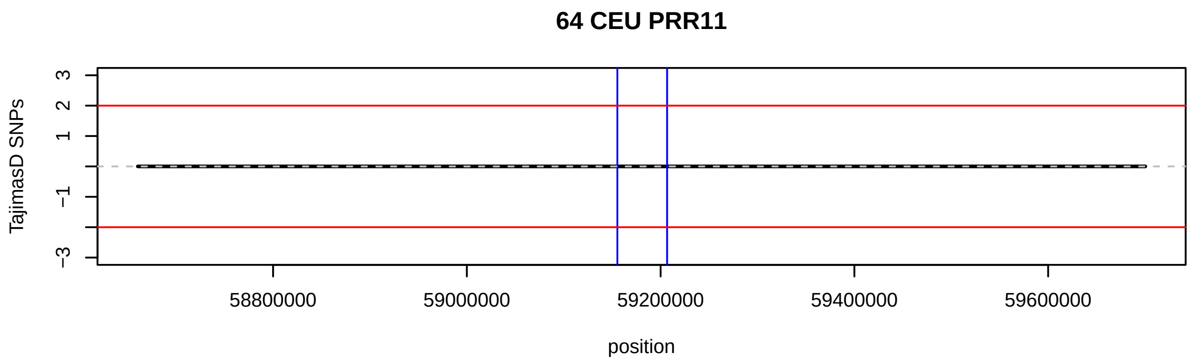
64 CEU PRR11
TajimasD SNPs
3
2
1
−1
−3
58800000
59000000
59200000
59400000
59600000
position

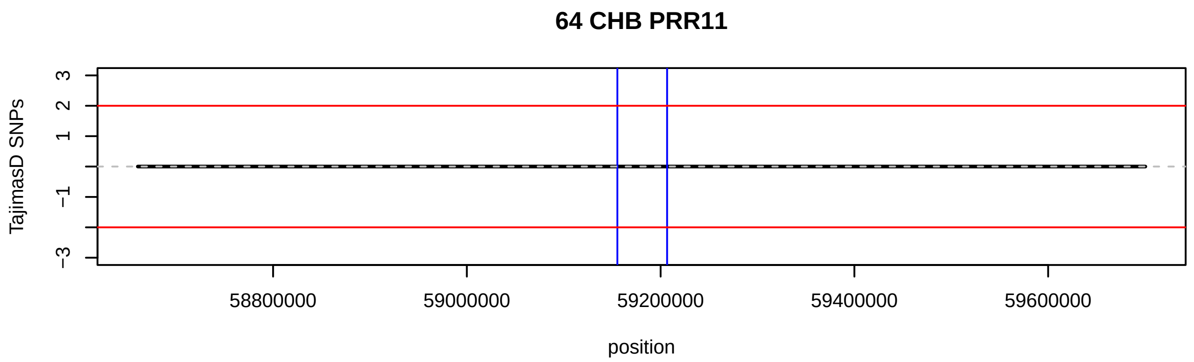
64 CHB PRR11
TajimasD SNPs
3
2
1
−1
−3
58800000
59000000
59200000
59400000
59600000
position

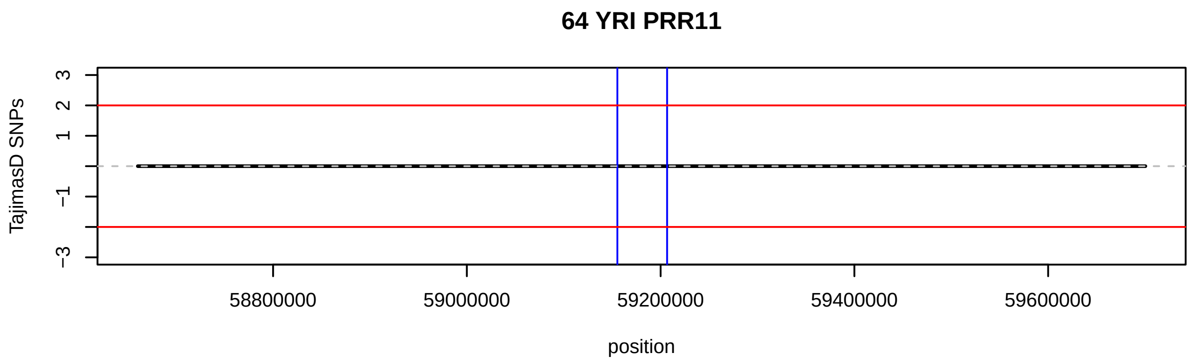
64 YRI PRR11
TajimasD SNPs
3
2
1
−1
−3
58800000
59000000
59200000
59400000
59600000
position

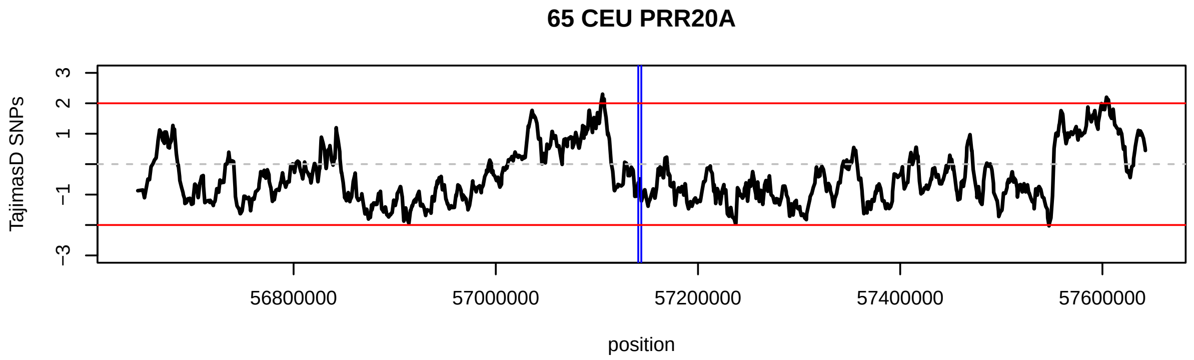
65 CEU PRR20A
TajimasD SNPs
3
2
1
−1
−3
56800000
57000000
57200000
57400000
57600000
position

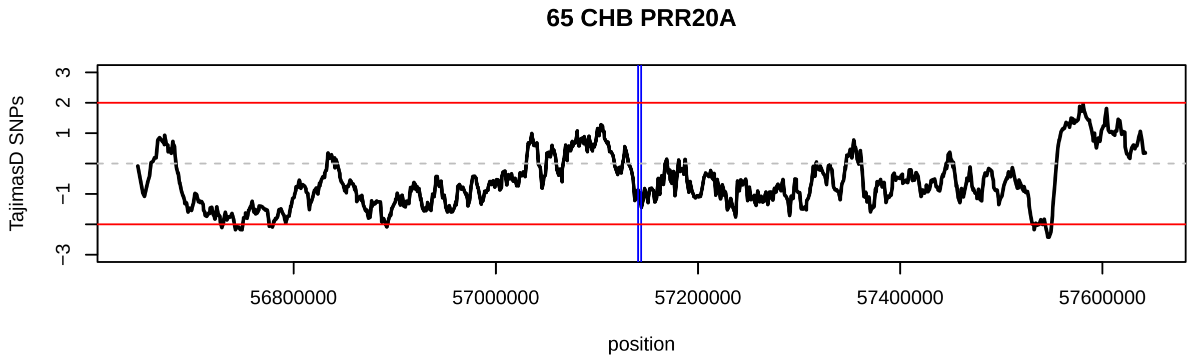
65 CHB PRR20A
TajimasD SNPs
3
2
1
−1
−3
56800000
57000000
57200000
57400000
57600000
position

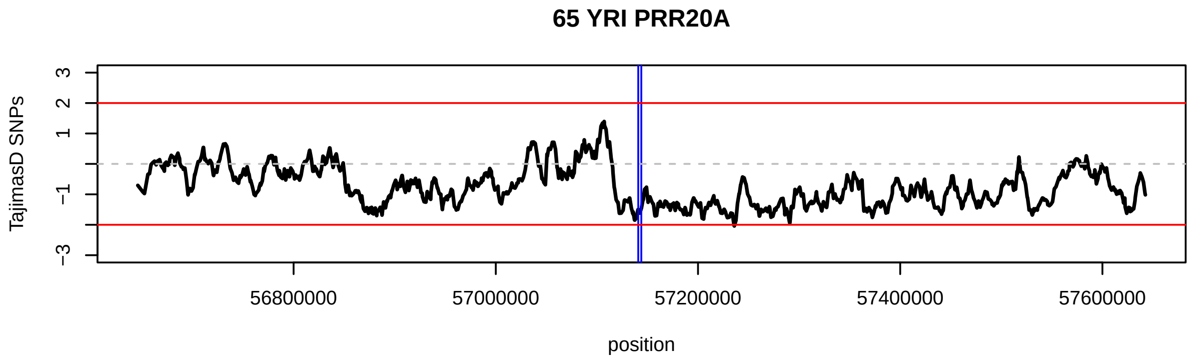
65 YRI PRR20A
TajimasD SNPs
3
2
1
−1
−3
56800000
57000000
57200000
57400000
57600000
position

66 CEU PRR20A

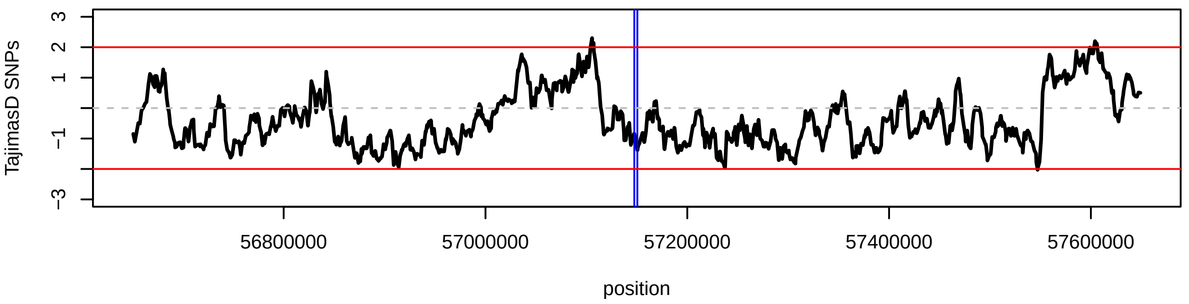

66 CHB PRR20A

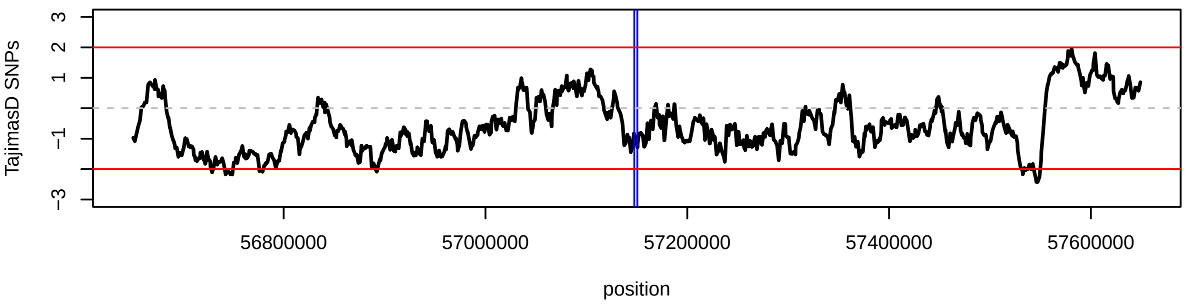

66 YRI PRR20A

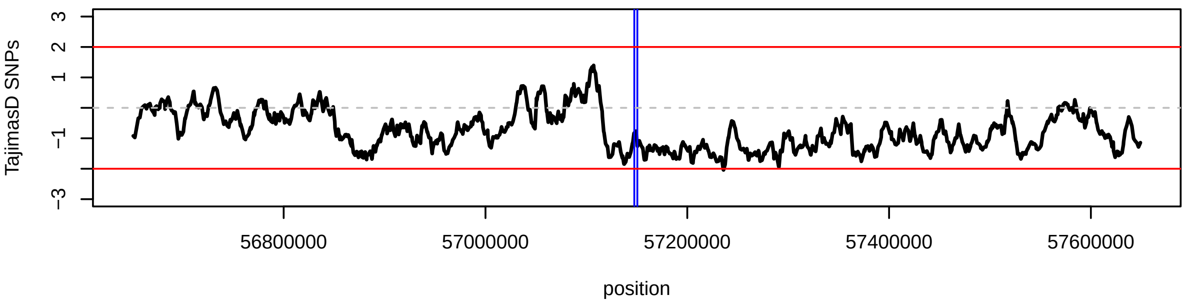

67 CEU PRR20A

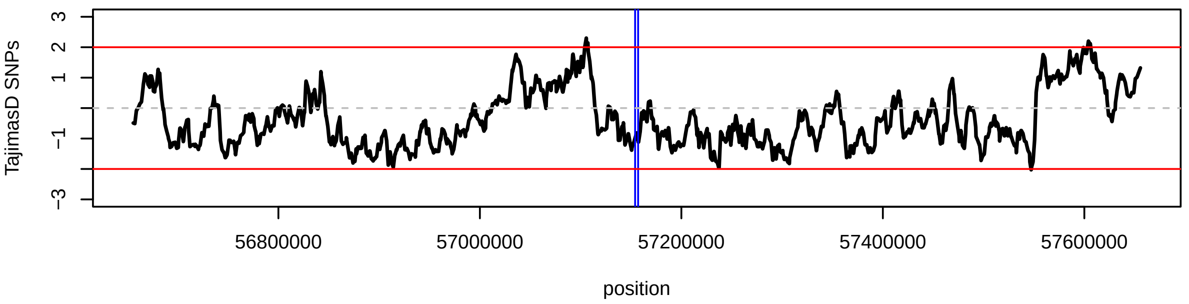

67 CHB PRR20A

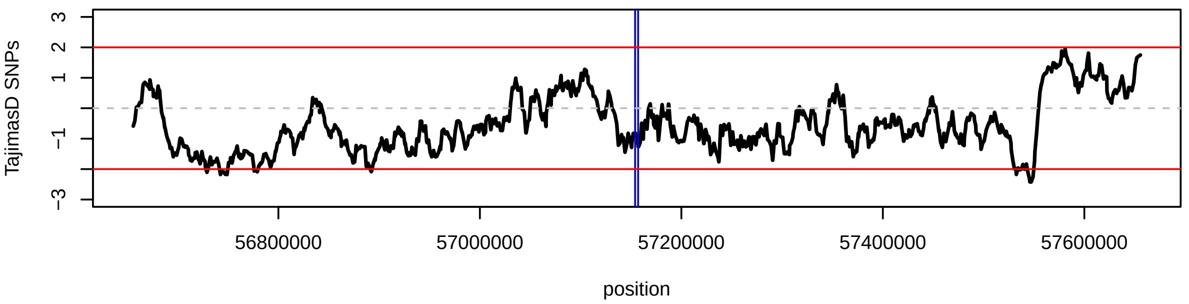

67 YRI PRR20A

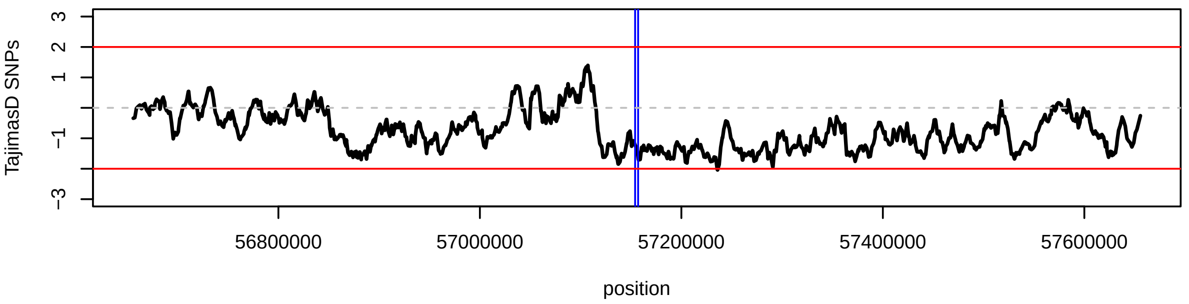

68 CEU PRR20A

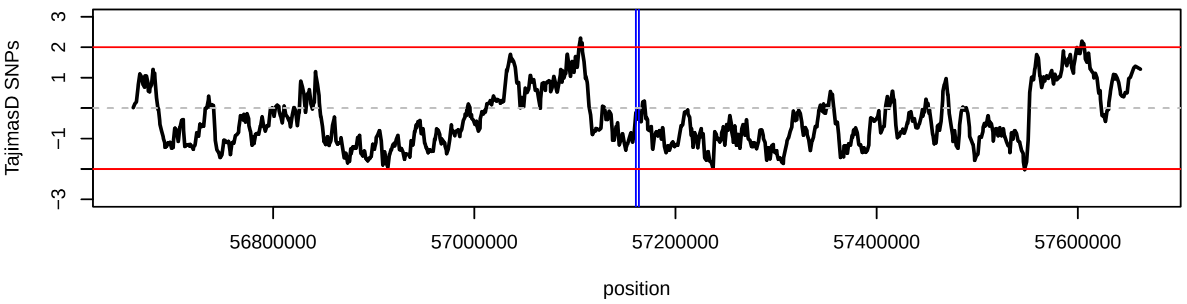

68 CHB PRR20A

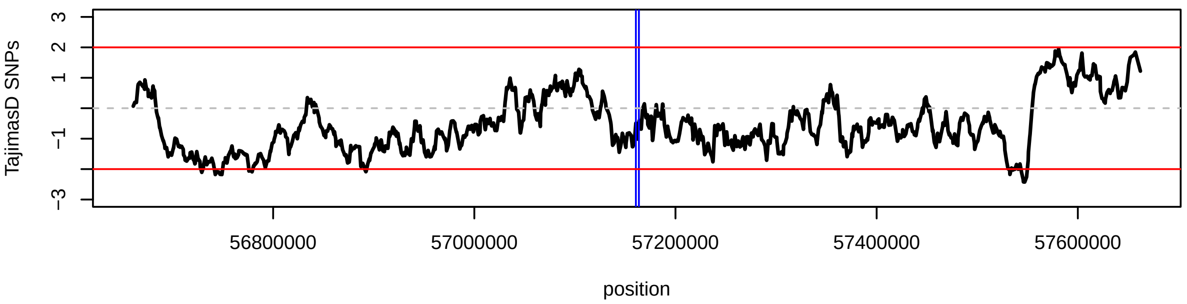

68 YRI PRR20A

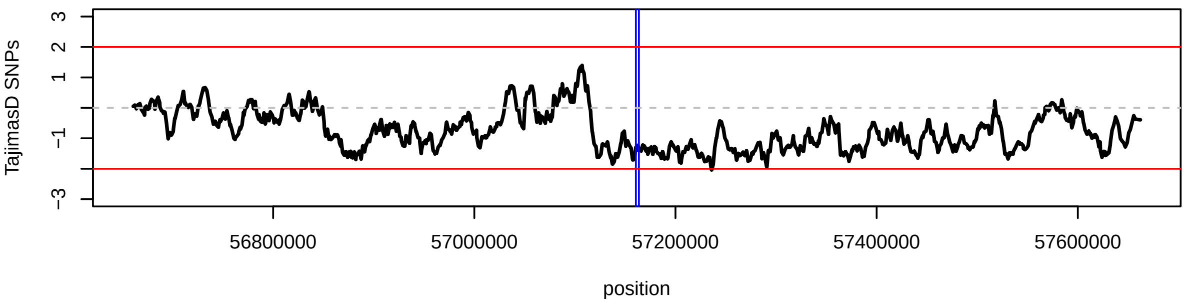

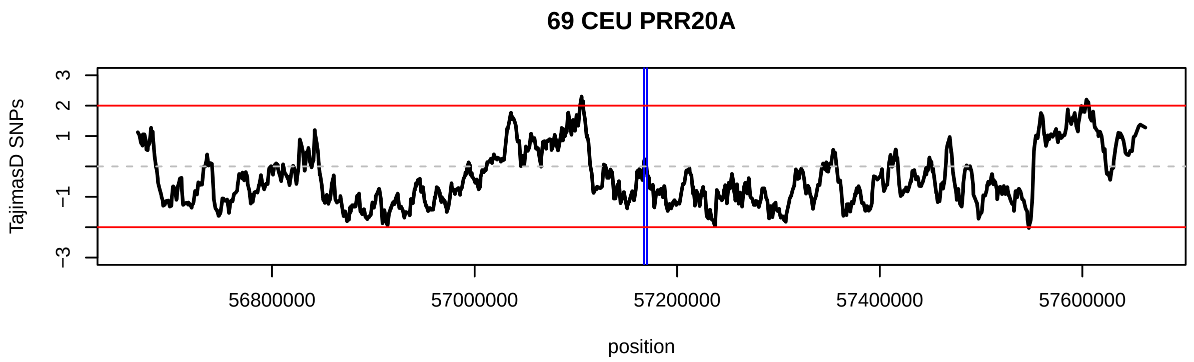
69 CEU PRR20A
TajimasD SNPs
3
2
1
−1
−3
56800000
57000000
57200000
57400000
57600000
position

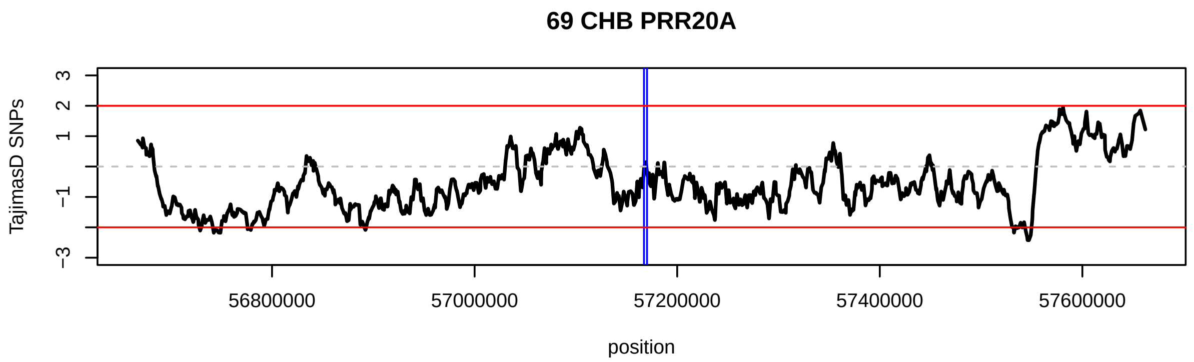
69 CHB PRR20A
TajimasD SNPs
3
2
1
−1
−3
56800000
57000000
57200000
57400000
57600000
position

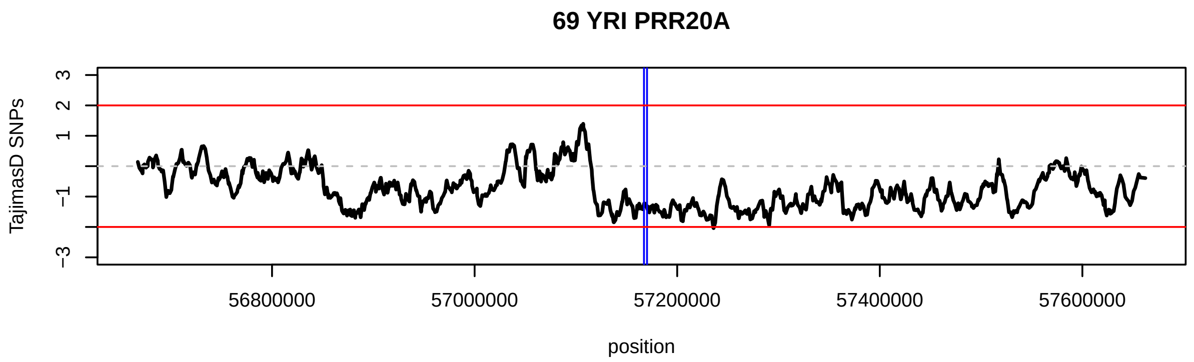
69 YRI PRR20A
TajimasD SNPs
3
2
1
−1
−3
56800000
57000000
57200000
57400000
57600000
position

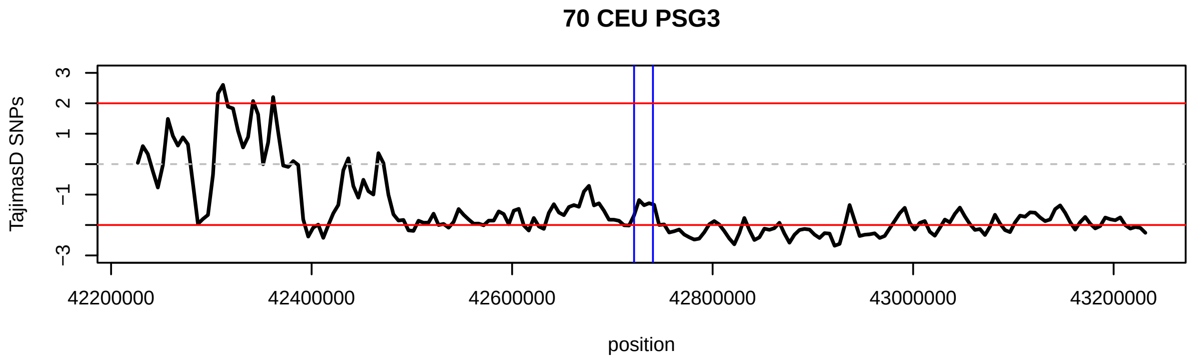

70 CEU PSG3
TajimasD SNPs
3
2
1
−1
−3
42200000
42400000
42600000
42800000
43000000
43200000
position

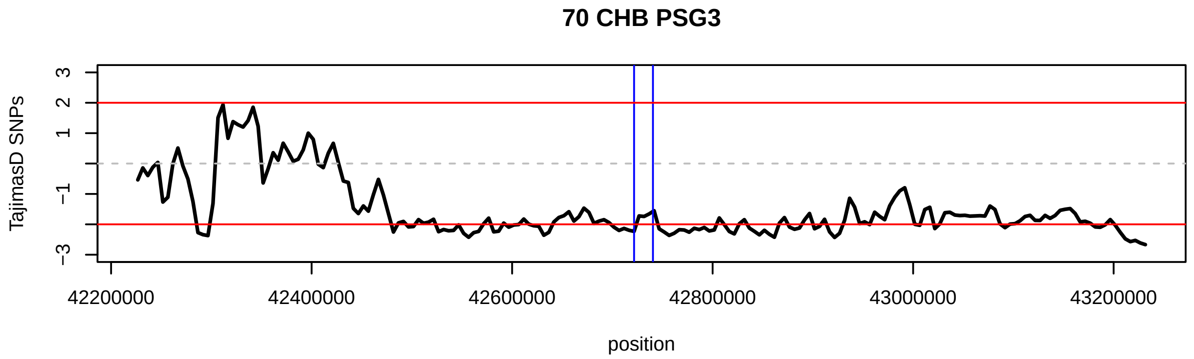

70 CHB PSG3
TajimasD SNPs
3
2
1
−1
−3
42200000
42400000
42600000
42800000
43000000
43200000
position

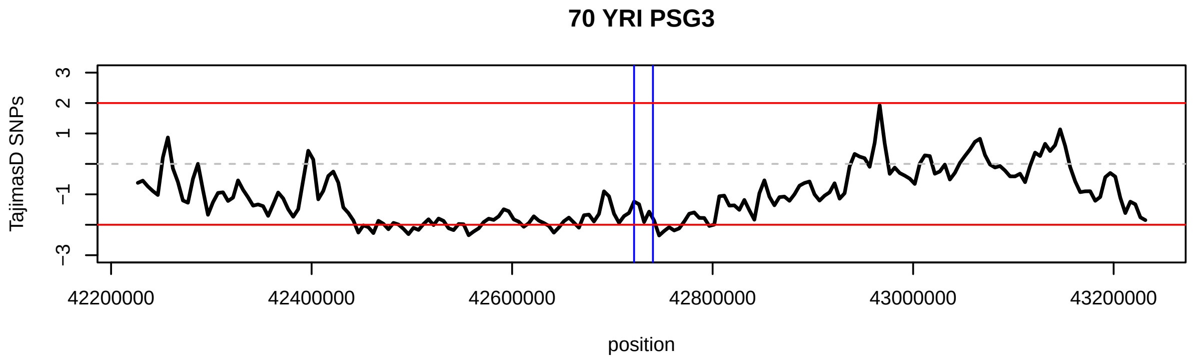

70 YRI PSG3
TajimasD SNPs
3
2
1
−1
−3
42200000
42400000
42600000
42800000
43000000
43200000
position

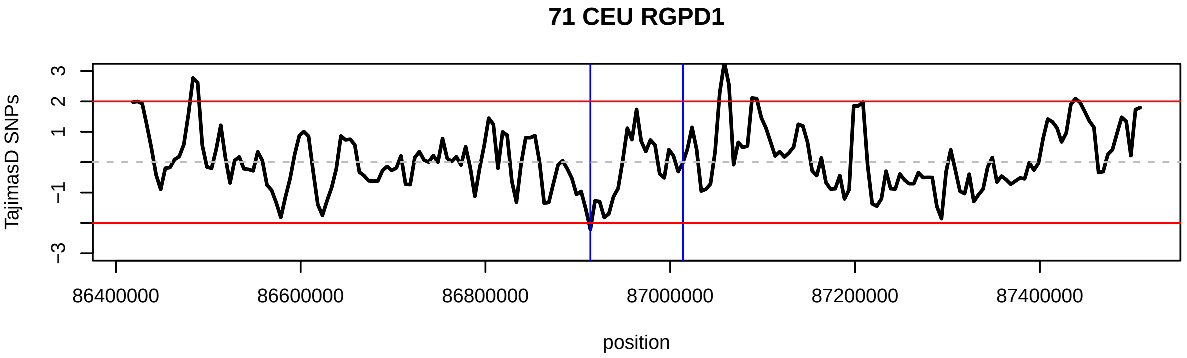
71 CEU RGPD1
TajimasD SNPs
3
2
1
−1
−3
86400000
86600000
86800000
87000000
87200000
87400000
position

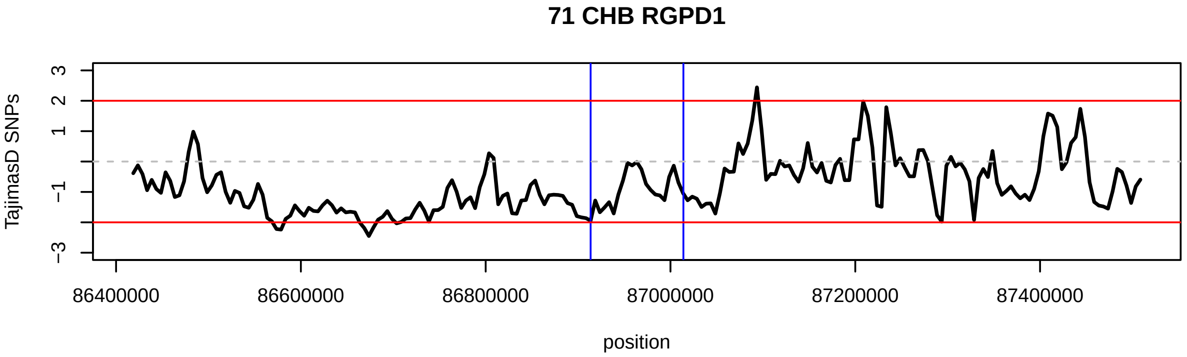
71 CHB RGPD1
TajimasD SNPs
3
2
1
−1
−3
86400000
86600000
86800000
87000000
87200000
87400000
position

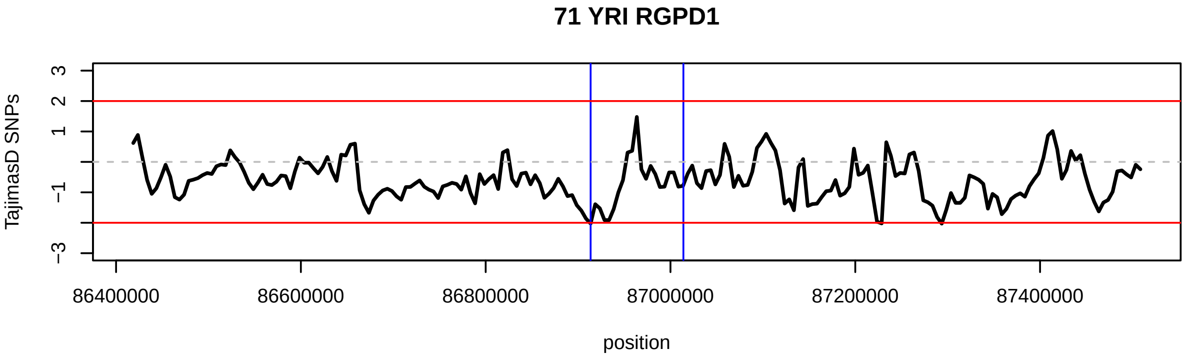
71 YRI RGPD1
TajimasD SNPs
3
2
1
−1
−3
86400000
86600000
86800000
87000000
87200000
87400000
position

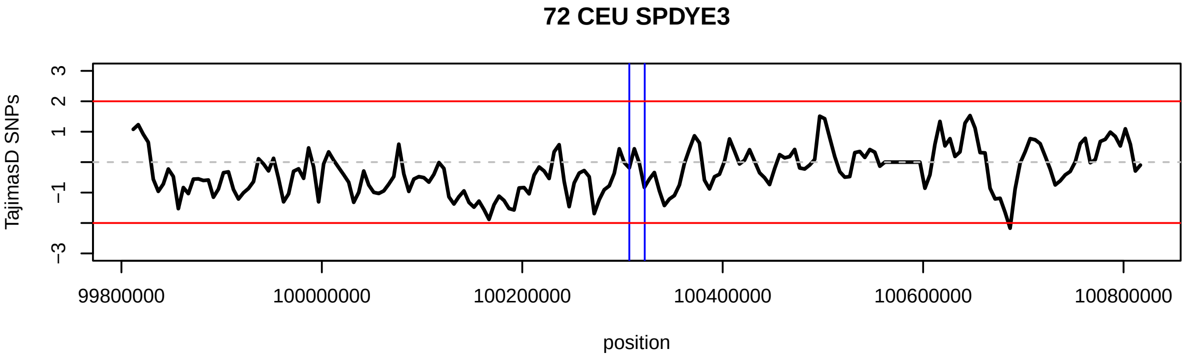
72 CEU SPDYE3
TajimasD SNPs
3
2
1
−1
−3
99800000
100000000
100200000
100400000
100600000
100800000
position

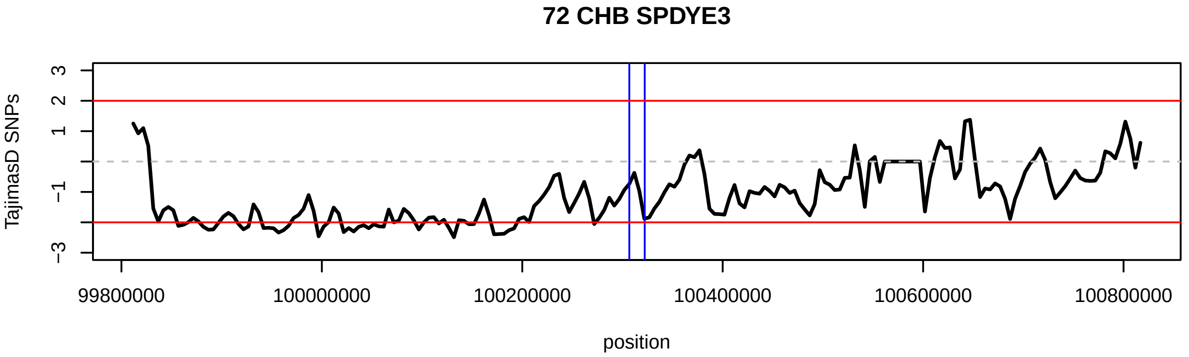
72 CHB SPDYE3
TajimasD SNPs
3
2
1
−1
−3
99800000
100000000
100200000
100400000
100600000
100800000
position

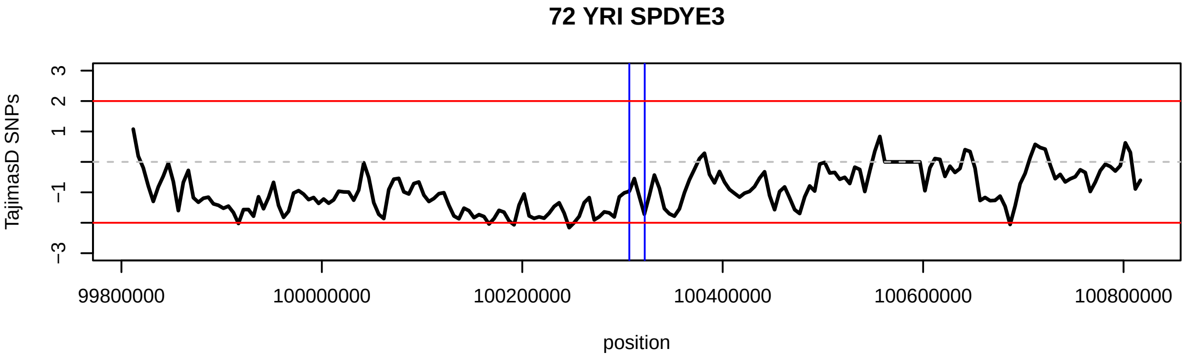
72 YRI SPDYE3
TajimasD SNPs
3
2
1
−1
−3
99800000
100000000
100200000
100400000
100600000
100800000
position

73 CEU SULT1A3

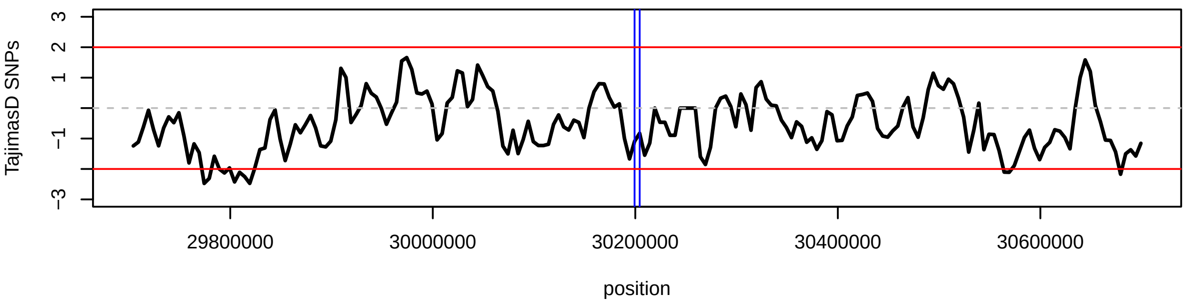

73 CHB SULT1A3

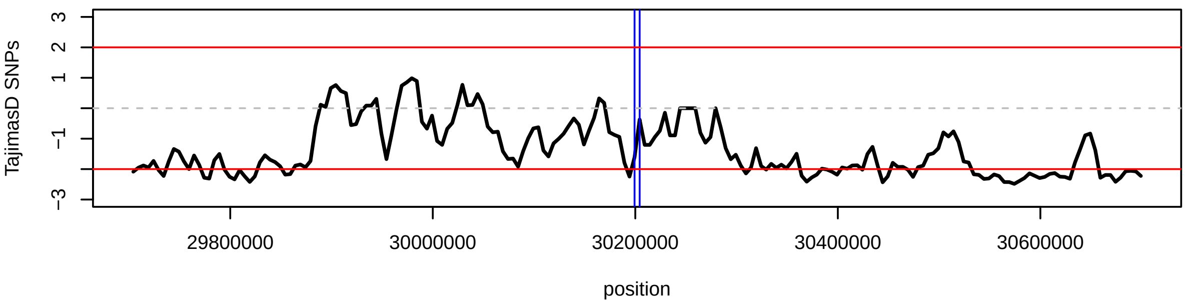

73 YRI SULT1A3

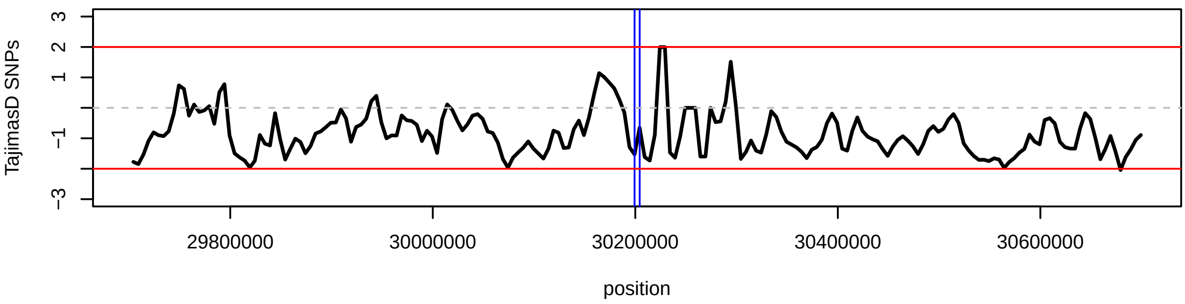

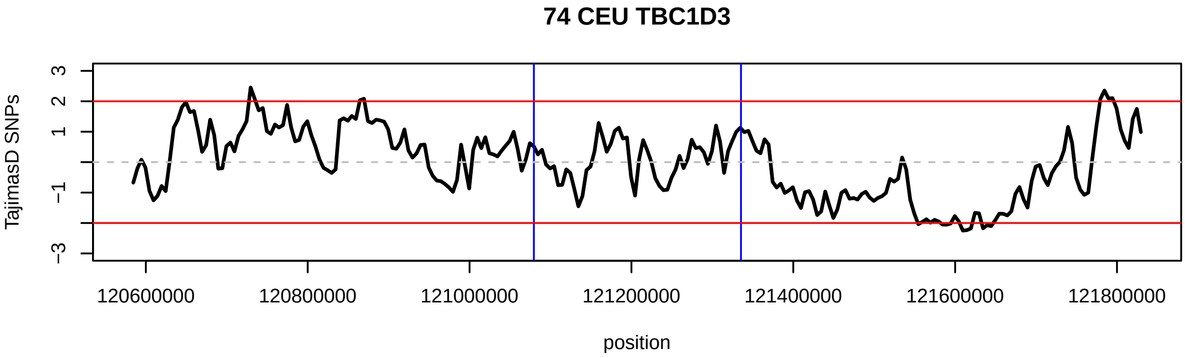
74 CEU TBC1D3
TajimasD SNPs
3
2
1
−1
−3
120600000
120800000
121000000
121200000
121400000
121600000
121800000
position

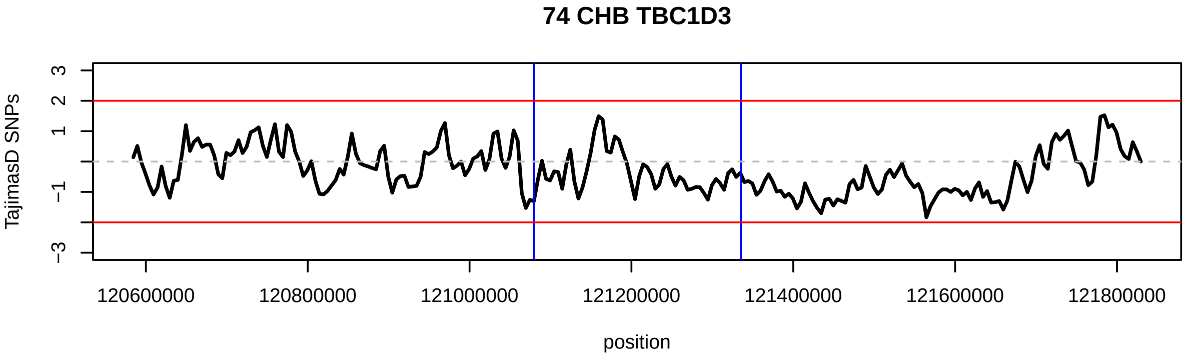
74 CHB TBC1D3
TajimasD SNPs
3
2
1
−1
−3
120600000
120800000
121000000
121200000
121400000
121600000
121800000
position

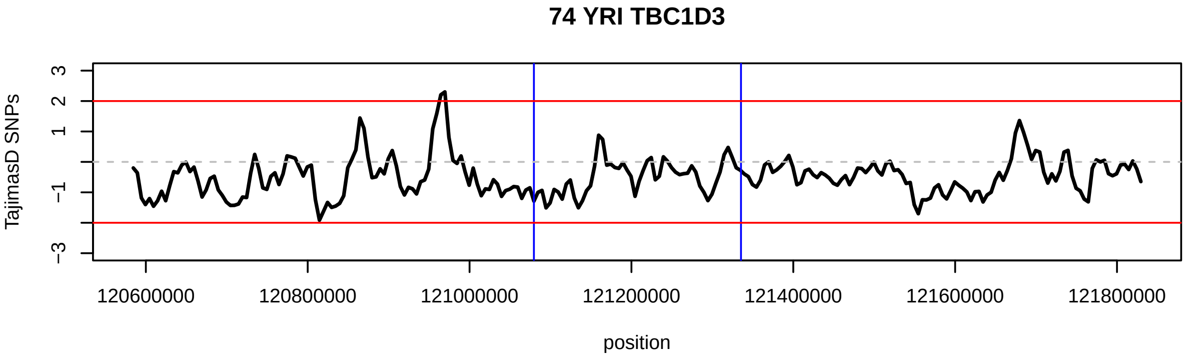
74 YRI TBC1D3
TajimasD SNPs
3
2
1
−1
−3
120600000
120800000
121000000
121200000
121400000
121600000
121800000
position

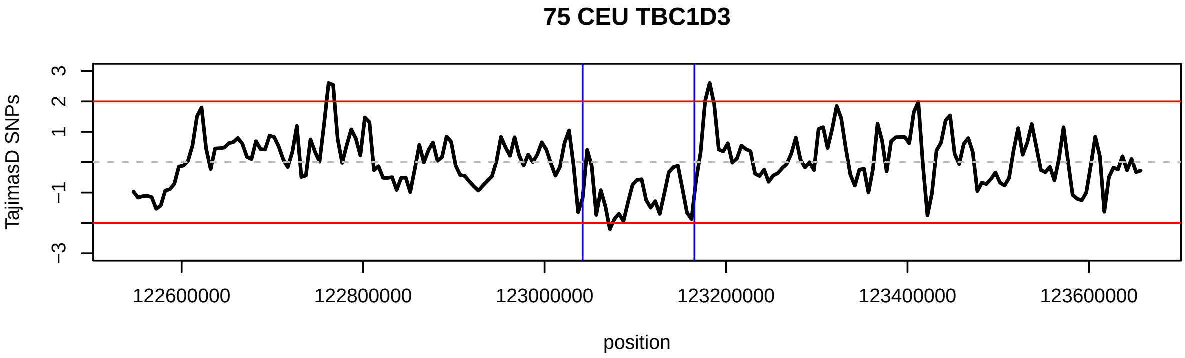
75 CEU TBC1D3
TajimasD SNPs
position
122600000
122800000
123000000
123200000
123400000
123600000

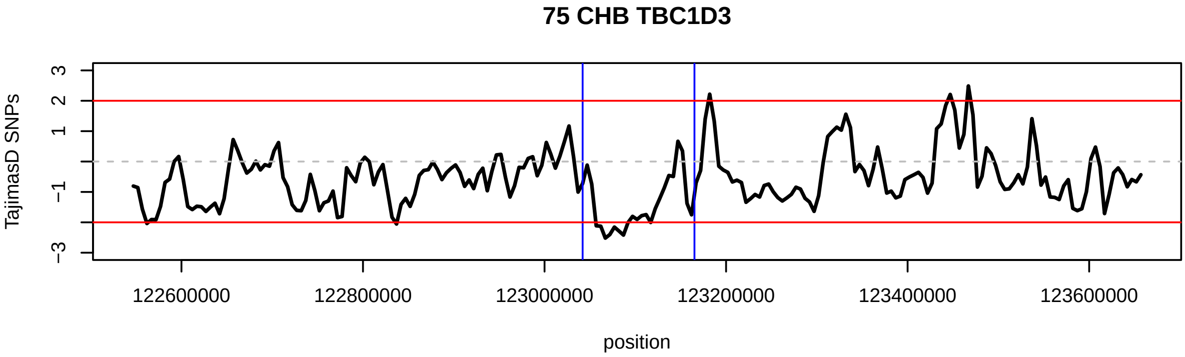
75 CHB TBC1D3
TajimasD SNPs
position
122600000
122800000
123000000
123200000
123400000
123600000

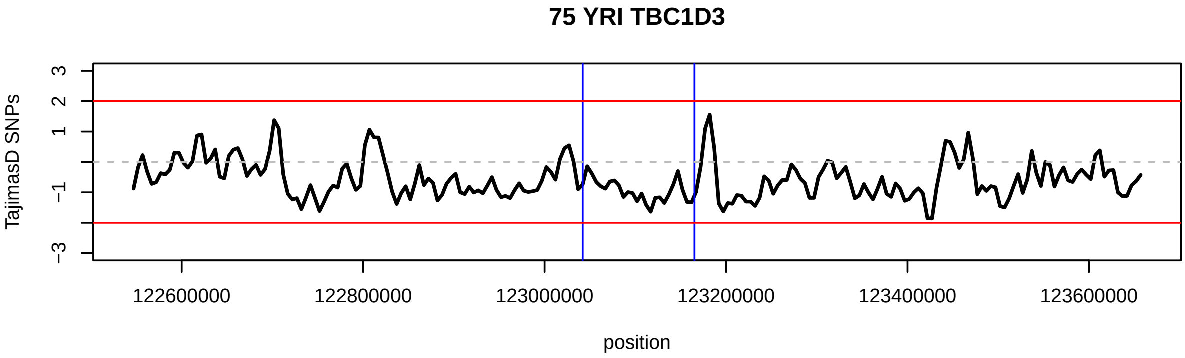
75 YRI TBC1D3
TajimasD SNPs
position
122600000
122800000
123000000
123200000
123400000
123600000

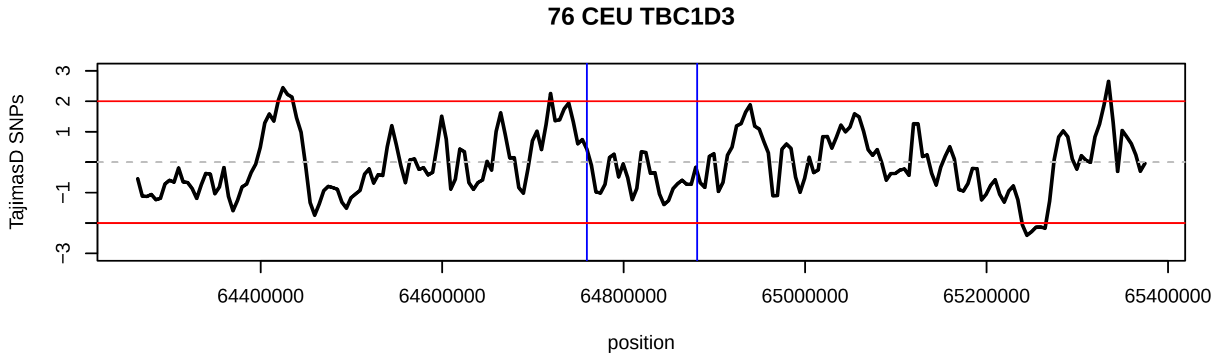
76 CEU TBC1D3
TajimasD SNPs
3
2
1
−1
−3
64400000
64600000
64800000
65000000
65200000
65400000
position

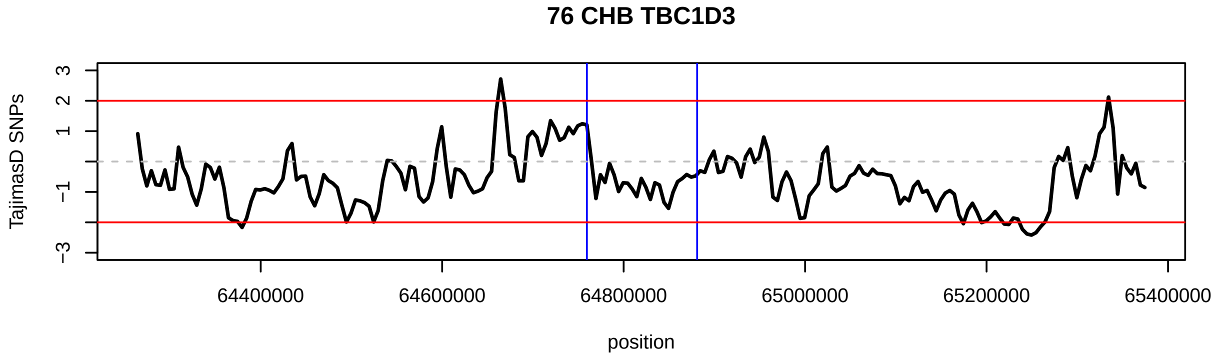
76 CHB TBC1D3
TajimasD SNPs
3
2
1
−1
−3
64400000
64600000
64800000
65000000
65200000
65400000
position

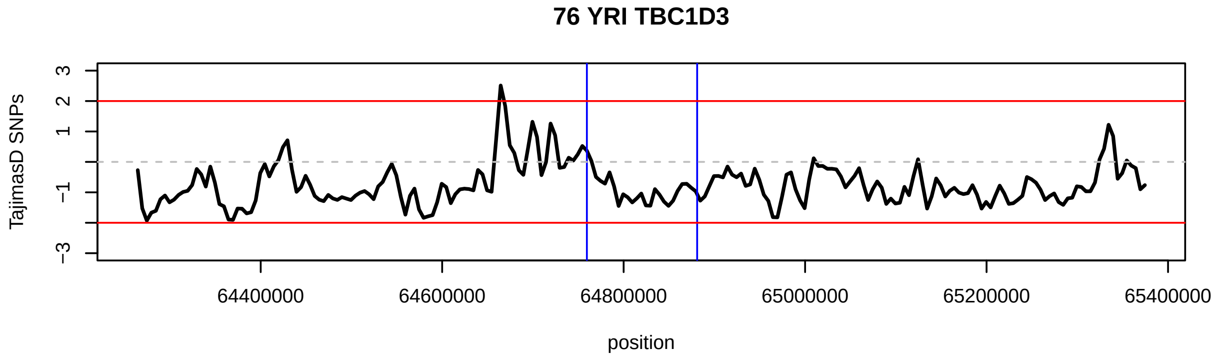
76 YRI TBC1D3
TajimasD SNPs
3
2
1
−1
−3
64400000
64600000
64800000
65000000
65200000
65400000
position

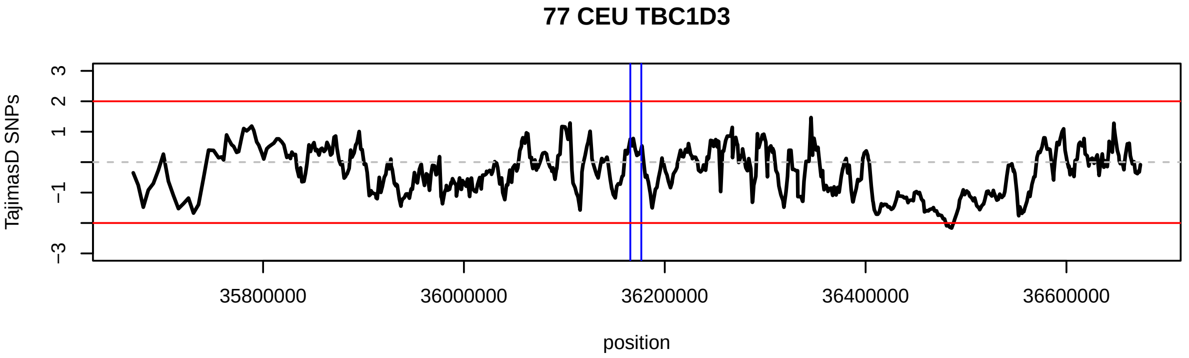
77 CEU TBC1D3
TajimasD SNPs
3
2
1
−1
−3
35800000
36000000
36200000
36400000
36600000
position

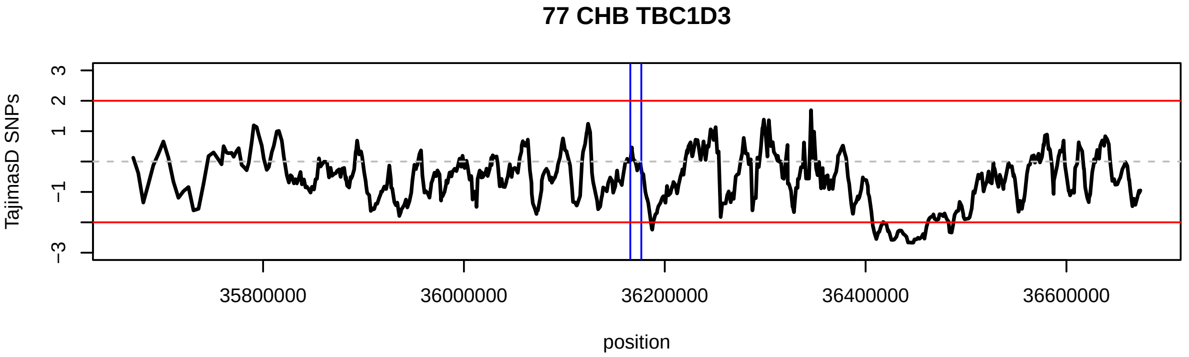
77 CHB TBC1D3
TajimasD SNPs
3
2
1
−1
−3
35800000
36000000
36200000
36400000
36600000
position

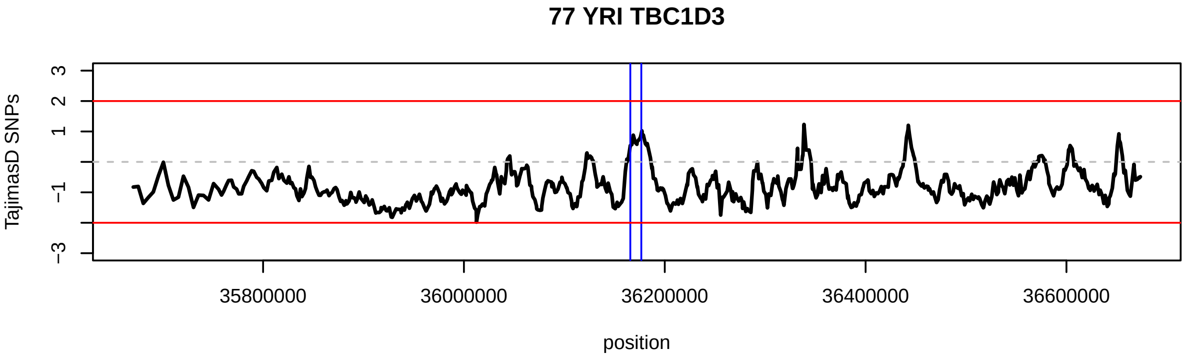
77 YRI TBC1D3
TajimasD SNPs
3
2
1
−1
−3
35800000
36000000
36200000
36400000
36600000
position

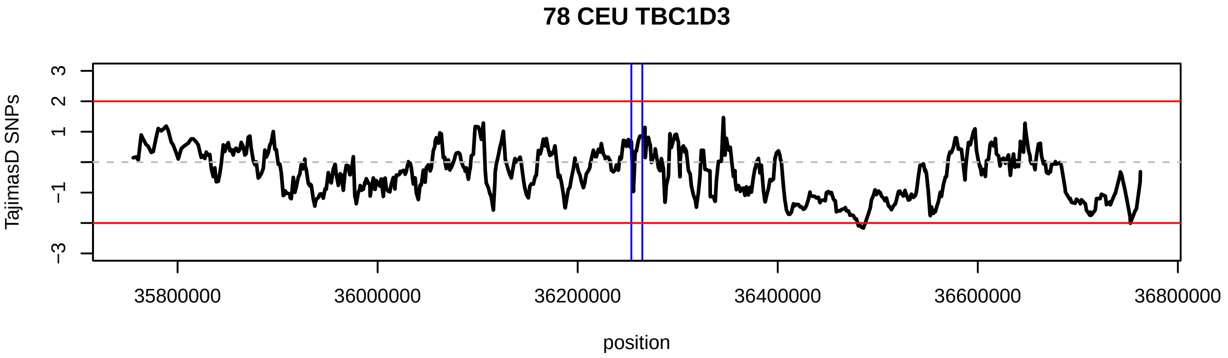
78 CEU TBC1D3
TajimasD SNPs
3
2
1
−1
−3
35800000
36000000
36200000
36400000
36600000
36800000
position

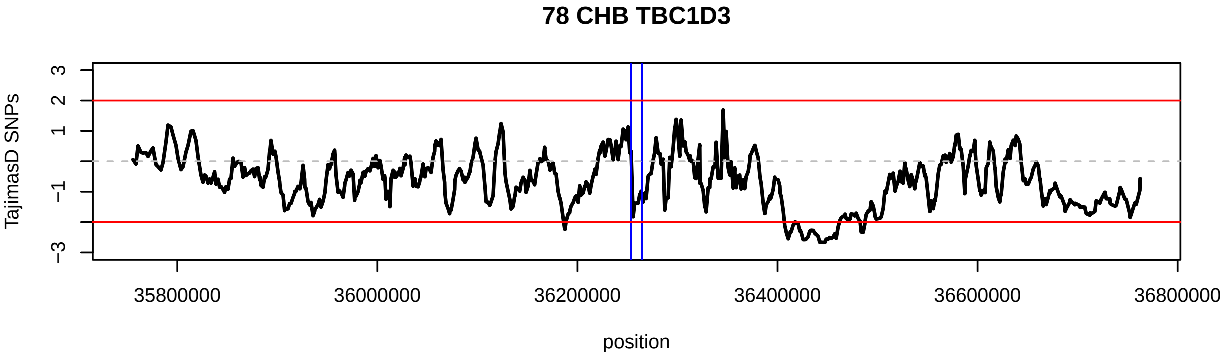
78 CHB TBC1D3
TajimasD SNPs
3
2
1
−1
−3
35800000
36000000
36200000
36400000
36600000
36800000
position

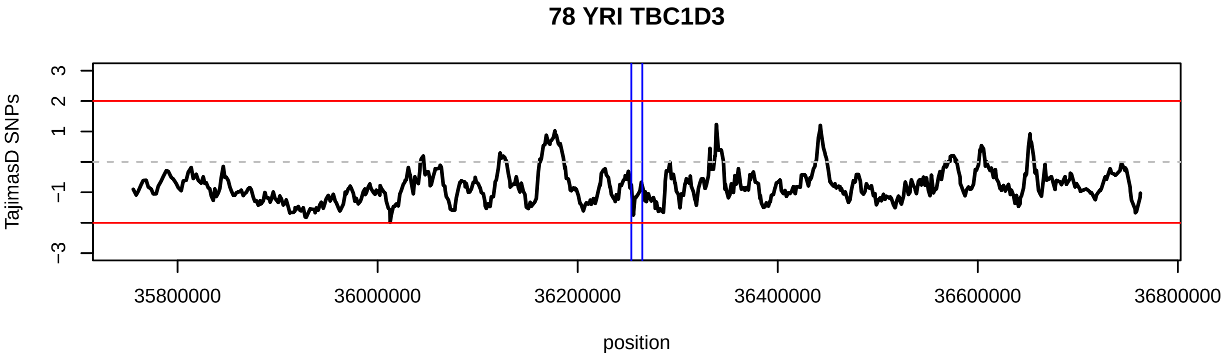
78 YRI TBC1D3
TajimasD SNPs
3
2
1
−1
−3
35800000
36000000
36200000
36400000
36600000
36800000
position

79 CEU TBC1D3

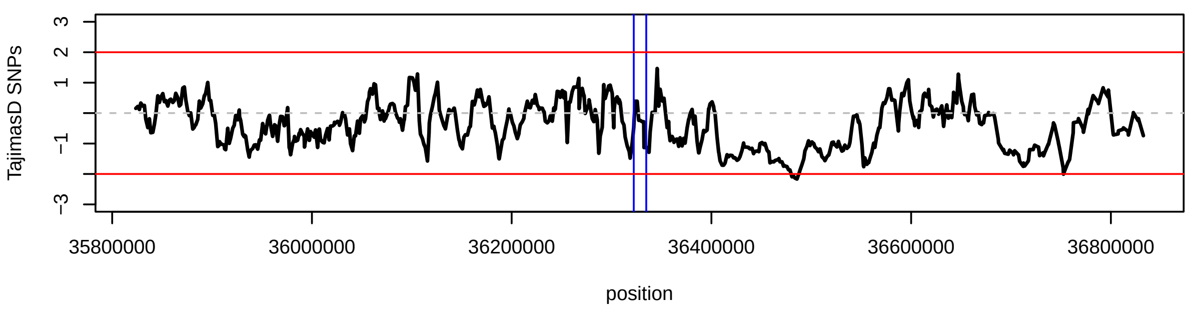

79 CHB TBC1D3

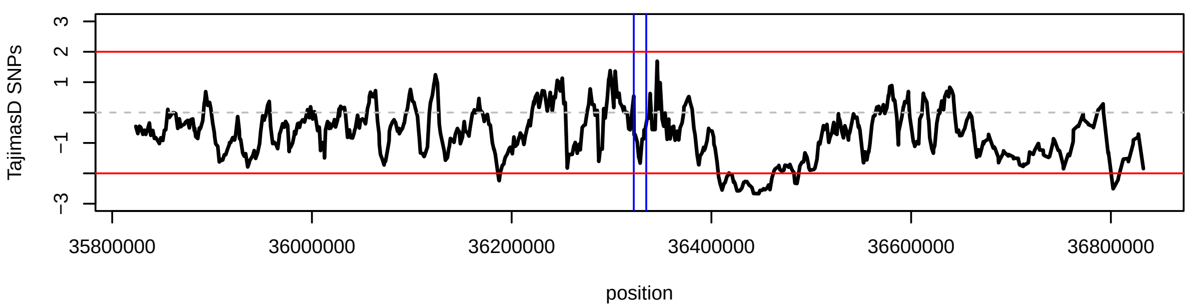

79 YRI TBC1D3

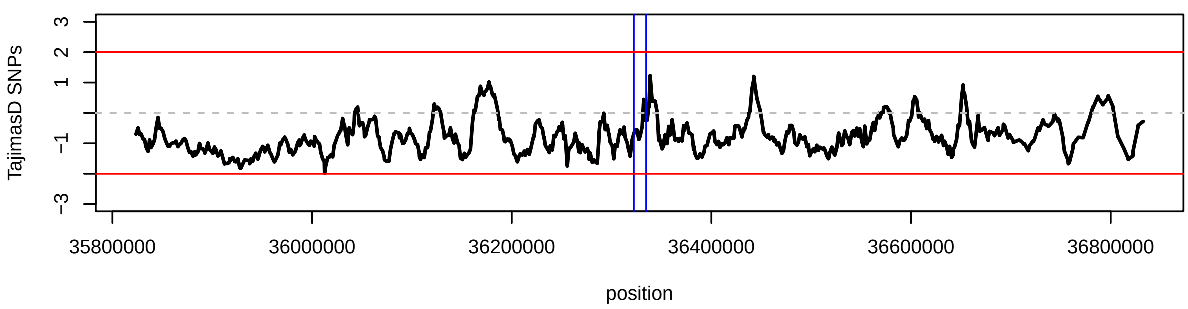

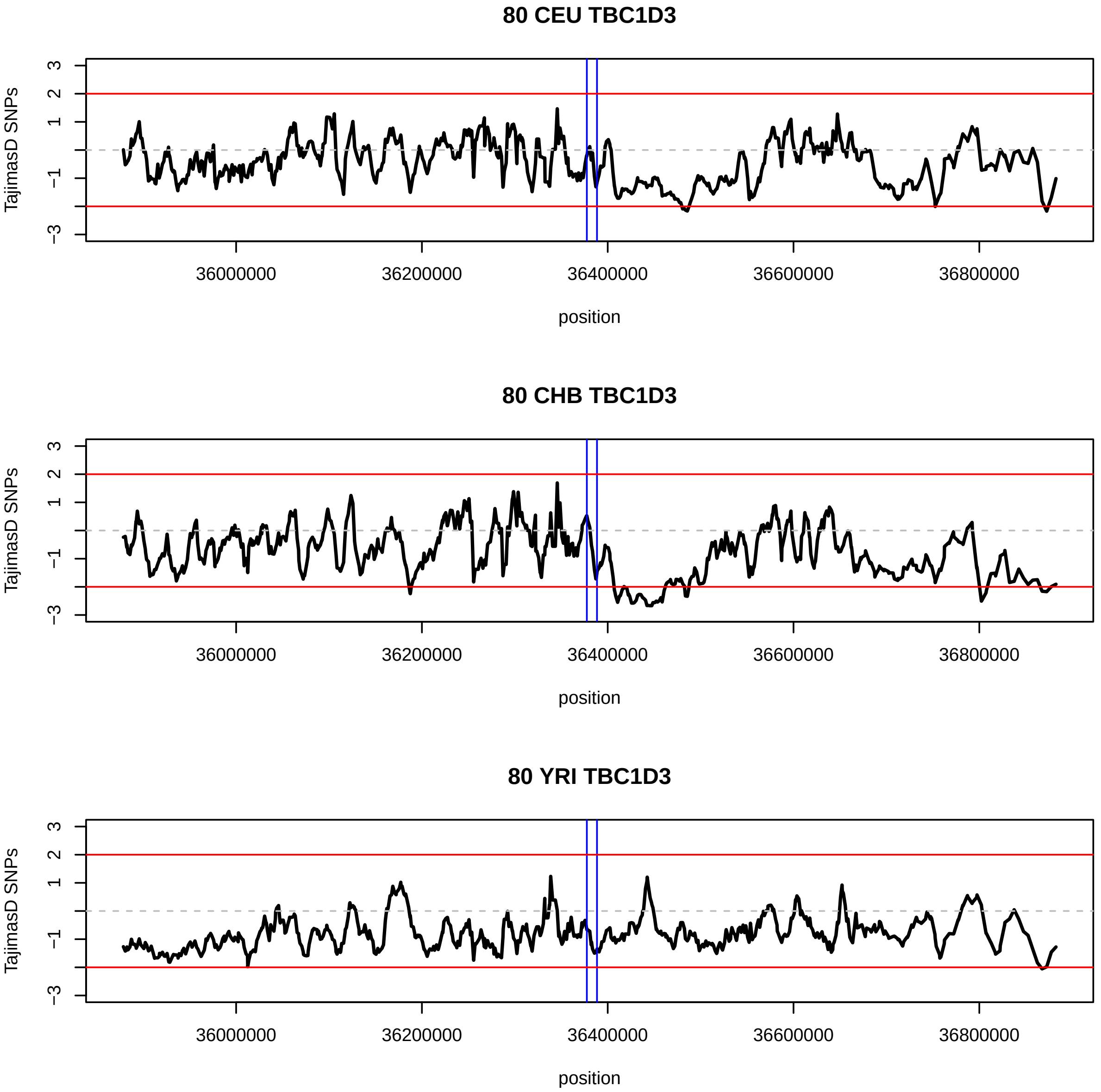
80 CEU TBC1D3
TajimasD SNPs
3
2
1
−1
−3
36000000
36200000
36400000
36600000
36800000
position
80 CHB TBC1D3
TajimasD SNPs
3
2
1
−1
−3
36000000
36200000
36400000
36600000
36800000
position
80 YRI TBC1D3
TajimasD SNPs
3
2
1
−1
−3
36000000
36200000
36400000
36600000
36800000
position

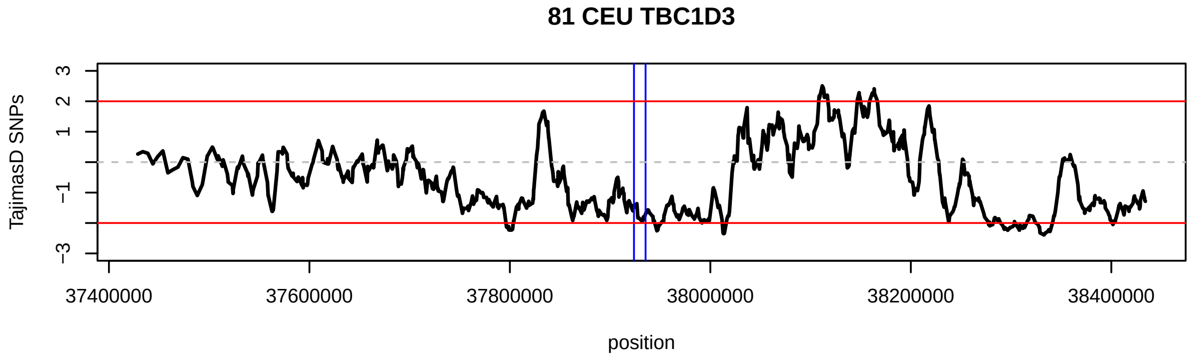
81 CEU TBC1D3
TajimasD SNPs
−3
−1
1
2
3
37400000
37600000
37800000
38000000
38200000
38400000
position

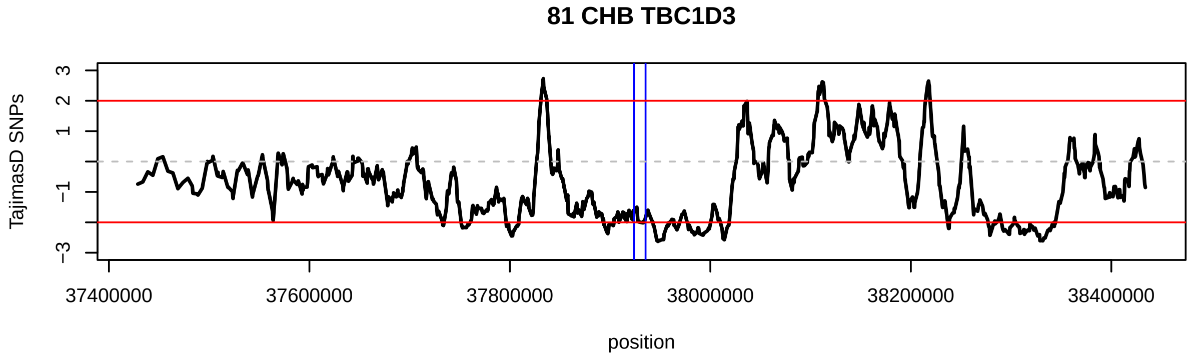
81 CHB TBC1D3
TajimasD SNPs
−3
−1
1
2
3
37400000
37600000
37800000
38000000
38200000
38400000
position

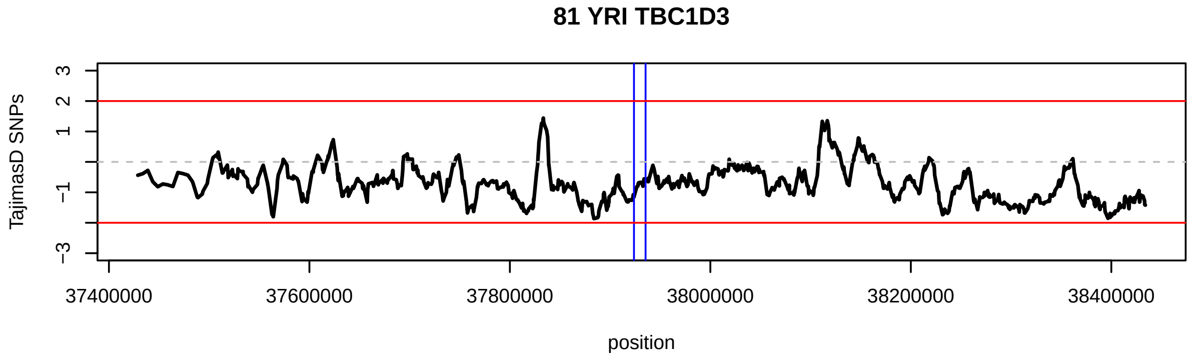
81 YRI TBC1D3
TajimasD SNPs
−3
−1
1
2
3
37400000
37600000
37800000
38000000
38200000
38400000
position

82 CEU TBC1D3

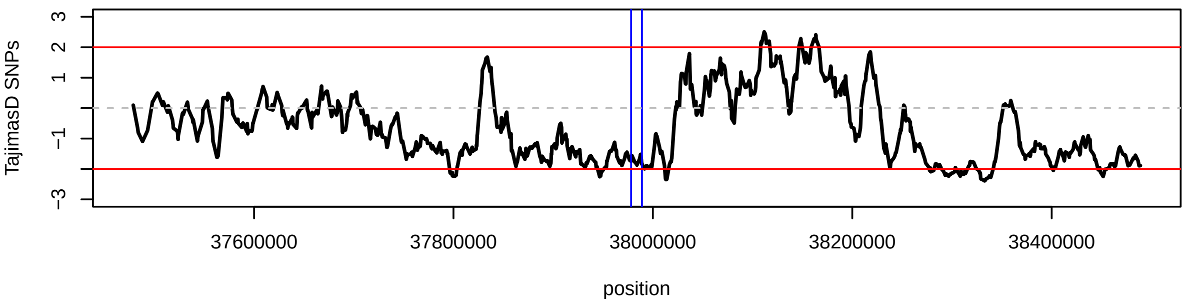

82 CHB TBC1D3

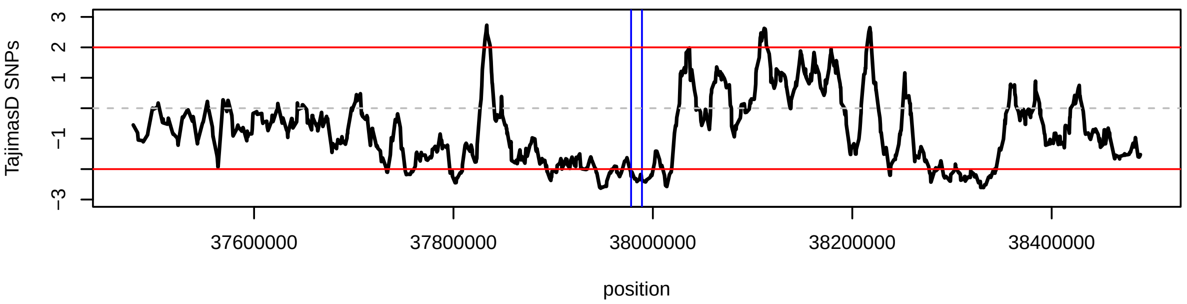

82 YRI TBC1D3

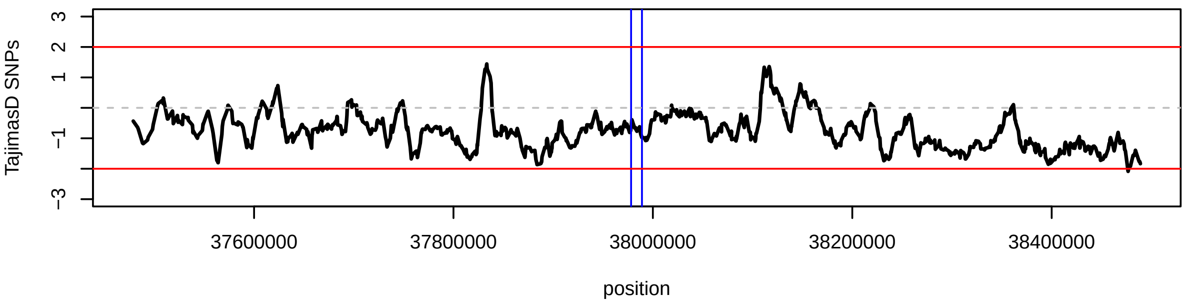

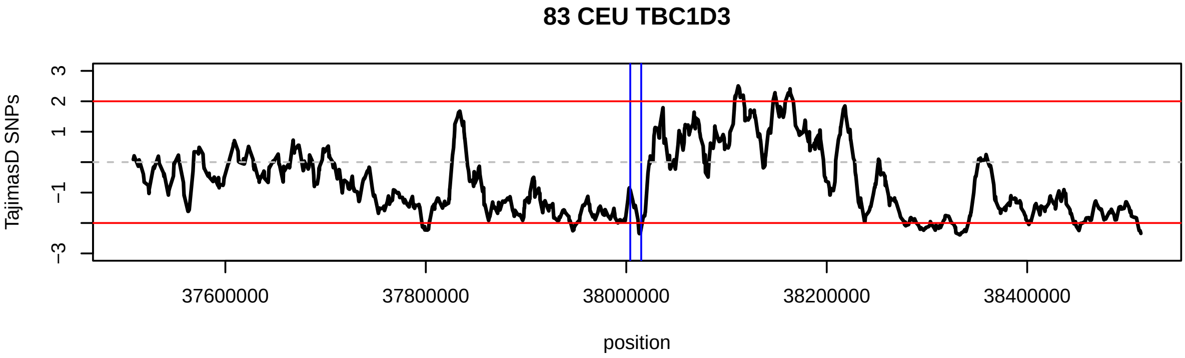
83 CEU TBC1D3
TajimasD SNPs
3
2
1
−1
−3
37600000
37800000
38000000
38200000
38400000
position

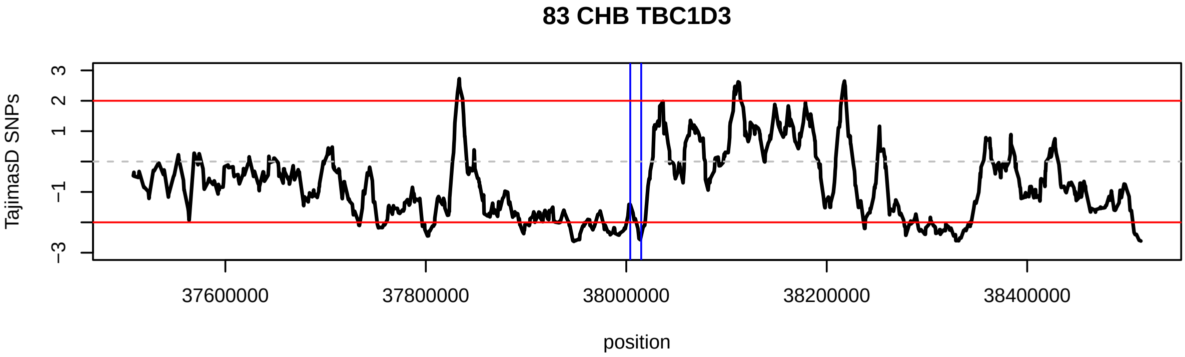
83 CHB TBC1D3
TajimasD SNPs
3
2
1
−1
−3
37600000
37800000
38000000
38200000
38400000
position

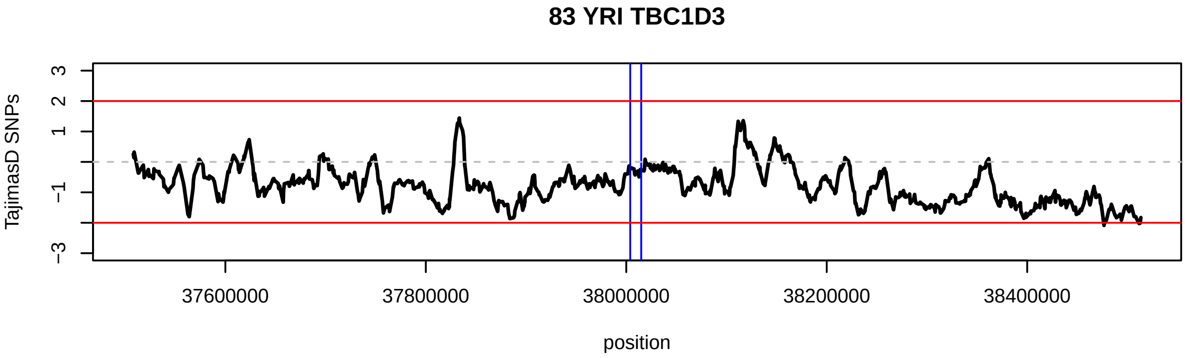
83 YRI TBC1D3
TajimasD SNPs
3
2
1
−1
−3
37600000
37800000
38000000
38200000
38400000
position

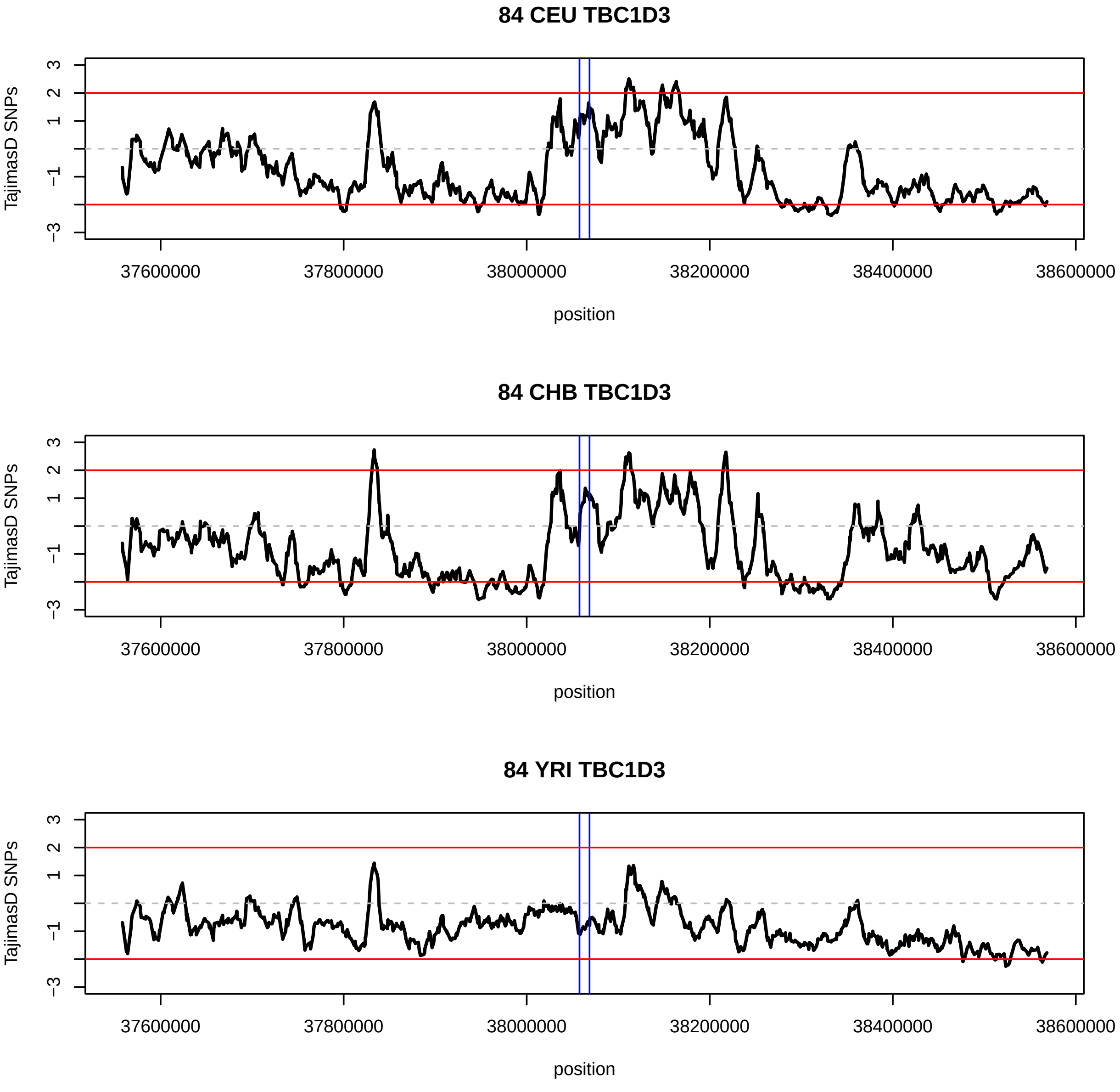
84 CEU TBC1D3
TajimasD SNPs
position
84 CHB TBC1D3
TajimasD SNPs
position
84 YRI TBC1D3
TajimasD SNPs
position
37600000
37800000
38000000
38200000
38400000
38600000

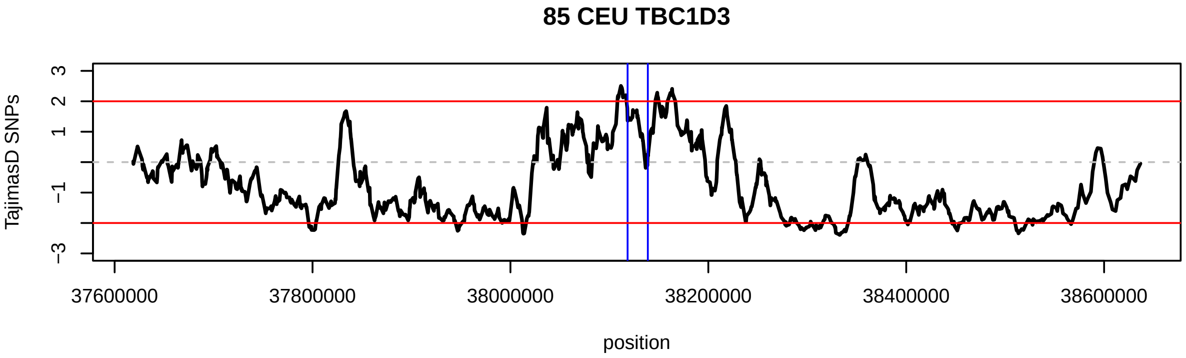
85 CEU TBC1D3
TajimasD SNPs
3
2
1
−1
−3
37600000
37800000
38000000
38200000
38400000
38600000
position

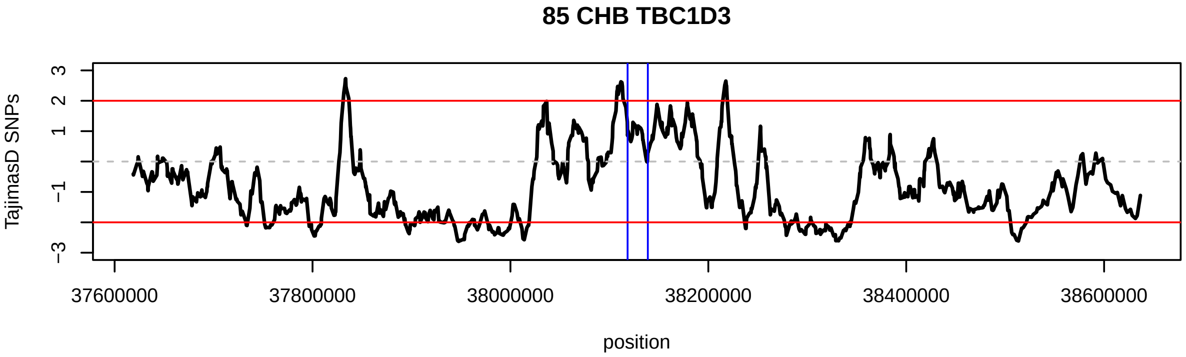
85 CHB TBC1D3
TajimasD SNPs
3
2
1
−1
−3
37600000
37800000
38000000
38200000
38400000
38600000
position

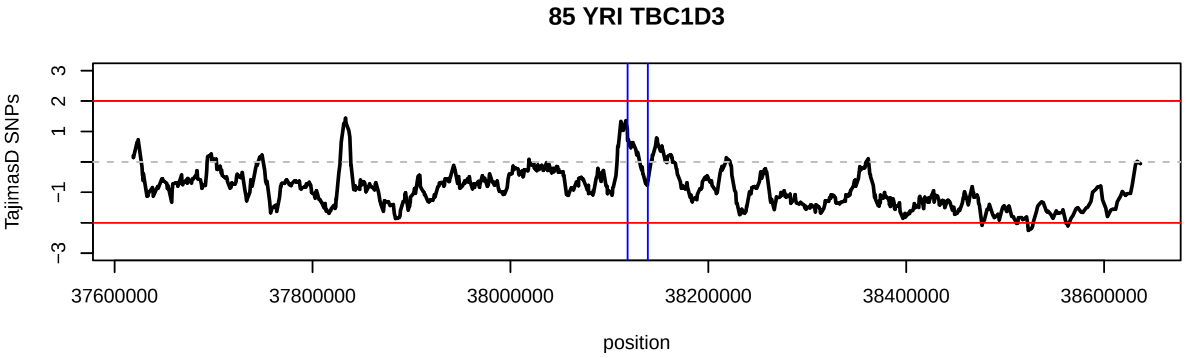
85 YRI TBC1D3
TajimasD SNPs
3
2
1
−1
−3
37600000
37800000
38000000
38200000
38400000
38600000
position

86 CEU TBC1D3

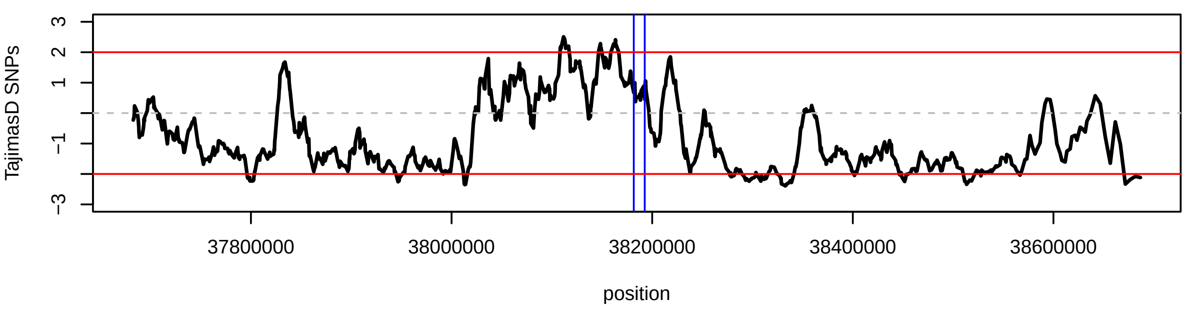

86 CHB TBC1D3

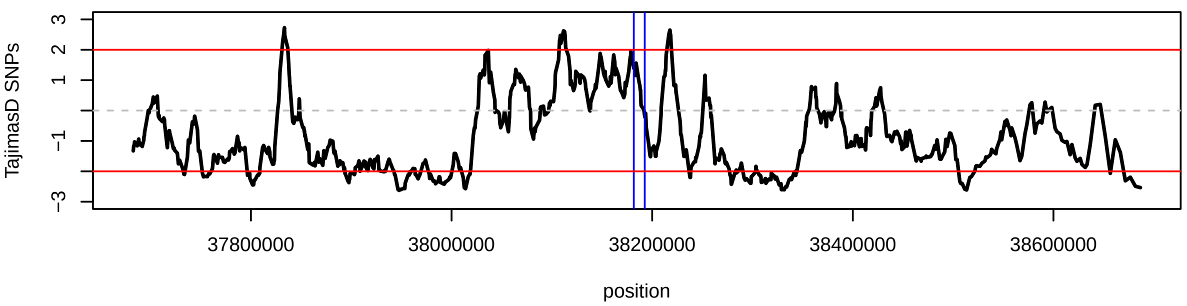

86 YRI TBC1D3

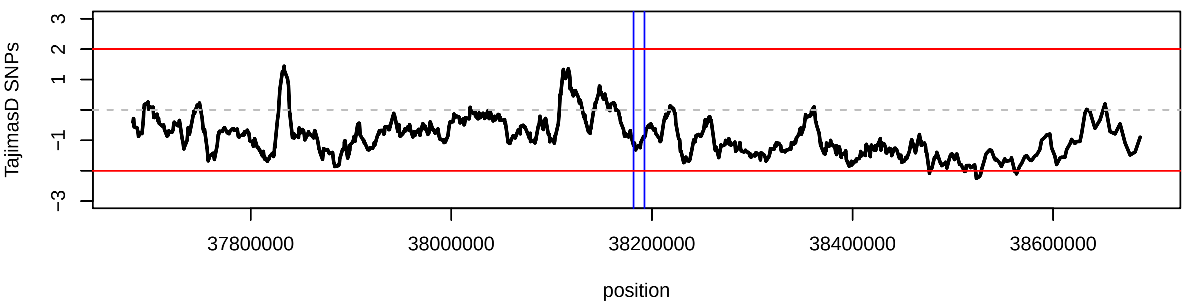

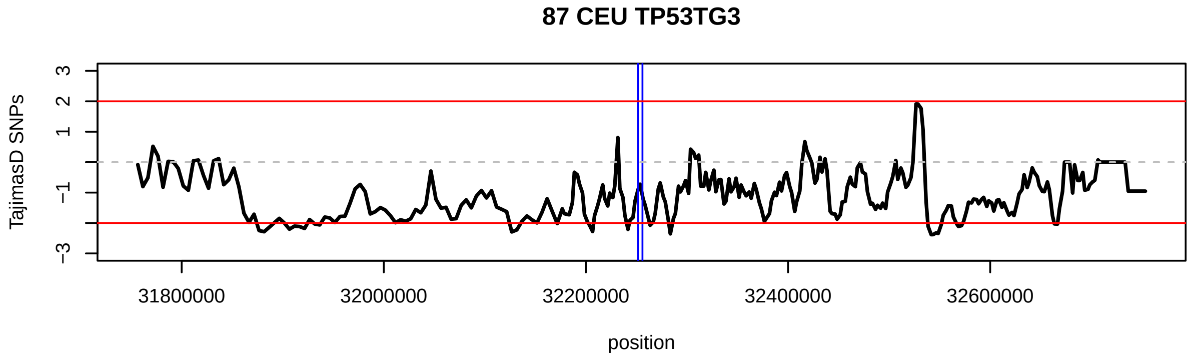
87 CEU TP53TG3
TajimasD SNPs
3
2
1
−1
−3
31800000
32000000
32200000
32400000
32600000
position

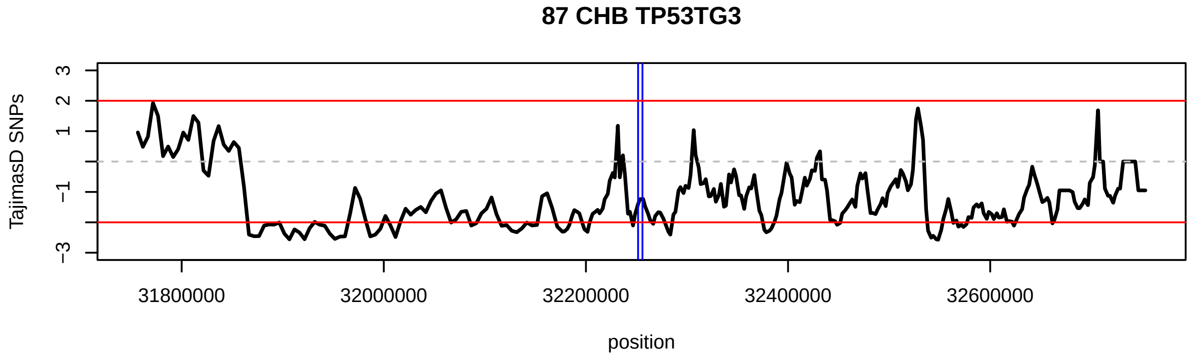
87 CHB TP53TG3
TajimasD SNPs
3
2
1
−1
−3
31800000
32000000
32200000
32400000
32600000
position

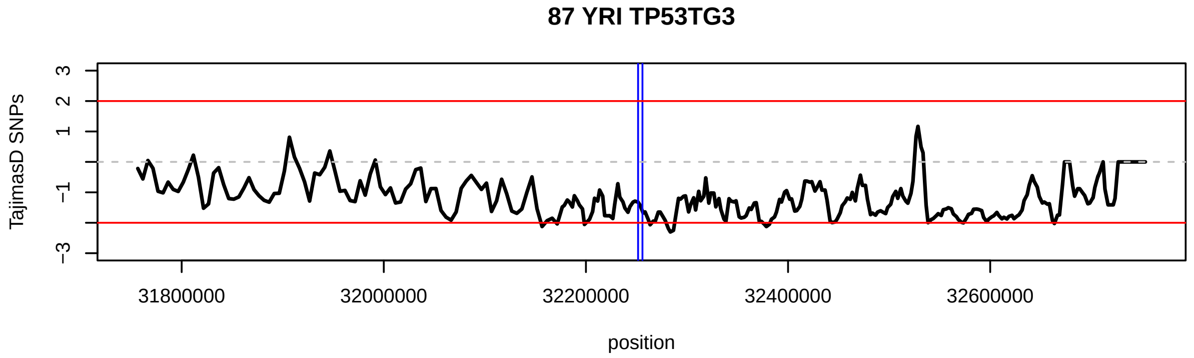
87 YRI TP53TG3
TajimasD SNPs
3
2
1
−1
−3
31800000
32000000
32200000
32400000
32600000
position

88 CEU TP53TG3

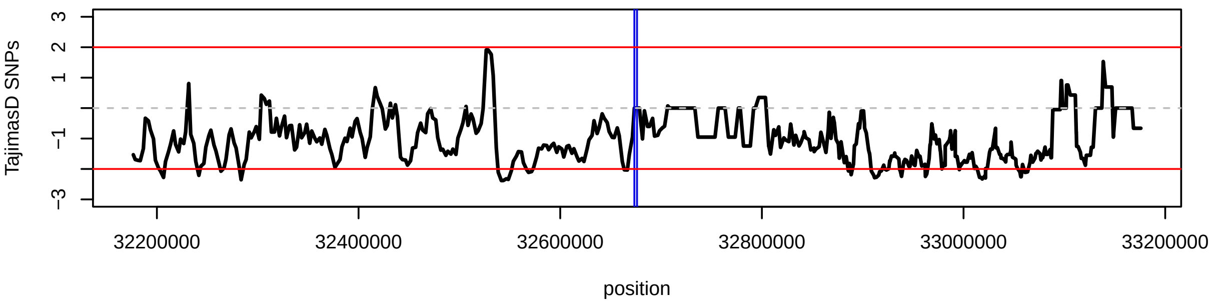

88 CHB TP53TG3

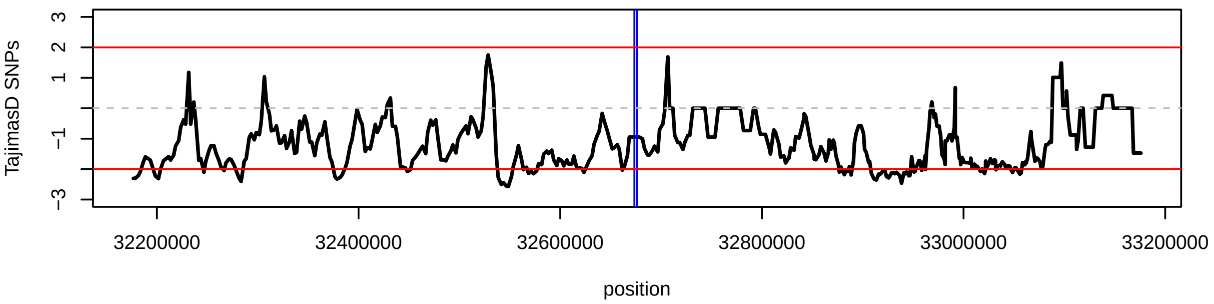

88 YRI TP53TG3

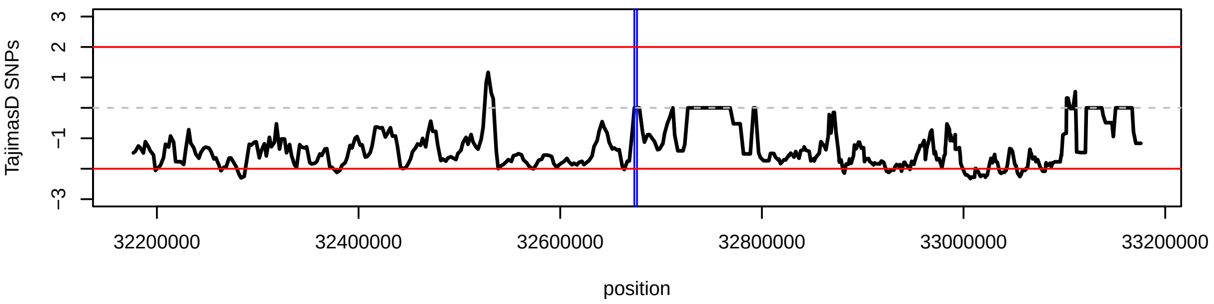

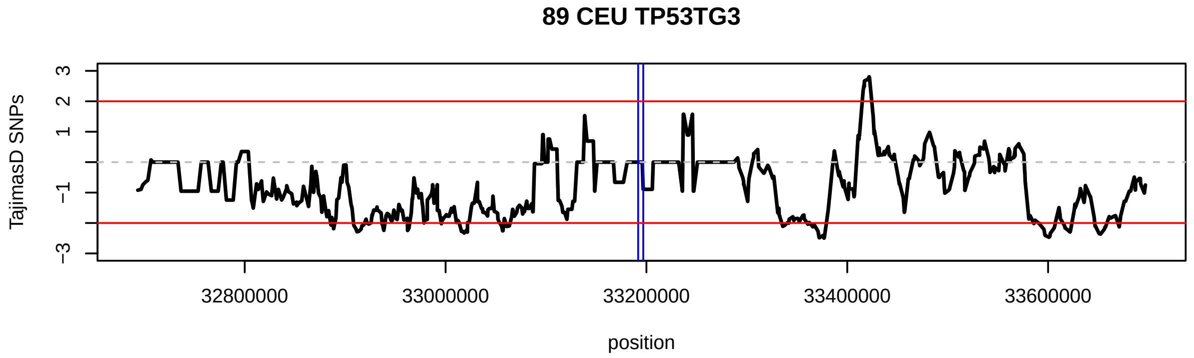
89 CEU TP53TG3
TajimasD SNPs
−3
−1
1
2
3
32800000
33000000
33200000
33400000
33600000
position

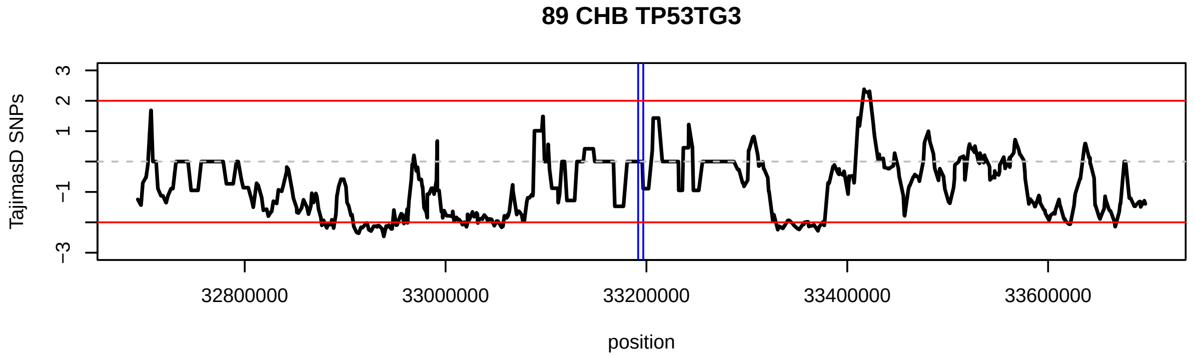
89 CHB TP53TG3
TajimasD SNPs
−3
−1
1
2
3
32800000
33000000
33200000
33400000
33600000
position

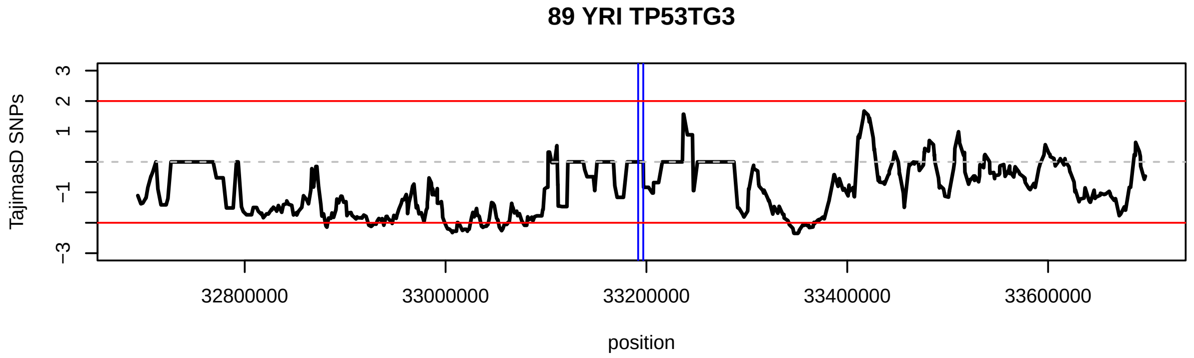
89 YRI TP53TG3
TajimasD SNPs
−3
−1
1
2
3
32800000
33000000
33200000
33400000
33600000
position

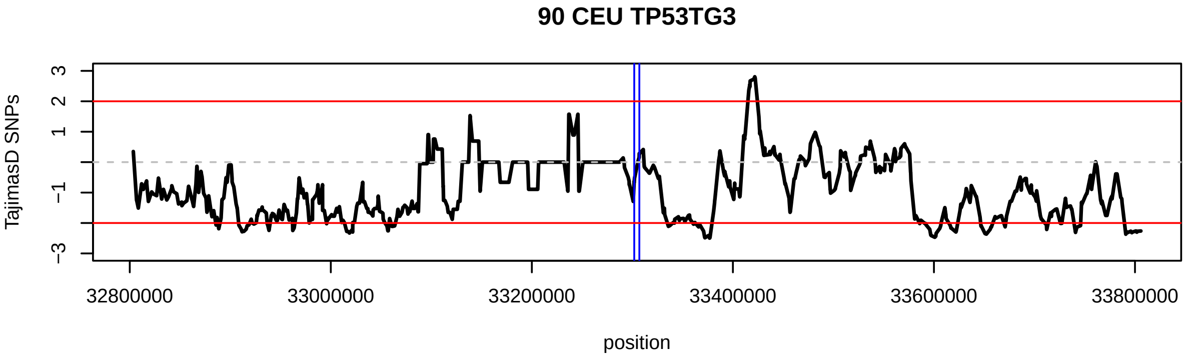
90 CEU TP53TG3
TajimasD SNPs
3
2
1
−1
−3
32800000
33000000
33200000
33400000
33600000
33800000
position

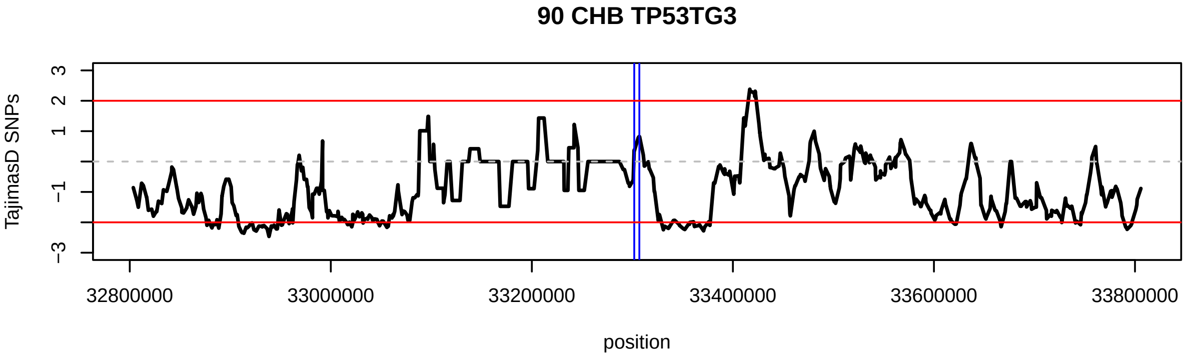
90 CHB TP53TG3
TajimasD SNPs
3
2
1
−1
−3
32800000
33000000
33200000
33400000
33600000
33800000
position

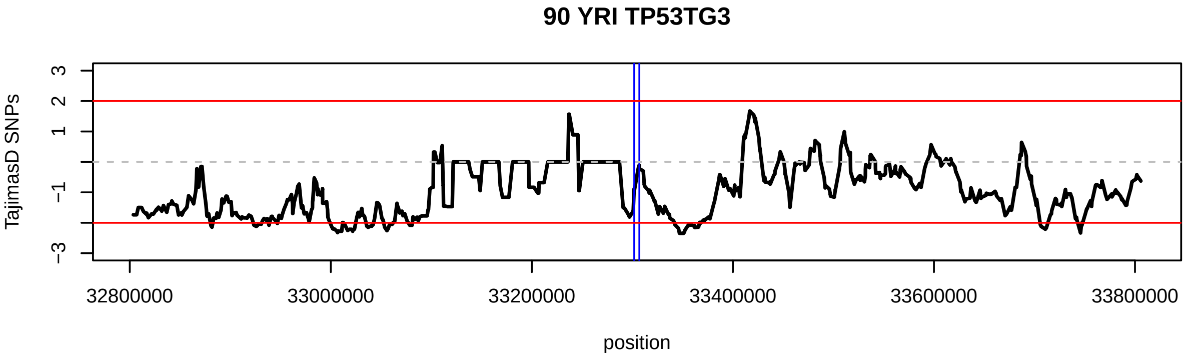
90 YRI TP53TG3
TajimasD SNPs
3
2
1
−1
−3
32800000
33000000
33200000
33400000
33600000
33800000
position

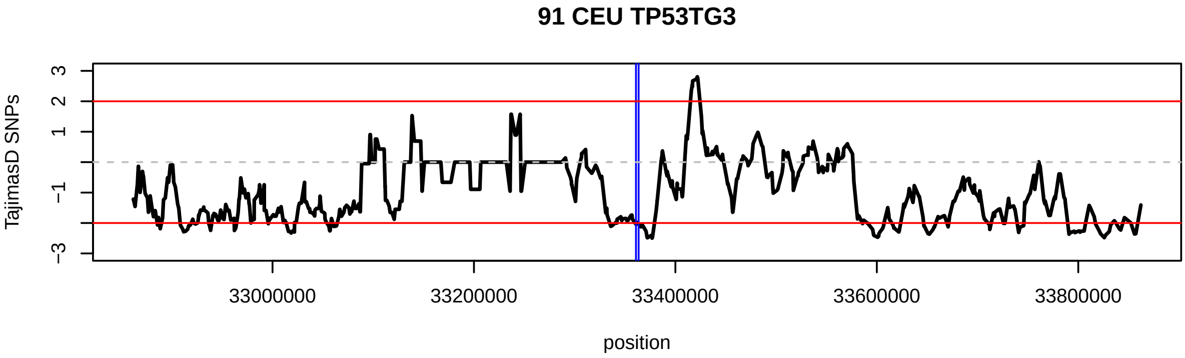
91 CEU TP53TG3
TajimasD SNPs
3
2
1
−1
−3
33000000
33200000
33400000
33600000
33800000
position

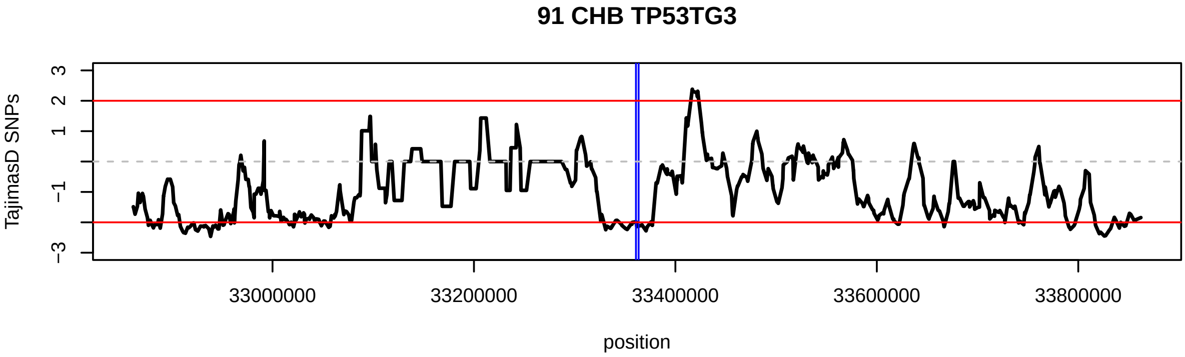
91 CHB TP53TG3
TajimasD SNPs
3
2
1
−1
−3
33000000
33200000
33400000
33600000
33800000
position

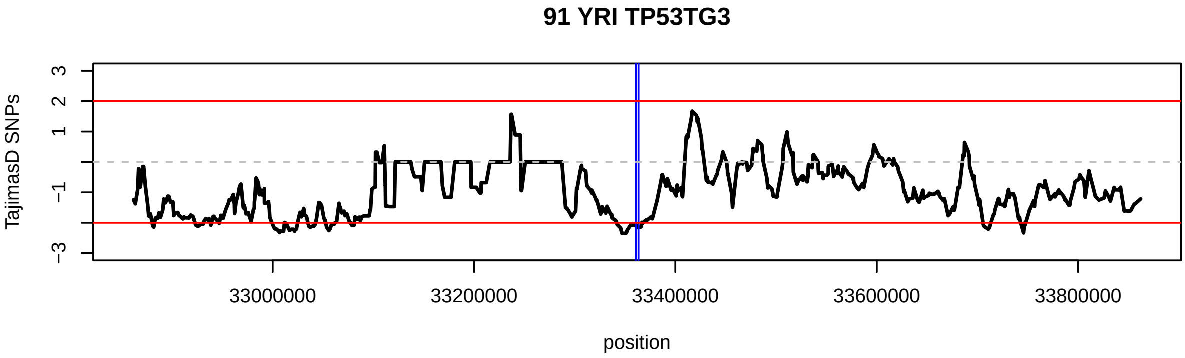
91 YRI TP53TG3
TajimasD SNPs
3
2
1
−1
−3
33000000
33200000
33400000
33600000
33800000
position

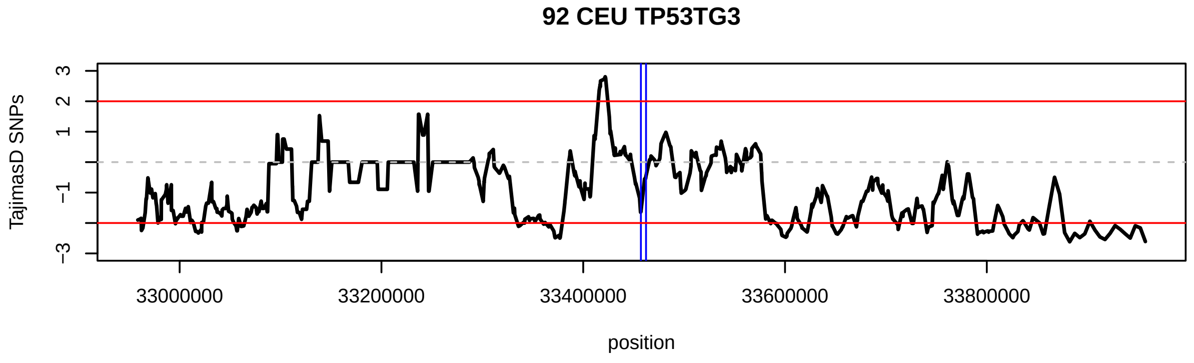
92 CEU TP53TG3
TajimasD SNPs
3
2
1
−1
−3
33000000
33200000
33400000
33600000
33800000
position

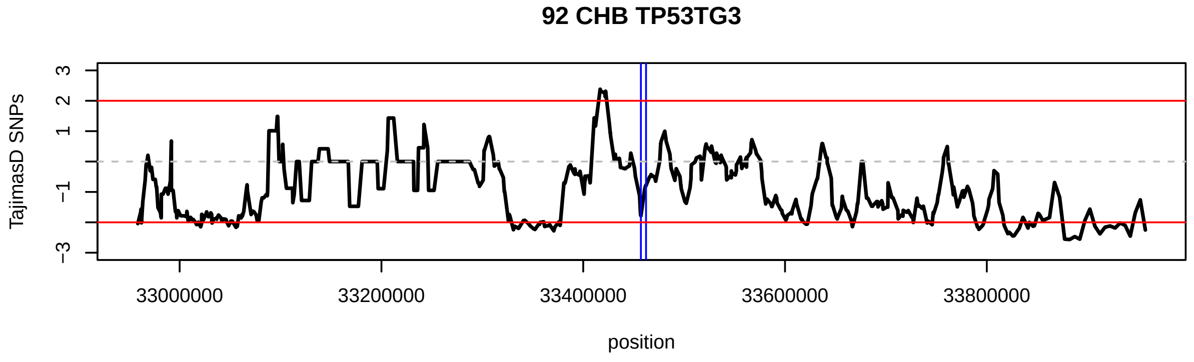
92 CHB TP53TG3
TajimasD SNPs
3
2
1
−1
−3
33000000
33200000
33400000
33600000
33800000
position

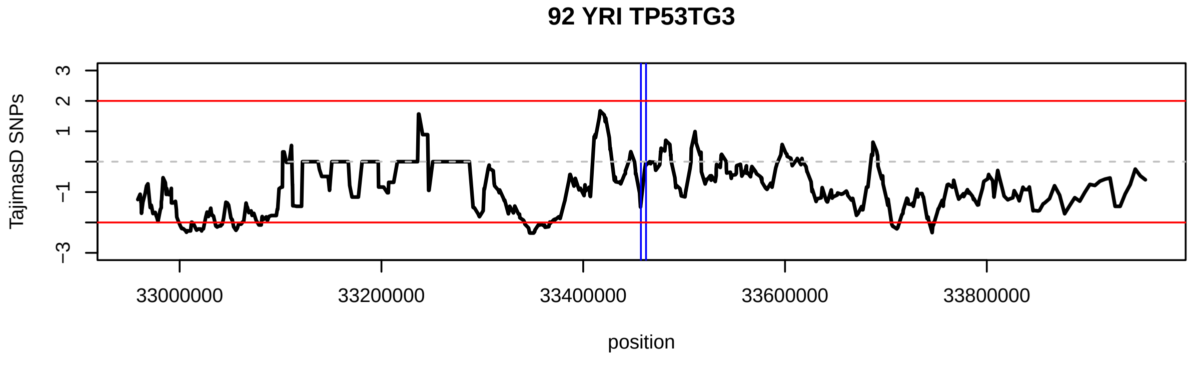
92 YRI TP53TG3
TajimasD SNPs
3
2
1
−1
−3
33000000
33200000
33400000
33600000
33800000
position

93 CEU TRIM49L1

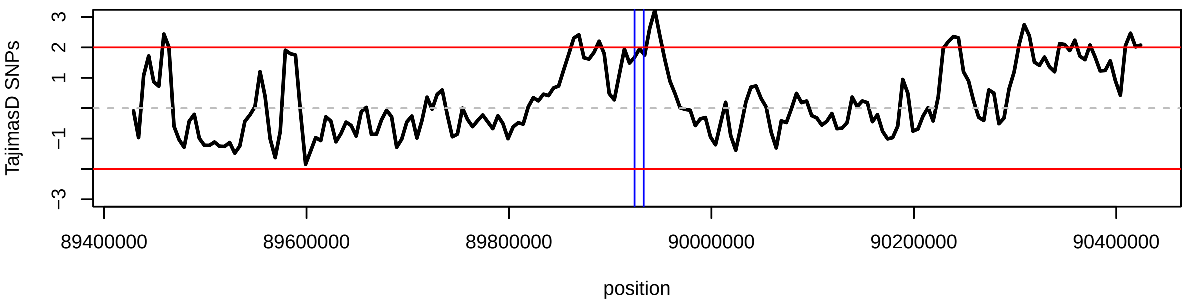

93 CHB TRIM49L1

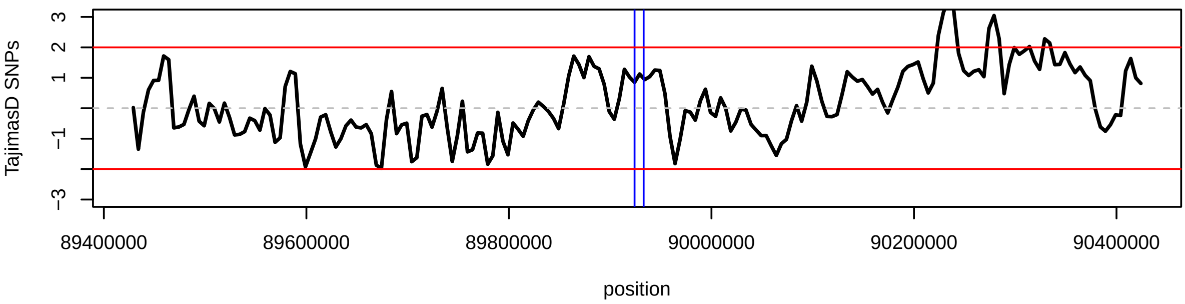

93 YRI TRIM49L1

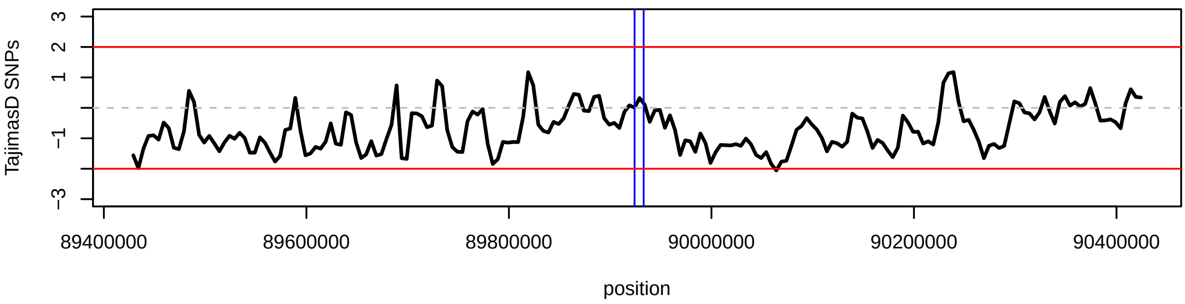

CEU chr1 AMY1A

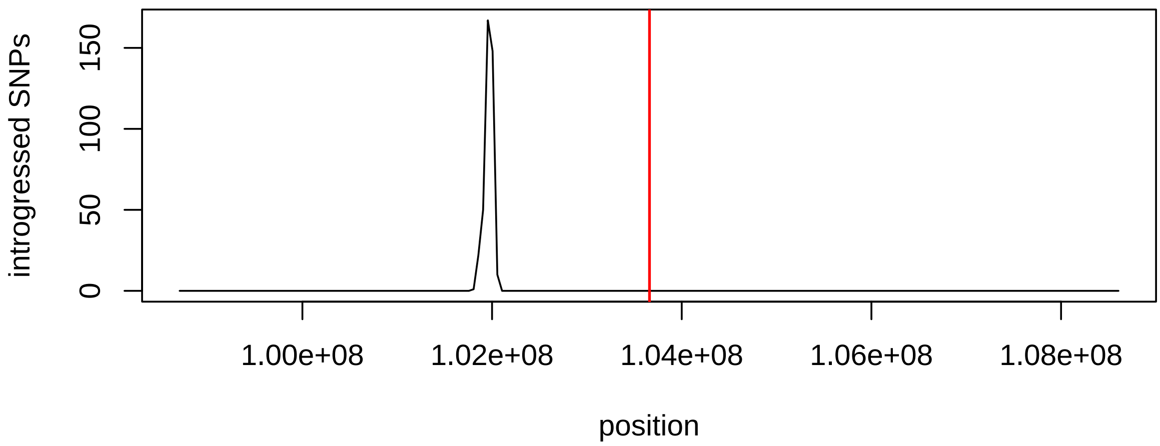

CHB chr1 AMY1A

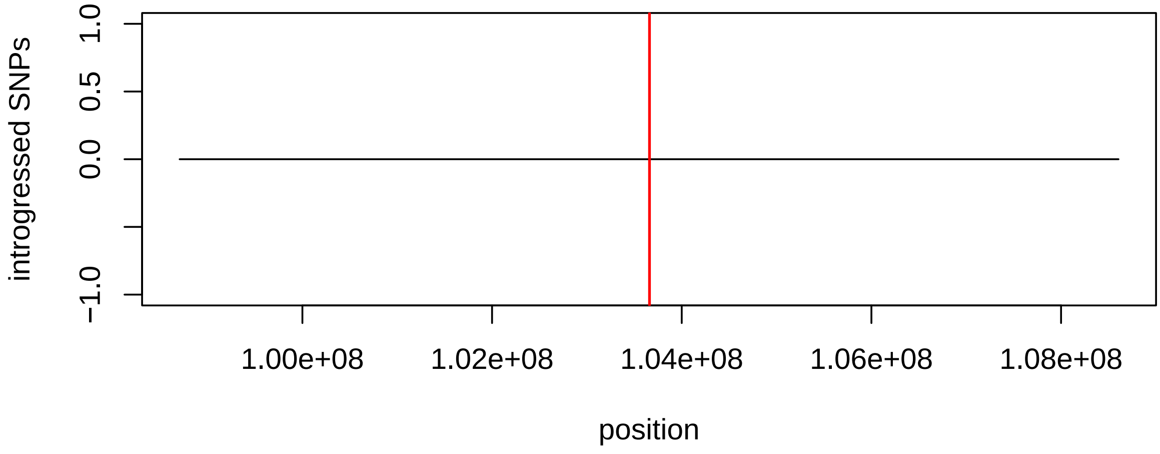

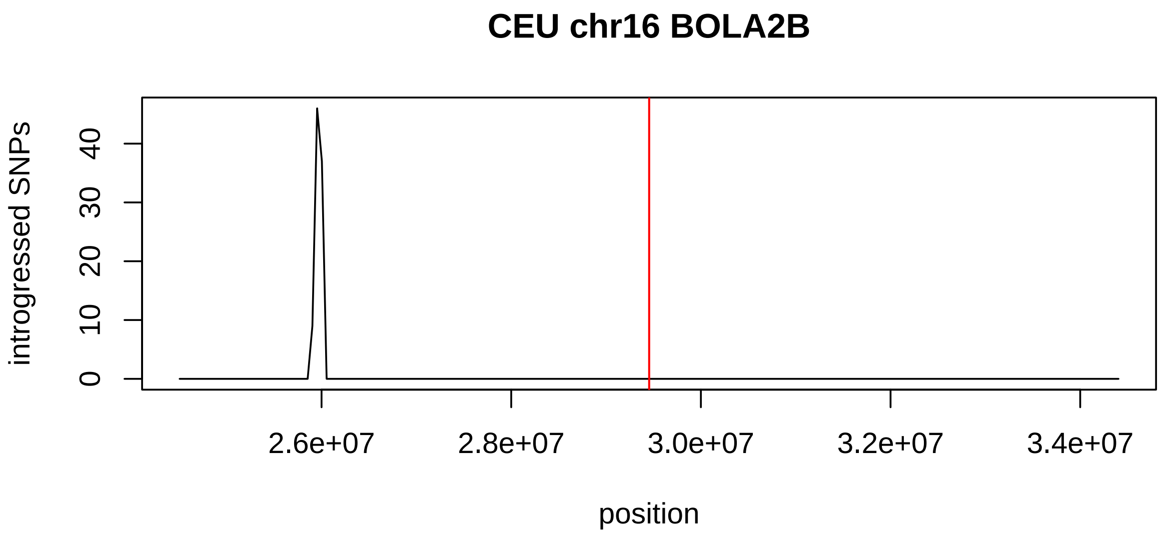
CEU chr16 BOLA2B
introgressed SNPs
0
10
20
30
40
2.6e+07
2.8e+07
3.0e+07
3.2e+07
3.4e+07
position

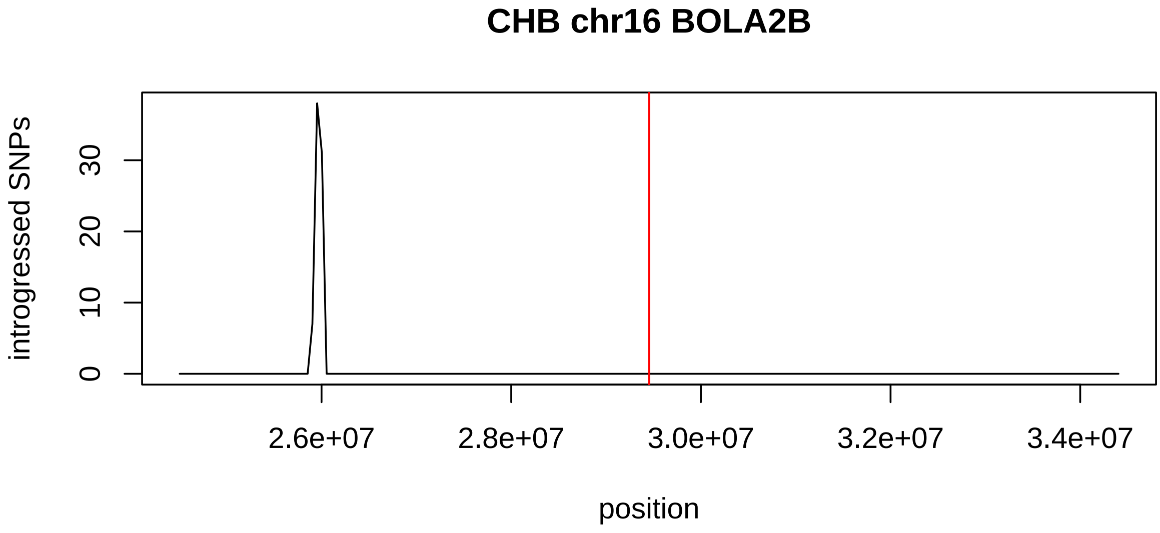
CHB chr16 BOLA2B
introgressed SNPs
0
10
20
30
2.6e+07
2.8e+07
3.0e+07
3.2e+07
3.4e+07
position

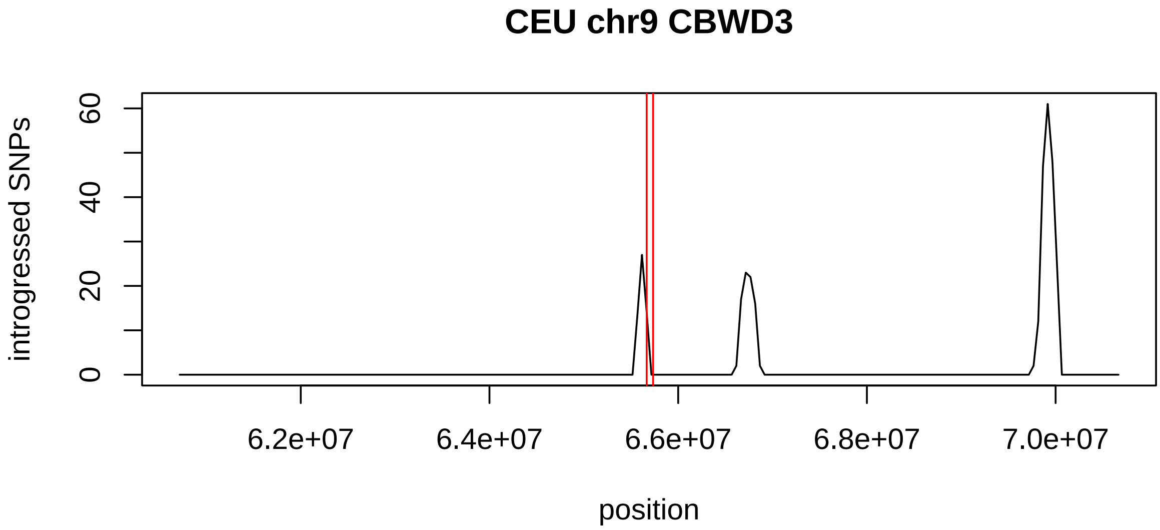
CEU chr9 CBWD3
introgressed SNPs
0
20
40
60
6.2e+07
6.4e+07
6.6e+07
6.8e+07
7.0e+07
position

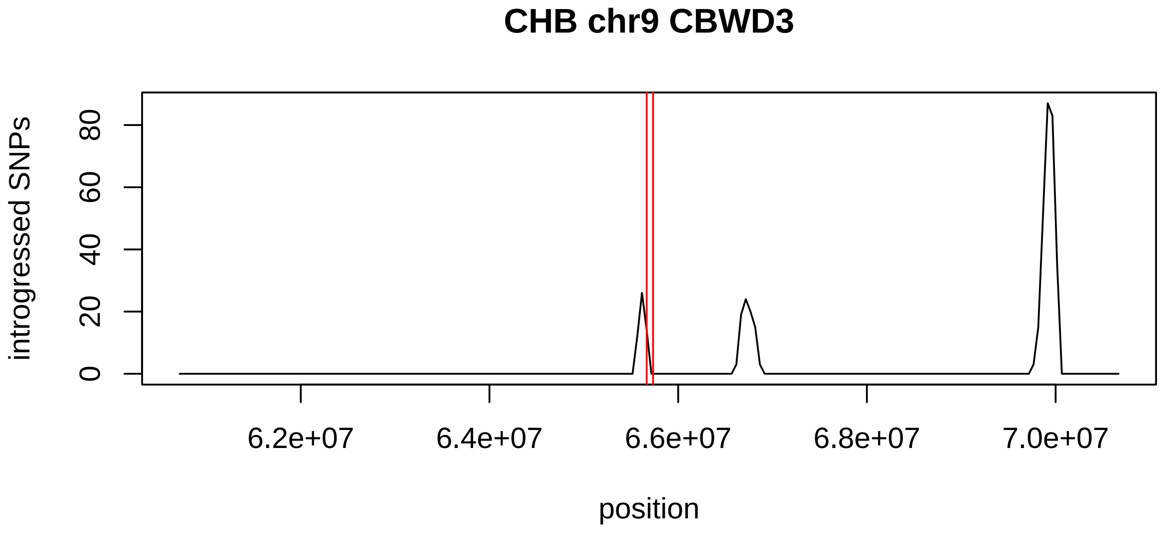
CHB chr9 CBWD3
introgressed SNPs
0
20
40
60
80
6.2e+07
6.4e+07
6.6e+07
6.8e+07
7.0e+07
position

CEU chr16 CLEC18A

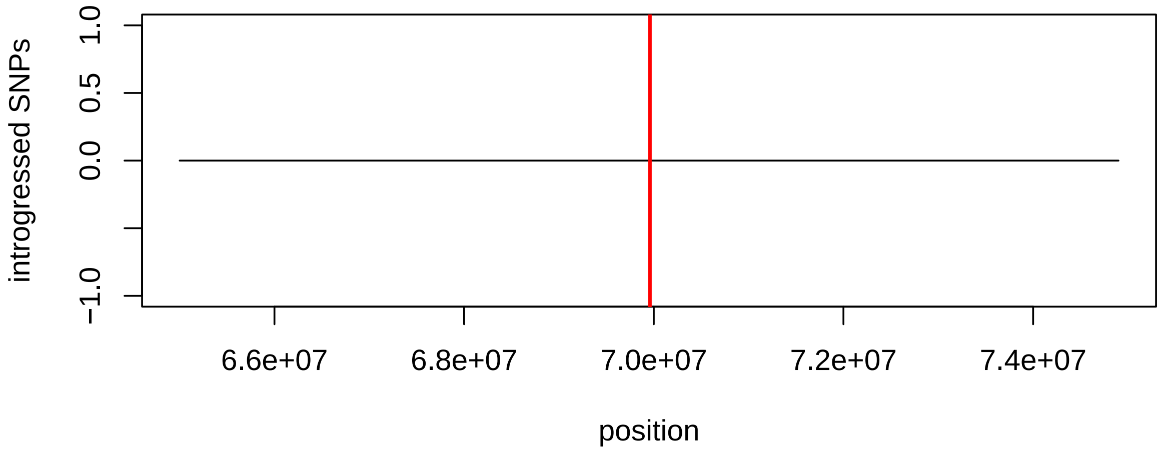

CHB chr16 CLEC18A

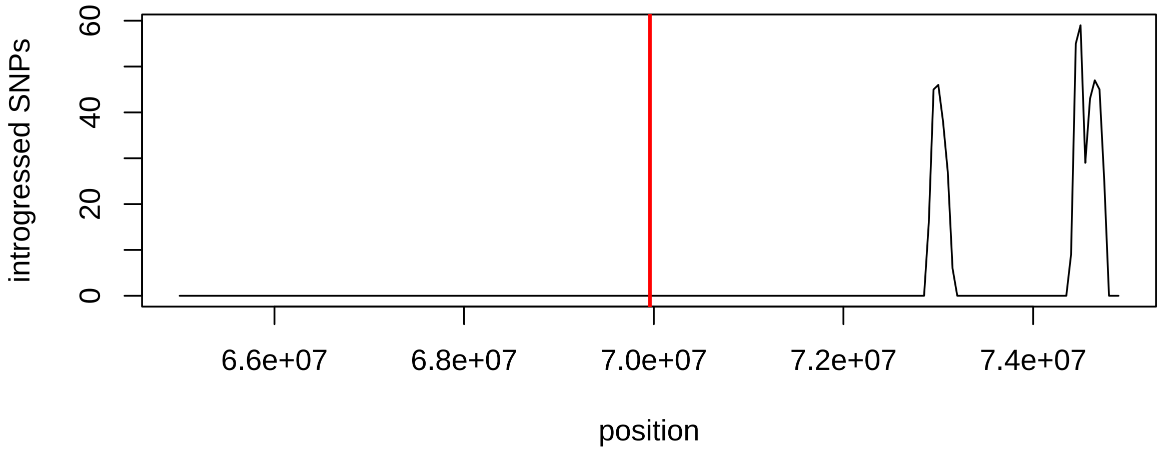

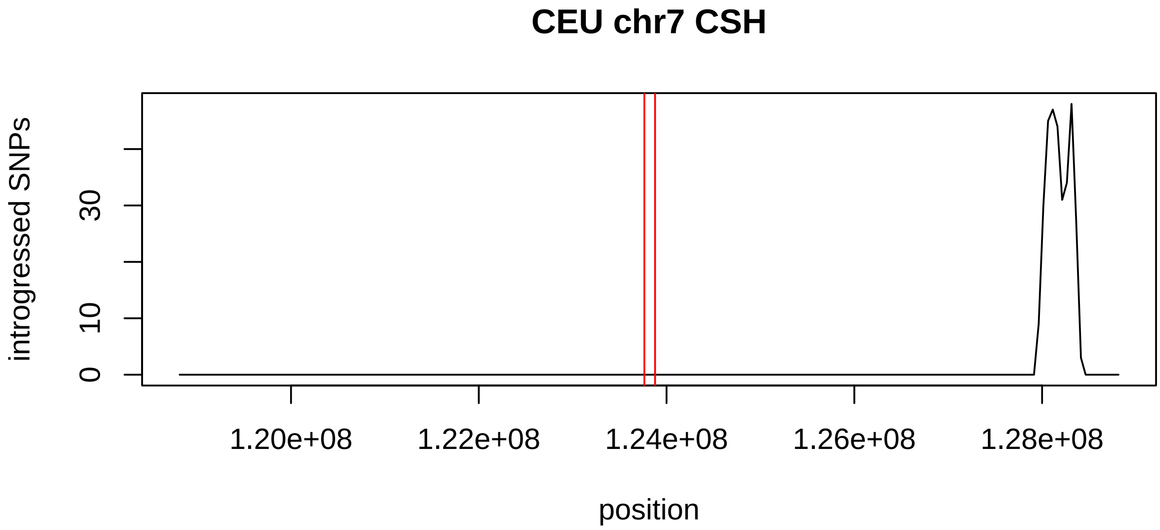
CEU chr7 CSH
introgressed SNPs
0
10
30
1.20e+08
1.22e+08
1.24e+08
1.26e+08
1.28e+08
position

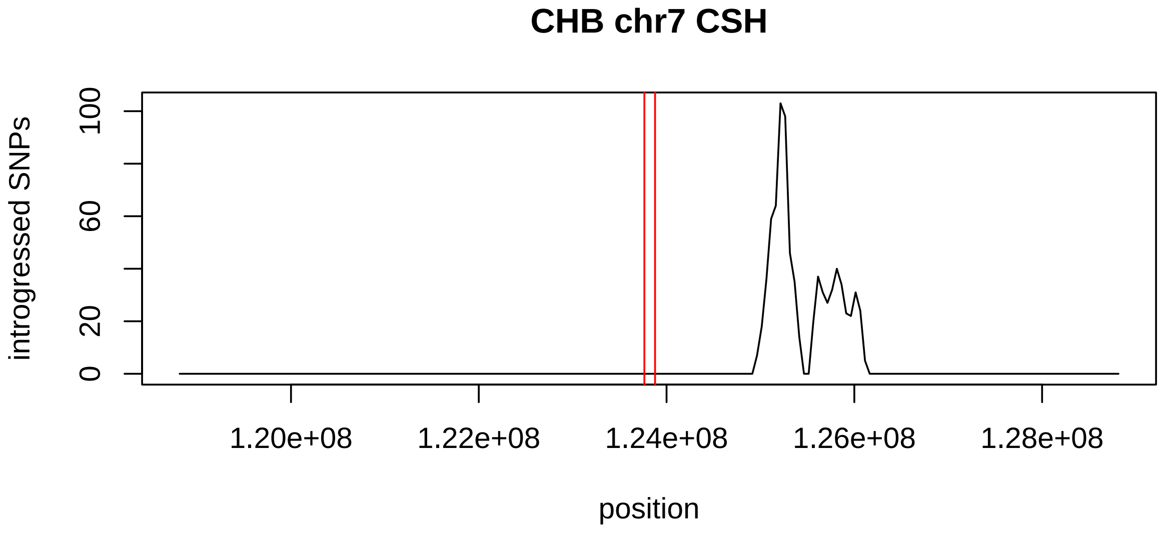
CHB chr7 CSH
introgressed SNPs
0
20
60
100
1.20e+08
1.22e+08
1.24e+08
1.26e+08
1.28e+08
position

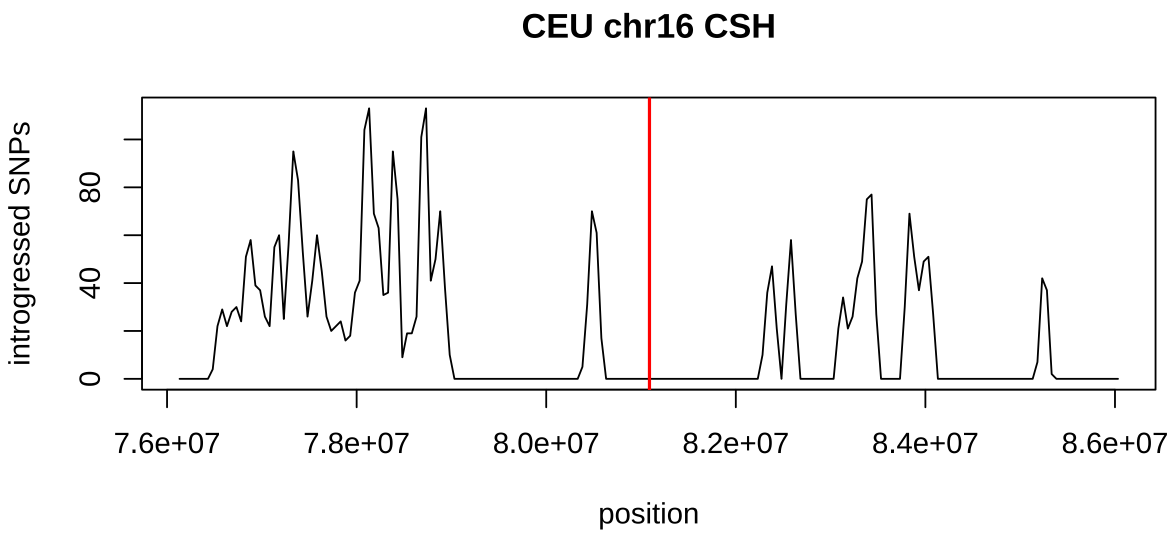
CEU chr16 CSH
introgressed SNPs
0
40
80
7.6e+07
7.8e+07
8.0e+07
8.2e+07
8.4e+07
8.6e+07
position

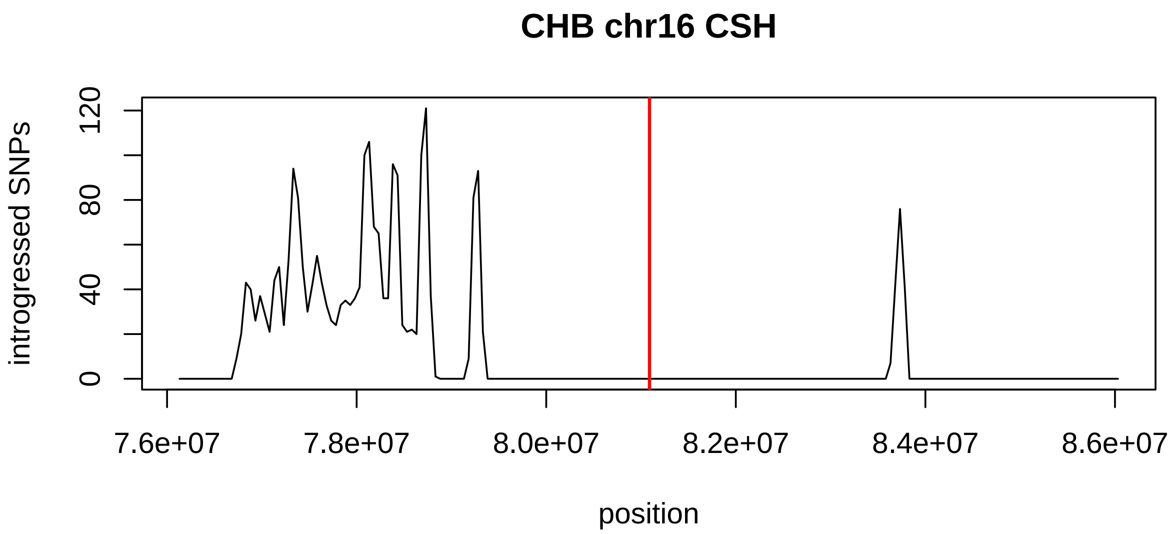
CHB chr16 CSH
introgressed SNPs
0
40
80
120
7.6e+07
7.8e+07
8.0e+07
8.2e+07
8.4e+07
8.6e+07
position

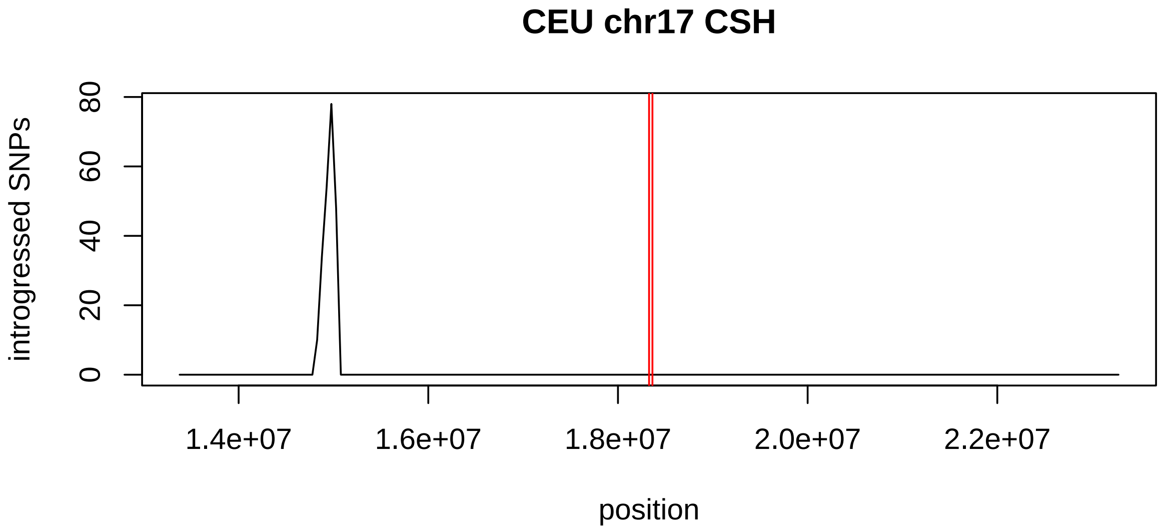
CEU chr17 CSH
introgressed SNPs
0
20
40
60
80
1.4e+07
1.6e+07
1.8e+07
2.0e+07
2.2e+07
position

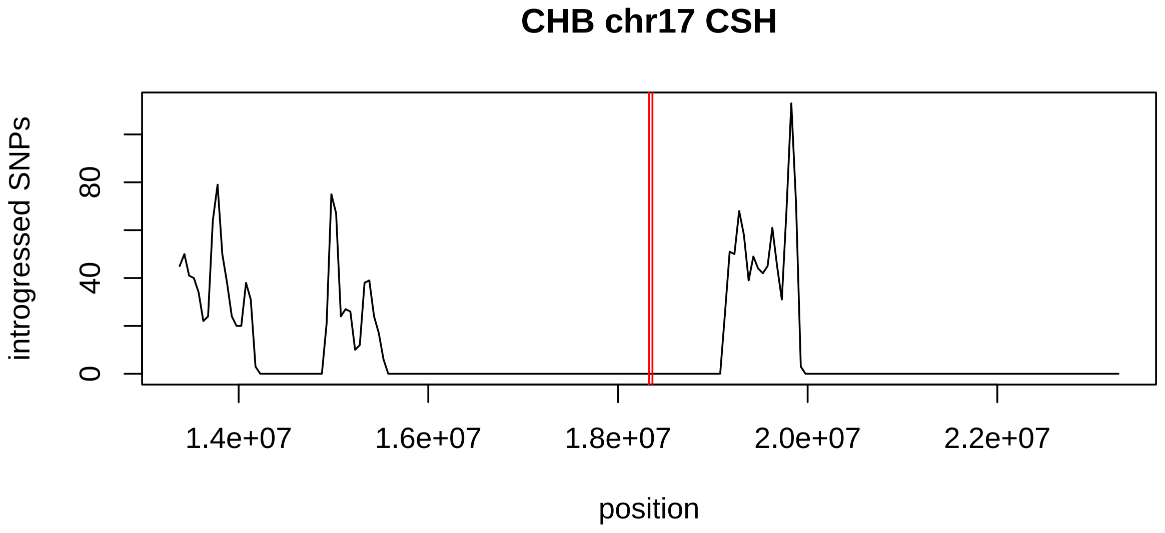
CHB chr17 CSH
introgressed SNPs
0
40
80
1.4e+07
1.6e+07
1.8e+07
2.0e+07
2.2e+07
position

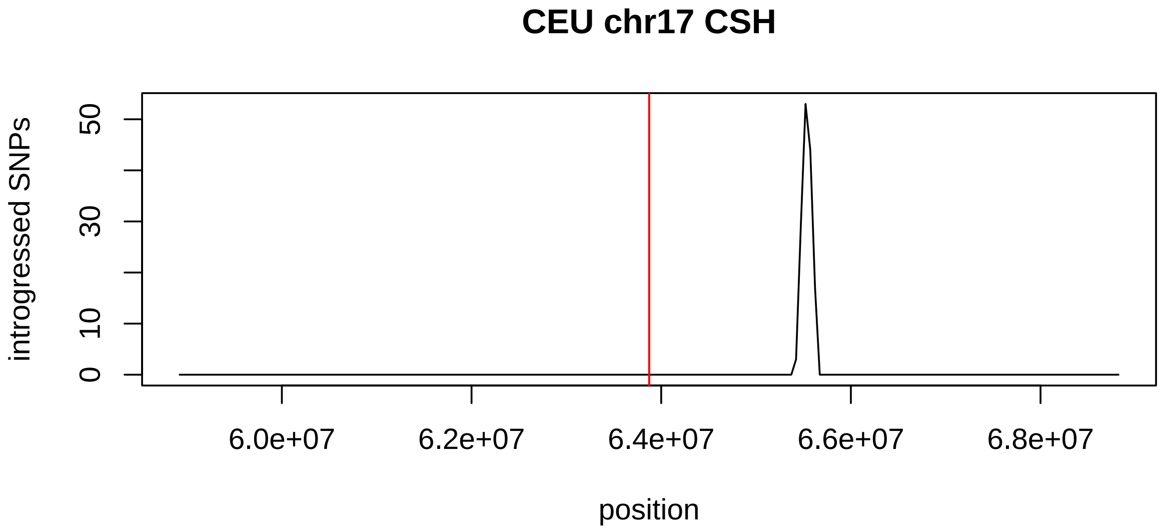
CEU chr17 CSH
introgressed SNPs
0
10
30
50
6.0e+07
6.2e+07
6.4e+07
6.6e+07
6.8e+07
position

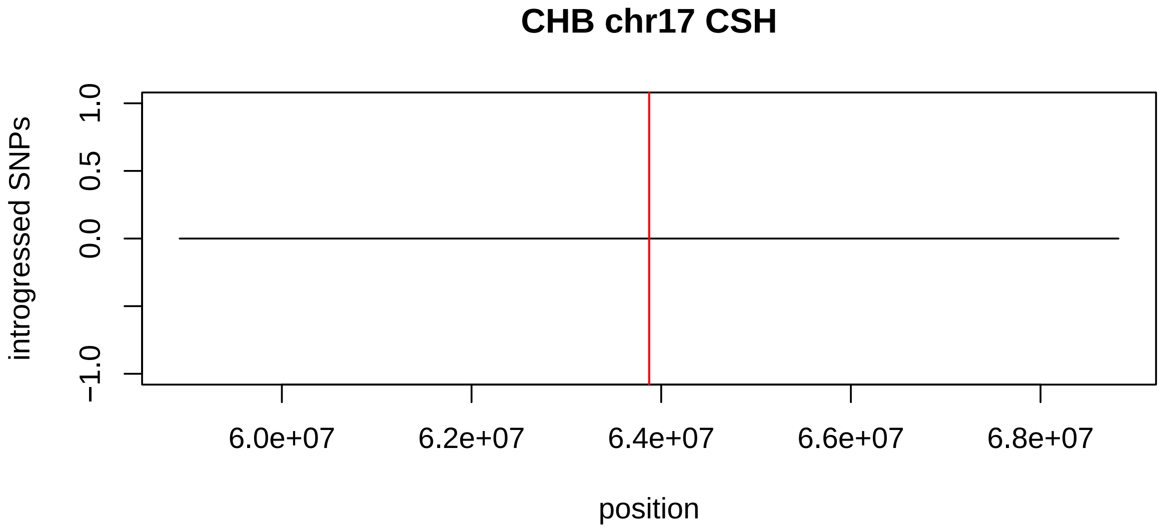
CHB chr17 CSH
introgressed SNPs
−1.0
0.0
0.5
1.0
6.0e+07
6.2e+07
6.4e+07
6.6e+07
6.8e+07
position

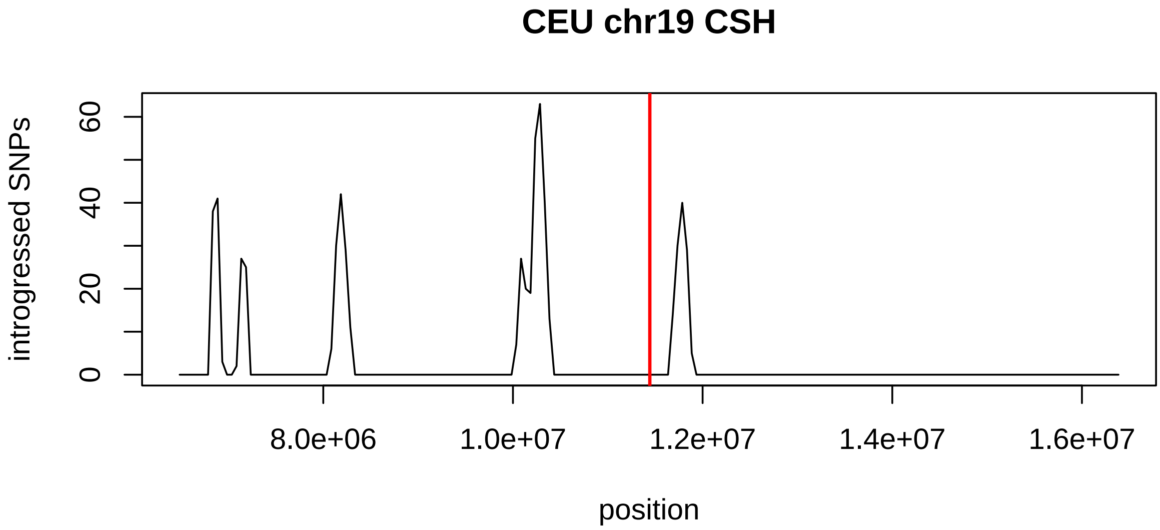
CEU chr19 CSH
introgressed SNPs
0
20
40
60
8.0e+06
1.0e+07
1.2e+07
1.4e+07
1.6e+07
position

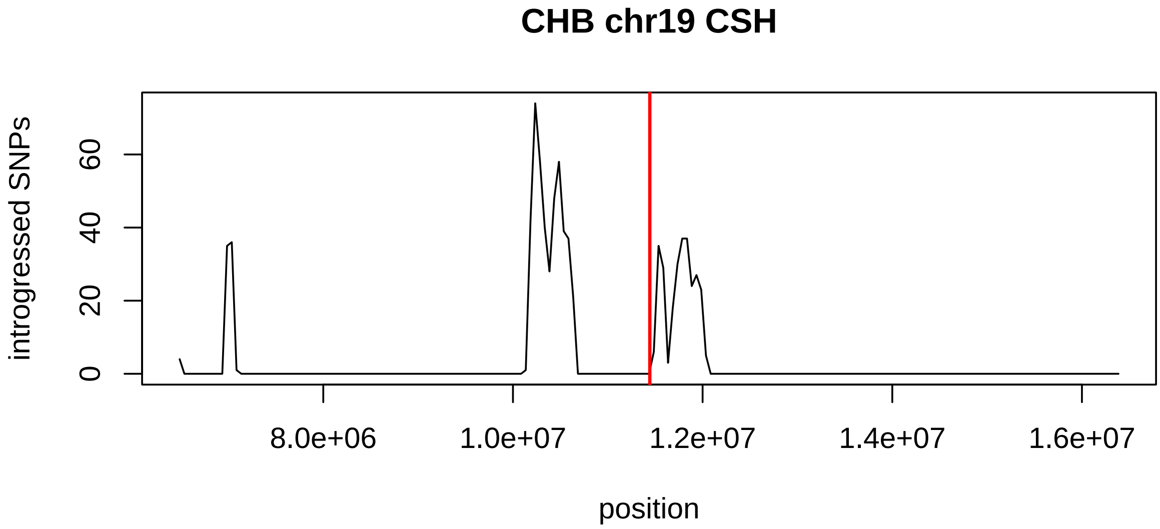
CHB chr19 CSH
introgressed SNPs
0
20
40
60
8.0e+06
1.0e+07
1.2e+07
1.4e+07
1.6e+07
position

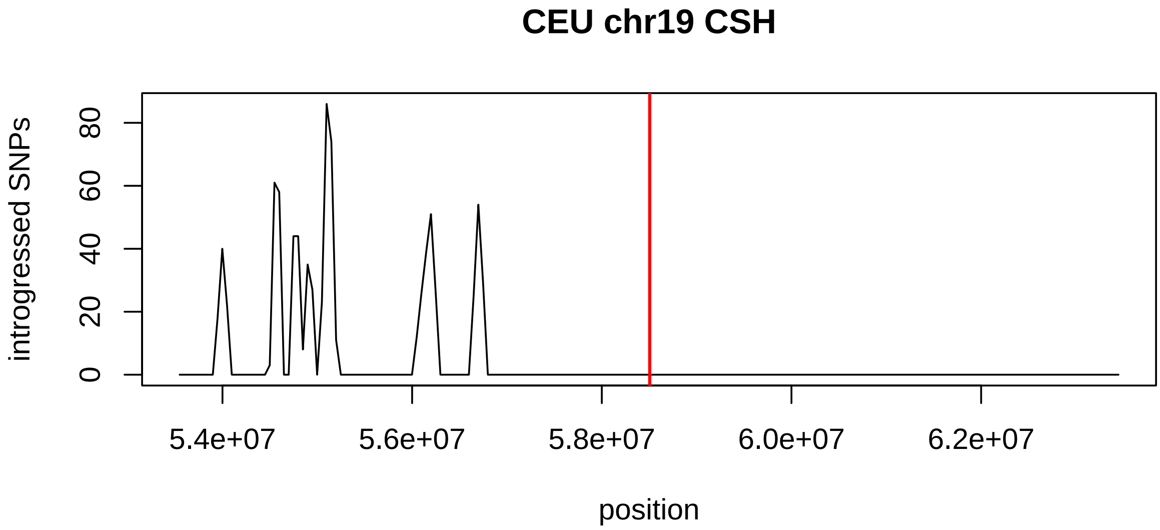
CEU chr19 CSH
introgressed SNPs
0
20
40
60
80
5.4e+07
5.6e+07
5.8e+07
6.0e+07
6.2e+07
position

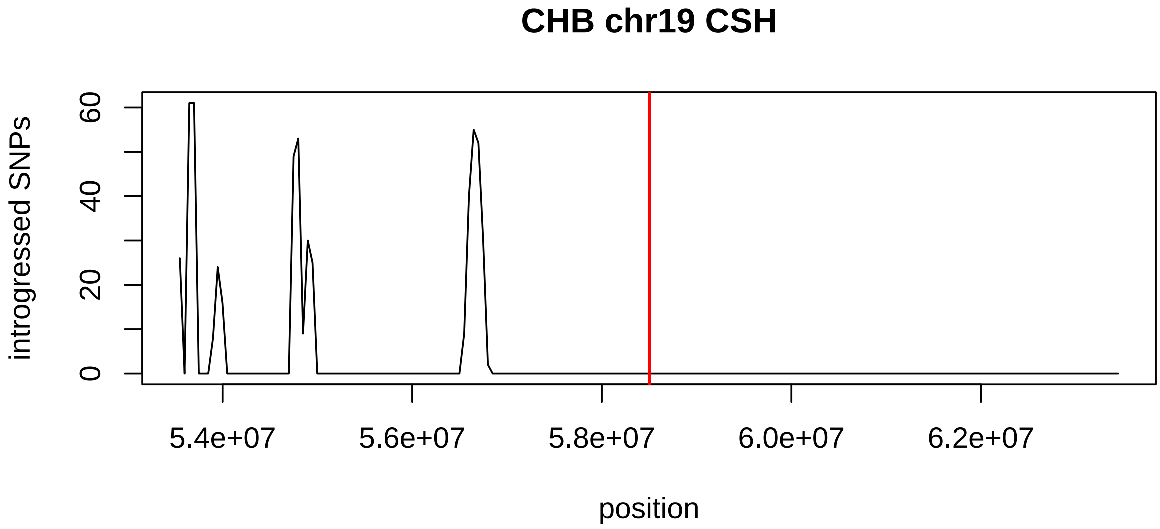
CHB chr19 CSH
introgressed SNPs
0
20
40
60
5.4e+07
5.6e+07
5.8e+07
6.0e+07
6.2e+07
position

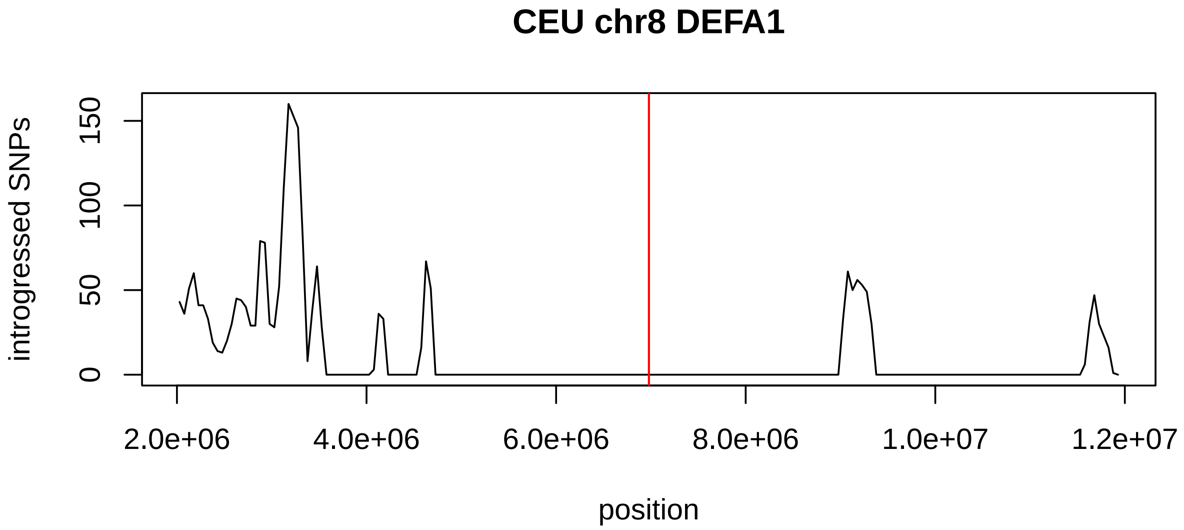
CEU chr8 DEFA1
introgressed SNPs
0
50
100
150
2.0e+06
4.0e+06
6.0e+06
8.0e+06
1.0e+07
1.2e+07
position

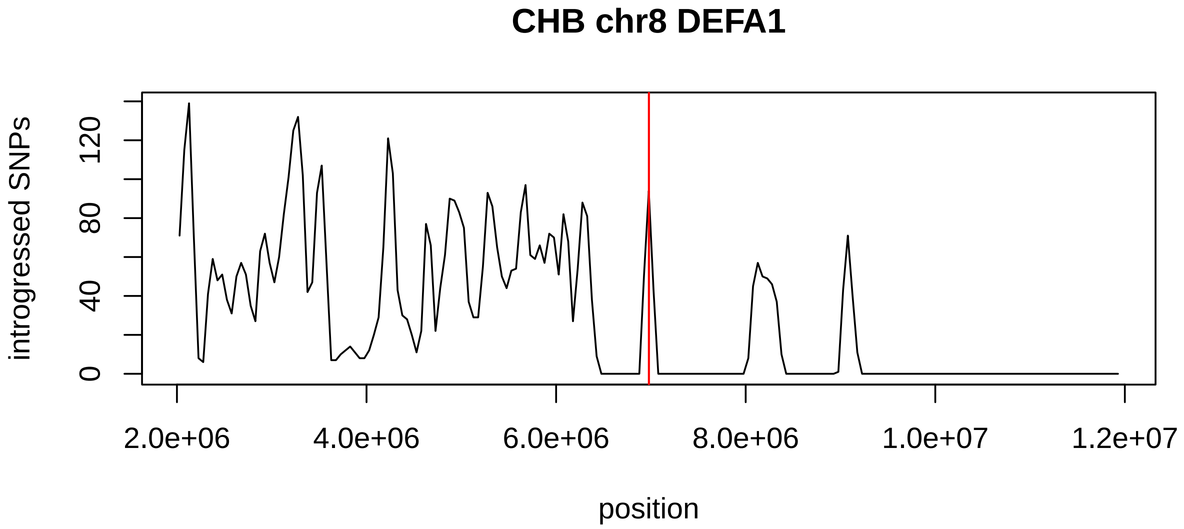
CHB chr8 DEFA1
introgressed SNPs
0
40
80
120
2.0e+06
4.0e+06
6.0e+06
8.0e+06
1.0e+07
1.2e+07
position

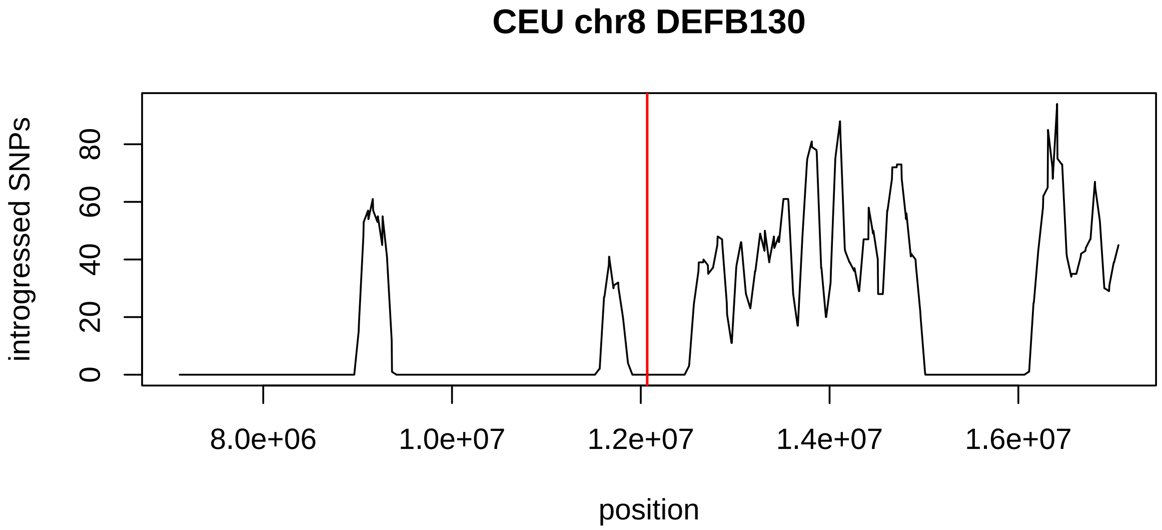
CEU chr8 DEFB130
introgressed SNPs
0
20
40
60
80
8.0e+06
1.0e+07
1.2e+07
1.4e+07
1.6e+07
position

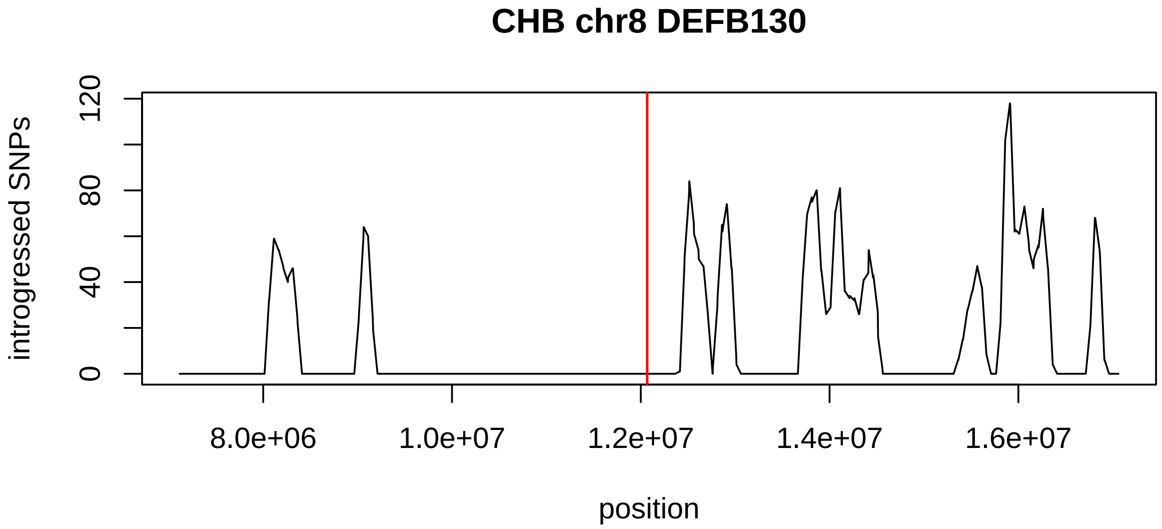
CHB chr8 DEFB130
introgressed SNPs
0
40
80
120
8.0e+06
1.0e+07
1.2e+07
1.4e+07
1.6e+07
position

CEU chr8 DEFB130

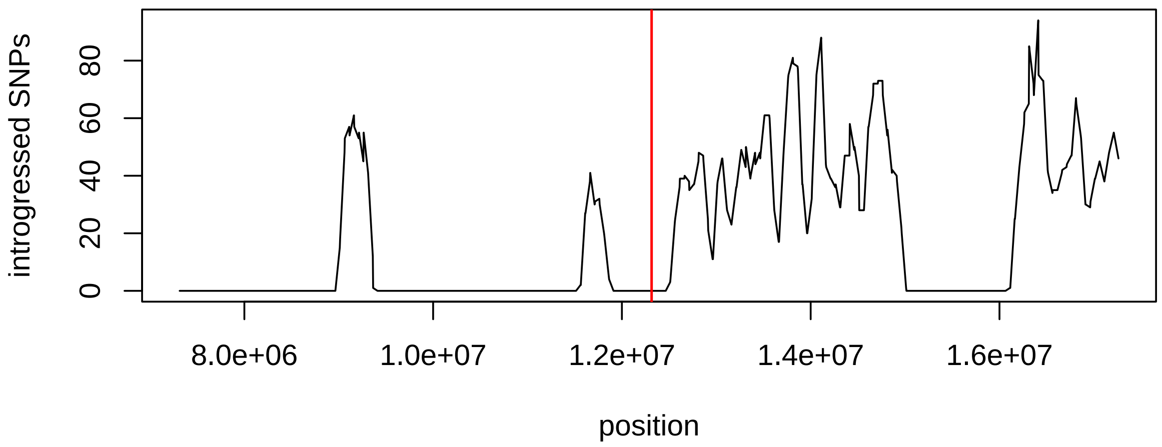

CHB chr8 DEFB130

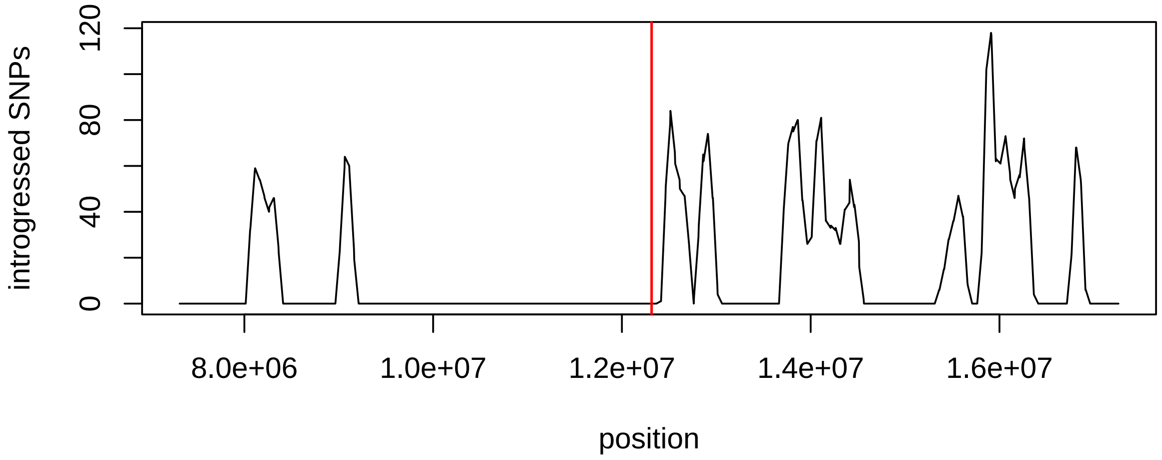

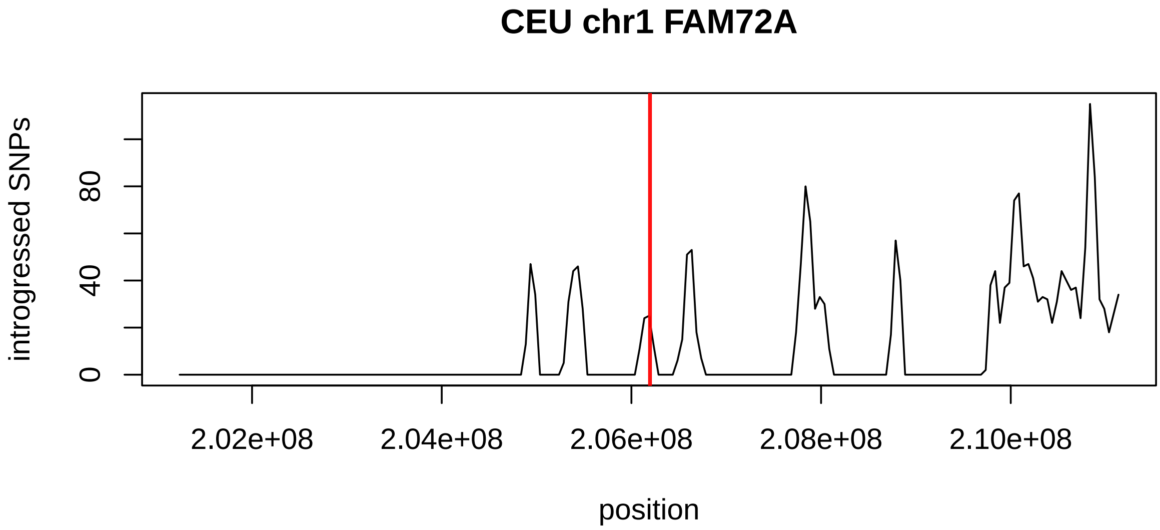
CEU chr1 FAM72A
introgressed SNPs
0
40
80
2.02e+08
2.04e+08
2.06e+08
2.08e+08
2.10e+08
position

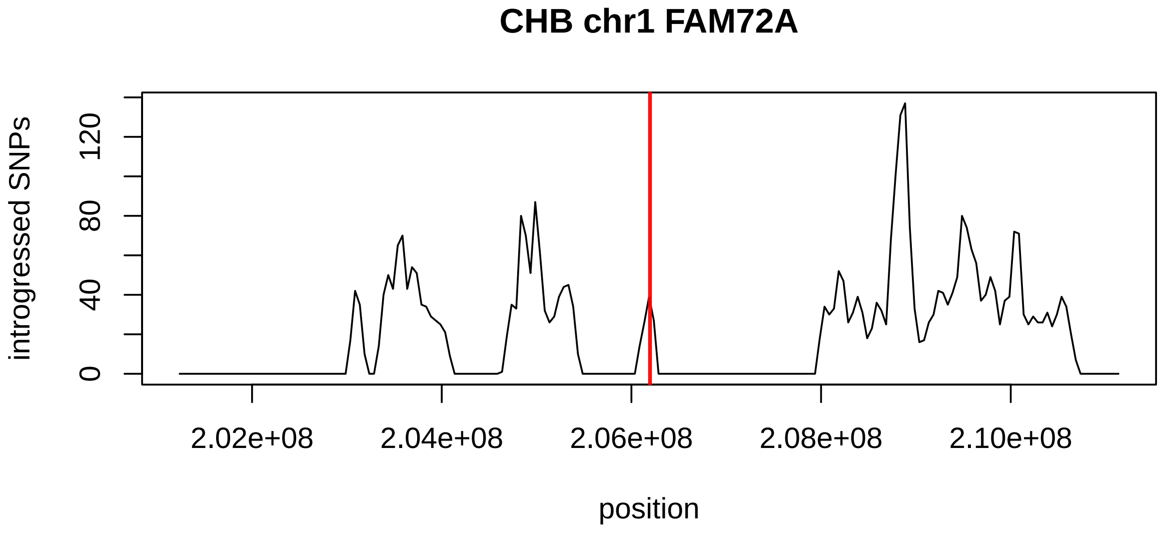
CHB chr1 FAM72A
introgressed SNPs
0
40
80
120
2.02e+08
2.04e+08
2.06e+08
2.08e+08
2.10e+08
position

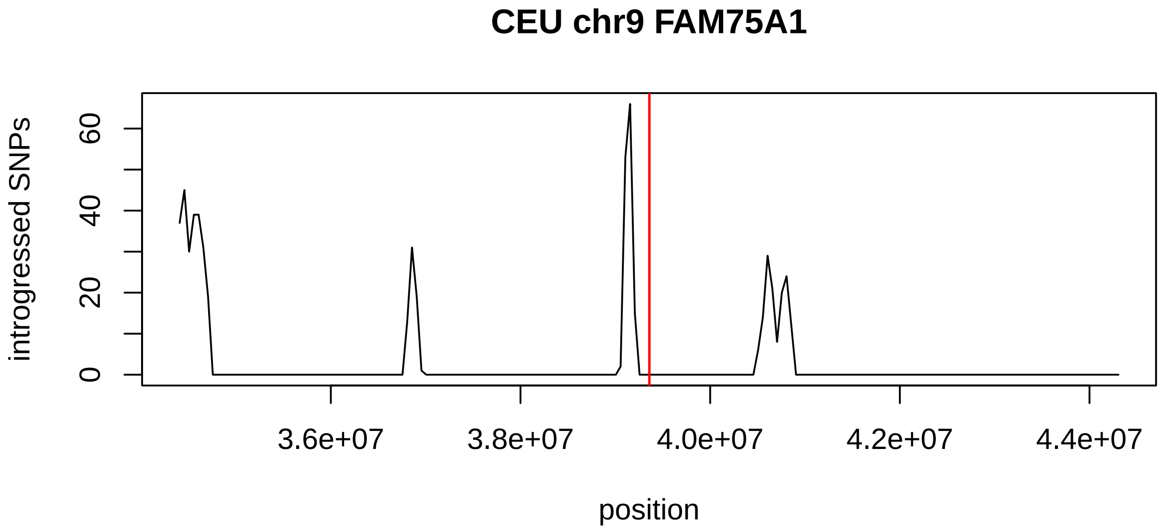
CEU chr9 FAM75A1
introgressed SNPs
0
20
40
60
3.6e+07
3.8e+07
4.0e+07
4.2e+07
4.4e+07
position

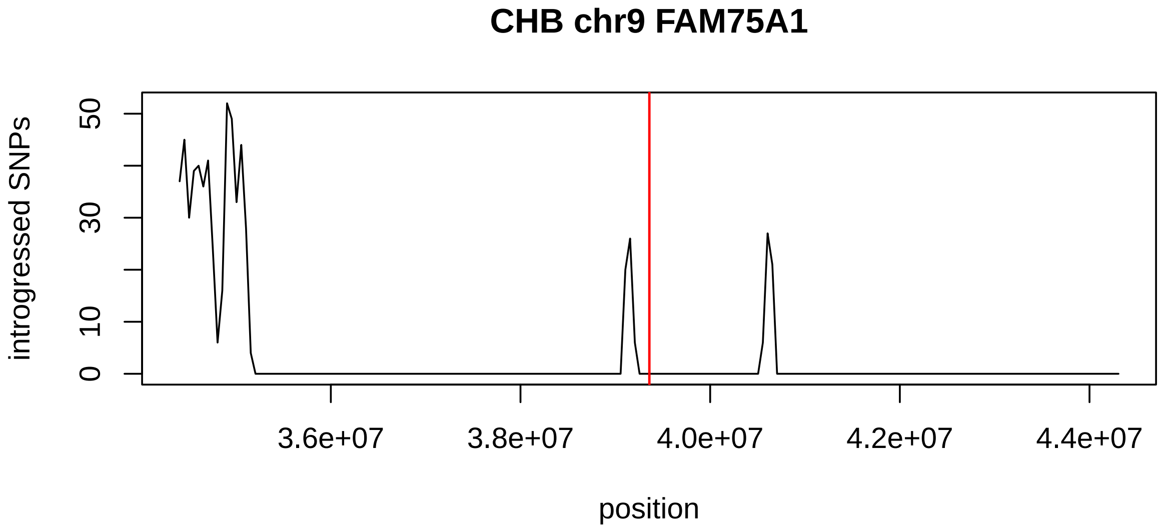
CHB chr9 FAM75A1
introgressed SNPs
0
10
30
50
3.6e+07
3.8e+07
4.0e+07
4.2e+07
4.4e+07
position

CEU chr9 FAM75A5

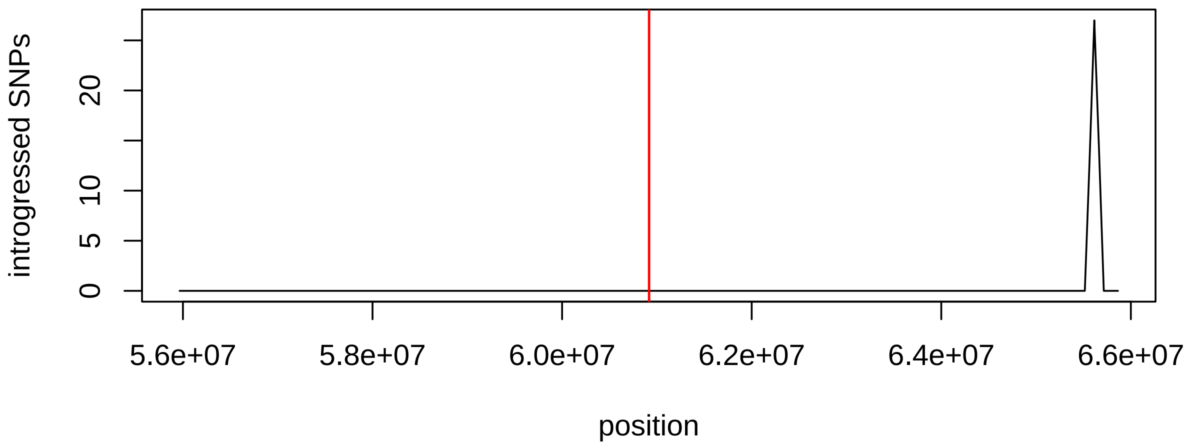

CHB chr9 FAM75A5

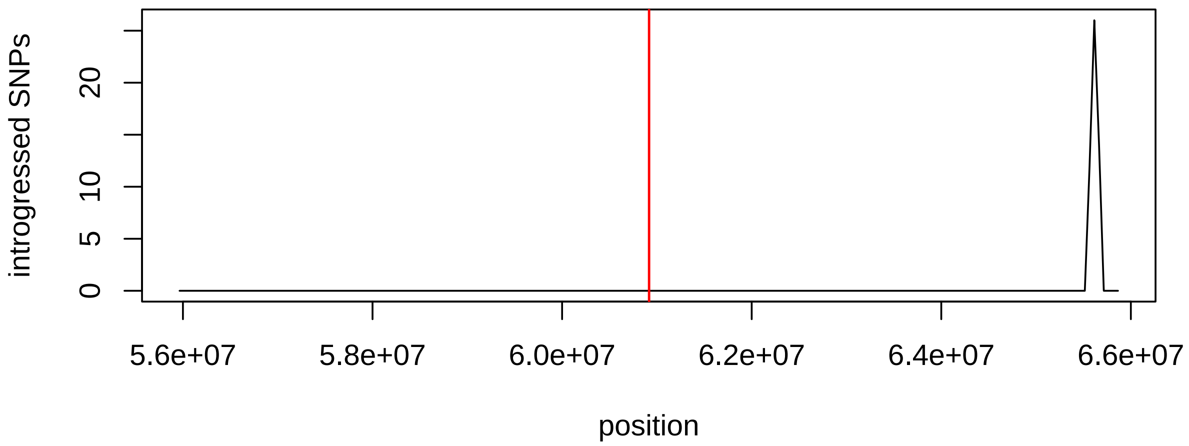

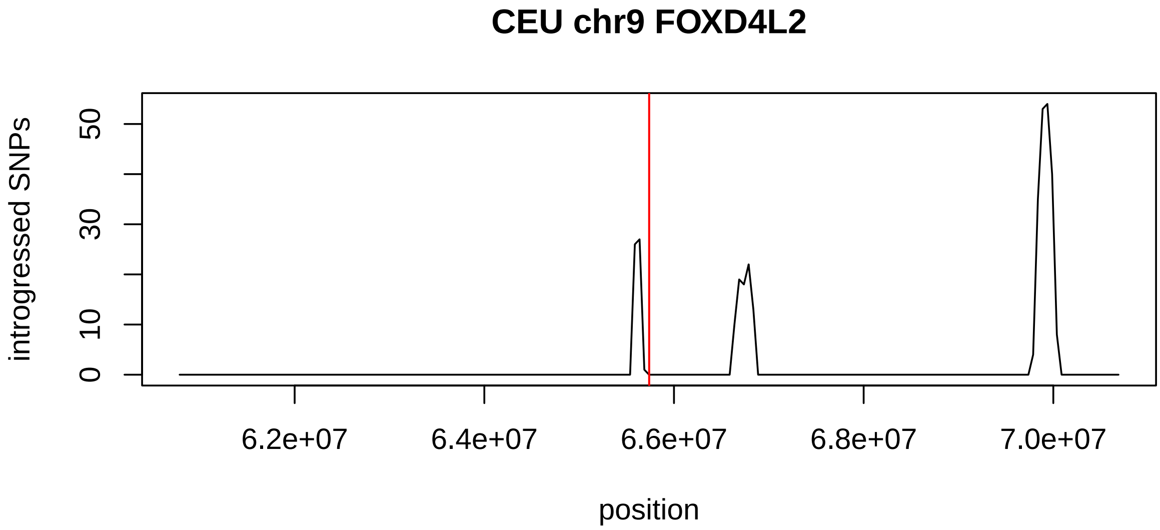
CEU chr9 FOXD4L2
introgressed SNPs
0
10
30
50
6.2e+07
6.4e+07
6.6e+07
6.8e+07
7.0e+07
position

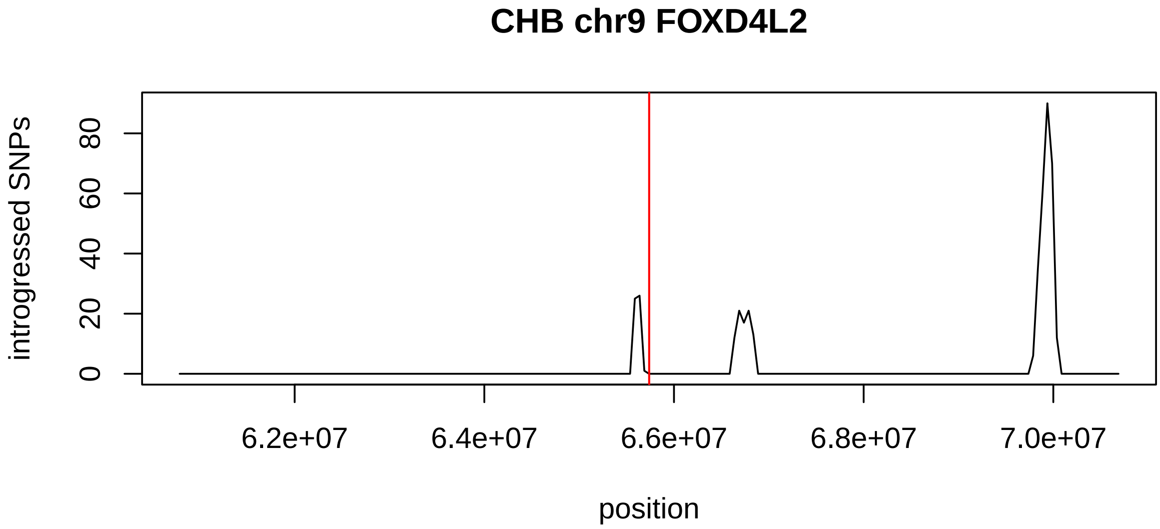
CHB chr9 FOXD4L2
introgressed SNPs
0
20
40
60
80
6.2e+07
6.4e+07
6.6e+07
6.8e+07
7.0e+07
position

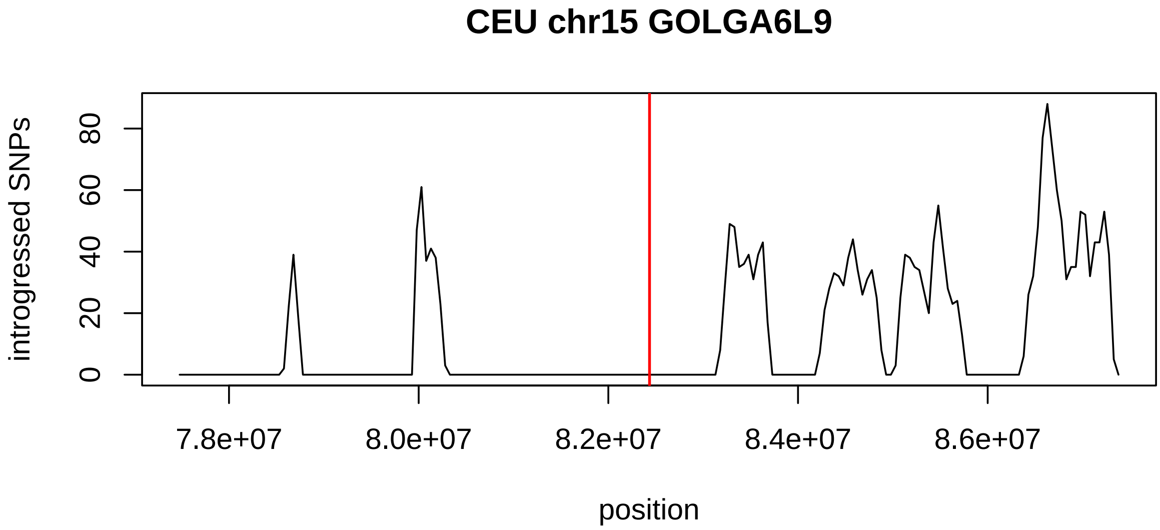
CEU chr15 GOLGA6L9
introgressed SNPs
0
20
40
60
80
7.8e+07
8.0e+07
8.2e+07
8.4e+07
8.6e+07
position

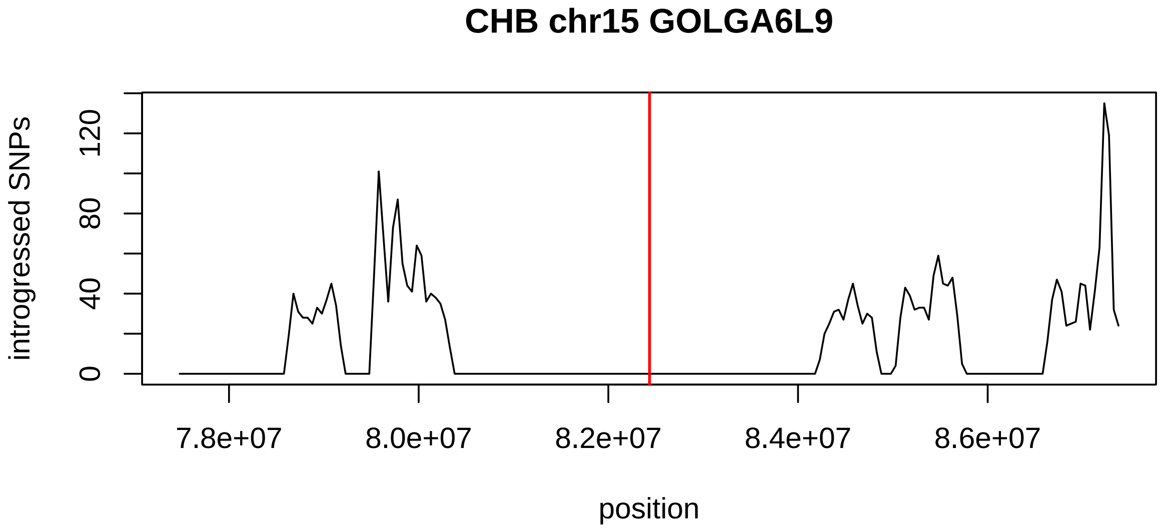
CHB chr15 GOLGA6L9
introgressed SNPs
0
40
80
120
7.8e+07
8.0e+07
8.2e+07
8.4e+07
8.6e+07
position

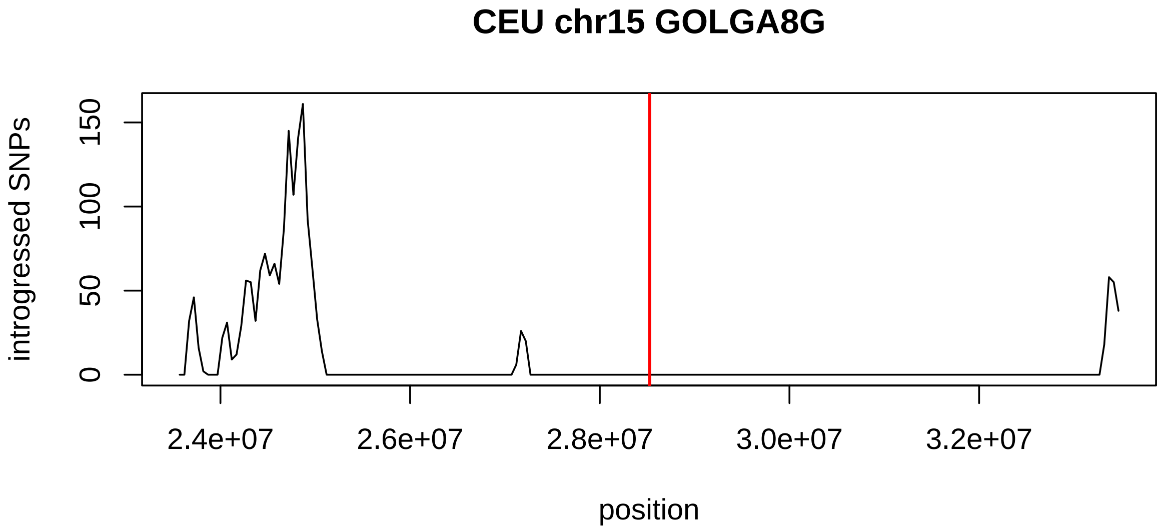
CEU chr15 GOLGA8G
introgressed SNPs
0
50
100
150
2.4e+07
2.6e+07
2.8e+07
3.0e+07
3.2e+07
position

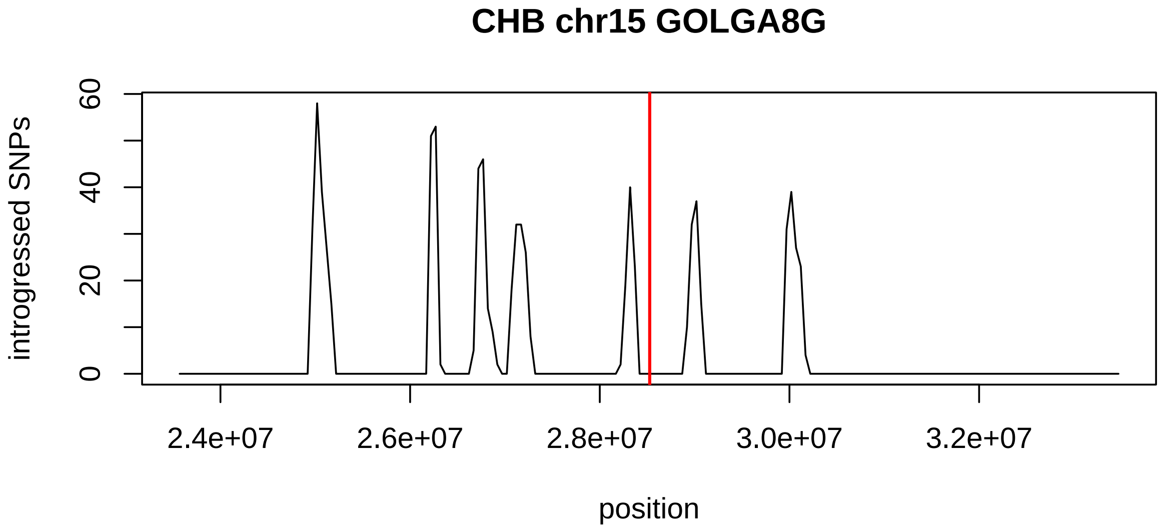
CHB chr15 GOLGA8G
introgressed SNPs
0
20
40
60
2.4e+07
2.6e+07
2.8e+07
3.0e+07
3.2e+07
position

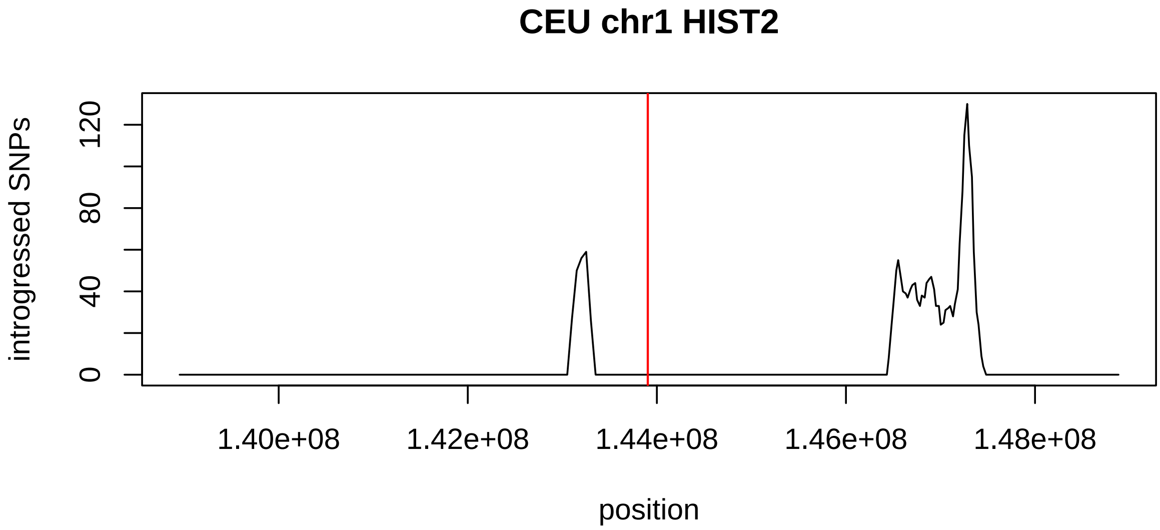

CEU chr1 HIST2
introgressed SNPs
0
40
80
120
1.40e+08
1.42e+08
1.44e+08
1.46e+08
1.48e+08
position

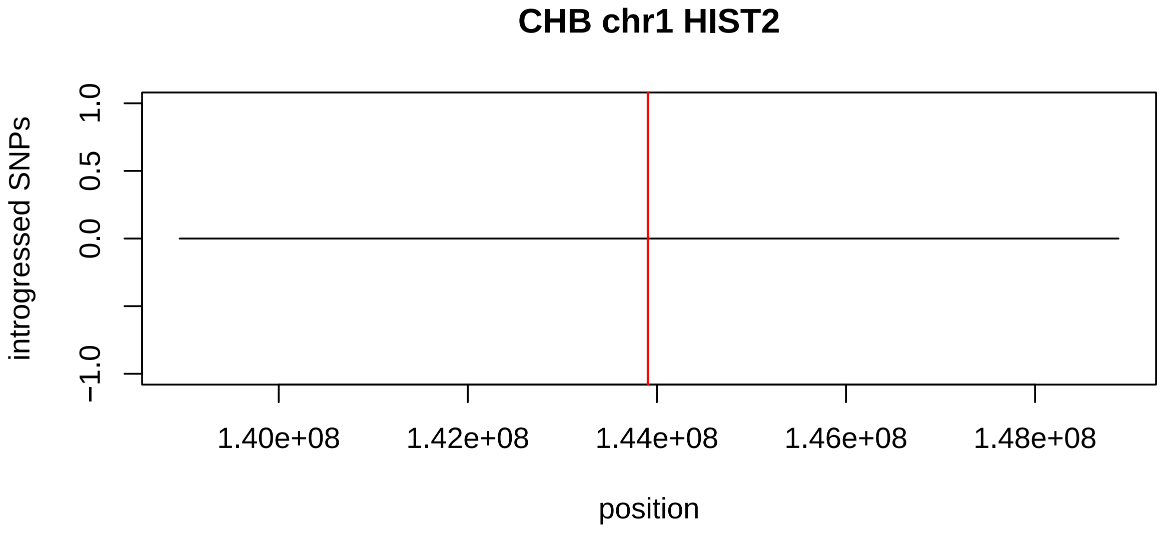

CHB chr1 HIST2
introgressed SNPs
−1.0
0.0
0.5
1.0
1.40e+08
1.42e+08
1.44e+08
1.46e+08
1.48e+08
position

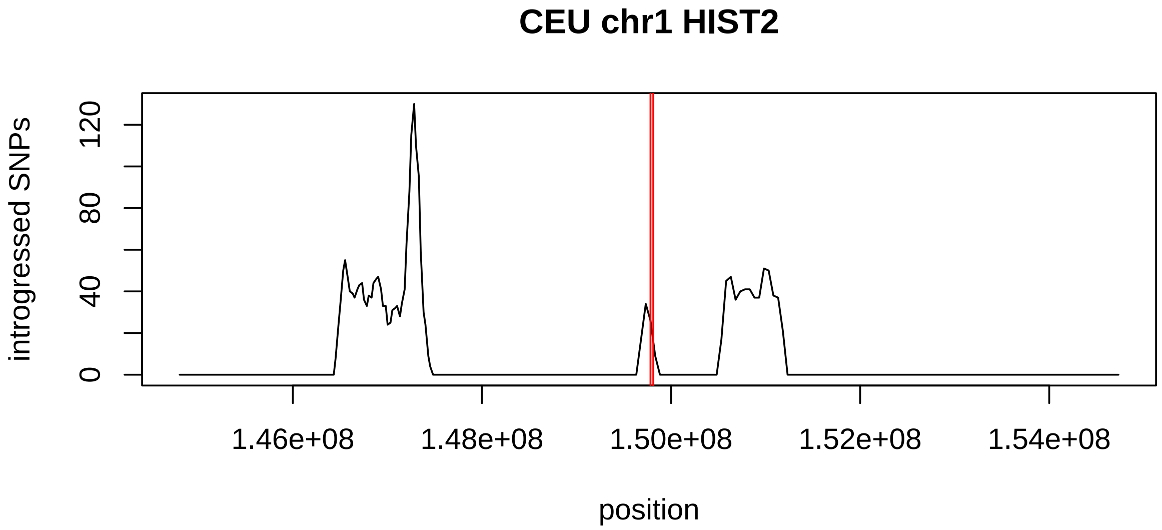
CEU chr1 HIST2
introgressed SNPs
0
40
80
120
1.46e+08
1.48e+08
1.50e+08
1.52e+08
1.54e+08
position

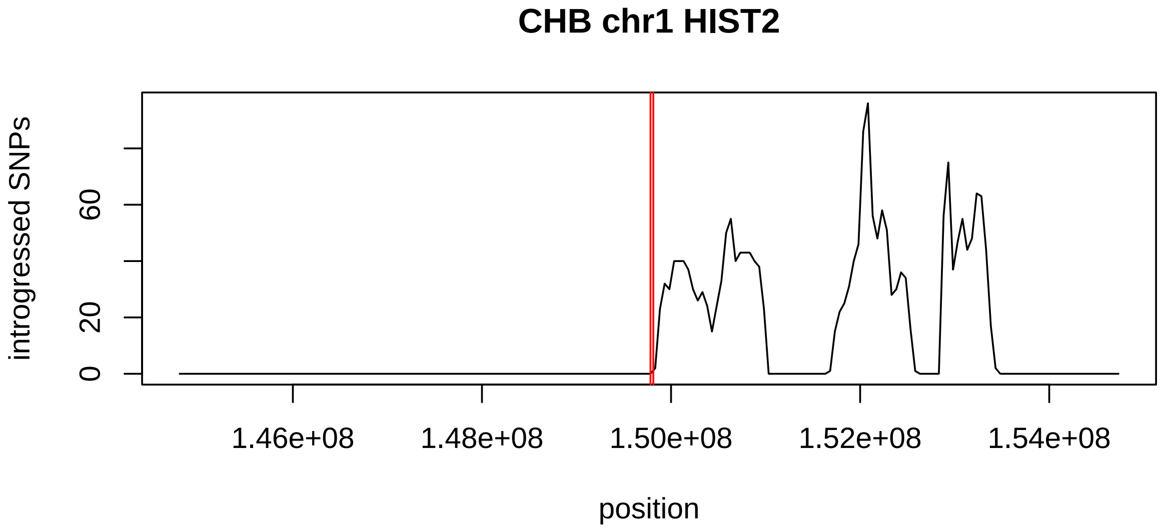
CHB chr1 HIST2
introgressed SNPs
0
20
60
1.46e+08
1.48e+08
1.50e+08
1.52e+08
1.54e+08
position

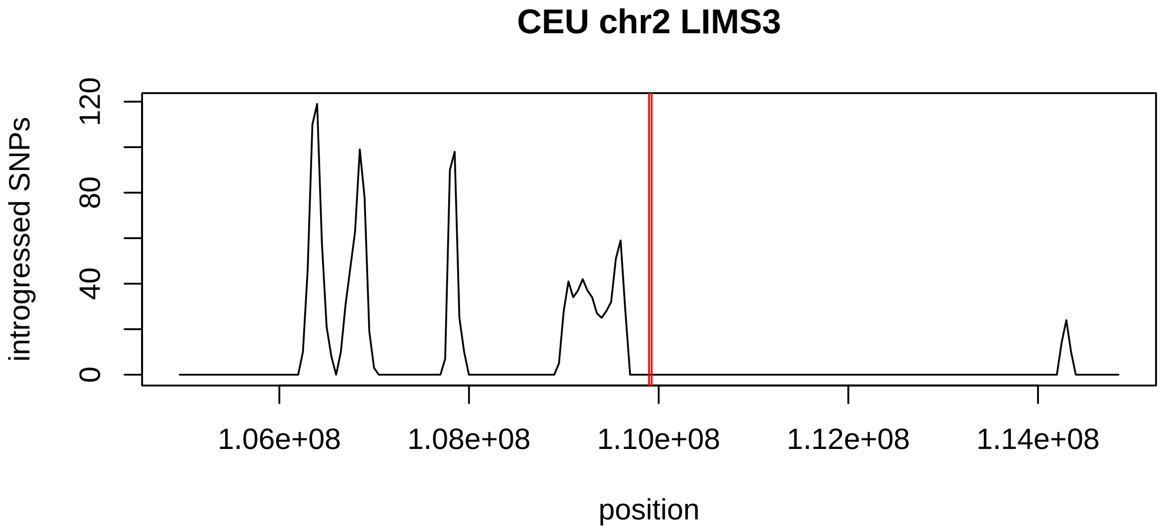
CEU chr2 LIMS3
introgressed SNPs
0
40
80
120
1.06e+08
1.08e+08
1.10e+08
1.12e+08
1.14e+08
position

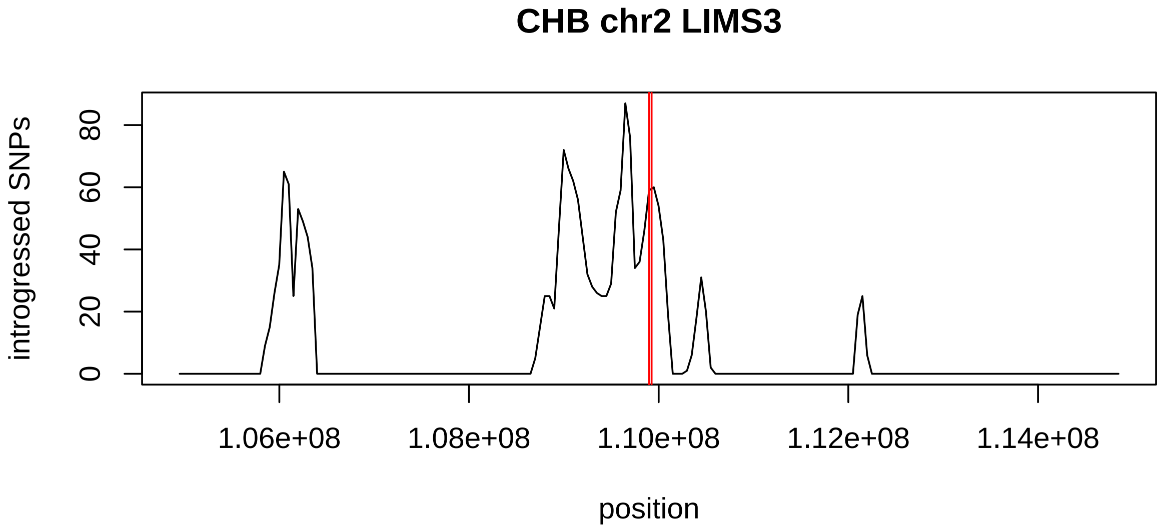
CHB chr2 LIMS3
introgressed SNPs
0
20
40
60
80
1.06e+08
1.08e+08
1.10e+08
1.12e+08
1.14e+08
position

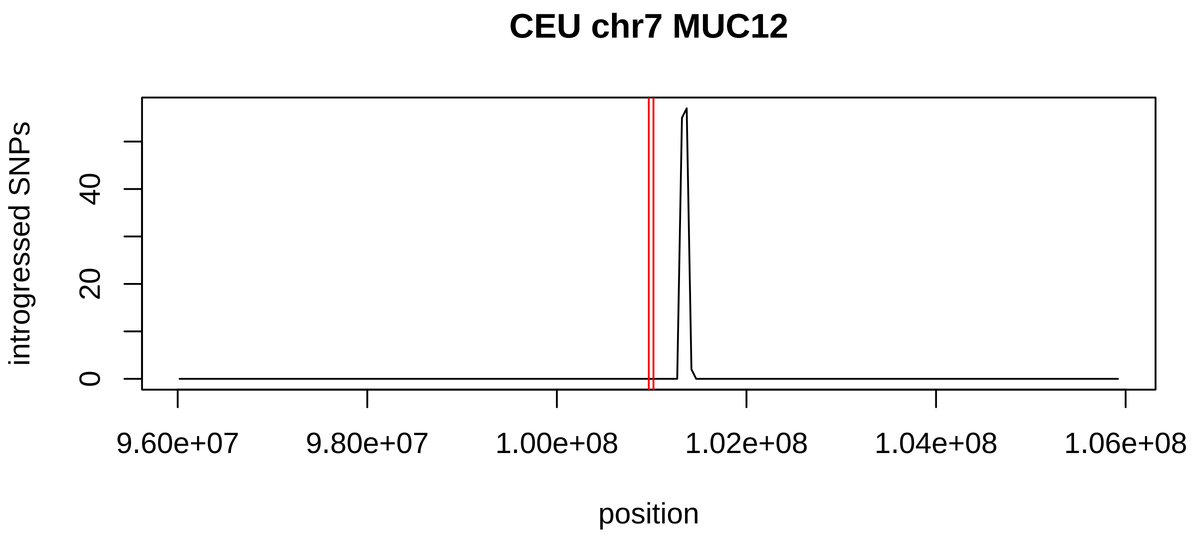
CEU chr7 MUC12
introgressed SNPs
0
20
40
9.60e+07
9.80e+07
1.00e+08
1.02e+08
1.04e+08
1.06e+08
position

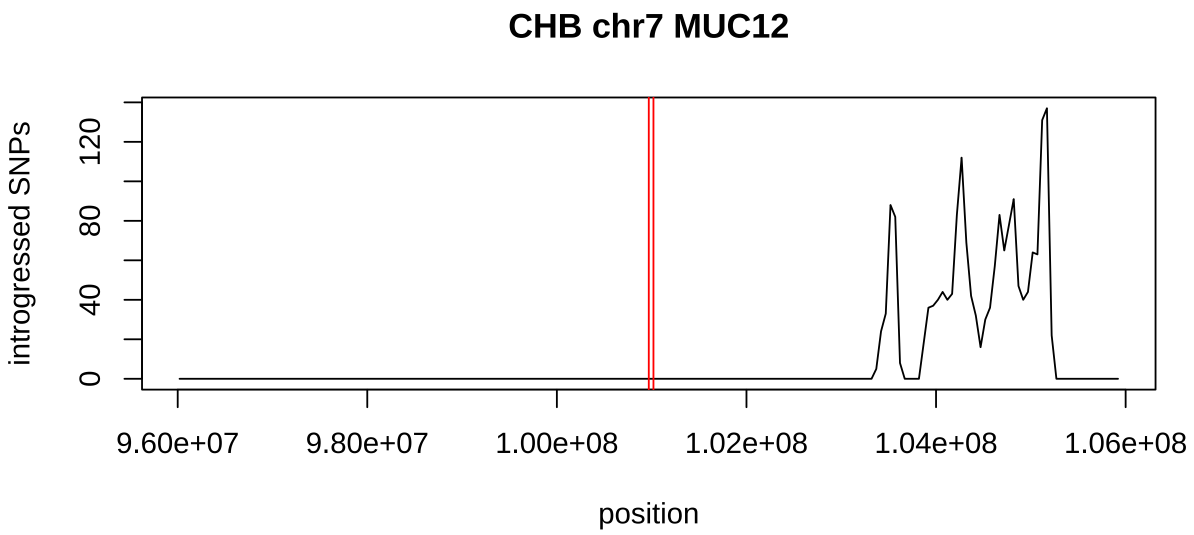
CHB chr7 MUC12
introgressed SNPs
0
40
80
120
9.60e+07
9.80e+07
1.00e+08
1.02e+08
1.04e+08
1.06e+08
position

CEU chr1 NBPF11

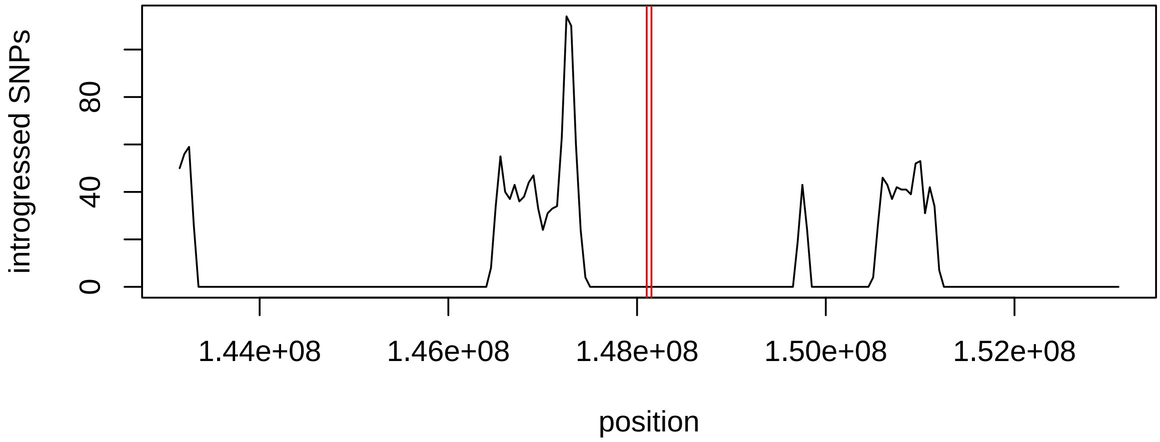

CHB chr1 NBPF11

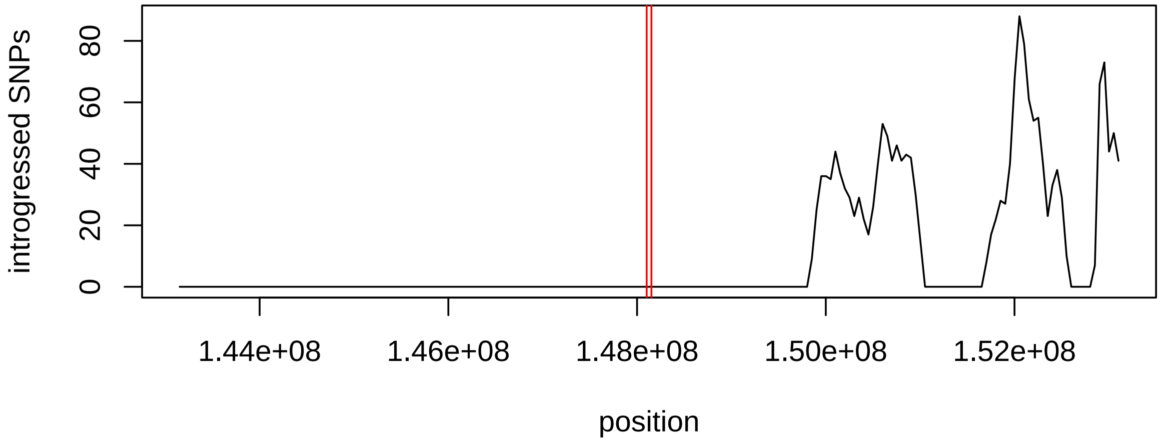

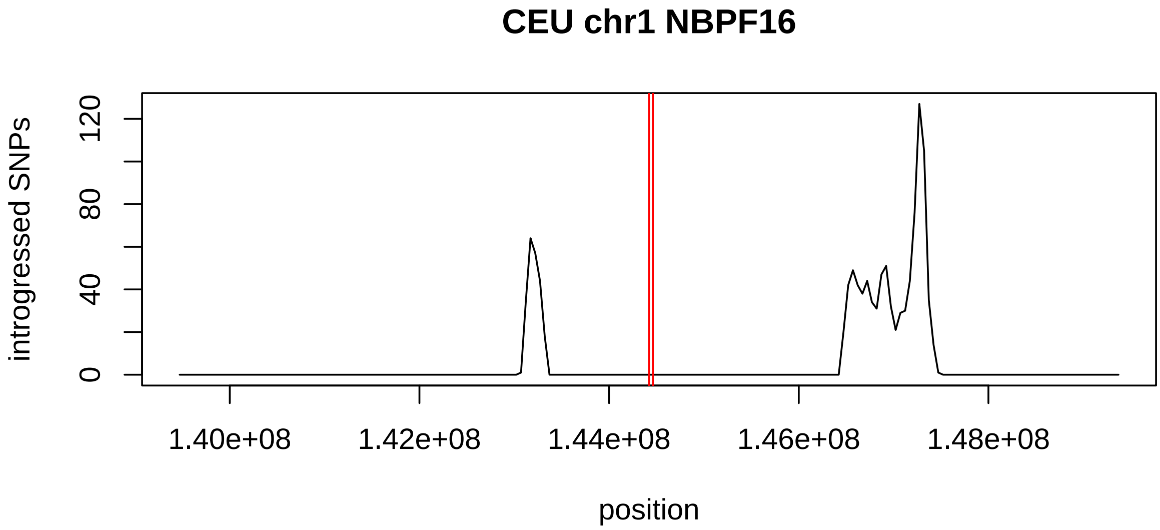
CEU chr1 NBPF16
introgressed SNPs
0
40
80
120
1.40e+08
1.42e+08
1.44e+08
1.46e+08
1.48e+08
position

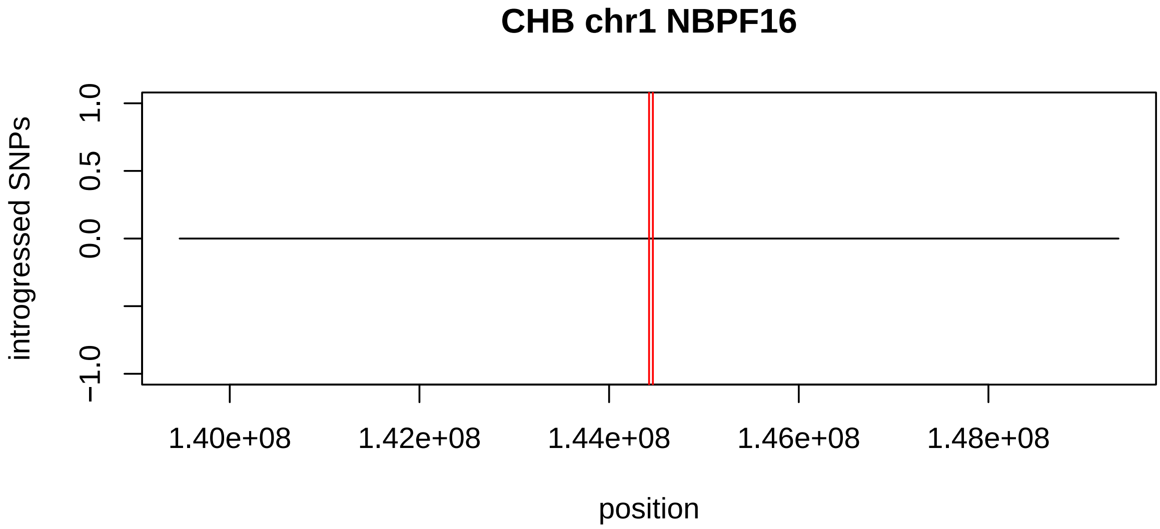
CHB chr1 NBPF16
introgressed SNPs
−1.0
0.0
0.5
1.0
1.40e+08
1.42e+08
1.44e+08
1.46e+08
1.48e+08
position

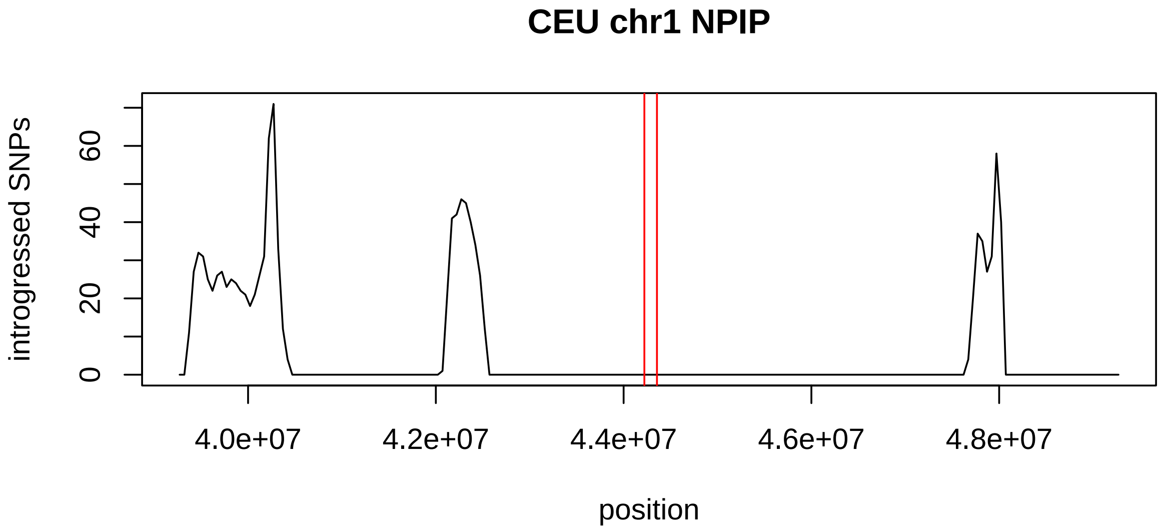
CEU chr1 NPIP
introgressed SNPs
0
20
40
60
4.0e+07
4.2e+07
4.4e+07
4.6e+07
4.8e+07
position

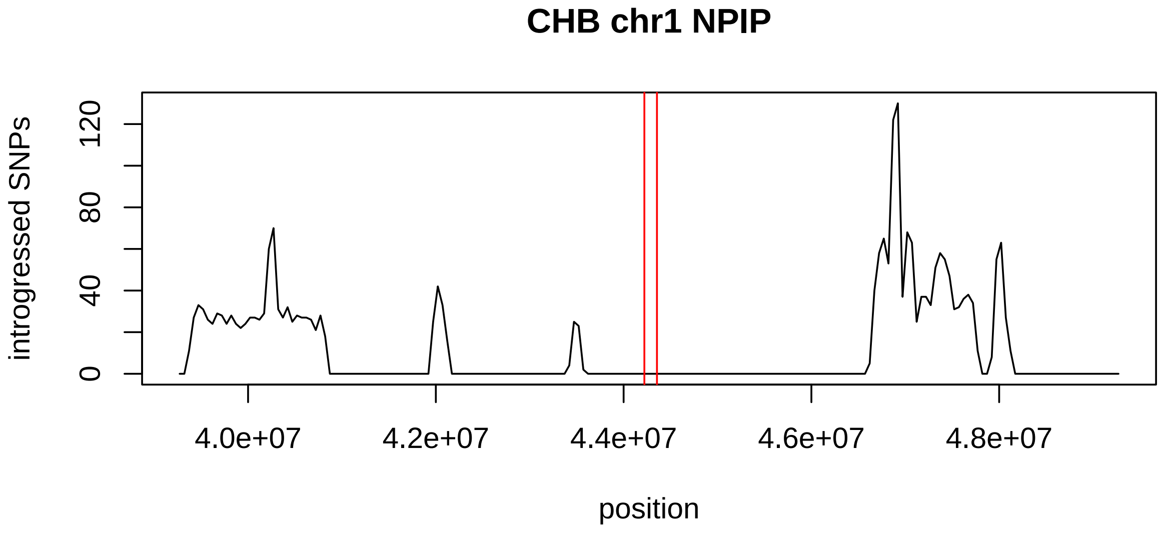
CHB chr1 NPIP
introgressed SNPs
0
40
80
120
4.0e+07
4.2e+07
4.4e+07
4.6e+07
4.8e+07
position

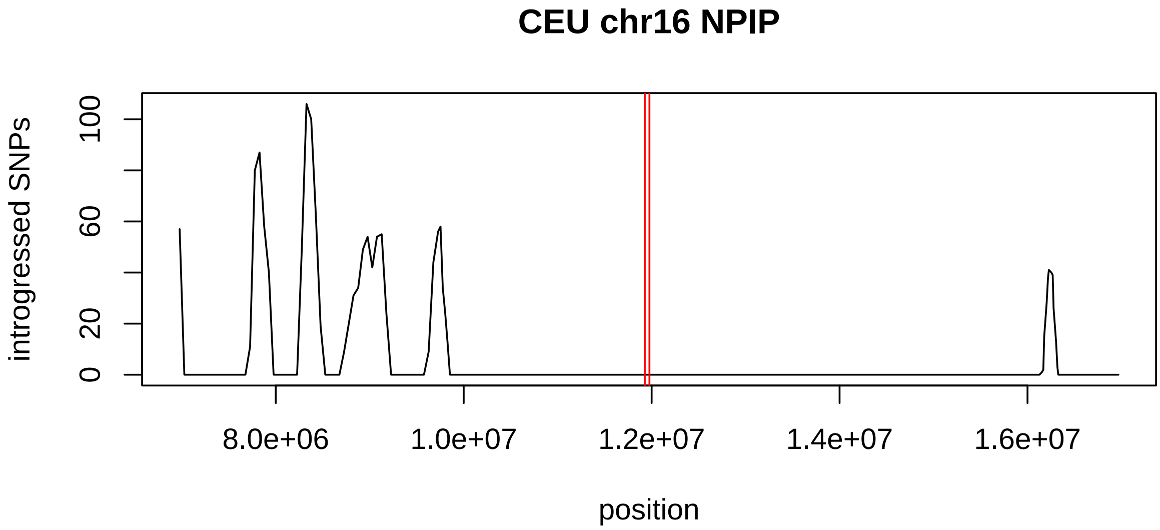
CEU chr16 NPIP
introgressed SNPs
0
20
60
100
8.0e+06
1.0e+07
1.2e+07
1.4e+07
1.6e+07
position

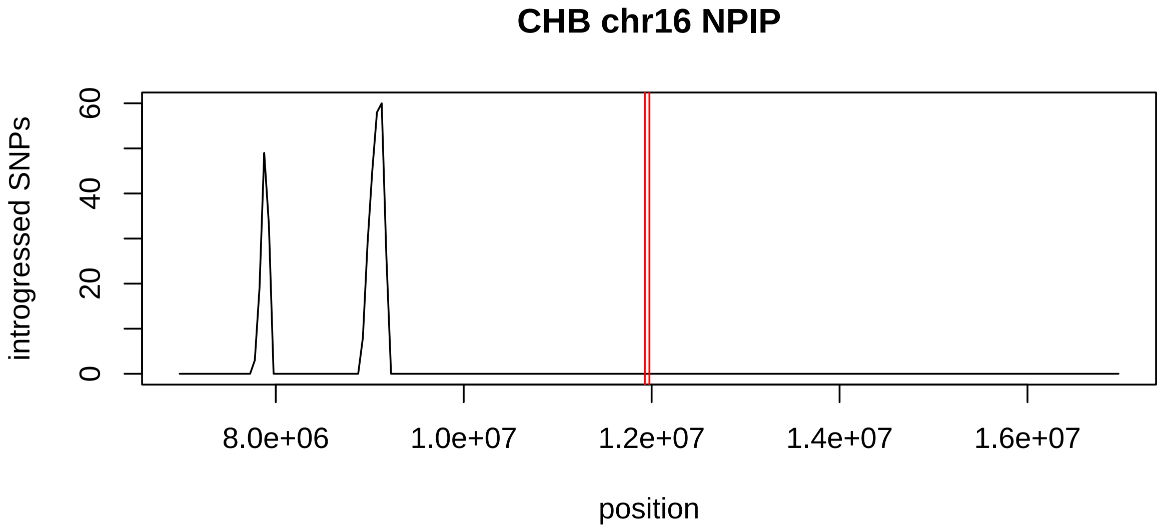
CHB chr16 NPIP
introgressed SNPs
0
20
40
60
8.0e+06
1.0e+07
1.2e+07
1.4e+07
1.6e+07
position

CEU chr16 NPIP

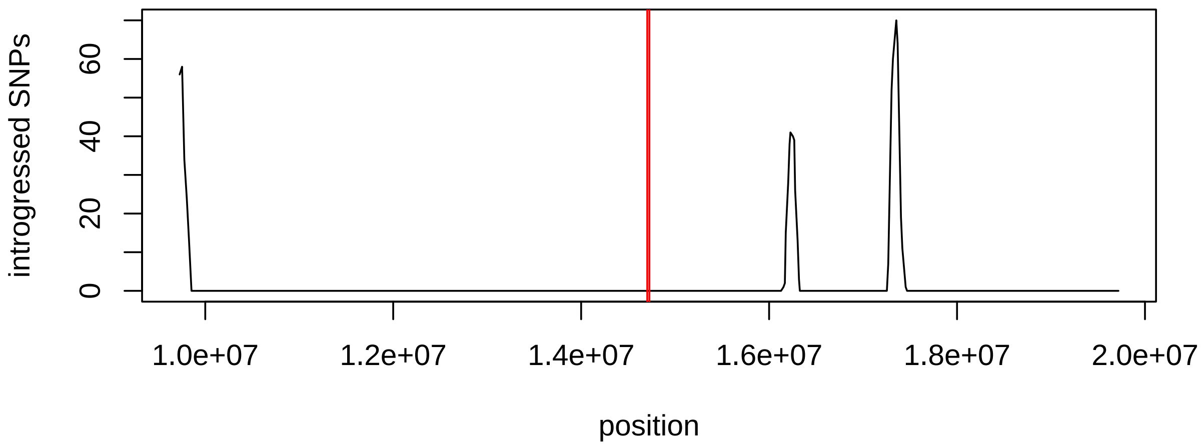

CHB chr16 NPIP

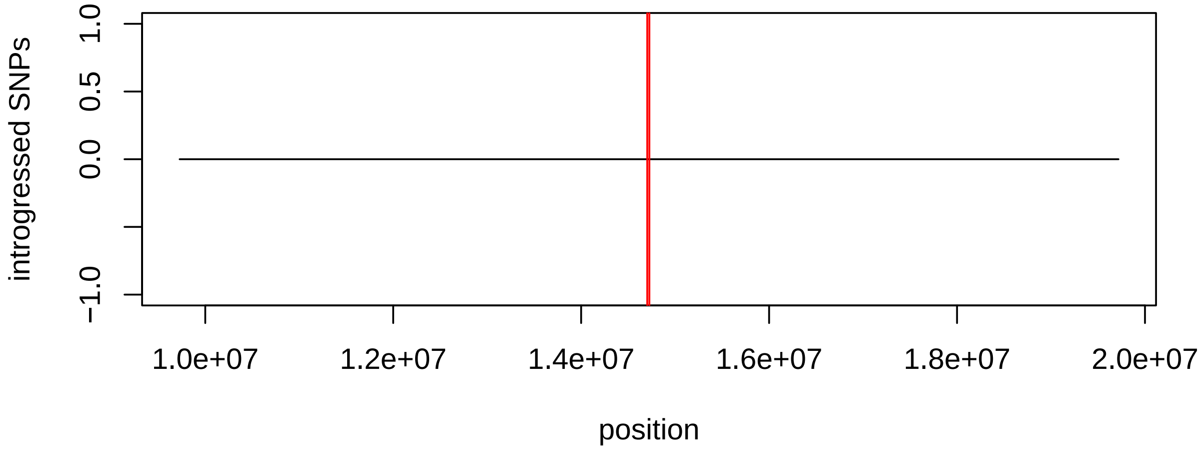

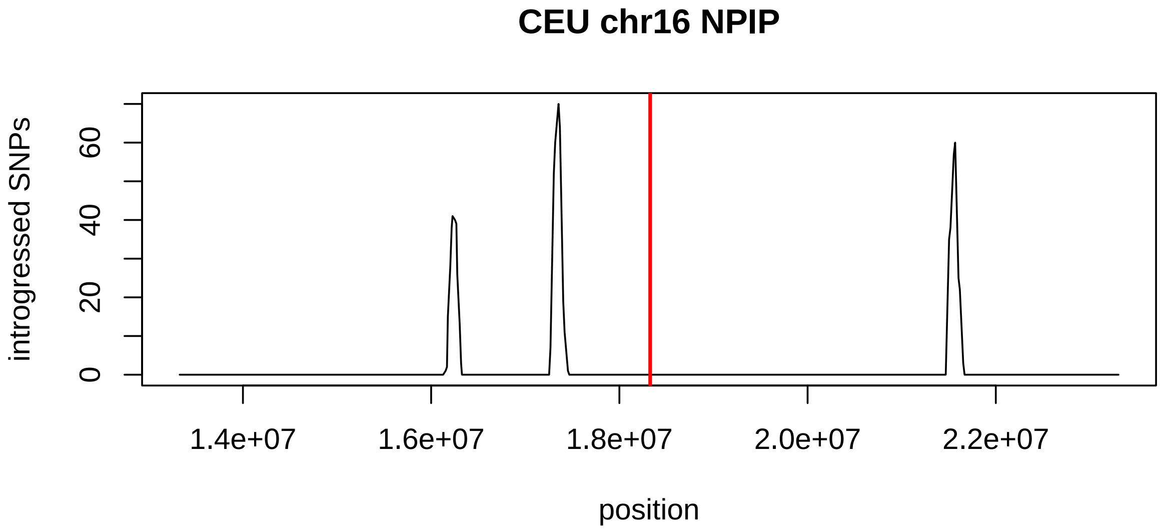
CEU chr16 NPIP
introgressed SNPs
0
20
40
60
1.4e+07
1.6e+07
1.8e+07
2.0e+07
2.2e+07
position

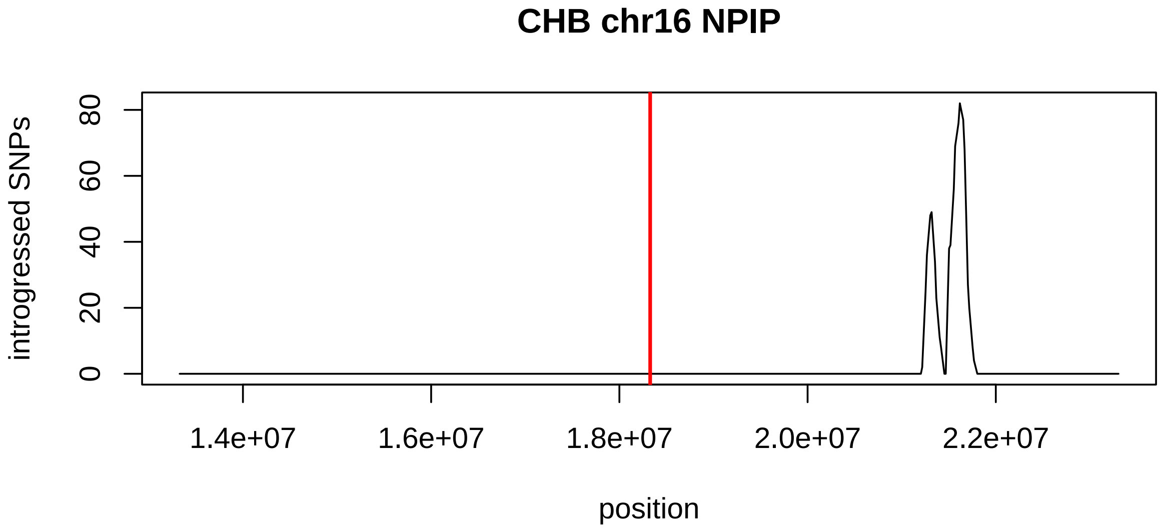
CHB chr16 NPIP
introgressed SNPs
0
20
40
60
80
1.4e+07
1.6e+07
1.8e+07
2.0e+07
2.2e+07
position

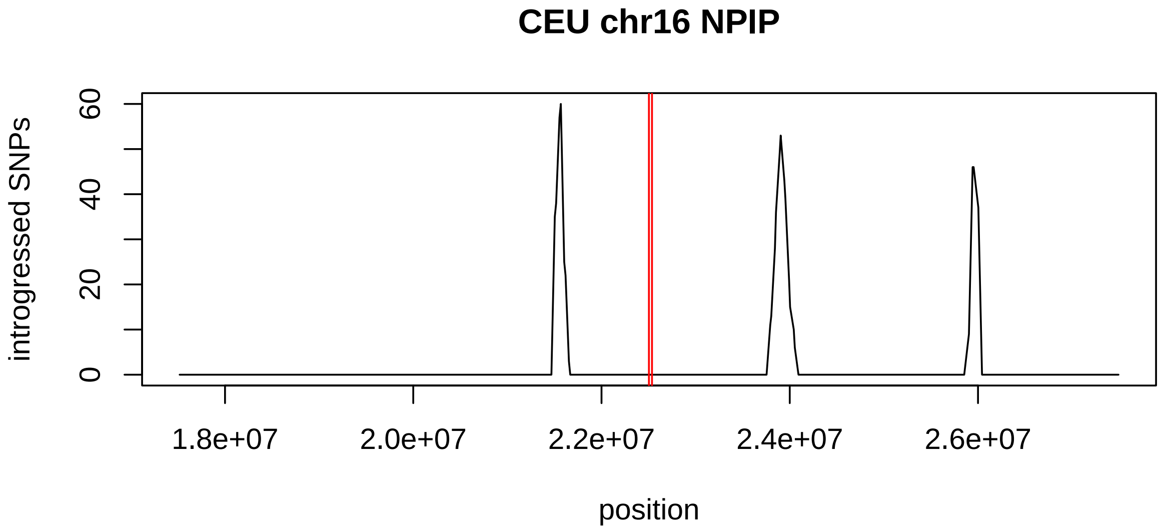
CEU chr16 NPIP
introgressed SNPs
0
20
40
60
1.8e+07
2.0e+07
2.2e+07
2.4e+07
2.6e+07
position

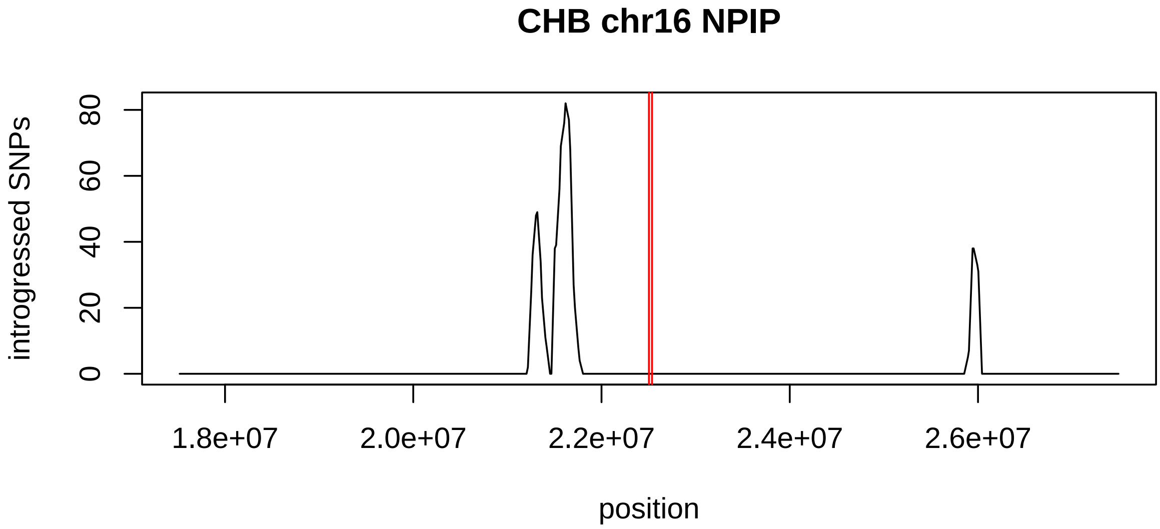
CHB chr16 NPIP
introgressed SNPs
0
20
40
60
80
1.8e+07
2.0e+07
2.2e+07
2.4e+07
2.6e+07
position

CEU chr16 NPIP

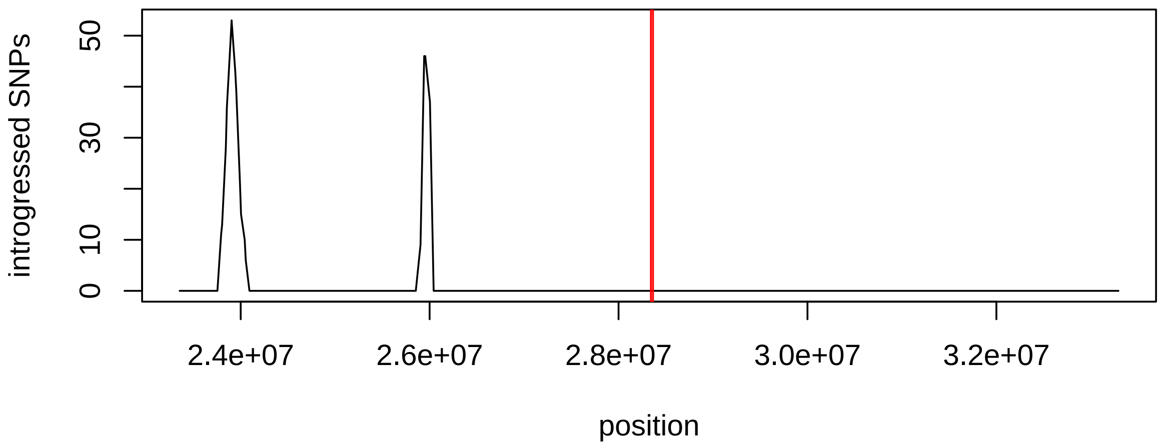

CHB chr16 NPIP

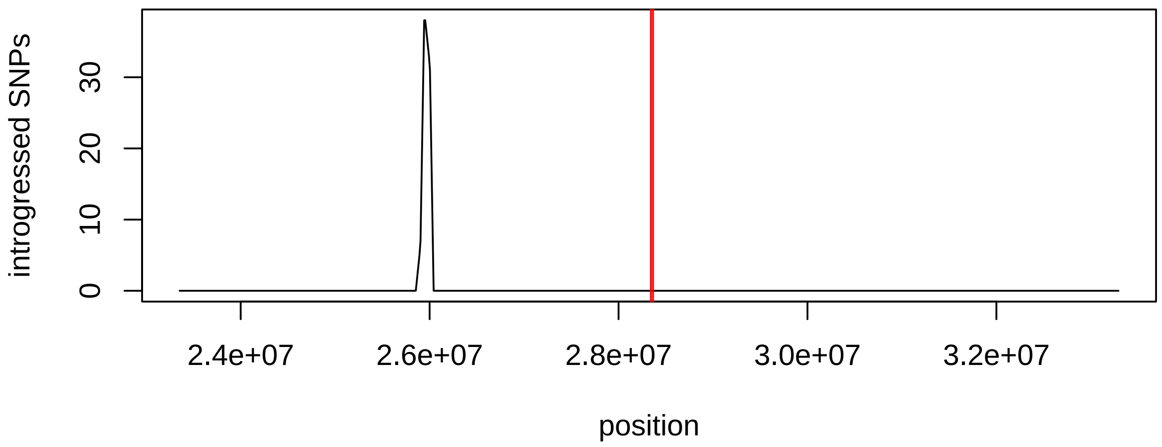

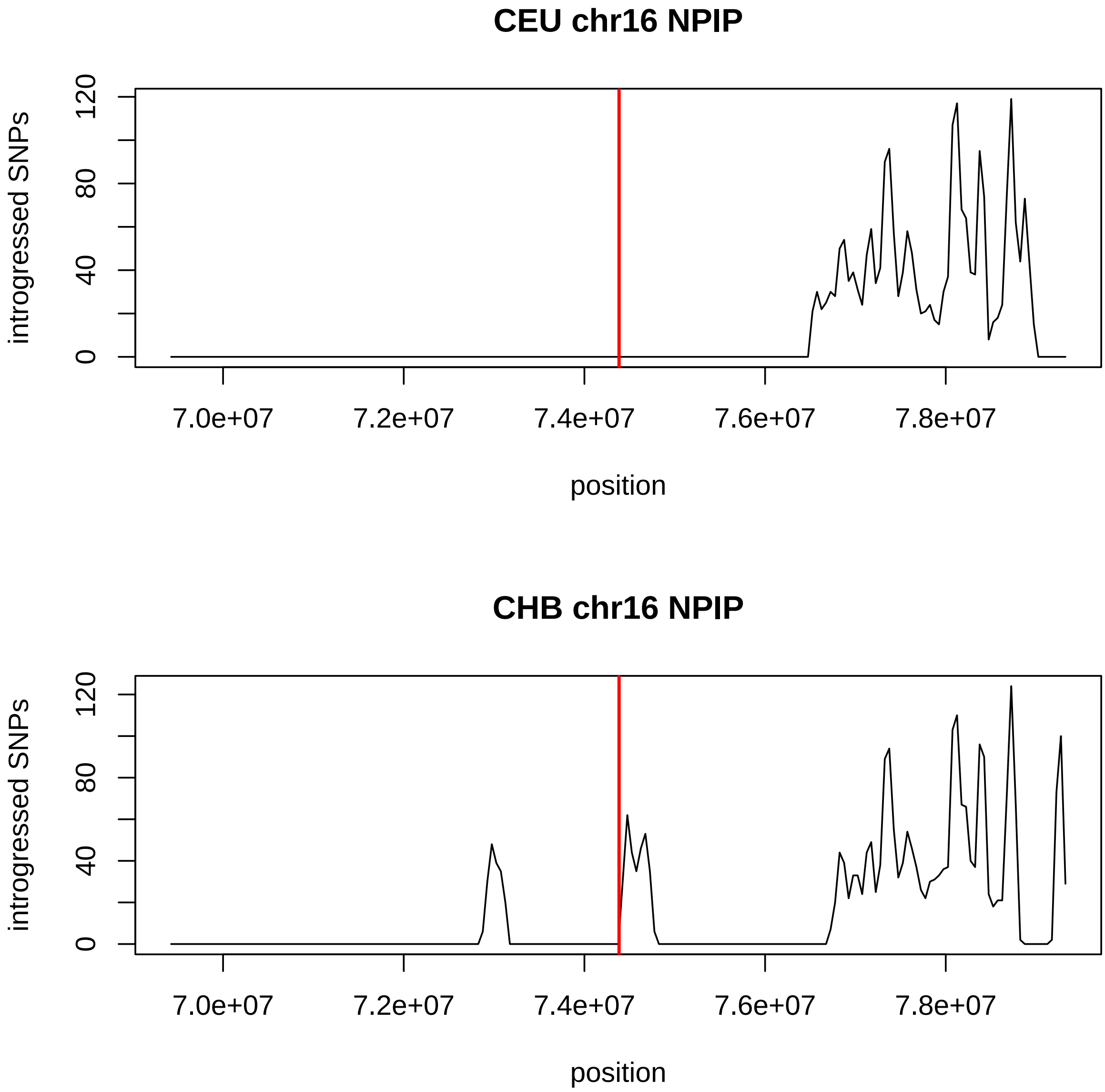
CEU chr16 NPIP
introgressed SNPs
0
40
80
120
7.0e+07
7.2e+07
7.4e+07
7.6e+07
7.8e+07
position
CHB chr16 NPIP
introgressed SNPs
0
40
80
120
7.0e+07
7.2e+07
7.4e+07
7.6e+07
7.8e+07
position

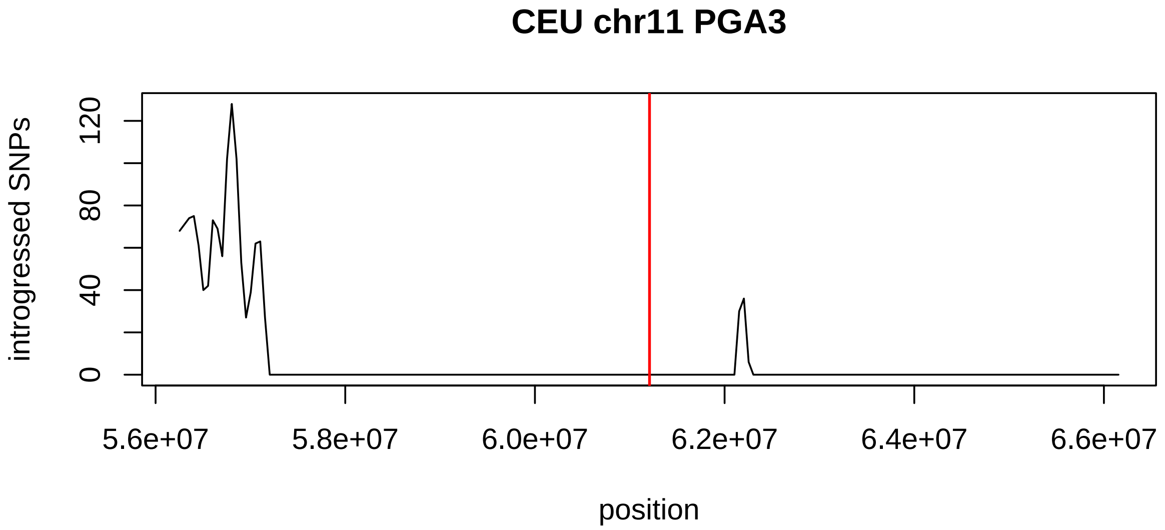
CEU chr11 PGA3
introgressed SNPs
0
40
80
120
5.6e+07
5.8e+07
6.0e+07
6.2e+07
6.4e+07
6.6e+07
position

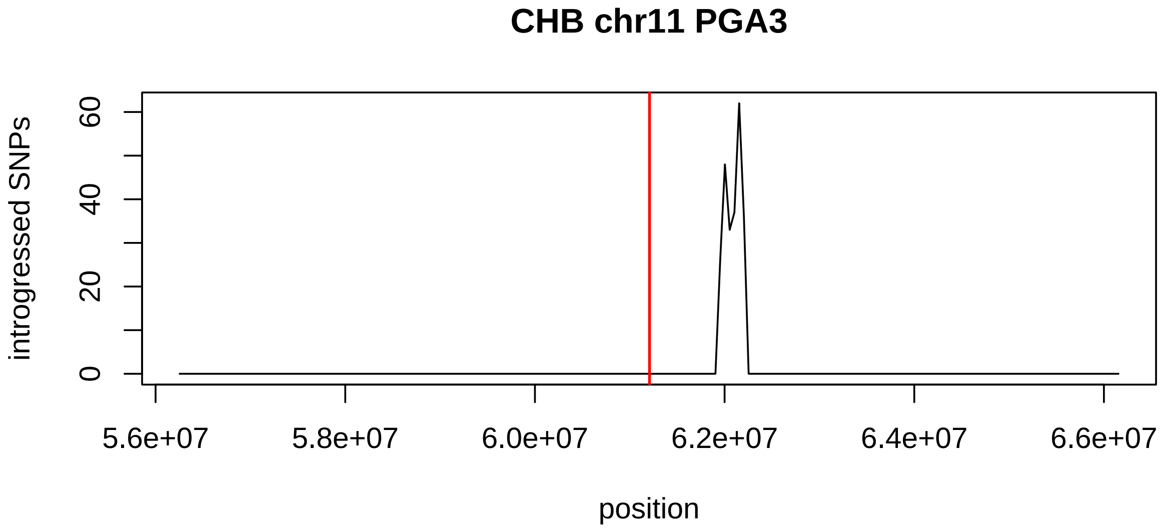
CHB chr11 PGA3
introgressed SNPs
0
20
40
60
5.6e+07
5.8e+07
6.0e+07
6.2e+07
6.4e+07
6.6e+07
position

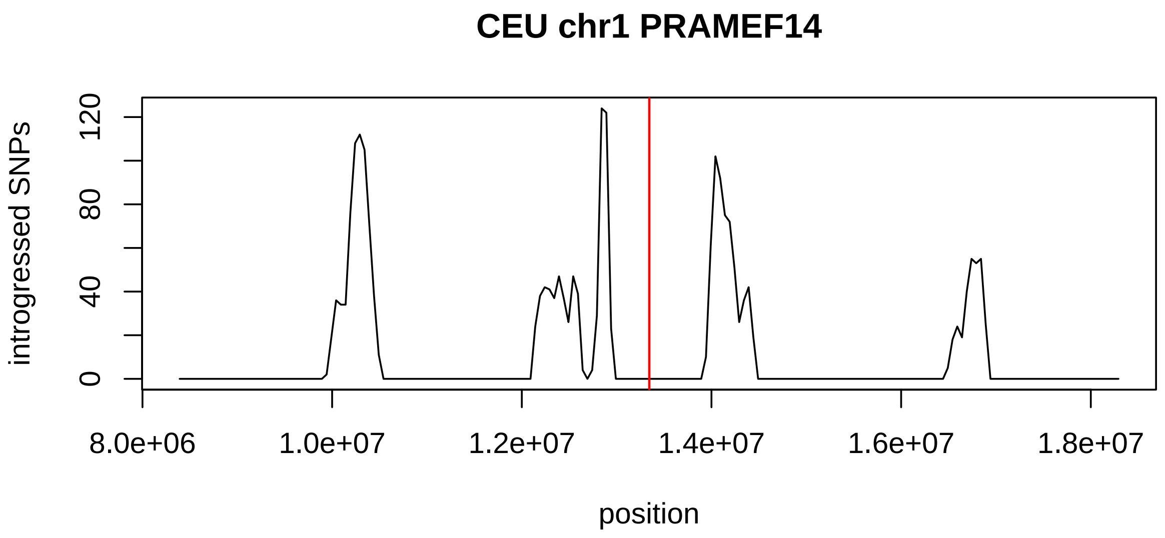
CEU chr1 PRAMEF14
introgressed SNPs
0
40
80
120
8.0e+06
1.0e+07
1.2e+07
1.4e+07
1.6e+07
1.8e+07
position

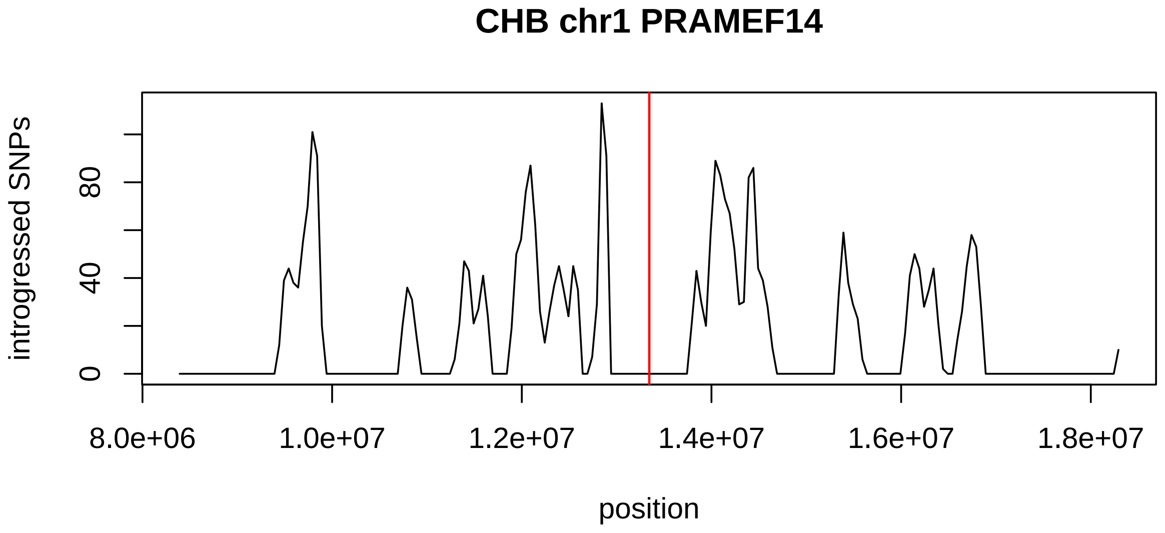
CHB chr1 PRAMEF14
introgressed SNPs
0
40
80
8.0e+06
1.0e+07
1.2e+07
1.4e+07
1.6e+07
1.8e+07
position

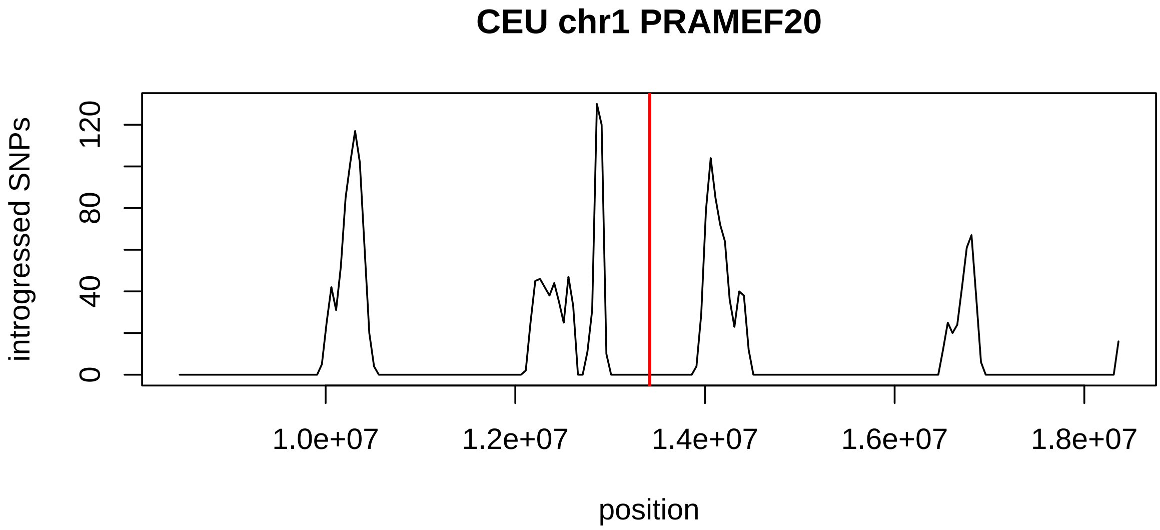
CEU chr1 PRAMEF20
introgressed SNPs
0
40
80
120
1.0e+07
1.2e+07
1.4e+07
1.6e+07
1.8e+07
position

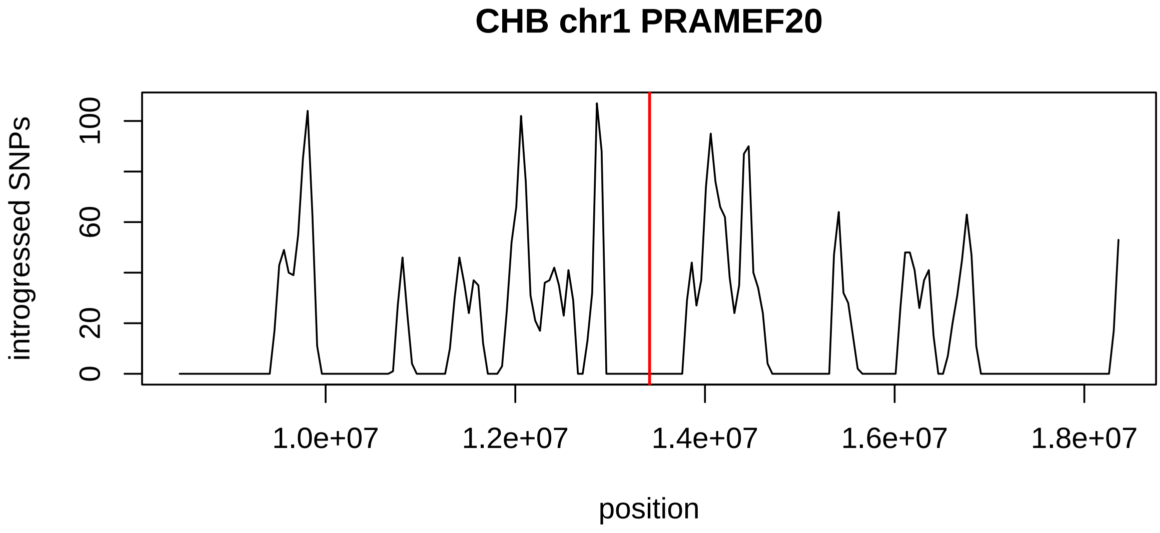
CHB chr1 PRAMEF20
introgressed SNPs
0
20
60
100
1.0e+07
1.2e+07
1.4e+07
1.6e+07
1.8e+07
position

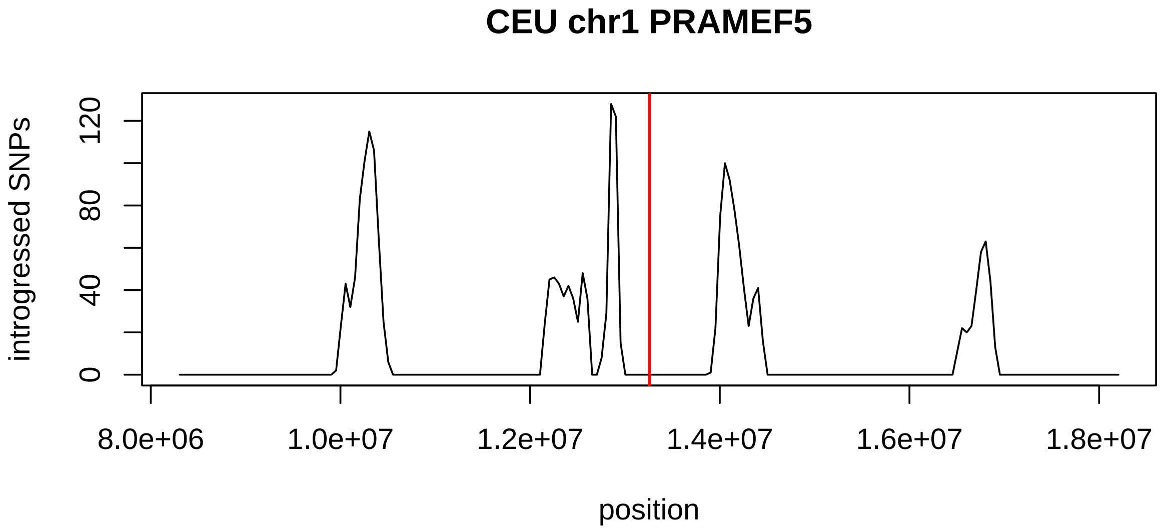
CEU chr1 PRAMEF5
introgressed SNPs
0
40
80
120
8.0e+06
1.0e+07
1.2e+07
1.4e+07
1.6e+07
1.8e+07
position

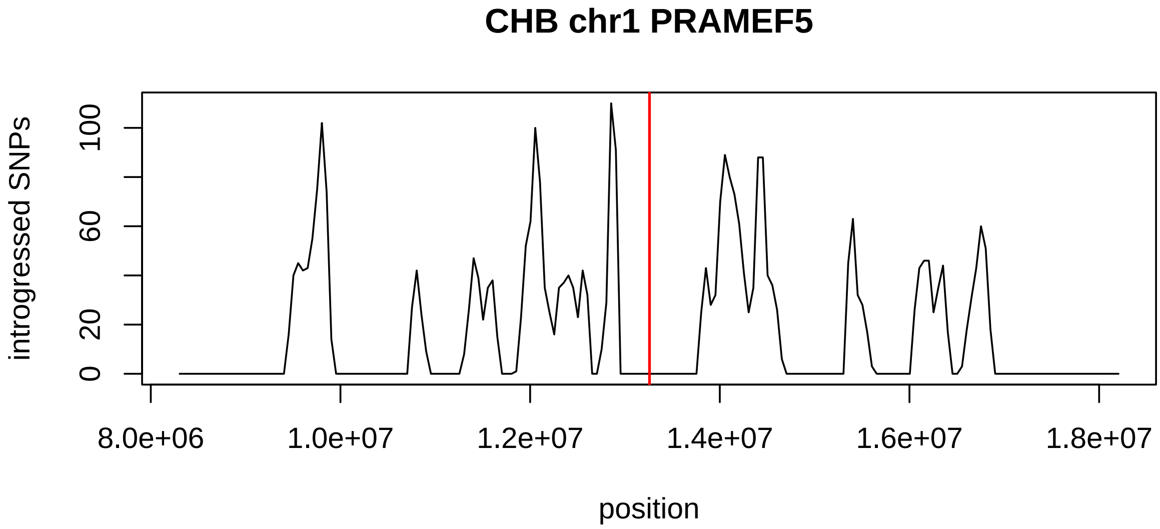
CHB chr1 PRAMEF5
introgressed SNPs
0
20
60
100
8.0e+06
1.0e+07
1.2e+07
1.4e+07
1.6e+07
1.8e+07
position

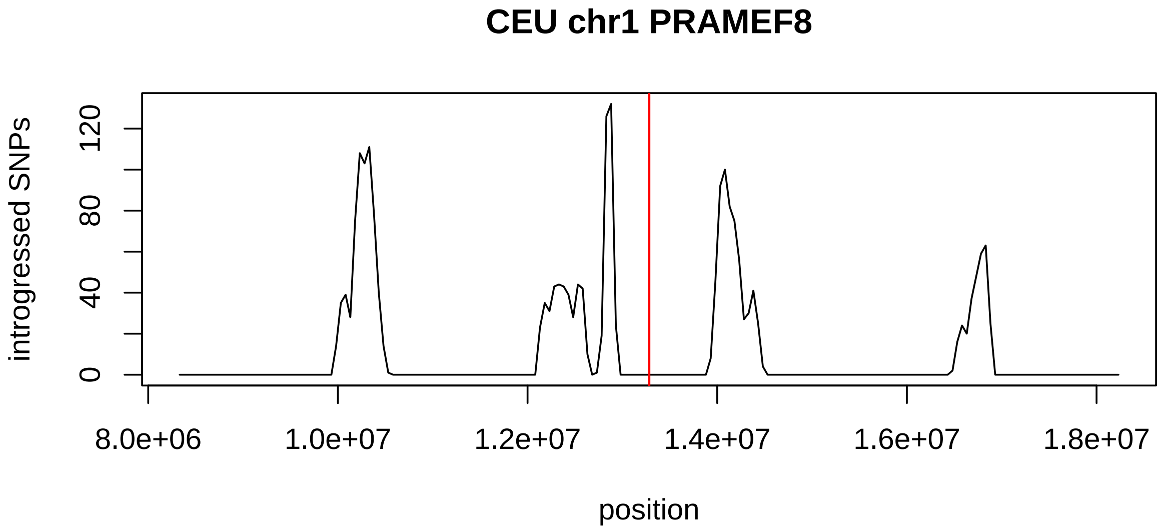
CEU chr1 PRAMEF8
introgressed SNPs
0
40
80
120
8.0e+06
1.0e+07
1.2e+07
1.4e+07
1.6e+07
1.8e+07
position

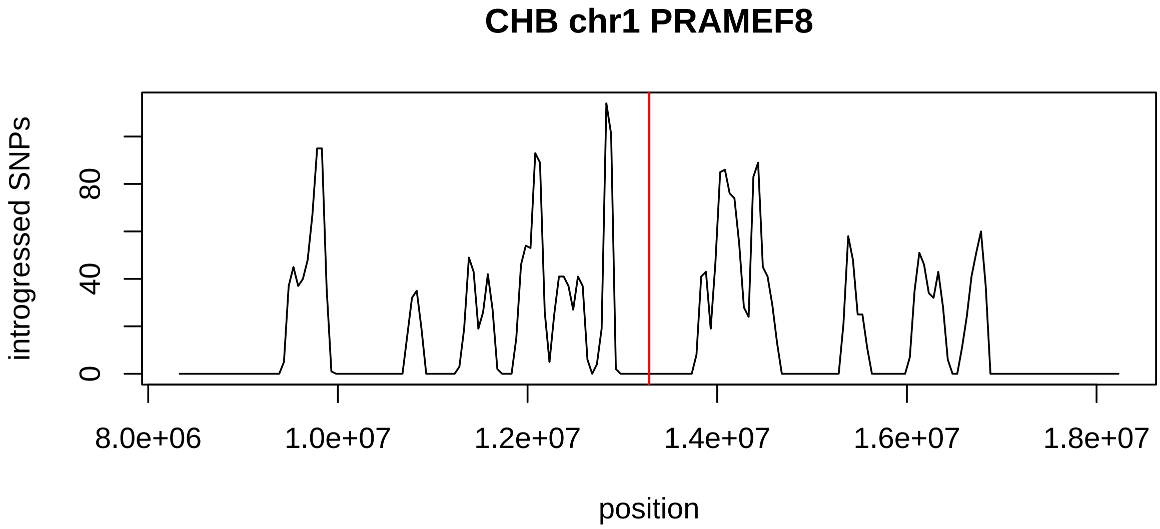
CHB chr1 PRAMEF8
introgressed SNPs
0
40
80
8.0e+06
1.0e+07
1.2e+07
1.4e+07
1.6e+07
1.8e+07
position

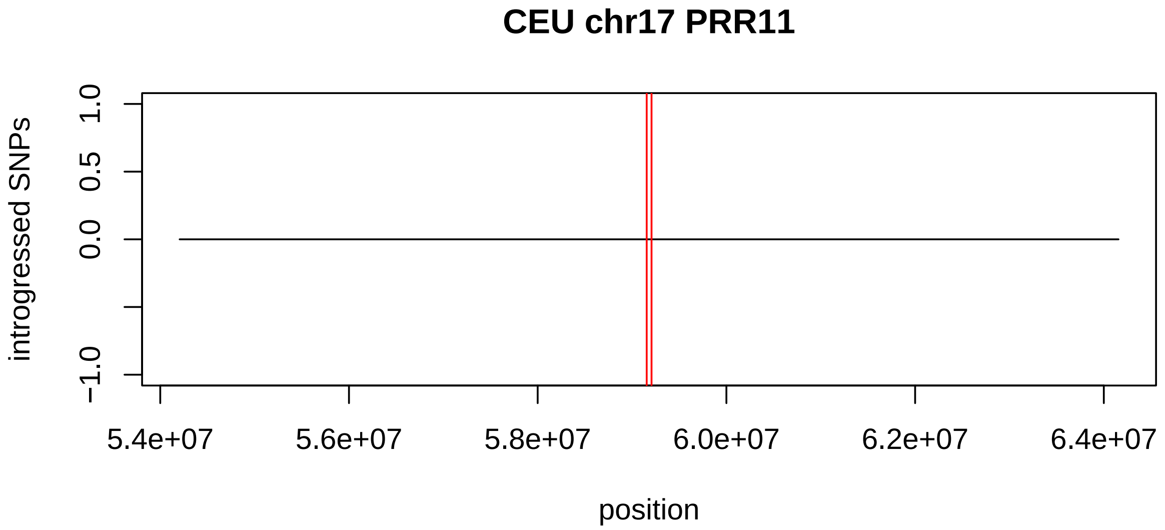
CEU chr17 PRR11
introgressed SNPs
1.0
0.5
0.0
−1.0
5.4e+07
5.6e+07
5.8e+07
6.0e+07
6.2e+07
6.4e+07
position

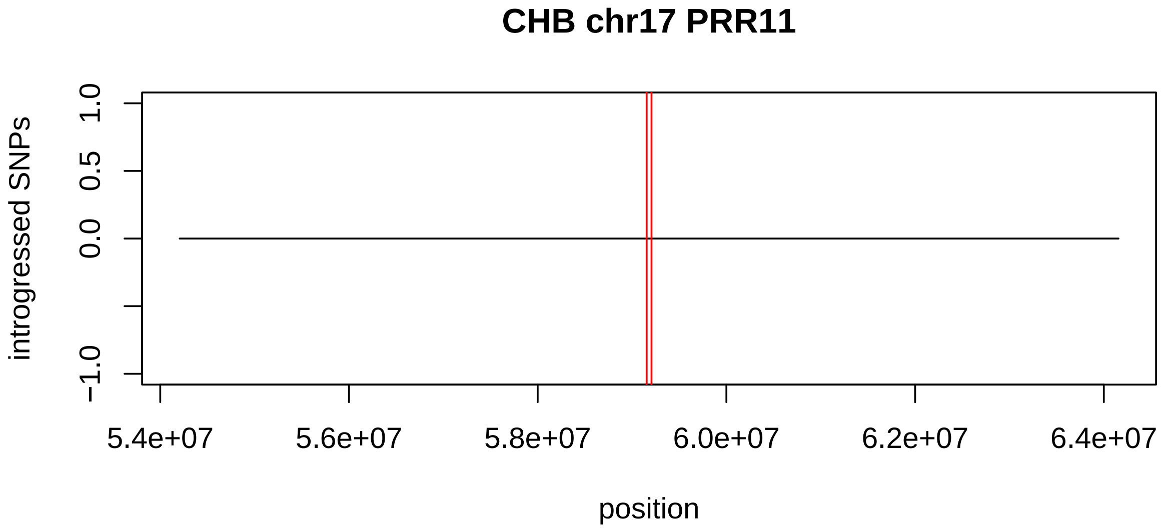
CHB chr17 PRR11
introgressed SNPs
1.0
0.5
0.0
−1.0
5.4e+07
5.6e+07
5.8e+07
6.0e+07
6.2e+07
6.4e+07
position

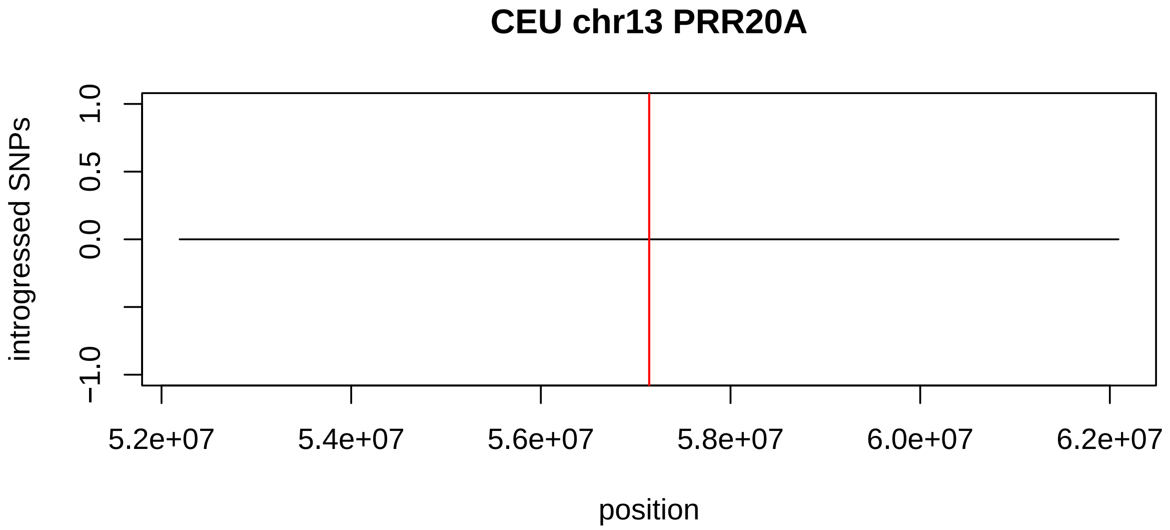
CEU chr13 PRR20A
introgressed SNPs
-1.0
0.0
0.5
1.0
5.2e+07
5.4e+07
5.6e+07
5.8e+07
6.0e+07
6.2e+07
position

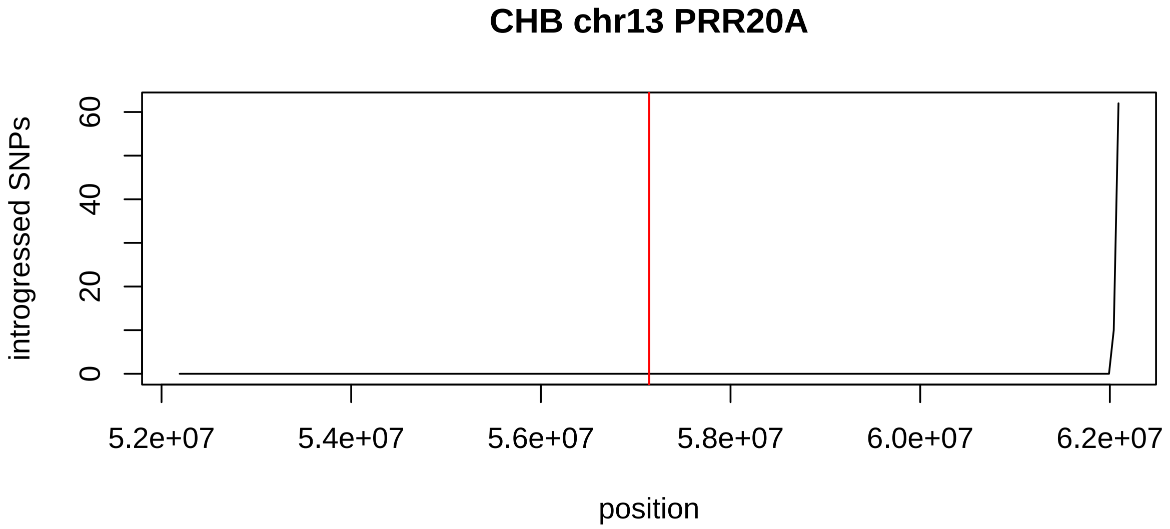
CHB chr13 PRR20A
introgressed SNPs
0
20
40
60
5.2e+07
5.4e+07
5.6e+07
5.8e+07
6.0e+07
6.2e+07
position

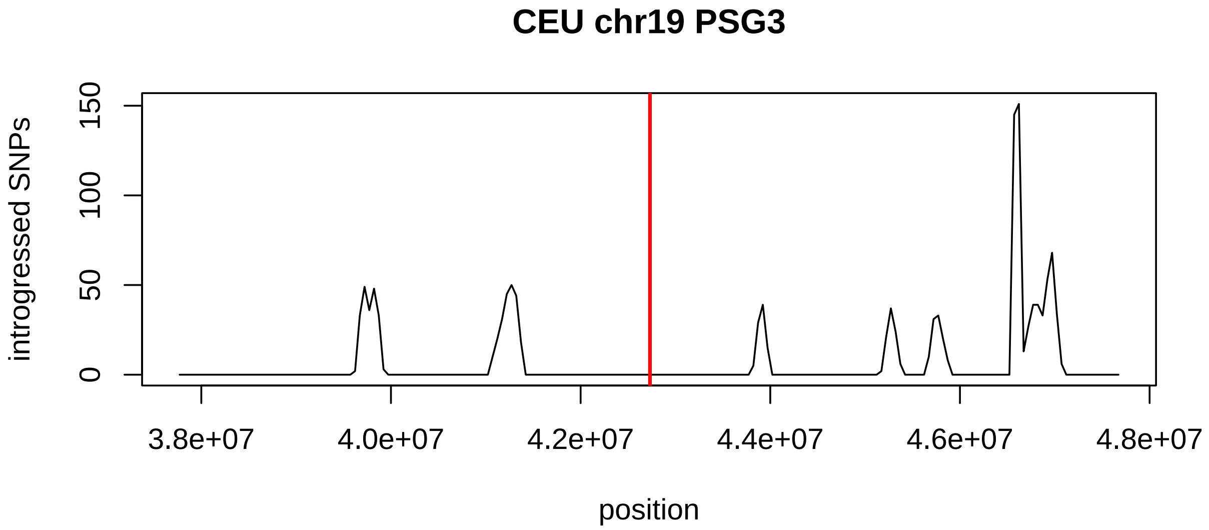
CEU chr19 PSG3
introgressed SNPs
0
50
100
150
3.8e+07
4.0e+07
4.2e+07
4.4e+07
4.6e+07
4.8e+07
position

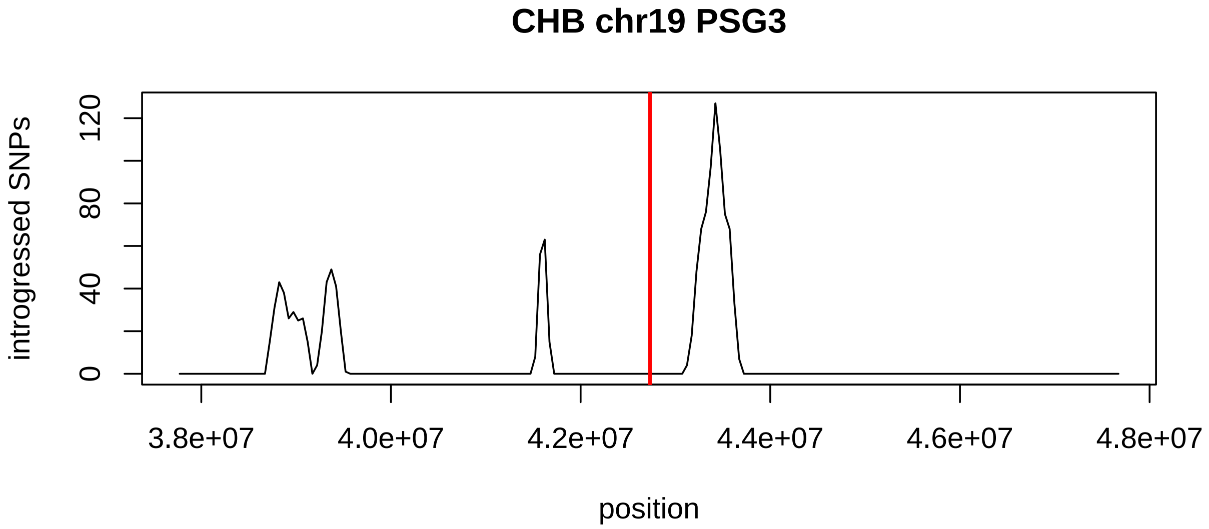
CHB chr19 PSG3
introgressed SNPs
0
40
80
120
3.8e+07
4.0e+07
4.2e+07
4.4e+07
4.6e+07
4.8e+07
position

CEU chr2 RGPD1

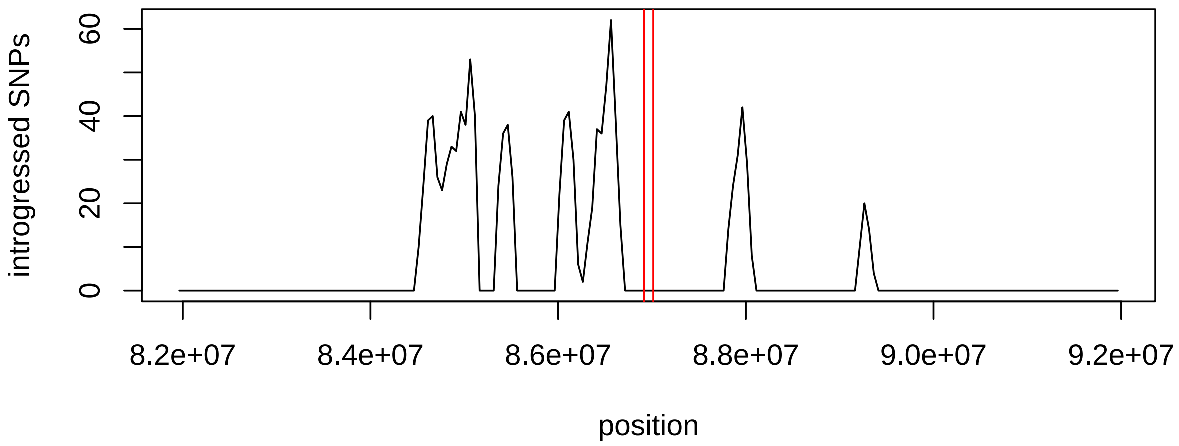

CHB chr2 RGPD1

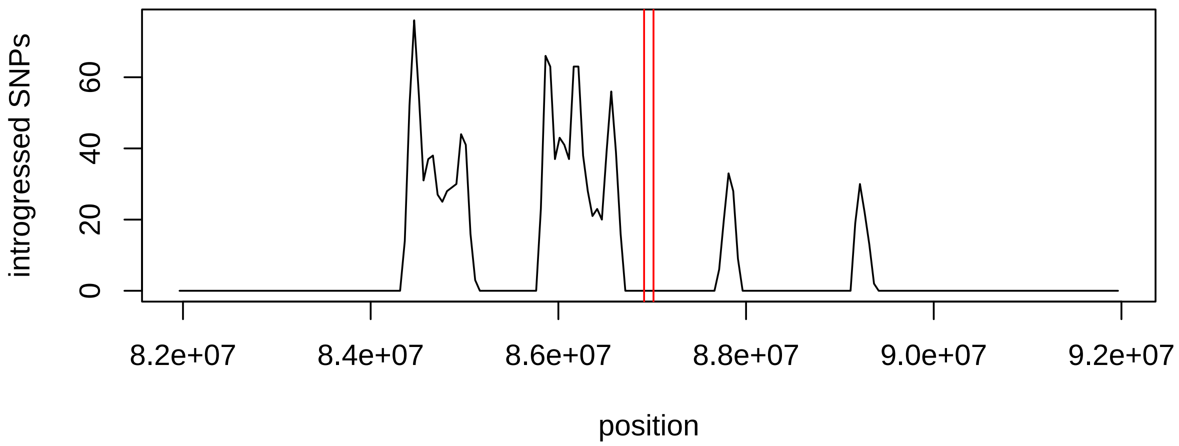

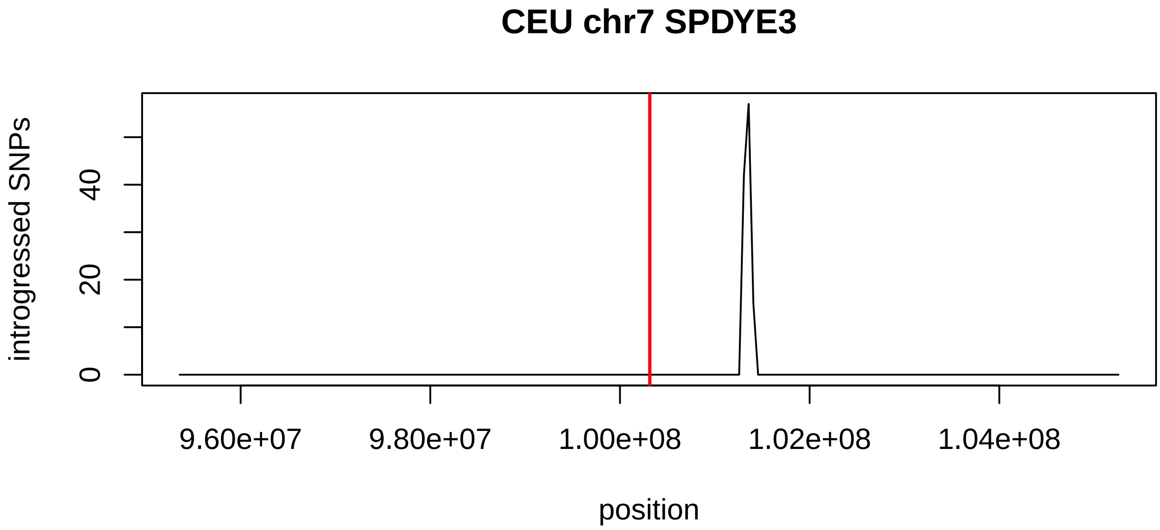
CEU chr7 SPDYE3
introgressed SNPs
0
20
40
9.60e+07
9.80e+07
1.00e+08
1.02e+08
1.04e+08
position

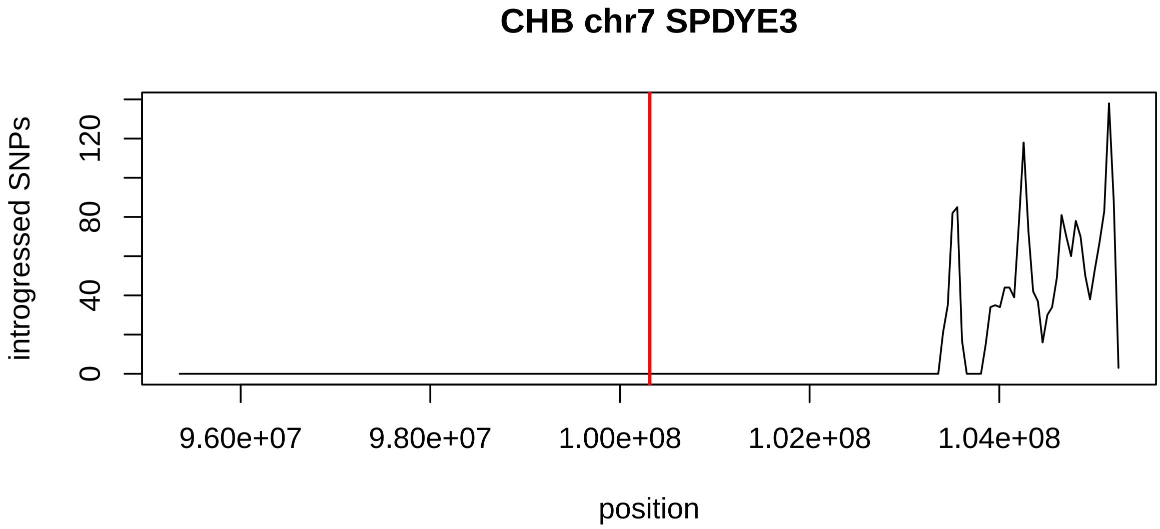
CHB chr7 SPDYE3
introgressed SNPs
0
40
80
120
9.60e+07
9.80e+07
1.00e+08
1.02e+08
1.04e+08
position

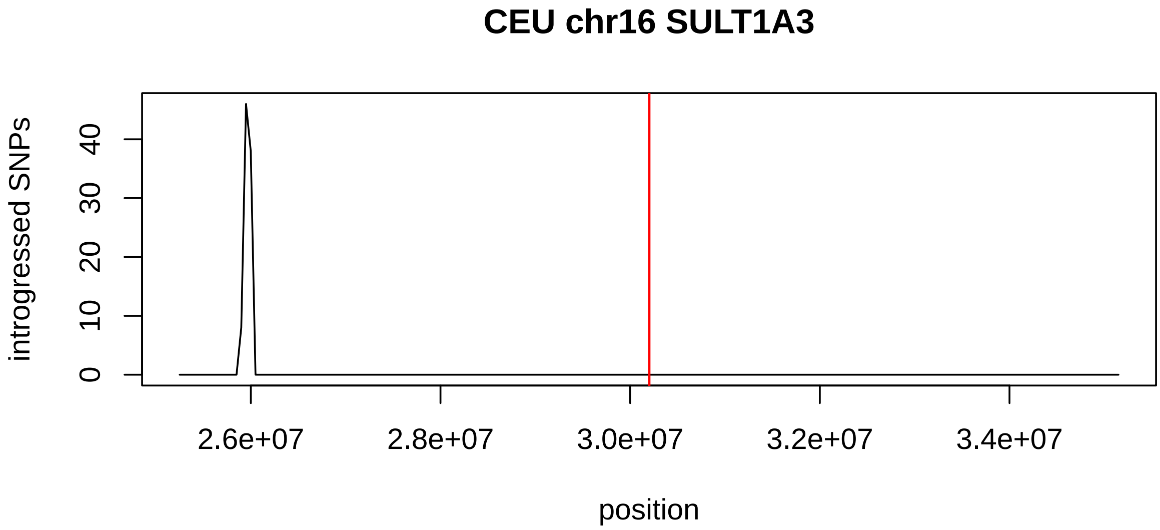
CEU chr16 SULT1A3
introgressed SNPs
0
10
20
30
40
2.6e+07
2.8e+07
3.0e+07
3.2e+07
3.4e+07
position

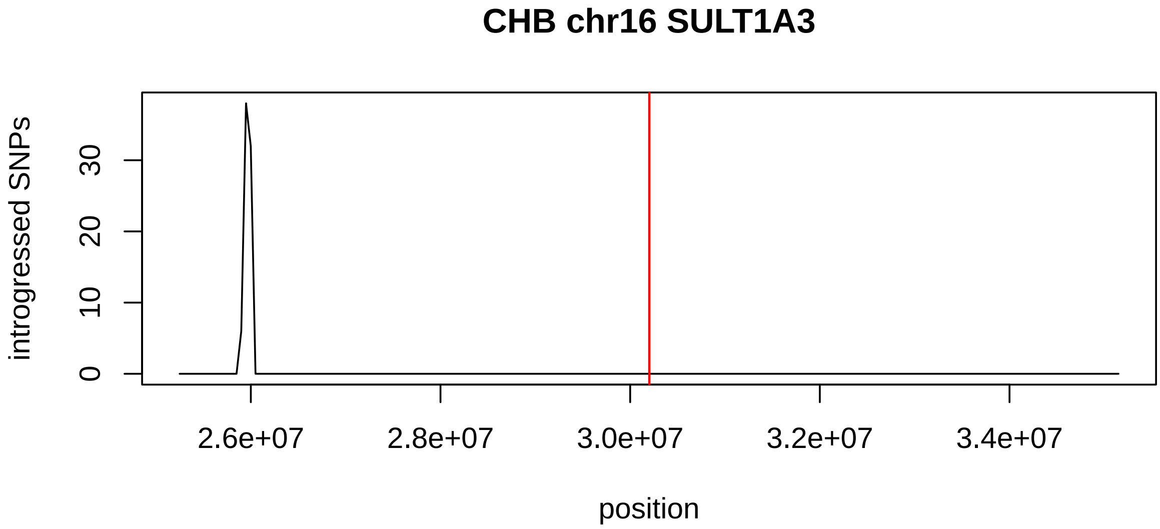
CHB chr16 SULT1A3
introgressed SNPs
0
10
20
30
2.6e+07
2.8e+07
3.0e+07
3.2e+07
3.4e+07
position

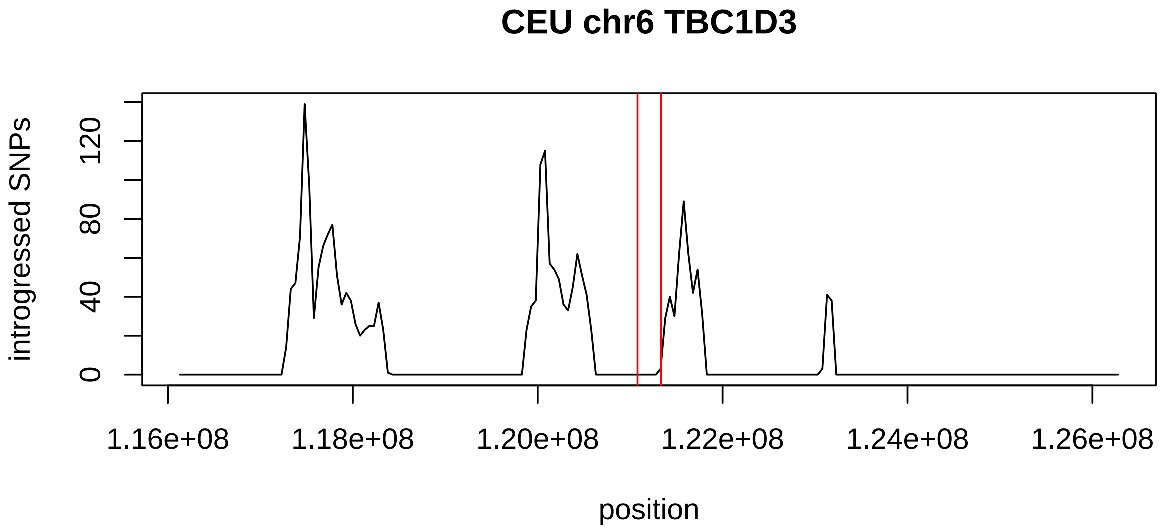
CEU chr6 TBC1D3
introgressed SNPs
0
40
80
120
1.16e+08
1.18e+08
1.20e+08
1.22e+08
1.24e+08
1.26e+08
position

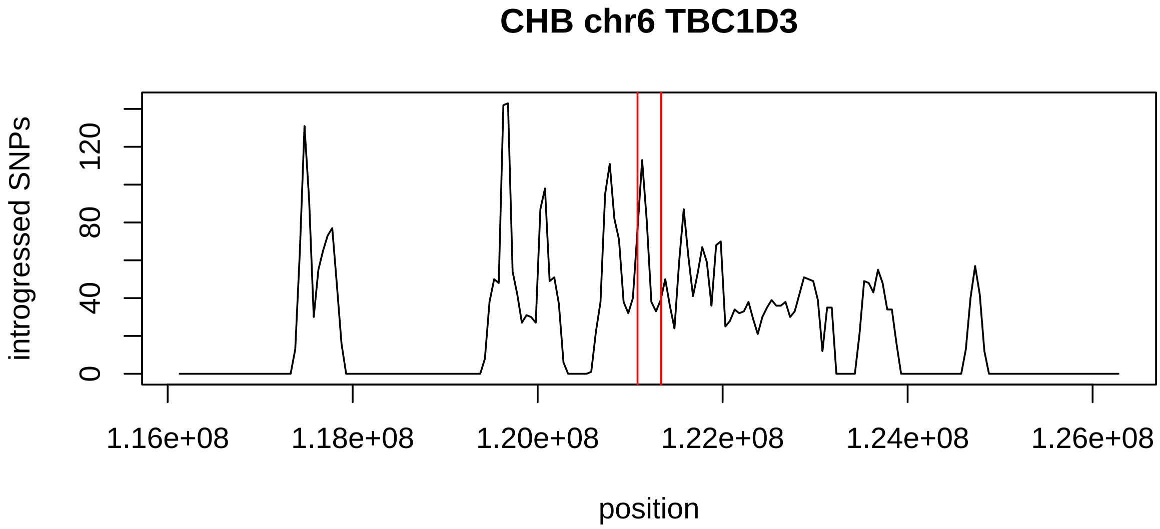
CHB chr6 TBC1D3
introgressed SNPs
0
40
80
120
1.16e+08
1.18e+08
1.20e+08
1.22e+08
1.24e+08
1.26e+08
position

CEU chr8 TBC1D3

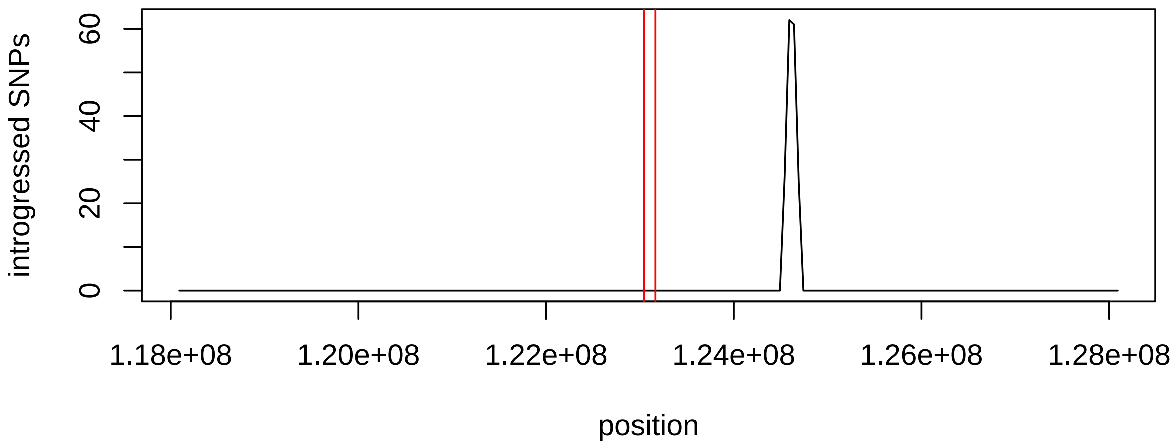

CHB chr8 TBC1D3

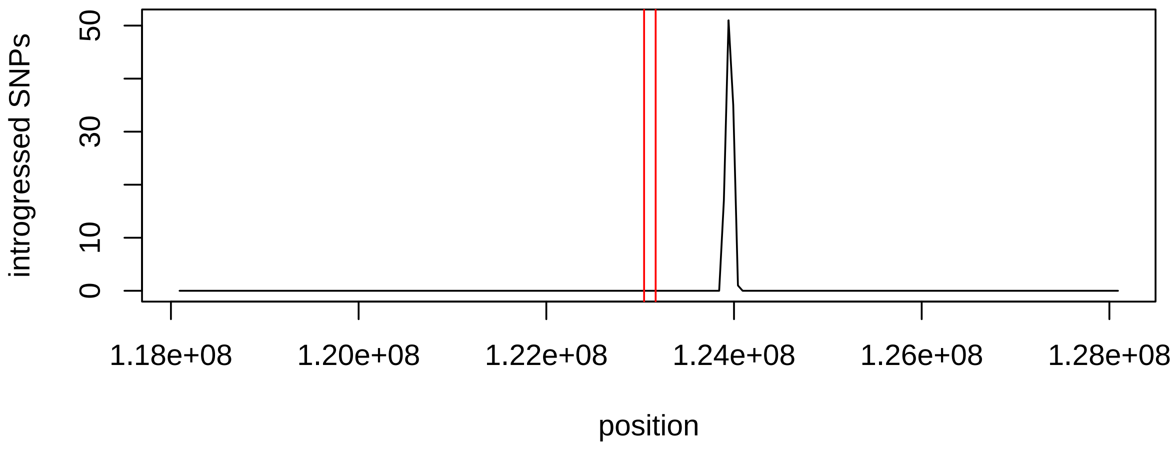

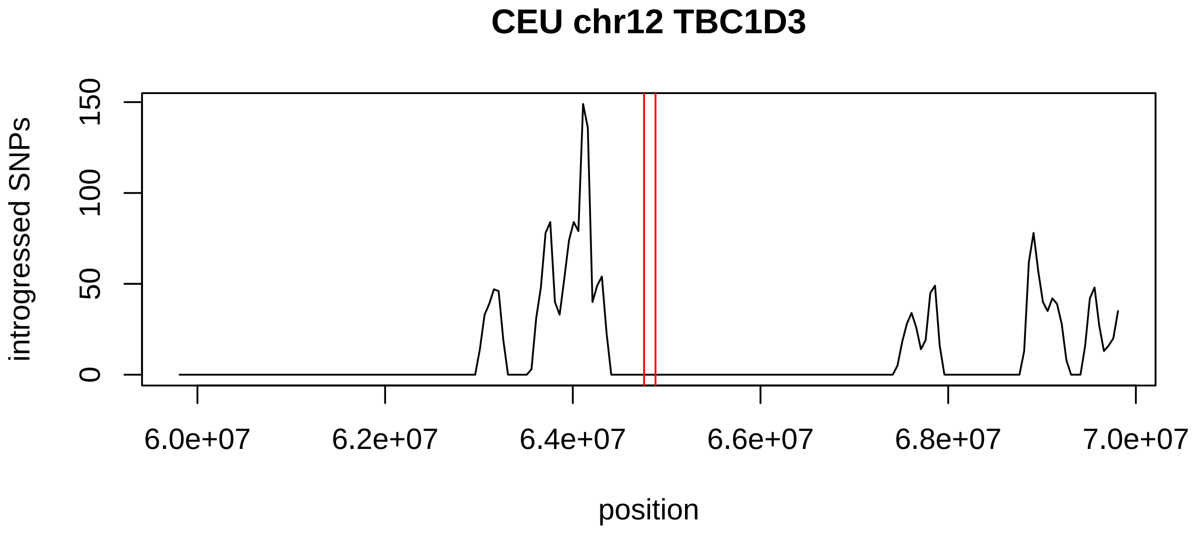
CEU chr12 TBC1D3
introgressed SNPs
0
50
100
150
6.0e+07
6.2e+07
6.4e+07
6.6e+07
6.8e+07
7.0e+07
position

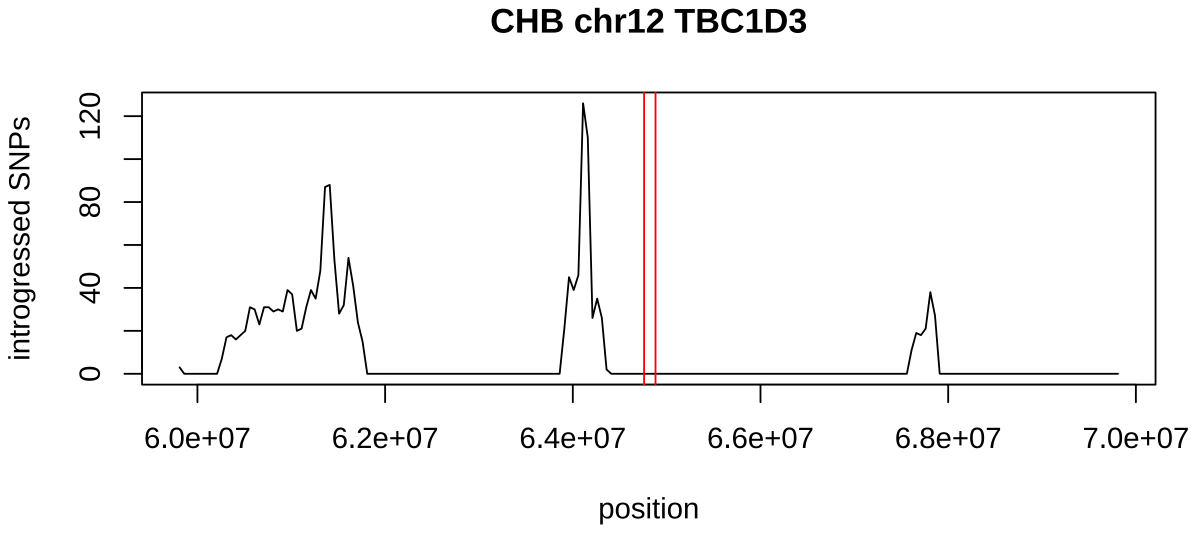
CHB chr12 TBC1D3
introgressed SNPs
0
40
80
120
6.0e+07
6.2e+07
6.4e+07
6.6e+07
6.8e+07
7.0e+07
position

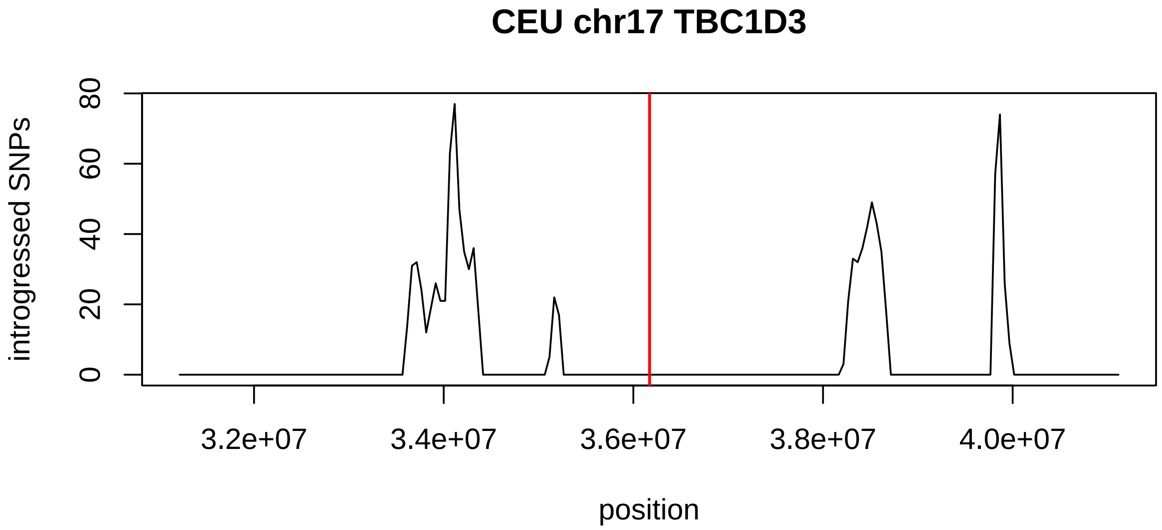
CEU chr17 TBC1D3
introgressed SNPs
0
20
40
60
80
3.2e+07
3.4e+07
3.6e+07
3.8e+07
4.0e+07
position

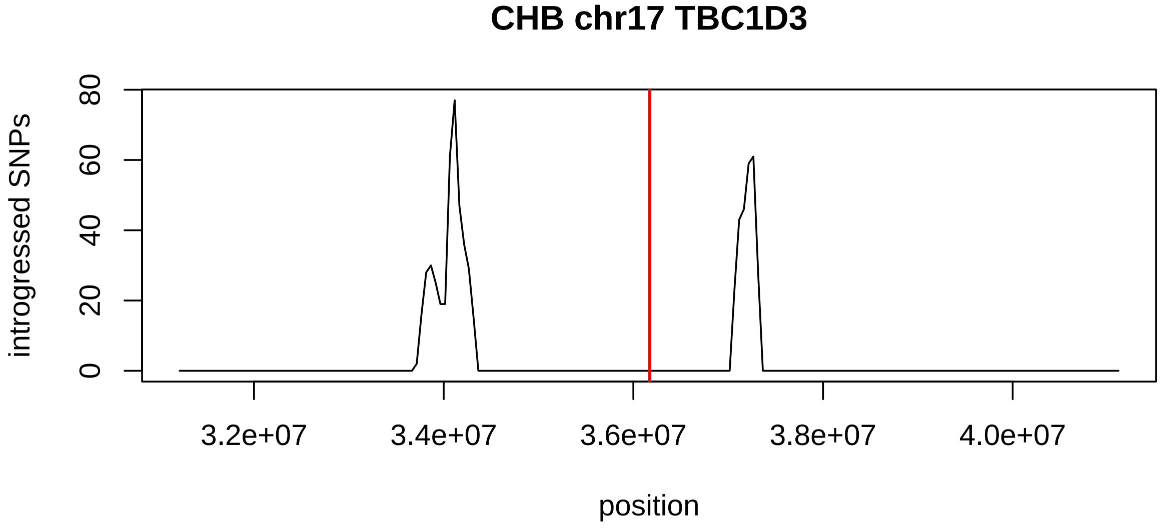
CHB chr17 TBC1D3
introgressed SNPs
0
20
40
60
80
3.2e+07
3.4e+07
3.6e+07
3.8e+07
4.0e+07
position

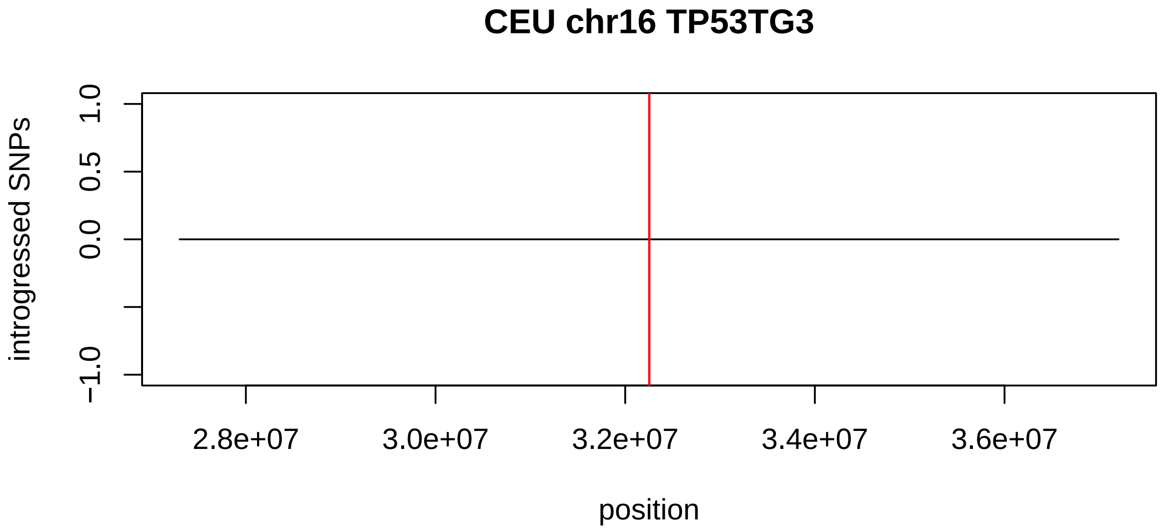
CEU chr16 TP53TG3
introgressed SNPs
−1.0
0.0
0.5
1.0
2.8e+07
3.0e+07
3.2e+07
3.4e+07
3.6e+07
position

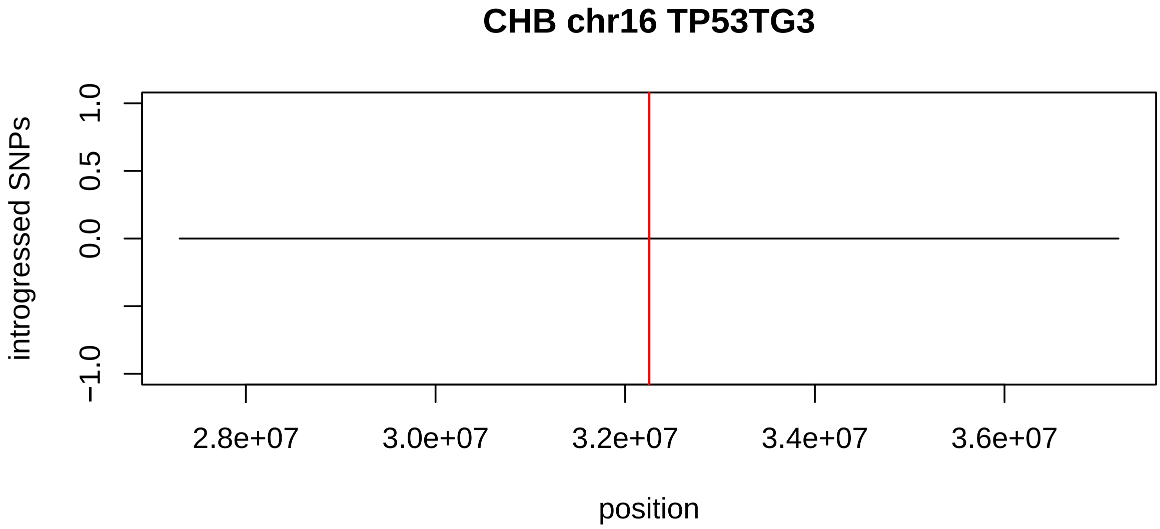
CHB chr16 TP53TG3
introgressed SNPs
−1.0
0.0
0.5
1.0
2.8e+07
3.0e+07
3.2e+07
3.4e+07
3.6e+07
position

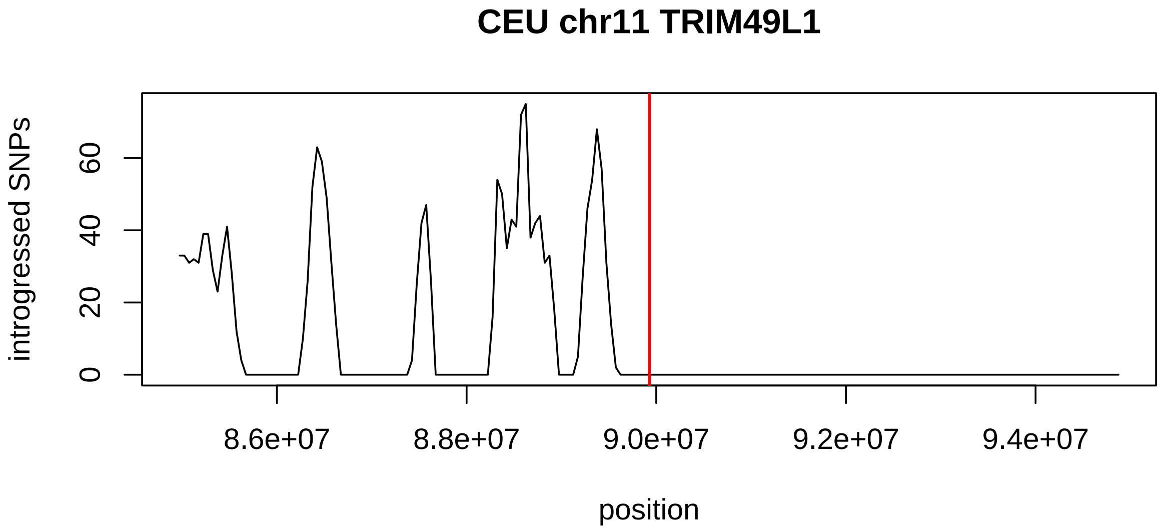
CEU chr11 TRIM49L1
introgressed SNPs
0
20
40
60
8.6e+07
8.8e+07
9.0e+07
9.2e+07
9.4e+07
position

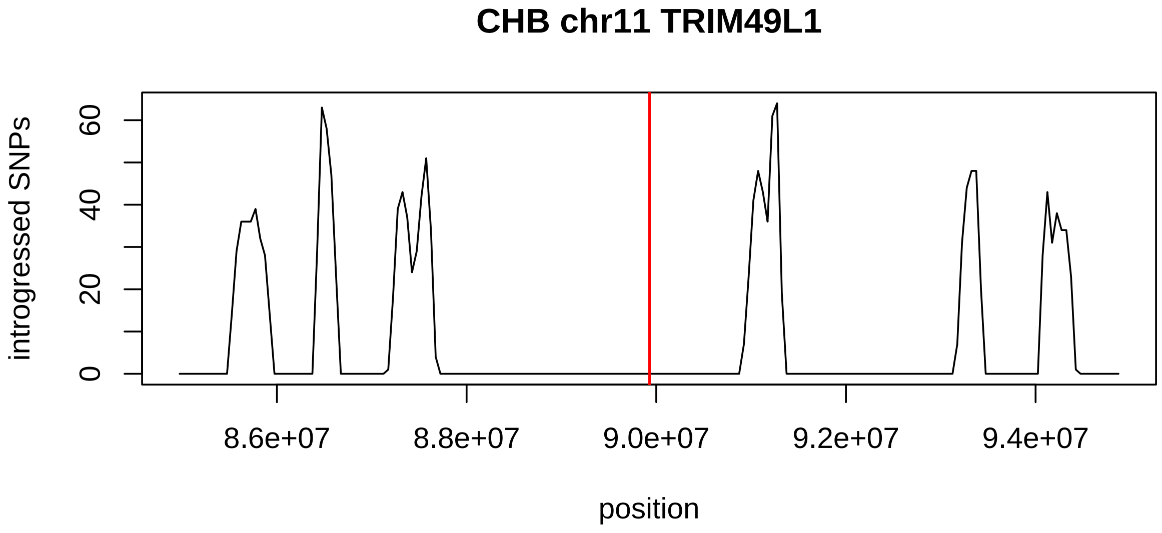
CHB chr11 TRIM49L1
introgressed SNPs
0
20
40
60
8.6e+07
8.8e+07
9.0e+07
9.2e+07
9.4e+07
position

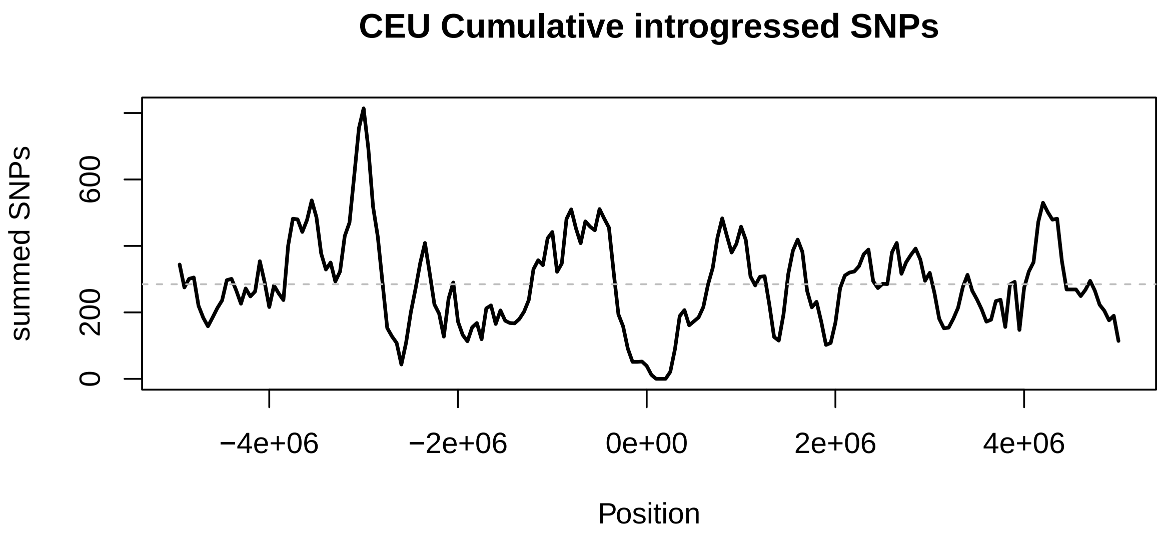
CEU Cumulative introgressed SNPs
summed SNPs
0
200
600
−4e+06
−2e+06
0e+00
2e+06
4e+06
Position

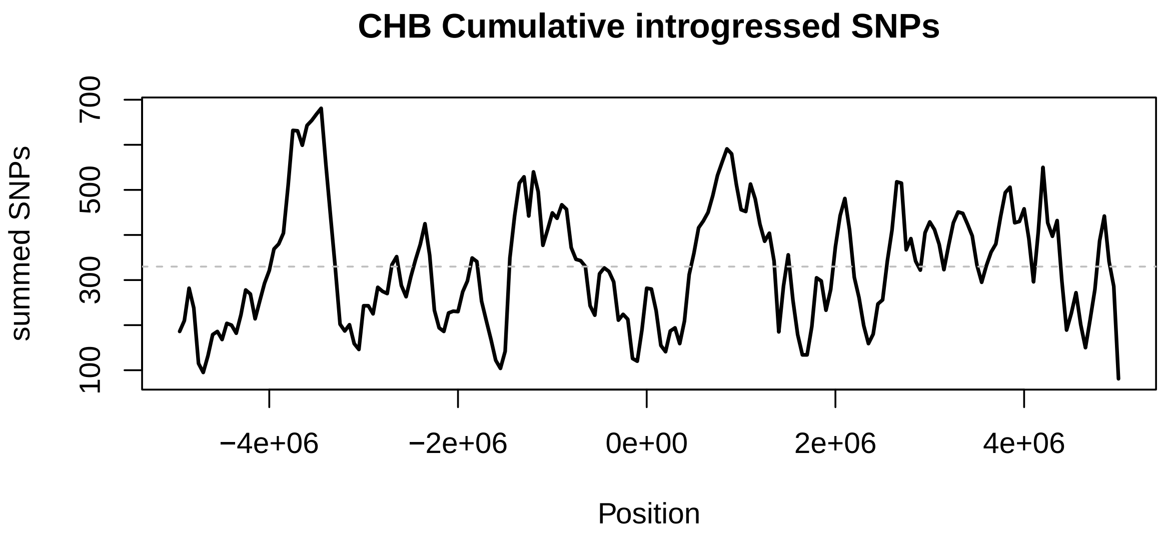
CHB Cumulative introgressed SNPs
summed SNPs
100
300
500
700
−4e+06
−2e+06
0e+00
2e+06
4e+06
Position
