# Supplementary material for: Detecting adaptive changes in gene copy number distribution accompanying the human out-of-Africa expansion
Source: Hum Genome Var. 2024 Sep 23;11:37. doi: 10.1038/s41439-024-00293-w (PMC11420239; doi:10.1038/s41439-024-00293-w)
Supplement: Supplementary file 3 — 3_Introgression_data [file 41439_2024_293_MOESM3_ESM.pdf]

POP	CHR	GENE	POS	INTRO_SNPS
CEU	chr1	AMY1A	98705519	0
CEU	chr1	AMY1A	98755519	0
CEU	chr1	AMY1A	98805519	0
CEU	chr1	AMY1A	98855519	0
CEU	chr1	AMY1A	98905519	0
CEU	chr1	AMY1A	98955519	0
CEU	chr1	AMY1A	99005519	0
CEU	chr1	AMY1A	99055519	0
CEU	chr1	AMY1A	99105519	0
CEU	chr1	AMY1A	99155519	0
CEU	chr1	AMY1A	99205519	0
CEU	chr1	AMY1A	99255519	0
CEU	chr1	AMY1A	99305519	0
CEU	chr1	AMY1A	99355519	0
CEU	chr1	AMY1A	99405519	0
CEU	chr1	AMY1A	99455519	0
CEU	chr1	AMY1A	99505519	0
CEU	chr1	AMY1A	99555519	0
CEU	chr1	AMY1A	99605519	0
CEU	chr1	AMY1A	99655519	0
CEU	chr1	AMY1A	99705519	0
CEU	chr1	AMY1A	99755519	0
CEU	chr1	AMY1A	99805519	0
CEU	chr1	AMY1A	99855519	0
CEU	chr1	AMY1A	99905519	0
CEU	chr1	AMY1A	99955519	0
CEU	chr1	AMY1A	100005519	0
CEU	chr1	AMY1A	100055519	0
CEU	chr1	AMY1A	100105519	0
CEU	chr1	AMY1A	100155519	0
CEU	chr1	AMY1A	100205519	0
CEU	chr1	AMY1A	100255519	0
CEU	chr1	AMY1A	100305519	0
CEU	chr1	AMY1A	100355519	0
CEU	chr1	AMY1A	100405519	0
CEU	chr1	AMY1A	100455519	0
CEU	chr1	AMY1A	100505519	0
CEU	chr1	AMY1A	100555519	0
CEU	chr1	AMY1A	100605519	0
CEU	chr1	AMY1A	100655519	0
CEU	chr1	AMY1A	100705519	0
CEU	chr1	AMY1A	100755519	0
CEU	chr1	AMY1A	100805519	0
CEU	chr1	AMY1A	100855519	0
CEU	chr1	AMY1A	100905519	0
CEU	chr1	AMY1A	100955519	0
CEU	chr1	AMY1A	101005519	0
CEU	chr1	AMY1A	101055519	0

CEU	chr1	AMY1A	101105519	0
CEU	chr1	AMY1A	101155519	0
CEU	chr1	AMY1A	101205519	0
CEU	chr1	AMY1A	101255519	0
CEU	chr1	AMY1A	101305519	0
CEU	chr1	AMY1A	101355519	0
CEU	chr1	AMY1A	101405519	0
CEU	chr1	AMY1A	101455519	0
CEU	chr1	AMY1A	101505519	0
CEU	chr1	AMY1A	101555519	0
CEU	chr1	AMY1A	101605519	0
CEU	chr1	AMY1A	101655519	0
CEU	chr1	AMY1A	101705519	0
CEU	chr1	AMY1A	101755519	0
CEU	chr1	AMY1A	101805519	1
CEU	chr1	AMY1A	101855519	22
CEU	chr1	AMY1A	101905519	50
CEU	chr1	AMY1A	101955519	167
CEU	chr1	AMY1A	102005519	148
CEU	chr1	AMY1A	102055519	10
CEU	chr1	AMY1A	102105519	0
CEU	chr1	AMY1A	102155519	0
CEU	chr1	AMY1A	102205519	0
CEU	chr1	AMY1A	102255519	0
CEU	chr1	AMY1A	102305519	0
CEU	chr1	AMY1A	102355519	0
CEU	chr1	AMY1A	102405519	0
CEU	chr1	AMY1A	102455519	0
CEU	chr1	AMY1A	102505519	0
CEU	chr1	AMY1A	102555519	0
CEU	chr1	AMY1A	102605519	0
CEU	chr1	AMY1A	102655519	0
CEU	chr1	AMY1A	102705519	0
CEU	chr1	AMY1A	102755519	0
CEU	chr1	AMY1A	102805519	0
CEU	chr1	AMY1A	102855519	0
CEU	chr1	AMY1A	102905519	0
CEU	chr1	AMY1A	102955519	0
CEU	chr1	AMY1A	103005519	0
CEU	chr1	AMY1A	103055519	0
CEU	chr1	AMY1A	103105519	0
CEU	chr1	AMY1A	103155519	0
CEU	chr1	AMY1A	103205519	0
CEU	chr1	AMY1A	103255519	0
CEU	chr1	AMY1A	103305519	0
CEU	chr1	AMY1A	103355519	0
CEU	chr1	AMY1A	103405519	0
CEU	chr1	AMY1A	103455519	0
CEU	chr1	AMY1A	103505519	0

CEU	chr1	AMY1A	103555519	0
CEU	chr1	AMY1A	103605519	0
CEU	chr1	AMY1A	103655519	0
CEU	chr1	AMY1A	103705519	0
CEU	chr1	AMY1A	103755519	0
CEU	chr1	AMY1A	103805519	0
CEU	chr1	AMY1A	103855519	0
CEU	chr1	AMY1A	103905519	0
CEU	chr1	AMY1A	103955519	0
CEU	chr1	AMY1A	104005519	0
CEU	chr1	AMY1A	104055519	0
CEU	chr1	AMY1A	104105519	0
CEU	chr1	AMY1A	104155519	0
CEU	chr1	AMY1A	104205519	0
CEU	chr1	AMY1A	104255519	0
CEU	chr1	AMY1A	104305519	0
CEU	chr1	AMY1A	104355519	0
CEU	chr1	AMY1A	104405519	0
CEU	chr1	AMY1A	104455519	0
CEU	chr1	AMY1A	104505519	0
CEU	chr1	AMY1A	104555519	0
CEU	chr1	AMY1A	104605519	0
CEU	chr1	AMY1A	104655519	0
CEU	chr1	AMY1A	104705519	0
CEU	chr1	AMY1A	104755519	0
CEU	chr1	AMY1A	104805519	0
CEU	chr1	AMY1A	104855519	0
CEU	chr1	AMY1A	104905519	0
CEU	chr1	AMY1A	104955519	0
CEU	chr1	AMY1A	105005519	0
CEU	chr1	AMY1A	105055519	0
CEU	chr1	AMY1A	105105519	0
CEU	chr1	AMY1A	105155519	0
CEU	chr1	AMY1A	105205519	0
CEU	chr1	AMY1A	105255519	0
CEU	chr1	AMY1A	105305519	0
CEU	chr1	AMY1A	105355519	0
CEU	chr1	AMY1A	105405519	0
CEU	chr1	AMY1A	105455519	0
CEU	chr1	AMY1A	105505519	0
CEU	chr1	AMY1A	105555519	0
CEU	chr1	AMY1A	105605519	0
CEU	chr1	AMY1A	105655519	0
CEU	chr1	AMY1A	105705519	0
CEU	chr1	AMY1A	105755519	0
CEU	chr1	AMY1A	105805519	0
CEU	chr1	AMY1A	105855519	0
CEU	chr1	AMY1A	105905519	0
CEU	chr1	AMY1A	105955519	0

CEU	chr1	AMY1A	106005519	0
CEU	chr1	AMY1A	106055519	0
CEU	chr1	AMY1A	106105519	0
CEU	chr1	AMY1A	106155519	0
CEU	chr1	AMY1A	106205519	0
CEU	chr1	AMY1A	106255519	0
CEU	chr1	AMY1A	106305519	0
CEU	chr1	AMY1A	106355519	0
CEU	chr1	AMY1A	106405519	0
CEU	chr1	AMY1A	106455519	0
CEU	chr1	AMY1A	106505519	0
CEU	chr1	AMY1A	106555519	0
CEU	chr1	AMY1A	106605519	0
CEU	chr1	AMY1A	106655519	0
CEU	chr1	AMY1A	106705519	0
CEU	chr1	AMY1A	106755519	0
CEU	chr1	AMY1A	106805519	0
CEU	chr1	AMY1A	106855519	0
CEU	chr1	AMY1A	106905519	0
CEU	chr1	AMY1A	106955519	0
CEU	chr1	AMY1A	107005519	0
CEU	chr1	AMY1A	107055519	0
CEU	chr1	AMY1A	107105519	0
CEU	chr1	AMY1A	107155519	0
CEU	chr1	AMY1A	107205519	0
CEU	chr1	AMY1A	107255519	0
CEU	chr1	AMY1A	107305519	0
CEU	chr1	AMY1A	107355519	0
CEU	chr1	AMY1A	107405519	0
CEU	chr1	AMY1A	107455519	0
CEU	chr1	AMY1A	107505519	0
CEU	chr1	AMY1A	107555519	0
CEU	chr1	AMY1A	107605519	0
CEU	chr1	AMY1A	107655519	0
CEU	chr1	AMY1A	107705519	0
CEU	chr1	AMY1A	107755519	0
CEU	chr1	AMY1A	107805519	0
CEU	chr1	AMY1A	107855519	0
CEU	chr1	AMY1A	107905519	0
CEU	chr1	AMY1A	107955519	0
CEU	chr1	AMY1A	108005519	0
CEU	chr1	AMY1A	108055519	0
CEU	chr1	AMY1A	108105519	0
CEU	chr1	AMY1A	108155519	0
CEU	chr1	AMY1A	108205519	0
CEU	chr1	AMY1A	108255519	0
CEU	chr1	AMY1A	108305519	0
CEU	chr1	AMY1A	108355519	0
CEU	chr1	AMY1A	108405519	0

CEU	chr1	AMY1A	108455519	0
CEU	chr1	AMY1A	108505519	0
CEU	chr1	AMY1A	108555519	0
CEU	chr1	AMY1A	108605519	0
CEU	chr16	BOLA2B	24503588	0
CEU	chr16	BOLA2B	24553588	0
CEU	chr16	BOLA2B	24603588	0
CEU	chr16	BOLA2B	24653588	0
CEU	chr16	BOLA2B	24703588	0
CEU	chr16	BOLA2B	24753588	0
CEU	chr16	BOLA2B	24803588	0
CEU	chr16	BOLA2B	24853588	0
CEU	chr16	BOLA2B	24903588	0
CEU	chr16	BOLA2B	24953588	0
CEU	chr16	BOLA2B	25003588	0
CEU	chr16	BOLA2B	25053588	0
CEU	chr16	BOLA2B	25103588	0
CEU	chr16	BOLA2B	25153588	0
CEU	chr16	BOLA2B	25203588	0
CEU	chr16	BOLA2B	25253588	0
CEU	chr16	BOLA2B	25303588	0
CEU	chr16	BOLA2B	25353588	0
CEU	chr16	BOLA2B	25403588	0
CEU	chr16	BOLA2B	25453588	0
CEU	chr16	BOLA2B	25503588	0
CEU	chr16	BOLA2B	25553588	0
CEU	chr16	BOLA2B	25603588	0
CEU	chr16	BOLA2B	25653588	0
CEU	chr16	BOLA2B	25703588	0
CEU	chr16	BOLA2B	25753588	0
CEU	chr16	BOLA2B	25803588	0
CEU	chr16	BOLA2B	25853588	0
CEU	chr16	BOLA2B	25903588	9
CEU	chr16	BOLA2B	25953588	46
CEU	chr16	BOLA2B	26003588	37
CEU	chr16	BOLA2B	26053588	0
CEU	chr16	BOLA2B	26103588	0
CEU	chr16	BOLA2B	26153588	0
CEU	chr16	BOLA2B	26203588	0
CEU	chr16	BOLA2B	26253588	0
CEU	chr16	BOLA2B	26303588	0
CEU	chr16	BOLA2B	26353588	0
CEU	chr16	BOLA2B	26403588	0
CEU	chr16	BOLA2B	26453588	0
CEU	chr16	BOLA2B	26503588	0
CEU	chr16	BOLA2B	26553588	0
CEU	chr16	BOLA2B	26603588	0
CEU	chr16	BOLA2B	26653588	0
CEU	chr16	BOLA2B	26703588	0

CEU	chr16	BOLA2B	26753588	0
CEU	chr16	BOLA2B	26803588	0
CEU	chr16	BOLA2B	26853588	0
CEU	chr16	BOLA2B	26903588	0
CEU	chr16	BOLA2B	26953588	0
CEU	chr16	BOLA2B	27003588	0
CEU	chr16	BOLA2B	27053588	0
CEU	chr16	BOLA2B	27103588	0
CEU	chr16	BOLA2B	27153588	0
CEU	chr16	BOLA2B	27203588	0
CEU	chr16	BOLA2B	27253588	0
CEU	chr16	BOLA2B	27303588	0
CEU	chr16	BOLA2B	27353588	0
CEU	chr16	BOLA2B	27403588	0
CEU	chr16	BOLA2B	27453588	0
CEU	chr16	BOLA2B	27503588	0
CEU	chr16	BOLA2B	27553588	0
CEU	chr16	BOLA2B	27603588	0
CEU	chr16	BOLA2B	27653588	0
CEU	chr16	BOLA2B	27703588	0
CEU	chr16	BOLA2B	27753588	0
CEU	chr16	BOLA2B	27803588	0
CEU	chr16	BOLA2B	27853588	0
CEU	chr16	BOLA2B	27903588	0
CEU	chr16	BOLA2B	27953588	0
CEU	chr16	BOLA2B	28003588	0
CEU	chr16	BOLA2B	28053588	0
CEU	chr16	BOLA2B	28103588	0
CEU	chr16	BOLA2B	28153588	0
CEU	chr16	BOLA2B	28203588	0
CEU	chr16	BOLA2B	28253588	0
CEU	chr16	BOLA2B	28303588	0
CEU	chr16	BOLA2B	28353588	0
CEU	chr16	BOLA2B	28403588	0
CEU	chr16	BOLA2B	28453588	0
CEU	chr16	BOLA2B	28503588	0
CEU	chr16	BOLA2B	28553588	0
CEU	chr16	BOLA2B	28603588	0
CEU	chr16	BOLA2B	28653588	0
CEU	chr16	BOLA2B	28703588	0
CEU	chr16	BOLA2B	28753588	0
CEU	chr16	BOLA2B	28803588	0
CEU	chr16	BOLA2B	28853588	0
CEU	chr16	BOLA2B	28903588	0
CEU	chr16	BOLA2B	28953588	0
CEU	chr16	BOLA2B	29003588	0
CEU	chr16	BOLA2B	29053588	0
CEU	chr16	BOLA2B	29103588	0
CEU	chr16	BOLA2B	29153588	0

CEU	chr16	BOLA2B	29203588	0
CEU	chr16	BOLA2B	29253588	0
CEU	chr16	BOLA2B	29303588	0
CEU	chr16	BOLA2B	29353588	0
CEU	chr16	BOLA2B	29403588	0
CEU	chr16	BOLA2B	29453588	0
CEU	chr16	BOLA2B	29503588	0
CEU	chr16	BOLA2B	29553588	0
CEU	chr16	BOLA2B	29603588	0
CEU	chr16	BOLA2B	29653588	0
CEU	chr16	BOLA2B	29703588	0
CEU	chr16	BOLA2B	29753588	0
CEU	chr16	BOLA2B	29803588	0
CEU	chr16	BOLA2B	29853588	0
CEU	chr16	BOLA2B	29903588	0
CEU	chr16	BOLA2B	29953588	0
CEU	chr16	BOLA2B	30003588	0
CEU	chr16	BOLA2B	30053588	0
CEU	chr16	BOLA2B	30103588	0
CEU	chr16	BOLA2B	30153588	0
CEU	chr16	BOLA2B	30203588	0
CEU	chr16	BOLA2B	30253588	0
CEU	chr16	BOLA2B	30303588	0
CEU	chr16	BOLA2B	30353588	0
CEU	chr16	BOLA2B	30403588	0
CEU	chr16	BOLA2B	30453588	0
CEU	chr16	BOLA2B	30503588	0
CEU	chr16	BOLA2B	30553588	0
CEU	chr16	BOLA2B	30603588	0
CEU	chr16	BOLA2B	30653588	0
CEU	chr16	BOLA2B	30703588	0
CEU	chr16	BOLA2B	30753588	0
CEU	chr16	BOLA2B	30803588	0
CEU	chr16	BOLA2B	30853588	0
CEU	chr16	BOLA2B	30903588	0
CEU	chr16	BOLA2B	30953588	0
CEU	chr16	BOLA2B	31003588	0
CEU	chr16	BOLA2B	31053588	0
CEU	chr16	BOLA2B	31103588	0
CEU	chr16	BOLA2B	31153588	0
CEU	chr16	BOLA2B	31203588	0
CEU	chr16	BOLA2B	31253588	0
CEU	chr16	BOLA2B	31303588	0
CEU	chr16	BOLA2B	31353588	0
CEU	chr16	BOLA2B	31403588	0
CEU	chr16	BOLA2B	31453588	0
CEU	chr16	BOLA2B	31503588	0
CEU	chr16	BOLA2B	31553588	0
CEU	chr16	BOLA2B	31603588	0

CEU	chr16	BOLA2B	31653588	0
CEU	chr16	BOLA2B	31703588	0
CEU	chr16	BOLA2B	31753588	0
CEU	chr16	BOLA2B	31803588	0
CEU	chr16	BOLA2B	31853588	0
CEU	chr16	BOLA2B	31903588	0
CEU	chr16	BOLA2B	31953588	0
CEU	chr16	BOLA2B	32003588	0
CEU	chr16	BOLA2B	32053588	0
CEU	chr16	BOLA2B	32103588	0
CEU	chr16	BOLA2B	32153588	0
CEU	chr16	BOLA2B	32203588	0
CEU	chr16	BOLA2B	32253588	0
CEU	chr16	BOLA2B	32303588	0
CEU	chr16	BOLA2B	32353588	0
CEU	chr16	BOLA2B	32403588	0
CEU	chr16	BOLA2B	32453588	0
CEU	chr16	BOLA2B	32503588	0
CEU	chr16	BOLA2B	32553588	0
CEU	chr16	BOLA2B	32603588	0
CEU	chr16	BOLA2B	32653588	0
CEU	chr16	BOLA2B	32703588	0
CEU	chr16	BOLA2B	32753588	0
CEU	chr16	BOLA2B	32803588	0
CEU	chr16	BOLA2B	32853588	0
CEU	chr16	BOLA2B	32903588	0
CEU	chr16	BOLA2B	32953588	0
CEU	chr16	BOLA2B	33003588	0
CEU	chr16	BOLA2B	33053588	0
CEU	chr16	BOLA2B	33103588	0
CEU	chr16	BOLA2B	33153588	0
CEU	chr16	BOLA2B	33203588	0
CEU	chr16	BOLA2B	33253588	0
CEU	chr16	BOLA2B	33303588	0
CEU	chr16	BOLA2B	33353588	0
CEU	chr16	BOLA2B	33403588	0
CEU	chr16	BOLA2B	33453588	0
CEU	chr16	BOLA2B	33503588	0
CEU	chr16	BOLA2B	33553588	0
CEU	chr16	BOLA2B	33603588	0
CEU	chr16	BOLA2B	33653588	0
CEU	chr16	BOLA2B	33703588	0
CEU	chr16	BOLA2B	33753588	0
CEU	chr16	BOLA2B	33803588	0
CEU	chr16	BOLA2B	33853588	0
CEU	chr16	BOLA2B	33903588	0
CEU	chr16	BOLA2B	33953588	0
CEU	chr16	BOLA2B	34003588	0
CEU	chr16	BOLA2B	34053588	0

CEU	chr16	BOLA2B	34103588	0
CEU	chr16	BOLA2B	34153588	0
CEU	chr16	BOLA2B	34203588	0
CEU	chr16	BOLA2B	34253588	0
CEU	chr16	BOLA2B	34303588	0
CEU	chr16	BOLA2B	34353588	0
CEU	chr16	BOLA2B	34403588	0
CEU	chr9	CBWD3	60716374	0
CEU	chr9	CBWD3	60766374	0
CEU	chr9	CBWD3	60816374	0
CEU	chr9	CBWD3	60866374	0
CEU	chr9	CBWD3	60916374	0
CEU	chr9	CBWD3	60966374	0
CEU	chr9	CBWD3	61016374	0
CEU	chr9	CBWD3	61066374	0
CEU	chr9	CBWD3	61116374	0
CEU	chr9	CBWD3	61166374	0
CEU	chr9	CBWD3	61216374	0
CEU	chr9	CBWD3	61266374	0
CEU	chr9	CBWD3	61316374	0
CEU	chr9	CBWD3	61366374	0
CEU	chr9	CBWD3	61416374	0
CEU	chr9	CBWD3	61466374	0
CEU	chr9	CBWD3	61516374	0
CEU	chr9	CBWD3	61566374	0
CEU	chr9	CBWD3	61616374	0
CEU	chr9	CBWD3	61666374	0
CEU	chr9	CBWD3	61716374	0
CEU	chr9	CBWD3	61766374	0
CEU	chr9	CBWD3	61816374	0
CEU	chr9	CBWD3	61866374	0
CEU	chr9	CBWD3	61916374	0
CEU	chr9	CBWD3	61966374	0
CEU	chr9	CBWD3	62016374	0
CEU	chr9	CBWD3	62066374	0
CEU	chr9	CBWD3	62116374	0
CEU	chr9	CBWD3	62166374	0
CEU	chr9	CBWD3	62216374	0
CEU	chr9	CBWD3	62266374	0
CEU	chr9	CBWD3	62316374	0
CEU	chr9	CBWD3	62366374	0
CEU	chr9	CBWD3	62416374	0
CEU	chr9	CBWD3	62466374	0
CEU	chr9	CBWD3	62516374	0
CEU	chr9	CBWD3	62566374	0
CEU	chr9	CBWD3	62616374	0
CEU	chr9	CBWD3	62666374	0
CEU	chr9	CBWD3	62716374	0
CEU	chr9	CBWD3	62766374	0

CEU	chr9	CBWD3	62816374	0
CEU	chr9	CBWD3	62866374	0
CEU	chr9	CBWD3	62916374	0
CEU	chr9	CBWD3	62966374	0
CEU	chr9	CBWD3	63016374	0
CEU	chr9	CBWD3	63066374	0
CEU	chr9	CBWD3	63116374	0
CEU	chr9	CBWD3	63166374	0
CEU	chr9	CBWD3	63216374	0
CEU	chr9	CBWD3	63266374	0
CEU	chr9	CBWD3	63316374	0
CEU	chr9	CBWD3	63366374	0
CEU	chr9	CBWD3	63416374	0
CEU	chr9	CBWD3	63466374	0
CEU	chr9	CBWD3	63516374	0
CEU	chr9	CBWD3	63566374	0
CEU	chr9	CBWD3	63616374	0
CEU	chr9	CBWD3	63666374	0
CEU	chr9	CBWD3	63716374	0
CEU	chr9	CBWD3	63766374	0
CEU	chr9	CBWD3	63816374	0
CEU	chr9	CBWD3	63866374	0
CEU	chr9	CBWD3	63916374	0
CEU	chr9	CBWD3	63966374	0
CEU	chr9	CBWD3	64016374	0
CEU	chr9	CBWD3	64066374	0
CEU	chr9	CBWD3	64116374	0
CEU	chr9	CBWD3	64166374	0
CEU	chr9	CBWD3	64216374	0
CEU	chr9	CBWD3	64266374	0
CEU	chr9	CBWD3	64316374	0
CEU	chr9	CBWD3	64366374	0
CEU	chr9	CBWD3	64416374	0
CEU	chr9	CBWD3	64466374	0
CEU	chr9	CBWD3	64516374	0
CEU	chr9	CBWD3	64566374	0
CEU	chr9	CBWD3	64616374	0
CEU	chr9	CBWD3	64666374	0
CEU	chr9	CBWD3	64716374	0
CEU	chr9	CBWD3	64766374	0
CEU	chr9	CBWD3	64816374	0
CEU	chr9	CBWD3	64866374	0
CEU	chr9	CBWD3	64916374	0
CEU	chr9	CBWD3	64966374	0
CEU	chr9	CBWD3	65016374	0
CEU	chr9	CBWD3	65066374	0
CEU	chr9	CBWD3	65116374	0
CEU	chr9	CBWD3	65166374	0
CEU	chr9	CBWD3	65216374	0

CEU	chr9	CBWD3	65266374	0
CEU	chr9	CBWD3	65316374	0
CEU	chr9	CBWD3	65366374	0
CEU	chr9	CBWD3	65416374	0
CEU	chr9	CBWD3	65466374	0
CEU	chr9	CBWD3	65516374	0
CEU	chr9	CBWD3	65566374	13
CEU	chr9	CBWD3	65616374	27
CEU	chr9	CBWD3	65666374	14
CEU	chr9	CBWD3	65716374	0
CEU	chr9	CBWD3	65766374	0
CEU	chr9	CBWD3	65816374	0
CEU	chr9	CBWD3	65866374	0
CEU	chr9	CBWD3	65916374	0
CEU	chr9	CBWD3	65966374	0
CEU	chr9	CBWD3	66016374	0
CEU	chr9	CBWD3	66066374	0
CEU	chr9	CBWD3	66116374	0
CEU	chr9	CBWD3	66166374	0
CEU	chr9	CBWD3	66216374	0
CEU	chr9	CBWD3	66266374	0
CEU	chr9	CBWD3	66316374	0
CEU	chr9	CBWD3	66366374	0
CEU	chr9	CBWD3	66416374	0
CEU	chr9	CBWD3	66466374	0
CEU	chr9	CBWD3	66516374	0
CEU	chr9	CBWD3	66566374	0
CEU	chr9	CBWD3	66616374	2
CEU	chr9	CBWD3	66666374	17
CEU	chr9	CBWD3	66716374	23
CEU	chr9	CBWD3	66766374	22
CEU	chr9	CBWD3	66816374	16
CEU	chr9	CBWD3	66866374	2
CEU	chr9	CBWD3	66916374	0
CEU	chr9	CBWD3	66966374	0
CEU	chr9	CBWD3	67016374	0
CEU	chr9	CBWD3	67066374	0
CEU	chr9	CBWD3	67116374	0
CEU	chr9	CBWD3	67166374	0
CEU	chr9	CBWD3	67216374	0
CEU	chr9	CBWD3	67266374	0
CEU	chr9	CBWD3	67316374	0
CEU	chr9	CBWD3	67366374	0
CEU	chr9	CBWD3	67416374	0
CEU	chr9	CBWD3	67466374	0
CEU	chr9	CBWD3	67516374	0
CEU	chr9	CBWD3	67566374	0
CEU	chr9	CBWD3	67616374	0
CEU	chr9	CBWD3	67666374	0

CEU	chr9	CBWD3	67716374	0
CEU	chr9	CBWD3	67766374	0
CEU	chr9	CBWD3	67816374	0
CEU	chr9	CBWD3	67866374	0
CEU	chr9	CBWD3	67916374	0
CEU	chr9	CBWD3	67966374	0
CEU	chr9	CBWD3	68016374	0
CEU	chr9	CBWD3	68066374	0
CEU	chr9	CBWD3	68116374	0
CEU	chr9	CBWD3	68166374	0
CEU	chr9	CBWD3	68216374	0
CEU	chr9	CBWD3	68266374	0
CEU	chr9	CBWD3	68316374	0
CEU	chr9	CBWD3	68366374	0
CEU	chr9	CBWD3	68416374	0
CEU	chr9	CBWD3	68466374	0
CEU	chr9	CBWD3	68516374	0
CEU	chr9	CBWD3	68566374	0
CEU	chr9	CBWD3	68616374	0
CEU	chr9	CBWD3	68666374	0
CEU	chr9	CBWD3	68716374	0
CEU	chr9	CBWD3	68766374	0
CEU	chr9	CBWD3	68816374	0
CEU	chr9	CBWD3	68866374	0
CEU	chr9	CBWD3	68916374	0
CEU	chr9	CBWD3	68966374	0
CEU	chr9	CBWD3	69016374	0
CEU	chr9	CBWD3	69066374	0
CEU	chr9	CBWD3	69116374	0
CEU	chr9	CBWD3	69166374	0
CEU	chr9	CBWD3	69216374	0
CEU	chr9	CBWD3	69266374	0
CEU	chr9	CBWD3	69316374	0
CEU	chr9	CBWD3	69366374	0
CEU	chr9	CBWD3	69416374	0
CEU	chr9	CBWD3	69466374	0
CEU	chr9	CBWD3	69516374	0
CEU	chr9	CBWD3	69566374	0
CEU	chr9	CBWD3	69616374	0
CEU	chr9	CBWD3	69666374	0
CEU	chr9	CBWD3	69716374	0
CEU	chr9	CBWD3	69766374	2
CEU	chr9	CBWD3	69816374	12
CEU	chr9	CBWD3	69866374	47
CEU	chr9	CBWD3	69916374	61
CEU	chr9	CBWD3	69966374	48
CEU	chr9	CBWD3	70016374	24
CEU	chr9	CBWD3	70066374	0
CEU	chr9	CBWD3	70116374	0

CEU	chr9	CBWD3	70166374	0
CEU	chr9	CBWD3	70216374	0
CEU	chr9	CBWD3	70266374	0
CEU	chr9	CBWD3	70316374	0
CEU	chr9	CBWD3	70366374	0
CEU	chr9	CBWD3	70416374	0
CEU	chr9	CBWD3	70466374	0
CEU	chr9	CBWD3	70516374	0
CEU	chr9	CBWD3	70566374	0
CEU	chr9	CBWD3	70616374	0
CEU	chr9	CBWD3	70666374	0
CEU	chr16	CLEC18A	65000232	0
CEU	chr16	CLEC18A	65050232	0
CEU	chr16	CLEC18A	65100232	0
CEU	chr16	CLEC18A	65150232	0
CEU	chr16	CLEC18A	65200232	0
CEU	chr16	CLEC18A	65250232	0
CEU	chr16	CLEC18A	65300232	0
CEU	chr16	CLEC18A	65350232	0
CEU	chr16	CLEC18A	65400232	0
CEU	chr16	CLEC18A	65450232	0
CEU	chr16	CLEC18A	65500232	0
CEU	chr16	CLEC18A	65550232	0
CEU	chr16	CLEC18A	65600232	0
CEU	chr16	CLEC18A	65650232	0
CEU	chr16	CLEC18A	65700232	0
CEU	chr16	CLEC18A	65750232	0
CEU	chr16	CLEC18A	65800232	0
CEU	chr16	CLEC18A	65850232	0
CEU	chr16	CLEC18A	65900232	0
CEU	chr16	CLEC18A	65950232	0
CEU	chr16	CLEC18A	66000232	0
CEU	chr16	CLEC18A	66050232	0
CEU	chr16	CLEC18A	66100232	0
CEU	chr16	CLEC18A	66150232	0
CEU	chr16	CLEC18A	66200232	0
CEU	chr16	CLEC18A	66250232	0
CEU	chr16	CLEC18A	66300232	0
CEU	chr16	CLEC18A	66350232	0
CEU	chr16	CLEC18A	66400232	0
CEU	chr16	CLEC18A	66450232	0
CEU	chr16	CLEC18A	66500232	0
CEU	chr16	CLEC18A	66550232	0
CEU	chr16	CLEC18A	66600232	0
CEU	chr16	CLEC18A	66650232	0
CEU	chr16	CLEC18A	66700232	0
CEU	chr16	CLEC18A	66750232	0
CEU	chr16	CLEC18A	66800232	0
CEU	chr16	CLEC18A	66850232	0

CEU	chr16	CLEC18A	66900232	0
CEU	chr16	CLEC18A	66950232	0
CEU	chr16	CLEC18A	67000232	0
CEU	chr16	CLEC18A	67050232	0
CEU	chr16	CLEC18A	67100232	0
CEU	chr16	CLEC18A	67150232	0
CEU	chr16	CLEC18A	67200232	0
CEU	chr16	CLEC18A	67250232	0
CEU	chr16	CLEC18A	67300232	0
CEU	chr16	CLEC18A	67350232	0
CEU	chr16	CLEC18A	67400232	0
CEU	chr16	CLEC18A	67450232	0
CEU	chr16	CLEC18A	67500232	0
CEU	chr16	CLEC18A	67550232	0
CEU	chr16	CLEC18A	67600232	0
CEU	chr16	CLEC18A	67650232	0
CEU	chr16	CLEC18A	67700232	0
CEU	chr16	CLEC18A	67750232	0
CEU	chr16	CLEC18A	67800232	0
CEU	chr16	CLEC18A	67850232	0
CEU	chr16	CLEC18A	67900232	0
CEU	chr16	CLEC18A	67950232	0
CEU	chr16	CLEC18A	68000232	0
CEU	chr16	CLEC18A	68050232	0
CEU	chr16	CLEC18A	68100232	0
CEU	chr16	CLEC18A	68150232	0
CEU	chr16	CLEC18A	68200232	0
CEU	chr16	CLEC18A	68250232	0
CEU	chr16	CLEC18A	68300232	0
CEU	chr16	CLEC18A	68350232	0
CEU	chr16	CLEC18A	68400232	0
CEU	chr16	CLEC18A	68450232	0
CEU	chr16	CLEC18A	68500232	0
CEU	chr16	CLEC18A	68550232	0
CEU	chr16	CLEC18A	68600232	0
CEU	chr16	CLEC18A	68650232	0
CEU	chr16	CLEC18A	68700232	0
CEU	chr16	CLEC18A	68750232	0
CEU	chr16	CLEC18A	68800232	0
CEU	chr16	CLEC18A	68850232	0
CEU	chr16	CLEC18A	68900232	0
CEU	chr16	CLEC18A	68950232	0
CEU	chr16	CLEC18A	69000232	0
CEU	chr16	CLEC18A	69050232	0
CEU	chr16	CLEC18A	69100232	0
CEU	chr16	CLEC18A	69150232	0
CEU	chr16	CLEC18A	69200232	0
CEU	chr16	CLEC18A	69250232	0
CEU	chr16	CLEC18A	69300232	0

CEU	chr16	CLEC18A	69350232	0
CEU	chr16	CLEC18A	69400232	0
CEU	chr16	CLEC18A	69450232	0
CEU	chr16	CLEC18A	69500232	0
CEU	chr16	CLEC18A	69550232	0
CEU	chr16	CLEC18A	69600232	0
CEU	chr16	CLEC18A	69650232	0
CEU	chr16	CLEC18A	69700232	0
CEU	chr16	CLEC18A	69750232	0
CEU	chr16	CLEC18A	69800232	0
CEU	chr16	CLEC18A	69850232	0
CEU	chr16	CLEC18A	69900232	0
CEU	chr16	CLEC18A	69950232	0
CEU	chr16	CLEC18A	70000232	0
CEU	chr16	CLEC18A	70050232	0
CEU	chr16	CLEC18A	70100232	0
CEU	chr16	CLEC18A	70150232	0
CEU	chr16	CLEC18A	70200232	0
CEU	chr16	CLEC18A	70250232	0
CEU	chr16	CLEC18A	70300232	0
CEU	chr16	CLEC18A	70350232	0
CEU	chr16	CLEC18A	70400232	0
CEU	chr16	CLEC18A	70450232	0
CEU	chr16	CLEC18A	70500232	0
CEU	chr16	CLEC18A	70550232	0
CEU	chr16	CLEC18A	70600232	0
CEU	chr16	CLEC18A	70650232	0
CEU	chr16	CLEC18A	70700232	0
CEU	chr16	CLEC18A	70750232	0
CEU	chr16	CLEC18A	70800232	0
CEU	chr16	CLEC18A	70850232	0
CEU	chr16	CLEC18A	70900232	0
CEU	chr16	CLEC18A	70950232	0
CEU	chr16	CLEC18A	71000232	0
CEU	chr16	CLEC18A	71050232	0
CEU	chr16	CLEC18A	71100232	0
CEU	chr16	CLEC18A	71150232	0
CEU	chr16	CLEC18A	71200232	0
CEU	chr16	CLEC18A	71250232	0
CEU	chr16	CLEC18A	71300232	0
CEU	chr16	CLEC18A	71350232	0
CEU	chr16	CLEC18A	71400232	0
CEU	chr16	CLEC18A	71450232	0
CEU	chr16	CLEC18A	71500232	0
CEU	chr16	CLEC18A	71550232	0
CEU	chr16	CLEC18A	71600232	0
CEU	chr16	CLEC18A	71650232	0
CEU	chr16	CLEC18A	71700232	0
CEU	chr16	CLEC18A	71750232	0

CEU	chr16	CLEC18A	71800232	0
CEU	chr16	CLEC18A	71850232	0
CEU	chr16	CLEC18A	71900232	0
CEU	chr16	CLEC18A	71950232	0
CEU	chr16	CLEC18A	72000232	0
CEU	chr16	CLEC18A	72050232	0
CEU	chr16	CLEC18A	72100232	0
CEU	chr16	CLEC18A	72150232	0
CEU	chr16	CLEC18A	72200232	0
CEU	chr16	CLEC18A	72250232	0
CEU	chr16	CLEC18A	72300232	0
CEU	chr16	CLEC18A	72350232	0
CEU	chr16	CLEC18A	72400232	0
CEU	chr16	CLEC18A	72450232	0
CEU	chr16	CLEC18A	72500232	0
CEU	chr16	CLEC18A	72550232	0
CEU	chr16	CLEC18A	72600232	0
CEU	chr16	CLEC18A	72650232	0
CEU	chr16	CLEC18A	72700232	0
CEU	chr16	CLEC18A	72750232	0
CEU	chr16	CLEC18A	72800232	0
CEU	chr16	CLEC18A	72850232	0
CEU	chr16	CLEC18A	72900232	0
CEU	chr16	CLEC18A	72950232	0
CEU	chr16	CLEC18A	73000232	0
CEU	chr16	CLEC18A	73050232	0
CEU	chr16	CLEC18A	73100232	0
CEU	chr16	CLEC18A	73150232	0
CEU	chr16	CLEC18A	73200232	0
CEU	chr16	CLEC18A	73250232	0
CEU	chr16	CLEC18A	73300232	0
CEU	chr16	CLEC18A	73350232	0
CEU	chr16	CLEC18A	73400232	0
CEU	chr16	CLEC18A	73450232	0
CEU	chr16	CLEC18A	73500232	0
CEU	chr16	CLEC18A	73550232	0
CEU	chr16	CLEC18A	73600232	0
CEU	chr16	CLEC18A	73650232	0
CEU	chr16	CLEC18A	73700232	0
CEU	chr16	CLEC18A	73750232	0
CEU	chr16	CLEC18A	73800232	0
CEU	chr16	CLEC18A	73850232	0
CEU	chr16	CLEC18A	73900232	0
CEU	chr16	CLEC18A	73950232	0
CEU	chr16	CLEC18A	74000232	0
CEU	chr16	CLEC18A	74050232	0
CEU	chr16	CLEC18A	74100232	0
CEU	chr16	CLEC18A	74150232	0
CEU	chr16	CLEC18A	74200232	0

CEU	chr16	CLEC18A	74250232	0
CEU	chr16	CLEC18A	74300232	0
CEU	chr16	CLEC18A	74350232	0
CEU	chr16	CLEC18A	74400232	0
CEU	chr16	CLEC18A	74450232	0
CEU	chr16	CLEC18A	74500232	0
CEU	chr16	CLEC18A	74550232	0
CEU	chr16	CLEC18A	74600232	0
CEU	chr16	CLEC18A	74650232	0
CEU	chr16	CLEC18A	74700232	0
CEU	chr16	CLEC18A	74750232	0
CEU	chr16	CLEC18A	74800232	0
CEU	chr16	CLEC18A	74850232	0
CEU	chr16	CLEC18A	74900232	0
CEU	chr7	CSH	118813708	0
CEU	chr7	CSH	118863708	0
CEU	chr7	CSH	118913708	0
CEU	chr7	CSH	118963708	0
CEU	chr7	CSH	119013708	0
CEU	chr7	CSH	119063708	0
CEU	chr7	CSH	119113708	0
CEU	chr7	CSH	119163708	0
CEU	chr7	CSH	119213708	0
CEU	chr7	CSH	119263708	0
CEU	chr7	CSH	119313708	0
CEU	chr7	CSH	119363708	0
CEU	chr7	CSH	119413708	0
CEU	chr7	CSH	119463708	0
CEU	chr7	CSH	119513708	0
CEU	chr7	CSH	119563708	0
CEU	chr7	CSH	119613708	0
CEU	chr7	CSH	119663708	0
CEU	chr7	CSH	119713708	0
CEU	chr7	CSH	119763708	0
CEU	chr7	CSH	119813708	0
CEU	chr7	CSH	119863708	0
CEU	chr7	CSH	119913708	0
CEU	chr7	CSH	119963708	0
CEU	chr7	CSH	120013708	0
CEU	chr7	CSH	120063708	0
CEU	chr7	CSH	120113708	0
CEU	chr7	CSH	120163708	0
CEU	chr7	CSH	120213708	0
CEU	chr7	CSH	120263708	0
CEU	chr7	CSH	120313708	0
CEU	chr7	CSH	120363708	0
CEU	chr7	CSH	120413708	0
CEU	chr7	CSH	120463708	0
CEU	chr7	CSH	120513708	0

CEU	chr7	CSH	120563708	0
CEU	chr7	CSH	120613708	0
CEU	chr7	CSH	120663708	0
CEU	chr7	CSH	120713708	0
CEU	chr7	CSH	120763708	0
CEU	chr7	CSH	120813708	0
CEU	chr7	CSH	120863708	0
CEU	chr7	CSH	120913708	0
CEU	chr7	CSH	120963708	0
CEU	chr7	CSH	121013708	0
CEU	chr7	CSH	121063708	0
CEU	chr7	CSH	121113708	0
CEU	chr7	CSH	121163708	0
CEU	chr7	CSH	121213708	0
CEU	chr7	CSH	121263708	0
CEU	chr7	CSH	121313708	0
CEU	chr7	CSH	121363708	0
CEU	chr7	CSH	121413708	0
CEU	chr7	CSH	121463708	0
CEU	chr7	CSH	121513708	0
CEU	chr7	CSH	121563708	0
CEU	chr7	CSH	121613708	0
CEU	chr7	CSH	121663708	0
CEU	chr7	CSH	121713708	0
CEU	chr7	CSH	121763708	0
CEU	chr7	CSH	121813708	0
CEU	chr7	CSH	121863708	0
CEU	chr7	CSH	121913708	0
CEU	chr7	CSH	121963708	0
CEU	chr7	CSH	122013708	0
CEU	chr7	CSH	122063708	0
CEU	chr7	CSH	122113708	0
CEU	chr7	CSH	122163708	0
CEU	chr7	CSH	122213708	0
CEU	chr7	CSH	122263708	0
CEU	chr7	CSH	122313708	0
CEU	chr7	CSH	122363708	0
CEU	chr7	CSH	122413708	0
CEU	chr7	CSH	122463708	0
CEU	chr7	CSH	122513708	0
CEU	chr7	CSH	122563708	0
CEU	chr7	CSH	122613708	0
CEU	chr7	CSH	122663708	0
CEU	chr7	CSH	122713708	0
CEU	chr7	CSH	122763708	0
CEU	chr7	CSH	122813708	0
CEU	chr7	CSH	122863708	0
CEU	chr7	CSH	122913708	0
CEU	chr7	CSH	122963708	0

CEU	chr7	CSH	123013708	0
CEU	chr7	CSH	123063708	0
CEU	chr7	CSH	123113708	0
CEU	chr7	CSH	123163708	0
CEU	chr7	CSH	123213708	0
CEU	chr7	CSH	123263708	0
CEU	chr7	CSH	123313708	0
CEU	chr7	CSH	123363708	0
CEU	chr7	CSH	123413708	0
CEU	chr7	CSH	123463708	0
CEU	chr7	CSH	123513708	0
CEU	chr7	CSH	123563708	0
CEU	chr7	CSH	123613708	0
CEU	chr7	CSH	123663708	0
CEU	chr7	CSH	123713708	0
CEU	chr7	CSH	123763708	0
CEU	chr7	CSH	123813708	0
CEU	chr7	CSH	123863708	0
CEU	chr7	CSH	123913708	0
CEU	chr7	CSH	123963708	0
CEU	chr7	CSH	124013708	0
CEU	chr7	CSH	124063708	0
CEU	chr7	CSH	124113708	0
CEU	chr7	CSH	124163708	0
CEU	chr7	CSH	124213708	0
CEU	chr7	CSH	124263708	0
CEU	chr7	CSH	124313708	0
CEU	chr7	CSH	124363708	0
CEU	chr7	CSH	124413708	0
CEU	chr7	CSH	124463708	0
CEU	chr7	CSH	124513708	0
CEU	chr7	CSH	124563708	0
CEU	chr7	CSH	124613708	0
CEU	chr7	CSH	124663708	0
CEU	chr7	CSH	124713708	0
CEU	chr7	CSH	124763708	0
CEU	chr7	CSH	124813708	0
CEU	chr7	CSH	124863708	0
CEU	chr7	CSH	124913708	0
CEU	chr7	CSH	124963708	0
CEU	chr7	CSH	125013708	0
CEU	chr7	CSH	125063708	0
CEU	chr7	CSH	125113708	0
CEU	chr7	CSH	125163708	0
CEU	chr7	CSH	125213708	0
CEU	chr7	CSH	125263708	0
CEU	chr7	CSH	125313708	0
CEU	chr7	CSH	125363708	0
CEU	chr7	CSH	125413708	0

CEU	chr7	CSH	125463708	0
CEU	chr7	CSH	125513708	0
CEU	chr7	CSH	125563708	0
CEU	chr7	CSH	125613708	0
CEU	chr7	CSH	125663708	0
CEU	chr7	CSH	125713708	0
CEU	chr7	CSH	125763708	0
CEU	chr7	CSH	125813708	0
CEU	chr7	CSH	125863708	0
CEU	chr7	CSH	125913708	0
CEU	chr7	CSH	125963708	0
CEU	chr7	CSH	126013708	0
CEU	chr7	CSH	126063708	0
CEU	chr7	CSH	126113708	0
CEU	chr7	CSH	126163708	0
CEU	chr7	CSH	126213708	0
CEU	chr7	CSH	126263708	0
CEU	chr7	CSH	126313708	0
CEU	chr7	CSH	126363708	0
CEU	chr7	CSH	126413708	0
CEU	chr7	CSH	126463708	0
CEU	chr7	CSH	126513708	0
CEU	chr7	CSH	126563708	0
CEU	chr7	CSH	126613708	0
CEU	chr7	CSH	126663708	0
CEU	chr7	CSH	126713708	0
CEU	chr7	CSH	126763708	0
CEU	chr7	CSH	126813708	0
CEU	chr7	CSH	126863708	0
CEU	chr7	CSH	126913708	0
CEU	chr7	CSH	126963708	0
CEU	chr7	CSH	127013708	0
CEU	chr7	CSH	127063708	0
CEU	chr7	CSH	127113708	0
CEU	chr7	CSH	127163708	0
CEU	chr7	CSH	127213708	0
CEU	chr7	CSH	127263708	0
CEU	chr7	CSH	127313708	0
CEU	chr7	CSH	127363708	0
CEU	chr7	CSH	127413708	0
CEU	chr7	CSH	127463708	0
CEU	chr7	CSH	127513708	0
CEU	chr7	CSH	127563708	0
CEU	chr7	CSH	127613708	0
CEU	chr7	CSH	127663708	0
CEU	chr7	CSH	127713708	0
CEU	chr7	CSH	127763708	0
CEU	chr7	CSH	127813708	0
CEU	chr7	CSH	127863708	0

CEU	chr7	CSH	127913708	0
CEU	chr7	CSH	127963708	9
CEU	chr7	CSH	128013708	30
CEU	chr7	CSH	128063708	45
CEU	chr7	CSH	128113708	47
CEU	chr7	CSH	128163708	44
CEU	chr7	CSH	128213708	31
CEU	chr7	CSH	128263708	34
CEU	chr7	CSH	128313708	48
CEU	chr7	CSH	128363708	27
CEU	chr7	CSH	128413708	3
CEU	chr7	CSH	128463708	0
CEU	chr7	CSH	128513708	0
CEU	chr7	CSH	128563708	0
CEU	chr7	CSH	128613708	0
CEU	chr7	CSH	128663708	0
CEU	chr7	CSH	128713708	0
CEU	chr7	CSH	128763708	0
CEU	chr7	CSH	128813708	0
CEU	chr16	CSH	76131945	0
CEU	chr16	CSH	76181945	0
CEU	chr16	CSH	76231945	0
CEU	chr16	CSH	76281945	0
CEU	chr16	CSH	76331945	0
CEU	chr16	CSH	76381945	0
CEU	chr16	CSH	76431945	0
CEU	chr16	CSH	76481945	4
CEU	chr16	CSH	76531945	22
CEU	chr16	CSH	76581945	29
CEU	chr16	CSH	76631945	22
CEU	chr16	CSH	76681945	28
CEU	chr16	CSH	76731945	30
CEU	chr16	CSH	76781945	24
CEU	chr16	CSH	76831945	51
CEU	chr16	CSH	76881945	58
CEU	chr16	CSH	76931945	39
CEU	chr16	CSH	76981945	37
CEU	chr16	CSH	77031945	26
CEU	chr16	CSH	77081945	22
CEU	chr16	CSH	77131945	55
CEU	chr16	CSH	77181945	60
CEU	chr16	CSH	77231945	25
CEU	chr16	CSH	77281945	56
CEU	chr16	CSH	77331945	95
CEU	chr16	CSH	77381945	83
CEU	chr16	CSH	77431945	53
CEU	chr16	CSH	77481945	26
CEU	chr16	CSH	77531945	41
CEU	chr16	CSH	77581945	60

CEU	chr16	CSH	77631945	45
CEU	chr16	CSH	77681945	26
CEU	chr16	CSH	77731945	20
CEU	chr16	CSH	77781945	22
CEU	chr16	CSH	77831945	24
CEU	chr16	CSH	77881945	16
CEU	chr16	CSH	77931945	18
CEU	chr16	CSH	77981945	36
CEU	chr16	CSH	78031945	41
CEU	chr16	CSH	78081945	104
CEU	chr16	CSH	78131945	113
CEU	chr16	CSH	78181945	69
CEU	chr16	CSH	78231945	63
CEU	chr16	CSH	78281945	35
CEU	chr16	CSH	78331945	36
CEU	chr16	CSH	78381945	95
CEU	chr16	CSH	78431945	75
CEU	chr16	CSH	78481945	9
CEU	chr16	CSH	78531945	19
CEU	chr16	CSH	78581945	19
CEU	chr16	CSH	78631945	26
CEU	chr16	CSH	78681945	101
CEU	chr16	CSH	78731945	113
CEU	chr16	CSH	78781945	41
CEU	chr16	CSH	78831945	50
CEU	chr16	CSH	78881945	70
CEU	chr16	CSH	78931945	38
CEU	chr16	CSH	78981945	10
CEU	chr16	CSH	79031945	0
CEU	chr16	CSH	79081945	0
CEU	chr16	CSH	79131945	0
CEU	chr16	CSH	79181945	0
CEU	chr16	CSH	79231945	0
CEU	chr16	CSH	79281945	0
CEU	chr16	CSH	79331945	0
CEU	chr16	CSH	79381945	0
CEU	chr16	CSH	79431945	0
CEU	chr16	CSH	79481945	0
CEU	chr16	CSH	79531945	0
CEU	chr16	CSH	79581945	0
CEU	chr16	CSH	79631945	0
CEU	chr16	CSH	79681945	0
CEU	chr16	CSH	79731945	0
CEU	chr16	CSH	79781945	0
CEU	chr16	CSH	79831945	0
CEU	chr16	CSH	79881945	0
CEU	chr16	CSH	79931945	0
CEU	chr16	CSH	79981945	0
CEU	chr16	CSH	80031945	0

CEU	chr16	CSH	80081945	0
CEU	chr16	CSH	80131945	0
CEU	chr16	CSH	80181945	0
CEU	chr16	CSH	80231945	0
CEU	chr16	CSH	80281945	0
CEU	chr16	CSH	80331945	0
CEU	chr16	CSH	80381945	5
CEU	chr16	CSH	80431945	31
CEU	chr16	CSH	80481945	70
CEU	chr16	CSH	80531945	61
CEU	chr16	CSH	80581945	17
CEU	chr16	CSH	80631945	0
CEU	chr16	CSH	80681945	0
CEU	chr16	CSH	80731945	0
CEU	chr16	CSH	80781945	0
CEU	chr16	CSH	80831945	0
CEU	chr16	CSH	80881945	0
CEU	chr16	CSH	80931945	0
CEU	chr16	CSH	80981945	0
CEU	chr16	CSH	81031945	0
CEU	chr16	CSH	81081945	0
CEU	chr16	CSH	81131945	0
CEU	chr16	CSH	81181945	0
CEU	chr16	CSH	81231945	0
CEU	chr16	CSH	81281945	0
CEU	chr16	CSH	81331945	0
CEU	chr16	CSH	81381945	0
CEU	chr16	CSH	81431945	0
CEU	chr16	CSH	81481945	0
CEU	chr16	CSH	81531945	0
CEU	chr16	CSH	81581945	0
CEU	chr16	CSH	81631945	0
CEU	chr16	CSH	81681945	0
CEU	chr16	CSH	81731945	0
CEU	chr16	CSH	81781945	0
CEU	chr16	CSH	81831945	0
CEU	chr16	CSH	81881945	0
CEU	chr16	CSH	81931945	0
CEU	chr16	CSH	81981945	0
CEU	chr16	CSH	82031945	0
CEU	chr16	CSH	82081945	0
CEU	chr16	CSH	82131945	0
CEU	chr16	CSH	82181945	0
CEU	chr16	CSH	82231945	0
CEU	chr16	CSH	82281945	10
CEU	chr16	CSH	82331945	36
CEU	chr16	CSH	82381945	47
CEU	chr16	CSH	82431945	21
CEU	chr16	CSH	82481945	0

CEU	chr16	CSH	82531945	31
CEU	chr16	CSH	82581945	58
CEU	chr16	CSH	82631945	27
CEU	chr16	CSH	82681945	0
CEU	chr16	CSH	82731945	0
CEU	chr16	CSH	82781945	0
CEU	chr16	CSH	82831945	0
CEU	chr16	CSH	82881945	0
CEU	chr16	CSH	82931945	0
CEU	chr16	CSH	82981945	0
CEU	chr16	CSH	83031945	0
CEU	chr16	CSH	83081945	21
CEU	chr16	CSH	83131945	34
CEU	chr16	CSH	83181945	21
CEU	chr16	CSH	83231945	26
CEU	chr16	CSH	83281945	42
CEU	chr16	CSH	83331945	49
CEU	chr16	CSH	83381945	75
CEU	chr16	CSH	83431945	77
CEU	chr16	CSH	83481945	27
CEU	chr16	CSH	83531945	0
CEU	chr16	CSH	83581945	0
CEU	chr16	CSH	83631945	0
CEU	chr16	CSH	83681945	0
CEU	chr16	CSH	83731945	0
CEU	chr16	CSH	83781945	30
CEU	chr16	CSH	83831945	69
CEU	chr16	CSH	83881945	51
CEU	chr16	CSH	83931945	37
CEU	chr16	CSH	83981945	49
CEU	chr16	CSH	84031945	51
CEU	chr16	CSH	84081945	27
CEU	chr16	CSH	84131945	0
CEU	chr16	CSH	84181945	0
CEU	chr16	CSH	84231945	0
CEU	chr16	CSH	84281945	0
CEU	chr16	CSH	84331945	0
CEU	chr16	CSH	84381945	0
CEU	chr16	CSH	84431945	0
CEU	chr16	CSH	84481945	0
CEU	chr16	CSH	84531945	0
CEU	chr16	CSH	84581945	0
CEU	chr16	CSH	84631945	0
CEU	chr16	CSH	84681945	0
CEU	chr16	CSH	84731945	0
CEU	chr16	CSH	84781945	0
CEU	chr16	CSH	84831945	0
CEU	chr16	CSH	84881945	0
CEU	chr16	CSH	84931945	0

CEU	chr16	CSH	84981945	0
CEU	chr16	CSH	85031945	0
CEU	chr16	CSH	85081945	0
CEU	chr16	CSH	85131945	0
CEU	chr16	CSH	85181945	7
CEU	chr16	CSH	85231945	42
CEU	chr16	CSH	85281945	37
CEU	chr16	CSH	85331945	2
CEU	chr16	CSH	85381945	0
CEU	chr16	CSH	85431945	0
CEU	chr16	CSH	85481945	0
CEU	chr16	CSH	85531945	0
CEU	chr16	CSH	85581945	0
CEU	chr16	CSH	85631945	0
CEU	chr16	CSH	85681945	0
CEU	chr16	CSH	85731945	0
CEU	chr16	CSH	85781945	0
CEU	chr16	CSH	85831945	0
CEU	chr16	CSH	85881945	0
CEU	chr16	CSH	85931945	0
CEU	chr16	CSH	85981945	0
CEU	chr16	CSH	86031945	0
CEU	chr17	CSH	13377873	0
CEU	chr17	CSH	13427873	0
CEU	chr17	CSH	13477873	0
CEU	chr17	CSH	13527873	0
CEU	chr17	CSH	13577873	0
CEU	chr17	CSH	13627873	0
CEU	chr17	CSH	13677873	0
CEU	chr17	CSH	13727873	0
CEU	chr17	CSH	13777873	0
CEU	chr17	CSH	13827873	0
CEU	chr17	CSH	13877873	0
CEU	chr17	CSH	13927873	0
CEU	chr17	CSH	13977873	0
CEU	chr17	CSH	14027873	0
CEU	chr17	CSH	14077873	0
CEU	chr17	CSH	14127873	0
CEU	chr17	CSH	14177873	0
CEU	chr17	CSH	14227873	0
CEU	chr17	CSH	14277873	0
CEU	chr17	CSH	14327873	0
CEU	chr17	CSH	14377873	0
CEU	chr17	CSH	14427873	0
CEU	chr17	CSH	14477873	0
CEU	chr17	CSH	14527873	0
CEU	chr17	CSH	14577873	0
CEU	chr17	CSH	14627873	0
CEU	chr17	CSH	14677873	0

CEU	chr17	CSH	14727873	0
CEU	chr17	CSH	14777873	0
CEU	chr17	CSH	14827873	10
CEU	chr17	CSH	14877873	34
CEU	chr17	CSH	14927873	54
CEU	chr17	CSH	14977873	78
CEU	chr17	CSH	15027873	48
CEU	chr17	CSH	15077873	0
CEU	chr17	CSH	15127873	0
CEU	chr17	CSH	15177873	0
CEU	chr17	CSH	15227873	0
CEU	chr17	CSH	15277873	0
CEU	chr17	CSH	15327873	0
CEU	chr17	CSH	15377873	0
CEU	chr17	CSH	15427873	0
CEU	chr17	CSH	15477873	0
CEU	chr17	CSH	15527873	0
CEU	chr17	CSH	15577873	0
CEU	chr17	CSH	15627873	0
CEU	chr17	CSH	15677873	0
CEU	chr17	CSH	15727873	0
CEU	chr17	CSH	15777873	0
CEU	chr17	CSH	15827873	0
CEU	chr17	CSH	15877873	0
CEU	chr17	CSH	15927873	0
CEU	chr17	CSH	15977873	0
CEU	chr17	CSH	16027873	0
CEU	chr17	CSH	16077873	0
CEU	chr17	CSH	16127873	0
CEU	chr17	CSH	16177873	0
CEU	chr17	CSH	16227873	0
CEU	chr17	CSH	16277873	0
CEU	chr17	CSH	16327873	0
CEU	chr17	CSH	16377873	0
CEU	chr17	CSH	16427873	0
CEU	chr17	CSH	16477873	0
CEU	chr17	CSH	16527873	0
CEU	chr17	CSH	16577873	0
CEU	chr17	CSH	16627873	0
CEU	chr17	CSH	16677873	0
CEU	chr17	CSH	16727873	0
CEU	chr17	CSH	16777873	0
CEU	chr17	CSH	16827873	0
CEU	chr17	CSH	16877873	0
CEU	chr17	CSH	16927873	0
CEU	chr17	CSH	16977873	0
CEU	chr17	CSH	17027873	0
CEU	chr17	CSH	17077873	0
CEU	chr17	CSH	17127873	0

CEU	chr17	CSH	17177873	0
CEU	chr17	CSH	17227873	0
CEU	chr17	CSH	17277873	0
CEU	chr17	CSH	17327873	0
CEU	chr17	CSH	17377873	0
CEU	chr17	CSH	17427873	0
CEU	chr17	CSH	17477873	0
CEU	chr17	CSH	17527873	0
CEU	chr17	CSH	17577873	0
CEU	chr17	CSH	17627873	0
CEU	chr17	CSH	17677873	0
CEU	chr17	CSH	17727873	0
CEU	chr17	CSH	17777873	0
CEU	chr17	CSH	17827873	0
CEU	chr17	CSH	17877873	0
CEU	chr17	CSH	17927873	0
CEU	chr17	CSH	17977873	0
CEU	chr17	CSH	18027873	0
CEU	chr17	CSH	18077873	0
CEU	chr17	CSH	18127873	0
CEU	chr17	CSH	18177873	0
CEU	chr17	CSH	18227873	0
CEU	chr17	CSH	18277873	0
CEU	chr17	CSH	18327873	0
CEU	chr17	CSH	18377873	0
CEU	chr17	CSH	18427873	0
CEU	chr17	CSH	18477873	0
CEU	chr17	CSH	18527873	0
CEU	chr17	CSH	18577873	0
CEU	chr17	CSH	18627873	0
CEU	chr17	CSH	18677873	0
CEU	chr17	CSH	18727873	0
CEU	chr17	CSH	18777873	0
CEU	chr17	CSH	18827873	0
CEU	chr17	CSH	18877873	0
CEU	chr17	CSH	18927873	0
CEU	chr17	CSH	18977873	0
CEU	chr17	CSH	19027873	0
CEU	chr17	CSH	19077873	0
CEU	chr17	CSH	19127873	0
CEU	chr17	CSH	19177873	0
CEU	chr17	CSH	19227873	0
CEU	chr17	CSH	19277873	0
CEU	chr17	CSH	19327873	0
CEU	chr17	CSH	19377873	0
CEU	chr17	CSH	19427873	0
CEU	chr17	CSH	19477873	0
CEU	chr17	CSH	19527873	0
CEU	chr17	CSH	19577873	0

CEU	chr17	CSH	19627873	0
CEU	chr17	CSH	19677873	0
CEU	chr17	CSH	19727873	0
CEU	chr17	CSH	19777873	0
CEU	chr17	CSH	19827873	0
CEU	chr17	CSH	19877873	0
CEU	chr17	CSH	19927873	0
CEU	chr17	CSH	19977873	0
CEU	chr17	CSH	20027873	0
CEU	chr17	CSH	20077873	0
CEU	chr17	CSH	20127873	0
CEU	chr17	CSH	20177873	0
CEU	chr17	CSH	20227873	0
CEU	chr17	CSH	20277873	0
CEU	chr17	CSH	20327873	0
CEU	chr17	CSH	20377873	0
CEU	chr17	CSH	20427873	0
CEU	chr17	CSH	20477873	0
CEU	chr17	CSH	20527873	0
CEU	chr17	CSH	20577873	0
CEU	chr17	CSH	20627873	0
CEU	chr17	CSH	20677873	0
CEU	chr17	CSH	20727873	0
CEU	chr17	CSH	20777873	0
CEU	chr17	CSH	20827873	0
CEU	chr17	CSH	20877873	0
CEU	chr17	CSH	20927873	0
CEU	chr17	CSH	20977873	0
CEU	chr17	CSH	21027873	0
CEU	chr17	CSH	21077873	0
CEU	chr17	CSH	21127873	0
CEU	chr17	CSH	21177873	0
CEU	chr17	CSH	21227873	0
CEU	chr17	CSH	21277873	0
CEU	chr17	CSH	21327873	0
CEU	chr17	CSH	21377873	0
CEU	chr17	CSH	21427873	0
CEU	chr17	CSH	21477873	0
CEU	chr17	CSH	21527873	0
CEU	chr17	CSH	21577873	0
CEU	chr17	CSH	21627873	0
CEU	chr17	CSH	21677873	0
CEU	chr17	CSH	21727873	0
CEU	chr17	CSH	21777873	0
CEU	chr17	CSH	21827873	0
CEU	chr17	CSH	21877873	0
CEU	chr17	CSH	21927873	0
CEU	chr17	CSH	21977873	0
CEU	chr17	CSH	22027873	0

CEU	chr17	CSH	22077873	0
CEU	chr17	CSH	22127873	0
CEU	chr17	CSH	22177873	0
CEU	chr17	CSH	22227873	0
CEU	chr17	CSH	22277873	0
CEU	chr17	CSH	22327873	0
CEU	chr17	CSH	22377873	0
CEU	chr17	CSH	22427873	0
CEU	chr17	CSH	22477873	0
CEU	chr17	CSH	22527873	0
CEU	chr17	CSH	22577873	0
CEU	chr17	CSH	22627873	0
CEU	chr17	CSH	22677873	0
CEU	chr17	CSH	22727873	0
CEU	chr17	CSH	22777873	0
CEU	chr17	CSH	22827873	0
CEU	chr17	CSH	22877873	0
CEU	chr17	CSH	22927873	0
CEU	chr17	CSH	22977873	0
CEU	chr17	CSH	23027873	0
CEU	chr17	CSH	23077873	0
CEU	chr17	CSH	23127873	0
CEU	chr17	CSH	23177873	0
CEU	chr17	CSH	23227873	0
CEU	chr17	CSH	23277873	0
CEU	chr17	CSH	58922012	0
CEU	chr17	CSH	58972012	0
CEU	chr17	CSH	59022012	0
CEU	chr17	CSH	59072012	0
CEU	chr17	CSH	59122012	0
CEU	chr17	CSH	59172012	0
CEU	chr17	CSH	59222012	0
CEU	chr17	CSH	59272012	0
CEU	chr17	CSH	59322012	0
CEU	chr17	CSH	59372012	0
CEU	chr17	CSH	59422012	0
CEU	chr17	CSH	59472012	0
CEU	chr17	CSH	59522012	0
CEU	chr17	CSH	59572012	0
CEU	chr17	CSH	59622012	0
CEU	chr17	CSH	59672012	0
CEU	chr17	CSH	59722012	0
CEU	chr17	CSH	59772012	0
CEU	chr17	CSH	59822012	0
CEU	chr17	CSH	59872012	0
CEU	chr17	CSH	59922012	0
CEU	chr17	CSH	59972012	0
CEU	chr17	CSH	60022012	0
CEU	chr17	CSH	60072012	0

CEU	chr17	CSH	60122012	0
CEU	chr17	CSH	60172012	0
CEU	chr17	CSH	60222012	0
CEU	chr17	CSH	60272012	0
CEU	chr17	CSH	60322012	0
CEU	chr17	CSH	60372012	0
CEU	chr17	CSH	60422012	0
CEU	chr17	CSH	60472012	0
CEU	chr17	CSH	60522012	0
CEU	chr17	CSH	60572012	0
CEU	chr17	CSH	60622012	0
CEU	chr17	CSH	60672012	0
CEU	chr17	CSH	60722012	0
CEU	chr17	CSH	60772012	0
CEU	chr17	CSH	60822012	0
CEU	chr17	CSH	60872012	0
CEU	chr17	CSH	60922012	0
CEU	chr17	CSH	60972012	0
CEU	chr17	CSH	61022012	0
CEU	chr17	CSH	61072012	0
CEU	chr17	CSH	61122012	0
CEU	chr17	CSH	61172012	0
CEU	chr17	CSH	61222012	0
CEU	chr17	CSH	61272012	0
CEU	chr17	CSH	61322012	0
CEU	chr17	CSH	61372012	0
CEU	chr17	CSH	61422012	0
CEU	chr17	CSH	61472012	0
CEU	chr17	CSH	61522012	0
CEU	chr17	CSH	61572012	0
CEU	chr17	CSH	61622012	0
CEU	chr17	CSH	61672012	0
CEU	chr17	CSH	61722012	0
CEU	chr17	CSH	61772012	0
CEU	chr17	CSH	61822012	0
CEU	chr17	CSH	61872012	0
CEU	chr17	CSH	61922012	0
CEU	chr17	CSH	61972012	0
CEU	chr17	CSH	62022012	0
CEU	chr17	CSH	62072012	0
CEU	chr17	CSH	62122012	0
CEU	chr17	CSH	62172012	0
CEU	chr17	CSH	62222012	0
CEU	chr17	CSH	62272012	0
CEU	chr17	CSH	62322012	0
CEU	chr17	CSH	62372012	0
CEU	chr17	CSH	62422012	0
CEU	chr17	CSH	62472012	0
CEU	chr17	CSH	62522012	0

CEU	chr17	CSH	62572012	0
CEU	chr17	CSH	62622012	0
CEU	chr17	CSH	62672012	0
CEU	chr17	CSH	62722012	0
CEU	chr17	CSH	62772012	0
CEU	chr17	CSH	62822012	0
CEU	chr17	CSH	62872012	0
CEU	chr17	CSH	62922012	0
CEU	chr17	CSH	62972012	0
CEU	chr17	CSH	63022012	0
CEU	chr17	CSH	63072012	0
CEU	chr17	CSH	63122012	0
CEU	chr17	CSH	63172012	0
CEU	chr17	CSH	63222012	0
CEU	chr17	CSH	63272012	0
CEU	chr17	CSH	63322012	0
CEU	chr17	CSH	63372012	0
CEU	chr17	CSH	63422012	0
CEU	chr17	CSH	63472012	0
CEU	chr17	CSH	63522012	0
CEU	chr17	CSH	63572012	0
CEU	chr17	CSH	63622012	0
CEU	chr17	CSH	63672012	0
CEU	chr17	CSH	63722012	0
CEU	chr17	CSH	63772012	0
CEU	chr17	CSH	63822012	0
CEU	chr17	CSH	63872012	0
CEU	chr17	CSH	63922012	0
CEU	chr17	CSH	63972012	0
CEU	chr17	CSH	64022012	0
CEU	chr17	CSH	64072012	0
CEU	chr17	CSH	64122012	0
CEU	chr17	CSH	64172012	0
CEU	chr17	CSH	64222012	0
CEU	chr17	CSH	64272012	0
CEU	chr17	CSH	64322012	0
CEU	chr17	CSH	64372012	0
CEU	chr17	CSH	64422012	0
CEU	chr17	CSH	64472012	0
CEU	chr17	CSH	64522012	0
CEU	chr17	CSH	64572012	0
CEU	chr17	CSH	64622012	0
CEU	chr17	CSH	64672012	0
CEU	chr17	CSH	64722012	0
CEU	chr17	CSH	64772012	0
CEU	chr17	CSH	64822012	0
CEU	chr17	CSH	64872012	0
CEU	chr17	CSH	64922012	0
CEU	chr17	CSH	64972012	0

CEU	chr17	CSH	65022012	0
CEU	chr17	CSH	65072012	0
CEU	chr17	CSH	65122012	0
CEU	chr17	CSH	65172012	0
CEU	chr17	CSH	65222012	0
CEU	chr17	CSH	65272012	0
CEU	chr17	CSH	65322012	0
CEU	chr17	CSH	65372012	0
CEU	chr17	CSH	65422012	3
CEU	chr17	CSH	65472012	29
CEU	chr17	CSH	65522012	53
CEU	chr17	CSH	65572012	44
CEU	chr17	CSH	65622012	17
CEU	chr17	CSH	65672012	0
CEU	chr17	CSH	65722012	0
CEU	chr17	CSH	65772012	0
CEU	chr17	CSH	65822012	0
CEU	chr17	CSH	65872012	0
CEU	chr17	CSH	65922012	0
CEU	chr17	CSH	65972012	0
CEU	chr17	CSH	66022012	0
CEU	chr17	CSH	66072012	0
CEU	chr17	CSH	66122012	0
CEU	chr17	CSH	66172012	0
CEU	chr17	CSH	66222012	0
CEU	chr17	CSH	66272012	0
CEU	chr17	CSH	66322012	0
CEU	chr17	CSH	66372012	0
CEU	chr17	CSH	66422012	0
CEU	chr17	CSH	66472012	0
CEU	chr17	CSH	66522012	0
CEU	chr17	CSH	66572012	0
CEU	chr17	CSH	66622012	0
CEU	chr17	CSH	66672012	0
CEU	chr17	CSH	66722012	0
CEU	chr17	CSH	66772012	0
CEU	chr17	CSH	66822012	0
CEU	chr17	CSH	66872012	0
CEU	chr17	CSH	66922012	0
CEU	chr17	CSH	66972012	0
CEU	chr17	CSH	67022012	0
CEU	chr17	CSH	67072012	0
CEU	chr17	CSH	67122012	0
CEU	chr17	CSH	67172012	0
CEU	chr17	CSH	67222012	0
CEU	chr17	CSH	67272012	0
CEU	chr17	CSH	67322012	0
CEU	chr17	CSH	67372012	0
CEU	chr17	CSH	67422012	0

CEU	chr17	CSH	67472012	0
CEU	chr17	CSH	67522012	0
CEU	chr17	CSH	67572012	0
CEU	chr17	CSH	67622012	0
CEU	chr17	CSH	67672012	0
CEU	chr17	CSH	67722012	0
CEU	chr17	CSH	67772012	0
CEU	chr17	CSH	67822012	0
CEU	chr17	CSH	67872012	0
CEU	chr17	CSH	67922012	0
CEU	chr17	CSH	67972012	0
CEU	chr17	CSH	68022012	0
CEU	chr17	CSH	68072012	0
CEU	chr17	CSH	68122012	0
CEU	chr17	CSH	68172012	0
CEU	chr17	CSH	68222012	0
CEU	chr17	CSH	68272012	0
CEU	chr17	CSH	68322012	0
CEU	chr17	CSH	68372012	0
CEU	chr17	CSH	68422012	0
CEU	chr17	CSH	68472012	0
CEU	chr17	CSH	68522012	0
CEU	chr17	CSH	68572012	0
CEU	chr17	CSH	68622012	0
CEU	chr17	CSH	68672012	0
CEU	chr17	CSH	68722012	0
CEU	chr17	CSH	68772012	0
CEU	chr17	CSH	68822012	0
CEU	chr19	CSH	6485290	0
CEU	chr19	CSH	6535290	0
CEU	chr19	CSH	6585290	0
CEU	chr19	CSH	6635290	0
CEU	chr19	CSH	6685290	0
CEU	chr19	CSH	6735290	0
CEU	chr19	CSH	6785290	0
CEU	chr19	CSH	6835290	38
CEU	chr19	CSH	6885290	41
CEU	chr19	CSH	6935290	3
CEU	chr19	CSH	6985290	0
CEU	chr19	CSH	7035290	0
CEU	chr19	CSH	7085290	2
CEU	chr19	CSH	7135290	27
CEU	chr19	CSH	7185290	25
CEU	chr19	CSH	7235290	0
CEU	chr19	CSH	7285290	0
CEU	chr19	CSH	7335290	0
CEU	chr19	CSH	7385290	0
CEU	chr19	CSH	7435290	0
CEU	chr19	CSH	7485290	0

CEU	chr19	CSH	7535290	0
CEU	chr19	CSH	7585290	0
CEU	chr19	CSH	7635290	0
CEU	chr19	CSH	7685290	0
CEU	chr19	CSH	7735290	0
CEU	chr19	CSH	7785290	0
CEU	chr19	CSH	7835290	0
CEU	chr19	CSH	7885290	0
CEU	chr19	CSH	7935290	0
CEU	chr19	CSH	7985290	0
CEU	chr19	CSH	8035290	0
CEU	chr19	CSH	8085290	6
CEU	chr19	CSH	8135290	30
CEU	chr19	CSH	8185290	42
CEU	chr19	CSH	8235290	29
CEU	chr19	CSH	8285290	11
CEU	chr19	CSH	8335290	0
CEU	chr19	CSH	8385290	0
CEU	chr19	CSH	8435290	0
CEU	chr19	CSH	8485290	0
CEU	chr19	CSH	8535290	0
CEU	chr19	CSH	8585290	0
CEU	chr19	CSH	8635290	0
CEU	chr19	CSH	8685290	0
CEU	chr19	CSH	8735290	0
CEU	chr19	CSH	8785290	0
CEU	chr19	CSH	8835290	0
CEU	chr19	CSH	8885290	0
CEU	chr19	CSH	8935290	0
CEU	chr19	CSH	8985290	0
CEU	chr19	CSH	9035290	0
CEU	chr19	CSH	9085290	0
CEU	chr19	CSH	9135290	0
CEU	chr19	CSH	9185290	0
CEU	chr19	CSH	9235290	0
CEU	chr19	CSH	9285290	0
CEU	chr19	CSH	9335290	0
CEU	chr19	CSH	9385290	0
CEU	chr19	CSH	9435290	0
CEU	chr19	CSH	9485290	0
CEU	chr19	CSH	9535290	0
CEU	chr19	CSH	9585290	0
CEU	chr19	CSH	9635290	0
CEU	chr19	CSH	9685290	0
CEU	chr19	CSH	9735290	0
CEU	chr19	CSH	9785290	0
CEU	chr19	CSH	9835290	0
CEU	chr19	CSH	9885290	0
CEU	chr19	CSH	9935290	0

CEU	chr19	CSH	9985290	0
CEU	chr19	CSH	10035290	7
CEU	chr19	CSH	10085290	27
CEU	chr19	CSH	10135290	20
CEU	chr19	CSH	10185290	19
CEU	chr19	CSH	10235290	55
CEU	chr19	CSH	10285290	63
CEU	chr19	CSH	10335290	40
CEU	chr19	CSH	10385290	13
CEU	chr19	CSH	10435290	0
CEU	chr19	CSH	10485290	0
CEU	chr19	CSH	10535290	0
CEU	chr19	CSH	10585290	0
CEU	chr19	CSH	10635290	0
CEU	chr19	CSH	10685290	0
CEU	chr19	CSH	10735290	0
CEU	chr19	CSH	10785290	0
CEU	chr19	CSH	10835290	0
CEU	chr19	CSH	10885290	0
CEU	chr19	CSH	10935290	0
CEU	chr19	CSH	10985290	0
CEU	chr19	CSH	11035290	0
CEU	chr19	CSH	11085290	0
CEU	chr19	CSH	11135290	0
CEU	chr19	CSH	11185290	0
CEU	chr19	CSH	11235290	0
CEU	chr19	CSH	11285290	0
CEU	chr19	CSH	11335290	0
CEU	chr19	CSH	11385290	0
CEU	chr19	CSH	11435290	0
CEU	chr19	CSH	11485290	0
CEU	chr19	CSH	11535290	0
CEU	chr19	CSH	11585290	0
CEU	chr19	CSH	11635290	0
CEU	chr19	CSH	11685290	14
CEU	chr19	CSH	11735290	30
CEU	chr19	CSH	11785290	40
CEU	chr19	CSH	11835290	29
CEU	chr19	CSH	11885290	5
CEU	chr19	CSH	11935290	0
CEU	chr19	CSH	11985290	0
CEU	chr19	CSH	12035290	0
CEU	chr19	CSH	12085290	0
CEU	chr19	CSH	12135290	0
CEU	chr19	CSH	12185290	0
CEU	chr19	CSH	12235290	0
CEU	chr19	CSH	12285290	0
CEU	chr19	CSH	12335290	0
CEU	chr19	CSH	12385290	0

CEU	chr19	CSH	12435290	0
CEU	chr19	CSH	12485290	0
CEU	chr19	CSH	12535290	0
CEU	chr19	CSH	12585290	0
CEU	chr19	CSH	12635290	0
CEU	chr19	CSH	12685290	0
CEU	chr19	CSH	12735290	0
CEU	chr19	CSH	12785290	0
CEU	chr19	CSH	12835290	0
CEU	chr19	CSH	12885290	0
CEU	chr19	CSH	12935290	0
CEU	chr19	CSH	12985290	0
CEU	chr19	CSH	13035290	0
CEU	chr19	CSH	13085290	0
CEU	chr19	CSH	13135290	0
CEU	chr19	CSH	13185290	0
CEU	chr19	CSH	13235290	0
CEU	chr19	CSH	13285290	0
CEU	chr19	CSH	13335290	0
CEU	chr19	CSH	13385290	0
CEU	chr19	CSH	13435290	0
CEU	chr19	CSH	13485290	0
CEU	chr19	CSH	13535290	0
CEU	chr19	CSH	13585290	0
CEU	chr19	CSH	13635290	0
CEU	chr19	CSH	13685290	0
CEU	chr19	CSH	13735290	0
CEU	chr19	CSH	13785290	0
CEU	chr19	CSH	13835290	0
CEU	chr19	CSH	13885290	0
CEU	chr19	CSH	13935290	0
CEU	chr19	CSH	13985290	0
CEU	chr19	CSH	14035290	0
CEU	chr19	CSH	14085290	0
CEU	chr19	CSH	14135290	0
CEU	chr19	CSH	14185290	0
CEU	chr19	CSH	14235290	0
CEU	chr19	CSH	14285290	0
CEU	chr19	CSH	14335290	0
CEU	chr19	CSH	14385290	0
CEU	chr19	CSH	14435290	0
CEU	chr19	CSH	14485290	0
CEU	chr19	CSH	14535290	0
CEU	chr19	CSH	14585290	0
CEU	chr19	CSH	14635290	0
CEU	chr19	CSH	14685290	0
CEU	chr19	CSH	14735290	0
CEU	chr19	CSH	14785290	0
CEU	chr19	CSH	14835290	0

CEU	chr19	CSH	14885290	0
CEU	chr19	CSH	14935290	0
CEU	chr19	CSH	14985290	0
CEU	chr19	CSH	15035290	0
CEU	chr19	CSH	15085290	0
CEU	chr19	CSH	15135290	0
CEU	chr19	CSH	15185290	0
CEU	chr19	CSH	15235290	0
CEU	chr19	CSH	15285290	0
CEU	chr19	CSH	15335290	0
CEU	chr19	CSH	15385290	0
CEU	chr19	CSH	15435290	0
CEU	chr19	CSH	15485290	0
CEU	chr19	CSH	15535290	0
CEU	chr19	CSH	15585290	0
CEU	chr19	CSH	15635290	0
CEU	chr19	CSH	15685290	0
CEU	chr19	CSH	15735290	0
CEU	chr19	CSH	15785290	0
CEU	chr19	CSH	15835290	0
CEU	chr19	CSH	15885290	0
CEU	chr19	CSH	15935290	0
CEU	chr19	CSH	15985290	0
CEU	chr19	CSH	16035290	0
CEU	chr19	CSH	16085290	0
CEU	chr19	CSH	16135290	0
CEU	chr19	CSH	16185290	0
CEU	chr19	CSH	16235290	0
CEU	chr19	CSH	16285290	0
CEU	chr19	CSH	16335290	0
CEU	chr19	CSH	16385290	0
CEU	chr19	CSH	53548333	0
CEU	chr19	CSH	53598333	0
CEU	chr19	CSH	53648333	0
CEU	chr19	CSH	53698333	0
CEU	chr19	CSH	53748333	0
CEU	chr19	CSH	53798333	0
CEU	chr19	CSH	53848333	0
CEU	chr19	CSH	53898333	0
CEU	chr19	CSH	53948333	18
CEU	chr19	CSH	53998333	40
CEU	chr19	CSH	54048333	22
CEU	chr19	CSH	54098333	0
CEU	chr19	CSH	54148333	0
CEU	chr19	CSH	54198333	0
CEU	chr19	CSH	54248333	0
CEU	chr19	CSH	54298333	0
CEU	chr19	CSH	54348333	0
CEU	chr19	CSH	54398333	0

CEU	chr19	CSH	54448333	0
CEU	chr19	CSH	54498333	3
CEU	chr19	CSH	54548333	61
CEU	chr19	CSH	54598333	58
CEU	chr19	CSH	54648333	0
CEU	chr19	CSH	54698333	0
CEU	chr19	CSH	54748333	44
CEU	chr19	CSH	54798333	44
CEU	chr19	CSH	54848333	8
CEU	chr19	CSH	54898333	35
CEU	chr19	CSH	54948333	27
CEU	chr19	CSH	54998333	0
CEU	chr19	CSH	55048333	23
CEU	chr19	CSH	55098333	86
CEU	chr19	CSH	55148333	74
CEU	chr19	CSH	55198333	11
CEU	chr19	CSH	55248333	0
CEU	chr19	CSH	55298333	0
CEU	chr19	CSH	55348333	0
CEU	chr19	CSH	55398333	0
CEU	chr19	CSH	55448333	0
CEU	chr19	CSH	55498333	0
CEU	chr19	CSH	55548333	0
CEU	chr19	CSH	55598333	0
CEU	chr19	CSH	55648333	0
CEU	chr19	CSH	55698333	0
CEU	chr19	CSH	55748333	0
CEU	chr19	CSH	55798333	0
CEU	chr19	CSH	55848333	0
CEU	chr19	CSH	55898333	0
CEU	chr19	CSH	55948333	0
CEU	chr19	CSH	55998333	0
CEU	chr19	CSH	56048333	12
CEU	chr19	CSH	56098333	26
CEU	chr19	CSH	56148333	39
CEU	chr19	CSH	56198333	51
CEU	chr19	CSH	56248333	26
CEU	chr19	CSH	56298333	0
CEU	chr19	CSH	56348333	0
CEU	chr19	CSH	56398333	0
CEU	chr19	CSH	56448333	0
CEU	chr19	CSH	56498333	0
CEU	chr19	CSH	56548333	0
CEU	chr19	CSH	56598333	0
CEU	chr19	CSH	56648333	25
CEU	chr19	CSH	56698333	54
CEU	chr19	CSH	56748333	29
CEU	chr19	CSH	56798333	0
CEU	chr19	CSH	56848333	0

CEU	chr19	CSH	56898333	0
CEU	chr19	CSH	56948333	0
CEU	chr19	CSH	56998333	0
CEU	chr19	CSH	57048333	0
CEU	chr19	CSH	57098333	0
CEU	chr19	CSH	57148333	0
CEU	chr19	CSH	57198333	0
CEU	chr19	CSH	57248333	0
CEU	chr19	CSH	57298333	0
CEU	chr19	CSH	57348333	0
CEU	chr19	CSH	57398333	0
CEU	chr19	CSH	57448333	0
CEU	chr19	CSH	57498333	0
CEU	chr19	CSH	57548333	0
CEU	chr19	CSH	57598333	0
CEU	chr19	CSH	57648333	0
CEU	chr19	CSH	57698333	0
CEU	chr19	CSH	57748333	0
CEU	chr19	CSH	57798333	0
CEU	chr19	CSH	57848333	0
CEU	chr19	CSH	57898333	0
CEU	chr19	CSH	57948333	0
CEU	chr19	CSH	57998333	0
CEU	chr19	CSH	58048333	0
CEU	chr19	CSH	58098333	0
CEU	chr19	CSH	58148333	0
CEU	chr19	CSH	58198333	0
CEU	chr19	CSH	58248333	0
CEU	chr19	CSH	58298333	0
CEU	chr19	CSH	58348333	0
CEU	chr19	CSH	58398333	0
CEU	chr19	CSH	58448333	0
CEU	chr19	CSH	58498333	0
CEU	chr19	CSH	58548333	0
CEU	chr19	CSH	58598333	0
CEU	chr19	CSH	58648333	0
CEU	chr19	CSH	58698333	0
CEU	chr19	CSH	58748333	0
CEU	chr19	CSH	58798333	0
CEU	chr19	CSH	58848333	0
CEU	chr19	CSH	58898333	0
CEU	chr19	CSH	58948333	0
CEU	chr19	CSH	58998333	0
CEU	chr19	CSH	59048333	0
CEU	chr19	CSH	59098333	0
CEU	chr19	CSH	59148333	0
CEU	chr19	CSH	59198333	0
CEU	chr19	CSH	59248333	0
CEU	chr19	CSH	59298333	0

CEU	chr19	CSH	59348333	0
CEU	chr19	CSH	59398333	0
CEU	chr19	CSH	59448333	0
CEU	chr19	CSH	59498333	0
CEU	chr19	CSH	59548333	0
CEU	chr19	CSH	59598333	0
CEU	chr19	CSH	59648333	0
CEU	chr19	CSH	59698333	0
CEU	chr19	CSH	59748333	0
CEU	chr19	CSH	59798333	0
CEU	chr19	CSH	59848333	0
CEU	chr19	CSH	59898333	0
CEU	chr19	CSH	59948333	0
CEU	chr19	CSH	59998333	0
CEU	chr19	CSH	60048333	0
CEU	chr19	CSH	60098333	0
CEU	chr19	CSH	60148333	0
CEU	chr19	CSH	60198333	0
CEU	chr19	CSH	60248333	0
CEU	chr19	CSH	60298333	0
CEU	chr19	CSH	60348333	0
CEU	chr19	CSH	60398333	0
CEU	chr19	CSH	60448333	0
CEU	chr19	CSH	60498333	0
CEU	chr19	CSH	60548333	0
CEU	chr19	CSH	60598333	0
CEU	chr19	CSH	60648333	0
CEU	chr19	CSH	60698333	0
CEU	chr19	CSH	60748333	0
CEU	chr19	CSH	60798333	0
CEU	chr19	CSH	60848333	0
CEU	chr19	CSH	60898333	0
CEU	chr19	CSH	60948333	0
CEU	chr19	CSH	60998333	0
CEU	chr19	CSH	61048333	0
CEU	chr19	CSH	61098333	0
CEU	chr19	CSH	61148333	0
CEU	chr19	CSH	61198333	0
CEU	chr19	CSH	61248333	0
CEU	chr19	CSH	61298333	0
CEU	chr19	CSH	61348333	0
CEU	chr19	CSH	61398333	0
CEU	chr19	CSH	61448333	0
CEU	chr19	CSH	61498333	0
CEU	chr19	CSH	61548333	0
CEU	chr19	CSH	61598333	0
CEU	chr19	CSH	61648333	0
CEU	chr19	CSH	61698333	0
CEU	chr19	CSH	61748333	0

CEU	chr19	CSH	61798333	0
CEU	chr19	CSH	61848333	0
CEU	chr19	CSH	61898333	0
CEU	chr19	CSH	61948333	0
CEU	chr19	CSH	61998333	0
CEU	chr19	CSH	62048333	0
CEU	chr19	CSH	62098333	0
CEU	chr19	CSH	62148333	0
CEU	chr19	CSH	62198333	0
CEU	chr19	CSH	62248333	0
CEU	chr19	CSH	62298333	0
CEU	chr19	CSH	62348333	0
CEU	chr19	CSH	62398333	0
CEU	chr19	CSH	62448333	0
CEU	chr19	CSH	62498333	0
CEU	chr19	CSH	62548333	0
CEU	chr19	CSH	62598333	0
CEU	chr19	CSH	62648333	0
CEU	chr19	CSH	62698333	0
CEU	chr19	CSH	62748333	0
CEU	chr19	CSH	62798333	0
CEU	chr19	CSH	62848333	0
CEU	chr19	CSH	62898333	0
CEU	chr19	CSH	62948333	0
CEU	chr19	CSH	62998333	0
CEU	chr19	CSH	63048333	0
CEU	chr19	CSH	63098333	0
CEU	chr19	CSH	63148333	0
CEU	chr19	CSH	63198333	0
CEU	chr19	CSH	63248333	0
CEU	chr19	CSH	63298333	0
CEU	chr19	CSH	63348333	0
CEU	chr19	CSH	63398333	0
CEU	chr19	CSH	63448333	0
CEU	chr8	DEFA1	2027649	43
CEU	chr8	DEFA1	2077649	36
CEU	chr8	DEFA1	2127649	51
CEU	chr8	DEFA1	2177649	60
CEU	chr8	DEFA1	2227649	41
CEU	chr8	DEFA1	2277649	41
CEU	chr8	DEFA1	2327649	33
CEU	chr8	DEFA1	2377649	19
CEU	chr8	DEFA1	2427649	14
CEU	chr8	DEFA1	2477649	13
CEU	chr8	DEFA1	2527649	20
CEU	chr8	DEFA1	2577649	30
CEU	chr8	DEFA1	2627649	45
CEU	chr8	DEFA1	2677649	44
CEU	chr8	DEFA1	2727649	40

CEU	chr8	DEFA1	2777649	29
CEU	chr8	DEFA1	2827649	29
CEU	chr8	DEFA1	2877649	79
CEU	chr8	DEFA1	2927649	78
CEU	chr8	DEFA1	2977649	30
CEU	chr8	DEFA1	3027649	28
CEU	chr8	DEFA1	3077649	52
CEU	chr8	DEFA1	3127649	111
CEU	chr8	DEFA1	3177649	160
CEU	chr8	DEFA1	3227649	153
CEU	chr8	DEFA1	3277649	146
CEU	chr8	DEFA1	3327649	80
CEU	chr8	DEFA1	3377649	8
CEU	chr8	DEFA1	3427649	38
CEU	chr8	DEFA1	3477649	64
CEU	chr8	DEFA1	3527649	28
CEU	chr8	DEFA1	3577649	0
CEU	chr8	DEFA1	3627649	0
CEU	chr8	DEFA1	3677649	0
CEU	chr8	DEFA1	3727649	0
CEU	chr8	DEFA1	3777649	0
CEU	chr8	DEFA1	3827649	0
CEU	chr8	DEFA1	3877649	0
CEU	chr8	DEFA1	3927649	0
CEU	chr8	DEFA1	3977649	0
CEU	chr8	DEFA1	4027649	0
CEU	chr8	DEFA1	4077649	3
CEU	chr8	DEFA1	4127649	36
CEU	chr8	DEFA1	4177649	33
CEU	chr8	DEFA1	4227649	0
CEU	chr8	DEFA1	4277649	0
CEU	chr8	DEFA1	4327649	0
CEU	chr8	DEFA1	4377649	0
CEU	chr8	DEFA1	4427649	0
CEU	chr8	DEFA1	4477649	0
CEU	chr8	DEFA1	4527649	0
CEU	chr8	DEFA1	4577649	16
CEU	chr8	DEFA1	4627649	67
CEU	chr8	DEFA1	4677649	51
CEU	chr8	DEFA1	4727649	0
CEU	chr8	DEFA1	4777649	0
CEU	chr8	DEFA1	4827649	0
CEU	chr8	DEFA1	4877649	0
CEU	chr8	DEFA1	4927649	0
CEU	chr8	DEFA1	4977649	0
CEU	chr8	DEFA1	5027649	0
CEU	chr8	DEFA1	5077649	0
CEU	chr8	DEFA1	5127649	0
CEU	chr8	DEFA1	5177649	0

CEU	chr8	DEFA1	5227649	0
CEU	chr8	DEFA1	5277649	0
CEU	chr8	DEFA1	5327649	0
CEU	chr8	DEFA1	5377649	0
CEU	chr8	DEFA1	5427649	0
CEU	chr8	DEFA1	5477649	0
CEU	chr8	DEFA1	5527649	0
CEU	chr8	DEFA1	5577649	0
CEU	chr8	DEFA1	5627649	0
CEU	chr8	DEFA1	5677649	0
CEU	chr8	DEFA1	5727649	0
CEU	chr8	DEFA1	5777649	0
CEU	chr8	DEFA1	5827649	0
CEU	chr8	DEFA1	5877649	0
CEU	chr8	DEFA1	5927649	0
CEU	chr8	DEFA1	5977649	0
CEU	chr8	DEFA1	6027649	0
CEU	chr8	DEFA1	6077649	0
CEU	chr8	DEFA1	6127649	0
CEU	chr8	DEFA1	6177649	0
CEU	chr8	DEFA1	6227649	0
CEU	chr8	DEFA1	6277649	0
CEU	chr8	DEFA1	6327649	0
CEU	chr8	DEFA1	6377649	0
CEU	chr8	DEFA1	6427649	0
CEU	chr8	DEFA1	6477649	0
CEU	chr8	DEFA1	6527649	0
CEU	chr8	DEFA1	6577649	0
CEU	chr8	DEFA1	6627649	0
CEU	chr8	DEFA1	6677649	0
CEU	chr8	DEFA1	6727649	0
CEU	chr8	DEFA1	6777649	0
CEU	chr8	DEFA1	6827649	0
CEU	chr8	DEFA1	6877649	0
CEU	chr8	DEFA1	6927649	0
CEU	chr8	DEFA1	6977649	0
CEU	chr8	DEFA1	7027649	0
CEU	chr8	DEFA1	7077649	0
CEU	chr8	DEFA1	7127649	0
CEU	chr8	DEFA1	7177649	0
CEU	chr8	DEFA1	7227649	0
CEU	chr8	DEFA1	7277649	0
CEU	chr8	DEFA1	7327649	0
CEU	chr8	DEFA1	7377649	0
CEU	chr8	DEFA1	7427649	0
CEU	chr8	DEFA1	7477649	0
CEU	chr8	DEFA1	7527649	0
CEU	chr8	DEFA1	7577649	0
CEU	chr8	DEFA1	7627649	0

CEU	chr8	DEFA1	7677649	0
CEU	chr8	DEFA1	7727649	0
CEU	chr8	DEFA1	7777649	0
CEU	chr8	DEFA1	7827649	0
CEU	chr8	DEFA1	7877649	0
CEU	chr8	DEFA1	7927649	0
CEU	chr8	DEFA1	7977649	0
CEU	chr8	DEFA1	8027649	0
CEU	chr8	DEFA1	8077649	0
CEU	chr8	DEFA1	8127649	0
CEU	chr8	DEFA1	8177649	0
CEU	chr8	DEFA1	8227649	0
CEU	chr8	DEFA1	8277649	0
CEU	chr8	DEFA1	8327649	0
CEU	chr8	DEFA1	8377649	0
CEU	chr8	DEFA1	8427649	0
CEU	chr8	DEFA1	8477649	0
CEU	chr8	DEFA1	8527649	0
CEU	chr8	DEFA1	8577649	0
CEU	chr8	DEFA1	8627649	0
CEU	chr8	DEFA1	8677649	0
CEU	chr8	DEFA1	8727649	0
CEU	chr8	DEFA1	8777649	0
CEU	chr8	DEFA1	8827649	0
CEU	chr8	DEFA1	8877649	0
CEU	chr8	DEFA1	8927649	0
CEU	chr8	DEFA1	8977649	0
CEU	chr8	DEFA1	9027649	33
CEU	chr8	DEFA1	9077649	61
CEU	chr8	DEFA1	9127649	50
CEU	chr8	DEFA1	9177649	56
CEU	chr8	DEFA1	9227649	53
CEU	chr8	DEFA1	9277649	49
CEU	chr8	DEFA1	9327649	30
CEU	chr8	DEFA1	9377649	0
CEU	chr8	DEFA1	9427649	0
CEU	chr8	DEFA1	9477649	0
CEU	chr8	DEFA1	9527649	0
CEU	chr8	DEFA1	9577649	0
CEU	chr8	DEFA1	9627649	0
CEU	chr8	DEFA1	9677649	0
CEU	chr8	DEFA1	9727649	0
CEU	chr8	DEFA1	9777649	0
CEU	chr8	DEFA1	9827649	0
CEU	chr8	DEFA1	9877649	0
CEU	chr8	DEFA1	9927649	0
CEU	chr8	DEFA1	9977649	0
CEU	chr8	DEFA1	10027649	0
CEU	chr8	DEFA1	10077649	0

CEU	chr8	DEFA1	10127649	0
CEU	chr8	DEFA1	10177649	0
CEU	chr8	DEFA1	10227649	0
CEU	chr8	DEFA1	10277649	0
CEU	chr8	DEFA1	10327649	0
CEU	chr8	DEFA1	10377649	0
CEU	chr8	DEFA1	10427649	0
CEU	chr8	DEFA1	10477649	0
CEU	chr8	DEFA1	10527649	0
CEU	chr8	DEFA1	10577649	0
CEU	chr8	DEFA1	10627649	0
CEU	chr8	DEFA1	10677649	0
CEU	chr8	DEFA1	10727649	0
CEU	chr8	DEFA1	10777649	0
CEU	chr8	DEFA1	10827649	0
CEU	chr8	DEFA1	10877649	0
CEU	chr8	DEFA1	10927649	0
CEU	chr8	DEFA1	10977649	0
CEU	chr8	DEFA1	11027649	0
CEU	chr8	DEFA1	11077649	0
CEU	chr8	DEFA1	11127649	0
CEU	chr8	DEFA1	11177649	0
CEU	chr8	DEFA1	11227649	0
CEU	chr8	DEFA1	11277649	0
CEU	chr8	DEFA1	11327649	0
CEU	chr8	DEFA1	11377649	0
CEU	chr8	DEFA1	11427649	0
CEU	chr8	DEFA1	11477649	0
CEU	chr8	DEFA1	11527649	0
CEU	chr8	DEFA1	11577649	6
CEU	chr8	DEFA1	11627649	31
CEU	chr8	DEFA1	11677649	47
CEU	chr8	DEFA1	11727649	30
CEU	chr8	DEFA1	11777649	23
CEU	chr8	DEFA1	11827649	16
CEU	chr8	DEFA1	11877649	1
CEU	chr8	DEFA1	11927649	0
CEU	chr8	DEFB130	7114389	0
CEU	chr8	DEFB130	7164389	0
CEU	chr8	DEFB130	7214389	0
CEU	chr8	DEFB130	7264389	0
CEU	chr8	DEFB130	7314389	0
CEU	chr8	DEFB130	7364389	0
CEU	chr8	DEFB130	7414389	0
CEU	chr8	DEFB130	7464389	0
CEU	chr8	DEFB130	7514389	0
CEU	chr8	DEFB130	7564389	0
CEU	chr8	DEFB130	7614389	0
CEU	chr8	DEFB130	7664389	0

CEU	chr8	DEFB130	7714389	0
CEU	chr8	DEFB130	7764389	0
CEU	chr8	DEFB130	7814389	0
CEU	chr8	DEFB130	7864389	0
CEU	chr8	DEFB130	7914389	0
CEU	chr8	DEFB130	7964389	0
CEU	chr8	DEFB130	8014389	0
CEU	chr8	DEFB130	8064389	0
CEU	chr8	DEFB130	8114389	0
CEU	chr8	DEFB130	8164389	0
CEU	chr8	DEFB130	8214389	0
CEU	chr8	DEFB130	8264389	0
CEU	chr8	DEFB130	8314389	0
CEU	chr8	DEFB130	8364389	0
CEU	chr8	DEFB130	8414389	0
CEU	chr8	DEFB130	8464389	0
CEU	chr8	DEFB130	8514389	0
CEU	chr8	DEFB130	8564389	0
CEU	chr8	DEFB130	8614389	0
CEU	chr8	DEFB130	8664389	0
CEU	chr8	DEFB130	8714389	0
CEU	chr8	DEFB130	8764389	0
CEU	chr8	DEFB130	8814389	0
CEU	chr8	DEFB130	8864389	0
CEU	chr8	DEFB130	8914389	0
CEU	chr8	DEFB130	8964389	0
CEU	chr8	DEFB130	9014389	18
CEU	chr8	DEFB130	9064389	53
CEU	chr8	DEFB130	9114389	54
CEU	chr8	DEFB130	9164389	57
CEU	chr8	DEFB130	9214389	55
CEU	chr8	DEFB130	9264389	55
CEU	chr8	DEFB130	9314389	39
CEU	chr8	DEFB130	9364389	1
CEU	chr8	DEFB130	9414389	0
CEU	chr8	DEFB130	9464389	0
CEU	chr8	DEFB130	9514389	0
CEU	chr8	DEFB130	9564389	0
CEU	chr8	DEFB130	9614389	0
CEU	chr8	DEFB130	9664389	0
CEU	chr8	DEFB130	9714389	0
CEU	chr8	DEFB130	9764389	0
CEU	chr8	DEFB130	9814389	0
CEU	chr8	DEFB130	9864389	0
CEU	chr8	DEFB130	9914389	0
CEU	chr8	DEFB130	9964389	0
CEU	chr8	DEFB130	10014389	0
CEU	chr8	DEFB130	10064389	0
CEU	chr8	DEFB130	10114389	0

CEU	chr8	DEFB130	10164389	0
CEU	chr8	DEFB130	10214389	0
CEU	chr8	DEFB130	10264389	0
CEU	chr8	DEFB130	10314389	0
CEU	chr8	DEFB130	10364389	0
CEU	chr8	DEFB130	10414389	0
CEU	chr8	DEFB130	10464389	0
CEU	chr8	DEFB130	10514389	0
CEU	chr8	DEFB130	10564389	0
CEU	chr8	DEFB130	10614389	0
CEU	chr8	DEFB130	10664389	0
CEU	chr8	DEFB130	10714389	0
CEU	chr8	DEFB130	10764389	0
CEU	chr8	DEFB130	10814389	0
CEU	chr8	DEFB130	10864389	0
CEU	chr8	DEFB130	10914389	0
CEU	chr8	DEFB130	10964389	0
CEU	chr8	DEFB130	11014389	0
CEU	chr8	DEFB130	11064389	0
CEU	chr8	DEFB130	11114389	0
CEU	chr8	DEFB130	11164389	0
CEU	chr8	DEFB130	11214389	0
CEU	chr8	DEFB130	11264389	0
CEU	chr8	DEFB130	11314389	0
CEU	chr8	DEFB130	11364389	0
CEU	chr8	DEFB130	11414389	0
CEU	chr8	DEFB130	11464389	0
CEU	chr8	DEFB130	11514389	0
CEU	chr8	DEFB130	11564389	2
CEU	chr8	DEFB130	11614389	27
CEU	chr8	DEFB130	11664389	41
CEU	chr8	DEFB130	11714389	31
CEU	chr8	DEFB130	11764389	30
CEU	chr8	DEFB130	11814389	19
CEU	chr8	DEFB130	11864389	4
CEU	chr8	DEFB130	11914389	0
CEU	chr8	DEFB130	11964389	0
CEU	chr8	DEFB130	12014389	0
CEU	chr8	DEFB130	12064389	0
CEU	chr8	DEFB130	12114389	0
CEU	chr8	DEFB130	12164389	0
CEU	chr8	DEFB130	12214389	0
CEU	chr8	DEFB130	12264389	0
CEU	chr8	DEFB130	12314389	0
CEU	chr8	DEFB130	12364389	0
CEU	chr8	DEFB130	12414389	0
CEU	chr8	DEFB130	12464389	0
CEU	chr8	DEFB130	12514389	4
CEU	chr8	DEFB130	12564389	25

CEU	chr8	DEFB130	12614389	39
CEU	chr8	DEFB130	12664389	40
CEU	chr8	DEFB130	12714389	35
CEU	chr8	DEFB130	12764389	37
CEU	chr8	DEFB130	12814389	48
CEU	chr8	DEFB130	12864389	45
CEU	chr8	DEFB130	12914389	21
CEU	chr8	DEFB130	12964389	11
CEU	chr8	DEFB130	13014389	38
CEU	chr8	DEFB130	13064389	46
CEU	chr8	DEFB130	13114389	28
CEU	chr8	DEFB130	13164389	24
CEU	chr8	DEFB130	13214389	36
CEU	chr8	DEFB130	13264389	49
CEU	chr8	DEFB130	13314389	50
CEU	chr8	DEFB130	13364389	40
CEU	chr8	DEFB130	13414389	44
CEU	chr8	DEFB130	13464389	46
CEU	chr8	DEFB130	13514389	61
CEU	chr8	DEFB130	13564389	60
CEU	chr8	DEFB130	13614389	28
CEU	chr8	DEFB130	13664389	17
CEU	chr8	DEFB130	13714389	49
CEU	chr8	DEFB130	13764389	75
CEU	chr8	DEFB130	13814389	79
CEU	chr8	DEFB130	13864389	77
CEU	chr8	DEFB130	13914389	37
CEU	chr8	DEFB130	13964389	20
CEU	chr8	DEFB130	14014389	36
CEU	chr8	DEFB130	14064389	76
CEU	chr8	DEFB130	14114389	84
CEU	chr8	DEFB130	14164389	43
CEU	chr8	DEFB130	14214389	39
CEU	chr8	DEFB130	14264389	37
CEU	chr8	DEFB130	14314389	29
CEU	chr8	DEFB130	14364389	47
CEU	chr8	DEFB130	14414389	58
CEU	chr8	DEFB130	14464389	50
CEU	chr8	DEFB130	14514389	28
CEU	chr8	DEFB130	14564389	28
CEU	chr8	DEFB130	14614389	57
CEU	chr8	DEFB130	14664389	72
CEU	chr8	DEFB130	14714389	73
CEU	chr8	DEFB130	14764389	68
CEU	chr8	DEFB130	14814389	56
CEU	chr8	DEFB130	14864389	42
CEU	chr8	DEFB130	14914389	38
CEU	chr8	DEFB130	14964389	20
CEU	chr8	DEFB130	15014389	0

CEU	chr8	DEFB130	15064389	0
CEU	chr8	DEFB130	15114389	0
CEU	chr8	DEFB130	15164389	0
CEU	chr8	DEFB130	15214389	0
CEU	chr8	DEFB130	15264389	0
CEU	chr8	DEFB130	15314389	0
CEU	chr8	DEFB130	15364389	0
CEU	chr8	DEFB130	15414389	0
CEU	chr8	DEFB130	15464389	0
CEU	chr8	DEFB130	15514389	0
CEU	chr8	DEFB130	15564389	0
CEU	chr8	DEFB130	15614389	0
CEU	chr8	DEFB130	15664389	0
CEU	chr8	DEFB130	15714389	0
CEU	chr8	DEFB130	15764389	0
CEU	chr8	DEFB130	15814389	0
CEU	chr8	DEFB130	15864389	0
CEU	chr8	DEFB130	15914389	0
CEU	chr8	DEFB130	15964389	0
CEU	chr8	DEFB130	16014389	0
CEU	chr8	DEFB130	16064389	0
CEU	chr8	DEFB130	16114389	1
CEU	chr8	DEFB130	16164389	25
CEU	chr8	DEFB130	16214389	44
CEU	chr8	DEFB130	16264389	62
CEU	chr8	DEFB130	16314389	85
CEU	chr8	DEFB130	16364389	68
CEU	chr8	DEFB130	16414389	75
CEU	chr8	DEFB130	16464389	73
CEU	chr8	DEFB130	16514389	41
CEU	chr8	DEFB130	16564389	35
CEU	chr8	DEFB130	16614389	35
CEU	chr8	DEFB130	16664389	42
CEU	chr8	DEFB130	16714389	44
CEU	chr8	DEFB130	16764389	47
CEU	chr8	DEFB130	16814389	65
CEU	chr8	DEFB130	16864389	53
CEU	chr8	DEFB130	16914389	30
CEU	chr8	DEFB130	16964389	31
CEU	chr8	DEFB130	17014389	39
CEU	chr8	DEFB130	7360962	0
CEU	chr8	DEFB130	7410962	0
CEU	chr8	DEFB130	7460962	0
CEU	chr8	DEFB130	7510962	0
CEU	chr8	DEFB130	7560962	0
CEU	chr8	DEFB130	7610962	0
CEU	chr8	DEFB130	7660962	0
CEU	chr8	DEFB130	7710962	0
CEU	chr8	DEFB130	7760962	0

CEU	chr8	DEFB130	7810962	0
CEU	chr8	DEFB130	7860962	0
CEU	chr8	DEFB130	7910962	0
CEU	chr8	DEFB130	7960962	0
CEU	chr8	DEFB130	8010962	0
CEU	chr8	DEFB130	8060962	0
CEU	chr8	DEFB130	8110962	0
CEU	chr8	DEFB130	8160962	0
CEU	chr8	DEFB130	8210962	0
CEU	chr8	DEFB130	8260962	0
CEU	chr8	DEFB130	8310962	0
CEU	chr8	DEFB130	8360962	0
CEU	chr8	DEFB130	8410962	0
CEU	chr8	DEFB130	8460962	0
CEU	chr8	DEFB130	8510962	0
CEU	chr8	DEFB130	8560962	0
CEU	chr8	DEFB130	8610962	0
CEU	chr8	DEFB130	8660962	0
CEU	chr8	DEFB130	8710962	0
CEU	chr8	DEFB130	8760962	0
CEU	chr8	DEFB130	8810962	0
CEU	chr8	DEFB130	8860962	0
CEU	chr8	DEFB130	8910962	0
CEU	chr8	DEFB130	8960962	0
CEU	chr8	DEFB130	9010962	15
CEU	chr8	DEFB130	9060962	48
CEU	chr8	DEFB130	9110962	57
CEU	chr8	DEFB130	9160962	61
CEU	chr8	DEFB130	9210962	53
CEU	chr8	DEFB130	9260962	45
CEU	chr8	DEFB130	9310962	41
CEU	chr8	DEFB130	9360962	12
CEU	chr8	DEFB130	9410962	0
CEU	chr8	DEFB130	9460962	0
CEU	chr8	DEFB130	9510962	0
CEU	chr8	DEFB130	9560962	0
CEU	chr8	DEFB130	9610962	0
CEU	chr8	DEFB130	9660962	0
CEU	chr8	DEFB130	9710962	0
CEU	chr8	DEFB130	9760962	0
CEU	chr8	DEFB130	9810962	0
CEU	chr8	DEFB130	9860962	0
CEU	chr8	DEFB130	9910962	0
CEU	chr8	DEFB130	9960962	0
CEU	chr8	DEFB130	10010962	0
CEU	chr8	DEFB130	10060962	0
CEU	chr8	DEFB130	10110962	0
CEU	chr8	DEFB130	10160962	0
CEU	chr8	DEFB130	10210962	0

CEU	chr8	DEFB130	10260962	0
CEU	chr8	DEFB130	10310962	0
CEU	chr8	DEFB130	10360962	0
CEU	chr8	DEFB130	10410962	0
CEU	chr8	DEFB130	10460962	0
CEU	chr8	DEFB130	10510962	0
CEU	chr8	DEFB130	10560962	0
CEU	chr8	DEFB130	10610962	0
CEU	chr8	DEFB130	10660962	0
CEU	chr8	DEFB130	10710962	0
CEU	chr8	DEFB130	10760962	0
CEU	chr8	DEFB130	10810962	0
CEU	chr8	DEFB130	10860962	0
CEU	chr8	DEFB130	10910962	0
CEU	chr8	DEFB130	10960962	0
CEU	chr8	DEFB130	11010962	0
CEU	chr8	DEFB130	11060962	0
CEU	chr8	DEFB130	11110962	0
CEU	chr8	DEFB130	11160962	0
CEU	chr8	DEFB130	11210962	0
CEU	chr8	DEFB130	11260962	0
CEU	chr8	DEFB130	11310962	0
CEU	chr8	DEFB130	11360962	0
CEU	chr8	DEFB130	11410962	0
CEU	chr8	DEFB130	11460962	0
CEU	chr8	DEFB130	11510962	0
CEU	chr8	DEFB130	11560962	2
CEU	chr8	DEFB130	11610962	27
CEU	chr8	DEFB130	11660962	38
CEU	chr8	DEFB130	11710962	30
CEU	chr8	DEFB130	11760962	32
CEU	chr8	DEFB130	11810962	20
CEU	chr8	DEFB130	11860962	5
CEU	chr8	DEFB130	11910962	0
CEU	chr8	DEFB130	11960962	0
CEU	chr8	DEFB130	12010962	0
CEU	chr8	DEFB130	12060962	0
CEU	chr8	DEFB130	12110962	0
CEU	chr8	DEFB130	12160962	0
CEU	chr8	DEFB130	12210962	0
CEU	chr8	DEFB130	12260962	0
CEU	chr8	DEFB130	12310962	0
CEU	chr8	DEFB130	12360962	0
CEU	chr8	DEFB130	12410962	0
CEU	chr8	DEFB130	12460962	0
CEU	chr8	DEFB130	12510962	3
CEU	chr8	DEFB130	12560962	24
CEU	chr8	DEFB130	12610962	36
CEU	chr8	DEFB130	12660962	39

CEU	chr8	DEFB130	12710962	38
CEU	chr8	DEFB130	12760962	37
CEU	chr8	DEFB130	12810962	45
CEU	chr8	DEFB130	12860962	47
CEU	chr8	DEFB130	12910962	25
CEU	chr8	DEFB130	12960962	11
CEU	chr8	DEFB130	13010962	37
CEU	chr8	DEFB130	13060962	46
CEU	chr8	DEFB130	13110962	29
CEU	chr8	DEFB130	13160962	23
CEU	chr8	DEFB130	13210962	36
CEU	chr8	DEFB130	13260962	48
CEU	chr8	DEFB130	13310962	43
CEU	chr8	DEFB130	13360962	39
CEU	chr8	DEFB130	13410962	48
CEU	chr8	DEFB130	13460962	48
CEU	chr8	DEFB130	13510962	61
CEU	chr8	DEFB130	13560962	61
CEU	chr8	DEFB130	13610962	30
CEU	chr8	DEFB130	13660962	17
CEU	chr8	DEFB130	13710962	47
CEU	chr8	DEFB130	13760962	74
CEU	chr8	DEFB130	13810962	81
CEU	chr8	DEFB130	13860962	78
CEU	chr8	DEFB130	13910962	37
CEU	chr8	DEFB130	13960962	20
CEU	chr8	DEFB130	14010962	32
CEU	chr8	DEFB130	14060962	75
CEU	chr8	DEFB130	14110962	88
CEU	chr8	DEFB130	14160962	44
CEU	chr8	DEFB130	14210962	39
CEU	chr8	DEFB130	14260962	36
CEU	chr8	DEFB130	14310962	29
CEU	chr8	DEFB130	14360962	47
CEU	chr8	DEFB130	14410962	47
CEU	chr8	DEFB130	14460962	49
CEU	chr8	DEFB130	14510962	40
CEU	chr8	DEFB130	14560962	28
CEU	chr8	DEFB130	14610962	57
CEU	chr8	DEFB130	14660962	68
CEU	chr8	DEFB130	14710962	72
CEU	chr8	DEFB130	14760962	73
CEU	chr8	DEFB130	14810962	54
CEU	chr8	DEFB130	14860962	41
CEU	chr8	DEFB130	14910962	40
CEU	chr8	DEFB130	14960962	22
CEU	chr8	DEFB130	15010962	1
CEU	chr8	DEFB130	15060962	0
CEU	chr8	DEFB130	15110962	0

CEU	chr8	DEFB130	15160962	0
CEU	chr8	DEFB130	15210962	0
CEU	chr8	DEFB130	15260962	0
CEU	chr8	DEFB130	15310962	0
CEU	chr8	DEFB130	15360962	0
CEU	chr8	DEFB130	15410962	0
CEU	chr8	DEFB130	15460962	0
CEU	chr8	DEFB130	15510962	0
CEU	chr8	DEFB130	15560962	0
CEU	chr8	DEFB130	15610962	0
CEU	chr8	DEFB130	15660962	0
CEU	chr8	DEFB130	15710962	0
CEU	chr8	DEFB130	15760962	0
CEU	chr8	DEFB130	15810962	0
CEU	chr8	DEFB130	15860962	0
CEU	chr8	DEFB130	15910962	0
CEU	chr8	DEFB130	15960962	0
CEU	chr8	DEFB130	16010962	0
CEU	chr8	DEFB130	16060962	0
CEU	chr8	DEFB130	16110962	1
CEU	chr8	DEFB130	16160962	25
CEU	chr8	DEFB130	16210962	43
CEU	chr8	DEFB130	16260962	58
CEU	chr8	DEFB130	16310962	65
CEU	chr8	DEFB130	16360962	72
CEU	chr8	DEFB130	16410962	94
CEU	chr8	DEFB130	16460962	73
CEU	chr8	DEFB130	16510962	42
CEU	chr8	DEFB130	16560962	34
CEU	chr8	DEFB130	16610962	35
CEU	chr8	DEFB130	16660962	41
CEU	chr8	DEFB130	16710962	43
CEU	chr8	DEFB130	16760962	47
CEU	chr8	DEFB130	16810962	67
CEU	chr8	DEFB130	16860962	54
CEU	chr8	DEFB130	16910962	30
CEU	chr8	DEFB130	16960962	29
CEU	chr8	DEFB130	17010962	39
CEU	chr8	DEFB130	17060962	45
CEU	chr8	DEFB130	17110962	38
CEU	chr8	DEFB130	17160962	48
CEU	chr8	DEFB130	17210962	55
CEU	chr8	DEFB130	17260962	46
CEU	chr1	FAM72A	201236179	0
CEU	chr1	FAM72A	201286179	0
CEU	chr1	FAM72A	201336179	0
CEU	chr1	FAM72A	201386179	0
CEU	chr1	FAM72A	201436179	0
CEU	chr1	FAM72A	201486179	0

CEU	chr1	FAM72A	201536179	0
CEU	chr1	FAM72A	201586179	0
CEU	chr1	FAM72A	201636179	0
CEU	chr1	FAM72A	201686179	0
CEU	chr1	FAM72A	201736179	0
CEU	chr1	FAM72A	201786179	0
CEU	chr1	FAM72A	201836179	0
CEU	chr1	FAM72A	201886179	0
CEU	chr1	FAM72A	201936179	0
CEU	chr1	FAM72A	201986179	0
CEU	chr1	FAM72A	202036179	0
CEU	chr1	FAM72A	202086179	0
CEU	chr1	FAM72A	202136179	0
CEU	chr1	FAM72A	202186179	0
CEU	chr1	FAM72A	202236179	0
CEU	chr1	FAM72A	202286179	0
CEU	chr1	FAM72A	202336179	0
CEU	chr1	FAM72A	202386179	0
CEU	chr1	FAM72A	202436179	0
CEU	chr1	FAM72A	202486179	0
CEU	chr1	FAM72A	202536179	0
CEU	chr1	FAM72A	202586179	0
CEU	chr1	FAM72A	202636179	0
CEU	chr1	FAM72A	202686179	0
CEU	chr1	FAM72A	202736179	0
CEU	chr1	FAM72A	202786179	0
CEU	chr1	FAM72A	202836179	0
CEU	chr1	FAM72A	202886179	0
CEU	chr1	FAM72A	202936179	0
CEU	chr1	FAM72A	202986179	0
CEU	chr1	FAM72A	203036179	0
CEU	chr1	FAM72A	203086179	0
CEU	chr1	FAM72A	203136179	0
CEU	chr1	FAM72A	203186179	0
CEU	chr1	FAM72A	203236179	0
CEU	chr1	FAM72A	203286179	0
CEU	chr1	FAM72A	203336179	0
CEU	chr1	FAM72A	203386179	0
CEU	chr1	FAM72A	203436179	0
CEU	chr1	FAM72A	203486179	0
CEU	chr1	FAM72A	203536179	0
CEU	chr1	FAM72A	203586179	0
CEU	chr1	FAM72A	203636179	0
CEU	chr1	FAM72A	203686179	0
CEU	chr1	FAM72A	203736179	0
CEU	chr1	FAM72A	203786179	0
CEU	chr1	FAM72A	203836179	0
CEU	chr1	FAM72A	203886179	0
CEU	chr1	FAM72A	203936179	0

CEU	chr1	FAM72A	203986179	0
CEU	chr1	FAM72A	204036179	0
CEU	chr1	FAM72A	204086179	0
CEU	chr1	FAM72A	204136179	0
CEU	chr1	FAM72A	204186179	0
CEU	chr1	FAM72A	204236179	0
CEU	chr1	FAM72A	204286179	0
CEU	chr1	FAM72A	204336179	0
CEU	chr1	FAM72A	204386179	0
CEU	chr1	FAM72A	204436179	0
CEU	chr1	FAM72A	204486179	0
CEU	chr1	FAM72A	204536179	0
CEU	chr1	FAM72A	204586179	0
CEU	chr1	FAM72A	204636179	0
CEU	chr1	FAM72A	204686179	0
CEU	chr1	FAM72A	204736179	0
CEU	chr1	FAM72A	204786179	0
CEU	chr1	FAM72A	204836179	0
CEU	chr1	FAM72A	204886179	13
CEU	chr1	FAM72A	204936179	47
CEU	chr1	FAM72A	204986179	34
CEU	chr1	FAM72A	205036179	0
CEU	chr1	FAM72A	205086179	0
CEU	chr1	FAM72A	205136179	0
CEU	chr1	FAM72A	205186179	0
CEU	chr1	FAM72A	205236179	0
CEU	chr1	FAM72A	205286179	5
CEU	chr1	FAM72A	205336179	31
CEU	chr1	FAM72A	205386179	44
CEU	chr1	FAM72A	205436179	46
CEU	chr1	FAM72A	205486179	28
CEU	chr1	FAM72A	205536179	0
CEU	chr1	FAM72A	205586179	0
CEU	chr1	FAM72A	205636179	0
CEU	chr1	FAM72A	205686179	0
CEU	chr1	FAM72A	205736179	0
CEU	chr1	FAM72A	205786179	0
CEU	chr1	FAM72A	205836179	0
CEU	chr1	FAM72A	205886179	0
CEU	chr1	FAM72A	205936179	0
CEU	chr1	FAM72A	205986179	0
CEU	chr1	FAM72A	206036179	0
CEU	chr1	FAM72A	206086179	11
CEU	chr1	FAM72A	206136179	24
CEU	chr1	FAM72A	206186179	25
CEU	chr1	FAM72A	206236179	12
CEU	chr1	FAM72A	206286179	0
CEU	chr1	FAM72A	206336179	0
CEU	chr1	FAM72A	206386179	0

CEU	chr1	FAM72A	206436179	0
CEU	chr1	FAM72A	206486179	6
CEU	chr1	FAM72A	206536179	15
CEU	chr1	FAM72A	206586179	51
CEU	chr1	FAM72A	206636179	53
CEU	chr1	FAM72A	206686179	18
CEU	chr1	FAM72A	206736179	7
CEU	chr1	FAM72A	206786179	0
CEU	chr1	FAM72A	206836179	0
CEU	chr1	FAM72A	206886179	0
CEU	chr1	FAM72A	206936179	0
CEU	chr1	FAM72A	206986179	0
CEU	chr1	FAM72A	207036179	0
CEU	chr1	FAM72A	207086179	0
CEU	chr1	FAM72A	207136179	0
CEU	chr1	FAM72A	207186179	0
CEU	chr1	FAM72A	207236179	0
CEU	chr1	FAM72A	207286179	0
CEU	chr1	FAM72A	207336179	0
CEU	chr1	FAM72A	207386179	0
CEU	chr1	FAM72A	207436179	0
CEU	chr1	FAM72A	207486179	0
CEU	chr1	FAM72A	207536179	0
CEU	chr1	FAM72A	207586179	0
CEU	chr1	FAM72A	207636179	0
CEU	chr1	FAM72A	207686179	0
CEU	chr1	FAM72A	207736179	18
CEU	chr1	FAM72A	207786179	47
CEU	chr1	FAM72A	207836179	80
CEU	chr1	FAM72A	207886179	65
CEU	chr1	FAM72A	207936179	28
CEU	chr1	FAM72A	207986179	33
CEU	chr1	FAM72A	208036179	30
CEU	chr1	FAM72A	208086179	11
CEU	chr1	FAM72A	208136179	0
CEU	chr1	FAM72A	208186179	0
CEU	chr1	FAM72A	208236179	0
CEU	chr1	FAM72A	208286179	0
CEU	chr1	FAM72A	208336179	0
CEU	chr1	FAM72A	208386179	0
CEU	chr1	FAM72A	208436179	0
CEU	chr1	FAM72A	208486179	0
CEU	chr1	FAM72A	208536179	0
CEU	chr1	FAM72A	208586179	0
CEU	chr1	FAM72A	208636179	0
CEU	chr1	FAM72A	208686179	0
CEU	chr1	FAM72A	208736179	17
CEU	chr1	FAM72A	208786179	57
CEU	chr1	FAM72A	208836179	40

CEU	chr1	FAM72A	208886179	0
CEU	chr1	FAM72A	208936179	0
CEU	chr1	FAM72A	208986179	0
CEU	chr1	FAM72A	209036179	0
CEU	chr1	FAM72A	209086179	0
CEU	chr1	FAM72A	209136179	0
CEU	chr1	FAM72A	209186179	0
CEU	chr1	FAM72A	209236179	0
CEU	chr1	FAM72A	209286179	0
CEU	chr1	FAM72A	209336179	0
CEU	chr1	FAM72A	209386179	0
CEU	chr1	FAM72A	209436179	0
CEU	chr1	FAM72A	209486179	0
CEU	chr1	FAM72A	209536179	0
CEU	chr1	FAM72A	209586179	0
CEU	chr1	FAM72A	209636179	0
CEU	chr1	FAM72A	209686179	0
CEU	chr1	FAM72A	209736179	2
CEU	chr1	FAM72A	209786179	38
CEU	chr1	FAM72A	209836179	44
CEU	chr1	FAM72A	209886179	22
CEU	chr1	FAM72A	209936179	37
CEU	chr1	FAM72A	209986179	39
CEU	chr1	FAM72A	210036179	74
CEU	chr1	FAM72A	210086179	77
CEU	chr1	FAM72A	210136179	46
CEU	chr1	FAM72A	210186179	47
CEU	chr1	FAM72A	210236179	41
CEU	chr1	FAM72A	210286179	31
CEU	chr1	FAM72A	210336179	33
CEU	chr1	FAM72A	210386179	32
CEU	chr1	FAM72A	210436179	22
CEU	chr1	FAM72A	210486179	31
CEU	chr1	FAM72A	210536179	44
CEU	chr1	FAM72A	210586179	40
CEU	chr1	FAM72A	210636179	36
CEU	chr1	FAM72A	210686179	37
CEU	chr1	FAM72A	210736179	24
CEU	chr1	FAM72A	210786179	54
CEU	chr1	FAM72A	210836179	115
CEU	chr1	FAM72A	210886179	84
CEU	chr1	FAM72A	210936179	32
CEU	chr1	FAM72A	210986179	28
CEU	chr1	FAM72A	211036179	18
CEU	chr1	FAM72A	211086179	26
CEU	chr1	FAM72A	211136179	34
CEU	chr9	FAM75A1	34405667	37
CEU	chr9	FAM75A1	34455667	45
CEU	chr9	FAM75A1	34505667	30

CEU	chr9	FAM75A1	34555667	39
CEU	chr9	FAM75A1	34605667	39
CEU	chr9	FAM75A1	34655667	31
CEU	chr9	FAM75A1	34705667	19
CEU	chr9	FAM75A1	34755667	0
CEU	chr9	FAM75A1	34805667	0
CEU	chr9	FAM75A1	34855667	0
CEU	chr9	FAM75A1	34905667	0
CEU	chr9	FAM75A1	34955667	0
CEU	chr9	FAM75A1	35005667	0
CEU	chr9	FAM75A1	35055667	0
CEU	chr9	FAM75A1	35105667	0
CEU	chr9	FAM75A1	35155667	0
CEU	chr9	FAM75A1	35205667	0
CEU	chr9	FAM75A1	35255667	0
CEU	chr9	FAM75A1	35305667	0
CEU	chr9	FAM75A1	35355667	0
CEU	chr9	FAM75A1	35405667	0
CEU	chr9	FAM75A1	35455667	0
CEU	chr9	FAM75A1	35505667	0
CEU	chr9	FAM75A1	35555667	0
CEU	chr9	FAM75A1	35605667	0
CEU	chr9	FAM75A1	35655667	0
CEU	chr9	FAM75A1	35705667	0
CEU	chr9	FAM75A1	35755667	0
CEU	chr9	FAM75A1	35805667	0
CEU	chr9	FAM75A1	35855667	0
CEU	chr9	FAM75A1	35905667	0
CEU	chr9	FAM75A1	35955667	0
CEU	chr9	FAM75A1	36005667	0
CEU	chr9	FAM75A1	36055667	0
CEU	chr9	FAM75A1	36105667	0
CEU	chr9	FAM75A1	36155667	0
CEU	chr9	FAM75A1	36205667	0
CEU	chr9	FAM75A1	36255667	0
CEU	chr9	FAM75A1	36305667	0
CEU	chr9	FAM75A1	36355667	0
CEU	chr9	FAM75A1	36405667	0
CEU	chr9	FAM75A1	36455667	0
CEU	chr9	FAM75A1	36505667	0
CEU	chr9	FAM75A1	36555667	0
CEU	chr9	FAM75A1	36605667	0
CEU	chr9	FAM75A1	36655667	0
CEU	chr9	FAM75A1	36705667	0
CEU	chr9	FAM75A1	36755667	0
CEU	chr9	FAM75A1	36805667	13
CEU	chr9	FAM75A1	36855667	31
CEU	chr9	FAM75A1	36905667	19
CEU	chr9	FAM75A1	36955667	1

CEU	chr9	FAM75A1	37005667	0
CEU	chr9	FAM75A1	37055667	0
CEU	chr9	FAM75A1	37105667	0
CEU	chr9	FAM75A1	37155667	0
CEU	chr9	FAM75A1	37205667	0
CEU	chr9	FAM75A1	37255667	0
CEU	chr9	FAM75A1	37305667	0
CEU	chr9	FAM75A1	37355667	0
CEU	chr9	FAM75A1	37405667	0
CEU	chr9	FAM75A1	37455667	0
CEU	chr9	FAM75A1	37505667	0
CEU	chr9	FAM75A1	37555667	0
CEU	chr9	FAM75A1	37605667	0
CEU	chr9	FAM75A1	37655667	0
CEU	chr9	FAM75A1	37705667	0
CEU	chr9	FAM75A1	37755667	0
CEU	chr9	FAM75A1	37805667	0
CEU	chr9	FAM75A1	37855667	0
CEU	chr9	FAM75A1	37905667	0
CEU	chr9	FAM75A1	37955667	0
CEU	chr9	FAM75A1	38005667	0
CEU	chr9	FAM75A1	38055667	0
CEU	chr9	FAM75A1	38105667	0
CEU	chr9	FAM75A1	38155667	0
CEU	chr9	FAM75A1	38205667	0
CEU	chr9	FAM75A1	38255667	0
CEU	chr9	FAM75A1	38305667	0
CEU	chr9	FAM75A1	38355667	0
CEU	chr9	FAM75A1	38405667	0
CEU	chr9	FAM75A1	38455667	0
CEU	chr9	FAM75A1	38505667	0
CEU	chr9	FAM75A1	38555667	0
CEU	chr9	FAM75A1	38605667	0
CEU	chr9	FAM75A1	38655667	0
CEU	chr9	FAM75A1	38705667	0
CEU	chr9	FAM75A1	38755667	0
CEU	chr9	FAM75A1	38805667	0
CEU	chr9	FAM75A1	38855667	0
CEU	chr9	FAM75A1	38905667	0
CEU	chr9	FAM75A1	38955667	0
CEU	chr9	FAM75A1	39005667	0
CEU	chr9	FAM75A1	39055667	2
CEU	chr9	FAM75A1	39105667	53
CEU	chr9	FAM75A1	39155667	66
CEU	chr9	FAM75A1	39205667	15
CEU	chr9	FAM75A1	39255667	0
CEU	chr9	FAM75A1	39305667	0
CEU	chr9	FAM75A1	39355667	0
CEU	chr9	FAM75A1	39405667	0

CEU	chr9	FAM75A1	39455667	0
CEU	chr9	FAM75A1	39505667	0
CEU	chr9	FAM75A1	39555667	0
CEU	chr9	FAM75A1	39605667	0
CEU	chr9	FAM75A1	39655667	0
CEU	chr9	FAM75A1	39705667	0
CEU	chr9	FAM75A1	39755667	0
CEU	chr9	FAM75A1	39805667	0
CEU	chr9	FAM75A1	39855667	0
CEU	chr9	FAM75A1	39905667	0
CEU	chr9	FAM75A1	39955667	0
CEU	chr9	FAM75A1	40005667	0
CEU	chr9	FAM75A1	40055667	0
CEU	chr9	FAM75A1	40105667	0
CEU	chr9	FAM75A1	40155667	0
CEU	chr9	FAM75A1	40205667	0
CEU	chr9	FAM75A1	40255667	0
CEU	chr9	FAM75A1	40305667	0
CEU	chr9	FAM75A1	40355667	0
CEU	chr9	FAM75A1	40405667	0
CEU	chr9	FAM75A1	40455667	0
CEU	chr9	FAM75A1	40505667	6
CEU	chr9	FAM75A1	40555667	14
CEU	chr9	FAM75A1	40605667	29
CEU	chr9	FAM75A1	40655667	21
CEU	chr9	FAM75A1	40705667	8
CEU	chr9	FAM75A1	40755667	20
CEU	chr9	FAM75A1	40805667	24
CEU	chr9	FAM75A1	40855667	12
CEU	chr9	FAM75A1	40905667	0
CEU	chr9	FAM75A1	40955667	0
CEU	chr9	FAM75A1	41005667	0
CEU	chr9	FAM75A1	41055667	0
CEU	chr9	FAM75A1	41105667	0
CEU	chr9	FAM75A1	41155667	0
CEU	chr9	FAM75A1	41205667	0
CEU	chr9	FAM75A1	41255667	0
CEU	chr9	FAM75A1	41305667	0
CEU	chr9	FAM75A1	41355667	0
CEU	chr9	FAM75A1	41405667	0
CEU	chr9	FAM75A1	41455667	0
CEU	chr9	FAM75A1	41505667	0
CEU	chr9	FAM75A1	41555667	0
CEU	chr9	FAM75A1	41605667	0
CEU	chr9	FAM75A1	41655667	0
CEU	chr9	FAM75A1	41705667	0
CEU	chr9	FAM75A1	41755667	0
CEU	chr9	FAM75A1	41805667	0
CEU	chr9	FAM75A1	41855667	0

CEU	chr9	FAM75A1	41905667	0
CEU	chr9	FAM75A1	41955667	0
CEU	chr9	FAM75A1	42005667	0
CEU	chr9	FAM75A1	42055667	0
CEU	chr9	FAM75A1	42105667	0
CEU	chr9	FAM75A1	42155667	0
CEU	chr9	FAM75A1	42205667	0
CEU	chr9	FAM75A1	42255667	0
CEU	chr9	FAM75A1	42305667	0
CEU	chr9	FAM75A1	42355667	0
CEU	chr9	FAM75A1	42405667	0
CEU	chr9	FAM75A1	42455667	0
CEU	chr9	FAM75A1	42505667	0
CEU	chr9	FAM75A1	42555667	0
CEU	chr9	FAM75A1	42605667	0
CEU	chr9	FAM75A1	42655667	0
CEU	chr9	FAM75A1	42705667	0
CEU	chr9	FAM75A1	42755667	0
CEU	chr9	FAM75A1	42805667	0
CEU	chr9	FAM75A1	42855667	0
CEU	chr9	FAM75A1	42905667	0
CEU	chr9	FAM75A1	42955667	0
CEU	chr9	FAM75A1	43005667	0
CEU	chr9	FAM75A1	43055667	0
CEU	chr9	FAM75A1	43105667	0
CEU	chr9	FAM75A1	43155667	0
CEU	chr9	FAM75A1	43205667	0
CEU	chr9	FAM75A1	43255667	0
CEU	chr9	FAM75A1	43305667	0
CEU	chr9	FAM75A1	43355667	0
CEU	chr9	FAM75A1	43405667	0
CEU	chr9	FAM75A1	43455667	0
CEU	chr9	FAM75A1	43505667	0
CEU	chr9	FAM75A1	43555667	0
CEU	chr9	FAM75A1	43605667	0
CEU	chr9	FAM75A1	43655667	0
CEU	chr9	FAM75A1	43705667	0
CEU	chr9	FAM75A1	43755667	0
CEU	chr9	FAM75A1	43805667	0
CEU	chr9	FAM75A1	43855667	0
CEU	chr9	FAM75A1	43905667	0
CEU	chr9	FAM75A1	43955667	0
CEU	chr9	FAM75A1	44005667	0
CEU	chr9	FAM75A1	44055667	0
CEU	chr9	FAM75A1	44105667	0
CEU	chr9	FAM75A1	44155667	0
CEU	chr9	FAM75A1	44205667	0
CEU	chr9	FAM75A1	44255667	0
CEU	chr9	FAM75A1	44305667	0

CEU	chr9	FAM75A5	55964372	0
CEU	chr9	FAM75A5	56014372	0
CEU	chr9	FAM75A5	56064372	0
CEU	chr9	FAM75A5	56114372	0
CEU	chr9	FAM75A5	56164372	0
CEU	chr9	FAM75A5	56214372	0
CEU	chr9	FAM75A5	56264372	0
CEU	chr9	FAM75A5	56314372	0
CEU	chr9	FAM75A5	56364372	0
CEU	chr9	FAM75A5	56414372	0
CEU	chr9	FAM75A5	56464372	0
CEU	chr9	FAM75A5	56514372	0
CEU	chr9	FAM75A5	56564372	0
CEU	chr9	FAM75A5	56614372	0
CEU	chr9	FAM75A5	56664372	0
CEU	chr9	FAM75A5	56714372	0
CEU	chr9	FAM75A5	56764372	0
CEU	chr9	FAM75A5	56814372	0
CEU	chr9	FAM75A5	56864372	0
CEU	chr9	FAM75A5	56914372	0
CEU	chr9	FAM75A5	56964372	0
CEU	chr9	FAM75A5	57014372	0
CEU	chr9	FAM75A5	57064372	0
CEU	chr9	FAM75A5	57114372	0
CEU	chr9	FAM75A5	57164372	0
CEU	chr9	FAM75A5	57214372	0
CEU	chr9	FAM75A5	57264372	0
CEU	chr9	FAM75A5	57314372	0
CEU	chr9	FAM75A5	57364372	0
CEU	chr9	FAM75A5	57414372	0
CEU	chr9	FAM75A5	57464372	0
CEU	chr9	FAM75A5	57514372	0
CEU	chr9	FAM75A5	57564372	0
CEU	chr9	FAM75A5	57614372	0
CEU	chr9	FAM75A5	57664372	0
CEU	chr9	FAM75A5	57714372	0
CEU	chr9	FAM75A5	57764372	0
CEU	chr9	FAM75A5	57814372	0
CEU	chr9	FAM75A5	57864372	0
CEU	chr9	FAM75A5	57914372	0
CEU	chr9	FAM75A5	57964372	0
CEU	chr9	FAM75A5	58014372	0
CEU	chr9	FAM75A5	58064372	0
CEU	chr9	FAM75A5	58114372	0
CEU	chr9	FAM75A5	58164372	0
CEU	chr9	FAM75A5	58214372	0
CEU	chr9	FAM75A5	58264372	0
CEU	chr9	FAM75A5	58314372	0
CEU	chr9	FAM75A5	58364372	0

CEU	chr9	FAM75A5	58414372	0
CEU	chr9	FAM75A5	58464372	0
CEU	chr9	FAM75A5	58514372	0
CEU	chr9	FAM75A5	58564372	0
CEU	chr9	FAM75A5	58614372	0
CEU	chr9	FAM75A5	58664372	0
CEU	chr9	FAM75A5	58714372	0
CEU	chr9	FAM75A5	58764372	0
CEU	chr9	FAM75A5	58814372	0
CEU	chr9	FAM75A5	58864372	0
CEU	chr9	FAM75A5	58914372	0
CEU	chr9	FAM75A5	58964372	0
CEU	chr9	FAM75A5	59014372	0
CEU	chr9	FAM75A5	59064372	0
CEU	chr9	FAM75A5	59114372	0
CEU	chr9	FAM75A5	59164372	0
CEU	chr9	FAM75A5	59214372	0
CEU	chr9	FAM75A5	59264372	0
CEU	chr9	FAM75A5	59314372	0
CEU	chr9	FAM75A5	59364372	0
CEU	chr9	FAM75A5	59414372	0
CEU	chr9	FAM75A5	59464372	0
CEU	chr9	FAM75A5	59514372	0
CEU	chr9	FAM75A5	59564372	0
CEU	chr9	FAM75A5	59614372	0
CEU	chr9	FAM75A5	59664372	0
CEU	chr9	FAM75A5	59714372	0
CEU	chr9	FAM75A5	59764372	0
CEU	chr9	FAM75A5	59814372	0
CEU	chr9	FAM75A5	59864372	0
CEU	chr9	FAM75A5	59914372	0
CEU	chr9	FAM75A5	59964372	0
CEU	chr9	FAM75A5	60014372	0
CEU	chr9	FAM75A5	60064372	0
CEU	chr9	FAM75A5	60114372	0
CEU	chr9	FAM75A5	60164372	0
CEU	chr9	FAM75A5	60214372	0
CEU	chr9	FAM75A5	60264372	0
CEU	chr9	FAM75A5	60314372	0
CEU	chr9	FAM75A5	60364372	0
CEU	chr9	FAM75A5	60414372	0
CEU	chr9	FAM75A5	60464372	0
CEU	chr9	FAM75A5	60514372	0
CEU	chr9	FAM75A5	60564372	0
CEU	chr9	FAM75A5	60614372	0
CEU	chr9	FAM75A5	60664372	0
CEU	chr9	FAM75A5	60714372	0
CEU	chr9	FAM75A5	60764372	0
CEU	chr9	FAM75A5	60814372	0

CEU	chr9	FAM75A5	60864372	0
CEU	chr9	FAM75A5	60914372	0
CEU	chr9	FAM75A5	60964372	0
CEU	chr9	FAM75A5	61014372	0
CEU	chr9	FAM75A5	61064372	0
CEU	chr9	FAM75A5	61114372	0
CEU	chr9	FAM75A5	61164372	0
CEU	chr9	FAM75A5	61214372	0
CEU	chr9	FAM75A5	61264372	0
CEU	chr9	FAM75A5	61314372	0
CEU	chr9	FAM75A5	61364372	0
CEU	chr9	FAM75A5	61414372	0
CEU	chr9	FAM75A5	61464372	0
CEU	chr9	FAM75A5	61514372	0
CEU	chr9	FAM75A5	61564372	0
CEU	chr9	FAM75A5	61614372	0
CEU	chr9	FAM75A5	61664372	0
CEU	chr9	FAM75A5	61714372	0
CEU	chr9	FAM75A5	61764372	0
CEU	chr9	FAM75A5	61814372	0
CEU	chr9	FAM75A5	61864372	0
CEU	chr9	FAM75A5	61914372	0
CEU	chr9	FAM75A5	61964372	0
CEU	chr9	FAM75A5	62014372	0
CEU	chr9	FAM75A5	62064372	0
CEU	chr9	FAM75A5	62114372	0
CEU	chr9	FAM75A5	62164372	0
CEU	chr9	FAM75A5	62214372	0
CEU	chr9	FAM75A5	62264372	0
CEU	chr9	FAM75A5	62314372	0
CEU	chr9	FAM75A5	62364372	0
CEU	chr9	FAM75A5	62414372	0
CEU	chr9	FAM75A5	62464372	0
CEU	chr9	FAM75A5	62514372	0
CEU	chr9	FAM75A5	62564372	0
CEU	chr9	FAM75A5	62614372	0
CEU	chr9	FAM75A5	62664372	0
CEU	chr9	FAM75A5	62714372	0
CEU	chr9	FAM75A5	62764372	0
CEU	chr9	FAM75A5	62814372	0
CEU	chr9	FAM75A5	62864372	0
CEU	chr9	FAM75A5	62914372	0
CEU	chr9	FAM75A5	62964372	0
CEU	chr9	FAM75A5	63014372	0
CEU	chr9	FAM75A5	63064372	0
CEU	chr9	FAM75A5	63114372	0
CEU	chr9	FAM75A5	63164372	0
CEU	chr9	FAM75A5	63214372	0
CEU	chr9	FAM75A5	63264372	0

CEU	chr9	FAM75A5	63314372	0
CEU	chr9	FAM75A5	63364372	0
CEU	chr9	FAM75A5	63414372	0
CEU	chr9	FAM75A5	63464372	0
CEU	chr9	FAM75A5	63514372	0
CEU	chr9	FAM75A5	63564372	0
CEU	chr9	FAM75A5	63614372	0
CEU	chr9	FAM75A5	63664372	0
CEU	chr9	FAM75A5	63714372	0
CEU	chr9	FAM75A5	63764372	0
CEU	chr9	FAM75A5	63814372	0
CEU	chr9	FAM75A5	63864372	0
CEU	chr9	FAM75A5	63914372	0
CEU	chr9	FAM75A5	63964372	0
CEU	chr9	FAM75A5	64014372	0
CEU	chr9	FAM75A5	64064372	0
CEU	chr9	FAM75A5	64114372	0
CEU	chr9	FAM75A5	64164372	0
CEU	chr9	FAM75A5	64214372	0
CEU	chr9	FAM75A5	64264372	0
CEU	chr9	FAM75A5	64314372	0
CEU	chr9	FAM75A5	64364372	0
CEU	chr9	FAM75A5	64414372	0
CEU	chr9	FAM75A5	64464372	0
CEU	chr9	FAM75A5	64514372	0
CEU	chr9	FAM75A5	64564372	0
CEU	chr9	FAM75A5	64614372	0
CEU	chr9	FAM75A5	64664372	0
CEU	chr9	FAM75A5	64714372	0
CEU	chr9	FAM75A5	64764372	0
CEU	chr9	FAM75A5	64814372	0
CEU	chr9	FAM75A5	64864372	0
CEU	chr9	FAM75A5	64914372	0
CEU	chr9	FAM75A5	64964372	0
CEU	chr9	FAM75A5	65014372	0
CEU	chr9	FAM75A5	65064372	0
CEU	chr9	FAM75A5	65114372	0
CEU	chr9	FAM75A5	65164372	0
CEU	chr9	FAM75A5	65214372	0
CEU	chr9	FAM75A5	65264372	0
CEU	chr9	FAM75A5	65314372	0
CEU	chr9	FAM75A5	65364372	0
CEU	chr9	FAM75A5	65414372	0
CEU	chr9	FAM75A5	65464372	0
CEU	chr9	FAM75A5	65514372	0
CEU	chr9	FAM75A5	65564372	13
CEU	chr9	FAM75A5	65614372	27
CEU	chr9	FAM75A5	65664372	14
CEU	chr9	FAM75A5	65714372	0

CEU	chr9	FAM75A5	65764372	0
CEU	chr9	FAM75A5	65814372	0
CEU	chr9	FAM75A5	65864372	0
CEU	chr9	FOXD4L2	60787146	0
CEU	chr9	FOXD4L2	60837146	0
CEU	chr9	FOXD4L2	60887146	0
CEU	chr9	FOXD4L2	60937146	0
CEU	chr9	FOXD4L2	60987146	0
CEU	chr9	FOXD4L2	61037146	0
CEU	chr9	FOXD4L2	61087146	0
CEU	chr9	FOXD4L2	61137146	0
CEU	chr9	FOXD4L2	61187146	0
CEU	chr9	FOXD4L2	61237146	0
CEU	chr9	FOXD4L2	61287146	0
CEU	chr9	FOXD4L2	61337146	0
CEU	chr9	FOXD4L2	61387146	0
CEU	chr9	FOXD4L2	61437146	0
CEU	chr9	FOXD4L2	61487146	0
CEU	chr9	FOXD4L2	61537146	0
CEU	chr9	FOXD4L2	61587146	0
CEU	chr9	FOXD4L2	61637146	0
CEU	chr9	FOXD4L2	61687146	0
CEU	chr9	FOXD4L2	61737146	0
CEU	chr9	FOXD4L2	61787146	0
CEU	chr9	FOXD4L2	61837146	0
CEU	chr9	FOXD4L2	61887146	0
CEU	chr9	FOXD4L2	61937146	0
CEU	chr9	FOXD4L2	61987146	0
CEU	chr9	FOXD4L2	62037146	0
CEU	chr9	FOXD4L2	62087146	0
CEU	chr9	FOXD4L2	62137146	0
CEU	chr9	FOXD4L2	62187146	0
CEU	chr9	FOXD4L2	62237146	0
CEU	chr9	FOXD4L2	62287146	0
CEU	chr9	FOXD4L2	62337146	0
CEU	chr9	FOXD4L2	62387146	0
CEU	chr9	FOXD4L2	62437146	0
CEU	chr9	FOXD4L2	62487146	0
CEU	chr9	FOXD4L2	62537146	0
CEU	chr9	FOXD4L2	62587146	0
CEU	chr9	FOXD4L2	62637146	0
CEU	chr9	FOXD4L2	62687146	0
CEU	chr9	FOXD4L2	62737146	0
CEU	chr9	FOXD4L2	62787146	0
CEU	chr9	FOXD4L2	62837146	0
CEU	chr9	FOXD4L2	62887146	0
CEU	chr9	FOXD4L2	62937146	0
CEU	chr9	FOXD4L2	62987146	0
CEU	chr9	FOXD4L2	63037146	0

CEU	chr9	FOXD4L2	63087146	0
CEU	chr9	FOXD4L2	63137146	0
CEU	chr9	FOXD4L2	63187146	0
CEU	chr9	FOXD4L2	63237146	0
CEU	chr9	FOXD4L2	63287146	0
CEU	chr9	FOXD4L2	63337146	0
CEU	chr9	FOXD4L2	63387146	0
CEU	chr9	FOXD4L2	63437146	0
CEU	chr9	FOXD4L2	63487146	0
CEU	chr9	FOXD4L2	63537146	0
CEU	chr9	FOXD4L2	63587146	0
CEU	chr9	FOXD4L2	63637146	0
CEU	chr9	FOXD4L2	63687146	0
CEU	chr9	FOXD4L2	63737146	0
CEU	chr9	FOXD4L2	63787146	0
CEU	chr9	FOXD4L2	63837146	0
CEU	chr9	FOXD4L2	63887146	0
CEU	chr9	FOXD4L2	63937146	0
CEU	chr9	FOXD4L2	63987146	0
CEU	chr9	FOXD4L2	64037146	0
CEU	chr9	FOXD4L2	64087146	0
CEU	chr9	FOXD4L2	64137146	0
CEU	chr9	FOXD4L2	64187146	0
CEU	chr9	FOXD4L2	64237146	0
CEU	chr9	FOXD4L2	64287146	0
CEU	chr9	FOXD4L2	64337146	0
CEU	chr9	FOXD4L2	64387146	0
CEU	chr9	FOXD4L2	64437146	0
CEU	chr9	FOXD4L2	64487146	0
CEU	chr9	FOXD4L2	64537146	0
CEU	chr9	FOXD4L2	64587146	0
CEU	chr9	FOXD4L2	64637146	0
CEU	chr9	FOXD4L2	64687146	0
CEU	chr9	FOXD4L2	64737146	0
CEU	chr9	FOXD4L2	64787146	0
CEU	chr9	FOXD4L2	64837146	0
CEU	chr9	FOXD4L2	64887146	0
CEU	chr9	FOXD4L2	64937146	0
CEU	chr9	FOXD4L2	64987146	0
CEU	chr9	FOXD4L2	65037146	0
CEU	chr9	FOXD4L2	65087146	0
CEU	chr9	FOXD4L2	65137146	0
CEU	chr9	FOXD4L2	65187146	0
CEU	chr9	FOXD4L2	65237146	0
CEU	chr9	FOXD4L2	65287146	0
CEU	chr9	FOXD4L2	65337146	0
CEU	chr9	FOXD4L2	65387146	0
CEU	chr9	FOXD4L2	65437146	0
CEU	chr9	FOXD4L2	65487146	0

CEU	chr9	FOXD4L2	65537146	0
CEU	chr9	FOXD4L2	65587146	26
CEU	chr9	FOXD4L2	65637146	27
CEU	chr9	FOXD4L2	65687146	1
CEU	chr9	FOXD4L2	65737146	0
CEU	chr9	FOXD4L2	65787146	0
CEU	chr9	FOXD4L2	65837146	0
CEU	chr9	FOXD4L2	65887146	0
CEU	chr9	FOXD4L2	65937146	0
CEU	chr9	FOXD4L2	65987146	0
CEU	chr9	FOXD4L2	66037146	0
CEU	chr9	FOXD4L2	66087146	0
CEU	chr9	FOXD4L2	66137146	0
CEU	chr9	FOXD4L2	66187146	0
CEU	chr9	FOXD4L2	66237146	0
CEU	chr9	FOXD4L2	66287146	0
CEU	chr9	FOXD4L2	66337146	0
CEU	chr9	FOXD4L2	66387146	0
CEU	chr9	FOXD4L2	66437146	0
CEU	chr9	FOXD4L2	66487146	0
CEU	chr9	FOXD4L2	66537146	0
CEU	chr9	FOXD4L2	66587146	0
CEU	chr9	FOXD4L2	66637146	10
CEU	chr9	FOXD4L2	66687146	19
CEU	chr9	FOXD4L2	66737146	18
CEU	chr9	FOXD4L2	66787146	22
CEU	chr9	FOXD4L2	66837146	13
CEU	chr9	FOXD4L2	66887146	0
CEU	chr9	FOXD4L2	66937146	0
CEU	chr9	FOXD4L2	66987146	0
CEU	chr9	FOXD4L2	67037146	0
CEU	chr9	FOXD4L2	67087146	0
CEU	chr9	FOXD4L2	67137146	0
CEU	chr9	FOXD4L2	67187146	0
CEU	chr9	FOXD4L2	67237146	0
CEU	chr9	FOXD4L2	67287146	0
CEU	chr9	FOXD4L2	67337146	0
CEU	chr9	FOXD4L2	67387146	0
CEU	chr9	FOXD4L2	67437146	0
CEU	chr9	FOXD4L2	67487146	0
CEU	chr9	FOXD4L2	67537146	0
CEU	chr9	FOXD4L2	67587146	0
CEU	chr9	FOXD4L2	67637146	0
CEU	chr9	FOXD4L2	67687146	0
CEU	chr9	FOXD4L2	67737146	0
CEU	chr9	FOXD4L2	67787146	0
CEU	chr9	FOXD4L2	67837146	0
CEU	chr9	FOXD4L2	67887146	0
CEU	chr9	FOXD4L2	67937146	0

CEU	chr9	FOXD4L2	67987146	0
CEU	chr9	FOXD4L2	68037146	0
CEU	chr9	FOXD4L2	68087146	0
CEU	chr9	FOXD4L2	68137146	0
CEU	chr9	FOXD4L2	68187146	0
CEU	chr9	FOXD4L2	68237146	0
CEU	chr9	FOXD4L2	68287146	0
CEU	chr9	FOXD4L2	68337146	0
CEU	chr9	FOXD4L2	68387146	0
CEU	chr9	FOXD4L2	68437146	0
CEU	chr9	FOXD4L2	68487146	0
CEU	chr9	FOXD4L2	68537146	0
CEU	chr9	FOXD4L2	68587146	0
CEU	chr9	FOXD4L2	68637146	0
CEU	chr9	FOXD4L2	68687146	0
CEU	chr9	FOXD4L2	68737146	0
CEU	chr9	FOXD4L2	68787146	0
CEU	chr9	FOXD4L2	68837146	0
CEU	chr9	FOXD4L2	68887146	0
CEU	chr9	FOXD4L2	68937146	0
CEU	chr9	FOXD4L2	68987146	0
CEU	chr9	FOXD4L2	69037146	0
CEU	chr9	FOXD4L2	69087146	0
CEU	chr9	FOXD4L2	69137146	0
CEU	chr9	FOXD4L2	69187146	0
CEU	chr9	FOXD4L2	69237146	0
CEU	chr9	FOXD4L2	69287146	0
CEU	chr9	FOXD4L2	69337146	0
CEU	chr9	FOXD4L2	69387146	0
CEU	chr9	FOXD4L2	69437146	0
CEU	chr9	FOXD4L2	69487146	0
CEU	chr9	FOXD4L2	69537146	0
CEU	chr9	FOXD4L2	69587146	0
CEU	chr9	FOXD4L2	69637146	0
CEU	chr9	FOXD4L2	69687146	0
CEU	chr9	FOXD4L2	69737146	0
CEU	chr9	FOXD4L2	69787146	4
CEU	chr9	FOXD4L2	69837146	35
CEU	chr9	FOXD4L2	69887146	53
CEU	chr9	FOXD4L2	69937146	54
CEU	chr9	FOXD4L2	69987146	40
CEU	chr9	FOXD4L2	70037146	8
CEU	chr9	FOXD4L2	70087146	0
CEU	chr9	FOXD4L2	70137146	0
CEU	chr9	FOXD4L2	70187146	0
CEU	chr9	FOXD4L2	70237146	0
CEU	chr9	FOXD4L2	70287146	0
CEU	chr9	FOXD4L2	70337146	0
CEU	chr9	FOXD4L2	70387146	0

CEU	chr9	FOXD4L2	70437146	0
CEU	chr9	FOXD4L2	70487146	0
CEU	chr9	FOXD4L2	70537146	0
CEU	chr9	FOXD4L2	70587146	0
CEU	chr9	FOXD4L2	70637146	0
CEU	chr9	FOXD4L2	70687146	0
CEU	chr15	GOLGA6L9	77479363	0
CEU	chr15	GOLGA6L9	77529363	0
CEU	chr15	GOLGA6L9	77579363	0
CEU	chr15	GOLGA6L9	77629363	0
CEU	chr15	GOLGA6L9	77679363	0
CEU	chr15	GOLGA6L9	77729363	0
CEU	chr15	GOLGA6L9	77779363	0
CEU	chr15	GOLGA6L9	77829363	0
CEU	chr15	GOLGA6L9	77879363	0
CEU	chr15	GOLGA6L9	77929363	0
CEU	chr15	GOLGA6L9	77979363	0
CEU	chr15	GOLGA6L9	78029363	0
CEU	chr15	GOLGA6L9	78079363	0
CEU	chr15	GOLGA6L9	78129363	0
CEU	chr15	GOLGA6L9	78179363	0
CEU	chr15	GOLGA6L9	78229363	0
CEU	chr15	GOLGA6L9	78279363	0
CEU	chr15	GOLGA6L9	78329363	0
CEU	chr15	GOLGA6L9	78379363	0
CEU	chr15	GOLGA6L9	78429363	0
CEU	chr15	GOLGA6L9	78479363	0
CEU	chr15	GOLGA6L9	78529363	0
CEU	chr15	GOLGA6L9	78579363	2
CEU	chr15	GOLGA6L9	78629363	22
CEU	chr15	GOLGA6L9	78679363	39
CEU	chr15	GOLGA6L9	78729363	19
CEU	chr15	GOLGA6L9	78779363	0
CEU	chr15	GOLGA6L9	78829363	0
CEU	chr15	GOLGA6L9	78879363	0
CEU	chr15	GOLGA6L9	78929363	0
CEU	chr15	GOLGA6L9	78979363	0
CEU	chr15	GOLGA6L9	79029363	0
CEU	chr15	GOLGA6L9	79079363	0
CEU	chr15	GOLGA6L9	79129363	0
CEU	chr15	GOLGA6L9	79179363	0
CEU	chr15	GOLGA6L9	79229363	0
CEU	chr15	GOLGA6L9	79279363	0
CEU	chr15	GOLGA6L9	79329363	0
CEU	chr15	GOLGA6L9	79379363	0
CEU	chr15	GOLGA6L9	79429363	0
CEU	chr15	GOLGA6L9	79479363	0
CEU	chr15	GOLGA6L9	79529363	0
CEU	chr15	GOLGA6L9	79579363	0

CEU	chr15	GOLGA6L9	79629363	0
CEU	chr15	GOLGA6L9	79679363	0
CEU	chr15	GOLGA6L9	79729363	0
CEU	chr15	GOLGA6L9	79779363	0
CEU	chr15	GOLGA6L9	79829363	0
CEU	chr15	GOLGA6L9	79879363	0
CEU	chr15	GOLGA6L9	79929363	0
CEU	chr15	GOLGA6L9	79979363	47
CEU	chr15	GOLGA6L9	80029363	61
CEU	chr15	GOLGA6L9	80079363	37
CEU	chr15	GOLGA6L9	80129363	41
CEU	chr15	GOLGA6L9	80179363	38
CEU	chr15	GOLGA6L9	80229363	23
CEU	chr15	GOLGA6L9	80279363	3
CEU	chr15	GOLGA6L9	80329363	0
CEU	chr15	GOLGA6L9	80379363	0
CEU	chr15	GOLGA6L9	80429363	0
CEU	chr15	GOLGA6L9	80479363	0
CEU	chr15	GOLGA6L9	80529363	0
CEU	chr15	GOLGA6L9	80579363	0
CEU	chr15	GOLGA6L9	80629363	0
CEU	chr15	GOLGA6L9	80679363	0
CEU	chr15	GOLGA6L9	80729363	0
CEU	chr15	GOLGA6L9	80779363	0
CEU	chr15	GOLGA6L9	80829363	0
CEU	chr15	GOLGA6L9	80879363	0
CEU	chr15	GOLGA6L9	80929363	0
CEU	chr15	GOLGA6L9	80979363	0
CEU	chr15	GOLGA6L9	81029363	0
CEU	chr15	GOLGA6L9	81079363	0
CEU	chr15	GOLGA6L9	81129363	0
CEU	chr15	GOLGA6L9	81179363	0
CEU	chr15	GOLGA6L9	81229363	0
CEU	chr15	GOLGA6L9	81279363	0
CEU	chr15	GOLGA6L9	81329363	0
CEU	chr15	GOLGA6L9	81379363	0
CEU	chr15	GOLGA6L9	81429363	0
CEU	chr15	GOLGA6L9	81479363	0
CEU	chr15	GOLGA6L9	81529363	0
CEU	chr15	GOLGA6L9	81579363	0
CEU	chr15	GOLGA6L9	81629363	0
CEU	chr15	GOLGA6L9	81679363	0
CEU	chr15	GOLGA6L9	81729363	0
CEU	chr15	GOLGA6L9	81779363	0
CEU	chr15	GOLGA6L9	81829363	0
CEU	chr15	GOLGA6L9	81879363	0
CEU	chr15	GOLGA6L9	81929363	0
CEU	chr15	GOLGA6L9	81979363	0
CEU	chr15	GOLGA6L9	82029363	0

CEU	chr15	GOLGA6L9	82079363	0
CEU	chr15	GOLGA6L9	82129363	0
CEU	chr15	GOLGA6L9	82179363	0
CEU	chr15	GOLGA6L9	82229363	0
CEU	chr15	GOLGA6L9	82279363	0
CEU	chr15	GOLGA6L9	82329363	0
CEU	chr15	GOLGA6L9	82379363	0
CEU	chr15	GOLGA6L9	82429363	0
CEU	chr15	GOLGA6L9	82479363	0
CEU	chr15	GOLGA6L9	82529363	0
CEU	chr15	GOLGA6L9	82579363	0
CEU	chr15	GOLGA6L9	82629363	0
CEU	chr15	GOLGA6L9	82679363	0
CEU	chr15	GOLGA6L9	82729363	0
CEU	chr15	GOLGA6L9	82779363	0
CEU	chr15	GOLGA6L9	82829363	0
CEU	chr15	GOLGA6L9	82879363	0
CEU	chr15	GOLGA6L9	82929363	0
CEU	chr15	GOLGA6L9	82979363	0
CEU	chr15	GOLGA6L9	83029363	0
CEU	chr15	GOLGA6L9	83079363	0
CEU	chr15	GOLGA6L9	83129363	0
CEU	chr15	GOLGA6L9	83179363	8
CEU	chr15	GOLGA6L9	83229363	29
CEU	chr15	GOLGA6L9	83279363	49
CEU	chr15	GOLGA6L9	83329363	48
CEU	chr15	GOLGA6L9	83379363	35
CEU	chr15	GOLGA6L9	83429363	36
CEU	chr15	GOLGA6L9	83479363	39
CEU	chr15	GOLGA6L9	83529363	31
CEU	chr15	GOLGA6L9	83579363	39
CEU	chr15	GOLGA6L9	83629363	43
CEU	chr15	GOLGA6L9	83679363	17
CEU	chr15	GOLGA6L9	83729363	0
CEU	chr15	GOLGA6L9	83779363	0
CEU	chr15	GOLGA6L9	83829363	0
CEU	chr15	GOLGA6L9	83879363	0
CEU	chr15	GOLGA6L9	83929363	0
CEU	chr15	GOLGA6L9	83979363	0
CEU	chr15	GOLGA6L9	84029363	0
CEU	chr15	GOLGA6L9	84079363	0
CEU	chr15	GOLGA6L9	84129363	0
CEU	chr15	GOLGA6L9	84179363	0
CEU	chr15	GOLGA6L9	84229363	7
CEU	chr15	GOLGA6L9	84279363	21
CEU	chr15	GOLGA6L9	84329363	28
CEU	chr15	GOLGA6L9	84379363	33
CEU	chr15	GOLGA6L9	84429363	32
CEU	chr15	GOLGA6L9	84479363	29

CEU	chr15	GOLGA6L9	84529363	38
CEU	chr15	GOLGA6L9	84579363	44
CEU	chr15	GOLGA6L9	84629363	34
CEU	chr15	GOLGA6L9	84679363	26
CEU	chr15	GOLGA6L9	84729363	31
CEU	chr15	GOLGA6L9	84779363	34
CEU	chr15	GOLGA6L9	84829363	25
CEU	chr15	GOLGA6L9	84879363	8
CEU	chr15	GOLGA6L9	84929363	0
CEU	chr15	GOLGA6L9	84979363	0
CEU	chr15	GOLGA6L9	85029363	3
CEU	chr15	GOLGA6L9	85079363	25
CEU	chr15	GOLGA6L9	85129363	39
CEU	chr15	GOLGA6L9	85179363	38
CEU	chr15	GOLGA6L9	85229363	35
CEU	chr15	GOLGA6L9	85279363	34
CEU	chr15	GOLGA6L9	85329363	27
CEU	chr15	GOLGA6L9	85379363	20
CEU	chr15	GOLGA6L9	85429363	43
CEU	chr15	GOLGA6L9	85479363	55
CEU	chr15	GOLGA6L9	85529363	41
CEU	chr15	GOLGA6L9	85579363	28
CEU	chr15	GOLGA6L9	85629363	23
CEU	chr15	GOLGA6L9	85679363	24
CEU	chr15	GOLGA6L9	85729363	13
CEU	chr15	GOLGA6L9	85779363	0
CEU	chr15	GOLGA6L9	85829363	0
CEU	chr15	GOLGA6L9	85879363	0
CEU	chr15	GOLGA6L9	85929363	0
CEU	chr15	GOLGA6L9	85979363	0
CEU	chr15	GOLGA6L9	86029363	0
CEU	chr15	GOLGA6L9	86079363	0
CEU	chr15	GOLGA6L9	86129363	0
CEU	chr15	GOLGA6L9	86179363	0
CEU	chr15	GOLGA6L9	86229363	0
CEU	chr15	GOLGA6L9	86279363	0
CEU	chr15	GOLGA6L9	86329363	0
CEU	chr15	GOLGA6L9	86379363	6
CEU	chr15	GOLGA6L9	86429363	26
CEU	chr15	GOLGA6L9	86479363	32
CEU	chr15	GOLGA6L9	86529363	48
CEU	chr15	GOLGA6L9	86579363	77
CEU	chr15	GOLGA6L9	86629363	88
CEU	chr15	GOLGA6L9	86679363	74
CEU	chr15	GOLGA6L9	86729363	60
CEU	chr15	GOLGA6L9	86779363	50
CEU	chr15	GOLGA6L9	86829363	31
CEU	chr15	GOLGA6L9	86879363	35
CEU	chr15	GOLGA6L9	86929363	35

CEU	chr15	GOLGA6L9	86979363	53
CEU	chr15	GOLGA6L9	87029363	52
CEU	chr15	GOLGA6L9	87079363	32
CEU	chr15	GOLGA6L9	87129363	43
CEU	chr15	GOLGA6L9	87179363	43
CEU	chr15	GOLGA6L9	87229363	53
CEU	chr15	GOLGA6L9	87279363	39
CEU	chr15	GOLGA6L9	87329363	5
CEU	chr15	GOLGA6L9	87379363	0
CEU	chr15	GOLGA8G	23569611	0
CEU	chr15	GOLGA8G	23619611	0
CEU	chr15	GOLGA8G	23669611	32
CEU	chr15	GOLGA8G	23719611	46
CEU	chr15	GOLGA8G	23769611	16
CEU	chr15	GOLGA8G	23819611	2
CEU	chr15	GOLGA8G	23869611	0
CEU	chr15	GOLGA8G	23919611	0
CEU	chr15	GOLGA8G	23969611	0
CEU	chr15	GOLGA8G	24019611	22
CEU	chr15	GOLGA8G	24069611	31
CEU	chr15	GOLGA8G	24119611	9
CEU	chr15	GOLGA8G	24169611	12
CEU	chr15	GOLGA8G	24219611	29
CEU	chr15	GOLGA8G	24269611	56
CEU	chr15	GOLGA8G	24319611	55
CEU	chr15	GOLGA8G	24369611	32
CEU	chr15	GOLGA8G	24419611	62
CEU	chr15	GOLGA8G	24469611	72
CEU	chr15	GOLGA8G	24519611	59
CEU	chr15	GOLGA8G	24569611	66
CEU	chr15	GOLGA8G	24619611	54
CEU	chr15	GOLGA8G	24669611	87
CEU	chr15	GOLGA8G	24719611	145
CEU	chr15	GOLGA8G	24769611	107
CEU	chr15	GOLGA8G	24819611	141
CEU	chr15	GOLGA8G	24869611	161
CEU	chr15	GOLGA8G	24919611	92
CEU	chr15	GOLGA8G	24969611	63
CEU	chr15	GOLGA8G	25019611	33
CEU	chr15	GOLGA8G	25069611	14
CEU	chr15	GOLGA8G	25119611	0
CEU	chr15	GOLGA8G	25169611	0
CEU	chr15	GOLGA8G	25219611	0
CEU	chr15	GOLGA8G	25269611	0
CEU	chr15	GOLGA8G	25319611	0
CEU	chr15	GOLGA8G	25369611	0
CEU	chr15	GOLGA8G	25419611	0
CEU	chr15	GOLGA8G	25469611	0
CEU	chr15	GOLGA8G	25519611	0

CEU	chr15	GOLGA8G	25569611	0
CEU	chr15	GOLGA8G	25619611	0
CEU	chr15	GOLGA8G	25669611	0
CEU	chr15	GOLGA8G	25719611	0
CEU	chr15	GOLGA8G	25769611	0
CEU	chr15	GOLGA8G	25819611	0
CEU	chr15	GOLGA8G	25869611	0
CEU	chr15	GOLGA8G	25919611	0
CEU	chr15	GOLGA8G	25969611	0
CEU	chr15	GOLGA8G	26019611	0
CEU	chr15	GOLGA8G	26069611	0
CEU	chr15	GOLGA8G	26119611	0
CEU	chr15	GOLGA8G	26169611	0
CEU	chr15	GOLGA8G	26219611	0
CEU	chr15	GOLGA8G	26269611	0
CEU	chr15	GOLGA8G	26319611	0
CEU	chr15	GOLGA8G	26369611	0
CEU	chr15	GOLGA8G	26419611	0
CEU	chr15	GOLGA8G	26469611	0
CEU	chr15	GOLGA8G	26519611	0
CEU	chr15	GOLGA8G	26569611	0
CEU	chr15	GOLGA8G	26619611	0
CEU	chr15	GOLGA8G	26669611	0
CEU	chr15	GOLGA8G	26719611	0
CEU	chr15	GOLGA8G	26769611	0
CEU	chr15	GOLGA8G	26819611	0
CEU	chr15	GOLGA8G	26869611	0
CEU	chr15	GOLGA8G	26919611	0
CEU	chr15	GOLGA8G	26969611	0
CEU	chr15	GOLGA8G	27019611	0
CEU	chr15	GOLGA8G	27069611	0
CEU	chr15	GOLGA8G	27119611	6
CEU	chr15	GOLGA8G	27169611	26
CEU	chr15	GOLGA8G	27219611	20
CEU	chr15	GOLGA8G	27269611	0
CEU	chr15	GOLGA8G	27319611	0
CEU	chr15	GOLGA8G	27369611	0
CEU	chr15	GOLGA8G	27419611	0
CEU	chr15	GOLGA8G	27469611	0
CEU	chr15	GOLGA8G	27519611	0
CEU	chr15	GOLGA8G	27569611	0
CEU	chr15	GOLGA8G	27619611	0
CEU	chr15	GOLGA8G	27669611	0
CEU	chr15	GOLGA8G	27719611	0
CEU	chr15	GOLGA8G	27769611	0
CEU	chr15	GOLGA8G	27819611	0
CEU	chr15	GOLGA8G	27869611	0
CEU	chr15	GOLGA8G	27919611	0
CEU	chr15	GOLGA8G	27969611	0

CEU	chr15	GOLGA8G	28019611	0
CEU	chr15	GOLGA8G	28069611	0
CEU	chr15	GOLGA8G	28119611	0
CEU	chr15	GOLGA8G	28169611	0
CEU	chr15	GOLGA8G	28219611	0
CEU	chr15	GOLGA8G	28269611	0
CEU	chr15	GOLGA8G	28319611	0
CEU	chr15	GOLGA8G	28369611	0
CEU	chr15	GOLGA8G	28419611	0
CEU	chr15	GOLGA8G	28469611	0
CEU	chr15	GOLGA8G	28519611	0
CEU	chr15	GOLGA8G	28569611	0
CEU	chr15	GOLGA8G	28619611	0
CEU	chr15	GOLGA8G	28669611	0
CEU	chr15	GOLGA8G	28719611	0
CEU	chr15	GOLGA8G	28769611	0
CEU	chr15	GOLGA8G	28819611	0
CEU	chr15	GOLGA8G	28869611	0
CEU	chr15	GOLGA8G	28919611	0
CEU	chr15	GOLGA8G	28969611	0
CEU	chr15	GOLGA8G	29019611	0
CEU	chr15	GOLGA8G	29069611	0
CEU	chr15	GOLGA8G	29119611	0
CEU	chr15	GOLGA8G	29169611	0
CEU	chr15	GOLGA8G	29219611	0
CEU	chr15	GOLGA8G	29269611	0
CEU	chr15	GOLGA8G	29319611	0
CEU	chr15	GOLGA8G	29369611	0
CEU	chr15	GOLGA8G	29419611	0
CEU	chr15	GOLGA8G	29469611	0
CEU	chr15	GOLGA8G	29519611	0
CEU	chr15	GOLGA8G	29569611	0
CEU	chr15	GOLGA8G	29619611	0
CEU	chr15	GOLGA8G	29669611	0
CEU	chr15	GOLGA8G	29719611	0
CEU	chr15	GOLGA8G	29769611	0
CEU	chr15	GOLGA8G	29819611	0
CEU	chr15	GOLGA8G	29869611	0
CEU	chr15	GOLGA8G	29919611	0
CEU	chr15	GOLGA8G	29969611	0
CEU	chr15	GOLGA8G	30019611	0
CEU	chr15	GOLGA8G	30069611	0
CEU	chr15	GOLGA8G	30119611	0
CEU	chr15	GOLGA8G	30169611	0
CEU	chr15	GOLGA8G	30219611	0
CEU	chr15	GOLGA8G	30269611	0
CEU	chr15	GOLGA8G	30319611	0
CEU	chr15	GOLGA8G	30369611	0
CEU	chr15	GOLGA8G	30419611	0

CEU	chr15	GOLGA8G	30469611	0
CEU	chr15	GOLGA8G	30519611	0
CEU	chr15	GOLGA8G	30569611	0
CEU	chr15	GOLGA8G	30619611	0
CEU	chr15	GOLGA8G	30669611	0
CEU	chr15	GOLGA8G	30719611	0
CEU	chr15	GOLGA8G	30769611	0
CEU	chr15	GOLGA8G	30819611	0
CEU	chr15	GOLGA8G	30869611	0
CEU	chr15	GOLGA8G	30919611	0
CEU	chr15	GOLGA8G	30969611	0
CEU	chr15	GOLGA8G	31019611	0
CEU	chr15	GOLGA8G	31069611	0
CEU	chr15	GOLGA8G	31119611	0
CEU	chr15	GOLGA8G	31169611	0
CEU	chr15	GOLGA8G	31219611	0
CEU	chr15	GOLGA8G	31269611	0
CEU	chr15	GOLGA8G	31319611	0
CEU	chr15	GOLGA8G	31369611	0
CEU	chr15	GOLGA8G	31419611	0
CEU	chr15	GOLGA8G	31469611	0
CEU	chr15	GOLGA8G	31519611	0
CEU	chr15	GOLGA8G	31569611	0
CEU	chr15	GOLGA8G	31619611	0
CEU	chr15	GOLGA8G	31669611	0
CEU	chr15	GOLGA8G	31719611	0
CEU	chr15	GOLGA8G	31769611	0
CEU	chr15	GOLGA8G	31819611	0
CEU	chr15	GOLGA8G	31869611	0
CEU	chr15	GOLGA8G	31919611	0
CEU	chr15	GOLGA8G	31969611	0
CEU	chr15	GOLGA8G	32019611	0
CEU	chr15	GOLGA8G	32069611	0
CEU	chr15	GOLGA8G	32119611	0
CEU	chr15	GOLGA8G	32169611	0
CEU	chr15	GOLGA8G	32219611	0
CEU	chr15	GOLGA8G	32269611	0
CEU	chr15	GOLGA8G	32319611	0
CEU	chr15	GOLGA8G	32369611	0
CEU	chr15	GOLGA8G	32419611	0
CEU	chr15	GOLGA8G	32469611	0
CEU	chr15	GOLGA8G	32519611	0
CEU	chr15	GOLGA8G	32569611	0
CEU	chr15	GOLGA8G	32619611	0
CEU	chr15	GOLGA8G	32669611	0
CEU	chr15	GOLGA8G	32719611	0
CEU	chr15	GOLGA8G	32769611	0
CEU	chr15	GOLGA8G	32819611	0
CEU	chr15	GOLGA8G	32869611	0

CEU	chr15	GOLGA8G	32919611	0
CEU	chr15	GOLGA8G	32969611	0
CEU	chr15	GOLGA8G	33019611	0
CEU	chr15	GOLGA8G	33069611	0
CEU	chr15	GOLGA8G	33119611	0
CEU	chr15	GOLGA8G	33169611	0
CEU	chr15	GOLGA8G	33219611	0
CEU	chr15	GOLGA8G	33269611	0
CEU	chr15	GOLGA8G	33319611	18
CEU	chr15	GOLGA8G	33369611	58
CEU	chr15	GOLGA8G	33419611	55
CEU	chr15	GOLGA8G	33469611	38
CEU	chr1	HIST2	138952386	0
CEU	chr1	HIST2	139002386	0
CEU	chr1	HIST2	139052386	0
CEU	chr1	HIST2	139102386	0
CEU	chr1	HIST2	139152386	0
CEU	chr1	HIST2	139202386	0
CEU	chr1	HIST2	139252386	0
CEU	chr1	HIST2	139302386	0
CEU	chr1	HIST2	139352386	0
CEU	chr1	HIST2	139402386	0
CEU	chr1	HIST2	139452386	0
CEU	chr1	HIST2	139502386	0
CEU	chr1	HIST2	139552386	0
CEU	chr1	HIST2	139602386	0
CEU	chr1	HIST2	139652386	0
CEU	chr1	HIST2	139702386	0
CEU	chr1	HIST2	139752386	0
CEU	chr1	HIST2	139802386	0
CEU	chr1	HIST2	139852386	0
CEU	chr1	HIST2	139902386	0
CEU	chr1	HIST2	139952386	0
CEU	chr1	HIST2	140002386	0
CEU	chr1	HIST2	140052386	0
CEU	chr1	HIST2	140102386	0
CEU	chr1	HIST2	140152386	0
CEU	chr1	HIST2	140202386	0
CEU	chr1	HIST2	140252386	0
CEU	chr1	HIST2	140302386	0
CEU	chr1	HIST2	140352386	0
CEU	chr1	HIST2	140402386	0
CEU	chr1	HIST2	140452386	0
CEU	chr1	HIST2	140502386	0
CEU	chr1	HIST2	140552386	0
CEU	chr1	HIST2	140602386	0
CEU	chr1	HIST2	140652386	0
CEU	chr1	HIST2	140702386	0
CEU	chr1	HIST2	140752386	0

CEU	chr1	HIST2	140802386	0
CEU	chr1	HIST2	140852386	0
CEU	chr1	HIST2	140902386	0
CEU	chr1	HIST2	140952386	0
CEU	chr1	HIST2	141002386	0
CEU	chr1	HIST2	141052386	0
CEU	chr1	HIST2	141102386	0
CEU	chr1	HIST2	141152386	0
CEU	chr1	HIST2	141202386	0
CEU	chr1	HIST2	141252386	0
CEU	chr1	HIST2	141302386	0
CEU	chr1	HIST2	141352386	0
CEU	chr1	HIST2	141402386	0
CEU	chr1	HIST2	141452386	0
CEU	chr1	HIST2	141502386	0
CEU	chr1	HIST2	141552386	0
CEU	chr1	HIST2	141602386	0
CEU	chr1	HIST2	141652386	0
CEU	chr1	HIST2	141702386	0
CEU	chr1	HIST2	141752386	0
CEU	chr1	HIST2	141802386	0
CEU	chr1	HIST2	141852386	0
CEU	chr1	HIST2	141902386	0
CEU	chr1	HIST2	141952386	0
CEU	chr1	HIST2	142002386	0
CEU	chr1	HIST2	142052386	0
CEU	chr1	HIST2	142102386	0
CEU	chr1	HIST2	142152386	0
CEU	chr1	HIST2	142202386	0
CEU	chr1	HIST2	142252386	0
CEU	chr1	HIST2	142302386	0
CEU	chr1	HIST2	142352386	0
CEU	chr1	HIST2	142402386	0
CEU	chr1	HIST2	142452386	0
CEU	chr1	HIST2	142502386	0
CEU	chr1	HIST2	142552386	0
CEU	chr1	HIST2	142602386	0
CEU	chr1	HIST2	142652386	0
CEU	chr1	HIST2	142702386	0
CEU	chr1	HIST2	142752386	0
CEU	chr1	HIST2	142802386	0
CEU	chr1	HIST2	142852386	0
CEU	chr1	HIST2	142902386	0
CEU	chr1	HIST2	142952386	0
CEU	chr1	HIST2	143002386	0
CEU	chr1	HIST2	143052386	0
CEU	chr1	HIST2	143102386	27
CEU	chr1	HIST2	143152386	50
CEU	chr1	HIST2	143202386	56

CEU	chr1	HIST2	143252386	59
CEU	chr1	HIST2	143302386	26
CEU	chr1	HIST2	143352386	0
CEU	chr1	HIST2	143402386	0
CEU	chr1	HIST2	143452386	0
CEU	chr1	HIST2	143502386	0
CEU	chr1	HIST2	143552386	0
CEU	chr1	HIST2	143602386	0
CEU	chr1	HIST2	143652386	0
CEU	chr1	HIST2	143702386	0
CEU	chr1	HIST2	143752386	0
CEU	chr1	HIST2	143802386	0
CEU	chr1	HIST2	143852386	0
CEU	chr1	HIST2	143902386	0
CEU	chr1	HIST2	143952386	0
CEU	chr1	HIST2	144002386	0
CEU	chr1	HIST2	144052386	0
CEU	chr1	HIST2	144102386	0
CEU	chr1	HIST2	144152386	0
CEU	chr1	HIST2	144202386	0
CEU	chr1	HIST2	144252386	0
CEU	chr1	HIST2	144302386	0
CEU	chr1	HIST2	144352386	0
CEU	chr1	HIST2	144402386	0
CEU	chr1	HIST2	144452386	0
CEU	chr1	HIST2	144502386	0
CEU	chr1	HIST2	144552386	0
CEU	chr1	HIST2	144602386	0
CEU	chr1	HIST2	144652386	0
CEU	chr1	HIST2	144702386	0
CEU	chr1	HIST2	144752386	0
CEU	chr1	HIST2	144802386	0
CEU	chr1	HIST2	144852386	0
CEU	chr1	HIST2	144902386	0
CEU	chr1	HIST2	144952386	0
CEU	chr1	HIST2	145002386	0
CEU	chr1	HIST2	145052386	0
CEU	chr1	HIST2	145102386	0
CEU	chr1	HIST2	145152386	0
CEU	chr1	HIST2	145202386	0
CEU	chr1	HIST2	145252386	0
CEU	chr1	HIST2	145302386	0
CEU	chr1	HIST2	145352386	0
CEU	chr1	HIST2	145402386	0
CEU	chr1	HIST2	145452386	0
CEU	chr1	HIST2	145502386	0
CEU	chr1	HIST2	145552386	0
CEU	chr1	HIST2	145602386	0
CEU	chr1	HIST2	145652386	0

CEU	chr1	HIST2	145702386	0
CEU	chr1	HIST2	145752386	0
CEU	chr1	HIST2	145802386	0
CEU	chr1	HIST2	145852386	0
CEU	chr1	HIST2	145902386	0
CEU	chr1	HIST2	145952386	0
CEU	chr1	HIST2	146002386	0
CEU	chr1	HIST2	146052386	0
CEU	chr1	HIST2	146102386	0
CEU	chr1	HIST2	146152386	0
CEU	chr1	HIST2	146202386	0
CEU	chr1	HIST2	146252386	0
CEU	chr1	HIST2	146302386	0
CEU	chr1	HIST2	146352386	0
CEU	chr1	HIST2	146402386	0
CEU	chr1	HIST2	146452386	8
CEU	chr1	HIST2	146502386	34
CEU	chr1	HIST2	146552386	55
CEU	chr1	HIST2	146602386	40
CEU	chr1	HIST2	146652386	37
CEU	chr1	HIST2	146702386	43
CEU	chr1	HIST2	146752386	36
CEU	chr1	HIST2	146802386	38
CEU	chr1	HIST2	146852386	44
CEU	chr1	HIST2	146902386	47
CEU	chr1	HIST2	146952386	33
CEU	chr1	HIST2	147002386	24
CEU	chr1	HIST2	147052386	31
CEU	chr1	HIST2	147102386	33
CEU	chr1	HIST2	147152386	34
CEU	chr1	HIST2	147202386	63
CEU	chr1	HIST2	147252386	115
CEU	chr1	HIST2	147302386	110
CEU	chr1	HIST2	147352386	59
CEU	chr1	HIST2	147402386	24
CEU	chr1	HIST2	147452386	4
CEU	chr1	HIST2	147502386	0
CEU	chr1	HIST2	147552386	0
CEU	chr1	HIST2	147602386	0
CEU	chr1	HIST2	147652386	0
CEU	chr1	HIST2	147702386	0
CEU	chr1	HIST2	147752386	0
CEU	chr1	HIST2	147802386	0
CEU	chr1	HIST2	147852386	0
CEU	chr1	HIST2	147902386	0
CEU	chr1	HIST2	147952386	0
CEU	chr1	HIST2	148002386	0
CEU	chr1	HIST2	148052386	0
CEU	chr1	HIST2	148102386	0

CEU	chr1	HIST2	148152386	0
CEU	chr1	HIST2	148202386	0
CEU	chr1	HIST2	148252386	0
CEU	chr1	HIST2	148302386	0
CEU	chr1	HIST2	148352386	0
CEU	chr1	HIST2	148402386	0
CEU	chr1	HIST2	148452386	0
CEU	chr1	HIST2	148502386	0
CEU	chr1	HIST2	148552386	0
CEU	chr1	HIST2	148602386	0
CEU	chr1	HIST2	148652386	0
CEU	chr1	HIST2	148702386	0
CEU	chr1	HIST2	148752386	0
CEU	chr1	HIST2	148802386	0
CEU	chr1	HIST2	148852386	0
CEU	chr1	HIST2	144832689	0
CEU	chr1	HIST2	144882689	0
CEU	chr1	HIST2	144932689	0
CEU	chr1	HIST2	144982689	0
CEU	chr1	HIST2	145032689	0
CEU	chr1	HIST2	145082689	0
CEU	chr1	HIST2	145132689	0
CEU	chr1	HIST2	145182689	0
CEU	chr1	HIST2	145232689	0
CEU	chr1	HIST2	145282689	0
CEU	chr1	HIST2	145332689	0
CEU	chr1	HIST2	145382689	0
CEU	chr1	HIST2	145432689	0
CEU	chr1	HIST2	145482689	0
CEU	chr1	HIST2	145532689	0
CEU	chr1	HIST2	145582689	0
CEU	chr1	HIST2	145632689	0
CEU	chr1	HIST2	145682689	0
CEU	chr1	HIST2	145732689	0
CEU	chr1	HIST2	145782689	0
CEU	chr1	HIST2	145832689	0
CEU	chr1	HIST2	145882689	0
CEU	chr1	HIST2	145932689	0
CEU	chr1	HIST2	145982689	0
CEU	chr1	HIST2	146032689	0
CEU	chr1	HIST2	146082689	0
CEU	chr1	HIST2	146132689	0
CEU	chr1	HIST2	146182689	0
CEU	chr1	HIST2	146232689	0
CEU	chr1	HIST2	146282689	0
CEU	chr1	HIST2	146332689	0
CEU	chr1	HIST2	146382689	0
CEU	chr1	HIST2	146432689	0
CEU	chr1	HIST2	146482689	24

CEU	chr1	HIST2	146532689	50
CEU	chr1	HIST2	146582689	46
CEU	chr1	HIST2	146632689	39
CEU	chr1	HIST2	146682689	41
CEU	chr1	HIST2	146732689	44
CEU	chr1	HIST2	146782689	33
CEU	chr1	HIST2	146832689	37
CEU	chr1	HIST2	146882689	46
CEU	chr1	HIST2	146932689	41
CEU	chr1	HIST2	146982689	33
CEU	chr1	HIST2	147032689	25
CEU	chr1	HIST2	147082689	32
CEU	chr1	HIST2	147132689	28
CEU	chr1	HIST2	147182689	41
CEU	chr1	HIST2	147232689	88
CEU	chr1	HIST2	147282689	130
CEU	chr1	HIST2	147332689	95
CEU	chr1	HIST2	147382689	30
CEU	chr1	HIST2	147432689	9
CEU	chr1	HIST2	147482689	0
CEU	chr1	HIST2	147532689	0
CEU	chr1	HIST2	147582689	0
CEU	chr1	HIST2	147632689	0
CEU	chr1	HIST2	147682689	0
CEU	chr1	HIST2	147732689	0
CEU	chr1	HIST2	147782689	0
CEU	chr1	HIST2	147832689	0
CEU	chr1	HIST2	147882689	0
CEU	chr1	HIST2	147932689	0
CEU	chr1	HIST2	147982689	0
CEU	chr1	HIST2	148032689	0
CEU	chr1	HIST2	148082689	0
CEU	chr1	HIST2	148132689	0
CEU	chr1	HIST2	148182689	0
CEU	chr1	HIST2	148232689	0
CEU	chr1	HIST2	148282689	0
CEU	chr1	HIST2	148332689	0
CEU	chr1	HIST2	148382689	0
CEU	chr1	HIST2	148432689	0
CEU	chr1	HIST2	148482689	0
CEU	chr1	HIST2	148532689	0
CEU	chr1	HIST2	148582689	0
CEU	chr1	HIST2	148632689	0
CEU	chr1	HIST2	148682689	0
CEU	chr1	HIST2	148732689	0
CEU	chr1	HIST2	148782689	0
CEU	chr1	HIST2	148832689	0
CEU	chr1	HIST2	148882689	0
CEU	chr1	HIST2	148932689	0

CEU	chr1	HIST2	148982689	0
CEU	chr1	HIST2	149032689	0
CEU	chr1	HIST2	149082689	0
CEU	chr1	HIST2	149132689	0
CEU	chr1	HIST2	149182689	0
CEU	chr1	HIST2	149232689	0
CEU	chr1	HIST2	149282689	0
CEU	chr1	HIST2	149332689	0
CEU	chr1	HIST2	149382689	0
CEU	chr1	HIST2	149432689	0
CEU	chr1	HIST2	149482689	0
CEU	chr1	HIST2	149532689	0
CEU	chr1	HIST2	149582689	0
CEU	chr1	HIST2	149632689	0
CEU	chr1	HIST2	149682689	17
CEU	chr1	HIST2	149732689	34
CEU	chr1	HIST2	149782689	26
CEU	chr1	HIST2	149832689	9
CEU	chr1	HIST2	149882689	0
CEU	chr1	HIST2	149932689	0
CEU	chr1	HIST2	149982689	0
CEU	chr1	HIST2	150032689	0
CEU	chr1	HIST2	150082689	0
CEU	chr1	HIST2	150132689	0
CEU	chr1	HIST2	150182689	0
CEU	chr1	HIST2	150232689	0
CEU	chr1	HIST2	150282689	0
CEU	chr1	HIST2	150332689	0
CEU	chr1	HIST2	150382689	0
CEU	chr1	HIST2	150432689	0
CEU	chr1	HIST2	150482689	0
CEU	chr1	HIST2	150532689	17
CEU	chr1	HIST2	150582689	45
CEU	chr1	HIST2	150632689	47
CEU	chr1	HIST2	150682689	36
CEU	chr1	HIST2	150732689	40
CEU	chr1	HIST2	150782689	41
CEU	chr1	HIST2	150832689	41
CEU	chr1	HIST2	150882689	37
CEU	chr1	HIST2	150932689	37
CEU	chr1	HIST2	150982689	51
CEU	chr1	HIST2	151032689	50
CEU	chr1	HIST2	151082689	38
CEU	chr1	HIST2	151132689	37
CEU	chr1	HIST2	151182689	21
CEU	chr1	HIST2	151232689	0
CEU	chr1	HIST2	151282689	0
CEU	chr1	HIST2	151332689	0
CEU	chr1	HIST2	151382689	0

CEU	chr1	HIST2	151432689	0
CEU	chr1	HIST2	151482689	0
CEU	chr1	HIST2	151532689	0
CEU	chr1	HIST2	151582689	0
CEU	chr1	HIST2	151632689	0
CEU	chr1	HIST2	151682689	0
CEU	chr1	HIST2	151732689	0
CEU	chr1	HIST2	151782689	0
CEU	chr1	HIST2	151832689	0
CEU	chr1	HIST2	151882689	0
CEU	chr1	HIST2	151932689	0
CEU	chr1	HIST2	151982689	0
CEU	chr1	HIST2	152032689	0
CEU	chr1	HIST2	152082689	0
CEU	chr1	HIST2	152132689	0
CEU	chr1	HIST2	152182689	0
CEU	chr1	HIST2	152232689	0
CEU	chr1	HIST2	152282689	0
CEU	chr1	HIST2	152332689	0
CEU	chr1	HIST2	152382689	0
CEU	chr1	HIST2	152432689	0
CEU	chr1	HIST2	152482689	0
CEU	chr1	HIST2	152532689	0
CEU	chr1	HIST2	152582689	0
CEU	chr1	HIST2	152632689	0
CEU	chr1	HIST2	152682689	0
CEU	chr1	HIST2	152732689	0
CEU	chr1	HIST2	152782689	0
CEU	chr1	HIST2	152832689	0
CEU	chr1	HIST2	152882689	0
CEU	chr1	HIST2	152932689	0
CEU	chr1	HIST2	152982689	0
CEU	chr1	HIST2	153032689	0
CEU	chr1	HIST2	153082689	0
CEU	chr1	HIST2	153132689	0
CEU	chr1	HIST2	153182689	0
CEU	chr1	HIST2	153232689	0
CEU	chr1	HIST2	153282689	0
CEU	chr1	HIST2	153332689	0
CEU	chr1	HIST2	153382689	0
CEU	chr1	HIST2	153432689	0
CEU	chr1	HIST2	153482689	0
CEU	chr1	HIST2	153532689	0
CEU	chr1	HIST2	153582689	0
CEU	chr1	HIST2	153632689	0
CEU	chr1	HIST2	153682689	0
CEU	chr1	HIST2	153732689	0
CEU	chr1	HIST2	153782689	0
CEU	chr1	HIST2	153832689	0

CEU	chr1	HIST2	153882689	0
CEU	chr1	HIST2	153932689	0
CEU	chr1	HIST2	153982689	0
CEU	chr1	HIST2	154032689	0
CEU	chr1	HIST2	154082689	0
CEU	chr1	HIST2	154132689	0
CEU	chr1	HIST2	154182689	0
CEU	chr1	HIST2	154232689	0
CEU	chr1	HIST2	154282689	0
CEU	chr1	HIST2	154332689	0
CEU	chr1	HIST2	154382689	0
CEU	chr1	HIST2	154432689	0
CEU	chr1	HIST2	154482689	0
CEU	chr1	HIST2	154532689	0
CEU	chr1	HIST2	154582689	0
CEU	chr1	HIST2	154632689	0
CEU	chr1	HIST2	154682689	0
CEU	chr1	HIST2	154732689	0
CEU	chr2	LIMS3	104948688	0
CEU	chr2	LIMS3	104998688	0
CEU	chr2	LIMS3	105048688	0
CEU	chr2	LIMS3	105098688	0
CEU	chr2	LIMS3	105148688	0
CEU	chr2	LIMS3	105198688	0
CEU	chr2	LIMS3	105248688	0
CEU	chr2	LIMS3	105298688	0
CEU	chr2	LIMS3	105348688	0
CEU	chr2	LIMS3	105398688	0
CEU	chr2	LIMS3	105448688	0
CEU	chr2	LIMS3	105498688	0
CEU	chr2	LIMS3	105548688	0
CEU	chr2	LIMS3	105598688	0
CEU	chr2	LIMS3	105648688	0
CEU	chr2	LIMS3	105698688	0
CEU	chr2	LIMS3	105748688	0
CEU	chr2	LIMS3	105798688	0
CEU	chr2	LIMS3	105848688	0
CEU	chr2	LIMS3	105898688	0
CEU	chr2	LIMS3	105948688	0
CEU	chr2	LIMS3	105998688	0
CEU	chr2	LIMS3	106048688	0
CEU	chr2	LIMS3	106098688	0
CEU	chr2	LIMS3	106148688	0
CEU	chr2	LIMS3	106198688	0
CEU	chr2	LIMS3	106248688	10
CEU	chr2	LIMS3	106298688	46
CEU	chr2	LIMS3	106348688	110
CEU	chr2	LIMS3	106398688	119
CEU	chr2	LIMS3	106448688	58

CEU	chr2	LIMS3	106498688	21
CEU	chr2	LIMS3	106548688	8
CEU	chr2	LIMS3	106598688	0
CEU	chr2	LIMS3	106648688	10
CEU	chr2	LIMS3	106698688	31
CEU	chr2	LIMS3	106748688	47
CEU	chr2	LIMS3	106798688	63
CEU	chr2	LIMS3	106848688	99
CEU	chr2	LIMS3	106898688	78
CEU	chr2	LIMS3	106948688	19
CEU	chr2	LIMS3	106998688	3
CEU	chr2	LIMS3	107048688	0
CEU	chr2	LIMS3	107098688	0
CEU	chr2	LIMS3	107148688	0
CEU	chr2	LIMS3	107198688	0
CEU	chr2	LIMS3	107248688	0
CEU	chr2	LIMS3	107298688	0
CEU	chr2	LIMS3	107348688	0
CEU	chr2	LIMS3	107398688	0
CEU	chr2	LIMS3	107448688	0
CEU	chr2	LIMS3	107498688	0
CEU	chr2	LIMS3	107548688	0
CEU	chr2	LIMS3	107598688	0
CEU	chr2	LIMS3	107648688	0
CEU	chr2	LIMS3	107698688	0
CEU	chr2	LIMS3	107748688	7
CEU	chr2	LIMS3	107798688	90
CEU	chr2	LIMS3	107848688	98
CEU	chr2	LIMS3	107898688	25
CEU	chr2	LIMS3	107948688	10
CEU	chr2	LIMS3	107998688	0
CEU	chr2	LIMS3	108048688	0
CEU	chr2	LIMS3	108098688	0
CEU	chr2	LIMS3	108148688	0
CEU	chr2	LIMS3	108198688	0
CEU	chr2	LIMS3	108248688	0
CEU	chr2	LIMS3	108298688	0
CEU	chr2	LIMS3	108348688	0
CEU	chr2	LIMS3	108398688	0
CEU	chr2	LIMS3	108448688	0
CEU	chr2	LIMS3	108498688	0
CEU	chr2	LIMS3	108548688	0
CEU	chr2	LIMS3	108598688	0
CEU	chr2	LIMS3	108648688	0
CEU	chr2	LIMS3	108698688	0
CEU	chr2	LIMS3	108748688	0
CEU	chr2	LIMS3	108798688	0
CEU	chr2	LIMS3	108848688	0
CEU	chr2	LIMS3	108898688	0

CEU	chr2	LIMS3	108948688	5
CEU	chr2	LIMS3	108998688	28
CEU	chr2	LIMS3	109048688	41
CEU	chr2	LIMS3	109098688	34
CEU	chr2	LIMS3	109148688	37
CEU	chr2	LIMS3	109198688	42
CEU	chr2	LIMS3	109248688	37
CEU	chr2	LIMS3	109298688	34
CEU	chr2	LIMS3	109348688	27
CEU	chr2	LIMS3	109398688	25
CEU	chr2	LIMS3	109448688	28
CEU	chr2	LIMS3	109498688	32
CEU	chr2	LIMS3	109548688	51
CEU	chr2	LIMS3	109598688	59
CEU	chr2	LIMS3	109648688	28
CEU	chr2	LIMS3	109698688	0
CEU	chr2	LIMS3	109748688	0
CEU	chr2	LIMS3	109798688	0
CEU	chr2	LIMS3	109848688	0
CEU	chr2	LIMS3	109898688	0
CEU	chr2	LIMS3	109948688	0
CEU	chr2	LIMS3	109998688	0
CEU	chr2	LIMS3	110048688	0
CEU	chr2	LIMS3	110098688	0
CEU	chr2	LIMS3	110148688	0
CEU	chr2	LIMS3	110198688	0
CEU	chr2	LIMS3	110248688	0
CEU	chr2	LIMS3	110298688	0
CEU	chr2	LIMS3	110348688	0
CEU	chr2	LIMS3	110398688	0
CEU	chr2	LIMS3	110448688	0
CEU	chr2	LIMS3	110498688	0
CEU	chr2	LIMS3	110548688	0
CEU	chr2	LIMS3	110598688	0
CEU	chr2	LIMS3	110648688	0
CEU	chr2	LIMS3	110698688	0
CEU	chr2	LIMS3	110748688	0
CEU	chr2	LIMS3	110798688	0
CEU	chr2	LIMS3	110848688	0
CEU	chr2	LIMS3	110898688	0
CEU	chr2	LIMS3	110948688	0
CEU	chr2	LIMS3	110998688	0
CEU	chr2	LIMS3	111048688	0
CEU	chr2	LIMS3	111098688	0
CEU	chr2	LIMS3	111148688	0
CEU	chr2	LIMS3	111198688	0
CEU	chr2	LIMS3	111248688	0
CEU	chr2	LIMS3	111298688	0
CEU	chr2	LIMS3	111348688	0

CEU	chr2	LIMS3	111398688	0
CEU	chr2	LIMS3	111448688	0
CEU	chr2	LIMS3	111498688	0
CEU	chr2	LIMS3	111548688	0
CEU	chr2	LIMS3	111598688	0
CEU	chr2	LIMS3	111648688	0
CEU	chr2	LIMS3	111698688	0
CEU	chr2	LIMS3	111748688	0
CEU	chr2	LIMS3	111798688	0
CEU	chr2	LIMS3	111848688	0
CEU	chr2	LIMS3	111898688	0
CEU	chr2	LIMS3	111948688	0
CEU	chr2	LIMS3	111998688	0
CEU	chr2	LIMS3	112048688	0
CEU	chr2	LIMS3	112098688	0
CEU	chr2	LIMS3	112148688	0
CEU	chr2	LIMS3	112198688	0
CEU	chr2	LIMS3	112248688	0
CEU	chr2	LIMS3	112298688	0
CEU	chr2	LIMS3	112348688	0
CEU	chr2	LIMS3	112398688	0
CEU	chr2	LIMS3	112448688	0
CEU	chr2	LIMS3	112498688	0
CEU	chr2	LIMS3	112548688	0
CEU	chr2	LIMS3	112598688	0
CEU	chr2	LIMS3	112648688	0
CEU	chr2	LIMS3	112698688	0
CEU	chr2	LIMS3	112748688	0
CEU	chr2	LIMS3	112798688	0
CEU	chr2	LIMS3	112848688	0
CEU	chr2	LIMS3	112898688	0
CEU	chr2	LIMS3	112948688	0
CEU	chr2	LIMS3	112998688	0
CEU	chr2	LIMS3	113048688	0
CEU	chr2	LIMS3	113098688	0
CEU	chr2	LIMS3	113148688	0
CEU	chr2	LIMS3	113198688	0
CEU	chr2	LIMS3	113248688	0
CEU	chr2	LIMS3	113298688	0
CEU	chr2	LIMS3	113348688	0
CEU	chr2	LIMS3	113398688	0
CEU	chr2	LIMS3	113448688	0
CEU	chr2	LIMS3	113498688	0
CEU	chr2	LIMS3	113548688	0
CEU	chr2	LIMS3	113598688	0
CEU	chr2	LIMS3	113648688	0
CEU	chr2	LIMS3	113698688	0
CEU	chr2	LIMS3	113748688	0
CEU	chr2	LIMS3	113798688	0

CEU	chr2	LIMS3	113848688	0
CEU	chr2	LIMS3	113898688	0
CEU	chr2	LIMS3	113948688	0
CEU	chr2	LIMS3	113998688	0
CEU	chr2	LIMS3	114048688	0
CEU	chr2	LIMS3	114098688	0
CEU	chr2	LIMS3	114148688	0
CEU	chr2	LIMS3	114198688	0
CEU	chr2	LIMS3	114248688	14
CEU	chr2	LIMS3	114298688	24
CEU	chr2	LIMS3	114348688	10
CEU	chr2	LIMS3	114398688	0
CEU	chr2	LIMS3	114448688	0
CEU	chr2	LIMS3	114498688	0
CEU	chr2	LIMS3	114548688	0
CEU	chr2	LIMS3	114598688	0
CEU	chr2	LIMS3	114648688	0
CEU	chr2	LIMS3	114698688	0
CEU	chr2	LIMS3	114748688	0
CEU	chr2	LIMS3	114798688	0
CEU	chr2	LIMS3	114848688	0
CEU	chr7	MUC12	96019565	0
CEU	chr7	MUC12	96069565	0
CEU	chr7	MUC12	96119565	0
CEU	chr7	MUC12	96169565	0
CEU	chr7	MUC12	96219565	0
CEU	chr7	MUC12	96269565	0
CEU	chr7	MUC12	96319565	0
CEU	chr7	MUC12	96369565	0
CEU	chr7	MUC12	96419565	0
CEU	chr7	MUC12	96469565	0
CEU	chr7	MUC12	96519565	0
CEU	chr7	MUC12	96569565	0
CEU	chr7	MUC12	96619565	0
CEU	chr7	MUC12	96669565	0
CEU	chr7	MUC12	96719565	0
CEU	chr7	MUC12	96769565	0
CEU	chr7	MUC12	96819565	0
CEU	chr7	MUC12	96869565	0
CEU	chr7	MUC12	96919565	0
CEU	chr7	MUC12	96969565	0
CEU	chr7	MUC12	97019565	0
CEU	chr7	MUC12	97069565	0
CEU	chr7	MUC12	97119565	0
CEU	chr7	MUC12	97169565	0
CEU	chr7	MUC12	97219565	0
CEU	chr7	MUC12	97269565	0
CEU	chr7	MUC12	97319565	0
CEU	chr7	MUC12	97369565	0

CEU	chr7	MUC12	97419565	0
CEU	chr7	MUC12	97469565	0
CEU	chr7	MUC12	97519565	0
CEU	chr7	MUC12	97569565	0
CEU	chr7	MUC12	97619565	0
CEU	chr7	MUC12	97669565	0
CEU	chr7	MUC12	97719565	0
CEU	chr7	MUC12	97769565	0
CEU	chr7	MUC12	97819565	0
CEU	chr7	MUC12	97869565	0
CEU	chr7	MUC12	97919565	0
CEU	chr7	MUC12	97969565	0
CEU	chr7	MUC12	98019565	0
CEU	chr7	MUC12	98069565	0
CEU	chr7	MUC12	98119565	0
CEU	chr7	MUC12	98169565	0
CEU	chr7	MUC12	98219565	0
CEU	chr7	MUC12	98269565	0
CEU	chr7	MUC12	98319565	0
CEU	chr7	MUC12	98369565	0
CEU	chr7	MUC12	98419565	0
CEU	chr7	MUC12	98469565	0
CEU	chr7	MUC12	98519565	0
CEU	chr7	MUC12	98569565	0
CEU	chr7	MUC12	98619565	0
CEU	chr7	MUC12	98669565	0
CEU	chr7	MUC12	98719565	0
CEU	chr7	MUC12	98769565	0
CEU	chr7	MUC12	98819565	0
CEU	chr7	MUC12	98869565	0
CEU	chr7	MUC12	98919565	0
CEU	chr7	MUC12	98969565	0
CEU	chr7	MUC12	99019565	0
CEU	chr7	MUC12	99069565	0
CEU	chr7	MUC12	99119565	0
CEU	chr7	MUC12	99169565	0
CEU	chr7	MUC12	99219565	0
CEU	chr7	MUC12	99269565	0
CEU	chr7	MUC12	99319565	0
CEU	chr7	MUC12	99369565	0
CEU	chr7	MUC12	99419565	0
CEU	chr7	MUC12	99469565	0
CEU	chr7	MUC12	99519565	0
CEU	chr7	MUC12	99569565	0
CEU	chr7	MUC12	99619565	0
CEU	chr7	MUC12	99669565	0
CEU	chr7	MUC12	99719565	0
CEU	chr7	MUC12	99769565	0
CEU	chr7	MUC12	99819565	0

CEU	chr7	MUC12	99869565	0
CEU	chr7	MUC12	99919565	0
CEU	chr7	MUC12	99969565	0
CEU	chr7	MUC12	100019565	0
CEU	chr7	MUC12	100069565	0
CEU	chr7	MUC12	100119565	0
CEU	chr7	MUC12	100169565	0
CEU	chr7	MUC12	100219565	0
CEU	chr7	MUC12	100269565	0
CEU	chr7	MUC12	100319565	0
CEU	chr7	MUC12	100369565	0
CEU	chr7	MUC12	100419565	0
CEU	chr7	MUC12	100469565	0
CEU	chr7	MUC12	100519565	0
CEU	chr7	MUC12	100569565	0
CEU	chr7	MUC12	100619565	0
CEU	chr7	MUC12	100669565	0
CEU	chr7	MUC12	100719565	0
CEU	chr7	MUC12	100769565	0
CEU	chr7	MUC12	100819565	0
CEU	chr7	MUC12	100869565	0
CEU	chr7	MUC12	100919565	0
CEU	chr7	MUC12	100969565	0
CEU	chr7	MUC12	101019565	0
CEU	chr7	MUC12	101069565	0
CEU	chr7	MUC12	101119565	0
CEU	chr7	MUC12	101169565	0
CEU	chr7	MUC12	101219565	0
CEU	chr7	MUC12	101269565	0
CEU	chr7	MUC12	101319565	55
CEU	chr7	MUC12	101369565	57
CEU	chr7	MUC12	101419565	2
CEU	chr7	MUC12	101469565	0
CEU	chr7	MUC12	101519565	0
CEU	chr7	MUC12	101569565	0
CEU	chr7	MUC12	101619565	0
CEU	chr7	MUC12	101669565	0
CEU	chr7	MUC12	101719565	0
CEU	chr7	MUC12	101769565	0
CEU	chr7	MUC12	101819565	0
CEU	chr7	MUC12	101869565	0
CEU	chr7	MUC12	101919565	0
CEU	chr7	MUC12	101969565	0
CEU	chr7	MUC12	102019565	0
CEU	chr7	MUC12	102069565	0
CEU	chr7	MUC12	102119565	0
CEU	chr7	MUC12	102169565	0
CEU	chr7	MUC12	102219565	0
CEU	chr7	MUC12	102269565	0

CEU	chr7	MUC12	102319565	0
CEU	chr7	MUC12	102369565	0
CEU	chr7	MUC12	102419565	0
CEU	chr7	MUC12	102469565	0
CEU	chr7	MUC12	102519565	0
CEU	chr7	MUC12	102569565	0
CEU	chr7	MUC12	102619565	0
CEU	chr7	MUC12	102669565	0
CEU	chr7	MUC12	102719565	0
CEU	chr7	MUC12	102769565	0
CEU	chr7	MUC12	102819565	0
CEU	chr7	MUC12	102869565	0
CEU	chr7	MUC12	102919565	0
CEU	chr7	MUC12	102969565	0
CEU	chr7	MUC12	103019565	0
CEU	chr7	MUC12	103069565	0
CEU	chr7	MUC12	103119565	0
CEU	chr7	MUC12	103169565	0
CEU	chr7	MUC12	103219565	0
CEU	chr7	MUC12	103269565	0
CEU	chr7	MUC12	103319565	0
CEU	chr7	MUC12	103369565	0
CEU	chr7	MUC12	103419565	0
CEU	chr7	MUC12	103469565	0
CEU	chr7	MUC12	103519565	0
CEU	chr7	MUC12	103569565	0
CEU	chr7	MUC12	103619565	0
CEU	chr7	MUC12	103669565	0
CEU	chr7	MUC12	103719565	0
CEU	chr7	MUC12	103769565	0
CEU	chr7	MUC12	103819565	0
CEU	chr7	MUC12	103869565	0
CEU	chr7	MUC12	103919565	0
CEU	chr7	MUC12	103969565	0
CEU	chr7	MUC12	104019565	0
CEU	chr7	MUC12	104069565	0
CEU	chr7	MUC12	104119565	0
CEU	chr7	MUC12	104169565	0
CEU	chr7	MUC12	104219565	0
CEU	chr7	MUC12	104269565	0
CEU	chr7	MUC12	104319565	0
CEU	chr7	MUC12	104369565	0
CEU	chr7	MUC12	104419565	0
CEU	chr7	MUC12	104469565	0
CEU	chr7	MUC12	104519565	0
CEU	chr7	MUC12	104569565	0
CEU	chr7	MUC12	104619565	0
CEU	chr7	MUC12	104669565	0
CEU	chr7	MUC12	104719565	0

CEU	chr7	MUC12	104769565	0
CEU	chr7	MUC12	104819565	0
CEU	chr7	MUC12	104869565	0
CEU	chr7	MUC12	104919565	0
CEU	chr7	MUC12	104969565	0
CEU	chr7	MUC12	105019565	0
CEU	chr7	MUC12	105069565	0
CEU	chr7	MUC12	105119565	0
CEU	chr7	MUC12	105169565	0
CEU	chr7	MUC12	105219565	0
CEU	chr7	MUC12	105269565	0
CEU	chr7	MUC12	105319565	0
CEU	chr7	MUC12	105369565	0
CEU	chr7	MUC12	105419565	0
CEU	chr7	MUC12	105469565	0
CEU	chr7	MUC12	105519565	0
CEU	chr7	MUC12	105569565	0
CEU	chr7	MUC12	105619565	0
CEU	chr7	MUC12	105669565	0
CEU	chr7	MUC12	105719565	0
CEU	chr7	MUC12	105769565	0
CEU	chr7	MUC12	105819565	0
CEU	chr7	MUC12	105869565	0
CEU	chr7	MUC12	105919565	0
CEU	chr1	NBPF11	143152151	50
CEU	chr1	NBPF11	143202151	56
CEU	chr1	NBPF11	143252151	59
CEU	chr1	NBPF11	143302151	26
CEU	chr1	NBPF11	143352151	0
CEU	chr1	NBPF11	143402151	0
CEU	chr1	NBPF11	143452151	0
CEU	chr1	NBPF11	143502151	0
CEU	chr1	NBPF11	143552151	0
CEU	chr1	NBPF11	143602151	0
CEU	chr1	NBPF11	143652151	0
CEU	chr1	NBPF11	143702151	0
CEU	chr1	NBPF11	143752151	0
CEU	chr1	NBPF11	143802151	0
CEU	chr1	NBPF11	143852151	0
CEU	chr1	NBPF11	143902151	0
CEU	chr1	NBPF11	143952151	0
CEU	chr1	NBPF11	144002151	0
CEU	chr1	NBPF11	144052151	0
CEU	chr1	NBPF11	144102151	0
CEU	chr1	NBPF11	144152151	0
CEU	chr1	NBPF11	144202151	0
CEU	chr1	NBPF11	144252151	0
CEU	chr1	NBPF11	144302151	0
CEU	chr1	NBPF11	144352151	0

CEU	chr1	NBPF11	144402151	0
CEU	chr1	NBPF11	144452151	0
CEU	chr1	NBPF11	144502151	0
CEU	chr1	NBPF11	144552151	0
CEU	chr1	NBPF11	144602151	0
CEU	chr1	NBPF11	144652151	0
CEU	chr1	NBPF11	144702151	0
CEU	chr1	NBPF11	144752151	0
CEU	chr1	NBPF11	144802151	0
CEU	chr1	NBPF11	144852151	0
CEU	chr1	NBPF11	144902151	0
CEU	chr1	NBPF11	144952151	0
CEU	chr1	NBPF11	145002151	0
CEU	chr1	NBPF11	145052151	0
CEU	chr1	NBPF11	145102151	0
CEU	chr1	NBPF11	145152151	0
CEU	chr1	NBPF11	145202151	0
CEU	chr1	NBPF11	145252151	0
CEU	chr1	NBPF11	145302151	0
CEU	chr1	NBPF11	145352151	0
CEU	chr1	NBPF11	145402151	0
CEU	chr1	NBPF11	145452151	0
CEU	chr1	NBPF11	145502151	0
CEU	chr1	NBPF11	145552151	0
CEU	chr1	NBPF11	145602151	0
CEU	chr1	NBPF11	145652151	0
CEU	chr1	NBPF11	145702151	0
CEU	chr1	NBPF11	145752151	0
CEU	chr1	NBPF11	145802151	0
CEU	chr1	NBPF11	145852151	0
CEU	chr1	NBPF11	145902151	0
CEU	chr1	NBPF11	145952151	0
CEU	chr1	NBPF11	146002151	0
CEU	chr1	NBPF11	146052151	0
CEU	chr1	NBPF11	146102151	0
CEU	chr1	NBPF11	146152151	0
CEU	chr1	NBPF11	146202151	0
CEU	chr1	NBPF11	146252151	0
CEU	chr1	NBPF11	146302151	0
CEU	chr1	NBPF11	146352151	0
CEU	chr1	NBPF11	146402151	0
CEU	chr1	NBPF11	146452151	8
CEU	chr1	NBPF11	146502151	34
CEU	chr1	NBPF11	146552151	55
CEU	chr1	NBPF11	146602151	40
CEU	chr1	NBPF11	146652151	37
CEU	chr1	NBPF11	146702151	43
CEU	chr1	NBPF11	146752151	36
CEU	chr1	NBPF11	146802151	38

CEU	chr1	NBPF11	146852151	44
CEU	chr1	NBPF11	146902151	47
CEU	chr1	NBPF11	146952151	33
CEU	chr1	NBPF11	147002151	24
CEU	chr1	NBPF11	147052151	31
CEU	chr1	NBPF11	147102151	33
CEU	chr1	NBPF11	147152151	34
CEU	chr1	NBPF11	147202151	63
CEU	chr1	NBPF11	147252151	114
CEU	chr1	NBPF11	147302151	110
CEU	chr1	NBPF11	147352151	60
CEU	chr1	NBPF11	147402151	24
CEU	chr1	NBPF11	147452151	4
CEU	chr1	NBPF11	147502151	0
CEU	chr1	NBPF11	147552151	0
CEU	chr1	NBPF11	147602151	0
CEU	chr1	NBPF11	147652151	0
CEU	chr1	NBPF11	147702151	0
CEU	chr1	NBPF11	147752151	0
CEU	chr1	NBPF11	147802151	0
CEU	chr1	NBPF11	147852151	0
CEU	chr1	NBPF11	147902151	0
CEU	chr1	NBPF11	147952151	0
CEU	chr1	NBPF11	148002151	0
CEU	chr1	NBPF11	148052151	0
CEU	chr1	NBPF11	148102151	0
CEU	chr1	NBPF11	148152151	0
CEU	chr1	NBPF11	148202151	0
CEU	chr1	NBPF11	148252151	0
CEU	chr1	NBPF11	148302151	0
CEU	chr1	NBPF11	148352151	0
CEU	chr1	NBPF11	148402151	0
CEU	chr1	NBPF11	148452151	0
CEU	chr1	NBPF11	148502151	0
CEU	chr1	NBPF11	148552151	0
CEU	chr1	NBPF11	148602151	0
CEU	chr1	NBPF11	148652151	0
CEU	chr1	NBPF11	148702151	0
CEU	chr1	NBPF11	148752151	0
CEU	chr1	NBPF11	148802151	0
CEU	chr1	NBPF11	148852151	0
CEU	chr1	NBPF11	148902151	0
CEU	chr1	NBPF11	148952151	0
CEU	chr1	NBPF11	149002151	0
CEU	chr1	NBPF11	149052151	0
CEU	chr1	NBPF11	149102151	0
CEU	chr1	NBPF11	149152151	0
CEU	chr1	NBPF11	149202151	0
CEU	chr1	NBPF11	149252151	0

CEU	chr1	NBPF11	149302151	0
CEU	chr1	NBPF11	149352151	0
CEU	chr1	NBPF11	149402151	0
CEU	chr1	NBPF11	149452151	0
CEU	chr1	NBPF11	149502151	0
CEU	chr1	NBPF11	149552151	0
CEU	chr1	NBPF11	149602151	0
CEU	chr1	NBPF11	149652151	0
CEU	chr1	NBPF11	149702151	19
CEU	chr1	NBPF11	149752151	43
CEU	chr1	NBPF11	149802151	24
CEU	chr1	NBPF11	149852151	0
CEU	chr1	NBPF11	149902151	0
CEU	chr1	NBPF11	149952151	0
CEU	chr1	NBPF11	150002151	0
CEU	chr1	NBPF11	150052151	0
CEU	chr1	NBPF11	150102151	0
CEU	chr1	NBPF11	150152151	0
CEU	chr1	NBPF11	150202151	0
CEU	chr1	NBPF11	150252151	0
CEU	chr1	NBPF11	150302151	0
CEU	chr1	NBPF11	150352151	0
CEU	chr1	NBPF11	150402151	0
CEU	chr1	NBPF11	150452151	0
CEU	chr1	NBPF11	150502151	4
CEU	chr1	NBPF11	150552151	26
CEU	chr1	NBPF11	150602151	46
CEU	chr1	NBPF11	150652151	43
CEU	chr1	NBPF11	150702151	37
CEU	chr1	NBPF11	150752151	42
CEU	chr1	NBPF11	150802151	41
CEU	chr1	NBPF11	150852151	41
CEU	chr1	NBPF11	150902151	39
CEU	chr1	NBPF11	150952151	52
CEU	chr1	NBPF11	151002151	53
CEU	chr1	NBPF11	151052151	31
CEU	chr1	NBPF11	151102151	42
CEU	chr1	NBPF11	151152151	34
CEU	chr1	NBPF11	151202151	7
CEU	chr1	NBPF11	151252151	0
CEU	chr1	NBPF11	151302151	0
CEU	chr1	NBPF11	151352151	0
CEU	chr1	NBPF11	151402151	0
CEU	chr1	NBPF11	151452151	0
CEU	chr1	NBPF11	151502151	0
CEU	chr1	NBPF11	151552151	0
CEU	chr1	NBPF11	151602151	0
CEU	chr1	NBPF11	151652151	0
CEU	chr1	NBPF11	151702151	0

CEU	chr1	NBPF11	151752151	0
CEU	chr1	NBPF11	151802151	0
CEU	chr1	NBPF11	151852151	0
CEU	chr1	NBPF11	151902151	0
CEU	chr1	NBPF11	151952151	0
CEU	chr1	NBPF11	152002151	0
CEU	chr1	NBPF11	152052151	0
CEU	chr1	NBPF11	152102151	0
CEU	chr1	NBPF11	152152151	0
CEU	chr1	NBPF11	152202151	0
CEU	chr1	NBPF11	152252151	0
CEU	chr1	NBPF11	152302151	0
CEU	chr1	NBPF11	152352151	0
CEU	chr1	NBPF11	152402151	0
CEU	chr1	NBPF11	152452151	0
CEU	chr1	NBPF11	152502151	0
CEU	chr1	NBPF11	152552151	0
CEU	chr1	NBPF11	152602151	0
CEU	chr1	NBPF11	152652151	0
CEU	chr1	NBPF11	152702151	0
CEU	chr1	NBPF11	152752151	0
CEU	chr1	NBPF11	152802151	0
CEU	chr1	NBPF11	152852151	0
CEU	chr1	NBPF11	152902151	0
CEU	chr1	NBPF11	152952151	0
CEU	chr1	NBPF11	153002151	0
CEU	chr1	NBPF11	153052151	0
CEU	chr1	NBPF11	153102151	0
CEU	chr1	NBPF16	139471390	0
CEU	chr1	NBPF16	139521390	0
CEU	chr1	NBPF16	139571390	0
CEU	chr1	NBPF16	139621390	0
CEU	chr1	NBPF16	139671390	0
CEU	chr1	NBPF16	139721390	0
CEU	chr1	NBPF16	139771390	0
CEU	chr1	NBPF16	139821390	0
CEU	chr1	NBPF16	139871390	0
CEU	chr1	NBPF16	139921390	0
CEU	chr1	NBPF16	139971390	0
CEU	chr1	NBPF16	140021390	0
CEU	chr1	NBPF16	140071390	0
CEU	chr1	NBPF16	140121390	0
CEU	chr1	NBPF16	140171390	0
CEU	chr1	NBPF16	140221390	0
CEU	chr1	NBPF16	140271390	0
CEU	chr1	NBPF16	140321390	0
CEU	chr1	NBPF16	140371390	0
CEU	chr1	NBPF16	140421390	0
CEU	chr1	NBPF16	140471390	0

CEU	chr1	NBPF16	140521390	0
CEU	chr1	NBPF16	140571390	0
CEU	chr1	NBPF16	140621390	0
CEU	chr1	NBPF16	140671390	0
CEU	chr1	NBPF16	140721390	0
CEU	chr1	NBPF16	140771390	0
CEU	chr1	NBPF16	140821390	0
CEU	chr1	NBPF16	140871390	0
CEU	chr1	NBPF16	140921390	0
CEU	chr1	NBPF16	140971390	0
CEU	chr1	NBPF16	141021390	0
CEU	chr1	NBPF16	141071390	0
CEU	chr1	NBPF16	141121390	0
CEU	chr1	NBPF16	141171390	0
CEU	chr1	NBPF16	141221390	0
CEU	chr1	NBPF16	141271390	0
CEU	chr1	NBPF16	141321390	0
CEU	chr1	NBPF16	141371390	0
CEU	chr1	NBPF16	141421390	0
CEU	chr1	NBPF16	141471390	0
CEU	chr1	NBPF16	141521390	0
CEU	chr1	NBPF16	141571390	0
CEU	chr1	NBPF16	141621390	0
CEU	chr1	NBPF16	141671390	0
CEU	chr1	NBPF16	141721390	0
CEU	chr1	NBPF16	141771390	0
CEU	chr1	NBPF16	141821390	0
CEU	chr1	NBPF16	141871390	0
CEU	chr1	NBPF16	141921390	0
CEU	chr1	NBPF16	141971390	0
CEU	chr1	NBPF16	142021390	0
CEU	chr1	NBPF16	142071390	0
CEU	chr1	NBPF16	142121390	0
CEU	chr1	NBPF16	142171390	0
CEU	chr1	NBPF16	142221390	0
CEU	chr1	NBPF16	142271390	0
CEU	chr1	NBPF16	142321390	0
CEU	chr1	NBPF16	142371390	0
CEU	chr1	NBPF16	142421390	0
CEU	chr1	NBPF16	142471390	0
CEU	chr1	NBPF16	142521390	0
CEU	chr1	NBPF16	142571390	0
CEU	chr1	NBPF16	142621390	0
CEU	chr1	NBPF16	142671390	0
CEU	chr1	NBPF16	142721390	0
CEU	chr1	NBPF16	142771390	0
CEU	chr1	NBPF16	142821390	0
CEU	chr1	NBPF16	142871390	0
CEU	chr1	NBPF16	142921390	0

CEU	chr1	NBPF16	142971390	0
CEU	chr1	NBPF16	143021390	0
CEU	chr1	NBPF16	143071390	1
CEU	chr1	NBPF16	143121390	34
CEU	chr1	NBPF16	143171390	64
CEU	chr1	NBPF16	143221390	57
CEU	chr1	NBPF16	143271390	44
CEU	chr1	NBPF16	143321390	18
CEU	chr1	NBPF16	143371390	0
CEU	chr1	NBPF16	143421390	0
CEU	chr1	NBPF16	143471390	0
CEU	chr1	NBPF16	143521390	0
CEU	chr1	NBPF16	143571390	0
CEU	chr1	NBPF16	143621390	0
CEU	chr1	NBPF16	143671390	0
CEU	chr1	NBPF16	143721390	0
CEU	chr1	NBPF16	143771390	0
CEU	chr1	NBPF16	143821390	0
CEU	chr1	NBPF16	143871390	0
CEU	chr1	NBPF16	143921390	0
CEU	chr1	NBPF16	143971390	0
CEU	chr1	NBPF16	144021390	0
CEU	chr1	NBPF16	144071390	0
CEU	chr1	NBPF16	144121390	0
CEU	chr1	NBPF16	144171390	0
CEU	chr1	NBPF16	144221390	0
CEU	chr1	NBPF16	144271390	0
CEU	chr1	NBPF16	144321390	0
CEU	chr1	NBPF16	144371390	0
CEU	chr1	NBPF16	144421390	0
CEU	chr1	NBPF16	144471390	0
CEU	chr1	NBPF16	144521390	0
CEU	chr1	NBPF16	144571390	0
CEU	chr1	NBPF16	144621390	0
CEU	chr1	NBPF16	144671390	0
CEU	chr1	NBPF16	144721390	0
CEU	chr1	NBPF16	144771390	0
CEU	chr1	NBPF16	144821390	0
CEU	chr1	NBPF16	144871390	0
CEU	chr1	NBPF16	144921390	0
CEU	chr1	NBPF16	144971390	0
CEU	chr1	NBPF16	145021390	0
CEU	chr1	NBPF16	145071390	0
CEU	chr1	NBPF16	145121390	0
CEU	chr1	NBPF16	145171390	0
CEU	chr1	NBPF16	145221390	0
CEU	chr1	NBPF16	145271390	0
CEU	chr1	NBPF16	145321390	0
CEU	chr1	NBPF16	145371390	0

CEU	chr1	NBPF16	145421390	0
CEU	chr1	NBPF16	145471390	0
CEU	chr1	NBPF16	145521390	0
CEU	chr1	NBPF16	145571390	0
CEU	chr1	NBPF16	145621390	0
CEU	chr1	NBPF16	145671390	0
CEU	chr1	NBPF16	145721390	0
CEU	chr1	NBPF16	145771390	0
CEU	chr1	NBPF16	145821390	0
CEU	chr1	NBPF16	145871390	0
CEU	chr1	NBPF16	145921390	0
CEU	chr1	NBPF16	145971390	0
CEU	chr1	NBPF16	146021390	0
CEU	chr1	NBPF16	146071390	0
CEU	chr1	NBPF16	146121390	0
CEU	chr1	NBPF16	146171390	0
CEU	chr1	NBPF16	146221390	0
CEU	chr1	NBPF16	146271390	0
CEU	chr1	NBPF16	146321390	0
CEU	chr1	NBPF16	146371390	0
CEU	chr1	NBPF16	146421390	0
CEU	chr1	NBPF16	146471390	20
CEU	chr1	NBPF16	146521390	42
CEU	chr1	NBPF16	146571390	49
CEU	chr1	NBPF16	146621390	42
CEU	chr1	NBPF16	146671390	38
CEU	chr1	NBPF16	146721390	44
CEU	chr1	NBPF16	146771390	34
CEU	chr1	NBPF16	146821390	31
CEU	chr1	NBPF16	146871390	47
CEU	chr1	NBPF16	146921390	51
CEU	chr1	NBPF16	146971390	32
CEU	chr1	NBPF16	147021390	21
CEU	chr1	NBPF16	147071390	29
CEU	chr1	NBPF16	147121390	30
CEU	chr1	NBPF16	147171390	44
CEU	chr1	NBPF16	147221390	76
CEU	chr1	NBPF16	147271390	127
CEU	chr1	NBPF16	147321390	105
CEU	chr1	NBPF16	147371390	35
CEU	chr1	NBPF16	147421390	14
CEU	chr1	NBPF16	147471390	1
CEU	chr1	NBPF16	147521390	0
CEU	chr1	NBPF16	147571390	0
CEU	chr1	NBPF16	147621390	0
CEU	chr1	NBPF16	147671390	0
CEU	chr1	NBPF16	147721390	0
CEU	chr1	NBPF16	147771390	0
CEU	chr1	NBPF16	147821390	0

CEU	chr1	NBPF16	147871390	0
CEU	chr1	NBPF16	147921390	0
CEU	chr1	NBPF16	147971390	0
CEU	chr1	NBPF16	148021390	0
CEU	chr1	NBPF16	148071390	0
CEU	chr1	NBPF16	148121390	0
CEU	chr1	NBPF16	148171390	0
CEU	chr1	NBPF16	148221390	0
CEU	chr1	NBPF16	148271390	0
CEU	chr1	NBPF16	148321390	0
CEU	chr1	NBPF16	148371390	0
CEU	chr1	NBPF16	148421390	0
CEU	chr1	NBPF16	148471390	0
CEU	chr1	NBPF16	148521390	0
CEU	chr1	NBPF16	148571390	0
CEU	chr1	NBPF16	148621390	0
CEU	chr1	NBPF16	148671390	0
CEU	chr1	NBPF16	148721390	0
CEU	chr1	NBPF16	148771390	0
CEU	chr1	NBPF16	148821390	0
CEU	chr1	NBPF16	148871390	0
CEU	chr1	NBPF16	148921390	0
CEU	chr1	NBPF16	148971390	0
CEU	chr1	NBPF16	149021390	0
CEU	chr1	NBPF16	149071390	0
CEU	chr1	NBPF16	149121390	0
CEU	chr1	NBPF16	149171390	0
CEU	chr1	NBPF16	149221390	0
CEU	chr1	NBPF16	149271390	0
CEU	chr1	NBPF16	149321390	0
CEU	chr1	NBPF16	149371390	0
CEU	chr1	NPIP	39271070	0
CEU	chr1	NPIP	39321070	0
CEU	chr1	NPIP	39371070	11
CEU	chr1	NPIP	39421070	27
CEU	chr1	NPIP	39471070	32
CEU	chr1	NPIP	39521070	31
CEU	chr1	NPIP	39571070	25
CEU	chr1	NPIP	39621070	22
CEU	chr1	NPIP	39671070	26
CEU	chr1	NPIP	39721070	27
CEU	chr1	NPIP	39771070	23
CEU	chr1	NPIP	39821070	25
CEU	chr1	NPIP	39871070	24
CEU	chr1	NPIP	39921070	22
CEU	chr1	NPIP	39971070	21
CEU	chr1	NPIP	40021070	18
CEU	chr1	NPIP	40071070	21
CEU	chr1	NPIP	40121070	26

CEU	chr1	NPIP	40171070	31
CEU	chr1	NPIP	40221070	62
CEU	chr1	NPIP	40271070	71
CEU	chr1	NPIP	40321070	33
CEU	chr1	NPIP	40371070	12
CEU	chr1	NPIP	40421070	4
CEU	chr1	NPIP	40471070	0
CEU	chr1	NPIP	40521070	0
CEU	chr1	NPIP	40571070	0
CEU	chr1	NPIP	40621070	0
CEU	chr1	NPIP	40671070	0
CEU	chr1	NPIP	40721070	0
CEU	chr1	NPIP	40771070	0
CEU	chr1	NPIP	40821070	0
CEU	chr1	NPIP	40871070	0
CEU	chr1	NPIP	40921070	0
CEU	chr1	NPIP	40971070	0
CEU	chr1	NPIP	41021070	0
CEU	chr1	NPIP	41071070	0
CEU	chr1	NPIP	41121070	0
CEU	chr1	NPIP	41171070	0
CEU	chr1	NPIP	41221070	0
CEU	chr1	NPIP	41271070	0
CEU	chr1	NPIP	41321070	0
CEU	chr1	NPIP	41371070	0
CEU	chr1	NPIP	41421070	0
CEU	chr1	NPIP	41471070	0
CEU	chr1	NPIP	41521070	0
CEU	chr1	NPIP	41571070	0
CEU	chr1	NPIP	41621070	0
CEU	chr1	NPIP	41671070	0
CEU	chr1	NPIP	41721070	0
CEU	chr1	NPIP	41771070	0
CEU	chr1	NPIP	41821070	0
CEU	chr1	NPIP	41871070	0
CEU	chr1	NPIP	41921070	0
CEU	chr1	NPIP	41971070	0
CEU	chr1	NPIP	42021070	0
CEU	chr1	NPIP	42071070	1
CEU	chr1	NPIP	42121070	21
CEU	chr1	NPIP	42171070	41
CEU	chr1	NPIP	42221070	42
CEU	chr1	NPIP	42271070	46
CEU	chr1	NPIP	42321070	45
CEU	chr1	NPIP	42371070	40
CEU	chr1	NPIP	42421070	34
CEU	chr1	NPIP	42471070	26
CEU	chr1	NPIP	42521070	12
CEU	chr1	NPIP	42571070	0

CEU	chr1	NPIP	42621070	0
CEU	chr1	NPIP	42671070	0
CEU	chr1	NPIP	42721070	0
CEU	chr1	NPIP	42771070	0
CEU	chr1	NPIP	42821070	0
CEU	chr1	NPIP	42871070	0
CEU	chr1	NPIP	42921070	0
CEU	chr1	NPIP	42971070	0
CEU	chr1	NPIP	43021070	0
CEU	chr1	NPIP	43071070	0
CEU	chr1	NPIP	43121070	0
CEU	chr1	NPIP	43171070	0
CEU	chr1	NPIP	43221070	0
CEU	chr1	NPIP	43271070	0
CEU	chr1	NPIP	43321070	0
CEU	chr1	NPIP	43371070	0
CEU	chr1	NPIP	43421070	0
CEU	chr1	NPIP	43471070	0
CEU	chr1	NPIP	43521070	0
CEU	chr1	NPIP	43571070	0
CEU	chr1	NPIP	43621070	0
CEU	chr1	NPIP	43671070	0
CEU	chr1	NPIP	43721070	0
CEU	chr1	NPIP	43771070	0
CEU	chr1	NPIP	43821070	0
CEU	chr1	NPIP	43871070	0
CEU	chr1	NPIP	43921070	0
CEU	chr1	NPIP	43971070	0
CEU	chr1	NPIP	44021070	0
CEU	chr1	NPIP	44071070	0
CEU	chr1	NPIP	44121070	0
CEU	chr1	NPIP	44171070	0
CEU	chr1	NPIP	44221070	0
CEU	chr1	NPIP	44271070	0
CEU	chr1	NPIP	44321070	0
CEU	chr1	NPIP	44371070	0
CEU	chr1	NPIP	44421070	0
CEU	chr1	NPIP	44471070	0
CEU	chr1	NPIP	44521070	0
CEU	chr1	NPIP	44571070	0
CEU	chr1	NPIP	44621070	0
CEU	chr1	NPIP	44671070	0
CEU	chr1	NPIP	44721070	0
CEU	chr1	NPIP	44771070	0
CEU	chr1	NPIP	44821070	0
CEU	chr1	NPIP	44871070	0
CEU	chr1	NPIP	44921070	0
CEU	chr1	NPIP	44971070	0
CEU	chr1	NPIP	45021070	0

CEU	chr1	NPIP	45071070	0
CEU	chr1	NPIP	45121070	0
CEU	chr1	NPIP	45171070	0
CEU	chr1	NPIP	45221070	0
CEU	chr1	NPIP	45271070	0
CEU	chr1	NPIP	45321070	0
CEU	chr1	NPIP	45371070	0
CEU	chr1	NPIP	45421070	0
CEU	chr1	NPIP	45471070	0
CEU	chr1	NPIP	45521070	0
CEU	chr1	NPIP	45571070	0
CEU	chr1	NPIP	45621070	0
CEU	chr1	NPIP	45671070	0
CEU	chr1	NPIP	45721070	0
CEU	chr1	NPIP	45771070	0
CEU	chr1	NPIP	45821070	0
CEU	chr1	NPIP	45871070	0
CEU	chr1	NPIP	45921070	0
CEU	chr1	NPIP	45971070	0
CEU	chr1	NPIP	46021070	0
CEU	chr1	NPIP	46071070	0
CEU	chr1	NPIP	46121070	0
CEU	chr1	NPIP	46171070	0
CEU	chr1	NPIP	46221070	0
CEU	chr1	NPIP	46271070	0
CEU	chr1	NPIP	46321070	0
CEU	chr1	NPIP	46371070	0
CEU	chr1	NPIP	46421070	0
CEU	chr1	NPIP	46471070	0
CEU	chr1	NPIP	46521070	0
CEU	chr1	NPIP	46571070	0
CEU	chr1	NPIP	46621070	0
CEU	chr1	NPIP	46671070	0
CEU	chr1	NPIP	46721070	0
CEU	chr1	NPIP	46771070	0
CEU	chr1	NPIP	46821070	0
CEU	chr1	NPIP	46871070	0
CEU	chr1	NPIP	46921070	0
CEU	chr1	NPIP	46971070	0
CEU	chr1	NPIP	47021070	0
CEU	chr1	NPIP	47071070	0
CEU	chr1	NPIP	47121070	0
CEU	chr1	NPIP	47171070	0
CEU	chr1	NPIP	47221070	0
CEU	chr1	NPIP	47271070	0
CEU	chr1	NPIP	47321070	0
CEU	chr1	NPIP	47371070	0
CEU	chr1	NPIP	47421070	0
CEU	chr1	NPIP	47471070	0

CEU	chr1	NPIP	47521070	0
CEU	chr1	NPIP	47571070	0
CEU	chr1	NPIP	47621070	0
CEU	chr1	NPIP	47671070	4
CEU	chr1	NPIP	47721070	20
CEU	chr1	NPIP	47771070	37
CEU	chr1	NPIP	47821070	35
CEU	chr1	NPIP	47871070	27
CEU	chr1	NPIP	47921070	31
CEU	chr1	NPIP	47971070	58
CEU	chr1	NPIP	48021070	40
CEU	chr1	NPIP	48071070	0
CEU	chr1	NPIP	48121070	0
CEU	chr1	NPIP	48171070	0
CEU	chr1	NPIP	48221070	0
CEU	chr1	NPIP	48271070	0
CEU	chr1	NPIP	48321070	0
CEU	chr1	NPIP	48371070	0
CEU	chr1	NPIP	48421070	0
CEU	chr1	NPIP	48471070	0
CEU	chr1	NPIP	48521070	0
CEU	chr1	NPIP	48571070	0
CEU	chr1	NPIP	48621070	0
CEU	chr1	NPIP	48671070	0
CEU	chr1	NPIP	48721070	0
CEU	chr1	NPIP	48771070	0
CEU	chr1	NPIP	48821070	0
CEU	chr1	NPIP	48871070	0
CEU	chr1	NPIP	48921070	0
CEU	chr1	NPIP	48971070	0
CEU	chr1	NPIP	49021070	0
CEU	chr1	NPIP	49071070	0
CEU	chr1	NPIP	49121070	0
CEU	chr1	NPIP	49171070	0
CEU	chr1	NPIP	49221070	0
CEU	chr1	NPIP	49271070	0
CEU	chr16	NPIP	6977263	57
CEU	chr16	NPIP	7027263	0
CEU	chr16	NPIP	7077263	0
CEU	chr16	NPIP	7127263	0
CEU	chr16	NPIP	7177263	0
CEU	chr16	NPIP	7227263	0
CEU	chr16	NPIP	7277263	0
CEU	chr16	NPIP	7327263	0
CEU	chr16	NPIP	7377263	0
CEU	chr16	NPIP	7427263	0
CEU	chr16	NPIP	7477263	0
CEU	chr16	NPIP	7527263	0
CEU	chr16	NPIP	7577263	0

CEU	chr16	NPIP	7627263	0
CEU	chr16	NPIP	7677263	0
CEU	chr16	NPIP	7727263	11
CEU	chr16	NPIP	7777263	80
CEU	chr16	NPIP	7827263	87
CEU	chr16	NPIP	7877263	58
CEU	chr16	NPIP	7927263	40
CEU	chr16	NPIP	7977263	0
CEU	chr16	NPIP	8027263	0
CEU	chr16	NPIP	8077263	0
CEU	chr16	NPIP	8127263	0
CEU	chr16	NPIP	8177263	0
CEU	chr16	NPIP	8227263	0
CEU	chr16	NPIP	8277263	49
CEU	chr16	NPIP	8327263	106
CEU	chr16	NPIP	8377263	100
CEU	chr16	NPIP	8427263	62
CEU	chr16	NPIP	8477263	19
CEU	chr16	NPIP	8527263	0
CEU	chr16	NPIP	8577263	0
CEU	chr16	NPIP	8627263	0
CEU	chr16	NPIP	8677263	0
CEU	chr16	NPIP	8727263	9
CEU	chr16	NPIP	8777263	20
CEU	chr16	NPIP	8827263	31
CEU	chr16	NPIP	8877263	34
CEU	chr16	NPIP	8927263	49
CEU	chr16	NPIP	8977263	54
CEU	chr16	NPIP	9027263	42
CEU	chr16	NPIP	9077263	54
CEU	chr16	NPIP	9127263	55
CEU	chr16	NPIP	9177263	24
CEU	chr16	NPIP	9227263	0
CEU	chr16	NPIP	9277263	0
CEU	chr16	NPIP	9327263	0
CEU	chr16	NPIP	9377263	0
CEU	chr16	NPIP	9427263	0
CEU	chr16	NPIP	9477263	0
CEU	chr16	NPIP	9527263	0
CEU	chr16	NPIP	9577263	0
CEU	chr16	NPIP	9627263	9
CEU	chr16	NPIP	9677263	44
CEU	chr16	NPIP	9727263	56
CEU	chr16	NPIP	9777263	34
CEU	chr16	NPIP	9827263	13
CEU	chr16	NPIP	9877263	0
CEU	chr16	NPIP	9927263	0
CEU	chr16	NPIP	9977263	0
CEU	chr16	NPIP	10027263	0

CEU	chr16	NPIP	10077263	0
CEU	chr16	NPIP	10127263	0
CEU	chr16	NPIP	10177263	0
CEU	chr16	NPIP	10227263	0
CEU	chr16	NPIP	10277263	0
CEU	chr16	NPIP	10327263	0
CEU	chr16	NPIP	10377263	0
CEU	chr16	NPIP	10427263	0
CEU	chr16	NPIP	10477263	0
CEU	chr16	NPIP	10527263	0
CEU	chr16	NPIP	10577263	0
CEU	chr16	NPIP	10627263	0
CEU	chr16	NPIP	10677263	0
CEU	chr16	NPIP	10727263	0
CEU	chr16	NPIP	10777263	0
CEU	chr16	NPIP	10827263	0
CEU	chr16	NPIP	10877263	0
CEU	chr16	NPIP	10927263	0
CEU	chr16	NPIP	10977263	0
CEU	chr16	NPIP	11027263	0
CEU	chr16	NPIP	11077263	0
CEU	chr16	NPIP	11127263	0
CEU	chr16	NPIP	11177263	0
CEU	chr16	NPIP	11227263	0
CEU	chr16	NPIP	11277263	0
CEU	chr16	NPIP	11327263	0
CEU	chr16	NPIP	11377263	0
CEU	chr16	NPIP	11427263	0
CEU	chr16	NPIP	11477263	0
CEU	chr16	NPIP	11527263	0
CEU	chr16	NPIP	11577263	0
CEU	chr16	NPIP	11627263	0
CEU	chr16	NPIP	11677263	0
CEU	chr16	NPIP	11727263	0
CEU	chr16	NPIP	11777263	0
CEU	chr16	NPIP	11827263	0
CEU	chr16	NPIP	11877263	0
CEU	chr16	NPIP	11927263	0
CEU	chr16	NPIP	11977263	0
CEU	chr16	NPIP	12027263	0
CEU	chr16	NPIP	12077263	0
CEU	chr16	NPIP	12127263	0
CEU	chr16	NPIP	12177263	0
CEU	chr16	NPIP	12227263	0
CEU	chr16	NPIP	12277263	0
CEU	chr16	NPIP	12327263	0
CEU	chr16	NPIP	12377263	0
CEU	chr16	NPIP	12427263	0
CEU	chr16	NPIP	12477263	0

CEU	chr16	NPIP	12527263	0
CEU	chr16	NPIP	12577263	0
CEU	chr16	NPIP	12627263	0
CEU	chr16	NPIP	12677263	0
CEU	chr16	NPIP	12727263	0
CEU	chr16	NPIP	12777263	0
CEU	chr16	NPIP	12827263	0
CEU	chr16	NPIP	12877263	0
CEU	chr16	NPIP	12927263	0
CEU	chr16	NPIP	12977263	0
CEU	chr16	NPIP	13027263	0
CEU	chr16	NPIP	13077263	0
CEU	chr16	NPIP	13127263	0
CEU	chr16	NPIP	13177263	0
CEU	chr16	NPIP	13227263	0
CEU	chr16	NPIP	13277263	0
CEU	chr16	NPIP	13327263	0
CEU	chr16	NPIP	13377263	0
CEU	chr16	NPIP	13427263	0
CEU	chr16	NPIP	13477263	0
CEU	chr16	NPIP	13527263	0
CEU	chr16	NPIP	13577263	0
CEU	chr16	NPIP	13627263	0
CEU	chr16	NPIP	13677263	0
CEU	chr16	NPIP	13727263	0
CEU	chr16	NPIP	13777263	0
CEU	chr16	NPIP	13827263	0
CEU	chr16	NPIP	13877263	0
CEU	chr16	NPIP	13927263	0
CEU	chr16	NPIP	13977263	0
CEU	chr16	NPIP	14027263	0
CEU	chr16	NPIP	14077263	0
CEU	chr16	NPIP	14127263	0
CEU	chr16	NPIP	14177263	0
CEU	chr16	NPIP	14227263	0
CEU	chr16	NPIP	14277263	0
CEU	chr16	NPIP	14327263	0
CEU	chr16	NPIP	14377263	0
CEU	chr16	NPIP	14427263	0
CEU	chr16	NPIP	14477263	0
CEU	chr16	NPIP	14527263	0
CEU	chr16	NPIP	14577263	0
CEU	chr16	NPIP	14627263	0
CEU	chr16	NPIP	14677263	0
CEU	chr16	NPIP	14727263	0
CEU	chr16	NPIP	14777263	0
CEU	chr16	NPIP	14827263	0
CEU	chr16	NPIP	14877263	0
CEU	chr16	NPIP	14927263	0

CEU	chr16	NPIP	14977263	0
CEU	chr16	NPIP	15027263	0
CEU	chr16	NPIP	15077263	0
CEU	chr16	NPIP	15127263	0
CEU	chr16	NPIP	15177263	0
CEU	chr16	NPIP	15227263	0
CEU	chr16	NPIP	15277263	0
CEU	chr16	NPIP	15327263	0
CEU	chr16	NPIP	15377263	0
CEU	chr16	NPIP	15427263	0
CEU	chr16	NPIP	15477263	0
CEU	chr16	NPIP	15527263	0
CEU	chr16	NPIP	15577263	0
CEU	chr16	NPIP	15627263	0
CEU	chr16	NPIP	15677263	0
CEU	chr16	NPIP	15727263	0
CEU	chr16	NPIP	15777263	0
CEU	chr16	NPIP	15827263	0
CEU	chr16	NPIP	15877263	0
CEU	chr16	NPIP	15927263	0
CEU	chr16	NPIP	15977263	0
CEU	chr16	NPIP	16027263	0
CEU	chr16	NPIP	16077263	0
CEU	chr16	NPIP	16127263	0
CEU	chr16	NPIP	16177263	15
CEU	chr16	NPIP	16227263	41
CEU	chr16	NPIP	16277263	26
CEU	chr16	NPIP	16327263	0
CEU	chr16	NPIP	16377263	0
CEU	chr16	NPIP	16427263	0
CEU	chr16	NPIP	16477263	0
CEU	chr16	NPIP	16527263	0
CEU	chr16	NPIP	16577263	0
CEU	chr16	NPIP	16627263	0
CEU	chr16	NPIP	16677263	0
CEU	chr16	NPIP	16727263	0
CEU	chr16	NPIP	16777263	0
CEU	chr16	NPIP	16827263	0
CEU	chr16	NPIP	16877263	0
CEU	chr16	NPIP	9753411	58
CEU	chr16	NPIP	9803411	24
CEU	chr16	NPIP	9853411	0
CEU	chr16	NPIP	9903411	0
CEU	chr16	NPIP	9953411	0
CEU	chr16	NPIP	10003411	0
CEU	chr16	NPIP	10053411	0
CEU	chr16	NPIP	10103411	0
CEU	chr16	NPIP	10153411	0
CEU	chr16	NPIP	10203411	0

CEU	chr16	NPIP	10253411	0
CEU	chr16	NPIP	10303411	0
CEU	chr16	NPIP	10353411	0
CEU	chr16	NPIP	10403411	0
CEU	chr16	NPIP	10453411	0
CEU	chr16	NPIP	10503411	0
CEU	chr16	NPIP	10553411	0
CEU	chr16	NPIP	10603411	0
CEU	chr16	NPIP	10653411	0
CEU	chr16	NPIP	10703411	0
CEU	chr16	NPIP	10753411	0
CEU	chr16	NPIP	10803411	0
CEU	chr16	NPIP	10853411	0
CEU	chr16	NPIP	10903411	0
CEU	chr16	NPIP	10953411	0
CEU	chr16	NPIP	11003411	0
CEU	chr16	NPIP	11053411	0
CEU	chr16	NPIP	11103411	0
CEU	chr16	NPIP	11153411	0
CEU	chr16	NPIP	11203411	0
CEU	chr16	NPIP	11253411	0
CEU	chr16	NPIP	11303411	0
CEU	chr16	NPIP	11353411	0
CEU	chr16	NPIP	11403411	0
CEU	chr16	NPIP	11453411	0
CEU	chr16	NPIP	11503411	0
CEU	chr16	NPIP	11553411	0
CEU	chr16	NPIP	11603411	0
CEU	chr16	NPIP	11653411	0
CEU	chr16	NPIP	11703411	0
CEU	chr16	NPIP	11753411	0
CEU	chr16	NPIP	11803411	0
CEU	chr16	NPIP	11853411	0
CEU	chr16	NPIP	11903411	0
CEU	chr16	NPIP	11953411	0
CEU	chr16	NPIP	12003411	0
CEU	chr16	NPIP	12053411	0
CEU	chr16	NPIP	12103411	0
CEU	chr16	NPIP	12153411	0
CEU	chr16	NPIP	12203411	0
CEU	chr16	NPIP	12253411	0
CEU	chr16	NPIP	12303411	0
CEU	chr16	NPIP	12353411	0
CEU	chr16	NPIP	12403411	0
CEU	chr16	NPIP	12453411	0
CEU	chr16	NPIP	12503411	0
CEU	chr16	NPIP	12553411	0
CEU	chr16	NPIP	12603411	0
CEU	chr16	NPIP	12653411	0

CEU	chr16	NPIP	12703411	0
CEU	chr16	NPIP	12753411	0
CEU	chr16	NPIP	12803411	0
CEU	chr16	NPIP	12853411	0
CEU	chr16	NPIP	12903411	0
CEU	chr16	NPIP	12953411	0
CEU	chr16	NPIP	13003411	0
CEU	chr16	NPIP	13053411	0
CEU	chr16	NPIP	13103411	0
CEU	chr16	NPIP	13153411	0
CEU	chr16	NPIP	13203411	0
CEU	chr16	NPIP	13253411	0
CEU	chr16	NPIP	13303411	0
CEU	chr16	NPIP	13353411	0
CEU	chr16	NPIP	13403411	0
CEU	chr16	NPIP	13453411	0
CEU	chr16	NPIP	13503411	0
CEU	chr16	NPIP	13553411	0
CEU	chr16	NPIP	13603411	0
CEU	chr16	NPIP	13653411	0
CEU	chr16	NPIP	13703411	0
CEU	chr16	NPIP	13753411	0
CEU	chr16	NPIP	13803411	0
CEU	chr16	NPIP	13853411	0
CEU	chr16	NPIP	13903411	0
CEU	chr16	NPIP	13953411	0
CEU	chr16	NPIP	14003411	0
CEU	chr16	NPIP	14053411	0
CEU	chr16	NPIP	14103411	0
CEU	chr16	NPIP	14153411	0
CEU	chr16	NPIP	14203411	0
CEU	chr16	NPIP	14253411	0
CEU	chr16	NPIP	14303411	0
CEU	chr16	NPIP	14353411	0
CEU	chr16	NPIP	14403411	0
CEU	chr16	NPIP	14453411	0
CEU	chr16	NPIP	14503411	0
CEU	chr16	NPIP	14553411	0
CEU	chr16	NPIP	14603411	0
CEU	chr16	NPIP	14653411	0
CEU	chr16	NPIP	14703411	0
CEU	chr16	NPIP	14753411	0
CEU	chr16	NPIP	14803411	0
CEU	chr16	NPIP	14853411	0
CEU	chr16	NPIP	14903411	0
CEU	chr16	NPIP	14953411	0
CEU	chr16	NPIP	15003411	0
CEU	chr16	NPIP	15053411	0
CEU	chr16	NPIP	15103411	0

CEU	chr16	NPIP	15153411	0
CEU	chr16	NPIP	15203411	0
CEU	chr16	NPIP	15253411	0
CEU	chr16	NPIP	15303411	0
CEU	chr16	NPIP	15353411	0
CEU	chr16	NPIP	15403411	0
CEU	chr16	NPIP	15453411	0
CEU	chr16	NPIP	15503411	0
CEU	chr16	NPIP	15553411	0
CEU	chr16	NPIP	15603411	0
CEU	chr16	NPIP	15653411	0
CEU	chr16	NPIP	15703411	0
CEU	chr16	NPIP	15753411	0
CEU	chr16	NPIP	15803411	0
CEU	chr16	NPIP	15853411	0
CEU	chr16	NPIP	15903411	0
CEU	chr16	NPIP	15953411	0
CEU	chr16	NPIP	16003411	0
CEU	chr16	NPIP	16053411	0
CEU	chr16	NPIP	16103411	0
CEU	chr16	NPIP	16153411	1
CEU	chr16	NPIP	16203411	28
CEU	chr16	NPIP	16253411	40
CEU	chr16	NPIP	16303411	13
CEU	chr16	NPIP	16353411	0
CEU	chr16	NPIP	16403411	0
CEU	chr16	NPIP	16453411	0
CEU	chr16	NPIP	16503411	0
CEU	chr16	NPIP	16553411	0
CEU	chr16	NPIP	16603411	0
CEU	chr16	NPIP	16653411	0
CEU	chr16	NPIP	16703411	0
CEU	chr16	NPIP	16753411	0
CEU	chr16	NPIP	16803411	0
CEU	chr16	NPIP	16853411	0
CEU	chr16	NPIP	16903411	0
CEU	chr16	NPIP	16953411	0
CEU	chr16	NPIP	17003411	0
CEU	chr16	NPIP	17053411	0
CEU	chr16	NPIP	17103411	0
CEU	chr16	NPIP	17153411	0
CEU	chr16	NPIP	17203411	0
CEU	chr16	NPIP	17253411	0
CEU	chr16	NPIP	17303411	52
CEU	chr16	NPIP	17353411	70
CEU	chr16	NPIP	17403411	19
CEU	chr16	NPIP	17453411	1
CEU	chr16	NPIP	17503411	0
CEU	chr16	NPIP	17553411	0

CEU	chr16	NPIP	17603411	0
CEU	chr16	NPIP	17653411	0
CEU	chr16	NPIP	17703411	0
CEU	chr16	NPIP	17753411	0
CEU	chr16	NPIP	17803411	0
CEU	chr16	NPIP	17853411	0
CEU	chr16	NPIP	17903411	0
CEU	chr16	NPIP	17953411	0
CEU	chr16	NPIP	18003411	0
CEU	chr16	NPIP	18053411	0
CEU	chr16	NPIP	18103411	0
CEU	chr16	NPIP	18153411	0
CEU	chr16	NPIP	18203411	0
CEU	chr16	NPIP	18253411	0
CEU	chr16	NPIP	18303411	0
CEU	chr16	NPIP	18353411	0
CEU	chr16	NPIP	18403411	0
CEU	chr16	NPIP	18453411	0
CEU	chr16	NPIP	18503411	0
CEU	chr16	NPIP	18553411	0
CEU	chr16	NPIP	18603411	0
CEU	chr16	NPIP	18653411	0
CEU	chr16	NPIP	18703411	0
CEU	chr16	NPIP	18753411	0
CEU	chr16	NPIP	18803411	0
CEU	chr16	NPIP	18853411	0
CEU	chr16	NPIP	18903411	0
CEU	chr16	NPIP	18953411	0
CEU	chr16	NPIP	19003411	0
CEU	chr16	NPIP	19053411	0
CEU	chr16	NPIP	19103411	0
CEU	chr16	NPIP	19153411	0
CEU	chr16	NPIP	19203411	0
CEU	chr16	NPIP	19253411	0
CEU	chr16	NPIP	19303411	0
CEU	chr16	NPIP	19353411	0
CEU	chr16	NPIP	19403411	0
CEU	chr16	NPIP	19453411	0
CEU	chr16	NPIP	19503411	0
CEU	chr16	NPIP	19553411	0
CEU	chr16	NPIP	19603411	0
CEU	chr16	NPIP	19653411	0
CEU	chr16	NPIP	13367919	0
CEU	chr16	NPIP	13417919	0
CEU	chr16	NPIP	13467919	0
CEU	chr16	NPIP	13517919	0
CEU	chr16	NPIP	13567919	0
CEU	chr16	NPIP	13617919	0
CEU	chr16	NPIP	13667919	0

CEU	chr16	NPIP	13717919	0
CEU	chr16	NPIP	13767919	0
CEU	chr16	NPIP	13817919	0
CEU	chr16	NPIP	13867919	0
CEU	chr16	NPIP	13917919	0
CEU	chr16	NPIP	13967919	0
CEU	chr16	NPIP	14017919	0
CEU	chr16	NPIP	14067919	0
CEU	chr16	NPIP	14117919	0
CEU	chr16	NPIP	14167919	0
CEU	chr16	NPIP	14217919	0
CEU	chr16	NPIP	14267919	0
CEU	chr16	NPIP	14317919	0
CEU	chr16	NPIP	14367919	0
CEU	chr16	NPIP	14417919	0
CEU	chr16	NPIP	14467919	0
CEU	chr16	NPIP	14517919	0
CEU	chr16	NPIP	14567919	0
CEU	chr16	NPIP	14617919	0
CEU	chr16	NPIP	14667919	0
CEU	chr16	NPIP	14717919	0
CEU	chr16	NPIP	14767919	0
CEU	chr16	NPIP	14817919	0
CEU	chr16	NPIP	14867919	0
CEU	chr16	NPIP	14917919	0
CEU	chr16	NPIP	14967919	0
CEU	chr16	NPIP	15017919	0
CEU	chr16	NPIP	15067919	0
CEU	chr16	NPIP	15117919	0
CEU	chr16	NPIP	15167919	0
CEU	chr16	NPIP	15217919	0
CEU	chr16	NPIP	15267919	0
CEU	chr16	NPIP	15317919	0
CEU	chr16	NPIP	15367919	0
CEU	chr16	NPIP	15417919	0
CEU	chr16	NPIP	15467919	0
CEU	chr16	NPIP	15517919	0
CEU	chr16	NPIP	15567919	0
CEU	chr16	NPIP	15617919	0
CEU	chr16	NPIP	15667919	0
CEU	chr16	NPIP	15717919	0
CEU	chr16	NPIP	15767919	0
CEU	chr16	NPIP	15817919	0
CEU	chr16	NPIP	15867919	0
CEU	chr16	NPIP	15917919	0
CEU	chr16	NPIP	15967919	0
CEU	chr16	NPIP	16017919	0
CEU	chr16	NPIP	16067919	0
CEU	chr16	NPIP	16117919	0

CEU	chr16	NPIP	16167919	2
CEU	chr16	NPIP	16217919	38
CEU	chr16	NPIP	16267919	39
CEU	chr16	NPIP	16317919	3
CEU	chr16	NPIP	16367919	0
CEU	chr16	NPIP	16417919	0
CEU	chr16	NPIP	16467919	0
CEU	chr16	NPIP	16517919	0
CEU	chr16	NPIP	16567919	0
CEU	chr16	NPIP	16617919	0
CEU	chr16	NPIP	16667919	0
CEU	chr16	NPIP	16717919	0
CEU	chr16	NPIP	16767919	0
CEU	chr16	NPIP	16817919	0
CEU	chr16	NPIP	16867919	0
CEU	chr16	NPIP	16917919	0
CEU	chr16	NPIP	16967919	0
CEU	chr16	NPIP	17017919	0
CEU	chr16	NPIP	17067919	0
CEU	chr16	NPIP	17117919	0
CEU	chr16	NPIP	17167919	0
CEU	chr16	NPIP	17217919	0
CEU	chr16	NPIP	17267919	7
CEU	chr16	NPIP	17317919	60
CEU	chr16	NPIP	17367919	64
CEU	chr16	NPIP	17417919	11
CEU	chr16	NPIP	17467919	0
CEU	chr16	NPIP	17517919	0
CEU	chr16	NPIP	17567919	0
CEU	chr16	NPIP	17617919	0
CEU	chr16	NPIP	17667919	0
CEU	chr16	NPIP	17717919	0
CEU	chr16	NPIP	17767919	0
CEU	chr16	NPIP	17817919	0
CEU	chr16	NPIP	17867919	0
CEU	chr16	NPIP	17917919	0
CEU	chr16	NPIP	17967919	0
CEU	chr16	NPIP	18017919	0
CEU	chr16	NPIP	18067919	0
CEU	chr16	NPIP	18117919	0
CEU	chr16	NPIP	18167919	0
CEU	chr16	NPIP	18217919	0
CEU	chr16	NPIP	18267919	0
CEU	chr16	NPIP	18317919	0
CEU	chr16	NPIP	18367919	0
CEU	chr16	NPIP	18417919	0
CEU	chr16	NPIP	18467919	0
CEU	chr16	NPIP	18517919	0
CEU	chr16	NPIP	18567919	0

CEU	chr16	NPIP	18617919	0
CEU	chr16	NPIP	18667919	0
CEU	chr16	NPIP	18717919	0
CEU	chr16	NPIP	18767919	0
CEU	chr16	NPIP	18817919	0
CEU	chr16	NPIP	18867919	0
CEU	chr16	NPIP	18917919	0
CEU	chr16	NPIP	18967919	0
CEU	chr16	NPIP	19017919	0
CEU	chr16	NPIP	19067919	0
CEU	chr16	NPIP	19117919	0
CEU	chr16	NPIP	19167919	0
CEU	chr16	NPIP	19217919	0
CEU	chr16	NPIP	19267919	0
CEU	chr16	NPIP	19317919	0
CEU	chr16	NPIP	19367919	0
CEU	chr16	NPIP	19417919	0
CEU	chr16	NPIP	19467919	0
CEU	chr16	NPIP	19517919	0
CEU	chr16	NPIP	19567919	0
CEU	chr16	NPIP	19617919	0
CEU	chr16	NPIP	19667919	0
CEU	chr16	NPIP	19717919	0
CEU	chr16	NPIP	19767919	0
CEU	chr16	NPIP	19817919	0
CEU	chr16	NPIP	19867919	0
CEU	chr16	NPIP	19917919	0
CEU	chr16	NPIP	19967919	0
CEU	chr16	NPIP	20017919	0
CEU	chr16	NPIP	20067919	0
CEU	chr16	NPIP	20117919	0
CEU	chr16	NPIP	20167919	0
CEU	chr16	NPIP	20217919	0
CEU	chr16	NPIP	20267919	0
CEU	chr16	NPIP	20317919	0
CEU	chr16	NPIP	20367919	0
CEU	chr16	NPIP	20417919	0
CEU	chr16	NPIP	20467919	0
CEU	chr16	NPIP	20517919	0
CEU	chr16	NPIP	20567919	0
CEU	chr16	NPIP	20617919	0
CEU	chr16	NPIP	20667919	0
CEU	chr16	NPIP	20717919	0
CEU	chr16	NPIP	20767919	0
CEU	chr16	NPIP	20817919	0
CEU	chr16	NPIP	20867919	0
CEU	chr16	NPIP	20917919	0
CEU	chr16	NPIP	20967919	0
CEU	chr16	NPIP	21017919	0

CEU	chr16	NPIP	21067919	0
CEU	chr16	NPIP	21117919	0
CEU	chr16	NPIP	21167919	0
CEU	chr16	NPIP	21217919	0
CEU	chr16	NPIP	21267919	0
CEU	chr16	NPIP	21317919	0
CEU	chr16	NPIP	21367919	0
CEU	chr16	NPIP	21417919	0
CEU	chr16	NPIP	21467919	0
CEU	chr16	NPIP	21517919	38
CEU	chr16	NPIP	21567919	60
CEU	chr16	NPIP	21617919	22
CEU	chr16	NPIP	21667919	0
CEU	chr16	NPIP	21717919	0
CEU	chr16	NPIP	21767919	0
CEU	chr16	NPIP	21817919	0
CEU	chr16	NPIP	21867919	0
CEU	chr16	NPIP	21917919	0
CEU	chr16	NPIP	21967919	0
CEU	chr16	NPIP	22017919	0
CEU	chr16	NPIP	22067919	0
CEU	chr16	NPIP	22117919	0
CEU	chr16	NPIP	22167919	0
CEU	chr16	NPIP	22217919	0
CEU	chr16	NPIP	22267919	0
CEU	chr16	NPIP	22317919	0
CEU	chr16	NPIP	22367919	0
CEU	chr16	NPIP	22417919	0
CEU	chr16	NPIP	22467919	0
CEU	chr16	NPIP	22517919	0
CEU	chr16	NPIP	22567919	0
CEU	chr16	NPIP	22617919	0
CEU	chr16	NPIP	22667919	0
CEU	chr16	NPIP	22717919	0
CEU	chr16	NPIP	22767919	0
CEU	chr16	NPIP	22817919	0
CEU	chr16	NPIP	22867919	0
CEU	chr16	NPIP	22917919	0
CEU	chr16	NPIP	22967919	0
CEU	chr16	NPIP	23017919	0
CEU	chr16	NPIP	23067919	0
CEU	chr16	NPIP	23117919	0
CEU	chr16	NPIP	23167919	0
CEU	chr16	NPIP	23217919	0
CEU	chr16	NPIP	23267919	0
CEU	chr16	NPIP	17553599	0
CEU	chr16	NPIP	17603599	0
CEU	chr16	NPIP	17653599	0
CEU	chr16	NPIP	17703599	0

CEU	chr16	NPIP	17753599	0
CEU	chr16	NPIP	17803599	0
CEU	chr16	NPIP	17853599	0
CEU	chr16	NPIP	17903599	0
CEU	chr16	NPIP	17953599	0
CEU	chr16	NPIP	18003599	0
CEU	chr16	NPIP	18053599	0
CEU	chr16	NPIP	18103599	0
CEU	chr16	NPIP	18153599	0
CEU	chr16	NPIP	18203599	0
CEU	chr16	NPIP	18253599	0
CEU	chr16	NPIP	18303599	0
CEU	chr16	NPIP	18353599	0
CEU	chr16	NPIP	18403599	0
CEU	chr16	NPIP	18453599	0
CEU	chr16	NPIP	18503599	0
CEU	chr16	NPIP	18553599	0
CEU	chr16	NPIP	18603599	0
CEU	chr16	NPIP	18653599	0
CEU	chr16	NPIP	18703599	0
CEU	chr16	NPIP	18753599	0
CEU	chr16	NPIP	18803599	0
CEU	chr16	NPIP	18853599	0
CEU	chr16	NPIP	18903599	0
CEU	chr16	NPIP	18953599	0
CEU	chr16	NPIP	19003599	0
CEU	chr16	NPIP	19053599	0
CEU	chr16	NPIP	19103599	0
CEU	chr16	NPIP	19153599	0
CEU	chr16	NPIP	19203599	0
CEU	chr16	NPIP	19253599	0
CEU	chr16	NPIP	19303599	0
CEU	chr16	NPIP	19353599	0
CEU	chr16	NPIP	19403599	0
CEU	chr16	NPIP	19453599	0
CEU	chr16	NPIP	19503599	0
CEU	chr16	NPIP	19553599	0
CEU	chr16	NPIP	19603599	0
CEU	chr16	NPIP	19653599	0
CEU	chr16	NPIP	19703599	0
CEU	chr16	NPIP	19753599	0
CEU	chr16	NPIP	19803599	0
CEU	chr16	NPIP	19853599	0
CEU	chr16	NPIP	19903599	0
CEU	chr16	NPIP	19953599	0
CEU	chr16	NPIP	20003599	0
CEU	chr16	NPIP	20053599	0
CEU	chr16	NPIP	20103599	0
CEU	chr16	NPIP	20153599	0

CEU	chr16	NPIP	20203599	0
CEU	chr16	NPIP	20253599	0
CEU	chr16	NPIP	20303599	0
CEU	chr16	NPIP	20353599	0
CEU	chr16	NPIP	20403599	0
CEU	chr16	NPIP	20453599	0
CEU	chr16	NPIP	20503599	0
CEU	chr16	NPIP	20553599	0
CEU	chr16	NPIP	20603599	0
CEU	chr16	NPIP	20653599	0
CEU	chr16	NPIP	20703599	0
CEU	chr16	NPIP	20753599	0
CEU	chr16	NPIP	20803599	0
CEU	chr16	NPIP	20853599	0
CEU	chr16	NPIP	20903599	0
CEU	chr16	NPIP	20953599	0
CEU	chr16	NPIP	21003599	0
CEU	chr16	NPIP	21053599	0
CEU	chr16	NPIP	21103599	0
CEU	chr16	NPIP	21153599	0
CEU	chr16	NPIP	21203599	0
CEU	chr16	NPIP	21253599	0
CEU	chr16	NPIP	21303599	0
CEU	chr16	NPIP	21353599	0
CEU	chr16	NPIP	21403599	0
CEU	chr16	NPIP	21453599	0
CEU	chr16	NPIP	21503599	35
CEU	chr16	NPIP	21553599	57
CEU	chr16	NPIP	21603599	25
CEU	chr16	NPIP	21653599	3
CEU	chr16	NPIP	21703599	0
CEU	chr16	NPIP	21753599	0
CEU	chr16	NPIP	21803599	0
CEU	chr16	NPIP	21853599	0
CEU	chr16	NPIP	21903599	0
CEU	chr16	NPIP	21953599	0
CEU	chr16	NPIP	22003599	0
CEU	chr16	NPIP	22053599	0
CEU	chr16	NPIP	22103599	0
CEU	chr16	NPIP	22153599	0
CEU	chr16	NPIP	22203599	0
CEU	chr16	NPIP	22253599	0
CEU	chr16	NPIP	22303599	0
CEU	chr16	NPIP	22353599	0
CEU	chr16	NPIP	22403599	0
CEU	chr16	NPIP	22453599	0
CEU	chr16	NPIP	22503599	0
CEU	chr16	NPIP	22553599	0
CEU	chr16	NPIP	22603599	0

CEU	chr16	NPIP	22653599	0
CEU	chr16	NPIP	22703599	0
CEU	chr16	NPIP	22753599	0
CEU	chr16	NPIP	22803599	0
CEU	chr16	NPIP	22853599	0
CEU	chr16	NPIP	22903599	0
CEU	chr16	NPIP	22953599	0
CEU	chr16	NPIP	23003599	0
CEU	chr16	NPIP	23053599	0
CEU	chr16	NPIP	23103599	0
CEU	chr16	NPIP	23153599	0
CEU	chr16	NPIP	23203599	0
CEU	chr16	NPIP	23253599	0
CEU	chr16	NPIP	23303599	0
CEU	chr16	NPIP	23353599	0
CEU	chr16	NPIP	23403599	0
CEU	chr16	NPIP	23453599	0
CEU	chr16	NPIP	23503599	0
CEU	chr16	NPIP	23553599	0
CEU	chr16	NPIP	23603599	0
CEU	chr16	NPIP	23653599	0
CEU	chr16	NPIP	23703599	0
CEU	chr16	NPIP	23753599	0
CEU	chr16	NPIP	23803599	13
CEU	chr16	NPIP	23853599	36
CEU	chr16	NPIP	23903599	53
CEU	chr16	NPIP	23953599	39
CEU	chr16	NPIP	24003599	15
CEU	chr16	NPIP	24053599	6
CEU	chr16	NPIP	24103599	0
CEU	chr16	NPIP	24153599	0
CEU	chr16	NPIP	24203599	0
CEU	chr16	NPIP	24253599	0
CEU	chr16	NPIP	24303599	0
CEU	chr16	NPIP	24353599	0
CEU	chr16	NPIP	24403599	0
CEU	chr16	NPIP	24453599	0
CEU	chr16	NPIP	24503599	0
CEU	chr16	NPIP	24553599	0
CEU	chr16	NPIP	24603599	0
CEU	chr16	NPIP	24653599	0
CEU	chr16	NPIP	24703599	0
CEU	chr16	NPIP	24753599	0
CEU	chr16	NPIP	24803599	0
CEU	chr16	NPIP	24853599	0
CEU	chr16	NPIP	24903599	0
CEU	chr16	NPIP	24953599	0
CEU	chr16	NPIP	25003599	0
CEU	chr16	NPIP	25053599	0

CEU	chr16	NPIP	25103599	0
CEU	chr16	NPIP	25153599	0
CEU	chr16	NPIP	25203599	0
CEU	chr16	NPIP	25253599	0
CEU	chr16	NPIP	25303599	0
CEU	chr16	NPIP	25353599	0
CEU	chr16	NPIP	25403599	0
CEU	chr16	NPIP	25453599	0
CEU	chr16	NPIP	25503599	0
CEU	chr16	NPIP	25553599	0
CEU	chr16	NPIP	25603599	0
CEU	chr16	NPIP	25653599	0
CEU	chr16	NPIP	25703599	0
CEU	chr16	NPIP	25753599	0
CEU	chr16	NPIP	25803599	0
CEU	chr16	NPIP	25853599	0
CEU	chr16	NPIP	25903599	9
CEU	chr16	NPIP	25953599	46
CEU	chr16	NPIP	26003599	37
CEU	chr16	NPIP	26053599	0
CEU	chr16	NPIP	26103599	0
CEU	chr16	NPIP	26153599	0
CEU	chr16	NPIP	26203599	0
CEU	chr16	NPIP	26253599	0
CEU	chr16	NPIP	26303599	0
CEU	chr16	NPIP	26353599	0
CEU	chr16	NPIP	26403599	0
CEU	chr16	NPIP	26453599	0
CEU	chr16	NPIP	26503599	0
CEU	chr16	NPIP	26553599	0
CEU	chr16	NPIP	26603599	0
CEU	chr16	NPIP	26653599	0
CEU	chr16	NPIP	26703599	0
CEU	chr16	NPIP	26753599	0
CEU	chr16	NPIP	26803599	0
CEU	chr16	NPIP	26853599	0
CEU	chr16	NPIP	26903599	0
CEU	chr16	NPIP	26953599	0
CEU	chr16	NPIP	27003599	0
CEU	chr16	NPIP	27053599	0
CEU	chr16	NPIP	27103599	0
CEU	chr16	NPIP	27153599	0
CEU	chr16	NPIP	27203599	0
CEU	chr16	NPIP	27253599	0
CEU	chr16	NPIP	27303599	0
CEU	chr16	NPIP	27353599	0
CEU	chr16	NPIP	27403599	0
CEU	chr16	NPIP	27453599	0
CEU	chr16	NPIP	23392517	0

CEU	chr16	NPIP	23442517	0
CEU	chr16	NPIP	23492517	0
CEU	chr16	NPIP	23542517	0
CEU	chr16	NPIP	23592517	0
CEU	chr16	NPIP	23642517	0
CEU	chr16	NPIP	23692517	0
CEU	chr16	NPIP	23742517	0
CEU	chr16	NPIP	23792517	11
CEU	chr16	NPIP	23842517	28
CEU	chr16	NPIP	23892517	49
CEU	chr16	NPIP	23942517	43
CEU	chr16	NPIP	23992517	21
CEU	chr16	NPIP	24042517	10
CEU	chr16	NPIP	24092517	0
CEU	chr16	NPIP	24142517	0
CEU	chr16	NPIP	24192517	0
CEU	chr16	NPIP	24242517	0
CEU	chr16	NPIP	24292517	0
CEU	chr16	NPIP	24342517	0
CEU	chr16	NPIP	24392517	0
CEU	chr16	NPIP	24442517	0
CEU	chr16	NPIP	24492517	0
CEU	chr16	NPIP	24542517	0
CEU	chr16	NPIP	24592517	0
CEU	chr16	NPIP	24642517	0
CEU	chr16	NPIP	24692517	0
CEU	chr16	NPIP	24742517	0
CEU	chr16	NPIP	24792517	0
CEU	chr16	NPIP	24842517	0
CEU	chr16	NPIP	24892517	0
CEU	chr16	NPIP	24942517	0
CEU	chr16	NPIP	24992517	0
CEU	chr16	NPIP	25042517	0
CEU	chr16	NPIP	25092517	0
CEU	chr16	NPIP	25142517	0
CEU	chr16	NPIP	25192517	0
CEU	chr16	NPIP	25242517	0
CEU	chr16	NPIP	25292517	0
CEU	chr16	NPIP	25342517	0
CEU	chr16	NPIP	25392517	0
CEU	chr16	NPIP	25442517	0
CEU	chr16	NPIP	25492517	0
CEU	chr16	NPIP	25542517	0
CEU	chr16	NPIP	25592517	0
CEU	chr16	NPIP	25642517	0
CEU	chr16	NPIP	25692517	0
CEU	chr16	NPIP	25742517	0
CEU	chr16	NPIP	25792517	0
CEU	chr16	NPIP	25842517	0

CEU	chr16	NPIP	25892517	7
CEU	chr16	NPIP	25942517	46
CEU	chr16	NPIP	25992517	39
CEU	chr16	NPIP	26042517	0
CEU	chr16	NPIP	26092517	0
CEU	chr16	NPIP	26142517	0
CEU	chr16	NPIP	26192517	0
CEU	chr16	NPIP	26242517	0
CEU	chr16	NPIP	26292517	0
CEU	chr16	NPIP	26342517	0
CEU	chr16	NPIP	26392517	0
CEU	chr16	NPIP	26442517	0
CEU	chr16	NPIP	26492517	0
CEU	chr16	NPIP	26542517	0
CEU	chr16	NPIP	26592517	0
CEU	chr16	NPIP	26642517	0
CEU	chr16	NPIP	26692517	0
CEU	chr16	NPIP	26742517	0
CEU	chr16	NPIP	26792517	0
CEU	chr16	NPIP	26842517	0
CEU	chr16	NPIP	26892517	0
CEU	chr16	NPIP	26942517	0
CEU	chr16	NPIP	26992517	0
CEU	chr16	NPIP	27042517	0
CEU	chr16	NPIP	27092517	0
CEU	chr16	NPIP	27142517	0
CEU	chr16	NPIP	27192517	0
CEU	chr16	NPIP	27242517	0
CEU	chr16	NPIP	27292517	0
CEU	chr16	NPIP	27342517	0
CEU	chr16	NPIP	27392517	0
CEU	chr16	NPIP	27442517	0
CEU	chr16	NPIP	27492517	0
CEU	chr16	NPIP	27542517	0
CEU	chr16	NPIP	27592517	0
CEU	chr16	NPIP	27642517	0
CEU	chr16	NPIP	27692517	0
CEU	chr16	NPIP	27742517	0
CEU	chr16	NPIP	27792517	0
CEU	chr16	NPIP	27842517	0
CEU	chr16	NPIP	27892517	0
CEU	chr16	NPIP	27942517	0
CEU	chr16	NPIP	27992517	0
CEU	chr16	NPIP	28042517	0
CEU	chr16	NPIP	28092517	0
CEU	chr16	NPIP	28142517	0
CEU	chr16	NPIP	28192517	0
CEU	chr16	NPIP	28242517	0
CEU	chr16	NPIP	28292517	0

CEU	chr16	NPIP	28342517	0
CEU	chr16	NPIP	28392517	0
CEU	chr16	NPIP	28442517	0
CEU	chr16	NPIP	28492517	0
CEU	chr16	NPIP	28542517	0
CEU	chr16	NPIP	28592517	0
CEU	chr16	NPIP	28642517	0
CEU	chr16	NPIP	28692517	0
CEU	chr16	NPIP	28742517	0
CEU	chr16	NPIP	28792517	0
CEU	chr16	NPIP	28842517	0
CEU	chr16	NPIP	28892517	0
CEU	chr16	NPIP	28942517	0
CEU	chr16	NPIP	28992517	0
CEU	chr16	NPIP	29042517	0
CEU	chr16	NPIP	29092517	0
CEU	chr16	NPIP	29142517	0
CEU	chr16	NPIP	29192517	0
CEU	chr16	NPIP	29242517	0
CEU	chr16	NPIP	29292517	0
CEU	chr16	NPIP	29342517	0
CEU	chr16	NPIP	29392517	0
CEU	chr16	NPIP	29442517	0
CEU	chr16	NPIP	29492517	0
CEU	chr16	NPIP	29542517	0
CEU	chr16	NPIP	29592517	0
CEU	chr16	NPIP	29642517	0
CEU	chr16	NPIP	29692517	0
CEU	chr16	NPIP	29742517	0
CEU	chr16	NPIP	29792517	0
CEU	chr16	NPIP	29842517	0
CEU	chr16	NPIP	29892517	0
CEU	chr16	NPIP	29942517	0
CEU	chr16	NPIP	29992517	0
CEU	chr16	NPIP	30042517	0
CEU	chr16	NPIP	30092517	0
CEU	chr16	NPIP	30142517	0
CEU	chr16	NPIP	30192517	0
CEU	chr16	NPIP	30242517	0
CEU	chr16	NPIP	30292517	0
CEU	chr16	NPIP	30342517	0
CEU	chr16	NPIP	30392517	0
CEU	chr16	NPIP	30442517	0
CEU	chr16	NPIP	30492517	0
CEU	chr16	NPIP	30542517	0
CEU	chr16	NPIP	30592517	0
CEU	chr16	NPIP	30642517	0
CEU	chr16	NPIP	30692517	0
CEU	chr16	NPIP	30742517	0

CEU	chr16	NPIP	30792517	0
CEU	chr16	NPIP	30842517	0
CEU	chr16	NPIP	30892517	0
CEU	chr16	NPIP	30942517	0
CEU	chr16	NPIP	30992517	0
CEU	chr16	NPIP	31042517	0
CEU	chr16	NPIP	31092517	0
CEU	chr16	NPIP	31142517	0
CEU	chr16	NPIP	31192517	0
CEU	chr16	NPIP	31242517	0
CEU	chr16	NPIP	31292517	0
CEU	chr16	NPIP	31342517	0
CEU	chr16	NPIP	31392517	0
CEU	chr16	NPIP	31442517	0
CEU	chr16	NPIP	31492517	0
CEU	chr16	NPIP	31542517	0
CEU	chr16	NPIP	31592517	0
CEU	chr16	NPIP	31642517	0
CEU	chr16	NPIP	31692517	0
CEU	chr16	NPIP	31742517	0
CEU	chr16	NPIP	31792517	0
CEU	chr16	NPIP	31842517	0
CEU	chr16	NPIP	31892517	0
CEU	chr16	NPIP	31942517	0
CEU	chr16	NPIP	31992517	0
CEU	chr16	NPIP	32042517	0
CEU	chr16	NPIP	32092517	0
CEU	chr16	NPIP	32142517	0
CEU	chr16	NPIP	32192517	0
CEU	chr16	NPIP	32242517	0
CEU	chr16	NPIP	32292517	0
CEU	chr16	NPIP	32342517	0
CEU	chr16	NPIP	32392517	0
CEU	chr16	NPIP	32442517	0
CEU	chr16	NPIP	32492517	0
CEU	chr16	NPIP	32542517	0
CEU	chr16	NPIP	32592517	0
CEU	chr16	NPIP	32642517	0
CEU	chr16	NPIP	32692517	0
CEU	chr16	NPIP	32742517	0
CEU	chr16	NPIP	32792517	0
CEU	chr16	NPIP	32842517	0
CEU	chr16	NPIP	32892517	0
CEU	chr16	NPIP	32942517	0
CEU	chr16	NPIP	32992517	0
CEU	chr16	NPIP	33042517	0
CEU	chr16	NPIP	33092517	0
CEU	chr16	NPIP	33142517	0
CEU	chr16	NPIP	33192517	0

CEU	chr16	NPIP	33242517	0
CEU	chr16	NPIP	33292517	0
CEU	chr16	NPIP	69424986	0
CEU	chr16	NPIP	69474986	0
CEU	chr16	NPIP	69524986	0
CEU	chr16	NPIP	69574986	0
CEU	chr16	NPIP	69624986	0
CEU	chr16	NPIP	69674986	0
CEU	chr16	NPIP	69724986	0
CEU	chr16	NPIP	69774986	0
CEU	chr16	NPIP	69824986	0
CEU	chr16	NPIP	69874986	0
CEU	chr16	NPIP	69924986	0
CEU	chr16	NPIP	69974986	0
CEU	chr16	NPIP	70024986	0
CEU	chr16	NPIP	70074986	0
CEU	chr16	NPIP	70124986	0
CEU	chr16	NPIP	70174986	0
CEU	chr16	NPIP	70224986	0
CEU	chr16	NPIP	70274986	0
CEU	chr16	NPIP	70324986	0
CEU	chr16	NPIP	70374986	0
CEU	chr16	NPIP	70424986	0
CEU	chr16	NPIP	70474986	0
CEU	chr16	NPIP	70524986	0
CEU	chr16	NPIP	70574986	0
CEU	chr16	NPIP	70624986	0
CEU	chr16	NPIP	70674986	0
CEU	chr16	NPIP	70724986	0
CEU	chr16	NPIP	70774986	0
CEU	chr16	NPIP	70824986	0
CEU	chr16	NPIP	70874986	0
CEU	chr16	NPIP	70924986	0
CEU	chr16	NPIP	70974986	0
CEU	chr16	NPIP	71024986	0
CEU	chr16	NPIP	71074986	0
CEU	chr16	NPIP	71124986	0
CEU	chr16	NPIP	71174986	0
CEU	chr16	NPIP	71224986	0
CEU	chr16	NPIP	71274986	0
CEU	chr16	NPIP	71324986	0
CEU	chr16	NPIP	71374986	0
CEU	chr16	NPIP	71424986	0
CEU	chr16	NPIP	71474986	0
CEU	chr16	NPIP	71524986	0
CEU	chr16	NPIP	71574986	0
CEU	chr16	NPIP	71624986	0
CEU	chr16	NPIP	71674986	0
CEU	chr16	NPIP	71724986	0

CEU	chr16	NPIP	71774986	0
CEU	chr16	NPIP	71824986	0
CEU	chr16	NPIP	71874986	0
CEU	chr16	NPIP	71924986	0
CEU	chr16	NPIP	71974986	0
CEU	chr16	NPIP	72024986	0
CEU	chr16	NPIP	72074986	0
CEU	chr16	NPIP	72124986	0
CEU	chr16	NPIP	72174986	0
CEU	chr16	NPIP	72224986	0
CEU	chr16	NPIP	72274986	0
CEU	chr16	NPIP	72324986	0
CEU	chr16	NPIP	72374986	0
CEU	chr16	NPIP	72424986	0
CEU	chr16	NPIP	72474986	0
CEU	chr16	NPIP	72524986	0
CEU	chr16	NPIP	72574986	0
CEU	chr16	NPIP	72624986	0
CEU	chr16	NPIP	72674986	0
CEU	chr16	NPIP	72724986	0
CEU	chr16	NPIP	72774986	0
CEU	chr16	NPIP	72824986	0
CEU	chr16	NPIP	72874986	0
CEU	chr16	NPIP	72924986	0
CEU	chr16	NPIP	72974986	0
CEU	chr16	NPIP	73024986	0
CEU	chr16	NPIP	73074986	0
CEU	chr16	NPIP	73124986	0
CEU	chr16	NPIP	73174986	0
CEU	chr16	NPIP	73224986	0
CEU	chr16	NPIP	73274986	0
CEU	chr16	NPIP	73324986	0
CEU	chr16	NPIP	73374986	0
CEU	chr16	NPIP	73424986	0
CEU	chr16	NPIP	73474986	0
CEU	chr16	NPIP	73524986	0
CEU	chr16	NPIP	73574986	0
CEU	chr16	NPIP	73624986	0
CEU	chr16	NPIP	73674986	0
CEU	chr16	NPIP	73724986	0
CEU	chr16	NPIP	73774986	0
CEU	chr16	NPIP	73824986	0
CEU	chr16	NPIP	73874986	0
CEU	chr16	NPIP	73924986	0
CEU	chr16	NPIP	73974986	0
CEU	chr16	NPIP	74024986	0
CEU	chr16	NPIP	74074986	0
CEU	chr16	NPIP	74124986	0
CEU	chr16	NPIP	74174986	0

CEU	chr16	NPIP	74224986	0
CEU	chr16	NPIP	74274986	0
CEU	chr16	NPIP	74324986	0
CEU	chr16	NPIP	74374986	0
CEU	chr16	NPIP	74424986	0
CEU	chr16	NPIP	74474986	0
CEU	chr16	NPIP	74524986	0
CEU	chr16	NPIP	74574986	0
CEU	chr16	NPIP	74624986	0
CEU	chr16	NPIP	74674986	0
CEU	chr16	NPIP	74724986	0
CEU	chr16	NPIP	74774986	0
CEU	chr16	NPIP	74824986	0
CEU	chr16	NPIP	74874986	0
CEU	chr16	NPIP	74924986	0
CEU	chr16	NPIP	74974986	0
CEU	chr16	NPIP	75024986	0
CEU	chr16	NPIP	75074986	0
CEU	chr16	NPIP	75124986	0
CEU	chr16	NPIP	75174986	0
CEU	chr16	NPIP	75224986	0
CEU	chr16	NPIP	75274986	0
CEU	chr16	NPIP	75324986	0
CEU	chr16	NPIP	75374986	0
CEU	chr16	NPIP	75424986	0
CEU	chr16	NPIP	75474986	0
CEU	chr16	NPIP	75524986	0
CEU	chr16	NPIP	75574986	0
CEU	chr16	NPIP	75624986	0
CEU	chr16	NPIP	75674986	0
CEU	chr16	NPIP	75724986	0
CEU	chr16	NPIP	75774986	0
CEU	chr16	NPIP	75824986	0
CEU	chr16	NPIP	75874986	0
CEU	chr16	NPIP	75924986	0
CEU	chr16	NPIP	75974986	0
CEU	chr16	NPIP	76024986	0
CEU	chr16	NPIP	76074986	0
CEU	chr16	NPIP	76124986	0
CEU	chr16	NPIP	76174986	0
CEU	chr16	NPIP	76224986	0
CEU	chr16	NPIP	76274986	0
CEU	chr16	NPIP	76324986	0
CEU	chr16	NPIP	76374986	0
CEU	chr16	NPIP	76424986	0
CEU	chr16	NPIP	76474986	0
CEU	chr16	NPIP	76524986	21
CEU	chr16	NPIP	76574986	30
CEU	chr16	NPIP	76624986	22

CEU	chr16	NPIP	76674986	25
CEU	chr16	NPIP	76724986	30
CEU	chr16	NPIP	76774986	28
CEU	chr16	NPIP	76824986	50
CEU	chr16	NPIP	76874986	54
CEU	chr16	NPIP	76924986	35
CEU	chr16	NPIP	76974986	39
CEU	chr16	NPIP	77024986	31
CEU	chr16	NPIP	77074986	24
CEU	chr16	NPIP	77124986	47
CEU	chr16	NPIP	77174986	59
CEU	chr16	NPIP	77224986	34
CEU	chr16	NPIP	77274986	41
CEU	chr16	NPIP	77324986	90
CEU	chr16	NPIP	77374986	96
CEU	chr16	NPIP	77424986	57
CEU	chr16	NPIP	77474986	28
CEU	chr16	NPIP	77524986	39
CEU	chr16	NPIP	77574986	58
CEU	chr16	NPIP	77624986	48
CEU	chr16	NPIP	77674986	31
CEU	chr16	NPIP	77724986	20
CEU	chr16	NPIP	77774986	21
CEU	chr16	NPIP	77824986	24
CEU	chr16	NPIP	77874986	17
CEU	chr16	NPIP	77924986	15
CEU	chr16	NPIP	77974986	30
CEU	chr16	NPIP	78024986	37
CEU	chr16	NPIP	78074986	107
CEU	chr16	NPIP	78124986	117
CEU	chr16	NPIP	78174986	68
CEU	chr16	NPIP	78224986	64
CEU	chr16	NPIP	78274986	39
CEU	chr16	NPIP	78324986	38
CEU	chr16	NPIP	78374986	95
CEU	chr16	NPIP	78424986	74
CEU	chr16	NPIP	78474986	8
CEU	chr16	NPIP	78524986	16
CEU	chr16	NPIP	78574986	18
CEU	chr16	NPIP	78624986	24
CEU	chr16	NPIP	78674986	74
CEU	chr16	NPIP	78724986	119
CEU	chr16	NPIP	78774986	62
CEU	chr16	NPIP	78824986	44
CEU	chr16	NPIP	78874986	73
CEU	chr16	NPIP	78924986	44
CEU	chr16	NPIP	78974986	15
CEU	chr16	NPIP	79024986	0
CEU	chr16	NPIP	79074986	0

CEU	chr16	NPIP	79124986	0
CEU	chr16	NPIP	79174986	0
CEU	chr16	NPIP	79224986	0
CEU	chr16	NPIP	79274986	0
CEU	chr16	NPIP	79324986	0
CEU	chr11	PGA3	56253515	68
CEU	chr11	PGA3	56303515	71
CEU	chr11	PGA3	56353515	74
CEU	chr11	PGA3	56403515	75
CEU	chr11	PGA3	56453515	61
CEU	chr11	PGA3	56503515	40
CEU	chr11	PGA3	56553515	42
CEU	chr11	PGA3	56603515	73
CEU	chr11	PGA3	56653515	69
CEU	chr11	PGA3	56703515	56
CEU	chr11	PGA3	56753515	102
CEU	chr11	PGA3	56803515	128
CEU	chr11	PGA3	56853515	102
CEU	chr11	PGA3	56903515	53
CEU	chr11	PGA3	56953515	27
CEU	chr11	PGA3	57003515	39
CEU	chr11	PGA3	57053515	62
CEU	chr11	PGA3	57103515	63
CEU	chr11	PGA3	57153515	27
CEU	chr11	PGA3	57203515	0
CEU	chr11	PGA3	57253515	0
CEU	chr11	PGA3	57303515	0
CEU	chr11	PGA3	57353515	0
CEU	chr11	PGA3	57403515	0
CEU	chr11	PGA3	57453515	0
CEU	chr11	PGA3	57503515	0
CEU	chr11	PGA3	57553515	0
CEU	chr11	PGA3	57603515	0
CEU	chr11	PGA3	57653515	0
CEU	chr11	PGA3	57703515	0
CEU	chr11	PGA3	57753515	0
CEU	chr11	PGA3	57803515	0
CEU	chr11	PGA3	57853515	0
CEU	chr11	PGA3	57903515	0
CEU	chr11	PGA3	57953515	0
CEU	chr11	PGA3	58003515	0
CEU	chr11	PGA3	58053515	0
CEU	chr11	PGA3	58103515	0
CEU	chr11	PGA3	58153515	0
CEU	chr11	PGA3	58203515	0
CEU	chr11	PGA3	58253515	0
CEU	chr11	PGA3	58303515	0
CEU	chr11	PGA3	58353515	0
CEU	chr11	PGA3	58403515	0

CEU	chr11	PGA3	58453515	0
CEU	chr11	PGA3	58503515	0
CEU	chr11	PGA3	58553515	0
CEU	chr11	PGA3	58603515	0
CEU	chr11	PGA3	58653515	0
CEU	chr11	PGA3	58703515	0
CEU	chr11	PGA3	58753515	0
CEU	chr11	PGA3	58803515	0
CEU	chr11	PGA3	58853515	0
CEU	chr11	PGA3	58903515	0
CEU	chr11	PGA3	58953515	0
CEU	chr11	PGA3	59003515	0
CEU	chr11	PGA3	59053515	0
CEU	chr11	PGA3	59103515	0
CEU	chr11	PGA3	59153515	0
CEU	chr11	PGA3	59203515	0
CEU	chr11	PGA3	59253515	0
CEU	chr11	PGA3	59303515	0
CEU	chr11	PGA3	59353515	0
CEU	chr11	PGA3	59403515	0
CEU	chr11	PGA3	59453515	0
CEU	chr11	PGA3	59503515	0
CEU	chr11	PGA3	59553515	0
CEU	chr11	PGA3	59603515	0
CEU	chr11	PGA3	59653515	0
CEU	chr11	PGA3	59703515	0
CEU	chr11	PGA3	59753515	0
CEU	chr11	PGA3	59803515	0
CEU	chr11	PGA3	59853515	0
CEU	chr11	PGA3	59903515	0
CEU	chr11	PGA3	59953515	0
CEU	chr11	PGA3	60003515	0
CEU	chr11	PGA3	60053515	0
CEU	chr11	PGA3	60103515	0
CEU	chr11	PGA3	60153515	0
CEU	chr11	PGA3	60203515	0
CEU	chr11	PGA3	60253515	0
CEU	chr11	PGA3	60303515	0
CEU	chr11	PGA3	60353515	0
CEU	chr11	PGA3	60403515	0
CEU	chr11	PGA3	60453515	0
CEU	chr11	PGA3	60503515	0
CEU	chr11	PGA3	60553515	0
CEU	chr11	PGA3	60603515	0
CEU	chr11	PGA3	60653515	0
CEU	chr11	PGA3	60703515	0
CEU	chr11	PGA3	60753515	0
CEU	chr11	PGA3	60803515	0
CEU	chr11	PGA3	60853515	0

CEU	chr11	PGA3	60903515	0
CEU	chr11	PGA3	60953515	0
CEU	chr11	PGA3	61003515	0
CEU	chr11	PGA3	61053515	0
CEU	chr11	PGA3	61103515	0
CEU	chr11	PGA3	61153515	0
CEU	chr11	PGA3	61203515	0
CEU	chr11	PGA3	61253515	0
CEU	chr11	PGA3	61303515	0
CEU	chr11	PGA3	61353515	0
CEU	chr11	PGA3	61403515	0
CEU	chr11	PGA3	61453515	0
CEU	chr11	PGA3	61503515	0
CEU	chr11	PGA3	61553515	0
CEU	chr11	PGA3	61603515	0
CEU	chr11	PGA3	61653515	0
CEU	chr11	PGA3	61703515	0
CEU	chr11	PGA3	61753515	0
CEU	chr11	PGA3	61803515	0
CEU	chr11	PGA3	61853515	0
CEU	chr11	PGA3	61903515	0
CEU	chr11	PGA3	61953515	0
CEU	chr11	PGA3	62003515	0
CEU	chr11	PGA3	62053515	0
CEU	chr11	PGA3	62103515	0
CEU	chr11	PGA3	62153515	30
CEU	chr11	PGA3	62203515	36
CEU	chr11	PGA3	62253515	6
CEU	chr11	PGA3	62303515	0
CEU	chr11	PGA3	62353515	0
CEU	chr11	PGA3	62403515	0
CEU	chr11	PGA3	62453515	0
CEU	chr11	PGA3	62503515	0
CEU	chr11	PGA3	62553515	0
CEU	chr11	PGA3	62603515	0
CEU	chr11	PGA3	62653515	0
CEU	chr11	PGA3	62703515	0
CEU	chr11	PGA3	62753515	0
CEU	chr11	PGA3	62803515	0
CEU	chr11	PGA3	62853515	0
CEU	chr11	PGA3	62903515	0
CEU	chr11	PGA3	62953515	0
CEU	chr11	PGA3	63003515	0
CEU	chr11	PGA3	63053515	0
CEU	chr11	PGA3	63103515	0
CEU	chr11	PGA3	63153515	0
CEU	chr11	PGA3	63203515	0
CEU	chr11	PGA3	63253515	0
CEU	chr11	PGA3	63303515	0

CEU	chr11	PGA3	63353515	0
CEU	chr11	PGA3	63403515	0
CEU	chr11	PGA3	63453515	0
CEU	chr11	PGA3	63503515	0
CEU	chr11	PGA3	63553515	0
CEU	chr11	PGA3	63603515	0
CEU	chr11	PGA3	63653515	0
CEU	chr11	PGA3	63703515	0
CEU	chr11	PGA3	63753515	0
CEU	chr11	PGA3	63803515	0
CEU	chr11	PGA3	63853515	0
CEU	chr11	PGA3	63903515	0
CEU	chr11	PGA3	63953515	0
CEU	chr11	PGA3	64003515	0
CEU	chr11	PGA3	64053515	0
CEU	chr11	PGA3	64103515	0
CEU	chr11	PGA3	64153515	0
CEU	chr11	PGA3	64203515	0
CEU	chr11	PGA3	64253515	0
CEU	chr11	PGA3	64303515	0
CEU	chr11	PGA3	64353515	0
CEU	chr11	PGA3	64403515	0
CEU	chr11	PGA3	64453515	0
CEU	chr11	PGA3	64503515	0
CEU	chr11	PGA3	64553515	0
CEU	chr11	PGA3	64603515	0
CEU	chr11	PGA3	64653515	0
CEU	chr11	PGA3	64703515	0
CEU	chr11	PGA3	64753515	0
CEU	chr11	PGA3	64803515	0
CEU	chr11	PGA3	64853515	0
CEU	chr11	PGA3	64903515	0
CEU	chr11	PGA3	64953515	0
CEU	chr11	PGA3	65003515	0
CEU	chr11	PGA3	65053515	0
CEU	chr11	PGA3	65103515	0
CEU	chr11	PGA3	65153515	0
CEU	chr11	PGA3	65203515	0
CEU	chr11	PGA3	65253515	0
CEU	chr11	PGA3	65303515	0
CEU	chr11	PGA3	65353515	0
CEU	chr11	PGA3	65403515	0
CEU	chr11	PGA3	65453515	0
CEU	chr11	PGA3	65503515	0
CEU	chr11	PGA3	65553515	0
CEU	chr11	PGA3	65603515	0
CEU	chr11	PGA3	65653515	0
CEU	chr11	PGA3	65703515	0
CEU	chr11	PGA3	65753515	0

CEU	chr11	PGA3	65803515	0
CEU	chr11	PGA3	65853515	0
CEU	chr11	PGA3	65903515	0
CEU	chr11	PGA3	65953515	0
CEU	chr11	PGA3	66003515	0
CEU	chr11	PGA3	66053515	0
CEU	chr11	PGA3	66103515	0
CEU	chr11	PGA3	66153515	0
CEU	chr1	PRAMEF14	8391892	0
CEU	chr1	PRAMEF14	8441892	0
CEU	chr1	PRAMEF14	8491892	0
CEU	chr1	PRAMEF14	8541892	0
CEU	chr1	PRAMEF14	8591892	0
CEU	chr1	PRAMEF14	8641892	0
CEU	chr1	PRAMEF14	8691892	0
CEU	chr1	PRAMEF14	8741892	0
CEU	chr1	PRAMEF14	8791892	0
CEU	chr1	PRAMEF14	8841892	0
CEU	chr1	PRAMEF14	8891892	0
CEU	chr1	PRAMEF14	8941892	0
CEU	chr1	PRAMEF14	8991892	0
CEU	chr1	PRAMEF14	9041892	0
CEU	chr1	PRAMEF14	9091892	0
CEU	chr1	PRAMEF14	9141892	0
CEU	chr1	PRAMEF14	9191892	0
CEU	chr1	PRAMEF14	9241892	0
CEU	chr1	PRAMEF14	9291892	0
CEU	chr1	PRAMEF14	9341892	0
CEU	chr1	PRAMEF14	9391892	0
CEU	chr1	PRAMEF14	9441892	0
CEU	chr1	PRAMEF14	9491892	0
CEU	chr1	PRAMEF14	9541892	0
CEU	chr1	PRAMEF14	9591892	0
CEU	chr1	PRAMEF14	9641892	0
CEU	chr1	PRAMEF14	9691892	0
CEU	chr1	PRAMEF14	9741892	0
CEU	chr1	PRAMEF14	9791892	0
CEU	chr1	PRAMEF14	9841892	0
CEU	chr1	PRAMEF14	9891892	0
CEU	chr1	PRAMEF14	9941892	2
CEU	chr1	PRAMEF14	9991892	19
CEU	chr1	PRAMEF14	10041892	36
CEU	chr1	PRAMEF14	10091892	34
CEU	chr1	PRAMEF14	10141892	34
CEU	chr1	PRAMEF14	10191892	76
CEU	chr1	PRAMEF14	10241892	108
CEU	chr1	PRAMEF14	10291892	112
CEU	chr1	PRAMEF14	10341892	105
CEU	chr1	PRAMEF14	10391892	71

CEU	chr1	PRAMEF14	10441892	38
CEU	chr1	PRAMEF14	10491892	11
CEU	chr1	PRAMEF14	10541892	0
CEU	chr1	PRAMEF14	10591892	0
CEU	chr1	PRAMEF14	10641892	0
CEU	chr1	PRAMEF14	10691892	0
CEU	chr1	PRAMEF14	10741892	0
CEU	chr1	PRAMEF14	10791892	0
CEU	chr1	PRAMEF14	10841892	0
CEU	chr1	PRAMEF14	10891892	0
CEU	chr1	PRAMEF14	10941892	0
CEU	chr1	PRAMEF14	10991892	0
CEU	chr1	PRAMEF14	11041892	0
CEU	chr1	PRAMEF14	11091892	0
CEU	chr1	PRAMEF14	11141892	0
CEU	chr1	PRAMEF14	11191892	0
CEU	chr1	PRAMEF14	11241892	0
CEU	chr1	PRAMEF14	11291892	0
CEU	chr1	PRAMEF14	11341892	0
CEU	chr1	PRAMEF14	11391892	0
CEU	chr1	PRAMEF14	11441892	0
CEU	chr1	PRAMEF14	11491892	0
CEU	chr1	PRAMEF14	11541892	0
CEU	chr1	PRAMEF14	11591892	0
CEU	chr1	PRAMEF14	11641892	0
CEU	chr1	PRAMEF14	11691892	0
CEU	chr1	PRAMEF14	11741892	0
CEU	chr1	PRAMEF14	11791892	0
CEU	chr1	PRAMEF14	11841892	0
CEU	chr1	PRAMEF14	11891892	0
CEU	chr1	PRAMEF14	11941892	0
CEU	chr1	PRAMEF14	11991892	0
CEU	chr1	PRAMEF14	12041892	0
CEU	chr1	PRAMEF14	12091892	0
CEU	chr1	PRAMEF14	12141892	24
CEU	chr1	PRAMEF14	12191892	38
CEU	chr1	PRAMEF14	12241892	42
CEU	chr1	PRAMEF14	12291892	41
CEU	chr1	PRAMEF14	12341892	37
CEU	chr1	PRAMEF14	12391892	47
CEU	chr1	PRAMEF14	12441892	37
CEU	chr1	PRAMEF14	12491892	26
CEU	chr1	PRAMEF14	12541892	47
CEU	chr1	PRAMEF14	12591892	39
CEU	chr1	PRAMEF14	12641892	4
CEU	chr1	PRAMEF14	12691892	0
CEU	chr1	PRAMEF14	12741892	4
CEU	chr1	PRAMEF14	12791892	29
CEU	chr1	PRAMEF14	12841892	124

CEU	chr1	PRAMEF14	12891892	122
CEU	chr1	PRAMEF14	12941892	23
CEU	chr1	PRAMEF14	12991892	0
CEU	chr1	PRAMEF14	13041892	0
CEU	chr1	PRAMEF14	13091892	0
CEU	chr1	PRAMEF14	13141892	0
CEU	chr1	PRAMEF14	13191892	0
CEU	chr1	PRAMEF14	13241892	0
CEU	chr1	PRAMEF14	13291892	0
CEU	chr1	PRAMEF14	13341892	0
CEU	chr1	PRAMEF14	13391892	0
CEU	chr1	PRAMEF14	13441892	0
CEU	chr1	PRAMEF14	13491892	0
CEU	chr1	PRAMEF14	13541892	0
CEU	chr1	PRAMEF14	13591892	0
CEU	chr1	PRAMEF14	13641892	0
CEU	chr1	PRAMEF14	13691892	0
CEU	chr1	PRAMEF14	13741892	0
CEU	chr1	PRAMEF14	13791892	0
CEU	chr1	PRAMEF14	13841892	0
CEU	chr1	PRAMEF14	13891892	0
CEU	chr1	PRAMEF14	13941892	10
CEU	chr1	PRAMEF14	13991892	61
CEU	chr1	PRAMEF14	14041892	102
CEU	chr1	PRAMEF14	14091892	92
CEU	chr1	PRAMEF14	14141892	75
CEU	chr1	PRAMEF14	14191892	72
CEU	chr1	PRAMEF14	14241892	51
CEU	chr1	PRAMEF14	14291892	26
CEU	chr1	PRAMEF14	14341892	36
CEU	chr1	PRAMEF14	14391892	42
CEU	chr1	PRAMEF14	14441892	19
CEU	chr1	PRAMEF14	14491892	0
CEU	chr1	PRAMEF14	14541892	0
CEU	chr1	PRAMEF14	14591892	0
CEU	chr1	PRAMEF14	14641892	0
CEU	chr1	PRAMEF14	14691892	0
CEU	chr1	PRAMEF14	14741892	0
CEU	chr1	PRAMEF14	14791892	0
CEU	chr1	PRAMEF14	14841892	0
CEU	chr1	PRAMEF14	14891892	0
CEU	chr1	PRAMEF14	14941892	0
CEU	chr1	PRAMEF14	14991892	0
CEU	chr1	PRAMEF14	15041892	0
CEU	chr1	PRAMEF14	15091892	0
CEU	chr1	PRAMEF14	15141892	0
CEU	chr1	PRAMEF14	15191892	0
CEU	chr1	PRAMEF14	15241892	0
CEU	chr1	PRAMEF14	15291892	0

CEU	chr1	PRAMEF14	15341892	0
CEU	chr1	PRAMEF14	15391892	0
CEU	chr1	PRAMEF14	15441892	0
CEU	chr1	PRAMEF14	15491892	0
CEU	chr1	PRAMEF14	15541892	0
CEU	chr1	PRAMEF14	15591892	0
CEU	chr1	PRAMEF14	15641892	0
CEU	chr1	PRAMEF14	15691892	0
CEU	chr1	PRAMEF14	15741892	0
CEU	chr1	PRAMEF14	15791892	0
CEU	chr1	PRAMEF14	15841892	0
CEU	chr1	PRAMEF14	15891892	0
CEU	chr1	PRAMEF14	15941892	0
CEU	chr1	PRAMEF14	15991892	0
CEU	chr1	PRAMEF14	16041892	0
CEU	chr1	PRAMEF14	16091892	0
CEU	chr1	PRAMEF14	16141892	0
CEU	chr1	PRAMEF14	16191892	0
CEU	chr1	PRAMEF14	16241892	0
CEU	chr1	PRAMEF14	16291892	0
CEU	chr1	PRAMEF14	16341892	0
CEU	chr1	PRAMEF14	16391892	0
CEU	chr1	PRAMEF14	16441892	0
CEU	chr1	PRAMEF14	16491892	5
CEU	chr1	PRAMEF14	16541892	18
CEU	chr1	PRAMEF14	16591892	24
CEU	chr1	PRAMEF14	16641892	19
CEU	chr1	PRAMEF14	16691892	40
CEU	chr1	PRAMEF14	16741892	55
CEU	chr1	PRAMEF14	16791892	53
CEU	chr1	PRAMEF14	16841892	55
CEU	chr1	PRAMEF14	16891892	25
CEU	chr1	PRAMEF14	16941892	0
CEU	chr1	PRAMEF14	16991892	0
CEU	chr1	PRAMEF14	17041892	0
CEU	chr1	PRAMEF14	17091892	0
CEU	chr1	PRAMEF14	17141892	0
CEU	chr1	PRAMEF14	17191892	0
CEU	chr1	PRAMEF14	17241892	0
CEU	chr1	PRAMEF14	17291892	0
CEU	chr1	PRAMEF14	17341892	0
CEU	chr1	PRAMEF14	17391892	0
CEU	chr1	PRAMEF14	17441892	0
CEU	chr1	PRAMEF14	17491892	0
CEU	chr1	PRAMEF14	17541892	0
CEU	chr1	PRAMEF14	17591892	0
CEU	chr1	PRAMEF14	17641892	0
CEU	chr1	PRAMEF14	17691892	0
CEU	chr1	PRAMEF14	17741892	0

CEU	chr1	PRAMEF14	17791892	0
CEU	chr1	PRAMEF14	17841892	0
CEU	chr1	PRAMEF14	17891892	0
CEU	chr1	PRAMEF14	17941892	0
CEU	chr1	PRAMEF14	17991892	0
CEU	chr1	PRAMEF14	18041892	0
CEU	chr1	PRAMEF14	18091892	0
CEU	chr1	PRAMEF14	18141892	0
CEU	chr1	PRAMEF14	18191892	0
CEU	chr1	PRAMEF14	18241892	0
CEU	chr1	PRAMEF14	18291892	0
CEU	chr1	PRAMEF20	8460450	0
CEU	chr1	PRAMEF20	8510450	0
CEU	chr1	PRAMEF20	8560450	0
CEU	chr1	PRAMEF20	8610450	0
CEU	chr1	PRAMEF20	8660450	0
CEU	chr1	PRAMEF20	8710450	0
CEU	chr1	PRAMEF20	8760450	0
CEU	chr1	PRAMEF20	8810450	0
CEU	chr1	PRAMEF20	8860450	0
CEU	chr1	PRAMEF20	8910450	0
CEU	chr1	PRAMEF20	8960450	0
CEU	chr1	PRAMEF20	9010450	0
CEU	chr1	PRAMEF20	9060450	0
CEU	chr1	PRAMEF20	9110450	0
CEU	chr1	PRAMEF20	9160450	0
CEU	chr1	PRAMEF20	9210450	0
CEU	chr1	PRAMEF20	9260450	0
CEU	chr1	PRAMEF20	9310450	0
CEU	chr1	PRAMEF20	9360450	0
CEU	chr1	PRAMEF20	9410450	0
CEU	chr1	PRAMEF20	9460450	0
CEU	chr1	PRAMEF20	9510450	0
CEU	chr1	PRAMEF20	9560450	0
CEU	chr1	PRAMEF20	9610450	0
CEU	chr1	PRAMEF20	9660450	0
CEU	chr1	PRAMEF20	9710450	0
CEU	chr1	PRAMEF20	9760450	0
CEU	chr1	PRAMEF20	9810450	0
CEU	chr1	PRAMEF20	9860450	0
CEU	chr1	PRAMEF20	9910450	0
CEU	chr1	PRAMEF20	9960450	5
CEU	chr1	PRAMEF20	10010450	25
CEU	chr1	PRAMEF20	10060450	42
CEU	chr1	PRAMEF20	10110450	31
CEU	chr1	PRAMEF20	10160450	52
CEU	chr1	PRAMEF20	10210450	85
CEU	chr1	PRAMEF20	10260450	102
CEU	chr1	PRAMEF20	10310450	117

CEU	chr1	PRAMEF20	10360450	102
CEU	chr1	PRAMEF20	10410450	61
CEU	chr1	PRAMEF20	10460450	20
CEU	chr1	PRAMEF20	10510450	4
CEU	chr1	PRAMEF20	10560450	0
CEU	chr1	PRAMEF20	10610450	0
CEU	chr1	PRAMEF20	10660450	0
CEU	chr1	PRAMEF20	10710450	0
CEU	chr1	PRAMEF20	10760450	0
CEU	chr1	PRAMEF20	10810450	0
CEU	chr1	PRAMEF20	10860450	0
CEU	chr1	PRAMEF20	10910450	0
CEU	chr1	PRAMEF20	10960450	0
CEU	chr1	PRAMEF20	11010450	0
CEU	chr1	PRAMEF20	11060450	0
CEU	chr1	PRAMEF20	11110450	0
CEU	chr1	PRAMEF20	11160450	0
CEU	chr1	PRAMEF20	11210450	0
CEU	chr1	PRAMEF20	11260450	0
CEU	chr1	PRAMEF20	11310450	0
CEU	chr1	PRAMEF20	11360450	0
CEU	chr1	PRAMEF20	11410450	0
CEU	chr1	PRAMEF20	11460450	0
CEU	chr1	PRAMEF20	11510450	0
CEU	chr1	PRAMEF20	11560450	0
CEU	chr1	PRAMEF20	11610450	0
CEU	chr1	PRAMEF20	11660450	0
CEU	chr1	PRAMEF20	11710450	0
CEU	chr1	PRAMEF20	11760450	0
CEU	chr1	PRAMEF20	11810450	0
CEU	chr1	PRAMEF20	11860450	0
CEU	chr1	PRAMEF20	11910450	0
CEU	chr1	PRAMEF20	11960450	0
CEU	chr1	PRAMEF20	12010450	0
CEU	chr1	PRAMEF20	12060450	0
CEU	chr1	PRAMEF20	12110450	2
CEU	chr1	PRAMEF20	12160450	25
CEU	chr1	PRAMEF20	12210450	45
CEU	chr1	PRAMEF20	12260450	46
CEU	chr1	PRAMEF20	12310450	42
CEU	chr1	PRAMEF20	12360450	38
CEU	chr1	PRAMEF20	12410450	44
CEU	chr1	PRAMEF20	12460450	35
CEU	chr1	PRAMEF20	12510450	25
CEU	chr1	PRAMEF20	12560450	47
CEU	chr1	PRAMEF20	12610450	33
CEU	chr1	PRAMEF20	12660450	0
CEU	chr1	PRAMEF20	12710450	0
CEU	chr1	PRAMEF20	12760450	11

CEU	chr1	PRAMEF20	12810450	31
CEU	chr1	PRAMEF20	12860450	130
CEU	chr1	PRAMEF20	12910450	120
CEU	chr1	PRAMEF20	12960450	10
CEU	chr1	PRAMEF20	13010450	0
CEU	chr1	PRAMEF20	13060450	0
CEU	chr1	PRAMEF20	13110450	0
CEU	chr1	PRAMEF20	13160450	0
CEU	chr1	PRAMEF20	13210450	0
CEU	chr1	PRAMEF20	13260450	0
CEU	chr1	PRAMEF20	13310450	0
CEU	chr1	PRAMEF20	13360450	0
CEU	chr1	PRAMEF20	13410450	0
CEU	chr1	PRAMEF20	13460450	0
CEU	chr1	PRAMEF20	13510450	0
CEU	chr1	PRAMEF20	13560450	0
CEU	chr1	PRAMEF20	13610450	0
CEU	chr1	PRAMEF20	13660450	0
CEU	chr1	PRAMEF20	13710450	0
CEU	chr1	PRAMEF20	13760450	0
CEU	chr1	PRAMEF20	13810450	0
CEU	chr1	PRAMEF20	13860450	0
CEU	chr1	PRAMEF20	13910450	4
CEU	chr1	PRAMEF20	13960450	29
CEU	chr1	PRAMEF20	14010450	79
CEU	chr1	PRAMEF20	14060450	104
CEU	chr1	PRAMEF20	14110450	85
CEU	chr1	PRAMEF20	14160450	72
CEU	chr1	PRAMEF20	14210450	64
CEU	chr1	PRAMEF20	14260450	36
CEU	chr1	PRAMEF20	14310450	23
CEU	chr1	PRAMEF20	14360450	40
CEU	chr1	PRAMEF20	14410450	38
CEU	chr1	PRAMEF20	14460450	12
CEU	chr1	PRAMEF20	14510450	0
CEU	chr1	PRAMEF20	14560450	0
CEU	chr1	PRAMEF20	14610450	0
CEU	chr1	PRAMEF20	14660450	0
CEU	chr1	PRAMEF20	14710450	0
CEU	chr1	PRAMEF20	14760450	0
CEU	chr1	PRAMEF20	14810450	0
CEU	chr1	PRAMEF20	14860450	0
CEU	chr1	PRAMEF20	14910450	0
CEU	chr1	PRAMEF20	14960450	0
CEU	chr1	PRAMEF20	15010450	0
CEU	chr1	PRAMEF20	15060450	0
CEU	chr1	PRAMEF20	15110450	0
CEU	chr1	PRAMEF20	15160450	0
CEU	chr1	PRAMEF20	15210450	0

CEU	chr1	PRAMEF20	15260450	0
CEU	chr1	PRAMEF20	15310450	0
CEU	chr1	PRAMEF20	15360450	0
CEU	chr1	PRAMEF20	15410450	0
CEU	chr1	PRAMEF20	15460450	0
CEU	chr1	PRAMEF20	15510450	0
CEU	chr1	PRAMEF20	15560450	0
CEU	chr1	PRAMEF20	15610450	0
CEU	chr1	PRAMEF20	15660450	0
CEU	chr1	PRAMEF20	15710450	0
CEU	chr1	PRAMEF20	15760450	0
CEU	chr1	PRAMEF20	15810450	0
CEU	chr1	PRAMEF20	15860450	0
CEU	chr1	PRAMEF20	15910450	0
CEU	chr1	PRAMEF20	15960450	0
CEU	chr1	PRAMEF20	16010450	0
CEU	chr1	PRAMEF20	16060450	0
CEU	chr1	PRAMEF20	16110450	0
CEU	chr1	PRAMEF20	16160450	0
CEU	chr1	PRAMEF20	16210450	0
CEU	chr1	PRAMEF20	16260450	0
CEU	chr1	PRAMEF20	16310450	0
CEU	chr1	PRAMEF20	16360450	0
CEU	chr1	PRAMEF20	16410450	0
CEU	chr1	PRAMEF20	16460450	0
CEU	chr1	PRAMEF20	16510450	12
CEU	chr1	PRAMEF20	16560450	25
CEU	chr1	PRAMEF20	16610450	20
CEU	chr1	PRAMEF20	16660450	24
CEU	chr1	PRAMEF20	16710450	42
CEU	chr1	PRAMEF20	16760450	61
CEU	chr1	PRAMEF20	16810450	67
CEU	chr1	PRAMEF20	16860450	37
CEU	chr1	PRAMEF20	16910450	6
CEU	chr1	PRAMEF20	16960450	0
CEU	chr1	PRAMEF20	17010450	0
CEU	chr1	PRAMEF20	17060450	0
CEU	chr1	PRAMEF20	17110450	0
CEU	chr1	PRAMEF20	17160450	0
CEU	chr1	PRAMEF20	17210450	0
CEU	chr1	PRAMEF20	17260450	0
CEU	chr1	PRAMEF20	17310450	0
CEU	chr1	PRAMEF20	17360450	0
CEU	chr1	PRAMEF20	17410450	0
CEU	chr1	PRAMEF20	17460450	0
CEU	chr1	PRAMEF20	17510450	0
CEU	chr1	PRAMEF20	17560450	0
CEU	chr1	PRAMEF20	17610450	0
CEU	chr1	PRAMEF20	17660450	0

CEU	chr1	PRAMEF20	17710450	0
CEU	chr1	PRAMEF20	17760450	0
CEU	chr1	PRAMEF20	17810450	0
CEU	chr1	PRAMEF20	17860450	0
CEU	chr1	PRAMEF20	17910450	0
CEU	chr1	PRAMEF20	17960450	0
CEU	chr1	PRAMEF20	18010450	0
CEU	chr1	PRAMEF20	18060450	0
CEU	chr1	PRAMEF20	18110450	0
CEU	chr1	PRAMEF20	18160450	0
CEU	chr1	PRAMEF20	18210450	0
CEU	chr1	PRAMEF20	18260450	0
CEU	chr1	PRAMEF20	18310450	0
CEU	chr1	PRAMEF20	18360450	16
CEU	chr1	PRAMEF5	8304198	0
CEU	chr1	PRAMEF5	8354198	0
CEU	chr1	PRAMEF5	8404198	0
CEU	chr1	PRAMEF5	8454198	0
CEU	chr1	PRAMEF5	8504198	0
CEU	chr1	PRAMEF5	8554198	0
CEU	chr1	PRAMEF5	8604198	0
CEU	chr1	PRAMEF5	8654198	0
CEU	chr1	PRAMEF5	8704198	0
CEU	chr1	PRAMEF5	8754198	0
CEU	chr1	PRAMEF5	8804198	0
CEU	chr1	PRAMEF5	8854198	0
CEU	chr1	PRAMEF5	8904198	0
CEU	chr1	PRAMEF5	8954198	0
CEU	chr1	PRAMEF5	9004198	0
CEU	chr1	PRAMEF5	9054198	0
CEU	chr1	PRAMEF5	9104198	0
CEU	chr1	PRAMEF5	9154198	0
CEU	chr1	PRAMEF5	9204198	0
CEU	chr1	PRAMEF5	9254198	0
CEU	chr1	PRAMEF5	9304198	0
CEU	chr1	PRAMEF5	9354198	0
CEU	chr1	PRAMEF5	9404198	0
CEU	chr1	PRAMEF5	9454198	0
CEU	chr1	PRAMEF5	9504198	0
CEU	chr1	PRAMEF5	9554198	0
CEU	chr1	PRAMEF5	9604198	0
CEU	chr1	PRAMEF5	9654198	0
CEU	chr1	PRAMEF5	9704198	0
CEU	chr1	PRAMEF5	9754198	0
CEU	chr1	PRAMEF5	9804198	0
CEU	chr1	PRAMEF5	9854198	0
CEU	chr1	PRAMEF5	9904198	0
CEU	chr1	PRAMEF5	9954198	2
CEU	chr1	PRAMEF5	10004198	23

CEU	chr1	PRAMEF5	10054198	43
CEU	chr1	PRAMEF5	10104198	32
CEU	chr1	PRAMEF5	10154198	46
CEU	chr1	PRAMEF5	10204198	83
CEU	chr1	PRAMEF5	10254198	101
CEU	chr1	PRAMEF5	10304198	115
CEU	chr1	PRAMEF5	10354198	106
CEU	chr1	PRAMEF5	10404198	64
CEU	chr1	PRAMEF5	10454198	25
CEU	chr1	PRAMEF5	10504198	6
CEU	chr1	PRAMEF5	10554198	0
CEU	chr1	PRAMEF5	10604198	0
CEU	chr1	PRAMEF5	10654198	0
CEU	chr1	PRAMEF5	10704198	0
CEU	chr1	PRAMEF5	10754198	0
CEU	chr1	PRAMEF5	10804198	0
CEU	chr1	PRAMEF5	10854198	0
CEU	chr1	PRAMEF5	10904198	0
CEU	chr1	PRAMEF5	10954198	0
CEU	chr1	PRAMEF5	11004198	0
CEU	chr1	PRAMEF5	11054198	0
CEU	chr1	PRAMEF5	11104198	0
CEU	chr1	PRAMEF5	11154198	0
CEU	chr1	PRAMEF5	11204198	0
CEU	chr1	PRAMEF5	11254198	0
CEU	chr1	PRAMEF5	11304198	0
CEU	chr1	PRAMEF5	11354198	0
CEU	chr1	PRAMEF5	11404198	0
CEU	chr1	PRAMEF5	11454198	0
CEU	chr1	PRAMEF5	11504198	0
CEU	chr1	PRAMEF5	11554198	0
CEU	chr1	PRAMEF5	11604198	0
CEU	chr1	PRAMEF5	11654198	0
CEU	chr1	PRAMEF5	11704198	0
CEU	chr1	PRAMEF5	11754198	0
CEU	chr1	PRAMEF5	11804198	0
CEU	chr1	PRAMEF5	11854198	0
CEU	chr1	PRAMEF5	11904198	0
CEU	chr1	PRAMEF5	11954198	0
CEU	chr1	PRAMEF5	12004198	0
CEU	chr1	PRAMEF5	12054198	0
CEU	chr1	PRAMEF5	12104198	0
CEU	chr1	PRAMEF5	12154198	24
CEU	chr1	PRAMEF5	12204198	45
CEU	chr1	PRAMEF5	12254198	46
CEU	chr1	PRAMEF5	12304198	43
CEU	chr1	PRAMEF5	12354198	37
CEU	chr1	PRAMEF5	12404198	42
CEU	chr1	PRAMEF5	12454198	36

CEU	chr1	PRAMEF5	12504198	25
CEU	chr1	PRAMEF5	12554198	48
CEU	chr1	PRAMEF5	12604198	36
CEU	chr1	PRAMEF5	12654198	0
CEU	chr1	PRAMEF5	12704198	0
CEU	chr1	PRAMEF5	12754198	8
CEU	chr1	PRAMEF5	12804198	29
CEU	chr1	PRAMEF5	12854198	128
CEU	chr1	PRAMEF5	12904198	122
CEU	chr1	PRAMEF5	12954198	15
CEU	chr1	PRAMEF5	13004198	0
CEU	chr1	PRAMEF5	13054198	0
CEU	chr1	PRAMEF5	13104198	0
CEU	chr1	PRAMEF5	13154198	0
CEU	chr1	PRAMEF5	13204198	0
CEU	chr1	PRAMEF5	13254198	0
CEU	chr1	PRAMEF5	13304198	0
CEU	chr1	PRAMEF5	13354198	0
CEU	chr1	PRAMEF5	13404198	0
CEU	chr1	PRAMEF5	13454198	0
CEU	chr1	PRAMEF5	13504198	0
CEU	chr1	PRAMEF5	13554198	0
CEU	chr1	PRAMEF5	13604198	0
CEU	chr1	PRAMEF5	13654198	0
CEU	chr1	PRAMEF5	13704198	0
CEU	chr1	PRAMEF5	13754198	0
CEU	chr1	PRAMEF5	13804198	0
CEU	chr1	PRAMEF5	13854198	0
CEU	chr1	PRAMEF5	13904198	1
CEU	chr1	PRAMEF5	13954198	22
CEU	chr1	PRAMEF5	14004198	75
CEU	chr1	PRAMEF5	14054198	100
CEU	chr1	PRAMEF5	14104198	92
CEU	chr1	PRAMEF5	14154198	78
CEU	chr1	PRAMEF5	14204198	61
CEU	chr1	PRAMEF5	14254198	41
CEU	chr1	PRAMEF5	14304198	23
CEU	chr1	PRAMEF5	14354198	36
CEU	chr1	PRAMEF5	14404198	41
CEU	chr1	PRAMEF5	14454198	16
CEU	chr1	PRAMEF5	14504198	0
CEU	chr1	PRAMEF5	14554198	0
CEU	chr1	PRAMEF5	14604198	0
CEU	chr1	PRAMEF5	14654198	0
CEU	chr1	PRAMEF5	14704198	0
CEU	chr1	PRAMEF5	14754198	0
CEU	chr1	PRAMEF5	14804198	0
CEU	chr1	PRAMEF5	14854198	0
CEU	chr1	PRAMEF5	14904198	0

CEU	chr1	PRAMEF5	14954198	0
CEU	chr1	PRAMEF5	15004198	0
CEU	chr1	PRAMEF5	15054198	0
CEU	chr1	PRAMEF5	15104198	0
CEU	chr1	PRAMEF5	15154198	0
CEU	chr1	PRAMEF5	15204198	0
CEU	chr1	PRAMEF5	15254198	0
CEU	chr1	PRAMEF5	15304198	0
CEU	chr1	PRAMEF5	15354198	0
CEU	chr1	PRAMEF5	15404198	0
CEU	chr1	PRAMEF5	15454198	0
CEU	chr1	PRAMEF5	15504198	0
CEU	chr1	PRAMEF5	15554198	0
CEU	chr1	PRAMEF5	15604198	0
CEU	chr1	PRAMEF5	15654198	0
CEU	chr1	PRAMEF5	15704198	0
CEU	chr1	PRAMEF5	15754198	0
CEU	chr1	PRAMEF5	15804198	0
CEU	chr1	PRAMEF5	15854198	0
CEU	chr1	PRAMEF5	15904198	0
CEU	chr1	PRAMEF5	15954198	0
CEU	chr1	PRAMEF5	16004198	0
CEU	chr1	PRAMEF5	16054198	0
CEU	chr1	PRAMEF5	16104198	0
CEU	chr1	PRAMEF5	16154198	0
CEU	chr1	PRAMEF5	16204198	0
CEU	chr1	PRAMEF5	16254198	0
CEU	chr1	PRAMEF5	16304198	0
CEU	chr1	PRAMEF5	16354198	0
CEU	chr1	PRAMEF5	16404198	0
CEU	chr1	PRAMEF5	16454198	0
CEU	chr1	PRAMEF5	16504198	11
CEU	chr1	PRAMEF5	16554198	22
CEU	chr1	PRAMEF5	16604198	20
CEU	chr1	PRAMEF5	16654198	23
CEU	chr1	PRAMEF5	16704198	40
CEU	chr1	PRAMEF5	16754198	58
CEU	chr1	PRAMEF5	16804198	63
CEU	chr1	PRAMEF5	16854198	44
CEU	chr1	PRAMEF5	16904198	13
CEU	chr1	PRAMEF5	16954198	0
CEU	chr1	PRAMEF5	17004198	0
CEU	chr1	PRAMEF5	17054198	0
CEU	chr1	PRAMEF5	17104198	0
CEU	chr1	PRAMEF5	17154198	0
CEU	chr1	PRAMEF5	17204198	0
CEU	chr1	PRAMEF5	17254198	0
CEU	chr1	PRAMEF5	17304198	0
CEU	chr1	PRAMEF5	17354198	0

CEU	chr1	PRAMEF5	17404198	0
CEU	chr1	PRAMEF5	17454198	0
CEU	chr1	PRAMEF5	17504198	0
CEU	chr1	PRAMEF5	17554198	0
CEU	chr1	PRAMEF5	17604198	0
CEU	chr1	PRAMEF5	17654198	0
CEU	chr1	PRAMEF5	17704198	0
CEU	chr1	PRAMEF5	17754198	0
CEU	chr1	PRAMEF5	17804198	0
CEU	chr1	PRAMEF5	17854198	0
CEU	chr1	PRAMEF5	17904198	0
CEU	chr1	PRAMEF5	17954198	0
CEU	chr1	PRAMEF5	18004198	0
CEU	chr1	PRAMEF5	18054198	0
CEU	chr1	PRAMEF5	18104198	0
CEU	chr1	PRAMEF5	18154198	0
CEU	chr1	PRAMEF5	18204198	0
CEU	chr1	PRAMEF8	8331223	0
CEU	chr1	PRAMEF8	8381223	0
CEU	chr1	PRAMEF8	8431223	0
CEU	chr1	PRAMEF8	8481223	0
CEU	chr1	PRAMEF8	8531223	0
CEU	chr1	PRAMEF8	8581223	0
CEU	chr1	PRAMEF8	8631223	0
CEU	chr1	PRAMEF8	8681223	0
CEU	chr1	PRAMEF8	8731223	0
CEU	chr1	PRAMEF8	8781223	0
CEU	chr1	PRAMEF8	8831223	0
CEU	chr1	PRAMEF8	8881223	0
CEU	chr1	PRAMEF8	8931223	0
CEU	chr1	PRAMEF8	8981223	0
CEU	chr1	PRAMEF8	9031223	0
CEU	chr1	PRAMEF8	9081223	0
CEU	chr1	PRAMEF8	9131223	0
CEU	chr1	PRAMEF8	9181223	0
CEU	chr1	PRAMEF8	9231223	0
CEU	chr1	PRAMEF8	9281223	0
CEU	chr1	PRAMEF8	9331223	0
CEU	chr1	PRAMEF8	9381223	0
CEU	chr1	PRAMEF8	9431223	0
CEU	chr1	PRAMEF8	9481223	0
CEU	chr1	PRAMEF8	9531223	0
CEU	chr1	PRAMEF8	9581223	0
CEU	chr1	PRAMEF8	9631223	0
CEU	chr1	PRAMEF8	9681223	0
CEU	chr1	PRAMEF8	9731223	0
CEU	chr1	PRAMEF8	9781223	0
CEU	chr1	PRAMEF8	9831223	0
CEU	chr1	PRAMEF8	9881223	0

CEU	chr1	PRAMEF8	9931223	0
CEU	chr1	PRAMEF8	9981223	14
CEU	chr1	PRAMEF8	10031223	35
CEU	chr1	PRAMEF8	10081223	39
CEU	chr1	PRAMEF8	10131223	28
CEU	chr1	PRAMEF8	10181223	75
CEU	chr1	PRAMEF8	10231223	108
CEU	chr1	PRAMEF8	10281223	103
CEU	chr1	PRAMEF8	10331223	111
CEU	chr1	PRAMEF8	10381223	78
CEU	chr1	PRAMEF8	10431223	40
CEU	chr1	PRAMEF8	10481223	14
CEU	chr1	PRAMEF8	10531223	1
CEU	chr1	PRAMEF8	10581223	0
CEU	chr1	PRAMEF8	10631223	0
CEU	chr1	PRAMEF8	10681223	0
CEU	chr1	PRAMEF8	10731223	0
CEU	chr1	PRAMEF8	10781223	0
CEU	chr1	PRAMEF8	10831223	0
CEU	chr1	PRAMEF8	10881223	0
CEU	chr1	PRAMEF8	10931223	0
CEU	chr1	PRAMEF8	10981223	0
CEU	chr1	PRAMEF8	11031223	0
CEU	chr1	PRAMEF8	11081223	0
CEU	chr1	PRAMEF8	11131223	0
CEU	chr1	PRAMEF8	11181223	0
CEU	chr1	PRAMEF8	11231223	0
CEU	chr1	PRAMEF8	11281223	0
CEU	chr1	PRAMEF8	11331223	0
CEU	chr1	PRAMEF8	11381223	0
CEU	chr1	PRAMEF8	11431223	0
CEU	chr1	PRAMEF8	11481223	0
CEU	chr1	PRAMEF8	11531223	0
CEU	chr1	PRAMEF8	11581223	0
CEU	chr1	PRAMEF8	11631223	0
CEU	chr1	PRAMEF8	11681223	0
CEU	chr1	PRAMEF8	11731223	0
CEU	chr1	PRAMEF8	11781223	0
CEU	chr1	PRAMEF8	11831223	0
CEU	chr1	PRAMEF8	11881223	0
CEU	chr1	PRAMEF8	11931223	0
CEU	chr1	PRAMEF8	11981223	0
CEU	chr1	PRAMEF8	12031223	0
CEU	chr1	PRAMEF8	12081223	0
CEU	chr1	PRAMEF8	12131223	23
CEU	chr1	PRAMEF8	12181223	35
CEU	chr1	PRAMEF8	12231223	31
CEU	chr1	PRAMEF8	12281223	43
CEU	chr1	PRAMEF8	12331223	44

CEU	chr1	PRAMEF8	12381223	43
CEU	chr1	PRAMEF8	12431223	39
CEU	chr1	PRAMEF8	12481223	28
CEU	chr1	PRAMEF8	12531223	44
CEU	chr1	PRAMEF8	12581223	42
CEU	chr1	PRAMEF8	12631223	10
CEU	chr1	PRAMEF8	12681223	0
CEU	chr1	PRAMEF8	12731223	1
CEU	chr1	PRAMEF8	12781223	19
CEU	chr1	PRAMEF8	12831223	126
CEU	chr1	PRAMEF8	12881223	132
CEU	chr1	PRAMEF8	12931223	24
CEU	chr1	PRAMEF8	12981223	0
CEU	chr1	PRAMEF8	13031223	0
CEU	chr1	PRAMEF8	13081223	0
CEU	chr1	PRAMEF8	13131223	0
CEU	chr1	PRAMEF8	13181223	0
CEU	chr1	PRAMEF8	13231223	0
CEU	chr1	PRAMEF8	13281223	0
CEU	chr1	PRAMEF8	13331223	0
CEU	chr1	PRAMEF8	13381223	0
CEU	chr1	PRAMEF8	13431223	0
CEU	chr1	PRAMEF8	13481223	0
CEU	chr1	PRAMEF8	13531223	0
CEU	chr1	PRAMEF8	13581223	0
CEU	chr1	PRAMEF8	13631223	0
CEU	chr1	PRAMEF8	13681223	0
CEU	chr1	PRAMEF8	13731223	0
CEU	chr1	PRAMEF8	13781223	0
CEU	chr1	PRAMEF8	13831223	0
CEU	chr1	PRAMEF8	13881223	0
CEU	chr1	PRAMEF8	13931223	8
CEU	chr1	PRAMEF8	13981223	46
CEU	chr1	PRAMEF8	14031223	92
CEU	chr1	PRAMEF8	14081223	100
CEU	chr1	PRAMEF8	14131223	82
CEU	chr1	PRAMEF8	14181223	75
CEU	chr1	PRAMEF8	14231223	56
CEU	chr1	PRAMEF8	14281223	27
CEU	chr1	PRAMEF8	14331223	30
CEU	chr1	PRAMEF8	14381223	41
CEU	chr1	PRAMEF8	14431223	25
CEU	chr1	PRAMEF8	14481223	4
CEU	chr1	PRAMEF8	14531223	0
CEU	chr1	PRAMEF8	14581223	0
CEU	chr1	PRAMEF8	14631223	0
CEU	chr1	PRAMEF8	14681223	0
CEU	chr1	PRAMEF8	14731223	0
CEU	chr1	PRAMEF8	14781223	0

CEU	chr1	PRAMEF8	14831223	0
CEU	chr1	PRAMEF8	14881223	0
CEU	chr1	PRAMEF8	14931223	0
CEU	chr1	PRAMEF8	14981223	0
CEU	chr1	PRAMEF8	15031223	0
CEU	chr1	PRAMEF8	15081223	0
CEU	chr1	PRAMEF8	15131223	0
CEU	chr1	PRAMEF8	15181223	0
CEU	chr1	PRAMEF8	15231223	0
CEU	chr1	PRAMEF8	15281223	0
CEU	chr1	PRAMEF8	15331223	0
CEU	chr1	PRAMEF8	15381223	0
CEU	chr1	PRAMEF8	15431223	0
CEU	chr1	PRAMEF8	15481223	0
CEU	chr1	PRAMEF8	15531223	0
CEU	chr1	PRAMEF8	15581223	0
CEU	chr1	PRAMEF8	15631223	0
CEU	chr1	PRAMEF8	15681223	0
CEU	chr1	PRAMEF8	15731223	0
CEU	chr1	PRAMEF8	15781223	0
CEU	chr1	PRAMEF8	15831223	0
CEU	chr1	PRAMEF8	15881223	0
CEU	chr1	PRAMEF8	15931223	0
CEU	chr1	PRAMEF8	15981223	0
CEU	chr1	PRAMEF8	16031223	0
CEU	chr1	PRAMEF8	16081223	0
CEU	chr1	PRAMEF8	16131223	0
CEU	chr1	PRAMEF8	16181223	0
CEU	chr1	PRAMEF8	16231223	0
CEU	chr1	PRAMEF8	16281223	0
CEU	chr1	PRAMEF8	16331223	0
CEU	chr1	PRAMEF8	16381223	0
CEU	chr1	PRAMEF8	16431223	0
CEU	chr1	PRAMEF8	16481223	2
CEU	chr1	PRAMEF8	16531223	16
CEU	chr1	PRAMEF8	16581223	24
CEU	chr1	PRAMEF8	16631223	20
CEU	chr1	PRAMEF8	16681223	37
CEU	chr1	PRAMEF8	16731223	48
CEU	chr1	PRAMEF8	16781223	59
CEU	chr1	PRAMEF8	16831223	63
CEU	chr1	PRAMEF8	16881223	25
CEU	chr1	PRAMEF8	16931223	0
CEU	chr1	PRAMEF8	16981223	0
CEU	chr1	PRAMEF8	17031223	0
CEU	chr1	PRAMEF8	17081223	0
CEU	chr1	PRAMEF8	17131223	0
CEU	chr1	PRAMEF8	17181223	0
CEU	chr1	PRAMEF8	17231223	0

CEU	chr1	PRAMEF8	17281223	0
CEU	chr1	PRAMEF8	17331223	0
CEU	chr1	PRAMEF8	17381223	0
CEU	chr1	PRAMEF8	17431223	0
CEU	chr1	PRAMEF8	17481223	0
CEU	chr1	PRAMEF8	17531223	0
CEU	chr1	PRAMEF8	17581223	0
CEU	chr1	PRAMEF8	17631223	0
CEU	chr1	PRAMEF8	17681223	0
CEU	chr1	PRAMEF8	17731223	0
CEU	chr1	PRAMEF8	17781223	0
CEU	chr1	PRAMEF8	17831223	0
CEU	chr1	PRAMEF8	17881223	0
CEU	chr1	PRAMEF8	17931223	0
CEU	chr1	PRAMEF8	17981223	0
CEU	chr1	PRAMEF8	18031223	0
CEU	chr1	PRAMEF8	18081223	0
CEU	chr1	PRAMEF8	18131223	0
CEU	chr1	PRAMEF8	18181223	0
CEU	chr1	PRAMEF8	18231223	0
CEU	chr17	PRR11	54205355	0
CEU	chr17	PRR11	54255355	0
CEU	chr17	PRR11	54305355	0
CEU	chr17	PRR11	54355355	0
CEU	chr17	PRR11	54405355	0
CEU	chr17	PRR11	54455355	0
CEU	chr17	PRR11	54505355	0
CEU	chr17	PRR11	54555355	0
CEU	chr17	PRR11	54605355	0
CEU	chr17	PRR11	54655355	0
CEU	chr17	PRR11	54705355	0
CEU	chr17	PRR11	54755355	0
CEU	chr17	PRR11	54805355	0
CEU	chr17	PRR11	54855355	0
CEU	chr17	PRR11	54905355	0
CEU	chr17	PRR11	54955355	0
CEU	chr17	PRR11	55005355	0
CEU	chr17	PRR11	55055355	0
CEU	chr17	PRR11	55105355	0
CEU	chr17	PRR11	55155355	0
CEU	chr17	PRR11	55205355	0
CEU	chr17	PRR11	55255355	0
CEU	chr17	PRR11	55305355	0
CEU	chr17	PRR11	55355355	0
CEU	chr17	PRR11	55405355	0
CEU	chr17	PRR11	55455355	0
CEU	chr17	PRR11	55505355	0
CEU	chr17	PRR11	55555355	0
CEU	chr17	PRR11	55605355	0

CEU	chr17	PRR11	55655355	0
CEU	chr17	PRR11	55705355	0
CEU	chr17	PRR11	55755355	0
CEU	chr17	PRR11	55805355	0
CEU	chr17	PRR11	55855355	0
CEU	chr17	PRR11	55905355	0
CEU	chr17	PRR11	55955355	0
CEU	chr17	PRR11	56005355	0
CEU	chr17	PRR11	56055355	0
CEU	chr17	PRR11	56105355	0
CEU	chr17	PRR11	56155355	0
CEU	chr17	PRR11	56205355	0
CEU	chr17	PRR11	56255355	0
CEU	chr17	PRR11	56305355	0
CEU	chr17	PRR11	56355355	0
CEU	chr17	PRR11	56405355	0
CEU	chr17	PRR11	56455355	0
CEU	chr17	PRR11	56505355	0
CEU	chr17	PRR11	56555355	0
CEU	chr17	PRR11	56605355	0
CEU	chr17	PRR11	56655355	0
CEU	chr17	PRR11	56705355	0
CEU	chr17	PRR11	56755355	0
CEU	chr17	PRR11	56805355	0
CEU	chr17	PRR11	56855355	0
CEU	chr17	PRR11	56905355	0
CEU	chr17	PRR11	56955355	0
CEU	chr17	PRR11	57005355	0
CEU	chr17	PRR11	57055355	0
CEU	chr17	PRR11	57105355	0
CEU	chr17	PRR11	57155355	0
CEU	chr17	PRR11	57205355	0
CEU	chr17	PRR11	57255355	0
CEU	chr17	PRR11	57305355	0
CEU	chr17	PRR11	57355355	0
CEU	chr17	PRR11	57405355	0
CEU	chr17	PRR11	57455355	0
CEU	chr17	PRR11	57505355	0
CEU	chr17	PRR11	57555355	0
CEU	chr17	PRR11	57605355	0
CEU	chr17	PRR11	57655355	0
CEU	chr17	PRR11	57705355	0
CEU	chr17	PRR11	57755355	0
CEU	chr17	PRR11	57805355	0
CEU	chr17	PRR11	57855355	0
CEU	chr17	PRR11	57905355	0
CEU	chr17	PRR11	57955355	0
CEU	chr17	PRR11	58005355	0
CEU	chr17	PRR11	58055355	0

CEU	chr17	PRR11	58105355	0
CEU	chr17	PRR11	58155355	0
CEU	chr17	PRR11	58205355	0
CEU	chr17	PRR11	58255355	0
CEU	chr17	PRR11	58305355	0
CEU	chr17	PRR11	58355355	0
CEU	chr17	PRR11	58405355	0
CEU	chr17	PRR11	58455355	0
CEU	chr17	PRR11	58505355	0
CEU	chr17	PRR11	58555355	0
CEU	chr17	PRR11	58605355	0
CEU	chr17	PRR11	58655355	0
CEU	chr17	PRR11	58705355	0
CEU	chr17	PRR11	58755355	0
CEU	chr17	PRR11	58805355	0
CEU	chr17	PRR11	58855355	0
CEU	chr17	PRR11	58905355	0
CEU	chr17	PRR11	58955355	0
CEU	chr17	PRR11	59005355	0
CEU	chr17	PRR11	59055355	0
CEU	chr17	PRR11	59105355	0
CEU	chr17	PRR11	59155355	0
CEU	chr17	PRR11	59205355	0
CEU	chr17	PRR11	59255355	0
CEU	chr17	PRR11	59305355	0
CEU	chr17	PRR11	59355355	0
CEU	chr17	PRR11	59405355	0
CEU	chr17	PRR11	59455355	0
CEU	chr17	PRR11	59505355	0
CEU	chr17	PRR11	59555355	0
CEU	chr17	PRR11	59605355	0
CEU	chr17	PRR11	59655355	0
CEU	chr17	PRR11	59705355	0
CEU	chr17	PRR11	59755355	0
CEU	chr17	PRR11	59805355	0
CEU	chr17	PRR11	59855355	0
CEU	chr17	PRR11	59905355	0
CEU	chr17	PRR11	59955355	0
CEU	chr17	PRR11	60005355	0
CEU	chr17	PRR11	60055355	0
CEU	chr17	PRR11	60105355	0
CEU	chr17	PRR11	60155355	0
CEU	chr17	PRR11	60205355	0
CEU	chr17	PRR11	60255355	0
CEU	chr17	PRR11	60305355	0
CEU	chr17	PRR11	60355355	0
CEU	chr17	PRR11	60405355	0
CEU	chr17	PRR11	60455355	0
CEU	chr17	PRR11	60505355	0

CEU	chr17	PRR11	60555355	0
CEU	chr17	PRR11	60605355	0
CEU	chr17	PRR11	60655355	0
CEU	chr17	PRR11	60705355	0
CEU	chr17	PRR11	60755355	0
CEU	chr17	PRR11	60805355	0
CEU	chr17	PRR11	60855355	0
CEU	chr17	PRR11	60905355	0
CEU	chr17	PRR11	60955355	0
CEU	chr17	PRR11	61005355	0
CEU	chr17	PRR11	61055355	0
CEU	chr17	PRR11	61105355	0
CEU	chr17	PRR11	61155355	0
CEU	chr17	PRR11	61205355	0
CEU	chr17	PRR11	61255355	0
CEU	chr17	PRR11	61305355	0
CEU	chr17	PRR11	61355355	0
CEU	chr17	PRR11	61405355	0
CEU	chr17	PRR11	61455355	0
CEU	chr17	PRR11	61505355	0
CEU	chr17	PRR11	61555355	0
CEU	chr17	PRR11	61605355	0
CEU	chr17	PRR11	61655355	0
CEU	chr17	PRR11	61705355	0
CEU	chr17	PRR11	61755355	0
CEU	chr17	PRR11	61805355	0
CEU	chr17	PRR11	61855355	0
CEU	chr17	PRR11	61905355	0
CEU	chr17	PRR11	61955355	0
CEU	chr17	PRR11	62005355	0
CEU	chr17	PRR11	62055355	0
CEU	chr17	PRR11	62105355	0
CEU	chr17	PRR11	62155355	0
CEU	chr17	PRR11	62205355	0
CEU	chr17	PRR11	62255355	0
CEU	chr17	PRR11	62305355	0
CEU	chr17	PRR11	62355355	0
CEU	chr17	PRR11	62405355	0
CEU	chr17	PRR11	62455355	0
CEU	chr17	PRR11	62505355	0
CEU	chr17	PRR11	62555355	0
CEU	chr17	PRR11	62605355	0
CEU	chr17	PRR11	62655355	0
CEU	chr17	PRR11	62705355	0
CEU	chr17	PRR11	62755355	0
CEU	chr17	PRR11	62805355	0
CEU	chr17	PRR11	62855355	0
CEU	chr17	PRR11	62905355	0
CEU	chr17	PRR11	62955355	0

CEU	chr17	PRR11	63005355	0
CEU	chr17	PRR11	63055355	0
CEU	chr17	PRR11	63105355	0
CEU	chr17	PRR11	63155355	0
CEU	chr17	PRR11	63205355	0
CEU	chr17	PRR11	63255355	0
CEU	chr17	PRR11	63305355	0
CEU	chr17	PRR11	63355355	0
CEU	chr17	PRR11	63405355	0
CEU	chr17	PRR11	63455355	0
CEU	chr17	PRR11	63505355	0
CEU	chr17	PRR11	63555355	0
CEU	chr17	PRR11	63605355	0
CEU	chr17	PRR11	63655355	0
CEU	chr17	PRR11	63705355	0
CEU	chr17	PRR11	63755355	0
CEU	chr17	PRR11	63805355	0
CEU	chr17	PRR11	63855355	0
CEU	chr17	PRR11	63905355	0
CEU	chr17	PRR11	63955355	0
CEU	chr17	PRR11	64005355	0
CEU	chr17	PRR11	64055355	0
CEU	chr17	PRR11	64105355	0
CEU	chr17	PRR11	64155355	0
CEU	chr13	PRR20A	52190918	0
CEU	chr13	PRR20A	52240918	0
CEU	chr13	PRR20A	52290918	0
CEU	chr13	PRR20A	52340918	0
CEU	chr13	PRR20A	52390918	0
CEU	chr13	PRR20A	52440918	0
CEU	chr13	PRR20A	52490918	0
CEU	chr13	PRR20A	52540918	0
CEU	chr13	PRR20A	52590918	0
CEU	chr13	PRR20A	52640918	0
CEU	chr13	PRR20A	52690918	0
CEU	chr13	PRR20A	52740918	0
CEU	chr13	PRR20A	52790918	0
CEU	chr13	PRR20A	52840918	0
CEU	chr13	PRR20A	52890918	0
CEU	chr13	PRR20A	52940918	0
CEU	chr13	PRR20A	52990918	0
CEU	chr13	PRR20A	53040918	0
CEU	chr13	PRR20A	53090918	0
CEU	chr13	PRR20A	53140918	0
CEU	chr13	PRR20A	53190918	0
CEU	chr13	PRR20A	53240918	0
CEU	chr13	PRR20A	53290918	0
CEU	chr13	PRR20A	53340918	0
CEU	chr13	PRR20A	53390918	0

CEU	chr13	PRR20A	53440918	0
CEU	chr13	PRR20A	53490918	0
CEU	chr13	PRR20A	53540918	0
CEU	chr13	PRR20A	53590918	0
CEU	chr13	PRR20A	53640918	0
CEU	chr13	PRR20A	53690918	0
CEU	chr13	PRR20A	53740918	0
CEU	chr13	PRR20A	53790918	0
CEU	chr13	PRR20A	53840918	0
CEU	chr13	PRR20A	53890918	0
CEU	chr13	PRR20A	53940918	0
CEU	chr13	PRR20A	53990918	0
CEU	chr13	PRR20A	54040918	0
CEU	chr13	PRR20A	54090918	0
CEU	chr13	PRR20A	54140918	0
CEU	chr13	PRR20A	54190918	0
CEU	chr13	PRR20A	54240918	0
CEU	chr13	PRR20A	54290918	0
CEU	chr13	PRR20A	54340918	0
CEU	chr13	PRR20A	54390918	0
CEU	chr13	PRR20A	54440918	0
CEU	chr13	PRR20A	54490918	0
CEU	chr13	PRR20A	54540918	0
CEU	chr13	PRR20A	54590918	0
CEU	chr13	PRR20A	54640918	0
CEU	chr13	PRR20A	54690918	0
CEU	chr13	PRR20A	54740918	0
CEU	chr13	PRR20A	54790918	0
CEU	chr13	PRR20A	54840918	0
CEU	chr13	PRR20A	54890918	0
CEU	chr13	PRR20A	54940918	0
CEU	chr13	PRR20A	54990918	0
CEU	chr13	PRR20A	55040918	0
CEU	chr13	PRR20A	55090918	0
CEU	chr13	PRR20A	55140918	0
CEU	chr13	PRR20A	55190918	0
CEU	chr13	PRR20A	55240918	0
CEU	chr13	PRR20A	55290918	0
CEU	chr13	PRR20A	55340918	0
CEU	chr13	PRR20A	55390918	0
CEU	chr13	PRR20A	55440918	0
CEU	chr13	PRR20A	55490918	0
CEU	chr13	PRR20A	55540918	0
CEU	chr13	PRR20A	55590918	0
CEU	chr13	PRR20A	55640918	0
CEU	chr13	PRR20A	55690918	0
CEU	chr13	PRR20A	55740918	0
CEU	chr13	PRR20A	55790918	0
CEU	chr13	PRR20A	55840918	0

CEU	chr13	PRR20A	55890918	0
CEU	chr13	PRR20A	55940918	0
CEU	chr13	PRR20A	55990918	0
CEU	chr13	PRR20A	56040918	0
CEU	chr13	PRR20A	56090918	0
CEU	chr13	PRR20A	56140918	0
CEU	chr13	PRR20A	56190918	0
CEU	chr13	PRR20A	56240918	0
CEU	chr13	PRR20A	56290918	0
CEU	chr13	PRR20A	56340918	0
CEU	chr13	PRR20A	56390918	0
CEU	chr13	PRR20A	56440918	0
CEU	chr13	PRR20A	56490918	0
CEU	chr13	PRR20A	56540918	0
CEU	chr13	PRR20A	56590918	0
CEU	chr13	PRR20A	56640918	0
CEU	chr13	PRR20A	56690918	0
CEU	chr13	PRR20A	56740918	0
CEU	chr13	PRR20A	56790918	0
CEU	chr13	PRR20A	56840918	0
CEU	chr13	PRR20A	56890918	0
CEU	chr13	PRR20A	56940918	0
CEU	chr13	PRR20A	56990918	0
CEU	chr13	PRR20A	57040918	0
CEU	chr13	PRR20A	57090918	0
CEU	chr13	PRR20A	57140918	0
CEU	chr13	PRR20A	57190918	0
CEU	chr13	PRR20A	57240918	0
CEU	chr13	PRR20A	57290918	0
CEU	chr13	PRR20A	57340918	0
CEU	chr13	PRR20A	57390918	0
CEU	chr13	PRR20A	57440918	0
CEU	chr13	PRR20A	57490918	0
CEU	chr13	PRR20A	57540918	0
CEU	chr13	PRR20A	57590918	0
CEU	chr13	PRR20A	57640918	0
CEU	chr13	PRR20A	57690918	0
CEU	chr13	PRR20A	57740918	0
CEU	chr13	PRR20A	57790918	0
CEU	chr13	PRR20A	57840918	0
CEU	chr13	PRR20A	57890918	0
CEU	chr13	PRR20A	57940918	0
CEU	chr13	PRR20A	57990918	0
CEU	chr13	PRR20A	58040918	0
CEU	chr13	PRR20A	58090918	0
CEU	chr13	PRR20A	58140918	0
CEU	chr13	PRR20A	58190918	0
CEU	chr13	PRR20A	58240918	0
CEU	chr13	PRR20A	58290918	0

CEU	chr13	PRR20A	58340918	0
CEU	chr13	PRR20A	58390918	0
CEU	chr13	PRR20A	58440918	0
CEU	chr13	PRR20A	58490918	0
CEU	chr13	PRR20A	58540918	0
CEU	chr13	PRR20A	58590918	0
CEU	chr13	PRR20A	58640918	0
CEU	chr13	PRR20A	58690918	0
CEU	chr13	PRR20A	58740918	0
CEU	chr13	PRR20A	58790918	0
CEU	chr13	PRR20A	58840918	0
CEU	chr13	PRR20A	58890918	0
CEU	chr13	PRR20A	58940918	0
CEU	chr13	PRR20A	58990918	0
CEU	chr13	PRR20A	59040918	0
CEU	chr13	PRR20A	59090918	0
CEU	chr13	PRR20A	59140918	0
CEU	chr13	PRR20A	59190918	0
CEU	chr13	PRR20A	59240918	0
CEU	chr13	PRR20A	59290918	0
CEU	chr13	PRR20A	59340918	0
CEU	chr13	PRR20A	59390918	0
CEU	chr13	PRR20A	59440918	0
CEU	chr13	PRR20A	59490918	0
CEU	chr13	PRR20A	59540918	0
CEU	chr13	PRR20A	59590918	0
CEU	chr13	PRR20A	59640918	0
CEU	chr13	PRR20A	59690918	0
CEU	chr13	PRR20A	59740918	0
CEU	chr13	PRR20A	59790918	0
CEU	chr13	PRR20A	59840918	0
CEU	chr13	PRR20A	59890918	0
CEU	chr13	PRR20A	59940918	0
CEU	chr13	PRR20A	59990918	0
CEU	chr13	PRR20A	60040918	0
CEU	chr13	PRR20A	60090918	0
CEU	chr13	PRR20A	60140918	0
CEU	chr13	PRR20A	60190918	0
CEU	chr13	PRR20A	60240918	0
CEU	chr13	PRR20A	60290918	0
CEU	chr13	PRR20A	60340918	0
CEU	chr13	PRR20A	60390918	0
CEU	chr13	PRR20A	60440918	0
CEU	chr13	PRR20A	60490918	0
CEU	chr13	PRR20A	60540918	0
CEU	chr13	PRR20A	60590918	0
CEU	chr13	PRR20A	60640918	0
CEU	chr13	PRR20A	60690918	0
CEU	chr13	PRR20A	60740918	0

CEU	chr13	PRR20A	60790918	0
CEU	chr13	PRR20A	60840918	0
CEU	chr13	PRR20A	60890918	0
CEU	chr13	PRR20A	60940918	0
CEU	chr13	PRR20A	60990918	0
CEU	chr13	PRR20A	61040918	0
CEU	chr13	PRR20A	61090918	0
CEU	chr13	PRR20A	61140918	0
CEU	chr13	PRR20A	61190918	0
CEU	chr13	PRR20A	61240918	0
CEU	chr13	PRR20A	61290918	0
CEU	chr13	PRR20A	61340918	0
CEU	chr13	PRR20A	61390918	0
CEU	chr13	PRR20A	61440918	0
CEU	chr13	PRR20A	61490918	0
CEU	chr13	PRR20A	61540918	0
CEU	chr13	PRR20A	61590918	0
CEU	chr13	PRR20A	61640918	0
CEU	chr13	PRR20A	61690918	0
CEU	chr13	PRR20A	61740918	0
CEU	chr13	PRR20A	61790918	0
CEU	chr13	PRR20A	61840918	0
CEU	chr13	PRR20A	61890918	0
CEU	chr13	PRR20A	61940918	0
CEU	chr13	PRR20A	61990918	0
CEU	chr13	PRR20A	62040918	0
CEU	chr13	PRR20A	62090918	0
CEU	chr19	PSG3	37771642	0
CEU	chr19	PSG3	37821642	0
CEU	chr19	PSG3	37871642	0
CEU	chr19	PSG3	37921642	0
CEU	chr19	PSG3	37971642	0
CEU	chr19	PSG3	38021642	0
CEU	chr19	PSG3	38071642	0
CEU	chr19	PSG3	38121642	0
CEU	chr19	PSG3	38171642	0
CEU	chr19	PSG3	38221642	0
CEU	chr19	PSG3	38271642	0
CEU	chr19	PSG3	38321642	0
CEU	chr19	PSG3	38371642	0
CEU	chr19	PSG3	38421642	0
CEU	chr19	PSG3	38471642	0
CEU	chr19	PSG3	38521642	0
CEU	chr19	PSG3	38571642	0
CEU	chr19	PSG3	38621642	0
CEU	chr19	PSG3	38671642	0
CEU	chr19	PSG3	38721642	0
CEU	chr19	PSG3	38771642	0
CEU	chr19	PSG3	38821642	0

CEU	chr19	PSG3	38871642	0
CEU	chr19	PSG3	38921642	0
CEU	chr19	PSG3	38971642	0
CEU	chr19	PSG3	39021642	0
CEU	chr19	PSG3	39071642	0
CEU	chr19	PSG3	39121642	0
CEU	chr19	PSG3	39171642	0
CEU	chr19	PSG3	39221642	0
CEU	chr19	PSG3	39271642	0
CEU	chr19	PSG3	39321642	0
CEU	chr19	PSG3	39371642	0
CEU	chr19	PSG3	39421642	0
CEU	chr19	PSG3	39471642	0
CEU	chr19	PSG3	39521642	0
CEU	chr19	PSG3	39571642	0
CEU	chr19	PSG3	39621642	2
CEU	chr19	PSG3	39671642	33
CEU	chr19	PSG3	39721642	49
CEU	chr19	PSG3	39771642	36
CEU	chr19	PSG3	39821642	48
CEU	chr19	PSG3	39871642	33
CEU	chr19	PSG3	39921642	3
CEU	chr19	PSG3	39971642	0
CEU	chr19	PSG3	40021642	0
CEU	chr19	PSG3	40071642	0
CEU	chr19	PSG3	40121642	0
CEU	chr19	PSG3	40171642	0
CEU	chr19	PSG3	40221642	0
CEU	chr19	PSG3	40271642	0
CEU	chr19	PSG3	40321642	0
CEU	chr19	PSG3	40371642	0
CEU	chr19	PSG3	40421642	0
CEU	chr19	PSG3	40471642	0
CEU	chr19	PSG3	40521642	0
CEU	chr19	PSG3	40571642	0
CEU	chr19	PSG3	40621642	0
CEU	chr19	PSG3	40671642	0
CEU	chr19	PSG3	40721642	0
CEU	chr19	PSG3	40771642	0
CEU	chr19	PSG3	40821642	0
CEU	chr19	PSG3	40871642	0
CEU	chr19	PSG3	40921642	0
CEU	chr19	PSG3	40971642	0
CEU	chr19	PSG3	41021642	0
CEU	chr19	PSG3	41071642	10
CEU	chr19	PSG3	41121642	20
CEU	chr19	PSG3	41171642	31
CEU	chr19	PSG3	41221642	45
CEU	chr19	PSG3	41271642	50

CEU	chr19	PSG3	41321642	44
CEU	chr19	PSG3	41371642	18
CEU	chr19	PSG3	41421642	0
CEU	chr19	PSG3	41471642	0
CEU	chr19	PSG3	41521642	0
CEU	chr19	PSG3	41571642	0
CEU	chr19	PSG3	41621642	0
CEU	chr19	PSG3	41671642	0
CEU	chr19	PSG3	41721642	0
CEU	chr19	PSG3	41771642	0
CEU	chr19	PSG3	41821642	0
CEU	chr19	PSG3	41871642	0
CEU	chr19	PSG3	41921642	0
CEU	chr19	PSG3	41971642	0
CEU	chr19	PSG3	42021642	0
CEU	chr19	PSG3	42071642	0
CEU	chr19	PSG3	42121642	0
CEU	chr19	PSG3	42171642	0
CEU	chr19	PSG3	42221642	0
CEU	chr19	PSG3	42271642	0
CEU	chr19	PSG3	42321642	0
CEU	chr19	PSG3	42371642	0
CEU	chr19	PSG3	42421642	0
CEU	chr19	PSG3	42471642	0
CEU	chr19	PSG3	42521642	0
CEU	chr19	PSG3	42571642	0
CEU	chr19	PSG3	42621642	0
CEU	chr19	PSG3	42671642	0
CEU	chr19	PSG3	42721642	0
CEU	chr19	PSG3	42771642	0
CEU	chr19	PSG3	42821642	0
CEU	chr19	PSG3	42871642	0
CEU	chr19	PSG3	42921642	0
CEU	chr19	PSG3	42971642	0
CEU	chr19	PSG3	43021642	0
CEU	chr19	PSG3	43071642	0
CEU	chr19	PSG3	43121642	0
CEU	chr19	PSG3	43171642	0
CEU	chr19	PSG3	43221642	0
CEU	chr19	PSG3	43271642	0
CEU	chr19	PSG3	43321642	0
CEU	chr19	PSG3	43371642	0
CEU	chr19	PSG3	43421642	0
CEU	chr19	PSG3	43471642	0
CEU	chr19	PSG3	43521642	0
CEU	chr19	PSG3	43571642	0
CEU	chr19	PSG3	43621642	0
CEU	chr19	PSG3	43671642	0
CEU	chr19	PSG3	43721642	0

CEU	chr19	PSG3	43771642	0
CEU	chr19	PSG3	43821642	5
CEU	chr19	PSG3	43871642	29
CEU	chr19	PSG3	43921642	39
CEU	chr19	PSG3	43971642	15
CEU	chr19	PSG3	44021642	0
CEU	chr19	PSG3	44071642	0
CEU	chr19	PSG3	44121642	0
CEU	chr19	PSG3	44171642	0
CEU	chr19	PSG3	44221642	0
CEU	chr19	PSG3	44271642	0
CEU	chr19	PSG3	44321642	0
CEU	chr19	PSG3	44371642	0
CEU	chr19	PSG3	44421642	0
CEU	chr19	PSG3	44471642	0
CEU	chr19	PSG3	44521642	0
CEU	chr19	PSG3	44571642	0
CEU	chr19	PSG3	44621642	0
CEU	chr19	PSG3	44671642	0
CEU	chr19	PSG3	44721642	0
CEU	chr19	PSG3	44771642	0
CEU	chr19	PSG3	44821642	0
CEU	chr19	PSG3	44871642	0
CEU	chr19	PSG3	44921642	0
CEU	chr19	PSG3	44971642	0
CEU	chr19	PSG3	45021642	0
CEU	chr19	PSG3	45071642	0
CEU	chr19	PSG3	45121642	0
CEU	chr19	PSG3	45171642	2
CEU	chr19	PSG3	45221642	21
CEU	chr19	PSG3	45271642	37
CEU	chr19	PSG3	45321642	24
CEU	chr19	PSG3	45371642	6
CEU	chr19	PSG3	45421642	0
CEU	chr19	PSG3	45471642	0
CEU	chr19	PSG3	45521642	0
CEU	chr19	PSG3	45571642	0
CEU	chr19	PSG3	45621642	0
CEU	chr19	PSG3	45671642	10
CEU	chr19	PSG3	45721642	31
CEU	chr19	PSG3	45771642	33
CEU	chr19	PSG3	45821642	20
CEU	chr19	PSG3	45871642	8
CEU	chr19	PSG3	45921642	0
CEU	chr19	PSG3	45971642	0
CEU	chr19	PSG3	46021642	0
CEU	chr19	PSG3	46071642	0
CEU	chr19	PSG3	46121642	0
CEU	chr19	PSG3	46171642	0

CEU	chr19	PSG3	46221642	0
CEU	chr19	PSG3	46271642	0
CEU	chr19	PSG3	46321642	0
CEU	chr19	PSG3	46371642	0
CEU	chr19	PSG3	46421642	0
CEU	chr19	PSG3	46471642	0
CEU	chr19	PSG3	46521642	0
CEU	chr19	PSG3	46571642	145
CEU	chr19	PSG3	46621642	151
CEU	chr19	PSG3	46671642	13
CEU	chr19	PSG3	46721642	27
CEU	chr19	PSG3	46771642	39
CEU	chr19	PSG3	46821642	39
CEU	chr19	PSG3	46871642	33
CEU	chr19	PSG3	46921642	53
CEU	chr19	PSG3	46971642	68
CEU	chr19	PSG3	47021642	34
CEU	chr19	PSG3	47071642	6
CEU	chr19	PSG3	47121642	0
CEU	chr19	PSG3	47171642	0
CEU	chr19	PSG3	47221642	0
CEU	chr19	PSG3	47271642	0
CEU	chr19	PSG3	47321642	0
CEU	chr19	PSG3	47371642	0
CEU	chr19	PSG3	47421642	0
CEU	chr19	PSG3	47471642	0
CEU	chr19	PSG3	47521642	0
CEU	chr19	PSG3	47571642	0
CEU	chr19	PSG3	47621642	0
CEU	chr19	PSG3	47671642	0
CEU	chr2	RGPD1	81963577	0
CEU	chr2	RGPD1	82013577	0
CEU	chr2	RGPD1	82063577	0
CEU	chr2	RGPD1	82113577	0
CEU	chr2	RGPD1	82163577	0
CEU	chr2	RGPD1	82213577	0
CEU	chr2	RGPD1	82263577	0
CEU	chr2	RGPD1	82313577	0
CEU	chr2	RGPD1	82363577	0
CEU	chr2	RGPD1	82413577	0
CEU	chr2	RGPD1	82463577	0
CEU	chr2	RGPD1	82513577	0
CEU	chr2	RGPD1	82563577	0
CEU	chr2	RGPD1	82613577	0
CEU	chr2	RGPD1	82663577	0
CEU	chr2	RGPD1	82713577	0
CEU	chr2	RGPD1	82763577	0
CEU	chr2	RGPD1	82813577	0
CEU	chr2	RGPD1	82863577	0

CEU	chr2	RGPD1	82913577	0
CEU	chr2	RGPD1	82963577	0
CEU	chr2	RGPD1	83013577	0
CEU	chr2	RGPD1	83063577	0
CEU	chr2	RGPD1	83113577	0
CEU	chr2	RGPD1	83163577	0
CEU	chr2	RGPD1	83213577	0
CEU	chr2	RGPD1	83263577	0
CEU	chr2	RGPD1	83313577	0
CEU	chr2	RGPD1	83363577	0
CEU	chr2	RGPD1	83413577	0
CEU	chr2	RGPD1	83463577	0
CEU	chr2	RGPD1	83513577	0
CEU	chr2	RGPD1	83563577	0
CEU	chr2	RGPD1	83613577	0
CEU	chr2	RGPD1	83663577	0
CEU	chr2	RGPD1	83713577	0
CEU	chr2	RGPD1	83763577	0
CEU	chr2	RGPD1	83813577	0
CEU	chr2	RGPD1	83863577	0
CEU	chr2	RGPD1	83913577	0
CEU	chr2	RGPD1	83963577	0
CEU	chr2	RGPD1	84013577	0
CEU	chr2	RGPD1	84063577	0
CEU	chr2	RGPD1	84113577	0
CEU	chr2	RGPD1	84163577	0
CEU	chr2	RGPD1	84213577	0
CEU	chr2	RGPD1	84263577	0
CEU	chr2	RGPD1	84313577	0
CEU	chr2	RGPD1	84363577	0
CEU	chr2	RGPD1	84413577	0
CEU	chr2	RGPD1	84463577	0
CEU	chr2	RGPD1	84513577	10
CEU	chr2	RGPD1	84563577	24
CEU	chr2	RGPD1	84613577	39
CEU	chr2	RGPD1	84663577	40
CEU	chr2	RGPD1	84713577	26
CEU	chr2	RGPD1	84763577	23
CEU	chr2	RGPD1	84813577	29
CEU	chr2	RGPD1	84863577	33
CEU	chr2	RGPD1	84913577	32
CEU	chr2	RGPD1	84963577	41
CEU	chr2	RGPD1	85013577	38
CEU	chr2	RGPD1	85063577	53
CEU	chr2	RGPD1	85113577	40
CEU	chr2	RGPD1	85163577	0
CEU	chr2	RGPD1	85213577	0
CEU	chr2	RGPD1	85263577	0
CEU	chr2	RGPD1	85313577	0

CEU	chr2	RGPD1	85363577	24
CEU	chr2	RGPD1	85413577	36
CEU	chr2	RGPD1	85463577	38
CEU	chr2	RGPD1	85513577	26
CEU	chr2	RGPD1	85563577	0
CEU	chr2	RGPD1	85613577	0
CEU	chr2	RGPD1	85663577	0
CEU	chr2	RGPD1	85713577	0
CEU	chr2	RGPD1	85763577	0
CEU	chr2	RGPD1	85813577	0
CEU	chr2	RGPD1	85863577	0
CEU	chr2	RGPD1	85913577	0
CEU	chr2	RGPD1	85963577	0
CEU	chr2	RGPD1	86013577	22
CEU	chr2	RGPD1	86063577	39
CEU	chr2	RGPD1	86113577	41
CEU	chr2	RGPD1	86163577	30
CEU	chr2	RGPD1	86213577	6
CEU	chr2	RGPD1	86263577	2
CEU	chr2	RGPD1	86313577	11
CEU	chr2	RGPD1	86363577	19
CEU	chr2	RGPD1	86413577	37
CEU	chr2	RGPD1	86463577	36
CEU	chr2	RGPD1	86513577	47
CEU	chr2	RGPD1	86563577	62
CEU	chr2	RGPD1	86613577	39
CEU	chr2	RGPD1	86663577	15
CEU	chr2	RGPD1	86713577	0
CEU	chr2	RGPD1	86763577	0
CEU	chr2	RGPD1	86813577	0
CEU	chr2	RGPD1	86863577	0
CEU	chr2	RGPD1	86913577	0
CEU	chr2	RGPD1	86963577	0
CEU	chr2	RGPD1	87013577	0
CEU	chr2	RGPD1	87063577	0
CEU	chr2	RGPD1	87113577	0
CEU	chr2	RGPD1	87163577	0
CEU	chr2	RGPD1	87213577	0
CEU	chr2	RGPD1	87263577	0
CEU	chr2	RGPD1	87313577	0
CEU	chr2	RGPD1	87363577	0
CEU	chr2	RGPD1	87413577	0
CEU	chr2	RGPD1	87463577	0
CEU	chr2	RGPD1	87513577	0
CEU	chr2	RGPD1	87563577	0
CEU	chr2	RGPD1	87613577	0
CEU	chr2	RGPD1	87663577	0
CEU	chr2	RGPD1	87713577	0
CEU	chr2	RGPD1	87763577	0

CEU	chr2	RGPD1	87813577	14
CEU	chr2	RGPD1	87863577	24
CEU	chr2	RGPD1	87913577	31
CEU	chr2	RGPD1	87963577	42
CEU	chr2	RGPD1	88013577	29
CEU	chr2	RGPD1	88063577	8
CEU	chr2	RGPD1	88113577	0
CEU	chr2	RGPD1	88163577	0
CEU	chr2	RGPD1	88213577	0
CEU	chr2	RGPD1	88263577	0
CEU	chr2	RGPD1	88313577	0
CEU	chr2	RGPD1	88363577	0
CEU	chr2	RGPD1	88413577	0
CEU	chr2	RGPD1	88463577	0
CEU	chr2	RGPD1	88513577	0
CEU	chr2	RGPD1	88563577	0
CEU	chr2	RGPD1	88613577	0
CEU	chr2	RGPD1	88663577	0
CEU	chr2	RGPD1	88713577	0
CEU	chr2	RGPD1	88763577	0
CEU	chr2	RGPD1	88813577	0
CEU	chr2	RGPD1	88863577	0
CEU	chr2	RGPD1	88913577	0
CEU	chr2	RGPD1	88963577	0
CEU	chr2	RGPD1	89013577	0
CEU	chr2	RGPD1	89063577	0
CEU	chr2	RGPD1	89113577	0
CEU	chr2	RGPD1	89163577	0
CEU	chr2	RGPD1	89213577	10
CEU	chr2	RGPD1	89263577	20
CEU	chr2	RGPD1	89313577	14
CEU	chr2	RGPD1	89363577	4
CEU	chr2	RGPD1	89413577	0
CEU	chr2	RGPD1	89463577	0
CEU	chr2	RGPD1	89513577	0
CEU	chr2	RGPD1	89563577	0
CEU	chr2	RGPD1	89613577	0
CEU	chr2	RGPD1	89663577	0
CEU	chr2	RGPD1	89713577	0
CEU	chr2	RGPD1	89763577	0
CEU	chr2	RGPD1	89813577	0
CEU	chr2	RGPD1	89863577	0
CEU	chr2	RGPD1	89913577	0
CEU	chr2	RGPD1	89963577	0
CEU	chr2	RGPD1	90013577	0
CEU	chr2	RGPD1	90063577	0
CEU	chr2	RGPD1	90113577	0
CEU	chr2	RGPD1	90163577	0
CEU	chr2	RGPD1	90213577	0

CEU	chr2	RGPD1	90263577	0
CEU	chr2	RGPD1	90313577	0
CEU	chr2	RGPD1	90363577	0
CEU	chr2	RGPD1	90413577	0
CEU	chr2	RGPD1	90463577	0
CEU	chr2	RGPD1	90513577	0
CEU	chr2	RGPD1	90563577	0
CEU	chr2	RGPD1	90613577	0
CEU	chr2	RGPD1	90663577	0
CEU	chr2	RGPD1	90713577	0
CEU	chr2	RGPD1	90763577	0
CEU	chr2	RGPD1	90813577	0
CEU	chr2	RGPD1	90863577	0
CEU	chr2	RGPD1	90913577	0
CEU	chr2	RGPD1	90963577	0
CEU	chr2	RGPD1	91013577	0
CEU	chr2	RGPD1	91063577	0
CEU	chr2	RGPD1	91113577	0
CEU	chr2	RGPD1	91163577	0
CEU	chr2	RGPD1	91213577	0
CEU	chr2	RGPD1	91263577	0
CEU	chr2	RGPD1	91313577	0
CEU	chr2	RGPD1	91363577	0
CEU	chr2	RGPD1	91413577	0
CEU	chr2	RGPD1	91463577	0
CEU	chr2	RGPD1	91513577	0
CEU	chr2	RGPD1	91563577	0
CEU	chr2	RGPD1	91613577	0
CEU	chr2	RGPD1	91663577	0
CEU	chr2	RGPD1	91713577	0
CEU	chr2	RGPD1	91763577	0
CEU	chr2	RGPD1	91813577	0
CEU	chr2	RGPD1	91863577	0
CEU	chr2	RGPD1	91913577	0
CEU	chr2	RGPD1	91963577	0
CEU	chr7	SPDYE3	95356798	0
CEU	chr7	SPDYE3	95406798	0
CEU	chr7	SPDYE3	95456798	0
CEU	chr7	SPDYE3	95506798	0
CEU	chr7	SPDYE3	95556798	0
CEU	chr7	SPDYE3	95606798	0
CEU	chr7	SPDYE3	95656798	0
CEU	chr7	SPDYE3	95706798	0
CEU	chr7	SPDYE3	95756798	0
CEU	chr7	SPDYE3	95806798	0
CEU	chr7	SPDYE3	95856798	0
CEU	chr7	SPDYE3	95906798	0
CEU	chr7	SPDYE3	95956798	0
CEU	chr7	SPDYE3	96006798	0

CEU	chr7	SPDYE3	96056798	0
CEU	chr7	SPDYE3	96106798	0
CEU	chr7	SPDYE3	96156798	0
CEU	chr7	SPDYE3	96206798	0
CEU	chr7	SPDYE3	96256798	0
CEU	chr7	SPDYE3	96306798	0
CEU	chr7	SPDYE3	96356798	0
CEU	chr7	SPDYE3	96406798	0
CEU	chr7	SPDYE3	96456798	0
CEU	chr7	SPDYE3	96506798	0
CEU	chr7	SPDYE3	96556798	0
CEU	chr7	SPDYE3	96606798	0
CEU	chr7	SPDYE3	96656798	0
CEU	chr7	SPDYE3	96706798	0
CEU	chr7	SPDYE3	96756798	0
CEU	chr7	SPDYE3	96806798	0
CEU	chr7	SPDYE3	96856798	0
CEU	chr7	SPDYE3	96906798	0
CEU	chr7	SPDYE3	96956798	0
CEU	chr7	SPDYE3	97006798	0
CEU	chr7	SPDYE3	97056798	0
CEU	chr7	SPDYE3	97106798	0
CEU	chr7	SPDYE3	97156798	0
CEU	chr7	SPDYE3	97206798	0
CEU	chr7	SPDYE3	97256798	0
CEU	chr7	SPDYE3	97306798	0
CEU	chr7	SPDYE3	97356798	0
CEU	chr7	SPDYE3	97406798	0
CEU	chr7	SPDYE3	97456798	0
CEU	chr7	SPDYE3	97506798	0
CEU	chr7	SPDYE3	97556798	0
CEU	chr7	SPDYE3	97606798	0
CEU	chr7	SPDYE3	97656798	0
CEU	chr7	SPDYE3	97706798	0
CEU	chr7	SPDYE3	97756798	0
CEU	chr7	SPDYE3	97806798	0
CEU	chr7	SPDYE3	97856798	0
CEU	chr7	SPDYE3	97906798	0
CEU	chr7	SPDYE3	97956798	0
CEU	chr7	SPDYE3	98006798	0
CEU	chr7	SPDYE3	98056798	0
CEU	chr7	SPDYE3	98106798	0
CEU	chr7	SPDYE3	98156798	0
CEU	chr7	SPDYE3	98206798	0
CEU	chr7	SPDYE3	98256798	0
CEU	chr7	SPDYE3	98306798	0
CEU	chr7	SPDYE3	98356798	0
CEU	chr7	SPDYE3	98406798	0
CEU	chr7	SPDYE3	98456798	0

CEU	chr7	SPDYE3	98506798	0
CEU	chr7	SPDYE3	98556798	0
CEU	chr7	SPDYE3	98606798	0
CEU	chr7	SPDYE3	98656798	0
CEU	chr7	SPDYE3	98706798	0
CEU	chr7	SPDYE3	98756798	0
CEU	chr7	SPDYE3	98806798	0
CEU	chr7	SPDYE3	98856798	0
CEU	chr7	SPDYE3	98906798	0
CEU	chr7	SPDYE3	98956798	0
CEU	chr7	SPDYE3	99006798	0
CEU	chr7	SPDYE3	99056798	0
CEU	chr7	SPDYE3	99106798	0
CEU	chr7	SPDYE3	99156798	0
CEU	chr7	SPDYE3	99206798	0
CEU	chr7	SPDYE3	99256798	0
CEU	chr7	SPDYE3	99306798	0
CEU	chr7	SPDYE3	99356798	0
CEU	chr7	SPDYE3	99406798	0
CEU	chr7	SPDYE3	99456798	0
CEU	chr7	SPDYE3	99506798	0
CEU	chr7	SPDYE3	99556798	0
CEU	chr7	SPDYE3	99606798	0
CEU	chr7	SPDYE3	99656798	0
CEU	chr7	SPDYE3	99706798	0
CEU	chr7	SPDYE3	99756798	0
CEU	chr7	SPDYE3	99806798	0
CEU	chr7	SPDYE3	99856798	0
CEU	chr7	SPDYE3	99906798	0
CEU	chr7	SPDYE3	99956798	0
CEU	chr7	SPDYE3	100006798	0
CEU	chr7	SPDYE3	100056798	0
CEU	chr7	SPDYE3	100106798	0
CEU	chr7	SPDYE3	100156798	0
CEU	chr7	SPDYE3	100206798	0
CEU	chr7	SPDYE3	100256798	0
CEU	chr7	SPDYE3	100306798	0
CEU	chr7	SPDYE3	100356798	0
CEU	chr7	SPDYE3	100406798	0
CEU	chr7	SPDYE3	100456798	0
CEU	chr7	SPDYE3	100506798	0
CEU	chr7	SPDYE3	100556798	0
CEU	chr7	SPDYE3	100606798	0
CEU	chr7	SPDYE3	100656798	0
CEU	chr7	SPDYE3	100706798	0
CEU	chr7	SPDYE3	100756798	0
CEU	chr7	SPDYE3	100806798	0
CEU	chr7	SPDYE3	100856798	0
CEU	chr7	SPDYE3	100906798	0

CEU	chr7	SPDYE3	100956798	0
CEU	chr7	SPDYE3	101006798	0
CEU	chr7	SPDYE3	101056798	0
CEU	chr7	SPDYE3	101106798	0
CEU	chr7	SPDYE3	101156798	0
CEU	chr7	SPDYE3	101206798	0
CEU	chr7	SPDYE3	101256798	0
CEU	chr7	SPDYE3	101306798	42
CEU	chr7	SPDYE3	101356798	57
CEU	chr7	SPDYE3	101406798	15
CEU	chr7	SPDYE3	101456798	0
CEU	chr7	SPDYE3	101506798	0
CEU	chr7	SPDYE3	101556798	0
CEU	chr7	SPDYE3	101606798	0
CEU	chr7	SPDYE3	101656798	0
CEU	chr7	SPDYE3	101706798	0
CEU	chr7	SPDYE3	101756798	0
CEU	chr7	SPDYE3	101806798	0
CEU	chr7	SPDYE3	101856798	0
CEU	chr7	SPDYE3	101906798	0
CEU	chr7	SPDYE3	101956798	0
CEU	chr7	SPDYE3	102006798	0
CEU	chr7	SPDYE3	102056798	0
CEU	chr7	SPDYE3	102106798	0
CEU	chr7	SPDYE3	102156798	0
CEU	chr7	SPDYE3	102206798	0
CEU	chr7	SPDYE3	102256798	0
CEU	chr7	SPDYE3	102306798	0
CEU	chr7	SPDYE3	102356798	0
CEU	chr7	SPDYE3	102406798	0
CEU	chr7	SPDYE3	102456798	0
CEU	chr7	SPDYE3	102506798	0
CEU	chr7	SPDYE3	102556798	0
CEU	chr7	SPDYE3	102606798	0
CEU	chr7	SPDYE3	102656798	0
CEU	chr7	SPDYE3	102706798	0
CEU	chr7	SPDYE3	102756798	0
CEU	chr7	SPDYE3	102806798	0
CEU	chr7	SPDYE3	102856798	0
CEU	chr7	SPDYE3	102906798	0
CEU	chr7	SPDYE3	102956798	0
CEU	chr7	SPDYE3	103006798	0
CEU	chr7	SPDYE3	103056798	0
CEU	chr7	SPDYE3	103106798	0
CEU	chr7	SPDYE3	103156798	0
CEU	chr7	SPDYE3	103206798	0
CEU	chr7	SPDYE3	103256798	0
CEU	chr7	SPDYE3	103306798	0
CEU	chr7	SPDYE3	103356798	0

CEU	chr7	SPDYE3	103406798	0
CEU	chr7	SPDYE3	103456798	0
CEU	chr7	SPDYE3	103506798	0
CEU	chr7	SPDYE3	103556798	0
CEU	chr7	SPDYE3	103606798	0
CEU	chr7	SPDYE3	103656798	0
CEU	chr7	SPDYE3	103706798	0
CEU	chr7	SPDYE3	103756798	0
CEU	chr7	SPDYE3	103806798	0
CEU	chr7	SPDYE3	103856798	0
CEU	chr7	SPDYE3	103906798	0
CEU	chr7	SPDYE3	103956798	0
CEU	chr7	SPDYE3	104006798	0
CEU	chr7	SPDYE3	104056798	0
CEU	chr7	SPDYE3	104106798	0
CEU	chr7	SPDYE3	104156798	0
CEU	chr7	SPDYE3	104206798	0
CEU	chr7	SPDYE3	104256798	0
CEU	chr7	SPDYE3	104306798	0
CEU	chr7	SPDYE3	104356798	0
CEU	chr7	SPDYE3	104406798	0
CEU	chr7	SPDYE3	104456798	0
CEU	chr7	SPDYE3	104506798	0
CEU	chr7	SPDYE3	104556798	0
CEU	chr7	SPDYE3	104606798	0
CEU	chr7	SPDYE3	104656798	0
CEU	chr7	SPDYE3	104706798	0
CEU	chr7	SPDYE3	104756798	0
CEU	chr7	SPDYE3	104806798	0
CEU	chr7	SPDYE3	104856798	0
CEU	chr7	SPDYE3	104906798	0
CEU	chr7	SPDYE3	104956798	0
CEU	chr7	SPDYE3	105006798	0
CEU	chr7	SPDYE3	105056798	0
CEU	chr7	SPDYE3	105106798	0
CEU	chr7	SPDYE3	105156798	0
CEU	chr7	SPDYE3	105206798	0
CEU	chr7	SPDYE3	105256798	0
CEU	chr16	SULT1A3	25249255	0
CEU	chr16	SULT1A3	25299255	0
CEU	chr16	SULT1A3	25349255	0
CEU	chr16	SULT1A3	25399255	0
CEU	chr16	SULT1A3	25449255	0
CEU	chr16	SULT1A3	25499255	0
CEU	chr16	SULT1A3	25549255	0
CEU	chr16	SULT1A3	25599255	0
CEU	chr16	SULT1A3	25649255	0
CEU	chr16	SULT1A3	25699255	0
CEU	chr16	SULT1A3	25749255	0

CEU	chr16	SULT1A3	25799255	0
CEU	chr16	SULT1A3	25849255	0
CEU	chr16	SULT1A3	25899255	8
CEU	chr16	SULT1A3	25949255	46
CEU	chr16	SULT1A3	25999255	38
CEU	chr16	SULT1A3	26049255	0
CEU	chr16	SULT1A3	26099255	0
CEU	chr16	SULT1A3	26149255	0
CEU	chr16	SULT1A3	26199255	0
CEU	chr16	SULT1A3	26249255	0
CEU	chr16	SULT1A3	26299255	0
CEU	chr16	SULT1A3	26349255	0
CEU	chr16	SULT1A3	26399255	0
CEU	chr16	SULT1A3	26449255	0
CEU	chr16	SULT1A3	26499255	0
CEU	chr16	SULT1A3	26549255	0
CEU	chr16	SULT1A3	26599255	0
CEU	chr16	SULT1A3	26649255	0
CEU	chr16	SULT1A3	26699255	0
CEU	chr16	SULT1A3	26749255	0
CEU	chr16	SULT1A3	26799255	0
CEU	chr16	SULT1A3	26849255	0
CEU	chr16	SULT1A3	26899255	0
CEU	chr16	SULT1A3	26949255	0
CEU	chr16	SULT1A3	26999255	0
CEU	chr16	SULT1A3	27049255	0
CEU	chr16	SULT1A3	27099255	0
CEU	chr16	SULT1A3	27149255	0
CEU	chr16	SULT1A3	27199255	0
CEU	chr16	SULT1A3	27249255	0
CEU	chr16	SULT1A3	27299255	0
CEU	chr16	SULT1A3	27349255	0
CEU	chr16	SULT1A3	27399255	0
CEU	chr16	SULT1A3	27449255	0
CEU	chr16	SULT1A3	27499255	0
CEU	chr16	SULT1A3	27549255	0
CEU	chr16	SULT1A3	27599255	0
CEU	chr16	SULT1A3	27649255	0
CEU	chr16	SULT1A3	27699255	0
CEU	chr16	SULT1A3	27749255	0
CEU	chr16	SULT1A3	27799255	0
CEU	chr16	SULT1A3	27849255	0
CEU	chr16	SULT1A3	27899255	0
CEU	chr16	SULT1A3	27949255	0
CEU	chr16	SULT1A3	27999255	0
CEU	chr16	SULT1A3	28049255	0
CEU	chr16	SULT1A3	28099255	0
CEU	chr16	SULT1A3	28149255	0
CEU	chr16	SULT1A3	28199255	0

CEU	chr16	SULT1A3	28249255	0
CEU	chr16	SULT1A3	28299255	0
CEU	chr16	SULT1A3	28349255	0
CEU	chr16	SULT1A3	28399255	0
CEU	chr16	SULT1A3	28449255	0
CEU	chr16	SULT1A3	28499255	0
CEU	chr16	SULT1A3	28549255	0
CEU	chr16	SULT1A3	28599255	0
CEU	chr16	SULT1A3	28649255	0
CEU	chr16	SULT1A3	28699255	0
CEU	chr16	SULT1A3	28749255	0
CEU	chr16	SULT1A3	28799255	0
CEU	chr16	SULT1A3	28849255	0
CEU	chr16	SULT1A3	28899255	0
CEU	chr16	SULT1A3	28949255	0
CEU	chr16	SULT1A3	28999255	0
CEU	chr16	SULT1A3	29049255	0
CEU	chr16	SULT1A3	29099255	0
CEU	chr16	SULT1A3	29149255	0
CEU	chr16	SULT1A3	29199255	0
CEU	chr16	SULT1A3	29249255	0
CEU	chr16	SULT1A3	29299255	0
CEU	chr16	SULT1A3	29349255	0
CEU	chr16	SULT1A3	29399255	0
CEU	chr16	SULT1A3	29449255	0
CEU	chr16	SULT1A3	29499255	0
CEU	chr16	SULT1A3	29549255	0
CEU	chr16	SULT1A3	29599255	0
CEU	chr16	SULT1A3	29649255	0
CEU	chr16	SULT1A3	29699255	0
CEU	chr16	SULT1A3	29749255	0
CEU	chr16	SULT1A3	29799255	0
CEU	chr16	SULT1A3	29849255	0
CEU	chr16	SULT1A3	29899255	0
CEU	chr16	SULT1A3	29949255	0
CEU	chr16	SULT1A3	29999255	0
CEU	chr16	SULT1A3	30049255	0
CEU	chr16	SULT1A3	30099255	0
CEU	chr16	SULT1A3	30149255	0
CEU	chr16	SULT1A3	30199255	0
CEU	chr16	SULT1A3	30249255	0
CEU	chr16	SULT1A3	30299255	0
CEU	chr16	SULT1A3	30349255	0
CEU	chr16	SULT1A3	30399255	0
CEU	chr16	SULT1A3	30449255	0
CEU	chr16	SULT1A3	30499255	0
CEU	chr16	SULT1A3	30549255	0
CEU	chr16	SULT1A3	30599255	0
CEU	chr16	SULT1A3	30649255	0

CEU	chr16	SULT1A3	30699255	0
CEU	chr16	SULT1A3	30749255	0
CEU	chr16	SULT1A3	30799255	0
CEU	chr16	SULT1A3	30849255	0
CEU	chr16	SULT1A3	30899255	0
CEU	chr16	SULT1A3	30949255	0
CEU	chr16	SULT1A3	30999255	0
CEU	chr16	SULT1A3	31049255	0
CEU	chr16	SULT1A3	31099255	0
CEU	chr16	SULT1A3	31149255	0
CEU	chr16	SULT1A3	31199255	0
CEU	chr16	SULT1A3	31249255	0
CEU	chr16	SULT1A3	31299255	0
CEU	chr16	SULT1A3	31349255	0
CEU	chr16	SULT1A3	31399255	0
CEU	chr16	SULT1A3	31449255	0
CEU	chr16	SULT1A3	31499255	0
CEU	chr16	SULT1A3	31549255	0
CEU	chr16	SULT1A3	31599255	0
CEU	chr16	SULT1A3	31649255	0
CEU	chr16	SULT1A3	31699255	0
CEU	chr16	SULT1A3	31749255	0
CEU	chr16	SULT1A3	31799255	0
CEU	chr16	SULT1A3	31849255	0
CEU	chr16	SULT1A3	31899255	0
CEU	chr16	SULT1A3	31949255	0
CEU	chr16	SULT1A3	31999255	0
CEU	chr16	SULT1A3	32049255	0
CEU	chr16	SULT1A3	32099255	0
CEU	chr16	SULT1A3	32149255	0
CEU	chr16	SULT1A3	32199255	0
CEU	chr16	SULT1A3	32249255	0
CEU	chr16	SULT1A3	32299255	0
CEU	chr16	SULT1A3	32349255	0
CEU	chr16	SULT1A3	32399255	0
CEU	chr16	SULT1A3	32449255	0
CEU	chr16	SULT1A3	32499255	0
CEU	chr16	SULT1A3	32549255	0
CEU	chr16	SULT1A3	32599255	0
CEU	chr16	SULT1A3	32649255	0
CEU	chr16	SULT1A3	32699255	0
CEU	chr16	SULT1A3	32749255	0
CEU	chr16	SULT1A3	32799255	0
CEU	chr16	SULT1A3	32849255	0
CEU	chr16	SULT1A3	32899255	0
CEU	chr16	SULT1A3	32949255	0
CEU	chr16	SULT1A3	32999255	0
CEU	chr16	SULT1A3	33049255	0
CEU	chr16	SULT1A3	33099255	0

CEU	chr16	SULT1A3	33149255	0
CEU	chr16	SULT1A3	33199255	0
CEU	chr16	SULT1A3	33249255	0
CEU	chr16	SULT1A3	33299255	0
CEU	chr16	SULT1A3	33349255	0
CEU	chr16	SULT1A3	33399255	0
CEU	chr16	SULT1A3	33449255	0
CEU	chr16	SULT1A3	33499255	0
CEU	chr16	SULT1A3	33549255	0
CEU	chr16	SULT1A3	33599255	0
CEU	chr16	SULT1A3	33649255	0
CEU	chr16	SULT1A3	33699255	0
CEU	chr16	SULT1A3	33749255	0
CEU	chr16	SULT1A3	33799255	0
CEU	chr16	SULT1A3	33849255	0
CEU	chr16	SULT1A3	33899255	0
CEU	chr16	SULT1A3	33949255	0
CEU	chr16	SULT1A3	33999255	0
CEU	chr16	SULT1A3	34049255	0
CEU	chr16	SULT1A3	34099255	0
CEU	chr16	SULT1A3	34149255	0
CEU	chr16	SULT1A3	34199255	0
CEU	chr16	SULT1A3	34249255	0
CEU	chr16	SULT1A3	34299255	0
CEU	chr16	SULT1A3	34349255	0
CEU	chr16	SULT1A3	34399255	0
CEU	chr16	SULT1A3	34449255	0
CEU	chr16	SULT1A3	34499255	0
CEU	chr16	SULT1A3	34549255	0
CEU	chr16	SULT1A3	34599255	0
CEU	chr16	SULT1A3	34649255	0
CEU	chr16	SULT1A3	34699255	0
CEU	chr16	SULT1A3	34749255	0
CEU	chr16	SULT1A3	34799255	0
CEU	chr16	SULT1A3	34849255	0
CEU	chr16	SULT1A3	34899255	0
CEU	chr16	SULT1A3	34949255	0
CEU	chr16	SULT1A3	34999255	0
CEU	chr16	SULT1A3	35049255	0
CEU	chr16	SULT1A3	35099255	0
CEU	chr16	SULT1A3	35149255	0
CEU	chr6	TBC1D3	116129494	0
CEU	chr6	TBC1D3	116179494	0
CEU	chr6	TBC1D3	116229494	0
CEU	chr6	TBC1D3	116279494	0
CEU	chr6	TBC1D3	116329494	0
CEU	chr6	TBC1D3	116379494	0
CEU	chr6	TBC1D3	116429494	0
CEU	chr6	TBC1D3	116479494	0

CEU	chr6	TBC1D3	116529494	0
CEU	chr6	TBC1D3	116579494	0
CEU	chr6	TBC1D3	116629494	0
CEU	chr6	TBC1D3	116679494	0
CEU	chr6	TBC1D3	116729494	0
CEU	chr6	TBC1D3	116779494	0
CEU	chr6	TBC1D3	116829494	0
CEU	chr6	TBC1D3	116879494	0
CEU	chr6	TBC1D3	116929494	0
CEU	chr6	TBC1D3	116979494	0
CEU	chr6	TBC1D3	117029494	0
CEU	chr6	TBC1D3	117079494	0
CEU	chr6	TBC1D3	117129494	0
CEU	chr6	TBC1D3	117179494	0
CEU	chr6	TBC1D3	117229494	0
CEU	chr6	TBC1D3	117279494	14
CEU	chr6	TBC1D3	117329494	44
CEU	chr6	TBC1D3	117379494	47
CEU	chr6	TBC1D3	117429494	71
CEU	chr6	TBC1D3	117479494	139
CEU	chr6	TBC1D3	117529494	97
CEU	chr6	TBC1D3	117579494	29
CEU	chr6	TBC1D3	117629494	55
CEU	chr6	TBC1D3	117679494	66
CEU	chr6	TBC1D3	117729494	72
CEU	chr6	TBC1D3	117779494	77
CEU	chr6	TBC1D3	117829494	51
CEU	chr6	TBC1D3	117879494	36
CEU	chr6	TBC1D3	117929494	42
CEU	chr6	TBC1D3	117979494	38
CEU	chr6	TBC1D3	118029494	26
CEU	chr6	TBC1D3	118079494	20
CEU	chr6	TBC1D3	118129494	23
CEU	chr6	TBC1D3	118179494	25
CEU	chr6	TBC1D3	118229494	25
CEU	chr6	TBC1D3	118279494	37
CEU	chr6	TBC1D3	118329494	23
CEU	chr6	TBC1D3	118379494	1
CEU	chr6	TBC1D3	118429494	0
CEU	chr6	TBC1D3	118479494	0
CEU	chr6	TBC1D3	118529494	0
CEU	chr6	TBC1D3	118579494	0
CEU	chr6	TBC1D3	118629494	0
CEU	chr6	TBC1D3	118679494	0
CEU	chr6	TBC1D3	118729494	0
CEU	chr6	TBC1D3	118779494	0
CEU	chr6	TBC1D3	118829494	0
CEU	chr6	TBC1D3	118879494	0
CEU	chr6	TBC1D3	118929494	0

CEU	chr6	TBC1D3	118979494	0
CEU	chr6	TBC1D3	119029494	0
CEU	chr6	TBC1D3	119079494	0
CEU	chr6	TBC1D3	119129494	0
CEU	chr6	TBC1D3	119179494	0
CEU	chr6	TBC1D3	119229494	0
CEU	chr6	TBC1D3	119279494	0
CEU	chr6	TBC1D3	119329494	0
CEU	chr6	TBC1D3	119379494	0
CEU	chr6	TBC1D3	119429494	0
CEU	chr6	TBC1D3	119479494	0
CEU	chr6	TBC1D3	119529494	0
CEU	chr6	TBC1D3	119579494	0
CEU	chr6	TBC1D3	119629494	0
CEU	chr6	TBC1D3	119679494	0
CEU	chr6	TBC1D3	119729494	0
CEU	chr6	TBC1D3	119779494	0
CEU	chr6	TBC1D3	119829494	0
CEU	chr6	TBC1D3	119879494	23
CEU	chr6	TBC1D3	119929494	35
CEU	chr6	TBC1D3	119979494	38
CEU	chr6	TBC1D3	120029494	108
CEU	chr6	TBC1D3	120079494	115
CEU	chr6	TBC1D3	120129494	57
CEU	chr6	TBC1D3	120179494	54
CEU	chr6	TBC1D3	120229494	49
CEU	chr6	TBC1D3	120279494	36
CEU	chr6	TBC1D3	120329494	33
CEU	chr6	TBC1D3	120379494	45
CEU	chr6	TBC1D3	120429494	62
CEU	chr6	TBC1D3	120479494	51
CEU	chr6	TBC1D3	120529494	41
CEU	chr6	TBC1D3	120579494	23
CEU	chr6	TBC1D3	120629494	0
CEU	chr6	TBC1D3	120679494	0
CEU	chr6	TBC1D3	120729494	0
CEU	chr6	TBC1D3	120779494	0
CEU	chr6	TBC1D3	120829494	0
CEU	chr6	TBC1D3	120879494	0
CEU	chr6	TBC1D3	120929494	0
CEU	chr6	TBC1D3	120979494	0
CEU	chr6	TBC1D3	121029494	0
CEU	chr6	TBC1D3	121079494	0
CEU	chr6	TBC1D3	121129494	0
CEU	chr6	TBC1D3	121179494	0
CEU	chr6	TBC1D3	121229494	0
CEU	chr6	TBC1D3	121279494	0
CEU	chr6	TBC1D3	121329494	3
CEU	chr6	TBC1D3	121379494	29

CEU	chr6	TBC1D3	121429494	40
CEU	chr6	TBC1D3	121479494	30
CEU	chr6	TBC1D3	121529494	62
CEU	chr6	TBC1D3	121579494	89
CEU	chr6	TBC1D3	121629494	62
CEU	chr6	TBC1D3	121679494	42
CEU	chr6	TBC1D3	121729494	54
CEU	chr6	TBC1D3	121779494	31
CEU	chr6	TBC1D3	121829494	0
CEU	chr6	TBC1D3	121879494	0
CEU	chr6	TBC1D3	121929494	0
CEU	chr6	TBC1D3	121979494	0
CEU	chr6	TBC1D3	122029494	0
CEU	chr6	TBC1D3	122079494	0
CEU	chr6	TBC1D3	122129494	0
CEU	chr6	TBC1D3	122179494	0
CEU	chr6	TBC1D3	122229494	0
CEU	chr6	TBC1D3	122279494	0
CEU	chr6	TBC1D3	122329494	0
CEU	chr6	TBC1D3	122379494	0
CEU	chr6	TBC1D3	122429494	0
CEU	chr6	TBC1D3	122479494	0
CEU	chr6	TBC1D3	122529494	0
CEU	chr6	TBC1D3	122579494	0
CEU	chr6	TBC1D3	122629494	0
CEU	chr6	TBC1D3	122679494	0
CEU	chr6	TBC1D3	122729494	0
CEU	chr6	TBC1D3	122779494	0
CEU	chr6	TBC1D3	122829494	0
CEU	chr6	TBC1D3	122879494	0
CEU	chr6	TBC1D3	122929494	0
CEU	chr6	TBC1D3	122979494	0
CEU	chr6	TBC1D3	123029494	0
CEU	chr6	TBC1D3	123079494	3
CEU	chr6	TBC1D3	123129494	41
CEU	chr6	TBC1D3	123179494	38
CEU	chr6	TBC1D3	123229494	0
CEU	chr6	TBC1D3	123279494	0
CEU	chr6	TBC1D3	123329494	0
CEU	chr6	TBC1D3	123379494	0
CEU	chr6	TBC1D3	123429494	0
CEU	chr6	TBC1D3	123479494	0
CEU	chr6	TBC1D3	123529494	0
CEU	chr6	TBC1D3	123579494	0
CEU	chr6	TBC1D3	123629494	0
CEU	chr6	TBC1D3	123679494	0
CEU	chr6	TBC1D3	123729494	0
CEU	chr6	TBC1D3	123779494	0
CEU	chr6	TBC1D3	123829494	0

CEU	chr6	TBC1D3	123879494	0
CEU	chr6	TBC1D3	123929494	0
CEU	chr6	TBC1D3	123979494	0
CEU	chr6	TBC1D3	124029494	0
CEU	chr6	TBC1D3	124079494	0
CEU	chr6	TBC1D3	124129494	0
CEU	chr6	TBC1D3	124179494	0
CEU	chr6	TBC1D3	124229494	0
CEU	chr6	TBC1D3	124279494	0
CEU	chr6	TBC1D3	124329494	0
CEU	chr6	TBC1D3	124379494	0
CEU	chr6	TBC1D3	124429494	0
CEU	chr6	TBC1D3	124479494	0
CEU	chr6	TBC1D3	124529494	0
CEU	chr6	TBC1D3	124579494	0
CEU	chr6	TBC1D3	124629494	0
CEU	chr6	TBC1D3	124679494	0
CEU	chr6	TBC1D3	124729494	0
CEU	chr6	TBC1D3	124779494	0
CEU	chr6	TBC1D3	124829494	0
CEU	chr6	TBC1D3	124879494	0
CEU	chr6	TBC1D3	124929494	0
CEU	chr6	TBC1D3	124979494	0
CEU	chr6	TBC1D3	125029494	0
CEU	chr6	TBC1D3	125079494	0
CEU	chr6	TBC1D3	125129494	0
CEU	chr6	TBC1D3	125179494	0
CEU	chr6	TBC1D3	125229494	0
CEU	chr6	TBC1D3	125279494	0
CEU	chr6	TBC1D3	125329494	0
CEU	chr6	TBC1D3	125379494	0
CEU	chr6	TBC1D3	125429494	0
CEU	chr6	TBC1D3	125479494	0
CEU	chr6	TBC1D3	125529494	0
CEU	chr6	TBC1D3	125579494	0
CEU	chr6	TBC1D3	125629494	0
CEU	chr6	TBC1D3	125679494	0
CEU	chr6	TBC1D3	125729494	0
CEU	chr6	TBC1D3	125779494	0
CEU	chr6	TBC1D3	125829494	0
CEU	chr6	TBC1D3	125879494	0
CEU	chr6	TBC1D3	125929494	0
CEU	chr6	TBC1D3	125979494	0
CEU	chr6	TBC1D3	126029494	0
CEU	chr6	TBC1D3	126079494	0
CEU	chr6	TBC1D3	126129494	0
CEU	chr6	TBC1D3	126179494	0
CEU	chr6	TBC1D3	126229494	0
CEU	chr6	TBC1D3	126279494	0

CEU	chr8	TBC1D3	118091961	0
CEU	chr8	TBC1D3	118141961	0
CEU	chr8	TBC1D3	118191961	0
CEU	chr8	TBC1D3	118241961	0
CEU	chr8	TBC1D3	118291961	0
CEU	chr8	TBC1D3	118341961	0
CEU	chr8	TBC1D3	118391961	0
CEU	chr8	TBC1D3	118441961	0
CEU	chr8	TBC1D3	118491961	0
CEU	chr8	TBC1D3	118541961	0
CEU	chr8	TBC1D3	118591961	0
CEU	chr8	TBC1D3	118641961	0
CEU	chr8	TBC1D3	118691961	0
CEU	chr8	TBC1D3	118741961	0
CEU	chr8	TBC1D3	118791961	0
CEU	chr8	TBC1D3	118841961	0
CEU	chr8	TBC1D3	118891961	0
CEU	chr8	TBC1D3	118941961	0
CEU	chr8	TBC1D3	118991961	0
CEU	chr8	TBC1D3	119041961	0
CEU	chr8	TBC1D3	119091961	0
CEU	chr8	TBC1D3	119141961	0
CEU	chr8	TBC1D3	119191961	0
CEU	chr8	TBC1D3	119241961	0
CEU	chr8	TBC1D3	119291961	0
CEU	chr8	TBC1D3	119341961	0
CEU	chr8	TBC1D3	119391961	0
CEU	chr8	TBC1D3	119441961	0
CEU	chr8	TBC1D3	119491961	0
CEU	chr8	TBC1D3	119541961	0
CEU	chr8	TBC1D3	119591961	0
CEU	chr8	TBC1D3	119641961	0
CEU	chr8	TBC1D3	119691961	0
CEU	chr8	TBC1D3	119741961	0
CEU	chr8	TBC1D3	119791961	0
CEU	chr8	TBC1D3	119841961	0
CEU	chr8	TBC1D3	119891961	0
CEU	chr8	TBC1D3	119941961	0
CEU	chr8	TBC1D3	119991961	0
CEU	chr8	TBC1D3	120041961	0
CEU	chr8	TBC1D3	120091961	0
CEU	chr8	TBC1D3	120141961	0
CEU	chr8	TBC1D3	120191961	0
CEU	chr8	TBC1D3	120241961	0
CEU	chr8	TBC1D3	120291961	0
CEU	chr8	TBC1D3	120341961	0
CEU	chr8	TBC1D3	120391961	0
CEU	chr8	TBC1D3	120441961	0
CEU	chr8	TBC1D3	120491961	0

CEU	chr8	TBC1D3	120541961	0
CEU	chr8	TBC1D3	120591961	0
CEU	chr8	TBC1D3	120641961	0
CEU	chr8	TBC1D3	120691961	0
CEU	chr8	TBC1D3	120741961	0
CEU	chr8	TBC1D3	120791961	0
CEU	chr8	TBC1D3	120841961	0
CEU	chr8	TBC1D3	120891961	0
CEU	chr8	TBC1D3	120941961	0
CEU	chr8	TBC1D3	120991961	0
CEU	chr8	TBC1D3	121041961	0
CEU	chr8	TBC1D3	121091961	0
CEU	chr8	TBC1D3	121141961	0
CEU	chr8	TBC1D3	121191961	0
CEU	chr8	TBC1D3	121241961	0
CEU	chr8	TBC1D3	121291961	0
CEU	chr8	TBC1D3	121341961	0
CEU	chr8	TBC1D3	121391961	0
CEU	chr8	TBC1D3	121441961	0
CEU	chr8	TBC1D3	121491961	0
CEU	chr8	TBC1D3	121541961	0
CEU	chr8	TBC1D3	121591961	0
CEU	chr8	TBC1D3	121641961	0
CEU	chr8	TBC1D3	121691961	0
CEU	chr8	TBC1D3	121741961	0
CEU	chr8	TBC1D3	121791961	0
CEU	chr8	TBC1D3	121841961	0
CEU	chr8	TBC1D3	121891961	0
CEU	chr8	TBC1D3	121941961	0
CEU	chr8	TBC1D3	121991961	0
CEU	chr8	TBC1D3	122041961	0
CEU	chr8	TBC1D3	122091961	0
CEU	chr8	TBC1D3	122141961	0
CEU	chr8	TBC1D3	122191961	0
CEU	chr8	TBC1D3	122241961	0
CEU	chr8	TBC1D3	122291961	0
CEU	chr8	TBC1D3	122341961	0
CEU	chr8	TBC1D3	122391961	0
CEU	chr8	TBC1D3	122441961	0
CEU	chr8	TBC1D3	122491961	0
CEU	chr8	TBC1D3	122541961	0
CEU	chr8	TBC1D3	122591961	0
CEU	chr8	TBC1D3	122641961	0
CEU	chr8	TBC1D3	122691961	0
CEU	chr8	TBC1D3	122741961	0
CEU	chr8	TBC1D3	122791961	0
CEU	chr8	TBC1D3	122841961	0
CEU	chr8	TBC1D3	122891961	0
CEU	chr8	TBC1D3	122941961	0

CEU	chr8	TBC1D3	122991961	0
CEU	chr8	TBC1D3	123041961	0
CEU	chr8	TBC1D3	123091961	0
CEU	chr8	TBC1D3	123141961	0
CEU	chr8	TBC1D3	123191961	0
CEU	chr8	TBC1D3	123241961	0
CEU	chr8	TBC1D3	123291961	0
CEU	chr8	TBC1D3	123341961	0
CEU	chr8	TBC1D3	123391961	0
CEU	chr8	TBC1D3	123441961	0
CEU	chr8	TBC1D3	123491961	0
CEU	chr8	TBC1D3	123541961	0
CEU	chr8	TBC1D3	123591961	0
CEU	chr8	TBC1D3	123641961	0
CEU	chr8	TBC1D3	123691961	0
CEU	chr8	TBC1D3	123741961	0
CEU	chr8	TBC1D3	123791961	0
CEU	chr8	TBC1D3	123841961	0
CEU	chr8	TBC1D3	123891961	0
CEU	chr8	TBC1D3	123941961	0
CEU	chr8	TBC1D3	123991961	0
CEU	chr8	TBC1D3	124041961	0
CEU	chr8	TBC1D3	124091961	0
CEU	chr8	TBC1D3	124141961	0
CEU	chr8	TBC1D3	124191961	0
CEU	chr8	TBC1D3	124241961	0
CEU	chr8	TBC1D3	124291961	0
CEU	chr8	TBC1D3	124341961	0
CEU	chr8	TBC1D3	124391961	0
CEU	chr8	TBC1D3	124441961	0
CEU	chr8	TBC1D3	124491961	0
CEU	chr8	TBC1D3	124541961	26
CEU	chr8	TBC1D3	124591961	62
CEU	chr8	TBC1D3	124641961	61
CEU	chr8	TBC1D3	124691961	25
CEU	chr8	TBC1D3	124741961	0
CEU	chr8	TBC1D3	124791961	0
CEU	chr8	TBC1D3	124841961	0
CEU	chr8	TBC1D3	124891961	0
CEU	chr8	TBC1D3	124941961	0
CEU	chr8	TBC1D3	124991961	0
CEU	chr8	TBC1D3	125041961	0
CEU	chr8	TBC1D3	125091961	0
CEU	chr8	TBC1D3	125141961	0
CEU	chr8	TBC1D3	125191961	0
CEU	chr8	TBC1D3	125241961	0
CEU	chr8	TBC1D3	125291961	0
CEU	chr8	TBC1D3	125341961	0
CEU	chr8	TBC1D3	125391961	0

CEU	chr8	TBC1D3	125441961	0
CEU	chr8	TBC1D3	125491961	0
CEU	chr8	TBC1D3	125541961	0
CEU	chr8	TBC1D3	125591961	0
CEU	chr8	TBC1D3	125641961	0
CEU	chr8	TBC1D3	125691961	0
CEU	chr8	TBC1D3	125741961	0
CEU	chr8	TBC1D3	125791961	0
CEU	chr8	TBC1D3	125841961	0
CEU	chr8	TBC1D3	125891961	0
CEU	chr8	TBC1D3	125941961	0
CEU	chr8	TBC1D3	125991961	0
CEU	chr8	TBC1D3	126041961	0
CEU	chr8	TBC1D3	126091961	0
CEU	chr8	TBC1D3	126141961	0
CEU	chr8	TBC1D3	126191961	0
CEU	chr8	TBC1D3	126241961	0
CEU	chr8	TBC1D3	126291961	0
CEU	chr8	TBC1D3	126341961	0
CEU	chr8	TBC1D3	126391961	0
CEU	chr8	TBC1D3	126441961	0
CEU	chr8	TBC1D3	126491961	0
CEU	chr8	TBC1D3	126541961	0
CEU	chr8	TBC1D3	126591961	0
CEU	chr8	TBC1D3	126641961	0
CEU	chr8	TBC1D3	126691961	0
CEU	chr8	TBC1D3	126741961	0
CEU	chr8	TBC1D3	126791961	0
CEU	chr8	TBC1D3	126841961	0
CEU	chr8	TBC1D3	126891961	0
CEU	chr8	TBC1D3	126941961	0
CEU	chr8	TBC1D3	126991961	0
CEU	chr8	TBC1D3	127041961	0
CEU	chr8	TBC1D3	127091961	0
CEU	chr8	TBC1D3	127141961	0
CEU	chr8	TBC1D3	127191961	0
CEU	chr8	TBC1D3	127241961	0
CEU	chr8	TBC1D3	127291961	0
CEU	chr8	TBC1D3	127341961	0
CEU	chr8	TBC1D3	127391961	0
CEU	chr8	TBC1D3	127441961	0
CEU	chr8	TBC1D3	127491961	0
CEU	chr8	TBC1D3	127541961	0
CEU	chr8	TBC1D3	127591961	0
CEU	chr8	TBC1D3	127641961	0
CEU	chr8	TBC1D3	127691961	0
CEU	chr8	TBC1D3	127741961	0
CEU	chr8	TBC1D3	127791961	0
CEU	chr8	TBC1D3	127841961	0

CEU	chr8	TBC1D3	127891961	0
CEU	chr8	TBC1D3	127941961	0
CEU	chr8	TBC1D3	127991961	0
CEU	chr8	TBC1D3	128041961	0
CEU	chr8	TBC1D3	128091961	0
CEU	chr12	TBC1D3	59809484	0
CEU	chr12	TBC1D3	59859484	0
CEU	chr12	TBC1D3	59909484	0
CEU	chr12	TBC1D3	59959484	0
CEU	chr12	TBC1D3	60009484	0
CEU	chr12	TBC1D3	60059484	0
CEU	chr12	TBC1D3	60109484	0
CEU	chr12	TBC1D3	60159484	0
CEU	chr12	TBC1D3	60209484	0
CEU	chr12	TBC1D3	60259484	0
CEU	chr12	TBC1D3	60309484	0
CEU	chr12	TBC1D3	60359484	0
CEU	chr12	TBC1D3	60409484	0
CEU	chr12	TBC1D3	60459484	0
CEU	chr12	TBC1D3	60509484	0
CEU	chr12	TBC1D3	60559484	0
CEU	chr12	TBC1D3	60609484	0
CEU	chr12	TBC1D3	60659484	0
CEU	chr12	TBC1D3	60709484	0
CEU	chr12	TBC1D3	60759484	0
CEU	chr12	TBC1D3	60809484	0
CEU	chr12	TBC1D3	60859484	0
CEU	chr12	TBC1D3	60909484	0
CEU	chr12	TBC1D3	60959484	0
CEU	chr12	TBC1D3	61009484	0
CEU	chr12	TBC1D3	61059484	0
CEU	chr12	TBC1D3	61109484	0
CEU	chr12	TBC1D3	61159484	0
CEU	chr12	TBC1D3	61209484	0
CEU	chr12	TBC1D3	61259484	0
CEU	chr12	TBC1D3	61309484	0
CEU	chr12	TBC1D3	61359484	0
CEU	chr12	TBC1D3	61409484	0
CEU	chr12	TBC1D3	61459484	0
CEU	chr12	TBC1D3	61509484	0
CEU	chr12	TBC1D3	61559484	0
CEU	chr12	TBC1D3	61609484	0
CEU	chr12	TBC1D3	61659484	0
CEU	chr12	TBC1D3	61709484	0
CEU	chr12	TBC1D3	61759484	0
CEU	chr12	TBC1D3	61809484	0
CEU	chr12	TBC1D3	61859484	0
CEU	chr12	TBC1D3	61909484	0
CEU	chr12	TBC1D3	61959484	0

CEU	chr12	TBC1D3	62009484	0
CEU	chr12	TBC1D3	62059484	0
CEU	chr12	TBC1D3	62109484	0
CEU	chr12	TBC1D3	62159484	0
CEU	chr12	TBC1D3	62209484	0
CEU	chr12	TBC1D3	62259484	0
CEU	chr12	TBC1D3	62309484	0
CEU	chr12	TBC1D3	62359484	0
CEU	chr12	TBC1D3	62409484	0
CEU	chr12	TBC1D3	62459484	0
CEU	chr12	TBC1D3	62509484	0
CEU	chr12	TBC1D3	62559484	0
CEU	chr12	TBC1D3	62609484	0
CEU	chr12	TBC1D3	62659484	0
CEU	chr12	TBC1D3	62709484	0
CEU	chr12	TBC1D3	62759484	0
CEU	chr12	TBC1D3	62809484	0
CEU	chr12	TBC1D3	62859484	0
CEU	chr12	TBC1D3	62909484	0
CEU	chr12	TBC1D3	62959484	0
CEU	chr12	TBC1D3	63009484	14
CEU	chr12	TBC1D3	63059484	33
CEU	chr12	TBC1D3	63109484	39
CEU	chr12	TBC1D3	63159484	47
CEU	chr12	TBC1D3	63209484	46
CEU	chr12	TBC1D3	63259484	19
CEU	chr12	TBC1D3	63309484	0
CEU	chr12	TBC1D3	63359484	0
CEU	chr12	TBC1D3	63409484	0
CEU	chr12	TBC1D3	63459484	0
CEU	chr12	TBC1D3	63509484	0
CEU	chr12	TBC1D3	63559484	3
CEU	chr12	TBC1D3	63609484	31
CEU	chr12	TBC1D3	63659484	48
CEU	chr12	TBC1D3	63709484	78
CEU	chr12	TBC1D3	63759484	84
CEU	chr12	TBC1D3	63809484	40
CEU	chr12	TBC1D3	63859484	33
CEU	chr12	TBC1D3	63909484	53
CEU	chr12	TBC1D3	63959484	74
CEU	chr12	TBC1D3	64009484	84
CEU	chr12	TBC1D3	64059484	79
CEU	chr12	TBC1D3	64109484	149
CEU	chr12	TBC1D3	64159484	136
CEU	chr12	TBC1D3	64209484	40
CEU	chr12	TBC1D3	64259484	49
CEU	chr12	TBC1D3	64309484	54
CEU	chr12	TBC1D3	64359484	23
CEU	chr12	TBC1D3	64409484	0

CEU	chr12	TBC1D3	64459484	0
CEU	chr12	TBC1D3	64509484	0
CEU	chr12	TBC1D3	64559484	0
CEU	chr12	TBC1D3	64609484	0
CEU	chr12	TBC1D3	64659484	0
CEU	chr12	TBC1D3	64709484	0
CEU	chr12	TBC1D3	64759484	0
CEU	chr12	TBC1D3	64809484	0
CEU	chr12	TBC1D3	64859484	0
CEU	chr12	TBC1D3	64909484	0
CEU	chr12	TBC1D3	64959484	0
CEU	chr12	TBC1D3	65009484	0
CEU	chr12	TBC1D3	65059484	0
CEU	chr12	TBC1D3	65109484	0
CEU	chr12	TBC1D3	65159484	0
CEU	chr12	TBC1D3	65209484	0
CEU	chr12	TBC1D3	65259484	0
CEU	chr12	TBC1D3	65309484	0
CEU	chr12	TBC1D3	65359484	0
CEU	chr12	TBC1D3	65409484	0
CEU	chr12	TBC1D3	65459484	0
CEU	chr12	TBC1D3	65509484	0
CEU	chr12	TBC1D3	65559484	0
CEU	chr12	TBC1D3	65609484	0
CEU	chr12	TBC1D3	65659484	0
CEU	chr12	TBC1D3	65709484	0
CEU	chr12	TBC1D3	65759484	0
CEU	chr12	TBC1D3	65809484	0
CEU	chr12	TBC1D3	65859484	0
CEU	chr12	TBC1D3	65909484	0
CEU	chr12	TBC1D3	65959484	0
CEU	chr12	TBC1D3	66009484	0
CEU	chr12	TBC1D3	66059484	0
CEU	chr12	TBC1D3	66109484	0
CEU	chr12	TBC1D3	66159484	0
CEU	chr12	TBC1D3	66209484	0
CEU	chr12	TBC1D3	66259484	0
CEU	chr12	TBC1D3	66309484	0
CEU	chr12	TBC1D3	66359484	0
CEU	chr12	TBC1D3	66409484	0
CEU	chr12	TBC1D3	66459484	0
CEU	chr12	TBC1D3	66509484	0
CEU	chr12	TBC1D3	66559484	0
CEU	chr12	TBC1D3	66609484	0
CEU	chr12	TBC1D3	66659484	0
CEU	chr12	TBC1D3	66709484	0
CEU	chr12	TBC1D3	66759484	0
CEU	chr12	TBC1D3	66809484	0
CEU	chr12	TBC1D3	66859484	0

CEU	chr12	TBC1D3	66909484	0
CEU	chr12	TBC1D3	66959484	0
CEU	chr12	TBC1D3	67009484	0
CEU	chr12	TBC1D3	67059484	0
CEU	chr12	TBC1D3	67109484	0
CEU	chr12	TBC1D3	67159484	0
CEU	chr12	TBC1D3	67209484	0
CEU	chr12	TBC1D3	67259484	0
CEU	chr12	TBC1D3	67309484	0
CEU	chr12	TBC1D3	67359484	0
CEU	chr12	TBC1D3	67409484	0
CEU	chr12	TBC1D3	67459484	5
CEU	chr12	TBC1D3	67509484	18
CEU	chr12	TBC1D3	67559484	28
CEU	chr12	TBC1D3	67609484	34
CEU	chr12	TBC1D3	67659484	26
CEU	chr12	TBC1D3	67709484	14
CEU	chr12	TBC1D3	67759484	19
CEU	chr12	TBC1D3	67809484	45
CEU	chr12	TBC1D3	67859484	49
CEU	chr12	TBC1D3	67909484	16
CEU	chr12	TBC1D3	67959484	0
CEU	chr12	TBC1D3	68009484	0
CEU	chr12	TBC1D3	68059484	0
CEU	chr12	TBC1D3	68109484	0
CEU	chr12	TBC1D3	68159484	0
CEU	chr12	TBC1D3	68209484	0
CEU	chr12	TBC1D3	68259484	0
CEU	chr12	TBC1D3	68309484	0
CEU	chr12	TBC1D3	68359484	0
CEU	chr12	TBC1D3	68409484	0
CEU	chr12	TBC1D3	68459484	0
CEU	chr12	TBC1D3	68509484	0
CEU	chr12	TBC1D3	68559484	0
CEU	chr12	TBC1D3	68609484	0
CEU	chr12	TBC1D3	68659484	0
CEU	chr12	TBC1D3	68709484	0
CEU	chr12	TBC1D3	68759484	0
CEU	chr12	TBC1D3	68809484	13
CEU	chr12	TBC1D3	68859484	62
CEU	chr12	TBC1D3	68909484	78
CEU	chr12	TBC1D3	68959484	57
CEU	chr12	TBC1D3	69009484	40
CEU	chr12	TBC1D3	69059484	35
CEU	chr12	TBC1D3	69109484	42
CEU	chr12	TBC1D3	69159484	39
CEU	chr12	TBC1D3	69209484	28
CEU	chr12	TBC1D3	69259484	8
CEU	chr12	TBC1D3	69309484	0

CEU	chr12	TBC1D3	69359484	0
CEU	chr12	TBC1D3	69409484	0
CEU	chr12	TBC1D3	69459484	16
CEU	chr12	TBC1D3	69509484	42
CEU	chr12	TBC1D3	69559484	48
CEU	chr12	TBC1D3	69609484	27
CEU	chr12	TBC1D3	69659484	13
CEU	chr12	TBC1D3	69709484	16
CEU	chr12	TBC1D3	69759484	20
CEU	chr12	TBC1D3	69809484	35
CEU	chr17	TBC1D3	31215683	0
CEU	chr17	TBC1D3	31265683	0
CEU	chr17	TBC1D3	31315683	0
CEU	chr17	TBC1D3	31365683	0
CEU	chr17	TBC1D3	31415683	0
CEU	chr17	TBC1D3	31465683	0
CEU	chr17	TBC1D3	31515683	0
CEU	chr17	TBC1D3	31565683	0
CEU	chr17	TBC1D3	31615683	0
CEU	chr17	TBC1D3	31665683	0
CEU	chr17	TBC1D3	31715683	0
CEU	chr17	TBC1D3	31765683	0
CEU	chr17	TBC1D3	31815683	0
CEU	chr17	TBC1D3	31865683	0
CEU	chr17	TBC1D3	31915683	0
CEU	chr17	TBC1D3	31965683	0
CEU	chr17	TBC1D3	32015683	0
CEU	chr17	TBC1D3	32065683	0
CEU	chr17	TBC1D3	32115683	0
CEU	chr17	TBC1D3	32165683	0
CEU	chr17	TBC1D3	32215683	0
CEU	chr17	TBC1D3	32265683	0
CEU	chr17	TBC1D3	32315683	0
CEU	chr17	TBC1D3	32365683	0
CEU	chr17	TBC1D3	32415683	0
CEU	chr17	TBC1D3	32465683	0
CEU	chr17	TBC1D3	32515683	0
CEU	chr17	TBC1D3	32565683	0
CEU	chr17	TBC1D3	32615683	0
CEU	chr17	TBC1D3	32665683	0
CEU	chr17	TBC1D3	32715683	0
CEU	chr17	TBC1D3	32765683	0
CEU	chr17	TBC1D3	32815683	0
CEU	chr17	TBC1D3	32865683	0
CEU	chr17	TBC1D3	32915683	0
CEU	chr17	TBC1D3	32965683	0
CEU	chr17	TBC1D3	33015683	0
CEU	chr17	TBC1D3	33065683	0
CEU	chr17	TBC1D3	33115683	0

CEU	chr17	TBC1D3	33165683	0
CEU	chr17	TBC1D3	33215683	0
CEU	chr17	TBC1D3	33265683	0
CEU	chr17	TBC1D3	33315683	0
CEU	chr17	TBC1D3	33365683	0
CEU	chr17	TBC1D3	33415683	0
CEU	chr17	TBC1D3	33465683	0
CEU	chr17	TBC1D3	33515683	0
CEU	chr17	TBC1D3	33565683	0
CEU	chr17	TBC1D3	33615683	14
CEU	chr17	TBC1D3	33665683	31
CEU	chr17	TBC1D3	33715683	32
CEU	chr17	TBC1D3	33765683	24
CEU	chr17	TBC1D3	33815683	12
CEU	chr17	TBC1D3	33865683	19
CEU	chr17	TBC1D3	33915683	26
CEU	chr17	TBC1D3	33965683	21
CEU	chr17	TBC1D3	34015683	21
CEU	chr17	TBC1D3	34065683	63
CEU	chr17	TBC1D3	34115683	77
CEU	chr17	TBC1D3	34165683	47
CEU	chr17	TBC1D3	34215683	35
CEU	chr17	TBC1D3	34265683	30
CEU	chr17	TBC1D3	34315683	36
CEU	chr17	TBC1D3	34365683	18
CEU	chr17	TBC1D3	34415683	0
CEU	chr17	TBC1D3	34465683	0
CEU	chr17	TBC1D3	34515683	0
CEU	chr17	TBC1D3	34565683	0
CEU	chr17	TBC1D3	34615683	0
CEU	chr17	TBC1D3	34665683	0
CEU	chr17	TBC1D3	34715683	0
CEU	chr17	TBC1D3	34765683	0
CEU	chr17	TBC1D3	34815683	0
CEU	chr17	TBC1D3	34865683	0
CEU	chr17	TBC1D3	34915683	0
CEU	chr17	TBC1D3	34965683	0
CEU	chr17	TBC1D3	35015683	0
CEU	chr17	TBC1D3	35065683	0
CEU	chr17	TBC1D3	35115683	5
CEU	chr17	TBC1D3	35165683	22
CEU	chr17	TBC1D3	35215683	17
CEU	chr17	TBC1D3	35265683	0
CEU	chr17	TBC1D3	35315683	0
CEU	chr17	TBC1D3	35365683	0
CEU	chr17	TBC1D3	35415683	0
CEU	chr17	TBC1D3	35465683	0
CEU	chr17	TBC1D3	35515683	0
CEU	chr17	TBC1D3	35565683	0

CEU	chr17	TBC1D3	35615683	0
CEU	chr17	TBC1D3	35665683	0
CEU	chr17	TBC1D3	35715683	0
CEU	chr17	TBC1D3	35765683	0
CEU	chr17	TBC1D3	35815683	0
CEU	chr17	TBC1D3	35865683	0
CEU	chr17	TBC1D3	35915683	0
CEU	chr17	TBC1D3	35965683	0
CEU	chr17	TBC1D3	36015683	0
CEU	chr17	TBC1D3	36065683	0
CEU	chr17	TBC1D3	36115683	0
CEU	chr17	TBC1D3	36165683	0
CEU	chr17	TBC1D3	36215683	0
CEU	chr17	TBC1D3	36265683	0
CEU	chr17	TBC1D3	36315683	0
CEU	chr17	TBC1D3	36365683	0
CEU	chr17	TBC1D3	36415683	0
CEU	chr17	TBC1D3	36465683	0
CEU	chr17	TBC1D3	36515683	0
CEU	chr17	TBC1D3	36565683	0
CEU	chr17	TBC1D3	36615683	0
CEU	chr17	TBC1D3	36665683	0
CEU	chr17	TBC1D3	36715683	0
CEU	chr17	TBC1D3	36765683	0
CEU	chr17	TBC1D3	36815683	0
CEU	chr17	TBC1D3	36865683	0
CEU	chr17	TBC1D3	36915683	0
CEU	chr17	TBC1D3	36965683	0
CEU	chr17	TBC1D3	37015683	0
CEU	chr17	TBC1D3	37065683	0
CEU	chr17	TBC1D3	37115683	0
CEU	chr17	TBC1D3	37165683	0
CEU	chr17	TBC1D3	37215683	0
CEU	chr17	TBC1D3	37265683	0
CEU	chr17	TBC1D3	37315683	0
CEU	chr17	TBC1D3	37365683	0
CEU	chr17	TBC1D3	37415683	0
CEU	chr17	TBC1D3	37465683	0
CEU	chr17	TBC1D3	37515683	0
CEU	chr17	TBC1D3	37565683	0
CEU	chr17	TBC1D3	37615683	0
CEU	chr17	TBC1D3	37665683	0
CEU	chr17	TBC1D3	37715683	0
CEU	chr17	TBC1D3	37765683	0
CEU	chr17	TBC1D3	37815683	0
CEU	chr17	TBC1D3	37865683	0
CEU	chr17	TBC1D3	37915683	0
CEU	chr17	TBC1D3	37965683	0
CEU	chr17	TBC1D3	38015683	0

CEU	chr17	TBC1D3	38065683	0
CEU	chr17	TBC1D3	38115683	0
CEU	chr17	TBC1D3	38165683	0
CEU	chr17	TBC1D3	38215683	3
CEU	chr17	TBC1D3	38265683	21
CEU	chr17	TBC1D3	38315683	33
CEU	chr17	TBC1D3	38365683	32
CEU	chr17	TBC1D3	38415683	36
CEU	chr17	TBC1D3	38465683	42
CEU	chr17	TBC1D3	38515683	49
CEU	chr17	TBC1D3	38565683	43
CEU	chr17	TBC1D3	38615683	35
CEU	chr17	TBC1D3	38665683	18
CEU	chr17	TBC1D3	38715683	0
CEU	chr17	TBC1D3	38765683	0
CEU	chr17	TBC1D3	38815683	0
CEU	chr17	TBC1D3	38865683	0
CEU	chr17	TBC1D3	38915683	0
CEU	chr17	TBC1D3	38965683	0
CEU	chr17	TBC1D3	39015683	0
CEU	chr17	TBC1D3	39065683	0
CEU	chr17	TBC1D3	39115683	0
CEU	chr17	TBC1D3	39165683	0
CEU	chr17	TBC1D3	39215683	0
CEU	chr17	TBC1D3	39265683	0
CEU	chr17	TBC1D3	39315683	0
CEU	chr17	TBC1D3	39365683	0
CEU	chr17	TBC1D3	39415683	0
CEU	chr17	TBC1D3	39465683	0
CEU	chr17	TBC1D3	39515683	0
CEU	chr17	TBC1D3	39565683	0
CEU	chr17	TBC1D3	39615683	0
CEU	chr17	TBC1D3	39665683	0
CEU	chr17	TBC1D3	39715683	0
CEU	chr17	TBC1D3	39765683	0
CEU	chr17	TBC1D3	39815683	57
CEU	chr17	TBC1D3	39865683	74
CEU	chr17	TBC1D3	39915683	26
CEU	chr17	TBC1D3	39965683	9
CEU	chr17	TBC1D3	40015683	0
CEU	chr17	TBC1D3	40065683	0
CEU	chr17	TBC1D3	40115683	0
CEU	chr17	TBC1D3	40165683	0
CEU	chr17	TBC1D3	40215683	0
CEU	chr17	TBC1D3	40265683	0
CEU	chr17	TBC1D3	40315683	0
CEU	chr17	TBC1D3	40365683	0
CEU	chr17	TBC1D3	40415683	0
CEU	chr17	TBC1D3	40465683	0

CEU	chr17	TBC1D3	40515683	0
CEU	chr17	TBC1D3	40565683	0
CEU	chr17	TBC1D3	40615683	0
CEU	chr17	TBC1D3	40665683	0
CEU	chr17	TBC1D3	40715683	0
CEU	chr17	TBC1D3	40765683	0
CEU	chr17	TBC1D3	40815683	0
CEU	chr17	TBC1D3	40865683	0
CEU	chr17	TBC1D3	40915683	0
CEU	chr17	TBC1D3	40965683	0
CEU	chr17	TBC1D3	41015683	0
CEU	chr17	TBC1D3	41065683	0
CEU	chr17	TBC1D3	41115683	0
CEU	chr16	TP53TG3	27301603	0
CEU	chr16	TP53TG3	27351603	0
CEU	chr16	TP53TG3	27401603	0
CEU	chr16	TP53TG3	27451603	0
CEU	chr16	TP53TG3	27501603	0
CEU	chr16	TP53TG3	27551603	0
CEU	chr16	TP53TG3	27601603	0
CEU	chr16	TP53TG3	27651603	0
CEU	chr16	TP53TG3	27701603	0
CEU	chr16	TP53TG3	27751603	0
CEU	chr16	TP53TG3	27801603	0
CEU	chr16	TP53TG3	27851603	0
CEU	chr16	TP53TG3	27901603	0
CEU	chr16	TP53TG3	27951603	0
CEU	chr16	TP53TG3	28001603	0
CEU	chr16	TP53TG3	28051603	0
CEU	chr16	TP53TG3	28101603	0
CEU	chr16	TP53TG3	28151603	0
CEU	chr16	TP53TG3	28201603	0
CEU	chr16	TP53TG3	28251603	0
CEU	chr16	TP53TG3	28301603	0
CEU	chr16	TP53TG3	28351603	0
CEU	chr16	TP53TG3	28401603	0
CEU	chr16	TP53TG3	28451603	0
CEU	chr16	TP53TG3	28501603	0
CEU	chr16	TP53TG3	28551603	0
CEU	chr16	TP53TG3	28601603	0
CEU	chr16	TP53TG3	28651603	0
CEU	chr16	TP53TG3	28701603	0
CEU	chr16	TP53TG3	28751603	0
CEU	chr16	TP53TG3	28801603	0
CEU	chr16	TP53TG3	28851603	0
CEU	chr16	TP53TG3	28901603	0
CEU	chr16	TP53TG3	28951603	0
CEU	chr16	TP53TG3	29001603	0
CEU	chr16	TP53TG3	29051603	0

CEU	chr16	TP53TG3	29101603	0
CEU	chr16	TP53TG3	29151603	0
CEU	chr16	TP53TG3	29201603	0
CEU	chr16	TP53TG3	29251603	0
CEU	chr16	TP53TG3	29301603	0
CEU	chr16	TP53TG3	29351603	0
CEU	chr16	TP53TG3	29401603	0
CEU	chr16	TP53TG3	29451603	0
CEU	chr16	TP53TG3	29501603	0
CEU	chr16	TP53TG3	29551603	0
CEU	chr16	TP53TG3	29601603	0
CEU	chr16	TP53TG3	29651603	0
CEU	chr16	TP53TG3	29701603	0
CEU	chr16	TP53TG3	29751603	0
CEU	chr16	TP53TG3	29801603	0
CEU	chr16	TP53TG3	29851603	0
CEU	chr16	TP53TG3	29901603	0
CEU	chr16	TP53TG3	29951603	0
CEU	chr16	TP53TG3	30001603	0
CEU	chr16	TP53TG3	30051603	0
CEU	chr16	TP53TG3	30101603	0
CEU	chr16	TP53TG3	30151603	0
CEU	chr16	TP53TG3	30201603	0
CEU	chr16	TP53TG3	30251603	0
CEU	chr16	TP53TG3	30301603	0
CEU	chr16	TP53TG3	30351603	0
CEU	chr16	TP53TG3	30401603	0
CEU	chr16	TP53TG3	30451603	0
CEU	chr16	TP53TG3	30501603	0
CEU	chr16	TP53TG3	30551603	0
CEU	chr16	TP53TG3	30601603	0
CEU	chr16	TP53TG3	30651603	0
CEU	chr16	TP53TG3	30701603	0
CEU	chr16	TP53TG3	30751603	0
CEU	chr16	TP53TG3	30801603	0
CEU	chr16	TP53TG3	30851603	0
CEU	chr16	TP53TG3	30901603	0
CEU	chr16	TP53TG3	30951603	0
CEU	chr16	TP53TG3	31001603	0
CEU	chr16	TP53TG3	31051603	0
CEU	chr16	TP53TG3	31101603	0
CEU	chr16	TP53TG3	31151603	0
CEU	chr16	TP53TG3	31201603	0
CEU	chr16	TP53TG3	31251603	0
CEU	chr16	TP53TG3	31301603	0
CEU	chr16	TP53TG3	31351603	0
CEU	chr16	TP53TG3	31401603	0
CEU	chr16	TP53TG3	31451603	0
CEU	chr16	TP53TG3	31501603	0

CEU	chr16	TP53TG3	31551603	0
CEU	chr16	TP53TG3	31601603	0
CEU	chr16	TP53TG3	31651603	0
CEU	chr16	TP53TG3	31701603	0
CEU	chr16	TP53TG3	31751603	0
CEU	chr16	TP53TG3	31801603	0
CEU	chr16	TP53TG3	31851603	0
CEU	chr16	TP53TG3	31901603	0
CEU	chr16	TP53TG3	31951603	0
CEU	chr16	TP53TG3	32001603	0
CEU	chr16	TP53TG3	32051603	0
CEU	chr16	TP53TG3	32101603	0
CEU	chr16	TP53TG3	32151603	0
CEU	chr16	TP53TG3	32201603	0
CEU	chr16	TP53TG3	32251603	0
CEU	chr16	TP53TG3	32301603	0
CEU	chr16	TP53TG3	32351603	0
CEU	chr16	TP53TG3	32401603	0
CEU	chr16	TP53TG3	32451603	0
CEU	chr16	TP53TG3	32501603	0
CEU	chr16	TP53TG3	32551603	0
CEU	chr16	TP53TG3	32601603	0
CEU	chr16	TP53TG3	32651603	0
CEU	chr16	TP53TG3	32701603	0
CEU	chr16	TP53TG3	32751603	0
CEU	chr16	TP53TG3	32801603	0
CEU	chr16	TP53TG3	32851603	0
CEU	chr16	TP53TG3	32901603	0
CEU	chr16	TP53TG3	32951603	0
CEU	chr16	TP53TG3	33001603	0
CEU	chr16	TP53TG3	33051603	0
CEU	chr16	TP53TG3	33101603	0
CEU	chr16	TP53TG3	33151603	0
CEU	chr16	TP53TG3	33201603	0
CEU	chr16	TP53TG3	33251603	0
CEU	chr16	TP53TG3	33301603	0
CEU	chr16	TP53TG3	33351603	0
CEU	chr16	TP53TG3	33401603	0
CEU	chr16	TP53TG3	33451603	0
CEU	chr16	TP53TG3	33501603	0
CEU	chr16	TP53TG3	33551603	0
CEU	chr16	TP53TG3	33601603	0
CEU	chr16	TP53TG3	33651603	0
CEU	chr16	TP53TG3	33701603	0
CEU	chr16	TP53TG3	33751603	0
CEU	chr16	TP53TG3	33801603	0
CEU	chr16	TP53TG3	33851603	0
CEU	chr16	TP53TG3	33901603	0
CEU	chr16	TP53TG3	33951603	0

CEU	chr16	TP53TG3	34001603	0
CEU	chr16	TP53TG3	34051603	0
CEU	chr16	TP53TG3	34101603	0
CEU	chr16	TP53TG3	34151603	0
CEU	chr16	TP53TG3	34201603	0
CEU	chr16	TP53TG3	34251603	0
CEU	chr16	TP53TG3	34301603	0
CEU	chr16	TP53TG3	34351603	0
CEU	chr16	TP53TG3	34401603	0
CEU	chr16	TP53TG3	34451603	0
CEU	chr16	TP53TG3	34501603	0
CEU	chr16	TP53TG3	34551603	0
CEU	chr16	TP53TG3	34601603	0
CEU	chr16	TP53TG3	34651603	0
CEU	chr16	TP53TG3	34701603	0
CEU	chr16	TP53TG3	34751603	0
CEU	chr16	TP53TG3	34801603	0
CEU	chr16	TP53TG3	34851603	0
CEU	chr16	TP53TG3	34901603	0
CEU	chr16	TP53TG3	34951603	0
CEU	chr16	TP53TG3	35001603	0
CEU	chr16	TP53TG3	35051603	0
CEU	chr16	TP53TG3	35101603	0
CEU	chr16	TP53TG3	35151603	0
CEU	chr16	TP53TG3	35201603	0
CEU	chr16	TP53TG3	35251603	0
CEU	chr16	TP53TG3	35301603	0
CEU	chr16	TP53TG3	35351603	0
CEU	chr16	TP53TG3	35401603	0
CEU	chr16	TP53TG3	35451603	0
CEU	chr16	TP53TG3	35501603	0
CEU	chr16	TP53TG3	35551603	0
CEU	chr16	TP53TG3	35601603	0
CEU	chr16	TP53TG3	35651603	0
CEU	chr16	TP53TG3	35701603	0
CEU	chr16	TP53TG3	35751603	0
CEU	chr16	TP53TG3	35801603	0
CEU	chr16	TP53TG3	35851603	0
CEU	chr16	TP53TG3	35901603	0
CEU	chr16	TP53TG3	35951603	0
CEU	chr16	TP53TG3	36001603	0
CEU	chr16	TP53TG3	36051603	0
CEU	chr16	TP53TG3	36101603	0
CEU	chr16	TP53TG3	36151603	0
CEU	chr16	TP53TG3	36201603	0
CEU	chr16	TP53TG3	36251603	0
CEU	chr16	TP53TG3	36301603	0
CEU	chr16	TP53TG3	36351603	0
CEU	chr16	TP53TG3	36401603	0

CEU	chr16	TP53TG3	36451603	0
CEU	chr16	TP53TG3	36501603	0
CEU	chr16	TP53TG3	36551603	0
CEU	chr16	TP53TG3	36601603	0
CEU	chr16	TP53TG3	36651603	0
CEU	chr16	TP53TG3	36701603	0
CEU	chr16	TP53TG3	36751603	0
CEU	chr16	TP53TG3	36801603	0
CEU	chr16	TP53TG3	36851603	0
CEU	chr16	TP53TG3	36901603	0
CEU	chr16	TP53TG3	36951603	0
CEU	chr16	TP53TG3	37001603	0
CEU	chr16	TP53TG3	37051603	0
CEU	chr16	TP53TG3	37101603	0
CEU	chr16	TP53TG3	37151603	0
CEU	chr16	TP53TG3	37201603	0
CEU	chr11	TRIM49L1	84974064	33
CEU	chr11	TRIM49L1	85024064	33
CEU	chr11	TRIM49L1	85074064	31
CEU	chr11	TRIM49L1	85124064	32
CEU	chr11	TRIM49L1	85174064	31
CEU	chr11	TRIM49L1	85224064	39
CEU	chr11	TRIM49L1	85274064	39
CEU	chr11	TRIM49L1	85324064	29
CEU	chr11	TRIM49L1	85374064	23
CEU	chr11	TRIM49L1	85424064	33
CEU	chr11	TRIM49L1	85474064	41
CEU	chr11	TRIM49L1	85524064	28
CEU	chr11	TRIM49L1	85574064	12
CEU	chr11	TRIM49L1	85624064	4
CEU	chr11	TRIM49L1	85674064	0
CEU	chr11	TRIM49L1	85724064	0
CEU	chr11	TRIM49L1	85774064	0
CEU	chr11	TRIM49L1	85824064	0
CEU	chr11	TRIM49L1	85874064	0
CEU	chr11	TRIM49L1	85924064	0
CEU	chr11	TRIM49L1	85974064	0
CEU	chr11	TRIM49L1	86024064	0
CEU	chr11	TRIM49L1	86074064	0
CEU	chr11	TRIM49L1	86124064	0
CEU	chr11	TRIM49L1	86174064	0
CEU	chr11	TRIM49L1	86224064	0
CEU	chr11	TRIM49L1	86274064	10
CEU	chr11	TRIM49L1	86324064	26
CEU	chr11	TRIM49L1	86374064	52
CEU	chr11	TRIM49L1	86424064	63
CEU	chr11	TRIM49L1	86474064	59
CEU	chr11	TRIM49L1	86524064	49
CEU	chr11	TRIM49L1	86574064	31

CEU	chr11	TRIM49L1	86624064	14
CEU	chr11	TRIM49L1	86674064	0
CEU	chr11	TRIM49L1	86724064	0
CEU	chr11	TRIM49L1	86774064	0
CEU	chr11	TRIM49L1	86824064	0
CEU	chr11	TRIM49L1	86874064	0
CEU	chr11	TRIM49L1	86924064	0
CEU	chr11	TRIM49L1	86974064	0
CEU	chr11	TRIM49L1	87024064	0
CEU	chr11	TRIM49L1	87074064	0
CEU	chr11	TRIM49L1	87124064	0
CEU	chr11	TRIM49L1	87174064	0
CEU	chr11	TRIM49L1	87224064	0
CEU	chr11	TRIM49L1	87274064	0
CEU	chr11	TRIM49L1	87324064	0
CEU	chr11	TRIM49L1	87374064	0
CEU	chr11	TRIM49L1	87424064	4
CEU	chr11	TRIM49L1	87474064	25
CEU	chr11	TRIM49L1	87524064	42
CEU	chr11	TRIM49L1	87574064	47
CEU	chr11	TRIM49L1	87624064	26
CEU	chr11	TRIM49L1	87674064	0
CEU	chr11	TRIM49L1	87724064	0
CEU	chr11	TRIM49L1	87774064	0
CEU	chr11	TRIM49L1	87824064	0
CEU	chr11	TRIM49L1	87874064	0
CEU	chr11	TRIM49L1	87924064	0
CEU	chr11	TRIM49L1	87974064	0
CEU	chr11	TRIM49L1	88024064	0
CEU	chr11	TRIM49L1	88074064	0
CEU	chr11	TRIM49L1	88124064	0
CEU	chr11	TRIM49L1	88174064	0
CEU	chr11	TRIM49L1	88224064	0
CEU	chr11	TRIM49L1	88274064	16
CEU	chr11	TRIM49L1	88324064	54
CEU	chr11	TRIM49L1	88374064	50
CEU	chr11	TRIM49L1	88424064	35
CEU	chr11	TRIM49L1	88474064	43
CEU	chr11	TRIM49L1	88524064	41
CEU	chr11	TRIM49L1	88574064	72
CEU	chr11	TRIM49L1	88624064	75
CEU	chr11	TRIM49L1	88674064	38
CEU	chr11	TRIM49L1	88724064	42
CEU	chr11	TRIM49L1	88774064	44
CEU	chr11	TRIM49L1	88824064	31
CEU	chr11	TRIM49L1	88874064	33
CEU	chr11	TRIM49L1	88924064	18
CEU	chr11	TRIM49L1	88974064	0
CEU	chr11	TRIM49L1	89024064	0

CEU	chr11	TRIM49L1	89074064	0
CEU	chr11	TRIM49L1	89124064	0
CEU	chr11	TRIM49L1	89174064	5
CEU	chr11	TRIM49L1	89224064	27
CEU	chr11	TRIM49L1	89274064	46
CEU	chr11	TRIM49L1	89324064	54
CEU	chr11	TRIM49L1	89374064	68
CEU	chr11	TRIM49L1	89424064	57
CEU	chr11	TRIM49L1	89474064	31
CEU	chr11	TRIM49L1	89524064	14
CEU	chr11	TRIM49L1	89574064	2
CEU	chr11	TRIM49L1	89624064	0
CEU	chr11	TRIM49L1	89674064	0
CEU	chr11	TRIM49L1	89724064	0
CEU	chr11	TRIM49L1	89774064	0
CEU	chr11	TRIM49L1	89824064	0
CEU	chr11	TRIM49L1	89874064	0
CEU	chr11	TRIM49L1	89924064	0
CEU	chr11	TRIM49L1	89974064	0
CEU	chr11	TRIM49L1	90024064	0
CEU	chr11	TRIM49L1	90074064	0
CEU	chr11	TRIM49L1	90124064	0
CEU	chr11	TRIM49L1	90174064	0
CEU	chr11	TRIM49L1	90224064	0
CEU	chr11	TRIM49L1	90274064	0
CEU	chr11	TRIM49L1	90324064	0
CEU	chr11	TRIM49L1	90374064	0
CEU	chr11	TRIM49L1	90424064	0
CEU	chr11	TRIM49L1	90474064	0
CEU	chr11	TRIM49L1	90524064	0
CEU	chr11	TRIM49L1	90574064	0
CEU	chr11	TRIM49L1	90624064	0
CEU	chr11	TRIM49L1	90674064	0
CEU	chr11	TRIM49L1	90724064	0
CEU	chr11	TRIM49L1	90774064	0
CEU	chr11	TRIM49L1	90824064	0
CEU	chr11	TRIM49L1	90874064	0
CEU	chr11	TRIM49L1	90924064	0
CEU	chr11	TRIM49L1	90974064	0
CEU	chr11	TRIM49L1	91024064	0
CEU	chr11	TRIM49L1	91074064	0
CEU	chr11	TRIM49L1	91124064	0
CEU	chr11	TRIM49L1	91174064	0
CEU	chr11	TRIM49L1	91224064	0
CEU	chr11	TRIM49L1	91274064	0
CEU	chr11	TRIM49L1	91324064	0
CEU	chr11	TRIM49L1	91374064	0
CEU	chr11	TRIM49L1	91424064	0
CEU	chr11	TRIM49L1	91474064	0

CEU	chr11	TRIM49L1	91524064	0
CEU	chr11	TRIM49L1	91574064	0
CEU	chr11	TRIM49L1	91624064	0
CEU	chr11	TRIM49L1	91674064	0
CEU	chr11	TRIM49L1	91724064	0
CEU	chr11	TRIM49L1	91774064	0
CEU	chr11	TRIM49L1	91824064	0
CEU	chr11	TRIM49L1	91874064	0
CEU	chr11	TRIM49L1	91924064	0
CEU	chr11	TRIM49L1	91974064	0
CEU	chr11	TRIM49L1	92024064	0
CEU	chr11	TRIM49L1	92074064	0
CEU	chr11	TRIM49L1	92124064	0
CEU	chr11	TRIM49L1	92174064	0
CEU	chr11	TRIM49L1	92224064	0
CEU	chr11	TRIM49L1	92274064	0
CEU	chr11	TRIM49L1	92324064	0
CEU	chr11	TRIM49L1	92374064	0
CEU	chr11	TRIM49L1	92424064	0
CEU	chr11	TRIM49L1	92474064	0
CEU	chr11	TRIM49L1	92524064	0
CEU	chr11	TRIM49L1	92574064	0
CEU	chr11	TRIM49L1	92624064	0
CEU	chr11	TRIM49L1	92674064	0
CEU	chr11	TRIM49L1	92724064	0
CEU	chr11	TRIM49L1	92774064	0
CEU	chr11	TRIM49L1	92824064	0
CEU	chr11	TRIM49L1	92874064	0
CEU	chr11	TRIM49L1	92924064	0
CEU	chr11	TRIM49L1	92974064	0
CEU	chr11	TRIM49L1	93024064	0
CEU	chr11	TRIM49L1	93074064	0
CEU	chr11	TRIM49L1	93124064	0
CEU	chr11	TRIM49L1	93174064	0
CEU	chr11	TRIM49L1	93224064	0
CEU	chr11	TRIM49L1	93274064	0
CEU	chr11	TRIM49L1	93324064	0
CEU	chr11	TRIM49L1	93374064	0
CEU	chr11	TRIM49L1	93424064	0
CEU	chr11	TRIM49L1	93474064	0
CEU	chr11	TRIM49L1	93524064	0
CEU	chr11	TRIM49L1	93574064	0
CEU	chr11	TRIM49L1	93624064	0
CEU	chr11	TRIM49L1	93674064	0
CEU	chr11	TRIM49L1	93724064	0
CEU	chr11	TRIM49L1	93774064	0
CEU	chr11	TRIM49L1	93824064	0
CEU	chr11	TRIM49L1	93874064	0
CEU	chr11	TRIM49L1	93924064	0

CEU	chr11	TRIM49L1	93974064	0
CEU	chr11	TRIM49L1	94024064	0
CEU	chr11	TRIM49L1	94074064	0
CEU	chr11	TRIM49L1	94124064	0
CEU	chr11	TRIM49L1	94174064	0
CEU	chr11	TRIM49L1	94224064	0
CEU	chr11	TRIM49L1	94274064	0
CEU	chr11	TRIM49L1	94324064	0
CEU	chr11	TRIM49L1	94374064	0
CEU	chr11	TRIM49L1	94424064	0
CEU	chr11	TRIM49L1	94474064	0
CEU	chr11	TRIM49L1	94524064	0
CEU	chr11	TRIM49L1	94574064	0
CEU	chr11	TRIM49L1	94624064	0
CEU	chr11	TRIM49L1	94674064	0
CEU	chr11	TRIM49L1	94724064	0
CEU	chr11	TRIM49L1	94774064	0
CEU	chr11	TRIM49L1	94824064	0
CEU	chr11	TRIM49L1	94874064	0
CHB	chr1	AMY1A	98705519	0
CHB	chr1	AMY1A	98755519	0
CHB	chr1	AMY1A	98805519	0
CHB	chr1	AMY1A	98855519	0
CHB	chr1	AMY1A	98905519	0
CHB	chr1	AMY1A	98955519	0
CHB	chr1	AMY1A	99005519	0
CHB	chr1	AMY1A	99055519	0
CHB	chr1	AMY1A	99105519	0
CHB	chr1	AMY1A	99155519	0
CHB	chr1	AMY1A	99205519	0
CHB	chr1	AMY1A	99255519	0
CHB	chr1	AMY1A	99305519	0
CHB	chr1	AMY1A	99355519	0
CHB	chr1	AMY1A	99405519	0
CHB	chr1	AMY1A	99455519	0
CHB	chr1	AMY1A	99505519	0
CHB	chr1	AMY1A	99555519	0
CHB	chr1	AMY1A	99605519	0
CHB	chr1	AMY1A	99655519	0
CHB	chr1	AMY1A	99705519	0
CHB	chr1	AMY1A	99755519	0
CHB	chr1	AMY1A	99805519	0
CHB	chr1	AMY1A	99855519	0
CHB	chr1	AMY1A	99905519	0
CHB	chr1	AMY1A	99955519	0
CHB	chr1	AMY1A	100005519	0
CHB	chr1	AMY1A	100055519	0
CHB	chr1	AMY1A	100105519	0
CHB	chr1	AMY1A	100155519	0

CHB	chr1	AMY1A	100205519	0
CHB	chr1	AMY1A	100255519	0
CHB	chr1	AMY1A	100305519	0
CHB	chr1	AMY1A	100355519	0
CHB	chr1	AMY1A	100405519	0
CHB	chr1	AMY1A	100455519	0
CHB	chr1	AMY1A	100505519	0
CHB	chr1	AMY1A	100555519	0
CHB	chr1	AMY1A	100605519	0
CHB	chr1	AMY1A	100655519	0
CHB	chr1	AMY1A	100705519	0
CHB	chr1	AMY1A	100755519	0
CHB	chr1	AMY1A	100805519	0
CHB	chr1	AMY1A	100855519	0
CHB	chr1	AMY1A	100905519	0
CHB	chr1	AMY1A	100955519	0
CHB	chr1	AMY1A	101005519	0
CHB	chr1	AMY1A	101055519	0
CHB	chr1	AMY1A	101105519	0
CHB	chr1	AMY1A	101155519	0
CHB	chr1	AMY1A	101205519	0
CHB	chr1	AMY1A	101255519	0
CHB	chr1	AMY1A	101305519	0
CHB	chr1	AMY1A	101355519	0
CHB	chr1	AMY1A	101405519	0
CHB	chr1	AMY1A	101455519	0
CHB	chr1	AMY1A	101505519	0
CHB	chr1	AMY1A	101555519	0
CHB	chr1	AMY1A	101605519	0
CHB	chr1	AMY1A	101655519	0
CHB	chr1	AMY1A	101705519	0
CHB	chr1	AMY1A	101755519	0
CHB	chr1	AMY1A	101805519	0
CHB	chr1	AMY1A	101855519	0
CHB	chr1	AMY1A	101905519	0
CHB	chr1	AMY1A	101955519	0
CHB	chr1	AMY1A	102005519	0
CHB	chr1	AMY1A	102055519	0
CHB	chr1	AMY1A	102105519	0
CHB	chr1	AMY1A	102155519	0
CHB	chr1	AMY1A	102205519	0
CHB	chr1	AMY1A	102255519	0
CHB	chr1	AMY1A	102305519	0
CHB	chr1	AMY1A	102355519	0
CHB	chr1	AMY1A	102405519	0
CHB	chr1	AMY1A	102455519	0
CHB	chr1	AMY1A	102505519	0
CHB	chr1	AMY1A	102555519	0
CHB	chr1	AMY1A	102605519	0

CHB	chr1	AMY1A	102655519	0
CHB	chr1	AMY1A	102705519	0
CHB	chr1	AMY1A	102755519	0
CHB	chr1	AMY1A	102805519	0
CHB	chr1	AMY1A	102855519	0
CHB	chr1	AMY1A	102905519	0
CHB	chr1	AMY1A	102955519	0
CHB	chr1	AMY1A	103005519	0
CHB	chr1	AMY1A	103055519	0
CHB	chr1	AMY1A	103105519	0
CHB	chr1	AMY1A	103155519	0
CHB	chr1	AMY1A	103205519	0
CHB	chr1	AMY1A	103255519	0
CHB	chr1	AMY1A	103305519	0
CHB	chr1	AMY1A	103355519	0
CHB	chr1	AMY1A	103405519	0
CHB	chr1	AMY1A	103455519	0
CHB	chr1	AMY1A	103505519	0
CHB	chr1	AMY1A	103555519	0
CHB	chr1	AMY1A	103605519	0
CHB	chr1	AMY1A	103655519	0
CHB	chr1	AMY1A	103705519	0
CHB	chr1	AMY1A	103755519	0
CHB	chr1	AMY1A	103805519	0
CHB	chr1	AMY1A	103855519	0
CHB	chr1	AMY1A	103905519	0
CHB	chr1	AMY1A	103955519	0
CHB	chr1	AMY1A	104005519	0
CHB	chr1	AMY1A	104055519	0
CHB	chr1	AMY1A	104105519	0
CHB	chr1	AMY1A	104155519	0
CHB	chr1	AMY1A	104205519	0
CHB	chr1	AMY1A	104255519	0
CHB	chr1	AMY1A	104305519	0
CHB	chr1	AMY1A	104355519	0
CHB	chr1	AMY1A	104405519	0
CHB	chr1	AMY1A	104455519	0
CHB	chr1	AMY1A	104505519	0
CHB	chr1	AMY1A	104555519	0
CHB	chr1	AMY1A	104605519	0
CHB	chr1	AMY1A	104655519	0
CHB	chr1	AMY1A	104705519	0
CHB	chr1	AMY1A	104755519	0
CHB	chr1	AMY1A	104805519	0
CHB	chr1	AMY1A	104855519	0
CHB	chr1	AMY1A	104905519	0
CHB	chr1	AMY1A	104955519	0
CHB	chr1	AMY1A	105005519	0
CHB	chr1	AMY1A	105055519	0

CHB	chr1	AMY1A	105105519	0
CHB	chr1	AMY1A	105155519	0
CHB	chr1	AMY1A	105205519	0
CHB	chr1	AMY1A	105255519	0
CHB	chr1	AMY1A	105305519	0
CHB	chr1	AMY1A	105355519	0
CHB	chr1	AMY1A	105405519	0
CHB	chr1	AMY1A	105455519	0
CHB	chr1	AMY1A	105505519	0
CHB	chr1	AMY1A	105555519	0
CHB	chr1	AMY1A	105605519	0
CHB	chr1	AMY1A	105655519	0
CHB	chr1	AMY1A	105705519	0
CHB	chr1	AMY1A	105755519	0
CHB	chr1	AMY1A	105805519	0
CHB	chr1	AMY1A	105855519	0
CHB	chr1	AMY1A	105905519	0
CHB	chr1	AMY1A	105955519	0
CHB	chr1	AMY1A	106005519	0
CHB	chr1	AMY1A	106055519	0
CHB	chr1	AMY1A	106105519	0
CHB	chr1	AMY1A	106155519	0
CHB	chr1	AMY1A	106205519	0
CHB	chr1	AMY1A	106255519	0
CHB	chr1	AMY1A	106305519	0
CHB	chr1	AMY1A	106355519	0
CHB	chr1	AMY1A	106405519	0
CHB	chr1	AMY1A	106455519	0
CHB	chr1	AMY1A	106505519	0
CHB	chr1	AMY1A	106555519	0
CHB	chr1	AMY1A	106605519	0
CHB	chr1	AMY1A	106655519	0
CHB	chr1	AMY1A	106705519	0
CHB	chr1	AMY1A	106755519	0
CHB	chr1	AMY1A	106805519	0
CHB	chr1	AMY1A	106855519	0
CHB	chr1	AMY1A	106905519	0
CHB	chr1	AMY1A	106955519	0
CHB	chr1	AMY1A	107005519	0
CHB	chr1	AMY1A	107055519	0
CHB	chr1	AMY1A	107105519	0
CHB	chr1	AMY1A	107155519	0
CHB	chr1	AMY1A	107205519	0
CHB	chr1	AMY1A	107255519	0
CHB	chr1	AMY1A	107305519	0
CHB	chr1	AMY1A	107355519	0
CHB	chr1	AMY1A	107405519	0
CHB	chr1	AMY1A	107455519	0
CHB	chr1	AMY1A	107505519	0

CHB	chr1	AMY1A	107555519	0
CHB	chr1	AMY1A	107605519	0
CHB	chr1	AMY1A	107655519	0
CHB	chr1	AMY1A	107705519	0
CHB	chr1	AMY1A	107755519	0
CHB	chr1	AMY1A	107805519	0
CHB	chr1	AMY1A	107855519	0
CHB	chr1	AMY1A	107905519	0
CHB	chr1	AMY1A	107955519	0
CHB	chr1	AMY1A	108005519	0
CHB	chr1	AMY1A	108055519	0
CHB	chr1	AMY1A	108105519	0
CHB	chr1	AMY1A	108155519	0
CHB	chr1	AMY1A	108205519	0
CHB	chr1	AMY1A	108255519	0
CHB	chr1	AMY1A	108305519	0
CHB	chr1	AMY1A	108355519	0
CHB	chr1	AMY1A	108405519	0
CHB	chr1	AMY1A	108455519	0
CHB	chr1	AMY1A	108505519	0
CHB	chr1	AMY1A	108555519	0
CHB	chr1	AMY1A	108605519	0
CHB	chr16	BOLA2B	24503588	0
CHB	chr16	BOLA2B	24553588	0
CHB	chr16	BOLA2B	24603588	0
CHB	chr16	BOLA2B	24653588	0
CHB	chr16	BOLA2B	24703588	0
CHB	chr16	BOLA2B	24753588	0
CHB	chr16	BOLA2B	24803588	0
CHB	chr16	BOLA2B	24853588	0
CHB	chr16	BOLA2B	24903588	0
CHB	chr16	BOLA2B	24953588	0
CHB	chr16	BOLA2B	25003588	0
CHB	chr16	BOLA2B	25053588	0
CHB	chr16	BOLA2B	25103588	0
CHB	chr16	BOLA2B	25153588	0
CHB	chr16	BOLA2B	25203588	0
CHB	chr16	BOLA2B	25253588	0
CHB	chr16	BOLA2B	25303588	0
CHB	chr16	BOLA2B	25353588	0
CHB	chr16	BOLA2B	25403588	0
CHB	chr16	BOLA2B	25453588	0
CHB	chr16	BOLA2B	25503588	0
CHB	chr16	BOLA2B	25553588	0
CHB	chr16	BOLA2B	25603588	0
CHB	chr16	BOLA2B	25653588	0
CHB	chr16	BOLA2B	25703588	0
CHB	chr16	BOLA2B	25753588	0
CHB	chr16	BOLA2B	25803588	0

CHB	chr16	BOLA2B	25853588	0
CHB	chr16	BOLA2B	25903588	7
CHB	chr16	BOLA2B	25953588	38
CHB	chr16	BOLA2B	26003588	31
CHB	chr16	BOLA2B	26053588	0
CHB	chr16	BOLA2B	26103588	0
CHB	chr16	BOLA2B	26153588	0
CHB	chr16	BOLA2B	26203588	0
CHB	chr16	BOLA2B	26253588	0
CHB	chr16	BOLA2B	26303588	0
CHB	chr16	BOLA2B	26353588	0
CHB	chr16	BOLA2B	26403588	0
CHB	chr16	BOLA2B	26453588	0
CHB	chr16	BOLA2B	26503588	0
CHB	chr16	BOLA2B	26553588	0
CHB	chr16	BOLA2B	26603588	0
CHB	chr16	BOLA2B	26653588	0
CHB	chr16	BOLA2B	26703588	0
CHB	chr16	BOLA2B	26753588	0
CHB	chr16	BOLA2B	26803588	0
CHB	chr16	BOLA2B	26853588	0
CHB	chr16	BOLA2B	26903588	0
CHB	chr16	BOLA2B	26953588	0
CHB	chr16	BOLA2B	27003588	0
CHB	chr16	BOLA2B	27053588	0
CHB	chr16	BOLA2B	27103588	0
CHB	chr16	BOLA2B	27153588	0
CHB	chr16	BOLA2B	27203588	0
CHB	chr16	BOLA2B	27253588	0
CHB	chr16	BOLA2B	27303588	0
CHB	chr16	BOLA2B	27353588	0
CHB	chr16	BOLA2B	27403588	0
CHB	chr16	BOLA2B	27453588	0
CHB	chr16	BOLA2B	27503588	0
CHB	chr16	BOLA2B	27553588	0
CHB	chr16	BOLA2B	27603588	0
CHB	chr16	BOLA2B	27653588	0
CHB	chr16	BOLA2B	27703588	0
CHB	chr16	BOLA2B	27753588	0
CHB	chr16	BOLA2B	27803588	0
CHB	chr16	BOLA2B	27853588	0
CHB	chr16	BOLA2B	27903588	0
CHB	chr16	BOLA2B	27953588	0
CHB	chr16	BOLA2B	28003588	0
CHB	chr16	BOLA2B	28053588	0
CHB	chr16	BOLA2B	28103588	0
CHB	chr16	BOLA2B	28153588	0
CHB	chr16	BOLA2B	28203588	0
CHB	chr16	BOLA2B	28253588	0

CHB	chr16	BOLA2B	28303588	0
CHB	chr16	BOLA2B	28353588	0
CHB	chr16	BOLA2B	28403588	0
CHB	chr16	BOLA2B	28453588	0
CHB	chr16	BOLA2B	28503588	0
CHB	chr16	BOLA2B	28553588	0
CHB	chr16	BOLA2B	28603588	0
CHB	chr16	BOLA2B	28653588	0
CHB	chr16	BOLA2B	28703588	0
CHB	chr16	BOLA2B	28753588	0
CHB	chr16	BOLA2B	28803588	0
CHB	chr16	BOLA2B	28853588	0
CHB	chr16	BOLA2B	28903588	0
CHB	chr16	BOLA2B	28953588	0
CHB	chr16	BOLA2B	29003588	0
CHB	chr16	BOLA2B	29053588	0
CHB	chr16	BOLA2B	29103588	0
CHB	chr16	BOLA2B	29153588	0
CHB	chr16	BOLA2B	29203588	0
CHB	chr16	BOLA2B	29253588	0
CHB	chr16	BOLA2B	29303588	0
CHB	chr16	BOLA2B	29353588	0
CHB	chr16	BOLA2B	29403588	0
CHB	chr16	BOLA2B	29453588	0
CHB	chr16	BOLA2B	29503588	0
CHB	chr16	BOLA2B	29553588	0
CHB	chr16	BOLA2B	29603588	0
CHB	chr16	BOLA2B	29653588	0
CHB	chr16	BOLA2B	29703588	0
CHB	chr16	BOLA2B	29753588	0
CHB	chr16	BOLA2B	29803588	0
CHB	chr16	BOLA2B	29853588	0
CHB	chr16	BOLA2B	29903588	0
CHB	chr16	BOLA2B	29953588	0
CHB	chr16	BOLA2B	30003588	0
CHB	chr16	BOLA2B	30053588	0
CHB	chr16	BOLA2B	30103588	0
CHB	chr16	BOLA2B	30153588	0
CHB	chr16	BOLA2B	30203588	0
CHB	chr16	BOLA2B	30253588	0
CHB	chr16	BOLA2B	30303588	0
CHB	chr16	BOLA2B	30353588	0
CHB	chr16	BOLA2B	30403588	0
CHB	chr16	BOLA2B	30453588	0
CHB	chr16	BOLA2B	30503588	0
CHB	chr16	BOLA2B	30553588	0
CHB	chr16	BOLA2B	30603588	0
CHB	chr16	BOLA2B	30653588	0
CHB	chr16	BOLA2B	30703588	0

CHB	chr16	BOLA2B	30753588	0
CHB	chr16	BOLA2B	30803588	0
CHB	chr16	BOLA2B	30853588	0
CHB	chr16	BOLA2B	30903588	0
CHB	chr16	BOLA2B	30953588	0
CHB	chr16	BOLA2B	31003588	0
CHB	chr16	BOLA2B	31053588	0
CHB	chr16	BOLA2B	31103588	0
CHB	chr16	BOLA2B	31153588	0
CHB	chr16	BOLA2B	31203588	0
CHB	chr16	BOLA2B	31253588	0
CHB	chr16	BOLA2B	31303588	0
CHB	chr16	BOLA2B	31353588	0
CHB	chr16	BOLA2B	31403588	0
CHB	chr16	BOLA2B	31453588	0
CHB	chr16	BOLA2B	31503588	0
CHB	chr16	BOLA2B	31553588	0
CHB	chr16	BOLA2B	31603588	0
CHB	chr16	BOLA2B	31653588	0
CHB	chr16	BOLA2B	31703588	0
CHB	chr16	BOLA2B	31753588	0
CHB	chr16	BOLA2B	31803588	0
CHB	chr16	BOLA2B	31853588	0
CHB	chr16	BOLA2B	31903588	0
CHB	chr16	BOLA2B	31953588	0
CHB	chr16	BOLA2B	32003588	0
CHB	chr16	BOLA2B	32053588	0
CHB	chr16	BOLA2B	32103588	0
CHB	chr16	BOLA2B	32153588	0
CHB	chr16	BOLA2B	32203588	0
CHB	chr16	BOLA2B	32253588	0
CHB	chr16	BOLA2B	32303588	0
CHB	chr16	BOLA2B	32353588	0
CHB	chr16	BOLA2B	32403588	0
CHB	chr16	BOLA2B	32453588	0
CHB	chr16	BOLA2B	32503588	0
CHB	chr16	BOLA2B	32553588	0
CHB	chr16	BOLA2B	32603588	0
CHB	chr16	BOLA2B	32653588	0
CHB	chr16	BOLA2B	32703588	0
CHB	chr16	BOLA2B	32753588	0
CHB	chr16	BOLA2B	32803588	0
CHB	chr16	BOLA2B	32853588	0
CHB	chr16	BOLA2B	32903588	0
CHB	chr16	BOLA2B	32953588	0
CHB	chr16	BOLA2B	33003588	0
CHB	chr16	BOLA2B	33053588	0
CHB	chr16	BOLA2B	33103588	0
CHB	chr16	BOLA2B	33153588	0

CHB	chr16	BOLA2B	33203588	0
CHB	chr16	BOLA2B	33253588	0
CHB	chr16	BOLA2B	33303588	0
CHB	chr16	BOLA2B	33353588	0
CHB	chr16	BOLA2B	33403588	0
CHB	chr16	BOLA2B	33453588	0
CHB	chr16	BOLA2B	33503588	0
CHB	chr16	BOLA2B	33553588	0
CHB	chr16	BOLA2B	33603588	0
CHB	chr16	BOLA2B	33653588	0
CHB	chr16	BOLA2B	33703588	0
CHB	chr16	BOLA2B	33753588	0
CHB	chr16	BOLA2B	33803588	0
CHB	chr16	BOLA2B	33853588	0
CHB	chr16	BOLA2B	33903588	0
CHB	chr16	BOLA2B	33953588	0
CHB	chr16	BOLA2B	34003588	0
CHB	chr16	BOLA2B	34053588	0
CHB	chr16	BOLA2B	34103588	0
CHB	chr16	BOLA2B	34153588	0
CHB	chr16	BOLA2B	34203588	0
CHB	chr16	BOLA2B	34253588	0
CHB	chr16	BOLA2B	34303588	0
CHB	chr16	BOLA2B	34353588	0
CHB	chr16	BOLA2B	34403588	0
CHB	chr9	CBWD3	60716374	0
CHB	chr9	CBWD3	60766374	0
CHB	chr9	CBWD3	60816374	0
CHB	chr9	CBWD3	60866374	0
CHB	chr9	CBWD3	60916374	0
CHB	chr9	CBWD3	60966374	0
CHB	chr9	CBWD3	61016374	0
CHB	chr9	CBWD3	61066374	0
CHB	chr9	CBWD3	61116374	0
CHB	chr9	CBWD3	61166374	0
CHB	chr9	CBWD3	61216374	0
CHB	chr9	CBWD3	61266374	0
CHB	chr9	CBWD3	61316374	0
CHB	chr9	CBWD3	61366374	0
CHB	chr9	CBWD3	61416374	0
CHB	chr9	CBWD3	61466374	0
CHB	chr9	CBWD3	61516374	0
CHB	chr9	CBWD3	61566374	0
CHB	chr9	CBWD3	61616374	0
CHB	chr9	CBWD3	61666374	0
CHB	chr9	CBWD3	61716374	0
CHB	chr9	CBWD3	61766374	0
CHB	chr9	CBWD3	61816374	0
CHB	chr9	CBWD3	61866374	0

CHB	chr9	CBWD3	61916374	0
CHB	chr9	CBWD3	61966374	0
CHB	chr9	CBWD3	62016374	0
CHB	chr9	CBWD3	62066374	0
CHB	chr9	CBWD3	62116374	0
CHB	chr9	CBWD3	62166374	0
CHB	chr9	CBWD3	62216374	0
CHB	chr9	CBWD3	62266374	0
CHB	chr9	CBWD3	62316374	0
CHB	chr9	CBWD3	62366374	0
CHB	chr9	CBWD3	62416374	0
CHB	chr9	CBWD3	62466374	0
CHB	chr9	CBWD3	62516374	0
CHB	chr9	CBWD3	62566374	0
CHB	chr9	CBWD3	62616374	0
CHB	chr9	CBWD3	62666374	0
CHB	chr9	CBWD3	62716374	0
CHB	chr9	CBWD3	62766374	0
CHB	chr9	CBWD3	62816374	0
CHB	chr9	CBWD3	62866374	0
CHB	chr9	CBWD3	62916374	0
CHB	chr9	CBWD3	62966374	0
CHB	chr9	CBWD3	63016374	0
CHB	chr9	CBWD3	63066374	0
CHB	chr9	CBWD3	63116374	0
CHB	chr9	CBWD3	63166374	0
CHB	chr9	CBWD3	63216374	0
CHB	chr9	CBWD3	63266374	0
CHB	chr9	CBWD3	63316374	0
CHB	chr9	CBWD3	63366374	0
CHB	chr9	CBWD3	63416374	0
CHB	chr9	CBWD3	63466374	0
CHB	chr9	CBWD3	63516374	0
CHB	chr9	CBWD3	63566374	0
CHB	chr9	CBWD3	63616374	0
CHB	chr9	CBWD3	63666374	0
CHB	chr9	CBWD3	63716374	0
CHB	chr9	CBWD3	63766374	0
CHB	chr9	CBWD3	63816374	0
CHB	chr9	CBWD3	63866374	0
CHB	chr9	CBWD3	63916374	0
CHB	chr9	CBWD3	63966374	0
CHB	chr9	CBWD3	64016374	0
CHB	chr9	CBWD3	64066374	0
CHB	chr9	CBWD3	64116374	0
CHB	chr9	CBWD3	64166374	0
CHB	chr9	CBWD3	64216374	0
CHB	chr9	CBWD3	64266374	0
CHB	chr9	CBWD3	64316374	0

CHB	chr9	CBWD3	64366374	0
CHB	chr9	CBWD3	64416374	0
CHB	chr9	CBWD3	64466374	0
CHB	chr9	CBWD3	64516374	0
CHB	chr9	CBWD3	64566374	0
CHB	chr9	CBWD3	64616374	0
CHB	chr9	CBWD3	64666374	0
CHB	chr9	CBWD3	64716374	0
CHB	chr9	CBWD3	64766374	0
CHB	chr9	CBWD3	64816374	0
CHB	chr9	CBWD3	64866374	0
CHB	chr9	CBWD3	64916374	0
CHB	chr9	CBWD3	64966374	0
CHB	chr9	CBWD3	65016374	0
CHB	chr9	CBWD3	65066374	0
CHB	chr9	CBWD3	65116374	0
CHB	chr9	CBWD3	65166374	0
CHB	chr9	CBWD3	65216374	0
CHB	chr9	CBWD3	65266374	0
CHB	chr9	CBWD3	65316374	0
CHB	chr9	CBWD3	65366374	0
CHB	chr9	CBWD3	65416374	0
CHB	chr9	CBWD3	65466374	0
CHB	chr9	CBWD3	65516374	0
CHB	chr9	CBWD3	65566374	12
CHB	chr9	CBWD3	65616374	26
CHB	chr9	CBWD3	65666374	14
CHB	chr9	CBWD3	65716374	0
CHB	chr9	CBWD3	65766374	0
CHB	chr9	CBWD3	65816374	0
CHB	chr9	CBWD3	65866374	0
CHB	chr9	CBWD3	65916374	0
CHB	chr9	CBWD3	65966374	0
CHB	chr9	CBWD3	66016374	0
CHB	chr9	CBWD3	66066374	0
CHB	chr9	CBWD3	66116374	0
CHB	chr9	CBWD3	66166374	0
CHB	chr9	CBWD3	66216374	0
CHB	chr9	CBWD3	66266374	0
CHB	chr9	CBWD3	66316374	0
CHB	chr9	CBWD3	66366374	0
CHB	chr9	CBWD3	66416374	0
CHB	chr9	CBWD3	66466374	0
CHB	chr9	CBWD3	66516374	0
CHB	chr9	CBWD3	66566374	0
CHB	chr9	CBWD3	66616374	3
CHB	chr9	CBWD3	66666374	19
CHB	chr9	CBWD3	66716374	24
CHB	chr9	CBWD3	66766374	20

CHB	chr9	CBWD3	66816374	15
CHB	chr9	CBWD3	66866374	3
CHB	chr9	CBWD3	66916374	0
CHB	chr9	CBWD3	66966374	0
CHB	chr9	CBWD3	67016374	0
CHB	chr9	CBWD3	67066374	0
CHB	chr9	CBWD3	67116374	0
CHB	chr9	CBWD3	67166374	0
CHB	chr9	CBWD3	67216374	0
CHB	chr9	CBWD3	67266374	0
CHB	chr9	CBWD3	67316374	0
CHB	chr9	CBWD3	67366374	0
CHB	chr9	CBWD3	67416374	0
CHB	chr9	CBWD3	67466374	0
CHB	chr9	CBWD3	67516374	0
CHB	chr9	CBWD3	67566374	0
CHB	chr9	CBWD3	67616374	0
CHB	chr9	CBWD3	67666374	0
CHB	chr9	CBWD3	67716374	0
CHB	chr9	CBWD3	67766374	0
CHB	chr9	CBWD3	67816374	0
CHB	chr9	CBWD3	67866374	0
CHB	chr9	CBWD3	67916374	0
CHB	chr9	CBWD3	67966374	0
CHB	chr9	CBWD3	68016374	0
CHB	chr9	CBWD3	68066374	0
CHB	chr9	CBWD3	68116374	0
CHB	chr9	CBWD3	68166374	0
CHB	chr9	CBWD3	68216374	0
CHB	chr9	CBWD3	68266374	0
CHB	chr9	CBWD3	68316374	0
CHB	chr9	CBWD3	68366374	0
CHB	chr9	CBWD3	68416374	0
CHB	chr9	CBWD3	68466374	0
CHB	chr9	CBWD3	68516374	0
CHB	chr9	CBWD3	68566374	0
CHB	chr9	CBWD3	68616374	0
CHB	chr9	CBWD3	68666374	0
CHB	chr9	CBWD3	68716374	0
CHB	chr9	CBWD3	68766374	0
CHB	chr9	CBWD3	68816374	0
CHB	chr9	CBWD3	68866374	0
CHB	chr9	CBWD3	68916374	0
CHB	chr9	CBWD3	68966374	0
CHB	chr9	CBWD3	69016374	0
CHB	chr9	CBWD3	69066374	0
CHB	chr9	CBWD3	69116374	0
CHB	chr9	CBWD3	69166374	0
CHB	chr9	CBWD3	69216374	0

CHB	chr9	CBWD3	69266374	0
CHB	chr9	CBWD3	69316374	0
CHB	chr9	CBWD3	69366374	0
CHB	chr9	CBWD3	69416374	0
CHB	chr9	CBWD3	69466374	0
CHB	chr9	CBWD3	69516374	0
CHB	chr9	CBWD3	69566374	0
CHB	chr9	CBWD3	69616374	0
CHB	chr9	CBWD3	69666374	0
CHB	chr9	CBWD3	69716374	0
CHB	chr9	CBWD3	69766374	3
CHB	chr9	CBWD3	69816374	15
CHB	chr9	CBWD3	69866374	51
CHB	chr9	CBWD3	69916374	87
CHB	chr9	CBWD3	69966374	83
CHB	chr9	CBWD3	70016374	35
CHB	chr9	CBWD3	70066374	0
CHB	chr9	CBWD3	70116374	0
CHB	chr9	CBWD3	70166374	0
CHB	chr9	CBWD3	70216374	0
CHB	chr9	CBWD3	70266374	0
CHB	chr9	CBWD3	70316374	0
CHB	chr9	CBWD3	70366374	0
CHB	chr9	CBWD3	70416374	0
CHB	chr9	CBWD3	70466374	0
CHB	chr9	CBWD3	70516374	0
CHB	chr9	CBWD3	70566374	0
CHB	chr9	CBWD3	70616374	0
CHB	chr9	CBWD3	70666374	0
CHB	chr16	CLEC18A	65000232	0
CHB	chr16	CLEC18A	65050232	0
CHB	chr16	CLEC18A	65100232	0
CHB	chr16	CLEC18A	65150232	0
CHB	chr16	CLEC18A	65200232	0
CHB	chr16	CLEC18A	65250232	0
CHB	chr16	CLEC18A	65300232	0
CHB	chr16	CLEC18A	65350232	0
CHB	chr16	CLEC18A	65400232	0
CHB	chr16	CLEC18A	65450232	0
CHB	chr16	CLEC18A	65500232	0
CHB	chr16	CLEC18A	65550232	0
CHB	chr16	CLEC18A	65600232	0
CHB	chr16	CLEC18A	65650232	0
CHB	chr16	CLEC18A	65700232	0
CHB	chr16	CLEC18A	65750232	0
CHB	chr16	CLEC18A	65800232	0
CHB	chr16	CLEC18A	65850232	0
CHB	chr16	CLEC18A	65900232	0
CHB	chr16	CLEC18A	65950232	0

CHB	chr16	CLEC18A	66000232	0
CHB	chr16	CLEC18A	66050232	0
CHB	chr16	CLEC18A	66100232	0
CHB	chr16	CLEC18A	66150232	0
CHB	chr16	CLEC18A	66200232	0
CHB	chr16	CLEC18A	66250232	0
CHB	chr16	CLEC18A	66300232	0
CHB	chr16	CLEC18A	66350232	0
CHB	chr16	CLEC18A	66400232	0
CHB	chr16	CLEC18A	66450232	0
CHB	chr16	CLEC18A	66500232	0
CHB	chr16	CLEC18A	66550232	0
CHB	chr16	CLEC18A	66600232	0
CHB	chr16	CLEC18A	66650232	0
CHB	chr16	CLEC18A	66700232	0
CHB	chr16	CLEC18A	66750232	0
CHB	chr16	CLEC18A	66800232	0
CHB	chr16	CLEC18A	66850232	0
CHB	chr16	CLEC18A	66900232	0
CHB	chr16	CLEC18A	66950232	0
CHB	chr16	CLEC18A	67000232	0
CHB	chr16	CLEC18A	67050232	0
CHB	chr16	CLEC18A	67100232	0
CHB	chr16	CLEC18A	67150232	0
CHB	chr16	CLEC18A	67200232	0
CHB	chr16	CLEC18A	67250232	0
CHB	chr16	CLEC18A	67300232	0
CHB	chr16	CLEC18A	67350232	0
CHB	chr16	CLEC18A	67400232	0
CHB	chr16	CLEC18A	67450232	0
CHB	chr16	CLEC18A	67500232	0
CHB	chr16	CLEC18A	67550232	0
CHB	chr16	CLEC18A	67600232	0
CHB	chr16	CLEC18A	67650232	0
CHB	chr16	CLEC18A	67700232	0
CHB	chr16	CLEC18A	67750232	0
CHB	chr16	CLEC18A	67800232	0
CHB	chr16	CLEC18A	67850232	0
CHB	chr16	CLEC18A	67900232	0
CHB	chr16	CLEC18A	67950232	0
CHB	chr16	CLEC18A	68000232	0
CHB	chr16	CLEC18A	68050232	0
CHB	chr16	CLEC18A	68100232	0
CHB	chr16	CLEC18A	68150232	0
CHB	chr16	CLEC18A	68200232	0
CHB	chr16	CLEC18A	68250232	0
CHB	chr16	CLEC18A	68300232	0
CHB	chr16	CLEC18A	68350232	0
CHB	chr16	CLEC18A	68400232	0

CHB	chr16	CLEC18A	68450232	0
CHB	chr16	CLEC18A	68500232	0
CHB	chr16	CLEC18A	68550232	0
CHB	chr16	CLEC18A	68600232	0
CHB	chr16	CLEC18A	68650232	0
CHB	chr16	CLEC18A	68700232	0
CHB	chr16	CLEC18A	68750232	0
CHB	chr16	CLEC18A	68800232	0
CHB	chr16	CLEC18A	68850232	0
CHB	chr16	CLEC18A	68900232	0
CHB	chr16	CLEC18A	68950232	0
CHB	chr16	CLEC18A	69000232	0
CHB	chr16	CLEC18A	69050232	0
CHB	chr16	CLEC18A	69100232	0
CHB	chr16	CLEC18A	69150232	0
CHB	chr16	CLEC18A	69200232	0
CHB	chr16	CLEC18A	69250232	0
CHB	chr16	CLEC18A	69300232	0
CHB	chr16	CLEC18A	69350232	0
CHB	chr16	CLEC18A	69400232	0
CHB	chr16	CLEC18A	69450232	0
CHB	chr16	CLEC18A	69500232	0
CHB	chr16	CLEC18A	69550232	0
CHB	chr16	CLEC18A	69600232	0
CHB	chr16	CLEC18A	69650232	0
CHB	chr16	CLEC18A	69700232	0
CHB	chr16	CLEC18A	69750232	0
CHB	chr16	CLEC18A	69800232	0
CHB	chr16	CLEC18A	69850232	0
CHB	chr16	CLEC18A	69900232	0
CHB	chr16	CLEC18A	69950232	0
CHB	chr16	CLEC18A	70000232	0
CHB	chr16	CLEC18A	70050232	0
CHB	chr16	CLEC18A	70100232	0
CHB	chr16	CLEC18A	70150232	0
CHB	chr16	CLEC18A	70200232	0
CHB	chr16	CLEC18A	70250232	0
CHB	chr16	CLEC18A	70300232	0
CHB	chr16	CLEC18A	70350232	0
CHB	chr16	CLEC18A	70400232	0
CHB	chr16	CLEC18A	70450232	0
CHB	chr16	CLEC18A	70500232	0
CHB	chr16	CLEC18A	70550232	0
CHB	chr16	CLEC18A	70600232	0
CHB	chr16	CLEC18A	70650232	0
CHB	chr16	CLEC18A	70700232	0
CHB	chr16	CLEC18A	70750232	0
CHB	chr16	CLEC18A	70800232	0
CHB	chr16	CLEC18A	70850232	0

CHB	chr16	CLEC18A	70900232	0
CHB	chr16	CLEC18A	70950232	0
CHB	chr16	CLEC18A	71000232	0
CHB	chr16	CLEC18A	71050232	0
CHB	chr16	CLEC18A	71100232	0
CHB	chr16	CLEC18A	71150232	0
CHB	chr16	CLEC18A	71200232	0
CHB	chr16	CLEC18A	71250232	0
CHB	chr16	CLEC18A	71300232	0
CHB	chr16	CLEC18A	71350232	0
CHB	chr16	CLEC18A	71400232	0
CHB	chr16	CLEC18A	71450232	0
CHB	chr16	CLEC18A	71500232	0
CHB	chr16	CLEC18A	71550232	0
CHB	chr16	CLEC18A	71600232	0
CHB	chr16	CLEC18A	71650232	0
CHB	chr16	CLEC18A	71700232	0
CHB	chr16	CLEC18A	71750232	0
CHB	chr16	CLEC18A	71800232	0
CHB	chr16	CLEC18A	71850232	0
CHB	chr16	CLEC18A	71900232	0
CHB	chr16	CLEC18A	71950232	0
CHB	chr16	CLEC18A	72000232	0
CHB	chr16	CLEC18A	72050232	0
CHB	chr16	CLEC18A	72100232	0
CHB	chr16	CLEC18A	72150232	0
CHB	chr16	CLEC18A	72200232	0
CHB	chr16	CLEC18A	72250232	0
CHB	chr16	CLEC18A	72300232	0
CHB	chr16	CLEC18A	72350232	0
CHB	chr16	CLEC18A	72400232	0
CHB	chr16	CLEC18A	72450232	0
CHB	chr16	CLEC18A	72500232	0
CHB	chr16	CLEC18A	72550232	0
CHB	chr16	CLEC18A	72600232	0
CHB	chr16	CLEC18A	72650232	0
CHB	chr16	CLEC18A	72700232	0
CHB	chr16	CLEC18A	72750232	0
CHB	chr16	CLEC18A	72800232	0
CHB	chr16	CLEC18A	72850232	0
CHB	chr16	CLEC18A	72900232	16
CHB	chr16	CLEC18A	72950232	45
CHB	chr16	CLEC18A	73000232	46
CHB	chr16	CLEC18A	73050232	38
CHB	chr16	CLEC18A	73100232	27
CHB	chr16	CLEC18A	73150232	6
CHB	chr16	CLEC18A	73200232	0
CHB	chr16	CLEC18A	73250232	0
CHB	chr16	CLEC18A	73300232	0

CHB	chr16	CLEC18A	73350232	0
CHB	chr16	CLEC18A	73400232	0
CHB	chr16	CLEC18A	73450232	0
CHB	chr16	CLEC18A	73500232	0
CHB	chr16	CLEC18A	73550232	0
CHB	chr16	CLEC18A	73600232	0
CHB	chr16	CLEC18A	73650232	0
CHB	chr16	CLEC18A	73700232	0
CHB	chr16	CLEC18A	73750232	0
CHB	chr16	CLEC18A	73800232	0
CHB	chr16	CLEC18A	73850232	0
CHB	chr16	CLEC18A	73900232	0
CHB	chr16	CLEC18A	73950232	0
CHB	chr16	CLEC18A	74000232	0
CHB	chr16	CLEC18A	74050232	0
CHB	chr16	CLEC18A	74100232	0
CHB	chr16	CLEC18A	74150232	0
CHB	chr16	CLEC18A	74200232	0
CHB	chr16	CLEC18A	74250232	0
CHB	chr16	CLEC18A	74300232	0
CHB	chr16	CLEC18A	74350232	0
CHB	chr16	CLEC18A	74400232	9
CHB	chr16	CLEC18A	74450232	55
CHB	chr16	CLEC18A	74500232	59
CHB	chr16	CLEC18A	74550232	29
CHB	chr16	CLEC18A	74600232	43
CHB	chr16	CLEC18A	74650232	47
CHB	chr16	CLEC18A	74700232	45
CHB	chr16	CLEC18A	74750232	25
CHB	chr16	CLEC18A	74800232	0
CHB	chr16	CLEC18A	74850232	0
CHB	chr16	CLEC18A	74900232	0
CHB	chr7	CSH	118813708	0
CHB	chr7	CSH	118863708	0
CHB	chr7	CSH	118913708	0
CHB	chr7	CSH	118963708	0
CHB	chr7	CSH	119013708	0
CHB	chr7	CSH	119063708	0
CHB	chr7	CSH	119113708	0
CHB	chr7	CSH	119163708	0
CHB	chr7	CSH	119213708	0
CHB	chr7	CSH	119263708	0
CHB	chr7	CSH	119313708	0
CHB	chr7	CSH	119363708	0
CHB	chr7	CSH	119413708	0
CHB	chr7	CSH	119463708	0
CHB	chr7	CSH	119513708	0
CHB	chr7	CSH	119563708	0
CHB	chr7	CSH	119613708	0

CHB	chr7	CSH	119663708	0
CHB	chr7	CSH	119713708	0
CHB	chr7	CSH	119763708	0
CHB	chr7	CSH	119813708	0
CHB	chr7	CSH	119863708	0
CHB	chr7	CSH	119913708	0
CHB	chr7	CSH	119963708	0
CHB	chr7	CSH	120013708	0
CHB	chr7	CSH	120063708	0
CHB	chr7	CSH	120113708	0
CHB	chr7	CSH	120163708	0
CHB	chr7	CSH	120213708	0
CHB	chr7	CSH	120263708	0
CHB	chr7	CSH	120313708	0
CHB	chr7	CSH	120363708	0
CHB	chr7	CSH	120413708	0
CHB	chr7	CSH	120463708	0
CHB	chr7	CSH	120513708	0
CHB	chr7	CSH	120563708	0
CHB	chr7	CSH	120613708	0
CHB	chr7	CSH	120663708	0
CHB	chr7	CSH	120713708	0
CHB	chr7	CSH	120763708	0
CHB	chr7	CSH	120813708	0
CHB	chr7	CSH	120863708	0
CHB	chr7	CSH	120913708	0
CHB	chr7	CSH	120963708	0
CHB	chr7	CSH	121013708	0
CHB	chr7	CSH	121063708	0
CHB	chr7	CSH	121113708	0
CHB	chr7	CSH	121163708	0
CHB	chr7	CSH	121213708	0
CHB	chr7	CSH	121263708	0
CHB	chr7	CSH	121313708	0
CHB	chr7	CSH	121363708	0
CHB	chr7	CSH	121413708	0
CHB	chr7	CSH	121463708	0
CHB	chr7	CSH	121513708	0
CHB	chr7	CSH	121563708	0
CHB	chr7	CSH	121613708	0
CHB	chr7	CSH	121663708	0
CHB	chr7	CSH	121713708	0
CHB	chr7	CSH	121763708	0
CHB	chr7	CSH	121813708	0
CHB	chr7	CSH	121863708	0
CHB	chr7	CSH	121913708	0
CHB	chr7	CSH	121963708	0
CHB	chr7	CSH	122013708	0
CHB	chr7	CSH	122063708	0

CHB	chr7	CSH	122113708	0
CHB	chr7	CSH	122163708	0
CHB	chr7	CSH	122213708	0
CHB	chr7	CSH	122263708	0
CHB	chr7	CSH	122313708	0
CHB	chr7	CSH	122363708	0
CHB	chr7	CSH	122413708	0
CHB	chr7	CSH	122463708	0
CHB	chr7	CSH	122513708	0
CHB	chr7	CSH	122563708	0
CHB	chr7	CSH	122613708	0
CHB	chr7	CSH	122663708	0
CHB	chr7	CSH	122713708	0
CHB	chr7	CSH	122763708	0
CHB	chr7	CSH	122813708	0
CHB	chr7	CSH	122863708	0
CHB	chr7	CSH	122913708	0
CHB	chr7	CSH	122963708	0
CHB	chr7	CSH	123013708	0
CHB	chr7	CSH	123063708	0
CHB	chr7	CSH	123113708	0
CHB	chr7	CSH	123163708	0
CHB	chr7	CSH	123213708	0
CHB	chr7	CSH	123263708	0
CHB	chr7	CSH	123313708	0
CHB	chr7	CSH	123363708	0
CHB	chr7	CSH	123413708	0
CHB	chr7	CSH	123463708	0
CHB	chr7	CSH	123513708	0
CHB	chr7	CSH	123563708	0
CHB	chr7	CSH	123613708	0
CHB	chr7	CSH	123663708	0
CHB	chr7	CSH	123713708	0
CHB	chr7	CSH	123763708	0
CHB	chr7	CSH	123813708	0
CHB	chr7	CSH	123863708	0
CHB	chr7	CSH	123913708	0
CHB	chr7	CSH	123963708	0
CHB	chr7	CSH	124013708	0
CHB	chr7	CSH	124063708	0
CHB	chr7	CSH	124113708	0
CHB	chr7	CSH	124163708	0
CHB	chr7	CSH	124213708	0
CHB	chr7	CSH	124263708	0
CHB	chr7	CSH	124313708	0
CHB	chr7	CSH	124363708	0
CHB	chr7	CSH	124413708	0
CHB	chr7	CSH	124463708	0
CHB	chr7	CSH	124513708	0

CHB	chr7	CSH	124563708	0
CHB	chr7	CSH	124613708	0
CHB	chr7	CSH	124663708	0
CHB	chr7	CSH	124713708	0
CHB	chr7	CSH	124763708	0
CHB	chr7	CSH	124813708	0
CHB	chr7	CSH	124863708	0
CHB	chr7	CSH	124913708	0
CHB	chr7	CSH	124963708	7
CHB	chr7	CSH	125013708	18
CHB	chr7	CSH	125063708	36
CHB	chr7	CSH	125113708	59
CHB	chr7	CSH	125163708	64
CHB	chr7	CSH	125213708	103
CHB	chr7	CSH	125263708	98
CHB	chr7	CSH	125313708	46
CHB	chr7	CSH	125363708	35
CHB	chr7	CSH	125413708	14
CHB	chr7	CSH	125463708	0
CHB	chr7	CSH	125513708	0
CHB	chr7	CSH	125563708	20
CHB	chr7	CSH	125613708	37
CHB	chr7	CSH	125663708	31
CHB	chr7	CSH	125713708	27
CHB	chr7	CSH	125763708	32
CHB	chr7	CSH	125813708	40
CHB	chr7	CSH	125863708	34
CHB	chr7	CSH	125913708	23
CHB	chr7	CSH	125963708	22
CHB	chr7	CSH	126013708	31
CHB	chr7	CSH	126063708	24
CHB	chr7	CSH	126113708	5
CHB	chr7	CSH	126163708	0
CHB	chr7	CSH	126213708	0
CHB	chr7	CSH	126263708	0
CHB	chr7	CSH	126313708	0
CHB	chr7	CSH	126363708	0
CHB	chr7	CSH	126413708	0
CHB	chr7	CSH	126463708	0
CHB	chr7	CSH	126513708	0
CHB	chr7	CSH	126563708	0
CHB	chr7	CSH	126613708	0
CHB	chr7	CSH	126663708	0
CHB	chr7	CSH	126713708	0
CHB	chr7	CSH	126763708	0
CHB	chr7	CSH	126813708	0
CHB	chr7	CSH	126863708	0
CHB	chr7	CSH	126913708	0
CHB	chr7	CSH	126963708	0

CHB	chr7	CSH	127013708	0
CHB	chr7	CSH	127063708	0
CHB	chr7	CSH	127113708	0
CHB	chr7	CSH	127163708	0
CHB	chr7	CSH	127213708	0
CHB	chr7	CSH	127263708	0
CHB	chr7	CSH	127313708	0
CHB	chr7	CSH	127363708	0
CHB	chr7	CSH	127413708	0
CHB	chr7	CSH	127463708	0
CHB	chr7	CSH	127513708	0
CHB	chr7	CSH	127563708	0
CHB	chr7	CSH	127613708	0
CHB	chr7	CSH	127663708	0
CHB	chr7	CSH	127713708	0
CHB	chr7	CSH	127763708	0
CHB	chr7	CSH	127813708	0
CHB	chr7	CSH	127863708	0
CHB	chr7	CSH	127913708	0
CHB	chr7	CSH	127963708	0
CHB	chr7	CSH	128013708	0
CHB	chr7	CSH	128063708	0
CHB	chr7	CSH	128113708	0
CHB	chr7	CSH	128163708	0
CHB	chr7	CSH	128213708	0
CHB	chr7	CSH	128263708	0
CHB	chr7	CSH	128313708	0
CHB	chr7	CSH	128363708	0
CHB	chr7	CSH	128413708	0
CHB	chr7	CSH	128463708	0
CHB	chr7	CSH	128513708	0
CHB	chr7	CSH	128563708	0
CHB	chr7	CSH	128613708	0
CHB	chr7	CSH	128663708	0
CHB	chr7	CSH	128713708	0
CHB	chr7	CSH	128763708	0
CHB	chr7	CSH	128813708	0
CHB	chr16	CSH	76131945	0
CHB	chr16	CSH	76181945	0
CHB	chr16	CSH	76231945	0
CHB	chr16	CSH	76281945	0
CHB	chr16	CSH	76331945	0
CHB	chr16	CSH	76381945	0
CHB	chr16	CSH	76431945	0
CHB	chr16	CSH	76481945	0
CHB	chr16	CSH	76531945	0
CHB	chr16	CSH	76581945	0
CHB	chr16	CSH	76631945	0
CHB	chr16	CSH	76681945	0

CHB	chr16	CSH	76731945	9
CHB	chr16	CSH	76781945	20
CHB	chr16	CSH	76831945	43
CHB	chr16	CSH	76881945	40
CHB	chr16	CSH	76931945	26
CHB	chr16	CSH	76981945	37
CHB	chr16	CSH	77031945	29
CHB	chr16	CSH	77081945	21
CHB	chr16	CSH	77131945	44
CHB	chr16	CSH	77181945	50
CHB	chr16	CSH	77231945	24
CHB	chr16	CSH	77281945	53
CHB	chr16	CSH	77331945	94
CHB	chr16	CSH	77381945	81
CHB	chr16	CSH	77431945	50
CHB	chr16	CSH	77481945	30
CHB	chr16	CSH	77531945	42
CHB	chr16	CSH	77581945	55
CHB	chr16	CSH	77631945	43
CHB	chr16	CSH	77681945	33
CHB	chr16	CSH	77731945	26
CHB	chr16	CSH	77781945	24
CHB	chr16	CSH	77831945	33
CHB	chr16	CSH	77881945	35
CHB	chr16	CSH	77931945	33
CHB	chr16	CSH	77981945	36
CHB	chr16	CSH	78031945	41
CHB	chr16	CSH	78081945	100
CHB	chr16	CSH	78131945	106
CHB	chr16	CSH	78181945	68
CHB	chr16	CSH	78231945	65
CHB	chr16	CSH	78281945	36
CHB	chr16	CSH	78331945	36
CHB	chr16	CSH	78381945	96
CHB	chr16	CSH	78431945	91
CHB	chr16	CSH	78481945	24
CHB	chr16	CSH	78531945	21
CHB	chr16	CSH	78581945	22
CHB	chr16	CSH	78631945	20
CHB	chr16	CSH	78681945	100
CHB	chr16	CSH	78731945	121
CHB	chr16	CSH	78781945	37
CHB	chr16	CSH	78831945	1
CHB	chr16	CSH	78881945	0
CHB	chr16	CSH	78931945	0
CHB	chr16	CSH	78981945	0
CHB	chr16	CSH	79031945	0
CHB	chr16	CSH	79081945	0
CHB	chr16	CSH	79131945	0

CHB	chr16	CSH	79181945	9
CHB	chr16	CSH	79231945	81
CHB	chr16	CSH	79281945	93
CHB	chr16	CSH	79331945	21
CHB	chr16	CSH	79381945	0
CHB	chr16	CSH	79431945	0
CHB	chr16	CSH	79481945	0
CHB	chr16	CSH	79531945	0
CHB	chr16	CSH	79581945	0
CHB	chr16	CSH	79631945	0
CHB	chr16	CSH	79681945	0
CHB	chr16	CSH	79731945	0
CHB	chr16	CSH	79781945	0
CHB	chr16	CSH	79831945	0
CHB	chr16	CSH	79881945	0
CHB	chr16	CSH	79931945	0
CHB	chr16	CSH	79981945	0
CHB	chr16	CSH	80031945	0
CHB	chr16	CSH	80081945	0
CHB	chr16	CSH	80131945	0
CHB	chr16	CSH	80181945	0
CHB	chr16	CSH	80231945	0
CHB	chr16	CSH	80281945	0
CHB	chr16	CSH	80331945	0
CHB	chr16	CSH	80381945	0
CHB	chr16	CSH	80431945	0
CHB	chr16	CSH	80481945	0
CHB	chr16	CSH	80531945	0
CHB	chr16	CSH	80581945	0
CHB	chr16	CSH	80631945	0
CHB	chr16	CSH	80681945	0
CHB	chr16	CSH	80731945	0
CHB	chr16	CSH	80781945	0
CHB	chr16	CSH	80831945	0
CHB	chr16	CSH	80881945	0
CHB	chr16	CSH	80931945	0
CHB	chr16	CSH	80981945	0
CHB	chr16	CSH	81031945	0
CHB	chr16	CSH	81081945	0
CHB	chr16	CSH	81131945	0
CHB	chr16	CSH	81181945	0
CHB	chr16	CSH	81231945	0
CHB	chr16	CSH	81281945	0
CHB	chr16	CSH	81331945	0
CHB	chr16	CSH	81381945	0
CHB	chr16	CSH	81431945	0
CHB	chr16	CSH	81481945	0
CHB	chr16	CSH	81531945	0
CHB	chr16	CSH	81581945	0

CHB	chr16	CSH	81631945	0
CHB	chr16	CSH	81681945	0
CHB	chr16	CSH	81731945	0
CHB	chr16	CSH	81781945	0
CHB	chr16	CSH	81831945	0
CHB	chr16	CSH	81881945	0
CHB	chr16	CSH	81931945	0
CHB	chr16	CSH	81981945	0
CHB	chr16	CSH	82031945	0
CHB	chr16	CSH	82081945	0
CHB	chr16	CSH	82131945	0
CHB	chr16	CSH	82181945	0
CHB	chr16	CSH	82231945	0
CHB	chr16	CSH	82281945	0
CHB	chr16	CSH	82331945	0
CHB	chr16	CSH	82381945	0
CHB	chr16	CSH	82431945	0
CHB	chr16	CSH	82481945	0
CHB	chr16	CSH	82531945	0
CHB	chr16	CSH	82581945	0
CHB	chr16	CSH	82631945	0
CHB	chr16	CSH	82681945	0
CHB	chr16	CSH	82731945	0
CHB	chr16	CSH	82781945	0
CHB	chr16	CSH	82831945	0
CHB	chr16	CSH	82881945	0
CHB	chr16	CSH	82931945	0
CHB	chr16	CSH	82981945	0
CHB	chr16	CSH	83031945	0
CHB	chr16	CSH	83081945	0
CHB	chr16	CSH	83131945	0
CHB	chr16	CSH	83181945	0
CHB	chr16	CSH	83231945	0
CHB	chr16	CSH	83281945	0
CHB	chr16	CSH	83331945	0
CHB	chr16	CSH	83381945	0
CHB	chr16	CSH	83431945	0
CHB	chr16	CSH	83481945	0
CHB	chr16	CSH	83531945	0
CHB	chr16	CSH	83581945	0
CHB	chr16	CSH	83631945	7
CHB	chr16	CSH	83681945	42
CHB	chr16	CSH	83731945	76
CHB	chr16	CSH	83781945	41
CHB	chr16	CSH	83831945	0
CHB	chr16	CSH	83881945	0
CHB	chr16	CSH	83931945	0
CHB	chr16	CSH	83981945	0
CHB	chr16	CSH	84031945	0

CHB	chr16	CSH	84081945	0
CHB	chr16	CSH	84131945	0
CHB	chr16	CSH	84181945	0
CHB	chr16	CSH	84231945	0
CHB	chr16	CSH	84281945	0
CHB	chr16	CSH	84331945	0
CHB	chr16	CSH	84381945	0
CHB	chr16	CSH	84431945	0
CHB	chr16	CSH	84481945	0
CHB	chr16	CSH	84531945	0
CHB	chr16	CSH	84581945	0
CHB	chr16	CSH	84631945	0
CHB	chr16	CSH	84681945	0
CHB	chr16	CSH	84731945	0
CHB	chr16	CSH	84781945	0
CHB	chr16	CSH	84831945	0
CHB	chr16	CSH	84881945	0
CHB	chr16	CSH	84931945	0
CHB	chr16	CSH	84981945	0
CHB	chr16	CSH	85031945	0
CHB	chr16	CSH	85081945	0
CHB	chr16	CSH	85131945	0
CHB	chr16	CSH	85181945	0
CHB	chr16	CSH	85231945	0
CHB	chr16	CSH	85281945	0
CHB	chr16	CSH	85331945	0
CHB	chr16	CSH	85381945	0
CHB	chr16	CSH	85431945	0
CHB	chr16	CSH	85481945	0
CHB	chr16	CSH	85531945	0
CHB	chr16	CSH	85581945	0
CHB	chr16	CSH	85631945	0
CHB	chr16	CSH	85681945	0
CHB	chr16	CSH	85731945	0
CHB	chr16	CSH	85781945	0
CHB	chr16	CSH	85831945	0
CHB	chr16	CSH	85881945	0
CHB	chr16	CSH	85931945	0
CHB	chr16	CSH	85981945	0
CHB	chr16	CSH	86031945	0
CHB	chr17	CSH	13377873	45
CHB	chr17	CSH	13427873	50
CHB	chr17	CSH	13477873	41
CHB	chr17	CSH	13527873	40
CHB	chr17	CSH	13577873	34
CHB	chr17	CSH	13627873	22
CHB	chr17	CSH	13677873	24
CHB	chr17	CSH	13727873	64
CHB	chr17	CSH	13777873	79

CHB	chr17	CSH	13827873	50
CHB	chr17	CSH	13877873	38
CHB	chr17	CSH	13927873	24
CHB	chr17	CSH	13977873	20
CHB	chr17	CSH	14027873	20
CHB	chr17	CSH	14077873	38
CHB	chr17	CSH	14127873	31
CHB	chr17	CSH	14177873	3
CHB	chr17	CSH	14227873	0
CHB	chr17	CSH	14277873	0
CHB	chr17	CSH	14327873	0
CHB	chr17	CSH	14377873	0
CHB	chr17	CSH	14427873	0
CHB	chr17	CSH	14477873	0
CHB	chr17	CSH	14527873	0
CHB	chr17	CSH	14577873	0
CHB	chr17	CSH	14627873	0
CHB	chr17	CSH	14677873	0
CHB	chr17	CSH	14727873	0
CHB	chr17	CSH	14777873	0
CHB	chr17	CSH	14827873	0
CHB	chr17	CSH	14877873	0
CHB	chr17	CSH	14927873	21
CHB	chr17	CSH	14977873	75
CHB	chr17	CSH	15027873	67
CHB	chr17	CSH	15077873	24
CHB	chr17	CSH	15127873	27
CHB	chr17	CSH	15177873	26
CHB	chr17	CSH	15227873	10
CHB	chr17	CSH	15277873	12
CHB	chr17	CSH	15327873	38
CHB	chr17	CSH	15377873	39
CHB	chr17	CSH	15427873	24
CHB	chr17	CSH	15477873	17
CHB	chr17	CSH	15527873	6
CHB	chr17	CSH	15577873	0
CHB	chr17	CSH	15627873	0
CHB	chr17	CSH	15677873	0
CHB	chr17	CSH	15727873	0
CHB	chr17	CSH	15777873	0
CHB	chr17	CSH	15827873	0
CHB	chr17	CSH	15877873	0
CHB	chr17	CSH	15927873	0
CHB	chr17	CSH	15977873	0
CHB	chr17	CSH	16027873	0
CHB	chr17	CSH	16077873	0
CHB	chr17	CSH	16127873	0
CHB	chr17	CSH	16177873	0
CHB	chr17	CSH	16227873	0

CHB	chr17	CSH	16277873	0
CHB	chr17	CSH	16327873	0
CHB	chr17	CSH	16377873	0
CHB	chr17	CSH	16427873	0
CHB	chr17	CSH	16477873	0
CHB	chr17	CSH	16527873	0
CHB	chr17	CSH	16577873	0
CHB	chr17	CSH	16627873	0
CHB	chr17	CSH	16677873	0
CHB	chr17	CSH	16727873	0
CHB	chr17	CSH	16777873	0
CHB	chr17	CSH	16827873	0
CHB	chr17	CSH	16877873	0
CHB	chr17	CSH	16927873	0
CHB	chr17	CSH	16977873	0
CHB	chr17	CSH	17027873	0
CHB	chr17	CSH	17077873	0
CHB	chr17	CSH	17127873	0
CHB	chr17	CSH	17177873	0
CHB	chr17	CSH	17227873	0
CHB	chr17	CSH	17277873	0
CHB	chr17	CSH	17327873	0
CHB	chr17	CSH	17377873	0
CHB	chr17	CSH	17427873	0
CHB	chr17	CSH	17477873	0
CHB	chr17	CSH	17527873	0
CHB	chr17	CSH	17577873	0
CHB	chr17	CSH	17627873	0
CHB	chr17	CSH	17677873	0
CHB	chr17	CSH	17727873	0
CHB	chr17	CSH	17777873	0
CHB	chr17	CSH	17827873	0
CHB	chr17	CSH	17877873	0
CHB	chr17	CSH	17927873	0
CHB	chr17	CSH	17977873	0
CHB	chr17	CSH	18027873	0
CHB	chr17	CSH	18077873	0
CHB	chr17	CSH	18127873	0
CHB	chr17	CSH	18177873	0
CHB	chr17	CSH	18227873	0
CHB	chr17	CSH	18277873	0
CHB	chr17	CSH	18327873	0
CHB	chr17	CSH	18377873	0
CHB	chr17	CSH	18427873	0
CHB	chr17	CSH	18477873	0
CHB	chr17	CSH	18527873	0
CHB	chr17	CSH	18577873	0
CHB	chr17	CSH	18627873	0
CHB	chr17	CSH	18677873	0

CHB	chr17	CSH	18727873	0
CHB	chr17	CSH	18777873	0
CHB	chr17	CSH	18827873	0
CHB	chr17	CSH	18877873	0
CHB	chr17	CSH	18927873	0
CHB	chr17	CSH	18977873	0
CHB	chr17	CSH	19027873	0
CHB	chr17	CSH	19077873	0
CHB	chr17	CSH	19127873	25
CHB	chr17	CSH	19177873	51
CHB	chr17	CSH	19227873	50
CHB	chr17	CSH	19277873	68
CHB	chr17	CSH	19327873	58
CHB	chr17	CSH	19377873	39
CHB	chr17	CSH	19427873	49
CHB	chr17	CSH	19477873	44
CHB	chr17	CSH	19527873	42
CHB	chr17	CSH	19577873	45
CHB	chr17	CSH	19627873	61
CHB	chr17	CSH	19677873	45
CHB	chr17	CSH	19727873	31
CHB	chr17	CSH	19777873	69
CHB	chr17	CSH	19827873	113
CHB	chr17	CSH	19877873	71
CHB	chr17	CSH	19927873	3
CHB	chr17	CSH	19977873	0
CHB	chr17	CSH	20027873	0
CHB	chr17	CSH	20077873	0
CHB	chr17	CSH	20127873	0
CHB	chr17	CSH	20177873	0
CHB	chr17	CSH	20227873	0
CHB	chr17	CSH	20277873	0
CHB	chr17	CSH	20327873	0
CHB	chr17	CSH	20377873	0
CHB	chr17	CSH	20427873	0
CHB	chr17	CSH	20477873	0
CHB	chr17	CSH	20527873	0
CHB	chr17	CSH	20577873	0
CHB	chr17	CSH	20627873	0
CHB	chr17	CSH	20677873	0
CHB	chr17	CSH	20727873	0
CHB	chr17	CSH	20777873	0
CHB	chr17	CSH	20827873	0
CHB	chr17	CSH	20877873	0
CHB	chr17	CSH	20927873	0
CHB	chr17	CSH	20977873	0
CHB	chr17	CSH	21027873	0
CHB	chr17	CSH	21077873	0
CHB	chr17	CSH	21127873	0

CHB	chr17	CSH	21177873	0
CHB	chr17	CSH	21227873	0
CHB	chr17	CSH	21277873	0
CHB	chr17	CSH	21327873	0
CHB	chr17	CSH	21377873	0
CHB	chr17	CSH	21427873	0
CHB	chr17	CSH	21477873	0
CHB	chr17	CSH	21527873	0
CHB	chr17	CSH	21577873	0
CHB	chr17	CSH	21627873	0
CHB	chr17	CSH	21677873	0
CHB	chr17	CSH	21727873	0
CHB	chr17	CSH	21777873	0
CHB	chr17	CSH	21827873	0
CHB	chr17	CSH	21877873	0
CHB	chr17	CSH	21927873	0
CHB	chr17	CSH	21977873	0
CHB	chr17	CSH	22027873	0
CHB	chr17	CSH	22077873	0
CHB	chr17	CSH	22127873	0
CHB	chr17	CSH	22177873	0
CHB	chr17	CSH	22227873	0
CHB	chr17	CSH	22277873	0
CHB	chr17	CSH	22327873	0
CHB	chr17	CSH	22377873	0
CHB	chr17	CSH	22427873	0
CHB	chr17	CSH	22477873	0
CHB	chr17	CSH	22527873	0
CHB	chr17	CSH	22577873	0
CHB	chr17	CSH	22627873	0
CHB	chr17	CSH	22677873	0
CHB	chr17	CSH	22727873	0
CHB	chr17	CSH	22777873	0
CHB	chr17	CSH	22827873	0
CHB	chr17	CSH	22877873	0
CHB	chr17	CSH	22927873	0
CHB	chr17	CSH	22977873	0
CHB	chr17	CSH	23027873	0
CHB	chr17	CSH	23077873	0
CHB	chr17	CSH	23127873	0
CHB	chr17	CSH	23177873	0
CHB	chr17	CSH	23227873	0
CHB	chr17	CSH	23277873	0
CHB	chr17	CSH	58922012	0
CHB	chr17	CSH	58972012	0
CHB	chr17	CSH	59022012	0
CHB	chr17	CSH	59072012	0
CHB	chr17	CSH	59122012	0
CHB	chr17	CSH	59172012	0

CHB	chr17	CSH	59222012	0
CHB	chr17	CSH	59272012	0
CHB	chr17	CSH	59322012	0
CHB	chr17	CSH	59372012	0
CHB	chr17	CSH	59422012	0
CHB	chr17	CSH	59472012	0
CHB	chr17	CSH	59522012	0
CHB	chr17	CSH	59572012	0
CHB	chr17	CSH	59622012	0
CHB	chr17	CSH	59672012	0
CHB	chr17	CSH	59722012	0
CHB	chr17	CSH	59772012	0
CHB	chr17	CSH	59822012	0
CHB	chr17	CSH	59872012	0
CHB	chr17	CSH	59922012	0
CHB	chr17	CSH	59972012	0
CHB	chr17	CSH	60022012	0
CHB	chr17	CSH	60072012	0
CHB	chr17	CSH	60122012	0
CHB	chr17	CSH	60172012	0
CHB	chr17	CSH	60222012	0
CHB	chr17	CSH	60272012	0
CHB	chr17	CSH	60322012	0
CHB	chr17	CSH	60372012	0
CHB	chr17	CSH	60422012	0
CHB	chr17	CSH	60472012	0
CHB	chr17	CSH	60522012	0
CHB	chr17	CSH	60572012	0
CHB	chr17	CSH	60622012	0
CHB	chr17	CSH	60672012	0
CHB	chr17	CSH	60722012	0
CHB	chr17	CSH	60772012	0
CHB	chr17	CSH	60822012	0
CHB	chr17	CSH	60872012	0
CHB	chr17	CSH	60922012	0
CHB	chr17	CSH	60972012	0
CHB	chr17	CSH	61022012	0
CHB	chr17	CSH	61072012	0
CHB	chr17	CSH	61122012	0
CHB	chr17	CSH	61172012	0
CHB	chr17	CSH	61222012	0
CHB	chr17	CSH	61272012	0
CHB	chr17	CSH	61322012	0
CHB	chr17	CSH	61372012	0
CHB	chr17	CSH	61422012	0
CHB	chr17	CSH	61472012	0
CHB	chr17	CSH	61522012	0
CHB	chr17	CSH	61572012	0
CHB	chr17	CSH	61622012	0

CHB	chr17	CSH	61672012	0
CHB	chr17	CSH	61722012	0
CHB	chr17	CSH	61772012	0
CHB	chr17	CSH	61822012	0
CHB	chr17	CSH	61872012	0
CHB	chr17	CSH	61922012	0
CHB	chr17	CSH	61972012	0
CHB	chr17	CSH	62022012	0
CHB	chr17	CSH	62072012	0
CHB	chr17	CSH	62122012	0
CHB	chr17	CSH	62172012	0
CHB	chr17	CSH	62222012	0
CHB	chr17	CSH	62272012	0
CHB	chr17	CSH	62322012	0
CHB	chr17	CSH	62372012	0
CHB	chr17	CSH	62422012	0
CHB	chr17	CSH	62472012	0
CHB	chr17	CSH	62522012	0
CHB	chr17	CSH	62572012	0
CHB	chr17	CSH	62622012	0
CHB	chr17	CSH	62672012	0
CHB	chr17	CSH	62722012	0
CHB	chr17	CSH	62772012	0
CHB	chr17	CSH	62822012	0
CHB	chr17	CSH	62872012	0
CHB	chr17	CSH	62922012	0
CHB	chr17	CSH	62972012	0
CHB	chr17	CSH	63022012	0
CHB	chr17	CSH	63072012	0
CHB	chr17	CSH	63122012	0
CHB	chr17	CSH	63172012	0
CHB	chr17	CSH	63222012	0
CHB	chr17	CSH	63272012	0
CHB	chr17	CSH	63322012	0
CHB	chr17	CSH	63372012	0
CHB	chr17	CSH	63422012	0
CHB	chr17	CSH	63472012	0
CHB	chr17	CSH	63522012	0
CHB	chr17	CSH	63572012	0
CHB	chr17	CSH	63622012	0
CHB	chr17	CSH	63672012	0
CHB	chr17	CSH	63722012	0
CHB	chr17	CSH	63772012	0
CHB	chr17	CSH	63822012	0
CHB	chr17	CSH	63872012	0
CHB	chr17	CSH	63922012	0
CHB	chr17	CSH	63972012	0
CHB	chr17	CSH	64022012	0
CHB	chr17	CSH	64072012	0

CHB	chr17	CSH	64122012	0
CHB	chr17	CSH	64172012	0
CHB	chr17	CSH	64222012	0
CHB	chr17	CSH	64272012	0
CHB	chr17	CSH	64322012	0
CHB	chr17	CSH	64372012	0
CHB	chr17	CSH	64422012	0
CHB	chr17	CSH	64472012	0
CHB	chr17	CSH	64522012	0
CHB	chr17	CSH	64572012	0
CHB	chr17	CSH	64622012	0
CHB	chr17	CSH	64672012	0
CHB	chr17	CSH	64722012	0
CHB	chr17	CSH	64772012	0
CHB	chr17	CSH	64822012	0
CHB	chr17	CSH	64872012	0
CHB	chr17	CSH	64922012	0
CHB	chr17	CSH	64972012	0
CHB	chr17	CSH	65022012	0
CHB	chr17	CSH	65072012	0
CHB	chr17	CSH	65122012	0
CHB	chr17	CSH	65172012	0
CHB	chr17	CSH	65222012	0
CHB	chr17	CSH	65272012	0
CHB	chr17	CSH	65322012	0
CHB	chr17	CSH	65372012	0
CHB	chr17	CSH	65422012	0
CHB	chr17	CSH	65472012	0
CHB	chr17	CSH	65522012	0
CHB	chr17	CSH	65572012	0
CHB	chr17	CSH	65622012	0
CHB	chr17	CSH	65672012	0
CHB	chr17	CSH	65722012	0
CHB	chr17	CSH	65772012	0
CHB	chr17	CSH	65822012	0
CHB	chr17	CSH	65872012	0
CHB	chr17	CSH	65922012	0
CHB	chr17	CSH	65972012	0
CHB	chr17	CSH	66022012	0
CHB	chr17	CSH	66072012	0
CHB	chr17	CSH	66122012	0
CHB	chr17	CSH	66172012	0
CHB	chr17	CSH	66222012	0
CHB	chr17	CSH	66272012	0
CHB	chr17	CSH	66322012	0
CHB	chr17	CSH	66372012	0
CHB	chr17	CSH	66422012	0
CHB	chr17	CSH	66472012	0
CHB	chr17	CSH	66522012	0

CHB	chr17	CSH	66572012	0
CHB	chr17	CSH	66622012	0
CHB	chr17	CSH	66672012	0
CHB	chr17	CSH	66722012	0
CHB	chr17	CSH	66772012	0
CHB	chr17	CSH	66822012	0
CHB	chr17	CSH	66872012	0
CHB	chr17	CSH	66922012	0
CHB	chr17	CSH	66972012	0
CHB	chr17	CSH	67022012	0
CHB	chr17	CSH	67072012	0
CHB	chr17	CSH	67122012	0
CHB	chr17	CSH	67172012	0
CHB	chr17	CSH	67222012	0
CHB	chr17	CSH	67272012	0
CHB	chr17	CSH	67322012	0
CHB	chr17	CSH	67372012	0
CHB	chr17	CSH	67422012	0
CHB	chr17	CSH	67472012	0
CHB	chr17	CSH	67522012	0
CHB	chr17	CSH	67572012	0
CHB	chr17	CSH	67622012	0
CHB	chr17	CSH	67672012	0
CHB	chr17	CSH	67722012	0
CHB	chr17	CSH	67772012	0
CHB	chr17	CSH	67822012	0
CHB	chr17	CSH	67872012	0
CHB	chr17	CSH	67922012	0
CHB	chr17	CSH	67972012	0
CHB	chr17	CSH	68022012	0
CHB	chr17	CSH	68072012	0
CHB	chr17	CSH	68122012	0
CHB	chr17	CSH	68172012	0
CHB	chr17	CSH	68222012	0
CHB	chr17	CSH	68272012	0
CHB	chr17	CSH	68322012	0
CHB	chr17	CSH	68372012	0
CHB	chr17	CSH	68422012	0
CHB	chr17	CSH	68472012	0
CHB	chr17	CSH	68522012	0
CHB	chr17	CSH	68572012	0
CHB	chr17	CSH	68622012	0
CHB	chr17	CSH	68672012	0
CHB	chr17	CSH	68722012	0
CHB	chr17	CSH	68772012	0
CHB	chr17	CSH	68822012	0
CHB	chr19	CSH	6485290	4
CHB	chr19	CSH	6535290	0
CHB	chr19	CSH	6585290	0

CHB	chr19	CSH	6635290	0
CHB	chr19	CSH	6685290	0
CHB	chr19	CSH	6735290	0
CHB	chr19	CSH	6785290	0
CHB	chr19	CSH	6835290	0
CHB	chr19	CSH	6885290	0
CHB	chr19	CSH	6935290	0
CHB	chr19	CSH	6985290	35
CHB	chr19	CSH	7035290	36
CHB	chr19	CSH	7085290	1
CHB	chr19	CSH	7135290	0
CHB	chr19	CSH	7185290	0
CHB	chr19	CSH	7235290	0
CHB	chr19	CSH	7285290	0
CHB	chr19	CSH	7335290	0
CHB	chr19	CSH	7385290	0
CHB	chr19	CSH	7435290	0
CHB	chr19	CSH	7485290	0
CHB	chr19	CSH	7535290	0
CHB	chr19	CSH	7585290	0
CHB	chr19	CSH	7635290	0
CHB	chr19	CSH	7685290	0
CHB	chr19	CSH	7735290	0
CHB	chr19	CSH	7785290	0
CHB	chr19	CSH	7835290	0
CHB	chr19	CSH	7885290	0
CHB	chr19	CSH	7935290	0
CHB	chr19	CSH	7985290	0
CHB	chr19	CSH	8035290	0
CHB	chr19	CSH	8085290	0
CHB	chr19	CSH	8135290	0
CHB	chr19	CSH	8185290	0
CHB	chr19	CSH	8235290	0
CHB	chr19	CSH	8285290	0
CHB	chr19	CSH	8335290	0
CHB	chr19	CSH	8385290	0
CHB	chr19	CSH	8435290	0
CHB	chr19	CSH	8485290	0
CHB	chr19	CSH	8535290	0
CHB	chr19	CSH	8585290	0
CHB	chr19	CSH	8635290	0
CHB	chr19	CSH	8685290	0
CHB	chr19	CSH	8735290	0
CHB	chr19	CSH	8785290	0
CHB	chr19	CSH	8835290	0
CHB	chr19	CSH	8885290	0
CHB	chr19	CSH	8935290	0
CHB	chr19	CSH	8985290	0
CHB	chr19	CSH	9035290	0

CHB	chr19	CSH	9085290	0
CHB	chr19	CSH	9135290	0
CHB	chr19	CSH	9185290	0
CHB	chr19	CSH	9235290	0
CHB	chr19	CSH	9285290	0
CHB	chr19	CSH	9335290	0
CHB	chr19	CSH	9385290	0
CHB	chr19	CSH	9435290	0
CHB	chr19	CSH	9485290	0
CHB	chr19	CSH	9535290	0
CHB	chr19	CSH	9585290	0
CHB	chr19	CSH	9635290	0
CHB	chr19	CSH	9685290	0
CHB	chr19	CSH	9735290	0
CHB	chr19	CSH	9785290	0
CHB	chr19	CSH	9835290	0
CHB	chr19	CSH	9885290	0
CHB	chr19	CSH	9935290	0
CHB	chr19	CSH	9985290	0
CHB	chr19	CSH	10035290	0
CHB	chr19	CSH	10085290	0
CHB	chr19	CSH	10135290	1
CHB	chr19	CSH	10185290	42
CHB	chr19	CSH	10235290	74
CHB	chr19	CSH	10285290	58
CHB	chr19	CSH	10335290	40
CHB	chr19	CSH	10385290	28
CHB	chr19	CSH	10435290	48
CHB	chr19	CSH	10485290	58
CHB	chr19	CSH	10535290	39
CHB	chr19	CSH	10585290	37
CHB	chr19	CSH	10635290	21
CHB	chr19	CSH	10685290	0
CHB	chr19	CSH	10735290	0
CHB	chr19	CSH	10785290	0
CHB	chr19	CSH	10835290	0
CHB	chr19	CSH	10885290	0
CHB	chr19	CSH	10935290	0
CHB	chr19	CSH	10985290	0
CHB	chr19	CSH	11035290	0
CHB	chr19	CSH	11085290	0
CHB	chr19	CSH	11135290	0
CHB	chr19	CSH	11185290	0
CHB	chr19	CSH	11235290	0
CHB	chr19	CSH	11285290	0
CHB	chr19	CSH	11335290	0
CHB	chr19	CSH	11385290	0
CHB	chr19	CSH	11435290	0
CHB	chr19	CSH	11485290	6

CHB	chr19	CSH	11535290	35
CHB	chr19	CSH	11585290	29
CHB	chr19	CSH	11635290	3
CHB	chr19	CSH	11685290	18
CHB	chr19	CSH	11735290	30
CHB	chr19	CSH	11785290	37
CHB	chr19	CSH	11835290	37
CHB	chr19	CSH	11885290	24
CHB	chr19	CSH	11935290	27
CHB	chr19	CSH	11985290	23
CHB	chr19	CSH	12035290	5
CHB	chr19	CSH	12085290	0
CHB	chr19	CSH	12135290	0
CHB	chr19	CSH	12185290	0
CHB	chr19	CSH	12235290	0
CHB	chr19	CSH	12285290	0
CHB	chr19	CSH	12335290	0
CHB	chr19	CSH	12385290	0
CHB	chr19	CSH	12435290	0
CHB	chr19	CSH	12485290	0
CHB	chr19	CSH	12535290	0
CHB	chr19	CSH	12585290	0
CHB	chr19	CSH	12635290	0
CHB	chr19	CSH	12685290	0
CHB	chr19	CSH	12735290	0
CHB	chr19	CSH	12785290	0
CHB	chr19	CSH	12835290	0
CHB	chr19	CSH	12885290	0
CHB	chr19	CSH	12935290	0
CHB	chr19	CSH	12985290	0
CHB	chr19	CSH	13035290	0
CHB	chr19	CSH	13085290	0
CHB	chr19	CSH	13135290	0
CHB	chr19	CSH	13185290	0
CHB	chr19	CSH	13235290	0
CHB	chr19	CSH	13285290	0
CHB	chr19	CSH	13335290	0
CHB	chr19	CSH	13385290	0
CHB	chr19	CSH	13435290	0
CHB	chr19	CSH	13485290	0
CHB	chr19	CSH	13535290	0
CHB	chr19	CSH	13585290	0
CHB	chr19	CSH	13635290	0
CHB	chr19	CSH	13685290	0
CHB	chr19	CSH	13735290	0
CHB	chr19	CSH	13785290	0
CHB	chr19	CSH	13835290	0
CHB	chr19	CSH	13885290	0
CHB	chr19	CSH	13935290	0

CHB	chr19	CSH	13985290	0
CHB	chr19	CSH	14035290	0
CHB	chr19	CSH	14085290	0
CHB	chr19	CSH	14135290	0
CHB	chr19	CSH	14185290	0
CHB	chr19	CSH	14235290	0
CHB	chr19	CSH	14285290	0
CHB	chr19	CSH	14335290	0
CHB	chr19	CSH	14385290	0
CHB	chr19	CSH	14435290	0
CHB	chr19	CSH	14485290	0
CHB	chr19	CSH	14535290	0
CHB	chr19	CSH	14585290	0
CHB	chr19	CSH	14635290	0
CHB	chr19	CSH	14685290	0
CHB	chr19	CSH	14735290	0
CHB	chr19	CSH	14785290	0
CHB	chr19	CSH	14835290	0
CHB	chr19	CSH	14885290	0
CHB	chr19	CSH	14935290	0
CHB	chr19	CSH	14985290	0
CHB	chr19	CSH	15035290	0
CHB	chr19	CSH	15085290	0
CHB	chr19	CSH	15135290	0
CHB	chr19	CSH	15185290	0
CHB	chr19	CSH	15235290	0
CHB	chr19	CSH	15285290	0
CHB	chr19	CSH	15335290	0
CHB	chr19	CSH	15385290	0
CHB	chr19	CSH	15435290	0
CHB	chr19	CSH	15485290	0
CHB	chr19	CSH	15535290	0
CHB	chr19	CSH	15585290	0
CHB	chr19	CSH	15635290	0
CHB	chr19	CSH	15685290	0
CHB	chr19	CSH	15735290	0
CHB	chr19	CSH	15785290	0
CHB	chr19	CSH	15835290	0
CHB	chr19	CSH	15885290	0
CHB	chr19	CSH	15935290	0
CHB	chr19	CSH	15985290	0
CHB	chr19	CSH	16035290	0
CHB	chr19	CSH	16085290	0
CHB	chr19	CSH	16135290	0
CHB	chr19	CSH	16185290	0
CHB	chr19	CSH	16235290	0
CHB	chr19	CSH	16285290	0
CHB	chr19	CSH	16335290	0
CHB	chr19	CSH	16385290	0

CHB	chr19	CSH	53548333	26
CHB	chr19	CSH	53598333	0
CHB	chr19	CSH	53648333	61
CHB	chr19	CSH	53698333	61
CHB	chr19	CSH	53748333	0
CHB	chr19	CSH	53798333	0
CHB	chr19	CSH	53848333	0
CHB	chr19	CSH	53898333	8
CHB	chr19	CSH	53948333	24
CHB	chr19	CSH	53998333	16
CHB	chr19	CSH	54048333	0
CHB	chr19	CSH	54098333	0
CHB	chr19	CSH	54148333	0
CHB	chr19	CSH	54198333	0
CHB	chr19	CSH	54248333	0
CHB	chr19	CSH	54298333	0
CHB	chr19	CSH	54348333	0
CHB	chr19	CSH	54398333	0
CHB	chr19	CSH	54448333	0
CHB	chr19	CSH	54498333	0
CHB	chr19	CSH	54548333	0
CHB	chr19	CSH	54598333	0
CHB	chr19	CSH	54648333	0
CHB	chr19	CSH	54698333	0
CHB	chr19	CSH	54748333	49
CHB	chr19	CSH	54798333	53
CHB	chr19	CSH	54848333	9
CHB	chr19	CSH	54898333	30
CHB	chr19	CSH	54948333	25
CHB	chr19	CSH	54998333	0
CHB	chr19	CSH	55048333	0
CHB	chr19	CSH	55098333	0
CHB	chr19	CSH	55148333	0
CHB	chr19	CSH	55198333	0
CHB	chr19	CSH	55248333	0
CHB	chr19	CSH	55298333	0
CHB	chr19	CSH	55348333	0
CHB	chr19	CSH	55398333	0
CHB	chr19	CSH	55448333	0
CHB	chr19	CSH	55498333	0
CHB	chr19	CSH	55548333	0
CHB	chr19	CSH	55598333	0
CHB	chr19	CSH	55648333	0
CHB	chr19	CSH	55698333	0
CHB	chr19	CSH	55748333	0
CHB	chr19	CSH	55798333	0
CHB	chr19	CSH	55848333	0
CHB	chr19	CSH	55898333	0
CHB	chr19	CSH	55948333	0

CHB	chr19	CSH	55998333	0
CHB	chr19	CSH	56048333	0
CHB	chr19	CSH	56098333	0
CHB	chr19	CSH	56148333	0
CHB	chr19	CSH	56198333	0
CHB	chr19	CSH	56248333	0
CHB	chr19	CSH	56298333	0
CHB	chr19	CSH	56348333	0
CHB	chr19	CSH	56398333	0
CHB	chr19	CSH	56448333	0
CHB	chr19	CSH	56498333	0
CHB	chr19	CSH	56548333	9
CHB	chr19	CSH	56598333	40
CHB	chr19	CSH	56648333	55
CHB	chr19	CSH	56698333	52
CHB	chr19	CSH	56748333	30
CHB	chr19	CSH	56798333	2
CHB	chr19	CSH	56848333	0
CHB	chr19	CSH	56898333	0
CHB	chr19	CSH	56948333	0
CHB	chr19	CSH	56998333	0
CHB	chr19	CSH	57048333	0
CHB	chr19	CSH	57098333	0
CHB	chr19	CSH	57148333	0
CHB	chr19	CSH	57198333	0
CHB	chr19	CSH	57248333	0
CHB	chr19	CSH	57298333	0
CHB	chr19	CSH	57348333	0
CHB	chr19	CSH	57398333	0
CHB	chr19	CSH	57448333	0
CHB	chr19	CSH	57498333	0
CHB	chr19	CSH	57548333	0
CHB	chr19	CSH	57598333	0
CHB	chr19	CSH	57648333	0
CHB	chr19	CSH	57698333	0
CHB	chr19	CSH	57748333	0
CHB	chr19	CSH	57798333	0
CHB	chr19	CSH	57848333	0
CHB	chr19	CSH	57898333	0
CHB	chr19	CSH	57948333	0
CHB	chr19	CSH	57998333	0
CHB	chr19	CSH	58048333	0
CHB	chr19	CSH	58098333	0
CHB	chr19	CSH	58148333	0
CHB	chr19	CSH	58198333	0
CHB	chr19	CSH	58248333	0
CHB	chr19	CSH	58298333	0
CHB	chr19	CSH	58348333	0
CHB	chr19	CSH	58398333	0

CHB	chr19	CSH	58448333	0
CHB	chr19	CSH	58498333	0
CHB	chr19	CSH	58548333	0
CHB	chr19	CSH	58598333	0
CHB	chr19	CSH	58648333	0
CHB	chr19	CSH	58698333	0
CHB	chr19	CSH	58748333	0
CHB	chr19	CSH	58798333	0
CHB	chr19	CSH	58848333	0
CHB	chr19	CSH	58898333	0
CHB	chr19	CSH	58948333	0
CHB	chr19	CSH	58998333	0
CHB	chr19	CSH	59048333	0
CHB	chr19	CSH	59098333	0
CHB	chr19	CSH	59148333	0
CHB	chr19	CSH	59198333	0
CHB	chr19	CSH	59248333	0
CHB	chr19	CSH	59298333	0
CHB	chr19	CSH	59348333	0
CHB	chr19	CSH	59398333	0
CHB	chr19	CSH	59448333	0
CHB	chr19	CSH	59498333	0
CHB	chr19	CSH	59548333	0
CHB	chr19	CSH	59598333	0
CHB	chr19	CSH	59648333	0
CHB	chr19	CSH	59698333	0
CHB	chr19	CSH	59748333	0
CHB	chr19	CSH	59798333	0
CHB	chr19	CSH	59848333	0
CHB	chr19	CSH	59898333	0
CHB	chr19	CSH	59948333	0
CHB	chr19	CSH	59998333	0
CHB	chr19	CSH	60048333	0
CHB	chr19	CSH	60098333	0
CHB	chr19	CSH	60148333	0
CHB	chr19	CSH	60198333	0
CHB	chr19	CSH	60248333	0
CHB	chr19	CSH	60298333	0
CHB	chr19	CSH	60348333	0
CHB	chr19	CSH	60398333	0
CHB	chr19	CSH	60448333	0
CHB	chr19	CSH	60498333	0
CHB	chr19	CSH	60548333	0
CHB	chr19	CSH	60598333	0
CHB	chr19	CSH	60648333	0
CHB	chr19	CSH	60698333	0
CHB	chr19	CSH	60748333	0
CHB	chr19	CSH	60798333	0
CHB	chr19	CSH	60848333	0

CHB	chr19	CSH	60898333	0
CHB	chr19	CSH	60948333	0
CHB	chr19	CSH	60998333	0
CHB	chr19	CSH	61048333	0
CHB	chr19	CSH	61098333	0
CHB	chr19	CSH	61148333	0
CHB	chr19	CSH	61198333	0
CHB	chr19	CSH	61248333	0
CHB	chr19	CSH	61298333	0
CHB	chr19	CSH	61348333	0
CHB	chr19	CSH	61398333	0
CHB	chr19	CSH	61448333	0
CHB	chr19	CSH	61498333	0
CHB	chr19	CSH	61548333	0
CHB	chr19	CSH	61598333	0
CHB	chr19	CSH	61648333	0
CHB	chr19	CSH	61698333	0
CHB	chr19	CSH	61748333	0
CHB	chr19	CSH	61798333	0
CHB	chr19	CSH	61848333	0
CHB	chr19	CSH	61898333	0
CHB	chr19	CSH	61948333	0
CHB	chr19	CSH	61998333	0
CHB	chr19	CSH	62048333	0
CHB	chr19	CSH	62098333	0
CHB	chr19	CSH	62148333	0
CHB	chr19	CSH	62198333	0
CHB	chr19	CSH	62248333	0
CHB	chr19	CSH	62298333	0
CHB	chr19	CSH	62348333	0
CHB	chr19	CSH	62398333	0
CHB	chr19	CSH	62448333	0
CHB	chr19	CSH	62498333	0
CHB	chr19	CSH	62548333	0
CHB	chr19	CSH	62598333	0
CHB	chr19	CSH	62648333	0
CHB	chr19	CSH	62698333	0
CHB	chr19	CSH	62748333	0
CHB	chr19	CSH	62798333	0
CHB	chr19	CSH	62848333	0
CHB	chr19	CSH	62898333	0
CHB	chr19	CSH	62948333	0
CHB	chr19	CSH	62998333	0
CHB	chr19	CSH	63048333	0
CHB	chr19	CSH	63098333	0
CHB	chr19	CSH	63148333	0
CHB	chr19	CSH	63198333	0
CHB	chr19	CSH	63248333	0
CHB	chr19	CSH	63298333	0

CHB	chr19	CSH	63348333	0
CHB	chr19	CSH	63398333	0
CHB	chr19	CSH	63448333	0
CHB	chr8	DEFA1	2027649	71
CHB	chr8	DEFA1	2077649	115
CHB	chr8	DEFA1	2127649	139
CHB	chr8	DEFA1	2177649	71
CHB	chr8	DEFA1	2227649	8
CHB	chr8	DEFA1	2277649	6
CHB	chr8	DEFA1	2327649	41
CHB	chr8	DEFA1	2377649	59
CHB	chr8	DEFA1	2427649	48
CHB	chr8	DEFA1	2477649	51
CHB	chr8	DEFA1	2527649	38
CHB	chr8	DEFA1	2577649	31
CHB	chr8	DEFA1	2627649	50
CHB	chr8	DEFA1	2677649	57
CHB	chr8	DEFA1	2727649	51
CHB	chr8	DEFA1	2777649	35
CHB	chr8	DEFA1	2827649	27
CHB	chr8	DEFA1	2877649	63
CHB	chr8	DEFA1	2927649	72
CHB	chr8	DEFA1	2977649	57
CHB	chr8	DEFA1	3027649	47
CHB	chr8	DEFA1	3077649	60
CHB	chr8	DEFA1	3127649	82
CHB	chr8	DEFA1	3177649	101
CHB	chr8	DEFA1	3227649	125
CHB	chr8	DEFA1	3277649	132
CHB	chr8	DEFA1	3327649	102
CHB	chr8	DEFA1	3377649	42
CHB	chr8	DEFA1	3427649	47
CHB	chr8	DEFA1	3477649	93
CHB	chr8	DEFA1	3527649	107
CHB	chr8	DEFA1	3577649	57
CHB	chr8	DEFA1	3627649	7
CHB	chr8	DEFA1	3677649	7
CHB	chr8	DEFA1	3727649	10
CHB	chr8	DEFA1	3777649	12
CHB	chr8	DEFA1	3827649	14
CHB	chr8	DEFA1	3877649	11
CHB	chr8	DEFA1	3927649	8
CHB	chr8	DEFA1	3977649	8
CHB	chr8	DEFA1	4027649	12
CHB	chr8	DEFA1	4077649	20
CHB	chr8	DEFA1	4127649	29
CHB	chr8	DEFA1	4177649	65
CHB	chr8	DEFA1	4227649	121
CHB	chr8	DEFA1	4277649	103

CHB	chr8	DEFA1	4327649	43
CHB	chr8	DEFA1	4377649	30
CHB	chr8	DEFA1	4427649	28
CHB	chr8	DEFA1	4477649	20
CHB	chr8	DEFA1	4527649	11
CHB	chr8	DEFA1	4577649	22
CHB	chr8	DEFA1	4627649	77
CHB	chr8	DEFA1	4677649	66
CHB	chr8	DEFA1	4727649	22
CHB	chr8	DEFA1	4777649	44
CHB	chr8	DEFA1	4827649	61
CHB	chr8	DEFA1	4877649	90
CHB	chr8	DEFA1	4927649	89
CHB	chr8	DEFA1	4977649	83
CHB	chr8	DEFA1	5027649	75
CHB	chr8	DEFA1	5077649	37
CHB	chr8	DEFA1	5127649	29
CHB	chr8	DEFA1	5177649	29
CHB	chr8	DEFA1	5227649	55
CHB	chr8	DEFA1	5277649	93
CHB	chr8	DEFA1	5327649	86
CHB	chr8	DEFA1	5377649	65
CHB	chr8	DEFA1	5427649	50
CHB	chr8	DEFA1	5477649	44
CHB	chr8	DEFA1	5527649	53
CHB	chr8	DEFA1	5577649	54
CHB	chr8	DEFA1	5627649	83
CHB	chr8	DEFA1	5677649	97
CHB	chr8	DEFA1	5727649	61
CHB	chr8	DEFA1	5777649	59
CHB	chr8	DEFA1	5827649	66
CHB	chr8	DEFA1	5877649	57
CHB	chr8	DEFA1	5927649	72
CHB	chr8	DEFA1	5977649	70
CHB	chr8	DEFA1	6027649	51
CHB	chr8	DEFA1	6077649	82
CHB	chr8	DEFA1	6127649	68
CHB	chr8	DEFA1	6177649	27
CHB	chr8	DEFA1	6227649	54
CHB	chr8	DEFA1	6277649	88
CHB	chr8	DEFA1	6327649	81
CHB	chr8	DEFA1	6377649	38
CHB	chr8	DEFA1	6427649	9
CHB	chr8	DEFA1	6477649	0
CHB	chr8	DEFA1	6527649	0
CHB	chr8	DEFA1	6577649	0
CHB	chr8	DEFA1	6627649	0
CHB	chr8	DEFA1	6677649	0
CHB	chr8	DEFA1	6727649	0

CHB	chr8	DEFA1	6777649	0
CHB	chr8	DEFA1	6827649	0
CHB	chr8	DEFA1	6877649	0
CHB	chr8	DEFA1	6927649	51
CHB	chr8	DEFA1	6977649	94
CHB	chr8	DEFA1	7027649	43
CHB	chr8	DEFA1	7077649	0
CHB	chr8	DEFA1	7127649	0
CHB	chr8	DEFA1	7177649	0
CHB	chr8	DEFA1	7227649	0
CHB	chr8	DEFA1	7277649	0
CHB	chr8	DEFA1	7327649	0
CHB	chr8	DEFA1	7377649	0
CHB	chr8	DEFA1	7427649	0
CHB	chr8	DEFA1	7477649	0
CHB	chr8	DEFA1	7527649	0
CHB	chr8	DEFA1	7577649	0
CHB	chr8	DEFA1	7627649	0
CHB	chr8	DEFA1	7677649	0
CHB	chr8	DEFA1	7727649	0
CHB	chr8	DEFA1	7777649	0
CHB	chr8	DEFA1	7827649	0
CHB	chr8	DEFA1	7877649	0
CHB	chr8	DEFA1	7927649	0
CHB	chr8	DEFA1	7977649	0
CHB	chr8	DEFA1	8027649	8
CHB	chr8	DEFA1	8077649	45
CHB	chr8	DEFA1	8127649	57
CHB	chr8	DEFA1	8177649	50
CHB	chr8	DEFA1	8227649	49
CHB	chr8	DEFA1	8277649	46
CHB	chr8	DEFA1	8327649	37
CHB	chr8	DEFA1	8377649	10
CHB	chr8	DEFA1	8427649	0
CHB	chr8	DEFA1	8477649	0
CHB	chr8	DEFA1	8527649	0
CHB	chr8	DEFA1	8577649	0
CHB	chr8	DEFA1	8627649	0
CHB	chr8	DEFA1	8677649	0
CHB	chr8	DEFA1	8727649	0
CHB	chr8	DEFA1	8777649	0
CHB	chr8	DEFA1	8827649	0
CHB	chr8	DEFA1	8877649	0
CHB	chr8	DEFA1	8927649	0
CHB	chr8	DEFA1	8977649	1
CHB	chr8	DEFA1	9027649	43
CHB	chr8	DEFA1	9077649	71
CHB	chr8	DEFA1	9127649	40
CHB	chr8	DEFA1	9177649	11

CHB	chr8	DEFA1	9227649	0
CHB	chr8	DEFA1	9277649	0
CHB	chr8	DEFA1	9327649	0
CHB	chr8	DEFA1	9377649	0
CHB	chr8	DEFA1	9427649	0
CHB	chr8	DEFA1	9477649	0
CHB	chr8	DEFA1	9527649	0
CHB	chr8	DEFA1	9577649	0
CHB	chr8	DEFA1	9627649	0
CHB	chr8	DEFA1	9677649	0
CHB	chr8	DEFA1	9727649	0
CHB	chr8	DEFA1	9777649	0
CHB	chr8	DEFA1	9827649	0
CHB	chr8	DEFA1	9877649	0
CHB	chr8	DEFA1	9927649	0
CHB	chr8	DEFA1	9977649	0
CHB	chr8	DEFA1	10027649	0
CHB	chr8	DEFA1	10077649	0
CHB	chr8	DEFA1	10127649	0
CHB	chr8	DEFA1	10177649	0
CHB	chr8	DEFA1	10227649	0
CHB	chr8	DEFA1	10277649	0
CHB	chr8	DEFA1	10327649	0
CHB	chr8	DEFA1	10377649	0
CHB	chr8	DEFA1	10427649	0
CHB	chr8	DEFA1	10477649	0
CHB	chr8	DEFA1	10527649	0
CHB	chr8	DEFA1	10577649	0
CHB	chr8	DEFA1	10627649	0
CHB	chr8	DEFA1	10677649	0
CHB	chr8	DEFA1	10727649	0
CHB	chr8	DEFA1	10777649	0
CHB	chr8	DEFA1	10827649	0
CHB	chr8	DEFA1	10877649	0
CHB	chr8	DEFA1	10927649	0
CHB	chr8	DEFA1	10977649	0
CHB	chr8	DEFA1	11027649	0
CHB	chr8	DEFA1	11077649	0
CHB	chr8	DEFA1	11127649	0
CHB	chr8	DEFA1	11177649	0
CHB	chr8	DEFA1	11227649	0
CHB	chr8	DEFA1	11277649	0
CHB	chr8	DEFA1	11327649	0
CHB	chr8	DEFA1	11377649	0
CHB	chr8	DEFA1	11427649	0
CHB	chr8	DEFA1	11477649	0
CHB	chr8	DEFA1	11527649	0
CHB	chr8	DEFA1	11577649	0
CHB	chr8	DEFA1	11627649	0

CHB	chr8	DEFA1	11677649	0
CHB	chr8	DEFA1	11727649	0
CHB	chr8	DEFA1	11777649	0
CHB	chr8	DEFA1	11827649	0
CHB	chr8	DEFA1	11877649	0
CHB	chr8	DEFA1	11927649	0
CHB	chr8	DEFB130	7114389	0
CHB	chr8	DEFB130	7164389	0
CHB	chr8	DEFB130	7214389	0
CHB	chr8	DEFB130	7264389	0
CHB	chr8	DEFB130	7314389	0
CHB	chr8	DEFB130	7364389	0
CHB	chr8	DEFB130	7414389	0
CHB	chr8	DEFB130	7464389	0
CHB	chr8	DEFB130	7514389	0
CHB	chr8	DEFB130	7564389	0
CHB	chr8	DEFB130	7614389	0
CHB	chr8	DEFB130	7664389	0
CHB	chr8	DEFB130	7714389	0
CHB	chr8	DEFB130	7764389	0
CHB	chr8	DEFB130	7814389	0
CHB	chr8	DEFB130	7864389	0
CHB	chr8	DEFB130	7914389	0
CHB	chr8	DEFB130	7964389	0
CHB	chr8	DEFB130	8014389	0
CHB	chr8	DEFB130	8064389	33
CHB	chr8	DEFB130	8114389	59
CHB	chr8	DEFB130	8164389	54
CHB	chr8	DEFB130	8214389	46
CHB	chr8	DEFB130	8264389	42
CHB	chr8	DEFB130	8314389	46
CHB	chr8	DEFB130	8364389	22
CHB	chr8	DEFB130	8414389	0
CHB	chr8	DEFB130	8464389	0
CHB	chr8	DEFB130	8514389	0
CHB	chr8	DEFB130	8564389	0
CHB	chr8	DEFB130	8614389	0
CHB	chr8	DEFB130	8664389	0
CHB	chr8	DEFB130	8714389	0
CHB	chr8	DEFB130	8764389	0
CHB	chr8	DEFB130	8814389	0
CHB	chr8	DEFB130	8864389	0
CHB	chr8	DEFB130	8914389	0
CHB	chr8	DEFB130	8964389	0
CHB	chr8	DEFB130	9014389	26
CHB	chr8	DEFB130	9064389	64
CHB	chr8	DEFB130	9114389	57
CHB	chr8	DEFB130	9164389	19
CHB	chr8	DEFB130	9214389	0

CHB	chr8	DEFB130	9264389	0
CHB	chr8	DEFB130	9314389	0
CHB	chr8	DEFB130	9364389	0
CHB	chr8	DEFB130	9414389	0
CHB	chr8	DEFB130	9464389	0
CHB	chr8	DEFB130	9514389	0
CHB	chr8	DEFB130	9564389	0
CHB	chr8	DEFB130	9614389	0
CHB	chr8	DEFB130	9664389	0
CHB	chr8	DEFB130	9714389	0
CHB	chr8	DEFB130	9764389	0
CHB	chr8	DEFB130	9814389	0
CHB	chr8	DEFB130	9864389	0
CHB	chr8	DEFB130	9914389	0
CHB	chr8	DEFB130	9964389	0
CHB	chr8	DEFB130	10014389	0
CHB	chr8	DEFB130	10064389	0
CHB	chr8	DEFB130	10114389	0
CHB	chr8	DEFB130	10164389	0
CHB	chr8	DEFB130	10214389	0
CHB	chr8	DEFB130	10264389	0
CHB	chr8	DEFB130	10314389	0
CHB	chr8	DEFB130	10364389	0
CHB	chr8	DEFB130	10414389	0
CHB	chr8	DEFB130	10464389	0
CHB	chr8	DEFB130	10514389	0
CHB	chr8	DEFB130	10564389	0
CHB	chr8	DEFB130	10614389	0
CHB	chr8	DEFB130	10664389	0
CHB	chr8	DEFB130	10714389	0
CHB	chr8	DEFB130	10764389	0
CHB	chr8	DEFB130	10814389	0
CHB	chr8	DEFB130	10864389	0
CHB	chr8	DEFB130	10914389	0
CHB	chr8	DEFB130	10964389	0
CHB	chr8	DEFB130	11014389	0
CHB	chr8	DEFB130	11064389	0
CHB	chr8	DEFB130	11114389	0
CHB	chr8	DEFB130	11164389	0
CHB	chr8	DEFB130	11214389	0
CHB	chr8	DEFB130	11264389	0
CHB	chr8	DEFB130	11314389	0
CHB	chr8	DEFB130	11364389	0
CHB	chr8	DEFB130	11414389	0
CHB	chr8	DEFB130	11464389	0
CHB	chr8	DEFB130	11514389	0
CHB	chr8	DEFB130	11564389	0
CHB	chr8	DEFB130	11614389	0
CHB	chr8	DEFB130	11664389	0

CHB	chr8	DEFB130	11714389	0
CHB	chr8	DEFB130	11764389	0
CHB	chr8	DEFB130	11814389	0
CHB	chr8	DEFB130	11864389	0
CHB	chr8	DEFB130	11914389	0
CHB	chr8	DEFB130	11964389	0
CHB	chr8	DEFB130	12014389	0
CHB	chr8	DEFB130	12064389	0
CHB	chr8	DEFB130	12114389	0
CHB	chr8	DEFB130	12164389	0
CHB	chr8	DEFB130	12214389	0
CHB	chr8	DEFB130	12264389	0
CHB	chr8	DEFB130	12314389	0
CHB	chr8	DEFB130	12364389	0
CHB	chr8	DEFB130	12414389	1
CHB	chr8	DEFB130	12464389	51
CHB	chr8	DEFB130	12514389	84
CHB	chr8	DEFB130	12564389	61
CHB	chr8	DEFB130	12614389	50
CHB	chr8	DEFB130	12664389	47
CHB	chr8	DEFB130	12714389	24
CHB	chr8	DEFB130	12764389	3
CHB	chr8	DEFB130	12814389	34
CHB	chr8	DEFB130	12864389	62
CHB	chr8	DEFB130	12914389	73
CHB	chr8	DEFB130	12964389	46
CHB	chr8	DEFB130	13014389	4
CHB	chr8	DEFB130	13064389	0
CHB	chr8	DEFB130	13114389	0
CHB	chr8	DEFB130	13164389	0
CHB	chr8	DEFB130	13214389	0
CHB	chr8	DEFB130	13264389	0
CHB	chr8	DEFB130	13314389	0
CHB	chr8	DEFB130	13364389	0
CHB	chr8	DEFB130	13414389	0
CHB	chr8	DEFB130	13464389	0
CHB	chr8	DEFB130	13514389	0
CHB	chr8	DEFB130	13564389	0
CHB	chr8	DEFB130	13614389	0
CHB	chr8	DEFB130	13664389	0
CHB	chr8	DEFB130	13714389	41
CHB	chr8	DEFB130	13764389	70
CHB	chr8	DEFB130	13814389	75
CHB	chr8	DEFB130	13864389	80
CHB	chr8	DEFB130	13914389	45
CHB	chr8	DEFB130	13964389	26
CHB	chr8	DEFB130	14014389	33
CHB	chr8	DEFB130	14064389	71
CHB	chr8	DEFB130	14114389	76

CHB	chr8	DEFB130	14164389	36
CHB	chr8	DEFB130	14214389	34
CHB	chr8	DEFB130	14264389	33
CHB	chr8	DEFB130	14314389	26
CHB	chr8	DEFB130	14364389	41
CHB	chr8	DEFB130	14414389	54
CHB	chr8	DEFB130	14464389	43
CHB	chr8	DEFB130	14514389	16
CHB	chr8	DEFB130	14564389	0
CHB	chr8	DEFB130	14614389	0
CHB	chr8	DEFB130	14664389	0
CHB	chr8	DEFB130	14714389	0
CHB	chr8	DEFB130	14764389	0
CHB	chr8	DEFB130	14814389	0
CHB	chr8	DEFB130	14864389	0
CHB	chr8	DEFB130	14914389	0
CHB	chr8	DEFB130	14964389	0
CHB	chr8	DEFB130	15014389	0
CHB	chr8	DEFB130	15064389	0
CHB	chr8	DEFB130	15114389	0
CHB	chr8	DEFB130	15164389	0
CHB	chr8	DEFB130	15214389	0
CHB	chr8	DEFB130	15264389	0
CHB	chr8	DEFB130	15314389	0
CHB	chr8	DEFB130	15364389	6
CHB	chr8	DEFB130	15414389	15
CHB	chr8	DEFB130	15464389	28
CHB	chr8	DEFB130	15514389	36
CHB	chr8	DEFB130	15564389	47
CHB	chr8	DEFB130	15614389	38
CHB	chr8	DEFB130	15664389	8
CHB	chr8	DEFB130	15714389	0
CHB	chr8	DEFB130	15764389	0
CHB	chr8	DEFB130	15814389	27
CHB	chr8	DEFB130	15864389	103
CHB	chr8	DEFB130	15914389	117
CHB	chr8	DEFB130	15964389	63
CHB	chr8	DEFB130	16014389	62
CHB	chr8	DEFB130	16064389	73
CHB	chr8	DEFB130	16114389	54
CHB	chr8	DEFB130	16164389	50
CHB	chr8	DEFB130	16214389	55
CHB	chr8	DEFB130	16264389	68
CHB	chr8	DEFB130	16314389	46
CHB	chr8	DEFB130	16364389	4
CHB	chr8	DEFB130	16414389	0
CHB	chr8	DEFB130	16464389	0
CHB	chr8	DEFB130	16514389	0
CHB	chr8	DEFB130	16564389	0

CHB	chr8	DEFB130	16614389	0
CHB	chr8	DEFB130	16664389	0
CHB	chr8	DEFB130	16714389	0
CHB	chr8	DEFB130	16764389	22
CHB	chr8	DEFB130	16814389	68
CHB	chr8	DEFB130	16864389	52
CHB	chr8	DEFB130	16914389	6
CHB	chr8	DEFB130	16964389	0
CHB	chr8	DEFB130	17014389	0
CHB	chr8	DEFB130	7360962	0
CHB	chr8	DEFB130	7410962	0
CHB	chr8	DEFB130	7460962	0
CHB	chr8	DEFB130	7510962	0
CHB	chr8	DEFB130	7560962	0
CHB	chr8	DEFB130	7610962	0
CHB	chr8	DEFB130	7660962	0
CHB	chr8	DEFB130	7710962	0
CHB	chr8	DEFB130	7760962	0
CHB	chr8	DEFB130	7810962	0
CHB	chr8	DEFB130	7860962	0
CHB	chr8	DEFB130	7910962	0
CHB	chr8	DEFB130	7960962	0
CHB	chr8	DEFB130	8010962	0
CHB	chr8	DEFB130	8060962	32
CHB	chr8	DEFB130	8110962	58
CHB	chr8	DEFB130	8160962	54
CHB	chr8	DEFB130	8210962	47
CHB	chr8	DEFB130	8260962	40
CHB	chr8	DEFB130	8310962	46
CHB	chr8	DEFB130	8360962	25
CHB	chr8	DEFB130	8410962	0
CHB	chr8	DEFB130	8460962	0
CHB	chr8	DEFB130	8510962	0
CHB	chr8	DEFB130	8560962	0
CHB	chr8	DEFB130	8610962	0
CHB	chr8	DEFB130	8660962	0
CHB	chr8	DEFB130	8710962	0
CHB	chr8	DEFB130	8760962	0
CHB	chr8	DEFB130	8810962	0
CHB	chr8	DEFB130	8860962	0
CHB	chr8	DEFB130	8910962	0
CHB	chr8	DEFB130	8960962	0
CHB	chr8	DEFB130	9010962	23
CHB	chr8	DEFB130	9060962	59
CHB	chr8	DEFB130	9110962	60
CHB	chr8	DEFB130	9160962	24
CHB	chr8	DEFB130	9210962	0
CHB	chr8	DEFB130	9260962	0
CHB	chr8	DEFB130	9310962	0

CHB	chr8	DEFB130	9360962	0
CHB	chr8	DEFB130	9410962	0
CHB	chr8	DEFB130	9460962	0
CHB	chr8	DEFB130	9510962	0
CHB	chr8	DEFB130	9560962	0
CHB	chr8	DEFB130	9610962	0
CHB	chr8	DEFB130	9660962	0
CHB	chr8	DEFB130	9710962	0
CHB	chr8	DEFB130	9760962	0
CHB	chr8	DEFB130	9810962	0
CHB	chr8	DEFB130	9860962	0
CHB	chr8	DEFB130	9910962	0
CHB	chr8	DEFB130	9960962	0
CHB	chr8	DEFB130	10010962	0
CHB	chr8	DEFB130	10060962	0
CHB	chr8	DEFB130	10110962	0
CHB	chr8	DEFB130	10160962	0
CHB	chr8	DEFB130	10210962	0
CHB	chr8	DEFB130	10260962	0
CHB	chr8	DEFB130	10310962	0
CHB	chr8	DEFB130	10360962	0
CHB	chr8	DEFB130	10410962	0
CHB	chr8	DEFB130	10460962	0
CHB	chr8	DEFB130	10510962	0
CHB	chr8	DEFB130	10560962	0
CHB	chr8	DEFB130	10610962	0
CHB	chr8	DEFB130	10660962	0
CHB	chr8	DEFB130	10710962	0
CHB	chr8	DEFB130	10760962	0
CHB	chr8	DEFB130	10810962	0
CHB	chr8	DEFB130	10860962	0
CHB	chr8	DEFB130	10910962	0
CHB	chr8	DEFB130	10960962	0
CHB	chr8	DEFB130	11010962	0
CHB	chr8	DEFB130	11060962	0
CHB	chr8	DEFB130	11110962	0
CHB	chr8	DEFB130	11160962	0
CHB	chr8	DEFB130	11210962	0
CHB	chr8	DEFB130	11260962	0
CHB	chr8	DEFB130	11310962	0
CHB	chr8	DEFB130	11360962	0
CHB	chr8	DEFB130	11410962	0
CHB	chr8	DEFB130	11460962	0
CHB	chr8	DEFB130	11510962	0
CHB	chr8	DEFB130	11560962	0
CHB	chr8	DEFB130	11610962	0
CHB	chr8	DEFB130	11660962	0
CHB	chr8	DEFB130	11710962	0
CHB	chr8	DEFB130	11760962	0

CHB	chr8	DEFB130	11810962	0
CHB	chr8	DEFB130	11860962	0
CHB	chr8	DEFB130	11910962	0
CHB	chr8	DEFB130	11960962	0
CHB	chr8	DEFB130	12010962	0
CHB	chr8	DEFB130	12060962	0
CHB	chr8	DEFB130	12110962	0
CHB	chr8	DEFB130	12160962	0
CHB	chr8	DEFB130	12210962	0
CHB	chr8	DEFB130	12260962	0
CHB	chr8	DEFB130	12310962	0
CHB	chr8	DEFB130	12360962	0
CHB	chr8	DEFB130	12410962	1
CHB	chr8	DEFB130	12460962	46
CHB	chr8	DEFB130	12510962	78
CHB	chr8	DEFB130	12560962	66
CHB	chr8	DEFB130	12610962	54
CHB	chr8	DEFB130	12660962	47
CHB	chr8	DEFB130	12710962	26
CHB	chr8	DEFB130	12760962	0
CHB	chr8	DEFB130	12810962	29
CHB	chr8	DEFB130	12860962	65
CHB	chr8	DEFB130	12910962	74
CHB	chr8	DEFB130	12960962	46
CHB	chr8	DEFB130	13010962	8
CHB	chr8	DEFB130	13060962	0
CHB	chr8	DEFB130	13110962	0
CHB	chr8	DEFB130	13160962	0
CHB	chr8	DEFB130	13210962	0
CHB	chr8	DEFB130	13260962	0
CHB	chr8	DEFB130	13310962	0
CHB	chr8	DEFB130	13360962	0
CHB	chr8	DEFB130	13410962	0
CHB	chr8	DEFB130	13460962	0
CHB	chr8	DEFB130	13510962	0
CHB	chr8	DEFB130	13560962	0
CHB	chr8	DEFB130	13610962	0
CHB	chr8	DEFB130	13660962	0
CHB	chr8	DEFB130	13710962	38
CHB	chr8	DEFB130	13760962	69
CHB	chr8	DEFB130	13810962	77
CHB	chr8	DEFB130	13860962	80
CHB	chr8	DEFB130	13910962	45
CHB	chr8	DEFB130	13960962	27
CHB	chr8	DEFB130	14010962	29
CHB	chr8	DEFB130	14060962	71
CHB	chr8	DEFB130	14110962	81
CHB	chr8	DEFB130	14160962	36
CHB	chr8	DEFB130	14210962	33

CHB	chr8	DEFB130	14260962	32
CHB	chr8	DEFB130	14310962	26
CHB	chr8	DEFB130	14360962	41
CHB	chr8	DEFB130	14410962	44
CHB	chr8	DEFB130	14460962	42
CHB	chr8	DEFB130	14510962	27
CHB	chr8	DEFB130	14560962	2
CHB	chr8	DEFB130	14610962	0
CHB	chr8	DEFB130	14660962	0
CHB	chr8	DEFB130	14710962	0
CHB	chr8	DEFB130	14760962	0
CHB	chr8	DEFB130	14810962	0
CHB	chr8	DEFB130	14860962	0
CHB	chr8	DEFB130	14910962	0
CHB	chr8	DEFB130	14960962	0
CHB	chr8	DEFB130	15010962	0
CHB	chr8	DEFB130	15060962	0
CHB	chr8	DEFB130	15110962	0
CHB	chr8	DEFB130	15160962	0
CHB	chr8	DEFB130	15210962	0
CHB	chr8	DEFB130	15260962	0
CHB	chr8	DEFB130	15310962	0
CHB	chr8	DEFB130	15360962	6
CHB	chr8	DEFB130	15410962	15
CHB	chr8	DEFB130	15460962	28
CHB	chr8	DEFB130	15510962	36
CHB	chr8	DEFB130	15560962	46
CHB	chr8	DEFB130	15610962	38
CHB	chr8	DEFB130	15660962	9
CHB	chr8	DEFB130	15710962	0
CHB	chr8	DEFB130	15760962	0
CHB	chr8	DEFB130	15810962	22
CHB	chr8	DEFB130	15860962	102
CHB	chr8	DEFB130	15910962	118
CHB	chr8	DEFB130	15960962	62
CHB	chr8	DEFB130	16010962	61
CHB	chr8	DEFB130	16060962	72
CHB	chr8	DEFB130	16110962	57
CHB	chr8	DEFB130	16160962	46
CHB	chr8	DEFB130	16210962	56
CHB	chr8	DEFB130	16260962	72
CHB	chr8	DEFB130	16310962	47
CHB	chr8	DEFB130	16360962	7
CHB	chr8	DEFB130	16410962	0
CHB	chr8	DEFB130	16460962	0
CHB	chr8	DEFB130	16510962	0
CHB	chr8	DEFB130	16560962	0
CHB	chr8	DEFB130	16610962	0
CHB	chr8	DEFB130	16660962	0

CHB	chr8	DEFB130	16710962	0
CHB	chr8	DEFB130	16760962	20
CHB	chr8	DEFB130	16810962	68
CHB	chr8	DEFB130	16860962	54
CHB	chr8	DEFB130	16910962	6
CHB	chr8	DEFB130	16960962	0
CHB	chr8	DEFB130	17010962	0
CHB	chr8	DEFB130	17060962	0
CHB	chr8	DEFB130	17110962	0
CHB	chr8	DEFB130	17160962	0
CHB	chr8	DEFB130	17210962	0
CHB	chr8	DEFB130	17260962	0
CHB	chr1	FAM72A	201236179	0
CHB	chr1	FAM72A	201286179	0
CHB	chr1	FAM72A	201336179	0
CHB	chr1	FAM72A	201386179	0
CHB	chr1	FAM72A	201436179	0
CHB	chr1	FAM72A	201486179	0
CHB	chr1	FAM72A	201536179	0
CHB	chr1	FAM72A	201586179	0
CHB	chr1	FAM72A	201636179	0
CHB	chr1	FAM72A	201686179	0
CHB	chr1	FAM72A	201736179	0
CHB	chr1	FAM72A	201786179	0
CHB	chr1	FAM72A	201836179	0
CHB	chr1	FAM72A	201886179	0
CHB	chr1	FAM72A	201936179	0
CHB	chr1	FAM72A	201986179	0
CHB	chr1	FAM72A	202036179	0
CHB	chr1	FAM72A	202086179	0
CHB	chr1	FAM72A	202136179	0
CHB	chr1	FAM72A	202186179	0
CHB	chr1	FAM72A	202236179	0
CHB	chr1	FAM72A	202286179	0
CHB	chr1	FAM72A	202336179	0
CHB	chr1	FAM72A	202386179	0
CHB	chr1	FAM72A	202436179	0
CHB	chr1	FAM72A	202486179	0
CHB	chr1	FAM72A	202536179	0
CHB	chr1	FAM72A	202586179	0
CHB	chr1	FAM72A	202636179	0
CHB	chr1	FAM72A	202686179	0
CHB	chr1	FAM72A	202736179	0
CHB	chr1	FAM72A	202786179	0
CHB	chr1	FAM72A	202836179	0
CHB	chr1	FAM72A	202886179	0
CHB	chr1	FAM72A	202936179	0
CHB	chr1	FAM72A	202986179	0
CHB	chr1	FAM72A	203036179	17

CHB	chr1	FAM72A	203086179	42
CHB	chr1	FAM72A	203136179	35
CHB	chr1	FAM72A	203186179	10
CHB	chr1	FAM72A	203236179	0
CHB	chr1	FAM72A	203286179	0
CHB	chr1	FAM72A	203336179	14
CHB	chr1	FAM72A	203386179	40
CHB	chr1	FAM72A	203436179	50
CHB	chr1	FAM72A	203486179	43
CHB	chr1	FAM72A	203536179	65
CHB	chr1	FAM72A	203586179	70
CHB	chr1	FAM72A	203636179	43
CHB	chr1	FAM72A	203686179	54
CHB	chr1	FAM72A	203736179	51
CHB	chr1	FAM72A	203786179	35
CHB	chr1	FAM72A	203836179	34
CHB	chr1	FAM72A	203886179	29
CHB	chr1	FAM72A	203936179	27
CHB	chr1	FAM72A	203986179	25
CHB	chr1	FAM72A	204036179	21
CHB	chr1	FAM72A	204086179	9
CHB	chr1	FAM72A	204136179	0
CHB	chr1	FAM72A	204186179	0
CHB	chr1	FAM72A	204236179	0
CHB	chr1	FAM72A	204286179	0
CHB	chr1	FAM72A	204336179	0
CHB	chr1	FAM72A	204386179	0
CHB	chr1	FAM72A	204436179	0
CHB	chr1	FAM72A	204486179	0
CHB	chr1	FAM72A	204536179	0
CHB	chr1	FAM72A	204586179	0
CHB	chr1	FAM72A	204636179	1
CHB	chr1	FAM72A	204686179	19
CHB	chr1	FAM72A	204736179	35
CHB	chr1	FAM72A	204786179	33
CHB	chr1	FAM72A	204836179	80
CHB	chr1	FAM72A	204886179	70
CHB	chr1	FAM72A	204936179	51
CHB	chr1	FAM72A	204986179	87
CHB	chr1	FAM72A	205036179	61
CHB	chr1	FAM72A	205086179	32
CHB	chr1	FAM72A	205136179	26
CHB	chr1	FAM72A	205186179	29
CHB	chr1	FAM72A	205236179	39
CHB	chr1	FAM72A	205286179	44
CHB	chr1	FAM72A	205336179	45
CHB	chr1	FAM72A	205386179	34
CHB	chr1	FAM72A	205436179	10
CHB	chr1	FAM72A	205486179	0

CHB	chr1	FAM72A	205536179	0
CHB	chr1	FAM72A	205586179	0
CHB	chr1	FAM72A	205636179	0
CHB	chr1	FAM72A	205686179	0
CHB	chr1	FAM72A	205736179	0
CHB	chr1	FAM72A	205786179	0
CHB	chr1	FAM72A	205836179	0
CHB	chr1	FAM72A	205886179	0
CHB	chr1	FAM72A	205936179	0
CHB	chr1	FAM72A	205986179	0
CHB	chr1	FAM72A	206036179	0
CHB	chr1	FAM72A	206086179	14
CHB	chr1	FAM72A	206136179	26
CHB	chr1	FAM72A	206186179	39
CHB	chr1	FAM72A	206236179	27
CHB	chr1	FAM72A	206286179	0
CHB	chr1	FAM72A	206336179	0
CHB	chr1	FAM72A	206386179	0
CHB	chr1	FAM72A	206436179	0
CHB	chr1	FAM72A	206486179	0
CHB	chr1	FAM72A	206536179	0
CHB	chr1	FAM72A	206586179	0
CHB	chr1	FAM72A	206636179	0
CHB	chr1	FAM72A	206686179	0
CHB	chr1	FAM72A	206736179	0
CHB	chr1	FAM72A	206786179	0
CHB	chr1	FAM72A	206836179	0
CHB	chr1	FAM72A	206886179	0
CHB	chr1	FAM72A	206936179	0
CHB	chr1	FAM72A	206986179	0
CHB	chr1	FAM72A	207036179	0
CHB	chr1	FAM72A	207086179	0
CHB	chr1	FAM72A	207136179	0
CHB	chr1	FAM72A	207186179	0
CHB	chr1	FAM72A	207236179	0
CHB	chr1	FAM72A	207286179	0
CHB	chr1	FAM72A	207336179	0
CHB	chr1	FAM72A	207386179	0
CHB	chr1	FAM72A	207436179	0
CHB	chr1	FAM72A	207486179	0
CHB	chr1	FAM72A	207536179	0
CHB	chr1	FAM72A	207586179	0
CHB	chr1	FAM72A	207636179	0
CHB	chr1	FAM72A	207686179	0
CHB	chr1	FAM72A	207736179	0
CHB	chr1	FAM72A	207786179	0
CHB	chr1	FAM72A	207836179	0
CHB	chr1	FAM72A	207886179	0
CHB	chr1	FAM72A	207936179	0

CHB	chr1	FAM72A	207986179	18
CHB	chr1	FAM72A	208036179	34
CHB	chr1	FAM72A	208086179	30
CHB	chr1	FAM72A	208136179	33
CHB	chr1	FAM72A	208186179	52
CHB	chr1	FAM72A	208236179	47
CHB	chr1	FAM72A	208286179	26
CHB	chr1	FAM72A	208336179	31
CHB	chr1	FAM72A	208386179	39
CHB	chr1	FAM72A	208436179	31
CHB	chr1	FAM72A	208486179	18
CHB	chr1	FAM72A	208536179	23
CHB	chr1	FAM72A	208586179	36
CHB	chr1	FAM72A	208636179	32
CHB	chr1	FAM72A	208686179	25
CHB	chr1	FAM72A	208736179	68
CHB	chr1	FAM72A	208786179	101
CHB	chr1	FAM72A	208836179	131
CHB	chr1	FAM72A	208886179	137
CHB	chr1	FAM72A	208936179	74
CHB	chr1	FAM72A	208986179	33
CHB	chr1	FAM72A	209036179	16
CHB	chr1	FAM72A	209086179	17
CHB	chr1	FAM72A	209136179	26
CHB	chr1	FAM72A	209186179	30
CHB	chr1	FAM72A	209236179	42
CHB	chr1	FAM72A	209286179	41
CHB	chr1	FAM72A	209336179	35
CHB	chr1	FAM72A	209386179	41
CHB	chr1	FAM72A	209436179	49
CHB	chr1	FAM72A	209486179	80
CHB	chr1	FAM72A	209536179	74
CHB	chr1	FAM72A	209586179	63
CHB	chr1	FAM72A	209636179	56
CHB	chr1	FAM72A	209686179	37
CHB	chr1	FAM72A	209736179	40
CHB	chr1	FAM72A	209786179	49
CHB	chr1	FAM72A	209836179	42
CHB	chr1	FAM72A	209886179	25
CHB	chr1	FAM72A	209936179	37
CHB	chr1	FAM72A	209986179	39
CHB	chr1	FAM72A	210036179	72
CHB	chr1	FAM72A	210086179	71
CHB	chr1	FAM72A	210136179	30
CHB	chr1	FAM72A	210186179	25
CHB	chr1	FAM72A	210236179	29
CHB	chr1	FAM72A	210286179	26
CHB	chr1	FAM72A	210336179	26
CHB	chr1	FAM72A	210386179	31

CHB	chr1	FAM72A	210436179	24
CHB	chr1	FAM72A	210486179	30
CHB	chr1	FAM72A	210536179	39
CHB	chr1	FAM72A	210586179	34
CHB	chr1	FAM72A	210636179	20
CHB	chr1	FAM72A	210686179	7
CHB	chr1	FAM72A	210736179	0
CHB	chr1	FAM72A	210786179	0
CHB	chr1	FAM72A	210836179	0
CHB	chr1	FAM72A	210886179	0
CHB	chr1	FAM72A	210936179	0
CHB	chr1	FAM72A	210986179	0
CHB	chr1	FAM72A	211036179	0
CHB	chr1	FAM72A	211086179	0
CHB	chr1	FAM72A	211136179	0
CHB	chr9	FAM75A1	34405667	37
CHB	chr9	FAM75A1	34455667	45
CHB	chr9	FAM75A1	34505667	30
CHB	chr9	FAM75A1	34555667	39
CHB	chr9	FAM75A1	34605667	40
CHB	chr9	FAM75A1	34655667	36
CHB	chr9	FAM75A1	34705667	41
CHB	chr9	FAM75A1	34755667	24
CHB	chr9	FAM75A1	34805667	6
CHB	chr9	FAM75A1	34855667	16
CHB	chr9	FAM75A1	34905667	52
CHB	chr9	FAM75A1	34955667	49
CHB	chr9	FAM75A1	35005667	33
CHB	chr9	FAM75A1	35055667	44
CHB	chr9	FAM75A1	35105667	28
CHB	chr9	FAM75A1	35155667	4
CHB	chr9	FAM75A1	35205667	0
CHB	chr9	FAM75A1	35255667	0
CHB	chr9	FAM75A1	35305667	0
CHB	chr9	FAM75A1	35355667	0
CHB	chr9	FAM75A1	35405667	0
CHB	chr9	FAM75A1	35455667	0
CHB	chr9	FAM75A1	35505667	0
CHB	chr9	FAM75A1	35555667	0
CHB	chr9	FAM75A1	35605667	0
CHB	chr9	FAM75A1	35655667	0
CHB	chr9	FAM75A1	35705667	0
CHB	chr9	FAM75A1	35755667	0
CHB	chr9	FAM75A1	35805667	0
CHB	chr9	FAM75A1	35855667	0
CHB	chr9	FAM75A1	35905667	0
CHB	chr9	FAM75A1	35955667	0
CHB	chr9	FAM75A1	36005667	0
CHB	chr9	FAM75A1	36055667	0

CHB	chr9	FAM75A1	36105667	0
CHB	chr9	FAM75A1	36155667	0
CHB	chr9	FAM75A1	36205667	0
CHB	chr9	FAM75A1	36255667	0
CHB	chr9	FAM75A1	36305667	0
CHB	chr9	FAM75A1	36355667	0
CHB	chr9	FAM75A1	36405667	0
CHB	chr9	FAM75A1	36455667	0
CHB	chr9	FAM75A1	36505667	0
CHB	chr9	FAM75A1	36555667	0
CHB	chr9	FAM75A1	36605667	0
CHB	chr9	FAM75A1	36655667	0
CHB	chr9	FAM75A1	36705667	0
CHB	chr9	FAM75A1	36755667	0
CHB	chr9	FAM75A1	36805667	0
CHB	chr9	FAM75A1	36855667	0
CHB	chr9	FAM75A1	36905667	0
CHB	chr9	FAM75A1	36955667	0
CHB	chr9	FAM75A1	37005667	0
CHB	chr9	FAM75A1	37055667	0
CHB	chr9	FAM75A1	37105667	0
CHB	chr9	FAM75A1	37155667	0
CHB	chr9	FAM75A1	37205667	0
CHB	chr9	FAM75A1	37255667	0
CHB	chr9	FAM75A1	37305667	0
CHB	chr9	FAM75A1	37355667	0
CHB	chr9	FAM75A1	37405667	0
CHB	chr9	FAM75A1	37455667	0
CHB	chr9	FAM75A1	37505667	0
CHB	chr9	FAM75A1	37555667	0
CHB	chr9	FAM75A1	37605667	0
CHB	chr9	FAM75A1	37655667	0
CHB	chr9	FAM75A1	37705667	0
CHB	chr9	FAM75A1	37755667	0
CHB	chr9	FAM75A1	37805667	0
CHB	chr9	FAM75A1	37855667	0
CHB	chr9	FAM75A1	37905667	0
CHB	chr9	FAM75A1	37955667	0
CHB	chr9	FAM75A1	38005667	0
CHB	chr9	FAM75A1	38055667	0
CHB	chr9	FAM75A1	38105667	0
CHB	chr9	FAM75A1	38155667	0
CHB	chr9	FAM75A1	38205667	0
CHB	chr9	FAM75A1	38255667	0
CHB	chr9	FAM75A1	38305667	0
CHB	chr9	FAM75A1	38355667	0
CHB	chr9	FAM75A1	38405667	0
CHB	chr9	FAM75A1	38455667	0
CHB	chr9	FAM75A1	38505667	0

CHB	chr9	FAM75A1	38555667	0
CHB	chr9	FAM75A1	38605667	0
CHB	chr9	FAM75A1	38655667	0
CHB	chr9	FAM75A1	38705667	0
CHB	chr9	FAM75A1	38755667	0
CHB	chr9	FAM75A1	38805667	0
CHB	chr9	FAM75A1	38855667	0
CHB	chr9	FAM75A1	38905667	0
CHB	chr9	FAM75A1	38955667	0
CHB	chr9	FAM75A1	39005667	0
CHB	chr9	FAM75A1	39055667	0
CHB	chr9	FAM75A1	39105667	20
CHB	chr9	FAM75A1	39155667	26
CHB	chr9	FAM75A1	39205667	6
CHB	chr9	FAM75A1	39255667	0
CHB	chr9	FAM75A1	39305667	0
CHB	chr9	FAM75A1	39355667	0
CHB	chr9	FAM75A1	39405667	0
CHB	chr9	FAM75A1	39455667	0
CHB	chr9	FAM75A1	39505667	0
CHB	chr9	FAM75A1	39555667	0
CHB	chr9	FAM75A1	39605667	0
CHB	chr9	FAM75A1	39655667	0
CHB	chr9	FAM75A1	39705667	0
CHB	chr9	FAM75A1	39755667	0
CHB	chr9	FAM75A1	39805667	0
CHB	chr9	FAM75A1	39855667	0
CHB	chr9	FAM75A1	39905667	0
CHB	chr9	FAM75A1	39955667	0
CHB	chr9	FAM75A1	40005667	0
CHB	chr9	FAM75A1	40055667	0
CHB	chr9	FAM75A1	40105667	0
CHB	chr9	FAM75A1	40155667	0
CHB	chr9	FAM75A1	40205667	0
CHB	chr9	FAM75A1	40255667	0
CHB	chr9	FAM75A1	40305667	0
CHB	chr9	FAM75A1	40355667	0
CHB	chr9	FAM75A1	40405667	0
CHB	chr9	FAM75A1	40455667	0
CHB	chr9	FAM75A1	40505667	0
CHB	chr9	FAM75A1	40555667	6
CHB	chr9	FAM75A1	40605667	27
CHB	chr9	FAM75A1	40655667	21
CHB	chr9	FAM75A1	40705667	0
CHB	chr9	FAM75A1	40755667	0
CHB	chr9	FAM75A1	40805667	0
CHB	chr9	FAM75A1	40855667	0
CHB	chr9	FAM75A1	40905667	0
CHB	chr9	FAM75A1	40955667	0

CHB	chr9	FAM75A1	41005667	0
CHB	chr9	FAM75A1	41055667	0
CHB	chr9	FAM75A1	41105667	0
CHB	chr9	FAM75A1	41155667	0
CHB	chr9	FAM75A1	41205667	0
CHB	chr9	FAM75A1	41255667	0
CHB	chr9	FAM75A1	41305667	0
CHB	chr9	FAM75A1	41355667	0
CHB	chr9	FAM75A1	41405667	0
CHB	chr9	FAM75A1	41455667	0
CHB	chr9	FAM75A1	41505667	0
CHB	chr9	FAM75A1	41555667	0
CHB	chr9	FAM75A1	41605667	0
CHB	chr9	FAM75A1	41655667	0
CHB	chr9	FAM75A1	41705667	0
CHB	chr9	FAM75A1	41755667	0
CHB	chr9	FAM75A1	41805667	0
CHB	chr9	FAM75A1	41855667	0
CHB	chr9	FAM75A1	41905667	0
CHB	chr9	FAM75A1	41955667	0
CHB	chr9	FAM75A1	42005667	0
CHB	chr9	FAM75A1	42055667	0
CHB	chr9	FAM75A1	42105667	0
CHB	chr9	FAM75A1	42155667	0
CHB	chr9	FAM75A1	42205667	0
CHB	chr9	FAM75A1	42255667	0
CHB	chr9	FAM75A1	42305667	0
CHB	chr9	FAM75A1	42355667	0
CHB	chr9	FAM75A1	42405667	0
CHB	chr9	FAM75A1	42455667	0
CHB	chr9	FAM75A1	42505667	0
CHB	chr9	FAM75A1	42555667	0
CHB	chr9	FAM75A1	42605667	0
CHB	chr9	FAM75A1	42655667	0
CHB	chr9	FAM75A1	42705667	0
CHB	chr9	FAM75A1	42755667	0
CHB	chr9	FAM75A1	42805667	0
CHB	chr9	FAM75A1	42855667	0
CHB	chr9	FAM75A1	42905667	0
CHB	chr9	FAM75A1	42955667	0
CHB	chr9	FAM75A1	43005667	0
CHB	chr9	FAM75A1	43055667	0
CHB	chr9	FAM75A1	43105667	0
CHB	chr9	FAM75A1	43155667	0
CHB	chr9	FAM75A1	43205667	0
CHB	chr9	FAM75A1	43255667	0
CHB	chr9	FAM75A1	43305667	0
CHB	chr9	FAM75A1	43355667	0
CHB	chr9	FAM75A1	43405667	0

CHB	chr9	FAM75A1	43455667	0
CHB	chr9	FAM75A1	43505667	0
CHB	chr9	FAM75A1	43555667	0
CHB	chr9	FAM75A1	43605667	0
CHB	chr9	FAM75A1	43655667	0
CHB	chr9	FAM75A1	43705667	0
CHB	chr9	FAM75A1	43755667	0
CHB	chr9	FAM75A1	43805667	0
CHB	chr9	FAM75A1	43855667	0
CHB	chr9	FAM75A1	43905667	0
CHB	chr9	FAM75A1	43955667	0
CHB	chr9	FAM75A1	44005667	0
CHB	chr9	FAM75A1	44055667	0
CHB	chr9	FAM75A1	44105667	0
CHB	chr9	FAM75A1	44155667	0
CHB	chr9	FAM75A1	44205667	0
CHB	chr9	FAM75A1	44255667	0
CHB	chr9	FAM75A1	44305667	0
CHB	chr9	FAM75A5	55964372	0
CHB	chr9	FAM75A5	56014372	0
CHB	chr9	FAM75A5	56064372	0
CHB	chr9	FAM75A5	56114372	0
CHB	chr9	FAM75A5	56164372	0
CHB	chr9	FAM75A5	56214372	0
CHB	chr9	FAM75A5	56264372	0
CHB	chr9	FAM75A5	56314372	0
CHB	chr9	FAM75A5	56364372	0
CHB	chr9	FAM75A5	56414372	0
CHB	chr9	FAM75A5	56464372	0
CHB	chr9	FAM75A5	56514372	0
CHB	chr9	FAM75A5	56564372	0
CHB	chr9	FAM75A5	56614372	0
CHB	chr9	FAM75A5	56664372	0
CHB	chr9	FAM75A5	56714372	0
CHB	chr9	FAM75A5	56764372	0
CHB	chr9	FAM75A5	56814372	0
CHB	chr9	FAM75A5	56864372	0
CHB	chr9	FAM75A5	56914372	0
CHB	chr9	FAM75A5	56964372	0
CHB	chr9	FAM75A5	57014372	0
CHB	chr9	FAM75A5	57064372	0
CHB	chr9	FAM75A5	57114372	0
CHB	chr9	FAM75A5	57164372	0
CHB	chr9	FAM75A5	57214372	0
CHB	chr9	FAM75A5	57264372	0
CHB	chr9	FAM75A5	57314372	0
CHB	chr9	FAM75A5	57364372	0
CHB	chr9	FAM75A5	57414372	0
CHB	chr9	FAM75A5	57464372	0

CHB	chr9	FAM75A5	57514372	0
CHB	chr9	FAM75A5	57564372	0
CHB	chr9	FAM75A5	57614372	0
CHB	chr9	FAM75A5	57664372	0
CHB	chr9	FAM75A5	57714372	0
CHB	chr9	FAM75A5	57764372	0
CHB	chr9	FAM75A5	57814372	0
CHB	chr9	FAM75A5	57864372	0
CHB	chr9	FAM75A5	57914372	0
CHB	chr9	FAM75A5	57964372	0
CHB	chr9	FAM75A5	58014372	0
CHB	chr9	FAM75A5	58064372	0
CHB	chr9	FAM75A5	58114372	0
CHB	chr9	FAM75A5	58164372	0
CHB	chr9	FAM75A5	58214372	0
CHB	chr9	FAM75A5	58264372	0
CHB	chr9	FAM75A5	58314372	0
CHB	chr9	FAM75A5	58364372	0
CHB	chr9	FAM75A5	58414372	0
CHB	chr9	FAM75A5	58464372	0
CHB	chr9	FAM75A5	58514372	0
CHB	chr9	FAM75A5	58564372	0
CHB	chr9	FAM75A5	58614372	0
CHB	chr9	FAM75A5	58664372	0
CHB	chr9	FAM75A5	58714372	0
CHB	chr9	FAM75A5	58764372	0
CHB	chr9	FAM75A5	58814372	0
CHB	chr9	FAM75A5	58864372	0
CHB	chr9	FAM75A5	58914372	0
CHB	chr9	FAM75A5	58964372	0
CHB	chr9	FAM75A5	59014372	0
CHB	chr9	FAM75A5	59064372	0
CHB	chr9	FAM75A5	59114372	0
CHB	chr9	FAM75A5	59164372	0
CHB	chr9	FAM75A5	59214372	0
CHB	chr9	FAM75A5	59264372	0
CHB	chr9	FAM75A5	59314372	0
CHB	chr9	FAM75A5	59364372	0
CHB	chr9	FAM75A5	59414372	0
CHB	chr9	FAM75A5	59464372	0
CHB	chr9	FAM75A5	59514372	0
CHB	chr9	FAM75A5	59564372	0
CHB	chr9	FAM75A5	59614372	0
CHB	chr9	FAM75A5	59664372	0
CHB	chr9	FAM75A5	59714372	0
CHB	chr9	FAM75A5	59764372	0
CHB	chr9	FAM75A5	59814372	0
CHB	chr9	FAM75A5	59864372	0
CHB	chr9	FAM75A5	59914372	0

CHB	chr9	FAM75A5	59964372	0
CHB	chr9	FAM75A5	60014372	0
CHB	chr9	FAM75A5	60064372	0
CHB	chr9	FAM75A5	60114372	0
CHB	chr9	FAM75A5	60164372	0
CHB	chr9	FAM75A5	60214372	0
CHB	chr9	FAM75A5	60264372	0
CHB	chr9	FAM75A5	60314372	0
CHB	chr9	FAM75A5	60364372	0
CHB	chr9	FAM75A5	60414372	0
CHB	chr9	FAM75A5	60464372	0
CHB	chr9	FAM75A5	60514372	0
CHB	chr9	FAM75A5	60564372	0
CHB	chr9	FAM75A5	60614372	0
CHB	chr9	FAM75A5	60664372	0
CHB	chr9	FAM75A5	60714372	0
CHB	chr9	FAM75A5	60764372	0
CHB	chr9	FAM75A5	60814372	0
CHB	chr9	FAM75A5	60864372	0
CHB	chr9	FAM75A5	60914372	0
CHB	chr9	FAM75A5	60964372	0
CHB	chr9	FAM75A5	61014372	0
CHB	chr9	FAM75A5	61064372	0
CHB	chr9	FAM75A5	61114372	0
CHB	chr9	FAM75A5	61164372	0
CHB	chr9	FAM75A5	61214372	0
CHB	chr9	FAM75A5	61264372	0
CHB	chr9	FAM75A5	61314372	0
CHB	chr9	FAM75A5	61364372	0
CHB	chr9	FAM75A5	61414372	0
CHB	chr9	FAM75A5	61464372	0
CHB	chr9	FAM75A5	61514372	0
CHB	chr9	FAM75A5	61564372	0
CHB	chr9	FAM75A5	61614372	0
CHB	chr9	FAM75A5	61664372	0
CHB	chr9	FAM75A5	61714372	0
CHB	chr9	FAM75A5	61764372	0
CHB	chr9	FAM75A5	61814372	0
CHB	chr9	FAM75A5	61864372	0
CHB	chr9	FAM75A5	61914372	0
CHB	chr9	FAM75A5	61964372	0
CHB	chr9	FAM75A5	62014372	0
CHB	chr9	FAM75A5	62064372	0
CHB	chr9	FAM75A5	62114372	0
CHB	chr9	FAM75A5	62164372	0
CHB	chr9	FAM75A5	62214372	0
CHB	chr9	FAM75A5	62264372	0
CHB	chr9	FAM75A5	62314372	0
CHB	chr9	FAM75A5	62364372	0

CHB	chr9	FAM75A5	62414372	0
CHB	chr9	FAM75A5	62464372	0
CHB	chr9	FAM75A5	62514372	0
CHB	chr9	FAM75A5	62564372	0
CHB	chr9	FAM75A5	62614372	0
CHB	chr9	FAM75A5	62664372	0
CHB	chr9	FAM75A5	62714372	0
CHB	chr9	FAM75A5	62764372	0
CHB	chr9	FAM75A5	62814372	0
CHB	chr9	FAM75A5	62864372	0
CHB	chr9	FAM75A5	62914372	0
CHB	chr9	FAM75A5	62964372	0
CHB	chr9	FAM75A5	63014372	0
CHB	chr9	FAM75A5	63064372	0
CHB	chr9	FAM75A5	63114372	0
CHB	chr9	FAM75A5	63164372	0
CHB	chr9	FAM75A5	63214372	0
CHB	chr9	FAM75A5	63264372	0
CHB	chr9	FAM75A5	63314372	0
CHB	chr9	FAM75A5	63364372	0
CHB	chr9	FAM75A5	63414372	0
CHB	chr9	FAM75A5	63464372	0
CHB	chr9	FAM75A5	63514372	0
CHB	chr9	FAM75A5	63564372	0
CHB	chr9	FAM75A5	63614372	0
CHB	chr9	FAM75A5	63664372	0
CHB	chr9	FAM75A5	63714372	0
CHB	chr9	FAM75A5	63764372	0
CHB	chr9	FAM75A5	63814372	0
CHB	chr9	FAM75A5	63864372	0
CHB	chr9	FAM75A5	63914372	0
CHB	chr9	FAM75A5	63964372	0
CHB	chr9	FAM75A5	64014372	0
CHB	chr9	FAM75A5	64064372	0
CHB	chr9	FAM75A5	64114372	0
CHB	chr9	FAM75A5	64164372	0
CHB	chr9	FAM75A5	64214372	0
CHB	chr9	FAM75A5	64264372	0
CHB	chr9	FAM75A5	64314372	0
CHB	chr9	FAM75A5	64364372	0
CHB	chr9	FAM75A5	64414372	0
CHB	chr9	FAM75A5	64464372	0
CHB	chr9	FAM75A5	64514372	0
CHB	chr9	FAM75A5	64564372	0
CHB	chr9	FAM75A5	64614372	0
CHB	chr9	FAM75A5	64664372	0
CHB	chr9	FAM75A5	64714372	0
CHB	chr9	FAM75A5	64764372	0
CHB	chr9	FAM75A5	64814372	0

CHB	chr9	FAM75A5	64864372	0
CHB	chr9	FAM75A5	64914372	0
CHB	chr9	FAM75A5	64964372	0
CHB	chr9	FAM75A5	65014372	0
CHB	chr9	FAM75A5	65064372	0
CHB	chr9	FAM75A5	65114372	0
CHB	chr9	FAM75A5	65164372	0
CHB	chr9	FAM75A5	65214372	0
CHB	chr9	FAM75A5	65264372	0
CHB	chr9	FAM75A5	65314372	0
CHB	chr9	FAM75A5	65364372	0
CHB	chr9	FAM75A5	65414372	0
CHB	chr9	FAM75A5	65464372	0
CHB	chr9	FAM75A5	65514372	0
CHB	chr9	FAM75A5	65564372	12
CHB	chr9	FAM75A5	65614372	26
CHB	chr9	FAM75A5	65664372	14
CHB	chr9	FAM75A5	65714372	0
CHB	chr9	FAM75A5	65764372	0
CHB	chr9	FAM75A5	65814372	0
CHB	chr9	FAM75A5	65864372	0
CHB	chr9	FOXD4L2	60787146	0
CHB	chr9	FOXD4L2	60837146	0
CHB	chr9	FOXD4L2	60887146	0
CHB	chr9	FOXD4L2	60937146	0
CHB	chr9	FOXD4L2	60987146	0
CHB	chr9	FOXD4L2	61037146	0
CHB	chr9	FOXD4L2	61087146	0
CHB	chr9	FOXD4L2	61137146	0
CHB	chr9	FOXD4L2	61187146	0
CHB	chr9	FOXD4L2	61237146	0
CHB	chr9	FOXD4L2	61287146	0
CHB	chr9	FOXD4L2	61337146	0
CHB	chr9	FOXD4L2	61387146	0
CHB	chr9	FOXD4L2	61437146	0
CHB	chr9	FOXD4L2	61487146	0
CHB	chr9	FOXD4L2	61537146	0
CHB	chr9	FOXD4L2	61587146	0
CHB	chr9	FOXD4L2	61637146	0
CHB	chr9	FOXD4L2	61687146	0
CHB	chr9	FOXD4L2	61737146	0
CHB	chr9	FOXD4L2	61787146	0
CHB	chr9	FOXD4L2	61837146	0
CHB	chr9	FOXD4L2	61887146	0
CHB	chr9	FOXD4L2	61937146	0
CHB	chr9	FOXD4L2	61987146	0
CHB	chr9	FOXD4L2	62037146	0
CHB	chr9	FOXD4L2	62087146	0
CHB	chr9	FOXD4L2	62137146	0

CHB	chr9	FOXD4L2	62187146	0
CHB	chr9	FOXD4L2	62237146	0
CHB	chr9	FOXD4L2	62287146	0
CHB	chr9	FOXD4L2	62337146	0
CHB	chr9	FOXD4L2	62387146	0
CHB	chr9	FOXD4L2	62437146	0
CHB	chr9	FOXD4L2	62487146	0
CHB	chr9	FOXD4L2	62537146	0
CHB	chr9	FOXD4L2	62587146	0
CHB	chr9	FOXD4L2	62637146	0
CHB	chr9	FOXD4L2	62687146	0
CHB	chr9	FOXD4L2	62737146	0
CHB	chr9	FOXD4L2	62787146	0
CHB	chr9	FOXD4L2	62837146	0
CHB	chr9	FOXD4L2	62887146	0
CHB	chr9	FOXD4L2	62937146	0
CHB	chr9	FOXD4L2	62987146	0
CHB	chr9	FOXD4L2	63037146	0
CHB	chr9	FOXD4L2	63087146	0
CHB	chr9	FOXD4L2	63137146	0
CHB	chr9	FOXD4L2	63187146	0
CHB	chr9	FOXD4L2	63237146	0
CHB	chr9	FOXD4L2	63287146	0
CHB	chr9	FOXD4L2	63337146	0
CHB	chr9	FOXD4L2	63387146	0
CHB	chr9	FOXD4L2	63437146	0
CHB	chr9	FOXD4L2	63487146	0
CHB	chr9	FOXD4L2	63537146	0
CHB	chr9	FOXD4L2	63587146	0
CHB	chr9	FOXD4L2	63637146	0
CHB	chr9	FOXD4L2	63687146	0
CHB	chr9	FOXD4L2	63737146	0
CHB	chr9	FOXD4L2	63787146	0
CHB	chr9	FOXD4L2	63837146	0
CHB	chr9	FOXD4L2	63887146	0
CHB	chr9	FOXD4L2	63937146	0
CHB	chr9	FOXD4L2	63987146	0
CHB	chr9	FOXD4L2	64037146	0
CHB	chr9	FOXD4L2	64087146	0
CHB	chr9	FOXD4L2	64137146	0
CHB	chr9	FOXD4L2	64187146	0
CHB	chr9	FOXD4L2	64237146	0
CHB	chr9	FOXD4L2	64287146	0
CHB	chr9	FOXD4L2	64337146	0
CHB	chr9	FOXD4L2	64387146	0
CHB	chr9	FOXD4L2	64437146	0
CHB	chr9	FOXD4L2	64487146	0
CHB	chr9	FOXD4L2	64537146	0
CHB	chr9	FOXD4L2	64587146	0

CHB	chr9	FOXD4L2	64637146	0
CHB	chr9	FOXD4L2	64687146	0
CHB	chr9	FOXD4L2	64737146	0
CHB	chr9	FOXD4L2	64787146	0
CHB	chr9	FOXD4L2	64837146	0
CHB	chr9	FOXD4L2	64887146	0
CHB	chr9	FOXD4L2	64937146	0
CHB	chr9	FOXD4L2	64987146	0
CHB	chr9	FOXD4L2	65037146	0
CHB	chr9	FOXD4L2	65087146	0
CHB	chr9	FOXD4L2	65137146	0
CHB	chr9	FOXD4L2	65187146	0
CHB	chr9	FOXD4L2	65237146	0
CHB	chr9	FOXD4L2	65287146	0
CHB	chr9	FOXD4L2	65337146	0
CHB	chr9	FOXD4L2	65387146	0
CHB	chr9	FOXD4L2	65437146	0
CHB	chr9	FOXD4L2	65487146	0
CHB	chr9	FOXD4L2	65537146	0
CHB	chr9	FOXD4L2	65587146	25
CHB	chr9	FOXD4L2	65637146	26
CHB	chr9	FOXD4L2	65687146	1
CHB	chr9	FOXD4L2	65737146	0
CHB	chr9	FOXD4L2	65787146	0
CHB	chr9	FOXD4L2	65837146	0
CHB	chr9	FOXD4L2	65887146	0
CHB	chr9	FOXD4L2	65937146	0
CHB	chr9	FOXD4L2	65987146	0
CHB	chr9	FOXD4L2	66037146	0
CHB	chr9	FOXD4L2	66087146	0
CHB	chr9	FOXD4L2	66137146	0
CHB	chr9	FOXD4L2	66187146	0
CHB	chr9	FOXD4L2	66237146	0
CHB	chr9	FOXD4L2	66287146	0
CHB	chr9	FOXD4L2	66337146	0
CHB	chr9	FOXD4L2	66387146	0
CHB	chr9	FOXD4L2	66437146	0
CHB	chr9	FOXD4L2	66487146	0
CHB	chr9	FOXD4L2	66537146	0
CHB	chr9	FOXD4L2	66587146	0
CHB	chr9	FOXD4L2	66637146	12
CHB	chr9	FOXD4L2	66687146	21
CHB	chr9	FOXD4L2	66737146	17
CHB	chr9	FOXD4L2	66787146	21
CHB	chr9	FOXD4L2	66837146	13
CHB	chr9	FOXD4L2	66887146	0
CHB	chr9	FOXD4L2	66937146	0
CHB	chr9	FOXD4L2	66987146	0
CHB	chr9	FOXD4L2	67037146	0

CHB	chr9	FOXD4L2	67087146	0
CHB	chr9	FOXD4L2	67137146	0
CHB	chr9	FOXD4L2	67187146	0
CHB	chr9	FOXD4L2	67237146	0
CHB	chr9	FOXD4L2	67287146	0
CHB	chr9	FOXD4L2	67337146	0
CHB	chr9	FOXD4L2	67387146	0
CHB	chr9	FOXD4L2	67437146	0
CHB	chr9	FOXD4L2	67487146	0
CHB	chr9	FOXD4L2	67537146	0
CHB	chr9	FOXD4L2	67587146	0
CHB	chr9	FOXD4L2	67637146	0
CHB	chr9	FOXD4L2	67687146	0
CHB	chr9	FOXD4L2	67737146	0
CHB	chr9	FOXD4L2	67787146	0
CHB	chr9	FOXD4L2	67837146	0
CHB	chr9	FOXD4L2	67887146	0
CHB	chr9	FOXD4L2	67937146	0
CHB	chr9	FOXD4L2	67987146	0
CHB	chr9	FOXD4L2	68037146	0
CHB	chr9	FOXD4L2	68087146	0
CHB	chr9	FOXD4L2	68137146	0
CHB	chr9	FOXD4L2	68187146	0
CHB	chr9	FOXD4L2	68237146	0
CHB	chr9	FOXD4L2	68287146	0
CHB	chr9	FOXD4L2	68337146	0
CHB	chr9	FOXD4L2	68387146	0
CHB	chr9	FOXD4L2	68437146	0
CHB	chr9	FOXD4L2	68487146	0
CHB	chr9	FOXD4L2	68537146	0
CHB	chr9	FOXD4L2	68587146	0
CHB	chr9	FOXD4L2	68637146	0
CHB	chr9	FOXD4L2	68687146	0
CHB	chr9	FOXD4L2	68737146	0
CHB	chr9	FOXD4L2	68787146	0
CHB	chr9	FOXD4L2	68837146	0
CHB	chr9	FOXD4L2	68887146	0
CHB	chr9	FOXD4L2	68937146	0
CHB	chr9	FOXD4L2	68987146	0
CHB	chr9	FOXD4L2	69037146	0
CHB	chr9	FOXD4L2	69087146	0
CHB	chr9	FOXD4L2	69137146	0
CHB	chr9	FOXD4L2	69187146	0
CHB	chr9	FOXD4L2	69237146	0
CHB	chr9	FOXD4L2	69287146	0
CHB	chr9	FOXD4L2	69337146	0
CHB	chr9	FOXD4L2	69387146	0
CHB	chr9	FOXD4L2	69437146	0
CHB	chr9	FOXD4L2	69487146	0

CHB	chr9	FOXD4L2	69537146	0
CHB	chr9	FOXD4L2	69587146	0
CHB	chr9	FOXD4L2	69637146	0
CHB	chr9	FOXD4L2	69687146	0
CHB	chr9	FOXD4L2	69737146	0
CHB	chr9	FOXD4L2	69787146	6
CHB	chr9	FOXD4L2	69837146	35
CHB	chr9	FOXD4L2	69887146	61
CHB	chr9	FOXD4L2	69937146	90
CHB	chr9	FOXD4L2	69987146	70
CHB	chr9	FOXD4L2	70037146	12
CHB	chr9	FOXD4L2	70087146	0
CHB	chr9	FOXD4L2	70137146	0
CHB	chr9	FOXD4L2	70187146	0
CHB	chr9	FOXD4L2	70237146	0
CHB	chr9	FOXD4L2	70287146	0
CHB	chr9	FOXD4L2	70337146	0
CHB	chr9	FOXD4L2	70387146	0
CHB	chr9	FOXD4L2	70437146	0
CHB	chr9	FOXD4L2	70487146	0
CHB	chr9	FOXD4L2	70537146	0
CHB	chr9	FOXD4L2	70587146	0
CHB	chr9	FOXD4L2	70637146	0
CHB	chr9	FOXD4L2	70687146	0
CHB	chr15	GOLGA6L9	77479363	0
CHB	chr15	GOLGA6L9	77529363	0
CHB	chr15	GOLGA6L9	77579363	0
CHB	chr15	GOLGA6L9	77629363	0
CHB	chr15	GOLGA6L9	77679363	0
CHB	chr15	GOLGA6L9	77729363	0
CHB	chr15	GOLGA6L9	77779363	0
CHB	chr15	GOLGA6L9	77829363	0
CHB	chr15	GOLGA6L9	77879363	0
CHB	chr15	GOLGA6L9	77929363	0
CHB	chr15	GOLGA6L9	77979363	0
CHB	chr15	GOLGA6L9	78029363	0
CHB	chr15	GOLGA6L9	78079363	0
CHB	chr15	GOLGA6L9	78129363	0
CHB	chr15	GOLGA6L9	78179363	0
CHB	chr15	GOLGA6L9	78229363	0
CHB	chr15	GOLGA6L9	78279363	0
CHB	chr15	GOLGA6L9	78329363	0
CHB	chr15	GOLGA6L9	78379363	0
CHB	chr15	GOLGA6L9	78429363	0
CHB	chr15	GOLGA6L9	78479363	0
CHB	chr15	GOLGA6L9	78529363	0
CHB	chr15	GOLGA6L9	78579363	0
CHB	chr15	GOLGA6L9	78629363	19
CHB	chr15	GOLGA6L9	78679363	40

CHB	chr15	GOLGA6L9	78729363	31
CHB	chr15	GOLGA6L9	78779363	28
CHB	chr15	GOLGA6L9	78829363	28
CHB	chr15	GOLGA6L9	78879363	25
CHB	chr15	GOLGA6L9	78929363	33
CHB	chr15	GOLGA6L9	78979363	30
CHB	chr15	GOLGA6L9	79029363	37
CHB	chr15	GOLGA6L9	79079363	45
CHB	chr15	GOLGA6L9	79129363	34
CHB	chr15	GOLGA6L9	79179363	14
CHB	chr15	GOLGA6L9	79229363	0
CHB	chr15	GOLGA6L9	79279363	0
CHB	chr15	GOLGA6L9	79329363	0
CHB	chr15	GOLGA6L9	79379363	0
CHB	chr15	GOLGA6L9	79429363	0
CHB	chr15	GOLGA6L9	79479363	0
CHB	chr15	GOLGA6L9	79529363	49
CHB	chr15	GOLGA6L9	79579363	101
CHB	chr15	GOLGA6L9	79629363	68
CHB	chr15	GOLGA6L9	79679363	36
CHB	chr15	GOLGA6L9	79729363	73
CHB	chr15	GOLGA6L9	79779363	87
CHB	chr15	GOLGA6L9	79829363	55
CHB	chr15	GOLGA6L9	79879363	44
CHB	chr15	GOLGA6L9	79929363	41
CHB	chr15	GOLGA6L9	79979363	64
CHB	chr15	GOLGA6L9	80029363	59
CHB	chr15	GOLGA6L9	80079363	36
CHB	chr15	GOLGA6L9	80129363	40
CHB	chr15	GOLGA6L9	80179363	38
CHB	chr15	GOLGA6L9	80229363	35
CHB	chr15	GOLGA6L9	80279363	27
CHB	chr15	GOLGA6L9	80329363	13
CHB	chr15	GOLGA6L9	80379363	0
CHB	chr15	GOLGA6L9	80429363	0
CHB	chr15	GOLGA6L9	80479363	0
CHB	chr15	GOLGA6L9	80529363	0
CHB	chr15	GOLGA6L9	80579363	0
CHB	chr15	GOLGA6L9	80629363	0
CHB	chr15	GOLGA6L9	80679363	0
CHB	chr15	GOLGA6L9	80729363	0
CHB	chr15	GOLGA6L9	80779363	0
CHB	chr15	GOLGA6L9	80829363	0
CHB	chr15	GOLGA6L9	80879363	0
CHB	chr15	GOLGA6L9	80929363	0
CHB	chr15	GOLGA6L9	80979363	0
CHB	chr15	GOLGA6L9	81029363	0
CHB	chr15	GOLGA6L9	81079363	0
CHB	chr15	GOLGA6L9	81129363	0

CHB	chr15	GOLGA6L9	81179363	0
CHB	chr15	GOLGA6L9	81229363	0
CHB	chr15	GOLGA6L9	81279363	0
CHB	chr15	GOLGA6L9	81329363	0
CHB	chr15	GOLGA6L9	81379363	0
CHB	chr15	GOLGA6L9	81429363	0
CHB	chr15	GOLGA6L9	81479363	0
CHB	chr15	GOLGA6L9	81529363	0
CHB	chr15	GOLGA6L9	81579363	0
CHB	chr15	GOLGA6L9	81629363	0
CHB	chr15	GOLGA6L9	81679363	0
CHB	chr15	GOLGA6L9	81729363	0
CHB	chr15	GOLGA6L9	81779363	0
CHB	chr15	GOLGA6L9	81829363	0
CHB	chr15	GOLGA6L9	81879363	0
CHB	chr15	GOLGA6L9	81929363	0
CHB	chr15	GOLGA6L9	81979363	0
CHB	chr15	GOLGA6L9	82029363	0
CHB	chr15	GOLGA6L9	82079363	0
CHB	chr15	GOLGA6L9	82129363	0
CHB	chr15	GOLGA6L9	82179363	0
CHB	chr15	GOLGA6L9	82229363	0
CHB	chr15	GOLGA6L9	82279363	0
CHB	chr15	GOLGA6L9	82329363	0
CHB	chr15	GOLGA6L9	82379363	0
CHB	chr15	GOLGA6L9	82429363	0
CHB	chr15	GOLGA6L9	82479363	0
CHB	chr15	GOLGA6L9	82529363	0
CHB	chr15	GOLGA6L9	82579363	0
CHB	chr15	GOLGA6L9	82629363	0
CHB	chr15	GOLGA6L9	82679363	0
CHB	chr15	GOLGA6L9	82729363	0
CHB	chr15	GOLGA6L9	82779363	0
CHB	chr15	GOLGA6L9	82829363	0
CHB	chr15	GOLGA6L9	82879363	0
CHB	chr15	GOLGA6L9	82929363	0
CHB	chr15	GOLGA6L9	82979363	0
CHB	chr15	GOLGA6L9	83029363	0
CHB	chr15	GOLGA6L9	83079363	0
CHB	chr15	GOLGA6L9	83129363	0
CHB	chr15	GOLGA6L9	83179363	0
CHB	chr15	GOLGA6L9	83229363	0
CHB	chr15	GOLGA6L9	83279363	0
CHB	chr15	GOLGA6L9	83329363	0
CHB	chr15	GOLGA6L9	83379363	0
CHB	chr15	GOLGA6L9	83429363	0
CHB	chr15	GOLGA6L9	83479363	0
CHB	chr15	GOLGA6L9	83529363	0
CHB	chr15	GOLGA6L9	83579363	0

CHB	chr15	GOLGA6L9	83629363	0
CHB	chr15	GOLGA6L9	83679363	0
CHB	chr15	GOLGA6L9	83729363	0
CHB	chr15	GOLGA6L9	83779363	0
CHB	chr15	GOLGA6L9	83829363	0
CHB	chr15	GOLGA6L9	83879363	0
CHB	chr15	GOLGA6L9	83929363	0
CHB	chr15	GOLGA6L9	83979363	0
CHB	chr15	GOLGA6L9	84029363	0
CHB	chr15	GOLGA6L9	84079363	0
CHB	chr15	GOLGA6L9	84129363	0
CHB	chr15	GOLGA6L9	84179363	0
CHB	chr15	GOLGA6L9	84229363	7
CHB	chr15	GOLGA6L9	84279363	20
CHB	chr15	GOLGA6L9	84329363	25
CHB	chr15	GOLGA6L9	84379363	31
CHB	chr15	GOLGA6L9	84429363	32
CHB	chr15	GOLGA6L9	84479363	27
CHB	chr15	GOLGA6L9	84529363	37
CHB	chr15	GOLGA6L9	84579363	45
CHB	chr15	GOLGA6L9	84629363	34
CHB	chr15	GOLGA6L9	84679363	25
CHB	chr15	GOLGA6L9	84729363	30
CHB	chr15	GOLGA6L9	84779363	28
CHB	chr15	GOLGA6L9	84829363	11
CHB	chr15	GOLGA6L9	84879363	0
CHB	chr15	GOLGA6L9	84929363	0
CHB	chr15	GOLGA6L9	84979363	0
CHB	chr15	GOLGA6L9	85029363	4
CHB	chr15	GOLGA6L9	85079363	28
CHB	chr15	GOLGA6L9	85129363	43
CHB	chr15	GOLGA6L9	85179363	39
CHB	chr15	GOLGA6L9	85229363	32
CHB	chr15	GOLGA6L9	85279363	33
CHB	chr15	GOLGA6L9	85329363	33
CHB	chr15	GOLGA6L9	85379363	27
CHB	chr15	GOLGA6L9	85429363	49
CHB	chr15	GOLGA6L9	85479363	59
CHB	chr15	GOLGA6L9	85529363	45
CHB	chr15	GOLGA6L9	85579363	44
CHB	chr15	GOLGA6L9	85629363	48
CHB	chr15	GOLGA6L9	85679363	29
CHB	chr15	GOLGA6L9	85729363	5
CHB	chr15	GOLGA6L9	85779363	0
CHB	chr15	GOLGA6L9	85829363	0
CHB	chr15	GOLGA6L9	85879363	0
CHB	chr15	GOLGA6L9	85929363	0
CHB	chr15	GOLGA6L9	85979363	0
CHB	chr15	GOLGA6L9	86029363	0

CHB	chr15	GOLGA6L9	86079363	0
CHB	chr15	GOLGA6L9	86129363	0
CHB	chr15	GOLGA6L9	86179363	0
CHB	chr15	GOLGA6L9	86229363	0
CHB	chr15	GOLGA6L9	86279363	0
CHB	chr15	GOLGA6L9	86329363	0
CHB	chr15	GOLGA6L9	86379363	0
CHB	chr15	GOLGA6L9	86429363	0
CHB	chr15	GOLGA6L9	86479363	0
CHB	chr15	GOLGA6L9	86529363	0
CHB	chr15	GOLGA6L9	86579363	0
CHB	chr15	GOLGA6L9	86629363	16
CHB	chr15	GOLGA6L9	86679363	37
CHB	chr15	GOLGA6L9	86729363	47
CHB	chr15	GOLGA6L9	86779363	41
CHB	chr15	GOLGA6L9	86829363	24
CHB	chr15	GOLGA6L9	86879363	25
CHB	chr15	GOLGA6L9	86929363	26
CHB	chr15	GOLGA6L9	86979363	45
CHB	chr15	GOLGA6L9	87029363	44
CHB	chr15	GOLGA6L9	87079363	22
CHB	chr15	GOLGA6L9	87129363	41
CHB	chr15	GOLGA6L9	87179363	63
CHB	chr15	GOLGA6L9	87229363	135
CHB	chr15	GOLGA6L9	87279363	119
CHB	chr15	GOLGA6L9	87329363	32
CHB	chr15	GOLGA6L9	87379363	24
CHB	chr15	GOLGA8G	23569611	0
CHB	chr15	GOLGA8G	23619611	0
CHB	chr15	GOLGA8G	23669611	0
CHB	chr15	GOLGA8G	23719611	0
CHB	chr15	GOLGA8G	23769611	0
CHB	chr15	GOLGA8G	23819611	0
CHB	chr15	GOLGA8G	23869611	0
CHB	chr15	GOLGA8G	23919611	0
CHB	chr15	GOLGA8G	23969611	0
CHB	chr15	GOLGA8G	24019611	0
CHB	chr15	GOLGA8G	24069611	0
CHB	chr15	GOLGA8G	24119611	0
CHB	chr15	GOLGA8G	24169611	0
CHB	chr15	GOLGA8G	24219611	0
CHB	chr15	GOLGA8G	24269611	0
CHB	chr15	GOLGA8G	24319611	0
CHB	chr15	GOLGA8G	24369611	0
CHB	chr15	GOLGA8G	24419611	0
CHB	chr15	GOLGA8G	24469611	0
CHB	chr15	GOLGA8G	24519611	0
CHB	chr15	GOLGA8G	24569611	0
CHB	chr15	GOLGA8G	24619611	0

CHB	chr15	GOLGA8G	24669611	0
CHB	chr15	GOLGA8G	24719611	0
CHB	chr15	GOLGA8G	24769611	0
CHB	chr15	GOLGA8G	24819611	0
CHB	chr15	GOLGA8G	24869611	0
CHB	chr15	GOLGA8G	24919611	0
CHB	chr15	GOLGA8G	24969611	31
CHB	chr15	GOLGA8G	25019611	58
CHB	chr15	GOLGA8G	25069611	39
CHB	chr15	GOLGA8G	25119611	27
CHB	chr15	GOLGA8G	25169611	15
CHB	chr15	GOLGA8G	25219611	0
CHB	chr15	GOLGA8G	25269611	0
CHB	chr15	GOLGA8G	25319611	0
CHB	chr15	GOLGA8G	25369611	0
CHB	chr15	GOLGA8G	25419611	0
CHB	chr15	GOLGA8G	25469611	0
CHB	chr15	GOLGA8G	25519611	0
CHB	chr15	GOLGA8G	25569611	0
CHB	chr15	GOLGA8G	25619611	0
CHB	chr15	GOLGA8G	25669611	0
CHB	chr15	GOLGA8G	25719611	0
CHB	chr15	GOLGA8G	25769611	0
CHB	chr15	GOLGA8G	25819611	0
CHB	chr15	GOLGA8G	25869611	0
CHB	chr15	GOLGA8G	25919611	0
CHB	chr15	GOLGA8G	25969611	0
CHB	chr15	GOLGA8G	26019611	0
CHB	chr15	GOLGA8G	26069611	0
CHB	chr15	GOLGA8G	26119611	0
CHB	chr15	GOLGA8G	26169611	0
CHB	chr15	GOLGA8G	26219611	51
CHB	chr15	GOLGA8G	26269611	53
CHB	chr15	GOLGA8G	26319611	2
CHB	chr15	GOLGA8G	26369611	0
CHB	chr15	GOLGA8G	26419611	0
CHB	chr15	GOLGA8G	26469611	0
CHB	chr15	GOLGA8G	26519611	0
CHB	chr15	GOLGA8G	26569611	0
CHB	chr15	GOLGA8G	26619611	0
CHB	chr15	GOLGA8G	26669611	5
CHB	chr15	GOLGA8G	26719611	44
CHB	chr15	GOLGA8G	26769611	46
CHB	chr15	GOLGA8G	26819611	14
CHB	chr15	GOLGA8G	26869611	9
CHB	chr15	GOLGA8G	26919611	2
CHB	chr15	GOLGA8G	26969611	0
CHB	chr15	GOLGA8G	27019611	0
CHB	chr15	GOLGA8G	27069611	18

CHB	chr15	GOLGA8G	27119611	32
CHB	chr15	GOLGA8G	27169611	32
CHB	chr15	GOLGA8G	27219611	26
CHB	chr15	GOLGA8G	27269611	8
CHB	chr15	GOLGA8G	27319611	0
CHB	chr15	GOLGA8G	27369611	0
CHB	chr15	GOLGA8G	27419611	0
CHB	chr15	GOLGA8G	27469611	0
CHB	chr15	GOLGA8G	27519611	0
CHB	chr15	GOLGA8G	27569611	0
CHB	chr15	GOLGA8G	27619611	0
CHB	chr15	GOLGA8G	27669611	0
CHB	chr15	GOLGA8G	27719611	0
CHB	chr15	GOLGA8G	27769611	0
CHB	chr15	GOLGA8G	27819611	0
CHB	chr15	GOLGA8G	27869611	0
CHB	chr15	GOLGA8G	27919611	0
CHB	chr15	GOLGA8G	27969611	0
CHB	chr15	GOLGA8G	28019611	0
CHB	chr15	GOLGA8G	28069611	0
CHB	chr15	GOLGA8G	28119611	0
CHB	chr15	GOLGA8G	28169611	0
CHB	chr15	GOLGA8G	28219611	2
CHB	chr15	GOLGA8G	28269611	19
CHB	chr15	GOLGA8G	28319611	40
CHB	chr15	GOLGA8G	28369611	23
CHB	chr15	GOLGA8G	28419611	0
CHB	chr15	GOLGA8G	28469611	0
CHB	chr15	GOLGA8G	28519611	0
CHB	chr15	GOLGA8G	28569611	0
CHB	chr15	GOLGA8G	28619611	0
CHB	chr15	GOLGA8G	28669611	0
CHB	chr15	GOLGA8G	28719611	0
CHB	chr15	GOLGA8G	28769611	0
CHB	chr15	GOLGA8G	28819611	0
CHB	chr15	GOLGA8G	28869611	0
CHB	chr15	GOLGA8G	28919611	10
CHB	chr15	GOLGA8G	28969611	32
CHB	chr15	GOLGA8G	29019611	37
CHB	chr15	GOLGA8G	29069611	15
CHB	chr15	GOLGA8G	29119611	0
CHB	chr15	GOLGA8G	29169611	0
CHB	chr15	GOLGA8G	29219611	0
CHB	chr15	GOLGA8G	29269611	0
CHB	chr15	GOLGA8G	29319611	0
CHB	chr15	GOLGA8G	29369611	0
CHB	chr15	GOLGA8G	29419611	0
CHB	chr15	GOLGA8G	29469611	0
CHB	chr15	GOLGA8G	29519611	0

CHB	chr15	GOLGA8G	29569611	0
CHB	chr15	GOLGA8G	29619611	0
CHB	chr15	GOLGA8G	29669611	0
CHB	chr15	GOLGA8G	29719611	0
CHB	chr15	GOLGA8G	29769611	0
CHB	chr15	GOLGA8G	29819611	0
CHB	chr15	GOLGA8G	29869611	0
CHB	chr15	GOLGA8G	29919611	0
CHB	chr15	GOLGA8G	29969611	31
CHB	chr15	GOLGA8G	30019611	39
CHB	chr15	GOLGA8G	30069611	27
CHB	chr15	GOLGA8G	30119611	23
CHB	chr15	GOLGA8G	30169611	4
CHB	chr15	GOLGA8G	30219611	0
CHB	chr15	GOLGA8G	30269611	0
CHB	chr15	GOLGA8G	30319611	0
CHB	chr15	GOLGA8G	30369611	0
CHB	chr15	GOLGA8G	30419611	0
CHB	chr15	GOLGA8G	30469611	0
CHB	chr15	GOLGA8G	30519611	0
CHB	chr15	GOLGA8G	30569611	0
CHB	chr15	GOLGA8G	30619611	0
CHB	chr15	GOLGA8G	30669611	0
CHB	chr15	GOLGA8G	30719611	0
CHB	chr15	GOLGA8G	30769611	0
CHB	chr15	GOLGA8G	30819611	0
CHB	chr15	GOLGA8G	30869611	0
CHB	chr15	GOLGA8G	30919611	0
CHB	chr15	GOLGA8G	30969611	0
CHB	chr15	GOLGA8G	31019611	0
CHB	chr15	GOLGA8G	31069611	0
CHB	chr15	GOLGA8G	31119611	0
CHB	chr15	GOLGA8G	31169611	0
CHB	chr15	GOLGA8G	31219611	0
CHB	chr15	GOLGA8G	31269611	0
CHB	chr15	GOLGA8G	31319611	0
CHB	chr15	GOLGA8G	31369611	0
CHB	chr15	GOLGA8G	31419611	0
CHB	chr15	GOLGA8G	31469611	0
CHB	chr15	GOLGA8G	31519611	0
CHB	chr15	GOLGA8G	31569611	0
CHB	chr15	GOLGA8G	31619611	0
CHB	chr15	GOLGA8G	31669611	0
CHB	chr15	GOLGA8G	31719611	0
CHB	chr15	GOLGA8G	31769611	0
CHB	chr15	GOLGA8G	31819611	0
CHB	chr15	GOLGA8G	31869611	0
CHB	chr15	GOLGA8G	31919611	0
CHB	chr15	GOLGA8G	31969611	0

CHB	chr15	GOLGA8G	32019611	0
CHB	chr15	GOLGA8G	32069611	0
CHB	chr15	GOLGA8G	32119611	0
CHB	chr15	GOLGA8G	32169611	0
CHB	chr15	GOLGA8G	32219611	0
CHB	chr15	GOLGA8G	32269611	0
CHB	chr15	GOLGA8G	32319611	0
CHB	chr15	GOLGA8G	32369611	0
CHB	chr15	GOLGA8G	32419611	0
CHB	chr15	GOLGA8G	32469611	0
CHB	chr15	GOLGA8G	32519611	0
CHB	chr15	GOLGA8G	32569611	0
CHB	chr15	GOLGA8G	32619611	0
CHB	chr15	GOLGA8G	32669611	0
CHB	chr15	GOLGA8G	32719611	0
CHB	chr15	GOLGA8G	32769611	0
CHB	chr15	GOLGA8G	32819611	0
CHB	chr15	GOLGA8G	32869611	0
CHB	chr15	GOLGA8G	32919611	0
CHB	chr15	GOLGA8G	32969611	0
CHB	chr15	GOLGA8G	33019611	0
CHB	chr15	GOLGA8G	33069611	0
CHB	chr15	GOLGA8G	33119611	0
CHB	chr15	GOLGA8G	33169611	0
CHB	chr15	GOLGA8G	33219611	0
CHB	chr15	GOLGA8G	33269611	0
CHB	chr15	GOLGA8G	33319611	0
CHB	chr15	GOLGA8G	33369611	0
CHB	chr15	GOLGA8G	33419611	0
CHB	chr15	GOLGA8G	33469611	0
CHB	chr1	HIST2	138952386	0
CHB	chr1	HIST2	139002386	0
CHB	chr1	HIST2	139052386	0
CHB	chr1	HIST2	139102386	0
CHB	chr1	HIST2	139152386	0
CHB	chr1	HIST2	139202386	0
CHB	chr1	HIST2	139252386	0
CHB	chr1	HIST2	139302386	0
CHB	chr1	HIST2	139352386	0
CHB	chr1	HIST2	139402386	0
CHB	chr1	HIST2	139452386	0
CHB	chr1	HIST2	139502386	0
CHB	chr1	HIST2	139552386	0
CHB	chr1	HIST2	139602386	0
CHB	chr1	HIST2	139652386	0
CHB	chr1	HIST2	139702386	0
CHB	chr1	HIST2	139752386	0
CHB	chr1	HIST2	139802386	0
CHB	chr1	HIST2	139852386	0

CHB	chr1	HIST2	139902386	0
CHB	chr1	HIST2	139952386	0
CHB	chr1	HIST2	140002386	0
CHB	chr1	HIST2	140052386	0
CHB	chr1	HIST2	140102386	0
CHB	chr1	HIST2	140152386	0
CHB	chr1	HIST2	140202386	0
CHB	chr1	HIST2	140252386	0
CHB	chr1	HIST2	140302386	0
CHB	chr1	HIST2	140352386	0
CHB	chr1	HIST2	140402386	0
CHB	chr1	HIST2	140452386	0
CHB	chr1	HIST2	140502386	0
CHB	chr1	HIST2	140552386	0
CHB	chr1	HIST2	140602386	0
CHB	chr1	HIST2	140652386	0
CHB	chr1	HIST2	140702386	0
CHB	chr1	HIST2	140752386	0
CHB	chr1	HIST2	140802386	0
CHB	chr1	HIST2	140852386	0
CHB	chr1	HIST2	140902386	0
CHB	chr1	HIST2	140952386	0
CHB	chr1	HIST2	141002386	0
CHB	chr1	HIST2	141052386	0
CHB	chr1	HIST2	141102386	0
CHB	chr1	HIST2	141152386	0
CHB	chr1	HIST2	141202386	0
CHB	chr1	HIST2	141252386	0
CHB	chr1	HIST2	141302386	0
CHB	chr1	HIST2	141352386	0
CHB	chr1	HIST2	141402386	0
CHB	chr1	HIST2	141452386	0
CHB	chr1	HIST2	141502386	0
CHB	chr1	HIST2	141552386	0
CHB	chr1	HIST2	141602386	0
CHB	chr1	HIST2	141652386	0
CHB	chr1	HIST2	141702386	0
CHB	chr1	HIST2	141752386	0
CHB	chr1	HIST2	141802386	0
CHB	chr1	HIST2	141852386	0
CHB	chr1	HIST2	141902386	0
CHB	chr1	HIST2	141952386	0
CHB	chr1	HIST2	142002386	0
CHB	chr1	HIST2	142052386	0
CHB	chr1	HIST2	142102386	0
CHB	chr1	HIST2	142152386	0
CHB	chr1	HIST2	142202386	0
CHB	chr1	HIST2	142252386	0
CHB	chr1	HIST2	142302386	0

CHB	chr1	HIST2	142352386	0
CHB	chr1	HIST2	142402386	0
CHB	chr1	HIST2	142452386	0
CHB	chr1	HIST2	142502386	0
CHB	chr1	HIST2	142552386	0
CHB	chr1	HIST2	142602386	0
CHB	chr1	HIST2	142652386	0
CHB	chr1	HIST2	142702386	0
CHB	chr1	HIST2	142752386	0
CHB	chr1	HIST2	142802386	0
CHB	chr1	HIST2	142852386	0
CHB	chr1	HIST2	142902386	0
CHB	chr1	HIST2	142952386	0
CHB	chr1	HIST2	143002386	0
CHB	chr1	HIST2	143052386	0
CHB	chr1	HIST2	143102386	0
CHB	chr1	HIST2	143152386	0
CHB	chr1	HIST2	143202386	0
CHB	chr1	HIST2	143252386	0
CHB	chr1	HIST2	143302386	0
CHB	chr1	HIST2	143352386	0
CHB	chr1	HIST2	143402386	0
CHB	chr1	HIST2	143452386	0
CHB	chr1	HIST2	143502386	0
CHB	chr1	HIST2	143552386	0
CHB	chr1	HIST2	143602386	0
CHB	chr1	HIST2	143652386	0
CHB	chr1	HIST2	143702386	0
CHB	chr1	HIST2	143752386	0
CHB	chr1	HIST2	143802386	0
CHB	chr1	HIST2	143852386	0
CHB	chr1	HIST2	143902386	0
CHB	chr1	HIST2	143952386	0
CHB	chr1	HIST2	144002386	0
CHB	chr1	HIST2	144052386	0
CHB	chr1	HIST2	144102386	0
CHB	chr1	HIST2	144152386	0
CHB	chr1	HIST2	144202386	0
CHB	chr1	HIST2	144252386	0
CHB	chr1	HIST2	144302386	0
CHB	chr1	HIST2	144352386	0
CHB	chr1	HIST2	144402386	0
CHB	chr1	HIST2	144452386	0
CHB	chr1	HIST2	144502386	0
CHB	chr1	HIST2	144552386	0
CHB	chr1	HIST2	144602386	0
CHB	chr1	HIST2	144652386	0
CHB	chr1	HIST2	144702386	0
CHB	chr1	HIST2	144752386	0

CHB	chr1	HIST2	144802386	0
CHB	chr1	HIST2	144852386	0
CHB	chr1	HIST2	144902386	0
CHB	chr1	HIST2	144952386	0
CHB	chr1	HIST2	145002386	0
CHB	chr1	HIST2	145052386	0
CHB	chr1	HIST2	145102386	0
CHB	chr1	HIST2	145152386	0
CHB	chr1	HIST2	145202386	0
CHB	chr1	HIST2	145252386	0
CHB	chr1	HIST2	145302386	0
CHB	chr1	HIST2	145352386	0
CHB	chr1	HIST2	145402386	0
CHB	chr1	HIST2	145452386	0
CHB	chr1	HIST2	145502386	0
CHB	chr1	HIST2	145552386	0
CHB	chr1	HIST2	145602386	0
CHB	chr1	HIST2	145652386	0
CHB	chr1	HIST2	145702386	0
CHB	chr1	HIST2	145752386	0
CHB	chr1	HIST2	145802386	0
CHB	chr1	HIST2	145852386	0
CHB	chr1	HIST2	145902386	0
CHB	chr1	HIST2	145952386	0
CHB	chr1	HIST2	146002386	0
CHB	chr1	HIST2	146052386	0
CHB	chr1	HIST2	146102386	0
CHB	chr1	HIST2	146152386	0
CHB	chr1	HIST2	146202386	0
CHB	chr1	HIST2	146252386	0
CHB	chr1	HIST2	146302386	0
CHB	chr1	HIST2	146352386	0
CHB	chr1	HIST2	146402386	0
CHB	chr1	HIST2	146452386	0
CHB	chr1	HIST2	146502386	0
CHB	chr1	HIST2	146552386	0
CHB	chr1	HIST2	146602386	0
CHB	chr1	HIST2	146652386	0
CHB	chr1	HIST2	146702386	0
CHB	chr1	HIST2	146752386	0
CHB	chr1	HIST2	146802386	0
CHB	chr1	HIST2	146852386	0
CHB	chr1	HIST2	146902386	0
CHB	chr1	HIST2	146952386	0
CHB	chr1	HIST2	147002386	0
CHB	chr1	HIST2	147052386	0
CHB	chr1	HIST2	147102386	0
CHB	chr1	HIST2	147152386	0
CHB	chr1	HIST2	147202386	0

CHB	chr1	HIST2	147252386	0
CHB	chr1	HIST2	147302386	0
CHB	chr1	HIST2	147352386	0
CHB	chr1	HIST2	147402386	0
CHB	chr1	HIST2	147452386	0
CHB	chr1	HIST2	147502386	0
CHB	chr1	HIST2	147552386	0
CHB	chr1	HIST2	147602386	0
CHB	chr1	HIST2	147652386	0
CHB	chr1	HIST2	147702386	0
CHB	chr1	HIST2	147752386	0
CHB	chr1	HIST2	147802386	0
CHB	chr1	HIST2	147852386	0
CHB	chr1	HIST2	147902386	0
CHB	chr1	HIST2	147952386	0
CHB	chr1	HIST2	148002386	0
CHB	chr1	HIST2	148052386	0
CHB	chr1	HIST2	148102386	0
CHB	chr1	HIST2	148152386	0
CHB	chr1	HIST2	148202386	0
CHB	chr1	HIST2	148252386	0
CHB	chr1	HIST2	148302386	0
CHB	chr1	HIST2	148352386	0
CHB	chr1	HIST2	148402386	0
CHB	chr1	HIST2	148452386	0
CHB	chr1	HIST2	148502386	0
CHB	chr1	HIST2	148552386	0
CHB	chr1	HIST2	148602386	0
CHB	chr1	HIST2	148652386	0
CHB	chr1	HIST2	148702386	0
CHB	chr1	HIST2	148752386	0
CHB	chr1	HIST2	148802386	0
CHB	chr1	HIST2	148852386	0
CHB	chr1	HIST2	144832689	0
CHB	chr1	HIST2	144882689	0
CHB	chr1	HIST2	144932689	0
CHB	chr1	HIST2	144982689	0
CHB	chr1	HIST2	145032689	0
CHB	chr1	HIST2	145082689	0
CHB	chr1	HIST2	145132689	0
CHB	chr1	HIST2	145182689	0
CHB	chr1	HIST2	145232689	0
CHB	chr1	HIST2	145282689	0
CHB	chr1	HIST2	145332689	0
CHB	chr1	HIST2	145382689	0
CHB	chr1	HIST2	145432689	0
CHB	chr1	HIST2	145482689	0
CHB	chr1	HIST2	145532689	0
CHB	chr1	HIST2	145582689	0

CHB	chr1	HIST2	145632689	0
CHB	chr1	HIST2	145682689	0
CHB	chr1	HIST2	145732689	0
CHB	chr1	HIST2	145782689	0
CHB	chr1	HIST2	145832689	0
CHB	chr1	HIST2	145882689	0
CHB	chr1	HIST2	145932689	0
CHB	chr1	HIST2	145982689	0
CHB	chr1	HIST2	146032689	0
CHB	chr1	HIST2	146082689	0
CHB	chr1	HIST2	146132689	0
CHB	chr1	HIST2	146182689	0
CHB	chr1	HIST2	146232689	0
CHB	chr1	HIST2	146282689	0
CHB	chr1	HIST2	146332689	0
CHB	chr1	HIST2	146382689	0
CHB	chr1	HIST2	146432689	0
CHB	chr1	HIST2	146482689	0
CHB	chr1	HIST2	146532689	0
CHB	chr1	HIST2	146582689	0
CHB	chr1	HIST2	146632689	0
CHB	chr1	HIST2	146682689	0
CHB	chr1	HIST2	146732689	0
CHB	chr1	HIST2	146782689	0
CHB	chr1	HIST2	146832689	0
CHB	chr1	HIST2	146882689	0
CHB	chr1	HIST2	146932689	0
CHB	chr1	HIST2	146982689	0
CHB	chr1	HIST2	147032689	0
CHB	chr1	HIST2	147082689	0
CHB	chr1	HIST2	147132689	0
CHB	chr1	HIST2	147182689	0
CHB	chr1	HIST2	147232689	0
CHB	chr1	HIST2	147282689	0
CHB	chr1	HIST2	147332689	0
CHB	chr1	HIST2	147382689	0
CHB	chr1	HIST2	147432689	0
CHB	chr1	HIST2	147482689	0
CHB	chr1	HIST2	147532689	0
CHB	chr1	HIST2	147582689	0
CHB	chr1	HIST2	147632689	0
CHB	chr1	HIST2	147682689	0
CHB	chr1	HIST2	147732689	0
CHB	chr1	HIST2	147782689	0
CHB	chr1	HIST2	147832689	0
CHB	chr1	HIST2	147882689	0
CHB	chr1	HIST2	147932689	0
CHB	chr1	HIST2	147982689	0
CHB	chr1	HIST2	148032689	0

CHB	chr1	HIST2	148082689	0
CHB	chr1	HIST2	148132689	0
CHB	chr1	HIST2	148182689	0
CHB	chr1	HIST2	148232689	0
CHB	chr1	HIST2	148282689	0
CHB	chr1	HIST2	148332689	0
CHB	chr1	HIST2	148382689	0
CHB	chr1	HIST2	148432689	0
CHB	chr1	HIST2	148482689	0
CHB	chr1	HIST2	148532689	0
CHB	chr1	HIST2	148582689	0
CHB	chr1	HIST2	148632689	0
CHB	chr1	HIST2	148682689	0
CHB	chr1	HIST2	148732689	0
CHB	chr1	HIST2	148782689	0
CHB	chr1	HIST2	148832689	0
CHB	chr1	HIST2	148882689	0
CHB	chr1	HIST2	148932689	0
CHB	chr1	HIST2	148982689	0
CHB	chr1	HIST2	149032689	0
CHB	chr1	HIST2	149082689	0
CHB	chr1	HIST2	149132689	0
CHB	chr1	HIST2	149182689	0
CHB	chr1	HIST2	149232689	0
CHB	chr1	HIST2	149282689	0
CHB	chr1	HIST2	149332689	0
CHB	chr1	HIST2	149382689	0
CHB	chr1	HIST2	149432689	0
CHB	chr1	HIST2	149482689	0
CHB	chr1	HIST2	149532689	0
CHB	chr1	HIST2	149582689	0
CHB	chr1	HIST2	149632689	0
CHB	chr1	HIST2	149682689	0
CHB	chr1	HIST2	149732689	0
CHB	chr1	HIST2	149782689	0
CHB	chr1	HIST2	149832689	2
CHB	chr1	HIST2	149882689	23
CHB	chr1	HIST2	149932689	32
CHB	chr1	HIST2	149982689	30
CHB	chr1	HIST2	150032689	40
CHB	chr1	HIST2	150082689	40
CHB	chr1	HIST2	150132689	40
CHB	chr1	HIST2	150182689	37
CHB	chr1	HIST2	150232689	30
CHB	chr1	HIST2	150282689	26
CHB	chr1	HIST2	150332689	29
CHB	chr1	HIST2	150382689	24
CHB	chr1	HIST2	150432689	15
CHB	chr1	HIST2	150482689	24

CHB	chr1	HIST2	150532689	33
CHB	chr1	HIST2	150582689	50
CHB	chr1	HIST2	150632689	55
CHB	chr1	HIST2	150682689	40
CHB	chr1	HIST2	150732689	43
CHB	chr1	HIST2	150782689	43
CHB	chr1	HIST2	150832689	43
CHB	chr1	HIST2	150882689	40
CHB	chr1	HIST2	150932689	38
CHB	chr1	HIST2	150982689	23
CHB	chr1	HIST2	151032689	0
CHB	chr1	HIST2	151082689	0
CHB	chr1	HIST2	151132689	0
CHB	chr1	HIST2	151182689	0
CHB	chr1	HIST2	151232689	0
CHB	chr1	HIST2	151282689	0
CHB	chr1	HIST2	151332689	0
CHB	chr1	HIST2	151382689	0
CHB	chr1	HIST2	151432689	0
CHB	chr1	HIST2	151482689	0
CHB	chr1	HIST2	151532689	0
CHB	chr1	HIST2	151582689	0
CHB	chr1	HIST2	151632689	0
CHB	chr1	HIST2	151682689	1
CHB	chr1	HIST2	151732689	15
CHB	chr1	HIST2	151782689	22
CHB	chr1	HIST2	151832689	25
CHB	chr1	HIST2	151882689	31
CHB	chr1	HIST2	151932689	40
CHB	chr1	HIST2	151982689	46
CHB	chr1	HIST2	152032689	86
CHB	chr1	HIST2	152082689	96
CHB	chr1	HIST2	152132689	56
CHB	chr1	HIST2	152182689	48
CHB	chr1	HIST2	152232689	58
CHB	chr1	HIST2	152282689	51
CHB	chr1	HIST2	152332689	28
CHB	chr1	HIST2	152382689	30
CHB	chr1	HIST2	152432689	36
CHB	chr1	HIST2	152482689	34
CHB	chr1	HIST2	152532689	16
CHB	chr1	HIST2	152582689	1
CHB	chr1	HIST2	152632689	0
CHB	chr1	HIST2	152682689	0
CHB	chr1	HIST2	152732689	0
CHB	chr1	HIST2	152782689	0
CHB	chr1	HIST2	152832689	0
CHB	chr1	HIST2	152882689	56
CHB	chr1	HIST2	152932689	75

CHB	chr1	HIST2	152982689	37
CHB	chr1	HIST2	153032689	47
CHB	chr1	HIST2	153082689	55
CHB	chr1	HIST2	153132689	44
CHB	chr1	HIST2	153182689	48
CHB	chr1	HIST2	153232689	64
CHB	chr1	HIST2	153282689	63
CHB	chr1	HIST2	153332689	44
CHB	chr1	HIST2	153382689	17
CHB	chr1	HIST2	153432689	2
CHB	chr1	HIST2	153482689	0
CHB	chr1	HIST2	153532689	0
CHB	chr1	HIST2	153582689	0
CHB	chr1	HIST2	153632689	0
CHB	chr1	HIST2	153682689	0
CHB	chr1	HIST2	153732689	0
CHB	chr1	HIST2	153782689	0
CHB	chr1	HIST2	153832689	0
CHB	chr1	HIST2	153882689	0
CHB	chr1	HIST2	153932689	0
CHB	chr1	HIST2	153982689	0
CHB	chr1	HIST2	154032689	0
CHB	chr1	HIST2	154082689	0
CHB	chr1	HIST2	154132689	0
CHB	chr1	HIST2	154182689	0
CHB	chr1	HIST2	154232689	0
CHB	chr1	HIST2	154282689	0
CHB	chr1	HIST2	154332689	0
CHB	chr1	HIST2	154382689	0
CHB	chr1	HIST2	154432689	0
CHB	chr1	HIST2	154482689	0
CHB	chr1	HIST2	154532689	0
CHB	chr1	HIST2	154582689	0
CHB	chr1	HIST2	154632689	0
CHB	chr1	HIST2	154682689	0
CHB	chr1	HIST2	154732689	0
CHB	chr2	LIMS3	104948688	0
CHB	chr2	LIMS3	104998688	0
CHB	chr2	LIMS3	105048688	0
CHB	chr2	LIMS3	105098688	0
CHB	chr2	LIMS3	105148688	0
CHB	chr2	LIMS3	105198688	0
CHB	chr2	LIMS3	105248688	0
CHB	chr2	LIMS3	105298688	0
CHB	chr2	LIMS3	105348688	0
CHB	chr2	LIMS3	105398688	0
CHB	chr2	LIMS3	105448688	0
CHB	chr2	LIMS3	105498688	0
CHB	chr2	LIMS3	105548688	0

CHB	chr2	LIMS3	105598688	0
CHB	chr2	LIMS3	105648688	0
CHB	chr2	LIMS3	105698688	0
CHB	chr2	LIMS3	105748688	0
CHB	chr2	LIMS3	105798688	0
CHB	chr2	LIMS3	105848688	9
CHB	chr2	LIMS3	105898688	15
CHB	chr2	LIMS3	105948688	26
CHB	chr2	LIMS3	105998688	35
CHB	chr2	LIMS3	106048688	65
CHB	chr2	LIMS3	106098688	61
CHB	chr2	LIMS3	106148688	25
CHB	chr2	LIMS3	106198688	53
CHB	chr2	LIMS3	106248688	49
CHB	chr2	LIMS3	106298688	44
CHB	chr2	LIMS3	106348688	34
CHB	chr2	LIMS3	106398688	0
CHB	chr2	LIMS3	106448688	0
CHB	chr2	LIMS3	106498688	0
CHB	chr2	LIMS3	106548688	0
CHB	chr2	LIMS3	106598688	0
CHB	chr2	LIMS3	106648688	0
CHB	chr2	LIMS3	106698688	0
CHB	chr2	LIMS3	106748688	0
CHB	chr2	LIMS3	106798688	0
CHB	chr2	LIMS3	106848688	0
CHB	chr2	LIMS3	106898688	0
CHB	chr2	LIMS3	106948688	0
CHB	chr2	LIMS3	106998688	0
CHB	chr2	LIMS3	107048688	0
CHB	chr2	LIMS3	107098688	0
CHB	chr2	LIMS3	107148688	0
CHB	chr2	LIMS3	107198688	0
CHB	chr2	LIMS3	107248688	0
CHB	chr2	LIMS3	107298688	0
CHB	chr2	LIMS3	107348688	0
CHB	chr2	LIMS3	107398688	0
CHB	chr2	LIMS3	107448688	0
CHB	chr2	LIMS3	107498688	0
CHB	chr2	LIMS3	107548688	0
CHB	chr2	LIMS3	107598688	0
CHB	chr2	LIMS3	107648688	0
CHB	chr2	LIMS3	107698688	0
CHB	chr2	LIMS3	107748688	0
CHB	chr2	LIMS3	107798688	0
CHB	chr2	LIMS3	107848688	0
CHB	chr2	LIMS3	107898688	0
CHB	chr2	LIMS3	107948688	0
CHB	chr2	LIMS3	107998688	0

CHB	chr2	LIMS3	108048688	0
CHB	chr2	LIMS3	108098688	0
CHB	chr2	LIMS3	108148688	0
CHB	chr2	LIMS3	108198688	0
CHB	chr2	LIMS3	108248688	0
CHB	chr2	LIMS3	108298688	0
CHB	chr2	LIMS3	108348688	0
CHB	chr2	LIMS3	108398688	0
CHB	chr2	LIMS3	108448688	0
CHB	chr2	LIMS3	108498688	0
CHB	chr2	LIMS3	108548688	0
CHB	chr2	LIMS3	108598688	0
CHB	chr2	LIMS3	108648688	0
CHB	chr2	LIMS3	108698688	5
CHB	chr2	LIMS3	108748688	15
CHB	chr2	LIMS3	108798688	25
CHB	chr2	LIMS3	108848688	25
CHB	chr2	LIMS3	108898688	21
CHB	chr2	LIMS3	108948688	47
CHB	chr2	LIMS3	108998688	72
CHB	chr2	LIMS3	109048688	66
CHB	chr2	LIMS3	109098688	62
CHB	chr2	LIMS3	109148688	56
CHB	chr2	LIMS3	109198688	44
CHB	chr2	LIMS3	109248688	32
CHB	chr2	LIMS3	109298688	28
CHB	chr2	LIMS3	109348688	26
CHB	chr2	LIMS3	109398688	25
CHB	chr2	LIMS3	109448688	25
CHB	chr2	LIMS3	109498688	29
CHB	chr2	LIMS3	109548688	52
CHB	chr2	LIMS3	109598688	59
CHB	chr2	LIMS3	109648688	87
CHB	chr2	LIMS3	109698688	76
CHB	chr2	LIMS3	109748688	34
CHB	chr2	LIMS3	109798688	36
CHB	chr2	LIMS3	109848688	46
CHB	chr2	LIMS3	109898688	59
CHB	chr2	LIMS3	109948688	60
CHB	chr2	LIMS3	109998688	54
CHB	chr2	LIMS3	110048688	43
CHB	chr2	LIMS3	110098688	19
CHB	chr2	LIMS3	110148688	0
CHB	chr2	LIMS3	110198688	0
CHB	chr2	LIMS3	110248688	0
CHB	chr2	LIMS3	110298688	1
CHB	chr2	LIMS3	110348688	6
CHB	chr2	LIMS3	110398688	18
CHB	chr2	LIMS3	110448688	31

CHB	chr2	LIMS3	110498688	20
CHB	chr2	LIMS3	110548688	2
CHB	chr2	LIMS3	110598688	0
CHB	chr2	LIMS3	110648688	0
CHB	chr2	LIMS3	110698688	0
CHB	chr2	LIMS3	110748688	0
CHB	chr2	LIMS3	110798688	0
CHB	chr2	LIMS3	110848688	0
CHB	chr2	LIMS3	110898688	0
CHB	chr2	LIMS3	110948688	0
CHB	chr2	LIMS3	110998688	0
CHB	chr2	LIMS3	111048688	0
CHB	chr2	LIMS3	111098688	0
CHB	chr2	LIMS3	111148688	0
CHB	chr2	LIMS3	111198688	0
CHB	chr2	LIMS3	111248688	0
CHB	chr2	LIMS3	111298688	0
CHB	chr2	LIMS3	111348688	0
CHB	chr2	LIMS3	111398688	0
CHB	chr2	LIMS3	111448688	0
CHB	chr2	LIMS3	111498688	0
CHB	chr2	LIMS3	111548688	0
CHB	chr2	LIMS3	111598688	0
CHB	chr2	LIMS3	111648688	0
CHB	chr2	LIMS3	111698688	0
CHB	chr2	LIMS3	111748688	0
CHB	chr2	LIMS3	111798688	0
CHB	chr2	LIMS3	111848688	0
CHB	chr2	LIMS3	111898688	0
CHB	chr2	LIMS3	111948688	0
CHB	chr2	LIMS3	111998688	0
CHB	chr2	LIMS3	112048688	0
CHB	chr2	LIMS3	112098688	19
CHB	chr2	LIMS3	112148688	25
CHB	chr2	LIMS3	112198688	6
CHB	chr2	LIMS3	112248688	0
CHB	chr2	LIMS3	112298688	0
CHB	chr2	LIMS3	112348688	0
CHB	chr2	LIMS3	112398688	0
CHB	chr2	LIMS3	112448688	0
CHB	chr2	LIMS3	112498688	0
CHB	chr2	LIMS3	112548688	0
CHB	chr2	LIMS3	112598688	0
CHB	chr2	LIMS3	112648688	0
CHB	chr2	LIMS3	112698688	0
CHB	chr2	LIMS3	112748688	0
CHB	chr2	LIMS3	112798688	0
CHB	chr2	LIMS3	112848688	0
CHB	chr2	LIMS3	112898688	0

CHB	chr2	LIMS3	112948688	0
CHB	chr2	LIMS3	112998688	0
CHB	chr2	LIMS3	113048688	0
CHB	chr2	LIMS3	113098688	0
CHB	chr2	LIMS3	113148688	0
CHB	chr2	LIMS3	113198688	0
CHB	chr2	LIMS3	113248688	0
CHB	chr2	LIMS3	113298688	0
CHB	chr2	LIMS3	113348688	0
CHB	chr2	LIMS3	113398688	0
CHB	chr2	LIMS3	113448688	0
CHB	chr2	LIMS3	113498688	0
CHB	chr2	LIMS3	113548688	0
CHB	chr2	LIMS3	113598688	0
CHB	chr2	LIMS3	113648688	0
CHB	chr2	LIMS3	113698688	0
CHB	chr2	LIMS3	113748688	0
CHB	chr2	LIMS3	113798688	0
CHB	chr2	LIMS3	113848688	0
CHB	chr2	LIMS3	113898688	0
CHB	chr2	LIMS3	113948688	0
CHB	chr2	LIMS3	113998688	0
CHB	chr2	LIMS3	114048688	0
CHB	chr2	LIMS3	114098688	0
CHB	chr2	LIMS3	114148688	0
CHB	chr2	LIMS3	114198688	0
CHB	chr2	LIMS3	114248688	0
CHB	chr2	LIMS3	114298688	0
CHB	chr2	LIMS3	114348688	0
CHB	chr2	LIMS3	114398688	0
CHB	chr2	LIMS3	114448688	0
CHB	chr2	LIMS3	114498688	0
CHB	chr2	LIMS3	114548688	0
CHB	chr2	LIMS3	114598688	0
CHB	chr2	LIMS3	114648688	0
CHB	chr2	LIMS3	114698688	0
CHB	chr2	LIMS3	114748688	0
CHB	chr2	LIMS3	114798688	0
CHB	chr2	LIMS3	114848688	0
CHB	chr7	MUC12	96019565	0
CHB	chr7	MUC12	96069565	0
CHB	chr7	MUC12	96119565	0
CHB	chr7	MUC12	96169565	0
CHB	chr7	MUC12	96219565	0
CHB	chr7	MUC12	96269565	0
CHB	chr7	MUC12	96319565	0
CHB	chr7	MUC12	96369565	0
CHB	chr7	MUC12	96419565	0
CHB	chr7	MUC12	96469565	0

CHB	chr7	MUC12	96519565	0
CHB	chr7	MUC12	96569565	0
CHB	chr7	MUC12	96619565	0
CHB	chr7	MUC12	96669565	0
CHB	chr7	MUC12	96719565	0
CHB	chr7	MUC12	96769565	0
CHB	chr7	MUC12	96819565	0
CHB	chr7	MUC12	96869565	0
CHB	chr7	MUC12	96919565	0
CHB	chr7	MUC12	96969565	0
CHB	chr7	MUC12	97019565	0
CHB	chr7	MUC12	97069565	0
CHB	chr7	MUC12	97119565	0
CHB	chr7	MUC12	97169565	0
CHB	chr7	MUC12	97219565	0
CHB	chr7	MUC12	97269565	0
CHB	chr7	MUC12	97319565	0
CHB	chr7	MUC12	97369565	0
CHB	chr7	MUC12	97419565	0
CHB	chr7	MUC12	97469565	0
CHB	chr7	MUC12	97519565	0
CHB	chr7	MUC12	97569565	0
CHB	chr7	MUC12	97619565	0
CHB	chr7	MUC12	97669565	0
CHB	chr7	MUC12	97719565	0
CHB	chr7	MUC12	97769565	0
CHB	chr7	MUC12	97819565	0
CHB	chr7	MUC12	97869565	0
CHB	chr7	MUC12	97919565	0
CHB	chr7	MUC12	97969565	0
CHB	chr7	MUC12	98019565	0
CHB	chr7	MUC12	98069565	0
CHB	chr7	MUC12	98119565	0
CHB	chr7	MUC12	98169565	0
CHB	chr7	MUC12	98219565	0
CHB	chr7	MUC12	98269565	0
CHB	chr7	MUC12	98319565	0
CHB	chr7	MUC12	98369565	0
CHB	chr7	MUC12	98419565	0
CHB	chr7	MUC12	98469565	0
CHB	chr7	MUC12	98519565	0
CHB	chr7	MUC12	98569565	0
CHB	chr7	MUC12	98619565	0
CHB	chr7	MUC12	98669565	0
CHB	chr7	MUC12	98719565	0
CHB	chr7	MUC12	98769565	0
CHB	chr7	MUC12	98819565	0
CHB	chr7	MUC12	98869565	0
CHB	chr7	MUC12	98919565	0

CHB	chr7	MUC12	98969565	0
CHB	chr7	MUC12	99019565	0
CHB	chr7	MUC12	99069565	0
CHB	chr7	MUC12	99119565	0
CHB	chr7	MUC12	99169565	0
CHB	chr7	MUC12	99219565	0
CHB	chr7	MUC12	99269565	0
CHB	chr7	MUC12	99319565	0
CHB	chr7	MUC12	99369565	0
CHB	chr7	MUC12	99419565	0
CHB	chr7	MUC12	99469565	0
CHB	chr7	MUC12	99519565	0
CHB	chr7	MUC12	99569565	0
CHB	chr7	MUC12	99619565	0
CHB	chr7	MUC12	99669565	0
CHB	chr7	MUC12	99719565	0
CHB	chr7	MUC12	99769565	0
CHB	chr7	MUC12	99819565	0
CHB	chr7	MUC12	99869565	0
CHB	chr7	MUC12	99919565	0
CHB	chr7	MUC12	99969565	0
CHB	chr7	MUC12	100019565	0
CHB	chr7	MUC12	100069565	0
CHB	chr7	MUC12	100119565	0
CHB	chr7	MUC12	100169565	0
CHB	chr7	MUC12	100219565	0
CHB	chr7	MUC12	100269565	0
CHB	chr7	MUC12	100319565	0
CHB	chr7	MUC12	100369565	0
CHB	chr7	MUC12	100419565	0
CHB	chr7	MUC12	100469565	0
CHB	chr7	MUC12	100519565	0
CHB	chr7	MUC12	100569565	0
CHB	chr7	MUC12	100619565	0
CHB	chr7	MUC12	100669565	0
CHB	chr7	MUC12	100719565	0
CHB	chr7	MUC12	100769565	0
CHB	chr7	MUC12	100819565	0
CHB	chr7	MUC12	100869565	0
CHB	chr7	MUC12	100919565	0
CHB	chr7	MUC12	100969565	0
CHB	chr7	MUC12	101019565	0
CHB	chr7	MUC12	101069565	0
CHB	chr7	MUC12	101119565	0
CHB	chr7	MUC12	101169565	0
CHB	chr7	MUC12	101219565	0
CHB	chr7	MUC12	101269565	0
CHB	chr7	MUC12	101319565	0
CHB	chr7	MUC12	101369565	0

CHB	chr7	MUC12	101419565	0
CHB	chr7	MUC12	101469565	0
CHB	chr7	MUC12	101519565	0
CHB	chr7	MUC12	101569565	0
CHB	chr7	MUC12	101619565	0
CHB	chr7	MUC12	101669565	0
CHB	chr7	MUC12	101719565	0
CHB	chr7	MUC12	101769565	0
CHB	chr7	MUC12	101819565	0
CHB	chr7	MUC12	101869565	0
CHB	chr7	MUC12	101919565	0
CHB	chr7	MUC12	101969565	0
CHB	chr7	MUC12	102019565	0
CHB	chr7	MUC12	102069565	0
CHB	chr7	MUC12	102119565	0
CHB	chr7	MUC12	102169565	0
CHB	chr7	MUC12	102219565	0
CHB	chr7	MUC12	102269565	0
CHB	chr7	MUC12	102319565	0
CHB	chr7	MUC12	102369565	0
CHB	chr7	MUC12	102419565	0
CHB	chr7	MUC12	102469565	0
CHB	chr7	MUC12	102519565	0
CHB	chr7	MUC12	102569565	0
CHB	chr7	MUC12	102619565	0
CHB	chr7	MUC12	102669565	0
CHB	chr7	MUC12	102719565	0
CHB	chr7	MUC12	102769565	0
CHB	chr7	MUC12	102819565	0
CHB	chr7	MUC12	102869565	0
CHB	chr7	MUC12	102919565	0
CHB	chr7	MUC12	102969565	0
CHB	chr7	MUC12	103019565	0
CHB	chr7	MUC12	103069565	0
CHB	chr7	MUC12	103119565	0
CHB	chr7	MUC12	103169565	0
CHB	chr7	MUC12	103219565	0
CHB	chr7	MUC12	103269565	0
CHB	chr7	MUC12	103319565	0
CHB	chr7	MUC12	103369565	5
CHB	chr7	MUC12	103419565	24
CHB	chr7	MUC12	103469565	33
CHB	chr7	MUC12	103519565	88
CHB	chr7	MUC12	103569565	82
CHB	chr7	MUC12	103619565	8
CHB	chr7	MUC12	103669565	0
CHB	chr7	MUC12	103719565	0
CHB	chr7	MUC12	103769565	0
CHB	chr7	MUC12	103819565	0

CHB	chr7	MUC12	103869565	18
CHB	chr7	MUC12	103919565	36
CHB	chr7	MUC12	103969565	37
CHB	chr7	MUC12	104019565	40
CHB	chr7	MUC12	104069565	44
CHB	chr7	MUC12	104119565	40
CHB	chr7	MUC12	104169565	43
CHB	chr7	MUC12	104219565	83
CHB	chr7	MUC12	104269565	112
CHB	chr7	MUC12	104319565	69
CHB	chr7	MUC12	104369565	42
CHB	chr7	MUC12	104419565	32
CHB	chr7	MUC12	104469565	16
CHB	chr7	MUC12	104519565	30
CHB	chr7	MUC12	104569565	36
CHB	chr7	MUC12	104619565	57
CHB	chr7	MUC12	104669565	83
CHB	chr7	MUC12	104719565	65
CHB	chr7	MUC12	104769565	78
CHB	chr7	MUC12	104819565	91
CHB	chr7	MUC12	104869565	47
CHB	chr7	MUC12	104919565	40
CHB	chr7	MUC12	104969565	44
CHB	chr7	MUC12	105019565	64
CHB	chr7	MUC12	105069565	63
CHB	chr7	MUC12	105119565	131
CHB	chr7	MUC12	105169565	137
CHB	chr7	MUC12	105219565	22
CHB	chr7	MUC12	105269565	0
CHB	chr7	MUC12	105319565	0
CHB	chr7	MUC12	105369565	0
CHB	chr7	MUC12	105419565	0
CHB	chr7	MUC12	105469565	0
CHB	chr7	MUC12	105519565	0
CHB	chr7	MUC12	105569565	0
CHB	chr7	MUC12	105619565	0
CHB	chr7	MUC12	105669565	0
CHB	chr7	MUC12	105719565	0
CHB	chr7	MUC12	105769565	0
CHB	chr7	MUC12	105819565	0
CHB	chr7	MUC12	105869565	0
CHB	chr7	MUC12	105919565	0
CHB	chr1	NBPF11	143152151	0
CHB	chr1	NBPF11	143202151	0
CHB	chr1	NBPF11	143252151	0
CHB	chr1	NBPF11	143302151	0
CHB	chr1	NBPF11	143352151	0
CHB	chr1	NBPF11	143402151	0
CHB	chr1	NBPF11	143452151	0

CHB	chr1	NBPF11	143502151	0
CHB	chr1	NBPF11	143552151	0
CHB	chr1	NBPF11	143602151	0
CHB	chr1	NBPF11	143652151	0
CHB	chr1	NBPF11	143702151	0
CHB	chr1	NBPF11	143752151	0
CHB	chr1	NBPF11	143802151	0
CHB	chr1	NBPF11	143852151	0
CHB	chr1	NBPF11	143902151	0
CHB	chr1	NBPF11	143952151	0
CHB	chr1	NBPF11	144002151	0
CHB	chr1	NBPF11	144052151	0
CHB	chr1	NBPF11	144102151	0
CHB	chr1	NBPF11	144152151	0
CHB	chr1	NBPF11	144202151	0
CHB	chr1	NBPF11	144252151	0
CHB	chr1	NBPF11	144302151	0
CHB	chr1	NBPF11	144352151	0
CHB	chr1	NBPF11	144402151	0
CHB	chr1	NBPF11	144452151	0
CHB	chr1	NBPF11	144502151	0
CHB	chr1	NBPF11	144552151	0
CHB	chr1	NBPF11	144602151	0
CHB	chr1	NBPF11	144652151	0
CHB	chr1	NBPF11	144702151	0
CHB	chr1	NBPF11	144752151	0
CHB	chr1	NBPF11	144802151	0
CHB	chr1	NBPF11	144852151	0
CHB	chr1	NBPF11	144902151	0
CHB	chr1	NBPF11	144952151	0
CHB	chr1	NBPF11	145002151	0
CHB	chr1	NBPF11	145052151	0
CHB	chr1	NBPF11	145102151	0
CHB	chr1	NBPF11	145152151	0
CHB	chr1	NBPF11	145202151	0
CHB	chr1	NBPF11	145252151	0
CHB	chr1	NBPF11	145302151	0
CHB	chr1	NBPF11	145352151	0
CHB	chr1	NBPF11	145402151	0
CHB	chr1	NBPF11	145452151	0
CHB	chr1	NBPF11	145502151	0
CHB	chr1	NBPF11	145552151	0
CHB	chr1	NBPF11	145602151	0
CHB	chr1	NBPF11	145652151	0
CHB	chr1	NBPF11	145702151	0
CHB	chr1	NBPF11	145752151	0
CHB	chr1	NBPF11	145802151	0
CHB	chr1	NBPF11	145852151	0
CHB	chr1	NBPF11	145902151	0

CHB	chr1	NBPF11	145952151	0
CHB	chr1	NBPF11	146002151	0
CHB	chr1	NBPF11	146052151	0
CHB	chr1	NBPF11	146102151	0
CHB	chr1	NBPF11	146152151	0
CHB	chr1	NBPF11	146202151	0
CHB	chr1	NBPF11	146252151	0
CHB	chr1	NBPF11	146302151	0
CHB	chr1	NBPF11	146352151	0
CHB	chr1	NBPF11	146402151	0
CHB	chr1	NBPF11	146452151	0
CHB	chr1	NBPF11	146502151	0
CHB	chr1	NBPF11	146552151	0
CHB	chr1	NBPF11	146602151	0
CHB	chr1	NBPF11	146652151	0
CHB	chr1	NBPF11	146702151	0
CHB	chr1	NBPF11	146752151	0
CHB	chr1	NBPF11	146802151	0
CHB	chr1	NBPF11	146852151	0
CHB	chr1	NBPF11	146902151	0
CHB	chr1	NBPF11	146952151	0
CHB	chr1	NBPF11	147002151	0
CHB	chr1	NBPF11	147052151	0
CHB	chr1	NBPF11	147102151	0
CHB	chr1	NBPF11	147152151	0
CHB	chr1	NBPF11	147202151	0
CHB	chr1	NBPF11	147252151	0
CHB	chr1	NBPF11	147302151	0
CHB	chr1	NBPF11	147352151	0
CHB	chr1	NBPF11	147402151	0
CHB	chr1	NBPF11	147452151	0
CHB	chr1	NBPF11	147502151	0
CHB	chr1	NBPF11	147552151	0
CHB	chr1	NBPF11	147602151	0
CHB	chr1	NBPF11	147652151	0
CHB	chr1	NBPF11	147702151	0
CHB	chr1	NBPF11	147752151	0
CHB	chr1	NBPF11	147802151	0
CHB	chr1	NBPF11	147852151	0
CHB	chr1	NBPF11	147902151	0
CHB	chr1	NBPF11	147952151	0
CHB	chr1	NBPF11	148002151	0
CHB	chr1	NBPF11	148052151	0
CHB	chr1	NBPF11	148102151	0
CHB	chr1	NBPF11	148152151	0
CHB	chr1	NBPF11	148202151	0
CHB	chr1	NBPF11	148252151	0
CHB	chr1	NBPF11	148302151	0
CHB	chr1	NBPF11	148352151	0

CHB	chr1	NBPF11	148402151	0
CHB	chr1	NBPF11	148452151	0
CHB	chr1	NBPF11	148502151	0
CHB	chr1	NBPF11	148552151	0
CHB	chr1	NBPF11	148602151	0
CHB	chr1	NBPF11	148652151	0
CHB	chr1	NBPF11	148702151	0
CHB	chr1	NBPF11	148752151	0
CHB	chr1	NBPF11	148802151	0
CHB	chr1	NBPF11	148852151	0
CHB	chr1	NBPF11	148902151	0
CHB	chr1	NBPF11	148952151	0
CHB	chr1	NBPF11	149002151	0
CHB	chr1	NBPF11	149052151	0
CHB	chr1	NBPF11	149102151	0
CHB	chr1	NBPF11	149152151	0
CHB	chr1	NBPF11	149202151	0
CHB	chr1	NBPF11	149252151	0
CHB	chr1	NBPF11	149302151	0
CHB	chr1	NBPF11	149352151	0
CHB	chr1	NBPF11	149402151	0
CHB	chr1	NBPF11	149452151	0
CHB	chr1	NBPF11	149502151	0
CHB	chr1	NBPF11	149552151	0
CHB	chr1	NBPF11	149602151	0
CHB	chr1	NBPF11	149652151	0
CHB	chr1	NBPF11	149702151	0
CHB	chr1	NBPF11	149752151	0
CHB	chr1	NBPF11	149802151	0
CHB	chr1	NBPF11	149852151	9
CHB	chr1	NBPF11	149902151	25
CHB	chr1	NBPF11	149952151	36
CHB	chr1	NBPF11	150002151	36
CHB	chr1	NBPF11	150052151	35
CHB	chr1	NBPF11	150102151	44
CHB	chr1	NBPF11	150152151	37
CHB	chr1	NBPF11	150202151	32
CHB	chr1	NBPF11	150252151	29
CHB	chr1	NBPF11	150302151	23
CHB	chr1	NBPF11	150352151	29
CHB	chr1	NBPF11	150402151	22
CHB	chr1	NBPF11	150452151	17
CHB	chr1	NBPF11	150502151	26
CHB	chr1	NBPF11	150552151	40
CHB	chr1	NBPF11	150602151	53
CHB	chr1	NBPF11	150652151	49
CHB	chr1	NBPF11	150702151	41
CHB	chr1	NBPF11	150752151	46
CHB	chr1	NBPF11	150802151	41

CHB	chr1	NBPF11	150852151	43
CHB	chr1	NBPF11	150902151	42
CHB	chr1	NBPF11	150952151	30
CHB	chr1	NBPF11	151002151	15
CHB	chr1	NBPF11	151052151	0
CHB	chr1	NBPF11	151102151	0
CHB	chr1	NBPF11	151152151	0
CHB	chr1	NBPF11	151202151	0
CHB	chr1	NBPF11	151252151	0
CHB	chr1	NBPF11	151302151	0
CHB	chr1	NBPF11	151352151	0
CHB	chr1	NBPF11	151402151	0
CHB	chr1	NBPF11	151452151	0
CHB	chr1	NBPF11	151502151	0
CHB	chr1	NBPF11	151552151	0
CHB	chr1	NBPF11	151602151	0
CHB	chr1	NBPF11	151652151	0
CHB	chr1	NBPF11	151702151	8
CHB	chr1	NBPF11	151752151	17
CHB	chr1	NBPF11	151802151	22
CHB	chr1	NBPF11	151852151	28
CHB	chr1	NBPF11	151902151	27
CHB	chr1	NBPF11	151952151	40
CHB	chr1	NBPF11	152002151	68
CHB	chr1	NBPF11	152052151	88
CHB	chr1	NBPF11	152102151	79
CHB	chr1	NBPF11	152152151	61
CHB	chr1	NBPF11	152202151	54
CHB	chr1	NBPF11	152252151	55
CHB	chr1	NBPF11	152302151	40
CHB	chr1	NBPF11	152352151	23
CHB	chr1	NBPF11	152402151	33
CHB	chr1	NBPF11	152452151	38
CHB	chr1	NBPF11	152502151	29
CHB	chr1	NBPF11	152552151	10
CHB	chr1	NBPF11	152602151	0
CHB	chr1	NBPF11	152652151	0
CHB	chr1	NBPF11	152702151	0
CHB	chr1	NBPF11	152752151	0
CHB	chr1	NBPF11	152802151	0
CHB	chr1	NBPF11	152852151	7
CHB	chr1	NBPF11	152902151	66
CHB	chr1	NBPF11	152952151	73
CHB	chr1	NBPF11	153002151	44
CHB	chr1	NBPF11	153052151	50
CHB	chr1	NBPF11	153102151	41
CHB	chr1	NBPF16	139471390	0
CHB	chr1	NBPF16	139521390	0
CHB	chr1	NBPF16	139571390	0

CHB	chr1	NBPF16	139621390	0
CHB	chr1	NBPF16	139671390	0
CHB	chr1	NBPF16	139721390	0
CHB	chr1	NBPF16	139771390	0
CHB	chr1	NBPF16	139821390	0
CHB	chr1	NBPF16	139871390	0
CHB	chr1	NBPF16	139921390	0
CHB	chr1	NBPF16	139971390	0
CHB	chr1	NBPF16	140021390	0
CHB	chr1	NBPF16	140071390	0
CHB	chr1	NBPF16	140121390	0
CHB	chr1	NBPF16	140171390	0
CHB	chr1	NBPF16	140221390	0
CHB	chr1	NBPF16	140271390	0
CHB	chr1	NBPF16	140321390	0
CHB	chr1	NBPF16	140371390	0
CHB	chr1	NBPF16	140421390	0
CHB	chr1	NBPF16	140471390	0
CHB	chr1	NBPF16	140521390	0
CHB	chr1	NBPF16	140571390	0
CHB	chr1	NBPF16	140621390	0
CHB	chr1	NBPF16	140671390	0
CHB	chr1	NBPF16	140721390	0
CHB	chr1	NBPF16	140771390	0
CHB	chr1	NBPF16	140821390	0
CHB	chr1	NBPF16	140871390	0
CHB	chr1	NBPF16	140921390	0
CHB	chr1	NBPF16	140971390	0
CHB	chr1	NBPF16	141021390	0
CHB	chr1	NBPF16	141071390	0
CHB	chr1	NBPF16	141121390	0
CHB	chr1	NBPF16	141171390	0
CHB	chr1	NBPF16	141221390	0
CHB	chr1	NBPF16	141271390	0
CHB	chr1	NBPF16	141321390	0
CHB	chr1	NBPF16	141371390	0
CHB	chr1	NBPF16	141421390	0
CHB	chr1	NBPF16	141471390	0
CHB	chr1	NBPF16	141521390	0
CHB	chr1	NBPF16	141571390	0
CHB	chr1	NBPF16	141621390	0
CHB	chr1	NBPF16	141671390	0
CHB	chr1	NBPF16	141721390	0
CHB	chr1	NBPF16	141771390	0
CHB	chr1	NBPF16	141821390	0
CHB	chr1	NBPF16	141871390	0
CHB	chr1	NBPF16	141921390	0
CHB	chr1	NBPF16	141971390	0
CHB	chr1	NBPF16	142021390	0

CHB	chr1	NBPF16	142071390	0
CHB	chr1	NBPF16	142121390	0
CHB	chr1	NBPF16	142171390	0
CHB	chr1	NBPF16	142221390	0
CHB	chr1	NBPF16	142271390	0
CHB	chr1	NBPF16	142321390	0
CHB	chr1	NBPF16	142371390	0
CHB	chr1	NBPF16	142421390	0
CHB	chr1	NBPF16	142471390	0
CHB	chr1	NBPF16	142521390	0
CHB	chr1	NBPF16	142571390	0
CHB	chr1	NBPF16	142621390	0
CHB	chr1	NBPF16	142671390	0
CHB	chr1	NBPF16	142721390	0
CHB	chr1	NBPF16	142771390	0
CHB	chr1	NBPF16	142821390	0
CHB	chr1	NBPF16	142871390	0
CHB	chr1	NBPF16	142921390	0
CHB	chr1	NBPF16	142971390	0
CHB	chr1	NBPF16	143021390	0
CHB	chr1	NBPF16	143071390	0
CHB	chr1	NBPF16	143121390	0
CHB	chr1	NBPF16	143171390	0
CHB	chr1	NBPF16	143221390	0
CHB	chr1	NBPF16	143271390	0
CHB	chr1	NBPF16	143321390	0
CHB	chr1	NBPF16	143371390	0
CHB	chr1	NBPF16	143421390	0
CHB	chr1	NBPF16	143471390	0
CHB	chr1	NBPF16	143521390	0
CHB	chr1	NBPF16	143571390	0
CHB	chr1	NBPF16	143621390	0
CHB	chr1	NBPF16	143671390	0
CHB	chr1	NBPF16	143721390	0
CHB	chr1	NBPF16	143771390	0
CHB	chr1	NBPF16	143821390	0
CHB	chr1	NBPF16	143871390	0
CHB	chr1	NBPF16	143921390	0
CHB	chr1	NBPF16	143971390	0
CHB	chr1	NBPF16	144021390	0
CHB	chr1	NBPF16	144071390	0
CHB	chr1	NBPF16	144121390	0
CHB	chr1	NBPF16	144171390	0
CHB	chr1	NBPF16	144221390	0
CHB	chr1	NBPF16	144271390	0
CHB	chr1	NBPF16	144321390	0
CHB	chr1	NBPF16	144371390	0
CHB	chr1	NBPF16	144421390	0
CHB	chr1	NBPF16	144471390	0

CHB	chr1	NBPF16	144521390	0
CHB	chr1	NBPF16	144571390	0
CHB	chr1	NBPF16	144621390	0
CHB	chr1	NBPF16	144671390	0
CHB	chr1	NBPF16	144721390	0
CHB	chr1	NBPF16	144771390	0
CHB	chr1	NBPF16	144821390	0
CHB	chr1	NBPF16	144871390	0
CHB	chr1	NBPF16	144921390	0
CHB	chr1	NBPF16	144971390	0
CHB	chr1	NBPF16	145021390	0
CHB	chr1	NBPF16	145071390	0
CHB	chr1	NBPF16	145121390	0
CHB	chr1	NBPF16	145171390	0
CHB	chr1	NBPF16	145221390	0
CHB	chr1	NBPF16	145271390	0
CHB	chr1	NBPF16	145321390	0
CHB	chr1	NBPF16	145371390	0
CHB	chr1	NBPF16	145421390	0
CHB	chr1	NBPF16	145471390	0
CHB	chr1	NBPF16	145521390	0
CHB	chr1	NBPF16	145571390	0
CHB	chr1	NBPF16	145621390	0
CHB	chr1	NBPF16	145671390	0
CHB	chr1	NBPF16	145721390	0
CHB	chr1	NBPF16	145771390	0
CHB	chr1	NBPF16	145821390	0
CHB	chr1	NBPF16	145871390	0
CHB	chr1	NBPF16	145921390	0
CHB	chr1	NBPF16	145971390	0
CHB	chr1	NBPF16	146021390	0
CHB	chr1	NBPF16	146071390	0
CHB	chr1	NBPF16	146121390	0
CHB	chr1	NBPF16	146171390	0
CHB	chr1	NBPF16	146221390	0
CHB	chr1	NBPF16	146271390	0
CHB	chr1	NBPF16	146321390	0
CHB	chr1	NBPF16	146371390	0
CHB	chr1	NBPF16	146421390	0
CHB	chr1	NBPF16	146471390	0
CHB	chr1	NBPF16	146521390	0
CHB	chr1	NBPF16	146571390	0
CHB	chr1	NBPF16	146621390	0
CHB	chr1	NBPF16	146671390	0
CHB	chr1	NBPF16	146721390	0
CHB	chr1	NBPF16	146771390	0
CHB	chr1	NBPF16	146821390	0
CHB	chr1	NBPF16	146871390	0
CHB	chr1	NBPF16	146921390	0

CHB	chr1	NBPF16	146971390	0
CHB	chr1	NBPF16	147021390	0
CHB	chr1	NBPF16	147071390	0
CHB	chr1	NBPF16	147121390	0
CHB	chr1	NBPF16	147171390	0
CHB	chr1	NBPF16	147221390	0
CHB	chr1	NBPF16	147271390	0
CHB	chr1	NBPF16	147321390	0
CHB	chr1	NBPF16	147371390	0
CHB	chr1	NBPF16	147421390	0
CHB	chr1	NBPF16	147471390	0
CHB	chr1	NBPF16	147521390	0
CHB	chr1	NBPF16	147571390	0
CHB	chr1	NBPF16	147621390	0
CHB	chr1	NBPF16	147671390	0
CHB	chr1	NBPF16	147721390	0
CHB	chr1	NBPF16	147771390	0
CHB	chr1	NBPF16	147821390	0
CHB	chr1	NBPF16	147871390	0
CHB	chr1	NBPF16	147921390	0
CHB	chr1	NBPF16	147971390	0
CHB	chr1	NBPF16	148021390	0
CHB	chr1	NBPF16	148071390	0
CHB	chr1	NBPF16	148121390	0
CHB	chr1	NBPF16	148171390	0
CHB	chr1	NBPF16	148221390	0
CHB	chr1	NBPF16	148271390	0
CHB	chr1	NBPF16	148321390	0
CHB	chr1	NBPF16	148371390	0
CHB	chr1	NBPF16	148421390	0
CHB	chr1	NBPF16	148471390	0
CHB	chr1	NBPF16	148521390	0
CHB	chr1	NBPF16	148571390	0
CHB	chr1	NBPF16	148621390	0
CHB	chr1	NBPF16	148671390	0
CHB	chr1	NBPF16	148721390	0
CHB	chr1	NBPF16	148771390	0
CHB	chr1	NBPF16	148821390	0
CHB	chr1	NBPF16	148871390	0
CHB	chr1	NBPF16	148921390	0
CHB	chr1	NBPF16	148971390	0
CHB	chr1	NBPF16	149021390	0
CHB	chr1	NBPF16	149071390	0
CHB	chr1	NBPF16	149121390	0
CHB	chr1	NBPF16	149171390	0
CHB	chr1	NBPF16	149221390	0
CHB	chr1	NBPF16	149271390	0
CHB	chr1	NBPF16	149321390	0
CHB	chr1	NBPF16	149371390	0

CHB	chr1	NPIP	39271070	0
CHB	chr1	NPIP	39321070	0
CHB	chr1	NPIP	39371070	11
CHB	chr1	NPIP	39421070	27
CHB	chr1	NPIP	39471070	33
CHB	chr1	NPIP	39521070	31
CHB	chr1	NPIP	39571070	26
CHB	chr1	NPIP	39621070	24
CHB	chr1	NPIP	39671070	29
CHB	chr1	NPIP	39721070	28
CHB	chr1	NPIP	39771070	24
CHB	chr1	NPIP	39821070	28
CHB	chr1	NPIP	39871070	24
CHB	chr1	NPIP	39921070	22
CHB	chr1	NPIP	39971070	24
CHB	chr1	NPIP	40021070	27
CHB	chr1	NPIP	40071070	27
CHB	chr1	NPIP	40121070	26
CHB	chr1	NPIP	40171070	29
CHB	chr1	NPIP	40221070	60
CHB	chr1	NPIP	40271070	70
CHB	chr1	NPIP	40321070	31
CHB	chr1	NPIP	40371070	27
CHB	chr1	NPIP	40421070	32
CHB	chr1	NPIP	40471070	25
CHB	chr1	NPIP	40521070	28
CHB	chr1	NPIP	40571070	27
CHB	chr1	NPIP	40621070	27
CHB	chr1	NPIP	40671070	26
CHB	chr1	NPIP	40721070	21
CHB	chr1	NPIP	40771070	28
CHB	chr1	NPIP	40821070	18
CHB	chr1	NPIP	40871070	0
CHB	chr1	NPIP	40921070	0
CHB	chr1	NPIP	40971070	0
CHB	chr1	NPIP	41021070	0
CHB	chr1	NPIP	41071070	0
CHB	chr1	NPIP	41121070	0
CHB	chr1	NPIP	41171070	0
CHB	chr1	NPIP	41221070	0
CHB	chr1	NPIP	41271070	0
CHB	chr1	NPIP	41321070	0
CHB	chr1	NPIP	41371070	0
CHB	chr1	NPIP	41421070	0
CHB	chr1	NPIP	41471070	0
CHB	chr1	NPIP	41521070	0
CHB	chr1	NPIP	41571070	0
CHB	chr1	NPIP	41621070	0
CHB	chr1	NPIP	41671070	0

CHB	chr1	NPIP	41721070	0
CHB	chr1	NPIP	41771070	0
CHB	chr1	NPIP	41821070	0
CHB	chr1	NPIP	41871070	0
CHB	chr1	NPIP	41921070	0
CHB	chr1	NPIP	41971070	25
CHB	chr1	NPIP	42021070	42
CHB	chr1	NPIP	42071070	33
CHB	chr1	NPIP	42121070	16
CHB	chr1	NPIP	42171070	0
CHB	chr1	NPIP	42221070	0
CHB	chr1	NPIP	42271070	0
CHB	chr1	NPIP	42321070	0
CHB	chr1	NPIP	42371070	0
CHB	chr1	NPIP	42421070	0
CHB	chr1	NPIP	42471070	0
CHB	chr1	NPIP	42521070	0
CHB	chr1	NPIP	42571070	0
CHB	chr1	NPIP	42621070	0
CHB	chr1	NPIP	42671070	0
CHB	chr1	NPIP	42721070	0
CHB	chr1	NPIP	42771070	0
CHB	chr1	NPIP	42821070	0
CHB	chr1	NPIP	42871070	0
CHB	chr1	NPIP	42921070	0
CHB	chr1	NPIP	42971070	0
CHB	chr1	NPIP	43021070	0
CHB	chr1	NPIP	43071070	0
CHB	chr1	NPIP	43121070	0
CHB	chr1	NPIP	43171070	0
CHB	chr1	NPIP	43221070	0
CHB	chr1	NPIP	43271070	0
CHB	chr1	NPIP	43321070	0
CHB	chr1	NPIP	43371070	0
CHB	chr1	NPIP	43421070	4
CHB	chr1	NPIP	43471070	25
CHB	chr1	NPIP	43521070	23
CHB	chr1	NPIP	43571070	2
CHB	chr1	NPIP	43621070	0
CHB	chr1	NPIP	43671070	0
CHB	chr1	NPIP	43721070	0
CHB	chr1	NPIP	43771070	0
CHB	chr1	NPIP	43821070	0
CHB	chr1	NPIP	43871070	0
CHB	chr1	NPIP	43921070	0
CHB	chr1	NPIP	43971070	0
CHB	chr1	NPIP	44021070	0
CHB	chr1	NPIP	44071070	0
CHB	chr1	NPIP	44121070	0

CHB	chr1	NPIP	44171070	0
CHB	chr1	NPIP	44221070	0
CHB	chr1	NPIP	44271070	0
CHB	chr1	NPIP	44321070	0
CHB	chr1	NPIP	44371070	0
CHB	chr1	NPIP	44421070	0
CHB	chr1	NPIP	44471070	0
CHB	chr1	NPIP	44521070	0
CHB	chr1	NPIP	44571070	0
CHB	chr1	NPIP	44621070	0
CHB	chr1	NPIP	44671070	0
CHB	chr1	NPIP	44721070	0
CHB	chr1	NPIP	44771070	0
CHB	chr1	NPIP	44821070	0
CHB	chr1	NPIP	44871070	0
CHB	chr1	NPIP	44921070	0
CHB	chr1	NPIP	44971070	0
CHB	chr1	NPIP	45021070	0
CHB	chr1	NPIP	45071070	0
CHB	chr1	NPIP	45121070	0
CHB	chr1	NPIP	45171070	0
CHB	chr1	NPIP	45221070	0
CHB	chr1	NPIP	45271070	0
CHB	chr1	NPIP	45321070	0
CHB	chr1	NPIP	45371070	0
CHB	chr1	NPIP	45421070	0
CHB	chr1	NPIP	45471070	0
CHB	chr1	NPIP	45521070	0
CHB	chr1	NPIP	45571070	0
CHB	chr1	NPIP	45621070	0
CHB	chr1	NPIP	45671070	0
CHB	chr1	NPIP	45721070	0
CHB	chr1	NPIP	45771070	0
CHB	chr1	NPIP	45821070	0
CHB	chr1	NPIP	45871070	0
CHB	chr1	NPIP	45921070	0
CHB	chr1	NPIP	45971070	0
CHB	chr1	NPIP	46021070	0
CHB	chr1	NPIP	46071070	0
CHB	chr1	NPIP	46121070	0
CHB	chr1	NPIP	46171070	0
CHB	chr1	NPIP	46221070	0
CHB	chr1	NPIP	46271070	0
CHB	chr1	NPIP	46321070	0
CHB	chr1	NPIP	46371070	0
CHB	chr1	NPIP	46421070	0
CHB	chr1	NPIP	46471070	0
CHB	chr1	NPIP	46521070	0
CHB	chr1	NPIP	46571070	0

CHB	chr1	NPIP	46621070	5
CHB	chr1	NPIP	46671070	40
CHB	chr1	NPIP	46721070	58
CHB	chr1	NPIP	46771070	65
CHB	chr1	NPIP	46821070	53
CHB	chr1	NPIP	46871070	122
CHB	chr1	NPIP	46921070	130
CHB	chr1	NPIP	46971070	37
CHB	chr1	NPIP	47021070	68
CHB	chr1	NPIP	47071070	63
CHB	chr1	NPIP	47121070	25
CHB	chr1	NPIP	47171070	37
CHB	chr1	NPIP	47221070	37
CHB	chr1	NPIP	47271070	33
CHB	chr1	NPIP	47321070	51
CHB	chr1	NPIP	47371070	58
CHB	chr1	NPIP	47421070	55
CHB	chr1	NPIP	47471070	47
CHB	chr1	NPIP	47521070	31
CHB	chr1	NPIP	47571070	32
CHB	chr1	NPIP	47621070	36
CHB	chr1	NPIP	47671070	38
CHB	chr1	NPIP	47721070	34
CHB	chr1	NPIP	47771070	11
CHB	chr1	NPIP	47821070	0
CHB	chr1	NPIP	47871070	0
CHB	chr1	NPIP	47921070	8
CHB	chr1	NPIP	47971070	55
CHB	chr1	NPIP	48021070	63
CHB	chr1	NPIP	48071070	27
CHB	chr1	NPIP	48121070	11
CHB	chr1	NPIP	48171070	0
CHB	chr1	NPIP	48221070	0
CHB	chr1	NPIP	48271070	0
CHB	chr1	NPIP	48321070	0
CHB	chr1	NPIP	48371070	0
CHB	chr1	NPIP	48421070	0
CHB	chr1	NPIP	48471070	0
CHB	chr1	NPIP	48521070	0
CHB	chr1	NPIP	48571070	0
CHB	chr1	NPIP	48621070	0
CHB	chr1	NPIP	48671070	0
CHB	chr1	NPIP	48721070	0
CHB	chr1	NPIP	48771070	0
CHB	chr1	NPIP	48821070	0
CHB	chr1	NPIP	48871070	0
CHB	chr1	NPIP	48921070	0
CHB	chr1	NPIP	48971070	0
CHB	chr1	NPIP	49021070	0

CHB	chr1	NPIP	49071070	0
CHB	chr1	NPIP	49121070	0
CHB	chr1	NPIP	49171070	0
CHB	chr1	NPIP	49221070	0
CHB	chr1	NPIP	49271070	0
CHB	chr16	NPIP	6977263	0
CHB	chr16	NPIP	7027263	0
CHB	chr16	NPIP	7077263	0
CHB	chr16	NPIP	7127263	0
CHB	chr16	NPIP	7177263	0
CHB	chr16	NPIP	7227263	0
CHB	chr16	NPIP	7277263	0
CHB	chr16	NPIP	7327263	0
CHB	chr16	NPIP	7377263	0
CHB	chr16	NPIP	7427263	0
CHB	chr16	NPIP	7477263	0
CHB	chr16	NPIP	7527263	0
CHB	chr16	NPIP	7577263	0
CHB	chr16	NPIP	7627263	0
CHB	chr16	NPIP	7677263	0
CHB	chr16	NPIP	7727263	0
CHB	chr16	NPIP	7777263	3
CHB	chr16	NPIP	7827263	19
CHB	chr16	NPIP	7877263	49
CHB	chr16	NPIP	7927263	33
CHB	chr16	NPIP	7977263	0
CHB	chr16	NPIP	8027263	0
CHB	chr16	NPIP	8077263	0
CHB	chr16	NPIP	8127263	0
CHB	chr16	NPIP	8177263	0
CHB	chr16	NPIP	8227263	0
CHB	chr16	NPIP	8277263	0
CHB	chr16	NPIP	8327263	0
CHB	chr16	NPIP	8377263	0
CHB	chr16	NPIP	8427263	0
CHB	chr16	NPIP	8477263	0
CHB	chr16	NPIP	8527263	0
CHB	chr16	NPIP	8577263	0
CHB	chr16	NPIP	8627263	0
CHB	chr16	NPIP	8677263	0
CHB	chr16	NPIP	8727263	0
CHB	chr16	NPIP	8777263	0
CHB	chr16	NPIP	8827263	0
CHB	chr16	NPIP	8877263	0
CHB	chr16	NPIP	8927263	8
CHB	chr16	NPIP	8977263	29
CHB	chr16	NPIP	9027263	45
CHB	chr16	NPIP	9077263	58
CHB	chr16	NPIP	9127263	60

CHB	chr16	NPIP	9177263	26
CHB	chr16	NPIP	9227263	0
CHB	chr16	NPIP	9277263	0
CHB	chr16	NPIP	9327263	0
CHB	chr16	NPIP	9377263	0
CHB	chr16	NPIP	9427263	0
CHB	chr16	NPIP	9477263	0
CHB	chr16	NPIP	9527263	0
CHB	chr16	NPIP	9577263	0
CHB	chr16	NPIP	9627263	0
CHB	chr16	NPIP	9677263	0
CHB	chr16	NPIP	9727263	0
CHB	chr16	NPIP	9777263	0
CHB	chr16	NPIP	9827263	0
CHB	chr16	NPIP	9877263	0
CHB	chr16	NPIP	9927263	0
CHB	chr16	NPIP	9977263	0
CHB	chr16	NPIP	10027263	0
CHB	chr16	NPIP	10077263	0
CHB	chr16	NPIP	10127263	0
CHB	chr16	NPIP	10177263	0
CHB	chr16	NPIP	10227263	0
CHB	chr16	NPIP	10277263	0
CHB	chr16	NPIP	10327263	0
CHB	chr16	NPIP	10377263	0
CHB	chr16	NPIP	10427263	0
CHB	chr16	NPIP	10477263	0
CHB	chr16	NPIP	10527263	0
CHB	chr16	NPIP	10577263	0
CHB	chr16	NPIP	10627263	0
CHB	chr16	NPIP	10677263	0
CHB	chr16	NPIP	10727263	0
CHB	chr16	NPIP	10777263	0
CHB	chr16	NPIP	10827263	0
CHB	chr16	NPIP	10877263	0
CHB	chr16	NPIP	10927263	0
CHB	chr16	NPIP	10977263	0
CHB	chr16	NPIP	11027263	0
CHB	chr16	NPIP	11077263	0
CHB	chr16	NPIP	11127263	0
CHB	chr16	NPIP	11177263	0
CHB	chr16	NPIP	11227263	0
CHB	chr16	NPIP	11277263	0
CHB	chr16	NPIP	11327263	0
CHB	chr16	NPIP	11377263	0
CHB	chr16	NPIP	11427263	0
CHB	chr16	NPIP	11477263	0
CHB	chr16	NPIP	11527263	0
CHB	chr16	NPIP	11577263	0

CHB	chr16	NPIP	11627263	0
CHB	chr16	NPIP	11677263	0
CHB	chr16	NPIP	11727263	0
CHB	chr16	NPIP	11777263	0
CHB	chr16	NPIP	11827263	0
CHB	chr16	NPIP	11877263	0
CHB	chr16	NPIP	11927263	0
CHB	chr16	NPIP	11977263	0
CHB	chr16	NPIP	12027263	0
CHB	chr16	NPIP	12077263	0
CHB	chr16	NPIP	12127263	0
CHB	chr16	NPIP	12177263	0
CHB	chr16	NPIP	12227263	0
CHB	chr16	NPIP	12277263	0
CHB	chr16	NPIP	12327263	0
CHB	chr16	NPIP	12377263	0
CHB	chr16	NPIP	12427263	0
CHB	chr16	NPIP	12477263	0
CHB	chr16	NPIP	12527263	0
CHB	chr16	NPIP	12577263	0
CHB	chr16	NPIP	12627263	0
CHB	chr16	NPIP	12677263	0
CHB	chr16	NPIP	12727263	0
CHB	chr16	NPIP	12777263	0
CHB	chr16	NPIP	12827263	0
CHB	chr16	NPIP	12877263	0
CHB	chr16	NPIP	12927263	0
CHB	chr16	NPIP	12977263	0
CHB	chr16	NPIP	13027263	0
CHB	chr16	NPIP	13077263	0
CHB	chr16	NPIP	13127263	0
CHB	chr16	NPIP	13177263	0
CHB	chr16	NPIP	13227263	0
CHB	chr16	NPIP	13277263	0
CHB	chr16	NPIP	13327263	0
CHB	chr16	NPIP	13377263	0
CHB	chr16	NPIP	13427263	0
CHB	chr16	NPIP	13477263	0
CHB	chr16	NPIP	13527263	0
CHB	chr16	NPIP	13577263	0
CHB	chr16	NPIP	13627263	0
CHB	chr16	NPIP	13677263	0
CHB	chr16	NPIP	13727263	0
CHB	chr16	NPIP	13777263	0
CHB	chr16	NPIP	13827263	0
CHB	chr16	NPIP	13877263	0
CHB	chr16	NPIP	13927263	0
CHB	chr16	NPIP	13977263	0
CHB	chr16	NPIP	14027263	0

CHB	chr16	NPIP	14077263	0
CHB	chr16	NPIP	14127263	0
CHB	chr16	NPIP	14177263	0
CHB	chr16	NPIP	14227263	0
CHB	chr16	NPIP	14277263	0
CHB	chr16	NPIP	14327263	0
CHB	chr16	NPIP	14377263	0
CHB	chr16	NPIP	14427263	0
CHB	chr16	NPIP	14477263	0
CHB	chr16	NPIP	14527263	0
CHB	chr16	NPIP	14577263	0
CHB	chr16	NPIP	14627263	0
CHB	chr16	NPIP	14677263	0
CHB	chr16	NPIP	14727263	0
CHB	chr16	NPIP	14777263	0
CHB	chr16	NPIP	14827263	0
CHB	chr16	NPIP	14877263	0
CHB	chr16	NPIP	14927263	0
CHB	chr16	NPIP	14977263	0
CHB	chr16	NPIP	15027263	0
CHB	chr16	NPIP	15077263	0
CHB	chr16	NPIP	15127263	0
CHB	chr16	NPIP	15177263	0
CHB	chr16	NPIP	15227263	0
CHB	chr16	NPIP	15277263	0
CHB	chr16	NPIP	15327263	0
CHB	chr16	NPIP	15377263	0
CHB	chr16	NPIP	15427263	0
CHB	chr16	NPIP	15477263	0
CHB	chr16	NPIP	15527263	0
CHB	chr16	NPIP	15577263	0
CHB	chr16	NPIP	15627263	0
CHB	chr16	NPIP	15677263	0
CHB	chr16	NPIP	15727263	0
CHB	chr16	NPIP	15777263	0
CHB	chr16	NPIP	15827263	0
CHB	chr16	NPIP	15877263	0
CHB	chr16	NPIP	15927263	0
CHB	chr16	NPIP	15977263	0
CHB	chr16	NPIP	16027263	0
CHB	chr16	NPIP	16077263	0
CHB	chr16	NPIP	16127263	0
CHB	chr16	NPIP	16177263	0
CHB	chr16	NPIP	16227263	0
CHB	chr16	NPIP	16277263	0
CHB	chr16	NPIP	16327263	0
CHB	chr16	NPIP	16377263	0
CHB	chr16	NPIP	16427263	0
CHB	chr16	NPIP	16477263	0

CHB	chr16	NPIP	16527263	0
CHB	chr16	NPIP	16577263	0
CHB	chr16	NPIP	16627263	0
CHB	chr16	NPIP	16677263	0
CHB	chr16	NPIP	16727263	0
CHB	chr16	NPIP	16777263	0
CHB	chr16	NPIP	16827263	0
CHB	chr16	NPIP	16877263	0
CHB	chr16	NPIP	9753411	0
CHB	chr16	NPIP	9803411	0
CHB	chr16	NPIP	9853411	0
CHB	chr16	NPIP	9903411	0
CHB	chr16	NPIP	9953411	0
CHB	chr16	NPIP	10003411	0
CHB	chr16	NPIP	10053411	0
CHB	chr16	NPIP	10103411	0
CHB	chr16	NPIP	10153411	0
CHB	chr16	NPIP	10203411	0
CHB	chr16	NPIP	10253411	0
CHB	chr16	NPIP	10303411	0
CHB	chr16	NPIP	10353411	0
CHB	chr16	NPIP	10403411	0
CHB	chr16	NPIP	10453411	0
CHB	chr16	NPIP	10503411	0
CHB	chr16	NPIP	10553411	0
CHB	chr16	NPIP	10603411	0
CHB	chr16	NPIP	10653411	0
CHB	chr16	NPIP	10703411	0
CHB	chr16	NPIP	10753411	0
CHB	chr16	NPIP	10803411	0
CHB	chr16	NPIP	10853411	0
CHB	chr16	NPIP	10903411	0
CHB	chr16	NPIP	10953411	0
CHB	chr16	NPIP	11003411	0
CHB	chr16	NPIP	11053411	0
CHB	chr16	NPIP	11103411	0
CHB	chr16	NPIP	11153411	0
CHB	chr16	NPIP	11203411	0
CHB	chr16	NPIP	11253411	0
CHB	chr16	NPIP	11303411	0
CHB	chr16	NPIP	11353411	0
CHB	chr16	NPIP	11403411	0
CHB	chr16	NPIP	11453411	0
CHB	chr16	NPIP	11503411	0
CHB	chr16	NPIP	11553411	0
CHB	chr16	NPIP	11603411	0
CHB	chr16	NPIP	11653411	0
CHB	chr16	NPIP	11703411	0
CHB	chr16	NPIP	11753411	0

CHB	chr16	NPIP	11803411	0
CHB	chr16	NPIP	11853411	0
CHB	chr16	NPIP	11903411	0
CHB	chr16	NPIP	11953411	0
CHB	chr16	NPIP	12003411	0
CHB	chr16	NPIP	12053411	0
CHB	chr16	NPIP	12103411	0
CHB	chr16	NPIP	12153411	0
CHB	chr16	NPIP	12203411	0
CHB	chr16	NPIP	12253411	0
CHB	chr16	NPIP	12303411	0
CHB	chr16	NPIP	12353411	0
CHB	chr16	NPIP	12403411	0
CHB	chr16	NPIP	12453411	0
CHB	chr16	NPIP	12503411	0
CHB	chr16	NPIP	12553411	0
CHB	chr16	NPIP	12603411	0
CHB	chr16	NPIP	12653411	0
CHB	chr16	NPIP	12703411	0
CHB	chr16	NPIP	12753411	0
CHB	chr16	NPIP	12803411	0
CHB	chr16	NPIP	12853411	0
CHB	chr16	NPIP	12903411	0
CHB	chr16	NPIP	12953411	0
CHB	chr16	NPIP	13003411	0
CHB	chr16	NPIP	13053411	0
CHB	chr16	NPIP	13103411	0
CHB	chr16	NPIP	13153411	0
CHB	chr16	NPIP	13203411	0
CHB	chr16	NPIP	13253411	0
CHB	chr16	NPIP	13303411	0
CHB	chr16	NPIP	13353411	0
CHB	chr16	NPIP	13403411	0
CHB	chr16	NPIP	13453411	0
CHB	chr16	NPIP	13503411	0
CHB	chr16	NPIP	13553411	0
CHB	chr16	NPIP	13603411	0
CHB	chr16	NPIP	13653411	0
CHB	chr16	NPIP	13703411	0
CHB	chr16	NPIP	13753411	0
CHB	chr16	NPIP	13803411	0
CHB	chr16	NPIP	13853411	0
CHB	chr16	NPIP	13903411	0
CHB	chr16	NPIP	13953411	0
CHB	chr16	NPIP	14003411	0
CHB	chr16	NPIP	14053411	0
CHB	chr16	NPIP	14103411	0
CHB	chr16	NPIP	14153411	0
CHB	chr16	NPIP	14203411	0

CHB	chr16	NPIP	14253411	0
CHB	chr16	NPIP	14303411	0
CHB	chr16	NPIP	14353411	0
CHB	chr16	NPIP	14403411	0
CHB	chr16	NPIP	14453411	0
CHB	chr16	NPIP	14503411	0
CHB	chr16	NPIP	14553411	0
CHB	chr16	NPIP	14603411	0
CHB	chr16	NPIP	14653411	0
CHB	chr16	NPIP	14703411	0
CHB	chr16	NPIP	14753411	0
CHB	chr16	NPIP	14803411	0
CHB	chr16	NPIP	14853411	0
CHB	chr16	NPIP	14903411	0
CHB	chr16	NPIP	14953411	0
CHB	chr16	NPIP	15003411	0
CHB	chr16	NPIP	15053411	0
CHB	chr16	NPIP	15103411	0
CHB	chr16	NPIP	15153411	0
CHB	chr16	NPIP	15203411	0
CHB	chr16	NPIP	15253411	0
CHB	chr16	NPIP	15303411	0
CHB	chr16	NPIP	15353411	0
CHB	chr16	NPIP	15403411	0
CHB	chr16	NPIP	15453411	0
CHB	chr16	NPIP	15503411	0
CHB	chr16	NPIP	15553411	0
CHB	chr16	NPIP	15603411	0
CHB	chr16	NPIP	15653411	0
CHB	chr16	NPIP	15703411	0
CHB	chr16	NPIP	15753411	0
CHB	chr16	NPIP	15803411	0
CHB	chr16	NPIP	15853411	0
CHB	chr16	NPIP	15903411	0
CHB	chr16	NPIP	15953411	0
CHB	chr16	NPIP	16003411	0
CHB	chr16	NPIP	16053411	0
CHB	chr16	NPIP	16103411	0
CHB	chr16	NPIP	16153411	0
CHB	chr16	NPIP	16203411	0
CHB	chr16	NPIP	16253411	0
CHB	chr16	NPIP	16303411	0
CHB	chr16	NPIP	16353411	0
CHB	chr16	NPIP	16403411	0
CHB	chr16	NPIP	16453411	0
CHB	chr16	NPIP	16503411	0
CHB	chr16	NPIP	16553411	0
CHB	chr16	NPIP	16603411	0
CHB	chr16	NPIP	16653411	0

CHB	chr16	NPIP	16703411	0
CHB	chr16	NPIP	16753411	0
CHB	chr16	NPIP	16803411	0
CHB	chr16	NPIP	16853411	0
CHB	chr16	NPIP	16903411	0
CHB	chr16	NPIP	16953411	0
CHB	chr16	NPIP	17003411	0
CHB	chr16	NPIP	17053411	0
CHB	chr16	NPIP	17103411	0
CHB	chr16	NPIP	17153411	0
CHB	chr16	NPIP	17203411	0
CHB	chr16	NPIP	17253411	0
CHB	chr16	NPIP	17303411	0
CHB	chr16	NPIP	17353411	0
CHB	chr16	NPIP	17403411	0
CHB	chr16	NPIP	17453411	0
CHB	chr16	NPIP	17503411	0
CHB	chr16	NPIP	17553411	0
CHB	chr16	NPIP	17603411	0
CHB	chr16	NPIP	17653411	0
CHB	chr16	NPIP	17703411	0
CHB	chr16	NPIP	17753411	0
CHB	chr16	NPIP	17803411	0
CHB	chr16	NPIP	17853411	0
CHB	chr16	NPIP	17903411	0
CHB	chr16	NPIP	17953411	0
CHB	chr16	NPIP	18003411	0
CHB	chr16	NPIP	18053411	0
CHB	chr16	NPIP	18103411	0
CHB	chr16	NPIP	18153411	0
CHB	chr16	NPIP	18203411	0
CHB	chr16	NPIP	18253411	0
CHB	chr16	NPIP	18303411	0
CHB	chr16	NPIP	18353411	0
CHB	chr16	NPIP	18403411	0
CHB	chr16	NPIP	18453411	0
CHB	chr16	NPIP	18503411	0
CHB	chr16	NPIP	18553411	0
CHB	chr16	NPIP	18603411	0
CHB	chr16	NPIP	18653411	0
CHB	chr16	NPIP	18703411	0
CHB	chr16	NPIP	18753411	0
CHB	chr16	NPIP	18803411	0
CHB	chr16	NPIP	18853411	0
CHB	chr16	NPIP	18903411	0
CHB	chr16	NPIP	18953411	0
CHB	chr16	NPIP	19003411	0
CHB	chr16	NPIP	19053411	0
CHB	chr16	NPIP	19103411	0

CHB	chr16	NPIP	19153411	0
CHB	chr16	NPIP	19203411	0
CHB	chr16	NPIP	19253411	0
CHB	chr16	NPIP	19303411	0
CHB	chr16	NPIP	19353411	0
CHB	chr16	NPIP	19403411	0
CHB	chr16	NPIP	19453411	0
CHB	chr16	NPIP	19503411	0
CHB	chr16	NPIP	19553411	0
CHB	chr16	NPIP	19603411	0
CHB	chr16	NPIP	19653411	0
CHB	chr16	NPIP	13367919	0
CHB	chr16	NPIP	13417919	0
CHB	chr16	NPIP	13467919	0
CHB	chr16	NPIP	13517919	0
CHB	chr16	NPIP	13567919	0
CHB	chr16	NPIP	13617919	0
CHB	chr16	NPIP	13667919	0
CHB	chr16	NPIP	13717919	0
CHB	chr16	NPIP	13767919	0
CHB	chr16	NPIP	13817919	0
CHB	chr16	NPIP	13867919	0
CHB	chr16	NPIP	13917919	0
CHB	chr16	NPIP	13967919	0
CHB	chr16	NPIP	14017919	0
CHB	chr16	NPIP	14067919	0
CHB	chr16	NPIP	14117919	0
CHB	chr16	NPIP	14167919	0
CHB	chr16	NPIP	14217919	0
CHB	chr16	NPIP	14267919	0
CHB	chr16	NPIP	14317919	0
CHB	chr16	NPIP	14367919	0
CHB	chr16	NPIP	14417919	0
CHB	chr16	NPIP	14467919	0
CHB	chr16	NPIP	14517919	0
CHB	chr16	NPIP	14567919	0
CHB	chr16	NPIP	14617919	0
CHB	chr16	NPIP	14667919	0
CHB	chr16	NPIP	14717919	0
CHB	chr16	NPIP	14767919	0
CHB	chr16	NPIP	14817919	0
CHB	chr16	NPIP	14867919	0
CHB	chr16	NPIP	14917919	0
CHB	chr16	NPIP	14967919	0
CHB	chr16	NPIP	15017919	0
CHB	chr16	NPIP	15067919	0
CHB	chr16	NPIP	15117919	0
CHB	chr16	NPIP	15167919	0
CHB	chr16	NPIP	15217919	0

CHB	chr16	NPIP	15267919	0
CHB	chr16	NPIP	15317919	0
CHB	chr16	NPIP	15367919	0
CHB	chr16	NPIP	15417919	0
CHB	chr16	NPIP	15467919	0
CHB	chr16	NPIP	15517919	0
CHB	chr16	NPIP	15567919	0
CHB	chr16	NPIP	15617919	0
CHB	chr16	NPIP	15667919	0
CHB	chr16	NPIP	15717919	0
CHB	chr16	NPIP	15767919	0
CHB	chr16	NPIP	15817919	0
CHB	chr16	NPIP	15867919	0
CHB	chr16	NPIP	15917919	0
CHB	chr16	NPIP	15967919	0
CHB	chr16	NPIP	16017919	0
CHB	chr16	NPIP	16067919	0
CHB	chr16	NPIP	16117919	0
CHB	chr16	NPIP	16167919	0
CHB	chr16	NPIP	16217919	0
CHB	chr16	NPIP	16267919	0
CHB	chr16	NPIP	16317919	0
CHB	chr16	NPIP	16367919	0
CHB	chr16	NPIP	16417919	0
CHB	chr16	NPIP	16467919	0
CHB	chr16	NPIP	16517919	0
CHB	chr16	NPIP	16567919	0
CHB	chr16	NPIP	16617919	0
CHB	chr16	NPIP	16667919	0
CHB	chr16	NPIP	16717919	0
CHB	chr16	NPIP	16767919	0
CHB	chr16	NPIP	16817919	0
CHB	chr16	NPIP	16867919	0
CHB	chr16	NPIP	16917919	0
CHB	chr16	NPIP	16967919	0
CHB	chr16	NPIP	17017919	0
CHB	chr16	NPIP	17067919	0
CHB	chr16	NPIP	17117919	0
CHB	chr16	NPIP	17167919	0
CHB	chr16	NPIP	17217919	0
CHB	chr16	NPIP	17267919	0
CHB	chr16	NPIP	17317919	0
CHB	chr16	NPIP	17367919	0
CHB	chr16	NPIP	17417919	0
CHB	chr16	NPIP	17467919	0
CHB	chr16	NPIP	17517919	0
CHB	chr16	NPIP	17567919	0
CHB	chr16	NPIP	17617919	0
CHB	chr16	NPIP	17667919	0

CHB	chr16	NPIP	17717919	0
CHB	chr16	NPIP	17767919	0
CHB	chr16	NPIP	17817919	0
CHB	chr16	NPIP	17867919	0
CHB	chr16	NPIP	17917919	0
CHB	chr16	NPIP	17967919	0
CHB	chr16	NPIP	18017919	0
CHB	chr16	NPIP	18067919	0
CHB	chr16	NPIP	18117919	0
CHB	chr16	NPIP	18167919	0
CHB	chr16	NPIP	18217919	0
CHB	chr16	NPIP	18267919	0
CHB	chr16	NPIP	18317919	0
CHB	chr16	NPIP	18367919	0
CHB	chr16	NPIP	18417919	0
CHB	chr16	NPIP	18467919	0
CHB	chr16	NPIP	18517919	0
CHB	chr16	NPIP	18567919	0
CHB	chr16	NPIP	18617919	0
CHB	chr16	NPIP	18667919	0
CHB	chr16	NPIP	18717919	0
CHB	chr16	NPIP	18767919	0
CHB	chr16	NPIP	18817919	0
CHB	chr16	NPIP	18867919	0
CHB	chr16	NPIP	18917919	0
CHB	chr16	NPIP	18967919	0
CHB	chr16	NPIP	19017919	0
CHB	chr16	NPIP	19067919	0
CHB	chr16	NPIP	19117919	0
CHB	chr16	NPIP	19167919	0
CHB	chr16	NPIP	19217919	0
CHB	chr16	NPIP	19267919	0
CHB	chr16	NPIP	19317919	0
CHB	chr16	NPIP	19367919	0
CHB	chr16	NPIP	19417919	0
CHB	chr16	NPIP	19467919	0
CHB	chr16	NPIP	19517919	0
CHB	chr16	NPIP	19567919	0
CHB	chr16	NPIP	19617919	0
CHB	chr16	NPIP	19667919	0
CHB	chr16	NPIP	19717919	0
CHB	chr16	NPIP	19767919	0
CHB	chr16	NPIP	19817919	0
CHB	chr16	NPIP	19867919	0
CHB	chr16	NPIP	19917919	0
CHB	chr16	NPIP	19967919	0
CHB	chr16	NPIP	20017919	0
CHB	chr16	NPIP	20067919	0
CHB	chr16	NPIP	20117919	0

CHB	chr16	NPIP	20167919	0
CHB	chr16	NPIP	20217919	0
CHB	chr16	NPIP	20267919	0
CHB	chr16	NPIP	20317919	0
CHB	chr16	NPIP	20367919	0
CHB	chr16	NPIP	20417919	0
CHB	chr16	NPIP	20467919	0
CHB	chr16	NPIP	20517919	0
CHB	chr16	NPIP	20567919	0
CHB	chr16	NPIP	20617919	0
CHB	chr16	NPIP	20667919	0
CHB	chr16	NPIP	20717919	0
CHB	chr16	NPIP	20767919	0
CHB	chr16	NPIP	20817919	0
CHB	chr16	NPIP	20867919	0
CHB	chr16	NPIP	20917919	0
CHB	chr16	NPIP	20967919	0
CHB	chr16	NPIP	21017919	0
CHB	chr16	NPIP	21067919	0
CHB	chr16	NPIP	21117919	0
CHB	chr16	NPIP	21167919	0
CHB	chr16	NPIP	21217919	2
CHB	chr16	NPIP	21267919	36
CHB	chr16	NPIP	21317919	49
CHB	chr16	NPIP	21367919	23
CHB	chr16	NPIP	21417919	8
CHB	chr16	NPIP	21467919	0
CHB	chr16	NPIP	21517919	39
CHB	chr16	NPIP	21567919	69
CHB	chr16	NPIP	21617919	82
CHB	chr16	NPIP	21667919	68
CHB	chr16	NPIP	21717919	20
CHB	chr16	NPIP	21767919	4
CHB	chr16	NPIP	21817919	0
CHB	chr16	NPIP	21867919	0
CHB	chr16	NPIP	21917919	0
CHB	chr16	NPIP	21967919	0
CHB	chr16	NPIP	22017919	0
CHB	chr16	NPIP	22067919	0
CHB	chr16	NPIP	22117919	0
CHB	chr16	NPIP	22167919	0
CHB	chr16	NPIP	22217919	0
CHB	chr16	NPIP	22267919	0
CHB	chr16	NPIP	22317919	0
CHB	chr16	NPIP	22367919	0
CHB	chr16	NPIP	22417919	0
CHB	chr16	NPIP	22467919	0
CHB	chr16	NPIP	22517919	0
CHB	chr16	NPIP	22567919	0

CHB	chr16	NPIP	22617919	0
CHB	chr16	NPIP	22667919	0
CHB	chr16	NPIP	22717919	0
CHB	chr16	NPIP	22767919	0
CHB	chr16	NPIP	22817919	0
CHB	chr16	NPIP	22867919	0
CHB	chr16	NPIP	22917919	0
CHB	chr16	NPIP	22967919	0
CHB	chr16	NPIP	23017919	0
CHB	chr16	NPIP	23067919	0
CHB	chr16	NPIP	23117919	0
CHB	chr16	NPIP	23167919	0
CHB	chr16	NPIP	23217919	0
CHB	chr16	NPIP	23267919	0
CHB	chr16	NPIP	17553599	0
CHB	chr16	NPIP	17603599	0
CHB	chr16	NPIP	17653599	0
CHB	chr16	NPIP	17703599	0
CHB	chr16	NPIP	17753599	0
CHB	chr16	NPIP	17803599	0
CHB	chr16	NPIP	17853599	0
CHB	chr16	NPIP	17903599	0
CHB	chr16	NPIP	17953599	0
CHB	chr16	NPIP	18003599	0
CHB	chr16	NPIP	18053599	0
CHB	chr16	NPIP	18103599	0
CHB	chr16	NPIP	18153599	0
CHB	chr16	NPIP	18203599	0
CHB	chr16	NPIP	18253599	0
CHB	chr16	NPIP	18303599	0
CHB	chr16	NPIP	18353599	0
CHB	chr16	NPIP	18403599	0
CHB	chr16	NPIP	18453599	0
CHB	chr16	NPIP	18503599	0
CHB	chr16	NPIP	18553599	0
CHB	chr16	NPIP	18603599	0
CHB	chr16	NPIP	18653599	0
CHB	chr16	NPIP	18703599	0
CHB	chr16	NPIP	18753599	0
CHB	chr16	NPIP	18803599	0
CHB	chr16	NPIP	18853599	0
CHB	chr16	NPIP	18903599	0
CHB	chr16	NPIP	18953599	0
CHB	chr16	NPIP	19003599	0
CHB	chr16	NPIP	19053599	0
CHB	chr16	NPIP	19103599	0
CHB	chr16	NPIP	19153599	0
CHB	chr16	NPIP	19203599	0
CHB	chr16	NPIP	19253599	0

CHB	chr16	NPIP	19303599	0
CHB	chr16	NPIP	19353599	0
CHB	chr16	NPIP	19403599	0
CHB	chr16	NPIP	19453599	0
CHB	chr16	NPIP	19503599	0
CHB	chr16	NPIP	19553599	0
CHB	chr16	NPIP	19603599	0
CHB	chr16	NPIP	19653599	0
CHB	chr16	NPIP	19703599	0
CHB	chr16	NPIP	19753599	0
CHB	chr16	NPIP	19803599	0
CHB	chr16	NPIP	19853599	0
CHB	chr16	NPIP	19903599	0
CHB	chr16	NPIP	19953599	0
CHB	chr16	NPIP	20003599	0
CHB	chr16	NPIP	20053599	0
CHB	chr16	NPIP	20103599	0
CHB	chr16	NPIP	20153599	0
CHB	chr16	NPIP	20203599	0
CHB	chr16	NPIP	20253599	0
CHB	chr16	NPIP	20303599	0
CHB	chr16	NPIP	20353599	0
CHB	chr16	NPIP	20403599	0
CHB	chr16	NPIP	20453599	0
CHB	chr16	NPIP	20503599	0
CHB	chr16	NPIP	20553599	0
CHB	chr16	NPIP	20603599	0
CHB	chr16	NPIP	20653599	0
CHB	chr16	NPIP	20703599	0
CHB	chr16	NPIP	20753599	0
CHB	chr16	NPIP	20803599	0
CHB	chr16	NPIP	20853599	0
CHB	chr16	NPIP	20903599	0
CHB	chr16	NPIP	20953599	0
CHB	chr16	NPIP	21003599	0
CHB	chr16	NPIP	21053599	0
CHB	chr16	NPIP	21103599	0
CHB	chr16	NPIP	21153599	0
CHB	chr16	NPIP	21203599	0
CHB	chr16	NPIP	21253599	25
CHB	chr16	NPIP	21303599	48
CHB	chr16	NPIP	21353599	34
CHB	chr16	NPIP	21403599	11
CHB	chr16	NPIP	21453599	0
CHB	chr16	NPIP	21503599	38
CHB	chr16	NPIP	21553599	56
CHB	chr16	NPIP	21603599	76
CHB	chr16	NPIP	21653599	77
CHB	chr16	NPIP	21703599	27

CHB	chr16	NPIP	21753599	8
CHB	chr16	NPIP	21803599	0
CHB	chr16	NPIP	21853599	0
CHB	chr16	NPIP	21903599	0
CHB	chr16	NPIP	21953599	0
CHB	chr16	NPIP	22003599	0
CHB	chr16	NPIP	22053599	0
CHB	chr16	NPIP	22103599	0
CHB	chr16	NPIP	22153599	0
CHB	chr16	NPIP	22203599	0
CHB	chr16	NPIP	22253599	0
CHB	chr16	NPIP	22303599	0
CHB	chr16	NPIP	22353599	0
CHB	chr16	NPIP	22403599	0
CHB	chr16	NPIP	22453599	0
CHB	chr16	NPIP	22503599	0
CHB	chr16	NPIP	22553599	0
CHB	chr16	NPIP	22603599	0
CHB	chr16	NPIP	22653599	0
CHB	chr16	NPIP	22703599	0
CHB	chr16	NPIP	22753599	0
CHB	chr16	NPIP	22803599	0
CHB	chr16	NPIP	22853599	0
CHB	chr16	NPIP	22903599	0
CHB	chr16	NPIP	22953599	0
CHB	chr16	NPIP	23003599	0
CHB	chr16	NPIP	23053599	0
CHB	chr16	NPIP	23103599	0
CHB	chr16	NPIP	23153599	0
CHB	chr16	NPIP	23203599	0
CHB	chr16	NPIP	23253599	0
CHB	chr16	NPIP	23303599	0
CHB	chr16	NPIP	23353599	0
CHB	chr16	NPIP	23403599	0
CHB	chr16	NPIP	23453599	0
CHB	chr16	NPIP	23503599	0
CHB	chr16	NPIP	23553599	0
CHB	chr16	NPIP	23603599	0
CHB	chr16	NPIP	23653599	0
CHB	chr16	NPIP	23703599	0
CHB	chr16	NPIP	23753599	0
CHB	chr16	NPIP	23803599	0
CHB	chr16	NPIP	23853599	0
CHB	chr16	NPIP	23903599	0
CHB	chr16	NPIP	23953599	0
CHB	chr16	NPIP	24003599	0
CHB	chr16	NPIP	24053599	0
CHB	chr16	NPIP	24103599	0
CHB	chr16	NPIP	24153599	0

CHB	chr16	NPIP	24203599	0
CHB	chr16	NPIP	24253599	0
CHB	chr16	NPIP	24303599	0
CHB	chr16	NPIP	24353599	0
CHB	chr16	NPIP	24403599	0
CHB	chr16	NPIP	24453599	0
CHB	chr16	NPIP	24503599	0
CHB	chr16	NPIP	24553599	0
CHB	chr16	NPIP	24603599	0
CHB	chr16	NPIP	24653599	0
CHB	chr16	NPIP	24703599	0
CHB	chr16	NPIP	24753599	0
CHB	chr16	NPIP	24803599	0
CHB	chr16	NPIP	24853599	0
CHB	chr16	NPIP	24903599	0
CHB	chr16	NPIP	24953599	0
CHB	chr16	NPIP	25003599	0
CHB	chr16	NPIP	25053599	0
CHB	chr16	NPIP	25103599	0
CHB	chr16	NPIP	25153599	0
CHB	chr16	NPIP	25203599	0
CHB	chr16	NPIP	25253599	0
CHB	chr16	NPIP	25303599	0
CHB	chr16	NPIP	25353599	0
CHB	chr16	NPIP	25403599	0
CHB	chr16	NPIP	25453599	0
CHB	chr16	NPIP	25503599	0
CHB	chr16	NPIP	25553599	0
CHB	chr16	NPIP	25603599	0
CHB	chr16	NPIP	25653599	0
CHB	chr16	NPIP	25703599	0
CHB	chr16	NPIP	25753599	0
CHB	chr16	NPIP	25803599	0
CHB	chr16	NPIP	25853599	0
CHB	chr16	NPIP	25903599	7
CHB	chr16	NPIP	25953599	38
CHB	chr16	NPIP	26003599	31
CHB	chr16	NPIP	26053599	0
CHB	chr16	NPIP	26103599	0
CHB	chr16	NPIP	26153599	0
CHB	chr16	NPIP	26203599	0
CHB	chr16	NPIP	26253599	0
CHB	chr16	NPIP	26303599	0
CHB	chr16	NPIP	26353599	0
CHB	chr16	NPIP	26403599	0
CHB	chr16	NPIP	26453599	0
CHB	chr16	NPIP	26503599	0
CHB	chr16	NPIP	26553599	0
CHB	chr16	NPIP	26603599	0

CHB	chr16	NPIP	26653599	0
CHB	chr16	NPIP	26703599	0
CHB	chr16	NPIP	26753599	0
CHB	chr16	NPIP	26803599	0
CHB	chr16	NPIP	26853599	0
CHB	chr16	NPIP	26903599	0
CHB	chr16	NPIP	26953599	0
CHB	chr16	NPIP	27003599	0
CHB	chr16	NPIP	27053599	0
CHB	chr16	NPIP	27103599	0
CHB	chr16	NPIP	27153599	0
CHB	chr16	NPIP	27203599	0
CHB	chr16	NPIP	27253599	0
CHB	chr16	NPIP	27303599	0
CHB	chr16	NPIP	27353599	0
CHB	chr16	NPIP	27403599	0
CHB	chr16	NPIP	27453599	0
CHB	chr16	NPIP	23392517	0
CHB	chr16	NPIP	23442517	0
CHB	chr16	NPIP	23492517	0
CHB	chr16	NPIP	23542517	0
CHB	chr16	NPIP	23592517	0
CHB	chr16	NPIP	23642517	0
CHB	chr16	NPIP	23692517	0
CHB	chr16	NPIP	23742517	0
CHB	chr16	NPIP	23792517	0
CHB	chr16	NPIP	23842517	0
CHB	chr16	NPIP	23892517	0
CHB	chr16	NPIP	23942517	0
CHB	chr16	NPIP	23992517	0
CHB	chr16	NPIP	24042517	0
CHB	chr16	NPIP	24092517	0
CHB	chr16	NPIP	24142517	0
CHB	chr16	NPIP	24192517	0
CHB	chr16	NPIP	24242517	0
CHB	chr16	NPIP	24292517	0
CHB	chr16	NPIP	24342517	0
CHB	chr16	NPIP	24392517	0
CHB	chr16	NPIP	24442517	0
CHB	chr16	NPIP	24492517	0
CHB	chr16	NPIP	24542517	0
CHB	chr16	NPIP	24592517	0
CHB	chr16	NPIP	24642517	0
CHB	chr16	NPIP	24692517	0
CHB	chr16	NPIP	24742517	0
CHB	chr16	NPIP	24792517	0
CHB	chr16	NPIP	24842517	0
CHB	chr16	NPIP	24892517	0
CHB	chr16	NPIP	24942517	0

CHB	chr16	NPIP	24992517	0
CHB	chr16	NPIP	25042517	0
CHB	chr16	NPIP	25092517	0
CHB	chr16	NPIP	25142517	0
CHB	chr16	NPIP	25192517	0
CHB	chr16	NPIP	25242517	0
CHB	chr16	NPIP	25292517	0
CHB	chr16	NPIP	25342517	0
CHB	chr16	NPIP	25392517	0
CHB	chr16	NPIP	25442517	0
CHB	chr16	NPIP	25492517	0
CHB	chr16	NPIP	25542517	0
CHB	chr16	NPIP	25592517	0
CHB	chr16	NPIP	25642517	0
CHB	chr16	NPIP	25692517	0
CHB	chr16	NPIP	25742517	0
CHB	chr16	NPIP	25792517	0
CHB	chr16	NPIP	25842517	0
CHB	chr16	NPIP	25892517	5
CHB	chr16	NPIP	25942517	38
CHB	chr16	NPIP	25992517	33
CHB	chr16	NPIP	26042517	0
CHB	chr16	NPIP	26092517	0
CHB	chr16	NPIP	26142517	0
CHB	chr16	NPIP	26192517	0
CHB	chr16	NPIP	26242517	0
CHB	chr16	NPIP	26292517	0
CHB	chr16	NPIP	26342517	0
CHB	chr16	NPIP	26392517	0
CHB	chr16	NPIP	26442517	0
CHB	chr16	NPIP	26492517	0
CHB	chr16	NPIP	26542517	0
CHB	chr16	NPIP	26592517	0
CHB	chr16	NPIP	26642517	0
CHB	chr16	NPIP	26692517	0
CHB	chr16	NPIP	26742517	0
CHB	chr16	NPIP	26792517	0
CHB	chr16	NPIP	26842517	0
CHB	chr16	NPIP	26892517	0
CHB	chr16	NPIP	26942517	0
CHB	chr16	NPIP	26992517	0
CHB	chr16	NPIP	27042517	0
CHB	chr16	NPIP	27092517	0
CHB	chr16	NPIP	27142517	0
CHB	chr16	NPIP	27192517	0
CHB	chr16	NPIP	27242517	0
CHB	chr16	NPIP	27292517	0
CHB	chr16	NPIP	27342517	0
CHB	chr16	NPIP	27392517	0

CHB	chr16	NPIP	27442517	0
CHB	chr16	NPIP	27492517	0
CHB	chr16	NPIP	27542517	0
CHB	chr16	NPIP	27592517	0
CHB	chr16	NPIP	27642517	0
CHB	chr16	NPIP	27692517	0
CHB	chr16	NPIP	27742517	0
CHB	chr16	NPIP	27792517	0
CHB	chr16	NPIP	27842517	0
CHB	chr16	NPIP	27892517	0
CHB	chr16	NPIP	27942517	0
CHB	chr16	NPIP	27992517	0
CHB	chr16	NPIP	28042517	0
CHB	chr16	NPIP	28092517	0
CHB	chr16	NPIP	28142517	0
CHB	chr16	NPIP	28192517	0
CHB	chr16	NPIP	28242517	0
CHB	chr16	NPIP	28292517	0
CHB	chr16	NPIP	28342517	0
CHB	chr16	NPIP	28392517	0
CHB	chr16	NPIP	28442517	0
CHB	chr16	NPIP	28492517	0
CHB	chr16	NPIP	28542517	0
CHB	chr16	NPIP	28592517	0
CHB	chr16	NPIP	28642517	0
CHB	chr16	NPIP	28692517	0
CHB	chr16	NPIP	28742517	0
CHB	chr16	NPIP	28792517	0
CHB	chr16	NPIP	28842517	0
CHB	chr16	NPIP	28892517	0
CHB	chr16	NPIP	28942517	0
CHB	chr16	NPIP	28992517	0
CHB	chr16	NPIP	29042517	0
CHB	chr16	NPIP	29092517	0
CHB	chr16	NPIP	29142517	0
CHB	chr16	NPIP	29192517	0
CHB	chr16	NPIP	29242517	0
CHB	chr16	NPIP	29292517	0
CHB	chr16	NPIP	29342517	0
CHB	chr16	NPIP	29392517	0
CHB	chr16	NPIP	29442517	0
CHB	chr16	NPIP	29492517	0
CHB	chr16	NPIP	29542517	0
CHB	chr16	NPIP	29592517	0
CHB	chr16	NPIP	29642517	0
CHB	chr16	NPIP	29692517	0
CHB	chr16	NPIP	29742517	0
CHB	chr16	NPIP	29792517	0
CHB	chr16	NPIP	29842517	0

CHB	chr16	NPIP	29892517	0
CHB	chr16	NPIP	29942517	0
CHB	chr16	NPIP	29992517	0
CHB	chr16	NPIP	30042517	0
CHB	chr16	NPIP	30092517	0
CHB	chr16	NPIP	30142517	0
CHB	chr16	NPIP	30192517	0
CHB	chr16	NPIP	30242517	0
CHB	chr16	NPIP	30292517	0
CHB	chr16	NPIP	30342517	0
CHB	chr16	NPIP	30392517	0
CHB	chr16	NPIP	30442517	0
CHB	chr16	NPIP	30492517	0
CHB	chr16	NPIP	30542517	0
CHB	chr16	NPIP	30592517	0
CHB	chr16	NPIP	30642517	0
CHB	chr16	NPIP	30692517	0
CHB	chr16	NPIP	30742517	0
CHB	chr16	NPIP	30792517	0
CHB	chr16	NPIP	30842517	0
CHB	chr16	NPIP	30892517	0
CHB	chr16	NPIP	30942517	0
CHB	chr16	NPIP	30992517	0
CHB	chr16	NPIP	31042517	0
CHB	chr16	NPIP	31092517	0
CHB	chr16	NPIP	31142517	0
CHB	chr16	NPIP	31192517	0
CHB	chr16	NPIP	31242517	0
CHB	chr16	NPIP	31292517	0
CHB	chr16	NPIP	31342517	0
CHB	chr16	NPIP	31392517	0
CHB	chr16	NPIP	31442517	0
CHB	chr16	NPIP	31492517	0
CHB	chr16	NPIP	31542517	0
CHB	chr16	NPIP	31592517	0
CHB	chr16	NPIP	31642517	0
CHB	chr16	NPIP	31692517	0
CHB	chr16	NPIP	31742517	0
CHB	chr16	NPIP	31792517	0
CHB	chr16	NPIP	31842517	0
CHB	chr16	NPIP	31892517	0
CHB	chr16	NPIP	31942517	0
CHB	chr16	NPIP	31992517	0
CHB	chr16	NPIP	32042517	0
CHB	chr16	NPIP	32092517	0
CHB	chr16	NPIP	32142517	0
CHB	chr16	NPIP	32192517	0
CHB	chr16	NPIP	32242517	0
CHB	chr16	NPIP	32292517	0

CHB	chr16	NPIP	32342517	0
CHB	chr16	NPIP	32392517	0
CHB	chr16	NPIP	32442517	0
CHB	chr16	NPIP	32492517	0
CHB	chr16	NPIP	32542517	0
CHB	chr16	NPIP	32592517	0
CHB	chr16	NPIP	32642517	0
CHB	chr16	NPIP	32692517	0
CHB	chr16	NPIP	32742517	0
CHB	chr16	NPIP	32792517	0
CHB	chr16	NPIP	32842517	0
CHB	chr16	NPIP	32892517	0
CHB	chr16	NPIP	32942517	0
CHB	chr16	NPIP	32992517	0
CHB	chr16	NPIP	33042517	0
CHB	chr16	NPIP	33092517	0
CHB	chr16	NPIP	33142517	0
CHB	chr16	NPIP	33192517	0
CHB	chr16	NPIP	33242517	0
CHB	chr16	NPIP	33292517	0
CHB	chr16	NPIP	69424986	0
CHB	chr16	NPIP	69474986	0
CHB	chr16	NPIP	69524986	0
CHB	chr16	NPIP	69574986	0
CHB	chr16	NPIP	69624986	0
CHB	chr16	NPIP	69674986	0
CHB	chr16	NPIP	69724986	0
CHB	chr16	NPIP	69774986	0
CHB	chr16	NPIP	69824986	0
CHB	chr16	NPIP	69874986	0
CHB	chr16	NPIP	69924986	0
CHB	chr16	NPIP	69974986	0
CHB	chr16	NPIP	70024986	0
CHB	chr16	NPIP	70074986	0
CHB	chr16	NPIP	70124986	0
CHB	chr16	NPIP	70174986	0
CHB	chr16	NPIP	70224986	0
CHB	chr16	NPIP	70274986	0
CHB	chr16	NPIP	70324986	0
CHB	chr16	NPIP	70374986	0
CHB	chr16	NPIP	70424986	0
CHB	chr16	NPIP	70474986	0
CHB	chr16	NPIP	70524986	0
CHB	chr16	NPIP	70574986	0
CHB	chr16	NPIP	70624986	0
CHB	chr16	NPIP	70674986	0
CHB	chr16	NPIP	70724986	0
CHB	chr16	NPIP	70774986	0
CHB	chr16	NPIP	70824986	0

CHB	chr16	NPIP	70874986	0
CHB	chr16	NPIP	70924986	0
CHB	chr16	NPIP	70974986	0
CHB	chr16	NPIP	71024986	0
CHB	chr16	NPIP	71074986	0
CHB	chr16	NPIP	71124986	0
CHB	chr16	NPIP	71174986	0
CHB	chr16	NPIP	71224986	0
CHB	chr16	NPIP	71274986	0
CHB	chr16	NPIP	71324986	0
CHB	chr16	NPIP	71374986	0
CHB	chr16	NPIP	71424986	0
CHB	chr16	NPIP	71474986	0
CHB	chr16	NPIP	71524986	0
CHB	chr16	NPIP	71574986	0
CHB	chr16	NPIP	71624986	0
CHB	chr16	NPIP	71674986	0
CHB	chr16	NPIP	71724986	0
CHB	chr16	NPIP	71774986	0
CHB	chr16	NPIP	71824986	0
CHB	chr16	NPIP	71874986	0
CHB	chr16	NPIP	71924986	0
CHB	chr16	NPIP	71974986	0
CHB	chr16	NPIP	72024986	0
CHB	chr16	NPIP	72074986	0
CHB	chr16	NPIP	72124986	0
CHB	chr16	NPIP	72174986	0
CHB	chr16	NPIP	72224986	0
CHB	chr16	NPIP	72274986	0
CHB	chr16	NPIP	72324986	0
CHB	chr16	NPIP	72374986	0
CHB	chr16	NPIP	72424986	0
CHB	chr16	NPIP	72474986	0
CHB	chr16	NPIP	72524986	0
CHB	chr16	NPIP	72574986	0
CHB	chr16	NPIP	72624986	0
CHB	chr16	NPIP	72674986	0
CHB	chr16	NPIP	72724986	0
CHB	chr16	NPIP	72774986	0
CHB	chr16	NPIP	72824986	0
CHB	chr16	NPIP	72874986	6
CHB	chr16	NPIP	72924986	30
CHB	chr16	NPIP	72974986	48
CHB	chr16	NPIP	73024986	39
CHB	chr16	NPIP	73074986	35
CHB	chr16	NPIP	73124986	20
CHB	chr16	NPIP	73174986	0
CHB	chr16	NPIP	73224986	0
CHB	chr16	NPIP	73274986	0

CHB	chr16	NPIP	73324986	0
CHB	chr16	NPIP	73374986	0
CHB	chr16	NPIP	73424986	0
CHB	chr16	NPIP	73474986	0
CHB	chr16	NPIP	73524986	0
CHB	chr16	NPIP	73574986	0
CHB	chr16	NPIP	73624986	0
CHB	chr16	NPIP	73674986	0
CHB	chr16	NPIP	73724986	0
CHB	chr16	NPIP	73774986	0
CHB	chr16	NPIP	73824986	0
CHB	chr16	NPIP	73874986	0
CHB	chr16	NPIP	73924986	0
CHB	chr16	NPIP	73974986	0
CHB	chr16	NPIP	74024986	0
CHB	chr16	NPIP	74074986	0
CHB	chr16	NPIP	74124986	0
CHB	chr16	NPIP	74174986	0
CHB	chr16	NPIP	74224986	0
CHB	chr16	NPIP	74274986	0
CHB	chr16	NPIP	74324986	0
CHB	chr16	NPIP	74374986	0
CHB	chr16	NPIP	74424986	31
CHB	chr16	NPIP	74474986	62
CHB	chr16	NPIP	74524986	44
CHB	chr16	NPIP	74574986	35
CHB	chr16	NPIP	74624986	46
CHB	chr16	NPIP	74674986	53
CHB	chr16	NPIP	74724986	35
CHB	chr16	NPIP	74774986	6
CHB	chr16	NPIP	74824986	0
CHB	chr16	NPIP	74874986	0
CHB	chr16	NPIP	74924986	0
CHB	chr16	NPIP	74974986	0
CHB	chr16	NPIP	75024986	0
CHB	chr16	NPIP	75074986	0
CHB	chr16	NPIP	75124986	0
CHB	chr16	NPIP	75174986	0
CHB	chr16	NPIP	75224986	0
CHB	chr16	NPIP	75274986	0
CHB	chr16	NPIP	75324986	0
CHB	chr16	NPIP	75374986	0
CHB	chr16	NPIP	75424986	0
CHB	chr16	NPIP	75474986	0
CHB	chr16	NPIP	75524986	0
CHB	chr16	NPIP	75574986	0
CHB	chr16	NPIP	75624986	0
CHB	chr16	NPIP	75674986	0
CHB	chr16	NPIP	75724986	0

CHB	chr16	NPIP	75774986	0
CHB	chr16	NPIP	75824986	0
CHB	chr16	NPIP	75874986	0
CHB	chr16	NPIP	75924986	0
CHB	chr16	NPIP	75974986	0
CHB	chr16	NPIP	76024986	0
CHB	chr16	NPIP	76074986	0
CHB	chr16	NPIP	76124986	0
CHB	chr16	NPIP	76174986	0
CHB	chr16	NPIP	76224986	0
CHB	chr16	NPIP	76274986	0
CHB	chr16	NPIP	76324986	0
CHB	chr16	NPIP	76374986	0
CHB	chr16	NPIP	76424986	0
CHB	chr16	NPIP	76474986	0
CHB	chr16	NPIP	76524986	0
CHB	chr16	NPIP	76574986	0
CHB	chr16	NPIP	76624986	0
CHB	chr16	NPIP	76674986	0
CHB	chr16	NPIP	76724986	7
CHB	chr16	NPIP	76774986	20
CHB	chr16	NPIP	76824986	44
CHB	chr16	NPIP	76874986	39
CHB	chr16	NPIP	76924986	22
CHB	chr16	NPIP	76974986	33
CHB	chr16	NPIP	77024986	33
CHB	chr16	NPIP	77074986	24
CHB	chr16	NPIP	77124986	44
CHB	chr16	NPIP	77174986	49
CHB	chr16	NPIP	77224986	25
CHB	chr16	NPIP	77274986	38
CHB	chr16	NPIP	77324986	89
CHB	chr16	NPIP	77374986	94
CHB	chr16	NPIP	77424986	55
CHB	chr16	NPIP	77474986	32
CHB	chr16	NPIP	77524986	39
CHB	chr16	NPIP	77574986	54
CHB	chr16	NPIP	77624986	46
CHB	chr16	NPIP	77674986	37
CHB	chr16	NPIP	77724986	26
CHB	chr16	NPIP	77774986	22
CHB	chr16	NPIP	77824986	30
CHB	chr16	NPIP	77874986	31
CHB	chr16	NPIP	77924986	33
CHB	chr16	NPIP	77974986	36
CHB	chr16	NPIP	78024986	37
CHB	chr16	NPIP	78074986	103
CHB	chr16	NPIP	78124986	110
CHB	chr16	NPIP	78174986	67

CHB	chr16	NPIP	78224986	66
CHB	chr16	NPIP	78274986	40
CHB	chr16	NPIP	78324986	37
CHB	chr16	NPIP	78374986	96
CHB	chr16	NPIP	78424986	90
CHB	chr16	NPIP	78474986	24
CHB	chr16	NPIP	78524986	18
CHB	chr16	NPIP	78574986	21
CHB	chr16	NPIP	78624986	21
CHB	chr16	NPIP	78674986	71
CHB	chr16	NPIP	78724986	124
CHB	chr16	NPIP	78774986	67
CHB	chr16	NPIP	78824986	2
CHB	chr16	NPIP	78874986	0
CHB	chr16	NPIP	78924986	0
CHB	chr16	NPIP	78974986	0
CHB	chr16	NPIP	79024986	0
CHB	chr16	NPIP	79074986	0
CHB	chr16	NPIP	79124986	0
CHB	chr16	NPIP	79174986	2
CHB	chr16	NPIP	79224986	73
CHB	chr16	NPIP	79274986	100
CHB	chr16	NPIP	79324986	29
CHB	chr11	PGA3	56253515	0
CHB	chr11	PGA3	56303515	0
CHB	chr11	PGA3	56353515	0
CHB	chr11	PGA3	56403515	0
CHB	chr11	PGA3	56453515	0
CHB	chr11	PGA3	56503515	0
CHB	chr11	PGA3	56553515	0
CHB	chr11	PGA3	56603515	0
CHB	chr11	PGA3	56653515	0
CHB	chr11	PGA3	56703515	0
CHB	chr11	PGA3	56753515	0
CHB	chr11	PGA3	56803515	0
CHB	chr11	PGA3	56853515	0
CHB	chr11	PGA3	56903515	0
CHB	chr11	PGA3	56953515	0
CHB	chr11	PGA3	57003515	0
CHB	chr11	PGA3	57053515	0
CHB	chr11	PGA3	57103515	0
CHB	chr11	PGA3	57153515	0
CHB	chr11	PGA3	57203515	0
CHB	chr11	PGA3	57253515	0
CHB	chr11	PGA3	57303515	0
CHB	chr11	PGA3	57353515	0
CHB	chr11	PGA3	57403515	0
CHB	chr11	PGA3	57453515	0
CHB	chr11	PGA3	57503515	0

CHB	chr11	PGA3	57553515	0
CHB	chr11	PGA3	57603515	0
CHB	chr11	PGA3	57653515	0
CHB	chr11	PGA3	57703515	0
CHB	chr11	PGA3	57753515	0
CHB	chr11	PGA3	57803515	0
CHB	chr11	PGA3	57853515	0
CHB	chr11	PGA3	57903515	0
CHB	chr11	PGA3	57953515	0
CHB	chr11	PGA3	58003515	0
CHB	chr11	PGA3	58053515	0
CHB	chr11	PGA3	58103515	0
CHB	chr11	PGA3	58153515	0
CHB	chr11	PGA3	58203515	0
CHB	chr11	PGA3	58253515	0
CHB	chr11	PGA3	58303515	0
CHB	chr11	PGA3	58353515	0
CHB	chr11	PGA3	58403515	0
CHB	chr11	PGA3	58453515	0
CHB	chr11	PGA3	58503515	0
CHB	chr11	PGA3	58553515	0
CHB	chr11	PGA3	58603515	0
CHB	chr11	PGA3	58653515	0
CHB	chr11	PGA3	58703515	0
CHB	chr11	PGA3	58753515	0
CHB	chr11	PGA3	58803515	0
CHB	chr11	PGA3	58853515	0
CHB	chr11	PGA3	58903515	0
CHB	chr11	PGA3	58953515	0
CHB	chr11	PGA3	59003515	0
CHB	chr11	PGA3	59053515	0
CHB	chr11	PGA3	59103515	0
CHB	chr11	PGA3	59153515	0
CHB	chr11	PGA3	59203515	0
CHB	chr11	PGA3	59253515	0
CHB	chr11	PGA3	59303515	0
CHB	chr11	PGA3	59353515	0
CHB	chr11	PGA3	59403515	0
CHB	chr11	PGA3	59453515	0
CHB	chr11	PGA3	59503515	0
CHB	chr11	PGA3	59553515	0
CHB	chr11	PGA3	59603515	0
CHB	chr11	PGA3	59653515	0
CHB	chr11	PGA3	59703515	0
CHB	chr11	PGA3	59753515	0
CHB	chr11	PGA3	59803515	0
CHB	chr11	PGA3	59853515	0
CHB	chr11	PGA3	59903515	0
CHB	chr11	PGA3	59953515	0

CHB	chr11	PGA3	60003515	0
CHB	chr11	PGA3	60053515	0
CHB	chr11	PGA3	60103515	0
CHB	chr11	PGA3	60153515	0
CHB	chr11	PGA3	60203515	0
CHB	chr11	PGA3	60253515	0
CHB	chr11	PGA3	60303515	0
CHB	chr11	PGA3	60353515	0
CHB	chr11	PGA3	60403515	0
CHB	chr11	PGA3	60453515	0
CHB	chr11	PGA3	60503515	0
CHB	chr11	PGA3	60553515	0
CHB	chr11	PGA3	60603515	0
CHB	chr11	PGA3	60653515	0
CHB	chr11	PGA3	60703515	0
CHB	chr11	PGA3	60753515	0
CHB	chr11	PGA3	60803515	0
CHB	chr11	PGA3	60853515	0
CHB	chr11	PGA3	60903515	0
CHB	chr11	PGA3	60953515	0
CHB	chr11	PGA3	61003515	0
CHB	chr11	PGA3	61053515	0
CHB	chr11	PGA3	61103515	0
CHB	chr11	PGA3	61153515	0
CHB	chr11	PGA3	61203515	0
CHB	chr11	PGA3	61253515	0
CHB	chr11	PGA3	61303515	0
CHB	chr11	PGA3	61353515	0
CHB	chr11	PGA3	61403515	0
CHB	chr11	PGA3	61453515	0
CHB	chr11	PGA3	61503515	0
CHB	chr11	PGA3	61553515	0
CHB	chr11	PGA3	61603515	0
CHB	chr11	PGA3	61653515	0
CHB	chr11	PGA3	61703515	0
CHB	chr11	PGA3	61753515	0
CHB	chr11	PGA3	61803515	0
CHB	chr11	PGA3	61853515	0
CHB	chr11	PGA3	61903515	0
CHB	chr11	PGA3	61953515	26
CHB	chr11	PGA3	62003515	48
CHB	chr11	PGA3	62053515	33
CHB	chr11	PGA3	62103515	37
CHB	chr11	PGA3	62153515	62
CHB	chr11	PGA3	62203515	36
CHB	chr11	PGA3	62253515	0
CHB	chr11	PGA3	62303515	0
CHB	chr11	PGA3	62353515	0
CHB	chr11	PGA3	62403515	0

CHB	chr11	PGA3	62453515	0
CHB	chr11	PGA3	62503515	0
CHB	chr11	PGA3	62553515	0
CHB	chr11	PGA3	62603515	0
CHB	chr11	PGA3	62653515	0
CHB	chr11	PGA3	62703515	0
CHB	chr11	PGA3	62753515	0
CHB	chr11	PGA3	62803515	0
CHB	chr11	PGA3	62853515	0
CHB	chr11	PGA3	62903515	0
CHB	chr11	PGA3	62953515	0
CHB	chr11	PGA3	63003515	0
CHB	chr11	PGA3	63053515	0
CHB	chr11	PGA3	63103515	0
CHB	chr11	PGA3	63153515	0
CHB	chr11	PGA3	63203515	0
CHB	chr11	PGA3	63253515	0
CHB	chr11	PGA3	63303515	0
CHB	chr11	PGA3	63353515	0
CHB	chr11	PGA3	63403515	0
CHB	chr11	PGA3	63453515	0
CHB	chr11	PGA3	63503515	0
CHB	chr11	PGA3	63553515	0
CHB	chr11	PGA3	63603515	0
CHB	chr11	PGA3	63653515	0
CHB	chr11	PGA3	63703515	0
CHB	chr11	PGA3	63753515	0
CHB	chr11	PGA3	63803515	0
CHB	chr11	PGA3	63853515	0
CHB	chr11	PGA3	63903515	0
CHB	chr11	PGA3	63953515	0
CHB	chr11	PGA3	64003515	0
CHB	chr11	PGA3	64053515	0
CHB	chr11	PGA3	64103515	0
CHB	chr11	PGA3	64153515	0
CHB	chr11	PGA3	64203515	0
CHB	chr11	PGA3	64253515	0
CHB	chr11	PGA3	64303515	0
CHB	chr11	PGA3	64353515	0
CHB	chr11	PGA3	64403515	0
CHB	chr11	PGA3	64453515	0
CHB	chr11	PGA3	64503515	0
CHB	chr11	PGA3	64553515	0
CHB	chr11	PGA3	64603515	0
CHB	chr11	PGA3	64653515	0
CHB	chr11	PGA3	64703515	0
CHB	chr11	PGA3	64753515	0
CHB	chr11	PGA3	64803515	0
CHB	chr11	PGA3	64853515	0

CHB	chr11	PGA3	64903515	0
CHB	chr11	PGA3	64953515	0
CHB	chr11	PGA3	65003515	0
CHB	chr11	PGA3	65053515	0
CHB	chr11	PGA3	65103515	0
CHB	chr11	PGA3	65153515	0
CHB	chr11	PGA3	65203515	0
CHB	chr11	PGA3	65253515	0
CHB	chr11	PGA3	65303515	0
CHB	chr11	PGA3	65353515	0
CHB	chr11	PGA3	65403515	0
CHB	chr11	PGA3	65453515	0
CHB	chr11	PGA3	65503515	0
CHB	chr11	PGA3	65553515	0
CHB	chr11	PGA3	65603515	0
CHB	chr11	PGA3	65653515	0
CHB	chr11	PGA3	65703515	0
CHB	chr11	PGA3	65753515	0
CHB	chr11	PGA3	65803515	0
CHB	chr11	PGA3	65853515	0
CHB	chr11	PGA3	65903515	0
CHB	chr11	PGA3	65953515	0
CHB	chr11	PGA3	66003515	0
CHB	chr11	PGA3	66053515	0
CHB	chr11	PGA3	66103515	0
CHB	chr11	PGA3	66153515	0
CHB	chr1	PRAMEF14	8391892	0
CHB	chr1	PRAMEF14	8441892	0
CHB	chr1	PRAMEF14	8491892	0
CHB	chr1	PRAMEF14	8541892	0
CHB	chr1	PRAMEF14	8591892	0
CHB	chr1	PRAMEF14	8641892	0
CHB	chr1	PRAMEF14	8691892	0
CHB	chr1	PRAMEF14	8741892	0
CHB	chr1	PRAMEF14	8791892	0
CHB	chr1	PRAMEF14	8841892	0
CHB	chr1	PRAMEF14	8891892	0
CHB	chr1	PRAMEF14	8941892	0
CHB	chr1	PRAMEF14	8991892	0
CHB	chr1	PRAMEF14	9041892	0
CHB	chr1	PRAMEF14	9091892	0
CHB	chr1	PRAMEF14	9141892	0
CHB	chr1	PRAMEF14	9191892	0
CHB	chr1	PRAMEF14	9241892	0
CHB	chr1	PRAMEF14	9291892	0
CHB	chr1	PRAMEF14	9341892	0
CHB	chr1	PRAMEF14	9391892	0
CHB	chr1	PRAMEF14	9441892	12
CHB	chr1	PRAMEF14	9491892	39

CHB	chr1	PRAMEF14	9541892	44
CHB	chr1	PRAMEF14	9591892	38
CHB	chr1	PRAMEF14	9641892	36
CHB	chr1	PRAMEF14	9691892	55
CHB	chr1	PRAMEF14	9741892	70
CHB	chr1	PRAMEF14	9791892	101
CHB	chr1	PRAMEF14	9841892	91
CHB	chr1	PRAMEF14	9891892	20
CHB	chr1	PRAMEF14	9941892	0
CHB	chr1	PRAMEF14	9991892	0
CHB	chr1	PRAMEF14	10041892	0
CHB	chr1	PRAMEF14	10091892	0
CHB	chr1	PRAMEF14	10141892	0
CHB	chr1	PRAMEF14	10191892	0
CHB	chr1	PRAMEF14	10241892	0
CHB	chr1	PRAMEF14	10291892	0
CHB	chr1	PRAMEF14	10341892	0
CHB	chr1	PRAMEF14	10391892	0
CHB	chr1	PRAMEF14	10441892	0
CHB	chr1	PRAMEF14	10491892	0
CHB	chr1	PRAMEF14	10541892	0
CHB	chr1	PRAMEF14	10591892	0
CHB	chr1	PRAMEF14	10641892	0
CHB	chr1	PRAMEF14	10691892	0
CHB	chr1	PRAMEF14	10741892	20
CHB	chr1	PRAMEF14	10791892	36
CHB	chr1	PRAMEF14	10841892	31
CHB	chr1	PRAMEF14	10891892	15
CHB	chr1	PRAMEF14	10941892	0
CHB	chr1	PRAMEF14	10991892	0
CHB	chr1	PRAMEF14	11041892	0
CHB	chr1	PRAMEF14	11091892	0
CHB	chr1	PRAMEF14	11141892	0
CHB	chr1	PRAMEF14	11191892	0
CHB	chr1	PRAMEF14	11241892	0
CHB	chr1	PRAMEF14	11291892	6
CHB	chr1	PRAMEF14	11341892	21
CHB	chr1	PRAMEF14	11391892	47
CHB	chr1	PRAMEF14	11441892	43
CHB	chr1	PRAMEF14	11491892	21
CHB	chr1	PRAMEF14	11541892	27
CHB	chr1	PRAMEF14	11591892	41
CHB	chr1	PRAMEF14	11641892	24
CHB	chr1	PRAMEF14	11691892	0
CHB	chr1	PRAMEF14	11741892	0
CHB	chr1	PRAMEF14	11791892	0
CHB	chr1	PRAMEF14	11841892	0
CHB	chr1	PRAMEF14	11891892	19
CHB	chr1	PRAMEF14	11941892	50

CHB	chr1	PRAMEF14	11991892	56
CHB	chr1	PRAMEF14	12041892	76
CHB	chr1	PRAMEF14	12091892	87
CHB	chr1	PRAMEF14	12141892	62
CHB	chr1	PRAMEF14	12191892	26
CHB	chr1	PRAMEF14	12241892	13
CHB	chr1	PRAMEF14	12291892	26
CHB	chr1	PRAMEF14	12341892	37
CHB	chr1	PRAMEF14	12391892	45
CHB	chr1	PRAMEF14	12441892	35
CHB	chr1	PRAMEF14	12491892	24
CHB	chr1	PRAMEF14	12541892	45
CHB	chr1	PRAMEF14	12591892	35
CHB	chr1	PRAMEF14	12641892	0
CHB	chr1	PRAMEF14	12691892	0
CHB	chr1	PRAMEF14	12741892	7
CHB	chr1	PRAMEF14	12791892	29
CHB	chr1	PRAMEF14	12841892	113
CHB	chr1	PRAMEF14	12891892	91
CHB	chr1	PRAMEF14	12941892	0
CHB	chr1	PRAMEF14	12991892	0
CHB	chr1	PRAMEF14	13041892	0
CHB	chr1	PRAMEF14	13091892	0
CHB	chr1	PRAMEF14	13141892	0
CHB	chr1	PRAMEF14	13191892	0
CHB	chr1	PRAMEF14	13241892	0
CHB	chr1	PRAMEF14	13291892	0
CHB	chr1	PRAMEF14	13341892	0
CHB	chr1	PRAMEF14	13391892	0
CHB	chr1	PRAMEF14	13441892	0
CHB	chr1	PRAMEF14	13491892	0
CHB	chr1	PRAMEF14	13541892	0
CHB	chr1	PRAMEF14	13591892	0
CHB	chr1	PRAMEF14	13641892	0
CHB	chr1	PRAMEF14	13691892	0
CHB	chr1	PRAMEF14	13741892	0
CHB	chr1	PRAMEF14	13791892	21
CHB	chr1	PRAMEF14	13841892	43
CHB	chr1	PRAMEF14	13891892	30
CHB	chr1	PRAMEF14	13941892	20
CHB	chr1	PRAMEF14	13991892	59
CHB	chr1	PRAMEF14	14041892	89
CHB	chr1	PRAMEF14	14091892	83
CHB	chr1	PRAMEF14	14141892	73
CHB	chr1	PRAMEF14	14191892	67
CHB	chr1	PRAMEF14	14241892	52
CHB	chr1	PRAMEF14	14291892	29
CHB	chr1	PRAMEF14	14341892	30
CHB	chr1	PRAMEF14	14391892	82

CHB	chr1	PRAMEF14	14441892	86
CHB	chr1	PRAMEF14	14491892	44
CHB	chr1	PRAMEF14	14541892	39
CHB	chr1	PRAMEF14	14591892	28
CHB	chr1	PRAMEF14	14641892	11
CHB	chr1	PRAMEF14	14691892	0
CHB	chr1	PRAMEF14	14741892	0
CHB	chr1	PRAMEF14	14791892	0
CHB	chr1	PRAMEF14	14841892	0
CHB	chr1	PRAMEF14	14891892	0
CHB	chr1	PRAMEF14	14941892	0
CHB	chr1	PRAMEF14	14991892	0
CHB	chr1	PRAMEF14	15041892	0
CHB	chr1	PRAMEF14	15091892	0
CHB	chr1	PRAMEF14	15141892	0
CHB	chr1	PRAMEF14	15191892	0
CHB	chr1	PRAMEF14	15241892	0
CHB	chr1	PRAMEF14	15291892	0
CHB	chr1	PRAMEF14	15341892	33
CHB	chr1	PRAMEF14	15391892	59
CHB	chr1	PRAMEF14	15441892	38
CHB	chr1	PRAMEF14	15491892	29
CHB	chr1	PRAMEF14	15541892	23
CHB	chr1	PRAMEF14	15591892	6
CHB	chr1	PRAMEF14	15641892	0
CHB	chr1	PRAMEF14	15691892	0
CHB	chr1	PRAMEF14	15741892	0
CHB	chr1	PRAMEF14	15791892	0
CHB	chr1	PRAMEF14	15841892	0
CHB	chr1	PRAMEF14	15891892	0
CHB	chr1	PRAMEF14	15941892	0
CHB	chr1	PRAMEF14	15991892	0
CHB	chr1	PRAMEF14	16041892	17
CHB	chr1	PRAMEF14	16091892	41
CHB	chr1	PRAMEF14	16141892	50
CHB	chr1	PRAMEF14	16191892	44
CHB	chr1	PRAMEF14	16241892	28
CHB	chr1	PRAMEF14	16291892	35
CHB	chr1	PRAMEF14	16341892	44
CHB	chr1	PRAMEF14	16391892	21
CHB	chr1	PRAMEF14	16441892	2
CHB	chr1	PRAMEF14	16491892	0
CHB	chr1	PRAMEF14	16541892	0
CHB	chr1	PRAMEF14	16591892	14
CHB	chr1	PRAMEF14	16641892	26
CHB	chr1	PRAMEF14	16691892	45
CHB	chr1	PRAMEF14	16741892	58
CHB	chr1	PRAMEF14	16791892	53
CHB	chr1	PRAMEF14	16841892	28

CHB	chr1	PRAMEF14	16891892	0
CHB	chr1	PRAMEF14	16941892	0
CHB	chr1	PRAMEF14	16991892	0
CHB	chr1	PRAMEF14	17041892	0
CHB	chr1	PRAMEF14	17091892	0
CHB	chr1	PRAMEF14	17141892	0
CHB	chr1	PRAMEF14	17191892	0
CHB	chr1	PRAMEF14	17241892	0
CHB	chr1	PRAMEF14	17291892	0
CHB	chr1	PRAMEF14	17341892	0
CHB	chr1	PRAMEF14	17391892	0
CHB	chr1	PRAMEF14	17441892	0
CHB	chr1	PRAMEF14	17491892	0
CHB	chr1	PRAMEF14	17541892	0
CHB	chr1	PRAMEF14	17591892	0
CHB	chr1	PRAMEF14	17641892	0
CHB	chr1	PRAMEF14	17691892	0
CHB	chr1	PRAMEF14	17741892	0
CHB	chr1	PRAMEF14	17791892	0
CHB	chr1	PRAMEF14	17841892	0
CHB	chr1	PRAMEF14	17891892	0
CHB	chr1	PRAMEF14	17941892	0
CHB	chr1	PRAMEF14	17991892	0
CHB	chr1	PRAMEF14	18041892	0
CHB	chr1	PRAMEF14	18091892	0
CHB	chr1	PRAMEF14	18141892	0
CHB	chr1	PRAMEF14	18191892	0
CHB	chr1	PRAMEF14	18241892	0
CHB	chr1	PRAMEF14	18291892	10
CHB	chr1	PRAMEF20	8460450	0
CHB	chr1	PRAMEF20	8510450	0
CHB	chr1	PRAMEF20	8560450	0
CHB	chr1	PRAMEF20	8610450	0
CHB	chr1	PRAMEF20	8660450	0
CHB	chr1	PRAMEF20	8710450	0
CHB	chr1	PRAMEF20	8760450	0
CHB	chr1	PRAMEF20	8810450	0
CHB	chr1	PRAMEF20	8860450	0
CHB	chr1	PRAMEF20	8910450	0
CHB	chr1	PRAMEF20	8960450	0
CHB	chr1	PRAMEF20	9010450	0
CHB	chr1	PRAMEF20	9060450	0
CHB	chr1	PRAMEF20	9110450	0
CHB	chr1	PRAMEF20	9160450	0
CHB	chr1	PRAMEF20	9210450	0
CHB	chr1	PRAMEF20	9260450	0
CHB	chr1	PRAMEF20	9310450	0
CHB	chr1	PRAMEF20	9360450	0
CHB	chr1	PRAMEF20	9410450	0

CHB	chr1	PRAMEF20	9460450	17
CHB	chr1	PRAMEF20	9510450	43
CHB	chr1	PRAMEF20	9560450	49
CHB	chr1	PRAMEF20	9610450	40
CHB	chr1	PRAMEF20	9660450	39
CHB	chr1	PRAMEF20	9710450	55
CHB	chr1	PRAMEF20	9760450	85
CHB	chr1	PRAMEF20	9810450	104
CHB	chr1	PRAMEF20	9860450	63
CHB	chr1	PRAMEF20	9910450	11
CHB	chr1	PRAMEF20	9960450	0
CHB	chr1	PRAMEF20	10010450	0
CHB	chr1	PRAMEF20	10060450	0
CHB	chr1	PRAMEF20	10110450	0
CHB	chr1	PRAMEF20	10160450	0
CHB	chr1	PRAMEF20	10210450	0
CHB	chr1	PRAMEF20	10260450	0
CHB	chr1	PRAMEF20	10310450	0
CHB	chr1	PRAMEF20	10360450	0
CHB	chr1	PRAMEF20	10410450	0
CHB	chr1	PRAMEF20	10460450	0
CHB	chr1	PRAMEF20	10510450	0
CHB	chr1	PRAMEF20	10560450	0
CHB	chr1	PRAMEF20	10610450	0
CHB	chr1	PRAMEF20	10660450	0
CHB	chr1	PRAMEF20	10710450	1
CHB	chr1	PRAMEF20	10760450	27
CHB	chr1	PRAMEF20	10810450	46
CHB	chr1	PRAMEF20	10860450	24
CHB	chr1	PRAMEF20	10910450	4
CHB	chr1	PRAMEF20	10960450	0
CHB	chr1	PRAMEF20	11010450	0
CHB	chr1	PRAMEF20	11060450	0
CHB	chr1	PRAMEF20	11110450	0
CHB	chr1	PRAMEF20	11160450	0
CHB	chr1	PRAMEF20	11210450	0
CHB	chr1	PRAMEF20	11260450	0
CHB	chr1	PRAMEF20	11310450	10
CHB	chr1	PRAMEF20	11360450	30
CHB	chr1	PRAMEF20	11410450	46
CHB	chr1	PRAMEF20	11460450	36
CHB	chr1	PRAMEF20	11510450	24
CHB	chr1	PRAMEF20	11560450	37
CHB	chr1	PRAMEF20	11610450	35
CHB	chr1	PRAMEF20	11660450	12
CHB	chr1	PRAMEF20	11710450	0
CHB	chr1	PRAMEF20	11760450	0
CHB	chr1	PRAMEF20	11810450	0
CHB	chr1	PRAMEF20	11860450	3

CHB	chr1	PRAMEF20	11910450	25
CHB	chr1	PRAMEF20	11960450	52
CHB	chr1	PRAMEF20	12010450	66
CHB	chr1	PRAMEF20	12060450	102
CHB	chr1	PRAMEF20	12110450	76
CHB	chr1	PRAMEF20	12160450	31
CHB	chr1	PRAMEF20	12210450	21
CHB	chr1	PRAMEF20	12260450	17
CHB	chr1	PRAMEF20	12310450	36
CHB	chr1	PRAMEF20	12360450	37
CHB	chr1	PRAMEF20	12410450	42
CHB	chr1	PRAMEF20	12460450	35
CHB	chr1	PRAMEF20	12510450	23
CHB	chr1	PRAMEF20	12560450	41
CHB	chr1	PRAMEF20	12610450	29
CHB	chr1	PRAMEF20	12660450	0
CHB	chr1	PRAMEF20	12710450	0
CHB	chr1	PRAMEF20	12760450	13
CHB	chr1	PRAMEF20	12810450	32
CHB	chr1	PRAMEF20	12860450	107
CHB	chr1	PRAMEF20	12910450	88
CHB	chr1	PRAMEF20	12960450	0
CHB	chr1	PRAMEF20	13010450	0
CHB	chr1	PRAMEF20	13060450	0
CHB	chr1	PRAMEF20	13110450	0
CHB	chr1	PRAMEF20	13160450	0
CHB	chr1	PRAMEF20	13210450	0
CHB	chr1	PRAMEF20	13260450	0
CHB	chr1	PRAMEF20	13310450	0
CHB	chr1	PRAMEF20	13360450	0
CHB	chr1	PRAMEF20	13410450	0
CHB	chr1	PRAMEF20	13460450	0
CHB	chr1	PRAMEF20	13510450	0
CHB	chr1	PRAMEF20	13560450	0
CHB	chr1	PRAMEF20	13610450	0
CHB	chr1	PRAMEF20	13660450	0
CHB	chr1	PRAMEF20	13710450	0
CHB	chr1	PRAMEF20	13760450	0
CHB	chr1	PRAMEF20	13810450	29
CHB	chr1	PRAMEF20	13860450	44
CHB	chr1	PRAMEF20	13910450	27
CHB	chr1	PRAMEF20	13960450	37
CHB	chr1	PRAMEF20	14010450	74
CHB	chr1	PRAMEF20	14060450	95
CHB	chr1	PRAMEF20	14110450	76
CHB	chr1	PRAMEF20	14160450	66
CHB	chr1	PRAMEF20	14210450	62
CHB	chr1	PRAMEF20	14260450	38
CHB	chr1	PRAMEF20	14310450	24

CHB	chr1	PRAMEF20	14360450	35
CHB	chr1	PRAMEF20	14410450	87
CHB	chr1	PRAMEF20	14460450	90
CHB	chr1	PRAMEF20	14510450	40
CHB	chr1	PRAMEF20	14560450	34
CHB	chr1	PRAMEF20	14610450	24
CHB	chr1	PRAMEF20	14660450	4
CHB	chr1	PRAMEF20	14710450	0
CHB	chr1	PRAMEF20	14760450	0
CHB	chr1	PRAMEF20	14810450	0
CHB	chr1	PRAMEF20	14860450	0
CHB	chr1	PRAMEF20	14910450	0
CHB	chr1	PRAMEF20	14960450	0
CHB	chr1	PRAMEF20	15010450	0
CHB	chr1	PRAMEF20	15060450	0
CHB	chr1	PRAMEF20	15110450	0
CHB	chr1	PRAMEF20	15160450	0
CHB	chr1	PRAMEF20	15210450	0
CHB	chr1	PRAMEF20	15260450	0
CHB	chr1	PRAMEF20	15310450	0
CHB	chr1	PRAMEF20	15360450	47
CHB	chr1	PRAMEF20	15410450	64
CHB	chr1	PRAMEF20	15460450	32
CHB	chr1	PRAMEF20	15510450	28
CHB	chr1	PRAMEF20	15560450	15
CHB	chr1	PRAMEF20	15610450	2
CHB	chr1	PRAMEF20	15660450	0
CHB	chr1	PRAMEF20	15710450	0
CHB	chr1	PRAMEF20	15760450	0
CHB	chr1	PRAMEF20	15810450	0
CHB	chr1	PRAMEF20	15860450	0
CHB	chr1	PRAMEF20	15910450	0
CHB	chr1	PRAMEF20	15960450	0
CHB	chr1	PRAMEF20	16010450	0
CHB	chr1	PRAMEF20	16060450	26
CHB	chr1	PRAMEF20	16110450	48
CHB	chr1	PRAMEF20	16160450	48
CHB	chr1	PRAMEF20	16210450	41
CHB	chr1	PRAMEF20	16260450	26
CHB	chr1	PRAMEF20	16310450	37
CHB	chr1	PRAMEF20	16360450	41
CHB	chr1	PRAMEF20	16410450	15
CHB	chr1	PRAMEF20	16460450	0
CHB	chr1	PRAMEF20	16510450	0
CHB	chr1	PRAMEF20	16560450	7
CHB	chr1	PRAMEF20	16610450	20
CHB	chr1	PRAMEF20	16660450	31
CHB	chr1	PRAMEF20	16710450	45
CHB	chr1	PRAMEF20	16760450	63

CHB	chr1	PRAMEF20	16810450	47
CHB	chr1	PRAMEF20	16860450	11
CHB	chr1	PRAMEF20	16910450	0
CHB	chr1	PRAMEF20	16960450	0
CHB	chr1	PRAMEF20	17010450	0
CHB	chr1	PRAMEF20	17060450	0
CHB	chr1	PRAMEF20	17110450	0
CHB	chr1	PRAMEF20	17160450	0
CHB	chr1	PRAMEF20	17210450	0
CHB	chr1	PRAMEF20	17260450	0
CHB	chr1	PRAMEF20	17310450	0
CHB	chr1	PRAMEF20	17360450	0
CHB	chr1	PRAMEF20	17410450	0
CHB	chr1	PRAMEF20	17460450	0
CHB	chr1	PRAMEF20	17510450	0
CHB	chr1	PRAMEF20	17560450	0
CHB	chr1	PRAMEF20	17610450	0
CHB	chr1	PRAMEF20	17660450	0
CHB	chr1	PRAMEF20	17710450	0
CHB	chr1	PRAMEF20	17760450	0
CHB	chr1	PRAMEF20	17810450	0
CHB	chr1	PRAMEF20	17860450	0
CHB	chr1	PRAMEF20	17910450	0
CHB	chr1	PRAMEF20	17960450	0
CHB	chr1	PRAMEF20	18010450	0
CHB	chr1	PRAMEF20	18060450	0
CHB	chr1	PRAMEF20	18110450	0
CHB	chr1	PRAMEF20	18160450	0
CHB	chr1	PRAMEF20	18210450	0
CHB	chr1	PRAMEF20	18260450	0
CHB	chr1	PRAMEF20	18310450	17
CHB	chr1	PRAMEF20	18360450	53
CHB	chr1	PRAMEF5	8304198	0
CHB	chr1	PRAMEF5	8354198	0
CHB	chr1	PRAMEF5	8404198	0
CHB	chr1	PRAMEF5	8454198	0
CHB	chr1	PRAMEF5	8504198	0
CHB	chr1	PRAMEF5	8554198	0
CHB	chr1	PRAMEF5	8604198	0
CHB	chr1	PRAMEF5	8654198	0
CHB	chr1	PRAMEF5	8704198	0
CHB	chr1	PRAMEF5	8754198	0
CHB	chr1	PRAMEF5	8804198	0
CHB	chr1	PRAMEF5	8854198	0
CHB	chr1	PRAMEF5	8904198	0
CHB	chr1	PRAMEF5	8954198	0
CHB	chr1	PRAMEF5	9004198	0
CHB	chr1	PRAMEF5	9054198	0
CHB	chr1	PRAMEF5	9104198	0

CHB	chr1	PRAMEF5	9154198	0
CHB	chr1	PRAMEF5	9204198	0
CHB	chr1	PRAMEF5	9254198	0
CHB	chr1	PRAMEF5	9304198	0
CHB	chr1	PRAMEF5	9354198	0
CHB	chr1	PRAMEF5	9404198	0
CHB	chr1	PRAMEF5	9454198	16
CHB	chr1	PRAMEF5	9504198	40
CHB	chr1	PRAMEF5	9554198	45
CHB	chr1	PRAMEF5	9604198	42
CHB	chr1	PRAMEF5	9654198	43
CHB	chr1	PRAMEF5	9704198	55
CHB	chr1	PRAMEF5	9754198	75
CHB	chr1	PRAMEF5	9804198	102
CHB	chr1	PRAMEF5	9854198	74
CHB	chr1	PRAMEF5	9904198	14
CHB	chr1	PRAMEF5	9954198	0
CHB	chr1	PRAMEF5	10004198	0
CHB	chr1	PRAMEF5	10054198	0
CHB	chr1	PRAMEF5	10104198	0
CHB	chr1	PRAMEF5	10154198	0
CHB	chr1	PRAMEF5	10204198	0
CHB	chr1	PRAMEF5	10254198	0
CHB	chr1	PRAMEF5	10304198	0
CHB	chr1	PRAMEF5	10354198	0
CHB	chr1	PRAMEF5	10404198	0
CHB	chr1	PRAMEF5	10454198	0
CHB	chr1	PRAMEF5	10504198	0
CHB	chr1	PRAMEF5	10554198	0
CHB	chr1	PRAMEF5	10604198	0
CHB	chr1	PRAMEF5	10654198	0
CHB	chr1	PRAMEF5	10704198	0
CHB	chr1	PRAMEF5	10754198	27
CHB	chr1	PRAMEF5	10804198	42
CHB	chr1	PRAMEF5	10854198	24
CHB	chr1	PRAMEF5	10904198	9
CHB	chr1	PRAMEF5	10954198	0
CHB	chr1	PRAMEF5	11004198	0
CHB	chr1	PRAMEF5	11054198	0
CHB	chr1	PRAMEF5	11104198	0
CHB	chr1	PRAMEF5	11154198	0
CHB	chr1	PRAMEF5	11204198	0
CHB	chr1	PRAMEF5	11254198	0
CHB	chr1	PRAMEF5	11304198	8
CHB	chr1	PRAMEF5	11354198	26
CHB	chr1	PRAMEF5	11404198	47
CHB	chr1	PRAMEF5	11454198	39
CHB	chr1	PRAMEF5	11504198	22
CHB	chr1	PRAMEF5	11554198	35

CHB	chr1	PRAMEF5	11604198	38
CHB	chr1	PRAMEF5	11654198	15
CHB	chr1	PRAMEF5	11704198	0
CHB	chr1	PRAMEF5	11754198	0
CHB	chr1	PRAMEF5	11804198	0
CHB	chr1	PRAMEF5	11854198	1
CHB	chr1	PRAMEF5	11904198	23
CHB	chr1	PRAMEF5	11954198	52
CHB	chr1	PRAMEF5	12004198	62
CHB	chr1	PRAMEF5	12054198	100
CHB	chr1	PRAMEF5	12104198	78
CHB	chr1	PRAMEF5	12154198	35
CHB	chr1	PRAMEF5	12204198	25
CHB	chr1	PRAMEF5	12254198	16
CHB	chr1	PRAMEF5	12304198	35
CHB	chr1	PRAMEF5	12354198	37
CHB	chr1	PRAMEF5	12404198	40
CHB	chr1	PRAMEF5	12454198	35
CHB	chr1	PRAMEF5	12504198	23
CHB	chr1	PRAMEF5	12554198	42
CHB	chr1	PRAMEF5	12604198	32
CHB	chr1	PRAMEF5	12654198	0
CHB	chr1	PRAMEF5	12704198	0
CHB	chr1	PRAMEF5	12754198	10
CHB	chr1	PRAMEF5	12804198	29
CHB	chr1	PRAMEF5	12854198	110
CHB	chr1	PRAMEF5	12904198	91
CHB	chr1	PRAMEF5	12954198	0
CHB	chr1	PRAMEF5	13004198	0
CHB	chr1	PRAMEF5	13054198	0
CHB	chr1	PRAMEF5	13104198	0
CHB	chr1	PRAMEF5	13154198	0
CHB	chr1	PRAMEF5	13204198	0
CHB	chr1	PRAMEF5	13254198	0
CHB	chr1	PRAMEF5	13304198	0
CHB	chr1	PRAMEF5	13354198	0
CHB	chr1	PRAMEF5	13404198	0
CHB	chr1	PRAMEF5	13454198	0
CHB	chr1	PRAMEF5	13504198	0
CHB	chr1	PRAMEF5	13554198	0
CHB	chr1	PRAMEF5	13604198	0
CHB	chr1	PRAMEF5	13654198	0
CHB	chr1	PRAMEF5	13704198	0
CHB	chr1	PRAMEF5	13754198	0
CHB	chr1	PRAMEF5	13804198	25
CHB	chr1	PRAMEF5	13854198	43
CHB	chr1	PRAMEF5	13904198	28
CHB	chr1	PRAMEF5	13954198	32
CHB	chr1	PRAMEF5	14004198	70

CHB	chr1	PRAMEF5	14054198	89
CHB	chr1	PRAMEF5	14104198	80
CHB	chr1	PRAMEF5	14154198	73
CHB	chr1	PRAMEF5	14204198	61
CHB	chr1	PRAMEF5	14254198	41
CHB	chr1	PRAMEF5	14304198	25
CHB	chr1	PRAMEF5	14354198	35
CHB	chr1	PRAMEF5	14404198	88
CHB	chr1	PRAMEF5	14454198	88
CHB	chr1	PRAMEF5	14504198	40
CHB	chr1	PRAMEF5	14554198	36
CHB	chr1	PRAMEF5	14604198	26
CHB	chr1	PRAMEF5	14654198	6
CHB	chr1	PRAMEF5	14704198	0
CHB	chr1	PRAMEF5	14754198	0
CHB	chr1	PRAMEF5	14804198	0
CHB	chr1	PRAMEF5	14854198	0
CHB	chr1	PRAMEF5	14904198	0
CHB	chr1	PRAMEF5	14954198	0
CHB	chr1	PRAMEF5	15004198	0
CHB	chr1	PRAMEF5	15054198	0
CHB	chr1	PRAMEF5	15104198	0
CHB	chr1	PRAMEF5	15154198	0
CHB	chr1	PRAMEF5	15204198	0
CHB	chr1	PRAMEF5	15254198	0
CHB	chr1	PRAMEF5	15304198	0
CHB	chr1	PRAMEF5	15354198	45
CHB	chr1	PRAMEF5	15404198	63
CHB	chr1	PRAMEF5	15454198	32
CHB	chr1	PRAMEF5	15504198	28
CHB	chr1	PRAMEF5	15554198	17
CHB	chr1	PRAMEF5	15604198	3
CHB	chr1	PRAMEF5	15654198	0
CHB	chr1	PRAMEF5	15704198	0
CHB	chr1	PRAMEF5	15754198	0
CHB	chr1	PRAMEF5	15804198	0
CHB	chr1	PRAMEF5	15854198	0
CHB	chr1	PRAMEF5	15904198	0
CHB	chr1	PRAMEF5	15954198	0
CHB	chr1	PRAMEF5	16004198	0
CHB	chr1	PRAMEF5	16054198	26
CHB	chr1	PRAMEF5	16104198	43
CHB	chr1	PRAMEF5	16154198	46
CHB	chr1	PRAMEF5	16204198	46
CHB	chr1	PRAMEF5	16254198	25
CHB	chr1	PRAMEF5	16304198	35
CHB	chr1	PRAMEF5	16354198	44
CHB	chr1	PRAMEF5	16404198	17
CHB	chr1	PRAMEF5	16454198	0

CHB	chr1	PRAMEF5	16504198	0
CHB	chr1	PRAMEF5	16554198	3
CHB	chr1	PRAMEF5	16604198	18
CHB	chr1	PRAMEF5	16654198	31
CHB	chr1	PRAMEF5	16704198	43
CHB	chr1	PRAMEF5	16754198	60
CHB	chr1	PRAMEF5	16804198	51
CHB	chr1	PRAMEF5	16854198	18
CHB	chr1	PRAMEF5	16904198	0
CHB	chr1	PRAMEF5	16954198	0
CHB	chr1	PRAMEF5	17004198	0
CHB	chr1	PRAMEF5	17054198	0
CHB	chr1	PRAMEF5	17104198	0
CHB	chr1	PRAMEF5	17154198	0
CHB	chr1	PRAMEF5	17204198	0
CHB	chr1	PRAMEF5	17254198	0
CHB	chr1	PRAMEF5	17304198	0
CHB	chr1	PRAMEF5	17354198	0
CHB	chr1	PRAMEF5	17404198	0
CHB	chr1	PRAMEF5	17454198	0
CHB	chr1	PRAMEF5	17504198	0
CHB	chr1	PRAMEF5	17554198	0
CHB	chr1	PRAMEF5	17604198	0
CHB	chr1	PRAMEF5	17654198	0
CHB	chr1	PRAMEF5	17704198	0
CHB	chr1	PRAMEF5	17754198	0
CHB	chr1	PRAMEF5	17804198	0
CHB	chr1	PRAMEF5	17854198	0
CHB	chr1	PRAMEF5	17904198	0
CHB	chr1	PRAMEF5	17954198	0
CHB	chr1	PRAMEF5	18004198	0
CHB	chr1	PRAMEF5	18054198	0
CHB	chr1	PRAMEF5	18104198	0
CHB	chr1	PRAMEF5	18154198	0
CHB	chr1	PRAMEF5	18204198	0
CHB	chr1	PRAMEF8	8331223	0
CHB	chr1	PRAMEF8	8381223	0
CHB	chr1	PRAMEF8	8431223	0
CHB	chr1	PRAMEF8	8481223	0
CHB	chr1	PRAMEF8	8531223	0
CHB	chr1	PRAMEF8	8581223	0
CHB	chr1	PRAMEF8	8631223	0
CHB	chr1	PRAMEF8	8681223	0
CHB	chr1	PRAMEF8	8731223	0
CHB	chr1	PRAMEF8	8781223	0
CHB	chr1	PRAMEF8	8831223	0
CHB	chr1	PRAMEF8	8881223	0
CHB	chr1	PRAMEF8	8931223	0
CHB	chr1	PRAMEF8	8981223	0

CHB	chr1	PRAMEF8	9031223	0
CHB	chr1	PRAMEF8	9081223	0
CHB	chr1	PRAMEF8	9131223	0
CHB	chr1	PRAMEF8	9181223	0
CHB	chr1	PRAMEF8	9231223	0
CHB	chr1	PRAMEF8	9281223	0
CHB	chr1	PRAMEF8	9331223	0
CHB	chr1	PRAMEF8	9381223	0
CHB	chr1	PRAMEF8	9431223	5
CHB	chr1	PRAMEF8	9481223	37
CHB	chr1	PRAMEF8	9531223	45
CHB	chr1	PRAMEF8	9581223	37
CHB	chr1	PRAMEF8	9631223	40
CHB	chr1	PRAMEF8	9681223	48
CHB	chr1	PRAMEF8	9731223	67
CHB	chr1	PRAMEF8	9781223	95
CHB	chr1	PRAMEF8	9831223	95
CHB	chr1	PRAMEF8	9881223	36
CHB	chr1	PRAMEF8	9931223	1
CHB	chr1	PRAMEF8	9981223	0
CHB	chr1	PRAMEF8	10031223	0
CHB	chr1	PRAMEF8	10081223	0
CHB	chr1	PRAMEF8	10131223	0
CHB	chr1	PRAMEF8	10181223	0
CHB	chr1	PRAMEF8	10231223	0
CHB	chr1	PRAMEF8	10281223	0
CHB	chr1	PRAMEF8	10331223	0
CHB	chr1	PRAMEF8	10381223	0
CHB	chr1	PRAMEF8	10431223	0
CHB	chr1	PRAMEF8	10481223	0
CHB	chr1	PRAMEF8	10531223	0
CHB	chr1	PRAMEF8	10581223	0
CHB	chr1	PRAMEF8	10631223	0
CHB	chr1	PRAMEF8	10681223	0
CHB	chr1	PRAMEF8	10731223	16
CHB	chr1	PRAMEF8	10781223	32
CHB	chr1	PRAMEF8	10831223	35
CHB	chr1	PRAMEF8	10881223	19
CHB	chr1	PRAMEF8	10931223	0
CHB	chr1	PRAMEF8	10981223	0
CHB	chr1	PRAMEF8	11031223	0
CHB	chr1	PRAMEF8	11081223	0
CHB	chr1	PRAMEF8	11131223	0
CHB	chr1	PRAMEF8	11181223	0
CHB	chr1	PRAMEF8	11231223	0
CHB	chr1	PRAMEF8	11281223	3
CHB	chr1	PRAMEF8	11331223	19
CHB	chr1	PRAMEF8	11381223	49
CHB	chr1	PRAMEF8	11431223	43

CHB	chr1	PRAMEF8	11481223	19
CHB	chr1	PRAMEF8	11531223	26
CHB	chr1	PRAMEF8	11581223	42
CHB	chr1	PRAMEF8	11631223	27
CHB	chr1	PRAMEF8	11681223	2
CHB	chr1	PRAMEF8	11731223	0
CHB	chr1	PRAMEF8	11781223	0
CHB	chr1	PRAMEF8	11831223	0
CHB	chr1	PRAMEF8	11881223	15
CHB	chr1	PRAMEF8	11931223	46
CHB	chr1	PRAMEF8	11981223	54
CHB	chr1	PRAMEF8	12031223	53
CHB	chr1	PRAMEF8	12081223	93
CHB	chr1	PRAMEF8	12131223	89
CHB	chr1	PRAMEF8	12181223	26
CHB	chr1	PRAMEF8	12231223	5
CHB	chr1	PRAMEF8	12281223	25
CHB	chr1	PRAMEF8	12331223	41
CHB	chr1	PRAMEF8	12381223	41
CHB	chr1	PRAMEF8	12431223	37
CHB	chr1	PRAMEF8	12481223	27
CHB	chr1	PRAMEF8	12531223	41
CHB	chr1	PRAMEF8	12581223	37
CHB	chr1	PRAMEF8	12631223	6
CHB	chr1	PRAMEF8	12681223	0
CHB	chr1	PRAMEF8	12731223	4
CHB	chr1	PRAMEF8	12781223	19
CHB	chr1	PRAMEF8	12831223	114
CHB	chr1	PRAMEF8	12881223	101
CHB	chr1	PRAMEF8	12931223	2
CHB	chr1	PRAMEF8	12981223	0
CHB	chr1	PRAMEF8	13031223	0
CHB	chr1	PRAMEF8	13081223	0
CHB	chr1	PRAMEF8	13131223	0
CHB	chr1	PRAMEF8	13181223	0
CHB	chr1	PRAMEF8	13231223	0
CHB	chr1	PRAMEF8	13281223	0
CHB	chr1	PRAMEF8	13331223	0
CHB	chr1	PRAMEF8	13381223	0
CHB	chr1	PRAMEF8	13431223	0
CHB	chr1	PRAMEF8	13481223	0
CHB	chr1	PRAMEF8	13531223	0
CHB	chr1	PRAMEF8	13581223	0
CHB	chr1	PRAMEF8	13631223	0
CHB	chr1	PRAMEF8	13681223	0
CHB	chr1	PRAMEF8	13731223	0
CHB	chr1	PRAMEF8	13781223	8
CHB	chr1	PRAMEF8	13831223	41
CHB	chr1	PRAMEF8	13881223	43

CHB	chr1	PRAMEF8	13931223	19
CHB	chr1	PRAMEF8	13981223	47
CHB	chr1	PRAMEF8	14031223	85
CHB	chr1	PRAMEF8	14081223	86
CHB	chr1	PRAMEF8	14131223	76
CHB	chr1	PRAMEF8	14181223	74
CHB	chr1	PRAMEF8	14231223	55
CHB	chr1	PRAMEF8	14281223	28
CHB	chr1	PRAMEF8	14331223	24
CHB	chr1	PRAMEF8	14381223	83
CHB	chr1	PRAMEF8	14431223	89
CHB	chr1	PRAMEF8	14481223	45
CHB	chr1	PRAMEF8	14531223	41
CHB	chr1	PRAMEF8	14581223	29
CHB	chr1	PRAMEF8	14631223	13
CHB	chr1	PRAMEF8	14681223	0
CHB	chr1	PRAMEF8	14731223	0
CHB	chr1	PRAMEF8	14781223	0
CHB	chr1	PRAMEF8	14831223	0
CHB	chr1	PRAMEF8	14881223	0
CHB	chr1	PRAMEF8	14931223	0
CHB	chr1	PRAMEF8	14981223	0
CHB	chr1	PRAMEF8	15031223	0
CHB	chr1	PRAMEF8	15081223	0
CHB	chr1	PRAMEF8	15131223	0
CHB	chr1	PRAMEF8	15181223	0
CHB	chr1	PRAMEF8	15231223	0
CHB	chr1	PRAMEF8	15281223	0
CHB	chr1	PRAMEF8	15331223	21
CHB	chr1	PRAMEF8	15381223	58
CHB	chr1	PRAMEF8	15431223	48
CHB	chr1	PRAMEF8	15481223	25
CHB	chr1	PRAMEF8	15531223	25
CHB	chr1	PRAMEF8	15581223	11
CHB	chr1	PRAMEF8	15631223	0
CHB	chr1	PRAMEF8	15681223	0
CHB	chr1	PRAMEF8	15731223	0
CHB	chr1	PRAMEF8	15781223	0
CHB	chr1	PRAMEF8	15831223	0
CHB	chr1	PRAMEF8	15881223	0
CHB	chr1	PRAMEF8	15931223	0
CHB	chr1	PRAMEF8	15981223	0
CHB	chr1	PRAMEF8	16031223	7
CHB	chr1	PRAMEF8	16081223	35
CHB	chr1	PRAMEF8	16131223	51
CHB	chr1	PRAMEF8	16181223	46
CHB	chr1	PRAMEF8	16231223	34
CHB	chr1	PRAMEF8	16281223	32
CHB	chr1	PRAMEF8	16331223	43

CHB	chr1	PRAMEF8	16381223	28
CHB	chr1	PRAMEF8	16431223	6
CHB	chr1	PRAMEF8	16481223	0
CHB	chr1	PRAMEF8	16531223	0
CHB	chr1	PRAMEF8	16581223	11
CHB	chr1	PRAMEF8	16631223	24
CHB	chr1	PRAMEF8	16681223	41
CHB	chr1	PRAMEF8	16731223	51
CHB	chr1	PRAMEF8	16781223	60
CHB	chr1	PRAMEF8	16831223	37
CHB	chr1	PRAMEF8	16881223	0
CHB	chr1	PRAMEF8	16931223	0
CHB	chr1	PRAMEF8	16981223	0
CHB	chr1	PRAMEF8	17031223	0
CHB	chr1	PRAMEF8	17081223	0
CHB	chr1	PRAMEF8	17131223	0
CHB	chr1	PRAMEF8	17181223	0
CHB	chr1	PRAMEF8	17231223	0
CHB	chr1	PRAMEF8	17281223	0
CHB	chr1	PRAMEF8	17331223	0
CHB	chr1	PRAMEF8	17381223	0
CHB	chr1	PRAMEF8	17431223	0
CHB	chr1	PRAMEF8	17481223	0
CHB	chr1	PRAMEF8	17531223	0
CHB	chr1	PRAMEF8	17581223	0
CHB	chr1	PRAMEF8	17631223	0
CHB	chr1	PRAMEF8	17681223	0
CHB	chr1	PRAMEF8	17731223	0
CHB	chr1	PRAMEF8	17781223	0
CHB	chr1	PRAMEF8	17831223	0
CHB	chr1	PRAMEF8	17881223	0
CHB	chr1	PRAMEF8	17931223	0
CHB	chr1	PRAMEF8	17981223	0
CHB	chr1	PRAMEF8	18031223	0
CHB	chr1	PRAMEF8	18081223	0
CHB	chr1	PRAMEF8	18131223	0
CHB	chr1	PRAMEF8	18181223	0
CHB	chr1	PRAMEF8	18231223	0
CHB	chr17	PRR11	54205355	0
CHB	chr17	PRR11	54255355	0
CHB	chr17	PRR11	54305355	0
CHB	chr17	PRR11	54355355	0
CHB	chr17	PRR11	54405355	0
CHB	chr17	PRR11	54455355	0
CHB	chr17	PRR11	54505355	0
CHB	chr17	PRR11	54555355	0
CHB	chr17	PRR11	54605355	0
CHB	chr17	PRR11	54655355	0
CHB	chr17	PRR11	54705355	0

CHB	chr17	PRR11	54755355	0
CHB	chr17	PRR11	54805355	0
CHB	chr17	PRR11	54855355	0
CHB	chr17	PRR11	54905355	0
CHB	chr17	PRR11	54955355	0
CHB	chr17	PRR11	55005355	0
CHB	chr17	PRR11	55055355	0
CHB	chr17	PRR11	55105355	0
CHB	chr17	PRR11	55155355	0
CHB	chr17	PRR11	55205355	0
CHB	chr17	PRR11	55255355	0
CHB	chr17	PRR11	55305355	0
CHB	chr17	PRR11	55355355	0
CHB	chr17	PRR11	55405355	0
CHB	chr17	PRR11	55455355	0
CHB	chr17	PRR11	55505355	0
CHB	chr17	PRR11	55555355	0
CHB	chr17	PRR11	55605355	0
CHB	chr17	PRR11	55655355	0
CHB	chr17	PRR11	55705355	0
CHB	chr17	PRR11	55755355	0
CHB	chr17	PRR11	55805355	0
CHB	chr17	PRR11	55855355	0
CHB	chr17	PRR11	55905355	0
CHB	chr17	PRR11	55955355	0
CHB	chr17	PRR11	56005355	0
CHB	chr17	PRR11	56055355	0
CHB	chr17	PRR11	56105355	0
CHB	chr17	PRR11	56155355	0
CHB	chr17	PRR11	56205355	0
CHB	chr17	PRR11	56255355	0
CHB	chr17	PRR11	56305355	0
CHB	chr17	PRR11	56355355	0
CHB	chr17	PRR11	56405355	0
CHB	chr17	PRR11	56455355	0
CHB	chr17	PRR11	56505355	0
CHB	chr17	PRR11	56555355	0
CHB	chr17	PRR11	56605355	0
CHB	chr17	PRR11	56655355	0
CHB	chr17	PRR11	56705355	0
CHB	chr17	PRR11	56755355	0
CHB	chr17	PRR11	56805355	0
CHB	chr17	PRR11	56855355	0
CHB	chr17	PRR11	56905355	0
CHB	chr17	PRR11	56955355	0
CHB	chr17	PRR11	57005355	0
CHB	chr17	PRR11	57055355	0
CHB	chr17	PRR11	57105355	0
CHB	chr17	PRR11	57155355	0

CHB	chr17	PRR11	57205355	0
CHB	chr17	PRR11	57255355	0
CHB	chr17	PRR11	57305355	0
CHB	chr17	PRR11	57355355	0
CHB	chr17	PRR11	57405355	0
CHB	chr17	PRR11	57455355	0
CHB	chr17	PRR11	57505355	0
CHB	chr17	PRR11	57555355	0
CHB	chr17	PRR11	57605355	0
CHB	chr17	PRR11	57655355	0
CHB	chr17	PRR11	57705355	0
CHB	chr17	PRR11	57755355	0
CHB	chr17	PRR11	57805355	0
CHB	chr17	PRR11	57855355	0
CHB	chr17	PRR11	57905355	0
CHB	chr17	PRR11	57955355	0
CHB	chr17	PRR11	58005355	0
CHB	chr17	PRR11	58055355	0
CHB	chr17	PRR11	58105355	0
CHB	chr17	PRR11	58155355	0
CHB	chr17	PRR11	58205355	0
CHB	chr17	PRR11	58255355	0
CHB	chr17	PRR11	58305355	0
CHB	chr17	PRR11	58355355	0
CHB	chr17	PRR11	58405355	0
CHB	chr17	PRR11	58455355	0
CHB	chr17	PRR11	58505355	0
CHB	chr17	PRR11	58555355	0
CHB	chr17	PRR11	58605355	0
CHB	chr17	PRR11	58655355	0
CHB	chr17	PRR11	58705355	0
CHB	chr17	PRR11	58755355	0
CHB	chr17	PRR11	58805355	0
CHB	chr17	PRR11	58855355	0
CHB	chr17	PRR11	58905355	0
CHB	chr17	PRR11	58955355	0
CHB	chr17	PRR11	59005355	0
CHB	chr17	PRR11	59055355	0
CHB	chr17	PRR11	59105355	0
CHB	chr17	PRR11	59155355	0
CHB	chr17	PRR11	59205355	0
CHB	chr17	PRR11	59255355	0
CHB	chr17	PRR11	59305355	0
CHB	chr17	PRR11	59355355	0
CHB	chr17	PRR11	59405355	0
CHB	chr17	PRR11	59455355	0
CHB	chr17	PRR11	59505355	0
CHB	chr17	PRR11	59555355	0
CHB	chr17	PRR11	59605355	0

CHB	chr17	PRR11	59655355	0
CHB	chr17	PRR11	59705355	0
CHB	chr17	PRR11	59755355	0
CHB	chr17	PRR11	59805355	0
CHB	chr17	PRR11	59855355	0
CHB	chr17	PRR11	59905355	0
CHB	chr17	PRR11	59955355	0
CHB	chr17	PRR11	60005355	0
CHB	chr17	PRR11	60055355	0
CHB	chr17	PRR11	60105355	0
CHB	chr17	PRR11	60155355	0
CHB	chr17	PRR11	60205355	0
CHB	chr17	PRR11	60255355	0
CHB	chr17	PRR11	60305355	0
CHB	chr17	PRR11	60355355	0
CHB	chr17	PRR11	60405355	0
CHB	chr17	PRR11	60455355	0
CHB	chr17	PRR11	60505355	0
CHB	chr17	PRR11	60555355	0
CHB	chr17	PRR11	60605355	0
CHB	chr17	PRR11	60655355	0
CHB	chr17	PRR11	60705355	0
CHB	chr17	PRR11	60755355	0
CHB	chr17	PRR11	60805355	0
CHB	chr17	PRR11	60855355	0
CHB	chr17	PRR11	60905355	0
CHB	chr17	PRR11	60955355	0
CHB	chr17	PRR11	61005355	0
CHB	chr17	PRR11	61055355	0
CHB	chr17	PRR11	61105355	0
CHB	chr17	PRR11	61155355	0
CHB	chr17	PRR11	61205355	0
CHB	chr17	PRR11	61255355	0
CHB	chr17	PRR11	61305355	0
CHB	chr17	PRR11	61355355	0
CHB	chr17	PRR11	61405355	0
CHB	chr17	PRR11	61455355	0
CHB	chr17	PRR11	61505355	0
CHB	chr17	PRR11	61555355	0
CHB	chr17	PRR11	61605355	0
CHB	chr17	PRR11	61655355	0
CHB	chr17	PRR11	61705355	0
CHB	chr17	PRR11	61755355	0
CHB	chr17	PRR11	61805355	0
CHB	chr17	PRR11	61855355	0
CHB	chr17	PRR11	61905355	0
CHB	chr17	PRR11	61955355	0
CHB	chr17	PRR11	62005355	0
CHB	chr17	PRR11	62055355	0

CHB	chr17	PRR11	62105355	0
CHB	chr17	PRR11	62155355	0
CHB	chr17	PRR11	62205355	0
CHB	chr17	PRR11	62255355	0
CHB	chr17	PRR11	62305355	0
CHB	chr17	PRR11	62355355	0
CHB	chr17	PRR11	62405355	0
CHB	chr17	PRR11	62455355	0
CHB	chr17	PRR11	62505355	0
CHB	chr17	PRR11	62555355	0
CHB	chr17	PRR11	62605355	0
CHB	chr17	PRR11	62655355	0
CHB	chr17	PRR11	62705355	0
CHB	chr17	PRR11	62755355	0
CHB	chr17	PRR11	62805355	0
CHB	chr17	PRR11	62855355	0
CHB	chr17	PRR11	62905355	0
CHB	chr17	PRR11	62955355	0
CHB	chr17	PRR11	63005355	0
CHB	chr17	PRR11	63055355	0
CHB	chr17	PRR11	63105355	0
CHB	chr17	PRR11	63155355	0
CHB	chr17	PRR11	63205355	0
CHB	chr17	PRR11	63255355	0
CHB	chr17	PRR11	63305355	0
CHB	chr17	PRR11	63355355	0
CHB	chr17	PRR11	63405355	0
CHB	chr17	PRR11	63455355	0
CHB	chr17	PRR11	63505355	0
CHB	chr17	PRR11	63555355	0
CHB	chr17	PRR11	63605355	0
CHB	chr17	PRR11	63655355	0
CHB	chr17	PRR11	63705355	0
CHB	chr17	PRR11	63755355	0
CHB	chr17	PRR11	63805355	0
CHB	chr17	PRR11	63855355	0
CHB	chr17	PRR11	63905355	0
CHB	chr17	PRR11	63955355	0
CHB	chr17	PRR11	64005355	0
CHB	chr17	PRR11	64055355	0
CHB	chr17	PRR11	64105355	0
CHB	chr17	PRR11	64155355	0
CHB	chr13	PRR20A	52190918	0
CHB	chr13	PRR20A	52240918	0
CHB	chr13	PRR20A	52290918	0
CHB	chr13	PRR20A	52340918	0
CHB	chr13	PRR20A	52390918	0
CHB	chr13	PRR20A	52440918	0
CHB	chr13	PRR20A	52490918	0

CHB	chr13	PRR20A	52540918	0
CHB	chr13	PRR20A	52590918	0
CHB	chr13	PRR20A	52640918	0
CHB	chr13	PRR20A	52690918	0
CHB	chr13	PRR20A	52740918	0
CHB	chr13	PRR20A	52790918	0
CHB	chr13	PRR20A	52840918	0
CHB	chr13	PRR20A	52890918	0
CHB	chr13	PRR20A	52940918	0
CHB	chr13	PRR20A	52990918	0
CHB	chr13	PRR20A	53040918	0
CHB	chr13	PRR20A	53090918	0
CHB	chr13	PRR20A	53140918	0
CHB	chr13	PRR20A	53190918	0
CHB	chr13	PRR20A	53240918	0
CHB	chr13	PRR20A	53290918	0
CHB	chr13	PRR20A	53340918	0
CHB	chr13	PRR20A	53390918	0
CHB	chr13	PRR20A	53440918	0
CHB	chr13	PRR20A	53490918	0
CHB	chr13	PRR20A	53540918	0
CHB	chr13	PRR20A	53590918	0
CHB	chr13	PRR20A	53640918	0
CHB	chr13	PRR20A	53690918	0
CHB	chr13	PRR20A	53740918	0
CHB	chr13	PRR20A	53790918	0
CHB	chr13	PRR20A	53840918	0
CHB	chr13	PRR20A	53890918	0
CHB	chr13	PRR20A	53940918	0
CHB	chr13	PRR20A	53990918	0
CHB	chr13	PRR20A	54040918	0
CHB	chr13	PRR20A	54090918	0
CHB	chr13	PRR20A	54140918	0
CHB	chr13	PRR20A	54190918	0
CHB	chr13	PRR20A	54240918	0
CHB	chr13	PRR20A	54290918	0
CHB	chr13	PRR20A	54340918	0
CHB	chr13	PRR20A	54390918	0
CHB	chr13	PRR20A	54440918	0
CHB	chr13	PRR20A	54490918	0
CHB	chr13	PRR20A	54540918	0
CHB	chr13	PRR20A	54590918	0
CHB	chr13	PRR20A	54640918	0
CHB	chr13	PRR20A	54690918	0
CHB	chr13	PRR20A	54740918	0
CHB	chr13	PRR20A	54790918	0
CHB	chr13	PRR20A	54840918	0
CHB	chr13	PRR20A	54890918	0
CHB	chr13	PRR20A	54940918	0

CHB	chr13	PRR20A	54990918	0
CHB	chr13	PRR20A	55040918	0
CHB	chr13	PRR20A	55090918	0
CHB	chr13	PRR20A	55140918	0
CHB	chr13	PRR20A	55190918	0
CHB	chr13	PRR20A	55240918	0
CHB	chr13	PRR20A	55290918	0
CHB	chr13	PRR20A	55340918	0
CHB	chr13	PRR20A	55390918	0
CHB	chr13	PRR20A	55440918	0
CHB	chr13	PRR20A	55490918	0
CHB	chr13	PRR20A	55540918	0
CHB	chr13	PRR20A	55590918	0
CHB	chr13	PRR20A	55640918	0
CHB	chr13	PRR20A	55690918	0
CHB	chr13	PRR20A	55740918	0
CHB	chr13	PRR20A	55790918	0
CHB	chr13	PRR20A	55840918	0
CHB	chr13	PRR20A	55890918	0
CHB	chr13	PRR20A	55940918	0
CHB	chr13	PRR20A	55990918	0
CHB	chr13	PRR20A	56040918	0
CHB	chr13	PRR20A	56090918	0
CHB	chr13	PRR20A	56140918	0
CHB	chr13	PRR20A	56190918	0
CHB	chr13	PRR20A	56240918	0
CHB	chr13	PRR20A	56290918	0
CHB	chr13	PRR20A	56340918	0
CHB	chr13	PRR20A	56390918	0
CHB	chr13	PRR20A	56440918	0
CHB	chr13	PRR20A	56490918	0
CHB	chr13	PRR20A	56540918	0
CHB	chr13	PRR20A	56590918	0
CHB	chr13	PRR20A	56640918	0
CHB	chr13	PRR20A	56690918	0
CHB	chr13	PRR20A	56740918	0
CHB	chr13	PRR20A	56790918	0
CHB	chr13	PRR20A	56840918	0
CHB	chr13	PRR20A	56890918	0
CHB	chr13	PRR20A	56940918	0
CHB	chr13	PRR20A	56990918	0
CHB	chr13	PRR20A	57040918	0
CHB	chr13	PRR20A	57090918	0
CHB	chr13	PRR20A	57140918	0
CHB	chr13	PRR20A	57190918	0
CHB	chr13	PRR20A	57240918	0
CHB	chr13	PRR20A	57290918	0
CHB	chr13	PRR20A	57340918	0
CHB	chr13	PRR20A	57390918	0

CHB	chr13	PRR20A	57440918	0
CHB	chr13	PRR20A	57490918	0
CHB	chr13	PRR20A	57540918	0
CHB	chr13	PRR20A	57590918	0
CHB	chr13	PRR20A	57640918	0
CHB	chr13	PRR20A	57690918	0
CHB	chr13	PRR20A	57740918	0
CHB	chr13	PRR20A	57790918	0
CHB	chr13	PRR20A	57840918	0
CHB	chr13	PRR20A	57890918	0
CHB	chr13	PRR20A	57940918	0
CHB	chr13	PRR20A	57990918	0
CHB	chr13	PRR20A	58040918	0
CHB	chr13	PRR20A	58090918	0
CHB	chr13	PRR20A	58140918	0
CHB	chr13	PRR20A	58190918	0
CHB	chr13	PRR20A	58240918	0
CHB	chr13	PRR20A	58290918	0
CHB	chr13	PRR20A	58340918	0
CHB	chr13	PRR20A	58390918	0
CHB	chr13	PRR20A	58440918	0
CHB	chr13	PRR20A	58490918	0
CHB	chr13	PRR20A	58540918	0
CHB	chr13	PRR20A	58590918	0
CHB	chr13	PRR20A	58640918	0
CHB	chr13	PRR20A	58690918	0
CHB	chr13	PRR20A	58740918	0
CHB	chr13	PRR20A	58790918	0
CHB	chr13	PRR20A	58840918	0
CHB	chr13	PRR20A	58890918	0
CHB	chr13	PRR20A	58940918	0
CHB	chr13	PRR20A	58990918	0
CHB	chr13	PRR20A	59040918	0
CHB	chr13	PRR20A	59090918	0
CHB	chr13	PRR20A	59140918	0
CHB	chr13	PRR20A	59190918	0
CHB	chr13	PRR20A	59240918	0
CHB	chr13	PRR20A	59290918	0
CHB	chr13	PRR20A	59340918	0
CHB	chr13	PRR20A	59390918	0
CHB	chr13	PRR20A	59440918	0
CHB	chr13	PRR20A	59490918	0
CHB	chr13	PRR20A	59540918	0
CHB	chr13	PRR20A	59590918	0
CHB	chr13	PRR20A	59640918	0
CHB	chr13	PRR20A	59690918	0
CHB	chr13	PRR20A	59740918	0
CHB	chr13	PRR20A	59790918	0
CHB	chr13	PRR20A	59840918	0

CHB	chr13	PRR20A	59890918	0
CHB	chr13	PRR20A	59940918	0
CHB	chr13	PRR20A	59990918	0
CHB	chr13	PRR20A	60040918	0
CHB	chr13	PRR20A	60090918	0
CHB	chr13	PRR20A	60140918	0
CHB	chr13	PRR20A	60190918	0
CHB	chr13	PRR20A	60240918	0
CHB	chr13	PRR20A	60290918	0
CHB	chr13	PRR20A	60340918	0
CHB	chr13	PRR20A	60390918	0
CHB	chr13	PRR20A	60440918	0
CHB	chr13	PRR20A	60490918	0
CHB	chr13	PRR20A	60540918	0
CHB	chr13	PRR20A	60590918	0
CHB	chr13	PRR20A	60640918	0
CHB	chr13	PRR20A	60690918	0
CHB	chr13	PRR20A	60740918	0
CHB	chr13	PRR20A	60790918	0
CHB	chr13	PRR20A	60840918	0
CHB	chr13	PRR20A	60890918	0
CHB	chr13	PRR20A	60940918	0
CHB	chr13	PRR20A	60990918	0
CHB	chr13	PRR20A	61040918	0
CHB	chr13	PRR20A	61090918	0
CHB	chr13	PRR20A	61140918	0
CHB	chr13	PRR20A	61190918	0
CHB	chr13	PRR20A	61240918	0
CHB	chr13	PRR20A	61290918	0
CHB	chr13	PRR20A	61340918	0
CHB	chr13	PRR20A	61390918	0
CHB	chr13	PRR20A	61440918	0
CHB	chr13	PRR20A	61490918	0
CHB	chr13	PRR20A	61540918	0
CHB	chr13	PRR20A	61590918	0
CHB	chr13	PRR20A	61640918	0
CHB	chr13	PRR20A	61690918	0
CHB	chr13	PRR20A	61740918	0
CHB	chr13	PRR20A	61790918	0
CHB	chr13	PRR20A	61840918	0
CHB	chr13	PRR20A	61890918	0
CHB	chr13	PRR20A	61940918	0
CHB	chr13	PRR20A	61990918	0
CHB	chr13	PRR20A	62040918	10
CHB	chr13	PRR20A	62090918	62
CHB	chr19	PSG3	37771642	0
CHB	chr19	PSG3	37821642	0
CHB	chr19	PSG3	37871642	0
CHB	chr19	PSG3	37921642	0

CHB	chr19	PSG3	37971642	0
CHB	chr19	PSG3	38021642	0
CHB	chr19	PSG3	38071642	0
CHB	chr19	PSG3	38121642	0
CHB	chr19	PSG3	38171642	0
CHB	chr19	PSG3	38221642	0
CHB	chr19	PSG3	38271642	0
CHB	chr19	PSG3	38321642	0
CHB	chr19	PSG3	38371642	0
CHB	chr19	PSG3	38421642	0
CHB	chr19	PSG3	38471642	0
CHB	chr19	PSG3	38521642	0
CHB	chr19	PSG3	38571642	0
CHB	chr19	PSG3	38621642	0
CHB	chr19	PSG3	38671642	0
CHB	chr19	PSG3	38721642	15
CHB	chr19	PSG3	38771642	31
CHB	chr19	PSG3	38821642	43
CHB	chr19	PSG3	38871642	38
CHB	chr19	PSG3	38921642	26
CHB	chr19	PSG3	38971642	29
CHB	chr19	PSG3	39021642	25
CHB	chr19	PSG3	39071642	26
CHB	chr19	PSG3	39121642	15
CHB	chr19	PSG3	39171642	0
CHB	chr19	PSG3	39221642	4
CHB	chr19	PSG3	39271642	20
CHB	chr19	PSG3	39321642	43
CHB	chr19	PSG3	39371642	49
CHB	chr19	PSG3	39421642	41
CHB	chr19	PSG3	39471642	20
CHB	chr19	PSG3	39521642	1
CHB	chr19	PSG3	39571642	0
CHB	chr19	PSG3	39621642	0
CHB	chr19	PSG3	39671642	0
CHB	chr19	PSG3	39721642	0
CHB	chr19	PSG3	39771642	0
CHB	chr19	PSG3	39821642	0
CHB	chr19	PSG3	39871642	0
CHB	chr19	PSG3	39921642	0
CHB	chr19	PSG3	39971642	0
CHB	chr19	PSG3	40021642	0
CHB	chr19	PSG3	40071642	0
CHB	chr19	PSG3	40121642	0
CHB	chr19	PSG3	40171642	0
CHB	chr19	PSG3	40221642	0
CHB	chr19	PSG3	40271642	0
CHB	chr19	PSG3	40321642	0
CHB	chr19	PSG3	40371642	0

CHB	chr19	PSG3	40421642	0
CHB	chr19	PSG3	40471642	0
CHB	chr19	PSG3	40521642	0
CHB	chr19	PSG3	40571642	0
CHB	chr19	PSG3	40621642	0
CHB	chr19	PSG3	40671642	0
CHB	chr19	PSG3	40721642	0
CHB	chr19	PSG3	40771642	0
CHB	chr19	PSG3	40821642	0
CHB	chr19	PSG3	40871642	0
CHB	chr19	PSG3	40921642	0
CHB	chr19	PSG3	40971642	0
CHB	chr19	PSG3	41021642	0
CHB	chr19	PSG3	41071642	0
CHB	chr19	PSG3	41121642	0
CHB	chr19	PSG3	41171642	0
CHB	chr19	PSG3	41221642	0
CHB	chr19	PSG3	41271642	0
CHB	chr19	PSG3	41321642	0
CHB	chr19	PSG3	41371642	0
CHB	chr19	PSG3	41421642	0
CHB	chr19	PSG3	41471642	0
CHB	chr19	PSG3	41521642	8
CHB	chr19	PSG3	41571642	56
CHB	chr19	PSG3	41621642	63
CHB	chr19	PSG3	41671642	15
CHB	chr19	PSG3	41721642	0
CHB	chr19	PSG3	41771642	0
CHB	chr19	PSG3	41821642	0
CHB	chr19	PSG3	41871642	0
CHB	chr19	PSG3	41921642	0
CHB	chr19	PSG3	41971642	0
CHB	chr19	PSG3	42021642	0
CHB	chr19	PSG3	42071642	0
CHB	chr19	PSG3	42121642	0
CHB	chr19	PSG3	42171642	0
CHB	chr19	PSG3	42221642	0
CHB	chr19	PSG3	42271642	0
CHB	chr19	PSG3	42321642	0
CHB	chr19	PSG3	42371642	0
CHB	chr19	PSG3	42421642	0
CHB	chr19	PSG3	42471642	0
CHB	chr19	PSG3	42521642	0
CHB	chr19	PSG3	42571642	0
CHB	chr19	PSG3	42621642	0
CHB	chr19	PSG3	42671642	0
CHB	chr19	PSG3	42721642	0
CHB	chr19	PSG3	42771642	0
CHB	chr19	PSG3	42821642	0

CHB	chr19	PSG3	42871642	0
CHB	chr19	PSG3	42921642	0
CHB	chr19	PSG3	42971642	0
CHB	chr19	PSG3	43021642	0
CHB	chr19	PSG3	43071642	0
CHB	chr19	PSG3	43121642	4
CHB	chr19	PSG3	43171642	18
CHB	chr19	PSG3	43221642	48
CHB	chr19	PSG3	43271642	68
CHB	chr19	PSG3	43321642	76
CHB	chr19	PSG3	43371642	97
CHB	chr19	PSG3	43421642	127
CHB	chr19	PSG3	43471642	105
CHB	chr19	PSG3	43521642	75
CHB	chr19	PSG3	43571642	68
CHB	chr19	PSG3	43621642	33
CHB	chr19	PSG3	43671642	7
CHB	chr19	PSG3	43721642	0
CHB	chr19	PSG3	43771642	0
CHB	chr19	PSG3	43821642	0
CHB	chr19	PSG3	43871642	0
CHB	chr19	PSG3	43921642	0
CHB	chr19	PSG3	43971642	0
CHB	chr19	PSG3	44021642	0
CHB	chr19	PSG3	44071642	0
CHB	chr19	PSG3	44121642	0
CHB	chr19	PSG3	44171642	0
CHB	chr19	PSG3	44221642	0
CHB	chr19	PSG3	44271642	0
CHB	chr19	PSG3	44321642	0
CHB	chr19	PSG3	44371642	0
CHB	chr19	PSG3	44421642	0
CHB	chr19	PSG3	44471642	0
CHB	chr19	PSG3	44521642	0
CHB	chr19	PSG3	44571642	0
CHB	chr19	PSG3	44621642	0
CHB	chr19	PSG3	44671642	0
CHB	chr19	PSG3	44721642	0
CHB	chr19	PSG3	44771642	0
CHB	chr19	PSG3	44821642	0
CHB	chr19	PSG3	44871642	0
CHB	chr19	PSG3	44921642	0
CHB	chr19	PSG3	44971642	0
CHB	chr19	PSG3	45021642	0
CHB	chr19	PSG3	45071642	0
CHB	chr19	PSG3	45121642	0
CHB	chr19	PSG3	45171642	0
CHB	chr19	PSG3	45221642	0
CHB	chr19	PSG3	45271642	0

CHB	chr19	PSG3	45321642	0
CHB	chr19	PSG3	45371642	0
CHB	chr19	PSG3	45421642	0
CHB	chr19	PSG3	45471642	0
CHB	chr19	PSG3	45521642	0
CHB	chr19	PSG3	45571642	0
CHB	chr19	PSG3	45621642	0
CHB	chr19	PSG3	45671642	0
CHB	chr19	PSG3	45721642	0
CHB	chr19	PSG3	45771642	0
CHB	chr19	PSG3	45821642	0
CHB	chr19	PSG3	45871642	0
CHB	chr19	PSG3	45921642	0
CHB	chr19	PSG3	45971642	0
CHB	chr19	PSG3	46021642	0
CHB	chr19	PSG3	46071642	0
CHB	chr19	PSG3	46121642	0
CHB	chr19	PSG3	46171642	0
CHB	chr19	PSG3	46221642	0
CHB	chr19	PSG3	46271642	0
CHB	chr19	PSG3	46321642	0
CHB	chr19	PSG3	46371642	0
CHB	chr19	PSG3	46421642	0
CHB	chr19	PSG3	46471642	0
CHB	chr19	PSG3	46521642	0
CHB	chr19	PSG3	46571642	0
CHB	chr19	PSG3	46621642	0
CHB	chr19	PSG3	46671642	0
CHB	chr19	PSG3	46721642	0
CHB	chr19	PSG3	46771642	0
CHB	chr19	PSG3	46821642	0
CHB	chr19	PSG3	46871642	0
CHB	chr19	PSG3	46921642	0
CHB	chr19	PSG3	46971642	0
CHB	chr19	PSG3	47021642	0
CHB	chr19	PSG3	47071642	0
CHB	chr19	PSG3	47121642	0
CHB	chr19	PSG3	47171642	0
CHB	chr19	PSG3	47221642	0
CHB	chr19	PSG3	47271642	0
CHB	chr19	PSG3	47321642	0
CHB	chr19	PSG3	47371642	0
CHB	chr19	PSG3	47421642	0
CHB	chr19	PSG3	47471642	0
CHB	chr19	PSG3	47521642	0
CHB	chr19	PSG3	47571642	0
CHB	chr19	PSG3	47621642	0
CHB	chr19	PSG3	47671642	0
CHB	chr2	RGPD1	81963577	0

CHB	chr2	RGPD1	82013577	0
CHB	chr2	RGPD1	82063577	0
CHB	chr2	RGPD1	82113577	0
CHB	chr2	RGPD1	82163577	0
CHB	chr2	RGPD1	82213577	0
CHB	chr2	RGPD1	82263577	0
CHB	chr2	RGPD1	82313577	0
CHB	chr2	RGPD1	82363577	0
CHB	chr2	RGPD1	82413577	0
CHB	chr2	RGPD1	82463577	0
CHB	chr2	RGPD1	82513577	0
CHB	chr2	RGPD1	82563577	0
CHB	chr2	RGPD1	82613577	0
CHB	chr2	RGPD1	82663577	0
CHB	chr2	RGPD1	82713577	0
CHB	chr2	RGPD1	82763577	0
CHB	chr2	RGPD1	82813577	0
CHB	chr2	RGPD1	82863577	0
CHB	chr2	RGPD1	82913577	0
CHB	chr2	RGPD1	82963577	0
CHB	chr2	RGPD1	83013577	0
CHB	chr2	RGPD1	83063577	0
CHB	chr2	RGPD1	83113577	0
CHB	chr2	RGPD1	83163577	0
CHB	chr2	RGPD1	83213577	0
CHB	chr2	RGPD1	83263577	0
CHB	chr2	RGPD1	83313577	0
CHB	chr2	RGPD1	83363577	0
CHB	chr2	RGPD1	83413577	0
CHB	chr2	RGPD1	83463577	0
CHB	chr2	RGPD1	83513577	0
CHB	chr2	RGPD1	83563577	0
CHB	chr2	RGPD1	83613577	0
CHB	chr2	RGPD1	83663577	0
CHB	chr2	RGPD1	83713577	0
CHB	chr2	RGPD1	83763577	0
CHB	chr2	RGPD1	83813577	0
CHB	chr2	RGPD1	83863577	0
CHB	chr2	RGPD1	83913577	0
CHB	chr2	RGPD1	83963577	0
CHB	chr2	RGPD1	84013577	0
CHB	chr2	RGPD1	84063577	0
CHB	chr2	RGPD1	84113577	0
CHB	chr2	RGPD1	84163577	0
CHB	chr2	RGPD1	84213577	0
CHB	chr2	RGPD1	84263577	0
CHB	chr2	RGPD1	84313577	0
CHB	chr2	RGPD1	84363577	14
CHB	chr2	RGPD1	84413577	52

CHB	chr2	RGPD1	84463577	76
CHB	chr2	RGPD1	84513577	55
CHB	chr2	RGPD1	84563577	31
CHB	chr2	RGPD1	84613577	37
CHB	chr2	RGPD1	84663577	38
CHB	chr2	RGPD1	84713577	27
CHB	chr2	RGPD1	84763577	25
CHB	chr2	RGPD1	84813577	28
CHB	chr2	RGPD1	84863577	29
CHB	chr2	RGPD1	84913577	30
CHB	chr2	RGPD1	84963577	44
CHB	chr2	RGPD1	85013577	41
CHB	chr2	RGPD1	85063577	16
CHB	chr2	RGPD1	85113577	3
CHB	chr2	RGPD1	85163577	0
CHB	chr2	RGPD1	85213577	0
CHB	chr2	RGPD1	85263577	0
CHB	chr2	RGPD1	85313577	0
CHB	chr2	RGPD1	85363577	0
CHB	chr2	RGPD1	85413577	0
CHB	chr2	RGPD1	85463577	0
CHB	chr2	RGPD1	85513577	0
CHB	chr2	RGPD1	85563577	0
CHB	chr2	RGPD1	85613577	0
CHB	chr2	RGPD1	85663577	0
CHB	chr2	RGPD1	85713577	0
CHB	chr2	RGPD1	85763577	0
CHB	chr2	RGPD1	85813577	23
CHB	chr2	RGPD1	85863577	66
CHB	chr2	RGPD1	85913577	63
CHB	chr2	RGPD1	85963577	37
CHB	chr2	RGPD1	86013577	43
CHB	chr2	RGPD1	86063577	41
CHB	chr2	RGPD1	86113577	37
CHB	chr2	RGPD1	86163577	63
CHB	chr2	RGPD1	86213577	63
CHB	chr2	RGPD1	86263577	38
CHB	chr2	RGPD1	86313577	28
CHB	chr2	RGPD1	86363577	21
CHB	chr2	RGPD1	86413577	23
CHB	chr2	RGPD1	86463577	20
CHB	chr2	RGPD1	86513577	39
CHB	chr2	RGPD1	86563577	56
CHB	chr2	RGPD1	86613577	39
CHB	chr2	RGPD1	86663577	16
CHB	chr2	RGPD1	86713577	0
CHB	chr2	RGPD1	86763577	0
CHB	chr2	RGPD1	86813577	0
CHB	chr2	RGPD1	86863577	0

CHB	chr2	RGPD1	86913577	0
CHB	chr2	RGPD1	86963577	0
CHB	chr2	RGPD1	87013577	0
CHB	chr2	RGPD1	87063577	0
CHB	chr2	RGPD1	87113577	0
CHB	chr2	RGPD1	87163577	0
CHB	chr2	RGPD1	87213577	0
CHB	chr2	RGPD1	87263577	0
CHB	chr2	RGPD1	87313577	0
CHB	chr2	RGPD1	87363577	0
CHB	chr2	RGPD1	87413577	0
CHB	chr2	RGPD1	87463577	0
CHB	chr2	RGPD1	87513577	0
CHB	chr2	RGPD1	87563577	0
CHB	chr2	RGPD1	87613577	0
CHB	chr2	RGPD1	87663577	0
CHB	chr2	RGPD1	87713577	6
CHB	chr2	RGPD1	87763577	20
CHB	chr2	RGPD1	87813577	33
CHB	chr2	RGPD1	87863577	28
CHB	chr2	RGPD1	87913577	9
CHB	chr2	RGPD1	87963577	0
CHB	chr2	RGPD1	88013577	0
CHB	chr2	RGPD1	88063577	0
CHB	chr2	RGPD1	88113577	0
CHB	chr2	RGPD1	88163577	0
CHB	chr2	RGPD1	88213577	0
CHB	chr2	RGPD1	88263577	0
CHB	chr2	RGPD1	88313577	0
CHB	chr2	RGPD1	88363577	0
CHB	chr2	RGPD1	88413577	0
CHB	chr2	RGPD1	88463577	0
CHB	chr2	RGPD1	88513577	0
CHB	chr2	RGPD1	88563577	0
CHB	chr2	RGPD1	88613577	0
CHB	chr2	RGPD1	88663577	0
CHB	chr2	RGPD1	88713577	0
CHB	chr2	RGPD1	88763577	0
CHB	chr2	RGPD1	88813577	0
CHB	chr2	RGPD1	88863577	0
CHB	chr2	RGPD1	88913577	0
CHB	chr2	RGPD1	88963577	0
CHB	chr2	RGPD1	89013577	0
CHB	chr2	RGPD1	89063577	0
CHB	chr2	RGPD1	89113577	0
CHB	chr2	RGPD1	89163577	19
CHB	chr2	RGPD1	89213577	30
CHB	chr2	RGPD1	89263577	22
CHB	chr2	RGPD1	89313577	13

CHB	chr2	RGPD1	89363577	2
CHB	chr2	RGPD1	89413577	0
CHB	chr2	RGPD1	89463577	0
CHB	chr2	RGPD1	89513577	0
CHB	chr2	RGPD1	89563577	0
CHB	chr2	RGPD1	89613577	0
CHB	chr2	RGPD1	89663577	0
CHB	chr2	RGPD1	89713577	0
CHB	chr2	RGPD1	89763577	0
CHB	chr2	RGPD1	89813577	0
CHB	chr2	RGPD1	89863577	0
CHB	chr2	RGPD1	89913577	0
CHB	chr2	RGPD1	89963577	0
CHB	chr2	RGPD1	90013577	0
CHB	chr2	RGPD1	90063577	0
CHB	chr2	RGPD1	90113577	0
CHB	chr2	RGPD1	90163577	0
CHB	chr2	RGPD1	90213577	0
CHB	chr2	RGPD1	90263577	0
CHB	chr2	RGPD1	90313577	0
CHB	chr2	RGPD1	90363577	0
CHB	chr2	RGPD1	90413577	0
CHB	chr2	RGPD1	90463577	0
CHB	chr2	RGPD1	90513577	0
CHB	chr2	RGPD1	90563577	0
CHB	chr2	RGPD1	90613577	0
CHB	chr2	RGPD1	90663577	0
CHB	chr2	RGPD1	90713577	0
CHB	chr2	RGPD1	90763577	0
CHB	chr2	RGPD1	90813577	0
CHB	chr2	RGPD1	90863577	0
CHB	chr2	RGPD1	90913577	0
CHB	chr2	RGPD1	90963577	0
CHB	chr2	RGPD1	91013577	0
CHB	chr2	RGPD1	91063577	0
CHB	chr2	RGPD1	91113577	0
CHB	chr2	RGPD1	91163577	0
CHB	chr2	RGPD1	91213577	0
CHB	chr2	RGPD1	91263577	0
CHB	chr2	RGPD1	91313577	0
CHB	chr2	RGPD1	91363577	0
CHB	chr2	RGPD1	91413577	0
CHB	chr2	RGPD1	91463577	0
CHB	chr2	RGPD1	91513577	0
CHB	chr2	RGPD1	91563577	0
CHB	chr2	RGPD1	91613577	0
CHB	chr2	RGPD1	91663577	0
CHB	chr2	RGPD1	91713577	0
CHB	chr2	RGPD1	91763577	0

CHB	chr2	RGPD1	91813577	0
CHB	chr2	RGPD1	91863577	0
CHB	chr2	RGPD1	91913577	0
CHB	chr2	RGPD1	91963577	0
CHB	chr7	SPDYE3	95356798	0
CHB	chr7	SPDYE3	95406798	0
CHB	chr7	SPDYE3	95456798	0
CHB	chr7	SPDYE3	95506798	0
CHB	chr7	SPDYE3	95556798	0
CHB	chr7	SPDYE3	95606798	0
CHB	chr7	SPDYE3	95656798	0
CHB	chr7	SPDYE3	95706798	0
CHB	chr7	SPDYE3	95756798	0
CHB	chr7	SPDYE3	95806798	0
CHB	chr7	SPDYE3	95856798	0
CHB	chr7	SPDYE3	95906798	0
CHB	chr7	SPDYE3	95956798	0
CHB	chr7	SPDYE3	96006798	0
CHB	chr7	SPDYE3	96056798	0
CHB	chr7	SPDYE3	96106798	0
CHB	chr7	SPDYE3	96156798	0
CHB	chr7	SPDYE3	96206798	0
CHB	chr7	SPDYE3	96256798	0
CHB	chr7	SPDYE3	96306798	0
CHB	chr7	SPDYE3	96356798	0
CHB	chr7	SPDYE3	96406798	0
CHB	chr7	SPDYE3	96456798	0
CHB	chr7	SPDYE3	96506798	0
CHB	chr7	SPDYE3	96556798	0
CHB	chr7	SPDYE3	96606798	0
CHB	chr7	SPDYE3	96656798	0
CHB	chr7	SPDYE3	96706798	0
CHB	chr7	SPDYE3	96756798	0
CHB	chr7	SPDYE3	96806798	0
CHB	chr7	SPDYE3	96856798	0
CHB	chr7	SPDYE3	96906798	0
CHB	chr7	SPDYE3	96956798	0
CHB	chr7	SPDYE3	97006798	0
CHB	chr7	SPDYE3	97056798	0
CHB	chr7	SPDYE3	97106798	0
CHB	chr7	SPDYE3	97156798	0
CHB	chr7	SPDYE3	97206798	0
CHB	chr7	SPDYE3	97256798	0
CHB	chr7	SPDYE3	97306798	0
CHB	chr7	SPDYE3	97356798	0
CHB	chr7	SPDYE3	97406798	0
CHB	chr7	SPDYE3	97456798	0
CHB	chr7	SPDYE3	97506798	0
CHB	chr7	SPDYE3	97556798	0

CHB	chr7	SPDYE3	97606798	0
CHB	chr7	SPDYE3	97656798	0
CHB	chr7	SPDYE3	97706798	0
CHB	chr7	SPDYE3	97756798	0
CHB	chr7	SPDYE3	97806798	0
CHB	chr7	SPDYE3	97856798	0
CHB	chr7	SPDYE3	97906798	0
CHB	chr7	SPDYE3	97956798	0
CHB	chr7	SPDYE3	98006798	0
CHB	chr7	SPDYE3	98056798	0
CHB	chr7	SPDYE3	98106798	0
CHB	chr7	SPDYE3	98156798	0
CHB	chr7	SPDYE3	98206798	0
CHB	chr7	SPDYE3	98256798	0
CHB	chr7	SPDYE3	98306798	0
CHB	chr7	SPDYE3	98356798	0
CHB	chr7	SPDYE3	98406798	0
CHB	chr7	SPDYE3	98456798	0
CHB	chr7	SPDYE3	98506798	0
CHB	chr7	SPDYE3	98556798	0
CHB	chr7	SPDYE3	98606798	0
CHB	chr7	SPDYE3	98656798	0
CHB	chr7	SPDYE3	98706798	0
CHB	chr7	SPDYE3	98756798	0
CHB	chr7	SPDYE3	98806798	0
CHB	chr7	SPDYE3	98856798	0
CHB	chr7	SPDYE3	98906798	0
CHB	chr7	SPDYE3	98956798	0
CHB	chr7	SPDYE3	99006798	0
CHB	chr7	SPDYE3	99056798	0
CHB	chr7	SPDYE3	99106798	0
CHB	chr7	SPDYE3	99156798	0
CHB	chr7	SPDYE3	99206798	0
CHB	chr7	SPDYE3	99256798	0
CHB	chr7	SPDYE3	99306798	0
CHB	chr7	SPDYE3	99356798	0
CHB	chr7	SPDYE3	99406798	0
CHB	chr7	SPDYE3	99456798	0
CHB	chr7	SPDYE3	99506798	0
CHB	chr7	SPDYE3	99556798	0
CHB	chr7	SPDYE3	99606798	0
CHB	chr7	SPDYE3	99656798	0
CHB	chr7	SPDYE3	99706798	0
CHB	chr7	SPDYE3	99756798	0
CHB	chr7	SPDYE3	99806798	0
CHB	chr7	SPDYE3	99856798	0
CHB	chr7	SPDYE3	99906798	0
CHB	chr7	SPDYE3	99956798	0
CHB	chr7	SPDYE3	100006798	0

CHB	chr7	SPDYE3	100056798	0
CHB	chr7	SPDYE3	100106798	0
CHB	chr7	SPDYE3	100156798	0
CHB	chr7	SPDYE3	100206798	0
CHB	chr7	SPDYE3	100256798	0
CHB	chr7	SPDYE3	100306798	0
CHB	chr7	SPDYE3	100356798	0
CHB	chr7	SPDYE3	100406798	0
CHB	chr7	SPDYE3	100456798	0
CHB	chr7	SPDYE3	100506798	0
CHB	chr7	SPDYE3	100556798	0
CHB	chr7	SPDYE3	100606798	0
CHB	chr7	SPDYE3	100656798	0
CHB	chr7	SPDYE3	100706798	0
CHB	chr7	SPDYE3	100756798	0
CHB	chr7	SPDYE3	100806798	0
CHB	chr7	SPDYE3	100856798	0
CHB	chr7	SPDYE3	100906798	0
CHB	chr7	SPDYE3	100956798	0
CHB	chr7	SPDYE3	101006798	0
CHB	chr7	SPDYE3	101056798	0
CHB	chr7	SPDYE3	101106798	0
CHB	chr7	SPDYE3	101156798	0
CHB	chr7	SPDYE3	101206798	0
CHB	chr7	SPDYE3	101256798	0
CHB	chr7	SPDYE3	101306798	0
CHB	chr7	SPDYE3	101356798	0
CHB	chr7	SPDYE3	101406798	0
CHB	chr7	SPDYE3	101456798	0
CHB	chr7	SPDYE3	101506798	0
CHB	chr7	SPDYE3	101556798	0
CHB	chr7	SPDYE3	101606798	0
CHB	chr7	SPDYE3	101656798	0
CHB	chr7	SPDYE3	101706798	0
CHB	chr7	SPDYE3	101756798	0
CHB	chr7	SPDYE3	101806798	0
CHB	chr7	SPDYE3	101856798	0
CHB	chr7	SPDYE3	101906798	0
CHB	chr7	SPDYE3	101956798	0
CHB	chr7	SPDYE3	102006798	0
CHB	chr7	SPDYE3	102056798	0
CHB	chr7	SPDYE3	102106798	0
CHB	chr7	SPDYE3	102156798	0
CHB	chr7	SPDYE3	102206798	0
CHB	chr7	SPDYE3	102256798	0
CHB	chr7	SPDYE3	102306798	0
CHB	chr7	SPDYE3	102356798	0
CHB	chr7	SPDYE3	102406798	0
CHB	chr7	SPDYE3	102456798	0

CHB	chr7	SPDYE3	102506798	0
CHB	chr7	SPDYE3	102556798	0
CHB	chr7	SPDYE3	102606798	0
CHB	chr7	SPDYE3	102656798	0
CHB	chr7	SPDYE3	102706798	0
CHB	chr7	SPDYE3	102756798	0
CHB	chr7	SPDYE3	102806798	0
CHB	chr7	SPDYE3	102856798	0
CHB	chr7	SPDYE3	102906798	0
CHB	chr7	SPDYE3	102956798	0
CHB	chr7	SPDYE3	103006798	0
CHB	chr7	SPDYE3	103056798	0
CHB	chr7	SPDYE3	103106798	0
CHB	chr7	SPDYE3	103156798	0
CHB	chr7	SPDYE3	103206798	0
CHB	chr7	SPDYE3	103256798	0
CHB	chr7	SPDYE3	103306798	0
CHB	chr7	SPDYE3	103356798	0
CHB	chr7	SPDYE3	103406798	21
CHB	chr7	SPDYE3	103456798	35
CHB	chr7	SPDYE3	103506798	82
CHB	chr7	SPDYE3	103556798	85
CHB	chr7	SPDYE3	103606798	17
CHB	chr7	SPDYE3	103656798	0
CHB	chr7	SPDYE3	103706798	0
CHB	chr7	SPDYE3	103756798	0
CHB	chr7	SPDYE3	103806798	0
CHB	chr7	SPDYE3	103856798	15
CHB	chr7	SPDYE3	103906798	34
CHB	chr7	SPDYE3	103956798	35
CHB	chr7	SPDYE3	104006798	34
CHB	chr7	SPDYE3	104056798	44
CHB	chr7	SPDYE3	104106798	44
CHB	chr7	SPDYE3	104156798	39
CHB	chr7	SPDYE3	104206798	77
CHB	chr7	SPDYE3	104256798	118
CHB	chr7	SPDYE3	104306798	73
CHB	chr7	SPDYE3	104356798	42
CHB	chr7	SPDYE3	104406798	37
CHB	chr7	SPDYE3	104456798	16
CHB	chr7	SPDYE3	104506798	30
CHB	chr7	SPDYE3	104556798	34
CHB	chr7	SPDYE3	104606798	49
CHB	chr7	SPDYE3	104656798	81
CHB	chr7	SPDYE3	104706798	70
CHB	chr7	SPDYE3	104756798	60
CHB	chr7	SPDYE3	104806798	78
CHB	chr7	SPDYE3	104856798	70
CHB	chr7	SPDYE3	104906798	50

CHB	chr7	SPDYE3	104956798	38
CHB	chr7	SPDYE3	105006798	53
CHB	chr7	SPDYE3	105056798	67
CHB	chr7	SPDYE3	105106798	83
CHB	chr7	SPDYE3	105156798	138
CHB	chr7	SPDYE3	105206798	88
CHB	chr7	SPDYE3	105256798	3
CHB	chr16	SULT1A3	25249255	0
CHB	chr16	SULT1A3	25299255	0
CHB	chr16	SULT1A3	25349255	0
CHB	chr16	SULT1A3	25399255	0
CHB	chr16	SULT1A3	25449255	0
CHB	chr16	SULT1A3	25499255	0
CHB	chr16	SULT1A3	25549255	0
CHB	chr16	SULT1A3	25599255	0
CHB	chr16	SULT1A3	25649255	0
CHB	chr16	SULT1A3	25699255	0
CHB	chr16	SULT1A3	25749255	0
CHB	chr16	SULT1A3	25799255	0
CHB	chr16	SULT1A3	25849255	0
CHB	chr16	SULT1A3	25899255	6
CHB	chr16	SULT1A3	25949255	38
CHB	chr16	SULT1A3	25999255	32
CHB	chr16	SULT1A3	26049255	0
CHB	chr16	SULT1A3	26099255	0
CHB	chr16	SULT1A3	26149255	0
CHB	chr16	SULT1A3	26199255	0
CHB	chr16	SULT1A3	26249255	0
CHB	chr16	SULT1A3	26299255	0
CHB	chr16	SULT1A3	26349255	0
CHB	chr16	SULT1A3	26399255	0
CHB	chr16	SULT1A3	26449255	0
CHB	chr16	SULT1A3	26499255	0
CHB	chr16	SULT1A3	26549255	0
CHB	chr16	SULT1A3	26599255	0
CHB	chr16	SULT1A3	26649255	0
CHB	chr16	SULT1A3	26699255	0
CHB	chr16	SULT1A3	26749255	0
CHB	chr16	SULT1A3	26799255	0
CHB	chr16	SULT1A3	26849255	0
CHB	chr16	SULT1A3	26899255	0
CHB	chr16	SULT1A3	26949255	0
CHB	chr16	SULT1A3	26999255	0
CHB	chr16	SULT1A3	27049255	0
CHB	chr16	SULT1A3	27099255	0
CHB	chr16	SULT1A3	27149255	0
CHB	chr16	SULT1A3	27199255	0
CHB	chr16	SULT1A3	27249255	0
CHB	chr16	SULT1A3	27299255	0

CHB	chr16	SULT1A3	27349255	0
CHB	chr16	SULT1A3	27399255	0
CHB	chr16	SULT1A3	27449255	0
CHB	chr16	SULT1A3	27499255	0
CHB	chr16	SULT1A3	27549255	0
CHB	chr16	SULT1A3	27599255	0
CHB	chr16	SULT1A3	27649255	0
CHB	chr16	SULT1A3	27699255	0
CHB	chr16	SULT1A3	27749255	0
CHB	chr16	SULT1A3	27799255	0
CHB	chr16	SULT1A3	27849255	0
CHB	chr16	SULT1A3	27899255	0
CHB	chr16	SULT1A3	27949255	0
CHB	chr16	SULT1A3	27999255	0
CHB	chr16	SULT1A3	28049255	0
CHB	chr16	SULT1A3	28099255	0
CHB	chr16	SULT1A3	28149255	0
CHB	chr16	SULT1A3	28199255	0
CHB	chr16	SULT1A3	28249255	0
CHB	chr16	SULT1A3	28299255	0
CHB	chr16	SULT1A3	28349255	0
CHB	chr16	SULT1A3	28399255	0
CHB	chr16	SULT1A3	28449255	0
CHB	chr16	SULT1A3	28499255	0
CHB	chr16	SULT1A3	28549255	0
CHB	chr16	SULT1A3	28599255	0
CHB	chr16	SULT1A3	28649255	0
CHB	chr16	SULT1A3	28699255	0
CHB	chr16	SULT1A3	28749255	0
CHB	chr16	SULT1A3	28799255	0
CHB	chr16	SULT1A3	28849255	0
CHB	chr16	SULT1A3	28899255	0
CHB	chr16	SULT1A3	28949255	0
CHB	chr16	SULT1A3	28999255	0
CHB	chr16	SULT1A3	29049255	0
CHB	chr16	SULT1A3	29099255	0
CHB	chr16	SULT1A3	29149255	0
CHB	chr16	SULT1A3	29199255	0
CHB	chr16	SULT1A3	29249255	0
CHB	chr16	SULT1A3	29299255	0
CHB	chr16	SULT1A3	29349255	0
CHB	chr16	SULT1A3	29399255	0
CHB	chr16	SULT1A3	29449255	0
CHB	chr16	SULT1A3	29499255	0
CHB	chr16	SULT1A3	29549255	0
CHB	chr16	SULT1A3	29599255	0
CHB	chr16	SULT1A3	29649255	0
CHB	chr16	SULT1A3	29699255	0
CHB	chr16	SULT1A3	29749255	0

CHB	chr16	SULT1A3	29799255	0
CHB	chr16	SULT1A3	29849255	0
CHB	chr16	SULT1A3	29899255	0
CHB	chr16	SULT1A3	29949255	0
CHB	chr16	SULT1A3	29999255	0
CHB	chr16	SULT1A3	30049255	0
CHB	chr16	SULT1A3	30099255	0
CHB	chr16	SULT1A3	30149255	0
CHB	chr16	SULT1A3	30199255	0
CHB	chr16	SULT1A3	30249255	0
CHB	chr16	SULT1A3	30299255	0
CHB	chr16	SULT1A3	30349255	0
CHB	chr16	SULT1A3	30399255	0
CHB	chr16	SULT1A3	30449255	0
CHB	chr16	SULT1A3	30499255	0
CHB	chr16	SULT1A3	30549255	0
CHB	chr16	SULT1A3	30599255	0
CHB	chr16	SULT1A3	30649255	0
CHB	chr16	SULT1A3	30699255	0
CHB	chr16	SULT1A3	30749255	0
CHB	chr16	SULT1A3	30799255	0
CHB	chr16	SULT1A3	30849255	0
CHB	chr16	SULT1A3	30899255	0
CHB	chr16	SULT1A3	30949255	0
CHB	chr16	SULT1A3	30999255	0
CHB	chr16	SULT1A3	31049255	0
CHB	chr16	SULT1A3	31099255	0
CHB	chr16	SULT1A3	31149255	0
CHB	chr16	SULT1A3	31199255	0
CHB	chr16	SULT1A3	31249255	0
CHB	chr16	SULT1A3	31299255	0
CHB	chr16	SULT1A3	31349255	0
CHB	chr16	SULT1A3	31399255	0
CHB	chr16	SULT1A3	31449255	0
CHB	chr16	SULT1A3	31499255	0
CHB	chr16	SULT1A3	31549255	0
CHB	chr16	SULT1A3	31599255	0
CHB	chr16	SULT1A3	31649255	0
CHB	chr16	SULT1A3	31699255	0
CHB	chr16	SULT1A3	31749255	0
CHB	chr16	SULT1A3	31799255	0
CHB	chr16	SULT1A3	31849255	0
CHB	chr16	SULT1A3	31899255	0
CHB	chr16	SULT1A3	31949255	0
CHB	chr16	SULT1A3	31999255	0
CHB	chr16	SULT1A3	32049255	0
CHB	chr16	SULT1A3	32099255	0
CHB	chr16	SULT1A3	32149255	0
CHB	chr16	SULT1A3	32199255	0

CHB	chr16	SULT1A3	32249255	0
CHB	chr16	SULT1A3	32299255	0
CHB	chr16	SULT1A3	32349255	0
CHB	chr16	SULT1A3	32399255	0
CHB	chr16	SULT1A3	32449255	0
CHB	chr16	SULT1A3	32499255	0
CHB	chr16	SULT1A3	32549255	0
CHB	chr16	SULT1A3	32599255	0
CHB	chr16	SULT1A3	32649255	0
CHB	chr16	SULT1A3	32699255	0
CHB	chr16	SULT1A3	32749255	0
CHB	chr16	SULT1A3	32799255	0
CHB	chr16	SULT1A3	32849255	0
CHB	chr16	SULT1A3	32899255	0
CHB	chr16	SULT1A3	32949255	0
CHB	chr16	SULT1A3	32999255	0
CHB	chr16	SULT1A3	33049255	0
CHB	chr16	SULT1A3	33099255	0
CHB	chr16	SULT1A3	33149255	0
CHB	chr16	SULT1A3	33199255	0
CHB	chr16	SULT1A3	33249255	0
CHB	chr16	SULT1A3	33299255	0
CHB	chr16	SULT1A3	33349255	0
CHB	chr16	SULT1A3	33399255	0
CHB	chr16	SULT1A3	33449255	0
CHB	chr16	SULT1A3	33499255	0
CHB	chr16	SULT1A3	33549255	0
CHB	chr16	SULT1A3	33599255	0
CHB	chr16	SULT1A3	33649255	0
CHB	chr16	SULT1A3	33699255	0
CHB	chr16	SULT1A3	33749255	0
CHB	chr16	SULT1A3	33799255	0
CHB	chr16	SULT1A3	33849255	0
CHB	chr16	SULT1A3	33899255	0
CHB	chr16	SULT1A3	33949255	0
CHB	chr16	SULT1A3	33999255	0
CHB	chr16	SULT1A3	34049255	0
CHB	chr16	SULT1A3	34099255	0
CHB	chr16	SULT1A3	34149255	0
CHB	chr16	SULT1A3	34199255	0
CHB	chr16	SULT1A3	34249255	0
CHB	chr16	SULT1A3	34299255	0
CHB	chr16	SULT1A3	34349255	0
CHB	chr16	SULT1A3	34399255	0
CHB	chr16	SULT1A3	34449255	0
CHB	chr16	SULT1A3	34499255	0
CHB	chr16	SULT1A3	34549255	0
CHB	chr16	SULT1A3	34599255	0
CHB	chr16	SULT1A3	34649255	0

CHB	chr16	SULT1A3	34699255	0
CHB	chr16	SULT1A3	34749255	0
CHB	chr16	SULT1A3	34799255	0
CHB	chr16	SULT1A3	34849255	0
CHB	chr16	SULT1A3	34899255	0
CHB	chr16	SULT1A3	34949255	0
CHB	chr16	SULT1A3	34999255	0
CHB	chr16	SULT1A3	35049255	0
CHB	chr16	SULT1A3	35099255	0
CHB	chr16	SULT1A3	35149255	0
CHB	chr6	TBC1D3	116129494	0
CHB	chr6	TBC1D3	116179494	0
CHB	chr6	TBC1D3	116229494	0
CHB	chr6	TBC1D3	116279494	0
CHB	chr6	TBC1D3	116329494	0
CHB	chr6	TBC1D3	116379494	0
CHB	chr6	TBC1D3	116429494	0
CHB	chr6	TBC1D3	116479494	0
CHB	chr6	TBC1D3	116529494	0
CHB	chr6	TBC1D3	116579494	0
CHB	chr6	TBC1D3	116629494	0
CHB	chr6	TBC1D3	116679494	0
CHB	chr6	TBC1D3	116729494	0
CHB	chr6	TBC1D3	116779494	0
CHB	chr6	TBC1D3	116829494	0
CHB	chr6	TBC1D3	116879494	0
CHB	chr6	TBC1D3	116929494	0
CHB	chr6	TBC1D3	116979494	0
CHB	chr6	TBC1D3	117029494	0
CHB	chr6	TBC1D3	117079494	0
CHB	chr6	TBC1D3	117129494	0
CHB	chr6	TBC1D3	117179494	0
CHB	chr6	TBC1D3	117229494	0
CHB	chr6	TBC1D3	117279494	0
CHB	chr6	TBC1D3	117329494	0
CHB	chr6	TBC1D3	117379494	13
CHB	chr6	TBC1D3	117429494	65
CHB	chr6	TBC1D3	117479494	131
CHB	chr6	TBC1D3	117529494	92
CHB	chr6	TBC1D3	117579494	30
CHB	chr6	TBC1D3	117629494	55
CHB	chr6	TBC1D3	117679494	65
CHB	chr6	TBC1D3	117729494	73
CHB	chr6	TBC1D3	117779494	77
CHB	chr6	TBC1D3	117829494	47
CHB	chr6	TBC1D3	117879494	16
CHB	chr6	TBC1D3	117929494	0
CHB	chr6	TBC1D3	117979494	0
CHB	chr6	TBC1D3	118029494	0

CHB	chr6	TBC1D3	118079494	0
CHB	chr6	TBC1D3	118129494	0
CHB	chr6	TBC1D3	118179494	0
CHB	chr6	TBC1D3	118229494	0
CHB	chr6	TBC1D3	118279494	0
CHB	chr6	TBC1D3	118329494	0
CHB	chr6	TBC1D3	118379494	0
CHB	chr6	TBC1D3	118429494	0
CHB	chr6	TBC1D3	118479494	0
CHB	chr6	TBC1D3	118529494	0
CHB	chr6	TBC1D3	118579494	0
CHB	chr6	TBC1D3	118629494	0
CHB	chr6	TBC1D3	118679494	0
CHB	chr6	TBC1D3	118729494	0
CHB	chr6	TBC1D3	118779494	0
CHB	chr6	TBC1D3	118829494	0
CHB	chr6	TBC1D3	118879494	0
CHB	chr6	TBC1D3	118929494	0
CHB	chr6	TBC1D3	118979494	0
CHB	chr6	TBC1D3	119029494	0
CHB	chr6	TBC1D3	119079494	0
CHB	chr6	TBC1D3	119129494	0
CHB	chr6	TBC1D3	119179494	0
CHB	chr6	TBC1D3	119229494	0
CHB	chr6	TBC1D3	119279494	0
CHB	chr6	TBC1D3	119329494	0
CHB	chr6	TBC1D3	119379494	0
CHB	chr6	TBC1D3	119429494	8
CHB	chr6	TBC1D3	119479494	38
CHB	chr6	TBC1D3	119529494	50
CHB	chr6	TBC1D3	119579494	48
CHB	chr6	TBC1D3	119629494	142
CHB	chr6	TBC1D3	119679494	143
CHB	chr6	TBC1D3	119729494	54
CHB	chr6	TBC1D3	119779494	42
CHB	chr6	TBC1D3	119829494	27
CHB	chr6	TBC1D3	119879494	31
CHB	chr6	TBC1D3	119929494	30
CHB	chr6	TBC1D3	119979494	27
CHB	chr6	TBC1D3	120029494	87
CHB	chr6	TBC1D3	120079494	98
CHB	chr6	TBC1D3	120129494	49
CHB	chr6	TBC1D3	120179494	51
CHB	chr6	TBC1D3	120229494	37
CHB	chr6	TBC1D3	120279494	6
CHB	chr6	TBC1D3	120329494	0
CHB	chr6	TBC1D3	120379494	0
CHB	chr6	TBC1D3	120429494	0
CHB	chr6	TBC1D3	120479494	0

CHB	chr6	TBC1D3	120529494	0
CHB	chr6	TBC1D3	120579494	1
CHB	chr6	TBC1D3	120629494	22
CHB	chr6	TBC1D3	120679494	38
CHB	chr6	TBC1D3	120729494	95
CHB	chr6	TBC1D3	120779494	111
CHB	chr6	TBC1D3	120829494	82
CHB	chr6	TBC1D3	120879494	71
CHB	chr6	TBC1D3	120929494	38
CHB	chr6	TBC1D3	120979494	32
CHB	chr6	TBC1D3	121029494	40
CHB	chr6	TBC1D3	121079494	76
CHB	chr6	TBC1D3	121129494	113
CHB	chr6	TBC1D3	121179494	81
CHB	chr6	TBC1D3	121229494	38
CHB	chr6	TBC1D3	121279494	33
CHB	chr6	TBC1D3	121329494	39
CHB	chr6	TBC1D3	121379494	50
CHB	chr6	TBC1D3	121429494	36
CHB	chr6	TBC1D3	121479494	24
CHB	chr6	TBC1D3	121529494	59
CHB	chr6	TBC1D3	121579494	87
CHB	chr6	TBC1D3	121629494	62
CHB	chr6	TBC1D3	121679494	41
CHB	chr6	TBC1D3	121729494	53
CHB	chr6	TBC1D3	121779494	67
CHB	chr6	TBC1D3	121829494	59
CHB	chr6	TBC1D3	121879494	36
CHB	chr6	TBC1D3	121929494	68
CHB	chr6	TBC1D3	121979494	70
CHB	chr6	TBC1D3	122029494	25
CHB	chr6	TBC1D3	122079494	28
CHB	chr6	TBC1D3	122129494	34
CHB	chr6	TBC1D3	122179494	32
CHB	chr6	TBC1D3	122229494	33
CHB	chr6	TBC1D3	122279494	38
CHB	chr6	TBC1D3	122329494	29
CHB	chr6	TBC1D3	122379494	21
CHB	chr6	TBC1D3	122429494	30
CHB	chr6	TBC1D3	122479494	35
CHB	chr6	TBC1D3	122529494	39
CHB	chr6	TBC1D3	122579494	36
CHB	chr6	TBC1D3	122629494	36
CHB	chr6	TBC1D3	122679494	38
CHB	chr6	TBC1D3	122729494	30
CHB	chr6	TBC1D3	122779494	33
CHB	chr6	TBC1D3	122829494	42
CHB	chr6	TBC1D3	122879494	51
CHB	chr6	TBC1D3	122929494	50

CHB	chr6	TBC1D3	122979494	49
CHB	chr6	TBC1D3	123029494	39
CHB	chr6	TBC1D3	123079494	12
CHB	chr6	TBC1D3	123129494	35
CHB	chr6	TBC1D3	123179494	35
CHB	chr6	TBC1D3	123229494	0
CHB	chr6	TBC1D3	123279494	0
CHB	chr6	TBC1D3	123329494	0
CHB	chr6	TBC1D3	123379494	0
CHB	chr6	TBC1D3	123429494	0
CHB	chr6	TBC1D3	123479494	21
CHB	chr6	TBC1D3	123529494	49
CHB	chr6	TBC1D3	123579494	48
CHB	chr6	TBC1D3	123629494	43
CHB	chr6	TBC1D3	123679494	55
CHB	chr6	TBC1D3	123729494	48
CHB	chr6	TBC1D3	123779494	34
CHB	chr6	TBC1D3	123829494	34
CHB	chr6	TBC1D3	123879494	16
CHB	chr6	TBC1D3	123929494	0
CHB	chr6	TBC1D3	123979494	0
CHB	chr6	TBC1D3	124029494	0
CHB	chr6	TBC1D3	124079494	0
CHB	chr6	TBC1D3	124129494	0
CHB	chr6	TBC1D3	124179494	0
CHB	chr6	TBC1D3	124229494	0
CHB	chr6	TBC1D3	124279494	0
CHB	chr6	TBC1D3	124329494	0
CHB	chr6	TBC1D3	124379494	0
CHB	chr6	TBC1D3	124429494	0
CHB	chr6	TBC1D3	124479494	0
CHB	chr6	TBC1D3	124529494	0
CHB	chr6	TBC1D3	124579494	0
CHB	chr6	TBC1D3	124629494	13
CHB	chr6	TBC1D3	124679494	40
CHB	chr6	TBC1D3	124729494	57
CHB	chr6	TBC1D3	124779494	42
CHB	chr6	TBC1D3	124829494	12
CHB	chr6	TBC1D3	124879494	0
CHB	chr6	TBC1D3	124929494	0
CHB	chr6	TBC1D3	124979494	0
CHB	chr6	TBC1D3	125029494	0
CHB	chr6	TBC1D3	125079494	0
CHB	chr6	TBC1D3	125129494	0
CHB	chr6	TBC1D3	125179494	0
CHB	chr6	TBC1D3	125229494	0
CHB	chr6	TBC1D3	125279494	0
CHB	chr6	TBC1D3	125329494	0
CHB	chr6	TBC1D3	125379494	0

CHB	chr6	TBC1D3	125429494	0
CHB	chr6	TBC1D3	125479494	0
CHB	chr6	TBC1D3	125529494	0
CHB	chr6	TBC1D3	125579494	0
CHB	chr6	TBC1D3	125629494	0
CHB	chr6	TBC1D3	125679494	0
CHB	chr6	TBC1D3	125729494	0
CHB	chr6	TBC1D3	125779494	0
CHB	chr6	TBC1D3	125829494	0
CHB	chr6	TBC1D3	125879494	0
CHB	chr6	TBC1D3	125929494	0
CHB	chr6	TBC1D3	125979494	0
CHB	chr6	TBC1D3	126029494	0
CHB	chr6	TBC1D3	126079494	0
CHB	chr6	TBC1D3	126129494	0
CHB	chr6	TBC1D3	126179494	0
CHB	chr6	TBC1D3	126229494	0
CHB	chr6	TBC1D3	126279494	0
CHB	chr8	TBC1D3	118091961	0
CHB	chr8	TBC1D3	118141961	0
CHB	chr8	TBC1D3	118191961	0
CHB	chr8	TBC1D3	118241961	0
CHB	chr8	TBC1D3	118291961	0
CHB	chr8	TBC1D3	118341961	0
CHB	chr8	TBC1D3	118391961	0
CHB	chr8	TBC1D3	118441961	0
CHB	chr8	TBC1D3	118491961	0
CHB	chr8	TBC1D3	118541961	0
CHB	chr8	TBC1D3	118591961	0
CHB	chr8	TBC1D3	118641961	0
CHB	chr8	TBC1D3	118691961	0
CHB	chr8	TBC1D3	118741961	0
CHB	chr8	TBC1D3	118791961	0
CHB	chr8	TBC1D3	118841961	0
CHB	chr8	TBC1D3	118891961	0
CHB	chr8	TBC1D3	118941961	0
CHB	chr8	TBC1D3	118991961	0
CHB	chr8	TBC1D3	119041961	0
CHB	chr8	TBC1D3	119091961	0
CHB	chr8	TBC1D3	119141961	0
CHB	chr8	TBC1D3	119191961	0
CHB	chr8	TBC1D3	119241961	0
CHB	chr8	TBC1D3	119291961	0
CHB	chr8	TBC1D3	119341961	0
CHB	chr8	TBC1D3	119391961	0
CHB	chr8	TBC1D3	119441961	0
CHB	chr8	TBC1D3	119491961	0
CHB	chr8	TBC1D3	119541961	0
CHB	chr8	TBC1D3	119591961	0

CHB	chr8	TBC1D3	119641961	0
CHB	chr8	TBC1D3	119691961	0
CHB	chr8	TBC1D3	119741961	0
CHB	chr8	TBC1D3	119791961	0
CHB	chr8	TBC1D3	119841961	0
CHB	chr8	TBC1D3	119891961	0
CHB	chr8	TBC1D3	119941961	0
CHB	chr8	TBC1D3	119991961	0
CHB	chr8	TBC1D3	120041961	0
CHB	chr8	TBC1D3	120091961	0
CHB	chr8	TBC1D3	120141961	0
CHB	chr8	TBC1D3	120191961	0
CHB	chr8	TBC1D3	120241961	0
CHB	chr8	TBC1D3	120291961	0
CHB	chr8	TBC1D3	120341961	0
CHB	chr8	TBC1D3	120391961	0
CHB	chr8	TBC1D3	120441961	0
CHB	chr8	TBC1D3	120491961	0
CHB	chr8	TBC1D3	120541961	0
CHB	chr8	TBC1D3	120591961	0
CHB	chr8	TBC1D3	120641961	0
CHB	chr8	TBC1D3	120691961	0
CHB	chr8	TBC1D3	120741961	0
CHB	chr8	TBC1D3	120791961	0
CHB	chr8	TBC1D3	120841961	0
CHB	chr8	TBC1D3	120891961	0
CHB	chr8	TBC1D3	120941961	0
CHB	chr8	TBC1D3	120991961	0
CHB	chr8	TBC1D3	121041961	0
CHB	chr8	TBC1D3	121091961	0
CHB	chr8	TBC1D3	121141961	0
CHB	chr8	TBC1D3	121191961	0
CHB	chr8	TBC1D3	121241961	0
CHB	chr8	TBC1D3	121291961	0
CHB	chr8	TBC1D3	121341961	0
CHB	chr8	TBC1D3	121391961	0
CHB	chr8	TBC1D3	121441961	0
CHB	chr8	TBC1D3	121491961	0
CHB	chr8	TBC1D3	121541961	0
CHB	chr8	TBC1D3	121591961	0
CHB	chr8	TBC1D3	121641961	0
CHB	chr8	TBC1D3	121691961	0
CHB	chr8	TBC1D3	121741961	0
CHB	chr8	TBC1D3	121791961	0
CHB	chr8	TBC1D3	121841961	0
CHB	chr8	TBC1D3	121891961	0
CHB	chr8	TBC1D3	121941961	0
CHB	chr8	TBC1D3	121991961	0
CHB	chr8	TBC1D3	122041961	0

CHB	chr8	TBC1D3	122091961	0
CHB	chr8	TBC1D3	122141961	0
CHB	chr8	TBC1D3	122191961	0
CHB	chr8	TBC1D3	122241961	0
CHB	chr8	TBC1D3	122291961	0
CHB	chr8	TBC1D3	122341961	0
CHB	chr8	TBC1D3	122391961	0
CHB	chr8	TBC1D3	122441961	0
CHB	chr8	TBC1D3	122491961	0
CHB	chr8	TBC1D3	122541961	0
CHB	chr8	TBC1D3	122591961	0
CHB	chr8	TBC1D3	122641961	0
CHB	chr8	TBC1D3	122691961	0
CHB	chr8	TBC1D3	122741961	0
CHB	chr8	TBC1D3	122791961	0
CHB	chr8	TBC1D3	122841961	0
CHB	chr8	TBC1D3	122891961	0
CHB	chr8	TBC1D3	122941961	0
CHB	chr8	TBC1D3	122991961	0
CHB	chr8	TBC1D3	123041961	0
CHB	chr8	TBC1D3	123091961	0
CHB	chr8	TBC1D3	123141961	0
CHB	chr8	TBC1D3	123191961	0
CHB	chr8	TBC1D3	123241961	0
CHB	chr8	TBC1D3	123291961	0
CHB	chr8	TBC1D3	123341961	0
CHB	chr8	TBC1D3	123391961	0
CHB	chr8	TBC1D3	123441961	0
CHB	chr8	TBC1D3	123491961	0
CHB	chr8	TBC1D3	123541961	0
CHB	chr8	TBC1D3	123591961	0
CHB	chr8	TBC1D3	123641961	0
CHB	chr8	TBC1D3	123691961	0
CHB	chr8	TBC1D3	123741961	0
CHB	chr8	TBC1D3	123791961	0
CHB	chr8	TBC1D3	123841961	0
CHB	chr8	TBC1D3	123891961	17
CHB	chr8	TBC1D3	123941961	51
CHB	chr8	TBC1D3	123991961	35
CHB	chr8	TBC1D3	124041961	1
CHB	chr8	TBC1D3	124091961	0
CHB	chr8	TBC1D3	124141961	0
CHB	chr8	TBC1D3	124191961	0
CHB	chr8	TBC1D3	124241961	0
CHB	chr8	TBC1D3	124291961	0
CHB	chr8	TBC1D3	124341961	0
CHB	chr8	TBC1D3	124391961	0
CHB	chr8	TBC1D3	124441961	0
CHB	chr8	TBC1D3	124491961	0

CHB	chr8	TBC1D3	124541961	0
CHB	chr8	TBC1D3	124591961	0
CHB	chr8	TBC1D3	124641961	0
CHB	chr8	TBC1D3	124691961	0
CHB	chr8	TBC1D3	124741961	0
CHB	chr8	TBC1D3	124791961	0
CHB	chr8	TBC1D3	124841961	0
CHB	chr8	TBC1D3	124891961	0
CHB	chr8	TBC1D3	124941961	0
CHB	chr8	TBC1D3	124991961	0
CHB	chr8	TBC1D3	125041961	0
CHB	chr8	TBC1D3	125091961	0
CHB	chr8	TBC1D3	125141961	0
CHB	chr8	TBC1D3	125191961	0
CHB	chr8	TBC1D3	125241961	0
CHB	chr8	TBC1D3	125291961	0
CHB	chr8	TBC1D3	125341961	0
CHB	chr8	TBC1D3	125391961	0
CHB	chr8	TBC1D3	125441961	0
CHB	chr8	TBC1D3	125491961	0
CHB	chr8	TBC1D3	125541961	0
CHB	chr8	TBC1D3	125591961	0
CHB	chr8	TBC1D3	125641961	0
CHB	chr8	TBC1D3	125691961	0
CHB	chr8	TBC1D3	125741961	0
CHB	chr8	TBC1D3	125791961	0
CHB	chr8	TBC1D3	125841961	0
CHB	chr8	TBC1D3	125891961	0
CHB	chr8	TBC1D3	125941961	0
CHB	chr8	TBC1D3	125991961	0
CHB	chr8	TBC1D3	126041961	0
CHB	chr8	TBC1D3	126091961	0
CHB	chr8	TBC1D3	126141961	0
CHB	chr8	TBC1D3	126191961	0
CHB	chr8	TBC1D3	126241961	0
CHB	chr8	TBC1D3	126291961	0
CHB	chr8	TBC1D3	126341961	0
CHB	chr8	TBC1D3	126391961	0
CHB	chr8	TBC1D3	126441961	0
CHB	chr8	TBC1D3	126491961	0
CHB	chr8	TBC1D3	126541961	0
CHB	chr8	TBC1D3	126591961	0
CHB	chr8	TBC1D3	126641961	0
CHB	chr8	TBC1D3	126691961	0
CHB	chr8	TBC1D3	126741961	0
CHB	chr8	TBC1D3	126791961	0
CHB	chr8	TBC1D3	126841961	0
CHB	chr8	TBC1D3	126891961	0
CHB	chr8	TBC1D3	126941961	0

CHB	chr8	TBC1D3	126991961	0
CHB	chr8	TBC1D3	127041961	0
CHB	chr8	TBC1D3	127091961	0
CHB	chr8	TBC1D3	127141961	0
CHB	chr8	TBC1D3	127191961	0
CHB	chr8	TBC1D3	127241961	0
CHB	chr8	TBC1D3	127291961	0
CHB	chr8	TBC1D3	127341961	0
CHB	chr8	TBC1D3	127391961	0
CHB	chr8	TBC1D3	127441961	0
CHB	chr8	TBC1D3	127491961	0
CHB	chr8	TBC1D3	127541961	0
CHB	chr8	TBC1D3	127591961	0
CHB	chr8	TBC1D3	127641961	0
CHB	chr8	TBC1D3	127691961	0
CHB	chr8	TBC1D3	127741961	0
CHB	chr8	TBC1D3	127791961	0
CHB	chr8	TBC1D3	127841961	0
CHB	chr8	TBC1D3	127891961	0
CHB	chr8	TBC1D3	127941961	0
CHB	chr8	TBC1D3	127991961	0
CHB	chr8	TBC1D3	128041961	0
CHB	chr8	TBC1D3	128091961	0
CHB	chr12	TBC1D3	59809484	3
CHB	chr12	TBC1D3	59859484	0
CHB	chr12	TBC1D3	59909484	0
CHB	chr12	TBC1D3	59959484	0
CHB	chr12	TBC1D3	60009484	0
CHB	chr12	TBC1D3	60059484	0
CHB	chr12	TBC1D3	60109484	0
CHB	chr12	TBC1D3	60159484	0
CHB	chr12	TBC1D3	60209484	0
CHB	chr12	TBC1D3	60259484	7
CHB	chr12	TBC1D3	60309484	17
CHB	chr12	TBC1D3	60359484	18
CHB	chr12	TBC1D3	60409484	16
CHB	chr12	TBC1D3	60459484	18
CHB	chr12	TBC1D3	60509484	20
CHB	chr12	TBC1D3	60559484	31
CHB	chr12	TBC1D3	60609484	30
CHB	chr12	TBC1D3	60659484	23
CHB	chr12	TBC1D3	60709484	31
CHB	chr12	TBC1D3	60759484	31
CHB	chr12	TBC1D3	60809484	29
CHB	chr12	TBC1D3	60859484	30
CHB	chr12	TBC1D3	60909484	29
CHB	chr12	TBC1D3	60959484	39
CHB	chr12	TBC1D3	61009484	37
CHB	chr12	TBC1D3	61059484	20

CHB	chr12	TBC1D3	61109484	21
CHB	chr12	TBC1D3	61159484	31
CHB	chr12	TBC1D3	61209484	39
CHB	chr12	TBC1D3	61259484	35
CHB	chr12	TBC1D3	61309484	48
CHB	chr12	TBC1D3	61359484	87
CHB	chr12	TBC1D3	61409484	88
CHB	chr12	TBC1D3	61459484	53
CHB	chr12	TBC1D3	61509484	28
CHB	chr12	TBC1D3	61559484	32
CHB	chr12	TBC1D3	61609484	54
CHB	chr12	TBC1D3	61659484	41
CHB	chr12	TBC1D3	61709484	24
CHB	chr12	TBC1D3	61759484	15
CHB	chr12	TBC1D3	61809484	0
CHB	chr12	TBC1D3	61859484	0
CHB	chr12	TBC1D3	61909484	0
CHB	chr12	TBC1D3	61959484	0
CHB	chr12	TBC1D3	62009484	0
CHB	chr12	TBC1D3	62059484	0
CHB	chr12	TBC1D3	62109484	0
CHB	chr12	TBC1D3	62159484	0
CHB	chr12	TBC1D3	62209484	0
CHB	chr12	TBC1D3	62259484	0
CHB	chr12	TBC1D3	62309484	0
CHB	chr12	TBC1D3	62359484	0
CHB	chr12	TBC1D3	62409484	0
CHB	chr12	TBC1D3	62459484	0
CHB	chr12	TBC1D3	62509484	0
CHB	chr12	TBC1D3	62559484	0
CHB	chr12	TBC1D3	62609484	0
CHB	chr12	TBC1D3	62659484	0
CHB	chr12	TBC1D3	62709484	0
CHB	chr12	TBC1D3	62759484	0
CHB	chr12	TBC1D3	62809484	0
CHB	chr12	TBC1D3	62859484	0
CHB	chr12	TBC1D3	62909484	0
CHB	chr12	TBC1D3	62959484	0
CHB	chr12	TBC1D3	63009484	0
CHB	chr12	TBC1D3	63059484	0
CHB	chr12	TBC1D3	63109484	0
CHB	chr12	TBC1D3	63159484	0
CHB	chr12	TBC1D3	63209484	0
CHB	chr12	TBC1D3	63259484	0
CHB	chr12	TBC1D3	63309484	0
CHB	chr12	TBC1D3	63359484	0
CHB	chr12	TBC1D3	63409484	0
CHB	chr12	TBC1D3	63459484	0
CHB	chr12	TBC1D3	63509484	0

CHB	chr12	TBC1D3	63559484	0
CHB	chr12	TBC1D3	63609484	0
CHB	chr12	TBC1D3	63659484	0
CHB	chr12	TBC1D3	63709484	0
CHB	chr12	TBC1D3	63759484	0
CHB	chr12	TBC1D3	63809484	0
CHB	chr12	TBC1D3	63859484	0
CHB	chr12	TBC1D3	63909484	21
CHB	chr12	TBC1D3	63959484	45
CHB	chr12	TBC1D3	64009484	39
CHB	chr12	TBC1D3	64059484	46
CHB	chr12	TBC1D3	64109484	126
CHB	chr12	TBC1D3	64159484	110
CHB	chr12	TBC1D3	64209484	26
CHB	chr12	TBC1D3	64259484	35
CHB	chr12	TBC1D3	64309484	26
CHB	chr12	TBC1D3	64359484	2
CHB	chr12	TBC1D3	64409484	0
CHB	chr12	TBC1D3	64459484	0
CHB	chr12	TBC1D3	64509484	0
CHB	chr12	TBC1D3	64559484	0
CHB	chr12	TBC1D3	64609484	0
CHB	chr12	TBC1D3	64659484	0
CHB	chr12	TBC1D3	64709484	0
CHB	chr12	TBC1D3	64759484	0
CHB	chr12	TBC1D3	64809484	0
CHB	chr12	TBC1D3	64859484	0
CHB	chr12	TBC1D3	64909484	0
CHB	chr12	TBC1D3	64959484	0
CHB	chr12	TBC1D3	65009484	0
CHB	chr12	TBC1D3	65059484	0
CHB	chr12	TBC1D3	65109484	0
CHB	chr12	TBC1D3	65159484	0
CHB	chr12	TBC1D3	65209484	0
CHB	chr12	TBC1D3	65259484	0
CHB	chr12	TBC1D3	65309484	0
CHB	chr12	TBC1D3	65359484	0
CHB	chr12	TBC1D3	65409484	0
CHB	chr12	TBC1D3	65459484	0
CHB	chr12	TBC1D3	65509484	0
CHB	chr12	TBC1D3	65559484	0
CHB	chr12	TBC1D3	65609484	0
CHB	chr12	TBC1D3	65659484	0
CHB	chr12	TBC1D3	65709484	0
CHB	chr12	TBC1D3	65759484	0
CHB	chr12	TBC1D3	65809484	0
CHB	chr12	TBC1D3	65859484	0
CHB	chr12	TBC1D3	65909484	0
CHB	chr12	TBC1D3	65959484	0

CHB	chr12	TBC1D3	66009484	0
CHB	chr12	TBC1D3	66059484	0
CHB	chr12	TBC1D3	66109484	0
CHB	chr12	TBC1D3	66159484	0
CHB	chr12	TBC1D3	66209484	0
CHB	chr12	TBC1D3	66259484	0
CHB	chr12	TBC1D3	66309484	0
CHB	chr12	TBC1D3	66359484	0
CHB	chr12	TBC1D3	66409484	0
CHB	chr12	TBC1D3	66459484	0
CHB	chr12	TBC1D3	66509484	0
CHB	chr12	TBC1D3	66559484	0
CHB	chr12	TBC1D3	66609484	0
CHB	chr12	TBC1D3	66659484	0
CHB	chr12	TBC1D3	66709484	0
CHB	chr12	TBC1D3	66759484	0
CHB	chr12	TBC1D3	66809484	0
CHB	chr12	TBC1D3	66859484	0
CHB	chr12	TBC1D3	66909484	0
CHB	chr12	TBC1D3	66959484	0
CHB	chr12	TBC1D3	67009484	0
CHB	chr12	TBC1D3	67059484	0
CHB	chr12	TBC1D3	67109484	0
CHB	chr12	TBC1D3	67159484	0
CHB	chr12	TBC1D3	67209484	0
CHB	chr12	TBC1D3	67259484	0
CHB	chr12	TBC1D3	67309484	0
CHB	chr12	TBC1D3	67359484	0
CHB	chr12	TBC1D3	67409484	0
CHB	chr12	TBC1D3	67459484	0
CHB	chr12	TBC1D3	67509484	0
CHB	chr12	TBC1D3	67559484	0
CHB	chr12	TBC1D3	67609484	11
CHB	chr12	TBC1D3	67659484	19
CHB	chr12	TBC1D3	67709484	18
CHB	chr12	TBC1D3	67759484	21
CHB	chr12	TBC1D3	67809484	38
CHB	chr12	TBC1D3	67859484	27
CHB	chr12	TBC1D3	67909484	0
CHB	chr12	TBC1D3	67959484	0
CHB	chr12	TBC1D3	68009484	0
CHB	chr12	TBC1D3	68059484	0
CHB	chr12	TBC1D3	68109484	0
CHB	chr12	TBC1D3	68159484	0
CHB	chr12	TBC1D3	68209484	0
CHB	chr12	TBC1D3	68259484	0
CHB	chr12	TBC1D3	68309484	0
CHB	chr12	TBC1D3	68359484	0
CHB	chr12	TBC1D3	68409484	0

CHB	chr12	TBC1D3	68459484	0
CHB	chr12	TBC1D3	68509484	0
CHB	chr12	TBC1D3	68559484	0
CHB	chr12	TBC1D3	68609484	0
CHB	chr12	TBC1D3	68659484	0
CHB	chr12	TBC1D3	68709484	0
CHB	chr12	TBC1D3	68759484	0
CHB	chr12	TBC1D3	68809484	0
CHB	chr12	TBC1D3	68859484	0
CHB	chr12	TBC1D3	68909484	0
CHB	chr12	TBC1D3	68959484	0
CHB	chr12	TBC1D3	69009484	0
CHB	chr12	TBC1D3	69059484	0
CHB	chr12	TBC1D3	69109484	0
CHB	chr12	TBC1D3	69159484	0
CHB	chr12	TBC1D3	69209484	0
CHB	chr12	TBC1D3	69259484	0
CHB	chr12	TBC1D3	69309484	0
CHB	chr12	TBC1D3	69359484	0
CHB	chr12	TBC1D3	69409484	0
CHB	chr12	TBC1D3	69459484	0
CHB	chr12	TBC1D3	69509484	0
CHB	chr12	TBC1D3	69559484	0
CHB	chr12	TBC1D3	69609484	0
CHB	chr12	TBC1D3	69659484	0
CHB	chr12	TBC1D3	69709484	0
CHB	chr12	TBC1D3	69759484	0
CHB	chr12	TBC1D3	69809484	0
CHB	chr17	TBC1D3	31215683	0
CHB	chr17	TBC1D3	31265683	0
CHB	chr17	TBC1D3	31315683	0
CHB	chr17	TBC1D3	31365683	0
CHB	chr17	TBC1D3	31415683	0
CHB	chr17	TBC1D3	31465683	0
CHB	chr17	TBC1D3	31515683	0
CHB	chr17	TBC1D3	31565683	0
CHB	chr17	TBC1D3	31615683	0
CHB	chr17	TBC1D3	31665683	0
CHB	chr17	TBC1D3	31715683	0
CHB	chr17	TBC1D3	31765683	0
CHB	chr17	TBC1D3	31815683	0
CHB	chr17	TBC1D3	31865683	0
CHB	chr17	TBC1D3	31915683	0
CHB	chr17	TBC1D3	31965683	0
CHB	chr17	TBC1D3	32015683	0
CHB	chr17	TBC1D3	32065683	0
CHB	chr17	TBC1D3	32115683	0
CHB	chr17	TBC1D3	32165683	0
CHB	chr17	TBC1D3	32215683	0

CHB	chr17	TBC1D3	32265683	0
CHB	chr17	TBC1D3	32315683	0
CHB	chr17	TBC1D3	32365683	0
CHB	chr17	TBC1D3	32415683	0
CHB	chr17	TBC1D3	32465683	0
CHB	chr17	TBC1D3	32515683	0
CHB	chr17	TBC1D3	32565683	0
CHB	chr17	TBC1D3	32615683	0
CHB	chr17	TBC1D3	32665683	0
CHB	chr17	TBC1D3	32715683	0
CHB	chr17	TBC1D3	32765683	0
CHB	chr17	TBC1D3	32815683	0
CHB	chr17	TBC1D3	32865683	0
CHB	chr17	TBC1D3	32915683	0
CHB	chr17	TBC1D3	32965683	0
CHB	chr17	TBC1D3	33015683	0
CHB	chr17	TBC1D3	33065683	0
CHB	chr17	TBC1D3	33115683	0
CHB	chr17	TBC1D3	33165683	0
CHB	chr17	TBC1D3	33215683	0
CHB	chr17	TBC1D3	33265683	0
CHB	chr17	TBC1D3	33315683	0
CHB	chr17	TBC1D3	33365683	0
CHB	chr17	TBC1D3	33415683	0
CHB	chr17	TBC1D3	33465683	0
CHB	chr17	TBC1D3	33515683	0
CHB	chr17	TBC1D3	33565683	0
CHB	chr17	TBC1D3	33615683	0
CHB	chr17	TBC1D3	33665683	0
CHB	chr17	TBC1D3	33715683	2
CHB	chr17	TBC1D3	33765683	16
CHB	chr17	TBC1D3	33815683	28
CHB	chr17	TBC1D3	33865683	30
CHB	chr17	TBC1D3	33915683	25
CHB	chr17	TBC1D3	33965683	19
CHB	chr17	TBC1D3	34015683	19
CHB	chr17	TBC1D3	34065683	61
CHB	chr17	TBC1D3	34115683	77
CHB	chr17	TBC1D3	34165683	47
CHB	chr17	TBC1D3	34215683	36
CHB	chr17	TBC1D3	34265683	29
CHB	chr17	TBC1D3	34315683	15
CHB	chr17	TBC1D3	34365683	0
CHB	chr17	TBC1D3	34415683	0
CHB	chr17	TBC1D3	34465683	0
CHB	chr17	TBC1D3	34515683	0
CHB	chr17	TBC1D3	34565683	0
CHB	chr17	TBC1D3	34615683	0
CHB	chr17	TBC1D3	34665683	0

CHB	chr17	TBC1D3	34715683	0
CHB	chr17	TBC1D3	34765683	0
CHB	chr17	TBC1D3	34815683	0
CHB	chr17	TBC1D3	34865683	0
CHB	chr17	TBC1D3	34915683	0
CHB	chr17	TBC1D3	34965683	0
CHB	chr17	TBC1D3	35015683	0
CHB	chr17	TBC1D3	35065683	0
CHB	chr17	TBC1D3	35115683	0
CHB	chr17	TBC1D3	35165683	0
CHB	chr17	TBC1D3	35215683	0
CHB	chr17	TBC1D3	35265683	0
CHB	chr17	TBC1D3	35315683	0
CHB	chr17	TBC1D3	35365683	0
CHB	chr17	TBC1D3	35415683	0
CHB	chr17	TBC1D3	35465683	0
CHB	chr17	TBC1D3	35515683	0
CHB	chr17	TBC1D3	35565683	0
CHB	chr17	TBC1D3	35615683	0
CHB	chr17	TBC1D3	35665683	0
CHB	chr17	TBC1D3	35715683	0
CHB	chr17	TBC1D3	35765683	0
CHB	chr17	TBC1D3	35815683	0
CHB	chr17	TBC1D3	35865683	0
CHB	chr17	TBC1D3	35915683	0
CHB	chr17	TBC1D3	35965683	0
CHB	chr17	TBC1D3	36015683	0
CHB	chr17	TBC1D3	36065683	0
CHB	chr17	TBC1D3	36115683	0
CHB	chr17	TBC1D3	36165683	0
CHB	chr17	TBC1D3	36215683	0
CHB	chr17	TBC1D3	36265683	0
CHB	chr17	TBC1D3	36315683	0
CHB	chr17	TBC1D3	36365683	0
CHB	chr17	TBC1D3	36415683	0
CHB	chr17	TBC1D3	36465683	0
CHB	chr17	TBC1D3	36515683	0
CHB	chr17	TBC1D3	36565683	0
CHB	chr17	TBC1D3	36615683	0
CHB	chr17	TBC1D3	36665683	0
CHB	chr17	TBC1D3	36715683	0
CHB	chr17	TBC1D3	36765683	0
CHB	chr17	TBC1D3	36815683	0
CHB	chr17	TBC1D3	36865683	0
CHB	chr17	TBC1D3	36915683	0
CHB	chr17	TBC1D3	36965683	0
CHB	chr17	TBC1D3	37015683	0
CHB	chr17	TBC1D3	37065683	23
CHB	chr17	TBC1D3	37115683	43

CHB	chr17	TBC1D3	37165683	46
CHB	chr17	TBC1D3	37215683	59
CHB	chr17	TBC1D3	37265683	61
CHB	chr17	TBC1D3	37315683	28
CHB	chr17	TBC1D3	37365683	0
CHB	chr17	TBC1D3	37415683	0
CHB	chr17	TBC1D3	37465683	0
CHB	chr17	TBC1D3	37515683	0
CHB	chr17	TBC1D3	37565683	0
CHB	chr17	TBC1D3	37615683	0
CHB	chr17	TBC1D3	37665683	0
CHB	chr17	TBC1D3	37715683	0
CHB	chr17	TBC1D3	37765683	0
CHB	chr17	TBC1D3	37815683	0
CHB	chr17	TBC1D3	37865683	0
CHB	chr17	TBC1D3	37915683	0
CHB	chr17	TBC1D3	37965683	0
CHB	chr17	TBC1D3	38015683	0
CHB	chr17	TBC1D3	38065683	0
CHB	chr17	TBC1D3	38115683	0
CHB	chr17	TBC1D3	38165683	0
CHB	chr17	TBC1D3	38215683	0
CHB	chr17	TBC1D3	38265683	0
CHB	chr17	TBC1D3	38315683	0
CHB	chr17	TBC1D3	38365683	0
CHB	chr17	TBC1D3	38415683	0
CHB	chr17	TBC1D3	38465683	0
CHB	chr17	TBC1D3	38515683	0
CHB	chr17	TBC1D3	38565683	0
CHB	chr17	TBC1D3	38615683	0
CHB	chr17	TBC1D3	38665683	0
CHB	chr17	TBC1D3	38715683	0
CHB	chr17	TBC1D3	38765683	0
CHB	chr17	TBC1D3	38815683	0
CHB	chr17	TBC1D3	38865683	0
CHB	chr17	TBC1D3	38915683	0
CHB	chr17	TBC1D3	38965683	0
CHB	chr17	TBC1D3	39015683	0
CHB	chr17	TBC1D3	39065683	0
CHB	chr17	TBC1D3	39115683	0
CHB	chr17	TBC1D3	39165683	0
CHB	chr17	TBC1D3	39215683	0
CHB	chr17	TBC1D3	39265683	0
CHB	chr17	TBC1D3	39315683	0
CHB	chr17	TBC1D3	39365683	0
CHB	chr17	TBC1D3	39415683	0
CHB	chr17	TBC1D3	39465683	0
CHB	chr17	TBC1D3	39515683	0
CHB	chr17	TBC1D3	39565683	0

CHB	chr17	TBC1D3	39615683	0
CHB	chr17	TBC1D3	39665683	0
CHB	chr17	TBC1D3	39715683	0
CHB	chr17	TBC1D3	39765683	0
CHB	chr17	TBC1D3	39815683	0
CHB	chr17	TBC1D3	39865683	0
CHB	chr17	TBC1D3	39915683	0
CHB	chr17	TBC1D3	39965683	0
CHB	chr17	TBC1D3	40015683	0
CHB	chr17	TBC1D3	40065683	0
CHB	chr17	TBC1D3	40115683	0
CHB	chr17	TBC1D3	40165683	0
CHB	chr17	TBC1D3	40215683	0
CHB	chr17	TBC1D3	40265683	0
CHB	chr17	TBC1D3	40315683	0
CHB	chr17	TBC1D3	40365683	0
CHB	chr17	TBC1D3	40415683	0
CHB	chr17	TBC1D3	40465683	0
CHB	chr17	TBC1D3	40515683	0
CHB	chr17	TBC1D3	40565683	0
CHB	chr17	TBC1D3	40615683	0
CHB	chr17	TBC1D3	40665683	0
CHB	chr17	TBC1D3	40715683	0
CHB	chr17	TBC1D3	40765683	0
CHB	chr17	TBC1D3	40815683	0
CHB	chr17	TBC1D3	40865683	0
CHB	chr17	TBC1D3	40915683	0
CHB	chr17	TBC1D3	40965683	0
CHB	chr17	TBC1D3	41015683	0
CHB	chr17	TBC1D3	41065683	0
CHB	chr17	TBC1D3	41115683	0
CHB	chr16	TP53TG3	27301603	0
CHB	chr16	TP53TG3	27351603	0
CHB	chr16	TP53TG3	27401603	0
CHB	chr16	TP53TG3	27451603	0
CHB	chr16	TP53TG3	27501603	0
CHB	chr16	TP53TG3	27551603	0
CHB	chr16	TP53TG3	27601603	0
CHB	chr16	TP53TG3	27651603	0
CHB	chr16	TP53TG3	27701603	0
CHB	chr16	TP53TG3	27751603	0
CHB	chr16	TP53TG3	27801603	0
CHB	chr16	TP53TG3	27851603	0
CHB	chr16	TP53TG3	27901603	0
CHB	chr16	TP53TG3	27951603	0
CHB	chr16	TP53TG3	28001603	0
CHB	chr16	TP53TG3	28051603	0
CHB	chr16	TP53TG3	28101603	0
CHB	chr16	TP53TG3	28151603	0

CHB	chr16	TP53TG3	28201603	0
CHB	chr16	TP53TG3	28251603	0
CHB	chr16	TP53TG3	28301603	0
CHB	chr16	TP53TG3	28351603	0
CHB	chr16	TP53TG3	28401603	0
CHB	chr16	TP53TG3	28451603	0
CHB	chr16	TP53TG3	28501603	0
CHB	chr16	TP53TG3	28551603	0
CHB	chr16	TP53TG3	28601603	0
CHB	chr16	TP53TG3	28651603	0
CHB	chr16	TP53TG3	28701603	0
CHB	chr16	TP53TG3	28751603	0
CHB	chr16	TP53TG3	28801603	0
CHB	chr16	TP53TG3	28851603	0
CHB	chr16	TP53TG3	28901603	0
CHB	chr16	TP53TG3	28951603	0
CHB	chr16	TP53TG3	29001603	0
CHB	chr16	TP53TG3	29051603	0
CHB	chr16	TP53TG3	29101603	0
CHB	chr16	TP53TG3	29151603	0
CHB	chr16	TP53TG3	29201603	0
CHB	chr16	TP53TG3	29251603	0
CHB	chr16	TP53TG3	29301603	0
CHB	chr16	TP53TG3	29351603	0
CHB	chr16	TP53TG3	29401603	0
CHB	chr16	TP53TG3	29451603	0
CHB	chr16	TP53TG3	29501603	0
CHB	chr16	TP53TG3	29551603	0
CHB	chr16	TP53TG3	29601603	0
CHB	chr16	TP53TG3	29651603	0
CHB	chr16	TP53TG3	29701603	0
CHB	chr16	TP53TG3	29751603	0
CHB	chr16	TP53TG3	29801603	0
CHB	chr16	TP53TG3	29851603	0
CHB	chr16	TP53TG3	29901603	0
CHB	chr16	TP53TG3	29951603	0
CHB	chr16	TP53TG3	30001603	0
CHB	chr16	TP53TG3	30051603	0
CHB	chr16	TP53TG3	30101603	0
CHB	chr16	TP53TG3	30151603	0
CHB	chr16	TP53TG3	30201603	0
CHB	chr16	TP53TG3	30251603	0
CHB	chr16	TP53TG3	30301603	0
CHB	chr16	TP53TG3	30351603	0
CHB	chr16	TP53TG3	30401603	0
CHB	chr16	TP53TG3	30451603	0
CHB	chr16	TP53TG3	30501603	0
CHB	chr16	TP53TG3	30551603	0
CHB	chr16	TP53TG3	30601603	0

CHB	chr16	TP53TG3	30651603	0
CHB	chr16	TP53TG3	30701603	0
CHB	chr16	TP53TG3	30751603	0
CHB	chr16	TP53TG3	30801603	0
CHB	chr16	TP53TG3	30851603	0
CHB	chr16	TP53TG3	30901603	0
CHB	chr16	TP53TG3	30951603	0
CHB	chr16	TP53TG3	31001603	0
CHB	chr16	TP53TG3	31051603	0
CHB	chr16	TP53TG3	31101603	0
CHB	chr16	TP53TG3	31151603	0
CHB	chr16	TP53TG3	31201603	0
CHB	chr16	TP53TG3	31251603	0
CHB	chr16	TP53TG3	31301603	0
CHB	chr16	TP53TG3	31351603	0
CHB	chr16	TP53TG3	31401603	0
CHB	chr16	TP53TG3	31451603	0
CHB	chr16	TP53TG3	31501603	0
CHB	chr16	TP53TG3	31551603	0
CHB	chr16	TP53TG3	31601603	0
CHB	chr16	TP53TG3	31651603	0
CHB	chr16	TP53TG3	31701603	0
CHB	chr16	TP53TG3	31751603	0
CHB	chr16	TP53TG3	31801603	0
CHB	chr16	TP53TG3	31851603	0
CHB	chr16	TP53TG3	31901603	0
CHB	chr16	TP53TG3	31951603	0
CHB	chr16	TP53TG3	32001603	0
CHB	chr16	TP53TG3	32051603	0
CHB	chr16	TP53TG3	32101603	0
CHB	chr16	TP53TG3	32151603	0
CHB	chr16	TP53TG3	32201603	0
CHB	chr16	TP53TG3	32251603	0
CHB	chr16	TP53TG3	32301603	0
CHB	chr16	TP53TG3	32351603	0
CHB	chr16	TP53TG3	32401603	0
CHB	chr16	TP53TG3	32451603	0
CHB	chr16	TP53TG3	32501603	0
CHB	chr16	TP53TG3	32551603	0
CHB	chr16	TP53TG3	32601603	0
CHB	chr16	TP53TG3	32651603	0
CHB	chr16	TP53TG3	32701603	0
CHB	chr16	TP53TG3	32751603	0
CHB	chr16	TP53TG3	32801603	0
CHB	chr16	TP53TG3	32851603	0
CHB	chr16	TP53TG3	32901603	0
CHB	chr16	TP53TG3	32951603	0
CHB	chr16	TP53TG3	33001603	0
CHB	chr16	TP53TG3	33051603	0

CHB	chr16	TP53TG3	33101603	0
CHB	chr16	TP53TG3	33151603	0
CHB	chr16	TP53TG3	33201603	0
CHB	chr16	TP53TG3	33251603	0
CHB	chr16	TP53TG3	33301603	0
CHB	chr16	TP53TG3	33351603	0
CHB	chr16	TP53TG3	33401603	0
CHB	chr16	TP53TG3	33451603	0
CHB	chr16	TP53TG3	33501603	0
CHB	chr16	TP53TG3	33551603	0
CHB	chr16	TP53TG3	33601603	0
CHB	chr16	TP53TG3	33651603	0
CHB	chr16	TP53TG3	33701603	0
CHB	chr16	TP53TG3	33751603	0
CHB	chr16	TP53TG3	33801603	0
CHB	chr16	TP53TG3	33851603	0
CHB	chr16	TP53TG3	33901603	0
CHB	chr16	TP53TG3	33951603	0
CHB	chr16	TP53TG3	34001603	0
CHB	chr16	TP53TG3	34051603	0
CHB	chr16	TP53TG3	34101603	0
CHB	chr16	TP53TG3	34151603	0
CHB	chr16	TP53TG3	34201603	0
CHB	chr16	TP53TG3	34251603	0
CHB	chr16	TP53TG3	34301603	0
CHB	chr16	TP53TG3	34351603	0
CHB	chr16	TP53TG3	34401603	0
CHB	chr16	TP53TG3	34451603	0
CHB	chr16	TP53TG3	34501603	0
CHB	chr16	TP53TG3	34551603	0
CHB	chr16	TP53TG3	34601603	0
CHB	chr16	TP53TG3	34651603	0
CHB	chr16	TP53TG3	34701603	0
CHB	chr16	TP53TG3	34751603	0
CHB	chr16	TP53TG3	34801603	0
CHB	chr16	TP53TG3	34851603	0
CHB	chr16	TP53TG3	34901603	0
CHB	chr16	TP53TG3	34951603	0
CHB	chr16	TP53TG3	35001603	0
CHB	chr16	TP53TG3	35051603	0
CHB	chr16	TP53TG3	35101603	0
CHB	chr16	TP53TG3	35151603	0
CHB	chr16	TP53TG3	35201603	0
CHB	chr16	TP53TG3	35251603	0
CHB	chr16	TP53TG3	35301603	0
CHB	chr16	TP53TG3	35351603	0
CHB	chr16	TP53TG3	35401603	0
CHB	chr16	TP53TG3	35451603	0
CHB	chr16	TP53TG3	35501603	0

CHB	chr16	TP53TG3	35551603	0
CHB	chr16	TP53TG3	35601603	0
CHB	chr16	TP53TG3	35651603	0
CHB	chr16	TP53TG3	35701603	0
CHB	chr16	TP53TG3	35751603	0
CHB	chr16	TP53TG3	35801603	0
CHB	chr16	TP53TG3	35851603	0
CHB	chr16	TP53TG3	35901603	0
CHB	chr16	TP53TG3	35951603	0
CHB	chr16	TP53TG3	36001603	0
CHB	chr16	TP53TG3	36051603	0
CHB	chr16	TP53TG3	36101603	0
CHB	chr16	TP53TG3	36151603	0
CHB	chr16	TP53TG3	36201603	0
CHB	chr16	TP53TG3	36251603	0
CHB	chr16	TP53TG3	36301603	0
CHB	chr16	TP53TG3	36351603	0
CHB	chr16	TP53TG3	36401603	0
CHB	chr16	TP53TG3	36451603	0
CHB	chr16	TP53TG3	36501603	0
CHB	chr16	TP53TG3	36551603	0
CHB	chr16	TP53TG3	36601603	0
CHB	chr16	TP53TG3	36651603	0
CHB	chr16	TP53TG3	36701603	0
CHB	chr16	TP53TG3	36751603	0
CHB	chr16	TP53TG3	36801603	0
CHB	chr16	TP53TG3	36851603	0
CHB	chr16	TP53TG3	36901603	0
CHB	chr16	TP53TG3	36951603	0
CHB	chr16	TP53TG3	37001603	0
CHB	chr16	TP53TG3	37051603	0
CHB	chr16	TP53TG3	37101603	0
CHB	chr16	TP53TG3	37151603	0
CHB	chr16	TP53TG3	37201603	0
CHB	chr11	TRIM49L1	84974064	0
CHB	chr11	TRIM49L1	85024064	0
CHB	chr11	TRIM49L1	85074064	0
CHB	chr11	TRIM49L1	85124064	0
CHB	chr11	TRIM49L1	85174064	0
CHB	chr11	TRIM49L1	85224064	0
CHB	chr11	TRIM49L1	85274064	0
CHB	chr11	TRIM49L1	85324064	0
CHB	chr11	TRIM49L1	85374064	0
CHB	chr11	TRIM49L1	85424064	0
CHB	chr11	TRIM49L1	85474064	0
CHB	chr11	TRIM49L1	85524064	14
CHB	chr11	TRIM49L1	85574064	29
CHB	chr11	TRIM49L1	85624064	36
CHB	chr11	TRIM49L1	85674064	36

CHB	chr11	TRIM49L1	85724064	36
CHB	chr11	TRIM49L1	85774064	39
CHB	chr11	TRIM49L1	85824064	32
CHB	chr11	TRIM49L1	85874064	28
CHB	chr11	TRIM49L1	85924064	14
CHB	chr11	TRIM49L1	85974064	0
CHB	chr11	TRIM49L1	86024064	0
CHB	chr11	TRIM49L1	86074064	0
CHB	chr11	TRIM49L1	86124064	0
CHB	chr11	TRIM49L1	86174064	0
CHB	chr11	TRIM49L1	86224064	0
CHB	chr11	TRIM49L1	86274064	0
CHB	chr11	TRIM49L1	86324064	0
CHB	chr11	TRIM49L1	86374064	0
CHB	chr11	TRIM49L1	86424064	29
CHB	chr11	TRIM49L1	86474064	63
CHB	chr11	TRIM49L1	86524064	58
CHB	chr11	TRIM49L1	86574064	47
CHB	chr11	TRIM49L1	86624064	23
CHB	chr11	TRIM49L1	86674064	0
CHB	chr11	TRIM49L1	86724064	0
CHB	chr11	TRIM49L1	86774064	0
CHB	chr11	TRIM49L1	86824064	0
CHB	chr11	TRIM49L1	86874064	0
CHB	chr11	TRIM49L1	86924064	0
CHB	chr11	TRIM49L1	86974064	0
CHB	chr11	TRIM49L1	87024064	0
CHB	chr11	TRIM49L1	87074064	0
CHB	chr11	TRIM49L1	87124064	0
CHB	chr11	TRIM49L1	87174064	1
CHB	chr11	TRIM49L1	87224064	18
CHB	chr11	TRIM49L1	87274064	39
CHB	chr11	TRIM49L1	87324064	43
CHB	chr11	TRIM49L1	87374064	37
CHB	chr11	TRIM49L1	87424064	24
CHB	chr11	TRIM49L1	87474064	29
CHB	chr11	TRIM49L1	87524064	42
CHB	chr11	TRIM49L1	87574064	51
CHB	chr11	TRIM49L1	87624064	34
CHB	chr11	TRIM49L1	87674064	4
CHB	chr11	TRIM49L1	87724064	0
CHB	chr11	TRIM49L1	87774064	0
CHB	chr11	TRIM49L1	87824064	0
CHB	chr11	TRIM49L1	87874064	0
CHB	chr11	TRIM49L1	87924064	0
CHB	chr11	TRIM49L1	87974064	0
CHB	chr11	TRIM49L1	88024064	0
CHB	chr11	TRIM49L1	88074064	0
CHB	chr11	TRIM49L1	88124064	0

CHB	chr11	TRIM49L1	88174064	0
CHB	chr11	TRIM49L1	88224064	0
CHB	chr11	TRIM49L1	88274064	0
CHB	chr11	TRIM49L1	88324064	0
CHB	chr11	TRIM49L1	88374064	0
CHB	chr11	TRIM49L1	88424064	0
CHB	chr11	TRIM49L1	88474064	0
CHB	chr11	TRIM49L1	88524064	0
CHB	chr11	TRIM49L1	88574064	0
CHB	chr11	TRIM49L1	88624064	0
CHB	chr11	TRIM49L1	88674064	0
CHB	chr11	TRIM49L1	88724064	0
CHB	chr11	TRIM49L1	88774064	0
CHB	chr11	TRIM49L1	88824064	0
CHB	chr11	TRIM49L1	88874064	0
CHB	chr11	TRIM49L1	88924064	0
CHB	chr11	TRIM49L1	88974064	0
CHB	chr11	TRIM49L1	89024064	0
CHB	chr11	TRIM49L1	89074064	0
CHB	chr11	TRIM49L1	89124064	0
CHB	chr11	TRIM49L1	89174064	0
CHB	chr11	TRIM49L1	89224064	0
CHB	chr11	TRIM49L1	89274064	0
CHB	chr11	TRIM49L1	89324064	0
CHB	chr11	TRIM49L1	89374064	0
CHB	chr11	TRIM49L1	89424064	0
CHB	chr11	TRIM49L1	89474064	0
CHB	chr11	TRIM49L1	89524064	0
CHB	chr11	TRIM49L1	89574064	0
CHB	chr11	TRIM49L1	89624064	0
CHB	chr11	TRIM49L1	89674064	0
CHB	chr11	TRIM49L1	89724064	0
CHB	chr11	TRIM49L1	89774064	0
CHB	chr11	TRIM49L1	89824064	0
CHB	chr11	TRIM49L1	89874064	0
CHB	chr11	TRIM49L1	89924064	0
CHB	chr11	TRIM49L1	89974064	0
CHB	chr11	TRIM49L1	90024064	0
CHB	chr11	TRIM49L1	90074064	0
CHB	chr11	TRIM49L1	90124064	0
CHB	chr11	TRIM49L1	90174064	0
CHB	chr11	TRIM49L1	90224064	0
CHB	chr11	TRIM49L1	90274064	0
CHB	chr11	TRIM49L1	90324064	0
CHB	chr11	TRIM49L1	90374064	0
CHB	chr11	TRIM49L1	90424064	0
CHB	chr11	TRIM49L1	90474064	0
CHB	chr11	TRIM49L1	90524064	0
CHB	chr11	TRIM49L1	90574064	0

CHB	chr11	TRIM49L1	90624064	0
CHB	chr11	TRIM49L1	90674064	0
CHB	chr11	TRIM49L1	90724064	0
CHB	chr11	TRIM49L1	90774064	0
CHB	chr11	TRIM49L1	90824064	0
CHB	chr11	TRIM49L1	90874064	0
CHB	chr11	TRIM49L1	90924064	7
CHB	chr11	TRIM49L1	90974064	23
CHB	chr11	TRIM49L1	91024064	41
CHB	chr11	TRIM49L1	91074064	48
CHB	chr11	TRIM49L1	91124064	43
CHB	chr11	TRIM49L1	91174064	36
CHB	chr11	TRIM49L1	91224064	61
CHB	chr11	TRIM49L1	91274064	64
CHB	chr11	TRIM49L1	91324064	19
CHB	chr11	TRIM49L1	91374064	0
CHB	chr11	TRIM49L1	91424064	0
CHB	chr11	TRIM49L1	91474064	0
CHB	chr11	TRIM49L1	91524064	0
CHB	chr11	TRIM49L1	91574064	0
CHB	chr11	TRIM49L1	91624064	0
CHB	chr11	TRIM49L1	91674064	0
CHB	chr11	TRIM49L1	91724064	0
CHB	chr11	TRIM49L1	91774064	0
CHB	chr11	TRIM49L1	91824064	0
CHB	chr11	TRIM49L1	91874064	0
CHB	chr11	TRIM49L1	91924064	0
CHB	chr11	TRIM49L1	91974064	0
CHB	chr11	TRIM49L1	92024064	0
CHB	chr11	TRIM49L1	92074064	0
CHB	chr11	TRIM49L1	92124064	0
CHB	chr11	TRIM49L1	92174064	0
CHB	chr11	TRIM49L1	92224064	0
CHB	chr11	TRIM49L1	92274064	0
CHB	chr11	TRIM49L1	92324064	0
CHB	chr11	TRIM49L1	92374064	0
CHB	chr11	TRIM49L1	92424064	0
CHB	chr11	TRIM49L1	92474064	0
CHB	chr11	TRIM49L1	92524064	0
CHB	chr11	TRIM49L1	92574064	0
CHB	chr11	TRIM49L1	92624064	0
CHB	chr11	TRIM49L1	92674064	0
CHB	chr11	TRIM49L1	92724064	0
CHB	chr11	TRIM49L1	92774064	0
CHB	chr11	TRIM49L1	92824064	0
CHB	chr11	TRIM49L1	92874064	0
CHB	chr11	TRIM49L1	92924064	0
CHB	chr11	TRIM49L1	92974064	0
CHB	chr11	TRIM49L1	93024064	0

CHB	chr11	TRIM49L1	93074064	0
CHB	chr11	TRIM49L1	93124064	0
CHB	chr11	TRIM49L1	93174064	7
CHB	chr11	TRIM49L1	93224064	31
CHB	chr11	TRIM49L1	93274064	44
CHB	chr11	TRIM49L1	93324064	48
CHB	chr11	TRIM49L1	93374064	48
CHB	chr11	TRIM49L1	93424064	20
CHB	chr11	TRIM49L1	93474064	0
CHB	chr11	TRIM49L1	93524064	0
CHB	chr11	TRIM49L1	93574064	0
CHB	chr11	TRIM49L1	93624064	0
CHB	chr11	TRIM49L1	93674064	0
CHB	chr11	TRIM49L1	93724064	0
CHB	chr11	TRIM49L1	93774064	0
CHB	chr11	TRIM49L1	93824064	0
CHB	chr11	TRIM49L1	93874064	0
CHB	chr11	TRIM49L1	93924064	0
CHB	chr11	TRIM49L1	93974064	0
CHB	chr11	TRIM49L1	94024064	0
CHB	chr11	TRIM49L1	94074064	28
CHB	chr11	TRIM49L1	94124064	43
CHB	chr11	TRIM49L1	94174064	31
CHB	chr11	TRIM49L1	94224064	38
CHB	chr11	TRIM49L1	94274064	34
CHB	chr11	TRIM49L1	94324064	34
CHB	chr11	TRIM49L1	94374064	23
CHB	chr11	TRIM49L1	94424064	1
CHB	chr11	TRIM49L1	94474064	0
CHB	chr11	TRIM49L1	94524064	0
CHB	chr11	TRIM49L1	94574064	0
CHB	chr11	TRIM49L1	94624064	0
CHB	chr11	TRIM49L1	94674064	0
CHB	chr11	TRIM49L1	94724064	0
CHB	chr11	TRIM49L1	94774064	0
CHB	chr11	TRIM49L1	94824064	0
CHB	chr11	TRIM49L1	94874064	0
