# Supplementary material for: Detecting adaptive changes in gene copy number distribution accompanying the human out-of-Africa expansion
Source: Hum Genome Var. 2024 Sep 23;11:37. doi: 10.1038/s41439-024-00293-w (PMC11420239; doi:10.1038/s41439-024-00293-w)
Supplement: Supplementary file 2 — 2_TajimasD_data [file 41439_2024_293_MOESM2_ESM.pdf]

POP	GENE	POS	theta_PI	theta_W	Tajimas_D
CEU	AMY1A	103160519	5.3368	9.7221	-1.3552
CEU	AMY1A	103165519	6.4319	10.916	-1.2437
CEU	AMY1A	103170519	5.4791	11.7688	-1.6255
CEU	AMY1A	103175519	5.8998	11.9394	-1.5398
CEU	AMY1A	103180519	6.7934	14.1567	-1.5979
CEU	AMY1A	103185519	7.3816	13.3039	-1.3632
CEU	AMY1A	103190519	6.1482	9.0398	-0.956
CEU	AMY1A	103195519	4.2663	7.6753	-1.3103
CEU	AMY1A	103200519	4.3362	7.8459	-1.3222
CEU	AMY1A	103205519	3.6663	8.0164	-1.6067
CEU	AMY1A	103210519	4.208	10.0632	-1.7522
CEU	AMY1A	103215519	7.972	11.7688	-0.9812
CEU	AMY1A	103220519	7.208	9.3809	-0.6942
CEU	AMY1A	103225519	6.1828	7.1636	-0.4015
CEU	AMY1A	103230519	7.665	7.3342	0.1325
CEU	AMY1A	103235519	9.9311	10.0632	-0.0395
CEU	AMY1A	103240519	10.3455	9.7221	0.1927
CEU	AMY1A	103245519	9.9046	8.5281	0.4803
CEU	AMY1A	103250519	11.1721	9.0398	0.705
CEU	AMY1A	103255519	9.8093	8.187	0.5877
CEU	AMY1A	103260519	10.0361	10.4043	-0.1068
CEU	AMY1A	103265519	9.3457	10.0632	-0.2147
CEU	AMY1A	103270519	9.1789	9.2104	-0.0102
CEU	AMY1A	103275519	9.0866	9.5515	-0.1461
CEU	AMY1A	103280519	9.3877	10.916	-0.4239
CEU	AMY1A	103285519	9.8158	11.5982	-0.467
CEU	AMY1A	103290519	7.5925	9.3809	-0.5713
CEU	AMY1A	103295519	10.5248	10.4043	0.035
CEU	AMY1A	103300519	10.1752	10.4043	-0.0665
CEU	AMY1A	103305519	6.6801	8.6987	-0.6916
CEU	AMY1A	103310519	7.597	8.3576	-0.2704
CEU	AMY1A	103315519	8.7326	9.8926	-0.3527
CEU	AMY1A	103320519	9.3731	11.9394	-0.6543
CEU	AMY1A	103325519	11.0973	13.3039	-0.5079
CEU	AMY1A	103330519	10.4391	11.9394	-0.3825
CEU	AMY1A	103335519	6.871	8.5281	-0.5782
CEU	AMY1A	103340519	5.3593	6.4814	-0.5029
CEU	AMY1A	103345519	6.6961	6.1402	0.2616
CEU	AMY1A	103350519	13.7085	9.8926	1.1603
CEU	AMY1A	103355519	13.8427	12.2805	0.3879
CEU	AMY1A	103360519	8.9955	12.4511	-0.8468
CEU	AMY1A	103365519	8.2379	10.7454	-0.7058
CEU	AMY1A	103370519	10.1418	12.6216	-0.6
CEU	AMY1A	103375519	12.1265	15.3506	-0.6478
CEU	AMY1A	103380519	12.9937	15.1801	-0.444
CEU	AMY1A	103385519	11.5982	13.8156	-0.4925
CEU	AMY1A	103390519	9.5329	12.9627	-0.8091
CEU	AMY1A	103395519	11.4471	14.3272	-0.6179

CEU	AMY1A	103400519	14.7006	15.3506	-0.1306
CEU	AMY1A	103405519	14.3811	12.6216	0.4257
CEU	AMY1A	103410519	10.0313	10.0632	-0.0095
CEU	AMY1A	103415519	9.0082	13.4744	-1.0157
CEU	AMY1A	103420519	9.3063	12.1099	-0.7053
CEU	AMY1A	103425519	10.0089	8.8692	0.3835
CEU	AMY1A	103430519	7.6413	8.3576	-0.2546
CEU	AMY1A	103435519	4.4543	7.8459	-1.2777
CEU	AMY1A	103440519	7.032	8.6987	-0.571
CEU	AMY1A	103445519	7.8273	8.8692	-0.3506
CEU	AMY1A	103450519	8.0451	9.0398	-0.3289
CEU	AMY1A	103455519	8.8355	10.7454	-0.5376
CEU	AMY1A	103460519	5.6555	10.2337	-1.3488
CEU	AMY1A	103465519	4.0324	9.7221	-1.7583
CEU	AMY1A	103470519	5.0437	9.8926	-1.4744
CEU	AMY1A	103475519	6.1216	10.5749	-1.2724
CEU	AMY1A	103480519	5.0054	9.7221	-1.4576
CEU	AMY1A	103485519	6.2441	12.2805	-1.4987
CEU	AMY1A	103490519	9.3593	16.7151	-1.3624
CEU	AMY1A	103495519	10.5338	16.8857	-1.1651
CEU	AMY1A	103500519	9.5924	16.0329	-1.2414
CEU	AMY1A	103505519	11.6117	16.5446	-0.9227
CEU	AMY1A	103510519	13.7116	16.8857	-0.5822
CEU	AMY1A	103515519	11.1976	14.3272	-0.6715
CEU	AMY1A	103520519	8.5706	11.0866	-0.6877
CEU	AMY1A	103525519	7.2995	9.8926	-0.7885
CEU	AMY1A	103530519	9.0082	12.7922	-0.9039
CEU	AMY1A	103535519	10.1712	13.4744	-0.7512
CEU	AMY1A	103540519	12.485	12.7922	-0.0734
CEU	AMY1A	103545519	13.2162	13.8156	-0.1331
CEU	AMY1A	103550519	18.4352	14.1567	0.9285
CEU	AMY1A	103555519	14.4545	10.916	0.9814
CEU	AMY1A	103560519	3.5772	6.1402	-1.2061
CEU	AMY1A	103565519	5.1123	7.6753	-0.9851
CEU	AMY1A	103570519	5.9988	8.8692	-0.9659
CEU	AMY1A	103575519	6.8297	8.0164	-0.4383
CEU	AMY1A	103580519	7.3673	8.6987	-0.4561
CEU	AMY1A	103585519	9.113	10.5749	-0.4177
CEU	AMY1A	103590519	20.1008	13.9861	1.3423
CEU	AMY1A	103595519	27.3352	17.0562	1.8674
CEU	AMY1A	103600519	25.0282	18.2502	1.154
CEU	AMY1A	103605519	19.6487	18.9324	0.1177
CEU	AMY1A	103610519	18.4556	19.4441	-0.1583
CEU	AMY1A	103615519	31.4051	19.9558	1.7887
CEU	AMY1A	103620519	32.578	16.8857	2.8783
CEU	AMY1A	103625519	20.9408	13.1333	1.8193
CEU	AMY1A	103630519	16.1927	13.8156	0.5279
CEU	AMY1A	103635519	16.6739	14.1567	0.5463
CEU	AMY1A	103640519	15.0155	11.7688	0.8391

CEU	AMY1A	103645519	13.3721	10.4043	0.8609
CEU	AMY1A	103650519	16.5382	11.7688	1.2326
CEU	AMY1A	103655519	16.9316	11.5982	1.3974
CEU	AMY1A	103660519	17.7737	11.2571	1.756
CEU	AMY1A	103665519	18.0837	12.4511	1.3804
CEU	AMY1A	103670519	16.4684	12.6216	0.9307
CEU	AMY1A	103675519	12.397	9.2104	1.0355
CEU	AMY1A	103680519	7.4824	6.1402	0.6316
CEU	AMY1A	103685519	6.5376	6.1402	0.187
CEU	AMY1A	103690519	6.8382	7.3342	-0.1987
CEU	AMY1A	103695519	6.3795	9.0398	-0.8796
CEU	AMY1A	103700519	5.8413	9.0398	-1.0575
CEU	AMY1A	103705519	4.2127	7.5047	-1.2916
CEU	AMY1A	103710519	3.1348	5.458	-1.2145
CEU	AMY1A	103715519	3.2381	6.4814	-1.4537
CEU	AMY1A	103720519	3.8466	8.5281	-1.6334
CEU	AMY1A	103725519	5.1938	9.2104	-1.3052
CEU	AMY1A	103730519	6.8666	10.5749	-1.0595
CEU	AMY1A	103735519	7.7054	12.4511	-1.163
CEU	AMY1A	103740519	7.8407	11.5982	-0.9845
CEU	AMY1A	103745519	10.1968	12.7922	-0.62
CEU	AMY1A	103750519	16.2872	19.2736	-0.4825
CEU	AMY1A	103755519	19.3753	17.9091	0.2542
CEU	AMY1A	103760519	16.918	12.7922	0.9856
CEU	AMY1A	103765519	12.1177	11.9394	0.0455
CEU	AMY1A	103770519	10.0202	14.6684	-0.9752
CEU	AMY1A	103775519	9.187	20.6381	-1.732
CEU	AMY1A	103780519	10.2406	19.9558	-1.5178
CEU	AMY1A	103785519	6.5584	8.0164	-0.5385
CEU	AMY1A	103790519	0	0	0
CEU	AMY1A	103795519	0.1833	0.1706	0.0754
CEU	AMY1A	103800519	0.1833	0.1706	0.0754
CEU	AMY1A	103805519	0	0	0
CEU	AMY1A	103810519	0	0	0
CEU	AMY1A	103815519	0	0	0
CEU	AMY1A	103820519	0	0	0
CEU	AMY1A	103825519	0	0	0
CEU	AMY1A	103830519	0	0	0
CEU	AMY1A	103835519	0	0	0
CEU	AMY1A	103840519	0	0	0
CEU	AMY1A	103845519	0	0	0
CEU	AMY1A	103850519	0	0	0
CEU	AMY1A	103855519	0	0	0
CEU	AMY1A	103860519	0	0	0
CEU	AMY1A	103865519	0	0	0
CEU	AMY1A	103870519	0	0	0
CEU	AMY1A	103875519	0	0	0
CEU	AMY1A	103880519	0	0	0
CEU	AMY1A	103885519	0	0	0

CEU	AMY1A	103890519	0	0	0
CEU	AMY1A	103895519	0	0	0
CEU	AMY1A	103900519	0	0	0
CEU	AMY1A	103905519	0	0	0
CEU	AMY1A	103910519	0.0402	0.5117	-1.4787
CEU	AMY1A	103915519	3.4987	3.9229	-0.2959
CEU	AMY1A	103920519	5.6514	7.1636	-0.619
CEU	AMY1A	103925519	2.1929	3.7524	-1.1298
CEU	AMY1A	103930519	0	0	0
CEU	AMY1A	103935519	0	0	0
CEU	AMY1A	103940519	0	0	0
CEU	AMY1A	103945519	0	0	0
CEU	AMY1A	103950519	0	0	0
CEU	AMY1A	103955519	0	0	0
CEU	AMY1A	103960519	0	0	0
CEU	AMY1A	103965519	0	0	0
CEU	AMY1A	103970519	0	0	0
CEU	AMY1A	103975519	0	0	0
CEU	AMY1A	103980519	0	0	0
CEU	AMY1A	103985519	0	0	0
CEU	AMY1A	103990519	0	0	0
CEU	AMY1A	103995519	0	0	0
CEU	AMY1A	104000519	0	0	0
CEU	AMY1A	104005519	0	0	0
CEU	AMY1A	104010519	0	0	0
CEU	AMY1A	104015519	0	0	0
CEU	AMY1A	104020519	0	0	0
CEU	AMY1A	104025519	0	0	0
CEU	AMY1A	104030519	0	0	0
CEU	AMY1A	104035519	0	0	0
CEU	AMY1A	104040519	0	0	0
CEU	AMY1A	104045519	0	0	0
CEU	AMY1A	104050519	0	0	0
CEU	AMY1A	104055519	0	0	0
CEU	AMY1A	104060519	0	0	0
CEU	AMY1A	104065519	0	0	0
CEU	AMY1A	104070519	0	0	0
CEU	AMY1A	104075519	0	0	0
CEU	AMY1A	104080519	0	0	0
CEU	AMY1A	104085519	0	0	0
CEU	AMY1A	104090519	0	0	0
CEU	AMY1A	104095519	0	0	0
CEU	AMY1A	104100519	0	0	0
CEU	AMY1A	104105519	0	0	0
CEU	AMY1A	104110519	0	0	0
CEU	AMY1A	104115519	0	0	0
CEU	AMY1A	104120519	0	0	0
CEU	AMY1A	104125519	0	0	0
CEU	AMY1A	104130519	0	0	0

CEU	AMY1A	104135519	0	0	0
CEU	AMY1A	104140519	0	0	0
CEU	AMY1A	104145519	0	0	0
CEU	AMY1A	104150519	0	0	0
CEU	AMY1A	104155519	0	0	0
CHB	AMY1A	103160519	8.72	12.3673	-0.897
CHB	AMY1A	103165519	9.2071	13.3838	-0.9532
CHB	AMY1A	103170519	7.8728	12.1979	-1.0776
CHB	AMY1A	103175519	7.5248	10.1649	-0.7804
CHB	AMY1A	103180519	8.3739	13.892	-1.2156
CHB	AMY1A	103185519	9.154	14.9085	-1.1854
CHB	AMY1A	103190519	8.5028	10.1649	-0.4913
CHB	AMY1A	103195519	7.2349	9.3178	-0.6676
CHB	AMY1A	103200519	5.6665	8.8096	-1.0612
CHB	AMY1A	103205519	4.1127	7.2848	-1.2751
CHB	AMY1A	103210519	4.6132	8.8096	-1.4168
CHB	AMY1A	103215519	8.739	10.8426	-0.5854
CHB	AMY1A	103220519	7.5684	9.3178	-0.5607
CHB	AMY1A	103225519	5.3375	7.9625	-0.9727
CHB	AMY1A	103230519	5.6472	8.1319	-0.9031
CHB	AMY1A	103235519	8.1593	9.6567	-0.4643
CHB	AMY1A	103240519	9.5283	9.3178	0.0675
CHB	AMY1A	103245519	8.8209	10.3343	-0.4405
CHB	AMY1A	103250519	9.3958	10.8426	-0.4027
CHB	AMY1A	103255519	7.5753	7.2848	0.1168
CHB	AMY1A	103260519	7.5538	8.6402	-0.3734
CHB	AMY1A	103265519	8.8224	10.3343	-0.4401
CHB	AMY1A	103270519	9.0923	10.8426	-0.4871
CHB	AMY1A	103275519	7.9143	11.012	-0.8497
CHB	AMY1A	103280519	8.0716	10.6731	-0.7348
CHB	AMY1A	103285519	8.2349	10.8426	-0.7257
CHB	AMY1A	103290519	6.1215	10.1649	-1.1953
CHB	AMY1A	103295519	8.9254	12.5367	-0.8768
CHB	AMY1A	103300519	10.0211	13.045	-0.7071
CHB	AMY1A	103305519	8.0849	9.8261	-0.5312
CHB	AMY1A	103310519	8.6764	9.4872	-0.2556
CHB	AMY1A	103315519	8.1637	9.9955	-0.5501
CHB	AMY1A	103320519	7.6872	10.6731	-0.8433
CHB	AMY1A	103325519	11.5038	12.1979	-0.1729
CHB	AMY1A	103330519	12.2346	10.8426	0.3874
CHB	AMY1A	103335519	8.3922	9.4872	-0.3452
CHB	AMY1A	103340519	6.4786	7.9625	-0.5499
CHB	AMY1A	103345519	7.2048	7.6237	-0.1615
CHB	AMY1A	103350519	14.2246	11.012	0.8812
CHB	AMY1A	103355519	14.0887	12.7061	0.3314
CHB	AMY1A	103360519	8.6771	11.5202	-0.7475
CHB	AMY1A	103365519	6.7531	10.3343	-1.0424
CHB	AMY1A	103370519	6.2671	13.2144	-1.6048
CHB	AMY1A	103375519	6.577	15.0779	-1.7324

CHB	AMY1A	103380519	6.917	16.2638	-1.772
CHB	AMY1A	103385519	6.4776	15.2474	-1.7683
CHB	AMY1A	103390519	5.4611	11.8591	-1.6369
CHB	AMY1A	103395519	6.3223	14.2309	-1.7028
CHB	AMY1A	103400519	7.7546	16.6027	-1.6447
CHB	AMY1A	103405519	7.8319	12.8755	-1.194
CHB	AMY1A	103410519	5.8677	11.6896	-1.5098
CHB	AMY1A	103415519	4.6427	14.0615	-2.0512
CHB	AMY1A	103420519	4.8985	12.3673	-1.8367
CHB	AMY1A	103425519	5.7852	10.3343	-1.3242
CHB	AMY1A	103430519	4.4711	9.8261	-1.6338
CHB	AMY1A	103435519	2.688	8.6402	-2.046
CHB	AMY1A	103440519	4.1696	8.979	-1.5954
CHB	AMY1A	103445519	5.1173	10.8426	-1.5934
CHB	AMY1A	103450519	4.9863	11.012	-1.6528
CHB	AMY1A	103455519	6.1574	12.0285	-1.4821
CHB	AMY1A	103460519	5.414	11.012	-1.5355
CHB	AMY1A	103465519	3.7988	8.4708	-1.6355
CHB	AMY1A	103470519	4.3857	9.4872	-1.6081
CHB	AMY1A	103475519	4.8736	9.9955	-1.538
CHB	AMY1A	103480519	3.7632	8.3013	-1.6185
CHB	AMY1A	103485519	6.6969	13.045	-1.4844
CHB	AMY1A	103490519	10.1704	16.4333	-1.1756
CHB	AMY1A	103495519	10.5917	16.0944	-1.0537
CHB	AMY1A	103500519	9.8618	16.7721	-1.2721
CHB	AMY1A	103505519	9.3868	16.6027	-1.3413
CHB	AMY1A	103510519	8.1871	16.9415	-1.5961
CHB	AMY1A	103515519	5.569	15.7556	-1.9907
CHB	AMY1A	103520519	4.3267	13.5532	-2.0808
CHB	AMY1A	103525519	5.0296	13.3838	-1.9066
CHB	AMY1A	103530519	6.9339	16.6027	-1.7973
CHB	AMY1A	103535519	7.379	16.6027	-1.7145
CHB	AMY1A	103540519	6.3523	14.9085	-1.7625
CHB	AMY1A	103545519	4.6651	13.2144	-1.9748
CHB	AMY1A	103550519	7.0845	14.9085	-1.6117
CHB	AMY1A	103555519	6.2138	13.892	-1.6915
CHB	AMY1A	103560519	2.2599	7.9625	-2.1132
CHB	AMY1A	103565519	2.8014	8.3013	-1.9615
CHB	AMY1A	103570519	2.9621	8.8096	-1.9743
CHB	AMY1A	103575519	2.455	8.3013	-2.0851
CHB	AMY1A	103580519	2.491	10.1649	-2.2685
CHB	AMY1A	103585519	3.1789	11.012	-2.1485
CHB	AMY1A	103590519	7.0366	13.7226	-1.4902
CHB	AMY1A	103595519	9.7747	16.9415	-1.3067
CHB	AMY1A	103600519	8.6431	16.7721	-1.4964
CHB	AMY1A	103605519	6.0982	19.4827	-2.1339
CHB	AMY1A	103610519	5.127	22.7016	-2.4174
CHB	AMY1A	103615519	10.5846	22.871	-1.6779
CHB	AMY1A	103620519	12.181	19.6521	-1.1812

CHB	AMY1A	103625519	8.3056	15.4168	-1.4188
CHB	AMY1A	103630519	6.1876	13.5532	-1.6611
CHB	AMY1A	103635519	6.7864	13.045	-1.4635
CHB	AMY1A	103640519	6.5629	13.045	-1.5157
CHB	AMY1A	103645519	5.2671	13.5532	-1.8687
CHB	AMY1A	103650519	6.4849	14.0615	-1.65
CHB	AMY1A	103655519	7.0573	13.5532	-1.465
CHB	AMY1A	103660519	7.5188	14.7391	-1.5036
CHB	AMY1A	103665519	7.1996	15.5862	-1.6559
CHB	AMY1A	103670519	6.2877	14.9085	-1.7758
CHB	AMY1A	103675519	5.059	13.3838	-1.8999
CHB	AMY1A	103680519	3.2817	11.3508	-2.1512
CHB	AMY1A	103685519	2.7944	9.9955	-2.1624
CHB	AMY1A	103690519	3.201	10.6731	-2.1104
CHB	AMY1A	103695519	3.474	12.0285	-2.1596
CHB	AMY1A	103700519	3.2442	12.1979	-2.2307
CHB	AMY1A	103705519	2.2119	10.8426	-2.402
CHB	AMY1A	103710519	1.7173	9.1484	-2.4228
CHB	AMY1A	103715519	1.9181	9.1484	-2.3574
CHB	AMY1A	103720519	1.648	10.1649	-2.5177
CHB	AMY1A	103725519	2.3221	10.8426	-2.3713
CHB	AMY1A	103730519	3.3628	12.5367	-2.2273
CHB	AMY1A	103735519	4.6156	14.2309	-2.0703
CHB	AMY1A	103740519	5.6955	13.2144	-1.7368
CHB	AMY1A	103745519	6.6077	13.3838	-1.5465
CHB	AMY1A	103750519	10.1932	17.6192	-1.304
CHB	AMY1A	103755519	12.4186	18.4662	-1.0151
CHB	AMY1A	103760519	11.6503	16.2638	-0.8746
CHB	AMY1A	103765519	8.6735	13.5532	-1.1005
CHB	AMY1A	103770519	7.0088	15.7556	-1.7093
CHB	AMY1A	103775519	6.316	21.8545	-2.2174
CHB	AMY1A	103780519	5.3823	19.6521	-2.2561
CHB	AMY1A	103785519	3.0407	7.9625	-1.8239
CHB	AMY1A	103790519	0	0	0
CHB	AMY1A	103795519	0.2128	0.3388	-0.5054
CHB	AMY1A	103800519	0.2128	0.3388	-0.5054
CHB	AMY1A	103805519	0	0	0
CHB	AMY1A	103810519	0	0	0
CHB	AMY1A	103815519	0	0	0
CHB	AMY1A	103820519	0	0	0
CHB	AMY1A	103825519	0	0	0
CHB	AMY1A	103830519	0	0	0
CHB	AMY1A	103835519	0	0	0
CHB	AMY1A	103840519	0	0	0
CHB	AMY1A	103845519	0	0	0
CHB	AMY1A	103850519	0	0	0
CHB	AMY1A	103855519	0	0	0
CHB	AMY1A	103860519	0	0	0
CHB	AMY1A	103865519	0	0	0

CHB	AMY1A	103870519	0	0	0
CHB	AMY1A	103875519	0	0	0
CHB	AMY1A	103880519	0	0	0
CHB	AMY1A	103885519	0	0	0
CHB	AMY1A	103890519	0	0	0
CHB	AMY1A	103895519	0	0	0
CHB	AMY1A	103900519	0	0	0
CHB	AMY1A	103905519	0	0	0
CHB	AMY1A	103910519	0.048	0.3388	-1.1665
CHB	AMY1A	103915519	3.0489	3.8965	-0.5928
CHB	AMY1A	103920519	4.763	6.2684	-0.6933
CHB	AMY1A	103925519	1.762	2.7106	-0.8989
CHB	AMY1A	103930519	0	0	0
CHB	AMY1A	103935519	0	0	0
CHB	AMY1A	103940519	0	0	0
CHB	AMY1A	103945519	0	0	0
CHB	AMY1A	103950519	0	0	0
CHB	AMY1A	103955519	0	0	0
CHB	AMY1A	103960519	0	0	0
CHB	AMY1A	103965519	0	0	0
CHB	AMY1A	103970519	0	0	0
CHB	AMY1A	103975519	0	0	0
CHB	AMY1A	103980519	0	0	0
CHB	AMY1A	103985519	0	0	0
CHB	AMY1A	103990519	0	0	0
CHB	AMY1A	103995519	0	0	0
CHB	AMY1A	104000519	0	0	0
CHB	AMY1A	104005519	0	0	0
CHB	AMY1A	104010519	0	0	0
CHB	AMY1A	104015519	0	0	0
CHB	AMY1A	104020519	0	0	0
CHB	AMY1A	104025519	0	0	0
CHB	AMY1A	104030519	0	0	0
CHB	AMY1A	104035519	0	0	0
CHB	AMY1A	104040519	0	0	0
CHB	AMY1A	104045519	0	0	0
CHB	AMY1A	104050519	0	0	0
CHB	AMY1A	104055519	0	0	0
CHB	AMY1A	104060519	0	0	0
CHB	AMY1A	104065519	0	0	0
CHB	AMY1A	104070519	0	0	0
CHB	AMY1A	104075519	0	0	0
CHB	AMY1A	104080519	0	0	0
CHB	AMY1A	104085519	0	0	0
CHB	AMY1A	104090519	0	0	0
CHB	AMY1A	104095519	0	0	0
CHB	AMY1A	104100519	0	0	0
CHB	AMY1A	104105519	0	0	0
CHB	AMY1A	104110519	0	0	0

CHB	AMY1A	104115519	0	0	0
CHB	AMY1A	104120519	0	0	0
CHB	AMY1A	104125519	0	0	0
CHB	AMY1A	104130519	0	0	0
CHB	AMY1A	104135519	0	0	0
CHB	AMY1A	104140519	0	0	0
CHB	AMY1A	104145519	0	0	0
CHB	AMY1A	104150519	0	0	0
CHB	AMY1A	104155519	0	0	0
YRI	AMY1A	103160519	22.0319	26.2177	-0.4991
YRI	AMY1A	103165519	21.759	28.5706	-0.747
YRI	AMY1A	103170519	19.9013	27.5622	-0.87
YRI	AMY1A	103175519	19.4212	26.3858	-0.8252
YRI	AMY1A	103180519	18.9714	27.8983	-1.0019
YRI	AMY1A	103185519	15.9857	23.3606	-0.9834
YRI	AMY1A	103190519	14.9098	18.3188	-0.5747
YRI	AMY1A	103195519	11.6637	16.134	-0.8511
YRI	AMY1A	103200519	8.1146	15.1256	-1.4197
YRI	AMY1A	103205519	7.9811	15.4617	-1.4833
YRI	AMY1A	103210519	8.7096	18.991	-1.6743
YRI	AMY1A	103215519	11.0738	20.1675	-1.3976
YRI	AMY1A	103220519	10.4641	16.9743	-1.1808
YRI	AMY1A	103225519	9.1141	16.4701	-1.3732
YRI	AMY1A	103230519	11.5351	15.7978	-0.8281
YRI	AMY1A	103235519	13.3987	15.4617	-0.4091
YRI	AMY1A	103240519	13.017	17.1423	-0.7412
YRI	AMY1A	103245519	13.6386	19.4952	-0.93
YRI	AMY1A	103250519	14.0483	20.3355	-0.9586
YRI	AMY1A	103255519	12.4478	16.8062	-0.7981
YRI	AMY1A	103260519	15.3011	16.9743	-0.3035
YRI	AMY1A	103265519	17.8881	19.8313	-0.3035
YRI	AMY1A	103270519	16.2171	18.6549	-0.4039
YRI	AMY1A	103275519	13.4284	15.2937	-0.3737
YRI	AMY1A	103280519	15.1888	17.1423	-0.351
YRI	AMY1A	103285519	16.607	19.8313	-0.5036
YRI	AMY1A	103290519	13.6109	17.9827	-0.7502
YRI	AMY1A	103295519	19.5821	22.6884	-0.4261
YRI	AMY1A	103300519	18.8936	21.3439	-0.3565
YRI	AMY1A	103305519	11.4404	17.3104	-1.0448
YRI	AMY1A	103310519	11.9419	18.6549	-1.1121
YRI	AMY1A	103315519	11.5799	17.8146	-1.0796
YRI	AMY1A	103320519	11.1224	18.823	-1.2648
YRI	AMY1A	103325519	15.6509	22.3523	-0.9326
YRI	AMY1A	103330519	15.9553	21.512	-0.8025
YRI	AMY1A	103335519	11.4879	16.9743	-0.9951
YRI	AMY1A	103340519	8.8023	13.9492	-1.1256
YRI	AMY1A	103345519	10.0032	13.9492	-0.863
YRI	AMY1A	103350519	13.7879	16.302	-0.474
YRI	AMY1A	103355519	15.6049	15.4617	0.0284

YRI	AMY1A	103360519	16.187	15.2937	0.179
YRI	AMY1A	103365519	14.0445	14.2853	-0.0515
YRI	AMY1A	103370519	12.6271	15.6298	-0.5893
YRI	AMY1A	103375519	11.7939	18.4868	-1.1185
YRI	AMY1A	103380519	12.0511	18.823	-1.1123
YRI	AMY1A	103385519	12.5537	17.3104	-0.8467
YRI	AMY1A	103390519	13.6568	17.3104	-0.6503
YRI	AMY1A	103395519	16.5314	19.3272	-0.4477
YRI	AMY1A	103400519	22.1693	20.1675	0.3077
YRI	AMY1A	103405519	21.2188	17.1423	0.7324
YRI	AMY1A	103410519	19.6707	19.4952	0.0279
YRI	AMY1A	103415519	19.6597	21.8481	-0.3113
YRI	AMY1A	103420519	15.1782	18.823	-0.5986
YRI	AMY1A	103425519	13.6723	19.4952	-0.9246
YRI	AMY1A	103430519	10.2326	17.9827	-1.33
YRI	AMY1A	103435519	8.5485	17.1423	-1.544
YRI	AMY1A	103440519	11.7732	17.9827	-1.0656
YRI	AMY1A	103445519	11.9045	17.4785	-0.983
YRI	AMY1A	103450519	12.1323	17.1423	-0.9001
YRI	AMY1A	103455519	14.5709	19.4952	-0.7819
YRI	AMY1A	103460519	12.3999	16.6382	-0.7836
YRI	AMY1A	103465519	9.742	14.2853	-0.9714
YRI	AMY1A	103470519	9.366	15.9659	-1.2692
YRI	AMY1A	103475519	9.2299	14.7895	-1.1501
YRI	AMY1A	103480519	7.9471	14.1172	-1.3341
YRI	AMY1A	103485519	13.6059	18.6549	-0.8365
YRI	AMY1A	103490519	16.931	21.0078	-0.6024
YRI	AMY1A	103495519	17.534	21.3439	-0.5544
YRI	AMY1A	103500519	16.2548	21.3439	-0.7405
YRI	AMY1A	103505519	17.404	20.1675	-0.4247
YRI	AMY1A	103510519	21.5318	21.1758	0.0522
YRI	AMY1A	103515519	18.6059	20.8397	-0.3326
YRI	AMY1A	103520519	15.0517	17.8146	-0.4784
YRI	AMY1A	103525519	14.2458	18.3188	-0.6867
YRI	AMY1A	103530519	18.0109	24.369	-0.8138
YRI	AMY1A	103535519	17.5628	25.0413	-0.9323
YRI	AMY1A	103540519	16.4056	23.0245	-0.8951
YRI	AMY1A	103545519	15.9226	21.68	-0.8252
YRI	AMY1A	103550519	18.0642	23.5287	-0.7236
YRI	AMY1A	103555519	14.5469	21.1758	-0.972
YRI	AMY1A	103560519	7.5798	15.4617	-1.5629
YRI	AMY1A	103565519	11.2521	16.134	-0.9295
YRI	AMY1A	103570519	11.4965	15.7978	-0.8356
YRI	AMY1A	103575519	11.7091	15.1256	-0.6918
YRI	AMY1A	103580519	13.1659	16.8062	-0.6666
YRI	AMY1A	103585519	12.7031	17.9827	-0.906
YRI	AMY1A	103590519	24.359	19.3272	0.8057
YRI	AMY1A	103595519	34.2078	22.8565	1.546
YRI	AMY1A	103600519	32.8547	25.3774	0.9201

YRI	AMY1A	103605519	34.1614	26.3858	0.9213
YRI	AMY1A	103610519	38.5935	26.5538	1.4178
YRI	AMY1A	103615519	42.86	27.058	1.8271
YRI	AMY1A	103620519	34.7611	24.7051	1.2701
YRI	AMY1A	103625519	20.3814	20.8397	-0.0682
YRI	AMY1A	103630519	17.8121	19.9994	-0.3389
YRI	AMY1A	103635519	21.5257	17.9827	0.608
YRI	AMY1A	103640519	19.2593	17.4785	0.3141
YRI	AMY1A	103645519	19.0688	18.6549	0.0686
YRI	AMY1A	103650519	21.9121	18.823	0.5074
YRI	AMY1A	103655519	19.4406	17.8146	0.2816
YRI	AMY1A	103660519	20.0986	17.4785	0.4621
YRI	AMY1A	103665519	23.1772	21.3439	0.2668
YRI	AMY1A	103670519	20.8294	21.0078	-0.0264
YRI	AMY1A	103675519	14.5023	14.9575	-0.0932
YRI	AMY1A	103680519	10.5188	13.1089	-0.6008
YRI	AMY1A	103685519	10.2142	15.4617	-1.0405
YRI	AMY1A	103690519	12.287	19.1591	-1.1097
YRI	AMY1A	103695519	15.2115	22.1842	-0.9775
YRI	AMY1A	103700519	14.196	21.512	-1.0565
YRI	AMY1A	103705519	10.6927	19.3272	-1.3826
YRI	AMY1A	103710519	9.7532	16.8062	-1.2915
YRI	AMY1A	103715519	8.7605	14.2853	-1.1813
YRI	AMY1A	103720519	8.8724	14.9575	-1.2454
YRI	AMY1A	103725519	12.2432	18.4868	-1.0434
YRI	AMY1A	103730519	15.7306	19.6633	-0.6193
YRI	AMY1A	103735519	16.1359	18.1507	-0.3427
YRI	AMY1A	103740519	15.988	17.1423	-0.2074
YRI	AMY1A	103745519	19.9243	18.991	0.152
YRI	AMY1A	103750519	30.349	24.5371	0.739
YRI	AMY1A	103755519	28.9091	25.5455	0.4113
YRI	AMY1A	103760519	17.9884	22.0162	-0.5688
YRI	AMY1A	103765519	11.9966	17.3104	-0.9458
YRI	AMY1A	103770519	10.9899	18.4868	-1.2528
YRI	AMY1A	103775519	13.0207	24.7051	-1.4758
YRI	AMY1A	103780519	13.1616	22.3523	-1.279
YRI	AMY1A	103785519	6.8118	9.0754	-0.741
YRI	AMY1A	103790519	0	0	0
YRI	AMY1A	103795519	0.4945	0.1681	1.9404
YRI	AMY1A	103800519	0.4945	0.1681	1.9404
YRI	AMY1A	103805519	0	0	0
YRI	AMY1A	103810519	0	0	0
YRI	AMY1A	103815519	0	0	0
YRI	AMY1A	103820519	0	0	0
YRI	AMY1A	103825519	0	0	0
YRI	AMY1A	103830519	0	0	0
YRI	AMY1A	103835519	0	0	0
YRI	AMY1A	103840519	0	0	0
YRI	AMY1A	103845519	0	0	0

YRI	AMY1A	103850519	0	0	0
YRI	AMY1A	103855519	0	0	0
YRI	AMY1A	103860519	0	0	0
YRI	AMY1A	103865519	0	0	0
YRI	AMY1A	103870519	0	0	0
YRI	AMY1A	103875519	0	0	0
YRI	AMY1A	103880519	0	0	0
YRI	AMY1A	103885519	0	0	0
YRI	AMY1A	103890519	0	0	0
YRI	AMY1A	103895519	0	0	0
YRI	AMY1A	103900519	0	0	0
YRI	AMY1A	103905519	0	0	0
YRI	AMY1A	103910519	0.3281	0.6722	-0.9008
YRI	AMY1A	103915519	2.8615	5.5461	-1.3748
YRI	AMY1A	103920519	4.7981	10.4199	-1.6183
YRI	AMY1A	103925519	2.2648	5.5461	-1.6803
YRI	AMY1A	103930519	0	0	0
YRI	AMY1A	103935519	0	0	0
YRI	AMY1A	103940519	0	0	0
YRI	AMY1A	103945519	0	0	0
YRI	AMY1A	103950519	0	0	0
YRI	AMY1A	103955519	0	0	0
YRI	AMY1A	103960519	0	0	0
YRI	AMY1A	103965519	0	0	0
YRI	AMY1A	103970519	0	0	0
YRI	AMY1A	103975519	0	0	0
YRI	AMY1A	103980519	0	0	0
YRI	AMY1A	103985519	0	0	0
YRI	AMY1A	103990519	0	0	0
YRI	AMY1A	103995519	0	0	0
YRI	AMY1A	104000519	0	0	0
YRI	AMY1A	104005519	0	0	0
YRI	AMY1A	104010519	0	0	0
YRI	AMY1A	104015519	0	0	0
YRI	AMY1A	104020519	0	0	0
YRI	AMY1A	104025519	0	0	0
YRI	AMY1A	104030519	0	0	0
YRI	AMY1A	104035519	0	0	0
YRI	AMY1A	104040519	0	0	0
YRI	AMY1A	104045519	0	0	0
YRI	AMY1A	104050519	0	0	0
YRI	AMY1A	104055519	0	0	0
YRI	AMY1A	104060519	0	0	0
YRI	AMY1A	104065519	0	0	0
YRI	AMY1A	104070519	0	0	0
YRI	AMY1A	104075519	0	0	0
YRI	AMY1A	104080519	0	0	0
YRI	AMY1A	104085519	0	0	0
YRI	AMY1A	104090519	0	0	0

YRI	AMY1A	104095519	0	0	0
YRI	AMY1A	104100519	0	0	0
YRI	AMY1A	104105519	0	0	0
YRI	AMY1A	104110519	0	0	0
YRI	AMY1A	104115519	0	0	0
YRI	AMY1A	104120519	0	0	0
YRI	AMY1A	104125519	0	0	0
YRI	AMY1A	104130519	0	0	0
YRI	AMY1A	104135519	0	0	0
YRI	AMY1A	104140519	0	0	0
YRI	AMY1A	104145519	0	0	0
YRI	AMY1A	104150519	0	0	0
YRI	AMY1A	104155519	0	0	0
CEU	BOLA2B	28958588	5.1	7.1636	-0.8447
CEU	BOLA2B	28963588	3.5677	6.1402	-1.2105
CEU	BOLA2B	28968588	3.8477	5.1169	-0.7025
CEU	BOLA2B	28973588	4.6588	5.2874	-0.338
CEU	BOLA2B	28978588	5.8248	6.6519	-0.3621
CEU	BOLA2B	28983588	6.0546	7.6753	-0.623
CEU	BOLA2B	28988588	5.0515	6.3108	-0.5782
CEU	BOLA2B	28993588	4.8291	5.9697	-0.5505
CEU	BOLA2B	28998588	4.0682	6.4814	-1.0816
CEU	BOLA2B	29003588	4.9847	8.0164	-1.1198
CEU	BOLA2B	29008588	6.657	9.7221	-0.9472
CEU	BOLA2B	29013588	6.2764	7.6753	-0.5377
CEU	BOLA2B	29018588	6.1029	6.3108	-0.0955
CEU	BOLA2B	29023588	5.6799	7.1636	-0.6074
CEU	BOLA2B	29028588	5.3676	6.6519	-0.5623
CEU	BOLA2B	29033588	5.6974	7.1636	-0.6002
CEU	BOLA2B	29038588	4.9204	6.9931	-0.8673
CEU	BOLA2B	29043588	5.0324	5.2874	-0.1371
CEU	BOLA2B	29048588	2.6819	2.729	-0.0445
CEU	BOLA2B	29053588	1.4886	2.0467	-0.6641
CEU	BOLA2B	29058588	4.4125	4.0935	0.2145
CEU	BOLA2B	29063588	5.4372	4.6052	0.5051
CEU	BOLA2B	29068588	3.9961	3.7524	0.1766
CEU	BOLA2B	29073588	3.5865	2.729	0.8105
CEU	BOLA2B	29078588	4.9819	4.4346	0.3434
CEU	BOLA2B	29083588	5.5945	5.7991	-0.1013
CEU	BOLA2B	29088588	6.37	5.7991	0.2828
CEU	BOLA2B	29093588	7.7555	7.1636	0.2423
CEU	BOLA2B	29098588	7.5582	7.5047	0.021
CEU	BOLA2B	29103588	7.2375	7.6753	-0.1683
CEU	BOLA2B	29108588	8.6479	7.8459	0.3021
CEU	BOLA2B	29113588	12.2356	8.187	1.4667
CEU	BOLA2B	29118588	13.0272	10.0632	0.887
CEU	BOLA2B	29123588	11.5088	9.7221	0.5521
CEU	BOLA2B	29128588	17.517	11.0866	1.7577
CEU	BOLA2B	29133588	21.4607	12.7922	2.0708

CEU	BOLA2B	29138588	22.5897	13.4744	2.073
CEU	BOLA2B	29143588	18.488	11.7688	1.7365
CEU	BOLA2B	29148588	19.7491	14.3272	1.1633
CEU	BOLA2B	29153588	26.4475	16.7151	1.8026
CEU	BOLA2B	29158588	22.6479	12.6216	2.4257
CEU	BOLA2B	29163588	17.1286	12.1099	1.2626
CEU	BOLA2B	29168588	10.3531	10.2337	0.0352
CEU	BOLA2B	29173588	5.5914	7.5047	-0.7507
CEU	BOLA2B	29178588	7.1777	7.5047	-0.1283
CEU	BOLA2B	29183588	11.9642	8.3576	1.282
CEU	BOLA2B	29188588	21.3215	12.2805	2.2447
CEU	BOLA2B	29193588	18.7714	12.2805	1.6115
CEU	BOLA2B	29198588	9.1796	8.3576	0.2922
CEU	BOLA2B	29203588	10.6881	10.0632	0.187
CEU	BOLA2B	29208588	11.188	11.4277	-0.0637
CEU	BOLA2B	29213588	11.8738	9.5515	0.7296
CEU	BOLA2B	29218588	10.0486	8.187	0.6744
CEU	BOLA2B	29223588	8.4972	7.3342	0.466
CEU	BOLA2B	29228588	12.9885	8.6987	1.4697
CEU	BOLA2B	29233588	15.7827	13.1333	0.6173
CEU	BOLA2B	29238588	16.2004	13.4744	0.62
CEU	BOLA2B	29243588	17.6704	12.6216	1.2215
CEU	BOLA2B	29248588	15.3148	11.2571	1.0934
CEU	BOLA2B	29253588	12.847	9.7221	0.9657
CEU	BOLA2B	29258588	15.5949	11.7688	0.9888
CEU	BOLA2B	29263588	22.3585	16.2034	1.1744
CEU	BOLA2B	29268588	26.8916	17.0562	1.7868
CEU	BOLA2B	29273588	34.733	16.8857	3.2736
CEU	BOLA2B	29278588	43.6731	18.9324	4.0662
CEU	BOLA2B	29283588	39.3886	17.7385	3.788
CEU	BOLA2B	29288588	26.6747	12.7922	3.3164
CEU	BOLA2B	29293588	19.8089	11.0866	2.3842
CEU	BOLA2B	29298588	21.1381	13.8156	1.6263
CEU	BOLA2B	29303588	16.1645	12.4511	0.91
CEU	BOLA2B	29308588	9.766	8.5281	0.4319
CEU	BOLA2B	29313588	11.3028	9.7221	0.4885
CEU	BOLA2B	29318588	12.6595	16.0329	-0.6502
CEU	BOLA2B	29323588	9.3436	15.0095	-1.163
CEU	BOLA2B	29328588	6.4659	9.8926	-1.042
CEU	BOLA2B	29333588	6.1293	9.3809	-1.0388
CEU	BOLA2B	29338588	6.6036	9.5515	-0.9261
CEU	BOLA2B	29343588	7.8602	10.2337	-0.6992
CEU	BOLA2B	29348588	8.3731	9.0398	-0.2204
CEU	BOLA2B	29353588	6.9878	7.3342	-0.1388
CEU	BOLA2B	29358588	5.2663	6.1402	-0.4112
CEU	BOLA2B	29363588	3.0785	4.0935	-0.6825
CEU	BOLA2B	29368588	2.4877	3.9229	-1.001
CEU	BOLA2B	29373588	1.7631	3.2407	-1.2116
CEU	BOLA2B	29378588	0.7172	2.5584	-1.8341

CEU	BOLA2B	29383588	1.6774	2.3879	-0.7482
CEU	BOLA2B	29388588	3.7462	2.5584	1.1832
CEU	BOLA2B	29393588	4.9052	4.4346	0.2953
CEU	BOLA2B	29398588	3.691	5.458	-0.9237
CEU	BOLA2B	29403588	3.1272	4.7757	-0.9695
CEU	BOLA2B	29408588	5.1478	6.4814	-0.5977
CEU	BOLA2B	29413588	5.4677	9.7221	-1.3147
CEU	BOLA2B	29418588	4.9324	8.6987	-1.2903
CEU	BOLA2B	29423588	7.3614	7.8459	-0.1825
CEU	BOLA2B	29428588	6.1717	8.0164	-0.6813
CEU	BOLA2B	29433588	5.6516	9.3809	-1.1914
CEU	BOLA2B	29438588	7.6745	9.5515	-0.5897
CEU	BOLA2B	29443588	4.6817	4.9463	-0.1509
CEU	BOLA2B	29448588	0.9221	2.0467	-1.3382
CEU	BOLA2B	29453588	0.0705	0.8528	-1.7617
CEU	BOLA2B	29458588	0.0402	0.3411	-1.2062
CEU	BOLA2B	29463588	0.0101	0.1706	-0.9534
CEU	BOLA2B	29468588	0.5362	0.5117	0.0768
CEU	BOLA2B	29473588	0.5955	0.6822	-0.2264
CEU	BOLA2B	29478588	0.1288	0.5117	-1.2009
CEU	BOLA2B	29483588	0.1192	0.5117	-1.231
CEU	BOLA2B	29488588	0.0497	0.1706	-0.7182
CEU	BOLA2B	29493588	1.0559	0.6822	0.9758
CEU	BOLA2B	29498588	1.0559	0.6822	0.9758
CEU	BOLA2B	29503588	0.1097	0.6822	-1.495
CEU	BOLA2B	29508588	4.6875	2.3879	2.4217
CEU	BOLA2B	29513588	5.0765	1.8762	4.0745
CEU	BOLA2B	29518588	0.7565	0.3411	1.6652
CEU	BOLA2B	29523588	0.2781	0.5117	-0.7326
CEU	BOLA2B	29528588	0.0303	0.5117	-1.5098
CEU	BOLA2B	29533588	0.0303	0.3411	-1.2459
CEU	BOLA2B	29538588	0.0505	0.6822	-1.6496
CEU	BOLA2B	29543588	1.0012	0.8528	0.3342
CEU	BOLA2B	29548588	0.981	0.5117	1.4718
CEU	BOLA2B	29553588	0.5683	0.5117	0.1775
CEU	BOLA2B	29558588	1.4368	1.3645	0.117
CEU	BOLA2B	29563588	1.2022	2.2173	-1.1341
CEU	BOLA2B	29568588	1.9029	2.729	-0.7808
CEU	BOLA2B	29573588	2.7485	3.2407	-0.4036
CEU	BOLA2B	29578588	3.6652	3.2407	0.3481
CEU	BOLA2B	29583588	4.235	3.0701	0.9992
CEU	BOLA2B	29588588	3.4387	4.9463	-0.8598
CEU	BOLA2B	29593588	2.8584	5.9697	-1.5016
CEU	BOLA2B	29598588	3.5741	5.2874	-0.9212
CEU	BOLA2B	29603588	5.9654	6.6519	-0.3006
CEU	BOLA2B	29608588	6.0827	7.6753	-0.6122
CEU	BOLA2B	29613588	5.5092	7.3342	-0.7312
CEU	BOLA2B	29618588	3.6885	5.2874	-0.8597
CEU	BOLA2B	29623588	3.3886	4.7757	-0.8158

CEU	BOLA2B	29628588	3.0517	5.9697	-1.4083
CEU	BOLA2B	29633588	1.128	4.4346	-2.0747
CEU	BOLA2B	29638588	6.2709	5.1169	0.6388
CEU	BOLA2B	29643588	11.4172	6.8225	1.9661
CEU	BOLA2B	29648588	8.0569	5.7991	1.1183
CEU	BOLA2B	29653588	6.0266	5.6286	0.2024
CEU	BOLA2B	29658588	7.6102	6.6519	0.4196
CEU	BOLA2B	29663588	5.7619	6.4814	-0.3225
CEU	BOLA2B	29668588	6.6863	7.1636	-0.1954
CEU	BOLA2B	29673588	8.1804	7.5047	0.2651
CEU	BOLA2B	29678588	8.851	7.6753	0.4519
CEU	BOLA2B	29683588	9.3043	8.187	0.4048
CEU	BOLA2B	29688588	8.6883	7.1636	0.6241
CEU	BOLA2B	29693588	7.6223	5.9697	0.7976
CEU	BOLA2B	29698588	6.4795	5.9697	0.246
CEU	BOLA2B	29703588	4.5516	6.3108	-0.8077
CEU	BOLA2B	29708588	3.5749	6.4814	-1.3027
CEU	BOLA2B	29713588	4.597	6.1402	-0.7262
CEU	BOLA2B	29718588	4.9629	5.2874	-0.1745
CEU	BOLA2B	29723588	6.0375	7.1636	-0.461
CEU	BOLA2B	29728588	5.6116	9.3809	-1.2041
CEU	BOLA2B	29733588	5.4655	7.5047	-0.8001
CEU	BOLA2B	29738588	5.9074	6.3108	-0.1852
CEU	BOLA2B	29743588	5.7926	6.9931	-0.5023
CEU	BOLA2B	29748588	7.3245	7.5047	-0.0707
CEU	BOLA2B	29753588	5.605	7.8459	-0.8442
CEU	BOLA2B	29758588	3.0029	8.0164	-1.8518
CEU	BOLA2B	29763588	5.1892	8.5281	-1.165
CEU	BOLA2B	29768588	3.7376	6.6519	-1.2759
CEU	BOLA2B	29773588	1.0287	6.6519	-2.4619
CEU	BOLA2B	29778588	1.3115	6.8225	-2.3581
CEU	BOLA2B	29783588	1.9359	5.2874	-1.8021
CEU	BOLA2B	29788588	2.0245	6.6519	-2.026
CEU	BOLA2B	29793588	2.0886	6.3108	-1.9385
CEU	BOLA2B	29798588	1.4539	4.9463	-1.9917
CEU	BOLA2B	29803588	0.522	4.6052	-2.4791
CEU	BOLA2B	29808588	0.8291	3.4112	-2.028
CEU	BOLA2B	29813588	0.5953	3.2407	-2.1692
CEU	BOLA2B	29818588	0.8139	5.9697	-2.4883
CEU	BOLA2B	29823588	1.8716	6.9931	-2.1429
CEU	BOLA2B	29828588	3.5773	6.9931	-1.4292
CEU	BOLA2B	29833588	4.4136	7.5047	-1.2128
CEU	BOLA2B	29838588	4.6842	5.6286	-0.4804
CEU	BOLA2B	29843588	4.1509	4.6052	-0.2758
CEU	BOLA2B	29848588	2.8023	4.2641	-0.9489
CEU	BOLA2B	29853588	2.3111	4.9463	-1.5029
CEU	BOLA2B	29858588	3.1387	5.1169	-1.095
CEU	BOLA2B	29863588	3.2548	4.4346	-0.7402
CEU	BOLA2B	29868588	2.8079	4.0935	-0.8645

CEU	BOLA2B	29873588	4.5764	5.1169	-0.2992
CEU	BOLA2B	29878588	5.0138	5.7991	-0.389
CEU	BOLA2B	29883588	3.9338	4.9463	-0.5774
CEU	BOLA2B	29888588	2.9501	5.1169	-1.1994
CEU	BOLA2B	29893588	3.3888	5.7991	-1.1938
CEU	BOLA2B	29898588	4.1033	6.4814	-1.0659
CEU	BOLA2B	29903588	4.2455	6.3108	-0.9482
CEU	BOLA2B	29908588	6.6261	4.7757	1.0883
CEU	BOLA2B	29913588	6.9781	4.7757	1.2953
CEU	BOLA2B	29918588	5.6366	5.9697	-0.1608
CEU	BOLA2B	29923588	5.9606	5.7991	0.08
CEU	BOLA2B	29928588	5.666	5.2874	0.2036
CEU	BOLA2B	29933588	7.6999	6.3108	0.6378
CEU	BOLA2B	29938588	8.5079	6.9931	0.6338
CEU	BOLA2B	29943588	6.65	6.3108	0.1557
CEU	BOLA2B	29948588	4.6924	4.9463	-0.1448
CHB	BOLA2B	28958588	6.6773	7.1154	-0.1799
CHB	BOLA2B	28963588	6.4967	7.1154	-0.2541
CHB	BOLA2B	28968588	6.4203	7.1154	-0.2855
CHB	BOLA2B	28973588	5.5509	7.7931	-0.8474
CHB	BOLA2B	28978588	5.8751	7.9625	-0.7735
CHB	BOLA2B	28983588	6.7866	8.4708	-0.5896
CHB	BOLA2B	28988588	5.9395	9.1484	-1.0462
CHB	BOLA2B	28993588	5.5102	7.6237	-0.815
CHB	BOLA2B	28998588	5.0274	6.4378	-0.6342
CHB	BOLA2B	29003588	6.7908	9.6567	-0.8887
CHB	BOLA2B	29008588	8.016	10.5037	-0.7132
CHB	BOLA2B	29013588	7.1108	7.9625	-0.3156
CHB	BOLA2B	29018588	7.0647	7.4543	-0.1534
CHB	BOLA2B	29023588	7.5743	8.4708	-0.3138
CHB	BOLA2B	29028588	8.4719	9.4872	-0.32
CHB	BOLA2B	29033588	8.1028	9.3178	-0.3895
CHB	BOLA2B	29038588	5.5863	7.4543	-0.7353
CHB	BOLA2B	29043588	4.2189	5.0825	-0.4795
CHB	BOLA2B	29048588	2.0306	2.3718	-0.3602
CHB	BOLA2B	29053588	1.5866	2.033	-0.5323
CHB	BOLA2B	29058588	4.3627	4.066	0.2001
CHB	BOLA2B	29063588	4.9513	4.7436	0.1225
CHB	BOLA2B	29068588	3.996	4.066	-0.0472
CHB	BOLA2B	29073588	3.9469	3.5577	0.2941
CHB	BOLA2B	29078588	4.739	3.5577	0.8928
CHB	BOLA2B	29083588	4.7521	5.0825	-0.1834
CHB	BOLA2B	29088588	4.4338	5.7601	-0.6589
CHB	BOLA2B	29093588	5.4578	6.946	-0.6247
CHB	BOLA2B	29098588	5.7866	8.8096	-1.0206
CHB	BOLA2B	29103588	5.8237	9.3178	-1.12
CHB	BOLA2B	29108588	6.6438	8.8096	-0.7312
CHB	BOLA2B	29113588	8.5585	8.8096	-0.0848
CHB	BOLA2B	29118588	8.8561	9.8261	-0.2959

CHB	BOLA2B	29123588	8.0158	8.979	-0.3195
CHB	BOLA2B	29128588	16.0114	11.1814	1.306
CHB	BOLA2B	29133588	22.9497	14.2309	1.8773
CHB	BOLA2B	29138588	24.7619	15.925	1.7094
CHB	BOLA2B	29143588	18.5682	14.0615	0.9815
CHB	BOLA2B	29148588	18.7226	15.4168	0.6596
CHB	BOLA2B	29153588	24.7803	17.2803	1.3417
CHB	BOLA2B	29158588	21.9566	14.9085	1.4519
CHB	BOLA2B	29163588	15.9885	16.0944	-0.0203
CHB	BOLA2B	29168588	7.3077	12.8755	-1.3181
CHB	BOLA2B	29173588	3.6288	7.9625	-1.6059
CHB	BOLA2B	29178588	5.0335	11.8591	-1.7463
CHB	BOLA2B	29183588	7.7954	16.2638	-1.6055
CHB	BOLA2B	29188588	17.9907	17.6192	0.0652
CHB	BOLA2B	29193588	16.558	15.5862	0.1919
CHB	BOLA2B	29198588	7.201	9.6567	-0.7615
CHB	BOLA2B	29203588	9.1776	10.3343	-0.3367
CHB	BOLA2B	29208588	10.2465	14.2309	-0.8579
CHB	BOLA2B	29213588	10.4662	16.2638	-1.0991
CHB	BOLA2B	29218588	8.7139	13.045	-1.0128
CHB	BOLA2B	29223588	8.1374	8.3013	-0.0585
CHB	BOLA2B	29228588	11.329	9.1484	0.711
CHB	BOLA2B	29233588	14.6743	14.4003	0.0583
CHB	BOLA2B	29238588	14.9762	16.6027	-0.3023
CHB	BOLA2B	29243588	12.918	13.5532	-0.1433
CHB	BOLA2B	29248588	12.3179	11.1814	0.3073
CHB	BOLA2B	29253588	14.1262	9.6567	1.3859
CHB	BOLA2B	29258588	16.2852	10.6731	1.5851
CHB	BOLA2B	29263588	20.7021	17.6192	0.5414
CHB	BOLA2B	29268588	24.7809	19.991	0.7449
CHB	BOLA2B	29273588	32.0339	21.5157	1.5238
CHB	BOLA2B	29278588	41.5412	25.2428	2.0228
CHB	BOLA2B	29283588	40.7064	24.0569	2.1651
CHB	BOLA2B	29288588	27.8093	17.958	1.6985
CHB	BOLA2B	29293588	20.172	14.4003	1.2288
CHB	BOLA2B	29298588	26.2417	20.3298	0.9047
CHB	BOLA2B	29303588	23.5283	22.1934	0.1877
CHB	BOLA2B	29308588	13.617	15.925	-0.4465
CHB	BOLA2B	29313588	12.1483	11.8591	0.074
CHB	BOLA2B	29318588	19.5302	15.925	0.6974
CHB	BOLA2B	29323588	18.2599	16.4333	0.3429
CHB	BOLA2B	29328588	8.948	13.2144	-0.9855
CHB	BOLA2B	29333588	7.2054	10.1649	-0.8749
CHB	BOLA2B	29338588	7.7072	7.6237	0.0322
CHB	BOLA2B	29343588	11.1013	9.6567	0.4479
CHB	BOLA2B	29348588	11.774	9.4872	0.7208
CHB	BOLA2B	29353588	9.044	7.6237	0.5477
CHB	BOLA2B	29358588	6.4264	6.2684	0.0728
CHB	BOLA2B	29363588	3.1123	5.5907	-1.2645

CHB	BOLA2B	29368588	3.0008	4.7436	-1.028
CHB	BOLA2B	29373588	2.26	2.2024	0.0645
CHB	BOLA2B	29378588	1.3145	2.3718	-1.1161
CHB	BOLA2B	29383588	2.3276	3.3883	-0.8353
CHB	BOLA2B	29388588	4.1576	4.7436	-0.3457
CHB	BOLA2B	29393588	4.9265	5.2519	-0.1755
CHB	BOLA2B	29398588	4.435	4.7436	-0.182
CHB	BOLA2B	29403588	4.5991	5.0825	-0.2684
CHB	BOLA2B	29408588	6.9221	8.3013	-0.4919
CHB	BOLA2B	29413588	7.5167	10.1649	-0.7828
CHB	BOLA2B	29418588	5.6244	8.3013	-0.9547
CHB	BOLA2B	29423588	7.2688	8.6402	-0.4714
CHB	BOLA2B	29428588	5.7916	7.2848	-0.6002
CHB	BOLA2B	29433588	4.7334	7.1154	-0.9782
CHB	BOLA2B	29438588	6.3864	8.3013	-0.6829
CHB	BOLA2B	29443588	3.8519	5.0825	-0.6832
CHB	BOLA2B	29448588	0.6785	1.5247	-1.2572
CHB	BOLA2B	29453588	0.0386	0.5082	-1.4741
CHB	BOLA2B	29458588	0.0958	0.5082	-1.2946
CHB	BOLA2B	29463588	0.0862	0.5082	-1.3247
CHB	BOLA2B	29468588	0.5121	0.6777	-0.4329
CHB	BOLA2B	29473588	0.5408	0.6777	-0.3579
CHB	BOLA2B	29478588	0.1133	0.6777	-1.4755
CHB	BOLA2B	29483588	0.2648	1.0165	-1.4959
CHB	BOLA2B	29488588	0.19	0.5082	-0.9989
CHB	BOLA2B	29493588	0.3564	1.1859	-1.4806
CHB	BOLA2B	29498588	0.3758	1.5247	-1.7069
CHB	BOLA2B	29503588	0.7002	1.1859	-0.867
CHB	BOLA2B	29508588	4.8346	2.5412	2.2901
CHB	BOLA2B	29513588	4.6568	1.8636	3.5637
CHB	BOLA2B	29518588	0.7249	0.3388	1.5488
CHB	BOLA2B	29523588	0.2701	0.6777	-1.0656
CHB	BOLA2B	29528588	0.0482	0.5082	-1.444
CHB	BOLA2B	29533588	0.0967	0.6777	-1.5189
CHB	BOLA2B	29538588	0.1064	0.8471	-1.6703
CHB	BOLA2B	29543588	0.8397	0.5082	1.0406
CHB	BOLA2B	29548588	0.83	0.3388	1.9704
CHB	BOLA2B	29553588	0.6611	0.6777	-0.0434
CHB	BOLA2B	29558588	1.3132	2.2024	-0.9957
CHB	BOLA2B	29563588	1.874	3.3883	-1.1925
CHB	BOLA2B	29568588	2.8662	3.0495	-0.1576
CHB	BOLA2B	29573588	2.9098	2.033	1.0456
CHB	BOLA2B	29578588	2.3998	2.033	0.4374
CHB	BOLA2B	29583588	2.6878	4.5742	-1.1487
CHB	BOLA2B	29588588	3.2376	5.7601	-1.2532
CHB	BOLA2B	29593588	2.8894	5.9295	-1.4718
CHB	BOLA2B	29598588	3.8612	7.4543	-1.4143
CHB	BOLA2B	29603588	5.4417	6.7766	-0.573
CHB	BOLA2B	29608588	3.8561	5.2519	-0.7528

CHB	BOLA2B	29613588	3.2465	6.4378	-1.4349
CHB	BOLA2B	29618588	2.5557	6.0989	-1.6725
CHB	BOLA2B	29623588	2.4359	4.913	-1.4169
CHB	BOLA2B	29628588	2.352	5.0825	-1.5159
CHB	BOLA2B	29633588	0.9802	4.7436	-2.2199
CHB	BOLA2B	29638588	3.1961	6.6072	-1.4982
CHB	BOLA2B	29643588	4.6214	7.6237	-1.1578
CHB	BOLA2B	29648588	4.5416	7.4543	-1.1465
CHB	BOLA2B	29653588	8.1361	7.7931	0.1296
CHB	BOLA2B	29658588	10.3265	7.9625	0.876
CHB	BOLA2B	29663588	8.5332	8.8096	-0.0933
CHB	BOLA2B	29668588	8.9166	8.8096	0.0361
CHB	BOLA2B	29673588	10.2576	7.4543	1.1035
CHB	BOLA2B	29678588	8.752	7.1154	0.6721
CHB	BOLA2B	29683588	7.3328	7.2848	0.0193
CHB	BOLA2B	29688588	7.8479	6.7766	0.4599
CHB	BOLA2B	29693588	7.7639	7.4543	0.1219
CHB	BOLA2B	29698588	5.1253	6.6072	-0.6509
CHB	BOLA2B	29703588	1.7933	5.4213	-1.9024
CHB	BOLA2B	29708588	1.8083	6.2684	-2.0542
CHB	BOLA2B	29713588	2.787	7.7931	-1.892
CHB	BOLA2B	29718588	3.3903	9.8261	-1.9636
CHB	BOLA2B	29723588	3.9103	9.4872	-1.758
CHB	BOLA2B	29728588	3.3086	9.4872	-1.9476
CHB	BOLA2B	29733588	2.6773	10.1649	-2.2134
CHB	BOLA2B	29738588	2.8968	7.7931	-1.8505
CHB	BOLA2B	29743588	3.3962	6.4378	-1.3676
CHB	BOLA2B	29748588	5.7889	10.8426	-1.4065
CHB	BOLA2B	29753588	5.0171	11.1814	-1.6668
CHB	BOLA2B	29758588	2.4495	7.7931	-2.0196
CHB	BOLA2B	29763588	4.7114	9.6567	-1.5334
CHB	BOLA2B	29768588	4.608	10.6731	-1.713
CHB	BOLA2B	29773588	2.4932	10.3343	-2.2824
CHB	BOLA2B	29778588	1.8993	9.4872	-2.3919
CHB	BOLA2B	29783588	2.3786	6.7766	-1.8879
CHB	BOLA2B	29788588	3.7184	7.6237	-1.506
CHB	BOLA2B	29793588	3.2972	9.1484	-1.9077
CHB	BOLA2B	29798588	1.9836	8.1319	-2.2347
CHB	BOLA2B	29803588	1.106	5.9295	-2.3351
CHB	BOLA2B	29808588	0.7872	3.5577	-2.0938
CHB	BOLA2B	29813588	1.0992	4.7436	-2.1497
CHB	BOLA2B	29818588	1.2378	6.946	-2.3961
CHB	BOLA2B	29823588	1.4801	6.946	-2.2944
CHB	BOLA2B	29828588	2.9214	7.6237	-1.8133
CHB	BOLA2B	29833588	3.1085	6.7766	-1.5746
CHB	BOLA2B	29838588	2.596	6.0989	-1.6535
CHB	BOLA2B	29843588	2.3318	6.4378	-1.8462
CHB	BOLA2B	29848588	1.6389	4.913	-1.8728
CHB	BOLA2B	29853588	1.4201	5.5907	-2.1279

CHB	BOLA2B	29858588	1.8239	6.946	-2.1501
CHB	BOLA2B	29863588	2.5854	7.4543	-1.9165
CHB	BOLA2B	29868588	2.0857	6.4378	-1.9568
CHB	BOLA2B	29873588	2.4064	6.946	-1.9055
CHB	BOLA2B	29878588	2.7243	6.6072	-1.7054
CHB	BOLA2B	29883588	3.8719	5.2519	-0.7443
CHB	BOLA2B	29888588	5.1228	5.4213	-0.1565
CHB	BOLA2B	29893588	5.6636	5.7601	-0.0479
CHB	BOLA2B	29898588	7.9364	6.2684	0.7682
CHB	BOLA2B	29903588	7.5711	5.9295	0.7947
CHB	BOLA2B	29908588	7.1485	6.0989	0.4955
CHB	BOLA2B	29913588	7.1658	5.9295	0.5985
CHB	BOLA2B	29918588	6.2994	6.6072	-0.1352
CHB	BOLA2B	29923588	5.9629	6.946	-0.4127
CHB	BOLA2B	29928588	5.6212	6.0989	-0.2255
CHB	BOLA2B	29933588	7.1972	7.4543	-0.1012
CHB	BOLA2B	29938588	7.5989	7.1154	0.1986
CHB	BOLA2B	29943588	6.3874	5.7601	0.3117
CHB	BOLA2B	29948588	4.3341	5.9295	-0.7724
YRI	BOLA2B	28958588	8.0622	9.7476	-0.5163
YRI	BOLA2B	28963588	7.7726	11.4282	-0.9651
YRI	BOLA2B	28968588	8.1207	11.4282	-0.8732
YRI	BOLA2B	28973588	6.9439	10.0837	-0.9319
YRI	BOLA2B	28978588	6.8357	10.4199	-1.0318
YRI	BOLA2B	28983588	8.8025	12.2685	-0.8559
YRI	BOLA2B	28988588	8.8949	13.1089	-0.9775
YRI	BOLA2B	28993588	6.891	9.5795	-0.837
YRI	BOLA2B	28998588	7.235	8.7392	-0.5099
YRI	BOLA2B	29003588	12.6551	13.445	-0.1789
YRI	BOLA2B	29008588	12.7541	14.9575	-0.4509
YRI	BOLA2B	29013588	8.0193	11.5963	-0.9315
YRI	BOLA2B	29018588	8.0279	10.2518	-0.65
YRI	BOLA2B	29023588	8.8918	11.0921	-0.5974
YRI	BOLA2B	29028588	9.9347	12.1005	-0.5418
YRI	BOLA2B	29033588	10.213	10.924	-0.1958
YRI	BOLA2B	29038588	6.7662	8.7392	-0.6688
YRI	BOLA2B	29043588	5.0361	9.4115	-1.3848
YRI	BOLA2B	29048588	2.4714	5.7141	-1.617
YRI	BOLA2B	29053588	1.9965	2.1848	-0.2114
YRI	BOLA2B	29058588	4.2444	4.2016	0.028
YRI	BOLA2B	29063588	5.6892	6.2183	-0.2446
YRI	BOLA2B	29068588	5.2812	6.3864	-0.4988
YRI	BOLA2B	29073588	3.549	5.2099	-0.899
YRI	BOLA2B	29078588	4.5209	5.7141	-0.595
YRI	BOLA2B	29083588	5.1621	7.5628	-0.9294
YRI	BOLA2B	29088588	6.0828	8.5712	-0.8588
YRI	BOLA2B	29093588	8.5599	11.0921	-0.6875
YRI	BOLA2B	29098588	7.7046	12.6047	-1.1796
YRI	BOLA2B	29103588	6.6989	11.7644	-1.3014

YRI	BOLA2B	29108588	9.1946	10.756	-0.4363
YRI	BOLA2B	29113588	12.127	11.9324	0.0493
YRI	BOLA2B	29118588	11.4679	14.7895	-0.6871
YRI	BOLA2B	29123588	10.8049	15.4617	-0.9234
YRI	BOLA2B	29128588	16.1031	16.302	-0.0375
YRI	BOLA2B	29133588	18.9053	16.8062	0.3844
YRI	BOLA2B	29138588	20.7801	20.1675	0.0942
YRI	BOLA2B	29143588	18.4842	20.6717	-0.3283
YRI	BOLA2B	29148588	16.8641	21.0078	-0.6123
YRI	BOLA2B	29153588	16.6197	21.68	-0.7253
YRI	BOLA2B	29158588	14.9345	18.4868	-0.5936
YRI	BOLA2B	29163588	17.9298	23.1926	-0.7067
YRI	BOLA2B	29168588	14.4919	24.201	-1.2511
YRI	BOLA2B	29173588	10.6263	18.823	-1.3463
YRI	BOLA2B	29178588	12.7074	17.4785	-0.8414
YRI	BOLA2B	29183588	15.9442	16.8062	-0.1578
YRI	BOLA2B	29188588	18.8011	18.3188	0.0813
YRI	BOLA2B	29193588	15.0852	17.6465	-0.4476
YRI	BOLA2B	29198588	10.0407	13.1089	-0.7117
YRI	BOLA2B	29203588	13.5439	13.2769	0.0612
YRI	BOLA2B	29208588	17.3191	17.1423	0.0318
YRI	BOLA2B	29213588	23.6319	22.3523	0.1781
YRI	BOLA2B	29218588	23.8645	20.1675	0.5682
YRI	BOLA2B	29223588	15.7368	14.7895	0.196
YRI	BOLA2B	29228588	14.5449	17.4785	-0.5174
YRI	BOLA2B	29233588	16.6537	21.3439	-0.6825
YRI	BOLA2B	29238588	17.6439	22.1842	-0.6365
YRI	BOLA2B	29243588	19.6373	20.3355	-0.1065
YRI	BOLA2B	29248588	17.6875	17.6465	0.0072
YRI	BOLA2B	29253588	14.5706	15.9659	-0.2683
YRI	BOLA2B	29258588	17.7523	16.9743	0.1411
YRI	BOLA2B	29263588	25.4461	22.8565	0.3527
YRI	BOLA2B	29268588	31.5816	26.5538	0.5921
YRI	BOLA2B	29273588	40.1235	27.3941	1.4543
YRI	BOLA2B	29278588	47.5042	28.7386	2.0462
YRI	BOLA2B	29283588	45.5556	29.2428	1.7488
YRI	BOLA2B	29288588	34.0826	24.0329	1.3038
YRI	BOLA2B	29293588	24.1633	19.9994	0.6451
YRI	BOLA2B	29298588	30.8824	24.369	0.8337
YRI	BOLA2B	29303588	35.8462	31.0915	0.4802
YRI	BOLA2B	29308588	23.5396	25.0413	-0.1872
YRI	BOLA2B	29313588	13.5074	14.7895	-0.2652
YRI	BOLA2B	29318588	21.7225	21.0078	0.1056
YRI	BOLA2B	29323588	22.191	22.6884	-0.0682
YRI	BOLA2B	29328588	12.2539	16.8062	-0.8336
YRI	BOLA2B	29333588	8.2151	13.7811	-1.2314
YRI	BOLA2B	29338588	8.2439	14.2853	-1.2917
YRI	BOLA2B	29343588	10.6909	16.4701	-1.0789
YRI	BOLA2B	29348588	11.6227	16.6382	-0.9273

YRI	BOLA2B	29353588	9.5837	13.613	-0.9018
YRI	BOLA2B	29358588	6.9763	9.4115	-0.7707
YRI	BOLA2B	29363588	6.388	9.2434	-0.9189
YRI	BOLA2B	29368588	4.7996	8.235	-1.2301
YRI	BOLA2B	29373588	2.1132	4.8738	-1.5847
YRI	BOLA2B	29378588	1.7641	3.5293	-1.3384
YRI	BOLA2B	29383588	2.5181	3.1932	-0.5568
YRI	BOLA2B	29388588	4.4029	4.5377	-0.0824
YRI	BOLA2B	29393588	5.1112	6.3864	-0.5755
YRI	BOLA2B	29398588	4.3559	7.7309	-1.2806
YRI	BOLA2B	29403588	4.0254	8.4031	-1.5386
YRI	BOLA2B	29408588	5.7287	11.4282	-1.5047
YRI	BOLA2B	29413588	7.3089	14.1172	-1.4721
YRI	BOLA2B	29418588	6.1262	12.4366	-1.5385
YRI	BOLA2B	29423588	5.607	11.5963	-1.5597
YRI	BOLA2B	29428588	4.3744	10.924	-1.8039
YRI	BOLA2B	29433588	5.7877	12.4366	-1.621
YRI	BOLA2B	29438588	8.8861	14.7895	-1.2212
YRI	BOLA2B	29443588	5.0323	8.5712	-1.2213
YRI	BOLA2B	29448588	0.246	1.6806	-1.9736
YRI	BOLA2B	29453588	0.0648	0.8403	-1.7516
YRI	BOLA2B	29458588	0.0278	0.3361	-1.2377
YRI	BOLA2B	29463588	0.0093	0.1681	-0.944
YRI	BOLA2B	29468588	0.5154	0.5042	0.0352
YRI	BOLA2B	29473588	0.6211	0.8403	-0.4951
YRI	BOLA2B	29478588	0.5323	0.8403	-0.6957
YRI	BOLA2B	29483588	0.6036	0.8403	-0.5346
YRI	BOLA2B	29488588	0.1771	0.3361	-0.6383
YRI	BOLA2B	29493588	0.1831	1.3445	-1.8863
YRI	BOLA2B	29498588	0.2291	1.8487	-2.0716
YRI	BOLA2B	29503588	0.346	1.5126	-1.7371
YRI	BOLA2B	29508588	4.3647	2.689	1.5925
YRI	BOLA2B	29513588	4.6604	2.3529	2.4426
YRI	BOLA2B	29518588	0.6502	0.8403	-0.4294
YRI	BOLA2B	29523588	0.1006	0.6722	-1.4964
YRI	BOLA2B	29528588	0.0554	0.6722	-1.6147
YRI	BOLA2B	29533588	0.0093	0.1681	-0.944
YRI	BOLA2B	29538588	0.2394	0.8403	-1.3572
YRI	BOLA2B	29543588	0.8969	1.3445	-0.727
YRI	BOLA2B	29548588	0.6939	0.8403	-0.3307
YRI	BOLA2B	29553588	0.4892	1.1764	-1.2291
YRI	BOLA2B	29558588	1.3441	4.5377	-1.9514
YRI	BOLA2B	29563588	1.7269	6.2183	-2.0763
YRI	BOLA2B	29568588	2.3405	4.2016	-1.2157
YRI	BOLA2B	29573588	2.5275	3.3612	-0.6587
YRI	BOLA2B	29578588	2.4417	4.3696	-1.2173
YRI	BOLA2B	29583588	3.4399	6.5544	-1.3731
YRI	BOLA2B	29588588	4.3013	7.8989	-1.3384
YRI	BOLA2B	29593588	4.0948	8.4031	-1.5142

YRI	BOLA2B	29598588	4.9926	10.0837	-1.5111
YRI	BOLA2B	29603588	6.7787	10.924	-1.1417
YRI	BOLA2B	29608588	5.0169	9.2434	-1.3602
YRI	BOLA2B	29613588	3.8802	8.4031	-1.5896
YRI	BOLA2B	29618588	3.7287	7.8989	-1.5514
YRI	BOLA2B	29623588	3.0543	9.0754	-1.971
YRI	BOLA2B	29628588	3.216	8.9073	-1.8955
YRI	BOLA2B	29633588	2.243	6.7225	-1.9302
YRI	BOLA2B	29638588	7.4627	9.4115	-0.6168
YRI	BOLA2B	29643588	11.3332	10.5879	0.2114
YRI	BOLA2B	29648588	8.0409	10.5879	-0.7223
YRI	BOLA2B	29653588	9.1338	11.7644	-0.6758
YRI	BOLA2B	29658588	11.6292	11.7644	-0.0347
YRI	BOLA2B	29663588	10.1236	11.4282	-0.3444
YRI	BOLA2B	29668588	11.1081	12.2685	-0.2866
YRI	BOLA2B	29673588	13.4124	13.2769	0.0311
YRI	BOLA2B	29678588	12.2016	14.2853	-0.4455
YRI	BOLA2B	29683588	10.4299	14.6214	-0.8766
YRI	BOLA2B	29688588	10.1057	12.6047	-0.6016
YRI	BOLA2B	29693588	8.6706	12.1005	-0.8581
YRI	BOLA2B	29698588	6.5837	11.7644	-1.331
YRI	BOLA2B	29703588	4.5621	10.0837	-1.6389
YRI	BOLA2B	29708588	3.5951	9.7476	-1.8847
YRI	BOLA2B	29713588	5.9684	12.7727	-1.6176
YRI	BOLA2B	29718588	7.5352	12.2685	-1.1689
YRI	BOLA2B	29723588	7.4566	10.0837	-0.7798
YRI	BOLA2B	29728588	9.9981	14.1172	-0.8907
YRI	BOLA2B	29733588	10.1761	14.4534	-0.9044
YRI	BOLA2B	29738588	7.5299	10.2518	-0.7955
YRI	BOLA2B	29743588	7.7046	8.5712	-0.2991
YRI	BOLA2B	29748588	17.2576	13.7811	0.7691
YRI	BOLA2B	29753588	19.0181	15.9659	0.587
YRI	BOLA2B	29758588	11.9286	13.1089	-0.2738
YRI	BOLA2B	29763588	15.5565	14.2853	0.2718
YRI	BOLA2B	29768588	14.9366	15.7978	-0.1673
YRI	BOLA2B	29773588	15.5987	16.302	-0.1326
YRI	BOLA2B	29778588	15.689	15.4617	0.0451
YRI	BOLA2B	29783588	9.777	12.1005	-0.5813
YRI	BOLA2B	29788588	12.9755	10.756	0.6202
YRI	BOLA2B	29793588	12.4509	9.9157	0.7644
YRI	BOLA2B	29798588	6.2764	8.4031	-0.7474
YRI	BOLA2B	29803588	3.8738	8.067	-1.5301
YRI	BOLA2B	29808588	3.4753	8.067	-1.6755
YRI	BOLA2B	29813588	4.0855	9.2434	-1.66
YRI	BOLA2B	29818588	4.6206	12.4366	-1.9055
YRI	BOLA2B	29823588	4.7183	12.4366	-1.8817
YRI	BOLA2B	29828588	6.8705	10.4199	-1.0218
YRI	BOLA2B	29833588	8.672	13.1089	-1.0292
YRI	BOLA2B	29838588	7.73	12.2685	-1.1208

YRI	BOLA2B	29843588	5.87	7.2267	-0.5475
YRI	BOLA2B	29848588	4.3924	6.7225	-1.004
YRI	BOLA2B	29853588	4.2596	8.4031	-1.4563
YRI	BOLA2B	29858588	5.3876	9.7476	-1.3356
YRI	BOLA2B	29863588	7.4765	12.1005	-1.1568
YRI	BOLA2B	29868588	6.2403	10.2518	-1.1725
YRI	BOLA2B	29873588	6.116	10.5879	-1.2682
YRI	BOLA2B	29878588	6.9065	12.2685	-1.3241
YRI	BOLA2B	29883588	6.7479	9.7476	-0.9189
YRI	BOLA2B	29888588	6.9275	9.5795	-0.8257
YRI	BOLA2B	29893588	7.7685	10.0837	-0.6872
YRI	BOLA2B	29898588	8.1875	9.4115	-0.3874
YRI	BOLA2B	29903588	7.4684	9.5795	-0.6573
YRI	BOLA2B	29908588	9.0464	9.9157	-0.2621
YRI	BOLA2B	29913588	7.7639	7.8989	-0.0502
YRI	BOLA2B	29918588	6.2738	8.235	-0.7022
YRI	BOLA2B	29923588	6.9871	8.9073	-0.6395
YRI	BOLA2B	29928588	7.2516	9.0754	-0.597
YRI	BOLA2B	29933588	9.0954	10.924	-0.5036
YRI	BOLA2B	29938588	9.6661	9.4115	0.0806
YRI	BOLA2B	29943588	9.1999	8.067	0.4134
YRI	BOLA2B	29948588	6.6833	7.8989	-0.4522
CEU	BOLA2B	29697932	6.5549	5.7991	0.3743
CEU	BOLA2B	29702932	4.5617	6.3108	-0.803
CEU	BOLA2B	29707932	3.5541	6.3108	-1.2657
CEU	BOLA2B	29712932	4.5768	5.9697	-0.6722
CEU	BOLA2B	29717932	4.9664	5.458	-0.257
CEU	BOLA2B	29722932	5.9483	6.9931	-0.4372
CEU	BOLA2B	29727932	4.7083	8.3576	-1.2972
CEU	BOLA2B	29732932	5.2919	7.5047	-0.8682
CEU	BOLA2B	29737932	6.2319	6.6519	-0.1839
CEU	BOLA2B	29742932	5.5253	6.9931	-0.6142
CEU	BOLA2B	29747932	7.7555	8.0164	-0.0964
CEU	BOLA2B	29752932	6.1253	7.8459	-0.6482
CEU	BOLA2B	29757932	3.0435	8.187	-1.8634
CEU	BOLA2B	29762932	5.2092	8.6987	-1.1955
CEU	BOLA2B	29767932	4.0851	6.4814	-1.0741
CEU	BOLA2B	29772932	1.0287	6.8225	-2.4792
CEU	BOLA2B	29777932	1.3113	6.9931	-2.3774
CEU	BOLA2B	29782932	1.9058	4.9463	-1.734
CEU	BOLA2B	29787932	2.0645	6.8225	-2.0359
CEU	BOLA2B	29792932	2.1288	6.8225	-2.0084
CEU	BOLA2B	29797932	1.4539	4.7757	-1.9537
CEU	BOLA2B	29802932	0.502	4.4346	-2.4674
CEU	BOLA2B	29807932	0.8291	3.4112	-2.028
CEU	BOLA2B	29812932	0.6052	3.0701	-2.1144
CEU	BOLA2B	29817932	0.7275	5.6286	-2.493
CEU	BOLA2B	29822932	1.8817	7.3342	-2.1847
CEU	BOLA2B	29827932	3.0626	6.9931	-1.6446

CEU	BOLA2B	29832932	4.4035	7.3342	-1.1742
CEU	BOLA2B	29837932	5.236	6.1402	-0.4255
CEU	BOLA2B	29842932	3.8571	4.2641	-0.2642
CEU	BOLA2B	29847932	2.8516	4.2641	-0.9169
CEU	BOLA2B	29852932	2.5949	5.2874	-1.4478
CEU	BOLA2B	29857932	3.1387	5.1169	-1.095
CEU	BOLA2B	29862932	3.2748	4.6052	-0.8077
CEU	BOLA2B	29867932	2.798	4.0935	-0.8711
CEU	BOLA2B	29872932	3.2581	4.6052	-0.8179
CEU	BOLA2B	29877932	5.0136	5.6286	-0.3128
CEU	BOLA2B	29882932	5.242	5.2874	-0.0244
CEU	BOLA2B	29887932	2.4708	5.1169	-1.4647
CEU	BOLA2B	29892932	2.9332	5.6286	-1.371
CEU	BOLA2B	29897932	4.5725	6.3108	-0.7981
CEU	BOLA2B	29902932	4.4877	6.3108	-0.837
CEU	BOLA2B	29907932	6.6564	5.2874	0.7361
CEU	BOLA2B	29912932	6.6832	4.6052	1.2616
CEU	BOLA2B	29917932	4.8701	5.2874	-0.2244
CEU	BOLA2B	29922932	6.0023	5.9697	0.0157
CEU	BOLA2B	29927932	6.4224	5.7991	0.3087
CEU	BOLA2B	29932932	8.1767	6.6519	0.6676
CEU	BOLA2B	29937932	7.4966	6.6519	0.3698
CEU	BOLA2B	29942932	6.0602	5.6286	0.2195
CEU	BOLA2B	29947932	5.6741	5.2874	0.2079
CEU	BOLA2B	29952932	3.3416	4.4346	-0.6858
CEU	BOLA2B	29957932	4.6933	5.2874	-0.3195
CEU	BOLA2B	29962932	5.7857	5.1169	0.3702
CEU	BOLA2B	29967932	6.7925	4.7757	1.1862
CEU	BOLA2B	29972932	6.0373	4.0935	1.3071
CEU	BOLA2B	29977932	5.8081	4.2641	1.0023
CEU	BOLA2B	29982932	8.2482	6.4814	0.7919
CEU	BOLA2B	29987932	5.2839	4.4346	0.5329
CEU	BOLA2B	29992932	5.2352	3.9229	0.9153
CEU	BOLA2B	29997932	6.0082	5.1169	0.4934
CEU	BOLA2B	30002932	3.3638	4.9463	-0.9025
CEU	BOLA2B	30007932	4.0804	6.1402	-0.9693
CEU	BOLA2B	30012932	7.9262	8.6987	-0.2647
CEU	BOLA2B	30017932	8.6331	7.8459	0.2965
CEU	BOLA2B	30022932	7.3191	4.9463	1.3532
CEU	BOLA2B	30027932	7.3529	5.2874	1.1106
CEU	BOLA2B	30032932	7.5958	7.1636	0.1769
CEU	BOLA2B	30037932	7.4529	6.9931	0.1924
CEU	BOLA2B	30042932	7.5448	5.1169	1.3439
CEU	BOLA2B	30047932	9.3781	5.9697	1.6449
CEU	BOLA2B	30052932	7.8081	5.7991	0.995
CEU	BOLA2B	30057932	5.5962	5.1169	0.2653
CEU	BOLA2B	30062932	5.608	6.1402	-0.2504
CEU	BOLA2B	30067932	4.4895	5.7991	-0.6486
CEU	BOLA2B	30072932	2.1697	4.2641	-1.3596

CEU	BOLA2B	30077932	2.2727	4.2641	-1.2927
CEU	BOLA2B	30082932	2.8771	4.7757	-1.1166
CEU	BOLA2B	30087932	3.0146	5.1169	-1.1637
CEU	BOLA2B	30092932	5.1789	6.6519	-0.6449
CEU	BOLA2B	30097932	5.0773	7.1636	-0.854
CEU	BOLA2B	30102932	3.7365	6.1402	-1.1311
CEU	BOLA2B	30107932	2.7654	4.9463	-1.2438
CEU	BOLA2B	30112932	2.3989	4.7757	-1.3979
CEU	BOLA2B	30117932	6.4869	7.8459	-0.512
CEU	BOLA2B	30122932	6.9615	7.3342	-0.1493
CEU	BOLA2B	30127932	3.3944	4.6052	-0.7351
CEU	BOLA2B	30132932	3.348	4.7757	-0.8397
CEU	BOLA2B	30137932	6.1276	6.4814	-0.1586
CEU	BOLA2B	30142932	6.1072	6.8225	-0.3061
CEU	BOLA2B	30147932	3.0787	5.2874	-1.1876
CEU	BOLA2B	30152932	5.5537	6.9931	-0.6023
CEU	BOLA2B	30157932	7.9839	7.3342	0.2603
CEU	BOLA2B	30162932	10.9984	7.6753	1.2773
CEU	BOLA2B	30167932	11.0763	8.0164	1.1302
CEU	BOLA2B	30172932	6.1082	5.7991	0.1531
CEU	BOLA2B	30177932	5.7947	5.2874	0.2728
CEU	BOLA2B	30182932	6.173	5.7991	0.1852
CEU	BOLA2B	30187932	3.5114	5.1169	-0.8887
CEU	BOLA2B	30192932	2.0614	5.458	-1.7757
CEU	BOLA2B	30197932	2.4461	4.9463	-1.4259
CEU	BOLA2B	30202932	1.1575	1.7056	-0.7507
CEU	BOLA2B	30207932	0.0499	0.5117	-1.4483
CEU	BOLA2B	30212932	0.1655	0.8528	-1.5478
CEU	BOLA2B	30217932	0.1156	0.3411	-0.904
CEU	BOLA2B	30222932	0.4937	0.5117	-0.0565
CEU	BOLA2B	30227932	0.5038	0.6822	-0.4659
CEU	BOLA2B	30232932	0.0303	0.3411	-1.2459
CEU	BOLA2B	30237932	0.0202	0.1706	-0.8934
CEU	BOLA2B	30242932	0	0	0
CEU	BOLA2B	30247932	0	0	0
CEU	BOLA2B	30252932	0	0	0
CEU	BOLA2B	30257932	0	0	0
CEU	BOLA2B	30262932	0.0499	0.3411	-1.1673
CEU	BOLA2B	30267932	0.0802	0.8528	-1.7399
CEU	BOLA2B	30272932	0.0404	0.6822	-1.6759
CEU	BOLA2B	30277932	0.0101	0.1706	-0.9534
CEU	BOLA2B	30282932	0.6151	0.5117	0.3243
CEU	BOLA2B	30287932	1.1631	0.8528	0.6988
CEU	BOLA2B	30292932	1.563	1.3645	0.3211
CEU	BOLA2B	30297932	1.0251	1.3645	-0.5491
CEU	BOLA2B	30302932	0.5187	0.6822	-0.4269
CEU	BOLA2B	30307932	1.0796	1.0234	0.1117
CEU	BOLA2B	30312932	1.0705	1.0234	0.0936
CEU	BOLA2B	30317932	1.5401	1.7056	-0.2267

CEU	BOLA2B	30322932	3.9025	3.4112	0.3859
CEU	BOLA2B	30327932	6.5723	4.7757	1.0567
CEU	BOLA2B	30332932	7.122	6.4814	0.2871
CEU	BOLA2B	30337932	6.0561	6.3108	-0.1169
CEU	BOLA2B	30342932	5.9314	6.1402	-0.0983
CEU	BOLA2B	30347932	5.6988	6.9931	-0.5416
CEU	BOLA2B	30352932	3.8633	5.9697	-1.0166
CEU	BOLA2B	30357932	4.4385	5.458	-0.533
CEU	BOLA2B	30362932	4.5203	5.9697	-0.6995
CEU	BOLA2B	30367932	2.4444	4.4346	-1.2487
CEU	BOLA2B	30372932	3.5529	4.6052	-0.6389
CEU	BOLA2B	30377932	4.3673	6.6519	-1.0002
CEU	BOLA2B	30382932	3.1472	6.1402	-1.4084
CEU	BOLA2B	30387932	3.8813	4.9463	-0.6074
CEU	BOLA2B	30392932	5.5545	5.9697	-0.2004
CEU	BOLA2B	30397932	5.1973	7.3342	-0.8562
CEU	BOLA2B	30402932	4.5143	6.9931	-1.0372
CEU	BOLA2B	30407932	4.3781	5.6286	-0.6361
CEU	BOLA2B	30412932	3.5121	3.5818	-0.0525
CEU	BOLA2B	30417932	2.9031	2.5584	0.3434
CEU	BOLA2B	30422932	2.8409	2.5584	0.2814
CEU	BOLA2B	30427932	3.6755	3.0701	0.5193
CEU	BOLA2B	30432932	4.556	4.6052	-0.0299
CEU	BOLA2B	30437932	3.9004	4.7757	-0.5148
CEU	BOLA2B	30442932	3.7846	4.6052	-0.4982
CEU	BOLA2B	30447932	4.5951	5.9697	-0.6634
CEU	BOLA2B	30452932	3.5253	5.6286	-1.0699
CEU	BOLA2B	30457932	4.691	6.4814	-0.8025
CEU	BOLA2B	30462932	5.7695	5.9697	-0.0966
CEU	BOLA2B	30467932	6.4686	5.9697	0.2408
CEU	BOLA2B	30472932	6.4395	7.5047	-0.4179
CEU	BOLA2B	30477932	5.1342	6.6519	-0.6645
CEU	BOLA2B	30482932	4.4842	5.1169	-0.3502
CEU	BOLA2B	30487932	4.8275	4.6052	0.135
CEU	BOLA2B	30492932	7.0959	5.2874	0.9724
CEU	BOLA2B	30497932	7.697	5.6286	1.0521
CEU	BOLA2B	30502932	7.4463	6.6519	0.3478
CEU	BOLA2B	30507932	8.7965	6.9931	0.7546
CEU	BOLA2B	30512932	11.859	8.3576	1.2446
CEU	BOLA2B	30517932	9.7018	8.3576	0.4778
CEU	BOLA2B	30522932	4.2126	4.9463	-0.4184
CEU	BOLA2B	30527932	2.7425	4.7757	-1.1958
CEU	BOLA2B	30532932	3.1635	5.2874	-1.142
CEU	BOLA2B	30537932	2.9225	3.0701	-0.1266
CEU	BOLA2B	30542932	2.2358	3.2407	-0.824
CEU	BOLA2B	30547932	3.4952	4.9463	-0.8276
CEU	BOLA2B	30552932	4.9254	6.8225	-0.8118
CEU	BOLA2B	30557932	4.0078	6.6519	-1.1576
CEU	BOLA2B	30562932	2.1338	5.6286	-1.7777

CEU	BOLA2B	30567932	1.6891	6.6519	-2.1728
CEU	BOLA2B	30572932	1.8472	5.6286	-1.9234
CEU	BOLA2B	30577932	1.4939	3.5818	-1.5736
CEU	BOLA2B	30582932	1.9652	3.4112	-1.1357
CEU	BOLA2B	30587932	3.0219	4.0935	-0.7206
CEU	BOLA2B	30592932	2.616	4.6052	-1.2077
CEU	BOLA2B	30597932	1.2773	4.0935	-1.8937
CEU	BOLA2B	30602932	1.8159	3.4112	-1.253
CEU	BOLA2B	30607932	2.0913	3.5818	-1.1234
CEU	BOLA2B	30612932	2.4234	3.5818	-0.8731
CEU	BOLA2B	30617932	1.9623	3.0701	-0.9503
CEU	BOLA2B	30622932	1.9027	3.0701	-1.0014
CEU	BOLA2B	30627932	2.3682	3.5818	-0.9147
CEU	BOLA2B	30632932	4.6343	4.0935	0.3636
CEU	BOLA2B	30637932	6.1929	4.9463	0.7109
CEU	BOLA2B	30642932	8.9202	6.6519	0.9931
CEU	BOLA2B	30647932	10.5813	7.5047	1.2071
CEU	BOLA2B	30652932	5.6867	4.6052	0.6566
CEU	BOLA2B	30657932	2.3331	2.2173	0.1294
CEU	BOLA2B	30662932	1.6976	2.729	-0.9749
CEU	BOLA2B	30667932	2.1855	2.8996	-0.6422
CEU	BOLA2B	30672932	1.3896	3.0701	-1.4415
CEU	BOLA2B	30677932	0.804	4.2641	-2.2461
CEU	BOLA2B	30682932	1.5244	4.2641	-1.7785
CEU	BOLA2B	30687932	2.0874	4.7757	-1.5811
CHB	BOLA2B	29697932	5.4569	6.4378	-0.441
CHB	BOLA2B	29702932	1.7933	5.2519	-1.8653
CHB	BOLA2B	29707932	1.7976	6.0989	-2.0304
CHB	BOLA2B	29712932	2.7579	7.4543	-1.8486
CHB	BOLA2B	29717932	3.4298	10.1649	-1.991
CHB	BOLA2B	29722932	3.9008	9.8261	-1.8078
CHB	BOLA2B	29727932	2.8978	7.7931	-1.8502
CHB	BOLA2B	29732932	2.5757	9.8261	-2.2121
CHB	BOLA2B	29737932	2.9922	8.979	-1.986
CHB	BOLA2B	29742932	3.0116	6.6072	-1.5792
CHB	BOLA2B	29747932	5.9539	10.5037	-1.3044
CHB	BOLA2B	29752932	5.4091	10.6731	-1.4868
CHB	BOLA2B	29757932	2.4234	7.9625	-2.0526
CHB	BOLA2B	29762932	4.806	10.3343	-1.6092
CHB	BOLA2B	29767932	4.5374	10.3343	-1.6874
CHB	BOLA2B	29772932	2.5315	10.5037	-2.2856
CHB	BOLA2B	29777932	2.2644	10.3343	-2.349
CHB	BOLA2B	29782932	2.0792	5.9295	-1.864
CHB	BOLA2B	29787932	3.6156	7.4543	-1.511
CHB	BOLA2B	29792932	3.558	9.4872	-1.869
CHB	BOLA2B	29797932	1.8597	7.6237	-2.2228
CHB	BOLA2B	29802932	1.1159	6.4378	-2.3929
CHB	BOLA2B	29807932	0.9122	4.2354	-2.1634
CHB	BOLA2B	29812932	1.0445	4.7436	-2.182

CHB	BOLA2B	29817932	1.2545	6.7766	-2.3704
CHB	BOLA2B	29822932	1.5635	6.946	-2.2594
CHB	BOLA2B	29827932	2.3322	7.2848	-1.9908
CHB	BOLA2B	29832932	3.0423	6.6072	-1.5657
CHB	BOLA2B	29837932	3.1942	6.4378	-1.4584
CHB	BOLA2B	29842932	2.268	6.2684	-1.8424
CHB	BOLA2B	29847932	1.7245	5.4213	-1.9385
CHB	BOLA2B	29852932	1.5599	6.0989	-2.1426
CHB	BOLA2B	29857932	1.7855	6.7766	-2.1425
CHB	BOLA2B	29862932	2.5854	7.4543	-1.9165
CHB	BOLA2B	29867932	2.1047	6.2684	-1.9177
CHB	BOLA2B	29872932	1.9429	6.0989	-1.9618
CHB	BOLA2B	29877932	2.7341	6.7766	-1.7353
CHB	BOLA2B	29882932	4.3257	5.9295	-0.7764
CHB	BOLA2B	29887932	4.2983	5.2519	-0.5143
CHB	BOLA2B	29892932	5.4059	5.5907	-0.0943
CHB	BOLA2B	29897932	7.8932	5.9295	0.9507
CHB	BOLA2B	29902932	7.3587	5.9295	0.6919
CHB	BOLA2B	29907932	8.0162	6.6072	0.6188
CHB	BOLA2B	29912932	7.1456	5.9295	0.5887
CHB	BOLA2B	29917932	5.3476	5.7601	-0.2049
CHB	BOLA2B	29922932	5.9558	6.7766	-0.3523
CHB	BOLA2B	29927932	6.4498	6.7766	-0.1403
CHB	BOLA2B	29932932	7.7044	7.9625	-0.0956
CHB	BOLA2B	29937932	6.7487	6.7766	-0.012
CHB	BOLA2B	29942932	5.8843	5.2519	0.3411
CHB	BOLA2B	29947932	5.288	6.0989	-0.3828
CHB	BOLA2B	29952932	2.9143	5.9295	-1.4597
CHB	BOLA2B	29957932	3.9006	6.6072	-1.1888
CHB	BOLA2B	29962932	5.1532	5.7601	-0.3015
CHB	BOLA2B	29967932	6.331	5.2519	0.582
CHB	BOLA2B	29972932	5.553	4.913	0.3661
CHB	BOLA2B	29977932	6.186	4.913	0.7282
CHB	BOLA2B	29982932	8.134	5.5907	1.2976
CHB	BOLA2B	29987932	4.6351	4.4048	0.1449
CHB	BOLA2B	29992932	4.7342	5.7601	-0.5097
CHB	BOLA2B	29997932	5.2324	6.0989	-0.409
CHB	BOLA2B	30002932	2.8117	4.7436	-1.1396
CHB	BOLA2B	30007932	3.2315	5.5907	-1.2037
CHB	BOLA2B	30012932	6.1112	8.1319	-0.7345
CHB	BOLA2B	30017932	6.8892	8.6402	-0.6019
CHB	BOLA2B	30022932	6.324	6.2684	0.0256
CHB	BOLA2B	30027932	6.3082	4.913	0.7981
CHB	BOLA2B	30032932	6.1108	5.4213	0.3616
CHB	BOLA2B	30037932	7.321	7.1154	0.0844
CHB	BOLA2B	30042932	8.0902	6.7766	0.5639
CHB	BOLA2B	30047932	8.1826	7.1154	0.4383
CHB	BOLA2B	30052932	6.1481	7.7931	-0.6217
CHB	BOLA2B	30057932	4.1519	6.2684	-0.9748

CHB	BOLA2B	30062932	4.7149	6.4378	-0.7747
CHB	BOLA2B	30067932	4.7105	7.2848	-1.0348
CHB	BOLA2B	30072932	2.9309	6.0989	-1.4954
CHB	BOLA2B	30077932	3.0192	7.6237	-1.7756
CHB	BOLA2B	30082932	2.7927	7.4543	-1.8349
CHB	BOLA2B	30087932	2.5243	5.5907	-1.5645
CHB	BOLA2B	30092932	5.1536	8.1319	-1.0825
CHB	BOLA2B	30097932	5.008	7.1154	-0.8654
CHB	BOLA2B	30102932	3.744	4.2354	-0.3199
CHB	BOLA2B	30107932	3.1296	5.4213	-1.2017
CHB	BOLA2B	30112932	2.0714	5.7601	-1.8326
CHB	BOLA2B	30117932	5.2968	7.9625	-0.9878
CHB	BOLA2B	30122932	5.7278	8.4708	-0.9602
CHB	BOLA2B	30127932	2.7849	5.0825	-1.2756
CHB	BOLA2B	30132932	3.7552	4.5742	-0.4987
CHB	BOLA2B	30137932	6.8468	6.2684	0.2664
CHB	BOLA2B	30142932	6.1685	6.946	-0.3264
CHB	BOLA2B	30147932	2.5799	5.4213	-1.4899
CHB	BOLA2B	30152932	3.6164	6.0989	-1.1718
CHB	BOLA2B	30157932	5.4517	7.1154	-0.6832
CHB	BOLA2B	30162932	8.478	7.4543	0.403
CHB	BOLA2B	30167932	8.1498	6.946	0.5053
CHB	BOLA2B	30172932	4.4786	6.2684	-0.8243
CHB	BOLA2B	30177932	4.8951	7.1154	-0.9118
CHB	BOLA2B	30182932	5.0044	7.1154	-0.8669
CHB	BOLA2B	30187932	3.0038	6.946	-1.6548
CHB	BOLA2B	30192932	1.218	5.5907	-2.231
CHB	BOLA2B	30197932	1.2254	3.7271	-1.8174
CHB	BOLA2B	30202932	0.8251	1.6942	-1.1927
CHB	BOLA2B	30207932	0.0097	0.1694	-0.949
CHB	BOLA2B	30212932	0.0386	0.3388	-1.2042
CHB	BOLA2B	30217932	0.0386	0.3388	-1.2042
CHB	BOLA2B	30222932	0.1542	0.3388	-0.7405
CHB	BOLA2B	30227932	0.1445	0.1694	-0.148
CHB	BOLA2B	30232932	0.0192	0.1694	-0.8925
CHB	BOLA2B	30237932	0.0192	0.1694	-0.8925
CHB	BOLA2B	30242932	0	0	0
CHB	BOLA2B	30247932	0	0	0
CHB	BOLA2B	30252932	0	0	0
CHB	BOLA2B	30257932	0	0	0
CHB	BOLA2B	30262932	0.1277	0.1694	-0.2478
CHB	BOLA2B	30267932	0.1472	0.5082	-1.1332
CHB	BOLA2B	30272932	0.0194	0.3388	-1.2812
CHB	BOLA2B	30277932	0	0	0
CHB	BOLA2B	30282932	0.5635	0.6777	-0.2986
CHB	BOLA2B	30287932	0.8327	1.3553	-0.847
CHB	BOLA2B	30292932	0.7049	2.2024	-1.6768
CHB	BOLA2B	30297932	0.4455	1.8636	-1.8093
CHB	BOLA2B	30302932	0.1776	1.1859	-1.7998

CHB	BOLA2B	30307932	0.3954	2.5412	-2.1427
CHB	BOLA2B	30312932	0.4217	2.3718	-2.0585
CHB	BOLA2B	30317932	0.4287	1.5247	-1.6283
CHB	BOLA2B	30322932	1.6657	5.2519	-1.9342
CHB	BOLA2B	30327932	2.9103	7.6237	-1.8176
CHB	BOLA2B	30332932	2.578	6.6072	-1.7697
CHB	BOLA2B	30337932	2.0346	6.946	-2.0616
CHB	BOLA2B	30342932	2.3362	6.0989	-1.7762
CHB	BOLA2B	30347932	2.107	5.9295	-1.8505
CHB	BOLA2B	30352932	2.0278	5.2519	-1.7389
CHB	BOLA2B	30357932	2.5115	5.2519	-1.478
CHB	BOLA2B	30362932	1.6722	6.946	-2.2137
CHB	BOLA2B	30367932	0.7101	5.7601	-2.509
CHB	BOLA2B	30372932	1.1123	5.4213	-2.2595
CHB	BOLA2B	30377932	1.7704	6.2684	-2.0716
CHB	BOLA2B	30382932	1.4734	5.2519	-2.0379
CHB	BOLA2B	30387932	1.5252	5.4213	-2.043
CHB	BOLA2B	30392932	1.8525	6.7766	-2.1137
CHB	BOLA2B	30397932	1.7729	6.946	-2.1715
CHB	BOLA2B	30402932	1.4795	5.5907	-2.0976
CHB	BOLA2B	30407932	1.3122	4.066	-1.857
CHB	BOLA2B	30412932	1.2675	3.3883	-1.6702
CHB	BOLA2B	30417932	1.0729	4.066	-2.0183
CHB	BOLA2B	30422932	1.4047	4.913	-2.0067
CHB	BOLA2B	30427932	1.8624	4.5742	-1.6513
CHB	BOLA2B	30432932	2.4963	4.7436	-1.3256
CHB	BOLA2B	30437932	2.4727	5.5907	-1.5909
CHB	BOLA2B	30442932	1.6846	7.4543	-2.2711
CHB	BOLA2B	30447932	2.2596	8.979	-2.229
CHB	BOLA2B	30452932	2.4168	7.7931	-2.032
CHB	BOLA2B	30457932	2.3137	6.7766	-1.9158
CHB	BOLA2B	30462932	2.6204	6.946	-1.8157
CHB	BOLA2B	30467932	2.2749	7.2848	-2.0138
CHB	BOLA2B	30472932	1.3552	6.2684	-2.2628
CHB	BOLA2B	30477932	1.2168	4.066	-1.9213
CHB	BOLA2B	30482932	1.3301	4.066	-1.8449
CHB	BOLA2B	30487932	2.8028	6.4378	-1.6344
CHB	BOLA2B	30492932	4.1907	9.3178	-1.6434
CHB	BOLA2B	30497932	4.657	8.8096	-1.402
CHB	BOLA2B	30502932	4.6292	7.4543	-1.112
CHB	BOLA2B	30507932	4.8841	6.7766	-0.8124
CHB	BOLA2B	30512932	7.2381	8.4708	-0.4315
CHB	BOLA2B	30517932	5.1472	8.3013	-1.1249
CHB	BOLA2B	30522932	2.1032	4.7436	-1.5575
CHB	BOLA2B	30527932	1.5399	4.066	-1.7034
CHB	BOLA2B	30532932	0.8532	4.4048	-2.2348
CHB	BOLA2B	30537932	0.7971	4.2354	-2.2383
CHB	BOLA2B	30542932	0.5378	3.7271	-2.317
CHB	BOLA2B	30547932	0.853	5.2519	-2.3725

CHB	BOLA2B	30552932	1.7537	6.0989	-2.0511
CHB	BOLA2B	30557932	1.4194	5.7601	-2.1566
CHB	BOLA2B	30562932	0.7472	5.7601	-2.4905
CHB	BOLA2B	30567932	0.8348	5.7601	-2.447
CHB	BOLA2B	30572932	0.6063	4.5742	-2.4161
CHB	BOLA2B	30577932	0.861	5.2519	-2.3682
CHB	BOLA2B	30582932	1.2104	6.2684	-2.3295
CHB	BOLA2B	30587932	1.47	5.5907	-2.1025
CHB	BOLA2B	30592932	1.1401	5.2519	-2.2176
CHB	BOLA2B	30597932	0.5673	3.8965	-2.3285
CHB	BOLA2B	30602932	0.7773	4.4048	-2.2826
CHB	BOLA2B	30607932	0.7188	4.066	-2.2571
CHB	BOLA2B	30612932	1.4436	5.5907	-2.1159
CHB	BOLA2B	30617932	1.3459	5.7601	-2.1931
CHB	BOLA2B	30622932	0.9259	5.5907	-2.3801
CHB	BOLA2B	30627932	1.3588	6.2684	-2.2612
CHB	BOLA2B	30632932	2.4089	6.2684	-1.7775
CHB	BOLA2B	30637932	3.012	6.6072	-1.5791
CHB	BOLA2B	30642932	3.7733	5.9295	-1.0438
CHB	BOLA2B	30647932	5.0596	6.6072	-0.6797
CHB	BOLA2B	30652932	3.1131	5.2519	-1.1535
CHB	BOLA2B	30657932	0.8782	4.2354	-2.1855
CHB	BOLA2B	30662932	1.1713	5.0825	-2.1714
CHB	BOLA2B	30667932	1.506	6.2684	-2.1934
CHB	BOLA2B	30672932	0.8713	5.7601	-2.4289
CHB	BOLA2B	30677932	0.763	4.066	-2.2273
CHB	BOLA2B	30682932	0.906	4.2354	-2.1674
CHB	BOLA2B	30687932	1.0583	5.0825	-2.2342
YRI	BOLA2B	29697932	6.9224	11.7644	-1.2439
YRI	BOLA2B	29702932	4.4302	10.2518	-1.7015
YRI	BOLA2B	29707932	3.5209	8.9073	-1.794
YRI	BOLA2B	29712932	5.9979	12.1005	-1.5267
YRI	BOLA2B	29717932	7.4137	13.1089	-1.3211
YRI	BOLA2B	29722932	7.5865	10.756	-0.8857
YRI	BOLA2B	29727932	7.8907	12.1005	-1.0532
YRI	BOLA2B	29732932	9.6737	14.1172	-0.9608
YRI	BOLA2B	29737932	8.3473	11.4282	-0.8134
YRI	BOLA2B	29742932	7.8548	8.9073	-0.3505
YRI	BOLA2B	29747932	17.295	14.2853	0.6435
YRI	BOLA2B	29752932	19.2376	15.7978	0.6682
YRI	BOLA2B	29757932	13.0797	12.9408	0.0326
YRI	BOLA2B	29762932	15.2446	13.9492	0.2833
YRI	BOLA2B	29767932	14.3692	15.4617	-0.2166
YRI	BOLA2B	29772932	15.5751	16.4701	-0.1671
YRI	BOLA2B	29777932	15.6639	15.2937	0.0742
YRI	BOLA2B	29782932	9.2227	12.2685	-0.7522
YRI	BOLA2B	29787932	13.4678	11.5963	0.4873
YRI	BOLA2B	29792932	13.429	10.2518	0.9286
YRI	BOLA2B	29797932	6.3555	7.8989	-0.5742

YRI	BOLA2B	29802932	3.9357	7.8989	-1.4744
YRI	BOLA2B	29807932	3.4297	8.4031	-1.748
YRI	BOLA2B	29812932	3.7304	9.5795	-1.821
YRI	BOLA2B	29817932	4.4151	11.7644	-1.8881
YRI	BOLA2B	29822932	5.1187	12.1005	-1.7467
YRI	BOLA2B	29827932	6.4279	10.756	-1.2095
YRI	BOLA2B	29832932	8.6355	12.9408	-1.0109
YRI	BOLA2B	29837932	8.8255	12.9408	-0.9663
YRI	BOLA2B	29842932	5.5425	7.3947	-0.7319
YRI	BOLA2B	29847932	4.3567	6.2183	-0.8606
YRI	BOLA2B	29852932	4.5801	8.7392	-1.4098
YRI	BOLA2B	29857932	5.3703	10.0837	-1.399
YRI	BOLA2B	29862932	7.4745	11.7644	-1.1021
YRI	BOLA2B	29867932	6.2497	10.2518	-1.1697
YRI	BOLA2B	29872932	5.0374	10.4199	-1.5495
YRI	BOLA2B	29877932	7.0135	12.6047	-1.346
YRI	BOLA2B	29882932	7.8122	10.0837	-0.6742
YRI	BOLA2B	29887932	6.0021	8.9073	-0.9676
YRI	BOLA2B	29892932	7.5091	9.7476	-0.6857
YRI	BOLA2B	29897932	8.4594	9.7476	-0.3946
YRI	BOLA2B	29902932	7.6085	9.5795	-0.6136
YRI	BOLA2B	29907932	9.7092	10.4199	-0.2046
YRI	BOLA2B	29912932	7.4686	8.4031	-0.3284
YRI	BOLA2B	29917932	5.3455	7.2267	-0.7591
YRI	BOLA2B	29922932	6.8697	8.5712	-0.5872
YRI	BOLA2B	29927932	8.0026	9.4115	-0.4459
YRI	BOLA2B	29932932	9.5601	11.0921	-0.4159
YRI	BOLA2B	29937932	8.5884	8.7392	-0.0511
YRI	BOLA2B	29942932	8.506	7.3947	0.4391
YRI	BOLA2B	29947932	7.9013	8.9073	-0.3351
YRI	BOLA2B	29952932	5.4524	7.7309	-0.8645
YRI	BOLA2B	29957932	5.9645	8.235	-0.813
YRI	BOLA2B	29962932	5.782	8.235	-0.8783
YRI	BOLA2B	29967932	7.2169	8.7392	-0.516
YRI	BOLA2B	29972932	7.2754	9.0754	-0.5892
YRI	BOLA2B	29977932	6.1289	7.8989	-0.6585
YRI	BOLA2B	29982932	7.7577	9.2434	-0.4781
YRI	BOLA2B	29987932	5.6971	8.067	-0.8648
YRI	BOLA2B	29992932	5.4564	7.3947	-0.7659
YRI	BOLA2B	29997932	5.7732	7.7309	-0.7428
YRI	BOLA2B	30002932	4.0994	7.2267	-1.2619
YRI	BOLA2B	30007932	6.8775	8.7392	-0.6311
YRI	BOLA2B	30012932	10.7363	10.756	-0.0055
YRI	BOLA2B	30017932	8.8666	9.0754	-0.0683
YRI	BOLA2B	30022932	6.1231	6.8906	-0.3234
YRI	BOLA2B	30027932	6.4209	8.235	-0.6495
YRI	BOLA2B	30032932	7.3274	9.7476	-0.7414
YRI	BOLA2B	30037932	7.9888	9.2434	-0.4038
YRI	BOLA2B	30042932	8.7323	9.0754	-0.1123

YRI	BOLA2B	30047932	9.5965	10.2518	-0.1915
YRI	BOLA2B	30052932	9.1369	11.5963	-0.6404
YRI	BOLA2B	30057932	8.4659	12.7727	-1.0239
YRI	BOLA2B	30062932	7.6134	12.4366	-1.1759
YRI	BOLA2B	30067932	6.068	10.2518	-1.2228
YRI	BOLA2B	30072932	3.1724	8.4031	-1.8384
YRI	BOLA2B	30077932	3.9363	10.0837	-1.8246
YRI	BOLA2B	30082932	5.2799	9.9157	-1.3977
YRI	BOLA2B	30087932	5.3808	9.5795	-1.3072
YRI	BOLA2B	30092932	7.258	11.7644	-1.1577
YRI	BOLA2B	30097932	5.5376	9.9157	-1.32
YRI	BOLA2B	30102932	3.422	6.8906	-1.4616
YRI	BOLA2B	30107932	3.5171	8.4031	-1.7172
YRI	BOLA2B	30112932	3.9093	8.9073	-1.6646
YRI	BOLA2B	30117932	7.9391	9.9157	-0.5959
YRI	BOLA2B	30122932	8.4192	10.2518	-0.5356
YRI	BOLA2B	30127932	5.1009	9.2434	-1.3332
YRI	BOLA2B	30132932	4.6229	8.235	-1.2933
YRI	BOLA2B	30137932	7.6366	9.7476	-0.6467
YRI	BOLA2B	30142932	7.6856	9.5795	-0.5896
YRI	BOLA2B	30147932	2.9103	4.7057	-1.0628
YRI	BOLA2B	30152932	5.1925	6.8906	-0.7155
YRI	BOLA2B	30157932	8.7311	8.7392	-0.0027
YRI	BOLA2B	30162932	12.4587	8.7392	1.2608
YRI	BOLA2B	30167932	12.8313	8.9073	1.3069
YRI	BOLA2B	30172932	8.1155	6.7225	0.6002
YRI	BOLA2B	30177932	8.4006	6.7225	0.7231
YRI	BOLA2B	30182932	9.116	7.5628	0.6013
YRI	BOLA2B	30187932	6.6339	6.3864	0.1117
YRI	BOLA2B	30192932	3.5185	6.5544	-1.3385
YRI	BOLA2B	30197932	2.5482	5.8822	-1.62
YRI	BOLA2B	30202932	1.4488	2.0167	-0.679
YRI	BOLA2B	30207932	0.179	0.6722	-1.2912
YRI	BOLA2B	30212932	0.2158	1.1764	-1.7181
YRI	BOLA2B	30217932	0.0369	0.5042	-1.4686
YRI	BOLA2B	30222932	0.5038	0.1681	1.9956
YRI	BOLA2B	30227932	0.5038	0.1681	1.9956
YRI	BOLA2B	30232932	0.0369	0.5042	-1.4686
YRI	BOLA2B	30237932	0.0369	0.5042	-1.4686
YRI	BOLA2B	30242932	0.0093	0.1681	-0.944
YRI	BOLA2B	30247932	0.0093	0.1681	-0.944
YRI	BOLA2B	30252932	0	0	0
YRI	BOLA2B	30257932	0	0	0
YRI	BOLA2B	30262932	0.1966	0.8403	-1.4539
YRI	BOLA2B	30267932	0.2059	1.0084	-1.5999
YRI	BOLA2B	30272932	0.0093	0.1681	-0.944
YRI	BOLA2B	30277932	0.0891	0.1681	-0.4696
YRI	BOLA2B	30282932	1.1364	1.1764	-0.0715
YRI	BOLA2B	30287932	2.3186	2.3529	-0.0363

YRI	BOLA2B	30292932	4.2576	3.1932	0.8778
YRI	BOLA2B	30297932	3.5639	3.1932	0.3057
YRI	BOLA2B	30302932	1.1959	2.3529	-1.2247
YRI	BOLA2B	30307932	1.4368	3.1932	-1.4485
YRI	BOLA2B	30312932	1.3584	2.689	-1.2646
YRI	BOLA2B	30317932	1.4136	3.1932	-1.4677
YRI	BOLA2B	30322932	3.4273	7.8989	-1.6635
YRI	BOLA2B	30327932	6.2004	9.2434	-0.9793
YRI	BOLA2B	30332932	7.4818	8.235	-0.2697
YRI	BOLA2B	30337932	6.2683	6.8906	-0.2622
YRI	BOLA2B	30342932	5.5157	6.3864	-0.393
YRI	BOLA2B	30347932	6.265	9.0754	-0.92
YRI	BOLA2B	30352932	4.1088	7.5628	-1.3371
YRI	BOLA2B	30357932	3.5727	6.8906	-1.3981
YRI	BOLA2B	30362932	4.5209	9.2434	-1.5198
YRI	BOLA2B	30367932	3.1575	7.2267	-1.642
YRI	BOLA2B	30372932	3.7059	6.8906	-1.3419
YRI	BOLA2B	30377932	4.7209	8.5712	-1.3288
YRI	BOLA2B	30382932	4.6663	6.7225	-0.886
YRI	BOLA2B	30387932	4.5275	5.378	-0.4476
YRI	BOLA2B	30392932	6.178	6.5544	-0.1659
YRI	BOLA2B	30397932	6.8902	8.235	-0.4815
YRI	BOLA2B	30402932	5.1051	9.7476	-1.4222
YRI	BOLA2B	30407932	4.8265	8.9073	-1.3591
YRI	BOLA2B	30412932	5.5518	7.8989	-0.8732
YRI	BOLA2B	30417932	5.0927	6.3864	-0.5839
YRI	BOLA2B	30422932	4.2249	5.0419	-0.4552
YRI	BOLA2B	30427932	3.585	6.0502	-1.168
YRI	BOLA2B	30432932	4.3761	7.3947	-1.1928
YRI	BOLA2B	30437932	4.416	7.2267	-1.1342
YRI	BOLA2B	30442932	5.105	8.235	-1.1207
YRI	BOLA2B	30447932	6.26	10.4199	-1.1975
YRI	BOLA2B	30452932	4.7342	9.9157	-1.5622
YRI	BOLA2B	30457932	5.7299	9.5795	-1.1985
YRI	BOLA2B	30462932	5.4928	8.235	-0.9819
YRI	BOLA2B	30467932	6.5706	9.9157	-1.0086
YRI	BOLA2B	30472932	6.8964	11.0921	-1.1392
YRI	BOLA2B	30477932	4.1288	7.7309	-1.3667
YRI	BOLA2B	30482932	3.946	7.2267	-1.3238
YRI	BOLA2B	30487932	6.4597	9.2434	-0.8959
YRI	BOLA2B	30492932	9.7429	11.2602	-0.4062
YRI	BOLA2B	30497932	8.2834	11.0921	-0.7626
YRI	BOLA2B	30502932	7.2494	10.2518	-0.8775
YRI	BOLA2B	30507932	9.5566	10.0837	-0.1565
YRI	BOLA2B	30512932	12.5506	12.9408	-0.0916
YRI	BOLA2B	30517932	10.3835	12.9408	-0.6005
YRI	BOLA2B	30522932	5.1796	9.0754	-1.2753
YRI	BOLA2B	30527932	3.7164	7.3947	-1.4535
YRI	BOLA2B	30532932	4.2996	8.4031	-1.4422

YRI	BOLA2B	30537932	4.9755	11.4282	-1.7035
YRI	BOLA2B	30542932	4.1284	9.2434	-1.6461
YRI	BOLA2B	30547932	3.7779	9.0754	-1.7341
YRI	BOLA2B	30552932	4.0229	9.4115	-1.7055
YRI	BOLA2B	30557932	4.0824	9.0754	-1.6344
YRI	BOLA2B	30562932	3.5981	10.0837	-1.925
YRI	BOLA2B	30567932	4.0997	10.2518	-1.7981
YRI	BOLA2B	30572932	4.1331	9.2434	-1.6446
YRI	BOLA2B	30577932	2.8454	5.8822	-1.4756
YRI	BOLA2B	30582932	2.8675	6.5544	-1.6255
YRI	BOLA2B	30587932	5.2471	8.4031	-1.1092
YRI	BOLA2B	30592932	6.5814	9.5795	-0.9334
YRI	BOLA2B	30597932	4.9406	8.4031	-1.2169
YRI	BOLA2B	30602932	5.74	6.8906	-0.4848
YRI	BOLA2B	30607932	5.3662	5.7141	-0.1735
YRI	BOLA2B	30612932	6.0883	7.3947	-0.5162
YRI	BOLA2B	30617932	5.8476	8.4031	-0.8982
YRI	BOLA2B	30622932	5.0517	7.5628	-0.9721
YRI	BOLA2B	30627932	4.9049	9.0754	-1.3652
YRI	BOLA2B	30632932	5.5298	10.756	-1.4604
YRI	BOLA2B	30637932	6.8328	9.4115	-0.8161
YRI	BOLA2B	30642932	7.4871	8.4031	-0.3219
YRI	BOLA2B	30647932	9.6902	10.924	-0.3398
YRI	BOLA2B	30652932	6.8228	9.0754	-0.7374
YRI	BOLA2B	30657932	3.0119	6.0502	-1.4396
YRI	BOLA2B	30662932	3.3568	6.7225	-1.4503
YRI	BOLA2B	30667932	4.9513	6.8906	-0.8172
YRI	BOLA2B	30672932	3.9805	7.0586	-1.2689
YRI	BOLA2B	30677932	2.6348	9.2434	-2.1268
YRI	BOLA2B	30682932	3.6773	9.5795	-1.8375
YRI	BOLA2B	30687932	4.9929	10.0837	-1.511
CEU	CBWD3	65171374	0	0	0
CEU	CBWD3	65176374	0	0	0
CEU	CBWD3	65181374	0	0	0
CEU	CBWD3	65186374	0	0	0
CEU	CBWD3	65191374	0	0	0
CEU	CBWD3	65196374	0	0	0
CEU	CBWD3	65201374	0	0	0
CEU	CBWD3	65206374	0	0	0
CEU	CBWD3	65211374	0	0	0
CEU	CBWD3	65216374	0	0	0
CEU	CBWD3	65221374	0	0	0
CEU	CBWD3	65226374	0	0	0
CEU	CBWD3	65231374	0	0	0
CEU	CBWD3	65236374	0	0	0
CEU	CBWD3	65241374	0	0	0
CEU	CBWD3	65246374	0	0	0
CEU	CBWD3	65251374	0	0	0
CEU	CBWD3	65256374	0	0	0

CEU	CBWD3	65261374	0	0	0
CEU	CBWD3	65266374	0	0	0
CEU	CBWD3	65271374	0	0	0
CEU	CBWD3	65276374	0	0	0
CEU	CBWD3	65281374	0	0	0
CEU	CBWD3	65286374	0	0	0
CEU	CBWD3	65291374	0	0	0
CEU	CBWD3	65296374	0	0	0
CEU	CBWD3	65301374	0	0	0
CEU	CBWD3	65306374	0	0	0
CEU	CBWD3	65311374	0	0	0
CEU	CBWD3	65316374	0	0	0
CEU	CBWD3	65321374	0	0	0
CEU	CBWD3	65326374	0	0	0
CEU	CBWD3	65331374	0	0	0
CEU	CBWD3	65336374	0	0	0
CEU	CBWD3	65341374	0	0	0
CEU	CBWD3	65346374	0	0	0
CEU	CBWD3	65351374	0	0	0
CEU	CBWD3	65356374	0	0	0
CEU	CBWD3	65361374	0	0	0
CEU	CBWD3	65366374	0	0	0
CEU	CBWD3	65371374	0	0	0
CEU	CBWD3	65376374	0	0	0
CEU	CBWD3	65381374	0	0	0
CEU	CBWD3	65386374	0	0	0
CEU	CBWD3	65391374	0	0	0
CEU	CBWD3	65396374	0	0	0
CEU	CBWD3	65401374	0	0	0
CEU	CBWD3	65406374	0	0	0
CEU	CBWD3	65411374	0	0	0
CEU	CBWD3	65416374	0	0	0
CEU	CBWD3	65421374	0	0	0
CEU	CBWD3	65426374	0	0	0
CEU	CBWD3	65431374	0	0	0
CEU	CBWD3	65436374	0	0	0
CEU	CBWD3	65441374	0	0	0
CEU	CBWD3	65446374	0	0	0
CEU	CBWD3	65451374	0	0	0
CEU	CBWD3	65456374	0	0	0
CEU	CBWD3	65461374	0	0	0
CEU	CBWD3	65466374	0	0	0
CEU	CBWD3	65471374	0.6937	0.6822	0.03
CEU	CBWD3	65476374	0.8955	1.3645	-0.7587
CEU	CBWD3	65481374	1.3539	1.5351	-0.2687
CEU	CBWD3	65486374	1.1521	0.8528	0.674
CEU	CBWD3	65491374	0	0	0
CEU	CBWD3	65496374	0	0	0
CEU	CBWD3	65501374	0	0	0

CEU	CBWD3	65506374	0.0404	0.6822	-1.6759
CEU	CBWD3	65511374	0.0404	0.6822	-1.6759
CEU	CBWD3	65516374	0	0	0
CEU	CBWD3	65521374	0	0	0
CEU	CBWD3	65526374	0	0	0
CEU	CBWD3	65531374	0	0	0
CEU	CBWD3	65536374	0.0101	0.1706	-0.9534
CEU	CBWD3	65541374	0.0101	0.1706	-0.9534
CEU	CBWD3	65546374	0.873	0.5117	1.1331
CEU	CBWD3	65551374	1.248	0.8528	0.89
CEU	CBWD3	65556374	0.9382	1.3645	-0.6897
CEU	CBWD3	65561374	0.5834	1.3645	-1.2637
CEU	CBWD3	65566374	0.0202	0.3411	-1.2864
CEU	CBWD3	65571374	0.0101	0.1706	-0.9534
CEU	CBWD3	65576374	1.3063	1.0234	0.5621
CEU	CBWD3	65581374	1.2962	0.8528	0.9985
CEU	CBWD3	65586374	0	0	0
CEU	CBWD3	65591374	1.5028	1.8762	-0.4754
CEU	CBWD3	65596374	3.8621	5.458	-0.8343
CEU	CBWD3	65601374	4.3185	9.3809	-1.6172
CEU	CBWD3	65606374	3.7846	8.3576	-1.6256
CEU	CBWD3	65611374	6.7862	8.6987	-0.6552
CEU	CBWD3	65616374	8.4875	11.2571	-0.7463
CEU	CBWD3	65621374	6.1843	8.8692	-0.9035
CEU	CBWD3	65626374	4.6458	8.187	-1.2829
CEU	CBWD3	65631374	6.1309	9.8926	-1.1438
CEU	CBWD3	65636374	4.8753	7.1636	-0.9367
CEU	CBWD3	65641374	0.8503	2.2173	-1.5272
CEU	CBWD3	65646374	0.1177	0.5117	-1.2357
CEU	CBWD3	65651374	0.0101	0.1706	-0.9534
CEU	CBWD3	65656374	0.0101	0.1706	-0.9534
CEU	CBWD3	65661374	0	0	0
CEU	CBWD3	65666374	0	0	0
CEU	CBWD3	65671374	0.04	0.1706	-0.7758
CEU	CBWD3	65676374	0.04	0.1706	-0.7758
CEU	CBWD3	65681374	0	0	0
CEU	CBWD3	65686374	0.1328	0.3411	-0.835
CEU	CBWD3	65691374	0.1328	0.3411	-0.835
CEU	CBWD3	65696374	0	0	0
CEU	CBWD3	65701374	0	0	0
CEU	CBWD3	65706374	0.02	0.1706	-0.8946
CEU	CBWD3	65711374	0.2216	0.5117	-0.9098
CEU	CBWD3	65716374	0.2016	0.3411	-0.5592
CEU	CBWD3	65721374	0	0	0
CEU	CBWD3	65726374	0	0	0
CEU	CBWD3	65731374	0.0202	0.1706	-0.8934
CEU	CBWD3	65736374	0.3871	0.5117	-0.3908
CEU	CBWD3	65741374	0.5852	0.8528	-0.6026
CEU	CBWD3	65746374	0.4098	0.6822	-0.7113

CEU	CBWD3	65751374	0.3445	0.5117	-0.5244
CEU	CBWD3	65756374	0.2228	0.8528	-1.4187
CEU	CBWD3	65761374	0.1	0.6822	-1.5203
CEU	CBWD3	65766374	0.7547	1.8762	-1.4279
CEU	CBWD3	65771374	1.1459	2.2173	-1.197
CEU	CBWD3	65776374	0.4995	0.6822	-0.4771
CEU	CBWD3	65781374	0.1078	0.3411	-0.9352
CEU	CBWD3	65786374	0.0398	0.3411	-1.2078
CEU	CBWD3	65791374	0.0303	0.3411	-1.2459
CEU	CBWD3	65796374	0.1651	0.5117	-1.087
CEU	CBWD3	65801374	0.1449	0.3411	-0.7865
CEU	CBWD3	65806374	0.0101	0.1706	-0.9534
CEU	CBWD3	65811374	0.0101	0.1706	-0.9534
CEU	CBWD3	65816374	0	0	0
CEU	CBWD3	65821374	0.4455	0.5117	-0.2076
CEU	CBWD3	65826374	0.4455	0.5117	-0.2076
CEU	CBWD3	65831374	0	0	0
CEU	CBWD3	65836374	0.0676	0.1706	-0.6118
CEU	CBWD3	65841374	0.0676	0.1706	-0.6118
CEU	CBWD3	65846374	0	0	0
CEU	CBWD3	65851374	0	0	0
CEU	CBWD3	65856374	0.4185	0.3411	0.3103
CEU	CBWD3	65861374	0.4185	0.5117	-0.2923
CEU	CBWD3	65866374	0.1348	0.5117	-1.1821
CEU	CBWD3	65871374	0.564	0.6822	-0.3087
CEU	CBWD3	65876374	1.5053	1.3645	0.2278
CEU	CBWD3	65881374	1.0761	1.0234	0.1047
CEU	CBWD3	65886374	0.4673	0.1706	1.7625
CEU	CBWD3	65891374	1.4797	0.8528	1.4118
CEU	CBWD3	65896374	1.6448	1.7056	-0.0833
CEU	CBWD3	65901374	0.6324	1.0234	-0.7769
CEU	CBWD3	65906374	0	0	0
CEU	CBWD3	65911374	0	0	0
CEU	CBWD3	65916374	1.8213	1.5351	0.4244
CEU	CBWD3	65921374	1.8213	1.5351	0.4244
CEU	CBWD3	65926374	0	0	0
CEU	CBWD3	65931374	0	0	0
CEU	CBWD3	65936374	0	0	0
CEU	CBWD3	65941374	0	0	0
CEU	CBWD3	65946374	0	0	0
CEU	CBWD3	65951374	0	0	0
CEU	CBWD3	65956374	0	0	0
CEU	CBWD3	65961374	0	0	0
CEU	CBWD3	65966374	0.0404	0.5117	-1.4781
CEU	CBWD3	65971374	2.0656	1.7056	0.4931
CEU	CBWD3	65976374	2.2773	1.7056	0.783
CEU	CBWD3	65981374	0.302	0.8528	-1.2404
CEU	CBWD3	65986374	0.1765	0.8528	-1.523
CEU	CBWD3	65991374	0.3434	1.7056	-1.8657

CEU	CBWD3	65996374	0.227	1.3645	-1.8402
CEU	CBWD3	66001374	0.2579	0.5117	-0.796
CEU	CBWD3	66006374	0.8932	1.5351	-0.9518
CEU	CBWD3	66011374	1.1857	1.8762	-0.8791
CEU	CBWD3	66016374	0.8108	1.7056	-1.2255
CEU	CBWD3	66021374	0.2705	1.0234	-1.496
CEU	CBWD3	66026374	0.0303	0.5117	-1.5098
CEU	CBWD3	66031374	0.1004	1.0234	-1.834
CEU	CBWD3	66036374	0.1771	0.8528	-1.5217
CEU	CBWD3	66041374	0.1171	0.5117	-1.2376
CEU	CBWD3	66046374	0.3317	1.0234	-1.3744
CEU	CBWD3	66051374	0.3418	1.1939	-1.5185
CEU	CBWD3	66056374	0.0202	0.3411	-1.2864
CEU	CBWD3	66061374	0	0	0
CEU	CBWD3	66066374	0	0	0
CEU	CBWD3	66071374	0.0101	0.1706	-0.9534
CEU	CBWD3	66076374	0.0101	0.1706	-0.9534
CEU	CBWD3	66081374	0.04	0.3411	-1.207
CEU	CBWD3	66086374	0.7912	0.8528	-0.1387
CEU	CBWD3	66091374	0.7512	0.5117	0.7511
CEU	CBWD3	66096374	0.1183	0.5117	-1.2338
CEU	CBWD3	66101374	0.6184	0.6822	-0.1666
CEU	CBWD3	66106374	0.5399	0.3411	0.7969
CEU	CBWD3	66111374	0.6613	1.3645	-1.1376
CEU	CBWD3	66116374	0.9928	2.3879	-1.4692
CEU	CBWD3	66121374	0.3713	1.1939	-1.4659
CEU	CBWD3	66126374	0.1002	0.6822	-1.5198
CEU	CBWD3	66131374	0.1002	0.6822	-1.5198
CEU	CBWD3	66136374	0.0992	0.6822	-1.5224
CEU	CBWD3	66141374	0.0992	0.6822	-1.5224
CEU	CBWD3	66146374	0	0	0
CEU	CBWD3	66151374	0.0101	0.1706	-0.9534
CEU	CBWD3	66156374	0.0101	0.1706	-0.9534
CEU	CBWD3	66161374	0.4745	0.1706	1.8052
CEU	CBWD3	66166374	0.4745	0.1706	1.8052
CEU	CBWD3	66171374	0	0	0
CEU	CBWD3	66176374	0	0	0
CEU	CBWD3	66181374	0	0	0
CEU	CBWD3	66186374	0	0	0
CEU	CBWD3	66191374	0	0	0
CEU	CBWD3	66196374	0	0	0
CEU	CBWD3	66201374	0	0	0
CEU	CBWD3	66206374	0	0	0
CEU	CBWD3	66211374	0	0	0
CEU	CBWD3	66216374	0	0	0
CEU	CBWD3	66221374	0	0	0
CEU	CBWD3	66226374	0	0	0
CHB	CBWD3	65171374	0	0	0
CHB	CBWD3	65176374	0	0	0

CHB	CBWD3	65181374	0	0	0
CHB	CBWD3	65186374	0	0	0
CHB	CBWD3	65191374	0	0	0
CHB	CBWD3	65196374	0	0	0
CHB	CBWD3	65201374	0	0	0
CHB	CBWD3	65206374	0	0	0
CHB	CBWD3	65211374	0	0	0
CHB	CBWD3	65216374	0	0	0
CHB	CBWD3	65221374	0	0	0
CHB	CBWD3	65226374	0	0	0
CHB	CBWD3	65231374	0	0	0
CHB	CBWD3	65236374	0	0	0
CHB	CBWD3	65241374	0	0	0
CHB	CBWD3	65246374	0	0	0
CHB	CBWD3	65251374	0	0	0
CHB	CBWD3	65256374	0	0	0
CHB	CBWD3	65261374	0	0	0
CHB	CBWD3	65266374	0	0	0
CHB	CBWD3	65271374	0	0	0
CHB	CBWD3	65276374	0	0	0
CHB	CBWD3	65281374	0	0	0
CHB	CBWD3	65286374	0	0	0
CHB	CBWD3	65291374	0	0	0
CHB	CBWD3	65296374	0	0	0
CHB	CBWD3	65301374	0	0	0
CHB	CBWD3	65306374	0	0	0
CHB	CBWD3	65311374	0	0	0
CHB	CBWD3	65316374	0	0	0
CHB	CBWD3	65321374	0	0	0
CHB	CBWD3	65326374	0	0	0
CHB	CBWD3	65331374	0	0	0
CHB	CBWD3	65336374	0	0	0
CHB	CBWD3	65341374	0	0	0
CHB	CBWD3	65346374	0	0	0
CHB	CBWD3	65351374	0	0	0
CHB	CBWD3	65356374	0	0	0
CHB	CBWD3	65361374	0	0	0
CHB	CBWD3	65366374	0	0	0
CHB	CBWD3	65371374	0	0	0
CHB	CBWD3	65376374	0	0	0
CHB	CBWD3	65381374	0	0	0
CHB	CBWD3	65386374	0	0	0
CHB	CBWD3	65391374	0	0	0
CHB	CBWD3	65396374	0	0	0
CHB	CBWD3	65401374	0	0	0
CHB	CBWD3	65406374	0	0	0
CHB	CBWD3	65411374	0	0	0
CHB	CBWD3	65416374	0	0	0
CHB	CBWD3	65421374	0	0	0

CHB	CBWD3	65426374	0	0	0
CHB	CBWD3	65431374	0	0	0
CHB	CBWD3	65436374	0	0	0
CHB	CBWD3	65441374	0	0	0
CHB	CBWD3	65446374	0	0	0
CHB	CBWD3	65451374	0	0	0
CHB	CBWD3	65456374	0	0	0
CHB	CBWD3	65461374	0	0	0
CHB	CBWD3	65466374	0	0	0
CHB	CBWD3	65471374	0.5772	0.8471	-0.6086
CHB	CBWD3	65476374	1.0893	1.1859	-0.1724
CHB	CBWD3	65481374	0.9219	0.8471	0.1687
CHB	CBWD3	65486374	0.4099	0.5082	-0.3086
CHB	CBWD3	65491374	0	0	0
CHB	CBWD3	65496374	0	0	0
CHB	CBWD3	65501374	0	0	0
CHB	CBWD3	65506374	0	0	0
CHB	CBWD3	65511374	0	0	0
CHB	CBWD3	65516374	0.0097	0.1694	-0.949
CHB	CBWD3	65521374	0.0097	0.1694	-0.949
CHB	CBWD3	65526374	0.0388	0.6777	-1.6703
CHB	CBWD3	65531374	0.0388	0.6777	-1.6703
CHB	CBWD3	65536374	0	0	0
CHB	CBWD3	65541374	0	0	0
CHB	CBWD3	65546374	0.5561	0.8471	-0.6562
CHB	CBWD3	65551374	0.8414	1.3553	-0.8329
CHB	CBWD3	65556374	0.4483	1.8636	-1.8057
CHB	CBWD3	65561374	0.163	1.3553	-1.9324
CHB	CBWD3	65566374	0	0	0
CHB	CBWD3	65571374	0	0	0
CHB	CBWD3	65576374	1.3939	0.8471	1.233
CHB	CBWD3	65581374	1.3939	0.8471	1.233
CHB	CBWD3	65586374	0	0	0
CHB	CBWD3	65591374	1.8165	2.3718	-0.5862
CHB	CBWD3	65596374	3.9001	5.7601	-0.9241
CHB	CBWD3	65601374	3.6292	6.4378	-1.2628
CHB	CBWD3	65606374	3.3722	5.0825	-0.9495
CHB	CBWD3	65611374	5.1135	7.7931	-1.0127
CHB	CBWD3	65616374	5.1424	10.6731	-1.5621
CHB	CBWD3	65621374	4.5142	7.2848	-1.1137
CHB	CBWD3	65626374	4.7291	6.946	-0.9306
CHB	CBWD3	65631374	6.1519	9.1484	-0.977
CHB	CBWD3	65636374	4.1668	5.0825	-0.5084
CHB	CBWD3	65641374	0.1047	0.6777	-1.498
CHB	CBWD3	65646374	0.0194	0.1694	-0.8913
CHB	CBWD3	65651374	0.0194	0.1694	-0.8913
CHB	CBWD3	65656374	0.0194	0.1694	-0.8913
CHB	CBWD3	65661374	0	0	0
CHB	CBWD3	65666374	0	0	0

CHB	CBWD3	65671374	0.0097	0.1694	-0.949
CHB	CBWD3	65676374	0.0097	0.1694	-0.949
CHB	CBWD3	65681374	0	0	0
CHB	CBWD3	65686374	0.1881	0.3388	-0.6045
CHB	CBWD3	65691374	0.2075	0.5082	-0.9439
CHB	CBWD3	65696374	0.0194	0.1694	-0.8913
CHB	CBWD3	65701374	0	0	0
CHB	CBWD3	65706374	0	0	0
CHB	CBWD3	65711374	0.3859	0.1694	1.2865
CHB	CBWD3	65716374	0.3859	0.1694	1.2865
CHB	CBWD3	65721374	0	0	0
CHB	CBWD3	65726374	0	0	0
CHB	CBWD3	65731374	0	0	0
CHB	CBWD3	65736374	0.7717	0.3388	1.7365
CHB	CBWD3	65741374	1.1342	0.6777	1.1934
CHB	CBWD3	65746374	0.6901	0.5082	0.571
CHB	CBWD3	65751374	0.6551	0.5082	0.4611
CHB	CBWD3	65756374	0.3468	0.5082	-0.5067
CHB	CBWD3	65761374	0.0194	0.1694	-0.8913
CHB	CBWD3	65766374	0.6141	1.0165	-0.8008
CHB	CBWD3	65771374	1.1371	1.5247	-0.5758
CHB	CBWD3	65776374	0.5422	0.6777	-0.3542
CHB	CBWD3	65781374	0.0482	0.5082	-1.444
CHB	CBWD3	65786374	0.0289	0.3388	-1.2431
CHB	CBWD3	65791374	0	0.1694	-1.0066
CHB	CBWD3	65796374	0.1498	0.6777	-1.3801
CHB	CBWD3	65801374	0.1498	0.5082	-1.1251
CHB	CBWD3	65806374	0	0	0
CHB	CBWD3	65811374	0.0579	0.5082	-1.4136
CHB	CBWD3	65816374	0.0579	0.5082	-1.4136
CHB	CBWD3	65821374	0.4139	0.5082	-0.296
CHB	CBWD3	65826374	0.4139	0.5082	-0.296
CHB	CBWD3	65831374	0	0	0
CHB	CBWD3	65836374	0.1272	0.1694	-0.2508
CHB	CBWD3	65841374	0.2031	0.5082	-0.9578
CHB	CBWD3	65846374	0.076	0.3388	-1.0542
CHB	CBWD3	65851374	0	0	0
CHB	CBWD3	65856374	0.1763	0.1694	0.041
CHB	CBWD3	65861374	0.1763	0.3388	-0.6518
CHB	CBWD3	65866374	0.0097	0.3388	-1.3201
CHB	CBWD3	65871374	0.4064	0.5082	-0.3196
CHB	CBWD3	65876374	1.2606	0.8471	0.9324
CHB	CBWD3	65881374	0.8639	0.5082	1.1166
CHB	CBWD3	65886374	0.3758	0.1694	1.2265
CHB	CBWD3	65891374	1.0484	0.8471	0.4539
CHB	CBWD3	65896374	1.0739	1.1859	-0.1999
CHB	CBWD3	65901374	0.4207	0.6777	-0.6719
CHB	CBWD3	65906374	0.0194	0.1694	-0.8913
CHB	CBWD3	65911374	0	0	0

CHB	CBWD3	65916374	1.1277	1.3553	-0.3689
CHB	CBWD3	65921374	1.1277	1.3553	-0.3689
CHB	CBWD3	65926374	0	0	0
CHB	CBWD3	65931374	0	0	0
CHB	CBWD3	65936374	0	0	0
CHB	CBWD3	65941374	0	0	0
CHB	CBWD3	65946374	0	0	0
CHB	CBWD3	65951374	0	0	0
CHB	CBWD3	65956374	0	0	0
CHB	CBWD3	65961374	0	0	0
CHB	CBWD3	65966374	0.1506	0.8471	-1.5706
CHB	CBWD3	65971374	2.1294	1.8636	0.3391
CHB	CBWD3	65976374	1.9983	1.3553	1.0421
CHB	CBWD3	65981374	0.0291	0.5082	-1.504
CHB	CBWD3	65986374	0.3128	0.3388	-0.1043
CHB	CBWD3	65991374	0.5285	0.6777	-0.3901
CHB	CBWD3	65996374	0.2825	0.6777	-1.0332
CHB	CBWD3	66001374	1.0436	0.5082	1.6807
CHB	CBWD3	66006374	1.7295	0.8471	1.9898
CHB	CBWD3	66011374	1.2046	1.8636	-0.8408
CHB	CBWD3	66016374	1.4097	2.5412	-1.1299
CHB	CBWD3	66021374	0.948	1.1859	-0.4246
CHB	CBWD3	66026374	0.0474	0.1694	-0.7249
CHB	CBWD3	66031374	0.4761	0.6777	-0.527
CHB	CBWD3	66036374	0.5351	1.3553	-1.3293
CHB	CBWD3	66041374	0.1161	1.0165	-1.7918
CHB	CBWD3	66046374	0.4959	0.6777	-0.4753
CHB	CBWD3	66051374	0.4862	0.5082	-0.0691
CHB	CBWD3	66056374	0	0	0
CHB	CBWD3	66061374	0	0	0
CHB	CBWD3	66066374	0.0388	0.3388	-1.2034
CHB	CBWD3	66071374	0.0388	0.3388	-1.2034
CHB	CBWD3	66076374	0	0	0
CHB	CBWD3	66081374	0	0	0
CHB	CBWD3	66086374	0.4323	0.1694	1.5622
CHB	CBWD3	66091374	0.4323	0.1694	1.5622
CHB	CBWD3	66096374	0.0485	0.5082	-1.4431
CHB	CBWD3	66101374	0.534	0.6777	-0.3757
CHB	CBWD3	66106374	0.4951	0.3388	0.627
CHB	CBWD3	66111374	0.9564	1.3553	-0.6465
CHB	CBWD3	66116374	1.8431	2.3718	-0.5581
CHB	CBWD3	66121374	0.9061	1.3553	-0.728
CHB	CBWD3	66126374	0.0485	0.6777	-1.6449
CHB	CBWD3	66131374	0.0388	0.5082	-1.4735
CHB	CBWD3	66136374	0.0554	0.1694	-0.6774
CHB	CBWD3	66141374	0.0554	0.1694	-0.6774
CHB	CBWD3	66146374	0	0	0
CHB	CBWD3	66151374	0	0	0
CHB	CBWD3	66156374	0	0	0

CHB	CBWD3	66161374	0.1769	0.1694	0.0446
CHB	CBWD3	66166374	0.1769	0.1694	0.0446
CHB	CBWD3	66171374	0	0	0
CHB	CBWD3	66176374	0	0	0
CHB	CBWD3	66181374	0.0097	0.1694	-0.949
CHB	CBWD3	66186374	0.0097	0.1694	-0.949
CHB	CBWD3	66191374	0	0	0
CHB	CBWD3	66196374	0	0	0
CHB	CBWD3	66201374	0	0	0
CHB	CBWD3	66206374	0	0	0
CHB	CBWD3	66211374	0	0	0
CHB	CBWD3	66216374	0	0	0
CHB	CBWD3	66221374	0	0	0
CHB	CBWD3	66226374	0	0	0
YRI	CBWD3	65171374	0	0	0
YRI	CBWD3	65176374	0	0	0
YRI	CBWD3	65181374	0	0	0
YRI	CBWD3	65186374	0	0	0
YRI	CBWD3	65191374	0	0	0
YRI	CBWD3	65196374	0	0	0
YRI	CBWD3	65201374	0	0	0
YRI	CBWD3	65206374	0	0	0
YRI	CBWD3	65211374	0	0	0
YRI	CBWD3	65216374	0	0	0
YRI	CBWD3	65221374	0	0	0
YRI	CBWD3	65226374	0	0	0
YRI	CBWD3	65231374	0	0	0
YRI	CBWD3	65236374	0	0	0
YRI	CBWD3	65241374	0	0	0
YRI	CBWD3	65246374	0	0	0
YRI	CBWD3	65251374	0	0	0
YRI	CBWD3	65256374	0	0	0
YRI	CBWD3	65261374	0	0	0
YRI	CBWD3	65266374	0	0	0
YRI	CBWD3	65271374	0	0	0
YRI	CBWD3	65276374	0	0	0
YRI	CBWD3	65281374	0	0	0
YRI	CBWD3	65286374	0	0	0
YRI	CBWD3	65291374	0	0	0
YRI	CBWD3	65296374	0	0	0
YRI	CBWD3	65301374	0	0	0
YRI	CBWD3	65306374	0	0	0
YRI	CBWD3	65311374	0	0	0
YRI	CBWD3	65316374	0	0	0
YRI	CBWD3	65321374	0	0	0
YRI	CBWD3	65326374	0	0	0
YRI	CBWD3	65331374	0	0	0
YRI	CBWD3	65336374	0	0	0
YRI	CBWD3	65341374	0	0	0

YRI	CBWD3	65346374	0	0	0
YRI	CBWD3	65351374	0	0	0
YRI	CBWD3	65356374	0	0	0
YRI	CBWD3	65361374	0	0	0
YRI	CBWD3	65366374	0	0	0
YRI	CBWD3	65371374	0	0	0
YRI	CBWD3	65376374	0	0	0
YRI	CBWD3	65381374	0	0	0
YRI	CBWD3	65386374	0	0	0
YRI	CBWD3	65391374	0	0	0
YRI	CBWD3	65396374	0	0	0
YRI	CBWD3	65401374	0	0	0
YRI	CBWD3	65406374	0	0	0
YRI	CBWD3	65411374	0	0	0
YRI	CBWD3	65416374	0	0	0
YRI	CBWD3	65421374	0	0	0
YRI	CBWD3	65426374	0	0	0
YRI	CBWD3	65431374	0	0	0
YRI	CBWD3	65436374	0	0	0
YRI	CBWD3	65441374	0	0	0
YRI	CBWD3	65446374	0	0	0
YRI	CBWD3	65451374	0	0	0
YRI	CBWD3	65456374	0	0	0
YRI	CBWD3	65461374	0	0	0
YRI	CBWD3	65466374	0	0	0
YRI	CBWD3	65471374	0.9335	1.5126	-0.8623
YRI	CBWD3	65476374	1.156	2.1848	-1.1551
YRI	CBWD3	65481374	0.9536	1.3445	-0.6349
YRI	CBWD3	65486374	0.7497	0.8403	-0.2046
YRI	CBWD3	65491374	0.0185	0.1681	-0.8893
YRI	CBWD3	65496374	0	0	0
YRI	CBWD3	65501374	0	0	0
YRI	CBWD3	65506374	0.2029	0.3361	-0.5347
YRI	CBWD3	65511374	0.2029	0.3361	-0.5347
YRI	CBWD3	65516374	0	0	0
YRI	CBWD3	65521374	0.0185	0.1681	-0.8893
YRI	CBWD3	65526374	0.2901	1.8487	-1.9936
YRI	CBWD3	65531374	0.2715	1.6806	-1.9385
YRI	CBWD3	65536374	0	0	0
YRI	CBWD3	65541374	0	0	0
YRI	CBWD3	65546374	0.6899	1.3445	-1.0632
YRI	CBWD3	65551374	1.5091	2.8571	-1.219
YRI	CBWD3	65556374	1.275	3.6974	-1.7656
YRI	CBWD3	65561374	0.4834	2.3529	-1.979
YRI	CBWD3	65566374	0.0276	0.1681	-0.8352
YRI	CBWD3	65571374	0.0093	0.1681	-0.944
YRI	CBWD3	65576374	0.7565	1.1764	-0.751
YRI	CBWD3	65581374	0.7472	1.0084	-0.5207
YRI	CBWD3	65586374	0	0	0

YRI	CBWD3	65591374	1.3191	3.6974	-1.7335
YRI	CBWD3	65596374	4.2391	8.9073	-1.5548
YRI	CBWD3	65601374	4.2741	9.0754	-1.5717
YRI	CBWD3	65606374	3.5053	7.5628	-1.5708
YRI	CBWD3	65611374	7.0987	11.9324	-1.2253
YRI	CBWD3	65616374	8.5476	13.9492	-1.1813
YRI	CBWD3	65621374	5.4186	10.2518	-1.4126
YRI	CBWD3	65626374	3.5619	9.9157	-1.9157
YRI	CBWD3	65631374	6.1178	11.9324	-1.474
YRI	CBWD3	65636374	4.8784	8.067	-1.1635
YRI	CBWD3	65641374	0.6676	2.3529	-1.784
YRI	CBWD3	65646374	0.1636	0.8403	-1.5284
YRI	CBWD3	65651374	0.072	0.1681	-0.5713
YRI	CBWD3	65656374	0.072	0.1681	-0.5713
YRI	CBWD3	65661374	0.0093	0.1681	-0.944
YRI	CBWD3	65666374	0.0093	0.1681	-0.944
YRI	CBWD3	65671374	0	0	0
YRI	CBWD3	65676374	0	0	0
YRI	CBWD3	65681374	0	0	0
YRI	CBWD3	65686374	0.0813	0.3361	-1.0229
YRI	CBWD3	65691374	0.0996	0.5042	-1.2715
YRI	CBWD3	65696374	0.0183	0.1681	-0.8905
YRI	CBWD3	65701374	0	0	0
YRI	CBWD3	65706374	0	0	0
YRI	CBWD3	65711374	0.1463	0.1681	-0.1296
YRI	CBWD3	65716374	0.1463	0.1681	-0.1296
YRI	CBWD3	65721374	0	0	0
YRI	CBWD3	65726374	0	0	0
YRI	CBWD3	65731374	0.0093	0.1681	-0.944
YRI	CBWD3	65736374	0.3019	0.5042	-0.6358
YRI	CBWD3	65741374	0.8473	0.8403	0.0158
YRI	CBWD3	65746374	0.776	1.1764	-0.7161
YRI	CBWD3	65751374	0.3025	1.0084	-1.4073
YRI	CBWD3	65756374	0.5291	1.1764	-1.1577
YRI	CBWD3	65761374	0.5757	1.5126	-1.3951
YRI	CBWD3	65766374	0.6255	2.689	-1.9611
YRI	CBWD3	65771374	0.8296	2.5209	-1.6938
YRI	CBWD3	65776374	0.3874	1.1764	-1.4112
YRI	CBWD3	65781374	0.0833	1.0084	-1.8444
YRI	CBWD3	65786374	0.0278	0.3361	-1.2377
YRI	CBWD3	65791374	0	0.1681	-0.9993
YRI	CBWD3	65796374	0.3386	0.8403	-1.1332
YRI	CBWD3	65801374	0.3662	0.8403	-1.0708
YRI	CBWD3	65806374	0.0369	0.3361	-1.2012
YRI	CBWD3	65811374	0.0278	0.3361	-1.2377
YRI	CBWD3	65816374	0.1603	0.5042	-1.0808
YRI	CBWD3	65821374	0.5879	0.8403	-0.5701
YRI	CBWD3	65826374	0.4462	0.5042	-0.1823
YRI	CBWD3	65831374	0.0093	0.1681	-0.944

YRI	CBWD3	65836374	0.0458	0.3361	-1.1654
YRI	CBWD3	65841374	0.0458	0.3361	-1.1654
YRI	CBWD3	65846374	0.0093	0.1681	-0.944
YRI	CBWD3	65851374	0	0	0
YRI	CBWD3	65856374	0.1314	0.3361	-0.8218
YRI	CBWD3	65861374	0.1587	0.5042	-1.0858
YRI	CBWD3	65866374	0.0458	0.3361	-1.1654
YRI	CBWD3	65871374	0.1377	1.0084	-1.7359
YRI	CBWD3	65876374	1.1716	2.1848	-1.1376
YRI	CBWD3	65881374	1.0524	1.3445	-0.4744
YRI	CBWD3	65886374	0.2066	0.1681	0.2289
YRI	CBWD3	65891374	0.6311	0.8403	-0.4725
YRI	CBWD3	65896374	0.9827	1.8487	-1.1077
YRI	CBWD3	65901374	0.5674	1.3445	-1.2622
YRI	CBWD3	65906374	0.0093	0.1681	-0.944
YRI	CBWD3	65911374	0	0	0
YRI	CBWD3	65916374	1.7041	1.8487	-0.185
YRI	CBWD3	65921374	1.7041	1.8487	-0.185
YRI	CBWD3	65926374	0	0	0
YRI	CBWD3	65931374	0	0	0
YRI	CBWD3	65936374	0	0	0
YRI	CBWD3	65941374	0	0	0
YRI	CBWD3	65946374	0	0	0
YRI	CBWD3	65951374	0	0	0
YRI	CBWD3	65956374	0	0	0
YRI	CBWD3	65961374	0	0	0
YRI	CBWD3	65966374	0.1997	0.8403	-1.4469
YRI	CBWD3	65971374	1.9839	2.0167	-0.0392
YRI	CBWD3	65976374	1.8483	1.6806	0.2307
YRI	CBWD3	65981374	0.5824	1.1764	-1.0624
YRI	CBWD3	65986374	0.6506	1.0084	-0.7133
YRI	CBWD3	65991374	0.6717	1.8487	-1.5055
YRI	CBWD3	65996374	0.724	2.3529	-1.7243
YRI	CBWD3	66001374	0.7442	1.5126	-1.1442
YRI	CBWD3	66006374	1.3696	2.8571	-1.3452
YRI	CBWD3	66011374	2.5039	3.6974	-0.8699
YRI	CBWD3	66016374	3.0739	3.1932	-0.0984
YRI	CBWD3	66021374	1.38	1.6806	-0.4135
YRI	CBWD3	66026374	0.474	0.3361	0.5536
YRI	CBWD3	66031374	1.5378	1.5126	0.0375
YRI	CBWD3	66036374	2.1512	2.3529	-0.2135
YRI	CBWD3	66041374	1.2129	1.8487	-0.8132
YRI	CBWD3	66046374	0.7947	1.3445	-0.893
YRI	CBWD3	66051374	0.6785	0.8403	-0.3654
YRI	CBWD3	66056374	0.2323	0.6722	-1.1516
YRI	CBWD3	66061374	0.2323	0.6722	-1.1516
YRI	CBWD3	66066374	0.6728	0.6722	0.0016
YRI	CBWD3	66071374	1.0562	0.8403	0.4876
YRI	CBWD3	66076374	0.3926	0.3361	0.2268

YRI	CBWD3	66081374	0.0185	0.3361	-1.275
YRI	CBWD3	66086374	0.4464	0.8403	-0.8897
YRI	CBWD3	66091374	0.4279	0.5042	-0.2398
YRI	CBWD3	66096374	0.3471	0.5042	-0.4937
YRI	CBWD3	66101374	0.8368	0.6722	0.4309
YRI	CBWD3	66106374	0.499	0.3361	0.654
YRI	CBWD3	66111374	0.2929	1.0084	-1.4265
YRI	CBWD3	66116374	1.1321	2.1848	-1.182
YRI	CBWD3	66121374	0.9706	1.5126	-0.8071
YRI	CBWD3	66126374	0.4837	1.0084	-1.0461
YRI	CBWD3	66131374	0.3615	0.8403	-1.0814
YRI	CBWD3	66136374	0.2837	0.3361	-0.2104
YRI	CBWD3	66141374	0.3802	0.5042	-0.3897
YRI	CBWD3	66146374	0.0965	0.1681	-0.4256
YRI	CBWD3	66151374	0.0093	0.1681	-0.944
YRI	CBWD3	66156374	0.0093	0.1681	-0.944
YRI	CBWD3	66161374	0.4565	0.1681	1.7145
YRI	CBWD3	66166374	0.4565	0.1681	1.7145
YRI	CBWD3	66171374	0	0	0
YRI	CBWD3	66176374	0	0	0
YRI	CBWD3	66181374	0.0273	0.1681	-0.837
YRI	CBWD3	66186374	0.0273	0.1681	-0.837
YRI	CBWD3	66191374	0	0	0
YRI	CBWD3	66196374	0	0	0
YRI	CBWD3	66201374	0	0	0
YRI	CBWD3	66206374	0	0	0
YRI	CBWD3	66211374	0	0	0
YRI	CBWD3	66216374	0	0	0
YRI	CBWD3	66221374	0	0	0
YRI	CBWD3	66226374	0	0	0
CEU	CBWD3	67746866	0.4172	0.3411	0.3051
CEU	CBWD3	67751866	0.404	0.1706	1.3864
CEU	CBWD3	67756866	0.404	0.1706	1.3864
CEU	CBWD3	67761866	0.0101	0.1706	-0.9534
CEU	CBWD3	67766866	0.7209	0.6822	0.1011
CEU	CBWD3	67771866	0.7108	0.5117	0.6244
CEU	CBWD3	67776866	0.0101	0.1706	-0.9534
CEU	CBWD3	67781866	0.0598	0.3411	-1.1277
CEU	CBWD3	67786866	0.5947	0.6822	-0.2285
CEU	CBWD3	67791866	0.7444	1.3645	-1.0032
CEU	CBWD3	67796866	0.8052	1.3645	-0.9048
CEU	CBWD3	67801866	0.6059	0.5117	0.2954
CEU	CBWD3	67806866	0.0101	0.1706	-0.9534
CEU	CBWD3	67811866	0.0101	0.1706	-0.9534
CEU	CBWD3	67816866	0	0	0
CEU	CBWD3	67821866	0.3191	0.3411	-0.0882
CEU	CBWD3	67826866	1.9955	1.5351	0.6827
CEU	CBWD3	67831866	2.1519	1.5351	0.9146
CEU	CBWD3	67836866	1.638	1.5351	0.1526

CEU	CBWD3	67841866	1.9629	1.7056	0.3524
CEU	CBWD3	67846866	1.9175	1.7056	0.2902
CEU	CBWD3	67851866	1.1569	1.3645	-0.3359
CEU	CBWD3	67856866	0.2224	0.3411	-0.4758
CEU	CBWD3	67861866	0.449	0.3411	0.4325
CEU	CBWD3	67866866	1.1495	0.5117	2.0003
CEU	CBWD3	67871866	0.8831	0.3411	2.1727
CEU	CBWD3	67876866	0.0101	0.1706	-0.9534
CEU	CBWD3	67881866	0.0303	0.3411	-1.2459
CEU	CBWD3	67886866	1.1668	1.0234	0.2849
CEU	CBWD3	67891866	1.3078	1.3645	-0.0917
CEU	CBWD3	67896866	4.7798	4.4346	0.2166
CEU	CBWD3	67901866	5.1847	4.6052	0.3518
CEU	CBWD3	67906866	0.5762	0.8528	-0.6229
CEU	CBWD3	67911866	2.1006	2.729	-0.594
CEU	CBWD3	67916866	3.9041	4.9463	-0.5944
CEU	CBWD3	67921866	2.241	3.5818	-1.0106
CEU	CBWD3	67926866	1.0326	2.0467	-1.2067
CEU	CBWD3	67931866	2.1713	2.729	-0.5271
CEU	CBWD3	67936866	2.5452	2.8996	-0.3187
CEU	CBWD3	67941866	1.0394	1.5351	-0.735
CEU	CBWD3	67946866	1.2731	2.0467	-0.9205
CEU	CBWD3	67951866	1.7229	2.0467	-0.3853
CEU	CBWD3	67956866	0.5201	0.6822	-0.4233
CEU	CBWD3	67961866	1.0493	1.3645	-0.5099
CEU	CBWD3	67966866	1.5038	1.8762	-0.4741
CEU	CBWD3	67971866	0.4646	0.6822	-0.5682
CEU	CBWD3	67976866	0.1973	0.6822	-1.2662
CEU	CBWD3	67981866	0.9878	1.0234	-0.0707
CEU	CBWD3	67986866	0.7906	0.3411	1.8019
CEU	CBWD3	67991866	0	0	0
CEU	CBWD3	67996866	0	0	0
CEU	CBWD3	68001866	0	0	0
CEU	CBWD3	68006866	0	0	0
CEU	CBWD3	68011866	0	0	0
CEU	CBWD3	68016866	0	0	0
CEU	CBWD3	68021866	0	0	0
CEU	CBWD3	68026866	0	0	0
CEU	CBWD3	68031866	0	0	0
CEU	CBWD3	68036866	0	0	0
CEU	CBWD3	68041866	0	0	0
CEU	CBWD3	68046866	0	0	0
CEU	CBWD3	68051866	0	0	0
CEU	CBWD3	68056866	0	0	0
CEU	CBWD3	68061866	0	0	0
CEU	CBWD3	68066866	0	0	0
CEU	CBWD3	68071866	0	0	0
CEU	CBWD3	68076866	0	0	0
CEU	CBWD3	68081866	0	0	0

CEU	CBWD3	68086866	0	0	0
CEU	CBWD3	68091866	0	0	0
CEU	CBWD3	68096866	0	0	0
CEU	CBWD3	68101866	0	0	0
CEU	CBWD3	68106866	0	0	0
CEU	CBWD3	68111866	0	0	0
CEU	CBWD3	68116866	0	0	0
CEU	CBWD3	68121866	0	0	0
CEU	CBWD3	68126866	0	0	0
CEU	CBWD3	68131866	0	0	0
CEU	CBWD3	68136866	1.1185	1.0234	0.189
CEU	CBWD3	68141866	2.4914	3.0701	-0.4964
CEU	CBWD3	68146866	2.6407	4.4346	-1.1256
CEU	CBWD3	68151866	3.4444	6.4814	-1.3612
CEU	CBWD3	68156866	3.4558	6.6519	-1.3993
CEU	CBWD3	68161866	2.3995	4.2641	-1.2104
CEU	CBWD3	68166866	2.9122	5.458	-1.3309
CEU	CBWD3	68171866	4.791	7.5047	-1.0647
CEU	CBWD3	68176866	5.6687	8.6987	-1.0381
CEU	CBWD3	68181866	4.1123	8.0164	-1.442
CEU	CBWD3	68186866	3.0954	5.2874	-1.1786
CEU	CBWD3	68191866	3.7805	5.458	-0.877
CEU	CBWD3	68196866	3.6782	6.3108	-1.2087
CEU	CBWD3	68201866	5.113	8.187	-1.1136
CEU	CBWD3	68206866	5.6836	9.7221	-1.248
CEU	CBWD3	68211866	3.9332	6.9931	-1.2803
CEU	CBWD3	68216866	4.4409	5.6286	-0.6041
CEU	CBWD3	68221866	4.8204	8.0164	-1.1805
CEU	CBWD3	68226866	3.7141	7.6753	-1.5226
CEU	CBWD3	68231866	2.4572	4.6052	-1.3041
CEU	CBWD3	68236866	3.5617	4.4346	-0.5477
CEU	CBWD3	68241866	3.6139	4.2641	-0.4221
CEU	CBWD3	68246866	2.8128	3.9229	-0.7743
CEU	CBWD3	68251866	5.382	5.7991	-0.2066
CEU	CBWD3	68256866	4.3851	5.1169	-0.4051
CEU	CBWD3	68261866	1.2573	3.7524	-1.8076
CEU	CBWD3	68266866	1.0175	3.7524	-1.9814
CEU	CBWD3	68271866	2.0486	4.9463	-1.6526
CEU	CBWD3	68276866	3.6392	6.8225	-1.3621
CEU	CBWD3	68281866	3.5861	6.3108	-1.251
CEU	CBWD3	68286866	5.0379	6.8225	-0.7636
CEU	CBWD3	68291866	5.2127	6.3108	-0.5042
CEU	CBWD3	68296866	3.4875	6.1402	-1.2483
CEU	CBWD3	68301866	3.7343	7.6753	-1.5148
CEU	CBWD3	68306866	3.1439	5.458	-1.2098
CEU	CBWD3	68311866	2.1045	4.0935	-1.3375
CEU	CBWD3	68316866	9.2319	9.2104	0.007
CEU	CBWD3	68321866	10.7269	11.2571	-0.1429
CEU	CBWD3	68326866	4.7838	7.3342	-1.0219

CEU	CBWD3	68331866	5.3647	7.5047	-0.8396
CEU	CBWD3	68336866	4.2459	8.187	-1.4278
CEU	CBWD3	68341866	4.2298	8.5281	-1.4997
CEU	CBWD3	68346866	5.223	9.3809	-1.3283
CEU	CBWD3	68351866	3.4912	8.0164	-1.6714
CEU	CBWD3	68356866	7.979	9.3809	-0.4479
CEU	CBWD3	68361866	10.9724	8.8692	0.7077
CEU	CBWD3	68366866	6.3968	6.3108	0.0395
CEU	CBWD3	68371866	5.1565	6.6519	-0.6547
CEU	CBWD3	68376866	5.0115	5.9697	-0.4624
CEU	CBWD3	68381866	12.2486	7.1636	2.0815
CEU	CBWD3	68386866	21.6935	10.916	2.9891
CEU	CBWD3	68391866	16.0266	10.5749	1.5576
CEU	CBWD3	68396866	7.0598	7.6753	-0.2366
CEU	CBWD3	68401866	4.3659	6.8225	-1.0512
CEU	CBWD3	68406866	4.6236	6.9931	-0.9914
CEU	CBWD3	68411866	25.0793	13.645	2.5696
CEU	CBWD3	68416866	36.8209	16.2034	3.9339
CEU	CBWD3	68421866	16.6734	8.8692	2.6261
CEU	CBWD3	68426866	6.0719	5.9697	0.0493
CEU	CBWD3	68431866	6.6473	7.6753	-0.3951
CEU	CBWD3	68436866	7.7858	11.2571	-0.9354
CEU	CBWD3	68441866	8.1936	12.4511	-1.0434
CEU	CBWD3	68446866	6.4923	10.2337	-1.1022
CEU	CBWD3	68451866	5.2618	9.2104	-1.2831
CEU	CBWD3	68456866	4.1214	10.0632	-1.7782
CEU	CBWD3	68461866	4.0206	10.0632	-1.8083
CEU	CBWD3	68466866	4.6059	8.187	-1.2974
CEU	CBWD3	68471866	5.4117	7.6753	-0.8701
CEU	CBWD3	68476866	8.6042	8.8692	-0.0892
CEU	CBWD3	68481866	7.9705	6.8225	0.4912
CEU	CBWD3	68486866	6.7497	7.3342	-0.2342
CEU	CBWD3	68491866	7.012	9.5515	-0.7978
CEU	CBWD3	68496866	4.5172	9.7221	-1.6085
CEU	CBWD3	68501866	4.1154	11.0866	-1.9056
CEU	CBWD3	68506866	4.4743	11.5982	-1.8665
CEU	CBWD3	68511866	3.275	7.1636	-1.5918
CEU	CBWD3	68516866	0.5391	1.1939	-1.1669
CEU	CBWD3	68521866	0	0	0
CEU	CBWD3	68526866	0	0	0
CEU	CBWD3	68531866	0	0	0
CEU	CBWD3	68536866	0	0	0
CEU	CBWD3	68541866	0	0	0
CEU	CBWD3	68546866	0	0	0
CEU	CBWD3	68551866	0	0	0
CEU	CBWD3	68556866	0	0	0
CEU	CBWD3	68561866	0	0	0
CEU	CBWD3	68566866	0	0	0
CEU	CBWD3	68571866	0	0	0

CEU	CBWD3	68576866	0	0	0
CEU	CBWD3	68581866	0	0	0
CEU	CBWD3	68586866	0	0	0
CEU	CBWD3	68591866	0	0	0
CEU	CBWD3	68596866	0	0	0
CEU	CBWD3	68601866	0	0	0
CEU	CBWD3	68606866	0	0	0
CEU	CBWD3	68611866	0	0	0
CEU	CBWD3	68616866	0	0	0
CEU	CBWD3	68621866	0	0	0
CEU	CBWD3	68626866	0	0	0
CEU	CBWD3	68631866	0	0	0
CEU	CBWD3	68636866	0	0	0
CEU	CBWD3	68641866	0	0	0
CEU	CBWD3	68646866	0	0	0
CEU	CBWD3	68651866	0	0	0
CEU	CBWD3	68656866	0	0	0
CEU	CBWD3	68661866	0.2544	1.1939	-1.6742
CEU	CBWD3	68666866	2.0511	4.4346	-1.4955
CEU	CBWD3	68671866	2.4597	4.6052	-1.3026
CEU	CBWD3	68676866	1.4424	2.5584	-1.1117
CEU	CBWD3	68681866	0.8299	1.8762	-1.3321
CEU	CBWD3	68686866	1.9151	2.8996	-0.8854
CEU	CBWD3	68691866	2.9755	3.5818	-0.457
CEU	CBWD3	68696866	2.3028	2.3879	-0.0896
CEU	CBWD3	68701866	1.3791	1.5351	-0.2313
CEU	CBWD3	68706866	0.8951	1.7056	-1.1101
CEU	CBWD3	68711866	0.7483	1.8762	-1.436
CEU	CBWD3	68716866	0.3589	1.7056	-1.8445
CEU	CBWD3	68721866	0.455	1.7056	-1.7128
CEU	CBWD3	68726866	0.6273	1.7056	-1.4769
CEU	CBWD3	68731866	2.4455	3.2407	-0.652
CEU	CBWD3	68736866	2.596	4.2641	-1.0828
CEU	CBWD3	68741866	4.5585	5.7991	-0.6145
CEU	CBWD3	68746866	4.5847	5.7991	-0.6015
CEU	CBWD3	68751866	2.6063	5.1169	-1.3897
CEU	CBWD3	68756866	3.268	5.1169	-1.0234
CEU	CBWD3	68761866	1.6696	3.2407	-1.2883
CEU	CBWD3	68766866	1.0952	3.2407	-1.7593
CEU	CBWD3	68771866	1.7304	3.4112	-1.3201
CEU	CBWD3	68776866	1.6799	3.2407	-1.2798
CEU	CBWD3	68781866	2.678	5.6286	-1.5009
CEU	CBWD3	68786866	3.3754	6.8225	-1.475
CEU	CBWD3	68791866	3.1657	5.7991	-1.3043
CHB	CBWD3	67746866	0.5899	0.5082	0.2565
CHB	CBWD3	67751866	0.3912	0.3388	0.2102
CHB	CBWD3	67756866	0.3912	0.3388	0.2102
CHB	CBWD3	67761866	0.0194	0.1694	-0.8913
CHB	CBWD3	67766866	0.7634	0.6777	0.224

CHB	CBWD3	67771866	0.744	0.5082	0.7402
CHB	CBWD3	67776866	0.2755	0.6777	-1.0515
CHB	CBWD3	67781866	0.2949	1.0165	-1.436
CHB	CBWD3	67786866	0.5785	1.1859	-1.0842
CHB	CBWD3	67791866	0.8102	1.5247	-1.0615
CHB	CBWD3	67796866	0.6773	1.1859	-0.9078
CHB	CBWD3	67801866	0.4262	0.5082	-0.2574
CHB	CBWD3	67806866	0	0	0
CHB	CBWD3	67811866	0	0	0
CHB	CBWD3	67816866	0.0097	0.1694	-0.949
CHB	CBWD3	67821866	0.6299	0.5082	0.382
CHB	CBWD3	67826866	2.3377	1.5247	1.2078
CHB	CBWD3	67831866	2.008	1.3553	1.0578
CHB	CBWD3	67836866	1.1133	1.1859	-0.1296
CHB	CBWD3	67841866	1.5049	1.5247	-0.0294
CHB	CBWD3	67846866	1.7822	1.6942	0.1208
CHB	CBWD3	67851866	1.1001	1.1859	-0.1532
CHB	CBWD3	67856866	0.1447	0.1694	-0.1468
CHB	CBWD3	67861866	0.4222	0.3388	0.3345
CHB	CBWD3	67866866	0.9564	0.5082	1.407
CHB	CBWD3	67871866	0.6789	0.3388	1.3643
CHB	CBWD3	67876866	0.0097	0.1694	-0.949
CHB	CBWD3	67881866	0.0194	0.3388	-1.2812
CHB	CBWD3	67886866	1.3219	1.5247	-0.3013
CHB	CBWD3	67891866	1.4679	1.8636	-0.5049
CHB	CBWD3	67896866	3.7478	3.2189	0.4348
CHB	CBWD3	67901866	4.1091	3.2189	0.7319
CHB	CBWD3	67906866	0.5654	1.0165	-0.8977
CHB	CBWD3	67911866	1.8903	1.6942	0.2691
CHB	CBWD3	67916866	3.2273	3.3883	-0.1268
CHB	CBWD3	67921866	1.5199	3.3883	-1.4714
CHB	CBWD3	67926866	0.7342	2.033	-1.5488
CHB	CBWD3	67931866	2.155	2.8801	-0.6538
CHB	CBWD3	67936866	2.3015	3.0495	-0.6433
CHB	CBWD3	67941866	0.8329	1.5247	-1.0278
CHB	CBWD3	67946866	0.525	1.3553	-1.3457
CHB	CBWD3	67951866	0.795	2.033	-1.4763
CHB	CBWD3	67956866	0.3566	1.1859	-1.4803
CHB	CBWD3	67961866	1.5069	1.8636	-0.4551
CHB	CBWD3	67966866	1.9735	2.5412	-0.5669
CHB	CBWD3	67971866	0.4666	0.6777	-0.5519
CHB	CBWD3	67976866	0.0097	0.1694	-0.949
CHB	CBWD3	67981866	1.1976	0.8471	0.7904
CHB	CBWD3	67986866	1.1976	0.8471	0.7904
CHB	CBWD3	67991866	0.0097	0.1694	-0.949
CHB	CBWD3	67996866	0	0	0
CHB	CBWD3	68001866	0	0	0
CHB	CBWD3	68006866	0	0	0
CHB	CBWD3	68011866	0	0	0

CHB	CBWD3	68016866	0	0	0
CHB	CBWD3	68021866	0	0	0
CHB	CBWD3	68026866	0	0	0
CHB	CBWD3	68031866	0	0	0
CHB	CBWD3	68036866	0	0	0
CHB	CBWD3	68041866	0	0	0
CHB	CBWD3	68046866	0	0	0
CHB	CBWD3	68051866	0	0	0
CHB	CBWD3	68056866	0	0	0
CHB	CBWD3	68061866	0	0	0
CHB	CBWD3	68066866	0	0	0
CHB	CBWD3	68071866	0	0	0
CHB	CBWD3	68076866	0	0	0
CHB	CBWD3	68081866	0	0	0
CHB	CBWD3	68086866	0	0	0
CHB	CBWD3	68091866	0	0	0
CHB	CBWD3	68096866	0	0	0
CHB	CBWD3	68101866	0	0	0
CHB	CBWD3	68106866	0	0	0
CHB	CBWD3	68111866	0	0	0
CHB	CBWD3	68116866	0	0	0
CHB	CBWD3	68121866	0	0	0
CHB	CBWD3	68126866	0	0	0
CHB	CBWD3	68131866	0	0	0
CHB	CBWD3	68136866	2.1289	1.8636	0.3385
CHB	CBWD3	68141866	5.2292	4.4048	0.5188
CHB	CBWD3	68146866	5.8667	5.2519	0.3316
CHB	CBWD3	68151866	7.7929	6.4378	0.6093
CHB	CBWD3	68156866	6.8985	6.6072	0.1279
CHB	CBWD3	68161866	3.0445	4.4048	-0.856
CHB	CBWD3	68166866	5.2056	4.7436	0.2725
CHB	CBWD3	68171866	8.2878	6.7766	0.6487
CHB	CBWD3	68176866	10.2935	8.3013	0.7105
CHB	CBWD3	68181866	10.3821	8.6402	0.5988
CHB	CBWD3	68186866	7.0782	7.2848	-0.083
CHB	CBWD3	68191866	7.4698	6.946	0.2199
CHB	CBWD3	68196866	10.0811	8.1319	0.7085
CHB	CBWD3	68201866	10.744	8.979	0.5855
CHB	CBWD3	68206866	9.3015	7.4543	0.7271
CHB	CBWD3	68211866	6.6465	5.4213	0.6425
CHB	CBWD3	68216866	7.0335	5.7601	0.6327
CHB	CBWD3	68221866	8.4581	7.7931	0.2513
CHB	CBWD3	68226866	7.71	8.6402	-0.3197
CHB	CBWD3	68231866	5.2753	6.6072	-0.585
CHB	CBWD3	68236866	3.9539	5.0825	-0.6266
CHB	CBWD3	68241866	4.411	5.0825	-0.3728
CHB	CBWD3	68246866	5.055	5.0825	-0.0153
CHB	CBWD3	68251866	8.7114	6.0989	1.2332
CHB	CBWD3	68256866	6.9328	4.5742	1.4362

CHB	CBWD3	68261866	1.7198	2.7106	-0.9388
CHB	CBWD3	68266866	1.4479	4.5742	-1.9037
CHB	CBWD3	68271866	3.4681	6.2684	-1.2897
CHB	CBWD3	68276866	6.505	7.2848	-0.3135
CHB	CBWD3	68281866	6.561	6.4378	0.0554
CHB	CBWD3	68286866	7.8233	5.7601	1.025
CHB	CBWD3	68291866	7.9846	6.2684	0.7904
CHB	CBWD3	68296866	6.9246	7.2848	-0.1448
CHB	CBWD3	68301866	7.1965	8.3013	-0.394
CHB	CBWD3	68306866	4.7872	6.946	-0.9062
CHB	CBWD3	68311866	4.0032	6.6072	-1.1437
CHB	CBWD3	68316866	14.6313	11.6896	0.7629
CHB	CBWD3	68321866	16.2589	13.5532	0.6102
CHB	CBWD3	68326866	6.3899	9.3178	-0.9385
CHB	CBWD3	68331866	6.9269	9.8261	-0.8845
CHB	CBWD3	68336866	7.1968	8.4708	-0.446
CHB	CBWD3	68341866	7.2591	6.7766	0.2071
CHB	CBWD3	68346866	10.6663	9.4872	0.3717
CHB	CBWD3	68351866	8.9242	8.8096	0.0387
CHB	CBWD3	68356866	10.9197	7.9625	1.0958
CHB	CBWD3	68361866	12.4738	6.4378	2.714
CHB	CBWD3	68366866	8.6069	4.5742	2.4556
CHB	CBWD3	68371866	7.7647	4.913	1.6312
CHB	CBWD3	68376866	6.7065	5.9295	0.3762
CHB	CBWD3	68381866	13.3554	9.6567	1.1469
CHB	CBWD3	68386866	23.0175	12.5367	2.5446
CHB	CBWD3	68391866	16.4013	9.4872	2.1795
CHB	CBWD3	68396866	6.7598	7.2848	-0.211
CHB	CBWD3	68401866	6.6189	8.4708	-0.6483
CHB	CBWD3	68406866	7.4485	9.1484	-0.5542
CHB	CBWD3	68411866	25.8505	14.9085	2.254
CHB	CBWD3	68416866	37.1403	17.1109	3.6172
CHB	CBWD3	68421866	17.4223	8.979	2.8009
CHB	CBWD3	68426866	7.5804	6.2684	0.6043
CHB	CBWD3	68431866	10.8804	9.6567	0.3794
CHB	CBWD3	68436866	13.2827	12.3673	0.2251
CHB	CBWD3	68441866	14.9397	13.3838	0.3551
CHB	CBWD3	68446866	12.5696	11.3508	0.3249
CHB	CBWD3	68451866	7.8968	9.1484	-0.4081
CHB	CBWD3	68456866	6.7792	8.979	-0.7297
CHB	CBWD3	68461866	7.7394	8.3013	-0.2004
CHB	CBWD3	68466866	8.5031	7.2848	0.4897
CHB	CBWD3	68471866	9.8648	8.1319	0.6299
CHB	CBWD3	68476866	11.6097	8.8096	0.9454
CHB	CBWD3	68481866	9.1466	8.4708	0.2366
CHB	CBWD3	68486866	8.3352	9.8261	-0.4549
CHB	CBWD3	68491866	9.3234	11.012	-0.4632
CHB	CBWD3	68496866	8.6948	10.6731	-0.5587
CHB	CBWD3	68501866	9.4316	9.9955	-0.1693

CHB	CBWD3	68506866	11.5064	11.8591	-0.0902
CHB	CBWD3	68511866	8.1294	8.979	-0.2818
CHB	CBWD3	68516866	0.6219	1.5247	-1.3412
CHB	CBWD3	68521866	0	0	0
CHB	CBWD3	68526866	0	0	0
CHB	CBWD3	68531866	0	0	0
CHB	CBWD3	68536866	0	0	0
CHB	CBWD3	68541866	0	0	0
CHB	CBWD3	68546866	0	0	0
CHB	CBWD3	68551866	0	0	0
CHB	CBWD3	68556866	0	0	0
CHB	CBWD3	68561866	0	0	0
CHB	CBWD3	68566866	0	0	0
CHB	CBWD3	68571866	0	0	0
CHB	CBWD3	68576866	0	0	0
CHB	CBWD3	68581866	0	0	0
CHB	CBWD3	68586866	0	0	0
CHB	CBWD3	68591866	0	0	0
CHB	CBWD3	68596866	0	0	0
CHB	CBWD3	68601866	0	0	0
CHB	CBWD3	68606866	0	0	0
CHB	CBWD3	68611866	0	0	0
CHB	CBWD3	68616866	0	0	0
CHB	CBWD3	68621866	0	0	0
CHB	CBWD3	68626866	0	0	0
CHB	CBWD3	68631866	0	0	0
CHB	CBWD3	68636866	0	0	0
CHB	CBWD3	68641866	0	0	0
CHB	CBWD3	68646866	0	0	0
CHB	CBWD3	68651866	0	0	0
CHB	CBWD3	68656866	0	0	0
CHB	CBWD3	68661866	0.1354	1.1859	-1.8751
CHB	CBWD3	68666866	1.119	3.5577	-1.843
CHB	CBWD3	68671866	1.6764	3.5577	-1.4218
CHB	CBWD3	68676866	1.1936	2.033	-1.001
CHB	CBWD3	68681866	0.8686	2.3718	-1.5867
CHB	CBWD3	68686866	2.0221	3.3883	-1.0759
CHB	CBWD3	68691866	2.7323	3.2189	-0.4001
CHB	CBWD3	68696866	2.0988	2.3718	-0.2882
CHB	CBWD3	68701866	1.2732	2.033	-0.9061
CHB	CBWD3	68706866	1.0482	2.3718	-1.3972
CHB	CBWD3	68711866	0.8249	1.8636	-1.3252
CHB	CBWD3	68716866	0.068	1.1859	-1.9954
CHB	CBWD3	68721866	0.1329	1.1859	-1.8796
CHB	CBWD3	68726866	0.5553	1.6942	-1.563
CHB	CBWD3	68731866	1.9518	3.7271	-1.2897
CHB	CBWD3	68736866	1.8803	5.2519	-1.8184
CHB	CBWD3	68741866	3.4607	6.2684	-1.2931
CHB	CBWD3	68746866	3.6206	6.6072	-1.3117

CHB	CBWD3	68751866	2.6254	6.0989	-1.6396
CHB	CBWD3	68756866	3.8578	4.7436	-0.5225
CHB	CBWD3	68761866	2.257	3.0495	-0.6816
CHB	CBWD3	68766866	1.1294	3.7271	-1.8872
CHB	CBWD3	68771866	1.4242	4.066	-1.7815
CHB	CBWD3	68776866	1.7637	4.5742	-1.7114
CHB	CBWD3	68781866	2.3023	6.0989	-1.7922
CHB	CBWD3	68786866	2.229	6.0989	-1.8268
CHB	CBWD3	68791866	2.3954	5.5907	-1.6303
YRI	CBWD3	67746866	0.5556	0.5042	0.1615
YRI	CBWD3	67751866	0.5145	0.3361	0.7162
YRI	CBWD3	67756866	0.5145	0.3361	0.7162
YRI	CBWD3	67761866	0.0806	0.1681	-0.5202
YRI	CBWD3	67766866	0.9597	0.6722	0.7527
YRI	CBWD3	67771866	0.8791	0.5042	1.1782
YRI	CBWD3	67776866	0.3541	0.6722	-0.8328
YRI	CBWD3	67781866	0.4079	0.8403	-0.9766
YRI	CBWD3	67786866	1.0138	2.0167	-1.1991
YRI	CBWD3	67791866	1.4538	2.8571	-1.2691
YRI	CBWD3	67796866	1.0042	1.6806	-0.9305
YRI	CBWD3	67801866	0.598	0.8403	-0.5473
YRI	CBWD3	67806866	0.1832	0.5042	-1.0088
YRI	CBWD3	67811866	0.0956	0.3361	-0.9655
YRI	CBWD3	67816866	0	0	0
YRI	CBWD3	67821866	0.2561	1.0084	-1.4998
YRI	CBWD3	67826866	2.1626	2.8571	-0.6281
YRI	CBWD3	67831866	2.3956	2.3529	0.0452
YRI	CBWD3	67836866	1.2714	2.0167	-0.8911
YRI	CBWD3	67841866	1.5611	2.1848	-0.7003
YRI	CBWD3	67846866	1.7994	1.5126	0.4271
YRI	CBWD3	67851866	1.0576	1.1764	-0.2125
YRI	CBWD3	67856866	0.5235	0.8403	-0.7155
YRI	CBWD3	67861866	0.8802	0.6722	0.5445
YRI	CBWD3	67866866	1.3122	0.5042	2.5393
YRI	CBWD3	67871866	0.9185	0.3361	2.3381
YRI	CBWD3	67876866	0.1782	0.3361	-0.6339
YRI	CBWD3	67881866	0.1874	0.5042	-0.9956
YRI	CBWD3	67886866	1.0175	2.1848	-1.3106
YRI	CBWD3	67891866	1.1964	2.689	-1.4185
YRI	CBWD3	67896866	5.0366	5.7141	-0.3378
YRI	CBWD3	67901866	5.3022	6.2183	-0.4235
YRI	CBWD3	67906866	0.5796	1.5126	-1.3893
YRI	CBWD3	67911866	1.9399	2.1848	-0.275
YRI	CBWD3	67916866	3.2992	3.5293	-0.1745
YRI	CBWD3	67921866	2.044	2.5209	-0.4776
YRI	CBWD3	67926866	1.3557	1.5126	-0.2336
YRI	CBWD3	67931866	2.347	2.1848	0.1821
YRI	CBWD3	67936866	2.4371	2.689	-0.2394
YRI	CBWD3	67941866	0.9332	2.0167	-1.2955

YRI	CBWD3	67946866	1.6292	3.0251	-1.2042
YRI	CBWD3	67951866	2.3664	3.3612	-0.7859
YRI	CBWD3	67956866	0.7835	1.1764	-0.7027
YRI	CBWD3	67961866	1.5137	2.1848	-0.7535
YRI	CBWD3	67966866	1.7606	2.8571	-0.9916
YRI	CBWD3	67971866	0.2931	1.1764	-1.5798
YRI	CBWD3	67976866	0.0646	0.8403	-1.752
YRI	CBWD3	67981866	0.7481	1.3445	-0.9687
YRI	CBWD3	67986866	0.7479	1.1764	-0.7664
YRI	CBWD3	67991866	0.0183	0.1681	-0.8905
YRI	CBWD3	67996866	0	0	0
YRI	CBWD3	68001866	0	0	0
YRI	CBWD3	68006866	0	0	0
YRI	CBWD3	68011866	0	0	0
YRI	CBWD3	68016866	0	0	0
YRI	CBWD3	68021866	0	0	0
YRI	CBWD3	68026866	0	0	0
YRI	CBWD3	68031866	0	0	0
YRI	CBWD3	68036866	0	0	0
YRI	CBWD3	68041866	0	0	0
YRI	CBWD3	68046866	0	0	0
YRI	CBWD3	68051866	0	0	0
YRI	CBWD3	68056866	0	0	0
YRI	CBWD3	68061866	0	0	0
YRI	CBWD3	68066866	0	0	0
YRI	CBWD3	68071866	0	0	0
YRI	CBWD3	68076866	0	0	0
YRI	CBWD3	68081866	0	0	0
YRI	CBWD3	68086866	0	0	0
YRI	CBWD3	68091866	0	0	0
YRI	CBWD3	68096866	0	0	0
YRI	CBWD3	68101866	0	0	0
YRI	CBWD3	68106866	0	0	0
YRI	CBWD3	68111866	0	0	0
YRI	CBWD3	68116866	0	0	0
YRI	CBWD3	68121866	0	0	0
YRI	CBWD3	68126866	0	0	0
YRI	CBWD3	68131866	0	0	0
YRI	CBWD3	68136866	1.7521	1.6806	0.0984
YRI	CBWD3	68141866	5.1558	5.378	-0.1169
YRI	CBWD3	68146866	7.8968	9.7476	-0.567
YRI	CBWD3	68151866	10.4052	11.9324	-0.3871
YRI	CBWD3	68156866	8.0186	9.4115	-0.4408
YRI	CBWD3	68161866	3.8902	6.3864	-1.1266
YRI	CBWD3	68166866	6.5986	8.4031	-0.6342
YRI	CBWD3	68171866	9.7379	11.0921	-0.3677
YRI	CBWD3	68176866	12.1905	12.2685	-0.0193
YRI	CBWD3	68181866	13.1574	12.6047	0.1331
YRI	CBWD3	68186866	8.7745	8.7392	0.012

YRI	CBWD3	68191866	7.8885	7.0586	0.3421
YRI	CBWD3	68196866	11.5904	9.4115	0.6896
YRI	CBWD3	68201866	12.864	10.756	0.5891
YRI	CBWD3	68206866	10.5823	9.7476	0.2557
YRI	CBWD3	68211866	7.3838	7.7309	-0.1317
YRI	CBWD3	68216866	7.4991	8.5712	-0.37
YRI	CBWD3	68221866	9.499	10.924	-0.3925
YRI	CBWD3	68226866	10.1376	11.2602	-0.3005
YRI	CBWD3	68231866	7.5305	8.5712	-0.3591
YRI	CBWD3	68236866	4.9789	6.2183	-0.573
YRI	CBWD3	68241866	5.4335	6.7225	-0.5554
YRI	CBWD3	68246866	6.7224	7.5628	-0.3253
YRI	CBWD3	68251866	9.7797	9.9157	-0.041
YRI	CBWD3	68256866	8.2723	9.4115	-0.3605
YRI	CBWD3	68261866	3.5449	5.8822	-1.1357
YRI	CBWD3	68266866	3.0774	5.7141	-1.3148
YRI	CBWD3	68271866	5.0947	7.5628	-0.9555
YRI	CBWD3	68276866	8.9376	11.0921	-0.585
YRI	CBWD3	68281866	9.4098	10.2518	-0.2461
YRI	CBWD3	68286866	9.597	10.0837	-0.1445
YRI	CBWD3	68291866	9.6251	11.4282	-0.476
YRI	CBWD3	68296866	10.0322	11.9324	-0.4817
YRI	CBWD3	68301866	10.5025	11.5963	-0.2848
YRI	CBWD3	68306866	7.0769	8.7392	-0.5635
YRI	CBWD3	68311866	5.6404	8.235	-0.929
YRI	CBWD3	68316866	18.062	16.134	0.3671
YRI	CBWD3	68321866	21.2808	18.6549	0.435
YRI	CBWD3	68326866	9.2261	12.1005	-0.7191
YRI	CBWD3	68331866	9.4566	12.6047	-0.7578
YRI	CBWD3	68336866	9.1623	11.5963	-0.6338
YRI	CBWD3	68341866	8.3562	11.5963	-0.8437
YRI	CBWD3	68346866	11.7087	13.7811	-0.4585
YRI	CBWD3	68351866	10.1229	11.7644	-0.4217
YRI	CBWD3	68356866	12.0807	10.924	0.3186
YRI	CBWD3	68361866	13.1223	9.5795	1.103
YRI	CBWD3	68366866	9.4487	8.4031	0.3675
YRI	CBWD3	68371866	8.1526	9.2434	-0.351
YRI	CBWD3	68376866	7.1841	9.5795	-0.7458
YRI	CBWD3	68381866	14.168	10.5879	1.0153
YRI	CBWD3	68386866	23.7604	12.1005	2.9171
YRI	CBWD3	68391866	17.8987	11.4282	1.7082
YRI	CBWD3	68396866	7.8731	10.0837	-0.6561
YRI	CBWD3	68401866	7.9808	12.1005	-1.0307
YRI	CBWD3	68406866	10.3897	13.1089	-0.6308
YRI	CBWD3	68411866	28.3128	16.4701	2.2108
YRI	CBWD3	68416866	38.2597	17.3104	3.7289
YRI	CBWD3	68421866	18.1404	10.924	1.9875
YRI	CBWD3	68426866	9.0228	9.5795	-0.1733
YRI	CBWD3	68431866	12.7673	13.2769	-0.1168

YRI	CBWD3	68436866	14.4831	15.6298	-0.225
YRI	CBWD3	68441866	15.8388	16.4701	-0.1179
YRI	CBWD3	68446866	13.7484	15.2937	-0.3096
YRI	CBWD3	68451866	8.974	12.2685	-0.8136
YRI	CBWD3	68456866	7.9519	11.5963	-0.949
YRI	CBWD3	68461866	9.1281	10.924	-0.4946
YRI	CBWD3	68466866	9.3637	10.2518	-0.2596
YRI	CBWD3	68471866	11.1385	12.2685	-0.2791
YRI	CBWD3	68476866	13.8298	12.9408	0.2088
YRI	CBWD3	68481866	10.858	9.7476	0.3402
YRI	CBWD3	68486866	9.9825	12.4366	-0.5983
YRI	CBWD3	68491866	10.9406	14.1172	-0.6869
YRI	CBWD3	68496866	9.5284	13.7811	-0.9408
YRI	CBWD3	68501866	11	15.9659	-0.955
YRI	CBWD3	68506866	13.7821	18.4868	-0.7862
YRI	CBWD3	68511866	9.5768	13.7811	-0.9301
YRI	CBWD3	68516866	1.1737	2.689	-1.4401
YRI	CBWD3	68521866	0	0	0
YRI	CBWD3	68526866	0	0	0
YRI	CBWD3	68531866	0	0	0
YRI	CBWD3	68536866	0	0	0
YRI	CBWD3	68541866	0	0	0
YRI	CBWD3	68546866	0	0	0
YRI	CBWD3	68551866	0	0	0
YRI	CBWD3	68556866	0	0	0
YRI	CBWD3	68561866	0	0	0
YRI	CBWD3	68566866	0	0	0
YRI	CBWD3	68571866	0	0	0
YRI	CBWD3	68576866	0	0	0
YRI	CBWD3	68581866	0	0	0
YRI	CBWD3	68586866	0	0	0
YRI	CBWD3	68591866	0	0	0
YRI	CBWD3	68596866	0	0	0
YRI	CBWD3	68601866	0	0	0
YRI	CBWD3	68606866	0	0	0
YRI	CBWD3	68611866	0	0	0
YRI	CBWD3	68616866	0	0	0
YRI	CBWD3	68621866	0	0	0
YRI	CBWD3	68626866	0	0	0
YRI	CBWD3	68631866	0	0	0
YRI	CBWD3	68636866	0	0	0
YRI	CBWD3	68641866	0	0	0
YRI	CBWD3	68646866	0	0	0
YRI	CBWD3	68651866	0	0	0
YRI	CBWD3	68656866	0	0	0
YRI	CBWD3	68661866	0.1464	1.3445	-1.9459
YRI	CBWD3	68666866	1.5378	5.2099	-1.9876
YRI	CBWD3	68671866	2.1106	5.7141	-1.7969
YRI	CBWD3	68676866	1.5687	3.6974	-1.5516

YRI	CBWD3	68681866	1.1047	3.1932	-1.7224
YRI	CBWD3	68686866	2.5445	3.8654	-0.927
YRI	CBWD3	68691866	3.2321	4.2016	-0.6333
YRI	CBWD3	68696866	2.4836	3.5293	-0.7929
YRI	CBWD3	68701866	1.7463	3.1932	-1.1933
YRI	CBWD3	68706866	1.1097	3.1932	-1.7183
YRI	CBWD3	68711866	1.0419	3.1932	-1.7742
YRI	CBWD3	68716866	0.1562	1.6806	-2.0971
YRI	CBWD3	68721866	0.173	1.5126	-1.9947
YRI	CBWD3	68726866	0.7739	2.0167	-1.486
YRI	CBWD3	68731866	2.7295	3.6974	-0.7055
YRI	CBWD3	68736866	2.9228	5.378	-1.2921
YRI	CBWD3	68741866	4.1517	7.2267	-1.2408
YRI	CBWD3	68746866	5.0221	7.8989	-1.0702
YRI	CBWD3	68751866	3.9614	7.2267	-1.3176
YRI	CBWD3	68756866	3.1629	6.5544	-1.4952
YRI	CBWD3	68761866	1.8086	4.3696	-1.6171
YRI	CBWD3	68766866	2.0718	4.2016	-1.3912
YRI	CBWD3	68771866	2.4153	5.2099	-1.5127
YRI	CBWD3	68776866	2.4765	5.8822	-1.6549
YRI	CBWD3	68781866	2.5496	6.3864	-1.7317
YRI	CBWD3	68786866	3.0416	7.3947	-1.7201
YRI	CBWD3	68791866	3.0102	7.0586	-1.669
CEU	CLEC18A	69455232	7.8924	8.0164	-0.0458
CEU	CLEC18A	69460232	11.8712	9.8926	0.6016
CEU	CLEC18A	69465232	11.1668	9.8926	0.3874
CEU	CLEC18A	69470232	10.3867	10.0632	0.0968
CEU	CLEC18A	69475232	12.7627	10.2337	0.7451
CEU	CLEC18A	69480232	11.1146	8.187	1.0606
CEU	CLEC18A	69485232	7.256	6.3108	0.434
CEU	CLEC18A	69490232	8.6207	6.6519	0.862
CEU	CLEC18A	69495232	7.8584	6.9931	0.3621
CEU	CLEC18A	69500232	8.3599	8.8692	-0.1714
CEU	CLEC18A	69505232	10.2274	10.5749	-0.0993
CEU	CLEC18A	69510232	9.5854	9.5515	0.0107
CEU	CLEC18A	69515232	9.355	8.187	0.4231
CEU	CLEC18A	69520232	8.6955	7.6753	0.3921
CEU	CLEC18A	69525232	6.8462	6.4814	0.1635
CEU	CLEC18A	69530232	9.162	8.3576	0.2859
CEU	CLEC18A	69535232	12.6184	11.9394	0.1731
CEU	CLEC18A	69540232	11.3315	10.5749	0.2162
CEU	CLEC18A	69545232	10.7949	8.5281	0.7909
CEU	CLEC18A	69550232	14.1789	11.2571	0.7873
CEU	CLEC18A	69555232	13.7217	12.1099	0.4055
CEU	CLEC18A	69560232	12.1173	9.7221	0.7402
CEU	CLEC18A	69565232	11.4583	8.8692	0.8712
CEU	CLEC18A	69570232	5.9903	6.9931	-0.4196
CEU	CLEC18A	69575232	5.5391	6.3108	-0.3543
CEU	CLEC18A	69580232	11.2286	8.8692	0.7939

CEU	CLEC18A	69585232	10.907	8.8692	0.6857
CEU	CLEC18A	69590232	6.4088	6.9931	-0.2445
CEU	CLEC18A	69595232	6.3315	7.3342	-0.4018
CEU	CLEC18A	69600232	6.9233	8.187	-0.4578
CEU	CLEC18A	69605232	9.0614	8.8692	0.0647
CEU	CLEC18A	69610232	7.9542	7.5047	0.1764
CEU	CLEC18A	69615232	4.8369	6.4814	-0.7371
CEU	CLEC18A	69620232	7.135	6.9931	0.0594
CEU	CLEC18A	69625232	8.0486	7.3342	0.2862
CEU	CLEC18A	69630232	8.37	8.187	0.0663
CEU	CLEC18A	69635232	7.22	7.3342	-0.0458
CEU	CLEC18A	69640232	4.3125	6.1402	-0.8601
CEU	CLEC18A	69645232	5.5584	7.1636	-0.6571
CEU	CLEC18A	69650232	7.0959	7.6753	-0.2227
CEU	CLEC18A	69655232	7.4162	7.6753	-0.0996
CEU	CLEC18A	69660232	6.9922	6.4814	0.2289
CEU	CLEC18A	69665232	7.0903	6.3108	0.3579
CEU	CLEC18A	69670232	6.6895	6.6519	0.0165
CEU	CLEC18A	69675232	4.3364	4.7757	-0.2584
CEU	CLEC18A	69680232	2.4803	4.2641	-1.1579
CEU	CLEC18A	69685232	4.8487	6.4814	-0.7318
CEU	CLEC18A	69690232	8.093	8.5281	-0.1518
CEU	CLEC18A	69695232	8.0752	7.6753	0.1537
CEU	CLEC18A	69700232	8.7337	7.5047	0.4822
CEU	CLEC18A	69705232	7.0049	7.5047	-0.1961
CEU	CLEC18A	69710232	4.8726	5.9697	-0.5295
CEU	CLEC18A	69715232	7.546	7.3342	0.0849
CEU	CLEC18A	69720232	8.1221	8.0164	0.039
CEU	CLEC18A	69725232	3.988	5.1169	-0.6249
CEU	CLEC18A	69730232	3.9965	4.6052	-0.3696
CEU	CLEC18A	69735232	7.081	6.8225	0.1106
CEU	CLEC18A	69740232	6.6681	6.3108	0.164
CEU	CLEC18A	69745232	5.16	5.9697	-0.3908
CEU	CLEC18A	69750232	5.4706	8.6987	-1.1059
CEU	CLEC18A	69755232	6.0437	9.3809	-1.0661
CEU	CLEC18A	69760232	6.0295	9.2104	-1.0336
CEU	CLEC18A	69765232	6.5422	8.8692	-0.783
CEU	CLEC18A	69770232	8.724	8.5281	0.0684
CEU	CLEC18A	69775232	7.2713	8.5281	-0.4385
CEU	CLEC18A	69780232	5.6316	8.0164	-0.8808
CEU	CLEC18A	69785232	7.0853	7.5047	-0.1646
CEU	CLEC18A	69790232	5.0066	6.8225	-0.777
CEU	CLEC18A	69795232	3.5929	6.4814	-1.2947
CEU	CLEC18A	69800232	5.7755	6.8225	-0.448
CEU	CLEC18A	69805232	5.568	6.4814	-0.4094
CEU	CLEC18A	69810232	5.9879	6.9931	-0.4206
CEU	CLEC18A	69815232	7.6122	8.5281	-0.3196
CEU	CLEC18A	69820232	5.9394	7.3342	-0.5589
CEU	CLEC18A	69825232	3.6151	5.7991	-1.0817

CEU	CLEC18A	69830232	6.6013	6.3108	0.1334
CEU	CLEC18A	69835232	8.2618	6.1402	0.9984
CEU	CLEC18A	69840232	4.8978	5.1169	-0.1213
CEU	CLEC18A	69845232	4.4721	5.458	-0.5154
CEU	CLEC18A	69850232	5.0893	7.5047	-0.9477
CEU	CLEC18A	69855232	7.129	8.5281	-0.4882
CEU	CLEC18A	69860232	11.032	9.2104	0.5919
CEU	CLEC18A	69865232	9.133	8.3576	0.2756
CEU	CLEC18A	69870232	6.2946	7.5047	-0.4748
CEU	CLEC18A	69875232	6.6228	8.0164	-0.5147
CEU	CLEC18A	69880232	12.637	10.0632	0.7702
CEU	CLEC18A	69885232	15.3501	11.4277	1.0421
CEU	CLEC18A	69890232	9.1345	7.8459	0.4854
CEU	CLEC18A	69895232	11.6395	6.6519	2.1837
CEU	CLEC18A	69900232	15.71	9.2104	2.112
CEU	CLEC18A	69905232	12.2672	8.5281	1.3046
CEU	CLEC18A	69910232	8.4923	8.3576	0.0479
CEU	CLEC18A	69915232	6.6193	9.7221	-0.9589
CEU	CLEC18A	69920232	4.8427	7.3342	-0.9983
CEU	CLEC18A	69925232	5.1115	4.6052	0.3074
CEU	CLEC18A	69930232	5.5267	4.7757	0.4417
CEU	CLEC18A	69935232	3.88	5.7991	-0.9505
CEU	CLEC18A	69940232	4.6368	6.9931	-0.9859
CEU	CLEC18A	69945232	11.5424	8.8692	0.8995
CEU	CLEC18A	69950232	11.3791	8.3576	1.0741
CEU	CLEC18A	69955232	8.2651	7.8459	0.1579
CEU	CLEC18A	69960232	10.6799	8.0164	0.9838
CEU	CLEC18A	69965232	13.3927	8.6987	1.6081
CEU	CLEC18A	69970232	12.9882	8.6987	1.4696
CEU	CLEC18A	69975232	11.2381	8.0164	1.1899
CEU	CLEC18A	69980232	7.5737	5.7991	0.8789
CEU	CLEC18A	69985232	8.8644	6.3108	1.1724
CEU	CLEC18A	69990232	8.9116	5.2874	1.9487
CEU	CLEC18A	69995232	6.3968	4.2641	1.3844
CEU	CLEC18A	70000232	9.1894	6.6519	1.111
CEU	CLEC18A	70005232	10.6753	7.3342	1.3387
CEU	CLEC18A	70010232	15.384	11.5982	0.9919
CEU	CLEC18A	70015232	17.3568	12.9627	1.0366
CEU	CLEC18A	70020232	17.6479	11.2571	1.7221
CEU	CLEC18A	70025232	15.3515	10.0632	1.5826
CEU	CLEC18A	70030232	13.8143	8.5281	1.8444
CEU	CLEC18A	70035232	15.6745	8.187	2.7126
CEU	CLEC18A	70040232	11.8794	7.3342	1.8211
CEU	CLEC18A	70045232	11.0921	8.187	1.0525
CEU	CLEC18A	70050232	12.1097	8.0164	1.5119
CEU	CLEC18A	70055232	8.067	7.3342	0.2936
CEU	CLEC18A	70060232	9.1721	8.5281	0.2247
CEU	CLEC18A	70065232	9.4212	6.9931	1.016
CEU	CLEC18A	70070232	6.5939	5.2874	0.7025

CEU	CLEC18A	70075232	7.5242	5.6286	0.9642
CEU	CLEC18A	70080232	11.5267	7.1636	1.786
CEU	CLEC18A	70085232	13.5531	7.6753	2.2593
CEU	CLEC18A	70090232	11.6015	7.5047	1.6074
CEU	CLEC18A	70095232	9.5201	7.1636	0.9646
CEU	CLEC18A	70100232	10.2393	7.3342	1.164
CEU	CLEC18A	70105232	13.141	9.2104	1.2772
CEU	CLEC18A	70110232	13.8093	9.7221	1.2631
CEU	CLEC18A	70115232	14.3513	9.3809	1.5879
CEU	CLEC18A	70120232	10.4162	6.6519	1.6481
CEU	CLEC18A	70125232	4.5354	3.5818	0.7187
CEU	CLEC18A	70130232	5.8341	5.6286	0.1045
CEU	CLEC18A	70135232	9.31	7.6753	0.6283
CEU	CLEC18A	70140232	10.054	6.9931	1.2807
CEU	CLEC18A	70145232	11.2548	6.8225	1.8966
CEU	CLEC18A	70150232	13.572	7.5047	2.3805
CEU	CLEC18A	70155232	11.4638	8.6987	0.9473
CEU	CLEC18A	70160232	8.207	7.5047	0.2755
CEU	CLEC18A	70165232	8.3581	6.8225	0.6571
CEU	CLEC18A	70170232	5.1309	6.4814	-0.6053
CEU	CLEC18A	70175232	3.1999	6.4814	-1.4708
CEU	CLEC18A	70180232	6.3834	7.1636	-0.3194
CEU	CLEC18A	70185232	11.23	8.187	1.1024
CEU	CLEC18A	70190232	12.7772	10.916	0.5162
CEU	CLEC18A	70195232	10.2224	11.5982	-0.3605
CEU	CLEC18A	70200232	5.3929	7.3342	-0.7778
CEU	CLEC18A	70205232	1.7613	3.4112	-1.2959
CEU	CLEC18A	70210232	2.0167	2.729	-0.6733
CEU	CLEC18A	70215232	1.1841	1.1939	-0.0175
CEU	CLEC18A	70220232	0.6479	1.7056	-1.4486
CEU	CLEC18A	70225232	2.1187	4.0935	-1.3279
CEU	CLEC18A	70230232	4.04	5.7991	-0.8713
CEU	CLEC18A	70235232	5.5209	9.5515	-1.2663
CEU	CLEC18A	70240232	5.4552	9.3809	-1.2541
CEU	CLEC18A	70245232	2.2791	5.1169	-1.5708
CEU	CLEC18A	70250232	1.3542	3.9229	-1.7916
CEU	CLEC18A	70255232	2.2816	2.8996	-0.5558
CEU	CLEC18A	70260232	1.5819	3.0701	-1.2766
CEU	CLEC18A	70265232	0.7951	3.7524	-2.1425
CEU	CLEC18A	70270232	1.3785	4.2641	-1.8732
CEU	CLEC18A	70275232	1.1431	3.5818	-1.838
CEU	CLEC18A	70280232	1.8642	3.4112	-1.215
CEU	CLEC18A	70285232	2.9138	4.9463	-1.1591
CEU	CLEC18A	70290232	2.361	6.4814	-1.8468
CEU	CLEC18A	70295232	2.638	6.1402	-1.648
CEU	CLEC18A	70300232	3.2397	4.4346	-0.7497
CEU	CLEC18A	70305232	3.1107	5.1169	-1.1105
CEU	CLEC18A	70310232	1.477	4.2641	-1.8092
CEU	CLEC18A	70315232	1.0664	4.0935	-2.0355

CEU	CLEC18A	70320232	2.0557	4.6052	-1.5479
CEU	CLEC18A	70325232	1.5104	3.0701	-1.3379
CEU	CLEC18A	70330232	0.952	3.5818	-1.9821
CEU	CLEC18A	70335232	1.5922	4.9463	-1.9128
CEU	CLEC18A	70340232	2.8299	5.2874	-1.3214
CEU	CLEC18A	70345232	2.1113	4.6052	-1.5142
CEU	CLEC18A	70350232	2.3948	4.4346	-1.2798
CEU	CLEC18A	70355232	2.2521	4.0935	-1.2382
CEU	CLEC18A	70360232	1.8334	3.9229	-1.4574
CEU	CLEC18A	70365232	2.416	4.7757	-1.3878
CEU	CLEC18A	70370232	1.3504	4.4346	-1.9351
CEU	CLEC18A	70375232	1.7559	4.6052	-1.7299
CEU	CLEC18A	70380232	3.8503	5.2874	-0.7727
CEU	CLEC18A	70385232	4.6654	5.1169	-0.2499
CEU	CLEC18A	70390232	4.3342	4.6052	-0.1645
CEU	CLEC18A	70395232	5.1315	4.4346	0.4373
CEU	CLEC18A	70400232	4.8743	4.9463	-0.0411
CEU	CLEC18A	70405232	3.7089	5.6286	-0.9765
CEU	CLEC18A	70410232	3.5663	7.3342	-1.5097
CEU	CLEC18A	70415232	2.8229	6.8225	-1.7114
CEU	CLEC18A	70420232	1.5389	4.2641	-1.769
CEU	CLEC18A	70425232	3.0748	4.0935	-0.685
CEU	CLEC18A	70430232	3.2484	4.4346	-0.7443
CEU	CLEC18A	70435232	2.3894	3.7524	-0.9875
CEU	CLEC18A	70440232	4.2902	4.4346	-0.0906
CEU	CLEC18A	70445232	4.1569	5.458	-0.6802
CEU	CLEC18A	70450232	2.3055	4.6052	-1.3963
CEU	CLEC18A	70455232	1.6001	2.8996	-1.1687
CEU	CLEC18A	70460232	1.6384	3.2407	-1.3139
CHB	CLEC18A	69455232	6.1365	9.1484	-0.982
CHB	CLEC18A	69460232	9.122	9.9955	-0.2623
CHB	CLEC18A	69465232	7.2534	9.1484	-0.6178
CHB	CLEC18A	69470232	6.1074	8.6402	-0.8706
CHB	CLEC18A	69475232	9.2479	10.3343	-0.3162
CHB	CLEC18A	69480232	8.248	9.3178	-0.3429
CHB	CLEC18A	69485232	5.4306	7.9625	-0.9382
CHB	CLEC18A	69490232	7.7986	9.9955	-0.6597
CHB	CLEC18A	69495232	6.92	8.3013	-0.4926
CHB	CLEC18A	69500232	6.9245	7.1154	-0.0784
CHB	CLEC18A	69505232	8.008	10.6731	-0.7527
CHB	CLEC18A	69510232	5.8572	11.012	-1.4139
CHB	CLEC18A	69515232	6.3621	8.4708	-0.7382
CHB	CLEC18A	69520232	8.1925	8.3013	-0.0388
CHB	CLEC18A	69525232	6.7502	7.4543	-0.2772
CHB	CLEC18A	69530232	7.3731	9.3178	-0.6234
CHB	CLEC18A	69535232	10.7668	11.1814	-0.1121
CHB	CLEC18A	69540232	9.5029	8.8096	0.2341
CHB	CLEC18A	69545232	7.7871	7.6237	0.063
CHB	CLEC18A	69550232	13.0166	10.5037	0.7204

CHB	CLEC18A	69555232	14.9949	10.8426	1.1556
CHB	CLEC18A	69560232	11.3373	9.3178	0.6473
CHB	CLEC18A	69565232	8.336	9.3178	-0.3147
CHB	CLEC18A	69570232	4.3019	6.4378	-0.9604
CHB	CLEC18A	69575232	4.242	6.6072	-1.0388
CHB	CLEC18A	69580232	8.162	9.4872	-0.4177
CHB	CLEC18A	69585232	7.6442	9.4872	-0.581
CHB	CLEC18A	69590232	4.2054	8.8096	-1.5545
CHB	CLEC18A	69595232	5.5761	8.4708	-1.0134
CHB	CLEC18A	69600232	7.3958	7.2848	0.0446
CHB	CLEC18A	69605232	9.0495	7.1154	0.7943
CHB	CLEC18A	69610232	7.2362	6.6072	0.2763
CHB	CLEC18A	69615232	5.0855	6.6072	-0.6683
CHB	CLEC18A	69620232	8.4274	7.7931	0.2397
CHB	CLEC18A	69625232	8.833	8.6402	0.0663
CHB	CLEC18A	69630232	9.3735	8.4708	0.316
CHB	CLEC18A	69635232	8.1744	7.2848	0.3576
CHB	CLEC18A	69640232	3.8085	7.7931	-1.506
CHB	CLEC18A	69645232	5.605	9.1484	-1.1553
CHB	CLEC18A	69650232	7.5553	8.979	-0.4723
CHB	CLEC18A	69655232	7.0084	8.3013	-0.4611
CHB	CLEC18A	69660232	6.7702	6.4378	0.1495
CHB	CLEC18A	69665232	7.538	7.2848	0.1018
CHB	CLEC18A	69670232	8.3646	8.1319	0.0846
CHB	CLEC18A	69675232	7.032	6.6072	0.1866
CHB	CLEC18A	69680232	4.0516	4.7436	-0.4082
CHB	CLEC18A	69685232	5.1551	5.5907	-0.2223
CHB	CLEC18A	69690232	9.3699	8.8096	0.1892
CHB	CLEC18A	69695232	9.9901	9.4872	0.1585
CHB	CLEC18A	69700232	9.8519	9.6567	0.0605
CHB	CLEC18A	69705232	9.0409	8.6402	0.1377
CHB	CLEC18A	69710232	6.4005	7.7931	-0.5263
CHB	CLEC18A	69715232	8.9542	10.1649	-0.3579
CHB	CLEC18A	69720232	11.2061	9.8261	0.421
CHB	CLEC18A	69725232	5.7636	5.5907	0.0882
CHB	CLEC18A	69730232	4.79	3.8965	0.6249
CHB	CLEC18A	69735232	8.2088	6.4378	0.7963
CHB	CLEC18A	69740232	7.9633	7.1154	0.3482
CHB	CLEC18A	69745232	6.1616	6.0989	0.0296
CHB	CLEC18A	69750232	8.2161	6.7766	0.6179
CHB	CLEC18A	69755232	9.2947	7.9625	0.4937
CHB	CLEC18A	69760232	8.7114	8.979	-0.0888
CHB	CLEC18A	69765232	10.2225	9.4872	0.2318
CHB	CLEC18A	69770232	11.1159	8.979	0.7089
CHB	CLEC18A	69775232	7.6659	9.1484	-0.4834
CHB	CLEC18A	69780232	7.3158	9.4872	-0.6845
CHB	CLEC18A	69785232	9.9621	7.6237	0.9018
CHB	CLEC18A	69790232	5.9254	6.2684	-0.158
CHB	CLEC18A	69795232	3.4161	5.4213	-1.0515

CHB	CLEC18A	69800232	6.4085	6.6072	-0.0873
CHB	CLEC18A	69805232	6.817	7.6237	-0.3111
CHB	CLEC18A	69810232	9.1002	9.1484	-0.0157
CHB	CLEC18A	69815232	12.3404	10.1649	0.6431
CHB	CLEC18A	69820232	9.787	8.4708	0.4608
CHB	CLEC18A	69825232	6.873	8.6402	-0.6075
CHB	CLEC18A	69830232	8.6419	10.6731	-0.5737
CHB	CLEC18A	69835232	9.6596	9.4872	0.0543
CHB	CLEC18A	69840232	5.9337	7.2848	-0.5431
CHB	CLEC18A	69845232	5.0694	8.6402	-1.2274
CHB	CLEC18A	69850232	4.7421	8.4708	-1.3053
CHB	CLEC18A	69855232	6.1993	8.3013	-0.7497
CHB	CLEC18A	69860232	12.4129	9.4872	0.9222
CHB	CLEC18A	69865232	13.5951	8.979	1.5313
CHB	CLEC18A	69870232	9.8886	7.2848	1.0466
CHB	CLEC18A	69875232	8.281	7.1154	0.4787
CHB	CLEC18A	69880232	14.7302	11.6896	0.7885
CHB	CLEC18A	69885232	19.4954	12.3673	1.753
CHB	CLEC18A	69890232	10.5087	8.979	0.5074
CHB	CLEC18A	69895232	5.7491	9.4872	-1.1783
CHB	CLEC18A	69900232	6.8323	11.1814	-1.176
CHB	CLEC18A	69905232	5.5288	10.3343	-1.3988
CHB	CLEC18A	69910232	4.446	8.8096	-1.4733
CHB	CLEC18A	69915232	2.9157	8.1319	-1.896
CHB	CLEC18A	69920232	2.4133	6.0989	-1.7398
CHB	CLEC18A	69925232	3.2972	4.913	-0.9242
CHB	CLEC18A	69930232	3.3398	5.7601	-1.2025
CHB	CLEC18A	69935232	3.6429	6.946	-1.3865
CHB	CLEC18A	69940232	5.1913	8.979	-1.2565
CHB	CLEC18A	69945232	5.9484	11.1814	-1.415
CHB	CLEC18A	69950232	4.8155	8.979	-1.3812
CHB	CLEC18A	69955232	5.4794	7.2848	-0.7257
CHB	CLEC18A	69960232	6.7355	8.3013	-0.5584
CHB	CLEC18A	69965232	5.9945	10.1649	-1.2328
CHB	CLEC18A	69970232	5.3977	11.3508	-1.5871
CHB	CLEC18A	69975232	6.1761	10.3343	-1.2104
CHB	CLEC18A	69980232	4.6865	6.2684	-0.7286
CHB	CLEC18A	69985232	5.1571	6.6072	-0.6369
CHB	CLEC18A	69990232	5.1963	5.7601	-0.2801
CHB	CLEC18A	69995232	4.2157	4.066	0.1009
CHB	CLEC18A	70000232	5.5616	6.7766	-0.5216
CHB	CLEC18A	70005232	6.6541	7.4543	-0.315
CHB	CLEC18A	70010232	10.3817	12.1979	-0.4525
CHB	CLEC18A	70015232	9.4358	14.7391	-1.1044
CHB	CLEC18A	70020232	8.6556	13.3838	-1.0791
CHB	CLEC18A	70025232	8.3151	11.8591	-0.9067
CHB	CLEC18A	70030232	5.6943	9.9955	-1.2916
CHB	CLEC18A	70035232	6.968	11.1814	-1.1393
CHB	CLEC18A	70040232	5.9585	10.8426	-1.3593

CHB	CLEC18A	70045232	5.0636	10.8426	-1.6083
CHB	CLEC18A	70050232	5.8597	12.1979	-1.5791
CHB	CLEC18A	70055232	3.8254	9.8261	-1.8308
CHB	CLEC18A	70060232	5.2096	9.1484	-1.2842
CHB	CLEC18A	70065232	5.8568	8.8096	-0.9969
CHB	CLEC18A	70070232	4.6469	7.7931	-1.1891
CHB	CLEC18A	70075232	4.5229	8.1319	-1.3118
CHB	CLEC18A	70080232	5.1359	10.1649	-1.4866
CHB	CLEC18A	70085232	7.2515	11.6896	-1.1509
CHB	CLEC18A	70090232	6.4108	9.4872	-0.9697
CHB	CLEC18A	70095232	5.2332	8.6402	-1.1711
CHB	CLEC18A	70100232	6.7516	10.8426	-1.1385
CHB	CLEC18A	70105232	7.4361	10.8426	-0.948
CHB	CLEC18A	70110232	6.7878	9.4872	-0.8509
CHB	CLEC18A	70115232	8.8381	10.3343	-0.4355
CHB	CLEC18A	70120232	8.206	9.6567	-0.4498
CHB	CLEC18A	70125232	4.1116	6.0989	-0.9381
CHB	CLEC18A	70130232	4.7885	6.0989	-0.6186
CHB	CLEC18A	70135232	7.281	7.4543	-0.0682
CHB	CLEC18A	70140232	7.3451	6.7766	0.244
CHB	CLEC18A	70145232	8.0213	8.4708	-0.1574
CHB	CLEC18A	70150232	10.2637	11.8591	-0.4082
CHB	CLEC18A	70155232	9.0497	10.6731	-0.4585
CHB	CLEC18A	70160232	6.8148	8.6402	-0.6275
CHB	CLEC18A	70165232	8.1022	10.8426	-0.7627
CHB	CLEC18A	70170232	6.1656	9.1484	-0.9725
CHB	CLEC18A	70175232	3.7333	7.7931	-1.5344
CHB	CLEC18A	70180232	5.398	8.979	-1.1879
CHB	CLEC18A	70185232	9.0526	9.8261	-0.236
CHB	CLEC18A	70190232	10.7716	11.3508	-0.1544
CHB	CLEC18A	70195232	8.8077	12.0285	-0.8131
CHB	CLEC18A	70200232	4.5705	7.2848	-1.0911
CHB	CLEC18A	70205232	1.8007	3.3883	-1.2503
CHB	CLEC18A	70210232	1.6195	3.3883	-1.393
CHB	CLEC18A	70215232	0.5271	1.5247	-1.4821
CHB	CLEC18A	70220232	0.6914	1.6942	-1.3762
CHB	CLEC18A	70225232	2.1626	5.0825	-1.6211
CHB	CLEC18A	70230232	3.1517	7.9625	-1.7827
CHB	CLEC18A	70235232	4.8972	10.6731	-1.6313
CHB	CLEC18A	70240232	5.7293	12.3673	-1.6324
CHB	CLEC18A	70245232	2.5974	8.4708	-2.0561
CHB	CLEC18A	70250232	1.5313	5.5907	-2.0712
CHB	CLEC18A	70255232	2.3507	5.5907	-1.6531
CHB	CLEC18A	70260232	1.7129	5.7601	-2.0107
CHB	CLEC18A	70265232	1.4184	6.4378	-2.2569
CHB	CLEC18A	70270232	1.8865	7.1154	-2.1474
CHB	CLEC18A	70275232	1.5148	7.2848	-2.3193
CHB	CLEC18A	70280232	2.1626	7.9625	-2.1493
CHB	CLEC18A	70285232	3.0579	7.9625	-1.8175

CHB	CLEC18A	70290232	2.4209	7.1154	-1.9279
CHB	CLEC18A	70295232	2.9465	7.6237	-1.8037
CHB	CLEC18A	70300232	3.5121	8.4708	-1.7359
CHB	CLEC18A	70305232	3.4198	10.8426	-2.0658
CHB	CLEC18A	70310232	2.3676	9.9955	-2.2905
CHB	CLEC18A	70315232	1.6973	7.9625	-2.3217
CHB	CLEC18A	70320232	2.3853	7.4543	-1.9953
CHB	CLEC18A	70325232	2.1782	7.4543	-2.0768
CHB	CLEC18A	70330232	1.7752	7.1154	-2.1931
CHB	CLEC18A	70335232	2.2907	6.2684	-1.832
CHB	CLEC18A	70340232	2.5611	8.1319	-2.0248
CHB	CLEC18A	70345232	1.7821	8.6402	-2.3574
CHB	CLEC18A	70350232	3.1382	7.9625	-1.7877
CHB	CLEC18A	70355232	3.3809	6.7766	-1.4577
CHB	CLEC18A	70360232	3.1352	7.6237	-1.7309
CHB	CLEC18A	70365232	3.1603	7.9625	-1.7795
CHB	CLEC18A	70370232	1.491	6.7766	-2.2689
CHB	CLEC18A	70375232	1.5245	6.0989	-2.1593
CHB	CLEC18A	70380232	3.2913	7.6237	-1.6707
CHB	CLEC18A	70385232	4.4724	8.6402	-1.4326
CHB	CLEC18A	70390232	4.346	6.6072	-0.9931
CHB	CLEC18A	70395232	4.3328	5.9295	-0.773
CHB	CLEC18A	70400232	4.1807	7.1154	-1.2052
CHB	CLEC18A	70405232	3.4514	7.6237	-1.609
CHB	CLEC18A	70410232	3.2477	6.4378	-1.4344
CHB	CLEC18A	70415232	2.6381	5.5907	-1.5065
CHB	CLEC18A	70420232	1.2751	5.2519	-2.1448
CHB	CLEC18A	70425232	2.2035	5.5907	-1.7282
CHB	CLEC18A	70430232	2.2859	5.9295	-1.7639
CHB	CLEC18A	70435232	2.5978	6.946	-1.8252
CHB	CLEC18A	70440232	4.7061	8.4708	-1.3179
CHB	CLEC18A	70445232	4.3543	10.8426	-1.8057
CHB	CLEC18A	70450232	2.3533	10.5037	-2.3367
CHB	CLEC18A	70455232	1.5646	7.9625	-2.3709
CHB	CLEC18A	70460232	1.9532	6.7766	-2.0705
YRI	CLEC18A	69455232	8.1326	11.7644	-0.933
YRI	CLEC18A	69460232	11.1871	13.1089	-0.4458
YRI	CLEC18A	69465232	11.2313	11.9324	-0.1777
YRI	CLEC18A	69470232	11.416	11.9324	-0.1309
YRI	CLEC18A	69475232	13.2491	14.2853	-0.2215
YRI	CLEC18A	69480232	10.8607	12.7727	-0.4545
YRI	CLEC18A	69485232	8.197	10.4199	-0.6399
YRI	CLEC18A	69490232	8.7743	11.5963	-0.7349
YRI	CLEC18A	69495232	6.7747	9.5795	-0.8732
YRI	CLEC18A	69500232	7.5088	10.0837	-0.7643
YRI	CLEC18A	69505232	9.6916	12.6047	-0.7013
YRI	CLEC18A	69510232	9.5684	11.9324	-0.5993
YRI	CLEC18A	69515232	9.893	12.7727	-0.6846
YRI	CLEC18A	69520232	9.704	13.613	-0.8749

YRI	CLEC18A	69525232	7.5032	11.9324	-1.1228
YRI	CLEC18A	69530232	11.7573	15.1256	-0.6821
YRI	CLEC18A	69535232	15.9047	16.302	-0.0749
YRI	CLEC18A	69540232	13.209	14.9575	-0.3578
YRI	CLEC18A	69545232	12.338	15.4617	-0.6194
YRI	CLEC18A	69550232	16.1328	16.9743	-0.1526
YRI	CLEC18A	69555232	14.625	15.2937	-0.134
YRI	CLEC18A	69560232	11.1792	12.7727	-0.3788
YRI	CLEC18A	69565232	10.9538	13.7811	-0.6255
YRI	CLEC18A	69570232	7.1449	10.5879	-0.9764
YRI	CLEC18A	69575232	7.8096	11.7644	-1.016
YRI	CLEC18A	69580232	14.1974	16.134	-0.3687
YRI	CLEC18A	69585232	13.588	15.1256	-0.3114
YRI	CLEC18A	69590232	8.0716	13.613	-1.2403
YRI	CLEC18A	69595232	8.0691	13.2769	-1.1936
YRI	CLEC18A	69600232	8.2312	11.0921	-0.7767
YRI	CLEC18A	69605232	9.6036	11.9324	-0.5903
YRI	CLEC18A	69610232	9.1263	12.1005	-0.7441
YRI	CLEC18A	69615232	6.2786	9.5795	-1.0277
YRI	CLEC18A	69620232	9.0534	10.5879	-0.4352
YRI	CLEC18A	69625232	10.8127	11.5963	-0.2041
YRI	CLEC18A	69630232	10.0139	11.0921	-0.2927
YRI	CLEC18A	69635232	9.5215	10.924	-0.3863
YRI	CLEC18A	69640232	7.7221	9.7476	-0.6205
YRI	CLEC18A	69645232	8.7696	9.4115	-0.2032
YRI	CLEC18A	69650232	10.2522	10.924	-0.185
YRI	CLEC18A	69655232	9.0139	11.4282	-0.6374
YRI	CLEC18A	69660232	7.5419	8.7392	-0.4059
YRI	CLEC18A	69665232	8.6894	9.9157	-0.3697
YRI	CLEC18A	69670232	11.2445	12.9408	-0.3983
YRI	CLEC18A	69675232	9.1415	10.0837	-0.2797
YRI	CLEC18A	69680232	5.5341	8.067	-0.9243
YRI	CLEC18A	69685232	7.7586	9.5795	-0.5669
YRI	CLEC18A	69690232	11.2405	12.4366	-0.2916
YRI	CLEC18A	69695232	10.7118	13.2769	-0.5879
YRI	CLEC18A	69700232	10.3531	12.4366	-0.508
YRI	CLEC18A	69705232	9.1929	10.5879	-0.3956
YRI	CLEC18A	69710232	7.328	8.9073	-0.526
YRI	CLEC18A	69715232	11.7442	10.756	0.2761
YRI	CLEC18A	69720232	13.3026	11.7644	0.3952
YRI	CLEC18A	69725232	6.2343	7.0586	-0.3398
YRI	CLEC18A	69730232	4.3886	5.2099	-0.4446
YRI	CLEC18A	69735232	8.1899	9.5795	-0.4326
YRI	CLEC18A	69740232	8.4455	10.4199	-0.5684
YRI	CLEC18A	69745232	7.4483	9.9157	-0.7439
YRI	CLEC18A	69750232	7.9953	10.0837	-0.6199
YRI	CLEC18A	69755232	8.9383	10.756	-0.508
YRI	CLEC18A	69760232	10.3066	14.7895	-0.9274
YRI	CLEC18A	69765232	9.534	14.7895	-1.0872

YRI	CLEC18A	69770232	9.594	12.4366	-0.693
YRI	CLEC18A	69775232	8.0943	12.2685	-1.0308
YRI	CLEC18A	69780232	7.0887	12.7727	-1.3513
YRI	CLEC18A	69785232	8.1107	11.4282	-0.8758
YRI	CLEC18A	69790232	6.7459	10.2518	-1.0247
YRI	CLEC18A	69795232	5.5038	11.5963	-1.5865
YRI	CLEC18A	69800232	6.5383	11.2602	-1.2641
YRI	CLEC18A	69805232	7.2715	8.7392	-0.4975
YRI	CLEC18A	69810232	10.4022	11.5963	-0.311
YRI	CLEC18A	69815232	13.0286	14.6214	-0.3331
YRI	CLEC18A	69820232	9.7169	12.9408	-0.757
YRI	CLEC18A	69825232	10.1205	14.9575	-0.9899
YRI	CLEC18A	69830232	16.3495	16.4701	-0.0225
YRI	CLEC18A	69835232	16.4447	13.9492	0.5458
YRI	CLEC18A	69840232	10.7798	11.4282	-0.1712
YRI	CLEC18A	69845232	10.1007	13.2769	-0.7279
YRI	CLEC18A	69850232	7.3589	12.4366	-1.2379
YRI	CLEC18A	69855232	7.7134	11.5963	-1.0111
YRI	CLEC18A	69860232	13.4766	14.7895	-0.2716
YRI	CLEC18A	69865232	13.1294	14.9575	-0.3741
YRI	CLEC18A	69870232	9.3239	11.4282	-0.5555
YRI	CLEC18A	69875232	7.6931	10.756	-0.8559
YRI	CLEC18A	69880232	15.3345	16.302	-0.1824
YRI	CLEC18A	69885232	18.6351	16.4701	0.4042
YRI	CLEC18A	69890232	13.6989	13.9492	-0.0547
YRI	CLEC18A	69895232	14.6944	14.9575	-0.0538
YRI	CLEC18A	69900232	15.3713	17.9827	-0.4481
YRI	CLEC18A	69905232	11.5966	17.6465	-1.0572
YRI	CLEC18A	69910232	10.0278	15.9659	-1.142
YRI	CLEC18A	69915232	11.2401	17.4785	-1.1002
YRI	CLEC18A	69920232	10.4245	16.302	-1.108
YRI	CLEC18A	69925232	13.0829	17.1423	-0.7293
YRI	CLEC18A	69930232	15.3367	17.9827	-0.4541
YRI	CLEC18A	69935232	10.5066	14.4534	-0.8345
YRI	CLEC18A	69940232	8.935	14.2853	-1.1439
YRI	CLEC18A	69945232	9.7633	15.4617	-1.13
YRI	CLEC18A	69950232	9.2739	12.1005	-0.7072
YRI	CLEC18A	69955232	10.6057	11.0921	-0.1321
YRI	CLEC18A	69960232	9.2323	12.1005	-0.7176
YRI	CLEC18A	69965232	9.0995	14.1172	-1.085
YRI	CLEC18A	69970232	10.3429	14.6214	-0.8948
YRI	CLEC18A	69975232	8.2174	11.7644	-0.9112
YRI	CLEC18A	69980232	5.2296	7.5628	-0.9032
YRI	CLEC18A	69985232	6.4166	9.0754	-0.8703
YRI	CLEC18A	69990232	5.99	7.8989	-0.7102
YRI	CLEC18A	69995232	4.8184	5.8822	-0.5169
YRI	CLEC18A	70000232	7.34	10.4199	-0.8866
YRI	CLEC18A	70005232	8.3423	11.7644	-0.8792
YRI	CLEC18A	70010232	12.189	16.134	-0.7511

YRI	CLEC18A	70015232	13.9732	17.6465	-0.6419
YRI	CLEC18A	70020232	10.6038	15.7978	-1.009
YRI	CLEC18A	70025232	7.067	15.1256	-1.6318
YRI	CLEC18A	70030232	5.1901	13.613	-1.8852
YRI	CLEC18A	70035232	8.8488	15.4617	-1.3113
YRI	CLEC18A	70040232	9.3917	13.613	-0.9448
YRI	CLEC18A	70045232	5.7757	10.924	-1.4179
YRI	CLEC18A	70050232	8.2049	12.4366	-1.0317
YRI	CLEC18A	70055232	8.8161	10.924	-0.5806
YRI	CLEC18A	70060232	11.1435	10.2518	0.2606
YRI	CLEC18A	70065232	12.4254	10.756	0.4665
YRI	CLEC18A	70070232	8.4594	10.0837	-0.4821
YRI	CLEC18A	70075232	8.675	9.5795	-0.2816
YRI	CLEC18A	70080232	14.7657	11.9324	0.7182
YRI	CLEC18A	70085232	15.9984	12.6047	0.817
YRI	CLEC18A	70090232	10.2728	11.2602	-0.2643
YRI	CLEC18A	70095232	9.2807	10.4199	-0.3279
YRI	CLEC18A	70100232	11.411	12.6047	-0.2874
YRI	CLEC18A	70105232	12.6002	15.2937	-0.5397
YRI	CLEC18A	70110232	12.6385	13.7811	-0.2528
YRI	CLEC18A	70115232	12.2478	13.7811	-0.3392
YRI	CLEC18A	70120232	10.8812	13.2769	-0.5491
YRI	CLEC18A	70125232	7.992	9.9157	-0.58
YRI	CLEC18A	70130232	6.4153	8.5712	-0.744
YRI	CLEC18A	70135232	7.0318	9.9157	-0.8695
YRI	CLEC18A	70140232	9.0539	11.0921	-0.5534
YRI	CLEC18A	70145232	12.2165	12.1005	0.029
YRI	CLEC18A	70150232	15.6096	14.2853	0.2831
YRI	CLEC18A	70155232	14.1009	16.9743	-0.5212
YRI	CLEC18A	70160232	9.6722	13.9492	-0.9354
YRI	CLEC18A	70165232	11.0366	11.9324	-0.2271
YRI	CLEC18A	70170232	8.8706	12.9408	-0.9557
YRI	CLEC18A	70175232	5.4889	10.5879	-1.4461
YRI	CLEC18A	70180232	7.9976	10.5879	-0.7346
YRI	CLEC18A	70185232	11.4218	14.4534	-0.641
YRI	CLEC18A	70190232	12.3169	16.8062	-0.822
YRI	CLEC18A	70195232	11.9067	16.6382	-0.8747
YRI	CLEC18A	70200232	6.6724	11.7644	-1.3082
YRI	CLEC18A	70205232	2.0288	6.0502	-1.9054
YRI	CLEC18A	70210232	2.4973	4.5377	-1.2468
YRI	CLEC18A	70215232	1.1516	2.3529	-1.2716
YRI	CLEC18A	70220232	2.7808	2.689	0.0872
YRI	CLEC18A	70225232	7.3298	8.4031	-0.3772
YRI	CLEC18A	70230232	10.9573	12.6047	-0.3966
YRI	CLEC18A	70235232	13.6751	15.4617	-0.3543
YRI	CLEC18A	70240232	15.3426	17.1423	-0.3233
YRI	CLEC18A	70245232	9.7229	12.9408	-0.7556
YRI	CLEC18A	70250232	6.0834	9.5795	-1.0884
YRI	CLEC18A	70255232	8.9187	9.9157	-0.3006

YRI	CLEC18A	70260232	8.9756	9.5795	-0.188
YRI	CLEC18A	70265232	8.8489	11.7644	-0.749
YRI	CLEC18A	70270232	9.3423	13.445	-0.9291
YRI	CLEC18A	70275232	10.1148	11.7644	-0.4238
YRI	CLEC18A	70280232	11.3604	12.4366	-0.2624
YRI	CLEC18A	70285232	10.3934	11.9324	-0.3901
YRI	CLEC18A	70290232	7.6369	10.2518	-0.7643
YRI	CLEC18A	70295232	8.5489	11.9324	-0.8577
YRI	CLEC18A	70300232	11.7519	11.7644	-0.0032
YRI	CLEC18A	70305232	13.1085	14.2853	-0.2516
YRI	CLEC18A	70310232	9.5896	14.1172	-0.979
YRI	CLEC18A	70315232	9.2498	10.5879	-0.3795
YRI	CLEC18A	70320232	10.8885	10.756	0.037
YRI	CLEC18A	70325232	11.3864	10.4199	0.2782
YRI	CLEC18A	70330232	11.6873	10.2518	0.4196
YRI	CLEC18A	70335232	9.3994	9.9157	-0.1557
YRI	CLEC18A	70340232	8.6005	10.2518	-0.4826
YRI	CLEC18A	70345232	9.4817	11.4282	-0.5139
YRI	CLEC18A	70350232	9.1044	11.4282	-0.6135
YRI	CLEC18A	70355232	6.0991	8.5712	-0.8531
YRI	CLEC18A	70360232	7.7671	9.9157	-0.6478
YRI	CLEC18A	70365232	8.4454	12.4366	-0.973
YRI	CLEC18A	70370232	7.3756	10.2518	-0.8406
YRI	CLEC18A	70375232	6.8858	9.9157	-0.9135
YRI	CLEC18A	70380232	6.5369	11.4282	-1.2913
YRI	CLEC18A	70385232	6.4459	9.9157	-1.0461
YRI	CLEC18A	70390232	7.5813	8.067	-0.1772
YRI	CLEC18A	70395232	8.0287	9.5795	-0.4828
YRI	CLEC18A	70400232	7.3271	10.756	-0.9582
YRI	CLEC18A	70405232	8.8929	12.7727	-0.9224
YRI	CLEC18A	70410232	7.1608	12.6047	-1.3105
YRI	CLEC18A	70415232	5.8911	8.7392	-0.9654
YRI	CLEC18A	70420232	5.5287	8.067	-0.9262
YRI	CLEC18A	70425232	5.4668	7.8989	-0.9048
YRI	CLEC18A	70430232	5.4878	7.0586	-0.6476
YRI	CLEC18A	70435232	6.4743	10.0837	-1.0713
YRI	CLEC18A	70440232	8.7255	12.4366	-0.9048
YRI	CLEC18A	70445232	12.6803	12.9408	-0.0612
YRI	CLEC18A	70450232	12.6295	11.7644	0.2222
YRI	CLEC18A	70455232	10.7134	9.0754	0.5362
YRI	CLEC18A	70460232	9.673	10.0837	-0.1219
CEU	CSH	123268708	7.3874	6.8225	0.2417
CEU	CSH	123273708	6.6348	6.1402	0.2327
CEU	CSH	123278708	2.4787	4.0935	-1.0858
CEU	CSH	123283708	2.2764	3.9229	-1.1484
CEU	CSH	123288708	5.0221	6.1402	-0.5261
CEU	CSH	123293708	6.6988	7.3342	-0.2546
CEU	CSH	123298708	6.1921	5.1169	0.5951
CEU	CSH	123303708	8.2801	6.1402	1.007

CEU	CSH	123308708	8.2863	7.3342	0.3815
CEU	CSH	123313708	8.2218	6.6519	0.6873
CEU	CSH	123318708	10.4933	7.8459	0.9973
CEU	CSH	123323708	10.6052	8.5281	0.7247
CEU	CSH	123328708	9.094	8.0164	0.398
CEU	CSH	123333708	7.7489	7.5047	0.0958
CEU	CSH	123338708	7.9505	6.9931	0.4006
CEU	CSH	123343708	7.94	6.4814	0.6538
CEU	CSH	123348708	7.1222	5.2874	0.9866
CEU	CSH	123353708	8.1872	6.1402	0.9633
CEU	CSH	123358708	9.4968	7.6753	0.7001
CEU	CSH	123363708	8.9431	7.3342	0.6446
CEU	CSH	123368708	8.3644	6.6519	0.7498
CEU	CSH	123373708	11.856	8.5281	1.1611
CEU	CSH	123378708	12.6419	9.8926	0.836
CEU	CSH	123383708	7.2447	7.5047	-0.102
CEU	CSH	123388708	5.6382	6.1402	-0.2362
CEU	CSH	123393708	7.6324	6.3108	0.6068
CEU	CSH	123398708	10.6582	7.6753	1.1465
CEU	CSH	123403708	10.6226	8.187	0.8824
CEU	CSH	123408708	6.805	7.3342	-0.212
CEU	CSH	123413708	6.074	7.6753	-0.6155
CEU	CSH	123418708	7.7809	7.3342	0.179
CEU	CSH	123423708	9.1909	8.187	0.3637
CEU	CSH	123428708	8.9025	8.6987	0.0698
CEU	CSH	123433708	5.5974	6.3108	-0.3275
CEU	CSH	123438708	6.0493	5.7991	0.1239
CEU	CSH	123443708	8.8707	7.3342	0.6156
CEU	CSH	123448708	5.3931	5.7991	-0.2011
CEU	CSH	123453708	3.6234	5.9697	-1.1324
CEU	CSH	123458708	4.3206	6.8225	-1.0706
CEU	CSH	123463708	4.5154	6.3108	-0.8243
CEU	CSH	123468708	3.7662	5.6286	-0.9473
CEU	CSH	123473708	4.0775	5.458	-0.7217
CEU	CSH	123478708	5.6597	6.6519	-0.4344
CEU	CSH	123483708	4.0899	5.7991	-0.8466
CEU	CSH	123488708	3.7609	5.2874	-0.8208
CEU	CSH	123493708	6.3791	6.6519	-0.1194
CEU	CSH	123498708	4.7223	5.458	-0.3846
CEU	CSH	123503708	2.6129	4.4346	-1.143
CEU	CSH	123508708	4.9316	6.8225	-0.8091
CEU	CSH	123513708	5.549	6.8225	-0.5449
CEU	CSH	123518708	3.952	5.2874	-0.7181
CEU	CSH	123523708	4.174	5.7991	-0.8049
CEU	CSH	123528708	6.6193	7.1636	-0.2228
CEU	CSH	123533708	8.4886	9.0398	-0.1822
CEU	CSH	123538708	9.4065	9.8926	-0.1478
CEU	CSH	123543708	7.3325	7.3342	-0.0007
CEU	CSH	123548708	5.5106	5.1169	0.2179

CEU	CSH	123553708	5.0973	4.9463	0.0861
CEU	CSH	123558708	2.5925	3.5818	-0.7456
CEU	CSH	123563708	1.5793	2.8996	-1.1874
CEU	CSH	123568708	3.174	4.0935	-0.6183
CEU	CSH	123573708	7.7572	8.0164	-0.0957
CEU	CSH	123578708	7.8957	8.8692	-0.3276
CEU	CSH	123583708	6.1435	5.7991	0.1706
CEU	CSH	123588708	7.0625	5.6286	0.7294
CEU	CSH	123593708	5.122	5.6286	-0.2577
CEU	CSH	123598708	6.5099	5.9697	0.2607
CEU	CSH	123603708	11.9813	9.7221	0.6982
CEU	CSH	123608708	12.5774	10.916	0.4608
CEU	CSH	123613708	7.4298	8.0164	-0.2167
CEU	CSH	123618708	4.3873	6.1402	-0.8249
CEU	CSH	123623708	5.4282	6.4814	-0.4721
CEU	CSH	123628708	4.6376	6.9931	-0.9856
CEU	CSH	123633708	3.3342	5.458	-1.1103
CEU	CSH	123638708	4.6112	5.9697	-0.6556
CEU	CSH	123643708	4.9594	6.9931	-0.8509
CEU	CSH	123648708	4.2109	5.9697	-0.8488
CEU	CSH	123653708	3.4378	4.6052	-0.7088
CEU	CSH	123658708	6.491	6.1402	0.1651
CEU	CSH	123663708	7.9295	6.4814	0.6491
CEU	CSH	123668708	5.3423	4.7757	0.3332
CEU	CSH	123673708	8.3214	7.6753	0.2483
CEU	CSH	123678708	9.8517	8.6987	0.395
CEU	CSH	123683708	9.1486	6.8225	0.9953
CEU	CSH	123688708	10.5326	7.8459	1.0121
CEU	CSH	123693708	10.2838	8.8692	0.476
CEU	CSH	123698708	12.6677	9.0398	1.1995
CEU	CSH	123703708	14.1643	10.5749	1.0256
CEU	CSH	123708708	10.1839	9.3809	0.2565
CEU	CSH	123713708	9.3537	7.3342	0.8092
CEU	CSH	123718708	7.2086	8.6987	-0.5105
CEU	CSH	123723708	1.8955	7.1636	-2.1565
CEU	CSH	123728708	2.2148	5.458	-1.6955
CEU	CSH	123733708	3.3593	6.6519	-1.4416
CEU	CSH	123738708	4.953	8.5281	-1.2474
CEU	CSH	123743708	6.046	10.4043	-1.2643
CEU	CSH	123748708	3.7392	7.1636	-1.4018
CEU	CSH	123753708	4.5479	5.458	-0.4758
CEU	CSH	123758708	6.7541	6.6519	0.0447
CEU	CSH	123763708	8.6236	6.6519	0.8632
CEU	CSH	123768708	20.2797	9.8926	3.1584
CEU	CSH	123773708	38.5914	15.3506	4.6694
CEU	CSH	123778708	42.9278	17.9091	4.3374
CEU	CSH	123783708	37.1193	17.7385	3.3909
CEU	CSH	123788708	34.0988	16.5446	3.2834
CEU	CSH	123793708	21.5995	11.0866	2.8737

CEU	CSH	123798708	10.8691	7.6753	1.2276
CEU	CSH	123803708	9.6964	8.3576	0.4759
CEU	CSH	123808708	8.1202	8.0164	0.0383
CEU	CSH	123813708	9.3612	7.5047	0.7284
CEU	CSH	123818708	10.4702	7.3342	1.2565
CEU	CSH	123823708	8.8955	7.5047	0.5457
CEU	CSH	123828708	8.1062	7.1636	0.3859
CEU	CSH	123833708	8.6803	6.6519	0.8881
CEU	CSH	123838708	11.6797	8.187	1.2653
CEU	CSH	123843708	13.2542	9.2104	1.314
CEU	CSH	123848708	13.1218	10.2337	0.8509
CEU	CSH	123853708	14.5071	11.0866	0.935
CEU	CSH	123858708	13.0006	10.5749	0.6931
CEU	CSH	123863708	12.1497	10.7454	0.3953
CEU	CSH	123868708	11.4929	11.4277	0.0173
CEU	CSH	123873708	10.4378	12.7922	-0.5624
CEU	CSH	123878708	13.2752	13.8156	-0.12
CEU	CSH	123883708	11.3354	11.2571	0.0211
CEU	CSH	123888708	7.8011	8.3576	-0.1978
CEU	CSH	123893708	10.1313	8.3576	0.6305
CEU	CSH	123898708	9.9705	8.3576	0.5733
CEU	CSH	123903708	12.8423	11.0866	0.4799
CEU	CSH	123908708	13.4758	11.2571	0.5979
CEU	CSH	123913708	10.6242	9.0398	0.5238
CEU	CSH	123918708	11.4082	10.0632	0.4025
CEU	CSH	123923708	11.6848	10.0632	0.4853
CEU	CSH	123928708	12.6749	10.4043	0.6587
CEU	CSH	123933708	11.4601	8.6987	0.946
CEU	CSH	123938708	10.0045	8.3576	0.5854
CEU	CSH	123943708	9.5867	9.3809	0.0657
CEU	CSH	123948708	8.0106	6.9931	0.4257
CEU	CSH	123953708	8.7315	8.8692	-0.0463
CEU	CSH	123958708	8.2055	9.5515	-0.4229
CEU	CSH	123963708	9.6786	8.187	0.5404
CEU	CSH	123968708	15.9163	11.0866	1.3202
CEU	CSH	123973708	19.5086	14.1567	1.1614
CEU	CSH	123978708	19.6178	15.1801	0.9011
CEU	CSH	123983708	16.1754	16.0329	0.0275
CEU	CSH	123988708	12.1612	12.9627	-0.1891
CEU	CSH	123993708	12.3633	10.7454	0.4554
CEU	CSH	123998708	21.5155	16.2034	1.0136
CEU	CSH	124003708	22.128	16.2034	1.1304
CEU	CSH	124008708	13.4273	11.2571	0.5848
CEU	CSH	124013708	9.7411	9.3809	0.1151
CEU	CSH	124018708	7.6621	8.6987	-0.3551
CEU	CSH	124023708	9.4367	9.3809	0.0178
CEU	CSH	124028708	15.6142	11.9394	0.9369
CEU	CSH	124033708	19.6574	15.3506	0.8653
CEU	CSH	124038708	13.7759	13.4744	0.0686

CEU	CSH	124043708	6.5564	10.0632	-1.0495
CEU	CSH	124048708	6.8837	10.5749	-1.0546
CEU	CSH	124053708	9.309	10.916	-0.4457
CEU	CSH	124058708	12.2641	13.4744	-0.2752
CEU	CSH	124063708	11.7741	13.4744	-0.3867
CEU	CSH	124068708	7.6461	9.5515	-0.5986
CEU	CSH	124073708	7.5828	9.7221	-0.6611
CEU	CSH	124078708	6.0604	9.0398	-0.9851
CEU	CSH	124083708	4.502	6.8225	-0.9929
CEU	CSH	124088708	4.1086	5.7991	-0.8373
CEU	CSH	124093708	4.4354	5.7991	-0.6754
CEU	CSH	124098708	8.4974	8.8692	-0.1251
CEU	CSH	124103708	10.6619	10.5749	0.0249
CEU	CSH	124108708	13.24	11.5982	0.4302
CEU	CSH	124113708	11.4952	10.916	0.1606
CEU	CSH	124118708	6.5704	7.8459	-0.4805
CEU	CSH	124123708	9.0897	8.6987	0.134
CEU	CSH	124128708	13.8355	10.5749	0.9316
CEU	CSH	124133708	19.5294	12.1099	1.8665
CEU	CSH	124138708	13.8724	11.0866	0.7615
CEU	CSH	124143708	5.2641	8.6987	-1.1767
CEU	CSH	124148708	13.4389	11.4277	0.5343
CEU	CSH	124153708	17.1117	12.2805	1.1995
CEU	CSH	124158708	12.2771	9.5515	0.8563
CEU	CSH	124163708	13.0499	9.0398	1.3258
CEU	CSH	124168708	14.778	9.8926	1.4855
CEU	CSH	124173708	16.1552	11.5982	1.194
CEU	CSH	124178708	14.1181	10.4043	1.0774
CEU	CSH	124183708	11.1325	8.5281	0.9087
CEU	CSH	124188708	10.826	8.0164	1.0377
CEU	CSH	124193708	10.6644	7.3342	1.3343
CEU	CSH	124198708	10.1486	6.4814	1.6437
CEU	CSH	124203708	11.5852	8.3576	1.1473
CEU	CSH	124208708	12.824	9.5515	1.0281
CEU	CSH	124213708	11.5312	7.1636	1.7879
CEU	CSH	124218708	10.8491	6.8225	1.723
CEU	CSH	124223708	9.717	8.0164	0.6281
CEU	CSH	124228708	10.1247	9.0398	0.3587
CEU	CSH	124233708	10.819	8.5281	0.7993
CEU	CSH	124238708	7.0765	6.6519	0.1859
CEU	CSH	124243708	5.8013	7.1636	-0.5577
CEU	CSH	124248708	8.8953	9.0398	-0.0478
CEU	CSH	124253708	10.1855	8.187	0.724
CEU	CSH	124258708	11.1577	7.5047	1.4333
CEU	CSH	124263708	10.8889	8.0164	1.061
CEU	CSH	124268708	17.3117	11.4277	1.5633
CEU	CSH	124273708	15.7489	10.2337	1.6248
CEU	CSH	124278708	6.1563	6.3108	-0.0709
CEU	CSH	124283708	6.9433	6.6519	0.1276

CEU	CSH	124288708	7.569	6.4814	0.4875
CEU	CSH	124293708	6.0231	6.9931	-0.4059
CEU	CSH	124298708	12.1453	12.9627	-0.1928
CEU	CSH	124303708	17.1095	15.0095	0.4311
CEU	CSH	124308708	11.1985	8.8692	0.7838
CEU	CSH	124313708	8.027	8.0164	0.0039
CEU	CSH	124318708	8.2062	8.187	0.007
CEU	CSH	124323708	8.9073	7.1636	0.7138
CEU	CSH	124328708	9.5259	7.3342	0.8781
CEU	CSH	124333708	8.7893	6.9931	0.7516
CEU	CSH	124338708	6.1697	6.4814	-0.1397
CEU	CSH	124343708	8.9812	8.8692	0.0377
CEU	CSH	124348708	11.4203	9.7221	0.5248
CEU	CSH	124353708	9.3243	8.187	0.412
CEU	CSH	124358708	7.9231	7.1636	0.3109
CEU	CSH	124363708	10.9004	8.3576	0.9039
CEU	CSH	124368708	19.9885	14.3272	1.2147
CHB	CSH	123268708	6.8888	6.6072	0.1237
CHB	CSH	123273708	6.2867	8.1319	-0.6707
CHB	CSH	123278708	1.9772	5.4213	-1.806
CHB	CSH	123283708	1.8045	2.7106	-0.8586
CHB	CSH	123288708	4.6596	4.5742	0.052
CHB	CSH	123293708	5.6994	5.9295	-0.1114
CHB	CSH	123298708	5.505	5.4213	0.0439
CHB	CSH	123303708	7.8862	5.7601	1.0563
CHB	CSH	123308708	7.2501	6.2684	0.4521
CHB	CSH	123313708	7.265	7.6237	-0.1383
CHB	CSH	123318708	9.4489	8.6402	0.278
CHB	CSH	123323708	9.395	7.6237	0.6831
CHB	CSH	123328708	8.1361	6.946	0.4996
CHB	CSH	123333708	7.2747	6.946	0.138
CHB	CSH	123338708	7.7384	7.4543	0.1118
CHB	CSH	123343708	7.6943	7.4543	0.0945
CHB	CSH	123348708	6.8233	7.2848	-0.1855
CHB	CSH	123353708	6.9315	6.2684	0.3054
CHB	CSH	123358708	8.3746	6.946	0.5997
CHB	CSH	123363708	8.3328	7.1154	0.4999
CHB	CSH	123368708	7.7698	6.4378	0.5989
CHB	CSH	123373708	10.997	8.6402	0.8101
CHB	CSH	123378708	11.0412	8.8096	0.7534
CHB	CSH	123383708	5.83	6.6072	-0.3414
CHB	CSH	123388708	5.0366	5.9295	-0.4323
CHB	CSH	123393708	7.0114	7.6237	-0.2361
CHB	CSH	123398708	9.285	11.1814	-0.5128
CHB	CSH	123403708	10.1055	11.3508	-0.332
CHB	CSH	123408708	7.1974	8.3013	-0.3937
CHB	CSH	123413708	6.0293	7.6237	-0.6148
CHB	CSH	123418708	7.5174	6.946	0.2399
CHB	CSH	123423708	8.506	7.4543	0.414

CHB	CSH	123428708	8.8317	9.8261	-0.3034
CHB	CSH	123433708	5.8487	7.6237	-0.6845
CHB	CSH	123438708	5.2248	6.2684	-0.4806
CHB	CSH	123443708	8.2269	8.6402	-0.1421
CHB	CSH	123448708	5.3699	6.2684	-0.4138
CHB	CSH	123453708	3.072	4.2354	-0.7574
CHB	CSH	123458708	4.1942	4.7436	-0.3241
CHB	CSH	123463708	4.6269	5.5907	-0.4918
CHB	CSH	123468708	3.6592	5.2519	-0.859
CHB	CSH	123473708	4.3417	5.0825	-0.4113
CHB	CSH	123478708	6.2648	7.9625	-0.6291
CHB	CSH	123483708	4.446	7.4543	-1.1841
CHB	CSH	123488708	3.6115	4.7436	-0.6678
CHB	CSH	123493708	5.8789	6.7766	-0.3854
CHB	CSH	123498708	4.1519	6.2684	-0.9748
CHB	CSH	123503708	1.8934	4.4048	-1.5803
CHB	CSH	123508708	1.917	5.0825	-1.7574
CHB	CSH	123513708	2.3815	4.7436	-1.3933
CHB	CSH	123518708	1.9669	3.7271	-1.2787
CHB	CSH	123523708	1.6196	3.7271	-1.5311
CHB	CSH	123528708	2.0891	4.5742	-1.5132
CHB	CSH	123533708	2.4076	5.5907	-1.6241
CHB	CSH	123538708	2.6313	5.2519	-1.4134
CHB	CSH	123543708	1.7668	3.3883	-1.277
CHB	CSH	123548708	1.2935	2.7106	-1.3428
CHB	CSH	123553708	1.9937	2.8801	-0.7992
CHB	CSH	123558708	1.9899	4.4048	-1.5196
CHB	CSH	123563708	1.0613	4.4048	-2.1039
CHB	CSH	123568708	2.2857	4.4048	-1.3334
CHB	CSH	123573708	5.8024	8.1319	-0.8467
CHB	CSH	123578708	6.1966	7.6237	-0.5503
CHB	CSH	123583708	5.2776	4.5742	0.4283
CHB	CSH	123588708	5.5818	5.7601	-0.0886
CHB	CSH	123593708	4.5471	6.2684	-0.7928
CHB	CSH	123598708	4.558	7.6237	-1.1822
CHB	CSH	123603708	5.5561	11.5202	-1.5681
CHB	CSH	123608708	4.2979	11.012	-1.8416
CHB	CSH	123613708	2.2416	7.2848	-2.0272
CHB	CSH	123618708	2.4485	6.946	-1.8879
CHB	CSH	123623708	3.3189	7.7931	-1.691
CHB	CSH	123628708	2.304	6.2684	-1.8259
CHB	CSH	123633708	1.9041	5.9295	-1.9488
CHB	CSH	123638708	2.9029	7.6237	-1.8205
CHB	CSH	123643708	2.1847	7.9625	-2.1411
CHB	CSH	123648708	1.605	6.6072	-2.197
CHB	CSH	123653708	1.5028	4.4048	-1.8261
CHB	CSH	123658708	2.1877	6.2684	-1.8794
CHB	CSH	123663708	2.9063	7.4543	-1.7902
CHB	CSH	123668708	2.0406	5.2519	-1.732

CHB	CSH	123673708	2.3115	6.6072	-1.8867
CHB	CSH	123678708	4.2067	8.6402	-1.524
CHB	CSH	123683708	6.846	7.4543	-0.2394
CHB	CSH	123688708	8.6504	7.7931	0.324
CHB	CSH	123693708	8.034	8.6402	-0.2084
CHB	CSH	123698708	9.0788	8.8096	0.0909
CHB	CSH	123703708	10.1114	9.6567	0.141
CHB	CSH	123708708	6.9429	8.3013	-0.4845
CHB	CSH	123713708	7.1723	7.4543	-0.111
CHB	CSH	123718708	6.0251	9.9955	-1.1922
CHB	CSH	123723708	1.3815	8.4708	-2.4818
CHB	CSH	123728708	1.8153	5.4213	-1.8909
CHB	CSH	123733708	2.7453	6.6072	-1.6962
CHB	CSH	123738708	3.7017	7.7931	-1.5463
CHB	CSH	123743708	4.5342	9.1484	-1.5044
CHB	CSH	123748708	2.9966	7.7931	-1.8128
CHB	CSH	123753708	3.8565	5.9295	-1.0036
CHB	CSH	123758708	5.3427	6.2684	-0.4263
CHB	CSH	123763708	7.6648	7.6237	0.0158
CHB	CSH	123768708	19.0213	11.1814	2.1198
CHB	CSH	123773708	35.6686	16.9415	3.4144
CHB	CSH	123778708	40.5266	18.6357	3.6425
CHB	CSH	123783708	34.1809	16.4333	3.3315
CHB	CSH	123788708	29.959	16.0944	2.6549
CHB	CSH	123793708	19.8907	11.3508	2.2767
CHB	CSH	123798708	10.4618	8.1319	0.8469
CHB	CSH	123803708	8.5713	8.3013	0.0963
CHB	CSH	123808708	7.3135	5.4213	0.9922
CHB	CSH	123813708	9.4165	6.4378	1.3393
CHB	CSH	123818708	10.4677	7.7931	1.0109
CHB	CSH	123823708	7.2397	6.7766	0.1988
CHB	CSH	123828708	5.9608	7.1154	-0.4742
CHB	CSH	123833708	6.739	7.2848	-0.2194
CHB	CSH	123838708	9.7048	9.4872	0.0686
CHB	CSH	123843708	11.7932	10.1649	0.4813
CHB	CSH	123848708	13.748	9.6567	1.2686
CHB	CSH	123853708	16.0813	9.6567	1.9921
CHB	CSH	123858708	13.1144	9.4872	1.1434
CHB	CSH	123863708	12.6056	10.1649	0.7215
CHB	CSH	123868708	14.4531	9.4872	1.5654
CHB	CSH	123873708	14.0742	9.9955	1.2248
CHB	CSH	123878708	16.5237	12.0285	1.1348
CHB	CSH	123883708	13.1468	9.3178	1.2273
CHB	CSH	123888708	7.1018	7.1154	-0.0056
CHB	CSH	123893708	9.3151	8.8096	0.1707
CHB	CSH	123898708	8.8888	9.4872	-0.1886
CHB	CSH	123903708	12.4718	11.6896	0.2028
CHB	CSH	123908708	14.9533	11.3508	0.9604
CHB	CSH	123913708	11.8254	9.6567	0.6725

CHB	CSH	123918708	12.6541	10.5037	0.6165
CHB	CSH	123923708	11.649	10.8426	0.2244
CHB	CSH	123928708	13.0067	11.8591	0.2936
CHB	CSH	123933708	13.4793	11.1814	0.6213
CHB	CSH	123938708	11.3914	9.6567	0.5379
CHB	CSH	123943708	10.4889	9.6567	0.258
CHB	CSH	123948708	8.6345	9.6567	-0.317
CHB	CSH	123953708	10.5246	12.3673	-0.4532
CHB	CSH	123958708	10.0873	11.8591	-0.4533
CHB	CSH	123963708	10.7196	10.5037	0.0619
CHB	CSH	123968708	17.0739	13.5532	0.794
CHB	CSH	123973708	19.9305	15.2474	0.9443
CHB	CSH	123978708	20.6486	15.4168	1.0438
CHB	CSH	123983708	20.0211	16.2638	0.7123
CHB	CSH	123988708	15.4447	13.7226	0.3838
CHB	CSH	123993708	15.9821	12.1979	0.9428
CHB	CSH	123998708	24.4779	16.2638	1.5573
CHB	CSH	124003708	22.3306	15.925	1.2391
CHB	CSH	124008708	13.7811	11.8591	0.4917
CHB	CSH	124013708	10.4746	8.6402	0.6306
CHB	CSH	124018708	9.0609	7.4543	0.6324
CHB	CSH	124023708	11.5087	8.3013	1.1439
CHB	CSH	124028708	17.57	11.6896	1.525
CHB	CSH	124033708	22.4135	16.4333	1.1226
CHB	CSH	124038708	18.2216	13.7226	1.0027
CHB	CSH	124043708	10.6943	9.4872	0.3805
CHB	CSH	124048708	11.4026	10.1649	0.3659
CHB	CSH	124053708	15.2621	10.1649	1.5068
CHB	CSH	124058708	22.4763	13.045	2.2054
CHB	CSH	124063708	21.4285	13.2144	1.8974
CHB	CSH	124068708	11.4493	9.3178	0.6832
CHB	CSH	124073708	9.9975	9.8261	0.0523
CHB	CSH	124078708	8.2452	9.6567	-0.4377
CHB	CSH	124083708	5.4693	7.2848	-0.7298
CHB	CSH	124088708	4.331	6.0989	-0.8345
CHB	CSH	124093708	5.2934	6.7766	-0.6367
CHB	CSH	124098708	9.1732	8.6402	0.1832
CHB	CSH	124103708	9.9347	11.3508	-0.3775
CHB	CSH	124108708	14.1826	11.5202	0.7
CHB	CSH	124113708	15.6366	8.979	2.2085
CHB	CSH	124118708	9.566	7.2848	0.917
CHB	CSH	124123708	10.7308	7.6237	1.1982
CHB	CSH	124128708	14.5237	9.3178	1.6687
CHB	CSH	124133708	19.9979	13.2144	1.567
CHB	CSH	124138708	16.5717	11.1814	1.4575
CHB	CSH	124143708	8.8134	5.7601	1.5169
CHB	CSH	124148708	9.2899	9.8261	-0.1636
CHB	CSH	124153708	12.4531	13.2144	-0.1759
CHB	CSH	124158708	12.0491	11.012	0.2845

CHB	CSH	124163708	9.9895	9.8261	0.0499
CHB	CSH	124168708	10.8865	10.5037	0.1097
CHB	CSH	124173708	13.8475	12.0285	0.4592
CHB	CSH	124178708	12.728	9.9955	0.8205
CHB	CSH	124183708	8.9968	8.4708	0.1841
CHB	CSH	124188708	8.6025	9.8261	-0.3733
CHB	CSH	124193708	9.0284	8.8096	0.0739
CHB	CSH	124198708	9.0739	8.1319	0.3424
CHB	CSH	124203708	11.8378	9.9955	0.5532
CHB	CSH	124208708	12.3735	10.1649	0.6529
CHB	CSH	124213708	9.1725	8.6402	0.183
CHB	CSH	124218708	9.8536	8.8096	0.3525
CHB	CSH	124223708	8.9642	7.1154	0.7592
CHB	CSH	124228708	9.0602	7.4543	0.6321
CHB	CSH	124233708	9.268	8.979	0.0959
CHB	CSH	124238708	6.7186	6.946	-0.0955
CHB	CSH	124243708	5.2854	8.3013	-1.0756
CHB	CSH	124248708	7.091	9.8261	-0.8345
CHB	CSH	124253708	8.5814	8.6402	-0.0202
CHB	CSH	124258708	9.7529	9.1484	0.1971
CHB	CSH	124263708	10.2507	9.3178	0.299
CHB	CSH	124268708	16.792	13.2144	0.8264
CHB	CSH	124273708	14.3787	11.3508	0.8072
CHB	CSH	124278708	5.3318	5.5907	-0.1321
CHB	CSH	124283708	7.0923	6.4378	0.2943
CHB	CSH	124288708	6.7651	5.7601	0.4993
CHB	CSH	124293708	5.7954	5.2519	0.2931
CHB	CSH	124298708	12.8241	10.6731	0.6075
CHB	CSH	124303708	18.0047	13.5532	1.0039
CHB	CSH	124308708	12.0622	8.6402	1.1763
CHB	CSH	124313708	9.6966	7.4543	0.8826
CHB	CSH	124318708	10.0692	8.6402	0.4912
CHB	CSH	124323708	9.6034	8.8096	0.268
CHB	CSH	124328708	9.4057	8.1319	0.463
CHB	CSH	124333708	7.5389	6.7766	0.3272
CHB	CSH	124338708	5.3122	5.5907	-0.1421
CHB	CSH	124343708	8.1734	6.946	0.5152
CHB	CSH	124348708	10.8136	8.1319	0.9747
CHB	CSH	124353708	8.909	7.7931	0.4218
CHB	CSH	124358708	7.1698	6.6072	0.2471
CHB	CSH	124363708	9.7769	6.0989	1.7362
CHB	CSH	124368708	16.8858	12.5367	1.0559
YRI	CSH	123268708	7.4349	10.2518	-0.8233
YRI	CSH	123273708	7.2769	9.7476	-0.7569
YRI	CSH	123278708	4.4058	9.7476	-1.6364
YRI	CSH	123283708	3.6749	9.4115	-1.8156
YRI	CSH	123288708	6.589	9.9157	-1.003
YRI	CSH	123293708	7.9026	11.0921	-0.866
YRI	CSH	123298708	6.7699	8.235	-0.5246

YRI	CSH	123303708	9.107	8.7392	0.1247
YRI	CSH	123308708	8.1512	10.2518	-0.6139
YRI	CSH	123313708	8.0275	11.7644	-0.96
YRI	CSH	123318708	10.8879	11.9324	-0.2648
YRI	CSH	123323708	11.0035	11.9324	-0.2355
YRI	CSH	123328708	10.1058	13.1089	-0.6966
YRI	CSH	123333708	8.1733	11.9324	-0.9529
YRI	CSH	123338708	7.5828	10.4199	-0.8167
YRI	CSH	123343708	7.762	8.7392	-0.3312
YRI	CSH	123348708	7.3935	9.2434	-0.5953
YRI	CSH	123353708	8.4875	11.7644	-0.8419
YRI	CSH	123358708	9.8393	12.9408	-0.7283
YRI	CSH	123363708	9.6461	13.7811	-0.9148
YRI	CSH	123368708	8.4789	12.2685	-0.9358
YRI	CSH	123373708	11.7937	12.2685	-0.1173
YRI	CSH	123378708	13.0331	13.7811	-0.1655
YRI	CSH	123383708	8.091	10.924	-0.7803
YRI	CSH	123388708	6.3917	7.7309	-0.5081
YRI	CSH	123393708	8.2549	9.5795	-0.4124
YRI	CSH	123398708	11.2363	13.9492	-0.5933
YRI	CSH	123403708	10.2343	14.6214	-0.9175
YRI	CSH	123408708	6.0864	13.445	-1.6665
YRI	CSH	123413708	4.8937	13.1089	-1.9056
YRI	CSH	123418708	6.2214	13.1089	-1.5977
YRI	CSH	123423708	7.6012	12.1005	-1.1256
YRI	CSH	123428708	8.4984	11.4282	-0.7735
YRI	CSH	123433708	6.6244	9.7476	-0.9568
YRI	CSH	123438708	6.4629	8.235	-0.6345
YRI	CSH	123443708	8.7987	10.2518	-0.4247
YRI	CSH	123448708	6.2558	12.7727	-1.5493
YRI	CSH	123453708	4.4654	11.5963	-1.8569
YRI	CSH	123458708	4.1798	7.3947	-1.2704
YRI	CSH	123463708	4.9842	7.8989	-1.0843
YRI	CSH	123468708	4.8766	8.067	-1.1642
YRI	CSH	123473708	5.3296	7.8989	-0.9558
YRI	CSH	123478708	7.3948	10.0837	-0.7981
YRI	CSH	123483708	6.3925	9.7476	-1.0278
YRI	CSH	123488708	6.0789	9.2434	-1.0184
YRI	CSH	123493708	8.3733	10.0837	-0.5077
YRI	CSH	123498708	8.4619	8.9073	-0.1483
YRI	CSH	123503708	5.8669	8.7392	-0.9736
YRI	CSH	123508708	5.0164	10.4199	-1.5555
YRI	CSH	123513708	5.952	11.5963	-1.4698
YRI	CSH	123518708	4.8781	10.5879	-1.6193
YRI	CSH	123523708	4.0794	9.0754	-1.6354
YRI	CSH	123528708	5.7068	9.9157	-1.269
YRI	CSH	123533708	7.5771	11.7644	-1.0757
YRI	CSH	123538708	8.8323	12.9408	-0.9647
YRI	CSH	123543708	7.4772	9.9157	-0.7352

YRI	CSH	123548708	6.3491	8.7392	-0.8102
YRI	CSH	123553708	7.1483	11.9324	-1.2128
YRI	CSH	123558708	4.9601	10.0837	-1.5208
YRI	CSH	123563708	2.8403	8.9073	-2.0207
YRI	CSH	123568708	3.6533	8.235	-1.6405
YRI	CSH	123573708	7.5033	8.5712	-0.3685
YRI	CSH	123578708	8.0166	9.9157	-0.5726
YRI	CSH	123583708	6.3474	7.2267	-0.3548
YRI	CSH	123588708	7.4727	7.8989	-0.1586
YRI	CSH	123593708	5.7324	8.7392	-1.0192
YRI	CSH	123598708	8.2953	10.5879	-0.6502
YRI	CSH	123603708	16.8197	13.7811	0.6722
YRI	CSH	123608708	18.9455	15.6298	0.6507
YRI	CSH	123613708	11.6187	13.1089	-0.3457
YRI	CSH	123618708	7.7452	11.0921	-0.9087
YRI	CSH	123623708	10.3186	13.9492	-0.794
YRI	CSH	123628708	8.697	13.445	-1.0753
YRI	CSH	123633708	6.3181	10.756	-1.2402
YRI	CSH	123638708	8.512	10.5879	-0.5887
YRI	CSH	123643708	8.83	10.924	-0.5767
YRI	CSH	123648708	7.8111	11.2602	-0.9233
YRI	CSH	123653708	8.7404	11.9324	-0.8092
YRI	CSH	123658708	12.0429	11.9324	0.028
YRI	CSH	123663708	12.6893	12.1005	0.1473
YRI	CSH	123668708	9.6717	11.7644	-0.5376
YRI	CSH	123673708	11.3387	11.0921	0.067
YRI	CSH	123678708	12.9948	12.6047	0.0939
YRI	CSH	123683708	12.0112	12.1005	-0.0223
YRI	CSH	123688708	10.7708	9.9157	0.2578
YRI	CSH	123693708	9.9881	10.4199	-0.1243
YRI	CSH	123698708	13.0488	13.2769	-0.0523
YRI	CSH	123703708	14.256	15.1256	-0.1761
YRI	CSH	123708708	9.8206	12.2685	-0.6045
YRI	CSH	123713708	9.922	11.2602	-0.3582
YRI	CSH	123718708	11.2552	13.2769	-0.4633
YRI	CSH	123723708	7.022	11.9324	-1.2448
YRI	CSH	123728708	5.7375	9.2434	-1.1283
YRI	CSH	123733708	7.9515	10.2518	-0.6723
YRI	CSH	123738708	10.8443	12.4366	-0.3882
YRI	CSH	123743708	12.1134	12.6047	-0.1183
YRI	CSH	123748708	9.1103	9.9157	-0.2428
YRI	CSH	123753708	7.8326	9.4115	-0.4997
YRI	CSH	123758708	10.0112	10.0837	-0.0215
YRI	CSH	123763708	10.9163	11.0921	-0.0477
YRI	CSH	123768708	19.863	15.1256	0.9593
YRI	CSH	123773708	38.1721	19.9994	2.8156
YRI	CSH	123778708	42.9804	21.68	3.053
YRI	CSH	123783708	36.1546	18.991	2.795
YRI	CSH	123788708	33.3974	18.1507	2.5932

YRI	CSH	123793708	21.4446	15.1256	1.2795
YRI	CSH	123798708	11.7175	13.9492	-0.4881
YRI	CSH	123803708	11.6244	14.7895	-0.6548
YRI	CSH	123808708	8.1729	11.9324	-0.953
YRI	CSH	123813708	8.8333	11.0921	-0.6133
YRI	CSH	123818708	10.338	11.2602	-0.2469
YRI	CSH	123823708	10.2152	11.9324	-0.4353
YRI	CSH	123828708	10.3673	13.445	-0.697
YRI	CSH	123833708	10.2462	12.4366	-0.534
YRI	CSH	123838708	12.542	12.9408	-0.0936
YRI	CSH	123843708	14.0305	14.9575	-0.1897
YRI	CSH	123848708	13.1451	16.4701	-0.6207
YRI	CSH	123853708	12.0198	16.4701	-0.8308
YRI	CSH	123858708	11.7248	17.8146	-1.0545
YRI	CSH	123863708	13.8402	16.9743	-0.5684
YRI	CSH	123868708	12.4799	16.302	-0.7205
YRI	CSH	123873708	10.4984	21.1758	-1.5656
YRI	CSH	123878708	15.2396	25.0413	-1.2219
YRI	CSH	123883708	13.286	21.3439	-1.1725
YRI	CSH	123888708	8.4965	13.7811	-1.1691
YRI	CSH	123893708	11.2042	13.445	-0.5075
YRI	CSH	123898708	12.3806	15.6298	-0.6377
YRI	CSH	123903708	15.8761	17.1423	-0.2275
YRI	CSH	123908708	16.3579	16.9743	-0.1118
YRI	CSH	123913708	13.6421	15.2937	-0.3309
YRI	CSH	123918708	14.0832	13.613	0.1052
YRI	CSH	123923708	14.1613	15.2937	-0.2269
YRI	CSH	123928708	15.4258	17.4785	-0.362
YRI	CSH	123933708	14.2306	13.9492	0.0615
YRI	CSH	123938708	11.4851	12.2685	-0.1935
YRI	CSH	123943708	11.5723	13.2769	-0.3907
YRI	CSH	123948708	14.0679	14.6214	-0.1158
YRI	CSH	123953708	18.5924	19.9994	-0.218
YRI	CSH	123958708	16.6561	19.3272	-0.4277
YRI	CSH	123963708	15.1732	15.2937	-0.0241
YRI	CSH	123968708	21.0106	15.4617	1.1003
YRI	CSH	123973708	26.0877	17.3104	1.5623
YRI	CSH	123978708	26.4053	19.4952	1.0973
YRI	CSH	123983708	21.5683	19.1591	0.389
YRI	CSH	123988708	16.9064	17.4785	-0.1009
YRI	CSH	123993708	16.3097	15.9659	0.0661
YRI	CSH	123998708	29.0164	18.3188	1.8035
YRI	CSH	124003708	32.6686	18.6549	2.3216
YRI	CSH	124008708	19.7224	15.2937	0.8874
YRI	CSH	124013708	11.8482	12.2685	-0.1038
YRI	CSH	124018708	9.5892	11.4282	-0.4855
YRI	CSH	124023708	12.0617	11.5963	0.1212
YRI	CSH	124028708	20.1066	14.4534	1.1953
YRI	CSH	124033708	25.7176	17.8146	1.3685

YRI	CSH	124038708	18.8864	15.2937	0.7199
YRI	CSH	124043708	9.8628	12.7727	-0.6918
YRI	CSH	124048708	10.7412	13.7811	-0.6725
YRI	CSH	124053708	14.679	15.1256	-0.0904
YRI	CSH	124058708	21.9334	17.9827	0.678
YRI	CSH	124063708	19.9784	15.9659	0.7717
YRI	CSH	124068708	9.357	11.5963	-0.5831
YRI	CSH	124073708	9.5562	12.7727	-0.7647
YRI	CSH	124078708	8.3784	11.4282	-0.8052
YRI	CSH	124083708	6.1652	9.0754	-0.9526
YRI	CSH	124088708	4.8291	8.235	-1.2195
YRI	CSH	124093708	5.9637	9.7476	-1.1592
YRI	CSH	124098708	10.4765	13.2769	-0.6418
YRI	CSH	124103708	11.5898	16.6382	-0.9333
YRI	CSH	124108708	14.3876	18.3188	-0.6627
YRI	CSH	124113708	14.1949	16.302	-0.3972
YRI	CSH	124118708	9.8142	13.2769	-0.7936
YRI	CSH	124123708	10.9148	11.7644	-0.2183
YRI	CSH	124128708	14.1389	13.1089	0.2389
YRI	CSH	124133708	16.8268	16.8062	0.0038
YRI	CSH	124138708	13.3245	16.6382	-0.6126
YRI	CSH	124143708	9.9055	16.302	-1.2059
YRI	CSH	124148708	18.267	19.4952	-0.195
YRI	CSH	124153708	21.0999	20.6717	0.0643
YRI	CSH	124158708	14.7731	17.6465	-0.5021
YRI	CSH	124163708	12.3078	14.1172	-0.3912
YRI	CSH	124168708	13.0607	15.2937	-0.4474
YRI	CSH	124173708	17.4704	16.4701	0.1867
YRI	CSH	124178708	15.1966	12.2685	0.7231
YRI	CSH	124183708	12.0854	10.924	0.3199
YRI	CSH	124188708	13.0615	13.445	-0.0869
YRI	CSH	124193708	10.1712	13.9492	-0.8262
YRI	CSH	124198708	9.4714	13.613	-0.927
YRI	CSH	124203708	12.589	15.4617	-0.5696
YRI	CSH	124208708	14.24	15.4617	-0.2423
YRI	CSH	124213708	11.4548	11.4282	0.007
YRI	CSH	124218708	9.3812	10.4199	-0.299
YRI	CSH	124223708	8.9139	11.0921	-0.5914
YRI	CSH	124228708	11.5537	13.1089	-0.3608
YRI	CSH	124233708	13.4883	14.4534	-0.2041
YRI	CSH	124238708	9.9994	12.1005	-0.5257
YRI	CSH	124243708	10.6882	13.613	-0.6546
YRI	CSH	124248708	12.5012	15.7978	-0.6404
YRI	CSH	124253708	12.5106	14.6214	-0.4414
YRI	CSH	124258708	14.5355	13.9492	0.1282
YRI	CSH	124263708	14.0022	14.6214	-0.1295
YRI	CSH	124268708	19.7978	18.6549	0.1893
YRI	CSH	124273708	17.571	17.1423	0.077
YRI	CSH	124278708	10.8619	13.445	-0.585

YRI	CSH	124283708	11.7322	14.2853	-0.5459
YRI	CSH	124288708	8.9775	12.4366	-0.8433
YRI	CSH	124293708	8.4656	11.0921	-0.7131
YRI	CSH	124298708	18.843	19.8313	-0.1544
YRI	CSH	124303708	23.2848	22.8565	0.0583
YRI	CSH	124308708	14.9033	14.7895	0.0235
YRI	CSH	124313708	11.8254	12.7727	-0.2252
YRI	CSH	124318708	11.7558	12.6047	-0.2044
YRI	CSH	124323708	12.5787	12.4366	0.0346
YRI	CSH	124328708	11.7426	11.9324	-0.0481
YRI	CSH	124333708	9.0592	11.0921	-0.5519
YRI	CSH	124338708	6.1991	11.5963	-1.4055
YRI	CSH	124343708	9.1911	13.1089	-0.9088
YRI	CSH	124348708	11.6453	13.9492	-0.5039
YRI	CSH	124353708	9.5582	13.9492	-0.9603
YRI	CSH	124358708	8.9911	14.7895	-1.1995
YRI	CSH	124363708	10.6477	15.9659	-1.0228
YRI	CSH	124368708	18.3023	23.1926	-0.6567
CEU	CSH	80586945	10.3585	12.6216	-0.5475
CEU	CSH	80591945	8.8089	11.9394	-0.7981
CEU	CSH	80596945	11.1833	10.7454	0.1233
CEU	CSH	80601945	8.4234	8.0164	0.1503
CEU	CSH	80606945	7.4113	8.6987	-0.4411
CEU	CSH	80611945	10.5723	11.4277	-0.2273
CEU	CSH	80616945	8.7396	8.6987	0.014
CEU	CSH	80621945	8.0854	7.3342	0.301
CEU	CSH	80626945	7.8597	8.0164	-0.0579
CEU	CSH	80631945	7.752	7.5047	0.097
CEU	CSH	80636945	14.7353	11.0866	0.9974
CEU	CSH	80641945	12.9227	12.1099	0.2045
CEU	CSH	80646945	7.1227	9.7221	-0.8033
CEU	CSH	80651945	9.334	10.2337	-0.2651
CEU	CSH	80656945	9.8975	10.2337	-0.099
CEU	CSH	80661945	8.303	9.7221	-0.4385
CEU	CSH	80666945	10.1828	12.7922	-0.6234
CEU	CSH	80671945	11.4315	14.1567	-0.5914
CEU	CSH	80676945	12.7785	12.2805	0.1236
CEU	CSH	80681945	11.7548	10.2337	0.4481
CEU	CSH	80686945	8.3913	9.5515	-0.3645
CEU	CSH	80691945	8.0757	9.0398	-0.3188
CEU	CSH	80696945	10.2261	9.7221	0.1558
CEU	CSH	80701945	17.2602	13.4744	0.861
CEU	CSH	80706945	17.395	16.7151	0.1259
CEU	CSH	80711945	11.8384	15.1801	-0.6786
CEU	CSH	80716945	10.8437	12.4511	-0.3939
CEU	CSH	80721945	10.494	12.9627	-0.5824
CEU	CSH	80726945	10.7991	11.5982	-0.2094
CEU	CSH	80731945	10.4137	10.0632	0.1049
CEU	CSH	80736945	7.0763	9.7221	-0.8176

CEU	CSH	80741945	8.719	12.2805	-0.8842
CEU	CSH	80746945	10.2705	12.2805	-0.499
CEU	CSH	80751945	8.6308	9.8926	-0.3837
CEU	CSH	80756945	9.7015	10.7454	-0.2938
CEU	CSH	80761945	14.4191	12.7922	0.3886
CEU	CSH	80766945	14.8035	11.7688	0.7843
CEU	CSH	80771945	9.6595	9.7221	-0.0193
CEU	CSH	80776945	8.7598	9.8926	-0.3444
CEU	CSH	80781945	11.5215	9.3809	0.6838
CEU	CSH	80786945	12.0237	10.4043	0.4698
CEU	CSH	80791945	9.4824	12.4511	-0.7275
CEU	CSH	80796945	7.9175	10.5749	-0.7593
CEU	CSH	80801945	6.0989	8.6987	-0.8907
CEU	CSH	80806945	6.9261	11.5982	-1.2241
CEU	CSH	80811945	7.9485	12.1099	-1.0469
CEU	CSH	80816945	6.4041	9.3809	-0.951
CEU	CSH	80821945	4.8093	7.3342	-1.0117
CEU	CSH	80826945	2.9759	6.9931	-1.6809
CEU	CSH	80831945	3.7557	7.8459	-1.5408
CEU	CSH	80836945	7.2523	10.2337	-0.8783
CEU	CSH	80841945	11.235	12.1099	-0.2201
CEU	CSH	80846945	9.9899	9.3809	0.1946
CEU	CSH	80851945	6.3016	6.6519	-0.1534
CEU	CSH	80856945	4.5611	6.9931	-1.0176
CEU	CSH	80861945	6.348	10.2337	-1.1448
CEU	CSH	80866945	7.5968	11.0866	-0.9539
CEU	CSH	80871945	5.7271	9.5515	-1.2015
CEU	CSH	80876945	5.691	9.0398	-1.1072
CEU	CSH	80881945	8.3302	8.5281	-0.069
CEU	CSH	80886945	13.4263	11.9394	0.3791
CEU	CSH	80891945	25.3354	16.7151	1.5966
CEU	CSH	80896945	32.5995	17.9091	2.5468
CEU	CSH	80901945	25.853	15.5212	2.054
CEU	CSH	80906945	17.9872	13.8156	0.9265
CEU	CSH	80911945	14.5537	13.4744	0.2455
CEU	CSH	80916945	13.0522	12.9627	0.0211
CEU	CSH	80921945	14.3228	12.7922	0.3656
CEU	CSH	80926945	17.2016	13.9861	0.7059
CEU	CSH	80931945	21.3501	17.0562	0.7801
CEU	CSH	80936945	21.1215	17.0562	0.7385
CEU	CSH	80941945	12.9229	14.6684	-0.3662
CEU	CSH	80946945	8.3865	15.5212	-1.4184
CEU	CSH	80951945	8.9437	14.3272	-1.155
CEU	CSH	80956945	9.7976	12.1099	-0.5817
CEU	CSH	80961945	13.0229	13.645	-0.1398
CEU	CSH	80966945	16.9891	14.3272	0.5711
CEU	CSH	80971945	12.6595	12.2805	0.0941
CEU	CSH	80976945	7.382	11.4277	-1.0749
CEU	CSH	80981945	7.9973	14.4978	-1.3791

CEU	CSH	80986945	19.6477	21.1497	-0.2219
CEU	CSH	80991945	23.8654	21.832	0.2913
CEU	CSH	80996945	20.482	20.2969	0.0284
CEU	CSH	81001945	23.3408	21.1497	0.3237
CEU	CSH	81006945	15.6444	15.6917	-0.0093
CEU	CSH	81011945	8.7941	12.4511	-0.8962
CEU	CSH	81016945	10.8048	13.3039	-0.5753
CEU	CSH	81021945	19.7924	16.5446	0.6075
CEU	CSH	81026945	23.0569	16.8857	1.1319
CEU	CSH	81031945	23.9532	18.0796	1.0091
CEU	CSH	81036945	22.0844	17.9091	0.7239
CEU	CSH	81041945	13.7321	12.7922	0.2245
CEU	CSH	81046945	12.6498	12.2805	0.0917
CEU	CSH	81051945	10.8773	11.2571	-0.1023
CEU	CSH	81056945	8.5842	10.2337	-0.486
CEU	CSH	81061945	8.4329	11.2571	-0.761
CEU	CSH	81066945	6.893	11.4277	-1.2048
CEU	CSH	81071945	4.0674	9.8926	-1.7713
CEU	CSH	81076945	4.0198	9.5515	-1.7378
CEU	CSH	81081945	5.0177	9.7221	-1.4538
CEU	CSH	81086945	7.4399	12.1099	-1.1748
CEU	CSH	81091945	7.4762	12.1099	-1.1657
CEU	CSH	81096945	5.4661	9.7221	-1.3152
CEU	CSH	81101945	5.9392	10.916	-1.3803
CEU	CSH	81106945	7.0369	11.5982	-1.1951
CEU	CSH	81111945	13.7105	17.0562	-0.6078
CEU	CSH	81116945	16.5095	20.1264	-0.5605
CEU	CSH	81121945	8.4605	13.3039	-1.1149
CEU	CSH	81126945	7.8728	14.8389	-1.4455
CEU	CSH	81131945	7.9419	14.1567	-1.3487
CEU	CSH	81136945	5.7854	9.0398	-1.076
CEU	CSH	81141945	15.1126	13.9861	0.2473
CEU	CSH	81146945	18.6539	19.4441	-0.1266
CEU	CSH	81151945	14.928	22.6848	-1.0708
CEU	CSH	81156945	24.8987	23.3671	0.2055
CEU	CSH	81161945	27.2278	21.1497	0.8978
CEU	CSH	81166945	24.0472	22.6848	0.1881
CEU	CSH	81171945	26.8598	24.2199	0.342
CEU	CSH	81176945	20.7394	20.9792	-0.0357
CEU	CSH	81181945	20.8103	25.7549	-0.6035
CEU	CSH	81186945	25.2136	30.1896	-0.5204
CEU	CSH	81191945	21.9584	21.832	0.0181
CEU	CSH	81196945	31.5632	19.103	2.0303
CEU	CSH	81201945	45.7918	23.8787	2.8784
CEU	CSH	81206945	39.2177	25.0727	1.7722
CEU	CSH	81211945	23.9546	22.0026	0.2775
CEU	CSH	81216945	15.3317	19.103	-0.6145
CEU	CSH	81221945	15.3865	17.2268	-0.3312
CEU	CSH	81226945	14.7835	16.7151	-0.3578

CEU	CSH	81231945	12.1429	16.374	-0.7993
CEU	CSH	81236945	31.2088	22.3437	1.2418
CEU	CSH	81241945	56.831	30.7012	2.6881
CEU	CSH	81246945	45.3506	23.0259	3.0376
CEU	CSH	81251945	32.4692	17.0562	2.8
CEU	CSH	81256945	36.4492	18.7619	2.9324
CEU	CSH	81261945	26.041	14.6684	2.386
CEU	CSH	81266945	11.6436	9.2104	0.7907
CEU	CSH	81271945	11.6858	10.4043	0.3718
CEU	CSH	81276945	19.2962	14.8389	0.9249
CEU	CSH	81281945	21.9256	16.5446	1.0065
CEU	CSH	81286945	15.3911	13.1333	0.5261
CEU	CSH	81291945	7.9897	7.8459	0.0542
CEU	CSH	81296945	8.1449	8.8692	-0.2437
CEU	CSH	81301945	11.0908	12.9627	-0.4416
CEU	CSH	81306945	12.3989	11.7688	0.1628
CEU	CSH	81311945	9.9296	8.8692	0.3568
CEU	CSH	81316945	7.4393	8.5281	-0.3799
CEU	CSH	81321945	5.658	7.6753	-0.7754
CEU	CSH	81326945	7.8932	8.187	-0.1064
CEU	CSH	81331945	10.8341	9.8926	0.2863
CEU	CSH	81336945	9.8563	9.5515	0.0958
CEU	CSH	81341945	7.1715	8.8692	-0.5713
CEU	CSH	81346945	5.3438	10.5749	-1.4946
CEU	CSH	81351945	6.6098	12.6216	-1.4545
CEU	CSH	81356945	8.6211	14.4978	-1.2468
CEU	CSH	81361945	10.3741	16.5446	-1.1541
CEU	CSH	81366945	13.9344	17.5679	-0.6417
CEU	CSH	81371945	16.1674	16.5446	-0.0706
CEU	CSH	81376945	11.757	13.3039	-0.3561
CEU	CSH	81381945	9.584	13.1333	-0.827
CEU	CSH	81386945	10.3878	14.4978	-0.8719
CEU	CSH	81391945	12.4993	16.2034	-0.7068
CEU	CSH	81396945	15.33	14.6684	0.1388
CEU	CSH	81401945	14.5733	13.4744	0.2499
CEU	CSH	81406945	13.3135	15.1801	-0.379
CEU	CSH	81411945	9.8604	13.1333	-0.7626
CEU	CSH	81416945	7.3552	10.7454	-0.9542
CEU	CSH	81421945	14.9167	13.9861	0.2043
CEU	CSH	81426945	22.0802	19.4441	0.4223
CEU	CSH	81431945	20.0297	16.5446	0.6519
CEU	CSH	81436945	20.0963	14.1567	1.2889
CEU	CSH	81441945	19.4036	16.374	0.5723
CEU	CSH	81446945	15.9361	17.5679	-0.2882
CEU	CSH	81451945	15.9524	16.8857	-0.1712
CEU	CSH	81456945	14.9592	14.6684	0.061
CEU	CSH	81461945	11.3774	12.7922	-0.338
CEU	CSH	81466945	8.9823	10.5749	-0.455
CEU	CSH	81471945	7.9738	9.8926	-0.5834

CEU	CSH	81476945	5.2412	8.3576	-1.1078
CEU	CSH	81481945	5.5861	7.8459	-0.8513
CEU	CSH	81486945	10.1909	11.0866	-0.2448
CEU	CSH	81491945	11.6142	13.1333	-0.354
CEU	CSH	81496945	12.6319	12.9627	-0.078
CEU	CSH	81501945	14.258	13.4744	0.1782
CEU	CSH	81506945	19.2361	16.5446	0.5034
CEU	CSH	81511945	22.364	17.2268	0.9244
CEU	CSH	81516945	20.5939	16.7151	0.7184
CEU	CSH	81521945	14.5051	12.9627	0.3639
CEU	CSH	81526945	11.659	10.2337	0.4199
CEU	CSH	81531945	12.6312	10.916	0.4757
CEU	CSH	81536945	10.1334	9.8926	0.0732
CEU	CSH	81541945	11.0169	12.2805	-0.3137
CEU	CSH	81546945	13.7044	13.645	0.0133
CEU	CSH	81551945	11.0425	10.916	0.0351
CEU	CSH	81556945	9.8081	11.7688	-0.5067
CEU	CSH	81561945	13.2171	13.645	-0.0962
CEU	CSH	81566945	13.2991	12.4511	0.2078
CEU	CSH	81571945	12.187	11.0866	0.3008
CEU	CSH	81576945	11.5885	10.4043	0.3435
CEU	CSH	81581945	9.8858	10.5749	-0.1969
CEU	CSH	81586945	16.9411	15.3506	0.3196
CHB	CSH	80586945	7.087	13.5532	-1.4583
CHB	CSH	80591945	4.8488	15.4168	-2.1085
CHB	CSH	80596945	6.4533	16.2638	-1.8599
CHB	CSH	80601945	4.9825	11.3508	-1.6978
CHB	CSH	80606945	4.0781	10.3343	-1.8211
CHB	CSH	80611945	6.1132	13.7226	-1.696
CHB	CSH	80616945	6.8526	13.3838	-1.4906
CHB	CSH	80621945	8.462	13.892	-1.1962
CHB	CSH	80626945	7.0855	13.045	-1.3935
CHB	CSH	80631945	6.416	11.1814	-1.2885
CHB	CSH	80636945	12.6469	16.0944	-0.6602
CHB	CSH	80641945	13.0809	15.7556	-0.5227
CHB	CSH	80646945	9.0883	13.3838	-0.9803
CHB	CSH	80651945	11.6398	14.5697	-0.6169
CHB	CSH	80656945	14.5184	15.4168	-0.1792
CHB	CSH	80661945	14.759	22.3628	-1.0613
CHB	CSH	80666945	19.8142	33.883	-1.3107
CHB	CSH	80671945	18.4837	34.3913	-1.4606
CHB	CSH	80676945	13.5244	20.6686	-1.0759
CHB	CSH	80681945	12.0469	13.5532	-0.3397
CHB	CSH	80686945	10.6665	17.2803	-1.1832
CHB	CSH	80691945	11.2715	18.2968	-1.1897
CHB	CSH	80696945	11.4101	14.7391	-0.6933
CHB	CSH	80701945	16.438	14.4003	0.4338
CHB	CSH	80706945	15.8072	17.4498	-0.2911
CHB	CSH	80711945	11.2859	17.1109	-1.052

CHB	CSH	80716945	11.526	16.7721	-0.9657
CHB	CSH	80721945	10.5339	16.2638	-1.0863
CHB	CSH	80726945	10.0658	13.3838	-0.7572
CHB	CSH	80731945	10.5128	13.045	-0.5921
CHB	CSH	80736945	8.7065	12.0285	-0.8386
CHB	CSH	80741945	11.3021	12.7061	-0.3366
CHB	CSH	80746945	10.6103	13.3838	-0.633
CHB	CSH	80751945	7.6202	11.3508	-0.9946
CHB	CSH	80756945	9.9375	11.8591	-0.4916
CHB	CSH	80761945	14.7016	15.2474	-0.1101
CHB	CSH	80766945	14.5365	14.4003	0.029
CHB	CSH	80771945	10.1966	13.7226	-0.7859
CHB	CSH	80776945	10.2852	12.5367	-0.5466
CHB	CSH	80781945	11.5399	12.3673	-0.2035
CHB	CSH	80786945	10.8405	14.2309	-0.73
CHB	CSH	80791945	9.1885	14.4003	-1.1096
CHB	CSH	80796945	8.9376	14.5697	-1.1858
CHB	CSH	80801945	6.6158	12.5367	-1.4375
CHB	CSH	80806945	6.1989	13.2144	-1.6205
CHB	CSH	80811945	7.7932	14.5697	-1.4268
CHB	CSH	80816945	8.453	14.5697	-1.2879
CHB	CSH	80821945	6.6766	12.5367	-1.4227
CHB	CSH	80826945	3.7472	9.8261	-1.8547
CHB	CSH	80831945	4.4569	9.8261	-1.6381
CHB	CSH	80836945	7.4634	10.1649	-0.7986
CHB	CSH	80841945	11.0234	10.3343	0.2006
CHB	CSH	80846945	10.1302	11.3508	-0.3254
CHB	CSH	80851945	7.0238	10.6731	-1.0307
CHB	CSH	80856945	4.5201	7.9625	-1.2756
CHB	CSH	80861945	4.9336	9.4872	-1.4354
CHB	CSH	80866945	7.1631	11.1814	-1.0865
CHB	CSH	80871945	5.9387	8.3013	-0.8426
CHB	CSH	80876945	5.1982	8.3013	-1.1067
CHB	CSH	80881945	7.9842	12.0285	-1.021
CHB	CSH	80886945	12.2758	13.892	-0.356
CHB	CSH	80891945	23.4191	17.4498	1.058
CHB	CSH	80896945	31.6222	20.6686	1.6497
CHB	CSH	80901945	25.5662	18.4662	1.1918
CHB	CSH	80906945	17.8051	16.9415	0.1575
CHB	CSH	80911945	15.2835	16.0944	-0.1553
CHB	CSH	80916945	13.415	12.7061	0.1699
CHB	CSH	80921945	14.8799	12.5367	0.5689
CHB	CSH	80926945	17.8806	17.2803	0.1074
CHB	CSH	80931945	21.5622	21.1769	0.0567
CHB	CSH	80936945	22.435	19.3133	0.5019
CHB	CSH	80941945	13.8761	14.4003	-0.1116
CHB	CSH	80946945	11.3828	14.7391	-0.6989
CHB	CSH	80951945	15.3626	16.9415	-0.2879
CHB	CSH	80956945	15.3815	16.4333	-0.1974

CHB	CSH	80961945	16.6104	15.0779	0.3123
CHB	CSH	80966945	20.188	16.4333	0.7048
CHB	CSH	80971945	15.1957	16.4333	-0.2323
CHB	CSH	80976945	10.4415	14.0615	-0.7884
CHB	CSH	80981945	14.8152	15.7556	-0.1838
CHB	CSH	80986945	26.5561	22.1934	0.6134
CHB	CSH	80991945	28.9468	25.4123	0.4358
CHB	CSH	80996945	26.4749	23.8875	0.3388
CHB	CSH	81001945	29.2215	21.3463	1.1497
CHB	CSH	81006945	20.4169	15.4168	0.9976
CHB	CSH	81011945	12.5328	14.0615	-0.3329
CHB	CSH	81016945	14.7405	15.4168	-0.1349
CHB	CSH	81021945	29.7452	19.1439	1.7189
CHB	CSH	81026945	35.247	21.1769	2.0699
CHB	CSH	81031945	33.1755	21.3463	1.7269
CHB	CSH	81036945	30.4799	21.3463	1.3334
CHB	CSH	81041945	20.7824	15.925	0.9396
CHB	CSH	81046945	18.4719	13.7226	1.0585
CHB	CSH	81051945	15.4575	14.4003	0.2251
CHB	CSH	81056945	12.4671	13.045	-0.1351
CHB	CSH	81061945	12.0521	12.3673	-0.0775
CHB	CSH	81066945	9.8014	11.6896	-0.4897
CHB	CSH	81071945	5.3925	10.3343	-1.4385
CHB	CSH	81076945	6.7841	12.0285	-1.3239
CHB	CSH	81081945	9.4163	13.2144	-0.8773
CHB	CSH	81086945	12.904	17.7886	-0.8499
CHB	CSH	81091945	12.8352	19.4827	-1.0598
CHB	CSH	81096945	8.249	14.0615	-1.2658
CHB	CSH	81101945	7.9751	12.3673	-1.0801
CHB	CSH	81106945	10.0581	13.892	-0.8446
CHB	CSH	81111945	23.2713	20.1604	0.4799
CHB	CSH	81116945	29.3718	28.8006	0.0624
CHB	CSH	81121945	16.5119	29.3088	-1.3735
CHB	CSH	81126945	10.4258	23.5487	-1.7422
CHB	CSH	81131945	8.6706	16.4333	-1.4572
CHB	CSH	81136945	6.6077	12.1979	-1.3928
CHB	CSH	81141945	20.0418	18.8051	0.204
CHB	CSH	81146945	28.9146	24.904	0.5043
CHB	CSH	81151945	21.3154	24.904	-0.4513
CHB	CSH	81156945	30.1746	25.0734	0.6373
CHB	CSH	81161945	32.2583	22.024	1.4496
CHB	CSH	81166945	23.6257	21.8545	0.2528
CHB	CSH	81171945	27.2288	25.7511	0.1799
CHB	CSH	81176945	28.5291	25.5817	0.3611
CHB	CSH	81181945	39.6708	30.4947	0.9475
CHB	CSH	81186945	47.6945	33.0359	1.3999
CHB	CSH	81191945	31.6614	23.8875	1.0179
CHB	CSH	81196945	25.5487	23.5487	0.2655
CHB	CSH	81201945	31.574	27.2758	0.4948

CHB	CSH	81206945	29.9621	27.1064	0.3307
CHB	CSH	81211945	19.8353	22.871	-0.4146
CHB	CSH	81216945	9.0402	19.3133	-1.6516
CHB	CSH	81221945	11.0152	18.9745	-1.3016
CHB	CSH	81226945	12.0817	17.6192	-0.9724
CHB	CSH	81231945	7.2707	14.7391	-1.5553
CHB	CSH	81236945	22.3476	22.1934	0.0217
CHB	CSH	81241945	46.6296	33.3748	1.2532
CHB	CSH	81246945	41.0707	25.0734	1.9985
CHB	CSH	81251945	32.8003	18.1274	2.5072
CHB	CSH	81256945	23.3406	19.4827	0.6151
CHB	CSH	81261945	10.1405	15.925	-1.1189
CHB	CSH	81266945	8.3101	10.6731	-0.6674
CHB	CSH	81271945	10.8528	11.8591	-0.2575
CHB	CSH	81276945	18.2583	17.2803	0.175
CHB	CSH	81281945	20.0508	17.2803	0.4956
CHB	CSH	81286945	13.799	12.8755	0.2186
CHB	CSH	81291945	6.9353	12.0285	-1.2858
CHB	CSH	81296945	5.1424	12.7061	-1.8132
CHB	CSH	81301945	8.5702	13.7226	-1.1484
CHB	CSH	81306945	10.2821	13.045	-0.6461
CHB	CSH	81311945	6.846	9.6567	-0.8715
CHB	CSH	81316945	4.7669	9.6567	-1.5162
CHB	CSH	81321945	3.6089	9.6567	-1.8753
CHB	CSH	81326945	7.2197	10.1649	-0.8706
CHB	CSH	81331945	8.4019	10.1649	-0.5212
CHB	CSH	81336945	5.6168	9.8261	-1.2843
CHB	CSH	81341945	5.2199	10.1649	-1.4618
CHB	CSH	81346945	5.068	9.4872	-1.393
CHB	CSH	81351945	5.7822	10.3343	-1.3251
CHB	CSH	81356945	10.1799	13.3838	-0.7312
CHB	CSH	81361945	14.3786	15.5862	-0.2384
CHB	CSH	81366945	15.4864	16.2638	-0.1474
CHB	CSH	81371945	15.7619	17.7886	-0.3526
CHB	CSH	81376945	11.7611	16.2638	-0.8536
CHB	CSH	81381945	10.6682	16.0944	-1.0391
CHB	CSH	81386945	12.1009	17.7886	-0.9896
CHB	CSH	81391945	14.9391	18.6357	-0.6151
CHB	CSH	81396945	13.9667	16.0944	-0.4074
CHB	CSH	81401945	11.3166	14.0615	-0.5978
CHB	CSH	81406945	13.0272	17.7886	-0.8285
CHB	CSH	81411945	10.9069	16.6027	-1.0588
CHB	CSH	81416945	8.5972	12.8755	-1.0129
CHB	CSH	81421945	11.0754	16.9415	-1.0695
CHB	CSH	81426945	15.5518	20.1604	-0.7109
CHB	CSH	81431945	19.8545	18.4662	0.233
CHB	CSH	81436945	22.3844	19.1439	0.5254
CHB	CSH	81441945	22.4386	21.0075	0.2122
CHB	CSH	81446945	18.7363	19.6521	-0.1448

CHB	CSH	81451945	16.5728	18.8051	-0.3682
CHB	CSH	81456945	16.0426	18.1274	-0.3562
CHB	CSH	81461945	12.7542	14.4003	-0.3505
CHB	CSH	81466945	10.0527	11.3508	-0.3461
CHB	CSH	81471945	8.9914	11.012	-0.5542
CHB	CSH	81476945	7.4736	9.3178	-0.5911
CHB	CSH	81481945	7.3531	9.8261	-0.7545
CHB	CSH	81486945	12.4091	14.9085	-0.5149
CHB	CSH	81491945	14.3872	16.6027	-0.4118
CHB	CSH	81496945	15.5385	16.2638	-0.1375
CHB	CSH	81501945	16.8401	15.2474	0.3211
CHB	CSH	81506945	19.2632	17.4498	0.3214
CHB	CSH	81511945	18.847	18.2968	0.0932
CHB	CSH	81516945	14.2098	14.9085	-0.1439
CHB	CSH	81521945	10.142	11.012	-0.2386
CHB	CSH	81526945	10.6217	11.012	-0.1071
CHB	CSH	81531945	11.7986	11.1814	0.1669
CHB	CSH	81536945	10.1289	10.8426	-0.1986
CHB	CSH	81541945	9.9874	12.5367	-0.6189
CHB	CSH	81546945	10.8538	12.5367	-0.4086
CHB	CSH	81551945	11.071	12.5367	-0.3558
CHB	CSH	81556945	12.0346	13.7226	-0.3762
CHB	CSH	81561945	13.4611	14.0615	-0.1308
CHB	CSH	81566945	12.9549	12.7061	0.0596
CHB	CSH	81571945	12.0851	12.3673	-0.0694
CHB	CSH	81576945	10.6524	13.5532	-0.6542
CHB	CSH	81581945	8.6465	13.2144	-1.0552
CHB	CSH	81586945	16.9518	16.7721	0.0331
YRI	CSH	80586945	17.7652	19.3272	-0.2501
YRI	CSH	80591945	16.6449	20.8397	-0.6246
YRI	CSH	80596945	16.1466	22.6884	-0.8973
YRI	CSH	80601945	15.1322	21.0078	-0.8682
YRI	CSH	80606945	16.1631	21.0078	-0.7158
YRI	CSH	80611945	16.7757	19.9994	-0.4995
YRI	CSH	80616945	18.5138	18.1507	0.0618
YRI	CSH	80621945	21.0887	19.6633	0.2245
YRI	CSH	80626945	16.1844	17.1423	-0.1721
YRI	CSH	80631945	12.9948	15.9659	-0.5714
YRI	CSH	80636945	23.1102	23.8648	-0.0986
YRI	CSH	80641945	23.0168	25.0413	-0.2524
YRI	CSH	80646945	19.7718	22.0162	-0.3169
YRI	CSH	80651945	19.2651	21.68	-0.3461
YRI	CSH	80656945	20.4998	22.1842	-0.2361
YRI	CSH	80661945	36.4306	28.4025	0.8855
YRI	CSH	80666945	55.6888	40.6711	1.166
YRI	CSH	80671945	51.2739	43.192	0.5915
YRI	CSH	80676945	27.6908	28.9067	-0.1318
YRI	CSH	80681945	13.7961	20.1675	-0.9792
YRI	CSH	80686945	15.1211	23.1926	-1.0839

YRI	CSH	80691945	17.4303	25.8816	-1.0203
YRI	CSH	80696945	14.8759	24.0329	-1.1879
YRI	CSH	80701945	17.491	24.5371	-0.8959
YRI	CSH	80706945	18.1863	25.2093	-0.8698
YRI	CSH	80711945	15.8122	21.8481	-0.8587
YRI	CSH	80716945	14.9556	20.1675	-0.801
YRI	CSH	80721945	14.571	23.0245	-1.1432
YRI	CSH	80726945	14.1362	23.8648	-1.2707
YRI	CSH	80731945	13.7214	21.0078	-1.0766
YRI	CSH	80736945	12.0915	20.1675	-1.2412
YRI	CSH	80741945	12.8666	23.3606	-1.3993
YRI	CSH	80746945	11.7153	23.3606	-1.5529
YRI	CSH	80751945	8.5826	20.3355	-1.7919
YRI	CSH	80756945	11.7603	20.5036	-1.3225
YRI	CSH	80761945	16.7045	24.5371	-0.9959
YRI	CSH	80766945	14.9881	23.1926	-1.1017
YRI	CSH	80771945	13.3591	23.6968	-1.3595
YRI	CSH	80776945	15.0347	23.8648	-1.1534
YRI	CSH	80781945	14.8358	19.4952	-0.7399
YRI	CSH	80786945	15.8127	18.991	-0.5176
YRI	CSH	80791945	16.0183	22.3523	-0.8815
YRI	CSH	80796945	14.8076	23.3606	-1.1405
YRI	CSH	80801945	12.8728	21.8481	-1.2769
YRI	CSH	80806945	12.7162	20.8397	-1.2097
YRI	CSH	80811945	14.9895	20.6717	-0.8528
YRI	CSH	80816945	14.5085	22.6884	-1.122
YRI	CSH	80821945	10.7267	21.1758	-1.5321
YRI	CSH	80826945	8.5949	17.4785	-1.5667
YRI	CSH	80831945	9.0607	13.445	-0.9929
YRI	CSH	80836945	11.086	11.7644	-0.1743
YRI	CSH	80841945	16.7337	13.9492	0.609
YRI	CSH	80846945	18.0423	17.4785	0.0994
YRI	CSH	80851945	13.9079	18.1507	-0.7216
YRI	CSH	80856945	12.2253	18.3188	-1.0273
YRI	CSH	80861945	13.4682	19.9994	-1.0119
YRI	CSH	80866945	12.8822	19.6633	-1.0679
YRI	CSH	80871945	10.9016	16.4701	-1.0395
YRI	CSH	80876945	10.5575	16.302	-1.083
YRI	CSH	80881945	11.7623	16.8062	-0.9236
YRI	CSH	80886945	14.6899	17.8146	-0.5411
YRI	CSH	80891945	27.4075	24.7051	0.3413
YRI	CSH	80896945	33.7282	26.7219	0.82
YRI	CSH	80901945	26.0154	24.8732	0.1433
YRI	CSH	80906945	21.0231	23.3606	-0.3117
YRI	CSH	80911945	20.4303	23.0245	-0.3508
YRI	CSH	80916945	25.3654	27.3941	-0.2318
YRI	CSH	80921945	26.3486	27.058	-0.082
YRI	CSH	80926945	22.035	25.0413	-0.3748
YRI	CSH	80931945	22.6293	27.5622	-0.5602

YRI	CSH	80936945	21.9952	27.058	-0.5854
YRI	CSH	80941945	15.649	21.8481	-0.8819
YRI	CSH	80946945	16.0884	21.68	-0.8015
YRI	CSH	80951945	22.5629	26.0496	-0.4183
YRI	CSH	80956945	19.272	23.8648	-0.5999
YRI	CSH	80961945	15.5834	20.5036	-0.7442
YRI	CSH	80966945	17.2582	20.1675	-0.4471
YRI	CSH	80971945	14.3602	18.1507	-0.6447
YRI	CSH	80976945	16.0993	15.7978	0.0586
YRI	CSH	80981945	25.9236	17.8146	1.4042
YRI	CSH	80986945	43.0054	24.7051	2.3114
YRI	CSH	80991945	46.142	26.0496	2.4105
YRI	CSH	80996945	42.6103	27.5622	1.709
YRI	CSH	81001945	43.1585	26.3858	1.9874
YRI	CSH	81006945	26.3834	22.0162	0.6167
YRI	CSH	81011945	14.8222	25.8816	-1.3352
YRI	CSH	81016945	19.0888	25.2093	-0.7581
YRI	CSH	81021945	33.0876	24.7051	1.0588
YRI	CSH	81026945	37.4259	26.0496	1.3648
YRI	CSH	81031945	36.1353	27.3941	0.9986
YRI	CSH	81036945	33.4425	28.5706	0.5343
YRI	CSH	81041945	20.9828	24.369	-0.4334
YRI	CSH	81046945	17.9686	23.0245	-0.6837
YRI	CSH	81051945	16.8243	19.9994	-0.4919
YRI	CSH	81056945	15.9894	16.302	-0.0589
YRI	CSH	81061945	16.1871	17.9827	-0.3081
YRI	CSH	81066945	13.6816	17.8146	-0.7157
YRI	CSH	81071945	10.4289	15.2937	-0.9748
YRI	CSH	81076945	11.5916	15.6298	-0.7925
YRI	CSH	81081945	14.0229	18.3188	-0.7242
YRI	CSH	81086945	22.6615	23.1926	-0.0713
YRI	CSH	81091945	24.3989	26.3858	-0.2354
YRI	CSH	81096945	16.7067	23.3606	-0.8873
YRI	CSH	81101945	14.3042	20.5036	-0.9377
YRI	CSH	81106945	13.4535	20.1675	-1.0319
YRI	CSH	81111945	24.8088	25.5455	-0.0901
YRI	CSH	81116945	36.1364	35.125	0.0907
YRI	CSH	81121945	31.7044	35.7973	-0.3601
YRI	CSH	81126945	24.6886	30.5873	-0.6053
YRI	CSH	81131945	16.1253	22.6884	-0.9003
YRI	CSH	81136945	11.9896	20.1675	-1.2569
YRI	CSH	81141945	28.2441	29.9151	-0.1752
YRI	CSH	81146945	36.918	33.9486	0.2752
YRI	CSH	81151945	27.7604	31.0915	-0.3364
YRI	CSH	81156945	28.607	33.2763	-0.4413
YRI	CSH	81161945	28.773	30.9234	-0.2183
YRI	CSH	81166945	29.7801	31.7638	-0.1962
YRI	CSH	81171945	33.1436	34.6208	-0.1343
YRI	CSH	81176945	28.9694	32.0999	-0.3064

YRI	CSH	81181945	36.3696	35.2931	0.096
YRI	CSH	81186945	45.4169	38.9904	0.5201
YRI	CSH	81191945	35.2463	35.6292	-0.0338
YRI	CSH	81196945	35.3927	35.125	0.024
YRI	CSH	81201945	46.3088	35.6292	0.944
YRI	CSH	81206945	44.9518	36.6376	0.7151
YRI	CSH	81211945	37.2681	38.6543	-0.1131
YRI	CSH	81216945	26.5874	34.6208	-0.7304
YRI	CSH	81221945	21.2554	28.4025	-0.7883
YRI	CSH	81226945	19.1855	24.5371	-0.6804
YRI	CSH	81231945	17.9224	25.7135	-0.9466
YRI	CSH	81236945	39.6734	35.4611	0.3741
YRI	CSH	81241945	64.3214	43.8642	1.4747
YRI	CSH	81246945	54.1986	37.6459	1.3864
YRI	CSH	81251945	42.8976	30.7554	1.2393
YRI	CSH	81256945	36.5715	27.2261	1.0741
YRI	CSH	81261945	24.2355	23.1926	0.14
YRI	CSH	81266945	15.7598	18.3188	-0.4314
YRI	CSH	81271945	17.6864	18.1507	-0.079
YRI	CSH	81276945	26.5173	23.1926	0.4464
YRI	CSH	81281945	29.0438	24.5371	0.573
YRI	CSH	81286945	23.3353	21.8481	0.2116
YRI	CSH	81291945	15.4937	20.5036	-0.7578
YRI	CSH	81296945	14.4566	18.6549	-0.6955
YRI	CSH	81301945	15.4916	17.3104	-0.3237
YRI	CSH	81306945	14.2878	18.3188	-0.6796
YRI	CSH	81311945	11.0245	15.6298	-0.9038
YRI	CSH	81316945	9.2673	13.613	-0.9727
YRI	CSH	81321945	7.2751	13.445	-1.3973
YRI	CSH	81326945	7.9739	13.9492	-1.3068
YRI	CSH	81331945	9.6695	14.7895	-1.0592
YRI	CSH	81336945	8.0537	13.7811	-1.2671
YRI	CSH	81341945	8.2324	13.445	-1.1805
YRI	CSH	81346945	9.3512	15.9659	-1.2721
YRI	CSH	81351945	10.2542	17.8146	-1.3092
YRI	CSH	81356945	14.5523	21.3439	-0.9883
YRI	CSH	81361945	18.4675	22.5203	-0.5599
YRI	CSH	81366945	18.2798	21.8481	-0.5076
YRI	CSH	81371945	18.1141	23.1926	-0.6819
YRI	CSH	81376945	13.0866	19.4952	-1.0176
YRI	CSH	81381945	11.966	18.823	-1.1262
YRI	CSH	81386945	14.7738	22.5203	-1.0702
YRI	CSH	81391945	16.5447	25.2093	-1.0731
YRI	CSH	81396945	18.4724	25.5455	-0.8648
YRI	CSH	81401945	16.1462	24.369	-1.0525
YRI	CSH	81406945	17.2485	26.3858	-1.0827
YRI	CSH	81411945	16.0876	22.5203	-0.8887
YRI	CSH	81416945	13.0645	18.823	-0.9458
YRI	CSH	81421945	18.8493	23.1926	-0.5832

YRI	CSH	81426945	22.5929	24.5371	-0.2472
YRI	CSH	81431945	23.3272	23.0245	0.0409
YRI	CSH	81436945	24.3232	24.5371	-0.0272
YRI	CSH	81441945	27.385	28.5706	-0.13
YRI	CSH	81446945	24.725	26.2177	-0.178
YRI	CSH	81451945	20.7997	24.0329	-0.4194
YRI	CSH	81456945	19.2548	22.1842	-0.4107
YRI	CSH	81461945	13.0221	19.8313	-1.0636
YRI	CSH	81466945	11.4215	19.4952	-1.282
YRI	CSH	81471945	12.0793	17.8146	-0.9931
YRI	CSH	81476945	9.99	15.4617	-1.085
YRI	CSH	81481945	9.0089	12.7727	-0.8948
YRI	CSH	81486945	11.0214	17.9827	-1.1946
YRI	CSH	81491945	11.7802	21.68	-1.419
YRI	CSH	81496945	12.7527	21.3439	-1.2501
YRI	CSH	81501945	13.7705	21.68	-1.1337
YRI	CSH	81506945	20.1014	24.5371	-0.564
YRI	CSH	81511945	21.7059	24.369	-0.3409
YRI	CSH	81516945	17.2418	22.0162	-0.6742
YRI	CSH	81521945	12.8925	18.823	-0.9741
YRI	CSH	81526945	13.4496	17.6465	-0.7334
YRI	CSH	81531945	14.2259	17.8146	-0.6214
YRI	CSH	81536945	11.5829	16.302	-0.8897
YRI	CSH	81541945	13.2221	17.8146	-0.7953
YRI	CSH	81546945	12.3123	19.4952	-1.1406
YRI	CSH	81551945	11.5477	18.4868	-1.1596
YRI	CSH	81556945	12.8026	18.1507	-0.9096
YRI	CSH	81561945	12.6477	19.1591	-1.0514
YRI	CSH	81566945	11.9607	16.4701	-0.8418
YRI	CSH	81571945	13.7137	16.4701	-0.5146
YRI	CSH	81576945	13.5212	19.8313	-0.9856
YRI	CSH	81581945	10.9367	19.9994	-1.4041
YRI	CSH	81586945	19.9545	23.6968	-0.4922
CEU	CSH	17832873	7.3686	6.4814	0.3977
CEU	CSH	17837873	4.6994	4.2641	0.2826
CEU	CSH	17842873	4.7669	4.9463	-0.1023
CEU	CSH	17847873	8.1705	6.4814	0.7571
CEU	CSH	17852873	9.6425	7.5047	0.8388
CEU	CSH	17857873	7.0738	7.1636	-0.0368
CEU	CSH	17862873	6.3393	6.1402	0.0937
CEU	CSH	17867873	7.053	6.3108	0.3408
CEU	CSH	17872873	8.2248	6.8225	0.6
CEU	CSH	17877873	7.5099	6.3108	0.5505
CEU	CSH	17882873	6.167	5.6286	0.2739
CEU	CSH	17887873	4.0491	3.4112	0.501
CEU	CSH	17892873	3.9262	3.9229	0.0023
CEU	CSH	17897873	5.8456	5.9697	-0.0599
CEU	CSH	17902873	7.1439	7.8459	-0.2645
CEU	CSH	17907873	6.0606	7.5047	-0.5666

CEU	CSH	17912873	3.7333	4.6052	-0.5294
CEU	CSH	17917873	3.6887	5.6286	-0.9868
CEU	CSH	17922873	3.4603	5.6286	-1.1029
CEU	CSH	17927873	3.1835	4.0935	-0.6119
CEU	CSH	17932873	2.6722	3.2407	-0.4662
CEU	CSH	17937873	3.7186	3.0701	0.5563
CEU	CSH	17942873	5.1983	3.7524	1.0475
CEU	CSH	17947873	7.9951	5.7991	1.0877
CEU	CSH	17952873	9.8233	7.1636	1.0887
CEU	CSH	17957873	7.269	5.7991	0.728
CEU	CSH	17962873	6.0202	5.9697	0.0244
CEU	CSH	17967873	5.4826	5.9697	-0.2351
CEU	CSH	17972873	7.4486	7.6753	-0.0871
CEU	CSH	17977873	9.1358	8.3576	0.2766
CEU	CSH	17982873	7.6842	5.7991	0.9337
CEU	CSH	17987873	9.1567	5.7991	1.663
CEU	CSH	17992873	8.5624	6.4814	0.9327
CEU	CSH	17997873	4.2612	5.7991	-0.7617
CEU	CSH	18002873	4.3781	4.9463	-0.324
CEU	CSH	18007873	7.9503	5.9697	0.9559
CEU	CSH	18012873	7.9336	7.8459	0.033
CEU	CSH	18017873	7.5976	7.5047	0.0364
CEU	CSH	18022873	7.5461	5.9697	0.7608
CEU	CSH	18027873	7.1375	5.9697	0.5636
CEU	CSH	18032873	5.2226	4.7757	0.2628
CEU	CSH	18037873	4.0282	4.7757	-0.4396
CEU	CSH	18042873	8.6768	7.8459	0.313
CEU	CSH	18047873	7.4682	6.8225	0.2763
CEU	CSH	18052873	5.2847	5.1169	0.0929
CEU	CSH	18057873	6.6246	4.7757	1.0874
CEU	CSH	18062873	7.3741	5.6286	0.8879
CEU	CSH	18067873	10.2045	8.6987	0.5159
CEU	CSH	18072873	9.8231	9.3809	0.1413
CEU	CSH	18077873	7.778	7.1636	0.2515
CEU	CSH	18082873	5.7763	4.7757	0.5885
CEU	CSH	18087873	6.0336	7.3342	-0.5211
CEU	CSH	18092873	8.3892	9.7221	-0.4119
CEU	CSH	18097873	7.7079	8.0164	-0.1139
CEU	CSH	18102873	10.6813	8.6987	0.6792
CEU	CSH	18107873	14.3709	10.4043	1.1507
CEU	CSH	18112873	11.9256	10.2337	0.4984
CEU	CSH	18117873	8.2224	9.2104	-0.321
CEU	CSH	18122873	8.9738	9.3809	-0.1301
CEU	CSH	18127873	11.9946	10.2337	0.5188
CEU	CSH	18132873	12.4816	10.4043	0.6026
CEU	CSH	18137873	9.7811	10.2337	-0.1333
CEU	CSH	18142873	6.5234	9.3809	-0.9129
CEU	CSH	18147873	5.5609	8.3576	-0.9941
CEU	CSH	18152873	6.1099	6.8225	-0.3049

CEU	CSH	18157873	4.6745	6.1402	-0.6897
CEU	CSH	18162873	4.8681	7.1636	-0.9397
CEU	CSH	18167873	7.168	8.6987	-0.5244
CEU	CSH	18172873	8.2707	9.7221	-0.4485
CEU	CSH	18177873	8.5834	9.7221	-0.3519
CEU	CSH	18182873	8.3836	9.5515	-0.3669
CEU	CSH	18187873	11.107	9.7221	0.428
CEU	CSH	18192873	9.8707	8.3576	0.5379
CEU	CSH	18197873	9.7489	8.5281	0.426
CEU	CSH	18202873	8.1055	8.187	-0.0295
CEU	CSH	18207873	8.7223	8.6987	0.0081
CEU	CSH	18212873	12.2791	10.5749	0.4869
CEU	CSH	18217873	11.5844	10.0632	0.4552
CEU	CSH	18222873	14.9608	10.916	1.1218
CEU	CSH	18227873	12.5925	9.0398	1.1746
CEU	CSH	18232873	6.3655	6.6519	-0.1254
CEU	CSH	18237873	7.7112	8.5281	-0.285
CEU	CSH	18242873	10.0992	8.8692	0.4139
CEU	CSH	18247873	8.9384	8.3576	0.2065
CEU	CSH	18252873	14.0113	12.1099	0.4783
CEU	CSH	18257873	16.2043	12.7922	0.8151
CEU	CSH	18262873	12.524	10.0632	0.7364
CEU	CSH	18267873	15.4534	11.2571	1.1307
CEU	CSH	18272873	11.9618	9.5515	0.7572
CEU	CSH	18277873	5.1841	6.1402	-0.4499
CEU	CSH	18282873	4.8767	6.8225	-0.8326
CEU	CSH	18287873	25.8551	13.3039	2.8891
CEU	CSH	18292873	35.6059	15.0095	4.2277
CEU	CSH	18297873	16.3232	8.8692	2.5083
CEU	CSH	18302873	9.0355	7.5047	0.6006
CEU	CSH	18307873	7.6001	7.3342	0.1065
CEU	CSH	18312873	4.8668	5.7991	-0.4618
CEU	CSH	18317873	3.86	5.458	-0.8354
CEU	CSH	18322873	4.2228	5.9697	-0.8431
CEU	CSH	18327873	4.1859	5.2874	-0.5923
CEU	CSH	18332873	3.2105	3.0701	0.1204
CEU	CSH	18337873	3.0167	1.5351	2.1969
CEU	CSH	18342873	4.7899	2.3879	2.5296
CEU	CSH	18347873	4.6976	4.0935	0.4062
CEU	CSH	18352873	11.3581	7.1636	1.717
CEU	CSH	18357873	12.8806	7.5047	2.1092
CEU	CSH	18362873	5.1148	5.458	-0.1794
CEU	CSH	18367873	4.9169	6.1402	-0.5757
CEU	CSH	18372873	2.7976	4.0935	-0.8714
CEU	CSH	18377873	1.7666	3.4112	-1.2917
CEU	CSH	18382873	2.6277	4.9463	-1.3223
CEU	CSH	18387873	2.6805	4.4346	-1.1006
CEU	CSH	18392873	5.5213	4.6052	0.5562
CEU	CSH	18397873	4.383	3.9229	0.3209

CEU	CSH	18402873	1.9105	2.729	-0.7737
CEU	CSH	18407873	1.5265	2.3879	-0.9071
CEU	CSH	18412873	1.6834	2.729	-0.9883
CEU	CSH	18417873	2.73	3.7524	-0.7407
CEU	CSH	18422873	1.9701	2.8996	-0.8359
CEU	CSH	18427873	1.0243	2.2173	-1.3328
CEU	CSH	18432873	0.6621	2.729	-1.9537
CEU	CSH	18437873	0.7205	2.3879	-1.7559
CEU	CSH	18442873	0.623	2.0467	-1.694
CEU	CSH	18447873	0.6768	1.7056	-1.4091
CEU	CSH	18452873	0.7238	1.3645	-1.0365
CEU	CSH	18457873	0.3847	1.3645	-1.5851
CEU	CSH	18462873	1.686	2.0467	-0.4292
CEU	CSH	18467873	2.1128	3.9229	-1.2625
CEU	CSH	18472873	2.67	5.458	-1.4575
CEU	CSH	18477873	3.2	3.7524	-0.4002
CEU	CSH	18482873	1.8992	2.2173	-0.3554
CEU	CSH	18487873	2.1	2.3879	-0.3032
CEU	CSH	18492873	3.8322	4.0935	-0.1757
CEU	CSH	18497873	5.5799	5.9697	-0.1881
CEU	CSH	18502873	5.6238	4.9463	0.3864
CEU	CSH	18507873	2.9895	3.2407	-0.206
CEU	CSH	18512873	1.8481	4.2641	-1.5683
CEU	CSH	18517873	2.8864	4.6052	-1.0436
CEU	CSH	18522873	3.2746	4.0935	-0.5507
CEU	CSH	18527873	4.946	5.2874	-0.1836
CEU	CSH	18532873	5.2338	4.9463	0.164
CEU	CSH	18537873	9.0981	7.1636	0.7919
CEU	CSH	18542873	12.1523	10.2337	0.5652
CEU	CSH	18547873	10.6617	10.7454	-0.0236
CEU	CSH	18552873	7.6696	8.6987	-0.3526
CEU	CSH	18557873	9.619	8.187	0.5188
CEU	CSH	18562873	9.4816	7.1636	0.9489
CEU	CSH	18567873	6.0303	5.2874	0.3995
CEU	CSH	18572873	10.7535	6.9931	1.5734
CEU	CSH	18577873	12.0293	10.916	0.3088
CEU	CSH	18582873	15.1853	12.9627	0.5243
CEU	CSH	18587873	20.2626	12.1099	2.051
CEU	CSH	18592873	17.1831	9.8926	2.2168
CEU	CSH	18597873	11.4669	7.8459	1.3641
CEU	CSH	18602873	7.9814	7.6753	0.1177
CEU	CSH	18607873	6.1119	6.3108	-0.0913
CEU	CSH	18612873	5.1717	5.7991	-0.3107
CEU	CSH	18617873	9.7619	9.5515	0.0661
CEU	CSH	18622873	10.4156	9.5515	0.2715
CEU	CSH	18627873	8.2191	7.1636	0.4321
CEU	CSH	18632873	9.1214	7.1636	0.8014
CEU	CSH	18637873	9.625	6.9931	1.1012
CEU	CSH	18642873	9.7289	7.1636	1.0501

CEU	CSH	18647873	7.7974	7.3342	0.1856
CEU	CSH	18652873	7.9182	7.8459	0.0272
CEU	CSH	18657873	8.7931	8.3576	0.1548
CEU	CSH	18662873	10.2901	9.5515	0.232
CEU	CSH	18667873	10.0773	8.8692	0.4065
CEU	CSH	18672873	9.6465	8.5281	0.3902
CEU	CSH	18677873	11.7667	9.0398	0.9016
CEU	CSH	18682873	15.4617	9.2104	2.0313
CEU	CSH	18687873	13.399	7.6753	2.2
CEU	CSH	18692873	7.8871	5.9697	0.9254
CEU	CSH	18697873	9.3348	7.8459	0.5609
CEU	CSH	18702873	12.0672	9.5515	0.7903
CEU	CSH	18707873	9.2109	8.0164	0.4412
CEU	CSH	18712873	7.7803	6.1402	0.7718
CEU	CSH	18717873	7.3603	4.9463	1.3767
CEU	CSH	18722873	7.3265	5.2874	1.0964
CEU	CSH	18727873	6.9114	5.6286	0.6525
CEU	CSH	18732873	3.3443	4.2641	-0.5971
CEU	CSH	18737873	4.7318	5.7991	-0.5286
CEU	CSH	18742873	4.737	5.2874	-0.296
CEU	CSH	18747873	5.1602	5.2874	-0.0684
CEU	CSH	18752873	8.4636	7.1636	0.5322
CEU	CSH	18757873	11.9584	7.5047	1.7474
CEU	CSH	18762873	17.0954	10.5749	1.863
CEU	CSH	18767873	18.8435	11.9394	1.7602
CEU	CSH	18772873	20.188	11.9394	2.103
CEU	CSH	18777873	16.733	9.7221	2.1666
CEU	CSH	18782873	13.6879	8.5281	1.8003
CEU	CSH	18787873	15.853	10.4043	1.5806
CEU	CSH	18792873	15.8189	10.2337	1.6454
CEU	CSH	18797873	13.1436	8.5281	1.6104
CEU	CSH	18802873	10.4063	7.6753	1.0497
CEU	CSH	18807873	11.529	8.3576	1.1273
CEU	CSH	18812873	12.1532	9.0398	1.0294
CEU	CSH	18817873	11.2694	10.0632	0.361
CEU	CSH	18822873	8.4787	9.2104	-0.2378
CEU	CSH	18827873	7.0161	8.0164	-0.3695
CEU	CSH	18832873	7.5807	8.187	-0.2196
CEU	CSH	18837873	7.9659	8.8692	-0.304
CEU	CSH	18842873	12.936	10.0632	0.8597
CEU	CSH	18847873	13.8582	10.4043	1.002
CEU	CSH	18852873	10.6277	11.2571	-0.1696
CEU	CSH	18857873	7.3523	9.8926	-0.7724
CHB	CSH	17832873	3.8578	7.9625	-1.5211
CHB	CSH	17837873	3.5186	6.6072	-1.3565
CHB	CSH	17842873	3.2153	6.4378	-1.4489
CHB	CSH	17847873	5.2509	9.8261	-1.3959
CHB	CSH	17852873	6.746	10.5037	-1.0773
CHB	CSH	17857873	4.961	8.4708	-1.2287

CHB	CSH	17862873	4.8835	8.3013	-1.2189
CHB	CSH	17867873	5.6948	7.2848	-0.6391
CHB	CSH	17872873	5.6525	7.4543	-0.7092
CHB	CSH	17877873	4.7096	7.9625	-1.2054
CHB	CSH	17882873	5.9516	8.8096	-0.9649
CHB	CSH	17887873	4.8985	7.4543	-1.006
CHB	CSH	17892873	2.7223	6.2684	-1.6332
CHB	CSH	17897873	4.2246	7.6237	-1.3108
CHB	CSH	17902873	5.4061	9.3178	-1.2538
CHB	CSH	17907873	4.2389	7.6237	-1.3053
CHB	CSH	17912873	3.1186	5.9295	-1.3608
CHB	CSH	17917873	3.1022	8.3013	-1.8542
CHB	CSH	17922873	2.4196	7.9625	-2.054
CHB	CSH	17927873	2.9591	6.2684	-1.5241
CHB	CSH	17932873	2.9602	5.4213	-1.2905
CHB	CSH	17937873	2.9109	6.0989	-1.5049
CHB	CSH	17942873	3.1569	6.6072	-1.5154
CHB	CSH	17947873	4.1721	7.6237	-1.331
CHB	CSH	17952873	5.8616	8.979	-1.0341
CHB	CSH	17957873	4.4555	7.2848	-1.1373
CHB	CSH	17962873	3.3143	6.7766	-1.4862
CHB	CSH	17967873	3.6792	8.8096	-1.7322
CHB	CSH	17972873	5.9852	10.3343	-1.266
CHB	CSH	17977873	8.0718	9.4872	-0.4462
CHB	CSH	17982873	7.7283	8.3013	-0.2044
CHB	CSH	17987873	9.9079	10.5037	-0.1708
CHB	CSH	17992873	8.853	11.1814	-0.6296
CHB	CSH	17997873	4.6613	7.1154	-1.0078
CHB	CSH	18002873	6.004	7.2848	-0.5148
CHB	CSH	18007873	7.688	9.4872	-0.5671
CHB	CSH	18012873	8.5856	9.6567	-0.3321
CHB	CSH	18017873	10.2393	8.6402	0.5497
CHB	CSH	18022873	8.9457	6.7766	0.9311
CHB	CSH	18027873	8.6804	8.1319	0.1994
CHB	CSH	18032873	6.0067	7.4543	-0.5698
CHB	CSH	18037873	4.2968	5.9295	-0.7904
CHB	CSH	18042873	9.4283	7.6237	0.6959
CHB	CSH	18047873	7.1354	6.7766	0.154
CHB	CSH	18052873	3.5328	5.7601	-1.1066
CHB	CSH	18057873	4.2857	5.7601	-0.7325
CHB	CSH	18062873	3.2243	7.1154	-1.598
CHB	CSH	18067873	4.8192	8.979	-1.3799
CHB	CSH	18072873	5.7188	7.6237	-0.7346
CHB	CSH	18077873	4.1072	7.1154	-1.2354
CHB	CSH	18082873	3.7373	5.7601	-1.005
CHB	CSH	18087873	4.3333	5.7601	-0.7089
CHB	CSH	18092873	5.0293	7.9625	-1.087
CHB	CSH	18097873	4.2178	6.0989	-0.888
CHB	CSH	18102873	3.657	8.6402	-1.7129

CHB	CSH	18107873	5.9661	11.8591	-1.5077
CHB	CSH	18112873	5.3695	7.9625	-0.9609
CHB	CSH	18117873	3.6288	5.5907	-1.001
CHB	CSH	18122873	4.3055	9.4872	-1.6334
CHB	CSH	18127873	7.6522	11.8591	-1.0763
CHB	CSH	18132873	11.1274	10.1649	0.2845
CHB	CSH	18137873	9.7521	9.6567	0.0296
CHB	CSH	18142873	6.8119	8.4708	-0.5807
CHB	CSH	18147873	5.5938	9.1484	-1.1589
CHB	CSH	18152873	6.5717	9.8261	-0.9929
CHB	CSH	18157873	4.8096	6.4378	-0.7321
CHB	CSH	18162873	3.984	5.5907	-0.8198
CHB	CSH	18167873	5.8176	8.3013	-0.8858
CHB	CSH	18172873	7.2435	11.1814	-1.0648
CHB	CSH	18177873	6.649	11.1814	-1.2255
CHB	CSH	18182873	4.8119	9.8261	-1.5298
CHB	CSH	18187873	7.0051	11.1814	-1.1292
CHB	CSH	18192873	6.8073	10.1649	-0.9925
CHB	CSH	18197873	6.4512	10.6731	-1.1924
CHB	CSH	18202873	5.3156	9.4872	-1.315
CHB	CSH	18207873	7.3194	9.8261	-0.7648
CHB	CSH	18212873	10.0282	12.3673	-0.5752
CHB	CSH	18217873	8.6242	9.9955	-0.4118
CHB	CSH	18222873	10.8094	9.9955	0.2444
CHB	CSH	18227873	8.1772	9.3178	-0.3656
CHB	CSH	18232873	3.7765	7.6237	-1.4836
CHB	CSH	18237873	5.0908	7.9625	-1.0642
CHB	CSH	18242873	7.0872	8.3013	-0.433
CHB	CSH	18247873	7.5168	8.3013	-0.2798
CHB	CSH	18252873	13.0482	11.5202	0.4017
CHB	CSH	18257873	15.648	12.5367	0.7554
CHB	CSH	18262873	11.6699	11.1814	0.1321
CHB	CSH	18267873	14.3879	13.5532	0.1882
CHB	CSH	18272873	12.4779	12.3673	0.0272
CHB	CSH	18277873	5.9792	9.8261	-1.1737
CHB	CSH	18282873	4.7004	9.4872	-1.5089
CHB	CSH	18287873	18.2096	15.7556	0.4796
CHB	CSH	18292873	24.2519	17.6192	1.1647
CHB	CSH	18297873	11.5952	9.6567	0.6011
CHB	CSH	18302873	7.1885	7.9625	-0.2868
CHB	CSH	18307873	5.5535	7.2848	-0.6959
CHB	CSH	18312873	3.9947	6.946	-1.2388
CHB	CSH	18317873	4.4578	8.3013	-1.3708
CHB	CSH	18322873	4.7314	8.1319	-1.236
CHB	CSH	18327873	3.8572	5.7601	-0.9454
CHB	CSH	18332873	3.0735	2.7106	0.3439
CHB	CSH	18337873	3.1696	1.8636	1.6663
CHB	CSH	18342873	4.7868	3.3883	1.1013
CHB	CSH	18347873	4.5761	4.4048	0.1078

CHB	CSH	18352873	10.2646	7.2848	1.1978
CHB	CSH	18357873	11.6402	9.3178	0.7444
CHB	CSH	18362873	5.7754	7.4543	-0.6609
CHB	CSH	18367873	5.2182	5.9295	-0.3444
CHB	CSH	18372873	2.2073	3.8965	-1.1815
CHB	CSH	18377873	0.833	3.3883	-2.0124
CHB	CSH	18382873	1.8496	3.8965	-1.4316
CHB	CSH	18387873	3.1192	6.0989	-1.4065
CHB	CSH	18392873	3.8024	8.4708	-1.6343
CHB	CSH	18397873	2.2547	6.6072	-1.9117
CHB	CSH	18402873	1.4622	3.7271	-1.6454
CHB	CSH	18407873	1.6212	2.8801	-1.135
CHB	CSH	18412873	1.5989	3.0495	-1.2475
CHB	CSH	18417873	1.4535	3.7271	-1.6517
CHB	CSH	18422873	1.2353	3.3883	-1.6955
CHB	CSH	18427873	0.8028	2.033	-1.467
CHB	CSH	18432873	0.0583	1.0165	-1.9069
CHB	CSH	18437873	0.7215	1.8636	-1.4572
CHB	CSH	18442873	0.77	2.7106	-1.8388
CHB	CSH	18447873	0.6686	3.2189	-2.0967
CHB	CSH	18452873	0.6973	3.2189	-2.0731
CHB	CSH	18457873	0.6469	2.033	-1.6529
CHB	CSH	18462873	1.8808	3.7271	-1.3413
CHB	CSH	18467873	1.9728	5.0825	-1.7264
CHB	CSH	18472873	1.2187	4.4048	-2.0049
CHB	CSH	18477873	0.9383	3.2189	-1.875
CHB	CSH	18482873	0.6608	2.5412	-1.8777
CHB	CSH	18487873	0.6358	3.0495	-2.0758
CHB	CSH	18492873	1.9023	3.7271	-1.3257
CHB	CSH	18497873	2.7481	4.066	-0.8887
CHB	CSH	18502873	2.3208	4.2354	-1.2464
CHB	CSH	18507873	1.2975	3.3883	-1.6466
CHB	CSH	18512873	0.7114	3.2189	-2.0615
CHB	CSH	18517873	1.1167	3.5577	-1.8448
CHB	CSH	18522873	2.4342	5.0825	-1.4703
CHB	CSH	18527873	2.6347	6.6072	-1.7448
CHB	CSH	18532873	2.0251	5.9295	-1.8902
CHB	CSH	18537873	3.3739	7.4543	-1.6062
CHB	CSH	18542873	3.7594	9.1484	-1.757
CHB	CSH	18547873	5.1173	11.012	-1.6169
CHB	CSH	18552873	7.1649	10.5037	-0.9572
CHB	CSH	18557873	10.9202	9.1484	0.5777
CHB	CSH	18562873	9.4896	11.012	-0.4176
CHB	CSH	18567873	6.4666	16.0944	-1.8436
CHB	CSH	18572873	11.0314	17.958	-1.1943
CHB	CSH	18577873	9.3179	15.925	-1.2781
CHB	CSH	18582873	9.596	14.7391	-1.071
CHB	CSH	18587873	16.0215	14.5697	0.3057
CHB	CSH	18592873	15.762	12.1979	0.888

CHB	CSH	18597873	12.3929	8.979	1.1325
CHB	CSH	18602873	9.9336	8.8096	0.3795
CHB	CSH	18607873	6.6929	7.1154	-0.1735
CHB	CSH	18612873	4.439	4.913	-0.2711
CHB	CSH	18617873	8.9001	7.7931	0.4184
CHB	CSH	18622873	8.7754	7.6237	0.4441
CHB	CSH	18627873	6.751	5.5907	0.592
CHB	CSH	18632873	8.4245	6.6072	0.7982
CHB	CSH	18637873	9.3307	7.7931	0.5811
CHB	CSH	18642873	10.6431	9.6567	0.3059
CHB	CSH	18647873	7.7171	7.7931	-0.0287
CHB	CSH	18652873	5.6507	5.7601	-0.0544
CHB	CSH	18657873	6.8076	6.4378	0.1663
CHB	CSH	18662873	9.3816	10.3343	-0.2773
CHB	CSH	18667873	8.6779	10.5037	-0.5235
CHB	CSH	18672873	7.7451	8.3013	-0.1984
CHB	CSH	18677873	11.4249	8.8096	0.883
CHB	CSH	18682873	14.2538	10.1649	1.2087
CHB	CSH	18687873	11.187	8.4708	0.9509
CHB	CSH	18692873	7.092	6.0989	0.4688
CHB	CSH	18697873	9.4289	8.3013	0.4022
CHB	CSH	18702873	12.9556	11.3508	0.4278
CHB	CSH	18707873	9.6183	10.1649	-0.1616
CHB	CSH	18712873	7.5842	7.2848	0.1203
CHB	CSH	18717873	6.7192	7.2848	-0.2274
CHB	CSH	18722873	5.7425	7.9625	-0.8227
CHB	CSH	18727873	5.5034	6.6072	-0.4848
CHB	CSH	18732873	2.3299	4.066	-1.1707
CHB	CSH	18737873	2.8917	5.9295	-1.4706
CHB	CSH	18742873	2.7204	5.5907	-1.4645
CHB	CSH	18747873	3.7373	4.913	-0.6725
CHB	CSH	18752873	7.0735	8.1319	-0.3847
CHB	CSH	18757873	10.8214	9.4872	0.4206
CHB	CSH	18762873	15.6408	11.8591	0.9675
CHB	CSH	18767873	18.7868	13.3838	1.2331
CHB	CSH	18772873	20.0058	14.0615	1.2945
CHB	CSH	18777873	14.8286	12.0285	0.7069
CHB	CSH	18782873	12.3105	10.8426	0.4085
CHB	CSH	18787873	14.0615	12.0285	0.5132
CHB	CSH	18792873	13.4224	11.3508	0.5523
CHB	CSH	18797873	10.8772	10.3343	0.158
CHB	CSH	18802873	9.6033	9.4872	0.0366
CHB	CSH	18807873	10.4274	9.1484	0.417
CHB	CSH	18812873	9.4268	8.6402	0.2704
CHB	CSH	18817873	9.7539	9.8261	-0.022
CHB	CSH	18822873	7.9022	9.4872	-0.4996
CHB	CSH	18827873	6.264	9.1484	-0.9404
CHB	CSH	18832873	6.1847	8.4708	-0.8003
CHB	CSH	18837873	6.1704	7.2848	-0.448

CHB	CSH	18842873	10.5385	9.1484	0.4532
CHB	CSH	18847873	10.8675	9.8261	0.3177
CHB	CSH	18852873	11.3156	9.3178	0.6404
CHB	CSH	18857873	10.0725	7.4543	1.0306
YRI	CSH	17832873	10.3284	11.2602	-0.2494
YRI	CSH	17837873	6.8799	9.0754	-0.7187
YRI	CSH	17842873	6.2785	8.5712	-0.7912
YRI	CSH	17847873	10.4134	10.4199	-0.0019
YRI	CSH	17852873	12.6228	11.0921	0.4156
YRI	CSH	17857873	9.6327	10.0837	-0.1339
YRI	CSH	17862873	9.3752	10.4199	-0.3007
YRI	CSH	17867873	10.8534	12.1005	-0.312
YRI	CSH	17872873	12.1346	11.2602	0.2341
YRI	CSH	17877873	10.8537	9.2434	0.5182
YRI	CSH	17882873	9.0003	10.4199	-0.4087
YRI	CSH	17887873	6.3971	10.0837	-1.0942
YRI	CSH	17892873	6.2354	9.4115	-1.0052
YRI	CSH	17897873	8.6801	9.7476	-0.327
YRI	CSH	17902873	10.1223	10.924	-0.2208
YRI	CSH	17907873	8.6376	10.0837	-0.4292
YRI	CSH	17912873	6.0642	8.067	-0.7308
YRI	CSH	17917873	7.0601	11.7644	-1.2086
YRI	CSH	17922873	6.5793	11.9324	-1.357
YRI	CSH	17927873	4.9699	7.8989	-1.0897
YRI	CSH	17932873	4.0604	6.8906	-1.1926
YRI	CSH	17937873	5.7989	8.4031	-0.9153
YRI	CSH	17942873	7.4263	9.0754	-0.5398
YRI	CSH	17947873	10.593	10.924	-0.0912
YRI	CSH	17952873	12.4161	11.5963	0.2135
YRI	CSH	17957873	8.3621	8.067	0.1077
YRI	CSH	17962873	7.1776	7.0586	0.0491
YRI	CSH	17967873	7.7606	10.2518	-0.7281
YRI	CSH	17972873	10.6354	13.1089	-0.5738
YRI	CSH	17977873	12.9218	12.7727	0.0354
YRI	CSH	17982873	11.1867	10.924	0.0724
YRI	CSH	17987873	11.8473	13.613	-0.3952
YRI	CSH	17992873	10.5871	13.7811	-0.7066
YRI	CSH	17997873	6.8306	10.0837	-0.9656
YRI	CSH	18002873	7.9836	12.7727	-1.1385
YRI	CSH	18007873	10.072	14.6214	-0.9514
YRI	CSH	18012873	10.1002	14.7895	-0.9701
YRI	CSH	18017873	10.5777	14.9575	-0.8964
YRI	CSH	18022873	10.0573	13.9492	-0.8512
YRI	CSH	18027873	9.8677	13.7811	-0.8658
YRI	CSH	18032873	7.0612	11.4282	-1.1529
YRI	CSH	18037873	5.018	8.9073	-1.2954
YRI	CSH	18042873	8.6011	10.0837	-0.4401
YRI	CSH	18047873	7.1848	8.9073	-0.5737
YRI	CSH	18052873	5.5897	7.2267	-0.6606

YRI	CSH	18057873	6.8208	9.2434	-0.7797
YRI	CSH	18062873	7.7561	10.756	-0.8383
YRI	CSH	18067873	11.407	12.4366	-0.251
YRI	CSH	18072873	10.7424	11.7644	-0.2626
YRI	CSH	18077873	8.3014	11.2602	-0.7921
YRI	CSH	18082873	6.865	11.9324	-1.2846
YRI	CSH	18087873	7.0776	12.2685	-1.2819
YRI	CSH	18092873	9.5221	13.445	-0.8884
YRI	CSH	18097873	8.4078	10.756	-0.6562
YRI	CSH	18102873	11.1948	11.9324	-0.187
YRI	CSH	18107873	14.944	14.2853	0.1408
YRI	CSH	18112873	11.8557	11.9324	-0.0194
YRI	CSH	18117873	7.724	10.0837	-0.7004
YRI	CSH	18122873	9.0694	12.6047	-0.851
YRI	CSH	18127873	12.6827	15.9659	-0.6314
YRI	CSH	18132873	12.9741	15.1256	-0.4357
YRI	CSH	18137873	9.9659	12.7727	-0.6673
YRI	CSH	18142873	7.5162	12.1005	-1.1469
YRI	CSH	18147873	7.2927	11.7644	-1.1488
YRI	CSH	18152873	7.5013	10.924	-0.9427
YRI	CSH	18157873	6.7349	9.5795	-0.8856
YRI	CSH	18162873	6.0567	10.2518	-1.2261
YRI	CSH	18167873	7.8743	12.9408	-1.1897
YRI	CSH	18172873	11.2837	14.6214	-0.698
YRI	CSH	18177873	13.215	14.4534	-0.2619
YRI	CSH	18182873	11.9122	11.5963	0.0823
YRI	CSH	18187873	14.1721	12.7727	0.3327
YRI	CSH	18192873	12.7688	13.7811	-0.224
YRI	CSH	18197873	12.0342	13.1089	-0.2493
YRI	CSH	18202873	10.5961	12.2685	-0.413
YRI	CSH	18207873	10.6795	12.7727	-0.4976
YRI	CSH	18212873	14.5077	14.1172	0.0844
YRI	CSH	18217873	13.0504	12.6047	0.1073
YRI	CSH	18222873	14.2164	13.2769	0.2153
YRI	CSH	18227873	13.4136	12.9408	0.111
YRI	CSH	18232873	10.7161	11.9324	-0.3083
YRI	CSH	18237873	11.7689	12.7727	-0.2386
YRI	CSH	18242873	12.3465	13.1089	-0.1769
YRI	CSH	18247873	10.8023	12.2685	-0.3621
YRI	CSH	18252873	14.1057	13.1089	0.2312
YRI	CSH	18257873	14.321	13.9492	0.0813
YRI	CSH	18262873	12.3457	15.4617	-0.6179
YRI	CSH	18267873	17.3201	19.3272	-0.3214
YRI	CSH	18272873	15.5478	16.8062	-0.2304
YRI	CSH	18277873	10.3203	13.613	-0.737
YRI	CSH	18282873	9.8383	13.445	-0.8168
YRI	CSH	18287873	28.6207	19.4952	1.4491
YRI	CSH	18292873	35.1227	19.6633	2.4346
YRI	CSH	18297873	17.3138	10.4199	1.9845

YRI	CSH	18302873	15.1714	10.4199	1.3678
YRI	CSH	18307873	14.3949	12.6047	0.431
YRI	CSH	18312873	9.5439	13.445	-0.8835
YRI	CSH	18317873	7.5817	12.9408	-1.2584
YRI	CSH	18322873	6.4815	10.4199	-1.1337
YRI	CSH	18327873	4.9467	7.2267	-0.92
YRI	CSH	18332873	3.3114	4.0335	-0.4886
YRI	CSH	18337873	3.5796	2.8571	0.6534
YRI	CSH	18342873	6.8554	5.5461	0.6705
YRI	CSH	18347873	7.2042	9.2434	-0.6563
YRI	CSH	18352873	11.7355	11.4282	0.0811
YRI	CSH	18357873	14.5869	13.445	0.2586
YRI	CSH	18362873	9.6039	12.1005	-0.6246
YRI	CSH	18367873	7.8297	11.5963	-0.9808
YRI	CSH	18372873	4.369	8.5712	-1.4502
YRI	CSH	18377873	3.4793	8.235	-1.7028
YRI	CSH	18382873	4.8416	9.5795	-1.4751
YRI	CSH	18387873	5.7814	8.5712	-0.9628
YRI	CSH	18392873	5.5224	9.5795	-1.2631
YRI	CSH	18397873	3.491	7.7309	-1.6087
YRI	CSH	18402873	3.1512	5.7141	-1.278
YRI	CSH	18407873	2.4414	5.8822	-1.6719
YRI	CSH	18412873	2.7995	7.5628	-1.844
YRI	CSH	18417873	3.616	8.5712	-1.7101
YRI	CSH	18422873	4.1179	6.8906	-1.1683
YRI	CSH	18427873	3.974	7.2267	-1.3125
YRI	CSH	18432873	4.0431	8.7392	-1.5919
YRI	CSH	18437873	4.7441	8.4031	-1.286
YRI	CSH	18442873	3.6666	7.2267	-1.4366
YRI	CSH	18447873	3.7658	7.0586	-1.3575
YRI	CSH	18452873	4.265	9.4115	-1.6288
YRI	CSH	18457873	3.0633	8.067	-1.8259
YRI	CSH	18462873	4.0453	7.7309	-1.3984
YRI	CSH	18467873	4.5271	8.5712	-1.3956
YRI	CSH	18472873	3.3657	8.7392	-1.8215
YRI	CSH	18477873	3.494	8.9073	-1.8029
YRI	CSH	18482873	2.983	7.2267	-1.7124
YRI	CSH	18487873	2.8677	6.8906	-1.6951
YRI	CSH	18492873	3.4841	6.3864	-1.3099
YRI	CSH	18497873	5.1089	8.067	-1.0794
YRI	CSH	18502873	6.1886	8.235	-0.7327
YRI	CSH	18507873	3.7763	7.0586	-1.3531
YRI	CSH	18512873	2.8449	9.5795	-2.0967
YRI	CSH	18517873	3.4307	8.5712	-1.774
YRI	CSH	18522873	4.1816	8.4031	-1.4837
YRI	CSH	18527873	5.0376	10.756	-1.598
YRI	CSH	18532873	4.2079	9.0754	-1.5934
YRI	CSH	18537873	7.218	11.2602	-1.0821
YRI	CSH	18542873	10.9634	14.4534	-0.7379

YRI	CSH	18547873	14.5592	16.134	-0.2998
YRI	CSH	18552873	13.9512	14.2853	-0.0714
YRI	CSH	18557873	13.3531	12.2685	0.2678
YRI	CSH	18562873	14.7574	13.7811	0.216
YRI	CSH	18567873	20.8823	16.4701	0.8237
YRI	CSH	18572873	26.5774	19.6633	1.0889
YRI	CSH	18577873	25.0178	19.6633	0.8433
YRI	CSH	18582873	23.1037	18.991	0.6697
YRI	CSH	18587873	20.7432	19.8313	0.1424
YRI	CSH	18592873	17.3227	17.8146	-0.0852
YRI	CSH	18597873	13.0411	13.613	-0.128
YRI	CSH	18602873	10.2735	10.5879	-0.0892
YRI	CSH	18607873	7.5665	9.2434	-0.5397
YRI	CSH	18612873	5.6971	8.9073	-1.0692
YRI	CSH	18617873	9.7464	10.5879	-0.2386
YRI	CSH	18622873	10.8062	11.7644	-0.2462
YRI	CSH	18627873	8.0144	11.7644	-0.9634
YRI	CSH	18632873	9.192	12.4366	-0.791
YRI	CSH	18637873	10.6878	12.4366	-0.4264
YRI	CSH	18642873	12.1799	13.445	-0.2865
YRI	CSH	18647873	10.4162	12.2685	-0.4574
YRI	CSH	18652873	8.4776	10.924	-0.6738
YRI	CSH	18657873	8.9884	10.4199	-0.4121
YRI	CSH	18662873	10.7591	11.5963	-0.218
YRI	CSH	18667873	10.6263	11.9324	-0.3311
YRI	CSH	18672873	11.1008	12.7727	-0.3975
YRI	CSH	18677873	14.3724	13.7811	0.1308
YRI	CSH	18682873	16.6213	13.7811	0.6283
YRI	CSH	18687873	12.9507	12.6047	0.0833
YRI	CSH	18692873	8.6469	10.5879	-0.5505
YRI	CSH	18697873	11.222	11.2602	-0.0102
YRI	CSH	18702873	13.6571	12.7727	0.2103
YRI	CSH	18707873	10.5284	11.7644	-0.3175
YRI	CSH	18712873	8.8529	10.924	-0.5704
YRI	CSH	18717873	9.1671	11.7644	-0.6673
YRI	CSH	18722873	9.3796	12.4366	-0.7453
YRI	CSH	18727873	8.0025	10.756	-0.7695
YRI	CSH	18732873	5.0366	9.0754	-1.3221
YRI	CSH	18737873	7.7506	10.2518	-0.731
YRI	CSH	18742873	7.0541	9.2434	-0.7046
YRI	CSH	18747873	6.2103	10.2518	-1.1812
YRI	CSH	18752873	9.3493	13.9492	-1.006
YRI	CSH	18757873	11.8228	13.7811	-0.4332
YRI	CSH	18762873	17.481	14.6214	0.598
YRI	CSH	18767873	20.0749	16.4701	0.6729
YRI	CSH	18772873	20.9489	16.9743	0.7209
YRI	CSH	18777873	17.412	16.134	0.2433
YRI	CSH	18782873	15.031	14.9575	0.015
YRI	CSH	18787873	16.9988	14.7895	0.457

YRI	CSH	18792873	16.6565	13.9492	0.5921
YRI	CSH	18797873	14.4161	13.1089	0.3032
YRI	CSH	18802873	12.287	13.9492	-0.3635
YRI	CSH	18807873	12.6361	14.2853	-0.3526
YRI	CSH	18812873	12.6828	11.7644	0.2359
YRI	CSH	18817873	12.5559	11.9324	0.1581
YRI	CSH	18822873	10.7969	12.9408	-0.5034
YRI	CSH	18827873	8.1658	10.4199	-0.6489
YRI	CSH	18832873	7.1977	10.2518	-0.8926
YRI	CSH	18837873	8.208	10.756	-0.712
YRI	CSH	18842873	13.3631	13.445	-0.0185
YRI	CSH	18847873	14.2668	14.7895	-0.1081
YRI	CSH	18852873	11.4687	14.2853	-0.6022
YRI	CSH	18857873	11.9885	14.7895	-0.5794
CEU	CSH	63377012	0	0	0
CEU	CSH	63382012	0	0	0
CEU	CSH	63387012	0	0	0
CEU	CSH	63392012	0	0	0
CEU	CSH	63397012	0	0	0
CEU	CSH	63402012	0	0	0
CEU	CSH	63407012	0	0	0
CEU	CSH	63412012	0	0	0
CEU	CSH	63417012	0	0	0
CEU	CSH	63422012	0	0	0
CEU	CSH	63427012	0	0	0
CEU	CSH	63432012	0	0	0
CEU	CSH	63437012	0	0	0
CEU	CSH	63442012	0	0	0
CEU	CSH	63447012	0	0	0
CEU	CSH	63452012	0	0	0
CEU	CSH	63457012	0	0	0
CEU	CSH	63462012	0	0	0
CEU	CSH	63467012	0	0	0
CEU	CSH	63472012	0	0	0
CEU	CSH	63477012	0	0	0
CEU	CSH	63482012	0	0	0
CEU	CSH	63487012	0	0	0
CEU	CSH	63492012	0	0	0
CEU	CSH	63497012	0	0	0
CEU	CSH	63502012	0	0	0
CEU	CSH	63507012	0	0	0
CEU	CSH	63512012	0	0	0
CEU	CSH	63517012	0	0	0
CEU	CSH	63522012	0	0	0
CEU	CSH	63527012	0	0	0
CEU	CSH	63532012	0	0	0
CEU	CSH	63537012	0	0	0
CEU	CSH	63542012	0	0	0
CEU	CSH	63547012	0	0	0

CEU	CSH	63552012	0	0	0
CEU	CSH	63557012	0	0	0
CEU	CSH	63562012	0	0	0
CEU	CSH	63567012	0	0	0
CEU	CSH	63572012	0	0	0
CEU	CSH	63577012	0	0	0
CEU	CSH	63582012	0	0	0
CEU	CSH	63587012	0	0	0
CEU	CSH	63592012	0	0	0
CEU	CSH	63597012	0	0	0
CEU	CSH	63602012	0	0	0
CEU	CSH	63607012	0	0	0
CEU	CSH	63612012	0	0	0
CEU	CSH	63617012	0	0	0
CEU	CSH	63622012	0	0	0
CEU	CSH	63627012	0	0	0
CEU	CSH	63632012	0	0	0
CEU	CSH	63637012	0	0	0
CEU	CSH	63642012	0	0	0
CEU	CSH	63647012	0	0	0
CEU	CSH	63652012	0	0	0
CEU	CSH	63657012	0	0	0
CEU	CSH	63662012	0	0	0
CEU	CSH	63667012	0	0	0
CEU	CSH	63672012	0	0	0
CEU	CSH	63677012	0	0	0
CEU	CSH	63682012	0	0	0
CEU	CSH	63687012	0	0	0
CEU	CSH	63692012	0	0	0
CEU	CSH	63697012	0	0	0
CEU	CSH	63702012	0	0	0
CEU	CSH	63707012	0	0	0
CEU	CSH	63712012	0	0	0
CEU	CSH	63717012	0	0	0
CEU	CSH	63722012	0	0	0
CEU	CSH	63727012	0	0	0
CEU	CSH	63732012	0	0	0
CEU	CSH	63737012	0	0	0
CEU	CSH	63742012	0	0	0
CEU	CSH	63747012	0	0	0
CEU	CSH	63752012	0	0	0
CEU	CSH	63757012	0	0	0
CEU	CSH	63762012	0	0	0
CEU	CSH	63767012	0	0	0
CEU	CSH	63772012	0	0	0
CEU	CSH	63777012	0	0	0
CEU	CSH	63782012	0	0	0
CEU	CSH	63787012	0	0	0
CEU	CSH	63792012	0	0	0

CEU	CSH	63797012	0	0	0
CEU	CSH	63802012	0	0	0
CEU	CSH	63807012	0	0	0
CEU	CSH	63812012	0	0	0
CEU	CSH	63817012	0	0	0
CEU	CSH	63822012	0	0	0
CEU	CSH	63827012	0	0	0
CEU	CSH	63832012	0	0	0
CEU	CSH	63837012	0	0	0
CEU	CSH	63842012	0	0	0
CEU	CSH	63847012	0	0	0
CEU	CSH	63852012	0	0	0
CEU	CSH	63857012	0	0	0
CEU	CSH	63862012	0	0	0
CEU	CSH	63867012	0	0	0
CEU	CSH	63872012	0	0	0
CEU	CSH	63877012	0	0	0
CEU	CSH	63882012	0	0	0
CEU	CSH	63887012	0	0	0
CEU	CSH	63892012	0	0	0
CEU	CSH	63897012	0	0	0
CEU	CSH	63902012	0	0	0
CEU	CSH	63907012	0	0	0
CEU	CSH	63912012	0	0	0
CEU	CSH	63917012	0	0	0
CEU	CSH	63922012	0	0	0
CEU	CSH	63927012	0	0	0
CEU	CSH	63932012	0	0	0
CEU	CSH	63937012	0	0	0
CEU	CSH	63942012	0	0	0
CEU	CSH	63947012	0	0	0
CEU	CSH	63952012	0	0	0
CEU	CSH	63957012	0	0	0
CEU	CSH	63962012	0	0	0
CEU	CSH	63967012	0	0	0
CEU	CSH	63972012	0	0	0
CEU	CSH	63977012	0	0	0
CEU	CSH	63982012	0	0	0
CEU	CSH	63987012	0	0	0
CEU	CSH	63992012	0	0	0
CEU	CSH	63997012	0	0	0
CEU	CSH	64002012	0	0	0
CEU	CSH	64007012	0	0	0
CEU	CSH	64012012	0	0	0
CEU	CSH	64017012	0	0	0
CEU	CSH	64022012	0	0	0
CEU	CSH	64027012	0	0	0
CEU	CSH	64032012	0	0	0
CEU	CSH	64037012	0	0	0

CEU	CSH	64042012	0	0	0
CEU	CSH	64047012	0	0	0
CEU	CSH	64052012	0	0	0
CEU	CSH	64057012	0	0	0
CEU	CSH	64062012	0	0	0
CEU	CSH	64067012	0	0	0
CEU	CSH	64072012	0	0	0
CEU	CSH	64077012	0	0	0
CEU	CSH	64082012	0	0	0
CEU	CSH	64087012	0	0	0
CEU	CSH	64092012	0	0	0
CEU	CSH	64097012	0	0	0
CEU	CSH	64102012	0	0	0
CEU	CSH	64107012	0	0	0
CEU	CSH	64112012	0	0	0
CEU	CSH	64117012	0	0	0
CEU	CSH	64122012	0	0	0
CEU	CSH	64127012	0	0	0
CEU	CSH	64132012	0	0	0
CEU	CSH	64137012	0	0	0
CEU	CSH	64142012	0	0	0
CEU	CSH	64147012	0	0	0
CEU	CSH	64152012	0	0	0
CEU	CSH	64157012	0	0	0
CEU	CSH	64162012	0	0	0
CEU	CSH	64167012	0	0	0
CEU	CSH	64172012	0	0	0
CEU	CSH	64177012	0	0	0
CEU	CSH	64182012	0	0	0
CEU	CSH	64187012	0	0	0
CEU	CSH	64192012	0	0	0
CEU	CSH	64197012	0	0	0
CEU	CSH	64202012	0	0	0
CEU	CSH	64207012	0	0	0
CEU	CSH	64212012	0	0	0
CEU	CSH	64217012	0	0	0
CEU	CSH	64222012	0	0	0
CEU	CSH	64227012	0	0	0
CEU	CSH	64232012	0	0	0
CEU	CSH	64237012	0	0	0
CEU	CSH	64242012	0	0	0
CEU	CSH	64247012	0	0	0
CEU	CSH	64252012	0	0	0
CEU	CSH	64257012	0	0	0
CEU	CSH	64262012	0	0	0
CEU	CSH	64267012	0	0	0
CEU	CSH	64272012	0	0	0
CEU	CSH	64277012	0	0	0
CEU	CSH	64282012	0	0	0

CEU	CSH	64287012	0	0	0
CEU	CSH	64292012	0	0	0
CEU	CSH	64297012	0	0	0
CEU	CSH	64302012	0	0	0
CEU	CSH	64307012	0	0	0
CEU	CSH	64312012	0	0	0
CEU	CSH	64317012	0	0	0
CEU	CSH	64322012	0	0	0
CEU	CSH	64327012	0	0	0
CEU	CSH	64332012	0	0	0
CEU	CSH	64337012	0	0	0
CEU	CSH	64342012	0	0	0
CEU	CSH	64347012	0	0	0
CEU	CSH	64352012	0	0	0
CEU	CSH	64357012	0	0	0
CEU	CSH	64362012	0	0	0
CEU	CSH	64367012	0	0	0
CHB	CSH	63377012	0	0	0
CHB	CSH	63382012	0	0	0
CHB	CSH	63387012	0	0	0
CHB	CSH	63392012	0	0	0
CHB	CSH	63397012	0	0	0
CHB	CSH	63402012	0	0	0
CHB	CSH	63407012	0	0	0
CHB	CSH	63412012	0	0	0
CHB	CSH	63417012	0	0	0
CHB	CSH	63422012	0	0	0
CHB	CSH	63427012	0	0	0
CHB	CSH	63432012	0	0	0
CHB	CSH	63437012	0	0	0
CHB	CSH	63442012	0	0	0
CHB	CSH	63447012	0	0	0
CHB	CSH	63452012	0	0	0
CHB	CSH	63457012	0	0	0
CHB	CSH	63462012	0	0	0
CHB	CSH	63467012	0	0	0
CHB	CSH	63472012	0	0	0
CHB	CSH	63477012	0	0	0
CHB	CSH	63482012	0	0	0
CHB	CSH	63487012	0	0	0
CHB	CSH	63492012	0	0	0
CHB	CSH	63497012	0	0	0
CHB	CSH	63502012	0	0	0
CHB	CSH	63507012	0	0	0
CHB	CSH	63512012	0	0	0
CHB	CSH	63517012	0	0	0
CHB	CSH	63522012	0	0	0
CHB	CSH	63527012	0	0	0
CHB	CSH	63532012	0	0	0

CHB	CSH	63537012	0	0	0
CHB	CSH	63542012	0	0	0
CHB	CSH	63547012	0	0	0
CHB	CSH	63552012	0	0	0
CHB	CSH	63557012	0	0	0
CHB	CSH	63562012	0	0	0
CHB	CSH	63567012	0	0	0
CHB	CSH	63572012	0	0	0
CHB	CSH	63577012	0	0	0
CHB	CSH	63582012	0	0	0
CHB	CSH	63587012	0	0	0
CHB	CSH	63592012	0	0	0
CHB	CSH	63597012	0	0	0
CHB	CSH	63602012	0	0	0
CHB	CSH	63607012	0	0	0
CHB	CSH	63612012	0	0	0
CHB	CSH	63617012	0	0	0
CHB	CSH	63622012	0	0	0
CHB	CSH	63627012	0	0	0
CHB	CSH	63632012	0	0	0
CHB	CSH	63637012	0	0	0
CHB	CSH	63642012	0	0	0
CHB	CSH	63647012	0	0	0
CHB	CSH	63652012	0	0	0
CHB	CSH	63657012	0	0	0
CHB	CSH	63662012	0	0	0
CHB	CSH	63667012	0	0	0
CHB	CSH	63672012	0	0	0
CHB	CSH	63677012	0	0	0
CHB	CSH	63682012	0	0	0
CHB	CSH	63687012	0	0	0
CHB	CSH	63692012	0	0	0
CHB	CSH	63697012	0	0	0
CHB	CSH	63702012	0	0	0
CHB	CSH	63707012	0	0	0
CHB	CSH	63712012	0	0	0
CHB	CSH	63717012	0	0	0
CHB	CSH	63722012	0	0	0
CHB	CSH	63727012	0	0	0
CHB	CSH	63732012	0	0	0
CHB	CSH	63737012	0	0	0
CHB	CSH	63742012	0	0	0
CHB	CSH	63747012	0	0	0
CHB	CSH	63752012	0	0	0
CHB	CSH	63757012	0	0	0
CHB	CSH	63762012	0	0	0
CHB	CSH	63767012	0	0	0
CHB	CSH	63772012	0	0	0
CHB	CSH	63777012	0	0	0

CHB	CSH	63782012	0	0	0
CHB	CSH	63787012	0	0	0
CHB	CSH	63792012	0	0	0
CHB	CSH	63797012	0	0	0
CHB	CSH	63802012	0	0	0
CHB	CSH	63807012	0	0	0
CHB	CSH	63812012	0	0	0
CHB	CSH	63817012	0	0	0
CHB	CSH	63822012	0	0	0
CHB	CSH	63827012	0	0	0
CHB	CSH	63832012	0	0	0
CHB	CSH	63837012	0	0	0
CHB	CSH	63842012	0	0	0
CHB	CSH	63847012	0	0	0
CHB	CSH	63852012	0	0	0
CHB	CSH	63857012	0	0	0
CHB	CSH	63862012	0	0	0
CHB	CSH	63867012	0	0	0
CHB	CSH	63872012	0	0	0
CHB	CSH	63877012	0	0	0
CHB	CSH	63882012	0	0	0
CHB	CSH	63887012	0	0	0
CHB	CSH	63892012	0	0	0
CHB	CSH	63897012	0	0	0
CHB	CSH	63902012	0	0	0
CHB	CSH	63907012	0	0	0
CHB	CSH	63912012	0	0	0
CHB	CSH	63917012	0	0	0
CHB	CSH	63922012	0	0	0
CHB	CSH	63927012	0	0	0
CHB	CSH	63932012	0	0	0
CHB	CSH	63937012	0	0	0
CHB	CSH	63942012	0	0	0
CHB	CSH	63947012	0	0	0
CHB	CSH	63952012	0	0	0
CHB	CSH	63957012	0	0	0
CHB	CSH	63962012	0	0	0
CHB	CSH	63967012	0	0	0
CHB	CSH	63972012	0	0	0
CHB	CSH	63977012	0	0	0
CHB	CSH	63982012	0	0	0
CHB	CSH	63987012	0	0	0
CHB	CSH	63992012	0	0	0
CHB	CSH	63997012	0	0	0
CHB	CSH	64002012	0	0	0
CHB	CSH	64007012	0	0	0
CHB	CSH	64012012	0	0	0
CHB	CSH	64017012	0	0	0
CHB	CSH	64022012	0	0	0

CHB	CSH	64027012	0	0	0
CHB	CSH	64032012	0	0	0
CHB	CSH	64037012	0	0	0
CHB	CSH	64042012	0	0	0
CHB	CSH	64047012	0	0	0
CHB	CSH	64052012	0	0	0
CHB	CSH	64057012	0	0	0
CHB	CSH	64062012	0	0	0
CHB	CSH	64067012	0	0	0
CHB	CSH	64072012	0	0	0
CHB	CSH	64077012	0	0	0
CHB	CSH	64082012	0	0	0
CHB	CSH	64087012	0	0	0
CHB	CSH	64092012	0	0	0
CHB	CSH	64097012	0	0	0
CHB	CSH	64102012	0	0	0
CHB	CSH	64107012	0	0	0
CHB	CSH	64112012	0	0	0
CHB	CSH	64117012	0	0	0
CHB	CSH	64122012	0	0	0
CHB	CSH	64127012	0	0	0
CHB	CSH	64132012	0	0	0
CHB	CSH	64137012	0	0	0
CHB	CSH	64142012	0	0	0
CHB	CSH	64147012	0	0	0
CHB	CSH	64152012	0	0	0
CHB	CSH	64157012	0	0	0
CHB	CSH	64162012	0	0	0
CHB	CSH	64167012	0	0	0
CHB	CSH	64172012	0	0	0
CHB	CSH	64177012	0	0	0
CHB	CSH	64182012	0	0	0
CHB	CSH	64187012	0	0	0
CHB	CSH	64192012	0	0	0
CHB	CSH	64197012	0	0	0
CHB	CSH	64202012	0	0	0
CHB	CSH	64207012	0	0	0
CHB	CSH	64212012	0	0	0
CHB	CSH	64217012	0	0	0
CHB	CSH	64222012	0	0	0
CHB	CSH	64227012	0	0	0
CHB	CSH	64232012	0	0	0
CHB	CSH	64237012	0	0	0
CHB	CSH	64242012	0	0	0
CHB	CSH	64247012	0	0	0
CHB	CSH	64252012	0	0	0
CHB	CSH	64257012	0	0	0
CHB	CSH	64262012	0	0	0
CHB	CSH	64267012	0	0	0

CHB	CSH	64272012	0	0	0
CHB	CSH	64277012	0	0	0
CHB	CSH	64282012	0	0	0
CHB	CSH	64287012	0	0	0
CHB	CSH	64292012	0	0	0
CHB	CSH	64297012	0	0	0
CHB	CSH	64302012	0	0	0
CHB	CSH	64307012	0	0	0
CHB	CSH	64312012	0	0	0
CHB	CSH	64317012	0	0	0
CHB	CSH	64322012	0	0	0
CHB	CSH	64327012	0	0	0
CHB	CSH	64332012	0	0	0
CHB	CSH	64337012	0	0	0
CHB	CSH	64342012	0	0	0
CHB	CSH	64347012	0	0	0
CHB	CSH	64352012	0	0	0
CHB	CSH	64357012	0	0	0
CHB	CSH	64362012	0	0	0
CHB	CSH	64367012	0	0	0
YRI	CSH	63377012	0	0	0
YRI	CSH	63382012	0	0	0
YRI	CSH	63387012	0	0	0
YRI	CSH	63392012	0	0	0
YRI	CSH	63397012	0	0	0
YRI	CSH	63402012	0	0	0
YRI	CSH	63407012	0	0	0
YRI	CSH	63412012	0	0	0
YRI	CSH	63417012	0	0	0
YRI	CSH	63422012	0	0	0
YRI	CSH	63427012	0	0	0
YRI	CSH	63432012	0	0	0
YRI	CSH	63437012	0	0	0
YRI	CSH	63442012	0	0	0
YRI	CSH	63447012	0	0	0
YRI	CSH	63452012	0	0	0
YRI	CSH	63457012	0	0	0
YRI	CSH	63462012	0	0	0
YRI	CSH	63467012	0	0	0
YRI	CSH	63472012	0	0	0
YRI	CSH	63477012	0	0	0
YRI	CSH	63482012	0	0	0
YRI	CSH	63487012	0	0	0
YRI	CSH	63492012	0	0	0
YRI	CSH	63497012	0	0	0
YRI	CSH	63502012	0	0	0
YRI	CSH	63507012	0	0	0
YRI	CSH	63512012	0	0	0
YRI	CSH	63517012	0	0	0

YRI	CSH	63522012	0	0	0
YRI	CSH	63527012	0	0	0
YRI	CSH	63532012	0	0	0
YRI	CSH	63537012	0	0	0
YRI	CSH	63542012	0	0	0
YRI	CSH	63547012	0	0	0
YRI	CSH	63552012	0	0	0
YRI	CSH	63557012	0	0	0
YRI	CSH	63562012	0	0	0
YRI	CSH	63567012	0	0	0
YRI	CSH	63572012	0	0	0
YRI	CSH	63577012	0	0	0
YRI	CSH	63582012	0	0	0
YRI	CSH	63587012	0	0	0
YRI	CSH	63592012	0	0	0
YRI	CSH	63597012	0	0	0
YRI	CSH	63602012	0	0	0
YRI	CSH	63607012	0	0	0
YRI	CSH	63612012	0	0	0
YRI	CSH	63617012	0	0	0
YRI	CSH	63622012	0	0	0
YRI	CSH	63627012	0	0	0
YRI	CSH	63632012	0	0	0
YRI	CSH	63637012	0	0	0
YRI	CSH	63642012	0	0	0
YRI	CSH	63647012	0	0	0
YRI	CSH	63652012	0	0	0
YRI	CSH	63657012	0	0	0
YRI	CSH	63662012	0	0	0
YRI	CSH	63667012	0	0	0
YRI	CSH	63672012	0	0	0
YRI	CSH	63677012	0	0	0
YRI	CSH	63682012	0	0	0
YRI	CSH	63687012	0	0	0
YRI	CSH	63692012	0	0	0
YRI	CSH	63697012	0	0	0
YRI	CSH	63702012	0	0	0
YRI	CSH	63707012	0	0	0
YRI	CSH	63712012	0	0	0
YRI	CSH	63717012	0	0	0
YRI	CSH	63722012	0	0	0
YRI	CSH	63727012	0	0	0
YRI	CSH	63732012	0	0	0
YRI	CSH	63737012	0	0	0
YRI	CSH	63742012	0	0	0
YRI	CSH	63747012	0	0	0
YRI	CSH	63752012	0	0	0
YRI	CSH	63757012	0	0	0
YRI	CSH	63762012	0	0	0

YRI	CSH	63767012	0	0	0
YRI	CSH	63772012	0	0	0
YRI	CSH	63777012	0	0	0
YRI	CSH	63782012	0	0	0
YRI	CSH	63787012	0	0	0
YRI	CSH	63792012	0	0	0
YRI	CSH	63797012	0	0	0
YRI	CSH	63802012	0	0	0
YRI	CSH	63807012	0	0	0
YRI	CSH	63812012	0	0	0
YRI	CSH	63817012	0	0	0
YRI	CSH	63822012	0	0	0
YRI	CSH	63827012	0	0	0
YRI	CSH	63832012	0	0	0
YRI	CSH	63837012	0	0	0
YRI	CSH	63842012	0	0	0
YRI	CSH	63847012	0	0	0
YRI	CSH	63852012	0	0	0
YRI	CSH	63857012	0	0	0
YRI	CSH	63862012	0	0	0
YRI	CSH	63867012	0	0	0
YRI	CSH	63872012	0	0	0
YRI	CSH	63877012	0	0	0
YRI	CSH	63882012	0	0	0
YRI	CSH	63887012	0	0	0
YRI	CSH	63892012	0	0	0
YRI	CSH	63897012	0	0	0
YRI	CSH	63902012	0	0	0
YRI	CSH	63907012	0	0	0
YRI	CSH	63912012	0	0	0
YRI	CSH	63917012	0	0	0
YRI	CSH	63922012	0	0	0
YRI	CSH	63927012	0	0	0
YRI	CSH	63932012	0	0	0
YRI	CSH	63937012	0	0	0
YRI	CSH	63942012	0	0	0
YRI	CSH	63947012	0	0	0
YRI	CSH	63952012	0	0	0
YRI	CSH	63957012	0	0	0
YRI	CSH	63962012	0	0	0
YRI	CSH	63967012	0	0	0
YRI	CSH	63972012	0	0	0
YRI	CSH	63977012	0	0	0
YRI	CSH	63982012	0	0	0
YRI	CSH	63987012	0	0	0
YRI	CSH	63992012	0	0	0
YRI	CSH	63997012	0	0	0
YRI	CSH	64002012	0	0	0
YRI	CSH	64007012	0	0	0

YRI	CSH	64012012	0	0	0
YRI	CSH	64017012	0	0	0
YRI	CSH	64022012	0	0	0
YRI	CSH	64027012	0	0	0
YRI	CSH	64032012	0	0	0
YRI	CSH	64037012	0	0	0
YRI	CSH	64042012	0	0	0
YRI	CSH	64047012	0	0	0
YRI	CSH	64052012	0	0	0
YRI	CSH	64057012	0	0	0
YRI	CSH	64062012	0	0	0
YRI	CSH	64067012	0	0	0
YRI	CSH	64072012	0	0	0
YRI	CSH	64077012	0	0	0
YRI	CSH	64082012	0	0	0
YRI	CSH	64087012	0	0	0
YRI	CSH	64092012	0	0	0
YRI	CSH	64097012	0	0	0
YRI	CSH	64102012	0	0	0
YRI	CSH	64107012	0	0	0
YRI	CSH	64112012	0	0	0
YRI	CSH	64117012	0	0	0
YRI	CSH	64122012	0	0	0
YRI	CSH	64127012	0	0	0
YRI	CSH	64132012	0	0	0
YRI	CSH	64137012	0	0	0
YRI	CSH	64142012	0	0	0
YRI	CSH	64147012	0	0	0
YRI	CSH	64152012	0	0	0
YRI	CSH	64157012	0	0	0
YRI	CSH	64162012	0	0	0
YRI	CSH	64167012	0	0	0
YRI	CSH	64172012	0	0	0
YRI	CSH	64177012	0	0	0
YRI	CSH	64182012	0	0	0
YRI	CSH	64187012	0	0	0
YRI	CSH	64192012	0	0	0
YRI	CSH	64197012	0	0	0
YRI	CSH	64202012	0	0	0
YRI	CSH	64207012	0	0	0
YRI	CSH	64212012	0	0	0
YRI	CSH	64217012	0	0	0
YRI	CSH	64222012	0	0	0
YRI	CSH	64227012	0	0	0
YRI	CSH	64232012	0	0	0
YRI	CSH	64237012	0	0	0
YRI	CSH	64242012	0	0	0
YRI	CSH	64247012	0	0	0
YRI	CSH	64252012	0	0	0

YRI	CSH	64257012	0	0	0
YRI	CSH	64262012	0	0	0
YRI	CSH	64267012	0	0	0
YRI	CSH	64272012	0	0	0
YRI	CSH	64277012	0	0	0
YRI	CSH	64282012	0	0	0
YRI	CSH	64287012	0	0	0
YRI	CSH	64292012	0	0	0
YRI	CSH	64297012	0	0	0
YRI	CSH	64302012	0	0	0
YRI	CSH	64307012	0	0	0
YRI	CSH	64312012	0	0	0
YRI	CSH	64317012	0	0	0
YRI	CSH	64322012	0	0	0
YRI	CSH	64327012	0	0	0
YRI	CSH	64332012	0	0	0
YRI	CSH	64337012	0	0	0
YRI	CSH	64342012	0	0	0
YRI	CSH	64347012	0	0	0
YRI	CSH	64352012	0	0	0
YRI	CSH	64357012	0	0	0
YRI	CSH	64362012	0	0	0
YRI	CSH	64367012	0	0	0
CEU	CSH	63399918	0	0	0
CEU	CSH	63404918	0	0	0
CEU	CSH	63409918	0	0	0
CEU	CSH	63414918	0	0	0
CEU	CSH	63419918	0	0	0
CEU	CSH	63424918	0	0	0
CEU	CSH	63429918	0	0	0
CEU	CSH	63434918	0	0	0
CEU	CSH	63439918	0	0	0
CEU	CSH	63444918	0	0	0
CEU	CSH	63449918	0	0	0
CEU	CSH	63454918	0	0	0
CEU	CSH	63459918	0	0	0
CEU	CSH	63464918	0	0	0
CEU	CSH	63469918	0	0	0
CEU	CSH	63474918	0	0	0
CEU	CSH	63479918	0	0	0
CEU	CSH	63484918	0	0	0
CEU	CSH	63489918	0	0	0
CEU	CSH	63494918	0	0	0
CEU	CSH	63499918	0	0	0
CEU	CSH	63504918	0	0	0
CEU	CSH	63509918	0	0	0
CEU	CSH	63514918	0	0	0
CEU	CSH	63519918	0	0	0
CEU	CSH	63524918	0	0	0

CEU	CSH	63529918	0	0	0
CEU	CSH	63534918	0	0	0
CEU	CSH	63539918	0	0	0
CEU	CSH	63544918	0	0	0
CEU	CSH	63549918	0	0	0
CEU	CSH	63554918	0	0	0
CEU	CSH	63559918	0	0	0
CEU	CSH	63564918	0	0	0
CEU	CSH	63569918	0	0	0
CEU	CSH	63574918	0	0	0
CEU	CSH	63579918	0	0	0
CEU	CSH	63584918	0	0	0
CEU	CSH	63589918	0	0	0
CEU	CSH	63594918	0	0	0
CEU	CSH	63599918	0	0	0
CEU	CSH	63604918	0	0	0
CEU	CSH	63609918	0	0	0
CEU	CSH	63614918	0	0	0
CEU	CSH	63619918	0	0	0
CEU	CSH	63624918	0	0	0
CEU	CSH	63629918	0	0	0
CEU	CSH	63634918	0	0	0
CEU	CSH	63639918	0	0	0
CEU	CSH	63644918	0	0	0
CEU	CSH	63649918	0	0	0
CEU	CSH	63654918	0	0	0
CEU	CSH	63659918	0	0	0
CEU	CSH	63664918	0	0	0
CEU	CSH	63669918	0	0	0
CEU	CSH	63674918	0	0	0
CEU	CSH	63679918	0	0	0
CEU	CSH	63684918	0	0	0
CEU	CSH	63689918	0	0	0
CEU	CSH	63694918	0	0	0
CEU	CSH	63699918	0	0	0
CEU	CSH	63704918	0	0	0
CEU	CSH	63709918	0	0	0
CEU	CSH	63714918	0	0	0
CEU	CSH	63719918	0	0	0
CEU	CSH	63724918	0	0	0
CEU	CSH	63729918	0	0	0
CEU	CSH	63734918	0	0	0
CEU	CSH	63739918	0	0	0
CEU	CSH	63744918	0	0	0
CEU	CSH	63749918	0	0	0
CEU	CSH	63754918	0	0	0
CEU	CSH	63759918	0	0	0
CEU	CSH	63764918	0	0	0
CEU	CSH	63769918	0	0	0

CEU	CSH	63774918	0	0	0
CEU	CSH	63779918	0	0	0
CEU	CSH	63784918	0	0	0
CEU	CSH	63789918	0	0	0
CEU	CSH	63794918	0	0	0
CEU	CSH	63799918	0	0	0
CEU	CSH	63804918	0	0	0
CEU	CSH	63809918	0	0	0
CEU	CSH	63814918	0	0	0
CEU	CSH	63819918	0	0	0
CEU	CSH	63824918	0	0	0
CEU	CSH	63829918	0	0	0
CEU	CSH	63834918	0	0	0
CEU	CSH	63839918	0	0	0
CEU	CSH	63844918	0	0	0
CEU	CSH	63849918	0	0	0
CEU	CSH	63854918	0	0	0
CEU	CSH	63859918	0	0	0
CEU	CSH	63864918	0	0	0
CEU	CSH	63869918	0	0	0
CEU	CSH	63874918	0	0	0
CEU	CSH	63879918	0	0	0
CEU	CSH	63884918	0	0	0
CEU	CSH	63889918	0	0	0
CEU	CSH	63894918	0	0	0
CEU	CSH	63899918	0	0	0
CEU	CSH	63904918	0	0	0
CEU	CSH	63909918	0	0	0
CEU	CSH	63914918	0	0	0
CEU	CSH	63919918	0	0	0
CEU	CSH	63924918	0	0	0
CEU	CSH	63929918	0	0	0
CEU	CSH	63934918	0	0	0
CEU	CSH	63939918	0	0	0
CEU	CSH	63944918	0	0	0
CEU	CSH	63949918	0	0	0
CEU	CSH	63954918	0	0	0
CEU	CSH	63959918	0	0	0
CEU	CSH	63964918	0	0	0
CEU	CSH	63969918	0	0	0
CEU	CSH	63974918	0	0	0
CEU	CSH	63979918	0	0	0
CEU	CSH	63984918	0	0	0
CEU	CSH	63989918	0	0	0
CEU	CSH	63994918	0	0	0
CEU	CSH	63999918	0	0	0
CEU	CSH	64004918	0	0	0
CEU	CSH	64009918	0	0	0
CEU	CSH	64014918	0	0	0

CEU	CSH	64019918	0	0	0
CEU	CSH	64024918	0	0	0
CEU	CSH	64029918	0	0	0
CEU	CSH	64034918	0	0	0
CEU	CSH	64039918	0	0	0
CEU	CSH	64044918	0	0	0
CEU	CSH	64049918	0	0	0
CEU	CSH	64054918	0	0	0
CEU	CSH	64059918	0	0	0
CEU	CSH	64064918	0	0	0
CEU	CSH	64069918	0	0	0
CEU	CSH	64074918	0	0	0
CEU	CSH	64079918	0	0	0
CEU	CSH	64084918	0	0	0
CEU	CSH	64089918	0	0	0
CEU	CSH	64094918	0	0	0
CEU	CSH	64099918	0	0	0
CEU	CSH	64104918	0	0	0
CEU	CSH	64109918	0	0	0
CEU	CSH	64114918	0	0	0
CEU	CSH	64119918	0	0	0
CEU	CSH	64124918	0	0	0
CEU	CSH	64129918	0	0	0
CEU	CSH	64134918	0	0	0
CEU	CSH	64139918	0	0	0
CEU	CSH	64144918	0	0	0
CEU	CSH	64149918	0	0	0
CEU	CSH	64154918	0	0	0
CEU	CSH	64159918	0	0	0
CEU	CSH	64164918	0	0	0
CEU	CSH	64169918	0	0	0
CEU	CSH	64174918	0	0	0
CEU	CSH	64179918	0	0	0
CEU	CSH	64184918	0	0	0
CEU	CSH	64189918	0	0	0
CEU	CSH	64194918	0	0	0
CEU	CSH	64199918	0	0	0
CEU	CSH	64204918	0	0	0
CEU	CSH	64209918	0	0	0
CEU	CSH	64214918	0	0	0
CEU	CSH	64219918	0	0	0
CEU	CSH	64224918	0	0	0
CEU	CSH	64229918	0	0	0
CEU	CSH	64234918	0	0	0
CEU	CSH	64239918	0	0	0
CEU	CSH	64244918	0	0	0
CEU	CSH	64249918	0	0	0
CEU	CSH	64254918	0	0	0
CEU	CSH	64259918	0	0	0

CEU	CSH	64264918	0	0	0
CEU	CSH	64269918	0	0	0
CEU	CSH	64274918	0	0	0
CEU	CSH	64279918	0	0	0
CEU	CSH	64284918	0	0	0
CEU	CSH	64289918	0	0	0
CEU	CSH	64294918	0	0	0
CEU	CSH	64299918	0	0	0
CEU	CSH	64304918	0	0	0
CEU	CSH	64309918	0	0	0
CEU	CSH	64314918	0	0	0
CEU	CSH	64319918	0	0	0
CEU	CSH	64324918	0	0	0
CEU	CSH	64329918	0	0	0
CEU	CSH	64334918	0	0	0
CEU	CSH	64339918	0	0	0
CEU	CSH	64344918	0	0	0
CEU	CSH	64349918	0	0	0
CEU	CSH	64354918	0	0	0
CEU	CSH	64359918	0	0	0
CEU	CSH	64364918	0	0	0
CEU	CSH	64369918	0	0	0
CEU	CSH	64374918	0	0	0
CEU	CSH	64379918	0	0	0
CEU	CSH	64384918	0	0	0
CEU	CSH	64389918	0	0	0
CHB	CSH	63399918	0	0	0
CHB	CSH	63404918	0	0	0
CHB	CSH	63409918	0	0	0
CHB	CSH	63414918	0	0	0
CHB	CSH	63419918	0	0	0
CHB	CSH	63424918	0	0	0
CHB	CSH	63429918	0	0	0
CHB	CSH	63434918	0	0	0
CHB	CSH	63439918	0	0	0
CHB	CSH	63444918	0	0	0
CHB	CSH	63449918	0	0	0
CHB	CSH	63454918	0	0	0
CHB	CSH	63459918	0	0	0
CHB	CSH	63464918	0	0	0
CHB	CSH	63469918	0	0	0
CHB	CSH	63474918	0	0	0
CHB	CSH	63479918	0	0	0
CHB	CSH	63484918	0	0	0
CHB	CSH	63489918	0	0	0
CHB	CSH	63494918	0	0	0
CHB	CSH	63499918	0	0	0
CHB	CSH	63504918	0	0	0
CHB	CSH	63509918	0	0	0

CHB	CSH	63514918	0	0	0
CHB	CSH	63519918	0	0	0
CHB	CSH	63524918	0	0	0
CHB	CSH	63529918	0	0	0
CHB	CSH	63534918	0	0	0
CHB	CSH	63539918	0	0	0
CHB	CSH	63544918	0	0	0
CHB	CSH	63549918	0	0	0
CHB	CSH	63554918	0	0	0
CHB	CSH	63559918	0	0	0
CHB	CSH	63564918	0	0	0
CHB	CSH	63569918	0	0	0
CHB	CSH	63574918	0	0	0
CHB	CSH	63579918	0	0	0
CHB	CSH	63584918	0	0	0
CHB	CSH	63589918	0	0	0
CHB	CSH	63594918	0	0	0
CHB	CSH	63599918	0	0	0
CHB	CSH	63604918	0	0	0
CHB	CSH	63609918	0	0	0
CHB	CSH	63614918	0	0	0
CHB	CSH	63619918	0	0	0
CHB	CSH	63624918	0	0	0
CHB	CSH	63629918	0	0	0
CHB	CSH	63634918	0	0	0
CHB	CSH	63639918	0	0	0
CHB	CSH	63644918	0	0	0
CHB	CSH	63649918	0	0	0
CHB	CSH	63654918	0	0	0
CHB	CSH	63659918	0	0	0
CHB	CSH	63664918	0	0	0
CHB	CSH	63669918	0	0	0
CHB	CSH	63674918	0	0	0
CHB	CSH	63679918	0	0	0
CHB	CSH	63684918	0	0	0
CHB	CSH	63689918	0	0	0
CHB	CSH	63694918	0	0	0
CHB	CSH	63699918	0	0	0
CHB	CSH	63704918	0	0	0
CHB	CSH	63709918	0	0	0
CHB	CSH	63714918	0	0	0
CHB	CSH	63719918	0	0	0
CHB	CSH	63724918	0	0	0
CHB	CSH	63729918	0	0	0
CHB	CSH	63734918	0	0	0
CHB	CSH	63739918	0	0	0
CHB	CSH	63744918	0	0	0
CHB	CSH	63749918	0	0	0
CHB	CSH	63754918	0	0	0

CHB	CSH	63759918	0	0	0
CHB	CSH	63764918	0	0	0
CHB	CSH	63769918	0	0	0
CHB	CSH	63774918	0	0	0
CHB	CSH	63779918	0	0	0
CHB	CSH	63784918	0	0	0
CHB	CSH	63789918	0	0	0
CHB	CSH	63794918	0	0	0
CHB	CSH	63799918	0	0	0
CHB	CSH	63804918	0	0	0
CHB	CSH	63809918	0	0	0
CHB	CSH	63814918	0	0	0
CHB	CSH	63819918	0	0	0
CHB	CSH	63824918	0	0	0
CHB	CSH	63829918	0	0	0
CHB	CSH	63834918	0	0	0
CHB	CSH	63839918	0	0	0
CHB	CSH	63844918	0	0	0
CHB	CSH	63849918	0	0	0
CHB	CSH	63854918	0	0	0
CHB	CSH	63859918	0	0	0
CHB	CSH	63864918	0	0	0
CHB	CSH	63869918	0	0	0
CHB	CSH	63874918	0	0	0
CHB	CSH	63879918	0	0	0
CHB	CSH	63884918	0	0	0
CHB	CSH	63889918	0	0	0
CHB	CSH	63894918	0	0	0
CHB	CSH	63899918	0	0	0
CHB	CSH	63904918	0	0	0
CHB	CSH	63909918	0	0	0
CHB	CSH	63914918	0	0	0
CHB	CSH	63919918	0	0	0
CHB	CSH	63924918	0	0	0
CHB	CSH	63929918	0	0	0
CHB	CSH	63934918	0	0	0
CHB	CSH	63939918	0	0	0
CHB	CSH	63944918	0	0	0
CHB	CSH	63949918	0	0	0
CHB	CSH	63954918	0	0	0
CHB	CSH	63959918	0	0	0
CHB	CSH	63964918	0	0	0
CHB	CSH	63969918	0	0	0
CHB	CSH	63974918	0	0	0
CHB	CSH	63979918	0	0	0
CHB	CSH	63984918	0	0	0
CHB	CSH	63989918	0	0	0
CHB	CSH	63994918	0	0	0
CHB	CSH	63999918	0	0	0

CHB	CSH	64004918	0	0	0
CHB	CSH	64009918	0	0	0
CHB	CSH	64014918	0	0	0
CHB	CSH	64019918	0	0	0
CHB	CSH	64024918	0	0	0
CHB	CSH	64029918	0	0	0
CHB	CSH	64034918	0	0	0
CHB	CSH	64039918	0	0	0
CHB	CSH	64044918	0	0	0
CHB	CSH	64049918	0	0	0
CHB	CSH	64054918	0	0	0
CHB	CSH	64059918	0	0	0
CHB	CSH	64064918	0	0	0
CHB	CSH	64069918	0	0	0
CHB	CSH	64074918	0	0	0
CHB	CSH	64079918	0	0	0
CHB	CSH	64084918	0	0	0
CHB	CSH	64089918	0	0	0
CHB	CSH	64094918	0	0	0
CHB	CSH	64099918	0	0	0
CHB	CSH	64104918	0	0	0
CHB	CSH	64109918	0	0	0
CHB	CSH	64114918	0	0	0
CHB	CSH	64119918	0	0	0
CHB	CSH	64124918	0	0	0
CHB	CSH	64129918	0	0	0
CHB	CSH	64134918	0	0	0
CHB	CSH	64139918	0	0	0
CHB	CSH	64144918	0	0	0
CHB	CSH	64149918	0	0	0
CHB	CSH	64154918	0	0	0
CHB	CSH	64159918	0	0	0
CHB	CSH	64164918	0	0	0
CHB	CSH	64169918	0	0	0
CHB	CSH	64174918	0	0	0
CHB	CSH	64179918	0	0	0
CHB	CSH	64184918	0	0	0
CHB	CSH	64189918	0	0	0
CHB	CSH	64194918	0	0	0
CHB	CSH	64199918	0	0	0
CHB	CSH	64204918	0	0	0
CHB	CSH	64209918	0	0	0
CHB	CSH	64214918	0	0	0
CHB	CSH	64219918	0	0	0
CHB	CSH	64224918	0	0	0
CHB	CSH	64229918	0	0	0
CHB	CSH	64234918	0	0	0
CHB	CSH	64239918	0	0	0
CHB	CSH	64244918	0	0	0

CHB	CSH	64249918	0	0	0
CHB	CSH	64254918	0	0	0
CHB	CSH	64259918	0	0	0
CHB	CSH	64264918	0	0	0
CHB	CSH	64269918	0	0	0
CHB	CSH	64274918	0	0	0
CHB	CSH	64279918	0	0	0
CHB	CSH	64284918	0	0	0
CHB	CSH	64289918	0	0	0
CHB	CSH	64294918	0	0	0
CHB	CSH	64299918	0	0	0
CHB	CSH	64304918	0	0	0
CHB	CSH	64309918	0	0	0
CHB	CSH	64314918	0	0	0
CHB	CSH	64319918	0	0	0
CHB	CSH	64324918	0	0	0
CHB	CSH	64329918	0	0	0
CHB	CSH	64334918	0	0	0
CHB	CSH	64339918	0	0	0
CHB	CSH	64344918	0	0	0
CHB	CSH	64349918	0	0	0
CHB	CSH	64354918	0	0	0
CHB	CSH	64359918	0	0	0
CHB	CSH	64364918	0	0	0
CHB	CSH	64369918	0	0	0
CHB	CSH	64374918	0	0	0
CHB	CSH	64379918	0	0	0
CHB	CSH	64384918	0	0	0
CHB	CSH	64389918	0	0	0
YRI	CSH	63399918	0	0	0
YRI	CSH	63404918	0	0	0
YRI	CSH	63409918	0	0	0
YRI	CSH	63414918	0	0	0
YRI	CSH	63419918	0	0	0
YRI	CSH	63424918	0	0	0
YRI	CSH	63429918	0	0	0
YRI	CSH	63434918	0	0	0
YRI	CSH	63439918	0	0	0
YRI	CSH	63444918	0	0	0
YRI	CSH	63449918	0	0	0
YRI	CSH	63454918	0	0	0
YRI	CSH	63459918	0	0	0
YRI	CSH	63464918	0	0	0
YRI	CSH	63469918	0	0	0
YRI	CSH	63474918	0	0	0
YRI	CSH	63479918	0	0	0
YRI	CSH	63484918	0	0	0
YRI	CSH	63489918	0	0	0
YRI	CSH	63494918	0	0	0

YRI	CSH	63499918	0	0	0
YRI	CSH	63504918	0	0	0
YRI	CSH	63509918	0	0	0
YRI	CSH	63514918	0	0	0
YRI	CSH	63519918	0	0	0
YRI	CSH	63524918	0	0	0
YRI	CSH	63529918	0	0	0
YRI	CSH	63534918	0	0	0
YRI	CSH	63539918	0	0	0
YRI	CSH	63544918	0	0	0
YRI	CSH	63549918	0	0	0
YRI	CSH	63554918	0	0	0
YRI	CSH	63559918	0	0	0
YRI	CSH	63564918	0	0	0
YRI	CSH	63569918	0	0	0
YRI	CSH	63574918	0	0	0
YRI	CSH	63579918	0	0	0
YRI	CSH	63584918	0	0	0
YRI	CSH	63589918	0	0	0
YRI	CSH	63594918	0	0	0
YRI	CSH	63599918	0	0	0
YRI	CSH	63604918	0	0	0
YRI	CSH	63609918	0	0	0
YRI	CSH	63614918	0	0	0
YRI	CSH	63619918	0	0	0
YRI	CSH	63624918	0	0	0
YRI	CSH	63629918	0	0	0
YRI	CSH	63634918	0	0	0
YRI	CSH	63639918	0	0	0
YRI	CSH	63644918	0	0	0
YRI	CSH	63649918	0	0	0
YRI	CSH	63654918	0	0	0
YRI	CSH	63659918	0	0	0
YRI	CSH	63664918	0	0	0
YRI	CSH	63669918	0	0	0
YRI	CSH	63674918	0	0	0
YRI	CSH	63679918	0	0	0
YRI	CSH	63684918	0	0	0
YRI	CSH	63689918	0	0	0
YRI	CSH	63694918	0	0	0
YRI	CSH	63699918	0	0	0
YRI	CSH	63704918	0	0	0
YRI	CSH	63709918	0	0	0
YRI	CSH	63714918	0	0	0
YRI	CSH	63719918	0	0	0
YRI	CSH	63724918	0	0	0
YRI	CSH	63729918	0	0	0
YRI	CSH	63734918	0	0	0
YRI	CSH	63739918	0	0	0

YRI	CSH	63744918	0	0	0
YRI	CSH	63749918	0	0	0
YRI	CSH	63754918	0	0	0
YRI	CSH	63759918	0	0	0
YRI	CSH	63764918	0	0	0
YRI	CSH	63769918	0	0	0
YRI	CSH	63774918	0	0	0
YRI	CSH	63779918	0	0	0
YRI	CSH	63784918	0	0	0
YRI	CSH	63789918	0	0	0
YRI	CSH	63794918	0	0	0
YRI	CSH	63799918	0	0	0
YRI	CSH	63804918	0	0	0
YRI	CSH	63809918	0	0	0
YRI	CSH	63814918	0	0	0
YRI	CSH	63819918	0	0	0
YRI	CSH	63824918	0	0	0
YRI	CSH	63829918	0	0	0
YRI	CSH	63834918	0	0	0
YRI	CSH	63839918	0	0	0
YRI	CSH	63844918	0	0	0
YRI	CSH	63849918	0	0	0
YRI	CSH	63854918	0	0	0
YRI	CSH	63859918	0	0	0
YRI	CSH	63864918	0	0	0
YRI	CSH	63869918	0	0	0
YRI	CSH	63874918	0	0	0
YRI	CSH	63879918	0	0	0
YRI	CSH	63884918	0	0	0
YRI	CSH	63889918	0	0	0
YRI	CSH	63894918	0	0	0
YRI	CSH	63899918	0	0	0
YRI	CSH	63904918	0	0	0
YRI	CSH	63909918	0	0	0
YRI	CSH	63914918	0	0	0
YRI	CSH	63919918	0	0	0
YRI	CSH	63924918	0	0	0
YRI	CSH	63929918	0	0	0
YRI	CSH	63934918	0	0	0
YRI	CSH	63939918	0	0	0
YRI	CSH	63944918	0	0	0
YRI	CSH	63949918	0	0	0
YRI	CSH	63954918	0	0	0
YRI	CSH	63959918	0	0	0
YRI	CSH	63964918	0	0	0
YRI	CSH	63969918	0	0	0
YRI	CSH	63974918	0	0	0
YRI	CSH	63979918	0	0	0
YRI	CSH	63984918	0	0	0

YRI	CSH	63989918	0	0	0
YRI	CSH	63994918	0	0	0
YRI	CSH	63999918	0	0	0
YRI	CSH	64004918	0	0	0
YRI	CSH	64009918	0	0	0
YRI	CSH	64014918	0	0	0
YRI	CSH	64019918	0	0	0
YRI	CSH	64024918	0	0	0
YRI	CSH	64029918	0	0	0
YRI	CSH	64034918	0	0	0
YRI	CSH	64039918	0	0	0
YRI	CSH	64044918	0	0	0
YRI	CSH	64049918	0	0	0
YRI	CSH	64054918	0	0	0
YRI	CSH	64059918	0	0	0
YRI	CSH	64064918	0	0	0
YRI	CSH	64069918	0	0	0
YRI	CSH	64074918	0	0	0
YRI	CSH	64079918	0	0	0
YRI	CSH	64084918	0	0	0
YRI	CSH	64089918	0	0	0
YRI	CSH	64094918	0	0	0
YRI	CSH	64099918	0	0	0
YRI	CSH	64104918	0	0	0
YRI	CSH	64109918	0	0	0
YRI	CSH	64114918	0	0	0
YRI	CSH	64119918	0	0	0
YRI	CSH	64124918	0	0	0
YRI	CSH	64129918	0	0	0
YRI	CSH	64134918	0	0	0
YRI	CSH	64139918	0	0	0
YRI	CSH	64144918	0	0	0
YRI	CSH	64149918	0	0	0
YRI	CSH	64154918	0	0	0
YRI	CSH	64159918	0	0	0
YRI	CSH	64164918	0	0	0
YRI	CSH	64169918	0	0	0
YRI	CSH	64174918	0	0	0
YRI	CSH	64179918	0	0	0
YRI	CSH	64184918	0	0	0
YRI	CSH	64189918	0	0	0
YRI	CSH	64194918	0	0	0
YRI	CSH	64199918	0	0	0
YRI	CSH	64204918	0	0	0
YRI	CSH	64209918	0	0	0
YRI	CSH	64214918	0	0	0
YRI	CSH	64219918	0	0	0
YRI	CSH	64224918	0	0	0
YRI	CSH	64229918	0	0	0

YRI	CSH	64234918	0	0	0
YRI	CSH	64239918	0	0	0
YRI	CSH	64244918	0	0	0
YRI	CSH	64249918	0	0	0
YRI	CSH	64254918	0	0	0
YRI	CSH	64259918	0	0	0
YRI	CSH	64264918	0	0	0
YRI	CSH	64269918	0	0	0
YRI	CSH	64274918	0	0	0
YRI	CSH	64279918	0	0	0
YRI	CSH	64284918	0	0	0
YRI	CSH	64289918	0	0	0
YRI	CSH	64294918	0	0	0
YRI	CSH	64299918	0	0	0
YRI	CSH	64304918	0	0	0
YRI	CSH	64309918	0	0	0
YRI	CSH	64314918	0	0	0
YRI	CSH	64319918	0	0	0
YRI	CSH	64324918	0	0	0
YRI	CSH	64329918	0	0	0
YRI	CSH	64334918	0	0	0
YRI	CSH	64339918	0	0	0
YRI	CSH	64344918	0	0	0
YRI	CSH	64349918	0	0	0
YRI	CSH	64354918	0	0	0
YRI	CSH	64359918	0	0	0
YRI	CSH	64364918	0	0	0
YRI	CSH	64369918	0	0	0
YRI	CSH	64374918	0	0	0
YRI	CSH	64379918	0	0	0
YRI	CSH	64384918	0	0	0
YRI	CSH	64389918	0	0	0
CEU	CSH	63414602	0	0	0
CEU	CSH	63419602	0	0	0
CEU	CSH	63424602	0	0	0
CEU	CSH	63429602	0	0	0
CEU	CSH	63434602	0	0	0
CEU	CSH	63439602	0	0	0
CEU	CSH	63444602	0	0	0
CEU	CSH	63449602	0	0	0
CEU	CSH	63454602	0	0	0
CEU	CSH	63459602	0	0	0
CEU	CSH	63464602	0	0	0
CEU	CSH	63469602	0	0	0
CEU	CSH	63474602	0	0	0
CEU	CSH	63479602	0	0	0
CEU	CSH	63484602	0	0	0
CEU	CSH	63489602	0	0	0
CEU	CSH	63494602	0	0	0

CEU	CSH	63499602	0	0	0
CEU	CSH	63504602	0	0	0
CEU	CSH	63509602	0	0	0
CEU	CSH	63514602	0	0	0
CEU	CSH	63519602	0	0	0
CEU	CSH	63524602	0	0	0
CEU	CSH	63529602	0	0	0
CEU	CSH	63534602	0	0	0
CEU	CSH	63539602	0	0	0
CEU	CSH	63544602	0	0	0
CEU	CSH	63549602	0	0	0
CEU	CSH	63554602	0	0	0
CEU	CSH	63559602	0	0	0
CEU	CSH	63564602	0	0	0
CEU	CSH	63569602	0	0	0
CEU	CSH	63574602	0	0	0
CEU	CSH	63579602	0	0	0
CEU	CSH	63584602	0	0	0
CEU	CSH	63589602	0	0	0
CEU	CSH	63594602	0	0	0
CEU	CSH	63599602	0	0	0
CEU	CSH	63604602	0	0	0
CEU	CSH	63609602	0	0	0
CEU	CSH	63614602	0	0	0
CEU	CSH	63619602	0	0	0
CEU	CSH	63624602	0	0	0
CEU	CSH	63629602	0	0	0
CEU	CSH	63634602	0	0	0
CEU	CSH	63639602	0	0	0
CEU	CSH	63644602	0	0	0
CEU	CSH	63649602	0	0	0
CEU	CSH	63654602	0	0	0
CEU	CSH	63659602	0	0	0
CEU	CSH	63664602	0	0	0
CEU	CSH	63669602	0	0	0
CEU	CSH	63674602	0	0	0
CEU	CSH	63679602	0	0	0
CEU	CSH	63684602	0	0	0
CEU	CSH	63689602	0	0	0
CEU	CSH	63694602	0	0	0
CEU	CSH	63699602	0	0	0
CEU	CSH	63704602	0	0	0
CEU	CSH	63709602	0	0	0
CEU	CSH	63714602	0	0	0
CEU	CSH	63719602	0	0	0
CEU	CSH	63724602	0	0	0
CEU	CSH	63729602	0	0	0
CEU	CSH	63734602	0	0	0
CEU	CSH	63739602	0	0	0

CEU	CSH	63744602	0	0	0
CEU	CSH	63749602	0	0	0
CEU	CSH	63754602	0	0	0
CEU	CSH	63759602	0	0	0
CEU	CSH	63764602	0	0	0
CEU	CSH	63769602	0	0	0
CEU	CSH	63774602	0	0	0
CEU	CSH	63779602	0	0	0
CEU	CSH	63784602	0	0	0
CEU	CSH	63789602	0	0	0
CEU	CSH	63794602	0	0	0
CEU	CSH	63799602	0	0	0
CEU	CSH	63804602	0	0	0
CEU	CSH	63809602	0	0	0
CEU	CSH	63814602	0	0	0
CEU	CSH	63819602	0	0	0
CEU	CSH	63824602	0	0	0
CEU	CSH	63829602	0	0	0
CEU	CSH	63834602	0	0	0
CEU	CSH	63839602	0	0	0
CEU	CSH	63844602	0	0	0
CEU	CSH	63849602	0	0	0
CEU	CSH	63854602	0	0	0
CEU	CSH	63859602	0	0	0
CEU	CSH	63864602	0	0	0
CEU	CSH	63869602	0	0	0
CEU	CSH	63874602	0	0	0
CEU	CSH	63879602	0	0	0
CEU	CSH	63884602	0	0	0
CEU	CSH	63889602	0	0	0
CEU	CSH	63894602	0	0	0
CEU	CSH	63899602	0	0	0
CEU	CSH	63904602	0	0	0
CEU	CSH	63909602	0	0	0
CEU	CSH	63914602	0	0	0
CEU	CSH	63919602	0	0	0
CEU	CSH	63924602	0	0	0
CEU	CSH	63929602	0	0	0
CEU	CSH	63934602	0	0	0
CEU	CSH	63939602	0	0	0
CEU	CSH	63944602	0	0	0
CEU	CSH	63949602	0	0	0
CEU	CSH	63954602	0	0	0
CEU	CSH	63959602	0	0	0
CEU	CSH	63964602	0	0	0
CEU	CSH	63969602	0	0	0
CEU	CSH	63974602	0	0	0
CEU	CSH	63979602	0	0	0
CEU	CSH	63984602	0	0	0

CEU	CSH	63989602	0	0	0
CEU	CSH	63994602	0	0	0
CEU	CSH	63999602	0	0	0
CEU	CSH	64004602	0	0	0
CEU	CSH	64009602	0	0	0
CEU	CSH	64014602	0	0	0
CEU	CSH	64019602	0	0	0
CEU	CSH	64024602	0	0	0
CEU	CSH	64029602	0	0	0
CEU	CSH	64034602	0	0	0
CEU	CSH	64039602	0	0	0
CEU	CSH	64044602	0	0	0
CEU	CSH	64049602	0	0	0
CEU	CSH	64054602	0	0	0
CEU	CSH	64059602	0	0	0
CEU	CSH	64064602	0	0	0
CEU	CSH	64069602	0	0	0
CEU	CSH	64074602	0	0	0
CEU	CSH	64079602	0	0	0
CEU	CSH	64084602	0	0	0
CEU	CSH	64089602	0	0	0
CEU	CSH	64094602	0	0	0
CEU	CSH	64099602	0	0	0
CEU	CSH	64104602	0	0	0
CEU	CSH	64109602	0	0	0
CEU	CSH	64114602	0	0	0
CEU	CSH	64119602	0	0	0
CEU	CSH	64124602	0	0	0
CEU	CSH	64129602	0	0	0
CEU	CSH	64134602	0	0	0
CEU	CSH	64139602	0	0	0
CEU	CSH	64144602	0	0	0
CEU	CSH	64149602	0	0	0
CEU	CSH	64154602	0	0	0
CEU	CSH	64159602	0	0	0
CEU	CSH	64164602	0	0	0
CEU	CSH	64169602	0	0	0
CEU	CSH	64174602	0	0	0
CEU	CSH	64179602	0	0	0
CEU	CSH	64184602	0	0	0
CEU	CSH	64189602	0	0	0
CEU	CSH	64194602	0	0	0
CEU	CSH	64199602	0	0	0
CEU	CSH	64204602	0	0	0
CEU	CSH	64209602	0	0	0
CEU	CSH	64214602	0	0	0
CEU	CSH	64219602	0	0	0
CEU	CSH	64224602	0	0	0
CEU	CSH	64229602	0	0	0

CEU	CSH	64234602	0	0	0
CEU	CSH	64239602	0	0	0
CEU	CSH	64244602	0	0	0
CEU	CSH	64249602	0	0	0
CEU	CSH	64254602	0	0	0
CEU	CSH	64259602	0	0	0
CEU	CSH	64264602	0	0	0
CEU	CSH	64269602	0	0	0
CEU	CSH	64274602	0	0	0
CEU	CSH	64279602	0	0	0
CEU	CSH	64284602	0	0	0
CEU	CSH	64289602	0	0	0
CEU	CSH	64294602	0	0	0
CEU	CSH	64299602	0	0	0
CEU	CSH	64304602	0	0	0
CEU	CSH	64309602	0	0	0
CEU	CSH	64314602	0	0	0
CEU	CSH	64319602	0	0	0
CEU	CSH	64324602	0	0	0
CEU	CSH	64329602	0	0	0
CEU	CSH	64334602	0	0	0
CEU	CSH	64339602	0	0	0
CEU	CSH	64344602	0	0	0
CEU	CSH	64349602	0	0	0
CEU	CSH	64354602	0	0	0
CEU	CSH	64359602	0	0	0
CEU	CSH	64364602	0	0	0
CEU	CSH	64369602	0	0	0
CEU	CSH	64374602	0	0	0
CEU	CSH	64379602	0	0	0
CEU	CSH	64384602	0	0	0
CEU	CSH	64389602	0	0	0
CEU	CSH	64394602	0	0	0
CEU	CSH	64399602	0	0	0
CEU	CSH	64404602	0	0	0
CHB	CSH	63414602	0	0	0
CHB	CSH	63419602	0	0	0
CHB	CSH	63424602	0	0	0
CHB	CSH	63429602	0	0	0
CHB	CSH	63434602	0	0	0
CHB	CSH	63439602	0	0	0
CHB	CSH	63444602	0	0	0
CHB	CSH	63449602	0	0	0
CHB	CSH	63454602	0	0	0
CHB	CSH	63459602	0	0	0
CHB	CSH	63464602	0	0	0
CHB	CSH	63469602	0	0	0
CHB	CSH	63474602	0	0	0
CHB	CSH	63479602	0	0	0

CHB	CSH	63484602	0	0	0
CHB	CSH	63489602	0	0	0
CHB	CSH	63494602	0	0	0
CHB	CSH	63499602	0	0	0
CHB	CSH	63504602	0	0	0
CHB	CSH	63509602	0	0	0
CHB	CSH	63514602	0	0	0
CHB	CSH	63519602	0	0	0
CHB	CSH	63524602	0	0	0
CHB	CSH	63529602	0	0	0
CHB	CSH	63534602	0	0	0
CHB	CSH	63539602	0	0	0
CHB	CSH	63544602	0	0	0
CHB	CSH	63549602	0	0	0
CHB	CSH	63554602	0	0	0
CHB	CSH	63559602	0	0	0
CHB	CSH	63564602	0	0	0
CHB	CSH	63569602	0	0	0
CHB	CSH	63574602	0	0	0
CHB	CSH	63579602	0	0	0
CHB	CSH	63584602	0	0	0
CHB	CSH	63589602	0	0	0
CHB	CSH	63594602	0	0	0
CHB	CSH	63599602	0	0	0
CHB	CSH	63604602	0	0	0
CHB	CSH	63609602	0	0	0
CHB	CSH	63614602	0	0	0
CHB	CSH	63619602	0	0	0
CHB	CSH	63624602	0	0	0
CHB	CSH	63629602	0	0	0
CHB	CSH	63634602	0	0	0
CHB	CSH	63639602	0	0	0
CHB	CSH	63644602	0	0	0
CHB	CSH	63649602	0	0	0
CHB	CSH	63654602	0	0	0
CHB	CSH	63659602	0	0	0
CHB	CSH	63664602	0	0	0
CHB	CSH	63669602	0	0	0
CHB	CSH	63674602	0	0	0
CHB	CSH	63679602	0	0	0
CHB	CSH	63684602	0	0	0
CHB	CSH	63689602	0	0	0
CHB	CSH	63694602	0	0	0
CHB	CSH	63699602	0	0	0
CHB	CSH	63704602	0	0	0
CHB	CSH	63709602	0	0	0
CHB	CSH	63714602	0	0	0
CHB	CSH	63719602	0	0	0
CHB	CSH	63724602	0	0	0

CHB	CSH	63729602	0	0	0
CHB	CSH	63734602	0	0	0
CHB	CSH	63739602	0	0	0
CHB	CSH	63744602	0	0	0
CHB	CSH	63749602	0	0	0
CHB	CSH	63754602	0	0	0
CHB	CSH	63759602	0	0	0
CHB	CSH	63764602	0	0	0
CHB	CSH	63769602	0	0	0
CHB	CSH	63774602	0	0	0
CHB	CSH	63779602	0	0	0
CHB	CSH	63784602	0	0	0
CHB	CSH	63789602	0	0	0
CHB	CSH	63794602	0	0	0
CHB	CSH	63799602	0	0	0
CHB	CSH	63804602	0	0	0
CHB	CSH	63809602	0	0	0
CHB	CSH	63814602	0	0	0
CHB	CSH	63819602	0	0	0
CHB	CSH	63824602	0	0	0
CHB	CSH	63829602	0	0	0
CHB	CSH	63834602	0	0	0
CHB	CSH	63839602	0	0	0
CHB	CSH	63844602	0	0	0
CHB	CSH	63849602	0	0	0
CHB	CSH	63854602	0	0	0
CHB	CSH	63859602	0	0	0
CHB	CSH	63864602	0	0	0
CHB	CSH	63869602	0	0	0
CHB	CSH	63874602	0	0	0
CHB	CSH	63879602	0	0	0
CHB	CSH	63884602	0	0	0
CHB	CSH	63889602	0	0	0
CHB	CSH	63894602	0	0	0
CHB	CSH	63899602	0	0	0
CHB	CSH	63904602	0	0	0
CHB	CSH	63909602	0	0	0
CHB	CSH	63914602	0	0	0
CHB	CSH	63919602	0	0	0
CHB	CSH	63924602	0	0	0
CHB	CSH	63929602	0	0	0
CHB	CSH	63934602	0	0	0
CHB	CSH	63939602	0	0	0
CHB	CSH	63944602	0	0	0
CHB	CSH	63949602	0	0	0
CHB	CSH	63954602	0	0	0
CHB	CSH	63959602	0	0	0
CHB	CSH	63964602	0	0	0
CHB	CSH	63969602	0	0	0

CHB	CSH	63974602	0	0	0
CHB	CSH	63979602	0	0	0
CHB	CSH	63984602	0	0	0
CHB	CSH	63989602	0	0	0
CHB	CSH	63994602	0	0	0
CHB	CSH	63999602	0	0	0
CHB	CSH	64004602	0	0	0
CHB	CSH	64009602	0	0	0
CHB	CSH	64014602	0	0	0
CHB	CSH	64019602	0	0	0
CHB	CSH	64024602	0	0	0
CHB	CSH	64029602	0	0	0
CHB	CSH	64034602	0	0	0
CHB	CSH	64039602	0	0	0
CHB	CSH	64044602	0	0	0
CHB	CSH	64049602	0	0	0
CHB	CSH	64054602	0	0	0
CHB	CSH	64059602	0	0	0
CHB	CSH	64064602	0	0	0
CHB	CSH	64069602	0	0	0
CHB	CSH	64074602	0	0	0
CHB	CSH	64079602	0	0	0
CHB	CSH	64084602	0	0	0
CHB	CSH	64089602	0	0	0
CHB	CSH	64094602	0	0	0
CHB	CSH	64099602	0	0	0
CHB	CSH	64104602	0	0	0
CHB	CSH	64109602	0	0	0
CHB	CSH	64114602	0	0	0
CHB	CSH	64119602	0	0	0
CHB	CSH	64124602	0	0	0
CHB	CSH	64129602	0	0	0
CHB	CSH	64134602	0	0	0
CHB	CSH	64139602	0	0	0
CHB	CSH	64144602	0	0	0
CHB	CSH	64149602	0	0	0
CHB	CSH	64154602	0	0	0
CHB	CSH	64159602	0	0	0
CHB	CSH	64164602	0	0	0
CHB	CSH	64169602	0	0	0
CHB	CSH	64174602	0	0	0
CHB	CSH	64179602	0	0	0
CHB	CSH	64184602	0	0	0
CHB	CSH	64189602	0	0	0
CHB	CSH	64194602	0	0	0
CHB	CSH	64199602	0	0	0
CHB	CSH	64204602	0	0	0
CHB	CSH	64209602	0	0	0
CHB	CSH	64214602	0	0	0

CHB	CSH	64219602	0	0	0
CHB	CSH	64224602	0	0	0
CHB	CSH	64229602	0	0	0
CHB	CSH	64234602	0	0	0
CHB	CSH	64239602	0	0	0
CHB	CSH	64244602	0	0	0
CHB	CSH	64249602	0	0	0
CHB	CSH	64254602	0	0	0
CHB	CSH	64259602	0	0	0
CHB	CSH	64264602	0	0	0
CHB	CSH	64269602	0	0	0
CHB	CSH	64274602	0	0	0
CHB	CSH	64279602	0	0	0
CHB	CSH	64284602	0	0	0
CHB	CSH	64289602	0	0	0
CHB	CSH	64294602	0	0	0
CHB	CSH	64299602	0	0	0
CHB	CSH	64304602	0	0	0
CHB	CSH	64309602	0	0	0
CHB	CSH	64314602	0	0	0
CHB	CSH	64319602	0	0	0
CHB	CSH	64324602	0	0	0
CHB	CSH	64329602	0	0	0
CHB	CSH	64334602	0	0	0
CHB	CSH	64339602	0	0	0
CHB	CSH	64344602	0	0	0
CHB	CSH	64349602	0	0	0
CHB	CSH	64354602	0	0	0
CHB	CSH	64359602	0	0	0
CHB	CSH	64364602	0	0	0
CHB	CSH	64369602	0	0	0
CHB	CSH	64374602	0	0	0
CHB	CSH	64379602	0	0	0
CHB	CSH	64384602	0	0	0
CHB	CSH	64389602	0	0	0
CHB	CSH	64394602	0	0	0
CHB	CSH	64399602	0	0	0
CHB	CSH	64404602	0	0	0
YRI	CSH	63414602	0	0	0
YRI	CSH	63419602	0	0	0
YRI	CSH	63424602	0	0	0
YRI	CSH	63429602	0	0	0
YRI	CSH	63434602	0	0	0
YRI	CSH	63439602	0	0	0
YRI	CSH	63444602	0	0	0
YRI	CSH	63449602	0	0	0
YRI	CSH	63454602	0	0	0
YRI	CSH	63459602	0	0	0
YRI	CSH	63464602	0	0	0

YRI	CSH	63469602	0	0	0
YRI	CSH	63474602	0	0	0
YRI	CSH	63479602	0	0	0
YRI	CSH	63484602	0	0	0
YRI	CSH	63489602	0	0	0
YRI	CSH	63494602	0	0	0
YRI	CSH	63499602	0	0	0
YRI	CSH	63504602	0	0	0
YRI	CSH	63509602	0	0	0
YRI	CSH	63514602	0	0	0
YRI	CSH	63519602	0	0	0
YRI	CSH	63524602	0	0	0
YRI	CSH	63529602	0	0	0
YRI	CSH	63534602	0	0	0
YRI	CSH	63539602	0	0	0
YRI	CSH	63544602	0	0	0
YRI	CSH	63549602	0	0	0
YRI	CSH	63554602	0	0	0
YRI	CSH	63559602	0	0	0
YRI	CSH	63564602	0	0	0
YRI	CSH	63569602	0	0	0
YRI	CSH	63574602	0	0	0
YRI	CSH	63579602	0	0	0
YRI	CSH	63584602	0	0	0
YRI	CSH	63589602	0	0	0
YRI	CSH	63594602	0	0	0
YRI	CSH	63599602	0	0	0
YRI	CSH	63604602	0	0	0
YRI	CSH	63609602	0	0	0
YRI	CSH	63614602	0	0	0
YRI	CSH	63619602	0	0	0
YRI	CSH	63624602	0	0	0
YRI	CSH	63629602	0	0	0
YRI	CSH	63634602	0	0	0
YRI	CSH	63639602	0	0	0
YRI	CSH	63644602	0	0	0
YRI	CSH	63649602	0	0	0
YRI	CSH	63654602	0	0	0
YRI	CSH	63659602	0	0	0
YRI	CSH	63664602	0	0	0
YRI	CSH	63669602	0	0	0
YRI	CSH	63674602	0	0	0
YRI	CSH	63679602	0	0	0
YRI	CSH	63684602	0	0	0
YRI	CSH	63689602	0	0	0
YRI	CSH	63694602	0	0	0
YRI	CSH	63699602	0	0	0
YRI	CSH	63704602	0	0	0
YRI	CSH	63709602	0	0	0

YRI	CSH	63714602	0	0	0
YRI	CSH	63719602	0	0	0
YRI	CSH	63724602	0	0	0
YRI	CSH	63729602	0	0	0
YRI	CSH	63734602	0	0	0
YRI	CSH	63739602	0	0	0
YRI	CSH	63744602	0	0	0
YRI	CSH	63749602	0	0	0
YRI	CSH	63754602	0	0	0
YRI	CSH	63759602	0	0	0
YRI	CSH	63764602	0	0	0
YRI	CSH	63769602	0	0	0
YRI	CSH	63774602	0	0	0
YRI	CSH	63779602	0	0	0
YRI	CSH	63784602	0	0	0
YRI	CSH	63789602	0	0	0
YRI	CSH	63794602	0	0	0
YRI	CSH	63799602	0	0	0
YRI	CSH	63804602	0	0	0
YRI	CSH	63809602	0	0	0
YRI	CSH	63814602	0	0	0
YRI	CSH	63819602	0	0	0
YRI	CSH	63824602	0	0	0
YRI	CSH	63829602	0	0	0
YRI	CSH	63834602	0	0	0
YRI	CSH	63839602	0	0	0
YRI	CSH	63844602	0	0	0
YRI	CSH	63849602	0	0	0
YRI	CSH	63854602	0	0	0
YRI	CSH	63859602	0	0	0
YRI	CSH	63864602	0	0	0
YRI	CSH	63869602	0	0	0
YRI	CSH	63874602	0	0	0
YRI	CSH	63879602	0	0	0
YRI	CSH	63884602	0	0	0
YRI	CSH	63889602	0	0	0
YRI	CSH	63894602	0	0	0
YRI	CSH	63899602	0	0	0
YRI	CSH	63904602	0	0	0
YRI	CSH	63909602	0	0	0
YRI	CSH	63914602	0	0	0
YRI	CSH	63919602	0	0	0
YRI	CSH	63924602	0	0	0
YRI	CSH	63929602	0	0	0
YRI	CSH	63934602	0	0	0
YRI	CSH	63939602	0	0	0
YRI	CSH	63944602	0	0	0
YRI	CSH	63949602	0	0	0
YRI	CSH	63954602	0	0	0

YRI	CSH	63959602	0	0	0
YRI	CSH	63964602	0	0	0
YRI	CSH	63969602	0	0	0
YRI	CSH	63974602	0	0	0
YRI	CSH	63979602	0	0	0
YRI	CSH	63984602	0	0	0
YRI	CSH	63989602	0	0	0
YRI	CSH	63994602	0	0	0
YRI	CSH	63999602	0	0	0
YRI	CSH	64004602	0	0	0
YRI	CSH	64009602	0	0	0
YRI	CSH	64014602	0	0	0
YRI	CSH	64019602	0	0	0
YRI	CSH	64024602	0	0	0
YRI	CSH	64029602	0	0	0
YRI	CSH	64034602	0	0	0
YRI	CSH	64039602	0	0	0
YRI	CSH	64044602	0	0	0
YRI	CSH	64049602	0	0	0
YRI	CSH	64054602	0	0	0
YRI	CSH	64059602	0	0	0
YRI	CSH	64064602	0	0	0
YRI	CSH	64069602	0	0	0
YRI	CSH	64074602	0	0	0
YRI	CSH	64079602	0	0	0
YRI	CSH	64084602	0	0	0
YRI	CSH	64089602	0	0	0
YRI	CSH	64094602	0	0	0
YRI	CSH	64099602	0	0	0
YRI	CSH	64104602	0	0	0
YRI	CSH	64109602	0	0	0
YRI	CSH	64114602	0	0	0
YRI	CSH	64119602	0	0	0
YRI	CSH	64124602	0	0	0
YRI	CSH	64129602	0	0	0
YRI	CSH	64134602	0	0	0
YRI	CSH	64139602	0	0	0
YRI	CSH	64144602	0	0	0
YRI	CSH	64149602	0	0	0
YRI	CSH	64154602	0	0	0
YRI	CSH	64159602	0	0	0
YRI	CSH	64164602	0	0	0
YRI	CSH	64169602	0	0	0
YRI	CSH	64174602	0	0	0
YRI	CSH	64179602	0	0	0
YRI	CSH	64184602	0	0	0
YRI	CSH	64189602	0	0	0
YRI	CSH	64194602	0	0	0
YRI	CSH	64199602	0	0	0

YRI	CSH	64204602	0	0	0
YRI	CSH	64209602	0	0	0
YRI	CSH	64214602	0	0	0
YRI	CSH	64219602	0	0	0
YRI	CSH	64224602	0	0	0
YRI	CSH	64229602	0	0	0
YRI	CSH	64234602	0	0	0
YRI	CSH	64239602	0	0	0
YRI	CSH	64244602	0	0	0
YRI	CSH	64249602	0	0	0
YRI	CSH	64254602	0	0	0
YRI	CSH	64259602	0	0	0
YRI	CSH	64264602	0	0	0
YRI	CSH	64269602	0	0	0
YRI	CSH	64274602	0	0	0
YRI	CSH	64279602	0	0	0
YRI	CSH	64284602	0	0	0
YRI	CSH	64289602	0	0	0
YRI	CSH	64294602	0	0	0
YRI	CSH	64299602	0	0	0
YRI	CSH	64304602	0	0	0
YRI	CSH	64309602	0	0	0
YRI	CSH	64314602	0	0	0
YRI	CSH	64319602	0	0	0
YRI	CSH	64324602	0	0	0
YRI	CSH	64329602	0	0	0
YRI	CSH	64334602	0	0	0
YRI	CSH	64339602	0	0	0
YRI	CSH	64344602	0	0	0
YRI	CSH	64349602	0	0	0
YRI	CSH	64354602	0	0	0
YRI	CSH	64359602	0	0	0
YRI	CSH	64364602	0	0	0
YRI	CSH	64369602	0	0	0
YRI	CSH	64374602	0	0	0
YRI	CSH	64379602	0	0	0
YRI	CSH	64384602	0	0	0
YRI	CSH	64389602	0	0	0
YRI	CSH	64394602	0	0	0
YRI	CSH	64399602	0	0	0
YRI	CSH	64404602	0	0	0
CEU	CSH	10940290	2.0291	4.9463	-1.6637
CEU	CSH	10945290	2.3393	4.2641	-1.2495
CEU	CSH	10950290	3.7615	4.4346	-0.4223
CEU	CSH	10955290	3.8866	5.6286	-0.8861
CEU	CSH	10960290	5.5088	6.1402	-0.2971
CEU	CSH	10965290	6.3131	6.6519	-0.1483
CEU	CSH	10970290	4.1886	6.1402	-0.9184
CEU	CSH	10975290	3.7196	5.7991	-1.03

CEU	CSH	10980290	3.5659	6.1402	-1.2114
CEU	CSH	10985290	4.0917	5.458	-0.7143
CEU	CSH	10990290	4.5568	5.9697	-0.6819
CEU	CSH	10995290	3.6116	5.1169	-0.8332
CEU	CSH	11000290	4.5203	6.9931	-1.0347
CEU	CSH	11005290	4.7749	7.6753	-1.1148
CEU	CSH	11010290	3.7833	5.7991	-0.9984
CEU	CSH	11015290	4.8835	5.7991	-0.4535
CEU	CSH	11020290	4.847	6.3108	-0.6721
CEU	CSH	11025290	4.329	7.8459	-1.3249
CEU	CSH	11030290	3.8382	7.1636	-1.3613
CEU	CSH	11035290	2.0726	5.1169	-1.6851
CEU	CSH	11040290	6.3395	7.8459	-0.5675
CEU	CSH	11045290	8.9705	9.2104	-0.078
CEU	CSH	11050290	7.4253	6.6519	0.3386
CEU	CSH	11055290	7.2593	7.6753	-0.1599
CEU	CSH	11060290	7.4527	9.7221	-0.7013
CEU	CSH	11065290	13.2059	11.4277	0.4724
CEU	CSH	11070290	13.377	10.4043	0.8624
CEU	CSH	11075290	7.3375	7.8459	-0.1915
CEU	CSH	11080290	6.2457	7.6753	-0.5495
CEU	CSH	11085290	6.4426	8.5281	-0.7277
CEU	CSH	11090290	4.4271	7.5047	-1.2075
CEU	CSH	11095290	3.1198	7.3342	-1.6886
CEU	CSH	11100290	3.8532	8.187	-1.57
CEU	CSH	11105290	4.8112	7.1636	-0.963
CEU	CSH	11110290	7.1614	9.0398	-0.621
CEU	CSH	11115290	7.0379	9.3809	-0.7485
CEU	CSH	11120290	3.7398	7.1636	-1.4015
CEU	CSH	11125290	5.2989	7.8459	-0.9595
CEU	CSH	11130290	6.1758	7.6753	-0.5764
CEU	CSH	11135290	6.1703	7.1636	-0.4066
CEU	CSH	11140290	4.9922	6.3108	-0.6054
CEU	CSH	11145290	4.176	7.1636	-1.223
CEU	CSH	11150290	6.0672	8.187	-0.768
CEU	CSH	11155290	6.6945	9.3809	-0.8582
CEU	CSH	11160290	9.9159	10.916	-0.2774
CEU	CSH	11165290	12.6751	11.4277	0.3314
CEU	CSH	11170290	13.6743	13.1333	0.1261
CEU	CSH	11175290	11.9779	12.2805	-0.0751
CEU	CSH	11180290	8.4158	8.8692	-0.1526
CEU	CSH	11185290	8.4566	10.7454	-0.6442
CEU	CSH	11190290	9.7926	15.1801	-1.094
CEU	CSH	11195290	6.2266	11.5982	-1.4074
CEU	CSH	11200290	2.7019	6.1402	-1.618
CEU	CSH	11205290	5.2923	8.5281	-1.129
CEU	CSH	11210290	5.7215	9.5515	-1.2032
CEU	CSH	11215290	4.1156	7.5047	-1.3297
CEU	CSH	11220290	7.0338	9.2104	-0.7073

CEU	CSH	11225290	17.7434	13.9861	0.8248
CEU	CSH	11230290	22.5624	16.374	1.169
CEU	CSH	11235290	20.5436	16.0329	0.8694
CEU	CSH	11240290	23.584	16.7151	1.2722
CEU	CSH	11245290	26.0074	17.2268	1.58
CEU	CSH	11250290	16.8891	14.3272	0.5497
CEU	CSH	11255290	9.2305	13.3039	-0.9376
CEU	CSH	11260290	15.4997	16.5446	-0.1954
CEU	CSH	11265290	18.7378	15.5212	0.6395
CEU	CSH	11270290	17.1132	12.4511	1.1425
CEU	CSH	11275290	14.557	12.2805	0.5652
CEU	CSH	11280290	10.0276	10.916	-0.2464
CEU	CSH	11285290	5.8907	8.0164	-0.7851
CEU	CSH	11290290	9.5152	11.7688	-0.5824
CEU	CSH	11295290	14.778	17.0562	-0.4139
CEU	CSH	11300290	14.4001	15.1801	-0.1584
CEU	CSH	11305290	11.5799	11.4277	0.0404
CEU	CSH	11310290	8.2041	8.5281	-0.113
CEU	CSH	11315290	8.8905	8.187	0.2549
CEU	CSH	11320290	9.8802	8.5281	0.4718
CEU	CSH	11325290	12.0214	9.0398	0.9858
CEU	CSH	11330290	9.9217	10.2337	-0.0919
CEU	CSH	11335290	4.1676	9.5515	-1.6914
CEU	CSH	11340290	4.7231	8.6987	-1.362
CEU	CSH	11345290	4.9808	8.0164	-1.1212
CEU	CSH	11350290	3.1767	6.3108	-1.4389
CEU	CSH	11355290	3.6267	5.6286	-1.0183
CEU	CSH	11360290	4.8761	5.2874	-0.2212
CEU	CSH	11365290	7.644	7.3342	0.1241
CEU	CSH	11370290	8.4343	9.0398	-0.2002
CEU	CSH	11375290	6.6096	7.1636	-0.2268
CEU	CSH	11380290	21.2696	13.645	1.7134
CEU	CSH	11385290	28.2523	18.7619	1.5734
CEU	CSH	11390290	20.1016	16.2034	0.7438
CEU	CSH	11395290	14.9953	16.5446	-0.2898
CEU	CSH	11400290	9.3405	15.5212	-1.2288
CEU	CSH	11405290	11.0645	11.2571	-0.0519
CEU	CSH	11410290	14.2061	10.2337	1.1703
CEU	CSH	11415290	8.7464	8.187	0.2027
CEU	CSH	11420290	4.6046	5.2874	-0.3671
CEU	CSH	11425290	6.4317	7.8459	-0.5327
CEU	CSH	11430290	6.1163	10.0632	-1.1812
CEU	CSH	11435290	4.7326	9.3809	-1.485
CEU	CSH	11440290	3.8792	8.3576	-1.5919
CEU	CSH	11445290	5.1171	10.0632	-1.4802
CEU	CSH	11450290	5.348	9.5515	-1.3206
CEU	CSH	11455290	3.6386	6.4814	-1.2742
CEU	CSH	11460290	3.2099	5.7991	-1.2824
CEU	CSH	11465290	4.2301	5.9697	-0.8396

CEU	CSH	11470290	4.3727	6.8225	-1.0483
CEU	CSH	11475290	2.4288	7.6753	-2.0166
CEU	CSH	11480290	2.6518	7.6753	-1.9309
CEU	CSH	11485290	2.176	5.2874	-1.673
CEU	CSH	11490290	0.7401	4.2641	-2.2876
CEU	CSH	11495290	2.1123	6.3108	-1.9276
CEU	CSH	11500290	3.8827	8.187	-1.5594
CEU	CSH	11505290	6.4766	13.645	-1.6109
CEU	CSH	11510290	6.3764	13.3039	-1.5946
CEU	CSH	11515290	2.8598	7.5047	-1.8224
CEU	CSH	11520290	3.2995	8.0164	-1.7422
CEU	CSH	11525290	5.4096	9.0398	-1.2002
CEU	CSH	11530290	3.785	7.5047	-1.4594
CEU	CSH	11535290	3.5327	10.0632	-1.9543
CEU	CSH	11540290	5.824	12.2805	-1.603
CEU	CSH	11545290	5.1936	10.0632	-1.4573
CEU	CSH	11550290	3.2181	8.187	-1.8001
CEU	CSH	11555290	3.827	8.6987	-1.669
CEU	CSH	11560290	4.5551	8.8692	-1.4517
CEU	CSH	11565290	5.3845	8.8692	-1.1726
CEU	CSH	11570290	7.3094	12.1099	-1.2077
CEU	CSH	11575290	7.0837	13.645	-1.4745
CEU	CSH	11580290	5.6895	11.7688	-1.5711
CEU	CSH	11585290	3.8227	8.187	-1.5811
CEU	CSH	11590290	2.3271	5.7991	-1.7197
CEU	CSH	11595290	3.8854	10.0632	-1.8488
CEU	CSH	11600290	4.9456	11.7688	-1.7633
CEU	CSH	11605290	3.6496	8.187	-1.6438
CEU	CSH	11610290	4.0557	8.8692	-1.6198
CEU	CSH	11615290	4.706	9.2104	-1.4637
CEU	CSH	11620290	4.0076	8.3576	-1.5463
CEU	CSH	11625290	4.3144	9.2104	-1.5909
CEU	CSH	11630290	3.746	10.0632	-1.8905
CEU	CSH	11635290	1.8462	7.1636	-2.1767
CEU	CSH	11640290	2.5214	6.8225	-1.8404
CEU	CSH	11645290	4.5416	10.5749	-1.7238
CEU	CSH	11650290	4.4661	11.7688	-1.8873
CEU	CSH	11655290	3.4106	10.5749	-2.047
CEU	CSH	11660290	4.3284	10.4043	-1.7626
CEU	CSH	11665290	4.2137	8.8692	-1.5666
CEU	CSH	11670290	4.0236	7.6753	-1.4036
CEU	CSH	11675290	7.5951	9.2104	-0.5249
CEU	CSH	11680290	12.1653	9.8926	0.6911
CEU	CSH	11685290	10.4834	8.0164	0.9112
CEU	CSH	11690290	9.1408	9.0398	0.0334
CEU	CSH	11695290	9.5323	10.916	-0.3838
CEU	CSH	11700290	10.1581	9.7221	0.1347
CEU	CSH	11705290	13.1655	9.7221	1.0641
CEU	CSH	11710290	14.8332	9.7221	1.5795

CEU	CSH	11715290	15.9619	9.2104	2.1939
CEU	CSH	11720290	12.4451	7.5047	1.9384
CEU	CSH	11725290	12.1325	7.5047	1.8157
CEU	CSH	11730290	20.1022	10.2337	2.9073
CEU	CSH	11735290	21.9152	12.7922	2.1794
CEU	CSH	11740290	11.9271	10.2337	0.4989
CEU	CSH	11745290	5.9503	7.6753	-0.663
CEU	CSH	11750290	8.9875	9.3809	-0.1257
CEU	CSH	11755290	11.165	9.2104	0.6351
CEU	CSH	11760290	9.6013	6.9931	1.0913
CEU	CSH	11765290	11.7273	9.2104	0.8179
CEU	CSH	11770290	18.946	12.2805	1.6549
CEU	CSH	11775290	17.1554	10.7454	1.8042
CEU	CSH	11780290	7.2731	8.0164	-0.2745
CEU	CSH	11785290	7.1579	10.916	-1.0423
CEU	CSH	11790290	7.6026	12.1099	-1.1339
CEU	CSH	11795290	4.9687	8.8692	-1.3125
CEU	CSH	11800290	5.4523	8.5281	-1.0732
CEU	CSH	11805290	5.0445	8.0164	-1.0977
CEU	CSH	11810290	5.0229	6.8225	-0.77
CEU	CSH	11815290	4.7166	5.7991	-0.5362
CEU	CSH	11820290	7.4995	8.187	-0.2491
CEU	CSH	11825290	7.3979	9.3809	-0.6335
CEU	CSH	11830290	4.176	7.5047	-1.306
CEU	CSH	11835290	6.1821	8.6987	-0.8622
CEU	CSH	11840290	7.0943	8.6987	-0.5497
CEU	CSH	11845290	7.0332	8.3576	-0.4708
CEU	CSH	11850290	6.2175	11.2571	-1.358
CEU	CSH	11855290	4.1537	15.6917	-2.27
CEU	CSH	11860290	3.9704	15.0095	-2.2659
CEU	CSH	11865290	2.9963	12.1099	-2.2927
CEU	CSH	11870290	14.1785	13.8156	0.0806
CEU	CSH	11875290	17.5499	10.2337	2.1554
CEU	CSH	11880290	21.1205	14.4978	1.405
CEU	CSH	11885290	22.4974	15.3506	1.4359
CEU	CSH	11890290	10.5673	7.1636	1.3933
CEU	CSH	11895290	6.0519	7.5047	-0.57
CEU	CSH	11900290	3.8994	8.0164	-1.5206
CEU	CSH	11905290	7.1142	7.8459	-0.2756
CEU	CSH	11910290	9.8435	7.6753	0.8334
CEU	CSH	11915290	6.8266	6.3108	0.2368
CEU	CSH	11920290	5.8229	6.4814	-0.2951
CEU	CSH	11925290	7.6287	8.3576	-0.2591
CEU	CSH	11930290	7.5108	8.5281	-0.3549
CEU	CSH	11935290	15.2723	10.0632	1.5589
CEU	CSH	11940290	21.9157	11.7688	2.6223
CEU	CSH	11945290	17.3185	10.5749	1.9268
CHB	CSH	10940290	6.3598	8.8096	-0.8271
CHB	CSH	10945290	5.3929	8.3013	-1.0373

CHB	CSH	10950290	6.4156	7.9625	-0.5732
CHB	CSH	10955290	6.2549	8.1319	-0.6822
CHB	CSH	10960290	7.7441	9.6567	-0.5931
CHB	CSH	10965290	7.6573	9.4872	-0.5768
CHB	CSH	10970290	6.1445	7.6237	-0.5704
CHB	CSH	10975290	6.5132	7.4543	-0.3704
CHB	CSH	10980290	5.1296	7.2848	-0.8663
CHB	CSH	10985290	4.4719	6.0989	-0.768
CHB	CSH	10990290	4.4948	6.4378	-0.8736
CHB	CSH	10995290	4.2147	7.4543	-1.2752
CHB	CSH	11000290	5.9109	9.1484	-1.0555
CHB	CSH	11005290	5.3826	9.6567	-1.3253
CHB	CSH	11010290	4.9218	9.9955	-1.5235
CHB	CSH	11015290	6.5939	9.3178	-0.8731
CHB	CSH	11020290	5.0474	6.7766	-0.7423
CHB	CSH	11025290	4.0044	6.2684	-1.0427
CHB	CSH	11030290	4.0204	7.2848	-1.3122
CHB	CSH	11035290	3.5955	7.7931	-1.5865
CHB	CSH	11040290	8.8519	9.8261	-0.2972
CHB	CSH	11045290	11.0636	10.3343	0.2123
CHB	CSH	11050290	8.0327	8.4708	-0.1534
CHB	CSH	11055290	6.6551	8.3013	-0.5871
CHB	CSH	11060290	6.6217	7.7931	-0.4427
CHB	CSH	11065290	11.6514	10.8426	0.2251
CHB	CSH	11070290	11.2115	10.3343	0.2553
CHB	CSH	11075290	6.616	8.1319	-0.551
CHB	CSH	11080290	6.0731	9.1484	-1.0027
CHB	CSH	11085290	5.7851	8.3013	-0.8974
CHB	CSH	11090290	3.57	7.2848	-1.4932
CHB	CSH	11095290	1.0257	5.5907	-2.3292
CHB	CSH	11100290	1.807	5.9295	-1.9958
CHB	CSH	11105290	3.9952	6.2684	-1.047
CHB	CSH	11110290	6.569	7.7931	-0.4626
CHB	CSH	11115290	5.909	8.6402	-0.9388
CHB	CSH	11120290	2.6269	7.1154	-1.8433
CHB	CSH	11125290	3.8072	7.4543	-1.4356
CHB	CSH	11130290	5.0202	6.6072	-0.697
CHB	CSH	11135290	6.1226	6.946	-0.3456
CHB	CSH	11140290	5.4121	6.6072	-0.5249
CHB	CSH	11145290	4.8534	7.2848	-0.9773
CHB	CSH	11150290	6.3023	8.1319	-0.665
CHB	CSH	11155290	5.9695	10.1649	-1.2402
CHB	CSH	11160290	7.2212	13.7226	-1.449
CHB	CSH	11165290	7.2496	13.5532	-1.4216
CHB	CSH	11170290	6.3284	14.5697	-1.7352
CHB	CSH	11175290	5.4407	14.5697	-1.9221
CHB	CSH	11180290	4.3636	11.5202	-1.8816
CHB	CSH	11185290	4.154	14.0615	-2.1576
CHB	CSH	11190290	4.4019	17.2803	-2.3039

CHB	CSH	11195290	3.2178	12.7061	-2.2746
CHB	CSH	11200290	1.8968	9.8261	-2.4192
CHB	CSH	11205290	4.5735	9.6567	-1.5762
CHB	CSH	11210290	6.4836	8.1319	-0.5991
CHB	CSH	11215290	6.8982	7.7931	-0.3382
CHB	CSH	11220290	8.5976	9.1484	-0.1796
CHB	CSH	11225290	18.0225	15.5862	0.481
CHB	CSH	11230290	21.608	17.7886	0.6646
CHB	CSH	11235290	19.5165	15.2474	0.8608
CHB	CSH	11240290	25.2277	16.4333	1.6508
CHB	CSH	11245290	27.4224	18.6357	1.462
CHB	CSH	11250290	18.1978	15.925	0.4396
CHB	CSH	11255290	11.6832	14.0615	-0.5179
CHB	CSH	11260290	18.8348	16.9415	0.3452
CHB	CSH	11265290	22.0253	17.958	0.7013
CHB	CSH	11270290	19.2621	19.3133	-0.0082
CHB	CSH	11275290	16.8172	17.958	-0.1967
CHB	CSH	11280290	12.4203	15.7556	-0.6518
CHB	CSH	11285290	8.2825	13.892	-1.2358
CHB	CSH	11290290	7.5858	13.892	-1.3893
CHB	CSH	11295290	9.8855	15.925	-1.1683
CHB	CSH	11300290	11.6131	16.2638	-0.8817
CHB	CSH	11305290	10.521	13.3838	-0.6534
CHB	CSH	11310290	8.8987	9.9955	-0.3293
CHB	CSH	11315290	10.8726	9.8261	0.3193
CHB	CSH	11320290	11.7306	10.1649	0.4628
CHB	CSH	11325290	13.3849	11.3508	0.5423
CHB	CSH	11330290	13.2239	11.3508	0.4994
CHB	CSH	11335290	8.2393	12.1979	-0.9863
CHB	CSH	11340290	9.1597	12.1979	-0.7569
CHB	CSH	11345290	10.186	10.8426	-0.1827
CHB	CSH	11350290	6.3524	10.5037	-1.1902
CHB	CSH	11355290	5.3982	8.8096	-1.1518
CHB	CSH	11360290	6.8283	7.9625	-0.4203
CHB	CSH	11365290	9.4131	9.9955	-0.1749
CHB	CSH	11370290	10.4373	10.6731	-0.0666
CHB	CSH	11375290	8.861	9.6567	-0.2467
CHB	CSH	11380290	24.4344	15.5862	1.7471
CHB	CSH	11385290	30.822	18.4662	2.074
CHB	CSH	11390290	18.8667	14.7391	0.8596
CHB	CSH	11395290	12.132	13.3838	-0.2857
CHB	CSH	11400290	7.3995	11.1814	-1.0226
CHB	CSH	11405290	10.0221	11.012	-0.2715
CHB	CSH	11410290	13.4401	11.8591	0.4045
CHB	CSH	11415290	8.4333	8.4708	-0.0131
CHB	CSH	11420290	4.7322	6.2684	-0.7075
CHB	CSH	11425290	6.8765	7.7931	-0.3464
CHB	CSH	11430290	7.8348	9.1484	-0.4283
CHB	CSH	11435290	5.9197	9.1484	-1.0527

CHB	CSH	11440290	4.2012	8.3013	-1.4623
CHB	CSH	11445290	3.9819	9.3178	-1.7104
CHB	CSH	11450290	3.1243	8.6402	-1.896
CHB	CSH	11455290	2.696	6.2684	-1.6453
CHB	CSH	11460290	2.4727	5.7601	-1.6333
CHB	CSH	11465290	3.2237	6.946	-1.5625
CHB	CSH	11470290	4.3718	8.1319	-1.3667
CHB	CSH	11475290	3.9983	7.1154	-1.2801
CHB	CSH	11480290	3.8315	7.1154	-1.3486
CHB	CSH	11485290	2.451	4.5742	-1.2929
CHB	CSH	11490290	0.7154	3.0495	-2.0073
CHB	CSH	11495290	2.9515	6.0989	-1.4857
CHB	CSH	11500290	6.1157	9.1484	-0.9888
CHB	CSH	11505290	13.2629	13.045	0.051
CHB	CSH	11510290	14.8308	12.5367	0.557
CHB	CSH	11515290	6.7559	8.3013	-0.5512
CHB	CSH	11520290	6.406	8.1319	-0.6273
CHB	CSH	11525290	7.4339	9.1484	-0.559
CHB	CSH	11530290	3.5452	8.6402	-1.7513
CHB	CSH	11535290	4.5387	16.4333	-2.2328
CHB	CSH	11540290	7.6891	16.9415	-1.6869
CHB	CSH	11545290	5.8338	10.3343	-1.31
CHB	CSH	11550290	3.151	11.1814	-2.1713
CHB	CSH	11555290	3.6004	11.3508	-2.0663
CHB	CSH	11560290	4.2658	9.9955	-1.7205
CHB	CSH	11565290	3.7333	9.8261	-1.8589
CHB	CSH	11570290	4.1371	12.5367	-2.0393
CHB	CSH	11575290	4.889	13.7226	-1.9688
CHB	CSH	11580290	5.6246	12.5367	-1.6781
CHB	CSH	11585290	4.8367	9.8261	-1.5223
CHB	CSH	11590290	3.0664	7.1154	-1.6628
CHB	CSH	11595290	4.3151	9.3178	-1.6036
CHB	CSH	11600290	5.6705	12.0285	-1.6051
CHB	CSH	11605290	5.6107	11.1814	-1.5063
CHB	CSH	11610290	6.7167	11.6896	-1.2896
CHB	CSH	11615290	6.7567	10.5037	-1.0743
CHB	CSH	11620290	5.2915	10.1649	-1.4406
CHB	CSH	11625290	5.8982	11.6896	-1.5019
CHB	CSH	11630290	4.4304	8.979	-1.5089
CHB	CSH	11635290	2.2905	6.6072	-1.8959
CHB	CSH	11640290	3.7864	8.3013	-1.6102
CHB	CSH	11645290	5.5791	10.6731	-1.4387
CHB	CSH	11650290	6.449	13.045	-1.5424
CHB	CSH	11655290	6.2411	13.892	-1.6855
CHB	CSH	11660290	7.762	14.0615	-1.3719
CHB	CSH	11665290	6.6322	10.6731	-1.1413
CHB	CSH	11670290	5.004	7.9625	-1.0963
CHB	CSH	11675290	8.0977	10.6731	-0.7274
CHB	CSH	11680290	11.8152	13.3838	-0.358

CHB	CSH	11685290	10.1308	12.3673	-0.55
CHB	CSH	11690290	9.8846	11.5202	-0.43
CHB	CSH	11695290	11.2842	12.8755	-0.3767
CHB	CSH	11700290	12.821	13.5532	-0.1651
CHB	CSH	11705290	14.378	11.6896	0.6972
CHB	CSH	11710290	12.3204	9.8261	0.761
CHB	CSH	11715290	12.0253	10.5037	0.4362
CHB	CSH	11720290	9.3491	9.4872	-0.0435
CHB	CSH	11725290	10.324	8.8096	0.5113
CHB	CSH	11730290	19.1864	11.8591	1.8746
CHB	CSH	11735290	18.8426	15.4168	0.6835
CHB	CSH	11740290	8.2871	11.012	-0.7474
CHB	CSH	11745290	3.6166	7.7931	-1.5785
CHB	CSH	11750290	5.5237	10.6731	-1.4544
CHB	CSH	11755290	6.6404	11.6896	-1.3094
CHB	CSH	11760290	5.5271	9.8261	-1.3116
CHB	CSH	11765290	7.8622	10.1649	-0.6807
CHB	CSH	11770290	14.6526	12.7061	0.4666
CHB	CSH	11775290	13.5618	11.5202	0.5368
CHB	CSH	11780290	5.7179	9.3178	-1.1539
CHB	CSH	11785290	5.3861	16.6027	-2.085
CHB	CSH	11790290	6.262	17.7886	-2.0056
CHB	CSH	11795290	5.7625	10.1649	-1.3014
CHB	CSH	11800290	5.9048	10.1649	-1.2593
CHB	CSH	11805290	5.5226	9.6567	-1.2819
CHB	CSH	11810290	5.5989	8.979	-1.1213
CHB	CSH	11815290	4.9952	8.6402	-1.2529
CHB	CSH	11820290	8.3497	9.6567	-0.4053
CHB	CSH	11825290	9.6097	9.8261	-0.066
CHB	CSH	11830290	6.7923	7.2848	-0.198
CHB	CSH	11835290	7.2989	9.6567	-0.7311
CHB	CSH	11840290	7.2983	9.4872	-0.69
CHB	CSH	11845290	7.0197	9.1484	-0.694
CHB	CSH	11850290	6.3369	12.7061	-1.5269
CHB	CSH	11855290	5.7725	17.4498	-2.0696
CHB	CSH	11860290	5.0815	16.4333	-2.1309
CHB	CSH	11865290	3.2893	11.8591	-2.1925
CHB	CSH	11870290	16.0919	15.0779	0.2066
CHB	CSH	11875290	18.1063	15.7556	0.4594
CHB	CSH	11880290	30.6989	20.3298	1.5867
CHB	CSH	11885290	33.087	18.8051	2.3558
CHB	CSH	11890290	10.7952	8.8096	0.6704
CHB	CSH	11895290	9.8796	8.3013	0.5629
CHB	CSH	11900290	10.1964	9.1484	0.3417
CHB	CSH	11905290	11.3107	7.7931	1.3295
CHB	CSH	11910290	12.633	9.4872	0.9916
CHB	CSH	11915290	8.1315	9.1484	-0.3315
CHB	CSH	11920290	6.2467	6.946	-0.2935
CHB	CSH	11925290	7.5431	8.6402	-0.3771

CHB	CSH	11930290	7.1007	10.1649	-0.9058
CHB	CSH	11935290	11.9642	12.1979	-0.0582
CHB	CSH	11940290	16.3666	13.892	0.5452
CHB	CSH	11945290	13.5856	12.5367	0.2547
YRI	CSH	10940290	4.2831	10.4199	-1.7666
YRI	CSH	10945290	4.9249	10.4199	-1.5818
YRI	CSH	10950290	5.9372	9.5795	-1.134
YRI	CSH	10955290	5.2265	8.235	-1.0772
YRI	CSH	10960290	6.0112	10.924	-1.3531
YRI	CSH	10965290	6.8912	12.2685	-1.3279
YRI	CSH	10970290	5.0072	12.1005	-1.7746
YRI	CSH	10975290	4.6501	11.9324	-1.846
YRI	CSH	10980290	4.5598	11.0921	-1.7736
YRI	CSH	10985290	5.0802	10.756	-1.5861
YRI	CSH	10990290	4.6012	10.924	-1.7414
YRI	CSH	10995290	3.7772	12.7727	-2.1385
YRI	CSH	11000290	4.9039	13.1089	-1.9033
YRI	CSH	11005290	4.9518	11.9324	-1.7696
YRI	CSH	11010290	4.4895	10.5879	-1.7295
YRI	CSH	11015290	4.6134	9.7476	-1.5728
YRI	CSH	11020290	4.891	10.0837	-1.5413
YRI	CSH	11025290	4.5162	10.0837	-1.6525
YRI	CSH	11030290	3.5067	10.2518	-1.9714
YRI	CSH	11035290	2.6473	10.5879	-2.2519
YRI	CSH	11040290	5.4888	13.1089	-1.7676
YRI	CSH	11045290	7.17	13.2769	-1.3996
YRI	CSH	11050290	6.5129	11.4282	-1.2977
YRI	CSH	11055290	6.3885	10.4199	-1.1605
YRI	CSH	11060290	6.9357	9.0754	-0.7004
YRI	CSH	11065290	12.7781	12.2685	0.1258
YRI	CSH	11070290	13.5132	12.9408	0.1344
YRI	CSH	11075290	7.9226	11.5963	-0.9567
YRI	CSH	11080290	6.0646	11.7644	-1.4643
YRI	CSH	11085290	6.8771	11.4282	-1.2015
YRI	CSH	11090290	5.6196	10.0837	-1.325
YRI	CSH	11095290	3.4245	10.2518	-1.9954
YRI	CSH	11100290	3.4112	11.2602	-2.1012
YRI	CSH	11105290	5.0696	10.756	-1.589
YRI	CSH	11110290	7.8883	12.4366	-1.1089
YRI	CSH	11115290	7.5639	11.4282	-1.0202
YRI	CSH	11120290	3.7493	9.2434	-1.7682
YRI	CSH	11125290	4.4297	10.756	-1.7679
YRI	CSH	11130290	6.365	9.7476	-1.0362
YRI	CSH	11135290	6.3832	9.2434	-0.9205
YRI	CSH	11140290	5.7388	10.924	-1.4281
YRI	CSH	11145290	5.3484	11.5963	-1.627
YRI	CSH	11150290	5.8489	11.2602	-1.4486
YRI	CSH	11155290	6.6702	13.7811	-1.5731
YRI	CSH	11160290	9.9207	16.9743	-1.2793

YRI	CSH	11165290	14.7345	18.991	-0.6932
YRI	CSH	11170290	15.3791	19.9994	-0.7159
YRI	CSH	11175290	13.6872	18.4868	-0.8021
YRI	CSH	11180290	11.4066	14.9575	-0.7267
YRI	CSH	11185290	15.9916	18.823	-0.465
YRI	CSH	11190290	22.6333	23.8648	-0.1609
YRI	CSH	11195290	14.9938	17.1423	-0.386
YRI	CSH	11200290	7.8015	13.1089	-1.2311
YRI	CSH	11205290	7.6297	14.9575	-1.4997
YRI	CSH	11210290	7.5828	14.2853	-1.4331
YRI	CSH	11215290	6.2663	10.5879	-1.2256
YRI	CSH	11220290	8.8179	12.6047	-0.9116
YRI	CSH	11225290	19.4995	19.8313	-0.0518
YRI	CSH	11230290	22.8421	21.512	0.1921
YRI	CSH	11235290	20.7492	19.1591	0.2568
YRI	CSH	11240290	24.0197	19.4952	0.7185
YRI	CSH	11245290	25.052	22.6884	0.3242
YRI	CSH	11250290	16.4771	18.823	-0.3853
YRI	CSH	11255290	13.7748	16.8062	-0.5551
YRI	CSH	11260290	21.7779	21.512	0.0384
YRI	CSH	11265290	23.8329	21.512	0.3352
YRI	CSH	11270290	21.9457	21.68	0.0381
YRI	CSH	11275290	19.4623	20.1675	-0.1084
YRI	CSH	11280290	15.1304	17.9827	-0.4895
YRI	CSH	11285290	10.9681	17.8146	-1.1856
YRI	CSH	11290290	12.184	19.1591	-1.1263
YRI	CSH	11295290	17.0754	21.0078	-0.581
YRI	CSH	11300290	15.8729	17.8146	-0.3362
YRI	CSH	11305290	11.625	14.1172	-0.5389
YRI	CSH	11310290	9.5144	13.1089	-0.8338
YRI	CSH	11315290	9.7431	13.1089	-0.7808
YRI	CSH	11320290	11.5882	13.7811	-0.4851
YRI	CSH	11325290	14.8501	15.2937	-0.0889
YRI	CSH	11330290	15.1593	14.1172	0.2253
YRI	CSH	11335290	11.4056	13.1089	-0.3951
YRI	CSH	11340290	11.9161	13.9492	-0.4446
YRI	CSH	11345290	12.7639	13.7811	-0.225
YRI	CSH	11350290	9.5819	14.7895	-1.0773
YRI	CSH	11355290	7.2007	13.1089	-1.3705
YRI	CSH	11360290	7.9104	12.2685	-1.0762
YRI	CSH	11365290	12.1305	14.7895	-0.5501
YRI	CSH	11370290	13.6766	14.4534	-0.1643
YRI	CSH	11375290	12.7547	13.9492	-0.2612
YRI	CSH	11380290	24.1752	21.0078	0.468
YRI	CSH	11385290	33.6675	24.5371	1.1609
YRI	CSH	11390290	29.4682	21.512	1.149
YRI	CSH	11395290	25.1386	21.8481	0.4681
YRI	CSH	11400290	22.9264	21.0078	0.2835
YRI	CSH	11405290	20.8019	18.4868	0.3869

YRI	CSH	11410290	19.9183	17.3104	0.4642
YRI	CSH	11415290	13.953	15.2937	-0.2686
YRI	CSH	11420290	7.4476	10.2518	-0.8196
YRI	CSH	11425290	9.4093	9.9157	-0.1527
YRI	CSH	11430290	14.242	15.1256	-0.1789
YRI	CSH	11435290	17.8576	17.6465	0.0369
YRI	CSH	11440290	14.9333	14.6214	0.0652
YRI	CSH	11445290	12.1732	14.2853	-0.4516
YRI	CSH	11450290	11.3964	15.4617	-0.8061
YRI	CSH	11455290	7.4144	12.2685	-1.1987
YRI	CSH	11460290	5.1741	9.7476	-1.401
YRI	CSH	11465290	5.8223	10.924	-1.4051
YRI	CSH	11470290	6.2899	12.4366	-1.4986
YRI	CSH	11475290	5.734	11.0921	-1.4548
YRI	CSH	11480290	5.6276	12.1005	-1.6194
YRI	CSH	11485290	3.943	10.0837	-1.8227
YRI	CSH	11490290	2.5011	7.7309	-1.9843
YRI	CSH	11495290	6.5583	9.9157	-1.0123
YRI	CSH	11500290	9.3658	11.7644	-0.6162
YRI	CSH	11505290	18.3886	16.134	0.4293
YRI	CSH	11510290	22.6354	17.4785	0.9095
YRI	CSH	11515290	10.78	14.1172	-0.7216
YRI	CSH	11520290	6.8229	13.613	-1.5198
YRI	CSH	11525290	7.8211	13.7811	-1.3185
YRI	CSH	11530290	6.7999	12.4366	-1.3742
YRI	CSH	11535290	21.5697	24.0329	-0.3196
YRI	CSH	11540290	24.9711	25.8816	-0.1099
YRI	CSH	11545290	10.1927	14.7895	-0.9509
YRI	CSH	11550290	5.2904	12.7727	-1.7788
YRI	CSH	11555290	5.0146	10.756	-1.6044
YRI	CSH	11560290	6.0633	10.756	-1.3114
YRI	CSH	11565290	10.4345	14.6214	-0.8756
YRI	CSH	11570290	19.4544	18.1507	0.2217
YRI	CSH	11575290	22.8879	18.823	0.6676
YRI	CSH	11580290	15.6195	16.8062	-0.2173
YRI	CSH	11585290	9.4213	12.4366	-0.7351
YRI	CSH	11590290	8.0211	9.0754	-0.3451
YRI	CSH	11595290	11.0474	12.9408	-0.4446
YRI	CSH	11600290	11.8918	16.134	-0.8077
YRI	CSH	11605290	10.3727	15.2937	-0.986
YRI	CSH	11610290	12.8767	16.302	-0.6457
YRI	CSH	11615290	11.6012	12.1005	-0.1249
YRI	CSH	11620290	12.0157	10.0837	0.5734
YRI	CSH	11625290	15.6335	14.4534	0.2495
YRI	CSH	11630290	12.2557	13.9492	-0.3704
YRI	CSH	11635290	7.2028	11.4282	-1.1155
YRI	CSH	11640290	9.2615	12.1005	-0.7103
YRI	CSH	11645290	10.963	14.2853	-0.7103
YRI	CSH	11650290	12.3764	17.9827	-0.9621

YRI	CSH	11655290	12.527	17.8146	-0.9156
YRI	CSH	11660290	12.6546	17.8146	-0.8935
YRI	CSH	11665290	10.9357	14.2853	-0.7162
YRI	CSH	11670290	9.8814	11.2602	-0.3691
YRI	CSH	11675290	12.3776	14.2853	-0.4079
YRI	CSH	11680290	14.3274	16.6382	-0.4272
YRI	CSH	11685290	12.0607	15.2937	-0.6478
YRI	CSH	11690290	10.2344	13.7811	-0.7846
YRI	CSH	11695290	9.8796	15.4617	-1.1069
YRI	CSH	11700290	12.5041	16.302	-0.716
YRI	CSH	11705290	16.7247	18.4868	-0.2945
YRI	CSH	11710290	15.6269	17.4785	-0.3265
YRI	CSH	11715290	14.5827	13.7811	0.1773
YRI	CSH	11720290	12.019	14.6214	-0.5442
YRI	CSH	11725290	12.3446	17.1423	-0.862
YRI	CSH	11730290	18.1722	18.823	-0.1069
YRI	CSH	11735290	21.8737	18.991	0.4694
YRI	CSH	11740290	13.8491	14.4534	-0.1278
YRI	CSH	11745290	7.6225	12.2685	-1.1473
YRI	CSH	11750290	12.0008	15.7978	-0.7376
YRI	CSH	11755290	14.6046	17.8146	-0.5559
YRI	CSH	11760290	12.8054	14.6214	-0.3798
YRI	CSH	11765290	11.9184	14.4534	-0.536
YRI	CSH	11770290	15.2214	18.4868	-0.5457
YRI	CSH	11775290	16.2276	16.4701	-0.0453
YRI	CSH	11780290	13.6423	14.1172	-0.1027
YRI	CSH	11785290	21.7787	21.68	0.0141
YRI	CSH	11790290	21.5108	22.0162	-0.0714
YRI	CSH	11795290	11.9485	14.7895	-0.5877
YRI	CSH	11800290	12.8875	14.7895	-0.3935
YRI	CSH	11805290	12.1554	14.6214	-0.5157
YRI	CSH	11810290	10.9659	13.1089	-0.4971
YRI	CSH	11815290	10.7165	12.7727	-0.4888
YRI	CSH	11820290	11.1701	14.2853	-0.6661
YRI	CSH	11825290	9.845	15.1256	-1.0693
YRI	CSH	11830290	6.3024	12.7727	-1.5382
YRI	CSH	11835290	12.1577	13.613	-0.3257
YRI	CSH	11840290	12.9293	14.2853	-0.2899
YRI	CSH	11845290	10.8539	14.1172	-0.7056
YRI	CSH	11850290	18.7371	17.1423	0.2865
YRI	CSH	11855290	30.8233	22.0162	1.2437
YRI	CSH	11860290	29.7846	19.6633	1.594
YRI	CSH	11865290	23.2247	15.4617	1.5393
YRI	CSH	11870290	29.3862	21.0078	1.238
YRI	CSH	11875290	24.5569	21.1758	0.4958
YRI	CSH	11880290	32.068	24.369	0.9854
YRI	CSH	11885290	29.2697	22.8565	0.8734
YRI	CSH	11890290	10.4884	13.2769	-0.6391
YRI	CSH	11895290	11.2853	13.2769	-0.4564

YRI	CSH	11900290	12.8405	13.2769	-0.1
YRI	CSH	11905290	13.777	11.0921	0.729
YRI	CSH	11910290	13.0123	11.5963	0.3687
YRI	CSH	11915290	8.6767	10.4199	-0.5018
YRI	CSH	11920290	7.881	10.0837	-0.6538
YRI	CSH	11925290	10.4412	12.4366	-0.4865
YRI	CSH	11930290	11.8099	14.9575	-0.6442
YRI	CSH	11935290	19.4178	18.1507	0.2155
YRI	CSH	11940290	24.0197	17.9827	1.036
YRI	CSH	11945290	19.8207	16.134	0.7019
CEU	CSH	58003333	0	0	0
CEU	CSH	58008333	0	0	0
CEU	CSH	58013333	0	0	0
CEU	CSH	58018333	0	0	0
CEU	CSH	58023333	0	0	0
CEU	CSH	58028333	0	0	0
CEU	CSH	58033333	0	0	0
CEU	CSH	58038333	0	0	0
CEU	CSH	58043333	0	0	0
CEU	CSH	58048333	0	0	0
CEU	CSH	58053333	0	0	0
CEU	CSH	58058333	0	0	0
CEU	CSH	58063333	0	0	0
CEU	CSH	58068333	0	0	0
CEU	CSH	58073333	0	0	0
CEU	CSH	58078333	0	0	0
CEU	CSH	58083333	0	0	0
CEU	CSH	58088333	0	0	0
CEU	CSH	58093333	0	0	0
CEU	CSH	58098333	0	0	0
CEU	CSH	58103333	0	0	0
CEU	CSH	58108333	0	0	0
CEU	CSH	58113333	0	0	0
CEU	CSH	58118333	0	0	0
CEU	CSH	58123333	0	0	0
CEU	CSH	58128333	0	0	0
CEU	CSH	58133333	0	0	0
CEU	CSH	58138333	0	0	0
CEU	CSH	58143333	0	0	0
CEU	CSH	58148333	0	0	0
CEU	CSH	58153333	0	0	0
CEU	CSH	58158333	0	0	0
CEU	CSH	58163333	0	0	0
CEU	CSH	58168333	0	0	0
CEU	CSH	58173333	0	0	0
CEU	CSH	58178333	0	0	0
CEU	CSH	58183333	0	0	0
CEU	CSH	58188333	0	0	0
CEU	CSH	58193333	0	0	0

CEU	CSH	58198333	0	0	0
CEU	CSH	58203333	0	0	0
CEU	CSH	58208333	0	0	0
CEU	CSH	58213333	0	0	0
CEU	CSH	58218333	0	0	0
CEU	CSH	58223333	0	0	0
CEU	CSH	58228333	0	0	0
CEU	CSH	58233333	0	0	0
CEU	CSH	58238333	0	0	0
CEU	CSH	58243333	0	0	0
CEU	CSH	58248333	0	0	0
CEU	CSH	58253333	0	0	0
CEU	CSH	58258333	0	0	0
CEU	CSH	58263333	0	0	0
CEU	CSH	58268333	0	0	0
CEU	CSH	58273333	0	0	0
CEU	CSH	58278333	0	0	0
CEU	CSH	58283333	0	0	0
CEU	CSH	58288333	0	0	0
CEU	CSH	58293333	0	0	0
CEU	CSH	58298333	0	0	0
CEU	CSH	58303333	0	0	0
CEU	CSH	58308333	0	0	0
CEU	CSH	58313333	0	0	0
CEU	CSH	58318333	0	0	0
CEU	CSH	58323333	0	0	0
CEU	CSH	58328333	0	0	0
CEU	CSH	58333333	0	0	0
CEU	CSH	58338333	0	0	0
CEU	CSH	58343333	0	0	0
CEU	CSH	58348333	0	0	0
CEU	CSH	58353333	0	0	0
CEU	CSH	58358333	0	0	0
CEU	CSH	58363333	0	0	0
CEU	CSH	58368333	0	0	0
CEU	CSH	58373333	0	0	0
CEU	CSH	58378333	0	0	0
CEU	CSH	58383333	0	0	0
CEU	CSH	58388333	0	0	0
CEU	CSH	58393333	0	0	0
CEU	CSH	58398333	0	0	0
CEU	CSH	58403333	0	0	0
CEU	CSH	58408333	0	0	0
CEU	CSH	58413333	0	0	0
CEU	CSH	58418333	0	0	0
CEU	CSH	58423333	0	0	0
CEU	CSH	58428333	0	0	0
CEU	CSH	58433333	0	0	0
CEU	CSH	58438333	0	0	0

CEU	CSH	58443333	0	0	0
CEU	CSH	58448333	0	0	0
CEU	CSH	58453333	0	0	0
CEU	CSH	58458333	0	0	0
CEU	CSH	58463333	0	0	0
CEU	CSH	58468333	0	0	0
CEU	CSH	58473333	0	0	0
CEU	CSH	58478333	0	0	0
CEU	CSH	58483333	0	0	0
CEU	CSH	58488333	0	0	0
CEU	CSH	58493333	0	0	0
CEU	CSH	58498333	0	0	0
CEU	CSH	58503333	0	0	0
CEU	CSH	58508333	0	0	0
CEU	CSH	58513333	0	0	0
CEU	CSH	58518333	0	0	0
CEU	CSH	58523333	0	0	0
CEU	CSH	58528333	0	0	0
CEU	CSH	58533333	0	0	0
CEU	CSH	58538333	0	0	0
CEU	CSH	58543333	0	0	0
CEU	CSH	58548333	0	0	0
CEU	CSH	58553333	0	0	0
CEU	CSH	58558333	0	0	0
CEU	CSH	58563333	0	0	0
CEU	CSH	58568333	0	0	0
CEU	CSH	58573333	0	0	0
CEU	CSH	58578333	0	0	0
CEU	CSH	58583333	0	0	0
CEU	CSH	58588333	0	0	0
CEU	CSH	58593333	0	0	0
CEU	CSH	58598333	0	0	0
CEU	CSH	58603333	0	0	0
CEU	CSH	58608333	0	0	0
CEU	CSH	58613333	0	0	0
CEU	CSH	58618333	0	0	0
CEU	CSH	58623333	0	0	0
CEU	CSH	58628333	0	0	0
CEU	CSH	58633333	0	0	0
CEU	CSH	58638333	0	0	0
CEU	CSH	58643333	0	0	0
CEU	CSH	58648333	0	0	0
CEU	CSH	58653333	0	0	0
CEU	CSH	58658333	0	0	0
CEU	CSH	58663333	0	0	0
CEU	CSH	58668333	0	0	0
CEU	CSH	58673333	0	0	0
CEU	CSH	58678333	0	0	0
CEU	CSH	58683333	0	0	0

CEU	CSH	58688333	0	0	0
CEU	CSH	58693333	0	0	0
CEU	CSH	58698333	0	0	0
CEU	CSH	58703333	0	0	0
CEU	CSH	58708333	0	0	0
CEU	CSH	58713333	0	0	0
CEU	CSH	58718333	0	0	0
CEU	CSH	58723333	0	0	0
CEU	CSH	58728333	0	0	0
CEU	CSH	58733333	0	0	0
CEU	CSH	58738333	0	0	0
CEU	CSH	58743333	0	0	0
CEU	CSH	58748333	0	0	0
CEU	CSH	58753333	0	0	0
CEU	CSH	58758333	0	0	0
CEU	CSH	58763333	0	0	0
CEU	CSH	58768333	0	0	0
CEU	CSH	58773333	0	0	0
CEU	CSH	58778333	0	0	0
CEU	CSH	58783333	0	0	0
CEU	CSH	58788333	0	0	0
CEU	CSH	58793333	0	0	0
CEU	CSH	58798333	0	0	0
CEU	CSH	58803333	0	0	0
CEU	CSH	58808333	0	0	0
CEU	CSH	58813333	0	0	0
CEU	CSH	58818333	0	0	0
CEU	CSH	58823333	0	0	0
CEU	CSH	58828333	0	0	0
CEU	CSH	58833333	0	0	0
CEU	CSH	58838333	0	0	0
CEU	CSH	58843333	0	0	0
CEU	CSH	58848333	0	0	0
CEU	CSH	58853333	0	0	0
CEU	CSH	58858333	0	0	0
CEU	CSH	58863333	0	0	0
CEU	CSH	58868333	0	0	0
CEU	CSH	58873333	0	0	0
CEU	CSH	58878333	0	0	0
CEU	CSH	58883333	0	0	0
CEU	CSH	58888333	0	0	0
CEU	CSH	58893333	0	0	0
CEU	CSH	58898333	0	0	0
CEU	CSH	58903333	0	0	0
CEU	CSH	58908333	0	0	0
CEU	CSH	58913333	0	0	0
CEU	CSH	58918333	0	0	0
CEU	CSH	58923333	0	0	0
CEU	CSH	58928333	0	0	0

CEU	CSH	58933333	0	0	0
CEU	CSH	58938333	0	0	0
CEU	CSH	58943333	0	0	0
CEU	CSH	58948333	0	0	0
CEU	CSH	58953333	0	0	0
CEU	CSH	58958333	0	0	0
CEU	CSH	58963333	0	0	0
CEU	CSH	58968333	0	0	0
CEU	CSH	58973333	0	0	0
CEU	CSH	58978333	0	0	0
CEU	CSH	58983333	0	0	0
CEU	CSH	58988333	0	0	0
CEU	CSH	58993333	0	0	0
CEU	CSH	58998333	0	0	0
CEU	CSH	59003333	0	0	0
CHB	CSH	58003333	0	0	0
CHB	CSH	58008333	0	0	0
CHB	CSH	58013333	0	0	0
CHB	CSH	58018333	0	0	0
CHB	CSH	58023333	0	0	0
CHB	CSH	58028333	0	0	0
CHB	CSH	58033333	0	0	0
CHB	CSH	58038333	0	0	0
CHB	CSH	58043333	0	0	0
CHB	CSH	58048333	0	0	0
CHB	CSH	58053333	0	0	0
CHB	CSH	58058333	0	0	0
CHB	CSH	58063333	0	0	0
CHB	CSH	58068333	0	0	0
CHB	CSH	58073333	0	0	0
CHB	CSH	58078333	0	0	0
CHB	CSH	58083333	0	0	0
CHB	CSH	58088333	0	0	0
CHB	CSH	58093333	0	0	0
CHB	CSH	58098333	0	0	0
CHB	CSH	58103333	0	0	0
CHB	CSH	58108333	0	0	0
CHB	CSH	58113333	0	0	0
CHB	CSH	58118333	0	0	0
CHB	CSH	58123333	0	0	0
CHB	CSH	58128333	0	0	0
CHB	CSH	58133333	0	0	0
CHB	CSH	58138333	0	0	0
CHB	CSH	58143333	0	0	0
CHB	CSH	58148333	0	0	0
CHB	CSH	58153333	0	0	0
CHB	CSH	58158333	0	0	0
CHB	CSH	58163333	0	0	0
CHB	CSH	58168333	0	0	0

CHB	CSH	58173333	0	0	0
CHB	CSH	58178333	0	0	0
CHB	CSH	58183333	0	0	0
CHB	CSH	58188333	0	0	0
CHB	CSH	58193333	0	0	0
CHB	CSH	58198333	0	0	0
CHB	CSH	58203333	0	0	0
CHB	CSH	58208333	0	0	0
CHB	CSH	58213333	0	0	0
CHB	CSH	58218333	0	0	0
CHB	CSH	58223333	0	0	0
CHB	CSH	58228333	0	0	0
CHB	CSH	58233333	0	0	0
CHB	CSH	58238333	0	0	0
CHB	CSH	58243333	0	0	0
CHB	CSH	58248333	0	0	0
CHB	CSH	58253333	0	0	0
CHB	CSH	58258333	0	0	0
CHB	CSH	58263333	0	0	0
CHB	CSH	58268333	0	0	0
CHB	CSH	58273333	0	0	0
CHB	CSH	58278333	0	0	0
CHB	CSH	58283333	0	0	0
CHB	CSH	58288333	0	0	0
CHB	CSH	58293333	0	0	0
CHB	CSH	58298333	0	0	0
CHB	CSH	58303333	0	0	0
CHB	CSH	58308333	0	0	0
CHB	CSH	58313333	0	0	0
CHB	CSH	58318333	0	0	0
CHB	CSH	58323333	0	0	0
CHB	CSH	58328333	0	0	0
CHB	CSH	58333333	0	0	0
CHB	CSH	58338333	0	0	0
CHB	CSH	58343333	0	0	0
CHB	CSH	58348333	0	0	0
CHB	CSH	58353333	0	0	0
CHB	CSH	58358333	0	0	0
CHB	CSH	58363333	0	0	0
CHB	CSH	58368333	0	0	0
CHB	CSH	58373333	0	0	0
CHB	CSH	58378333	0	0	0
CHB	CSH	58383333	0	0	0
CHB	CSH	58388333	0	0	0
CHB	CSH	58393333	0	0	0
CHB	CSH	58398333	0	0	0
CHB	CSH	58403333	0	0	0
CHB	CSH	58408333	0	0	0
CHB	CSH	58413333	0	0	0

CHB	CSH	58418333	0	0	0
CHB	CSH	58423333	0	0	0
CHB	CSH	58428333	0	0	0
CHB	CSH	58433333	0	0	0
CHB	CSH	58438333	0	0	0
CHB	CSH	58443333	0	0	0
CHB	CSH	58448333	0	0	0
CHB	CSH	58453333	0	0	0
CHB	CSH	58458333	0	0	0
CHB	CSH	58463333	0	0	0
CHB	CSH	58468333	0	0	0
CHB	CSH	58473333	0	0	0
CHB	CSH	58478333	0	0	0
CHB	CSH	58483333	0	0	0
CHB	CSH	58488333	0	0	0
CHB	CSH	58493333	0	0	0
CHB	CSH	58498333	0	0	0
CHB	CSH	58503333	0	0	0
CHB	CSH	58508333	0	0	0
CHB	CSH	58513333	0	0	0
CHB	CSH	58518333	0	0	0
CHB	CSH	58523333	0	0	0
CHB	CSH	58528333	0	0	0
CHB	CSH	58533333	0	0	0
CHB	CSH	58538333	0	0	0
CHB	CSH	58543333	0	0	0
CHB	CSH	58548333	0	0	0
CHB	CSH	58553333	0	0	0
CHB	CSH	58558333	0	0	0
CHB	CSH	58563333	0	0	0
CHB	CSH	58568333	0	0	0
CHB	CSH	58573333	0	0	0
CHB	CSH	58578333	0	0	0
CHB	CSH	58583333	0	0	0
CHB	CSH	58588333	0	0	0
CHB	CSH	58593333	0	0	0
CHB	CSH	58598333	0	0	0
CHB	CSH	58603333	0	0	0
CHB	CSH	58608333	0	0	0
CHB	CSH	58613333	0	0	0
CHB	CSH	58618333	0	0	0
CHB	CSH	58623333	0	0	0
CHB	CSH	58628333	0	0	0
CHB	CSH	58633333	0	0	0
CHB	CSH	58638333	0	0	0
CHB	CSH	58643333	0	0	0
CHB	CSH	58648333	0	0	0
CHB	CSH	58653333	0	0	0
CHB	CSH	58658333	0	0	0

CHB	CSH	58663333	0	0	0
CHB	CSH	58668333	0	0	0
CHB	CSH	58673333	0	0	0
CHB	CSH	58678333	0	0	0
CHB	CSH	58683333	0	0	0
CHB	CSH	58688333	0	0	0
CHB	CSH	58693333	0	0	0
CHB	CSH	58698333	0	0	0
CHB	CSH	58703333	0	0	0
CHB	CSH	58708333	0	0	0
CHB	CSH	58713333	0	0	0
CHB	CSH	58718333	0	0	0
CHB	CSH	58723333	0	0	0
CHB	CSH	58728333	0	0	0
CHB	CSH	58733333	0	0	0
CHB	CSH	58738333	0	0	0
CHB	CSH	58743333	0	0	0
CHB	CSH	58748333	0	0	0
CHB	CSH	58753333	0	0	0
CHB	CSH	58758333	0	0	0
CHB	CSH	58763333	0	0	0
CHB	CSH	58768333	0	0	0
CHB	CSH	58773333	0	0	0
CHB	CSH	58778333	0	0	0
CHB	CSH	58783333	0	0	0
CHB	CSH	58788333	0	0	0
CHB	CSH	58793333	0	0	0
CHB	CSH	58798333	0	0	0
CHB	CSH	58803333	0	0	0
CHB	CSH	58808333	0	0	0
CHB	CSH	58813333	0	0	0
CHB	CSH	58818333	0	0	0
CHB	CSH	58823333	0	0	0
CHB	CSH	58828333	0	0	0
CHB	CSH	58833333	0	0	0
CHB	CSH	58838333	0	0	0
CHB	CSH	58843333	0	0	0
CHB	CSH	58848333	0	0	0
CHB	CSH	58853333	0	0	0
CHB	CSH	58858333	0	0	0
CHB	CSH	58863333	0	0	0
CHB	CSH	58868333	0	0	0
CHB	CSH	58873333	0	0	0
CHB	CSH	58878333	0	0	0
CHB	CSH	58883333	0	0	0
CHB	CSH	58888333	0	0	0
CHB	CSH	58893333	0	0	0
CHB	CSH	58898333	0	0	0
CHB	CSH	58903333	0	0	0

CHB	CSH	58908333	0	0	0
CHB	CSH	58913333	0	0	0
CHB	CSH	58918333	0	0	0
CHB	CSH	58923333	0	0	0
CHB	CSH	58928333	0	0	0
CHB	CSH	58933333	0	0	0
CHB	CSH	58938333	0	0	0
CHB	CSH	58943333	0	0	0
CHB	CSH	58948333	0	0	0
CHB	CSH	58953333	0	0	0
CHB	CSH	58958333	0	0	0
CHB	CSH	58963333	0	0	0
CHB	CSH	58968333	0	0	0
CHB	CSH	58973333	0	0	0
CHB	CSH	58978333	0	0	0
CHB	CSH	58983333	0	0	0
CHB	CSH	58988333	0	0	0
CHB	CSH	58993333	0	0	0
CHB	CSH	58998333	0	0	0
CHB	CSH	59003333	0	0	0
YRI	CSH	58003333	0	0	0
YRI	CSH	58008333	0	0	0
YRI	CSH	58013333	0	0	0
YRI	CSH	58018333	0	0	0
YRI	CSH	58023333	0	0	0
YRI	CSH	58028333	0	0	0
YRI	CSH	58033333	0	0	0
YRI	CSH	58038333	0	0	0
YRI	CSH	58043333	0	0	0
YRI	CSH	58048333	0	0	0
YRI	CSH	58053333	0	0	0
YRI	CSH	58058333	0	0	0
YRI	CSH	58063333	0	0	0
YRI	CSH	58068333	0	0	0
YRI	CSH	58073333	0	0	0
YRI	CSH	58078333	0	0	0
YRI	CSH	58083333	0	0	0
YRI	CSH	58088333	0	0	0
YRI	CSH	58093333	0	0	0
YRI	CSH	58098333	0	0	0
YRI	CSH	58103333	0	0	0
YRI	CSH	58108333	0	0	0
YRI	CSH	58113333	0	0	0
YRI	CSH	58118333	0	0	0
YRI	CSH	58123333	0	0	0
YRI	CSH	58128333	0	0	0
YRI	CSH	58133333	0	0	0
YRI	CSH	58138333	0	0	0
YRI	CSH	58143333	0	0	0

YRI	CSH	58148333	0	0	0
YRI	CSH	58153333	0	0	0
YRI	CSH	58158333	0	0	0
YRI	CSH	58163333	0	0	0
YRI	CSH	58168333	0	0	0
YRI	CSH	58173333	0	0	0
YRI	CSH	58178333	0	0	0
YRI	CSH	58183333	0	0	0
YRI	CSH	58188333	0	0	0
YRI	CSH	58193333	0	0	0
YRI	CSH	58198333	0	0	0
YRI	CSH	58203333	0	0	0
YRI	CSH	58208333	0	0	0
YRI	CSH	58213333	0	0	0
YRI	CSH	58218333	0	0	0
YRI	CSH	58223333	0	0	0
YRI	CSH	58228333	0	0	0
YRI	CSH	58233333	0	0	0
YRI	CSH	58238333	0	0	0
YRI	CSH	58243333	0	0	0
YRI	CSH	58248333	0	0	0
YRI	CSH	58253333	0	0	0
YRI	CSH	58258333	0	0	0
YRI	CSH	58263333	0	0	0
YRI	CSH	58268333	0	0	0
YRI	CSH	58273333	0	0	0
YRI	CSH	58278333	0	0	0
YRI	CSH	58283333	0	0	0
YRI	CSH	58288333	0	0	0
YRI	CSH	58293333	0	0	0
YRI	CSH	58298333	0	0	0
YRI	CSH	58303333	0	0	0
YRI	CSH	58308333	0	0	0
YRI	CSH	58313333	0	0	0
YRI	CSH	58318333	0	0	0
YRI	CSH	58323333	0	0	0
YRI	CSH	58328333	0	0	0
YRI	CSH	58333333	0	0	0
YRI	CSH	58338333	0	0	0
YRI	CSH	58343333	0	0	0
YRI	CSH	58348333	0	0	0
YRI	CSH	58353333	0	0	0
YRI	CSH	58358333	0	0	0
YRI	CSH	58363333	0	0	0
YRI	CSH	58368333	0	0	0
YRI	CSH	58373333	0	0	0
YRI	CSH	58378333	0	0	0
YRI	CSH	58383333	0	0	0
YRI	CSH	58388333	0	0	0

YRI	CSH	58393333	0	0	0
YRI	CSH	58398333	0	0	0
YRI	CSH	58403333	0	0	0
YRI	CSH	58408333	0	0	0
YRI	CSH	58413333	0	0	0
YRI	CSH	58418333	0	0	0
YRI	CSH	58423333	0	0	0
YRI	CSH	58428333	0	0	0
YRI	CSH	58433333	0	0	0
YRI	CSH	58438333	0	0	0
YRI	CSH	58443333	0	0	0
YRI	CSH	58448333	0	0	0
YRI	CSH	58453333	0	0	0
YRI	CSH	58458333	0	0	0
YRI	CSH	58463333	0	0	0
YRI	CSH	58468333	0	0	0
YRI	CSH	58473333	0	0	0
YRI	CSH	58478333	0	0	0
YRI	CSH	58483333	0	0	0
YRI	CSH	58488333	0	0	0
YRI	CSH	58493333	0	0	0
YRI	CSH	58498333	0	0	0
YRI	CSH	58503333	0	0	0
YRI	CSH	58508333	0	0	0
YRI	CSH	58513333	0	0	0
YRI	CSH	58518333	0	0	0
YRI	CSH	58523333	0	0	0
YRI	CSH	58528333	0	0	0
YRI	CSH	58533333	0	0	0
YRI	CSH	58538333	0	0	0
YRI	CSH	58543333	0	0	0
YRI	CSH	58548333	0	0	0
YRI	CSH	58553333	0	0	0
YRI	CSH	58558333	0	0	0
YRI	CSH	58563333	0	0	0
YRI	CSH	58568333	0	0	0
YRI	CSH	58573333	0	0	0
YRI	CSH	58578333	0	0	0
YRI	CSH	58583333	0	0	0
YRI	CSH	58588333	0	0	0
YRI	CSH	58593333	0	0	0
YRI	CSH	58598333	0	0	0
YRI	CSH	58603333	0	0	0
YRI	CSH	58608333	0	0	0
YRI	CSH	58613333	0	0	0
YRI	CSH	58618333	0	0	0
YRI	CSH	58623333	0	0	0
YRI	CSH	58628333	0	0	0
YRI	CSH	58633333	0	0	0

YRI	CSH	58638333	0	0	0
YRI	CSH	58643333	0	0	0
YRI	CSH	58648333	0	0	0
YRI	CSH	58653333	0	0	0
YRI	CSH	58658333	0	0	0
YRI	CSH	58663333	0	0	0
YRI	CSH	58668333	0	0	0
YRI	CSH	58673333	0	0	0
YRI	CSH	58678333	0	0	0
YRI	CSH	58683333	0	0	0
YRI	CSH	58688333	0	0	0
YRI	CSH	58693333	0	0	0
YRI	CSH	58698333	0	0	0
YRI	CSH	58703333	0	0	0
YRI	CSH	58708333	0	0	0
YRI	CSH	58713333	0	0	0
YRI	CSH	58718333	0	0	0
YRI	CSH	58723333	0	0	0
YRI	CSH	58728333	0	0	0
YRI	CSH	58733333	0	0	0
YRI	CSH	58738333	0	0	0
YRI	CSH	58743333	0	0	0
YRI	CSH	58748333	0	0	0
YRI	CSH	58753333	0	0	0
YRI	CSH	58758333	0	0	0
YRI	CSH	58763333	0	0	0
YRI	CSH	58768333	0	0	0
YRI	CSH	58773333	0	0	0
YRI	CSH	58778333	0	0	0
YRI	CSH	58783333	0	0	0
YRI	CSH	58788333	0	0	0
YRI	CSH	58793333	0	0	0
YRI	CSH	58798333	0	0	0
YRI	CSH	58803333	0	0	0
YRI	CSH	58808333	0	0	0
YRI	CSH	58813333	0	0	0
YRI	CSH	58818333	0	0	0
YRI	CSH	58823333	0	0	0
YRI	CSH	58828333	0	0	0
YRI	CSH	58833333	0	0	0
YRI	CSH	58838333	0	0	0
YRI	CSH	58843333	0	0	0
YRI	CSH	58848333	0	0	0
YRI	CSH	58853333	0	0	0
YRI	CSH	58858333	0	0	0
YRI	CSH	58863333	0	0	0
YRI	CSH	58868333	0	0	0
YRI	CSH	58873333	0	0	0
YRI	CSH	58878333	0	0	0

YRI	CSH	58883333	0	0	0
YRI	CSH	58888333	0	0	0
YRI	CSH	58893333	0	0	0
YRI	CSH	58898333	0	0	0
YRI	CSH	58903333	0	0	0
YRI	CSH	58908333	0	0	0
YRI	CSH	58913333	0	0	0
YRI	CSH	58918333	0	0	0
YRI	CSH	58923333	0	0	0
YRI	CSH	58928333	0	0	0
YRI	CSH	58933333	0	0	0
YRI	CSH	58938333	0	0	0
YRI	CSH	58943333	0	0	0
YRI	CSH	58948333	0	0	0
YRI	CSH	58953333	0	0	0
YRI	CSH	58958333	0	0	0
YRI	CSH	58963333	0	0	0
YRI	CSH	58968333	0	0	0
YRI	CSH	58973333	0	0	0
YRI	CSH	58978333	0	0	0
YRI	CSH	58983333	0	0	0
YRI	CSH	58988333	0	0	0
YRI	CSH	58993333	0	0	0
YRI	CSH	58998333	0	0	0
YRI	CSH	59003333	0	0	0
CEU	DEFA1	6482649	13.4754	15.5212	-0.4067
CEU	DEFA1	6487649	16.6397	14.1567	0.5388
CEU	DEFA1	6492649	18.7572	15.1801	0.7264
CEU	DEFA1	6497649	11.8169	11.9394	-0.0312
CEU	DEFA1	6502649	9.5963	11.4277	-0.4866
CEU	DEFA1	6507649	13.8632	15.5212	-0.3296
CEU	DEFA1	6512649	20.8192	17.2268	0.6464
CEU	DEFA1	6517649	21.0388	17.3974	0.6491
CEU	DEFA1	6522649	13.8895	15.8623	-0.3842
CEU	DEFA1	6527649	13.1338	13.9861	-0.1871
CEU	DEFA1	6532649	15.3651	14.3272	0.2227
CEU	DEFA1	6537649	11.6726	15.8623	-0.8158
CEU	DEFA1	6542649	6.8151	15.1801	-1.6986
CEU	DEFA1	6547649	7.3579	15.5212	-1.6229
CEU	DEFA1	6552649	14.2181	16.7151	-0.4625
CEU	DEFA1	6557649	18.6533	15.5212	0.6227
CEU	DEFA1	6562649	15.3933	15.5212	-0.0254
CEU	DEFA1	6567649	14.5817	15.3506	-0.1545
CEU	DEFA1	6572649	13.7877	12.4511	0.3276
CEU	DEFA1	6577649	12.7972	10.916	0.5218
CEU	DEFA1	6582649	13.5846	10.2337	0.9872
CEU	DEFA1	6587649	11.9555	10.4043	0.45
CEU	DEFA1	6592649	10.255	10.5749	-0.0914
CEU	DEFA1	6597649	7.659	8.0164	-0.132

CEU	DEFA1	6602649	8.0322	8.187	-0.0561
CEU	DEFA1	6607649	9.3927	8.6987	0.2378
CEU	DEFA1	6612649	6.5797	6.1402	0.2068
CEU	DEFA1	6617649	5.867	7.1636	-0.5308
CEU	DEFA1	6622649	7.5217	9.7221	-0.68
CEU	DEFA1	6627649	17.6922	15.0095	0.5507
CEU	DEFA1	6632649	25.5523	21.3203	0.6203
CEU	DEFA1	6637649	20.3496	18.9324	0.2329
CEU	DEFA1	6642649	21.3558	15.8623	1.0697
CEU	DEFA1	6647649	23.8586	15.5212	1.6575
CEU	DEFA1	6652649	16.9021	12.1099	1.2056
CEU	DEFA1	6657649	12.6543	11.0866	0.4285
CEU	DEFA1	6662649	13.7481	14.3272	-0.1242
CEU	DEFA1	6667649	9.3795	13.3039	-0.9033
CEU	DEFA1	6672649	8.733	11.5982	-0.7507
CEU	DEFA1	6677649	11.7351	11.7688	-0.0087
CEU	DEFA1	6682649	11.3777	10.7454	0.178
CEU	DEFA1	6687649	12.0509	11.5982	0.1186
CEU	DEFA1	6692649	17.0456	14.1567	0.6269
CEU	DEFA1	6697649	19.4739	15.5212	0.7858
CEU	DEFA1	6702649	16.7415	14.6684	0.4349
CEU	DEFA1	6707649	18.888	14.8389	0.8402
CEU	DEFA1	6712649	34.5561	24.3904	1.3082
CEU	DEFA1	6717649	41.0641	27.4606	1.5601
CEU	DEFA1	6722649	37.2237	22.5142	2.0454
CEU	DEFA1	6727649	40.6333	26.7783	1.6283
CEU	DEFA1	6732649	39.8578	26.9489	1.5078
CEU	DEFA1	6737649	38.0344	23.5376	1.931
CEU	DEFA1	6742649	25.0103	22.0026	0.4276
CEU	DEFA1	6747649	15.3368	20.2969	-0.7624
CEU	DEFA1	6752649	18.9493	19.2736	-0.0524
CEU	DEFA1	6757649	25.1563	22.1731	0.421
CEU	DEFA1	6762649	45.3649	28.9956	1.7805
CEU	DEFA1	6767649	33.8788	21.1497	1.8803
CEU	DEFA1	6772649	7.8559	11.0866	-0.8831
CEU	DEFA1	6777649	12.9202	14.4978	-0.3347
CEU	DEFA1	6782649	16.5707	15.0095	0.3205
CEU	DEFA1	6787649	22.1341	17.0562	0.9225
CEU	DEFA1	6792649	22.2635	17.7385	0.7917
CEU	DEFA1	6797649	17.1449	14.3272	0.6045
CEU	DEFA1	6802649	14.3342	12.1099	0.5596
CEU	DEFA1	6807649	14.1826	12.9627	0.2878
CEU	DEFA1	6812649	13.8844	12.6216	0.3055
CEU	DEFA1	6817649	12.6889	11.2571	0.3858
CEU	DEFA1	6822649	20.6764	15.6917	0.9807
CEU	DEFA1	6827649	20.5277	14.8389	1.1805
CEU	DEFA1	6832649	8.9546	6.6519	1.0082
CEU	DEFA1	6837649	4.6733	3.4112	0.9913
CEU	DEFA1	6842649	5.3805	3.5818	1.3557

CEU	DEFA1	6847649	2.5501	1.7056	1.1566
CEU	DEFA1	6852649	1.5194	1.3645	0.2506
CEU	DEFA1	6857649	4.4947	2.729	1.669
CEU	DEFA1	6862649	4.5306	2.5584	1.9646
CEU	DEFA1	6867649	1.5504	0.8528	1.571
CEU	DEFA1	6872649	9.2688	7.8459	0.536
CEU	DEFA1	6877649	16.0395	16.0329	0.0013
CEU	DEFA1	6882649	11.8923	13.3039	-0.3249
CEU	DEFA1	6887649	12.3603	11.7688	0.1529
CEU	DEFA1	6892649	23.8623	17.3974	1.1524
CEU	DEFA1	6897649	38.8999	24.0493	1.9373
CEU	DEFA1	6902649	40.1443	24.3904	2.0273
CEU	DEFA1	6907649	28.9238	19.2736	1.559
CEU	DEFA1	6912649	17.3838	13.9861	0.7459
CEU	DEFA1	6917649	9.2161	11.0866	-0.5113
CEU	DEFA1	6922649	9.407	13.4744	-0.925
CEU	DEFA1	6927649	13.3486	16.2034	-0.5447
CEU	DEFA1	6932649	13.7993	17.5679	-0.6655
CEU	DEFA1	6937649	15.3585	25.0727	-1.217
CEU	DEFA1	6942649	22.8008	27.1194	-0.5013
CEU	DEFA1	6947649	32.0404	27.29	0.5481
CEU	DEFA1	6952649	30.7001	28.4839	0.2453
CEU	DEFA1	6957649	27.9021	25.4138	0.3077
CEU	DEFA1	6962649	26.3469	22.3437	0.5608
CEU	DEFA1	6967649	17.4552	18.7619	-0.2166
CEU	DEFA1	6972649	15.7877	17.7385	-0.3413
CEU	DEFA1	6977649	23.007	19.4441	0.5707
CEU	DEFA1	6982649	31.2177	28.9956	0.2417
CEU	DEFA1	6987649	31.9101	29.3367	0.2767
CEU	DEFA1	6992649	27.0808	21.3203	0.8444
CEU	DEFA1	6997649	28.6197	26.0961	0.3041
CEU	DEFA1	7002649	31.1882	29.8484	0.1417
CEU	DEFA1	7007649	32.7392	26.4372	0.7499
CEU	DEFA1	7012649	19.7168	16.7151	0.556
CEU	DEFA1	7017649	4.3882	7.3342	-1.1804
CEU	DEFA1	7022649	9.1808	7.8459	0.5029
CEU	DEFA1	7027649	11.679	9.0398	0.8726
CEU	DEFA1	7032649	5.1872	9.8926	-1.4308
CEU	DEFA1	7037649	1.8852	8.0164	-2.2646
CEU	DEFA1	7042649	4.3597	6.8225	-1.0538
CEU	DEFA1	7047649	6.3671	7.1636	-0.326
CEU	DEFA1	7052649	8.1016	7.8459	0.0963
CEU	DEFA1	7057649	14.1429	11.0866	0.8354
CEU	DEFA1	7062649	15.4989	11.0866	1.2061
CEU	DEFA1	7067649	6.7124	5.9697	0.3584
CEU	DEFA1	7072649	1.5026	1.7056	-0.278
CEU	DEFA1	7077649	3.9981	3.2407	0.6211
CEU	DEFA1	7082649	7.0882	5.2874	0.9683
CEU	DEFA1	7087649	4.6285	3.0701	1.3368

CEU	DEFA1	7092649	0.6883	0.8528	-0.3704
CEU	DEFA1	7097649	0.2041	0.5117	-0.9647
CEU	DEFA1	7102649	0.1552	0.5117	-1.1181
CEU	DEFA1	7107649	1.8433	2.3879	-0.5735
CEU	DEFA1	7112649	1.6881	1.8762	-0.2395
CEU	DEFA1	7117649	0	0	0
CEU	DEFA1	7122649	0	0	0
CEU	DEFA1	7127649	2.6009	1.5351	1.5804
CEU	DEFA1	7132649	2.6009	1.5351	1.5804
CEU	DEFA1	7137649	0	0	0
CEU	DEFA1	7142649	0.0392	0.1706	-0.7805
CEU	DEFA1	7147649	0.8489	1.0234	-0.3467
CEU	DEFA1	7152649	7.5154	15.8623	-1.6253
CEU	DEFA1	7157649	7.5485	16.2034	-1.6514
CEU	DEFA1	7162649	1.6213	2.729	-1.047
CEU	DEFA1	7167649	1.2896	3.2407	-1.5999
CEU	DEFA1	7172649	1.4704	4.7757	-1.944
CEU	DEFA1	7177649	1.6726	4.9463	-1.867
CEU	DEFA1	7182649	1.5209	4.2641	-1.7807
CEU	DEFA1	7187649	1.8396	7.1636	-2.1794
CEU	DEFA1	7192649	2.4026	8.3576	-2.1168
CEU	DEFA1	7197649	2.852	9.0398	-2.0458
CEU	DEFA1	7202649	2.3298	8.8692	-2.2005
CEU	DEFA1	7207649	3.0793	7.5047	-1.7363
CEU	DEFA1	7212649	4.8647	10.2337	-1.5817
CEU	DEFA1	7217649	4.9542	11.7688	-1.7611
CEU	DEFA1	7222649	4.6042	11.9394	-1.8702
CEU	DEFA1	7227649	4.4267	9.0398	-1.5252
CEU	DEFA1	7232649	3.5844	5.7991	-1.0969
CEU	DEFA1	7237649	2.7594	6.4814	-1.6683
CEU	DEFA1	7242649	3.5024	7.5047	-1.5703
CEU	DEFA1	7247649	2.9149	6.8225	-1.6721
CEU	DEFA1	7252649	3.5065	6.3108	-1.2875
CEU	DEFA1	7257649	5.4313	5.9697	-0.2598
CEU	DEFA1	7262649	5.1408	5.2874	-0.0788
CEU	DEFA1	7267649	3.477	4.6052	-0.685
CEU	DEFA1	7272649	3.4607	6.4814	-1.3539
CEU	DEFA1	7277649	2.9971	6.8225	-1.6369
CEU	DEFA1	7282649	1.0738	2.8996	-1.642
CEU	DEFA1	7287649	2.0107	2.3879	-0.3972
CEU	DEFA1	7292649	3.1911	2.729	0.4368
CEU	DEFA1	7297649	1.9944	1.8762	0.1505
CEU	DEFA1	7302649	0.3774	1.3645	-1.5969
CEU	DEFA1	7307649	0.9652	1.7056	-1.0141
CEU	DEFA1	7312649	1.2847	2.3879	-1.1618
CEU	DEFA1	7317649	0.4389	1.5351	-1.6254
CEU	DEFA1	7322649	0.529	0.5117	0.0543
CEU	DEFA1	7327649	2.7547	2.729	0.0243
CEU	DEFA1	7332649	2.3941	3.7524	-0.9841

CEU	DEFA1	7337649	1.3208	3.5818	-1.7041
CEU	DEFA1	7342649	1.6871	2.5584	-0.868
CEU	DEFA1	7347649	0.6378	0.5117	0.3955
CEU	DEFA1	7352649	0.9992	2.2173	-1.3608
CEU	DEFA1	7357649	0.926	2.2173	-1.4426
CEU	DEFA1	7362649	1.5067	1.1939	0.5574
CEU	DEFA1	7367649	1.8141	1.8762	-0.0791
CEU	DEFA1	7372649	1.9695	2.0467	-0.0919
CEU	DEFA1	7377649	1.6221	1.1939	0.7631
CEU	DEFA1	7382649	1.1406	0.5117	1.9724
CEU	DEFA1	7387649	1.9326	1.0234	1.8066
CEU	DEFA1	7392649	0.9322	0.6822	0.6528
CEU	DEFA1	7397649	0.1503	0.3411	-0.7649
CEU	DEFA1	7402649	1.9163	2.5584	-0.6396
CEU	DEFA1	7407649	1.9062	2.3879	-0.5073
CEU	DEFA1	7412649	0	0	0
CEU	DEFA1	7417649	0	0	0
CEU	DEFA1	7422649	0.04	0.3411	-1.207
CEU	DEFA1	7427649	0.12	0.6822	-1.4681
CEU	DEFA1	7432649	0.08	0.3411	-1.0467
CEU	DEFA1	7437649	0.0101	0.1706	-0.9534
CEU	DEFA1	7442649	0.5135	0.8528	-0.7641
CEU	DEFA1	7447649	1.4587	2.2173	-0.8475
CEU	DEFA1	7452649	1.5993	2.2173	-0.6904
CEU	DEFA1	7457649	0.6743	1.1939	-0.9259
CEU	DEFA1	7462649	0.784	1.8762	-1.3906
CEU	DEFA1	7467649	1.5001	2.2173	-0.8012
CEU	DEFA1	7472649	1.322	1.7056	-0.5254
CHB	DEFA1	6482649	14.0772	14.5697	-0.1037
CHB	DEFA1	6487649	13.6936	13.892	-0.0437
CHB	DEFA1	6492649	16.3468	17.958	-0.2778
CHB	DEFA1	6497649	9.3381	14.7391	-1.1247
CHB	DEFA1	6502649	7.0833	11.3508	-1.1377
CHB	DEFA1	6507649	11.3751	16.6027	-0.9717
CHB	DEFA1	6512649	16.809	24.2264	-0.958
CHB	DEFA1	6517649	17.0372	23.2099	-0.8311
CHB	DEFA1	6522649	13.3071	17.6192	-0.7572
CHB	DEFA1	6527649	13.999	16.2638	-0.4294
CHB	DEFA1	6532649	15.85	17.958	-0.3635
CHB	DEFA1	6537649	11.958	17.6192	-0.9941
CHB	DEFA1	6542649	8.0388	15.7556	-1.508
CHB	DEFA1	6547649	7.6273	15.7556	-1.5884
CHB	DEFA1	6552649	9.2012	16.6027	-1.3758
CHB	DEFA1	6557649	15.7763	19.1439	-0.546
CHB	DEFA1	6562649	17.9764	18.1274	-0.0258
CHB	DEFA1	6567649	19.0525	17.7886	0.2199
CHB	DEFA1	6572649	16.7512	18.4662	-0.2879
CHB	DEFA1	6577649	12.6242	15.2474	-0.5289
CHB	DEFA1	6582649	12.6895	13.892	-0.2649

CHB	DEFA1	6587649	11.6589	13.2144	-0.3593
CHB	DEFA1	6592649	10.8547	12.7061	-0.4438
CHB	DEFA1	6597649	8.8188	11.6896	-0.7445
CHB	DEFA1	6602649	8.2555	10.6731	-0.6828
CHB	DEFA1	6607649	10.8608	13.892	-0.6678
CHB	DEFA1	6612649	10.9191	16.4333	-1.0351
CHB	DEFA1	6617649	10.7226	14.4003	-0.783
CHB	DEFA1	6622649	9.7131	12.8755	-0.7487
CHB	DEFA1	6627649	17.8174	17.958	-0.0242
CHB	DEFA1	6632649	29.2486	22.1934	0.992
CHB	DEFA1	6637649	30.8273	23.3793	0.9957
CHB	DEFA1	6642649	29.6716	22.7016	0.9587
CHB	DEFA1	6647649	26.7632	20.8381	0.8853
CHB	DEFA1	6652649	19.1315	18.8051	0.0538
CHB	DEFA1	6657649	16.0268	14.9085	0.2304
CHB	DEFA1	6662649	21.2538	15.5862	1.1191
CHB	DEFA1	6667649	13.7443	12.3673	0.3386
CHB	DEFA1	6672649	9.4616	10.6731	-0.3422
CHB	DEFA1	6677649	13.1064	12.3673	0.1818
CHB	DEFA1	6682649	12.9729	12.3673	0.1489
CHB	DEFA1	6687649	14.5892	17.6192	-0.5321
CHB	DEFA1	6692649	22.8874	24.2264	-0.1729
CHB	DEFA1	6697649	25.1432	23.3793	0.2358
CHB	DEFA1	6702649	17.7474	19.1439	-0.2264
CHB	DEFA1	6707649	19.4139	21.3463	-0.2821
CHB	DEFA1	6712649	27.3501	26.7676	0.0683
CHB	DEFA1	6717649	27.7076	27.9535	-0.0276
CHB	DEFA1	6722649	30.6552	26.937	0.4332
CHB	DEFA1	6727649	35.6383	27.6147	0.9126
CHB	DEFA1	6732649	33.8709	26.7676	0.8328
CHB	DEFA1	6737649	31.9208	23.8875	1.0518
CHB	DEFA1	6742649	22.1376	20.4992	0.2487
CHB	DEFA1	6747649	15.9098	18.2968	-0.4042
CHB	DEFA1	6752649	18.6387	18.4662	0.029
CHB	DEFA1	6757649	23.2804	22.3628	0.1281
CHB	DEFA1	6762649	44.304	27.9535	1.8377
CHB	DEFA1	6767649	36.7794	21.8545	2.1299
CHB	DEFA1	6772649	10.9132	14.0615	-0.6856
CHB	DEFA1	6777649	14.9634	15.0779	-0.0233
CHB	DEFA1	6782649	18.4711	16.4333	0.3825
CHB	DEFA1	6787649	23.8409	20.4992	0.5073
CHB	DEFA1	6792649	22.47	17.6192	0.8518
CHB	DEFA1	6797649	15.5871	14.4003	0.2527
CHB	DEFA1	6802649	12.7834	14.7391	-0.4073
CHB	DEFA1	6807649	9.9332	12.0285	-0.529
CHB	DEFA1	6812649	9.0228	11.1814	-0.5837
CHB	DEFA1	6817649	10.8795	12.7061	-0.4379
CHB	DEFA1	6822649	17.7478	18.8051	-0.1744
CHB	DEFA1	6827649	18.7485	19.8216	-0.1683

CHB	DEFA1	6832649	8.5778	9.9955	-0.4257
CHB	DEFA1	6837649	1.7794	3.7271	-1.415
CHB	DEFA1	6842649	3.2305	3.0495	0.1557
CHB	DEFA1	6847649	2.2878	1.6942	0.8146
CHB	DEFA1	6852649	0.3116	1.3553	-1.6915
CHB	DEFA1	6857649	2.6271	2.7106	-0.0791
CHB	DEFA1	6862649	3.344	2.7106	0.6002
CHB	DEFA1	6867649	1.367	1.1859	0.3233
CHB	DEFA1	6872649	6.3956	5.5907	0.4107
CHB	DEFA1	6877649	13.1302	13.7226	-0.132
CHB	DEFA1	6882649	12.0264	14.4003	-0.5054
CHB	DEFA1	6887649	12.4682	12.3673	0.0248
CHB	DEFA1	6892649	28.9863	19.1439	1.5959
CHB	DEFA1	6897649	44.7263	26.937	2.0728
CHB	DEFA1	6902649	39.0327	23.5487	2.0557
CHB	DEFA1	6907649	26.3301	16.2638	1.9084
CHB	DEFA1	6912649	14.9435	11.6896	0.8438
CHB	DEFA1	6917649	8.5393	11.5202	-0.7837
CHB	DEFA1	6922649	11.5705	16.0944	-0.8663
CHB	DEFA1	6927649	16.0197	18.4662	-0.4107
CHB	DEFA1	6932649	15.6795	18.8051	-0.5156
CHB	DEFA1	6937649	17.4275	24.2264	-0.8781
CHB	DEFA1	6942649	21.5372	27.7841	-0.7063
CHB	DEFA1	6947649	30.0515	31.5112	-0.146
CHB	DEFA1	6952649	29.1772	30.8335	-0.1692
CHB	DEFA1	6957649	21.741	26.7676	-0.5893
CHB	DEFA1	6962649	18.5019	25.9205	-0.8973
CHB	DEFA1	6967649	11.7801	22.3628	-1.477
CHB	DEFA1	6972649	12.3343	21.3463	-1.3156
CHB	DEFA1	6977649	18.2402	23.0404	-0.6509
CHB	DEFA1	6982649	27.0204	30.4947	-0.3588
CHB	DEFA1	6987649	30.3074	29.8171	0.0517
CHB	DEFA1	6992649	26.1784	22.1934	0.5603
CHB	DEFA1	6997649	25.4698	25.2428	0.0282
CHB	DEFA1	7002649	30.6944	30.3253	0.0383
CHB	DEFA1	7007649	38.8762	32.0194	0.6751
CHB	DEFA1	7012649	24.4889	21.8545	0.3759
CHB	DEFA1	7017649	5.2039	8.8096	-1.2174
CHB	DEFA1	7022649	8.5201	8.3013	0.078
CHB	DEFA1	7027649	12.2945	8.4708	1.3386
CHB	DEFA1	7032649	8.6731	7.9625	0.2633
CHB	DEFA1	7037649	3.6244	6.4378	-1.265
CHB	DEFA1	7042649	5.118	7.2848	-0.871
CHB	DEFA1	7047649	8.4422	8.6402	-0.0681
CHB	DEFA1	7052649	11.5852	9.6567	0.598
CHB	DEFA1	7057649	16.0539	11.6896	1.1318
CHB	DEFA1	7062649	15.2686	11.5202	0.9855
CHB	DEFA1	7067649	7.2421	7.1154	0.052
CHB	DEFA1	7072649	2.0933	3.5577	-1.1067

CHB	DEFA1	7077649	4.5551	8.6402	-1.4042
CHB	DEFA1	7082649	7.4365	8.1319	-0.2528
CHB	DEFA1	7087649	4.849	2.5412	2.3045
CHB	DEFA1	7092649	0.8643	0.6777	0.4878
CHB	DEFA1	7097649	0.3859	0.1694	1.2865
CHB	DEFA1	7102649	0.446	0.5082	-0.1953
CHB	DEFA1	7107649	2.6326	2.7106	-0.0739
CHB	DEFA1	7112649	2.1866	2.2024	-0.0177
CHB	DEFA1	7117649	0	0	0
CHB	DEFA1	7122649	0	0	0
CHB	DEFA1	7127649	1.2524	1.3553	-0.1668
CHB	DEFA1	7132649	1.2621	1.5247	-0.3901
CHB	DEFA1	7137649	0.0097	0.1694	-0.949
CHB	DEFA1	7142649	0.0659	0.1694	-0.615
CHB	DEFA1	7147649	0.7006	0.6777	0.0599
CHB	DEFA1	7152649	7.1462	15.925	-1.6981
CHB	DEFA1	7157649	7.1715	16.9415	-1.7813
CHB	DEFA1	7162649	0.8321	2.8801	-1.8465
CHB	DEFA1	7167649	0.4205	3.2189	-2.3007
CHB	DEFA1	7172649	0.6602	4.7436	-2.4087
CHB	DEFA1	7177649	0.518	4.066	-2.3925
CHB	DEFA1	7182649	0.6054	2.2024	-1.7882
CHB	DEFA1	7187649	1.5574	5.5907	-2.0579
CHB	DEFA1	7192649	2.0182	7.4543	-2.1398
CHB	DEFA1	7197649	2.8406	8.3013	-1.9475
CHB	DEFA1	7202649	3.7451	12.7061	-2.1482
CHB	DEFA1	7207649	4.3061	12.3673	-1.9824
CHB	DEFA1	7212649	4.5389	8.6402	-1.4098
CHB	DEFA1	7217649	4.3573	9.3178	-1.59
CHB	DEFA1	7222649	3.9254	10.1649	-1.8445
CHB	DEFA1	7227649	3.4457	5.5907	-1.0944
CHB	DEFA1	7232649	3.003	3.5577	-0.4192
CHB	DEFA1	7237649	2.509	6.4378	-1.7665
CHB	DEFA1	7242649	3.7687	7.9625	-1.5541
CHB	DEFA1	7247649	3.1129	7.1154	-1.6437
CHB	DEFA1	7252649	3.5521	7.1154	-1.4633
CHB	DEFA1	7257649	5.1845	6.0989	-0.4316
CHB	DEFA1	7262649	4.8197	5.0825	-0.1459
CHB	DEFA1	7267649	3.7799	5.2519	-0.7939
CHB	DEFA1	7272649	2.7411	7.1154	-1.7964
CHB	DEFA1	7277649	1.9187	7.7931	-2.2202
CHB	DEFA1	7282649	0.6585	3.2189	-2.105
CHB	DEFA1	7287649	1.206	1.6942	-0.67
CHB	DEFA1	7292649	2.6849	2.5412	0.1435
CHB	DEFA1	7297649	1.7756	1.6942	0.1117
CHB	DEFA1	7302649	0.1249	1.1859	-1.8939
CHB	DEFA1	7307649	0.8469	2.033	-1.4144
CHB	DEFA1	7312649	0.8934	2.033	-1.359
CHB	DEFA1	7317649	0.1711	1.0165	-1.6824

CHB	DEFA1	7322649	0.3356	1.0165	-1.355
CHB	DEFA1	7327649	2.1317	2.5412	-0.4089
CHB	DEFA1	7332649	2.333	4.4048	-1.3037
CHB	DEFA1	7337649	1.4778	3.0495	-1.3517
CHB	DEFA1	7342649	1.5406	1.5247	0.0236
CHB	DEFA1	7347649	0.6632	1.1859	-0.933
CHB	DEFA1	7352649	0.7613	2.3718	-1.7
CHB	DEFA1	7357649	0.831	2.5412	-1.7078
CHB	DEFA1	7362649	1.841	2.033	-0.229
CHB	DEFA1	7367649	1.9457	3.0495	-0.9493
CHB	DEFA1	7372649	1.6276	2.7106	-1.0262
CHB	DEFA1	7377649	1.351	1.1859	0.2947
CHB	DEFA1	7382649	1.0529	0.8471	0.4641
CHB	DEFA1	7387649	1.6688	1.6942	-0.0349
CHB	DEFA1	7392649	0.7122	1.8636	-1.469
CHB	DEFA1	7397649	0.1157	1.3553	-2.009
CHB	DEFA1	7402649	1.9393	2.2024	-0.2946
CHB	DEFA1	7407649	1.9199	1.8636	0.0718
CHB	DEFA1	7412649	0	0	0
CHB	DEFA1	7417649	0	0	0
CHB	DEFA1	7422649	0	0	0
CHB	DEFA1	7427649	0.0097	0.1694	-0.949
CHB	DEFA1	7432649	0.0097	0.1694	-0.949
CHB	DEFA1	7437649	0.134	0.1694	-0.2104
CHB	DEFA1	7442649	0.6669	1.0165	-0.6957
CHB	DEFA1	7447649	1.8987	2.2024	-0.3401
CHB	DEFA1	7452649	1.9585	2.3718	-0.4363
CHB	DEFA1	7457649	0.612	1.1859	-1.0244
CHB	DEFA1	7462649	0.5089	1.5247	-1.5091
CHB	DEFA1	7467649	1.0417	2.7106	-1.5814
CHB	DEFA1	7472649	1.056	1.5247	-0.6963
YRI	DEFA1	6482649	18.8686	30.7554	-1.2132
YRI	DEFA1	6487649	18.9227	25.7135	-0.8251
YRI	DEFA1	6492649	20.0849	24.8732	-0.6008
YRI	DEFA1	6497649	14.0208	22.5203	-1.1743
YRI	DEFA1	6502649	11.4621	20.3355	-1.3529
YRI	DEFA1	6507649	15.2039	25.3774	-1.2519
YRI	DEFA1	6512649	25.2336	35.4611	-0.9083
YRI	DEFA1	6517649	27.5771	33.4444	-0.5518
YRI	DEFA1	6522649	19.7617	23.5287	-0.4988
YRI	DEFA1	6527649	18.1825	22.1842	-0.561
YRI	DEFA1	6532649	20.2625	27.3941	-0.8147
YRI	DEFA1	6537649	18.803	28.7386	-1.0834
YRI	DEFA1	6542649	14.1635	23.1926	-1.2124
YRI	DEFA1	6547649	12.31	25.5455	-1.6183
YRI	DEFA1	6552649	15.1388	27.3941	-1.4001
YRI	DEFA1	6557649	20.058	23.0245	-0.4012
YRI	DEFA1	6562649	21.6777	22.8565	-0.1605
YRI	DEFA1	6567649	20.2303	26.89	-0.7747

YRI	DEFA1	6572649	19.4544	30.4193	-1.1312
YRI	DEFA1	6577649	20.2901	30.0831	-1.0213
YRI	DEFA1	6582649	19.3186	27.058	-0.8949
YRI	DEFA1	6587649	14.1623	21.68	-1.0775
YRI	DEFA1	6592649	13.6407	19.9994	-0.9852
YRI	DEFA1	6597649	12.4535	20.6717	-1.2334
YRI	DEFA1	6602649	11.3294	20.1675	-1.3583
YRI	DEFA1	6607649	13.9857	19.8313	-0.9131
YRI	DEFA1	6612649	13.3356	18.6549	-0.8812
YRI	DEFA1	6617649	14.2085	20.8397	-0.9874
YRI	DEFA1	6622649	13.2586	21.68	-1.2071
YRI	DEFA1	6627649	22.1358	28.9067	-0.7341
YRI	DEFA1	6632649	35.3697	36.8056	-0.123
YRI	DEFA1	6637649	40.1643	32.6041	0.7289
YRI	DEFA1	6642649	36.2388	27.5622	0.9854
YRI	DEFA1	6647649	28.4601	26.3858	0.2458
YRI	DEFA1	6652649	19.7249	23.0245	-0.4462
YRI	DEFA1	6657649	15.3827	21.3439	-0.8674
YRI	DEFA1	6662649	23.6953	25.2093	-0.1875
YRI	DEFA1	6667649	19.6409	23.1926	-0.4769
YRI	DEFA1	6672649	14.1476	22.0162	-1.1112
YRI	DEFA1	6677649	16.5205	22.1842	-0.794
YRI	DEFA1	6682649	17.7735	22.6884	-0.6742
YRI	DEFA1	6687649	22.9099	27.8983	-0.5599
YRI	DEFA1	6692649	32.5306	34.2847	-0.161
YRI	DEFA1	6697649	32.0889	34.2847	-0.2015
YRI	DEFA1	6702649	23.7436	31.2596	-0.755
YRI	DEFA1	6707649	27.7157	36.6376	-0.7674
YRI	DEFA1	6712649	49.3491	43.5281	0.4228
YRI	DEFA1	6717649	56.2682	42.8559	0.9892
YRI	DEFA1	6722649	44.7397	40.1669	0.3594
YRI	DEFA1	6727649	37.7014	42.0156	-0.3244
YRI	DEFA1	6732649	33.0659	40.8391	-0.6011
YRI	DEFA1	6737649	35.0029	37.1417	-0.1815
YRI	DEFA1	6742649	28.7421	33.1083	-0.4147
YRI	DEFA1	6747649	21.2409	32.6041	-1.0955
YRI	DEFA1	6752649	20.9097	30.5873	-0.993
YRI	DEFA1	6757649	33.7259	33.9486	-0.0206
YRI	DEFA1	6762649	53.4136	41.6794	0.8894
YRI	DEFA1	6767649	37.2749	33.1083	0.3957
YRI	DEFA1	6772649	14.6033	23.3606	-1.1677
YRI	DEFA1	6777649	17.1853	25.0413	-0.9793
YRI	DEFA1	6782649	19.6796	26.3858	-0.7946
YRI	DEFA1	6787649	23.0519	27.2261	-0.4797
YRI	DEFA1	6792649	21.2222	26.89	-0.6593
YRI	DEFA1	6797649	16.002	26.89	-1.2666
YRI	DEFA1	6802649	14.2471	24.7051	-1.3209
YRI	DEFA1	6807649	14.0294	21.512	-1.0806
YRI	DEFA1	6812649	14.8413	23.6968	-1.1646

YRI	DEFA1	6817649	17.2059	24.201	-0.9014
YRI	DEFA1	6822649	23.6394	28.2345	-0.5098
YRI	DEFA1	6827649	22.6097	26.0496	-0.4127
YRI	DEFA1	6832649	11.3332	11.7644	-0.1108
YRI	DEFA1	6837649	4.7804	5.7141	-0.4656
YRI	DEFA1	6842649	4.9347	5.5461	-0.3131
YRI	DEFA1	6847649	3.4184	2.8571	0.5076
YRI	DEFA1	6852649	1.3512	2.5209	-1.1714
YRI	DEFA1	6857649	3.9575	5.2099	-0.6779
YRI	DEFA1	6862649	5.1205	5.378	-0.1355
YRI	DEFA1	6867649	2.1398	2.689	-0.5219
YRI	DEFA1	6872649	7.9984	9.7476	-0.5358
YRI	DEFA1	6877649	17.922	24.5371	-0.8411
YRI	DEFA1	6882649	16.5678	27.3941	-1.2368
YRI	DEFA1	6887649	13.7046	23.8648	-1.3271
YRI	DEFA1	6892649	29.028	31.0915	-0.2084
YRI	DEFA1	6897649	45.7533	38.8224	0.5633
YRI	DEFA1	6902649	41.3769	34.1166	0.6696
YRI	DEFA1	6907649	27.9571	27.3941	0.0643
YRI	DEFA1	6912649	15.5806	22.3523	-0.9424
YRI	DEFA1	6917649	12.4665	20.5036	-1.2157
YRI	DEFA1	6922649	19.3963	29.4109	-1.0677
YRI	DEFA1	6927649	24.3819	35.4611	-0.9839
YRI	DEFA1	6932649	22.899	34.7889	-1.0759
YRI	DEFA1	6937649	21.0881	34.9569	-1.249
YRI	DEFA1	6942649	22.498	35.7973	-1.1702
YRI	DEFA1	6947649	32.5973	42.0156	-0.7083
YRI	DEFA1	6952649	33.1663	41.5114	-0.6351
YRI	DEFA1	6957649	26.4933	35.9653	-0.8296
YRI	DEFA1	6962649	23.1577	32.7721	-0.9223
YRI	DEFA1	6967649	16.688	29.4109	-1.3564
YRI	DEFA1	6972649	17.9316	31.5957	-1.3584
YRI	DEFA1	6977649	23.877	31.9318	-0.7925
YRI	DEFA1	6982649	29.3527	39.9988	-0.8402
YRI	DEFA1	6987649	28.5245	39.8307	-0.896
YRI	DEFA1	6992649	23.0741	28.7386	-0.6177
YRI	DEFA1	6997649	26.9312	34.7889	-0.711
YRI	DEFA1	7002649	38.6209	41.0072	-0.1838
YRI	DEFA1	7007649	45.393	41.8475	0.2677
YRI	DEFA1	7012649	27.9497	28.4025	-0.0499
YRI	DEFA1	7017649	9.8027	13.2769	-0.7962
YRI	DEFA1	7022649	12.1483	12.6047	-0.1099
YRI	DEFA1	7027649	13.1542	12.6047	0.1323
YRI	DEFA1	7032649	11.9106	13.2769	-0.3131
YRI	DEFA1	7037649	8.1468	9.7476	-0.4904
YRI	DEFA1	7042649	8.0455	10.5879	-0.721
YRI	DEFA1	7047649	11.894	13.1089	-0.2818
YRI	DEFA1	7052649	15.99	16.6382	-0.1198
YRI	DEFA1	7057649	21.5738	20.1675	0.2161

YRI	DEFA1	7062649	19.5686	16.302	0.6158
YRI	DEFA1	7067649	8.9983	8.4031	0.2092
YRI	DEFA1	7072649	2.5808	3.6974	-0.8139
YRI	DEFA1	7077649	4.3475	7.3947	-1.2041
YRI	DEFA1	7082649	6.4872	7.7309	-0.4719
YRI	DEFA1	7087649	4.4196	4.0335	0.2612
YRI	DEFA1	7092649	0.9667	1.5126	-0.8129
YRI	DEFA1	7097649	0.2903	1.0084	-1.4317
YRI	DEFA1	7102649	0.764	2.3529	-1.6819
YRI	DEFA1	7107649	2.2594	5.8822	-1.7604
YRI	DEFA1	7112649	1.6968	4.3696	-1.6877
YRI	DEFA1	7117649	0	0	0
YRI	DEFA1	7122649	0	0	0
YRI	DEFA1	7127649	1.6231	1.5126	0.1645
YRI	DEFA1	7132649	1.6783	1.8487	-0.218
YRI	DEFA1	7137649	0.0552	0.3361	-1.1277
YRI	DEFA1	7142649	0.1681	0.1681	0
YRI	DEFA1	7147649	1.6615	1.0084	1.3021
YRI	DEFA1	7152649	11.4297	18.991	-1.2313
YRI	DEFA1	7157649	10.8502	21.0078	-1.5009
YRI	DEFA1	7162649	3.2063	5.2099	-1.0845
YRI	DEFA1	7167649	5.0123	6.3864	-0.6202
YRI	DEFA1	7172649	4.703	8.4031	-1.3004
YRI	DEFA1	7177649	3.6383	6.2183	-1.1927
YRI	DEFA1	7182649	2.3609	3.8654	-1.0558
YRI	DEFA1	7187649	5.1223	9.7476	-1.4169
YRI	DEFA1	7192649	7.3507	11.4282	-1.0765
YRI	DEFA1	7197649	10.3949	11.5963	-0.3129
YRI	DEFA1	7202649	13.4241	17.9827	-0.7823
YRI	DEFA1	7207649	10.6011	16.4701	-1.0956
YRI	DEFA1	7212649	8.6394	14.1172	-1.1844
YRI	DEFA1	7217649	8.0842	16.4701	-1.5655
YRI	DEFA1	7222649	8.0033	18.3188	-1.7391
YRI	DEFA1	7227649	6.662	12.9408	-1.4744
YRI	DEFA1	7232649	4.1183	5.7141	-0.7958
YRI	DEFA1	7237649	4.2027	8.7392	-1.5378
YRI	DEFA1	7242649	6.3645	14.2853	-1.6935
YRI	DEFA1	7247649	5.3532	13.613	-1.8487
YRI	DEFA1	7252649	5.6303	12.1005	-1.6187
YRI	DEFA1	7257649	6.9081	9.9157	-0.9068
YRI	DEFA1	7262649	5.0813	6.2183	-0.5256
YRI	DEFA1	7267649	3.5136	6.3864	-1.2966
YRI	DEFA1	7272649	3.7943	10.924	-1.9637
YRI	DEFA1	7277649	4.1374	12.9408	-2.0672
YRI	DEFA1	7282649	2.1944	6.5544	-1.9222
YRI	DEFA1	7287649	1.816	2.689	-0.8297
YRI	DEFA1	7292649	2.9284	4.0335	-0.7477
YRI	DEFA1	7297649	2.1385	5.0419	-1.6177
YRI	DEFA1	7302649	1.1899	6.2183	-2.3246

YRI	DEFA1	7307649	1.6533	6.2183	-2.1104
YRI	DEFA1	7312649	1.4905	4.3696	-1.8179
YRI	DEFA1	7317649	0.365	1.8487	-1.8978
YRI	DEFA1	7322649	0.7639	1.1764	-0.7378
YRI	DEFA1	7327649	2.908	4.5377	-0.9958
YRI	DEFA1	7332649	3.5086	7.8989	-1.6333
YRI	DEFA1	7337649	2.2474	6.5544	-1.8989
YRI	DEFA1	7342649	1.8812	3.5293	-1.2496
YRI	DEFA1	7347649	1.0442	2.0167	-1.1628
YRI	DEFA1	7352649	2.093	3.6974	-1.1694
YRI	DEFA1	7357649	2.4375	3.5293	-0.8278
YRI	DEFA1	7362649	1.7831	2.0167	-0.2793
YRI	DEFA1	7367649	3.0507	3.3612	-0.2453
YRI	DEFA1	7372649	3.7848	3.6974	0.0637
YRI	DEFA1	7377649	2.0897	1.6806	0.5628
YRI	DEFA1	7382649	0.6723	1.1764	-0.9016
YRI	DEFA1	7387649	1.3384	2.1848	-0.9503
YRI	DEFA1	7392649	0.8767	2.689	-1.7223
YRI	DEFA1	7397649	0.3182	2.0167	-2.0308
YRI	DEFA1	7402649	2.0421	6.5544	-1.9894
YRI	DEFA1	7407649	1.9345	6.2183	-1.9804
YRI	DEFA1	7412649	0	0	0
YRI	DEFA1	7417649	0	0	0
YRI	DEFA1	7422649	0.0369	0.3361	-1.2012
YRI	DEFA1	7427649	0.1648	1.3445	-1.9161
YRI	DEFA1	7432649	0.1557	1.5126	-2.0205
YRI	DEFA1	7437649	0.1527	1.1764	-1.8309
YRI	DEFA1	7442649	0.9824	2.1848	-1.35
YRI	DEFA1	7447649	1.7621	3.0251	-1.0895
YRI	DEFA1	7452649	2.5801	3.1932	-0.5056
YRI	DEFA1	7457649	1.7125	2.1848	-0.5303
YRI	DEFA1	7462649	1.4165	3.3612	-1.5364
YRI	DEFA1	7467649	2.887	4.3696	-0.9362
YRI	DEFA1	7472649	1.7622	2.0167	-0.3043
CEU	DEFA1	6501766	8.9047	11.7688	-0.7402
CEU	DEFA1	6506766	13.736	16.0329	-0.4427
CEU	DEFA1	6511766	17.6553	16.0329	0.3127
CEU	DEFA1	6516766	19.9852	16.0329	0.7618
CEU	DEFA1	6521766	17.4341	17.2268	0.0373
CEU	DEFA1	6526766	13.0872	14.1567	-0.2321
CEU	DEFA1	6531766	16.4045	14.3272	0.4457
CEU	DEFA1	6536766	13.2604	15.0095	-0.359
CEU	DEFA1	6541766	6.0495	14.6684	-1.8083
CEU	DEFA1	6546766	6.7207	16.7151	-1.8511
CEU	DEFA1	6551766	13.8248	17.0562	-0.587
CEU	DEFA1	6556766	18.471	15.3506	0.6269
CEU	DEFA1	6561766	14.6953	15.3506	-0.1317
CEU	DEFA1	6566766	13.9025	14.8389	-0.1943
CEU	DEFA1	6571766	14.2834	12.7922	0.3562

CEU	DEFA1	6576766	12.5624	11.5982	0.2526
CEU	DEFA1	6581766	13.2208	10.5749	0.756
CEU	DEFA1	6586766	12.2117	9.7221	0.7694
CEU	DEFA1	6591766	11.6026	10.7454	0.2413
CEU	DEFA1	6596766	9.1965	9.2104	-0.0045
CEU	DEFA1	6601766	7.4927	7.8459	-0.1331
CEU	DEFA1	6606766	9.1315	8.8692	0.0883
CEU	DEFA1	6611766	7.5945	6.8225	0.3303
CEU	DEFA1	6616766	5.2059	6.4814	-0.5717
CEU	DEFA1	6621766	7.0752	9.2104	-0.6938
CEU	DEFA1	6626766	14.2014	13.8156	0.0857
CEU	DEFA1	6631766	25.335	21.4909	0.5591
CEU	DEFA1	6636766	24.5199	20.8086	0.5569
CEU	DEFA1	6641766	18.5094	14.8389	0.7617
CEU	DEFA1	6646766	22.9353	15.1801	1.5748
CEU	DEFA1	6651766	18.1262	11.9394	1.5774
CEU	DEFA1	6656766	13.3886	10.7454	0.744
CEU	DEFA1	6661766	15.2608	15.5212	-0.0518
CEU	DEFA1	6666766	9.7019	12.9627	-0.7693
CEU	DEFA1	6671766	7.0299	10.4043	-0.9789
CEU	DEFA1	6676766	11.8052	12.4511	-0.1583
CEU	DEFA1	6681766	13.0058	11.9394	0.2719
CEU	DEFA1	6686766	11.6085	11.7688	-0.0414
CEU	DEFA1	6691766	14.3348	12.6216	0.4145
CEU	DEFA1	6696766	19.4527	15.6917	0.74
CEU	DEFA1	6701766	19.3226	15.8623	0.6738
CEU	DEFA1	6706766	18.8562	14.3272	0.9717
CEU	DEFA1	6711766	33.622	23.5376	1.3432
CEU	DEFA1	6716766	36.7333	26.0961	1.2819
CEU	DEFA1	6721766	36.5609	22.8554	1.8783
CEU	DEFA1	6726766	41.8097	26.6077	1.7978
CEU	DEFA1	6731766	38.2432	26.0961	1.4639
CEU	DEFA1	6736766	38.9161	23.7082	2.0116
CEU	DEFA1	6741766	28.8934	23.5376	0.7134
CEU	DEFA1	6746766	15.8211	20.1264	-0.6671
CEU	DEFA1	6751766	16.9287	17.5679	-0.1129
CEU	DEFA1	6756766	25.2212	22.5142	0.3764
CEU	DEFA1	6761766	46.1954	30.7012	1.5939
CEU	DEFA1	6766766	36.102	23.0259	1.7792
CEU	DEFA1	6771766	9.1769	11.4277	-0.598
CEU	DEFA1	6776766	11.7376	13.8156	-0.4615
CEU	DEFA1	6781766	14.6565	14.4978	0.0337
CEU	DEFA1	6786766	20.4157	16.5446	0.7241
CEU	DEFA1	6791766	23.6448	18.4207	0.8815
CEU	DEFA1	6796766	17.7722	14.1567	0.7846
CEU	DEFA1	6801766	14.0326	11.4277	0.6921
CEU	DEFA1	6806766	14.6817	13.1333	0.3608
CEU	DEFA1	6811766	14.3579	12.6216	0.4201
CEU	DEFA1	6816766	12.8306	11.5982	0.3229

CEU	DEFA1	6821766	18.9592	15.5212	0.6835
CEU	DEFA1	6826766	21.8322	15.8623	1.1625
CEU	DEFA1	6831766	10.1373	7.1636	1.2173
CEU	DEFA1	6836766	5.1175	3.5818	1.1575
CEU	DEFA1	6841766	5.7506	4.0935	1.1143
CEU	DEFA1	6846766	2.5501	1.7056	1.1566
CEU	DEFA1	6851766	1.232	1.0234	0.4145
CEU	DEFA1	6856766	4.4947	2.729	1.669
CEU	DEFA1	6861766	4.8458	2.729	2.0008
CEU	DEFA1	6866766	1.1723	0.6822	1.2798
CEU	DEFA1	6871766	7.5347	6.4814	0.4721
CEU	DEFA1	6876766	15.7484	15.0095	0.1517
CEU	DEFA1	6881766	13.7193	14.6684	-0.1991
CEU	DEFA1	6886766	11.7027	11.9394	-0.0603
CEU	DEFA1	6891766	22.8279	16.374	1.2192
CEU	DEFA1	6896766	36.6233	22.3437	2.0003
CEU	DEFA1	6901766	37.4935	23.5376	1.8589
CEU	DEFA1	6906766	31.4557	21.1497	1.5224
CEU	DEFA1	6911766	21.2387	15.8623	1.0469
CEU	DEFA1	6916766	9.8577	10.7454	-0.2499
CEU	DEFA1	6921766	8.8146	12.4511	-0.8912
CEU	DEFA1	6926766	11.9889	15.8623	-0.7542
CEU	DEFA1	6931766	13.5723	17.7385	-0.7289
CEU	DEFA1	6936766	15.8064	24.561	-1.119
CEU	DEFA1	6941766	22.4886	26.2666	-0.4524
CEU	DEFA1	6946766	30.9371	27.6311	0.3769
CEU	DEFA1	6951766	31.0689	29.1662	0.2058
CEU	DEFA1	6956766	27.59	25.2432	0.2921
CEU	DEFA1	6961766	26.45	22.5142	0.5473
CEU	DEFA1	6966766	18.9194	18.7619	0.0261
CEU	DEFA1	6971766	16.5347	17.3974	-0.1538
CEU	DEFA1	6976766	22.7706	19.6147	0.5013
CEU	DEFA1	6981766	30.2554	29.3367	0.0988
CEU	DEFA1	6986766	30.4997	29.6779	0.0874
CEU	DEFA1	6991766	25.2016	20.2969	0.7539
CEU	DEFA1	6996766	30.545	25.9255	0.5603
CEU	DEFA1	7001766	30.7454	28.8251	0.2101
CEU	DEFA1	7006766	31.0148	26.0961	0.5928
CEU	DEFA1	7011766	23.0237	18.9324	0.6724
CEU	DEFA1	7016766	7.1169	9.0398	-0.6358
CEU	DEFA1	7021766	8.966	8.5281	0.1528
CEU	DEFA1	7026766	11.3321	9.0398	0.7579
CEU	DEFA1	7031766	5.6036	9.5515	-1.2403
CEU	DEFA1	7036766	1.7796	7.5047	-2.2463
CEU	DEFA1	7041766	4.5184	7.5047	-1.1717
CEU	DEFA1	7046766	6.6961	7.5047	-0.3173
CEU	DEFA1	7051766	6.035	7.1636	-0.462
CEU	DEFA1	7056766	12.2787	10.0632	0.663
CEU	DEFA1	7061766	17.5756	11.9394	1.437

CEU	DEFA1	7066766	8.7001	7.1636	0.629
CEU	DEFA1	7071766	1.5026	1.7056	-0.278
CEU	DEFA1	7076766	3.9485	3.0701	0.7535
CEU	DEFA1	7081766	7.0882	5.2874	0.9683
CEU	DEFA1	7086766	4.6883	3.4112	1.0031
CEU	DEFA1	7091766	0.6782	0.6822	-0.0104
CEU	DEFA1	7096766	0.2041	0.5117	-0.9647
CEU	DEFA1	7101766	0.1653	0.6822	-1.3498
CEU	DEFA1	7106766	1.8433	2.3879	-0.5735
CEU	DEFA1	7111766	1.6881	1.8762	-0.2395
CEU	DEFA1	7116766	0	0	0
CEU	DEFA1	7121766	0	0	0
CEU	DEFA1	7126766	2.6009	1.5351	1.5804
CEU	DEFA1	7131766	2.6009	1.5351	1.5804
CEU	DEFA1	7136766	0	0	0
CEU	DEFA1	7141766	0.0392	0.1706	-0.7805
CEU	DEFA1	7146766	0.8489	1.0234	-0.3467
CEU	DEFA1	7151766	7.3243	15.6917	-1.6462
CEU	DEFA1	7156766	7.5384	16.0329	-1.6373
CEU	DEFA1	7161766	1.8124	2.8996	-0.9778
CEU	DEFA1	7166766	1.2997	3.4112	-1.6584
CEU	DEFA1	7171766	1.0606	3.5818	-1.9002
CEU	DEFA1	7176766	1.6726	4.9463	-1.867
CEU	DEFA1	7181766	1.8512	4.9463	-1.7651
CEU	DEFA1	7186766	1.3067	5.7991	-2.2251
CEU	DEFA1	7191766	2.442	8.5281	-2.1235
CEU	DEFA1	7196766	3.1004	9.2104	-1.9854
CEU	DEFA1	7201766	2.37	9.2104	-2.2228
CEU	DEFA1	7206766	2.3032	7.5047	-2.0408
CEU	DEFA1	7211766	4.1277	9.0398	-1.6241
CEU	DEFA1	7216766	5.3322	11.4277	-1.6195
CEU	DEFA1	7221766	4.7728	12.2805	-1.864
CEU	DEFA1	7226766	4.8545	10.2337	-1.5847
CEU	DEFA1	7231766	3.5405	5.7991	-1.1187
CEU	DEFA1	7236766	2.8994	6.6519	-1.6429
CEU	DEFA1	7241766	3.8757	7.6753	-1.4605
CEU	DEFA1	7246766	2.9029	6.6519	-1.6414
CEU	DEFA1	7251766	3.7454	6.9931	-1.3589
CEU	DEFA1	7256766	4.0503	4.6052	-0.3369
CEU	DEFA1	7261766	4.6792	5.1169	-0.2423
CEU	DEFA1	7266766	4.9443	5.6286	-0.3481
CEU	DEFA1	7271766	3.1694	5.9697	-1.3515
CEU	DEFA1	7276766	3.0375	7.5047	-1.7527
CEU	DEFA1	7281766	1.8266	3.5818	-1.3229
CEU	DEFA1	7286766	1.5805	2.0467	-0.5547
CEU	DEFA1	7291766	3.1911	2.729	0.4368
CEU	DEFA1	7296766	2.4146	2.0467	0.4378
CEU	DEFA1	7301766	0.3774	1.3645	-1.5969
CEU	DEFA1	7306766	0.9753	1.8762	-1.147

CEU	DEFA1	7311766	1.2445	2.0467	-0.9545
CEU	DEFA1	7316766	0.4389	1.5351	-1.6254
CEU	DEFA1	7321766	0.4644	0.6822	-0.5687
CEU	DEFA1	7326766	2.2189	2.0467	0.2049
CEU	DEFA1	7331766	2.4989	3.9229	-0.9932
CEU	DEFA1	7336766	1.8464	4.0935	-1.511
CEU	DEFA1	7341766	1.1624	2.2173	-1.1785
CEU	DEFA1	7346766	0.6479	0.6822	-0.0896
CEU	DEFA1	7351766	1.5238	2.5584	-1.0306
CEU	DEFA1	7356766	0.926	2.2173	-1.4426
CEU	DEFA1	7361766	0.5955	0.8528	-0.5794
CEU	DEFA1	7366766	1.699	1.7056	-0.009
CEU	DEFA1	7371766	2.8806	2.3879	0.5189
CEU	DEFA1	7376766	1.7372	1.3645	0.6029
CEU	DEFA1	7381766	1.1406	0.5117	1.9724
CEU	DEFA1	7386766	1.9126	0.8528	2.3866
CEU	DEFA1	7391766	0.9322	0.6822	0.6528
CEU	DEFA1	7396766	0.1703	0.5117	-1.0707
CEU	DEFA1	7401766	1.9163	2.5584	-0.6396
CEU	DEFA1	7406766	1.9062	2.3879	-0.5073
CEU	DEFA1	7411766	0	0	0
CEU	DEFA1	7416766	0	0	0
CEU	DEFA1	7421766	0	0	0
CEU	DEFA1	7426766	0.04	0.3411	-1.207
CEU	DEFA1	7431766	0.12	0.6822	-1.4681
CEU	DEFA1	7436766	0.0901	0.5117	-1.3222
CEU	DEFA1	7441766	0.5034	0.6822	-0.4669
CEU	DEFA1	7446766	1.4587	2.2173	-0.8475
CEU	DEFA1	7451766	1.6094	2.3879	-0.8198
CEU	DEFA1	7456766	0.6642	1.0234	-0.7137
CEU	DEFA1	7461766	0.3496	1.7056	-1.8572
CEU	DEFA1	7466766	1.5001	2.2173	-0.8012
CEU	DEFA1	7471766	1.7563	1.8762	-0.1527
CEU	DEFA1	7476766	0.5857	1.0234	-0.8697
CEU	DEFA1	7481766	0	0	0
CEU	DEFA1	7486766	0	0	0
CEU	DEFA1	7491766	0	0	0
CHB	DEFA1	6501766	6.425	11.3508	-1.3132
CHB	DEFA1	6506766	11.4684	16.6027	-0.9544
CHB	DEFA1	6511766	14.7515	22.5322	-1.078
CHB	DEFA1	6516766	15.3685	22.1934	-0.9596
CHB	DEFA1	6521766	15.4436	18.6357	-0.5311
CHB	DEFA1	6526766	13.704	16.4333	-0.5123
CHB	DEFA1	6531766	16.9345	18.8051	-0.3086
CHB	DEFA1	6536766	13.977	17.958	-0.6864
CHB	DEFA1	6541766	6.8726	15.2474	-1.6886
CHB	DEFA1	6546766	7.4316	15.925	-1.6429
CHB	DEFA1	6551766	9.8433	15.925	-1.1764
CHB	DEFA1	6556766	14.7754	18.2968	-0.5964

CHB	DEFA1	6561766	16.9119	18.9745	-0.3373
CHB	DEFA1	6566766	18.5871	16.6027	0.3689
CHB	DEFA1	6571766	17.139	17.7886	-0.113
CHB	DEFA1	6576766	13.1176	17.4498	-0.7678
CHB	DEFA1	6581766	12.9709	15.0779	-0.4294
CHB	DEFA1	6586766	11.185	12.5367	-0.3282
CHB	DEFA1	6591766	11.8561	12.8755	-0.2413
CHB	DEFA1	6596766	10.6429	13.045	-0.5617
CHB	DEFA1	6601766	7.7988	9.9955	-0.6596
CHB	DEFA1	6606766	10.1551	12.8755	-0.644
CHB	DEFA1	6611766	11.7105	17.2803	-0.9964
CHB	DEFA1	6616766	9.8176	14.5697	-1.0006
CHB	DEFA1	6621766	9.588	12.3673	-0.6835
CHB	DEFA1	6626766	14.6246	16.6027	-0.3677
CHB	DEFA1	6631766	28.7596	22.1934	0.9232
CHB	DEFA1	6636766	35.1557	24.904	1.2891
CHB	DEFA1	6641766	27.3573	21.8545	0.7853
CHB	DEFA1	6646766	25.7436	20.6686	0.7643
CHB	DEFA1	6651766	19.6872	18.9745	0.1166
CHB	DEFA1	6656766	15.7569	14.7391	0.212
CHB	DEFA1	6661766	22.8717	16.4333	1.2086
CHB	DEFA1	6666766	15.4898	13.2144	0.5256
CHB	DEFA1	6671766	7.7657	9.9955	-0.6696
CHB	DEFA1	6676766	12.292	11.6896	0.1562
CHB	DEFA1	6681766	14.3321	12.7061	0.3898
CHB	DEFA1	6686766	14.6484	16.9415	-0.4181
CHB	DEFA1	6691766	19.7294	22.7016	-0.4088
CHB	DEFA1	6696766	25.2642	23.7181	0.2038
CHB	DEFA1	6701766	21.0621	20.3298	0.1121
CHB	DEFA1	6706766	19.42	21.8545	-0.3474
CHB	DEFA1	6711766	26.8915	25.7511	0.1388
CHB	DEFA1	6716766	25.8919	26.4288	-0.0637
CHB	DEFA1	6721766	29.6023	27.4452	0.2468
CHB	DEFA1	6726766	35.2846	28.1229	0.8002
CHB	DEFA1	6731766	32.5648	26.937	0.6558
CHB	DEFA1	6736766	32.6212	24.0569	1.1137
CHB	DEFA1	6741766	25.379	20.6686	0.7094
CHB	DEFA1	6746766	16.3495	17.958	-0.2773
CHB	DEFA1	6751766	16.7101	17.958	-0.2152
CHB	DEFA1	6756766	22.8841	23.2099	-0.0439
CHB	DEFA1	6761766	45.4017	28.97	1.7837
CHB	DEFA1	6766766	38.8523	22.871	2.1825
CHB	DEFA1	6771766	11.957	14.7391	-0.5794
CHB	DEFA1	6776766	13.9282	15.0779	-0.2343
CHB	DEFA1	6781766	16.8229	15.7556	0.2086
CHB	DEFA1	6786766	22.1221	19.6521	0.3905
CHB	DEFA1	6791766	24.0786	18.6357	0.9057
CHB	DEFA1	6796766	15.9838	14.4003	0.3371
CHB	DEFA1	6801766	12.5321	14.2309	-0.3658

CHB	DEFA1	6806766	10.6301	11.8591	-0.3144
CHB	DEFA1	6811766	9.1041	11.012	-0.5233
CHB	DEFA1	6816766	10.4417	12.0285	-0.4006
CHB	DEFA1	6821766	16.3619	18.1274	-0.3017
CHB	DEFA1	6826766	20.407	21.8545	-0.2066
CHB	DEFA1	6831766	10.0571	10.5037	-0.128
CHB	DEFA1	6836766	2.2482	3.8965	-1.1529
CHB	DEFA1	6841766	3.1437	3.8965	-0.5265
CHB	DEFA1	6846766	2.2878	1.6942	0.8146
CHB	DEFA1	6851766	0.8498	1.5247	-1.0027
CHB	DEFA1	6856766	2.6271	2.7106	-0.0791
CHB	DEFA1	6861766	2.999	2.5412	0.4571
CHB	DEFA1	6866766	0.9994	0.8471	0.3434
CHB	DEFA1	6871766	4.6156	4.7436	-0.0755
CHB	DEFA1	6876766	12.6811	12.8755	-0.046
CHB	DEFA1	6881766	13.4385	14.5697	-0.2382
CHB	DEFA1	6886766	11.9915	12.3673	-0.0924
CHB	DEFA1	6891766	28.6038	18.6357	1.6586
CHB	DEFA1	6896766	41.9539	25.7511	1.9724
CHB	DEFA1	6901766	36.4634	22.5322	1.9302
CHB	DEFA1	6906766	29.0421	17.4498	2.0545
CHB	DEFA1	6911766	17.8252	13.7226	0.9144
CHB	DEFA1	6916766	9.7959	11.012	-0.3336
CHB	DEFA1	6921766	10.2366	14.5697	-0.9123
CHB	DEFA1	6926766	14.644	18.9745	-0.7082
CHB	DEFA1	6931766	17.0623	18.2968	-0.2091
CHB	DEFA1	6936766	17.7853	23.7181	-0.7822
CHB	DEFA1	6941766	21.0036	27.6147	-0.7519
CHB	DEFA1	6946766	29.0266	31.003	-0.2008
CHB	DEFA1	6951766	30.1942	32.5277	-0.2262
CHB	DEFA1	6956766	21.5566	26.4288	-0.5783
CHB	DEFA1	6961766	17.8575	24.7346	-0.8705
CHB	DEFA1	6966766	13.4463	22.871	-1.2871
CHB	DEFA1	6971766	13.1963	21.3463	-1.1898
CHB	DEFA1	6976766	17.6155	23.5487	-0.7877
CHB	DEFA1	6981766	25.4868	30.6641	-0.5317
CHB	DEFA1	6986766	29.2286	29.6476	-0.0445
CHB	DEFA1	6991766	24.6391	22.3628	0.3177
CHB	DEFA1	6996766	26.5907	25.7511	0.1022
CHB	DEFA1	7001766	29.901	29.3088	0.0636
CHB	DEFA1	7006766	35.0934	29.1394	0.6427
CHB	DEFA1	7011766	28.0509	22.871	0.7074
CHB	DEFA1	7016766	10.2081	12.5367	-0.5653
CHB	DEFA1	7021766	8.554	9.6567	-0.3419
CHB	DEFA1	7026766	11.8142	8.6402	1.091
CHB	DEFA1	7031766	8.5062	7.4543	0.4141
CHB	DEFA1	7036766	3.9478	5.9295	-0.9594
CHB	DEFA1	7041766	5.7476	8.1319	-0.8666
CHB	DEFA1	7046766	8.1022	8.3013	-0.071

CHB	DEFA1	7051766	9.5566	8.6402	0.315
CHB	DEFA1	7056766	14.582	11.3508	0.8614
CHB	DEFA1	7061766	17.3069	12.7061	1.1029
CHB	DEFA1	7066766	9.2303	8.4708	0.2659
CHB	DEFA1	7071766	2.0642	3.0495	-0.8474
CHB	DEFA1	7076766	4.4586	7.6237	-1.2205
CHB	DEFA1	7081766	7.4656	8.6402	-0.4038
CHB	DEFA1	7086766	4.9456	3.5577	1.0489
CHB	DEFA1	7091766	0.8643	0.6777	0.4878
CHB	DEFA1	7096766	0.3859	0.1694	1.2865
CHB	DEFA1	7101766	0.446	0.5082	-0.1953
CHB	DEFA1	7106766	2.6326	2.7106	-0.0739
CHB	DEFA1	7111766	2.1866	2.2024	-0.0177
CHB	DEFA1	7116766	0	0	0
CHB	DEFA1	7121766	0	0	0
CHB	DEFA1	7126766	1.2524	1.3553	-0.1668
CHB	DEFA1	7131766	1.2621	1.5247	-0.3901
CHB	DEFA1	7136766	0.0097	0.1694	-0.949
CHB	DEFA1	7141766	0.0659	0.1694	-0.615
CHB	DEFA1	7146766	0.7006	0.6777	0.0599
CHB	DEFA1	7151766	7.1365	15.7556	-1.6844
CHB	DEFA1	7156766	7.1715	16.9415	-1.7813
CHB	DEFA1	7161766	0.7756	2.8801	-1.8974
CHB	DEFA1	7166766	0.4205	3.2189	-2.3007
CHB	DEFA1	7171766	0.5635	3.8965	-2.3312
CHB	DEFA1	7176766	0.518	4.066	-2.3925
CHB	DEFA1	7181766	0.7586	3.0495	-1.9702
CHB	DEFA1	7186766	0.8707	4.2354	-2.1904
CHB	DEFA1	7191766	2.0182	7.4543	-2.1398
CHB	DEFA1	7196766	3.1391	8.3013	-1.8411
CHB	DEFA1	7201766	3.5368	11.3508	-2.0832
CHB	DEFA1	7206766	3.5753	12.1979	-2.1483
CHB	DEFA1	7211766	4.2098	9.1484	-1.6102
CHB	DEFA1	7216766	4.9966	9.9955	-1.5011
CHB	DEFA1	7221766	3.9084	10.6731	-1.9106
CHB	DEFA1	7226766	3.9254	6.4378	-1.1297
CHB	DEFA1	7231766	2.897	2.3718	0.5544
CHB	DEFA1	7236766	2.4702	5.7601	-1.6345
CHB	DEFA1	7241766	4.0086	8.8096	-1.6209
CHB	DEFA1	7246766	3.142	7.6237	-1.7283
CHB	DEFA1	7251766	3.9823	7.9625	-1.4749
CHB	DEFA1	7256766	3.8083	4.913	-0.6319
CHB	DEFA1	7261766	4.3063	4.7436	-0.258
CHB	DEFA1	7266766	5.098	5.7601	-0.3289
CHB	DEFA1	7271766	2.7915	6.6072	-1.6759
CHB	DEFA1	7276766	1.8995	7.6237	-2.2074
CHB	DEFA1	7281766	1.1215	4.066	-1.9856
CHB	DEFA1	7286766	0.8787	2.2024	-1.4822
CHB	DEFA1	7291766	2.6849	2.5412	0.1435

CHB	DEFA1	7296766	2.1801	2.033	0.1754
CHB	DEFA1	7301766	0.1249	1.1859	-1.8939
CHB	DEFA1	7306766	0.8469	2.033	-1.4144
CHB	DEFA1	7311766	0.8934	2.033	-1.359
CHB	DEFA1	7316766	0.1808	1.1859	-1.7941
CHB	DEFA1	7321766	0.3259	0.8471	-1.1753
CHB	DEFA1	7326766	1.665	1.8636	-0.2534
CHB	DEFA1	7331766	2.333	4.4048	-1.3037
CHB	DEFA1	7336766	1.9446	3.7271	-1.2949
CHB	DEFA1	7341766	0.999	1.0165	-0.0348
CHB	DEFA1	7346766	0.6632	1.1859	-0.933
CHB	DEFA1	7351766	1.3126	3.0495	-1.4937
CHB	DEFA1	7356766	0.831	2.5412	-1.7078
CHB	DEFA1	7361766	0.8479	1.3553	-0.8223
CHB	DEFA1	7366766	1.898	2.8801	-0.8855
CHB	DEFA1	7371766	2.6208	3.3883	-0.6044
CHB	DEFA1	7376766	1.3988	1.3553	0.0705
CHB	DEFA1	7381766	1.0529	0.8471	0.4641
CHB	DEFA1	7386766	1.6688	1.6942	-0.0349
CHB	DEFA1	7391766	0.7122	1.8636	-1.469
CHB	DEFA1	7396766	0.106	1.1859	-1.9276
CHB	DEFA1	7401766	1.9393	2.2024	-0.2946
CHB	DEFA1	7406766	1.9296	2.033	-0.1233
CHB	DEFA1	7411766	0	0	0
CHB	DEFA1	7416766	0	0	0
CHB	DEFA1	7421766	0	0	0
CHB	DEFA1	7426766	0.0097	0.1694	-0.949
CHB	DEFA1	7431766	0.0097	0.1694	-0.949
CHB	DEFA1	7436766	0.134	0.1694	-0.2104
CHB	DEFA1	7441766	0.6669	1.0165	-0.6957
CHB	DEFA1	7446766	1.8987	2.2024	-0.3401
CHB	DEFA1	7451766	1.9585	2.3718	-0.4363
CHB	DEFA1	7456766	0.5926	1.0165	-0.8436
CHB	DEFA1	7461766	0.2178	1.3553	-1.8435
CHB	DEFA1	7466766	1.0417	2.7106	-1.5814
CHB	DEFA1	7471766	1.347	1.6942	-0.4765
CHB	DEFA1	7476766	0.5231	0.3388	0.7393
CHB	DEFA1	7481766	0	0	0
CHB	DEFA1	7486766	0	0	0
CHB	DEFA1	7491766	0	0	0
YRI	DEFA1	6501766	10.988	20.1675	-1.4108
YRI	DEFA1	6506766	15.4367	26.0496	-1.2733
YRI	DEFA1	6511766	21.5507	33.4444	-1.1185
YRI	DEFA1	6516766	25.0959	31.4276	-0.6327
YRI	DEFA1	6521766	22.9545	25.3774	-0.2982
YRI	DEFA1	6526766	18.3587	22.5203	-0.575
YRI	DEFA1	6531766	21.7015	27.3941	-0.6504
YRI	DEFA1	6536766	20.089	28.4025	-0.9169
YRI	DEFA1	6541766	13.2122	23.6968	-1.3789

YRI	DEFA1	6546766	13.0577	26.3858	-1.5792
YRI	DEFA1	6551766	14.5317	26.5538	-1.4157
YRI	DEFA1	6556766	18.5035	23.1926	-0.6297
YRI	DEFA1	6561766	22.0661	24.0329	-0.2552
YRI	DEFA1	6566766	20.9048	25.8816	-0.6008
YRI	DEFA1	6571766	20.5299	29.747	-0.9718
YRI	DEFA1	6576766	20.5954	31.2596	-1.0713
YRI	DEFA1	6581766	18.794	27.2261	-0.9691
YRI	DEFA1	6586766	13.535	21.1758	-1.1203
YRI	DEFA1	6591766	13.8667	20.5036	-1.0039
YRI	DEFA1	6596766	14.2136	21.512	-1.054
YRI	DEFA1	6601766	11.4079	19.9994	-1.3311
YRI	DEFA1	6606766	13.6481	19.8313	-0.9658
YRI	DEFA1	6611766	13.7903	19.6633	-0.9249
YRI	DEFA1	6616766	12.6475	18.991	-1.033
YRI	DEFA1	6621766	13.3396	20.1675	-1.0494
YRI	DEFA1	6626766	19.0719	28.2345	-1.0164
YRI	DEFA1	6631766	35.0368	37.1417	-0.1786
YRI	DEFA1	6636766	44.6732	34.7889	0.8944
YRI	DEFA1	6641766	34.3911	26.7219	0.8976
YRI	DEFA1	6646766	28.0068	26.5538	0.1711
YRI	DEFA1	6651766	20.5219	23.0245	-0.3384
YRI	DEFA1	6656766	14.396	20.5036	-0.9238
YRI	DEFA1	6661766	24.8476	26.2177	-0.1634
YRI	DEFA1	6666766	20.6816	23.5287	-0.377
YRI	DEFA1	6671766	12.6433	21.68	-1.2952
YRI	DEFA1	6676766	16.9157	22.8565	-0.8091
YRI	DEFA1	6681766	18.2994	22.6884	-0.602
YRI	DEFA1	6686766	22.0286	26.2177	-0.4994
YRI	DEFA1	6691766	30.1861	33.1083	-0.2775
YRI	DEFA1	6696766	32.6373	34.7889	-0.1947
YRI	DEFA1	6701766	26.2831	31.5957	-0.5281
YRI	DEFA1	6706766	27.5403	36.1334	-0.7492
YRI	DEFA1	6711766	45.8215	43.0239	0.2055
YRI	DEFA1	6716766	51.5717	42.1836	0.7033
YRI	DEFA1	6721766	46.7506	41.0072	0.4423
YRI	DEFA1	6726766	41.4846	42.5197	-0.0769
YRI	DEFA1	6731766	32.7927	40.6711	-0.6117
YRI	DEFA1	6736766	34.3429	36.9737	-0.2243
YRI	DEFA1	6741766	31.0376	33.6124	-0.241
YRI	DEFA1	6746766	21.7786	33.1083	-1.076
YRI	DEFA1	6751766	20.6488	30.5873	-1.0198
YRI	DEFA1	6756766	32.9123	33.1083	-0.0186
YRI	DEFA1	6761766	52.9746	41.8475	0.8401
YRI	DEFA1	6766766	40.0197	35.2931	0.4217
YRI	DEFA1	6771766	15.8247	24.5371	-1.1077
YRI	DEFA1	6776766	16.5618	25.0413	-1.0571
YRI	DEFA1	6781766	18.4352	25.7135	-0.8843
YRI	DEFA1	6786766	21.4649	25.8816	-0.5332

YRI	DEFA1	6791766	22.8998	27.058	-0.4808
YRI	DEFA1	6796766	16.8463	26.7219	-1.1558
YRI	DEFA1	6801766	13.3183	24.369	-1.4144
YRI	DEFA1	6806766	14.0523	22.6884	-1.1846
YRI	DEFA1	6811766	15.0406	24.201	-1.1804
YRI	DEFA1	6816766	16.8337	24.0329	-0.934
YRI	DEFA1	6821766	22.3846	27.7303	-0.6035
YRI	DEFA1	6826766	24.3358	27.8983	-0.3998
YRI	DEFA1	6831766	11.5238	12.2685	-0.1839
YRI	DEFA1	6836766	5.1313	5.8822	-0.3649
YRI	DEFA1	6841766	6.3125	6.5544	-0.1066
YRI	DEFA1	6846766	3.4277	3.0251	0.3473
YRI	DEFA1	6851766	1.8816	2.1848	-0.3404
YRI	DEFA1	6856766	3.9575	5.2099	-0.6779
YRI	DEFA1	6861766	4.7219	5.5461	-0.4221
YRI	DEFA1	6866766	1.7998	1.6806	0.164
YRI	DEFA1	6871766	6.1326	8.235	-0.7528
YRI	DEFA1	6876766	17.2471	23.8648	-0.8644
YRI	DEFA1	6881766	17.8149	27.3941	-1.0944
YRI	DEFA1	6886766	13.6625	23.1926	-1.2797
YRI	DEFA1	6891766	28.265	30.4193	-0.2222
YRI	DEFA1	6896766	42.9058	36.9737	0.5057
YRI	DEFA1	6901766	39.2234	33.7805	0.5069
YRI	DEFA1	6906766	31.0789	30.5873	0.0504
YRI	DEFA1	6911766	19.2356	24.5371	-0.6741
YRI	DEFA1	6916766	10.5634	19.3272	-1.4033
YRI	DEFA1	6921766	16.4294	27.3941	-1.2527
YRI	DEFA1	6926766	25.1268	34.9569	-0.8853
YRI	DEFA1	6931766	23.8214	34.1166	-0.9495
YRI	DEFA1	6936766	22.163	35.6292	-1.1904
YRI	DEFA1	6941766	22.4908	35.9653	-1.1802
YRI	DEFA1	6946766	30.6481	40.8391	-0.7881
YRI	DEFA1	6951766	35.1211	43.3601	-0.6007
YRI	DEFA1	6956766	26.7518	36.9737	-0.8714
YRI	DEFA1	6961766	22.6198	31.5957	-0.8923
YRI	DEFA1	6966766	18.028	28.9067	-1.1795
YRI	DEFA1	6971766	17.462	32.2679	-1.4419
YRI	DEFA1	6976766	24.1084	32.2679	-0.7947
YRI	DEFA1	6981766	30.0002	38.8224	-0.717
YRI	DEFA1	6986766	27.6224	39.9988	-0.9768
YRI	DEFA1	6991766	22.4547	30.2512	-0.8087
YRI	DEFA1	6996766	27.3478	35.125	-0.6971
YRI	DEFA1	7001766	36.0627	39.6627	-0.2865
YRI	DEFA1	7006766	41.5664	39.3266	0.1797
YRI	DEFA1	7011766	30.641	28.9067	0.188
YRI	DEFA1	7016766	14.1804	16.134	-0.372
YRI	DEFA1	7021766	13.1128	14.4534	-0.2835
YRI	DEFA1	7026766	13.5294	13.7811	-0.0557
YRI	DEFA1	7031766	11.4194	13.445	-0.4587

YRI	DEFA1	7036766	7.7202	9.2434	-0.4902
YRI	DEFA1	7041766	8.9617	11.0921	-0.5784
YRI	DEFA1	7046766	11.6805	12.6047	-0.2225
YRI	DEFA1	7051766	13.5603	14.9575	-0.2859
YRI	DEFA1	7056766	20.6814	20.1675	0.079
YRI	DEFA1	7061766	22.3043	18.3188	0.6719
YRI	DEFA1	7066766	11.0479	9.5795	0.4572
YRI	DEFA1	7071766	2.5808	3.6974	-0.8139
YRI	DEFA1	7076766	4.3288	7.2267	-1.1694
YRI	DEFA1	7081766	6.4872	7.7309	-0.4719
YRI	DEFA1	7086766	4.4656	4.3696	0.0606
YRI	DEFA1	7091766	0.9667	1.5126	-0.8129
YRI	DEFA1	7096766	0.2903	1.0084	-1.4317
YRI	DEFA1	7101766	0.764	2.3529	-1.6819
YRI	DEFA1	7106766	2.2594	5.8822	-1.7604
YRI	DEFA1	7111766	1.6968	4.3696	-1.6877
YRI	DEFA1	7116766	0	0	0
YRI	DEFA1	7121766	0	0	0
YRI	DEFA1	7126766	1.6231	1.5126	0.1645
YRI	DEFA1	7131766	1.6783	1.8487	-0.218
YRI	DEFA1	7136766	0.0552	0.3361	-1.1277
YRI	DEFA1	7141766	0.1681	0.1681	0
YRI	DEFA1	7146766	1.6615	1.0084	1.3021
YRI	DEFA1	7151766	11.175	18.823	-1.2561
YRI	DEFA1	7156766	10.8409	20.8397	-1.4889
YRI	DEFA1	7161766	3.4242	5.0419	-0.9013
YRI	DEFA1	7166766	5.0215	6.5544	-0.6758
YRI	DEFA1	7171766	4.1957	7.3947	-1.2641
YRI	DEFA1	7176766	3.6383	6.2183	-1.1927
YRI	DEFA1	7181766	2.6581	4.7057	-1.2121
YRI	DEFA1	7186766	4.1205	8.4031	-1.5052
YRI	DEFA1	7191766	7.1548	11.5963	-1.1566
YRI	DEFA1	7196766	10.6021	11.2602	-0.1762
YRI	DEFA1	7201766	13.0225	15.9659	-0.5661
YRI	DEFA1	7206766	9.9641	16.9743	-1.2715
YRI	DEFA1	7211766	8.8005	15.2937	-1.301
YRI	DEFA1	7216766	8.2451	15.6298	-1.4493
YRI	DEFA1	7221766	7.7904	17.4785	-1.7086
YRI	DEFA1	7226766	7.7811	14.4534	-1.4108
YRI	DEFA1	7231766	4.1423	6.7225	-1.1118
YRI	DEFA1	7236766	4.1936	8.4031	-1.4795
YRI	DEFA1	7241766	7.0628	14.1172	-1.5254
YRI	DEFA1	7246766	5.2253	13.2769	-1.8453
YRI	DEFA1	7251766	5.8043	13.2769	-1.7126
YRI	DEFA1	7256766	5.5512	9.0754	-1.1536
YRI	DEFA1	7261766	4.718	5.8822	-0.5657
YRI	DEFA1	7266766	4.8547	7.0586	-0.9086
YRI	DEFA1	7271766	3.6422	10.756	-1.9879
YRI	DEFA1	7276766	3.5357	11.7644	-2.114

YRI	DEFA1	7281766	2.7098	7.0586	-1.7928
YRI	DEFA1	7286766	2.3109	4.7057	-1.4176
YRI	DEFA1	7291766	2.8648	3.6974	-0.6069
YRI	DEFA1	7296766	2.5311	5.2099	-1.45
YRI	DEFA1	7301766	1.1443	6.0502	-2.3244
YRI	DEFA1	7306766	1.5523	6.3864	-2.1818
YRI	DEFA1	7311766	1.5904	4.7057	-1.8441
YRI	DEFA1	7316766	0.4846	2.0167	-1.8319
YRI	DEFA1	7321766	0.651	1.1764	-0.9397
YRI	DEFA1	7326766	2.533	3.1932	-0.5445
YRI	DEFA1	7331766	3.6308	8.067	-1.6188
YRI	DEFA1	7336766	2.6132	7.7309	-1.9418
YRI	DEFA1	7341766	1.0648	3.1932	-1.7553
YRI	DEFA1	7346766	1.035	1.8487	-1.0408
YRI	DEFA1	7351766	2.9094	4.0335	-0.7606
YRI	DEFA1	7356766	2.456	3.8654	-0.9891
YRI	DEFA1	7361766	1.213	1.6806	-0.6433
YRI	DEFA1	7366766	2.6877	3.1932	-0.4169
YRI	DEFA1	7371766	4.355	4.0335	0.2175
YRI	DEFA1	7376766	2.4528	1.8487	0.7727
YRI	DEFA1	7381766	0.6447	1.0084	-0.7251
YRI	DEFA1	7386766	1.3384	2.1848	-0.9503
YRI	DEFA1	7391766	0.9043	2.8571	-1.766
YRI	DEFA1	7396766	0.2997	1.8487	-1.9813
YRI	DEFA1	7401766	2.0421	6.5544	-1.9894
YRI	DEFA1	7406766	1.953	6.3864	-2.0009
YRI	DEFA1	7411766	0	0	0
YRI	DEFA1	7416766	0	0	0
YRI	DEFA1	7421766	0	0	0
YRI	DEFA1	7426766	0.0914	0.6722	-1.5205
YRI	DEFA1	7431766	0.1833	1.6806	-2.0598
YRI	DEFA1	7436766	0.2261	1.8487	-2.0755
YRI	DEFA1	7441766	0.808	2.0167	-1.4452
YRI	DEFA1	7446766	1.7621	3.0251	-1.0895
YRI	DEFA1	7451766	2.7455	3.3612	-0.4864
YRI	DEFA1	7456766	1.7125	2.1848	-0.5303
YRI	DEFA1	7461766	1.4165	3.3612	-1.5364
YRI	DEFA1	7466766	2.7501	4.2016	-0.9482
YRI	DEFA1	7471766	1.7805	2.1848	-0.4539
YRI	DEFA1	7476766	0.3916	0.6722	-0.7346
YRI	DEFA1	7481766	0	0	0
YRI	DEFA1	7486766	0	0	0
YRI	DEFA1	7491766	0	0	0
CEU	DEFB130	11569389	4.496	6.6519	-0.9439
CEU	DEFB130	11574389	3.9458	6.8225	-1.2309
CEU	DEFB130	11579389	6.7642	8.8692	-0.7083
CEU	DEFB130	11584389	10.2406	9.7221	0.1602
CEU	DEFB130	11589389	10.7904	9.0398	0.5788
CEU	DEFB130	11594389	10.3581	8.5281	0.6385

CEU	DEFB130	11599389	10.4705	8.8692	0.5388
CEU	DEFB130	11604389	14.1691	10.2337	1.1594
CEU	DEFB130	11609389	22.335	14.1567	1.7748
CEU	DEFB130	11614389	24.7462	16.7151	1.4875
CEU	DEFB130	11619389	20.1043	15.3506	0.9551
CEU	DEFB130	11624389	20.8586	15.3506	1.1066
CEU	DEFB130	11629389	20.1111	15.6917	0.8695
CEU	DEFB130	11634389	13.5336	15.0095	-0.3029
CEU	DEFB130	11639389	11.6649	14.1567	-0.5407
CEU	DEFB130	11644389	19.5271	15.8623	0.7136
CEU	DEFB130	11649389	26.4897	22.5142	0.5528
CEU	DEFB130	11654389	24.5346	23.8787	0.0862
CEU	DEFB130	11659389	19.6316	19.103	0.0861
CEU	DEFB130	11664389	20.4554	19.103	0.2204
CEU	DEFB130	11669389	21.1022	20.1264	0.1512
CEU	DEFB130	11674389	21.1274	19.2736	0.2995
CEU	DEFB130	11679389	20.7046	19.4441	0.2019
CEU	DEFB130	11684389	19.5426	19.7852	-0.0382
CEU	DEFB130	11689389	20.0198	18.0796	0.3333
CEU	DEFB130	11694389	15.5743	16.0329	-0.0884
CEU	DEFB130	11699389	20.5939	19.6147	0.1555
CEU	DEFB130	11704389	28.6578	22.8554	0.7952
CEU	DEFB130	11709389	23.8287	18.7619	0.84
CEU	DEFB130	11714389	12.1323	11.7688	0.0939
CEU	DEFB130	11719389	8.2245	10.4043	-0.6323
CEU	DEFB130	11724389	8.9751	11.5982	-0.6873
CEU	DEFB130	11729389	10.8458	12.9627	-0.4994
CEU	DEFB130	11734389	18.1093	15.5212	0.5145
CEU	DEFB130	11739389	19.6731	14.4978	1.098
CEU	DEFB130	11744389	21.6909	16.374	1.0044
CEU	DEFB130	11749389	23.9679	15.6917	1.6283
CEU	DEFB130	11754389	17.2061	10.7454	1.8185
CEU	DEFB130	11759389	8.4168	9.5515	-0.3565
CEU	DEFB130	11764389	24.222	17.0562	1.3018
CEU	DEFB130	11769389	43.1055	24.0493	2.4859
CEU	DEFB130	11774389	32.9464	24.0493	1.1606
CEU	DEFB130	11779389	43.6205	29.8484	1.4563
CEU	DEFB130	11784389	64.1099	33.9419	2.8137
CEU	DEFB130	11789389	74.2839	37.6943	3.0797
CEU	DEFB130	11794389	65.2346	34.1125	2.8885
CEU	DEFB130	11799389	31.4016	20.8086	1.5895
CEU	DEFB130	11804389	16.677	15.0095	0.3423
CEU	DEFB130	11809389	17.5308	13.645	0.8732
CEU	DEFB130	11814389	21.8076	14.6684	1.4978
CEU	DEFB130	11819389	21.775	13.4744	1.8877
CEU	DEFB130	11824389	17.3327	12.1099	1.3139
CEU	DEFB130	11829389	16.3791	12.2805	1.0176
CEU	DEFB130	11834389	14.4879	11.5982	0.7571
CEU	DEFB130	11839389	9.4735	10.7454	-0.358

CEU	DEFB130	11844389	7.9878	8.5281	-0.1885
CEU	DEFB130	11849389	11.7442	8.8692	0.9674
CEU	DEFB130	11854389	13.1785	10.916	0.6275
CEU	DEFB130	11859389	11.2329	11.5982	-0.0957
CEU	DEFB130	11864389	9.0635	11.9394	-0.7332
CEU	DEFB130	11869389	20.1284	19.103	0.1671
CEU	DEFB130	11874389	21.7817	20.2969	0.2282
CEU	DEFB130	11879389	12.8231	16.2034	-0.645
CEU	DEFB130	11884389	13.8569	17.7385	-0.6791
CEU	DEFB130	11889389	22.4224	18.4207	0.6752
CEU	DEFB130	11894389	28.0305	16.2034	2.2567
CEU	DEFB130	11899389	12.4545	6.8225	2.4099
CEU	DEFB130	11904389	2.1492	1.8762	0.3476
CEU	DEFB130	11909389	3.5331	2.5584	0.971
CEU	DEFB130	11914389	2.7458	2.0467	0.8319
CEU	DEFB130	11919389	2.1664	1.7056	0.6311
CEU	DEFB130	11924389	1.267	0.8528	0.9328
CEU	DEFB130	11929389	1.7518	1.1939	0.9942
CEU	DEFB130	11934389	2.4566	1.5351	1.3664
CEU	DEFB130	11939389	1.1674	0.8528	0.7085
CEU	DEFB130	11944389	2.7899	2.0467	0.8843
CEU	DEFB130	11949389	6.1453	3.7524	1.7336
CEU	DEFB130	11954389	3.981	2.2173	1.9704
CEU	DEFB130	11959389	1.0196	0.8528	0.3756
CEU	DEFB130	11964389	0.5026	1.3645	-1.3944
CEU	DEFB130	11969389	1.5861	1.8762	-0.3693
CEU	DEFB130	11974389	9.6054	5.9697	1.7546
CEU	DEFB130	11979389	12.6685	9.3809	1.0503
CEU	DEFB130	11984389	11.2707	12.4511	-0.2893
CEU	DEFB130	11989389	18.5784	20.6381	-0.3115
CEU	DEFB130	11994389	27.4574	24.7316	0.3461
CEU	DEFB130	11999389	25.4329	19.4441	0.9593
CEU	DEFB130	12004389	13.9796	12.6216	0.3285
CEU	DEFB130	12009389	5.765	6.6519	-0.3883
CEU	DEFB130	12014389	2.2195	2.3879	-0.1773
CEU	DEFB130	12019389	0.872	1.5351	-0.9832
CEU	DEFB130	12024389	0.342	1.1939	-1.5181
CEU	DEFB130	12029389	3.8075	4.2641	-0.2964
CEU	DEFB130	12034389	10.7085	10.7454	-0.0104
CEU	DEFB130	12039389	9.3148	9.5515	-0.0744
CEU	DEFB130	12044389	2.5955	3.7524	-0.8381
CEU	DEFB130	12049389	1.5358	2.729	-1.1278
CEU	DEFB130	12054389	2.6514	2.8996	-0.2232
CEU	DEFB130	12059389	2.9569	2.5584	0.397
CEU	DEFB130	12064389	2.4807	2.3879	0.0977
CEU	DEFB130	12069389	3.4768	3.4112	0.0515
CEU	DEFB130	12074389	4.3729	3.4112	0.7553
CEU	DEFB130	12079389	3.1754	2.8996	0.248
CEU	DEFB130	12084389	8.3624	9.7221	-0.4202

CEU	DEFB130	12089389	7.2645	9.5515	-0.7185
CEU	DEFB130	12094389	0.2797	1.3645	-1.755
CEU	DEFB130	12099389	0	0	0
CEU	DEFB130	12104389	0	0	0
CEU	DEFB130	12109389	0	0	0
CEU	DEFB130	12114389	0	0	0
CEU	DEFB130	12119389	0	0	0
CEU	DEFB130	12124389	0	0	0
CEU	DEFB130	12129389	0	0	0
CEU	DEFB130	12134389	0	0	0
CEU	DEFB130	12139389	3.059	1.5351	2.2596
CEU	DEFB130	12144389	8.2109	4.6052	2.1892
CEU	DEFB130	12149389	5.3826	3.9229	1.0181
CEU	DEFB130	12154389	0.7986	2.3879	-1.6737
CEU	DEFB130	12159389	0.5679	1.5351	-1.4342
CEU	DEFB130	12164389	4.4679	1.8762	3.2997
CEU	DEFB130	12169389	5.3816	2.3879	3.1527
CEU	DEFB130	12174389	4.7535	4.2641	0.3177
CEU	DEFB130	12179389	13.2933	9.8926	1.034
CEU	DEFB130	12184389	16.0138	10.4043	1.6273
CEU	DEFB130	12189389	8.6479	6.4814	0.9711
CEU	DEFB130	12194389	3.5156	3.7524	-0.1716
CEU	DEFB130	12199389	3.8905	3.0701	0.7037
CEU	DEFB130	12204389	2.9287	2.3879	0.5695
CEU	DEFB130	12209389	0.8231	1.3645	-0.8759
CEU	DEFB130	12214389	2.4898	3.0701	-0.4978
CEU	DEFB130	12219389	2.7972	3.5818	-0.5914
CEU	DEFB130	12224389	3.9126	3.7524	0.1161
CEU	DEFB130	12229389	12.4997	9.3809	0.9963
CEU	DEFB130	12234389	24.8153	19.6147	0.8261
CEU	DEFB130	12239389	26.476	24.0493	0.3166
CEU	DEFB130	12244389	19.899	18.7619	0.1885
CEU	DEFB130	12249389	10.2503	9.0398	0.4002
CEU	DEFB130	12254389	1.9705	2.3879	-0.4396
CEU	DEFB130	12259389	2.0019	2.5584	-0.5544
CEU	DEFB130	12264389	1.335	2.0467	-0.8468
CEU	DEFB130	12269389	1.1831	2.8996	-1.5437
CEU	DEFB130	12274389	6.8343	9.2104	-0.7721
CEU	DEFB130	12279389	7.852	9.2104	-0.4414
CEU	DEFB130	12284389	7.2552	7.5047	-0.0979
CEU	DEFB130	12289389	7.7706	7.3342	0.1749
CEU	DEFB130	12294389	4.5951	3.5818	0.7637
CEU	DEFB130	12299389	3.5055	2.729	0.734
CEU	DEFB130	12304389	5.4954	3.9229	1.0968
CEU	DEFB130	12309389	5.3076	4.2641	0.6774
CEU	DEFB130	12314389	1.792	2.8996	-0.9961
CEU	DEFB130	12319389	0.6999	2.2173	-1.6952
CEU	DEFB130	12324389	0.3463	1.3645	-1.6472
CEU	DEFB130	12329389	0.6446	0.8528	-0.4689

CEU	DEFB130	12334389	1.3783	1.3645	0.0223
CEU	DEFB130	12339389	6.9297	4.6052	1.4113
CEU	DEFB130	12344389	7.1637	5.6286	0.7808
CEU	DEFB130	12349389	1.7592	2.8996	-1.0256
CEU	DEFB130	12354389	0.9118	1.5351	-0.9242
CEU	DEFB130	12359389	0.7681	1.7056	-1.284
CEU	DEFB130	12364389	0.6679	1.8762	-1.5384
CEU	DEFB130	12369389	0.4783	2.0467	-1.8662
CEU	DEFB130	12374389	2.3315	2.8996	-0.5109
CEU	DEFB130	12379389	3.8479	3.7524	0.0692
CEU	DEFB130	12384389	2.6671	3.4112	-0.5844
CEU	DEFB130	12389389	1.3054	1.3645	-0.0956
CEU	DEFB130	12394389	6.0092	4.0935	1.2882
CEU	DEFB130	12399389	11.3886	7.5047	1.5239
CEU	DEFB130	12404389	10.0748	5.9697	1.9812
CEU	DEFB130	12409389	6.012	3.9229	1.4571
CEU	DEFB130	12414389	6.6056	4.4346	1.3622
CEU	DEFB130	12419389	8.8705	5.2874	1.9266
CEU	DEFB130	12424389	8.1748	6.6519	0.6668
CEU	DEFB130	12429389	8.8602	7.3342	0.6114
CEU	DEFB130	12434389	8.6289	6.9931	0.6844
CEU	DEFB130	12439389	6.031	5.9697	0.0296
CEU	DEFB130	12444389	4.2493	5.1169	-0.4802
CEU	DEFB130	12449389	5.5824	6.6519	-0.4682
CEU	DEFB130	12454389	6.0787	6.3108	-0.1066
CEU	DEFB130	12459389	2.6411	3.2407	-0.4917
CEU	DEFB130	12464389	3.894	4.2641	-0.2402
CEU	DEFB130	12469389	12.1556	11.5982	0.146
CEU	DEFB130	12474389	19.3221	17.2268	0.377
CEU	DEFB130	12479389	20.9638	20.8086	0.0233
CEU	DEFB130	12484389	18.9215	19.6147	-0.1101
CEU	DEFB130	12489389	23.8084	19.103	0.7667
CEU	DEFB130	12494389	23.8358	19.9558	0.6062
CEU	DEFB130	12499389	17.9798	17.2268	0.1355
CEU	DEFB130	12504389	19.3562	18.0796	0.2193
CEU	DEFB130	12509389	17.6776	16.374	0.2463
CEU	DEFB130	12514389	20.899	18.7619	0.3543
CEU	DEFB130	12519389	20.3211	21.4909	-0.1702
CEU	DEFB130	12524389	16.6099	17.3974	-0.1404
CEU	DEFB130	12529389	21.5121	18.5913	0.4885
CEU	DEFB130	12534389	24.9239	21.3203	0.5282
CEU	DEFB130	12539389	26.5483	19.2736	1.1753
CEU	DEFB130	12544389	21.9011	22.0026	-0.0144
CEU	DEFB130	12549389	15.8718	22.6848	-0.9405
CEU	DEFB130	12554389	11.7301	17.3974	-1.0102
CEU	DEFB130	12559389	6.6457	14.6684	-1.6832
CEU	DEFB130	12564389	6.1646	16.8857	-1.9665
CHB	DEFB130	11569389	3.8886	7.4543	-1.4036
CHB	DEFB130	11574389	2.5884	6.4378	-1.7308

CHB	DEFB130	11579389	5.2273	8.4708	-1.1355
CHB	DEFB130	11584389	9.0607	11.6896	-0.6818
CHB	DEFB130	11589389	8.446	10.1649	-0.5081
CHB	DEFB130	11594389	6.4434	9.9955	-1.0666
CHB	DEFB130	11599389	6.6616	12.7061	-1.449
CHB	DEFB130	11604389	10.2106	15.0779	-0.9919
CHB	DEFB130	11609389	16.8523	19.1439	-0.3716
CHB	DEFB130	11614389	19.9804	21.5157	-0.2224
CHB	DEFB130	11619389	17.4776	18.8051	-0.219
CHB	DEFB130	11624389	15.8791	17.7886	-0.3322
CHB	DEFB130	11629389	14.9054	16.4333	-0.2868
CHB	DEFB130	11634389	14.5128	15.2474	-0.1481
CHB	DEFB130	11639389	14.8961	16.2638	-0.2593
CHB	DEFB130	11644389	20.9908	19.6521	0.2117
CHB	DEFB130	11649389	23.7928	23.0404	0.102
CHB	DEFB130	11654389	19.8161	21.6851	-0.2687
CHB	DEFB130	11659389	17.7424	20.4992	-0.4185
CHB	DEFB130	11664389	19.9027	21.6851	-0.2563
CHB	DEFB130	11669389	15.5802	22.1934	-0.9298
CHB	DEFB130	11674389	10.6486	22.1934	-1.6232
CHB	DEFB130	11679389	12.4627	22.3628	-1.3817
CHB	DEFB130	11684389	12.887	22.7016	-1.35
CHB	DEFB130	11689389	15.0784	21.1769	-0.8972
CHB	DEFB130	11694389	12.5003	20.8381	-1.2459
CHB	DEFB130	11699389	12.2075	25.5817	-1.6385
CHB	DEFB130	11704389	20.1614	28.1229	-0.8896
CHB	DEFB130	11709389	16.3463	22.871	-0.8911
CHB	DEFB130	11714389	6.1748	14.4003	-1.7513
CHB	DEFB130	11719389	7.2909	10.8426	-0.9885
CHB	DEFB130	11724389	8.6067	10.3343	-0.5029
CHB	DEFB130	11729389	11.0617	10.6731	0.1098
CHB	DEFB130	11734389	16.2294	12.1979	1.0044
CHB	DEFB130	11739389	15.0933	13.045	0.479
CHB	DEFB130	11744389	17.3394	16.6027	0.1369
CHB	DEFB130	11749389	21.6238	17.2803	0.777
CHB	DEFB130	11754389	13.2686	13.3838	-0.0263
CHB	DEFB130	11759389	4.9261	12.1979	-1.8117
CHB	DEFB130	11764389	23.3607	19.4827	0.6183
CHB	DEFB130	11769389	40.9284	25.7511	1.8476
CHB	DEFB130	11774389	26.3855	26.7676	-0.0448
CHB	DEFB130	11779389	11.3147	33.3748	-2.0858
CHB	DEFB130	11784389	12.3762	35.5772	-2.0607
CHB	DEFB130	11789389	15.4852	38.1184	-1.8789
CHB	DEFB130	11794389	11.877	34.3913	-2.0672
CHB	DEFB130	11799389	6.34	20.6686	-2.1579
CHB	DEFB130	11804389	4.4055	15.925	-2.2283
CHB	DEFB130	11809389	8.4991	15.2474	-1.3607
CHB	DEFB130	11814389	18.3344	16.9415	0.254
CHB	DEFB130	11819389	20.1777	16.6027	0.6645

CHB	DEFB130	11824389	14.617	15.5862	-0.1914
CHB	DEFB130	11829389	10.0042	16.9415	-1.2648
CHB	DEFB130	11834389	6.3345	15.925	-1.8552
CHB	DEFB130	11839389	4.0432	14.0615	-2.1818
CHB	DEFB130	11844389	3.314	12.5367	-2.2391
CHB	DEFB130	11849389	4.7568	11.6896	-1.7979
CHB	DEFB130	11854389	5.2352	13.2144	-1.8432
CHB	DEFB130	11859389	4.6961	16.6027	-2.2132
CHB	DEFB130	11864389	4.5724	18.8051	-2.3477
CHB	DEFB130	11869389	7.8025	26.4288	-2.2109
CHB	DEFB130	11874389	7.3134	26.2593	-2.2629
CHB	DEFB130	11879389	4.329	24.3958	-2.5744
CHB	DEFB130	11884389	5.3883	27.9535	-2.5362
CHB	DEFB130	11889389	6.4212	28.2923	-2.4296
CHB	DEFB130	11894389	5.441	23.8875	-2.4153
CHB	DEFB130	11899389	2.119	9.1484	-2.2919
CHB	DEFB130	11904389	0.7601	4.2354	-2.2624
CHB	DEFB130	11909389	1.202	5.7601	-2.2646
CHB	DEFB130	11914389	1.3263	5.7601	-2.2028
CHB	DEFB130	11919389	1.3529	7.2848	-2.3844
CHB	DEFB130	11924389	0.6408	4.4048	-2.3685
CHB	DEFB130	11929389	0.8745	2.8801	-1.8083
CHB	DEFB130	11934389	1.2545	2.3718	-1.1794
CHB	DEFB130	11939389	0.533	0.6777	-0.3783
CHB	DEFB130	11944389	1.5334	2.2024	-0.7491
CHB	DEFB130	11949389	5	3.5577	1.09
CHB	DEFB130	11954389	4.0259	1.8636	2.7588
CHB	DEFB130	11959389	1.0367	0.8471	0.4275
CHB	DEFB130	11964389	1.127	1.5247	-0.5908
CHB	DEFB130	11969389	2.0647	1.6942	0.5085
CHB	DEFB130	11974389	9.8964	5.2519	2.5049
CHB	DEFB130	11979389	12.8574	10.1649	0.7959
CHB	DEFB130	11984389	10.7843	14.7391	-0.8236
CHB	DEFB130	11989389	15.8131	21.1769	-0.7891
CHB	DEFB130	11994389	25.1272	25.5817	-0.0557
CHB	DEFB130	11999389	24.842	20.6686	0.6285
CHB	DEFB130	12004389	13.1393	12.0285	0.2804
CHB	DEFB130	12009389	5.1058	6.6072	-0.6594
CHB	DEFB130	12014389	2.0291	3.7271	-1.2336
CHB	DEFB130	12019389	1.0685	2.5412	-1.4706
CHB	DEFB130	12024389	0.174	0.8471	-1.5178
CHB	DEFB130	12029389	2.3371	6.0989	-1.7757
CHB	DEFB130	12034389	9.4108	11.1814	-0.4788
CHB	DEFB130	12039389	9.5795	8.979	0.1992
CHB	DEFB130	12044389	2.7896	4.4048	-1.0164
CHB	DEFB130	12049389	1.1148	2.3718	-1.3269
CHB	DEFB130	12054389	1.585	3.3883	-1.4201
CHB	DEFB130	12059389	2.4576	3.0495	-0.509
CHB	DEFB130	12064389	2.3251	1.8636	0.5888

CHB	DEFB130	12069389	2.8171	3.0495	-0.1999
CHB	DEFB130	12074389	4.4483	3.3883	0.8348
CHB	DEFB130	12079389	3.1397	2.2024	1.0495
CHB	DEFB130	12084389	3.1445	10.3343	-2.0928
CHB	DEFB130	12089389	2.7921	12.8755	-2.3872
CHB	DEFB130	12094389	0.5151	3.7271	-2.3334
CHB	DEFB130	12099389	0	0	0
CHB	DEFB130	12104389	0	0	0
CHB	DEFB130	12109389	0	0	0
CHB	DEFB130	12114389	0	0	0
CHB	DEFB130	12119389	0	0	0
CHB	DEFB130	12124389	0	0	0
CHB	DEFB130	12129389	0	0	0
CHB	DEFB130	12134389	0	0	0
CHB	DEFB130	12139389	0.3225	2.5412	-2.2155
CHB	DEFB130	12144389	1.4346	6.0989	-2.2018
CHB	DEFB130	12149389	1.2073	4.4048	-2.012
CHB	DEFB130	12154389	0.7464	1.8636	-1.4254
CHB	DEFB130	12159389	0.6804	1.5247	-1.2543
CHB	DEFB130	12164389	1.2745	4.2354	-1.9276
CHB	DEFB130	12169389	2.0025	4.7436	-1.6169
CHB	DEFB130	12174389	1.9577	6.7766	-2.0686
CHB	DEFB130	12179389	3.0156	14.5697	-2.4327
CHB	DEFB130	12184389	3.0853	13.892	-2.3807
CHB	DEFB130	12189389	1.7352	7.7931	-2.2896
CHB	DEFB130	12194389	1.3071	5.2519	-2.1276
CHB	DEFB130	12199389	1.3623	3.7271	-1.718
CHB	DEFB130	12204389	0.5395	1.5247	-1.4637
CHB	DEFB130	12209389	0.0485	0.6777	-1.6449
CHB	DEFB130	12214389	0.9134	2.3718	-1.5395
CHB	DEFB130	12219389	1.1317	3.5577	-1.8334
CHB	DEFB130	12224389	0.5176	4.2354	-2.4203
CHB	DEFB130	12229389	4.5957	9.9955	-1.6215
CHB	DEFB130	12234389	14.0725	18.4662	-0.7375
CHB	DEFB130	12239389	19.1938	22.1934	-0.4217
CHB	DEFB130	12244389	15.2895	18.6357	-0.5568
CHB	DEFB130	12249389	6.4434	9.8261	-1.0321
CHB	DEFB130	12254389	1.1152	2.7106	-1.5117
CHB	DEFB130	12259389	1.7655	2.033	-0.319
CHB	DEFB130	12264389	1.2509	1.5247	-0.4068
CHB	DEFB130	12269389	1.1843	2.2024	-1.14
CHB	DEFB130	12274389	5.824	8.1319	-0.8389
CHB	DEFB130	12279389	7.3391	8.6402	-0.4472
CHB	DEFB130	12284389	7.9374	7.2848	0.2623
CHB	DEFB130	12289389	6.5656	6.2684	0.1369
CHB	DEFB130	12294389	2.478	3.3883	-0.7169
CHB	DEFB130	12299389	2.4238	2.8801	-0.4114
CHB	DEFB130	12304389	4.8989	3.5577	1.0136
CHB	DEFB130	12309389	4.819	3.3883	1.1267

CHB	DEFB130	12314389	1.8058	1.3553	0.7301
CHB	DEFB130	12319389	0.8247	1.0165	-0.3817
CHB	DEFB130	12324389	0.3063	1.0165	-1.4133
CHB	DEFB130	12329389	0.624	1.1859	-1.003
CHB	DEFB130	12334389	1.3394	1.8636	-0.6688
CHB	DEFB130	12339389	6.6337	5.5907	0.5322
CHB	DEFB130	12344389	6.2779	6.2684	0.0044
CHB	DEFB130	12349389	1.5673	3.0495	-1.2747
CHB	DEFB130	12354389	1.2199	2.3718	-1.2159
CHB	DEFB130	12359389	0.7291	2.033	-1.5549
CHB	DEFB130	12364389	0.7729	1.5247	-1.1169
CHB	DEFB130	12369389	0.684	1.0165	-0.6617
CHB	DEFB130	12374389	2.3493	2.2024	0.1645
CHB	DEFB130	12379389	3.7639	3.3883	0.2958
CHB	DEFB130	12384389	2.4708	2.5412	-0.0703
CHB	DEFB130	12389389	1.2462	1.6942	-0.6148
CHB	DEFB130	12394389	5.59	5.0825	0.2818
CHB	DEFB130	12399389	10.5321	7.7931	1.0352
CHB	DEFB130	12404389	9.747	6.7766	1.2751
CHB	DEFB130	12409389	5.8629	5.0825	0.4333
CHB	DEFB130	12414389	6.3937	5.2519	0.6158
CHB	DEFB130	12419389	8.0624	6.4378	0.7305
CHB	DEFB130	12424389	7.4687	8.6402	-0.4027
CHB	DEFB130	12429389	8.8742	9.1484	-0.0894
CHB	DEFB130	12434389	8.8009	9.3178	-0.1657
CHB	DEFB130	12439389	6.2448	7.6237	-0.5317
CHB	DEFB130	12444389	4.2269	4.066	0.1085
CHB	DEFB130	12449389	6.0261	5.4213	0.3171
CHB	DEFB130	12454389	6.3488	6.2684	0.037
CHB	DEFB130	12459389	2.3994	3.5577	-0.8754
CHB	DEFB130	12464389	3.5431	4.7436	-0.7081
CHB	DEFB130	12469389	10.7342	13.045	-0.5403
CHB	DEFB130	12474389	16.6687	19.8216	-0.4944
CHB	DEFB130	12479389	18.1511	26.7676	-1.0102
CHB	DEFB130	12484389	17.0626	27.4452	-1.188
CHB	DEFB130	12489389	21.0072	28.8006	-0.8509
CHB	DEFB130	12494389	22.1559	31.5112	-0.9356
CHB	DEFB130	12499389	18.333	26.4288	-0.961
CHB	DEFB130	12504389	19.2658	28.2923	-1.0027
CHB	DEFB130	12509389	20.0704	29.3088	-0.9916
CHB	DEFB130	12514389	24.2928	29.3088	-0.5384
CHB	DEFB130	12519389	23.8245	32.3583	-0.8316
CHB	DEFB130	12524389	21.0533	31.6806	-1.0573
CHB	DEFB130	12529389	24.7523	30.8335	-0.6212
CHB	DEFB130	12534389	27.2367	29.9865	-0.2886
CHB	DEFB130	12539389	30.2529	31.3418	-0.1095
CHB	DEFB130	12544389	25.9749	34.0524	-0.7489
CHB	DEFB130	12549389	20.4262	28.6311	-0.9009
CHB	DEFB130	12554389	13.7459	24.904	-1.4031

CHB	DEFB130	12559389	6.2939	24.904	-2.3402
CHB	DEFB130	12564389	7.151	27.2758	-2.3166
YRI	DEFB130	11569389	8.2131	13.1089	-1.1357
YRI	DEFB130	11574389	7.911	11.2602	-0.8966
YRI	DEFB130	11579389	10.4157	16.134	-1.0888
YRI	DEFB130	11584389	12.267	20.8397	-1.2765
YRI	DEFB130	11589389	13.1051	18.4868	-0.8994
YRI	DEFB130	11594389	15.0888	16.8062	-0.3145
YRI	DEFB130	11599389	13.6805	18.1507	-0.7603
YRI	DEFB130	11604389	14.8798	19.3272	-0.7121
YRI	DEFB130	11609389	25.3892	24.0329	0.176
YRI	DEFB130	11614389	27.9517	27.2261	0.0834
YRI	DEFB130	11619389	20.9967	22.8565	-0.2533
YRI	DEFB130	11624389	22.9293	24.5371	-0.2044
YRI	DEFB130	11629389	21.815	24.369	-0.3269
YRI	DEFB130	11634389	13.8011	21.0078	-1.0649
YRI	DEFB130	11639389	13.6392	21.512	-1.1369
YRI	DEFB130	11644389	20.8666	24.369	-0.4483
YRI	DEFB130	11649389	26.7457	31.7638	-0.4963
YRI	DEFB130	11654389	25.6474	34.9569	-0.8384
YRI	DEFB130	11659389	21.0562	30.2512	-0.9537
YRI	DEFB130	11664389	21.4323	25.7135	-0.5201
YRI	DEFB130	11669389	23.0025	26.3858	-0.4009
YRI	DEFB130	11674389	24.8754	28.5706	-0.4052
YRI	DEFB130	11679389	25.2653	29.747	-0.4725
YRI	DEFB130	11684389	26.8498	33.4444	-0.6202
YRI	DEFB130	11689389	25.4496	32.2679	-0.664
YRI	DEFB130	11694389	18.5153	28.9067	-1.1266
YRI	DEFB130	11699389	23.0952	34.7889	-1.0581
YRI	DEFB130	11704389	30.8647	36.8056	-0.5087
YRI	DEFB130	11709389	23.7613	28.9067	-0.5579
YRI	DEFB130	11714389	12.2321	20.8397	-1.2817
YRI	DEFB130	11719389	11.2548	19.1591	-1.2763
YRI	DEFB130	11724389	13.3911	19.4952	-0.9693
YRI	DEFB130	11729389	13.0956	17.9827	-0.8387
YRI	DEFB130	11734389	15.7562	20.5036	-0.7181
YRI	DEFB130	11739389	17.6649	20.6717	-0.4512
YRI	DEFB130	11744389	22.4025	20.8397	0.2327
YRI	DEFB130	11749389	26.3705	22.6884	0.5051
YRI	DEFB130	11754389	20.7916	21.8481	-0.1503
YRI	DEFB130	11759389	14.9672	23.8648	-1.1622
YRI	DEFB130	11764389	32.6912	34.1166	-0.1315
YRI	DEFB130	11769389	48.5666	38.9904	0.7749
YRI	DEFB130	11774389	43.6106	39.4946	0.3289
YRI	DEFB130	11779389	60.3292	48.4019	0.7805
YRI	DEFB130	11784389	65.2191	51.5951	0.8372
YRI	DEFB130	11789389	61.2047	56.805	0.2459
YRI	DEFB130	11794389	59.1567	52.7715	0.3837
YRI	DEFB130	11799389	34.5308	35.4611	-0.0826

YRI	DEFB130	11804389	21.2753	26.5538	-0.6216
YRI	DEFB130	11809389	21.8505	24.7051	-0.3606
YRI	DEFB130	11814389	24.381	25.3774	-0.1226
YRI	DEFB130	11819389	24.4477	23.0245	0.1925
YRI	DEFB130	11824389	24.2368	23.0245	0.1639
YRI	DEFB130	11829389	22.6951	23.6968	-0.1317
YRI	DEFB130	11834389	17.8519	21.3439	-0.5081
YRI	DEFB130	11839389	17.8812	20.5036	-0.3967
YRI	DEFB130	11844389	17.2809	18.991	-0.2785
YRI	DEFB130	11849389	15.437	17.6465	-0.3861
YRI	DEFB130	11854389	16.8414	19.8313	-0.467
YRI	DEFB130	11859389	18.1544	24.369	-0.7954
YRI	DEFB130	11864389	20.3603	30.7554	-1.061
YRI	DEFB130	11869389	32.0489	45.3768	-0.9293
YRI	DEFB130	11874389	30.7072	43.8642	-0.9485
YRI	DEFB130	11879389	21.4187	33.2763	-1.1206
YRI	DEFB130	11884389	28.8992	36.8056	-0.677
YRI	DEFB130	11889389	32.3846	37.3098	-0.4162
YRI	DEFB130	11894389	29.2221	32.436	-0.3114
YRI	DEFB130	11899389	15.0079	15.6298	-0.1221
YRI	DEFB130	11904389	7.3473	6.8906	0.1924
YRI	DEFB130	11909389	8.1741	9.2434	-0.3441
YRI	DEFB130	11914389	7.8227	8.9073	-0.3612
YRI	DEFB130	11919389	10.1587	9.2434	0.2946
YRI	DEFB130	11924389	4.9645	4.8738	0.0521
YRI	DEFB130	11929389	3.7476	4.2016	-0.2966
YRI	DEFB130	11934389	5.1938	5.7141	-0.2595
YRI	DEFB130	11939389	2.9372	3.3612	-0.335
YRI	DEFB130	11944389	5.2003	3.6974	1.0954
YRI	DEFB130	11949389	6.9875	5.7141	0.635
YRI	DEFB130	11954389	3.4667	4.2016	-0.4801
YRI	DEFB130	11959389	1.4226	1.8487	-0.545
YRI	DEFB130	11964389	1.0755	2.1848	-1.2455
YRI	DEFB130	11969389	2.249	3.6974	-1.0557
YRI	DEFB130	11974389	9.7669	8.9073	0.2863
YRI	DEFB130	11979389	13.0726	14.6214	-0.3239
YRI	DEFB130	11984389	15.8209	23.5287	-1.0207
YRI	DEFB130	11989389	26.1283	34.4528	-0.7604
YRI	DEFB130	11994389	31.6603	37.814	-0.5132
YRI	DEFB130	11999389	25.0852	32.436	-0.7123
YRI	DEFB130	12004389	14.187	20.3355	-0.9374
YRI	DEFB130	12009389	8.4995	9.7476	-0.3823
YRI	DEFB130	12014389	4.278	5.0419	-0.4256
YRI	DEFB130	12019389	2.0513	3.5293	-1.1206
YRI	DEFB130	12024389	0.9843	2.1848	-1.3479
YRI	DEFB130	12029389	5.29	7.5628	-0.8799
YRI	DEFB130	12034389	13.3648	15.1256	-0.3565
YRI	DEFB130	12039389	12.4292	12.9408	-0.1201
YRI	DEFB130	12044389	5.3658	6.7225	-0.5846

YRI	DEFB130	12049389	3.6571	5.5461	-0.9673
YRI	DEFB130	12054389	4.2159	6.3864	-0.9796
YRI	DEFB130	12059389	3.4491	5.0419	-0.8875
YRI	DEFB130	12064389	2.5584	4.5377	-1.2094
YRI	DEFB130	12069389	3.6292	5.5461	-0.9816
YRI	DEFB130	12074389	3.8222	3.8654	-0.0303
YRI	DEFB130	12079389	2.8816	2.3529	0.5597
YRI	DEFB130	12084389	16.1756	19.8313	-0.571
YRI	DEFB130	12089389	21.1568	24.8732	-0.4663
YRI	DEFB130	12094389	6.7102	6.3864	0.1461
YRI	DEFB130	12099389	0	0	0
YRI	DEFB130	12104389	0	0	0
YRI	DEFB130	12109389	0	0	0
YRI	DEFB130	12114389	0	0	0
YRI	DEFB130	12119389	0	0	0
YRI	DEFB130	12124389	0	0	0
YRI	DEFB130	12129389	0	0	0
YRI	DEFB130	12134389	0	0	0
YRI	DEFB130	12139389	3.4109	4.2016	-0.5165
YRI	DEFB130	12144389	10.9207	8.7392	0.7395
YRI	DEFB130	12149389	8.8729	6.5544	1.0222
YRI	DEFB130	12154389	2.6285	3.1932	-0.4657
YRI	DEFB130	12159389	2.444	2.3529	0.0964
YRI	DEFB130	12164389	7.8984	5.378	1.3264
YRI	DEFB130	12169389	7.6445	5.5461	1.0746
YRI	DEFB130	12174389	8.6495	10.924	-0.6264
YRI	DEFB130	12179389	15.7719	20.1675	-0.6756
YRI	DEFB130	12184389	13.3944	17.3104	-0.697
YRI	DEFB130	12189389	8.0192	10.5879	-0.7285
YRI	DEFB130	12194389	4.9909	6.8906	-0.8005
YRI	DEFB130	12199389	4.6558	6.0502	-0.6607
YRI	DEFB130	12204389	3.0813	4.0335	-0.6443
YRI	DEFB130	12209389	1.1774	1.5126	-0.4991
YRI	DEFB130	12214389	3.8452	4.0335	-0.1274
YRI	DEFB130	12219389	5.1063	6.3864	-0.5777
YRI	DEFB130	12224389	6.3765	7.3947	-0.4023
YRI	DEFB130	12229389	17.2068	17.6465	-0.0768
YRI	DEFB130	12234389	35.0714	34.6208	0.041
YRI	DEFB130	12239389	36.146	38.3182	-0.1788
YRI	DEFB130	12244389	25.5021	27.7303	-0.2516
YRI	DEFB130	12249389	15.2325	15.7978	-0.1098
YRI	DEFB130	12254389	4.4845	7.3947	-1.15
YRI	DEFB130	12259389	2.7625	4.8738	-1.212
YRI	DEFB130	12264389	1.9004	3.5293	-1.235
YRI	DEFB130	12269389	2.9122	5.2099	-1.2437
YRI	DEFB130	12274389	9.7421	14.6214	-1.0204
YRI	DEFB130	12279389	11.3232	14.7895	-0.7171
YRI	DEFB130	12284389	12.3746	12.4366	-0.0151
YRI	DEFB130	12289389	10.7381	11.0921	-0.0961

YRI	DEFB130	12294389	4.357	5.378	-0.5373
YRI	DEFB130	12299389	3.5331	4.7057	-0.6941
YRI	DEFB130	12304389	6.109	6.2183	-0.0505
YRI	DEFB130	12309389	5.828	6.8906	-0.4477
YRI	DEFB130	12314389	2.2839	4.7057	-1.4336
YRI	DEFB130	12319389	1.9562	3.0251	-0.9221
YRI	DEFB130	12324389	1.8149	3.1932	-1.1367
YRI	DEFB130	12329389	1.5948	4.2016	-1.7028
YRI	DEFB130	12334389	1.7703	4.8738	-1.7815
YRI	DEFB130	12339389	7.9837	8.067	-0.0304
YRI	DEFB130	12344389	9.2461	8.4031	0.2963
YRI	DEFB130	12349389	2.7411	4.5377	-1.0978
YRI	DEFB130	12354389	0.9774	1.8487	-1.1145
YRI	DEFB130	12359389	2.2693	2.1848	0.0949
YRI	DEFB130	12364389	2.0574	2.8571	-0.7232
YRI	DEFB130	12369389	1.4209	1.8487	-0.5472
YRI	DEFB130	12374389	3.9843	3.6974	0.2091
YRI	DEFB130	12379389	5.1864	5.378	-0.1008
YRI	DEFB130	12384389	2.9049	3.5293	-0.4734
YRI	DEFB130	12389389	1.2811	1.3445	-0.103
YRI	DEFB130	12394389	6.0429	6.0502	-0.0035
YRI	DEFB130	12399389	11.1155	9.7476	0.419
YRI	DEFB130	12404389	10.9199	8.4031	0.8846
YRI	DEFB130	12409389	6.9504	7.3947	-0.1756
YRI	DEFB130	12414389	6.2741	8.067	-0.6542
YRI	DEFB130	12419389	7.557	7.8989	-0.1272
YRI	DEFB130	12424389	8.1694	7.3947	0.3061
YRI	DEFB130	12429389	9.6879	9.0754	0.2005
YRI	DEFB130	12434389	10.4444	10.756	-0.0871
YRI	DEFB130	12439389	9.0334	10.5879	-0.4408
YRI	DEFB130	12444389	6.3715	8.5712	-0.7591
YRI	DEFB130	12449389	7.5904	8.7392	-0.3894
YRI	DEFB130	12454389	7.6589	8.5712	-0.3148
YRI	DEFB130	12459389	4.1896	5.0419	-0.4749
YRI	DEFB130	12464389	6.4155	9.2434	-0.9101
YRI	DEFB130	12469389	13.5864	21.68	-1.1601
YRI	DEFB130	12474389	19.1947	26.7219	-0.881
YRI	DEFB130	12479389	23.7531	32.6041	-0.8533
YRI	DEFB130	12484389	23.7802	37.814	-1.1703
YRI	DEFB130	12489389	31.1582	43.3601	-0.8897
YRI	DEFB130	12494389	31.1972	41.3433	-0.7752
YRI	DEFB130	12499389	23.4494	34.7889	-1.0261
YRI	DEFB130	12504389	25.241	37.6459	-1.039
YRI	DEFB130	12509389	23.2966	37.1417	-1.175
YRI	DEFB130	12514389	25.9193	44.2004	-1.308
YRI	DEFB130	12519389	25.8293	49.7464	-1.5234
YRI	DEFB130	12524389	25.3811	44.8726	-1.3741
YRI	DEFB130	12529389	30.6192	42.5197	-0.8845
YRI	DEFB130	12534389	30.1407	39.4946	-0.7475

YRI	DEFB130	12539389	31.776	38.9904	-0.5838
YRI	DEFB130	12544389	33.5315	44.7045	-0.7906
YRI	DEFB130	12549389	29.2015	41.1752	-0.9185
YRI	DEFB130	12554389	26.0377	34.6208	-0.7803
YRI	DEFB130	12559389	24.2719	34.1166	-0.908
YRI	DEFB130	12564389	25.8642	35.2931	-0.8413
CEU	DEFB130	11815962	21.138	14.4978	1.4087
CEU	DEFB130	11820962	19.3714	12.1099	1.8268
CEU	DEFB130	11825962	18.2272	13.3039	1.1333
CEU	DEFB130	11830962	14.9342	11.4277	0.9316
CEU	DEFB130	11835962	12.6607	11.2571	0.3782
CEU	DEFB130	11840962	9.8155	10.916	-0.3052
CEU	DEFB130	11845962	9.5986	8.5281	0.3735
CEU	DEFB130	11850962	11.9138	9.5515	0.7421
CEU	DEFB130	11855962	13.2373	10.916	0.6438
CEU	DEFB130	11860962	10.7135	11.0866	-0.102
CEU	DEFB130	11865962	10.0996	13.9861	-0.8532
CEU	DEFB130	11870962	20.2142	19.2736	0.152
CEU	DEFB130	11875962	20.6131	19.2736	0.2164
CEU	DEFB130	11880962	13.3941	17.7385	-0.7601
CEU	DEFB130	11885962	14.8343	16.5446	-0.3199
CEU	DEFB130	11890962	30.1474	19.7852	1.6323
CEU	DEFB130	11895962	24.1115	14.3272	2.0993
CEU	DEFB130	11900962	4.2039	3.4112	0.6226
CEU	DEFB130	11905962	2.9314	2.2173	0.7978
CEU	DEFB130	11910962	1.9476	1.5351	0.6117
CEU	DEFB130	11915962	1.9637	1.7056	0.3535
CEU	DEFB130	11920962	1.8883	1.5351	0.5237
CEU	DEFB130	11925962	1.7549	1.0234	1.4535
CEU	DEFB130	11930962	2.86	1.7056	1.5811
CEU	DEFB130	11935962	1.9687	1.5351	0.6429
CEU	DEFB130	11940962	0.0592	0.3411	-1.1301
CEU	DEFB130	11945962	3.0359	2.3879	0.6824
CEU	DEFB130	11950962	6.1453	3.7524	1.7336
CEU	DEFB130	11955962	3.7351	1.7056	2.7796
CEU	DEFB130	11960962	1.0596	1.1939	-0.2393
CEU	DEFB130	11965962	0.8771	1.7056	-1.1347
CEU	DEFB130	11970962	3.523	2.5584	0.9609
CEU	DEFB130	11975962	11.1538	6.9931	1.7409
CEU	DEFB130	11980962	11.5279	10.0632	0.4383
CEU	DEFB130	11985962	12.0087	14.4978	-0.5281
CEU	DEFB130	11990962	20.7607	21.3203	-0.082
CEU	DEFB130	11995962	27.0282	23.0259	0.5446
CEU	DEFB130	12000962	24.8802	19.4441	0.8708
CEU	DEFB130	12005962	12.972	11.9394	0.2633
CEU	DEFB130	12010962	3.3288	4.4346	-0.6938
CEU	DEFB130	12015962	1.0744	1.7056	-0.8645
CEU	DEFB130	12020962	0.8423	1.3645	-0.8448
CEU	DEFB130	12025962	1.3502	2.2173	-0.9687

CEU	DEFB130	12030962	5.7368	5.6286	0.055
CEU	DEFB130	12035962	10.7067	10.4043	0.0877
CEU	DEFB130	12040962	7.6073	8.8692	-0.4246
CEU	DEFB130	12045962	1.5102	2.729	-1.152
CEU	DEFB130	12050962	1.7984	2.5584	-0.7571
CEU	DEFB130	12055962	4.1903	3.5818	0.4586
CEU	DEFB130	12060962	3.2373	2.8996	0.3037
CEU	DEFB130	12065962	1.4442	1.8762	-0.55
CEU	DEFB130	12070962	3.9658	3.2407	0.5946
CEU	DEFB130	12075962	4.597	3.7524	0.6119
CEU	DEFB130	12080962	4.5234	5.6286	-0.5622
CEU	DEFB130	12085962	7.9056	10.4043	-0.7249
CEU	DEFB130	12090962	4.6627	6.1402	-0.6953
CEU	DEFB130	12095962	0.0101	0.1706	-0.9534
CEU	DEFB130	12100962	0	0	0
CEU	DEFB130	12105962	0	0	0
CEU	DEFB130	12110962	0	0	0
CEU	DEFB130	12115962	0	0	0
CEU	DEFB130	12120962	0	0	0
CEU	DEFB130	12125962	0	0	0
CEU	DEFB130	12130962	0	0	0
CEU	DEFB130	12135962	0	0	0
CEU	DEFB130	12140962	3.7271	2.3879	1.4103
CEU	DEFB130	12145962	8.221	4.7757	2.0263
CEU	DEFB130	12150962	5.242	4.0935	0.7723
CEU	DEFB130	12155962	0.7885	2.2173	-1.5962
CEU	DEFB130	12160962	2.0635	1.5351	0.7835
CEU	DEFB130	12165962	4.4679	1.8762	3.2997
CEU	DEFB130	12170962	3.4084	1.7056	2.3322
CEU	DEFB130	12175962	8.6562	6.3108	1.0768
CEU	DEFB130	12180962	17.5106	11.9394	1.4204
CEU	DEFB130	12185962	12.3335	8.5281	1.3278
CEU	DEFB130	12190962	4.9087	4.6052	0.1843
CEU	DEFB130	12195962	4.315	4.2641	0.033
CEU	DEFB130	12200962	3.3727	2.729	0.6084
CEU	DEFB130	12205962	1.9068	1.7056	0.2756
CEU	DEFB130	12210962	1.5191	2.0467	-0.6278
CEU	DEFB130	12215962	2.4999	3.2407	-0.6074
CEU	DEFB130	12220962	2.9503	3.5818	-0.476
CEU	DEFB130	12225962	5.223	4.2641	0.6225
CEU	DEFB130	12230962	17.7322	14.4978	0.6862
CEU	DEFB130	12235962	28.5182	22.8554	0.7761
CEU	DEFB130	12240962	24.0853	21.1497	0.4336
CEU	DEFB130	12245962	15.3177	15.5212	-0.0405
CEU	DEFB130	12250962	7.2461	6.8225	0.1813
CEU	DEFB130	12255962	2.4721	2.3879	0.0887
CEU	DEFB130	12260962	1.3249	1.8762	-0.7019
CEU	DEFB130	12265962	0.3913	1.3645	-1.5744
CEU	DEFB130	12270962	3.1047	5.6286	-1.2838

CEU	DEFB130	12275962	7.8481	9.8926	-0.6217
CEU	DEFB130	12280962	8.3212	8.3576	-0.0129
CEU	DEFB130	12285962	6.2414	6.8225	-0.2487
CEU	DEFB130	12290962	5.7751	5.6286	0.0745
CEU	DEFB130	12295962	4.6052	3.7524	0.6178
CEU	DEFB130	12300962	3.5793	2.729	0.8037
CEU	DEFB130	12305962	6.4459	4.0935	1.5818
CEU	DEFB130	12310962	5.1142	4.7757	0.1991
CEU	DEFB130	12315962	0.8314	2.5584	-1.7204
CEU	DEFB130	12320962	0.424	1.5351	-1.6475
CEU	DEFB130	12325962	0.8439	1.5351	-1.0249
CEU	DEFB130	12330962	0.6246	0.6822	-0.1504
CEU	DEFB130	12335962	2.2412	2.0467	0.2314
CEU	DEFB130	12340962	7.2422	5.7991	0.7148
CEU	DEFB130	12345962	5.8031	4.7757	0.6043
CEU	DEFB130	12350962	1.4568	1.8762	-0.534
CEU	DEFB130	12355962	1.2288	1.8762	-0.8242
CEU	DEFB130	12360962	0.7681	1.7056	-1.284
CEU	DEFB130	12365962	0.361	1.7056	-1.8416
CEU	DEFB130	12370962	1.3474	2.2173	-0.9718
CEU	DEFB130	12375962	3.2787	3.7524	-0.3432
CEU	DEFB130	12380962	3.1938	3.9229	-0.5085
CEU	DEFB130	12385962	2.2827	2.5584	-0.2746
CEU	DEFB130	12390962	4.0619	2.2173	2.0608
CEU	DEFB130	12395962	6.428	4.4346	1.2507
CEU	DEFB130	12400962	9.9645	6.8225	1.3445
CEU	DEFB130	12405962	10.4463	6.1402	2.0263
CEU	DEFB130	12410962	5.4989	3.9229	1.0992
CEU	DEFB130	12415962	7.2964	4.6052	1.6339
CEU	DEFB130	12420962	10.3267	7.1636	1.2948
CEU	DEFB130	12425962	7.8215	6.4814	0.6007
CEU	DEFB130	12430962	6.3696	5.1169	0.6934
CEU	DEFB130	12435962	6.9586	6.8225	0.0582
CEU	DEFB130	12440962	6.0807	6.4814	-0.1796
CEU	DEFB130	12445962	4.5635	5.458	-0.4676
CEU	DEFB130	12450962	6.4418	7.1636	-0.2955
CEU	DEFB130	12455962	5.8716	5.9697	-0.0473
CEU	DEFB130	12460962	1.8108	2.2173	-0.4541
CEU	DEFB130	12465962	6.4168	6.8225	-0.1736
CEU	DEFB130	12470962	16.1722	14.4978	0.3552
CEU	DEFB130	12475962	18.5016	16.374	0.4019
CEU	DEFB130	12480962	18.7396	19.9558	-0.19
CEU	DEFB130	12485962	22.1435	20.1264	0.3126
CEU	DEFB130	12490962	23.9787	19.6147	0.6932
CEU	DEFB130	12495962	21.6099	20.4675	0.1742
CEU	DEFB130	12500962	19.5207	16.7151	0.5196
CEU	DEFB130	12505962	17.9182	16.374	0.2917
CEU	DEFB130	12510962	20.0909	17.9091	0.3782
CEU	DEFB130	12515962	21.282	19.6147	0.2648

CEU	DEFB130	12520962	17.0551	20.6381	-0.5419
CEU	DEFB130	12525962	17.1038	18.2502	-0.1952
CEU	DEFB130	12530962	23.1802	19.4441	0.5985
CEU	DEFB130	12535962	27.4199	20.6381	1.0257
CEU	DEFB130	12540962	27.0635	20.1264	1.0749
CEU	DEFB130	12545962	20.8351	23.3671	-0.3396
CEU	DEFB130	12550962	11.9546	20.1264	-1.2663
CEU	DEFB130	12555962	8.7165	16.2034	-1.4285
CEU	DEFB130	12560962	6.4101	14.1567	-1.6811
CEU	DEFB130	12565962	7.6744	18.0796	-1.7876
CEU	DEFB130	12570962	10.781	20.6381	-1.4909
CEU	DEFB130	12575962	8.1408	12.6216	-1.0841
CEU	DEFB130	12580962	6.3496	9.0398	-0.8894
CEU	DEFB130	12585962	7.0936	8.5281	-0.5005
CEU	DEFB130	12590962	5.6926	8.8692	-1.0689
CEU	DEFB130	12595962	4.8477	10.2337	-1.5868
CEU	DEFB130	12600962	9.3243	14.4978	-1.0976
CEU	DEFB130	12605962	9.3676	13.4744	-0.934
CEU	DEFB130	12610962	5.8839	9.2104	-1.0809
CEU	DEFB130	12615962	9.9676	10.4043	-0.1267
CEU	DEFB130	12620962	11.874	9.5515	0.7296
CEU	DEFB130	12625962	18.1086	10.4043	2.235
CEU	DEFB130	12630962	22.632	13.4744	2.0826
CEU	DEFB130	12635962	18.858	12.2805	1.633
CEU	DEFB130	12640962	18.182	11.2571	1.866
CEU	DEFB130	12645962	14.4649	10.5749	1.1114
CEU	DEFB130	12650962	11.0676	9.5515	0.4763
CEU	DEFB130	12655962	10.5531	10.2337	0.0941
CEU	DEFB130	12660962	14.1579	12.7922	0.3262
CEU	DEFB130	12665962	13.76	12.1099	0.4151
CEU	DEFB130	12670962	11.234	11.9394	-0.1798
CEU	DEFB130	12675962	12.2373	12.4511	-0.0524
CEU	DEFB130	12680962	13.2249	12.2805	0.2345
CEU	DEFB130	12685962	16.9219	15.3506	0.3157
CEU	DEFB130	12690962	16.9853	15.5212	0.2911
CEU	DEFB130	12695962	11.8366	14.6684	-0.5941
CEU	DEFB130	12700962	13.4073	16.7151	-0.6127
CEU	DEFB130	12705962	14.8786	15.6917	-0.16
CEU	DEFB130	12710962	13.5382	16.0329	-0.4808
CEU	DEFB130	12715962	13.7551	16.374	-0.4947
CEU	DEFB130	12720962	10.3647	12.6216	-0.546
CEU	DEFB130	12725962	8.4317	11.7688	-0.8624
CEU	DEFB130	12730962	8.4244	11.9394	-0.8962
CEU	DEFB130	12735962	9.2834	12.6216	-0.8076
CEU	DEFB130	12740962	9.8637	13.3039	-0.7919
CEU	DEFB130	12745962	9.9002	14.4978	-0.9754
CEU	DEFB130	12750962	8.5199	13.8156	-1.1761
CEU	DEFB130	12755962	7.51	11.9394	-1.1293
CEU	DEFB130	12760962	11.2336	12.9627	-0.4079

CEU	DEFB130	12765962	12.9058	13.645	-0.1661
CEU	DEFB130	12770962	11.3459	12.4511	-0.2708
CEU	DEFB130	12775962	11.3958	11.9394	-0.1386
CEU	DEFB130	12780962	10.8732	11.7688	-0.2315
CEU	DEFB130	12785962	13.1016	11.7688	0.3444
CEU	DEFB130	12790962	16.2509	14.3272	0.4127
CEU	DEFB130	12795962	16.2731	14.3272	0.4175
CEU	DEFB130	12800962	13.068	10.7454	0.6537
CEU	DEFB130	12805962	13.6675	11.9394	0.4406
CEU	DEFB130	12810962	15.6801	15.0095	0.1376
CHB	DEFB130	11815962	19.6371	17.1109	0.4562
CHB	DEFB130	11820962	18.6793	16.0944	0.495
CHB	DEFB130	11825962	14.1447	17.4498	-0.5858
CHB	DEFB130	11830962	7.3889	14.9085	-1.549
CHB	DEFB130	11835962	5.347	15.0779	-1.9831
CHB	DEFB130	11840962	4.0235	15.2474	-2.2631
CHB	DEFB130	11845962	3.6565	11.6896	-2.0832
CHB	DEFB130	11850962	4.8086	10.8426	-1.6793
CHB	DEFB130	11855962	5.3146	14.2309	-1.9198
CHB	DEFB130	11860962	5.347	17.2803	-2.1348
CHB	DEFB130	11865962	4.853	19.991	-2.3543
CHB	DEFB130	11870962	7.0047	26.2593	-2.2998
CHB	DEFB130	11875962	6.8783	27.4452	-2.3533
CHB	DEFB130	11880962	4.7274	26.937	-2.5879
CHB	DEFB130	11885962	5.2223	26.4288	-2.5172
CHB	DEFB130	11890962	7.1035	28.6311	-2.3638
CHB	DEFB130	11895962	4.7876	20.4992	-2.3851
CHB	DEFB130	11900962	0.9998	6.2684	-2.4265
CHB	DEFB130	11905962	0.9526	4.913	-2.2653
CHB	DEFB130	11910962	0.7834	3.7271	-2.1385
CHB	DEFB130	11915962	1.2022	6.7766	-2.3929
CHB	DEFB130	11920962	1.3061	6.946	-2.3674
CHB	DEFB130	11925962	1.0685	3.5577	-1.8812
CHB	DEFB130	11930962	1.0402	3.0495	-1.728
CHB	DEFB130	11935962	0.7489	1.5247	-1.1526
CHB	DEFB130	11940962	0.3385	0.3388	-0.0012
CHB	DEFB130	11945962	1.5142	2.2024	-0.7706
CHB	DEFB130	11950962	5	3.5577	1.09
CHB	DEFB130	11955962	4.0259	1.8636	2.7588
CHB	DEFB130	11960962	1.0853	1.3553	-0.4376
CHB	DEFB130	11965962	1.6128	1.5247	0.1309
CHB	DEFB130	11970962	4.1494	2.2024	2.1801
CHB	DEFB130	11975962	10.858	7.1154	1.537
CHB	DEFB130	11980962	11.4651	10.8426	0.1732
CHB	DEFB130	11985962	11.3908	16.9415	-1.012
CHB	DEFB130	11990962	17.9813	23.2099	-0.704
CHB	DEFB130	11995962	25.0773	23.5487	0.2029
CHB	DEFB130	12000962	24.2136	18.6357	0.9281
CHB	DEFB130	12005962	11.9918	11.012	0.2687

CHB	DEFB130	12010962	2.9027	6.0989	-1.5087
CHB	DEFB130	12015962	1.3078	2.8801	-1.4176
CHB	DEFB130	12020962	0.9909	1.3553	-0.5906
CHB	DEFB130	12025962	0.4647	2.5412	-2.0735
CHB	DEFB130	12030962	4.2816	7.2848	-1.2072
CHB	DEFB130	12035962	9.944	10.3343	-0.1136
CHB	DEFB130	12040962	7.9286	8.4708	-0.1898
CHB	DEFB130	12045962	1.8791	4.066	-1.4747
CHB	DEFB130	12050962	1.2404	2.8801	-1.4784
CHB	DEFB130	12055962	3.1917	3.5577	-0.2766
CHB	DEFB130	12060962	2.6794	2.5412	0.138
CHB	DEFB130	12065962	1.1649	1.0165	0.2953
CHB	DEFB130	12070962	3.9591	3.0495	0.7823
CHB	DEFB130	12075962	4.3202	3.8965	0.2963
CHB	DEFB130	12080962	2.2633	5.2519	-1.6118
CHB	DEFB130	12085962	3.2734	13.2144	-2.2963
CHB	DEFB130	12090962	1.8856	9.1484	-2.368
CHB	DEFB130	12095962	0.0097	0.1694	-0.949
CHB	DEFB130	12100962	0	0	0
CHB	DEFB130	12105962	0	0	0
CHB	DEFB130	12110962	0	0	0
CHB	DEFB130	12115962	0	0	0
CHB	DEFB130	12120962	0	0	0
CHB	DEFB130	12125962	0	0	0
CHB	DEFB130	12130962	0	0	0
CHB	DEFB130	12135962	0	0	0
CHB	DEFB130	12140962	0.5926	3.3883	-2.2017
CHB	DEFB130	12145962	1.4443	6.2684	-2.2218
CHB	DEFB130	12150962	1.5222	4.4048	-1.8139
CHB	DEFB130	12155962	0.7367	1.6942	-1.3141
CHB	DEFB130	12160962	0.7401	2.8801	-1.9294
CHB	DEFB130	12165962	1.2745	4.2354	-1.9276
CHB	DEFB130	12170962	1.4809	3.3883	-1.5021
CHB	DEFB130	12175962	2.5553	9.3178	-2.1676
CHB	DEFB130	12180962	3.554	16.9415	-2.4408
CHB	DEFB130	12185962	2.5915	12.0285	-2.3824
CHB	DEFB130	12190962	1.1609	5.7601	-2.285
CHB	DEFB130	12195962	1.4407	5.0825	-2.0219
CHB	DEFB130	12200962	1.2751	2.5412	-1.2643
CHB	DEFB130	12205962	0.3021	1.0165	-1.4217
CHB	DEFB130	12210962	0.5543	1.0165	-0.9198
CHB	DEFB130	12215962	0.9425	2.7106	-1.6754
CHB	DEFB130	12220962	0.6745	3.7271	-2.2176
CHB	DEFB130	12225962	0.6387	4.5742	-2.3964
CHB	DEFB130	12230962	8.004	13.892	-1.2971
CHB	DEFB130	12235962	18.3499	22.1934	-0.5404
CHB	DEFB130	12240962	18.2166	21.3463	-0.4569
CHB	DEFB130	12245962	11.0114	15.0779	-0.8287
CHB	DEFB130	12250962	4.4782	6.7766	-0.9866

CHB	DEFB130	12255962	1.8296	2.7106	-0.8348
CHB	DEFB130	12260962	1.2509	1.5247	-0.4068
CHB	DEFB130	12265962	0.3967	0.8471	-1.0156
CHB	DEFB130	12270962	2.6617	4.5742	-1.1646
CHB	DEFB130	12275962	6.9346	8.979	-0.6782
CHB	DEFB130	12280962	8.204	8.3013	-0.0347
CHB	DEFB130	12285962	6.8268	6.4378	0.1749
CHB	DEFB130	12290962	4.3076	4.5742	-0.1623
CHB	DEFB130	12295962	2.478	3.3883	-0.7169
CHB	DEFB130	12300962	2.8144	2.7106	0.0984
CHB	DEFB130	12305962	5.7339	3.7271	1.4579
CHB	DEFB130	12310962	4.7716	3.3883	1.0894
CHB	DEFB130	12315962	0.9612	1.0165	-0.1101
CHB	DEFB130	12320962	0.4814	1.0165	-1.0649
CHB	DEFB130	12325962	0.7974	1.1859	-0.6935
CHB	DEFB130	12330962	0.5494	1.1859	-1.1361
CHB	DEFB130	12335962	2.657	2.8801	-0.2011
CHB	DEFB130	12340962	6.7843	6.6072	0.0778
CHB	DEFB130	12345962	4.4693	5.0825	-0.3404
CHB	DEFB130	12350962	1.4455	2.033	-0.7006
CHB	DEFB130	12355962	1.3878	2.7106	-1.2534
CHB	DEFB130	12360962	0.831	2.2024	-1.5356
CHB	DEFB130	12365962	0.605	1.1859	-1.0369
CHB	DEFB130	12370962	1.3579	1.1859	0.307
CHB	DEFB130	12375962	3.3503	2.8801	0.4239
CHB	DEFB130	12380962	3.0723	3.3883	-0.2489
CHB	DEFB130	12385962	2.1939	2.7106	-0.4896
CHB	DEFB130	12390962	3.5969	2.5412	1.0542
CHB	DEFB130	12395962	5.8768	5.4213	0.2389
CHB	DEFB130	12400962	9.6107	7.6237	0.7662
CHB	DEFB130	12405962	9.9684	6.2684	1.7041
CHB	DEFB130	12410962	5.2947	4.4048	0.56
CHB	DEFB130	12415962	6.699	5.7601	0.4665
CHB	DEFB130	12420962	9.4194	8.3013	0.3988
CHB	DEFB130	12425962	7.7717	8.6402	-0.2985
CHB	DEFB130	12430962	6.6014	7.7931	-0.4504
CHB	DEFB130	12435962	6.9602	8.3013	-0.4783
CHB	DEFB130	12440962	6.206	7.1154	-0.3735
CHB	DEFB130	12445962	4.6362	4.7436	-0.0634
CHB	DEFB130	12450962	6.9353	6.0989	0.3948
CHB	DEFB130	12455962	6.0798	5.7601	0.1588
CHB	DEFB130	12460962	1.5281	2.7106	-1.1205
CHB	DEFB130	12465962	5.8035	7.1154	-0.5388
CHB	DEFB130	12470962	14.5489	15.5862	-0.2048
CHB	DEFB130	12475962	15.6677	20.1604	-0.6931
CHB	DEFB130	12480962	16.0215	26.7676	-1.2598
CHB	DEFB130	12485962	19.3908	29.8171	-1.1005
CHB	DEFB130	12490962	21.8424	29.4782	-0.815
CHB	DEFB130	12495962	21.6973	30.3253	-0.8958

CHB	DEFB130	12500962	18.9918	27.4452	-0.9672
CHB	DEFB130	12505962	18.3602	27.1064	-1.0129
CHB	DEFB130	12510962	23.0899	31.003	-0.804
CHB	DEFB130	12515962	23.9733	29.9865	-0.6312
CHB	DEFB130	12520962	20.6063	31.6806	-1.1017
CHB	DEFB130	12525962	21.2412	32.3583	-1.0834
CHB	DEFB130	12530962	26.1336	29.8171	-0.3888
CHB	DEFB130	12535962	30.4138	29.8171	0.063
CHB	DEFB130	12540962	30.6753	33.7136	-0.2844
CHB	DEFB130	12545962	25.3394	33.7136	-0.784
CHB	DEFB130	12550962	16.3147	25.4123	-1.1218
CHB	DEFB130	12555962	9.505	24.2264	-1.9014
CHB	DEFB130	12560962	6.1407	24.904	-2.3595
CHB	DEFB130	12565962	8.3935	28.6311	-2.2222
CHB	DEFB130	12570962	11.0946	26.4288	-1.8201
CHB	DEFB130	12575962	7.9614	16.4333	-1.5903
CHB	DEFB130	12580962	4.895	14.2309	-2.0102
CHB	DEFB130	12585962	6.0385	13.045	-1.6384
CHB	DEFB130	12590962	6.1622	11.3508	-1.3833
CHB	DEFB130	12595962	4.5854	12.3673	-1.9137
CHB	DEFB130	12600962	6.7955	15.7556	-1.751
CHB	DEFB130	12605962	7.2539	15.0779	-1.5945
CHB	DEFB130	12610962	4.1085	10.5037	-1.8335
CHB	DEFB130	12615962	7.333	10.3343	-0.8736
CHB	DEFB130	12620962	9.1038	10.6731	-0.4432
CHB	DEFB130	12625962	15.6149	14.0615	0.3383
CHB	DEFB130	12630962	23.1483	16.6027	1.2167
CHB	DEFB130	12635962	20.9585	15.5862	1.0608
CHB	DEFB130	12640962	17.4148	13.5532	0.8709
CHB	DEFB130	12645962	12.1264	10.3343	0.5217
CHB	DEFB130	12650962	9.2427	11.5202	-0.5988
CHB	DEFB130	12655962	9.3923	15.0779	-1.1587
CHB	DEFB130	12660962	13.7068	15.7556	-0.4004
CHB	DEFB130	12665962	13.7044	14.2309	-0.1134
CHB	DEFB130	12670962	11.5387	14.0615	-0.5494
CHB	DEFB130	12675962	13.4363	14.2309	-0.1711
CHB	DEFB130	12680962	13.901	14.7391	-0.1745
CHB	DEFB130	12685962	17.1354	16.6027	0.099
CHB	DEFB130	12690962	17.5636	16.9415	0.1134
CHB	DEFB130	12695962	11.7437	15.7556	-0.784
CHB	DEFB130	12700962	13.1397	17.7886	-0.8089
CHB	DEFB130	12705962	15.3484	16.4333	-0.2037
CHB	DEFB130	12710962	13.8643	14.7391	-0.1822
CHB	DEFB130	12715962	12.5106	16.0944	-0.6863
CHB	DEFB130	12720962	8.4763	13.7226	-1.1693
CHB	DEFB130	12725962	7.7607	10.1649	-0.7107
CHB	DEFB130	12730962	10.8233	9.9955	0.2486
CHB	DEFB130	12735962	12.2269	13.5532	-0.2991
CHB	DEFB130	12740962	12.3708	13.892	-0.3351

CHB	DEFB130	12745962	13.973	12.8755	0.2598
CHB	DEFB130	12750962	13.7392	13.7226	0.0037
CHB	DEFB130	12755962	12.3352	13.892	-0.343
CHB	DEFB130	12760962	14.1785	14.9085	-0.1504
CHB	DEFB130	12765962	20.0572	14.5697	1.1554
CHB	DEFB130	12770962	16.5047	12.7061	0.9106
CHB	DEFB130	12775962	10.5111	11.1814	-0.1812
CHB	DEFB130	12780962	10.9884	9.1484	0.5999
CHB	DEFB130	12785962	13.916	10.5037	0.9783
CHB	DEFB130	12790962	15.4959	14.5697	0.195
CHB	DEFB130	12795962	12.0992	13.5532	-0.3279
CHB	DEFB130	12800962	9.1032	12.0285	-0.7385
CHB	DEFB130	12805962	10.9768	13.892	-0.6422
CHB	DEFB130	12810962	14.2006	14.0615	0.0303
YRI	DEFB130	11815962	23.3406	23.8648	-0.0685
YRI	DEFB130	11820962	23.2688	20.8397	0.3617
YRI	DEFB130	11825962	24.9336	24.5371	0.0504
YRI	DEFB130	11830962	19.1198	22.3523	-0.4498
YRI	DEFB130	11835962	18.3635	20.1675	-0.2773
YRI	DEFB130	11840962	19.3058	21.68	-0.3403
YRI	DEFB130	11845962	16.2621	18.6549	-0.3964
YRI	DEFB130	11850962	15.3123	18.3188	-0.5069
YRI	DEFB130	11855962	16.7315	20.3355	-0.5495
YRI	DEFB130	11860962	20.2536	25.5455	-0.6471
YRI	DEFB130	11865962	22.7388	34.6208	-1.0803
YRI	DEFB130	11870962	30.3784	45.5449	-1.0537
YRI	DEFB130	11875962	29.616	42.8559	-0.9765
YRI	DEFB130	11880962	23.494	34.4528	-1.0011
YRI	DEFB130	11885962	29.4925	36.6376	-0.6146
YRI	DEFB130	11890962	36.4933	38.6543	-0.1764
YRI	DEFB130	11895962	24.7764	27.058	-0.2638
YRI	DEFB130	11900962	10.3597	11.0921	-0.1989
YRI	DEFB130	11905962	7.9868	7.3947	0.234
YRI	DEFB130	11910962	4.6056	7.0586	-1.0112
YRI	DEFB130	11915962	9.7048	9.4115	0.0928
YRI	DEFB130	11920962	9.0643	8.235	0.2969
YRI	DEFB130	11925962	3.2347	4.7057	-0.8708
YRI	DEFB130	11930962	5.1416	5.5461	-0.2071
YRI	DEFB130	11935962	4.6448	4.7057	-0.0361
YRI	DEFB130	11940962	1.5432	2.0167	-0.5661
YRI	DEFB130	11945962	4.6986	3.5293	0.8866
YRI	DEFB130	11950962	7.0151	5.8822	0.5505
YRI	DEFB130	11955962	3.3596	3.8654	-0.3549
YRI	DEFB130	11960962	1.5046	2.3529	-0.898
YRI	DEFB130	11965962	1.1437	2.1848	-1.1689
YRI	DEFB130	11970962	3.7917	4.5377	-0.4558
YRI	DEFB130	11975962	11.3523	11.4282	-0.02
YRI	DEFB130	11980962	12.8622	16.302	-0.6485
YRI	DEFB130	11985962	19.0227	25.8816	-0.8281

YRI	DEFB130	11990962	28.3129	37.4779	-0.771
YRI	DEFB130	11995962	29.693	36.4695	-0.5855
YRI	DEFB130	12000962	24.5777	30.0831	-0.5741
YRI	DEFB130	12005962	13.398	18.1507	-0.8084
YRI	DEFB130	12010962	6.0813	7.2267	-0.4622
YRI	DEFB130	12015962	2.6055	4.2016	-1.0426
YRI	DEFB130	12020962	1.3506	2.1848	-0.9366
YRI	DEFB130	12025962	2.122	4.0335	-1.2933
YRI	DEFB130	12030962	6.8519	9.5795	-0.8492
YRI	DEFB130	12035962	12.6282	13.613	-0.2204
YRI	DEFB130	12040962	11.8381	12.1005	-0.0656
YRI	DEFB130	12045962	4.8957	6.3864	-0.6728
YRI	DEFB130	12050962	3.3527	6.0502	-1.2781
YRI	DEFB130	12055962	5.3287	6.3864	-0.4774
YRI	DEFB130	12060962	3.6704	5.8822	-1.0747
YRI	DEFB130	12065962	1.559	4.3696	-1.7747
YRI	DEFB130	12070962	3.5745	3.6974	-0.0896
YRI	DEFB130	12075962	4.2847	3.8654	0.2942
YRI	DEFB130	12080962	8.5128	8.9073	-0.1314
YRI	DEFB130	12085962	21.7265	25.2093	-0.4314
YRI	DEFB130	12090962	14.6926	17.6465	-0.5162
YRI	DEFB130	12095962	0.1802	0.5042	-1.0182
YRI	DEFB130	12100962	0	0	0
YRI	DEFB130	12105962	0	0	0
YRI	DEFB130	12110962	0	0	0
YRI	DEFB130	12115962	0	0	0
YRI	DEFB130	12120962	0	0	0
YRI	DEFB130	12125962	0	0	0
YRI	DEFB130	12130962	0	0	0
YRI	DEFB130	12135962	0	0	0
YRI	DEFB130	12140962	4.9144	4.8738	0.0233
YRI	DEFB130	12145962	11.4207	9.0754	0.7677
YRI	DEFB130	12150962	8.1069	6.5544	0.6845
YRI	DEFB130	12155962	2.4465	3.5293	-0.821
YRI	DEFB130	12160962	5.5174	3.8654	1.1593
YRI	DEFB130	12165962	7.5804	4.7057	1.7017
YRI	DEFB130	12170962	3.9683	4.2016	-0.1524
YRI	DEFB130	12175962	12.0405	14.2853	-0.48
YRI	DEFB130	12180962	18.2294	22.8565	-0.6302
YRI	DEFB130	12185962	10.4749	15.2937	-0.9655
YRI	DEFB130	12190962	6.7929	8.5712	-0.6137
YRI	DEFB130	12195962	5.3633	6.7225	-0.5857
YRI	DEFB130	12200962	3.3629	4.8738	-0.8673
YRI	DEFB130	12205962	2.2375	2.8571	-0.5603
YRI	DEFB130	12210962	1.8062	2.8571	-0.9504
YRI	DEFB130	12215962	4.2002	5.2099	-0.5465
YRI	DEFB130	12220962	5.8557	6.3864	-0.2395
YRI	DEFB130	12225962	7.8738	7.5628	0.1204
YRI	DEFB130	12230962	25.6553	23.6968	0.2576

YRI	DEFB130	12235962	39.3353	39.3266	0.0007
YRI	DEFB130	12240962	30.6039	34.1166	-0.324
YRI	DEFB130	12245962	20.6286	24.8732	-0.5326
YRI	DEFB130	12250962	11.8287	13.2769	-0.3319
YRI	DEFB130	12255962	3.8087	5.2099	-0.7584
YRI	DEFB130	12260962	1.8086	3.8654	-1.4434
YRI	DEFB130	12265962	1.406	2.8571	-1.3123
YRI	DEFB130	12270962	5.857	9.5795	-1.1589
YRI	DEFB130	12275962	11.1243	16.134	-0.9538
YRI	DEFB130	12280962	12.3559	12.7727	-0.0991
YRI	DEFB130	12285962	10.9995	10.756	0.068
YRI	DEFB130	12290962	7.2922	9.4115	-0.6707
YRI	DEFB130	12295962	4.4919	6.2183	-0.7981
YRI	DEFB130	12300962	3.2467	4.8738	-0.934
YRI	DEFB130	12305962	6.7325	6.2183	0.2377
YRI	DEFB130	12310962	5.9567	7.5628	-0.6218
YRI	DEFB130	12315962	1.446	3.6974	-1.641
YRI	DEFB130	12320962	1.8101	2.689	-0.8353
YRI	DEFB130	12325962	2.375	4.0335	-1.1222
YRI	DEFB130	12330962	1.4408	3.8654	-1.7015
YRI	DEFB130	12335962	2.8487	5.7141	-1.4288
YRI	DEFB130	12340962	8.9941	8.9073	0.0289
YRI	DEFB130	12345962	7.6076	6.7225	0.3814
YRI	DEFB130	12350962	1.6568	2.8571	-1.0855
YRI	DEFB130	12355962	1.1642	2.3529	-1.2583
YRI	DEFB130	12360962	2.3694	2.689	-0.3037
YRI	DEFB130	12365962	1.8705	2.3529	-0.5106
YRI	DEFB130	12370962	1.9328	2.0167	-0.1003
YRI	DEFB130	12375962	4.4436	4.2016	0.1581
YRI	DEFB130	12380962	4.744	5.2099	-0.2522
YRI	DEFB130	12385962	3.2985	3.3612	-0.0495
YRI	DEFB130	12390962	3.4696	2.1848	1.4426
YRI	DEFB130	12395962	6.1428	6.3864	-0.1099
YRI	DEFB130	12400962	10.8453	10.0837	0.2261
YRI	DEFB130	12405962	11.0754	9.4115	0.5266
YRI	DEFB130	12410962	5.8678	6.7225	-0.3683
YRI	DEFB130	12415962	6.4228	7.5628	-0.4413
YRI	DEFB130	12420962	9.1488	9.5795	-0.1341
YRI	DEFB130	12425962	8.4885	7.8989	0.2193
YRI	DEFB130	12430962	7.4259	7.7309	-0.1157
YRI	DEFB130	12435962	8.9951	9.7476	-0.2305
YRI	DEFB130	12440962	9.2521	10.5879	-0.3788
YRI	DEFB130	12445962	7.1256	9.2434	-0.6816
YRI	DEFB130	12450962	8.0419	8.9073	-0.2882
YRI	DEFB130	12455962	7.3409	7.8989	-0.2076
YRI	DEFB130	12460962	3.4643	4.7057	-0.7349
YRI	DEFB130	12465962	9.194	13.613	-0.9891
YRI	DEFB130	12470962	17.0062	25.3774	-1.0301
YRI	DEFB130	12475962	19.1551	26.5538	-0.8713

YRI	DEFB130	12480962	22.3448	31.7638	-0.9315
YRI	DEFB130	12485962	28.1456	41.0072	-0.9906
YRI	DEFB130	12490962	30.9402	43.8642	-0.9317
YRI	DEFB130	12495962	26.3583	37.6459	-0.9454
YRI	DEFB130	12500962	24.5429	35.9653	-1.0005
YRI	DEFB130	12505962	25.2128	39.4946	-1.1413
YRI	DEFB130	12510962	26.5446	40.1669	-1.0707
YRI	DEFB130	12515962	24.6605	43.0239	-1.3492
YRI	DEFB130	12520962	23.472	47.3935	-1.5981
YRI	DEFB130	12525962	27.9131	46.7213	-1.2743
YRI	DEFB130	12530962	30.8612	41.1752	-0.7912
YRI	DEFB130	12535962	31.984	38.9904	-0.567
YRI	DEFB130	12540962	32.2686	42.3517	-0.7524
YRI	DEFB130	12545962	33.0117	43.8642	-0.7823
YRI	DEFB130	12550962	27.0413	36.8056	-0.8361
YRI	DEFB130	12555962	23.5625	33.2763	-0.918
YRI	DEFB130	12560962	24.7773	34.9569	-0.9168
YRI	DEFB130	12565962	27.971	37.3098	-0.7891
YRI	DEFB130	12570962	25.6637	33.9486	-0.7678
YRI	DEFB130	12575962	12.5717	23.0245	-1.4135
YRI	DEFB130	12580962	10.0036	16.9743	-1.2643
YRI	DEFB130	12585962	12.2649	16.302	-0.7611
YRI	DEFB130	12590962	9.9654	15.2937	-1.0676
YRI	DEFB130	12595962	10.9565	15.1256	-0.8442
YRI	DEFB130	12600962	18.8883	21.512	-0.3789
YRI	DEFB130	12605962	18.4769	22.6884	-0.5777
YRI	DEFB130	12610962	11.222	16.134	-0.9352
YRI	DEFB130	12615962	10.6516	16.6382	-1.1068
YRI	DEFB130	12620962	10.3913	20.5036	-1.5296
YRI	DEFB130	12625962	19.6359	23.5287	-0.5155
YRI	DEFB130	12630962	25.9525	23.3606	0.3456
YRI	DEFB130	12635962	22.452	21.512	0.1357
YRI	DEFB130	12640962	19.7304	21.0078	-0.1887
YRI	DEFB130	12645962	13.747	19.4952	-0.9128
YRI	DEFB130	12650962	11.4069	20.6717	-1.3904
YRI	DEFB130	12655962	12.2305	24.369	-1.5537
YRI	DEFB130	12660962	16.2561	24.8732	-1.0813
YRI	DEFB130	12665962	16.8722	19.8313	-0.4622
YRI	DEFB130	12670962	15.1896	17.9827	-0.4793
YRI	DEFB130	12675962	14.3155	17.3104	-0.5331
YRI	DEFB130	12680962	14.4565	18.6549	-0.6955
YRI	DEFB130	12685962	20.2482	22.5203	-0.3139
YRI	DEFB130	12690962	20.9595	21.512	-0.0798
YRI	DEFB130	12695962	16.8556	21.68	-0.6915
YRI	DEFB130	12700962	21.8572	25.3774	-0.4332
YRI	DEFB130	12705962	21.9245	23.3606	-0.1915
YRI	DEFB130	12710962	17.7047	22.3523	-0.6468
YRI	DEFB130	12715962	17.8545	26.0496	-0.9832
YRI	DEFB130	12720962	14.5645	23.5287	-1.1871

YRI	DEFB130	12725962	12.9138	21.1758	-1.2114
YRI	DEFB130	12730962	14.4809	22.6884	-1.1258
YRI	DEFB130	12735962	16.6931	23.8648	-0.9367
YRI	DEFB130	12740962	15.3083	21.1758	-0.8603
YRI	DEFB130	12745962	14.7099	19.8313	-0.8
YRI	DEFB130	12750962	16.2736	20.3355	-0.6193
YRI	DEFB130	12755962	14.3589	20.6717	-0.9474
YRI	DEFB130	12760962	14.0871	22.3523	-1.1502
YRI	DEFB130	12765962	21.0522	22.5203	-0.2028
YRI	DEFB130	12770962	20.25	22.3523	-0.2926
YRI	DEFB130	12775962	13.4983	19.3272	-0.9333
YRI	DEFB130	12780962	11.8351	17.3104	-0.9746
YRI	DEFB130	12785962	12.5835	20.1675	-1.1656
YRI	DEFB130	12790962	15.4033	21.8481	-0.9169
YRI	DEFB130	12795962	16.304	21.68	-0.7706
YRI	DEFB130	12800962	15.4176	22.0162	-0.9318
YRI	DEFB130	12805962	17.9598	22.6884	-0.6486
YRI	DEFB130	12810962	20.9005	23.8648	-0.3872
CEU	FAM72A	205691179	5.2791	7.8459	-0.9669
CEU	FAM72A	205696179	4.6545	8.187	-1.2797
CEU	FAM72A	205701179	3.6263	6.8225	-1.3676
CEU	FAM72A	205706179	3.57	6.6519	-1.3493
CEU	FAM72A	205711179	7.1744	8.6987	-0.5222
CEU	FAM72A	205716179	8.5409	9.3809	-0.2683
CEU	FAM72A	205721179	7.9763	8.187	-0.0763
CEU	FAM72A	205726179	6.7388	8.0164	-0.4719
CEU	FAM72A	205731179	5.0202	8.6987	-1.2602
CEU	FAM72A	205736179	7.5558	10.916	-0.932
CEU	FAM72A	205741179	9.388	10.4043	-0.2948
CEU	FAM72A	205746179	7.153	8.3576	-0.4282
CEU	FAM72A	205751179	10.3292	10.0632	0.0796
CEU	FAM72A	205756179	11.482	9.7221	0.5439
CEU	FAM72A	205761179	7.6342	8.6987	-0.3647
CEU	FAM72A	205766179	5.7954	7.5047	-0.6706
CEU	FAM72A	205771179	6.0954	7.8459	-0.6594
CEU	FAM72A	205776179	5.826	8.3576	-0.8999
CEU	FAM72A	205781179	4.0099	5.2874	-0.6869
CEU	FAM72A	205786179	6.3457	6.1402	0.0967
CEU	FAM72A	205791179	8.6265	8.187	0.1592
CEU	FAM72A	205796179	7.9588	8.0164	-0.0213
CEU	FAM72A	205801179	4.8303	5.7991	-0.4798
CEU	FAM72A	205806179	4.6291	5.458	-0.4333
CEU	FAM72A	205811179	6.1699	7.5047	-0.5237
CEU	FAM72A	205816179	7.8922	8.5281	-0.2219
CEU	FAM72A	205821179	8.4677	11.4277	-0.7864
CEU	FAM72A	205826179	6.4451	10.916	-1.24
CEU	FAM72A	205831179	5.6698	8.0164	-0.8667
CEU	FAM72A	205836179	5.7641	7.1636	-0.5729
CEU	FAM72A	205841179	7.6903	9.0398	-0.4462

CEU	FAM72A	205846179	9.4616	11.2571	-0.4838
CEU	FAM72A	205851179	7.791	10.916	-0.8667
CEU	FAM72A	205856179	6.2004	10.2337	-1.1882
CEU	FAM72A	205861179	5.2016	8.0164	-1.0397
CEU	FAM72A	205866179	5.2321	7.5047	-0.8917
CEU	FAM72A	205871179	5.4593	6.8225	-0.5833
CEU	FAM72A	205876179	4.2804	5.458	-0.6156
CEU	FAM72A	205881179	5.3799	8.187	-1.017
CEU	FAM72A	205886179	8.9392	11.9394	-0.7649
CEU	FAM72A	205891179	11.462	11.5982	-0.0357
CEU	FAM72A	205896179	11.4172	11.4277	-0.0028
CEU	FAM72A	205901179	8.1037	10.5749	-0.7061
CEU	FAM72A	205906179	11.9854	11.0866	0.2457
CEU	FAM72A	205911179	15.96	11.5982	1.1428
CEU	FAM72A	205916179	11.6855	9.3809	0.7362
CEU	FAM72A	205921179	6.5237	6.1402	0.1805
CEU	FAM72A	205926179	1.7621	1.7056	0.0774
CEU	FAM72A	205931179	0	0	0
CEU	FAM72A	205936179	0	0	0
CEU	FAM72A	205941179	0	0	0
CEU	FAM72A	205946179	0	0	0
CEU	FAM72A	205951179	0	0	0
CEU	FAM72A	205956179	0	0	0
CEU	FAM72A	205961179	0	0	0
CEU	FAM72A	205966179	0	0	0
CEU	FAM72A	205971179	0	0	0
CEU	FAM72A	205976179	0	0	0
CEU	FAM72A	205981179	0	0	0
CEU	FAM72A	205986179	0	0	0
CEU	FAM72A	205991179	0	0	0
CEU	FAM72A	205996179	0	0	0
CEU	FAM72A	206001179	0	0	0
CEU	FAM72A	206006179	0	0	0
CEU	FAM72A	206011179	0	0	0
CEU	FAM72A	206016179	0	0	0
CEU	FAM72A	206021179	0	0	0
CEU	FAM72A	206026179	0	0	0
CEU	FAM72A	206031179	0	0	0
CEU	FAM72A	206036179	0	0	0
CEU	FAM72A	206041179	0	0	0
CEU	FAM72A	206046179	0	0	0
CEU	FAM72A	206051179	0	0	0
CEU	FAM72A	206056179	0	0	0
CEU	FAM72A	206061179	0	0	0
CEU	FAM72A	206066179	0	0	0
CEU	FAM72A	206071179	1.2531	1.5351	-0.4182
CEU	FAM72A	206076179	2.9272	3.2407	-0.2571
CEU	FAM72A	206081179	6.046	3.9229	1.4808
CEU	FAM72A	206086179	5.3898	3.4112	1.554

CEU	FAM72A	206091179	2.683	2.5584	0.1241
CEU	FAM72A	206096179	4.4548	3.5818	0.658
CEU	FAM72A	206101179	4.3401	4.0935	0.1658
CEU	FAM72A	206106179	1.8771	2.8996	-0.9196
CEU	FAM72A	206111179	0.6392	1.8762	-1.5749
CEU	FAM72A	206116179	1.9845	2.729	-0.7037
CEU	FAM72A	206121179	1.831	3.0701	-1.0629
CEU	FAM72A	206126179	0.5329	3.2407	-2.2203
CEU	FAM72A	206131179	0.7947	2.8996	-1.893
CEU	FAM72A	206136179	1.5972	1.8762	-0.3552
CEU	FAM72A	206141179	1.4842	2.729	-1.1766
CEU	FAM72A	206146179	0.7405	2.5584	-1.8109
CEU	FAM72A	206151179	0.5747	1.1939	-1.1034
CEU	FAM72A	206156179	0.5886	1.7056	-1.5299
CEU	FAM72A	206161179	0.5165	1.7056	-1.6286
CEU	FAM72A	206166179	0.3381	1.3645	-1.6605
CEU	FAM72A	206171179	2.2472	3.5818	-1.0059
CEU	FAM72A	206176179	2.289	3.5818	-0.9744
CEU	FAM72A	206181179	0.7108	2.5584	-1.8405
CEU	FAM72A	206186179	1.5158	4.6052	-1.8757
CEU	FAM72A	206191179	2.3329	6.8225	-1.9211
CEU	FAM72A	206196179	2.6999	8.3576	-2.0111
CEU	FAM72A	206201179	2.6957	8.6987	-2.0566
CEU	FAM72A	206206179	6.7553	11.2571	-1.2131
CEU	FAM72A	206211179	9.8683	13.3039	-0.7908
CEU	FAM72A	206216179	8.4554	11.7688	-0.8563
CEU	FAM72A	206221179	6.6003	9.0398	-0.8066
CEU	FAM72A	206226179	7.2832	10.5749	-0.9405
CEU	FAM72A	206231179	8.5027	11.5982	-0.811
CEU	FAM72A	206236179	6.2603	9.7221	-1.0698
CEU	FAM72A	206241179	4.2999	8.8692	-1.5376
CEU	FAM72A	206246179	2.9485	5.9697	-1.4581
CEU	FAM72A	206251179	1.5271	3.9229	-1.671
CEU	FAM72A	206256179	2.1905	4.4346	-1.408
CEU	FAM72A	206261179	3.2125	4.9463	-0.9888
CEU	FAM72A	206266179	2.7345	4.7757	-1.2005
CEU	FAM72A	206271179	2.0557	5.1169	-1.6944
CEU	FAM72A	206276179	2.2426	5.7991	-1.7615
CEU	FAM72A	206281179	6.4376	8.187	-0.6338
CEU	FAM72A	206286179	6.9817	9.0398	-0.6805
CEU	FAM72A	206291179	4.2323	8.187	-1.4327
CEU	FAM72A	206296179	2.6751	8.3576	-2.02
CEU	FAM72A	206301179	1.4525	9.2104	-2.5209
CEU	FAM72A	206306179	1.7471	9.8926	-2.4768
CEU	FAM72A	206311179	1.529	10.916	-2.6035
CEU	FAM72A	206316179	1.2845	9.5515	-2.5972
CEU	FAM72A	206321179	3.5553	6.8225	-1.398
CEU	FAM72A	206326179	6.6448	5.9697	0.3258
CEU	FAM72A	206331179	3.8693	3.5818	0.2167

CEU	FAM72A	206336179	0.153	0.6822	-1.3819
CEU	FAM72A	206341179	0	0	0
CEU	FAM72A	206346179	0	0	0
CEU	FAM72A	206351179	0	0	0
CEU	FAM72A	206356179	0	0	0
CEU	FAM72A	206361179	0	0	0
CEU	FAM72A	206366179	0	0	0
CEU	FAM72A	206371179	0	0	0
CEU	FAM72A	206376179	0	0	0
CEU	FAM72A	206381179	0	0	0
CEU	FAM72A	206386179	0	0	0
CEU	FAM72A	206391179	0	0	0
CEU	FAM72A	206396179	0	0	0
CEU	FAM72A	206401179	0	0	0
CEU	FAM72A	206406179	0	0	0
CEU	FAM72A	206411179	0	0	0
CEU	FAM72A	206416179	0	0	0
CEU	FAM72A	206421179	0	0	0
CEU	FAM72A	206426179	0	0	0
CEU	FAM72A	206431179	0	0	0
CEU	FAM72A	206436179	0	0	0
CEU	FAM72A	206441179	0	0	0
CEU	FAM72A	206446179	0	0	0
CEU	FAM72A	206451179	0	0	0
CEU	FAM72A	206456179	0	0	0
CEU	FAM72A	206461179	0	0	0
CEU	FAM72A	206466179	0	0	0
CEU	FAM72A	206471179	0	0	0
CEU	FAM72A	206476179	0	0	0
CEU	FAM72A	206481179	0.5321	1.5351	-1.4873
CEU	FAM72A	206486179	1.8258	3.4112	-1.2452
CEU	FAM72A	206491179	1.9171	2.8996	-0.8836
CEU	FAM72A	206496179	1.3851	2.2173	-0.9297
CEU	FAM72A	206501179	0.8211	1.3645	-0.8791
CEU	FAM72A	206506179	0.3364	1.8762	-1.9604
CEU	FAM72A	206511179	1.9476	3.7524	-1.3075
CEU	FAM72A	206516179	2.7992	4.0935	-0.8703
CEU	FAM72A	206521179	2.6947	3.7524	-0.7663
CEU	FAM72A	206526179	2.8984	4.0935	-0.8036
CEU	FAM72A	206531179	1.8064	4.0935	-1.5379
CEU	FAM72A	206536179	2.2946	3.5818	-0.9702
CEU	FAM72A	206541179	4.0159	4.4346	-0.2627
CEU	FAM72A	206546179	2.3845	3.9229	-1.073
CEU	FAM72A	206551179	0.8598	2.5584	-1.6921
CEU	FAM72A	206556179	1.4135	4.0935	-1.8021
CEU	FAM72A	206561179	1.3723	4.0935	-1.8298
CEU	FAM72A	206566179	1.6465	3.4112	-1.386
CEU	FAM72A	206571179	1.2915	3.0701	-1.5257
CEU	FAM72A	206576179	0.7718	1.1939	-0.7522

CEU	FAM72A	206581179	1.3434	3.4112	-1.6241
CEU	FAM72A	206586179	1.3228	8.187	-2.4868
CEU	FAM72A	206591179	2.484	8.6987	-2.1291
CEU	FAM72A	206596179	4.1713	10.4043	-1.8082
CEU	FAM72A	206601179	4.3935	11.0866	-1.8295
CEU	FAM72A	206606179	3.497	7.8459	-1.6383
CEU	FAM72A	206611179	2.8617	7.3342	-1.792
CEU	FAM72A	206616179	5.06	6.8225	-0.7542
CEU	FAM72A	206621179	11.8128	9.2104	0.8456
CEU	FAM72A	206626179	11.3634	8.8692	0.8393
CEU	FAM72A	206631179	6.1828	6.4814	-0.1338
CEU	FAM72A	206636179	8.3059	8.8692	-0.1896
CEU	FAM72A	206641179	9.3307	10.2337	-0.266
CEU	FAM72A	206646179	10.4964	11.0866	-0.1613
CEU	FAM72A	206651179	10.3412	10.916	-0.1594
CEU	FAM72A	206656179	7.1534	8.6987	-0.5294
CEU	FAM72A	206661179	4.4047	7.1636	-1.1294
CEU	FAM72A	206666179	8.5741	8.8692	-0.0993
CEU	FAM72A	206671179	12.0225	8.8692	1.0611
CEU	FAM72A	206676179	11.3894	7.8459	1.3349
CEU	FAM72A	206681179	9.8233	6.6519	1.3885
CEU	FAM72A	206686179	5.0177	4.7757	0.1423
CEU	FAM72A	206691179	6.1785	6.3108	-0.0607
CEU	FAM72A	206696179	6.6955	7.1636	-0.1916
CHB	FAM72A	205691179	3.9193	5.7601	-0.9146
CHB	FAM72A	205696179	5.6467	5.9295	-0.1369
CHB	FAM72A	205701179	3.146	5.0825	-1.0751
CHB	FAM72A	205706179	1.7299	5.2519	-1.8995
CHB	FAM72A	205711179	6.984	6.946	0.016
CHB	FAM72A	205716179	8.5991	5.9295	1.2924
CHB	FAM72A	205721179	9.4904	6.7766	1.1649
CHB	FAM72A	205726179	9.9753	7.7931	0.8248
CHB	FAM72A	205731179	9.3516	6.946	1.0098
CHB	FAM72A	205736179	12.9777	9.6567	1.0298
CHB	FAM72A	205741179	10.9025	8.8096	0.7066
CHB	FAM72A	205746179	6.2621	6.0989	0.077
CHB	FAM72A	205751179	13.0841	8.979	1.3618
CHB	FAM72A	205756179	14.0409	8.4708	1.9499
CHB	FAM72A	205761179	7.6737	6.6072	0.4684
CHB	FAM72A	205766179	6.055	7.1154	-0.4355
CHB	FAM72A	205771179	7.2753	7.6237	-0.1344
CHB	FAM72A	205776179	7.6354	6.2684	0.6296
CHB	FAM72A	205781179	4.6189	4.7436	-0.0736
CHB	FAM72A	205786179	4.248	6.7766	-1.0854
CHB	FAM72A	205791179	6.344	7.7931	-0.5477
CHB	FAM72A	205796179	6.6926	6.946	-0.1064
CHB	FAM72A	205801179	3.0093	5.0825	-1.151
CHB	FAM72A	205806179	3.5924	4.2354	-0.4186
CHB	FAM72A	205811179	6.331	6.0989	0.1096

CHB	FAM72A	205816179	9.4272	8.6402	0.2705
CHB	FAM72A	205821179	11.9417	9.3178	0.8411
CHB	FAM72A	205826179	9.7881	8.979	0.2684
CHB	FAM72A	205831179	6.9212	7.4543	-0.2098
CHB	FAM72A	205836179	6.083	7.2848	-0.4831
CHB	FAM72A	205841179	7.9838	8.979	-0.3301
CHB	FAM72A	205846179	9.7472	9.6567	0.0281
CHB	FAM72A	205851179	7.4752	8.8096	-0.4505
CHB	FAM72A	205856179	6.8747	7.9625	-0.4031
CHB	FAM72A	205861179	7.0339	6.946	0.0369
CHB	FAM72A	205866179	6.569	7.2848	-0.2877
CHB	FAM72A	205871179	6.7017	7.2848	-0.2344
CHB	FAM72A	205876179	5.0849	7.1154	-0.8339
CHB	FAM72A	205881179	6.0941	9.1484	-0.9958
CHB	FAM72A	205886179	7.9517	12.3673	-1.0859
CHB	FAM72A	205891179	11.0961	13.892	-0.6159
CHB	FAM72A	205896179	12.8938	11.8591	0.2647
CHB	FAM72A	205901179	8.695	9.6567	-0.2982
CHB	FAM72A	205906179	8.1753	11.1814	-0.8128
CHB	FAM72A	205911179	9.3918	11.012	-0.4444
CHB	FAM72A	205916179	6.4769	9.3178	-0.9106
CHB	FAM72A	205921179	3.3373	6.6072	-1.4362
CHB	FAM72A	205926179	1.0683	1.5247	-0.678
CHB	FAM72A	205931179	0	0	0
CHB	FAM72A	205936179	0	0	0
CHB	FAM72A	205941179	0	0	0
CHB	FAM72A	205946179	0	0	0
CHB	FAM72A	205951179	0	0	0
CHB	FAM72A	205956179	0	0	0
CHB	FAM72A	205961179	0	0	0
CHB	FAM72A	205966179	0	0	0
CHB	FAM72A	205971179	0	0	0
CHB	FAM72A	205976179	0	0	0
CHB	FAM72A	205981179	0	0	0
CHB	FAM72A	205986179	0	0	0
CHB	FAM72A	205991179	0	0	0
CHB	FAM72A	205996179	0	0	0
CHB	FAM72A	206001179	0	0	0
CHB	FAM72A	206006179	0	0	0
CHB	FAM72A	206011179	0	0	0
CHB	FAM72A	206016179	0	0	0
CHB	FAM72A	206021179	0	0	0
CHB	FAM72A	206026179	0	0	0
CHB	FAM72A	206031179	0	0	0
CHB	FAM72A	206036179	0	0	0
CHB	FAM72A	206041179	0	0	0
CHB	FAM72A	206046179	0	0	0
CHB	FAM72A	206051179	0	0	0
CHB	FAM72A	206056179	0	0	0

CHB	FAM72A	206061179	0	0	0
CHB	FAM72A	206066179	0	0	0
CHB	FAM72A	206071179	1.2085	1.8636	-0.8358
CHB	FAM72A	206076179	2.7716	3.5577	-0.5941
CHB	FAM72A	206081179	5.5745	4.913	0.3784
CHB	FAM72A	206086179	5.0742	5.4213	-0.182
CHB	FAM72A	206091179	2.4824	3.5577	-0.8127
CHB	FAM72A	206096179	3.6503	3.8965	-0.1722
CHB	FAM72A	206101179	3.2658	4.066	-0.5396
CHB	FAM72A	206106179	1.2935	2.8801	-1.4305
CHB	FAM72A	206111179	0.7531	3.5577	-2.1196
CHB	FAM72A	206116179	2.1618	4.066	-1.2841
CHB	FAM72A	206121179	1.981	3.0495	-0.9189
CHB	FAM72A	206126179	0.5722	3.0495	-2.1305
CHB	FAM72A	206131179	0.7874	3.8965	-2.1746
CHB	FAM72A	206136179	1.6566	3.3883	-1.3638
CHB	FAM72A	206141179	1.4609	3.2189	-1.4453
CHB	FAM72A	206146179	0.6179	3.5577	-2.2218
CHB	FAM72A	206151179	0.6297	2.5412	-1.9088
CHB	FAM72A	206156179	0.5322	1.5247	-1.4745
CHB	FAM72A	206161179	0.4952	2.8801	-2.1502
CHB	FAM72A	206166179	0.5637	3.5577	-2.2627
CHB	FAM72A	206171179	1.8627	3.7271	-1.3544
CHB	FAM72A	206176179	2.5707	5.9295	-1.626
CHB	FAM72A	206181179	1.6859	5.7601	-2.0242
CHB	FAM72A	206186179	1.6421	5.7601	-2.0459
CHB	FAM72A	206191179	2.2102	6.7766	-1.9602
CHB	FAM72A	206196179	2.2498	7.1154	-1.9982
CHB	FAM72A	206201179	2.7512	7.7931	-1.9056
CHB	FAM72A	206206179	9.663	13.045	-0.7908
CHB	FAM72A	206211179	14.0899	18.1274	-0.6899
CHB	FAM72A	206216179	10.4234	16.4333	-1.1281
CHB	FAM72A	206221179	5.9701	12.1979	-1.5516
CHB	FAM72A	206226179	5.6472	12.3673	-1.6526
CHB	FAM72A	206231179	7.0238	15.7556	-1.7064
CHB	FAM72A	206236179	7.252	15.2474	-1.6121
CHB	FAM72A	206241179	7.6891	14.0615	-1.3878
CHB	FAM72A	206246179	5.4633	10.8426	-1.4971
CHB	FAM72A	206251179	2.5606	7.9625	-2.0018
CHB	FAM72A	206256179	2.8959	7.4543	-1.7943
CHB	FAM72A	206261179	3.9534	7.1154	-1.2985
CHB	FAM72A	206266179	3.6092	8.4708	-1.7019
CHB	FAM72A	206271179	2.6221	8.8096	-2.0891
CHB	FAM72A	206276179	3.3969	9.6567	-1.941
CHB	FAM72A	206281179	7.8205	11.012	-0.8754
CHB	FAM72A	206286179	6.3533	11.012	-1.2778
CHB	FAM72A	206291179	2.0649	8.6402	-2.2602
CHB	FAM72A	206296179	1.4911	7.7931	-2.3818
CHB	FAM72A	206301179	1.5696	11.012	-2.59

CHB	FAM72A	206306179	1.6364	11.6896	-2.6071
CHB	FAM72A	206311179	0.9861	8.6402	-2.631
CHB	FAM72A	206316179	0.7818	6.4378	-2.5431
CHB	FAM72A	206321179	1.7624	7.6237	-2.2603
CHB	FAM72A	206326179	2.2985	8.4708	-2.1608
CHB	FAM72A	206331179	1.0367	4.4048	-2.1194
CHB	FAM72A	206336179	0.0386	0.5082	-1.4741
CHB	FAM72A	206341179	0	0	0
CHB	FAM72A	206346179	0	0	0
CHB	FAM72A	206351179	0	0	0
CHB	FAM72A	206356179	0	0	0
CHB	FAM72A	206361179	0	0	0
CHB	FAM72A	206366179	0	0	0
CHB	FAM72A	206371179	0	0	0
CHB	FAM72A	206376179	0	0	0
CHB	FAM72A	206381179	0	0	0
CHB	FAM72A	206386179	0	0	0
CHB	FAM72A	206391179	0	0	0
CHB	FAM72A	206396179	0	0	0
CHB	FAM72A	206401179	0	0	0
CHB	FAM72A	206406179	0	0	0
CHB	FAM72A	206411179	0	0	0
CHB	FAM72A	206416179	0	0	0
CHB	FAM72A	206421179	0	0	0
CHB	FAM72A	206426179	0	0	0
CHB	FAM72A	206431179	0	0	0
CHB	FAM72A	206436179	0	0	0
CHB	FAM72A	206441179	0	0	0
CHB	FAM72A	206446179	0	0	0
CHB	FAM72A	206451179	0	0	0
CHB	FAM72A	206456179	0	0	0
CHB	FAM72A	206461179	0	0	0
CHB	FAM72A	206466179	0	0	0
CHB	FAM72A	206471179	0	0	0
CHB	FAM72A	206476179	0	0	0
CHB	FAM72A	206481179	0.5722	2.2024	-1.8254
CHB	FAM72A	206486179	1.7658	3.5577	-1.3542
CHB	FAM72A	206491179	1.8915	3.2189	-1.0913
CHB	FAM72A	206496179	1.422	3.3883	-1.5485
CHB	FAM72A	206501179	0.7719	1.6942	-1.2657
CHB	FAM72A	206506179	0.3219	1.8636	-1.967
CHB	FAM72A	206511179	1.712	4.913	-1.831
CHB	FAM72A	206516179	2.6598	6.0989	-1.6234
CHB	FAM72A	206521179	2.5024	4.4048	-1.1971
CHB	FAM72A	206526179	2.5085	3.8965	-0.9708
CHB	FAM72A	206531179	1.6269	5.2519	-1.9551
CHB	FAM72A	206536179	2.4289	4.7436	-1.3654
CHB	FAM72A	206541179	3.6073	3.7271	-0.087
CHB	FAM72A	206546179	1.9341	3.7271	-1.3026

CHB	FAM72A	206551179	0.9328	2.8801	-1.7557
CHB	FAM72A	206556179	1.0095	3.3883	-1.8734
CHB	FAM72A	206561179	1.1342	3.7271	-1.8837
CHB	FAM72A	206566179	1.4997	3.8965	-1.6764
CHB	FAM72A	206571179	0.8766	3.5577	-2.0262
CHB	FAM72A	206576179	0.7245	2.2024	-1.6548
CHB	FAM72A	206581179	1.6929	3.5577	-1.4093
CHB	FAM72A	206586179	1.7381	6.6072	-2.1386
CHB	FAM72A	206591179	2.7986	9.8261	-2.1441
CHB	FAM72A	206596179	4.6699	11.8591	-1.8393
CHB	FAM72A	206601179	4.7719	11.3508	-1.7539
CHB	FAM72A	206606179	3.2688	8.6402	-1.8463
CHB	FAM72A	206611179	3.0415	7.7931	-1.7959
CHB	FAM72A	206616179	4.2256	8.6402	-1.5175
CHB	FAM72A	206621179	9.8799	11.012	-0.3105
CHB	FAM72A	206626179	10.2283	8.979	0.4144
CHB	FAM72A	206631179	5.0434	5.5907	-0.2792
CHB	FAM72A	206636179	6.4578	8.6402	-0.7502
CHB	FAM72A	206641179	8.4118	10.8426	-0.6765
CHB	FAM72A	206646179	10.1359	12.1979	-0.5137
CHB	FAM72A	206651179	10.2956	13.2144	-0.6742
CHB	FAM72A	206656179	7.6918	10.3343	-0.7692
CHB	FAM72A	206661179	7.1976	8.1319	-0.3396
CHB	FAM72A	206666179	10.6345	10.1649	0.1388
CHB	FAM72A	206671179	10.9372	11.012	-0.0205
CHB	FAM72A	206676179	10.5309	10.3343	0.0572
CHB	FAM72A	206681179	9.9713	9.1484	0.2683
CHB	FAM72A	206686179	5.348	7.1154	-0.7258
CHB	FAM72A	206691179	6.2145	8.6402	-0.8338
CHB	FAM72A	206696179	7.4215	9.9955	-0.7729
YRI	FAM72A	205691179	6.3972	9.2434	-0.916
YRI	FAM72A	205696179	7.2374	11.4282	-1.1064
YRI	FAM72A	205701179	5.4874	10.4199	-1.4199
YRI	FAM72A	205706179	4.8223	10.2518	-1.5869
YRI	FAM72A	205711179	10.472	13.613	-0.703
YRI	FAM72A	205716179	11.6001	13.9492	-0.5137
YRI	FAM72A	205721179	11.6408	13.445	-0.4086
YRI	FAM72A	205726179	12.6886	12.9408	-0.0592
YRI	FAM72A	205731179	13.1591	11.9324	0.311
YRI	FAM72A	205736179	17.6538	13.9492	0.8102
YRI	FAM72A	205741179	15.4729	15.1256	0.0703
YRI	FAM72A	205746179	8.3958	15.1256	-1.3627
YRI	FAM72A	205751179	13.0368	16.134	-0.5897
YRI	FAM72A	205756179	14.6495	14.1172	0.1151
YRI	FAM72A	205761179	10.9677	12.1005	-0.2834
YRI	FAM72A	205766179	8.7016	10.5879	-0.5349
YRI	FAM72A	205771179	9.248	10.924	-0.4616
YRI	FAM72A	205776179	9.8531	12.2685	-0.5965
YRI	FAM72A	205781179	5.953	11.5963	-1.4695

YRI	FAM72A	205786179	7.2499	10.756	-0.9798
YRI	FAM72A	205791179	10.5264	9.7476	0.2386
YRI	FAM72A	205796179	10.1927	9.5795	0.1909
YRI	FAM72A	205801179	5.4176	7.8989	-0.9231
YRI	FAM72A	205806179	5.7035	8.4031	-0.9488
YRI	FAM72A	205811179	8.4756	11.9324	-0.8763
YRI	FAM72A	205816179	9.992	14.1172	-0.892
YRI	FAM72A	205821179	10.5247	16.4701	-1.1099
YRI	FAM72A	205826179	9.2265	14.9575	-1.1729
YRI	FAM72A	205831179	7.0183	12.1005	-1.2715
YRI	FAM72A	205836179	6.6565	14.4534	-1.6486
YRI	FAM72A	205841179	9.5026	15.9659	-1.243
YRI	FAM72A	205846179	12.398	16.302	-0.736
YRI	FAM72A	205851179	11.5734	16.6382	-0.9364
YRI	FAM72A	205856179	10.398	15.6298	-1.0268
YRI	FAM72A	205861179	9.8268	13.613	-0.8474
YRI	FAM72A	205866179	9.8467	11.7644	-0.4927
YRI	FAM72A	205871179	9.5456	11.7644	-0.57
YRI	FAM72A	205876179	8.0951	13.7811	-1.2579
YRI	FAM72A	205881179	9.8066	14.9575	-1.0542
YRI	FAM72A	205886179	13.9249	17.3104	-0.6026
YRI	FAM72A	205891179	15.9716	17.4785	-0.2658
YRI	FAM72A	205896179	15.8718	16.134	-0.0499
YRI	FAM72A	205901179	11.7501	14.4534	-0.5716
YRI	FAM72A	205906179	13.6772	16.6382	-0.5474
YRI	FAM72A	205911179	16.5439	17.3104	-0.1364
YRI	FAM72A	205916179	12.231	13.613	-0.3093
YRI	FAM72A	205921179	7.4183	9.0754	-0.5424
YRI	FAM72A	205926179	2.183	2.1848	-0.002
YRI	FAM72A	205931179	0	0	0
YRI	FAM72A	205936179	0	0	0
YRI	FAM72A	205941179	0	0	0
YRI	FAM72A	205946179	0	0	0
YRI	FAM72A	205951179	0	0	0
YRI	FAM72A	205956179	0	0	0
YRI	FAM72A	205961179	0	0	0
YRI	FAM72A	205966179	0	0	0
YRI	FAM72A	205971179	0	0	0
YRI	FAM72A	205976179	0	0	0
YRI	FAM72A	205981179	0	0	0
YRI	FAM72A	205986179	0	0	0
YRI	FAM72A	205991179	0	0	0
YRI	FAM72A	205996179	0	0	0
YRI	FAM72A	206001179	0	0	0
YRI	FAM72A	206006179	0	0	0
YRI	FAM72A	206011179	0	0	0
YRI	FAM72A	206016179	0	0	0
YRI	FAM72A	206021179	0	0	0
YRI	FAM72A	206026179	0	0	0

YRI	FAM72A	206031179	0	0	0
YRI	FAM72A	206036179	0	0	0
YRI	FAM72A	206041179	0	0	0
YRI	FAM72A	206046179	0	0	0
YRI	FAM72A	206051179	0	0	0
YRI	FAM72A	206056179	0	0	0
YRI	FAM72A	206061179	0	0	0
YRI	FAM72A	206066179	0	0	0
YRI	FAM72A	206071179	3.0707	3.0251	0.0393
YRI	FAM72A	206076179	5.2291	5.7141	-0.2418
YRI	FAM72A	206081179	7.7016	7.8989	-0.0734
YRI	FAM72A	206086179	7.3602	8.067	-0.2579
YRI	FAM72A	206091179	4.0151	5.5461	-0.784
YRI	FAM72A	206096179	6.528	6.7225	-0.0838
YRI	FAM72A	206101179	6.6791	7.2267	-0.221
YRI	FAM72A	206106179	2.981	5.0419	-1.1483
YRI	FAM72A	206111179	1.9645	5.7141	-1.8698
YRI	FAM72A	206116179	3.6179	7.0586	-1.4184
YRI	FAM72A	206121179	2.9357	5.378	-1.2853
YRI	FAM72A	206126179	2.0392	4.7057	-1.5784
YRI	FAM72A	206131179	3.2414	5.2099	-1.0655
YRI	FAM72A	206136179	3.0967	5.0419	-1.0838
YRI	FAM72A	206141179	2.3912	4.5377	-1.3116
YRI	FAM72A	206146179	2.171	4.0335	-1.2602
YRI	FAM72A	206151179	1.3334	3.5293	-1.6649
YRI	FAM72A	206156179	1.2739	3.6974	-1.7664
YRI	FAM72A	206161179	1.9631	5.378	-1.7972
YRI	FAM72A	206166179	2.1481	5.378	-1.6998
YRI	FAM72A	206171179	4.0015	6.7225	-1.1725
YRI	FAM72A	206176179	5.2905	8.5712	-1.1322
YRI	FAM72A	206181179	3.8321	6.3864	-1.1528
YRI	FAM72A	206186179	4.4746	6.7225	-0.9686
YRI	FAM72A	206191179	5.8529	10.0837	-1.2558
YRI	FAM72A	206196179	8.0121	13.2769	-1.2066
YRI	FAM72A	206201179	11.6314	15.4617	-0.7595
YRI	FAM72A	206206179	23.6469	21.512	0.3083
YRI	FAM72A	206211179	31.3647	24.7051	0.8411
YRI	FAM72A	206216179	25.0518	20.5036	0.688
YRI	FAM72A	206221179	18.4437	16.9743	0.2665
YRI	FAM72A	206226179	21.2442	17.4785	0.6641
YRI	FAM72A	206231179	20.6198	18.1507	0.42
YRI	FAM72A	206236179	17.2667	18.4868	-0.2039
YRI	FAM72A	206241179	17.0003	18.1507	-0.1957
YRI	FAM72A	206246179	11.26	13.7811	-0.5577
YRI	FAM72A	206251179	6.8344	10.756	-1.0959
YRI	FAM72A	206256179	7.1353	10.924	-1.0435
YRI	FAM72A	206261179	7.892	10.924	-0.8351
YRI	FAM72A	206266179	9.7516	12.4366	-0.6546
YRI	FAM72A	206271179	10.1258	12.9408	-0.661

YRI	FAM72A	206276179	11.567	15.4617	-0.7723
YRI	FAM72A	206281179	14.0591	16.302	-0.4228
YRI	FAM72A	206286179	11.3635	15.1256	-0.7618
YRI	FAM72A	206291179	11.3665	16.134	-0.9077
YRI	FAM72A	206296179	12.9889	13.613	-0.1397
YRI	FAM72A	206301179	18.2289	14.7895	0.7115
YRI	FAM72A	206306179	19.2802	16.4701	0.5246
YRI	FAM72A	206311179	14.7582	16.302	-0.291
YRI	FAM72A	206316179	11.3358	14.9575	-0.7412
YRI	FAM72A	206321179	10.6324	14.7895	-0.86
YRI	FAM72A	206326179	12.1674	16.9743	-0.8718
YRI	FAM72A	206331179	7.1205	10.5879	-0.9833
YRI	FAM72A	206336179	0.8795	1.8487	-1.2397
YRI	FAM72A	206341179	0	0	0
YRI	FAM72A	206346179	0	0	0
YRI	FAM72A	206351179	0	0	0
YRI	FAM72A	206356179	0	0	0
YRI	FAM72A	206361179	0	0	0
YRI	FAM72A	206366179	0	0	0
YRI	FAM72A	206371179	0	0	0
YRI	FAM72A	206376179	0	0	0
YRI	FAM72A	206381179	0	0	0
YRI	FAM72A	206386179	0	0	0
YRI	FAM72A	206391179	0	0	0
YRI	FAM72A	206396179	0	0	0
YRI	FAM72A	206401179	0	0	0
YRI	FAM72A	206406179	0	0	0
YRI	FAM72A	206411179	0	0	0
YRI	FAM72A	206416179	0	0	0
YRI	FAM72A	206421179	0	0	0
YRI	FAM72A	206426179	0	0	0
YRI	FAM72A	206431179	0	0	0
YRI	FAM72A	206436179	0	0	0
YRI	FAM72A	206441179	0	0	0
YRI	FAM72A	206446179	0	0	0
YRI	FAM72A	206451179	0	0	0
YRI	FAM72A	206456179	0	0	0
YRI	FAM72A	206461179	0	0	0
YRI	FAM72A	206466179	0	0	0
YRI	FAM72A	206471179	0	0	0
YRI	FAM72A	206476179	0	0	0
YRI	FAM72A	206481179	2.7929	5.8822	-1.5011
YRI	FAM72A	206486179	5.3107	8.9073	-1.1979
YRI	FAM72A	206491179	5.2542	5.378	-0.0652
YRI	FAM72A	206496179	4.33	5.0419	-0.3966
YRI	FAM72A	206501179	1.8072	2.8571	-0.9495
YRI	FAM72A	206506179	1.55	2.5209	-0.9723
YRI	FAM72A	206511179	4.9017	7.3947	-0.9851
YRI	FAM72A	206516179	5.2226	8.7392	-1.192

YRI	FAM72A	206521179	4.2763	6.5544	-1.0044
YRI	FAM72A	206526179	4.5594	7.3947	-1.1204
YRI	FAM72A	206531179	3.3218	9.4115	-1.9273
YRI	FAM72A	206536179	3.5144	8.067	-1.6613
YRI	FAM72A	206541179	5.8015	6.8906	-0.4589
YRI	FAM72A	206546179	5.1114	6.3864	-0.5754
YRI	FAM72A	206551179	2.5579	5.378	-1.4842
YRI	FAM72A	206556179	3.715	7.5628	-1.4896
YRI	FAM72A	206561179	3.9029	7.8989	-1.4866
YRI	FAM72A	206566179	4.0399	7.0586	-1.2445
YRI	FAM72A	206571179	3.8389	6.2183	-1.1
YRI	FAM72A	206576179	1.962	3.5293	-1.1883
YRI	FAM72A	206581179	3.825	5.7141	-0.942
YRI	FAM72A	206586179	8.2785	11.9324	-0.9263
YRI	FAM72A	206591179	11.3397	15.2937	-0.7923
YRI	FAM72A	206596179	12.0349	16.9743	-0.8959
YRI	FAM72A	206601179	9.8339	17.1423	-1.3131
YRI	FAM72A	206606179	6.6334	13.445	-1.5426
YRI	FAM72A	206611179	5.1564	10.4199	-1.5152
YRI	FAM72A	206616179	6.6123	10.924	-1.1875
YRI	FAM72A	206621179	14.478	15.4617	-0.1951
YRI	FAM72A	206626179	13.2936	12.9408	0.0828
YRI	FAM72A	206631179	7.169	9.2434	-0.6676
YRI	FAM72A	206636179	9.8636	12.7727	-0.6916
YRI	FAM72A	206641179	11.0087	15.4617	-0.883
YRI	FAM72A	206646179	12.0552	18.1507	-1.0368
YRI	FAM72A	206651179	12.9808	17.6465	-0.8153
YRI	FAM72A	206656179	9.2881	13.445	-0.9414
YRI	FAM72A	206661179	7.6475	12.2685	-1.1411
YRI	FAM72A	206666179	15.2299	14.9575	0.0557
YRI	FAM72A	206671179	16.6816	13.9492	0.5976
YRI	FAM72A	206676179	13.6847	13.1089	0.1336
YRI	FAM72A	206681179	12.1631	12.7727	-0.1449
YRI	FAM72A	206686179	7.8596	10.0837	-0.6601
YRI	FAM72A	206691179	8.6672	12.1005	-0.8589
YRI	FAM72A	206696179	10.2958	14.7895	-0.9296
CEU	FAM75A1	38860667	0.1093	0.6822	-1.496
CEU	FAM75A1	38865667	0.4422	1.1939	-1.3395
CEU	FAM75A1	38870667	1.5473	1.3645	0.2957
CEU	FAM75A1	38875667	4.455	2.729	1.6314
CEU	FAM75A1	38880667	3.7417	2.5584	1.1788
CEU	FAM75A1	38885667	1.3465	1.5351	-0.2797
CEU	FAM75A1	38890667	0.8555	1.0234	-0.3336
CEU	FAM75A1	38895667	0.7355	0.8528	-0.2642
CEU	FAM75A1	38900667	0.7456	1.0234	-0.552
CEU	FAM75A1	38905667	0.0101	0.1706	-0.9534
CEU	FAM75A1	38910667	1.9256	1.1939	1.3039
CEU	FAM75A1	38915667	5.8487	5.6286	0.112
CEU	FAM75A1	38920667	6.5912	7.6753	-0.4167

CEU	FAM75A1	38925667	3.1394	3.7524	-0.4441
CEU	FAM75A1	38930667	2.6162	2.3879	0.2404
CEU	FAM75A1	38935667	2.4426	2.5584	-0.1154
CEU	FAM75A1	38940667	0.2977	0.6822	-1.004
CEU	FAM75A1	38945667	0.7374	1.0234	-0.5683
CEU	FAM75A1	38950667	1.2212	2.0467	-0.9823
CEU	FAM75A1	38955667	2.3715	2.729	-0.3379
CEU	FAM75A1	38960667	2.9942	2.8996	0.0851
CEU	FAM75A1	38965667	1.638	2.0467	-0.4863
CEU	FAM75A1	38970667	0.6982	1.0234	-0.6462
CEU	FAM75A1	38975667	0.1971	0.6822	-1.2667
CEU	FAM75A1	38980667	0.0701	0.6822	-1.5984
CEU	FAM75A1	38985667	0.1488	0.6822	-1.3929
CEU	FAM75A1	38990667	1.1177	2.5584	-1.4352
CEU	FAM75A1	38995667	3.7188	5.458	-0.9092
CEU	FAM75A1	39000667	6.6298	7.6753	-0.4019
CEU	FAM75A1	39005667	10.0429	11.9394	-0.4835
CEU	FAM75A1	39010667	12.5141	14.6684	-0.452
CEU	FAM75A1	39015667	12.0534	13.3039	-0.2878
CEU	FAM75A1	39020667	11.4467	12.6216	-0.2843
CEU	FAM75A1	39025667	7.7963	9.8926	-0.6374
CEU	FAM75A1	39030667	2.6502	5.7991	-1.5596
CEU	FAM75A1	39035667	2.6667	6.1402	-1.6345
CEU	FAM75A1	39040667	3.6482	7.6753	-1.5479
CEU	FAM75A1	39045667	3.5062	6.8225	-1.419
CEU	FAM75A1	39050667	5.3521	8.187	-1.027
CEU	FAM75A1	39055667	8.1657	12.2805	-1.0216
CEU	FAM75A1	39060667	5.8442	9.2104	-1.0938
CEU	FAM75A1	39065667	1.2905	2.3879	-1.1557
CEU	FAM75A1	39070667	4.7108	4.6052	0.0641
CEU	FAM75A1	39075667	11.0818	10.5749	0.1448
CEU	FAM75A1	39080667	17.1004	14.8389	0.4693
CEU	FAM75A1	39085667	18.7225	15.6917	0.5963
CEU	FAM75A1	39090667	18.5945	13.9861	1.0116
CEU	FAM75A1	39095667	23.2033	14.8389	1.7357
CEU	FAM75A1	39100667	17.7627	15.3506	0.4846
CEU	FAM75A1	39105667	7.2587	11.9394	-1.1934
CEU	FAM75A1	39110667	5.7007	10.4043	-1.3645
CEU	FAM75A1	39115667	8.2094	11.7688	-0.9199
CEU	FAM75A1	39120667	7.6871	11.4277	-0.9938
CEU	FAM75A1	39125667	5.5005	9.8926	-1.3355
CEU	FAM75A1	39130667	6.0237	9.3809	-1.0725
CEU	FAM75A1	39135667	7.3452	10.916	-0.9904
CEU	FAM75A1	39140667	7.7148	9.2104	-0.486
CEU	FAM75A1	39145667	9.6584	10.2337	-0.1695
CEU	FAM75A1	39150667	10.9817	14.3272	-0.7178
CEU	FAM75A1	39155667	7.3449	12.2805	-1.2254
CEU	FAM75A1	39160667	4.6271	9.7221	-1.5745
CEU	FAM75A1	39165667	4.2397	8.3576	-1.4638

CEU	FAM75A1	39170667	2.6054	6.4814	-1.7373
CEU	FAM75A1	39175667	2.0971	6.6519	-1.9942
CEU	FAM75A1	39180667	2.3286	5.9697	-1.7572
CEU	FAM75A1	39185667	1.0293	2.2173	-1.3272
CEU	FAM75A1	39190667	0	0	0
CEU	FAM75A1	39195667	0	0	0
CEU	FAM75A1	39200667	0.3562	0.3411	0.0605
CEU	FAM75A1	39205667	0.3562	0.3411	0.0605
CEU	FAM75A1	39210667	0.3004	0.1706	0.771
CEU	FAM75A1	39215667	0.4585	0.3411	0.4706
CEU	FAM75A1	39220667	0.1581	0.1706	-0.0743
CEU	FAM75A1	39225667	0.8543	0.5117	1.0745
CEU	FAM75A1	39230667	0.8543	0.5117	1.0745
CEU	FAM75A1	39235667	0	0	0
CEU	FAM75A1	39240667	0.3131	0.3411	-0.1122
CEU	FAM75A1	39245667	1.3179	0.6822	1.66
CEU	FAM75A1	39250667	1.3051	0.5117	2.4883
CEU	FAM75A1	39255667	1.3051	0.5117	2.4883
CEU	FAM75A1	39260667	1.0047	0.3411	2.6602
CEU	FAM75A1	39265667	0.9528	0.6822	0.7066
CEU	FAM75A1	39270667	0.9728	0.8528	0.2702
CEU	FAM75A1	39275667	0.4343	0.5117	-0.2427
CEU	FAM75A1	39280667	0.4143	0.3411	0.2934
CEU	FAM75A1	39285667	1.3121	0.6822	1.6449
CEU	FAM75A1	39290667	1.3222	0.8528	1.0571
CEU	FAM75A1	39295667	0.9458	1.8762	-1.1846
CEU	FAM75A1	39300667	1.2426	1.8762	-0.8067
CEU	FAM75A1	39305667	0.3069	0.1706	0.8097
CEU	FAM75A1	39310667	0	0	0
CEU	FAM75A1	39315667	0	0	0
CEU	FAM75A1	39320667	0	0	0
CEU	FAM75A1	39325667	0	0	0
CEU	FAM75A1	39330667	0	0	0
CEU	FAM75A1	39335667	0	0	0
CEU	FAM75A1	39340667	0	0	0
CEU	FAM75A1	39345667	0	0	0
CEU	FAM75A1	39350667	1.2158	1.0234	0.3823
CEU	FAM75A1	39355667	2.1101	2.3879	-0.2926
CEU	FAM75A1	39360667	0.8942	1.3645	-0.7608
CEU	FAM75A1	39365667	0.0101	0.1706	-0.9534
CEU	FAM75A1	39370667	0.2406	0.5117	-0.8502
CEU	FAM75A1	39375667	0.5086	0.5117	-0.0097
CEU	FAM75A1	39380667	0.2781	0.1706	0.6386
CEU	FAM75A1	39385667	0.0101	0.1706	-0.9534
CEU	FAM75A1	39390667	0.0101	0.1706	-0.9534
CEU	FAM75A1	39395667	0	0	0
CEU	FAM75A1	39400667	0	0	0
CEU	FAM75A1	39405667	0.0101	0.1706	-0.9534
CEU	FAM75A1	39410667	0.0303	0.3411	-1.2459

CEU	FAM75A1	39415667	0.0202	0.1706	-0.8934
CEU	FAM75A1	39420667	0	0	0
CEU	FAM75A1	39425667	0	0	0
CEU	FAM75A1	39430667	0	0	0
CEU	FAM75A1	39435667	0	0	0
CEU	FAM75A1	39440667	0	0	0
CEU	FAM75A1	39445667	0.0977	0.3411	-0.9757
CEU	FAM75A1	39450667	0.128	0.6822	-1.4472
CEU	FAM75A1	39455667	0.1583	0.6822	-1.3681
CEU	FAM75A1	39460667	0.2882	0.6822	-1.0289
CEU	FAM75A1	39465667	0.1602	0.3411	-0.7252
CEU	FAM75A1	39470667	0	0	0
CEU	FAM75A1	39475667	0.0202	0.3411	-1.2864
CEU	FAM75A1	39480667	0.2639	0.8528	-1.3262
CEU	FAM75A1	39485667	0.3034	0.8528	-1.2372
CEU	FAM75A1	39490667	0.0598	0.3411	-1.1277
CEU	FAM75A1	39495667	0	0	0
CEU	FAM75A1	39500667	0	0	0
CEU	FAM75A1	39505667	0	0	0
CEU	FAM75A1	39510667	0	0	0
CEU	FAM75A1	39515667	0	0	0
CEU	FAM75A1	39520667	0	0	0
CEU	FAM75A1	39525667	0	0	0
CEU	FAM75A1	39530667	0	0	0
CEU	FAM75A1	39535667	0	0	0
CEU	FAM75A1	39540667	0	0	0
CEU	FAM75A1	39545667	0	0	0
CEU	FAM75A1	39550667	0.9858	1.3645	-0.6127
CEU	FAM75A1	39555667	2.283	2.8996	-0.5545
CEU	FAM75A1	39560667	1.2973	1.5351	-0.3526
CEU	FAM75A1	39565667	0.0961	0.1706	-0.4425
CEU	FAM75A1	39570667	0.1361	0.3411	-0.8218
CEU	FAM75A1	39575667	0.0903	0.8528	-1.7171
CEU	FAM75A1	39580667	0.1375	0.8528	-1.6108
CEU	FAM75A1	39585667	0.1173	0.5117	-1.2369
CEU	FAM75A1	39590667	0.5321	0.6822	-0.392
CEU	FAM75A1	39595667	0.5121	0.5117	0.0013
CEU	FAM75A1	39600667	0.0101	0.1706	-0.9534
CEU	FAM75A1	39605667	0	0	0
CEU	FAM75A1	39610667	0.5032	0.1706	1.9757
CEU	FAM75A1	39615667	0.5032	0.1706	1.9757
CEU	FAM75A1	39620667	0.0101	0.1706	-0.9534
CEU	FAM75A1	39625667	0.0101	0.1706	-0.9534
CEU	FAM75A1	39630667	0.0684	0.1706	-0.6071
CEU	FAM75A1	39635667	0.0684	0.1706	-0.6071
CEU	FAM75A1	39640667	0.0101	0.1706	-0.9534
CEU	FAM75A1	39645667	0.0101	0.1706	-0.9534
CEU	FAM75A1	39650667	0	0	0
CEU	FAM75A1	39655667	0	0	0

CEU	FAM75A1	39660667	0.1573	0.1706	-0.079
CEU	FAM75A1	39665667	0.1573	0.1706	-0.079
CEU	FAM75A1	39670667	0	0	0
CEU	FAM75A1	39675667	0	0	0
CEU	FAM75A1	39680667	0	0	0
CEU	FAM75A1	39685667	0	0	0
CEU	FAM75A1	39690667	0	0	0
CEU	FAM75A1	39695667	0	0	0
CEU	FAM75A1	39700667	0	0	0
CEU	FAM75A1	39705667	0	0	0
CEU	FAM75A1	39710667	0	0	0
CEU	FAM75A1	39715667	0	0	0
CEU	FAM75A1	39720667	0	0	0
CEU	FAM75A1	39725667	0.04	0.3411	-1.207
CEU	FAM75A1	39730667	0.8233	1.5351	-1.0555
CEU	FAM75A1	39735667	0.7934	1.3645	-0.9239
CEU	FAM75A1	39740667	0.3861	2.0467	-1.9759
CEU	FAM75A1	39745667	0.6704	2.5584	-1.8808
CEU	FAM75A1	39750667	0.2944	0.6822	-1.0127
CEU	FAM75A1	39755667	0.1833	0.1706	0.0754
CEU	FAM75A1	39760667	0.2235	0.5117	-0.9039
CEU	FAM75A1	39765667	0.1109	1.0234	-1.8132
CEU	FAM75A1	39770667	0.7196	1.7056	-1.3504
CEU	FAM75A1	39775667	2.368	4.4346	-1.2967
CEU	FAM75A1	39780667	2.6316	5.1169	-1.3757
CEU	FAM75A1	39785667	1.1492	2.3879	-1.3045
CEU	FAM75A1	39790667	0.5969	1.3645	-1.2418
CEU	FAM75A1	39795667	0.6641	1.7056	-1.4265
CEU	FAM75A1	39800667	0.7227	2.0467	-1.5754
CEU	FAM75A1	39805667	0.556	1.5351	-1.4518
CEU	FAM75A1	39810667	0.2696	0.6822	-1.0774
CEU	FAM75A1	39815667	0.5986	0.6822	-0.2183
CEU	FAM75A1	39820667	0.545	0.8528	-0.6932
CEU	FAM75A1	39825667	0.1391	0.6822	-1.4182
CEU	FAM75A1	39830667	0.0602	0.3411	-1.126
CEU	FAM75A1	39835667	0	0	0
CEU	FAM75A1	39840667	0	0	0
CEU	FAM75A1	39845667	0.0202	0.1706	-0.8934
CEU	FAM75A1	39850667	0.0202	0.1706	-0.8934
CEU	FAM75A1	39855667	0	0	0
CHB	FAM75A1	38860667	0.0773	1.0165	-1.8691
CHB	FAM75A1	38865667	0.1927	1.6942	-2.0606
CHB	FAM75A1	38870667	0.688	1.3553	-1.0815
CHB	FAM75A1	38875667	2.4498	2.7106	-0.2471
CHB	FAM75A1	38880667	2.4911	2.5412	-0.05
CHB	FAM75A1	38885667	1.0476	1.1859	-0.2469
CHB	FAM75A1	38890667	0.4531	1.0165	-1.1212
CHB	FAM75A1	38895667	0.7476	1.1859	-0.7824
CHB	FAM75A1	38900667	0.8389	1.0165	-0.3534

CHB	FAM75A1	38905667	0.1108	0.1694	-0.3482
CHB	FAM75A1	38910667	1.3705	1.0165	0.7045
CHB	FAM75A1	38915667	3.5102	4.4048	-0.5629
CHB	FAM75A1	38920667	4.0725	6.6072	-1.1133
CHB	FAM75A1	38925667	2.4304	4.066	-1.1029
CHB	FAM75A1	38930667	2.4866	2.033	0.5409
CHB	FAM75A1	38935667	2.0461	1.5247	0.7746
CHB	FAM75A1	38940667	0.0571	0.3388	-1.13
CHB	FAM75A1	38945667	0.5069	0.5082	-0.0041
CHB	FAM75A1	38950667	1.0491	1.0165	0.0649
CHB	FAM75A1	38955667	2.8561	2.2024	0.732
CHB	FAM75A1	38960667	3.442	2.7106	0.693
CHB	FAM75A1	38965667	1.6615	1.6942	-0.0449
CHB	FAM75A1	38970667	0.9781	1.0165	-0.0764
CHB	FAM75A1	38975667	0.5578	1.0165	-0.9128
CHB	FAM75A1	38980667	0.1131	0.6777	-1.476
CHB	FAM75A1	38985667	0.2519	0.5082	-0.8046
CHB	FAM75A1	38990667	3.1949	2.8801	0.2838
CHB	FAM75A1	38995667	6.0461	5.9295	0.0564
CHB	FAM75A1	39000667	12.5184	10.5037	0.5776
CHB	FAM75A1	39005667	23.6598	16.4333	1.3565
CHB	FAM75A1	39010667	29.0276	19.8216	1.4435
CHB	FAM75A1	39015667	27.4469	19.6521	1.2324
CHB	FAM75A1	39020667	24.2733	16.2638	1.5185
CHB	FAM75A1	39025667	15.5113	11.6896	0.9911
CHB	FAM75A1	39030667	5.1211	6.946	-0.766
CHB	FAM75A1	39035667	4.8789	6.0989	-0.5759
CHB	FAM75A1	39040667	7.3713	6.946	0.1785
CHB	FAM75A1	39045667	7.5236	7.6237	-0.0386
CHB	FAM75A1	39050667	9.6056	8.979	0.2079
CHB	FAM75A1	39055667	15.185	10.6731	1.2743
CHB	FAM75A1	39060667	11.3817	8.3013	1.0986
CHB	FAM75A1	39065667	2.1611	2.3718	-0.2224
CHB	FAM75A1	39070667	5.5205	4.7436	0.4583
CHB	FAM75A1	39075667	12.8928	9.4872	1.0735
CHB	FAM75A1	39080667	20.2064	13.2144	1.6151
CHB	FAM75A1	39085667	22.5049	15.4168	1.4142
CHB	FAM75A1	39090667	22.0281	13.892	1.7924
CHB	FAM75A1	39095667	27.0706	13.892	2.9032
CHB	FAM75A1	39100667	20.0394	13.892	1.3543
CHB	FAM75A1	39105667	7.4776	10.6731	-0.9025
CHB	FAM75A1	39110667	7.6419	10.3343	-0.7837
CHB	FAM75A1	39115667	11.059	12.5367	-0.3588
CHB	FAM75A1	39120667	8.6857	10.1649	-0.4373
CHB	FAM75A1	39125667	6.3887	8.979	-0.8593
CHB	FAM75A1	39130667	7.321	9.4872	-0.6828
CHB	FAM75A1	39135667	8.349	8.6402	-0.1001
CHB	FAM75A1	39140667	8.286	7.2848	0.4024
CHB	FAM75A1	39145667	9.6224	8.6402	0.3376

CHB	FAM75A1	39150667	11.1619	11.5202	-0.0942
CHB	FAM75A1	39155667	8.7582	11.5202	-0.7262
CHB	FAM75A1	39160667	7.91	11.012	-0.8508
CHB	FAM75A1	39165667	6.8664	8.979	-0.7008
CHB	FAM75A1	39170667	3.246	5.9295	-1.2991
CHB	FAM75A1	39175667	2.4704	4.913	-1.3972
CHB	FAM75A1	39180667	2.6621	4.4048	-1.0966
CHB	FAM75A1	39185667	1.0779	1.8636	-1.0024
CHB	FAM75A1	39190667	0	0	0
CHB	FAM75A1	39195667	0	0	0
CHB	FAM75A1	39200667	0.0388	0.3388	-1.2034
CHB	FAM75A1	39205667	0.0388	0.3388	-1.2034
CHB	FAM75A1	39210667	0	0	0
CHB	FAM75A1	39215667	0	0	0
CHB	FAM75A1	39220667	0	0	0
CHB	FAM75A1	39225667	0.4877	0.3388	0.5973
CHB	FAM75A1	39230667	0.4877	0.3388	0.5973
CHB	FAM75A1	39235667	0	0	0
CHB	FAM75A1	39240667	0.0097	0.3388	-1.3201
CHB	FAM75A1	39245667	1.015	0.8471	0.3786
CHB	FAM75A1	39250667	1.0053	0.5082	1.5605
CHB	FAM75A1	39255667	1.0048	0.3388	2.6715
CHB	FAM75A1	39260667	1.0048	0.3388	2.6715
CHB	FAM75A1	39265667	0.1108	0.1694	-0.3482
CHB	FAM75A1	39270667	0.1108	0.1694	-0.3482
CHB	FAM75A1	39275667	0.2975	0.1694	0.7612
CHB	FAM75A1	39280667	0.2975	0.1694	0.7612
CHB	FAM75A1	39285667	1.2227	0.8471	0.847
CHB	FAM75A1	39290667	1.2324	1.0165	0.4297
CHB	FAM75A1	39295667	0.4923	1.1859	-1.2381
CHB	FAM75A1	39300667	0.4826	1.0165	-1.0625
CHB	FAM75A1	39305667	0	0	0
CHB	FAM75A1	39310667	0	0	0
CHB	FAM75A1	39315667	0	0	0
CHB	FAM75A1	39320667	0	0	0
CHB	FAM75A1	39325667	0	0	0
CHB	FAM75A1	39330667	0	0	0
CHB	FAM75A1	39335667	0	0	0
CHB	FAM75A1	39340667	0	0	0
CHB	FAM75A1	39345667	0	0	0
CHB	FAM75A1	39350667	0.7259	1.0165	-0.5783
CHB	FAM75A1	39355667	1.2629	1.6942	-0.5919
CHB	FAM75A1	39360667	0.537	0.6777	-0.3678
CHB	FAM75A1	39365667	0	0	0
CHB	FAM75A1	39370667	0.3208	0.6777	-0.933
CHB	FAM75A1	39375667	0.4885	1.0165	-1.0507
CHB	FAM75A1	39380667	0.1677	0.3388	-0.6863
CHB	FAM75A1	39385667	0.0194	0.3388	-1.2812
CHB	FAM75A1	39390667	0.0194	0.3388	-1.2812

CHB	FAM75A1	39395667	0	0	0
CHB	FAM75A1	39400667	0	0	0
CHB	FAM75A1	39405667	0.0097	0.1694	-0.949
CHB	FAM75A1	39410667	0.0771	0.5082	-1.3533
CHB	FAM75A1	39415667	0.0674	0.3388	-1.0887
CHB	FAM75A1	39420667	0	0	0
CHB	FAM75A1	39425667	0	0	0
CHB	FAM75A1	39430667	0	0	0
CHB	FAM75A1	39435667	0	0	0
CHB	FAM75A1	39440667	0	0	0
CHB	FAM75A1	39445667	0.0097	0.1694	-0.949
CHB	FAM75A1	39450667	0.0097	0.1694	-0.949
CHB	FAM75A1	39455667	0.102	0.1694	-0.4005
CHB	FAM75A1	39460667	0.1403	0.5082	-1.1549
CHB	FAM75A1	39465667	0.0383	0.3388	-1.2054
CHB	FAM75A1	39470667	0	0	0
CHB	FAM75A1	39475667	0.0097	0.1694	-0.949
CHB	FAM75A1	39480667	0.1319	0.5082	-1.1813
CHB	FAM75A1	39485667	0.1416	0.6777	-1.4015
CHB	FAM75A1	39490667	0.0194	0.3388	-1.2812
CHB	FAM75A1	39495667	0	0	0
CHB	FAM75A1	39500667	0	0	0
CHB	FAM75A1	39505667	0	0	0
CHB	FAM75A1	39510667	0	0	0
CHB	FAM75A1	39515667	0	0	0
CHB	FAM75A1	39520667	0	0	0
CHB	FAM75A1	39525667	0	0	0
CHB	FAM75A1	39530667	0	0	0
CHB	FAM75A1	39535667	0	0	0
CHB	FAM75A1	39540667	0	0	0
CHB	FAM75A1	39545667	0	0	0
CHB	FAM75A1	39550667	1.5612	1.1859	0.6699
CHB	FAM75A1	39555667	3.4034	2.2024	1.3448
CHB	FAM75A1	39560667	1.8519	1.1859	1.1888
CHB	FAM75A1	39565667	0.3859	0.3388	0.1889
CHB	FAM75A1	39570667	0.3954	0.3388	0.227
CHB	FAM75A1	39575667	0.1437	0.8471	-1.5862
CHB	FAM75A1	39580667	0.5056	0.8471	-0.7701
CHB	FAM75A1	39585667	0.4005	0.6777	-0.7247
CHB	FAM75A1	39590667	0.5262	1.0165	-0.9757
CHB	FAM75A1	39595667	0.5165	0.6777	-0.4214
CHB	FAM75A1	39600667	0.0097	0.1694	-0.949
CHB	FAM75A1	39605667	0	0	0
CHB	FAM75A1	39610667	0.4891	0.1694	1.8997
CHB	FAM75A1	39615667	0.4891	0.1694	1.8997
CHB	FAM75A1	39620667	0	0	0
CHB	FAM75A1	39625667	0	0	0
CHB	FAM75A1	39630667	0	0	0
CHB	FAM75A1	39635667	0	0	0

CHB	FAM75A1	39640667	0.0097	0.1694	-0.949
CHB	FAM75A1	39645667	0.0097	0.1694	-0.949
CHB	FAM75A1	39650667	0	0	0
CHB	FAM75A1	39655667	0	0	0
CHB	FAM75A1	39660667	0.0097	0.1694	-0.949
CHB	FAM75A1	39665667	0.0097	0.1694	-0.949
CHB	FAM75A1	39670667	0	0	0
CHB	FAM75A1	39675667	0	0	0
CHB	FAM75A1	39680667	0	0	0
CHB	FAM75A1	39685667	0	0	0
CHB	FAM75A1	39690667	0	0	0
CHB	FAM75A1	39695667	0	0	0
CHB	FAM75A1	39700667	0	0	0
CHB	FAM75A1	39705667	0	0	0
CHB	FAM75A1	39710667	0	0	0
CHB	FAM75A1	39715667	0	0	0
CHB	FAM75A1	39720667	0.0194	0.3388	-1.2812
CHB	FAM75A1	39725667	0.0194	0.3388	-1.2812
CHB	FAM75A1	39730667	0.665	0.8471	-0.4106
CHB	FAM75A1	39735667	0.6939	1.0165	-0.642
CHB	FAM75A1	39740667	0.4862	1.1859	-1.249
CHB	FAM75A1	39745667	0.5235	1.1859	-1.1824
CHB	FAM75A1	39750667	0.0662	0.1694	-0.6132
CHB	FAM75A1	39755667	0.0097	0.1694	-0.949
CHB	FAM75A1	39760667	0.0291	0.5082	-1.504
CHB	FAM75A1	39765667	0.0194	0.3388	-1.2812
CHB	FAM75A1	39770667	1.0282	1.3553	-0.5301
CHB	FAM75A1	39775667	3.9707	3.7271	0.177
CHB	FAM75A1	39780667	3.5641	3.8965	-0.2325
CHB	FAM75A1	39785667	1.1196	2.5412	-1.4196
CHB	FAM75A1	39790667	0.7906	1.8636	-1.369
CHB	FAM75A1	39795667	0.5168	1.3553	-1.359
CHB	FAM75A1	39800667	0.7582	1.1859	-0.7634
CHB	FAM75A1	39805667	1.3225	2.3718	-1.1076
CHB	FAM75A1	39810667	0.8369	2.2024	-1.529
CHB	FAM75A1	39815667	1.2254	1.5247	-0.4447
CHB	FAM75A1	39820667	1.4832	1.3553	0.2073
CHB	FAM75A1	39825667	0.5273	0.6777	-0.3932
CHB	FAM75A1	39830667	0.2212	0.3388	-0.4717
CHB	FAM75A1	39835667	0	0	0
CHB	FAM75A1	39840667	0	0	0
CHB	FAM75A1	39845667	0.0097	0.1694	-0.949
CHB	FAM75A1	39850667	0.0097	0.1694	-0.949
CHB	FAM75A1	39855667	0	0	0
YRI	FAM75A1	38860667	0.8499	1.0084	-0.316
YRI	FAM75A1	38865667	0.9679	1.6806	-0.9805
YRI	FAM75A1	38870667	0.5503	1.5126	-1.4329
YRI	FAM75A1	38875667	2.5025	3.6974	-0.8709
YRI	FAM75A1	38880667	2.5828	3.6974	-0.8124

YRI	FAM75A1	38885667	1.2195	2.0167	-0.9532
YRI	FAM75A1	38890667	0.7068	1.1764	-0.8399
YRI	FAM75A1	38895667	0.3789	1.0084	-1.255
YRI	FAM75A1	38900667	0.5242	1.1764	-1.1665
YRI	FAM75A1	38905667	0.1638	0.3361	-0.6917
YRI	FAM75A1	38910667	1.4102	2.5209	-1.1123
YRI	FAM75A1	38915667	5.7443	8.9073	-1.0535
YRI	FAM75A1	38920667	7.2414	9.7476	-0.7677
YRI	FAM75A1	38925667	3.3109	4.5377	-0.7496
YRI	FAM75A1	38930667	2.2065	3.8654	-1.1641
YRI	FAM75A1	38935667	1.9689	3.3612	-1.1
YRI	FAM75A1	38940667	0.2031	1.1764	-1.7408
YRI	FAM75A1	38945667	0.6183	1.5126	-1.3317
YRI	FAM75A1	38950667	1.0629	2.689	-1.5454
YRI	FAM75A1	38955667	2.5123	4.2016	-1.1035
YRI	FAM75A1	38960667	3.5068	4.5377	-0.6299
YRI	FAM75A1	38965667	2.9316	3.1932	-0.2157
YRI	FAM75A1	38970667	1.6279	1.5126	0.1717
YRI	FAM75A1	38975667	0.3313	0.8403	-1.1497
YRI	FAM75A1	38980667	0.1772	0.6722	-1.2959
YRI	FAM75A1	38985667	0.8437	1.1764	-0.5951
YRI	FAM75A1	38990667	5.545	3.8654	1.1787
YRI	FAM75A1	38995667	9.3024	7.2267	0.8376
YRI	FAM75A1	39000667	15.7488	12.1005	0.9127
YRI	FAM75A1	39005667	27.4127	19.1591	1.3327
YRI	FAM75A1	39010667	33.6685	23.0245	1.4394
YRI	FAM75A1	39015667	30.7945	21.8481	1.2728
YRI	FAM75A1	39020667	25.2885	21.0078	0.6325
YRI	FAM75A1	39025667	17.9732	16.8062	0.2137
YRI	FAM75A1	39030667	8.0726	9.0754	-0.3283
YRI	FAM75A1	39035667	6.2024	9.9157	-1.1196
YRI	FAM75A1	39040667	7.9095	15.1256	-1.4612
YRI	FAM75A1	39045667	8.7557	15.9659	-1.3866
YRI	FAM75A1	39050667	12.5434	14.9575	-0.4941
YRI	FAM75A1	39055667	18.4181	16.4701	0.3637
YRI	FAM75A1	39060667	13.5778	13.613	-0.0079
YRI	FAM75A1	39065667	3.1793	4.7057	-0.9036
YRI	FAM75A1	39070667	6.7154	6.0502	0.3152
YRI	FAM75A1	39075667	14.1036	13.2769	0.1895
YRI	FAM75A1	39080667	20.2779	18.3188	0.3303
YRI	FAM75A1	39085667	23.2816	22.8565	0.0579
YRI	FAM75A1	39090667	22.4506	22.5203	-0.0096
YRI	FAM75A1	39095667	23.9271	19.4952	0.7038
YRI	FAM75A1	39100667	19.2576	19.4952	-0.0377
YRI	FAM75A1	39105667	10.6996	18.823	-1.3342
YRI	FAM75A1	39110667	11.0274	20.5036	-1.4334
YRI	FAM75A1	39115667	13.0799	22.5203	-1.3043
YRI	FAM75A1	39120667	9.7063	19.9994	-1.5948
YRI	FAM75A1	39125667	8.0856	17.4785	-1.6565

YRI	FAM75A1	39130667	9.0354	15.4617	-1.2743
YRI	FAM75A1	39135667	10.6386	16.302	-1.0677
YRI	FAM75A1	39140667	9.2404	16.9743	-1.4027
YRI	FAM75A1	39145667	9.4959	19.1591	-1.5603
YRI	FAM75A1	39150667	11.9036	19.6633	-1.222
YRI	FAM75A1	39155667	11.6757	17.4785	-1.0234
YRI	FAM75A1	39160667	11.4624	17.1423	-1.0205
YRI	FAM75A1	39165667	8.5787	15.9659	-1.4206
YRI	FAM75A1	39170667	7.5405	17.6465	-1.766
YRI	FAM75A1	39175667	7.7715	17.4785	-1.7119
YRI	FAM75A1	39180667	6.4296	13.613	-1.6078
YRI	FAM75A1	39185667	3.1814	6.0502	-1.3593
YRI	FAM75A1	39190667	0.0185	0.3361	-1.275
YRI	FAM75A1	39195667	0.0185	0.3361	-1.275
YRI	FAM75A1	39200667	0.9887	1.5126	-0.7801
YRI	FAM75A1	39205667	1.1432	1.8487	-0.9024
YRI	FAM75A1	39210667	0.2187	0.6722	-1.1872
YRI	FAM75A1	39215667	0.0641	0.3361	-1.092
YRI	FAM75A1	39220667	0.0185	0.1681	-0.8893
YRI	FAM75A1	39225667	0.5102	0.5042	0.0189
YRI	FAM75A1	39230667	0.8378	0.5042	1.0484
YRI	FAM75A1	39235667	0.3461	0.1681	1.0582
YRI	FAM75A1	39240667	0.7134	0.6722	0.1079
YRI	FAM75A1	39245667	1.3911	1.1764	0.384
YRI	FAM75A1	39250667	1.0918	1.1764	-0.1513
YRI	FAM75A1	39255667	1.5869	1.8487	-0.3349
YRI	FAM75A1	39260667	1.1727	1.1764	-0.0066
YRI	FAM75A1	39265667	0.1003	0.6722	-1.4972
YRI	FAM75A1	39270667	0.7529	1.6806	-1.2762
YRI	FAM75A1	39275667	1.2128	1.5126	-0.4464
YRI	FAM75A1	39280667	0.5601	0.5042	0.1757
YRI	FAM75A1	39285667	1.6255	1.3445	0.4564
YRI	FAM75A1	39290667	1.7067	1.6806	0.0359
YRI	FAM75A1	39295667	0.7217	2.0167	-1.5484
YRI	FAM75A1	39300667	0.6588	1.8487	-1.522
YRI	FAM75A1	39305667	0.0183	0.1681	-0.8905
YRI	FAM75A1	39310667	0	0	0
YRI	FAM75A1	39315667	0	0	0
YRI	FAM75A1	39320667	0	0	0
YRI	FAM75A1	39325667	0	0	0
YRI	FAM75A1	39330667	0	0	0
YRI	FAM75A1	39335667	0	0	0
YRI	FAM75A1	39340667	0	0	0
YRI	FAM75A1	39345667	0	0	0
YRI	FAM75A1	39350667	1.2085	1.0084	0.3989
YRI	FAM75A1	39355667	1.8867	1.8487	0.0486
YRI	FAM75A1	39360667	0.7058	1.1764	-0.8417
YRI	FAM75A1	39365667	0.1071	0.5042	-1.248
YRI	FAM75A1	39370667	0.3718	0.8403	-1.0582

YRI	FAM75A1	39375667	0.4776	1.0084	-1.0582
YRI	FAM75A1	39380667	0.231	0.5042	-0.8586
YRI	FAM75A1	39385667	0.1351	0.5042	-1.16
YRI	FAM75A1	39390667	0.0895	0.3361	-0.99
YRI	FAM75A1	39395667	0	0	0
YRI	FAM75A1	39400667	0	0	0
YRI	FAM75A1	39405667	0	0	0
YRI	FAM75A1	39410667	0.3253	0.6722	-0.9082
YRI	FAM75A1	39415667	0.3253	0.6722	-0.9082
YRI	FAM75A1	39420667	0	0	0
YRI	FAM75A1	39425667	0	0	0
YRI	FAM75A1	39430667	0	0	0
YRI	FAM75A1	39435667	0	0	0
YRI	FAM75A1	39440667	0	0	0
YRI	FAM75A1	39445667	0.0638	0.3361	-1.0932
YRI	FAM75A1	39450667	0.073	0.5042	-1.3551
YRI	FAM75A1	39455667	0.0895	0.3361	-0.99
YRI	FAM75A1	39460667	0.163	1.0084	-1.6855
YRI	FAM75A1	39465667	0.2536	1.1764	-1.6505
YRI	FAM75A1	39470667	0.1708	0.3361	-0.6636
YRI	FAM75A1	39475667	0.1083	0.6722	-1.4762
YRI	FAM75A1	39480667	0.1452	1.1764	-1.8444
YRI	FAM75A1	39485667	0.2419	1.0084	-1.5282
YRI	FAM75A1	39490667	0.205	0.5042	-0.9403
YRI	FAM75A1	39495667	0	0	0
YRI	FAM75A1	39500667	0	0	0
YRI	FAM75A1	39505667	0	0	0
YRI	FAM75A1	39510667	0	0	0
YRI	FAM75A1	39515667	0	0	0
YRI	FAM75A1	39520667	0	0	0
YRI	FAM75A1	39525667	0	0	0
YRI	FAM75A1	39530667	0	0	0
YRI	FAM75A1	39535667	0	0	0
YRI	FAM75A1	39540667	0	0	0
YRI	FAM75A1	39545667	0	0	0
YRI	FAM75A1	39550667	2.5017	1.8487	0.8352
YRI	FAM75A1	39555667	4.1909	3.6974	0.3597
YRI	FAM75A1	39560667	1.7612	2.0167	-0.3055
YRI	FAM75A1	39565667	0.2183	0.3361	-0.4729
YRI	FAM75A1	39570667	0.1739	0.3361	-0.6512
YRI	FAM75A1	39575667	1.1476	1.0084	0.2775
YRI	FAM75A1	39580667	1.3114	1.1764	0.2415
YRI	FAM75A1	39585667	0.4752	1.0084	-1.063
YRI	FAM75A1	39590667	0.857	1.3445	-0.7918
YRI	FAM75A1	39595667	0.5918	0.8403	-0.5613
YRI	FAM75A1	39600667	0.0185	0.1681	-0.8893
YRI	FAM75A1	39605667	0.0093	0.1681	-0.944
YRI	FAM75A1	39610667	0.237	0.3361	-0.3978
YRI	FAM75A1	39615667	0.2278	0.1681	0.3549

YRI	FAM75A1	39620667	0.1258	0.5042	-1.1892
YRI	FAM75A1	39625667	0.1258	0.5042	-1.1892
YRI	FAM75A1	39630667	0.0093	0.1681	-0.944
YRI	FAM75A1	39635667	0.037	0.6722	-1.6629
YRI	FAM75A1	39640667	0.0554	0.6722	-1.6147
YRI	FAM75A1	39645667	0.0276	0.1681	-0.8352
YRI	FAM75A1	39650667	0	0	0
YRI	FAM75A1	39655667	0	0	0
YRI	FAM75A1	39660667	0.2541	0.1681	0.5112
YRI	FAM75A1	39665667	0.2541	0.1681	0.5112
YRI	FAM75A1	39670667	0	0	0
YRI	FAM75A1	39675667	0	0	0
YRI	FAM75A1	39680667	0	0	0
YRI	FAM75A1	39685667	0	0	0
YRI	FAM75A1	39690667	0	0	0
YRI	FAM75A1	39695667	0	0	0
YRI	FAM75A1	39700667	0	0	0
YRI	FAM75A1	39705667	0	0	0
YRI	FAM75A1	39710667	0	0	0
YRI	FAM75A1	39715667	0.1616	0.1681	-0.0386
YRI	FAM75A1	39720667	0.1616	0.1681	-0.0386
YRI	FAM75A1	39725667	0	0	0
YRI	FAM75A1	39730667	0.3205	1.5126	-1.7751
YRI	FAM75A1	39735667	0.4121	2.0167	-1.9185
YRI	FAM75A1	39740667	0.8923	2.5209	-1.631
YRI	FAM75A1	39745667	1.609	4.0335	-1.6404
YRI	FAM75A1	39750667	1.123	2.1848	-1.1922
YRI	FAM75A1	39755667	0.3332	0.5042	-0.5374
YRI	FAM75A1	39760667	0.3647	1.0084	-1.2833
YRI	FAM75A1	39765667	0.411	1.6806	-1.7466
YRI	FAM75A1	39770667	1.0999	2.689	-1.5102
YRI	FAM75A1	39775667	4.6388	5.7141	-0.5362
YRI	FAM75A1	39780667	5.1645	6.3864	-0.5515
YRI	FAM75A1	39785667	2.6113	4.0335	-0.9623
YRI	FAM75A1	39790667	1.3153	2.3529	-1.0983
YRI	FAM75A1	39795667	0.7971	1.3445	-0.8891
YRI	FAM75A1	39800667	0.932	1.6806	-1.0299
YRI	FAM75A1	39805667	1.0721	2.0167	-1.1294
YRI	FAM75A1	39810667	0.7181	1.3445	-1.0174
YRI	FAM75A1	39815667	1.1289	2.3529	-1.2957
YRI	FAM75A1	39820667	1.233	2.3529	-1.1855
YRI	FAM75A1	39825667	0.3616	1.0084	-1.2895
YRI	FAM75A1	39830667	0.4172	0.8403	-0.9556
YRI	FAM75A1	39835667	0.2148	0.3361	-0.487
YRI	FAM75A1	39840667	0.0093	0.1681	-0.944
YRI	FAM75A1	39845667	0	0	0
YRI	FAM75A1	39850667	0	0	0
YRI	FAM75A1	39855667	0	0	0
CEU	FAM75A5	60419372	0	0	0

CEU	FAM75A5	60424372	0	0	0
CEU	FAM75A5	60429372	0	0	0
CEU	FAM75A5	60434372	0	0	0
CEU	FAM75A5	60439372	0	0	0
CEU	FAM75A5	60444372	0	0	0
CEU	FAM75A5	60449372	0	0	0
CEU	FAM75A5	60454372	0	0	0
CEU	FAM75A5	60459372	0	0	0
CEU	FAM75A5	60464372	0	0	0
CEU	FAM75A5	60469372	0	0	0
CEU	FAM75A5	60474372	0	0	0
CEU	FAM75A5	60479372	0	0	0
CEU	FAM75A5	60484372	0	0	0
CEU	FAM75A5	60489372	0	0	0
CEU	FAM75A5	60494372	0	0	0
CEU	FAM75A5	60499372	0	0	0
CEU	FAM75A5	60504372	0	0	0
CEU	FAM75A5	60509372	0	0	0
CEU	FAM75A5	60514372	0	0	0
CEU	FAM75A5	60519372	0	0	0
CEU	FAM75A5	60524372	0	0	0
CEU	FAM75A5	60529372	0	0	0
CEU	FAM75A5	60534372	0	0	0
CEU	FAM75A5	60539372	0	0	0
CEU	FAM75A5	60544372	0	0	0
CEU	FAM75A5	60549372	0	0	0
CEU	FAM75A5	60554372	0	0	0
CEU	FAM75A5	60559372	0	0	0
CEU	FAM75A5	60564372	0	0	0
CEU	FAM75A5	60569372	0	0	0
CEU	FAM75A5	60574372	0	0	0
CEU	FAM75A5	60579372	0	0	0
CEU	FAM75A5	60584372	0	0	0
CEU	FAM75A5	60589372	0	0	0
CEU	FAM75A5	60594372	0	0	0
CEU	FAM75A5	60599372	0	0	0
CEU	FAM75A5	60604372	0	0	0
CEU	FAM75A5	60609372	0	0	0
CEU	FAM75A5	60614372	0	0	0
CEU	FAM75A5	60619372	0	0	0
CEU	FAM75A5	60624372	0	0	0
CEU	FAM75A5	60629372	0	0	0
CEU	FAM75A5	60634372	0	0	0
CEU	FAM75A5	60639372	0	0	0
CEU	FAM75A5	60644372	0	0	0
CEU	FAM75A5	60649372	0	0	0
CEU	FAM75A5	60654372	0	0	0
CEU	FAM75A5	60659372	0	0	0
CEU	FAM75A5	60664372	0	0	0

CEU	FAM75A5	60669372	0	0	0
CEU	FAM75A5	60674372	0	0	0
CEU	FAM75A5	60679372	0	0	0
CEU	FAM75A5	60684372	0	0	0
CEU	FAM75A5	60689372	0	0	0
CEU	FAM75A5	60694372	0	0	0
CEU	FAM75A5	60699372	0	0	0
CEU	FAM75A5	60704372	0	0	0
CEU	FAM75A5	60709372	0	0	0
CEU	FAM75A5	60714372	0	0	0
CEU	FAM75A5	60719372	0	0	0
CEU	FAM75A5	60724372	0	0	0
CEU	FAM75A5	60729372	0	0	0
CEU	FAM75A5	60734372	0	0	0
CEU	FAM75A5	60739372	0	0	0
CEU	FAM75A5	60744372	0	0	0
CEU	FAM75A5	60749372	0	0	0
CEU	FAM75A5	60754372	0	0	0
CEU	FAM75A5	60759372	0	0	0
CEU	FAM75A5	60764372	0	0	0
CEU	FAM75A5	60769372	0	0	0
CEU	FAM75A5	60774372	0	0	0
CEU	FAM75A5	60779372	0	0	0
CEU	FAM75A5	60784372	0	0	0
CEU	FAM75A5	60789372	0	0	0
CEU	FAM75A5	60794372	0	0	0
CEU	FAM75A5	60799372	0	0	0
CEU	FAM75A5	60804372	0	0	0
CEU	FAM75A5	60809372	0	0	0
CEU	FAM75A5	60814372	0	0	0
CEU	FAM75A5	60819372	0	0	0
CEU	FAM75A5	60824372	0	0	0
CEU	FAM75A5	60829372	0	0	0
CEU	FAM75A5	60834372	0	0	0
CEU	FAM75A5	60839372	0	0	0
CEU	FAM75A5	60844372	0	0	0
CEU	FAM75A5	60849372	0	0	0
CEU	FAM75A5	60854372	0	0	0
CEU	FAM75A5	60859372	0	0	0
CEU	FAM75A5	60864372	0	0	0
CEU	FAM75A5	60869372	0	0	0
CEU	FAM75A5	60874372	0	0	0
CEU	FAM75A5	60879372	0	0	0
CEU	FAM75A5	60884372	0	0	0
CEU	FAM75A5	60889372	0	0	0
CEU	FAM75A5	60894372	0	0	0
CEU	FAM75A5	60899372	0	0	0
CEU	FAM75A5	60904372	0	0	0
CEU	FAM75A5	60909372	0	0	0

CEU	FAM75A5	60914372	0	0	0
CEU	FAM75A5	60919372	0	0	0
CEU	FAM75A5	60924372	0	0	0
CEU	FAM75A5	60929372	0	0	0
CEU	FAM75A5	60934372	0	0	0
CEU	FAM75A5	60939372	0	0	0
CEU	FAM75A5	60944372	0	0	0
CEU	FAM75A5	60949372	0	0	0
CEU	FAM75A5	60954372	0	0	0
CEU	FAM75A5	60959372	0	0	0
CEU	FAM75A5	60964372	0	0	0
CEU	FAM75A5	60969372	0	0	0
CEU	FAM75A5	60974372	0	0	0
CEU	FAM75A5	60979372	0	0	0
CEU	FAM75A5	60984372	0	0	0
CEU	FAM75A5	60989372	0	0	0
CEU	FAM75A5	60994372	0	0	0
CEU	FAM75A5	60999372	0	0	0
CEU	FAM75A5	61004372	0	0	0
CEU	FAM75A5	61009372	0	0	0
CEU	FAM75A5	61014372	0	0	0
CEU	FAM75A5	61019372	0	0	0
CEU	FAM75A5	61024372	0	0	0
CEU	FAM75A5	61029372	0	0	0
CEU	FAM75A5	61034372	0	0	0
CEU	FAM75A5	61039372	0	0	0
CEU	FAM75A5	61044372	0	0	0
CEU	FAM75A5	61049372	0	0	0
CEU	FAM75A5	61054372	0	0	0
CEU	FAM75A5	61059372	0	0	0
CEU	FAM75A5	61064372	0	0	0
CEU	FAM75A5	61069372	0	0	0
CEU	FAM75A5	61074372	0	0	0
CEU	FAM75A5	61079372	0	0	0
CEU	FAM75A5	61084372	0	0	0
CEU	FAM75A5	61089372	0	0	0
CEU	FAM75A5	61094372	0	0	0
CEU	FAM75A5	61099372	0	0	0
CEU	FAM75A5	61104372	0	0	0
CEU	FAM75A5	61109372	0	0	0
CEU	FAM75A5	61114372	0	0	0
CEU	FAM75A5	61119372	0	0	0
CEU	FAM75A5	61124372	0	0	0
CEU	FAM75A5	61129372	0	0	0
CEU	FAM75A5	61134372	0	0	0
CEU	FAM75A5	61139372	0	0	0
CEU	FAM75A5	61144372	0	0	0
CEU	FAM75A5	61149372	0	0	0
CEU	FAM75A5	61154372	0	0	0

CEU	FAM75A5	61159372	0	0	0
CEU	FAM75A5	61164372	0	0	0
CEU	FAM75A5	61169372	0	0	0
CEU	FAM75A5	61174372	0	0	0
CEU	FAM75A5	61179372	0	0	0
CEU	FAM75A5	61184372	0	0	0
CEU	FAM75A5	61189372	0	0	0
CEU	FAM75A5	61194372	0	0	0
CEU	FAM75A5	61199372	0	0	0
CEU	FAM75A5	61204372	0	0	0
CEU	FAM75A5	61209372	0	0	0
CEU	FAM75A5	61214372	0	0	0
CEU	FAM75A5	61219372	0	0	0
CEU	FAM75A5	61224372	0	0	0
CEU	FAM75A5	61229372	0	0	0
CEU	FAM75A5	61234372	0	0	0
CEU	FAM75A5	61239372	0	0	0
CEU	FAM75A5	61244372	0	0	0
CEU	FAM75A5	61249372	0	0	0
CEU	FAM75A5	61254372	0	0	0
CEU	FAM75A5	61259372	0	0	0
CEU	FAM75A5	61264372	0	0	0
CEU	FAM75A5	61269372	0	0	0
CEU	FAM75A5	61274372	0	0	0
CEU	FAM75A5	61279372	0	0	0
CEU	FAM75A5	61284372	0	0	0
CEU	FAM75A5	61289372	0	0	0
CEU	FAM75A5	61294372	0	0	0
CEU	FAM75A5	61299372	0	0	0
CEU	FAM75A5	61304372	0	0	0
CEU	FAM75A5	61309372	0	0	0
CEU	FAM75A5	61314372	0	0	0
CEU	FAM75A5	61319372	0	0	0
CEU	FAM75A5	61324372	0	0	0
CEU	FAM75A5	61329372	0	0	0
CEU	FAM75A5	61334372	0	0	0
CEU	FAM75A5	61339372	0	0	0
CEU	FAM75A5	61344372	0	0	0
CEU	FAM75A5	61349372	0	0	0
CEU	FAM75A5	61354372	0	0	0
CEU	FAM75A5	61359372	0	0	0
CEU	FAM75A5	61364372	0	0	0
CEU	FAM75A5	61369372	0	0	0
CEU	FAM75A5	61374372	0	0	0
CEU	FAM75A5	61379372	0	0	0
CEU	FAM75A5	61384372	0	0	0
CEU	FAM75A5	61389372	0	0	0
CEU	FAM75A5	61394372	0	0	0
CEU	FAM75A5	61399372	0	0	0

CEU	FAM75A5	61404372	0	0	0
CEU	FAM75A5	61409372	0	0	0
CEU	FAM75A5	61414372	0	0	0
CHB	FAM75A5	60419372	0	0	0
CHB	FAM75A5	60424372	0	0	0
CHB	FAM75A5	60429372	0	0	0
CHB	FAM75A5	60434372	0	0	0
CHB	FAM75A5	60439372	0	0	0
CHB	FAM75A5	60444372	0	0	0
CHB	FAM75A5	60449372	0	0	0
CHB	FAM75A5	60454372	0	0	0
CHB	FAM75A5	60459372	0	0	0
CHB	FAM75A5	60464372	0	0	0
CHB	FAM75A5	60469372	0	0	0
CHB	FAM75A5	60474372	0	0	0
CHB	FAM75A5	60479372	0	0	0
CHB	FAM75A5	60484372	0	0	0
CHB	FAM75A5	60489372	0	0	0
CHB	FAM75A5	60494372	0	0	0
CHB	FAM75A5	60499372	0	0	0
CHB	FAM75A5	60504372	0	0	0
CHB	FAM75A5	60509372	0	0	0
CHB	FAM75A5	60514372	0	0	0
CHB	FAM75A5	60519372	0	0	0
CHB	FAM75A5	60524372	0	0	0
CHB	FAM75A5	60529372	0	0	0
CHB	FAM75A5	60534372	0	0	0
CHB	FAM75A5	60539372	0	0	0
CHB	FAM75A5	60544372	0	0	0
CHB	FAM75A5	60549372	0	0	0
CHB	FAM75A5	60554372	0	0	0
CHB	FAM75A5	60559372	0	0	0
CHB	FAM75A5	60564372	0	0	0
CHB	FAM75A5	60569372	0	0	0
CHB	FAM75A5	60574372	0	0	0
CHB	FAM75A5	60579372	0	0	0
CHB	FAM75A5	60584372	0	0	0
CHB	FAM75A5	60589372	0	0	0
CHB	FAM75A5	60594372	0	0	0
CHB	FAM75A5	60599372	0	0	0
CHB	FAM75A5	60604372	0	0	0
CHB	FAM75A5	60609372	0	0	0
CHB	FAM75A5	60614372	0	0	0
CHB	FAM75A5	60619372	0	0	0
CHB	FAM75A5	60624372	0	0	0
CHB	FAM75A5	60629372	0	0	0
CHB	FAM75A5	60634372	0	0	0
CHB	FAM75A5	60639372	0	0	0
CHB	FAM75A5	60644372	0	0	0

CHB	FAM75A5	60649372	0	0	0
CHB	FAM75A5	60654372	0	0	0
CHB	FAM75A5	60659372	0	0	0
CHB	FAM75A5	60664372	0	0	0
CHB	FAM75A5	60669372	0	0	0
CHB	FAM75A5	60674372	0	0	0
CHB	FAM75A5	60679372	0	0	0
CHB	FAM75A5	60684372	0	0	0
CHB	FAM75A5	60689372	0	0	0
CHB	FAM75A5	60694372	0	0	0
CHB	FAM75A5	60699372	0	0	0
CHB	FAM75A5	60704372	0	0	0
CHB	FAM75A5	60709372	0	0	0
CHB	FAM75A5	60714372	0	0	0
CHB	FAM75A5	60719372	0	0	0
CHB	FAM75A5	60724372	0	0	0
CHB	FAM75A5	60729372	0	0	0
CHB	FAM75A5	60734372	0	0	0
CHB	FAM75A5	60739372	0	0	0
CHB	FAM75A5	60744372	0	0	0
CHB	FAM75A5	60749372	0	0	0
CHB	FAM75A5	60754372	0	0	0
CHB	FAM75A5	60759372	0	0	0
CHB	FAM75A5	60764372	0	0	0
CHB	FAM75A5	60769372	0	0	0
CHB	FAM75A5	60774372	0	0	0
CHB	FAM75A5	60779372	0	0	0
CHB	FAM75A5	60784372	0	0	0
CHB	FAM75A5	60789372	0	0	0
CHB	FAM75A5	60794372	0	0	0
CHB	FAM75A5	60799372	0	0	0
CHB	FAM75A5	60804372	0	0	0
CHB	FAM75A5	60809372	0	0	0
CHB	FAM75A5	60814372	0	0	0
CHB	FAM75A5	60819372	0	0	0
CHB	FAM75A5	60824372	0	0	0
CHB	FAM75A5	60829372	0	0	0
CHB	FAM75A5	60834372	0	0	0
CHB	FAM75A5	60839372	0	0	0
CHB	FAM75A5	60844372	0	0	0
CHB	FAM75A5	60849372	0	0	0
CHB	FAM75A5	60854372	0	0	0
CHB	FAM75A5	60859372	0	0	0
CHB	FAM75A5	60864372	0	0	0
CHB	FAM75A5	60869372	0	0	0
CHB	FAM75A5	60874372	0	0	0
CHB	FAM75A5	60879372	0	0	0
CHB	FAM75A5	60884372	0	0	0
CHB	FAM75A5	60889372	0	0	0

CHB	FAM75A5	60894372	0	0	0
CHB	FAM75A5	60899372	0	0	0
CHB	FAM75A5	60904372	0	0	0
CHB	FAM75A5	60909372	0	0	0
CHB	FAM75A5	60914372	0	0	0
CHB	FAM75A5	60919372	0	0	0
CHB	FAM75A5	60924372	0	0	0
CHB	FAM75A5	60929372	0	0	0
CHB	FAM75A5	60934372	0	0	0
CHB	FAM75A5	60939372	0	0	0
CHB	FAM75A5	60944372	0	0	0
CHB	FAM75A5	60949372	0	0	0
CHB	FAM75A5	60954372	0	0	0
CHB	FAM75A5	60959372	0	0	0
CHB	FAM75A5	60964372	0	0	0
CHB	FAM75A5	60969372	0	0	0
CHB	FAM75A5	60974372	0	0	0
CHB	FAM75A5	60979372	0	0	0
CHB	FAM75A5	60984372	0	0	0
CHB	FAM75A5	60989372	0	0	0
CHB	FAM75A5	60994372	0	0	0
CHB	FAM75A5	60999372	0	0	0
CHB	FAM75A5	61004372	0	0	0
CHB	FAM75A5	61009372	0	0	0
CHB	FAM75A5	61014372	0	0	0
CHB	FAM75A5	61019372	0	0	0
CHB	FAM75A5	61024372	0	0	0
CHB	FAM75A5	61029372	0	0	0
CHB	FAM75A5	61034372	0	0	0
CHB	FAM75A5	61039372	0	0	0
CHB	FAM75A5	61044372	0	0	0
CHB	FAM75A5	61049372	0	0	0
CHB	FAM75A5	61054372	0	0	0
CHB	FAM75A5	61059372	0	0	0
CHB	FAM75A5	61064372	0	0	0
CHB	FAM75A5	61069372	0	0	0
CHB	FAM75A5	61074372	0	0	0
CHB	FAM75A5	61079372	0	0	0
CHB	FAM75A5	61084372	0	0	0
CHB	FAM75A5	61089372	0	0	0
CHB	FAM75A5	61094372	0	0	0
CHB	FAM75A5	61099372	0	0	0
CHB	FAM75A5	61104372	0	0	0
CHB	FAM75A5	61109372	0	0	0
CHB	FAM75A5	61114372	0	0	0
CHB	FAM75A5	61119372	0	0	0
CHB	FAM75A5	61124372	0	0	0
CHB	FAM75A5	61129372	0	0	0
CHB	FAM75A5	61134372	0	0	0

CHB	FAM75A5	61139372	0	0	0
CHB	FAM75A5	61144372	0	0	0
CHB	FAM75A5	61149372	0	0	0
CHB	FAM75A5	61154372	0	0	0
CHB	FAM75A5	61159372	0	0	0
CHB	FAM75A5	61164372	0	0	0
CHB	FAM75A5	61169372	0	0	0
CHB	FAM75A5	61174372	0	0	0
CHB	FAM75A5	61179372	0	0	0
CHB	FAM75A5	61184372	0	0	0
CHB	FAM75A5	61189372	0	0	0
CHB	FAM75A5	61194372	0	0	0
CHB	FAM75A5	61199372	0	0	0
CHB	FAM75A5	61204372	0	0	0
CHB	FAM75A5	61209372	0	0	0
CHB	FAM75A5	61214372	0	0	0
CHB	FAM75A5	61219372	0	0	0
CHB	FAM75A5	61224372	0	0	0
CHB	FAM75A5	61229372	0	0	0
CHB	FAM75A5	61234372	0	0	0
CHB	FAM75A5	61239372	0	0	0
CHB	FAM75A5	61244372	0	0	0
CHB	FAM75A5	61249372	0	0	0
CHB	FAM75A5	61254372	0	0	0
CHB	FAM75A5	61259372	0	0	0
CHB	FAM75A5	61264372	0	0	0
CHB	FAM75A5	61269372	0	0	0
CHB	FAM75A5	61274372	0	0	0
CHB	FAM75A5	61279372	0	0	0
CHB	FAM75A5	61284372	0	0	0
CHB	FAM75A5	61289372	0	0	0
CHB	FAM75A5	61294372	0	0	0
CHB	FAM75A5	61299372	0	0	0
CHB	FAM75A5	61304372	0	0	0
CHB	FAM75A5	61309372	0	0	0
CHB	FAM75A5	61314372	0	0	0
CHB	FAM75A5	61319372	0	0	0
CHB	FAM75A5	61324372	0	0	0
CHB	FAM75A5	61329372	0	0	0
CHB	FAM75A5	61334372	0	0	0
CHB	FAM75A5	61339372	0	0	0
CHB	FAM75A5	61344372	0	0	0
CHB	FAM75A5	61349372	0	0	0
CHB	FAM75A5	61354372	0	0	0
CHB	FAM75A5	61359372	0	0	0
CHB	FAM75A5	61364372	0	0	0
CHB	FAM75A5	61369372	0	0	0
CHB	FAM75A5	61374372	0	0	0
CHB	FAM75A5	61379372	0	0	0

CHB	FAM75A5	61384372	0	0	0
CHB	FAM75A5	61389372	0	0	0
CHB	FAM75A5	61394372	0	0	0
CHB	FAM75A5	61399372	0	0	0
CHB	FAM75A5	61404372	0	0	0
CHB	FAM75A5	61409372	0	0	0
CHB	FAM75A5	61414372	0	0	0
YRI	FAM75A5	60419372	0	0	0
YRI	FAM75A5	60424372	0	0	0
YRI	FAM75A5	60429372	0	0	0
YRI	FAM75A5	60434372	0	0	0
YRI	FAM75A5	60439372	0	0	0
YRI	FAM75A5	60444372	0	0	0
YRI	FAM75A5	60449372	0	0	0
YRI	FAM75A5	60454372	0	0	0
YRI	FAM75A5	60459372	0	0	0
YRI	FAM75A5	60464372	0	0	0
YRI	FAM75A5	60469372	0	0	0
YRI	FAM75A5	60474372	0	0	0
YRI	FAM75A5	60479372	0	0	0
YRI	FAM75A5	60484372	0	0	0
YRI	FAM75A5	60489372	0	0	0
YRI	FAM75A5	60494372	0	0	0
YRI	FAM75A5	60499372	0	0	0
YRI	FAM75A5	60504372	0	0	0
YRI	FAM75A5	60509372	0	0	0
YRI	FAM75A5	60514372	0	0	0
YRI	FAM75A5	60519372	0	0	0
YRI	FAM75A5	60524372	0	0	0
YRI	FAM75A5	60529372	0	0	0
YRI	FAM75A5	60534372	0	0	0
YRI	FAM75A5	60539372	0	0	0
YRI	FAM75A5	60544372	0	0	0
YRI	FAM75A5	60549372	0	0	0
YRI	FAM75A5	60554372	0	0	0
YRI	FAM75A5	60559372	0	0	0
YRI	FAM75A5	60564372	0	0	0
YRI	FAM75A5	60569372	0	0	0
YRI	FAM75A5	60574372	0	0	0
YRI	FAM75A5	60579372	0	0	0
YRI	FAM75A5	60584372	0	0	0
YRI	FAM75A5	60589372	0	0	0
YRI	FAM75A5	60594372	0	0	0
YRI	FAM75A5	60599372	0	0	0
YRI	FAM75A5	60604372	0	0	0
YRI	FAM75A5	60609372	0	0	0
YRI	FAM75A5	60614372	0	0	0
YRI	FAM75A5	60619372	0	0	0
YRI	FAM75A5	60624372	0	0	0

YRI	FAM75A5	60629372	0	0	0
YRI	FAM75A5	60634372	0	0	0
YRI	FAM75A5	60639372	0	0	0
YRI	FAM75A5	60644372	0	0	0
YRI	FAM75A5	60649372	0	0	0
YRI	FAM75A5	60654372	0	0	0
YRI	FAM75A5	60659372	0	0	0
YRI	FAM75A5	60664372	0	0	0
YRI	FAM75A5	60669372	0	0	0
YRI	FAM75A5	60674372	0	0	0
YRI	FAM75A5	60679372	0	0	0
YRI	FAM75A5	60684372	0	0	0
YRI	FAM75A5	60689372	0	0	0
YRI	FAM75A5	60694372	0	0	0
YRI	FAM75A5	60699372	0	0	0
YRI	FAM75A5	60704372	0	0	0
YRI	FAM75A5	60709372	0	0	0
YRI	FAM75A5	60714372	0	0	0
YRI	FAM75A5	60719372	0	0	0
YRI	FAM75A5	60724372	0	0	0
YRI	FAM75A5	60729372	0	0	0
YRI	FAM75A5	60734372	0	0	0
YRI	FAM75A5	60739372	0	0	0
YRI	FAM75A5	60744372	0	0	0
YRI	FAM75A5	60749372	0	0	0
YRI	FAM75A5	60754372	0	0	0
YRI	FAM75A5	60759372	0	0	0
YRI	FAM75A5	60764372	0	0	0
YRI	FAM75A5	60769372	0	0	0
YRI	FAM75A5	60774372	0	0	0
YRI	FAM75A5	60779372	0	0	0
YRI	FAM75A5	60784372	0	0	0
YRI	FAM75A5	60789372	0	0	0
YRI	FAM75A5	60794372	0	0	0
YRI	FAM75A5	60799372	0	0	0
YRI	FAM75A5	60804372	0	0	0
YRI	FAM75A5	60809372	0	0	0
YRI	FAM75A5	60814372	0	0	0
YRI	FAM75A5	60819372	0	0	0
YRI	FAM75A5	60824372	0	0	0
YRI	FAM75A5	60829372	0	0	0
YRI	FAM75A5	60834372	0	0	0
YRI	FAM75A5	60839372	0	0	0
YRI	FAM75A5	60844372	0	0	0
YRI	FAM75A5	60849372	0	0	0
YRI	FAM75A5	60854372	0	0	0
YRI	FAM75A5	60859372	0	0	0
YRI	FAM75A5	60864372	0	0	0
YRI	FAM75A5	60869372	0	0	0

YRI	FAM75A5	60874372	0	0	0
YRI	FAM75A5	60879372	0	0	0
YRI	FAM75A5	60884372	0	0	0
YRI	FAM75A5	60889372	0	0	0
YRI	FAM75A5	60894372	0	0	0
YRI	FAM75A5	60899372	0	0	0
YRI	FAM75A5	60904372	0	0	0
YRI	FAM75A5	60909372	0	0	0
YRI	FAM75A5	60914372	0	0	0
YRI	FAM75A5	60919372	0	0	0
YRI	FAM75A5	60924372	0	0	0
YRI	FAM75A5	60929372	0	0	0
YRI	FAM75A5	60934372	0	0	0
YRI	FAM75A5	60939372	0	0	0
YRI	FAM75A5	60944372	0	0	0
YRI	FAM75A5	60949372	0	0	0
YRI	FAM75A5	60954372	0	0	0
YRI	FAM75A5	60959372	0	0	0
YRI	FAM75A5	60964372	0	0	0
YRI	FAM75A5	60969372	0	0	0
YRI	FAM75A5	60974372	0	0	0
YRI	FAM75A5	60979372	0	0	0
YRI	FAM75A5	60984372	0	0	0
YRI	FAM75A5	60989372	0	0	0
YRI	FAM75A5	60994372	0	0	0
YRI	FAM75A5	60999372	0	0	0
YRI	FAM75A5	61004372	0	0	0
YRI	FAM75A5	61009372	0	0	0
YRI	FAM75A5	61014372	0	0	0
YRI	FAM75A5	61019372	0	0	0
YRI	FAM75A5	61024372	0	0	0
YRI	FAM75A5	61029372	0	0	0
YRI	FAM75A5	61034372	0	0	0
YRI	FAM75A5	61039372	0	0	0
YRI	FAM75A5	61044372	0	0	0
YRI	FAM75A5	61049372	0	0	0
YRI	FAM75A5	61054372	0	0	0
YRI	FAM75A5	61059372	0	0	0
YRI	FAM75A5	61064372	0	0	0
YRI	FAM75A5	61069372	0	0	0
YRI	FAM75A5	61074372	0	0	0
YRI	FAM75A5	61079372	0	0	0
YRI	FAM75A5	61084372	0	0	0
YRI	FAM75A5	61089372	0	0	0
YRI	FAM75A5	61094372	0	0	0
YRI	FAM75A5	61099372	0	0	0
YRI	FAM75A5	61104372	0	0	0
YRI	FAM75A5	61109372	0	0	0
YRI	FAM75A5	61114372	0	0	0

YRI	FAM75A5	61119372	0	0	0
YRI	FAM75A5	61124372	0	0	0
YRI	FAM75A5	61129372	0	0	0
YRI	FAM75A5	61134372	0	0	0
YRI	FAM75A5	61139372	0	0	0
YRI	FAM75A5	61144372	0	0	0
YRI	FAM75A5	61149372	0	0	0
YRI	FAM75A5	61154372	0	0	0
YRI	FAM75A5	61159372	0	0	0
YRI	FAM75A5	61164372	0	0	0
YRI	FAM75A5	61169372	0	0	0
YRI	FAM75A5	61174372	0	0	0
YRI	FAM75A5	61179372	0	0	0
YRI	FAM75A5	61184372	0	0	0
YRI	FAM75A5	61189372	0	0	0
YRI	FAM75A5	61194372	0	0	0
YRI	FAM75A5	61199372	0	0	0
YRI	FAM75A5	61204372	0	0	0
YRI	FAM75A5	61209372	0	0	0
YRI	FAM75A5	61214372	0	0	0
YRI	FAM75A5	61219372	0	0	0
YRI	FAM75A5	61224372	0	0	0
YRI	FAM75A5	61229372	0	0	0
YRI	FAM75A5	61234372	0	0	0
YRI	FAM75A5	61239372	0	0	0
YRI	FAM75A5	61244372	0	0	0
YRI	FAM75A5	61249372	0	0	0
YRI	FAM75A5	61254372	0	0	0
YRI	FAM75A5	61259372	0	0	0
YRI	FAM75A5	61264372	0	0	0
YRI	FAM75A5	61269372	0	0	0
YRI	FAM75A5	61274372	0	0	0
YRI	FAM75A5	61279372	0	0	0
YRI	FAM75A5	61284372	0	0	0
YRI	FAM75A5	61289372	0	0	0
YRI	FAM75A5	61294372	0	0	0
YRI	FAM75A5	61299372	0	0	0
YRI	FAM75A5	61304372	0	0	0
YRI	FAM75A5	61309372	0	0	0
YRI	FAM75A5	61314372	0	0	0
YRI	FAM75A5	61319372	0	0	0
YRI	FAM75A5	61324372	0	0	0
YRI	FAM75A5	61329372	0	0	0
YRI	FAM75A5	61334372	0	0	0
YRI	FAM75A5	61339372	0	0	0
YRI	FAM75A5	61344372	0	0	0
YRI	FAM75A5	61349372	0	0	0
YRI	FAM75A5	61354372	0	0	0
YRI	FAM75A5	61359372	0	0	0

YRI	FAM75A5	61364372	0	0	0
YRI	FAM75A5	61369372	0	0	0
YRI	FAM75A5	61374372	0	0	0
YRI	FAM75A5	61379372	0	0	0
YRI	FAM75A5	61384372	0	0	0
YRI	FAM75A5	61389372	0	0	0
YRI	FAM75A5	61394372	0	0	0
YRI	FAM75A5	61399372	0	0	0
YRI	FAM75A5	61404372	0	0	0
YRI	FAM75A5	61409372	0	0	0
YRI	FAM75A5	61414372	0	0	0
CEU	FOXD4L2	65242146	0	0	0
CEU	FOXD4L2	65247146	0	0	0
CEU	FOXD4L2	65252146	0	0	0
CEU	FOXD4L2	65257146	0	0	0
CEU	FOXD4L2	65262146	0	0	0
CEU	FOXD4L2	65267146	0	0	0
CEU	FOXD4L2	65272146	0	0	0
CEU	FOXD4L2	65277146	0	0	0
CEU	FOXD4L2	65282146	0	0	0
CEU	FOXD4L2	65287146	0	0	0
CEU	FOXD4L2	65292146	0	0	0
CEU	FOXD4L2	65297146	0	0	0
CEU	FOXD4L2	65302146	0	0	0
CEU	FOXD4L2	65307146	0	0	0
CEU	FOXD4L2	65312146	0	0	0
CEU	FOXD4L2	65317146	0	0	0
CEU	FOXD4L2	65322146	0	0	0
CEU	FOXD4L2	65327146	0	0	0
CEU	FOXD4L2	65332146	0	0	0
CEU	FOXD4L2	65337146	0	0	0
CEU	FOXD4L2	65342146	0	0	0
CEU	FOXD4L2	65347146	0	0	0
CEU	FOXD4L2	65352146	0	0	0
CEU	FOXD4L2	65357146	0	0	0
CEU	FOXD4L2	65362146	0	0	0
CEU	FOXD4L2	65367146	0	0	0
CEU	FOXD4L2	65372146	0	0	0
CEU	FOXD4L2	65377146	0	0	0
CEU	FOXD4L2	65382146	0	0	0
CEU	FOXD4L2	65387146	0	0	0
CEU	FOXD4L2	65392146	0	0	0
CEU	FOXD4L2	65397146	0	0	0
CEU	FOXD4L2	65402146	0	0	0
CEU	FOXD4L2	65407146	0	0	0
CEU	FOXD4L2	65412146	0	0	0
CEU	FOXD4L2	65417146	0	0	0
CEU	FOXD4L2	65422146	0	0	0
CEU	FOXD4L2	65427146	0	0	0

CEU	FOXD4L2	65432146	0	0	0
CEU	FOXD4L2	65437146	0	0	0
CEU	FOXD4L2	65442146	0	0	0
CEU	FOXD4L2	65447146	0	0	0
CEU	FOXD4L2	65452146	0	0	0
CEU	FOXD4L2	65457146	0	0	0
CEU	FOXD4L2	65462146	0	0	0
CEU	FOXD4L2	65467146	0.1585	0.1706	-0.0719
CEU	FOXD4L2	65472146	0.6937	0.6822	0.03
CEU	FOXD4L2	65477146	0.7761	1.3645	-0.9519
CEU	FOXD4L2	65482146	1.3539	1.5351	-0.2687
CEU	FOXD4L2	65487146	1.113	0.6822	1.1249
CEU	FOXD4L2	65492146	0	0	0
CEU	FOXD4L2	65497146	0	0	0
CEU	FOXD4L2	65502146	0	0	0
CEU	FOXD4L2	65507146	0.0404	0.6822	-1.6759
CEU	FOXD4L2	65512146	0.0404	0.6822	-1.6759
CEU	FOXD4L2	65517146	0	0	0
CEU	FOXD4L2	65522146	0	0	0
CEU	FOXD4L2	65527146	0	0	0
CEU	FOXD4L2	65532146	0	0	0
CEU	FOXD4L2	65537146	0.0101	0.1706	-0.9534
CEU	FOXD4L2	65542146	0.0101	0.1706	-0.9534
CEU	FOXD4L2	65547146	0.873	0.5117	1.1331
CEU	FOXD4L2	65552146	1.248	0.8528	0.89
CEU	FOXD4L2	65557146	0.9382	1.3645	-0.6897
CEU	FOXD4L2	65562146	0.5834	1.3645	-1.2637
CEU	FOXD4L2	65567146	0.0202	0.3411	-1.2864
CEU	FOXD4L2	65572146	1.0396	0.8528	0.4207
CEU	FOXD4L2	65577146	1.3063	1.0234	0.5621
CEU	FOXD4L2	65582146	0.2667	0.1706	0.5709
CEU	FOXD4L2	65587146	0	0	0
CEU	FOXD4L2	65592146	1.523	2.0467	-0.6231
CEU	FOXD4L2	65597146	4.4308	6.3108	-0.8631
CEU	FOXD4L2	65602146	4.3875	9.5515	-1.6223
CEU	FOXD4L2	65607146	3.3554	8.3576	-1.7781
CEU	FOXD4L2	65612146	7.1383	9.0398	-0.6287
CEU	FOXD4L2	65617146	8.5768	10.916	-0.6488
CEU	FOXD4L2	65622146	5.7429	8.187	-0.8854
CEU	FOXD4L2	65627146	6.122	9.3809	-1.0411
CEU	FOXD4L2	65632146	6.6063	10.5749	-1.1339
CEU	FOXD4L2	65637146	3.1903	5.6286	-1.2403
CEU	FOXD4L2	65642146	0.375	1.5351	-1.7202
CEU	FOXD4L2	65647146	0.1078	0.5117	-1.2667
CEU	FOXD4L2	65652146	0.0101	0.1706	-0.9534
CEU	FOXD4L2	65657146	0	0	0
CEU	FOXD4L2	65662146	0	0	0
CEU	FOXD4L2	65667146	0	0	0
CEU	FOXD4L2	65672146	0.04	0.1706	-0.7758

CEU	FOXD4L2	65677146	0.04	0.1706	-0.7758
CEU	FOXD4L2	65682146	0	0	0
CEU	FOXD4L2	65687146	0.1328	0.3411	-0.835
CEU	FOXD4L2	65692146	0.1328	0.3411	-0.835
CEU	FOXD4L2	65697146	0	0	0
CEU	FOXD4L2	65702146	0.02	0.1706	-0.8946
CEU	FOXD4L2	65707146	0.0301	0.3411	-1.2467
CEU	FOXD4L2	65712146	0.2016	0.3411	-0.5592
CEU	FOXD4L2	65717146	0.1915	0.1706	0.1242
CEU	FOXD4L2	65722146	0	0	0
CEU	FOXD4L2	65727146	0.0202	0.1706	-0.8934
CEU	FOXD4L2	65732146	0.0202	0.1706	-0.8934
CEU	FOXD4L2	65737146	0.3669	0.3411	0.1034
CEU	FOXD4L2	65742146	0.5852	0.8528	-0.6026
CEU	FOXD4L2	65747146	0.4098	0.6822	-0.7113
CEU	FOXD4L2	65752146	0.3445	0.5117	-0.5244
CEU	FOXD4L2	65757146	0.2228	0.8528	-1.4187
CEU	FOXD4L2	65762146	0.2764	1.1939	-1.635
CEU	FOXD4L2	65767146	0.7547	1.8762	-1.4279
CEU	FOXD4L2	65772146	0.9695	1.7056	-1.0082
CEU	FOXD4L2	65777146	0.4995	0.6822	-0.4771
CEU	FOXD4L2	65782146	0.1078	0.3411	-0.9352
CEU	FOXD4L2	65787146	0.0398	0.3411	-1.2078
CEU	FOXD4L2	65792146	0.0303	0.3411	-1.2459
CEU	FOXD4L2	65797146	0.1651	0.5117	-1.087
CEU	FOXD4L2	65802146	0.1449	0.3411	-0.7865
CEU	FOXD4L2	65807146	0.0101	0.1706	-0.9534
CEU	FOXD4L2	65812146	0.0101	0.1706	-0.9534
CEU	FOXD4L2	65817146	0	0	0
CEU	FOXD4L2	65822146	0.4455	0.5117	-0.2076
CEU	FOXD4L2	65827146	0.4455	0.5117	-0.2076
CEU	FOXD4L2	65832146	0	0	0
CEU	FOXD4L2	65837146	0.0676	0.1706	-0.6118
CEU	FOXD4L2	65842146	0.0676	0.1706	-0.6118
CEU	FOXD4L2	65847146	0	0	0
CEU	FOXD4L2	65852146	0	0	0
CEU	FOXD4L2	65857146	0.4185	0.5117	-0.2923
CEU	FOXD4L2	65862146	0.5533	0.8528	-0.6745
CEU	FOXD4L2	65867146	0.5143	0.5117	0.0082
CEU	FOXD4L2	65872146	0.4292	0.3411	0.3532
CEU	FOXD4L2	65877146	1.1257	1.1939	-0.1215
CEU	FOXD4L2	65882146	1.0761	1.0234	0.1047
CEU	FOXD4L2	65887146	0.4673	0.1706	1.7625
CEU	FOXD4L2	65892146	2.0035	1.3645	1.0338
CEU	FOXD4L2	65897146	1.6448	1.7056	-0.0833
CEU	FOXD4L2	65902146	0.1086	0.5117	-1.2642
CEU	FOXD4L2	65907146	0	0	0
CEU	FOXD4L2	65912146	0.2111	0.3411	-0.5211
CEU	FOXD4L2	65917146	1.8213	1.5351	0.4244

CEU	FOXD4L2	65922146	1.6102	1.1939	0.7419
CEU	FOXD4L2	65927146	0	0	0
CEU	FOXD4L2	65932146	0	0	0
CEU	FOXD4L2	65937146	0	0	0
CEU	FOXD4L2	65942146	0	0	0
CEU	FOXD4L2	65947146	0	0	0
CEU	FOXD4L2	65952146	0	0	0
CEU	FOXD4L2	65957146	0	0	0
CEU	FOXD4L2	65962146	0	0	0
CEU	FOXD4L2	65967146	0.5246	0.6822	-0.4115
CEU	FOXD4L2	65972146	2.0656	1.7056	0.4931
CEU	FOXD4L2	65977146	1.793	1.5351	0.3824
CEU	FOXD4L2	65982146	0.302	0.8528	-1.2404
CEU	FOXD4L2	65987146	0.1765	0.8528	-1.523
CEU	FOXD4L2	65992146	0.3434	1.7056	-1.8657
CEU	FOXD4L2	65997146	0.227	1.3645	-1.8402
CEU	FOXD4L2	66002146	0.2579	0.5117	-0.796
CEU	FOXD4L2	66007146	1.0781	1.8762	-1.0161
CEU	FOXD4L2	66012146	1.396	2.2173	-0.9175
CEU	FOXD4L2	66017146	0.6259	1.3645	-1.1949
CEU	FOXD4L2	66022146	0.0804	1.0234	-1.8738
CEU	FOXD4L2	66027146	0.0303	0.5117	-1.5098
CEU	FOXD4L2	66032146	0.1771	0.8528	-1.5217
CEU	FOXD4L2	66037146	0.1771	0.8528	-1.5217
CEU	FOXD4L2	66042146	0.0202	0.3411	-1.2864
CEU	FOXD4L2	66047146	0.3418	1.1939	-1.5185
CEU	FOXD4L2	66052146	0.3418	1.1939	-1.5185
CEU	FOXD4L2	66057146	0.0101	0.1706	-0.9534
CEU	FOXD4L2	66062146	0	0	0
CEU	FOXD4L2	66067146	0	0	0
CEU	FOXD4L2	66072146	0.0101	0.1706	-0.9534
CEU	FOXD4L2	66077146	0.0101	0.1706	-0.9534
CEU	FOXD4L2	66082146	0.04	0.3411	-1.207
CEU	FOXD4L2	66087146	0.7912	0.8528	-0.1387
CEU	FOXD4L2	66092146	0.7512	0.5117	0.7511
CEU	FOXD4L2	66097146	0.6184	0.6822	-0.1666
CEU	FOXD4L2	66102146	0.6184	0.6822	-0.1666
CEU	FOXD4L2	66107146	0.0398	0.1706	-0.777
CEU	FOXD4L2	66112146	0.6613	1.3645	-1.1376
CEU	FOXD4L2	66117146	0.9928	2.3879	-1.4692
CEU	FOXD4L2	66122146	0.3713	1.1939	-1.4659
CEU	FOXD4L2	66127146	0.1002	0.6822	-1.5198
CEU	FOXD4L2	66132146	0.1002	0.6822	-1.5198
CEU	FOXD4L2	66137146	0.0992	0.6822	-1.5224
CEU	FOXD4L2	66142146	0.0992	0.6822	-1.5224
CEU	FOXD4L2	66147146	0.0101	0.1706	-0.9534
CEU	FOXD4L2	66152146	0.0101	0.1706	-0.9534
CEU	FOXD4L2	66157146	0	0	0
CEU	FOXD4L2	66162146	0.4745	0.1706	1.8052

CEU	FOXD4L2	66167146	0.4745	0.1706	1.8052
CEU	FOXD4L2	66172146	0	0	0
CEU	FOXD4L2	66177146	0	0	0
CEU	FOXD4L2	66182146	0	0	0
CEU	FOXD4L2	66187146	0	0	0
CEU	FOXD4L2	66192146	0	0	0
CEU	FOXD4L2	66197146	0	0	0
CEU	FOXD4L2	66202146	0	0	0
CEU	FOXD4L2	66207146	0	0	0
CEU	FOXD4L2	66212146	0	0	0
CEU	FOXD4L2	66217146	0	0	0
CEU	FOXD4L2	66222146	0	0	0
CEU	FOXD4L2	66227146	0	0	0
CEU	FOXD4L2	66232146	0	0	0
CHB	FOXD4L2	65242146	0	0	0
CHB	FOXD4L2	65247146	0	0	0
CHB	FOXD4L2	65252146	0	0	0
CHB	FOXD4L2	65257146	0	0	0
CHB	FOXD4L2	65262146	0	0	0
CHB	FOXD4L2	65267146	0	0	0
CHB	FOXD4L2	65272146	0	0	0
CHB	FOXD4L2	65277146	0	0	0
CHB	FOXD4L2	65282146	0	0	0
CHB	FOXD4L2	65287146	0	0	0
CHB	FOXD4L2	65292146	0	0	0
CHB	FOXD4L2	65297146	0	0	0
CHB	FOXD4L2	65302146	0	0	0
CHB	FOXD4L2	65307146	0	0	0
CHB	FOXD4L2	65312146	0	0	0
CHB	FOXD4L2	65317146	0	0	0
CHB	FOXD4L2	65322146	0	0	0
CHB	FOXD4L2	65327146	0	0	0
CHB	FOXD4L2	65332146	0	0	0
CHB	FOXD4L2	65337146	0	0	0
CHB	FOXD4L2	65342146	0	0	0
CHB	FOXD4L2	65347146	0	0	0
CHB	FOXD4L2	65352146	0	0	0
CHB	FOXD4L2	65357146	0	0	0
CHB	FOXD4L2	65362146	0	0	0
CHB	FOXD4L2	65367146	0	0	0
CHB	FOXD4L2	65372146	0	0	0
CHB	FOXD4L2	65377146	0	0	0
CHB	FOXD4L2	65382146	0	0	0
CHB	FOXD4L2	65387146	0	0	0
CHB	FOXD4L2	65392146	0	0	0
CHB	FOXD4L2	65397146	0	0	0
CHB	FOXD4L2	65402146	0	0	0
CHB	FOXD4L2	65407146	0	0	0
CHB	FOXD4L2	65412146	0	0	0

CHB	FOXD4L2	65417146	0	0	0
CHB	FOXD4L2	65422146	0	0	0
CHB	FOXD4L2	65427146	0	0	0
CHB	FOXD4L2	65432146	0	0	0
CHB	FOXD4L2	65437146	0	0	0
CHB	FOXD4L2	65442146	0	0	0
CHB	FOXD4L2	65447146	0	0	0
CHB	FOXD4L2	65452146	0	0	0
CHB	FOXD4L2	65457146	0	0	0
CHB	FOXD4L2	65462146	0	0	0
CHB	FOXD4L2	65467146	0.0674	0.3388	-1.0887
CHB	FOXD4L2	65472146	0.5772	0.8471	-0.6086
CHB	FOXD4L2	65477146	1.0219	0.8471	0.3942
CHB	FOXD4L2	65482146	0.9219	0.8471	0.1687
CHB	FOXD4L2	65487146	0.4099	0.5082	-0.3086
CHB	FOXD4L2	65492146	0	0	0
CHB	FOXD4L2	65497146	0	0	0
CHB	FOXD4L2	65502146	0	0	0
CHB	FOXD4L2	65507146	0	0	0
CHB	FOXD4L2	65512146	0.0097	0.1694	-0.949
CHB	FOXD4L2	65517146	0.0097	0.1694	-0.949
CHB	FOXD4L2	65522146	0	0	0
CHB	FOXD4L2	65527146	0.0388	0.6777	-1.6703
CHB	FOXD4L2	65532146	0.0388	0.6777	-1.6703
CHB	FOXD4L2	65537146	0	0	0
CHB	FOXD4L2	65542146	0	0	0
CHB	FOXD4L2	65547146	0.5561	0.8471	-0.6562
CHB	FOXD4L2	65552146	0.8608	1.6942	-1.1437
CHB	FOXD4L2	65557146	0.4483	1.8636	-1.8057
CHB	FOXD4L2	65562146	0.1435	1.0165	-1.7373
CHB	FOXD4L2	65567146	0	0	0
CHB	FOXD4L2	65572146	1.0781	0.6777	1.0468
CHB	FOXD4L2	65577146	1.3939	0.8471	1.233
CHB	FOXD4L2	65582146	0.3158	0.1694	0.8699
CHB	FOXD4L2	65587146	0	0	0
CHB	FOXD4L2	65592146	1.8262	2.5412	-0.714
CHB	FOXD4L2	65597146	4.379	5.9295	-0.7506
CHB	FOXD4L2	65602146	3.6581	6.6072	-1.2953
CHB	FOXD4L2	65607146	2.9417	5.4213	-1.3002
CHB	FOXD4L2	65612146	5.2551	7.7931	-0.9592
CHB	FOXD4L2	65617146	5.4748	10.3343	-1.4145
CHB	FOXD4L2	65622146	4.4017	7.4543	-1.2016
CHB	FOXD4L2	65627146	6.0029	7.9625	-0.7262
CHB	FOXD4L2	65632146	6.1694	9.1484	-0.9713
CHB	FOXD4L2	65637146	2.5315	4.066	-1.0348
CHB	FOXD4L2	65642146	0.0194	0.1694	-0.8913
CHB	FOXD4L2	65647146	0.0194	0.1694	-0.8913
CHB	FOXD4L2	65652146	0.0194	0.1694	-0.8913
CHB	FOXD4L2	65657146	0	0	0

CHB	FOXD4L2	65662146	0	0	0
CHB	FOXD4L2	65667146	0	0	0
CHB	FOXD4L2	65672146	0.0097	0.1694	-0.949
CHB	FOXD4L2	65677146	0.0097	0.1694	-0.949
CHB	FOXD4L2	65682146	0	0	0
CHB	FOXD4L2	65687146	0.1881	0.3388	-0.6045
CHB	FOXD4L2	65692146	0.2075	0.5082	-0.9439
CHB	FOXD4L2	65697146	0.0194	0.1694	-0.8913
CHB	FOXD4L2	65702146	0	0	0
CHB	FOXD4L2	65707146	0	0	0
CHB	FOXD4L2	65712146	0.3859	0.1694	1.2865
CHB	FOXD4L2	65717146	0.3859	0.1694	1.2865
CHB	FOXD4L2	65722146	0	0	0
CHB	FOXD4L2	65727146	0	0	0
CHB	FOXD4L2	65732146	0	0	0
CHB	FOXD4L2	65737146	0.7717	0.3388	1.7365
CHB	FOXD4L2	65742146	1.1342	0.6777	1.1934
CHB	FOXD4L2	65747146	0.6901	0.5082	0.571
CHB	FOXD4L2	65752146	0.6551	0.5082	0.4611
CHB	FOXD4L2	65757146	0.3468	0.5082	-0.5067
CHB	FOXD4L2	65762146	0.301	0.6777	-0.9848
CHB	FOXD4L2	65767146	0.6141	1.0165	-0.8008
CHB	FOXD4L2	65772146	0.8555	1.0165	-0.3204
CHB	FOXD4L2	65777146	0.5422	0.6777	-0.3542
CHB	FOXD4L2	65782146	0.0482	0.5082	-1.444
CHB	FOXD4L2	65787146	0.0289	0.3388	-1.2431
CHB	FOXD4L2	65792146	0	0.1694	-1.0066
CHB	FOXD4L2	65797146	0.1498	0.6777	-1.3801
CHB	FOXD4L2	65802146	0.1498	0.5082	-1.1251
CHB	FOXD4L2	65807146	0.0097	0.1694	-0.949
CHB	FOXD4L2	65812146	0.0579	0.5082	-1.4136
CHB	FOXD4L2	65817146	0.0482	0.3388	-1.1657
CHB	FOXD4L2	65822146	0.4139	0.5082	-0.296
CHB	FOXD4L2	65827146	0.4139	0.5082	-0.296
CHB	FOXD4L2	65832146	0	0	0
CHB	FOXD4L2	65837146	0.1272	0.1694	-0.2508
CHB	FOXD4L2	65842146	0.2031	0.5082	-0.9578
CHB	FOXD4L2	65847146	0.076	0.3388	-1.0542
CHB	FOXD4L2	65852146	0	0	0
CHB	FOXD4L2	65857146	0.1763	0.3388	-0.6518
CHB	FOXD4L2	65862146	0.186	0.5082	-1.0114
CHB	FOXD4L2	65867146	0.17	0.3388	-0.6771
CHB	FOXD4L2	65872146	0.3967	0.3388	0.2323
CHB	FOXD4L2	65877146	1.1003	0.6777	1.1048
CHB	FOXD4L2	65882146	0.8639	0.5082	1.1166
CHB	FOXD4L2	65887146	0.3758	0.1694	1.2265
CHB	FOXD4L2	65892146	1.4019	1.1859	0.3856
CHB	FOXD4L2	65897146	1.0739	1.1859	-0.1999
CHB	FOXD4L2	65902146	0.0672	0.3388	-1.0895

CHB	FOXD4L2	65907146	0.0194	0.1694	-0.8913
CHB	FOXD4L2	65912146	0.1668	0.1694	-0.0154
CHB	FOXD4L2	65917146	1.1277	1.3553	-0.3689
CHB	FOXD4L2	65922146	0.961	1.1859	-0.4014
CHB	FOXD4L2	65927146	0	0	0
CHB	FOXD4L2	65932146	0	0	0
CHB	FOXD4L2	65937146	0	0	0
CHB	FOXD4L2	65942146	0	0	0
CHB	FOXD4L2	65947146	0	0	0
CHB	FOXD4L2	65952146	0	0	0
CHB	FOXD4L2	65957146	0	0	0
CHB	FOXD4L2	65962146	0	0	0
CHB	FOXD4L2	65967146	0.6537	1.0165	-0.722
CHB	FOXD4L2	65972146	2.1294	1.8636	0.3391
CHB	FOXD4L2	65977146	1.4951	1.1859	0.5519
CHB	FOXD4L2	65982146	0.0291	0.5082	-1.504
CHB	FOXD4L2	65987146	0.3128	0.3388	-0.1043
CHB	FOXD4L2	65992146	0.5285	0.6777	-0.3901
CHB	FOXD4L2	65997146	0.2825	0.6777	-1.0332
CHB	FOXD4L2	66002146	1.0436	0.5082	1.6807
CHB	FOXD4L2	66007146	1.8066	1.5247	0.4188
CHB	FOXD4L2	66012146	1.6644	2.5412	-0.8755
CHB	FOXD4L2	66017146	1.3326	1.8636	-0.6775
CHB	FOXD4L2	66022146	0.5357	0.6777	-0.3712
CHB	FOXD4L2	66027146	0.0474	0.1694	-0.7249
CHB	FOXD4L2	66032146	0.4866	0.8471	-0.8129
CHB	FOXD4L2	66037146	0.5351	1.3553	-1.3293
CHB	FOXD4L2	66042146	0.0583	0.6777	-1.6193
CHB	FOXD4L2	66047146	0.4959	0.6777	-0.4753
CHB	FOXD4L2	66052146	0.4862	0.5082	-0.0691
CHB	FOXD4L2	66057146	0	0	0
CHB	FOXD4L2	66062146	0	0	0
CHB	FOXD4L2	66067146	0.0388	0.3388	-1.2034
CHB	FOXD4L2	66072146	0.0388	0.3388	-1.2034
CHB	FOXD4L2	66077146	0	0	0
CHB	FOXD4L2	66082146	0	0	0
CHB	FOXD4L2	66087146	0.4323	0.1694	1.5622
CHB	FOXD4L2	66092146	0.4323	0.1694	1.5622
CHB	FOXD4L2	66097146	0.534	0.6777	-0.3757
CHB	FOXD4L2	66102146	0.534	0.6777	-0.3757
CHB	FOXD4L2	66107146	0.0097	0.1694	-0.949
CHB	FOXD4L2	66112146	0.9564	1.3553	-0.6465
CHB	FOXD4L2	66117146	1.8431	2.3718	-0.5581
CHB	FOXD4L2	66122146	0.9159	1.5247	-0.9045
CHB	FOXD4L2	66127146	0.0485	0.6777	-1.6449
CHB	FOXD4L2	66132146	0.0291	0.3388	-1.2423
CHB	FOXD4L2	66137146	0.0554	0.1694	-0.6774
CHB	FOXD4L2	66142146	0.0554	0.1694	-0.6774
CHB	FOXD4L2	66147146	0	0	0

CHB	FOXD4L2	66152146	0	0	0
CHB	FOXD4L2	66157146	0	0	0
CHB	FOXD4L2	66162146	0.1769	0.1694	0.0446
CHB	FOXD4L2	66167146	0.1769	0.1694	0.0446
CHB	FOXD4L2	66172146	0	0	0
CHB	FOXD4L2	66177146	0.0097	0.1694	-0.949
CHB	FOXD4L2	66182146	0.0097	0.1694	-0.949
CHB	FOXD4L2	66187146	0	0	0
CHB	FOXD4L2	66192146	0	0	0
CHB	FOXD4L2	66197146	0	0	0
CHB	FOXD4L2	66202146	0	0	0
CHB	FOXD4L2	66207146	0	0	0
CHB	FOXD4L2	66212146	0	0	0
CHB	FOXD4L2	66217146	0	0	0
CHB	FOXD4L2	66222146	0	0	0
CHB	FOXD4L2	66227146	0	0	0
CHB	FOXD4L2	66232146	0	0	0
YRI	FOXD4L2	65242146	0	0	0
YRI	FOXD4L2	65247146	0	0	0
YRI	FOXD4L2	65252146	0	0	0
YRI	FOXD4L2	65257146	0	0	0
YRI	FOXD4L2	65262146	0	0	0
YRI	FOXD4L2	65267146	0	0	0
YRI	FOXD4L2	65272146	0	0	0
YRI	FOXD4L2	65277146	0	0	0
YRI	FOXD4L2	65282146	0	0	0
YRI	FOXD4L2	65287146	0	0	0
YRI	FOXD4L2	65292146	0	0	0
YRI	FOXD4L2	65297146	0	0	0
YRI	FOXD4L2	65302146	0	0	0
YRI	FOXD4L2	65307146	0	0	0
YRI	FOXD4L2	65312146	0	0	0
YRI	FOXD4L2	65317146	0	0	0
YRI	FOXD4L2	65322146	0	0	0
YRI	FOXD4L2	65327146	0	0	0
YRI	FOXD4L2	65332146	0	0	0
YRI	FOXD4L2	65337146	0	0	0
YRI	FOXD4L2	65342146	0	0	0
YRI	FOXD4L2	65347146	0	0	0
YRI	FOXD4L2	65352146	0	0	0
YRI	FOXD4L2	65357146	0	0	0
YRI	FOXD4L2	65362146	0	0	0
YRI	FOXD4L2	65367146	0	0	0
YRI	FOXD4L2	65372146	0	0	0
YRI	FOXD4L2	65377146	0	0	0
YRI	FOXD4L2	65382146	0	0	0
YRI	FOXD4L2	65387146	0	0	0
YRI	FOXD4L2	65392146	0	0	0
YRI	FOXD4L2	65397146	0	0	0

YRI	FOXD4L2	65402146	0	0	0
YRI	FOXD4L2	65407146	0	0	0
YRI	FOXD4L2	65412146	0	0	0
YRI	FOXD4L2	65417146	0	0	0
YRI	FOXD4L2	65422146	0	0	0
YRI	FOXD4L2	65427146	0	0	0
YRI	FOXD4L2	65432146	0	0	0
YRI	FOXD4L2	65437146	0	0	0
YRI	FOXD4L2	65442146	0	0	0
YRI	FOXD4L2	65447146	0	0	0
YRI	FOXD4L2	65452146	0	0	0
YRI	FOXD4L2	65457146	0	0	0
YRI	FOXD4L2	65462146	0	0	0
YRI	FOXD4L2	65467146	0.0093	0.1681	-0.944
YRI	FOXD4L2	65472146	0.9335	1.5126	-0.8623
YRI	FOXD4L2	65477146	1.1468	2.0167	-1.0401
YRI	FOXD4L2	65482146	0.9536	1.3445	-0.6349
YRI	FOXD4L2	65487146	0.7497	0.8403	-0.2046
YRI	FOXD4L2	65492146	0.0185	0.1681	-0.8893
YRI	FOXD4L2	65497146	0	0	0
YRI	FOXD4L2	65502146	0.1224	0.1681	-0.2717
YRI	FOXD4L2	65507146	0.2029	0.3361	-0.5347
YRI	FOXD4L2	65512146	0.0806	0.1681	-0.5202
YRI	FOXD4L2	65517146	0.0185	0.1681	-0.8893
YRI	FOXD4L2	65522146	0.0185	0.1681	-0.8893
YRI	FOXD4L2	65527146	0.2715	1.6806	-1.9385
YRI	FOXD4L2	65532146	0.2715	1.6806	-1.9385
YRI	FOXD4L2	65537146	0	0	0
YRI	FOXD4L2	65542146	0	0	0
YRI	FOXD4L2	65547146	0.6899	1.3445	-1.0632
YRI	FOXD4L2	65552146	1.5276	3.0251	-1.2918
YRI	FOXD4L2	65557146	1.275	3.6974	-1.7656
YRI	FOXD4L2	65562146	0.4649	2.1848	-1.9311
YRI	FOXD4L2	65567146	0.0276	0.1681	-0.8352
YRI	FOXD4L2	65572146	0.4326	0.8403	-0.9208
YRI	FOXD4L2	65577146	0.7565	1.1764	-0.751
YRI	FOXD4L2	65582146	0.3239	0.3361	-0.049
YRI	FOXD4L2	65587146	0	0	0
YRI	FOXD4L2	65592146	1.6402	4.8738	-1.8562
YRI	FOXD4L2	65597146	4.6935	9.2434	-1.4643
YRI	FOXD4L2	65602146	3.953	7.8989	-1.468
YRI	FOXD4L2	65607146	3.125	8.067	-1.8034
YRI	FOXD4L2	65612146	7.7175	12.6047	-1.1765
YRI	FOXD4L2	65617146	8.5911	13.445	-1.0993
YRI	FOXD4L2	65622146	4.8887	9.7476	-1.4885
YRI	FOXD4L2	65627146	5.5585	11.5963	-1.5723
YRI	FOXD4L2	65632146	6.3335	12.1005	-1.4428
YRI	FOXD4L2	65637146	2.8287	6.5544	-1.6426
YRI	FOXD4L2	65642146	0.3629	2.0167	-1.9774

YRI	FOXD4L2	65647146	0.1711	0.5042	-1.0468
YRI	FOXD4L2	65652146	0.072	0.1681	-0.5713
YRI	FOXD4L2	65657146	0.0093	0.1681	-0.944
YRI	FOXD4L2	65662146	0.0093	0.1681	-0.944
YRI	FOXD4L2	65667146	0	0	0
YRI	FOXD4L2	65672146	0	0	0
YRI	FOXD4L2	65677146	0	0	0
YRI	FOXD4L2	65682146	0	0	0
YRI	FOXD4L2	65687146	0.0813	0.3361	-1.0229
YRI	FOXD4L2	65692146	0.0996	0.5042	-1.2715
YRI	FOXD4L2	65697146	0.0183	0.1681	-0.8905
YRI	FOXD4L2	65702146	0	0	0
YRI	FOXD4L2	65707146	0	0	0
YRI	FOXD4L2	65712146	0.1463	0.1681	-0.1296
YRI	FOXD4L2	65717146	0.1463	0.1681	-0.1296
YRI	FOXD4L2	65722146	0	0	0
YRI	FOXD4L2	65727146	0.0093	0.1681	-0.944
YRI	FOXD4L2	65732146	0.0093	0.1681	-0.944
YRI	FOXD4L2	65737146	0.2927	0.3361	-0.1742
YRI	FOXD4L2	65742146	0.8473	0.8403	0.0158
YRI	FOXD4L2	65747146	0.776	1.1764	-0.7161
YRI	FOXD4L2	65752146	0.3118	1.1764	-1.5464
YRI	FOXD4L2	65757146	0.5291	1.1764	-1.1577
YRI	FOXD4L2	65762146	0.7274	1.8487	-1.4342
YRI	FOXD4L2	65767146	0.6255	2.689	-1.9611
YRI	FOXD4L2	65772146	0.6872	2.3529	-1.7632
YRI	FOXD4L2	65777146	0.4059	1.3445	-1.5245
YRI	FOXD4L2	65782146	0.0648	0.6722	-1.5901
YRI	FOXD4L2	65787146	0.0093	0.1681	-0.944
YRI	FOXD4L2	65792146	0	0.1681	-0.9993
YRI	FOXD4L2	65797146	0.3386	0.8403	-1.1332
YRI	FOXD4L2	65802146	0.3662	0.8403	-1.0708
YRI	FOXD4L2	65807146	0.0369	0.3361	-1.2012
YRI	FOXD4L2	65812146	0.1695	0.6722	-1.316
YRI	FOXD4L2	65817146	0.1603	0.5042	-1.0808
YRI	FOXD4L2	65822146	0.4462	0.5042	-0.1823
YRI	FOXD4L2	65827146	0.4462	0.5042	-0.1823
YRI	FOXD4L2	65832146	0.0093	0.1681	-0.944
YRI	FOXD4L2	65837146	0.0458	0.3361	-1.1654
YRI	FOXD4L2	65842146	0.0458	0.3361	-1.1654
YRI	FOXD4L2	65847146	0.0093	0.1681	-0.944
YRI	FOXD4L2	65852146	0	0	0
YRI	FOXD4L2	65857146	0.1587	0.5042	-1.0858
YRI	FOXD4L2	65862146	0.1587	0.5042	-1.0858
YRI	FOXD4L2	65867146	0.1006	0.5042	-1.2684
YRI	FOXD4L2	65872146	0.1377	1.0084	-1.7359
YRI	FOXD4L2	65877146	1.0895	1.8487	-0.9711
YRI	FOXD4L2	65882146	1.0524	1.3445	-0.4744
YRI	FOXD4L2	65887146	0.2066	0.1681	0.2289

YRI	FOXD4L2	65892146	0.9229	1.3445	-0.6848
YRI	FOXD4L2	65897146	0.9827	1.8487	-1.1077
YRI	FOXD4L2	65902146	0.2756	0.8403	-1.2755
YRI	FOXD4L2	65907146	0.0093	0.1681	-0.944
YRI	FOXD4L2	65912146	0.0713	0.1681	-0.5754
YRI	FOXD4L2	65917146	1.7041	1.8487	-0.185
YRI	FOXD4L2	65922146	1.6328	1.6806	-0.0658
YRI	FOXD4L2	65927146	0	0	0
YRI	FOXD4L2	65932146	0	0	0
YRI	FOXD4L2	65937146	0	0	0
YRI	FOXD4L2	65942146	0	0	0
YRI	FOXD4L2	65947146	0	0	0
YRI	FOXD4L2	65952146	0	0	0
YRI	FOXD4L2	65957146	0	0	0
YRI	FOXD4L2	65962146	0	0	0
YRI	FOXD4L2	65967146	0.6725	1.0084	-0.6697
YRI	FOXD4L2	65972146	1.9839	2.0167	-0.0392
YRI	FOXD4L2	65977146	1.3756	1.5126	-0.204
YRI	FOXD4L2	65982146	0.5824	1.1764	-1.0624
YRI	FOXD4L2	65987146	0.7558	1.1764	-0.7523
YRI	FOXD4L2	65992146	0.6995	2.1848	-1.6677
YRI	FOXD4L2	65997146	0.6187	2.1848	-1.7584
YRI	FOXD4L2	66002146	0.7164	1.1764	-0.8227
YRI	FOXD4L2	66007146	1.9609	3.5293	-1.1892
YRI	FOXD4L2	66012146	2.9692	4.3696	-0.8842
YRI	FOXD4L2	66017146	2.4826	2.5209	-0.0384
YRI	FOXD4L2	66022146	1.2767	1.1764	0.1794
YRI	FOXD4L2	66027146	0.474	0.3361	0.5536
YRI	FOXD4L2	66032146	1.5245	1.6806	-0.2147
YRI	FOXD4L2	66037146	2.1512	2.3529	-0.2135
YRI	FOXD4L2	66042146	0.8642	1.5126	-0.9655
YRI	FOXD4L2	66047146	0.804	1.5126	-1.0551
YRI	FOXD4L2	66052146	0.6785	0.8403	-0.3654
YRI	FOXD4L2	66057146	0.223	0.5042	-0.8837
YRI	FOXD4L2	66062146	0.2323	0.6722	-1.1516
YRI	FOXD4L2	66067146	0.6728	0.6722	0.0016
YRI	FOXD4L2	66072146	1.0562	0.8403	0.4876
YRI	FOXD4L2	66077146	0.4019	0.5042	-0.3215
YRI	FOXD4L2	66082146	0.0185	0.3361	-1.275
YRI	FOXD4L2	66087146	0.4372	0.6722	-0.6152
YRI	FOXD4L2	66092146	0.4279	0.5042	-0.2398
YRI	FOXD4L2	66097146	0.8368	0.6722	0.4309
YRI	FOXD4L2	66102146	0.8368	0.6722	0.4309
YRI	FOXD4L2	66107146	0.0093	0.1681	-0.944
YRI	FOXD4L2	66112146	0.2929	1.0084	-1.4265
YRI	FOXD4L2	66117146	1.1321	2.1848	-1.182
YRI	FOXD4L2	66122146	1.2587	1.8487	-0.7547
YRI	FOXD4L2	66127146	0.4837	1.0084	-1.0461
YRI	FOXD4L2	66132146	0.0734	0.5042	-1.3539

YRI	FOXD4L2	66137146	0.3802	0.5042	-0.3897
YRI	FOXD4L2	66142146	0.3802	0.5042	-0.3897
YRI	FOXD4L2	66147146	0.0093	0.1681	-0.944
YRI	FOXD4L2	66152146	0.0093	0.1681	-0.944
YRI	FOXD4L2	66157146	0	0	0
YRI	FOXD4L2	66162146	0.4565	0.1681	1.7145
YRI	FOXD4L2	66167146	0.4565	0.1681	1.7145
YRI	FOXD4L2	66172146	0	0	0
YRI	FOXD4L2	66177146	0.0273	0.1681	-0.837
YRI	FOXD4L2	66182146	0.0273	0.1681	-0.837
YRI	FOXD4L2	66187146	0	0	0
YRI	FOXD4L2	66192146	0	0	0
YRI	FOXD4L2	66197146	0	0	0
YRI	FOXD4L2	66202146	0	0	0
YRI	FOXD4L2	66207146	0	0	0
YRI	FOXD4L2	66212146	0	0	0
YRI	FOXD4L2	66217146	0	0	0
YRI	FOXD4L2	66222146	0	0	0
YRI	FOXD4L2	66227146	0	0	0
YRI	FOXD4L2	66232146	0	0	0
CEU	FOXD4L2	67807867	0.0101	0.1706	-0.9534
CEU	FOXD4L2	67812867	0.0101	0.1706	-0.9534
CEU	FOXD4L2	67817867	0	0	0
CEU	FOXD4L2	67822867	0.3191	0.3411	-0.0882
CEU	FOXD4L2	67827867	1.9955	1.5351	0.6827
CEU	FOXD4L2	67832867	2.1519	1.5351	0.9146
CEU	FOXD4L2	67837867	1.638	1.5351	0.1526
CEU	FOXD4L2	67842867	1.993	1.8762	0.1487
CEU	FOXD4L2	67847867	1.9175	1.7056	0.2902
CEU	FOXD4L2	67852867	1.1268	1.1939	-0.1196
CEU	FOXD4L2	67857867	0.2224	0.3411	-0.4758
CEU	FOXD4L2	67862867	0.8631	0.5117	1.1021
CEU	FOXD4L2	67867867	1.1495	0.5117	2.0003
CEU	FOXD4L2	67872867	0.469	0.1706	1.7726
CEU	FOXD4L2	67877867	0.0101	0.1706	-0.9534
CEU	FOXD4L2	67882867	0.1692	0.5117	-1.0742
CEU	FOXD4L2	67887867	1.1668	1.0234	0.2849
CEU	FOXD4L2	67892867	1.3729	1.5351	-0.2405
CEU	FOXD4L2	67897867	4.7899	4.6052	0.1121
CEU	FOXD4L2	67902867	4.9806	4.2641	0.4651
CEU	FOXD4L2	67907867	0.5762	0.8528	-0.6229
CEU	FOXD4L2	67912867	2.1806	3.2407	-0.8693
CEU	FOXD4L2	67917867	4.2031	5.6286	-0.7251
CEU	FOXD4L2	67922867	2.161	3.0701	-0.7798
CEU	FOXD4L2	67927867	0.7236	1.1939	-0.8381
CEU	FOXD4L2	67932867	2.1713	2.729	-0.5271
CEU	FOXD4L2	67937867	2.5452	2.8996	-0.3187
CEU	FOXD4L2	67942867	1.0394	1.5351	-0.735
CEU	FOXD4L2	67947867	1.7831	2.5584	-0.7723

CEU	FOXD4L2	67952867	1.7229	2.0467	-0.3853
CEU	FOXD4L2	67957867	0.0202	0.3411	-1.2864
CEU	FOXD4L2	67962867	1.0493	1.3645	-0.5099
CEU	FOXD4L2	67967867	1.4937	1.7056	-0.2902
CEU	FOXD4L2	67972867	0.4646	0.6822	-0.5682
CEU	FOXD4L2	67977867	0.1973	0.6822	-1.2662
CEU	FOXD4L2	67982867	0.9878	1.0234	-0.0707
CEU	FOXD4L2	67987867	0.7906	0.3411	1.8019
CEU	FOXD4L2	67992867	0	0	0
CEU	FOXD4L2	67997867	0	0	0
CEU	FOXD4L2	68002867	0	0	0
CEU	FOXD4L2	68007867	0	0	0
CEU	FOXD4L2	68012867	0	0	0
CEU	FOXD4L2	68017867	0	0	0
CEU	FOXD4L2	68022867	0	0	0
CEU	FOXD4L2	68027867	0	0	0
CEU	FOXD4L2	68032867	0	0	0
CEU	FOXD4L2	68037867	0	0	0
CEU	FOXD4L2	68042867	0	0	0
CEU	FOXD4L2	68047867	0	0	0
CEU	FOXD4L2	68052867	0	0	0
CEU	FOXD4L2	68057867	0	0	0
CEU	FOXD4L2	68062867	0	0	0
CEU	FOXD4L2	68067867	0	0	0
CEU	FOXD4L2	68072867	0	0	0
CEU	FOXD4L2	68077867	0	0	0
CEU	FOXD4L2	68082867	0	0	0
CEU	FOXD4L2	68087867	0	0	0
CEU	FOXD4L2	68092867	0	0	0
CEU	FOXD4L2	68097867	0	0	0
CEU	FOXD4L2	68102867	0	0	0
CEU	FOXD4L2	68107867	0	0	0
CEU	FOXD4L2	68112867	0	0	0
CEU	FOXD4L2	68117867	0	0	0
CEU	FOXD4L2	68122867	0	0	0
CEU	FOXD4L2	68127867	0	0	0
CEU	FOXD4L2	68132867	0	0	0
CEU	FOXD4L2	68137867	1.1185	1.0234	0.189
CEU	FOXD4L2	68142867	2.7943	3.4112	-0.4845
CEU	FOXD4L2	68147867	3.0843	5.1169	-1.1251
CEU	FOXD4L2	68152867	3.3342	6.8225	-1.4926
CEU	FOXD4L2	68157867	3.1614	6.1402	-1.4017
CEU	FOXD4L2	68162867	2.7988	4.4346	-1.0264
CEU	FOXD4L2	68167867	3.9674	6.1402	-1.0225
CEU	FOXD4L2	68172867	4.7199	7.5047	-1.0926
CEU	FOXD4L2	68177867	4.7495	8.5281	-1.3184
CEU	FOXD4L2	68182867	4.161	7.6753	-1.3508
CEU	FOXD4L2	68187867	3.1499	5.458	-1.2066
CEU	FOXD4L2	68192867	3.2509	5.1169	-1.0329

CEU	FOXD4L2	68197867	3.4896	5.7991	-1.1439
CEU	FOXD4L2	68202867	5.4469	9.0398	-1.1879
CEU	FOXD4L2	68207867	5.7939	9.7221	-1.2139
CEU	FOXD4L2	68212867	3.9099	6.3108	-1.1023
CEU	FOXD4L2	68217867	4.7953	6.1402	-0.6329
CEU	FOXD4L2	68222867	4.9105	8.8692	-1.3321
CEU	FOXD4L2	68227867	3.1084	6.9931	-1.6254
CEU	FOXD4L2	68232867	2.1319	3.9229	-1.2492
CEU	FOXD4L2	68237867	3.906	4.9463	-0.5933
CEU	FOXD4L2	68242867	3.8794	4.4346	-0.3484
CEU	FOXD4L2	68247867	3.323	3.9229	-0.4184
CEU	FOXD4L2	68252867	5.0113	5.2874	-0.1485
CEU	FOXD4L2	68257867	3.5508	4.7757	-0.7204
CEU	FOXD4L2	68262867	1.2674	3.9229	-1.8521
CEU	FOXD4L2	68267867	1.2727	3.9229	-1.8484
CEU	FOXD4L2	68272867	2.2948	4.7757	-1.4591
CEU	FOXD4L2	68277867	3.4267	6.9931	-1.4922
CEU	FOXD4L2	68282867	3.9716	7.1636	-1.3066
CEU	FOXD4L2	68287867	4.7897	5.9697	-0.5695
CEU	FOXD4L2	68292867	4.571	5.458	-0.4637
CEU	FOXD4L2	68297867	3.9219	7.3342	-1.3672
CEU	FOXD4L2	68302867	4.1723	8.0164	-1.4198
CEU	FOXD4L2	68307867	2.8161	4.6052	-1.0862
CEU	FOXD4L2	68312867	1.9367	4.0935	-1.4503
CEU	FOXD4L2	68317867	9.4158	9.7221	-0.0947
CEU	FOXD4L2	68322867	11.0109	12.1099	-0.2765
CEU	FOXD4L2	68327867	5.2319	7.1636	-0.7907
CEU	FOXD4L2	68332867	5.356	6.9931	-0.685
CEU	FOXD4L2	68337867	4.5376	8.0164	-1.2849
CEU	FOXD4L2	68342867	3.9959	8.187	-1.5183
CEU	FOXD4L2	68347867	4.7972	10.2337	-1.6016
CEU	FOXD4L2	68352867	3.5417	8.6987	-1.7668
CEU	FOXD4L2	68357867	8.2245	8.5281	-0.1059
CEU	FOXD4L2	68362867	10.8301	8.187	0.9575
CEU	FOXD4L2	68367867	6.2935	6.6519	-0.1569
CEU	FOXD4L2	68372867	5.0977	6.8225	-0.738
CEU	FOXD4L2	68377867	4.4261	5.7991	-0.68
CEU	FOXD4L2	68382867	14.8365	8.0164	2.519
CEU	FOXD4L2	68387867	22.1325	10.916	3.1109
CEU	FOXD4L2	68392867	14.2397	9.3809	1.5522
CEU	FOXD4L2	68397867	6.6706	7.8459	-0.4428
CEU	FOXD4L2	68402867	3.4137	6.8225	-1.4586
CEU	FOXD4L2	68407867	8.3814	8.187	0.0704
CEU	FOXD4L2	68412867	26.7946	14.1567	2.7425
CEU	FOXD4L2	68417867	32.8922	14.6684	3.8234
CEU	FOXD4L2	68422867	16.7311	9.0398	2.5429
CEU	FOXD4L2	68427867	6.6854	6.4814	0.0914
CEU	FOXD4L2	68432867	6.032	7.6753	-0.6316
CEU	FOXD4L2	68437867	7.9239	11.7688	-0.9937

CEU	FOXD4L2	68442867	7.6656	11.9394	-1.0896
CEU	FOXD4L2	68447867	6.1251	9.7221	-1.1116
CEU	FOXD4L2	68452867	6.0697	10.0632	-1.1951
CEU	FOXD4L2	68457867	4.1789	9.7221	-1.713
CEU	FOXD4L2	68462867	2.8549	9.2104	-2.0652
CEU	FOXD4L2	68467867	4.6634	9.0398	-1.4469
CEU	FOXD4L2	68472867	5.6961	7.5047	-0.7096
CEU	FOXD4L2	68477867	8.1971	7.5047	0.2717
CEU	FOXD4L2	68482867	9.4094	7.8459	0.589
CEU	FOXD4L2	68487867	7.2894	8.3576	-0.3797
CEU	FOXD4L2	68492867	5.3088	9.0398	-1.2336
CEU	FOXD4L2	68497867	4.093	9.3809	-1.6893
CEU	FOXD4L2	68502867	3.9839	10.7454	-1.9032
CEU	FOXD4L2	68507867	4.2564	11.0866	-1.867
CEU	FOXD4L2	68512867	3.0942	6.6519	-1.5576
CEU	FOXD4L2	68517867	0.509	0.8528	-0.7742
CEU	FOXD4L2	68522867	0	0	0
CEU	FOXD4L2	68527867	0	0	0
CEU	FOXD4L2	68532867	0	0	0
CEU	FOXD4L2	68537867	0	0	0
CEU	FOXD4L2	68542867	0	0	0
CEU	FOXD4L2	68547867	0	0	0
CEU	FOXD4L2	68552867	0	0	0
CEU	FOXD4L2	68557867	0	0	0
CEU	FOXD4L2	68562867	0	0	0
CEU	FOXD4L2	68567867	0	0	0
CEU	FOXD4L2	68572867	0	0	0
CEU	FOXD4L2	68577867	0	0	0
CEU	FOXD4L2	68582867	0	0	0
CEU	FOXD4L2	68587867	0	0	0
CEU	FOXD4L2	68592867	0	0	0
CEU	FOXD4L2	68597867	0	0	0
CEU	FOXD4L2	68602867	0	0	0
CEU	FOXD4L2	68607867	0	0	0
CEU	FOXD4L2	68612867	0	0	0
CEU	FOXD4L2	68617867	0	0	0
CEU	FOXD4L2	68622867	0	0	0
CEU	FOXD4L2	68627867	0	0	0
CEU	FOXD4L2	68632867	0	0	0
CEU	FOXD4L2	68637867	0	0	0
CEU	FOXD4L2	68642867	0	0	0
CEU	FOXD4L2	68647867	0	0	0
CEU	FOXD4L2	68652867	0	0	0
CEU	FOXD4L2	68657867	0	0	0
CEU	FOXD4L2	68662867	0.2744	1.3645	-1.7635
CEU	FOXD4L2	68667867	2.3564	4.7757	-1.4229
CEU	FOXD4L2	68672867	3.1787	4.9463	-1.0081
CEU	FOXD4L2	68677867	1.1371	2.2173	-1.2068
CEU	FOXD4L2	68682867	0.101	1.5351	-2.1265

CEU	FOXD4L2	68687867	2.4069	3.0701	-0.5689
CEU	FOXD4L2	68692867	2.9755	3.5818	-0.457
CEU	FOXD4L2	68697867	1.811	2.2173	-0.4539
CEU	FOXD4L2	68702867	1.369	1.3645	0.0073
CEU	FOXD4L2	68707867	0.9052	1.8762	-1.2362
CEU	FOXD4L2	68712867	0.7483	1.8762	-1.436
CEU	FOXD4L2	68717867	0.3488	1.5351	-1.7591
CEU	FOXD4L2	68722867	0.4651	1.8762	-1.7966
CEU	FOXD4L2	68727867	0.6273	1.7056	-1.4769
CEU	FOXD4L2	68732867	2.5459	3.9229	-0.9604
CEU	FOXD4L2	68737867	5.3427	6.1402	-0.3753
CEU	FOXD4L2	68742867	4.438	4.7757	-0.1986
CEU	FOXD4L2	68747867	1.8973	4.2641	-1.5364
CEU	FOXD4L2	68752867	2.8891	5.458	-1.343
CEU	FOXD4L2	68757867	3.2086	4.7757	-0.9217
CEU	FOXD4L2	68762867	1.3867	2.8996	-1.3606
CEU	FOXD4L2	68767867	1.0952	3.2407	-1.7593
CEU	FOXD4L2	68772867	1.7405	3.5818	-1.3878
CEU	FOXD4L2	68777867	2.2146	4.2641	-1.3304
CEU	FOXD4L2	68782867	2.6679	5.458	-1.4586
CEU	FOXD4L2	68787867	3.4343	6.1402	-1.2733
CEU	FOXD4L2	68792867	3.2061	6.1402	-1.3807
CEU	FOXD4L2	68797867	2.4166	4.6052	-1.3288
CHB	FOXD4L2	67807867	0	0	0
CHB	FOXD4L2	67812867	0	0	0
CHB	FOXD4L2	67817867	0.0097	0.1694	-0.949
CHB	FOXD4L2	67822867	0.6396	0.6777	-0.0996
CHB	FOXD4L2	67827867	2.3377	1.5247	1.2078
CHB	FOXD4L2	67832867	1.9983	1.1859	1.4501
CHB	FOXD4L2	67837867	1.1133	1.1859	-0.1296
CHB	FOXD4L2	67842867	1.7898	1.8636	-0.0942
CHB	FOXD4L2	67847867	1.7822	1.6942	0.1208
CHB	FOXD4L2	67852867	0.8152	0.8471	-0.0719
CHB	FOXD4L2	67857867	0.1447	0.1694	-0.1468
CHB	FOXD4L2	67862867	0.6448	0.5082	0.4288
CHB	FOXD4L2	67867867	0.9564	0.5082	1.407
CHB	FOXD4L2	67872867	0.4563	0.1694	1.7048
CHB	FOXD4L2	67877867	0.0097	0.1694	-0.949
CHB	FOXD4L2	67882867	0.2842	0.5082	-0.7032
CHB	FOXD4L2	67887867	1.3219	1.5247	-0.3013
CHB	FOXD4L2	67892867	1.2602	2.033	-0.9216
CHB	FOXD4L2	67897867	3.7575	3.3883	0.2908
CHB	FOXD4L2	67902867	4.052	2.8801	1.0566
CHB	FOXD4L2	67907867	0.5751	1.0165	-0.8784
CHB	FOXD4L2	67912867	1.9098	2.033	-0.1469
CHB	FOXD4L2	67917867	3.2941	4.066	-0.5205
CHB	FOXD4L2	67922867	1.5005	3.0495	-1.3321
CHB	FOXD4L2	67927867	0.6674	1.5247	-1.2736
CHB	FOXD4L2	67932867	2.155	2.8801	-0.6538

CHB	FOXD4L2	67937867	2.2821	2.7106	-0.406
CHB	FOXD4L2	67942867	0.8329	1.5247	-1.0278
CHB	FOXD4L2	67947867	0.8816	2.5412	-1.6572
CHB	FOXD4L2	67952867	0.795	2.033	-1.4763
CHB	FOXD4L2	67957867	0	0	0
CHB	FOXD4L2	67962867	1.5069	1.8636	-0.4551
CHB	FOXD4L2	67967867	1.9735	2.5412	-0.5669
CHB	FOXD4L2	67972867	0.4666	0.6777	-0.5519
CHB	FOXD4L2	67977867	0.0097	0.1694	-0.949
CHB	FOXD4L2	67982867	1.2073	1.0165	0.3797
CHB	FOXD4L2	67987867	1.1976	0.8471	0.7904
CHB	FOXD4L2	67992867	0	0	0
CHB	FOXD4L2	67997867	0	0	0
CHB	FOXD4L2	68002867	0	0	0
CHB	FOXD4L2	68007867	0	0	0
CHB	FOXD4L2	68012867	0	0	0
CHB	FOXD4L2	68017867	0	0	0
CHB	FOXD4L2	68022867	0	0	0
CHB	FOXD4L2	68027867	0	0	0
CHB	FOXD4L2	68032867	0	0	0
CHB	FOXD4L2	68037867	0	0	0
CHB	FOXD4L2	68042867	0	0	0
CHB	FOXD4L2	68047867	0	0	0
CHB	FOXD4L2	68052867	0	0	0
CHB	FOXD4L2	68057867	0	0	0
CHB	FOXD4L2	68062867	0	0	0
CHB	FOXD4L2	68067867	0	0	0
CHB	FOXD4L2	68072867	0	0	0
CHB	FOXD4L2	68077867	0	0	0
CHB	FOXD4L2	68082867	0	0	0
CHB	FOXD4L2	68087867	0	0	0
CHB	FOXD4L2	68092867	0	0	0
CHB	FOXD4L2	68097867	0	0	0
CHB	FOXD4L2	68102867	0	0	0
CHB	FOXD4L2	68107867	0	0	0
CHB	FOXD4L2	68112867	0	0	0
CHB	FOXD4L2	68117867	0	0	0
CHB	FOXD4L2	68122867	0	0	0
CHB	FOXD4L2	68127867	0	0	0
CHB	FOXD4L2	68132867	0	0	0
CHB	FOXD4L2	68137867	2.1289	1.8636	0.3385
CHB	FOXD4L2	68142867	5.4158	4.7436	0.3965
CHB	FOXD4L2	68147867	6.6876	6.0989	0.2779
CHB	FOXD4L2	68152867	7.9296	6.6072	0.5808
CHB	FOXD4L2	68157867	6.5387	6.0989	0.2076
CHB	FOXD4L2	68162867	4.4887	4.7436	-0.1504
CHB	FOXD4L2	68167867	6.3507	5.5907	0.3878
CHB	FOXD4L2	68172867	7.9644	7.1154	0.3487
CHB	FOXD4L2	68177867	9.6242	7.6237	0.7714

CHB	FOXD4L2	68182867	9.9101	8.3013	0.5738
CHB	FOXD4L2	68187867	7.067	7.2848	-0.0875
CHB	FOXD4L2	68192867	6.5075	6.2684	0.1101
CHB	FOXD4L2	68197867	9.6368	7.9625	0.6204
CHB	FOXD4L2	68202867	11.9155	9.3178	0.8327
CHB	FOXD4L2	68207867	8.9971	7.6237	0.5296
CHB	FOXD4L2	68212867	6.4278	5.7601	0.3317
CHB	FOXD4L2	68217867	7.7179	5.9295	0.8658
CHB	FOXD4L2	68222867	8.4831	8.4708	0.0043
CHB	FOXD4L2	68227867	6.9236	8.3013	-0.4914
CHB	FOXD4L2	68232867	4.4517	5.5907	-0.5811
CHB	FOXD4L2	68237867	4.9785	5.4213	-0.2322
CHB	FOXD4L2	68242867	5.3031	5.4213	-0.062
CHB	FOXD4L2	68247867	5.0177	4.913	0.0599
CHB	FOXD4L2	68252867	7.6651	5.4213	1.1766
CHB	FOXD4L2	68257867	5.8654	4.066	1.2134
CHB	FOXD4L2	68262867	1.7868	3.0495	-1.0859
CHB	FOXD4L2	68267867	1.9315	5.2519	-1.7908
CHB	FOXD4L2	68272867	3.6254	6.4378	-1.2645
CHB	FOXD4L2	68277867	6.1658	6.946	-0.3275
CHB	FOXD4L2	68282867	7.1266	6.2684	0.3953
CHB	FOXD4L2	68287867	7.2383	5.5907	0.8406
CHB	FOXD4L2	68292867	7.2511	6.0989	0.5439
CHB	FOXD4L2	68297867	7.5665	7.9625	-0.1467
CHB	FOXD4L2	68302867	7.3992	8.8096	-0.4762
CHB	FOXD4L2	68307867	4.1453	6.2684	-0.9778
CHB	FOXD4L2	68312867	4.2692	6.6072	-1.0269
CHB	FOXD4L2	68317867	15.09	11.6896	0.8818
CHB	FOXD4L2	68322867	16.6368	13.5532	0.6954
CHB	FOXD4L2	68327867	6.8469	9.8261	-0.909
CHB	FOXD4L2	68332867	6.8239	9.9955	-0.9524
CHB	FOXD4L2	68337867	7.2989	8.4708	-0.4102
CHB	FOXD4L2	68342867	7.4097	6.2684	0.5256
CHB	FOXD4L2	68347867	11.502	9.6567	0.5722
CHB	FOXD4L2	68352867	8.8957	8.8096	0.0291
CHB	FOXD4L2	68357867	9.9417	7.6237	0.8939
CHB	FOXD4L2	68362867	12.0023	6.0989	2.7867
CHB	FOXD4L2	68367867	8.1502	4.4048	2.3568
CHB	FOXD4L2	68372867	7.7955	5.2519	1.3718
CHB	FOXD4L2	68377867	6.7664	5.9295	0.4052
CHB	FOXD4L2	68382867	15.7221	10.6731	1.426
CHB	FOXD4L2	68387867	23.028	12.5367	2.5471
CHB	FOXD4L2	68392867	14.4276	8.3013	2.1849
CHB	FOXD4L2	68397867	6.7885	7.9625	-0.435
CHB	FOXD4L2	68402867	5.8026	8.8096	-1.0152
CHB	FOXD4L2	68407867	11.636	10.1649	0.4349
CHB	FOXD4L2	68412867	27.5314	15.0779	2.5379
CHB	FOXD4L2	68417867	32.4346	15.2474	3.4655
CHB	FOXD4L2	68422867	18.1413	9.4872	2.728

CHB	FOXD4L2	68427867	8.5035	6.6072	0.8329
CHB	FOXD4L2	68432867	9.6377	9.4872	0.0474
CHB	FOXD4L2	68437867	15.2656	13.7226	0.3439
CHB	FOXD4L2	68442867	14.5639	13.045	0.3552
CHB	FOXD4L2	68447867	10.114	9.6567	0.1418
CHB	FOXD4L2	68452867	8.7379	9.4872	-0.2362
CHB	FOXD4L2	68457867	7.2404	9.8261	-0.7889
CHB	FOXD4L2	68462867	7.0836	7.6237	-0.2083
CHB	FOXD4L2	68467867	8.3	6.946	0.5684
CHB	FOXD4L2	68472867	9.3547	8.1319	0.4445
CHB	FOXD4L2	68477867	11.2037	8.4708	0.9567
CHB	FOXD4L2	68482867	10.8586	9.4872	0.4323
CHB	FOXD4L2	68487867	8.6493	11.012	-0.6481
CHB	FOXD4L2	68492867	8.7588	10.5037	-0.5003
CHB	FOXD4L2	68497867	8.5903	9.9955	-0.422
CHB	FOXD4L2	68502867	8.4413	9.6567	-0.3769
CHB	FOXD4L2	68507867	10.9943	11.1814	-0.0506
CHB	FOXD4L2	68512867	7.4483	8.3013	-0.3042
CHB	FOXD4L2	68517867	0.6025	1.1859	-1.0414
CHB	FOXD4L2	68522867	0	0	0
CHB	FOXD4L2	68527867	0	0	0
CHB	FOXD4L2	68532867	0	0	0
CHB	FOXD4L2	68537867	0	0	0
CHB	FOXD4L2	68542867	0	0	0
CHB	FOXD4L2	68547867	0	0	0
CHB	FOXD4L2	68552867	0	0	0
CHB	FOXD4L2	68557867	0	0	0
CHB	FOXD4L2	68562867	0	0	0
CHB	FOXD4L2	68567867	0	0	0
CHB	FOXD4L2	68572867	0	0	0
CHB	FOXD4L2	68577867	0	0	0
CHB	FOXD4L2	68582867	0	0	0
CHB	FOXD4L2	68587867	0	0	0
CHB	FOXD4L2	68592867	0	0	0
CHB	FOXD4L2	68597867	0	0	0
CHB	FOXD4L2	68602867	0	0	0
CHB	FOXD4L2	68607867	0	0	0
CHB	FOXD4L2	68612867	0	0	0
CHB	FOXD4L2	68617867	0	0	0
CHB	FOXD4L2	68622867	0	0	0
CHB	FOXD4L2	68627867	0	0	0
CHB	FOXD4L2	68632867	0	0	0
CHB	FOXD4L2	68637867	0	0	0
CHB	FOXD4L2	68642867	0	0	0
CHB	FOXD4L2	68647867	0	0	0
CHB	FOXD4L2	68652867	0	0	0
CHB	FOXD4L2	68657867	0	0	0
CHB	FOXD4L2	68662867	0.174	1.5247	-2.0067
CHB	FOXD4L2	68667867	1.3954	4.066	-1.8009

CHB	FOXD4L2	68672867	2.0999	3.5577	-1.1017
CHB	FOXD4L2	68677867	0.9366	1.8636	-1.1827
CHB	FOXD4L2	68682867	0.4161	2.2024	-2.0002
CHB	FOXD4L2	68687867	2.505	3.2189	-0.5869
CHB	FOXD4L2	68692867	2.7226	3.0495	-0.2811
CHB	FOXD4L2	68697867	1.5964	2.2024	-0.6786
CHB	FOXD4L2	68702867	1.2732	2.033	-0.9061
CHB	FOXD4L2	68707867	1.0482	2.3718	-1.3972
CHB	FOXD4L2	68712867	0.8249	1.8636	-1.3252
CHB	FOXD4L2	68717867	0.068	1.1859	-1.9954
CHB	FOXD4L2	68722867	0.1426	1.3553	-1.9654
CHB	FOXD4L2	68727867	0.5553	1.6942	-1.563
CHB	FOXD4L2	68732867	2.047	4.5742	-1.5389
CHB	FOXD4L2	68737867	3.9732	7.6237	-1.4077
CHB	FOXD4L2	68742867	3.3558	5.2519	-1.0226
CHB	FOXD4L2	68747867	1.5277	4.2354	-1.7627
CHB	FOXD4L2	68752867	2.6351	6.2684	-1.6734
CHB	FOXD4L2	68757867	3.8578	4.7436	-0.5225
CHB	FOXD4L2	68762867	2.2665	3.0495	-0.6734
CHB	FOXD4L2	68767867	1.1294	3.7271	-1.8872
CHB	FOXD4L2	68772867	1.4438	4.5742	-1.9062
CHB	FOXD4L2	68777867	2.0532	5.5907	-1.8049
CHB	FOXD4L2	68782867	2.2732	5.5907	-1.6927
CHB	FOXD4L2	68787867	2.4698	5.5907	-1.5924
CHB	FOXD4L2	68792867	2.4243	5.9295	-1.6969
CHB	FOXD4L2	68797867	2.2587	5.2519	-1.6143
YRI	FOXD4L2	67807867	0.1832	0.5042	-1.0088
YRI	FOXD4L2	67812867	0.0864	0.1681	-0.4857
YRI	FOXD4L2	67817867	0	0	0
YRI	FOXD4L2	67822867	0.3291	1.3445	-1.6492
YRI	FOXD4L2	67827867	2.1626	2.8571	-0.6281
YRI	FOXD4L2	67832867	2.3319	2.1848	0.1652
YRI	FOXD4L2	67837867	1.2714	2.0167	-0.8911
YRI	FOXD4L2	67842867	1.5887	2.3529	-0.8089
YRI	FOXD4L2	67847867	1.8179	1.6806	0.1889
YRI	FOXD4L2	67852867	1.0208	0.8403	0.4077
YRI	FOXD4L2	67857867	0.505	0.6722	-0.4377
YRI	FOXD4L2	67862867	1.3129	0.8403	1.0674
YRI	FOXD4L2	67867867	1.3122	0.5042	2.5393
YRI	FOXD4L2	67872867	0.4858	0.1681	1.8886
YRI	FOXD4L2	67877867	0.1782	0.3361	-0.6339
YRI	FOXD4L2	67882867	0.2497	0.6722	-1.1061
YRI	FOXD4L2	67887867	1.0267	2.3529	-1.4038
YRI	FOXD4L2	67892867	1.1983	2.8571	-1.5001
YRI	FOXD4L2	67897867	5.0366	5.7141	-0.3378
YRI	FOXD4L2	67902867	5.2381	5.8822	-0.313
YRI	FOXD4L2	67907867	0.5704	1.3445	-1.2573
YRI	FOXD4L2	67912867	1.9584	2.5209	-0.5633
YRI	FOXD4L2	67917867	3.785	4.0335	-0.1681

YRI	FOXD4L2	67922867	2.0255	2.1848	-0.1789
YRI	FOXD4L2	67927867	0.8699	1.0084	-0.2761
YRI	FOXD4L2	67932867	2.347	2.1848	0.1821
YRI	FOXD4L2	67937867	2.4371	2.689	-0.2394
YRI	FOXD4L2	67942867	0.9332	2.0167	-1.2955
YRI	FOXD4L2	67947867	2.4127	4.2016	-1.1686
YRI	FOXD4L2	67952867	2.3664	3.3612	-0.7859
YRI	FOXD4L2	67957867	0	0	0
YRI	FOXD4L2	67962867	1.5137	2.1848	-0.7535
YRI	FOXD4L2	67967867	1.7606	2.8571	-0.9916
YRI	FOXD4L2	67972867	0.2931	1.1764	-1.5798
YRI	FOXD4L2	67977867	0.0646	0.8403	-1.752
YRI	FOXD4L2	67982867	0.7664	1.5126	-1.1111
YRI	FOXD4L2	67987867	0.7479	1.1764	-0.7664
YRI	FOXD4L2	67992867	0	0	0
YRI	FOXD4L2	67997867	0	0	0
YRI	FOXD4L2	68002867	0	0	0
YRI	FOXD4L2	68007867	0	0	0
YRI	FOXD4L2	68012867	0	0	0
YRI	FOXD4L2	68017867	0	0	0
YRI	FOXD4L2	68022867	0	0	0
YRI	FOXD4L2	68027867	0	0	0
YRI	FOXD4L2	68032867	0	0	0
YRI	FOXD4L2	68037867	0	0	0
YRI	FOXD4L2	68042867	0	0	0
YRI	FOXD4L2	68047867	0	0	0
YRI	FOXD4L2	68052867	0	0	0
YRI	FOXD4L2	68057867	0	0	0
YRI	FOXD4L2	68062867	0	0	0
YRI	FOXD4L2	68067867	0	0	0
YRI	FOXD4L2	68072867	0	0	0
YRI	FOXD4L2	68077867	0	0	0
YRI	FOXD4L2	68082867	0	0	0
YRI	FOXD4L2	68087867	0	0	0
YRI	FOXD4L2	68092867	0	0	0
YRI	FOXD4L2	68097867	0	0	0
YRI	FOXD4L2	68102867	0	0	0
YRI	FOXD4L2	68107867	0	0	0
YRI	FOXD4L2	68112867	0	0	0
YRI	FOXD4L2	68117867	0	0	0
YRI	FOXD4L2	68122867	0	0	0
YRI	FOXD4L2	68127867	0	0	0
YRI	FOXD4L2	68132867	0	0	0
YRI	FOXD4L2	68137867	1.7797	2.0167	-0.2834
YRI	FOXD4L2	68142867	5.3599	6.2183	-0.3968
YRI	FOXD4L2	68147867	8.8868	10.5879	-0.4824
YRI	FOXD4L2	68152867	10.5442	11.4282	-0.2334
YRI	FOXD4L2	68157867	7.4417	8.5712	-0.3898
YRI	FOXD4L2	68162867	4.5669	7.2267	-1.0733

YRI	FOXD4L2	68167867	7.34	9.7476	-0.7375
YRI	FOXD4L2	68172867	10.5513	11.2602	-0.1898
YRI	FOXD4L2	68177867	12.109	11.4282	0.1797
YRI	FOXD4L2	68182867	12.4871	12.2685	0.054
YRI	FOXD4L2	68187867	8.6314	8.235	0.1419
YRI	FOXD4L2	68192867	6.7349	6.2183	0.2388
YRI	FOXD4L2	68197867	11.2014	9.7476	0.4454
YRI	FOXD4L2	68202867	13.4456	11.0921	0.639
YRI	FOXD4L2	68207867	10.239	9.7476	0.1505
YRI	FOXD4L2	68212867	8.0243	8.4031	-0.1331
YRI	FOXD4L2	68217867	8.1185	8.5712	-0.1562
YRI	FOXD4L2	68222867	9.8117	11.5963	-0.4647
YRI	FOXD4L2	68227867	9.3023	10.756	-0.4062
YRI	FOXD4L2	68232867	6.0954	7.2267	-0.4565
YRI	FOXD4L2	68237867	5.9546	6.8906	-0.3944
YRI	FOXD4L2	68242867	6.5862	7.0586	-0.1947
YRI	FOXD4L2	68247867	6.8671	8.067	-0.4378
YRI	FOXD4L2	68252867	8.6963	9.4115	-0.2264
YRI	FOXD4L2	68257867	7.1603	8.4031	-0.4368
YRI	FOXD4L2	68262867	3.4753	5.8822	-1.1695
YRI	FOXD4L2	68267867	3.561	5.8822	-1.1279
YRI	FOXD4L2	68272867	5.2754	8.235	-1.0597
YRI	FOXD4L2	68277867	8.4369	10.924	-0.685
YRI	FOXD4L2	68282867	10.2183	10.0837	0.04
YRI	FOXD4L2	68287867	9.2016	10.0837	-0.2618
YRI	FOXD4L2	68292867	8.6759	10.924	-0.6192
YRI	FOXD4L2	68297867	10.8998	12.2685	-0.338
YRI	FOXD4L2	68302867	10.706	11.9324	-0.3109
YRI	FOXD4L2	68307867	6.1109	8.067	-0.7138
YRI	FOXD4L2	68312867	5.8844	8.4031	-0.8852
YRI	FOXD4L2	68317867	18.7927	16.6382	0.3983
YRI	FOXD4L2	68322867	21.8614	19.1591	0.4363
YRI	FOXD4L2	68327867	9.4907	12.6047	-0.7496
YRI	FOXD4L2	68332867	8.5138	12.4366	-0.9564
YRI	FOXD4L2	68337867	9.33	11.4282	-0.5539
YRI	FOXD4L2	68342867	9.546	11.9324	-0.6049
YRI	FOXD4L2	68347867	12.757	14.9575	-0.4503
YRI	FOXD4L2	68352867	9.7335	11.9324	-0.5574
YRI	FOXD4L2	68357867	10.8629	9.5795	0.3996
YRI	FOXD4L2	68362867	12.6439	8.9073	1.2445
YRI	FOXD4L2	68367867	8.8009	8.9073	-0.0354
YRI	FOXD4L2	68372867	8.5447	10.2518	-0.4989
YRI	FOXD4L2	68377867	7.5536	8.5712	-0.3512
YRI	FOXD4L2	68382867	16.4483	10.4199	1.7354
YRI	FOXD4L2	68387867	23.8632	12.7727	2.6366
YRI	FOXD4L2	68392867	15.6546	10.0837	1.6535
YRI	FOXD4L2	68397867	7.846	10.0837	-0.6642
YRI	FOXD4L2	68402867	7.3646	12.4366	-1.2365
YRI	FOXD4L2	68407867	14.1247	14.1172	0.0016

YRI	FOXD4L2	68412867	29.7503	16.4701	2.4792
YRI	FOXD4L2	68417867	33.8969	16.302	3.317
YRI	FOXD4L2	68422867	18.8255	11.5963	1.8825
YRI	FOXD4L2	68427867	9.7811	9.7476	0.0103
YRI	FOXD4L2	68432867	11.6001	12.7727	-0.2788
YRI	FOXD4L2	68437867	16.5515	16.4701	0.0152
YRI	FOXD4L2	68442867	15.2013	17.1423	-0.3487
YRI	FOXD4L2	68447867	11.4728	14.7895	-0.6861
YRI	FOXD4L2	68452867	9.9016	11.5963	-0.4413
YRI	FOXD4L2	68457867	8.043	11.0921	-0.8278
YRI	FOXD4L2	68462867	8.5766	10.756	-0.609
YRI	FOXD4L2	68467867	9.705	10.924	-0.3357
YRI	FOXD4L2	68472867	10.6698	12.1005	-0.3579
YRI	FOXD4L2	68477867	13.1809	11.9324	0.3165
YRI	FOXD4L2	68482867	12.581	11.2602	0.3536
YRI	FOXD4L2	68487867	10.0652	13.445	-0.7654
YRI	FOXD4L2	68492867	9.7521	13.1089	-0.7787
YRI	FOXD4L2	68497867	9.507	13.2769	-0.864
YRI	FOXD4L2	68502867	10.6093	15.7978	-1.0079
YRI	FOXD4L2	68507867	13.2866	17.9827	-0.8059
YRI	FOXD4L2	68512867	8.8393	12.7727	-0.9351
YRI	FOXD4L2	68517867	1.1006	2.0167	-1.0953
YRI	FOXD4L2	68522867	0	0	0
YRI	FOXD4L2	68527867	0	0	0
YRI	FOXD4L2	68532867	0	0	0
YRI	FOXD4L2	68537867	0	0	0
YRI	FOXD4L2	68542867	0	0	0
YRI	FOXD4L2	68547867	0	0	0
YRI	FOXD4L2	68552867	0	0	0
YRI	FOXD4L2	68557867	0	0	0
YRI	FOXD4L2	68562867	0	0	0
YRI	FOXD4L2	68567867	0	0	0
YRI	FOXD4L2	68572867	0	0	0
YRI	FOXD4L2	68577867	0	0	0
YRI	FOXD4L2	68582867	0	0	0
YRI	FOXD4L2	68587867	0	0	0
YRI	FOXD4L2	68592867	0	0	0
YRI	FOXD4L2	68597867	0	0	0
YRI	FOXD4L2	68602867	0	0	0
YRI	FOXD4L2	68607867	0	0	0
YRI	FOXD4L2	68612867	0	0	0
YRI	FOXD4L2	68617867	0	0	0
YRI	FOXD4L2	68622867	0	0	0
YRI	FOXD4L2	68627867	0	0	0
YRI	FOXD4L2	68632867	0	0	0
YRI	FOXD4L2	68637867	0	0	0
YRI	FOXD4L2	68642867	0	0	0
YRI	FOXD4L2	68647867	0	0	0
YRI	FOXD4L2	68652867	0	0	0

YRI	FOXD4L2	68657867	0	0	0
YRI	FOXD4L2	68662867	0.3709	1.6806	-1.8018
YRI	FOXD4L2	68667867	1.714	5.7141	-1.9947
YRI	FOXD4L2	68672867	2.4316	6.3864	-1.7849
YRI	FOXD4L2	68677867	1.5829	3.6974	-1.5412
YRI	FOXD4L2	68682867	0.5953	2.3529	-1.8605
YRI	FOXD4L2	68687867	2.8475	3.6974	-0.6195
YRI	FOXD4L2	68692867	3.3284	4.5377	-0.7389
YRI	FOXD4L2	68697867	1.9901	3.1932	-0.9922
YRI	FOXD4L2	68702867	1.614	2.689	-1.0216
YRI	FOXD4L2	68707867	1.1461	3.1932	-1.6883
YRI	FOXD4L2	68712867	1.0419	3.1932	-1.7742
YRI	FOXD4L2	68717867	0.1106	1.5126	-2.0876
YRI	FOXD4L2	68722867	0.2006	1.6806	-2.036
YRI	FOXD4L2	68727867	0.8017	2.3529	-1.642
YRI	FOXD4L2	68732867	3.1765	5.2099	-1.1006
YRI	FOXD4L2	68737867	5.2792	7.5628	-0.884
YRI	FOXD4L2	68742867	3.8724	6.0502	-1.0319
YRI	FOXD4L2	68747867	2.6841	5.7141	-1.5109
YRI	FOXD4L2	68752867	3.7753	6.8906	-1.3127
YRI	FOXD4L2	68757867	3.1353	6.3864	-1.4673
YRI	FOXD4L2	68762867	1.7993	4.2016	-1.5693
YRI	FOXD4L2	68767867	2.0626	4.2016	-1.3973
YRI	FOXD4L2	68772867	2.521	5.378	-1.5036
YRI	FOXD4L2	68777867	2.7049	6.5544	-1.6972
YRI	FOXD4L2	68782867	2.7781	6.7225	-1.6996
YRI	FOXD4L2	68787867	3.3334	7.0586	-1.5357
YRI	FOXD4L2	68792867	2.8489	7.8989	-1.8787
YRI	FOXD4L2	68797867	2.6694	7.0586	-1.8094
CEU	GOLGA6L9	81934363	0	0	0
CEU	GOLGA6L9	81939363	0	0	0
CEU	GOLGA6L9	81944363	0	0	0
CEU	GOLGA6L9	81949363	0	0	0
CEU	GOLGA6L9	81954363	0	0	0
CEU	GOLGA6L9	81959363	0	0	0
CEU	GOLGA6L9	81964363	0	0	0
CEU	GOLGA6L9	81969363	0	0	0
CEU	GOLGA6L9	81974363	0	0	0
CEU	GOLGA6L9	81979363	0	0	0
CEU	GOLGA6L9	81984363	0	0	0
CEU	GOLGA6L9	81989363	0	0	0
CEU	GOLGA6L9	81994363	0	0	0
CEU	GOLGA6L9	81999363	0	0	0
CEU	GOLGA6L9	82004363	0	0	0
CEU	GOLGA6L9	82009363	0	0	0
CEU	GOLGA6L9	82014363	0	0	0
CEU	GOLGA6L9	82019363	0	0	0
CEU	GOLGA6L9	82024363	0	0	0
CEU	GOLGA6L9	82029363	0	0	0

CEU	GOLGA6L9	82034363	0	0	0
CEU	GOLGA6L9	82039363	0	0	0
CEU	GOLGA6L9	82044363	0	0	0
CEU	GOLGA6L9	82049363	0	0	0
CEU	GOLGA6L9	82054363	0	0	0
CEU	GOLGA6L9	82059363	0	0	0
CEU	GOLGA6L9	82064363	0	0	0
CEU	GOLGA6L9	82069363	0	0	0
CEU	GOLGA6L9	82074363	0	0	0
CEU	GOLGA6L9	82079363	0	0	0
CEU	GOLGA6L9	82084363	0	0	0
CEU	GOLGA6L9	82089363	0	0	0
CEU	GOLGA6L9	82094363	0	0	0
CEU	GOLGA6L9	82099363	0	0	0
CEU	GOLGA6L9	82104363	0	0	0
CEU	GOLGA6L9	82109363	0	0	0
CEU	GOLGA6L9	82114363	0	0	0
CEU	GOLGA6L9	82119363	0	0	0
CEU	GOLGA6L9	82124363	0	0	0
CEU	GOLGA6L9	82129363	0	0	0
CEU	GOLGA6L9	82134363	0	0	0
CEU	GOLGA6L9	82139363	0	0	0
CEU	GOLGA6L9	82144363	0	0	0
CEU	GOLGA6L9	82149363	0	0	0
CEU	GOLGA6L9	82154363	0	0	0
CEU	GOLGA6L9	82159363	0	0	0
CEU	GOLGA6L9	82164363	0	0	0
CEU	GOLGA6L9	82169363	0	0	0
CEU	GOLGA6L9	82174363	0	0	0
CEU	GOLGA6L9	82179363	0	0	0
CEU	GOLGA6L9	82184363	0	0	0
CEU	GOLGA6L9	82189363	0	0	0
CEU	GOLGA6L9	82194363	0	0	0
CEU	GOLGA6L9	82199363	0	0	0
CEU	GOLGA6L9	82204363	0	0	0
CEU	GOLGA6L9	82209363	0	0	0
CEU	GOLGA6L9	82214363	0	0	0
CEU	GOLGA6L9	82219363	0	0	0
CEU	GOLGA6L9	82224363	0	0	0
CEU	GOLGA6L9	82229363	0	0	0
CEU	GOLGA6L9	82234363	0	0	0
CEU	GOLGA6L9	82239363	0	0	0
CEU	GOLGA6L9	82244363	0	0	0
CEU	GOLGA6L9	82249363	0	0	0
CEU	GOLGA6L9	82254363	0	0	0
CEU	GOLGA6L9	82259363	0	0	0
CEU	GOLGA6L9	82264363	0	0	0
CEU	GOLGA6L9	82269363	0	0	0
CEU	GOLGA6L9	82274363	0	0	0

CEU	GOLGA6L9	82279363	0	0	0
CEU	GOLGA6L9	82284363	0	0	0
CEU	GOLGA6L9	82289363	0	0	0
CEU	GOLGA6L9	82294363	0	0	0
CEU	GOLGA6L9	82299363	0	0	0
CEU	GOLGA6L9	82304363	0	0	0
CEU	GOLGA6L9	82309363	0	0	0
CEU	GOLGA6L9	82314363	0	0	0
CEU	GOLGA6L9	82319363	0	0	0
CEU	GOLGA6L9	82324363	0	0	0
CEU	GOLGA6L9	82329363	0	0	0
CEU	GOLGA6L9	82334363	0	0	0
CEU	GOLGA6L9	82339363	0	0	0
CEU	GOLGA6L9	82344363	0	0	0
CEU	GOLGA6L9	82349363	0	0	0
CEU	GOLGA6L9	82354363	0	0	0
CEU	GOLGA6L9	82359363	0	0	0
CEU	GOLGA6L9	82364363	0	0	0
CEU	GOLGA6L9	82369363	0	0	0
CEU	GOLGA6L9	82374363	0	0	0
CEU	GOLGA6L9	82379363	0	0	0
CEU	GOLGA6L9	82384363	0	0	0
CEU	GOLGA6L9	82389363	0	0	0
CEU	GOLGA6L9	82394363	0	0	0
CEU	GOLGA6L9	82399363	0	0	0
CEU	GOLGA6L9	82404363	0	0	0
CEU	GOLGA6L9	82409363	0	0	0
CEU	GOLGA6L9	82414363	0	0	0
CEU	GOLGA6L9	82419363	0	0	0
CEU	GOLGA6L9	82424363	0	0	0
CEU	GOLGA6L9	82429363	0	0	0
CEU	GOLGA6L9	82434363	0	0	0
CEU	GOLGA6L9	82439363	0	0	0
CEU	GOLGA6L9	82444363	0	0	0
CEU	GOLGA6L9	82449363	0	0	0
CEU	GOLGA6L9	82454363	0	0	0
CEU	GOLGA6L9	82459363	0	0	0
CEU	GOLGA6L9	82464363	0	0	0
CEU	GOLGA6L9	82469363	0	0	0
CEU	GOLGA6L9	82474363	0	0	0
CEU	GOLGA6L9	82479363	0	0	0
CEU	GOLGA6L9	82484363	0	0	0
CEU	GOLGA6L9	82489363	0	0	0
CEU	GOLGA6L9	82494363	0	0	0
CEU	GOLGA6L9	82499363	0	0	0
CEU	GOLGA6L9	82504363	0	0	0
CEU	GOLGA6L9	82509363	0	0	0
CEU	GOLGA6L9	82514363	0	0	0
CEU	GOLGA6L9	82519363	0	0	0

CEU	GOLGA6L9	82524363	0	0	0
CEU	GOLGA6L9	82529363	0	0	0
CEU	GOLGA6L9	82534363	0	0	0
CEU	GOLGA6L9	82539363	0	0	0
CEU	GOLGA6L9	82544363	0	0	0
CEU	GOLGA6L9	82549363	0	0	0
CEU	GOLGA6L9	82554363	0	0	0
CEU	GOLGA6L9	82559363	0	0	0
CEU	GOLGA6L9	82564363	0	0	0
CEU	GOLGA6L9	82569363	0	0	0
CEU	GOLGA6L9	82574363	0	0	0
CEU	GOLGA6L9	82579363	0	0	0
CEU	GOLGA6L9	82584363	0	0	0
CEU	GOLGA6L9	82589363	0	0	0
CEU	GOLGA6L9	82594363	0	0	0
CEU	GOLGA6L9	82599363	0	0	0
CEU	GOLGA6L9	82604363	0	0	0
CEU	GOLGA6L9	82609363	0	0	0
CEU	GOLGA6L9	82614363	0	0	0
CEU	GOLGA6L9	82619363	0	0	0
CEU	GOLGA6L9	82624363	0	0	0
CEU	GOLGA6L9	82629363	0	0	0
CEU	GOLGA6L9	82634363	0	0	0
CEU	GOLGA6L9	82639363	0	0	0
CEU	GOLGA6L9	82644363	0	0	0
CEU	GOLGA6L9	82649363	0	0	0
CEU	GOLGA6L9	82654363	0	0	0
CEU	GOLGA6L9	82659363	0	0	0
CEU	GOLGA6L9	82664363	0	0	0
CEU	GOLGA6L9	82669363	0	0	0
CEU	GOLGA6L9	82674363	0	0	0
CEU	GOLGA6L9	82679363	0	0	0
CEU	GOLGA6L9	82684363	0	0	0
CEU	GOLGA6L9	82689363	0	0	0
CEU	GOLGA6L9	82694363	0	0	0
CEU	GOLGA6L9	82699363	0	0	0
CEU	GOLGA6L9	82704363	0	0	0
CEU	GOLGA6L9	82709363	0	0	0
CEU	GOLGA6L9	82714363	0	0	0
CEU	GOLGA6L9	82719363	0	0	0
CEU	GOLGA6L9	82724363	0	0	0
CEU	GOLGA6L9	82729363	0	0	0
CEU	GOLGA6L9	82734363	0	0	0
CEU	GOLGA6L9	82739363	0	0	0
CEU	GOLGA6L9	82744363	0	0	0
CEU	GOLGA6L9	82749363	0	0	0
CEU	GOLGA6L9	82754363	0	0	0
CEU	GOLGA6L9	82759363	0	0	0
CEU	GOLGA6L9	82764363	0	0	0

CEU	GOLGA6L9	82769363	0	0	0
CEU	GOLGA6L9	82774363	0	0	0
CEU	GOLGA6L9	82779363	0	0	0
CEU	GOLGA6L9	82784363	0	0	0
CEU	GOLGA6L9	82789363	0	0	0
CEU	GOLGA6L9	82794363	0	0	0
CEU	GOLGA6L9	82799363	0	0	0
CEU	GOLGA6L9	82804363	0	0	0
CEU	GOLGA6L9	82809363	0	0	0
CEU	GOLGA6L9	82814363	0	0	0
CEU	GOLGA6L9	82819363	0	0	0
CEU	GOLGA6L9	82824363	0	0	0
CEU	GOLGA6L9	82829363	0	0	0
CEU	GOLGA6L9	82834363	0	0	0
CEU	GOLGA6L9	82839363	0	0	0
CEU	GOLGA6L9	82844363	0	0	0
CEU	GOLGA6L9	82849363	0	0	0
CEU	GOLGA6L9	82854363	0	0	0
CEU	GOLGA6L9	82859363	0	0	0
CEU	GOLGA6L9	82864363	0	0	0
CEU	GOLGA6L9	82869363	0	0	0
CEU	GOLGA6L9	82874363	0	0	0
CEU	GOLGA6L9	82879363	0	0	0
CEU	GOLGA6L9	82884363	0	0	0
CEU	GOLGA6L9	82889363	0	0	0
CEU	GOLGA6L9	82894363	0	0	0
CEU	GOLGA6L9	82899363	0	0	0
CEU	GOLGA6L9	82904363	0	0	0
CEU	GOLGA6L9	82909363	0	0	0
CEU	GOLGA6L9	82914363	0	0	0
CEU	GOLGA6L9	82919363	0	0	0
CEU	GOLGA6L9	82924363	0	0	0
CEU	GOLGA6L9	82929363	0	0	0
CHB	GOLGA6L9	81934363	0	0	0
CHB	GOLGA6L9	81939363	0	0	0
CHB	GOLGA6L9	81944363	0	0	0
CHB	GOLGA6L9	81949363	0	0	0
CHB	GOLGA6L9	81954363	0	0	0
CHB	GOLGA6L9	81959363	0	0	0
CHB	GOLGA6L9	81964363	0	0	0
CHB	GOLGA6L9	81969363	0	0	0
CHB	GOLGA6L9	81974363	0	0	0
CHB	GOLGA6L9	81979363	0	0	0
CHB	GOLGA6L9	81984363	0	0	0
CHB	GOLGA6L9	81989363	0	0	0
CHB	GOLGA6L9	81994363	0	0	0
CHB	GOLGA6L9	81999363	0	0	0
CHB	GOLGA6L9	82004363	0	0	0
CHB	GOLGA6L9	82009363	0	0	0

CHB	GOLGA6L9	82014363	0	0	0
CHB	GOLGA6L9	82019363	0	0	0
CHB	GOLGA6L9	82024363	0	0	0
CHB	GOLGA6L9	82029363	0	0	0
CHB	GOLGA6L9	82034363	0	0	0
CHB	GOLGA6L9	82039363	0	0	0
CHB	GOLGA6L9	82044363	0	0	0
CHB	GOLGA6L9	82049363	0	0	0
CHB	GOLGA6L9	82054363	0	0	0
CHB	GOLGA6L9	82059363	0	0	0
CHB	GOLGA6L9	82064363	0	0	0
CHB	GOLGA6L9	82069363	0	0	0
CHB	GOLGA6L9	82074363	0	0	0
CHB	GOLGA6L9	82079363	0	0	0
CHB	GOLGA6L9	82084363	0	0	0
CHB	GOLGA6L9	82089363	0	0	0
CHB	GOLGA6L9	82094363	0	0	0
CHB	GOLGA6L9	82099363	0	0	0
CHB	GOLGA6L9	82104363	0	0	0
CHB	GOLGA6L9	82109363	0	0	0
CHB	GOLGA6L9	82114363	0	0	0
CHB	GOLGA6L9	82119363	0	0	0
CHB	GOLGA6L9	82124363	0	0	0
CHB	GOLGA6L9	82129363	0	0	0
CHB	GOLGA6L9	82134363	0	0	0
CHB	GOLGA6L9	82139363	0	0	0
CHB	GOLGA6L9	82144363	0	0	0
CHB	GOLGA6L9	82149363	0	0	0
CHB	GOLGA6L9	82154363	0	0	0
CHB	GOLGA6L9	82159363	0	0	0
CHB	GOLGA6L9	82164363	0	0	0
CHB	GOLGA6L9	82169363	0	0	0
CHB	GOLGA6L9	82174363	0	0	0
CHB	GOLGA6L9	82179363	0	0	0
CHB	GOLGA6L9	82184363	0	0	0
CHB	GOLGA6L9	82189363	0	0	0
CHB	GOLGA6L9	82194363	0	0	0
CHB	GOLGA6L9	82199363	0	0	0
CHB	GOLGA6L9	82204363	0	0	0
CHB	GOLGA6L9	82209363	0	0	0
CHB	GOLGA6L9	82214363	0	0	0
CHB	GOLGA6L9	82219363	0	0	0
CHB	GOLGA6L9	82224363	0	0	0
CHB	GOLGA6L9	82229363	0	0	0
CHB	GOLGA6L9	82234363	0	0	0
CHB	GOLGA6L9	82239363	0	0	0
CHB	GOLGA6L9	82244363	0	0	0
CHB	GOLGA6L9	82249363	0	0	0
CHB	GOLGA6L9	82254363	0	0	0

CHB	GOLGA6L9	82259363	0	0	0
CHB	GOLGA6L9	82264363	0	0	0
CHB	GOLGA6L9	82269363	0	0	0
CHB	GOLGA6L9	82274363	0	0	0
CHB	GOLGA6L9	82279363	0	0	0
CHB	GOLGA6L9	82284363	0	0	0
CHB	GOLGA6L9	82289363	0	0	0
CHB	GOLGA6L9	82294363	0	0	0
CHB	GOLGA6L9	82299363	0	0	0
CHB	GOLGA6L9	82304363	0	0	0
CHB	GOLGA6L9	82309363	0	0	0
CHB	GOLGA6L9	82314363	0	0	0
CHB	GOLGA6L9	82319363	0	0	0
CHB	GOLGA6L9	82324363	0	0	0
CHB	GOLGA6L9	82329363	0	0	0
CHB	GOLGA6L9	82334363	0	0	0
CHB	GOLGA6L9	82339363	0	0	0
CHB	GOLGA6L9	82344363	0	0	0
CHB	GOLGA6L9	82349363	0	0	0
CHB	GOLGA6L9	82354363	0	0	0
CHB	GOLGA6L9	82359363	0	0	0
CHB	GOLGA6L9	82364363	0	0	0
CHB	GOLGA6L9	82369363	0	0	0
CHB	GOLGA6L9	82374363	0	0	0
CHB	GOLGA6L9	82379363	0	0	0
CHB	GOLGA6L9	82384363	0	0	0
CHB	GOLGA6L9	82389363	0	0	0
CHB	GOLGA6L9	82394363	0	0	0
CHB	GOLGA6L9	82399363	0	0	0
CHB	GOLGA6L9	82404363	0	0	0
CHB	GOLGA6L9	82409363	0	0	0
CHB	GOLGA6L9	82414363	0	0	0
CHB	GOLGA6L9	82419363	0	0	0
CHB	GOLGA6L9	82424363	0	0	0
CHB	GOLGA6L9	82429363	0	0	0
CHB	GOLGA6L9	82434363	0	0	0
CHB	GOLGA6L9	82439363	0	0	0
CHB	GOLGA6L9	82444363	0	0	0
CHB	GOLGA6L9	82449363	0	0	0
CHB	GOLGA6L9	82454363	0	0	0
CHB	GOLGA6L9	82459363	0	0	0
CHB	GOLGA6L9	82464363	0	0	0
CHB	GOLGA6L9	82469363	0	0	0
CHB	GOLGA6L9	82474363	0	0	0
CHB	GOLGA6L9	82479363	0	0	0
CHB	GOLGA6L9	82484363	0	0	0
CHB	GOLGA6L9	82489363	0	0	0
CHB	GOLGA6L9	82494363	0	0	0
CHB	GOLGA6L9	82499363	0	0	0

CHB	GOLGA6L9	82504363	0	0	0
CHB	GOLGA6L9	82509363	0	0	0
CHB	GOLGA6L9	82514363	0	0	0
CHB	GOLGA6L9	82519363	0	0	0
CHB	GOLGA6L9	82524363	0	0	0
CHB	GOLGA6L9	82529363	0	0	0
CHB	GOLGA6L9	82534363	0	0	0
CHB	GOLGA6L9	82539363	0	0	0
CHB	GOLGA6L9	82544363	0	0	0
CHB	GOLGA6L9	82549363	0	0	0
CHB	GOLGA6L9	82554363	0	0	0
CHB	GOLGA6L9	82559363	0	0	0
CHB	GOLGA6L9	82564363	0	0	0
CHB	GOLGA6L9	82569363	0	0	0
CHB	GOLGA6L9	82574363	0	0	0
CHB	GOLGA6L9	82579363	0	0	0
CHB	GOLGA6L9	82584363	0	0	0
CHB	GOLGA6L9	82589363	0	0	0
CHB	GOLGA6L9	82594363	0	0	0
CHB	GOLGA6L9	82599363	0	0	0
CHB	GOLGA6L9	82604363	0	0	0
CHB	GOLGA6L9	82609363	0	0	0
CHB	GOLGA6L9	82614363	0	0	0
CHB	GOLGA6L9	82619363	0	0	0
CHB	GOLGA6L9	82624363	0	0	0
CHB	GOLGA6L9	82629363	0	0	0
CHB	GOLGA6L9	82634363	0	0	0
CHB	GOLGA6L9	82639363	0	0	0
CHB	GOLGA6L9	82644363	0	0	0
CHB	GOLGA6L9	82649363	0	0	0
CHB	GOLGA6L9	82654363	0	0	0
CHB	GOLGA6L9	82659363	0	0	0
CHB	GOLGA6L9	82664363	0	0	0
CHB	GOLGA6L9	82669363	0	0	0
CHB	GOLGA6L9	82674363	0	0	0
CHB	GOLGA6L9	82679363	0	0	0
CHB	GOLGA6L9	82684363	0	0	0
CHB	GOLGA6L9	82689363	0	0	0
CHB	GOLGA6L9	82694363	0	0	0
CHB	GOLGA6L9	82699363	0	0	0
CHB	GOLGA6L9	82704363	0	0	0
CHB	GOLGA6L9	82709363	0	0	0
CHB	GOLGA6L9	82714363	0	0	0
CHB	GOLGA6L9	82719363	0	0	0
CHB	GOLGA6L9	82724363	0	0	0
CHB	GOLGA6L9	82729363	0	0	0
CHB	GOLGA6L9	82734363	0	0	0
CHB	GOLGA6L9	82739363	0	0	0
CHB	GOLGA6L9	82744363	0	0	0

CHB	GOLGA6L9	82749363	0	0	0
CHB	GOLGA6L9	82754363	0	0	0
CHB	GOLGA6L9	82759363	0	0	0
CHB	GOLGA6L9	82764363	0	0	0
CHB	GOLGA6L9	82769363	0	0	0
CHB	GOLGA6L9	82774363	0	0	0
CHB	GOLGA6L9	82779363	0	0	0
CHB	GOLGA6L9	82784363	0	0	0
CHB	GOLGA6L9	82789363	0	0	0
CHB	GOLGA6L9	82794363	0	0	0
CHB	GOLGA6L9	82799363	0	0	0
CHB	GOLGA6L9	82804363	0	0	0
CHB	GOLGA6L9	82809363	0	0	0
CHB	GOLGA6L9	82814363	0	0	0
CHB	GOLGA6L9	82819363	0	0	0
CHB	GOLGA6L9	82824363	0	0	0
CHB	GOLGA6L9	82829363	0	0	0
CHB	GOLGA6L9	82834363	0	0	0
CHB	GOLGA6L9	82839363	0	0	0
CHB	GOLGA6L9	82844363	0	0	0
CHB	GOLGA6L9	82849363	0	0	0
CHB	GOLGA6L9	82854363	0	0	0
CHB	GOLGA6L9	82859363	0	0	0
CHB	GOLGA6L9	82864363	0	0	0
CHB	GOLGA6L9	82869363	0	0	0
CHB	GOLGA6L9	82874363	0	0	0
CHB	GOLGA6L9	82879363	0	0	0
CHB	GOLGA6L9	82884363	0	0	0
CHB	GOLGA6L9	82889363	0	0	0
CHB	GOLGA6L9	82894363	0	0	0
CHB	GOLGA6L9	82899363	0	0	0
CHB	GOLGA6L9	82904363	0	0	0
CHB	GOLGA6L9	82909363	0	0	0
CHB	GOLGA6L9	82914363	0	0	0
CHB	GOLGA6L9	82919363	0	0	0
CHB	GOLGA6L9	82924363	0	0	0
CHB	GOLGA6L9	82929363	0	0	0
YRI	GOLGA6L9	81934363	0	0	0
YRI	GOLGA6L9	81939363	0	0	0
YRI	GOLGA6L9	81944363	0	0	0
YRI	GOLGA6L9	81949363	0	0	0
YRI	GOLGA6L9	81954363	0	0	0
YRI	GOLGA6L9	81959363	0	0	0
YRI	GOLGA6L9	81964363	0	0	0
YRI	GOLGA6L9	81969363	0	0	0
YRI	GOLGA6L9	81974363	0	0	0
YRI	GOLGA6L9	81979363	0	0	0
YRI	GOLGA6L9	81984363	0	0	0
YRI	GOLGA6L9	81989363	0	0	0

YRI	GOLGA6L9	81994363	0	0	0
YRI	GOLGA6L9	81999363	0	0	0
YRI	GOLGA6L9	82004363	0	0	0
YRI	GOLGA6L9	82009363	0	0	0
YRI	GOLGA6L9	82014363	0	0	0
YRI	GOLGA6L9	82019363	0	0	0
YRI	GOLGA6L9	82024363	0	0	0
YRI	GOLGA6L9	82029363	0	0	0
YRI	GOLGA6L9	82034363	0	0	0
YRI	GOLGA6L9	82039363	0	0	0
YRI	GOLGA6L9	82044363	0	0	0
YRI	GOLGA6L9	82049363	0	0	0
YRI	GOLGA6L9	82054363	0	0	0
YRI	GOLGA6L9	82059363	0	0	0
YRI	GOLGA6L9	82064363	0	0	0
YRI	GOLGA6L9	82069363	0	0	0
YRI	GOLGA6L9	82074363	0	0	0
YRI	GOLGA6L9	82079363	0	0	0
YRI	GOLGA6L9	82084363	0	0	0
YRI	GOLGA6L9	82089363	0	0	0
YRI	GOLGA6L9	82094363	0	0	0
YRI	GOLGA6L9	82099363	0	0	0
YRI	GOLGA6L9	82104363	0	0	0
YRI	GOLGA6L9	82109363	0	0	0
YRI	GOLGA6L9	82114363	0	0	0
YRI	GOLGA6L9	82119363	0	0	0
YRI	GOLGA6L9	82124363	0	0	0
YRI	GOLGA6L9	82129363	0	0	0
YRI	GOLGA6L9	82134363	0	0	0
YRI	GOLGA6L9	82139363	0	0	0
YRI	GOLGA6L9	82144363	0	0	0
YRI	GOLGA6L9	82149363	0	0	0
YRI	GOLGA6L9	82154363	0	0	0
YRI	GOLGA6L9	82159363	0	0	0
YRI	GOLGA6L9	82164363	0	0	0
YRI	GOLGA6L9	82169363	0	0	0
YRI	GOLGA6L9	82174363	0	0	0
YRI	GOLGA6L9	82179363	0	0	0
YRI	GOLGA6L9	82184363	0	0	0
YRI	GOLGA6L9	82189363	0	0	0
YRI	GOLGA6L9	82194363	0	0	0
YRI	GOLGA6L9	82199363	0	0	0
YRI	GOLGA6L9	82204363	0	0	0
YRI	GOLGA6L9	82209363	0	0	0
YRI	GOLGA6L9	82214363	0	0	0
YRI	GOLGA6L9	82219363	0	0	0
YRI	GOLGA6L9	82224363	0	0	0
YRI	GOLGA6L9	82229363	0	0	0
YRI	GOLGA6L9	82234363	0	0	0

YRI	GOLGA6L9	82239363	0	0	0
YRI	GOLGA6L9	82244363	0	0	0
YRI	GOLGA6L9	82249363	0	0	0
YRI	GOLGA6L9	82254363	0	0	0
YRI	GOLGA6L9	82259363	0	0	0
YRI	GOLGA6L9	82264363	0	0	0
YRI	GOLGA6L9	82269363	0	0	0
YRI	GOLGA6L9	82274363	0	0	0
YRI	GOLGA6L9	82279363	0	0	0
YRI	GOLGA6L9	82284363	0	0	0
YRI	GOLGA6L9	82289363	0	0	0
YRI	GOLGA6L9	82294363	0	0	0
YRI	GOLGA6L9	82299363	0	0	0
YRI	GOLGA6L9	82304363	0	0	0
YRI	GOLGA6L9	82309363	0	0	0
YRI	GOLGA6L9	82314363	0	0	0
YRI	GOLGA6L9	82319363	0	0	0
YRI	GOLGA6L9	82324363	0	0	0
YRI	GOLGA6L9	82329363	0	0	0
YRI	GOLGA6L9	82334363	0	0	0
YRI	GOLGA6L9	82339363	0	0	0
YRI	GOLGA6L9	82344363	0	0	0
YRI	GOLGA6L9	82349363	0	0	0
YRI	GOLGA6L9	82354363	0	0	0
YRI	GOLGA6L9	82359363	0	0	0
YRI	GOLGA6L9	82364363	0	0	0
YRI	GOLGA6L9	82369363	0	0	0
YRI	GOLGA6L9	82374363	0	0	0
YRI	GOLGA6L9	82379363	0	0	0
YRI	GOLGA6L9	82384363	0	0	0
YRI	GOLGA6L9	82389363	0	0	0
YRI	GOLGA6L9	82394363	0	0	0
YRI	GOLGA6L9	82399363	0	0	0
YRI	GOLGA6L9	82404363	0	0	0
YRI	GOLGA6L9	82409363	0	0	0
YRI	GOLGA6L9	82414363	0	0	0
YRI	GOLGA6L9	82419363	0	0	0
YRI	GOLGA6L9	82424363	0	0	0
YRI	GOLGA6L9	82429363	0	0	0
YRI	GOLGA6L9	82434363	0	0	0
YRI	GOLGA6L9	82439363	0	0	0
YRI	GOLGA6L9	82444363	0	0	0
YRI	GOLGA6L9	82449363	0	0	0
YRI	GOLGA6L9	82454363	0	0	0
YRI	GOLGA6L9	82459363	0	0	0
YRI	GOLGA6L9	82464363	0	0	0
YRI	GOLGA6L9	82469363	0	0	0
YRI	GOLGA6L9	82474363	0	0	0
YRI	GOLGA6L9	82479363	0	0	0

YRI	GOLGA6L9	82484363	0	0	0
YRI	GOLGA6L9	82489363	0	0	0
YRI	GOLGA6L9	82494363	0	0	0
YRI	GOLGA6L9	82499363	0	0	0
YRI	GOLGA6L9	82504363	0	0	0
YRI	GOLGA6L9	82509363	0	0	0
YRI	GOLGA6L9	82514363	0	0	0
YRI	GOLGA6L9	82519363	0	0	0
YRI	GOLGA6L9	82524363	0	0	0
YRI	GOLGA6L9	82529363	0	0	0
YRI	GOLGA6L9	82534363	0	0	0
YRI	GOLGA6L9	82539363	0	0	0
YRI	GOLGA6L9	82544363	0	0	0
YRI	GOLGA6L9	82549363	0	0	0
YRI	GOLGA6L9	82554363	0	0	0
YRI	GOLGA6L9	82559363	0	0	0
YRI	GOLGA6L9	82564363	0	0	0
YRI	GOLGA6L9	82569363	0	0	0
YRI	GOLGA6L9	82574363	0	0	0
YRI	GOLGA6L9	82579363	0	0	0
YRI	GOLGA6L9	82584363	0	0	0
YRI	GOLGA6L9	82589363	0	0	0
YRI	GOLGA6L9	82594363	0	0	0
YRI	GOLGA6L9	82599363	0	0	0
YRI	GOLGA6L9	82604363	0	0	0
YRI	GOLGA6L9	82609363	0	0	0
YRI	GOLGA6L9	82614363	0	0	0
YRI	GOLGA6L9	82619363	0	0	0
YRI	GOLGA6L9	82624363	0	0	0
YRI	GOLGA6L9	82629363	0	0	0
YRI	GOLGA6L9	82634363	0	0	0
YRI	GOLGA6L9	82639363	0	0	0
YRI	GOLGA6L9	82644363	0	0	0
YRI	GOLGA6L9	82649363	0	0	0
YRI	GOLGA6L9	82654363	0	0	0
YRI	GOLGA6L9	82659363	0	0	0
YRI	GOLGA6L9	82664363	0	0	0
YRI	GOLGA6L9	82669363	0	0	0
YRI	GOLGA6L9	82674363	0	0	0
YRI	GOLGA6L9	82679363	0	0	0
YRI	GOLGA6L9	82684363	0	0	0
YRI	GOLGA6L9	82689363	0	0	0
YRI	GOLGA6L9	82694363	0	0	0
YRI	GOLGA6L9	82699363	0	0	0
YRI	GOLGA6L9	82704363	0	0	0
YRI	GOLGA6L9	82709363	0	0	0
YRI	GOLGA6L9	82714363	0	0	0
YRI	GOLGA6L9	82719363	0	0	0
YRI	GOLGA6L9	82724363	0	0	0

YRI	GOLGA6L9	82729363	0	0	0
YRI	GOLGA6L9	82734363	0	0	0
YRI	GOLGA6L9	82739363	0	0	0
YRI	GOLGA6L9	82744363	0	0	0
YRI	GOLGA6L9	82749363	0	0	0
YRI	GOLGA6L9	82754363	0	0	0
YRI	GOLGA6L9	82759363	0	0	0
YRI	GOLGA6L9	82764363	0	0	0
YRI	GOLGA6L9	82769363	0	0	0
YRI	GOLGA6L9	82774363	0	0	0
YRI	GOLGA6L9	82779363	0	0	0
YRI	GOLGA6L9	82784363	0	0	0
YRI	GOLGA6L9	82789363	0	0	0
YRI	GOLGA6L9	82794363	0	0	0
YRI	GOLGA6L9	82799363	0	0	0
YRI	GOLGA6L9	82804363	0	0	0
YRI	GOLGA6L9	82809363	0	0	0
YRI	GOLGA6L9	82814363	0	0	0
YRI	GOLGA6L9	82819363	0	0	0
YRI	GOLGA6L9	82824363	0	0	0
YRI	GOLGA6L9	82829363	0	0	0
YRI	GOLGA6L9	82834363	0	0	0
YRI	GOLGA6L9	82839363	0	0	0
YRI	GOLGA6L9	82844363	0	0	0
YRI	GOLGA6L9	82849363	0	0	0
YRI	GOLGA6L9	82854363	0	0	0
YRI	GOLGA6L9	82859363	0	0	0
YRI	GOLGA6L9	82864363	0	0	0
YRI	GOLGA6L9	82869363	0	0	0
YRI	GOLGA6L9	82874363	0	0	0
YRI	GOLGA6L9	82879363	0	0	0
YRI	GOLGA6L9	82884363	0	0	0
YRI	GOLGA6L9	82889363	0	0	0
YRI	GOLGA6L9	82894363	0	0	0
YRI	GOLGA6L9	82899363	0	0	0
YRI	GOLGA6L9	82904363	0	0	0
YRI	GOLGA6L9	82909363	0	0	0
YRI	GOLGA6L9	82914363	0	0	0
YRI	GOLGA6L9	82919363	0	0	0
YRI	GOLGA6L9	82924363	0	0	0
YRI	GOLGA6L9	82929363	0	0	0
CEU	GOLGA8G	28024611	6.1876	6.4814	-0.1317
CEU	GOLGA8G	28029611	4.1839	4.7757	-0.3481
CEU	GOLGA8G	28034611	5.7046	5.1169	0.3253
CEU	GOLGA8G	28039611	9.5813	6.3108	1.5016
CEU	GOLGA8G	28044611	14.7918	7.6753	2.7354
CEU	GOLGA8G	28049611	17.5168	9.0398	2.8027
CEU	GOLGA8G	28054611	15.4832	9.7221	1.7804
CEU	GOLGA8G	28059611	13.032	10.0632	0.8885

CEU	GOLGA8G	28064611	20.9843	11.9394	2.3061
CEU	GOLGA8G	28069611	30.4908	14.6684	3.3196
CEU	GOLGA8G	28074611	22.3972	12.4511	2.4374
CEU	GOLGA8G	28079611	14.4139	9.7221	1.4499
CEU	GOLGA8G	28084611	13.1097	12.1099	0.2515
CEU	GOLGA8G	28089611	14.223	13.9861	0.052
CEU	GOLGA8G	28094611	14.5199	11.2571	0.8792
CEU	GOLGA8G	28099611	8.8231	7.6753	0.4412
CEU	GOLGA8G	28104611	8.316	6.9931	0.5535
CEU	GOLGA8G	28109611	6.82	5.7991	0.5056
CEU	GOLGA8G	28114611	7.0423	6.9931	0.0206
CEU	GOLGA8G	28119611	9.1218	8.3576	0.2717
CEU	GOLGA8G	28124611	7.9542	6.6519	0.5702
CEU	GOLGA8G	28129611	8.1591	6.6519	0.6599
CEU	GOLGA8G	28134611	8.1317	6.4814	0.7397
CEU	GOLGA8G	28139611	9.2657	7.5047	0.6909
CEU	GOLGA8G	28144611	15.9732	12.1099	0.9719
CEU	GOLGA8G	28149611	21.7957	12.2805	2.3624
CEU	GOLGA8G	28154611	21.4475	10.916	2.9209
CEU	GOLGA8G	28159611	16.503	9.8926	2.01
CEU	GOLGA8G	28164611	14.3335	7.8459	2.444
CEU	GOLGA8G	28169611	16.2624	8.6987	2.5913
CEU	GOLGA8G	28174611	18.5671	10.4043	2.368
CEU	GOLGA8G	28179611	16.1684	10.5749	1.5982
CEU	GOLGA8G	28184611	11.2078	8.6987	0.8596
CEU	GOLGA8G	28189611	6.1043	5.2874	0.4393
CEU	GOLGA8G	28194611	3.0674	3.9229	-0.5967
CEU	GOLGA8G	28199611	5.2713	5.6286	-0.1817
CEU	GOLGA8G	28204611	5.0377	6.4814	-0.6471
CEU	GOLGA8G	28209611	4.8345	6.1402	-0.6144
CEU	GOLGA8G	28214611	7.9977	6.6519	0.5892
CEU	GOLGA8G	28219611	10.1894	7.3342	1.144
CEU	GOLGA8G	28224611	8.3859	7.3342	0.4214
CEU	GOLGA8G	28229611	7.3785	7.5047	-0.0495
CEU	GOLGA8G	28234611	8.5442	7.8459	0.2631
CEU	GOLGA8G	28239611	14.7172	10.7454	1.1179
CEU	GOLGA8G	28244611	11.4962	9.2104	0.7428
CEU	GOLGA8G	28249611	2.1505	4.6052	-1.4904
CEU	GOLGA8G	28254611	1.2935	3.4112	-1.6633
CEU	GOLGA8G	28259611	1.8011	4.2641	-1.5988
CEU	GOLGA8G	28264611	3.945	6.1402	-1.033
CEU	GOLGA8G	28269611	3.8248	5.7991	-0.9779
CEU	GOLGA8G	28274611	3.0289	4.9463	-1.0935
CEU	GOLGA8G	28279611	3.9629	7.1636	-1.3102
CEU	GOLGA8G	28284611	6.6864	9.0398	-0.7781
CEU	GOLGA8G	28289611	6.7132	7.6753	-0.3698
CEU	GOLGA8G	28294611	7.2181	8.8692	-0.5556
CEU	GOLGA8G	28299611	9.0339	11.0866	-0.5611
CEU	GOLGA8G	28304611	6.0448	8.187	-0.7761

CEU	GOLGA8G	28309611	3.8889	6.1402	-1.0594
CEU	GOLGA8G	28314611	3.2441	4.9463	-0.9708
CEU	GOLGA8G	28319611	3.4251	4.2641	-0.5446
CEU	GOLGA8G	28324611	4.6607	7.1636	-1.0246
CEU	GOLGA8G	28329611	4.5467	6.6519	-0.9217
CEU	GOLGA8G	28334611	3.2119	4.9463	-0.9891
CEU	GOLGA8G	28339611	1.9654	4.9463	-1.7
CEU	GOLGA8G	28344611	2.033	6.8225	-2.0494
CEU	GOLGA8G	28349611	4.5514	12.7922	-1.9686
CEU	GOLGA8G	28354611	4.5343	12.2805	-1.9232
CEU	GOLGA8G	28359611	2.7811	7.5047	-1.8533
CEU	GOLGA8G	28364611	2.5042	6.9931	-1.8782
CEU	GOLGA8G	28369611	2.0472	6.8225	-2.0433
CEU	GOLGA8G	28374611	2.0303	6.4814	-1.9951
CEU	GOLGA8G	28379611	3.2185	9.0398	-1.9247
CEU	GOLGA8G	28384611	2.9136	7.5047	-1.8013
CEU	GOLGA8G	28389611	1.7159	5.7991	-2.0224
CEU	GOLGA8G	28394611	1.8732	8.3576	-2.305
CEU	GOLGA8G	28399611	2.1901	8.3576	-2.1924
CEU	GOLGA8G	28404611	1.5069	5.7991	-2.1259
CEU	GOLGA8G	28409611	1.068	4.6052	-2.1476
CEU	GOLGA8G	28414611	3.2301	9.5515	-1.9859
CEU	GOLGA8G	28419611	3.1901	9.2104	-1.9563
CEU	GOLGA8G	28424611	1.7192	5.2874	-1.9186
CEU	GOLGA8G	28429611	1.5492	5.7991	-2.1049
CEU	GOLGA8G	28434611	1.3801	6.3108	-2.2638
CEU	GOLGA8G	28439611	1.3888	6.4814	-2.2826
CEU	GOLGA8G	28444611	1.0099	5.6286	-2.3494
CEU	GOLGA8G	28449611	1.3587	5.458	-2.143
CEU	GOLGA8G	28454611	2.1575	6.1402	-1.8741
CEU	GOLGA8G	28459611	2.2369	7.1636	-2.0167
CEU	GOLGA8G	28464611	2.6654	7.8459	-1.9516
CEU	GOLGA8G	28469611	3.2259	8.6987	-1.875
CEU	GOLGA8G	28474611	2.7279	8.0164	-1.9533
CEU	GOLGA8G	28479611	2.6999	8.6987	-2.0552
CEU	GOLGA8G	28484611	2.8087	8.5281	-1.9956
CEU	GOLGA8G	28489611	1.6329	4.6052	-1.8046
CEU	GOLGA8G	28494611	2.2599	6.1402	-1.826
CEU	GOLGA8G	28499611	3.0276	6.8225	-1.6238
CEU	GOLGA8G	28504611	2.2746	5.1169	-1.5733
CEU	GOLGA8G	28509611	2.0909	5.7991	-1.8366
CEU	GOLGA8G	28514611	1.8605	4.7757	-1.7145
CEU	GOLGA8G	28519611	1.4808	4.2641	-1.8068
CEU	GOLGA8G	28524611	1.3379	5.6286	-2.1825
CEU	GOLGA8G	28529611	3.585	8.3576	-1.6965
CEU	GOLGA8G	28534611	4.3438	8.5281	-1.46
CEU	GOLGA8G	28539611	3.1956	8.0164	-1.7806
CEU	GOLGA8G	28544611	2.9301	7.6753	-1.8239
CEU	GOLGA8G	28549611	1.5731	5.2874	-1.9972

CEU	GOLGA8G	28554611	0.9695	5.458	-2.3465
CEU	GOLGA8G	28559611	2.1453	7.5047	-2.1028
CEU	GOLGA8G	28564611	2.4646	7.1636	-1.9235
CEU	GOLGA8G	28569611	1.3517	3.5818	-1.6808
CEU	GOLGA8G	28574611	1.5492	1.7056	-0.2142
CEU	GOLGA8G	28579611	1.079	0.8528	0.5094
CEU	GOLGA8G	28584611	0.0101	0.3411	-1.3269
CEU	GOLGA8G	28589611	0.0501	0.5117	-1.4477
CEU	GOLGA8G	28594611	0.2995	0.3411	-0.1668
CEU	GOLGA8G	28599611	0.2797	0.5117	-0.7276
CEU	GOLGA8G	28604611	0.2626	0.6822	-1.0957
CEU	GOLGA8G	28609611	1.0831	1.0234	0.1186
CEU	GOLGA8G	28614611	2.2307	3.0701	-0.72
CEU	GOLGA8G	28619611	1.5739	2.8996	-1.1922
CEU	GOLGA8G	28624611	0.2527	0.6822	-1.1216
CEU	GOLGA8G	28629611	2.1606	2.729	-0.5373
CEU	GOLGA8G	28634611	2.0917	2.5584	-0.4649
CEU	GOLGA8G	28639611	0.2307	0.3411	-0.4426
CEU	GOLGA8G	28644611	0.4313	2.0467	-1.9221
CEU	GOLGA8G	28649611	0.9798	2.729	-1.6534
CEU	GOLGA8G	28654611	0.7792	1.1939	-0.739
CEU	GOLGA8G	28659611	0.3156	1.7056	-1.9038
CEU	GOLGA8G	28664611	0.3558	2.0467	-2.012
CEU	GOLGA8G	28669611	0.5267	1.1939	-1.189
CEU	GOLGA8G	28674611	2.4381	2.729	-0.275
CEU	GOLGA8G	28679611	2.0606	2.3879	-0.3447
CEU	GOLGA8G	28684611	0.5867	1.8762	-1.6418
CEU	GOLGA8G	28689611	0.5576	2.0467	-1.7719
CEU	GOLGA8G	28694611	0.8431	2.2173	-1.5352
CEU	GOLGA8G	28699611	4.8679	5.1169	-0.1378
CEU	GOLGA8G	28704611	10.3884	8.6987	0.5789
CEU	GOLGA8G	28709611	7.3156	6.3108	0.4613
CEU	GOLGA8G	28714611	1.1216	1.7056	-0.7999
CEU	GOLGA8G	28719611	0.3727	1.1939	-1.4634
CEU	GOLGA8G	28724611	0.51	0.8528	-0.772
CEU	GOLGA8G	28729611	0.5857	1.5351	-1.4078
CEU	GOLGA8G	28734611	0.6776	2.729	-1.939
CEU	GOLGA8G	28739611	1.236	3.5818	-1.768
CEU	GOLGA8G	28744611	2.3533	2.729	-0.3551
CEU	GOLGA8G	28749611	1.7988	1.5351	0.391
CEU	GOLGA8G	28754611	1.4632	2.0467	-0.6943
CEU	GOLGA8G	28759611	1.6011	1.5351	0.0979
CEU	GOLGA8G	28764611	1.0111	2.729	-1.6238
CEU	GOLGA8G	28769611	0.551	3.0701	-2.1608
CEU	GOLGA8G	28774611	0.0802	0.8528	-1.7399
CEU	GOLGA8G	28779611	0.7337	0.6822	0.1345
CEU	GOLGA8G	28784611	1.2358	1.1939	0.0747
CEU	GOLGA8G	28789611	0.572	0.8528	-0.6324
CEU	GOLGA8G	28794611	0.0301	0.1706	-0.8346

CEU	GOLGA8G	28799611	0.2719	0.1706	0.6017
CEU	GOLGA8G	28804611	0.3787	0.5117	-0.4171
CEU	GOLGA8G	28809611	1.2086	2.3879	-1.2419
CEU	GOLGA8G	28814611	1.142	2.3879	-1.3121
CEU	GOLGA8G	28819611	0.0402	0.3411	-1.2062
CEU	GOLGA8G	28824611	0	0	0
CEU	GOLGA8G	28829611	1.1006	0.8528	0.558
CEU	GOLGA8G	28834611	1.308	1.7056	-0.5446
CEU	GOLGA8G	28839611	0.2773	1.3645	-1.7589
CEU	GOLGA8G	28844611	0.22	1.8762	-2.1086
CEU	GOLGA8G	28849611	0.22	1.8762	-2.1086
CEU	GOLGA8G	28854611	1.2253	3.2407	-1.6526
CEU	GOLGA8G	28859611	1.1554	2.729	-1.4874
CEU	GOLGA8G	28864611	0.0699	0.5117	-1.3856
CEU	GOLGA8G	28869611	0.16	1.1939	-1.8424
CEU	GOLGA8G	28874611	1.3655	1.8762	-0.6502
CEU	GOLGA8G	28879611	1.2754	1.1939	0.1452
CEU	GOLGA8G	28884611	0.4457	0.6822	-0.6176
CEU	GOLGA8G	28889611	0.7782	1.5351	-1.1223
CEU	GOLGA8G	28894611	1.2981	3.7524	-1.7781
CEU	GOLGA8G	28899611	2.4698	6.4814	-1.7981
CEU	GOLGA8G	28904611	1.5042	3.5818	-1.5659
CEU	GOLGA8G	28909611	0.2789	1.5351	-1.8627
CEU	GOLGA8G	28914611	0.4879	2.5584	-2.0626
CEU	GOLGA8G	28919611	1.8611	3.9229	-1.438
CEU	GOLGA8G	28924611	2.2538	5.458	-1.6751
CEU	GOLGA8G	28929611	2.3084	6.8225	-1.9316
CEU	GOLGA8G	28934611	2.826	7.6753	-1.8639
CEU	GOLGA8G	28939611	3.2	8.3576	-1.8334
CEU	GOLGA8G	28944611	6.7225	13.8156	-1.5753
CEU	GOLGA8G	28949611	5.5407	11.2571	-1.5403
CEU	GOLGA8G	28954611	2.3931	6.8225	-1.8953
CEU	GOLGA8G	28959611	4.0715	9.8926	-1.77
CEU	GOLGA8G	28964611	4.6759	9.5515	-1.5317
CEU	GOLGA8G	28969611	3.7574	9.2104	-1.7719
CEU	GOLGA8G	28974611	3.2235	10.0632	-2.0469
CEU	GOLGA8G	28979611	3.7609	10.7454	-1.9659
CEU	GOLGA8G	28984611	4.5364	12.2805	-1.9227
CEU	GOLGA8G	28989611	7.2103	13.1333	-1.3801
CEU	GOLGA8G	28994611	7.1152	9.3809	-0.7238
CEU	GOLGA8G	28999611	2.2756	3.0701	-0.6815
CEU	GOLGA8G	29004611	0.0303	0.3411	-1.2459
CEU	GOLGA8G	29009611	0	0	0
CEU	GOLGA8G	29014611	0	0	0
CEU	GOLGA8G	29019611	0	0	0
CEU	GOLGA8G	29024611	0	0	0
CHB	GOLGA8G	28024611	5.902	6.0989	-0.0929
CHB	GOLGA8G	28029611	4.4441	4.4048	0.0247
CHB	GOLGA8G	28034611	4.7935	5.5907	-0.4067

CHB	GOLGA8G	28039611	10.2802	7.9625	0.8589
CHB	GOLGA8G	28044611	13.5616	9.4872	1.2843
CHB	GOLGA8G	28049611	8.4607	10.8426	-0.6629
CHB	GOLGA8G	28054611	6.6071	10.6731	-1.1484
CHB	GOLGA8G	28059611	11.4925	9.8261	0.5084
CHB	GOLGA8G	28064611	20.6868	12.8755	1.8493
CHB	GOLGA8G	28069611	30.6889	15.925	2.8559
CHB	GOLGA8G	28074611	22.2557	12.1979	2.5058
CHB	GOLGA8G	28079611	10.5958	8.4708	0.7439
CHB	GOLGA8G	28084611	8.9935	7.6237	0.5282
CHB	GOLGA8G	28089611	11.9665	9.1484	0.9188
CHB	GOLGA8G	28094611	14.016	9.9955	1.2073
CHB	GOLGA8G	28099611	9.5071	7.1154	0.9822
CHB	GOLGA8G	28104611	8.6263	6.0989	1.193
CHB	GOLGA8G	28109611	6.2758	6.0989	0.0835
CHB	GOLGA8G	28114611	5.7777	7.4543	-0.66
CHB	GOLGA8G	28119611	8.5585	9.1484	-0.1923
CHB	GOLGA8G	28124611	7.5342	7.4543	0.0315
CHB	GOLGA8G	28129611	8.2381	6.2684	0.9072
CHB	GOLGA8G	28134611	8.8435	6.4378	1.0817
CHB	GOLGA8G	28139611	10.3737	7.9625	0.8935
CHB	GOLGA8G	28144611	15.3548	12.7061	0.635
CHB	GOLGA8G	28149611	14.9339	13.2144	0.3972
CHB	GOLGA8G	28154611	12.6789	11.012	0.4572
CHB	GOLGA8G	28159611	11.8271	9.9955	0.55
CHB	GOLGA8G	28164611	10.7561	8.4708	0.8
CHB	GOLGA8G	28169611	12.996	8.1319	1.768
CHB	GOLGA8G	28174611	17.1671	9.4872	2.4209
CHB	GOLGA8G	28179611	13.2418	10.5037	0.785
CHB	GOLGA8G	28184611	6.4769	9.4872	-0.9489
CHB	GOLGA8G	28189611	3.3581	6.2684	-1.3404
CHB	GOLGA8G	28194611	2.6966	10.6731	-2.2529
CHB	GOLGA8G	28199611	3.7034	14.2309	-2.2667
CHB	GOLGA8G	28204611	2.9917	9.3178	-2.0278
CHB	GOLGA8G	28209611	3.2227	7.7931	-1.7274
CHB	GOLGA8G	28214611	4.9892	8.3013	-1.1812
CHB	GOLGA8G	28219611	5.5646	10.3343	-1.3884
CHB	GOLGA8G	28224611	4.7034	10.3343	-1.6391
CHB	GOLGA8G	28229611	6.7788	9.1484	-0.7726
CHB	GOLGA8G	28234611	11.7049	9.4872	0.6991
CHB	GOLGA8G	28239611	20.2069	10.8426	2.6061
CHB	GOLGA8G	28244611	14.9825	10.6731	1.2171
CHB	GOLGA8G	28249611	2.7797	6.7766	-1.7157
CHB	GOLGA8G	28254611	0.7476	3.8965	-2.2024
CHB	GOLGA8G	28259611	1.5125	4.5742	-1.8643
CHB	GOLGA8G	28264611	3.7068	6.7766	-1.3178
CHB	GOLGA8G	28269611	4.454	6.946	-1.046
CHB	GOLGA8G	28274611	4.3861	6.946	-1.0745
CHB	GOLGA8G	28279611	4.9962	9.1484	-1.3538

CHB	GOLGA8G	28284611	8.0898	10.5037	-0.6921
CHB	GOLGA8G	28289611	7.9125	9.3178	-0.4505
CHB	GOLGA8G	28294611	6.375	11.8591	-1.4031
CHB	GOLGA8G	28299611	5.6949	15.7556	-1.9661
CHB	GOLGA8G	28304611	3.623	13.045	-2.2032
CHB	GOLGA8G	28309611	3.6878	10.1649	-1.9147
CHB	GOLGA8G	28314611	4.1502	9.1484	-1.6296
CHB	GOLGA8G	28319611	6.3044	9.8261	-1.0745
CHB	GOLGA8G	28324611	7.2123	15.4168	-1.6369
CHB	GOLGA8G	28329611	6.541	15.0779	-1.7397
CHB	GOLGA8G	28334611	5.7026	10.1649	-1.3191
CHB	GOLGA8G	28339611	4.0249	8.3013	-1.5252
CHB	GOLGA8G	28344611	4.4283	8.8096	-1.4792
CHB	GOLGA8G	28349611	8.4438	13.7226	-1.1765
CHB	GOLGA8G	28354611	7.6003	12.0285	-1.1179
CHB	GOLGA8G	28359611	3.724	8.4708	-1.6617
CHB	GOLGA8G	28364611	3.6362	10.3343	-1.9497
CHB	GOLGA8G	28369611	3.5568	9.9955	-1.9334
CHB	GOLGA8G	28374611	3.3429	8.1319	-1.7407
CHB	GOLGA8G	28379611	6.7179	11.6896	-1.2893
CHB	GOLGA8G	28384611	5.4386	9.6567	-1.3079
CHB	GOLGA8G	28389611	1.8496	8.1319	-2.2835
CHB	GOLGA8G	28394611	4.3467	11.8591	-1.922
CHB	GOLGA8G	28399611	5.6691	10.6731	-1.4133
CHB	GOLGA8G	28404611	3.4344	10.1649	-1.9896
CHB	GOLGA8G	28409611	2.2795	8.8096	-2.2047
CHB	GOLGA8G	28414611	8.0253	12.1979	-1.0396
CHB	GOLGA8G	28419611	8.4072	13.7226	-1.1847
CHB	GOLGA8G	28424611	4.743	9.8261	-1.5509
CHB	GOLGA8G	28429611	4.0805	8.979	-1.625
CHB	GOLGA8G	28434611	3.8763	9.6567	-1.7924
CHB	GOLGA8G	28439611	4.1769	9.9955	-1.7472
CHB	GOLGA8G	28444611	2.7038	8.8096	-2.0615
CHB	GOLGA8G	28449611	4.5347	8.979	-1.4743
CHB	GOLGA8G	28454611	6.7855	9.4872	-0.8516
CHB	GOLGA8G	28459611	6.9029	10.8426	-1.0964
CHB	GOLGA8G	28464611	6.3082	10.8426	-1.2619
CHB	GOLGA8G	28469611	7.5964	10.3343	-0.797
CHB	GOLGA8G	28474611	8.0999	9.1484	-0.3419
CHB	GOLGA8G	28479611	7.4986	9.9955	-0.7498
CHB	GOLGA8G	28484611	7.3076	11.1814	-1.0474
CHB	GOLGA8G	28489611	4.5159	6.946	-1.0201
CHB	GOLGA8G	28494611	5.0824	8.3013	-1.148
CHB	GOLGA8G	28499611	6.3765	8.4708	-0.7332
CHB	GOLGA8G	28504611	6.2056	5.7601	0.2213
CHB	GOLGA8G	28509611	5.4062	7.4543	-0.8062
CHB	GOLGA8G	28514611	4.1047	7.4543	-1.3185
CHB	GOLGA8G	28519611	4.1776	6.6072	-1.0671
CHB	GOLGA8G	28524611	3.5926	7.1154	-1.4467

CHB	GOLGA8G	28529611	5.3992	9.1484	-1.2224
CHB	GOLGA8G	28534611	8.1856	12.3673	-1.0284
CHB	GOLGA8G	28539611	8.1668	11.6896	-0.9136
CHB	GOLGA8G	28544611	6.7708	9.8261	-0.9322
CHB	GOLGA8G	28549611	4.5365	8.4708	-1.3773
CHB	GOLGA8G	28554611	2.6809	7.4543	-1.8789
CHB	GOLGA8G	28559611	4.8131	9.6567	-1.5019
CHB	GOLGA8G	28564611	7.1519	8.1319	-0.3562
CHB	GOLGA8G	28569611	4.193	2.8801	1.1837
CHB	GOLGA8G	28574611	1.9039	1.6942	0.2878
CHB	GOLGA8G	28579611	0.9855	1.1859	-0.3577
CHB	GOLGA8G	28584611	0.0484	0.5082	-1.4434
CHB	GOLGA8G	28589611	0.0386	0.3388	-1.2042
CHB	GOLGA8G	28594611	0.2781	0.1694	0.6459
CHB	GOLGA8G	28599611	0.2781	0.1694	0.6459
CHB	GOLGA8G	28604611	0.5195	0.3388	0.7248
CHB	GOLGA8G	28609611	1.9659	1.3553	0.9896
CHB	GOLGA8G	28614611	3.312	2.7106	0.5699
CHB	GOLGA8G	28619611	2.2859	2.2024	0.0935
CHB	GOLGA8G	28624611	0.752	0.8471	-0.2144
CHB	GOLGA8G	28629611	2.9431	3.2189	-0.2267
CHB	GOLGA8G	28634611	2.6115	2.8801	-0.2422
CHB	GOLGA8G	28639611	0.335	0.3388	-0.0152
CHB	GOLGA8G	28644611	1.396	2.2024	-0.903
CHB	GOLGA8G	28649611	1.8528	3.2189	-1.1231
CHB	GOLGA8G	28654611	0.7919	1.5247	-1.0887
CHB	GOLGA8G	28659611	0.9629	2.3718	-1.4872
CHB	GOLGA8G	28664611	1.0015	2.7106	-1.6195
CHB	GOLGA8G	28669611	0.1887	1.0165	-1.6474
CHB	GOLGA8G	28674611	3.0775	2.3718	0.7449
CHB	GOLGA8G	28679611	2.9566	2.2024	0.8445
CHB	GOLGA8G	28684611	0.3874	1.8636	-1.8834
CHB	GOLGA8G	28689611	0.3969	2.033	-1.9511
CHB	GOLGA8G	28694611	1.2519	1.8636	-0.7804
CHB	GOLGA8G	28699611	5.248	6.7766	-0.6562
CHB	GOLGA8G	28704611	9.4032	10.3343	-0.271
CHB	GOLGA8G	28709611	6.3478	5.5907	0.3863
CHB	GOLGA8G	28714611	0.9989	1.0165	-0.035
CHB	GOLGA8G	28719611	0.1321	0.8471	-1.6123
CHB	GOLGA8G	28724611	0.189	1.0165	-1.6468
CHB	GOLGA8G	28729611	1.3628	1.3553	0.0122
CHB	GOLGA8G	28734611	1.8146	1.5247	0.4307
CHB	GOLGA8G	28739611	1.3526	1.8636	-0.652
CHB	GOLGA8G	28744611	1.4019	1.6942	-0.4011
CHB	GOLGA8G	28749611	1.5899	1.3553	0.3802
CHB	GOLGA8G	28754611	1.6893	1.5247	0.2445
CHB	GOLGA8G	28759611	0.913	1.0165	-0.206
CHB	GOLGA8G	28764611	0.8555	1.5247	-0.9942
CHB	GOLGA8G	28769611	1.0202	1.8636	-1.0761

CHB	GOLGA8G	28774611	0.43	1.0165	-1.1672
CHB	GOLGA8G	28779611	1.2817	1.1859	0.171
CHB	GOLGA8G	28784611	1.5998	1.5247	0.1116
CHB	GOLGA8G	28789611	0.3569	0.8471	-1.1054
CHB	GOLGA8G	28794611	0.1601	0.5082	-1.0927
CHB	GOLGA8G	28799611	0.47	0.6777	-0.543
CHB	GOLGA8G	28804611	0.75	0.5082	0.759
CHB	GOLGA8G	28809611	2.5315	2.7106	-0.1697
CHB	GOLGA8G	28814611	2.1205	2.8801	-0.6849
CHB	GOLGA8G	28819611	0.0194	0.3388	-1.2812
CHB	GOLGA8G	28824611	0	0	0
CHB	GOLGA8G	28829611	1.0478	0.6777	0.9676
CHB	GOLGA8G	28834611	1.7775	1.8636	-0.1099
CHB	GOLGA8G	28839611	0.9181	1.6942	-1.0651
CHB	GOLGA8G	28844611	0.2267	0.8471	-1.399
CHB	GOLGA8G	28849611	0.2283	0.8471	-1.3954
CHB	GOLGA8G	28854611	2.5376	2.033	0.6017
CHB	GOLGA8G	28859611	2.3573	1.6942	0.91
CHB	GOLGA8G	28864611	0.534	0.6777	-0.3757
CHB	GOLGA8G	28869611	0.8167	1.1859	-0.659
CHB	GOLGA8G	28874611	2.11	2.7106	-0.5691
CHB	GOLGA8G	28879611	1.8176	2.033	-0.2569
CHB	GOLGA8G	28884611	1.031	1.3553	-0.5256
CHB	GOLGA8G	28889611	1.9718	1.8636	0.138
CHB	GOLGA8G	28894611	6.4677	4.5742	1.153
CHB	GOLGA8G	28899611	10.7362	8.1319	0.9466
CHB	GOLGA8G	28904611	5.2092	4.066	0.7709
CHB	GOLGA8G	28909611	2.3922	2.3718	0.0215
CHB	GOLGA8G	28914611	3.4605	3.5577	-0.0735
CHB	GOLGA8G	28919611	5.1028	3.8965	0.8437
CHB	GOLGA8G	28924611	10.5254	6.0989	2.0895
CHB	GOLGA8G	28929611	11.5365	7.6237	1.5089
CHB	GOLGA8G	28934611	8.5814	7.7931	0.2979
CHB	GOLGA8G	28939611	9.3678	8.8096	0.1885
CHB	GOLGA8G	28944611	18.4466	14.2309	0.9077
CHB	GOLGA8G	28949611	15.7004	11.3508	1.1596
CHB	GOLGA8G	28954611	9.8058	7.6237	0.8415
CHB	GOLGA8G	28959611	15.5523	12.7061	0.6823
CHB	GOLGA8G	28964611	15.0775	13.5532	0.3438
CHB	GOLGA8G	28969611	13.0131	12.8755	0.0326
CHB	GOLGA8G	28974611	14.7032	12.5367	0.526
CHB	GOLGA8G	28979611	15.7674	12.8755	0.6846
CHB	GOLGA8G	28984611	18.7933	14.7391	0.8443
CHB	GOLGA8G	28989611	17.8791	16.4333	0.2714
CHB	GOLGA8G	28994611	10.3151	11.6896	-0.3565
CHB	GOLGA8G	28999611	3.6406	3.7271	-0.0628
CHB	GOLGA8G	29004611	0.2433	0.8471	-1.3616
CHB	GOLGA8G	29009611	0	0	0
CHB	GOLGA8G	29014611	0	0	0

CHB	GOLGA8G	29019611	0	0	0
CHB	GOLGA8G	29024611	0	0	0
YRI	GOLGA8G	28024611	8.2439	11.2602	-0.8075
YRI	GOLGA8G	28029611	6.1905	9.9157	-1.1232
YRI	GOLGA8G	28034611	8.143	14.7895	-1.375
YRI	GOLGA8G	28039611	12.6879	17.6465	-0.8665
YRI	GOLGA8G	28044611	15.2356	15.4617	-0.0448
YRI	GOLGA8G	28049611	16.1171	15.1256	0.2008
YRI	GOLGA8G	28054611	14.6027	14.1172	0.105
YRI	GOLGA8G	28059611	13.3168	13.2769	0.0091
YRI	GOLGA8G	28064611	21.7807	18.3188	0.5836
YRI	GOLGA8G	28069611	31.6585	21.0078	1.5737
YRI	GOLGA8G	28074611	24.0741	18.823	0.8625
YRI	GOLGA8G	28079611	15.479	16.9743	-0.2712
YRI	GOLGA8G	28084611	14.3731	16.8062	-0.4455
YRI	GOLGA8G	28089611	16.689	19.9994	-0.5129
YRI	GOLGA8G	28094611	17.5426	17.9827	-0.0755
YRI	GOLGA8G	28099611	11.7363	12.6047	-0.209
YRI	GOLGA8G	28104611	10.4316	12.6047	-0.5231
YRI	GOLGA8G	28109611	9.9921	14.4534	-0.9433
YRI	GOLGA8G	28114611	13.6154	21.68	-1.1559
YRI	GOLGA8G	28119611	15.8069	23.5287	-1.0225
YRI	GOLGA8G	28124611	12.2851	19.3272	-1.1276
YRI	GOLGA8G	28129611	12.34	17.6465	-0.9273
YRI	GOLGA8G	28134611	14.1166	17.8146	-0.6404
YRI	GOLGA8G	28139611	14.6451	18.6549	-0.6643
YRI	GOLGA8G	28144611	21.1539	19.3272	0.2925
YRI	GOLGA8G	28149611	26.1341	18.3188	1.3176
YRI	GOLGA8G	28154611	28.0474	17.6465	1.8175
YRI	GOLGA8G	28159611	26.9313	17.3104	1.7125
YRI	GOLGA8G	28164611	20.6151	14.9575	1.1579
YRI	GOLGA8G	28169611	19.7284	15.1256	0.932
YRI	GOLGA8G	28174611	17.9946	16.302	0.3191
YRI	GOLGA8G	28179611	15.655	15.9659	-0.0598
YRI	GOLGA8G	28184611	13.3218	14.1172	-0.172
YRI	GOLGA8G	28189611	7.4448	13.9492	-1.4225
YRI	GOLGA8G	28194611	6.7657	16.8062	-1.8385
YRI	GOLGA8G	28199611	8.2046	16.9743	-1.5906
YRI	GOLGA8G	28204611	7.7757	13.2769	-1.2608
YRI	GOLGA8G	28209611	7.9708	10.4199	-0.705
YRI	GOLGA8G	28214611	8.9759	12.1005	-0.7817
YRI	GOLGA8G	28219611	11.2629	14.2853	-0.6462
YRI	GOLGA8G	28224611	10.7568	13.2769	-0.5776
YRI	GOLGA8G	28229611	10.4973	12.7727	-0.5409
YRI	GOLGA8G	28234611	11.1659	15.1256	-0.8018
YRI	GOLGA8G	28239611	14.25	17.4785	-0.5694
YRI	GOLGA8G	28244611	14.1139	16.302	-0.4125
YRI	GOLGA8G	28249611	8.6611	16.6382	-1.4748
YRI	GOLGA8G	28254611	7.3941	18.3188	-1.8418

YRI	GOLGA8G	28259611	6.9091	17.3104	-1.8514
YRI	GOLGA8G	28264611	6.2026	12.4366	-1.5198
YRI	GOLGA8G	28269611	6.7114	12.7727	-1.441
YRI	GOLGA8G	28274611	8.9613	13.7811	-1.0663
YRI	GOLGA8G	28279611	11.8417	13.445	-0.3631
YRI	GOLGA8G	28284611	11.6209	15.2937	-0.7359
YRI	GOLGA8G	28289611	11.5347	12.9408	-0.3302
YRI	GOLGA8G	28294611	14.4174	15.1256	-0.1434
YRI	GOLGA8G	28299611	17.9262	18.6549	-0.1207
YRI	GOLGA8G	28304611	15.9283	16.9743	-0.1897
YRI	GOLGA8G	28309611	11.0211	14.6214	-0.7529
YRI	GOLGA8G	28314611	9.9617	11.9324	-0.4996
YRI	GOLGA8G	28319611	12.3136	12.7727	-0.1091
YRI	GOLGA8G	28324611	12.7373	16.9743	-0.7685
YRI	GOLGA8G	28329611	10.6084	15.6298	-0.9855
YRI	GOLGA8G	28334611	10.443	12.4366	-0.486
YRI	GOLGA8G	28339611	9.6046	10.756	-0.3218
YRI	GOLGA8G	28344611	11.2487	11.7644	-0.1325
YRI	GOLGA8G	28349611	20.2959	21.3439	-0.1525
YRI	GOLGA8G	28354611	18.0652	21.8481	-0.5382
YRI	GOLGA8G	28359611	11.4657	16.4701	-0.9342
YRI	GOLGA8G	28364611	12.8076	15.7978	-0.5809
YRI	GOLGA8G	28369611	11.9924	13.7811	-0.3957
YRI	GOLGA8G	28374611	10.6674	11.2602	-0.1587
YRI	GOLGA8G	28379611	16.8723	15.6298	0.2439
YRI	GOLGA8G	28384611	15.1791	17.3104	-0.3794
YRI	GOLGA8G	28389611	9.3897	15.1256	-1.1615
YRI	GOLGA8G	28394611	11.1827	17.9827	-1.167
YRI	GOLGA8G	28399611	12.4234	16.4701	-0.7554
YRI	GOLGA8G	28404611	11.1886	15.6298	-0.8716
YRI	GOLGA8G	28409611	7.8491	14.9575	-1.4548
YRI	GOLGA8G	28414611	14.8718	18.4868	-0.6041
YRI	GOLGA8G	28419611	15.4781	16.9743	-0.2714
YRI	GOLGA8G	28424611	9.5641	10.924	-0.3745
YRI	GOLGA8G	28429611	10.6742	14.1172	-0.7445
YRI	GOLGA8G	28434611	13.6242	15.6298	-0.3936
YRI	GOLGA8G	28439611	15.0176	14.2853	0.1566
YRI	GOLGA8G	28444611	12.1738	14.4534	-0.482
YRI	GOLGA8G	28449611	13.0015	16.9743	-0.7206
YRI	GOLGA8G	28454611	12.1505	19.1591	-1.1317
YRI	GOLGA8G	28459611	14.4058	20.8397	-0.9581
YRI	GOLGA8G	28464611	14.4668	17.9827	-0.6034
YRI	GOLGA8G	28469611	11.3188	16.134	-0.9168
YRI	GOLGA8G	28474611	9.8371	14.2853	-0.9511
YRI	GOLGA8G	28479611	8.9534	13.445	-1.0172
YRI	GOLGA8G	28484611	10.4621	16.134	-1.0799
YRI	GOLGA8G	28489611	7.7905	12.9408	-1.2094
YRI	GOLGA8G	28494611	9.8283	13.613	-0.8471
YRI	GOLGA8G	28499611	9.1207	13.2769	-0.9525

YRI	GOLGA8G	28504611	6.8705	9.9157	-0.9181
YRI	GOLGA8G	28509611	6.9677	11.9324	-1.2585
YRI	GOLGA8G	28514611	6.2097	13.1089	-1.6004
YRI	GOLGA8G	28519611	6.7131	11.5963	-1.2716
YRI	GOLGA8G	28524611	7.6688	14.2853	-1.4147
YRI	GOLGA8G	28529611	10.2301	17.1423	-1.2419
YRI	GOLGA8G	28534611	11.5826	20.1675	-1.3194
YRI	GOLGA8G	28539611	9.8562	18.6549	-1.4577
YRI	GOLGA8G	28544611	10.806	16.302	-1.0361
YRI	GOLGA8G	28549611	11.0356	16.8062	-1.0566
YRI	GOLGA8G	28554611	8.8787	15.2937	-1.2854
YRI	GOLGA8G	28559611	11.7101	19.3272	-1.2197
YRI	GOLGA8G	28564611	9.5991	15.6298	-1.1836
YRI	GOLGA8G	28569611	2.9752	5.7141	-1.3658
YRI	GOLGA8G	28574611	1.9253	3.0251	-0.9488
YRI	GOLGA8G	28579611	1.237	1.8487	-0.7824
YRI	GOLGA8G	28584611	0.1006	0.8403	-1.6707
YRI	GOLGA8G	28589611	0.0914	0.6722	-1.5205
YRI	GOLGA8G	28594611	0.1552	0.3361	-0.7262
YRI	GOLGA8G	28599611	0.1552	0.3361	-0.7262
YRI	GOLGA8G	28604611	0.4844	0.3361	0.5954
YRI	GOLGA8G	28609611	1.9509	1.6806	0.3719
YRI	GOLGA8G	28614611	2.8744	2.689	0.1762
YRI	GOLGA8G	28619611	1.6427	2.3529	-0.7518
YRI	GOLGA8G	28624611	0.615	2.0167	-1.6759
YRI	GOLGA8G	28629611	3.7901	4.8738	-0.6221
YRI	GOLGA8G	28634611	3.4099	3.8654	-0.3197
YRI	GOLGA8G	28639611	1.0202	0.6722	0.911
YRI	GOLGA8G	28644611	2.5485	3.8654	-0.9241
YRI	GOLGA8G	28649611	2.8216	4.7057	-1.1153
YRI	GOLGA8G	28654611	1.3572	1.8487	-0.6287
YRI	GOLGA8G	28659611	2.9335	4.0335	-0.7443
YRI	GOLGA8G	28664611	3.2523	4.3696	-0.7055
YRI	GOLGA8G	28669611	1.2553	1.8487	-0.759
YRI	GOLGA8G	28674611	4.9193	4.2016	0.4688
YRI	GOLGA8G	28679611	4.3241	3.8654	0.3219
YRI	GOLGA8G	28684611	2.3924	3.0251	-0.5458
YRI	GOLGA8G	28689611	2.6739	3.3612	-0.543
YRI	GOLGA8G	28694611	2.4895	3.5293	-0.7884
YRI	GOLGA8G	28699611	7.8609	10.4199	-0.7367
YRI	GOLGA8G	28704611	10.8011	15.1256	-0.8757
YRI	GOLGA8G	28709611	6.2005	8.9073	-0.9015
YRI	GOLGA8G	28714611	1.7367	3.8654	-1.4938
YRI	GOLGA8G	28719611	0.5408	2.5209	-1.983
YRI	GOLGA8G	28724611	0.3982	1.0084	-1.2165
YRI	GOLGA8G	28729611	1.0891	2.5209	-1.4339
YRI	GOLGA8G	28734611	1.5172	5.0419	-1.9639
YRI	GOLGA8G	28739611	2.7995	5.378	-1.357
YRI	GOLGA8G	28744611	3.515	3.3612	0.1215

YRI	GOLGA8G	28749611	2.239	2.1848	0.0609
YRI	GOLGA8G	28754611	1.7779	2.689	-0.8659
YRI	GOLGA8G	28759611	1.4555	2.8571	-1.2675
YRI	GOLGA8G	28764611	2.0922	3.8654	-1.2444
YRI	GOLGA8G	28769611	1.7364	3.0251	-1.1117
YRI	GOLGA8G	28774611	0.3525	1.6806	-1.8271
YRI	GOLGA8G	28779611	1.6552	2.689	-0.9825
YRI	GOLGA8G	28784611	2.0791	2.5209	-0.4424
YRI	GOLGA8G	28789611	0.7845	1.1764	-0.7009
YRI	GOLGA8G	28794611	0.1976	0.5042	-0.9635
YRI	GOLGA8G	28799611	0.5381	0.6722	-0.3511
YRI	GOLGA8G	28804611	0.6815	0.8403	-0.3587
YRI	GOLGA8G	28809611	2.2614	2.5209	-0.2599
YRI	GOLGA8G	28814611	2.1444	2.3529	-0.2207
YRI	GOLGA8G	28819611	0.0631	0.3361	-1.096
YRI	GOLGA8G	28824611	0.1804	0.5042	-1.0176
YRI	GOLGA8G	28829611	1.5364	1.1764	0.6439
YRI	GOLGA8G	28834611	2.9061	3.0251	-0.1027
YRI	GOLGA8G	28839611	1.7593	4.0335	-1.5388
YRI	GOLGA8G	28844611	0.8347	3.0251	-1.8896
YRI	GOLGA8G	28849611	1.0355	2.689	-1.5714
YRI	GOLGA8G	28854611	3.1376	3.5293	-0.297
YRI	GOLGA8G	28859611	2.7369	2.3529	0.4065
YRI	GOLGA8G	28864611	0.2082	0.8403	-1.4277
YRI	GOLGA8G	28869611	0.2816	1.5126	-1.833
YRI	GOLGA8G	28874611	2.1107	4.5377	-1.483
YRI	GOLGA8G	28879611	2.0738	4.0335	-1.326
YRI	GOLGA8G	28884611	0.9711	2.3529	-1.4627
YRI	GOLGA8G	28889611	1.6956	3.0251	-1.1469
YRI	GOLGA8G	28894611	6.1465	5.7141	0.2156
YRI	GOLGA8G	28899611	11.3305	11.2602	0.0188
YRI	GOLGA8G	28904611	5.9542	6.5544	-0.2646
YRI	GOLGA8G	28909611	1.9708	4.2016	-1.4572
YRI	GOLGA8G	28914611	3.3798	7.0586	-1.5166
YRI	GOLGA8G	28919611	7.1155	9.2434	-0.6848
YRI	GOLGA8G	28924611	10.2312	10.924	-0.1908
YRI	GOLGA8G	28929611	10.2301	10.924	-0.1911
YRI	GOLGA8G	28934611	10.3073	14.7895	-0.9272
YRI	GOLGA8G	28939611	13.321	18.6549	-0.8837
YRI	GOLGA8G	28944611	19.9282	22.3523	-0.3373
YRI	GOLGA8G	28949611	15.9817	17.9827	-0.3434
YRI	GOLGA8G	28954611	12.2082	14.6214	-0.5047
YRI	GOLGA8G	28959611	15.3474	19.8313	-0.7004
YRI	GOLGA8G	28964611	13.7523	17.9827	-0.726
YRI	GOLGA8G	28969611	12.5313	13.9492	-0.3101
YRI	GOLGA8G	28974611	11.7304	14.4534	-0.5758
YRI	GOLGA8G	28979611	11.9665	17.6465	-0.9926
YRI	GOLGA8G	28984611	14.6183	19.4952	-0.7744
YRI	GOLGA8G	28989611	16.6192	20.1675	-0.5453

YRI	GOLGA8G	28994611	12.6134	15.7978	-0.6186
YRI	GOLGA8G	28999611	4.7177	5.7141	-0.4969
YRI	GOLGA8G	29004611	1.0322	0.8403	0.4334
YRI	GOLGA8G	29009611	0	0	0
YRI	GOLGA8G	29014611	0	0	0
YRI	GOLGA8G	29019611	0	0	0
YRI	GOLGA8G	29024611	0	0	0
CEU	HIST2	143407386	2.4331	3.7524	-0.9558
CEU	HIST2	143412386	3.7633	4.9463	-0.6747
CEU	HIST2	143417386	3.7411	4.2641	-0.3395
CEU	HIST2	143422386	3.663	3.7524	-0.0648
CEU	HIST2	143427386	2.4238	3.2407	-0.6698
CEU	HIST2	143432386	0.0503	0.6822	-1.6501
CEU	HIST2	143437386	1.4853	1.1939	0.5193
CEU	HIST2	143442386	2.1128	2.3879	-0.2897
CEU	HIST2	143447386	1.6283	1.8762	-0.3156
CEU	HIST2	143452386	1.5395	1.1939	0.6159
CEU	HIST2	143457386	1.2272	1.8762	-0.8263
CEU	HIST2	143462386	2.0583	4.2641	-1.4319
CEU	HIST2	143467386	3.4865	8.187	-1.7029
CEU	HIST2	143472386	4.2144	8.6987	-1.5363
CEU	HIST2	143477386	5.0787	6.4814	-0.6287
CEU	HIST2	143482386	3.8666	4.7757	-0.5347
CEU	HIST2	143487386	3.2115	5.458	-1.1744
CEU	HIST2	143492386	7.6314	10.2337	-0.7667
CEU	HIST2	143497386	10.6658	11.2571	-0.1593
CEU	HIST2	143502386	11.8762	9.3809	0.7972
CEU	HIST2	143507386	11.0347	10.0632	0.2907
CEU	HIST2	143512386	8.356	9.0398	-0.2261
CEU	HIST2	143517386	9.9101	10.0632	-0.0458
CEU	HIST2	143522386	11.5488	13.645	-0.4711
CEU	HIST2	143527386	9.5803	11.0866	-0.4117
CEU	HIST2	143532386	9.1175	7.1636	0.7998
CEU	HIST2	143537386	14.3548	8.8692	1.8459
CEU	HIST2	143542386	10.2931	7.8459	0.9219
CEU	HIST2	143547386	0.9514	2.2173	-1.4142
CEU	HIST2	143552386	0	0	0
CEU	HIST2	143557386	0	0	0
CEU	HIST2	143562386	0	0	0
CEU	HIST2	143567386	0	0	0
CEU	HIST2	143572386	0	0	0
CEU	HIST2	143577386	0	0	0
CEU	HIST2	143582386	0	0	0
CEU	HIST2	143587386	0	0	0
CEU	HIST2	143592386	0	0	0
CEU	HIST2	143597386	0	0	0
CEU	HIST2	143602386	0	0	0
CEU	HIST2	143607386	0	0	0
CEU	HIST2	143612386	0	0	0

CEU	HIST2	143617386	0	0	0
CEU	HIST2	143622386	0	0	0
CEU	HIST2	143627386	0	0	0
CEU	HIST2	143632386	0	0	0
CEU	HIST2	143637386	0	0	0
CEU	HIST2	143642386	0.3356	0.3411	-0.022
CEU	HIST2	143647386	0.3653	0.5117	-0.4591
CEU	HIST2	143652386	0.0297	0.1706	-0.837
CEU	HIST2	143657386	0.02	0.1706	-0.8946
CEU	HIST2	143662386	0.7778	2.2173	-1.6082
CEU	HIST2	143667386	1.0952	2.8996	-1.6228
CEU	HIST2	143672386	0.3375	0.8528	-1.1604
CEU	HIST2	143677386	0	0	0
CEU	HIST2	143682386	0	0	0
CEU	HIST2	143687386	0	0	0
CEU	HIST2	143692386	0	0	0
CEU	HIST2	143697386	0	0	0
CEU	HIST2	143702386	0	0	0
CEU	HIST2	143707386	0.7743	0.3411	1.7366
CEU	HIST2	143712386	0.7743	0.3411	1.7366
CEU	HIST2	143717386	0.5477	0.8528	-0.6871
CEU	HIST2	143722386	0.7312	1.0234	-0.5806
CEU	HIST2	143727386	0.7714	0.8528	-0.1833
CEU	HIST2	143732386	1.9887	2.2173	-0.2554
CEU	HIST2	143737386	1.4007	1.5351	-0.1993
CEU	HIST2	143742386	1.0812	1.3645	-0.4583
CEU	HIST2	143747386	1.4418	2.5584	-1.1123
CEU	HIST2	143752386	1.2538	2.729	-1.3944
CEU	HIST2	143757386	1.8984	4.2641	-1.5357
CEU	HIST2	143762386	3.7833	4.9463	-0.6633
CEU	HIST2	143767386	6.4071	5.9697	0.2111
CEU	HIST2	143772386	3.6289	3.7524	-0.0895
CEU	HIST2	143777386	0	0	0
CEU	HIST2	143782386	0	0	0
CEU	HIST2	143787386	0	0	0
CEU	HIST2	143792386	0	0	0
CEU	HIST2	143797386	0	0	0
CEU	HIST2	143802386	0	0	0
CEU	HIST2	143807386	0	0	0
CEU	HIST2	143812386	0	0	0
CEU	HIST2	143817386	0	0	0
CEU	HIST2	143822386	0	0	0
CEU	HIST2	143827386	0	0	0
CEU	HIST2	143832386	0	0	0
CEU	HIST2	143837386	0	0	0
CEU	HIST2	143842386	0	0	0
CEU	HIST2	143847386	0	0	0
CEU	HIST2	143852386	0	0	0
CEU	HIST2	143857386	0	0	0

CEU	HIST2	143862386	0	0	0
CEU	HIST2	143867386	0	0	0
CEU	HIST2	143872386	0.0404	0.5117	-1.4781
CEU	HIST2	143877386	0.5564	1.1939	-1.136
CEU	HIST2	143882386	0.516	0.6822	-0.434
CEU	HIST2	143887386	0	0	0
CEU	HIST2	143892386	0	0	0
CEU	HIST2	143897386	0.2872	0.1706	0.6926
CEU	HIST2	143902386	0.3863	0.8528	-1.0505
CEU	HIST2	143907386	0.1396	1.1939	-1.8788
CEU	HIST2	143912386	0.2756	1.1939	-1.6364
CEU	HIST2	143917386	0.3552	1.3645	-1.6328
CEU	HIST2	143922386	0.8202	2.2173	-1.5608
CEU	HIST2	143927386	1.7679	3.5818	-1.3671
CEU	HIST2	143932386	1.1758	2.5584	-1.3773
CEU	HIST2	143937386	1.1233	1.5351	-0.6106
CEU	HIST2	143942386	1.6102	2.2173	-0.6782
CEU	HIST2	143947386	0.8318	2.8996	-1.8596
CEU	HIST2	143952386	0.2569	2.0467	-2.1297
CEU	HIST2	143957386	0.0998	0.8528	-1.6957
CEU	HIST2	143962386	0.1301	1.1939	-1.8957
CEU	HIST2	143967386	0.6067	1.5351	-1.3766
CEU	HIST2	143972386	0.9157	1.7056	-1.0819
CEU	HIST2	143977386	0.3595	0.8528	-1.1109
CEU	HIST2	143982386	2.6096	2.2173	0.4383
CEU	HIST2	143987386	3.1517	3.4112	-0.2038
CEU	HIST2	143992386	2.1581	2.5584	-0.3988
CEU	HIST2	143997386	3.0565	3.0701	-0.0117
CEU	HIST2	144002386	2.2981	3.7524	-1.0536
CEU	HIST2	144007386	5.9082	5.1169	0.438
CEU	HIST2	144012386	7.453	7.3342	0.0476
CEU	HIST2	144017386	3.7339	6.4814	-1.2315
CEU	HIST2	144022386	3.2667	3.7524	-0.3519
CEU	HIST2	144027386	3.8326	3.7524	0.0581
CEU	HIST2	144032386	2.0272	3.7524	-1.2499
CEU	HIST2	144037386	0.3964	2.5584	-2.1537
CEU	HIST2	144042386	3.2672	3.9229	-0.4573
CEU	HIST2	144047386	4.2051	4.7757	-0.3356
CEU	HIST2	144052386	1.2948	2.8996	-1.4432
CEU	HIST2	144057386	0.3379	2.3879	-2.1589
CEU	HIST2	144062386	1.2641	2.8996	-1.4709
CEU	HIST2	144067386	2.0651	2.5584	-0.4914
CEU	HIST2	144072386	1.8499	1.7056	0.1976
CEU	HIST2	144077386	0.9351	1.3645	-0.6947
CEU	HIST2	144082386	0.2637	1.7056	-1.9749
CEU	HIST2	144087386	0.1602	1.3645	-1.9483
CEU	HIST2	144092386	0.0808	0.8528	-1.7385
CEU	HIST2	144097386	0.0505	0.5117	-1.4464
CEU	HIST2	144102386	0	0	0

CEU	HIST2	144107386	0	0	0
CEU	HIST2	144112386	0	0	0
CEU	HIST2	144117386	0	0	0
CEU	HIST2	144122386	0	0	0
CEU	HIST2	144127386	0	0	0
CEU	HIST2	144132386	0	0	0
CEU	HIST2	144137386	0	0	0
CEU	HIST2	144142386	0.02	0.1706	-0.8946
CEU	HIST2	144147386	0.02	0.1706	-0.8946
CEU	HIST2	144152386	0.0101	0.1706	-0.9534
CEU	HIST2	144157386	0.2325	1.0234	-1.5716
CEU	HIST2	144162386	0.2325	1.0234	-1.5716
CEU	HIST2	144167386	1.0183	0.6822	0.8777
CEU	HIST2	144172386	1.0082	0.5117	1.5572
CEU	HIST2	144177386	0.4945	0.1706	1.924
CEU	HIST2	144182386	0.4945	0.1706	1.924
CEU	HIST2	144187386	0	0	0
CEU	HIST2	144192386	0	0	0
CEU	HIST2	144197386	0.0594	0.3411	-1.1293
CEU	HIST2	144202386	0.0594	0.3411	-1.1293
CEU	HIST2	144207386	0	0	0
CEU	HIST2	144212386	0	0	0
CEU	HIST2	144217386	0.8332	1.1939	-0.6428
CEU	HIST2	144222386	0.8433	1.3645	-0.8432
CEU	HIST2	144227386	0.0101	0.1706	-0.9534
CEU	HIST2	144232386	0	0	0
CEU	HIST2	144237386	0	0	0
CEU	HIST2	144242386	0	0	0
CEU	HIST2	144247386	0	0	0
CEU	HIST2	144252386	0	0	0
CEU	HIST2	144257386	0	0	0
CEU	HIST2	144262386	0	0	0
CEU	HIST2	144267386	0	0	0
CEU	HIST2	144272386	0.4686	0.3411	0.5111
CEU	HIST2	144277386	0.4686	0.3411	0.5111
CEU	HIST2	144282386	0.4585	0.1706	1.7102
CEU	HIST2	144287386	0.4585	0.1706	1.7102
CEU	HIST2	144292386	0	0	0
CEU	HIST2	144297386	0	0	0
CEU	HIST2	144302386	1.4576	0.8528	1.362
CEU	HIST2	144307386	1.4576	0.8528	1.362
CEU	HIST2	144312386	0.0101	0.1706	-0.9534
CEU	HIST2	144317386	0.1977	0.8528	-1.4753
CEU	HIST2	144322386	0.2078	0.8528	-1.4525
CEU	HIST2	144327386	0.0505	0.6822	-1.6496
CEU	HIST2	144332386	0.0303	0.5117	-1.5098
CEU	HIST2	144337386	0.0202	0.3411	-1.2864
CEU	HIST2	144342386	0.0891	0.5117	-1.3254
CEU	HIST2	144347386	0.0689	0.1706	-0.6041

CEU	HIST2	144352386	0	0	0
CEU	HIST2	144357386	0	0	0
CEU	HIST2	144362386	0.2591	0.5117	-0.7922
CEU	HIST2	144367386	0.2591	0.5117	-0.7922
CEU	HIST2	144372386	0	0	0
CEU	HIST2	144377386	0	0	0
CEU	HIST2	144382386	0	0	0
CEU	HIST2	144387386	0	0	0
CEU	HIST2	144392386	0	0	0
CEU	HIST2	144397386	0	0	0
CHB	HIST2	143407386	2.6196	4.4048	-1.1233
CHB	HIST2	143412386	4.1296	5.5907	-0.7455
CHB	HIST2	143417386	4.1038	5.2519	-0.6192
CHB	HIST2	143422386	3.767	4.913	-0.6555
CHB	HIST2	143427386	2.3015	3.0495	-0.6433
CHB	HIST2	143432386	0.0097	0.1694	-0.949
CHB	HIST2	143437386	1.6986	1.1859	0.9152
CHB	HIST2	143442386	2.1795	2.2024	-0.0256
CHB	HIST2	143447386	1.4599	1.6942	-0.3215
CHB	HIST2	143452386	1.5696	1.6942	-0.171
CHB	HIST2	143457386	1.3255	3.0495	-1.4826
CHB	HIST2	143462386	1.9185	5.4213	-1.8368
CHB	HIST2	143467386	3.1673	7.6237	-1.7185
CHB	HIST2	143472386	3.8045	7.6237	-1.4728
CHB	HIST2	143477386	5.1051	8.1319	-1.1002
CHB	HIST2	143482386	4.5433	8.8096	-1.4404
CHB	HIST2	143487386	3.5443	7.7931	-1.6058
CHB	HIST2	143492386	7.6067	10.8426	-0.9006
CHB	HIST2	143497386	11.02	11.6896	-0.1736
CHB	HIST2	143502386	12.2582	9.6567	0.8067
CHB	HIST2	143507386	10.0776	8.979	0.3644
CHB	HIST2	143512386	7.1972	8.8096	-0.5444
CHB	HIST2	143517386	10.5547	11.1814	-0.1695
CHB	HIST2	143522386	10.9671	12.8755	-0.4518
CHB	HIST2	143527386	7.1736	10.1649	-0.8843
CHB	HIST2	143532386	7.0944	7.6237	-0.2041
CHB	HIST2	143537386	12.7493	10.8426	0.5306
CHB	HIST2	143542386	9.7561	8.4708	0.4499
CHB	HIST2	143547386	1.0192	1.6942	-0.9264
CHB	HIST2	143552386	0	0	0
CHB	HIST2	143557386	0	0	0
CHB	HIST2	143562386	0	0	0
CHB	HIST2	143567386	0	0	0
CHB	HIST2	143572386	0	0	0
CHB	HIST2	143577386	0	0	0
CHB	HIST2	143582386	0	0	0
CHB	HIST2	143587386	0	0	0
CHB	HIST2	143592386	0	0	0
CHB	HIST2	143597386	0	0	0

CHB	HIST2	143602386	0	0	0
CHB	HIST2	143607386	0	0	0
CHB	HIST2	143612386	0	0	0
CHB	HIST2	143617386	0	0	0
CHB	HIST2	143622386	0	0	0
CHB	HIST2	143627386	0	0	0
CHB	HIST2	143632386	0	0	0
CHB	HIST2	143637386	0	0	0
CHB	HIST2	143642386	0	0	0
CHB	HIST2	143647386	0.0192	0.1694	-0.8925
CHB	HIST2	143652386	0.0192	0.1694	-0.8925
CHB	HIST2	143657386	0.3705	0.1694	1.195
CHB	HIST2	143662386	1.1017	2.033	-1.1106
CHB	HIST2	143667386	1.6033	3.2189	-1.3282
CHB	HIST2	143672386	0.8721	1.3553	-0.7831
CHB	HIST2	143677386	0	0	0
CHB	HIST2	143682386	0	0	0
CHB	HIST2	143687386	0.0097	0.1694	-0.949
CHB	HIST2	143692386	0.0097	0.1694	-0.949
CHB	HIST2	143697386	0	0	0
CHB	HIST2	143702386	0	0	0
CHB	HIST2	143707386	0.7843	0.3388	1.787
CHB	HIST2	143712386	0.7843	0.3388	1.787
CHB	HIST2	143717386	0.4784	0.6777	-0.521
CHB	HIST2	143722386	0.9044	1.8636	-1.2238
CHB	HIST2	143727386	0.5313	2.033	-1.7908
CHB	HIST2	143732386	1.3145	2.3718	-1.1161
CHB	HIST2	143737386	1.2092	1.5247	-0.4687
CHB	HIST2	143742386	1.0977	1.1859	-0.1574
CHB	HIST2	143747386	1.5688	2.5412	-0.971
CHB	HIST2	143752386	1.3095	2.5412	-1.2299
CHB	HIST2	143757386	1.0626	2.3718	-1.382
CHB	HIST2	143762386	2.715	3.5577	-0.6369
CHB	HIST2	143767386	6.292	6.6072	-0.1384
CHB	HIST2	143772386	3.8013	4.2354	-0.2826
CHB	HIST2	143777386	0	0	0
CHB	HIST2	143782386	0	0	0
CHB	HIST2	143787386	0	0	0
CHB	HIST2	143792386	0	0	0
CHB	HIST2	143797386	0	0	0
CHB	HIST2	143802386	0	0	0
CHB	HIST2	143807386	0	0	0
CHB	HIST2	143812386	0	0	0
CHB	HIST2	143817386	0	0	0
CHB	HIST2	143822386	0	0	0
CHB	HIST2	143827386	0	0	0
CHB	HIST2	143832386	0	0	0
CHB	HIST2	143837386	0	0	0
CHB	HIST2	143842386	0	0	0

CHB	HIST2	143847386	0	0	0
CHB	HIST2	143852386	0	0	0
CHB	HIST2	143857386	0	0	0
CHB	HIST2	143862386	0	0	0
CHB	HIST2	143867386	0	0	0
CHB	HIST2	143872386	0.0097	0.1694	-0.949
CHB	HIST2	143877386	0.5924	1.5247	-1.3851
CHB	HIST2	143882386	0.5827	1.3553	-1.2521
CHB	HIST2	143887386	0	0	0
CHB	HIST2	143892386	0.0659	0.1694	-0.615
CHB	HIST2	143897386	0.3564	0.3388	0.0706
CHB	HIST2	143902386	0.5534	1.0165	-0.9216
CHB	HIST2	143907386	0.4156	2.033	-1.9288
CHB	HIST2	143912386	0.3398	2.2024	-2.0856
CHB	HIST2	143917386	0.3132	2.5412	-2.2248
CHB	HIST2	143922386	0.8717	2.2024	-1.49
CHB	HIST2	143927386	1.8987	1.8636	0.0448
CHB	HIST2	143932386	1.3259	2.5412	-1.2136
CHB	HIST2	143937386	1.2347	2.8801	-1.4835
CHB	HIST2	143942386	1.6134	2.2024	-0.6595
CHB	HIST2	143947386	0.6486	2.2024	-1.7398
CHB	HIST2	143952386	0.1262	1.8636	-2.2167
CHB	HIST2	143957386	0.0777	1.1859	-1.9781
CHB	HIST2	143962386	0.1163	1.8636	-2.2293
CHB	HIST2	143967386	0.592	1.5247	-1.3857
CHB	HIST2	143972386	0.9427	1.3553	-0.6687
CHB	HIST2	143977386	0.4377	1.0165	-1.1518
CHB	HIST2	143982386	2.7283	2.3718	0.3763
CHB	HIST2	143987386	3.3114	3.3883	-0.0606
CHB	HIST2	143992386	2.3048	2.5412	-0.2361
CHB	HIST2	143997386	2.9636	3.3883	-0.3345
CHB	HIST2	144002386	1.8563	4.066	-1.4901
CHB	HIST2	144007386	5.5533	5.4213	0.0692
CHB	HIST2	144012386	7.5729	8.3013	-0.2598
CHB	HIST2	144017386	3.5328	6.6072	-1.3503
CHB	HIST2	144022386	2.4666	2.8801	-0.3728
CHB	HIST2	144027386	2.9566	2.7106	0.2331
CHB	HIST2	144032386	1.7211	3.0495	-1.1424
CHB	HIST2	144037386	0.4373	2.3718	-2.042
CHB	HIST2	144042386	3.0813	3.5577	-0.36
CHB	HIST2	144047386	4.264	4.5742	-0.1889
CHB	HIST2	144052386	1.4803	3.3883	-1.5026
CHB	HIST2	144057386	0.3061	2.2024	-2.1233
CHB	HIST2	144062386	0.9088	2.7106	-1.7073
CHB	HIST2	144067386	1.6511	3.0495	-1.2026
CHB	HIST2	144072386	1.0841	2.3718	-1.3593
CHB	HIST2	144077386	0.344	3.0495	-2.3267
CHB	HIST2	144082386	0.414	3.0495	-2.2665
CHB	HIST2	144087386	0.3371	2.033	-2.0224

CHB	HIST2	144092386	0.2911	1.5247	-1.8327
CHB	HIST2	144097386	0.233	0.8471	-1.3848
CHB	HIST2	144102386	0	0	0
CHB	HIST2	144107386	0	0	0
CHB	HIST2	144112386	0	0	0
CHB	HIST2	144117386	0	0	0
CHB	HIST2	144122386	0	0	0
CHB	HIST2	144127386	0	0	0
CHB	HIST2	144132386	0	0	0
CHB	HIST2	144137386	0	0	0
CHB	HIST2	144142386	0	0	0
CHB	HIST2	144147386	0	0	0
CHB	HIST2	144152386	0.0289	0.1694	-0.8349
CHB	HIST2	144157386	0.6709	0.5082	0.5107
CHB	HIST2	144162386	0.6901	0.8471	-0.354
CHB	HIST2	144167386	1.0135	1.0165	-0.006
CHB	HIST2	144172386	0.9654	0.5082	1.4352
CHB	HIST2	144177386	0.4934	0.1694	1.9253
CHB	HIST2	144182386	0.4934	0.1694	1.9253
CHB	HIST2	144187386	0	0	0
CHB	HIST2	144192386	0.0097	0.1694	-0.949
CHB	HIST2	144197386	0.1376	0.3388	-0.8071
CHB	HIST2	144202386	0.1279	0.1694	-0.2466
CHB	HIST2	144207386	0	0	0
CHB	HIST2	144212386	0	0	0
CHB	HIST2	144217386	0.5108	1.1859	-1.205
CHB	HIST2	144222386	0.5108	1.1859	-1.205
CHB	HIST2	144227386	0	0	0
CHB	HIST2	144232386	0	0	0
CHB	HIST2	144237386	0	0	0
CHB	HIST2	144242386	0	0	0
CHB	HIST2	144247386	0	0	0
CHB	HIST2	144252386	0	0	0
CHB	HIST2	144257386	0	0	0
CHB	HIST2	144262386	0	0	0
CHB	HIST2	144267386	0	0	0
CHB	HIST2	144272386	0.3031	0.1694	0.7945
CHB	HIST2	144277386	0.3031	0.1694	0.7945
CHB	HIST2	144282386	0.3031	0.1694	0.7945
CHB	HIST2	144287386	0.3031	0.1694	0.7945
CHB	HIST2	144292386	0	0	0
CHB	HIST2	144297386	0	0	0
CHB	HIST2	144302386	0.8692	0.8471	0.0498
CHB	HIST2	144307386	0.8692	0.8471	0.0498
CHB	HIST2	144312386	0	0.1694	-1.0066
CHB	HIST2	144317386	0.2496	0.6777	-1.1192
CHB	HIST2	144322386	0.3065	0.6777	-0.9704
CHB	HIST2	144327386	0.0666	0.3388	-1.0919
CHB	HIST2	144332386	0.0097	0.1694	-0.949

CHB	HIST2	144337386	0.0192	0.1694	-0.8925
CHB	HIST2	144342386	0.0577	0.3388	-1.1276
CHB	HIST2	144347386	0.0385	0.1694	-0.7778
CHB	HIST2	144352386	0	0	0
CHB	HIST2	144357386	0	0	0
CHB	HIST2	144362386	0.1239	0.5082	-1.2064
CHB	HIST2	144367386	0.1239	0.5082	-1.2064
CHB	HIST2	144372386	0	0	0
CHB	HIST2	144377386	0	0	0
CHB	HIST2	144382386	0	0	0
CHB	HIST2	144387386	0.0097	0.1694	-0.949
CHB	HIST2	144392386	0.0097	0.1694	-0.949
CHB	HIST2	144397386	0	0	0
YRI	HIST2	143407386	2.6805	6.7225	-1.7417
YRI	HIST2	143412386	4.4176	8.5712	-1.4334
YRI	HIST2	143417386	4.5031	8.7392	-1.4359
YRI	HIST2	143422386	4.3184	7.7309	-1.2948
YRI	HIST2	143427386	2.5742	4.5377	-1.1998
YRI	HIST2	143432386	0.1725	1.0084	-1.6665
YRI	HIST2	143437386	0.6372	1.5126	-1.3035
YRI	HIST2	143442386	1.8666	2.5209	-0.6553
YRI	HIST2	143447386	2.6342	2.3529	0.2978
YRI	HIST2	143452386	1.5768	1.5126	0.0956
YRI	HIST2	143457386	2.1789	4.0335	-1.2548
YRI	HIST2	143462386	2.7601	8.7392	-2.0268
YRI	HIST2	143467386	1.9548	13.7811	-2.6163
YRI	HIST2	143472386	2.5691	13.9492	-2.4888
YRI	HIST2	143477386	3.7722	12.7727	-2.1397
YRI	HIST2	143482386	3.4752	12.2685	-2.1715
YRI	HIST2	143487386	3.1726	10.924	-2.1349
YRI	HIST2	143492386	8.8184	14.1172	-1.1457
YRI	HIST2	143497386	10.9744	14.6214	-0.7627
YRI	HIST2	143502386	9.1533	11.4282	-0.6006
YRI	HIST2	143507386	10.4027	12.6047	-0.5301
YRI	HIST2	143512386	10.0613	14.7895	-0.9781
YRI	HIST2	143517386	10.9798	14.7895	-0.7881
YRI	HIST2	143522386	13.1785	17.8146	-0.8028
YRI	HIST2	143527386	10.979	14.9575	-0.8142
YRI	HIST2	143532386	9.3211	10.756	-0.401
YRI	HIST2	143537386	13.0918	13.1089	-0.004
YRI	HIST2	143542386	9.6054	10.756	-0.3215
YRI	HIST2	143547386	1.6449	3.3612	-1.3559
YRI	HIST2	143552386	0	0	0
YRI	HIST2	143557386	0	0	0
YRI	HIST2	143562386	0	0	0
YRI	HIST2	143567386	0	0	0
YRI	HIST2	143572386	0	0	0
YRI	HIST2	143577386	0	0	0
YRI	HIST2	143582386	0	0	0

YRI	HIST2	143587386	0	0	0
YRI	HIST2	143592386	0	0	0
YRI	HIST2	143597386	0	0	0
YRI	HIST2	143602386	0	0	0
YRI	HIST2	143607386	0	0	0
YRI	HIST2	143612386	0	0	0
YRI	HIST2	143617386	0	0	0
YRI	HIST2	143622386	0	0	0
YRI	HIST2	143627386	0	0	0
YRI	HIST2	143632386	0	0	0
YRI	HIST2	143637386	0	0	0
YRI	HIST2	143642386	0	0	0
YRI	HIST2	143647386	0	0	0
YRI	HIST2	143652386	0.0278	0.3361	-1.2377
YRI	HIST2	143657386	0.2998	0.5042	-0.6424
YRI	HIST2	143662386	2.5627	4.0335	-0.9952
YRI	HIST2	143667386	3.2208	6.7225	-1.5089
YRI	HIST2	143672386	0.93	2.8571	-1.7428
YRI	HIST2	143677386	0	0	0
YRI	HIST2	143682386	0	0	0
YRI	HIST2	143687386	0.7359	0.6722	0.1668
YRI	HIST2	143692386	0.7359	0.6722	0.1668
YRI	HIST2	143697386	0	0	0
YRI	HIST2	143702386	0	0	0
YRI	HIST2	143707386	0.4924	0.6722	-0.4707
YRI	HIST2	143712386	0.4924	0.6722	-0.4707
YRI	HIST2	143717386	0.8239	1.3445	-0.8456
YRI	HIST2	143722386	0.9714	2.8571	-1.7053
YRI	HIST2	143727386	0.5687	2.689	-2.0151
YRI	HIST2	143732386	1.4673	2.689	-1.1611
YRI	HIST2	143737386	1.0826	1.6806	-0.8227
YRI	HIST2	143742386	0.6066	2.689	-1.979
YRI	HIST2	143747386	2.2516	6.5544	-1.897
YRI	HIST2	143752386	2.7569	6.3864	-1.6381
YRI	HIST2	143757386	1.9095	7.0586	-2.1227
YRI	HIST2	143762386	3.2209	8.7392	-1.8706
YRI	HIST2	143767386	6.129	8.7392	-0.8848
YRI	HIST2	143772386	3.7422	4.7057	-0.5703
YRI	HIST2	143777386	0	0	0
YRI	HIST2	143782386	0	0	0
YRI	HIST2	143787386	0	0	0
YRI	HIST2	143792386	0	0	0
YRI	HIST2	143797386	0	0	0
YRI	HIST2	143802386	0	0	0
YRI	HIST2	143807386	0	0	0
YRI	HIST2	143812386	0	0	0
YRI	HIST2	143817386	0	0	0
YRI	HIST2	143822386	0	0	0
YRI	HIST2	143827386	0	0	0

YRI	HIST2	143832386	0	0	0
YRI	HIST2	143837386	0	0	0
YRI	HIST2	143842386	0	0	0
YRI	HIST2	143847386	0	0	0
YRI	HIST2	143852386	0	0	0
YRI	HIST2	143857386	0	0	0
YRI	HIST2	143862386	0	0	0
YRI	HIST2	143867386	0	0	0
YRI	HIST2	143872386	0.4454	1.1764	-1.3074
YRI	HIST2	143877386	1.4515	3.3612	-1.5087
YRI	HIST2	143882386	1.0061	2.1848	-1.3234
YRI	HIST2	143887386	0.0718	0.1681	-0.5725
YRI	HIST2	143892386	0.0718	0.1681	-0.5725
YRI	HIST2	143897386	0.2783	0.5042	-0.7099
YRI	HIST2	143902386	0.3779	1.0084	-1.257
YRI	HIST2	143907386	0.4659	2.0167	-1.8542
YRI	HIST2	143912386	0.9369	2.8571	-1.7365
YRI	HIST2	143917386	0.6169	2.0167	-1.6737
YRI	HIST2	143922386	1.1127	2.5209	-1.4103
YRI	HIST2	143927386	2.433	4.3696	-1.2228
YRI	HIST2	143932386	2.3113	4.3696	-1.2997
YRI	HIST2	143937386	2.3952	3.3612	-0.7632
YRI	HIST2	143942386	2.3116	2.5209	-0.2096
YRI	HIST2	143947386	1.2472	2.689	-1.3702
YRI	HIST2	143952386	0.5504	2.689	-2.0324
YRI	HIST2	143957386	0.6371	2.689	-1.95
YRI	HIST2	143962386	0.9082	3.8654	-2.0752
YRI	HIST2	143967386	0.8757	3.1932	-1.9113
YRI	HIST2	143972386	1.0502	2.0167	-1.1556
YRI	HIST2	143977386	0.736	1.6806	-1.2995
YRI	HIST2	143982386	3.1308	3.6974	-0.413
YRI	HIST2	143987386	4.2257	4.8738	-0.372
YRI	HIST2	143992386	3.5532	5.0419	-0.8295
YRI	HIST2	143997386	4.1897	5.2099	-0.5522
YRI	HIST2	144002386	3.4764	5.2099	-0.9383
YRI	HIST2	144007386	7.3358	8.9073	-0.5234
YRI	HIST2	144012386	8.797	13.1089	-1.0002
YRI	HIST2	144017386	4.5092	11.2602	-1.8073
YRI	HIST2	144022386	2.9238	5.7141	-1.3914
YRI	HIST2	144027386	3.19	4.7057	-0.8972
YRI	HIST2	144032386	2.2247	4.8738	-1.5207
YRI	HIST2	144037386	0.9351	3.8654	-2.0564
YRI	HIST2	144042386	3.781	5.7141	-0.964
YRI	HIST2	144047386	4.8642	7.2267	-0.9533
YRI	HIST2	144052386	2.1861	6.0502	-1.8308
YRI	HIST2	144057386	1.0654	4.5377	-2.1217
YRI	HIST2	144062386	1.9046	4.8738	-1.7044
YRI	HIST2	144067386	2.7733	6.0502	-1.5526
YRI	HIST2	144072386	1.7407	4.7057	-1.7551

YRI	HIST2	144077386	0.9994	3.3612	-1.8659
YRI	HIST2	144082386	1.6202	3.3612	-1.3755
YRI	HIST2	144087386	1.3293	2.1848	-0.9605
YRI	HIST2	144092386	0.516	1.6806	-1.6021
YRI	HIST2	144097386	0.3113	1.1764	-1.5473
YRI	HIST2	144102386	0	0	0
YRI	HIST2	144107386	0	0	0
YRI	HIST2	144112386	0	0	0
YRI	HIST2	144117386	0	0	0
YRI	HIST2	144122386	0	0	0
YRI	HIST2	144127386	0	0	0
YRI	HIST2	144132386	0	0	0
YRI	HIST2	144137386	0	0	0
YRI	HIST2	144142386	0.0276	0.3361	-1.2385
YRI	HIST2	144147386	0.0276	0.3361	-1.2385
YRI	HIST2	144152386	0.0276	0.1681	-0.8352
YRI	HIST2	144157386	0.6035	1.0084	-0.8072
YRI	HIST2	144162386	0.8103	1.3445	-0.8676
YRI	HIST2	144167386	1.2144	1.0084	0.4107
YRI	HIST2	144172386	0.98	0.5042	1.4953
YRI	HIST2	144177386	0.4914	0.1681	1.9219
YRI	HIST2	144182386	0.4914	0.1681	1.9219
YRI	HIST2	144187386	0.0093	0.1681	-0.944
YRI	HIST2	144192386	0.0185	0.3361	-1.275
YRI	HIST2	144197386	0.1234	0.3361	-0.8539
YRI	HIST2	144202386	0.1141	0.1681	-0.321
YRI	HIST2	144207386	0	0	0
YRI	HIST2	144212386	0	0	0
YRI	HIST2	144217386	0.6969	1.5126	-1.2146
YRI	HIST2	144222386	0.6969	1.5126	-1.2146
YRI	HIST2	144227386	0	0	0
YRI	HIST2	144232386	0	0	0
YRI	HIST2	144237386	0	0	0
YRI	HIST2	144242386	0	0	0
YRI	HIST2	144247386	0	0	0
YRI	HIST2	144252386	0	0	0
YRI	HIST2	144257386	0	0	0
YRI	HIST2	144262386	0	0	0
YRI	HIST2	144267386	0	0	0
YRI	HIST2	144272386	0.2983	0.1681	0.774
YRI	HIST2	144277386	0.2983	0.1681	0.774
YRI	HIST2	144282386	0.2983	0.1681	0.774
YRI	HIST2	144287386	0.2983	0.1681	0.774
YRI	HIST2	144292386	0	0	0
YRI	HIST2	144297386	0	0	0
YRI	HIST2	144302386	1.6516	1.0084	1.2823
YRI	HIST2	144307386	1.6516	1.0084	1.2823
YRI	HIST2	144312386	0	0.1681	-0.9993
YRI	HIST2	144317386	0.3371	1.0084	-1.3384

YRI	HIST2	144322386	0.3827	1.0084	-1.2474
YRI	HIST2	144327386	0.0549	0.3361	-1.1289
YRI	HIST2	144332386	0.0093	0.1681	-0.944
YRI	HIST2	144337386	0.5042	0.1681	1.998
YRI	HIST2	144342386	0.5042	0.1681	1.998
YRI	HIST2	144347386	0	0	0
YRI	HIST2	144352386	0	0	0
YRI	HIST2	144357386	0.0093	0.1681	-0.944
YRI	HIST2	144362386	0.6053	0.6722	-0.1751
YRI	HIST2	144367386	0.5961	0.5042	0.2888
YRI	HIST2	144372386	0	0	0
YRI	HIST2	144377386	0.0093	0.1681	-0.944
YRI	HIST2	144382386	0.0093	0.1681	-0.944
YRI	HIST2	144387386	0	0	0
YRI	HIST2	144392386	0	0	0
YRI	HIST2	144397386	0	0	0
CEU	HIST2	149287689	3.9555	2.8996	0.9496
CEU	HIST2	149292689	4.4016	4.2641	0.0893
CEU	HIST2	149297689	4.7409	4.7757	-0.0205
CEU	HIST2	149302689	2.3857	2.3879	-0.0023
CEU	HIST2	149307689	3.8181	2.8996	0.826
CEU	HIST2	149312689	3.2485	3.2407	0.0064
CEU	HIST2	149317689	2.3018	2.2173	0.0944
CEU	HIST2	149322689	3.0649	1.7056	1.8617
CEU	HIST2	149327689	2.8089	2.8996	-0.0816
CEU	HIST2	149332689	6.0913	6.8225	-0.3129
CEU	HIST2	149337689	7.1923	8.3576	-0.4142
CEU	HIST2	149342689	6.0491	5.7991	0.1238
CEU	HIST2	149347689	6.6485	5.6286	0.5188
CEU	HIST2	149352689	5.6024	6.1402	-0.2531
CEU	HIST2	149357689	5.7143	5.9697	-0.1233
CEU	HIST2	149362689	5.215	5.1169	0.0543
CEU	HIST2	149367689	2.5683	3.2407	-0.5514
CEU	HIST2	149372689	3.4358	4.7757	-0.788
CEU	HIST2	149377689	4.537	5.458	-0.4815
CEU	HIST2	149382689	3.7069	4.0935	-0.26
CEU	HIST2	149387689	5.2144	4.4346	0.4893
CEU	HIST2	149392689	6.3327	5.9697	0.1752
CEU	HIST2	149397689	5.2449	5.7991	-0.2745
CEU	HIST2	149402689	5.9522	5.9697	-0.0084
CEU	HIST2	149407689	4.525	4.6052	-0.0487
CEU	HIST2	149412689	4.3368	3.4112	0.727
CEU	HIST2	149417689	4.1068	3.2407	0.7102
CEU	HIST2	149422689	2.0064	1.8762	0.1658
CEU	HIST2	149427689	2.7172	2.729	-0.0112
CEU	HIST2	149432689	2.2086	2.3879	-0.1888
CEU	HIST2	149437689	0.5232	0.6822	-0.4152
CEU	HIST2	149442689	0	0	0
CEU	HIST2	149447689	0.08	0.5117	-1.3539

CEU	HIST2	149452689	0.1394	0.8528	-1.6066
CEU	HIST2	149457689	0.128	0.6822	-1.4472
CEU	HIST2	149462689	0.0686	0.3411	-1.0924
CEU	HIST2	149467689	0	0	0
CEU	HIST2	149472689	0	0	0
CEU	HIST2	149477689	0	0	0
CEU	HIST2	149482689	0	0	0
CEU	HIST2	149487689	0	0	0
CEU	HIST2	149492689	0	0	0
CEU	HIST2	149497689	0	0	0
CEU	HIST2	149502689	0	0	0
CEU	HIST2	149507689	0.1363	0.5117	-1.1774
CEU	HIST2	149512689	0.4605	1.1939	-1.3069
CEU	HIST2	149517689	0.3243	0.6822	-0.9346
CEU	HIST2	149522689	0	0	0
CEU	HIST2	149527689	0.0101	0.1706	-0.9534
CEU	HIST2	149532689	0.0101	0.1706	-0.9534
CEU	HIST2	149537689	0	0	0
CEU	HIST2	149542689	0	0	0
CEU	HIST2	149547689	0	0	0
CEU	HIST2	149552689	0	0	0
CEU	HIST2	149557689	0	0	0
CEU	HIST2	149562689	0	0	0
CEU	HIST2	149567689	0.5038	0.1706	1.9793
CEU	HIST2	149572689	0.885	0.6822	0.5296
CEU	HIST2	149577689	0.4805	1.0234	-1.0788
CEU	HIST2	149582689	2.8182	3.2407	-0.3464
CEU	HIST2	149587689	3.7007	3.2407	0.3772
CEU	HIST2	149592689	2.1459	2.0467	0.118
CEU	HIST2	149597689	2.4286	2.8996	-0.4236
CEU	HIST2	149602689	5.8841	4.4346	0.9095
CEU	HIST2	149607689	7.5463	4.7757	1.6295
CEU	HIST2	149612689	3.7706	2.2173	1.7353
CEU	HIST2	149617689	0.8439	0.5117	1.0419
CEU	HIST2	149622689	0	0	0
CEU	HIST2	149627689	0.0301	0.1706	-0.8346
CEU	HIST2	149632689	0.0301	0.1706	-0.8346
CEU	HIST2	149637689	2.3997	1.7056	0.9507
CEU	HIST2	149642689	3.7413	3.5818	0.1202
CEU	HIST2	149647689	7.4811	6.6519	0.363
CEU	HIST2	149652689	7.906	6.1402	0.8309
CEU	HIST2	149657689	4.3752	3.5818	0.598
CEU	HIST2	149662689	6.0134	6.3108	-0.1365
CEU	HIST2	149667689	5.4199	9.3809	-1.2654
CEU	HIST2	149672689	5.4801	9.5515	-1.2791
CEU	HIST2	149677689	5.2674	6.9931	-0.7221
CEU	HIST2	149682689	2.4492	4.4346	-1.2457
CEU	HIST2	149687689	0.9485	3.4112	-1.9342
CEU	HIST2	149692689	1.3814	3.4112	-1.5942

CEU	HIST2	149697689	2.8858	5.1169	-1.235
CEU	HIST2	149702689	5.2948	6.9931	-0.7106
CEU	HIST2	149707689	8.1801	7.3342	0.3389
CEU	HIST2	149712689	9.3602	7.8459	0.5705
CEU	HIST2	149717689	10.1014	8.0164	0.7701
CEU	HIST2	149722689	8.69	6.8225	0.7991
CEU	HIST2	149727689	7.3047	6.8225	0.2063
CEU	HIST2	149732689	5.9689	5.458	0.2671
CEU	HIST2	149737689	2.8054	3.2407	-0.3569
CEU	HIST2	149742689	2.4663	3.4112	-0.7421
CEU	HIST2	149747689	4.6718	4.0935	0.3889
CEU	HIST2	149752689	3.3781	3.0701	0.2642
CEU	HIST2	149757689	1.8277	3.2407	-1.1586
CEU	HIST2	149762689	2.309	5.1169	-1.5542
CEU	HIST2	149767689	2.5848	5.458	-1.502
CEU	HIST2	149772689	3.2146	5.9697	-1.3296
CEU	HIST2	149777689	4.4694	6.6519	-0.9555
CEU	HIST2	149782689	5.437	7.6753	-0.8603
CEU	HIST2	149787689	3.731	6.9931	-1.3649
CEU	HIST2	149792689	1.4871	3.0701	-1.3579
CEU	HIST2	149797689	0.1235	0.1706	-0.2798
CEU	HIST2	149802689	0	0	0
CEU	HIST2	149807689	0	0	0
CEU	HIST2	149812689	1.5739	2.0467	-0.5626
CEU	HIST2	149817689	2.7071	4.2641	-1.0107
CEU	HIST2	149822689	1.6324	2.3879	-0.7956
CEU	HIST2	149827689	0.4993	0.1706	1.9525
CEU	HIST2	149832689	0	0	0
CEU	HIST2	149837689	0	0	0
CEU	HIST2	149842689	1.0373	1.5351	-0.7381
CEU	HIST2	149847689	2.0711	4.0935	-1.3599
CEU	HIST2	149852689	2.2626	5.2874	-1.6264
CEU	HIST2	149857689	3.3267	6.8225	-1.4958
CEU	HIST2	149862689	4.2778	8.0164	-1.3809
CEU	HIST2	149867689	4.5095	8.187	-1.3323
CEU	HIST2	149872689	4.3408	6.8225	-1.0619
CEU	HIST2	149877689	3.9984	6.4814	-1.1129
CEU	HIST2	149882689	2.7013	5.2874	-1.3906
CEU	HIST2	149887689	2.3034	3.5818	-0.9635
CEU	HIST2	149892689	3.6869	4.9463	-0.7182
CEU	HIST2	149897689	3.5384	5.6286	-1.0632
CEU	HIST2	149902689	3.7885	5.9697	-1.0527
CEU	HIST2	149907689	3.8075	5.458	-0.8628
CEU	HIST2	149912689	1.6186	3.9229	-1.6072
CEU	HIST2	149917689	1.7209	4.0935	-1.5954
CEU	HIST2	149922689	3.7238	5.2874	-0.8408
CEU	HIST2	149927689	4.1686	5.6286	-0.7426
CEU	HIST2	149932689	2.617	5.458	-1.4852
CEU	HIST2	149937689	2.0439	4.6052	-1.5551

CEU	HIST2	149942689	2.6215	3.7524	-0.8193
CEU	HIST2	149947689	2.2977	4.4346	-1.3408
CEU	HIST2	149952689	2.9812	5.9697	-1.4423
CEU	HIST2	149957689	2.1946	4.4346	-1.4055
CEU	HIST2	149962689	1.1688	2.5584	-1.3843
CEU	HIST2	149967689	2.1791	3.2407	-0.8705
CEU	HIST2	149972689	2.1826	3.9229	-1.2138
CEU	HIST2	149977689	2.2919	4.6052	-1.4045
CEU	HIST2	149982689	3.1297	4.9463	-1.036
CEU	HIST2	149987689	3.8433	5.7991	-0.9687
CEU	HIST2	149992689	5.8895	7.6753	-0.6864
CEU	HIST2	149997689	9.3993	8.5281	0.304
CEU	HIST2	150002689	8.1973	6.8225	0.5883
CEU	HIST2	150007689	7.2562	6.9931	0.1101
CEU	HIST2	150012689	11.1618	9.7221	0.4449
CEU	HIST2	150017689	10.6663	8.6987	0.6741
CEU	HIST2	150022689	8.8505	7.3342	0.6075
CEU	HIST2	150027689	6.0136	6.1402	-0.0596
CEU	HIST2	150032689	1.9462	4.2641	-1.5046
CEU	HIST2	150037689	2.7005	5.7991	-1.5347
CEU	HIST2	150042689	4.1932	6.3108	-0.9722
CEU	HIST2	150047689	4.4902	5.458	-0.5059
CEU	HIST2	150052689	3.1923	4.9463	-1.0003
CEU	HIST2	150057689	2.4185	4.6052	-1.3276
CEU	HIST2	150062689	3.0056	4.7757	-1.0411
CEU	HIST2	150067689	1.987	3.2407	-1.028
CEU	HIST2	150072689	5.9037	5.7991	0.0518
CEU	HIST2	150077689	7.4368	7.6753	-0.0917
CEU	HIST2	150082689	3.1187	5.7991	-1.3276
CEU	HIST2	150087689	3.1278	6.9931	-1.6173
CEU	HIST2	150092689	4.008	6.3108	-1.0573
CEU	HIST2	150097689	3.9445	4.7757	-0.4889
CEU	HIST2	150102689	5.1649	6.8225	-0.7093
CEU	HIST2	150107689	6.5529	6.9931	-0.1842
CEU	HIST2	150112689	5.2084	5.6286	-0.2137
CEU	HIST2	150117689	2.9899	4.9463	-1.1157
CEU	HIST2	150122689	3.5481	6.1402	-1.2198
CEU	HIST2	150127689	5.9969	8.3576	-0.8392
CEU	HIST2	150132689	6.0233	8.6987	-0.9166
CEU	HIST2	150137689	6.0427	11.4277	-1.4307
CEU	HIST2	150142689	6.8081	9.7221	-0.9005
CEU	HIST2	150147689	5.0326	6.1402	-0.5212
CEU	HIST2	150152689	2.9186	7.1636	-1.7377
CEU	HIST2	150157689	5.2408	9.5515	-1.3542
CEU	HIST2	150162689	9.0115	9.7221	-0.2196
CEU	HIST2	150167689	10.4014	7.8459	0.9627
CEU	HIST2	150172689	8.5935	6.8225	0.7578
CEU	HIST2	150177689	6.1723	5.2874	0.4758
CEU	HIST2	150182689	7.6821	6.1402	0.7256

CEU	HIST2	150187689	6.2696	6.8225	-0.2366
CEU	HIST2	150192689	5.329	5.9697	-0.3092
CEU	HIST2	150197689	8.2754	6.4814	0.8041
CEU	HIST2	150202689	9.3094	7.5047	0.7081
CEU	HIST2	150207689	9.1612	8.187	0.3529
CEU	HIST2	150212689	9.5529	7.1636	0.9781
CEU	HIST2	150217689	8.6856	6.3108	1.0903
CEU	HIST2	150222689	6.4754	6.1402	0.1577
CEU	HIST2	150227689	3.2977	4.2641	-0.6273
CEU	HIST2	150232689	2.6716	3.4112	-0.5809
CEU	HIST2	150237689	3.665	3.2407	0.3479
CEU	HIST2	150242689	3.0375	4.2641	-0.7962
CEU	HIST2	150247689	4.9192	5.2874	-0.198
CEU	HIST2	150252689	5.5411	5.1169	0.2348
CEU	HIST2	150257689	9.2591	8.0164	0.459
CEU	HIST2	150262689	14.1268	8.6987	1.8596
CEU	HIST2	150267689	18.2255	10.0632	2.4427
CEU	HIST2	150272689	17.2191	11.9394	1.3461
CEU	HIST2	150277689	13.2507	10.7454	0.7052
CEU	HIST2	150282689	14.9365	10.5749	1.2462
CEU	HIST2	150287689	13.2278	8.5281	1.6398
CEU	HIST2	150292689	10.5173	7.5047	1.182
CEU	HIST2	150297689	8.1028	7.1636	0.3845
CEU	HIST2	150302689	7.8524	7.3342	0.2076
CHB	HIST2	149287689	3.9103	2.7106	1.1368
CHB	HIST2	149292689	4.0881	3.5577	0.4008
CHB	HIST2	149297689	4.5445	4.066	0.3227
CHB	HIST2	149302689	2.2473	3.3883	-0.8986
CHB	HIST2	149307689	3.5359	4.2354	-0.4554
CHB	HIST2	149312689	3.2448	4.4048	-0.7299
CHB	HIST2	149317689	1.9838	2.7106	-0.6887
CHB	HIST2	149322689	2.3238	2.7106	-0.3665
CHB	HIST2	149327689	2.5713	5.4213	-1.4945
CHB	HIST2	149332689	5.8686	8.1319	-0.8227
CHB	HIST2	149337689	6.3626	7.9625	-0.5929
CHB	HIST2	149342689	5.7942	7.1154	-0.5426
CHB	HIST2	149347689	6.0312	7.6237	-0.6141
CHB	HIST2	149352689	4.5502	6.7766	-0.9557
CHB	HIST2	149357689	5.151	5.2519	-0.0544
CHB	HIST2	149362689	4.5073	4.066	0.2976
CHB	HIST2	149367689	2.2054	3.8965	-1.1828
CHB	HIST2	149372689	2.4304	5.2519	-1.5217
CHB	HIST2	149377689	3.2667	4.913	-0.9417
CHB	HIST2	149382689	3.0484	3.7271	-0.4931
CHB	HIST2	149387689	4.0811	5.5907	-0.7702
CHB	HIST2	149392689	4.9347	5.7601	-0.4101
CHB	HIST2	149397689	4.4259	5.4213	-0.522
CHB	HIST2	149402689	4.9065	6.7766	-0.8028
CHB	HIST2	149407689	3.0881	4.7436	-0.9765

CHB	HIST2	149412689	3.8726	4.4048	-0.3349
CHB	HIST2	149417689	4.2707	4.5742	-0.1848
CHB	HIST2	149422689	1.5395	3.0495	-1.2986
CHB	HIST2	149427689	1.9899	3.7271	-1.262
CHB	HIST2	149432689	1.886	3.0495	-1.0006
CHB	HIST2	149437689	0.399	0.8471	-1.0105
CHB	HIST2	149442689	0	0	0
CHB	HIST2	149447689	0.0386	0.5082	-1.4741
CHB	HIST2	149452689	0.1065	1.3553	-2.0239
CHB	HIST2	149457689	0.0873	1.1859	-1.961
CHB	HIST2	149462689	0.0194	0.3388	-1.2812
CHB	HIST2	149467689	0	0	0
CHB	HIST2	149472689	0	0	0
CHB	HIST2	149477689	0	0	0
CHB	HIST2	149482689	0	0	0
CHB	HIST2	149487689	0	0	0
CHB	HIST2	149492689	0	0	0
CHB	HIST2	149497689	0	0	0
CHB	HIST2	149502689	0	0	0
CHB	HIST2	149507689	0.6372	0.6777	-0.1059
CHB	HIST2	149512689	0.8968	1.0165	-0.2382
CHB	HIST2	149517689	0.2694	0.5082	-0.7496
CHB	HIST2	149522689	0.0097	0.1694	-0.949
CHB	HIST2	149527689	0.2578	0.1694	0.5253
CHB	HIST2	149532689	0.2578	0.1694	0.5253
CHB	HIST2	149537689	0	0	0
CHB	HIST2	149542689	0	0	0
CHB	HIST2	149547689	0	0	0
CHB	HIST2	149552689	0	0	0
CHB	HIST2	149557689	0	0	0
CHB	HIST2	149562689	0	0	0
CHB	HIST2	149567689	0.4601	0.3388	0.4866
CHB	HIST2	149572689	0.9039	0.8471	0.1281
CHB	HIST2	149577689	1.095	1.0165	0.1562
CHB	HIST2	149582689	3.3082	3.3883	-0.0631
CHB	HIST2	149587689	3.4287	3.5577	-0.0975
CHB	HIST2	149592689	2.0525	1.6942	0.4917
CHB	HIST2	149597689	2.9082	2.2024	0.7903
CHB	HIST2	149602689	6.534	4.2354	1.4964
CHB	HIST2	149607689	7.0862	5.4213	0.873
CHB	HIST2	149612689	2.7537	2.8801	-0.114
CHB	HIST2	149617689	0.574	0.5082	0.2066
CHB	HIST2	149622689	0	0	0
CHB	HIST2	149627689	0.0097	0.1694	-0.949
CHB	HIST2	149632689	0.0097	0.1694	-0.949
CHB	HIST2	149637689	2.8808	1.5247	2.0147
CHB	HIST2	149642689	4.7697	4.2354	0.3478
CHB	HIST2	149647689	7.5328	6.7766	0.3246
CHB	HIST2	149652689	6.6267	5.4213	0.6321

CHB	HIST2	149657689	3.0921	4.5742	-0.9025
CHB	HIST2	149662689	5.9966	5.4213	0.3017
CHB	HIST2	149667689	8.2781	6.946	0.5592
CHB	HIST2	149672689	8.1593	8.6402	-0.1653
CHB	HIST2	149677689	5.5639	6.0989	-0.2525
CHB	HIST2	149682689	2.9461	4.7436	-1.0603
CHB	HIST2	149687689	2.256	4.2354	-1.2886
CHB	HIST2	149692689	3.1883	4.066	-0.5919
CHB	HIST2	149697689	4.2125	4.5742	-0.2202
CHB	HIST2	149702689	6.1365	6.946	-0.3398
CHB	HIST2	149707689	8.0221	7.9625	0.0221
CHB	HIST2	149712689	7.1178	6.4378	0.3058
CHB	HIST2	149717689	7.4384	8.1319	-0.2521
CHB	HIST2	149722689	7.2953	8.6402	-0.4623
CHB	HIST2	149727689	7.0956	8.8096	-0.5787
CHB	HIST2	149732689	5.754	7.2848	-0.6153
CHB	HIST2	149737689	2.2852	4.066	-1.2009
CHB	HIST2	149742689	1.5443	3.0495	-1.2945
CHB	HIST2	149747689	3.8666	2.8801	0.8894
CHB	HIST2	149752689	3.2732	2.8801	0.3544
CHB	HIST2	149757689	1.1856	5.5907	-2.2476
CHB	HIST2	149762689	1.9924	6.6072	-2.0269
CHB	HIST2	149767689	2.1599	4.7436	-1.5241
CHB	HIST2	149772689	1.3202	5.9295	-2.2314
CHB	HIST2	149777689	0.7826	4.913	-2.3626
CHB	HIST2	149782689	1.0246	4.4048	-2.127
CHB	HIST2	149787689	0.744	5.7601	-2.4921
CHB	HIST2	149792689	0.4026	3.3883	-2.3513
CHB	HIST2	149797689	0.1513	0.5082	-1.1204
CHB	HIST2	149802689	0	0	0
CHB	HIST2	149807689	0	0	0
CHB	HIST2	149812689	0.4879	2.2024	-1.9198
CHB	HIST2	149817689	1.276	5.4213	-2.1737
CHB	HIST2	149822689	1.1542	3.3883	-1.7594
CHB	HIST2	149827689	0.3661	0.1694	1.1688
CHB	HIST2	149832689	0	0	0
CHB	HIST2	149837689	0	0	0
CHB	HIST2	149842689	0.2878	1.8636	-2.0105
CHB	HIST2	149847689	0.6405	4.913	-2.4439
CHB	HIST2	149852689	0.8979	7.1154	-2.5533
CHB	HIST2	149857689	1.5561	6.4378	-2.195
CHB	HIST2	149862689	1.4534	6.6072	-2.2636
CHB	HIST2	149867689	0.9816	6.946	-2.5036
CHB	HIST2	149872689	1.0817	5.5907	-2.3006
CHB	HIST2	149877689	1.6012	6.6072	-2.1987
CHB	HIST2	149882689	1.7603	7.1154	-2.1992
CHB	HIST2	149887689	1.2762	5.7601	-2.2277
CHB	HIST2	149892689	1.5239	6.7766	-2.2548
CHB	HIST2	149897689	1.1626	7.2848	-2.4609

CHB	HIST2	149902689	1.4296	6.946	-2.3156
CHB	HIST2	149907689	2.6127	8.1319	-2.0061
CHB	HIST2	149912689	1.5418	6.2684	-2.1769
CHB	HIST2	149917689	1.4919	6.7766	-2.2685
CHB	HIST2	149922689	3.5157	8.979	-1.8123
CHB	HIST2	149927689	3.9733	10.3343	-1.8516
CHB	HIST2	149932689	2.1807	10.3343	-2.3734
CHB	HIST2	149937689	2.0573	9.3178	-2.3273
CHB	HIST2	149942689	2.9528	7.9625	-1.8564
CHB	HIST2	149947689	2.0617	6.2684	-1.9375
CHB	HIST2	149952689	1.9979	8.3013	-2.2481
CHB	HIST2	149957689	1.6149	7.7931	-2.335
CHB	HIST2	149962689	0.988	5.9295	-2.3923
CHB	HIST2	149967689	1.1896	6.4378	-2.3598
CHB	HIST2	149972689	1.4017	5.2519	-2.0765
CHB	HIST2	149977689	1.9513	4.913	-1.6941
CHB	HIST2	149982689	2.2138	7.7931	-2.1087
CHB	HIST2	149987689	2.2511	9.9955	-2.3255
CHB	HIST2	149992689	3.9113	9.6567	-1.7815
CHB	HIST2	149997689	6.7367	10.5037	-1.08
CHB	HIST2	150002689	5.34	9.9955	-1.398
CHB	HIST2	150007689	2.9031	9.6567	-2.0941
CHB	HIST2	150012689	2.2269	10.5037	-2.3729
CHB	HIST2	150017689	1.4828	8.6402	-2.4603
CHB	HIST2	150022689	1.732	8.8096	-2.3896
CHB	HIST2	150027689	1.3954	8.6402	-2.4903
CHB	HIST2	150032689	1.1041	6.4378	-2.3982
CHB	HIST2	150037689	1.8289	6.6072	-2.0987
CHB	HIST2	150042689	1.9001	6.7766	-2.0933
CHB	HIST2	150047689	0.9341	6.7766	-2.508
CHB	HIST2	150052689	1.0657	6.6072	-2.4339
CHB	HIST2	150057689	1.1677	6.0989	-2.3277
CHB	HIST2	150062689	0.6227	5.0825	-2.476
CHB	HIST2	150067689	0.5625	4.913	-2.4885
CHB	HIST2	150072689	1.3189	7.4543	-2.4151
CHB	HIST2	150077689	1.3231	7.4543	-2.4134
CHB	HIST2	150082689	0.8818	5.2519	-2.3569
CHB	HIST2	150087689	1.351	6.2684	-2.2648
CHB	HIST2	150092689	1.5734	6.7766	-2.2335
CHB	HIST2	150097689	1.4826	6.2684	-2.2042
CHB	HIST2	150102689	1.6471	7.9625	-2.3403
CHB	HIST2	150107689	1.8193	7.9625	-2.2765
CHB	HIST2	150112689	1.419	6.2684	-2.2335
CHB	HIST2	150117689	1.3644	6.2684	-2.2586
CHB	HIST2	150122689	2.2237	7.6237	-2.0824
CHB	HIST2	150127689	2.64	8.1319	-1.9962
CHB	HIST2	150132689	2.1415	8.3013	-2.1969
CHB	HIST2	150137689	2.0311	9.4872	-2.3503
CHB	HIST2	150142689	2.6756	9.3178	-2.1291

CHB	HIST2	150147689	2.7567	8.4708	-2.0003
CHB	HIST2	150152689	1.9176	8.8096	-2.3269
CHB	HIST2	150157689	2.7124	12.7061	-2.3957
CHB	HIST2	150162689	6.6899	12.5367	-1.4195
CHB	HIST2	150167689	10.2798	9.8261	0.1384
CHB	HIST2	150172689	7.3381	10.1649	-0.8356
CHB	HIST2	150177689	5.1327	9.9955	-1.4602
CHB	HIST2	150182689	7.0729	9.3178	-0.7196
CHB	HIST2	150187689	4.4826	6.7766	-0.9847
CHB	HIST2	150192689	4.0677	6.7766	-1.1628
CHB	HIST2	150197689	8.1986	9.4872	-0.4062
CHB	HIST2	150202689	9.2675	10.1649	-0.2653
CHB	HIST2	150207689	8.6322	10.8426	-0.6152
CHB	HIST2	150212689	8.9313	11.8591	-0.7491
CHB	HIST2	150217689	7.8589	9.9955	-0.6416
CHB	HIST2	150222689	5.5802	8.3013	-0.9705
CHB	HIST2	150227689	2.9787	6.4378	-1.5553
CHB	HIST2	150232689	2.5696	4.4048	-1.1548
CHB	HIST2	150237689	3.3891	4.5742	-0.7216
CHB	HIST2	150242689	3.4205	5.7601	-1.1624
CHB	HIST2	150247689	5.0069	7.2848	-0.9156
CHB	HIST2	150252689	4.7007	7.7931	-1.1688
CHB	HIST2	150257689	7.4337	8.4708	-0.3631
CHB	HIST2	150262689	10.3708	9.9955	0.1127
CHB	HIST2	150267689	12.8469	11.6896	0.3001
CHB	HIST2	150272689	11.7183	11.1814	0.1452
CHB	HIST2	150277689	8.2082	10.3343	-0.6189
CHB	HIST2	150282689	10.3698	12.0285	-0.4187
CHB	HIST2	150287689	10.2166	10.8426	-0.1742
CHB	HIST2	150292689	7.4507	8.6402	-0.4089
CHB	HIST2	150297689	4.8423	7.9625	-1.1562
CHB	HIST2	150302689	5.5199	8.3013	-0.992
YRI	HIST2	149287689	4.1276	4.7057	-0.3422
YRI	HIST2	149292689	4.3613	5.7141	-0.6746
YRI	HIST2	149297689	4.6328	5.5461	-0.4677
YRI	HIST2	149302689	2.7627	4.7057	-1.1502
YRI	HIST2	149307689	4.4592	5.2099	-0.4063
YRI	HIST2	149312689	3.6981	4.2016	-0.3289
YRI	HIST2	149317689	2.1341	2.8571	-0.6538
YRI	HIST2	149322689	3.3407	4.8738	-0.88
YRI	HIST2	149327689	3.4906	6.8906	-1.4327
YRI	HIST2	149332689	6.5221	8.067	-0.5637
YRI	HIST2	149337689	7.7992	10.4199	-0.7544
YRI	HIST2	149342689	6.5663	8.7392	-0.7366
YRI	HIST2	149347689	7.3879	8.9073	-0.506
YRI	HIST2	149352689	6.5304	8.4031	-0.6582
YRI	HIST2	149357689	5.9701	6.5544	-0.2576
YRI	HIST2	149362689	4.8751	7.0586	-0.9001
YRI	HIST2	149367689	3.0712	6.3864	-1.4962

YRI	HIST2	149372689	3.3885	7.3947	-1.5831
YRI	HIST2	149377689	4.0781	7.5628	-1.349
YRI	HIST2	149382689	4.4672	5.7141	-0.6218
YRI	HIST2	149387689	6.215	6.0502	0.0781
YRI	HIST2	149392689	6.7938	7.2267	-0.1747
YRI	HIST2	149397689	5.7216	7.3947	-0.6611
YRI	HIST2	149402689	7.2413	8.9073	-0.5549
YRI	HIST2	149407689	5.7873	8.5712	-0.9607
YRI	HIST2	149412689	5.3418	7.5628	-0.8598
YRI	HIST2	149417689	5.4578	5.7141	-0.1278
YRI	HIST2	149422689	2.9383	3.3612	-0.3341
YRI	HIST2	149427689	3.1793	4.2016	-0.6678
YRI	HIST2	149432689	2.5221	3.3612	-0.6629
YRI	HIST2	149437689	0.5529	1.0084	-0.9081
YRI	HIST2	149442689	0.0185	0.1681	-0.8893
YRI	HIST2	149447689	0.1568	0.5042	-1.0918
YRI	HIST2	149452689	0.4181	1.1764	-1.3563
YRI	HIST2	149457689	0.5682	1.5126	-1.4063
YRI	HIST2	149462689	0.3069	0.8403	-1.2048
YRI	HIST2	149467689	0	0	0
YRI	HIST2	149472689	0	0	0
YRI	HIST2	149477689	0	0	0
YRI	HIST2	149482689	0	0	0
YRI	HIST2	149487689	0	0	0
YRI	HIST2	149492689	0	0	0
YRI	HIST2	149497689	0	0	0
YRI	HIST2	149502689	0	0	0
YRI	HIST2	149507689	0.4018	0.5042	-0.3218
YRI	HIST2	149512689	0.6674	2.0167	-1.6133
YRI	HIST2	149517689	0.4991	2.0167	-1.8145
YRI	HIST2	149522689	0.2335	0.5042	-0.8507
YRI	HIST2	149527689	0.2339	0.5042	-0.8495
YRI	HIST2	149532689	0.2339	0.5042	-0.8495
YRI	HIST2	149537689	0	0	0
YRI	HIST2	149542689	0	0	0
YRI	HIST2	149547689	0	0	0
YRI	HIST2	149552689	0	0	0
YRI	HIST2	149557689	0	0	0
YRI	HIST2	149562689	0	0	0
YRI	HIST2	149567689	0.5133	0.5042	0.0286
YRI	HIST2	149572689	1.1098	1.6806	-0.7853
YRI	HIST2	149577689	0.9289	1.6806	-1.0341
YRI	HIST2	149582689	2.3949	5.0419	-1.4748
YRI	HIST2	149587689	2.5004	5.5461	-1.5597
YRI	HIST2	149592689	1.2767	2.5209	-1.246
YRI	HIST2	149597689	2.5434	3.0251	-0.4155
YRI	HIST2	149602689	5.5452	5.8822	-0.1638
YRI	HIST2	149607689	5.9104	7.2267	-0.5312
YRI	HIST2	149612689	2.6056	3.6974	-0.7958

YRI	HIST2	149617689	0.5357	0.8403	-0.688
YRI	HIST2	149622689	0	0	0
YRI	HIST2	149627689	0.0093	0.1681	-0.944
YRI	HIST2	149632689	0.3282	0.6722	-0.9006
YRI	HIST2	149637689	3.1238	2.1848	1.0543
YRI	HIST2	149642689	4.4903	4.7057	-0.1275
YRI	HIST2	149647689	8.5788	9.2434	-0.2139
YRI	HIST2	149652689	9.4155	10.4199	-0.2891
YRI	HIST2	149657689	5.7279	7.2267	-0.6048
YRI	HIST2	149662689	7.8662	8.067	-0.0733
YRI	HIST2	149667689	11.1589	14.1172	-0.6397
YRI	HIST2	149672689	10.1832	14.1172	-0.8506
YRI	HIST2	149677689	6.5119	9.5795	-0.955
YRI	HIST2	149682689	4.4768	9.0754	-1.5053
YRI	HIST2	149687689	2.4816	8.067	-2.0381
YRI	HIST2	149692689	3.0043	6.5544	-1.5652
YRI	HIST2	149697689	4.7508	7.2267	-0.9991
YRI	HIST2	149702689	8.2867	11.4282	-0.8294
YRI	HIST2	149707689	10.2015	13.613	-0.7636
YRI	HIST2	149712689	7.4268	10.924	-0.9632
YRI	HIST2	149717689	7.7001	11.7644	-1.0441
YRI	HIST2	149722689	8.826	12.6047	-0.9096
YRI	HIST2	149727689	9.2535	12.9408	-0.8658
YRI	HIST2	149732689	7.7238	12.4366	-1.149
YRI	HIST2	149737689	3.838	7.7309	-1.4771
YRI	HIST2	149742689	4.376	6.3864	-0.9074
YRI	HIST2	149747689	6.9734	7.7309	-0.2874
YRI	HIST2	149752689	5.9506	7.3947	-0.5706
YRI	HIST2	149757689	6.3799	9.7476	-1.0317
YRI	HIST2	149762689	6.0755	10.756	-1.308
YRI	HIST2	149767689	3.2155	9.2434	-1.9399
YRI	HIST2	149772689	5.4029	10.924	-1.5206
YRI	HIST2	149777689	6.5576	12.1005	-1.3867
YRI	HIST2	149782689	4.9324	13.2769	-1.9124
YRI	HIST2	149787689	4.1861	11.4282	-1.9119
YRI	HIST2	149792689	2.1767	5.2099	-1.6418
YRI	HIST2	149797689	0.2934	0.8403	-1.2353
YRI	HIST2	149802689	0	0	0
YRI	HIST2	149807689	0	0	0
YRI	HIST2	149812689	2.8529	5.2099	-1.2758
YRI	HIST2	149817689	5.0549	8.5712	-1.2135
YRI	HIST2	149822689	2.6837	3.5293	-0.6411
YRI	HIST2	149827689	0.4817	0.1681	1.8643
YRI	HIST2	149832689	0	0	0
YRI	HIST2	149837689	0.0183	0.1681	-0.8905
YRI	HIST2	149842689	1.4735	3.1932	-1.4183
YRI	HIST2	149847689	4.215	9.5795	-1.6701
YRI	HIST2	149852689	5.1135	13.445	-1.8868
YRI	HIST2	149857689	4.1301	11.9324	-1.9779

YRI	HIST2	149862689	5.5321	11.5963	-1.5792
YRI	HIST2	149867689	7.5245	12.4366	-1.1976
YRI	HIST2	149872689	5.8643	10.756	-1.367
YRI	HIST2	149877689	7.0118	11.2602	-1.1373
YRI	HIST2	149882689	8.5046	11.7644	-0.8375
YRI	HIST2	149887689	5.7786	11.9324	-1.56
YRI	HIST2	149892689	5.2314	12.9408	-1.8103
YRI	HIST2	149897689	4.7192	13.1089	-1.9461
YRI	HIST2	149902689	3.7105	11.2602	-2.0211
YRI	HIST2	149907689	6.6539	11.5963	-1.287
YRI	HIST2	149912689	6.3756	12.1005	-1.4323
YRI	HIST2	149917689	5.752	12.6047	-1.6496
YRI	HIST2	149922689	7.5794	14.1172	-1.4137
YRI	HIST2	149927689	8.7358	13.7811	-1.1162
YRI	HIST2	149932689	9.4913	13.613	-0.9225
YRI	HIST2	149937689	8.7695	12.2685	-0.8641
YRI	HIST2	149942689	5.9611	8.9073	-0.9813
YRI	HIST2	149947689	4.4553	8.235	-1.3533
YRI	HIST2	149952689	8.4325	12.1005	-0.9177
YRI	HIST2	149957689	7.4836	11.2602	-1.011
YRI	HIST2	149962689	4.2266	8.5712	-1.4993
YRI	HIST2	149967689	6.5968	9.7476	-0.9652
YRI	HIST2	149972689	6.8664	9.2434	-0.765
YRI	HIST2	149977689	7.2876	9.7476	-0.7536
YRI	HIST2	149982689	9.4432	12.4366	-0.7298
YRI	HIST2	149987689	9.1385	11.9324	-0.7082
YRI	HIST2	149992689	8.9371	13.2769	-0.9946
YRI	HIST2	149997689	13.5133	15.4617	-0.3864
YRI	HIST2	150002689	13.5325	13.613	-0.018
YRI	HIST2	150007689	11.4365	15.7978	-0.8472
YRI	HIST2	150012689	15.8248	17.9827	-0.3703
YRI	HIST2	150017689	15.4784	15.1256	0.0714
YRI	HIST2	150022689	13.2064	15.1256	-0.3886
YRI	HIST2	150027689	11.8021	14.1172	-0.5006
YRI	HIST2	150032689	8.8688	11.2602	-0.6402
YRI	HIST2	150037689	8.303	12.7727	-1.0626
YRI	HIST2	150042689	11.6426	14.7895	-0.651
YRI	HIST2	150047689	11.6029	14.7895	-0.6592
YRI	HIST2	150052689	7.9602	13.1089	-1.1943
YRI	HIST2	150057689	7.4345	12.6047	-1.2446
YRI	HIST2	150062689	8.7149	13.1089	-1.0193
YRI	HIST2	150067689	8.5175	12.2685	-0.9263
YRI	HIST2	150072689	10.401	14.4534	-0.8569
YRI	HIST2	150077689	12.0651	16.8062	-0.8681
YRI	HIST2	150082689	10.2687	15.1256	-0.9835
YRI	HIST2	150087689	13.0869	15.4617	-0.4709
YRI	HIST2	150092689	14.5781	15.7978	-0.2369
YRI	HIST2	150097689	11.3238	13.9492	-0.5742
YRI	HIST2	150102689	11.5213	15.1256	-0.7298

YRI	HIST2	150107689	11.9195	16.8062	-0.8948
YRI	HIST2	150112689	9.0986	14.6214	-1.155
YRI	HIST2	150117689	7.3768	13.7811	-1.4168
YRI	HIST2	150122689	8.5138	15.2937	-1.3585
YRI	HIST2	150127689	10.358	15.2937	-0.989
YRI	HIST2	150132689	11.0102	15.6298	-0.9066
YRI	HIST2	150137689	12.5614	17.4785	-0.8672
YRI	HIST2	150142689	11.6407	15.9659	-0.8318
YRI	HIST2	150147689	7.9015	11.7644	-0.9924
YRI	HIST2	150152689	10.1963	13.613	-0.7647
YRI	HIST2	150157689	17.2033	19.3272	-0.3401
YRI	HIST2	150162689	16.2985	18.823	-0.4146
YRI	HIST2	150167689	13.4431	16.134	-0.5123
YRI	HIST2	150172689	12.7297	15.7978	-0.596
YRI	HIST2	150177689	11.0689	14.6214	-0.7429
YRI	HIST2	150182689	11.6681	14.1172	-0.5296
YRI	HIST2	150187689	9.2865	12.1005	-0.704
YRI	HIST2	150192689	8.856	11.0921	-0.6071
YRI	HIST2	150197689	10.8715	11.2602	-0.1041
YRI	HIST2	150202689	11.4618	11.2602	0.054
YRI	HIST2	150207689	12.7787	13.613	-0.1867
YRI	HIST2	150212689	13.5415	14.9575	-0.2898
YRI	HIST2	150217689	12.1199	13.2769	-0.2652
YRI	HIST2	150222689	8.8104	10.2518	-0.4213
YRI	HIST2	150227689	4.4688	8.4031	-1.3827
YRI	HIST2	150232689	3.5175	7.7309	-1.5987
YRI	HIST2	150237689	3.6382	6.7225	-1.329
YRI	HIST2	150242689	4.4049	9.2434	-1.5572
YRI	HIST2	150247689	6.5472	11.0921	-1.234
YRI	HIST2	150252689	6.678	10.2518	-1.0445
YRI	HIST2	150257689	10.2802	10.5879	-0.0873
YRI	HIST2	150262689	14.7909	13.1089	0.3902
YRI	HIST2	150267689	18.5391	16.9743	0.2838
YRI	HIST2	150272689	16.8153	16.9743	-0.0288
YRI	HIST2	150277689	13.3488	14.9575	-0.3292
YRI	HIST2	150282689	12.6911	14.4534	-0.3726
YRI	HIST2	150287689	10.99	13.1089	-0.4915
YRI	HIST2	150292689	10.3046	11.9324	-0.4126
YRI	HIST2	150297689	9.3925	12.1005	-0.6775
YRI	HIST2	150302689	10.5637	11.9324	-0.347
CEU	HIST2	149318225	2.3018	2.2173	0.0944
CEU	HIST2	149323225	3.1142	1.7056	1.9292
CEU	HIST2	149328225	3.3034	3.0701	0.2001
CEU	HIST2	149333225	6.0917	6.9931	-0.3772
CEU	HIST2	149338225	7.1942	8.3576	-0.4136
CEU	HIST2	149343225	5.9994	5.6286	0.1886
CEU	HIST2	149348225	6.2315	5.7991	0.2142
CEU	HIST2	149353225	5.6125	6.3108	-0.3206
CEU	HIST2	149358225	5.6349	5.6286	0.0032

CEU	HIST2	149363225	5.699	4.9463	0.4293
CEU	HIST2	149368225	3.5393	3.5818	-0.032
CEU	HIST2	149373225	2.9417	4.7757	-1.0786
CEU	HIST2	149378225	3.5661	5.1169	-0.8584
CEU	HIST2	149383225	4.4593	4.4346	0.0155
CEU	HIST2	149388225	5.8295	5.1169	0.3944
CEU	HIST2	149393225	5.5702	5.458	0.0587
CEU	HIST2	149398225	4.6601	5.6286	-0.4926
CEU	HIST2	149403225	6.0439	6.1402	-0.0453
CEU	HIST2	149408225	5.0012	4.4346	0.3555
CEU	HIST2	149413225	4.7126	3.4112	1.0221
CEU	HIST2	149418225	4.449	3.2407	0.9908
CEU	HIST2	149423225	1.9637	1.8762	0.1114
CEU	HIST2	149428225	2.3816	2.8996	-0.4659
CEU	HIST2	149433225	1.7838	2.2173	-0.4843
CEU	HIST2	149438225	0.0101	0.1706	-0.9534
CEU	HIST2	149443225	0	0	0
CEU	HIST2	149448225	0.08	0.5117	-1.3539
CEU	HIST2	149453225	0.1979	1.0234	-1.6403
CEU	HIST2	149458225	0.128	0.6822	-1.4472
CEU	HIST2	149463225	0.0101	0.1706	-0.9534
CEU	HIST2	149468225	0	0	0
CEU	HIST2	149473225	0	0	0
CEU	HIST2	149478225	0	0	0
CEU	HIST2	149483225	0	0	0
CEU	HIST2	149488225	0	0	0
CEU	HIST2	149493225	0	0	0
CEU	HIST2	149498225	0	0	0
CEU	HIST2	149503225	0	0	0
CEU	HIST2	149508225	0.1363	0.5117	-1.1774
CEU	HIST2	149513225	0.4605	1.1939	-1.3069
CEU	HIST2	149518225	0.3243	0.6822	-0.9346
CEU	HIST2	149523225	0	0	0
CEU	HIST2	149528225	0.0101	0.1706	-0.9534
CEU	HIST2	149533225	0.0101	0.1706	-0.9534
CEU	HIST2	149538225	0	0	0
CEU	HIST2	149543225	0	0	0
CEU	HIST2	149548225	0	0	0
CEU	HIST2	149553225	0	0	0
CEU	HIST2	149558225	0	0	0
CEU	HIST2	149563225	0	0	0
CEU	HIST2	149568225	0.6005	0.3411	1.0399
CEU	HIST2	149573225	0.885	0.6822	0.5296
CEU	HIST2	149578225	0.3939	1.0234	-1.2508
CEU	HIST2	149583225	2.8182	3.2407	-0.3464
CEU	HIST2	149588225	3.6906	3.0701	0.5323
CEU	HIST2	149593225	2.1459	2.0467	0.118
CEU	HIST2	149598225	3.4073	3.2407	0.1366
CEU	HIST2	149603225	6.8606	4.9463	1.0917

CEU	HIST2	149608225	6.5675	4.4346	1.3383
CEU	HIST2	149613225	2.7941	1.7056	1.4908
CEU	HIST2	149618225	0.8439	0.5117	1.0419
CEU	HIST2	149623225	0	0	0
CEU	HIST2	149628225	0.0301	0.1706	-0.8346
CEU	HIST2	149633225	0.0503	0.5117	-1.4471
CEU	HIST2	149638225	2.3997	1.7056	0.9507
CEU	HIST2	149643225	4.7056	3.9229	0.5459
CEU	HIST2	149648225	7.9742	6.8225	0.4928
CEU	HIST2	149653225	8.1868	6.1402	0.9631
CEU	HIST2	149658225	4.5382	3.9229	0.4292
CEU	HIST2	149663225	4.7986	6.1402	-0.6313
CEU	HIST2	149668225	5.228	10.0632	-1.447
CEU	HIST2	149673225	5.4397	9.2104	-1.2253
CEU	HIST2	149678225	4.8332	6.1402	-0.615
CEU	HIST2	149683225	2.4888	4.4346	-1.2209
CEU	HIST2	149688225	0.9285	3.2407	-1.896
CEU	HIST2	149693225	1.362	3.5818	-1.6731
CEU	HIST2	149698225	3.4795	5.7991	-1.1489
CEU	HIST2	149703225	5.2746	6.9931	-0.7191
CEU	HIST2	149708225	7.6066	6.9931	0.2567
CEU	HIST2	149713225	10.7902	8.3576	0.8647
CEU	HIST2	149718225	10.9326	8.187	0.9947
CEU	HIST2	149723225	7.2799	6.3108	0.4449
CEU	HIST2	149728225	6.4432	6.1402	0.1426
CEU	HIST2	149733225	6.1517	5.7991	0.1746
CEU	HIST2	149738225	3.1814	3.7524	-0.4137
CEU	HIST2	149743225	2.2533	2.729	-0.4496
CEU	HIST2	149748225	4.2958	3.5818	0.5381
CEU	HIST2	149753225	3.5908	3.4112	0.1411
CEU	HIST2	149758225	1.8874	3.5818	-1.2771
CEU	HIST2	149763225	2.1659	5.1169	-1.6334
CEU	HIST2	149768225	2.5351	5.2874	-1.4799
CEU	HIST2	149773225	3.7594	6.3108	-1.1714
CEU	HIST2	149778225	5.0441	7.5047	-0.9654
CEU	HIST2	149783225	5.1115	7.8459	-1.0301
CEU	HIST2	149788225	3.2696	6.1402	-1.3508
CEU	HIST2	149793225	1.1981	2.2173	-1.1386
CEU	HIST2	149798225	0	0	0
CEU	HIST2	149803225	0	0	0
CEU	HIST2	149808225	0	0	0
CEU	HIST2	149813225	1.7827	2.3879	-0.6373
CEU	HIST2	149818225	2.7071	4.2641	-1.0107
CEU	HIST2	149823225	1.4236	2.0467	-0.7414
CEU	HIST2	149828225	0.4993	0.1706	1.9525
CEU	HIST2	149833225	0	0	0
CEU	HIST2	149838225	0	0	0
CEU	HIST2	149843225	1.2872	2.2173	-1.0391
CEU	HIST2	149848225	2.3686	4.4346	-1.2963

CEU	HIST2	149853225	2.053	5.1169	-1.6959
CEU	HIST2	149858225	3.0495	6.8225	-1.6145
CEU	HIST2	149863225	4.548	7.8459	-1.2424
CEU	HIST2	149868225	4.9739	8.5281	-1.2401
CEU	HIST2	149873225	4.0303	6.4814	-1.0986
CEU	HIST2	149878225	3.7207	6.1402	-1.1385
CEU	HIST2	149883225	2.7013	5.2874	-1.3906
CEU	HIST2	149888225	2.603	3.5818	-0.7377
CEU	HIST2	149893225	3.8856	5.6286	-0.8866
CEU	HIST2	149898225	3.3659	5.6286	-1.1509
CEU	HIST2	149903225	4.126	5.9697	-0.8898
CEU	HIST2	149908225	3.4735	5.2874	-0.9753
CEU	HIST2	149913225	1.2511	3.5818	-1.7566
CEU	HIST2	149918225	2.2224	4.2641	-1.3254
CEU	HIST2	149923225	3.7423	5.2874	-0.8308
CEU	HIST2	149928225	3.8543	5.7991	-0.9632
CEU	HIST2	149933225	2.45	5.458	-1.5725
CEU	HIST2	149938225	2.0338	4.4346	-1.5063
CEU	HIST2	149943225	2.7784	3.7524	-0.7056
CEU	HIST2	149948225	2.1206	4.0935	-1.3266
CEU	HIST2	149953225	2.8042	5.6286	-1.4367
CEU	HIST2	149958225	2.2919	4.7757	-1.4608
CEU	HIST2	149963225	1.2185	2.729	-1.4277
CEU	HIST2	149968225	2.1701	3.2407	-0.8779
CEU	HIST2	149973225	2.2014	3.9229	-1.2007
CEU	HIST2	149978225	2.2138	4.4346	-1.3934
CEU	HIST2	149983225	3.5459	5.6286	-1.0594
CEU	HIST2	149988225	4.0167	5.7991	-0.8828
CEU	HIST2	149993225	5.4251	7.1636	-0.7117
CEU	HIST2	149998225	9.7349	8.6987	0.355
CEU	HIST2	150003225	8.577	6.6519	0.8428
CEU	HIST2	150008225	7.9959	7.5047	0.1927
CEU	HIST2	150013225	11.0676	9.7221	0.4158
CEU	HIST2	150018225	10.0734	8.3576	0.6099
CEU	HIST2	150023225	8.5648	7.3342	0.4931
CEU	HIST2	150028225	5.368	5.9697	-0.2904
CEU	HIST2	150033225	2.2319	4.4346	-1.382
CEU	HIST2	150038225	2.8551	5.6286	-1.4108
CEU	HIST2	150043225	4.2235	6.6519	-1.0632
CEU	HIST2	150048225	4.3154	5.2874	-0.5226
CEU	HIST2	150053225	2.8961	4.4346	-0.9653
CEU	HIST2	150058225	2.4185	4.6052	-1.3276
CEU	HIST2	150063225	3.2917	5.1169	-1.0103
CEU	HIST2	150068225	1.987	3.2407	-1.028
CEU	HIST2	150073225	5.6081	5.6286	-0.0104
CEU	HIST2	150078225	7.457	8.0164	-0.2066
CEU	HIST2	150083225	3.3999	5.6286	-1.1337
CEU	HIST2	150088225	3.6172	6.9931	-1.4125
CEU	HIST2	150093225	4.3137	6.4814	-0.9716

CEU	HIST2	150098225	3.435	4.4346	-0.6272
CEU	HIST2	150103225	4.5578	6.6519	-0.9168
CEU	HIST2	150108225	6.563	7.3342	-0.309
CEU	HIST2	150113225	5.1983	5.6286	-0.2189
CEU	HIST2	150118225	3.0588	4.9463	-1.0764
CEU	HIST2	150123225	3.6353	6.3108	-1.2284
CEU	HIST2	150128225	5.9878	8.5281	-0.8863
CEU	HIST2	150133225	6.5949	9.2104	-0.8499
CEU	HIST2	150138225	6.6069	11.2571	-1.253
CEU	HIST2	150143225	6.4964	9.3809	-0.9215
CEU	HIST2	150148225	4.8918	6.3108	-0.6515
CEU	HIST2	150153225	3.6945	7.3342	-1.4583
CEU	HIST2	150158225	4.7576	9.2104	-1.4469
CEU	HIST2	150163225	9.1387	9.5515	-0.1297
CEU	HIST2	150168225	10.4315	8.187	0.8131
CEU	HIST2	150173225	7.3634	6.6519	0.3115
CEU	HIST2	150178225	6.643	5.2874	0.7289
CEU	HIST2	150183225	7.6821	6.1402	0.7256
CEU	HIST2	150188225	6.0486	6.4814	-0.194
CEU	HIST2	150193225	5.3088	5.6286	-0.1627
CEU	HIST2	150198225	8.3972	6.4814	0.8587
CEU	HIST2	150203225	9.3395	7.8459	0.5627
CEU	HIST2	150208225	9.1899	8.3576	0.2959
CEU	HIST2	150213225	9.8248	7.3342	0.9979
CEU	HIST2	150218225	8.2554	6.1402	0.9954
CEU	HIST2	150223225	6.1936	5.9697	0.1081
CEU	HIST2	150228225	3.3274	4.4346	-0.6947
CEU	HIST2	150233225	2.6514	3.0701	-0.3592
CEU	HIST2	150238225	3.6852	3.5818	0.0779
CEU	HIST2	150243225	3.3667	4.4346	-0.67
CEU	HIST2	150248225	5.3434	4.7757	0.3339
CEU	HIST2	150253225	6.5502	5.9697	0.2802
CEU	HIST2	150258225	8.7749	7.8459	0.35
CEU	HIST2	150263225	13.2997	8.0164	1.9514
CEU	HIST2	150268225	18.5523	10.5749	2.2793
CEU	HIST2	150273225	16.718	11.7688	1.279
CEU	HIST2	150278225	14.8289	11.0866	1.0229
CEU	HIST2	150283225	15.4127	10.5749	1.3822
CEU	HIST2	150288225	12.5706	8.3576	1.4976
CEU	HIST2	150293225	10.4645	7.8459	0.9865
CEU	HIST2	150298225	6.855	6.4814	0.1675
CEU	HIST2	150303225	8.1971	7.3342	0.3457
CEU	HIST2	150308225	10.564	9.7221	0.2602
CHB	HIST2	149318225	1.9838	2.7106	-0.6887
CHB	HIST2	149323225	2.3238	2.7106	-0.3665
CHB	HIST2	149328225	3.1131	5.9295	-1.3635
CHB	HIST2	149333225	5.8784	8.3013	-0.8641
CHB	HIST2	149338225	6.2412	7.9625	-0.6379
CHB	HIST2	149343225	5.8039	7.1154	-0.5386

CHB	HIST2	149348225	5.6497	7.6237	-0.7612
CHB	HIST2	149353225	4.5307	6.6072	-0.912
CHB	HIST2	149358225	5.1121	4.7436	0.2174
CHB	HIST2	149363225	5.0194	4.4048	0.3867
CHB	HIST2	149368225	2.8193	4.5742	-1.0686
CHB	HIST2	149373225	1.9473	5.0825	-1.7406
CHB	HIST2	149378225	2.6528	4.2354	-1.0303
CHB	HIST2	149383225	3.6038	4.2354	-0.4112
CHB	HIST2	149388225	4.5578	5.9295	-0.6641
CHB	HIST2	149393225	4.3503	5.0825	-0.4065
CHB	HIST2	149398225	3.9874	5.2519	-0.682
CHB	HIST2	149403225	4.9162	6.946	-0.852
CHB	HIST2	149408225	3.5174	4.7436	-0.7233
CHB	HIST2	149413225	4.3154	4.5742	-0.1576
CHB	HIST2	149418225	4.2381	4.913	-0.386
CHB	HIST2	149423225	1.3975	3.2189	-1.4974
CHB	HIST2	149428225	1.9442	3.8965	-1.3655
CHB	HIST2	149433225	1.5755	2.5412	-0.9643
CHB	HIST2	149438225	0.0097	0.1694	-0.949
CHB	HIST2	149443225	0	0	0
CHB	HIST2	149448225	0.0386	0.5082	-1.4741
CHB	HIST2	149453225	0.1162	1.5247	-2.0925
CHB	HIST2	149458225	0.0873	1.1859	-1.961
CHB	HIST2	149463225	0.0097	0.1694	-0.949
CHB	HIST2	149468225	0	0	0
CHB	HIST2	149473225	0	0	0
CHB	HIST2	149478225	0	0	0
CHB	HIST2	149483225	0	0	0
CHB	HIST2	149488225	0	0	0
CHB	HIST2	149493225	0	0	0
CHB	HIST2	149498225	0	0	0
CHB	HIST2	149503225	0	0	0
CHB	HIST2	149508225	0.6372	0.6777	-0.1059
CHB	HIST2	149513225	0.9065	1.1859	-0.4987
CHB	HIST2	149518225	0.2694	0.5082	-0.7496
CHB	HIST2	149523225	0	0	0
CHB	HIST2	149528225	0.2578	0.1694	0.5253
CHB	HIST2	149533225	0.2578	0.1694	0.5253
CHB	HIST2	149538225	0	0	0
CHB	HIST2	149543225	0	0	0
CHB	HIST2	149548225	0	0	0
CHB	HIST2	149553225	0	0	0
CHB	HIST2	149558225	0	0	0
CHB	HIST2	149563225	0	0	0
CHB	HIST2	149568225	0.4891	0.5082	-0.06
CHB	HIST2	149573225	0.9039	0.8471	0.1281
CHB	HIST2	149578225	1.4257	1.1859	0.428
CHB	HIST2	149583225	3.3082	3.3883	-0.0631
CHB	HIST2	149588225	3.0691	3.2189	-0.1232

CHB	HIST2	149593225	2.0525	1.6942	0.4917
CHB	HIST2	149598225	3.8962	2.5412	1.3531
CHB	HIST2	149603225	7.5197	4.5742	1.7936
CHB	HIST2	149608225	6.0982	5.0825	0.5639
CHB	HIST2	149613225	1.7679	2.5412	-0.7722
CHB	HIST2	149618225	0.574	0.5082	0.2066
CHB	HIST2	149623225	0	0	0
CHB	HIST2	149628225	0.0097	0.1694	-0.949
CHB	HIST2	149633225	0.0097	0.1694	-0.949
CHB	HIST2	149638225	2.8808	1.5247	2.0147
CHB	HIST2	149643225	5.534	4.7436	0.4662
CHB	HIST2	149648225	7.8298	7.2848	0.2191
CHB	HIST2	149653225	6.9298	5.9295	0.4843
CHB	HIST2	149658225	3.3567	4.4048	-0.6595
CHB	HIST2	149663225	5.8429	5.0825	0.4222
CHB	HIST2	149668225	8.4805	7.6237	0.3304
CHB	HIST2	149673225	7.5292	8.1319	-0.2191
CHB	HIST2	149678225	4.7999	5.0825	-0.1569
CHB	HIST2	149683225	2.6722	4.7436	-1.2219
CHB	HIST2	149688225	2.3229	4.5742	-1.3709
CHB	HIST2	149693225	3.497	4.4048	-0.5712
CHB	HIST2	149698225	5.2147	4.913	0.1726
CHB	HIST2	149703225	5.8565	6.6072	-0.3297
CHB	HIST2	149708225	6.9815	7.4543	-0.1861
CHB	HIST2	149713225	8.423	7.2848	0.4575
CHB	HIST2	149718225	8.151	8.4708	-0.112
CHB	HIST2	149723225	5.9903	8.1319	-0.7784
CHB	HIST2	149728225	6.3545	8.3013	-0.6943
CHB	HIST2	149733225	5.8551	7.2848	-0.5747
CHB	HIST2	149738225	2.396	4.2354	-1.1975
CHB	HIST2	149743225	1.4142	2.7106	-1.2284
CHB	HIST2	149748225	3.7558	2.7106	0.9904
CHB	HIST2	149753225	3.2829	3.0495	0.2007
CHB	HIST2	149758225	1.2983	6.0989	-2.2661
CHB	HIST2	149763225	1.973	6.2684	-1.9783
CHB	HIST2	149768225	2.1232	4.5742	-1.4925
CHB	HIST2	149773225	1.5264	6.0989	-2.1584
CHB	HIST2	149778225	1.016	5.2519	-2.2846
CHB	HIST2	149783225	0.8184	4.2354	-2.2245
CHB	HIST2	149788225	0.586	5.5907	-2.5535
CHB	HIST2	149793225	0.4026	3.3883	-2.3513
CHB	HIST2	149798225	0	0	0
CHB	HIST2	149803225	0	0	0
CHB	HIST2	149808225	0	0	0
CHB	HIST2	149813225	0.4976	2.3718	-1.9784
CHB	HIST2	149818225	1.276	5.4213	-2.1737
CHB	HIST2	149823225	1.1445	3.2189	-1.7054
CHB	HIST2	149828225	0.3661	0.1694	1.1688
CHB	HIST2	149833225	0	0	0

CHB	HIST2	149838225	0	0	0
CHB	HIST2	149843225	0.3553	2.3718	-2.1286
CHB	HIST2	149848225	0.6599	5.4213	-2.4967
CHB	HIST2	149853225	0.8304	6.6072	-2.5372
CHB	HIST2	149858225	1.5658	6.4378	-2.1906
CHB	HIST2	149863225	1.7519	7.2848	-2.224
CHB	HIST2	149868225	0.9816	6.946	-2.5036
CHB	HIST2	149873225	0.7929	5.0825	-2.3815
CHB	HIST2	149878225	1.6206	6.7766	-2.2133
CHB	HIST2	149883225	1.7603	7.1154	-2.1992
CHB	HIST2	149888225	1.7257	5.7601	-2.0044
CHB	HIST2	149893225	1.5433	7.1154	-2.2883
CHB	HIST2	149898225	0.7034	7.1154	-2.6332
CHB	HIST2	149903225	1.8057	7.1154	-2.1805
CHB	HIST2	149908225	2.5933	7.9625	-1.9897
CHB	HIST2	149913225	1.4991	6.2684	-2.1966
CHB	HIST2	149918225	1.9937	6.946	-2.0788
CHB	HIST2	149923225	3.1725	8.6402	-1.8794
CHB	HIST2	149928225	3.4908	10.1649	-1.9729
CHB	HIST2	149933225	2.1807	10.3343	-2.3734
CHB	HIST2	149938225	2.0284	9.1484	-2.3214
CHB	HIST2	149943225	2.9916	8.4708	-1.9181
CHB	HIST2	149948225	2.1917	6.7766	-1.9681
CHB	HIST2	149953225	1.9785	8.1319	-2.2366
CHB	HIST2	149958225	1.5429	7.9625	-2.3789
CHB	HIST2	149963225	0.9977	6.0989	-2.408
CHB	HIST2	149968225	1.1218	5.5907	-2.2801
CHB	HIST2	149973225	1.3725	4.7436	-1.9885
CHB	HIST2	149978225	1.9992	5.2519	-1.7543
CHB	HIST2	149983225	2.4022	8.4708	-2.1244
CHB	HIST2	149988225	2.2322	9.9955	-2.3312
CHB	HIST2	149993225	3.7422	9.3178	-1.7872
CHB	HIST2	149998225	7.1146	10.8426	-1.0375
CHB	HIST2	150003225	5.8317	9.8261	-1.2187
CHB	HIST2	150008225	2.6309	9.8261	-2.1953
CHB	HIST2	150013225	1.7255	10.3343	-2.5059
CHB	HIST2	150018225	1.7489	8.3013	-2.3369
CHB	HIST2	150023225	1.7805	9.8261	-2.4547
CHB	HIST2	150028225	1.0042	8.3013	-2.6025
CHB	HIST2	150033225	1.0459	5.5907	-2.3189
CHB	HIST2	150038225	2.1462	6.6072	-1.9593
CHB	HIST2	150043225	1.9676	7.1154	-2.114
CHB	HIST2	150048225	0.636	6.7766	-2.6359
CHB	HIST2	150053225	0.9884	5.9295	-2.3921
CHB	HIST2	150058225	1.1485	6.0989	-2.3368
CHB	HIST2	150063225	0.7002	6.0989	-2.5484
CHB	HIST2	150068225	0.6195	5.0825	-2.4778
CHB	HIST2	150073225	1.2414	6.4378	-2.3365
CHB	HIST2	150078225	1.2661	7.2848	-2.4193

CHB	HIST2	150083225	0.9775	5.7601	-2.3761
CHB	HIST2	150088225	1.8403	6.2684	-2.0394
CHB	HIST2	150093225	1.5445	6.6072	-2.2236
CHB	HIST2	150098225	0.9933	6.2684	-2.4295
CHB	HIST2	150103225	1.6092	7.7931	-2.3372
CHB	HIST2	150108225	1.8386	8.1319	-2.2875
CHB	HIST2	150113225	1.4287	6.6072	-2.2745
CHB	HIST2	150118225	1.4759	6.7766	-2.2754
CHB	HIST2	150123225	2.2237	7.6237	-2.0824
CHB	HIST2	150128225	2.5092	7.4543	-1.9465
CHB	HIST2	150133225	2.1606	8.3013	-2.1901
CHB	HIST2	150138225	2.089	10.1649	-2.3873
CHB	HIST2	150143225	2.6663	9.3178	-2.1321
CHB	HIST2	150148225	3.0685	8.1319	-1.8404
CHB	HIST2	150153225	2.0584	8.8096	-2.2794
CHB	HIST2	150158225	2.3716	12.5367	-2.4679
CHB	HIST2	150163225	7.6231	13.045	-1.2678
CHB	HIST2	150168225	10.2798	9.8261	0.1384
CHB	HIST2	150173225	6.2543	9.4872	-1.0191
CHB	HIST2	150178225	5.5844	9.8261	-1.2941
CHB	HIST2	150183225	7.0824	9.3178	-0.7165
CHB	HIST2	150188225	4.0306	6.7766	-1.1788
CHB	HIST2	150193225	4.0195	6.4378	-1.0873
CHB	HIST2	150198225	8.6675	9.6567	-0.3067
CHB	HIST2	150203225	9.2966	10.6731	-0.3888
CHB	HIST2	150208225	8.6404	11.012	-0.6505
CHB	HIST2	150213225	8.9794	12.1979	-0.8019
CHB	HIST2	150218225	7.3725	9.8261	-0.7486
CHB	HIST2	150223225	5.5319	7.7931	-0.8546
CHB	HIST2	150228225	2.9593	6.2684	-1.5241
CHB	HIST2	150233225	2.5791	4.2354	-1.0783
CHB	HIST2	150238225	3.6267	5.0825	-0.8082
CHB	HIST2	150243225	3.5481	6.2684	-1.2529
CHB	HIST2	150248225	5.1123	6.946	-0.7697
CHB	HIST2	150253225	5.5913	8.1319	-0.9234
CHB	HIST2	150258225	7.1003	8.4708	-0.4798
CHB	HIST2	150263225	9.6421	9.1484	0.161
CHB	HIST2	150268225	12.8372	11.5202	0.3463
CHB	HIST2	150273225	11.4002	11.1814	0.0592
CHB	HIST2	150278225	9.6291	11.012	-0.3793
CHB	HIST2	150283225	10.725	12.1979	-0.367
CHB	HIST2	150288225	9.5454	10.6731	-0.3185
CHB	HIST2	150293225	7.1532	8.979	-0.6057
CHB	HIST2	150298225	4.0926	7.4543	-1.3233
CHB	HIST2	150303225	5.9437	8.1319	-0.7954
CHB	HIST2	150308225	7.8534	10.6731	-0.7964
YRI	HIST2	149318225	2.0704	2.5209	-0.4512
YRI	HIST2	149323225	3.3407	4.8738	-0.88
YRI	HIST2	149328225	4.0129	7.3947	-1.3363

YRI	HIST2	149333225	6.6138	8.7392	-0.7205
YRI	HIST2	149338225	7.769	10.0837	-0.687
YRI	HIST2	149343225	6.4838	8.235	-0.627
YRI	HIST2	149348225	7.0928	9.2434	-0.6921
YRI	HIST2	149353225	6.5672	8.7392	-0.7362
YRI	HIST2	149358225	5.7731	6.0502	-0.1313
YRI	HIST2	149363225	5.3881	7.2267	-0.7419
YRI	HIST2	149368225	3.5455	7.2267	-1.4854
YRI	HIST2	149373225	2.9023	7.2267	-1.745
YRI	HIST2	149378225	3.6038	6.7225	-1.3439
YRI	HIST2	149383225	5.2329	5.5461	-0.1604
YRI	HIST2	149388225	6.9174	7.0586	-0.0582
YRI	HIST2	149393225	5.9553	6.8906	-0.3941
YRI	HIST2	149398225	5.1599	6.7225	-0.6733
YRI	HIST2	149403225	7.3933	9.9157	-0.7605
YRI	HIST2	149408225	6.2346	8.7392	-0.849
YRI	HIST2	149413225	5.9953	7.0586	-0.4383
YRI	HIST2	149418225	5.7145	5.5461	0.0862
YRI	HIST2	149423225	2.4603	3.3612	-0.7117
YRI	HIST2	149428225	2.8504	4.3696	-0.9593
YRI	HIST2	149433225	2.1945	2.8571	-0.5992
YRI	HIST2	149438225	0.037	0.5042	-1.4683
YRI	HIST2	149443225	0.0185	0.1681	-0.8893
YRI	HIST2	149448225	0.1661	0.6722	-1.3249
YRI	HIST2	149453225	0.6607	1.5126	-1.2685
YRI	HIST2	149458225	0.5589	1.3445	-1.276
YRI	HIST2	149463225	0.0643	0.5042	-1.3825
YRI	HIST2	149468225	0	0	0
YRI	HIST2	149473225	0	0	0
YRI	HIST2	149478225	0	0	0
YRI	HIST2	149483225	0	0	0
YRI	HIST2	149488225	0	0	0
YRI	HIST2	149493225	0	0	0
YRI	HIST2	149498225	0	0	0
YRI	HIST2	149503225	0	0	0
YRI	HIST2	149508225	0.411	0.6722	-0.6838
YRI	HIST2	149513225	0.8557	2.3529	-1.5849
YRI	HIST2	149518225	0.4899	1.8487	-1.738
YRI	HIST2	149523225	0.0453	0.1681	-0.73
YRI	HIST2	149528225	0.2339	0.5042	-0.8495
YRI	HIST2	149533225	0.2339	0.5042	-0.8495
YRI	HIST2	149538225	0	0	0
YRI	HIST2	149543225	0	0	0
YRI	HIST2	149548225	0	0	0
YRI	HIST2	149553225	0	0	0
YRI	HIST2	149558225	0	0	0
YRI	HIST2	149563225	0	0	0
YRI	HIST2	149568225	0.6355	0.6722	-0.0961
YRI	HIST2	149573225	1.1098	1.6806	-0.7853

YRI	HIST2	149578225	1.0245	2.0167	-1.1863
YRI	HIST2	149583225	2.3949	5.0419	-1.4748
YRI	HIST2	149588225	2.2919	5.2099	-1.5795
YRI	HIST2	149593225	1.2767	2.5209	-1.246
YRI	HIST2	149598225	3.5693	3.5293	0.0303
YRI	HIST2	149603225	6.2413	6.3864	-0.0655
YRI	HIST2	149608225	4.9205	6.7225	-0.7765
YRI	HIST2	149613225	1.9094	3.1932	-1.0588
YRI	HIST2	149618225	0.4905	0.6722	-0.4757
YRI	HIST2	149623225	0	0	0
YRI	HIST2	149628225	0.0093	0.1681	-0.944
YRI	HIST2	149633225	0.3282	0.6722	-0.9006
YRI	HIST2	149638225	3.1238	2.1848	1.0543
YRI	HIST2	149643225	6.1235	5.7141	0.2041
YRI	HIST2	149648225	9.6677	10.4199	-0.2165
YRI	HIST2	149653225	9.2435	10.4199	-0.3386
YRI	HIST2	149658225	5.0448	6.3864	-0.6055
YRI	HIST2	149663225	7.5524	7.7309	-0.0677
YRI	HIST2	149668225	11.5005	14.9575	-0.7075
YRI	HIST2	149673225	9.1905	13.7811	-1.0156
YRI	HIST2	149678225	5.8105	8.9073	-1.0314
YRI	HIST2	149683225	4.3956	9.4115	-1.5875
YRI	HIST2	149688225	2.5513	8.067	-2.0127
YRI	HIST2	149693225	3.0862	6.2183	-1.4479
YRI	HIST2	149698225	6.3608	7.8989	-0.5722
YRI	HIST2	149703225	8.2581	11.5963	-0.8693
YRI	HIST2	149708225	8.6828	13.2769	-1.0529
YRI	HIST2	149713225	8.9771	11.7644	-0.7161
YRI	HIST2	149718225	8.3639	11.5963	-0.8417
YRI	HIST2	149723225	7.1768	11.5963	-1.1509
YRI	HIST2	149728225	8.4011	12.6047	-1.0119
YRI	HIST2	149733225	8.0828	12.4366	-1.0614
YRI	HIST2	149738225	4.6484	8.235	-1.2842
YRI	HIST2	149743225	4.3186	6.2183	-0.8782
YRI	HIST2	149748225	6.322	7.2267	-0.3651
YRI	HIST2	149753225	6.2614	7.8989	-0.6092
YRI	HIST2	149758225	6.3939	10.4199	-1.159
YRI	HIST2	149763225	5.4444	10.2518	-1.4051
YRI	HIST2	149768225	3.1474	8.5712	-1.8718
YRI	HIST2	149773225	5.4939	11.5963	-1.5891
YRI	HIST2	149778225	6.9407	12.9408	-1.4089
YRI	HIST2	149783225	4.8686	12.7727	-1.8791
YRI	HIST2	149788225	3.9274	10.924	-1.927
YRI	HIST2	149793225	2.1402	4.8738	-1.5692
YRI	HIST2	149798225	0.0456	0.1681	-0.7282
YRI	HIST2	149803225	0	0	0
YRI	HIST2	149808225	0	0	0
YRI	HIST2	149813225	2.9777	5.7141	-1.3645
YRI	HIST2	149818225	5.0549	8.5712	-1.2135

YRI	HIST2	149823225	2.5589	3.0251	-0.4022
YRI	HIST2	149828225	0.4817	0.1681	1.8643
YRI	HIST2	149833225	0	0	0
YRI	HIST2	149838225	0.0183	0.1681	-0.8905
YRI	HIST2	149843225	2.537	4.2016	-1.0874
YRI	HIST2	149848225	4.6542	10.5879	-1.6828
YRI	HIST2	149853225	4.0685	12.7727	-2.0693
YRI	HIST2	149858225	3.81	11.7644	-2.0435
YRI	HIST2	149863225	6.3146	11.9324	-1.4241
YRI	HIST2	149868225	7.8463	12.6047	-1.1455
YRI	HIST2	149873225	5.2619	10.924	-1.5594
YRI	HIST2	149878225	7.4703	11.5963	-1.0744
YRI	HIST2	149883225	8.5321	11.4282	-0.7646
YRI	HIST2	149888225	5.4249	11.9324	-1.6496
YRI	HIST2	149893225	5.1862	13.1089	-1.8378
YRI	HIST2	149898225	4.378	12.6047	-1.9804
YRI	HIST2	149903225	5.1857	12.1005	-1.7299
YRI	HIST2	149908225	6.7262	11.0921	-1.1854
YRI	HIST2	149913225	5.1674	11.9324	-1.7149
YRI	HIST2	149918225	6.2594	13.613	-1.6459
YRI	HIST2	149923225	7.2406	13.445	-1.4051
YRI	HIST2	149928225	8.4768	12.7727	-1.0213
YRI	HIST2	149933225	9.6141	13.445	-0.8676
YRI	HIST2	149938225	8.3499	12.2685	-0.9677
YRI	HIST2	149943225	6.3429	9.2434	-0.9335
YRI	HIST2	149948225	4.9525	8.9073	-1.3172
YRI	HIST2	149953225	8.6647	12.6047	-0.9485
YRI	HIST2	149958225	7.2806	10.924	-1.0035
YRI	HIST2	149963225	3.7702	7.7309	-1.5028
YRI	HIST2	149968225	6.4047	9.4115	-0.9516
YRI	HIST2	149973225	6.9166	9.4115	-0.7896
YRI	HIST2	149978225	7.1072	9.5795	-0.7697
YRI	HIST2	149983225	10.3106	12.9408	-0.6176
YRI	HIST2	149988225	9.2029	12.7727	-0.8487
YRI	HIST2	149993225	8.8764	13.1089	-0.9818
YRI	HIST2	149998225	14.1567	15.4617	-0.2588
YRI	HIST2	150003225	13.1306	13.9492	-0.179
YRI	HIST2	150008225	12.5625	16.134	-0.68
YRI	HIST2	150013225	15.4155	17.1423	-0.3103
YRI	HIST2	150018225	14.3006	14.9575	-0.1344
YRI	HIST2	150023225	14.1563	16.134	-0.3765
YRI	HIST2	150028225	11.3176	13.1089	-0.4155
YRI	HIST2	150033225	7.6112	10.5879	-0.8442
YRI	HIST2	150038225	8.9277	13.445	-1.023
YRI	HIST2	150043225	12.7063	14.9575	-0.4607
YRI	HIST2	150048225	11.1573	14.6214	-0.7245
YRI	HIST2	150053225	6.8504	12.6047	-1.3852
YRI	HIST2	150058225	7.4823	12.7727	-1.2577
YRI	HIST2	150063225	9.4442	13.445	-0.9061

YRI	HIST2	150068225	8.429	11.7644	-0.8569
YRI	HIST2	150073225	9.8846	14.1172	-0.9152
YRI	HIST2	150078225	11.9997	17.1423	-0.924
YRI	HIST2	150083225	11.6259	16.134	-0.8583
YRI	HIST2	150088225	14.1893	16.134	-0.3703
YRI	HIST2	150093225	14.225	15.4617	-0.2452
YRI	HIST2	150098225	10.556	13.2769	-0.6236
YRI	HIST2	150103225	10.6994	14.7895	-0.8461
YRI	HIST2	150108225	12.1453	16.8062	-0.8534
YRI	HIST2	150113225	9.628	15.2937	-1.1352
YRI	HIST2	150118225	7.5142	14.6214	-1.4864
YRI	HIST2	150123225	7.9899	14.7895	-1.4066
YRI	HIST2	150128225	10.0548	14.7895	-0.9795
YRI	HIST2	150133225	11.5888	15.7978	-0.8177
YRI	HIST2	150138225	12.7201	17.3104	-0.8171
YRI	HIST2	150143225	11.5205	16.134	-0.8784
YRI	HIST2	150148225	7.9123	11.5963	-0.9593
YRI	HIST2	150153225	10.4622	13.1089	-0.6139
YRI	HIST2	150158225	16.656	19.1591	-0.4042
YRI	HIST2	150163225	16.7745	19.3272	-0.4087
YRI	HIST2	150168225	13.8711	17.1423	-0.5877
YRI	HIST2	150173225	11.5174	15.1256	-0.7306
YRI	HIST2	150178225	10.8839	13.7811	-0.6409
YRI	HIST2	150183225	11.7537	14.4534	-0.5708
YRI	HIST2	150188225	9.2996	11.9324	-0.6674
YRI	HIST2	150193225	8.5916	10.5879	-0.5661
YRI	HIST2	150198225	11.1994	11.2602	-0.0163
YRI	HIST2	150203225	11.9021	11.5963	0.0796
YRI	HIST2	150208225	13.1178	14.1172	-0.2161
YRI	HIST2	150213225	13.4481	14.9575	-0.3089
YRI	HIST2	150218225	11.256	12.7727	-0.3606
YRI	HIST2	150223225	8.6042	10.0837	-0.4391
YRI	HIST2	150228225	4.3385	8.4031	-1.4285
YRI	HIST2	150233225	3.1394	7.0586	-1.6157
YRI	HIST2	150238225	4.1905	7.2267	-1.2252
YRI	HIST2	150243225	4.8219	10.2518	-1.587
YRI	HIST2	150248225	6.1037	10.924	-1.3276
YRI	HIST2	150253225	7.1965	9.9157	-0.8198
YRI	HIST2	150258225	10.1715	10.2518	-0.0235
YRI	HIST2	150263225	14.0328	12.9408	0.2564
YRI	HIST2	150268225	18.5669	17.3104	0.2237
YRI	HIST2	150273225	16.7098	16.6382	0.0132
YRI	HIST2	150278225	13.7164	15.1256	-0.2854
YRI	HIST2	150283225	13.1092	14.6214	-0.3163
YRI	HIST2	150288225	11.1294	12.7727	-0.3907
YRI	HIST2	150293225	10.3933	12.4366	-0.4982
YRI	HIST2	150298225	9.0042	12.1005	-0.7746
YRI	HIST2	150303225	10.8396	11.9324	-0.277
YRI	HIST2	150308225	12.5138	13.9492	-0.3139

CEU	HIST2	149337657	7.1923	8.3576	-0.4142
CEU	HIST2	149342657	6.5479	5.9697	0.279
CEU	HIST2	149347657	6.6485	5.6286	0.5188
CEU	HIST2	149352657	5.6024	6.1402	-0.2531
CEU	HIST2	149357657	5.7143	5.9697	-0.1233
CEU	HIST2	149362657	5.215	5.1169	0.0543
CEU	HIST2	149367657	2.5683	3.2407	-0.5514
CEU	HIST2	149372657	3.4358	4.7757	-0.788
CEU	HIST2	149377657	4.537	5.458	-0.4815
CEU	HIST2	149382657	3.7069	4.0935	-0.26
CEU	HIST2	149387657	5.2144	4.4346	0.4893
CEU	HIST2	149392657	6.3327	5.9697	0.1752
CEU	HIST2	149397657	5.2449	5.7991	-0.2745
CEU	HIST2	149402657	5.9522	5.9697	-0.0084
CEU	HIST2	149407657	4.525	4.6052	-0.0487
CEU	HIST2	149412657	4.3368	3.4112	0.727
CEU	HIST2	149417657	4.1068	3.2407	0.7102
CEU	HIST2	149422657	2.0064	1.8762	0.1658
CEU	HIST2	149427657	2.7172	2.729	-0.0112
CEU	HIST2	149432657	2.2086	2.3879	-0.1888
CEU	HIST2	149437657	0.5232	0.6822	-0.4152
CEU	HIST2	149442657	0	0	0
CEU	HIST2	149447657	0.08	0.5117	-1.3539
CEU	HIST2	149452657	0.1394	0.8528	-1.6066
CEU	HIST2	149457657	0.128	0.6822	-1.4472
CEU	HIST2	149462657	0.0686	0.3411	-1.0924
CEU	HIST2	149467657	0	0	0
CEU	HIST2	149472657	0	0	0
CEU	HIST2	149477657	0	0	0
CEU	HIST2	149482657	0	0	0
CEU	HIST2	149487657	0	0	0
CEU	HIST2	149492657	0	0	0
CEU	HIST2	149497657	0	0	0
CEU	HIST2	149502657	0	0	0
CEU	HIST2	149507657	0.1363	0.5117	-1.1774
CEU	HIST2	149512657	0.4605	1.1939	-1.3069
CEU	HIST2	149517657	0.3243	0.6822	-0.9346
CEU	HIST2	149522657	0	0	0
CEU	HIST2	149527657	0.0101	0.1706	-0.9534
CEU	HIST2	149532657	0.0101	0.1706	-0.9534
CEU	HIST2	149537657	0	0	0
CEU	HIST2	149542657	0	0	0
CEU	HIST2	149547657	0	0	0
CEU	HIST2	149552657	0	0	0
CEU	HIST2	149557657	0	0	0
CEU	HIST2	149562657	0	0	0
CEU	HIST2	149567657	0.5038	0.1706	1.9793
CEU	HIST2	149572657	0.885	0.6822	0.5296
CEU	HIST2	149577657	0.4805	1.0234	-1.0788

CEU	HIST2	149582657	2.8182	3.2407	-0.3464
CEU	HIST2	149587657	3.7007	3.2407	0.3772
CEU	HIST2	149592657	2.1459	2.0467	0.118
CEU	HIST2	149597657	2.4286	2.8996	-0.4236
CEU	HIST2	149602657	5.8841	4.4346	0.9095
CEU	HIST2	149607657	7.5463	4.7757	1.6295
CEU	HIST2	149612657	3.7706	2.2173	1.7353
CEU	HIST2	149617657	0.8439	0.5117	1.0419
CEU	HIST2	149622657	0	0	0
CEU	HIST2	149627657	0.0301	0.1706	-0.8346
CEU	HIST2	149632657	0.0301	0.1706	-0.8346
CEU	HIST2	149637657	2.3997	1.7056	0.9507
CEU	HIST2	149642657	3.7413	3.5818	0.1202
CEU	HIST2	149647657	7.4811	6.6519	0.363
CEU	HIST2	149652657	7.906	6.1402	0.8309
CEU	HIST2	149657657	4.3752	3.5818	0.598
CEU	HIST2	149662657	6.0134	6.3108	-0.1365
CEU	HIST2	149667657	5.4199	9.3809	-1.2654
CEU	HIST2	149672657	5.4801	9.5515	-1.2791
CEU	HIST2	149677657	5.2674	6.9931	-0.7221
CEU	HIST2	149682657	2.4492	4.4346	-1.2457
CEU	HIST2	149687657	0.9485	3.4112	-1.9342
CEU	HIST2	149692657	1.3814	3.4112	-1.5942
CEU	HIST2	149697657	2.8858	5.1169	-1.235
CEU	HIST2	149702657	5.2948	6.9931	-0.7106
CEU	HIST2	149707657	8.1801	7.3342	0.3389
CEU	HIST2	149712657	9.3602	7.8459	0.5705
CEU	HIST2	149717657	10.1014	8.0164	0.7701
CEU	HIST2	149722657	8.6799	6.6519	0.8879
CEU	HIST2	149727657	6.7996	6.6519	0.0647
CEU	HIST2	149732657	5.979	5.6286	0.1782
CEU	HIST2	149737657	3.3105	3.4112	-0.0791
CEU	HIST2	149742657	2.4663	3.4112	-0.7421
CEU	HIST2	149747657	4.6718	4.0935	0.3889
CEU	HIST2	149752657	3.3781	3.0701	0.2642
CEU	HIST2	149757657	1.8277	3.2407	-1.1586
CEU	HIST2	149762657	2.309	5.1169	-1.5542
CEU	HIST2	149767657	2.5848	5.458	-1.502
CEU	HIST2	149772657	3.2146	5.9697	-1.3296
CEU	HIST2	149777657	4.2707	6.4814	-0.9909
CEU	HIST2	149782657	5.437	7.6753	-0.8603
CEU	HIST2	149787657	3.9297	7.1636	-1.3238
CEU	HIST2	149792657	1.4871	3.0701	-1.3579
CEU	HIST2	149797657	0.1235	0.1706	-0.2798
CEU	HIST2	149802657	0	0	0
CEU	HIST2	149807657	0	0	0
CEU	HIST2	149812657	1.5739	2.0467	-0.5626
CEU	HIST2	149817657	2.7071	4.2641	-1.0107
CEU	HIST2	149822657	1.6324	2.3879	-0.7956

CEU	HIST2	149827657	0.4993	0.1706	1.9525
CEU	HIST2	149832657	0	0	0
CEU	HIST2	149837657	0	0	0
CEU	HIST2	149842657	1.0373	1.5351	-0.7381
CEU	HIST2	149847657	2.0711	4.0935	-1.3599
CEU	HIST2	149852657	2.2626	5.2874	-1.6264
CEU	HIST2	149857657	3.1433	6.6519	-1.5361
CEU	HIST2	149862657	4.2778	8.0164	-1.3809
CEU	HIST2	149867657	4.6929	8.3576	-1.3027
CEU	HIST2	149872657	4.3408	6.8225	-1.0619
CEU	HIST2	149877657	3.9984	6.4814	-1.1129
CEU	HIST2	149882657	2.7013	5.2874	-1.3906
CEU	HIST2	149887657	2.3034	3.5818	-0.9635
CEU	HIST2	149892657	3.6869	4.9463	-0.7182
CEU	HIST2	149897657	3.5384	5.6286	-1.0632
CEU	HIST2	149902657	3.7885	5.9697	-1.0527
CEU	HIST2	149907657	3.8075	5.458	-0.8628
CEU	HIST2	149912657	1.6186	3.9229	-1.6072
CEU	HIST2	149917657	1.7209	4.0935	-1.5954
CEU	HIST2	149922657	3.7238	5.2874	-0.8408
CEU	HIST2	149927657	4.1686	5.6286	-0.7426
CEU	HIST2	149932657	2.617	5.458	-1.4852
CEU	HIST2	149937657	2.0439	4.6052	-1.5551
CEU	HIST2	149942657	2.6215	3.7524	-0.8193
CEU	HIST2	149947657	2.2977	4.4346	-1.3408
CEU	HIST2	149952657	2.9812	5.9697	-1.4423
CEU	HIST2	149957657	2.1946	4.4346	-1.4055
CEU	HIST2	149962657	1.1688	2.5584	-1.3843
CEU	HIST2	149967657	2.1791	3.2407	-0.8705
CEU	HIST2	149972657	2.1826	3.9229	-1.2138
CEU	HIST2	149977657	2.2919	4.6052	-1.4045
CEU	HIST2	149982657	3.1297	4.9463	-1.036
CEU	HIST2	149987657	3.8433	5.7991	-0.9687
CEU	HIST2	149992657	5.8895	7.6753	-0.6864
CEU	HIST2	149997657	9.3993	8.5281	0.304
CEU	HIST2	150002657	8.1973	6.8225	0.5883
CEU	HIST2	150007657	7.1431	6.8225	0.1372
CEU	HIST2	150012657	11.1517	9.5515	0.5027
CEU	HIST2	150017657	10.7794	8.8692	0.6428
CEU	HIST2	150022657	8.8606	7.5047	0.532
CEU	HIST2	150027657	6.0136	6.1402	-0.0596
CEU	HIST2	150032657	1.9462	4.2641	-1.5046
CEU	HIST2	150037657	2.7005	5.7991	-1.5347
CEU	HIST2	150042657	4.1932	6.3108	-0.9722
CEU	HIST2	150047657	4.4902	5.458	-0.5059
CEU	HIST2	150052657	3.1923	4.9463	-1.0003
CEU	HIST2	150057657	2.4185	4.6052	-1.3276
CEU	HIST2	150062657	3.0056	4.7757	-1.0411
CEU	HIST2	150067657	1.987	3.2407	-1.028

CEU	HIST2	150072657	5.9037	5.7991	0.0518
CEU	HIST2	150077657	7.4368	7.6753	-0.0917
CEU	HIST2	150082657	3.1187	5.7991	-1.3276
CEU	HIST2	150087657	3.1278	6.9931	-1.6173
CEU	HIST2	150092657	4.008	6.3108	-1.0573
CEU	HIST2	150097657	3.9445	4.7757	-0.4889
CEU	HIST2	150102657	5.1649	6.8225	-0.7093
CEU	HIST2	150107657	6.5529	6.9931	-0.1842
CEU	HIST2	150112657	5.2084	5.6286	-0.2137
CEU	HIST2	150117657	2.9899	4.9463	-1.1157
CEU	HIST2	150122657	3.5481	6.1402	-1.2198
CEU	HIST2	150127657	5.9571	8.187	-0.8078
CEU	HIST2	150132657	6.0233	8.6987	-0.9166
CEU	HIST2	150137657	6.0825	11.5982	-1.4452
CEU	HIST2	150142657	6.8081	9.7221	-0.9005
CEU	HIST2	150147657	5.0326	6.1402	-0.5212
CEU	HIST2	150152657	2.9186	7.1636	-1.7377
CEU	HIST2	150157657	5.2408	9.5515	-1.3542
CEU	HIST2	150162657	9.0115	9.7221	-0.2196
CEU	HIST2	150167657	10.4014	7.8459	0.9627
CEU	HIST2	150172657	8.5935	6.8225	0.7578
CEU	HIST2	150177657	6.1723	5.2874	0.4758
CEU	HIST2	150182657	7.6821	6.1402	0.7256
CEU	HIST2	150187657	6.2696	6.8225	-0.2366
CEU	HIST2	150192657	5.329	5.9697	-0.3092
CEU	HIST2	150197657	8.2754	6.4814	0.8041
CEU	HIST2	150202657	9.3094	7.5047	0.7081
CEU	HIST2	150207657	9.1214	7.8459	0.4805
CEU	HIST2	150212657	9.5529	7.1636	0.9781
CEU	HIST2	150217657	8.7254	6.6519	0.9078
CEU	HIST2	150222657	6.4754	6.1402	0.1577
CEU	HIST2	150227657	3.2977	4.2641	-0.6273
CEU	HIST2	150232657	2.6716	3.4112	-0.5809
CEU	HIST2	150237657	3.665	3.2407	0.3479
CEU	HIST2	150242657	3.0375	4.2641	-0.7962
CEU	HIST2	150247657	4.9192	5.2874	-0.198
CEU	HIST2	150252657	5.5411	5.1169	0.2348
CEU	HIST2	150257657	9.2591	8.0164	0.459
CEU	HIST2	150262657	14.1268	8.6987	1.8596
CEU	HIST2	150267657	18.2255	10.0632	2.4427
CEU	HIST2	150272657	17.2191	11.9394	1.3461
CEU	HIST2	150277657	13.2507	10.7454	0.7052
CEU	HIST2	150282657	14.9365	10.5749	1.2462
CEU	HIST2	150287657	13.2278	8.5281	1.6398
CEU	HIST2	150292657	10.5173	7.5047	1.182
CEU	HIST2	150297657	8.1028	7.1636	0.3845
CEU	HIST2	150302657	7.8524	7.3342	0.2076
CEU	HIST2	150307657	10.5539	9.5515	0.3149
CEU	HIST2	150312657	10.9722	9.7221	0.3863

CEU	HIST2	150317657	13.53	10.4043	0.9067
CEU	HIST2	150322657	13.6643	10.0632	1.0777
CEU	HIST2	150327657	12.6128	9.3809	1.0325
CHB	HIST2	149337657	6.3626	7.9625	-0.5929
CHB	HIST2	149342657	6.1856	7.2848	-0.4418
CHB	HIST2	149347657	6.0312	7.6237	-0.6141
CHB	HIST2	149352657	4.5502	6.7766	-0.9557
CHB	HIST2	149357657	5.151	5.2519	-0.0544
CHB	HIST2	149362657	4.5073	4.066	0.2976
CHB	HIST2	149367657	2.1862	3.7271	-1.1194
CHB	HIST2	149372657	2.4304	5.2519	-1.5217
CHB	HIST2	149377657	3.2859	5.0825	-0.9974
CHB	HIST2	149382657	3.0484	3.7271	-0.4931
CHB	HIST2	149387657	4.0811	5.5907	-0.7702
CHB	HIST2	149392657	4.9347	5.7601	-0.4101
CHB	HIST2	149397657	4.4259	5.4213	-0.522
CHB	HIST2	149402657	4.9065	6.7766	-0.8028
CHB	HIST2	149407657	3.0881	4.7436	-0.9765
CHB	HIST2	149412657	3.8726	4.4048	-0.3349
CHB	HIST2	149417657	4.2707	4.5742	-0.1848
CHB	HIST2	149422657	1.5395	3.0495	-1.2986
CHB	HIST2	149427657	1.9899	3.7271	-1.262
CHB	HIST2	149432657	1.886	3.0495	-1.0006
CHB	HIST2	149437657	0.399	0.8471	-1.0105
CHB	HIST2	149442657	0	0	0
CHB	HIST2	149447657	0.0386	0.5082	-1.4741
CHB	HIST2	149452657	0.1065	1.3553	-2.0239
CHB	HIST2	149457657	0.0873	1.1859	-1.961
CHB	HIST2	149462657	0.0194	0.3388	-1.2812
CHB	HIST2	149467657	0	0	0
CHB	HIST2	149472657	0	0	0
CHB	HIST2	149477657	0	0	0
CHB	HIST2	149482657	0	0	0
CHB	HIST2	149487657	0	0	0
CHB	HIST2	149492657	0	0	0
CHB	HIST2	149497657	0	0	0
CHB	HIST2	149502657	0	0	0
CHB	HIST2	149507657	0.6372	0.6777	-0.1059
CHB	HIST2	149512657	0.8968	1.0165	-0.2382
CHB	HIST2	149517657	0.2694	0.5082	-0.7496
CHB	HIST2	149522657	0.0097	0.1694	-0.949
CHB	HIST2	149527657	0.2578	0.1694	0.5253
CHB	HIST2	149532657	0.2578	0.1694	0.5253
CHB	HIST2	149537657	0	0	0
CHB	HIST2	149542657	0	0	0
CHB	HIST2	149547657	0	0	0
CHB	HIST2	149552657	0	0	0
CHB	HIST2	149557657	0	0	0
CHB	HIST2	149562657	0	0	0

CHB	HIST2	149567657	0.4601	0.3388	0.4866
CHB	HIST2	149572657	0.9039	0.8471	0.1281
CHB	HIST2	149577657	1.095	1.0165	0.1562
CHB	HIST2	149582657	3.3082	3.3883	-0.0631
CHB	HIST2	149587657	3.4287	3.5577	-0.0975
CHB	HIST2	149592657	2.0525	1.6942	0.4917
CHB	HIST2	149597657	2.9082	2.2024	0.7903
CHB	HIST2	149602657	6.534	4.2354	1.4964
CHB	HIST2	149607657	7.0862	5.4213	0.873
CHB	HIST2	149612657	2.7537	2.8801	-0.114
CHB	HIST2	149617657	0.574	0.5082	0.2066
CHB	HIST2	149622657	0	0	0
CHB	HIST2	149627657	0.0097	0.1694	-0.949
CHB	HIST2	149632657	0.0097	0.1694	-0.949
CHB	HIST2	149637657	2.8808	1.5247	2.0147
CHB	HIST2	149642657	4.7697	4.2354	0.3478
CHB	HIST2	149647657	7.5328	6.7766	0.3246
CHB	HIST2	149652657	6.6267	5.4213	0.6321
CHB	HIST2	149657657	3.0921	4.5742	-0.9025
CHB	HIST2	149662657	5.9966	5.4213	0.3017
CHB	HIST2	149667657	8.2781	6.946	0.5592
CHB	HIST2	149672657	8.1593	8.6402	-0.1653
CHB	HIST2	149677657	5.5639	6.0989	-0.2525
CHB	HIST2	149682657	2.9461	4.7436	-1.0603
CHB	HIST2	149687657	2.256	4.2354	-1.2886
CHB	HIST2	149692657	3.1883	4.066	-0.5919
CHB	HIST2	149697657	4.2125	4.5742	-0.2202
CHB	HIST2	149702657	6.1365	6.946	-0.3398
CHB	HIST2	149707657	8.0221	7.9625	0.0221
CHB	HIST2	149712657	7.1081	6.2684	0.3867
CHB	HIST2	149717657	7.4384	8.1319	-0.2521
CHB	HIST2	149722657	7.305	8.8096	-0.508
CHB	HIST2	149727657	6.5926	8.6402	-0.7038
CHB	HIST2	149732657	5.754	7.2848	-0.6153
CHB	HIST2	149737657	2.7881	4.2354	-0.9422
CHB	HIST2	149742657	1.5443	3.0495	-1.2945
CHB	HIST2	149747657	3.8666	2.8801	0.8894
CHB	HIST2	149752657	3.2732	2.8801	0.3544
CHB	HIST2	149757657	1.1856	5.5907	-2.2476
CHB	HIST2	149762657	1.9924	6.6072	-2.0269
CHB	HIST2	149767657	2.1599	4.7436	-1.5241
CHB	HIST2	149772657	1.3202	5.9295	-2.2314
CHB	HIST2	149777657	0.7826	4.913	-2.3626
CHB	HIST2	149782657	1.0246	4.4048	-2.127
CHB	HIST2	149787657	0.744	5.7601	-2.4921
CHB	HIST2	149792657	0.4026	3.3883	-2.3513
CHB	HIST2	149797657	0.1513	0.5082	-1.1204
CHB	HIST2	149802657	0	0	0
CHB	HIST2	149807657	0	0	0

CHB	HIST2	149812657	0.4879	2.2024	-1.9198
CHB	HIST2	149817657	1.276	5.4213	-2.1737
CHB	HIST2	149822657	1.1542	3.3883	-1.7594
CHB	HIST2	149827657	0.3661	0.1694	1.1688
CHB	HIST2	149832657	0	0	0
CHB	HIST2	149837657	0	0	0
CHB	HIST2	149842657	0.2878	1.8636	-2.0105
CHB	HIST2	149847657	0.6405	4.913	-2.4439
CHB	HIST2	149852657	0.8979	7.1154	-2.5533
CHB	HIST2	149857657	1.5464	6.2684	-2.1748
CHB	HIST2	149862657	1.4534	6.6072	-2.2636
CHB	HIST2	149867657	0.9913	7.1154	-2.515
CHB	HIST2	149872657	1.0914	5.7601	-2.3195
CHB	HIST2	149877657	1.6012	6.6072	-2.1987
CHB	HIST2	149882657	1.7603	7.1154	-2.1992
CHB	HIST2	149887657	1.2762	5.7601	-2.2277
CHB	HIST2	149892657	1.5239	6.7766	-2.2548
CHB	HIST2	149897657	1.1626	7.2848	-2.4609
CHB	HIST2	149902657	1.4296	6.946	-2.3156
CHB	HIST2	149907657	2.6127	8.1319	-2.0061
CHB	HIST2	149912657	1.5418	6.2684	-2.1769
CHB	HIST2	149917657	1.4919	6.7766	-2.2685
CHB	HIST2	149922657	3.5157	8.979	-1.8123
CHB	HIST2	149927657	3.9733	10.3343	-1.8516
CHB	HIST2	149932657	2.1807	10.3343	-2.3734
CHB	HIST2	149937657	2.0573	9.3178	-2.3273
CHB	HIST2	149942657	2.9528	7.9625	-1.8564
CHB	HIST2	149947657	2.0617	6.2684	-1.9375
CHB	HIST2	149952657	1.9882	8.1319	-2.2331
CHB	HIST2	149957657	1.6149	7.7931	-2.335
CHB	HIST2	149962657	0.9977	6.0989	-2.408
CHB	HIST2	149967657	1.1896	6.4378	-2.3598
CHB	HIST2	149972657	1.4017	5.2519	-2.0765
CHB	HIST2	149977657	1.9513	4.913	-1.6941
CHB	HIST2	149982657	2.2138	7.7931	-2.1087
CHB	HIST2	149987657	2.2511	9.9955	-2.3255
CHB	HIST2	149992657	3.9113	9.6567	-1.7815
CHB	HIST2	149997657	6.7367	10.5037	-1.08
CHB	HIST2	150002657	5.34	9.9955	-1.398
CHB	HIST2	150007657	2.8742	9.4872	-2.0846
CHB	HIST2	150012657	2.2269	10.5037	-2.3729
CHB	HIST2	150017657	1.5117	8.8096	-2.464
CHB	HIST2	150022657	1.732	8.8096	-2.3896
CHB	HIST2	150027657	1.3954	8.6402	-2.4903
CHB	HIST2	150032657	1.1041	6.4378	-2.3982
CHB	HIST2	150037657	1.8289	6.6072	-2.0987
CHB	HIST2	150042657	1.9001	6.7766	-2.0933
CHB	HIST2	150047657	0.9341	6.7766	-2.508
CHB	HIST2	150052657	1.0657	6.6072	-2.4339

CHB	HIST2	150057657	1.1677	6.0989	-2.3277
CHB	HIST2	150062657	0.6227	5.0825	-2.476
CHB	HIST2	150067657	0.5625	4.913	-2.4885
CHB	HIST2	150072657	1.3189	7.4543	-2.4151
CHB	HIST2	150077657	1.3231	7.4543	-2.4134
CHB	HIST2	150082657	0.8818	5.2519	-2.3569
CHB	HIST2	150087657	1.351	6.2684	-2.2648
CHB	HIST2	150092657	1.5734	6.7766	-2.2335
CHB	HIST2	150097657	1.4826	6.2684	-2.2042
CHB	HIST2	150102657	1.6471	7.9625	-2.3403
CHB	HIST2	150107657	1.8193	7.9625	-2.2765
CHB	HIST2	150112657	1.419	6.2684	-2.2335
CHB	HIST2	150117657	1.3644	6.2684	-2.2586
CHB	HIST2	150122657	2.2237	7.6237	-2.0824
CHB	HIST2	150127657	2.6016	7.9625	-1.9866
CHB	HIST2	150132657	2.1415	8.3013	-2.1969
CHB	HIST2	150137657	2.0696	9.6567	-2.3526
CHB	HIST2	150142657	2.6756	9.3178	-2.1291
CHB	HIST2	150147657	2.7567	8.4708	-2.0003
CHB	HIST2	150152657	1.9176	8.8096	-2.3269
CHB	HIST2	150157657	2.7124	12.7061	-2.3957
CHB	HIST2	150162657	6.6802	12.3673	-1.3986
CHB	HIST2	150167657	10.2798	9.8261	0.1384
CHB	HIST2	150172657	7.3478	10.3343	-0.8693
CHB	HIST2	150177657	5.1327	9.9955	-1.4602
CHB	HIST2	150182657	7.0729	9.3178	-0.7196
CHB	HIST2	150187657	4.4826	6.7766	-0.9847
CHB	HIST2	150192657	4.0677	6.7766	-1.1628
CHB	HIST2	150197657	8.1889	9.3178	-0.3619
CHB	HIST2	150202657	9.2675	10.1649	-0.2653
CHB	HIST2	150207657	8.6419	11.012	-0.6501
CHB	HIST2	150212657	8.9313	11.8591	-0.7491
CHB	HIST2	150217657	7.8589	9.9955	-0.6416
CHB	HIST2	150222657	5.5802	8.3013	-0.9705
CHB	HIST2	150227657	2.9787	6.4378	-1.5553
CHB	HIST2	150232657	2.5696	4.4048	-1.1548
CHB	HIST2	150237657	3.3891	4.5742	-0.7216
CHB	HIST2	150242657	3.4205	5.7601	-1.1624
CHB	HIST2	150247657	5.0069	7.2848	-0.9156
CHB	HIST2	150252657	4.7007	7.7931	-1.1688
CHB	HIST2	150257657	7.4337	8.4708	-0.3631
CHB	HIST2	150262657	10.3708	9.9955	0.1127
CHB	HIST2	150267657	12.8469	11.6896	0.3001
CHB	HIST2	150272657	11.7183	11.1814	0.1452
CHB	HIST2	150277657	8.2082	10.3343	-0.6189
CHB	HIST2	150282657	10.3698	12.0285	-0.4187
CHB	HIST2	150287657	10.2166	10.8426	-0.1742
CHB	HIST2	150292657	7.4507	8.6402	-0.4089
CHB	HIST2	150297657	4.8423	7.9625	-1.1562

CHB	HIST2	150302657	5.5199	8.3013	-0.992
CHB	HIST2	150307657	7.8342	10.6731	-0.8018
CHB	HIST2	150312657	7.9126	9.6567	-0.5408
CHB	HIST2	150317657	9.4946	10.1649	-0.1981
CHB	HIST2	150322657	8.8245	11.3508	-0.6735
CHB	HIST2	150327657	8.6257	11.5202	-0.761
YRI	HIST2	149337657	7.79	10.2518	-0.7195
YRI	HIST2	149342657	7.0609	8.9073	-0.615
YRI	HIST2	149347657	7.3971	9.0754	-0.5494
YRI	HIST2	149352657	6.5304	8.4031	-0.6582
YRI	HIST2	149357657	5.9701	6.5544	-0.2576
YRI	HIST2	149362657	4.8751	7.0586	-0.9001
YRI	HIST2	149367657	3.0712	6.3864	-1.4962
YRI	HIST2	149372657	3.3885	7.3947	-1.5831
YRI	HIST2	149377657	4.0781	7.5628	-1.349
YRI	HIST2	149382657	4.4672	5.7141	-0.6218
YRI	HIST2	149387657	6.215	6.0502	0.0781
YRI	HIST2	149392657	6.7938	7.2267	-0.1747
YRI	HIST2	149397657	5.7216	7.3947	-0.6611
YRI	HIST2	149402657	7.2413	8.9073	-0.5549
YRI	HIST2	149407657	5.7873	8.5712	-0.9607
YRI	HIST2	149412657	5.3418	7.5628	-0.8598
YRI	HIST2	149417657	5.4578	5.7141	-0.1278
YRI	HIST2	149422657	2.9383	3.3612	-0.3341
YRI	HIST2	149427657	3.1793	4.2016	-0.6678
YRI	HIST2	149432657	2.5221	3.3612	-0.6629
YRI	HIST2	149437657	0.5529	1.0084	-0.9081
YRI	HIST2	149442657	0.0185	0.1681	-0.8893
YRI	HIST2	149447657	0.1568	0.5042	-1.0918
YRI	HIST2	149452657	0.4181	1.1764	-1.3563
YRI	HIST2	149457657	0.5682	1.5126	-1.4063
YRI	HIST2	149462657	0.3069	0.8403	-1.2048
YRI	HIST2	149467657	0	0	0
YRI	HIST2	149472657	0	0	0
YRI	HIST2	149477657	0	0	0
YRI	HIST2	149482657	0	0	0
YRI	HIST2	149487657	0	0	0
YRI	HIST2	149492657	0	0	0
YRI	HIST2	149497657	0	0	0
YRI	HIST2	149502657	0	0	0
YRI	HIST2	149507657	0.4018	0.5042	-0.3218
YRI	HIST2	149512657	0.6674	2.0167	-1.6133
YRI	HIST2	149517657	0.4991	2.0167	-1.8145
YRI	HIST2	149522657	0.2335	0.5042	-0.8507
YRI	HIST2	149527657	0.2339	0.5042	-0.8495
YRI	HIST2	149532657	0.2339	0.5042	-0.8495
YRI	HIST2	149537657	0	0	0
YRI	HIST2	149542657	0	0	0
YRI	HIST2	149547657	0	0	0

YRI	HIST2	149552657	0	0	0
YRI	HIST2	149557657	0	0	0
YRI	HIST2	149562657	0	0	0
YRI	HIST2	149567657	0.5133	0.5042	0.0286
YRI	HIST2	149572657	1.1098	1.6806	-0.7853
YRI	HIST2	149577657	0.9289	1.6806	-1.0341
YRI	HIST2	149582657	2.3949	5.0419	-1.4748
YRI	HIST2	149587657	2.5004	5.5461	-1.5597
YRI	HIST2	149592657	1.2767	2.5209	-1.246
YRI	HIST2	149597657	2.5434	3.0251	-0.4155
YRI	HIST2	149602657	5.5452	5.8822	-0.1638
YRI	HIST2	149607657	5.9012	7.0586	-0.4771
YRI	HIST2	149612657	2.6056	3.6974	-0.7958
YRI	HIST2	149617657	0.545	1.0084	-0.9239
YRI	HIST2	149622657	0	0	0
YRI	HIST2	149627657	0.0093	0.1681	-0.944
YRI	HIST2	149632657	0.3282	0.6722	-0.9006
YRI	HIST2	149637657	3.1238	2.1848	1.0543
YRI	HIST2	149642657	4.4903	4.7057	-0.1275
YRI	HIST2	149647657	8.5788	9.2434	-0.2139
YRI	HIST2	149652657	9.4155	10.4199	-0.2891
YRI	HIST2	149657657	5.7279	7.2267	-0.6048
YRI	HIST2	149662657	7.8662	8.067	-0.0733
YRI	HIST2	149667657	11.1589	14.1172	-0.6397
YRI	HIST2	149672657	10.1832	14.1172	-0.8506
YRI	HIST2	149677657	6.4663	9.4115	-0.9321
YRI	HIST2	149682657	4.4768	9.0754	-1.5053
YRI	HIST2	149687657	2.5272	8.235	-2.0437
YRI	HIST2	149692657	3.0043	6.5544	-1.5652
YRI	HIST2	149697657	4.7508	7.2267	-0.9991
YRI	HIST2	149702657	8.2867	11.4282	-0.8294
YRI	HIST2	149707657	10.0844	13.2769	-0.7317
YRI	HIST2	149712657	7.4268	10.924	-0.9632
YRI	HIST2	149717657	7.8172	12.1005	-1.0716
YRI	HIST2	149722657	8.7893	12.4366	-0.8892
YRI	HIST2	149727657	8.7538	12.7727	-0.9554
YRI	HIST2	149732657	7.7605	12.6047	-1.1661
YRI	HIST2	149737657	4.3377	7.8989	-1.3248
YRI	HIST2	149742657	4.376	6.3864	-0.9074
YRI	HIST2	149747657	6.9642	7.5628	-0.2317
YRI	HIST2	149752657	5.9506	7.3947	-0.5706
YRI	HIST2	149757657	6.3891	9.9157	-1.0633
YRI	HIST2	149762657	6.0755	10.756	-1.308
YRI	HIST2	149767657	3.2155	9.2434	-1.9399
YRI	HIST2	149772657	5.4029	10.924	-1.5206
YRI	HIST2	149777657	6.5304	11.9324	-1.3694
YRI	HIST2	149782657	4.9324	13.2769	-1.9124
YRI	HIST2	149787657	4.2133	11.5963	-1.9226
YRI	HIST2	149792657	2.1767	5.2099	-1.6418

YRI	HIST2	149797657	0.2934	0.8403	-1.2353
YRI	HIST2	149802657	0	0	0
YRI	HIST2	149807657	0	0	0
YRI	HIST2	149812657	2.8529	5.2099	-1.2758
YRI	HIST2	149817657	5.0549	8.5712	-1.2135
YRI	HIST2	149822657	2.6837	3.5293	-0.6411
YRI	HIST2	149827657	0.4817	0.1681	1.8643
YRI	HIST2	149832657	0	0	0
YRI	HIST2	149837657	0.0183	0.1681	-0.8905
YRI	HIST2	149842657	1.4735	3.1932	-1.4183
YRI	HIST2	149847657	4.215	9.5795	-1.6701
YRI	HIST2	149852657	5.1135	13.445	-1.8868
YRI	HIST2	149857657	4.0582	11.7644	-1.9798
YRI	HIST2	149862657	5.5321	11.5963	-1.5792
YRI	HIST2	149867657	7.5965	12.6047	-1.2056
YRI	HIST2	149872657	5.9272	10.924	-1.3762
YRI	HIST2	149877657	7.0118	11.2602	-1.1373
YRI	HIST2	149882657	8.3506	11.5963	-0.8452
YRI	HIST2	149887657	5.7786	11.9324	-1.56
YRI	HIST2	149892657	5.3854	13.1089	-1.7916
YRI	HIST2	149897657	4.7192	13.1089	-1.9461
YRI	HIST2	149902657	3.7105	11.2602	-2.0211
YRI	HIST2	149907657	6.6539	11.5963	-1.287
YRI	HIST2	149912657	6.3756	12.1005	-1.4323
YRI	HIST2	149917657	5.7244	12.4366	-1.6364
YRI	HIST2	149922657	7.5794	14.1172	-1.4137
YRI	HIST2	149927657	8.7634	13.9492	-1.1341
YRI	HIST2	149932657	9.4913	13.613	-0.9225
YRI	HIST2	149937657	8.733	12.1005	-0.8425
YRI	HIST2	149942657	5.9611	8.9073	-0.9813
YRI	HIST2	149947657	4.4918	8.4031	-1.3747
YRI	HIST2	149952657	8.3324	11.7644	-0.8817
YRI	HIST2	149957657	7.4743	11.0921	-0.9823
YRI	HIST2	149962657	4.3268	8.9073	-1.5256
YRI	HIST2	149967657	6.6061	9.9157	-0.9978
YRI	HIST2	149972657	6.8664	9.2434	-0.765
YRI	HIST2	149977657	7.2876	9.7476	-0.7536
YRI	HIST2	149982657	9.3457	12.2685	-0.7218
YRI	HIST2	149987657	9.1385	11.9324	-0.7082
YRI	HIST2	149992657	9.0346	13.445	-0.9988
YRI	HIST2	149997657	13.5133	15.4617	-0.3864
YRI	HIST2	150002657	13.5325	13.613	-0.018
YRI	HIST2	150007657	11.3554	15.4617	-0.8142
YRI	HIST2	150012657	15.8248	17.9827	-0.3703
YRI	HIST2	150017657	15.5594	15.4617	0.0194
YRI	HIST2	150022657	13.2064	15.1256	-0.3886
YRI	HIST2	150027657	11.992	14.1172	-0.4595
YRI	HIST2	150032657	8.8688	11.2602	-0.6402
YRI	HIST2	150037657	8.3122	12.9408	-1.0869

YRI	HIST2	150042657	11.6243	14.6214	-0.6268
YRI	HIST2	150047657	11.6029	14.7895	-0.6592
YRI	HIST2	150052657	7.9785	13.2769	-1.2143
YRI	HIST2	150057657	7.4345	12.6047	-1.2446
YRI	HIST2	150062657	8.7149	13.1089	-1.0193
YRI	HIST2	150067657	8.5175	12.2685	-0.9263
YRI	HIST2	150072657	10.2018	14.2853	-0.8731
YRI	HIST2	150077657	12.0651	16.8062	-0.8681
YRI	HIST2	150082657	10.2687	15.1256	-0.9835
YRI	HIST2	150087657	13.0869	15.4617	-0.4709
YRI	HIST2	150092657	14.5688	15.6298	-0.2082
YRI	HIST2	150097657	11.3238	13.9492	-0.5742
YRI	HIST2	150102657	11.5305	15.2937	-0.754
YRI	HIST2	150107657	11.9195	16.8062	-0.8948
YRI	HIST2	150112657	9.0986	14.6214	-1.155
YRI	HIST2	150117657	7.3768	13.7811	-1.4168
YRI	HIST2	150122657	8.5138	15.2937	-1.3585
YRI	HIST2	150127657	9.9576	15.1256	-1.0465
YRI	HIST2	150132657	11.0102	15.6298	-0.9066
YRI	HIST2	150137657	12.9618	17.6465	-0.8186
YRI	HIST2	150142657	11.6407	15.9659	-0.8318
YRI	HIST2	150147657	7.9015	11.7644	-0.9924
YRI	HIST2	150152657	10.1871	13.445	-0.7378
YRI	HIST2	150157657	17.2033	19.3272	-0.3401
YRI	HIST2	150162657	16.2272	18.823	-0.4263
YRI	HIST2	150167657	13.4431	16.134	-0.5123
YRI	HIST2	150172657	12.8102	15.9659	-0.6069
YRI	HIST2	150177657	11.0689	14.6214	-0.7429
YRI	HIST2	150182657	11.6681	14.1172	-0.5296
YRI	HIST2	150187657	9.2865	12.1005	-0.704
YRI	HIST2	150192657	8.856	11.0921	-0.6071
YRI	HIST2	150197657	10.6518	11.0921	-0.1195
YRI	HIST2	150202657	11.4618	11.2602	0.054
YRI	HIST2	150207657	12.9984	13.7811	-0.1732
YRI	HIST2	150212657	13.5415	14.9575	-0.2898
YRI	HIST2	150217657	12.1199	13.2769	-0.2652
YRI	HIST2	150222657	8.8104	10.2518	-0.4213
YRI	HIST2	150227657	4.4688	8.4031	-1.3827
YRI	HIST2	150232657	3.5175	7.7309	-1.5987
YRI	HIST2	150237657	3.6382	6.7225	-1.329
YRI	HIST2	150242657	4.4049	9.2434	-1.5572
YRI	HIST2	150247657	6.5472	11.0921	-1.234
YRI	HIST2	150252657	6.678	10.2518	-1.0445
YRI	HIST2	150257657	10.2802	10.5879	-0.0873
YRI	HIST2	150262657	14.7909	13.1089	0.3902
YRI	HIST2	150267657	18.5391	16.9743	0.2838
YRI	HIST2	150272657	16.8153	16.9743	-0.0288
YRI	HIST2	150277657	13.3488	14.9575	-0.3292
YRI	HIST2	150282657	12.6911	14.4534	-0.3726

YRI	HIST2	150287657	10.99	13.1089	-0.4915
YRI	HIST2	150292657	10.3046	11.9324	-0.4126
YRI	HIST2	150297657	9.3925	12.1005	-0.6775
YRI	HIST2	150302657	10.5637	11.9324	-0.347
YRI	HIST2	150307657	12.6786	14.4534	-0.3753
YRI	HIST2	150312657	12.748	13.7811	-0.2286
YRI	HIST2	150317657	15.2769	13.9492	0.2904
YRI	HIST2	150322657	13.47	15.1256	-0.3352
YRI	HIST2	150327657	11.6829	14.1172	-0.5264
CEU	HIST2	149345687	4.6832	5.1169	-0.2401
CEU	HIST2	149350687	6.5281	5.9697	0.2695
CEU	HIST2	149355687	7.3962	6.3108	0.4983
CEU	HIST2	149360687	5.355	6.1402	-0.3695
CEU	HIST2	149365687	3.6021	4.6052	-0.609
CEU	HIST2	149370687	3.1136	3.2407	-0.1042
CEU	HIST2	149375687	4.8237	5.7991	-0.4831
CEU	HIST2	149380687	4.1051	5.1169	-0.56
CEU	HIST2	149385687	4.4073	3.9229	0.3379
CEU	HIST2	149390687	7.1274	5.9697	0.5587
CEU	HIST2	149395687	4.1111	4.9463	-0.4763
CEU	HIST2	149400687	5.7411	6.3108	-0.2616
CEU	HIST2	149405687	6.1134	5.6286	0.2466
CEU	HIST2	149410687	3.8971	3.2407	0.5382
CEU	HIST2	149415687	4.2911	3.4112	0.6911
CEU	HIST2	149420687	2.5547	2.0467	0.6045
CEU	HIST2	149425687	2.7926	2.729	0.0601
CEU	HIST2	149430687	2.3717	2.8996	-0.4748
CEU	HIST2	149435687	0.8755	1.0234	-0.2939
CEU	HIST2	149440687	0	0	0
CEU	HIST2	149445687	0.08	0.5117	-1.3539
CEU	HIST2	149450687	0.1394	0.8528	-1.6066
CEU	HIST2	149455687	0.128	0.6822	-1.4472
CEU	HIST2	149460687	0.0686	0.3411	-1.0924
CEU	HIST2	149465687	0	0	0
CEU	HIST2	149470687	0	0	0
CEU	HIST2	149475687	0	0	0
CEU	HIST2	149480687	0	0	0
CEU	HIST2	149485687	0	0	0
CEU	HIST2	149490687	0	0	0
CEU	HIST2	149495687	0	0	0
CEU	HIST2	149500687	0	0	0
CEU	HIST2	149505687	0.1161	0.3411	-0.902
CEU	HIST2	149510687	0.4605	1.1939	-1.3069
CEU	HIST2	149515687	0.3445	0.8528	-1.1447
CEU	HIST2	149520687	0	0	0
CEU	HIST2	149525687	0	0	0
CEU	HIST2	149530687	0.0101	0.1706	-0.9534
CEU	HIST2	149535687	0.0101	0.1706	-0.9534
CEU	HIST2	149540687	0	0	0

CEU	HIST2	149545687	0	0	0
CEU	HIST2	149550687	0	0	0
CEU	HIST2	149555687	0	0	0
CEU	HIST2	149560687	0	0	0
CEU	HIST2	149565687	0	0	0
CEU	HIST2	149570687	0.6005	0.3411	1.0399
CEU	HIST2	149575687	0.885	0.6822	0.5296
CEU	HIST2	149580687	2.026	2.729	-0.6645
CEU	HIST2	149585687	3.8	3.7524	0.0345
CEU	HIST2	149590687	2.8122	2.2173	0.6646
CEU	HIST2	149595687	1.5782	2.5584	-0.9764
CEU	HIST2	149600687	3.3799	3.2407	0.1141
CEU	HIST2	149605687	6.5329	4.0935	1.6403
CEU	HIST2	149610687	6.6852	4.0935	1.7427
CEU	HIST2	149615687	2.7077	1.5351	1.7387
CEU	HIST2	149620687	0	0	0
CEU	HIST2	149625687	0	0	0
CEU	HIST2	149630687	0.0301	0.1706	-0.8346
CEU	HIST2	149635687	0.0503	0.5117	-1.4471
CEU	HIST2	149640687	2.8157	2.0467	0.915
CEU	HIST2	149645687	6.906	5.9697	0.4519
CEU	HIST2	149650687	8.1224	6.9931	0.4725
CEU	HIST2	149655687	6.993	4.7757	1.3041
CEU	HIST2	149660687	6.1709	6.1402	0.0144
CEU	HIST2	149665687	5.0033	8.6987	-1.266
CEU	HIST2	149670687	5.1422	8.5281	-1.1814
CEU	HIST2	149675687	5.5077	7.3342	-0.7318
CEU	HIST2	149680687	2.8672	5.6286	-1.4046
CEU	HIST2	149685687	1.3911	3.7524	-1.7107
CEU	HIST2	149690687	0.8437	3.2407	-1.9655
CEU	HIST2	149695687	2.7873	4.9463	-1.2313
CEU	HIST2	149700687	4.9128	6.3108	-0.6418
CEU	HIST2	149705687	6.2715	6.8225	-0.2358
CEU	HIST2	149710687	7.2794	7.1636	0.0474
CEU	HIST2	149715687	9.5118	8.0164	0.5523
CEU	HIST2	149720687	11.0752	7.8459	1.2165
CEU	HIST2	149725687	7.7065	6.3108	0.6408
CEU	HIST2	149730687	5.8685	5.7991	0.0344
CEU	HIST2	149735687	4.9041	4.7757	0.0755
CEU	HIST2	149740687	2.8264	3.2407	-0.3397
CEU	HIST2	149745687	4.7582	3.9229	0.5826
CEU	HIST2	149750687	4.0045	3.7524	0.1826
CEU	HIST2	149755687	0.7846	2.2173	-1.6006
CEU	HIST2	149760687	2.4591	5.1169	-1.4711
CEU	HIST2	149765687	2.2538	5.458	-1.6751
CEU	HIST2	149770687	3.0021	5.1169	-1.1706
CEU	HIST2	149775687	4.8458	7.1636	-0.9488
CEU	HIST2	149780687	5.0361	7.8459	-1.0585
CEU	HIST2	149785687	4.4255	7.3342	-1.1654

CEU	HIST2	149790687	2.1509	4.0935	-1.3063
CEU	HIST2	149795687	0.5071	1.0234	-1.0259
CEU	HIST2	149800687	0	0	0
CEU	HIST2	149805687	0	0	0
CEU	HIST2	149810687	0.8023	1.0234	-0.4393
CEU	HIST2	149815687	2.4158	3.4112	-0.7818
CEU	HIST2	149820687	2.404	3.4112	-0.7911
CEU	HIST2	149825687	0.7906	1.0234	-0.4626
CEU	HIST2	149830687	0	0	0
CEU	HIST2	149835687	0	0	0
CEU	HIST2	149840687	0.3923	0.5117	-0.3745
CEU	HIST2	149845687	1.78	3.2407	-1.1977
CEU	HIST2	149850687	2.4545	5.458	-1.5702
CEU	HIST2	149855687	1.8877	5.458	-1.8665
CEU	HIST2	149860687	3.3262	6.3108	-1.3703
CEU	HIST2	149865687	5.3475	8.8692	-1.1851
CEU	HIST2	149870687	5.1039	8.5281	-1.1947
CEU	HIST2	149875687	3.7314	6.4814	-1.2326
CEU	HIST2	149880687	3.0142	5.458	-1.2776
CEU	HIST2	149885687	2.5149	3.7524	-0.8965
CEU	HIST2	149890687	3.1824	4.2641	-0.7022
CEU	HIST2	149895687	4.2787	6.3108	-0.933
CEU	HIST2	149900687	3.2051	5.7991	-1.2848
CEU	HIST2	149905687	3.2783	4.9463	-0.9513
CEU	HIST2	149910687	2.9746	4.7757	-1.0593
CEU	HIST2	149915687	1.8485	4.4346	-1.6226
CEU	HIST2	149920687	3.0089	4.9463	-1.1049
CEU	HIST2	149925687	4.3426	5.2874	-0.508
CEU	HIST2	149930687	3.2931	5.7991	-1.2412
CEU	HIST2	149935687	1.8936	4.9463	-1.741
CEU	HIST2	149940687	2.3709	3.9229	-1.0825
CEU	HIST2	149945687	2.3931	4.2641	-1.2145
CEU	HIST2	149950687	2.7341	5.6286	-1.4723
CEU	HIST2	149955687	2.4113	4.7757	-1.3906
CEU	HIST2	149960687	1.2548	2.729	-1.3934
CEU	HIST2	149965687	1.5325	3.0701	-1.3189
CEU	HIST2	149970687	2.5166	4.2641	-1.1344
CEU	HIST2	149975687	2.4527	4.2641	-1.1759
CEU	HIST2	149980687	2.2981	4.2641	-1.2762
CEU	HIST2	149985687	3.2799	5.458	-1.1387
CEU	HIST2	149990687	5.4222	6.4814	-0.4747
CEU	HIST2	149995687	7.8707	8.0164	-0.0538
CEU	HIST2	150000687	9.3315	8.187	0.4146
CEU	HIST2	150005687	8.1569	6.6519	0.6589
CEU	HIST2	150010687	10.0975	9.0398	0.3497
CEU	HIST2	150015687	11.7246	10.4043	0.383
CEU	HIST2	150020687	8.189	7.1636	0.4197
CEU	HIST2	150025687	5.9483	5.9697	-0.0103
CEU	HIST2	150030687	3.5411	5.458	-1.0021

CEU	HIST2	150035687	2.6452	4.9463	-1.3123
CEU	HIST2	150040687	3.5801	5.6286	-1.042
CEU	HIST2	150045687	5.0503	6.8225	-0.7583
CEU	HIST2	150050687	3.9221	5.2874	-0.7341
CEU	HIST2	150055687	2.5551	4.2641	-1.1094
CEU	HIST2	150060687	3.0258	5.1169	-1.1575
CEU	HIST2	150065687	1.9971	3.7524	-1.2717
CEU	HIST2	150070687	4.2647	4.6052	-0.2067
CEU	HIST2	150075687	6.9497	6.6519	0.1304
CEU	HIST2	150080687	4.2303	5.6286	-0.7113
CEU	HIST2	150085687	3.2529	6.9931	-1.565
CEU	HIST2	150090687	4.2043	7.1636	-1.2114
CEU	HIST2	150095687	3.9647	5.1169	-0.6378
CEU	HIST2	150100687	4.0254	5.7991	-0.8785
CEU	HIST2	150105687	5.6617	6.8225	-0.4967
CEU	HIST2	150110687	6.3793	6.4814	-0.0458
CEU	HIST2	150115687	4.2647	5.1169	-0.4717
CEU	HIST2	150120687	3.5875	5.9697	-1.1497
CEU	HIST2	150125687	3.9645	6.9931	-1.2672
CEU	HIST2	150130687	6.5492	9.2104	-0.8647
CEU	HIST2	150135687	7.5696	11.0866	-0.9614
CEU	HIST2	150140687	5.6677	9.3809	-1.1862
CEU	HIST2	150145687	5.093	7.5047	-0.9462
CEU	HIST2	150150687	4.0992	7.3342	-1.2962
CEU	HIST2	150155687	5.4238	9.2104	-1.2304
CEU	HIST2	150160687	6.4822	9.2104	-0.8865
CEU	HIST2	150165687	9.0563	7.8459	0.456
CEU	HIST2	150170687	9.5343	6.8225	1.1604
CEU	HIST2	150175687	6.3542	5.9697	0.1856
CEU	HIST2	150180687	8.0293	6.3108	0.789
CEU	HIST2	150185687	7.8027	6.6519	0.5038
CEU	HIST2	150190687	5.6038	6.3108	-0.3246
CEU	HIST2	150195687	6.0748	5.9697	0.0507
CEU	HIST2	150200687	8.4333	6.8225	0.6893
CEU	HIST2	150205687	9.4444	7.6753	0.68
CEU	HIST2	150210687	9.356	7.6753	0.646
CEU	HIST2	150215687	8.6153	6.8225	0.7671
CEU	HIST2	150220687	7.7638	6.3108	0.6671
CEU	HIST2	150225687	4.9643	5.2874	-0.1737
CEU	HIST2	150230687	2.3593	3.7524	-1.0093
CEU	HIST2	150235687	2.919	2.729	0.1796
CEU	HIST2	150240687	3.0823	3.9229	-0.5863
CEU	HIST2	150245687	5.0921	5.7991	-0.3502
CEU	HIST2	150250687	5.711	5.1169	0.3288
CEU	HIST2	150255687	6.4828	5.9697	0.2476
CEU	HIST2	150260687	13.1734	8.8692	1.4484
CEU	HIST2	150265687	18.7497	10.4043	2.4209
CEU	HIST2	150270687	15.7213	10.916	1.3328
CEU	HIST2	150275687	13.4298	10.916	0.6972

CEU	HIST2	150280687	17.3247	11.5982	1.5004
CEU	HIST2	150285687	13.5617	8.6987	1.666
CEU	HIST2	150290687	9.0524	6.3108	1.2587
CEU	HIST2	150295687	9.6066	7.8459	0.6633
CEU	HIST2	150300687	8.1362	7.6753	0.1772
CEU	HIST2	150305687	9.2167	8.5281	0.2403
CEU	HIST2	150310687	10.6481	9.3809	0.4048
CEU	HIST2	150315687	11.8046	10.2337	0.4628
CEU	HIST2	150320687	14.108	10.4043	1.0744
CEU	HIST2	150325687	14.2341	9.7221	1.3943
CEU	HIST2	150330687	12.7087	9.2104	1.1368
CEU	HIST2	150335687	11.0529	9.2104	0.5987
CHB	HIST2	149345687	4.5724	7.1154	-1.0443
CHB	HIST2	149350687	5.6311	7.2848	-0.6647
CHB	HIST2	149355687	6.0879	6.0989	-0.0052
CHB	HIST2	149360687	4.6375	4.2354	0.2618
CHB	HIST2	149365687	3.221	3.5577	-0.2545
CHB	HIST2	149370687	2.7124	4.5742	-1.1337
CHB	HIST2	149375687	3.3482	5.9295	-1.2496
CHB	HIST2	149380687	3.0503	4.5742	-0.9279
CHB	HIST2	149385687	3.5802	4.913	-0.7624
CHB	HIST2	149390687	5.4818	6.2684	-0.3623
CHB	HIST2	149395687	3.443	4.913	-0.8408
CHB	HIST2	149400687	4.6943	5.9295	-0.598
CHB	HIST2	149405687	4.6316	5.7601	-0.5607
CHB	HIST2	149410687	3.5648	4.913	-0.7712
CHB	HIST2	149415687	4.3147	4.2354	0.0516
CHB	HIST2	149420687	2.0497	3.0495	-0.8598
CHB	HIST2	149425687	1.641	3.3883	-1.376
CHB	HIST2	149430687	1.9248	3.5577	-1.2341
CHB	HIST2	149435687	1.051	2.033	-1.171
CHB	HIST2	149440687	0	0	0
CHB	HIST2	149445687	0.0386	0.5082	-1.4741
CHB	HIST2	149450687	0.0774	1.0165	-1.8689
CHB	HIST2	149455687	0.0873	1.1859	-1.961
CHB	HIST2	149460687	0.0485	0.6777	-1.6449
CHB	HIST2	149465687	0	0	0
CHB	HIST2	149470687	0	0	0
CHB	HIST2	149475687	0	0	0
CHB	HIST2	149480687	0	0	0
CHB	HIST2	149485687	0	0	0
CHB	HIST2	149490687	0	0	0
CHB	HIST2	149495687	0	0	0
CHB	HIST2	149500687	0	0	0
CHB	HIST2	149505687	0.3379	0.3388	-0.0036
CHB	HIST2	149510687	0.8968	1.0165	-0.2382
CHB	HIST2	149515687	0.5686	0.8471	-0.628
CHB	HIST2	149520687	0.0097	0.1694	-0.949
CHB	HIST2	149525687	0	0	0

CHB	HIST2	149530687	0.2578	0.1694	0.5253
CHB	HIST2	149535687	0.2578	0.1694	0.5253
CHB	HIST2	149540687	0	0	0
CHB	HIST2	149545687	0	0	0
CHB	HIST2	149550687	0	0	0
CHB	HIST2	149555687	0	0	0
CHB	HIST2	149560687	0	0	0
CHB	HIST2	149565687	0.0097	0.1694	-0.949
CHB	HIST2	149570687	0.4891	0.5082	-0.06
CHB	HIST2	149575687	0.9039	0.8471	0.1281
CHB	HIST2	149580687	2.7101	3.0495	-0.2919
CHB	HIST2	149585687	4.0702	3.8965	0.1215
CHB	HIST2	149590687	2.2336	1.8636	0.4721
CHB	HIST2	149595687	2.0855	1.8636	0.2831
CHB	HIST2	149600687	4.4603	2.5412	1.9164
CHB	HIST2	149605687	6.8024	4.7436	1.2144
CHB	HIST2	149610687	5.6592	5.0825	0.3202
CHB	HIST2	149615687	1.6806	1.5247	0.2316
CHB	HIST2	149620687	0	0	0
CHB	HIST2	149625687	0	0	0
CHB	HIST2	149630687	0.0097	0.1694	-0.949
CHB	HIST2	149635687	0.0769	0.6777	-1.5707
CHB	HIST2	149640687	3.3649	2.3718	1.0483
CHB	HIST2	149645687	7.6918	5.9295	0.8532
CHB	HIST2	149650687	7.8726	6.946	0.3889
CHB	HIST2	149655687	5.2022	5.7601	-0.2772
CHB	HIST2	149660687	5.0558	5.0825	-0.0148
CHB	HIST2	149665687	7.7653	5.5907	1.1095
CHB	HIST2	149670687	8.0883	7.9625	0.0466
CHB	HIST2	149675687	6.3802	7.2848	-0.3636
CHB	HIST2	149680687	3.6421	5.2519	-0.8682
CHB	HIST2	149685687	2.1883	4.5742	-1.4528
CHB	HIST2	149690687	2.3844	4.2354	-1.205
CHB	HIST2	149695687	4.4445	4.5742	-0.079
CHB	HIST2	149700687	6.0059	6.2684	-0.1209
CHB	HIST2	149705687	6.3994	6.6072	-0.0913
CHB	HIST2	149710687	6.15	5.5907	0.2854
CHB	HIST2	149715687	7.6463	7.7931	-0.0555
CHB	HIST2	149720687	8.6215	9.6567	-0.321
CHB	HIST2	149725687	6.38	9.1484	-0.9026
CHB	HIST2	149730687	5.727	7.2848	-0.6262
CHB	HIST2	149735687	4.4731	5.5907	-0.5702
CHB	HIST2	149740687	2.0782	3.3883	-1.0317
CHB	HIST2	149745687	3.8763	3.0495	0.7111
CHB	HIST2	149750687	3.6467	3.2189	0.3517
CHB	HIST2	149755687	0.5134	3.5577	-2.3007
CHB	HIST2	149760687	2.1626	7.1154	-2.034
CHB	HIST2	149765687	1.8871	5.2519	-1.8147
CHB	HIST2	149770687	1.2717	4.913	-2.0828

CHB	HIST2	149775687	1.5793	5.9295	-2.106
CHB	HIST2	149780687	1.044	4.913	-2.2131
CHB	HIST2	149785687	0.8346	5.5907	-2.4267
CHB	HIST2	149790687	0.4318	3.8965	-2.4233
CHB	HIST2	149795687	0.2187	1.3553	-1.8421
CHB	HIST2	149800687	0	0	0
CHB	HIST2	149805687	0	0	0
CHB	HIST2	149810687	0.3718	0.8471	-1.0718
CHB	HIST2	149815687	1.1116	3.7271	-1.9001
CHB	HIST2	149820687	1.2703	4.7436	-2.0488
CHB	HIST2	149825687	0.5306	1.8636	-1.7007
CHB	HIST2	149830687	0	0	0
CHB	HIST2	149835687	0	0	0
CHB	HIST2	149840687	0.0192	0.1694	-0.8925
CHB	HIST2	149845687	0.4614	3.5577	-2.34
CHB	HIST2	149850687	1.0985	7.6237	-2.5163
CHB	HIST2	149855687	1.0963	6.6072	-2.4204
CHB	HIST2	149860687	1.3382	5.9295	-2.2227
CHB	HIST2	149865687	1.5333	7.1154	-2.2924
CHB	HIST2	149870687	0.9307	6.6072	-2.4932
CHB	HIST2	149875687	1.3126	5.7601	-2.2096
CHB	HIST2	149880687	1.6813	6.6072	-2.1635
CHB	HIST2	149885687	1.1645	6.4378	-2.371
CHB	HIST2	149890687	1.7339	5.7601	-2.0003
CHB	HIST2	149895687	1.6018	7.9625	-2.3571
CHB	HIST2	149900687	1.0234	7.2848	-2.5169
CHB	HIST2	149905687	1.7475	6.4378	-2.1089
CHB	HIST2	149910687	2.1027	7.7931	-2.1507
CHB	HIST2	149915687	1.8938	6.6072	-2.0702
CHB	HIST2	149920687	2.7419	8.4708	-2.0055
CHB	HIST2	149925687	3.7428	10.5037	-1.9383
CHB	HIST2	149930687	2.8961	9.4872	-2.0777
CHB	HIST2	149935687	2.3034	10.1649	-2.324
CHB	HIST2	149940687	2.5589	8.6402	-2.0904
CHB	HIST2	149945687	2.2937	6.2684	-1.8306
CHB	HIST2	149950687	2.0594	8.1319	-2.2072
CHB	HIST2	149955687	1.5694	8.1319	-2.3853
CHB	HIST2	149960687	1.34	6.0989	-2.2464
CHB	HIST2	149965687	1.0556	6.2684	-2.4008
CHB	HIST2	149970687	1.3541	5.5907	-2.1616
CHB	HIST2	149975687	1.9322	5.0825	-1.749
CHB	HIST2	149980687	1.5441	6.7766	-2.2461
CHB	HIST2	149985687	2.2104	8.6402	-2.2102
CHB	HIST2	149990687	3.5818	9.1484	-1.8149
CHB	HIST2	149995687	6.1018	10.5037	-1.262
CHB	HIST2	150000687	6.3783	11.8591	-1.4022
CHB	HIST2	150005687	3.3267	9.6567	-1.9628
CHB	HIST2	150010687	2.1784	9.9955	-2.3473
CHB	HIST2	150015687	2.0027	9.8261	-2.3869

CHB	HIST2	150020687	1.6358	7.9625	-2.3445
CHB	HIST2	150025687	1.5127	8.8096	-2.4636
CHB	HIST2	150030687	0.8881	6.946	-2.5429
CHB	HIST2	150035687	1.1696	5.7601	-2.2807
CHB	HIST2	150040687	2.1565	6.946	-2.0104
CHB	HIST2	150045687	1.6029	7.4543	-2.3033
CHB	HIST2	150050687	1.0029	6.6072	-2.4615
CHB	HIST2	150055687	1.0727	5.5907	-2.3052
CHB	HIST2	150060687	0.8376	5.7601	-2.4456
CHB	HIST2	150065687	0.6575	5.4213	-2.498
CHB	HIST2	150070687	1.1749	6.7766	-2.4046
CHB	HIST2	150075687	1.3139	7.6237	-2.4332
CHB	HIST2	150080687	0.6514	5.2519	-2.4812
CHB	HIST2	150085687	1.284	6.0989	-2.2728
CHB	HIST2	150090687	1.8519	6.7766	-2.114
CHB	HIST2	150095687	1.4727	6.0989	-2.1838
CHB	HIST2	150100687	1.4289	7.1154	-2.3353
CHB	HIST2	150105687	1.8089	7.4543	-2.2222
CHB	HIST2	150110687	1.3745	7.1154	-2.3576
CHB	HIST2	150115687	1.0927	6.7766	-2.4399
CHB	HIST2	150120687	1.8646	7.4543	-2.2003
CHB	HIST2	150125687	2.1251	7.7931	-2.1422
CHB	HIST2	150130687	2.8204	8.8096	-2.0221
CHB	HIST2	150135687	2.7987	9.1484	-2.0702
CHB	HIST2	150140687	2.1439	9.1484	-2.2837
CHB	HIST2	150145687	2.355	8.6402	-2.1605
CHB	HIST2	150150687	2.4295	8.3013	-2.0942
CHB	HIST2	150155687	3.0849	12.1979	-2.2704
CHB	HIST2	150160687	4.1354	12.3673	-2.0244
CHB	HIST2	150165687	8.7834	10.5037	-0.4932
CHB	HIST2	150170687	8.739	9.9955	-0.3773
CHB	HIST2	150175687	5.2113	9.6567	-1.3784
CHB	HIST2	150180687	6.8188	9.6567	-0.88
CHB	HIST2	150185687	6.084	8.4708	-0.8356
CHB	HIST2	150190687	4.4631	7.2848	-1.1342
CHB	HIST2	150195687	5.8497	6.946	-0.4602
CHB	HIST2	150200687	8.5654	9.4872	-0.2906
CHB	HIST2	150205687	9.3992	10.8426	-0.4017
CHB	HIST2	150210687	8.6789	11.1814	-0.6767
CHB	HIST2	150215687	7.9109	11.012	-0.8506
CHB	HIST2	150220687	6.8538	8.6402	-0.6141
CHB	HIST2	150225687	4.09	7.6237	-1.3627
CHB	HIST2	150230687	2.1388	5.7601	-1.7991
CHB	HIST2	150235687	2.7594	3.8965	-0.7953
CHB	HIST2	150240687	3.8892	5.0825	-0.6625
CHB	HIST2	150245687	5.4599	7.1154	-0.6799
CHB	HIST2	150250687	4.8018	7.9625	-1.1713
CHB	HIST2	150255687	4.8943	7.9625	-1.137
CHB	HIST2	150260687	9.53	9.4872	0.0135

CHB	HIST2	150265687	13.7822	11.1814	0.7032
CHB	HIST2	150270687	10.9701	10.5037	0.1337
CHB	HIST2	150275687	8.6457	10.3343	-0.4915
CHB	HIST2	150280687	11.9424	13.2144	-0.2938
CHB	HIST2	150285687	9.4067	10.6731	-0.3577
CHB	HIST2	150290687	6.5976	8.3013	-0.6076
CHB	HIST2	150295687	6.5739	9.3178	-0.8795
CHB	HIST2	150300687	5.6868	8.4708	-0.9746
CHB	HIST2	150305687	7.1887	10.1649	-0.8798
CHB	HIST2	150310687	7.1759	9.3178	-0.6866
CHB	HIST2	150315687	7.3663	8.979	-0.535
CHB	HIST2	150320687	10.0327	11.3508	-0.3514
CHB	HIST2	150325687	10.672	12.3673	-0.4169
CHB	HIST2	150330687	8.5642	11.5202	-0.7772
CHB	HIST2	150335687	6.9526	8.4708	-0.5315
YRI	HIST2	149345687	5.5964	8.235	-0.9448
YRI	HIST2	149350687	7.6952	9.0754	-0.4518
YRI	HIST2	149355687	7.8101	7.2267	0.2354
YRI	HIST2	149360687	5.4838	6.8906	-0.5928
YRI	HIST2	149365687	3.6761	7.3947	-1.4694
YRI	HIST2	149370687	3.0298	6.0502	-1.4311
YRI	HIST2	149375687	4.6279	8.235	-1.2915
YRI	HIST2	149380687	4.0569	7.0586	-1.2375
YRI	HIST2	149385687	5.5465	5.2099	0.1822
YRI	HIST2	149390687	7.9415	7.5628	0.1466
YRI	HIST2	149395687	5.1813	7.2267	-0.8254
YRI	HIST2	149400687	6.5498	8.4031	-0.6514
YRI	HIST2	149405687	6.9294	9.0754	-0.7025
YRI	HIST2	149410687	5.6369	8.4031	-0.9722
YRI	HIST2	149415687	5.0759	5.8822	-0.3918
YRI	HIST2	149420687	3.4411	3.0251	0.3589
YRI	HIST2	149425687	3.4028	4.0335	-0.4267
YRI	HIST2	149430687	2.5774	4.0335	-0.9852
YRI	HIST2	149435687	1.1793	1.8487	-0.8562
YRI	HIST2	149440687	0.0278	0.3361	-1.2377
YRI	HIST2	149445687	0.1568	0.5042	-1.0918
YRI	HIST2	149450687	0.2572	1.0084	-1.4977
YRI	HIST2	149455687	0.5682	1.5126	-1.4063
YRI	HIST2	149460687	0.4678	1.0084	-1.0778
YRI	HIST2	149465687	0	0	0
YRI	HIST2	149470687	0	0	0
YRI	HIST2	149475687	0	0	0
YRI	HIST2	149480687	0	0	0
YRI	HIST2	149485687	0	0	0
YRI	HIST2	149490687	0	0	0
YRI	HIST2	149495687	0	0	0
YRI	HIST2	149500687	0	0	0
YRI	HIST2	149505687	0.3128	0.3361	-0.0935
YRI	HIST2	149510687	0.6674	2.0167	-1.6133

YRI	HIST2	149515687	0.5881	2.1848	-1.7927
YRI	HIST2	149520687	0.2335	0.5042	-0.8507
YRI	HIST2	149525687	0.073	0.3361	-1.0562
YRI	HIST2	149530687	0.2339	0.5042	-0.8495
YRI	HIST2	149535687	0.1609	0.1681	-0.0428
YRI	HIST2	149540687	0	0	0
YRI	HIST2	149545687	0	0	0
YRI	HIST2	149550687	0	0	0
YRI	HIST2	149555687	0	0	0
YRI	HIST2	149560687	0	0	0
YRI	HIST2	149565687	0.0278	0.3361	-1.2377
YRI	HIST2	149570687	0.6448	0.8403	-0.4416
YRI	HIST2	149575687	1.082	1.3445	-0.4263
YRI	HIST2	149580687	2.3976	5.2099	-1.5222
YRI	HIST2	149585687	2.8328	6.0502	-1.5244
YRI	HIST2	149590687	1.2306	2.5209	-1.2922
YRI	HIST2	149595687	1.8514	2.5209	-0.6705
YRI	HIST2	149600687	3.8506	3.8654	-0.0104
YRI	HIST2	149605687	5.3659	6.0502	-0.3242
YRI	HIST2	149610687	4.8085	6.3864	-0.7122
YRI	HIST2	149615687	1.7722	2.5209	-0.7498
YRI	HIST2	149620687	0	0	0
YRI	HIST2	149625687	0	0	0
YRI	HIST2	149630687	0.0093	0.1681	-0.944
YRI	HIST2	149635687	0.3558	0.8403	-1.0943
YRI	HIST2	149640687	3.6547	2.8571	0.7213
YRI	HIST2	149645687	8.4209	8.067	0.1291
YRI	HIST2	149650687	9.9648	11.2602	-0.3468
YRI	HIST2	149655687	8.3394	8.7392	-0.1355
YRI	HIST2	149660687	7.1161	8.067	-0.347
YRI	HIST2	149665687	9.7576	11.7644	-0.5156
YRI	HIST2	149670687	10.5671	14.1172	-0.7676
YRI	HIST2	149675687	7.2322	11.0921	-1.048
YRI	HIST2	149680687	4.6278	9.2434	-1.4854
YRI	HIST2	149685687	3.2238	9.0754	-1.9155
YRI	HIST2	149690687	2.5511	6.3864	-1.731
YRI	HIST2	149695687	4.3393	7.0586	-1.121
YRI	HIST2	149700687	7.644	10.4199	-0.7991
YRI	HIST2	149705687	8.8619	11.7644	-0.7457
YRI	HIST2	149710687	7.1213	10.756	-1.0157
YRI	HIST2	149715687	8.7912	11.5963	-0.7305
YRI	HIST2	149720687	9.7433	13.2769	-0.8098
YRI	HIST2	149725687	7.8402	13.7811	-1.3143
YRI	HIST2	149730687	7.4415	12.7727	-1.2674
YRI	HIST2	149735687	6.0543	8.7392	-0.9101
YRI	HIST2	149740687	4.2073	6.0502	-0.8732
YRI	HIST2	149745687	6.7209	7.2267	-0.2041
YRI	HIST2	149750687	7.0974	8.5712	-0.5086
YRI	HIST2	149755687	4.927	8.067	-1.1458

YRI	HIST2	149760687	6.5569	10.924	-1.2028
YRI	HIST2	149765687	3.9928	9.2434	-1.6898
YRI	HIST2	149770687	4.6881	9.4115	-1.4949
YRI	HIST2	149775687	6.6059	13.9492	-1.606
YRI	HIST2	149780687	5.4091	13.9492	-1.8677
YRI	HIST2	149785687	4.6848	11.7644	-1.8188
YRI	HIST2	149790687	2.5421	6.7225	-1.8013
YRI	HIST2	149795687	0.794	2.1848	-1.5616
YRI	HIST2	149800687	0	0	0
YRI	HIST2	149805687	0	0	0
YRI	HIST2	149810687	1.771	2.0167	-0.2938
YRI	HIST2	149815687	4.0118	6.8906	-1.213
YRI	HIST2	149820687	3.7656	6.7225	-1.2741
YRI	HIST2	149825687	1.5248	1.8487	-0.4143
YRI	HIST2	149830687	0	0	0
YRI	HIST2	149835687	0	0	0
YRI	HIST2	149840687	0.3604	0.8403	-1.0839
YRI	HIST2	149845687	3.0805	6.7225	-1.5693
YRI	HIST2	149850687	5.8194	14.1172	-1.7942
YRI	HIST2	149855687	3.9958	11.9324	-2.0119
YRI	HIST2	149860687	3.8139	10.4199	-1.9017
YRI	HIST2	149865687	7.9343	13.2769	-1.2244
YRI	HIST2	149870687	7.8361	12.6047	-1.1479
YRI	HIST2	149875687	5.392	11.4282	-1.5936
YRI	HIST2	149880687	7.045	11.0921	-1.0988
YRI	HIST2	149885687	7.3283	11.7644	-1.1397
YRI	HIST2	149890687	5.8105	12.2685	-1.5948
YRI	HIST2	149895687	5.1829	12.6047	-1.7866
YRI	HIST2	149900687	3.6505	11.7644	-2.0845
YRI	HIST2	149905687	5.0059	11.0921	-1.6524
YRI	HIST2	149910687	7.2868	12.4366	-1.2555
YRI	HIST2	149915687	5.952	12.7727	-1.6215
YRI	HIST2	149920687	6.3749	12.9408	-1.5418
YRI	HIST2	149925687	8.9958	14.1172	-1.1074
YRI	HIST2	149930687	9.4189	13.9492	-0.9908
YRI	HIST2	149935687	8.8358	12.4366	-0.8779
YRI	HIST2	149940687	6.8566	10.756	-1.0897
YRI	HIST2	149945687	4.9945	8.9073	-1.3032
YRI	HIST2	149950687	7.1591	10.2518	-0.9039
YRI	HIST2	149955687	8.1103	11.4282	-0.8759
YRI	HIST2	149960687	5.1239	9.5795	-1.3872
YRI	HIST2	149965687	5.1618	8.9073	-1.2475
YRI	HIST2	149970687	6.9734	9.2434	-0.7305
YRI	HIST2	149975687	7.4001	10.0837	-0.7965
YRI	HIST2	149980687	7.1825	10.4199	-0.9319
YRI	HIST2	149985687	9.275	11.9324	-0.6736
YRI	HIST2	149990687	9.5777	13.2769	-0.8478
YRI	HIST2	149995687	11.7908	15.1256	-0.6753
YRI	HIST2	150000687	14.6298	14.7895	-0.033

YRI	HIST2	150005687	11.7842	14.1172	-0.5045
YRI	HIST2	150010687	14.8799	17.6465	-0.4835
YRI	HIST2	150015687	16.5702	16.8062	-0.0432
YRI	HIST2	150020687	12.3487	14.6214	-0.4753
YRI	HIST2	150025687	12.6453	15.1256	-0.5022
YRI	HIST2	150030687	10.6271	12.6047	-0.4761
YRI	HIST2	150035687	6.6803	9.7476	-0.9396
YRI	HIST2	150040687	10.3483	14.1172	-0.8149
YRI	HIST2	150045687	13.0111	16.302	-0.6204
YRI	HIST2	150050687	8.9108	13.2769	-1.0006
YRI	HIST2	150055687	7.464	13.1089	-1.3094
YRI	HIST2	150060687	9.8941	13.613	-0.8324
YRI	HIST2	150065687	8.4815	12.2685	-0.9352
YRI	HIST2	150070687	7.9696	13.613	-1.2631
YRI	HIST2	150075687	11.9678	15.7978	-0.744
YRI	HIST2	150080687	10.8321	15.2937	-0.894
YRI	HIST2	150085687	12.3291	16.302	-0.749
YRI	HIST2	150090687	14.1904	15.1256	-0.1894
YRI	HIST2	150095687	11.748	14.2853	-0.5425
YRI	HIST2	150100687	12.2606	14.7895	-0.5232
YRI	HIST2	150105687	12.2568	16.9743	-0.8556
YRI	HIST2	150110687	9.807	15.4617	-1.1213
YRI	HIST2	150115687	7.898	13.2769	-1.2328
YRI	HIST2	150120687	8.5946	14.9575	-1.3022
YRI	HIST2	150125687	8.636	13.9492	-1.162
YRI	HIST2	150130687	11.6966	16.8062	-0.9356
YRI	HIST2	150135687	13.6467	18.991	-0.8703
YRI	HIST2	150140687	10.6132	15.7978	-1.0072
YRI	HIST2	150145687	8.2985	12.9408	-1.0901
YRI	HIST2	150150687	10.0282	12.9408	-0.6839
YRI	HIST2	150155687	15.6957	16.8062	-0.2033
YRI	HIST2	150160687	15.7906	18.6549	-0.4745
YRI	HIST2	150165687	13.9296	16.9743	-0.5522
YRI	HIST2	150170687	12.2884	16.302	-0.7566
YRI	HIST2	150175687	11.0381	14.7895	-0.7761
YRI	HIST2	150180687	12.6705	14.2853	-0.3453
YRI	HIST2	150185687	10.9232	14.1172	-0.6906
YRI	HIST2	150190687	9.3911	11.5963	-0.5742
YRI	HIST2	150195687	9.1269	9.9157	-0.2378
YRI	HIST2	150200687	9.6844	10.0837	-0.1185
YRI	HIST2	150205687	11.4763	12.2685	-0.1956
YRI	HIST2	150210687	13.2729	14.9575	-0.3448
YRI	HIST2	150215687	13.8174	14.7895	-0.2011
YRI	HIST2	150220687	10.9399	10.924	0.0044
YRI	HIST2	150225687	6.2934	8.9073	-0.8706
YRI	HIST2	150230687	3.7123	8.7392	-1.704
YRI	HIST2	150235687	2.8731	6.3864	-1.5857
YRI	HIST2	150240687	4.2078	7.8989	-1.3732
YRI	HIST2	150245687	6.2699	10.924	-1.2818

YRI	HIST2	150250687	6.3516	10.5879	-1.2014
YRI	HIST2	150255687	7.0664	9.4115	-0.7422
YRI	HIST2	150260687	14.1165	12.9408	0.2761
YRI	HIST2	150265687	19.3635	16.6382	0.5038
YRI	HIST2	150270687	15.2574	15.4617	-0.0405
YRI	HIST2	150275687	13.9662	15.7978	-0.3558
YRI	HIST2	150280687	15.4575	16.302	-0.1592
YRI	HIST2	150285687	11.2587	12.2685	-0.2494
YRI	HIST2	150290687	8.8346	11.4282	-0.6847
YRI	HIST2	150295687	10.1552	13.1089	-0.6852
YRI	HIST2	150300687	10.7939	12.4366	-0.4005
YRI	HIST2	150305687	12.0064	14.2853	-0.4873
YRI	HIST2	150310687	12.2611	13.445	-0.2681
YRI	HIST2	150315687	13.7686	13.1089	0.153
YRI	HIST2	150320687	14.4169	15.4617	-0.2072
YRI	HIST2	150325687	13.1102	14.9575	-0.3781
YRI	HIST2	150330687	12.7934	13.2769	-0.1108
YRI	HIST2	150335687	10.758	12.2685	-0.373
CEU	HIST2	149347218	6.0408	5.458	0.3047
CEU	HIST2	149352218	6.0998	6.3108	-0.0969
CEU	HIST2	149357218	5.3072	5.6286	-0.1635
CEU	HIST2	149362218	4.7357	5.1169	-0.211
CEU	HIST2	149367218	3.5933	3.9229	-0.2299
CEU	HIST2	149372218	3.9252	4.9463	-0.5823
CEU	HIST2	149377218	4.537	5.458	-0.4815
CEU	HIST2	149382218	3.6968	3.9229	-0.1577
CEU	HIST2	149387218	5.1558	4.2641	0.5788
CEU	HIST2	149392218	6.3327	5.9697	0.1752
CEU	HIST2	149397218	4.8079	5.7991	-0.4909
CEU	HIST2	149402218	5.9221	5.7991	0.0609
CEU	HIST2	149407218	5.0206	4.7757	0.144
CEU	HIST2	149412218	4.3467	3.4112	0.7348
CEU	HIST2	149417218	4.1068	3.2407	0.7102
CEU	HIST2	149422218	2.0165	1.8762	0.1786
CEU	HIST2	149427218	2.7172	2.729	-0.0112
CEU	HIST2	149432218	2.2187	2.5584	-0.3384
CEU	HIST2	149437218	0.5232	0.6822	-0.4152
CEU	HIST2	149442218	0	0	0
CEU	HIST2	149447218	0.08	0.5117	-1.3539
CEU	HIST2	149452218	0.1394	0.8528	-1.6066
CEU	HIST2	149457218	0.128	0.6822	-1.4472
CEU	HIST2	149462218	0.0686	0.3411	-1.0924
CEU	HIST2	149467218	0	0	0
CEU	HIST2	149472218	0	0	0
CEU	HIST2	149477218	0	0	0
CEU	HIST2	149482218	0	0	0
CEU	HIST2	149487218	0	0	0
CEU	HIST2	149492218	0	0	0
CEU	HIST2	149497218	0	0	0

CEU	HIST2	149502218	0	0	0
CEU	HIST2	149507218	0.1363	0.5117	-1.1774
CEU	HIST2	149512218	0.4605	1.1939	-1.3069
CEU	HIST2	149517218	0.3243	0.6822	-0.9346
CEU	HIST2	149522218	0	0	0
CEU	HIST2	149527218	0	0	0
CEU	HIST2	149532218	0.0101	0.1706	-0.9534
CEU	HIST2	149537218	0.0101	0.1706	-0.9534
CEU	HIST2	149542218	0	0	0
CEU	HIST2	149547218	0	0	0
CEU	HIST2	149552218	0	0	0
CEU	HIST2	149557218	0	0	0
CEU	HIST2	149562218	0	0	0
CEU	HIST2	149567218	0.5038	0.1706	1.9793
CEU	HIST2	149572218	0.885	0.6822	0.5296
CEU	HIST2	149577218	0.3812	0.5117	-0.4093
CEU	HIST2	149582218	2.8182	3.2407	-0.3464
CEU	HIST2	149587218	3.8	3.7524	0.0345
CEU	HIST2	149592218	1.7355	1.3645	0.6002
CEU	HIST2	149597218	2.4286	2.8996	-0.4236
CEU	HIST2	149602218	6.2946	5.1169	0.6519
CEU	HIST2	149607218	7.5261	4.6052	1.7734
CEU	HIST2	149612218	3.7706	2.2173	1.7353
CEU	HIST2	149617218	0.8642	0.6822	0.4753
CEU	HIST2	149622218	0	0	0
CEU	HIST2	149627218	0.0301	0.1706	-0.8346
CEU	HIST2	149632218	0.0301	0.1706	-0.8346
CEU	HIST2	149637218	2.3997	1.7056	0.9507
CEU	HIST2	149642218	3.1884	2.8996	0.2597
CEU	HIST2	149647218	7.4811	6.6519	0.363
CEU	HIST2	149652218	8.4589	6.8225	0.7002
CEU	HIST2	149657218	4.3451	3.2407	0.9056
CEU	HIST2	149662218	6.0134	6.3108	-0.1365
CEU	HIST2	149667218	5.1579	9.2104	-1.3168
CEU	HIST2	149672218	5.47	9.3809	-1.2494
CEU	HIST2	149677218	5.5494	7.3342	-0.7151
CEU	HIST2	149682218	2.4492	4.4346	-1.2457
CEU	HIST2	149687218	0.9386	3.4112	-1.942
CEU	HIST2	149692218	1.3713	3.2407	-1.5329
CEU	HIST2	149697218	2.8957	5.1169	-1.2295
CEU	HIST2	149702218	5.0012	6.8225	-0.7793
CEU	HIST2	149707218	7.6804	6.9931	0.2876
CEU	HIST2	149712218	8.5538	7.5047	0.4116
CEU	HIST2	149717218	10.6112	8.5281	0.7268
CEU	HIST2	149722218	9.7798	7.1636	1.0709
CEU	HIST2	149727218	6.7895	6.4814	0.1381
CEU	HIST2	149732218	5.9891	5.7991	0.0941
CEU	HIST2	149737218	3.3206	3.5818	-0.1969
CEU	HIST2	149742218	2.2494	3.2407	-0.8128

CEU	HIST2	149747218	4.6417	3.9229	0.5013
CEU	HIST2	149752218	3.605	3.4112	0.1522
CEU	HIST2	149757218	1.7541	3.0701	-1.1288
CEU	HIST2	149762218	2.309	5.1169	-1.5542
CEU	HIST2	149767218	2.269	5.458	-1.6671
CEU	HIST2	149772218	3.0989	5.6286	-1.2868
CEU	HIST2	149777218	4.6702	6.6519	-0.8676
CEU	HIST2	149782218	5.5527	8.0164	-0.91
CEU	HIST2	149787218	3.9497	7.3342	-1.3561
CEU	HIST2	149792218	1.4871	3.0701	-1.3579
CEU	HIST2	149797218	0.1235	0.1706	-0.2798
CEU	HIST2	149802218	0	0	0
CEU	HIST2	149807218	0	0	0
CEU	HIST2	149812218	1.3752	1.8762	-0.6379
CEU	HIST2	149817218	2.6199	4.0935	-0.9909
CEU	HIST2	149822218	1.8312	2.5584	-0.7244
CEU	HIST2	149827218	0.5865	0.3411	0.9837
CEU	HIST2	149832218	0	0	0
CEU	HIST2	149837218	0	0	0
CEU	HIST2	149842218	0.8703	1.3645	-0.7995
CEU	HIST2	149847218	2.0313	3.9229	-1.3193
CEU	HIST2	149852218	2.3896	5.2874	-1.5582
CEU	HIST2	149857218	2.8143	6.3108	-1.6053
CEU	HIST2	149862218	3.9306	7.5047	-1.4023
CEU	HIST2	149867218	4.9634	8.5281	-1.2438
CEU	HIST2	149872218	4.7279	7.5047	-1.0895
CEU	HIST2	149877218	4.0565	6.4814	-1.0869
CEU	HIST2	149882218	2.6912	5.1169	-1.3427
CEU	HIST2	149887218	2.3436	3.9229	-1.1015
CEU	HIST2	149892218	3.5081	4.6052	-0.6661
CEU	HIST2	149897218	3.3513	5.2874	-1.041
CEU	HIST2	149902218	3.5549	5.7991	-1.1115
CEU	HIST2	149907218	3.9946	5.7991	-0.8938
CEU	HIST2	149912218	2.021	4.4346	-1.5144
CEU	HIST2	149917218	1.3774	3.9229	-1.7754
CEU	HIST2	149922218	3.7236	5.1169	-0.7712
CEU	HIST2	149927218	4.4919	5.458	-0.5051
CEU	HIST2	149932218	2.6372	5.7991	-1.5661
CEU	HIST2	149937218	2.0641	4.9463	-1.6437
CEU	HIST2	149942218	2.6215	3.7524	-0.8193
CEU	HIST2	149947218	2.2478	4.0935	-1.2411
CEU	HIST2	149952218	2.9812	5.9697	-1.4423
CEU	HIST2	149957218	2.1484	4.4346	-1.4344
CEU	HIST2	149962218	1.0019	2.3879	-1.4596
CEU	HIST2	149967218	1.9435	3.4112	-1.1528
CEU	HIST2	149972218	2.1725	3.7524	-1.1446
CEU	HIST2	149977218	2.0581	3.9229	-1.3006
CEU	HIST2	149982218	2.6574	4.9463	-1.3054
CEU	HIST2	149987218	3.6749	5.9697	-1.1075

CEU	HIST2	149992218	6.3636	7.8459	-0.5584
CEU	HIST2	149997218	9.5217	8.6987	0.282
CEU	HIST2	150002218	8.3725	6.9931	0.5772
CEU	HIST2	150007218	7.7545	7.3342	0.1684
CEU	HIST2	150012218	10.5403	9.0398	0.4961
CEU	HIST2	150017218	10.7794	8.8692	0.6428
CEU	HIST2	150022218	9.1606	7.8459	0.4953
CEU	HIST2	150027218	5.7079	5.9697	-0.1263
CEU	HIST2	150032218	1.9462	4.2641	-1.5046
CEU	HIST2	150037218	3.0062	5.9697	-1.4302
CEU	HIST2	150042218	4.5046	6.4814	-0.886
CEU	HIST2	150047218	4.4902	5.458	-0.5059
CEU	HIST2	150052218	3.1923	4.9463	-1.0003
CEU	HIST2	150057218	2.1128	4.4346	-1.4568
CEU	HIST2	150062218	3.0056	4.7757	-1.0411
CEU	HIST2	150067218	1.987	3.2407	-1.028
CEU	HIST2	150072218	5.5576	5.2874	0.1453
CEU	HIST2	150077218	7.7425	7.8459	-0.039
CEU	HIST2	150082218	3.4447	5.7991	-1.1661
CEU	HIST2	150087218	2.7961	6.6519	-1.6881
CEU	HIST2	150092218	3.7225	6.6519	-1.2825
CEU	HIST2	150097218	4.2762	5.1169	-0.4653
CEU	HIST2	150102218	5.1591	6.8225	-0.7118
CEU	HIST2	150107218	6.2472	6.8225	-0.2462
CEU	HIST2	150112218	5.381	5.458	-0.0403
CEU	HIST2	150117218	3.2653	4.6052	-0.8135
CEU	HIST2	150122218	3.677	6.3108	-1.2092
CEU	HIST2	150127218	4.8244	8.0164	-1.179
CEU	HIST2	150132218	6.0334	8.8692	-0.9543
CEU	HIST2	150137218	7.2057	11.9394	-1.2069
CEU	HIST2	150142218	6.4966	9.5515	-0.9597
CEU	HIST2	150147218	5.0724	6.3108	-0.5686
CEU	HIST2	150152218	3.22	7.1636	-1.6143
CEU	HIST2	150157218	5.1905	9.0398	-1.2727
CEU	HIST2	150162218	8.7335	9.5515	-0.257
CEU	HIST2	150167218	9.7998	8.0164	0.6587
CEU	HIST2	150172218	8.5918	6.9931	0.6689
CEU	HIST2	150177218	6.8242	5.7991	0.5077
CEU	HIST2	150182218	7.4803	6.1402	0.6306
CEU	HIST2	150187218	6.154	6.4814	-0.1467
CEU	HIST2	150192218	5.8207	6.1402	-0.1503
CEU	HIST2	150197218	8.0969	6.4814	0.7241
CEU	HIST2	150202218	9.269	6.9931	0.9523
CEU	HIST2	150207218	9.4055	8.0164	0.5131
CEU	HIST2	150212218	8.7671	7.1636	0.6564
CEU	HIST2	150217218	8.7254	6.6519	0.9078
CEU	HIST2	150222218	7.2614	6.3108	0.4364
CEU	HIST2	150227218	3.3078	4.4346	-0.707
CEU	HIST2	150232218	2.2224	3.5818	-1.0246

CEU	HIST2	150237218	3.1212	2.8996	0.1993
CEU	HIST2	150242218	2.7774	3.9229	-0.799
CEU	HIST2	150247218	4.9716	5.458	-0.2543
CEU	HIST2	150252218	5.4418	5.1169	0.1798
CEU	HIST2	150257218	8.0536	7.1636	0.3643
CEU	HIST2	150262218	14.4719	9.0398	1.796
CEU	HIST2	150267218	19.1497	10.7454	2.3656
CEU	HIST2	150272218	17.2167	11.7688	1.4079
CEU	HIST2	150277218	13.6498	10.7454	0.8175
CEU	HIST2	150282218	15.1563	10.7454	1.2415
CEU	HIST2	150287218	12.5504	8.187	1.5808
CEU	HIST2	150292218	10.1462	7.1636	1.2209
CEU	HIST2	150297218	9.1539	7.8459	0.4927
CEU	HIST2	150302218	8.0121	7.5047	0.1991
CEU	HIST2	150307218	10.0324	8.8692	0.3914
CEU	HIST2	150312218	10.6691	9.3809	0.4115
CEU	HIST2	150317218	12.6151	10.4043	0.6413
CEU	HIST2	150322218	14.0898	10.0632	1.205
CEU	HIST2	150327218	13.2813	9.5515	1.1718
CEU	HIST2	150332218	12.0168	9.3809	0.8421
CEU	HIST2	150337218	10.1044	8.8692	0.4156
CHB	HIST2	149347218	5.6195	7.4543	-0.7222
CHB	HIST2	149352218	5.0348	6.7766	-0.7477
CHB	HIST2	149357218	4.5631	5.2519	-0.3715
CHB	HIST2	149362218	4.1312	4.066	0.044
CHB	HIST2	149367218	3.1955	4.066	-0.587
CHB	HIST2	149372218	2.7969	5.0825	-1.2689
CHB	HIST2	149377218	3.2859	5.0825	-0.9974
CHB	HIST2	149382218	3.0581	3.8965	-0.5864
CHB	HIST2	149387218	4.0619	5.4213	-0.7128
CHB	HIST2	149392218	4.9155	5.7601	-0.4196
CHB	HIST2	149397218	4.0552	5.4213	-0.7163
CHB	HIST2	149402218	4.8488	6.4378	-0.7145
CHB	HIST2	149407218	3.478	4.913	-0.8208
CHB	HIST2	149412218	3.9303	4.7436	-0.4797
CHB	HIST2	149417218	4.2513	4.2354	0.0104
CHB	HIST2	149422218	1.5298	2.8801	-1.2174
CHB	HIST2	149427218	1.9996	3.8965	-1.3267
CHB	HIST2	149432218	1.8957	3.2189	-1.0879
CHB	HIST2	149437218	0.4087	1.0165	-1.2096
CHB	HIST2	149442218	0	0	0
CHB	HIST2	149447218	0.0386	0.5082	-1.4741
CHB	HIST2	149452218	0.1065	1.3553	-2.0239
CHB	HIST2	149457218	0.0873	1.1859	-1.961
CHB	HIST2	149462218	0.0194	0.3388	-1.2812
CHB	HIST2	149467218	0	0	0
CHB	HIST2	149472218	0	0	0
CHB	HIST2	149477218	0	0	0
CHB	HIST2	149482218	0	0	0

CHB	HIST2	149487218	0	0	0
CHB	HIST2	149492218	0	0	0
CHB	HIST2	149497218	0	0	0
CHB	HIST2	149502218	0	0	0
CHB	HIST2	149507218	0.6275	0.5082	0.3745
CHB	HIST2	149512218	0.8968	1.0165	-0.2382
CHB	HIST2	149517218	0.2791	0.6777	-1.0421
CHB	HIST2	149522218	0.0097	0.1694	-0.949
CHB	HIST2	149527218	0	0	0
CHB	HIST2	149532218	0.2578	0.1694	0.5253
CHB	HIST2	149537218	0.2578	0.1694	0.5253
CHB	HIST2	149542218	0	0	0
CHB	HIST2	149547218	0	0	0
CHB	HIST2	149552218	0	0	0
CHB	HIST2	149557218	0	0	0
CHB	HIST2	149562218	0	0	0
CHB	HIST2	149567218	0.4601	0.3388	0.4866
CHB	HIST2	149572218	0.9039	0.8471	0.1281
CHB	HIST2	149577218	0.4535	0.6777	-0.5861
CHB	HIST2	149582218	3.3082	3.3883	-0.0631
CHB	HIST2	149587218	4.0702	3.8965	0.1215
CHB	HIST2	149592218	1.2206	1.1859	0.0619
CHB	HIST2	149597218	2.9082	2.2024	0.7903
CHB	HIST2	149602218	7.3659	4.7436	1.5468
CHB	HIST2	149607218	7.0862	5.4213	0.873
CHB	HIST2	149612218	2.7537	2.8801	-0.114
CHB	HIST2	149617218	0.574	0.5082	0.2066
CHB	HIST2	149622218	0	0	0
CHB	HIST2	149627218	0.0097	0.1694	-0.949
CHB	HIST2	149632218	0.0097	0.1694	-0.949
CHB	HIST2	149637218	2.8808	1.5247	2.0147
CHB	HIST2	149642218	4.2753	3.7271	0.3983
CHB	HIST2	149647218	7.5328	6.7766	0.3246
CHB	HIST2	149652218	7.1211	5.9295	0.5769
CHB	HIST2	149657218	3.0921	4.5742	-0.9025
CHB	HIST2	149662218	5.9966	5.4213	0.3017
CHB	HIST2	149667218	7.7836	6.4378	0.6051
CHB	HIST2	149672218	8.1593	8.6402	-0.1653
CHB	HIST2	149677218	6.0198	6.2684	-0.1145
CHB	HIST2	149682218	2.9364	4.5742	-0.9973
CHB	HIST2	149687218	2.285	4.4048	-1.3339
CHB	HIST2	149692218	3.198	4.2354	-0.6753
CHB	HIST2	149697218	4.2222	4.7436	-0.3076
CHB	HIST2	149702218	5.9206	6.6072	-0.3016
CHB	HIST2	149707218	7.5389	7.6237	-0.0327
CHB	HIST2	149712218	6.6324	6.0989	0.2518
CHB	HIST2	149717218	7.9216	8.4708	-0.1923
CHB	HIST2	149722218	7.9966	9.3178	-0.4235
CHB	HIST2	149727218	6.5829	8.4708	-0.6609

CHB	HIST2	149732218	5.7443	7.1154	-0.5631
CHB	HIST2	149737218	2.7881	4.2354	-0.9422
CHB	HIST2	149742218	1.5443	3.0495	-1.2945
CHB	HIST2	149747218	3.8763	3.0495	0.7111
CHB	HIST2	149752218	3.2829	3.0495	0.2007
CHB	HIST2	149757218	1.0577	5.2519	-2.2621
CHB	HIST2	149762218	1.9924	6.6072	-2.0269
CHB	HIST2	149767218	2.2781	4.5742	-1.3981
CHB	HIST2	149772218	1.3008	5.4213	-2.1607
CHB	HIST2	149777218	0.7826	5.2519	-2.4104
CHB	HIST2	149782218	1.0246	4.5742	-2.1614
CHB	HIST2	149787218	0.744	5.7601	-2.4921
CHB	HIST2	149792218	0.422	3.7271	-2.4011
CHB	HIST2	149797218	0.1611	0.6777	-1.3505
CHB	HIST2	149802218	0	0	0
CHB	HIST2	149807218	0	0	0
CHB	HIST2	149812218	0.4588	1.6942	-1.6954
CHB	HIST2	149817218	1.2566	5.2519	-2.1548
CHB	HIST2	149822218	1.1833	3.8965	-1.8977
CHB	HIST2	149827218	0.3855	0.3388	0.1873
CHB	HIST2	149832218	0	0	0
CHB	HIST2	149837218	0	0	0
CHB	HIST2	149842218	0.2393	1.1859	-1.6897
CHB	HIST2	149847218	0.5099	4.4048	-2.4509
CHB	HIST2	149852218	0.9464	7.7931	-2.5877
CHB	HIST2	149857218	1.4982	6.4378	-2.221
CHB	HIST2	149862218	1.3862	6.0989	-2.2246
CHB	HIST2	149867218	1.1507	7.1154	-2.4495
CHB	HIST2	149872218	1.1392	5.9295	-2.3191
CHB	HIST2	149877218	1.6109	6.7766	-2.2175
CHB	HIST2	149882218	1.7217	6.946	-2.193
CHB	HIST2	149887218	1.2667	5.7601	-2.2324
CHB	HIST2	149892218	1.5431	6.6072	-2.2242
CHB	HIST2	149897218	1.1624	7.1154	-2.4447
CHB	HIST2	149902218	1.4296	7.1154	-2.335
CHB	HIST2	149907218	2.6127	8.1319	-2.0061
CHB	HIST2	149912218	1.5806	6.7766	-2.2305
CHB	HIST2	149917218	1.1064	6.2684	-2.3774
CHB	HIST2	149922218	3.506	8.8096	-1.7906
CHB	HIST2	149927218	4.3588	10.8426	-1.8045
CHB	HIST2	149932218	2.1807	10.3343	-2.3734
CHB	HIST2	149937218	2.0573	9.3178	-2.3273
CHB	HIST2	149942218	2.9625	8.1319	-1.8789
CHB	HIST2	149947218	2.0714	6.4378	-1.9633
CHB	HIST2	149952218	1.9591	7.6237	-2.1844
CHB	HIST2	149957218	1.4021	7.7931	-2.4155
CHB	HIST2	149962218	1.0171	6.4378	-2.4373
CHB	HIST2	149967218	1.0364	6.2684	-2.4097
CHB	HIST2	149972218	1.3438	4.913	-2.0416

CHB	HIST2	149977218	1.9513	4.913	-1.6941
CHB	HIST2	149982218	1.8032	7.6237	-2.2445
CHB	HIST2	149987218	2.5589	9.1484	-2.1484
CHB	HIST2	149992218	4.3701	9.9955	-1.6892
CHB	HIST2	149997218	6.7369	11.012	-1.1726
CHB	HIST2	150002218	5.3594	10.3343	-1.4481
CHB	HIST2	150007218	2.9322	9.9955	-2.121
CHB	HIST2	150012218	2.1689	9.9955	-2.3502
CHB	HIST2	150017218	1.5214	8.979	-2.4739
CHB	HIST2	150022218	1.7038	8.6402	-2.3843
CHB	HIST2	150027218	1.3857	8.4708	-2.4803
CHB	HIST2	150032218	1.0946	6.6072	-2.4212
CHB	HIST2	150037218	1.8289	6.6072	-2.0987
CHB	HIST2	150042218	1.9958	7.2848	-2.126
CHB	HIST2	150047218	0.9147	6.4378	-2.4834
CHB	HIST2	150052218	1.0657	6.6072	-2.4339
CHB	HIST2	150057218	1.158	6.0989	-2.3323
CHB	HIST2	150062218	0.613	4.913	-2.4596
CHB	HIST2	150067218	0.5722	4.913	-2.4829
CHB	HIST2	150072218	1.3189	7.4543	-2.4151
CHB	HIST2	150077218	1.3522	7.9625	-2.4496
CHB	HIST2	150082218	0.8626	5.0825	-2.3428
CHB	HIST2	150087218	1.2939	6.0989	-2.2682
CHB	HIST2	150092218	1.5829	6.7766	-2.2295
CHB	HIST2	150097218	1.5108	6.2684	-2.1912
CHB	HIST2	150102218	1.5997	7.9625	-2.3579
CHB	HIST2	150107218	1.8289	7.7931	-2.2542
CHB	HIST2	150112218	1.4472	6.0989	-2.1958
CHB	HIST2	150117218	1.3547	6.0989	-2.2395
CHB	HIST2	150122218	2.2429	7.7931	-2.0977
CHB	HIST2	150127218	2.2012	7.7931	-2.1134
CHB	HIST2	150132218	2.1033	8.1319	-2.1912
CHB	HIST2	150137218	2.4602	9.8261	-2.2473
CHB	HIST2	150142218	2.7236	9.6567	-2.1498
CHB	HIST2	150147218	2.7567	8.6402	-2.0224
CHB	HIST2	150152218	1.9176	8.8096	-2.3269
CHB	HIST2	150157218	2.7124	12.5367	-2.3852
CHB	HIST2	150162218	6.518	12.0285	-1.3911
CHB	HIST2	150167218	9.8096	9.9955	-0.0558
CHB	HIST2	150172218	7.4525	10.3343	-0.8389
CHB	HIST2	150177218	5.6126	9.9955	-1.3161
CHB	HIST2	150182218	6.6275	9.4872	-0.9014
CHB	HIST2	150187218	4.5117	7.1154	-1.0693
CHB	HIST2	150192218	4.5803	7.1154	-1.0411
CHB	HIST2	150197218	8.1406	8.8096	-0.2259
CHB	HIST2	150202218	9.248	9.8261	-0.1764
CHB	HIST2	150207218	8.6806	11.3508	-0.7119
CHB	HIST2	150212218	7.9743	11.5202	-0.9323
CHB	HIST2	150217218	7.8589	9.9955	-0.6416

CHB	HIST2	150222218	6.5275	8.4708	-0.6803
CHB	HIST2	150227218	2.9787	6.4378	-1.5553
CHB	HIST2	150232218	2.1346	4.7436	-1.539
CHB	HIST2	150237218	3.2612	4.2354	-0.6342
CHB	HIST2	150242218	3.414	5.5907	-1.1106
CHB	HIST2	150247218	4.5978	7.4543	-1.1244
CHB	HIST2	150252218	4.7691	7.4543	-1.057
CHB	HIST2	150257218	6.7619	8.1319	-0.498
CHB	HIST2	150262218	10.4298	10.3343	0.0278
CHB	HIST2	150267218	13.5743	11.8591	0.4388
CHB	HIST2	150272218	11.6577	10.5037	0.3308
CHB	HIST2	150277218	8.6513	10.3343	-0.4899
CHB	HIST2	150282218	10.7638	12.8755	-0.4999
CHB	HIST2	150287218	9.3432	10.5037	-0.3327
CHB	HIST2	150292218	6.6992	8.3013	-0.5714
CHB	HIST2	150297218	5.754	8.6402	-0.9921
CHB	HIST2	150302218	5.8606	8.6402	-0.9554
CHB	HIST2	150307218	7.4055	10.5037	-0.8882
CHB	HIST2	150312218	7.8418	9.3178	-0.4731
CHB	HIST2	150317218	9.1085	9.6567	-0.17
CHB	HIST2	150322218	9.2867	11.3508	-0.5503
CHB	HIST2	150327218	9.107	12.0285	-0.7375
CHB	HIST2	150332218	8.5134	11.5202	-0.7905
CHB	HIST2	150337218	6.4127	8.1319	-0.6249
YRI	HIST2	149347218	6.9479	8.9073	-0.6526
YRI	HIST2	149352218	6.9164	8.4031	-0.5225
YRI	HIST2	149357218	5.8626	7.0586	-0.4931
YRI	HIST2	149362218	4.6115	6.8906	-0.9603
YRI	HIST2	149367218	4.07	6.7225	-1.143
YRI	HIST2	149372218	3.7664	7.7309	-1.5042
YRI	HIST2	149377218	4.0781	7.5628	-1.349
YRI	HIST2	149382218	4.3368	5.5461	-0.6193
YRI	HIST2	149387218	6.2057	5.8822	0.1572
YRI	HIST2	149392218	6.8524	7.2267	-0.151
YRI	HIST2	149397218	5.2869	7.2267	-0.7827
YRI	HIST2	149402218	7.196	8.7392	-0.5231
YRI	HIST2	149407218	6.2313	8.9073	-0.8913
YRI	HIST2	149412218	5.3871	7.7309	-0.8893
YRI	HIST2	149417218	5.4485	5.5461	-0.05
YRI	HIST2	149422218	2.9105	2.8571	0.0483
YRI	HIST2	149427218	3.035	4.2016	-0.7621
YRI	HIST2	149432218	2.5498	3.8654	-0.9232
YRI	HIST2	149437218	0.7064	1.1764	-0.8406
YRI	HIST2	149442218	0.0185	0.1681	-0.8893
YRI	HIST2	149447218	0.1568	0.5042	-1.0918
YRI	HIST2	149452218	0.4181	1.1764	-1.3563
YRI	HIST2	149457218	0.5682	1.5126	-1.4063
YRI	HIST2	149462218	0.3069	0.8403	-1.2048
YRI	HIST2	149467218	0	0	0

YRI	HIST2	149472218	0	0	0
YRI	HIST2	149477218	0	0	0
YRI	HIST2	149482218	0	0	0
YRI	HIST2	149487218	0	0	0
YRI	HIST2	149492218	0	0	0
YRI	HIST2	149497218	0	0	0
YRI	HIST2	149502218	0	0	0
YRI	HIST2	149507218	0.4018	0.5042	-0.3218
YRI	HIST2	149512218	0.6674	2.0167	-1.6133
YRI	HIST2	149517218	0.4991	2.0167	-1.8145
YRI	HIST2	149522218	0.2335	0.5042	-0.8507
YRI	HIST2	149527218	0.073	0.3361	-1.0562
YRI	HIST2	149532218	0.2339	0.5042	-0.8495
YRI	HIST2	149537218	0.1609	0.1681	-0.0428
YRI	HIST2	149542218	0	0	0
YRI	HIST2	149547218	0	0	0
YRI	HIST2	149552218	0	0	0
YRI	HIST2	149557218	0	0	0
YRI	HIST2	149562218	0	0	0
YRI	HIST2	149567218	0.5133	0.5042	0.0286
YRI	HIST2	149572218	1.1098	1.6806	-0.7853
YRI	HIST2	149577218	0.5965	1.1764	-1.0372
YRI	HIST2	149582218	2.3949	5.0419	-1.4748
YRI	HIST2	149587218	2.8328	6.0502	-1.5244
YRI	HIST2	149592218	0.7683	1.8487	-1.3819
YRI	HIST2	149597218	2.5434	3.0251	-0.4155
YRI	HIST2	149602218	6.0536	6.5544	-0.2208
YRI	HIST2	149607218	5.8919	6.8906	-0.4208
YRI	HIST2	149612218	2.6056	3.6974	-0.7958
YRI	HIST2	149617218	0.5543	1.1764	-1.1127
YRI	HIST2	149622218	0	0	0
YRI	HIST2	149627218	0.0093	0.1681	-0.944
YRI	HIST2	149632218	0.2214	0.3361	-0.4605
YRI	HIST2	149637218	3.1146	2.0167	1.3127
YRI	HIST2	149642218	4.1004	4.2016	-0.0661
YRI	HIST2	149647218	8.5605	9.0754	-0.1686
YRI	HIST2	149652218	9.903	11.0921	-0.3228
YRI	HIST2	149657218	5.7555	7.5628	-0.6997
YRI	HIST2	149662218	7.8571	8.067	-0.0766
YRI	HIST2	149667218	10.8331	12.9408	-0.4949
YRI	HIST2	149672218	10.1832	14.1172	-0.8506
YRI	HIST2	149677218	6.2483	10.0837	-1.1384
YRI	HIST2	149682218	4.4674	8.9073	-1.4787
YRI	HIST2	149687218	3.0618	8.5712	-1.9013
YRI	HIST2	149692218	3.0228	6.7225	-1.5942
YRI	HIST2	149697218	4.7415	7.0586	-0.9552
YRI	HIST2	149702218	7.7985	10.756	-0.8265
YRI	HIST2	149707218	9.61	13.445	-0.8685
YRI	HIST2	149712218	7.4347	11.2602	-1.0241

YRI	HIST2	149717218	8.0637	11.2602	-0.8557
YRI	HIST2	149722218	9.2788	12.9408	-0.8599
YRI	HIST2	149727218	8.8999	13.1089	-0.9763
YRI	HIST2	149732218	7.7159	12.4366	-1.1509
YRI	HIST2	149737218	4.4014	8.235	-1.3726
YRI	HIST2	149742218	3.6835	6.0502	-1.1214
YRI	HIST2	149747218	6.839	7.3947	-0.2196
YRI	HIST2	149752218	6.6334	7.7309	-0.4164
YRI	HIST2	149757218	6.2109	9.9157	-1.117
YRI	HIST2	149762218	6.0842	10.756	-1.3055
YRI	HIST2	149767218	3.4367	8.9073	-1.822
YRI	HIST2	149772218	5.4209	10.756	-1.4909
YRI	HIST2	149777218	6.534	12.2685	-1.4161
YRI	HIST2	149782218	4.9148	13.445	-1.9318
YRI	HIST2	149787218	4.2145	11.9324	-1.9565
YRI	HIST2	149792218	2.222	5.378	-1.6609
YRI	HIST2	149797218	0.4072	1.0084	-1.1986
YRI	HIST2	149802218	0	0	0
YRI	HIST2	149807218	0	0	0
YRI	HIST2	149812218	2.4624	3.6974	-0.9002
YRI	HIST2	149817218	4.7635	8.4031	-1.2792
YRI	HIST2	149822218	3.0742	5.0419	-1.0963
YRI	HIST2	149827218	0.7731	0.3361	1.7544
YRI	HIST2	149832218	0	0	0
YRI	HIST2	149837218	0	0	0
YRI	HIST2	149842218	0.7311	2.0167	-1.5371
YRI	HIST2	149847218	3.9361	9.4115	-1.7329
YRI	HIST2	149852218	5.8281	14.2853	-1.8082
YRI	HIST2	149857218	4.2471	11.5963	-1.9138
YRI	HIST2	149862218	4.8498	11.4282	-1.7367
YRI	HIST2	149867218	7.6956	12.9408	-1.2317
YRI	HIST2	149872218	6.6281	11.2602	-1.24
YRI	HIST2	149877218	6.9578	11.2602	-1.1518
YRI	HIST2	149882218	7.7874	11.4282	-0.9612
YRI	HIST2	149887218	5.8326	11.9324	-1.5463
YRI	HIST2	149892218	5.6857	12.6047	-1.6656
YRI	HIST2	149897218	4.555	12.7727	-1.9536
YRI	HIST2	149902218	3.9827	12.1005	-2.0309
YRI	HIST2	149907218	6.6628	11.5963	-1.2847
YRI	HIST2	149912218	6.3571	11.7644	-1.3892
YRI	HIST2	149917218	5.032	11.9324	-1.7492
YRI	HIST2	149922218	7.461	14.2853	-1.4591
YRI	HIST2	149927218	9.1921	14.2853	-1.089
YRI	HIST2	149932218	9.6005	13.445	-0.8707
YRI	HIST2	149937218	8.8322	12.2685	-0.8486
YRI	HIST2	149942218	5.9889	9.2434	-1.0474
YRI	HIST2	149947218	4.8024	8.7392	-1.3345
YRI	HIST2	149952218	7.4519	10.756	-0.9233
YRI	HIST2	149957218	7.465	11.0921	-0.9848

YRI	HIST2	149962218	4.9213	9.4115	-1.4211
YRI	HIST2	149967218	5.8211	9.4115	-1.1363
YRI	HIST2	149972218	6.3678	9.0754	-0.8863
YRI	HIST2	149977218	7.4583	9.9157	-0.7409
YRI	HIST2	149982218	8.5253	11.5963	-0.7997
YRI	HIST2	149987218	9.3239	11.4282	-0.5555
YRI	HIST2	149992218	10.1035	14.2853	-0.8941
YRI	HIST2	149997218	13.2451	15.9659	-0.5232
YRI	HIST2	150002218	13.9151	13.9492	-0.0075
YRI	HIST2	150007218	12.0523	15.6298	-0.7021
YRI	HIST2	150012218	14.9534	17.3104	-0.4195
YRI	HIST2	150017218	15.5687	15.4617	0.0212
YRI	HIST2	150022218	13.236	14.9575	-0.3523
YRI	HIST2	150027218	11.4588	13.9492	-0.5447
YRI	HIST2	150032218	8.7145	11.2602	-0.6815
YRI	HIST2	150037218	8.8002	13.1089	-0.9995
YRI	HIST2	150042218	12.0729	15.1256	-0.6181
YRI	HIST2	150047218	11.473	14.2853	-0.6013
YRI	HIST2	150052218	8.4284	13.445	-1.1361
YRI	HIST2	150057218	7.1816	12.7727	-1.3292
YRI	HIST2	150062218	8.6603	12.7727	-0.9777
YRI	HIST2	150067218	8.7662	12.1005	-0.8342
YRI	HIST2	150072218	9.9093	14.1172	-0.9099
YRI	HIST2	150077218	12.0361	17.1423	-0.9174
YRI	HIST2	150082218	10.1377	15.1256	-1.01
YRI	HIST2	150087218	12.4534	15.2937	-0.5691
YRI	HIST2	150092218	14.7377	15.4617	-0.1436
YRI	HIST2	150097218	11.7857	14.2853	-0.5344
YRI	HIST2	150102218	11.6516	15.6298	-0.7808
YRI	HIST2	150107218	12.0909	16.6382	-0.8407
YRI	HIST2	150112218	9.5465	14.9575	-1.1074
YRI	HIST2	150117218	7.3261	13.1089	-1.3414
YRI	HIST2	150122218	8.5138	15.2937	-1.3585
YRI	HIST2	150127218	9.5921	14.6214	-1.0518
YRI	HIST2	150132218	11.0011	15.6298	-0.9084
YRI	HIST2	150137218	13.477	18.4868	-0.8372
YRI	HIST2	150142218	11.3877	15.6298	-0.8325
YRI	HIST2	150147218	7.6196	12.1005	-1.121
YRI	HIST2	150152218	10.3245	13.9492	-0.7927
YRI	HIST2	150157218	16.6305	18.6549	-0.3354
YRI	HIST2	150162218	16.1314	18.1507	-0.3435
YRI	HIST2	150167218	13.6272	16.302	-0.5043
YRI	HIST2	150172218	12.0446	16.134	-0.7786
YRI	HIST2	150177218	11.8676	15.6298	-0.7384
YRI	HIST2	150182218	12.1707	14.4534	-0.4827
YRI	HIST2	150187218	9.2495	11.7644	-0.6461
YRI	HIST2	150192218	9.3765	11.4282	-0.5417
YRI	HIST2	150197218	10.2786	10.924	-0.1778
YRI	HIST2	150202218	11.0233	10.756	0.0747

YRI	HIST2	150207218	13.2454	13.9492	-0.1539
YRI	HIST2	150212218	12.7696	14.6214	-0.3873
YRI	HIST2	150217218	12.0709	12.9408	-0.2043
YRI	HIST2	150222218	9.3845	10.4199	-0.2981
YRI	HIST2	150227218	4.6446	8.9073	-1.4197
YRI	HIST2	150232218	3.8797	8.235	-1.5594
YRI	HIST2	150237218	3.5922	6.3864	-1.2611
YRI	HIST2	150242218	4.4458	8.7392	-1.4553
YRI	HIST2	150247218	5.7601	10.924	-1.4222
YRI	HIST2	150252218	6.3576	10.4199	-1.1694
YRI	HIST2	150257218	9.4516	10.5879	-0.3222
YRI	HIST2	150262218	15.0424	12.9408	0.4935
YRI	HIST2	150267218	19.3435	16.9743	0.4297
YRI	HIST2	150272218	16.2061	16.8062	-0.1099
YRI	HIST2	150277218	13.7827	15.1256	-0.2719
YRI	HIST2	150282218	13.4605	14.7895	-0.2749
YRI	HIST2	150287218	10.3363	12.9408	-0.6116
YRI	HIST2	150292218	9.4936	11.7644	-0.5834
YRI	HIST2	150297218	10.2339	12.1005	-0.467
YRI	HIST2	150302218	11.0884	12.4366	-0.3287
YRI	HIST2	150307218	12.8151	14.6214	-0.3778
YRI	HIST2	150312218	12.6707	13.445	-0.1754
YRI	HIST2	150317218	13.9021	13.2769	0.1433
YRI	HIST2	150322218	13.6148	14.9575	-0.2748
YRI	HIST2	150327218	12.9435	14.7895	-0.3819
YRI	HIST2	150332218	12.5213	14.2853	-0.3772
YRI	HIST2	150337218	10.7833	12.6047	-0.4385
CEU	HIST2	149356061	6.4378	6.3108	0.0583
CEU	HIST2	149361061	4.798	5.6286	-0.4225
CEU	HIST2	149366061	3.5523	4.0935	-0.3639
CEU	HIST2	149371061	3.4556	3.9229	-0.3259
CEU	HIST2	149376061	4.8237	5.7991	-0.4831
CEU	HIST2	149381061	3.7631	4.4346	-0.4213
CEU	HIST2	149386061	4.9029	4.0935	0.5443
CEU	HIST2	149391061	7.1274	5.9697	0.5587
CEU	HIST2	149396061	3.6256	4.9463	-0.7532
CEU	HIST2	149401061	5.7411	6.3108	-0.2616
CEU	HIST2	149406061	6.5902	5.6286	0.4891
CEU	HIST2	149411061	3.8971	3.2407	0.5382
CEU	HIST2	149416061	3.8042	3.2407	0.4621
CEU	HIST2	149421061	2.5547	2.0467	0.6045
CEU	HIST2	149426061	2.8027	2.8996	-0.0871
CEU	HIST2	149431061	2.3717	2.8996	-0.4748
CEU	HIST2	149436061	0.8654	0.8528	0.0284
CEU	HIST2	149441061	0	0	0
CEU	HIST2	149446061	0.08	0.5117	-1.3539
CEU	HIST2	149451061	0.1394	0.8528	-1.6066
CEU	HIST2	149456061	0.128	0.6822	-1.4472
CEU	HIST2	149461061	0.0686	0.3411	-1.0924

CEU	HIST2	149466061	0	0	0
CEU	HIST2	149471061	0	0	0
CEU	HIST2	149476061	0	0	0
CEU	HIST2	149481061	0	0	0
CEU	HIST2	149486061	0	0	0
CEU	HIST2	149491061	0	0	0
CEU	HIST2	149496061	0	0	0
CEU	HIST2	149501061	0	0	0
CEU	HIST2	149506061	0.1161	0.3411	-0.902
CEU	HIST2	149511061	0.4605	1.1939	-1.3069
CEU	HIST2	149516061	0.3445	0.8528	-1.1447
CEU	HIST2	149521061	0	0	0
CEU	HIST2	149526061	0	0	0
CEU	HIST2	149531061	0.0101	0.1706	-0.9534
CEU	HIST2	149536061	0.0101	0.1706	-0.9534
CEU	HIST2	149541061	0	0	0
CEU	HIST2	149546061	0	0	0
CEU	HIST2	149551061	0	0	0
CEU	HIST2	149556061	0	0	0
CEU	HIST2	149561061	0	0	0
CEU	HIST2	149566061	0	0	0
CEU	HIST2	149571061	0.6005	0.3411	1.0399
CEU	HIST2	149576061	0.885	0.6822	0.5296
CEU	HIST2	149581061	2.6434	3.0701	-0.366
CEU	HIST2	149586061	3.8	3.7524	0.0345
CEU	HIST2	149591061	2.1948	1.8762	0.4056
CEU	HIST2	149596061	2.012	2.729	-0.6777
CEU	HIST2	149601061	4.7794	4.2641	0.3345
CEU	HIST2	149606061	7.0796	4.2641	1.8277
CEU	HIST2	149611061	5.2857	3.0701	1.9005
CEU	HIST2	149616061	1.7273	1.1939	0.9505
CEU	HIST2	149621061	0	0	0
CEU	HIST2	149626061	0	0	0
CEU	HIST2	149631061	0.0301	0.1706	-0.8346
CEU	HIST2	149636061	1.5059	1.3645	0.2288
CEU	HIST2	149641061	2.9559	2.2173	0.8252
CEU	HIST2	149646061	5.7737	5.458	0.165
CEU	HIST2	149651061	8.0025	7.1636	0.3434
CEU	HIST2	149656061	6.69	4.6052	1.2658
CEU	HIST2	149661061	6.1608	5.9697	0.0922
CEU	HIST2	149666061	5.0033	8.6987	-1.266
CEU	HIST2	149671061	5.1422	8.6987	-1.2184
CEU	HIST2	149676061	5.8806	7.3342	-0.5824
CEU	HIST2	149681061	2.8672	5.458	-1.3544
CEU	HIST2	149686061	0.9979	3.5818	-1.9475
CEU	HIST2	149691061	1.3179	3.2407	-1.5767
CEU	HIST2	149696061	2.7873	4.9463	-1.2313
CEU	HIST2	149701061	4.4286	6.1402	-0.8054
CEU	HIST2	149706061	6.7262	6.9931	-0.1117

CEU	HIST2	149711061	7.6835	7.3342	0.14
CEU	HIST2	149716061	9.9725	8.3576	0.574
CEU	HIST2	149721061	11.173	7.8459	1.2534
CEU	HIST2	149726061	6.8407	6.1402	0.3296
CEU	HIST2	149731061	5.3667	5.6286	-0.1332
CEU	HIST2	149736061	5.0111	4.7757	0.1384
CEU	HIST2	149741061	2.9128	3.4112	-0.3915
CEU	HIST2	149746061	4.6015	3.5818	0.7685
CEU	HIST2	149751061	3.9283	3.7524	0.1274
CEU	HIST2	149756061	1.7943	3.4112	-1.2699
CEU	HIST2	149761061	2.4993	5.2874	-1.4992
CEU	HIST2	149766061	1.2942	4.6052	-2.0103
CEU	HIST2	149771061	2.9518	4.7757	-1.0727
CEU	HIST2	149776061	5.2746	7.1636	-0.7733
CEU	HIST2	149781061	5.0462	8.0164	-1.0971
CEU	HIST2	149786061	4.0701	7.1636	-1.2663
CEU	HIST2	149791061	2.1408	3.9229	-1.243
CEU	HIST2	149796061	0.3836	0.8528	-1.0566
CEU	HIST2	149801061	0	0	0
CEU	HIST2	149806061	0	0	0
CEU	HIST2	149811061	1.1563	1.3645	-0.3368
CEU	HIST2	149816061	2.503	3.5818	-0.8131
CEU	HIST2	149821061	2.0501	3.0701	-0.8749
CEU	HIST2	149826061	0.7034	0.8528	-0.3364
CEU	HIST2	149831061	0	0	0
CEU	HIST2	149836061	0	0	0
CEU	HIST2	149841061	0.3923	0.5117	-0.3745
CEU	HIST2	149846061	1.9915	3.5818	-1.1986
CEU	HIST2	149851061	2.4545	5.458	-1.5702
CEU	HIST2	149856061	1.6761	5.1169	-1.9045
CEU	HIST2	149861061	3.5198	6.6519	-1.3713
CEU	HIST2	149866061	5.7144	9.2104	-1.136
CEU	HIST2	149871061	4.9204	8.3576	-1.2218
CEU	HIST2	149876061	3.5312	6.3108	-1.2762
CEU	HIST2	149881061	3.1727	5.6286	-1.2492
CEU	HIST2	149886061	2.5168	3.9229	-0.9807
CEU	HIST2	149891061	3.0138	3.9229	-0.6341
CEU	HIST2	149896061	4.1303	6.3108	-1.0011
CEU	HIST2	149901061	3.2051	5.7991	-1.2848
CEU	HIST2	149906061	3.491	4.9463	-0.83
CEU	HIST2	149911061	2.9746	4.7757	-1.0593
CEU	HIST2	149916061	1.6155	4.0935	-1.6663
CEU	HIST2	149921061	3.0489	5.1169	-1.1447
CEU	HIST2	149926061	4.3426	5.2874	-0.508
CEU	HIST2	149931061	3.2531	5.6286	-1.2083
CEU	HIST2	149936061	2.1371	5.458	-1.7361
CEU	HIST2	149941061	2.3709	3.9229	-1.0825
CEU	HIST2	149946061	2.1497	3.7524	-1.1611
CEU	HIST2	149951061	2.7442	5.7991	-1.5131

CEU	HIST2	149956061	2.4113	4.7757	-1.3906
CEU	HIST2	149961061	1.2548	2.729	-1.3934
CEU	HIST2	149966061	1.5325	3.0701	-1.3189
CEU	HIST2	149971061	2.5065	4.0935	-1.0671
CEU	HIST2	149976061	2.4527	4.2641	-1.1759
CEU	HIST2	149981061	2.3082	4.4346	-1.3342
CEU	HIST2	149986061	3.2799	5.458	-1.1387
CEU	HIST2	149991061	5.7891	6.8225	-0.4422
CEU	HIST2	149996061	8.1765	8.3576	-0.0644
CEU	HIST2	150001061	8.9544	7.8459	0.4176
CEU	HIST2	150006061	7.8714	6.6519	0.5339
CEU	HIST2	150011061	10.4032	9.2104	0.3876
CEU	HIST2	150016061	12.0101	10.2337	0.5233
CEU	HIST2	150021061	8.2729	7.1636	0.4541
CEU	HIST2	150026061	5.6425	5.7991	-0.0776
CEU	HIST2	150031061	3.1717	5.2874	-1.1376
CEU	HIST2	150036061	2.6553	5.1169	-1.3625
CEU	HIST2	150041061	3.57	5.6286	-1.0471
CEU	HIST2	150046061	5.4927	6.9931	-0.6278
CEU	HIST2	150051061	3.9221	5.2874	-0.7341
CEU	HIST2	150056061	2.1027	3.9229	-1.2695
CEU	HIST2	150061061	3.0258	5.1169	-1.1575
CEU	HIST2	150066061	1.9971	3.7524	-1.2717
CEU	HIST2	150071061	4.2546	4.4346	-0.1129
CEU	HIST2	150076061	6.9598	6.8225	0.0588
CEU	HIST2	150081061	4.5764	6.3108	-0.7963
CEU	HIST2	150086061	3.5586	7.1636	-1.4757
CEU	HIST2	150091061	4.1697	6.6519	-1.0868
CEU	HIST2	150096061	3.659	4.9463	-0.7341
CEU	HIST2	150101061	3.7442	6.1402	-1.1275
CEU	HIST2	150106061	5.6516	6.6519	-0.438
CEU	HIST2	150111061	6.6848	6.4814	0.0912
CEU	HIST2	150116061	4.2647	5.1169	-0.4717
CEU	HIST2	150121061	3.2517	5.6286	-1.209
CEU	HIST2	150126061	5.0276	7.8459	-1.0617
CEU	HIST2	150131061	6.5593	9.2104	-0.8615
CEU	HIST2	150136061	7.1653	11.2571	-1.1026
CEU	HIST2	150141061	5.9015	9.5515	-1.1467
CEU	HIST2	150146061	4.6846	6.9931	-0.9659
CEU	HIST2	150151061	3.8654	7.5047	-1.4279
CEU	HIST2	150156061	5.1734	8.6987	-1.2077
CEU	HIST2	150161061	7.3892	9.0398	-0.5457
CEU	HIST2	150166061	9.3344	8.0164	0.4868
CEU	HIST2	150171061	9.1189	6.8225	0.9826
CEU	HIST2	150176061	6.0761	5.7991	0.1372
CEU	HIST2	150181061	7.5974	6.4814	0.5002
CEU	HIST2	150186061	7.8027	6.6519	0.5038
CEU	HIST2	150191061	6.0346	6.1402	-0.0497
CEU	HIST2	150196061	6.4914	6.1402	0.1653

CEU	HIST2	150201061	8.4232	6.6519	0.7755
CEU	HIST2	150206061	9.0678	7.8459	0.4603
CEU	HIST2	150211061	8.8654	7.5047	0.5339
CEU	HIST2	150216061	8.5753	6.4814	0.9385
CEU	HIST2	150221061	7.7838	6.4814	0.5838
CEU	HIST2	150226061	5.0878	5.458	-0.1935
CEU	HIST2	150231061	2.3393	3.5818	-0.9365
CEU	HIST2	150236061	2.7955	2.5584	0.2362
CEU	HIST2	150241061	3.0823	3.9229	-0.5863
CEU	HIST2	150246061	5.3715	5.9697	-0.2887
CEU	HIST2	150251061	5.7211	5.2874	0.2332
CEU	HIST2	150256061	6.6999	6.1402	0.2634
CEU	HIST2	150261061	13.6526	8.8692	1.6096
CEU	HIST2	150266061	18.6572	10.2337	2.4816
CEU	HIST2	150271061	15.7421	11.0866	1.2726
CEU	HIST2	150276061	14.0435	11.2571	0.7508
CEU	HIST2	150281061	17.242	11.5982	1.4787
CEU	HIST2	150286061	13.7757	8.8692	1.651
CEU	HIST2	150291061	9.316	6.4814	1.2705
CEU	HIST2	150296061	9.3435	7.6753	0.6412
CEU	HIST2	150301061	8.2621	7.6753	0.2255
CEU	HIST2	150306061	8.7949	8.187	0.2202
CEU	HIST2	150311061	10.416	9.2104	0.3918
CEU	HIST2	150316061	12.4321	10.7454	0.4748
CEU	HIST2	150321061	13.523	10.0632	1.0354
CEU	HIST2	150326061	13.0498	8.8692	1.4068
CEU	HIST2	150331061	12.7776	9.3809	1.0851
CEU	HIST2	150336061	11.5536	9.3809	0.6941
CEU	HIST2	150341061	9.3917	9.2104	0.0589
CEU	HIST2	150346061	8.6794	8.0164	0.2449
CHB	HIST2	149356061	5.3149	5.9295	-0.2975
CHB	HIST2	149361061	4.2311	3.8965	0.234
CHB	HIST2	149366061	3.2501	3.8965	-0.4521
CHB	HIST2	149371061	2.7796	5.0825	-1.2785
CHB	HIST2	149376061	3.5393	5.5907	-1.0467
CHB	HIST2	149381061	2.9831	4.066	-0.7302
CHB	HIST2	149386061	3.7443	4.913	-0.6685
CHB	HIST2	149391061	5.4818	6.2684	-0.3623
CHB	HIST2	149396061	3.0779	4.913	-1.0497
CHB	HIST2	149401061	4.704	6.0989	-0.6585
CHB	HIST2	149406061	4.9303	5.7601	-0.4123
CHB	HIST2	149411061	3.5551	4.7436	-0.7011
CHB	HIST2	149416061	3.9871	4.066	-0.0532
CHB	HIST2	149421061	2.0497	3.0495	-0.8598
CHB	HIST2	149426061	1.9568	3.8965	-1.3567
CHB	HIST2	149431061	1.9248	3.5577	-1.2341
CHB	HIST2	149436061	0.7352	1.5247	-1.1729
CHB	HIST2	149441061	0	0	0
CHB	HIST2	149446061	0.0386	0.5082	-1.4741

CHB	HIST2	149451061	0.0871	1.1859	-1.9613
CHB	HIST2	149456061	0.0873	1.1859	-1.961
CHB	HIST2	149461061	0.0388	0.5082	-1.4735
CHB	HIST2	149466061	0	0	0
CHB	HIST2	149471061	0	0	0
CHB	HIST2	149476061	0	0	0
CHB	HIST2	149481061	0	0	0
CHB	HIST2	149486061	0	0	0
CHB	HIST2	149491061	0	0	0
CHB	HIST2	149496061	0	0	0
CHB	HIST2	149501061	0	0	0
CHB	HIST2	149506061	0.3379	0.3388	-0.0036
CHB	HIST2	149511061	0.8968	1.0165	-0.2382
CHB	HIST2	149516061	0.5686	0.8471	-0.628
CHB	HIST2	149521061	0.0097	0.1694	-0.949
CHB	HIST2	149526061	0	0	0
CHB	HIST2	149531061	0.2578	0.1694	0.5253
CHB	HIST2	149536061	0.2578	0.1694	0.5253
CHB	HIST2	149541061	0	0	0
CHB	HIST2	149546061	0	0	0
CHB	HIST2	149551061	0	0	0
CHB	HIST2	149556061	0	0	0
CHB	HIST2	149561061	0	0	0
CHB	HIST2	149566061	0.0097	0.1694	-0.949
CHB	HIST2	149571061	0.4891	0.5082	-0.06
CHB	HIST2	149576061	0.9039	0.8471	0.1281
CHB	HIST2	149581061	3.1039	3.3883	-0.224
CHB	HIST2	149586061	4.0702	3.8965	0.1215
CHB	HIST2	149591061	1.8397	1.5247	0.468
CHB	HIST2	149596061	2.4835	2.033	0.5372
CHB	HIST2	149601061	6.0246	3.7271	1.6691
CHB	HIST2	149606061	6.6937	4.913	1.0186
CHB	HIST2	149611061	4.095	3.8965	0.1388
CHB	HIST2	149616061	1.3912	1.1859	0.3665
CHB	HIST2	149621061	0	0	0
CHB	HIST2	149626061	0	0	0
CHB	HIST2	149631061	0.0097	0.1694	-0.949
CHB	HIST2	149636061	1.9596	1.3553	0.9794
CHB	HIST2	149641061	3.5026	2.7106	0.7505
CHB	HIST2	149646061	5.9046	5.5907	0.1602
CHB	HIST2	149651061	7.735	6.6072	0.4953
CHB	HIST2	149656061	5.4565	5.5907	-0.0685
CHB	HIST2	149661061	5.3882	5.2519	0.0735
CHB	HIST2	149666061	7.7929	5.7601	1.0099
CHB	HIST2	149671061	8.0493	8.1319	-0.03
CHB	HIST2	149676061	6.1386	7.1154	-0.4011
CHB	HIST2	149681061	3.3585	5.0825	-0.9571
CHB	HIST2	149686061	2.0524	4.4048	-1.4802
CHB	HIST2	149691061	2.8776	4.2354	-0.8839

CHB	HIST2	149696061	4.4445	4.5742	-0.079
CHB	HIST2	149701061	5.503	6.0989	-0.2813
CHB	HIST2	149706061	6.799	6.7766	0.0096
CHB	HIST2	149711061	6.55	5.9295	0.3004
CHB	HIST2	149716061	7.762	8.3013	-0.1923
CHB	HIST2	149721061	8.7421	9.8261	-0.3307
CHB	HIST2	149726061	5.8646	8.4708	-0.9124
CHB	HIST2	149731061	5.2065	6.7766	-0.674
CHB	HIST2	149736061	4.4731	5.5907	-0.5702
CHB	HIST2	149741061	2.0879	3.5577	-1.1108
CHB	HIST2	149746061	3.8763	3.0495	0.7111
CHB	HIST2	149751061	3.7139	3.3883	0.2564
CHB	HIST2	149756061	0.8115	4.2354	-2.229
CHB	HIST2	149761061	2.0857	6.7766	-2.0136
CHB	HIST2	149766061	1.6082	4.7436	-1.8495
CHB	HIST2	149771061	1.2717	4.913	-2.0828
CHB	HIST2	149776061	1.6794	5.9295	-2.0575
CHB	HIST2	149781061	1.0537	5.0825	-2.2367
CHB	HIST2	149786061	0.7438	5.5907	-2.473
CHB	HIST2	149791061	0.422	3.7271	-2.4011
CHB	HIST2	149796061	0.1902	1.1859	-1.7773
CHB	HIST2	149801061	0	0	0
CHB	HIST2	149806061	0	0	0
CHB	HIST2	149811061	0.4297	1.3553	-1.5001
CHB	HIST2	149816061	1.1502	4.066	-1.9662
CHB	HIST2	149821061	1.2125	4.2354	-1.9679
CHB	HIST2	149826061	0.4919	1.5247	-1.5344
CHB	HIST2	149831061	0	0	0
CHB	HIST2	149836061	0	0	0
CHB	HIST2	149841061	0.0289	0.3388	-1.2431
CHB	HIST2	149846061	0.5002	4.2354	-2.4316
CHB	HIST2	149851061	1.1179	7.9625	-2.5364
CHB	HIST2	149856061	1.0769	6.0989	-2.3706
CHB	HIST2	149861061	1.3091	5.4213	-2.1563
CHB	HIST2	149866061	1.5333	7.1154	-2.2924
CHB	HIST2	149871061	0.9307	6.6072	-2.4932
CHB	HIST2	149876061	1.3607	6.2684	-2.2603
CHB	HIST2	149881061	1.7582	7.2848	-2.2215
CHB	HIST2	149886061	1.1163	6.0989	-2.352
CHB	HIST2	149891061	1.6861	5.4213	-1.9586
CHB	HIST2	149896061	1.5823	7.6237	-2.3297
CHB	HIST2	149901061	1.0137	7.1154	-2.5058
CHB	HIST2	149906061	2.1499	7.1154	-2.0392
CHB	HIST2	149911061	2.1122	7.7931	-2.1471
CHB	HIST2	149916061	1.5108	6.2684	-2.1912
CHB	HIST2	149921061	2.7226	8.4708	-2.0123
CHB	HIST2	149926061	3.7428	10.5037	-1.9383
CHB	HIST2	149931061	2.8961	9.4872	-2.0777
CHB	HIST2	149936061	2.3324	10.3343	-2.3292

CHB	HIST2	149941061	2.5686	8.8096	-2.1071
CHB	HIST2	149946061	2.2648	6.0989	-1.8099
CHB	HIST2	149951061	2.04	7.7931	-2.1744
CHB	HIST2	149956061	1.5597	7.9625	-2.3727
CHB	HIST2	149961061	1.34	6.0989	-2.2464
CHB	HIST2	149966061	1.0556	6.2684	-2.4008
CHB	HIST2	149971061	1.4213	5.9295	-2.1825
CHB	HIST2	149976061	1.9322	5.0825	-1.749
CHB	HIST2	149981061	1.4866	6.6072	-2.249
CHB	HIST2	149986061	2.2292	8.6402	-2.2037
CHB	HIST2	149991061	3.6107	9.4872	-1.8524
CHB	HIST2	149996061	6.1119	10.6731	-1.2883
CHB	HIST2	150001061	6.3588	11.5202	-1.357
CHB	HIST2	150006061	3.288	9.3178	-1.9328
CHB	HIST2	150011061	2.1883	10.1649	-2.358
CHB	HIST2	150016061	2.0415	10.3343	-2.4139
CHB	HIST2	150021061	1.6832	8.1319	-2.3439
CHB	HIST2	150026061	1.4738	8.3013	-2.435
CHB	HIST2	150031061	0.8212	6.6072	-2.5413
CHB	HIST2	150036061	1.189	5.9295	-2.2949
CHB	HIST2	150041061	2.1468	6.7766	-1.9874
CHB	HIST2	150046061	1.6223	7.9625	-2.3495
CHB	HIST2	150051061	1.0413	6.7766	-2.462
CHB	HIST2	150056061	1.0436	5.0825	-2.2423
CHB	HIST2	150061061	0.7992	5.5907	-2.4447
CHB	HIST2	150066061	0.6478	5.2519	-2.4831
CHB	HIST2	150071061	1.1749	6.7766	-2.4046
CHB	HIST2	150076061	1.3236	7.7931	-2.4451
CHB	HIST2	150081061	0.6806	5.5907	-2.5052
CHB	HIST2	150086061	1.3324	6.6072	-2.3168
CHB	HIST2	150091061	1.8993	6.946	-2.1184
CHB	HIST2	150096061	1.434	5.7601	-2.1493
CHB	HIST2	150101061	1.4005	7.1154	-2.3469
CHB	HIST2	150106061	1.8186	7.4543	-2.2184
CHB	HIST2	150111061	1.3842	7.1154	-2.3536
CHB	HIST2	150116061	1.0733	6.6072	-2.4306
CHB	HIST2	150121061	1.8549	7.4543	-2.2041
CHB	HIST2	150126061	2.5888	8.3013	-2.0373
CHB	HIST2	150131061	2.7722	8.3013	-1.9719
CHB	HIST2	150136061	2.3924	8.979	-2.185
CHB	HIST2	150141061	2.5599	9.4872	-2.1836
CHB	HIST2	150146061	2.533	8.4708	-2.0787
CHB	HIST2	150151061	2.0617	8.4708	-2.2436
CHB	HIST2	150156061	2.8397	11.8591	-2.3076
CHB	HIST2	150161061	5.0817	12.3673	-1.7917
CHB	HIST2	150166061	8.9528	11.012	-0.5648
CHB	HIST2	150171061	8.2659	9.8261	-0.476
CHB	HIST2	150176061	5.0807	9.8261	-1.4478
CHB	HIST2	150181061	6.3457	9.8261	-1.0619

CHB	HIST2	150186061	6.0451	7.7931	-0.6607
CHB	HIST2	150191061	4.911	6.946	-0.8542
CHB	HIST2	150196061	6.4069	7.6237	-0.4692
CHB	HIST2	150201061	8.5789	9.8261	-0.3805
CHB	HIST2	150206061	8.8808	10.8426	-0.546
CHB	HIST2	150211061	8.2269	11.1814	-0.7989
CHB	HIST2	150216061	7.8818	10.5037	-0.7517
CHB	HIST2	150221061	6.8812	8.6402	-0.6046
CHB	HIST2	150226061	4.0803	7.4543	-1.3281
CHB	HIST2	150231061	2.0535	5.2519	-1.725
CHB	HIST2	150236061	2.7594	3.8965	-0.7953
CHB	HIST2	150241061	3.8989	5.2519	-0.7297
CHB	HIST2	150246061	5.4891	7.4543	-0.7736
CHB	HIST2	150251061	4.7921	7.7931	-1.1342
CHB	HIST2	150256061	5.2857	7.7931	-0.9477
CHB	HIST2	150261061	9.9897	9.8261	0.0499
CHB	HIST2	150266061	13.6783	11.5202	0.5674
CHB	HIST2	150271061	10.8496	10.3343	0.15
CHB	HIST2	150276061	9.0219	10.3343	-0.382
CHB	HIST2	150281061	11.8653	13.5532	-0.3807
CHB	HIST2	150286061	9.9702	10.8426	-0.2428
CHB	HIST2	150291061	6.4022	8.3013	-0.6773
CHB	HIST2	150296061	6.148	9.3178	-1.016
CHB	HIST2	150301061	6.0429	8.4708	-0.8499
CHB	HIST2	150306061	6.7982	9.9955	-0.9601
CHB	HIST2	150311061	7.5165	9.3178	-0.5774
CHB	HIST2	150316061	7.8782	9.4872	-0.5072
CHB	HIST2	150321061	9.2692	10.8426	-0.4379
CHB	HIST2	150326061	9.7202	11.3508	-0.4347
CHB	HIST2	150331061	8.574	11.6896	-0.808
CHB	HIST2	150336061	7.2781	8.8096	-0.5171
CHB	HIST2	150341061	6.5481	6.7766	-0.0981
CHB	HIST2	150346061	5.9178	7.1154	-0.4918
YRI	HIST2	149356061	6.8644	7.3947	-0.2096
YRI	HIST2	149361061	5.0016	6.7225	-0.7415
YRI	HIST2	149366061	3.8247	7.3947	-1.4107
YRI	HIST2	149371061	3.2879	7.0586	-1.5545
YRI	HIST2	149376061	4.4699	7.8989	-1.2757
YRI	HIST2	149381061	3.7988	6.0502	-1.0667
YRI	HIST2	149386061	6.0587	5.378	0.3582
YRI	HIST2	149391061	7.9415	7.5628	0.1466
YRI	HIST2	149396061	4.6509	7.2267	-1.0394
YRI	HIST2	149401061	6.7805	8.9073	-0.7083
YRI	HIST2	149406061	7.3143	8.9073	-0.5306
YRI	HIST2	149411061	5.4248	8.067	-0.9642
YRI	HIST2	149416061	4.6725	5.7141	-0.5194
YRI	HIST2	149421061	3.4411	3.0251	0.3589
YRI	HIST2	149426061	3.4213	4.3696	-0.5988
YRI	HIST2	149431061	2.5589	3.8654	-0.9168

YRI	HIST2	149436061	1.1608	1.5126	-0.5238
YRI	HIST2	149441061	0.0278	0.3361	-1.2377
YRI	HIST2	149446061	0.1568	0.5042	-1.0918
YRI	HIST2	149451061	0.4181	1.1764	-1.3563
YRI	HIST2	149456061	0.5682	1.5126	-1.4063
YRI	HIST2	149461061	0.3069	0.8403	-1.2048
YRI	HIST2	149466061	0	0	0
YRI	HIST2	149471061	0	0	0
YRI	HIST2	149476061	0	0	0
YRI	HIST2	149481061	0	0	0
YRI	HIST2	149486061	0	0	0
YRI	HIST2	149491061	0	0	0
YRI	HIST2	149496061	0	0	0
YRI	HIST2	149501061	0	0	0
YRI	HIST2	149506061	0.3128	0.3361	-0.0935
YRI	HIST2	149511061	0.6674	2.0167	-1.6133
YRI	HIST2	149516061	0.5881	2.1848	-1.7927
YRI	HIST2	149521061	0.2335	0.5042	-0.8507
YRI	HIST2	149526061	0.073	0.3361	-1.0562
YRI	HIST2	149531061	0.2339	0.5042	-0.8495
YRI	HIST2	149536061	0.1609	0.1681	-0.0428
YRI	HIST2	149541061	0	0	0
YRI	HIST2	149546061	0	0	0
YRI	HIST2	149551061	0	0	0
YRI	HIST2	149556061	0	0	0
YRI	HIST2	149561061	0	0	0
YRI	HIST2	149566061	0.0278	0.3361	-1.2377
YRI	HIST2	149571061	0.6993	1.1764	-0.8533
YRI	HIST2	149576061	1.082	1.3445	-0.4263
YRI	HIST2	149581061	2.57	5.2099	-1.4289
YRI	HIST2	149586061	2.8328	6.0502	-1.5244
YRI	HIST2	149591061	1.0037	2.1848	-1.3261
YRI	HIST2	149596061	2.1545	2.689	-0.508
YRI	HIST2	149601061	5.0294	5.7141	-0.3414
YRI	HIST2	149606061	5.4612	6.2183	-0.35
YRI	HIST2	149611061	3.6297	4.5377	-0.5548
YRI	HIST2	149616061	1.3738	2.1848	-0.9106
YRI	HIST2	149621061	0	0	0
YRI	HIST2	149626061	0	0	0
YRI	HIST2	149631061	0.0093	0.1681	-0.944
YRI	HIST2	149636061	2.0972	1.5126	0.8705
YRI	HIST2	149641061	3.6907	3.0251	0.5742
YRI	HIST2	149646061	6.9691	7.7309	-0.289
YRI	HIST2	149651061	10.0256	11.4282	-0.3703
YRI	HIST2	149656061	8.1974	8.7392	-0.1837
YRI	HIST2	149661061	7.1932	8.067	-0.3189
YRI	HIST2	149666061	10.2637	11.7644	-0.3855
YRI	HIST2	149671061	10.6195	14.2853	-0.7838
YRI	HIST2	149676061	6.802	11.0921	-1.1648

YRI	HIST2	149681061	4.7524	9.0754	-1.4151
YRI	HIST2	149686061	3.0003	8.7392	-1.9453
YRI	HIST2	149691061	2.7131	6.3864	-1.6579
YRI	HIST2	149696061	4.4725	7.3947	-1.1547
YRI	HIST2	149701061	7.1493	10.2518	-0.9068
YRI	HIST2	149706061	9.2796	12.1005	-0.7057
YRI	HIST2	149711061	7.5691	10.756	-0.8906
YRI	HIST2	149716061	8.2773	11.4282	-0.8318
YRI	HIST2	149721061	9.7818	13.2769	-0.801
YRI	HIST2	149726061	7.8031	13.2769	-1.2545
YRI	HIST2	149731061	6.9369	12.6047	-1.3644
YRI	HIST2	149736061	6.1002	9.0754	-0.9739
YRI	HIST2	149741061	4.3387	6.3864	-0.9242
YRI	HIST2	149746061	6.6841	6.8906	-0.087
YRI	HIST2	149751061	6.9753	8.4031	-0.5018
YRI	HIST2	149756061	5.4039	9.0754	-1.2019
YRI	HIST2	149761061	6.6294	11.2602	-1.2397
YRI	HIST2	149766061	3.5886	8.5712	-1.7195
YRI	HIST2	149771061	4.6249	9.0754	-1.4569
YRI	HIST2	149776061	6.6878	13.9492	-1.5881
YRI	HIST2	149781061	5.4871	13.9492	-1.8507
YRI	HIST2	149786061	4.5396	11.5963	-1.8376
YRI	HIST2	149791061	2.4457	6.5544	-1.8114
YRI	HIST2	149796061	0.7663	1.8487	-1.3845
YRI	HIST2	149801061	0	0	0
YRI	HIST2	149806061	0	0	0
YRI	HIST2	149811061	2.3101	2.8571	-0.4947
YRI	HIST2	149816061	4.2558	7.3947	-1.2403
YRI	HIST2	149821061	3.2265	5.8822	-1.2904
YRI	HIST2	149826061	1.2808	1.3445	-0.1035
YRI	HIST2	149831061	0	0	0
YRI	HIST2	149836061	0	0	0
YRI	HIST2	149841061	0.405	1.0084	-1.203
YRI	HIST2	149846061	3.6241	7.8989	-1.5903
YRI	HIST2	149851061	5.9907	14.2853	-1.7735
YRI	HIST2	149856061	3.8384	11.4282	-2.0037
YRI	HIST2	149861061	3.7767	10.4199	-1.9124
YRI	HIST2	149866061	7.5852	13.2769	-1.3045
YRI	HIST2	149871061	7.6574	12.2685	-1.1387
YRI	HIST2	149876061	6.1207	11.2602	-1.3759
YRI	HIST2	149881061	7.9393	11.9324	-1.0122
YRI	HIST2	149886061	6.7343	11.7644	-1.2923
YRI	HIST2	149891061	4.9618	11.5963	-1.7277
YRI	HIST2	149896061	5.0849	12.7727	-1.8277
YRI	HIST2	149901061	3.8197	11.9324	-2.0566
YRI	HIST2	149906061	5.6663	11.5963	-1.5442
YRI	HIST2	149911061	7.0905	12.4366	-1.3034
YRI	HIST2	149916061	5.3547	11.7644	-1.6467
YRI	HIST2	149921061	7.0787	13.9492	-1.5026

YRI	HIST2	149926061	8.9319	14.2853	-1.1446
YRI	HIST2	149931061	8.8564	12.7727	-0.931
YRI	HIST2	149936061	9.3951	12.7727	-0.803
YRI	HIST2	149941061	6.6968	10.5879	-1.1035
YRI	HIST2	149946061	4.7258	8.9073	-1.3927
YRI	HIST2	149951061	7.1591	10.2518	-0.9039
YRI	HIST2	149956061	7.7652	11.0921	-0.9033
YRI	HIST2	149961061	5.1332	9.7476	-1.4136
YRI	HIST2	149966061	5.526	8.9073	-1.1262
YRI	HIST2	149971061	7.0876	9.4115	-0.7355
YRI	HIST2	149976061	7.0174	9.7476	-0.8364
YRI	HIST2	149981061	7.3634	10.5879	-0.9145
YRI	HIST2	149986061	9.275	11.9324	-0.6736
YRI	HIST2	149991061	9.6697	13.2769	-0.8267
YRI	HIST2	149996061	12.3367	15.6298	-0.6463
YRI	HIST2	150001061	14.6841	15.2937	-0.1221
YRI	HIST2	150006061	11.4374	13.7811	-0.5185
YRI	HIST2	150011061	14.8816	17.1423	-0.4062
YRI	HIST2	150016061	17.0874	17.6465	-0.0977
YRI	HIST2	150021061	12.5468	14.6214	-0.4339
YRI	HIST2	150026061	12.0264	14.2853	-0.483
YRI	HIST2	150031061	9.9952	12.4366	-0.5952
YRI	HIST2	150036061	6.9393	10.5879	-1.0347
YRI	HIST2	150041061	10.3481	13.9492	-0.7876
YRI	HIST2	150046061	13.2483	16.302	-0.5757
YRI	HIST2	150051061	9.4244	13.613	-0.9375
YRI	HIST2	150056061	7.3917	12.7727	-1.2793
YRI	HIST2	150061061	9.3714	13.2769	-0.8951
YRI	HIST2	150066061	7.9601	11.5963	-0.9469
YRI	HIST2	150071061	8.1596	13.613	-1.2206
YRI	HIST2	150076061	12.3311	16.9743	-0.8421
YRI	HIST2	150081061	10.6607	15.4617	-0.952
YRI	HIST2	150086061	12.495	16.302	-0.7177
YRI	HIST2	150091061	15.2376	15.4617	-0.0444
YRI	HIST2	150096061	11.59	13.7811	-0.4847
YRI	HIST2	150101061	11.5804	15.2937	-0.744
YRI	HIST2	150106061	12.1751	16.8062	-0.848
YRI	HIST2	150111061	10.3858	14.9575	-0.9356
YRI	HIST2	150116061	7.6362	13.1089	-1.2695
YRI	HIST2	150121061	7.9616	15.1256	-1.4506
YRI	HIST2	150126061	9.9335	14.7895	-1.0046
YRI	HIST2	150131061	11.393	16.302	-0.9255
YRI	HIST2	150136061	13.1365	18.823	-0.934
YRI	HIST2	150141061	11.2407	15.9659	-0.9087
YRI	HIST2	150146061	7.7856	12.2685	-1.107
YRI	HIST2	150151061	10.1005	12.9408	-0.6669
YRI	HIST2	150156061	15.3937	16.4701	-0.2009
YRI	HIST2	150161061	15.5659	18.4868	-0.4881
YRI	HIST2	150166061	13.9665	17.3104	-0.5952

YRI	HIST2	150171061	12.5194	16.4701	-0.7375
YRI	HIST2	150176061	12.2169	15.9659	-0.721
YRI	HIST2	150181061	12.4549	14.2853	-0.3914
YRI	HIST2	150186061	9.7074	12.6047	-0.6975
YRI	HIST2	150191061	9.3604	11.4282	-0.5459
YRI	HIST2	150196061	9.7021	10.2518	-0.1607
YRI	HIST2	150201061	9.7953	10.5879	-0.2248
YRI	HIST2	150206061	11.5683	12.4366	-0.2117
YRI	HIST2	150211061	12.906	14.6214	-0.3587
YRI	HIST2	150216061	13.1502	14.2853	-0.2427
YRI	HIST2	150221061	10.7085	10.5879	0.0342
YRI	HIST2	150226061	6.3478	9.0754	-0.8929
YRI	HIST2	150231061	3.7215	8.7392	-1.7009
YRI	HIST2	150236061	2.8188	6.2183	-1.5716
YRI	HIST2	150241061	4.3541	8.067	-1.3549
YRI	HIST2	150246061	6.3342	11.4282	-1.3448
YRI	HIST2	150251061	6.3345	10.5879	-1.2062
YRI	HIST2	150256061	7.6761	9.5795	-0.5926
YRI	HIST2	150261061	14.3801	13.1089	0.2949
YRI	HIST2	150266061	18.8856	16.6382	0.4155
YRI	HIST2	150271061	15.277	15.7978	-0.1012
YRI	HIST2	150276061	14.314	15.4617	-0.2276
YRI	HIST2	150281061	15.6301	15.9659	-0.0646
YRI	HIST2	150286061	11.3395	12.9408	-0.376
YRI	HIST2	150291061	8.9155	11.7644	-0.7319
YRI	HIST2	150296061	10.736	12.7727	-0.4842
YRI	HIST2	150301061	10.8452	12.2685	-0.3515
YRI	HIST2	150306061	11.5184	14.2853	-0.5916
YRI	HIST2	150311061	12.2546	13.613	-0.304
YRI	HIST2	150316061	14.7263	13.7811	0.2091
YRI	HIST2	150321061	13.6878	14.6214	-0.1952
YRI	HIST2	150326061	11.4443	13.7811	-0.517
YRI	HIST2	150331061	12.9611	13.613	-0.1459
YRI	HIST2	150336061	11.3785	12.6047	-0.2952
YRI	HIST2	150341061	10.8981	12.9408	-0.4797
YRI	HIST2	150346061	10.6406	11.7644	-0.2887
CEU	HIST2	149357608	5.9862	6.1402	-0.0725
CEU	HIST2	149362608	5.215	5.1169	0.0543
CEU	HIST2	149367608	2.5683	3.2407	-0.5514
CEU	HIST2	149372608	3.4358	4.7757	-0.788
CEU	HIST2	149377608	4.537	5.458	-0.4815
CEU	HIST2	149382608	3.7069	4.0935	-0.26
CEU	HIST2	149387608	5.2144	4.4346	0.4893
CEU	HIST2	149392608	6.3327	5.9697	0.1752
CEU	HIST2	149397608	5.2449	5.7991	-0.2745
CEU	HIST2	149402608	5.9522	5.9697	-0.0084
CEU	HIST2	149407608	4.525	4.6052	-0.0487
CEU	HIST2	149412608	4.3368	3.4112	0.727
CEU	HIST2	149417608	4.1068	3.2407	0.7102

CEU	HIST2	149422608	2.0064	1.8762	0.1658
CEU	HIST2	149427608	2.7172	2.729	-0.0112
CEU	HIST2	149432608	2.2086	2.3879	-0.1888
CEU	HIST2	149437608	0.5232	0.6822	-0.4152
CEU	HIST2	149442608	0	0	0
CEU	HIST2	149447608	0.08	0.5117	-1.3539
CEU	HIST2	149452608	0.1394	0.8528	-1.6066
CEU	HIST2	149457608	0.128	0.6822	-1.4472
CEU	HIST2	149462608	0.0686	0.3411	-1.0924
CEU	HIST2	149467608	0	0	0
CEU	HIST2	149472608	0	0	0
CEU	HIST2	149477608	0	0	0
CEU	HIST2	149482608	0	0	0
CEU	HIST2	149487608	0	0	0
CEU	HIST2	149492608	0	0	0
CEU	HIST2	149497608	0	0	0
CEU	HIST2	149502608	0	0	0
CEU	HIST2	149507608	0.1363	0.5117	-1.1774
CEU	HIST2	149512608	0.4605	1.1939	-1.3069
CEU	HIST2	149517608	0.3243	0.6822	-0.9346
CEU	HIST2	149522608	0	0	0
CEU	HIST2	149527608	0.0101	0.1706	-0.9534
CEU	HIST2	149532608	0.0101	0.1706	-0.9534
CEU	HIST2	149537608	0	0	0
CEU	HIST2	149542608	0	0	0
CEU	HIST2	149547608	0	0	0
CEU	HIST2	149552608	0	0	0
CEU	HIST2	149557608	0	0	0
CEU	HIST2	149562608	0	0	0
CEU	HIST2	149567608	0.5038	0.1706	1.9793
CEU	HIST2	149572608	0.885	0.6822	0.5296
CEU	HIST2	149577608	0.4805	1.0234	-1.0788
CEU	HIST2	149582608	2.8182	3.2407	-0.3464
CEU	HIST2	149587608	3.7007	3.2407	0.3772
CEU	HIST2	149592608	2.1459	2.0467	0.118
CEU	HIST2	149597608	2.4286	2.8996	-0.4236
CEU	HIST2	149602608	5.8841	4.4346	0.9095
CEU	HIST2	149607608	7.5261	4.6052	1.7734
CEU	HIST2	149612608	3.7706	2.2173	1.7353
CEU	HIST2	149617608	0.8642	0.6822	0.4753
CEU	HIST2	149622608	0	0	0
CEU	HIST2	149627608	0.0301	0.1706	-0.8346
CEU	HIST2	149632608	0.0301	0.1706	-0.8346
CEU	HIST2	149637608	2.3997	1.7056	0.9507
CEU	HIST2	149642608	3.7413	3.5818	0.1202
CEU	HIST2	149647608	7.4811	6.6519	0.363
CEU	HIST2	149652608	7.906	6.1402	0.8309
CEU	HIST2	149657608	4.3752	3.5818	0.598
CEU	HIST2	149662608	6.0134	6.3108	-0.1365

CEU	HIST2	149667608	5.1379	9.0398	-1.2901
CEU	HIST2	149672608	5.4801	9.5515	-1.2791
CEU	HIST2	149677608	5.5494	7.3342	-0.7151
CEU	HIST2	149682608	2.4492	4.4346	-1.2457
CEU	HIST2	149687608	0.9285	3.2407	-1.896
CEU	HIST2	149692608	1.3814	3.4112	-1.5942
CEU	HIST2	149697608	2.9058	5.2874	-1.2806
CEU	HIST2	149702608	5.2948	6.9931	-0.7106
CEU	HIST2	149707608	8.1801	7.3342	0.3389
CEU	HIST2	149712608	8.9034	7.3342	0.6287
CEU	HIST2	149717608	10.1014	8.0164	0.7701
CEU	HIST2	149722608	9.1266	6.9931	0.8927
CEU	HIST2	149727608	6.7996	6.6519	0.0647
CEU	HIST2	149732608	5.9891	5.7991	0.0941
CEU	HIST2	149737608	3.3105	3.4112	-0.0791
CEU	HIST2	149742608	2.4663	3.4112	-0.7421
CEU	HIST2	149747608	4.6718	4.0935	0.3889
CEU	HIST2	149752608	3.3781	3.0701	0.2642
CEU	HIST2	149757608	1.8277	3.2407	-1.1586
CEU	HIST2	149762608	2.309	5.1169	-1.5542
CEU	HIST2	149767608	2.5848	5.458	-1.502
CEU	HIST2	149772608	3.2146	5.9697	-1.3296
CEU	HIST2	149777608	4.2707	6.4814	-0.9909
CEU	HIST2	149782608	5.437	7.6753	-0.8603
CEU	HIST2	149787608	3.9297	7.1636	-1.3238
CEU	HIST2	149792608	1.4871	3.0701	-1.3579
CEU	HIST2	149797608	0.1235	0.1706	-0.2798
CEU	HIST2	149802608	0	0	0
CEU	HIST2	149807608	0	0	0
CEU	HIST2	149812608	1.5739	2.0467	-0.5626
CEU	HIST2	149817608	2.7071	4.2641	-1.0107
CEU	HIST2	149822608	1.6324	2.3879	-0.7956
CEU	HIST2	149827608	0.4993	0.1706	1.9525
CEU	HIST2	149832608	0	0	0
CEU	HIST2	149837608	0	0	0
CEU	HIST2	149842608	1.0373	1.5351	-0.7381
CEU	HIST2	149847608	2.0711	4.0935	-1.3599
CEU	HIST2	149852608	2.2626	5.2874	-1.6264
CEU	HIST2	149857608	3.1433	6.6519	-1.5361
CEU	HIST2	149862608	4.2778	8.0164	-1.3809
CEU	HIST2	149867608	4.6148	8.187	-1.2941
CEU	HIST2	149872608	4.3408	6.8225	-1.0619
CEU	HIST2	149877608	4.0765	6.6519	-1.1276
CEU	HIST2	149882608	2.7013	5.2874	-1.3906
CEU	HIST2	149887608	2.3034	3.5818	-0.9635
CEU	HIST2	149892608	3.6768	4.7757	-0.6463
CEU	HIST2	149897608	3.5384	5.6286	-1.0632
CEU	HIST2	149902608	3.7986	6.1402	-1.1019
CEU	HIST2	149907608	3.8075	5.458	-0.8628

CEU	HIST2	149912608	1.6186	3.9229	-1.6072
CEU	HIST2	149917608	1.7209	4.0935	-1.5954
CEU	HIST2	149922608	3.7137	5.1169	-0.7767
CEU	HIST2	149927608	4.1686	5.6286	-0.7426
CEU	HIST2	149932608	2.6271	5.6286	-1.5267
CEU	HIST2	149937608	2.0439	4.6052	-1.5551
CEU	HIST2	149942608	2.6215	3.7524	-0.8193
CEU	HIST2	149947608	2.2977	4.4346	-1.3408
CEU	HIST2	149952608	2.9812	5.9697	-1.4423
CEU	HIST2	149957608	2.1946	4.4346	-1.4055
CEU	HIST2	149962608	1.1688	2.5584	-1.3843
CEU	HIST2	149967608	2.1791	3.2407	-0.8705
CEU	HIST2	149972608	2.1725	3.7524	-1.1446
CEU	HIST2	149977608	1.94	4.2641	-1.5087
CEU	HIST2	149982608	3.1398	5.1169	-1.0944
CEU	HIST2	149987608	4.1952	6.1402	-0.9153
CEU	HIST2	149992608	5.8895	7.6753	-0.6864
CEU	HIST2	149997608	9.3993	8.5281	0.304
CEU	HIST2	150002608	8.1973	6.8225	0.5883
CEU	HIST2	150007608	7.1431	6.8225	0.1372
CEU	HIST2	150012608	11.1517	9.3809	0.5657
CEU	HIST2	150017608	10.7794	8.8692	0.6428
CEU	HIST2	150022608	8.8606	7.6753	0.4556
CEU	HIST2	150027608	6.0136	6.1402	-0.0596
CEU	HIST2	150032608	1.9462	4.2641	-1.5046
CEU	HIST2	150037608	2.7005	5.7991	-1.5347
CEU	HIST2	150042608	4.1932	6.3108	-0.9722
CEU	HIST2	150047608	4.4902	5.458	-0.5059
CEU	HIST2	150052608	3.1923	4.9463	-1.0003
CEU	HIST2	150057608	2.1128	4.4346	-1.4568
CEU	HIST2	150062608	3.0056	4.7757	-1.0411
CEU	HIST2	150067608	2.2927	3.4112	-0.8785
CEU	HIST2	150072608	5.9037	5.7991	0.0518
CEU	HIST2	150077608	7.4368	7.6753	-0.0917
CEU	HIST2	150082608	3.0985	5.458	-1.2335
CEU	HIST2	150087608	3.1278	6.9931	-1.6173
CEU	HIST2	150092608	4.0282	6.6519	-1.1487
CEU	HIST2	150097608	3.9445	4.7757	-0.4889
CEU	HIST2	150102608	5.1649	6.8225	-0.7093
CEU	HIST2	150107608	6.5529	6.9931	-0.1842
CEU	HIST2	150112608	5.0695	5.458	-0.2031
CEU	HIST2	150117608	2.9899	4.9463	-1.1157
CEU	HIST2	150122608	3.677	6.1402	-1.1591
CEU	HIST2	150127608	5.6514	8.0164	-0.8735
CEU	HIST2	150132608	6.0334	8.8692	-0.9543
CEU	HIST2	150137608	6.3882	11.7688	-1.3905
CEU	HIST2	150142608	6.8081	9.7221	-0.9005
CEU	HIST2	150147608	5.0326	6.1402	-0.5212
CEU	HIST2	150152608	2.9186	7.1636	-1.7377

CEU	HIST2	150157608	5.2208	9.3809	-1.329
CEU	HIST2	150162608	9.0115	9.7221	-0.2196
CEU	HIST2	150167608	10.0012	7.8459	0.8119
CEU	HIST2	150172608	8.5935	6.8225	0.7578
CEU	HIST2	150177608	6.5925	5.458	0.5931
CEU	HIST2	150182608	7.6821	6.1402	0.7256
CEU	HIST2	150187608	6.2696	6.8225	-0.2366
CEU	HIST2	150192608	5.329	5.9697	-0.3092
CEU	HIST2	150197608	7.9913	6.3108	0.7716
CEU	HIST2	150202608	9.3094	7.5047	0.7081
CEU	HIST2	150207608	9.4055	8.0164	0.5131
CEU	HIST2	150212608	9.5529	7.1636	0.9781
CEU	HIST2	150217608	8.7254	6.6519	0.9078
CEU	HIST2	150222608	6.4754	6.1402	0.1577
CEU	HIST2	150227608	3.2977	4.2641	-0.6273
CEU	HIST2	150232608	2.1822	3.2407	-0.8679
CEU	HIST2	150237608	3.665	3.2407	0.3479
CEU	HIST2	150242608	3.5269	4.4346	-0.5695
CEU	HIST2	150247608	4.9192	5.2874	-0.198
CEU	HIST2	150252608	5.5411	5.1169	0.2348
CEU	HIST2	150257608	9.2389	7.6753	0.601
CEU	HIST2	150262608	14.1268	8.6987	1.8596
CEU	HIST2	150267608	17.9592	10.2337	2.276
CEU	HIST2	150272608	17.1991	11.7688	1.4034
CEU	HIST2	150277608	13.5372	10.916	0.727
CEU	HIST2	150282608	14.9565	10.7454	1.1853
CEU	HIST2	150287608	13.2278	8.5281	1.6398
CEU	HIST2	150292608	10.5072	7.3342	1.2713
CEU	HIST2	150297608	8.1028	7.1636	0.3845
CEU	HIST2	150302608	7.8625	7.5047	0.1404
CEU	HIST2	150307608	10.5539	9.5515	0.3149
CEU	HIST2	150312608	10.9722	9.7221	0.3863
CEU	HIST2	150317608	13.53	10.4043	0.9067
CEU	HIST2	150322608	13.6542	9.8926	1.1438
CEU	HIST2	150327608	12.6128	9.3809	1.0325
CEU	HIST2	150332608	11.9378	9.0398	0.9581
CEU	HIST2	150337608	9.3365	8.3576	0.348
CEU	HIST2	150342608	10.478	9.0398	0.4755
CEU	HIST2	150347608	10.2558	8.187	0.7495
CHB	HIST2	149357608	5.32	5.4213	-0.0531
CHB	HIST2	149362608	4.5073	4.066	0.2976
CHB	HIST2	149367608	2.1862	3.7271	-1.1194
CHB	HIST2	149372608	2.4207	5.0825	-1.4778
CHB	HIST2	149377608	3.2859	5.0825	-0.9974
CHB	HIST2	149382608	3.0581	3.8965	-0.5864
CHB	HIST2	149387608	4.0811	5.5907	-0.7702
CHB	HIST2	149392608	4.9347	5.7601	-0.4101
CHB	HIST2	149397608	4.4259	5.4213	-0.522
CHB	HIST2	149402608	4.8871	6.6072	-0.7555

CHB	HIST2	149407608	3.0881	4.7436	-0.9765
CHB	HIST2	149412608	3.8921	4.5742	-0.4153
CHB	HIST2	149417608	4.2707	4.5742	-0.1848
CHB	HIST2	149422608	1.5298	2.8801	-1.2174
CHB	HIST2	149427608	1.9899	3.7271	-1.262
CHB	HIST2	149432608	1.8957	3.2189	-1.0879
CHB	HIST2	149437608	0.399	0.8471	-1.0105
CHB	HIST2	149442608	0	0	0
CHB	HIST2	149447608	0.0386	0.5082	-1.4741
CHB	HIST2	149452608	0.1065	1.3553	-2.0239
CHB	HIST2	149457608	0.0873	1.1859	-1.961
CHB	HIST2	149462608	0.0194	0.3388	-1.2812
CHB	HIST2	149467608	0	0	0
CHB	HIST2	149472608	0	0	0
CHB	HIST2	149477608	0	0	0
CHB	HIST2	149482608	0	0	0
CHB	HIST2	149487608	0	0	0
CHB	HIST2	149492608	0	0	0
CHB	HIST2	149497608	0	0	0
CHB	HIST2	149502608	0	0	0
CHB	HIST2	149507608	0.6372	0.6777	-0.1059
CHB	HIST2	149512608	0.8968	1.0165	-0.2382
CHB	HIST2	149517608	0.2694	0.5082	-0.7496
CHB	HIST2	149522608	0.0097	0.1694	-0.949
CHB	HIST2	149527608	0.2578	0.1694	0.5253
CHB	HIST2	149532608	0.2578	0.1694	0.5253
CHB	HIST2	149537608	0	0	0
CHB	HIST2	149542608	0	0	0
CHB	HIST2	149547608	0	0	0
CHB	HIST2	149552608	0	0	0
CHB	HIST2	149557608	0	0	0
CHB	HIST2	149562608	0	0	0
CHB	HIST2	149567608	0.4601	0.3388	0.4866
CHB	HIST2	149572608	0.9039	0.8471	0.1281
CHB	HIST2	149577608	1.095	1.0165	0.1562
CHB	HIST2	149582608	3.3082	3.3883	-0.0631
CHB	HIST2	149587608	3.4287	3.5577	-0.0975
CHB	HIST2	149592608	2.0525	1.6942	0.4917
CHB	HIST2	149597608	2.9082	2.2024	0.7903
CHB	HIST2	149602608	6.534	4.2354	1.4964
CHB	HIST2	149607608	7.0862	5.4213	0.873
CHB	HIST2	149612608	2.7537	2.8801	-0.114
CHB	HIST2	149617608	0.574	0.5082	0.2066
CHB	HIST2	149622608	0	0	0
CHB	HIST2	149627608	0.0097	0.1694	-0.949
CHB	HIST2	149632608	0.0097	0.1694	-0.949
CHB	HIST2	149637608	2.8808	1.5247	2.0147
CHB	HIST2	149642608	4.7697	4.2354	0.3478
CHB	HIST2	149647608	7.5328	6.7766	0.3246

CHB	HIST2	149652608	6.6267	5.4213	0.6321
CHB	HIST2	149657608	3.0921	4.5742	-0.9025
CHB	HIST2	149662608	5.9966	5.4213	0.3017
CHB	HIST2	149667608	7.7933	6.6072	0.5209
CHB	HIST2	149672608	8.1593	8.6402	-0.1653
CHB	HIST2	149677608	6.0487	6.4378	-0.175
CHB	HIST2	149682608	2.9461	4.7436	-1.0603
CHB	HIST2	149687608	2.256	4.2354	-1.2886
CHB	HIST2	149692608	3.1883	4.066	-0.5919
CHB	HIST2	149697608	4.2125	4.5742	-0.2202
CHB	HIST2	149702608	6.1365	6.946	-0.3398
CHB	HIST2	149707608	8.0221	7.9625	0.0221
CHB	HIST2	149712608	6.7044	6.0989	0.2858
CHB	HIST2	149717608	7.4384	8.1319	-0.2521
CHB	HIST2	149722608	7.7087	8.979	-0.4214
CHB	HIST2	149727608	6.5926	8.6402	-0.7038
CHB	HIST2	149732608	5.754	7.2848	-0.6153
CHB	HIST2	149737608	2.7881	4.2354	-0.9422
CHB	HIST2	149742608	1.5443	3.0495	-1.2945
CHB	HIST2	149747608	3.8666	2.8801	0.8894
CHB	HIST2	149752608	3.2732	2.8801	0.3544
CHB	HIST2	149757608	1.1856	5.5907	-2.2476
CHB	HIST2	149762608	1.9924	6.6072	-2.0269
CHB	HIST2	149767608	2.1599	4.7436	-1.5241
CHB	HIST2	149772608	1.3105	5.7601	-2.2107
CHB	HIST2	149777608	0.7826	4.913	-2.3626
CHB	HIST2	149782608	1.0343	4.5742	-2.1555
CHB	HIST2	149787608	0.7342	5.5907	-2.4779
CHB	HIST2	149792608	0.4026	3.3883	-2.3513
CHB	HIST2	149797608	0.1611	0.6777	-1.3505
CHB	HIST2	149802608	0	0	0
CHB	HIST2	149807608	0	0	0
CHB	HIST2	149812608	0.4782	2.033	-1.8541
CHB	HIST2	149817608	1.276	5.4213	-2.1737
CHB	HIST2	149822608	1.1639	3.5577	-1.8091
CHB	HIST2	149827608	0.3661	0.1694	1.1688
CHB	HIST2	149832608	0	0	0
CHB	HIST2	149837608	0	0	0
CHB	HIST2	149842608	0.2781	1.6942	-1.9434
CHB	HIST2	149847608	0.6405	4.913	-2.4439
CHB	HIST2	149852608	0.9076	7.2848	-2.5634
CHB	HIST2	149857608	1.5464	6.2684	-2.1748
CHB	HIST2	149862608	1.4534	6.6072	-2.2636
CHB	HIST2	149867608	0.9816	6.946	-2.5036
CHB	HIST2	149872608	1.0914	5.7601	-2.3195
CHB	HIST2	149877608	1.6109	6.7766	-2.2175
CHB	HIST2	149882608	1.7603	7.1154	-2.1992
CHB	HIST2	149887608	1.2762	5.7601	-2.2277
CHB	HIST2	149892608	1.5239	6.7766	-2.2548

CHB	HIST2	149897608	1.1626	7.2848	-2.4609
CHB	HIST2	149902608	1.4296	6.946	-2.3156
CHB	HIST2	149907608	2.603	7.9625	-1.9861
CHB	HIST2	149912608	1.5418	6.2684	-2.1769
CHB	HIST2	149917608	1.5016	6.946	-2.2853
CHB	HIST2	149922608	3.5157	8.979	-1.8123
CHB	HIST2	149927608	3.9733	10.3343	-1.8516
CHB	HIST2	149932608	2.1807	10.3343	-2.3734
CHB	HIST2	149937608	2.0573	9.3178	-2.3273
CHB	HIST2	149942608	2.9528	7.9625	-1.8564
CHB	HIST2	149947608	2.0617	6.2684	-1.9375
CHB	HIST2	149952608	1.9785	7.9625	-2.2175
CHB	HIST2	149957608	1.6149	7.7931	-2.335
CHB	HIST2	149962608	1.0074	6.2684	-2.423
CHB	HIST2	149967608	1.1896	6.4378	-2.3598
CHB	HIST2	149972608	1.4017	5.2519	-2.0765
CHB	HIST2	149977608	1.5852	4.7436	-1.8631
CHB	HIST2	149982608	2.2138	7.7931	-2.1087
CHB	HIST2	149987608	2.6172	10.1649	-2.2312
CHB	HIST2	149992608	3.9113	9.6567	-1.7815
CHB	HIST2	149997608	6.7367	10.5037	-1.08
CHB	HIST2	150002608	5.34	9.9955	-1.398
CHB	HIST2	150007608	2.8742	9.4872	-2.0846
CHB	HIST2	150012608	2.2077	10.3343	-2.3655
CHB	HIST2	150017608	1.5117	8.8096	-2.464
CHB	HIST2	150022608	1.7512	8.979	-2.3977
CHB	HIST2	150027608	1.3954	8.6402	-2.4903
CHB	HIST2	150032608	1.1041	6.4378	-2.3982
CHB	HIST2	150037608	1.8192	6.4378	-2.0767
CHB	HIST2	150042608	1.9001	6.7766	-2.0933
CHB	HIST2	150047608	0.9438	6.946	-2.5195
CHB	HIST2	150052608	1.0657	6.6072	-2.4339
CHB	HIST2	150057608	1.1483	5.9295	-2.3146
CHB	HIST2	150062608	0.6227	5.0825	-2.476
CHB	HIST2	150067608	0.582	5.0825	-2.4986
CHB	HIST2	150072608	1.3189	7.4543	-2.4151
CHB	HIST2	150077608	1.3231	7.4543	-2.4134
CHB	HIST2	150082608	0.8818	5.2519	-2.3569
CHB	HIST2	150087608	1.351	6.2684	-2.2648
CHB	HIST2	150092608	1.5734	6.7766	-2.2335
CHB	HIST2	150097608	1.4826	6.2684	-2.2042
CHB	HIST2	150102608	1.6471	7.9625	-2.3403
CHB	HIST2	150107608	1.8193	7.9625	-2.2765
CHB	HIST2	150112608	1.419	6.2684	-2.2335
CHB	HIST2	150117608	1.3644	6.2684	-2.2586
CHB	HIST2	150122608	2.214	7.4543	-2.0627
CHB	HIST2	150127608	2.5919	7.7931	-1.9658
CHB	HIST2	150132608	2.1512	8.4708	-2.2123
CHB	HIST2	150137608	2.0793	9.8261	-2.3636

CHB	HIST2	150142608	2.6756	9.3178	-2.1291
CHB	HIST2	150147608	2.7567	8.4708	-2.0003
CHB	HIST2	150152608	1.9176	8.8096	-2.3269
CHB	HIST2	150157608	2.6929	12.5367	-2.3899
CHB	HIST2	150162608	6.6802	12.3673	-1.3986
CHB	HIST2	150167608	9.7999	9.8261	-0.008
CHB	HIST2	150172608	7.3478	10.3343	-0.8693
CHB	HIST2	150177608	5.632	10.1649	-1.34
CHB	HIST2	150182608	7.0729	9.3178	-0.7196
CHB	HIST2	150187608	4.4826	6.7766	-0.9847
CHB	HIST2	150192608	4.0677	6.7766	-1.1628
CHB	HIST2	150197608	8.1792	9.1484	-0.316
CHB	HIST2	150202608	9.2675	10.1649	-0.2653
CHB	HIST2	150207608	8.6516	11.1814	-0.684
CHB	HIST2	150212608	8.9216	11.6896	-0.7178
CHB	HIST2	150217608	7.8589	9.9955	-0.6416
CHB	HIST2	150222608	5.5899	8.4708	-1.0085
CHB	HIST2	150227608	2.9787	6.4378	-1.5553
CHB	HIST2	150232608	2.1055	4.2354	-1.3866
CHB	HIST2	150237608	3.3891	4.5742	-0.7216
CHB	HIST2	150242608	3.8846	5.9295	-0.99
CHB	HIST2	150247608	5.0069	7.2848	-0.9156
CHB	HIST2	150252608	4.7007	7.7931	-1.1688
CHB	HIST2	150257608	7.4337	8.4708	-0.3631
CHB	HIST2	150262608	10.3708	9.9955	0.1127
CHB	HIST2	150267608	12.8372	11.5202	0.3463
CHB	HIST2	150272608	11.6988	10.8426	0.2383
CHB	HIST2	150277608	8.2179	10.5037	-0.6553
CHB	HIST2	150282608	10.3892	12.3673	-0.4865
CHB	HIST2	150287608	10.2166	10.8426	-0.1742
CHB	HIST2	150292608	7.4507	8.6402	-0.4089
CHB	HIST2	150297608	4.8423	7.9625	-1.1562
CHB	HIST2	150302608	5.5199	8.3013	-0.992
CHB	HIST2	150307608	7.8342	10.6731	-0.8018
CHB	HIST2	150312608	7.9126	9.6567	-0.5408
CHB	HIST2	150317608	9.4849	9.9955	-0.1533
CHB	HIST2	150322608	8.8245	11.3508	-0.6735
CHB	HIST2	150327608	8.6354	11.6896	-0.792
CHB	HIST2	150332608	8.5037	11.3508	-0.759
CHB	HIST2	150337608	6.0792	7.9625	-0.6979
CHB	HIST2	150342608	7.1215	6.4378	0.3074
CHB	HIST2	150347608	7.0495	7.6237	-0.2214
YRI	HIST2	149357608	6.3206	6.7225	-0.1732
YRI	HIST2	149362608	4.8751	7.0586	-0.9001
YRI	HIST2	149367608	3.0712	6.3864	-1.4962
YRI	HIST2	149372608	3.3885	7.3947	-1.5831
YRI	HIST2	149377608	4.0781	7.5628	-1.349
YRI	HIST2	149382608	4.4672	5.7141	-0.6218
YRI	HIST2	149387608	6.215	6.0502	0.0781

YRI	HIST2	149392608	6.7938	7.2267	-0.1747
YRI	HIST2	149397608	5.7216	7.3947	-0.6611
YRI	HIST2	149402608	7.2413	8.9073	-0.5549
YRI	HIST2	149407608	5.7873	8.5712	-0.9607
YRI	HIST2	149412608	5.3418	7.5628	-0.8598
YRI	HIST2	149417608	5.4578	5.7141	-0.1278
YRI	HIST2	149422608	2.9383	3.3612	-0.3341
YRI	HIST2	149427608	3.1793	4.2016	-0.6678
YRI	HIST2	149432608	2.5221	3.3612	-0.6629
YRI	HIST2	149437608	0.5529	1.0084	-0.9081
YRI	HIST2	149442608	0.0185	0.1681	-0.8893
YRI	HIST2	149447608	0.1568	0.5042	-1.0918
YRI	HIST2	149452608	0.4181	1.1764	-1.3563
YRI	HIST2	149457608	0.5682	1.5126	-1.4063
YRI	HIST2	149462608	0.3069	0.8403	-1.2048
YRI	HIST2	149467608	0	0	0
YRI	HIST2	149472608	0	0	0
YRI	HIST2	149477608	0	0	0
YRI	HIST2	149482608	0	0	0
YRI	HIST2	149487608	0	0	0
YRI	HIST2	149492608	0	0	0
YRI	HIST2	149497608	0	0	0
YRI	HIST2	149502608	0	0	0
YRI	HIST2	149507608	0.4018	0.5042	-0.3218
YRI	HIST2	149512608	0.6674	2.0167	-1.6133
YRI	HIST2	149517608	0.4991	2.0167	-1.8145
YRI	HIST2	149522608	0.2335	0.5042	-0.8507
YRI	HIST2	149527608	0.2339	0.5042	-0.8495
YRI	HIST2	149532608	0.2339	0.5042	-0.8495
YRI	HIST2	149537608	0	0	0
YRI	HIST2	149542608	0	0	0
YRI	HIST2	149547608	0	0	0
YRI	HIST2	149552608	0	0	0
YRI	HIST2	149557608	0	0	0
YRI	HIST2	149562608	0	0	0
YRI	HIST2	149567608	0.5133	0.5042	0.0286
YRI	HIST2	149572608	1.1098	1.6806	-0.7853
YRI	HIST2	149577608	0.9289	1.6806	-1.0341
YRI	HIST2	149582608	2.3949	5.0419	-1.4748
YRI	HIST2	149587608	2.5004	5.5461	-1.5597
YRI	HIST2	149592608	1.2221	2.3529	-1.197
YRI	HIST2	149597608	2.5434	3.0251	-0.4155
YRI	HIST2	149602608	5.5997	6.0502	-0.2134
YRI	HIST2	149607608	5.8919	6.8906	-0.4208
YRI	HIST2	149612608	2.6056	3.6974	-0.7958
YRI	HIST2	149617608	0.5543	1.1764	-1.1127
YRI	HIST2	149622608	0	0	0
YRI	HIST2	149627608	0.0093	0.1681	-0.944
YRI	HIST2	149632608	0.3282	0.6722	-0.9006

YRI	HIST2	149637608	3.1238	2.1848	1.0543
YRI	HIST2	149642608	4.4903	4.7057	-0.1275
YRI	HIST2	149647608	8.5788	9.2434	-0.2139
YRI	HIST2	149652608	9.4155	10.4199	-0.2891
YRI	HIST2	149657608	5.7279	7.2267	-0.6048
YRI	HIST2	149662608	7.8662	8.067	-0.0733
YRI	HIST2	149667608	10.9669	13.613	-0.5922
YRI	HIST2	149672608	10.1832	14.1172	-0.8506
YRI	HIST2	149677608	6.6127	9.7476	-0.9603
YRI	HIST2	149682608	4.4768	9.0754	-1.5053
YRI	HIST2	149687608	2.5728	8.4031	-2.0491
YRI	HIST2	149692608	2.9951	6.3864	-1.5306
YRI	HIST2	149697608	4.7508	7.2267	-0.9991
YRI	HIST2	149702608	8.2867	11.4282	-0.8294
YRI	HIST2	149707608	10.0844	13.2769	-0.7317
YRI	HIST2	149712608	6.965	10.924	-1.0904
YRI	HIST2	149717608	7.7259	11.7644	-1.0375
YRI	HIST2	149722608	9.2603	12.6047	-0.8051
YRI	HIST2	149727608	8.845	13.1089	-0.9891
YRI	HIST2	149732608	7.7605	12.6047	-1.1661
YRI	HIST2	149737608	4.3377	7.8989	-1.3248
YRI	HIST2	149742608	4.2459	6.2183	-0.9118
YRI	HIST2	149747608	6.9642	7.5628	-0.2317
YRI	HIST2	149752608	6.0807	7.5628	-0.5738
YRI	HIST2	149757608	6.3891	9.9157	-1.0633
YRI	HIST2	149762608	6.0755	10.756	-1.308
YRI	HIST2	149767608	3.2155	9.2434	-1.9399
YRI	HIST2	149772608	5.4029	10.924	-1.5206
YRI	HIST2	149777608	6.5211	11.7644	-1.347
YRI	HIST2	149782608	4.9324	13.2769	-1.9124
YRI	HIST2	149787608	4.1088	11.5963	-1.9498
YRI	HIST2	149792608	2.1767	5.2099	-1.6418
YRI	HIST2	149797608	0.4072	1.0084	-1.1986
YRI	HIST2	149802608	0	0	0
YRI	HIST2	149807608	0	0	0
YRI	HIST2	149812608	2.8529	5.2099	-1.2758
YRI	HIST2	149817608	5.0549	8.5712	-1.2135
YRI	HIST2	149822608	2.6837	3.5293	-0.6411
YRI	HIST2	149827608	0.4817	0.1681	1.8643
YRI	HIST2	149832608	0	0	0
YRI	HIST2	149837608	0.0183	0.1681	-0.8905
YRI	HIST2	149842608	1.3352	3.0251	-1.4578
YRI	HIST2	149847608	4.1965	9.4115	-1.6505
YRI	HIST2	149852608	5.2333	13.445	-1.8597
YRI	HIST2	149857608	4.0767	11.9324	-1.9914
YRI	HIST2	149862608	5.5506	11.7644	-1.5964
YRI	HIST2	149867608	7.5872	12.4366	-1.1823
YRI	HIST2	149872608	5.9272	10.924	-1.3762
YRI	HIST2	149877608	7.021	11.4282	-1.1635

YRI	HIST2	149882608	8.3506	11.5963	-0.8452
YRI	HIST2	149887608	5.7786	11.9324	-1.56
YRI	HIST2	149892608	5.2319	12.9408	-1.8102
YRI	HIST2	149897608	4.7192	13.1089	-1.9461
YRI	HIST2	149902608	3.8641	11.4282	-1.9969
YRI	HIST2	149907608	6.6539	11.5963	-1.287
YRI	HIST2	149912608	6.3756	12.1005	-1.4323
YRI	HIST2	149917608	5.7244	12.4366	-1.6364
YRI	HIST2	149922608	7.5794	14.1172	-1.4137
YRI	HIST2	149927608	8.7634	13.9492	-1.1341
YRI	HIST2	149932608	9.4821	13.445	-0.8975
YRI	HIST2	149937608	8.733	12.1005	-0.8425
YRI	HIST2	149942608	5.9704	9.0754	-1.0164
YRI	HIST2	149947608	4.4918	8.4031	-1.3747
YRI	HIST2	149952608	8.1726	11.5963	-0.8916
YRI	HIST2	149957608	7.4743	11.0921	-0.9823
YRI	HIST2	149962608	4.4682	8.9073	-1.4785
YRI	HIST2	149967608	6.6061	9.9157	-0.9978
YRI	HIST2	149972608	6.8847	9.4115	-0.7997
YRI	HIST2	149977608	6.8085	9.5795	-0.8627
YRI	HIST2	149982608	9.3457	12.2685	-0.7218
YRI	HIST2	149987608	9.6175	12.1005	-0.6212
YRI	HIST2	149992608	9.0346	13.445	-0.9988
YRI	HIST2	149997608	13.5133	15.4617	-0.3864
YRI	HIST2	150002608	13.5325	13.613	-0.018
YRI	HIST2	150007608	11.3461	15.2937	-0.791
YRI	HIST2	150012608	15.6615	17.6465	-0.3469
YRI	HIST2	150017608	15.5687	15.6298	-0.012
YRI	HIST2	150022608	13.3697	15.4617	-0.4148
YRI	HIST2	150027608	11.7443	13.9492	-0.4822
YRI	HIST2	150032608	8.6696	11.0921	-0.6577
YRI	HIST2	150037608	8.56	13.1089	-1.0552
YRI	HIST2	150042608	11.8235	14.7895	-0.6136
YRI	HIST2	150047608	11.6029	14.7895	-0.6592
YRI	HIST2	150052608	7.881	13.1089	-1.2127
YRI	HIST2	150057608	7.0524	12.4366	-1.3127
YRI	HIST2	150062608	8.8031	13.1089	-0.9988
YRI	HIST2	150067608	8.8995	12.4366	-0.8623
YRI	HIST2	150072608	10.2111	14.4534	-0.897
YRI	HIST2	150077608	12.0651	16.8062	-0.8681
YRI	HIST2	150082608	10.4679	15.2937	-0.9669
YRI	HIST2	150087608	13.0869	15.4617	-0.4709
YRI	HIST2	150092608	14.5688	15.6298	-0.2082
YRI	HIST2	150097608	11.3238	13.9492	-0.5742
YRI	HIST2	150102608	11.5122	15.1256	-0.7317
YRI	HIST2	150107608	11.9195	16.8062	-0.8948
YRI	HIST2	150112608	9.1169	14.7895	-1.1735
YRI	HIST2	150117608	7.3768	13.7811	-1.4168
YRI	HIST2	150122608	8.5138	15.2937	-1.3585

YRI	HIST2	150127608	9.9391	14.9575	-1.0271
YRI	HIST2	150132608	11.0102	15.6298	-0.9066
YRI	HIST2	150137608	12.9711	17.6465	-0.817
YRI	HIST2	150142608	11.6407	15.9659	-0.8318
YRI	HIST2	150147608	7.9108	11.9324	-1.0195
YRI	HIST2	150152608	10.1871	13.445	-0.7378
YRI	HIST2	150157608	16.8299	19.1591	-0.3761
YRI	HIST2	150162608	16.2272	18.823	-0.4263
YRI	HIST2	150167608	13.501	16.134	-0.5013
YRI	HIST2	150172608	12.8102	15.9659	-0.6069
YRI	HIST2	150177608	11.3844	14.7895	-0.7044
YRI	HIST2	150182608	11.6681	14.1172	-0.5296
YRI	HIST2	150187608	9.2865	12.1005	-0.704
YRI	HIST2	150192608	8.856	11.0921	-0.6071
YRI	HIST2	150197608	10.6242	10.924	-0.0826
YRI	HIST2	150202608	11.4618	11.2602	0.054
YRI	HIST2	150207608	13.026	13.9492	-0.2019
YRI	HIST2	150212608	13.3002	14.7895	-0.3081
YRI	HIST2	150217608	12.1107	13.1089	-0.2315
YRI	HIST2	150222608	9.0517	10.4199	-0.3939
YRI	HIST2	150227608	4.478	8.5712	-1.4126
YRI	HIST2	150232608	3.2434	7.5628	-1.6722
YRI	HIST2	150237608	3.6382	6.7225	-1.329
YRI	HIST2	150242608	4.6251	9.2434	-1.4863
YRI	HIST2	150247608	6.5472	11.0921	-1.234
YRI	HIST2	150252608	6.7318	10.4199	-1.0617
YRI	HIST2	150257608	10.2802	10.5879	-0.0873
YRI	HIST2	150262608	14.7817	12.9408	0.4323
YRI	HIST2	150267608	18.4065	16.6382	0.3269
YRI	HIST2	150272608	16.6557	16.9743	-0.0578
YRI	HIST2	150277608	13.4815	15.2937	-0.3631
YRI	HIST2	150282608	12.86	14.6214	-0.3684
YRI	HIST2	150287608	10.99	13.1089	-0.4915
YRI	HIST2	150292608	10.3046	11.9324	-0.4126
YRI	HIST2	150297608	9.3833	11.9324	-0.6462
YRI	HIST2	150302608	10.5637	11.9324	-0.347
YRI	HIST2	150307608	12.6879	14.6214	-0.4044
YRI	HIST2	150312608	12.7388	13.613	-0.1957
YRI	HIST2	150317608	15.1552	13.7811	0.304
YRI	HIST2	150322608	13.4792	15.2937	-0.3636
YRI	HIST2	150327608	11.8046	14.2853	-0.5304
YRI	HIST2	150332608	12.2157	13.9492	-0.3791
YRI	HIST2	150337608	10.6826	12.6047	-0.4627
YRI	HIST2	150342608	11.9996	12.1005	-0.0252
YRI	HIST2	150347608	10.1839	10.924	-0.2038
CEU	HIST2	149358262	5.6349	5.6286	0.0032
CEU	HIST2	149363262	5.699	4.9463	0.4293
CEU	HIST2	149368262	3.5393	3.5818	-0.032
CEU	HIST2	149373262	2.9417	4.7757	-1.0786

CEU	HIST2	149378262	3.5661	5.1169	-0.8584
CEU	HIST2	149383262	4.4593	4.4346	0.0155
CEU	HIST2	149388262	5.8396	5.2874	0.2969
CEU	HIST2	149393262	5.9612	5.6286	0.1692
CEU	HIST2	149398262	4.65	5.458	-0.4224
CEU	HIST2	149403262	5.6529	5.9697	-0.1529
CEU	HIST2	149408262	5.0012	4.4346	0.3555
CEU	HIST2	149413262	4.7126	3.4112	1.0221
CEU	HIST2	149418262	4.449	3.2407	0.9908
CEU	HIST2	149423262	1.9637	1.8762	0.1114
CEU	HIST2	149428262	2.3816	2.8996	-0.4659
CEU	HIST2	149433262	1.7838	2.2173	-0.4843
CEU	HIST2	149438262	0.0101	0.1706	-0.9534
CEU	HIST2	149443262	0	0	0
CEU	HIST2	149448262	0.08	0.5117	-1.3539
CEU	HIST2	149453262	0.1979	1.0234	-1.6403
CEU	HIST2	149458262	0.128	0.6822	-1.4472
CEU	HIST2	149463262	0.0101	0.1706	-0.9534
CEU	HIST2	149468262	0	0	0
CEU	HIST2	149473262	0	0	0
CEU	HIST2	149478262	0	0	0
CEU	HIST2	149483262	0	0	0
CEU	HIST2	149488262	0	0	0
CEU	HIST2	149493262	0	0	0
CEU	HIST2	149498262	0	0	0
CEU	HIST2	149503262	0	0	0
CEU	HIST2	149508262	0.1363	0.5117	-1.1774
CEU	HIST2	149513262	0.4605	1.1939	-1.3069
CEU	HIST2	149518262	0.3243	0.6822	-0.9346
CEU	HIST2	149523262	0	0	0
CEU	HIST2	149528262	0.0101	0.1706	-0.9534
CEU	HIST2	149533262	0.0101	0.1706	-0.9534
CEU	HIST2	149538262	0	0	0
CEU	HIST2	149543262	0	0	0
CEU	HIST2	149548262	0	0	0
CEU	HIST2	149553262	0	0	0
CEU	HIST2	149558262	0	0	0
CEU	HIST2	149563262	0	0	0
CEU	HIST2	149568262	0.6005	0.3411	1.0399
CEU	HIST2	149573262	0.885	0.6822	0.5296
CEU	HIST2	149578262	0.3939	1.0234	-1.2508
CEU	HIST2	149583262	2.8182	3.2407	-0.3464
CEU	HIST2	149588262	3.6906	3.0701	0.5323
CEU	HIST2	149593262	2.1459	2.0467	0.118
CEU	HIST2	149598262	3.4073	3.2407	0.1366
CEU	HIST2	149603262	6.8606	4.9463	1.0917
CEU	HIST2	149608262	7.033	4.6052	1.474
CEU	HIST2	149613262	2.7941	1.7056	1.4908
CEU	HIST2	149618262	0.3785	0.3411	0.1499

CEU	HIST2	149623262	0	0	0
CEU	HIST2	149628262	0.0301	0.1706	-0.8346
CEU	HIST2	149633262	0.0503	0.5117	-1.4471
CEU	HIST2	149638262	2.4197	1.8762	0.692
CEU	HIST2	149643262	4.7056	3.9229	0.5459
CEU	HIST2	149648262	7.9542	6.6519	0.5702
CEU	HIST2	149653262	8.2068	6.3108	0.8705
CEU	HIST2	149658262	4.5382	3.9229	0.4292
CEU	HIST2	149663262	4.7786	5.9697	-0.5748
CEU	HIST2	149668262	5.228	10.0632	-1.447
CEU	HIST2	149673262	5.4397	9.2104	-1.2253
CEU	HIST2	149678262	4.8332	6.1402	-0.615
CEU	HIST2	149683262	2.4888	4.4346	-1.2209
CEU	HIST2	149688262	0.9285	3.2407	-1.896
CEU	HIST2	149693262	1.8536	3.7524	-1.3756
CEU	HIST2	149698262	3.4795	5.7991	-1.1489
CEU	HIST2	149703262	4.7829	6.8225	-0.8727
CEU	HIST2	149708262	7.6066	6.9931	0.2567
CEU	HIST2	149713262	10.7902	8.3576	0.8647
CEU	HIST2	149718262	10.9326	8.187	0.9947
CEU	HIST2	149723262	7.2799	6.3108	0.4449
CEU	HIST2	149728262	6.945	6.3108	0.2912
CEU	HIST2	149733262	6.1517	5.7991	0.1746
CEU	HIST2	149738262	2.6797	3.5818	-0.6799
CEU	HIST2	149743262	2.2533	2.729	-0.4496
CEU	HIST2	149748262	4.2958	3.5818	0.5381
CEU	HIST2	149753262	3.5908	3.4112	0.1411
CEU	HIST2	149758262	1.8874	3.5818	-1.2771
CEU	HIST2	149763262	2.1659	5.1169	-1.6334
CEU	HIST2	149768262	2.5351	5.2874	-1.4799
CEU	HIST2	149773262	3.7594	6.3108	-1.1714
CEU	HIST2	149778262	5.0441	7.5047	-0.9654
CEU	HIST2	149783262	5.1115	7.8459	-1.0301
CEU	HIST2	149788262	3.2696	6.1402	-1.3508
CEU	HIST2	149793262	1.1981	2.2173	-1.1386
CEU	HIST2	149798262	0	0	0
CEU	HIST2	149803262	0	0	0
CEU	HIST2	149808262	0	0	0
CEU	HIST2	149813262	1.7827	2.3879	-0.6373
CEU	HIST2	149818262	2.7071	4.2641	-1.0107
CEU	HIST2	149823262	1.4236	2.0467	-0.7414
CEU	HIST2	149828262	0.4993	0.1706	1.9525
CEU	HIST2	149833262	0	0	0
CEU	HIST2	149838262	0	0	0
CEU	HIST2	149843262	1.2872	2.2173	-1.0391
CEU	HIST2	149848262	2.3686	4.4346	-1.2963
CEU	HIST2	149853262	2.053	5.1169	-1.6959
CEU	HIST2	149858262	3.0596	6.9931	-1.6458
CEU	HIST2	149863262	4.548	7.8459	-1.2424

CEU	HIST2	149868262	4.9638	8.3576	-1.2064
CEU	HIST2	149873262	4.0303	6.4814	-1.0986
CEU	HIST2	149878262	3.7207	6.1402	-1.1385
CEU	HIST2	149883262	2.7013	5.2874	-1.3906
CEU	HIST2	149888262	2.603	3.5818	-0.7377
CEU	HIST2	149893262	3.8856	5.6286	-0.8866
CEU	HIST2	149898262	3.3659	5.6286	-1.1509
CEU	HIST2	149903262	4.126	5.9697	-0.8898
CEU	HIST2	149908262	3.4735	5.2874	-0.9753
CEU	HIST2	149913262	1.2511	3.5818	-1.7566
CEU	HIST2	149918262	2.2224	4.2641	-1.3254
CEU	HIST2	149923262	3.7423	5.2874	-0.8308
CEU	HIST2	149928262	3.8543	5.7991	-0.9632
CEU	HIST2	149933262	2.45	5.458	-1.5725
CEU	HIST2	149938262	2.0338	4.4346	-1.5063
CEU	HIST2	149943262	2.7784	3.7524	-0.7056
CEU	HIST2	149948262	2.1206	4.0935	-1.3266
CEU	HIST2	149953262	2.8042	5.6286	-1.4367
CEU	HIST2	149958262	2.2919	4.7757	-1.4608
CEU	HIST2	149963262	1.2185	2.729	-1.4277
CEU	HIST2	149968262	2.1701	3.2407	-0.8779
CEU	HIST2	149973262	2.2014	3.9229	-1.2007
CEU	HIST2	149978262	2.2138	4.4346	-1.3934
CEU	HIST2	149983262	3.5459	5.6286	-1.0594
CEU	HIST2	149988262	4.0167	5.7991	-0.8828
CEU	HIST2	149993262	5.4251	7.1636	-0.7117
CEU	HIST2	149998262	9.7349	8.6987	0.355
CEU	HIST2	150003262	8.577	6.6519	0.8428
CEU	HIST2	150008262	7.9959	7.5047	0.1927
CEU	HIST2	150013262	11.0676	9.7221	0.4158
CEU	HIST2	150018262	10.0734	8.3576	0.6099
CEU	HIST2	150023262	8.5648	7.3342	0.4931
CEU	HIST2	150028262	5.368	5.9697	-0.2904
CEU	HIST2	150033262	2.2319	4.4346	-1.382
CEU	HIST2	150038262	2.8551	5.6286	-1.4108
CEU	HIST2	150043262	4.2235	6.6519	-1.0632
CEU	HIST2	150048262	4.3154	5.2874	-0.5226
CEU	HIST2	150053262	2.8961	4.4346	-0.9653
CEU	HIST2	150058262	2.4185	4.6052	-1.3276
CEU	HIST2	150063262	3.2917	5.1169	-1.0103
CEU	HIST2	150068262	2.2927	3.4112	-0.8785
CEU	HIST2	150073262	5.6081	5.6286	-0.0104
CEU	HIST2	150078262	7.1513	7.8459	-0.2617
CEU	HIST2	150083262	3.3799	5.458	-1.0864
CEU	HIST2	150088262	3.6172	6.9931	-1.4125
CEU	HIST2	150093262	4.3137	6.4814	-0.9716
CEU	HIST2	150098262	3.5584	4.6052	-0.6356
CEU	HIST2	150103262	4.5578	6.6519	-0.9168
CEU	HIST2	150108262	6.4395	7.1636	-0.2964

CEU	HIST2	150113262	5.1983	5.6286	-0.2189
CEU	HIST2	150118262	3.0588	4.9463	-1.0764
CEU	HIST2	150123262	3.6353	6.3108	-1.2284
CEU	HIST2	150128262	5.9878	8.5281	-0.8863
CEU	HIST2	150133262	6.5949	9.2104	-0.8499
CEU	HIST2	150138262	6.6069	11.2571	-1.253
CEU	HIST2	150143262	6.4964	9.3809	-0.9215
CEU	HIST2	150148262	4.8918	6.3108	-0.6515
CEU	HIST2	150153262	3.6945	7.3342	-1.4583
CEU	HIST2	150158262	4.7576	9.2104	-1.4469
CEU	HIST2	150163262	9.1387	9.5515	-0.1297
CEU	HIST2	150168262	10.4315	8.187	0.8131
CEU	HIST2	150173262	7.3634	6.6519	0.3115
CEU	HIST2	150178262	6.643	5.2874	0.7289
CEU	HIST2	150183262	7.6821	6.1402	0.7256
CEU	HIST2	150188262	6.0486	6.4814	-0.194
CEU	HIST2	150193262	5.3088	5.6286	-0.1627
CEU	HIST2	150198262	8.4269	6.6519	0.7771
CEU	HIST2	150203262	9.3395	7.8459	0.5627
CEU	HIST2	150208262	9.1703	8.3576	0.2889
CEU	HIST2	150213262	10.2542	7.5047	1.0788
CEU	HIST2	150218262	8.2453	5.9697	1.0982
CEU	HIST2	150223262	5.7642	5.7991	-0.0173
CEU	HIST2	150228262	3.3274	4.4346	-0.6947
CEU	HIST2	150233262	2.6514	3.0701	-0.3592
CEU	HIST2	150238262	3.7153	3.7524	-0.0269
CEU	HIST2	150243262	3.3667	4.4346	-0.67
CEU	HIST2	150248262	5.3133	4.6052	0.4299
CEU	HIST2	150253262	6.5502	5.9697	0.2802
CEU	HIST2	150258262	8.8621	8.0164	0.3124
CEU	HIST2	150263262	13.2997	8.0164	1.9514
CEU	HIST2	150268262	18.4651	10.4043	2.3384
CEU	HIST2	150273262	16.718	11.7688	1.279
CEU	HIST2	150278262	14.8289	11.0866	1.0229
CEU	HIST2	150283262	15.4127	10.5749	1.3822
CEU	HIST2	150288262	12.5706	8.3576	1.4976
CEU	HIST2	150293262	10.4645	7.8459	0.9865
CEU	HIST2	150298262	6.9238	6.6519	0.119
CEU	HIST2	150303262	8.5154	7.5047	0.3965
CEU	HIST2	150308262	10.4952	9.5515	0.2965
CEU	HIST2	150313262	9.9742	9.5515	0.1328
CEU	HIST2	150318262	14.0132	10.4043	1.0469
CEU	HIST2	150323262	14.1021	10.4043	1.0727
CEU	HIST2	150328262	13.0786	9.5515	1.1081
CEU	HIST2	150333262	11.687	8.5281	1.1022
CEU	HIST2	150338262	8.8754	8.3576	0.1841
CEU	HIST2	150343262	10.6739	9.0398	0.5403
CEU	HIST2	150348262	10.3913	8.6987	0.5799
CEU	HIST2	150353262	12.5539	9.7221	0.8751

CHB	HIST2	149358262	5.1121	4.7436	0.2174
CHB	HIST2	149363262	5.0579	4.5742	0.2945
CHB	HIST2	149368262	2.8193	4.5742	-1.0686
CHB	HIST2	149373262	1.9088	4.913	-1.7184
CHB	HIST2	149378262	2.6528	4.2354	-1.0303
CHB	HIST2	149383262	3.6038	4.2354	-0.4112
CHB	HIST2	149388262	4.5578	5.9295	-0.6641
CHB	HIST2	149393262	4.5998	5.2519	-0.3517
CHB	HIST2	149398262	3.9874	5.2519	-0.682
CHB	HIST2	149403262	4.6667	6.7766	-0.9057
CHB	HIST2	149408262	3.5174	4.7436	-0.7233
CHB	HIST2	149413262	4.3154	4.5742	-0.1576
CHB	HIST2	149418262	4.2381	4.913	-0.386
CHB	HIST2	149423262	1.3975	3.2189	-1.4974
CHB	HIST2	149428262	1.9442	3.8965	-1.3655
CHB	HIST2	149433262	1.5755	2.5412	-0.9643
CHB	HIST2	149438262	0.0097	0.1694	-0.949
CHB	HIST2	149443262	0	0	0
CHB	HIST2	149448262	0.0386	0.5082	-1.4741
CHB	HIST2	149453262	0.1162	1.5247	-2.0925
CHB	HIST2	149458262	0.0873	1.1859	-1.961
CHB	HIST2	149463262	0.0097	0.1694	-0.949
CHB	HIST2	149468262	0	0	0
CHB	HIST2	149473262	0	0	0
CHB	HIST2	149478262	0	0	0
CHB	HIST2	149483262	0	0	0
CHB	HIST2	149488262	0	0	0
CHB	HIST2	149493262	0	0	0
CHB	HIST2	149498262	0	0	0
CHB	HIST2	149503262	0	0	0
CHB	HIST2	149508262	0.6372	0.6777	-0.1059
CHB	HIST2	149513262	0.9065	1.1859	-0.4987
CHB	HIST2	149518262	0.2694	0.5082	-0.7496
CHB	HIST2	149523262	0	0	0
CHB	HIST2	149528262	0.2578	0.1694	0.5253
CHB	HIST2	149533262	0.2578	0.1694	0.5253
CHB	HIST2	149538262	0	0	0
CHB	HIST2	149543262	0	0	0
CHB	HIST2	149548262	0	0	0
CHB	HIST2	149553262	0	0	0
CHB	HIST2	149558262	0	0	0
CHB	HIST2	149563262	0	0	0
CHB	HIST2	149568262	0.4891	0.5082	-0.06
CHB	HIST2	149573262	0.9039	0.8471	0.1281
CHB	HIST2	149578262	1.4257	1.1859	0.428
CHB	HIST2	149583262	3.3082	3.3883	-0.0631
CHB	HIST2	149588262	3.0691	3.2189	-0.1232
CHB	HIST2	149593262	2.0525	1.6942	0.4917
CHB	HIST2	149598262	3.8962	2.5412	1.3531

CHB	HIST2	149603262	7.5197	4.5742	1.7936
CHB	HIST2	149608262	6.226	5.2519	0.5254
CHB	HIST2	149613262	1.7679	2.5412	-0.7722
CHB	HIST2	149618262	0.4462	0.3388	0.4308
CHB	HIST2	149623262	0	0	0
CHB	HIST2	149628262	0.0097	0.1694	-0.949
CHB	HIST2	149633262	0.0194	0.3388	-1.2812
CHB	HIST2	149638262	2.8905	1.6942	1.6418
CHB	HIST2	149643262	5.5243	4.5742	0.5785
CHB	HIST2	149648262	7.8201	7.1154	0.2894
CHB	HIST2	149653262	6.9298	5.9295	0.4843
CHB	HIST2	149658262	3.3567	4.4048	-0.6595
CHB	HIST2	149663262	5.8429	5.0825	0.4222
CHB	HIST2	149668262	8.4805	7.6237	0.3304
CHB	HIST2	149673262	7.5292	8.1319	-0.2191
CHB	HIST2	149678262	4.7999	5.0825	-0.1569
CHB	HIST2	149683262	2.6722	4.7436	-1.2219
CHB	HIST2	149688262	2.3229	4.5742	-1.3709
CHB	HIST2	149693262	3.984	4.5742	-0.3594
CHB	HIST2	149698262	5.2147	4.913	0.1726
CHB	HIST2	149703262	5.3695	6.4378	-0.4803
CHB	HIST2	149708262	6.9815	7.4543	-0.1861
CHB	HIST2	149713262	8.423	7.2848	0.4575
CHB	HIST2	149718262	8.151	8.4708	-0.112
CHB	HIST2	149723262	5.9903	8.1319	-0.7784
CHB	HIST2	149728262	6.8462	8.4708	-0.5687
CHB	HIST2	149733262	5.8551	7.2848	-0.5747
CHB	HIST2	149738262	1.9042	4.066	-1.4578
CHB	HIST2	149743262	1.4142	2.7106	-1.2284
CHB	HIST2	149748262	3.7558	2.7106	0.9904
CHB	HIST2	149753262	3.2829	3.0495	0.2007
CHB	HIST2	149758262	1.308	6.2684	-2.2846
CHB	HIST2	149763262	1.973	6.2684	-1.9783
CHB	HIST2	149768262	2.1135	4.4048	-1.4418
CHB	HIST2	149773262	1.5264	6.0989	-2.1584
CHB	HIST2	149778262	1.016	5.2519	-2.2846
CHB	HIST2	149783262	0.8184	4.2354	-2.2245
CHB	HIST2	149788262	0.586	5.5907	-2.5535
CHB	HIST2	149793262	0.4026	3.3883	-2.3513
CHB	HIST2	149798262	0	0	0
CHB	HIST2	149803262	0	0	0
CHB	HIST2	149808262	0	0	0
CHB	HIST2	149813262	0.4976	2.3718	-1.9784
CHB	HIST2	149818262	1.276	5.4213	-2.1737
CHB	HIST2	149823262	1.1445	3.2189	-1.7054
CHB	HIST2	149828262	0.3661	0.1694	1.1688
CHB	HIST2	149833262	0	0	0
CHB	HIST2	149838262	0	0	0
CHB	HIST2	149843262	0.3553	2.3718	-2.1286

CHB	HIST2	149848262	0.6599	5.4213	-2.4967
CHB	HIST2	149853262	0.8304	6.6072	-2.5372
CHB	HIST2	149858262	1.5658	6.4378	-2.1906
CHB	HIST2	149863262	1.7519	7.2848	-2.224
CHB	HIST2	149868262	0.9816	6.946	-2.5036
CHB	HIST2	149873262	0.7929	5.0825	-2.3815
CHB	HIST2	149878262	1.64	7.1154	-2.2486
CHB	HIST2	149883262	1.77	7.2848	-2.2168
CHB	HIST2	149888262	1.7063	5.4213	-1.948
CHB	HIST2	149893262	1.5336	6.946	-2.2719
CHB	HIST2	149898262	0.7034	7.1154	-2.6332
CHB	HIST2	149903262	1.8057	7.1154	-2.1805
CHB	HIST2	149908262	2.5933	7.9625	-1.9897
CHB	HIST2	149913262	1.4991	6.2684	-2.1966
CHB	HIST2	149918262	2.0129	7.1154	-2.0954
CHB	HIST2	149923262	3.1725	8.6402	-1.8794
CHB	HIST2	149928262	3.4715	9.9955	-1.959
CHB	HIST2	149933262	2.1807	10.3343	-2.3734
CHB	HIST2	149938262	2.0284	9.1484	-2.3214
CHB	HIST2	149943262	2.9916	8.4708	-1.9181
CHB	HIST2	149948262	2.1917	6.7766	-1.9681
CHB	HIST2	149953262	1.9785	8.1319	-2.2366
CHB	HIST2	149958262	1.5429	7.9625	-2.3789
CHB	HIST2	149963262	0.9977	6.0989	-2.408
CHB	HIST2	149968262	1.1218	5.5907	-2.2801
CHB	HIST2	149973262	1.3725	4.7436	-1.9885
CHB	HIST2	149978262	1.9992	5.2519	-1.7543
CHB	HIST2	149983262	2.4022	8.4708	-2.1244
CHB	HIST2	149988262	2.2322	9.9955	-2.3312
CHB	HIST2	149993262	3.7422	9.3178	-1.7872
CHB	HIST2	149998262	7.1146	10.8426	-1.0375
CHB	HIST2	150003262	5.8317	9.8261	-1.2187
CHB	HIST2	150008262	2.6309	9.8261	-2.1953
CHB	HIST2	150013262	1.7255	10.3343	-2.5059
CHB	HIST2	150018262	1.7489	8.3013	-2.3369
CHB	HIST2	150023262	1.7805	9.8261	-2.4547
CHB	HIST2	150028262	1.0042	8.3013	-2.6025
CHB	HIST2	150033262	1.0459	5.5907	-2.3189
CHB	HIST2	150038262	2.1462	6.6072	-1.9593
CHB	HIST2	150043262	1.9676	7.1154	-2.114
CHB	HIST2	150048262	0.636	6.7766	-2.6359
CHB	HIST2	150053262	0.9884	5.9295	-2.3921
CHB	HIST2	150058262	1.1485	6.0989	-2.3368
CHB	HIST2	150063262	0.7002	6.0989	-2.5484
CHB	HIST2	150068262	0.6389	5.2519	-2.4879
CHB	HIST2	150073262	1.2511	6.6072	-2.3525
CHB	HIST2	150078262	1.2467	7.1154	-2.4101
CHB	HIST2	150083262	0.9678	5.5907	-2.3587
CHB	HIST2	150088262	1.8403	6.2684	-2.0394

CHB	HIST2	150093262	1.5445	6.6072	-2.2236
CHB	HIST2	150098262	0.9933	6.2684	-2.4295
CHB	HIST2	150103262	1.6092	7.7931	-2.3372
CHB	HIST2	150108262	1.8386	8.1319	-2.2875
CHB	HIST2	150113262	1.4287	6.6072	-2.2745
CHB	HIST2	150118262	1.4759	6.7766	-2.2754
CHB	HIST2	150123262	2.2237	7.6237	-2.0824
CHB	HIST2	150128262	2.5092	7.4543	-1.9465
CHB	HIST2	150133262	2.1606	8.3013	-2.1901
CHB	HIST2	150138262	2.089	10.1649	-2.3873
CHB	HIST2	150143262	2.676	9.4872	-2.147
CHB	HIST2	150148262	3.0685	8.1319	-1.8404
CHB	HIST2	150153262	2.0872	8.8096	-2.2697
CHB	HIST2	150158262	2.3716	12.5367	-2.4679
CHB	HIST2	150163262	7.5846	12.8755	-1.2526
CHB	HIST2	150168262	10.2798	9.8261	0.1384
CHB	HIST2	150173262	6.2543	9.4872	-1.0191
CHB	HIST2	150178262	5.5844	9.8261	-1.2941
CHB	HIST2	150183262	7.0824	9.3178	-0.7165
CHB	HIST2	150188262	4.0306	6.7766	-1.1788
CHB	HIST2	150193262	4.0195	6.4378	-1.0873
CHB	HIST2	150198262	8.6772	9.8261	-0.3505
CHB	HIST2	150203262	9.2966	10.6731	-0.3888
CHB	HIST2	150208262	8.6307	10.8426	-0.6156
CHB	HIST2	150213262	9.4733	12.3673	-0.7117
CHB	HIST2	150218262	7.3725	9.8261	-0.7486
CHB	HIST2	150223262	5.0381	7.6237	-0.9971
CHB	HIST2	150228262	2.9593	6.2684	-1.5241
CHB	HIST2	150233262	2.5888	4.4048	-1.1427
CHB	HIST2	150238262	4.0061	5.2519	-0.6719
CHB	HIST2	150243262	3.5384	6.0989	-1.2087
CHB	HIST2	150248262	4.7329	6.7766	-0.8773
CHB	HIST2	150253262	5.5913	8.1319	-0.9234
CHB	HIST2	150258262	7.1003	8.4708	-0.4798
CHB	HIST2	150263262	9.6421	9.1484	0.161
CHB	HIST2	150268262	12.8372	11.5202	0.3463
CHB	HIST2	150273262	11.4002	11.1814	0.0592
CHB	HIST2	150278262	9.6291	11.012	-0.3793
CHB	HIST2	150283262	10.725	12.1979	-0.367
CHB	HIST2	150288262	9.5454	10.6731	-0.3185
CHB	HIST2	150293262	7.1532	8.979	-0.6057
CHB	HIST2	150298262	4.2285	7.7931	-1.3472
CHB	HIST2	150303262	6.2768	8.3013	-0.722
CHB	HIST2	150308262	7.7175	10.3343	-0.7617
CHB	HIST2	150313262	7.1367	9.6567	-0.7814
CHB	HIST2	150318262	9.9189	10.6731	-0.213
CHB	HIST2	150323262	9.2399	11.1814	-0.525
CHB	HIST2	150328262	8.969	11.6896	-0.7055
CHB	HIST2	150333262	8.0592	11.012	-0.8099

CHB	HIST2	150338262	5.6434	7.2848	-0.6598
CHB	HIST2	150343262	7.4455	6.6072	0.3682
CHB	HIST2	150348262	6.8536	8.1319	-0.4646
CHB	HIST2	150353262	7.3474	11.6896	-1.1261
YRI	HIST2	149358262	5.7731	6.0502	-0.1313
YRI	HIST2	149363262	5.4157	7.3947	-0.782
YRI	HIST2	149368262	3.5455	7.2267	-1.4854
YRI	HIST2	149373262	2.8747	7.0586	-1.7248
YRI	HIST2	149378262	3.6038	6.7225	-1.3439
YRI	HIST2	149383262	5.2329	5.5461	-0.1604
YRI	HIST2	149388262	6.9174	7.0586	-0.0582
YRI	HIST2	149393262	6.4411	7.0586	-0.2546
YRI	HIST2	149398262	5.1599	6.7225	-0.6733
YRI	HIST2	149403262	6.9075	9.7476	-0.87
YRI	HIST2	149408262	6.2346	8.7392	-0.849
YRI	HIST2	149413262	6.0138	7.2267	-0.4894
YRI	HIST2	149418262	5.7145	5.5461	0.0862
YRI	HIST2	149423262	2.4418	3.1932	-0.6197
YRI	HIST2	149428262	2.8504	4.3696	-0.9593
YRI	HIST2	149433262	2.1945	2.8571	-0.5992
YRI	HIST2	149438262	0.037	0.5042	-1.4683
YRI	HIST2	149443262	0.0185	0.1681	-0.8893
YRI	HIST2	149448262	0.1661	0.6722	-1.3249
YRI	HIST2	149453262	0.6607	1.5126	-1.2685
YRI	HIST2	149458262	0.5589	1.3445	-1.276
YRI	HIST2	149463262	0.0643	0.5042	-1.3825
YRI	HIST2	149468262	0	0	0
YRI	HIST2	149473262	0	0	0
YRI	HIST2	149478262	0	0	0
YRI	HIST2	149483262	0	0	0
YRI	HIST2	149488262	0	0	0
YRI	HIST2	149493262	0	0	0
YRI	HIST2	149498262	0	0	0
YRI	HIST2	149503262	0	0	0
YRI	HIST2	149508262	0.411	0.6722	-0.6838
YRI	HIST2	149513262	0.8557	2.3529	-1.5849
YRI	HIST2	149518262	0.4899	1.8487	-1.738
YRI	HIST2	149523262	0.0453	0.1681	-0.73
YRI	HIST2	149528262	0.2339	0.5042	-0.8495
YRI	HIST2	149533262	0.2339	0.5042	-0.8495
YRI	HIST2	149538262	0	0	0
YRI	HIST2	149543262	0	0	0
YRI	HIST2	149548262	0	0	0
YRI	HIST2	149553262	0	0	0
YRI	HIST2	149558262	0	0	0
YRI	HIST2	149563262	0	0	0
YRI	HIST2	149568262	0.6355	0.6722	-0.0961
YRI	HIST2	149573262	1.1098	1.6806	-0.7853
YRI	HIST2	149578262	1.0245	2.0167	-1.1863

YRI	HIST2	149583262	2.3949	5.0419	-1.4748
YRI	HIST2	149588262	2.2919	5.2099	-1.5795
YRI	HIST2	149593262	1.2767	2.5209	-1.246
YRI	HIST2	149598262	3.5693	3.5293	0.0303
YRI	HIST2	149603262	6.2413	6.3864	-0.0655
YRI	HIST2	149608262	5.1044	6.8906	-0.7526
YRI	HIST2	149613262	1.9094	3.1932	-1.0588
YRI	HIST2	149618262	0.3065	0.5042	-0.6213
YRI	HIST2	149623262	0	0	0
YRI	HIST2	149628262	0.0093	0.1681	-0.944
YRI	HIST2	149633262	0.3282	0.6722	-0.9006
YRI	HIST2	149638262	3.1603	2.3529	0.8547
YRI	HIST2	149643262	6.1235	5.7141	0.2041
YRI	HIST2	149648262	9.6312	10.2518	-0.1814
YRI	HIST2	149653262	9.2435	10.4199	-0.3386
YRI	HIST2	149658262	5.0448	6.3864	-0.6055
YRI	HIST2	149663262	7.5524	7.7309	-0.0677
YRI	HIST2	149668262	11.5005	14.9575	-0.7075
YRI	HIST2	149673262	9.1905	13.7811	-1.0156
YRI	HIST2	149678262	5.8105	8.9073	-1.0314
YRI	HIST2	149683262	4.3956	9.4115	-1.5875
YRI	HIST2	149688262	2.5697	8.235	-2.0285
YRI	HIST2	149693262	3.4596	6.3864	-1.321
YRI	HIST2	149698262	6.3424	7.7309	-0.5268
YRI	HIST2	149703262	7.8847	11.4282	-0.9355
YRI	HIST2	149708262	8.7188	13.445	-1.0703
YRI	HIST2	149713262	8.9771	11.7644	-0.7161
YRI	HIST2	149718262	8.4155	11.5963	-0.8283
YRI	HIST2	149723262	7.1768	11.5963	-1.1509
YRI	HIST2	149728262	8.8147	12.6047	-0.9124
YRI	HIST2	149733262	8.0828	12.4366	-1.0614
YRI	HIST2	149738262	4.1472	8.067	-1.4304
YRI	HIST2	149743262	4.3186	6.2183	-0.8782
YRI	HIST2	149748262	6.322	7.2267	-0.3651
YRI	HIST2	149753262	6.2614	7.8989	-0.6092
YRI	HIST2	149758262	6.3939	10.4199	-1.159
YRI	HIST2	149763262	5.4444	10.2518	-1.4051
YRI	HIST2	149768262	3.1474	8.5712	-1.8718
YRI	HIST2	149773262	5.5032	11.7644	-1.6085
YRI	HIST2	149778262	6.9407	12.9408	-1.4089
YRI	HIST2	149783262	4.8594	12.6047	-1.8645
YRI	HIST2	149788262	3.9274	10.924	-1.927
YRI	HIST2	149793262	2.1402	4.8738	-1.5692
YRI	HIST2	149798262	0.0456	0.1681	-0.7282
YRI	HIST2	149803262	0	0	0
YRI	HIST2	149808262	0	0	0
YRI	HIST2	149813262	2.9777	5.7141	-1.3645
YRI	HIST2	149818262	5.0549	8.5712	-1.2135
YRI	HIST2	149823262	2.5589	3.0251	-0.4022

YRI	HIST2	149828262	0.4817	0.1681	1.8643
YRI	HIST2	149833262	0	0	0
YRI	HIST2	149838262	0.0183	0.1681	-0.8905
YRI	HIST2	149843262	2.537	4.2016	-1.0874
YRI	HIST2	149848262	4.6542	10.5879	-1.6828
YRI	HIST2	149853262	4.0685	12.7727	-2.0693
YRI	HIST2	149858262	3.8285	11.9324	-2.0543
YRI	HIST2	149863262	6.3146	11.9324	-1.4241
YRI	HIST2	149868262	7.8278	12.4366	-1.1236
YRI	HIST2	149873262	5.2619	10.924	-1.5594
YRI	HIST2	149878262	7.4796	11.7644	-1.1008
YRI	HIST2	149883262	8.5321	11.4282	-0.7646
YRI	HIST2	149888262	5.4156	11.7644	-1.631
YRI	HIST2	149893262	5.1862	13.1089	-1.8378
YRI	HIST2	149898262	4.378	12.6047	-1.9804
YRI	HIST2	149903262	5.195	12.2685	-1.7468
YRI	HIST2	149908262	6.7355	11.2602	-1.2113
YRI	HIST2	149913262	5.1582	11.7644	-1.6972
YRI	HIST2	149918262	6.2502	13.445	-1.6294
YRI	HIST2	149923262	7.2406	13.445	-1.4051
YRI	HIST2	149928262	8.4768	12.7727	-1.0213
YRI	HIST2	149933262	9.6141	13.445	-0.8676
YRI	HIST2	149938262	8.3499	12.2685	-0.9677
YRI	HIST2	149943262	6.3429	9.2434	-0.9335
YRI	HIST2	149948262	4.9525	8.9073	-1.3172
YRI	HIST2	149953262	8.6647	12.6047	-0.9485
YRI	HIST2	149958262	7.2806	10.924	-1.0035
YRI	HIST2	149963262	3.7702	7.7309	-1.5028
YRI	HIST2	149968262	6.4047	9.4115	-0.9516
YRI	HIST2	149973262	6.9166	9.4115	-0.7896
YRI	HIST2	149978262	7.1072	9.5795	-0.7697
YRI	HIST2	149983262	10.3106	12.9408	-0.6176
YRI	HIST2	149988262	9.2029	12.7727	-0.8487
YRI	HIST2	149993262	8.8764	13.1089	-0.9818
YRI	HIST2	149998262	14.1567	15.4617	-0.2588
YRI	HIST2	150003262	13.1306	13.9492	-0.179
YRI	HIST2	150008262	12.5625	16.134	-0.68
YRI	HIST2	150013262	15.4155	17.1423	-0.3103
YRI	HIST2	150018262	14.3006	14.9575	-0.1344
YRI	HIST2	150023262	14.1563	16.134	-0.3765
YRI	HIST2	150028262	11.3176	13.1089	-0.4155
YRI	HIST2	150033262	7.6112	10.5879	-0.8442
YRI	HIST2	150038262	8.9277	13.445	-1.023
YRI	HIST2	150043262	12.7246	15.1256	-0.4862
YRI	HIST2	150048262	11.1573	14.6214	-0.7245
YRI	HIST2	150053262	6.832	12.4366	-1.3664
YRI	HIST2	150058262	7.5799	12.9408	-1.2588
YRI	HIST2	150063262	9.4442	13.445	-0.9061
YRI	HIST2	150068262	8.7135	11.7644	-0.7838

YRI	HIST2	150073262	9.9749	14.4534	-0.947
YRI	HIST2	150078262	11.7244	17.3104	-0.9943
YRI	HIST2	150083262	11.5356	15.7978	-0.828
YRI	HIST2	150088262	14.0826	15.7978	-0.3332
YRI	HIST2	150093262	14.225	15.4617	-0.2452
YRI	HIST2	150098262	10.556	13.2769	-0.6236
YRI	HIST2	150103262	10.6994	14.7895	-0.8461
YRI	HIST2	150108262	12.1453	16.8062	-0.8534
YRI	HIST2	150113262	9.628	15.2937	-1.1352
YRI	HIST2	150118262	7.5142	14.6214	-1.4864
YRI	HIST2	150123262	7.9899	14.7895	-1.4066
YRI	HIST2	150128262	10.0548	14.7895	-0.9795
YRI	HIST2	150133262	11.5888	15.7978	-0.8177
YRI	HIST2	150138262	12.7201	17.3104	-0.8171
YRI	HIST2	150143262	11.5205	16.134	-0.8784
YRI	HIST2	150148262	7.9488	11.7644	-0.9802
YRI	HIST2	150153262	10.4622	13.1089	-0.6139
YRI	HIST2	150158262	16.6195	18.991	-0.3862
YRI	HIST2	150163262	16.7745	19.3272	-0.4087
YRI	HIST2	150168262	13.8711	17.1423	-0.5877
YRI	HIST2	150173262	11.5174	15.1256	-0.7306
YRI	HIST2	150178262	10.8839	13.7811	-0.6409
YRI	HIST2	150183262	11.7537	14.4534	-0.5708
YRI	HIST2	150188262	9.2996	11.9324	-0.6674
YRI	HIST2	150193262	8.5916	10.5879	-0.5661
YRI	HIST2	150198262	11.1994	11.2602	-0.0163
YRI	HIST2	150203262	11.9021	11.5963	0.0796
YRI	HIST2	150208262	13.1178	14.1172	-0.2161
YRI	HIST2	150213262	13.8995	15.2937	-0.2794
YRI	HIST2	150218262	11.256	12.7727	-0.3606
YRI	HIST2	150223262	8.162	9.9157	-0.5287
YRI	HIST2	150228262	4.3385	8.4031	-1.4285
YRI	HIST2	150233262	3.1579	7.2267	-1.6418
YRI	HIST2	150238262	4.1905	7.2267	-1.2252
YRI	HIST2	150243262	4.8034	10.0837	-1.5673
YRI	HIST2	150248262	6.1037	10.924	-1.3276
YRI	HIST2	150253262	7.1965	9.9157	-0.8198
YRI	HIST2	150258262	10.1715	10.2518	-0.0235
YRI	HIST2	150263262	14.0328	12.9408	0.2564
YRI	HIST2	150268262	18.5669	17.3104	0.2237
YRI	HIST2	150273262	16.7098	16.6382	0.0132
YRI	HIST2	150278262	13.7164	15.1256	-0.2854
YRI	HIST2	150283262	13.1092	14.6214	-0.3163
YRI	HIST2	150288262	11.1294	12.7727	-0.3907
YRI	HIST2	150293262	10.3933	12.4366	-0.4982
YRI	HIST2	150298262	9.4527	12.6047	-0.7588
YRI	HIST2	150303262	11.1764	12.1005	-0.2312
YRI	HIST2	150308262	12.0652	13.445	-0.3125
YRI	HIST2	150313262	12.1713	14.1172	-0.4208

YRI	HIST2	150318262	15.7792	14.6214	0.2421
YRI	HIST2	150323262	13.6168	14.9575	-0.2744
YRI	HIST2	150328262	12.1122	14.4534	-0.495
YRI	HIST2	150333262	11.4728	13.1089	-0.3795
YRI	HIST2	150338262	10.2635	11.9324	-0.4231
YRI	HIST2	150343262	12.3647	12.1005	0.0661
YRI	HIST2	150348262	10.0257	11.4282	-0.3703
YRI	HIST2	150353262	12.4942	13.1089	-0.1426
CEU	HIST2	149389459	6.7359	5.9697	0.3698
CEU	HIST2	149394459	4.5343	5.458	-0.4829
CEU	HIST2	149399459	5.9819	6.6519	-0.2933
CEU	HIST2	149404459	5.8442	5.6286	0.1097
CEU	HIST2	149409459	2.7594	2.2173	0.6056
CEU	HIST2	149414459	4.5403	3.4112	0.8868
CEU	HIST2	149419459	3.9518	3.0701	0.7563
CEU	HIST2	149424459	1.9445	1.8762	0.087
CEU	HIST2	149429459	2.3917	3.0701	-0.5819
CEU	HIST2	149434459	1.7537	2.0467	-0.3486
CEU	HIST2	149439459	0	0	0
CEU	HIST2	149444459	0.08	0.5117	-1.3539
CEU	HIST2	149449459	0.1097	0.6822	-1.495
CEU	HIST2	149454459	0.128	0.6822	-1.4472
CEU	HIST2	149459459	0.0983	0.5117	-1.2965
CEU	HIST2	149464459	0	0	0
CEU	HIST2	149469459	0	0	0
CEU	HIST2	149474459	0	0	0
CEU	HIST2	149479459	0	0	0
CEU	HIST2	149484459	0	0	0
CEU	HIST2	149489459	0	0	0
CEU	HIST2	149494459	0	0	0
CEU	HIST2	149499459	0	0	0
CEU	HIST2	149504459	0	0	0
CEU	HIST2	149509459	0.4605	1.1939	-1.3069
CEU	HIST2	149514459	0.4605	1.1939	-1.3069
CEU	HIST2	149519459	0	0	0
CEU	HIST2	149524459	0	0	0
CEU	HIST2	149529459	0.0101	0.1706	-0.9534
CEU	HIST2	149534459	0.0101	0.1706	-0.9534
CEU	HIST2	149539459	0	0	0
CEU	HIST2	149544459	0	0	0
CEU	HIST2	149549459	0	0	0
CEU	HIST2	149554459	0	0	0
CEU	HIST2	149559459	0	0	0
CEU	HIST2	149564459	0	0	0
CEU	HIST2	149569459	0.6005	0.3411	1.0399
CEU	HIST2	149574459	0.885	0.6822	0.5296
CEU	HIST2	149579459	1.1946	1.7056	-0.6999
CEU	HIST2	149584459	2.8182	3.2407	-0.3464
CEU	HIST2	149589459	3.6436	3.2407	0.3304

CEU	HIST2	149594459	2.5601	3.0701	-0.4375
CEU	HIST2	149599459	2.6537	2.3879	0.2799
CEU	HIST2	149604459	6.5329	4.0935	1.6403
CEU	HIST2	149609459	7.4115	4.9463	1.4059
CEU	HIST2	149614459	2.7077	1.5351	1.7387
CEU	HIST2	149619459	0	0	0
CEU	HIST2	149624459	0	0	0
CEU	HIST2	149629459	0.0301	0.1706	-0.8346
CEU	HIST2	149634459	0.0503	0.5117	-1.4471
CEU	HIST2	149639459	2.4197	1.8762	0.692
CEU	HIST2	149644459	5.8895	5.2874	0.3238
CEU	HIST2	149649459	7.9742	6.8225	0.4928
CEU	HIST2	149654459	7.0229	4.9463	1.1843
CEU	HIST2	149659459	5.1544	4.7757	0.2227
CEU	HIST2	149664459	5.181	7.1636	-0.8116
CEU	HIST2	149669459	5.0812	9.3809	-1.3736
CEU	HIST2	149674459	5.0775	8.3576	-1.166
CEU	HIST2	149679459	4.4789	6.3108	-0.8411
CEU	HIST2	149684459	2.59	4.2641	-1.0867
CEU	HIST2	149689459	0.8336	3.0701	-1.9184
CEU	HIST2	149694459	2.3156	4.6052	-1.3901
CEU	HIST2	149699459	3.4494	5.6286	-1.1085
CEU	HIST2	149704459	6.2375	7.1636	-0.3791
CEU	HIST2	149709459	7.6464	7.1636	0.1976
CEU	HIST2	149714459	9.2695	7.8459	0.5363
CEU	HIST2	149719459	11.4108	8.187	1.1679
CEU	HIST2	149724459	8.444	6.3108	0.9794
CEU	HIST2	149729459	6.5995	6.1402	0.2161
CEU	HIST2	149734459	4.9445	5.2874	-0.1844
CEU	HIST2	149739459	2.497	3.4112	-0.718
CEU	HIST2	149744459	4.2909	3.7524	0.3901
CEU	HIST2	149749459	4.3739	3.7524	0.4503
CEU	HIST2	149754459	1.0794	2.3879	-1.378
CEU	HIST2	149759459	2.0045	4.6052	-1.579
CEU	HIST2	149764459	2.4263	5.458	-1.5849
CEU	HIST2	149769459	2.698	4.7757	-1.222
CEU	HIST2	149774459	4.4282	6.4814	-0.9203
CEU	HIST2	149779459	5.0936	7.3342	-0.8977
CEU	HIST2	149784459	4.7163	7.8459	-1.179
CEU	HIST2	149789459	2.8621	5.6286	-1.4072
CEU	HIST2	149794459	0.644	1.3645	-1.1656
CEU	HIST2	149799459	0	0	0
CEU	HIST2	149804459	0	0	0
CEU	HIST2	149809459	0	0	0
CEU	HIST2	149814459	1.9779	2.8996	-0.8289
CEU	HIST2	149819459	2.7071	4.2641	-1.0107
CEU	HIST2	149824459	1.2284	1.5351	-0.4548
CEU	HIST2	149829459	0.4993	0.1706	1.9525
CEU	HIST2	149834459	0	0	0

CEU	HIST2	149839459	0.2012	0.3411	-0.5608
CEU	HIST2	149844459	1.7025	3.0701	-1.1731
CEU	HIST2	149849459	2.4467	4.7757	-1.3698
CEU	HIST2	149854459	1.698	4.9463	-1.8525
CEU	HIST2	149859459	3.1481	6.6519	-1.534
CEU	HIST2	149864459	5.1378	8.6987	-1.2199
CEU	HIST2	149869459	5.0006	8.5281	-1.2308
CEU	HIST2	149874459	3.4304	5.458	-1.06
CEU	HIST2	149879459	3.336	5.7991	-1.22
CEU	HIST2	149884459	3.1241	5.2874	-1.1632
CEU	HIST2	149889459	3.1824	4.2641	-0.7022
CEU	HIST2	149894459	3.7693	5.7991	-1.0053
CEU	HIST2	149899459	2.9536	5.2874	-1.2549
CEU	HIST2	149904459	3.7792	5.458	-0.8776
CEU	HIST2	149909459	3.3746	5.1169	-0.9644
CEU	HIST2	149914459	1.7974	4.2641	-1.6012
CEU	HIST2	149919459	2.4978	4.4346	-1.2152
CEU	HIST2	149924459	4.3737	5.2874	-0.4913
CEU	HIST2	149929459	3.5704	5.7991	-1.1039
CEU	HIST2	149934459	1.8611	5.2874	-1.8423
CEU	HIST2	149939459	2.1715	4.2641	-1.3584
CEU	HIST2	149944459	2.3768	3.7524	-0.9966
CEU	HIST2	149949459	2.3469	4.7757	-1.4285
CEU	HIST2	149954459	2.617	5.1169	-1.3837
CEU	HIST2	149959459	1.8481	3.7524	-1.3796
CEU	HIST2	149964459	1.3956	3.0701	-1.4364
CEU	HIST2	149969459	2.22	3.7524	-1.1102
CEU	HIST2	149974459	2.5533	4.2641	-1.1106
CEU	HIST2	149979459	2.1841	4.2641	-1.3502
CEU	HIST2	149984459	3.316	5.2874	-1.06
CEU	HIST2	149989459	4.7126	6.3108	-0.7338
CEU	HIST2	149994459	7.1295	8.187	-0.3831
CEU	HIST2	149999459	10.0827	8.5281	0.5424
CEU	HIST2	150004459	7.9911	5.9697	0.9756
CEU	HIST2	150009459	9.4409	8.8692	0.1924
CEU	HIST2	150014459	11.8881	10.5749	0.3752
CEU	HIST2	150019459	8.1927	6.8225	0.5863
CEU	HIST2	150024459	6.4265	6.6519	-0.0987
CEU	HIST2	150029459	4.7699	5.9697	-0.579
CEU	HIST2	150034459	2.6294	4.7757	-1.2623
CEU	HIST2	150039459	3.4766	5.9697	-1.2032
CEU	HIST2	150044459	4.1018	5.9697	-0.9015
CEU	HIST2	150049459	3.7922	4.9463	-0.6582
CEU	HIST2	150054459	3.7751	4.9463	-0.6679
CEU	HIST2	150059459	2.31	4.4346	-1.333
CEU	HIST2	150064459	2.027	3.9229	-1.3223
CEU	HIST2	150069459	3.6792	4.4346	-0.474
CEU	HIST2	150074459	5.7254	6.1402	-0.1952
CEU	HIST2	150079459	5.7547	6.6519	-0.3928

CEU	HIST2	150084459	3.9699	5.9697	-0.9651
CEU	HIST2	150089459	3.9388	7.3342	-1.3604
CEU	HIST2	150094459	4.1577	6.4814	-1.0415
CEU	HIST2	150099459	4.2117	5.458	-0.6515
CEU	HIST2	150104459	5.2463	6.8225	-0.6745
CEU	HIST2	150109459	5.7805	6.3108	-0.2435
CEU	HIST2	150114459	4.6889	5.2874	-0.3218
CEU	HIST2	150119459	3.0992	5.458	-1.2331
CEU	HIST2	150124459	3.5883	6.6519	-1.3413
CEU	HIST2	150129459	6.7664	9.0398	-0.7516
CEU	HIST2	150134459	7.8642	9.8926	-0.6168
CEU	HIST2	150139459	5.8427	10.2337	-1.2936
CEU	HIST2	150144459	5.4302	8.5281	-1.0809
CEU	HIST2	150149459	4.8528	6.8225	-0.8428
CEU	HIST2	150154459	3.7067	7.8459	-1.5593
CEU	HIST2	150159459	5.6559	9.7221	-1.2566
CEU	HIST2	150164459	9.1921	8.8692	0.1087
CEU	HIST2	150169459	9.7535	6.9931	1.155
CEU	HIST2	150174459	7.589	6.9931	0.2493
CEU	HIST2	150179459	7.6423	5.7991	0.9129
CEU	HIST2	150184459	7.6252	6.1402	0.6988
CEU	HIST2	150189459	5.437	6.4814	-0.4681
CEU	HIST2	150194459	5.6015	5.6286	-0.0138
CEU	HIST2	150199459	9.2808	6.8225	1.0519
CEU	HIST2	150204459	8.4469	7.1636	0.5253
CEU	HIST2	150209459	9.1062	8.0164	0.4025
CEU	HIST2	150214459	9.804	7.8459	0.7376
CEU	HIST2	150219459	7.2171	5.7991	0.7023
CEU	HIST2	150224459	5.8103	5.2874	0.2812
CEU	HIST2	150229459	3.1051	4.4346	-0.8342
CEU	HIST2	150234459	2.8266	3.0701	-0.2089
CEU	HIST2	150239459	3.2463	3.5818	-0.2529
CEU	HIST2	150244459	4.229	5.458	-0.6425
CEU	HIST2	150249459	5.3032	4.4346	0.545
CEU	HIST2	150254459	6.9219	5.6286	0.6579
CEU	HIST2	150259459	9.9767	8.5281	0.5054
CEU	HIST2	150264459	15.1144	9.0398	2.0084
CEU	HIST2	150269459	18.4865	11.4277	1.8754
CEU	HIST2	150274459	15.8481	11.4277	1.1744
CEU	HIST2	150279459	16.1664	11.0866	1.3886
CEU	HIST2	150284459	13.5457	9.5515	1.2548
CEU	HIST2	150289459	10.147	7.1636	1.2213
CEU	HIST2	150294459	10.9838	8.187	1.0132
CEU	HIST2	150299459	8.7981	7.8459	0.3587
CEU	HIST2	150304459	8.5962	8.0164	0.2142
CEU	HIST2	150309459	9.9116	8.6987	0.4155
CEU	HIST2	150314459	9.4723	8.6987	0.265
CEU	HIST2	150319459	13.4572	10.2337	0.9497
CEU	HIST2	150324459	13.9644	10.4043	1.0328

CEU	HIST2	150329459	12.7874	9.2104	1.1623
CEU	HIST2	150334459	12.7375	9.5515	1.0009
CEU	HIST2	150339459	9.6156	9.0398	0.1904
CEU	HIST2	150344459	9.3276	8.0164	0.4843
CEU	HIST2	150349459	10.1779	8.6987	0.5068
CEU	HIST2	150354459	13.1525	10.0632	0.9245
CEU	HIST2	150359459	15.3976	10.5749	1.3779
CEU	HIST2	150364459	13.1202	10.4043	0.7879
CEU	HIST2	150369459	11.3063	10.916	0.1082
CEU	HIST2	150374459	13.987	11.4277	0.68
CEU	HIST2	150379459	14.7563	10.916	1.0651
CHB	HIST2	149389459	5.0986	6.6072	-0.6626
CHB	HIST2	149394459	3.6448	5.4213	-0.9315
CHB	HIST2	149399459	5.0101	6.2684	-0.5795
CHB	HIST2	149404459	4.5336	6.0989	-0.7389
CHB	HIST2	149409459	1.9524	3.2189	-1.0412
CHB	HIST2	149414459	4.1706	4.2354	-0.0422
CHB	HIST2	149419459	3.7875	4.7436	-0.564
CHB	HIST2	149424459	1.3878	3.0495	-1.4291
CHB	HIST2	149429459	1.9345	3.7271	-1.3023
CHB	HIST2	149434459	1.5755	2.5412	-0.9643
CHB	HIST2	149439459	0	0	0
CHB	HIST2	149444459	0.0289	0.3388	-1.2431
CHB	HIST2	149449459	0.0386	0.5082	-1.4741
CHB	HIST2	149454459	0.097	1.3553	-2.0393
CHB	HIST2	149459459	0.0873	1.1859	-1.961
CHB	HIST2	149464459	0	0	0
CHB	HIST2	149469459	0	0	0
CHB	HIST2	149474459	0	0	0
CHB	HIST2	149479459	0	0	0
CHB	HIST2	149484459	0	0	0
CHB	HIST2	149489459	0	0	0
CHB	HIST2	149494459	0	0	0
CHB	HIST2	149499459	0	0	0
CHB	HIST2	149504459	0	0	0
CHB	HIST2	149509459	0.8968	1.0165	-0.2382
CHB	HIST2	149514459	0.9065	1.1859	-0.4987
CHB	HIST2	149519459	0.0097	0.1694	-0.949
CHB	HIST2	149524459	0	0	0
CHB	HIST2	149529459	0.2578	0.1694	0.5253
CHB	HIST2	149534459	0.2578	0.1694	0.5253
CHB	HIST2	149539459	0	0	0
CHB	HIST2	149544459	0	0	0
CHB	HIST2	149549459	0	0	0
CHB	HIST2	149554459	0	0	0
CHB	HIST2	149559459	0	0	0
CHB	HIST2	149564459	0.0097	0.1694	-0.949
CHB	HIST2	149569459	0.4891	0.5082	-0.06
CHB	HIST2	149574459	0.8942	0.6777	0.566

CHB	HIST2	149579459	1.624	2.033	-0.4877
CHB	HIST2	149584459	3.6655	3.7271	-0.0448
CHB	HIST2	149589459	3.3196	2.8801	0.3963
CHB	HIST2	149594459	2.4999	2.2024	0.3331
CHB	HIST2	149599459	3.4474	2.033	1.6867
CHB	HIST2	149604459	6.7636	4.4048	1.4843
CHB	HIST2	149609459	6.6722	5.5907	0.5518
CHB	HIST2	149614459	1.7194	1.8636	-0.184
CHB	HIST2	149619459	0	0	0
CHB	HIST2	149624459	0	0	0
CHB	HIST2	149629459	0.0097	0.1694	-0.949
CHB	HIST2	149634459	0.0769	0.6777	-1.5707
CHB	HIST2	149639459	2.9195	2.033	1.0572
CHB	HIST2	149644459	6.6878	5.2519	0.7744
CHB	HIST2	149649459	7.8009	6.946	0.3589
CHB	HIST2	149654459	5.7282	5.2519	0.2569
CHB	HIST2	149659459	3.8761	4.5742	-0.4251
CHB	HIST2	149664459	6.4864	5.4213	0.5585
CHB	HIST2	149669459	8.3809	7.6237	0.292
CHB	HIST2	149674459	6.8664	7.4543	-0.2314
CHB	HIST2	149679459	4.7181	5.5907	-0.4452
CHB	HIST2	149684459	3.15	5.2519	-1.1336
CHB	HIST2	149689459	2.4038	4.5742	-1.3216
CHB	HIST2	149694459	4.2829	4.7436	-0.2718
CHB	HIST2	149699459	4.8056	4.2354	0.3712
CHB	HIST2	149704459	6.3931	6.6072	-0.094
CHB	HIST2	149709459	6.9815	7.4543	-0.1861
CHB	HIST2	149714459	7.1251	7.2848	-0.0642
CHB	HIST2	149719459	8.6398	9.1484	-0.1658
CHB	HIST2	149724459	7.1888	8.8096	-0.5472
CHB	HIST2	149729459	6.3573	7.9625	-0.5948
CHB	HIST2	149734459	4.6429	6.4378	-0.807
CHB	HIST2	149739459	1.8848	3.7271	-1.3384
CHB	HIST2	149744459	3.4293	3.0495	0.3266
CHB	HIST2	149749459	3.8595	3.2189	0.5267
CHB	HIST2	149754459	0.8839	2.8801	-1.7998
CHB	HIST2	149759459	2.1334	6.7766	-1.9932
CHB	HIST2	149764459	1.9448	6.0989	-1.9609
CHB	HIST2	149769459	1.2619	4.5742	-2.0169
CHB	HIST2	149774459	1.6078	5.9295	-2.0922
CHB	HIST2	149779459	1.0154	4.913	-2.2294
CHB	HIST2	149784459	0.7089	4.4048	-2.3256
CHB	HIST2	149789459	0.5089	4.7436	-2.4979
CHB	HIST2	149794459	0.3541	2.7106	-2.2329
CHB	HIST2	149799459	0	0	0
CHB	HIST2	149804459	0	0	0
CHB	HIST2	149809459	0	0	0
CHB	HIST2	149814459	0.5846	3.2189	-2.1658
CHB	HIST2	149819459	1.276	5.4213	-2.1737

CHB	HIST2	149824459	1.0575	2.3718	-1.3873
CHB	HIST2	149829459	0.3661	0.1694	1.1688
CHB	HIST2	149834459	0	0	0
CHB	HIST2	149839459	0.0192	0.1694	-0.8925
CHB	HIST2	149844459	0.4419	3.2189	-2.2831
CHB	HIST2	149849459	0.6989	6.0989	-2.549
CHB	HIST2	149854459	0.7824	6.0989	-2.5096
CHB	HIST2	149859459	1.6797	6.7766	-2.1879
CHB	HIST2	149864459	1.8187	7.6237	-2.2386
CHB	HIST2	149869459	0.858	6.6072	-2.5251
CHB	HIST2	149874459	0.6971	4.5742	-2.3608
CHB	HIST2	149879459	1.6206	6.6072	-2.1902
CHB	HIST2	149884459	1.7893	7.4543	-2.2299
CHB	HIST2	149889459	1.906	6.0989	-1.9792
CHB	HIST2	149894459	1.5627	7.2848	-2.3001
CHB	HIST2	149899459	0.6136	6.6072	-2.6325
CHB	HIST2	149904459	1.7671	6.7766	-2.1504
CHB	HIST2	149909459	2.4931	8.1319	-2.0496
CHB	HIST2	149914459	1.8749	6.6072	-2.0785
CHB	HIST2	149919459	2.4125	7.7931	-2.0336
CHB	HIST2	149924459	3.7421	10.1649	-1.8987
CHB	HIST2	149929459	3.0817	9.4872	-2.0192
CHB	HIST2	149934459	1.9947	9.6567	-2.3758
CHB	HIST2	149939459	2.3731	9.3178	-2.226
CHB	HIST2	149944459	2.4479	7.6237	-1.9959
CHB	HIST2	149949459	1.924	7.4543	-2.1769
CHB	HIST2	149954459	1.7725	7.9625	-2.2938
CHB	HIST2	149959459	1.4464	6.7766	-2.2881
CHB	HIST2	149964459	1.0267	6.2684	-2.4141
CHB	HIST2	149969459	1.1121	5.4213	-2.2596
CHB	HIST2	149974459	1.7386	4.913	-1.8157
CHB	HIST2	149979459	2.0187	5.5907	-1.8225
CHB	HIST2	149984459	2.3748	8.1319	-2.0926
CHB	HIST2	149989459	2.2899	10.3343	-2.3416
CHB	HIST2	149994459	5.6202	10.6731	-1.4271
CHB	HIST2	149999459	7.5326	11.3508	-1.0179
CHB	HIST2	150004459	3.7986	9.4872	-1.7932
CHB	HIST2	150009459	2.3642	10.1649	-2.306
CHB	HIST2	150014459	1.6198	10.1649	-2.526
CHB	HIST2	150019459	1.6077	7.7931	-2.3378
CHB	HIST2	150024459	1.897	9.1484	-2.3642
CHB	HIST2	150029459	0.9937	7.6237	-2.5567
CHB	HIST2	150034459	1.1982	5.9295	-2.2905
CHB	HIST2	150039459	2.147	7.1154	-2.0404
CHB	HIST2	150044459	1.6018	6.7766	-2.2214
CHB	HIST2	150049459	0.6539	6.4378	-2.6006
CHB	HIST2	150054459	1.102	6.0989	-2.3588
CHB	HIST2	150059459	1.0721	5.4213	-2.2806
CHB	HIST2	150064459	0.5477	5.2519	-2.5371

CHB	HIST2	150069459	1.2126	6.4378	-2.3494
CHB	HIST2	150074459	1.2802	7.1154	-2.3963
CHB	HIST2	150079459	0.6728	5.9295	-2.5448
CHB	HIST2	150084459	1.0249	5.5907	-2.3296
CHB	HIST2	150089459	1.8978	6.7766	-2.0943
CHB	HIST2	150094459	1.8374	7.2848	-2.1897
CHB	HIST2	150099459	1.127	6.946	-2.4426
CHB	HIST2	150104459	1.8089	7.4543	-2.2222
CHB	HIST2	150109459	1.6577	7.4543	-2.2817
CHB	HIST2	150114459	0.9918	6.4378	-2.4487
CHB	HIST2	150119459	1.5049	7.1154	-2.3041
CHB	HIST2	150124459	2.2345	7.7931	-2.1009
CHB	HIST2	150129459	2.6314	8.1319	-1.9993
CHB	HIST2	150134459	2.7703	8.979	-2.0596
CHB	HIST2	150139459	1.967	9.6567	-2.3844
CHB	HIST2	150144459	2.3647	8.8096	-2.176
CHB	HIST2	150149459	3.0868	7.7931	-1.7787
CHB	HIST2	150154459	1.7704	8.979	-2.3913
CHB	HIST2	150159459	3.2751	13.3838	-2.3071
CHB	HIST2	150164459	8.5075	13.045	-1.061
CHB	HIST2	150169459	9.3674	9.1484	0.0714
CHB	HIST2	150174459	6.3406	10.3343	-1.1625
CHB	HIST2	150179459	6.5998	9.9955	-1.0197
CHB	HIST2	150184459	6.5448	8.4708	-0.6742
CHB	HIST2	150189459	3.9913	6.6072	-1.1489
CHB	HIST2	150194459	4.9538	6.4378	-0.6673
CHB	HIST2	150199459	9.4986	10.1649	-0.197
CHB	HIST2	150204459	8.3413	10.6731	-0.6586
CHB	HIST2	150209459	8.2692	11.1814	-0.7874
CHB	HIST2	150214459	8.9364	11.6896	-0.714
CHB	HIST2	150219459	6.3823	8.8096	-0.8195
CHB	HIST2	150224459	5.0286	7.7931	-1.0448
CHB	HIST2	150229459	2.9499	6.0989	-1.4865
CHB	HIST2	150234459	2.5597	3.8965	-0.935
CHB	HIST2	150239459	3.6457	5.0825	-0.7977
CHB	HIST2	150244459	4.8837	7.1154	-0.9165
CHB	HIST2	150249459	4.7634	7.1154	-0.9659
CHB	HIST2	150254459	5.3335	7.6237	-0.8832
CHB	HIST2	150259459	7.5113	8.6402	-0.388
CHB	HIST2	150264459	10.913	10.1649	0.2211
CHB	HIST2	150269459	13.0615	11.5202	0.4052
CHB	HIST2	150274459	10.7015	11.012	-0.0852
CHB	HIST2	150279459	10.2984	11.8591	-0.3993
CHB	HIST2	150284459	9.5655	11.1814	-0.4369
CHB	HIST2	150289459	8.241	9.8261	-0.4836
CHB	HIST2	150294459	7.448	9.3178	-0.5993
CHB	HIST2	150299459	5.6961	8.8096	-1.0512
CHB	HIST2	150304459	6.2947	9.3178	-0.969
CHB	HIST2	150309459	6.9379	9.4872	-0.8036

CHB	HIST2	150314459	6.5543	8.6402	-0.717
CHB	HIST2	150319459	9.6168	10.5037	-0.2543
CHB	HIST2	150324459	9.5155	11.5202	-0.5271
CHB	HIST2	150329459	8.9638	11.3508	-0.6364
CHB	HIST2	150334459	8.7974	10.6731	-0.5298
CHB	HIST2	150339459	6.4319	7.6237	-0.4596
CHB	HIST2	150344459	6.3941	6.4378	-0.0196
CHB	HIST2	150349459	6.0097	8.3013	-0.8173
CHB	HIST2	150354459	7.6448	12.1979	-1.1344
CHB	HIST2	150359459	9.1793	12.8755	-0.875
CHB	HIST2	150364459	8.2857	11.1814	-0.783
CHB	HIST2	150369459	8.3099	12.8755	-1.0809
CHB	HIST2	150374459	10.4287	15.4168	-0.9952
CHB	HIST2	150379459	10.0878	15.4168	-1.0632
YRI	HIST2	149389459	7.5779	7.5628	0.0058
YRI	HIST2	149394459	5.0741	6.7225	-0.7103
YRI	HIST2	149399459	6.8782	8.7392	-0.6308
YRI	HIST2	149404459	7.1835	9.4115	-0.7051
YRI	HIST2	149409459	4.0802	6.3864	-1.0409
YRI	HIST2	149414459	5.4012	6.5544	-0.5084
YRI	HIST2	149419459	5.2146	5.0419	0.0962
YRI	HIST2	149424459	2.6292	3.3612	-0.5783
YRI	HIST2	149429459	2.8596	4.5377	-1.0254
YRI	HIST2	149434459	1.9528	2.5209	-0.5689
YRI	HIST2	149439459	0.0278	0.3361	-1.2377
YRI	HIST2	149444459	0.1661	0.5042	-1.0625
YRI	HIST2	149449459	0.1661	0.6722	-1.3249
YRI	HIST2	149454459	0.5774	1.6806	-1.5177
YRI	HIST2	149459459	0.5589	1.3445	-1.276
YRI	HIST2	149464459	0	0	0
YRI	HIST2	149469459	0	0	0
YRI	HIST2	149474459	0	0	0
YRI	HIST2	149479459	0	0	0
YRI	HIST2	149484459	0	0	0
YRI	HIST2	149489459	0	0	0
YRI	HIST2	149494459	0	0	0
YRI	HIST2	149499459	0	0	0
YRI	HIST2	149504459	0	0	0
YRI	HIST2	149509459	0.6582	1.8487	-1.5228
YRI	HIST2	149514459	0.8557	2.3529	-1.5849
YRI	HIST2	149519459	0.2427	0.6722	-1.1244
YRI	HIST2	149524459	0.0453	0.1681	-0.73
YRI	HIST2	149529459	0.2339	0.5042	-0.8495
YRI	HIST2	149534459	0.2339	0.5042	-0.8495
YRI	HIST2	149539459	0	0	0
YRI	HIST2	149544459	0	0	0
YRI	HIST2	149549459	0	0	0
YRI	HIST2	149554459	0	0	0
YRI	HIST2	149559459	0	0	0

YRI	HIST2	149564459	0.0093	0.1681	-0.944
YRI	HIST2	149569459	0.6355	0.6722	-0.0961
YRI	HIST2	149574459	1.1006	1.5126	-0.6135
YRI	HIST2	149579459	1.4095	3.6974	-1.6676
YRI	HIST2	149584459	2.5283	5.378	-1.4997
YRI	HIST2	149589459	2.228	4.2016	-1.2892
YRI	HIST2	149594459	2.1559	3.1932	-0.8555
YRI	HIST2	149599459	3.294	3.1932	0.0831
YRI	HIST2	149604459	5.2471	5.5461	-0.1531
YRI	HIST2	149609459	5.3652	7.0586	-0.6981
YRI	HIST2	149614459	1.8911	3.0251	-0.9783
YRI	HIST2	149619459	0	0	0
YRI	HIST2	149624459	0	0	0
YRI	HIST2	149629459	0.0093	0.1681	-0.944
YRI	HIST2	149634459	0.3558	0.8403	-1.0943
YRI	HIST2	149639459	3.2063	2.689	0.4916
YRI	HIST2	149644459	7.2436	7.2267	0.0068
YRI	HIST2	149649459	9.8555	10.756	-0.2516
YRI	HIST2	149654459	8.1051	8.9073	-0.2672
YRI	HIST2	149659459	5.9006	6.5544	-0.2882
YRI	HIST2	149664459	8.7336	9.0754	-0.1119
YRI	HIST2	149669459	10.9207	14.7895	-0.8003
YRI	HIST2	149674459	8.0915	12.7727	-1.1129
YRI	HIST2	149679459	5.6964	9.5795	-1.2089
YRI	HIST2	149684459	4.4759	9.4115	-1.5621
YRI	HIST2	149689459	2.7485	7.3947	-1.836
YRI	HIST2	149694459	4.0113	7.3947	-1.337
YRI	HIST2	149699459	6.144	7.8989	-0.6529
YRI	HIST2	149704459	8.7003	11.5963	-0.7541
YRI	HIST2	149709459	8.3155	12.7727	-1.0596
YRI	HIST2	149714459	8.0815	11.5963	-0.9153
YRI	HIST2	149719459	9.2653	12.7727	-0.8338
YRI	HIST2	149724459	9.0132	12.9408	-0.9223
YRI	HIST2	149729459	8.0748	12.4366	-1.0634
YRI	HIST2	149734459	6.3229	10.4199	-1.1794
YRI	HIST2	149739459	4.0556	7.2267	-1.2796
YRI	HIST2	149744459	6.3476	7.2267	-0.3547
YRI	HIST2	149749459	7.2433	8.4031	-0.4076
YRI	HIST2	149754459	3.8133	6.5544	-1.2085
YRI	HIST2	149759459	6.4196	10.2518	-1.1201
YRI	HIST2	149764459	5.3634	10.2518	-1.4288
YRI	HIST2	149769459	3.7942	9.2434	-1.7537
YRI	HIST2	149774459	6.5177	13.1089	-1.5289
YRI	HIST2	149779459	5.5616	12.2685	-1.6563
YRI	HIST2	149784459	3.7895	12.2685	-2.0939
YRI	HIST2	149789459	3.685	9.5795	-1.8351
YRI	HIST2	149794459	1.9758	3.3612	-1.0945
YRI	HIST2	149799459	0.0456	0.1681	-0.7282
YRI	HIST2	149804459	0	0	0

YRI	HIST2	149809459	0.0641	0.3361	-1.092
YRI	HIST2	149814459	3.5538	6.3864	-1.2784
YRI	HIST2	149819459	4.9907	8.235	-1.1616
YRI	HIST2	149824459	1.9828	2.3529	-0.3918
YRI	HIST2	149829459	0.4817	0.1681	1.8643
YRI	HIST2	149834459	0	0	0
YRI	HIST2	149839459	0.229	0.5042	-0.8649
YRI	HIST2	149844459	2.8358	5.5461	-1.3879
YRI	HIST2	149849459	4.9206	11.5963	-1.7384
YRI	HIST2	149854459	3.8866	12.1005	-2.055
YRI	HIST2	149859459	4.2297	11.7644	-1.9357
YRI	HIST2	149864459	7.48	12.9408	-1.2823
YRI	HIST2	149869459	7.7677	12.9408	-1.2147
YRI	HIST2	149874459	4.2439	10.0837	-1.7333
YRI	HIST2	149879459	7.4204	11.4282	-1.0581
YRI	HIST2	149884459	8.7712	12.6047	-0.9228
YRI	HIST2	149889459	5.9315	12.6047	-1.6064
YRI	HIST2	149894459	5.2168	11.9324	-1.7024
YRI	HIST2	149899459	3.3737	11.0921	-2.0956
YRI	HIST2	149904459	5.0117	12.1005	-1.7735
YRI	HIST2	149909459	7.5325	12.4366	-1.1956
YRI	HIST2	149914459	5.6678	12.6047	-1.6699
YRI	HIST2	149919459	6.1358	12.9408	-1.5979
YRI	HIST2	149924459	8.9531	13.9492	-1.0926
YRI	HIST2	149929459	8.2737	13.445	-1.1711
YRI	HIST2	149934459	8.5618	12.6047	-0.9732
YRI	HIST2	149939459	8.1615	11.2602	-0.8295
YRI	HIST2	149944459	5.6624	9.2434	-1.1525
YRI	HIST2	149949459	6.0382	9.9157	-1.1691
YRI	HIST2	149954459	7.9667	11.9324	-1.0053
YRI	HIST2	149959459	6.2213	10.2518	-1.178
YRI	HIST2	149964459	4.5833	8.7392	-1.4087
YRI	HIST2	149969459	6.345	8.9073	-0.8534
YRI	HIST2	149974459	7.9168	9.7476	-0.5608
YRI	HIST2	149979459	7.1759	9.9157	-0.8261
YRI	HIST2	149984459	8.7389	11.7644	-0.7773
YRI	HIST2	149989459	9.2394	13.2769	-0.9253
YRI	HIST2	149994459	12.1353	14.9575	-0.5776
YRI	HIST2	149999459	14.8793	15.2937	-0.083
YRI	HIST2	150004459	10.8959	13.1089	-0.5133
YRI	HIST2	150009459	13.8234	17.1423	-0.5963
YRI	HIST2	150014459	16.3391	17.8146	-0.2555
YRI	HIST2	150019459	13.0491	14.2853	-0.2643
YRI	HIST2	150024459	13.1477	15.2937	-0.43
YRI	HIST2	150029459	11.2213	12.9408	-0.4038
YRI	HIST2	150034459	7.6433	10.756	-0.8698
YRI	HIST2	150039459	10.394	14.7895	-0.9093
YRI	HIST2	150044459	12.7762	15.2937	-0.5044
YRI	HIST2	150049459	8.6399	12.1005	-0.8658

YRI	HIST2	150054459	6.9081	12.7727	-1.3942
YRI	HIST2	150059459	8.5104	13.7811	-1.166
YRI	HIST2	150064459	8.5689	12.1005	-0.8835
YRI	HIST2	150069459	9.3262	13.2769	-0.9054
YRI	HIST2	150074459	10.1852	15.2937	-1.0236
YRI	HIST2	150079459	10.7654	15.9659	-1.0001
YRI	HIST2	150084459	12.5056	15.6298	-0.6131
YRI	HIST2	150089459	14.3191	15.6298	-0.2572
YRI	HIST2	150094459	13.3286	15.9659	-0.5072
YRI	HIST2	150099459	12.296	14.2853	-0.4253
YRI	HIST2	150104459	12.2694	16.134	-0.7358
YRI	HIST2	150109459	10.1388	15.6298	-1.0777
YRI	HIST2	150114459	8.7173	14.1172	-1.1676
YRI	HIST2	150119459	7.6213	14.4534	-1.4446
YRI	HIST2	150124459	7.8355	13.9492	-1.3371
YRI	HIST2	150129459	10.6789	16.134	-1.0386
YRI	HIST2	150134459	12.2996	17.8146	-0.955
YRI	HIST2	150139459	12.0414	17.3104	-0.9379
YRI	HIST2	150144459	10.0938	14.2853	-0.8962
YRI	HIST2	150149459	9.2867	12.2685	-0.7363
YRI	HIST2	150154459	11.4496	13.2769	-0.4188
YRI	HIST2	150159459	16.6998	19.3272	-0.4207
YRI	HIST2	150164459	16.9757	19.4952	-0.4001
YRI	HIST2	150169459	12.5241	15.9659	-0.6619
YRI	HIST2	150174459	11.9003	15.4617	-0.7062
YRI	HIST2	150179459	12.3152	13.445	-0.2559
YRI	HIST2	150184459	11.3191	14.4534	-0.6627
YRI	HIST2	150189459	8.8071	12.1005	-0.8239
YRI	HIST2	150194459	8.4306	9.7476	-0.4034
YRI	HIST2	150199459	10.4735	10.756	-0.0789
YRI	HIST2	150204459	10.6374	11.0921	-0.1235
YRI	HIST2	150209459	13.4833	14.9575	-0.3017
YRI	HIST2	150214459	14.2423	15.2937	-0.2107
YRI	HIST2	150219459	10.5734	11.2602	-0.1839
YRI	HIST2	150224459	7.723	9.7476	-0.6202
YRI	HIST2	150229459	4.0713	8.4031	-1.5225
YRI	HIST2	150234459	2.9267	6.8906	-1.6703
YRI	HIST2	150239459	4.1672	7.3947	-1.2754
YRI	HIST2	150244459	5.1575	10.5879	-1.54
YRI	HIST2	150249459	5.9107	10.756	-1.354
YRI	HIST2	150254459	7.4465	9.5795	-0.6641
YRI	HIST2	150259459	11.3215	10.756	0.158
YRI	HIST2	150264459	16.0956	14.4534	0.3472
YRI	HIST2	150269459	18.2561	17.6465	0.1065
YRI	HIST2	150274459	15.8191	17.3104	-0.2654
YRI	HIST2	150279459	14.1404	15.4617	-0.262
YRI	HIST2	150284459	12.4261	12.6047	-0.043
YRI	HIST2	150289459	10.0521	11.7644	-0.4399
YRI	HIST2	150294459	10.7521	13.445	-0.6099

YRI	HIST2	150299459	11.7498	13.9492	-0.481
YRI	HIST2	150304459	10.8566	12.7727	-0.4555
YRI	HIST2	150309459	10.9874	12.9408	-0.4587
YRI	HIST2	150314459	12.4443	13.445	-0.2266
YRI	HIST2	150319459	15.0185	14.9575	0.0125
YRI	HIST2	150324459	12.9782	14.4534	-0.3119
YRI	HIST2	150329459	11.987	13.1089	-0.2602
YRI	HIST2	150334459	12.213	13.7811	-0.3469
YRI	HIST2	150339459	10.752	12.7727	-0.4804
YRI	HIST2	150344459	11.873	11.4282	0.1174
YRI	HIST2	150349459	9.9633	11.5963	-0.4252
YRI	HIST2	150354459	12.4912	14.1172	-0.3516
YRI	HIST2	150359459	15.7362	14.9575	0.1594
YRI	HIST2	150364459	13.8457	13.445	0.0907
YRI	HIST2	150369459	13.8327	15.7978	-0.3817
YRI	HIST2	150374459	15.494	16.9743	-0.2685
YRI	HIST2	150379459	15.7388	16.302	-0.1062
CEU	HIST2	149391918	6.3327	5.9697	0.1752
CEU	HIST2	149396918	4.7877	5.458	-0.3504
CEU	HIST2	149401918	5.9322	5.9697	-0.0181
CEU	HIST2	149406918	5.0408	5.1169	-0.0421
CEU	HIST2	149411918	3.6555	2.8996	0.6798
CEU	HIST2	149416918	4.1068	3.2407	0.7102
CEU	HIST2	149421918	2.5749	2.2173	0.3995
CEU	HIST2	149426918	2.7172	2.729	-0.0112
CEU	HIST2	149431918	2.3515	2.729	-0.3568
CEU	HIST2	149436918	0.5232	0.6822	-0.4152
CEU	HIST2	149441918	0	0	0
CEU	HIST2	149446918	0.08	0.5117	-1.3539
CEU	HIST2	149451918	0.1394	0.8528	-1.6066
CEU	HIST2	149456918	0.128	0.6822	-1.4472
CEU	HIST2	149461918	0.0686	0.3411	-1.0924
CEU	HIST2	149466918	0	0	0
CEU	HIST2	149471918	0	0	0
CEU	HIST2	149476918	0	0	0
CEU	HIST2	149481918	0	0	0
CEU	HIST2	149486918	0	0	0
CEU	HIST2	149491918	0	0	0
CEU	HIST2	149496918	0	0	0
CEU	HIST2	149501918	0	0	0
CEU	HIST2	149506918	0.1363	0.5117	-1.1774
CEU	HIST2	149511918	0.4605	1.1939	-1.3069
CEU	HIST2	149516918	0.3243	0.6822	-0.9346
CEU	HIST2	149521918	0	0	0
CEU	HIST2	149526918	0	0	0
CEU	HIST2	149531918	0.0101	0.1706	-0.9534
CEU	HIST2	149536918	0.0101	0.1706	-0.9534
CEU	HIST2	149541918	0	0	0
CEU	HIST2	149546918	0	0	0

CEU	HIST2	149551918	0	0	0
CEU	HIST2	149556918	0	0	0
CEU	HIST2	149561918	0	0	0
CEU	HIST2	149566918	0	0	0
CEU	HIST2	149571918	0.885	0.6822	0.5296
CEU	HIST2	149576918	0.885	0.6822	0.5296
CEU	HIST2	149581918	2.8182	3.2407	-0.3464
CEU	HIST2	149586918	3.8	3.7524	0.0345
CEU	HIST2	149591918	1.7355	1.3645	0.6002
CEU	HIST2	149596918	2.012	2.729	-0.6777
CEU	HIST2	149601918	6.1911	4.9463	0.7099
CEU	HIST2	149606918	7.4409	4.6052	1.7217
CEU	HIST2	149611918	3.874	2.3879	1.565
CEU	HIST2	149616918	1.3659	0.8528	1.1555
CEU	HIST2	149621918	0	0	0
CEU	HIST2	149626918	0.0301	0.1706	-0.8346
CEU	HIST2	149631918	0.0301	0.1706	-0.8346
CEU	HIST2	149636918	2.3997	1.7056	0.9507
CEU	HIST2	149641918	3.1783	2.729	0.4247
CEU	HIST2	149646918	5.8648	5.9697	-0.0506
CEU	HIST2	149651918	8.469	6.9931	0.6175
CEU	HIST2	149656918	5.9615	3.9229	1.4219
CEU	HIST2	149661918	6.0134	6.3108	-0.1365
CEU	HIST2	149666918	5.1377	9.0398	-1.2901
CEU	HIST2	149671918	4.6613	8.8692	-1.416
CEU	HIST2	149676918	5.5595	7.3342	-0.7111
CEU	HIST2	149681918	3.2579	4.9463	-0.9629
CEU	HIST2	149686918	0.9285	3.2407	-1.896
CEU	HIST2	149691918	1.3713	3.2407	-1.5329
CEU	HIST2	149696918	2.9058	5.2874	-1.2806
CEU	HIST2	149701918	4.5174	6.3108	-0.8234
CEU	HIST2	149706918	7.1126	6.8225	0.1241
CEU	HIST2	149711918	8.3743	7.6753	0.2687
CEU	HIST2	149716918	11.1891	8.8692	0.7807
CEU	HIST2	149721918	10.4432	7.5047	1.1529
CEU	HIST2	149726918	6.2684	5.9697	0.1442
CEU	HIST2	149731918	5.9891	5.7991	0.0941
CEU	HIST2	149736918	3.8417	4.0935	-0.1693
CEU	HIST2	149741918	1.746	2.8996	-1.0375
CEU	HIST2	149746918	4.6417	3.9229	0.5013
CEU	HIST2	149751918	3.9182	3.5818	0.2535
CEU	HIST2	149756918	1.7541	3.0701	-1.1288
CEU	HIST2	149761918	2.4993	5.2874	-1.4992
CEU	HIST2	149766918	1.7405	5.1169	-1.8689
CEU	HIST2	149771918	3.0118	5.458	-1.2788
CEU	HIST2	149776918	4.9843	6.8225	-0.7866
CEU	HIST2	149781918	5.411	7.8459	-0.9173
CEU	HIST2	149786918	4.1148	7.3342	-1.2899
CEU	HIST2	149791918	1.7159	3.4112	-1.3315

CEU	HIST2	149796918	0.1727	0.3411	-0.6751
CEU	HIST2	149801918	0	0	0
CEU	HIST2	149806918	0	0	0
CEU	HIST2	149811918	1.1765	1.7056	-0.7247
CEU	HIST2	149816918	2.6098	3.9229	-0.9158
CEU	HIST2	149821918	2.0299	2.729	-0.6608
CEU	HIST2	149826918	0.5966	0.5117	0.2663
CEU	HIST2	149831918	0	0	0
CEU	HIST2	149836918	0	0	0
CEU	HIST2	149841918	0.6976	1.1939	-0.8844
CEU	HIST2	149846918	2.0016	3.7524	-1.2684
CEU	HIST2	149851918	2.5624	5.458	-1.5138
CEU	HIST2	149856918	2.4302	5.9697	-1.7082
CEU	HIST2	149861918	3.7472	7.3342	-1.4372
CEU	HIST2	149866918	5.3771	9.0398	-1.211
CEU	HIST2	149871918	4.9114	7.6753	-1.0624
CEU	HIST2	149876918	3.898	6.3108	-1.1078
CEU	HIST2	149881918	2.6912	5.1169	-1.3427
CEU	HIST2	149886918	2.5022	4.0935	-1.07
CEU	HIST2	149891918	3.1911	4.2641	-0.6965
CEU	HIST2	149896918	3.3513	5.2874	-1.041
CEU	HIST2	149901918	3.5364	5.6286	-1.0642
CEU	HIST2	149906918	3.5805	5.458	-0.9815
CEU	HIST2	149911918	2.3566	4.9463	-1.4769
CEU	HIST2	149916918	1.7032	3.9229	-1.5482
CEU	HIST2	149921918	3.0085	4.6052	-0.9694
CEU	HIST2	149926918	4.4133	5.6286	-0.6182
CEU	HIST2	149931918	3.3523	6.3108	-1.3583
CEU	HIST2	149936918	2.221	4.9463	-1.5542
CEU	HIST2	149941918	2.6215	3.7524	-0.8193
CEU	HIST2	149946918	2.2579	4.2641	-1.3023
CEU	HIST2	149951918	2.9711	5.7991	-1.4007
CEU	HIST2	149956918	2.1484	4.4346	-1.4344
CEU	HIST2	149961918	1.012	2.5584	-1.5405
CEU	HIST2	149966918	1.7765	3.2407	-1.2006
CEU	HIST2	149971918	2.1725	3.7524	-1.1446
CEU	HIST2	149976918	2.215	3.9229	-1.1912
CEU	HIST2	149981918	2.6574	4.9463	-1.3054
CEU	HIST2	149986918	3.665	5.9697	-1.1123
CEU	HIST2	149991918	5.9705	7.3342	-0.5464
CEU	HIST2	149996918	9.5316	8.6987	0.2853
CEU	HIST2	150001918	8.4541	7.3342	0.4487
CEU	HIST2	150006918	7.4292	7.1636	0.1087
CEU	HIST2	150011918	10.6844	8.8692	0.6108
CEU	HIST2	150016918	11.1148	9.2104	0.6188
CEU	HIST2	150021918	9.0223	8.0164	0.3715
CEU	HIST2	150026918	5.7079	5.9697	-0.1263
CEU	HIST2	150031918	2.2519	4.4346	-1.3695
CEU	HIST2	150036918	3.0062	5.7991	-1.3833

CEU	HIST2	150041918	3.7549	5.9697	-1.0689
CEU	HIST2	150046918	4.4902	5.6286	-0.5791
CEU	HIST2	150051918	3.912	5.2874	-0.7396
CEU	HIST2	150056918	2.1128	4.4346	-1.4568
CEU	HIST2	150061918	3.0357	4.9463	-1.0896
CEU	HIST2	150066918	1.987	3.2407	-1.028
CEU	HIST2	150071918	5.2461	5.1169	0.0715
CEU	HIST2	150076918	7.7223	7.5047	0.0854
CEU	HIST2	150081918	3.2894	5.7991	-1.243
CEU	HIST2	150086918	2.8062	6.6519	-1.6837
CEU	HIST2	150091918	4.1495	6.4814	-1.0452
CEU	HIST2	150096918	4.2863	5.458	-0.6125
CEU	HIST2	150101918	4.8773	6.8225	-0.8323
CEU	HIST2	150106918	5.9415	6.6519	-0.311
CEU	HIST2	150111918	5.7025	5.7991	-0.0478
CEU	HIST2	150116918	3.3577	4.6052	-0.7574
CEU	HIST2	150121918	3.1707	5.7991	-1.3018
CEU	HIST2	150126918	5.0276	8.0164	-1.1039
CEU	HIST2	150131918	6.5195	9.0398	-0.8333
CEU	HIST2	150136918	7.2158	11.9394	-1.2043
CEU	HIST2	150141918	6.1897	9.5515	-1.0561
CEU	HIST2	150146918	5.0423	6.3108	-0.5824
CEU	HIST2	150151918	3.5471	7.3342	-1.5174
CEU	HIST2	150156918	4.9324	8.8692	-1.3247
CEU	HIST2	150161918	8.7335	9.7221	-0.3055
CEU	HIST2	150166918	9.5873	8.0164	0.5802
CEU	HIST2	150171918	7.8149	6.6519	0.5092
CEU	HIST2	150176918	6.9083	5.9697	0.453
CEU	HIST2	150181918	8.2573	6.4814	0.796
CEU	HIST2	150186918	6.5504	6.3108	0.11
CEU	HIST2	150191918	5.8106	5.9697	-0.0768
CEU	HIST2	150196918	7.2738	6.3108	0.4421
CEU	HIST2	150201918	9.2791	7.1636	0.866
CEU	HIST2	150206918	10.2387	8.3576	0.6687
CEU	HIST2	150211918	8.2839	6.9931	0.5401
CEU	HIST2	150216918	8.296	6.4814	0.8133
CEU	HIST2	150221918	7.7446	6.4814	0.5662
CEU	HIST2	150226918	3.5176	4.6052	-0.6603
CEU	HIST2	150231918	2.2224	3.5818	-1.0246
CEU	HIST2	150236918	2.9215	2.8996	0.0197
CEU	HIST2	150241918	2.7673	3.7524	-0.7137
CEU	HIST2	150246918	5.4009	5.6286	-0.1158
CEU	HIST2	150251918	5.4519	5.2874	0.0885
CEU	HIST2	150256918	6.9371	6.6519	0.1249
CEU	HIST2	150261918	14.4618	8.8692	1.8819
CEU	HIST2	150266918	19.5147	10.916	2.3848
CEU	HIST2	150271918	16.9402	11.7688	1.3365
CEU	HIST2	150276918	14.3911	10.916	0.9638
CEU	HIST2	150281918	15.4428	10.916	1.2555

CEU	HIST2	150286918	12.0742	8.187	1.4082
CEU	HIST2	150291918	10.1462	7.1636	1.2209
CEU	HIST2	150296918	9.6301	7.8459	0.6721
CEU	HIST2	150301918	7.9921	7.3342	0.2636
CEU	HIST2	150306918	9.4628	8.3576	0.3929
CEU	HIST2	150311918	10.6891	9.5515	0.3574
CEU	HIST2	150316918	12.7236	10.916	0.5013
CEU	HIST2	150321918	13.1306	9.7221	1.0533
CEU	HIST2	150326918	13.4522	9.3809	1.3006
CEU	HIST2	150331918	12.9558	9.5515	1.0695
CEU	HIST2	150336918	10.4048	9.2104	0.3881
CEU	HIST2	150341918	9.3972	8.8692	0.1777
CEU	HIST2	150346918	9.2597	7.6753	0.609
CEU	HIST2	150351918	11.3979	9.2104	0.7108
CEU	HIST2	150356918	14.2175	10.5749	1.0408
CEU	HIST2	150361918	13.766	10.2337	1.0406
CEU	HIST2	150366918	12.2263	10.5749	0.4718
CEU	HIST2	150371918	13.0328	10.7454	0.6438
CEU	HIST2	150376918	14.7998	11.5982	0.8388
CEU	HIST2	150381918	13.7603	10.916	0.7889
CHB	HIST2	149391918	4.9252	5.9295	-0.4862
CHB	HIST2	149396918	4.0455	5.2519	-0.6506
CHB	HIST2	149401918	4.8778	6.6072	-0.7596
CHB	HIST2	149406918	3.4877	5.0825	-0.8854
CHB	HIST2	149411918	3.3813	4.2354	-0.556
CHB	HIST2	149416918	4.2416	4.066	0.1184
CHB	HIST2	149421918	2.0788	3.3883	-1.0313
CHB	HIST2	149426918	2.0093	4.066	-1.3869
CHB	HIST2	149431918	1.8957	3.2189	-1.0879
CHB	HIST2	149436918	0.4087	1.0165	-1.2096
CHB	HIST2	149441918	0	0	0
CHB	HIST2	149446918	0.0386	0.5082	-1.4741
CHB	HIST2	149451918	0.1065	1.3553	-2.0239
CHB	HIST2	149456918	0.0873	1.1859	-1.961
CHB	HIST2	149461918	0.0194	0.3388	-1.2812
CHB	HIST2	149466918	0	0	0
CHB	HIST2	149471918	0	0	0
CHB	HIST2	149476918	0	0	0
CHB	HIST2	149481918	0	0	0
CHB	HIST2	149486918	0	0	0
CHB	HIST2	149491918	0	0	0
CHB	HIST2	149496918	0	0	0
CHB	HIST2	149501918	0	0	0
CHB	HIST2	149506918	0.6275	0.5082	0.3745
CHB	HIST2	149511918	0.8968	1.0165	-0.2382
CHB	HIST2	149516918	0.2791	0.6777	-1.0421
CHB	HIST2	149521918	0.0097	0.1694	-0.949
CHB	HIST2	149526918	0	0	0
CHB	HIST2	149531918	0.2578	0.1694	0.5253

CHB	HIST2	149536918	0.2578	0.1694	0.5253
CHB	HIST2	149541918	0	0	0
CHB	HIST2	149546918	0	0	0
CHB	HIST2	149551918	0	0	0
CHB	HIST2	149556918	0	0	0
CHB	HIST2	149561918	0	0	0
CHB	HIST2	149566918	0.0097	0.1694	-0.949
CHB	HIST2	149571918	0.9039	0.8471	0.1281
CHB	HIST2	149576918	0.9039	0.8471	0.1281
CHB	HIST2	149581918	3.3082	3.3883	-0.0631
CHB	HIST2	149586918	4.0702	3.8965	0.1215
CHB	HIST2	149591918	1.2206	1.1859	0.0619
CHB	HIST2	149596918	2.4835	2.033	0.5372
CHB	HIST2	149601918	7.3181	4.5742	1.6708
CHB	HIST2	149606918	7.0409	5.4213	0.8493
CHB	HIST2	149611918	2.8014	3.0495	-0.2134
CHB	HIST2	149616918	1.044	0.6777	0.9576
CHB	HIST2	149621918	0	0	0
CHB	HIST2	149626918	0.0097	0.1694	-0.949
CHB	HIST2	149631918	0.0097	0.1694	-0.949
CHB	HIST2	149636918	2.8808	1.5247	2.0147
CHB	HIST2	149641918	3.9928	3.5577	0.3288
CHB	HIST2	149646918	5.9671	5.7601	0.1028
CHB	HIST2	149651918	7.4036	6.0989	0.6159
CHB	HIST2	149656918	4.6482	5.4213	-0.4054
CHB	HIST2	149661918	5.9966	5.4213	0.3017
CHB	HIST2	149666918	7.7933	6.6072	0.5209
CHB	HIST2	149671918	7.8681	7.9625	-0.035
CHB	HIST2	149676918	5.9629	6.0989	-0.0642
CHB	HIST2	149681918	3.2277	5.2519	-1.0917
CHB	HIST2	149686918	2.0524	4.4048	-1.4802
CHB	HIST2	149691918	3.198	4.2354	-0.6753
CHB	HIST2	149696918	4.4445	4.5742	-0.079
CHB	HIST2	149701918	5.1584	6.2684	-0.5112
CHB	HIST2	149706918	7.3682	7.6237	-0.0985
CHB	HIST2	149711918	7.0874	5.9295	0.5606
CHB	HIST2	149716918	8.1498	8.6402	-0.1686
CHB	HIST2	149721918	8.3038	9.8261	-0.4645
CHB	HIST2	149726918	6.0784	8.3013	-0.7928
CHB	HIST2	149731918	5.7443	7.1154	-0.5631
CHB	HIST2	149736918	3.2926	4.4048	-0.6999
CHB	HIST2	149741918	1.0297	2.7106	-1.5927
CHB	HIST2	149746918	3.886	3.2189	0.5484
CHB	HIST2	149751918	3.7042	3.2189	0.399
CHB	HIST2	149756918	1.0577	5.2519	-2.2621
CHB	HIST2	149761918	2.0857	6.7766	-2.0136
CHB	HIST2	149766918	1.7815	4.4048	-1.6507
CHB	HIST2	149771918	1.3008	5.4213	-2.1607
CHB	HIST2	149776918	1.2696	5.2519	-2.1478

CHB	HIST2	149781918	1.0246	4.5742	-2.1614
CHB	HIST2	149786918	0.7342	5.5907	-2.4779
CHB	HIST2	149791918	0.422	3.7271	-2.4011
CHB	HIST2	149796918	0.1805	1.0165	-1.6637
CHB	HIST2	149801918	0	0	0
CHB	HIST2	149806918	0	0	0
CHB	HIST2	149811918	0.4491	1.5247	-1.598
CHB	HIST2	149816918	1.2566	5.2519	-2.1548
CHB	HIST2	149821918	1.193	4.066	-1.9374
CHB	HIST2	149826918	0.3855	0.3388	0.1873
CHB	HIST2	149831918	0	0	0
CHB	HIST2	149836918	0	0	0
CHB	HIST2	149841918	0.0771	0.8471	-1.7363
CHB	HIST2	149846918	0.5099	4.4048	-2.4509
CHB	HIST2	149851918	1.1086	8.1319	-2.5528
CHB	HIST2	149856918	1.0769	6.0989	-2.3706
CHB	HIST2	149861918	1.3765	5.9295	-2.2042
CHB	HIST2	149866918	1.5623	7.2848	-2.3003
CHB	HIST2	149871918	1.0129	5.9295	-2.3802
CHB	HIST2	149876918	1.6109	6.7766	-2.2175
CHB	HIST2	149881918	1.8576	7.1154	-2.1592
CHB	HIST2	149886918	1.2475	5.7601	-2.242
CHB	HIST2	149891918	1.4856	6.2684	-2.2028
CHB	HIST2	149896918	1.1913	7.2848	-2.4494
CHB	HIST2	149901918	1.458	7.1154	-2.3233
CHB	HIST2	149906918	2.1598	7.6237	-2.107
CHB	HIST2	149911918	1.6097	7.1154	-2.261
CHB	HIST2	149916918	1.5205	6.2684	-2.1867
CHB	HIST2	149921918	2.7129	8.3013	-1.9931
CHB	HIST2	149926918	4.359	11.012	-1.8249
CHB	HIST2	149931918	2.9446	10.3343	-2.151
CHB	HIST2	149936918	2.0959	9.6567	-2.3444
CHB	HIST2	149941918	2.9341	8.3013	-1.9142
CHB	HIST2	149946918	2.0714	6.4378	-1.9633
CHB	HIST2	149951918	2.0166	7.9625	-2.2034
CHB	HIST2	149956918	1.4021	7.7931	-2.4155
CHB	HIST2	149961918	0.9977	6.0989	-2.408
CHB	HIST2	149966918	1.0267	6.0989	-2.3943
CHB	HIST2	149971918	1.3632	5.2519	-2.0973
CHB	HIST2	149976918	1.961	5.0825	-1.733
CHB	HIST2	149981918	1.7935	7.4543	-2.2282
CHB	HIST2	149986918	2.5492	8.979	-2.133
CHB	HIST2	149991918	3.6819	9.4872	-1.83
CHB	HIST2	149996918	6.7369	11.012	-1.1726
CHB	HIST2	150001918	6.0002	10.8426	-1.3477
CHB	HIST2	150006918	2.9031	9.6567	-2.0941
CHB	HIST2	150011918	2.226	10.1649	-2.3468
CHB	HIST2	150016918	1.5603	9.4872	-2.4987
CHB	HIST2	150021918	1.6747	8.3013	-2.3634

CHB	HIST2	150026918	1.3857	8.4708	-2.4803
CHB	HIST2	150031918	1.1237	6.946	-2.444
CHB	HIST2	150036918	1.8096	6.4378	-2.081
CHB	HIST2	150041918	1.8247	6.4378	-2.0742
CHB	HIST2	150046918	0.9339	6.6072	-2.4918
CHB	HIST2	150051918	1.0897	7.1154	-2.4746
CHB	HIST2	150056918	1.1483	5.9295	-2.3146
CHB	HIST2	150061918	0.7601	5.2519	-2.4226
CHB	HIST2	150066918	0.5625	4.7436	-2.4663
CHB	HIST2	150071918	1.252	7.1154	-2.4079
CHB	HIST2	150076918	1.3716	8.3013	-2.4715
CHB	HIST2	150081918	0.5846	5.2519	-2.5172
CHB	HIST2	150086918	1.265	5.7601	-2.2333
CHB	HIST2	150091918	1.918	6.7766	-2.0856
CHB	HIST2	150096918	1.5203	6.4378	-2.2111
CHB	HIST2	150101918	1.5425	7.7931	-2.3624
CHB	HIST2	150106918	1.8386	7.7931	-2.2505
CHB	HIST2	150111918	1.514	6.4378	-2.2139
CHB	HIST2	150116918	0.9865	5.9295	-2.393
CHB	HIST2	150121918	1.7411	7.6237	-2.2685
CHB	HIST2	150126918	2.5791	8.1319	-2.0183
CHB	HIST2	150131918	2.5859	8.1319	-2.0158
CHB	HIST2	150136918	2.4311	9.4872	-2.2242
CHB	HIST2	150141918	2.695	9.6567	-2.1587
CHB	HIST2	150146918	2.7569	8.8096	-2.0436
CHB	HIST2	150151918	1.9459	8.6402	-2.3011
CHB	HIST2	150156918	2.6927	12.1979	-2.3681
CHB	HIST2	150161918	6.5277	12.1979	-1.4127
CHB	HIST2	150166918	9.356	10.1649	-0.2391
CHB	HIST2	150171918	6.9408	10.1649	-0.9531
CHB	HIST2	150176918	5.5677	9.9955	-1.3296
CHB	HIST2	150181918	7.1489	9.8261	-0.8168
CHB	HIST2	150186918	5.0491	7.2848	-0.8987
CHB	HIST2	150191918	4.5609	6.7766	-0.9511
CHB	HIST2	150196918	7.1516	8.6402	-0.5117
CHB	HIST2	150201918	9.2675	10.1649	-0.2653
CHB	HIST2	150206918	9.6792	11.6896	-0.5214
CHB	HIST2	150211918	7.4653	11.1814	-1.0048
CHB	HIST2	150216918	7.3554	9.6567	-0.7136
CHB	HIST2	150221918	6.8858	8.3013	-0.5048
CHB	HIST2	150226918	3.0442	6.2684	-1.485
CHB	HIST2	150231918	2.2854	5.2519	-1.5999
CHB	HIST2	150236918	3.2071	4.5742	-0.8325
CHB	HIST2	150241918	3.2781	5.2519	-1.0645
CHB	HIST2	150246918	5.0704	7.2848	-0.8901
CHB	HIST2	150251918	4.8388	7.6237	-1.0739
CHB	HIST2	150256918	5.8228	7.9625	-0.7929
CHB	HIST2	150261918	10.4672	10.1649	0.0894
CHB	HIST2	150266918	14.0217	11.6896	0.6048

CHB	HIST2	150271918	11.6672	10.5037	0.3336
CHB	HIST2	150276918	9.1625	11.012	-0.5073
CHB	HIST2	150281918	10.7638	12.8755	-0.4999
CHB	HIST2	150286918	8.9783	10.1649	-0.3508
CHB	HIST2	150291918	6.7186	8.6402	-0.6605
CHB	HIST2	150296918	6.1092	8.8096	-0.9117
CHB	HIST2	150301918	5.8606	8.6402	-0.9554
CHB	HIST2	150306918	7.0588	10.3343	-0.9535
CHB	HIST2	150311918	7.8418	9.3178	-0.4731
CHB	HIST2	150316918	9.1314	9.8261	-0.212
CHB	HIST2	150321918	8.5193	10.8426	-0.6466
CHB	HIST2	150326918	9.238	11.5202	-0.6
CHB	HIST2	150331918	9.2711	11.8591	-0.6621
CHB	HIST2	150336918	6.6153	8.8096	-0.7409
CHB	HIST2	150341918	6.126	6.2684	-0.0656
CHB	HIST2	150346918	6.2258	6.7766	-0.2364
CHB	HIST2	150351918	6.9638	10.6731	-1.0476
CHB	HIST2	150356918	8.4662	13.2144	-1.0968
CHB	HIST2	150361918	8.0091	11.012	-0.8237
CHB	HIST2	150366918	8.5387	11.8591	-0.8495
CHB	HIST2	150371918	9.8892	14.5697	-0.9855
CHB	HIST2	150376918	10.6242	15.2474	-0.9322
CHB	HIST2	150381918	8.9733	15.2474	-1.2651
YRI	HIST2	149391918	6.955	7.0586	-0.0427
YRI	HIST2	149396918	5.2869	7.2267	-0.7827
YRI	HIST2	149401918	7.2956	9.2434	-0.6269
YRI	HIST2	149406918	6.2313	8.9073	-0.8913
YRI	HIST2	149411918	4.8382	7.3947	-1.0102
YRI	HIST2	149416918	5.2805	5.378	-0.0513
YRI	HIST2	149421918	3.4594	3.1932	0.2195
YRI	HIST2	149426918	3.1755	4.2016	-0.6703
YRI	HIST2	149431918	2.5498	3.8654	-0.9232
YRI	HIST2	149436918	0.734	1.3445	-0.9916
YRI	HIST2	149441918	0.0185	0.1681	-0.8893
YRI	HIST2	149446918	0.1568	0.5042	-1.0918
YRI	HIST2	149451918	0.4181	1.1764	-1.3563
YRI	HIST2	149456918	0.5682	1.5126	-1.4063
YRI	HIST2	149461918	0.3069	0.8403	-1.2048
YRI	HIST2	149466918	0	0	0
YRI	HIST2	149471918	0	0	0
YRI	HIST2	149476918	0	0	0
YRI	HIST2	149481918	0	0	0
YRI	HIST2	149486918	0	0	0
YRI	HIST2	149491918	0	0	0
YRI	HIST2	149496918	0	0	0
YRI	HIST2	149501918	0	0	0
YRI	HIST2	149506918	0.4018	0.5042	-0.3218
YRI	HIST2	149511918	0.6674	2.0167	-1.6133
YRI	HIST2	149516918	0.4991	2.0167	-1.8145

YRI	HIST2	149521918	0.2335	0.5042	-0.8507
YRI	HIST2	149526918	0.073	0.3361	-1.0562
YRI	HIST2	149531918	0.2339	0.5042	-0.8495
YRI	HIST2	149536918	0.1609	0.1681	-0.0428
YRI	HIST2	149541918	0	0	0
YRI	HIST2	149546918	0	0	0
YRI	HIST2	149551918	0	0	0
YRI	HIST2	149556918	0	0	0
YRI	HIST2	149561918	0	0	0
YRI	HIST2	149566918	0.0278	0.3361	-1.2377
YRI	HIST2	149571918	1.1098	1.6806	-0.7853
YRI	HIST2	149576918	1.082	1.3445	-0.4263
YRI	HIST2	149581918	2.3949	5.0419	-1.4748
YRI	HIST2	149586918	2.8328	6.0502	-1.5244
YRI	HIST2	149591918	0.7683	1.8487	-1.3819
YRI	HIST2	149596918	2.1637	2.8571	-0.6271
YRI	HIST2	149601918	6.0443	6.3864	-0.1544
YRI	HIST2	149606918	5.8024	6.8906	-0.4585
YRI	HIST2	149611918	2.6148	3.8654	-0.8776
YRI	HIST2	149616918	1.0235	1.3445	-0.5214
YRI	HIST2	149621918	0	0	0
YRI	HIST2	149626918	0.0093	0.1681	-0.944
YRI	HIST2	149631918	0.0093	0.1681	-0.944
YRI	HIST2	149636918	3.1146	2.0167	1.3127
YRI	HIST2	149641918	4.1673	4.2016	-0.0224
YRI	HIST2	149646918	7.0209	8.235	-0.4347
YRI	HIST2	149651918	10.0483	11.2602	-0.3244
YRI	HIST2	149656918	7.2859	8.235	-0.3398
YRI	HIST2	149661918	7.8571	8.067	-0.0766
YRI	HIST2	149666918	10.778	12.6047	-0.4397
YRI	HIST2	149671918	9.844	13.7811	-0.871
YRI	HIST2	149676918	6.2212	10.0837	-1.1464
YRI	HIST2	149681918	4.8066	9.2434	-1.4279
YRI	HIST2	149686918	2.964	8.7392	-1.9576
YRI	HIST2	149691918	3.0136	6.5544	-1.5611
YRI	HIST2	149696918	4.4269	7.2267	-1.1298
YRI	HIST2	149701918	6.8933	10.2518	-0.9816
YRI	HIST2	149706918	10.0404	12.9408	-0.6811
YRI	HIST2	149711918	8.1616	11.2602	-0.8295
YRI	HIST2	149716918	7.9476	11.4282	-0.9189
YRI	HIST2	149721918	9.3344	13.2769	-0.9036
YRI	HIST2	149726918	8.5864	13.445	-1.1003
YRI	HIST2	149731918	7.8295	12.6047	-1.1495
YRI	HIST2	149736918	4.8859	8.067	-1.1608
YRI	HIST2	149741918	3.1465	5.8822	-1.3293
YRI	HIST2	149746918	6.8576	7.7309	-0.3314
YRI	HIST2	149751918	6.698	7.8989	-0.4468
YRI	HIST2	149756918	6.0847	9.5795	-1.088
YRI	HIST2	149761918	6.5749	10.924	-1.1978

YRI	HIST2	149766918	3.4078	8.4031	-1.7556
YRI	HIST2	149771918	5.2975	10.4199	-1.4746
YRI	HIST2	149776918	6.5051	12.7727	-1.49
YRI	HIST2	149781918	4.8875	13.2769	-1.9227
YRI	HIST2	149786918	4.2685	12.1005	-1.9594
YRI	HIST2	149791918	2.3726	5.8822	-1.7054
YRI	HIST2	149796918	0.5373	1.1764	-1.1431
YRI	HIST2	149801918	0	0	0
YRI	HIST2	149806918	0	0	0
YRI	HIST2	149811918	2.3469	3.3612	-0.8013
YRI	HIST2	149816918	4.7635	8.4031	-1.2792
YRI	HIST2	149821918	3.1897	5.378	-1.1517
YRI	HIST2	149826918	0.7731	0.3361	1.7544
YRI	HIST2	149831918	0	0	0
YRI	HIST2	149836918	0	0	0
YRI	HIST2	149841918	0.7039	1.8487	-1.4643
YRI	HIST2	149846918	3.827	8.7392	-1.6651
YRI	HIST2	149851918	5.8461	14.2853	-1.8044
YRI	HIST2	149856918	3.9012	11.9324	-2.0359
YRI	HIST2	149861918	4.7065	11.0921	-1.7337
YRI	HIST2	149866918	7.9347	12.9408	-1.1755
YRI	HIST2	149871918	6.7001	11.5963	-1.275
YRI	HIST2	149876918	7.1644	11.4282	-1.1257
YRI	HIST2	149881918	7.8587	11.4282	-0.9424
YRI	HIST2	149886918	5.8142	11.7644	-1.5286
YRI	HIST2	149891918	5.0434	12.1005	-1.7655
YRI	HIST2	149896918	4.5016	12.7727	-1.9663
YRI	HIST2	149901918	3.9427	12.1005	-2.0409
YRI	HIST2	149906918	5.8398	11.2602	-1.4511
YRI	HIST2	149911918	7.021	12.1005	-1.2708
YRI	HIST2	149916918	5.6254	11.9324	-1.5988
YRI	HIST2	149921918	7.2442	14.1172	-1.4861
YRI	HIST2	149926918	8.8809	14.6214	-1.2005
YRI	HIST2	149931918	9.3388	13.2769	-0.9026
YRI	HIST2	149936918	9.4264	12.4366	-0.7339
YRI	HIST2	149941918	6.495	9.9157	-1.0313
YRI	HIST2	149946918	4.83	8.9073	-1.358
YRI	HIST2	149951918	7.3377	10.5879	-0.9217
YRI	HIST2	149956918	7.465	11.0921	-0.9848
YRI	HIST2	149961918	5.0128	9.4115	-1.3922
YRI	HIST2	149966918	5.7236	9.2434	-1.1328
YRI	HIST2	149971918	6.5439	9.4115	-0.9076
YRI	HIST2	149976918	7.4242	9.7476	-0.7117
YRI	HIST2	149981918	8.4613	11.2602	-0.7493
YRI	HIST2	149986918	9.0638	11.5963	-0.6595
YRI	HIST2	149991918	9.6696	13.2769	-0.8267
YRI	HIST2	149996918	13.4448	15.9659	-0.4848
YRI	HIST2	150001918	13.9501	14.9575	-0.2062
YRI	HIST2	150006918	11.7445	14.9575	-0.6576

YRI	HIST2	150011918	15.2079	17.3104	-0.3742
YRI	HIST2	150016918	16.0318	16.134	-0.0195
YRI	HIST2	150021918	12.8877	14.7895	-0.3934
YRI	HIST2	150026918	11.4955	14.1172	-0.5669
YRI	HIST2	150031918	9.2713	11.7644	-0.6405
YRI	HIST2	150036918	8.5099	12.2685	-0.9282
YRI	HIST2	150041918	11.1871	14.6214	-0.7182
YRI	HIST2	150046918	11.2786	14.7895	-0.7263
YRI	HIST2	150051918	8.9208	13.445	-1.0246
YRI	HIST2	150056918	7.5279	12.9408	-1.271
YRI	HIST2	150061918	8.947	12.9408	-0.9378
YRI	HIST2	150066918	8.6188	11.7644	-0.8081
YRI	HIST2	150071918	9.2924	13.9492	-1.0184
YRI	HIST2	150076918	12.3127	17.4785	-0.911
YRI	HIST2	150081918	9.9794	15.1256	-1.0421
YRI	HIST2	150086918	11.7293	14.9575	-0.6607
YRI	HIST2	150091918	15.6197	15.9659	-0.0666
YRI	HIST2	150096918	12.5007	14.6214	-0.4435
YRI	HIST2	150101918	11.1174	15.2937	-0.8368
YRI	HIST2	150106918	11.8366	16.4701	-0.865
YRI	HIST2	150111918	10.0344	14.6214	-0.9593
YRI	HIST2	150116918	7.2743	12.9408	-1.3306
YRI	HIST2	150121918	8.1495	15.4617	-1.45
YRI	HIST2	150126918	9.9072	14.9575	-1.0336
YRI	HIST2	150131918	11.4117	16.134	-0.8991
YRI	HIST2	150136918	12.9766	18.3188	-0.9006
YRI	HIST2	150141918	10.8039	15.1256	-0.8751
YRI	HIST2	150146918	8.1292	12.4366	-1.0501
YRI	HIST2	150151918	10.5053	13.9492	-0.7532
YRI	HIST2	150156918	15.9743	17.6465	-0.2922
YRI	HIST2	150161918	16.5344	18.6549	-0.3513
YRI	HIST2	150166918	13.6607	16.6382	-0.5505
YRI	HIST2	150171918	11.708	15.7978	-0.7945
YRI	HIST2	150176918	12.1766	15.9659	-0.7287
YRI	HIST2	150181918	12.4342	14.4534	-0.427
YRI	HIST2	150186918	9.3999	11.7644	-0.6075
YRI	HIST2	150191918	9.2298	11.5963	-0.6163
YRI	HIST2	150196918	9.9879	10.924	-0.2578
YRI	HIST2	150201918	11.2337	10.756	0.1335
YRI	HIST2	150206918	13.3994	13.9492	-0.1202
YRI	HIST2	150211918	12.0565	14.2853	-0.4765
YRI	HIST2	150216918	11.9225	13.1089	-0.2752
YRI	HIST2	150221918	10.1069	10.924	-0.225
YRI	HIST2	150226918	5.0016	8.5712	-1.2319
YRI	HIST2	150231918	3.8797	8.235	-1.5594
YRI	HIST2	150236918	3.0906	6.3864	-1.4875
YRI	HIST2	150241918	4.1562	8.4031	-1.4926
YRI	HIST2	150246918	6.3439	11.2602	-1.3161
YRI	HIST2	150251918	6.6472	10.756	-1.1482

YRI	HIST2	150256918	8.2844	9.9157	-0.4918
YRI	HIST2	150261918	14.8358	12.7727	0.4905
YRI	HIST2	150266918	20.1074	17.1423	0.5327
YRI	HIST2	150271918	16.3573	16.4701	-0.0211
YRI	HIST2	150276918	14.1861	15.6298	-0.2833
YRI	HIST2	150281918	13.4973	14.9575	-0.2988
YRI	HIST2	150286918	9.8463	12.6047	-0.664
YRI	HIST2	150291918	9.4845	11.7644	-0.5857
YRI	HIST2	150296918	10.3939	11.9324	-0.39
YRI	HIST2	150301918	11.0701	12.4366	-0.3332
YRI	HIST2	150306918	12.6043	14.4534	-0.391
YRI	HIST2	150311918	12.5842	13.445	-0.1949
YRI	HIST2	150316918	14.3613	13.613	0.1675
YRI	HIST2	150321918	13.066	14.7895	-0.3565
YRI	HIST2	150326918	12.5787	14.4534	-0.3964
YRI	HIST2	150331918	12.9667	14.2853	-0.2819
YRI	HIST2	150336918	11.1487	12.9408	-0.4208
YRI	HIST2	150341918	10.8111	12.1005	-0.3226
YRI	HIST2	150346918	9.2952	10.756	-0.4082
YRI	HIST2	150351918	11.6979	11.9324	-0.0594
YRI	HIST2	150356918	15.7291	15.4617	0.053
YRI	HIST2	150361918	14.1042	14.1172	-0.0028
YRI	HIST2	150366918	13.2521	13.613	-0.0808
YRI	HIST2	150371918	15.206	17.3104	-0.3746
YRI	HIST2	150376918	15.8402	17.4785	-0.2889
YRI	HIST2	150381918	16.5979	15.2937	0.2613
CEU	HIST2	149392469	6.3428	6.1402	0.0953
CEU	HIST2	149397469	5.3034	5.9697	-0.3216
CEU	HIST2	149402469	5.9522	5.9697	-0.0084
CEU	HIST2	149407469	4.525	4.6052	-0.0487
CEU	HIST2	149412469	4.3368	3.4112	0.727
CEU	HIST2	149417469	4.1068	3.2407	0.7102
CEU	HIST2	149422469	1.9963	1.7056	0.3981
CEU	HIST2	149427469	2.7172	2.729	-0.0112
CEU	HIST2	149432469	2.2187	2.5584	-0.3384
CEU	HIST2	149437469	0.5232	0.6822	-0.4152
CEU	HIST2	149442469	0	0	0
CEU	HIST2	149447469	0.08	0.5117	-1.3539
CEU	HIST2	149452469	0.1394	0.8528	-1.6066
CEU	HIST2	149457469	0.128	0.6822	-1.4472
CEU	HIST2	149462469	0.0686	0.3411	-1.0924
CEU	HIST2	149467469	0	0	0
CEU	HIST2	149472469	0	0	0
CEU	HIST2	149477469	0	0	0
CEU	HIST2	149482469	0	0	0
CEU	HIST2	149487469	0	0	0
CEU	HIST2	149492469	0	0	0
CEU	HIST2	149497469	0	0	0
CEU	HIST2	149502469	0	0	0

CEU	HIST2	149507469	0.1363	0.5117	-1.1774
CEU	HIST2	149512469	0.4605	1.1939	-1.3069
CEU	HIST2	149517469	0.3243	0.6822	-0.9346
CEU	HIST2	149522469	0	0	0
CEU	HIST2	149527469	0.0101	0.1706	-0.9534
CEU	HIST2	149532469	0.0101	0.1706	-0.9534
CEU	HIST2	149537469	0	0	0
CEU	HIST2	149542469	0	0	0
CEU	HIST2	149547469	0	0	0
CEU	HIST2	149552469	0	0	0
CEU	HIST2	149557469	0	0	0
CEU	HIST2	149562469	0	0	0
CEU	HIST2	149567469	0.5038	0.1706	1.9793
CEU	HIST2	149572469	0.885	0.6822	0.5296
CEU	HIST2	149577469	0.4405	0.8528	-0.9285
CEU	HIST2	149582469	2.8182	3.2407	-0.3464
CEU	HIST2	149587469	3.7407	3.4112	0.2588
CEU	HIST2	149592469	2.1358	1.8762	0.3305
CEU	HIST2	149597469	2.4286	2.8996	-0.4236
CEU	HIST2	149602469	5.8942	4.6052	0.7826
CEU	HIST2	149607469	7.5261	4.6052	1.7734
CEU	HIST2	149612469	3.7706	2.2173	1.7353
CEU	HIST2	149617469	0.8642	0.6822	0.4753
CEU	HIST2	149622469	0	0	0
CEU	HIST2	149627469	0.0301	0.1706	-0.8346
CEU	HIST2	149632469	0.0301	0.1706	-0.8346
CEU	HIST2	149637469	2.3997	1.7056	0.9507
CEU	HIST2	149642469	3.2488	3.4112	-0.1276
CEU	HIST2	149647469	7.4811	6.6519	0.363
CEU	HIST2	149652469	8.3985	6.3108	0.9585
CEU	HIST2	149657469	4.3752	3.5818	0.598
CEU	HIST2	149662469	6.0134	6.3108	-0.1365
CEU	HIST2	149667469	5.1379	9.0398	-1.2901
CEU	HIST2	149672469	5.47	9.3809	-1.2494
CEU	HIST2	149677469	5.5393	7.1636	-0.6649
CEU	HIST2	149682469	2.4492	4.4346	-1.2457
CEU	HIST2	149687469	0.9386	3.4112	-1.942
CEU	HIST2	149692469	1.3915	3.5818	-1.6508
CEU	HIST2	149697469	2.9058	5.2874	-1.2806
CEU	HIST2	149702469	5.0212	6.8225	-0.7708
CEU	HIST2	149707469	8.17	7.1636	0.412
CEU	HIST2	149712469	8.8453	7.3342	0.6055
CEU	HIST2	149717469	10.1115	8.187	0.6972
CEU	HIST2	149722469	9.4482	6.9931	1.0273
CEU	HIST2	149727469	6.7996	6.6519	0.0647
CEU	HIST2	149732469	5.9992	5.9697	0.0142
CEU	HIST2	149737469	3.3105	3.4112	-0.0791
CEU	HIST2	149742469	2.4663	3.4112	-0.7421
CEU	HIST2	149747469	4.6718	4.0935	0.3889

CEU	HIST2	149752469	3.3781	3.0701	0.2642
CEU	HIST2	149757469	1.8176	3.0701	-1.0744
CEU	HIST2	149762469	2.309	5.1169	-1.5542
CEU	HIST2	149767469	2.1754	5.2874	-1.6733
CEU	HIST2	149772469	3.2146	5.9697	-1.3296
CEU	HIST2	149777469	4.6902	6.8225	-0.9124
CEU	HIST2	149782469	5.437	7.6753	-0.8603
CEU	HIST2	149787469	3.9297	7.1636	-1.3238
CEU	HIST2	149792469	1.4871	3.0701	-1.3579
CEU	HIST2	149797469	0.1235	0.1706	-0.2798
CEU	HIST2	149802469	0	0	0
CEU	HIST2	149807469	0	0	0
CEU	HIST2	149812469	1.3752	1.8762	-0.6379
CEU	HIST2	149817469	2.7071	4.2641	-1.0107
CEU	HIST2	149822469	1.8312	2.5584	-0.7244
CEU	HIST2	149827469	0.4993	0.1706	1.9525
CEU	HIST2	149832469	0	0	0
CEU	HIST2	149837469	0	0	0
CEU	HIST2	149842469	1.0373	1.5351	-0.7381
CEU	HIST2	149847469	2.0711	4.0935	-1.3599
CEU	HIST2	149852469	2.2226	5.1169	-1.602
CEU	HIST2	149857469	2.9598	6.4814	-1.5784
CEU	HIST2	149862469	4.1343	8.0164	-1.4339
CEU	HIST2	149867469	4.7781	8.187	-1.235
CEU	HIST2	149872469	4.5242	6.9931	-1.033
CEU	HIST2	149877469	4.0866	6.6519	-1.1231
CEU	HIST2	149882469	2.6912	5.1169	-1.3427
CEU	HIST2	149887469	2.3135	3.7524	-1.0425
CEU	HIST2	149892469	3.5283	4.7757	-0.7336
CEU	HIST2	149897469	3.3715	5.458	-1.0908
CEU	HIST2	149902469	3.9571	6.3108	-1.0806
CEU	HIST2	149907469	3.9744	5.6286	-0.8414
CEU	HIST2	149912469	1.6186	3.9229	-1.6072
CEU	HIST2	149917469	1.7209	4.0935	-1.5954
CEU	HIST2	149922469	3.7137	5.1169	-0.7767
CEU	HIST2	149927469	4.1585	5.458	-0.6793
CEU	HIST2	149932469	2.6271	5.6286	-1.5267
CEU	HIST2	149937469	2.054	4.7757	-1.6007
CEU	HIST2	149942469	2.6215	3.7524	-0.8193
CEU	HIST2	149947469	2.2977	4.4346	-1.3408
CEU	HIST2	149952469	2.9812	5.9697	-1.4423
CEU	HIST2	149957469	2.1282	4.2641	-1.3865
CEU	HIST2	149962469	1.1688	2.5584	-1.3843
CEU	HIST2	149967469	2.2455	3.4112	-0.9156
CEU	HIST2	149972469	2.0056	3.5818	-1.188
CEU	HIST2	149977469	1.7365	3.9229	-1.5249
CEU	HIST2	149982469	3.1398	5.1169	-1.0944
CEU	HIST2	149987469	4.2152	6.3108	-0.9621
CEU	HIST2	149992469	5.7143	7.5047	-0.7025

CEU	HIST2	149997469	9.5828	8.6987	0.3029
CEU	HIST2	150002469	8.3725	6.9931	0.5772
CEU	HIST2	150007469	7.1431	6.8225	0.1372
CEU	HIST2	150012469	11.1517	9.3809	0.5657
CEU	HIST2	150017469	10.7794	8.8692	0.6428
CEU	HIST2	150022469	8.5492	7.5047	0.4098
CEU	HIST2	150027469	6.0136	6.1402	-0.0596
CEU	HIST2	150032469	1.9462	4.2641	-1.5046
CEU	HIST2	150037469	2.7005	5.7991	-1.5347
CEU	HIST2	150042469	4.5046	6.4814	-0.886
CEU	HIST2	150047469	4.4902	5.458	-0.5059
CEU	HIST2	150052469	3.1923	4.9463	-1.0003
CEU	HIST2	150057469	2.1128	4.4346	-1.4568
CEU	HIST2	150062469	3.0056	4.7757	-1.0411
CEU	HIST2	150067469	1.987	3.2407	-1.028
CEU	HIST2	150072469	5.8835	5.6286	0.1297
CEU	HIST2	150077469	7.7425	7.8459	-0.039
CEU	HIST2	150082469	3.1187	5.458	-1.2229
CEU	HIST2	150087469	3.1076	6.8225	-1.5896
CEU	HIST2	150092469	3.7225	6.6519	-1.2825
CEU	HIST2	150097469	3.9647	4.9463	-0.5598
CEU	HIST2	150102469	5.1591	6.8225	-0.7118
CEU	HIST2	150107469	6.2472	6.8225	-0.2462
CEU	HIST2	150112469	5.381	5.458	-0.0403
CEU	HIST2	150117469	3.2754	4.7757	-0.8824
CEU	HIST2	150122469	3.677	6.3108	-1.2092
CEU	HIST2	150127469	5.6716	8.3576	-0.9548
CEU	HIST2	150132469	6.0334	8.8692	-0.9543
CEU	HIST2	150137469	6.3882	11.7688	-1.3905
CEU	HIST2	150142469	6.4966	9.5515	-0.9597
CEU	HIST2	150147469	5.0326	5.9697	-0.4523
CEU	HIST2	150152469	3.2301	7.3342	-1.6444
CEU	HIST2	150157469	5.2107	9.3809	-1.3322
CEU	HIST2	150162469	9.0014	9.5515	-0.1728
CEU	HIST2	150167469	9.7796	7.6753	0.8088
CEU	HIST2	150172469	8.6036	6.9931	0.6739
CEU	HIST2	150177469	6.8242	5.7991	0.5077
CEU	HIST2	150182469	7.6821	6.1402	0.7256
CEU	HIST2	150187469	6.2595	6.6519	-0.1718
CEU	HIST2	150192469	5.329	5.9697	-0.3092
CEU	HIST2	150197469	8.0014	6.4814	0.6813
CEU	HIST2	150202469	9.2993	7.3342	0.7874
CEU	HIST2	150207469	9.4055	8.0164	0.5131
CEU	HIST2	150212469	8.7469	6.9931	0.7338
CEU	HIST2	150217469	8.7153	6.4814	1.0013
CEU	HIST2	150222469	7.2513	6.1402	0.5229
CEU	HIST2	150227469	3.3078	4.4346	-0.707
CEU	HIST2	150232469	2.2224	3.5818	-1.0246
CEU	HIST2	150237469	3.3931	3.0701	0.2771

CEU	HIST2	150242469	3.5269	4.4346	-0.5695
CEU	HIST2	150247469	5.1911	5.458	-0.1395
CEU	HIST2	150252469	5.5411	5.1169	0.2348
CEU	HIST2	150257469	9.2389	7.6753	0.601
CEU	HIST2	150262469	14.1268	8.6987	1.8596
CEU	HIST2	150267469	17.9592	10.2337	2.276
CEU	HIST2	150272469	17.1991	11.7688	1.4034
CEU	HIST2	150277469	13.45	10.7454	0.7613
CEU	HIST2	150282469	14.67	10.5749	1.17
CEU	HIST2	150287469	13.3049	8.5281	1.6667
CEU	HIST2	150292469	10.7938	7.5047	1.2905
CEU	HIST2	150297469	8.1129	7.3342	0.312
CEU	HIST2	150302469	7.8625	7.5047	0.1404
CEU	HIST2	150307469	10.5438	9.3809	0.3715
CEU	HIST2	150312469	10.6992	9.5515	0.3606
CEU	HIST2	150317469	12.5809	10.2337	0.6915
CEU	HIST2	150322469	13.8482	9.7221	1.2751
CEU	HIST2	150327469	13.5618	9.5515	1.2599
CEU	HIST2	150332469	12.0168	9.3809	0.8421
CEU	HIST2	150337469	9.3466	8.5281	0.2856
CEU	HIST2	150342469	10.0664	8.8692	0.4029
CEU	HIST2	150347469	9.7679	8.0164	0.6469
CEU	HIST2	150352469	12.6992	10.0632	0.7889
CEU	HIST2	150357469	14.7213	10.7454	1.1191
CEU	HIST2	150362469	12.0299	9.7221	0.7132
CEU	HIST2	150367469	12.4471	10.7454	0.479
CEU	HIST2	150372469	13.6547	10.916	0.7596
CEU	HIST2	150377469	14.0484	11.2571	0.7521
CEU	HIST2	150382469	13.6518	10.7454	0.8181
CHB	HIST2	149392469	4.9347	5.7601	-0.4101
CHB	HIST2	149397469	4.4259	5.4213	-0.522
CHB	HIST2	149402469	4.8871	6.6072	-0.7555
CHB	HIST2	149407469	3.0881	4.7436	-0.9765
CHB	HIST2	149412469	3.8921	4.5742	-0.4153
CHB	HIST2	149417469	4.261	4.4048	-0.0905
CHB	HIST2	149422469	1.5298	2.8801	-1.2174
CHB	HIST2	149427469	1.9996	3.8965	-1.3267
CHB	HIST2	149432469	1.8957	3.2189	-1.0879
CHB	HIST2	149437469	0.399	0.8471	-1.0105
CHB	HIST2	149442469	0	0	0
CHB	HIST2	149447469	0.0386	0.5082	-1.4741
CHB	HIST2	149452469	0.1065	1.3553	-2.0239
CHB	HIST2	149457469	0.0873	1.1859	-1.961
CHB	HIST2	149462469	0.0194	0.3388	-1.2812
CHB	HIST2	149467469	0	0	0
CHB	HIST2	149472469	0	0	0
CHB	HIST2	149477469	0	0	0
CHB	HIST2	149482469	0	0	0
CHB	HIST2	149487469	0	0	0

CHB	HIST2	149492469	0	0	0
CHB	HIST2	149497469	0	0	0
CHB	HIST2	149502469	0	0	0
CHB	HIST2	149507469	0.6372	0.6777	-0.1059
CHB	HIST2	149512469	0.8968	1.0165	-0.2382
CHB	HIST2	149517469	0.2694	0.5082	-0.7496
CHB	HIST2	149522469	0.0097	0.1694	-0.949
CHB	HIST2	149527469	0.2578	0.1694	0.5253
CHB	HIST2	149532469	0.2578	0.1694	0.5253
CHB	HIST2	149537469	0	0	0
CHB	HIST2	149542469	0	0	0
CHB	HIST2	149547469	0	0	0
CHB	HIST2	149552469	0	0	0
CHB	HIST2	149557469	0	0	0
CHB	HIST2	149562469	0	0	0
CHB	HIST2	149567469	0.4601	0.3388	0.4866
CHB	HIST2	149572469	0.9039	0.8471	0.1281
CHB	HIST2	149577469	1.095	1.0165	0.1562
CHB	HIST2	149582469	3.3082	3.3883	-0.0631
CHB	HIST2	149587469	3.4287	3.5577	-0.0975
CHB	HIST2	149592469	2.0525	1.6942	0.4917
CHB	HIST2	149597469	2.9082	2.2024	0.7903
CHB	HIST2	149602469	6.534	4.2354	1.4964
CHB	HIST2	149607469	7.0862	5.4213	0.873
CHB	HIST2	149612469	2.7537	2.8801	-0.114
CHB	HIST2	149617469	0.574	0.5082	0.2066
CHB	HIST2	149622469	0	0	0
CHB	HIST2	149627469	0.0097	0.1694	-0.949
CHB	HIST2	149632469	0.0097	0.1694	-0.949
CHB	HIST2	149637469	2.8808	1.5247	2.0147
CHB	HIST2	149642469	4.3038	3.8965	0.2849
CHB	HIST2	149647469	7.5328	6.7766	0.3246
CHB	HIST2	149652469	7.0925	5.7601	0.662
CHB	HIST2	149657469	3.0921	4.5742	-0.9025
CHB	HIST2	149662469	5.9966	5.4213	0.3017
CHB	HIST2	149667469	7.7933	6.6072	0.5209
CHB	HIST2	149672469	8.1593	8.6402	-0.1653
CHB	HIST2	149677469	6.0101	6.0989	-0.0419
CHB	HIST2	149682469	2.9364	4.5742	-0.9973
CHB	HIST2	149687469	2.2947	4.5742	-1.388
CHB	HIST2	149692469	3.198	4.2354	-0.6753
CHB	HIST2	149697469	4.2125	4.5742	-0.2202
CHB	HIST2	149702469	5.94	6.7766	-0.3591
CHB	HIST2	149707469	8.0221	7.9625	0.0221
CHB	HIST2	149712469	6.7569	6.0989	0.3106
CHB	HIST2	149717469	7.4384	8.1319	-0.2521
CHB	HIST2	149722469	7.8527	9.1484	-0.4224
CHB	HIST2	149727469	6.5829	8.4708	-0.6609
CHB	HIST2	149732469	5.754	7.2848	-0.6153

CHB	HIST2	149737469	2.7978	4.4048	-1.0112
CHB	HIST2	149742469	1.5346	2.8801	-1.2131
CHB	HIST2	149747469	3.8666	2.8801	0.8894
CHB	HIST2	149752469	3.2829	3.0495	0.2007
CHB	HIST2	149757469	1.1759	5.4213	-2.2262
CHB	HIST2	149762469	1.9924	6.6072	-2.0269
CHB	HIST2	149767469	2.1696	4.5742	-1.4642
CHB	HIST2	149772469	1.3105	5.7601	-2.2107
CHB	HIST2	149777469	0.7729	5.0825	-2.3926
CHB	HIST2	149782469	1.0246	4.4048	-2.127
CHB	HIST2	149787469	0.744	5.7601	-2.4921
CHB	HIST2	149792469	0.4123	3.5577	-2.3771
CHB	HIST2	149797469	0.1611	0.6777	-1.3505
CHB	HIST2	149802469	0	0	0
CHB	HIST2	149807469	0	0	0
CHB	HIST2	149812469	0.4782	2.033	-1.8541
CHB	HIST2	149817469	1.276	5.4213	-2.1737
CHB	HIST2	149822469	1.1639	3.5577	-1.8091
CHB	HIST2	149827469	0.3661	0.1694	1.1688
CHB	HIST2	149832469	0	0	0
CHB	HIST2	149837469	0	0	0
CHB	HIST2	149842469	0.249	1.3553	-1.793
CHB	HIST2	149847469	0.6308	4.7436	-2.426
CHB	HIST2	149852469	0.9367	7.6237	-2.5787
CHB	HIST2	149857469	1.5464	6.2684	-2.1748
CHB	HIST2	149862469	1.4339	6.2684	-2.2266
CHB	HIST2	149867469	0.9913	7.1154	-2.515
CHB	HIST2	149872469	1.1011	5.9295	-2.3375
CHB	HIST2	149877469	1.6012	6.6072	-2.1987
CHB	HIST2	149882469	1.7603	7.1154	-2.1992
CHB	HIST2	149887469	1.2859	5.9295	-2.248
CHB	HIST2	149892469	1.5239	6.7766	-2.2548
CHB	HIST2	149897469	1.1529	7.1154	-2.4486
CHB	HIST2	149902469	1.4393	7.1154	-2.331
CHB	HIST2	149907469	2.6127	8.1319	-2.0061
CHB	HIST2	149912469	1.5418	6.2684	-2.1769
CHB	HIST2	149917469	1.4919	6.7766	-2.2685
CHB	HIST2	149922469	3.5157	8.979	-1.8123
CHB	HIST2	149927469	3.9733	10.3343	-1.8516
CHB	HIST2	149932469	2.1709	10.1649	-2.3631
CHB	HIST2	149937469	2.0573	9.3178	-2.3273
CHB	HIST2	149942469	2.9625	8.1319	-1.8789
CHB	HIST2	149947469	2.0617	6.2684	-1.9375
CHB	HIST2	149952469	1.9688	7.7931	-2.2013
CHB	HIST2	149957469	1.3923	7.6237	-2.403
CHB	HIST2	149962469	1.0171	6.4378	-2.4373
CHB	HIST2	149967469	1.4121	6.6072	-2.2817
CHB	HIST2	149972469	1.3341	4.7436	-2.0112
CHB	HIST2	149977469	1.5755	4.5742	-1.826

CHB	HIST2	149982469	2.233	7.7931	-2.1014
CHB	HIST2	149987469	2.6075	9.9955	-2.2185
CHB	HIST2	149992469	3.921	9.8261	-1.8017
CHB	HIST2	149997469	6.7561	10.8426	-1.1373
CHB	HIST2	150002469	5.3497	10.1649	-1.4234
CHB	HIST2	150007469	2.8742	9.4872	-2.0846
CHB	HIST2	150012469	2.2077	10.3343	-2.3655
CHB	HIST2	150017469	1.5117	8.8096	-2.464
CHB	HIST2	150022469	1.6941	8.8096	-2.4024
CHB	HIST2	150027469	1.3954	8.6402	-2.4903
CHB	HIST2	150032469	1.0944	6.2684	-2.383
CHB	HIST2	150037469	1.8192	6.4378	-2.0767
CHB	HIST2	150042469	1.9669	7.1154	-2.1143
CHB	HIST2	150047469	0.9244	6.6072	-2.4959
CHB	HIST2	150052469	1.0657	6.6072	-2.4339
CHB	HIST2	150057469	1.1483	5.9295	-2.3146
CHB	HIST2	150062469	0.613	4.913	-2.4596
CHB	HIST2	150067469	0.5917	5.2519	-2.5134
CHB	HIST2	150072469	1.3286	7.6237	-2.4276
CHB	HIST2	150077469	1.3328	7.6237	-2.4259
CHB	HIST2	150082469	0.8626	5.0825	-2.3428
CHB	HIST2	150087469	1.351	6.2684	-2.2648
CHB	HIST2	150092469	1.5829	6.7766	-2.2295
CHB	HIST2	150097469	1.4826	6.2684	-2.2042
CHB	HIST2	150102469	1.5997	7.9625	-2.3579
CHB	HIST2	150107469	1.8096	7.7931	-2.2614
CHB	HIST2	150112469	1.4567	6.0989	-2.1913
CHB	HIST2	150117469	1.3547	6.0989	-2.2395
CHB	HIST2	150122469	2.2334	7.7931	-2.1013
CHB	HIST2	150127469	2.6113	8.1319	-2.0066
CHB	HIST2	150132469	2.1033	8.1319	-2.1912
CHB	HIST2	150137469	2.0793	9.8261	-2.3636
CHB	HIST2	150142469	2.7139	9.4872	-2.1351
CHB	HIST2	150147469	2.7179	8.1319	-1.9678
CHB	HIST2	150152469	1.9273	8.979	-2.3393
CHB	HIST2	150157469	2.7318	12.8755	-2.4014
CHB	HIST2	150162469	6.5275	12.0285	-1.3887
CHB	HIST2	150167469	9.7902	9.6567	0.0414
CHB	HIST2	150172469	7.4908	10.5037	-0.8638
CHB	HIST2	150177469	5.6417	10.3343	-1.3659
CHB	HIST2	150182469	7.0632	9.3178	-0.7227
CHB	HIST2	150187469	4.4826	6.7766	-0.9847
CHB	HIST2	150192469	4.0871	6.946	-1.2001
CHB	HIST2	150197469	8.1695	8.979	-0.2685
CHB	HIST2	150202469	9.2578	9.9955	-0.2215
CHB	HIST2	150207469	8.6613	11.3508	-0.717
CHB	HIST2	150212469	7.9743	11.5202	-0.9323
CHB	HIST2	150217469	7.8589	9.9955	-0.6416
CHB	HIST2	150222469	6.5469	8.8096	-0.7639

CHB	HIST2	150227469	2.969	6.2684	-1.5196
CHB	HIST2	150232469	2.1055	4.2354	-1.3866
CHB	HIST2	150237469	3.3891	4.5742	-0.7216
CHB	HIST2	150242469	3.8846	5.9295	-0.99
CHB	HIST2	150247469	5.0166	7.4543	-0.9595
CHB	HIST2	150252469	4.6815	7.6237	-1.1346
CHB	HIST2	150257469	7.4337	8.4708	-0.3631
CHB	HIST2	150262469	10.3901	10.1649	0.0666
CHB	HIST2	150267469	12.8372	11.5202	0.3463
CHB	HIST2	150272469	11.6891	10.6731	0.287
CHB	HIST2	150277469	8.1893	10.3343	-0.6244
CHB	HIST2	150282469	10.3892	12.3673	-0.4865
CHB	HIST2	150287469	10.2355	10.8426	-0.169
CHB	HIST2	150292469	7.4604	8.8096	-0.4555
CHB	HIST2	150297469	4.852	8.1319	-1.1922
CHB	HIST2	150302469	5.5199	8.3013	-0.992
CHB	HIST2	150307469	7.7236	10.5037	-0.797
CHB	HIST2	150312469	7.8652	9.4872	-0.5113
CHB	HIST2	150317469	8.8378	9.8261	-0.3015
CHB	HIST2	150322469	8.8622	11.3508	-0.6635
CHB	HIST2	150327469	9.3931	12.0285	-0.6653
CHB	HIST2	150332469	8.5134	11.5202	-0.7905
CHB	HIST2	150337469	6.0792	7.9625	-0.6979
CHB	HIST2	150342469	6.8915	6.2684	0.287
CHB	HIST2	150347469	6.6887	7.4543	-0.3014
CHB	HIST2	150352469	7.558	11.5202	-1.0417
CHB	HIST2	150357469	8.9225	12.8755	-0.9358
CHB	HIST2	150362469	6.9539	9.8261	-0.8763
CHB	HIST2	150367469	8.3628	12.3673	-0.9848
CHB	HIST2	150372469	10.004	15.4168	-1.08
CHB	HIST2	150377469	10.2816	15.0779	-0.9774
CHB	HIST2	150382469	9.4824	15.2474	-1.1624
YRI	HIST2	149392469	6.7938	7.2267	-0.1747
YRI	HIST2	149397469	5.7309	7.5628	-0.7092
YRI	HIST2	149402469	7.2413	8.9073	-0.5549
YRI	HIST2	149407469	5.7873	8.5712	-0.9607
YRI	HIST2	149412469	5.3418	7.5628	-0.8598
YRI	HIST2	149417469	5.4485	5.5461	-0.05
YRI	HIST2	149422469	2.9383	3.3612	-0.3341
YRI	HIST2	149427469	3.1886	4.3696	-0.7457
YRI	HIST2	149432469	2.5221	3.3612	-0.6629
YRI	HIST2	149437469	0.5529	1.0084	-0.9081
YRI	HIST2	149442469	0.0185	0.1681	-0.8893
YRI	HIST2	149447469	0.1568	0.5042	-1.0918
YRI	HIST2	149452469	0.4181	1.1764	-1.3563
YRI	HIST2	149457469	0.5682	1.5126	-1.4063
YRI	HIST2	149462469	0.3069	0.8403	-1.2048
YRI	HIST2	149467469	0	0	0
YRI	HIST2	149472469	0	0	0

YRI	HIST2	149477469	0	0	0
YRI	HIST2	149482469	0	0	0
YRI	HIST2	149487469	0	0	0
YRI	HIST2	149492469	0	0	0
YRI	HIST2	149497469	0	0	0
YRI	HIST2	149502469	0	0	0
YRI	HIST2	149507469	0.4018	0.5042	-0.3218
YRI	HIST2	149512469	0.6674	2.0167	-1.6133
YRI	HIST2	149517469	0.4991	2.0167	-1.8145
YRI	HIST2	149522469	0.2335	0.5042	-0.8507
YRI	HIST2	149527469	0.2339	0.5042	-0.8495
YRI	HIST2	149532469	0.2339	0.5042	-0.8495
YRI	HIST2	149537469	0	0	0
YRI	HIST2	149542469	0	0	0
YRI	HIST2	149547469	0	0	0
YRI	HIST2	149552469	0	0	0
YRI	HIST2	149557469	0	0	0
YRI	HIST2	149562469	0	0	0
YRI	HIST2	149567469	0.5133	0.5042	0.0286
YRI	HIST2	149572469	1.1098	1.6806	-0.7853
YRI	HIST2	149577469	0.9196	1.5126	-0.883
YRI	HIST2	149582469	2.3949	5.0419	-1.4748
YRI	HIST2	149587469	2.5097	5.7141	-1.5979
YRI	HIST2	149592469	1.2129	2.1848	-1.0912
YRI	HIST2	149597469	2.5434	3.0251	-0.4155
YRI	HIST2	149602469	5.6089	6.2183	-0.2817
YRI	HIST2	149607469	5.8919	6.8906	-0.4208
YRI	HIST2	149612469	2.6056	3.6974	-0.7958
YRI	HIST2	149617469	0.5543	1.1764	-1.1127
YRI	HIST2	149622469	0	0	0
YRI	HIST2	149627469	0.0093	0.1681	-0.944
YRI	HIST2	149632469	0.3282	0.6722	-0.9006
YRI	HIST2	149637469	3.1146	2.0167	1.3127
YRI	HIST2	149642469	4.0305	4.2016	-0.1118
YRI	HIST2	149647469	8.5881	9.4115	-0.2606
YRI	HIST2	149652469	9.8754	10.924	-0.2888
YRI	HIST2	149657469	5.7279	7.2267	-0.6048
YRI	HIST2	149662469	7.8479	7.8989	-0.019
YRI	HIST2	149667469	10.8516	13.1089	-0.5236
YRI	HIST2	149672469	10.2015	14.2853	-0.8732
YRI	HIST2	149677469	6.239	10.0837	-1.1412
YRI	HIST2	149682469	4.449	8.7392	-1.4543
YRI	HIST2	149687469	3.0618	8.5712	-1.9013
YRI	HIST2	149692469	3.0228	6.7225	-1.5942
YRI	HIST2	149697469	4.7415	7.0586	-0.9552
YRI	HIST2	149702469	8.2414	11.0921	-0.774
YRI	HIST2	149707469	10.0936	13.445	-0.759
YRI	HIST2	149712469	7.001	11.0921	-1.1108
YRI	HIST2	149717469	7.6164	11.2602	-0.9755

YRI	HIST2	149722469	9.2696	12.7727	-0.8328
YRI	HIST2	149727469	8.9001	13.2769	-1.0031
YRI	HIST2	149732469	7.7605	12.6047	-1.1661
YRI	HIST2	149737469	4.3649	8.067	-1.3509
YRI	HIST2	149742469	4.2459	6.2183	-0.9118
YRI	HIST2	149747469	6.9196	7.5628	-0.249
YRI	HIST2	149752469	6.0807	7.5628	-0.5738
YRI	HIST2	149757469	6.1394	9.7476	-1.1053
YRI	HIST2	149762469	6.0299	10.5879	-1.2926
YRI	HIST2	149767469	3.4277	8.9073	-1.825
YRI	HIST2	149772469	5.4485	11.0921	-1.5323
YRI	HIST2	149777469	6.5247	12.1005	-1.395
YRI	HIST2	149782469	4.9324	13.2769	-1.9124
YRI	HIST2	149787469	4.2145	11.9324	-1.9565
YRI	HIST2	149792469	2.1767	5.2099	-1.6418
YRI	HIST2	149797469	0.4072	1.0084	-1.1986
YRI	HIST2	149802469	0	0	0
YRI	HIST2	149807469	0	0	0
YRI	HIST2	149812469	2.7381	4.8738	-1.226
YRI	HIST2	149817469	5.0549	8.5712	-1.2135
YRI	HIST2	149822469	2.7985	3.8654	-0.7487
YRI	HIST2	149827469	0.4817	0.1681	1.8643
YRI	HIST2	149832469	0	0	0
YRI	HIST2	149837469	0.0183	0.1681	-0.8905
YRI	HIST2	149842469	0.8756	2.689	-1.7234
YRI	HIST2	149847469	4.1965	9.4115	-1.6505
YRI	HIST2	149852469	5.6836	13.613	-1.7748
YRI	HIST2	149857469	4.0674	11.7644	-1.9774
YRI	HIST2	149862469	5.3263	11.5963	-1.6327
YRI	HIST2	149867469	7.5965	12.6047	-1.2056
YRI	HIST2	149872469	6.1608	11.2602	-1.3651
YRI	HIST2	149877469	7.021	11.4282	-1.1635
YRI	HIST2	149882469	8.3506	11.5963	-0.8452
YRI	HIST2	149887469	5.7786	11.9324	-1.56
YRI	HIST2	149892469	5.1776	12.7727	-1.8056
YRI	HIST2	149897469	4.6919	12.9408	-1.937
YRI	HIST2	149902469	3.9184	11.5963	-1.9994
YRI	HIST2	149907469	6.6719	11.5963	-1.2823
YRI	HIST2	149912469	6.3664	11.9324	-1.411
YRI	HIST2	149917469	5.7153	12.4366	-1.6386
YRI	HIST2	149922469	7.5886	14.2853	-1.4318
YRI	HIST2	149927469	8.3536	13.445	-1.1531
YRI	HIST2	149932469	9.4821	13.445	-0.8975
YRI	HIST2	149937469	8.992	12.4366	-0.8398
YRI	HIST2	149942469	5.9704	9.0754	-1.0164
YRI	HIST2	149947469	4.6609	8.7392	-1.3824
YRI	HIST2	149952469	7.7748	11.0921	-0.9007
YRI	HIST2	149957469	7.4467	10.924	-0.9577
YRI	HIST2	149962469	4.866	9.4115	-1.4386

YRI	HIST2	149967469	6.4652	9.7476	-1.0055
YRI	HIST2	149972469	6.2536	8.9073	-0.8838
YRI	HIST2	149977469	6.8142	9.5795	-0.8609
YRI	HIST2	149982469	9.1001	12.2685	-0.7824
YRI	HIST2	149987469	9.771	12.2685	-0.6168
YRI	HIST2	149992469	9.44	13.613	-0.934
YRI	HIST2	149997469	13.5226	15.6298	-0.4136
YRI	HIST2	150002469	13.5417	13.7811	-0.053
YRI	HIST2	150007469	11.3461	15.2937	-0.791
YRI	HIST2	150012469	15.6615	17.6465	-0.3469
YRI	HIST2	150017469	15.5594	15.4617	0.0194
YRI	HIST2	150022469	12.9184	15.1256	-0.4469
YRI	HIST2	150027469	11.7535	14.1172	-0.5111
YRI	HIST2	150032469	8.6821	11.2602	-0.6902
YRI	HIST2	150037469	8.5055	12.9408	-1.0415
YRI	HIST2	150042469	11.9886	14.7895	-0.5794
YRI	HIST2	150047469	11.5099	14.6214	-0.6507
YRI	HIST2	150052469	8.1547	13.2769	-1.1739
YRI	HIST2	150057469	7.1448	12.4366	-1.2901
YRI	HIST2	150062469	8.7056	12.9408	-0.9945
YRI	HIST2	150067469	8.7754	12.2685	-0.8626
YRI	HIST2	150072469	10.0818	14.2853	-0.8987
YRI	HIST2	150077469	12.226	17.1423	-0.8833
YRI	HIST2	150082469	9.9292	14.9575	-1.0291
YRI	HIST2	150087469	13.1052	15.6298	-0.4955
YRI	HIST2	150092469	15.3158	16.134	-0.1558
YRI	HIST2	150097469	11.3238	13.9492	-0.5742
YRI	HIST2	150102469	11.0643	14.7895	-0.7706
YRI	HIST2	150107469	11.6925	16.302	-0.869
YRI	HIST2	150112469	9.5648	15.1256	-1.126
YRI	HIST2	150117469	7.3353	13.2769	-1.3617
YRI	HIST2	150122469	8.5324	15.4617	-1.374
YRI	HIST2	150127469	10.02	15.4617	-1.079
YRI	HIST2	150132469	10.9919	15.4617	-0.8863
YRI	HIST2	150137469	13.1586	18.1507	-0.8491
YRI	HIST2	150142469	11.6313	15.7978	-0.8094
YRI	HIST2	150147469	7.5008	11.4282	-1.0368
YRI	HIST2	150152469	10.0533	13.613	-0.7967
YRI	HIST2	150157469	17.0935	19.4952	-0.3814
YRI	HIST2	150162469	16.3703	18.6549	-0.3785
YRI	HIST2	150167469	13.1734	15.6298	-0.4821
YRI	HIST2	150172469	12.4708	15.9659	-0.6721
YRI	HIST2	150177469	11.8583	15.4617	-0.7145
YRI	HIST2	150182469	12.0261	14.4534	-0.5132
YRI	HIST2	150187469	9.268	11.9324	-0.6754
YRI	HIST2	150192469	8.856	11.0921	-0.6071
YRI	HIST2	150197469	10.6334	10.924	-0.08
YRI	HIST2	150202469	11.243	10.924	0.0879
YRI	HIST2	150207469	13.0353	14.1172	-0.2339

YRI	HIST2	150212469	12.7722	14.7895	-0.4173
YRI	HIST2	150217469	11.9443	12.7727	-0.1969
YRI	HIST2	150222469	9.6681	10.5879	-0.2609
YRI	HIST2	150227469	4.6079	8.7392	-1.4004
YRI	HIST2	150232469	3.3738	7.7309	-1.6532
YRI	HIST2	150237469	3.6291	6.7225	-1.3329
YRI	HIST2	150242469	4.6251	9.2434	-1.4863
YRI	HIST2	150247469	6.5836	11.0921	-1.2241
YRI	HIST2	150252469	6.7318	10.4199	-1.0617
YRI	HIST2	150257469	10.2895	10.756	-0.1304
YRI	HIST2	150262469	14.7724	12.7727	0.4754
YRI	HIST2	150267469	18.4065	16.6382	0.3269
YRI	HIST2	150272469	16.5249	16.8062	-0.0515
YRI	HIST2	150277469	13.0402	14.9575	-0.3924
YRI	HIST2	150282469	12.9815	14.7895	-0.374
YRI	HIST2	150287469	11.4314	13.445	-0.456
YRI	HIST2	150292469	10.3231	12.1005	-0.4447
YRI	HIST2	150297469	9.3289	11.7644	-0.6257
YRI	HIST2	150302469	10.5637	11.9324	-0.347
YRI	HIST2	150307469	12.6697	14.4534	-0.3772
YRI	HIST2	150312469	12.7295	13.445	-0.162
YRI	HIST2	150317469	14.5579	13.613	0.2115
YRI	HIST2	150322469	13.2606	15.1256	-0.3776
YRI	HIST2	150327469	12.4745	14.7895	-0.4789
YRI	HIST2	150332469	12.4071	14.1172	-0.3698
YRI	HIST2	150337469	10.6826	12.6047	-0.4627
YRI	HIST2	150342469	11.544	12.1005	-0.1392
YRI	HIST2	150347469	9.6714	10.5879	-0.2599
YRI	HIST2	150352469	13.1122	12.9408	0.0402
YRI	HIST2	150357469	15.7741	15.4617	0.0619
YRI	HIST2	150362469	11.8663	13.2769	-0.3233
YRI	HIST2	150367469	13.1349	14.4534	-0.2788
YRI	HIST2	150372469	16.3624	17.8146	-0.2515
YRI	HIST2	150377469	15.7318	16.8062	-0.1967
YRI	HIST2	150382469	16.4967	15.1256	0.2776
CEU	LIMS3	109403688	4.7098	5.7991	-0.5395
CEU	LIMS3	109408688	4.9192	6.4814	-0.7002
CEU	LIMS3	109413688	5.2564	6.1402	-0.4159
CEU	LIMS3	109418688	4.6246	5.6286	-0.5107
CEU	LIMS3	109423688	4.9114	7.3342	-0.9707
CEU	LIMS3	109428688	4.2839	7.1636	-1.1788
CEU	LIMS3	109433688	5.2597	8.0164	-1.0182
CEU	LIMS3	109438688	5.5638	8.5281	-1.0343
CEU	LIMS3	109443688	5.8885	8.3576	-0.8777
CEU	LIMS3	109448688	5.3193	7.1636	-0.755
CEU	LIMS3	109453688	3.3234	5.7991	-1.2262
CEU	LIMS3	109458688	4.5762	6.9931	-1.0113
CEU	LIMS3	109463688	3.8551	6.3108	-1.1275
CEU	LIMS3	109468688	2.6794	4.9463	-1.2928

CEU	LIMS3	109473688	3.7275	5.1169	-0.7691
CEU	LIMS3	109478688	5.0606	7.6753	-1.005
CEU	LIMS3	109483688	3.8866	6.9931	-1.2998
CEU	LIMS3	109488688	4.9128	7.1636	-0.9214
CEU	LIMS3	109493688	7.4005	10.0632	-0.7968
CEU	LIMS3	109498688	7.1305	9.8926	-0.8399
CEU	LIMS3	109503688	6.5887	9.8926	-1.0046
CEU	LIMS3	109508688	5.6197	8.8692	-1.0935
CEU	LIMS3	109513688	6.9163	9.5515	-0.8279
CEU	LIMS3	109518688	6.0045	9.3809	-1.0786
CEU	LIMS3	109523688	5.1233	8.8692	-1.2605
CEU	LIMS3	109528688	4.8437	8.3576	-1.2491
CEU	LIMS3	109533688	3.6693	6.4814	-1.2604
CEU	LIMS3	109538688	4.2321	7.6753	-1.3235
CEU	LIMS3	109543688	4.1901	9.5515	-1.6843
CEU	LIMS3	109548688	3.2132	9.8926	-2.031
CEU	LIMS3	109553688	5.4181	13.8156	-1.865
CEU	LIMS3	109558688	5.6364	11.5982	-1.562
CEU	LIMS3	109563688	2.7537	6.4814	-1.6708
CEU	LIMS3	109568688	3.2319	8.5281	-1.8479
CEU	LIMS3	109573688	3.3533	8.187	-1.7511
CEU	LIMS3	109578688	4.0524	8.187	-1.4979
CEU	LIMS3	109583688	3.959	8.6987	-1.6238
CEU	LIMS3	109588688	6.846	9.7221	-0.8888
CEU	LIMS3	109593688	13.4372	14.3272	-0.191
CEU	LIMS3	109598688	12.0418	14.1567	-0.459
CEU	LIMS3	109603688	7.0394	11.0866	-1.1063
CEU	LIMS3	109608688	6.1567	10.0632	-1.1691
CEU	LIMS3	109613688	6.5108	8.3576	-0.6565
CEU	LIMS3	109618688	5.361	5.7991	-0.217
CEU	LIMS3	109623688	6.3364	6.6519	-0.1381
CEU	LIMS3	109628688	8.6265	11.0866	-0.6725
CEU	LIMS3	109633688	8.5883	11.0866	-0.6829
CEU	LIMS3	109638688	6.2554	7.3342	-0.4322
CEU	LIMS3	109643688	4.0765	5.1169	-0.5759
CEU	LIMS3	109648688	3.4834	4.7757	-0.7601
CEU	LIMS3	109653688	2.3102	4.6052	-1.3934
CEU	LIMS3	109658688	1.7444	5.6286	-1.9757
CEU	LIMS3	109663688	2.8563	6.3108	-1.586
CEU	LIMS3	109668688	4.2045	6.9931	-1.1668
CEU	LIMS3	109673688	7.9041	8.8692	-0.3248
CEU	LIMS3	109678688	11.4296	10.2337	0.3523
CEU	LIMS3	109683688	9.9371	8.6987	0.4243
CEU	LIMS3	109688688	10.2416	8.3576	0.6697
CEU	LIMS3	109693688	12.173	11.2571	0.2468
CEU	LIMS3	109698688	11.0544	10.4043	0.1886
CEU	LIMS3	109703688	7.9722	8.0164	-0.0163
CEU	LIMS3	109708688	7.5869	7.5047	0.0323
CEU	LIMS3	109713688	6.3758	5.7991	0.2856

CEU	LIMS3	109718688	3.7969	4.7757	-0.5757
CEU	LIMS3	109723688	2.5345	4.6052	-1.2572
CEU	LIMS3	109728688	2.0765	3.9229	-1.2878
CEU	LIMS3	109733688	2.4045	4.4346	-1.2738
CEU	LIMS3	109738688	3.1752	5.9697	-1.3487
CEU	LIMS3	109743688	6.4776	7.1636	-0.2808
CEU	LIMS3	109748688	8.9291	8.5281	0.1399
CEU	LIMS3	109753688	8.9309	8.6987	0.0796
CEU	LIMS3	109758688	7.0202	7.3342	-0.1258
CEU	LIMS3	109763688	8.8005	7.5047	0.5084
CEU	LIMS3	109768688	10.6207	8.187	0.8817
CEU	LIMS3	109773688	10.3659	8.6987	0.5712
CEU	LIMS3	109778688	10.9415	9.3809	0.4986
CEU	LIMS3	109783688	11.6726	10.2337	0.4239
CEU	LIMS3	109788688	9.329	8.3576	0.3453
CEU	LIMS3	109793688	6.0781	6.8225	-0.3185
CEU	LIMS3	109798688	6.8606	8.0164	-0.4269
CEU	LIMS3	109803688	11.6657	11.2571	0.1101
CEU	LIMS3	109808688	11.7863	10.5749	0.3461
CEU	LIMS3	109813688	8.2472	7.8459	0.1512
CEU	LIMS3	109818688	11.6994	9.7221	0.611
CEU	LIMS3	109823688	13.227	11.5982	0.4268
CEU	LIMS3	109828688	10.9328	10.7454	0.0527
CEU	LIMS3	109833688	9.7636	9.7221	0.0128
CEU	LIMS3	109838688	7.8658	9.2104	-0.4369
CEU	LIMS3	109843688	5.0959	8.6987	-1.2343
CEU	LIMS3	109848688	3.8411	7.8459	-1.5087
CEU	LIMS3	109853688	5.2599	6.4814	-0.5475
CEU	LIMS3	109858688	6.7737	7.6753	-0.3466
CEU	LIMS3	109863688	5.4616	8.0164	-0.9436
CEU	LIMS3	109868688	4.5706	6.6519	-0.9112
CEU	LIMS3	109873688	8.9862	8.3576	0.2234
CEU	LIMS3	109878688	12.4261	9.5515	0.9031
CEU	LIMS3	109883688	9.5916	7.5047	0.8188
CEU	LIMS3	109888688	6.9555	6.3108	0.296
CEU	LIMS3	109893688	9.4391	6.8225	1.1196
CEU	LIMS3	109898688	8.8066	7.1636	0.6726
CEU	LIMS3	109903688	4.5178	4.9463	-0.2444
CEU	LIMS3	109908688	3.6261	5.1169	-0.8252
CEU	LIMS3	109913688	4.5875	6.1402	-0.7307
CEU	LIMS3	109918688	16.6767	10.7454	1.6695
CEU	LIMS3	109923688	23.327	13.645	2.1758
CEU	LIMS3	109928688	37.5088	20.2969	2.6455
CEU	LIMS3	109933688	37.4306	21.1497	2.405
CEU	LIMS3	109938688	15.5859	9.8926	1.7312
CEU	LIMS3	109943688	9.0258	7.3342	0.6778
CEU	LIMS3	109948688	9.9289	9.3809	0.1751
CEU	LIMS3	109953688	10.804	9.2104	0.5178
CEU	LIMS3	109958688	9.6252	8.5281	0.3828

CEU	LIMS3	109963688	9.3329	9.2104	0.0398
CEU	LIMS3	109968688	5.8462	9.3809	-1.1292
CEU	LIMS3	109973688	7.0089	10.2337	-0.95
CEU	LIMS3	109978688	7.0231	11.0866	-1.1107
CEU	LIMS3	109983688	7.263	12.1099	-1.2193
CEU	LIMS3	109988688	10.8005	14.8389	-0.838
CEU	LIMS3	109993688	10.6242	14.6684	-0.8485
CEU	LIMS3	109998688	12.6887	12.1099	0.1456
CEU	LIMS3	110003688	19.2179	12.2805	1.7224
CEU	LIMS3	110008688	14.7704	9.7221	1.5601
CEU	LIMS3	110013688	11.7233	8.3576	1.1964
CEU	LIMS3	110018688	15.822	12.1099	0.9339
CEU	LIMS3	110023688	15.92	14.4978	0.3017
CEU	LIMS3	110028688	13.3408	11.5982	0.4566
CEU	LIMS3	110033688	7.7404	8.8692	-0.3798
CEU	LIMS3	110038688	10.4079	10.5749	-0.0477
CEU	LIMS3	110043688	12.9819	10.5749	0.6877
CEU	LIMS3	110048688	7.8192	8.0164	-0.0728
CEU	LIMS3	110053688	6.5947	6.6519	-0.025
CEU	LIMS3	110058688	6.1641	7.1636	-0.4091
CEU	LIMS3	110063688	4.0668	7.1636	-1.2677
CEU	LIMS3	110068688	3.7355	5.7991	-1.0221
CEU	LIMS3	110073688	3.8621	6.1402	-1.072
CEU	LIMS3	110078688	5.5545	7.6753	-0.8152
CEU	LIMS3	110083688	5.4824	6.9931	-0.6321
CEU	LIMS3	110088688	4.4085	7.5047	-1.2148
CEU	LIMS3	110093688	5.0288	7.5047	-0.9714
CEU	LIMS3	110098688	5.1148	5.7991	-0.3389
CEU	LIMS3	110103688	6.462	8.0164	-0.5741
CEU	LIMS3	110108688	5.2665	5.9697	-0.3394
CEU	LIMS3	110113688	1.2816	1.0234	0.5131
CEU	LIMS3	110118688	0	0	0
CEU	LIMS3	110123688	0	0	0
CEU	LIMS3	110128688	0	0	0
CEU	LIMS3	110133688	0	0	0
CEU	LIMS3	110138688	0	0	0
CEU	LIMS3	110143688	0	0	0
CEU	LIMS3	110148688	0	0	0
CEU	LIMS3	110153688	0	0	0
CEU	LIMS3	110158688	0	0	0
CEU	LIMS3	110163688	0	0	0
CEU	LIMS3	110168688	0	0	0
CEU	LIMS3	110173688	0	0	0
CEU	LIMS3	110178688	0	0	0
CEU	LIMS3	110183688	0	0	0
CEU	LIMS3	110188688	0	0	0
CEU	LIMS3	110193688	0	0	0
CEU	LIMS3	110198688	0	0	0
CEU	LIMS3	110203688	0	0	0

CEU	LIMS3	110208688	0	0	0
CEU	LIMS3	110213688	0	0	0
CEU	LIMS3	110218688	0	0	0
CEU	LIMS3	110223688	0	0	0
CEU	LIMS3	110228688	0	0	0
CEU	LIMS3	110233688	0	0	0
CEU	LIMS3	110238688	0	0	0
CEU	LIMS3	110243688	0	0	0
CEU	LIMS3	110248688	7.8068	4.6052	1.9438
CEU	LIMS3	110253688	22.6996	13.4744	2.098
CEU	LIMS3	110258688	17.4795	12.7922	1.1197
CEU	LIMS3	110263688	7.5506	9.0398	-0.4924
CEU	LIMS3	110268688	10.357	9.5515	0.2531
CEU	LIMS3	110273688	9.3548	9.8926	-0.1635
CEU	LIMS3	110278688	7.5259	10.0632	-0.7593
CEU	LIMS3	110283688	6.1288	8.0164	-0.6972
CEU	LIMS3	110288688	5.7671	6.1402	-0.1756
CEU	LIMS3	110293688	7.1538	7.5047	-0.1377
CEU	LIMS3	110298688	6.2814	9.3809	-0.9902
CEU	LIMS3	110303688	6.5475	8.187	-0.594
CEU	LIMS3	110308688	6.4696	6.3108	0.0729
CEU	LIMS3	110313688	6.2915	6.1402	0.0712
CEU	LIMS3	110318688	7.604	6.9931	0.2556
CEU	LIMS3	110323688	7.0614	8.0164	-0.3527
CEU	LIMS3	110328688	8.9567	9.8926	-0.2846
CEU	LIMS3	110333688	9.3948	10.4043	-0.2928
CEU	LIMS3	110338688	5.8448	8.5281	-0.9362
CEU	LIMS3	110343688	6.2501	8.3576	-0.7492
CEU	LIMS3	110348688	8.5753	8.0164	0.2064
CEU	LIMS3	110353688	7.9893	6.1402	0.8701
CEU	LIMS3	110358688	6.7041	6.9931	-0.1209
CEU	LIMS3	110363688	8.1021	8.6987	-0.2044
CEU	LIMS3	110368688	8.838	8.187	0.2358
CEU	LIMS3	110373688	8.0499	6.9931	0.4422
CEU	LIMS3	110378688	8.7456	7.6753	0.4114
CEU	LIMS3	110383688	10.4108	9.2104	0.3901
CEU	LIMS3	110388688	13.826	11.4277	0.6372
CEU	LIMS3	110393688	16.3519	13.3039	0.7016
CEU	LIMS3	110398688	15.0707	12.1099	0.7449
CEU	LIMS3	110403688	13.0031	11.0866	0.5239
CEU	LIMS3	110408688	9.2402	9.5515	-0.0978
CEU	LIMS3	110413688	7.9817	8.187	-0.0744
CEU	LIMS3	110418688	10.8163	8.5281	0.7984
CHB	LIMS3	109403688	1.3699	6.946	-2.3406
CHB	LIMS3	109408688	1.894	7.7931	-2.2295
CHB	LIMS3	109413688	2.1224	7.9625	-2.1642
CHB	LIMS3	109418688	1.6242	6.7766	-2.2117
CHB	LIMS3	109423688	1.6855	5.5907	-1.9925
CHB	LIMS3	109428688	1.7832	5.9295	-2.0073

CHB	LIMS3	109433688	1.9417	6.946	-2.1006
CHB	LIMS3	109438688	1.9924	7.7931	-2.1924
CHB	LIMS3	109443688	2.6887	10.6731	-2.2551
CHB	LIMS3	109448688	3.1814	11.1814	-2.1631
CHB	LIMS3	109453688	2.3688	9.8261	-2.2752
CHB	LIMS3	109458688	2.4098	9.8261	-2.2627
CHB	LIMS3	109463688	1.8605	6.946	-2.1347
CHB	LIMS3	109468688	1.3628	5.9295	-2.2108
CHB	LIMS3	109473688	2.2686	8.3013	-2.1515
CHB	LIMS3	109478688	2.7534	8.979	-2.0652
CHB	LIMS3	109483688	1.8676	8.1319	-2.2769
CHB	LIMS3	109488688	2.4194	9.9955	-2.275
CHB	LIMS3	109493688	3.4942	12.3673	-2.1821
CHB	LIMS3	109498688	3.0862	11.3508	-2.2033
CHB	LIMS3	109503688	3.016	10.6731	-2.1627
CHB	LIMS3	109508688	2.8883	9.8261	-2.1167
CHB	LIMS3	109513688	3.4194	11.012	-2.0826
CHB	LIMS3	109518688	2.9132	11.012	-2.2214
CHB	LIMS3	109523688	2.2522	10.5037	-2.3657
CHB	LIMS3	109528688	2.2066	10.1649	-2.3526
CHB	LIMS3	109533688	1.6938	7.9625	-2.323
CHB	LIMS3	109538688	1.7935	7.7931	-2.2675
CHB	LIMS3	109543688	2.0029	8.4708	-2.2642
CHB	LIMS3	109548688	1.9355	9.3178	-2.3663
CHB	LIMS3	109553688	2.4273	14.2309	-2.5415
CHB	LIMS3	109558688	2.1783	13.045	-2.541
CHB	LIMS3	109563688	1.649	8.4708	-2.3881
CHB	LIMS3	109568688	1.7592	9.3178	-2.4228
CHB	LIMS3	109573688	1.7504	8.979	-2.398
CHB	LIMS3	109578688	2.2743	9.8261	-2.3041
CHB	LIMS3	109583688	2.6126	10.1649	-2.2325
CHB	LIMS3	109588688	2.8875	10.5037	-2.1835
CHB	LIMS3	109593688	5.9191	13.892	-1.7564
CHB	LIMS3	109598688	6.3982	13.892	-1.6509
CHB	LIMS3	109603688	4.2012	11.8591	-1.9592
CHB	LIMS3	109608688	3.3611	10.5037	-2.0478
CHB	LIMS3	109613688	3.023	9.6567	-2.057
CHB	LIMS3	109618688	3.0876	8.6402	-1.9086
CHB	LIMS3	109623688	2.9534	8.8096	-1.9772
CHB	LIMS3	109628688	4.075	11.8591	-1.9915
CHB	LIMS3	109633688	7.4319	11.6896	-1.1042
CHB	LIMS3	109638688	7.095	8.8096	-0.5789
CHB	LIMS3	109643688	4.1041	7.6237	-1.3573
CHB	LIMS3	109648688	4.3053	9.9955	-1.7087
CHB	LIMS3	109653688	6.2732	15.5862	-1.8388
CHB	LIMS3	109658688	8.5622	15.7556	-1.4057
CHB	LIMS3	109663688	6.2086	10.1649	-1.1695
CHB	LIMS3	109668688	4.6284	7.7931	-1.1961
CHB	LIMS3	109673688	9.0244	9.3178	-0.094

CHB	LIMS3	109678688	10.819	10.5037	0.0904
CHB	LIMS3	109683688	8.3912	9.3178	-0.297
CHB	LIMS3	109688688	8.0811	8.6402	-0.1922
CHB	LIMS3	109693688	10.6052	10.6731	-0.0192
CHB	LIMS3	109698688	10.3225	11.1814	-0.2322
CHB	LIMS3	109703688	7.7419	8.8096	-0.3605
CHB	LIMS3	109708688	7.3606	8.6402	-0.4398
CHB	LIMS3	109713688	5.4184	6.6072	-0.5221
CHB	LIMS3	109718688	4.3324	5.2519	-0.4959
CHB	LIMS3	109723688	3.2046	6.0989	-1.3662
CHB	LIMS3	109728688	2.5877	5.4213	-1.4859
CHB	LIMS3	109733688	3.7061	7.1154	-1.4001
CHB	LIMS3	109738688	3.5096	7.7931	-1.6189
CHB	LIMS3	109743688	5.6737	7.1154	-0.5921
CHB	LIMS3	109748688	6.7843	6.7766	0.0033
CHB	LIMS3	109753688	6.5427	6.7766	-0.1004
CHB	LIMS3	109758688	7.0689	9.3178	-0.7209
CHB	LIMS3	109763688	8.2644	8.979	-0.2371
CHB	LIMS3	109768688	9.474	7.6237	0.7135
CHB	LIMS3	109773688	9.667	8.1319	0.558
CHB	LIMS3	109778688	10.0737	11.012	-0.2574
CHB	LIMS3	109783688	12.6945	13.5532	-0.1937
CHB	LIMS3	109788688	10.6082	10.3343	0.0797
CHB	LIMS3	109793688	7.0284	8.1319	-0.4011
CHB	LIMS3	109798688	7.5486	8.8096	-0.4257
CHB	LIMS3	109803688	11.7122	11.8591	-0.0376
CHB	LIMS3	109808688	12.3653	12.3673	-0.0005
CHB	LIMS3	109813688	9.143	10.6731	-0.4322
CHB	LIMS3	109818688	11.4853	11.6896	-0.053
CHB	LIMS3	109823688	13.0703	12.1979	0.2174
CHB	LIMS3	109828688	10.5094	10.3343	0.051
CHB	LIMS3	109833688	8.9503	8.979	-0.0095
CHB	LIMS3	109838688	11.0063	9.1484	0.6057
CHB	LIMS3	109843688	10.4279	10.5037	-0.0217
CHB	LIMS3	109848688	6.5637	10.6731	-1.1607
CHB	LIMS3	109853688	6.6975	11.5202	-1.268
CHB	LIMS3	109858688	7.4156	12.7061	-1.2683
CHB	LIMS3	109863688	6.521	12.0285	-1.3904
CHB	LIMS3	109868688	6.732	12.0285	-1.3371
CHB	LIMS3	109873688	10.3903	10.8426	-0.1259
CHB	LIMS3	109878688	12.1687	10.1649	0.5923
CHB	LIMS3	109883688	8.6349	8.4708	0.0574
CHB	LIMS3	109888688	7.2753	8.8096	-0.518
CHB	LIMS3	109893688	8.6288	8.8096	-0.061
CHB	LIMS3	109898688	7.4584	6.2684	0.5481
CHB	LIMS3	109903688	4.7929	5.5907	-0.4071
CHB	LIMS3	109908688	5.1163	9.8261	-1.437
CHB	LIMS3	109913688	5.4297	12.3673	-1.7061
CHB	LIMS3	109918688	14.6509	14.7391	-0.0184

CHB	LIMS3	109923688	22.6194	15.925	1.2949
CHB	LIMS3	109928688	41.1528	21.5157	2.8449
CHB	LIMS3	109933688	41.2879	23.8875	2.2783
CHB	LIMS3	109938688	14.8202	14.0615	0.1652
CHB	LIMS3	109943688	6.9223	9.9955	-0.9228
CHB	LIMS3	109948688	8.7124	9.8261	-0.3398
CHB	LIMS3	109953688	8.8643	9.8261	-0.2934
CHB	LIMS3	109958688	5.5644	9.8261	-1.3002
CHB	LIMS3	109963688	4.8704	8.8096	-1.33
CHB	LIMS3	109968688	3.6031	9.3178	-1.8318
CHB	LIMS3	109973688	3.1578	11.1814	-2.1695
CHB	LIMS3	109978688	3.3745	11.5202	-2.1416
CHB	LIMS3	109983688	3.2935	12.5367	-2.2441
CHB	LIMS3	109988688	5.6014	16.6027	-2.045
CHB	LIMS3	109993688	6.8523	16.2638	-1.7843
CHB	LIMS3	109998688	9.0034	13.892	-1.077
CHB	LIMS3	110003688	16.8943	15.0779	0.3702
CHB	LIMS3	110008688	14.6196	13.892	0.1603
CHB	LIMS3	110013688	11.3138	13.3838	-0.4724
CHB	LIMS3	110018688	15.4562	15.2474	0.0421
CHB	LIMS3	110023688	17.5582	17.2803	0.0497
CHB	LIMS3	110028688	14.5304	14.0615	0.1021
CHB	LIMS3	110033688	7.8188	10.3343	-0.7322
CHB	LIMS3	110038688	11.0432	10.8426	0.0558
CHB	LIMS3	110043688	13.4043	10.8426	0.7129
CHB	LIMS3	110048688	8.2172	9.9955	-0.534
CHB	LIMS3	110053688	6.7432	8.4708	-0.6048
CHB	LIMS3	110058688	5.7436	7.2848	-0.6195
CHB	LIMS3	110063688	3.8028	5.7601	-0.9724
CHB	LIMS3	110068688	3.8125	5.0825	-0.7051
CHB	LIMS3	110073688	3.5315	4.913	-0.7902
CHB	LIMS3	110078688	5.0921	5.7601	-0.3319
CHB	LIMS3	110083688	5.1656	6.0989	-0.4406
CHB	LIMS3	110088688	3.9415	6.7766	-1.217
CHB	LIMS3	110093688	4.5946	7.4543	-1.1257
CHB	LIMS3	110098688	4.8905	7.7931	-1.097
CHB	LIMS3	110103688	7.246	10.8426	-1.001
CHB	LIMS3	110108688	6.4951	7.6237	-0.4352
CHB	LIMS3	110113688	1.6526	1.1859	0.8331
CHB	LIMS3	110118688	0	0	0
CHB	LIMS3	110123688	0	0	0
CHB	LIMS3	110128688	0	0	0
CHB	LIMS3	110133688	0	0	0
CHB	LIMS3	110138688	0	0	0
CHB	LIMS3	110143688	0	0	0
CHB	LIMS3	110148688	0	0	0
CHB	LIMS3	110153688	0	0	0
CHB	LIMS3	110158688	0	0	0
CHB	LIMS3	110163688	0	0	0

CHB	LIMS3	110168688	0	0	0
CHB	LIMS3	110173688	0	0	0
CHB	LIMS3	110178688	0	0	0
CHB	LIMS3	110183688	0	0	0
CHB	LIMS3	110188688	0	0	0
CHB	LIMS3	110193688	0	0	0
CHB	LIMS3	110198688	0	0	0
CHB	LIMS3	110203688	0	0	0
CHB	LIMS3	110208688	0	0	0
CHB	LIMS3	110213688	0	0	0
CHB	LIMS3	110218688	0	0	0
CHB	LIMS3	110223688	0	0	0
CHB	LIMS3	110228688	0	0	0
CHB	LIMS3	110233688	0	0	0
CHB	LIMS3	110238688	0	0	0
CHB	LIMS3	110243688	0	0	0
CHB	LIMS3	110248688	10.916	6.0989	2.2739
CHB	LIMS3	110253688	29.3543	18.2968	1.8726
CHB	LIMS3	110258688	21.3265	17.4498	0.6871
CHB	LIMS3	110263688	6.4418	12.0285	-1.4104
CHB	LIMS3	110268688	10.3099	11.6896	-0.3578
CHB	LIMS3	110273688	15.1458	16.9415	-0.3274
CHB	LIMS3	110278688	15.5967	20.1604	-0.704
CHB	LIMS3	110283688	10.4714	11.5202	-0.2757
CHB	LIMS3	110288688	5.6313	6.6072	-0.4286
CHB	LIMS3	110293688	6.1498	7.6237	-0.5684
CHB	LIMS3	110298688	6.4232	8.979	-0.8478
CHB	LIMS3	110303688	6.9467	7.6237	-0.2611
CHB	LIMS3	110308688	6.3914	6.4378	-0.0209
CHB	LIMS3	110313688	5.264	8.3013	-1.0832
CHB	LIMS3	110318688	7.4123	8.979	-0.5197
CHB	LIMS3	110323688	9.9061	8.979	0.3075
CHB	LIMS3	110328688	10.8964	8.6402	0.7755
CHB	LIMS3	110333688	9.4445	9.9955	-0.1655
CHB	LIMS3	110338688	6.9425	9.9955	-0.9168
CHB	LIMS3	110343688	7.3234	9.4872	-0.6821
CHB	LIMS3	110348688	8.3036	9.6567	-0.4196
CHB	LIMS3	110353688	7.4243	7.7931	-0.1394
CHB	LIMS3	110358688	7.3941	7.2848	0.0439
CHB	LIMS3	110363688	8.7712	7.9625	0.2997
CHB	LIMS3	110368688	8.2972	9.4872	-0.3751
CHB	LIMS3	110373688	6.5667	9.8261	-0.9944
CHB	LIMS3	110378688	7.8749	9.6567	-0.5525
CHB	LIMS3	110383688	11.0786	10.8426	0.0657
CHB	LIMS3	110388688	14.6339	12.1979	0.6069
CHB	LIMS3	110393688	17.0603	13.5532	0.7909
CHB	LIMS3	110398688	18.3044	13.3838	1.123
CHB	LIMS3	110403688	16.0341	11.6896	1.1267
CHB	LIMS3	110408688	8.6903	11.5202	-0.744

CHB	LIMS3	110413688	7.3352	10.5037	-0.9084
CHB	LIMS3	110418688	10.7428	9.9955	0.2244
YRI	LIMS3	109403688	10.2462	11.4282	-0.3121
YRI	LIMS3	109408688	9.5071	12.6047	-0.7457
YRI	LIMS3	109413688	10.4508	12.9408	-0.5847
YRI	LIMS3	109418688	8.051	9.4115	-0.4306
YRI	LIMS3	109423688	7.1032	9.5795	-0.771
YRI	LIMS3	109428688	8.9073	11.7644	-0.734
YRI	LIMS3	109433688	10.032	13.1089	-0.7137
YRI	LIMS3	109438688	8.9362	11.5963	-0.6927
YRI	LIMS3	109443688	12.1735	13.9492	-0.3883
YRI	LIMS3	109448688	12.8588	15.6298	-0.5438
YRI	LIMS3	109453688	9.2939	12.1005	-0.7022
YRI	LIMS3	109458688	8.9131	10.924	-0.5538
YRI	LIMS3	109463688	8.0749	10.4199	-0.6751
YRI	LIMS3	109468688	8.1583	11.9324	-0.9567
YRI	LIMS3	109473688	9.9983	13.445	-0.7806
YRI	LIMS3	109478688	10.8117	13.445	-0.5964
YRI	LIMS3	109483688	7.2058	11.9324	-1.1982
YRI	LIMS3	109488688	10.9506	12.7727	-0.4332
YRI	LIMS3	109493688	16.7036	16.302	0.0757
YRI	LIMS3	109498688	16.5098	16.134	0.0716
YRI	LIMS3	109503688	16.0582	14.2853	0.3791
YRI	LIMS3	109508688	13.7439	13.2769	0.107
YRI	LIMS3	109513688	16.3573	15.2937	0.2131
YRI	LIMS3	109518688	16.5395	16.134	0.0772
YRI	LIMS3	109523688	11.6083	14.7895	-0.6581
YRI	LIMS3	109528688	8.3429	11.9324	-0.9099
YRI	LIMS3	109533688	9.6131	12.9408	-0.7814
YRI	LIMS3	109538688	11.9757	15.7978	-0.7425
YRI	LIMS3	109543688	10.0747	16.134	-1.1537
YRI	LIMS3	109548688	7.6984	15.9659	-1.5899
YRI	LIMS3	109553688	13.0488	18.6549	-0.9287
YRI	LIMS3	109558688	13.5402	16.134	-0.4939
YRI	LIMS3	109563688	7.6423	10.4199	-0.7996
YRI	LIMS3	109568688	7.2009	11.0921	-1.0565
YRI	LIMS3	109573688	10.2687	13.613	-0.7485
YRI	LIMS3	109578688	12.716	15.4617	-0.5445
YRI	LIMS3	109583688	11.0878	13.445	-0.5338
YRI	LIMS3	109588688	10.6443	13.613	-0.6645
YRI	LIMS3	109593688	15.5453	15.7978	-0.0491
YRI	LIMS3	109598688	16.126	15.4617	0.1317
YRI	LIMS3	109603688	11.2913	13.613	-0.5196
YRI	LIMS3	109608688	9.2621	12.4366	-0.7739
YRI	LIMS3	109613688	9.6901	11.9324	-0.5684
YRI	LIMS3	109618688	7.3346	10.2518	-0.8526
YRI	LIMS3	109623688	6.2979	10.0837	-1.1237
YRI	LIMS3	109628688	10.1193	14.4534	-0.9164
YRI	LIMS3	109633688	12.5476	15.9659	-0.6574

YRI	LIMS3	109638688	10.3389	13.9492	-0.7896
YRI	LIMS3	109643688	7.8825	15.1256	-1.4667
YRI	LIMS3	109648688	9.0241	15.9659	-1.335
YRI	LIMS3	109653688	9.2043	20.3355	-1.6971
YRI	LIMS3	109658688	11.9256	21.8481	-1.4116
YRI	LIMS3	109663688	11.3809	18.991	-1.2393
YRI	LIMS3	109668688	10.5806	19.6633	-1.4304
YRI	LIMS3	109673688	15.7119	19.9994	-0.6643
YRI	LIMS3	109678688	16.081	17.9827	-0.3264
YRI	LIMS3	109683688	11.9012	15.6298	-0.7318
YRI	LIMS3	109688688	10.9804	16.134	-0.9812
YRI	LIMS3	109693688	12.8007	17.4785	-0.825
YRI	LIMS3	109698688	11.3576	14.6214	-0.6826
YRI	LIMS3	109703688	10.5993	12.9408	-0.5498
YRI	LIMS3	109708688	10.9996	12.2685	-0.3134
YRI	LIMS3	109713688	7.6664	8.9073	-0.4133
YRI	LIMS3	109718688	5.5552	8.9073	-1.1164
YRI	LIMS3	109723688	4.0778	8.067	-1.4557
YRI	LIMS3	109728688	3.639	7.0586	-1.4097
YRI	LIMS3	109733688	5.9228	10.0837	-1.235
YRI	LIMS3	109738688	6.2703	10.5879	-1.2245
YRI	LIMS3	109743688	6.4891	9.5795	-0.9621
YRI	LIMS3	109748688	6.7286	10.5879	-1.0945
YRI	LIMS3	109753688	7.9027	9.5795	-0.522
YRI	LIMS3	109758688	10.0102	10.756	-0.2084
YRI	LIMS3	109763688	10.7966	12.4366	-0.3998
YRI	LIMS3	109768688	10.9688	13.7811	-0.6222
YRI	LIMS3	109773688	12.4671	14.7895	-0.4804
YRI	LIMS3	109778688	15.5252	15.6298	-0.0205
YRI	LIMS3	109783688	16.256	18.1507	-0.3223
YRI	LIMS3	109788688	11.8453	13.9492	-0.4601
YRI	LIMS3	109793688	9.1261	10.0837	-0.2842
YRI	LIMS3	109798688	10.0207	11.9324	-0.4846
YRI	LIMS3	109803688	14.9352	14.9575	-0.0046
YRI	LIMS3	109808688	15.2534	15.9659	-0.137
YRI	LIMS3	109813688	11.94	16.302	-0.8223
YRI	LIMS3	109818688	15.6142	17.6465	-0.3551
YRI	LIMS3	109823688	18.1763	17.1423	0.1858
YRI	LIMS3	109828688	15.6856	15.6298	0.011
YRI	LIMS3	109833688	14.2624	13.9492	0.0685
YRI	LIMS3	109838688	15.4763	14.6214	0.1788
YRI	LIMS3	109843688	12.6196	16.4701	-0.7188
YRI	LIMS3	109848688	8.7929	16.8062	-1.4673
YRI	LIMS3	109853688	8.7272	16.9743	-1.4958
YRI	LIMS3	109858688	10.9545	16.8062	-1.0715
YRI	LIMS3	109863688	11.6075	16.9743	-0.9734
YRI	LIMS3	109868688	10.2845	15.6298	-1.0491
YRI	LIMS3	109873688	12.2656	12.9408	-0.1585
YRI	LIMS3	109878688	12.828	13.1089	-0.0652

YRI	LIMS3	109883688	10.3764	13.7811	-0.7532
YRI	LIMS3	109888688	10.6591	15.4617	-0.9523
YRI	LIMS3	109893688	11.4189	14.4534	-0.6416
YRI	LIMS3	109898688	9.7945	11.7644	-0.5061
YRI	LIMS3	109903688	7.8133	12.7727	-1.179
YRI	LIMS3	109908688	9.5131	15.2937	-1.1583
YRI	LIMS3	109913688	9.6739	15.2937	-1.126
YRI	LIMS3	109918688	20.5375	16.4701	0.7593
YRI	LIMS3	109923688	30.9061	17.9827	2.2178
YRI	LIMS3	109928688	49.5907	25.8816	2.8624
YRI	LIMS3	109933688	46.562	26.2177	2.4256
YRI	LIMS3	109938688	16.3681	17.3104	-0.1677
YRI	LIMS3	109943688	8.1026	13.1089	-1.1613
YRI	LIMS3	109948688	8.6455	12.4366	-0.9243
YRI	LIMS3	109953688	8.2242	13.7811	-1.2294
YRI	LIMS3	109958688	10.2441	13.445	-0.7249
YRI	LIMS3	109963688	13.3643	14.1172	-0.1628
YRI	LIMS3	109968688	10.8729	13.9492	-0.6728
YRI	LIMS3	109973688	10.4928	15.2937	-0.962
YRI	LIMS3	109978688	11.0256	17.1423	-1.099
YRI	LIMS3	109983688	13.3021	17.3104	-0.7135
YRI	LIMS3	109988688	15.1899	19.1591	-0.6409
YRI	LIMS3	109993688	13.3823	18.3188	-0.8322
YRI	LIMS3	109998688	19.2825	18.3188	0.1625
YRI	LIMS3	110003688	25.6778	20.6717	0.7513
YRI	LIMS3	110008688	17.4118	18.991	-0.2572
YRI	LIMS3	110013688	17.1399	19.4952	-0.374
YRI	LIMS3	110018688	21.7571	20.3355	0.2167
YRI	LIMS3	110023688	21.228	20.5036	0.1096
YRI	LIMS3	110028688	17.8583	17.9827	-0.0213
YRI	LIMS3	110033688	10.7088	15.2937	-0.9187
YRI	LIMS3	110038688	14.8808	18.991	-0.6693
YRI	LIMS3	110043688	18.0202	22.6884	-0.6403
YRI	LIMS3	110048688	13.9618	20.5036	-0.9895
YRI	LIMS3	110053688	12.4512	16.134	-0.7012
YRI	LIMS3	110058688	11.7359	14.9575	-0.6593
YRI	LIMS3	110063688	10.2298	14.6214	-0.9184
YRI	LIMS3	110068688	8.8489	13.9492	-1.1154
YRI	LIMS3	110073688	8.3069	13.2769	-1.1391
YRI	LIMS3	110078688	10.9008	15.2937	-0.8802
YRI	LIMS3	110083688	11.3647	15.7978	-0.8612
YRI	LIMS3	110088688	10.1988	14.7895	-0.9497
YRI	LIMS3	110093688	8.6067	13.613	-1.1205
YRI	LIMS3	110098688	6.5389	11.7644	-1.3425
YRI	LIMS3	110103688	9.6631	16.302	-1.2516
YRI	LIMS3	110108688	7.2763	11.4282	-1.0961
YRI	LIMS3	110113688	1.0744	1.1764	-0.1824
YRI	LIMS3	110118688	0	0	0
YRI	LIMS3	110123688	0	0	0

YRI	LIMS3	110128688	0	0	0
YRI	LIMS3	110133688	0	0	0
YRI	LIMS3	110138688	0	0	0
YRI	LIMS3	110143688	0	0	0
YRI	LIMS3	110148688	0	0	0
YRI	LIMS3	110153688	0	0	0
YRI	LIMS3	110158688	0	0	0
YRI	LIMS3	110163688	0	0	0
YRI	LIMS3	110168688	0	0	0
YRI	LIMS3	110173688	0	0	0
YRI	LIMS3	110178688	0	0	0
YRI	LIMS3	110183688	0	0	0
YRI	LIMS3	110188688	0	0	0
YRI	LIMS3	110193688	0	0	0
YRI	LIMS3	110198688	0	0	0
YRI	LIMS3	110203688	0	0	0
YRI	LIMS3	110208688	0	0	0
YRI	LIMS3	110213688	0	0	0
YRI	LIMS3	110218688	0	0	0
YRI	LIMS3	110223688	0	0	0
YRI	LIMS3	110228688	0	0	0
YRI	LIMS3	110233688	0	0	0
YRI	LIMS3	110238688	0	0	0
YRI	LIMS3	110243688	0	0	0
YRI	LIMS3	110248688	8.8105	7.2267	0.6391
YRI	LIMS3	110253688	24.6908	21.68	0.4315
YRI	LIMS3	110258688	20.1877	22.8565	-0.3635
YRI	LIMS3	110263688	10.2133	17.8146	-1.3163
YRI	LIMS3	110268688	12.2439	15.9659	-0.7158
YRI	LIMS3	110273688	23.476	21.1758	0.3373
YRI	LIMS3	110278688	33.2145	27.7303	0.6192
YRI	LIMS3	110283688	27.4618	23.6968	0.4951
YRI	LIMS3	110288688	16.7417	15.1256	0.3272
YRI	LIMS3	110293688	9.2002	10.924	-0.4748
YRI	LIMS3	110298688	7.2365	11.7644	-1.1632
YRI	LIMS3	110303688	7.0103	10.924	-1.0779
YRI	LIMS3	110308688	6.5215	11.4282	-1.2954
YRI	LIMS3	110313688	7.8664	10.4199	-0.7351
YRI	LIMS3	110318688	10.1567	11.9324	-0.4501
YRI	LIMS3	110323688	10.2731	13.7811	-0.7761
YRI	LIMS3	110328688	11.9782	12.2685	-0.0717
YRI	LIMS3	110333688	14.8641	13.445	0.3214
YRI	LIMS3	110338688	12.1812	13.1089	-0.2152
YRI	LIMS3	110343688	10.1115	12.1005	-0.4976
YRI	LIMS3	110348688	10.7275	12.6047	-0.4519
YRI	LIMS3	110353688	8.5496	11.0921	-0.6903
YRI	LIMS3	110358688	8.1983	10.756	-0.7147
YRI	LIMS3	110363688	9.0093	10.924	-0.5273
YRI	LIMS3	110368688	8.5221	11.2602	-0.733

YRI	LIMS3	110373688	9.1018	10.756	-0.4623
YRI	LIMS3	110378688	10.1952	11.4282	-0.3255
YRI	LIMS3	110383688	11.5073	14.9575	-0.7061
YRI	LIMS3	110388688	13.2402	16.8062	-0.653
YRI	LIMS3	110393688	17.1629	18.4868	-0.2212
YRI	LIMS3	110398688	18.0754	19.1591	-0.175
YRI	LIMS3	110403688	13.6937	16.8062	-0.5699
YRI	LIMS3	110408688	10.2859	14.2853	-0.8551
YRI	LIMS3	110413688	10.4084	14.7895	-0.9063
YRI	LIMS3	110418688	11.2707	15.1256	-0.7806
CEU	LIMS3	109891341	8.6722	6.6519	0.8845
CEU	LIMS3	109896341	8.7163	6.9931	0.721
CEU	LIMS3	109901341	5.3414	5.458	-0.061
CEU	LIMS3	109906341	3.7001	5.1169	-0.7842
CEU	LIMS3	109911341	2.8194	5.458	-1.3794
CEU	LIMS3	109916341	11.3819	9.0398	0.7744
CEU	LIMS3	109921341	20.3149	12.2805	1.9947
CEU	LIMS3	109926341	26.496	14.4978	2.5455
CEU	LIMS3	109931341	40.5459	22.0026	2.6365
CEU	LIMS3	109936341	31.556	17.5679	2.4702
CEU	LIMS3	109941341	9.1029	7.5047	0.6271
CEU	LIMS3	109946341	9.7848	8.5281	0.4385
CEU	LIMS3	109951341	11.5302	9.0398	0.8234
CEU	LIMS3	109956341	8.2033	8.6987	-0.1697
CEU	LIMS3	109961341	10.0802	9.2104	0.2826
CEU	LIMS3	109966341	8.3447	9.8926	-0.4707
CEU	LIMS3	109971341	5.9052	9.5515	-1.1455
CEU	LIMS3	109976341	5.6928	8.8692	-1.0689
CEU	LIMS3	109981341	6.3717	11.0866	-1.2888
CEU	LIMS3	109986341	9.3643	14.1567	-1.04
CEU	LIMS3	109991341	12.0674	16.0329	-0.7643
CEU	LIMS3	109996341	11.8015	14.6684	-0.6015
CEU	LIMS3	110001341	18.3119	12.6216	1.3767
CEU	LIMS3	110006341	17.5356	10.2337	2.1512
CEU	LIMS3	110011341	8.0289	7.1636	0.3542
CEU	LIMS3	110016341	13.4373	9.7221	1.1481
CEU	LIMS3	110021341	16.7261	13.3039	0.7877
CEU	LIMS3	110026341	15.5327	13.4744	0.4681
CEU	LIMS3	110031341	11.5056	10.5749	0.2659
CEU	LIMS3	110036341	7.6614	9.3809	-0.5493
CEU	LIMS3	110041341	11.5877	10.916	0.1863
CEU	LIMS3	110046341	11.2632	8.8692	0.8056
CEU	LIMS3	110051341	6.7605	6.8225	-0.0265
CEU	LIMS3	110056341	5.8134	7.6753	-0.7157
CEU	LIMS3	110061341	4.6889	7.3342	-1.0599
CEU	LIMS3	110066341	3.672	6.3108	-1.2115
CEU	LIMS3	110071341	4.376	6.4814	-0.9437
CEU	LIMS3	110076341	5.8526	6.4814	-0.2818
CEU	LIMS3	110081341	6.2187	8.0164	-0.664

CEU	LIMS3	110086341	4.8237	8.0164	-1.1792
CEU	LIMS3	110091341	5.4134	6.9931	-0.661
CEU	LIMS3	110096341	5.2213	6.6519	-0.6263
CEU	LIMS3	110101341	4.5228	6.4814	-0.8779
CEU	LIMS3	110106341	6.0796	7.1636	-0.4437
CEU	LIMS3	110111341	3.3894	3.5818	-0.145
CEU	LIMS3	110116341	0	0	0
CEU	LIMS3	110121341	0	0	0
CEU	LIMS3	110126341	0	0	0
CEU	LIMS3	110131341	0	0	0
CEU	LIMS3	110136341	0	0	0
CEU	LIMS3	110141341	0	0	0
CEU	LIMS3	110146341	0	0	0
CEU	LIMS3	110151341	0	0	0
CEU	LIMS3	110156341	0	0	0
CEU	LIMS3	110161341	0	0	0
CEU	LIMS3	110166341	0	0	0
CEU	LIMS3	110171341	0	0	0
CEU	LIMS3	110176341	0	0	0
CEU	LIMS3	110181341	0	0	0
CEU	LIMS3	110186341	0	0	0
CEU	LIMS3	110191341	0	0	0
CEU	LIMS3	110196341	0	0	0
CEU	LIMS3	110201341	0	0	0
CEU	LIMS3	110206341	0	0	0
CEU	LIMS3	110211341	0	0	0
CEU	LIMS3	110216341	0	0	0
CEU	LIMS3	110221341	0	0	0
CEU	LIMS3	110226341	0	0	0
CEU	LIMS3	110231341	0	0	0
CEU	LIMS3	110236341	0	0	0
CEU	LIMS3	110241341	0	0	0
CEU	LIMS3	110246341	0	0	0
CEU	LIMS3	110251341	17.7613	10.0632	2.3038
CEU	LIMS3	110256341	23.8404	15.5212	1.6539
CEU	LIMS3	110261341	11.0328	10.2337	0.2354
CEU	LIMS3	110266341	8.4867	8.8692	-0.1287
CEU	LIMS3	110271341	8.7965	9.7221	-0.286
CEU	LIMS3	110276341	9.0043	10.0632	-0.3169
CEU	LIMS3	110281341	7.4649	9.0398	-0.5207
CEU	LIMS3	110286341	6.4892	8.0164	-0.5641
CEU	LIMS3	110291341	6.43	6.3108	0.0547
CEU	LIMS3	110296341	6.0427	7.6753	-0.6275
CEU	LIMS3	110301341	5.791	9.0398	-1.0741
CEU	LIMS3	110306341	7.3181	8.3576	-0.3695
CEU	LIMS3	110311341	6.5275	5.9697	0.2692
CEU	LIMS3	110316341	6.4444	5.6286	0.415
CEU	LIMS3	110321341	7.2134	7.3342	-0.0484
CEU	LIMS3	110326341	8.267	8.3576	-0.0322

CEU	LIMS3	110331341	9.033	10.4043	-0.3978
CEU	LIMS3	110336341	7.1416	10.0632	-0.8743
CEU	LIMS3	110341341	6.3412	8.5281	-0.763
CEU	LIMS3	110346341	7.2773	8.187	-0.3296
CEU	LIMS3	110351341	8.4752	7.3342	0.4572
CEU	LIMS3	110356341	7.9938	7.5047	0.1919
CEU	LIMS3	110361341	6.5732	7.1636	-0.2417
CEU	LIMS3	110366341	8.1681	7.6753	0.1894
CEU	LIMS3	110371341	10.174	8.187	0.7198
CEU	LIMS3	110376341	7.5832	6.6519	0.4077
CEU	LIMS3	110381341	8.255	8.3576	-0.0365
CEU	LIMS3	110386341	12.6007	11.0866	0.4139
CEU	LIMS3	110391341	14.7223	12.2805	0.6062
CEU	LIMS3	110396341	17.3208	12.4511	1.1934
CEU	LIMS3	110401341	14.5504	11.9394	0.6657
CEU	LIMS3	110406341	9.973	10.5749	-0.172
CEU	LIMS3	110411341	9.61	9.0398	0.1885
CEU	LIMS3	110416341	9.6986	8.187	0.5476
CEU	LIMS3	110421341	7.8108	7.6753	0.0521
CEU	LIMS3	110426341	4.8393	5.9697	-0.5455
CEU	LIMS3	110431341	3.1501	5.2874	-1.1492
CEU	LIMS3	110436341	7.5998	10.0632	-0.7372
CEU	LIMS3	110441341	10.2092	11.0866	-0.2398
CEU	LIMS3	110446341	10.8547	10.7454	0.0308
CEU	LIMS3	110451341	10.3575	10.4043	-0.0136
CEU	LIMS3	110456341	6.8965	8.5281	-0.5693
CEU	LIMS3	110461341	7.0173	9.5515	-0.7961
CEU	LIMS3	110466341	9.1051	10.0632	-0.2867
CEU	LIMS3	110471341	8.5209	7.6753	0.325
CEU	LIMS3	110476341	5.3799	5.458	-0.0408
CEU	LIMS3	110481341	4.411	5.9697	-0.7523
CEU	LIMS3	110486341	4.4688	7.3342	-1.1481
CEU	LIMS3	110491341	5.2486	8.187	-1.0645
CEU	LIMS3	110496341	5.5775	5.9697	-0.1893
CEU	LIMS3	110501341	3.2273	3.4112	-0.1444
CEU	LIMS3	110506341	1.3036	1.7056	-0.5506
CEU	LIMS3	110511341	1.001	0.6822	0.8325
CEU	LIMS3	110516341	0.5964	0.8528	-0.5774
CEU	LIMS3	110521341	1.4758	2.2173	-0.8284
CEU	LIMS3	110526341	0.9398	1.8762	-1.1922
CEU	LIMS3	110531341	0.1678	1.0234	-1.7001
CEU	LIMS3	110536341	0.1375	0.6822	-1.4224
CEU	LIMS3	110541341	0	0	0
CEU	LIMS3	110546341	0	0	0
CEU	LIMS3	110551341	0	0	0
CEU	LIMS3	110556341	0	0	0
CEU	LIMS3	110561341	0	0	0
CEU	LIMS3	110566341	0	0	0
CEU	LIMS3	110571341	0	0	0

CEU	LIMS3	110576341	0	0	0
CEU	LIMS3	110581341	0	0	0
CEU	LIMS3	110586341	0	0	0
CEU	LIMS3	110591341	0	0	0
CEU	LIMS3	110596341	0	0	0
CEU	LIMS3	110601341	0	0	0
CEU	LIMS3	110606341	0	0	0
CEU	LIMS3	110611341	0	0	0
CEU	LIMS3	110616341	0	0	0
CEU	LIMS3	110621341	0	0	0
CEU	LIMS3	110626341	0	0	0
CEU	LIMS3	110631341	0	0	0
CEU	LIMS3	110636341	0	0	0
CEU	LIMS3	110641341	0	0	0
CEU	LIMS3	110646341	0	0	0
CEU	LIMS3	110651341	0	0	0
CEU	LIMS3	110656341	0	0	0
CEU	LIMS3	110661341	0	0	0
CEU	LIMS3	110666341	0	0	0
CEU	LIMS3	110671341	0	0	0
CEU	LIMS3	110676341	0	0	0
CEU	LIMS3	110681341	0	0	0
CEU	LIMS3	110686341	0	0	0
CEU	LIMS3	110691341	0	0	0
CEU	LIMS3	110696341	0	0	0
CEU	LIMS3	110701341	0.0785	0.3411	-1.0527
CEU	LIMS3	110706341	0.0785	0.3411	-1.0527
CEU	LIMS3	110711341	0	0	0
CEU	LIMS3	110716341	0	0	0
CEU	LIMS3	110721341	0.02	0.1706	-0.8946
CEU	LIMS3	110726341	0.02	0.1706	-0.8946
CEU	LIMS3	110731341	0.1187	0.5117	-1.2326
CEU	LIMS3	110736341	0.6578	1.1939	-0.9553
CEU	LIMS3	110741341	1.6658	1.8762	-0.2679
CEU	LIMS3	110746341	1.167	1.5351	-0.5458
CEU	LIMS3	110751341	0.544	0.5117	0.1013
CEU	LIMS3	110756341	0.5541	0.8528	-0.6727
CEU	LIMS3	110761341	0.9423	1.8762	-1.189
CEU	LIMS3	110766341	1.2624	2.5584	-1.291
CEU	LIMS3	110771341	0.5075	2.2173	-1.9102
CEU	LIMS3	110776341	0.3513	1.0234	-1.3355
CEU	LIMS3	110781341	0.7234	1.3645	-1.0372
CEU	LIMS3	110786341	0.5867	1.3645	-1.2583
CEU	LIMS3	110791341	0.8487	0.8528	-0.0092
CEU	LIMS3	110796341	1.2319	1.3645	-0.2145
CEU	LIMS3	110801341	1.1354	1.8762	-0.9432
CEU	LIMS3	110806341	2.0792	2.729	-0.6142
CEU	LIMS3	110811341	1.4636	1.7056	-0.3314
CEU	LIMS3	110816341	0.1095	0.5117	-1.2614

CEU	LIMS3	110821341	1.0091	2.0467	-1.2346
CEU	LIMS3	110826341	0.979	1.8762	-1.1423
CEU	LIMS3	110831341	0.1587	0.8528	-1.5631
CEU	LIMS3	110836341	0.1385	0.6822	-1.4198
CEU	LIMS3	110841341	0	0	0
CEU	LIMS3	110846341	0.2132	0.8528	-1.4404
CEU	LIMS3	110851341	1.5906	4.2641	-1.7355
CEU	LIMS3	110856341	2.8351	8.3576	-1.9631
CEU	LIMS3	110861341	3.7984	8.0164	-1.5579
CEU	LIMS3	110866341	3.5362	4.7757	-0.729
CEU	LIMS3	110871341	3.7786	4.6052	-0.5019
CEU	LIMS3	110876341	5.3154	5.2874	0.0151
CEU	LIMS3	110881341	5.5553	5.7991	-0.1208
CEU	LIMS3	110886341	6.1878	6.1402	0.0224
CEU	LIMS3	110891341	7.1666	5.7991	0.6773
CEU	LIMS3	110896341	7.6162	5.9697	0.7946
CEU	LIMS3	110901341	6.5182	4.9463	0.8965
CEU	LIMS3	110906341	6.4688	5.458	0.5284
CEU	LIMS3	110911341	7.0981	6.1402	0.4508
CEU	LIMS3	110916341	5.2643	4.0935	0.7873
CEU	LIMS3	110921341	3.4558	3.0701	0.3308
CEU	LIMS3	110926341	3.3709	3.7524	-0.2764
CEU	LIMS3	110931341	4.5321	4.4346	0.0612
CEU	LIMS3	110936341	4.7906	4.9463	-0.0888
CEU	LIMS3	110941341	7.2653	5.9697	0.6253
CEU	LIMS3	110946341	8.946	8.6987	0.0847
CEU	LIMS3	110951341	6.7275	8.3576	-0.5795
CEU	LIMS3	110956341	5.7984	5.9697	-0.0827
CEU	LIMS3	110961341	4.5768	5.6286	-0.535
CEU	LIMS3	110966341	3.4475	6.1402	-1.2671
CHB	LIMS3	109891341	8.4129	8.979	-0.1878
CHB	LIMS3	109896341	7.3482	5.9295	0.6868
CHB	LIMS3	109901341	4.9463	5.5907	-0.3288
CHB	LIMS3	109906341	4.5585	8.3013	-1.3349
CHB	LIMS3	109911341	4.1563	10.1649	-1.7762
CHB	LIMS3	109916341	10.0803	13.892	-0.8397
CHB	LIMS3	109921341	18.0653	15.4168	0.5284
CHB	LIMS3	109926341	27.9514	16.2638	2.2158
CHB	LIMS3	109931341	45.245	24.7346	2.5963
CHB	LIMS3	109936341	32.7701	21.3463	1.6677
CHB	LIMS3	109941341	7.1763	9.9955	-0.8466
CHB	LIMS3	109946341	9.4102	10.1649	-0.2231
CHB	LIMS3	109951341	9.91	10.6731	-0.2155
CHB	LIMS3	109956341	5.5802	9.3178	-1.198
CHB	LIMS3	109961341	5.5612	8.8096	-1.0967
CHB	LIMS3	109966341	4.4847	9.3178	-1.5492
CHB	LIMS3	109971341	2.8231	10.6731	-2.2171
CHB	LIMS3	109976341	3.0051	10.6731	-2.1657
CHB	LIMS3	109981341	3.3274	12.0285	-2.1966

CHB	LIMS3	109986341	3.9962	15.7556	-2.298
CHB	LIMS3	109991341	6.6809	16.9415	-1.8707
CHB	LIMS3	109996341	8.23	15.0779	-1.3955
CHB	LIMS3	110001341	16.1116	14.9085	0.2478
CHB	LIMS3	110006341	15.9364	14.2309	0.3672
CHB	LIMS3	110011341	8.6591	12.3673	-0.9119
CHB	LIMS3	110016341	12.7465	13.7226	-0.2176
CHB	LIMS3	110021341	16.1086	15.7556	0.069
CHB	LIMS3	110026341	17.4544	16.4333	0.1917
CHB	LIMS3	110031341	12.5755	12.3673	0.0512
CHB	LIMS3	110036341	8.1249	9.6567	-0.475
CHB	LIMS3	110041341	11.981	11.012	0.2658
CHB	LIMS3	110046341	11.57	11.1814	0.1051
CHB	LIMS3	110051341	6.9722	8.6402	-0.5734
CHB	LIMS3	110056341	5.4028	7.4543	-0.8075
CHB	LIMS3	110061341	4.1464	6.946	-1.1752
CHB	LIMS3	110066341	3.7737	5.2519	-0.7972
CHB	LIMS3	110071341	4.3328	5.4213	-0.5708
CHB	LIMS3	110076341	5.506	5.4213	0.0444
CHB	LIMS3	110081341	5.7249	6.4378	-0.3205
CHB	LIMS3	110086341	4.3607	7.4543	-1.2177
CHB	LIMS3	110091341	4.9704	6.7766	-0.7753
CHB	LIMS3	110096341	4.5763	6.7766	-0.9445
CHB	LIMS3	110101341	5.5326	9.6567	-1.2788
CHB	LIMS3	110106341	7.5858	9.8261	-0.6835
CHB	LIMS3	110111341	3.6128	3.8965	-0.1984
CHB	LIMS3	110116341	0	0	0
CHB	LIMS3	110121341	0	0	0
CHB	LIMS3	110126341	0	0	0
CHB	LIMS3	110131341	0	0	0
CHB	LIMS3	110136341	0	0	0
CHB	LIMS3	110141341	0	0	0
CHB	LIMS3	110146341	0	0	0
CHB	LIMS3	110151341	0	0	0
CHB	LIMS3	110156341	0	0	0
CHB	LIMS3	110161341	0	0	0
CHB	LIMS3	110166341	0	0	0
CHB	LIMS3	110171341	0	0	0
CHB	LIMS3	110176341	0	0	0
CHB	LIMS3	110181341	0	0	0
CHB	LIMS3	110186341	0	0	0
CHB	LIMS3	110191341	0	0	0
CHB	LIMS3	110196341	0	0	0
CHB	LIMS3	110201341	0	0	0
CHB	LIMS3	110206341	0	0	0
CHB	LIMS3	110211341	0	0	0
CHB	LIMS3	110216341	0	0	0
CHB	LIMS3	110221341	0	0	0
CHB	LIMS3	110226341	0	0	0

CHB	LIMS3	110231341	0	0	0
CHB	LIMS3	110236341	0	0	0
CHB	LIMS3	110241341	0	0	0
CHB	LIMS3	110246341	0	0	0
CHB	LIMS3	110251341	22.9143	12.8755	2.3766
CHB	LIMS3	110256341	30.6874	20.8381	1.4717
CHB	LIMS3	110261341	11.6238	14.2309	-0.5613
CHB	LIMS3	110266341	7.3082	11.6896	-1.1362
CHB	LIMS3	110271341	12.4314	12.7061	-0.0659
CHB	LIMS3	110276341	17.1366	19.8216	-0.421
CHB	LIMS3	110281341	13.3222	17.958	-0.7993
CHB	LIMS3	110286341	7.7855	8.8096	-0.3458
CHB	LIMS3	110291341	5.1875	6.4378	-0.5622
CHB	LIMS3	110296341	6.1336	7.2848	-0.4627
CHB	LIMS3	110301341	7.2787	8.8096	-0.5169
CHB	LIMS3	110306341	7.0202	8.3013	-0.4569
CHB	LIMS3	110311341	5.5922	6.7766	-0.5084
CHB	LIMS3	110316341	6.4569	7.2848	-0.3328
CHB	LIMS3	110321341	8.2105	8.4708	-0.0911
CHB	LIMS3	110326341	9.6297	9.3178	0.1
CHB	LIMS3	110331341	11.0689	10.1649	0.2672
CHB	LIMS3	110336341	8.5974	9.4872	-0.2805
CHB	LIMS3	110341341	6.7013	9.1484	-0.7978
CHB	LIMS3	110346341	7.6857	9.6567	-0.6112
CHB	LIMS3	110351341	8.0527	9.4872	-0.4522
CHB	LIMS3	110356341	7.2271	7.7931	-0.2139
CHB	LIMS3	110361341	7.2294	6.7766	0.1944
CHB	LIMS3	110366341	8.5818	8.979	-0.1318
CHB	LIMS3	110371341	8.5425	11.012	-0.6774
CHB	LIMS3	110376341	7.0714	9.6567	-0.8016
CHB	LIMS3	110381341	8.5679	9.3178	-0.2404
CHB	LIMS3	110386341	12.8528	11.3508	0.4004
CHB	LIMS3	110391341	15.8471	12.7061	0.753
CHB	LIMS3	110396341	18.5736	13.045	1.2928
CHB	LIMS3	110401341	17.6834	12.7061	1.1932
CHB	LIMS3	110406341	11.8835	12.8755	-0.2348
CHB	LIMS3	110411341	8.7864	11.8591	-0.7861
CHB	LIMS3	110416341	9.0541	9.3178	-0.0845
CHB	LIMS3	110421341	9.6988	10.8426	-0.3183
CHB	LIMS3	110426341	7.1862	9.6567	-0.766
CHB	LIMS3	110431341	4.8999	6.6072	-0.7499
CHB	LIMS3	110436341	10.1209	10.6731	-0.156
CHB	LIMS3	110441341	12.1847	12.8755	-0.1635
CHB	LIMS3	110446341	10.0705	12.5367	-0.5988
CHB	LIMS3	110451341	8.6249	11.1814	-0.6913
CHB	LIMS3	110456341	6.5007	9.3178	-0.903
CHB	LIMS3	110461341	6.2932	9.6567	-1.0429
CHB	LIMS3	110466341	7.6286	8.8096	-0.3987
CHB	LIMS3	110471341	7.2915	6.7766	0.221

CHB	LIMS3	110476341	5.202	5.7601	-0.2773
CHB	LIMS3	110481341	4.0109	6.4378	-1.0912
CHB	LIMS3	110486341	3.7691	7.4543	-1.4506
CHB	LIMS3	110491341	4.7687	8.1319	-1.2224
CHB	LIMS3	110496341	5.8181	7.2848	-0.5896
CHB	LIMS3	110501341	3.9685	4.913	-0.5403
CHB	LIMS3	110506341	1.5941	2.5412	-0.9457
CHB	LIMS3	110511341	0.8228	0.8471	-0.0548
CHB	LIMS3	110516341	0.5254	0.5082	0.054
CHB	LIMS3	110521341	1.5869	2.2024	-0.6892
CHB	LIMS3	110526341	1.2631	2.2024	-1.0518
CHB	LIMS3	110531341	0.4575	0.5082	-0.1592
CHB	LIMS3	110536341	0.2844	0.1694	0.6833
CHB	LIMS3	110541341	0	0	0
CHB	LIMS3	110546341	0	0	0
CHB	LIMS3	110551341	0	0	0
CHB	LIMS3	110556341	0	0	0
CHB	LIMS3	110561341	0	0	0
CHB	LIMS3	110566341	0	0	0
CHB	LIMS3	110571341	0	0	0
CHB	LIMS3	110576341	0	0	0
CHB	LIMS3	110581341	0	0	0
CHB	LIMS3	110586341	0	0	0
CHB	LIMS3	110591341	0	0	0
CHB	LIMS3	110596341	0	0	0
CHB	LIMS3	110601341	0	0	0
CHB	LIMS3	110606341	0	0	0
CHB	LIMS3	110611341	0	0	0
CHB	LIMS3	110616341	0	0	0
CHB	LIMS3	110621341	0	0	0
CHB	LIMS3	110626341	0	0	0
CHB	LIMS3	110631341	0	0	0
CHB	LIMS3	110636341	0	0	0
CHB	LIMS3	110641341	0	0	0
CHB	LIMS3	110646341	0	0	0
CHB	LIMS3	110651341	0	0	0
CHB	LIMS3	110656341	0	0	0
CHB	LIMS3	110661341	0	0	0
CHB	LIMS3	110666341	0	0	0
CHB	LIMS3	110671341	0.0097	0.1694	-0.949
CHB	LIMS3	110676341	0.0097	0.1694	-0.949
CHB	LIMS3	110681341	0	0	0
CHB	LIMS3	110686341	0	0	0
CHB	LIMS3	110691341	0	0	0
CHB	LIMS3	110696341	0	0	0
CHB	LIMS3	110701341	0.1314	0.3388	-0.8319
CHB	LIMS3	110706341	0.1314	0.3388	-0.8319
CHB	LIMS3	110711341	0	0	0
CHB	LIMS3	110716341	0	0	0

CHB	LIMS3	110721341	0.0194	0.1694	-0.8913
CHB	LIMS3	110726341	0.0194	0.1694	-0.8913
CHB	LIMS3	110731341	0.0666	0.3388	-1.0919
CHB	LIMS3	110736341	0.6469	1.6942	-1.4373
CHB	LIMS3	110741341	2.0402	3.3883	-1.0617
CHB	LIMS3	110746341	1.5641	2.5412	-0.9757
CHB	LIMS3	110751341	0.6183	0.8471	-0.5159
CHB	LIMS3	110756341	0.601	0.8471	-0.555
CHB	LIMS3	110761341	0.9343	1.8636	-1.1857
CHB	LIMS3	110766341	1.3476	3.8965	-1.7828
CHB	LIMS3	110771341	0.885	3.5577	-2.0199
CHB	LIMS3	110776341	0.7599	1.1859	-0.7604
CHB	LIMS3	110781341	1.4755	1.6942	-0.3001
CHB	LIMS3	110786341	1.1856	1.8636	-0.865
CHB	LIMS3	110791341	1.2781	1.6942	-0.571
CHB	LIMS3	110796341	1.8125	2.5412	-0.7277
CHB	LIMS3	110801341	1.23	2.2024	-1.0888
CHB	LIMS3	110806341	2.1296	3.0495	-0.7911
CHB	LIMS3	110811341	1.5852	2.5412	-0.9546
CHB	LIMS3	110816341	0.0758	0.6777	-1.5735
CHB	LIMS3	110821341	1.205	1.3553	-0.2436
CHB	LIMS3	110826341	1.2717	1.5247	-0.3759
CHB	LIMS3	110831341	0.4247	0.8471	-0.9525
CHB	LIMS3	110836341	0.3581	0.6777	-0.8355
CHB	LIMS3	110841341	0.0097	0.1694	-0.949
CHB	LIMS3	110846341	0.3097	1.0165	-1.4066
CHB	LIMS3	110851341	2.2696	5.5907	-1.6945
CHB	LIMS3	110856341	4.2465	9.6567	-1.6776
CHB	LIMS3	110861341	5.1614	9.1484	-1.2999
CHB	LIMS3	110866341	4.1114	7.4543	-1.3159
CHB	LIMS3	110871341	4.2267	7.1154	-1.1863
CHB	LIMS3	110876341	5.703	7.4543	-0.6894
CHB	LIMS3	110881341	5.4704	6.7766	-0.5607
CHB	LIMS3	110886341	6.4022	5.5907	0.414
CHB	LIMS3	110891341	7.835	6.6072	0.5393
CHB	LIMS3	110896341	8.5464	7.6237	0.3558
CHB	LIMS3	110901341	7.6857	6.7766	0.3902
CHB	LIMS3	110906341	7.7658	7.2848	0.1933
CHB	LIMS3	110911341	7.9722	6.7766	0.5132
CHB	LIMS3	110916341	5.8306	6.0989	-0.1266
CHB	LIMS3	110921341	4.3185	6.7766	-1.0552
CHB	LIMS3	110926341	3.7388	6.6072	-1.2598
CHB	LIMS3	110931341	4.7657	5.9295	-0.5634
CHB	LIMS3	110936341	5.7023	6.6072	-0.3974
CHB	LIMS3	110941341	8.3813	7.7931	0.2223
CHB	LIMS3	110946341	9.724	8.1319	0.5787
CHB	LIMS3	110951341	7.2298	7.2848	-0.0221
CHB	LIMS3	110956341	6.2985	5.9295	0.1786
CHB	LIMS3	110961341	5.2104	4.7436	0.2754

CHB	LIMS3	110966341	3.9543	4.2354	-0.183
YRI	LIMS3	109891341	11.3651	15.7978	-0.8611
YRI	LIMS3	109896341	9.9899	12.2685	-0.5627
YRI	LIMS3	109901341	7.747	12.1005	-1.0892
YRI	LIMS3	109906341	7.0589	12.2685	-1.2865
YRI	LIMS3	109911341	8.1945	13.445	-1.1891
YRI	LIMS3	109916341	16.9667	17.6465	-0.1188
YRI	LIMS3	109921341	26.6802	18.3188	1.4096
YRI	LIMS3	109926341	34.4798	19.1591	2.4739
YRI	LIMS3	109931341	51.2419	27.3941	2.7245
YRI	LIMS3	109936341	37.7446	24.7051	1.647
YRI	LIMS3	109941341	9.2922	13.7811	-0.9931
YRI	LIMS3	109946341	8.0244	12.9408	-1.1544
YRI	LIMS3	109951341	7.9734	13.7811	-1.2848
YRI	LIMS3	109956341	9.3058	13.2769	-0.9101
YRI	LIMS3	109961341	13.6728	14.9575	-0.2629
YRI	LIMS3	109966341	12.3294	14.6214	-0.4793
YRI	LIMS3	109971341	9.9211	14.1172	-0.9073
YRI	LIMS3	109976341	8.4529	13.7811	-1.1788
YRI	LIMS3	109981341	10.756	15.6298	-0.9565
YRI	LIMS3	109986341	16.0604	20.3355	-0.6518
YRI	LIMS3	109991341	15.6603	20.1675	-0.6927
YRI	LIMS3	109996341	16.7431	18.6549	-0.3167
YRI	LIMS3	110001341	25.275	20.1675	0.785
YRI	LIMS3	110006341	20.1069	19.3272	0.1248
YRI	LIMS3	110011341	14.3366	18.1507	-0.6487
YRI	LIMS3	110016341	19.671	19.6633	0.0012
YRI	LIMS3	110021341	19.7018	18.991	0.1158
YRI	LIMS3	110026341	21.1038	19.3272	0.2845
YRI	LIMS3	110031341	15.037	17.4785	-0.4306
YRI	LIMS3	110036341	10.8708	17.1423	-1.1268
YRI	LIMS3	110041341	17.2536	21.512	-0.615
YRI	LIMS3	110046341	16.4764	21.3439	-0.7083
YRI	LIMS3	110051341	12.0013	17.3104	-0.945
YRI	LIMS3	110056341	12.0872	15.2937	-0.6425
YRI	LIMS3	110061341	11.1429	15.4617	-0.8564
YRI	LIMS3	110066341	9.8282	14.9575	-1.0498
YRI	LIMS3	110071341	9.0708	13.613	-1.0166
YRI	LIMS3	110076341	9.7158	13.2769	-0.8162
YRI	LIMS3	110081341	12.3844	16.8062	-0.8097
YRI	LIMS3	110086341	10.331	16.4701	-1.1461
YRI	LIMS3	110091341	8.886	13.2769	-1.0063
YRI	LIMS3	110096341	8.3101	11.5963	-0.8557
YRI	LIMS3	110101341	7.5617	13.445	-1.3324
YRI	LIMS3	110106341	8.6338	14.9575	-1.2942
YRI	LIMS3	110111341	4.0029	6.3864	-1.0757
YRI	LIMS3	110116341	0	0	0
YRI	LIMS3	110121341	0	0	0
YRI	LIMS3	110126341	0	0	0

YRI	LIMS3	110131341	0	0	0
YRI	LIMS3	110136341	0	0	0
YRI	LIMS3	110141341	0	0	0
YRI	LIMS3	110146341	0	0	0
YRI	LIMS3	110151341	0	0	0
YRI	LIMS3	110156341	0	0	0
YRI	LIMS3	110161341	0	0	0
YRI	LIMS3	110166341	0	0	0
YRI	LIMS3	110171341	0	0	0
YRI	LIMS3	110176341	0	0	0
YRI	LIMS3	110181341	0	0	0
YRI	LIMS3	110186341	0	0	0
YRI	LIMS3	110191341	0	0	0
YRI	LIMS3	110196341	0	0	0
YRI	LIMS3	110201341	0	0	0
YRI	LIMS3	110206341	0	0	0
YRI	LIMS3	110211341	0	0	0
YRI	LIMS3	110216341	0	0	0
YRI	LIMS3	110221341	0	0	0
YRI	LIMS3	110226341	0	0	0
YRI	LIMS3	110231341	0	0	0
YRI	LIMS3	110236341	0	0	0
YRI	LIMS3	110241341	0	0	0
YRI	LIMS3	110246341	0	0	0
YRI	LIMS3	110251341	19.0588	14.6214	0.928
YRI	LIMS3	110256341	26.1911	26.3858	-0.0231
YRI	LIMS3	110261341	13.4369	19.8313	-0.9988
YRI	LIMS3	110266341	11.0395	15.7978	-0.9244
YRI	LIMS3	110271341	15.0776	17.1423	-0.371
YRI	LIMS3	110276341	29.068	25.3774	0.4542
YRI	LIMS3	110281341	36.9836	29.9151	0.7412
YRI	LIMS3	110286341	23.7234	19.9994	0.577
YRI	LIMS3	110291341	8.8321	11.2602	-0.65
YRI	LIMS3	110296341	6.8226	10.756	-1.0992
YRI	LIMS3	110301341	6.9467	10.756	-1.0645
YRI	LIMS3	110306341	7.188	11.7644	-1.1757
YRI	LIMS3	110311341	6.7563	10.5879	-1.0866
YRI	LIMS3	110316341	8.6678	9.9157	-0.3762
YRI	LIMS3	110321341	10.1432	12.7727	-0.6251
YRI	LIMS3	110326341	10.5368	13.613	-0.6885
YRI	LIMS3	110331341	12.8747	13.1089	-0.0543
YRI	LIMS3	110336341	14.0391	13.1089	0.2158
YRI	LIMS3	110341341	11.3823	12.4366	-0.257
YRI	LIMS3	110346341	10.113	12.2685	-0.5323
YRI	LIMS3	110351341	10.4647	12.2685	-0.4454
YRI	LIMS3	110356341	8.9474	11.2602	-0.6191
YRI	LIMS3	110361341	7.9452	10.5879	-0.7495
YRI	LIMS3	110366341	8.3969	10.2518	-0.5421
YRI	LIMS3	110371341	10.5286	11.2602	-0.1959

YRI	LIMS3	110376341	9.8691	12.4366	-0.626
YRI	LIMS3	110381341	10.483	13.2769	-0.6403
YRI	LIMS3	110386341	11.915	15.7978	-0.7543
YRI	LIMS3	110391341	13.6449	17.6465	-0.6993
YRI	LIMS3	110396341	18.8211	18.3188	0.0847
YRI	LIMS3	110401341	17.5643	18.1507	-0.0997
YRI	LIMS3	110406341	11.5555	16.4701	-0.9175
YRI	LIMS3	110411341	10.0834	14.7895	-0.9736
YRI	LIMS3	110416341	10.7313	13.613	-0.645
YRI	LIMS3	110421341	10.0297	13.613	-0.802
YRI	LIMS3	110426341	7.4663	12.2685	-1.1859
YRI	LIMS3	110431341	6.0066	11.9324	-1.5022
YRI	LIMS3	110436341	11.5669	16.302	-0.8927
YRI	LIMS3	110441341	13.0087	19.1591	-0.9931
YRI	LIMS3	110446341	13.1308	20.3355	-1.0985
YRI	LIMS3	110451341	13.0714	15.6298	-0.5021
YRI	LIMS3	110456341	8.5346	11.2602	-0.7296
YRI	LIMS3	110461341	9.2737	12.7727	-0.8318
YRI	LIMS3	110466341	11.0409	12.2685	-0.3032
YRI	LIMS3	110471341	7.5304	9.2434	-0.5513
YRI	LIMS3	110476341	3.8244	6.0502	-1.0546
YRI	LIMS3	110481341	4.9463	7.0586	-0.8708
YRI	LIMS3	110486341	5.8124	7.8989	-0.7762
YRI	LIMS3	110491341	5.5568	7.5628	-0.7766
YRI	LIMS3	110496341	5.4916	7.3947	-0.752
YRI	LIMS3	110501341	3.6267	5.0419	-0.7885
YRI	LIMS3	110506341	1.4785	2.3529	-0.9256
YRI	LIMS3	110511341	1.1019	1.6806	-0.7961
YRI	LIMS3	110516341	0.8392	1.1764	-0.6031
YRI	LIMS3	110521341	2.0927	1.6806	0.5669
YRI	LIMS3	110526341	1.8885	1.8487	0.0509
YRI	LIMS3	110531341	0.2948	1.0084	-1.4227
YRI	LIMS3	110536341	0.0461	0.5042	-1.4397
YRI	LIMS3	110541341	0	0	0
YRI	LIMS3	110546341	0	0	0
YRI	LIMS3	110551341	0	0	0
YRI	LIMS3	110556341	0	0	0
YRI	LIMS3	110561341	0	0	0
YRI	LIMS3	110566341	0	0	0
YRI	LIMS3	110571341	0	0	0
YRI	LIMS3	110576341	0	0	0
YRI	LIMS3	110581341	0	0	0
YRI	LIMS3	110586341	0	0	0
YRI	LIMS3	110591341	0	0	0
YRI	LIMS3	110596341	0	0	0
YRI	LIMS3	110601341	0	0	0
YRI	LIMS3	110606341	0	0	0
YRI	LIMS3	110611341	0	0	0
YRI	LIMS3	110616341	0	0	0

YRI	LIMS3	110621341	0	0	0
YRI	LIMS3	110626341	0	0	0
YRI	LIMS3	110631341	0	0	0
YRI	LIMS3	110636341	0	0	0
YRI	LIMS3	110641341	0	0	0
YRI	LIMS3	110646341	0	0	0
YRI	LIMS3	110651341	0	0	0
YRI	LIMS3	110656341	0	0	0
YRI	LIMS3	110661341	0	0	0
YRI	LIMS3	110666341	0	0	0
YRI	LIMS3	110671341	0.1057	0.1681	-0.3709
YRI	LIMS3	110676341	0.1057	0.1681	-0.3709
YRI	LIMS3	110681341	0.0545	0.3361	-1.1305
YRI	LIMS3	110686341	0.0545	0.3361	-1.1305
YRI	LIMS3	110691341	0	0	0
YRI	LIMS3	110696341	0.0278	0.3361	-1.2377
YRI	LIMS3	110701341	0.694	0.6722	0.0571
YRI	LIMS3	110706341	0.6662	0.3361	1.3252
YRI	LIMS3	110711341	0	0	0
YRI	LIMS3	110716341	0.1181	0.6722	-1.4506
YRI	LIMS3	110721341	0.3551	1.0084	-1.3025
YRI	LIMS3	110726341	0.2463	0.5042	-0.8105
YRI	LIMS3	110731341	0.5965	0.6722	-0.1982
YRI	LIMS3	110736341	1.8551	2.0167	-0.1932
YRI	LIMS3	110741341	3.8467	3.6974	0.1088
YRI	LIMS3	110746341	2.9262	2.5209	0.4059
YRI	LIMS3	110751341	0.9068	0.8403	0.1502
YRI	LIMS3	110756341	1.1758	1.3445	-0.274
YRI	LIMS3	110761341	2.3984	3.0251	-0.5406
YRI	LIMS3	110766341	3.6121	5.5461	-0.9904
YRI	LIMS3	110771341	3.1657	4.8738	-0.9805
YRI	LIMS3	110776341	1.9096	2.3529	-0.4693
YRI	LIMS3	110781341	2.7044	3.5293	-0.6254
YRI	LIMS3	110786341	2.1934	3.0251	-0.7175
YRI	LIMS3	110791341	1.862	2.689	-0.7859
YRI	LIMS3	110796341	3.1469	4.3696	-0.772
YRI	LIMS3	110801341	2.3616	3.6974	-0.9736
YRI	LIMS3	110806341	2.947	5.0419	-1.1672
YRI	LIMS3	110811341	2.2928	5.2099	-1.579
YRI	LIMS3	110816341	0.4054	2.1848	-1.9979
YRI	LIMS3	110821341	1.3157	3.0251	-1.4746
YRI	LIMS3	110826341	1.7675	3.1932	-1.1758
YRI	LIMS3	110831341	1.7408	2.0167	-0.3299
YRI	LIMS3	110836341	1.4048	2.1848	-0.8758
YRI	LIMS3	110841341	0.1618	0.6722	-1.3362
YRI	LIMS3	110846341	1.0588	0.6722	1.0121
YRI	LIMS3	110851341	5.132	5.0419	0.0502
YRI	LIMS3	110856341	11.0042	11.0921	-0.0239
YRI	LIMS3	110861341	12.299	12.1005	0.0497

YRI	LIMS3	110866341	6.7551	8.7392	-0.6726
YRI	LIMS3	110871341	6.6124	10.5879	-1.1274
YRI	LIMS3	110876341	9.3148	13.7811	-0.9881
YRI	LIMS3	110881341	7.2867	9.9157	-0.7926
YRI	LIMS3	110886341	7.0178	8.9073	-0.6293
YRI	LIMS3	110891341	9.953	12.7727	-0.6703
YRI	LIMS3	110896341	11.249	12.7727	-0.3622
YRI	LIMS3	110901341	9.1351	10.0837	-0.2816
YRI	LIMS3	110906341	9.6257	10.2518	-0.183
YRI	LIMS3	110911341	10.8602	12.2685	-0.3478
YRI	LIMS3	110916341	8.8383	11.7644	-0.7517
YRI	LIMS3	110921341	6.4794	10.2518	-1.1026
YRI	LIMS3	110926341	6.5152	10.2518	-1.0921
YRI	LIMS3	110931341	8.0057	10.2518	-0.6565
YRI	LIMS3	110936341	8.924	10.5879	-0.4719
YRI	LIMS3	110941341	10.8617	12.4366	-0.384
YRI	LIMS3	110946341	12.3746	14.7895	-0.4996
YRI	LIMS3	110951341	11.4308	14.7895	-0.6948
YRI	LIMS3	110956341	9.2942	12.7727	-0.827
YRI	LIMS3	110961341	7.0343	9.0754	-0.6681
YRI	LIMS3	110966341	7.5157	8.067	-0.2012
CEU	MUC12	100474565	8.7386	8.3576	0.1354
CEU	MUC12	100479565	7.5333	7.6753	-0.0546
CEU	MUC12	100484565	6.9666	7.3342	-0.1473
CEU	MUC12	100489565	6.8959	7.3342	-0.1756
CEU	MUC12	100494565	10.4778	8.0164	0.9091
CEU	MUC12	100499565	12.9303	8.6987	1.4497
CEU	MUC12	100504565	14.5211	9.7221	1.483
CEU	MUC12	100509565	11.9713	9.5515	0.7602
CEU	MUC12	100514565	6.1379	7.1636	-0.4199
CEU	MUC12	100519565	6.8747	8.187	-0.4754
CEU	MUC12	100524565	9.4323	11.4277	-0.5301
CEU	MUC12	100529565	11.7621	12.2805	-0.1287
CEU	MUC12	100534565	14.1002	13.1333	0.2253
CEU	MUC12	100539565	12.5148	11.4277	0.2888
CEU	MUC12	100544565	11.218	10.5749	0.1837
CEU	MUC12	100549565	25.691	22.8554	0.3886
CEU	MUC12	100554565	20.2393	18.0796	0.371
CEU	MUC12	100559565	0.8821	1.3645	-0.7804
CEU	MUC12	100564565	0	0	0
CEU	MUC12	100569565	0	0	0
CEU	MUC12	100574565	0	0	0
CEU	MUC12	100579565	0	0	0
CEU	MUC12	100584565	0	0	0
CEU	MUC12	100589565	0	0	0
CEU	MUC12	100594565	0	0	0
CEU	MUC12	100599565	0	0	0
CEU	MUC12	100604565	6.4168	6.9931	-0.2411
CEU	MUC12	100609565	12.2538	12.7922	-0.1286

CEU	MUC12	100614565	14.9874	12.4511	0.6216
CEU	MUC12	100619565	15.7188	11.4277	1.1401
CEU	MUC12	100624565	11.9344	8.5281	1.1885
CEU	MUC12	100629565	11.4143	9.8926	0.4627
CEU	MUC12	100634565	23.0858	20.6381	0.3702
CEU	MUC12	100639565	18.5269	15.8623	0.5189
CEU	MUC12	100644565	14.811	9.5515	1.6523
CEU	MUC12	100649565	16.9907	11.5982	1.4129
CEU	MUC12	100654565	5.9971	5.1169	0.4872
CEU	MUC12	100659565	5.0944	4.9463	0.0845
CEU	MUC12	100664565	5.4444	6.9931	-0.648
CEU	MUC12	100669565	5.3962	8.187	-1.011
CEU	MUC12	100674565	7.4559	12.1099	-1.1708
CEU	MUC12	100679565	8.19	15.3506	-1.4387
CEU	MUC12	100684565	3.9023	10.7454	-1.9261
CEU	MUC12	100689565	4.7112	8.187	-1.2592
CEU	MUC12	100694565	7.9033	9.8926	-0.6049
CEU	MUC12	100699565	9.9959	9.7221	0.0846
CEU	MUC12	100704565	15.3362	10.916	1.2259
CEU	MUC12	100709565	16.7879	13.3039	0.802
CEU	MUC12	100714565	17.0297	14.4978	0.5371
CEU	MUC12	100719565	16.5928	14.4978	0.4445
CEU	MUC12	100724565	11.4703	11.5982	-0.0335
CEU	MUC12	100729565	7.4661	9.0398	-0.5203
CEU	MUC12	100734565	8.2338	11.7688	-0.9136
CEU	MUC12	100739565	9.4993	11.2571	-0.4737
CEU	MUC12	100744565	9.0876	9.7221	-0.1961
CEU	MUC12	100749565	8.7161	10.0632	-0.4031
CEU	MUC12	100754565	14.5366	12.6216	0.4633
CEU	MUC12	100759565	15.657	13.645	0.4521
CEU	MUC12	100764565	11.0499	10.0632	0.2953
CEU	MUC12	100769565	10.5088	10.0632	0.1334
CEU	MUC12	100774565	12.2425	11.0866	0.316
CEU	MUC12	100779565	13.7616	10.4043	0.9739
CEU	MUC12	100784565	13.3286	9.8926	1.0448
CEU	MUC12	100789565	13.0577	10.0632	0.8961
CEU	MUC12	100794565	12.4261	10.5749	0.5289
CEU	MUC12	100799565	15.6155	11.4277	1.1126
CEU	MUC12	100804565	11.6366	8.8692	0.9312
CEU	MUC12	100809565	6.3894	6.8225	-0.1853
CEU	MUC12	100814565	10.9015	11.2571	-0.0958
CEU	MUC12	100819565	9.9287	11.7688	-0.4755
CEU	MUC12	100824565	7.2127	10.0632	-0.853
CEU	MUC12	100829565	7.3791	10.2337	-0.841
CEU	MUC12	100834565	7.0544	9.5515	-0.7845
CEU	MUC12	100839565	7.0649	8.6987	-0.5597
CEU	MUC12	100844565	7.5962	10.4043	-0.8146
CEU	MUC12	100849565	7.1138	10.7454	-1.0222
CEU	MUC12	100854565	4.4978	8.187	-1.3365

CEU	MUC12	100859565	6.6456	8.3576	-0.6086
CEU	MUC12	100864565	6.6054	7.3342	-0.292
CEU	MUC12	100869565	3.7095	6.4814	-1.2424
CEU	MUC12	100874565	4.3067	6.9931	-1.124
CEU	MUC12	100879565	5.1177	7.3342	-0.8881
CEU	MUC12	100884565	4.9779	7.6753	-1.0368
CEU	MUC12	100889565	5.8031	9.2104	-1.1072
CEU	MUC12	100894565	10.126	13.4744	-0.7615
CEU	MUC12	100899565	8.9421	16.8857	-1.457
CEU	MUC12	100904565	5.5865	16.5446	-2.0496
CEU	MUC12	100909565	5.5009	16.8857	-2.0882
CEU	MUC12	100914565	5.9491	13.9861	-1.7643
CEU	MUC12	100919565	9.214	10.0632	-0.2541
CEU	MUC12	100924565	10.6555	10.0632	0.1773
CEU	MUC12	100929565	15.1159	15.5212	-0.0806
CEU	MUC12	100934565	16.0196	16.374	-0.0669
CEU	MUC12	100939565	11.0577	10.916	0.0393
CEU	MUC12	100944565	9.651	8.6987	0.3263
CEU	MUC12	100949565	12.8932	9.8926	0.9124
CEU	MUC12	100954565	13.5053	11.0866	0.6611
CEU	MUC12	100959565	12.7151	11.9394	0.1978
CEU	MUC12	100964565	13.9592	12.9627	0.2351
CEU	MUC12	100969565	11.8627	10.4043	0.4231
CEU	MUC12	100974565	13.5523	10.7454	0.7901
CEU	MUC12	100979565	17.8581	12.9627	1.1549
CEU	MUC12	100984565	15.3425	12.4511	0.7086
CEU	MUC12	100989565	14.8986	13.645	0.2817
CEU	MUC12	100994565	23.0076	16.374	1.2531
CEU	MUC12	100999565	23.7899	13.4744	2.346
CEU	MUC12	101004565	37.6246	17.0562	3.7366
CEU	MUC12	101009565	55.153	24.2199	4.0077
CEU	MUC12	101014565	42.7114	19.6147	3.6689
CEU	MUC12	101019565	18.9751	14.8389	0.8583
CEU	MUC12	101024565	10.7246	16.7151	-1.1095
CEU	MUC12	101029565	13.4811	18.4207	-0.8335
CEU	MUC12	101034565	10.4517	15.0095	-0.9355
CEU	MUC12	101039565	9.7932	11.2571	-0.3945
CEU	MUC12	101044565	14.5784	13.3039	0.2934
CEU	MUC12	101049565	13.1024	12.9627	0.033
CEU	MUC12	101054565	11.9038	11.7688	0.0349
CEU	MUC12	101059565	13.9804	12.4511	0.3748
CEU	MUC12	101064565	13.6772	11.7688	0.4932
CEU	MUC12	101069565	11.8027	11.2571	0.147
CEU	MUC12	101074565	11.9002	11.0866	0.2224
CEU	MUC12	101079565	13.712	12.9627	0.1768
CEU	MUC12	101084565	13.8973	12.7922	0.264
CEU	MUC12	101089565	11.4131	10.5749	0.2395
CEU	MUC12	101094565	15.4556	13.645	0.4069
CEU	MUC12	101099565	31.9834	19.7852	1.9216

CEU	MUC12	101104565	39.0298	20.2969	2.8793
CEU	MUC12	101109565	24.7005	14.4978	2.1645
CEU	MUC12	101114565	14.4743	11.7688	0.6992
CEU	MUC12	101119565	16.6889	13.8156	0.6381
CEU	MUC12	101124565	19.4564	13.645	1.306
CEU	MUC12	101129565	21.3078	14.6684	1.393
CEU	MUC12	101134565	16.9843	14.4978	0.5275
CEU	MUC12	101139565	13.5675	14.3272	-0.163
CEU	MUC12	101144565	11.0194	14.6684	-0.7656
CEU	MUC12	101149565	10.8	16.0329	-1.0086
CEU	MUC12	101154565	17.6855	18.7619	-0.1785
CEU	MUC12	101159565	21.0875	18.9324	0.3542
CEU	MUC12	101164565	16.4164	17.2268	-0.1458
CEU	MUC12	101169565	13.9992	15.6917	-0.333
CEU	MUC12	101174565	13.7166	15.6917	-0.3886
CEU	MUC12	101179565	11.8241	14.4978	-0.5672
CEU	MUC12	101184565	12.4322	12.6216	-0.0458
CEU	MUC12	101189565	17.4395	13.3039	0.9519
CEU	MUC12	101194565	15.9821	13.1333	0.6638
CEU	MUC12	101199565	16.3859	14.1567	0.4838
CEU	MUC12	101204565	18.1171	14.8389	0.6803
CEU	MUC12	101209565	9.9454	9.7221	0.069
CEU	MUC12	101214565	7.0957	8.0164	-0.3401
CEU	MUC12	101219565	7.5912	7.3342	0.103
CEU	MUC12	101224565	10.5253	7.8459	1.0094
CEU	MUC12	101229565	11.9988	9.0398	0.9783
CEU	MUC12	101234565	12.0276	9.3809	0.8455
CEU	MUC12	101239565	11.3688	8.6987	0.9148
CEU	MUC12	101244565	13.0167	9.2104	1.2368
CEU	MUC12	101249565	15.8452	10.5749	1.5058
CEU	MUC12	101254565	12.4454	9.2104	1.0512
CEU	MUC12	101259565	9.4097	9.2104	0.0648
CEU	MUC12	101264565	8.5502	8.6987	-0.0509
CEU	MUC12	101269565	12.0431	10.7454	0.3653
CEU	MUC12	101274565	14.4857	13.3039	0.272
CEU	MUC12	101279565	15.1892	12.7922	0.5726
CEU	MUC12	101284565	16.2175	12.1099	1.0334
CEU	MUC12	101289565	9.334	6.8225	1.0747
CEU	MUC12	101294565	2.7215	2.2173	0.5633
CEU	MUC12	101299565	2.2855	1.7056	0.7942
CEU	MUC12	101304565	2.2659	1.7056	0.7674
CEU	MUC12	101309565	1.8707	1.8762	-0.007
CEU	MUC12	101314565	0.6302	0.8528	-0.5013
CEU	MUC12	101319565	0.0101	0.1706	-0.9534
CEU	MUC12	101324565	0.7904	1.1939	-0.719
CEU	MUC12	101329565	6.3861	7.3342	-0.3799
CEU	MUC12	101334565	11.7464	11.4277	0.0847
CEU	MUC12	101339565	12.5108	11.2571	0.3378
CEU	MUC12	101344565	16.7025	15.5212	0.2349

CEU	MUC12	101349565	18.5758	15.3506	0.648
CEU	MUC12	101354565	15.8821	11.5982	1.1224
CEU	MUC12	101359565	12.3535	10.7454	0.4526
CEU	MUC12	101364565	11.1746	10.5749	0.1713
CEU	MUC12	101369565	13.4799	10.916	0.7111
CEU	MUC12	101374565	13.7017	10.5749	0.8934
CEU	MUC12	101379565	13.8553	10.7454	0.8753
CEU	MUC12	101384565	12.5331	10.5749	0.5595
CEU	MUC12	101389565	11.0651	10.7454	0.09
CEU	MUC12	101394565	7.8662	8.6987	-0.2852
CEU	MUC12	101399565	7.4449	6.8225	0.2663
CEU	MUC12	101404565	11.6698	7.8459	1.4405
CEU	MUC12	101409565	15.8928	9.2104	2.1714
CEU	MUC12	101414565	13.5871	8.8692	1.5876
CEU	MUC12	101419565	9.168	8.187	0.3554
CEU	MUC12	101424565	10.1381	9.5515	0.1843
CEU	MUC12	101429565	11.7155	10.7454	0.2731
CEU	MUC12	101434565	17.3898	11.7688	1.4527
CEU	MUC12	101439565	17.598	11.0866	1.7799
CEU	MUC12	101444565	13.2653	10.4043	0.83
CEU	MUC12	101449565	9.665	8.5281	0.3967
CEU	MUC12	101454565	5.0334	5.6286	-0.3028
CEU	MUC12	101459565	3.0016	3.9229	-0.6426
CEU	MUC12	101464565	4.221	5.6286	-0.716
CEU	MUC12	101469565	7.1647	7.6753	-0.1963
CEU	MUC12	101474565	7.3337	8.187	-0.3091
CEU	MUC12	101479565	6.6682	8.3576	-0.6005
CEU	MUC12	101484565	4.8991	6.3108	-0.6481
CEU	MUC12	101489565	3.7289	6.3108	-1.1854
CEU	MUC12	101494565	7.375	8.6987	-0.4535
CEU	MUC12	101499565	6.4884	7.3342	-0.3389
CEU	MUC12	101504565	5.4684	6.1402	-0.3161
CEU	MUC12	101509565	7.9594	7.1636	0.3258
CHB	MUC12	100474565	6.9859	10.5037	-1.0085
CHB	MUC12	100479565	5.4234	9.3178	-1.2483
CHB	MUC12	100484565	4.28	9.8261	-1.6921
CHB	MUC12	100489565	4.0242	9.6567	-1.7465
CHB	MUC12	100494565	6.7605	9.9955	-0.9714
CHB	MUC12	100499565	9.1121	11.5202	-0.6331
CHB	MUC12	100504565	10.2397	11.1814	-0.2546
CHB	MUC12	100509565	9.3853	11.012	-0.4462
CHB	MUC12	100514565	6.0434	10.1649	-1.2184
CHB	MUC12	100519565	7.193	10.8426	-1.0157
CHB	MUC12	100524565	10.9806	13.045	-0.4827
CHB	MUC12	100529565	12.4367	12.3673	0.0171
CHB	MUC12	100534565	12.869	12.8755	-0.0015
CHB	MUC12	100539565	9.7531	13.2144	-0.7995
CHB	MUC12	100544565	7.3223	12.3673	-1.2407
CHB	MUC12	100549565	26.2095	25.2428	0.12

CHB	MUC12	100554565	22.9243	21.3463	0.2304
CHB	MUC12	100559565	0.9282	2.3718	-1.5238
CHB	MUC12	100564565	0	0	0
CHB	MUC12	100569565	0	0	0
CHB	MUC12	100574565	0	0	0
CHB	MUC12	100579565	0	0	0
CHB	MUC12	100584565	0	0	0
CHB	MUC12	100589565	0	0	0
CHB	MUC12	100594565	0	0	0
CHB	MUC12	100599565	0	0	0
CHB	MUC12	100604565	4.7036	5.5907	-0.4526
CHB	MUC12	100609565	10.8395	13.7226	-0.6426
CHB	MUC12	100614565	15.3666	14.5697	0.1678
CHB	MUC12	100619565	15.5134	11.5202	1.0499
CHB	MUC12	100624565	11.2562	9.3178	0.6213
CHB	MUC12	100629565	9.5397	9.1484	0.1276
CHB	MUC12	100634565	20.644	21.1769	-0.0784
CHB	MUC12	100639565	17.6507	17.7886	-0.024
CHB	MUC12	100644565	14.541	9.3178	1.6742
CHB	MUC12	100649565	16.0653	12.1979	0.9635
CHB	MUC12	100654565	5.2935	8.4708	-1.1123
CHB	MUC12	100659565	4.8921	8.979	-1.3558
CHB	MUC12	100664565	6.3449	9.3178	-0.9529
CHB	MUC12	100669565	8.3541	10.5037	-0.6163
CHB	MUC12	100674565	12.0352	15.5862	-0.7011
CHB	MUC12	100679565	14.1763	21.0075	-1.0128
CHB	MUC12	100684565	7.7396	15.5862	-1.5493
CHB	MUC12	100689565	4.8848	9.4872	-1.4508
CHB	MUC12	100694565	7.8903	12.0285	-1.0447
CHB	MUC12	100699565	10.2176	13.045	-0.6611
CHB	MUC12	100704565	14.2294	14.2309	-0.0003
CHB	MUC12	100709565	15.9515	16.2638	-0.0592
CHB	MUC12	100714565	18.7701	16.7721	0.3678
CHB	MUC12	100719565	18.7922	16.2638	0.4793
CHB	MUC12	100724565	12.2817	13.892	-0.3547
CHB	MUC12	100729565	7.9625	11.8591	-0.9969
CHB	MUC12	100734565	8.099	13.7226	-1.2534
CHB	MUC12	100739565	9.414	13.3838	-0.906
CHB	MUC12	100744565	9.8726	12.1979	-0.5793
CHB	MUC12	100749565	9.1076	11.8591	-0.704
CHB	MUC12	100754565	12.628	14.0615	-0.3122
CHB	MUC12	100759565	12.4719	16.2638	-0.7189
CHB	MUC12	100764565	9.4812	12.7061	-0.7731
CHB	MUC12	100769565	9.9905	11.5202	-0.4022
CHB	MUC12	100774565	11.1445	13.3838	-0.5111
CHB	MUC12	100779565	12.3347	11.6896	0.1673
CHB	MUC12	100784565	11.3705	10.8426	0.1469
CHB	MUC12	100789565	11.3767	11.5202	-0.0377
CHB	MUC12	100794565	13.0826	10.6731	0.6805

CHB	MUC12	100799565	16.5974	12.0285	1.1534
CHB	MUC12	100804565	12.2117	9.4872	0.8588
CHB	MUC12	100809565	7.3038	6.7766	0.2263
CHB	MUC12	100814565	11.6556	9.9955	0.4985
CHB	MUC12	100819565	10.6267	9.9955	0.1895
CHB	MUC12	100824565	8.0516	9.1484	-0.3576
CHB	MUC12	100829565	7.7314	9.9955	-0.6799
CHB	MUC12	100834565	7.9739	10.5037	-0.7253
CHB	MUC12	100839565	8.2918	8.979	-0.228
CHB	MUC12	100844565	9.2321	9.4872	-0.0804
CHB	MUC12	100849565	14.6004	16.9415	-0.4268
CHB	MUC12	100854565	13.3889	17.2803	-0.6962
CHB	MUC12	100859565	9.4297	11.5202	-0.5496
CHB	MUC12	100864565	5.8923	7.9625	-0.7672
CHB	MUC12	100869565	2.3103	6.7766	-1.9172
CHB	MUC12	100874565	2.7729	6.4378	-1.6479
CHB	MUC12	100879565	3.8725	6.7766	-1.2466
CHB	MUC12	100884565	4.3198	7.7931	-1.3127
CHB	MUC12	100889565	3.5009	9.6567	-1.9088
CHB	MUC12	100894565	4.4308	14.4003	-2.1226
CHB	MUC12	100899565	3.8209	17.1109	-2.4001
CHB	MUC12	100904565	2.5656	15.2474	-2.557
CHB	MUC12	100909565	2.5397	14.2309	-2.5173
CHB	MUC12	100914565	4.4508	13.2144	-2.0243
CHB	MUC12	100919565	8.055	9.9955	-0.5827
CHB	MUC12	100924565	8.6552	9.3178	-0.2124
CHB	MUC12	100929565	15.8633	15.925	-0.0119
CHB	MUC12	100934565	17.1144	16.9415	0.0315
CHB	MUC12	100939565	10.0965	11.8591	-0.4509
CHB	MUC12	100944565	7.9273	10.8426	-0.8113
CHB	MUC12	100949565	8.9003	11.6896	-0.7234
CHB	MUC12	100954565	12.2009	13.892	-0.3725
CHB	MUC12	100959565	12.5572	15.2474	-0.5424
CHB	MUC12	100964565	10.7604	14.5697	-0.8021
CHB	MUC12	100969565	9.6919	11.6896	-0.5181
CHB	MUC12	100974565	10.9433	11.5202	-0.1517
CHB	MUC12	100979565	18.5103	14.7391	0.7853
CHB	MUC12	100984565	20.2723	14.2309	1.3008
CHB	MUC12	100989565	16.8677	16.4333	0.0815
CHB	MUC12	100994565	20.4222	20.4992	-0.0117
CHB	MUC12	100999565	19.3771	14.2309	1.1081
CHB	MUC12	101004565	30.3221	16.0944	2.7245
CHB	MUC12	101009565	44.5714	23.3793	2.8332
CHB	MUC12	101014565	35.3485	21.1769	2.0848
CHB	MUC12	101019565	19.2105	18.8051	0.0669
CHB	MUC12	101024565	14.052	18.9745	-0.805
CHB	MUC12	101029565	16.6048	21.1769	-0.6726
CHB	MUC12	101034565	13.2867	16.7721	-0.6416
CHB	MUC12	101039565	8.8947	12.0285	-0.7911

CHB	MUC12	101044565	10.1957	13.2144	-0.6973
CHB	MUC12	101049565	8.6543	12.3673	-0.9131
CHB	MUC12	101054565	6.035	12.1979	-1.5354
CHB	MUC12	101059565	7.1466	10.8426	-1.0286
CHB	MUC12	101064565	10.4019	9.9955	0.122
CHB	MUC12	101069565	11.6328	10.1649	0.4339
CHB	MUC12	101074565	11.9812	10.3343	0.4794
CHB	MUC12	101079565	13.0664	11.8591	0.3089
CHB	MUC12	101084565	12.894	12.7061	0.045
CHB	MUC12	101089565	9.5536	11.5202	-0.517
CHB	MUC12	101094565	7.9181	13.5532	-1.2708
CHB	MUC12	101099565	13.1524	18.8051	-0.9324
CHB	MUC12	101104565	18.0703	19.1439	-0.1741
CHB	MUC12	101109565	14.2065	13.7226	0.1079
CHB	MUC12	101114565	9.6796	12.1979	-0.6274
CHB	MUC12	101119565	10.2414	13.892	-0.8042
CHB	MUC12	101124565	11.4599	13.5532	-0.4721
CHB	MUC12	101129565	12.38	14.7391	-0.4913
CHB	MUC12	101134565	9.5985	14.0615	-0.9719
CHB	MUC12	101139565	7.7816	14.5697	-1.4292
CHB	MUC12	101144565	6.6591	14.7391	-1.6826
CHB	MUC12	101149565	7.3518	13.892	-1.4408
CHB	MUC12	101154565	10.604	16.6027	-1.1151
CHB	MUC12	101159565	11.039	17.1109	-1.0965
CHB	MUC12	101164565	8.173	13.5532	-1.2133
CHB	MUC12	101169565	5.5671	12.1979	-1.652
CHB	MUC12	101174565	5.562	13.7226	-1.8188
CHB	MUC12	101179565	5.4982	13.3838	-1.7997
CHB	MUC12	101184565	4.8671	11.1814	-1.7073
CHB	MUC12	101189565	5.9204	11.8591	-1.5194
CHB	MUC12	101194565	5.5385	11.8591	-1.6171
CHB	MUC12	101199565	5.8539	12.3673	-1.6018
CHB	MUC12	101204565	6.1697	12.1979	-1.5019
CHB	MUC12	101209565	4.1892	7.7931	-1.3621
CHB	MUC12	101214565	3.9661	7.2848	-1.334
CHB	MUC12	101219565	5.0672	7.4543	-0.9396
CHB	MUC12	101224565	7.4797	8.8096	-0.449
CHB	MUC12	101229565	8.028	9.1484	-0.3653
CHB	MUC12	101234565	8.2817	9.8261	-0.4712
CHB	MUC12	101239565	8.1749	11.5202	-0.8795
CHB	MUC12	101244565	7.9791	10.6731	-0.7609
CHB	MUC12	101249565	8.7773	9.6567	-0.2727
CHB	MUC12	101254565	7.2924	8.4708	-0.4125
CHB	MUC12	101259565	6.5109	9.3178	-0.8997
CHB	MUC12	101264565	5.7219	10.1649	-1.3134
CHB	MUC12	101269565	7.7106	12.5367	-1.1717
CHB	MUC12	101274565	11.0394	15.5862	-0.8978
CHB	MUC12	101279565	13.6511	15.4168	-0.3523
CHB	MUC12	101284565	14.2846	13.892	0.0865

CHB	MUC12	101289565	7.8325	7.6237	0.0805
CHB	MUC12	101294565	3.0645	1.8636	1.5322
CHB	MUC12	101299565	2.1401	1.5247	0.9143
CHB	MUC12	101304565	2.0919	1.5247	0.8427
CHB	MUC12	101309565	2.0769	1.5247	0.8204
CHB	MUC12	101314565	0.5405	0.6777	-0.3587
CHB	MUC12	101319565	0.2764	0.5082	-0.7277
CHB	MUC12	101324565	1.2722	2.033	-0.9073
CHB	MUC12	101329565	4.3102	7.9625	-1.3534
CHB	MUC12	101334565	8.2184	12.1979	-0.9915
CHB	MUC12	101339565	9.0845	11.8591	-0.7099
CHB	MUC12	101344565	8.4755	12.8755	-1.0417
CHB	MUC12	101349565	11.0337	12.1979	-0.2901
CHB	MUC12	101354565	13.253	10.5037	0.7882
CHB	MUC12	101359565	10.0358	8.4708	0.5479
CHB	MUC12	101364565	8.3499	7.1154	0.507
CHB	MUC12	101369565	8.9315	9.3178	-0.1238
CHB	MUC12	101374565	8.4	12.1979	-0.9462
CHB	MUC12	101379565	8.5652	12.0285	-0.8743
CHB	MUC12	101384565	8.131	10.3343	-0.6413
CHB	MUC12	101389565	7.8677	10.1649	-0.6791
CHB	MUC12	101394565	5.8896	8.3013	-0.8601
CHB	MUC12	101399565	6.1654	7.6237	-0.5624
CHB	MUC12	101404565	8.3975	9.1484	-0.2448
CHB	MUC12	101409565	10.2248	11.8591	-0.4181
CHB	MUC12	101414565	9.2201	12.8755	-0.8654
CHB	MUC12	101419565	7.08	12.7061	-1.3487
CHB	MUC12	101424565	7.458	11.3508	-1.0378
CHB	MUC12	101429565	8.3326	8.8096	-0.161
CHB	MUC12	101434565	11.1513	12.5367	-0.3364
CHB	MUC12	101439565	11.9951	13.2144	-0.2817
CHB	MUC12	101444565	10.351	9.9955	0.1068
CHB	MUC12	101449565	7.4963	8.6402	-0.3932
CHB	MUC12	101454565	3.7799	6.2684	-1.1461
CHB	MUC12	101459565	3.0114	4.066	-0.7112
CHB	MUC12	101464565	4.1673	5.9295	-0.8531
CHB	MUC12	101469565	4.2753	7.6237	-1.2912
CHB	MUC12	101474565	4.1751	7.6237	-1.3299
CHB	MUC12	101479565	3.2207	8.979	-1.9102
CHB	MUC12	101484565	2.1046	7.6237	-2.1283
CHB	MUC12	101489565	3.0051	6.6072	-1.5821
CHB	MUC12	101494565	6.1682	7.4543	-0.5062
CHB	MUC12	101499565	5.7635	5.4213	0.1794
CHB	MUC12	101504565	3.4377	5.5907	-1.0985
CHB	MUC12	101509565	4.0013	6.6072	-1.1445
YRI	MUC12	100474565	7.2653	15.6298	-1.6416
YRI	MUC12	100479565	7.5173	13.445	-1.3425
YRI	MUC12	100484565	7.578	10.5879	-0.8536
YRI	MUC12	100489565	7.3423	11.2602	-1.0488

YRI	MUC12	100494565	10.8204	14.2853	-0.7408
YRI	MUC12	100499565	15.0074	14.9575	0.0102
YRI	MUC12	100504565	14.7367	13.7811	0.2114
YRI	MUC12	100509565	14.6321	15.6298	-0.1958
YRI	MUC12	100514565	12.9288	15.7978	-0.5573
YRI	MUC12	100519565	11.6256	14.7895	-0.6545
YRI	MUC12	100524565	13.5732	16.9743	-0.6169
YRI	MUC12	100529565	13.8472	16.6382	-0.516
YRI	MUC12	100534565	14.0211	14.7895	-0.159
YRI	MUC12	100539565	11.8848	14.1172	-0.4827
YRI	MUC12	100544565	9.2378	14.7895	-1.1485
YRI	MUC12	100549565	30.7276	28.2345	0.2766
YRI	MUC12	100554565	27.4434	22.5203	0.6802
YRI	MUC12	100559565	1.8344	2.689	-0.8122
YRI	MUC12	100564565	0	0	0
YRI	MUC12	100569565	0	0	0
YRI	MUC12	100574565	0	0	0
YRI	MUC12	100579565	0	0	0
YRI	MUC12	100584565	0	0	0
YRI	MUC12	100589565	0	0	0
YRI	MUC12	100594565	0	0	0
YRI	MUC12	100599565	0	0	0
YRI	MUC12	100604565	5.3879	5.5461	-0.081
YRI	MUC12	100609565	13.7929	15.9659	-0.4179
YRI	MUC12	100614565	17.3253	17.9827	-0.1128
YRI	MUC12	100619565	15.7474	13.9492	0.3933
YRI	MUC12	100624565	11.4284	12.4366	-0.2458
YRI	MUC12	100629565	11.0807	13.2769	-0.5033
YRI	MUC12	100634565	24.6913	25.0413	-0.0436
YRI	MUC12	100639565	20.4782	20.8397	-0.0538
YRI	MUC12	100644565	14.9772	12.7727	0.5241
YRI	MUC12	100649565	16.9071	15.2937	0.3233
YRI	MUC12	100654565	7.3332	10.924	-0.989
YRI	MUC12	100659565	7.0844	13.7811	-1.4815
YRI	MUC12	100664565	8.8219	15.7978	-1.3552
YRI	MUC12	100669565	10.0839	15.9659	-1.1312
YRI	MUC12	100674565	13.9139	22.1842	-1.1594
YRI	MUC12	100679565	15.9064	27.3941	-1.3124
YRI	MUC12	100684565	8.8239	19.4952	-1.6945
YRI	MUC12	100689565	5.6977	12.2685	-1.6226
YRI	MUC12	100694565	9.5582	14.2853	-1.0107
YRI	MUC12	100699565	11.8266	15.2937	-0.6947
YRI	MUC12	100704565	17.2587	17.3104	-0.0092
YRI	MUC12	100709565	20.449	17.6465	0.4897
YRI	MUC12	100714565	21.8081	18.3188	0.5882
YRI	MUC12	100719565	24.3702	20.6717	0.5551
YRI	MUC12	100724565	17.4242	16.6382	0.1453
YRI	MUC12	100729565	10.6178	13.445	-0.6403
YRI	MUC12	100734565	14.4881	18.1507	-0.623

YRI	MUC12	100739565	14.6124	18.823	-0.6916
YRI	MUC12	100744565	12.4419	14.4534	-0.4253
YRI	MUC12	100749565	12.4715	14.7895	-0.4795
YRI	MUC12	100754565	16.964	18.6549	-0.2801
YRI	MUC12	100759565	17.5533	18.823	-0.2085
YRI	MUC12	100764565	11.8316	15.7978	-0.7705
YRI	MUC12	100769565	13.1134	17.3104	-0.7471
YRI	MUC12	100774565	15.2602	18.4868	-0.5392
YRI	MUC12	100779565	14.5213	15.7978	-0.248
YRI	MUC12	100784565	13.7075	15.1256	-0.2872
YRI	MUC12	100789565	13.4793	14.7895	-0.271
YRI	MUC12	100794565	13.9351	14.7895	-0.1768
YRI	MUC12	100799565	16.6571	14.1172	0.5492
YRI	MUC12	100804565	12.4297	10.4199	0.5786
YRI	MUC12	100809565	7.583	9.7476	-0.6631
YRI	MUC12	100814565	10.6016	12.9408	-0.5493
YRI	MUC12	100819565	10.3419	13.1089	-0.6418
YRI	MUC12	100824565	9.3048	13.7811	-0.9903
YRI	MUC12	100829565	9.8165	19.3272	-1.5229
YRI	MUC12	100834565	11.3276	20.3355	-1.3734
YRI	MUC12	100839565	11.1955	15.6298	-0.8703
YRI	MUC12	100844565	11.5095	18.1507	-1.1296
YRI	MUC12	100849565	12.9363	22.8565	-1.3511
YRI	MUC12	100854565	8.5608	19.8313	-1.7605
YRI	MUC12	100859565	9.7863	15.9659	-1.1884
YRI	MUC12	100864565	9.938	13.445	-0.7942
YRI	MUC12	100869565	6.5047	13.2769	-1.5521
YRI	MUC12	100874565	6.8551	11.7644	-1.2612
YRI	MUC12	100879565	8.0988	11.5963	-0.9108
YRI	MUC12	100884565	8.5484	14.1172	-1.2041
YRI	MUC12	100889565	10.0099	14.6214	-0.9644
YRI	MUC12	100894565	18.8724	18.6549	0.036
YRI	MUC12	100899565	25.5942	22.0162	0.5053
YRI	MUC12	100904565	23.7911	20.1675	0.5569
YRI	MUC12	100909565	24.0104	20.5036	0.5304
YRI	MUC12	100914565	20.6096	22.8565	-0.306
YRI	MUC12	100919565	14.5179	24.201	-1.2477
YRI	MUC12	100924565	12.1584	19.8313	-1.1985
YRI	MUC12	100929565	19.1213	21.68	-0.3667
YRI	MUC12	100934565	22.7373	22.6884	0.0067
YRI	MUC12	100939565	15.1618	14.1172	0.2259
YRI	MUC12	100944565	12.7639	13.613	-0.19
YRI	MUC12	100949565	16.2354	17.6465	-0.2466
YRI	MUC12	100954565	17.5905	17.4785	0.0198
YRI	MUC12	100959565	16.9224	16.134	0.1501
YRI	MUC12	100964565	17.1499	16.9743	0.0318
YRI	MUC12	100969565	14.6639	15.2937	-0.1262
YRI	MUC12	100974565	15.9552	15.4617	0.0979
YRI	MUC12	100979565	21.7052	18.823	0.4734

YRI	MUC12	100984565	21.1171	17.8146	0.5719
YRI	MUC12	100989565	19.9081	20.3355	-0.0652
YRI	MUC12	100994565	24.765	24.369	0.0507
YRI	MUC12	100999565	19.9663	17.8146	0.3726
YRI	MUC12	101004565	24.0788	21.3439	0.398
YRI	MUC12	101009565	36.5274	30.2512	0.651
YRI	MUC12	101014565	29.4276	24.8732	0.5715
YRI	MUC12	101019565	21.0301	20.3355	0.1059
YRI	MUC12	101024565	22.2124	24.0329	-0.2362
YRI	MUC12	101029565	26.2189	28.9067	-0.2914
YRI	MUC12	101034565	22.4036	24.369	-0.2516
YRI	MUC12	101039565	16.6582	17.3104	-0.1161
YRI	MUC12	101044565	17.9962	19.8313	-0.2866
YRI	MUC12	101049565	14.2854	19.1591	-0.787
YRI	MUC12	101054565	10.7747	15.4617	-0.9294
YRI	MUC12	101059565	10.4264	15.6298	-1.0212
YRI	MUC12	101064565	11.9746	17.4785	-0.9706
YRI	MUC12	101069565	12.5296	17.3104	-0.851
YRI	MUC12	101074565	12.0537	14.9575	-0.5943
YRI	MUC12	101079565	15.6933	16.8062	-0.2038
YRI	MUC12	101084565	15.1444	16.9743	-0.3319
YRI	MUC12	101089565	10.7729	14.1172	-0.7231
YRI	MUC12	101094565	16.6906	18.4868	-0.3002
YRI	MUC12	101099565	31.7989	25.7135	0.7394
YRI	MUC12	101104565	38.3195	25.2093	1.6238
YRI	MUC12	101109565	27.3719	20.8397	0.9727
YRI	MUC12	101114565	20.2226	20.6717	-0.0674
YRI	MUC12	101119565	21.2449	19.6633	0.2491
YRI	MUC12	101124565	19.8465	18.1507	0.2884
YRI	MUC12	101129565	23.5646	23.5287	0.0048
YRI	MUC12	101134565	25.3313	24.369	0.1232
YRI	MUC12	101139565	25.1704	22.0162	0.4454
YRI	MUC12	101144565	21.7009	19.8313	0.292
YRI	MUC12	101149565	22.2631	20.3355	0.2939
YRI	MUC12	101154565	30.5601	24.7051	0.7395
YRI	MUC12	101159565	30.6141	26.5538	0.4781
YRI	MUC12	101164565	20.7912	24.0329	-0.4205
YRI	MUC12	101169565	14.8892	19.4952	-0.7314
YRI	MUC12	101174565	18.4174	19.1591	-0.1198
YRI	MUC12	101179565	16.908	18.991	-0.3392
YRI	MUC12	101184565	13.028	15.7978	-0.5381
YRI	MUC12	101189565	18.6545	17.4785	0.2074
YRI	MUC12	101194565	17.7153	16.4701	0.2325
YRI	MUC12	101199565	15.9354	16.6382	-0.1299
YRI	MUC12	101204565	17.1522	19.1591	-0.3241
YRI	MUC12	101209565	11.138	14.2853	-0.6729
YRI	MUC12	101214565	8.435	12.4366	-0.9756
YRI	MUC12	101219565	8.4727	13.445	-1.1261
YRI	MUC12	101224565	12.1385	15.2937	-0.6322

YRI	MUC12	101229565	13.1923	15.2937	-0.4211
YRI	MUC12	101234565	11.9602	16.134	-0.7947
YRI	MUC12	101239565	9.8451	15.1256	-1.0693
YRI	MUC12	101244565	10.2844	14.2853	-0.8554
YRI	MUC12	101249565	14.5029	15.2937	-0.1585
YRI	MUC12	101254565	12.7105	12.9408	-0.0541
YRI	MUC12	101259565	9.2916	13.7811	-0.9932
YRI	MUC12	101264565	9.6439	17.3104	-1.3646
YRI	MUC12	101269565	15.3507	19.4952	-0.6581
YRI	MUC12	101274565	18.1227	18.991	-0.1414
YRI	MUC12	101279565	17.6148	18.823	-0.1984
YRI	MUC12	101284565	17.4457	17.4785	-0.0058
YRI	MUC12	101289565	8.9942	9.0754	-0.0266
YRI	MUC12	101294565	2.6597	3.1932	-0.44
YRI	MUC12	101299565	2.999	3.3612	-0.2862
YRI	MUC12	101304565	3.3676	3.3612	0.0051
YRI	MUC12	101309565	3.6263	4.3696	-0.4693
YRI	MUC12	101314565	1.5395	2.689	-1.0924
YRI	MUC12	101319565	0.5652	1.0084	-0.8836
YRI	MUC12	101324565	4.0251	3.1932	0.6861
YRI	MUC12	101329565	9.3737	11.0921	-0.4666
YRI	MUC12	101334565	14.3806	18.3188	-0.6639
YRI	MUC12	101339565	15.6218	18.1507	-0.4301
YRI	MUC12	101344565	16.9996	21.512	-0.6517
YRI	MUC12	101349565	18.5286	21.8481	-0.4723
YRI	MUC12	101354565	14.157	16.9743	-0.511
YRI	MUC12	101359565	11.3584	17.1423	-1.0392
YRI	MUC12	101364565	11.481	16.9743	-0.9963
YRI	MUC12	101369565	13.5491	14.4534	-0.1912
YRI	MUC12	101374565	15.6111	15.6298	-0.0037
YRI	MUC12	101379565	16.8265	18.1507	-0.2252
YRI	MUC12	101384565	17.1293	17.1423	-0.0023
YRI	MUC12	101389565	17.3039	15.7978	0.2926
YRI	MUC12	101394565	14.199	12.7727	0.3391
YRI	MUC12	101399565	9.8047	13.1089	-0.7665
YRI	MUC12	101404565	10.4669	14.7895	-0.8942
YRI	MUC12	101409565	13.3358	16.8062	-0.6355
YRI	MUC12	101414565	16.4502	19.6633	-0.506
YRI	MUC12	101419565	16.0832	18.3188	-0.3769
YRI	MUC12	101424565	14.5429	15.6298	-0.2133
YRI	MUC12	101429565	17.2137	16.4701	0.1388
YRI	MUC12	101434565	20.6291	17.1423	0.6265
YRI	MUC12	101439565	17.9977	14.2853	0.7937
YRI	MUC12	101444565	14.6512	14.1172	0.1155
YRI	MUC12	101449565	10.9691	14.7895	-0.7903
YRI	MUC12	101454565	7.1796	13.7811	-1.4605
YRI	MUC12	101459565	5.3181	9.7476	-1.3569
YRI	MUC12	101464565	4.6793	10.2518	-1.6287
YRI	MUC12	101469565	7.0588	12.9408	-1.3812

YRI	MUC12	101474565	7.702	11.0921	-0.9204
YRI	MUC12	101479565	7.1844	10.756	-0.9981
YRI	MUC12	101484565	5.5411	9.4115	-1.225
YRI	MUC12	101489565	7.7414	9.5795	-0.5723
YRI	MUC12	101494565	12.465	12.7727	-0.0732
YRI	MUC12	101499565	9.2911	10.756	-0.4094
YRI	MUC12	101504565	5.5637	8.5712	-1.0379
YRI	MUC12	101509565	6.2593	10.4199	-1.1977
CEU	NBPF11	147607151	0.2119	0.1706	0.2453
CEU	NBPF11	147612151	0	0	0
CEU	NBPF11	147617151	0	0	0
CEU	NBPF11	147622151	0	0	0
CEU	NBPF11	147627151	2.2855	2.5584	-0.2719
CEU	NBPF11	147632151	2.2855	2.5584	-0.2719
CEU	NBPF11	147637151	0	0	0
CEU	NBPF11	147642151	0	0	0
CEU	NBPF11	147647151	0	0	0
CEU	NBPF11	147652151	0	0	0
CEU	NBPF11	147657151	0	0	0
CEU	NBPF11	147662151	0	0	0
CEU	NBPF11	147667151	0.0202	0.3411	-1.2864
CEU	NBPF11	147672151	0.0202	0.3411	-1.2864
CEU	NBPF11	147677151	0	0	0
CEU	NBPF11	147682151	0	0	0
CEU	NBPF11	147687151	0	0	0
CEU	NBPF11	147692151	0.3723	0.5117	-0.4372
CEU	NBPF11	147697151	0.3925	0.8528	-1.0366
CEU	NBPF11	147702151	1.0447	1.1939	-0.2659
CEU	NBPF11	147707151	1.5366	2.5584	-1.0179
CEU	NBPF11	147712151	1.5409	4.0935	-1.7164
CEU	NBPF11	147717151	2.1653	4.6052	-1.4814
CEU	NBPF11	147722151	2.691	5.1169	-1.3428
CEU	NBPF11	147727151	3.2136	5.9697	-1.3301
CEU	NBPF11	147732151	6.0111	10.2337	-1.244
CEU	NBPF11	147737151	5.786	10.0632	-1.28
CEU	NBPF11	147742151	3.8757	8.0164	-1.5294
CEU	NBPF11	147747151	5.2719	9.5515	-1.3445
CEU	NBPF11	147752151	4.1538	6.6519	-1.0937
CEU	NBPF11	147757151	6.1612	8.0164	-0.6852
CEU	NBPF11	147762151	6.2224	8.3576	-0.759
CEU	NBPF11	147767151	3.3177	5.9697	-1.2799
CEU	NBPF11	147772151	3.5854	4.7757	-0.7001
CEU	NBPF11	147777151	4.9235	5.2874	-0.1957
CEU	NBPF11	147782151	8.2923	8.5281	-0.0823
CEU	NBPF11	147787151	7.4051	8.6987	-0.4432
CEU	NBPF11	147792151	6.0759	9.0398	-0.9799
CEU	NBPF11	147797151	6.5422	9.7221	-0.9827
CEU	NBPF11	147802151	6.6432	10.4043	-1.0911
CEU	NBPF11	147807151	6.5735	10.916	-1.2044

CEU	NBPF11	147812151	5.4278	9.7221	-1.3271
CEU	NBPF11	147817151	8.407	11.0866	-0.7325
CEU	NBPF11	147822151	10.3521	12.9627	-0.6159
CEU	NBPF11	147827151	6.851	9.3809	-0.8082
CEU	NBPF11	147832151	2.9749	3.9229	-0.6612
CEU	NBPF11	147837151	0.8491	1.0234	-0.3463
CEU	NBPF11	147842151	0.5079	0.6822	-0.4551
CEU	NBPF11	147847151	0.5079	0.6822	-0.4551
CEU	NBPF11	147852151	0.2847	0.6822	-1.038
CEU	NBPF11	147857151	0.4193	1.1939	-1.3804
CEU	NBPF11	147862151	0.2672	0.6822	-1.0837
CEU	NBPF11	147867151	0.2589	0.1706	0.5245
CEU	NBPF11	147872151	0.2589	0.1706	0.5245
CEU	NBPF11	147877151	0	0	0
CEU	NBPF11	147882151	0	0	0
CEU	NBPF11	147887151	0	0	0
CEU	NBPF11	147892151	0	0	0
CEU	NBPF11	147897151	0.451	0.1706	1.6656
CEU	NBPF11	147902151	0.451	0.1706	1.6656
CEU	NBPF11	147907151	0.0101	0.1706	-0.9534
CEU	NBPF11	147912151	0.1328	0.3411	-0.835
CEU	NBPF11	147917151	1.214	0.8528	0.8134
CEU	NBPF11	147922151	2.0998	1.5351	0.8373
CEU	NBPF11	147927151	1.0186	1.0234	-0.0095
CEU	NBPF11	147932151	0.0101	0.1706	-0.9534
CEU	NBPF11	147937151	0	0	0
CEU	NBPF11	147942151	0.5727	0.5117	0.1913
CEU	NBPF11	147947151	1.1093	1.1939	-0.1508
CEU	NBPF11	147952151	3.4374	2.8996	0.4837
CEU	NBPF11	147957151	3.2684	3.2407	0.0227
CEU	NBPF11	147962151	0.3676	1.0234	-1.3031
CEU	NBPF11	147967151	0.0594	0.3411	-1.1293
CEU	NBPF11	147972151	1.1266	1.8762	-0.9544
CEU	NBPF11	147977151	1.0872	1.7056	-0.847
CEU	NBPF11	147982151	0.02	0.1706	-0.8946
CEU	NBPF11	147987151	0	0	0
CEU	NBPF11	147992151	2.1769	1.7056	0.6455
CEU	NBPF11	147997151	6.6677	6.3108	0.1639
CEU	NBPF11	148002151	10.1849	10.916	-0.2028
CEU	NBPF11	148007151	7.3034	9.8926	-0.7873
CEU	NBPF11	148012151	3.1723	6.3108	-1.4409
CEU	NBPF11	148017151	4.9231	5.9697	-0.5051
CEU	NBPF11	148022151	8.2422	9.7221	-0.4573
CEU	NBPF11	148027151	4.8821	6.4814	-0.7168
CEU	NBPF11	148032151	0	0	0
CEU	NBPF11	148037151	0	0	0
CEU	NBPF11	148042151	0	0	0
CEU	NBPF11	148047151	0	0	0
CEU	NBPF11	148052151	0	0	0

CEU	NBPF11	148057151	0	0	0
CEU	NBPF11	148062151	0	0	0
CEU	NBPF11	148067151	0	0	0
CEU	NBPF11	148072151	0	0	0
CEU	NBPF11	148077151	0	0	0
CEU	NBPF11	148082151	0	0	0
CEU	NBPF11	148087151	0	0	0
CEU	NBPF11	148092151	0	0	0
CEU	NBPF11	148097151	0	0	0
CEU	NBPF11	148102151	0	0	0
CEU	NBPF11	148107151	0	0	0
CEU	NBPF11	148112151	0	0	0
CEU	NBPF11	148117151	0	0	0
CEU	NBPF11	148122151	0	0	0
CEU	NBPF11	148127151	0	0	0
CEU	NBPF11	148132151	0	0	0
CEU	NBPF11	148137151	0	0	0
CEU	NBPF11	148142151	0	0	0
CEU	NBPF11	148147151	0	0	0
CEU	NBPF11	148152151	0	0	0
CEU	NBPF11	148157151	0	0	0
CEU	NBPF11	148162151	0	0	0
CEU	NBPF11	148167151	0	0	0
CEU	NBPF11	148172151	0.0202	0.3411	-1.2864
CEU	NBPF11	148177151	0.2585	1.7056	-1.982
CEU	NBPF11	148182151	0.8664	3.0701	-1.8903
CEU	NBPF11	148187151	0.7969	2.5584	-1.7547
CEU	NBPF11	148192151	0.3824	2.0467	-1.9803
CEU	NBPF11	148197151	0.2136	1.1939	-1.7469
CEU	NBPF11	148202151	0.5294	1.0234	-0.9816
CEU	NBPF11	148207151	1.7258	2.5584	-0.8294
CEU	NBPF11	148212151	1.7425	2.0467	-0.362
CEU	NBPF11	148217151	1.3024	2.0467	-0.8856
CEU	NBPF11	148222151	1.2801	2.729	-1.3695
CEU	NBPF11	148227151	1.1323	2.0467	-1.088
CEU	NBPF11	148232151	1.3055	2.5584	-1.2481
CEU	NBPF11	148237151	2.885	8.187	-1.9208
CEU	NBPF11	148242151	3.7034	7.5047	-1.4914
CEU	NBPF11	148247151	2.1148	3.9229	-1.2611
CEU	NBPF11	148252151	1.4319	5.458	-2.1047
CEU	NBPF11	148257151	0.8916	2.729	-1.7367
CEU	NBPF11	148262151	0.0592	0.1706	-0.6617
CEU	NBPF11	148267151	0	0	0
CEU	NBPF11	148272151	0	0	0
CEU	NBPF11	148277151	0.0493	0.1706	-0.7205
CEU	NBPF11	148282151	0.0493	0.1706	-0.7205
CEU	NBPF11	148287151	0	0	0
CEU	NBPF11	148292151	0	0	0
CEU	NBPF11	148297151	0	0	0

CEU	NBPF11	148302151	0.1944	0.1706	0.1414
CEU	NBPF11	148307151	0.2144	0.3411	-0.5079
CEU	NBPF11	148312151	0.02	0.1706	-0.8946
CEU	NBPF11	148317151	0.1276	0.3411	-0.8559
CEU	NBPF11	148322151	0.1276	0.3411	-0.8559
CEU	NBPF11	148327151	0.4877	0.1706	1.8836
CEU	NBPF11	148332151	0.4877	0.1706	1.8836
CEU	NBPF11	148337151	0.4787	0.5117	-0.1035
CEU	NBPF11	148342151	2.6232	5.1169	-1.3803
CEU	NBPF11	148347151	2.7543	6.6519	-1.7064
CEU	NBPF11	148352151	1.906	3.5818	-1.263
CEU	NBPF11	148357151	1.4265	2.5584	-1.1276
CEU	NBPF11	148362151	0.1303	1.0234	-1.7746
CEU	NBPF11	148367151	0	0	0
CEU	NBPF11	148372151	0	0	0
CEU	NBPF11	148377151	0	0	0
CEU	NBPF11	148382151	0	0	0
CEU	NBPF11	148387151	0	0	0
CEU	NBPF11	148392151	0	0	0
CEU	NBPF11	148397151	0	0	0
CEU	NBPF11	148402151	0	0	0
CEU	NBPF11	148407151	0	0	0
CEU	NBPF11	148412151	0	0	0
CEU	NBPF11	148417151	0	0	0
CEU	NBPF11	148422151	0	0	0
CEU	NBPF11	148427151	0	0	0
CEU	NBPF11	148432151	0	0	0
CEU	NBPF11	148437151	0	0	0
CEU	NBPF11	148442151	0	0	0
CEU	NBPF11	148447151	0	0	0
CEU	NBPF11	148452151	0	0	0
CEU	NBPF11	148457151	0	0	0
CEU	NBPF11	148462151	0	0	0
CEU	NBPF11	148467151	0	0	0
CEU	NBPF11	148472151	0	0	0
CEU	NBPF11	148477151	0	0	0
CEU	NBPF11	148482151	0	0	0
CEU	NBPF11	148487151	0	0	0
CEU	NBPF11	148492151	0	0	0
CEU	NBPF11	148497151	0	0	0
CEU	NBPF11	148502151	0	0	0
CEU	NBPF11	148507151	0.0202	0.3411	-1.2864
CEU	NBPF11	148512151	1.4613	2.5584	-1.0929
CEU	NBPF11	148517151	2.0616	4.0935	-1.3663
CEU	NBPF11	148522151	1.6597	4.9463	-1.8743
CEU	NBPF11	148527151	2.187	5.7991	-1.789
CEU	NBPF11	148532151	3.4632	7.1636	-1.5148
CEU	NBPF11	148537151	2.9243	6.4814	-1.5943
CEU	NBPF11	148542151	1.1181	4.7757	-2.1512

CEU	NBPF11	148547151	4.1148	7.5047	-1.33
CEU	NBPF11	148552151	4.8571	8.5281	-1.2809
CEU	NBPF11	148557151	1.757	5.9697	-2.0331
CEU	NBPF11	148562151	1.6516	5.9697	-2.084
CEU	NBPF11	148567151	1.6164	5.6286	-2.0408
CEU	NBPF11	148572151	1.1934	3.0701	-1.6098
CEU	NBPF11	148577151	1.0188	2.3879	-1.4418
CEU	NBPF11	148582151	0.8153	1.7056	-1.2194
CEU	NBPF11	148587151	0.6003	1.5351	-1.3861
CEU	NBPF11	148592151	1.2569	3.2407	-1.6267
CEU	NBPF11	148597151	1.8044	3.5818	-1.3396
CEU	NBPF11	148602151	0.8281	2.5584	-1.7237
CEU	NBPF11	148607151	0.7671	2.3879	-1.7069
CEU	NBPF11	148612151	0.5772	1.3645	-1.2737
CEU	NBPF11	148617151	0.0101	0.1706	-0.9534
CEU	NBPF11	148622151	0.61	1.3645	-1.2206
CEU	NBPF11	148627151	2.7924	3.7524	-0.6955
CEU	NBPF11	148632151	2.8221	3.4112	-0.4627
CEU	NBPF11	148637151	1.6487	1.5351	0.1684
CEU	NBPF11	148642151	1.5098	0.6822	2.1611
CEU	NBPF11	148647151	0.8765	0.5117	1.1441
CHB	NBPF11	147607151	0.3162	0.1694	0.8723
CHB	NBPF11	147612151	0	0	0
CHB	NBPF11	147617151	0	0	0
CHB	NBPF11	147622151	0	0	0
CHB	NBPF11	147627151	2.9212	2.8801	0.0371
CHB	NBPF11	147632151	2.9212	2.8801	0.0371
CHB	NBPF11	147637151	0	0	0
CHB	NBPF11	147642151	0	0	0
CHB	NBPF11	147647151	0	0	0
CHB	NBPF11	147652151	0	0	0
CHB	NBPF11	147657151	0	0	0
CHB	NBPF11	147662151	0	0	0
CHB	NBPF11	147667151	0.0097	0.1694	-0.949
CHB	NBPF11	147672151	0.0097	0.1694	-0.949
CHB	NBPF11	147677151	0	0	0
CHB	NBPF11	147682151	0	0	0
CHB	NBPF11	147687151	0	0	0
CHB	NBPF11	147692151	0	0	0
CHB	NBPF11	147697151	0.1765	0.3388	-0.651
CHB	NBPF11	147702151	1.1588	1.5247	-0.5436
CHB	NBPF11	147707151	1.4767	2.033	-0.6634
CHB	NBPF11	147712151	1.019	1.8636	-1.0776
CHB	NBPF11	147717151	2.3109	3.3883	-0.8485
CHB	NBPF11	147722151	3.6107	5.2519	-0.8852
CHB	NBPF11	147727151	3.8028	6.6072	-1.2317
CHB	NBPF11	147732151	7.321	11.6896	-1.1329
CHB	NBPF11	147737151	7.5978	13.7226	-1.3651
CHB	NBPF11	147742151	5.1196	10.3343	-1.5179

CHB	NBPF11	147747151	6.4415	9.3178	-0.922
CHB	NBPF11	147752151	5.0571	6.4378	-0.6208
CHB	NBPF11	147757151	8.2789	13.045	-1.1145
CHB	NBPF11	147762151	7.9973	15.7556	-1.5161
CHB	NBPF11	147767151	3.7474	9.8261	-1.8546
CHB	NBPF11	147772151	3.8923	8.1319	-1.541
CHB	NBPF11	147777151	4.9075	7.6237	-1.0474
CHB	NBPF11	147782151	9.1081	8.979	0.0428
CHB	NBPF11	147787151	8.7379	9.6567	-0.2849
CHB	NBPF11	147792151	7.7723	9.9955	-0.6676
CHB	NBPF11	147797151	8.1932	10.5037	-0.6624
CHB	NBPF11	147802151	8.9575	12.1979	-0.8073
CHB	NBPF11	147807151	8.8291	11.1814	-0.636
CHB	NBPF11	147812151	7.0293	9.4872	-0.7748
CHB	NBPF11	147817151	12.2157	12.7061	-0.1176
CHB	NBPF11	147822151	14.6745	15.925	-0.2419
CHB	NBPF11	147827151	8.419	11.8591	-0.8801
CHB	NBPF11	147832151	2.9185	4.913	-1.1408
CHB	NBPF11	147837151	0.7264	1.0165	-0.5773
CHB	NBPF11	147842151	0.8784	1.1859	-0.5489
CHB	NBPF11	147847151	0.9754	2.2024	-1.3739
CHB	NBPF11	147852151	0.5414	2.7106	-2.0554
CHB	NBPF11	147857151	0.6393	2.033	-1.662
CHB	NBPF11	147862151	0.42	0.6777	-0.6737
CHB	NBPF11	147867151	0.3448	0.1694	1.0423
CHB	NBPF11	147872151	0.3448	0.1694	1.0423
CHB	NBPF11	147877151	0	0	0
CHB	NBPF11	147882151	0.0097	0.1694	-0.949
CHB	NBPF11	147887151	0.0097	0.1694	-0.949
CHB	NBPF11	147892151	0	0	0
CHB	NBPF11	147897151	0.3992	0.3388	0.2423
CHB	NBPF11	147902151	0.4184	0.5082	-0.2819
CHB	NBPF11	147907151	0.0192	0.1694	-0.8925
CHB	NBPF11	147912151	0.0097	0.1694	-0.949
CHB	NBPF11	147917151	1.9425	1.0165	1.8428
CHB	NBPF11	147922151	2.953	1.6942	1.7276
CHB	NBPF11	147927151	1.1045	1.1859	-0.1453
CHB	NBPF11	147932151	0.222	0.6777	-1.1913
CHB	NBPF11	147937151	0.1571	0.6777	-1.361
CHB	NBPF11	147942151	0.1215	0.5082	-1.2139
CHB	NBPF11	147947151	1.0912	1.1859	-0.169
CHB	NBPF11	147952151	3.0676	3.7271	-0.4791
CHB	NBPF11	147957151	2.8178	3.5577	-0.5592
CHB	NBPF11	147962151	0.7491	1.0165	-0.5321
CHB	NBPF11	147967151	0.5802	1.0165	-0.8683
CHB	NBPF11	147972151	0.9876	1.8636	-1.1177
CHB	NBPF11	147977151	0.4171	1.0165	-1.1928
CHB	NBPF11	147982151	0.0097	0.1694	-0.949
CHB	NBPF11	147987151	0.0834	0.3388	-1.0245

CHB	NBPF11	147992151	2.0857	3.3883	-1.0258
CHB	NBPF11	147997151	7.06	7.6237	-0.2174
CHB	NBPF11	148002151	9.7843	10.6731	-0.251
CHB	NBPF11	148007151	6.4953	10.6731	-1.18
CHB	NBPF11	148012151	5.1274	8.3013	-1.132
CHB	NBPF11	148017151	6.4632	7.1154	-0.2678
CHB	NBPF11	148022151	8.7299	9.6567	-0.2874
CHB	NBPF11	148027151	5.6351	6.4378	-0.3609
CHB	NBPF11	148032151	0	0	0
CHB	NBPF11	148037151	0	0	0
CHB	NBPF11	148042151	0	0	0
CHB	NBPF11	148047151	0	0	0
CHB	NBPF11	148052151	0	0	0
CHB	NBPF11	148057151	0	0	0
CHB	NBPF11	148062151	0	0	0
CHB	NBPF11	148067151	0	0	0
CHB	NBPF11	148072151	0	0	0
CHB	NBPF11	148077151	0	0	0
CHB	NBPF11	148082151	0	0	0
CHB	NBPF11	148087151	0	0	0
CHB	NBPF11	148092151	0	0	0
CHB	NBPF11	148097151	0	0	0
CHB	NBPF11	148102151	0	0	0
CHB	NBPF11	148107151	0	0	0
CHB	NBPF11	148112151	0	0	0
CHB	NBPF11	148117151	0	0	0
CHB	NBPF11	148122151	0	0	0
CHB	NBPF11	148127151	0	0	0
CHB	NBPF11	148132151	0	0	0
CHB	NBPF11	148137151	0	0	0
CHB	NBPF11	148142151	0	0	0
CHB	NBPF11	148147151	0	0	0
CHB	NBPF11	148152151	0	0	0
CHB	NBPF11	148157151	0	0	0
CHB	NBPF11	148162151	0	0	0
CHB	NBPF11	148167151	0	0	0
CHB	NBPF11	148172151	0.0097	0.1694	-0.949
CHB	NBPF11	148177151	0.0777	1.3553	-2.0706
CHB	NBPF11	148182151	0.3265	2.3718	-2.159
CHB	NBPF11	148187151	0.6802	2.3718	-1.7856
CHB	NBPF11	148192151	0.6503	2.5412	-1.8882
CHB	NBPF11	148197151	0.2286	1.3553	-1.826
CHB	NBPF11	148202151	0.3644	1.0165	-1.2977
CHB	NBPF11	148207151	2.0602	2.8801	-0.7392
CHB	NBPF11	148212151	2.2656	2.8801	-0.554
CHB	NBPF11	148217151	1.0388	3.0495	-1.7292
CHB	NBPF11	148222151	0.7906	3.5577	-2.0912
CHB	NBPF11	148227151	0.8509	2.2024	-1.5133
CHB	NBPF11	148232151	1.1036	1.8636	-0.9697

CHB	NBPF11	148237151	2.1642	6.946	-2.0072
CHB	NBPF11	148242151	3.1474	7.1154	-1.6295
CHB	NBPF11	148247151	2.2349	5.2519	-1.6272
CHB	NBPF11	148252151	1.7006	7.9625	-2.3205
CHB	NBPF11	148257151	1.023	4.066	-2.052
CHB	NBPF11	148262151	0	0	0
CHB	NBPF11	148267151	0	0	0
CHB	NBPF11	148272151	0	0	0
CHB	NBPF11	148277151	0.0097	0.1694	-0.949
CHB	NBPF11	148282151	0.0097	0.1694	-0.949
CHB	NBPF11	148287151	0	0	0
CHB	NBPF11	148292151	0	0	0
CHB	NBPF11	148297151	0	0	0
CHB	NBPF11	148302151	0.102	0.1694	-0.4005
CHB	NBPF11	148307151	0.23	0.3388	-0.4364
CHB	NBPF11	148312151	0.1279	0.1694	-0.2466
CHB	NBPF11	148317151	0.0383	0.1694	-0.779
CHB	NBPF11	148322151	0.0383	0.1694	-0.779
CHB	NBPF11	148327151	0.4647	0.1694	1.7547
CHB	NBPF11	148332151	0.4647	0.1694	1.7547
CHB	NBPF11	148337151	0.5848	1.1859	-1.073
CHB	NBPF11	148342151	3.0293	6.2684	-1.4918
CHB	NBPF11	148347151	2.944	8.3013	-1.9107
CHB	NBPF11	148352151	1.815	5.4213	-1.891
CHB	NBPF11	148357151	1.3834	3.3883	-1.5789
CHB	NBPF11	148362151	0.068	1.1859	-1.9954
CHB	NBPF11	148367151	0	0	0
CHB	NBPF11	148372151	0	0	0
CHB	NBPF11	148377151	0	0	0
CHB	NBPF11	148382151	0	0	0
CHB	NBPF11	148387151	0	0	0
CHB	NBPF11	148392151	0	0	0
CHB	NBPF11	148397151	0	0	0
CHB	NBPF11	148402151	0	0	0
CHB	NBPF11	148407151	0	0	0
CHB	NBPF11	148412151	0	0	0
CHB	NBPF11	148417151	0	0	0
CHB	NBPF11	148422151	0	0	0
CHB	NBPF11	148427151	0	0	0
CHB	NBPF11	148432151	0	0	0
CHB	NBPF11	148437151	0	0	0
CHB	NBPF11	148442151	0	0	0
CHB	NBPF11	148447151	0	0	0
CHB	NBPF11	148452151	0	0	0
CHB	NBPF11	148457151	0	0	0
CHB	NBPF11	148462151	0	0	0
CHB	NBPF11	148467151	0	0	0
CHB	NBPF11	148472151	0	0	0
CHB	NBPF11	148477151	0	0	0

CHB	NBPF11	148482151	0	0	0
CHB	NBPF11	148487151	0	0	0
CHB	NBPF11	148492151	0	0	0
CHB	NBPF11	148497151	0	0	0
CHB	NBPF11	148502151	0	0	0
CHB	NBPF11	148507151	0.0674	0.3388	-1.0887
CHB	NBPF11	148512151	1.2404	4.7436	-2.0665
CHB	NBPF11	148517151	2.099	7.9625	-2.1728
CHB	NBPF11	148522151	1.8801	7.1154	-2.15
CHB	NBPF11	148527151	1.778	6.2684	-2.0681
CHB	NBPF11	148532151	3.1024	7.1154	-1.648
CHB	NBPF11	148537151	2.7868	6.7766	-1.7127
CHB	NBPF11	148542151	1.0238	4.913	-2.2246
CHB	NBPF11	148547151	3.8688	6.4378	-1.1551
CHB	NBPF11	148552151	4.5998	7.6237	-1.1661
CHB	NBPF11	148557151	1.902	8.6402	-2.3162
CHB	NBPF11	148562151	1.7959	9.9955	-2.4622
CHB	NBPF11	148567151	1.3678	7.1154	-2.3604
CHB	NBPF11	148572151	0.7836	2.7106	-1.8259
CHB	NBPF11	148577151	0.8414	1.1859	-0.6149
CHB	NBPF11	148582151	0.798	0.8471	-0.1107
CHB	NBPF11	148587151	0.6861	1.8636	-1.5023
CHB	NBPF11	148592151	0.9172	4.2354	-2.1602
CHB	NBPF11	148597151	1.3387	3.7271	-1.7351
CHB	NBPF11	148602151	0.9762	2.5412	-1.5628
CHB	NBPF11	148607151	0.9829	2.7106	-1.6371
CHB	NBPF11	148612151	0.6766	1.3553	-1.1
CHB	NBPF11	148617151	0.0946	0.5082	-1.2984
CHB	NBPF11	148622151	0.2774	0.6777	-1.0465
CHB	NBPF11	148627151	1.9048	2.8801	-0.8793
CHB	NBPF11	148632151	2.0691	3.5577	-1.125
CHB	NBPF11	148637151	1.392	1.5247	-0.1971
CHB	NBPF11	148642151	1.2878	1.1859	0.1819
CHB	NBPF11	148647151	0.4203	1.0165	-1.1865
YRI	NBPF11	147607151	0.3319	0.1681	0.9737
YRI	NBPF11	147612151	0	0	0
YRI	NBPF11	147617151	0	0	0
YRI	NBPF11	147622151	0.089	0.1681	-0.4702
YRI	NBPF11	147627151	4.1108	3.3612	0.5922
YRI	NBPF11	147632151	4.0218	3.1932	0.6834
YRI	NBPF11	147637151	0	0	0
YRI	NBPF11	147642151	0	0	0
YRI	NBPF11	147647151	0	0	0
YRI	NBPF11	147652151	0	0	0
YRI	NBPF11	147657151	0	0	0
YRI	NBPF11	147662151	0	0	0
YRI	NBPF11	147667151	0	0	0
YRI	NBPF11	147672151	0	0	0
YRI	NBPF11	147677151	0	0	0

YRI	NBPF11	147682151	0	0	0
YRI	NBPF11	147687151	0	0	0
YRI	NBPF11	147692151	0.574	0.5042	0.2194
YRI	NBPF11	147697151	0.8193	1.6806	-1.1849
YRI	NBPF11	147702151	1.0571	2.689	-1.5509
YRI	NBPF11	147707151	1.6359	4.8738	-1.8587
YRI	NBPF11	147712151	3.2109	8.5712	-1.8499
YRI	NBPF11	147717151	5.3765	11.0921	-1.5518
YRI	NBPF11	147722151	5.9303	10.924	-1.3754
YRI	NBPF11	147727151	5.1547	10.2518	-1.4897
YRI	NBPF11	147732151	10.4686	17.8146	-1.2721
YRI	NBPF11	147737151	12.6803	20.3355	-1.1672
YRI	NBPF11	147742151	8.852	15.6298	-1.3302
YRI	NBPF11	147747151	9.3838	18.991	-1.5645
YRI	NBPF11	147752151	6.9894	14.7895	-1.6136
YRI	NBPF11	147757151	12.7813	17.3104	-0.8062
YRI	NBPF11	147762151	14.0438	21.512	-1.0785
YRI	NBPF11	147767151	7.9399	16.8062	-1.6235
YRI	NBPF11	147772151	6.9563	12.2685	-1.3118
YRI	NBPF11	147777151	6.9798	10.756	-1.0552
YRI	NBPF11	147782151	10.9094	15.2937	-0.8785
YRI	NBPF11	147787151	13.3085	15.2937	-0.3978
YRI	NBPF11	147792151	13.7021	13.613	0.0199
YRI	NBPF11	147797151	12.3498	18.3188	-1.0063
YRI	NBPF11	147802151	16.5264	26.3858	-1.1682
YRI	NBPF11	147807151	19.6419	27.2261	-0.8717
YRI	NBPF11	147812151	18.2899	26.2177	-0.9452
YRI	NBPF11	147817151	23.4529	25.7135	-0.2747
YRI	NBPF11	147822151	24.3774	22.3523	0.2818
YRI	NBPF11	147827151	16.4326	18.4868	-0.3433
YRI	NBPF11	147832151	7.0854	9.5795	-0.7765
YRI	NBPF11	147837151	1.6243	1.8487	-0.287
YRI	NBPF11	147842151	1.3219	1.6806	-0.4935
YRI	NBPF11	147847151	2.3739	2.8571	-0.437
YRI	NBPF11	147852151	4.3217	4.8738	-0.3169
YRI	NBPF11	147857151	4.2474	4.2016	0.0299
YRI	NBPF11	147862151	1.5662	1.3445	0.3601
YRI	NBPF11	147867151	0.246	0.1681	0.4631
YRI	NBPF11	147872151	0.2553	0.3361	-0.3244
YRI	NBPF11	147877151	0.0093	0.1681	-0.944
YRI	NBPF11	147882151	0.089	0.1681	-0.4702
YRI	NBPF11	147887151	0.089	0.1681	-0.4702
YRI	NBPF11	147892151	0	0	0
YRI	NBPF11	147897151	0.4308	0.3361	0.3802
YRI	NBPF11	147902151	0.4668	0.5042	-0.1175
YRI	NBPF11	147907151	0.1099	1.1764	-1.9075
YRI	NBPF11	147912151	0.5647	1.6806	-1.5351
YRI	NBPF11	147917151	1.6988	1.8487	-0.1917
YRI	NBPF11	147922151	2.3333	1.8487	0.6198

YRI	NBPF11	147927151	1.479	1.0084	0.9382
YRI	NBPF11	147932151	0.4836	0.5042	-0.0647
YRI	NBPF11	147937151	0.2442	0.3361	-0.3689
YRI	NBPF11	147942151	0.5838	0.5042	0.2502
YRI	NBPF11	147947151	1.4998	1.8487	-0.4463
YRI	NBPF11	147952151	5.7155	5.378	0.1776
YRI	NBPF11	147957151	5.5659	5.7141	-0.0739
YRI	NBPF11	147962151	1.0029	2.0167	-1.2122
YRI	NBPF11	147967151	0.6264	0.6722	-0.1199
YRI	NBPF11	147972151	1.6012	1.6806	-0.1092
YRI	NBPF11	147977151	1.0971	1.1764	-0.1418
YRI	NBPF11	147982151	0	0	0
YRI	NBPF11	147987151	0	0	0
YRI	NBPF11	147992151	1.7907	2.689	-0.8537
YRI	NBPF11	147997151	6.9843	8.7392	-0.5949
YRI	NBPF11	148002151	14.2284	13.2769	0.2181
YRI	NBPF11	148007151	12.0665	12.7727	-0.1679
YRI	NBPF11	148012151	8.1835	10.0837	-0.564
YRI	NBPF11	148017151	9.2475	9.0754	0.0563
YRI	NBPF11	148022151	11.0404	11.4282	-0.1024
YRI	NBPF11	148027151	6.9447	6.8906	0.0228
YRI	NBPF11	148032151	0	0	0
YRI	NBPF11	148037151	0	0	0
YRI	NBPF11	148042151	0	0	0
YRI	NBPF11	148047151	0	0	0
YRI	NBPF11	148052151	0	0	0
YRI	NBPF11	148057151	0	0	0
YRI	NBPF11	148062151	0	0	0
YRI	NBPF11	148067151	0	0	0
YRI	NBPF11	148072151	0	0	0
YRI	NBPF11	148077151	0	0	0
YRI	NBPF11	148082151	0	0	0
YRI	NBPF11	148087151	0	0	0
YRI	NBPF11	148092151	0	0	0
YRI	NBPF11	148097151	0	0	0
YRI	NBPF11	148102151	0	0	0
YRI	NBPF11	148107151	0	0	0
YRI	NBPF11	148112151	0	0	0
YRI	NBPF11	148117151	0	0	0
YRI	NBPF11	148122151	0	0	0
YRI	NBPF11	148127151	0	0	0
YRI	NBPF11	148132151	0	0	0
YRI	NBPF11	148137151	0	0	0
YRI	NBPF11	148142151	0	0	0
YRI	NBPF11	148147151	0	0	0
YRI	NBPF11	148152151	0	0	0
YRI	NBPF11	148157151	0	0	0
YRI	NBPF11	148162151	0	0	0
YRI	NBPF11	148167151	0	0	0

YRI	NBPF11	148172151	0.0549	0.3361	-1.1289
YRI	NBPF11	148177151	0.3149	1.1764	-1.5408
YRI	NBPF11	148182151	0.8728	2.5209	-1.6505
YRI	NBPF11	148187151	1.058	2.689	-1.55
YRI	NBPF11	148192151	0.9785	3.6974	-1.9817
YRI	NBPF11	148197151	0.5332	2.689	-2.0488
YRI	NBPF11	148202151	0.0554	0.8403	-1.7728
YRI	NBPF11	148207151	1.6634	3.5293	-1.4147
YRI	NBPF11	148212151	2.1785	4.0335	-1.2551
YRI	NBPF11	148217151	0.9457	2.8571	-1.7286
YRI	NBPF11	148222151	1.164	3.0251	-1.6055
YRI	NBPF11	148227151	1.4765	2.3529	-0.9277
YRI	NBPF11	148232151	2.2105	2.689	-0.4547
YRI	NBPF11	148237151	5.6002	10.4199	-1.3874
YRI	NBPF11	148242151	5.5393	11.7644	-1.5993
YRI	NBPF11	148247151	2.2663	7.2267	-2.0016
YRI	NBPF11	148252151	2.4911	7.3947	-1.9377
YRI	NBPF11	148257151	1.7052	3.6974	-1.4521
YRI	NBPF11	148262151	0.0185	0.3361	-1.275
YRI	NBPF11	148267151	0	0	0
YRI	NBPF11	148272151	0	0	0
YRI	NBPF11	148277151	0.1761	0.1681	0.0476
YRI	NBPF11	148282151	0.1761	0.1681	0.0476
YRI	NBPF11	148287151	0	0	0
YRI	NBPF11	148292151	0	0	0
YRI	NBPF11	148297151	0	0	0
YRI	NBPF11	148302151	0.2276	0.1681	0.3537
YRI	NBPF11	148307151	0.6734	0.3361	1.3541
YRI	NBPF11	148312151	0.4458	0.1681	1.6508
YRI	NBPF11	148317151	0.0273	0.1681	-0.837
YRI	NBPF11	148322151	0.0273	0.1681	-0.837
YRI	NBPF11	148327151	0.4907	0.5042	-0.0424
YRI	NBPF11	148332151	0.4907	0.5042	-0.0424
YRI	NBPF11	148337151	0.6931	1.3445	-1.058
YRI	NBPF11	148342151	3.8834	6.8906	-1.2671
YRI	NBPF11	148347151	4.999	9.4115	-1.3965
YRI	NBPF11	148352151	3.3055	6.5544	-1.4324
YRI	NBPF11	148357151	2.1495	4.5377	-1.4593
YRI	NBPF11	148362151	0.6528	1.8487	-1.5297
YRI	NBPF11	148367151	0	0	0
YRI	NBPF11	148372151	0	0	0
YRI	NBPF11	148377151	0	0	0
YRI	NBPF11	148382151	0	0	0
YRI	NBPF11	148387151	0	0	0
YRI	NBPF11	148392151	0	0	0
YRI	NBPF11	148397151	0	0	0
YRI	NBPF11	148402151	0	0	0
YRI	NBPF11	148407151	0	0	0
YRI	NBPF11	148412151	0	0	0

YRI	NBPF11	148417151	0	0	0
YRI	NBPF11	148422151	0	0	0
YRI	NBPF11	148427151	0	0	0
YRI	NBPF11	148432151	0	0	0
YRI	NBPF11	148437151	0	0	0
YRI	NBPF11	148442151	0	0	0
YRI	NBPF11	148447151	0	0	0
YRI	NBPF11	148452151	0	0	0
YRI	NBPF11	148457151	0	0	0
YRI	NBPF11	148462151	0	0	0
YRI	NBPF11	148467151	0	0	0
YRI	NBPF11	148472151	0	0	0
YRI	NBPF11	148477151	0	0	0
YRI	NBPF11	148482151	0	0	0
YRI	NBPF11	148487151	0	0	0
YRI	NBPF11	148492151	0	0	0
YRI	NBPF11	148497151	0	0	0
YRI	NBPF11	148502151	0	0	0
YRI	NBPF11	148507151	0.0369	0.3361	-1.2012
YRI	NBPF11	148512151	1.878	4.8738	-1.7197
YRI	NBPF11	148517151	5.7485	9.7476	-1.2251
YRI	NBPF11	148522151	6.2555	9.7476	-1.0698
YRI	NBPF11	148527151	4.4836	8.5712	-1.4106
YRI	NBPF11	148532151	6.3638	8.7392	-0.8052
YRI	NBPF11	148537151	6.1938	9.2434	-0.9814
YRI	NBPF11	148542151	3.9817	8.7392	-1.6127
YRI	NBPF11	148547151	7.1161	9.5795	-0.7669
YRI	NBPF11	148552151	7.5005	10.756	-0.9097
YRI	NBPF11	148557151	5.4438	11.2602	-1.5571
YRI	NBPF11	148562151	7.985	12.9408	-1.1637
YRI	NBPF11	148567151	6.4323	10.756	-1.2082
YRI	NBPF11	148572151	2.8151	5.7141	-1.4456
YRI	NBPF11	148577151	1.9312	3.1932	-1.0408
YRI	NBPF11	148582151	1.4171	2.3529	-0.9906
YRI	NBPF11	148587151	0.8936	2.0167	-1.3428
YRI	NBPF11	148592151	1.5314	4.3696	-1.7921
YRI	NBPF11	148597151	2.0051	4.7057	-1.5986
YRI	NBPF11	148602151	0.9855	3.3612	-1.8769
YRI	NBPF11	148607151	0.5664	3.5293	-2.2465
YRI	NBPF11	148612151	0.1562	1.5126	-2.0197
YRI	NBPF11	148617151	0.2185	0.6722	-1.1878
YRI	NBPF11	148622151	0.7157	2.5209	-1.8078
YRI	NBPF11	148627151	2.2519	5.0419	-1.5545
YRI	NBPF11	148632151	1.8997	4.0335	-1.4438
YRI	NBPF11	148637151	1.1091	1.3445	-0.3823
YRI	NBPF11	148642151	1.2523	1.0084	0.4863
YRI	NBPF11	148647151	0.4683	0.8403	-0.8402
CEU	NBPF16	143926390	1.7578	3.4112	-1.2986
CEU	NBPF16	143931390	1.1676	2.729	-1.4759

CEU	NBPF16	143936390	1.1132	1.3645	-0.4065
CEU	NBPF16	143941390	1.5984	1.8762	-0.3537
CEU	NBPF16	143946390	0.7825	2.729	-1.8399
CEU	NBPF16	143951390	0.317	2.5584	-2.2328
CEU	NBPF16	143956390	0.1389	1.0234	-1.7575
CEU	NBPF16	143961390	0.1301	1.1939	-1.8957
CEU	NBPF16	143966390	0.6269	1.7056	-1.4774
CEU	NBPF16	143971390	0.8658	1.3645	-0.8068
CEU	NBPF16	143976390	0.3696	1.0234	-1.2991
CEU	NBPF16	143981390	2.1229	1.7056	0.5715
CEU	NBPF16	143986390	3.0812	2.5584	0.5208
CEU	NBPF16	143991390	2.1909	3.0701	-0.7542
CEU	NBPF16	143996390	3.0777	3.7524	-0.4888
CEU	NBPF16	144001390	2.2078	3.2407	-0.847
CEU	NBPF16	144006390	4.4382	4.6052	-0.1014
CEU	NBPF16	144011390	7.9764	7.5047	0.1851
CEU	NBPF16	144016390	4.8421	6.6519	-0.7924
CEU	NBPF16	144021390	2.8314	4.0935	-0.8487
CEU	NBPF16	144026390	4.2237	4.0935	0.0876
CEU	NBPF16	144031390	2.4941	3.9229	-0.9965
CEU	NBPF16	144036390	0.3863	2.5584	-2.1638
CEU	NBPF16	144041390	3.2962	3.9229	-0.4371
CEU	NBPF16	144046390	3.6842	4.6052	-0.5592
CEU	NBPF16	144051390	1.2847	2.729	-1.3652
CEU	NBPF16	144056390	0.7922	2.5584	-1.7594
CEU	NBPF16	144061390	1.2542	3.0701	-1.5577
CEU	NBPF16	144066390	2.1618	2.729	-0.5361
CEU	NBPF16	144071390	1.4995	1.7056	-0.2823
CEU	NBPF16	144076390	0.905	1.1939	-0.5148
CEU	NBPF16	144081390	0.5949	1.7056	-1.5212
CEU	NBPF16	144086390	0.1903	1.5351	-1.9941
CEU	NBPF16	144091390	0.1301	1.0234	-1.775
CEU	NBPF16	144096390	0.0505	0.5117	-1.4464
CEU	NBPF16	144101390	0	0	0
CEU	NBPF16	144106390	0	0	0
CEU	NBPF16	144111390	0	0	0
CEU	NBPF16	144116390	0	0	0
CEU	NBPF16	144121390	0	0	0
CEU	NBPF16	144126390	0	0	0
CEU	NBPF16	144131390	0	0	0
CEU	NBPF16	144136390	0	0	0
CEU	NBPF16	144141390	0.02	0.1706	-0.8946
CEU	NBPF16	144146390	0.02	0.1706	-0.8946
CEU	NBPF16	144151390	0	0	0
CEU	NBPF16	144156390	0.0202	0.3411	-1.2864
CEU	NBPF16	144161390	0.2325	1.0234	-1.5716
CEU	NBPF16	144166390	1.2307	1.3645	-0.2165
CEU	NBPF16	144171390	1.0183	0.6822	0.8777
CEU	NBPF16	144176390	0.4945	0.1706	1.924

CEU	NBPF16	144181390	0.4945	0.1706	1.924
CEU	NBPF16	144186390	0	0	0
CEU	NBPF16	144191390	0	0	0
CEU	NBPF16	144196390	0.0594	0.3411	-1.1293
CEU	NBPF16	144201390	0.0594	0.3411	-1.1293
CEU	NBPF16	144206390	0	0	0
CEU	NBPF16	144211390	0	0	0
CEU	NBPF16	144216390	0.8332	1.1939	-0.6428
CEU	NBPF16	144221390	0.8433	1.3645	-0.8432
CEU	NBPF16	144226390	0.0101	0.1706	-0.9534
CEU	NBPF16	144231390	0	0	0
CEU	NBPF16	144236390	0	0	0
CEU	NBPF16	144241390	0	0	0
CEU	NBPF16	144246390	0	0	0
CEU	NBPF16	144251390	0	0	0
CEU	NBPF16	144256390	0	0	0
CEU	NBPF16	144261390	0	0	0
CEU	NBPF16	144266390	0	0	0
CEU	NBPF16	144271390	0.4686	0.3411	0.5111
CEU	NBPF16	144276390	0.4686	0.3411	0.5111
CEU	NBPF16	144281390	0.4585	0.1706	1.7102
CEU	NBPF16	144286390	0.4585	0.1706	1.7102
CEU	NBPF16	144291390	0	0	0
CEU	NBPF16	144296390	0	0	0
CEU	NBPF16	144301390	1.4576	0.8528	1.362
CEU	NBPF16	144306390	1.4576	0.8528	1.362
CEU	NBPF16	144311390	0	0	0
CEU	NBPF16	144316390	0.1876	0.6822	-1.2915
CEU	NBPF16	144321390	0.2179	1.0234	-1.6006
CEU	NBPF16	144326390	0.0606	0.8528	-1.784
CEU	NBPF16	144331390	0.0303	0.5117	-1.5098
CEU	NBPF16	144336390	0.0202	0.3411	-1.2864
CEU	NBPF16	144341390	0.0891	0.5117	-1.3254
CEU	NBPF16	144346390	0.0689	0.1706	-0.6041
CEU	NBPF16	144351390	0	0	0
CEU	NBPF16	144356390	0	0	0
CEU	NBPF16	144361390	0.2591	0.5117	-0.7922
CEU	NBPF16	144366390	0.2591	0.5117	-0.7922
CEU	NBPF16	144371390	0	0	0
CEU	NBPF16	144376390	0	0	0
CEU	NBPF16	144381390	0	0	0
CEU	NBPF16	144386390	0	0	0
CEU	NBPF16	144391390	0	0	0
CEU	NBPF16	144396390	0	0	0
CEU	NBPF16	144401390	0	0	0
CEU	NBPF16	144406390	0	0	0
CEU	NBPF16	144411390	0	0	0
CEU	NBPF16	144416390	0	0	0
CEU	NBPF16	144421390	0	0	0

CEU	NBPF16	144426390	0	0	0
CEU	NBPF16	144431390	0	0	0
CEU	NBPF16	144436390	0	0	0
CEU	NBPF16	144441390	0	0	0
CEU	NBPF16	144446390	0	0	0
CEU	NBPF16	144451390	0.9093	0.5117	1.247
CEU	NBPF16	144456390	0.9093	0.5117	1.247
CEU	NBPF16	144461390	0.483	0.3411	0.5688
CEU	NBPF16	144466390	1.4102	1.7056	-0.4046
CEU	NBPF16	144471390	2.2754	2.5584	-0.2819
CEU	NBPF16	144476390	2.5958	2.729	-0.1259
CEU	NBPF16	144481390	1.2476	1.5351	-0.4263
CEU	NBPF16	144486390	0.0202	0.1706	-0.8934
CEU	NBPF16	144491390	0.0202	0.1706	-0.8934
CEU	NBPF16	144496390	0	0	0
CEU	NBPF16	144501390	0.0493	0.1706	-0.7205
CEU	NBPF16	144506390	0.3092	1.0234	-1.4191
CEU	NBPF16	144511390	0.9685	1.7056	-1.0095
CEU	NBPF16	144516390	5.4628	3.7524	1.2391
CEU	NBPF16	144521390	9.4407	7.3342	0.844
CEU	NBPF16	144526390	12.9931	10.5749	0.6909
CEU	NBPF16	144531390	8.8913	8.187	0.2552
CEU	NBPF16	144536390	0.6947	3.0701	-2.0376
CEU	NBPF16	144541390	1.6712	3.9229	-1.5705
CEU	NBPF16	144546390	3.3655	6.1402	-1.3057
CEU	NBPF16	144551390	4.498	7.3342	-1.1364
CEU	NBPF16	144556390	6.2112	7.8459	-0.6158
CEU	NBPF16	144561390	8.049	8.187	-0.05
CEU	NBPF16	144566390	10.3847	9.0398	0.4447
CEU	NBPF16	144571390	8.813	6.8225	0.8517
CEU	NBPF16	144576390	9.008	6.8225	0.9352
CEU	NBPF16	144581390	9.0183	7.3342	0.6748
CEU	NBPF16	144586390	6.9627	6.3108	0.2993
CEU	NBPF16	144591390	9.0066	8.3576	0.2307
CEU	NBPF16	144596390	8.2948	8.187	0.0391
CEU	NBPF16	144601390	7.7747	7.5047	0.1059
CEU	NBPF16	144606390	8.8668	8.0164	0.3141
CEU	NBPF16	144611390	11.4036	9.2104	0.7127
CEU	NBPF16	144616390	13.4602	10.5749	0.8244
CEU	NBPF16	144621390	7.5412	7.5047	0.0143
CEU	NBPF16	144626390	1.1117	2.2173	-1.2352
CEU	NBPF16	144631390	0	0	0
CEU	NBPF16	144636390	0	0	0
CEU	NBPF16	144641390	0	0	0
CEU	NBPF16	144646390	0	0	0
CEU	NBPF16	144651390	0	0	0
CEU	NBPF16	144656390	0	0	0
CEU	NBPF16	144661390	0	0	0
CEU	NBPF16	144666390	0	0	0

CEU	NBPF16	144671390	0.7355	1.7056	-1.3287
CEU	NBPF16	144676390	1.216	4.7757	-2.0936
CEU	NBPF16	144681390	1.2228	4.0935	-1.9303
CEU	NBPF16	144686390	2.8415	3.9229	-0.7542
CEU	NBPF16	144691390	2.9559	3.7524	-0.577
CEU	NBPF16	144696390	2.2517	3.5818	-1.0025
CEU	NBPF16	144701390	2.5869	6.3108	-1.7097
CEU	NBPF16	144706390	1.2828	4.9463	-2.0893
CEU	NBPF16	144711390	0.0909	1.3645	-2.0604
CEU	NBPF16	144716390	0	0	0
CEU	NBPF16	144721390	0	0	0
CEU	NBPF16	144726390	0	0	0
CEU	NBPF16	144731390	0	0	0
CEU	NBPF16	144736390	0	0	0
CEU	NBPF16	144741390	0	0	0
CEU	NBPF16	144746390	0	0	0
CEU	NBPF16	144751390	0	0	0
CEU	NBPF16	144756390	0	0	0
CEU	NBPF16	144761390	0	0	0
CEU	NBPF16	144766390	0	0	0
CEU	NBPF16	144771390	0	0	0
CEU	NBPF16	144776390	0	0	0
CEU	NBPF16	144781390	0	0	0
CEU	NBPF16	144786390	0	0	0
CEU	NBPF16	144791390	0	0	0
CEU	NBPF16	144796390	0	0	0
CEU	NBPF16	144801390	0	0	0
CEU	NBPF16	144806390	0.9598	0.8528	0.241
CEU	NBPF16	144811390	6.7809	8.5281	-0.6096
CEU	NBPF16	144816390	7.2779	11.4277	-1.1025
CEU	NBPF16	144821390	5.7215	8.8692	-1.0592
CEU	NBPF16	144826390	6.5879	7.8459	-0.4739
CEU	NBPF16	144831390	3.3309	4.6052	-0.7737
CEU	NBPF16	144836390	3.1255	6.4814	-1.5042
CEU	NBPF16	144841390	3.3634	7.5047	-1.6248
CEU	NBPF16	144846390	5.4442	5.9697	-0.2536
CEU	NBPF16	144851390	11.2488	8.5281	0.9493
CEU	NBPF16	144856390	12.7722	10.916	0.5148
CEU	NBPF16	144861390	9.2342	9.2104	0.0077
CEU	NBPF16	144866390	8.2554	8.5281	-0.0951
CEU	NBPF16	144871390	6.4884	7.8459	-0.5114
CEU	NBPF16	144876390	4.6322	6.4814	-0.8288
CEU	NBPF16	144881390	4.6322	6.3108	-0.7707
CEU	NBPF16	144886390	4.7699	6.3108	-0.7075
CEU	NBPF16	144891390	4.8538	6.6519	-0.7872
CEU	NBPF16	144896390	2.18	4.0935	-1.2867
CEU	NBPF16	144901390	0.6778	2.5584	-1.8734
CEU	NBPF16	144906390	4.2447	4.9463	-0.4001
CEU	NBPF16	144911390	9.9502	7.8459	0.7927

CEU	NBPF16	144916390	9.7435	8.8692	0.2942
CEU	NBPF16	144921390	5.3995	6.4814	-0.4849
CEU	NBPF16	144926390	5.6089	6.1402	-0.25
CEU	NBPF16	144931390	9.8682	8.187	0.6091
CEU	NBPF16	144936390	8.9517	8.3576	0.2112
CEU	NBPF16	144941390	5.867	7.6753	-0.6951
CEU	NBPF16	144946390	5.9858	6.9931	-0.4215
CEU	NBPF16	144951390	5.2711	5.9697	-0.3372
CEU	NBPF16	144956390	3.8872	4.4346	-0.3435
CHB	NBPF16	143926390	1.8987	1.8636	0.0448
CHB	NBPF16	143931390	1.3259	2.5412	-1.2136
CHB	NBPF16	143936390	1.2056	2.3718	-1.231
CHB	NBPF16	143941390	1.565	1.6942	-0.1773
CHB	NBPF16	143946390	0.668	2.5412	-1.8705
CHB	NBPF16	143951390	0.1746	2.3718	-2.3193
CHB	NBPF16	143956390	0.068	1.0165	-1.8876
CHB	NBPF16	143961390	0.1163	1.8636	-2.2293
CHB	NBPF16	143966390	0.6115	1.8636	-1.5975
CHB	NBPF16	143971390	0.7719	1.0165	-0.4868
CHB	NBPF16	143976390	0.4377	1.0165	-1.1518
CHB	NBPF16	143981390	2.4051	2.5412	-0.1359
CHB	NBPF16	143986390	3.2345	2.8801	0.3195
CHB	NBPF16	143991390	2.3339	2.5412	-0.207
CHB	NBPF16	143996390	3.0019	3.3883	-0.3043
CHB	NBPF16	144001390	1.794	3.5577	-1.3329
CHB	NBPF16	144006390	4.0793	5.0825	-0.557
CHB	NBPF16	144011390	8.0517	8.3013	-0.089
CHB	NBPF16	144016390	4.8207	6.946	-0.8921
CHB	NBPF16	144021390	2.2808	3.3883	-0.8722
CHB	NBPF16	144026390	3.1814	3.2189	-0.0308
CHB	NBPF16	144031390	1.8627	3.0495	-1.0207
CHB	NBPF16	144036390	0.4276	2.2024	-1.9873
CHB	NBPF16	144041390	3.1544	3.3883	-0.1842
CHB	NBPF16	144046390	3.5269	3.8965	-0.2585
CHB	NBPF16	144051390	1.4803	3.5577	-1.57
CHB	NBPF16	144056390	0.9958	2.8801	-1.6989
CHB	NBPF16	144061390	0.9088	2.5412	-1.6301
CHB	NBPF16	144066390	1.7082	3.2189	-1.242
CHB	NBPF16	144071390	1.0088	2.2024	-1.3365
CHB	NBPF16	144076390	0.2962	2.8801	-2.3297
CHB	NBPF16	144081390	0.4894	3.2189	-2.244
CHB	NBPF16	144086390	0.3752	2.033	-1.9769
CHB	NBPF16	144091390	0.3105	1.8636	-1.9815
CHB	NBPF16	144096390	0.2427	1.0165	-1.5399
CHB	NBPF16	144101390	0	0	0
CHB	NBPF16	144106390	0	0	0
CHB	NBPF16	144111390	0	0	0
CHB	NBPF16	144116390	0	0	0
CHB	NBPF16	144121390	0	0	0

CHB	NBPF16	144126390	0	0	0
CHB	NBPF16	144131390	0	0	0
CHB	NBPF16	144136390	0	0	0
CHB	NBPF16	144141390	0	0	0
CHB	NBPF16	144146390	0	0	0
CHB	NBPF16	144151390	0	0	0
CHB	NBPF16	144156390	0.0289	0.1694	-0.8349
CHB	NBPF16	144161390	0.6709	0.5082	0.5107
CHB	NBPF16	144166390	1.6554	1.3553	0.4864
CHB	NBPF16	144171390	1.0135	1.0165	-0.006
CHB	NBPF16	144176390	0.4934	0.1694	1.9253
CHB	NBPF16	144181390	0.4934	0.1694	1.9253
CHB	NBPF16	144186390	0	0	0
CHB	NBPF16	144191390	0	0	0
CHB	NBPF16	144196390	0.1376	0.3388	-0.8071
CHB	NBPF16	144201390	0.1376	0.3388	-0.8071
CHB	NBPF16	144206390	0	0	0
CHB	NBPF16	144211390	0	0	0
CHB	NBPF16	144216390	0.5108	1.1859	-1.205
CHB	NBPF16	144221390	0.5108	1.1859	-1.205
CHB	NBPF16	144226390	0	0	0
CHB	NBPF16	144231390	0	0	0
CHB	NBPF16	144236390	0	0	0
CHB	NBPF16	144241390	0	0	0
CHB	NBPF16	144246390	0	0	0
CHB	NBPF16	144251390	0	0	0
CHB	NBPF16	144256390	0	0	0
CHB	NBPF16	144261390	0	0	0
CHB	NBPF16	144266390	0	0	0
CHB	NBPF16	144271390	0.3031	0.1694	0.7945
CHB	NBPF16	144276390	0.3031	0.1694	0.7945
CHB	NBPF16	144281390	0.3031	0.1694	0.7945
CHB	NBPF16	144286390	0.3031	0.1694	0.7945
CHB	NBPF16	144291390	0	0	0
CHB	NBPF16	144296390	0	0	0
CHB	NBPF16	144301390	0.8692	0.8471	0.0498
CHB	NBPF16	144306390	0.8692	0.8471	0.0498
CHB	NBPF16	144311390	0	0	0
CHB	NBPF16	144316390	0.2496	0.6777	-1.1192
CHB	NBPF16	144321390	0.3065	0.8471	-1.219
CHB	NBPF16	144326390	0.0666	0.3388	-1.0919
CHB	NBPF16	144331390	0.0097	0.1694	-0.949
CHB	NBPF16	144336390	0.0192	0.1694	-0.8925
CHB	NBPF16	144341390	0.0577	0.3388	-1.1276
CHB	NBPF16	144346390	0.0385	0.1694	-0.7778
CHB	NBPF16	144351390	0	0	0
CHB	NBPF16	144356390	0	0	0
CHB	NBPF16	144361390	0.1239	0.5082	-1.2064
CHB	NBPF16	144366390	0.1239	0.5082	-1.2064

CHB	NBPF16	144371390	0	0	0
CHB	NBPF16	144376390	0	0	0
CHB	NBPF16	144381390	0	0	0
CHB	NBPF16	144386390	0.0097	0.1694	-0.949
CHB	NBPF16	144391390	0.0097	0.1694	-0.949
CHB	NBPF16	144396390	0	0	0
CHB	NBPF16	144401390	0	0	0
CHB	NBPF16	144406390	0	0	0
CHB	NBPF16	144411390	0	0	0
CHB	NBPF16	144416390	0	0	0
CHB	NBPF16	144421390	0	0	0
CHB	NBPF16	144426390	0	0	0
CHB	NBPF16	144431390	0	0	0
CHB	NBPF16	144436390	0	0	0
CHB	NBPF16	144441390	0	0	0
CHB	NBPF16	144446390	0	0	0
CHB	NBPF16	144451390	0.8382	0.8471	-0.0201
CHB	NBPF16	144456390	0.8382	0.8471	-0.0201
CHB	NBPF16	144461390	0.5378	0.8471	-0.6975
CHB	NBPF16	144466390	1.0702	2.2024	-1.2678
CHB	NBPF16	144471390	1.0527	3.2189	-1.7809
CHB	NBPF16	144476390	1.6078	3.5577	-1.4736
CHB	NBPF16	144481390	1.0973	1.8636	-0.9777
CHB	NBPF16	144486390	0.0097	0.1694	-0.949
CHB	NBPF16	144491390	0.0097	0.1694	-0.949
CHB	NBPF16	144496390	0.0097	0.1694	-0.949
CHB	NBPF16	144501390	0.0579	0.3388	-1.1268
CHB	NBPF16	144506390	0.7192	1.1859	-0.8331
CHB	NBPF16	144511390	1.2557	1.6942	-0.6018
CHB	NBPF16	144516390	4.9562	4.7436	0.1254
CHB	NBPF16	144521390	8.7542	8.4708	0.0992
CHB	NBPF16	144526390	13.3449	11.3508	0.5316
CHB	NBPF16	144531390	9.6002	9.9955	-0.1187
CHB	NBPF16	144536390	1.0228	4.5742	-2.1625
CHB	NBPF16	144541390	2.2515	4.7436	-1.47
CHB	NBPF16	144546390	3.4925	7.2848	-1.5244
CHB	NBPF16	144551390	4.502	7.6237	-1.2038
CHB	NBPF16	144556390	5.9779	7.4543	-0.5812
CHB	NBPF16	144561390	7.7382	9.4872	-0.5513
CHB	NBPF16	144566390	10.6198	11.1814	-0.1519
CHB	NBPF16	144571390	8.4649	8.4708	-0.0021
CHB	NBPF16	144576390	7.434	8.8096	-0.4644
CHB	NBPF16	144581390	8.3173	8.8096	-0.1662
CHB	NBPF16	144586390	8.2109	7.4543	0.2978
CHB	NBPF16	144591390	9.1022	8.6402	0.1588
CHB	NBPF16	144596390	6.9758	8.3013	-0.4727
CHB	NBPF16	144601390	6.6834	6.4378	0.1104
CHB	NBPF16	144606390	8.9724	6.6072	1.0388
CHB	NBPF16	144611390	12.4634	10.5037	0.5618

CHB	NBPF16	144616390	13.6497	14.7391	-0.2269
CHB	NBPF16	144621390	6.9787	10.1649	-0.9419
CHB	NBPF16	144626390	0.7933	1.6942	-1.2364
CHB	NBPF16	144631390	0	0	0
CHB	NBPF16	144636390	0	0	0
CHB	NBPF16	144641390	0	0	0
CHB	NBPF16	144646390	0	0	0
CHB	NBPF16	144651390	0	0	0
CHB	NBPF16	144656390	0	0	0
CHB	NBPF16	144661390	0	0	0
CHB	NBPF16	144666390	0	0	0
CHB	NBPF16	144671390	0.7561	1.8636	-1.413
CHB	NBPF16	144676390	1.7308	5.9295	-2.0327
CHB	NBPF16	144681390	1.7091	6.4378	-2.1262
CHB	NBPF16	144686390	2.7173	6.0989	-1.5963
CHB	NBPF16	144691390	2.7183	5.7601	-1.5112
CHB	NBPF16	144696390	2.6223	5.2519	-1.4182
CHB	NBPF16	144701390	2.8797	7.4543	-1.8007
CHB	NBPF16	144706390	1.3141	5.7601	-2.2089
CHB	NBPF16	144711390	0.3213	1.5247	-1.7878
CHB	NBPF16	144716390	0	0	0
CHB	NBPF16	144721390	0	0	0
CHB	NBPF16	144726390	0	0	0
CHB	NBPF16	144731390	0	0	0
CHB	NBPF16	144736390	0	0	0
CHB	NBPF16	144741390	0	0	0
CHB	NBPF16	144746390	0	0	0
CHB	NBPF16	144751390	0	0	0
CHB	NBPF16	144756390	0	0	0
CHB	NBPF16	144761390	0	0	0
CHB	NBPF16	144766390	0	0	0
CHB	NBPF16	144771390	0	0	0
CHB	NBPF16	144776390	0	0	0
CHB	NBPF16	144781390	0	0	0
CHB	NBPF16	144786390	0	0	0
CHB	NBPF16	144791390	0	0	0
CHB	NBPF16	144796390	0	0	0
CHB	NBPF16	144801390	0	0	0
CHB	NBPF16	144806390	1.8597	1.5247	0.4977
CHB	NBPF16	144811390	11.1342	10.6731	0.1302
CHB	NBPF16	144816390	14.1862	13.045	0.2669
CHB	NBPF16	144821390	10.6678	9.6567	0.3135
CHB	NBPF16	144826390	7.7308	7.7931	-0.0235
CHB	NBPF16	144831390	3.1416	3.8965	-0.528
CHB	NBPF16	144836390	3.4209	5.7601	-1.1622
CHB	NBPF16	144841390	2.6484	7.9625	-1.9692
CHB	NBPF16	144846390	4.9126	8.6402	-1.2813
CHB	NBPF16	144851390	11.846	12.3673	-0.1282
CHB	NBPF16	144856390	12.5279	13.2144	-0.1586

CHB	NBPF16	144861390	8.7838	9.3178	-0.1712
CHB	NBPF16	144866390	7.8903	7.9625	-0.0268
CHB	NBPF16	144871390	6.3432	7.7931	-0.548
CHB	NBPF16	144876390	4.8494	7.1154	-0.9306
CHB	NBPF16	144881390	5.0383	7.7931	-1.0412
CHB	NBPF16	144886390	5.3866	7.9625	-0.9545
CHB	NBPF16	144891390	5.4298	7.7931	-0.8932
CHB	NBPF16	144896390	3.3758	5.2519	-1.0118
CHB	NBPF16	144901390	2.252	4.2354	-1.2912
CHB	NBPF16	144906390	5.371	8.1319	-1.0035
CHB	NBPF16	144911390	10.3151	11.1814	-0.2342
CHB	NBPF16	144916390	9.3743	10.5037	-0.3238
CHB	NBPF16	144921390	4.7917	7.7931	-1.1344
CHB	NBPF16	144926390	5.2284	7.9625	-1.0132
CHB	NBPF16	144931390	9.8758	9.8261	0.0152
CHB	NBPF16	144936390	9.2297	9.4872	-0.0812
CHB	NBPF16	144941390	6.7124	9.1484	-0.7942
CHB	NBPF16	144946390	6.6354	8.979	-0.7774
CHB	NBPF16	144951390	5.1245	7.1154	-0.8176
CHB	NBPF16	144956390	4.2909	5.5907	-0.6632
YRI	NBPF16	143926390	2.433	4.3696	-1.2228
YRI	NBPF16	143931390	2.2034	4.0335	-1.2383
YRI	NBPF16	143936390	2.3952	3.3612	-0.7632
YRI	NBPF16	143941390	2.0898	2.3529	-0.2785
YRI	NBPF16	143946390	1.1742	2.1848	-1.1347
YRI	NBPF16	143951390	0.8801	3.1932	-1.9077
YRI	NBPF16	143956390	0.5328	2.8571	-2.102
YRI	NBPF16	143961390	0.8898	3.6974	-2.0464
YRI	NBPF16	143966390	1.053	3.5293	-1.8775
YRI	NBPF16	143971390	0.8293	1.6806	-1.1711
YRI	NBPF16	143976390	0.7176	1.5126	-1.1838
YRI	NBPF16	143981390	2.6817	3.0251	-0.2962
YRI	NBPF16	143986390	3.8228	4.3696	-0.3453
YRI	NBPF16	143991390	3.6944	5.7141	-1.0071
YRI	NBPF16	143996390	4.5837	5.7141	-0.5637
YRI	NBPF16	144001390	3.3824	4.7057	-0.7833
YRI	NBPF16	144006390	5.7728	8.067	-0.8372
YRI	NBPF16	144011390	9.2444	13.7811	-1.0037
YRI	NBPF16	144016390	5.6022	11.2602	-1.5147
YRI	NBPF16	144021390	3.0632	5.8822	-1.3698
YRI	NBPF16	144026390	3.6688	5.378	-0.8995
YRI	NBPF16	144031390	2.2513	4.5377	-1.3971
YRI	NBPF16	144036390	0.9444	4.0335	-2.0901
YRI	NBPF16	144041390	3.5951	5.5461	-0.9991
YRI	NBPF16	144046390	4.3349	6.7225	-1.0288
YRI	NBPF16	144051390	2.2484	6.3864	-1.8676
YRI	NBPF16	144056390	1.5229	4.8738	-1.9235
YRI	NBPF16	144061390	2.0102	4.8738	-1.6438
YRI	NBPF16	144066390	2.799	5.7141	-1.4536

YRI	NBPF16	144071390	1.4017	4.3696	-1.874
YRI	NBPF16	144076390	0.9919	3.8654	-2.0165
YRI	NBPF16	144081390	1.8077	3.6974	-1.3774
YRI	NBPF16	144086390	1.3922	2.3529	-1.0169
YRI	NBPF16	144091390	0.7132	2.0167	-1.5585
YRI	NBPF16	144096390	0.3113	1.1764	-1.5473
YRI	NBPF16	144101390	0	0	0
YRI	NBPF16	144106390	0	0	0
YRI	NBPF16	144111390	0	0	0
YRI	NBPF16	144116390	0	0	0
YRI	NBPF16	144121390	0	0	0
YRI	NBPF16	144126390	0	0	0
YRI	NBPF16	144131390	0	0	0
YRI	NBPF16	144136390	0	0	0
YRI	NBPF16	144141390	0.0276	0.3361	-1.2385
YRI	NBPF16	144146390	0.0276	0.3361	-1.2385
YRI	NBPF16	144151390	0	0	0
YRI	NBPF16	144156390	0.0369	0.3361	-1.2012
YRI	NBPF16	144161390	0.6035	1.0084	-0.8072
YRI	NBPF16	144166390	1.7811	1.6806	0.1383
YRI	NBPF16	144171390	1.2144	1.0084	0.4107
YRI	NBPF16	144176390	0.4914	0.1681	1.9219
YRI	NBPF16	144181390	0.4914	0.1681	1.9219
YRI	NBPF16	144186390	0.0093	0.1681	-0.944
YRI	NBPF16	144191390	0.0185	0.3361	-1.275
YRI	NBPF16	144196390	0.1234	0.3361	-0.8539
YRI	NBPF16	144201390	0.1141	0.1681	-0.321
YRI	NBPF16	144206390	0	0	0
YRI	NBPF16	144211390	0	0	0
YRI	NBPF16	144216390	0.6969	1.5126	-1.2146
YRI	NBPF16	144221390	0.6969	1.5126	-1.2146
YRI	NBPF16	144226390	0	0	0
YRI	NBPF16	144231390	0	0	0
YRI	NBPF16	144236390	0	0	0
YRI	NBPF16	144241390	0	0	0
YRI	NBPF16	144246390	0	0	0
YRI	NBPF16	144251390	0	0	0
YRI	NBPF16	144256390	0	0	0
YRI	NBPF16	144261390	0	0	0
YRI	NBPF16	144266390	0	0	0
YRI	NBPF16	144271390	0.2983	0.1681	0.774
YRI	NBPF16	144276390	0.2983	0.1681	0.774
YRI	NBPF16	144281390	0.2983	0.1681	0.774
YRI	NBPF16	144286390	0.2983	0.1681	0.774
YRI	NBPF16	144291390	0	0	0
YRI	NBPF16	144296390	0	0	0
YRI	NBPF16	144301390	1.6516	1.0084	1.2823
YRI	NBPF16	144306390	1.6516	1.0084	1.2823
YRI	NBPF16	144311390	0	0	0

YRI	NBPF16	144316390	0.2915	0.8403	-1.2395
YRI	NBPF16	144321390	0.3827	1.1764	-1.4196
YRI	NBPF16	144326390	0.1005	0.5042	-1.2687
YRI	NBPF16	144331390	0.0093	0.1681	-0.944
YRI	NBPF16	144336390	0.5042	0.1681	1.998
YRI	NBPF16	144341390	0.5042	0.1681	1.998
YRI	NBPF16	144346390	0	0	0
YRI	NBPF16	144351390	0	0	0
YRI	NBPF16	144356390	0.0093	0.1681	-0.944
YRI	NBPF16	144361390	0.6053	0.6722	-0.1751
YRI	NBPF16	144366390	0.5961	0.5042	0.2888
YRI	NBPF16	144371390	0	0	0
YRI	NBPF16	144376390	0.0093	0.1681	-0.944
YRI	NBPF16	144381390	0.0093	0.1681	-0.944
YRI	NBPF16	144386390	0	0	0
YRI	NBPF16	144391390	0	0	0
YRI	NBPF16	144396390	0	0	0
YRI	NBPF16	144401390	0	0	0
YRI	NBPF16	144406390	0	0	0
YRI	NBPF16	144411390	0	0	0
YRI	NBPF16	144416390	0	0	0
YRI	NBPF16	144421390	0	0	0
YRI	NBPF16	144426390	0	0	0
YRI	NBPF16	144431390	0	0	0
YRI	NBPF16	144436390	0	0	0
YRI	NBPF16	144441390	0	0	0
YRI	NBPF16	144446390	0	0	0
YRI	NBPF16	144451390	0.7028	0.8403	-0.3106
YRI	NBPF16	144456390	0.7028	0.8403	-0.3106
YRI	NBPF16	144461390	0.8775	1.1764	-0.5346
YRI	NBPF16	144466390	2.6992	4.5377	-1.1234
YRI	NBPF16	144471390	3.0513	5.5461	-1.2776
YRI	NBPF16	144476390	2.8798	4.2016	-0.8634
YRI	NBPF16	144481390	1.7136	2.1848	-0.5291
YRI	NBPF16	144486390	0.1892	0.6722	-1.2645
YRI	NBPF16	144491390	0.1623	0.6722	-1.3349
YRI	NBPF16	144496390	0.0365	0.1681	-0.7823
YRI	NBPF16	144501390	0.0185	0.3361	-1.275
YRI	NBPF16	144506390	0.2261	1.1764	-1.6997
YRI	NBPF16	144511390	1.0273	1.6806	-0.8987
YRI	NBPF16	144516390	5.0217	5.7141	-0.3453
YRI	NBPF16	144521390	9.0116	10.924	-0.5267
YRI	NBPF16	144526390	14.37	15.2937	-0.1851
YRI	NBPF16	144531390	13.8969	14.6214	-0.1515
YRI	NBPF16	144536390	5.6131	7.3947	-0.704
YRI	NBPF16	144541390	6.5549	6.8906	-0.1415
YRI	NBPF16	144546390	10.1572	12.7727	-0.6218
YRI	NBPF16	144551390	8.2651	13.7811	-1.2203
YRI	NBPF16	144556390	9.4001	11.0921	-0.4594

YRI	NBPF16	144561390	12.6806	13.613	-0.2087
YRI	NBPF16	144566390	15.3568	16.302	-0.1782
YRI	NBPF16	144571390	11.9521	11.9324	0.005
YRI	NBPF16	144576390	9.9961	11.4282	-0.3781
YRI	NBPF16	144581390	10.5001	11.0921	-0.1607
YRI	NBPF16	144586390	11.4925	10.756	0.2058
YRI	NBPF16	144591390	12.5259	13.7811	-0.2777
YRI	NBPF16	144596390	9.0731	12.1005	-0.7574
YRI	NBPF16	144601390	6.9564	9.9157	-0.8922
YRI	NBPF16	144606390	8.3905	8.9073	-0.1721
YRI	NBPF16	144611390	13.1512	14.2853	-0.2425
YRI	NBPF16	144616390	14.5111	19.6633	-0.8114
YRI	NBPF16	144621390	7.6314	11.5963	-1.0325
YRI	NBPF16	144626390	0.5634	1.8487	-1.644
YRI	NBPF16	144631390	0	0	0
YRI	NBPF16	144636390	0	0	0
YRI	NBPF16	144641390	0	0	0
YRI	NBPF16	144646390	0	0	0
YRI	NBPF16	144651390	0	0	0
YRI	NBPF16	144656390	0	0	0
YRI	NBPF16	144661390	0	0	0
YRI	NBPF16	144666390	0	0	0
YRI	NBPF16	144671390	1.4173	4.2016	-1.8188
YRI	NBPF16	144676390	5.0858	11.4282	-1.6744
YRI	NBPF16	144681390	5.0165	10.756	-1.6039
YRI	NBPF16	144686390	3.8765	7.8989	-1.4964
YRI	NBPF16	144691390	3.9594	8.067	-1.4989
YRI	NBPF16	144696390	4.2834	8.7392	-1.5104
YRI	NBPF16	144701390	4.835	11.0921	-1.6988
YRI	NBPF16	144706390	3.1434	10.2518	-2.0776
YRI	NBPF16	144711390	1.1609	4.2016	-1.9863
YRI	NBPF16	144716390	0	0	0
YRI	NBPF16	144721390	0	0	0
YRI	NBPF16	144726390	0	0	0
YRI	NBPF16	144731390	0	0	0
YRI	NBPF16	144736390	0	0	0
YRI	NBPF16	144741390	0	0	0
YRI	NBPF16	144746390	0	0	0
YRI	NBPF16	144751390	0	0	0
YRI	NBPF16	144756390	0	0	0
YRI	NBPF16	144761390	0	0	0
YRI	NBPF16	144766390	0	0	0
YRI	NBPF16	144771390	0	0	0
YRI	NBPF16	144776390	0	0	0
YRI	NBPF16	144781390	0	0	0
YRI	NBPF16	144786390	0	0	0
YRI	NBPF16	144791390	0	0	0
YRI	NBPF16	144796390	0	0	0
YRI	NBPF16	144801390	0	0	0

YRI	NBPF16	144806390	1.9423	1.6806	0.36
YRI	NBPF16	144811390	9.2198	13.1089	-0.9021
YRI	NBPF16	144816390	10.8736	16.8062	-1.0863
YRI	NBPF16	144821390	8.9937	13.1089	-0.9546
YRI	NBPF16	144826390	7.8094	10.756	-0.8234
YRI	NBPF16	144831390	4.0291	5.7141	-0.8402
YRI	NBPF16	144836390	4.0455	8.235	-1.5001
YRI	NBPF16	144841390	4.1267	10.4199	-1.8116
YRI	NBPF16	144846390	6.727	11.5963	-1.268
YRI	NBPF16	144851390	12.7619	14.7895	-0.4195
YRI	NBPF16	144856390	14.0073	15.2937	-0.2578
YRI	NBPF16	144861390	10.3531	12.7727	-0.5752
YRI	NBPF16	144866390	9.8507	13.613	-0.8421
YRI	NBPF16	144871390	8.7652	12.1005	-0.8344
YRI	NBPF16	144876390	6.3705	8.7392	-0.8029
YRI	NBPF16	144881390	6.8743	11.2602	-1.1741
YRI	NBPF16	144886390	7.0213	11.0921	-1.1052
YRI	NBPF16	144891390	6.0627	8.4031	-0.8226
YRI	NBPF16	144896390	3.4921	6.8906	-1.432
YRI	NBPF16	144901390	2.1918	6.7225	-1.9523
YRI	NBPF16	144906390	5.9296	9.9157	-1.2018
YRI	NBPF16	144911390	11.1671	12.4366	-0.3095
YRI	NBPF16	144916390	11.1783	12.4366	-0.3068
YRI	NBPF16	144921390	6.2233	10.756	-1.2667
YRI	NBPF16	144926390	6.5138	10.4199	-1.1244
YRI	NBPF16	144931390	12.0402	13.1089	-0.2479
YRI	NBPF16	144936390	11.5786	12.4366	-0.2092
YRI	NBPF16	144941390	9.039	11.2602	-0.5946
YRI	NBPF16	144946390	8.3262	11.7644	-0.8833
YRI	NBPF16	144951390	6.4627	9.9157	-1.0411
YRI	NBPF16	144956390	4.8533	7.0586	-0.9091
CEU	NPIP	43726070	7.4185	8.187	-0.2784
CEU	NPIP	43731070	7.1115	10.0632	-0.8833
CEU	NPIP	43736070	6.1793	9.5515	-1.0594
CEU	NPIP	43741070	6.6118	8.3576	-0.6206
CEU	NPIP	43746070	6.9623	6.9931	-0.0129
CEU	NPIP	43751070	5.9744	7.1636	-0.4868
CEU	NPIP	43756070	7.9728	8.187	-0.0776
CEU	NPIP	43761070	8.6141	7.3342	0.5128
CEU	NPIP	43766070	7.5927	6.6519	0.4119
CEU	NPIP	43771070	9.3424	5.9697	1.6277
CEU	NPIP	43776070	6.3537	4.2641	1.3564
CEU	NPIP	43781070	2.5026	2.729	-0.214
CEU	NPIP	43786070	4.2222	4.2641	-0.0272
CEU	NPIP	43791070	5.1995	6.1402	-0.4427
CEU	NPIP	43796070	2.8277	4.7757	-1.1457
CEU	NPIP	43801070	2.853	3.5818	-0.5493
CEU	NPIP	43806070	4.046	3.2407	0.6603
CEU	NPIP	43811070	3.5269	3.0701	0.3918

CEU	NPIP	43816070	2.7943	4.2641	-0.9541
CEU	NPIP	43821070	5.0744	4.9463	0.0731
CEU	NPIP	43826070	4.8155	4.0935	0.4855
CEU	NPIP	43831070	3.0511	4.0935	-0.7009
CEU	NPIP	43836070	4.0719	4.2641	-0.1248
CEU	NPIP	43841070	8.5595	5.9697	1.2499
CEU	NPIP	43846070	7.3335	6.8225	0.2187
CEU	NPIP	43851070	2.6324	4.4346	-1.1308
CEU	NPIP	43856070	5.303	5.1169	0.103
CEU	NPIP	43861070	5.786	5.458	0.1715
CEU	NPIP	43866070	3.0703	4.2641	-0.7749
CEU	NPIP	43871070	4.8097	4.9463	-0.0779
CEU	NPIP	43876070	9.342	6.9931	0.9828
CEU	NPIP	43881070	8.343	6.4814	0.8344
CEU	NPIP	43886070	5.2144	4.6052	0.3699
CEU	NPIP	43891070	2.7091	3.5818	-0.6578
CEU	NPIP	43896070	2.0328	3.2407	-0.9905
CEU	NPIP	43901070	4.4939	4.7757	-0.1657
CEU	NPIP	43906070	5.4189	4.6052	0.494
CEU	NPIP	43911070	3.6976	3.5818	0.0873
CEU	NPIP	43916070	4.7328	5.1169	-0.2126
CEU	NPIP	43921070	5.0781	5.458	-0.1986
CEU	NPIP	43926070	7.1484	5.6286	0.7731
CEU	NPIP	43931070	6.4721	5.458	0.5301
CEU	NPIP	43936070	1.7508	3.5818	-1.38
CEU	NPIP	43941070	3.9876	4.0935	-0.0712
CEU	NPIP	43946070	6.241	4.9463	0.7384
CEU	NPIP	43951070	4.5463	4.7757	-0.1349
CEU	NPIP	43956070	5.0608	4.9463	0.0653
CEU	NPIP	43961070	5.5675	5.1169	0.2494
CEU	NPIP	43966070	4.0635	4.6052	-0.3289
CEU	NPIP	43971070	4.4879	4.9463	-0.2614
CEU	NPIP	43976070	8.3111	6.6519	0.7264
CEU	NPIP	43981070	11.0643	7.5047	1.3966
CEU	NPIP	43986070	7.0478	6.4814	0.2539
CEU	NPIP	43991070	3.5743	5.6286	-1.0449
CEU	NPIP	43996070	2.7479	4.0935	-0.9048
CEU	NPIP	44001070	2.5387	4.7757	-1.3157
CEU	NPIP	44006070	4.167	5.9697	-0.87
CEU	NPIP	44011070	7.9499	6.1402	0.8516
CEU	NPIP	44016070	7.7267	5.7991	0.9547
CEU	NPIP	44021070	8.0109	6.9931	0.4259
CEU	NPIP	44026070	8.1239	6.8225	0.5569
CEU	NPIP	44031070	6.8668	5.6286	0.6298
CEU	NPIP	44036070	10.8862	8.187	0.9779
CEU	NPIP	44041070	11.737	9.5515	0.6866
CEU	NPIP	44046070	10.8287	9.2104	0.5259
CEU	NPIP	44051070	15.5434	9.7221	1.799
CEU	NPIP	44056070	21.1476	11.7688	2.4238

CEU	NPIP	44061070	19.476	11.9394	1.9215
CEU	NPIP	44066070	13.6955	8.6987	1.7119
CEU	NPIP	44071070	9.2006	6.3108	1.3268
CEU	NPIP	44076070	8.3212	6.4814	0.8246
CEU	NPIP	44081070	10.6425	7.5047	1.2311
CEU	NPIP	44086070	9.7745	7.3342	0.9778
CEU	NPIP	44091070	7.9386	7.1636	0.3172
CEU	NPIP	44096070	11.9235	9.0398	0.9534
CEU	NPIP	44101070	16.5941	10.4043	1.7956
CEU	NPIP	44106070	15.2876	9.7221	1.7199
CEU	NPIP	44111070	15.7724	10.2337	1.6317
CEU	NPIP	44116070	12.6417	9.2104	1.115
CEU	NPIP	44121070	8.7056	8.3576	0.1237
CEU	NPIP	44126070	10.422	8.3576	0.7338
CEU	NPIP	44131070	7.8194	6.1402	0.7902
CEU	NPIP	44136070	4.3816	4.2641	0.0763
CEU	NPIP	44141070	2.7318	2.8996	-0.1509
CEU	NPIP	44146070	3.9363	3.0701	0.743
CEU	NPIP	44151070	7.5217	4.4346	1.937
CEU	NPIP	44156070	5.9897	5.2874	0.3776
CEU	NPIP	44161070	3.49	4.2641	-0.5025
CEU	NPIP	44166070	2.9167	4.2641	-0.8747
CEU	NPIP	44171070	3.1202	4.9463	-1.0414
CEU	NPIP	44176070	4.7543	4.0935	0.4443
CEU	NPIP	44181070	7.2797	5.1169	1.1971
CEU	NPIP	44186070	7.9641	6.6519	0.5745
CEU	NPIP	44191070	7.5238	6.9931	0.2221
CEU	NPIP	44196070	10.7974	8.8692	0.6488
CEU	NPIP	44201070	8.9518	7.1636	0.732
CEU	NPIP	44206070	8.1587	6.4814	0.7518
CEU	NPIP	44211070	14.3836	9.3809	1.5982
CEU	NPIP	44216070	14.4682	8.5281	2.0726
CEU	NPIP	44221070	13.3455	8.0164	1.9683
CEU	NPIP	44226070	11.3455	6.4814	2.1802
CEU	NPIP	44231070	9.1697	6.4814	1.2049
CEU	NPIP	44236070	10.1507	8.0164	0.7883
CEU	NPIP	44241070	10.7081	7.5047	1.2569
CEU	NPIP	44246070	11.6069	7.8459	1.4168
CEU	NPIP	44251070	11.0987	7.8459	1.2254
CEU	NPIP	44256070	11.0954	7.8459	1.2241
CEU	NPIP	44261070	10.1962	6.9931	1.3402
CEU	NPIP	44266070	11.6481	7.6753	1.527
CEU	NPIP	44271070	12.8551	9.0398	1.2614
CEU	NPIP	44276070	12.5372	9.0398	1.1563
CEU	NPIP	44281070	10.3867	8.3576	0.7213
CEU	NPIP	44286070	7.9924	6.6519	0.5869
CEU	NPIP	44291070	7.4209	5.458	1.0262
CEU	NPIP	44296070	5.806	6.1402	-0.1573
CEU	NPIP	44301070	4.724	5.9697	-0.6012

CEU	NPIP	44306070	5.5156	5.9697	-0.2192
CEU	NPIP	44311070	6.7069	6.8225	-0.0495
CEU	NPIP	44316070	7.2154	8.0164	-0.2959
CEU	NPIP	44321070	8.3185	9.2104	-0.2898
CEU	NPIP	44326070	6.9013	8.3576	-0.5177
CEU	NPIP	44331070	4.2117	6.4814	-1.0173
CEU	NPIP	44336070	3.6693	5.6286	-0.9966
CEU	NPIP	44341070	4.0579	6.8225	-1.183
CEU	NPIP	44346070	4.9808	7.3342	-0.9429
CEU	NPIP	44351070	5.5638	6.9931	-0.598
CEU	NPIP	44356070	3.8427	6.1402	-1.0811
CEU	NPIP	44361070	4.0158	7.6753	-1.4066
CEU	NPIP	44366070	6.896	8.8692	-0.664
CEU	NPIP	44371070	6.5762	8.5281	-0.681
CEU	NPIP	44376070	6.1763	9.8926	-1.13
CEU	NPIP	44381070	6.5321	8.5281	-0.6964
CEU	NPIP	44386070	6.1746	7.3342	-0.4646
CEU	NPIP	44391070	6.6019	7.8459	-0.4686
CEU	NPIP	44396070	5.1173	7.5047	-0.9367
CEU	NPIP	44401070	4.6646	6.8225	-0.9234
CEU	NPIP	44406070	5.3191	6.3108	-0.4553
CEU	NPIP	44411070	4.1123	5.7991	-0.8355
CEU	NPIP	44416070	3.0282	3.9229	-0.624
CEU	NPIP	44421070	3.0043	3.2407	-0.1938
CEU	NPIP	44426070	3.0924	4.2641	-0.7606
CEU	NPIP	44431070	2.738	3.5818	-0.636
CEU	NPIP	44436070	2.7549	3.5818	-0.6232
CEU	NPIP	44441070	2.6636	4.9463	-1.3018
CEU	NPIP	44446070	2.6504	5.1169	-1.3653
CEU	NPIP	44451070	4.1616	5.7991	-0.811
CEU	NPIP	44456070	5.2041	6.8225	-0.6925
CEU	NPIP	44461070	3.611	6.6519	-1.3314
CEU	NPIP	44466070	2.2255	6.9931	-1.9948
CEU	NPIP	44471070	3.5275	9.2104	-1.8466
CEU	NPIP	44476070	3.6487	8.0164	-1.6132
CEU	NPIP	44481070	2.3212	5.9697	-1.7608
CEU	NPIP	44486070	3.1676	6.9931	-1.6007
CEU	NPIP	44491070	3.9765	6.1402	-1.0182
CEU	NPIP	44496070	5.9571	6.3108	-0.1624
CEU	NPIP	44501070	8.5172	6.9931	0.6377
CEU	NPIP	44506070	9.2641	9.2104	0.0174
CEU	NPIP	44511070	10.2336	10.2337	0
CEU	NPIP	44516070	9.5951	7.5047	0.8202
CEU	NPIP	44521070	7.7271	6.4814	0.5583
CEU	NPIP	44526070	7.3836	5.458	1.0067
CEU	NPIP	44531070	13.6059	8.6987	1.6812
CEU	NPIP	44536070	18.2899	13.3039	1.1477
CEU	NPIP	44541070	13.0852	10.7454	0.6586
CEU	NPIP	44546070	8.9295	8.3576	0.2033

CEU	NPIP	44551070	7.4362	8.6987	-0.4325
CEU	NPIP	44556070	14.905	12.2805	0.6516
CEU	NPIP	44561070	35.2766	21.1497	2.0868
CEU	NPIP	44566070	41.4698	23.3671	2.4283
CEU	NPIP	44571070	23.2197	16.2034	1.3387
CEU	NPIP	44576070	8.8439	11.4277	-0.6865
CEU	NPIP	44581070	8.1165	11.2571	-0.8463
CEU	NPIP	44586070	8.8683	10.7454	-0.5283
CEU	NPIP	44591070	10.5737	12.2805	-0.4238
CEU	NPIP	44596070	11.2725	10.7454	0.1484
CEU	NPIP	44601070	12.1123	9.0398	1.0158
CEU	NPIP	44606070	13.449	11.4277	0.537
CEU	NPIP	44611070	8.3744	9.8926	-0.4616
CEU	NPIP	44616070	4.3441	6.4814	-0.958
CEU	NPIP	44621070	5.2049	5.1169	0.0487
CEU	NPIP	44626070	5.0754	4.9463	0.0736
CEU	NPIP	44631070	4.9668	5.1169	-0.0831
CEU	NPIP	44636070	5.135	6.3108	-0.5398
CEU	NPIP	44641070	3.1499	6.4814	-1.4932
CEU	NPIP	44646070	6.4038	7.5047	-0.4319
CEU	NPIP	44651070	7.631	7.6753	-0.017
CEU	NPIP	44656070	3.228	6.4814	-1.4582
CEU	NPIP	44661070	3.8534	5.7991	-0.9637
CEU	NPIP	44666070	4.7374	5.2874	-0.2957
CEU	NPIP	44671070	4.1672	6.1402	-0.9284
CEU	NPIP	44676070	4.0538	6.3108	-1.0362
CEU	NPIP	44681070	4.0138	5.7991	-0.8842
CEU	NPIP	44686070	2.899	5.9697	-1.482
CEU	NPIP	44691070	2.9988	5.7991	-1.387
CEU	NPIP	44696070	3.384	5.2874	-1.0235
CEU	NPIP	44701070	2.8464	5.7991	-1.4625
CEU	NPIP	44706070	4.4552	5.1169	-0.3663
CEU	NPIP	44711070	3.7454	3.5818	0.1233
CEU	NPIP	44716070	2.0169	3.9229	-1.3294
CEU	NPIP	44721070	4.0825	5.6286	-0.7864
CEU	NPIP	44726070	5.9549	6.4814	-0.236
CEU	NPIP	44731070	4.1313	4.7757	-0.379
CEU	NPIP	44736070	3.4327	4.6052	-0.7119
CEU	NPIP	44741070	4.6038	6.3108	-0.7837
CEU	NPIP	44746070	4.0008	5.9697	-0.9502
CEU	NPIP	44751070	5.7289	5.9697	-0.1162
CEU	NPIP	44756070	6.1639	5.458	0.369
CEU	NPIP	44761070	4.039	4.4346	-0.2482
CEU	NPIP	44766070	5.4933	5.6286	-0.0688
CEU	NPIP	44771070	5.7481	6.9931	-0.5209
CEU	NPIP	44776070	4.3737	6.9931	-1.096
CEU	NPIP	44781070	3.8699	5.458	-0.8302
CEU	NPIP	44786070	2.6824	5.458	-1.451
CEU	NPIP	44791070	2.2512	5.1169	-1.5862

CEU	NPIP	44796070	3.3222	4.9463	-0.9262
CEU	NPIP	44801070	6.1169	6.9931	-0.3666
CEU	NPIP	44806070	8.2979	7.6753	0.2393
CEU	NPIP	44811070	5.0915	5.6286	-0.2732
CEU	NPIP	44816070	2.7421	4.4346	-1.0619
CEU	NPIP	44821070	3.9847	5.9697	-0.958
CEU	NPIP	44826070	5.4515	7.3342	-0.7543
CEU	NPIP	44831070	5.5803	7.6753	-0.8053
CEU	NPIP	44836070	4.6932	8.6987	-1.3723
CEU	NPIP	44841070	4.9282	9.0398	-1.3594
CEU	NPIP	44846070	4.0206	8.0164	-1.4759
CHB	NPIP	43726070	6.9798	8.6402	-0.5707
CHB	NPIP	43731070	6.2298	9.1484	-0.9516
CHB	NPIP	43736070	6.5842	7.7931	-0.4569
CHB	NPIP	43741070	6.5835	7.4543	-0.3428
CHB	NPIP	43746070	5.8498	7.7931	-0.7345
CHB	NPIP	43751070	5.7306	6.4378	-0.318
CHB	NPIP	43756070	7.6684	7.9625	-0.109
CHB	NPIP	43761070	8.1289	8.6402	-0.1758
CHB	NPIP	43766070	6.6153	7.4543	-0.3303
CHB	NPIP	43771070	6.8879	8.1319	-0.4522
CHB	NPIP	43776070	4.3415	7.1154	-1.1392
CHB	NPIP	43781070	2.0758	6.0989	-1.8991
CHB	NPIP	43786070	3.3914	6.6072	-1.4124
CHB	NPIP	43791070	4.2286	7.2848	-1.2285
CHB	NPIP	43796070	2.4575	5.9295	-1.6808
CHB	NPIP	43801070	2.3946	6.0989	-1.7486
CHB	NPIP	43806070	2.6535	6.946	-1.8018
CHB	NPIP	43811070	1.9617	5.9295	-1.9209
CHB	NPIP	43816070	1.944	5.7601	-1.8959
CHB	NPIP	43821070	3.4386	6.0989	-1.2558
CHB	NPIP	43826070	3.1317	4.913	-1.0189
CHB	NPIP	43831070	1.608	3.7271	-1.5395
CHB	NPIP	43836070	3.2555	5.5907	-1.1915
CHB	NPIP	43841070	6.6469	7.4543	-0.3178
CHB	NPIP	43846070	4.916	6.0989	-0.5584
CHB	NPIP	43851070	1.6975	4.7436	-1.7968
CHB	NPIP	43856070	4.0948	7.2848	-1.2823
CHB	NPIP	43861070	4.3617	8.3013	-1.405
CHB	NPIP	43866070	2.217	5.5907	-1.7213
CHB	NPIP	43871070	2.5479	5.4213	-1.5067
CHB	NPIP	43876070	5.2996	6.6072	-0.5743
CHB	NPIP	43881070	6.2949	5.9295	0.1769
CHB	NPIP	43886070	3.8675	5.7601	-0.9403
CHB	NPIP	43891070	1.6585	5.4213	-1.9731
CHB	NPIP	43896070	1.2144	4.913	-2.1156
CHB	NPIP	43901070	2.3044	6.0989	-1.7912
CHB	NPIP	43906070	3.2583	7.1154	-1.584
CHB	NPIP	43911070	2.8942	6.4378	-1.5933

CHB	NPIP	43916070	3.6185	7.6237	-1.5445
CHB	NPIP	43921070	3.3501	7.4543	-1.6155
CHB	NPIP	43926070	3.8686	6.4378	-1.1552
CHB	NPIP	43931070	3.8399	6.4378	-1.1681
CHB	NPIP	43936070	1.5454	6.2684	-2.1752
CHB	NPIP	43941070	3.3651	6.946	-1.5031
CHB	NPIP	43946070	4.06	6.4378	-1.0691
CHB	NPIP	43951070	2.9905	5.2519	-1.2196
CHB	NPIP	43956070	4.9515	5.5907	-0.3261
CHB	NPIP	43961070	5.5003	6.2684	-0.3538
CHB	NPIP	43966070	4.3069	5.9295	-0.7855
CHB	NPIP	43971070	4.3859	6.6072	-0.9756
CHB	NPIP	43976070	8.9172	8.4708	0.1563
CHB	NPIP	43981070	10.933	9.3178	0.5177
CHB	NPIP	43986070	6.209	9.6567	-1.0691
CHB	NPIP	43991070	3.5913	7.7931	-1.5881
CHB	NPIP	43996070	3.3678	6.0989	-1.2892
CHB	NPIP	44001070	3.6196	7.6237	-1.5441
CHB	NPIP	44006070	4.8639	9.1484	-1.3969
CHB	NPIP	44011070	7.7906	10.6731	-0.8141
CHB	NPIP	44016070	7.6552	10.6731	-0.8524
CHB	NPIP	44021070	8.2939	10.3343	-0.5939
CHB	NPIP	44026070	8.2515	9.6567	-0.4357
CHB	NPIP	44031070	7.4047	8.6402	-0.4247
CHB	NPIP	44036070	11.5867	9.9955	0.4778
CHB	NPIP	44041070	12.2026	12.1979	0.0012
CHB	NPIP	44046070	10.8841	10.8426	0.0115
CHB	NPIP	44051070	15.3267	10.3343	1.4532
CHB	NPIP	44056070	21.1744	13.3838	1.778
CHB	NPIP	44061070	20.0842	12.8755	1.7066
CHB	NPIP	44066070	13.6928	8.3013	1.9229
CHB	NPIP	44071070	8.7919	7.2848	0.6058
CHB	NPIP	44076070	8.426	8.1319	0.1069
CHB	NPIP	44081070	10.6937	9.1484	0.5038
CHB	NPIP	44086070	9.6446	9.8261	-0.0554
CHB	NPIP	44091070	7.9239	7.7931	0.0494
CHB	NPIP	44096070	12.0506	9.9955	0.6171
CHB	NPIP	44101070	16.3451	12.8755	0.8214
CHB	NPIP	44106070	14.7096	11.1814	0.954
CHB	NPIP	44111070	14.9763	10.1649	1.4223
CHB	NPIP	44116070	12.0491	7.2848	1.9151
CHB	NPIP	44121070	8.6096	7.2848	0.5325
CHB	NPIP	44126070	10.0902	10.5037	-0.1185
CHB	NPIP	44131070	7.6541	9.1484	-0.4872
CHB	NPIP	44136070	4.5125	6.4378	-0.8657
CHB	NPIP	44141070	2.7533	5.0825	-1.2931
CHB	NPIP	44146070	3.289	4.5742	-0.7826
CHB	NPIP	44151070	6.3554	5.2519	0.5952
CHB	NPIP	44156070	5.2854	5.4213	-0.0713

CHB	NPIP	44161070	3.2043	5.2519	-1.1043
CHB	NPIP	44166070	2.7881	5.2519	-1.3288
CHB	NPIP	44171070	2.774	4.4048	-1.0262
CHB	NPIP	44176070	4.6859	5.9295	-0.602
CHB	NPIP	44181070	7.206	7.9625	-0.2803
CHB	NPIP	44186070	7.7028	7.6237	0.0305
CHB	NPIP	44191070	7.6453	6.946	0.2935
CHB	NPIP	44196070	10.4812	8.1319	0.8539
CHB	NPIP	44201070	8.5778	7.6237	0.3679
CHB	NPIP	44206070	7.9136	7.4543	0.1808
CHB	NPIP	44211070	14.2014	9.8261	1.3349
CHB	NPIP	44216070	13.9229	8.8096	1.7264
CHB	NPIP	44221070	11.9298	8.4708	1.2109
CHB	NPIP	44226070	10.3052	7.7931	0.9494
CHB	NPIP	44231070	8.5128	7.2848	0.4936
CHB	NPIP	44236070	9.8837	9.1484	0.2397
CHB	NPIP	44241070	10.0392	7.4543	1.0175
CHB	NPIP	44246070	9.3952	7.2848	0.8483
CHB	NPIP	44251070	9.4597	8.979	0.1595
CHB	NPIP	44256070	10.6903	8.979	0.5677
CHB	NPIP	44261070	9.6292	7.6237	0.7734
CHB	NPIP	44266070	10.9694	7.2848	1.4811
CHB	NPIP	44271070	12.13	7.9625	1.5443
CHB	NPIP	44276070	11.0484	7.6237	1.3207
CHB	NPIP	44281070	9.0154	8.1319	0.3211
CHB	NPIP	44286070	7.305	7.9625	-0.2436
CHB	NPIP	44291070	6.6254	7.2848	-0.2651
CHB	NPIP	44296070	6.191	6.6072	-0.1828
CHB	NPIP	44301070	5.7309	6.6072	-0.3849
CHB	NPIP	44306070	5.802	6.4378	-0.2859
CHB	NPIP	44311070	7.5877	6.946	0.2694
CHB	NPIP	44316070	9.0089	9.3178	-0.099
CHB	NPIP	44321070	9.3775	9.3178	0.0191
CHB	NPIP	44326070	7.0345	6.7766	0.1107
CHB	NPIP	44331070	4.3767	5.2519	-0.472
CHB	NPIP	44336070	4.0588	6.4378	-1.0697
CHB	NPIP	44341070	4.6235	5.9295	-0.6323
CHB	NPIP	44346070	4.898	4.7436	0.0911
CHB	NPIP	44351070	5.7283	6.4378	-0.319
CHB	NPIP	44356070	4.4769	6.946	-1.0364
CHB	NPIP	44361070	4.8223	6.946	-0.8914
CHB	NPIP	44366070	8.1819	7.6237	0.2153
CHB	NPIP	44371070	7.1283	9.1484	-0.6586
CHB	NPIP	44376070	7.5667	10.8426	-0.9117
CHB	NPIP	44381070	8.8837	9.8261	-0.2875
CHB	NPIP	44386070	8.217	9.4872	-0.4004
CHB	NPIP	44391070	9.5665	10.3343	-0.2235
CHB	NPIP	44396070	7.2886	8.979	-0.5608
CHB	NPIP	44401070	6.5928	7.4543	-0.3391

CHB	NPIP	44406070	8.032	7.2848	0.3003
CHB	NPIP	44411070	6.3345	6.946	-0.2567
CHB	NPIP	44416070	4.7192	6.4378	-0.7727
CHB	NPIP	44421070	4.1622	5.2519	-0.5877
CHB	NPIP	44426070	4.5652	6.0989	-0.724
CHB	NPIP	44431070	4.3863	6.7766	-1.0261
CHB	NPIP	44436070	4.3373	5.5907	-0.6395
CHB	NPIP	44441070	3.8877	5.9295	-0.9885
CHB	NPIP	44446070	4.2578	7.1154	-1.1735
CHB	NPIP	44451070	6.2258	7.4543	-0.4836
CHB	NPIP	44456070	6.6526	5.5907	0.5418
CHB	NPIP	44461070	4.826	5.9295	-0.5342
CHB	NPIP	44466070	3.9414	8.1319	-1.5231
CHB	NPIP	44471070	4.8812	8.6402	-1.2921
CHB	NPIP	44476070	4.5452	7.7931	-1.2275
CHB	NPIP	44481070	3.1359	8.4708	-1.8676
CHB	NPIP	44486070	3.9155	10.6731	-1.9086
CHB	NPIP	44491070	5.2802	9.9955	-1.4159
CHB	NPIP	44496070	6.3975	8.3013	-0.679
CHB	NPIP	44501070	7.331	7.7931	-0.1746
CHB	NPIP	44506070	6.8469	8.4708	-0.5685
CHB	NPIP	44511070	8.0527	8.4708	-0.1464
CHB	NPIP	44516070	8.1582	7.1154	0.4282
CHB	NPIP	44521070	6.3805	7.2848	-0.3635
CHB	NPIP	44526070	6.7782	6.7766	0.0007
CHB	NPIP	44531070	9.9229	9.8261	0.0295
CHB	NPIP	44536070	11.528	14.4003	-0.6115
CHB	NPIP	44541070	9.1874	11.6896	-0.6489
CHB	NPIP	44546070	7.2503	8.4708	-0.4273
CHB	NPIP	44551070	6.2374	8.3013	-0.7361
CHB	NPIP	44556070	14.1527	11.8591	0.5868
CHB	NPIP	44561070	35.3219	21.1769	2.0809
CHB	NPIP	44566070	42.6712	24.3958	2.3446
CHB	NPIP	44571070	24.198	16.9415	1.323
CHB	NPIP	44576070	10.1513	12.0285	-0.4739
CHB	NPIP	44581070	9.2994	11.8591	-0.6549
CHB	NPIP	44586070	9.2496	11.5202	-0.597
CHB	NPIP	44591070	11.4558	13.3838	-0.44
CHB	NPIP	44596070	11.6157	12.5367	-0.2236
CHB	NPIP	44601070	12.0289	10.3343	0.4933
CHB	NPIP	44606070	13.8852	12.5367	0.3274
CHB	NPIP	44611070	8.585	11.012	-0.6657
CHB	NPIP	44616070	4.3644	7.2848	-1.1739
CHB	NPIP	44621070	5.2385	7.4543	-0.8722
CHB	NPIP	44626070	4.775	7.9625	-1.1812
CHB	NPIP	44631070	4.7611	8.1319	-1.2252
CHB	NPIP	44636070	5.2184	7.1154	-0.779
CHB	NPIP	44641070	3.3168	5.7601	-1.2139
CHB	NPIP	44646070	6.4245	8.4708	-0.7164

CHB	NPIP	44651070	7.0345	8.3013	-0.4518
CHB	NPIP	44656070	2.9109	5.7601	-1.4155
CHB	NPIP	44661070	3.957	5.7601	-0.8958
CHB	NPIP	44666070	5.047	5.5907	-0.2774
CHB	NPIP	44671070	4.7095	6.6072	-0.8335
CHB	NPIP	44676070	3.795	7.2848	-1.4028
CHB	NPIP	44681070	3.699	6.6072	-1.2773
CHB	NPIP	44686070	3.7651	7.4543	-1.4522
CHB	NPIP	44691070	3.5391	7.1154	-1.4687
CHB	NPIP	44696070	2.8947	6.2684	-1.5538
CHB	NPIP	44701070	2.9048	7.6237	-1.8197
CHB	NPIP	44706070	4.9928	8.1319	-1.141
CHB	NPIP	44711070	3.9673	6.7766	-1.2059
CHB	NPIP	44716070	2.0978	5.9295	-1.855
CHB	NPIP	44721070	4.1921	6.4378	-1.0097
CHB	NPIP	44726070	5.6514	7.2848	-0.6566
CHB	NPIP	44731070	3.9549	7.2848	-1.3385
CHB	NPIP	44736070	3.985	6.4378	-1.1029
CHB	NPIP	44741070	5.0314	7.1154	-0.8558
CHB	NPIP	44746070	3.4417	5.9295	-1.2044
CHB	NPIP	44751070	4.8068	5.5907	-0.4
CHB	NPIP	44756070	6.3248	6.4378	-0.0508
CHB	NPIP	44761070	4.3969	6.0989	-0.8034
CHB	NPIP	44766070	5.6307	8.4708	-0.9942
CHB	NPIP	44771070	6.1673	8.4708	-0.8064
CHB	NPIP	44776070	4.5989	6.6072	-0.8821
CHB	NPIP	44781070	4.0512	5.4213	-0.7184
CHB	NPIP	44786070	2.7276	5.4213	-1.4125
CHB	NPIP	44791070	2.1949	6.0989	-1.8429
CHB	NPIP	44796070	3.1279	6.6072	-1.5281
CHB	NPIP	44801070	5.752	8.3013	-0.9092
CHB	NPIP	44806070	7.5957	8.3013	-0.2517
CHB	NPIP	44811070	4.7333	6.4378	-0.7664
CHB	NPIP	44816070	2.7771	5.0825	-1.2799
CHB	NPIP	44821070	4.056	6.2684	-1.019
CHB	NPIP	44826070	5.462	7.6237	-0.8336
CHB	NPIP	44831070	5.0293	8.3013	-1.1669
CHB	NPIP	44836070	4.2183	10.6731	-1.8231
CHB	NPIP	44841070	4.3319	11.5202	-1.8899
CHB	NPIP	44846070	3.3036	9.3178	-1.9278
YRI	NPIP	43726070	8.4142	14.4534	-1.277
YRI	NPIP	43731070	7.7697	12.7727	-1.1894
YRI	NPIP	43736070	7.0628	11.9324	-1.2344
YRI	NPIP	43741070	9.5423	12.2685	-0.6732
YRI	NPIP	43746070	10.8128	12.7727	-0.4659
YRI	NPIP	43751070	9.2904	11.9324	-0.6697
YRI	NPIP	43756070	9.1034	12.1005	-0.7498
YRI	NPIP	43761070	9.1849	11.5963	-0.6279
YRI	NPIP	43766070	7.7114	10.756	-0.8508

YRI	NPIP	43771070	7.3162	11.4282	-1.0856
YRI	NPIP	43776070	5.4804	10.756	-1.4743
YRI	NPIP	43781070	3.1523	7.8989	-1.7658
YRI	NPIP	43786070	4.2553	8.067	-1.3909
YRI	NPIP	43791070	4.7238	9.2434	-1.4545
YRI	NPIP	43796070	2.6401	7.8989	-1.9564
YRI	NPIP	43801070	3.9406	9.2434	-1.7066
YRI	NPIP	43806070	4.7393	10.5879	-1.6586
YRI	NPIP	43811070	2.8714	7.5628	-1.8162
YRI	NPIP	43816070	1.9419	5.378	-1.8083
YRI	NPIP	43821070	4.5152	9.0754	-1.4928
YRI	NPIP	43826070	4.7464	9.2434	-1.4473
YRI	NPIP	43831070	2.5895	7.2267	-1.8712
YRI	NPIP	43836070	3.5871	7.8989	-1.6041
YRI	NPIP	43841070	6.543	9.4115	-0.9079
YRI	NPIP	43846070	5.9176	8.4031	-0.8736
YRI	NPIP	43851070	2.5794	4.8738	-1.3171
YRI	NPIP	43856070	4.982	6.8906	-0.8042
YRI	NPIP	43861070	6.0874	9.9157	-1.1542
YRI	NPIP	43866070	3.7378	7.8989	-1.548
YRI	NPIP	43871070	3.5022	7.3947	-1.5381
YRI	NPIP	43876070	6.3013	10.4199	-1.1856
YRI	NPIP	43881070	6.8523	11.0921	-1.1511
YRI	NPIP	43886070	5.1235	11.2602	-1.6428
YRI	NPIP	43891070	3.3247	8.7392	-1.8354
YRI	NPIP	43896070	2.4553	7.3947	-1.9518
YRI	NPIP	43901070	3.7322	8.9073	-1.7236
YRI	NPIP	43906070	3.3605	9.0754	-1.8707
YRI	NPIP	43911070	2.3612	7.8989	-2.0602
YRI	NPIP	43916070	4.7605	8.4031	-1.2802
YRI	NPIP	43921070	6.0808	9.9157	-1.1562
YRI	NPIP	43926070	6.876	9.9157	-0.9165
YRI	NPIP	43931070	5.7801	9.7476	-1.2154
YRI	NPIP	43936070	2.3745	7.7309	-2.0324
YRI	NPIP	43941070	3.6376	8.7392	-1.7293
YRI	NPIP	43946070	5.5059	10.5879	-1.4412
YRI	NPIP	43951070	5.7651	10.5879	-1.3677
YRI	NPIP	43956070	8.2492	10.5879	-0.6632
YRI	NPIP	43961070	8.6726	10.0837	-0.4188
YRI	NPIP	43966070	7.195	11.7644	-1.1739
YRI	NPIP	43971070	7.4109	11.5963	-1.0899
YRI	NPIP	43976070	11.6332	12.6047	-0.2339
YRI	NPIP	43981070	15.4489	14.7895	0.1364
YRI	NPIP	43986070	12.7723	13.7811	-0.2232
YRI	NPIP	43991070	8.7972	13.613	-1.0779
YRI	NPIP	43996070	6.2913	13.7811	-1.657
YRI	NPIP	44001070	4.5273	13.613	-2.0336
YRI	NPIP	44006070	5.5391	14.4534	-1.8849
YRI	NPIP	44011070	9.6851	16.9743	-1.3221

YRI	NPIP	44016070	10.9594	17.6465	-1.1685
YRI	NPIP	44021070	12.2609	16.6382	-0.8093
YRI	NPIP	44026070	10.5828	13.7811	-0.7076
YRI	NPIP	44031070	7.4486	11.0921	-0.9892
YRI	NPIP	44036070	8.8342	12.6047	-0.9077
YRI	NPIP	44041070	10.6037	13.2769	-0.6127
YRI	NPIP	44046070	11.6067	14.6214	-0.6305
YRI	NPIP	44051070	11.5774	17.8146	-1.0801
YRI	NPIP	44056070	14.425	18.4868	-0.6788
YRI	NPIP	44061070	13.5305	18.3188	-0.8072
YRI	NPIP	44066070	9.1128	14.9575	-1.1962
YRI	NPIP	44071070	7.1599	11.7644	-1.1829
YRI	NPIP	44076070	7.9828	13.1089	-1.1891
YRI	NPIP	44081070	9.827	14.4534	-0.9782
YRI	NPIP	44086070	9.3587	13.7811	-0.9784
YRI	NPIP	44091070	8.9431	12.6047	-0.8815
YRI	NPIP	44096070	10.4691	14.9575	-0.9186
YRI	NPIP	44101070	12.3438	16.134	-0.7216
YRI	NPIP	44106070	10.5521	14.6214	-0.851
YRI	NPIP	44111070	10.5284	14.6214	-0.856
YRI	NPIP	44116070	9.8115	12.2685	-0.6068
YRI	NPIP	44121070	7.6579	12.4366	-1.165
YRI	NPIP	44126070	10.0692	14.2853	-0.9014
YRI	NPIP	44131070	8.484	11.5963	-0.8105
YRI	NPIP	44136070	4.4451	9.4115	-1.5718
YRI	NPIP	44141070	3.6615	8.235	-1.6376
YRI	NPIP	44146070	3.7919	9.0754	-1.7295
YRI	NPIP	44151070	6.8364	11.9324	-1.2918
YRI	NPIP	44156070	6.7705	9.7476	-0.912
YRI	NPIP	44161070	5.1957	8.9073	-1.2362
YRI	NPIP	44166070	6.6109	11.0921	-1.2167
YRI	NPIP	44171070	7.508	11.7644	-1.0935
YRI	NPIP	44176070	8.2872	11.2602	-0.7959
YRI	NPIP	44181070	11.028	12.7727	-0.4148
YRI	NPIP	44186070	10.4895	14.6214	-0.8641
YRI	NPIP	44191070	8.9854	11.5963	-0.6799
YRI	NPIP	44196070	12.9895	13.2769	-0.0659
YRI	NPIP	44201070	11.571	14.9575	-0.6931
YRI	NPIP	44206070	10.2977	13.9492	-0.7986
YRI	NPIP	44211070	15.1471	17.6465	-0.4368
YRI	NPIP	44216070	14.7798	16.9743	-0.398
YRI	NPIP	44221070	13.16	16.8062	-0.6676
YRI	NPIP	44226070	10.9342	16.134	-0.99
YRI	NPIP	44231070	11.6053	15.1256	-0.7128
YRI	NPIP	44236070	11.4144	14.9575	-0.7251
YRI	NPIP	44241070	8.7786	13.1089	-1.0045
YRI	NPIP	44246070	11.2087	14.9575	-0.7672
YRI	NPIP	44251070	11.9386	14.7895	-0.5898
YRI	NPIP	44256070	11.8281	12.6047	-0.1869

YRI	NPIP	44261070	10.2341	10.0837	0.0446
YRI	NPIP	44266070	9.4677	10.2518	-0.2292
YRI	NPIP	44271070	11.1153	11.9324	-0.2071
YRI	NPIP	44276070	11.5391	11.9324	-0.0997
YRI	NPIP	44281070	11.2655	12.1005	-0.2089
YRI	NPIP	44286070	9.3449	10.756	-0.3943
YRI	NPIP	44291070	7.6584	8.9073	-0.416
YRI	NPIP	44296070	7.1872	9.0754	-0.6181
YRI	NPIP	44301070	7.7381	10.0837	-0.6962
YRI	NPIP	44306070	7.2029	9.2434	-0.6567
YRI	NPIP	44311070	7.9585	9.5795	-0.5047
YRI	NPIP	44316070	8.8778	12.6047	-0.8972
YRI	NPIP	44321070	9.4339	12.6047	-0.7633
YRI	NPIP	44326070	10.0792	10.756	-0.1891
YRI	NPIP	44331070	9.7718	11.0921	-0.3585
YRI	NPIP	44336070	8.4826	11.4282	-0.7776
YRI	NPIP	44341070	6.9181	11.2602	-1.1624
YRI	NPIP	44346070	7.4291	12.6047	-1.2459
YRI	NPIP	44351070	7.266	11.5963	-1.1276
YRI	NPIP	44356070	6.4876	9.9157	-1.0336
YRI	NPIP	44361070	6.9464	9.9157	-0.8952
YRI	NPIP	44366070	7.6134	10.5879	-0.8436
YRI	NPIP	44371070	6.9677	12.7727	-1.3801
YRI	NPIP	44376070	9.1412	16.302	-1.35
YRI	NPIP	44381070	10.7759	15.6298	-0.9526
YRI	NPIP	44386070	11.319	13.7811	-0.5447
YRI	NPIP	44391070	11.4621	13.613	-0.4814
YRI	NPIP	44396070	9.0184	14.1172	-1.1025
YRI	NPIP	44401070	7.6974	13.2769	-1.2787
YRI	NPIP	44406070	8.3318	11.5963	-0.8501
YRI	NPIP	44411070	7.0382	9.7476	-0.83
YRI	NPIP	44416070	5.4133	9.4115	-1.2654
YRI	NPIP	44421070	4.8176	9.5795	-1.4825
YRI	NPIP	44426070	5.2259	8.5712	-1.1545
YRI	NPIP	44431070	5.8713	9.7476	-1.1875
YRI	NPIP	44436070	5.539	8.7392	-1.0848
YRI	NPIP	44441070	6.0173	7.8989	-0.7
YRI	NPIP	44446070	6.5133	9.4115	-0.9173
YRI	NPIP	44451070	7.175	12.1005	-1.2323
YRI	NPIP	44456070	7.062	12.9408	-1.3804
YRI	NPIP	44461070	5.7345	11.0921	-1.4546
YRI	NPIP	44466070	7.2776	11.0921	-1.0357
YRI	NPIP	44471070	9.856	12.9408	-0.7244
YRI	NPIP	44476070	7.3638	11.9324	-1.1581
YRI	NPIP	44481070	7.0713	11.2602	-1.1214
YRI	NPIP	44486070	9.0226	11.5963	-0.6702
YRI	NPIP	44491070	8.6173	11.5963	-0.7757
YRI	NPIP	44496070	7.9746	12.4366	-1.0878
YRI	NPIP	44501070	8.108	12.4366	-1.0553

YRI	NPIP	44506070	8.9117	12.2685	-0.829
YRI	NPIP	44511070	9.6618	11.2602	-0.4279
YRI	NPIP	44516070	10.1254	9.5795	0.17
YRI	NPIP	44521070	8.3553	10.4199	-0.5943
YRI	NPIP	44526070	7.6305	10.0837	-0.7281
YRI	NPIP	44531070	14.1839	13.1089	0.2494
YRI	NPIP	44536070	18.1105	18.4868	-0.0629
YRI	NPIP	44541070	13.3296	15.4617	-0.4228
YRI	NPIP	44546070	10.266	12.1005	-0.459
YRI	NPIP	44551070	8.0931	11.0921	-0.8142
YRI	NPIP	44556070	13.0582	15.1256	-0.4186
YRI	NPIP	44561070	25.7818	25.3774	0.0498
YRI	NPIP	44566070	28.3731	27.8983	0.0533
YRI	NPIP	44571070	17.166	20.8397	-0.547
YRI	NPIP	44576070	9.9581	15.4617	-1.0913
YRI	NPIP	44581070	10.0773	14.6214	-0.9503
YRI	NPIP	44586070	9.019	14.6214	-1.1716
YRI	NPIP	44591070	11.6859	16.9743	-0.9592
YRI	NPIP	44596070	13.3247	17.3104	-0.7094
YRI	NPIP	44601070	12.5512	15.2937	-0.5495
YRI	NPIP	44606070	14.2394	14.7895	-0.1138
YRI	NPIP	44611070	11.0057	15.9659	-0.9539
YRI	NPIP	44616070	5.9712	14.1172	-1.7614
YRI	NPIP	44621070	6.3924	12.2685	-1.4511
YRI	NPIP	44626070	6.2051	11.7644	-1.4282
YRI	NPIP	44631070	5.8731	10.2518	-1.2798
YRI	NPIP	44636070	6.2012	10.0837	-1.1524
YRI	NPIP	44641070	5.0287	7.8989	-1.0678
YRI	NPIP	44646070	8.9894	9.5795	-0.1837
YRI	NPIP	44651070	9.4617	10.0837	-0.1846
YRI	NPIP	44656070	4.8916	8.067	-1.1587
YRI	NPIP	44661070	4.9759	9.0754	-1.342
YRI	NPIP	44666070	5.4185	8.9073	-1.162
YRI	NPIP	44671070	5.231	9.0754	-1.2584
YRI	NPIP	44676070	4.3501	9.5795	-1.6281
YRI	NPIP	44681070	4.1069	10.0837	-1.774
YRI	NPIP	44686070	4.1031	11.2602	-1.916
YRI	NPIP	44691070	4.2553	10.924	-1.8367
YRI	NPIP	44696070	3.648	8.7392	-1.7258
YRI	NPIP	44701070	2.6523	7.3947	-1.874
YRI	NPIP	44706070	4.2758	7.7309	-1.311
YRI	NPIP	44711070	4.1898	8.7392	-1.5421
YRI	NPIP	44716070	2.6546	8.5712	-2.0418
YRI	NPIP	44721070	5.2155	7.5628	-0.9087
YRI	NPIP	44726070	7.418	7.8989	-0.1789
YRI	NPIP	44731070	5.3215	8.067	-1.0018
YRI	NPIP	44736070	4.1896	8.235	-1.4485
YRI	NPIP	44741070	5.2919	9.5795	-1.3349
YRI	NPIP	44746070	4.6688	8.5712	-1.3467

YRI	NPIP	44751070	6.1518	7.5628	-0.5462
YRI	NPIP	44756070	7.1811	9.2434	-0.6637
YRI	NPIP	44761070	5.3017	10.756	-1.5242
YRI	NPIP	44766070	7.6842	12.1005	-1.1049
YRI	NPIP	44771070	7.7704	10.924	-0.8686
YRI	NPIP	44776070	4.8539	9.0754	-1.3819
YRI	NPIP	44781070	3.9932	10.0837	-1.8078
YRI	NPIP	44786070	3.4502	8.7392	-1.7928
YRI	NPIP	44791070	2.5783	6.5544	-1.753
YRI	NPIP	44796070	3.8688	9.4115	-1.7542
YRI	NPIP	44801070	7.2895	11.2602	-1.063
YRI	NPIP	44806070	8.8319	10.0837	-0.3716
YRI	NPIP	44811070	6.5455	8.4031	-0.6529
YRI	NPIP	44816070	4.169	5.5461	-0.7052
YRI	NPIP	44821070	5.0036	6.3864	-0.6241
YRI	NPIP	44826070	5.8959	9.2434	-1.0773
YRI	NPIP	44831070	6.3589	9.7476	-1.0381
YRI	NPIP	44836070	6.4589	10.0837	-1.0759
YRI	NPIP	44841070	5.6045	10.0837	-1.3295
YRI	NPIP	44846070	5.4928	10.0837	-1.3626
CEU	NPIP	11432263	19.966	12.6216	1.7769
CEU	NPIP	11437263	21.3389	13.3039	1.8495
CEU	NPIP	11442263	22.8246	14.1567	1.881
CEU	NPIP	11447263	16.7616	12.6216	1.0016
CEU	NPIP	11452263	10.9785	11.7688	-0.2042
CEU	NPIP	11457263	13.7308	13.4744	0.0583
CEU	NPIP	11462263	19.2801	15.6917	0.706
CEU	NPIP	11467263	24.0891	17.0562	1.2776
CEU	NPIP	11472263	32.1884	19.9558	1.9111
CEU	NPIP	11477263	31.5692	19.9558	1.8144
CEU	NPIP	11482263	32.4613	23.7082	1.1578
CEU	NPIP	11487263	29.4218	24.9021	0.57
CEU	NPIP	11492263	14.2773	14.4978	-0.0468
CEU	NPIP	11497263	15.4529	12.9627	0.5875
CEU	NPIP	11502263	25.8103	18.9324	1.1304
CEU	NPIP	11507263	29.4096	21.6614	1.1184
CEU	NPIP	11512263	27.4205	20.2969	1.0949
CEU	NPIP	11517263	21.686	16.5446	0.9617
CEU	NPIP	11522263	17.7246	15.0095	0.5573
CEU	NPIP	11527263	13.6716	11.2571	0.6506
CEU	NPIP	11532263	15.0266	10.0632	1.4854
CEU	NPIP	11537263	16.9206	11.9394	1.27
CEU	NPIP	11542263	14.0515	13.3039	0.1721
CEU	NPIP	11547263	18.967	16.5446	0.4531
CEU	NPIP	11552263	20.0806	16.0329	0.7802
CEU	NPIP	11557263	20.7619	16.8857	0.711
CEU	NPIP	11562263	22.985	18.5913	0.7349
CEU	NPIP	11567263	23.2358	16.374	1.2962
CEU	NPIP	11572263	20.7273	14.6684	1.2712

CEU	NPIP	11577263	18.9732	16.2034	0.5285
CEU	NPIP	11582263	22.2989	17.9091	0.761
CEU	NPIP	11587263	19.0074	17.2268	0.3204
CEU	NPIP	11592263	24.9128	18.9324	0.9829
CEU	NPIP	11597263	22.0348	14.6684	1.5455
CEU	NPIP	11602263	19.7084	12.4511	1.7785
CEU	NPIP	11607263	26.1438	15.0095	2.2855
CEU	NPIP	11612263	14.0639	9.7221	1.3418
CEU	NPIP	11617263	11.2979	8.8692	0.8173
CEU	NPIP	11622263	13.6009	11.4277	0.5774
CEU	NPIP	11627263	12.2698	12.4511	-0.0444
CEU	NPIP	11632263	21.3239	14.6684	1.3964
CEU	NPIP	11637263	20.6984	13.8156	1.5286
CEU	NPIP	11642263	13.4255	12.2805	0.2843
CEU	NPIP	11647263	14.1427	11.7688	0.6135
CEU	NPIP	11652263	12.5584	9.3809	1.0151
CEU	NPIP	11657263	14.0499	9.7221	1.3374
CEU	NPIP	11662263	14.7786	10.916	1.0713
CEU	NPIP	11667263	17.2378	12.2805	1.2308
CEU	NPIP	11672263	21.2873	14.4978	1.4404
CEU	NPIP	11677263	19.0886	13.1333	1.3877
CEU	NPIP	11682263	23.9619	14.8389	1.8931
CEU	NPIP	11687263	29.6823	17.7385	2.0897
CEU	NPIP	11692263	21.7143	14.6684	1.4783
CEU	NPIP	11697263	15.0443	11.5982	0.9029
CEU	NPIP	11702263	17.3715	11.7688	1.4479
CEU	NPIP	11707263	15.6731	11.0866	1.2537
CEU	NPIP	11712263	9.5446	9.2104	0.1086
CEU	NPIP	11717263	7.0864	8.6987	-0.5524
CEU	NPIP	11722263	7.9534	8.0164	-0.0233
CEU	NPIP	11727263	12.7807	10.2337	0.7504
CEU	NPIP	11732263	21.1324	14.4978	1.4075
CEU	NPIP	11737263	24.6137	15.0095	1.9714
CEU	NPIP	11742263	19.9154	12.6216	1.7646
CEU	NPIP	11747263	15.0346	10.4043	1.3432
CEU	NPIP	11752263	14.8722	10.7454	1.1616
CEU	NPIP	11757263	11.5092	10.0632	0.4327
CEU	NPIP	11762263	11.0295	9.0398	0.6578
CEU	NPIP	11767263	13.1752	11.0866	0.5709
CEU	NPIP	11772263	9.2047	9.0398	0.0545
CEU	NPIP	11777263	7.8858	7.8459	0.015
CEU	NPIP	11782263	7.322	9.0398	-0.5679
CEU	NPIP	11787263	6.3671	8.5281	-0.754
CEU	NPIP	11792263	7.7798	9.5515	-0.5566
CEU	NPIP	11797263	7.4451	9.3809	-0.6184
CEU	NPIP	11802263	10.1348	9.7221	0.1275
CEU	NPIP	11807263	11.9301	9.2104	0.8838
CEU	NPIP	11812263	11.6083	8.5281	1.0747
CEU	NPIP	11817263	12.4682	9.3809	0.9863

CEU	NPIP	11822263	10.0585	8.187	0.678
CEU	NPIP	11827263	8.3523	6.8225	0.6546
CEU	NPIP	11832263	10.7994	7.6753	1.2008
CEU	NPIP	11837263	10.9957	8.5281	0.861
CEU	NPIP	11842263	8.8916	8.5281	0.1268
CEU	NPIP	11847263	9.2152	9.2104	0.0016
CEU	NPIP	11852263	9.4733	8.8692	0.2033
CEU	NPIP	11857263	6.5871	6.1402	0.2103
CEU	NPIP	11862263	5.8662	6.1402	-0.1289
CEU	NPIP	11867263	7.3752	10.4043	-0.8787
CEU	NPIP	11872263	6.9132	11.2571	-1.1705
CEU	NPIP	11877263	7.6364	10.5749	-0.8396
CEU	NPIP	11882263	9.9536	9.8926	0.0185
CEU	NPIP	11887263	9.329	8.187	0.4137
CEU	NPIP	11892263	11.1373	9.5515	0.4982
CEU	NPIP	11897263	17.7686	11.7688	1.5505
CEU	NPIP	11902263	18.8001	15.3506	0.693
CEU	NPIP	11907263	17.9913	16.2034	0.3411
CEU	NPIP	11912263	14.7458	13.645	0.2474
CEU	NPIP	11917263	13.8598	13.8156	0.0098
CEU	NPIP	11922263	13.8586	12.9627	0.2114
CEU	NPIP	11927263	9.4919	10.0632	-0.171
CEU	NPIP	11932263	10.2962	8.6987	0.5473
CEU	NPIP	11937263	8.6918	7.1636	0.6256
CEU	NPIP	11942263	6.5988	9.0398	-0.8071
CEU	NPIP	11947263	8.4708	13.8156	-1.187
CEU	NPIP	11952263	9.8219	12.6216	-0.6773
CEU	NPIP	11957263	9.0858	10.0632	-0.2925
CEU	NPIP	11962263	6.0967	8.3576	-0.8037
CEU	NPIP	11967263	5.7716	7.5047	-0.68
CEU	NPIP	11972263	9.3519	9.7221	-0.1144
CEU	NPIP	11977263	9.6098	9.0398	0.1885
CEU	NPIP	11982263	8.4735	8.3576	0.0412
CEU	NPIP	11987263	7.8092	8.8692	-0.3567
CEU	NPIP	11992263	8.3571	9.2104	-0.2773
CEU	NPIP	11997263	6.8571	9.3809	-0.8063
CEU	NPIP	12002263	7.8658	10.0632	-0.6576
CEU	NPIP	12007263	10.7947	11.2571	-0.1246
CEU	NPIP	12012263	6.6223	9.8926	-0.9944
CEU	NPIP	12017263	8.1412	10.916	-0.7696
CEU	NPIP	12022263	7.4372	9.0398	-0.5299
CEU	NPIP	12027263	4.1956	6.3108	-0.9711
CEU	NPIP	12032263	6.2845	8.0164	-0.6397
CEU	NPIP	12037263	8.8481	9.7221	-0.2701
CEU	NPIP	12042263	10.9246	12.7922	-0.4461
CEU	NPIP	12047263	8.1311	11.4277	-0.8758
CEU	NPIP	12052263	5.3822	9.3809	-1.2774
CEU	NPIP	12057263	7.793	11.0866	-0.9003
CEU	NPIP	12062263	9.0295	11.0866	-0.5623

CEU	NPIP	12067263	12.4958	11.2571	0.3338
CEU	NPIP	12072263	16.9786	13.8156	0.7025
CEU	NPIP	12077263	16.1198	13.645	0.5562
CEU	NPIP	12082263	11.1808	11.4277	-0.0656
CEU	NPIP	12087263	5.6386	10.0632	-1.3241
CEU	NPIP	12092263	6.737	8.3576	-0.5761
CEU	NPIP	12097263	9.3484	9.2104	0.0448
CEU	NPIP	12102263	10.9728	9.5515	0.4465
CEU	NPIP	12107263	9.0847	8.5281	0.1942
CEU	NPIP	12112263	5.9013	7.1636	-0.5167
CEU	NPIP	12117263	7.4519	5.7991	0.8186
CEU	NPIP	12122263	8.4572	6.1402	1.0903
CEU	NPIP	12127263	11.578	8.187	1.2285
CEU	NPIP	12132263	14.7368	10.0632	1.3986
CEU	NPIP	12137263	21.6277	13.645	1.7939
CEU	NPIP	12142263	20.7015	13.4744	1.6436
CEU	NPIP	12147263	19.0676	12.2805	1.6851
CEU	NPIP	12152263	19.9727	13.645	1.422
CEU	NPIP	12157263	14.3767	11.4277	0.7835
CEU	NPIP	12162263	14.7982	11.5982	0.8384
CEU	NPIP	12167263	16.2977	14.8389	0.3027
CEU	NPIP	12172263	15.6467	15.6917	-0.0089
CEU	NPIP	12177263	18.0251	15.0095	0.619
CEU	NPIP	12182263	17.4335	12.9627	1.0547
CEU	NPIP	12187263	10.7912	8.5281	0.7896
CEU	NPIP	12192263	7.2099	7.5047	-0.1157
CEU	NPIP	12197263	6.858	8.3576	-0.5331
CEU	NPIP	12202263	14.0835	10.916	0.8785
CEU	NPIP	12207263	18.2362	13.1333	1.189
CEU	NPIP	12212263	14.8167	12.1099	0.681
CEU	NPIP	12217263	13.1299	11.9394	0.3035
CEU	NPIP	12222263	10.9905	10.7454	0.069
CEU	NPIP	12227263	10.5863	10.4043	0.0528
CEU	NPIP	12232263	13.14	12.7922	0.0831
CEU	NPIP	12237263	14.0095	13.3039	0.1624
CEU	NPIP	12242263	13.3793	12.2805	0.2728
CEU	NPIP	12247263	15.7777	13.4744	0.5238
CEU	NPIP	12252263	15.816	12.7922	0.7223
CEU	NPIP	12257263	11.2393	9.7221	0.4689
CEU	NPIP	12262263	6.2746	7.6753	-0.5384
CEU	NPIP	12267263	3.8223	6.1402	-1.0907
CEU	NPIP	12272263	4.4793	5.7991	-0.6537
CEU	NPIP	12277263	5.7666	6.6519	-0.3876
CEU	NPIP	12282263	7.1163	7.8459	-0.2749
CEU	NPIP	12287263	6.811	9.2104	-0.7797
CEU	NPIP	12292263	7.0398	10.2337	-0.9409
CEU	NPIP	12297263	7.1233	10.5749	-0.9862
CEU	NPIP	12302263	10.1268	12.4511	-0.5696
CEU	NPIP	12307263	10.8176	14.3272	-0.753

CEU	NPIP	12312263	6.2119	12.1099	-1.4838
CEU	NPIP	12317263	3.5525	8.0164	-1.6488
CEU	NPIP	12322263	6.0095	7.6753	-0.6403
CEU	NPIP	12327263	6.5387	8.0164	-0.5458
CEU	NPIP	12332263	4.4084	8.3576	-1.4038
CEU	NPIP	12337263	3.3195	7.1636	-1.5736
CEU	NPIP	12342263	3.2072	5.7991	-1.2837
CEU	NPIP	12347263	5.704	7.5047	-0.7065
CEU	NPIP	12352263	11.1462	12.2805	-0.2816
CEU	NPIP	12357263	13.3979	14.3272	-0.1994
CEU	NPIP	12362263	12.4822	12.9627	-0.1134
CEU	NPIP	12367263	10.0338	12.7922	-0.6589
CEU	NPIP	12372263	6.9959	12.4511	-1.3369
CEU	NPIP	12377263	6.0375	10.4043	-1.2668
CEU	NPIP	12382263	6.6242	9.8926	-0.9938
CEU	NPIP	12387263	6.4341	10.916	-1.2431
CEU	NPIP	12392263	3.8	11.2571	-2.0094
CEU	NPIP	12397263	4.6766	10.5749	-1.6852
CEU	NPIP	12402263	8.1402	10.2337	-0.6168
CEU	NPIP	12407263	7.5644	9.2104	-0.5349
CEU	NPIP	12412263	6.1538	6.8225	-0.2861
CEU	NPIP	12417263	8.5424	9.5515	-0.317
CEU	NPIP	12422263	8.5242	12.2805	-0.9326
CEU	NPIP	12427263	5.9526	11.7688	-1.5031
CEU	NPIP	12432263	4.3317	9.3809	-1.613
CEU	NPIP	12437263	7.317	10.4043	-0.8956
CEU	NPIP	12442263	8.482	11.0866	-0.712
CEU	NPIP	12447263	9.4554	11.4277	-0.524
CEU	NPIP	12452263	15.4192	15.0095	0.0841
CEU	NPIP	12457263	13.942	13.9861	-0.0097
CEU	NPIP	12462263	10.8077	13.4744	-0.6065
CEU	NPIP	12467263	11.6438	15.5212	-0.7709
CHB	NPIP	11432263	16.8639	12.8755	0.9442
CHB	NPIP	11437263	15.4496	13.2144	0.5163
CHB	NPIP	11442263	12.8039	15.0779	-0.4634
CHB	NPIP	11447263	11.1201	13.2144	-0.4838
CHB	NPIP	11452263	10.4445	13.7226	-0.7306
CHB	NPIP	11457263	17.3805	18.6357	-0.2089
CHB	NPIP	11462263	20.956	18.9745	0.324
CHB	NPIP	11467263	19.2557	17.6192	0.2874
CHB	NPIP	11472263	19.8145	18.9745	0.1374
CHB	NPIP	11477263	19.1568	18.8051	0.058
CHB	NPIP	11482263	26.8945	22.7016	0.5767
CHB	NPIP	11487263	26.7639	20.6686	0.918
CHB	NPIP	11492263	14.1418	14.7391	-0.1244
CHB	NPIP	11497263	15.845	18.2968	-0.4152
CHB	NPIP	11502263	26.3417	20.3298	0.92
CHB	NPIP	11507263	27.339	25.0734	0.283
CHB	NPIP	11512263	22.9638	24.904	-0.244

CHB	NPIP	11517263	21.92	17.6192	0.7552
CHB	NPIP	11522263	20.94	15.2474	1.1478
CHB	NPIP	11527263	16.5127	12.5367	0.9653
CHB	NPIP	11532263	17.8576	13.045	1.1253
CHB	NPIP	11537263	21.091	15.7556	1.0427
CHB	NPIP	11542263	20.8134	14.5697	1.3146
CHB	NPIP	11547263	22.7283	15.4168	1.4588
CHB	NPIP	11552263	20.0748	16.0944	0.7622
CHB	NPIP	11557263	20.3533	17.6192	0.4801
CHB	NPIP	11562263	24.8167	19.991	0.7505
CHB	NPIP	11567263	26.0379	18.4662	1.271
CHB	NPIP	11572263	23.609	16.9415	1.2156
CHB	NPIP	11577263	28.9924	20.4992	1.2893
CHB	NPIP	11582263	31.913	24.2264	0.9928
CHB	NPIP	11587263	22.8997	19.6521	0.5135
CHB	NPIP	11592263	26.6141	19.1439	1.2112
CHB	NPIP	11597263	22.0697	20.8381	0.184
CHB	NPIP	11602263	18.8389	18.1274	0.1216
CHB	NPIP	11607263	25.4991	17.4498	1.4266
CHB	NPIP	11612263	14.3274	13.2144	0.2571
CHB	NPIP	11617263	11.0699	10.3343	0.2141
CHB	NPIP	11622263	10.5111	11.1814	-0.1812
CHB	NPIP	11627263	8.9551	14.5697	-1.1822
CHB	NPIP	11632263	23.0672	19.3133	0.6035
CHB	NPIP	11637263	22.696	16.4333	1.1756
CHB	NPIP	11642263	11.9939	14.4003	-0.5123
CHB	NPIP	11647263	13.2105	14.2309	-0.2197
CHB	NPIP	11652263	9.8791	10.8426	-0.2681
CHB	NPIP	11657263	10.2747	11.012	-0.2022
CHB	NPIP	11662263	13.8456	14.2309	-0.083
CHB	NPIP	11667263	16.2084	14.9085	0.2678
CHB	NPIP	11672263	15.0121	14.5697	0.0931
CHB	NPIP	11677263	10.2283	13.2144	-0.6898
CHB	NPIP	11682263	19.2393	13.892	1.178
CHB	NPIP	11687263	27.9863	16.4333	2.1687
CHB	NPIP	11692263	20.6354	14.0615	1.4317
CHB	NPIP	11697263	12.7405	12.0285	0.1797
CHB	NPIP	11702263	14.9907	13.7226	0.2826
CHB	NPIP	11707263	13.5618	12.5367	0.2489
CHB	NPIP	11712263	8.0036	9.8261	-0.556
CHB	NPIP	11717263	6.3088	9.6567	-1.0381
CHB	NPIP	11722263	6.2802	10.5037	-1.2109
CHB	NPIP	11727263	7.8277	12.8755	-1.195
CHB	NPIP	11732263	12.7634	15.4168	-0.5294
CHB	NPIP	11737263	18.2029	17.958	0.0422
CHB	NPIP	11742263	16.4297	16.6027	-0.0322
CHB	NPIP	11747263	11.962	11.8591	0.0263
CHB	NPIP	11752263	14.1444	14.4003	-0.0545
CHB	NPIP	11757263	14.708	17.2803	-0.4602

CHB	NPIP	11762263	13.0897	11.6896	0.3631
CHB	NPIP	11767263	15.0563	11.8591	0.818
CHB	NPIP	11772263	11.4689	10.8426	0.1743
CHB	NPIP	11777263	8.0518	7.1154	0.3846
CHB	NPIP	11782263	8.8924	8.6402	0.0867
CHB	NPIP	11787263	10.0874	10.3343	-0.0719
CHB	NPIP	11792263	9.8382	11.5202	-0.4422
CHB	NPIP	11797263	7.5254	9.6567	-0.6609
CHB	NPIP	11802263	9.8667	10.1649	-0.0882
CHB	NPIP	11807263	10.7074	10.6731	0.0097
CHB	NPIP	11812263	10.4872	9.3178	0.3748
CHB	NPIP	11817263	11.3746	8.979	0.7947
CHB	NPIP	11822263	9.0206	8.1319	0.323
CHB	NPIP	11827263	7.6265	7.1154	0.2099
CHB	NPIP	11832263	10.249	9.1484	0.3588
CHB	NPIP	11837263	11.0373	11.1814	-0.039
CHB	NPIP	11842263	9.6349	9.9955	-0.1083
CHB	NPIP	11847263	9.0215	10.5037	-0.4249
CHB	NPIP	11852263	7.7523	9.3178	-0.5018
CHB	NPIP	11857263	4.9448	7.2848	-0.9406
CHB	NPIP	11862263	5.1383	8.3013	-1.1281
CHB	NPIP	11867263	7.0622	8.979	-0.6359
CHB	NPIP	11872263	7.7908	11.1814	-0.9168
CHB	NPIP	11877263	8.3651	12.5367	-1.0128
CHB	NPIP	11882263	9.6964	11.1814	-0.4015
CHB	NPIP	11887263	10.7211	10.5037	0.0623
CHB	NPIP	11892263	13.4674	12.0285	0.3632
CHB	NPIP	11897263	21.5025	15.5862	1.1682
CHB	NPIP	11902263	24.2075	18.4662	0.9637
CHB	NPIP	11907263	21.0492	17.1109	0.7112
CHB	NPIP	11912263	16.2089	14.0615	0.4677
CHB	NPIP	11917263	13.6407	13.7226	-0.0183
CHB	NPIP	11922263	12.4509	13.2144	-0.1764
CHB	NPIP	11927263	9.5219	12.5367	-0.7319
CHB	NPIP	11932263	9.4348	12.0285	-0.6548
CHB	NPIP	11937263	8.2281	8.979	-0.2491
CHB	NPIP	11942263	6.6825	9.8261	-0.9591
CHB	NPIP	11947263	6.4099	13.5532	-1.611
CHB	NPIP	11952263	6.1742	11.8591	-1.4544
CHB	NPIP	11957263	5.7599	10.1649	-1.3022
CHB	NPIP	11962263	3.8721	8.6402	-1.639
CHB	NPIP	11967263	3.3501	8.1319	-1.7381
CHB	NPIP	11972263	6.0781	11.012	-1.3533
CHB	NPIP	11977263	7.0282	10.6731	-1.0295
CHB	NPIP	11982263	6.0188	9.9955	-1.1941
CHB	NPIP	11987263	6.2875	10.6731	-1.2387
CHB	NPIP	11992263	7.7617	10.6731	-0.8223
CHB	NPIP	11997263	6.762	9.1484	-0.7781
CHB	NPIP	12002263	7.6726	10.5037	-0.8117

CHB	NPIP	12007263	8.9968	12.5367	-0.8594
CHB	NPIP	12012263	5.3055	10.3343	-1.4638
CHB	NPIP	12017263	6.5759	10.1649	-1.061
CHB	NPIP	12022263	6.3227	7.9625	-0.6077
CHB	NPIP	12027263	5.0297	7.4543	-0.9544
CHB	NPIP	12032263	7.1007	8.979	-0.6231
CHB	NPIP	12037263	6.9842	9.4872	-0.789
CHB	NPIP	12042263	10.7769	12.3673	-0.3911
CHB	NPIP	12047263	10.4013	11.5202	-0.2942
CHB	NPIP	12052263	6.8884	10.8426	-1.1005
CHB	NPIP	12057263	9.7709	11.1814	-0.3814
CHB	NPIP	12062263	11.9877	12.8755	-0.2102
CHB	NPIP	12067263	14.403	16.0944	-0.3239
CHB	NPIP	12072263	16.9212	14.9085	0.4146
CHB	NPIP	12077263	16.6876	14.0615	0.5719
CHB	NPIP	12082263	11.2688	13.045	-0.4153
CHB	NPIP	12087263	5.4529	10.1649	-1.3929
CHB	NPIP	12092263	4.6052	9.8261	-1.5929
CHB	NPIP	12097263	4.0202	10.5037	-1.8588
CHB	NPIP	12102263	3.5182	9.8261	-1.9245
CHB	NPIP	12107263	3.1038	8.1319	-1.8276
CHB	NPIP	12112263	2.2368	7.2848	-2.0291
CHB	NPIP	12117263	3.4691	8.3013	-1.7234
CHB	NPIP	12122263	5.8631	7.7931	-0.7294
CHB	NPIP	12127263	7.929	10.1649	-0.661
CHB	NPIP	12132263	10.0944	13.045	-0.6899
CHB	NPIP	12137263	9.9433	14.2309	-0.9232
CHB	NPIP	12142263	7.9859	12.5367	-1.1049
CHB	NPIP	12147263	10.4997	11.5202	-0.2683
CHB	NPIP	12152263	14.1761	13.5532	0.1405
CHB	NPIP	12157263	14.8915	14.4003	0.1046
CHB	NPIP	12162263	16.384	14.4003	0.4223
CHB	NPIP	12167263	20.5591	13.045	1.757
CHB	NPIP	12172263	20.8962	13.3838	1.7145
CHB	NPIP	12177263	20.2482	14.9085	1.1
CHB	NPIP	12182263	17.7455	13.5532	0.9454
CHB	NPIP	12187263	9.8572	9.9955	-0.0415
CHB	NPIP	12192263	6.1993	8.4708	-0.7952
CHB	NPIP	12197263	6.3269	7.9625	-0.6061
CHB	NPIP	12202263	12.6368	13.892	-0.2765
CHB	NPIP	12207263	16.2732	16.6027	-0.0612
CHB	NPIP	12212263	13.6474	13.045	0.1409
CHB	NPIP	12217263	13.613	11.6896	0.4988
CHB	NPIP	12222263	11.9111	10.8426	0.2974
CHB	NPIP	12227263	10.5226	12.5367	-0.489
CHB	NPIP	12232263	10.7936	13.3838	-0.5911
CHB	NPIP	12237263	13.418	14.0615	-0.1401
CHB	NPIP	12242263	14.4312	13.5532	0.198
CHB	NPIP	12247263	17.7512	14.4003	0.7134

CHB	NPIP	12252263	18.9694	14.0615	1.0688
CHB	NPIP	12257263	13.2939	13.2144	0.0184
CHB	NPIP	12262263	7.9084	12.3673	-1.0965
CHB	NPIP	12267263	5.4097	9.4872	-1.2853
CHB	NPIP	12272263	5.8511	11.012	-1.4156
CHB	NPIP	12277263	6.6775	9.9955	-0.9963
CHB	NPIP	12282263	7.7948	8.1319	-0.1225
CHB	NPIP	12287263	7.0464	9.3178	-0.7281
CHB	NPIP	12292263	7.9528	11.6896	-0.9691
CHB	NPIP	12297263	8.5901	12.5367	-0.9582
CHB	NPIP	12302263	12.6686	13.5532	-0.1995
CHB	NPIP	12307263	13.0118	16.7721	-0.6922
CHB	NPIP	12312263	7.1104	16.6027	-1.7645
CHB	NPIP	12317263	4.6438	14.2309	-2.0642
CHB	NPIP	12322263	7.1333	16.4333	-1.7458
CHB	NPIP	12327263	7.3588	15.2474	-1.5906
CHB	NPIP	12332263	5.1812	12.5367	-1.7858
CHB	NPIP	12337263	5.4483	14.5697	-1.9205
CHB	NPIP	12342263	4.5972	11.8591	-1.8579
CHB	NPIP	12347263	5.4637	9.1484	-1.2014
CHB	NPIP	12352263	10.7946	14.4003	-0.7677
CHB	NPIP	12357263	13.635	17.2803	-0.6521
CHB	NPIP	12362263	13.6453	17.4498	-0.6743
CHB	NPIP	12367263	10.7706	15.2474	-0.9027
CHB	NPIP	12372263	7.5298	12.0285	-1.1357
CHB	NPIP	12377263	7.4213	12.1979	-1.1901
CHB	NPIP	12382263	8.7641	13.2144	-1.028
CHB	NPIP	12387263	7.1199	12.7061	-1.3392
CHB	NPIP	12392263	4.4371	11.8591	-1.8989
CHB	NPIP	12397263	7.2823	12.8755	-1.3241
CHB	NPIP	12402263	9.3457	13.2144	-0.8937
CHB	NPIP	12407263	7.5924	12.0285	-1.1199
CHB	NPIP	12412263	7.2894	8.6402	-0.4643
CHB	NPIP	12417263	9.1748	8.8096	0.1233
CHB	NPIP	12422263	9.1687	12.3673	-0.7866
CHB	NPIP	12427263	7.5214	13.5532	-1.3603
CHB	NPIP	12432263	5.5934	11.5202	-1.5582
CHB	NPIP	12437263	9.3389	13.892	-1.003
CHB	NPIP	12442263	10.4952	14.9085	-0.9091
CHB	NPIP	12447263	10.85	13.2144	-0.5462
CHB	NPIP	12452263	17.084	16.4333	0.1221
CHB	NPIP	12457263	16.5511	17.7886	-0.2153
CHB	NPIP	12462263	14.0651	17.1109	-0.5501
CHB	NPIP	12467263	14.9033	16.6027	-0.3159
YRI	NPIP	11432263	19.7182	18.1507	0.2666
YRI	NPIP	11437263	21.3468	16.9743	0.793
YRI	NPIP	11442263	24.1009	19.8313	0.6669
YRI	NPIP	11447263	20.4789	19.8313	0.1012
YRI	NPIP	11452263	18.8935	22.5203	-0.5011

YRI	NPIP	11457263	26.3563	26.7219	-0.0428
YRI	NPIP	11462263	28.1552	28.0664	0.0099
YRI	NPIP	11467263	25.3097	26.7219	-0.1653
YRI	NPIP	11472263	34.2478	31.2596	0.3002
YRI	NPIP	11477263	34.4312	33.6124	0.0766
YRI	NPIP	11482263	37.6994	33.6124	0.3825
YRI	NPIP	11487263	37.3767	33.2763	0.3875
YRI	NPIP	11492263	23.3556	29.747	-0.6739
YRI	NPIP	11497263	21.4561	30.5873	-0.937
YRI	NPIP	11502263	28.4145	30.5873	-0.223
YRI	NPIP	11507263	41.967	36.4695	0.475
YRI	NPIP	11512263	41.3307	38.3182	0.248
YRI	NPIP	11517263	26.1158	28.7386	-0.286
YRI	NPIP	11522263	22.1402	24.201	-0.2655
YRI	NPIP	11527263	19.8287	19.9994	-0.0264
YRI	NPIP	11532263	19.487	19.3272	0.0256
YRI	NPIP	11537263	20.0899	21.0078	-0.1356
YRI	NPIP	11542263	17.9811	22.0162	-0.5698
YRI	NPIP	11547263	25.1277	27.058	-0.2232
YRI	NPIP	11552263	25.3475	27.2261	-0.2159
YRI	NPIP	11557263	25.5295	26.7219	-0.1396
YRI	NPIP	11562263	32.0816	28.7386	0.3645
YRI	NPIP	11567263	31.3232	28.9067	0.262
YRI	NPIP	11572263	26.6344	27.8983	-0.1419
YRI	NPIP	11577263	28.2734	29.5789	-0.1384
YRI	NPIP	11582263	30.4646	32.2679	-0.1756
YRI	NPIP	11587263	24.5716	29.2428	-0.5008
YRI	NPIP	11592263	30.3903	30.9234	-0.0541
YRI	NPIP	11597263	37.5972	32.436	0.5001
YRI	NPIP	11602263	36.5244	26.89	1.1207
YRI	NPIP	11607263	33.9288	26.7219	0.8435
YRI	NPIP	11612263	21.1925	26.3858	-0.6153
YRI	NPIP	11617263	18.0913	25.3774	-0.8966
YRI	NPIP	11622263	18.5202	22.3523	-0.5333
YRI	NPIP	11627263	20.4278	25.0413	-0.5751
YRI	NPIP	11632263	33.7293	31.9318	0.1769
YRI	NPIP	11637263	27.7748	26.5538	0.1438
YRI	NPIP	11642263	19.2767	22.0162	-0.3869
YRI	NPIP	11647263	19.9464	20.6717	-0.1089
YRI	NPIP	11652263	14.9992	20.1675	-0.7943
YRI	NPIP	11657263	15.1329	21.512	-0.9212
YRI	NPIP	11662263	14.8632	19.8313	-0.776
YRI	NPIP	11667263	13.9959	21.512	-1.0854
YRI	NPIP	11672263	16.5919	24.201	-0.9805
YRI	NPIP	11677263	14.6628	19.8313	-0.8073
YRI	NPIP	11682263	20.9131	18.3188	0.4374
YRI	NPIP	11687263	27.5848	20.6717	1.0375
YRI	NPIP	11692263	20.4829	18.3188	0.3648
YRI	NPIP	11697263	14.4042	17.9827	-0.6141

YRI	NPIP	11702263	14.9443	20.5036	-0.8409
YRI	NPIP	11707263	13.8604	18.823	-0.8151
YRI	NPIP	11712263	11.3341	16.9743	-1.023
YRI	NPIP	11717263	11.5129	17.1423	-1.0114
YRI	NPIP	11722263	11.2888	16.6382	-0.989
YRI	NPIP	11727263	18.6619	17.6465	0.1774
YRI	NPIP	11732263	27.8401	20.8397	1.0424
YRI	NPIP	11737263	29.3758	24.5371	0.6152
YRI	NPIP	11742263	24.7371	22.8565	0.2561
YRI	NPIP	11747263	19.0106	18.991	0.0032
YRI	NPIP	11752263	21.2953	22.3523	-0.1471
YRI	NPIP	11757263	22.6365	25.2093	-0.3187
YRI	NPIP	11762263	16.9906	20.3355	-0.51
YRI	NPIP	11767263	15.639	19.1591	-0.5684
YRI	NPIP	11772263	14.1126	17.1423	-0.5443
YRI	NPIP	11777263	10.7502	14.4534	-0.783
YRI	NPIP	11782263	11.492	15.7978	-0.8365
YRI	NPIP	11787263	13.8381	16.134	-0.4371
YRI	NPIP	11792263	13.2859	17.8146	-0.7842
YRI	NPIP	11797263	9.6894	18.823	-1.5002
YRI	NPIP	11802263	11.36	16.8062	-0.9972
YRI	NPIP	11807263	11.8648	14.4534	-0.5473
YRI	NPIP	11812263	12.1173	15.9659	-0.7401
YRI	NPIP	11817263	12.647	15.4617	-0.5581
YRI	NPIP	11822263	9.8586	12.2685	-0.5951
YRI	NPIP	11827263	8.5241	13.1089	-1.0635
YRI	NPIP	11832263	10.8467	17.8146	-1.2066
YRI	NPIP	11837263	13.3994	17.3104	-0.6961
YRI	NPIP	11842263	12.4072	13.7811	-0.3039
YRI	NPIP	11847263	13.1271	15.2937	-0.4341
YRI	NPIP	11852263	11.1571	14.7895	-0.7514
YRI	NPIP	11857263	7.6252	13.2769	-1.2953
YRI	NPIP	11862263	9.9648	13.7811	-0.8443
YRI	NPIP	11867263	10.3438	14.2853	-0.8427
YRI	NPIP	11872263	10.2774	16.8062	-1.1955
YRI	NPIP	11877263	10.7414	17.3104	-1.1693
YRI	NPIP	11882263	10.4855	16.9743	-1.1769
YRI	NPIP	11887263	12.1422	18.1507	-1.022
YRI	NPIP	11892263	16.5617	19.3272	-0.4428
YRI	NPIP	11897263	22.1442	22.1842	-0.0056
YRI	NPIP	11902263	23.5074	22.5203	0.1364
YRI	NPIP	11907263	21.601	19.9994	0.2481
YRI	NPIP	11912263	18.1663	22.8565	-0.6388
YRI	NPIP	11917263	17.6534	22.8565	-0.7086
YRI	NPIP	11922263	17.3872	18.1507	-0.1299
YRI	NPIP	11927263	13.8134	15.7978	-0.3855
YRI	NPIP	11932263	11.7188	14.6214	-0.607
YRI	NPIP	11937263	8.0526	12.1005	-1.0127
YRI	NPIP	11942263	8.5466	13.2769	-1.0841

YRI	NPIP	11947263	12.142	18.1507	-1.022
YRI	NPIP	11952263	11.3769	15.7978	-0.8588
YRI	NPIP	11957263	10.3295	14.1172	-0.819
YRI	NPIP	11962263	6.7891	12.9408	-1.4445
YRI	NPIP	11967263	5.5729	10.756	-1.4484
YRI	NPIP	11972263	9.9945	14.4534	-0.9428
YRI	NPIP	11977263	11.0297	15.2937	-0.8544
YRI	NPIP	11982263	9.6751	12.9408	-0.7668
YRI	NPIP	11987263	8.7315	11.0921	-0.6409
YRI	NPIP	11992263	9.6863	11.4282	-0.4599
YRI	NPIP	11997263	9.0536	12.4366	-0.8248
YRI	NPIP	12002263	11.3909	14.6214	-0.6756
YRI	NPIP	12007263	14.2721	18.3188	-0.6822
YRI	NPIP	12012263	8.8843	14.7895	-1.2216
YRI	NPIP	12017263	8.9906	15.6298	-1.303
YRI	NPIP	12022263	9.1484	13.445	-0.9731
YRI	NPIP	12027263	6.2546	8.5712	-0.7995
YRI	NPIP	12032263	9.0472	11.7644	-0.6981
YRI	NPIP	12037263	11.6072	15.4617	-0.7643
YRI	NPIP	12042263	14.934	18.823	-0.6387
YRI	NPIP	12047263	13.6636	17.6465	-0.696
YRI	NPIP	12052263	9.8183	14.9575	-1.0518
YRI	NPIP	12057263	12.6461	16.302	-0.6892
YRI	NPIP	12062263	15.7025	19.1591	-0.5581
YRI	NPIP	12067263	18.893	19.9994	-0.1714
YRI	NPIP	12072263	21.2416	17.6465	0.6282
YRI	NPIP	12077263	20.0856	16.6382	0.6373
YRI	NPIP	12082263	17.3676	17.4785	-0.0196
YRI	NPIP	12087263	12.0007	13.445	-0.3271
YRI	NPIP	12092263	9.3707	12.4366	-0.7475
YRI	NPIP	12097263	10.3422	16.302	-1.1235
YRI	NPIP	12102263	11.5839	18.1507	-1.1169
YRI	NPIP	12107263	9.8876	16.8062	-1.2669
YRI	NPIP	12112263	7.1718	16.302	-1.7212
YRI	NPIP	12117263	9.3742	17.4785	-1.4292
YRI	NPIP	12122263	10.9288	17.4785	-1.1551
YRI	NPIP	12127263	14.402	17.9827	-0.6145
YRI	NPIP	12132263	16.4635	18.823	-0.3875
YRI	NPIP	12137263	21.7909	21.512	0.0403
YRI	NPIP	12142263	20.7664	20.5036	0.0398
YRI	NPIP	12147263	17.574	19.4952	-0.3051
YRI	NPIP	12152263	19.1386	19.8313	-0.1082
YRI	NPIP	12157263	17.736	19.3272	-0.2548
YRI	NPIP	12162263	19.9769	21.3439	-0.1989
YRI	NPIP	12167263	22.046	21.1758	0.1276
YRI	NPIP	12172263	22.2544	18.4868	0.6296
YRI	NPIP	12177263	23.6489	18.4868	0.8627
YRI	NPIP	12182263	21.665	17.9827	0.6319
YRI	NPIP	12187263	13.1275	14.1172	-0.214

YRI	NPIP	12192263	8.0522	13.1089	-1.173
YRI	NPIP	12197263	8.6957	12.9408	-0.9968
YRI	NPIP	12202263	13.8977	16.6382	-0.5067
YRI	NPIP	12207263	16.112	20.8397	-0.704
YRI	NPIP	12212263	14.6065	19.6633	-0.7964
YRI	NPIP	12217263	14.522	17.4785	-0.5214
YRI	NPIP	12222263	13.1112	15.7978	-0.5219
YRI	NPIP	12227263	12.652	15.2937	-0.5293
YRI	NPIP	12232263	15.7242	18.3188	-0.4374
YRI	NPIP	12237263	16.5619	20.3355	-0.5754
YRI	NPIP	12242263	13.884	17.9827	-0.7034
YRI	NPIP	12247263	15.2409	20.3355	-0.7768
YRI	NPIP	12252263	15.1332	19.9994	-0.754
YRI	NPIP	12257263	10.5586	14.2853	-0.7968
YRI	NPIP	12262263	8.9606	12.6047	-0.8772
YRI	NPIP	12267263	8.0305	12.9408	-1.153
YRI	NPIP	12272263	6.9498	13.445	-1.471
YRI	NPIP	12277263	7.5138	12.4366	-1.2002
YRI	NPIP	12282263	7.8515	9.9157	-0.6224
YRI	NPIP	12287263	7.4881	9.5795	-0.6511
YRI	NPIP	12292263	10.6267	14.2853	-0.7822
YRI	NPIP	12297263	10.9065	17.3104	-1.1399
YRI	NPIP	12302263	12.9447	17.4785	-0.7996
YRI	NPIP	12307263	18.7328	19.8313	-0.1716
YRI	NPIP	12312263	16.9927	22.1842	-0.7278
YRI	NPIP	12317263	12.6976	21.3439	-1.2581
YRI	NPIP	12322263	16.0427	21.512	-0.7898
YRI	NPIP	12327263	16.2445	19.9994	-0.5818
YRI	NPIP	12332263	13.4043	18.823	-0.89
YRI	NPIP	12337263	13.9127	20.6717	-1.0144
YRI	NPIP	12342263	11.3583	18.6549	-1.2088
YRI	NPIP	12347263	9.5538	16.8062	-1.328
YRI	NPIP	12352263	14.5658	20.1675	-0.8609
YRI	NPIP	12357263	17.9545	22.3523	-0.612
YRI	NPIP	12362263	17.9877	23.1926	-0.6989
YRI	NPIP	12367263	15.8609	22.8565	-0.9527
YRI	NPIP	12372263	14.4462	18.823	-0.7189
YRI	NPIP	12377263	14.8599	16.4701	-0.3006
YRI	NPIP	12382263	12.7489	16.8062	-0.7429
YRI	NPIP	12387263	10.4763	17.1423	-1.1977
YRI	NPIP	12392263	10.2452	17.3104	-1.2576
YRI	NPIP	12397263	11.0022	16.8062	-1.0628
YRI	NPIP	12402263	10.5803	17.9827	-1.2703
YRI	NPIP	12407263	10.1864	18.823	-1.4185
YRI	NPIP	12412263	9.1551	17.1423	-1.435
YRI	NPIP	12417263	11.1655	17.6465	-1.1325
YRI	NPIP	12422263	11.959	17.1423	-0.9313
YRI	NPIP	12427263	10.866	14.4534	-0.7585
YRI	NPIP	12432263	9.3154	13.445	-0.9352

YRI	NPIP	12437263	15.3024	19.3272	-0.6445
YRI	NPIP	12442263	18.5427	20.8397	-0.342
YRI	NPIP	12447263	16.451	19.3272	-0.4605
YRI	NPIP	12452263	20.8244	22.8565	-0.2768
YRI	NPIP	12457263	18.0657	21.68	-0.518
YRI	NPIP	12462263	16.7485	22.8565	-0.8319
YRI	NPIP	12467263	18.3389	24.201	-0.7554
CEU	NPIP	14208411	6.0647	9.8926	-1.1639
CEU	NPIP	14213411	5.5904	9.0398	-1.1405
CEU	NPIP	14218411	3.3368	6.3108	-1.3654
CEU	NPIP	14223411	4.227	6.9931	-1.1574
CEU	NPIP	14228411	4.1948	7.1636	-1.2153
CEU	NPIP	14233411	3.4352	6.9931	-1.4887
CEU	NPIP	14238411	4.0113	8.187	-1.5128
CEU	NPIP	14243411	2.6065	5.9697	-1.6231
CEU	NPIP	14248411	3.2208	5.9697	-1.3267
CEU	NPIP	14253411	4.5304	8.0164	-1.2876
CEU	NPIP	14258411	3.3533	6.6519	-1.4442
CEU	NPIP	14263411	2.6471	6.9931	-1.8184
CEU	NPIP	14268411	2.7054	6.6519	-1.7279
CEU	NPIP	14273411	4.2088	7.5047	-1.2932
CEU	NPIP	14278411	3.5966	6.8225	-1.3804
CEU	NPIP	14283411	1.4725	3.9229	-1.7091
CEU	NPIP	14288411	2.4024	4.4346	-1.2751
CEU	NPIP	14293411	2.4752	3.7524	-0.9253
CEU	NPIP	14298411	1.9006	3.7524	-1.3416
CEU	NPIP	14303411	1.7809	4.6052	-1.7148
CEU	NPIP	14308411	1.9295	4.2641	-1.5155
CEU	NPIP	14313411	3.3375	5.9697	-1.2703
CEU	NPIP	14318411	4.9217	9.3809	-1.4245
CEU	NPIP	14323411	4.7394	9.5515	-1.5118
CEU	NPIP	14328411	3.6009	8.5281	-1.7192
CEU	NPIP	14333411	3.9184	9.5515	-1.7697
CEU	NPIP	14338411	3.8477	9.3809	-1.7676
CEU	NPIP	14343411	3.0524	6.9931	-1.6489
CEU	NPIP	14348411	3.0078	6.3108	-1.5165
CEU	NPIP	14353411	2.7969	6.3108	-1.6133
CEU	NPIP	14358411	3.082	6.1402	-1.4391
CEU	NPIP	14363411	4.6535	8.187	-1.2801
CEU	NPIP	14368411	5.1556	9.2104	-1.3176
CEU	NPIP	14373411	8.0544	9.7221	-0.5154
CEU	NPIP	14378411	10.0647	8.8692	0.4023
CEU	NPIP	14383411	6.9619	8.187	-0.4438
CEU	NPIP	14388411	5.7594	10.5749	-1.3759
CEU	NPIP	14393411	9.8967	10.4043	-0.1473
CEU	NPIP	14398411	12.216	8.0164	1.5511
CEU	NPIP	14403411	11.8732	9.2104	0.8653
CEU	NPIP	14408411	10.9528	9.5515	0.4402
CEU	NPIP	14413411	9.0641	8.5281	0.187

CEU	NPIP	14418411	7.5935	8.0164	-0.1562
CEU	NPIP	14423411	8.8013	7.5047	0.5087
CEU	NPIP	14428411	11.523	8.5281	1.045
CEU	NPIP	14433411	8.6825	6.6519	0.889
CEU	NPIP	14438411	9.0672	5.9697	1.4949
CEU	NPIP	14443411	10.0254	9.3809	0.2059
CEU	NPIP	14448411	9.9773	9.7221	0.0789
CEU	NPIP	14453411	15.055	10.7454	1.213
CEU	NPIP	14458411	15.4671	11.2571	1.1344
CEU	NPIP	14463411	13.4313	10.4043	0.8781
CEU	NPIP	14468411	15.0122	14.3272	0.147
CEU	NPIP	14473411	14.0062	14.6684	-0.1389
CEU	NPIP	14478411	6.871	8.187	-0.4768
CEU	NPIP	14483411	3.3201	4.9463	-0.9274
CEU	NPIP	14488411	4.3181	5.7991	-0.7335
CEU	NPIP	14493411	5.9134	6.3108	-0.1825
CEU	NPIP	14498411	5.9866	5.7991	0.0929
CEU	NPIP	14503411	4.4714	5.1169	-0.3573
CEU	NPIP	14508411	4.6623	6.1402	-0.6955
CEU	NPIP	14513411	3.0812	5.458	-1.2425
CEU	NPIP	14518411	1.8369	4.7757	-1.7284
CEU	NPIP	14523411	3.3088	6.4814	-1.422
CEU	NPIP	14528411	4.0994	6.9931	-1.2108
CEU	NPIP	14533411	4.0744	6.1402	-0.9721
CEU	NPIP	14538411	3.8946	5.7991	-0.9433
CEU	NPIP	14543411	3.7294	6.4814	-1.2335
CEU	NPIP	14548411	3.1156	5.7991	-1.3291
CEU	NPIP	14553411	2.1738	4.6052	-1.4762
CEU	NPIP	14558411	2.2371	4.7757	-1.4931
CEU	NPIP	14563411	3.7526	6.3108	-1.1745
CEU	NPIP	14568411	2.7718	5.458	-1.4043
CEU	NPIP	14573411	2.4618	5.9697	-1.693
CEU	NPIP	14578411	2.8526	6.9931	-1.7325
CEU	NPIP	14583411	1.4785	4.7757	-1.9392
CEU	NPIP	14588411	1.9351	5.9697	-1.9472
CEU	NPIP	14593411	2.5013	7.5047	-1.9631
CEU	NPIP	14598411	3.4958	5.9697	-1.1939
CEU	NPIP	14603411	2.3692	3.5818	-0.9139
CEU	NPIP	14608411	1.471	3.9229	-1.7101
CEU	NPIP	14613411	2.8759	5.7991	-1.4478
CEU	NPIP	14618411	3.108	4.7757	-0.9808
CEU	NPIP	14623411	8.0181	6.3108	0.7839
CEU	NPIP	14628411	9.5112	7.5047	0.7873
CEU	NPIP	14633411	9.2569	8.187	0.3876
CEU	NPIP	14638411	9.8308	8.5281	0.4545
CEU	NPIP	14643411	5.0814	5.458	-0.1969
CEU	NPIP	14648411	4.5428	5.9697	-0.6886
CEU	NPIP	14653411	5.0664	6.3108	-0.5713
CEU	NPIP	14658411	3.818	6.1402	-1.0928

CEU	NPIP	14663411	3.1686	7.3342	-1.669
CEU	NPIP	14668411	3.6731	7.5047	-1.5033
CEU	NPIP	14673411	5.2643	5.9697	-0.3404
CEU	NPIP	14678411	4.8186	4.7757	0.0252
CEU	NPIP	14683411	3.4197	5.6286	-1.1236
CEU	NPIP	14688411	2.5558	5.9697	-1.6476
CEU	NPIP	14693411	1.9977	6.3108	-1.9802
CEU	NPIP	14698411	3.9732	7.1636	-1.306
CEU	NPIP	14703411	4.9518	6.8225	-0.8005
CEU	NPIP	14708411	4.9079	7.1636	-0.9234
CEU	NPIP	14713411	3.1773	6.1402	-1.3943
CEU	NPIP	14718411	0.6505	3.7524	-2.2473
CEU	NPIP	14723411	0.9038	3.7524	-2.0638
CEU	NPIP	14728411	2.3668	4.6052	-1.359
CEU	NPIP	14733411	3.8521	5.1169	-0.7001
CEU	NPIP	14738411	3.5958	4.9463	-0.7702
CEU	NPIP	14743411	1.7481	4.2641	-1.6332
CEU	NPIP	14748411	1.7868	4.7757	-1.7579
CEU	NPIP	14753411	2.9276	5.1169	-1.2118
CEU	NPIP	14758411	2.7547	5.1169	-1.3075
CEU	NPIP	14763411	2.3026	5.7991	-1.7318
CEU	NPIP	14768411	1.9701	5.2874	-1.7837
CEU	NPIP	14773411	3.0179	4.6052	-0.9637
CEU	NPIP	14778411	3.8887	5.6286	-0.885
CEU	NPIP	14783411	1.9656	3.7524	-1.2945
CEU	NPIP	14788411	0.0602	0.3411	-1.126
CEU	NPIP	14793411	0.0101	0.1706	-0.9534
CEU	NPIP	14798411	0.0101	0.1706	-0.9534
CEU	NPIP	14803411	0	0	0
CEU	NPIP	14808411	0	0	0
CEU	NPIP	14813411	0	0	0
CEU	NPIP	14818411	0.1309	0.1706	-0.2358
CEU	NPIP	14823411	0.5803	1.0234	-0.8805
CEU	NPIP	14828411	0.4494	0.8528	-0.9084
CEU	NPIP	14833411	0.095	0.1706	-0.4491
CEU	NPIP	14838411	0.095	0.1706	-0.4491
CEU	NPIP	14843411	0.0202	0.1706	-0.8934
CEU	NPIP	14848411	0.1441	0.3411	-0.7897
CEU	NPIP	14853411	0.1239	0.1706	-0.2774
CEU	NPIP	14858411	1.0208	1.5351	-0.7626
CEU	NPIP	14863411	3.9676	4.7757	-0.4753
CEU	NPIP	14868411	5.6162	6.8225	-0.5162
CEU	NPIP	14873411	2.9542	4.2641	-0.8503
CEU	NPIP	14878411	0.8417	1.5351	-1.0282
CEU	NPIP	14883411	1.0025	1.0234	-0.0415
CEU	NPIP	14888411	0.8955	0.6822	0.557
CEU	NPIP	14893411	0.7318	1.7056	-1.3337
CEU	NPIP	14898411	1.9105	3.4112	-1.1787
CEU	NPIP	14903411	1.7873	3.7524	-1.4237

CEU	NPIP	14908411	0.959	3.0701	-1.8109
CEU	NPIP	14913411	1.8516	3.4112	-1.2249
CEU	NPIP	14918411	1.1515	2.5584	-1.4015
CEU	NPIP	14923411	0.9248	2.0467	-1.3349
CEU	NPIP	14928411	2.2674	3.4112	-0.8984
CEU	NPIP	14933411	2.214	3.0701	-0.7343
CEU	NPIP	14938411	1.3983	2.2173	-0.915
CEU	NPIP	14943411	1.3181	2.729	-1.3336
CEU	NPIP	14948411	1.8021	3.2407	-1.1796
CEU	NPIP	14953411	1.6075	3.4112	-1.4167
CEU	NPIP	14958411	2.2165	4.4346	-1.3917
CEU	NPIP	14963411	4.7633	5.2874	-0.2818
CEU	NPIP	14968411	4.9204	4.7757	0.0851
CEU	NPIP	14973411	2.2742	3.5818	-0.9855
CEU	NPIP	14978411	1.1325	3.5818	-1.846
CEU	NPIP	14983411	1.4117	3.9229	-1.7515
CEU	NPIP	14988411	0.8932	3.0701	-1.8673
CEU	NPIP	14993411	0.6423	3.0701	-2.0825
CEU	NPIP	14998411	0.4651	1.8762	-1.7966
CEU	NPIP	15003411	0.3834	0.6822	-0.7803
CEU	NPIP	15008411	0.3537	0.6822	-0.8578
CEU	NPIP	15013411	0.0998	0.8528	-1.6957
CEU	NPIP	15018411	1.536	2.2173	-0.7611
CEU	NPIP	15023411	2.3593	2.5584	-0.1983
CEU	NPIP	15028411	1.4475	1.7056	-0.3535
CEU	NPIP	15033411	0.9948	1.8762	-1.1222
CEU	NPIP	15038411	0.9091	1.7056	-1.0909
CEU	NPIP	15043411	2.1816	2.729	-0.5174
CEU	NPIP	15048411	4.6071	6.4814	-0.8401
CEU	NPIP	15053411	6.0375	7.5047	-0.5757
CEU	NPIP	15058411	6.5943	8.0164	-0.5253
CEU	NPIP	15063411	4.7539	7.3342	-1.0338
CEU	NPIP	15068411	3.6557	5.458	-0.9422
CEU	NPIP	15073411	5.1837	5.7991	-0.3048
CEU	NPIP	15078411	5.2356	7.3342	-0.8408
CEU	NPIP	15083411	6.2653	8.5281	-0.7895
CEU	NPIP	15088411	6.7116	7.3342	-0.2495
CEU	NPIP	15093411	4.9349	6.8225	-0.8077
CEU	NPIP	15098411	4.035	6.4814	-1.0965
CEU	NPIP	15103411	4.7675	7.1636	-0.9808
CEU	NPIP	15108411	4.6226	6.6519	-0.8885
CEU	NPIP	15113411	5.106	6.4814	-0.6165
CEU	NPIP	15118411	6.5613	8.0164	-0.5374
CEU	NPIP	15123411	6.3676	7.8459	-0.5569
CEU	NPIP	15128411	10.6895	9.0398	0.5454
CEU	NPIP	15133411	12.1507	10.7454	0.3956
CEU	NPIP	15138411	9.9922	9.2104	0.254
CEU	NPIP	15143411	9.8042	7.1636	1.0809
CEU	NPIP	15148411	7.925	6.6519	0.5574

CEU	NPIP	15153411	6.4482	7.6753	-0.4717
CEU	NPIP	15158411	5.5991	6.4814	-0.3955
CEU	NPIP	15163411	4.7982	4.6052	0.1172
CEU	NPIP	15168411	7.4799	7.5047	-0.0097
CEU	NPIP	15173411	9.7846	8.8692	0.308
CEU	NPIP	15178411	6.5351	6.8225	-0.123
CEU	NPIP	15183411	4.6966	6.4814	-0.8
CEU	NPIP	15188411	3.733	6.3108	-1.1835
CEU	NPIP	15193411	2.671	6.6519	-1.7429
CEU	NPIP	15198411	4.5826	6.8225	-0.9584
CEU	NPIP	15203411	5.0233	5.9697	-0.4567
CEU	NPIP	15208411	3.2665	4.6052	-0.8128
CEU	NPIP	15213411	1.7143	2.729	-0.9591
CEU	NPIP	15218411	3.7464	7.1636	-1.3988
CHB	NPIP	14208411	13.9318	10.5037	0.9828
CHB	NPIP	14213411	11.9876	9.8261	0.6595
CHB	NPIP	14218411	7.1095	7.2848	-0.0705
CHB	NPIP	14223411	9.8867	9.9955	-0.0327
CHB	NPIP	14228411	10.1786	10.6731	-0.1397
CHB	NPIP	14233411	8.925	9.9955	-0.3215
CHB	NPIP	14238411	8.5871	10.1649	-0.4664
CHB	NPIP	14243411	4.9398	6.6072	-0.7323
CHB	NPIP	14248411	7.5285	7.2848	0.098
CHB	NPIP	14253411	9.9339	9.9955	-0.0185
CHB	NPIP	14258411	6.2218	7.6237	-0.5406
CHB	NPIP	14263411	4.189	6.6072	-1.0621
CHB	NPIP	14268411	3.6802	7.4543	-1.4856
CHB	NPIP	14273411	4.7443	8.979	-1.4048
CHB	NPIP	14278411	3.9646	8.6402	-1.6072
CHB	NPIP	14283411	1.5188	6.4378	-2.2117
CHB	NPIP	14288411	2.5271	6.946	-1.8549
CHB	NPIP	14293411	3.2201	6.0989	-1.3589
CHB	NPIP	14298411	2.9825	5.4213	-1.2788
CHB	NPIP	14303411	3.0444	6.946	-1.6377
CHB	NPIP	14308411	3.237	5.9295	-1.3035
CHB	NPIP	14313411	4.6345	7.2848	-1.0653
CHB	NPIP	14318411	7.7907	11.8591	-1.0409
CHB	NPIP	14323411	6.2719	12.0285	-1.4532
CHB	NPIP	14328411	2.6019	10.1649	-2.2357
CHB	NPIP	14333411	3.9661	11.1814	-1.951
CHB	NPIP	14338411	4.4274	9.9955	-1.672
CHB	NPIP	14343411	4.5267	7.9625	-1.2732
CHB	NPIP	14348411	4.194	7.7931	-1.3603
CHB	NPIP	14353411	4.6235	6.946	-0.9749
CHB	NPIP	14358411	6.1643	6.2684	-0.0479
CHB	NPIP	14363411	8.2336	7.2848	0.3814
CHB	NPIP	14368411	8.9437	9.4872	-0.1713
CHB	NPIP	14373411	9.8418	11.3508	-0.4023
CHB	NPIP	14378411	10.4694	9.6567	0.252

CHB	NPIP	14383411	6.7786	7.2848	-0.2035
CHB	NPIP	14388411	5.0253	7.6237	-1.002
CHB	NPIP	14393411	10.0846	8.6402	0.4965
CHB	NPIP	14398411	11.367	9.1484	0.7233
CHB	NPIP	14403411	10.4073	12.1979	-0.4461
CHB	NPIP	14408411	11.2728	11.3508	-0.0208
CHB	NPIP	14413411	9.9815	9.4872	0.1558
CHB	NPIP	14418411	8.185	9.9955	-0.5437
CHB	NPIP	14423411	8.7984	9.3178	-0.1665
CHB	NPIP	14428411	11.5094	8.3013	1.1442
CHB	NPIP	14433411	8.7816	6.4378	1.0538
CHB	NPIP	14438411	9.3398	8.6402	0.2405
CHB	NPIP	14443411	10.2283	10.3343	-0.0309
CHB	NPIP	14448411	9.8441	9.3178	0.1687
CHB	NPIP	14453411	14.9223	10.6731	1.2001
CHB	NPIP	14458411	15.0082	11.8591	0.8057
CHB	NPIP	14463411	11.3331	12.3673	-0.2543
CHB	NPIP	14468411	11.9962	14.2309	-0.4812
CHB	NPIP	14473411	11.6589	13.3838	-0.3937
CHB	NPIP	14478411	5.9037	7.7931	-0.7141
CHB	NPIP	14483411	2.6393	5.0825	-1.3564
CHB	NPIP	14488411	2.7689	6.4378	-1.6497
CHB	NPIP	14493411	3.238	6.6072	-1.4798
CHB	NPIP	14498411	3.5865	6.2684	-1.2352
CHB	NPIP	14503411	3.3322	5.4213	-1.0955
CHB	NPIP	14508411	4.3013	5.9295	-0.7882
CHB	NPIP	14513411	3.0505	6.2684	-1.482
CHB	NPIP	14518411	0.9861	5.9295	-2.3932
CHB	NPIP	14523411	0.7811	6.4378	-2.5434
CHB	NPIP	14528411	1.8816	6.0989	-1.9907
CHB	NPIP	14533411	3.5808	4.913	-0.762
CHB	NPIP	14538411	4.4784	5.4213	-0.4944
CHB	NPIP	14543411	5.9657	7.4543	-0.586
CHB	NPIP	14548411	4.8279	7.2848	-0.9876
CHB	NPIP	14553411	2.5239	5.7601	-1.6078
CHB	NPIP	14558411	1.6452	3.8965	-1.5746
CHB	NPIP	14563411	2.2083	4.4048	-1.3821
CHB	NPIP	14568411	2.0256	6.4378	-1.9839
CHB	NPIP	14573411	1.1766	5.7601	-2.2772
CHB	NPIP	14578411	1.7887	5.2519	-1.8678
CHB	NPIP	14583411	1.1799	4.913	-2.1353
CHB	NPIP	14588411	1.3745	5.5907	-2.1512
CHB	NPIP	14593411	1.3377	6.4378	-2.2932
CHB	NPIP	14598411	0.878	5.5907	-2.4045
CHB	NPIP	14603411	0.9872	4.5742	-2.1842
CHB	NPIP	14608411	0.5346	4.066	-2.3813
CHB	NPIP	14613411	0.857	4.7436	-2.2926
CHB	NPIP	14618411	1.0822	4.913	-2.1912
CHB	NPIP	14623411	1.6337	8.6402	-2.4084

CHB	NPIP	14628411	2.1157	9.8261	-2.3525
CHB	NPIP	14633411	3.4076	7.6237	-1.6258
CHB	NPIP	14638411	3.5865	7.1154	-1.4492
CHB	NPIP	14643411	1.9126	6.2684	-2.0061
CHB	NPIP	14648411	1.6097	7.7931	-2.337
CHB	NPIP	14653411	2.119	8.6402	-2.2416
CHB	NPIP	14658411	2.016	7.1154	-2.0942
CHB	NPIP	14663411	1.0754	5.9295	-2.3499
CHB	NPIP	14668411	2.1235	6.7766	-1.9974
CHB	NPIP	14673411	2.4453	6.946	-1.8892
CHB	NPIP	14678411	1.3457	5.2519	-2.1067
CHB	NPIP	14683411	1.8852	5.0825	-1.7751
CHB	NPIP	14688411	2.5435	7.9625	-2.0081
CHB	NPIP	14693411	2.0638	8.6402	-2.2605
CHB	NPIP	14698411	2.8726	6.4378	-1.603
CHB	NPIP	14703411	3.6724	6.6072	-1.289
CHB	NPIP	14708411	3.5125	7.9625	-1.649
CHB	NPIP	14713411	2.2975	6.6072	-1.8929
CHB	NPIP	14718411	0.7002	4.2354	-2.3014
CHB	NPIP	14723411	0.7874	4.066	-2.2109
CHB	NPIP	14728411	1.3617	4.066	-1.8236
CHB	NPIP	14733411	2.6988	4.7436	-1.2062
CHB	NPIP	14738411	3.0249	4.913	-1.08
CHB	NPIP	14743411	2.0613	4.913	-1.6312
CHB	NPIP	14748411	2.0148	6.6072	-2.017
CHB	NPIP	14753411	2.0619	5.9295	-1.8724
CHB	NPIP	14758411	1.751	5.4213	-1.9246
CHB	NPIP	14763411	1.3501	5.7601	-2.191
CHB	NPIP	14768411	1.5159	4.7436	-1.9039
CHB	NPIP	14773411	2.5709	5.5907	-1.5408
CHB	NPIP	14778411	3.4392	6.7766	-1.4326
CHB	NPIP	14783411	2.2107	4.2354	-1.3181
CHB	NPIP	14788411	0.2915	0.6777	-1.0096
CHB	NPIP	14793411	0	0	0
CHB	NPIP	14798411	0	0	0
CHB	NPIP	14803411	0	0	0
CHB	NPIP	14808411	0	0	0
CHB	NPIP	14813411	0.0097	0.1694	-0.949
CHB	NPIP	14818411	0.0194	0.3388	-1.2812
CHB	NPIP	14823411	0.5071	0.8471	-0.7667
CHB	NPIP	14828411	0.4974	0.6777	-0.4714
CHB	NPIP	14833411	0.0377	0.1694	-0.7826
CHB	NPIP	14838411	0.0377	0.1694	-0.7826
CHB	NPIP	14843411	0	0	0
CHB	NPIP	14848411	0.2142	0.1694	0.2662
CHB	NPIP	14853411	0.2142	0.1694	0.2662
CHB	NPIP	14858411	1.3944	1.1859	0.3722
CHB	NPIP	14863411	4.1315	4.4048	-0.172
CHB	NPIP	14868411	6.5858	6.4378	0.0665

CHB	NPIP	14873411	4.4447	3.8965	0.3834
CHB	NPIP	14878411	1.1542	1.3553	-0.3259
CHB	NPIP	14883411	0.7087	0.8471	-0.3121
CHB	NPIP	14888411	0.1989	0.6777	-1.2517
CHB	NPIP	14893411	0.3628	2.2024	-2.0599
CHB	NPIP	14898411	1.9823	2.5412	-0.5581
CHB	NPIP	14903411	1.9115	1.5247	0.5746
CHB	NPIP	14908411	1.4064	1.6942	-0.395
CHB	NPIP	14913411	2.4626	1.8636	0.7642
CHB	NPIP	14918411	1.4156	1.8636	-0.5716
CHB	NPIP	14923411	0.7472	2.033	-1.5333
CHB	NPIP	14928411	1.8382	3.2189	-1.1351
CHB	NPIP	14933411	2.234	3.2189	-0.8097
CHB	NPIP	14938411	1.0952	2.2024	-1.2398
CHB	NPIP	14943411	1.0364	2.3718	-1.4096
CHB	NPIP	14948411	2.2075	3.0495	-0.7241
CHB	NPIP	14953411	1.3263	3.0495	-1.482
CHB	NPIP	14958411	1.3842	3.7271	-1.7021
CHB	NPIP	14963411	4.9743	5.0825	-0.0601
CHB	NPIP	14968411	5.7379	4.913	0.4718
CHB	NPIP	14973411	2.7021	5.0825	-1.3216
CHB	NPIP	14978411	2.0621	4.7436	-1.5818
CHB	NPIP	14983411	2.5155	3.3883	-0.6873
CHB	NPIP	14988411	1.1727	2.7106	-1.4572
CHB	NPIP	14993411	0.7588	2.7106	-1.8494
CHB	NPIP	14998411	0.6908	1.5247	-1.2389
CHB	NPIP	15003411	0.7936	0.6777	0.303
CHB	NPIP	15008411	0.7936	0.6777	0.303
CHB	NPIP	15013411	0.1043	0.6777	-1.499
CHB	NPIP	15018411	1.6682	1.6942	-0.0357
CHB	NPIP	15023411	2.9482	3.2189	-0.2226
CHB	NPIP	15028411	1.7489	3.0495	-1.1185
CHB	NPIP	15033411	0.4892	1.6942	-1.6537
CHB	NPIP	15038411	0.5711	1.0165	-0.8864
CHB	NPIP	15043411	2.7683	3.2189	-0.3705
CHB	NPIP	15048411	5.9299	6.6072	-0.2975
CHB	NPIP	15053411	6.139	7.1154	-0.401
CHB	NPIP	15058411	6.8869	7.1154	-0.0938
CHB	NPIP	15063411	5.6889	6.7766	-0.4669
CHB	NPIP	15068411	3.6526	5.5907	-0.9889
CHB	NPIP	15073411	5.3217	5.7601	-0.2178
CHB	NPIP	15078411	5.1536	5.9295	-0.3756
CHB	NPIP	15083411	5.6697	6.2684	-0.2757
CHB	NPIP	15088411	6.6501	6.946	-0.1242
CHB	NPIP	15093411	5.1211	6.6072	-0.6527
CHB	NPIP	15098411	4.2998	6.7766	-1.0632
CHB	NPIP	15103411	4.6238	6.6072	-0.8711
CHB	NPIP	15108411	4.5397	5.7601	-0.6063
CHB	NPIP	15113411	5.6587	5.7601	-0.0504

CHB	NPIP	15118411	6.1523	5.5907	0.2865
CHB	NPIP	15123411	6.0598	5.0825	0.5426
CHB	NPIP	15128411	11.8557	8.3013	1.2677
CHB	NPIP	15133411	13.2977	10.3343	0.8626
CHB	NPIP	15138411	10.8818	8.4708	0.844
CHB	NPIP	15143411	11.3217	7.2848	1.6227
CHB	NPIP	15148411	8.4049	6.0989	1.0885
CHB	NPIP	15153411	7.1788	7.1154	0.026
CHB	NPIP	15158411	7.134	6.6072	0.2314
CHB	NPIP	15163411	5.5005	5.4213	0.0415
CHB	NPIP	15168411	8.2599	7.4543	0.3171
CHB	NPIP	15173411	10.6185	8.3013	0.8264
CHB	NPIP	15178411	7.4335	6.6072	0.3629
CHB	NPIP	15183411	5.4833	6.2684	-0.3616
CHB	NPIP	15188411	3.9753	5.2519	-0.6885
CHB	NPIP	15193411	3.5871	5.2519	-0.8979
CHB	NPIP	15198411	4.9402	6.4378	-0.6734
CHB	NPIP	15203411	4.8915	4.913	-0.0123
CHB	NPIP	15208411	4.0695	3.5577	0.3868
CHB	NPIP	15213411	2.3499	2.5412	-0.191
CHB	NPIP	15218411	5.8388	6.7766	-0.4026
YRI	NPIP	14208411	15.3227	18.1507	-0.481
YRI	NPIP	14213411	16.3256	16.134	0.0365
YRI	NPIP	14218411	10.0332	11.2602	-0.3285
YRI	NPIP	14223411	15.4585	14.6214	0.1751
YRI	NPIP	14228411	16.5069	16.9743	-0.0848
YRI	NPIP	14233411	13.5105	14.2853	-0.1657
YRI	NPIP	14238411	17.0548	14.2853	0.5921
YRI	NPIP	14243411	12.9135	13.1089	-0.0453
YRI	NPIP	14248411	12.2526	11.9324	0.0812
YRI	NPIP	14253411	16.4247	14.6214	0.3771
YRI	NPIP	14258411	12.6207	14.1172	-0.3236
YRI	NPIP	14263411	10.0172	11.0921	-0.2918
YRI	NPIP	14268411	10.0993	10.5879	-0.1386
YRI	NPIP	14273411	11.3949	11.7644	-0.0949
YRI	NPIP	14278411	11.6607	10.5879	0.3042
YRI	NPIP	14283411	7.8666	9.7476	-0.5762
YRI	NPIP	14288411	6.3401	9.9157	-1.078
YRI	NPIP	14293411	6.3629	8.9073	-0.8474
YRI	NPIP	14298411	5.4014	8.235	-1.0146
YRI	NPIP	14303411	6.0414	7.8989	-0.691
YRI	NPIP	14308411	5.5682	8.9073	-1.1121
YRI	NPIP	14313411	6.7116	10.4199	-1.0675
YRI	NPIP	14318411	14.2337	15.2937	-0.2124
YRI	NPIP	14323411	16.8369	17.6465	-0.1415
YRI	NPIP	14328411	14.4537	14.2853	0.036
YRI	NPIP	14333411	15.5628	14.1172	0.3126
YRI	NPIP	14338411	13.8947	15.4617	-0.3107
YRI	NPIP	14343411	9.8294	16.302	-1.2202

YRI	NPIP	14348411	9.6436	15.6298	-1.1748
YRI	NPIP	14353411	9.3366	13.2769	-0.9031
YRI	NPIP	14358411	9.0461	13.1089	-0.9424
YRI	NPIP	14363411	12.1456	13.1089	-0.2235
YRI	NPIP	14368411	15.0569	13.9492	0.2423
YRI	NPIP	14373411	17.2755	16.8062	0.0859
YRI	NPIP	14378411	15.0849	15.7978	-0.1385
YRI	NPIP	14383411	11.3519	15.6298	-0.8396
YRI	NPIP	14388411	12.3101	17.8146	-0.9532
YRI	NPIP	14393411	14.2923	15.4617	-0.2319
YRI	NPIP	14398411	14.1752	14.1172	0.0125
YRI	NPIP	14403411	16.0668	17.8146	-0.3027
YRI	NPIP	14408411	13.947	18.823	-0.8009
YRI	NPIP	14413411	9.0181	17.3104	-1.476
YRI	NPIP	14418411	7.1494	14.9575	-1.598
YRI	NPIP	14423411	9.1816	13.2769	-0.9386
YRI	NPIP	14428411	12.2854	14.4534	-0.4584
YRI	NPIP	14433411	9.6076	13.613	-0.8965
YRI	NPIP	14438411	10.7626	15.7978	-0.9782
YRI	NPIP	14443411	10.8756	16.4701	-1.0444
YRI	NPIP	14448411	9.2945	12.7727	-0.8269
YRI	NPIP	14453411	13.57	15.1256	-0.315
YRI	NPIP	14458411	14.0146	18.1507	-0.7035
YRI	NPIP	14463411	11.6534	16.302	-0.8764
YRI	NPIP	14468411	14.6438	16.6382	-0.3687
YRI	NPIP	14473411	15.4322	15.7978	-0.071
YRI	NPIP	14478411	8.4428	11.4282	-0.7882
YRI	NPIP	14483411	6.3593	9.9157	-1.0723
YRI	NPIP	14488411	7.1036	10.4199	-0.9547
YRI	NPIP	14493411	7.1025	10.5879	-0.9884
YRI	NPIP	14498411	7.2817	9.0754	-0.5872
YRI	NPIP	14503411	5.9575	7.0586	-0.4539
YRI	NPIP	14508411	8.3072	9.7476	-0.4412
YRI	NPIP	14513411	7.0422	9.9157	-0.8664
YRI	NPIP	14518411	4.9454	6.8906	-0.8196
YRI	NPIP	14523411	5.8381	7.7309	-0.7182
YRI	NPIP	14528411	5.3103	7.5628	-0.872
YRI	NPIP	14533411	4.6782	6.7225	-0.8809
YRI	NPIP	14538411	4.3294	7.5628	-1.2517
YRI	NPIP	14543411	4.9038	8.067	-1.1543
YRI	NPIP	14548411	3.9279	6.8906	-1.2484
YRI	NPIP	14553411	2.7675	6.5544	-1.6696
YRI	NPIP	14558411	2.8066	7.5628	-1.8413
YRI	NPIP	14563411	4.4624	8.235	-1.3508
YRI	NPIP	14568411	4.7275	6.5544	-0.8054
YRI	NPIP	14573411	3.0407	6.7225	-1.5865
YRI	NPIP	14578411	2.2169	8.235	-2.1548
YRI	NPIP	14583411	1.9799	7.2267	-2.1172
YRI	NPIP	14588411	3.1019	6.3864	-1.4824

YRI	NPIP	14593411	3.6885	6.7225	-1.3074
YRI	NPIP	14598411	5.0635	6.8906	-0.7699
YRI	NPIP	14603411	3.8512	6.3864	-1.1442
YRI	NPIP	14608411	1.5861	7.2267	-2.2761
YRI	NPIP	14613411	2.1481	7.7309	-2.1183
YRI	NPIP	14618411	2.1398	6.2183	-1.8855
YRI	NPIP	14623411	4.1974	9.0754	-1.5968
YRI	NPIP	14628411	4.5687	9.7476	-1.5865
YRI	NPIP	14633411	4.8343	8.235	-1.2176
YRI	NPIP	14638411	4.7912	9.2434	-1.4328
YRI	NPIP	14643411	2.9407	8.5712	-1.9431
YRI	NPIP	14648411	3.5024	8.9073	-1.8001
YRI	NPIP	14653411	3.8354	8.5712	-1.6343
YRI	NPIP	14658411	3.1739	8.067	-1.7855
YRI	NPIP	14663411	2.8388	8.4031	-1.9556
YRI	NPIP	14668411	3.1687	8.4031	-1.8397
YRI	NPIP	14673411	3.2677	8.067	-1.7513
YRI	NPIP	14678411	2.7635	7.3947	-1.83
YRI	NPIP	14683411	2.0995	6.3864	-1.9348
YRI	NPIP	14688411	2.4792	7.5628	-1.968
YRI	NPIP	14693411	2.8237	9.5795	-2.1033
YRI	NPIP	14698411	3.6498	8.067	-1.6119
YRI	NPIP	14703411	4.2562	9.0754	-1.5775
YRI	NPIP	14708411	4.1747	10.2518	-1.7762
YRI	NPIP	14713411	2.6465	6.8906	-1.7883
YRI	NPIP	14718411	1.1256	7.8989	-2.5198
YRI	NPIP	14723411	1.2375	7.5628	-2.4487
YRI	NPIP	14728411	2.1024	5.8822	-1.8366
YRI	NPIP	14733411	3.2034	7.8989	-1.7468
YRI	NPIP	14738411	3.1034	7.0586	-1.6305
YRI	NPIP	14743411	2.3156	5.8822	-1.733
YRI	NPIP	14748411	2.3919	7.8989	-2.0487
YRI	NPIP	14753411	2.2879	8.235	-2.1294
YRI	NPIP	14758411	1.7167	6.8906	-2.1801
YRI	NPIP	14763411	2.0778	8.235	-2.2046
YRI	NPIP	14768411	2.3875	7.7309	-2.0274
YRI	NPIP	14773411	2.6989	7.2267	-1.8271
YRI	NPIP	14778411	3.143	8.5712	-1.8733
YRI	NPIP	14783411	1.7065	4.8738	-1.8181
YRI	NPIP	14788411	0.0278	0.5042	-1.4972
YRI	NPIP	14793411	0	0	0
YRI	NPIP	14798411	0	0	0
YRI	NPIP	14803411	0	0	0
YRI	NPIP	14808411	0	0	0
YRI	NPIP	14813411	0	0	0
YRI	NPIP	14818411	0.1063	0.5042	-1.2505
YRI	NPIP	14823411	0.4422	1.1764	-1.3132
YRI	NPIP	14828411	0.3359	0.6722	-0.8804
YRI	NPIP	14833411	0.1057	0.1681	-0.3709

YRI	NPIP	14838411	0.1057	0.1681	-0.3709
YRI	NPIP	14843411	0	0	0
YRI	NPIP	14848411	0.358	0.3361	0.0879
YRI	NPIP	14853411	0.358	0.3361	0.0879
YRI	NPIP	14858411	0.2761	1.5126	-1.8412
YRI	NPIP	14863411	2.2475	5.7141	-1.7286
YRI	NPIP	14868411	6.3168	7.8989	-0.5886
YRI	NPIP	14873411	4.7767	4.8738	-0.0557
YRI	NPIP	14878411	0.8738	2.0167	-1.3665
YRI	NPIP	14883411	0.4702	1.0084	-1.073
YRI	NPIP	14888411	0.3258	1.1764	-1.5213
YRI	NPIP	14893411	0.8727	2.8571	-1.7946
YRI	NPIP	14898411	2.2977	5.0419	-1.529
YRI	NPIP	14903411	2.6614	5.0419	-1.3263
YRI	NPIP	14908411	2.5386	5.378	-1.4943
YRI	NPIP	14913411	2.8532	5.8822	-1.4718
YRI	NPIP	14918411	1.4826	4.5377	-1.8668
YRI	NPIP	14923411	1.5492	5.8822	-2.1055
YRI	NPIP	14928411	2.8816	7.5628	-1.8122
YRI	NPIP	14933411	2.6018	5.8822	-1.594
YRI	NPIP	14938411	1.4097	3.6974	-1.6674
YRI	NPIP	14943411	1.4261	4.7057	-1.9414
YRI	NPIP	14948411	2.3656	6.7225	-1.8774
YRI	NPIP	14953411	1.6516	5.7141	-2.0258
YRI	NPIP	14958411	2.6928	6.8906	-1.7688
YRI	NPIP	14963411	5.2209	10.5879	-1.5221
YRI	NPIP	14968411	4.6096	8.7392	-1.3998
YRI	NPIP	14973411	2.6888	5.5461	-1.4632
YRI	NPIP	14978411	1.713	5.5461	-1.9629
YRI	NPIP	14983411	2.0169	6.3864	-1.9721
YRI	NPIP	14988411	1.6143	5.5461	-2.0134
YRI	NPIP	14993411	0.9595	4.5377	-2.1864
YRI	NPIP	14998411	0.5983	2.3529	-1.8573
YRI	NPIP	15003411	0.5258	0.6722	-0.3833
YRI	NPIP	15008411	0.7435	1.6806	-1.2892
YRI	NPIP	15013411	0.5191	3.0251	-2.1618
YRI	NPIP	15018411	2.6625	5.5461	-1.4767
YRI	NPIP	15023411	3.5133	6.0502	-1.202
YRI	NPIP	15028411	1.3829	3.8654	-1.7421
YRI	NPIP	15033411	0.4942	3.0251	-2.1833
YRI	NPIP	15038411	0.9518	2.689	-1.651
YRI	NPIP	15043411	2.5985	4.5377	-1.1849
YRI	NPIP	15048411	6.6233	10.0837	-1.0271
YRI	NPIP	15053411	8.5663	11.4282	-0.7556
YRI	NPIP	15058411	7.6069	9.0754	-0.4807
YRI	NPIP	15063411	4.9518	7.0586	-0.8685
YRI	NPIP	15068411	3.2875	6.5544	-1.4403
YRI	NPIP	15073411	5.3009	8.4031	-1.0903
YRI	NPIP	15078411	5.3323	9.2434	-1.2587

YRI	NPIP	15083411	5.8752	9.7476	-1.1863
YRI	NPIP	15088411	6.0857	9.7476	-1.1218
YRI	NPIP	15093411	4.2439	7.8989	-1.3597
YRI	NPIP	15098411	4.2828	7.7309	-1.3083
YRI	NPIP	15103411	5.5434	9.0754	-1.1562
YRI	NPIP	15108411	5.0886	9.0754	-1.3051
YRI	NPIP	15113411	5.0267	10.0837	-1.501
YRI	NPIP	15118411	6.6693	12.6047	-1.4288
YRI	NPIP	15123411	6.2567	11.4282	-1.3653
YRI	NPIP	15128411	8.5971	10.2518	-0.4836
YRI	NPIP	15133411	9.3885	11.0921	-0.4625
YRI	NPIP	15138411	7.1154	10.756	-1.0174
YRI	NPIP	15143411	7.2291	9.2434	-0.6483
YRI	NPIP	15148411	6.1166	8.4031	-0.8036
YRI	NPIP	15153411	6.7193	10.0837	-0.9986
YRI	NPIP	15158411	6.6319	9.2434	-0.8405
YRI	NPIP	15163411	5.05	8.4031	-1.1785
YRI	NPIP	15168411	7.3418	9.9157	-0.776
YRI	NPIP	15173411	9.0562	10.2518	-0.3494
YRI	NPIP	15178411	6.3055	8.7392	-0.825
YRI	NPIP	15183411	4.857	8.4031	-1.2463
YRI	NPIP	15188411	3.9497	7.2267	-1.3223
YRI	NPIP	15193411	3.7103	8.235	-1.6201
YRI	NPIP	15198411	4.5117	9.5795	-1.5778
YRI	NPIP	15203411	4.5293	7.2267	-1.0885
YRI	NPIP	15208411	4.1688	5.0419	-0.4865
YRI	NPIP	15213411	2.6186	4.5377	-1.1727
YRI	NPIP	15218411	7.069	11.4282	-1.1508
CEU	NPIP	14247533	3.0163	5.2874	-1.2212
CEU	NPIP	14252533	3.8897	7.1636	-1.3402
CEU	NPIP	14257533	3.4611	7.1636	-1.5156
CEU	NPIP	14262533	3.1701	7.5047	-1.7007
CEU	NPIP	14267533	2.6949	6.3108	-1.6601
CEU	NPIP	14272533	3.1709	7.1636	-1.6344
CEU	NPIP	14277533	3.9264	7.5047	-1.404
CEU	NPIP	14282533	2.182	3.9229	-1.2142
CEU	NPIP	14287533	2.4321	4.4346	-1.2564
CEU	NPIP	14292533	2.7464	4.2641	-0.9852
CEU	NPIP	14297533	1.7939	3.4112	-1.2703
CEU	NPIP	14302533	1.9557	4.7757	-1.6586
CEU	NPIP	14307533	1.8802	4.6052	-1.6545
CEU	NPIP	14312533	3.0573	5.6286	-1.3079
CEU	NPIP	14317533	4.2004	8.187	-1.4443
CEU	NPIP	14322533	4.8545	9.7221	-1.5042
CEU	NPIP	14327533	3.9639	9.0398	-1.6782
CEU	NPIP	14332533	3.7133	8.8692	-1.735
CEU	NPIP	14337533	4.2031	9.5515	-1.6803
CEU	NPIP	14342533	3.2266	7.8459	-1.7402
CEU	NPIP	14347533	2.713	6.3108	-1.6518

CEU	NPIP	14352533	3.0031	6.4814	-1.559
CEU	NPIP	14357533	3.1447	6.1402	-1.4096
CEU	NPIP	14362533	4.2296	6.8225	-1.1095
CEU	NPIP	14367533	5.1866	9.5515	-1.3713
CEU	NPIP	14372533	6.5368	10.0632	-1.0553
CEU	NPIP	14377533	9.784	8.3576	0.507
CEU	NPIP	14382533	8.2729	7.8459	0.1609
CEU	NPIP	14387533	5.6737	10.4043	-1.3723
CEU	NPIP	14392533	9.0454	10.2337	-0.3501
CEU	NPIP	14397533	12.9326	8.6987	1.4505
CEU	NPIP	14402533	12.8576	10.4043	0.7117
CEU	NPIP	14407533	9.7065	9.2104	0.1612
CEU	NPIP	14412533	8.8378	8.3576	0.1707
CEU	NPIP	14417533	7.813	7.8459	-0.0124
CEU	NPIP	14422533	6.1954	6.4814	-0.1282
CEU	NPIP	14427533	11.9942	8.5281	1.2094
CEU	NPIP	14432533	11.5395	8.0164	1.3013
CEU	NPIP	14437533	8.2286	5.9697	1.0902
CEU	NPIP	14442533	10.3311	8.3576	0.7015
CEU	NPIP	14447533	10.0169	9.3809	0.2032
CEU	NPIP	14452533	11.9604	10.0632	0.5678
CEU	NPIP	14457533	14.9913	11.4277	0.9468
CEU	NPIP	14462533	16.6726	11.9394	1.2068
CEU	NPIP	14467533	16.2653	14.3272	0.4158
CEU	NPIP	14472533	14.0062	14.6684	-0.1389
CEU	NPIP	14477533	7.4583	9.0398	-0.5229
CEU	NPIP	14482533	2.2927	4.2641	-1.2797
CEU	NPIP	14487533	4.2092	5.1169	-0.5024
CEU	NPIP	14492533	6.961	7.3342	-0.1495
CEU	NPIP	14497533	6.0853	6.4814	-0.1775
CEU	NPIP	14502533	3.3049	4.4346	-0.7088
CEU	NPIP	14507533	4.4772	5.6286	-0.5857
CEU	NPIP	14512533	4.1427	5.7991	-0.8204
CEU	NPIP	14517533	1.9371	5.1169	-1.7601
CEU	NPIP	14522533	3.3535	6.1402	-1.3113
CEU	NPIP	14527533	3.7796	6.6519	-1.2575
CEU	NPIP	14532533	3.1235	5.6286	-1.2742
CEU	NPIP	14537533	4.2419	6.3108	-0.9499
CEU	NPIP	14542533	4.5163	7.3342	-1.129
CEU	NPIP	14547533	2.9641	5.458	-1.3038
CEU	NPIP	14552533	1.845	4.0935	-1.512
CEU	NPIP	14557533	2.3288	4.6052	-1.3821
CEU	NPIP	14562533	4.0074	6.6519	-1.1578
CEU	NPIP	14567533	2.8487	5.458	-1.3641
CEU	NPIP	14572533	1.5341	5.2874	-2.0182
CEU	NPIP	14577533	2.8536	7.1636	-1.7643
CEU	NPIP	14582533	2.6143	5.2874	-1.4373
CEU	NPIP	14587533	1.9742	6.1402	-1.9604
CEU	NPIP	14592533	2.5613	7.5047	-1.9395

CEU	NPIP	14597533	2.7326	5.7991	-1.5188
CEU	NPIP	14602533	2.3793	4.0935	-1.1527
CEU	NPIP	14607533	2.2544	4.4346	-1.3679
CEU	NPIP	14612533	2.1789	5.458	-1.7142
CEU	NPIP	14617533	3.0878	4.4346	-0.845
CEU	NPIP	14622533	7.5112	5.9697	0.7439
CEU	NPIP	14627533	9.1678	7.5047	0.6525
CEU	NPIP	14632533	9.3956	8.0164	0.5094
CEU	NPIP	14637533	10.1643	8.6987	0.5021
CEU	NPIP	14642533	5.7345	6.1402	-0.1909
CEU	NPIP	14647533	3.7304	5.1169	-0.7675
CEU	NPIP	14652533	4.8456	5.9697	-0.5425
CEU	NPIP	14657533	4.5415	6.1402	-0.7523
CEU	NPIP	14662533	3.5677	7.1636	-1.472
CEU	NPIP	14667533	3.792	8.5281	-1.6525
CEU	NPIP	14672533	4.941	6.3108	-0.6289
CEU	NPIP	14677533	4.364	4.2641	0.0648
CEU	NPIP	14682533	3.9567	5.7991	-0.9125
CEU	NPIP	14687533	2.9318	6.1402	-1.5098
CEU	NPIP	14692533	1.8734	6.3108	-2.0373
CEU	NPIP	14697533	4.0016	6.9931	-1.2517
CEU	NPIP	14702533	5.066	6.6519	-0.6943
CEU	NPIP	14707533	4.5364	7.1636	-1.0754
CEU	NPIP	14712533	3.169	6.4814	-1.4847
CEU	NPIP	14717533	1.0518	3.9229	-2.0025
CEU	NPIP	14722533	0.9321	3.4112	-1.9471
CEU	NPIP	14727533	2.1172	4.6052	-1.5106
CEU	NPIP	14732533	2.9205	5.1169	-1.2157
CEU	NPIP	14737533	3.8258	5.1169	-0.7146
CEU	NPIP	14742533	2.5539	4.6052	-1.2454
CEU	NPIP	14747533	1.274	4.2641	-1.941
CEU	NPIP	14752533	2.9951	4.7757	-1.0472
CEU	NPIP	14757533	3.2088	5.458	-1.1758
CEU	NPIP	14762533	2.3913	6.3108	-1.7995
CEU	NPIP	14767533	1.6386	4.9463	-1.8864
CEU	NPIP	14772533	2.8613	4.0935	-0.8286
CEU	NPIP	14777533	4.3187	6.3108	-0.9146
CEU	NPIP	14782533	2.1222	4.2641	-1.3904
CEU	NPIP	14787533	0.0602	0.3411	-1.126
CEU	NPIP	14792533	0.0101	0.1706	-0.9534
CEU	NPIP	14797533	0.0101	0.1706	-0.9534
CEU	NPIP	14802533	0	0	0
CEU	NPIP	14807533	0	0	0
CEU	NPIP	14812533	0	0	0
CEU	NPIP	14817533	0.1309	0.1706	-0.2358
CEU	NPIP	14822533	0.5803	1.0234	-0.8805
CEU	NPIP	14827533	0.4494	0.8528	-0.9084
CEU	NPIP	14832533	0.095	0.1706	-0.4491
CEU	NPIP	14837533	0.095	0.1706	-0.4491

CEU	NPIP	14842533	0.0202	0.1706	-0.8934
CEU	NPIP	14847533	0.1441	0.3411	-0.7897
CEU	NPIP	14852533	0.1239	0.1706	-0.2774
CEU	NPIP	14857533	0.873	0.6822	0.4982
CEU	NPIP	14862533	2.772	4.2641	-0.9686
CEU	NPIP	14867533	5.6197	7.1636	-0.632
CEU	NPIP	14872533	4.1101	4.6052	-0.3006
CEU	NPIP	14877533	0.986	2.0467	-1.2621
CEU	NPIP	14882533	1.0423	1.1939	-0.2702
CEU	NPIP	14887533	0.8854	0.5117	1.172
CEU	NPIP	14892533	0.7318	1.7056	-1.3337
CEU	NPIP	14897533	1.4985	3.4112	-1.5023
CEU	NPIP	14902533	1.7772	3.5818	-1.3601
CEU	NPIP	14907533	0.9229	2.8996	-1.7777
CEU	NPIP	14912533	1.8617	3.5818	-1.2964
CEU	NPIP	14917533	1.3882	2.729	-1.2673
CEU	NPIP	14922533	0.9147	1.8762	-1.2242
CEU	NPIP	14927533	1.9079	2.8996	-0.8919
CEU	NPIP	14932533	1.8592	2.8996	-0.9357
CEU	NPIP	14937533	1.7578	2.729	-0.918
CEU	NPIP	14942533	1.683	3.0701	-1.1898
CEU	NPIP	14947533	1.7128	2.8996	-1.0673
CEU	NPIP	14952533	1.5673	3.0701	-1.2891
CEU	NPIP	14957533	2.2956	4.6052	-1.4023
CEU	NPIP	14962533	2.9223	4.2641	-0.871
CEU	NPIP	14967533	4.9305	4.9463	-0.009
CEU	NPIP	14972533	4.0952	4.4346	-0.213
CEU	NPIP	14977533	0.6178	2.5584	-1.9332
CEU	NPIP	14982533	1.402	3.9229	-1.7583
CEU	NPIP	14987533	1.408	4.0935	-1.8058
CEU	NPIP	14992533	0.6157	3.2407	-2.1524
CEU	NPIP	14997533	0.4651	1.8762	-1.7966
CEU	NPIP	15002533	0.4599	0.8528	-0.8848
CEU	NPIP	15007533	0.3537	0.6822	-0.8578
CEU	NPIP	15012533	0.0604	0.8528	-1.7845
CEU	NPIP	15017533	1.3729	1.5351	-0.2405
CEU	NPIP	15022533	2.4086	2.5584	-0.1492
CEU	NPIP	15027533	1.6106	2.3879	-0.8186
CEU	NPIP	15032533	1.0049	2.0467	-1.2396
CEU	NPIP	15037533	0.9091	1.7056	-1.0909
CEU	NPIP	15042533	1.1888	2.2173	-1.149
CEU	NPIP	15047533	3.4051	5.2874	-1.0121
CEU	NPIP	15052533	6.5271	7.5047	-0.3836
CEU	NPIP	15057533	7.5869	8.8692	-0.4315
CEU	NPIP	15062533	5.247	7.6753	-0.9334
CEU	NPIP	15067533	3.396	5.6286	-1.1356
CEU	NPIP	15072533	4.1593	5.2874	-0.6066
CEU	NPIP	15077533	5.5358	6.4814	-0.4238
CEU	NPIP	15082533	7.2997	9.2104	-0.6209

CEU	NPIP	15087533	6.3269	7.5047	-0.4621
CEU	NPIP	15092533	4.5467	6.4814	-0.8672
CEU	NPIP	15097533	4.5887	7.3342	-1.1
CEU	NPIP	15102533	4.1266	6.6519	-1.1056
CEU	NPIP	15107533	4.6226	6.6519	-0.8885
CEU	NPIP	15112533	5.0841	6.6519	-0.6864
CEU	NPIP	15117533	6.4061	7.3342	-0.3719
CEU	NPIP	15122533	6.2865	7.3342	-0.4198
CEU	NPIP	15127533	10.2105	9.3809	0.265
CEU	NPIP	15132533	13.1641	11.0866	0.5679
CEU	NPIP	15137533	9.631	8.8692	0.2563
CEU	NPIP	15142533	8.6063	7.3342	0.5097
CEU	NPIP	15147533	8.1431	6.6519	0.6529
CEU	NPIP	15152533	7.0963	7.1636	-0.0275
CEU	NPIP	15157533	6.3562	6.9931	-0.2665
CEU	NPIP	15162533	5.4263	5.458	-0.0166
CEU	NPIP	15167533	6.5236	6.8225	-0.1279
CEU	NPIP	15172533	8.0614	8.0164	0.0166
CEU	NPIP	15177533	7.4914	7.3342	0.063
CEU	NPIP	15182533	5.7642	7.3342	-0.6291
CEU	NPIP	15187533	3.6207	6.4814	-1.2822
CEU	NPIP	15192533	2.7726	5.7991	-1.499
CEU	NPIP	15197533	4.5382	6.6519	-0.9254
CEU	NPIP	15202533	5.6174	7.1636	-0.6329
CEU	NPIP	15207533	3.1503	4.6052	-0.8833
CEU	NPIP	15212533	1.6347	2.2173	-0.6509
CEU	NPIP	15217533	3.7462	6.8225	-1.3163
CEU	NPIP	15222533	6.4401	11.9394	-1.4021
CEU	NPIP	15227533	6.2635	10.2337	-1.1696
CEU	NPIP	15232533	4.4921	7.6753	-1.2235
CEU	NPIP	15237533	2.511	7.1636	-1.9045
CEU	NPIP	15242533	2.4094	4.9463	-1.4468
CEU	NPIP	15247533	3.6319	4.6052	-0.5909
CEU	NPIP	15252533	4.3081	7.1636	-1.1689
CEU	NPIP	15257533	2.8011	5.9697	-1.5292
CHB	NPIP	14247533	7.1163	6.946	0.0715
CHB	NPIP	14252533	8.5321	8.979	-0.1483
CHB	NPIP	14257533	6.2581	7.6237	-0.5266
CHB	NPIP	14262533	5.5911	7.6237	-0.7838
CHB	NPIP	14267533	4.0048	7.2848	-1.3185
CHB	NPIP	14272533	3.5496	8.3013	-1.6947
CHB	NPIP	14277533	4.0662	8.979	-1.6297
CHB	NPIP	14282533	2.6752	6.7766	-1.7606
CHB	NPIP	14287533	2.8865	7.2848	-1.768
CHB	NPIP	14292533	2.8058	5.9295	-1.5122
CHB	NPIP	14297533	2.62	5.5907	-1.5157
CHB	NPIP	14302533	3.5161	7.6237	-1.584
CHB	NPIP	14307533	3.2511	5.9295	-1.2967
CHB	NPIP	14312533	4.1235	5.9295	-0.8743

CHB	NPIP	14317533	5.6453	10.3343	-1.3649
CHB	NPIP	14322533	6.6866	12.5367	-1.4203
CHB	NPIP	14327533	4.6	10.8426	-1.7373
CHB	NPIP	14332533	3.5172	10.5037	-2.003
CHB	NPIP	14337533	4.9317	10.5037	-1.5975
CHB	NPIP	14342533	4.625	8.979	-1.4444
CHB	NPIP	14347533	3.7708	7.9625	-1.5533
CHB	NPIP	14352533	5.051	7.2848	-0.8979
CHB	NPIP	14357533	6.043	5.9295	0.0549
CHB	NPIP	14362533	7.3689	6.7766	0.2543
CHB	NPIP	14367533	8.4784	9.3178	-0.2691
CHB	NPIP	14372533	9.0011	10.8426	-0.5125
CHB	NPIP	14377533	10.9937	9.9955	0.2997
CHB	NPIP	14382533	7.9596	7.7931	0.0629
CHB	NPIP	14387533	4.83	7.4543	-1.033
CHB	NPIP	14392533	9.2318	8.1319	0.3998
CHB	NPIP	14397533	11.9629	8.979	0.9899
CHB	NPIP	14402533	10.9573	12.5367	-0.3835
CHB	NPIP	14407533	10.1917	11.6896	-0.3885
CHB	NPIP	14412533	10.0765	9.6567	0.1302
CHB	NPIP	14417533	8.2566	9.8261	-0.4789
CHB	NPIP	14422533	6.1376	8.3013	-0.7717
CHB	NPIP	14427533	12.0208	8.6402	1.162
CHB	NPIP	14432533	11.6766	7.4543	1.662
CHB	NPIP	14437533	8.5222	8.4708	0.018
CHB	NPIP	14442533	10.6688	10.3343	0.0974
CHB	NPIP	14447533	10.1249	9.1484	0.3184
CHB	NPIP	14452533	12.1053	9.3178	0.8935
CHB	NPIP	14457533	14.0051	11.012	0.821
CHB	NPIP	14462533	14.2271	14.4003	-0.0369
CHB	NPIP	14467533	13.667	15.4168	-0.3491
CHB	NPIP	14472533	11.6103	12.5367	-0.2249
CHB	NPIP	14477533	6.4043	8.1319	-0.6279
CHB	NPIP	14482533	1.7839	4.913	-1.7898
CHB	NPIP	14487533	2.6168	5.2519	-1.4212
CHB	NPIP	14492533	4.1321	7.4543	-1.3077
CHB	NPIP	14497533	3.4702	7.2848	-1.5333
CHB	NPIP	14502533	2.455	5.0825	-1.4587
CHB	NPIP	14507533	3.9335	5.4213	-0.7802
CHB	NPIP	14512533	3.8699	6.2684	-1.1047
CHB	NPIP	14517533	1.4485	6.2684	-2.2199
CHB	NPIP	14522533	0.8389	6.7766	-2.5488
CHB	NPIP	14527533	1.5372	6.2684	-2.179
CHB	NPIP	14532533	3.1864	4.913	-0.9876
CHB	NPIP	14537533	4.9869	5.5907	-0.3081
CHB	NPIP	14542533	5.984	7.2848	-0.5229
CHB	NPIP	14547533	4.4569	6.946	-1.0448
CHB	NPIP	14552533	2.2913	5.0825	-1.5496
CHB	NPIP	14557533	2.0356	4.4048	-1.4908

CHB	NPIP	14562533	2.2559	4.7436	-1.4674
CHB	NPIP	14567533	1.9483	5.5907	-1.8584
CHB	NPIP	14572533	1.623	5.4213	-1.9917
CHB	NPIP	14577533	1.8369	5.5907	-1.9153
CHB	NPIP	14582533	1.2656	5.5907	-2.2068
CHB	NPIP	14587533	1.0529	5.7601	-2.3386
CHB	NPIP	14592533	1.3668	6.4378	-2.2801
CHB	NPIP	14597533	1.1805	5.5907	-2.2502
CHB	NPIP	14602533	0.997	4.913	-2.2399
CHB	NPIP	14607533	0.5827	4.4048	-2.4051
CHB	NPIP	14612533	0.6539	4.2354	-2.3316
CHB	NPIP	14617533	1.0531	4.4048	-2.1091
CHB	NPIP	14622533	1.6551	7.9625	-2.3373
CHB	NPIP	14627533	2.1255	9.9955	-2.3632
CHB	NPIP	14632533	3.57	8.4708	-1.7156
CHB	NPIP	14637533	3.5865	7.1154	-1.4492
CHB	NPIP	14642533	1.8117	6.2684	-2.0526
CHB	NPIP	14647533	1.5614	7.4543	-2.3196
CHB	NPIP	14652533	1.5494	8.1319	-2.3926
CHB	NPIP	14657533	2.0548	7.6237	-2.1475
CHB	NPIP	14662533	1.7556	6.6072	-2.1309
CHB	NPIP	14667533	2.1525	6.946	-2.0121
CHB	NPIP	14672533	2.1136	6.7766	-2.0017
CHB	NPIP	14677533	1.3263	5.0825	-2.0854
CHB	NPIP	14682533	2.1591	4.7436	-1.5245
CHB	NPIP	14687533	2.3489	7.7931	-2.0576
CHB	NPIP	14692533	1.771	9.1484	-2.4053
CHB	NPIP	14697533	3.0769	6.6072	-1.5505
CHB	NPIP	14702533	4.0131	6.4378	-1.0902
CHB	NPIP	14707533	3.5028	7.7931	-1.6215
CHB	NPIP	14712533	2.317	6.946	-1.9431
CHB	NPIP	14717533	0.7196	4.5742	-2.3471
CHB	NPIP	14722533	0.7582	3.7271	-2.1568
CHB	NPIP	14727533	1.352	3.8965	-1.7797
CHB	NPIP	14732533	1.814	4.066	-1.5186
CHB	NPIP	14737533	3.0057	4.913	-1.091
CHB	NPIP	14742533	2.668	5.5907	-1.4912
CHB	NPIP	14747533	1.9665	5.9295	-1.9185
CHB	NPIP	14752533	2.2638	5.5907	-1.6975
CHB	NPIP	14757533	1.8283	6.2684	-2.045
CHB	NPIP	14762533	1.3973	5.7601	-2.1675
CHB	NPIP	14767533	1.13	4.5742	-2.0972
CHB	NPIP	14772533	2.5418	5.0825	-1.4105
CHB	NPIP	14777533	3.8348	7.1154	-1.3472
CHB	NPIP	14782533	2.2501	5.2519	-1.619
CHB	NPIP	14787533	0.2915	0.6777	-1.0096
CHB	NPIP	14792533	0.0478	0.1694	-0.7226
CHB	NPIP	14797533	0	0	0
CHB	NPIP	14802533	0	0	0

CHB	NPIP	14807533	0	0	0
CHB	NPIP	14812533	0	0	0
CHB	NPIP	14817533	0.0194	0.3388	-1.2812
CHB	NPIP	14822533	0.5168	1.0165	-0.9944
CHB	NPIP	14827533	0.4974	0.6777	-0.4714
CHB	NPIP	14832533	0.0377	0.1694	-0.7826
CHB	NPIP	14837533	0.0377	0.1694	-0.7826
CHB	NPIP	14842533	0	0	0
CHB	NPIP	14847533	0.2142	0.1694	0.2662
CHB	NPIP	14852533	0.2142	0.1694	0.2662
CHB	NPIP	14857533	1.3556	0.6777	1.7722
CHB	NPIP	14862533	3.1196	3.7271	-0.4413
CHB	NPIP	14867533	6.2349	6.6072	-0.1635
CHB	NPIP	14872533	5.4278	4.4048	0.6437
CHB	NPIP	14877533	1.5342	1.5247	0.0141
CHB	NPIP	14882533	0.7377	1.0165	-0.5548
CHB	NPIP	14887533	0.1989	0.6777	-1.2517
CHB	NPIP	14892533	0.3337	1.6942	-1.8671
CHB	NPIP	14897533	1.992	2.7106	-0.6809
CHB	NPIP	14902533	1.9406	2.033	-0.1102
CHB	NPIP	14907533	0.5231	1.3553	-1.3487
CHB	NPIP	14912533	2.4626	1.8636	0.7642
CHB	NPIP	14917533	1.8161	1.8636	-0.0606
CHB	NPIP	14922533	0.7472	2.033	-1.5333
CHB	NPIP	14927533	1.3678	2.7106	-1.2724
CHB	NPIP	14932533	1.7491	2.8801	-1.0197
CHB	NPIP	14937533	1.5945	2.8801	-1.1591
CHB	NPIP	14942533	1.5212	2.7106	-1.127
CHB	NPIP	14947533	2.1978	2.8801	-0.6152
CHB	NPIP	14952533	1.3069	2.8801	-1.4184
CHB	NPIP	14957533	1.3647	3.3883	-1.5936
CHB	NPIP	14962533	2.4359	3.8965	-1.0216
CHB	NPIP	14967533	5.6341	4.913	0.4125
CHB	NPIP	14972533	5.2599	6.4378	-0.5296
CHB	NPIP	14977533	1.1947	4.7436	-2.0934
CHB	NPIP	14982533	2.4493	3.2189	-0.6327
CHB	NPIP	14987533	2.1729	3.2189	-0.86
CHB	NPIP	14992533	0.6777	2.2024	-1.7073
CHB	NPIP	14997533	0.6908	1.5247	-1.2389
CHB	NPIP	15002533	0.941	1.3553	-0.6715
CHB	NPIP	15007533	0.7839	0.5082	0.8655
CHB	NPIP	15012533	0.048	0.5082	-1.4446
CHB	NPIP	15017533	1.525	1.6942	-0.2322
CHB	NPIP	15022533	3.0046	3.3883	-0.3022
CHB	NPIP	15027533	1.9018	3.2189	-1.0828
CHB	NPIP	15032533	0.4892	1.6942	-1.6537
CHB	NPIP	15037533	0.5711	1.0165	-0.8864
CHB	NPIP	15042533	1.9505	2.2024	-0.2821
CHB	NPIP	15047533	4.4773	5.2519	-0.4178

CHB	NPIP	15052533	6.28	7.4543	-0.4622
CHB	NPIP	15057533	8.0425	8.3013	-0.0923
CHB	NPIP	15062533	6.3657	7.4543	-0.4285
CHB	NPIP	15067533	3.4908	5.2519	-0.9498
CHB	NPIP	15072533	4.5298	4.7436	-0.1261
CHB	NPIP	15077533	5.5932	6.0989	-0.2387
CHB	NPIP	15082533	6.4036	6.6072	-0.0894
CHB	NPIP	15087533	5.9461	6.0989	-0.0721
CHB	NPIP	15092533	4.64	6.4378	-0.8083
CHB	NPIP	15097533	4.8766	7.4543	-1.0147
CHB	NPIP	15102533	4.205	6.6072	-1.0551
CHB	NPIP	15107533	4.6766	6.0989	-0.6714
CHB	NPIP	15112533	5.7653	6.0989	-0.1575
CHB	NPIP	15117533	5.8812	5.0825	0.4434
CHB	NPIP	15122533	5.3385	4.7436	0.3509
CHB	NPIP	15127533	11.1603	8.1319	1.1007
CHB	NPIP	15132533	14.8509	10.8426	1.1155
CHB	NPIP	15137533	10.9377	8.979	0.6498
CHB	NPIP	15142533	9.5494	6.946	1.0928
CHB	NPIP	15147533	8.1725	5.9295	1.0859
CHB	NPIP	15152533	8.2336	7.4543	0.3068
CHB	NPIP	15157533	8.2867	7.1154	0.481
CHB	NPIP	15162533	6.3286	5.7601	0.2824
CHB	NPIP	15167533	6.9063	6.7766	0.0557
CHB	NPIP	15172533	8.3194	7.6237	0.2683
CHB	NPIP	15177533	8.7773	7.1154	0.6825
CHB	NPIP	15182533	6.9772	6.2684	0.3264
CHB	NPIP	15187533	3.985	5.4213	-0.7532
CHB	NPIP	15192533	3.4654	5.0825	-0.8978
CHB	NPIP	15197533	4.8924	6.2684	-0.6337
CHB	NPIP	15202533	5.8378	5.9295	-0.0444
CHB	NPIP	15207533	3.5772	3.2189	0.2946
CHB	NPIP	15212533	2.1793	2.2024	-0.0259
CHB	NPIP	15217533	5.9899	6.6072	-0.2711
CHB	NPIP	15222533	10.3606	11.6896	-0.3447
CHB	NPIP	15227533	8.3463	8.979	-0.2099
CHB	NPIP	15232533	4.9764	6.2684	-0.595
CHB	NPIP	15237533	3.1064	7.2848	-1.6796
CHB	NPIP	15242533	2.8947	6.7766	-1.6664
CHB	NPIP	15247533	5.6115	7.2848	-0.6726
CHB	NPIP	15252533	5.5608	7.1154	-0.6384
CHB	NPIP	15257533	2.6745	4.913	-1.2804
YRI	NPIP	14247533	12.4338	11.5963	0.2181
YRI	NPIP	14252533	15.3592	13.7811	0.3491
YRI	NPIP	14257533	12.9555	14.6214	-0.3484
YRI	NPIP	14262533	10.9129	12.2685	-0.3348
YRI	NPIP	14267533	9.5075	10.4199	-0.2627
YRI	NPIP	14272533	10.8494	11.4282	-0.1528
YRI	NPIP	14277533	12.0611	10.756	0.3647

YRI	NPIP	14282533	8.5099	9.2434	-0.2361
YRI	NPIP	14287533	7.5064	10.4199	-0.8387
YRI	NPIP	14292533	5.8171	9.2434	-1.1027
YRI	NPIP	14297533	5.5448	8.5712	-1.0444
YRI	NPIP	14302533	6.9672	8.9073	-0.6462
YRI	NPIP	14307533	5.3422	8.7392	-1.1515
YRI	NPIP	14312533	5.7864	9.5795	-1.1809
YRI	NPIP	14317533	11.6211	14.1172	-0.5397
YRI	NPIP	14322533	16.1425	16.9743	-0.1509
YRI	NPIP	14327533	15.4567	14.9575	0.1022
YRI	NPIP	14332533	15.6286	14.2853	0.2872
YRI	NPIP	14337533	14.2699	14.6214	-0.0735
YRI	NPIP	14342533	10.8187	16.4701	-1.055
YRI	NPIP	14347533	10.0727	16.8062	-1.233
YRI	NPIP	14352533	10.0003	13.9492	-0.8636
YRI	NPIP	14357533	9.1388	12.6047	-0.8343
YRI	NPIP	14362533	10.9463	12.6047	-0.3992
YRI	NPIP	14367533	14.7623	14.1172	0.1395
YRI	NPIP	14372533	16.8983	16.8062	0.0169
YRI	NPIP	14377533	15.1178	15.6298	-0.1005
YRI	NPIP	14382533	11.6494	14.4534	-0.5929
YRI	NPIP	14387533	12.5396	17.9827	-0.9341
YRI	NPIP	14392533	13.5118	16.6382	-0.578
YRI	NPIP	14397533	14.3911	13.9492	0.0966
YRI	NPIP	14402533	17.2526	17.9827	-0.1253
YRI	NPIP	14407533	13.9814	19.1591	-0.8361
YRI	NPIP	14412533	9.4271	16.4701	-1.3148
YRI	NPIP	14417533	6.8313	13.613	-1.5179
YRI	NPIP	14422533	6.6071	13.613	-1.5681
YRI	NPIP	14427533	12.5229	15.7978	-0.6362
YRI	NPIP	14432533	11.7795	13.613	-0.4104
YRI	NPIP	14437533	9.6192	14.7895	-1.0696
YRI	NPIP	14442533	11.6581	16.302	-0.8755
YRI	NPIP	14447533	10.4906	13.9492	-0.7564
YRI	NPIP	14452533	11.3101	13.9492	-0.5772
YRI	NPIP	14457533	12.4155	16.8062	-0.804
YRI	NPIP	14462533	14.0702	18.991	-0.8013
YRI	NPIP	14467533	16.4907	17.8146	-0.2293
YRI	NPIP	14472533	15.4505	15.7978	-0.0675
YRI	NPIP	14477533	8.6948	11.2602	-0.6868
YRI	NPIP	14482533	4.947	8.9073	-1.319
YRI	NPIP	14487533	6.9784	9.5795	-0.8098
YRI	NPIP	14492533	8.4234	11.0921	-0.7246
YRI	NPIP	14497533	7.4136	10.924	-0.9668
YRI	NPIP	14502533	4.587	6.5544	-0.8674
YRI	NPIP	14507533	7.5169	8.4031	-0.3115
YRI	NPIP	14512533	7.58	10.2518	-0.7809
YRI	NPIP	14517533	5.5055	8.067	-0.9347
YRI	NPIP	14522533	6.291	7.7309	-0.5463

YRI	NPIP	14527533	5.3525	7.7309	-0.9024
YRI	NPIP	14532533	4.6594	7.2267	-1.036
YRI	NPIP	14537533	4.6902	7.3947	-1.0687
YRI	NPIP	14542533	5.1242	8.235	-1.1138
YRI	NPIP	14547533	3.6449	6.5544	-1.2827
YRI	NPIP	14552533	2.1481	5.2099	-1.6573
YRI	NPIP	14557533	3.1315	7.3947	-1.6846
YRI	NPIP	14562533	5.1993	9.0754	-1.2688
YRI	NPIP	14567533	4.1303	6.7225	-1.117
YRI	NPIP	14572533	2.9155	6.3864	-1.5665
YRI	NPIP	14577533	2.7615	8.067	-1.936
YRI	NPIP	14582533	1.9688	7.7309	-2.1863
YRI	NPIP	14587533	3.228	7.0586	-1.5792
YRI	NPIP	14592533	3.9054	6.8906	-1.2579
YRI	NPIP	14597533	4.5961	6.5544	-0.8634
YRI	NPIP	14602533	3.7857	6.0502	-1.0729
YRI	NPIP	14607533	1.9432	6.5544	-2.033
YRI	NPIP	14612533	2.0072	7.2267	-2.1062
YRI	NPIP	14617533	2.268	7.2267	-2.0009
YRI	NPIP	14622533	3.8906	8.9073	-1.6709
YRI	NPIP	14627533	4.4347	9.4115	-1.5751
YRI	NPIP	14632533	5.1961	8.7392	-1.201
YRI	NPIP	14637533	4.6907	8.7392	-1.3723
YRI	NPIP	14642533	2.765	8.4031	-1.9816
YRI	NPIP	14647533	3.3339	8.7392	-1.8323
YRI	NPIP	14652533	3.4437	7.8989	-1.6574
YRI	NPIP	14657533	3.3966	8.4031	-1.7596
YRI	NPIP	14662533	3.5648	9.4115	-1.8504
YRI	NPIP	14667533	3.3583	9.2434	-1.894
YRI	NPIP	14672533	2.9984	7.8989	-1.8231
YRI	NPIP	14677533	2.6809	6.7225	-1.7415
YRI	NPIP	14682533	2.2973	6.5544	-1.8769
YRI	NPIP	14687533	2.3565	7.7309	-2.0392
YRI	NPIP	14692533	2.6376	9.4115	-2.1439
YRI	NPIP	14697533	3.8368	8.4031	-1.6049
YRI	NPIP	14702533	4.4588	8.9073	-1.4816
YRI	NPIP	14707533	3.9685	9.4115	-1.7227
YRI	NPIP	14712533	2.7564	7.7309	-1.8875
YRI	NPIP	14717533	1.3132	8.235	-2.4784
YRI	NPIP	14722533	1.0934	7.2267	-2.4749
YRI	NPIP	14727533	1.9231	5.5461	-1.8553
YRI	NPIP	14732533	2.6049	7.7309	-1.9449
YRI	NPIP	14737533	3.1597	7.5628	-1.7046
YRI	NPIP	14742533	2.8964	5.8822	-1.4508
YRI	NPIP	14747533	2.1012	7.3947	-2.0917
YRI	NPIP	14752533	2.3003	8.5712	-2.1641
YRI	NPIP	14757533	1.9719	7.2267	-2.1204
YRI	NPIP	14762533	2.1364	8.067	-2.1641
YRI	NPIP	14767533	1.9997	7.7309	-2.1746

YRI	NPIP	14772533	2.4261	6.5544	-1.8201
YRI	NPIP	14777533	3.7264	9.2434	-1.7755
YRI	NPIP	14782533	2.0887	6.0502	-1.877
YRI	NPIP	14787533	0.0278	0.5042	-1.4972
YRI	NPIP	14792533	0	0	0
YRI	NPIP	14797533	0	0	0
YRI	NPIP	14802533	0	0	0
YRI	NPIP	14807533	0	0	0
YRI	NPIP	14812533	0	0	0
YRI	NPIP	14817533	0.1063	0.5042	-1.2505
YRI	NPIP	14822533	0.4422	1.1764	-1.3132
YRI	NPIP	14827533	0.3359	0.6722	-0.8804
YRI	NPIP	14832533	0.1057	0.1681	-0.3709
YRI	NPIP	14837533	0.1057	0.1681	-0.3709
YRI	NPIP	14842533	0	0	0
YRI	NPIP	14847533	0.358	0.3361	0.0879
YRI	NPIP	14852533	0.358	0.3361	0.0879
YRI	NPIP	14857533	0.2118	0.8403	-1.4196
YRI	NPIP	14862533	1.6827	4.8738	-1.8318
YRI	NPIP	14867533	5.8325	7.7309	-0.7203
YRI	NPIP	14872533	5.3415	5.7141	-0.1858
YRI	NPIP	14877533	1.4132	2.689	-1.2125
YRI	NPIP	14882533	0.4702	1.0084	-1.073
YRI	NPIP	14887533	0.3165	1.1764	-1.538
YRI	NPIP	14892533	0.733	2.5209	-1.7905
YRI	NPIP	14897533	2.2444	5.0419	-1.5587
YRI	NPIP	14902533	2.738	5.0419	-1.2837
YRI	NPIP	14907533	1.6146	5.0419	-1.9096
YRI	NPIP	14912533	2.8798	5.8822	-1.4589
YRI	NPIP	14917533	1.9839	4.8738	-1.6589
YRI	NPIP	14922533	1.5765	6.0502	-2.1197
YRI	NPIP	14927533	2.7354	6.5544	-1.6837
YRI	NPIP	14932533	2.2124	5.8822	-1.7832
YRI	NPIP	14937533	1.556	4.7057	-1.8645
YRI	NPIP	14942533	1.8155	4.7057	-1.7109
YRI	NPIP	14947533	2.3197	6.3864	-1.8354
YRI	NPIP	14952533	1.562	5.5461	-2.0402
YRI	NPIP	14957533	2.5198	6.0502	-1.6727
YRI	NPIP	14962533	3.52	8.4031	-1.7162
YRI	NPIP	14967533	4.7105	9.2434	-1.4588
YRI	NPIP	14972533	4.4516	7.5628	-1.2044
YRI	NPIP	14977533	1.2973	5.5461	-2.1758
YRI	NPIP	14982533	2.0261	6.3864	-1.9679
YRI	NPIP	14987533	2.1389	6.0502	-1.8532
YRI	NPIP	14992533	0.7104	4.3696	-2.3105
YRI	NPIP	14997533	0.6076	2.5209	-1.9161
YRI	NPIP	15002533	0.8027	1.3445	-0.88
YRI	NPIP	15007533	0.6526	1.3445	-1.1238
YRI	NPIP	15012533	0.4202	2.689	-2.1562

YRI	NPIP	15017533	1.999	4.7057	-1.6022
YRI	NPIP	15022533	3.6122	6.3864	-1.2521
YRI	NPIP	15027533	2.1372	5.0419	-1.6184
YRI	NPIP	15032533	0.4942	3.0251	-2.1833
YRI	NPIP	15037533	0.9518	2.689	-1.651
YRI	NPIP	15042533	1.7885	2.8571	-0.9664
YRI	NPIP	15047533	4.6752	8.5712	-1.3445
YRI	NPIP	15052533	8.4374	11.9324	-0.886
YRI	NPIP	15057533	9.2442	10.0837	-0.2492
YRI	NPIP	15062533	5.863	8.067	-0.8043
YRI	NPIP	15067533	3.1102	6.5544	-1.5185
YRI	NPIP	15072533	4.5612	7.7309	-1.2027
YRI	NPIP	15077533	5.8454	9.2434	-1.0936
YRI	NPIP	15082533	6.6149	10.4199	-1.0953
YRI	NPIP	15087533	5.5327	9.2434	-1.1942
YRI	NPIP	15092533	4.0304	7.7309	-1.4041
YRI	NPIP	15097533	4.8267	8.4031	-1.257
YRI	NPIP	15102533	4.8705	7.8989	-1.1266
YRI	NPIP	15107533	4.9834	8.7392	-1.2731
YRI	NPIP	15112533	4.6765	10.2518	-1.6295
YRI	NPIP	15117533	6.4383	12.1005	-1.4166
YRI	NPIP	15122533	6.4801	11.9324	-1.3821
YRI	NPIP	15127533	8.1244	10.5879	-0.6986
YRI	NPIP	15132533	10.3646	11.2602	-0.2398
YRI	NPIP	15137533	7.3968	10.756	-0.9387
YRI	NPIP	15142533	5.6655	8.5712	-1.0028
YRI	NPIP	15147533	5.6385	8.4031	-0.9716
YRI	NPIP	15152533	7.3843	9.9157	-0.7632
YRI	NPIP	15157533	7.6107	9.7476	-0.6546
YRI	NPIP	15162533	5.8583	8.7392	-0.9765
YRI	NPIP	15167533	6.2644	9.0754	-0.9202
YRI	NPIP	15172533	7.4066	10.0837	-0.7946
YRI	NPIP	15177533	7.321	9.4115	-0.6616
YRI	NPIP	15182533	5.5327	8.067	-0.9248
YRI	NPIP	15187533	4.0659	7.3947	-1.3154
YRI	NPIP	15192533	3.9526	8.067	-1.5014
YRI	NPIP	15197533	4.4203	9.4115	-1.5797
YRI	NPIP	15202533	5.3881	8.9073	-1.1721
YRI	NPIP	15207533	3.764	5.2099	-0.7826
YRI	NPIP	15212533	2.1279	3.6974	-1.144
YRI	NPIP	15217533	6.3332	10.2518	-1.1453
YRI	NPIP	15222533	11.5447	18.823	-1.1954
YRI	NPIP	15227533	9.0016	16.302	-1.3763
YRI	NPIP	15232533	5.5217	10.924	-1.4879
YRI	NPIP	15237533	4.0692	10.4199	-1.8282
YRI	NPIP	15242533	3.6693	9.5795	-1.84
YRI	NPIP	15247533	6.789	9.7476	-0.9063
YRI	NPIP	15252533	6.239	8.067	-0.667
YRI	NPIP	15257533	3.1625	5.5461	-1.2206

CEU	NPIP	14442443	10.3311	8.3576	0.7015
CEU	NPIP	14447443	9.6364	9.2104	0.1384
CEU	NPIP	14452443	11.9604	10.0632	0.5678
CEU	NPIP	14457443	15.3719	11.5982	0.9887
CEU	NPIP	14462443	16.6625	11.7688	1.2647
CEU	NPIP	14467443	15.7825	13.9861	0.3943
CEU	NPIP	14472443	13.7638	14.6684	-0.1898
CEU	NPIP	14477443	7.941	9.3809	-0.46
CEU	NPIP	14482443	2.5452	4.4346	-1.1855
CEU	NPIP	14487443	4.2092	5.1169	-0.5024
CEU	NPIP	14492443	6.961	7.3342	-0.1495
CEU	NPIP	14497443	6.0853	6.4814	-0.1775
CEU	NPIP	14502443	3.3049	4.4346	-0.7088
CEU	NPIP	14507443	4.4772	5.6286	-0.5857
CEU	NPIP	14512443	4.1325	5.6286	-0.761
CEU	NPIP	14517443	1.9371	5.1169	-1.7601
CEU	NPIP	14522443	3.3636	6.3108	-1.3531
CEU	NPIP	14527443	3.7796	6.6519	-1.2575
CEU	NPIP	14532443	3.1235	5.6286	-1.2742
CEU	NPIP	14537443	4.2419	6.3108	-0.9499
CEU	NPIP	14542443	4.4476	6.9931	-1.0651
CEU	NPIP	14547443	2.9641	5.458	-1.3038
CEU	NPIP	14552443	1.8551	4.2641	-1.5638
CEU	NPIP	14557443	2.3086	4.2641	-1.2694
CEU	NPIP	14562443	3.9973	6.4814	-1.1134
CEU	NPIP	14567443	2.8689	5.7991	-1.4513
CEU	NPIP	14572443	1.5242	5.2874	-2.0235
CEU	NPIP	14577443	2.8536	7.1636	-1.7643
CEU	NPIP	14582443	2.6343	5.2874	-1.4266
CEU	NPIP	14587443	1.9742	6.1402	-1.9604
CEU	NPIP	14592443	2.5613	7.6753	-1.9657
CEU	NPIP	14597443	2.7326	5.7991	-1.5188
CEU	NPIP	14602443	2.3793	4.0935	-1.1527
CEU	NPIP	14607443	2.1373	4.0935	-1.3154
CEU	NPIP	14612443	2.1789	5.458	-1.7142
CEU	NPIP	14617443	3.2049	4.7757	-0.9239
CEU	NPIP	14622443	7.5112	5.9697	0.7439
CEU	NPIP	14627443	8.7864	7.3342	0.5819
CEU	NPIP	14632443	9.3956	8.0164	0.5094
CEU	NPIP	14637443	10.5457	8.8692	0.5641
CEU	NPIP	14642443	5.7244	5.9697	-0.1184
CEU	NPIP	14647443	3.7304	5.1169	-0.7675
CEU	NPIP	14652443	4.8557	6.1402	-0.6044
CEU	NPIP	14657443	4.5213	5.7991	-0.6329
CEU	NPIP	14662443	3.5677	7.1636	-1.472
CEU	NPIP	14667443	3.6578	8.6987	-1.727
CEU	NPIP	14672443	4.941	6.3108	-0.6289
CEU	NPIP	14677443	4.5184	4.4346	0.0526
CEU	NPIP	14682443	3.9567	5.7991	-0.9125

CEU	NPIP	14687443	2.9318	6.1402	-1.5098
CEU	NPIP	14692443	1.8734	6.3108	-2.0373
CEU	NPIP	14697443	4.0016	6.9931	-1.2517
CEU	NPIP	14702443	5.066	6.6519	-0.6943
CEU	NPIP	14707443	4.5364	7.1636	-1.0754
CEU	NPIP	14712443	3.169	6.4814	-1.4847
CEU	NPIP	14717443	1.0518	3.9229	-2.0025
CEU	NPIP	14722443	0.9321	3.4112	-1.9471
CEU	NPIP	14727443	2.1172	4.6052	-1.5106
CEU	NPIP	14732443	2.9205	5.1169	-1.2157
CEU	NPIP	14737443	3.8258	5.1169	-0.7146
CEU	NPIP	14742443	2.5539	4.6052	-1.2454
CEU	NPIP	14747443	1.274	4.2641	-1.941
CEU	NPIP	14752443	2.9951	4.7757	-1.0472
CEU	NPIP	14757443	3.1886	5.2874	-1.1285
CEU	NPIP	14762443	2.3913	6.3108	-1.7995
CEU	NPIP	14767443	1.6588	5.1169	-1.9141
CEU	NPIP	14772443	2.8613	4.0935	-0.8286
CEU	NPIP	14777443	4.1952	6.1402	-0.9153
CEU	NPIP	14782443	2.1222	4.2641	-1.3904
CEU	NPIP	14787443	0.1837	0.5117	-1.0287
CEU	NPIP	14792443	0.0101	0.1706	-0.9534
CEU	NPIP	14797443	0.0101	0.1706	-0.9534
CEU	NPIP	14802443	0	0	0
CEU	NPIP	14807443	0	0	0
CEU	NPIP	14812443	0	0	0
CEU	NPIP	14817443	0.1309	0.1706	-0.2358
CEU	NPIP	14822443	0.5803	1.0234	-0.8805
CEU	NPIP	14827443	0.4494	0.8528	-0.9084
CEU	NPIP	14832443	0.095	0.1706	-0.4491
CEU	NPIP	14837443	0.095	0.1706	-0.4491
CEU	NPIP	14842443	0.0202	0.1706	-0.8934
CEU	NPIP	14847443	0.1441	0.3411	-0.7897
CEU	NPIP	14852443	0.1239	0.1706	-0.2774
CEU	NPIP	14857443	0.873	0.6822	0.4982
CEU	NPIP	14862443	2.772	4.2641	-0.9686
CEU	NPIP	14867443	5.6197	7.1636	-0.632
CEU	NPIP	14872443	4.08	4.4346	-0.2225
CEU	NPIP	14877443	0.986	2.0467	-1.2621
CEU	NPIP	14882443	1.0724	1.3645	-0.4726
CEU	NPIP	14887443	0.8854	0.5117	1.172
CEU	NPIP	14892443	0.7318	1.7056	-1.3337
CEU	NPIP	14897443	1.4585	3.2407	-1.4614
CEU	NPIP	14902443	1.7772	3.5818	-1.3601
CEU	NPIP	14907443	0.9629	3.0701	-1.8075
CEU	NPIP	14912443	1.8516	3.4112	-1.2249
CEU	NPIP	14917443	1.6098	2.8996	-1.16
CEU	NPIP	14922443	0.9248	2.0467	-1.3349
CEU	NPIP	14927443	1.8978	2.729	-0.7857

CEU	NPIP	14932443	1.8592	2.8996	-0.9357
CEU	NPIP	14937443	1.7679	2.8996	-1.0178
CEU	NPIP	14942443	1.683	3.0701	-1.1898
CEU	NPIP	14947443	1.7128	2.8996	-1.0673
CEU	NPIP	14952443	1.5673	3.0701	-1.2891
CEU	NPIP	14957443	2.2956	4.6052	-1.4023
CEU	NPIP	14962443	2.9223	4.2641	-0.871
CEU	NPIP	14967443	4.9305	4.9463	-0.009
CEU	NPIP	14972443	4.0952	4.4346	-0.213
CEU	NPIP	14977443	0.6178	2.5584	-1.9332
CEU	NPIP	14982443	1.402	3.9229	-1.7583
CEU	NPIP	14987443	1.408	4.0935	-1.8058
CEU	NPIP	14992443	0.6157	3.2407	-2.1524
CEU	NPIP	14997443	0.4651	1.8762	-1.7966
CEU	NPIP	15002443	0.4599	0.8528	-0.8848
CEU	NPIP	15007443	0.3436	0.5117	-0.5272
CEU	NPIP	15012443	0.0503	0.6822	-1.6501
CEU	NPIP	15017443	1.383	1.7056	-0.4418
CEU	NPIP	15022443	2.4187	2.729	-0.2933
CEU	NPIP	15027443	1.6106	2.3879	-0.8186
CEU	NPIP	15032443	1.0049	2.0467	-1.2396
CEU	NPIP	15037443	0.9091	1.7056	-1.0909
CEU	NPIP	15042443	1.1012	2.0467	-1.125
CEU	NPIP	15047443	3.4051	5.2874	-1.0121
CEU	NPIP	15052443	6.6147	7.6753	-0.4077
CEU	NPIP	15057443	7.5869	8.8692	-0.4315
CEU	NPIP	15062443	5.247	7.6753	-0.9334
CEU	NPIP	15067443	3.3859	5.458	-1.0832
CEU	NPIP	15072443	4.1593	5.2874	-0.6066
CEU	NPIP	15077443	5.5257	6.3108	-0.3605
CEU	NPIP	15082443	7.2505	9.0398	-0.5916
CEU	NPIP	15087443	6.3471	7.8459	-0.5646
CEU	NPIP	15092443	4.596	6.6519	-0.9001
CEU	NPIP	15097443	4.5395	7.1636	-1.0742
CEU	NPIP	15102443	4.1266	6.6519	-1.1056
CEU	NPIP	15107443	4.6718	6.8225	-0.9203
CEU	NPIP	15112443	5.0441	6.4814	-0.6442
CEU	NPIP	15117443	6.4061	7.3342	-0.3719
CEU	NPIP	15122443	6.1892	7.3342	-0.4588
CEU	NPIP	15127443	10.2004	9.2104	0.3217
CEU	NPIP	15132443	12.4238	10.916	0.4182
CEU	NPIP	15137443	9.6411	9.0398	0.1988
CEU	NPIP	15142443	9.4838	7.6753	0.6951
CEU	NPIP	15147443	8.1431	6.6519	0.6529
CEU	NPIP	15152443	7.0963	7.1636	-0.0275
CEU	NPIP	15157443	6.3461	6.8225	-0.2039
CEU	NPIP	15162443	5.4263	5.458	-0.0166
CEU	NPIP	15167443	6.5337	6.9931	-0.1922
CEU	NPIP	15172443	8.0614	8.0164	0.0166

CEU	NPIP	15177443	7.4914	7.3342	0.063
CEU	NPIP	15182443	5.7642	7.3342	-0.6291
CEU	NPIP	15187443	3.5622	6.3108	-1.2619
CEU	NPIP	15192443	2.7726	5.7991	-1.499
CEU	NPIP	15197443	4.5968	6.8225	-0.9524
CEU	NPIP	15202443	5.6174	7.1636	-0.6329
CEU	NPIP	15207443	3.1503	4.6052	-0.8833
CEU	NPIP	15212443	1.6347	2.2173	-0.6509
CEU	NPIP	15217443	3.7361	6.6519	-1.2766
CEU	NPIP	15222443	6.4401	11.9394	-1.4021
CEU	NPIP	15227443	6.2736	10.4043	-1.1983
CEU	NPIP	15232443	4.2944	7.5047	-1.2596
CEU	NPIP	15237443	2.511	7.1636	-1.9045
CEU	NPIP	15242443	2.6071	5.1169	-1.3892
CEU	NPIP	15247443	3.6319	4.6052	-0.5909
CEU	NPIP	15252443	4.3081	7.1636	-1.1689
CEU	NPIP	15257443	2.791	5.7991	-1.4899
CEU	NPIP	15262443	2.2408	4.2641	-1.3134
CEU	NPIP	15267443	2.0915	4.4346	-1.4701
CEU	NPIP	15272443	1.6494	3.7524	-1.5236
CEU	NPIP	15277443	4.368	3.9229	0.3104
CEU	NPIP	15282443	4.4729	4.4346	0.024
CEU	NPIP	15287443	2.1808	4.0935	-1.2862
CEU	NPIP	15292443	1.9922	4.2641	-1.4748
CEU	NPIP	15297443	4.3088	6.4814	-0.9738
CEU	NPIP	15302443	5.8231	5.9697	-0.0708
CEU	NPIP	15307443	5.3731	3.7524	1.1742
CEU	NPIP	15312443	6.6557	4.6052	1.245
CEU	NPIP	15317443	4.8545	4.4346	0.2635
CEU	NPIP	15322443	6.261	6.6519	-0.1711
CEU	NPIP	15327443	6	5.458	0.2833
CEU	NPIP	15332443	1.2622	1.5351	-0.4047
CEU	NPIP	15337443	0.7376	1.5351	-1.1825
CEU	NPIP	15342443	0.8252	1.1939	-0.657
CEU	NPIP	15347443	1.3929	1.5351	-0.2109
CEU	NPIP	15352443	1.9458	2.2173	-0.3033
CEU	NPIP	15357443	2.7403	3.7524	-0.7332
CEU	NPIP	15362443	2.0377	3.9229	-1.3149
CEU	NPIP	15367443	2.2416	2.8996	-0.5918
CEU	NPIP	15372443	2.1497	2.3879	-0.2508
CEU	NPIP	15377443	6.5459	5.458	0.5687
CEU	NPIP	15382443	9.5479	7.1636	0.976
CEU	NPIP	15387443	5.0588	4.2641	0.5159
CEU	NPIP	15392443	5.1144	5.6286	-0.2616
CEU	NPIP	15397443	8.1589	8.187	-0.0102
CEU	NPIP	15402443	7.079	6.8225	0.1098
CEU	NPIP	15407443	6.3894	5.9697	0.2026
CEU	NPIP	15412443	7.6145	6.1402	0.6938
CEU	NPIP	15417443	6.4374	6.1402	0.1399

CEU	NPIP	15422443	3.4682	3.9229	-0.3171
CEU	NPIP	15427443	0.6735	1.3645	-1.1179
CEU	NPIP	15432443	0.6226	1.3645	-1.2002
CEU	NPIP	15437443	1.0532	1.1939	-0.2507
CEU	NPIP	15442443	1.5424	1.1939	0.621
CHB	NPIP	14442443	10.6688	10.3343	0.0974
CHB	NPIP	14447443	9.7264	8.979	0.2479
CHB	NPIP	14452443	12.0575	9.1484	0.9485
CHB	NPIP	14457443	14.3939	11.012	0.9276
CHB	NPIP	14462443	14.1909	14.4003	-0.0446
CHB	NPIP	14467443	13.1841	15.4168	-0.4455
CHB	NPIP	14472443	11.6364	12.3673	-0.1797
CHB	NPIP	14477443	6.897	8.3013	-0.5008
CHB	NPIP	14482443	1.8418	5.2519	-1.8392
CHB	NPIP	14487443	2.6168	5.2519	-1.4212
CHB	NPIP	14492443	4.1321	7.4543	-1.3077
CHB	NPIP	14497443	3.4702	7.2848	-1.5333
CHB	NPIP	14502443	2.455	5.0825	-1.4587
CHB	NPIP	14507443	3.9335	5.4213	-0.7802
CHB	NPIP	14512443	3.8699	6.2684	-1.1047
CHB	NPIP	14517443	1.4485	6.2684	-2.2199
CHB	NPIP	14522443	0.8292	6.6072	-2.5378
CHB	NPIP	14527443	1.5372	6.2684	-2.179
CHB	NPIP	14532443	3.1961	5.0825	-1.0473
CHB	NPIP	14537443	4.9772	5.4213	-0.2329
CHB	NPIP	14542443	5.9743	7.1154	-0.4686
CHB	NPIP	14547443	4.4666	7.1154	-1.0878
CHB	NPIP	14552443	2.301	5.2519	-1.5915
CHB	NPIP	14557443	2.0356	4.4048	-1.4908
CHB	NPIP	14562443	2.2559	4.7436	-1.4674
CHB	NPIP	14567443	1.9386	5.4213	-1.8262
CHB	NPIP	14572443	1.623	5.4213	-1.9917
CHB	NPIP	14577443	1.8369	5.5907	-1.9153
CHB	NPIP	14582443	1.1812	5.4213	-2.2234
CHB	NPIP	14587443	1.0626	5.9295	-2.3561
CHB	NPIP	14592443	1.4512	6.6072	-2.2646
CHB	NPIP	14597443	1.1805	5.5907	-2.2502
CHB	NPIP	14602443	0.997	4.913	-2.2399
CHB	NPIP	14607443	0.573	4.2354	-2.3842
CHB	NPIP	14612443	0.6442	4.066	-2.3074
CHB	NPIP	14617443	1.0628	4.5742	-2.1382
CHB	NPIP	14622443	1.6648	8.1319	-2.3506
CHB	NPIP	14627443	2.1157	9.8261	-2.3525
CHB	NPIP	14632443	3.57	8.4708	-1.7156
CHB	NPIP	14637443	3.5962	7.2848	-1.4827
CHB	NPIP	14642443	1.802	6.0989	-2.0283
CHB	NPIP	14647443	1.5614	7.4543	-2.3196
CHB	NPIP	14652443	1.5591	8.3013	-2.4046
CHB	NPIP	14657443	2.0548	7.6237	-2.1475

CHB	NPIP	14662443	1.7556	6.6072	-2.1309
CHB	NPIP	14667443	1.7915	6.7766	-2.1399
CHB	NPIP	14672443	2.1039	6.6072	-1.9779
CHB	NPIP	14677443	1.6775	5.0825	-1.8904
CHB	NPIP	14682443	2.1689	4.913	-1.5696
CHB	NPIP	14687443	2.3587	7.9625	-2.0766
CHB	NPIP	14692443	1.771	9.1484	-2.4053
CHB	NPIP	14697443	3.0769	6.6072	-1.5505
CHB	NPIP	14702443	4.0131	6.4378	-1.0902
CHB	NPIP	14707443	3.5028	7.7931	-1.6215
CHB	NPIP	14712443	2.317	6.946	-1.9431
CHB	NPIP	14717443	0.7196	4.5742	-2.3471
CHB	NPIP	14722443	0.7485	3.5577	-2.1231
CHB	NPIP	14727443	1.352	3.8965	-1.7797
CHB	NPIP	14732443	1.8237	4.2354	-1.57
CHB	NPIP	14737443	2.996	4.7436	-1.0309
CHB	NPIP	14742443	2.668	5.5907	-1.4912
CHB	NPIP	14747443	1.9762	6.0989	-1.9461
CHB	NPIP	14752443	2.2638	5.5907	-1.6975
CHB	NPIP	14757443	1.8186	6.0989	-2.0205
CHB	NPIP	14762443	1.3973	5.7601	-2.1675
CHB	NPIP	14767443	1.13	4.5742	-2.0972
CHB	NPIP	14772443	2.5418	5.0825	-1.4105
CHB	NPIP	14777443	3.7514	7.1154	-1.3815
CHB	NPIP	14782443	2.2501	5.2519	-1.619
CHB	NPIP	14787443	0.3845	0.8471	-1.0432
CHB	NPIP	14792443	0.0478	0.1694	-0.7226
CHB	NPIP	14797443	0	0	0
CHB	NPIP	14802443	0	0	0
CHB	NPIP	14807443	0	0	0
CHB	NPIP	14812443	0	0	0
CHB	NPIP	14817443	0.0194	0.3388	-1.2812
CHB	NPIP	14822443	0.5168	1.0165	-0.9944
CHB	NPIP	14827443	0.4974	0.6777	-0.4714
CHB	NPIP	14832443	0.0377	0.1694	-0.7826
CHB	NPIP	14837443	0.0377	0.1694	-0.7826
CHB	NPIP	14842443	0	0	0
CHB	NPIP	14847443	0.2142	0.1694	0.2662
CHB	NPIP	14852443	0.2142	0.1694	0.2662
CHB	NPIP	14857443	1.3556	0.6777	1.7722
CHB	NPIP	14862443	3.1196	3.7271	-0.4413
CHB	NPIP	14867443	6.2157	6.4378	-0.0999
CHB	NPIP	14872443	5.4278	4.4048	0.6437
CHB	NPIP	14877443	1.5534	1.6942	-0.1932
CHB	NPIP	14882443	0.7377	1.0165	-0.5548
CHB	NPIP	14887443	0.1989	0.6777	-1.2517
CHB	NPIP	14892443	0.3337	1.6942	-1.8671
CHB	NPIP	14897443	1.992	2.7106	-0.6809
CHB	NPIP	14902443	1.9406	2.033	-0.1102

CHB	NPIP	14907443	0.5231	1.3553	-1.3487
CHB	NPIP	14912443	2.4626	1.8636	0.7642
CHB	NPIP	14917443	2.2699	2.033	0.2825
CHB	NPIP	14922443	0.7472	2.033	-1.5333
CHB	NPIP	14927443	1.3678	2.7106	-1.2724
CHB	NPIP	14932443	1.7491	2.8801	-1.0197
CHB	NPIP	14937443	1.5945	2.8801	-1.1591
CHB	NPIP	14942443	1.3221	2.5412	-1.2174
CHB	NPIP	14947443	2.1978	2.8801	-0.6152
CHB	NPIP	14952443	1.506	3.0495	-1.3274
CHB	NPIP	14957443	1.3647	3.3883	-1.5936
CHB	NPIP	14962443	2.4359	3.8965	-1.0216
CHB	NPIP	14967443	5.6341	4.913	0.4125
CHB	NPIP	14972443	5.2501	6.2684	-0.469
CHB	NPIP	14977443	1.0927	4.5742	-2.12
CHB	NPIP	14982443	2.459	3.3883	-0.7318
CHB	NPIP	14987443	2.2749	3.3883	-0.8768
CHB	NPIP	14992443	0.6777	2.2024	-1.7073
CHB	NPIP	14997443	0.6908	1.5247	-1.2389
CHB	NPIP	15002443	0.9025	1.1859	-0.5059
CHB	NPIP	15007443	0.7839	0.5082	0.8655
CHB	NPIP	15012443	0.0864	0.6777	-1.5458
CHB	NPIP	15017443	1.5153	1.5247	-0.014
CHB	NPIP	15022443	3.0046	3.3883	-0.3022
CHB	NPIP	15027443	1.9018	3.2189	-1.0828
CHB	NPIP	15032443	0.4892	1.6942	-1.6537
CHB	NPIP	15037443	0.5711	1.0165	-0.8864
CHB	NPIP	15042443	1.9505	2.2024	-0.2821
CHB	NPIP	15047443	4.4773	5.2519	-0.4178
CHB	NPIP	15052443	6.28	7.4543	-0.4622
CHB	NPIP	15057443	8.023	8.1319	-0.0396
CHB	NPIP	15062443	6.356	7.2848	-0.3733
CHB	NPIP	15067443	3.5102	5.4213	-1.0021
CHB	NPIP	15072443	4.5395	4.913	-0.2136
CHB	NPIP	15077443	5.5932	6.0989	-0.2387
CHB	NPIP	15082443	6.4036	6.6072	-0.0894
CHB	NPIP	15087443	5.9461	6.0989	-0.0721
CHB	NPIP	15092443	4.64	6.4378	-0.8083
CHB	NPIP	15097443	4.8108	7.2848	-0.9945
CHB	NPIP	15102443	4.205	6.6072	-1.0551
CHB	NPIP	15107443	4.6678	6.0989	-0.6755
CHB	NPIP	15112443	5.6994	5.9295	-0.1114
CHB	NPIP	15117443	5.9558	5.2519	0.3796
CHB	NPIP	15122443	5.2195	4.7436	0.2807
CHB	NPIP	15127443	11.1603	8.1319	1.1007
CHB	NPIP	15132443	14.0752	10.6731	0.9609
CHB	NPIP	15137443	10.9377	8.979	0.6498
CHB	NPIP	15142443	10.51	7.2848	1.2964
CHB	NPIP	15147443	8.1725	5.9295	1.0859

CHB	NPIP	15152443	8.2239	7.2848	0.3775
CHB	NPIP	15157443	8.2867	7.1154	0.481
CHB	NPIP	15162443	6.3383	5.9295	0.1979
CHB	NPIP	15167443	6.9063	6.7766	0.0557
CHB	NPIP	15172443	8.3194	7.6237	0.2683
CHB	NPIP	15177443	8.7773	7.1154	0.6825
CHB	NPIP	15182443	6.9772	6.2684	0.3264
CHB	NPIP	15187443	3.985	5.4213	-0.7532
CHB	NPIP	15192443	3.4654	5.0825	-0.8978
CHB	NPIP	15197443	4.8924	6.2684	-0.6337
CHB	NPIP	15202443	5.8378	5.9295	-0.0444
CHB	NPIP	15207443	3.5772	3.2189	0.2946
CHB	NPIP	15212443	2.1793	2.2024	-0.0259
CHB	NPIP	15217443	5.9516	6.4378	-0.2186
CHB	NPIP	15222443	10.3606	11.6896	-0.3447
CHB	NPIP	15227443	8.3845	9.1484	-0.2491
CHB	NPIP	15232443	4.6111	5.9295	-0.6383
CHB	NPIP	15237443	3.1064	7.2848	-1.6796
CHB	NPIP	15242443	3.26	7.1154	-1.5833
CHB	NPIP	15247443	5.5543	7.1154	-0.6411
CHB	NPIP	15252443	5.5608	7.1154	-0.6384
CHB	NPIP	15257443	2.7316	5.0825	-1.3052
CHB	NPIP	15262443	3.6442	4.913	-0.7257
CHB	NPIP	15267443	3.5894	5.5907	-1.0211
CHB	NPIP	15272443	2.9524	5.7601	-1.3949
CHB	NPIP	15277443	5.2597	5.7601	-0.2486
CHB	NPIP	15282443	4.6298	4.4048	0.1416
CHB	NPIP	15287443	2.4956	3.7271	-0.8947
CHB	NPIP	15292443	2.2157	4.5742	-1.4361
CHB	NPIP	15297443	5.6229	6.4378	-0.3664
CHB	NPIP	15302443	6.9777	5.2519	0.9308
CHB	NPIP	15307443	5.992	3.8965	1.4656
CHB	NPIP	15312443	7.0645	5.5907	0.752
CHB	NPIP	15317443	4.7626	4.4048	0.2251
CHB	NPIP	15322443	6.7824	5.2519	0.8254
CHB	NPIP	15327443	6.2488	4.913	0.7641
CHB	NPIP	15332443	1.1858	1.3553	-0.2747
CHB	NPIP	15337443	0.7072	1.5247	-1.2145
CHB	NPIP	15342443	0.6433	1.3553	-1.1539
CHB	NPIP	15347443	1.6261	1.6942	-0.0935
CHB	NPIP	15352443	2.1531	2.2024	-0.0552
CHB	NPIP	15357443	2.2275	2.8801	-0.5884
CHB	NPIP	15362443	1.3413	3.3883	-1.6121
CHB	NPIP	15367443	1.973	3.3883	-1.1146
CHB	NPIP	15372443	2.1862	2.7106	-0.4969
CHB	NPIP	15377443	4.9307	4.7436	0.1104
CHB	NPIP	15382443	7.4671	6.946	0.2187
CHB	NPIP	15387443	4.0073	4.066	-0.0396
CHB	NPIP	15392443	5.2244	3.8965	0.9288

CHB	NPIP	15397443	7.7416	7.1154	0.2572
CHB	NPIP	15402443	5.1756	6.0989	-0.4358
CHB	NPIP	15407443	4.6693	4.7436	-0.0438
CHB	NPIP	15412443	6.238	6.2684	-0.014
CHB	NPIP	15417443	5.3686	5.5907	-0.1133
CHB	NPIP	15422443	2.9949	2.7106	0.2694
CHB	NPIP	15427443	0.9608	0.8471	0.2564
CHB	NPIP	15432443	0.692	0.6777	0.0374
CHB	NPIP	15437443	0.9663	0.8471	0.2688
CHB	NPIP	15442443	1.1097	0.8471	0.5922
YRI	NPIP	14442443	11.6581	16.302	-0.8755
YRI	NPIP	14447443	10.0517	13.7811	-0.8251
YRI	NPIP	14452443	11.3101	13.9492	-0.5772
YRI	NPIP	14457443	12.8544	16.9743	-0.7472
YRI	NPIP	14462443	14.0702	18.991	-0.8013
YRI	NPIP	14467443	15.9449	17.3104	-0.2431
YRI	NPIP	14472443	15.1461	15.6298	-0.0949
YRI	NPIP	14477443	9.2406	11.7644	-0.6484
YRI	NPIP	14482443	5.2514	9.0754	-1.2518
YRI	NPIP	14487443	6.9784	9.5795	-0.8098
YRI	NPIP	14492443	8.4234	11.0921	-0.7246
YRI	NPIP	14497443	7.4136	10.924	-0.9668
YRI	NPIP	14502443	4.587	6.5544	-0.8674
YRI	NPIP	14507443	7.5169	8.4031	-0.3115
YRI	NPIP	14512443	7.58	10.2518	-0.7809
YRI	NPIP	14517443	5.469	7.8989	-0.904
YRI	NPIP	14522443	6.291	7.7309	-0.5463
YRI	NPIP	14527443	5.3434	7.7309	-0.9059
YRI	NPIP	14532443	4.6594	7.2267	-1.036
YRI	NPIP	14537443	4.7358	7.5628	-1.0944
YRI	NPIP	14542443	5.1242	8.235	-1.1138
YRI	NPIP	14547443	3.6449	6.5544	-1.2827
YRI	NPIP	14552443	2.1481	5.2099	-1.6573
YRI	NPIP	14557443	3.1315	7.3947	-1.6846
YRI	NPIP	14562443	5.1993	9.0754	-1.2688
YRI	NPIP	14567443	3.984	6.5544	-1.1332
YRI	NPIP	14572443	2.9155	6.3864	-1.5665
YRI	NPIP	14577443	2.9078	8.235	-1.9074
YRI	NPIP	14582443	1.9688	7.5628	-2.1656
YRI	NPIP	14587443	3.228	7.0586	-1.5792
YRI	NPIP	14592443	3.9054	7.0586	-1.2999
YRI	NPIP	14597443	4.5961	6.5544	-0.8634
YRI	NPIP	14602443	3.7857	6.0502	-1.0729
YRI	NPIP	14607443	1.9064	6.2183	-1.9934
YRI	NPIP	14612443	2.0072	7.2267	-2.1062
YRI	NPIP	14617443	2.3049	7.5628	-2.0355
YRI	NPIP	14622443	3.8906	8.9073	-1.6709
YRI	NPIP	14627443	4.3371	9.2434	-1.579
YRI	NPIP	14632443	5.1961	8.7392	-1.201

YRI	NPIP	14637443	4.7882	8.9073	-1.3719
YRI	NPIP	14642443	2.765	8.4031	-1.9816
YRI	NPIP	14647443	3.3339	8.7392	-1.8323
YRI	NPIP	14652443	3.4437	7.8989	-1.6574
YRI	NPIP	14657443	3.3325	8.067	-1.7276
YRI	NPIP	14662443	3.5648	9.4115	-1.8504
YRI	NPIP	14667443	3.3771	9.2434	-1.8879
YRI	NPIP	14672443	2.9984	7.8989	-1.8231
YRI	NPIP	14677443	2.7261	7.0586	-1.7861
YRI	NPIP	14682443	2.2973	6.5544	-1.8769
YRI	NPIP	14687443	2.3565	7.7309	-2.0392
YRI	NPIP	14692443	2.6376	9.4115	-2.1439
YRI	NPIP	14697443	3.8368	8.4031	-1.6049
YRI	NPIP	14702443	4.4223	8.7392	-1.4633
YRI	NPIP	14707443	3.9685	9.4115	-1.7227
YRI	NPIP	14712443	2.7929	7.8989	-1.8995
YRI	NPIP	14717443	1.3039	8.067	-2.4679
YRI	NPIP	14722443	1.0934	7.2267	-2.4749
YRI	NPIP	14727443	1.9323	5.7141	-1.8858
YRI	NPIP	14732443	2.6049	7.7309	-1.9449
YRI	NPIP	14737443	3.1597	7.5628	-1.7046
YRI	NPIP	14742443	2.8688	5.5461	-1.371
YRI	NPIP	14747443	2.1012	7.3947	-2.0917
YRI	NPIP	14752443	2.3279	8.9073	-2.1913
YRI	NPIP	14757443	1.9719	7.2267	-2.1204
YRI	NPIP	14762443	2.1364	8.067	-2.1641
YRI	NPIP	14767443	1.9997	7.7309	-2.1746
YRI	NPIP	14772443	2.4261	6.5544	-1.8201
YRI	NPIP	14777443	3.7171	9.0754	-1.754
YRI	NPIP	14782443	2.0887	6.0502	-1.877
YRI	NPIP	14787443	0.037	0.6722	-1.6629
YRI	NPIP	14792443	0	0	0
YRI	NPIP	14797443	0	0	0
YRI	NPIP	14802443	0	0	0
YRI	NPIP	14807443	0	0	0
YRI	NPIP	14812443	0	0	0
YRI	NPIP	14817443	0.1063	0.5042	-1.2505
YRI	NPIP	14822443	0.4422	1.1764	-1.3132
YRI	NPIP	14827443	0.3359	0.6722	-0.8804
YRI	NPIP	14832443	0.1057	0.1681	-0.3709
YRI	NPIP	14837443	0.1057	0.1681	-0.3709
YRI	NPIP	14842443	0	0	0
YRI	NPIP	14847443	0.358	0.3361	0.0879
YRI	NPIP	14852443	0.358	0.3361	0.0879
YRI	NPIP	14857443	0.2118	0.8403	-1.4196
YRI	NPIP	14862443	1.6827	4.8738	-1.8318
YRI	NPIP	14867443	5.8325	7.7309	-0.7203
YRI	NPIP	14872443	5.3415	5.7141	-0.1858
YRI	NPIP	14877443	1.4132	2.689	-1.2125

YRI	NPIP	14882443	0.4702	1.0084	-1.073
YRI	NPIP	14887443	0.3165	1.1764	-1.538
YRI	NPIP	14892443	0.733	2.5209	-1.7905
YRI	NPIP	14897443	2.2351	4.8738	-1.5147
YRI	NPIP	14902443	2.738	5.0419	-1.2837
YRI	NPIP	14907443	1.6238	5.2099	-1.9411
YRI	NPIP	14912443	2.8798	5.8822	-1.4589
YRI	NPIP	14917443	2.4785	5.0419	-1.4282
YRI	NPIP	14922443	1.5765	6.0502	-2.1197
YRI	NPIP	14927443	2.7261	6.3864	-1.652
YRI	NPIP	14932443	2.1491	5.7141	-1.7777
YRI	NPIP	14937443	1.5652	4.8738	-1.8992
YRI	NPIP	14942443	1.8604	4.7057	-1.6843
YRI	NPIP	14947443	2.3197	6.3864	-1.8354
YRI	NPIP	14952443	1.5803	5.7141	-2.0613
YRI	NPIP	14957443	2.5013	5.8822	-1.6428
YRI	NPIP	14962443	3.52	8.4031	-1.7162
YRI	NPIP	14967443	4.729	9.4115	-1.482
YRI	NPIP	14972443	4.2791	7.2267	-1.1894
YRI	NPIP	14977443	1.2973	5.5461	-2.1758
YRI	NPIP	14982443	2.1987	6.7225	-1.9493
YRI	NPIP	14987443	2.1389	6.0502	-1.8532
YRI	NPIP	14992443	0.7104	4.3696	-2.3105
YRI	NPIP	14997443	0.6076	2.5209	-1.9161
YRI	NPIP	15002443	0.8027	1.3445	-0.88
YRI	NPIP	15007443	0.6526	1.3445	-1.1238
YRI	NPIP	15012443	0.411	2.5209	-2.113
YRI	NPIP	15017443	1.9898	4.5377	-1.5569
YRI	NPIP	15022443	3.6215	6.5544	-1.293
YRI	NPIP	15027443	2.1372	5.0419	-1.6184
YRI	NPIP	15032443	0.4942	3.0251	-2.1833
YRI	NPIP	15037443	0.9518	2.689	-1.651
YRI	NPIP	15042443	1.7609	2.689	-0.882
YRI	NPIP	15047443	4.6387	8.4031	-1.323
YRI	NPIP	15052443	8.4926	12.2685	-0.9325
YRI	NPIP	15057443	9.2807	10.2518	-0.2838
YRI	NPIP	15062443	5.863	8.067	-0.8043
YRI	NPIP	15067443	3.0552	6.2183	-1.4623
YRI	NPIP	15072443	4.5612	7.7309	-1.2027
YRI	NPIP	15077443	5.864	9.4115	-1.1228
YRI	NPIP	15082443	6.6149	10.4199	-1.0953
YRI	NPIP	15087443	5.5692	9.4115	-1.2161
YRI	NPIP	15092443	4.0304	7.7309	-1.4041
YRI	NPIP	15097443	4.7809	8.067	-1.1991
YRI	NPIP	15102443	4.8705	7.8989	-1.1266
YRI	NPIP	15107443	5.0016	8.7392	-1.2669
YRI	NPIP	15112443	4.589	10.0837	-1.6309
YRI	NPIP	15117443	6.4292	12.2685	-1.442
YRI	NPIP	15122443	6.4945	11.7644	-1.3539

YRI	NPIP	15127443	8.161	10.756	-0.7252
YRI	NPIP	15132443	9.8329	11.2602	-0.3821
YRI	NPIP	15137443	7.3968	10.756	-0.9387
YRI	NPIP	15142443	6.2704	8.9073	-0.8782
YRI	NPIP	15147443	5.6385	8.4031	-0.9716
YRI	NPIP	15152443	7.3109	9.4115	-0.6648
YRI	NPIP	15157443	7.6107	9.7476	-0.6546
YRI	NPIP	15162443	5.9317	9.2434	-1.0658
YRI	NPIP	15167443	6.2644	9.0754	-0.9202
YRI	NPIP	15172443	7.4066	10.0837	-0.7946
YRI	NPIP	15177443	7.3118	9.2434	-0.6216
YRI	NPIP	15182443	5.5327	8.067	-0.9248
YRI	NPIP	15187443	4.0752	7.5628	-1.3502
YRI	NPIP	15192443	3.9526	8.067	-1.5014
YRI	NPIP	15197443	4.4203	9.4115	-1.5797
YRI	NPIP	15202443	5.3881	8.9073	-1.1721
YRI	NPIP	15207443	3.764	5.2099	-0.7826
YRI	NPIP	15212443	2.1279	3.6974	-1.144
YRI	NPIP	15217443	6.3239	10.0837	-1.116
YRI	NPIP	15222443	11.5078	18.4868	-1.1663
YRI	NPIP	15227443	9.0108	16.4701	-1.3925
YRI	NPIP	15232443	5.0567	11.0921	-1.6386
YRI	NPIP	15237443	4.0692	10.4199	-1.8282
YRI	NPIP	15242443	4.1713	9.7476	-1.7082
YRI	NPIP	15247443	6.7431	9.4115	-0.8445
YRI	NPIP	15252443	6.239	8.067	-0.667
YRI	NPIP	15257443	3.2085	5.8822	-1.2992
YRI	NPIP	15262443	3.6733	6.3864	-1.2245
YRI	NPIP	15267443	3.5662	7.3947	-1.5128
YRI	NPIP	15272443	3.1303	7.0586	-1.6194
YRI	NPIP	15277443	4.0737	6.5544	-1.0937
YRI	NPIP	15282443	3.9071	5.378	-0.7741
YRI	NPIP	15287443	3.1658	5.378	-1.1642
YRI	NPIP	15292443	2.4949	6.3864	-1.7563
YRI	NPIP	15297443	5.3994	6.8906	-0.6283
YRI	NPIP	15302443	5.8556	6.5544	-0.3081
YRI	NPIP	15307443	3.7108	5.5461	-0.9398
YRI	NPIP	15312443	5.1486	6.3864	-0.5587
YRI	NPIP	15317443	4.066	6.2183	-0.995
YRI	NPIP	15322443	6.6268	8.5712	-0.671
YRI	NPIP	15327443	6.0776	6.7225	-0.2779
YRI	NPIP	15332443	0.6835	0.8403	-0.3542
YRI	NPIP	15337443	0.5508	2.5209	-1.973
YRI	NPIP	15342443	0.5003	3.0251	-2.178
YRI	NPIP	15347443	1.2004	3.0251	-1.5741
YRI	NPIP	15352443	1.7452	3.3612	-1.2767
YRI	NPIP	15357443	2.3249	4.0335	-1.1561
YRI	NPIP	15362443	1.8368	4.8738	-1.7433
YRI	NPIP	15367443	1.6676	5.378	-1.9527

YRI	NPIP	15372443	1.7017	4.3696	-1.6846
YRI	NPIP	15377443	5.015	7.7309	-1.0305
YRI	NPIP	15382443	7.7549	10.5879	-0.8034
YRI	NPIP	15387443	3.7614	6.7225	-1.2759
YRI	NPIP	15392443	4.5136	7.8989	-1.2594
YRI	NPIP	15397443	6.329	9.5795	-1.012
YRI	NPIP	15402443	4.5291	8.7392	-1.4271
YRI	NPIP	15407443	5.0238	7.2267	-0.8889
YRI	NPIP	15412443	6.9695	7.5628	-0.2297
YRI	NPIP	15417443	5.206	7.3947	-0.8649
YRI	NPIP	15422443	2.0358	4.3696	-1.4736
YRI	NPIP	15427443	0.8056	2.3529	-1.6379
YRI	NPIP	15432443	0.5354	2.3529	-1.9239
YRI	NPIP	15437443	0.7802	2.8571	-1.8782
YRI	NPIP	15442443	0.6867	2.8571	-1.9628
CEU	NPIP	14868613	5.5071	6.3108	-0.369
CEU	NPIP	14873613	2.8365	3.7524	-0.6636
CEU	NPIP	14878613	0.8316	1.3645	-0.8621
CEU	NPIP	14883613	1.0025	1.0234	-0.0415
CEU	NPIP	14888613	0.8955	0.6822	0.557
CEU	NPIP	14893613	1.1235	2.0467	-1.0985
CEU	NPIP	14898613	1.9105	3.4112	-1.1787
CEU	NPIP	14903613	1.4057	3.5818	-1.6401
CEU	NPIP	14908613	0.959	3.0701	-1.8109
CEU	NPIP	14913613	1.8415	3.2407	-1.1473
CEU	NPIP	14918613	1.1915	2.729	-1.4533
CEU	NPIP	14923613	1.3785	2.3879	-1.063
CEU	NPIP	14928613	2.2274	3.2407	-0.8309
CEU	NPIP	14933613	1.7603	2.729	-0.9156
CEU	NPIP	14938613	1.3983	2.2173	-0.915
CEU	NPIP	14943613	1.7137	2.8996	-1.0665
CEU	NPIP	14948613	1.8021	3.2407	-1.1796
CEU	NPIP	14953613	1.4238	3.4112	-1.5609
CEU	NPIP	14958613	2.2165	4.4346	-1.3917
CEU	NPIP	14963613	4.5514	5.1169	-0.313
CEU	NPIP	14968613	4.9604	4.9463	0.008
CEU	NPIP	14973613	2.2742	3.5818	-0.9855
CEU	NPIP	14978613	1.0926	3.4112	-1.8211
CEU	NPIP	14983613	1.4418	4.0935	-1.7831
CEU	NPIP	14988613	0.8932	3.0701	-1.8673
CEU	NPIP	14993613	0.652	3.0701	-2.0742
CEU	NPIP	14998613	0.4651	1.8762	-1.7966
CEU	NPIP	15003613	0.3436	0.5117	-0.5272
CEU	NPIP	15008613	0.3537	0.6822	-0.8578
CEU	NPIP	15013613	0.0998	0.8528	-1.6957
CEU	NPIP	15018613	1.536	2.2173	-0.7611
CEU	NPIP	15023613	2.3593	2.5584	-0.1983
CEU	NPIP	15028613	1.4475	1.7056	-0.3535
CEU	NPIP	15033613	0.9948	1.8762	-1.1222

CEU	NPIP	15038613	0.9192	1.8762	-1.2184
CEU	NPIP	15043613	2.1816	2.729	-0.5174
CEU	NPIP	15048613	4.6271	6.6519	-0.8865
CEU	NPIP	15053613	6.5471	7.8459	-0.4893
CEU	NPIP	15058613	7.0416	8.0164	-0.36
CEU	NPIP	15063613	4.2443	6.9931	-1.1501
CEU	NPIP	15068613	3.1884	5.2874	-1.1286
CEU	NPIP	15073613	5.1938	5.9697	-0.3745
CEU	NPIP	15078613	5.9079	7.6753	-0.6793
CEU	NPIP	15083613	6.2954	8.6987	-0.8234
CEU	NPIP	15088613	6.0394	6.9931	-0.399
CEU	NPIP	15093613	4.8947	6.4814	-0.7112
CEU	NPIP	15098613	4.035	6.4814	-1.0965
CEU	NPIP	15103613	4.7776	7.3342	-1.0244
CEU	NPIP	15108613	4.6226	6.6519	-0.8885
CEU	NPIP	15113613	5.106	6.4814	-0.6165
CEU	NPIP	15118613	6.5914	8.3576	-0.6278
CEU	NPIP	15123613	6.7355	7.8459	-0.4183
CEU	NPIP	15128613	10.6595	8.6987	0.6718
CEU	NPIP	15133613	12.6314	10.916	0.4758
CEU	NPIP	15138613	10.0023	9.3809	0.1985
CEU	NPIP	15143613	8.9555	6.9931	0.8211
CEU	NPIP	15148613	7.9149	6.4814	0.6425
CEU	NPIP	15153613	6.4381	7.5047	-0.4185
CEU	NPIP	15158613	5.5991	6.4814	-0.3955
CEU	NPIP	15163613	4.7982	4.6052	0.1172
CEU	NPIP	15168613	8.5584	8.187	0.1346
CEU	NPIP	15173613	9.7846	8.8692	0.308
CEU	NPIP	15178613	5.6741	6.4814	-0.3618
CEU	NPIP	15183613	4.7365	6.6519	-0.8386
CEU	NPIP	15188613	3.3253	5.7991	-1.2253
CEU	NPIP	15193613	3.5477	7.1636	-1.4802
CEU	NPIP	15198613	4.5927	6.9931	-1.0044
CEU	NPIP	15203613	4.1066	5.2874	-0.6349
CEU	NPIP	15208613	3.2564	4.4346	-0.7392
CEU	NPIP	15213613	2.1156	3.0701	-0.8188
CEU	NPIP	15218613	3.8359	7.5047	-1.4395
CEU	NPIP	15223613	6.1971	11.5982	-1.4151
CEU	NPIP	15228613	5.8705	9.0398	-1.0478
CEU	NPIP	15233613	4.4976	7.3342	-1.1365
CEU	NPIP	15238613	2.5413	7.6753	-1.9734
CEU	NPIP	15243613	2.4012	5.2874	-1.5519
CEU	NPIP	15248613	4.0832	5.1169	-0.5722
CEU	NPIP	15253613	4.298	7.1636	-1.173
CEU	NPIP	15258613	2.3789	5.1169	-1.5155
CEU	NPIP	15263613	2.0359	4.2641	-1.4464
CEU	NPIP	15268613	2.3346	4.9463	-1.4895
CEU	NPIP	15273613	2.4199	3.7524	-0.9654
CEU	NPIP	15278613	4.69	4.2641	0.2765

CEU	NPIP	15283613	3.946	4.2641	-0.2065
CEU	NPIP	15288613	1.6553	3.4112	-1.3791
CEU	NPIP	15293613	2.2348	4.6052	-1.4392
CEU	NPIP	15298613	6.0254	7.3342	-0.5244
CEU	NPIP	15303613	5.3855	5.458	-0.0379
CEU	NPIP	15308613	3.5677	2.729	0.7927
CEU	NPIP	15313613	7.0266	4.7757	1.3238
CEU	NPIP	15318613	5.7992	5.2874	0.2752
CEU	NPIP	15323613	6.2292	6.4814	-0.113
CEU	NPIP	15328613	5.0552	4.6052	0.2732
CEU	NPIP	15333613	0.9027	1.5351	-0.9377
CEU	NPIP	15338613	1.176	1.7056	-0.7253
CEU	NPIP	15343613	0.7868	1.1939	-0.7255
CEU	NPIP	15348613	0.9443	1.1939	-0.4448
CEU	NPIP	15353613	2.1128	2.3879	-0.2897
CEU	NPIP	15358613	2.9664	4.0935	-0.7579
CEU	NPIP	15363613	1.8707	3.7524	-1.3633
CEU	NPIP	15368613	2.1715	2.729	-0.527
CEU	NPIP	15373613	3.3952	3.2407	0.1267
CEU	NPIP	15378613	6.6042	6.1402	0.2183
CEU	NPIP	15383613	8.4465	6.6519	0.7857
CEU	NPIP	15388613	5.7174	4.0935	1.092
CEU	NPIP	15393613	5.7217	5.9697	-0.1197
CEU	NPIP	15398613	7.5296	8.6987	-0.4005
CEU	NPIP	15403613	8.5586	7.3342	0.4906
CEU	NPIP	15408613	7.5143	5.6286	0.9592
CEU	NPIP	15413613	6.4168	5.6286	0.4009
CEU	NPIP	15418613	5.0689	5.2874	-0.1175
CEU	NPIP	15423613	2.4247	3.0701	-0.5536
CEU	NPIP	15428613	1.1657	1.7056	-0.7395
CEU	NPIP	15433613	0.6226	1.3645	-1.2002
CEU	NPIP	15438613	0.571	1.0234	-0.8989
CEU	NPIP	15443613	1.8013	1.3645	0.7066
CEU	NPIP	15448613	2.5471	3.0701	-0.4486
CEU	NPIP	15453613	2.3657	3.7524	-1.0046
CEU	NPIP	15458613	2.726	3.2407	-0.422
CEU	NPIP	15463613	4.2313	4.2641	-0.0213
CEU	NPIP	15468613	6.1653	5.7991	0.1814
CEU	NPIP	15473613	6.4873	6.9931	-0.2116
CEU	NPIP	15478613	5.4133	6.9931	-0.661
CEU	NPIP	15483613	6.3137	6.9931	-0.2843
CEU	NPIP	15488613	7.9695	7.6753	0.1131
CEU	NPIP	15493613	6.739	7.6753	-0.3599
CEU	NPIP	15498613	8.1923	8.6987	-0.1735
CEU	NPIP	15503613	9.0926	9.8926	-0.2433
CEU	NPIP	15508613	8.8573	10.0632	-0.3609
CEU	NPIP	15513613	11.9977	9.8926	0.6401
CEU	NPIP	15518613	12.2927	9.8926	0.7298
CEU	NPIP	15523613	7.5376	8.0164	-0.1768

CEU	NPIP	15528613	4.9375	5.7991	-0.4267
CEU	NPIP	15533613	6.4306	6.8225	-0.1677
CEU	NPIP	15538613	8.4768	7.6753	0.3081
CEU	NPIP	15543613	8.8369	7.1636	0.685
CEU	NPIP	15548613	7.3878	6.6519	0.3222
CEU	NPIP	15553613	8.7001	8.3576	0.1217
CEU	NPIP	15558613	6.5931	7.6753	-0.416
CEU	NPIP	15563613	4.3772	6.1402	-0.8296
CEU	NPIP	15568613	3.6823	6.3108	-1.2068
CEU	NPIP	15573613	3.2713	5.9697	-1.3023
CEU	NPIP	15578613	3.5428	5.458	-1.0012
CEU	NPIP	15583613	3.2884	4.4346	-0.7192
CEU	NPIP	15588613	3.241	3.5818	-0.2569
CEU	NPIP	15593613	4.7287	5.6286	-0.4577
CEU	NPIP	15598613	7.8273	8.3576	-0.1885
CEU	NPIP	15603613	5.3335	6.1402	-0.3796
CEU	NPIP	15608613	4.3105	5.9697	-0.8008
CEU	NPIP	15613613	5.3007	6.6519	-0.5916
CEU	NPIP	15618613	3.4293	4.7757	-0.7919
CEU	NPIP	15623613	4.9685	5.2874	-0.1715
CEU	NPIP	15628613	7.0196	5.6286	0.7075
CEU	NPIP	15633613	4.2072	4.4346	-0.1427
CEU	NPIP	15638613	3.2604	5.1169	-1.0276
CEU	NPIP	15643613	3.8648	6.1402	-1.0707
CEU	NPIP	15648613	2.907	4.2641	-0.881
CEU	NPIP	15653613	2.6533	3.0701	-0.3575
CEU	NPIP	15658613	3.1853	4.4346	-0.7839
CEU	NPIP	15663613	3.5446	6.6519	-1.3604
CEU	NPIP	15668613	3.1288	8.0164	-1.8053
CEU	NPIP	15673613	2.7755	8.187	-1.9605
CEU	NPIP	15678613	3.898	7.5047	-1.4151
CEU	NPIP	15683613	7.7469	8.5281	-0.2726
CEU	NPIP	15688613	5.6848	5.7991	-0.0566
CEU	NPIP	15693613	3.5702	3.9229	-0.246
CEU	NPIP	15698613	5.3857	8.0164	-0.9717
CEU	NPIP	15703613	6.1175	8.3576	-0.7963
CEU	NPIP	15708613	4.6974	5.6286	-0.4737
CEU	NPIP	15713613	2.442	5.458	-1.5767
CEU	NPIP	15718613	3.2973	6.9931	-1.5464
CEU	NPIP	15723613	4.456	7.3342	-1.1532
CEU	NPIP	15728613	3.9128	5.6286	-0.8728
CEU	NPIP	15733613	3.3117	4.2641	-0.6182
CEU	NPIP	15738613	3.5065	4.9463	-0.8211
CEU	NPIP	15743613	3.5207	5.2874	-0.95
CEU	NPIP	15748613	3.7633	5.9697	-1.0648
CEU	NPIP	15753613	4.6184	8.3576	-1.3292
CEU	NPIP	15758613	8.545	9.2104	-0.2162
CEU	NPIP	15763613	9.2117	8.0164	0.4415
CEU	NPIP	15768613	6.9903	8.3576	-0.486

CEU	NPIP	15773613	5.7452	8.5281	-0.971
CEU	NPIP	15778613	4.384	6.9931	-1.0917
CEU	NPIP	15783613	6.9293	7.8459	-0.3453
CEU	NPIP	15788613	9.0992	9.7221	-0.1925
CEU	NPIP	15793613	8.2531	10.5749	-0.6634
CEU	NPIP	15798613	7.8058	10.0632	-0.6756
CEU	NPIP	15803613	7.6481	9.7221	-0.6409
CEU	NPIP	15808613	7.0074	9.5515	-0.7993
CEU	NPIP	15813613	6.8081	8.6987	-0.6477
CEU	NPIP	15818613	6.9487	9.2104	-0.7349
CEU	NPIP	15823613	5.5859	8.0164	-0.8977
CEU	NPIP	15828613	3.6865	6.1402	-1.1546
CEU	NPIP	15833613	5.1742	6.8225	-0.7053
CEU	NPIP	15838613	6.576	7.6753	-0.4225
CEU	NPIP	15843613	7.1055	7.6753	-0.219
CEU	NPIP	15848613	7.4595	8.5281	-0.3728
CEU	NPIP	15853613	3.8176	7.3342	-1.409
CEU	NPIP	15858613	5.6953	6.8225	-0.4823
CEU	NPIP	15863613	10.6745	9.2104	0.4758
CEU	NPIP	15868613	15.1835	12.1099	0.7732
CEU	NPIP	15873613	13.8592	11.4277	0.646
CHB	NPIP	14868613	6.6874	6.6072	0.0352
CHB	NPIP	14873613	4.2077	3.7271	0.3491
CHB	NPIP	14878613	1.0525	1.1859	-0.2381
CHB	NPIP	14883613	0.7087	0.8471	-0.3121
CHB	NPIP	14888613	0.1989	0.6777	-1.2517
CHB	NPIP	14893613	0.9101	2.5412	-1.6288
CHB	NPIP	14898613	1.9823	2.5412	-0.5581
CHB	NPIP	14903613	1.3642	1.1859	0.3183
CHB	NPIP	14908613	1.4064	1.6942	-0.395
CHB	NPIP	14913613	2.4626	1.8636	0.7642
CHB	NPIP	14918613	1.4253	2.033	-0.7247
CHB	NPIP	14923613	1.2376	2.2024	-1.0803
CHB	NPIP	14928613	1.8285	3.0495	-1.0501
CHB	NPIP	14933613	1.7436	3.0495	-1.1231
CHB	NPIP	14938613	1.0952	2.2024	-1.2398
CHB	NPIP	14943613	1.5353	2.5412	-1.0045
CHB	NPIP	14948613	2.2267	3.2189	-0.8157
CHB	NPIP	14953613	0.8273	2.8801	-1.8508
CHB	NPIP	14958613	1.3649	3.5577	-1.6572
CHB	NPIP	14963613	4.984	5.2519	-0.1445
CHB	NPIP	14968613	5.7476	5.0825	0.3693
CHB	NPIP	14973613	2.6924	4.913	-1.2702
CHB	NPIP	14978613	2.0524	4.5742	-1.5356
CHB	NPIP	14983613	2.5349	3.7271	-0.8661
CHB	NPIP	14988613	1.1727	2.7106	-1.4572
CHB	NPIP	14993613	0.7394	2.3718	-1.7231
CHB	NPIP	14998613	0.6908	1.5247	-1.2389
CHB	NPIP	15003613	0.7936	0.6777	0.303

CHB	NPIP	15008613	0.7936	0.6777	0.303
CHB	NPIP	15013613	0.1043	0.6777	-1.499
CHB	NPIP	15018613	1.6682	1.6942	-0.0357
CHB	NPIP	15023613	2.9482	3.2189	-0.2226
CHB	NPIP	15028613	1.7489	3.0495	-1.1185
CHB	NPIP	15033613	0.4892	1.6942	-1.6537
CHB	NPIP	15038613	0.5711	1.0165	-0.8864
CHB	NPIP	15043613	2.7683	3.2189	-0.3705
CHB	NPIP	15048613	5.9299	6.6072	-0.2975
CHB	NPIP	15053613	6.6412	7.4543	-0.3201
CHB	NPIP	15058613	7.1243	7.4543	-0.1299
CHB	NPIP	15063613	5.1868	6.4378	-0.5625
CHB	NPIP	15068613	3.4152	5.2519	-0.9906
CHB	NPIP	15073613	5.3217	5.7601	-0.2178
CHB	NPIP	15078613	5.584	6.4378	-0.3839
CHB	NPIP	15083613	5.6794	6.4378	-0.341
CHB	NPIP	15088613	6.2294	6.6072	-0.1659
CHB	NPIP	15093613	5.1114	6.4378	-0.5964
CHB	NPIP	15098613	4.2901	6.6072	-1.0177
CHB	NPIP	15103613	4.6238	6.6072	-0.8711
CHB	NPIP	15108613	4.5494	5.9295	-0.6681
CHB	NPIP	15113613	5.6587	5.7601	-0.0504
CHB	NPIP	15118613	6.1523	5.5907	0.2865
CHB	NPIP	15123613	6.2829	5.7601	0.2597
CHB	NPIP	15128613	11.8557	8.3013	1.2677
CHB	NPIP	15133613	14.2903	10.3343	1.1515
CHB	NPIP	15138613	10.8721	8.3013	0.9169
CHB	NPIP	15143613	10.2166	6.7766	1.4767
CHB	NPIP	15148613	8.4049	6.0989	1.0885
CHB	NPIP	15153613	7.1788	7.1154	0.026
CHB	NPIP	15158613	7.134	6.6072	0.2314
CHB	NPIP	15163613	5.5102	5.5907	-0.0411
CHB	NPIP	15168613	9.7592	7.9625	0.6658
CHB	NPIP	15173613	10.6088	8.1319	0.9003
CHB	NPIP	15178613	5.9438	6.2684	-0.1495
CHB	NPIP	15183613	5.4641	6.0989	-0.2997
CHB	NPIP	15188613	3.9655	5.0825	-0.6201
CHB	NPIP	15193613	4.1043	5.7601	-0.8226
CHB	NPIP	15198613	4.9402	6.4378	-0.6734
CHB	NPIP	15203613	4.3743	4.4048	-0.0192
CHB	NPIP	15208613	4.0695	3.5577	0.3868
CHB	NPIP	15213613	2.8544	2.7106	0.1363
CHB	NPIP	15218613	5.9996	6.946	-0.3973
CHB	NPIP	15223613	10.2427	11.6896	-0.3752
CHB	NPIP	15228613	7.8353	8.4708	-0.2225
CHB	NPIP	15233613	5.1538	7.4543	-0.9055
CHB	NPIP	15238613	3.2545	7.9625	-1.7446
CHB	NPIP	15243613	2.5717	5.4213	-1.4942
CHB	NPIP	15248613	5.9766	6.946	-0.4069

CHB	NPIP	15253613	5.5092	7.1154	-0.6596
CHB	NPIP	15258613	2.2934	4.5742	-1.3888
CHB	NPIP	15263613	3.3328	5.0825	-0.9714
CHB	NPIP	15268613	3.9446	5.5907	-0.8399
CHB	NPIP	15273613	3.8559	5.7601	-0.9461
CHB	NPIP	15278613	5.5921	6.0989	-0.2392
CHB	NPIP	15283613	4.2441	4.4048	-0.1011
CHB	NPIP	15288613	1.6849	3.5577	-1.4154
CHB	NPIP	15293613	2.1218	4.5742	-1.4933
CHB	NPIP	15298613	7.4409	7.1154	0.1337
CHB	NPIP	15303613	6.5149	4.913	0.9163
CHB	NPIP	15308613	4.174	3.0495	0.9671
CHB	NPIP	15313613	7.5287	5.7601	0.8787
CHB	NPIP	15318613	5.7925	5.0825	0.3942
CHB	NPIP	15323613	6.8163	5.2519	0.8437
CHB	NPIP	15328613	5.1807	4.066	0.7517
CHB	NPIP	15333613	0.7263	1.5247	-1.1861
CHB	NPIP	15338613	1.2635	1.8636	-0.7656
CHB	NPIP	15343613	0.6145	1.1859	-1.0199
CHB	NPIP	15348613	1.0602	1.1859	-0.2244
CHB	NPIP	15353613	2.2254	2.2024	0.0258
CHB	NPIP	15358613	2.3566	3.2189	-0.7089
CHB	NPIP	15363613	1.3158	3.3883	-1.6321
CHB	NPIP	15368613	2.0482	3.5577	-1.1408
CHB	NPIP	15373613	2.6585	3.0495	-0.3363
CHB	NPIP	15378613	5.1186	4.913	0.1176
CHB	NPIP	15383613	6.9382	6.4378	0.225
CHB	NPIP	15388613	4.5731	4.066	0.342
CHB	NPIP	15393613	5.862	4.7436	0.6597
CHB	NPIP	15398613	6.7836	6.4378	0.1555
CHB	NPIP	15403613	5.8863	6.4378	-0.248
CHB	NPIP	15408613	5.5167	5.9295	-0.1998
CHB	NPIP	15413613	5.8894	5.4213	0.2455
CHB	NPIP	15418613	4.5212	4.4048	0.0732
CHB	NPIP	15423613	1.9855	2.2024	-0.2429
CHB	NPIP	15428613	1.4466	1.0165	0.8559
CHB	NPIP	15433613	0.692	0.6777	0.0374
CHB	NPIP	15438613	0.4805	0.6777	-0.5155
CHB	NPIP	15443613	1.4201	1.1859	0.418
CHB	NPIP	15448613	2.6162	2.3718	0.258
CHB	NPIP	15453613	2.9471	3.2189	-0.2235
CHB	NPIP	15458613	2.5831	3.2189	-0.5227
CHB	NPIP	15463613	4.1984	3.5577	0.4842
CHB	NPIP	15468613	6.6669	4.066	1.7539
CHB	NPIP	15473613	6.9107	5.4213	0.781
CHB	NPIP	15478613	6.3461	6.0989	0.1167
CHB	NPIP	15483613	8.625	6.2684	1.0854
CHB	NPIP	15488613	10.989	6.946	1.6971
CHB	NPIP	15493613	8.3368	6.946	0.5838

CHB	NPIP	15498613	9.4894	10.6731	-0.3343
CHB	NPIP	15503613	9.8679	10.1649	-0.0878
CHB	NPIP	15508613	9.522	9.1484	0.1218
CHB	NPIP	15513613	12.2601	11.1814	0.2917
CHB	NPIP	15518613	12.4227	10.5037	0.5502
CHB	NPIP	15523613	8.4358	8.4708	-0.0123
CHB	NPIP	15528613	4.8685	7.6237	-1.0625
CHB	NPIP	15533613	4.482	8.6402	-1.4293
CHB	NPIP	15538613	6.1713	9.1484	-0.9706
CHB	NPIP	15543613	7.5722	8.8096	-0.4178
CHB	NPIP	15548613	6.4894	7.4543	-0.3798
CHB	NPIP	15553613	9.0426	7.9625	0.4003
CHB	NPIP	15558613	7.4883	6.6072	0.387
CHB	NPIP	15563613	4.6267	4.913	-0.1638
CHB	NPIP	15568613	3.6863	5.5907	-0.9717
CHB	NPIP	15573613	2.9221	6.4378	-1.5808
CHB	NPIP	15578613	3.3785	6.0989	-1.2841
CHB	NPIP	15583613	4.3432	6.4378	-0.9418
CHB	NPIP	15588613	4.1382	6.0989	-0.9255
CHB	NPIP	15593613	5.5037	6.0989	-0.281
CHB	NPIP	15598613	9.6627	7.7931	0.7066
CHB	NPIP	15603613	7.0177	8.8096	-0.605
CHB	NPIP	15608613	5.8273	10.6731	-1.3686
CHB	NPIP	15613613	7.2363	10.6731	-0.9707
CHB	NPIP	15618613	4.9259	10.1649	-1.5487
CHB	NPIP	15623613	6.436	11.012	-1.2552
CHB	NPIP	15628613	8.8289	9.3178	-0.1567
CHB	NPIP	15633613	5.3781	6.4378	-0.4765
CHB	NPIP	15638613	5.0067	6.946	-0.814
CHB	NPIP	15643613	6.0543	9.3178	-1.0461
CHB	NPIP	15648613	4.343	8.4708	-1.445
CHB	NPIP	15653613	4.4118	6.946	-1.0638
CHB	NPIP	15658613	5.0247	7.1154	-0.8586
CHB	NPIP	15663613	4.8812	9.1484	-1.3913
CHB	NPIP	15668613	4.9269	10.8426	-1.6464
CHB	NPIP	15673613	4.0187	10.1649	-1.8169
CHB	NPIP	15678613	3.747	8.1319	-1.5938
CHB	NPIP	15683613	8.0807	8.8096	-0.2461
CHB	NPIP	15688613	6.6752	9.3178	-0.8471
CHB	NPIP	15693613	4.0417	8.6402	-1.5807
CHB	NPIP	15698613	4.668	8.8096	-1.3983
CHB	NPIP	15703613	5.3552	9.3178	-1.2702
CHB	NPIP	15708613	5.4428	9.3178	-1.2421
CHB	NPIP	15713613	3.0461	7.4543	-1.7352
CHB	NPIP	15718613	3.1772	7.2848	-1.6511
CHB	NPIP	15723613	4.4776	7.6237	-1.2132
CHB	NPIP	15728613	4.9345	6.4378	-0.6759
CHB	NPIP	15733613	5.3278	6.946	-0.6793
CHB	NPIP	15738613	5.9282	7.9625	-0.7538

CHB	NPIP	15743613	5.6996	8.6402	-1.0108
CHB	NPIP	15748613	4.696	7.7931	-1.1705
CHB	NPIP	15753613	4.6836	7.9625	-1.2151
CHB	NPIP	15758613	8.3328	9.6567	-0.4105
CHB	NPIP	15763613	8.5686	9.6567	-0.3374
CHB	NPIP	15768613	7.1639	9.8261	-0.8122
CHB	NPIP	15773613	6.4864	8.8096	-0.7844
CHB	NPIP	15778613	4.1392	6.6072	-1.084
CHB	NPIP	15783613	6.335	7.4543	-0.4406
CHB	NPIP	15788613	9.0714	11.3508	-0.6077
CHB	NPIP	15793613	9.2336	12.7061	-0.8324
CHB	NPIP	15798613	8.0836	10.6731	-0.7314
CHB	NPIP	15803613	8.1949	12.1979	-0.9973
CHB	NPIP	15808613	7.4514	13.892	-1.4189
CHB	NPIP	15813613	7.3973	11.8591	-1.1415
CHB	NPIP	15818613	7.8858	10.5037	-0.7505
CHB	NPIP	15823613	6.139	9.4872	-1.0554
CHB	NPIP	15828613	4.522	7.6237	-1.1961
CHB	NPIP	15833613	5.2905	5.5907	-0.1532
CHB	NPIP	15838613	7.495	7.7931	-0.1127
CHB	NPIP	15843613	9.5633	10.1649	-0.1778
CHB	NPIP	15848613	10.3512	9.8261	0.1602
CHB	NPIP	15853613	6.2119	8.4708	-0.7908
CHB	NPIP	15858613	6.8626	8.8096	-0.6574
CHB	NPIP	15863613	12.0156	10.5037	0.4335
CHB	NPIP	15868613	16.7611	12.5367	1.0256
CHB	NPIP	15873613	15.6101	13.2144	0.5534
YRI	NPIP	14868613	6.3444	8.067	-0.6286
YRI	NPIP	14873613	4.2863	4.8738	-0.3372
YRI	NPIP	14878613	0.837	1.6806	-1.1605
YRI	NPIP	14883613	0.6398	1.0084	-0.7349
YRI	NPIP	14888613	0.3258	1.1764	-1.5213
YRI	NPIP	14893613	1.3857	3.0251	-1.4142
YRI	NPIP	14898613	2.2977	5.0419	-1.529
YRI	NPIP	14903613	1.9512	4.5377	-1.5805
YRI	NPIP	14908613	2.5386	5.378	-1.4943
YRI	NPIP	14913613	2.8532	5.8822	-1.4718
YRI	NPIP	14918613	1.5099	4.7057	-1.8918
YRI	NPIP	14923613	1.9926	6.0502	-1.9225
YRI	NPIP	14928613	2.8727	7.5628	-1.8157
YRI	NPIP	14933613	2.1584	5.7141	-1.7731
YRI	NPIP	14938613	1.3914	3.5293	-1.621
YRI	NPIP	14943613	1.8036	5.0419	-1.8043
YRI	NPIP	14948613	2.3656	6.7225	-1.8774
YRI	NPIP	14953613	1.4512	5.7141	-2.1257
YRI	NPIP	14958613	2.6928	6.8906	-1.7688
YRI	NPIP	14963613	5.0439	10.2518	-1.5221
YRI	NPIP	14968613	4.6282	8.9073	-1.4252
YRI	NPIP	14973613	2.7073	5.7141	-1.4994

YRI	NPIP	14978613	1.7588	5.7141	-1.9723
YRI	NPIP	14983613	2.0621	6.5544	-1.9806
YRI	NPIP	14988613	1.55	5.2099	-1.981
YRI	NPIP	14993613	0.8957	4.2016	-2.1595
YRI	NPIP	14998613	0.5983	2.3529	-1.8573
YRI	NPIP	15003613	0.5258	0.6722	-0.3833
YRI	NPIP	15008613	0.7435	1.6806	-1.2892
YRI	NPIP	15013613	0.5284	3.1932	-2.1977
YRI	NPIP	15018613	2.6625	5.5461	-1.4767
YRI	NPIP	15023613	3.5041	5.8822	-1.1555
YRI	NPIP	15028613	1.3829	3.8654	-1.7421
YRI	NPIP	15033613	0.4942	3.0251	-2.1833
YRI	NPIP	15038613	1.0689	3.1932	-1.752
YRI	NPIP	15043613	2.5985	4.5377	-1.1849
YRI	NPIP	15048613	6.5775	9.7476	-0.9711
YRI	NPIP	15053613	9.338	11.9324	-0.6577
YRI	NPIP	15058613	7.9554	9.7476	-0.549
YRI	NPIP	15063613	4.1893	6.7225	-1.0916
YRI	NPIP	15068613	2.9389	5.8822	-1.4302
YRI	NPIP	15073613	5.2916	8.235	-1.0539
YRI	NPIP	15078613	5.7925	9.7476	-1.2116
YRI	NPIP	15083613	5.9028	10.0837	-1.241
YRI	NPIP	15088613	5.6255	9.2434	-1.1643
YRI	NPIP	15093613	4.2625	8.067	-1.3883
YRI	NPIP	15098613	4.3377	8.067	-1.3608
YRI	NPIP	15103613	5.4972	8.5712	-1.0608
YRI	NPIP	15108613	5.0337	8.7392	-1.2561
YRI	NPIP	15113613	5.0267	10.0837	-1.501
YRI	NPIP	15118613	6.6785	12.7727	-1.4488
YRI	NPIP	15123613	6.4632	11.5963	-1.3367
YRI	NPIP	15128613	8.5878	10.0837	-0.444
YRI	NPIP	15133613	9.7173	11.2602	-0.413
YRI	NPIP	15138613	7.1154	10.756	-1.0174
YRI	NPIP	15143613	6.7214	9.0754	-0.7706
YRI	NPIP	15148613	6.1259	8.5712	-0.8439
YRI	NPIP	15153613	6.6917	9.9157	-0.972
YRI	NPIP	15158613	6.6226	9.0754	-0.8029
YRI	NPIP	15163613	5.05	8.4031	-1.1785
YRI	NPIP	15168613	8.6178	10.5879	-0.5587
YRI	NPIP	15173613	9.0562	10.2518	-0.3494
YRI	NPIP	15178613	5.1121	8.7392	-1.2295
YRI	NPIP	15183613	4.857	8.4031	-1.2463
YRI	NPIP	15188613	3.8671	6.5544	-1.1848
YRI	NPIP	15193613	4.1269	9.2434	-1.6466
YRI	NPIP	15198613	4.5024	9.4115	-1.5537
YRI	NPIP	15203613	4.1128	6.2183	-0.9734
YRI	NPIP	15208613	4.1688	5.0419	-0.4865
YRI	NPIP	15213613	2.9807	5.0419	-1.1484
YRI	NPIP	15218613	7.4114	11.7644	-1.1183

YRI	NPIP	15223613	10.9856	18.991	-1.3037
YRI	NPIP	15228613	7.3994	14.2853	-1.4723
YRI	NPIP	15233613	5.7392	10.2518	-1.3189
YRI	NPIP	15238613	4.2244	11.5963	-1.9197
YRI	NPIP	15243613	3.2479	9.2434	-1.9295
YRI	NPIP	15248613	7.0807	9.7476	-0.817
YRI	NPIP	15253613	6.7026	8.067	-0.4979
YRI	NPIP	15258613	2.9648	5.2099	-1.2152
YRI	NPIP	15263613	3.4459	6.7225	-1.4119
YRI	NPIP	15268613	3.767	7.0586	-1.357
YRI	NPIP	15273613	3.4757	7.0586	-1.4771
YRI	NPIP	15278613	4.7243	6.7225	-0.861
YRI	NPIP	15283613	3.4958	5.378	-0.9906
YRI	NPIP	15288613	2.4542	5.5461	-1.5833
YRI	NPIP	15293613	2.693	5.8822	-1.5497
YRI	NPIP	15298613	6.2205	7.8989	-0.6244
YRI	NPIP	15303613	5.4345	6.7225	-0.555
YRI	NPIP	15308613	2.5649	4.2016	-1.0691
YRI	NPIP	15313613	5.6003	6.5544	-0.4206
YRI	NPIP	15318613	5.0106	7.3947	-0.9421
YRI	NPIP	15323613	6.6156	8.067	-0.5296
YRI	NPIP	15328613	5.069	5.0419	0.0151
YRI	NPIP	15333613	0.2705	1.6806	-1.9399
YRI	NPIP	15338613	0.6422	3.1932	-2.1039
YRI	NPIP	15343613	0.5543	2.689	-2.0287
YRI	NPIP	15348613	1.109	2.3529	-1.3167
YRI	NPIP	15353613	1.6904	3.1932	-1.2394
YRI	NPIP	15358613	2.3897	4.7057	-1.371
YRI	NPIP	15363613	1.8653	5.378	-1.8487
YRI	NPIP	15368613	1.6121	4.8738	-1.8723
YRI	NPIP	15373613	2.6329	5.2099	-1.3949
YRI	NPIP	15378613	5.8091	8.7392	-0.9932
YRI	NPIP	15383613	6.9714	10.0837	-0.9238
YRI	NPIP	15388613	3.6387	6.2183	-1.1925
YRI	NPIP	15393613	4.5877	7.7309	-1.1926
YRI	NPIP	15398613	5.8088	9.9157	-1.2382
YRI	NPIP	15403613	5.9488	8.7392	-0.9459
YRI	NPIP	15408613	5.8944	7.0586	-0.4799
YRI	NPIP	15413613	5.911	7.2267	-0.5309
YRI	NPIP	15418613	4.175	6.5544	-1.049
YRI	NPIP	15423613	1.3971	3.6974	-1.6766
YRI	NPIP	15428613	1.0487	2.8571	-1.6354
YRI	NPIP	15433613	0.4989	2.1848	-1.8929
YRI	NPIP	15438613	0.5738	2.689	-2.0102
YRI	NPIP	15443613	0.9413	3.0251	-1.7976
YRI	NPIP	15448613	1.4574	4.0335	-1.743
YRI	NPIP	15453613	1.8158	4.7057	-1.7107
YRI	NPIP	15458613	2.5518	5.2099	-1.4388
YRI	NPIP	15463613	3.3881	6.7225	-1.4368

YRI	NPIP	15468613	4.7679	8.5712	-1.3125
YRI	NPIP	15473613	6.6583	10.0837	-1.0167
YRI	NPIP	15478613	6.2816	10.756	-1.2504
YRI	NPIP	15483613	5.2837	9.9157	-1.3966
YRI	NPIP	15488613	7.4899	10.5879	-0.8786
YRI	NPIP	15493613	7.1362	11.2602	-1.104
YRI	NPIP	15498613	8.0639	12.4366	-1.0661
YRI	NPIP	15503613	9.6203	13.2769	-0.838
YRI	NPIP	15508613	10.0432	13.1089	-0.7111
YRI	NPIP	15513613	11.2903	13.1089	-0.4219
YRI	NPIP	15518613	11.4271	13.2769	-0.4239
YRI	NPIP	15523613	9.6186	15.6298	-1.1797
YRI	NPIP	15528613	6.384	15.2937	-1.7852
YRI	NPIP	15533613	5.9236	12.6047	-1.6083
YRI	NPIP	15538613	6.9742	12.1005	-1.2825
YRI	NPIP	15543613	8.6479	13.445	-1.0864
YRI	NPIP	15548613	9.5229	13.7811	-0.942
YRI	NPIP	15553613	10.0781	12.7727	-0.6406
YRI	NPIP	15558613	9.0055	10.756	-0.4892
YRI	NPIP	15563613	8.5529	11.9324	-0.8567
YRI	NPIP	15568613	7.8694	10.5879	-0.771
YRI	NPIP	15573613	6.5663	10.756	-1.1708
YRI	NPIP	15578613	5.4762	11.2602	-1.5484
YRI	NPIP	15583613	7.6845	9.4115	-0.5466
YRI	NPIP	15588613	8.6531	11.4282	-0.7326
YRI	NPIP	15593613	6.8788	13.613	-1.5072
YRI	NPIP	15598613	10.6267	13.9492	-0.7266
YRI	NPIP	15603613	10.3358	14.2853	-0.8444
YRI	NPIP	15608613	9.0058	17.1423	-1.4619
YRI	NPIP	15613613	10.0642	16.6382	-1.2154
YRI	NPIP	15618613	9.7799	13.7811	-0.8852
YRI	NPIP	15623613	12.1568	13.613	-0.3259
YRI	NPIP	15628613	12.1709	14.9575	-0.5703
YRI	NPIP	15633613	8.9436	13.2769	-0.9931
YRI	NPIP	15638613	8.81	11.4282	-0.6912
YRI	NPIP	15643613	9.1838	12.7727	-0.8532
YRI	NPIP	15648613	7.6625	12.9408	-1.2394
YRI	NPIP	15653613	7.985	11.5963	-0.9404
YRI	NPIP	15658613	9.4979	10.756	-0.3516
YRI	NPIP	15663613	10.0187	11.5963	-0.4108
YRI	NPIP	15668613	11.2454	15.2937	-0.8112
YRI	NPIP	15673613	11.8711	15.2937	-0.6858
YRI	NPIP	15678613	8.7619	11.4282	-0.7039
YRI	NPIP	15683613	9.1079	12.7727	-0.8713
YRI	NPIP	15688613	7.0737	12.4366	-1.3075
YRI	NPIP	15693613	4.4553	9.7476	-1.6212
YRI	NPIP	15698613	5.8589	10.4199	-1.313
YRI	NPIP	15703613	8.4748	12.1005	-0.9071
YRI	NPIP	15708613	8.1781	10.2518	-0.6061

YRI	NPIP	15713613	5.0113	8.4031	-1.1921
YRI	NPIP	15718613	5.8575	10.4199	-1.3134
YRI	NPIP	15723613	5.6953	9.2434	-1.1419
YRI	NPIP	15728613	5.5382	7.2267	-0.6813
YRI	NPIP	15733613	6.0751	9.2434	-1.0196
YRI	NPIP	15738613	5.6721	10.924	-1.4465
YRI	NPIP	15743613	5.393	13.1089	-1.7898
YRI	NPIP	15748613	4.5907	12.2685	-1.896
YRI	NPIP	15753613	5.2239	12.1005	-1.7204
YRI	NPIP	15758613	8.0588	15.6298	-1.4859
YRI	NPIP	15763613	9.0409	15.1256	-1.2321
YRI	NPIP	15768613	8.0819	13.7811	-1.2608
YRI	NPIP	15773613	8.3889	12.6047	-1.0149
YRI	NPIP	15778613	7.4995	11.2602	-1.0067
YRI	NPIP	15783613	7.5013	11.7644	-1.0952
YRI	NPIP	15788613	12.2618	15.9659	-0.7123
YRI	NPIP	15793613	13.4391	17.6465	-0.7352
YRI	NPIP	15798613	11.4258	14.4534	-0.6402
YRI	NPIP	15803613	12.3591	15.6298	-0.6419
YRI	NPIP	15808613	11.8052	16.4701	-0.8709
YRI	NPIP	15813613	11.1411	15.7978	-0.9046
YRI	NPIP	15818613	13.0337	17.4785	-0.7839
YRI	NPIP	15823613	12.1755	17.9827	-0.9966
YRI	NPIP	15828613	7.9934	14.2853	-1.3453
YRI	NPIP	15833613	7.1161	11.9324	-1.2209
YRI	NPIP	15838613	10.7041	12.9408	-0.5252
YRI	NPIP	15843613	12.9266	13.445	-0.1174
YRI	NPIP	15848613	12.0262	14.7895	-0.5716
YRI	NPIP	15853613	9.65	12.2685	-0.6466
YRI	NPIP	15858613	10.0807	12.6047	-0.6076
YRI	NPIP	15863613	11.2808	15.1256	-0.7785
YRI	NPIP	15868613	15.3683	17.4785	-0.3721
YRI	NPIP	15873613	15.1715	17.4785	-0.4069
CEU	NPIP	15884140	14.884	13.645	0.2784
CEU	NPIP	15889140	12.7221	10.0632	0.7957
CEU	NPIP	15894140	8.5436	7.5047	0.4076
CEU	NPIP	15899140	10.7721	9.5515	0.3835
CEU	NPIP	15904140	12.5068	11.0866	0.3882
CEU	NPIP	15909140	18.0852	15.1801	0.5899
CEU	NPIP	15914140	32.1735	21.832	1.4815
CEU	NPIP	15919140	26.2782	18.7619	1.2461
CEU	NPIP	15924140	13.5493	10.7454	0.7892
CEU	NPIP	15929140	13.5102	9.8926	1.1
CEU	NPIP	15934140	7.9765	7.6753	0.1158
CEU	NPIP	15939140	10.0495	7.5047	0.9985
CEU	NPIP	15944140	15.7444	9.0398	2.2167
CEU	NPIP	15949140	18.3884	11.5982	1.7791
CEU	NPIP	15954140	14.9029	12.4511	0.6009
CEU	NPIP	15959140	15.7588	16.2034	-0.0848

CEU	NPIP	15964140	18.7906	19.7852	-0.1567
CEU	NPIP	15969140	15.1045	18.0796	-0.5111
CEU	NPIP	15974140	18.4593	19.7852	-0.2089
CEU	NPIP	15979140	20.2449	17.5679	0.4727
CEU	NPIP	15984140	14.987	11.5982	0.8879
CEU	NPIP	15989140	9.6219	8.5281	0.3816
CEU	NPIP	15994140	7.8613	7.6753	0.0715
CEU	NPIP	15999140	10.6273	8.3576	0.8068
CEU	NPIP	16004140	10.4098	9.0398	0.453
CEU	NPIP	16009140	6.7739	9.7221	-0.9111
CEU	NPIP	16014140	4.0695	9.0398	-1.6433
CEU	NPIP	16019140	2.4028	7.8459	-2.0505
CEU	NPIP	16024140	2.9695	5.2874	-1.2463
CEU	NPIP	16029140	3.5935	3.0701	0.449
CEU	NPIP	16034140	5.7887	5.1169	0.3719
CEU	NPIP	16039140	9.2171	7.8459	0.5165
CEU	NPIP	16044140	9.2863	8.6987	0.2013
CEU	NPIP	16049140	8.1575	7.3342	0.3299
CEU	NPIP	16054140	7.3898	6.1402	0.588
CEU	NPIP	16059140	9.3385	8.3576	0.3487
CEU	NPIP	16064140	9.6001	8.8692	0.2459
CEU	NPIP	16069140	8.6914	8.6987	-0.0025
CEU	NPIP	16074140	11.8376	11.4277	0.1089
CEU	NPIP	16079140	11.8765	11.7688	0.0278
CEU	NPIP	16084140	10.4271	11.4277	-0.2658
CEU	NPIP	16089140	9.5968	11.0866	-0.4072
CEU	NPIP	16094140	21.3426	13.9861	1.6149
CEU	NPIP	16099140	27.8907	15.3506	2.5195
CEU	NPIP	16104140	29.195	17.7385	2.0045
CEU	NPIP	16109140	23.061	15.8623	1.4018
CEU	NPIP	16114140	9.5883	10.2337	-0.1901
CEU	NPIP	16119140	8.8167	10.5749	-0.5023
CEU	NPIP	16124140	10.7739	9.7221	0.325
CEU	NPIP	16129140	12.0616	9.7221	0.723
CEU	NPIP	16134140	10.2463	9.7221	0.162
CEU	NPIP	16139140	9.9633	10.2337	-0.0797
CEU	NPIP	16144140	10.6514	10.5749	0.0219
CEU	NPIP	16149140	9.0693	9.3809	-0.0995
CEU	NPIP	16154140	6.8879	7.8459	-0.3609
CEU	NPIP	16159140	11.5918	10.4043	0.3445
CEU	NPIP	16164140	13.4968	11.4277	0.5497
CEU	NPIP	16169140	12.0008	11.2571	0.2004
CEU	NPIP	16174140	11.3236	11.5982	-0.0719
CEU	NPIP	16179140	11.1519	9.8926	0.3829
CEU	NPIP	16184140	12.45	10.2337	0.6529
CEU	NPIP	16189140	10.2261	9.2104	0.33
CEU	NPIP	16194140	10.8194	12.6216	-0.436
CEU	NPIP	16199140	14.2268	15.1801	-0.1936
CEU	NPIP	16204140	17.5281	15.0095	0.517

CEU	NPIP	16209140	11.872	12.1099	-0.0598
CEU	NPIP	16214140	3.5584	6.6519	-1.3544
CEU	NPIP	16219140	4.9877	8.0164	-1.1187
CEU	NPIP	16224140	8.5604	11.0866	-0.6905
CEU	NPIP	16229140	9.8881	10.5749	-0.1962
CEU	NPIP	16234140	8.7685	8.187	0.2107
CEU	NPIP	16239140	8.2552	8.3576	-0.0364
CEU	NPIP	16244140	9.1235	11.4277	-0.6122
CEU	NPIP	16249140	11.3059	12.7922	-0.3551
CEU	NPIP	16254140	10.1499	11.0866	-0.256
CEU	NPIP	16259140	11.7295	12.2805	-0.1368
CEU	NPIP	16264140	14.4585	13.1333	0.3088
CEU	NPIP	16269140	11.3251	11.2571	0.0183
CEU	NPIP	16274140	12.2527	13.8156	-0.3471
CEU	NPIP	16279140	14.9602	12.7922	0.5179
CEU	NPIP	16284140	16.3577	11.5982	1.247
CEU	NPIP	16289140	24.1864	15.5212	1.7227
CEU	NPIP	16294140	21.7215	14.1567	1.6416
CEU	NPIP	16299140	7.9316	9.3809	-0.463
CEU	NPIP	16304140	5.4016	9.7221	-1.3352
CEU	NPIP	16309140	5.651	9.3809	-1.1916
CEU	NPIP	16314140	2.7469	5.6286	-1.4658
CEU	NPIP	16319140	2.6491	5.9697	-1.6026
CEU	NPIP	16324140	4.0631	7.3342	-1.3106
CEU	NPIP	16329140	3.024	5.9697	-1.4216
CEU	NPIP	16334140	1.1413	2.729	-1.5007
CEU	NPIP	16339140	1.1521	2.0467	-1.0645
CEU	NPIP	16344140	1.8126	3.0701	-1.0787
CEU	NPIP	16349140	3.2338	5.2874	-1.1042
CEU	NPIP	16354140	4.9274	8.8692	-1.3264
CEU	NPIP	16359140	5.2632	9.2104	-1.2826
CEU	NPIP	16364140	4.9528	5.7991	-0.4192
CEU	NPIP	16369140	2.9423	1.8762	1.3573
CEU	NPIP	16374140	0.5314	0.5117	0.0618
CEU	NPIP	16379140	0.0501	0.5117	-1.4477
CEU	NPIP	16384140	0.3531	0.3411	0.0481
CEU	NPIP	16389140	0.343	0.1706	1.0241
CEU	NPIP	16394140	0	0	0
CEU	NPIP	16399140	0	0	0
CEU	NPIP	16404140	0	0	0
CEU	NPIP	16409140	0	0	0
CEU	NPIP	16414140	0.0901	0.5117	-1.3222
CEU	NPIP	16419140	0.5366	1.3645	-1.3394
CEU	NPIP	16424140	0.584	1.5351	-1.4103
CEU	NPIP	16429140	0.1476	0.8528	-1.5881
CEU	NPIP	16434140	0.0202	0.3411	-1.2864
CEU	NPIP	16439140	0.0101	0.1706	-0.9534
CEU	NPIP	16444140	0	0	0
CEU	NPIP	16449140	1.2946	1.8762	-0.7405

CEU	NPIP	16454140	1.698	3.2407	-1.265
CEU	NPIP	16459140	0.7838	4.6052	-2.3201
CEU	NPIP	16464140	1.7788	6.4814	-2.1078
CEU	NPIP	16469140	1.4477	3.4112	-1.5422
CEU	NPIP	16474140	0.0493	0.1706	-0.7205
CEU	NPIP	16479140	0.5129	0.8528	-0.7654
CEU	NPIP	16484140	0.5129	0.8528	-0.7654
CEU	NPIP	16489140	0	0	0
CEU	NPIP	16494140	0.4919	0.6822	-0.4969
CEU	NPIP	16499140	1.1575	1.1939	-0.0649
CEU	NPIP	16504140	0.7351	0.8528	-0.2651
CEU	NPIP	16509140	2.0355	2.0467	-0.0133
CEU	NPIP	16514140	3.9173	2.729	1.1232
CEU	NPIP	16519140	2.0416	2.0467	-0.0061
CEU	NPIP	16524140	0.1105	1.3645	-2.0287
CEU	NPIP	16529140	0.5902	1.0234	-0.8608
CEU	NPIP	16534140	2.9021	2.5584	0.3424
CEU	NPIP	16539140	4.1229	4.6052	-0.2928
CEU	NPIP	16544140	3.4323	3.9229	-0.3422
CEU	NPIP	16549140	2.1548	1.5351	0.9189
CEU	NPIP	16554140	3.6007	3.4112	0.1488
CEU	NPIP	16559140	6.2113	8.3576	-0.7629
CEU	NPIP	16564140	7.5799	11.0866	-0.9585
CEU	NPIP	16569140	8.9817	9.0398	-0.0192
CEU	NPIP	16574140	7.4133	5.2874	1.1431
CEU	NPIP	16579140	4.7815	3.4112	1.0763
CEU	NPIP	16584140	5.932	4.6052	0.8056
CEU	NPIP	16589140	5.6632	4.4346	0.7709
CEU	NPIP	16594140	2.6217	2.0467	0.6842
CEU	NPIP	16599140	1.1606	1.5351	-0.5553
CEU	NPIP	16604140	0.9546	1.8762	-1.1734
CEU	NPIP	16609140	0.8009	1.3645	-0.9118
CEU	NPIP	16614140	2.545	2.5584	-0.0133
CEU	NPIP	16619140	4.5774	4.7757	-0.1166
CEU	NPIP	16624140	5.06	5.9697	-0.439
CEU	NPIP	16629140	11.1371	8.5281	0.9103
CEU	NPIP	16634140	17.4471	10.7454	1.8863
CEU	NPIP	16639140	13.637	10.7454	0.8139
CEU	NPIP	16644140	10.126	10.4043	-0.0807
CEU	NPIP	16649140	6.76	8.0164	-0.4641
CEU	NPIP	16654140	4.0771	5.9697	-0.9134
CEU	NPIP	16659140	4.3673	6.4814	-0.9476
CEU	NPIP	16664140	2.1849	5.2874	-1.6682
CEU	NPIP	16669140	5.4494	6.8225	-0.5875
CEU	NPIP	16674140	8.9507	13.1333	-0.9746
CEU	NPIP	16679140	8.7734	14.1567	-1.1682
CEU	NPIP	16684140	8.7277	10.2337	-0.4437
CEU	NPIP	16689140	6.1084	7.1636	-0.4319
CEU	NPIP	16694140	6.7516	5.9697	0.3774

CEU	NPIP	16699140	7.6428	5.9697	0.8075
CEU	NPIP	16704140	5.4108	5.458	-0.0247
CEU	NPIP	16709140	7.3185	6.8225	0.2122
CEU	NPIP	16714140	8.0324	7.5047	0.207
CEU	NPIP	16719140	5.4842	6.4814	-0.447
CEU	NPIP	16724140	6.4288	8.8692	-0.8212
CEU	NPIP	16729140	10.0831	11.9394	-0.4733
CEU	NPIP	16734140	7.4583	8.5281	-0.3733
CEU	NPIP	16739140	3.5345	4.7757	-0.73
CEU	NPIP	16744140	4.9437	5.6286	-0.3484
CEU	NPIP	16749140	7.6984	7.1636	0.2189
CEU	NPIP	16754140	5.8986	5.1169	0.4327
CEU	NPIP	16759140	2.1758	2.0467	0.1536
CEU	NPIP	16764140	1.1608	1.0234	0.273
CEU	NPIP	16769140	1.4826	1.7056	-0.3054
CEU	NPIP	16774140	2.1356	2.0467	0.1058
CEU	NPIP	16779140	7.6753	4.0935	2.4085
CEU	NPIP	16784140	10.1282	6.3108	1.7526
CEU	NPIP	16789140	4.2507	3.9229	0.2286
CEU	NPIP	16794140	3.375	3.2407	0.1101
CEU	NPIP	16799140	3.6139	2.729	0.8364
CEU	NPIP	16804140	2.0247	1.5351	0.726
CEU	NPIP	16809140	3.4609	3.0701	0.3352
CEU	NPIP	16814140	2.5067	2.2173	0.3233
CEU	NPIP	16819140	0.7227	1.0234	-0.5975
CEU	NPIP	16824140	0.719	1.3645	-1.0443
CEU	NPIP	16829140	4.4334	3.9229	0.3561
CEU	NPIP	16834140	8.3252	6.8225	0.643
CEU	NPIP	16839140	7.2577	6.6519	0.2652
CEU	NPIP	16844140	8.8417	7.8459	0.3751
CEU	NPIP	16849140	10.7821	8.0164	1.0215
CEU	NPIP	16854140	9.568	8.187	0.5003
CEU	NPIP	16859140	8.1669	9.8926	-0.5247
CEU	NPIP	16864140	10.9032	12.1099	-0.3036
CEU	NPIP	16869140	15.473	13.645	0.4108
CEU	NPIP	16874140	11.785	9.8926	0.5754
CEU	NPIP	16879140	5.9184	7.5047	-0.6224
CEU	NPIP	16884140	5.2422	6.6519	-0.6172
CHB	NPIP	15884140	10.5448	13.5532	-0.6785
CHB	NPIP	15889140	8.3255	11.8591	-0.904
CHB	NPIP	15894140	6.8003	9.1484	-0.7656
CHB	NPIP	15899140	10.3432	10.6731	-0.0932
CHB	NPIP	15904140	12.1932	13.7226	-0.3409
CHB	NPIP	15909140	16.7116	16.7721	-0.0111
CHB	NPIP	15914140	29.5907	23.0404	0.8882
CHB	NPIP	15919140	23.6623	21.6851	0.2843
CHB	NPIP	15924140	12.3899	12.7061	-0.0758
CHB	NPIP	15929140	12.7719	8.4708	1.5057
CHB	NPIP	15934140	8.0996	7.6237	0.1835

CHB	NPIP	15939140	11.8679	8.979	0.9583
CHB	NPIP	15944140	18.913	10.6731	2.3273
CHB	NPIP	15949140	23.2696	13.5532	2.1912
CHB	NPIP	15954140	21.5361	13.5532	1.8003
CHB	NPIP	15959140	27.0179	15.7556	2.2009
CHB	NPIP	15964140	33.2749	18.2968	2.5366
CHB	NPIP	15969140	29.5035	16.7721	2.3437
CHB	NPIP	15974140	33.337	18.9745	2.3488
CHB	NPIP	15979140	29.9086	17.1109	2.3112
CHB	NPIP	15984140	17.8398	12.3673	1.3458
CHB	NPIP	15989140	9.5876	8.979	0.2019
CHB	NPIP	15994140	6.5496	7.4543	-0.3561
CHB	NPIP	15999140	8.8145	8.1319	0.2481
CHB	NPIP	16004140	8.2502	9.6567	-0.4361
CHB	NPIP	16009140	5.4059	11.5202	-1.6075
CHB	NPIP	16014140	3.4019	9.8261	-1.96
CHB	NPIP	16019140	2.0348	8.3013	-2.2349
CHB	NPIP	16024140	3.719	7.7931	-1.5398
CHB	NPIP	16029140	4.5254	5.9295	-0.6797
CHB	NPIP	16034140	6.0857	6.7766	-0.2966
CHB	NPIP	16039140	9.7474	8.979	0.2549
CHB	NPIP	16044140	10.1708	10.6731	-0.1419
CHB	NPIP	16049140	9.1738	10.6731	-0.4235
CHB	NPIP	16054140	8.2962	8.4708	-0.0611
CHB	NPIP	16059140	10.5698	10.8426	-0.0759
CHB	NPIP	16064140	11.3377	11.6896	-0.0913
CHB	NPIP	16069140	9.7702	11.1814	-0.3816
CHB	NPIP	16074140	15.5077	13.892	0.3559
CHB	NPIP	16079140	14.5747	14.7391	-0.0342
CHB	NPIP	16084140	10.0967	14.0615	-0.8634
CHB	NPIP	16089140	9.8951	12.0285	-0.5386
CHB	NPIP	16094140	21.4754	15.4168	1.2088
CHB	NPIP	16099140	27.4475	16.2638	2.1203
CHB	NPIP	16104140	23.056	17.1109	1.0736
CHB	NPIP	16109140	17.6484	14.4003	0.6915
CHB	NPIP	16114140	9.8521	8.8096	0.352
CHB	NPIP	16119140	8.2452	9.4872	-0.3915
CHB	NPIP	16124140	9.087	8.4708	0.2157
CHB	NPIP	16129140	12.2469	9.1484	1.0102
CHB	NPIP	16134140	12.8778	10.3343	0.7404
CHB	NPIP	16139140	11.6162	11.012	0.1657
CHB	NPIP	16144140	9.3849	9.8261	-0.1346
CHB	NPIP	16149140	8.3788	8.4708	-0.0322
CHB	NPIP	16154140	7.2604	9.3178	-0.6595
CHB	NPIP	16159140	11.9933	11.6896	0.0788
CHB	NPIP	16164140	13.8688	11.5202	0.6175
CHB	NPIP	16169140	11.4279	11.3508	0.0206
CHB	NPIP	16174140	11.2121	11.6896	-0.1238
CHB	NPIP	16179140	11.4457	10.3343	0.3235

CHB	NPIP	16184140	13.1952	10.5037	0.7716
CHB	NPIP	16189140	10.91	9.8261	0.3307
CHB	NPIP	16194140	10.9846	13.045	-0.4818
CHB	NPIP	16199140	14.0626	15.7556	-0.3308
CHB	NPIP	16204140	15.5037	16.6027	-0.2043
CHB	NPIP	16209140	11.2001	14.4003	-0.6813
CHB	NPIP	16214140	5.7674	9.1484	-1.1023
CHB	NPIP	16219140	5.9409	7.7931	-0.7
CHB	NPIP	16224140	8.4642	10.1649	-0.5027
CHB	NPIP	16229140	9.6722	10.6731	-0.2827
CHB	NPIP	16234140	8.2846	8.8096	-0.1773
CHB	NPIP	16239140	6.6827	9.6567	-0.9222
CHB	NPIP	16244140	7.517	10.8426	-0.9255
CHB	NPIP	16249140	11.3782	11.3508	0.0073
CHB	NPIP	16254140	9.9519	9.6567	0.0915
CHB	NPIP	16259140	9.1316	11.1814	-0.5542
CHB	NPIP	16264140	12.3005	13.3838	-0.2472
CHB	NPIP	16269140	12.3042	11.5202	0.2061
CHB	NPIP	16274140	12.148	14.4003	-0.4795
CHB	NPIP	16279140	10.8253	13.3838	-0.5839
CHB	NPIP	16284140	16.015	11.3508	1.2435
CHB	NPIP	16289140	23.6648	15.4168	1.6456
CHB	NPIP	16294140	18.1498	12.8755	1.2487
CHB	NPIP	16299140	6.3044	9.3178	-0.9659
CHB	NPIP	16304140	2.4562	11.1814	-2.3592
CHB	NPIP	16309140	3.6119	9.3178	-1.829
CHB	NPIP	16314140	2.771	5.0825	-1.2833
CHB	NPIP	16319140	2.5347	4.913	-1.3604
CHB	NPIP	16324140	2.7984	6.0989	-1.558
CHB	NPIP	16329140	1.5926	5.4213	-2.0077
CHB	NPIP	16334140	0.5652	3.2189	-2.1817
CHB	NPIP	16339140	0.4862	1.8636	-1.7574
CHB	NPIP	16344140	0.6314	1.8636	-1.5721
CHB	NPIP	16349140	1.6621	4.7436	-1.8177
CHB	NPIP	16354140	2.6433	6.946	-1.8061
CHB	NPIP	16359140	2.3981	7.2848	-1.9643
CHB	NPIP	16364140	2.3516	5.5907	-1.6527
CHB	NPIP	16369140	1.83	1.6942	0.1864
CHB	NPIP	16374140	0.4872	0.1694	1.8884
CHB	NPIP	16379140	0	0	0
CHB	NPIP	16384140	0.1611	0.1694	-0.0493
CHB	NPIP	16389140	0.1611	0.1694	-0.0493
CHB	NPIP	16394140	0	0	0
CHB	NPIP	16399140	0	0	0
CHB	NPIP	16404140	0	0	0
CHB	NPIP	16409140	0	0	0
CHB	NPIP	16414140	0.8506	1.0165	-0.3302
CHB	NPIP	16419140	1.3838	1.6942	-0.426
CHB	NPIP	16424140	0.6967	1.8636	-1.4888

CHB	NPIP	16429140	0.1732	1.3553	-1.9158
CHB	NPIP	16434140	0.0097	0.1694	-0.949
CHB	NPIP	16439140	0	0	0
CHB	NPIP	16444140	0	0	0
CHB	NPIP	16449140	1.5366	2.033	-0.592
CHB	NPIP	16454140	1.6967	2.8801	-1.067
CHB	NPIP	16459140	0.4116	4.066	-2.4643
CHB	NPIP	16464140	1.5842	4.7436	-1.8637
CHB	NPIP	16469140	1.3328	1.5247	-0.2851
CHB	NPIP	16474140	0	0	0
CHB	NPIP	16479140	0.1321	0.5082	-1.1806
CHB	NPIP	16484140	0.1321	0.5082	-1.1806
CHB	NPIP	16489140	0.0377	0.1694	-0.7826
CHB	NPIP	16494140	0.565	0.6777	-0.2946
CHB	NPIP	16499140	0.8104	1.0165	-0.4102
CHB	NPIP	16504140	0.2831	0.5082	-0.7066
CHB	NPIP	16509140	2.1732	1.5247	0.9634
CHB	NPIP	16514140	4.0927	3.3883	0.5547
CHB	NPIP	16519140	1.968	2.7106	-0.7037
CHB	NPIP	16524140	0.1797	1.5247	-1.9982
CHB	NPIP	16529140	0.6353	0.8471	-0.4776
CHB	NPIP	16534140	2.8016	1.5247	1.897
CHB	NPIP	16539140	4.1858	3.2189	0.7949
CHB	NPIP	16544140	3.5146	3.5577	-0.0326
CHB	NPIP	16549140	2.2024	2.5412	-0.3383
CHB	NPIP	16554140	3.5644	2.7106	0.809
CHB	NPIP	16559140	5.7912	4.7436	0.618
CHB	NPIP	16564140	7.1348	6.7766	0.1538
CHB	NPIP	16569140	8.8035	6.7766	0.8701
CHB	NPIP	16574140	6.9334	4.7436	1.2917
CHB	NPIP	16579140	4.1127	3.0495	0.9144
CHB	NPIP	16584140	5.071	4.4048	0.4192
CHB	NPIP	16589140	5.036	4.4048	0.3972
CHB	NPIP	16594140	2.6037	1.8636	0.9443
CHB	NPIP	16599140	1.0449	1.0165	0.0565
CHB	NPIP	16604140	1.0272	1.1859	-0.2833
CHB	NPIP	16609140	1.0088	1.1859	-0.3161
CHB	NPIP	16614140	2.0838	2.2024	-0.1328
CHB	NPIP	16619140	4.3634	4.066	0.2005
CHB	NPIP	16624140	5.7997	4.7436	0.623
CHB	NPIP	16629140	12.985	8.1319	1.764
CHB	NPIP	16634140	19.2001	10.8426	2.3259
CHB	NPIP	16639140	14.8542	10.5037	1.2473
CHB	NPIP	16644140	11.8626	10.5037	0.3896
CHB	NPIP	16649140	8.0944	8.4708	-0.1318
CHB	NPIP	16654140	4.3308	7.4543	-1.2295
CHB	NPIP	16659140	3.9812	7.6237	-1.4046
CHB	NPIP	16664140	1.9572	5.5907	-1.8539
CHB	NPIP	16669140	4.9391	6.4378	-0.6739

CHB	NPIP	16674140	8.3569	13.7226	-1.1959
CHB	NPIP	16679140	8.6528	15.2474	-1.3297
CHB	NPIP	16684140	8.5743	10.6731	-0.5928
CHB	NPIP	16689140	6.2229	7.2848	-0.4268
CHB	NPIP	16694140	7.9657	6.7766	0.5104
CHB	NPIP	16699140	8.9916	7.4543	0.6051
CHB	NPIP	16704140	5.7556	5.7601	-0.0022
CHB	NPIP	16709140	7.2677	6.4378	0.3732
CHB	NPIP	16714140	9.1226	7.7931	0.5025
CHB	NPIP	16719140	6.372	6.2684	0.0477
CHB	NPIP	16724140	5.7445	6.6072	-0.3789
CHB	NPIP	16729140	7.6172	11.012	-0.9312
CHB	NPIP	16734140	6.2463	9.3178	-0.9845
CHB	NPIP	16739140	4.754	5.2519	-0.2685
CHB	NPIP	16744140	5.6465	4.913	0.4196
CHB	NPIP	16749140	10.419	5.5907	2.4635
CHB	NPIP	16754140	8.6731	4.913	2.1508
CHB	NPIP	16759140	2.9654	2.3718	0.6266
CHB	NPIP	16764140	1.7179	1.1859	0.9496
CHB	NPIP	16769140	1.2303	1.5247	-0.4374
CHB	NPIP	16774140	1.8915	2.033	-0.1687
CHB	NPIP	16779140	5.3139	4.2354	0.7021
CHB	NPIP	16784140	6.6077	6.0989	0.2402
CHB	NPIP	16789140	3.1392	3.7271	-0.4271
CHB	NPIP	16794140	3.1234	3.2189	-0.0785
CHB	NPIP	16799140	3.9183	3.0495	0.7472
CHB	NPIP	16804140	2.6225	1.5247	1.6309
CHB	NPIP	16809140	4.9785	2.7106	2.149
CHB	NPIP	16814140	3.8355	2.033	2.1495
CHB	NPIP	16819140	0.945	0.8471	0.2208
CHB	NPIP	16824140	1.1443	1.0165	0.2543
CHB	NPIP	16829140	5.0453	3.5577	1.1243
CHB	NPIP	16834140	9.6547	6.6072	1.3385
CHB	NPIP	16839140	8.7853	6.6072	0.9566
CHB	NPIP	16844140	10.3429	8.8096	0.5177
CHB	NPIP	16849140	12.4847	10.5037	0.5679
CHB	NPIP	16854140	10.0366	11.8591	-0.4663
CHB	NPIP	16859140	7.2663	13.2144	-1.374
CHB	NPIP	16864140	13.1018	13.045	0.0133
CHB	NPIP	16869140	18.9485	15.0779	0.7888
CHB	NPIP	16874140	12.5293	12.1979	0.0826
CHB	NPIP	16879140	5.965	7.2848	-0.5305
CHB	NPIP	16884140	5.0891	7.1154	-0.8321
YRI	NPIP	15884140	18.0708	21.512	-0.497
YRI	NPIP	15889140	17.5142	20.5036	-0.4522
YRI	NPIP	15894140	14.722	18.1507	-0.5832
YRI	NPIP	15899140	17.4581	19.3272	-0.2993
YRI	NPIP	15904140	18.5016	19.9994	-0.2321
YRI	NPIP	15909140	20.4765	24.5371	-0.5163

YRI	NPIP	15914140	29.079	30.0831	-0.1047
YRI	NPIP	15919140	26.5232	25.7135	0.0984
YRI	NPIP	15924140	18.7485	18.6549	0.0155
YRI	NPIP	15929140	19.1627	19.3272	-0.0263
YRI	NPIP	15934140	14.9483	18.6549	-0.6141
YRI	NPIP	15939140	11.9114	16.8062	-0.8963
YRI	NPIP	15944140	11.2863	17.1423	-1.0521
YRI	NPIP	15949140	13.0608	18.6549	-0.9268
YRI	NPIP	15954140	14.9784	17.3104	-0.4151
YRI	NPIP	15959140	20.7559	20.3355	0.0641
YRI	NPIP	15964140	25.4491	23.6968	0.2305
YRI	NPIP	15969140	22.0792	22.6884	-0.0836
YRI	NPIP	15974140	26.8721	25.0413	0.2282
YRI	NPIP	15979140	31.0684	22.6884	1.1495
YRI	NPIP	15984140	21.698	15.9659	1.1024
YRI	NPIP	15989140	11.6532	13.613	-0.4386
YRI	NPIP	15994140	10.4288	14.9575	-0.9268
YRI	NPIP	15999140	12.9996	17.6465	-0.812
YRI	NPIP	16004140	13.4615	18.823	-0.8806
YRI	NPIP	16009140	12.0649	18.6549	-1.0917
YRI	NPIP	16014140	9.6254	13.9492	-0.9456
YRI	NPIP	16019140	8.8377	14.6214	-1.2096
YRI	NPIP	16024140	9.6237	15.6298	-1.1787
YRI	NPIP	16029140	7.9059	12.7727	-1.157
YRI	NPIP	16034140	9.2153	13.9492	-1.0353
YRI	NPIP	16039140	11.3651	14.1172	-0.5951
YRI	NPIP	16044140	11.414	15.1256	-0.7516
YRI	NPIP	16049140	11.6359	13.2769	-0.3761
YRI	NPIP	16054140	10.8195	13.445	-0.5946
YRI	NPIP	16059140	13.7079	18.1507	-0.7557
YRI	NPIP	16064140	15.8378	19.1591	-0.5363
YRI	NPIP	16069140	15.4424	17.9827	-0.4359
YRI	NPIP	16074140	18.7895	19.1591	-0.0597
YRI	NPIP	16079140	19.2183	20.3355	-0.1703
YRI	NPIP	16084140	17.2829	18.991	-0.2782
YRI	NPIP	16089140	15.4802	17.6465	-0.3786
YRI	NPIP	16094140	24.4904	21.0078	0.5146
YRI	NPIP	16099140	28.7818	21.8481	0.9864
YRI	NPIP	16104140	34.1555	22.6884	1.5729
YRI	NPIP	16109140	28.5013	18.1507	1.7605
YRI	NPIP	16114140	12.3226	12.2685	0.0134
YRI	NPIP	16119140	11.0135	12.6047	-0.383
YRI	NPIP	16124140	11.1984	13.1089	-0.4432
YRI	NPIP	16129140	15.1141	15.2937	-0.036
YRI	NPIP	16134140	15.8947	17.1423	-0.2242
YRI	NPIP	16139140	13.8688	17.8146	-0.6833
YRI	NPIP	16144140	13.8439	18.1507	-0.7325
YRI	NPIP	16149140	12.7197	17.6465	-0.8609
YRI	NPIP	16154140	10.7182	16.134	-1.0312

YRI	NPIP	16159140	14.4683	18.1507	-0.6263
YRI	NPIP	16164140	15.5814	17.4785	-0.3346
YRI	NPIP	16169140	15.6542	17.3104	-0.2948
YRI	NPIP	16174140	15.485	17.4785	-0.3516
YRI	NPIP	16179140	14.2434	16.134	-0.36
YRI	NPIP	16184140	14.239	17.6465	-0.5954
YRI	NPIP	16189140	10.4141	16.6382	-1.1507
YRI	NPIP	16194140	14.8744	22.0162	-1.0085
YRI	NPIP	16199140	20.7467	23.3606	-0.3486
YRI	NPIP	16204140	21.8414	20.5036	0.2024
YRI	NPIP	16209140	17.0299	19.1591	-0.3438
YRI	NPIP	16214140	7.5422	14.2853	-1.4417
YRI	NPIP	16219140	6.5707	13.9492	-1.6137
YRI	NPIP	16224140	9.8666	15.4617	-1.1095
YRI	NPIP	16229140	10.6051	14.9575	-0.8908
YRI	NPIP	16234140	9.6894	13.7811	-0.9052
YRI	NPIP	16239140	9.5431	15.9659	-1.2352
YRI	NPIP	16244140	10.3699	18.1507	-1.3234
YRI	NPIP	16249140	10.2651	16.302	-1.1381
YRI	NPIP	16254140	9.5415	14.6214	-1.0624
YRI	NPIP	16259140	16.4836	17.6465	-0.2032
YRI	NPIP	16264140	19.1194	18.6549	0.077
YRI	NPIP	16269140	14.5924	15.7978	-0.2342
YRI	NPIP	16274140	17.1784	18.6549	-0.2446
YRI	NPIP	16279140	17.2132	19.3272	-0.3385
YRI	NPIP	16284140	19.4179	20.3355	-0.1399
YRI	NPIP	16289140	25.7945	22.0162	0.5336
YRI	NPIP	16294140	19.3329	16.8062	0.4627
YRI	NPIP	16299140	8.4485	13.9492	-1.203
YRI	NPIP	16304140	5.8704	14.4534	-1.8148
YRI	NPIP	16309140	5.676	11.7644	-1.5641
YRI	NPIP	16314140	3.8588	7.8989	-1.503
YRI	NPIP	16319140	3.305	7.7309	-1.6793
YRI	NPIP	16324140	5.6777	10.4199	-1.3651
YRI	NPIP	16329140	5.3576	9.7476	-1.3448
YRI	NPIP	16334140	3.7777	6.5544	-1.2242
YRI	NPIP	16339140	3.1029	5.0419	-1.0804
YRI	NPIP	16344140	2.2836	5.0419	-1.5368
YRI	NPIP	16349140	4.5102	7.8989	-1.2607
YRI	NPIP	16354140	6.0961	10.756	-1.3022
YRI	NPIP	16359140	6.7882	10.4199	-1.0455
YRI	NPIP	16364140	6.0715	6.5544	-0.2129
YRI	NPIP	16369140	2.6224	2.1848	0.4913
YRI	NPIP	16374140	0.4597	0.1681	1.7335
YRI	NPIP	16379140	0.0367	0.1681	-0.7811
YRI	NPIP	16384140	0.3029	0.3361	-0.1333
YRI	NPIP	16389140	0.2662	0.1681	0.5832
YRI	NPIP	16394140	0.0185	0.1681	-0.8893
YRI	NPIP	16399140	0.0185	0.1681	-0.8893

YRI	NPIP	16404140	0	0	0
YRI	NPIP	16409140	0	0	0
YRI	NPIP	16414140	0.2719	1.6806	-1.938
YRI	NPIP	16419140	1.0127	2.8571	-1.668
YRI	NPIP	16424140	1.2003	3.0251	-1.5742
YRI	NPIP	16429140	0.496	2.0167	-1.8182
YRI	NPIP	16434140	0.0549	0.3361	-1.1289
YRI	NPIP	16439140	0.0183	0.1681	-0.8905
YRI	NPIP	16444140	0.055	0.5042	-1.4117
YRI	NPIP	16449140	1.306	3.0251	-1.483
YRI	NPIP	16454140	1.5228	4.2016	-1.7499
YRI	NPIP	16459140	1.5095	7.3947	-2.3256
YRI	NPIP	16464140	2.5358	10.2518	-2.2552
YRI	NPIP	16469140	1.326	4.8738	-2.0365
YRI	NPIP	16474140	0.0278	0.3361	-1.2377
YRI	NPIP	16479140	0.1833	1.6806	-2.0598
YRI	NPIP	16484140	0.1833	1.6806	-2.0598
YRI	NPIP	16489140	0	0	0
YRI	NPIP	16494140	0.4192	0.3361	0.3336
YRI	NPIP	16499140	0.6893	1.3445	-1.0642
YRI	NPIP	16504140	0.4318	2.0167	-1.895
YRI	NPIP	16509140	2.5108	3.6974	-0.8649
YRI	NPIP	16514140	5.1528	5.5461	-0.2014
YRI	NPIP	16519140	3.1363	4.3696	-0.7787
YRI	NPIP	16524140	0.9501	3.6974	-2.0024
YRI	NPIP	16529140	1.2094	3.6974	-1.8134
YRI	NPIP	16534140	2.4731	4.3696	-1.1975
YRI	NPIP	16539140	4.2505	5.7141	-0.7298
YRI	NPIP	16544140	5.1893	5.7141	-0.2617
YRI	NPIP	16549140	3.5728	4.2016	-0.4108
YRI	NPIP	16554140	4.2658	4.3696	-0.0655
YRI	NPIP	16559140	6.8244	8.5712	-0.6028
YRI	NPIP	16564140	7.9441	11.2602	-0.8877
YRI	NPIP	16569140	9.3415	8.7392	0.2042
YRI	NPIP	16574140	7.6621	6.0502	0.7637
YRI	NPIP	16579140	5.1989	5.0419	0.0875
YRI	NPIP	16584140	4.6147	5.378	-0.4017
YRI	NPIP	16589140	4.0275	4.7057	-0.4015
YRI	NPIP	16594140	2.4984	2.5209	-0.0225
YRI	NPIP	16599140	1.0444	1.8487	-1.0288
YRI	NPIP	16604140	1.1245	2.3529	-1.3003
YRI	NPIP	16609140	1.0302	2.689	-1.5765
YRI	NPIP	16614140	2.2476	3.5293	-0.9718
YRI	NPIP	16619140	5.1447	5.5461	-0.2056
YRI	NPIP	16624140	6.5276	6.8906	-0.153
YRI	NPIP	16629140	12.8829	12.6047	0.067
YRI	NPIP	16634140	19.1283	15.7978	0.647
YRI	NPIP	16639140	16.0073	14.6214	0.2898
YRI	NPIP	16644140	13.6968	15.6298	-0.3794

YRI	NPIP	16649140	9.3854	12.6047	-0.775
YRI	NPIP	16654140	6.1336	11.0921	-1.3463
YRI	NPIP	16659140	6.6309	11.4282	-1.2665
YRI	NPIP	16664140	4.1711	9.5795	-1.6838
YRI	NPIP	16669140	5.7267	12.4366	-1.6359
YRI	NPIP	16674140	9.9573	19.6633	-1.5286
YRI	NPIP	16679140	10.5215	19.1591	-1.3947
YRI	NPIP	16684140	8.2577	13.7811	-1.2219
YRI	NPIP	16689140	6.2356	12.7727	-1.5541
YRI	NPIP	16694140	8.0157	13.1089	-1.1814
YRI	NPIP	16699140	8.6165	11.5963	-0.776
YRI	NPIP	16704140	6.3602	8.9073	-0.8483
YRI	NPIP	16709140	9.2535	10.0837	-0.2464
YRI	NPIP	16714140	10.2209	11.4282	-0.3187
YRI	NPIP	16719140	6.1983	10.5879	-1.2449
YRI	NPIP	16724140	8.4839	13.613	-1.148
YRI	NPIP	16729140	15.0462	21.1758	-0.8988
YRI	NPIP	16734140	11.4951	16.302	-0.9062
YRI	NPIP	16739140	6.0613	6.7225	-0.2849
YRI	NPIP	16744140	6.8471	7.5628	-0.2771
YRI	NPIP	16749140	10.4311	8.067	0.8627
YRI	NPIP	16754140	10.1762	7.5628	1.0117
YRI	NPIP	16759140	4.7911	5.8822	-0.5302
YRI	NPIP	16764140	1.7041	2.5209	-0.818
YRI	NPIP	16769140	1.4652	3.0251	-1.3457
YRI	NPIP	16774140	2.2704	4.3696	-1.3255
YRI	NPIP	16779140	5.4029	6.0502	-0.3067
YRI	NPIP	16784140	7.276	7.5628	-0.111
YRI	NPIP	16789140	4.1063	5.0419	-0.5213
YRI	NPIP	16794140	5.957	6.8906	-0.3934
YRI	NPIP	16799140	6.2035	6.3864	-0.0825
YRI	NPIP	16804140	2.1726	2.8571	-0.619
YRI	NPIP	16809140	4.2316	4.8738	-0.3686
YRI	NPIP	16814140	3.8451	3.5293	0.2394
YRI	NPIP	16819140	1.3106	1.3445	-0.0551
YRI	NPIP	16824140	1.4138	1.5126	-0.1471
YRI	NPIP	16829140	5.2751	6.8906	-0.6807
YRI	NPIP	16834140	9.7006	12.1005	-0.6004
YRI	NPIP	16839140	9.5884	9.9157	-0.0987
YRI	NPIP	16844140	11.954	12.2685	-0.0777
YRI	NPIP	16849140	14.2603	15.1256	-0.1752
YRI	NPIP	16854140	13.0683	14.6214	-0.3248
YRI	NPIP	16859140	10.3606	14.9575	-0.9408
YRI	NPIP	16864140	13.4835	15.6298	-0.4212
YRI	NPIP	16869140	18.6806	17.6465	0.1807
YRI	NPIP	16874140	13.6716	14.6214	-0.1986
YRI	NPIP	16879140	7.6731	10.0837	-0.7155
YRI	NPIP	16884140	8.0979	11.5963	-0.911
CEU	NPIP	17822919	10.859	9.5515	0.4108

CEU	NPIP	17827919	15.765	11.4277	1.1523
CEU	NPIP	17832919	14.0891	10.7454	0.9412
CEU	NPIP	17837919	9.1812	8.8692	0.105
CEU	NPIP	17842919	9.9728	8.8692	0.3714
CEU	NPIP	17847919	18.7135	11.5982	1.8643
CEU	NPIP	17852919	19.645	12.1099	1.8956
CEU	NPIP	17857919	14.022	10.2337	1.1161
CEU	NPIP	17862919	13.7381	9.0398	1.5534
CEU	NPIP	17867919	12.812	8.8692	1.3268
CEU	NPIP	17872919	13.303	7.5047	2.275
CEU	NPIP	17877919	12.2284	8.3576	1.376
CEU	NPIP	17882919	9.9029	10.0632	-0.048
CEU	NPIP	17887919	11.1699	9.7221	0.4474
CEU	NPIP	17892919	15.2152	11.5982	0.9477
CEU	NPIP	17897919	16.0478	12.1099	0.9907
CEU	NPIP	17902919	14.115	9.8926	1.2839
CEU	NPIP	17907919	14.3469	9.2104	1.6691
CEU	NPIP	17912919	17.8064	11.0866	1.8368
CEU	NPIP	17917919	18.9641	13.1333	1.3587
CEU	NPIP	17922919	17.7227	14.6684	0.6408
CEU	NPIP	17927919	16.4364	16.8857	-0.0824
CEU	NPIP	17932919	19.5986	21.832	-0.3199
CEU	NPIP	17937919	20.6615	20.8086	-0.0221
CEU	NPIP	17942919	14.4303	13.4744	0.2174
CEU	NPIP	17947919	12.4052	13.645	-0.2786
CEU	NPIP	17952919	11.5191	15.8623	-0.8457
CEU	NPIP	17957919	9.4663	14.4978	-1.0674
CEU	NPIP	17962919	9.9048	12.6216	-0.6573
CEU	NPIP	17967919	9.5927	10.2337	-0.1888
CEU	NPIP	17972919	7.0377	8.5281	-0.52
CEU	NPIP	17977919	7.531	10.4043	-0.8335
CEU	NPIP	17982919	9.1528	12.7922	-0.8694
CEU	NPIP	17987919	12.4212	12.6216	-0.0485
CEU	NPIP	17992919	20.6984	12.6216	1.9541
CEU	NPIP	17997919	21.6193	12.4511	2.2468
CEU	NPIP	18002919	18.7666	12.1099	1.6746
CEU	NPIP	18007919	13.4001	9.5515	1.2091
CEU	NPIP	18012919	7.475	7.6753	-0.077
CEU	NPIP	18017919	8.0262	7.3342	0.2773
CEU	NPIP	18022919	10.7807	8.3576	0.8613
CEU	NPIP	18027919	9.0763	8.187	0.3222
CEU	NPIP	18032919	4.1509	5.9697	-0.8778
CEU	NPIP	18037919	3.8802	6.3108	-1.1159
CEU	NPIP	18042919	7.4451	8.0164	-0.211
CEU	NPIP	18047919	8.7798	8.6987	0.0278
CEU	NPIP	18052919	7.6042	12.4511	-1.1878
CEU	NPIP	18057919	6.6865	12.6216	-1.4359
CEU	NPIP	18062919	3.8227	6.4814	-1.1917
CEU	NPIP	18067919	6.3255	6.1402	0.0872

CEU	NPIP	18072919	8.2872	7.3342	0.3818
CEU	NPIP	18077919	4.5821	5.6286	-0.5323
CEU	NPIP	18082919	3.0552	4.7757	-1.0119
CEU	NPIP	18087919	3.3766	4.7757	-0.8229
CEU	NPIP	18092919	4.4275	5.1169	-0.3816
CEU	NPIP	18097919	4.8033	5.7991	-0.4932
CEU	NPIP	18102919	4.9944	5.6286	-0.3226
CEU	NPIP	18107919	10.221	8.6987	0.5215
CEU	NPIP	18112919	11.7928	11.4277	0.097
CEU	NPIP	18117919	6.858	10.4043	-1.0288
CEU	NPIP	18122919	7.926	8.8692	-0.3174
CEU	NPIP	18127919	10.8	8.8692	0.6497
CEU	NPIP	18132919	12.3838	12.4511	-0.0165
CEU	NPIP	18137919	12.7598	11.7688	0.2561
CEU	NPIP	18142919	11.2187	8.3576	1.017
CEU	NPIP	18147919	15.3067	11.0866	1.1536
CEU	NPIP	18152919	18.3933	14.1567	0.9194
CEU	NPIP	18157919	12.4725	12.9627	-0.1156
CEU	NPIP	18162919	6.1532	9.2104	-0.9934
CEU	NPIP	18167919	10.2259	8.3576	0.6641
CEU	NPIP	18172919	13.4014	9.3809	1.2844
CEU	NPIP	18177919	9.8192	8.3576	0.5196
CEU	NPIP	18182919	7.7462	6.9931	0.3151
CEU	NPIP	18187919	4.5345	4.2641	0.1755
CEU	NPIP	18192919	4.4857	4.0935	0.2637
CEU	NPIP	18197919	5.7685	5.1169	0.3607
CEU	NPIP	18202919	3.133	3.0701	0.054
CEU	NPIP	18207919	3.7231	4.4346	-0.4464
CEU	NPIP	18212919	5.2344	5.7991	-0.2797
CEU	NPIP	18217919	3.223	2.5584	0.6621
CEU	NPIP	18222919	3.8792	2.2173	1.8566
CEU	NPIP	18227919	10.2727	5.2874	2.6806
CEU	NPIP	18232919	16.8819	9.0398	2.5928
CEU	NPIP	18237919	14.8586	8.6987	2.1104
CEU	NPIP	18242919	10.1864	6.6519	1.5475
CEU	NPIP	18247919	11.2113	8.5281	0.9362
CEU	NPIP	18252919	13.1147	12.9627	0.0359
CEU	NPIP	18257919	11.3431	13.3039	-0.4513
CEU	NPIP	18262919	12.4224	10.7454	0.472
CEU	NPIP	18267919	10.2865	8.0164	0.8385
CEU	NPIP	18272919	4.7864	4.0935	0.4659
CEU	NPIP	18277919	4.2375	2.729	1.4259
CEU	NPIP	18282919	7.6549	4.2641	2.2011
CEU	NPIP	18287919	11.5884	7.1636	1.8113
CEU	NPIP	18292919	10.9708	9.2104	0.572
CEU	NPIP	18297919	7.9567	7.8459	0.0417
CEU	NPIP	18302919	7.3537	7.3342	0.0078
CEU	NPIP	18307919	8.885	8.8692	0.0053
CEU	NPIP	18312919	9.3014	9.5515	-0.0786

CEU	NPIP	18317919	6.0992	6.9931	-0.374
CEU	NPIP	18322919	6.7287	5.6286	0.5596
CEU	NPIP	18327919	7.9806	7.3342	0.259
CEU	NPIP	18332919	4.3728	5.6286	-0.6388
CEU	NPIP	18337919	6.5864	5.9697	0.2976
CEU	NPIP	18342919	5.5749	5.458	0.0611
CEU	NPIP	18347919	4.5269	4.0935	0.2914
CEU	NPIP	18352919	7.6298	5.1169	1.3909
CEU	NPIP	18357919	5.2078	4.6052	0.3659
CEU	NPIP	18362919	2.5525	3.5818	-0.7758
CEU	NPIP	18367919	2.3206	2.729	-0.386
CEU	NPIP	18372919	1.8677	2.3879	-0.5478
CEU	NPIP	18377919	1.6155	2.8996	-1.1548
CEU	NPIP	18382919	0.7405	3.2407	-2.0501
CEU	NPIP	18387919	0.3206	2.0467	-2.0539
CEU	NPIP	18392919	0.142	0.3411	-0.7981
CEU	NPIP	18397919	0.0404	0.6822	-1.6759
CEU	NPIP	18402919	0.4613	2.0467	-1.8865
CEU	NPIP	18407919	0.7108	2.2173	-1.683
CEU	NPIP	18412919	0.2898	0.8528	-1.2679
CEU	NPIP	18417919	0.1262	0.3411	-0.8615
CEU	NPIP	18422919	0.1262	0.3411	-0.8615
CEU	NPIP	18427919	0.357	0.5117	-0.4852
CEU	NPIP	18432919	0.6836	1.0234	-0.6752
CEU	NPIP	18437919	0.5772	0.8528	-0.6206
CEU	NPIP	18442919	0.2507	0.3411	-0.3624
CEU	NPIP	18447919	0.3313	0.1706	0.9546
CEU	NPIP	18452919	0.3313	0.1706	0.9546
CEU	NPIP	18457919	0	0.1706	-1.0134
CEU	NPIP	18462919	0.0404	0.6822	-1.6759
CEU	NPIP	18467919	0.281	1.8762	-2.031
CEU	NPIP	18472919	0.9639	2.3879	-1.4996
CEU	NPIP	18477919	0.824	2.2173	-1.5566
CEU	NPIP	18482919	0.7213	2.2173	-1.6713
CEU	NPIP	18487919	0.8023	1.5351	-1.0866
CEU	NPIP	18492919	0.1816	0.5117	-1.0353
CEU	NPIP	18497919	0.4832	0.1706	1.8569
CEU	NPIP	18502919	0.4832	0.1706	1.8569
CEU	NPIP	18507919	0	0	0
CEU	NPIP	18512919	0.2253	0.3411	-0.4642
CEU	NPIP	18517919	0.2253	0.3411	-0.4642
CEU	NPIP	18522919	0	0	0
CEU	NPIP	18527919	0.3645	0.3411	0.0938
CEU	NPIP	18532919	0.5007	1.0234	-1.0386
CEU	NPIP	18537919	0.9551	2.8996	-1.7487
CEU	NPIP	18542919	1.1736	4.4346	-2.0461
CEU	NPIP	18547919	0.6112	4.0935	-2.3416
CEU	NPIP	18552919	1.463	2.729	-1.1966
CEU	NPIP	18557919	1.9582	2.3879	-0.4525

CEU	NPIP	18562919	1.1433	3.0701	-1.6528
CEU	NPIP	18567919	1.1342	4.0935	-1.9899
CEU	NPIP	18572919	2.7809	4.9463	-1.2349
CEU	NPIP	18577919	3.5863	4.2641	-0.44
CEU	NPIP	18582919	2.0954	4.6052	-1.5238
CEU	NPIP	18587919	1.2663	5.1169	-2.1314
CEU	NPIP	18592919	1.3851	5.2874	-2.0983
CEU	NPIP	18597919	2.4465	5.2874	-1.5276
CEU	NPIP	18602919	3.4298	5.6286	-1.1184
CEU	NPIP	18607919	2.9528	5.2874	-1.2553
CEU	NPIP	18612919	2.4596	3.9229	-1.0206
CEU	NPIP	18617919	1.3928	3.0701	-1.4388
CEU	NPIP	18622919	0.7638	2.3879	-1.7103
CEU	NPIP	18627919	1.363	3.2407	-1.5397
CEU	NPIP	18632919	1.9448	3.7524	-1.3096
CEU	NPIP	18637919	1.1872	1.8762	-0.8772
CEU	NPIP	18642919	0.1185	0.8528	-1.6536
CEU	NPIP	18647919	2.8293	2.5584	0.2699
CEU	NPIP	18652919	2.8489	2.5584	0.2894
CEU	NPIP	18657919	0.06	0.6822	-1.6248
CEU	NPIP	18662919	2.4304	1.8762	0.7056
CEU	NPIP	18667919	3.985	3.5818	0.3039
CEU	NPIP	18672919	2.941	3.0701	-0.1107
CEU	NPIP	18677919	2.1779	2.5584	-0.379
CEU	NPIP	18682919	1.0144	2.0467	-1.2283
CEU	NPIP	18687919	0.5902	1.5351	-1.4011
CEU	NPIP	18692919	0.4364	1.0234	-1.1664
CEU	NPIP	18697919	0.665	0.8528	-0.4229
CEU	NPIP	18702919	3.0763	2.3879	0.725
CEU	NPIP	18707919	3.5912	3.9229	-0.2314
CEU	NPIP	18712919	2.777	3.9229	-0.7992
CEU	NPIP	18717919	2.3018	2.8996	-0.5376
CEU	NPIP	18722919	5.1093	4.4346	0.4233
CEU	NPIP	18727919	10.9507	7.8459	1.1696
CEU	NPIP	18732919	12.5461	8.8692	1.2373
CEU	NPIP	18737919	9.0398	7.6753	0.5245
CEU	NPIP	18742919	10.5582	9.5515	0.3163
CEU	NPIP	18747919	11.1591	9.0398	0.7007
CEU	NPIP	18752919	12.4846	7.6753	1.8486
CEU	NPIP	18757919	22.2284	10.5749	3.3296
CEU	NPIP	18762919	26.3115	13.645	2.8465
CEU	NPIP	18767919	17.6384	13.8156	0.849
CEU	NPIP	18772919	8.2356	9.5515	-0.4134
CEU	NPIP	18777919	9.2968	8.8692	0.1439
CEU	NPIP	18782919	13.3026	11.2571	0.5512
CEU	NPIP	18787919	15.1878	11.9394	0.8282
CEU	NPIP	18792919	10.1919	8.6987	0.5116
CEU	NPIP	18797919	5.5469	6.6519	-0.4838
CEU	NPIP	18802919	7.0132	8.0164	-0.3705

CEU	NPIP	18807919	6.4568	8.0164	-0.576
CEU	NPIP	18812919	5.4232	7.5047	-0.8167
CEU	NPIP	18817919	5.3698	6.6519	-0.5613
CEU	NPIP	18822919	10.6195	9.7221	0.2773
CEU	NPIP	18827919	13.6432	12.4511	0.2921
CHB	NPIP	17822919	9.8745	12.0285	-0.5438
CHB	NPIP	17827919	14.7461	14.5697	0.0371
CHB	NPIP	17832919	13.828	12.8755	0.2255
CHB	NPIP	17837919	9.9313	12.1979	-0.5647
CHB	NPIP	17842919	11.0655	13.5532	-0.561
CHB	NPIP	17847919	17.9692	14.2309	0.8049
CHB	NPIP	17852919	18.0261	14.9085	0.6422
CHB	NPIP	17857919	14.3179	15.2474	-0.1874
CHB	NPIP	17862919	13.5052	12.5367	0.2351
CHB	NPIP	17867919	10.964	9.1484	0.592
CHB	NPIP	17872919	11.873	9.6567	0.6872
CHB	NPIP	17877919	12.3021	11.3508	0.2536
CHB	NPIP	17882919	9.6857	10.8426	-0.322
CHB	NPIP	17887919	8.8854	9.9955	-0.3333
CHB	NPIP	17892919	11.3705	13.2144	-0.4259
CHB	NPIP	17897919	10.9482	14.0615	-0.678
CHB	NPIP	17902919	9.8268	10.3343	-0.1477
CHB	NPIP	17907919	13.7287	10.3343	0.9881
CHB	NPIP	17912919	18.1399	12.0285	1.5428
CHB	NPIP	17917919	18.6691	12.1979	1.6122
CHB	NPIP	17922919	19.3581	12.3673	1.7192
CHB	NPIP	17927919	22.1449	13.2144	2.0629
CHB	NPIP	17932919	29.6368	16.9415	2.3146
CHB	NPIP	17937919	31.8132	18.1274	2.3385
CHB	NPIP	17942919	23.3391	15.0779	1.6835
CHB	NPIP	17947919	16.4806	14.0615	0.5268
CHB	NPIP	17952919	12.3292	14.7391	-0.5019
CHB	NPIP	17957919	9.2755	13.892	-1.017
CHB	NPIP	17962919	9.577	13.2144	-0.8402
CHB	NPIP	17967919	9.0626	11.6896	-0.6813
CHB	NPIP	17972919	8.4165	8.979	-0.1866
CHB	NPIP	17977919	10.8839	9.4872	0.4403
CHB	NPIP	17982919	11.9689	11.6896	0.0724
CHB	NPIP	17987919	14.42	13.045	0.3215
CHB	NPIP	17992919	22.6288	14.4003	1.7519
CHB	NPIP	17997919	22.8595	15.7556	1.3883
CHB	NPIP	18002919	19.8534	14.7391	1.065
CHB	NPIP	18007919	14.8841	10.5037	1.2558
CHB	NPIP	18012919	7.8755	7.4543	0.1658
CHB	NPIP	18017919	9.6581	7.4543	0.8675
CHB	NPIP	18022919	13.641	10.8426	0.7788
CHB	NPIP	18027919	10.5066	11.012	-0.1386
CHB	NPIP	18032919	5.948	8.979	-1.0055
CHB	NPIP	18037919	6.1812	8.3013	-0.7561

CHB	NPIP	18042919	9.7365	9.1484	0.1917
CHB	NPIP	18047919	11.1664	11.8591	-0.1772
CHB	NPIP	18052919	9.7763	17.6192	-1.3772
CHB	NPIP	18057919	8.0866	16.6027	-1.583
CHB	NPIP	18062919	4.431	9.8261	-1.6461
CHB	NPIP	18067919	7.9994	8.979	-0.325
CHB	NPIP	18072919	9.6577	8.6402	0.3498
CHB	NPIP	18077919	5.0815	6.4378	-0.6098
CHB	NPIP	18082919	3.9145	6.2684	-1.0841
CHB	NPIP	18087919	3.6429	8.4708	-1.6901
CHB	NPIP	18092919	2.8697	10.6731	-2.204
CHB	NPIP	18097919	2.5114	10.5037	-2.2914
CHB	NPIP	18102919	4.9962	7.6237	-1.0132
CHB	NPIP	18107919	11.9273	8.8096	1.0526
CHB	NPIP	18112919	12.541	9.4872	0.9626
CHB	NPIP	18117919	7.3625	9.1484	-0.5823
CHB	NPIP	18122919	8.2251	10.6731	-0.6914
CHB	NPIP	18127919	11.0418	12.7061	-0.399
CHB	NPIP	18132919	12.3583	13.7226	-0.3041
CHB	NPIP	18137919	12.4337	12.5367	-0.025
CHB	NPIP	18142919	11.3939	11.3508	0.0115
CHB	NPIP	18147919	16.3703	14.5697	0.3791
CHB	NPIP	18152919	18.9935	16.2638	0.5175
CHB	NPIP	18157919	12.3678	14.2309	-0.4012
CHB	NPIP	18162919	6.7542	10.5037	-1.075
CHB	NPIP	18167919	10.9328	8.979	0.6481
CHB	NPIP	18172919	13.0788	9.3178	1.2055
CHB	NPIP	18177919	9.5863	8.8096	0.2622
CHB	NPIP	18182919	8.7299	9.9955	-0.38
CHB	NPIP	18187919	5.0845	6.4378	-0.6085
CHB	NPIP	18192919	4.2353	3.2189	0.8356
CHB	NPIP	18197919	5.2564	3.5577	1.2838
CHB	NPIP	18202919	2.8359	3.2189	-0.3149
CHB	NPIP	18207919	3.5167	5.2519	-0.9358
CHB	NPIP	18212919	5.0946	7.6237	-0.9753
CHB	NPIP	18217919	3.2553	4.5742	-0.8031
CHB	NPIP	18222919	3.754	2.8801	0.7879
CHB	NPIP	18227919	10.2142	6.2684	1.8173
CHB	NPIP	18232919	16.4397	9.6567	2.1033
CHB	NPIP	18237919	14.4894	8.8096	1.9177
CHB	NPIP	18242919	9.9528	7.6237	0.8982
CHB	NPIP	18247919	10.7247	12.0285	-0.3291
CHB	NPIP	18252919	11.9241	15.925	-0.7739
CHB	NPIP	18257919	10.0007	13.3838	-0.7721
CHB	NPIP	18262919	12.0981	13.3838	-0.2934
CHB	NPIP	18267919	10.0768	10.6731	-0.1684
CHB	NPIP	18272919	4.5136	4.2354	0.1811
CHB	NPIP	18277919	4.2355	3.2189	0.8358
CHB	NPIP	18282919	7.6886	4.7436	1.7372

CHB	NPIP	18287919	11.6947	7.1154	1.8806
CHB	NPIP	18292919	11.0133	8.4708	0.8901
CHB	NPIP	18297919	7.9035	7.4543	0.1768
CHB	NPIP	18302919	6.9674	6.2684	0.3219
CHB	NPIP	18307919	8.6377	8.8096	-0.058
CHB	NPIP	18312919	9.5743	9.9955	-0.1265
CHB	NPIP	18317919	6.5448	7.1154	-0.2343
CHB	NPIP	18322919	7.2835	6.7766	0.2176
CHB	NPIP	18327919	8.3781	8.1319	0.0895
CHB	NPIP	18332919	4.4186	7.4543	-1.1949
CHB	NPIP	18337919	5.8348	8.4708	-0.9228
CHB	NPIP	18342919	4.5875	6.0989	-0.7134
CHB	NPIP	18347919	3.8049	3.2189	0.4818
CHB	NPIP	18352919	7.8037	5.4213	1.2493
CHB	NPIP	18357919	5.514	5.2519	0.1414
CHB	NPIP	18362919	2.2705	3.3883	-0.8803
CHB	NPIP	18367919	2.4713	2.8801	-0.3686
CHB	NPIP	18372919	1.9709	1.6942	0.3797
CHB	NPIP	18377919	1.1848	1.5247	-0.505
CHB	NPIP	18382919	0.6311	2.7106	-1.9704
CHB	NPIP	18387919	0.4238	2.3718	-2.0563
CHB	NPIP	18392919	0.0581	0.8471	-1.7792
CHB	NPIP	18397919	0.0672	0.5082	-1.3844
CHB	NPIP	18402919	0.4624	1.5247	-1.5782
CHB	NPIP	18407919	0.7599	2.5412	-1.7788
CHB	NPIP	18412919	0.355	1.3553	-1.6212
CHB	NPIP	18417919	0.0773	1.0165	-1.8691
CHB	NPIP	18422919	0.0676	0.8471	-1.7578
CHB	NPIP	18427919	0.5075	1.0165	-1.0129
CHB	NPIP	18432919	0.8544	2.5412	-1.6844
CHB	NPIP	18437919	0.4569	1.6942	-1.698
CHB	NPIP	18442919	0.11	0.1694	-0.353
CHB	NPIP	18447919	0.1936	0.6777	-1.2656
CHB	NPIP	18452919	0.1936	0.6777	-1.2656
CHB	NPIP	18457919	0.0383	0.3388	-1.2054
CHB	NPIP	18462919	0.0769	0.8471	-1.7368
CHB	NPIP	18467919	0.2461	1.3553	-1.7977
CHB	NPIP	18472919	0.5254	2.2024	-1.8778
CHB	NPIP	18477919	0.434	2.8801	-2.2054
CHB	NPIP	18482919	0.712	2.5412	-1.8266
CHB	NPIP	18487919	0.7318	1.1859	-0.8106
CHB	NPIP	18492919	0.1456	0.3388	-0.775
CHB	NPIP	18497919	0.4967	0.3388	0.6334
CHB	NPIP	18502919	0.5161	0.6777	-0.4225
CHB	NPIP	18507919	0.0291	0.5082	-1.504
CHB	NPIP	18512919	0.0383	0.1694	-0.779
CHB	NPIP	18517919	0.0383	0.1694	-0.779
CHB	NPIP	18522919	0	0	0
CHB	NPIP	18527919	0.2941	0.6777	-1.0028

CHB	NPIP	18532919	0.6634	1.3553	-1.1214
CHB	NPIP	18537919	1.2138	2.7106	-1.4183
CHB	NPIP	18542919	1.4177	3.7271	-1.6777
CHB	NPIP	18547919	0.7548	3.0495	-1.9734
CHB	NPIP	18552919	1.2172	2.7106	-1.4151
CHB	NPIP	18557919	1.4474	2.8801	-1.2917
CHB	NPIP	18562919	0.688	2.8801	-1.9764
CHB	NPIP	18567919	1.0925	3.5577	-1.8631
CHB	NPIP	18572919	2.2311	3.8965	-1.1648
CHB	NPIP	18577919	2.9989	4.4048	-0.8847
CHB	NPIP	18582919	2.237	5.5907	-1.7111
CHB	NPIP	18587919	1.4969	4.2354	-1.7828
CHB	NPIP	18592919	1.7276	3.8965	-1.517
CHB	NPIP	18597919	2.0923	5.4213	-1.7456
CHB	NPIP	18602919	2.6343	6.6072	-1.7449
CHB	NPIP	18607919	2.6261	5.2519	-1.4162
CHB	NPIP	18612919	2.2867	3.8965	-1.1259
CHB	NPIP	18617919	1.2597	3.7271	-1.7925
CHB	NPIP	18622919	0.3368	2.8801	-2.2931
CHB	NPIP	18627919	0.8452	3.8965	-2.1341
CHB	NPIP	18632919	1.4516	3.2189	-1.453
CHB	NPIP	18637919	0.881	1.1859	-0.5442
CHB	NPIP	18642919	0.1502	0.8471	-1.5715
CHB	NPIP	18647919	3.0746	2.3718	0.7419
CHB	NPIP	18652919	3.1841	2.2024	1.0992
CHB	NPIP	18657919	0.1483	0.3388	-0.7642
CHB	NPIP	18662919	2.9587	2.2024	0.8469
CHB	NPIP	18667919	4.6777	3.7271	0.6906
CHB	NPIP	18672919	3.1125	3.0495	0.0542
CHB	NPIP	18677919	2.0192	3.2189	-0.9863
CHB	NPIP	18682919	1.1713	3.0495	-1.6153
CHB	NPIP	18687919	1.1175	2.2024	-1.2148
CHB	NPIP	18692919	0.5816	1.0165	-0.8655
CHB	NPIP	18697919	0.4976	0.5082	-0.0333
CHB	NPIP	18702919	2.6204	2.7106	-0.0855
CHB	NPIP	18707919	3.1751	4.066	-0.6008
CHB	NPIP	18712919	2.5561	3.8965	-0.9375
CHB	NPIP	18717919	2.1117	3.2189	-0.9103
CHB	NPIP	18722919	5.6246	3.7271	1.3785
CHB	NPIP	18727919	9.8999	7.1154	1.1435
CHB	NPIP	18732919	9.442	8.8096	0.2135
CHB	NPIP	18737919	7.9054	7.2848	0.2495
CHB	NPIP	18742919	8.6784	7.7931	0.3346
CHB	NPIP	18747919	9.0995	7.4543	0.6476
CHB	NPIP	18752919	9.4603	7.4543	0.7896
CHB	NPIP	18757919	15.0796	10.3343	1.3813
CHB	NPIP	18762919	18.8018	12.5367	1.5211
CHB	NPIP	18767919	13.5403	13.3838	0.0357
CHB	NPIP	18772919	6.3459	9.9955	-1.0959

CHB	NPIP	18777919	5.4104	7.9625	-0.9457
CHB	NPIP	18782919	6.9431	9.3178	-0.7612
CHB	NPIP	18787919	7.0124	9.9955	-0.8958
CHB	NPIP	18792919	4.3331	7.7931	-1.3077
CHB	NPIP	18797919	2.1848	6.7766	-1.9711
CHB	NPIP	18802919	3.6478	8.3013	-1.6597
CHB	NPIP	18807919	3.3531	7.6237	-1.6469
CHB	NPIP	18812919	2.3025	6.4378	-1.8594
CHB	NPIP	18817919	2.6467	6.0989	-1.6296
CHB	NPIP	18822919	4.89	7.7931	-1.0972
CHB	NPIP	18827919	6.9294	8.979	-0.6799
YRI	NPIP	17822919	13.4586	16.6382	-0.5878
YRI	NPIP	17827919	20.4403	24.0329	-0.4661
YRI	NPIP	17832919	20.2887	25.2093	-0.6094
YRI	NPIP	17837919	15.6481	21.512	-0.8468
YRI	NPIP	17842919	16.5868	19.6633	-0.4845
YRI	NPIP	17847919	22.7857	20.6717	0.3173
YRI	NPIP	17852919	21.1648	17.6465	0.6148
YRI	NPIP	17857919	18.267	16.134	0.4061
YRI	NPIP	17862919	16.9599	17.9827	-0.1755
YRI	NPIP	17867919	13.4172	15.9659	-0.4901
YRI	NPIP	17872919	13.8258	14.2853	-0.0982
YRI	NPIP	17877919	13.8977	14.7895	-0.1845
YRI	NPIP	17882919	12.5813	15.2937	-0.5435
YRI	NPIP	17887919	13.6057	16.6382	-0.5606
YRI	NPIP	17892919	20.1719	21.0078	-0.1235
YRI	NPIP	17897919	22.4708	22.3523	0.0165
YRI	NPIP	17902919	17.8966	18.823	-0.1522
YRI	NPIP	17907919	16.4904	16.302	0.0355
YRI	NPIP	17912919	17.9518	16.6382	0.2429
YRI	NPIP	17917919	17.6694	17.3104	0.0639
YRI	NPIP	17922919	19.4623	19.9994	-0.0832
YRI	NPIP	17927919	23.8249	21.1758	0.3884
YRI	NPIP	17932919	33.0196	26.0496	0.8362
YRI	NPIP	17937919	34.6355	27.5622	0.8033
YRI	NPIP	17942919	25.2082	18.823	1.0487
YRI	NPIP	17947919	23.3019	19.6633	0.573
YRI	NPIP	17952919	20.6531	21.8481	-0.17
YRI	NPIP	17957919	14.8938	18.1507	-0.554
YRI	NPIP	17962919	14.6063	17.4785	-0.5065
YRI	NPIP	17967919	10.9323	15.1256	-0.8491
YRI	NPIP	17972919	9.1988	13.445	-0.9616
YRI	NPIP	17977919	13.7298	14.9575	-0.2513
YRI	NPIP	17982919	19.0598	17.1423	0.3445
YRI	NPIP	17987919	23.9638	20.5036	0.5234
YRI	NPIP	17992919	25.6768	20.6717	0.7511
YRI	NPIP	17997919	23.3717	19.8313	0.553
YRI	NPIP	18002919	21.6543	20.3355	0.2011
YRI	NPIP	18007919	17.3217	14.4534	0.6065

YRI	NPIP	18012919	10.7719	9.2434	0.4919
YRI	NPIP	18017919	11.0061	11.5963	-0.1537
YRI	NPIP	18022919	16.4093	15.1256	0.2599
YRI	NPIP	18027919	12.7728	14.4534	-0.3554
YRI	NPIP	18032919	7.2497	12.9408	-1.3364
YRI	NPIP	18037919	7.5472	13.613	-1.3576
YRI	NPIP	18042919	9.0425	13.7811	-1.0483
YRI	NPIP	18047919	15.0161	15.7978	-0.1519
YRI	NPIP	18052919	23.5532	21.1758	0.3486
YRI	NPIP	18057919	19.5024	19.6633	-0.0253
YRI	NPIP	18062919	10.7183	14.6214	-0.8163
YRI	NPIP	18067919	11.9901	13.9492	-0.4285
YRI	NPIP	18072919	11.2198	14.7895	-0.7385
YRI	NPIP	18077919	7.0903	14.2853	-1.5384
YRI	NPIP	18082919	5.8223	11.9324	-1.5489
YRI	NPIP	18087919	7.8298	13.1089	-1.2246
YRI	NPIP	18092919	9.5536	15.1256	-1.1283
YRI	NPIP	18097919	11.3863	14.4534	-0.6485
YRI	NPIP	18102919	11.661	12.9408	-0.3005
YRI	NPIP	18107919	13.5493	13.7811	-0.0513
YRI	NPIP	18112919	13.7034	13.2769	0.0977
YRI	NPIP	18117919	11.1338	14.6214	-0.7294
YRI	NPIP	18122919	10.7901	15.7978	-0.9728
YRI	NPIP	18127919	12.6134	16.4701	-0.72
YRI	NPIP	18132919	14.5269	18.6549	-0.6839
YRI	NPIP	18137919	12.7034	17.3104	-0.82
YRI	NPIP	18142919	11.0686	15.7978	-0.9187
YRI	NPIP	18147919	16.7617	17.3104	-0.0977
YRI	NPIP	18152919	20.9556	20.1675	0.1211
YRI	NPIP	18157919	17.0277	19.6633	-0.4151
YRI	NPIP	18162919	12.0805	17.4785	-0.952
YRI	NPIP	18167919	12.7921	14.4534	-0.3513
YRI	NPIP	18172919	15.1135	14.2853	0.1771
YRI	NPIP	18177919	14.4875	16.4701	-0.3701
YRI	NPIP	18182919	12.1883	15.9659	-0.7265
YRI	NPIP	18187919	6.5722	10.0837	-1.0423
YRI	NPIP	18192919	3.7735	5.7141	-0.9677
YRI	NPIP	18197919	4.2216	7.8989	-1.368
YRI	NPIP	18202919	2.7894	6.7225	-1.6948
YRI	NPIP	18207919	4.6193	8.5712	-1.3638
YRI	NPIP	18212919	6.3593	10.5879	-1.1992
YRI	NPIP	18217919	3.1443	6.0502	-1.3768
YRI	NPIP	18222919	3.1594	4.8738	-0.9841
YRI	NPIP	18227919	6.5373	6.8906	-0.1489
YRI	NPIP	18232919	13.9277	12.1005	0.4571
YRI	NPIP	18237919	15.198	13.445	0.397
YRI	NPIP	18242919	11.6019	13.1089	-0.3496
YRI	NPIP	18247919	13.2609	16.134	-0.547
YRI	NPIP	18252919	18.1008	18.6549	-0.0918

YRI	NPIP	18257919	15.0105	16.6382	-0.3009
YRI	NPIP	18262919	11.6834	15.6298	-0.7745
YRI	NPIP	18267919	10.8053	15.6298	-0.9468
YRI	NPIP	18272919	5.666	10.0837	-1.3112
YRI	NPIP	18277919	4.4406	5.378	-0.4933
YRI	NPIP	18282919	7.3786	6.2183	0.5364
YRI	NPIP	18287919	12.8236	12.6047	0.0527
YRI	NPIP	18292919	13.9276	16.8062	-0.5271
YRI	NPIP	18297919	10.8274	15.2937	-0.8949
YRI	NPIP	18302919	9.8037	12.7727	-0.7058
YRI	NPIP	18307919	10.692	13.7811	-0.6834
YRI	NPIP	18312919	12.0834	15.4617	-0.6699
YRI	NPIP	18317919	9.3871	12.2685	-0.7116
YRI	NPIP	18322919	9.4361	10.0837	-0.1922
YRI	NPIP	18327919	10.7231	9.5795	0.356
YRI	NPIP	18332919	10.3715	9.2434	0.3631
YRI	NPIP	18337919	14.1254	12.4366	0.4117
YRI	NPIP	18342919	10.1216	9.5795	0.1688
YRI	NPIP	18347919	6.7369	5.7141	0.51
YRI	NPIP	18352919	8.7225	6.3864	1.0544
YRI	NPIP	18357919	6.518	6.5544	-0.016
YRI	NPIP	18362919	4.1892	6.2183	-0.938
YRI	NPIP	18367919	3.6629	4.5377	-0.5345
YRI	NPIP	18372919	2.7861	3.6974	-0.6642
YRI	NPIP	18377919	1.7456	4.2016	-1.6043
YRI	NPIP	18382919	1.101	4.0335	-1.9842
YRI	NPIP	18387919	0.8209	2.3529	-1.6217
YRI	NPIP	18392919	0.1003	0.8403	-1.6714
YRI	NPIP	18397919	0.1414	0.8403	-1.5786
YRI	NPIP	18402919	0.4844	1.6806	-1.6456
YRI	NPIP	18407919	0.5625	2.3529	-1.8952
YRI	NPIP	18412919	0.1917	1.0084	-1.6282
YRI	NPIP	18417919	0.1087	0.6722	-1.4752
YRI	NPIP	18422919	0.1087	0.6722	-1.4752
YRI	NPIP	18427919	0.6779	1.1764	-0.8916
YRI	NPIP	18432919	1.4803	2.689	-1.1487
YRI	NPIP	18437919	1.2644	2.1848	-1.0334
YRI	NPIP	18442919	0.462	0.6722	-0.5503
YRI	NPIP	18447919	0.0369	0.5042	-1.4686
YRI	NPIP	18452919	0.0369	0.5042	-1.4686
YRI	NPIP	18457919	0.0093	0.1681	-0.944
YRI	NPIP	18462919	0.289	0.6722	-1.0032
YRI	NPIP	18467919	0.5482	2.0167	-1.7558
YRI	NPIP	18472919	1.0173	3.1932	-1.7945
YRI	NPIP	18477919	0.958	3.3612	-1.8986
YRI	NPIP	18482919	0.7476	2.8571	-1.9077
YRI	NPIP	18487919	0.6989	1.5126	-1.2116
YRI	NPIP	18492919	0.188	0.6722	-1.2676
YRI	NPIP	18497919	0.5301	0.5042	0.0814

YRI	NPIP	18502919	0.6121	0.6722	-0.1573
YRI	NPIP	18507919	0.1096	0.5042	-1.2401
YRI	NPIP	18512919	0.2481	0.1681	0.4756
YRI	NPIP	18517919	0.3287	0.5042	-0.5515
YRI	NPIP	18522919	0.0806	0.3361	-1.0257
YRI	NPIP	18527919	0.3884	0.6722	-0.743
YRI	NPIP	18532919	0.4803	1.5126	-1.5371
YRI	NPIP	18537919	1.5616	5.5461	-2.0404
YRI	NPIP	18542919	2.4778	9.7476	-2.227
YRI	NPIP	18547919	1.7743	8.235	-2.3133
YRI	NPIP	18552919	2.3692	5.378	-1.5835
YRI	NPIP	18557919	2.4347	5.0419	-1.4527
YRI	NPIP	18562919	1.5091	6.7225	-2.2465
YRI	NPIP	18567919	2.5151	8.7392	-2.1098
YRI	NPIP	18572919	3.9186	8.9073	-1.6615
YRI	NPIP	18577919	4.1207	8.7392	-1.5655
YRI	NPIP	18582919	2.8655	8.7392	-1.991
YRI	NPIP	18587919	1.669	6.7225	-2.1776
YRI	NPIP	18592919	1.9698	7.2267	-2.1213
YRI	NPIP	18597919	3.5543	10.4199	-1.9764
YRI	NPIP	18602919	4.0252	10.5879	-1.8612
YRI	NPIP	18607919	2.8221	7.7309	-1.8625
YRI	NPIP	18612919	2.5704	6.2183	-1.6864
YRI	NPIP	18617919	2.8673	8.235	-1.9219
YRI	NPIP	18622919	2.6797	9.4115	-2.1306
YRI	NPIP	18627919	2.4361	8.9073	-2.1553
YRI	NPIP	18632919	2.0116	6.5544	-2.0028
YRI	NPIP	18637919	1.1165	2.689	-1.4944
YRI	NPIP	18642919	0.7252	2.5209	-1.7983
YRI	NPIP	18647919	1.2001	3.8654	-1.8704
YRI	NPIP	18652919	1.4064	3.6974	-1.6698
YRI	NPIP	18657919	0.604	2.3529	-1.8513
YRI	NPIP	18662919	1.7601	3.8654	-1.4774
YRI	NPIP	18667919	2.7058	5.8822	-1.5434
YRI	NPIP	18672919	2.7311	5.8822	-1.5312
YRI	NPIP	18677919	2.9238	6.7225	-1.6369
YRI	NPIP	18682919	2.274	5.0419	-1.5422
YRI	NPIP	18687919	1.9134	3.1932	-1.0555
YRI	NPIP	18692919	1.1806	2.3529	-1.2409
YRI	NPIP	18697919	1.5909	1.6806	-0.1234
YRI	NPIP	18702919	5.5404	4.8738	0.3826
YRI	NPIP	18707919	6.7812	7.2267	-0.1798
YRI	NPIP	18712919	5.5675	8.7392	-1.0751
YRI	NPIP	18717919	4.6659	8.067	-1.2411
YRI	NPIP	18722919	9.0173	9.2434	-0.0728
YRI	NPIP	18727919	17.4855	12.4366	1.2309
YRI	NPIP	18732919	19.3235	14.2853	1.0772
YRI	NPIP	18737919	14.0124	12.9408	0.2516
YRI	NPIP	18742919	11.4522	13.9492	-0.5461

YRI	NPIP	18747919	11.473	16.302	-0.9104
YRI	NPIP	18752919	12.8226	12.7727	0.0119
YRI	NPIP	18757919	19.2451	13.1089	1.4234
YRI	NPIP	18762919	24.0403	16.302	1.4588
YRI	NPIP	18767919	21.3403	18.1507	0.5425
YRI	NPIP	18772919	14.0942	15.6298	-0.3014
YRI	NPIP	18777919	11.9129	14.7895	-0.5951
YRI	NPIP	18782919	12.2307	16.9743	-0.8604
YRI	NPIP	18787919	11.732	16.8062	-0.9291
YRI	NPIP	18792919	8.7573	13.2769	-1.0358
YRI	NPIP	18797919	6.0986	12.4366	-1.5452
YRI	NPIP	18802919	7.4044	12.6047	-1.2519
YRI	NPIP	18807919	7.102	9.7476	-0.8104
YRI	NPIP	18812919	6.1662	8.4031	-0.7862
YRI	NPIP	18817919	5.9033	8.9073	-1.0005
YRI	NPIP	18822919	9.4744	12.9408	-0.814
YRI	NPIP	18827919	11.6443	14.4534	-0.594
CEU	NPIP	21339568	1.7675	5.1169	-1.854
CEU	NPIP	21344568	1.5912	4.6052	-1.8299
CEU	NPIP	21349568	2.4739	6.3108	-1.7616
CEU	NPIP	21354568	2.5982	6.3108	-1.7045
CEU	NPIP	21359568	2.0866	5.458	-1.7625
CEU	NPIP	21364568	3.0868	6.9931	-1.6345
CEU	NPIP	21369568	2.6452	6.1402	-1.6446
CEU	NPIP	21374568	1.9112	3.7524	-1.3339
CEU	NPIP	21379568	3.8165	5.2874	-0.7909
CEU	NPIP	21384568	4.2618	7.5047	-1.2724
CEU	NPIP	21389568	3.9518	6.3108	-1.0831
CEU	NPIP	21394568	2.8093	4.0935	-0.8635
CEU	NPIP	21399568	1.4494	2.8996	-1.3042
CEU	NPIP	21404568	1.3867	2.5584	-1.1672
CEU	NPIP	21409568	1.1315	2.2173	-1.213
CEU	NPIP	21414568	0.7361	1.3645	-1.0166
CEU	NPIP	21419568	0.2632	0.5117	-0.7794
CEU	NPIP	21424568	0.02	0.1706	-0.8946
CEU	NPIP	21429568	0.2552	0.5117	-0.8045
CEU	NPIP	21434568	0.2352	0.3411	-0.4245
CEU	NPIP	21439568	0	0	0
CEU	NPIP	21444568	0	0	0
CEU	NPIP	21449568	0	0	0
CEU	NPIP	21454568	0	0	0
CEU	NPIP	21459568	0.0301	0.1706	-0.8346
CEU	NPIP	21464568	0.109	0.5117	-1.263
CEU	NPIP	21469568	0.3987	1.0234	-1.2413
CEU	NPIP	21474568	0.6392	0.8528	-0.481
CEU	NPIP	21479568	0.3397	0.5117	-0.5394
CEU	NPIP	21484568	0.3076	2.0467	-2.0693
CEU	NPIP	21489568	0.5199	2.5584	-2.0307
CEU	NPIP	21494568	0.4661	1.7056	-1.6976

CEU	NPIP	21499568	0.2437	1.0234	-1.5493
CEU	NPIP	21504568	0.0402	0.5117	-1.4787
CEU	NPIP	21509568	0.7743	3.0701	-1.9693
CEU	NPIP	21514568	2.1791	5.7991	-1.793
CEU	NPIP	21519568	3.8576	8.5281	-1.6296
CEU	NPIP	21524568	4.0804	10.7454	-1.876
CEU	NPIP	21529568	2.2249	9.2104	-2.2699
CEU	NPIP	21534568	1.7407	8.3576	-2.3521
CEU	NPIP	21539568	3.3101	8.0164	-1.7383
CEU	NPIP	21544568	3.1337	7.5047	-1.715
CEU	NPIP	21549568	2.7083	8.3576	-2.0082
CEU	NPIP	21554568	2.1457	7.3342	-2.0789
CEU	NPIP	21559568	1.4989	5.6286	-2.1006
CEU	NPIP	21564568	2.167	6.3108	-1.9025
CEU	NPIP	21569568	3.7377	8.0164	-1.5804
CEU	NPIP	21574568	4.3126	8.3576	-1.4379
CEU	NPIP	21579568	2.9037	7.6753	-1.8341
CEU	NPIP	21584568	3.1123	7.8459	-1.7832
CEU	NPIP	21589568	2.4593	6.9931	-1.897
CEU	NPIP	21594568	2.6131	5.7991	-1.578
CEU	NPIP	21599568	2.5733	5.1169	-1.4079
CEU	NPIP	21604568	1.8561	4.4346	-1.6178
CEU	NPIP	21609568	4.3092	5.2874	-0.526
CEU	NPIP	21614568	3.3144	4.2641	-0.6165
CEU	NPIP	21619568	2.3026	3.5818	-0.9641
CEU	NPIP	21624568	3.9709	4.2641	-0.1903
CEU	NPIP	21629568	3.4364	5.1169	-0.9302
CEU	NPIP	21634568	2.5234	5.2874	-1.4862
CEU	NPIP	21639568	3.1692	4.9463	-1.0135
CEU	NPIP	21644568	3.5516	5.2874	-0.9333
CEU	NPIP	21649568	2.6662	4.4346	-1.1096
CEU	NPIP	21654568	2.4557	4.0935	-1.1013
CEU	NPIP	21659568	2.0981	4.2641	-1.406
CEU	NPIP	21664568	2.4084	4.7757	-1.3923
CEU	NPIP	21669568	3.1618	4.7757	-0.9492
CEU	NPIP	21674568	4.7514	5.6286	-0.4462
CEU	NPIP	21679568	5.0418	5.9697	-0.4478
CEU	NPIP	21684568	4.1989	5.6286	-0.7272
CEU	NPIP	21689568	4.8561	6.4814	-0.7285
CEU	NPIP	21694568	3.1655	5.458	-1.1985
CEU	NPIP	21699568	1.0853	4.7757	-2.1705
CEU	NPIP	21704568	2.9217	4.7757	-1.0904
CEU	NPIP	21709568	6.1944	4.7757	0.8344
CEU	NPIP	21714568	5.8363	5.458	0.1978
CEU	NPIP	21719568	3.4162	5.9697	-1.2324
CEU	NPIP	21724568	3.8874	5.9697	-1.0049
CEU	NPIP	21729568	8.2783	7.6753	0.2318
CEU	NPIP	21734568	12.4589	9.5515	0.9134
CEU	NPIP	21739568	9.5743	7.3342	0.8975

CEU	NPIP	21744568	6.7848	6.1402	0.3033
CEU	NPIP	21749568	5.7829	4.2641	0.9859
CEU	NPIP	21754568	1.8411	1.0234	1.6248
CEU	NPIP	21759568	0.7691	1.5351	-1.1358
CEU	NPIP	21764568	2.3618	2.5584	-0.1958
CEU	NPIP	21769568	4.4824	4.2641	0.1417
CEU	NPIP	21774568	4.8384	5.7991	-0.4758
CEU	NPIP	21779568	3.2989	4.9463	-0.9395
CEU	NPIP	21784568	2.2391	4.9463	-1.5439
CEU	NPIP	21789568	1.429	3.7524	-1.6833
CEU	NPIP	21794568	1.67	2.2173	-0.6114
CEU	NPIP	21799568	1.4642	1.7056	-0.3306
CEU	NPIP	21804568	2.3556	3.9229	-1.0931
CEU	NPIP	21809568	2.8456	5.1169	-1.2572
CEU	NPIP	21814568	2.5327	4.7757	-1.3192
CEU	NPIP	21819568	3.6516	6.8225	-1.3568
CEU	NPIP	21824568	3.3416	6.1402	-1.3169
CEU	NPIP	21829568	2.9118	5.9697	-1.4758
CEU	NPIP	21834568	2.0686	6.9931	-2.0605
CEU	NPIP	21839568	2.1241	6.3108	-1.9222
CEU	NPIP	21844568	2.1705	4.4346	-1.4206
CEU	NPIP	21849568	0.7745	2.0467	-1.5138
CEU	NPIP	21854568	0.417	0.6822	-0.6925
CEU	NPIP	21859568	0.2744	0.3411	-0.2674
CEU	NPIP	21864568	0.0101	0.1706	-0.9534
CEU	NPIP	21869568	0.0301	0.1706	-0.8346
CEU	NPIP	21874568	0.0301	0.1706	-0.8346
CEU	NPIP	21879568	0	0	0
CEU	NPIP	21884568	0.0505	0.6822	-1.6496
CEU	NPIP	21889568	0.0907	1.1939	-1.9659
CEU	NPIP	21894568	0.6407	1.8762	-1.573
CEU	NPIP	21899568	1.1793	2.8996	-1.5471
CEU	NPIP	21904568	1.7058	3.0701	-1.1703
CEU	NPIP	21909568	1.6961	2.5584	-0.859
CEU	NPIP	21914568	0.5692	1.0234	-0.9025
CEU	NPIP	21919568	0.4368	0.1706	1.5813
CEU	NPIP	21924568	0.5351	0.8528	-0.7154
CEU	NPIP	21929568	0.3896	1.1939	-1.4333
CEU	NPIP	21934568	0.6686	0.8528	-0.4148
CEU	NPIP	21939568	0.5849	1.5351	-1.409
CEU	NPIP	21944568	1.5718	4.4346	-1.7962
CEU	NPIP	21949568	1.7986	4.4346	-1.6539
CEU	NPIP	21954568	1.6436	3.4112	-1.3883
CEU	NPIP	21959568	1.6802	4.6052	-1.7759
CEU	NPIP	21964568	2.1296	4.4346	-1.4462
CEU	NPIP	21969568	2.1006	4.7757	-1.5733
CEU	NPIP	21974568	1.879	5.7991	-1.9416
CEU	NPIP	21979568	2.4117	6.1402	-1.7545
CEU	NPIP	21984568	1.1678	4.0935	-1.9673

CEU	NPIP	21989568	1.6118	3.4112	-1.4133
CEU	NPIP	21994568	2.3257	4.2641	-1.2583
CEU	NPIP	21999568	1.5083	3.7524	-1.6258
CEU	NPIP	22004568	0.7518	3.5818	-2.133
CEU	NPIP	22009568	2.054	4.6052	-1.5489
CEU	NPIP	22014568	3.7155	6.1402	-1.141
CEU	NPIP	22019568	3.4335	5.458	-1.0584
CEU	NPIP	22024568	2.451	3.9229	-1.0266
CEU	NPIP	22029568	1.4352	2.729	-1.2229
CEU	NPIP	22034568	1.8916	3.7524	-1.3481
CEU	NPIP	22039568	1.9382	4.9463	-1.7155
CEU	NPIP	22044568	1.328	3.0701	-1.4943
CEU	NPIP	22049568	1.4119	2.0467	-0.7553
CEU	NPIP	22054568	2.0917	3.2407	-0.9422
CEU	NPIP	22059568	3.2088	5.458	-1.1758
CEU	NPIP	22064568	2.3387	5.2874	-1.5855
CEU	NPIP	22069568	2.5088	4.7757	-1.3333
CEU	NPIP	22074568	2.6469	4.7757	-1.252
CEU	NPIP	22079568	2.831	4.6052	-1.0772
CEU	NPIP	22084568	2.7658	4.6052	-1.1168
CEU	NPIP	22089568	1.5502	3.9229	-1.6549
CEU	NPIP	22094568	1.3902	3.4112	-1.5873
CEU	NPIP	22099568	0.4591	2.3879	-2.0312
CEU	NPIP	22104568	1.9017	4.4346	-1.5892
CEU	NPIP	22109568	2.3239	5.2874	-1.5935
CEU	NPIP	22114568	1.0072	3.4112	-1.8881
CEU	NPIP	22119568	1.5601	4.0935	-1.7035
CEU	NPIP	22124568	3.5584	5.9697	-1.1637
CEU	NPIP	22129568	3.8547	6.6519	-1.2247
CEU	NPIP	22134568	5.0942	7.5047	-0.9458
CEU	NPIP	22139568	7.0903	7.6753	-0.2249
CEU	NPIP	22144568	6.1156	6.8225	-0.3025
CEU	NPIP	22149568	3.846	5.7991	-0.9674
CEU	NPIP	22154568	3.1396	5.7991	-1.3172
CEU	NPIP	22159568	2.8207	5.6286	-1.4283
CEU	NPIP	22164568	1.6283	4.9463	-1.8923
CEU	NPIP	22169568	2.9755	5.458	-1.2978
CEU	NPIP	22174568	3.2107	5.7991	-1.282
CEU	NPIP	22179568	2.6223	5.458	-1.4824
CEU	NPIP	22184568	3.6656	5.2874	-0.872
CEU	NPIP	22189568	4.0633	5.6286	-0.7962
CEU	NPIP	22194568	4.9897	5.6286	-0.325
CEU	NPIP	22199568	4.3966	4.9463	-0.3135
CEU	NPIP	22204568	4.912	4.7757	0.0802
CEU	NPIP	22209568	5.8887	5.9697	-0.0391
CEU	NPIP	22214568	3.4583	5.1169	-0.9181
CEU	NPIP	22219568	4.6528	6.1402	-0.6999
CEU	NPIP	22224568	7.4495	9.7221	-0.7023
CEU	NPIP	22229568	8.1845	10.4043	-0.644

CEU	NPIP	22234568	7.4234	8.3576	-0.3321
CEU	NPIP	22239568	6.6805	7.3342	-0.2619
CEU	NPIP	22244568	6.6242	8.187	-0.5662
CEU	NPIP	22249568	5.6052	7.6753	-0.7957
CEU	NPIP	22254568	4.9858	6.1402	-0.5432
CEU	NPIP	22259568	4.6114	5.458	-0.4426
CEU	NPIP	22264568	4.679	6.4814	-0.8079
CEU	NPIP	22269568	4.4591	7.1636	-1.1071
CEU	NPIP	22274568	3.1459	5.7991	-1.3141
CEU	NPIP	22279568	2.2987	5.7991	-1.7337
CEU	NPIP	22284568	2.569	6.9931	-1.8511
CEU	NPIP	22289568	3.055	5.9697	-1.4067
CEU	NPIP	22294568	3.6275	5.458	-0.9569
CEU	NPIP	22299568	3.9262	6.4814	-1.1453
CEU	NPIP	22304568	3.0136	6.8225	-1.6298
CEU	NPIP	22309568	2.2424	7.6753	-2.0883
CEU	NPIP	22314568	2.6294	8.5281	-2.0581
CEU	NPIP	22319568	1.8116	5.2874	-1.869
CEU	NPIP	22324568	1.5838	3.9229	-1.6315
CEU	NPIP	22329568	2.2562	5.9697	-1.7922
CEU	NPIP	22334568	1.5217	4.6052	-1.8721
CEU	NPIP	22339568	1.6042	4.4346	-1.7759
CEU	NPIP	22344568	2.2367	4.7757	-1.4933
CEU	NPIP	22349568	1.4539	3.0701	-1.3864
CHB	NPIP	21339568	2.3697	7.7931	-2.0498
CHB	NPIP	21344568	3.0788	7.2848	-1.6907
CHB	NPIP	21349568	3.9806	6.7766	-1.2002
CHB	NPIP	21354568	4.7725	7.9625	-1.1821
CHB	NPIP	21359568	3.3735	8.979	-1.8595
CHB	NPIP	21364568	3.1784	8.3013	-1.8271
CHB	NPIP	21369568	3.368	7.6237	-1.6411
CHB	NPIP	21374568	2.1702	7.1154	-2.0308
CHB	NPIP	21379568	3.3436	9.1484	-1.8926
CHB	NPIP	21384568	3.8426	9.1484	-1.7299
CHB	NPIP	21389568	3.2039	6.7766	-1.5336
CHB	NPIP	21394568	2.3522	4.5742	-1.353
CHB	NPIP	21399568	1.0668	2.033	-1.1522
CHB	NPIP	21404568	0.4428	2.033	-1.8963
CHB	NPIP	21409568	0.3278	2.033	-2.0335
CHB	NPIP	21414568	0.4546	1.3553	-1.4598
CHB	NPIP	21419568	0.1946	0.3388	-0.5784
CHB	NPIP	21424568	0	0	0
CHB	NPIP	21429568	0.2397	0.3388	-0.3975
CHB	NPIP	21434568	0.2397	0.3388	-0.3975
CHB	NPIP	21439568	0	0	0
CHB	NPIP	21444568	0	0	0
CHB	NPIP	21449568	0	0	0
CHB	NPIP	21454568	0	0	0
CHB	NPIP	21459568	0	0	0

CHB	NPIP	21464568	0	0	0
CHB	NPIP	21469568	0.7377	0.8471	-0.2467
CHB	NPIP	21474568	1.3002	1.1859	0.204
CHB	NPIP	21479568	0.8458	0.6777	0.4395
CHB	NPIP	21484568	1.5898	2.033	-0.5285
CHB	NPIP	21489568	1.7407	2.5412	-0.7994
CHB	NPIP	21494568	0.9063	1.8636	-1.2214
CHB	NPIP	21499568	0.5195	1.1859	-1.1895
CHB	NPIP	21504568	0.0474	0.1694	-0.7249
CHB	NPIP	21509568	0.4679	1.8636	-1.7807
CHB	NPIP	21514568	2.5955	7.2848	-1.8849
CHB	NPIP	21519568	7.9815	10.1649	-0.6454
CHB	NPIP	21524568	11.0659	10.3343	0.213
CHB	NPIP	21529568	7.2718	9.4872	-0.6983
CHB	NPIP	21534568	7.1494	9.9955	-0.8546
CHB	NPIP	21539568	8.4394	10.5037	-0.5918
CHB	NPIP	21544568	5.3655	8.1319	-1.0055
CHB	NPIP	21549568	5.0352	6.7766	-0.7475
CHB	NPIP	21554568	4.0468	6.4378	-1.0751
CHB	NPIP	21559568	2.3684	7.6237	-2.0266
CHB	NPIP	21564568	2.7856	6.946	-1.7464
CHB	NPIP	21569568	4.9964	6.6072	-0.7075
CHB	NPIP	21574568	6.8214	6.7766	0.0192
CHB	NPIP	21579568	5.1262	6.946	-0.7639
CHB	NPIP	21584568	4.6882	7.9625	-1.2134
CHB	NPIP	21589568	4.7794	7.2848	-1.0071
CHB	NPIP	21594568	5.7569	6.4378	-0.3062
CHB	NPIP	21599568	5.6478	6.2684	-0.2858
CHB	NPIP	21604568	4.2505	5.7601	-0.75
CHB	NPIP	21609568	6.1881	6.4378	-0.1123
CHB	NPIP	21614568	5.3773	6.2684	-0.4104
CHB	NPIP	21619568	4.8711	6.6072	-0.7625
CHB	NPIP	21624568	5.7026	6.6072	-0.3973
CHB	NPIP	21629568	5.4938	6.2684	-0.3568
CHB	NPIP	21634568	5.6924	6.946	-0.5262
CHB	NPIP	21639568	6.5667	8.4708	-0.6666
CHB	NPIP	21644568	7.6486	10.5037	-0.8185
CHB	NPIP	21649568	7.082	8.8096	-0.5833
CHB	NPIP	21654568	5.0817	6.0989	-0.4802
CHB	NPIP	21659568	2.8106	4.5742	-1.0739
CHB	NPIP	21664568	3.9317	5.9295	-0.9672
CHB	NPIP	21669568	6.4439	8.1319	-0.6135
CHB	NPIP	21674568	6.8429	8.4708	-0.5699
CHB	NPIP	21679568	6.1232	7.6237	-0.5786
CHB	NPIP	21684568	8.0392	7.1154	0.3794
CHB	NPIP	21689568	10.6427	7.2848	1.3498
CHB	NPIP	21694568	6.8785	6.0989	0.368
CHB	NPIP	21699568	3.6514	5.9295	-1.1029
CHB	NPIP	21704568	6.0659	7.6237	-0.6007

CHB	NPIP	21709568	7.6419	7.7931	-0.0571
CHB	NPIP	21714568	6.5532	7.6237	-0.4128
CHB	NPIP	21719568	4.6777	7.4543	-1.0929
CHB	NPIP	21724568	6.0832	7.6237	-0.5941
CHB	NPIP	21729568	9.494	9.1484	0.1127
CHB	NPIP	21734568	13.0942	9.9955	0.9305
CHB	NPIP	21739568	10.8698	7.2848	1.4411
CHB	NPIP	21744568	7.7021	5.7601	0.9648
CHB	NPIP	21749568	6.7984	4.5742	1.3544
CHB	NPIP	21754568	3.2564	2.033	1.4589
CHB	NPIP	21759568	2.5395	1.8636	0.8624
CHB	NPIP	21764568	3.4274	3.2189	0.1714
CHB	NPIP	21769568	5.8528	5.2519	0.3241
CHB	NPIP	21774568	7.8355	5.9295	0.9227
CHB	NPIP	21779568	6.8894	5.7601	0.5611
CHB	NPIP	21784568	5.9501	5.4213	0.2773
CHB	NPIP	21789568	4.3802	3.5577	0.6216
CHB	NPIP	21794568	2.5711	2.2024	0.4128
CHB	NPIP	21799568	1.9404	1.8636	0.098
CHB	NPIP	21804568	4.6701	4.066	0.4074
CHB	NPIP	21809568	5.1083	5.5907	-0.2461
CHB	NPIP	21814568	5.0029	7.1154	-0.8675
CHB	NPIP	21819568	7.2943	8.4708	-0.4119
CHB	NPIP	21824568	5.5757	7.1154	-0.6323
CHB	NPIP	21829568	5.8464	8.4708	-0.9187
CHB	NPIP	21834568	5.0621	9.3178	-1.3641
CHB	NPIP	21839568	3.6562	7.4543	-1.495
CHB	NPIP	21844568	3.23	4.7436	-0.8928
CHB	NPIP	21849568	1.0805	1.8636	-0.9991
CHB	NPIP	21854568	0.8696	0.5082	1.1345
CHB	NPIP	21859568	0.6318	0.5082	0.388
CHB	NPIP	21864568	0.0192	0.1694	-0.8925
CHB	NPIP	21869568	0.0097	0.1694	-0.949
CHB	NPIP	21874568	0.0097	0.1694	-0.949
CHB	NPIP	21879568	0	0	0
CHB	NPIP	21884568	0.0672	0.3388	-1.0895
CHB	NPIP	21889568	0.4967	0.5082	-0.0361
CHB	NPIP	21894568	1.5898	1.3553	0.3801
CHB	NPIP	21899568	1.7807	3.5577	-1.343
CHB	NPIP	21904568	2.8157	4.5742	-1.0708
CHB	NPIP	21909568	4.0211	3.8965	0.0871
CHB	NPIP	21914568	1.8355	1.8636	-0.0359
CHB	NPIP	21919568	0.4799	0.3388	0.566
CHB	NPIP	21924568	0.538	1.1859	-1.1565
CHB	NPIP	21929568	0.6629	1.5247	-1.2803
CHB	NPIP	21934568	0.6821	1.3553	-1.091
CHB	NPIP	21939568	0.3779	2.2024	-2.0429
CHB	NPIP	21944568	2.1146	3.8965	-1.2463
CHB	NPIP	21949568	2.4727	4.7436	-1.3395

CHB	NPIP	21954568	1.8709	4.066	-1.4802
CHB	NPIP	21959568	3.0381	4.913	-1.0724
CHB	NPIP	21964568	3.8959	5.9295	-0.9845
CHB	NPIP	21969568	2.8907	4.913	-1.1567
CHB	NPIP	21974568	3.352	4.7436	-0.8209
CHB	NPIP	21979568	4.8123	6.7766	-0.8432
CHB	NPIP	21984568	2.4259	5.9295	-1.6961
CHB	NPIP	21989568	1.8132	5.2519	-1.8546
CHB	NPIP	21994568	3.49	5.4213	-1.0127
CHB	NPIP	21999568	2.7508	4.7436	-1.1755
CHB	NPIP	22004568	1.1551	4.5742	-2.082
CHB	NPIP	22009568	2.1656	4.7436	-1.5207
CHB	NPIP	22014568	4.3031	5.4213	-0.5864
CHB	NPIP	22019568	4.933	4.2354	0.4541
CHB	NPIP	22024568	3.2621	4.066	-0.5421
CHB	NPIP	22029568	1.4034	3.8965	-1.7437
CHB	NPIP	22034568	3.1449	3.8965	-0.5257
CHB	NPIP	22039568	3.723	3.8965	-0.1213
CHB	NPIP	22044568	1.8159	3.2189	-1.1535
CHB	NPIP	22049568	1.447	2.7106	-1.1973
CHB	NPIP	22054568	2.7249	4.2354	-0.9833
CHB	NPIP	22059568	4.613	5.9295	-0.6373
CHB	NPIP	22064568	3.9338	4.4048	-0.2964
CHB	NPIP	22069568	3.8572	5.2519	-0.7522
CHB	NPIP	22074568	3.5542	4.913	-0.7772
CHB	NPIP	22079568	3.5439	4.2354	-0.4502
CHB	NPIP	22084568	3.3017	4.913	-0.9217
CHB	NPIP	22089568	1.7864	4.066	-1.5372
CHB	NPIP	22094568	1.8289	3.3883	-1.2281
CHB	NPIP	22099568	0.7	2.2024	-1.6823
CHB	NPIP	22104568	2.3733	4.066	-1.1414
CHB	NPIP	22109568	3.1997	4.2354	-0.6742
CHB	NPIP	22114568	2.107	5.5907	-1.7775
CHB	NPIP	22119568	2.7805	7.1154	-1.7802
CHB	NPIP	22124568	4.4418	5.9295	-0.7202
CHB	NPIP	22129568	4.3817	6.946	-1.0764
CHB	NPIP	22134568	5.8963	7.7931	-0.7169
CHB	NPIP	22139568	7.6879	8.6402	-0.3273
CHB	NPIP	22144568	6.089	7.4543	-0.5374
CHB	NPIP	22149568	3.5297	5.5907	-1.0516
CHB	NPIP	22154568	3.1308	5.7601	-1.3063
CHB	NPIP	22159568	3.8346	6.6072	-1.2178
CHB	NPIP	22164568	3.1936	6.7766	-1.5381
CHB	NPIP	22169568	4.2922	6.4378	-0.9647
CHB	NPIP	22174568	3.6532	6.2684	-1.2045
CHB	NPIP	22179568	2.9747	6.946	-1.667
CHB	NPIP	22184568	4.3	7.4543	-1.2416
CHB	NPIP	22189568	5.2658	7.7931	-0.9552
CHB	NPIP	22194568	5.5285	8.3013	-0.9889

CHB	NPIP	22199568	4.3583	7.9625	-1.3356
CHB	NPIP	22204568	6.1296	9.1484	-0.9842
CHB	NPIP	22209568	7.301	9.1484	-0.6023
CHB	NPIP	22214568	4.4072	7.1154	-1.1122
CHB	NPIP	22219568	6.8119	7.6237	-0.3131
CHB	NPIP	22224568	11.0699	9.3178	0.5616
CHB	NPIP	22229568	10.4353	8.4708	0.6877
CHB	NPIP	22234568	8.0591	6.6072	0.6377
CHB	NPIP	22239568	8.9684	7.7931	0.4442
CHB	NPIP	22244568	8.7468	7.9625	0.2906
CHB	NPIP	22249568	5.2208	6.6072	-0.6089
CHB	NPIP	22254568	5.4312	7.2848	-0.7451
CHB	NPIP	22259568	5.3739	6.4378	-0.4784
CHB	NPIP	22264568	3.7243	6.7766	-1.3102
CHB	NPIP	22269568	2.6912	7.7931	-1.9283
CHB	NPIP	22274568	2.3779	6.6072	-1.8576
CHB	NPIP	22279568	1.763	6.946	-2.1756
CHB	NPIP	22284568	1.7085	7.2848	-2.2415
CHB	NPIP	22289568	2.1799	7.4543	-2.0761
CHB	NPIP	22294568	3.1496	7.1154	-1.6286
CHB	NPIP	22299568	5.3442	9.1484	-1.2403
CHB	NPIP	22304568	5.3587	11.1814	-1.5744
CHB	NPIP	22309568	3.7647	9.4872	-1.8039
CHB	NPIP	22314568	3.5462	7.9625	-1.6365
CHB	NPIP	22319568	3.3211	6.6072	-1.4433
CHB	NPIP	22324568	4.0394	7.1154	-1.2632
CHB	NPIP	22329568	4.3409	7.6237	-1.2659
CHB	NPIP	22334568	3.5212	7.1154	-1.476
CHB	NPIP	22339568	4.1711	7.6237	-1.3314
CHB	NPIP	22344568	3.8315	6.6072	-1.2191
CHB	NPIP	22349568	2.3638	5.5907	-1.6464
YRI	NPIP	21339568	4.5369	10.4199	-1.6935
YRI	NPIP	21344568	4.1475	11.5963	-1.9397
YRI	NPIP	21349568	3.9524	10.756	-1.9012
YRI	NPIP	21354568	5.155	11.0921	-1.612
YRI	NPIP	21359568	4.7644	11.5963	-1.7791
YRI	NPIP	21364568	5.7805	13.2769	-1.7181
YRI	NPIP	21369568	4.285	11.2602	-1.8673
YRI	NPIP	21374568	2.8633	10.0837	-2.1431
YRI	NPIP	21379568	4.54	12.6047	-1.9414
YRI	NPIP	21384568	3.9554	10.756	-1.9004
YRI	NPIP	21389568	3.1143	8.5712	-1.8832
YRI	NPIP	21394568	2.1758	5.8822	-1.801
YRI	NPIP	21399568	1.1652	2.689	-1.4482
YRI	NPIP	21404568	1.7998	3.3612	-1.2336
YRI	NPIP	21409568	1.5992	3.6974	-1.5293
YRI	NPIP	21414568	0.7333	2.0167	-1.5345
YRI	NPIP	21419568	0.3612	0.3361	0.1008
YRI	NPIP	21424568	0.0456	0.1681	-0.7282

YRI	NPIP	21429568	0.2217	0.5042	-0.8878
YRI	NPIP	21434568	0.1854	0.5042	-1.0019
YRI	NPIP	21439568	0.0093	0.1681	-0.944
YRI	NPIP	21444568	0	0	0
YRI	NPIP	21449568	0	0	0
YRI	NPIP	21454568	0	0	0
YRI	NPIP	21459568	0	0	0
YRI	NPIP	21464568	0.0638	0.5042	-1.384
YRI	NPIP	21469568	0.6097	1.6806	-1.4732
YRI	NPIP	21474568	0.9953	2.1848	-1.3356
YRI	NPIP	21479568	0.4586	1.1764	-1.2838
YRI	NPIP	21484568	0.2626	1.5126	-1.8613
YRI	NPIP	21489568	0.5563	2.0167	-1.7461
YRI	NPIP	21494568	0.7517	2.689	-1.8411
YRI	NPIP	21499568	0.4488	2.0167	-1.8747
YRI	NPIP	21504568	0	0	0
YRI	NPIP	21509568	0.9907	3.3612	-1.8728
YRI	NPIP	21514568	3.17	8.9073	-1.9109
YRI	NPIP	21519568	4.7857	13.1089	-1.9307
YRI	NPIP	21524568	5.5592	14.9575	-1.9234
YRI	NPIP	21529568	4.1316	12.2685	-2.0094
YRI	NPIP	21534568	3.4214	12.6047	-2.2107
YRI	NPIP	21539568	4.1558	14.9575	-2.2107
YRI	NPIP	21544568	3.4292	13.1089	-2.2454
YRI	NPIP	21549568	3.4517	10.924	-2.058
YRI	NPIP	21554568	3.0678	8.9073	-1.9449
YRI	NPIP	21559568	2.6946	9.4115	-2.1258
YRI	NPIP	21564568	2.7246	9.7476	-2.1514
YRI	NPIP	21569568	4.3977	10.4199	-1.7336
YRI	NPIP	21574568	5.2443	10.5879	-1.5154
YRI	NPIP	21579568	3.7625	8.5712	-1.6595
YRI	NPIP	21584568	3.4837	9.0754	-1.8304
YRI	NPIP	21589568	3.0854	9.7476	-2.0409
YRI	NPIP	21594568	3.427	9.7476	-1.9362
YRI	NPIP	21599568	2.8569	8.067	-1.9012
YRI	NPIP	21604568	2.3335	6.5544	-1.8609
YRI	NPIP	21609568	3.8835	7.3947	-1.3875
YRI	NPIP	21614568	3.1521	6.5544	-1.5
YRI	NPIP	21619568	2.8429	6.2183	-1.5604
YRI	NPIP	21624568	3.9046	6.7225	-1.2142
YRI	NPIP	21629568	3.4835	7.0586	-1.4738
YRI	NPIP	21634568	3.787	7.3947	-1.4256
YRI	NPIP	21639568	4.8927	8.9073	-1.3371
YRI	NPIP	21644568	4.4476	10.5879	-1.7414
YRI	NPIP	21649568	2.8288	7.5628	-1.8327
YRI	NPIP	21654568	1.9335	4.8738	-1.6878
YRI	NPIP	21659568	1.7159	5.0419	-1.8531
YRI	NPIP	21664568	2.2055	6.0502	-1.8216
YRI	NPIP	21669568	4.2612	6.8906	-1.1079

YRI	NPIP	21674568	6.1342	8.4031	-0.7974
YRI	NPIP	21679568	6.4651	9.5795	-0.9696
YRI	NPIP	21684568	9.0774	9.9157	-0.2527
YRI	NPIP	21689568	10.7749	9.7476	0.3147
YRI	NPIP	21694568	6.7452	8.9073	-0.7201
YRI	NPIP	21699568	3.8967	7.7309	-1.4548
YRI	NPIP	21704568	5.5236	7.2267	-0.6872
YRI	NPIP	21709568	7.1346	7.0586	0.0313
YRI	NPIP	21714568	6.6251	7.2267	-0.2428
YRI	NPIP	21719568	5.5053	7.8989	-0.8905
YRI	NPIP	21724568	6.2651	7.5628	-0.5024
YRI	NPIP	21729568	8.6343	8.9073	-0.0909
YRI	NPIP	21734568	8.8269	10.4199	-0.4586
YRI	NPIP	21739568	5.1669	8.235	-1.0986
YRI	NPIP	21744568	3.564	6.2183	-1.2271
YRI	NPIP	21749568	3.58	4.2016	-0.406
YRI	NPIP	21754568	1.3817	1.8487	-0.5973
YRI	NPIP	21759568	1.0344	2.1848	-1.2916
YRI	NPIP	21764568	2.0852	3.5293	-1.0949
YRI	NPIP	21769568	2.4271	5.0419	-1.4569
YRI	NPIP	21774568	3.248	6.3864	-1.4165
YRI	NPIP	21779568	4.1936	7.8989	-1.3785
YRI	NPIP	21784568	4.6372	7.3947	-1.0896
YRI	NPIP	21789568	2.7246	4.0335	-0.8856
YRI	NPIP	21794568	1.0532	3.5293	-1.8774
YRI	NPIP	21799568	0.8566	3.1932	-1.9271
YRI	NPIP	21804568	3.2984	5.5461	-1.151
YRI	NPIP	21809568	4.0375	8.5712	-1.5646
YRI	NPIP	21814568	4.0397	8.5712	-1.5638
YRI	NPIP	21819568	5.7964	10.2518	-1.3022
YRI	NPIP	21824568	4.2907	9.0754	-1.5663
YRI	NPIP	21829568	3.7806	9.4115	-1.7821
YRI	NPIP	21834568	3.3391	10.0837	-2.0019
YRI	NPIP	21839568	2.7636	7.8989	-1.9104
YRI	NPIP	21844568	3.3132	5.8822	-1.2483
YRI	NPIP	21849568	1.7824	2.5209	-0.7396
YRI	NPIP	21854568	0.2558	0.6722	-1.0901
YRI	NPIP	21859568	0.3808	1.1764	-1.423
YRI	NPIP	21864568	0.1615	0.6722	-1.337
YRI	NPIP	21869568	0.0734	0.6722	-1.5676
YRI	NPIP	21874568	0.0826	0.8403	-1.7114
YRI	NPIP	21879568	0.0093	0.1681	-0.944
YRI	NPIP	21884568	0.4369	0.3361	0.4047
YRI	NPIP	21889568	0.7217	0.6722	0.1296
YRI	NPIP	21894568	1.0818	2.1848	-1.2384
YRI	NPIP	21899568	2.2291	5.5461	-1.6986
YRI	NPIP	21904568	3.4065	6.3864	-1.3449
YRI	NPIP	21909568	3.1244	4.7057	-0.9361
YRI	NPIP	21914568	1.1778	2.5209	-1.3451

YRI	NPIP	21919568	0.3918	0.6722	-0.7341
YRI	NPIP	21924568	0.4282	0.6722	-0.6388
YRI	NPIP	21929568	0.6409	1.1764	-0.9578
YRI	NPIP	21934568	0.8498	1.5126	-0.9869
YRI	NPIP	21939568	1.1846	1.8487	-0.8494
YRI	NPIP	21944568	3.6066	4.8738	-0.7274
YRI	NPIP	21949568	3.8198	6.3864	-1.1584
YRI	NPIP	21954568	3.2913	5.2099	-1.0385
YRI	NPIP	21959568	5.5055	7.2267	-0.6945
YRI	NPIP	21964568	6.3904	8.4031	-0.7074
YRI	NPIP	21969568	3.8204	7.5628	-1.4488
YRI	NPIP	21974568	2.3295	7.7309	-2.0494
YRI	NPIP	21979568	3.2519	8.067	-1.7571
YRI	NPIP	21984568	2.3564	6.3864	-1.8189
YRI	NPIP	21989568	1.7929	6.7225	-2.1242
YRI	NPIP	21994568	2.9928	7.0586	-1.6761
YRI	NPIP	21999568	2.4829	6.0502	-1.6902
YRI	NPIP	22004568	1.3253	5.0419	-2.0708
YRI	NPIP	22009568	2.7816	5.5461	-1.4157
YRI	NPIP	22014568	4.5846	8.235	-1.307
YRI	NPIP	22019568	4.8641	7.5628	-1.0447
YRI	NPIP	22024568	2.854	6.5544	-1.6314
YRI	NPIP	22029568	2.7561	6.5544	-1.6746
YRI	NPIP	22034568	4.1571	6.7225	-1.1054
YRI	NPIP	22039568	3.4046	6.0502	-1.2535
YRI	NPIP	22044568	3.1354	6.7225	-1.5457
YRI	NPIP	22049568	2.8079	7.2267	-1.7831
YRI	NPIP	22054568	3.5405	7.3947	-1.523
YRI	NPIP	22059568	3.8349	6.7225	-1.2443
YRI	NPIP	22064568	2.7945	5.0419	-1.2522
YRI	NPIP	22069568	3.0319	5.7141	-1.3375
YRI	NPIP	22074568	1.8266	4.0335	-1.4932
YRI	NPIP	22079568	3.2724	4.7057	-0.8484
YRI	NPIP	22084568	3.5503	5.5461	-1.022
YRI	NPIP	22089568	1.4378	6.0502	-2.1854
YRI	NPIP	22094568	1.9843	7.0586	-2.0919
YRI	NPIP	22099568	1.9031	4.8738	-1.7053
YRI	NPIP	22104568	3.3569	5.7141	-1.1754
YRI	NPIP	22109568	3.3754	6.3864	-1.359
YRI	NPIP	22114568	2.8111	7.0586	-1.751
YRI	NPIP	22119568	2.7836	7.8989	-1.903
YRI	NPIP	22124568	4.1363	7.3947	-1.2876
YRI	NPIP	22129568	4.8179	7.2267	-0.972
YRI	NPIP	22134568	5.532	9.0754	-1.1599
YRI	NPIP	22139568	6.2581	9.5795	-1.0341
YRI	NPIP	22144568	4.5678	7.0586	-1.0268
YRI	NPIP	22149568	4.3416	6.5544	-0.9756
YRI	NPIP	22154568	4.2558	7.2267	-1.1988
YRI	NPIP	22159568	3.519	9.0754	-1.8189

YRI	NPIP	22164568	3.0999	9.4115	-1.9976
YRI	NPIP	22169568	5.1075	9.4115	-1.3622
YRI	NPIP	22174568	5.634	10.924	-1.457
YRI	NPIP	22179568	3.8218	8.235	-1.5802
YRI	NPIP	22184568	5.9219	7.3947	-0.582
YRI	NPIP	22189568	7.9142	10.4199	-0.7213
YRI	NPIP	22194568	8.2093	9.9157	-0.5145
YRI	NPIP	22199568	6.4404	8.235	-0.6426
YRI	NPIP	22204568	7.8673	9.5795	-0.5331
YRI	NPIP	22209568	9.498	11.0921	-0.4328
YRI	NPIP	22214568	5.717	8.9073	-1.0626
YRI	NPIP	22219568	6.6861	8.235	-0.5546
YRI	NPIP	22224568	9.371	10.5879	-0.3451
YRI	NPIP	22229568	10.3648	11.0921	-0.1975
YRI	NPIP	22234568	9.2705	9.4115	-0.0446
YRI	NPIP	22239568	8.6796	9.2434	-0.1814
YRI	NPIP	22244568	9.1413	11.0921	-0.5297
YRI	NPIP	22249568	8.0982	10.4199	-0.6683
YRI	NPIP	22254568	8.5806	10.5879	-0.5693
YRI	NPIP	22259568	7.8544	10.5879	-0.7752
YRI	NPIP	22264568	7.0812	8.5712	-0.5142
YRI	NPIP	22269568	6.5974	8.9073	-0.7693
YRI	NPIP	22274568	7.1291	9.7476	-0.8021
YRI	NPIP	22279568	8.9525	9.0754	-0.0402
YRI	NPIP	22284568	9.3867	9.4115	-0.0078
YRI	NPIP	22289568	8.4825	10.924	-0.6724
YRI	NPIP	22294568	8.9122	10.2518	-0.3915
YRI	NPIP	22299568	11.6263	12.6047	-0.2355
YRI	NPIP	22304568	10.1963	13.7811	-0.7931
YRI	NPIP	22309568	8.5489	10.4199	-0.5386
YRI	NPIP	22314568	8.2536	10.0837	-0.5432
YRI	NPIP	22319568	5.5836	9.4115	-1.2115
YRI	NPIP	22324568	5.2209	10.0837	-1.4434
YRI	NPIP	22329568	5.5681	10.756	-1.4497
YRI	NPIP	22334568	4.8126	9.7476	-1.5118
YRI	NPIP	22339568	4.4785	11.4282	-1.8347
YRI	NPIP	22344568	3.9425	9.9157	-1.8009
YRI	NPIP	22349568	3.3444	6.5544	-1.4152
CEU	NPIP	22008599	2.0641	4.7757	-1.5948
CEU	NPIP	22013599	3.5067	5.9697	-1.1887
CEU	NPIP	22018599	3.1148	5.1169	-1.1082
CEU	NPIP	22023599	2.4929	4.0935	-1.0763
CEU	NPIP	22028599	1.7539	3.0701	-1.129
CEU	NPIP	22033599	1.8499	3.7524	-1.3783
CEU	NPIP	22038599	1.9081	4.6052	-1.6375
CEU	NPIP	22043599	1.3581	3.2407	-1.5437
CEU	NPIP	22048599	1.3006	2.2173	-1.0241
CEU	NPIP	22053599	1.9365	2.8996	-0.8661
CEU	NPIP	22058599	2.5455	4.9463	-1.3692

CEU	NPIP	22063599	2.6423	5.2874	-1.4223
CEU	NPIP	22068599	2.818	4.7757	-1.1514
CEU	NPIP	22073599	2.3527	4.6052	-1.3676
CEU	NPIP	22078599	2.3593	4.6052	-1.3636
CEU	NPIP	22083599	3.1103	5.2874	-1.1706
CEU	NPIP	22088599	2.3849	4.4346	-1.2861
CEU	NPIP	22093599	1.2847	3.2407	-1.6039
CEU	NPIP	22098599	0.5714	2.2173	-1.8388
CEU	NPIP	22103599	2.0072	4.6052	-1.5774
CEU	NPIP	22108599	1.9907	4.9463	-1.6856
CEU	NPIP	22113599	1.0072	3.4112	-1.8881
CEU	NPIP	22118599	1.752	4.4346	-1.6832
CEU	NPIP	22123599	3.135	5.6286	-1.2684
CEU	NPIP	22128599	3.8343	6.1402	-1.0851
CEU	NPIP	22133599	4.6545	7.3342	-1.0737
CEU	NPIP	22138599	6.8289	7.8459	-0.3831
CEU	NPIP	22143599	6.8136	6.6519	0.0708
CEU	NPIP	22148599	4.2191	5.7991	-0.7826
CEU	NPIP	22153599	2.9388	6.3108	-1.5482
CEU	NPIP	22158599	2.8705	5.9697	-1.4957
CEU	NPIP	22163599	1.8112	4.4346	-1.646
CEU	NPIP	22168599	2.8093	5.2874	-1.3325
CEU	NPIP	22173599	3.2476	5.9697	-1.3137
CEU	NPIP	22178599	2.6751	5.6286	-1.5023
CEU	NPIP	22183599	3.5302	5.1169	-0.8783
CEU	NPIP	22188599	3.8021	5.2874	-0.7987
CEU	NPIP	22193599	3.8299	5.458	-0.8511
CEU	NPIP	22198599	4.4659	5.2874	-0.4417
CEU	NPIP	22203599	5.9423	5.458	0.2532
CEU	NPIP	22208599	5.7829	5.7991	-0.008
CEU	NPIP	22213599	3.5374	4.6052	-0.6483
CEU	NPIP	22218599	4.8723	6.1402	-0.5966
CEU	NPIP	22223599	6.8797	9.5515	-0.8394
CEU	NPIP	22228599	6.8701	9.7221	-0.8814
CEU	NPIP	22233599	8.2556	8.6987	-0.1518
CEU	NPIP	22238599	7.6065	7.6753	-0.0264
CEU	NPIP	22243599	5.4925	7.3342	-0.7379
CEU	NPIP	22248599	6.1851	8.187	-0.7252
CEU	NPIP	22253599	5.3115	6.4814	-0.5244
CEU	NPIP	22258599	4.6316	5.2874	-0.3526
CEU	NPIP	22263599	5.4745	6.9931	-0.6354
CEU	NPIP	22268599	4.1344	6.8225	-1.1502
CEU	NPIP	22273599	3.1204	6.1402	-1.421
CEU	NPIP	22278599	2.7759	6.3108	-1.6229
CEU	NPIP	22283599	2.5947	6.6519	-1.7763
CEU	NPIP	22288599	2.9365	5.9697	-1.4639
CEU	NPIP	22293599	3.207	5.458	-1.1768
CEU	NPIP	22298599	3.7271	5.7991	-1.0262
CEU	NPIP	22303599	3.3156	6.6519	-1.4607

CEU	NPIP	22308599	2.7671	8.187	-1.9635
CEU	NPIP	22313599	2.6011	8.3576	-2.0463
CEU	NPIP	22318599	1.8221	5.7991	-1.9698
CEU	NPIP	22323599	1.5937	3.9229	-1.6245
CEU	NPIP	22328599	2.0695	5.2874	-1.7303
CEU	NPIP	22333599	1.7182	5.1169	-1.8812
CEU	NPIP	22338599	1.751	4.7757	-1.7789
CEU	NPIP	22343599	2.267	5.2874	-1.6241
CEU	NPIP	22348599	1.5339	3.5818	-1.5435
CEU	NPIP	22353599	1.3255	2.3879	-1.1188
CEU	NPIP	22358599	1.1936	2.5584	-1.3596
CEU	NPIP	22363599	0.5312	2.0467	-1.8033
CEU	NPIP	22368599	0.9829	2.2173	-1.3791
CEU	NPIP	22373599	1.9031	3.4112	-1.1845
CEU	NPIP	22378599	2.4232	3.5818	-0.8732
CEU	NPIP	22383599	1.7137	2.8996	-1.0665
CEU	NPIP	22388599	1.4026	3.7524	-1.7024
CEU	NPIP	22393599	1.7328	4.7757	-1.7897
CEU	NPIP	22398599	1.2723	4.7757	-2.0605
CEU	NPIP	22403599	2.0095	5.1169	-1.72
CEU	NPIP	22408599	1.8011	4.2641	-1.5988
CEU	NPIP	22413599	0.9384	3.9229	-2.0816
CEU	NPIP	22418599	1.0066	4.7757	-2.2168
CEU	NPIP	22423599	1.1958	5.2874	-2.2001
CEU	NPIP	22428599	0.9518	4.7757	-2.249
CEU	NPIP	22433599	1.6388	4.9463	-1.8863
CEU	NPIP	22438599	2.0289	4.4346	-1.5094
CEU	NPIP	22443599	0.9893	2.5584	-1.5631
CEU	NPIP	22448599	0.4999	1.1939	-1.2367
CEU	NPIP	22453599	0.2995	0.3411	-0.1668
CEU	NPIP	22458599	0.1616	0.5117	-1.098
CEU	NPIP	22463599	0.0897	0.6822	-1.5472
CEU	NPIP	22468599	0.1843	0.5117	-1.0268
CEU	NPIP	22473599	0.2698	0.8528	-1.3129
CEU	NPIP	22478599	0.155	0.6822	-1.3767
CEU	NPIP	22483599	0.2942	1.0234	-1.449
CEU	NPIP	22488599	0.7767	1.5351	-1.1246
CEU	NPIP	22493599	0.4826	0.5117	-0.0913
CEU	NPIP	22498599	0.0684	0.3411	-1.0932
CEU	NPIP	22503599	0.0684	0.3411	-1.0932
CEU	NPIP	22508599	0.0101	0.1706	-0.9534
CEU	NPIP	22513599	0.1148	0.3411	-0.9072
CEU	NPIP	22518599	0.1047	0.1706	-0.3915
CEU	NPIP	22523599	0	0.3411	-1.3674
CEU	NPIP	22528599	0.0202	0.6822	-1.7287
CEU	NPIP	22533599	0.1616	0.5117	-1.098
CEU	NPIP	22538599	0.3226	0.5117	-0.5931
CEU	NPIP	22543599	0.2311	0.6822	-1.178
CEU	NPIP	22548599	0.2002	2.0467	-2.1971

CEU	NPIP	22553599	1.235	5.2874	-2.179
CEU	NPIP	22558599	1.9812	7.6753	-2.1887
CEU	NPIP	22563599	1.0767	5.7991	-2.339
CEU	NPIP	22568599	0.1802	1.7056	-2.0892
CEU	NPIP	22573599	0.1189	0.6822	-1.4709
CEU	NPIP	22578599	1.2432	2.3879	-1.2055
CEU	NPIP	22583599	2.1884	5.2874	-1.6663
CEU	NPIP	22588599	1.7831	6.1402	-2.0503
CEU	NPIP	22593599	1.5706	5.7991	-2.0943
CEU	NPIP	22598599	2.1433	6.6519	-1.974
CEU	NPIP	22603599	1.7881	3.5818	-1.3519
CEU	NPIP	22608599	1.3966	1.7056	-0.4232
CEU	NPIP	22613599	1.2402	2.0467	-0.9596
CEU	NPIP	22618599	1.0239	4.2641	-2.1034
CEU	NPIP	22623599	1.4356	7.3342	-2.3634
CEU	NPIP	22628599	1.9833	6.8225	-2.0707
CEU	NPIP	22633599	4.3106	7.8459	-1.3318
CEU	NPIP	22638599	5.5142	8.5281	-1.0516
CEU	NPIP	22643599	5.073	8.6987	-1.2421
CEU	NPIP	22648599	5.853	8.3576	-0.8903
CEU	NPIP	22653599	7.4094	7.8459	-0.1644
CEU	NPIP	22658599	6.8182	8.187	-0.4959
CEU	NPIP	22663599	5.1455	8.0164	-1.0604
CEU	NPIP	22668599	4.0058	6.4814	-1.1096
CEU	NPIP	22673599	3.8141	5.1169	-0.7211
CEU	NPIP	22678599	4.073	6.6519	-1.1291
CEU	NPIP	22683599	3.0216	6.6519	-1.5894
CEU	NPIP	22688599	2.4482	5.7991	-1.6597
CEU	NPIP	22693599	3.6174	8.8692	-1.7672
CEU	NPIP	22698599	4.8116	9.3809	-1.4597
CEU	NPIP	22703599	3.6337	6.1402	-1.1795
CEU	NPIP	22708599	2.939	5.2874	-1.2627
CEU	NPIP	22713599	3.8435	6.6519	-1.2296
CEU	NPIP	22718599	5.6832	7.8459	-0.8147
CEU	NPIP	22723599	7.133	8.5281	-0.4868
CEU	NPIP	22728599	13.0616	10.0632	0.8973
CEU	NPIP	22733599	15.9641	10.5749	1.5398
CEU	NPIP	22738599	8.1152	7.3342	0.3129
CEU	NPIP	22743599	5.0703	6.1402	-0.5035
CEU	NPIP	22748599	5.1319	6.6519	-0.6655
CEU	NPIP	22753599	4.4702	7.1636	-1.1025
CEU	NPIP	22758599	6.2593	8.8692	-0.8782
CEU	NPIP	22763599	5.9876	8.187	-0.7968
CEU	NPIP	22768599	7.1216	7.3342	-0.0852
CEU	NPIP	22773599	7.7724	6.6519	0.4906
CEU	NPIP	22778599	5.5077	5.6286	-0.0615
CEU	NPIP	22783599	5.3694	6.4814	-0.4984
CEU	NPIP	22788599	8.5925	8.5281	0.0225
CEU	NPIP	22793599	9.8277	8.3576	0.5226

CEU	NPIP	22798599	9.699	8.5281	0.4085
CEU	NPIP	22803599	13.5683	9.5515	1.2619
CEU	NPIP	22808599	13.1206	9.2104	1.2706
CEU	NPIP	22813599	7.1915	7.5047	-0.1229
CEU	NPIP	22818599	7.0542	7.5047	-0.1768
CEU	NPIP	22823599	8.8062	8.187	0.2243
CEU	NPIP	22828599	6.5088	6.1402	0.1735
CEU	NPIP	22833599	15.1154	11.9394	0.8097
CEU	NPIP	22838599	18.0104	14.1567	0.8363
CEU	NPIP	22843599	12.1038	9.5515	0.8018
CEU	NPIP	22848599	10.122	8.5281	0.5561
CEU	NPIP	22853599	8.831	8.0164	0.3009
CEU	NPIP	22858599	8.2962	7.8459	0.1696
CEU	NPIP	22863599	5.7374	7.1636	-0.5838
CEU	NPIP	22868599	8.6533	9.0398	-0.1278
CEU	NPIP	22873599	9.6564	11.2571	-0.4313
CEU	NPIP	22878599	5.7588	8.6987	-1.0072
CEU	NPIP	22883599	5.4888	7.5047	-0.7909
CEU	NPIP	22888599	7.032	8.8692	-0.6182
CEU	NPIP	22893599	6.9064	7.5047	-0.2347
CEU	NPIP	22898599	9.2391	6.9931	0.9398
CEU	NPIP	22903599	9.3203	6.6519	1.1683
CEU	NPIP	22908599	5.5114	5.2874	0.1204
CEU	NPIP	22913599	6.3609	5.458	0.472
CEU	NPIP	22918599	7.628	6.6519	0.4274
CEU	NPIP	22923599	7.4185	8.8692	-0.4882
CEU	NPIP	22928599	9.7471	11.7688	-0.5225
CEU	NPIP	22933599	14.2305	14.6684	-0.0919
CEU	NPIP	22938599	12.8382	12.6216	0.0524
CEU	NPIP	22943599	10.7306	10.916	-0.0514
CEU	NPIP	22948599	14.1847	13.4744	0.1615
CEU	NPIP	22953599	10.4572	10.2337	0.0658
CEU	NPIP	22958599	11.0759	10.0632	0.3031
CEU	NPIP	22963599	11.8609	10.7454	0.314
CEU	NPIP	22968599	11.4985	9.2104	0.7435
CEU	NPIP	22973599	11.8561	9.0398	0.9311
CEU	NPIP	22978599	9.8503	8.5281	0.4613
CEU	NPIP	22983599	13.1752	10.0632	0.9313
CEU	NPIP	22988599	13.5238	10.5749	0.8426
CEU	NPIP	22993599	10.7343	9.3809	0.4324
CEU	NPIP	22998599	7.1727	7.8459	-0.2536
CEU	NPIP	23003599	5.5279	7.1636	-0.6696
CEU	NPIP	23008599	17.0219	12.4511	1.1201
CEU	NPIP	23013599	24.9375	14.4978	2.2148
CEU	NPIP	23018599	18.6865	11.0866	2.0774
CEU	NPIP	23023599	18.1212	13.3039	1.1089
CEU	NPIP	23028599	12.572	10.916	0.4593
CHB	NPIP	22008599	2.2237	5.2519	-1.6332
CHB	NPIP	22013599	3.8945	4.913	-0.5826

CHB	NPIP	22018599	3.9783	4.066	-0.0591
CHB	NPIP	22023599	3.5248	4.4048	-0.5537
CHB	NPIP	22028599	2.3581	4.066	-1.1517
CHB	NPIP	22033599	2.9966	3.8965	-0.6294
CHB	NPIP	22038599	3.0887	3.5577	-0.3544
CHB	NPIP	22043599	1.8062	3.0495	-1.0692
CHB	NPIP	22048599	1.9547	3.0495	-0.9415
CHB	NPIP	22053599	2.2296	3.7271	-1.0879
CHB	NPIP	22058599	3.999	5.4213	-0.7458
CHB	NPIP	22063599	4.7426	5.2519	-0.2747
CHB	NPIP	22068599	4.02	4.913	-0.5108
CHB	NPIP	22073599	3.3876	4.913	-0.8725
CHB	NPIP	22078599	2.9834	4.4048	-0.8944
CHB	NPIP	22083599	3.4586	4.913	-0.8319
CHB	NPIP	22088599	2.9151	4.7436	-1.0786
CHB	NPIP	22093599	1.81	3.2189	-1.1583
CHB	NPIP	22098599	0.7192	2.3718	-1.7444
CHB	NPIP	22103599	2.4019	4.2354	-1.1936
CHB	NPIP	22108599	2.8565	3.8965	-0.7274
CHB	NPIP	22113599	1.9661	4.913	-1.6856
CHB	NPIP	22118599	2.4767	6.946	-1.876
CHB	NPIP	22123599	3.9551	6.4378	-1.1163
CHB	NPIP	22128599	4.8068	6.6072	-0.7908
CHB	NPIP	22133599	5.8935	7.6237	-0.6672
CHB	NPIP	22138599	7.4202	8.6402	-0.4194
CHB	NPIP	22143599	6.7001	7.6237	-0.3562
CHB	NPIP	22148599	3.9517	5.9295	-0.9575
CHB	NPIP	22153599	2.6094	5.7601	-1.5653
CHB	NPIP	22158599	3.8441	6.4378	-1.1662
CHB	NPIP	22163599	3.2861	6.4378	-1.4171
CHB	NPIP	22168599	4.3309	6.946	-1.0977
CHB	NPIP	22173599	4.0249	6.2684	-1.0333
CHB	NPIP	22178599	2.4331	6.2684	-1.7664
CHB	NPIP	22183599	4.2836	7.6237	-1.288
CHB	NPIP	22188599	4.9507	7.7931	-1.0743
CHB	NPIP	22193599	4.8003	7.6237	-1.0888
CHB	NPIP	22198599	4.6811	7.9625	-1.216
CHB	NPIP	22203599	6.6722	9.6567	-0.9254
CHB	NPIP	22208599	7.6122	9.4872	-0.591
CHB	NPIP	22213599	4.1416	7.1154	-1.2213
CHB	NPIP	22218599	6.6493	7.7931	-0.4323
CHB	NPIP	22223599	10.0133	8.4708	0.54
CHB	NPIP	22228599	9.0616	7.9625	0.4073
CHB	NPIP	22233599	9.4912	7.4543	0.8018
CHB	NPIP	22238599	10.0552	8.1319	0.6991
CHB	NPIP	22243599	7.7177	7.6237	0.0362
CHB	NPIP	22248599	5.8542	6.4378	-0.2624
CHB	NPIP	22253599	6.028	7.7931	-0.6671
CHB	NPIP	22258599	5.3857	6.7766	-0.5971

CHB	NPIP	22263599	4.2574	6.2684	-0.9262
CHB	NPIP	22268599	2.9311	7.6237	-1.8096
CHB	NPIP	22273599	2.4262	7.2848	-1.953
CHB	NPIP	22278599	1.9052	7.4543	-2.1843
CHB	NPIP	22283599	1.7377	7.6237	-2.2698
CHB	NPIP	22288599	1.9844	6.7766	-2.0571
CHB	NPIP	22293599	2.6718	6.7766	-1.762
CHB	NPIP	22298599	5.234	8.4708	-1.1331
CHB	NPIP	22303599	5.6539	10.5037	-1.3904
CHB	NPIP	22308599	4.1354	10.8426	-1.8666
CHB	NPIP	22313599	3.6806	8.6402	-1.7048
CHB	NPIP	22318599	3.1239	6.2684	-1.4482
CHB	NPIP	22323599	3.2286	6.2684	-1.4
CHB	NPIP	22328599	3.8768	7.2848	-1.3699
CHB	NPIP	22333599	3.9615	7.7931	-1.4481
CHB	NPIP	22338599	4.5207	7.9625	-1.2754
CHB	NPIP	22343599	4.1138	6.946	-1.1888
CHB	NPIP	22348599	2.6659	5.7601	-1.5373
CHB	NPIP	22353599	2.5071	5.5907	-1.5733
CHB	NPIP	22358599	1.5783	4.2354	-1.7298
CHB	NPIP	22363599	0.6728	3.7271	-2.2189
CHB	NPIP	22368599	1.3998	4.066	-1.7979
CHB	NPIP	22373599	2.387	4.5742	-1.3318
CHB	NPIP	22378599	2.4803	5.5907	-1.587
CHB	NPIP	22383599	1.858	5.4213	-1.8685
CHB	NPIP	22388599	2.2383	6.0989	-1.8224
CHB	NPIP	22393599	2.7318	5.9295	-1.5481
CHB	NPIP	22398599	1.7217	4.2354	-1.6364
CHB	NPIP	22403599	3.2353	6.0989	-1.3517
CHB	NPIP	22408599	3.7205	7.7931	-1.5392
CHB	NPIP	22413599	2.261	6.946	-1.9666
CHB	NPIP	22418599	2.9958	6.7766	-1.623
CHB	NPIP	22423599	2.351	6.6072	-1.8694
CHB	NPIP	22428599	1.4963	5.7601	-2.1184
CHB	NPIP	22433599	2.9444	5.9295	-1.4451
CHB	NPIP	22438599	3.5151	6.6072	-1.3581
CHB	NPIP	22443599	2.1346	5.4213	-1.7234
CHB	NPIP	22448599	0.8586	3.0495	-1.8842
CHB	NPIP	22453599	0.4375	1.0165	-1.1522
CHB	NPIP	22458599	0.4375	1.0165	-1.1522
CHB	NPIP	22463599	0.1064	1.3553	-2.0241
CHB	NPIP	22468599	0.4278	0.6777	-0.6533
CHB	NPIP	22473599	1.0556	1.5247	-0.6969
CHB	NPIP	22478599	0.6762	1.3553	-1.1006
CHB	NPIP	22483599	0.186	1.0165	-1.6527
CHB	NPIP	22488599	0.8441	1.6942	-1.1667
CHB	NPIP	22493599	0.6775	0.8471	-0.3824
CHB	NPIP	22498599	0.1038	0.5082	-1.2695
CHB	NPIP	22503599	0.0843	0.3388	-1.0209

CHB	NPIP	22508599	0	0	0
CHB	NPIP	22513599	0.1609	0.1694	-0.0505
CHB	NPIP	22518599	0.1609	0.1694	-0.0505
CHB	NPIP	22523599	0	0.3388	-1.359
CHB	NPIP	22528599	0.048	0.6777	-1.6462
CHB	NPIP	22533599	0.0577	0.5082	-1.4142
CHB	NPIP	22538599	0.2088	0.5082	-0.9399
CHB	NPIP	22543599	0.5016	1.1859	-1.2215
CHB	NPIP	22548599	0.7139	2.7106	-1.892
CHB	NPIP	22553599	3.0278	4.913	-1.0783
CHB	NPIP	22558599	5.1279	8.6402	-1.2073
CHB	NPIP	22563599	3.5197	7.4543	-1.5488
CHB	NPIP	22568599	1.0276	2.2024	-1.3155
CHB	NPIP	22573599	0.0874	1.3553	-2.0549
CHB	NPIP	22578599	1.633	3.0495	-1.2182
CHB	NPIP	22583599	4.3484	6.4378	-0.9395
CHB	NPIP	22588599	3.6636	6.6072	-1.2929
CHB	NPIP	22593599	1.8757	4.5742	-1.6432
CHB	NPIP	22598599	3.222	7.7931	-1.7276
CHB	NPIP	22603599	2.7371	5.7601	-1.5019
CHB	NPIP	22608599	1.8666	2.033	-0.1984
CHB	NPIP	22613599	1.4335	2.5412	-1.1061
CHB	NPIP	22618599	1.8021	2.7106	-0.8609
CHB	NPIP	22623599	4.5948	6.2684	-0.7708
CHB	NPIP	22628599	6.3366	9.1484	-0.9168
CHB	NPIP	22633599	7.2161	10.3343	-0.9077
CHB	NPIP	22638599	5.202	8.3013	-1.1054
CHB	NPIP	22643599	4.9625	7.4543	-0.9808
CHB	NPIP	22648599	6.5492	7.6237	-0.4144
CHB	NPIP	22653599	7.3415	7.9625	-0.2301
CHB	NPIP	22658599	8.0051	10.3343	-0.678
CHB	NPIP	22663599	6.1673	10.5037	-1.2432
CHB	NPIP	22668599	3.9844	9.4872	-1.7346
CHB	NPIP	22673599	5.011	8.3013	-1.1735
CHB	NPIP	22678599	5.8567	8.979	-1.0358
CHB	NPIP	22683599	4.5069	8.1319	-1.3176
CHB	NPIP	22688599	3.8016	7.2848	-1.4001
CHB	NPIP	22693599	4.2977	7.7931	-1.3211
CHB	NPIP	22698599	5.6044	7.9625	-0.8738
CHB	NPIP	22703599	4.5664	7.9625	-1.2585
CHB	NPIP	22708599	4.0357	8.1319	-1.4889
CHB	NPIP	22713599	4.9271	7.9625	-1.1248
CHB	NPIP	22718599	5.6221	6.946	-0.5557
CHB	NPIP	22723599	7.6284	9.8261	-0.6705
CHB	NPIP	22728599	15.3695	14.7391	0.1313
CHB	NPIP	22733599	18.261	15.925	0.4519
CHB	NPIP	22738599	8.8936	11.3508	-0.6551
CHB	NPIP	22743599	5.1467	9.3178	-1.337
CHB	NPIP	22748599	5.7397	11.012	-1.4461

CHB	NPIP	22753599	5.1664	11.1814	-1.6264
CHB	NPIP	22758599	6.1125	8.6402	-0.8689
CHB	NPIP	22763599	4.9225	6.0989	-0.5553
CHB	NPIP	22768599	5.3895	8.1319	-0.9968
CHB	NPIP	22773599	5.4761	7.7931	-0.8757
CHB	NPIP	22778599	4.015	6.2684	-1.0378
CHB	NPIP	22783599	4.2003	6.7766	-1.1059
CHB	NPIP	22788599	6.9406	8.1319	-0.433
CHB	NPIP	22793599	8.7314	10.5037	-0.5081
CHB	NPIP	22798599	9.348	12.3673	-0.7425
CHB	NPIP	22803599	13.5351	12.3673	0.2872
CHB	NPIP	22808599	13.0169	9.8261	0.9735
CHB	NPIP	22813599	7.6113	8.8096	-0.4046
CHB	NPIP	22818599	7.6236	10.8426	-0.8959
CHB	NPIP	22823599	8.7221	11.6896	-0.7696
CHB	NPIP	22828599	6.1725	8.8096	-0.8904
CHB	NPIP	22833599	14.8578	12.5367	0.5635
CHB	NPIP	22838599	18.8908	15.4168	0.6931
CHB	NPIP	22843599	13.4564	11.8591	0.4087
CHB	NPIP	22848599	10.7655	11.5202	-0.1984
CHB	NPIP	22853599	10.2701	12.3673	-0.5157
CHB	NPIP	22858599	10.32	12.0285	-0.4313
CHB	NPIP	22863599	7.7131	10.3343	-0.763
CHB	NPIP	22868599	11.3695	12.0285	-0.1664
CHB	NPIP	22873599	13.799	13.7226	0.017
CHB	NPIP	22878599	10.8679	12.0285	-0.293
CHB	NPIP	22883599	10.2334	11.6896	-0.3776
CHB	NPIP	22888599	9.5243	10.8426	-0.3669
CHB	NPIP	22893599	7.4342	7.9625	-0.1958
CHB	NPIP	22898599	10.0982	8.1319	0.7147
CHB	NPIP	22903599	8.8822	7.6237	0.4853
CHB	NPIP	22908599	4.5517	6.0989	-0.7303
CHB	NPIP	22913599	6.3065	6.6072	-0.1321
CHB	NPIP	22918599	8.0524	8.1319	-0.0289
CHB	NPIP	22923599	7.6208	9.9955	-0.7131
CHB	NPIP	22928599	12.5985	13.045	-0.1044
CHB	NPIP	22933599	18.1443	15.0779	0.6249
CHB	NPIP	22938599	13.1776	13.2144	-0.0085
CHB	NPIP	22943599	9.9989	12.3673	-0.5824
CHB	NPIP	22948599	12.8976	13.045	-0.0345
CHB	NPIP	22953599	9.2393	11.1814	-0.5251
CHB	NPIP	22958599	11.6136	13.3838	-0.404
CHB	NPIP	22963599	16.8344	17.2803	-0.0798
CHB	NPIP	22968599	17.2951	17.7886	-0.0859
CHB	NPIP	22973599	16.0194	18.4662	-0.4107
CHB	NPIP	22978599	12.7714	16.4333	-0.6874
CHB	NPIP	22983599	13.8576	13.2144	0.1486
CHB	NPIP	22988599	14.213	13.3838	0.1892
CHB	NPIP	22993599	11.8555	12.8755	-0.2415

CHB	NPIP	22998599	8.9989	11.3508	-0.627
CHB	NPIP	23003599	6.5106	9.6567	-0.9755
CHB	NPIP	23008599	14.5686	10.8426	1.037
CHB	NPIP	23013599	20.6439	12.8755	1.8391
CHB	NPIP	23018599	18.8733	11.012	2.1563
CHB	NPIP	23023599	19.5446	12.3673	1.7651
CHB	NPIP	23028599	11.9604	11.1814	0.2106
YRI	NPIP	22008599	2.7175	5.2099	-1.3491
YRI	NPIP	22013599	4.2615	7.3947	-1.2381
YRI	NPIP	22018599	4.4146	7.5628	-1.2188
YRI	NPIP	22023599	3.0407	6.7225	-1.5865
YRI	NPIP	22028599	2.5663	6.5544	-1.7583
YRI	NPIP	22033599	4.266	7.2267	-1.1947
YRI	NPIP	22038599	3.3782	6.0502	-1.266
YRI	NPIP	22043599	3.0893	6.0502	-1.4029
YRI	NPIP	22048599	3.4098	6.8906	-1.4667
YRI	NPIP	22053599	3.0744	7.2267	-1.6755
YRI	NPIP	22058599	3.5031	7.2267	-1.5025
YRI	NPIP	22063599	3.2438	5.5461	-1.179
YRI	NPIP	22068599	2.9575	5.378	-1.2739
YRI	NPIP	22073599	1.9171	4.5377	-1.6013
YRI	NPIP	22078599	2.7013	4.2016	-0.98
YRI	NPIP	22083599	3.5227	5.2099	-0.9132
YRI	NPIP	22088599	2.4799	6.5544	-1.7964
YRI	NPIP	22093599	1.7249	6.5544	-2.1292
YRI	NPIP	22098599	1.9026	5.2099	-1.7902
YRI	NPIP	22103599	3.6347	6.5544	-1.2872
YRI	NPIP	22108599	3.3423	6.5544	-1.4161
YRI	NPIP	22113599	2.3796	6.7225	-1.8714
YRI	NPIP	22118599	2.6196	7.8989	-1.964
YRI	NPIP	22123599	4.5225	7.5628	-1.177
YRI	NPIP	22128599	4.6409	6.7225	-0.897
YRI	NPIP	22133599	5.356	8.7392	-1.1468
YRI	NPIP	22138599	6.2934	9.9157	-1.0921
YRI	NPIP	22143599	4.7981	7.5628	-1.0703
YRI	NPIP	22148599	4.4383	6.3864	-0.8792
YRI	NPIP	22153599	4.0884	6.8906	-1.1808
YRI	NPIP	22158599	3.7332	8.7392	-1.6969
YRI	NPIP	22163599	3.0081	9.4115	-2.0266
YRI	NPIP	22168599	4.9047	9.7476	-1.4836
YRI	NPIP	22173599	5.6922	11.0921	-1.4661
YRI	NPIP	22178599	3.7458	8.5712	-1.6653
YRI	NPIP	22183599	5.6805	6.7225	-0.449
YRI	NPIP	22188599	7.6908	9.9157	-0.6708
YRI	NPIP	22193599	6.7962	9.5795	-0.8665
YRI	NPIP	22198599	6.4388	8.4031	-0.6904
YRI	NPIP	22203599	9.1391	10.0837	-0.2804
YRI	NPIP	22208599	9.7804	11.0921	-0.3561
YRI	NPIP	22213599	5.9153	8.4031	-0.8744

YRI	NPIP	22218599	6.8834	8.4031	-0.5341
YRI	NPIP	22223599	8.5526	10.5879	-0.5772
YRI	NPIP	22228599	8.7643	10.5879	-0.5172
YRI	NPIP	22233599	10.4559	10.2518	0.0597
YRI	NPIP	22238599	9.6177	9.4115	0.0653
YRI	NPIP	22243599	7.8952	10.0837	-0.6496
YRI	NPIP	22248599	8.7329	11.0921	-0.6405
YRI	NPIP	22253599	8.765	10.924	-0.5946
YRI	NPIP	22258599	8.1199	10.5879	-0.6999
YRI	NPIP	22263599	7.9146	8.7392	-0.2795
YRI	NPIP	22268599	6.1793	8.235	-0.7361
YRI	NPIP	22273599	6.9474	9.9157	-0.8949
YRI	NPIP	22278599	9.2361	9.7476	-0.1567
YRI	NPIP	22283599	9.7424	9.7476	-0.0016
YRI	NPIP	22288599	7.3928	8.7392	-0.4564
YRI	NPIP	22293599	7.989	9.9157	-0.5809
YRI	NPIP	22298599	12.9056	14.1172	-0.262
YRI	NPIP	22303599	10.6931	13.7811	-0.6832
YRI	NPIP	22308599	8.8192	11.0921	-0.6171
YRI	NPIP	22313599	8.6532	9.9157	-0.3806
YRI	NPIP	22318599	4.9724	8.7392	-1.2768
YRI	NPIP	22323599	4.6826	9.5795	-1.5246
YRI	NPIP	22328599	5.0706	10.4199	-1.5399
YRI	NPIP	22333599	5.3216	9.9157	-1.3851
YRI	NPIP	22338599	5.3896	11.5963	-1.6163
YRI	NPIP	22343599	4.0347	10.756	-1.8782
YRI	NPIP	22348599	3.4672	7.5628	-1.5855
YRI	NPIP	22353599	3.419	6.2183	-1.2941
YRI	NPIP	22358599	2.773	5.2099	-1.319
YRI	NPIP	22363599	1.4007	4.8738	-1.9937
YRI	NPIP	22368599	2.4154	6.8906	-1.8857
YRI	NPIP	22373599	3.3445	7.5628	-1.633
YRI	NPIP	22378599	2.7118	6.8906	-1.7608
YRI	NPIP	22383599	3.2236	6.8906	-1.5452
YRI	NPIP	22388599	4.1332	6.7225	-1.1157
YRI	NPIP	22393599	3.8217	6.8906	-1.2931
YRI	NPIP	22398599	2.5281	6.2183	-1.706
YRI	NPIP	22403599	4.6842	7.2267	-1.0259
YRI	NPIP	22408599	5.701	8.4031	-0.9497
YRI	NPIP	22413599	3.0563	9.0754	-1.9703
YRI	NPIP	22418599	3.3804	8.5712	-1.7914
YRI	NPIP	22423599	4.2574	8.5712	-1.4887
YRI	NPIP	22428599	2.9949	8.067	-1.8508
YRI	NPIP	22433599	3.121	8.067	-1.8048
YRI	NPIP	22438599	2.8048	8.9073	-2.0325
YRI	NPIP	22443599	1.4778	6.0502	-2.1664
YRI	NPIP	22448599	0.7241	3.0251	-1.985
YRI	NPIP	22453599	0.3841	1.1764	-1.4171
YRI	NPIP	22458599	0.4604	0.8403	-0.8581

YRI	NPIP	22463599	0.1409	0.6722	-1.3909
YRI	NPIP	22468599	0.1255	0.5042	-1.1901
YRI	NPIP	22473599	0.3956	1.8487	-1.8586
YRI	NPIP	22478599	0.343	1.8487	-1.9259
YRI	NPIP	22483599	0.5154	1.3445	-1.3466
YRI	NPIP	22488599	0.948	2.1848	-1.3887
YRI	NPIP	22493599	0.4962	1.1764	-1.2166
YRI	NPIP	22498599	0.0365	0.5042	-1.4698
YRI	NPIP	22503599	0.0365	0.5042	-1.4698
YRI	NPIP	22508599	0	0	0
YRI	NPIP	22513599	0.0884	0.1681	-0.4738
YRI	NPIP	22518599	0.0884	0.1681	-0.4738
YRI	NPIP	22523599	0	0.3361	-1.3493
YRI	NPIP	22528599	0.0093	0.6722	-1.7354
YRI	NPIP	22533599	0.0093	0.3361	-1.312
YRI	NPIP	22538599	0.5059	0.3361	0.6817
YRI	NPIP	22543599	1.3359	1.6806	-0.4742
YRI	NPIP	22548599	1.096	4.0335	-1.9875
YRI	NPIP	22553599	1.8336	8.4031	-2.3089
YRI	NPIP	22558599	3.3846	12.1005	-2.1805
YRI	NPIP	22563599	2.1222	8.4031	-2.2075
YRI	NPIP	22568599	0.3963	2.3529	-2.0712
YRI	NPIP	22573599	0.3226	1.5126	-1.772
YRI	NPIP	22578599	1.5505	3.6974	-1.5648
YRI	NPIP	22583599	3.0262	8.235	-1.865
YRI	NPIP	22588599	2.3228	8.5712	-2.1563
YRI	NPIP	22593599	1.8368	6.3864	-2.0534
YRI	NPIP	22598599	3.548	9.0754	-1.8094
YRI	NPIP	22603599	2.7773	6.3864	-1.6289
YRI	NPIP	22608599	1.2741	2.1848	-1.0225
YRI	NPIP	22613599	0.8606	2.0167	-1.3823
YRI	NPIP	22618599	0.4573	4.0335	-2.4197
YRI	NPIP	22623599	2.351	9.4115	-2.2346
YRI	NPIP	22628599	4.7265	13.7811	-2.0032
YRI	NPIP	22633599	7.4103	14.9575	-1.5446
YRI	NPIP	22638599	6.7652	13.1089	-1.4715
YRI	NPIP	22643599	5.477	10.924	-1.5002
YRI	NPIP	22648599	6.6948	10.756	-1.1349
YRI	NPIP	22653599	8.7134	13.2769	-1.0459
YRI	NPIP	22658599	8.3247	14.2853	-1.2744
YRI	NPIP	22663599	6.0763	12.2685	-1.5292
YRI	NPIP	22668599	4.8939	11.2602	-1.7043
YRI	NPIP	22673599	4.5157	11.2602	-1.8055
YRI	NPIP	22678599	4.9396	10.5879	-1.6018
YRI	NPIP	22683599	4.7489	10.0837	-1.5835
YRI	NPIP	22688599	3.9834	10.0837	-1.8107
YRI	NPIP	22693599	4.7477	11.9324	-1.8213
YRI	NPIP	22698599	6.661	12.1005	-1.3609
YRI	NPIP	22703599	6.2669	12.1005	-1.4595

YRI	NPIP	22708599	4.8466	11.4282	-1.7376
YRI	NPIP	22713599	5.2646	7.8989	-0.98
YRI	NPIP	22718599	6.8931	8.235	-0.4805
YRI	NPIP	22723599	9.163	11.9324	-0.702
YRI	NPIP	22728599	16.3083	18.3188	-0.3389
YRI	NPIP	22733599	17.5352	18.823	-0.2115
YRI	NPIP	22738599	9.3884	12.2685	-0.7112
YRI	NPIP	22743599	8.7611	11.5963	-0.7383
YRI	NPIP	22748599	10.6358	14.9575	-0.8845
YRI	NPIP	22753599	10.0039	14.9575	-1.0138
YRI	NPIP	22758599	9.6861	11.9324	-0.5694
YRI	NPIP	22763599	7.4822	9.2434	-0.5668
YRI	NPIP	22768599	8.2306	8.7392	-0.1724
YRI	NPIP	22773599	8.0056	10.2518	-0.6565
YRI	NPIP	22778599	5.6204	10.4199	-1.3816
YRI	NPIP	22783599	5.4978	10.0837	-1.3612
YRI	NPIP	22788599	8.9162	11.5963	-0.6979
YRI	NPIP	22793599	9.6144	13.2769	-0.8394
YRI	NPIP	22798599	7.5892	14.7895	-1.4895
YRI	NPIP	22803599	12.5977	15.6298	-0.5951
YRI	NPIP	22808599	13.9901	12.4366	0.3787
YRI	NPIP	22813599	9.8043	9.9157	-0.0336
YRI	NPIP	22818599	9.6594	13.613	-0.8849
YRI	NPIP	22823599	12.3435	15.7978	-0.671
YRI	NPIP	22828599	11.936	12.9408	-0.2359
YRI	NPIP	22833599	17.0693	16.4701	0.1119
YRI	NPIP	22838599	17.9302	18.1507	-0.0375
YRI	NPIP	22843599	14.2571	14.9575	-0.1433
YRI	NPIP	22848599	15.6719	16.302	-0.1188
YRI	NPIP	22853599	14.7435	16.9743	-0.4046
YRI	NPIP	22858599	13.2956	16.134	-0.5404
YRI	NPIP	22863599	11.5296	15.7978	-0.8292
YRI	NPIP	22868599	12.9245	15.9659	-0.5849
YRI	NPIP	22873599	15.567	17.9827	-0.4146
YRI	NPIP	22878599	12.1043	15.7978	-0.7175
YRI	NPIP	22883599	9.3801	13.7811	-0.9736
YRI	NPIP	22888599	11.0557	15.6298	-0.8977
YRI	NPIP	22893599	9.0299	12.1005	-0.7682
YRI	NPIP	22898599	8.7036	9.2434	-0.1737
YRI	NPIP	22903599	7.9404	9.4115	-0.4656
YRI	NPIP	22908599	5.387	8.5712	-1.0989
YRI	NPIP	22913599	6.7908	9.7476	-0.9058
YRI	NPIP	22918599	8.3737	11.7644	-0.8711
YRI	NPIP	22923599	11.2571	11.9324	-0.1712
YRI	NPIP	22928599	18.259	14.1172	0.8956
YRI	NPIP	22933599	24.3366	16.8062	1.3789
YRI	NPIP	22938599	19.3666	17.6465	0.3006
YRI	NPIP	22943599	13.5144	16.134	-0.4988
YRI	NPIP	22948599	14.3236	14.9575	-0.1297

YRI	NPIP	22953599	10.9067	13.613	-0.6057
YRI	NPIP	22958599	9.5731	15.1256	-1.1243
YRI	NPIP	22963599	10.0504	19.8313	-1.5278
YRI	NPIP	22968599	10.9395	21.512	-1.5268
YRI	NPIP	22973599	11.2069	20.5036	-1.4062
YRI	NPIP	22978599	9.0629	17.1423	-1.4516
YRI	NPIP	22983599	9.5382	15.2937	-1.1532
YRI	NPIP	22988599	11.6751	16.6382	-0.9176
YRI	NPIP	22993599	10.788	16.134	-1.0179
YRI	NPIP	22998599	8.2748	14.1172	-1.2633
YRI	NPIP	23003599	6.0877	11.5963	-1.4345
YRI	NPIP	23008599	11.1411	14.2853	-0.6723
YRI	NPIP	23013599	14.1875	17.4785	-0.5804
YRI	NPIP	23018599	8.2074	15.4617	-1.4385
YRI	NPIP	23023599	8.7839	16.134	-1.3994
YRI	NPIP	23028599	6.9063	12.1005	-1.2995
CEU	NPIP	27847517	12.0019	8.187	1.3821
CEU	NPIP	27852517	18.9672	10.5749	2.3978
CEU	NPIP	27857517	17.4688	10.4043	2.0494
CEU	NPIP	27862517	9.5351	7.8459	0.6363
CEU	NPIP	27867517	6.536	6.1402	0.1863
CEU	NPIP	27872517	7.9973	6.3108	0.7743
CEU	NPIP	27877517	6.7155	6.9931	-0.1162
CEU	NPIP	27882517	5.2272	7.1636	-0.7927
CEU	NPIP	27887517	10.7421	8.8692	0.6302
CEU	NPIP	27892517	19.1991	12.6216	1.5913
CEU	NPIP	27897517	18.8769	14.1567	1.0243
CEU	NPIP	27902517	12.0994	12.2805	-0.045
CEU	NPIP	27907517	9.7988	11.4277	-0.4328
CEU	NPIP	27912517	9.4164	11.0866	-0.4565
CEU	NPIP	27917517	8.6642	10.2337	-0.4624
CEU	NPIP	27922517	10.8178	10.916	-0.0272
CEU	NPIP	27927517	10.4071	10.7454	-0.0952
CEU	NPIP	27932517	9.9336	9.2104	0.235
CEU	NPIP	27937517	12.657	10.2337	0.7139
CEU	NPIP	27942517	15.6019	11.9394	0.9338
CEU	NPIP	27947517	18.2902	13.645	1.0439
CEU	NPIP	27952517	15.4743	11.5982	1.0156
CEU	NPIP	27957517	11.8283	9.2104	0.8507
CEU	NPIP	27962517	11.5393	9.2104	0.7568
CEU	NPIP	27967517	11.4047	9.0398	0.7819
CEU	NPIP	27972517	12.075	9.0398	1.0035
CEU	NPIP	27977517	11.3239	7.1636	1.703
CEU	NPIP	27982517	9.4605	6.8225	1.1288
CEU	NPIP	27987517	12.9643	10.7454	0.6246
CEU	NPIP	27992517	14.0705	10.0632	1.1992
CEU	NPIP	27997517	11.7481	8.0164	1.3783
CEU	NPIP	28002517	13.7532	10.4043	0.9715
CEU	NPIP	28007517	10.8312	8.5281	0.8036

CEU	NPIP	28012517	9.9711	8.8692	0.3708
CEU	NPIP	28017517	10.1622	10.916	-0.2091
CEU	NPIP	28022517	9.5848	9.3809	0.0651
CEU	NPIP	28027517	6.1408	6.8225	-0.2917
CEU	NPIP	28032517	2.8194	5.458	-1.3794
CEU	NPIP	28037517	4.9971	5.7991	-0.3972
CEU	NPIP	28042517	5.3719	5.9697	-0.2885
CEU	NPIP	28047517	6.6461	5.9697	0.3264
CEU	NPIP	28052517	9.3711	6.8225	1.0905
CEU	NPIP	28057517	12.4626	9.3809	0.9845
CEU	NPIP	28062517	14.3857	10.5749	1.0888
CEU	NPIP	28067517	12.2455	9.0398	1.0599
CEU	NPIP	28072517	14.2358	9.2104	1.633
CEU	NPIP	28077517	13.369	7.8459	2.0806
CEU	NPIP	28082517	12.8844	8.6987	1.434
CEU	NPIP	28087517	9.9598	10.2337	-0.0807
CEU	NPIP	28092517	4.287	8.0164	-1.3775
CEU	NPIP	28097517	6.1293	6.9931	-0.3614
CEU	NPIP	28102517	4.9913	5.6286	-0.3242
CEU	NPIP	28107517	2.9528	5.1169	-1.1979
CEU	NPIP	28112517	3.3072	6.6519	-1.4644
CEU	NPIP	28117517	3.954	7.5047	-1.3931
CEU	NPIP	28122517	3.7904	5.7991	-0.9949
CEU	NPIP	28127517	2.9672	4.4346	-0.9207
CEU	NPIP	28132517	2.7681	5.1169	-1.3001
CEU	NPIP	28137517	2.7236	5.7991	-1.5233
CEU	NPIP	28142517	2.6141	5.1169	-1.3853
CEU	NPIP	28147517	5.4622	5.6286	-0.0846
CEU	NPIP	28152517	4.9975	5.9697	-0.4692
CEU	NPIP	28157517	1.7017	4.0935	-1.6083
CEU	NPIP	28162517	2.5203	3.7524	-0.8926
CEU	NPIP	28167517	3.8677	4.4346	-0.3557
CEU	NPIP	28172517	4.8075	5.7991	-0.4911
CEU	NPIP	28177517	4.8885	7.1636	-0.9313
CEU	NPIP	28182517	2.4869	5.9697	-1.6808
CEU	NPIP	28187517	2.1936	5.458	-1.7066
CEU	NPIP	28192517	3.3597	5.6286	-1.1541
CEU	NPIP	28197517	5.8712	6.8225	-0.4071
CEU	NPIP	28202517	5.4075	7.3342	-0.772
CEU	NPIP	28207517	3.8736	6.8225	-1.2618
CEU	NPIP	28212517	5.0594	6.9931	-0.8091
CEU	NPIP	28217517	4.718	6.3108	-0.7313
CEU	NPIP	28222517	4.5706	6.1402	-0.7386
CEU	NPIP	28227517	4.7336	6.6519	-0.8399
CEU	NPIP	28232517	6.3352	7.5047	-0.4589
CEU	NPIP	28237517	6.9851	8.0164	-0.3809
CEU	NPIP	28242517	7.1624	8.6987	-0.5263
CEU	NPIP	28247517	11.589	12.1099	-0.131
CEU	NPIP	28252517	12.1599	11.7688	0.1011

CEU	NPIP	28257517	9.4227	8.6987	0.248
CEU	NPIP	28262517	9.8128	8.6987	0.3817
CEU	NPIP	28267517	8.1616	7.6753	0.1869
CEU	NPIP	28272517	6.1563	7.3342	-0.4719
CEU	NPIP	28277517	5.5933	8.5281	-1.024
CEU	NPIP	28282517	5.2968	8.187	-1.0471
CEU	NPIP	28287517	7.3987	8.187	-0.2856
CEU	NPIP	28292517	9.3707	8.0164	0.5002
CEU	NPIP	28297517	7.4276	7.3342	0.0374
CEU	NPIP	28302517	4.5539	6.1402	-0.7465
CEU	NPIP	28307517	3.6157	4.9463	-0.7588
CEU	NPIP	28312517	2.3951	3.5818	-0.8944
CEU	NPIP	28317517	2.1902	4.0935	-1.2798
CEU	NPIP	28322517	4.6318	6.1402	-0.7098
CEU	NPIP	28327517	4.8724	6.3108	-0.6604
CEU	NPIP	28332517	5.8462	6.3108	-0.2133
CEU	NPIP	28337517	8.3291	8.0164	0.1155
CEU	NPIP	28342517	6.5254	8.0164	-0.5507
CEU	NPIP	28347517	5.7124	6.1402	-0.2013
CEU	NPIP	28352517	4.9751	4.9463	0.0164
CEU	NPIP	28357517	1.9687	2.3879	-0.4415
CEU	NPIP	28362517	0.9068	0.8528	0.1216
CEU	NPIP	28367517	0.9264	0.5117	1.3006
CEU	NPIP	28372517	2.6761	2.2173	0.5126
CEU	NPIP	28377517	3.0445	3.0701	-0.022
CEU	NPIP	28382517	3.4137	4.2641	-0.552
CEU	NPIP	28387517	4.0171	4.9463	-0.5299
CEU	NPIP	28392517	1.7386	2.2173	-0.5348
CEU	NPIP	28397517	0.7326	1.1939	-0.822
CEU	NPIP	28402517	0.9571	1.5351	-0.8571
CEU	NPIP	28407517	0.4605	0.6822	-0.5789
CEU	NPIP	28412517	1.6182	1.1939	0.7561
CEU	NPIP	28417517	3.2507	2.8996	0.3158
CEU	NPIP	28422517	3.0445	4.0935	-0.7054
CEU	NPIP	28427517	1.4222	2.5584	-1.1318
CEU	NPIP	28432517	0.6112	0.8528	-0.5441
CEU	NPIP	28437517	0.6213	1.0234	-0.799
CEU	NPIP	28442517	0.4251	1.0234	-1.1889
CEU	NPIP	28447517	0.9911	2.0467	-1.256
CEU	NPIP	28452517	1.7763	3.4112	-1.2841
CEU	NPIP	28457517	1.1901	2.0467	-1.0193
CEU	NPIP	28462517	0.7285	1.5351	-1.196
CEU	NPIP	28467517	0.9691	1.8762	-1.1549
CEU	NPIP	28472517	0.3292	0.8528	-1.1791
CEU	NPIP	28477517	0.2428	1.1939	-1.6949
CEU	NPIP	28482517	2.1725	2.729	-0.526
CEU	NPIP	28487517	5.8046	5.2874	0.2781
CEU	NPIP	28492517	6.3094	6.3108	-0.0006
CEU	NPIP	28497517	4.8035	6.6519	-0.8093

CEU	NPIP	28502517	4.167	5.9697	-0.87
CEU	NPIP	28507517	6.6583	6.3108	0.1595
CEU	NPIP	28512517	10.2168	9.3809	0.267
CEU	NPIP	28517517	8.4335	8.3576	0.027
CEU	NPIP	28522517	6.5683	6.4814	0.0389
CEU	NPIP	28527517	8.8998	8.3576	0.1927
CEU	NPIP	28532517	10.3337	8.6987	0.5601
CEU	NPIP	28537517	11.2229	8.6987	0.8648
CEU	NPIP	28542517	8.0417	6.9931	0.4388
CEU	NPIP	28547517	3.8064	5.6286	-0.9269
CEU	NPIP	28552517	5.8825	6.9931	-0.4647
CEU	NPIP	28557517	8.5929	7.3342	0.5043
CEU	NPIP	28562517	8.7207	7.5047	0.4771
CEU	NPIP	28567517	6.2189	7.1636	-0.3867
CEU	NPIP	28572517	6.1002	7.5047	-0.5511
CEU	NPIP	28577517	8.247	8.0164	0.0852
CEU	NPIP	28582517	8.7932	8.0164	0.2869
CEU	NPIP	28587517	9.1394	8.0164	0.4148
CEU	NPIP	28592517	8.6382	7.8459	0.2985
CEU	NPIP	28597517	6.0074	6.4814	-0.2125
CEU	NPIP	28602517	8.2964	9.2104	-0.297
CEU	NPIP	28607517	11.4548	13.4744	-0.4593
CEU	NPIP	28612517	12.4659	12.1099	0.0896
CEU	NPIP	28617517	27.6415	18.0796	1.6427
CEU	NPIP	28622517	24.6246	17.0562	1.3749
CEU	NPIP	28627517	11.3319	10.2337	0.3235
CEU	NPIP	28632517	7.6524	8.3576	-0.2507
CEU	NPIP	28637517	1.9716	4.4346	-1.5454
CEU	NPIP	28642517	4.1775	6.6519	-1.0833
CEU	NPIP	28647517	9.2525	10.916	-0.4614
CEU	NPIP	28652517	10.1101	10.2337	-0.0364
CEU	NPIP	28657517	3.6403	4.0935	-0.3047
CEU	NPIP	28662517	0.0202	0.3411	-1.2864
CEU	NPIP	28667517	0.0101	0.1706	-0.9534
CEU	NPIP	28672517	0.3795	0.1706	1.2409
CEU	NPIP	28677517	0.4937	0.3411	0.6117
CEU	NPIP	28682517	0.6155	0.5117	0.3255
CEU	NPIP	28687517	0.9625	0.5117	1.4138
CEU	NPIP	28692517	0.8023	0.6822	0.3136
CEU	NPIP	28697517	0.5368	1.1939	-1.171
CEU	NPIP	28702517	1.8973	1.3645	0.862
CEU	NPIP	28707517	3.7223	3.4112	0.2443
CEU	NPIP	28712517	2.1676	3.5818	-1.0659
CEU	NPIP	28717517	0.147	0.8528	-1.5894
CEU	NPIP	28722517	0.5813	0.8528	-0.6114
CEU	NPIP	28727517	0.5813	0.8528	-0.6114
CEU	NPIP	28732517	0.49	0.8528	-0.817
CEU	NPIP	28737517	1.1806	1.8762	-0.8856
CEU	NPIP	28742517	1.2255	1.3645	-0.2249

CEU	NPIP	28747517	2.7155	4.0935	-0.9266
CEU	NPIP	28752517	7.3123	7.3342	-0.0088
CEU	NPIP	28757517	5.3886	4.0935	0.8709
CEU	NPIP	28762517	3.2738	2.729	0.515
CEU	NPIP	28767517	4.0381	3.0701	0.8303
CEU	NPIP	28772517	1.0313	1.0234	0.0157
CEU	NPIP	28777517	0.9773	0.6822	0.7706
CEU	NPIP	28782517	1.0361	0.6822	0.9241
CEU	NPIP	28787517	0.0689	0.1706	-0.6041
CEU	NPIP	28792517	0	0	0
CEU	NPIP	28797517	1.7811	1.5351	0.3648
CEU	NPIP	28802517	2.5189	2.5584	-0.0393
CEU	NPIP	28807517	2.7098	2.3879	0.339
CEU	NPIP	28812517	2.6384	3.2407	-0.4939
CEU	NPIP	28817517	1.6392	2.8996	-1.1335
CEU	NPIP	28822517	7.7135	5.458	1.1791
CEU	NPIP	28827517	9.8868	7.1636	1.1147
CEU	NPIP	28832517	8.0911	6.1402	0.918
CEU	NPIP	28837517	8.9481	7.8459	0.4152
CEU	NPIP	28842517	10.1534	9.3809	0.2468
CEU	NPIP	28847517	8.6947	7.6753	0.3918
CEU	NPIP	28852517	5.1072	4.4346	0.422
CEU	NPIP	28857517	5.6221	4.7757	0.4978
CHB	NPIP	27847517	11.4917	8.8096	0.9055
CHB	NPIP	27852517	18.518	12.7061	1.3933
CHB	NPIP	27857517	16.7021	12.8755	0.9059
CHB	NPIP	27862517	9.0693	8.6402	0.1475
CHB	NPIP	27867517	6.3453	6.946	-0.2521
CHB	NPIP	27872517	7.7853	7.7931	-0.0029
CHB	NPIP	27877517	7.577	7.6237	-0.018
CHB	NPIP	27882517	5.8671	6.946	-0.4529
CHB	NPIP	27887517	9.6979	8.8096	0.2999
CHB	NPIP	27892517	17.833	12.1979	1.4039
CHB	NPIP	27897517	18.9334	14.0615	1.061
CHB	NPIP	27902517	13.1395	12.5367	0.1464
CHB	NPIP	27907517	9.9912	10.6731	-0.1926
CHB	NPIP	27912517	8.7399	11.012	-0.6232
CHB	NPIP	27917517	6.6944	10.3343	-1.0595
CHB	NPIP	27922517	7.6281	9.9955	-0.7109
CHB	NPIP	27927517	6.69	10.5037	-1.0934
CHB	NPIP	27932517	8.1399	8.979	-0.2784
CHB	NPIP	27937517	13.5519	9.6567	1.2078
CHB	NPIP	27942517	15.5134	14.0615	0.3162
CHB	NPIP	27947517	18.4906	15.925	0.4963
CHB	NPIP	27952517	14.9579	12.5367	0.5878
CHB	NPIP	27957517	9.3034	10.8426	-0.4284
CHB	NPIP	27962517	10.1266	9.9955	0.0394
CHB	NPIP	27967517	10.3562	10.3343	0.0064
CHB	NPIP	27972517	10.6573	10.1649	0.1456

CHB	NPIP	27977517	11.9674	7.9625	1.4841
CHB	NPIP	27982517	9.7807	8.1319	0.5993
CHB	NPIP	27987517	9.3807	9.4872	-0.0336
CHB	NPIP	27992517	9.4576	8.6402	0.281
CHB	NPIP	27997517	7.502	8.979	-0.49
CHB	NPIP	28002517	8.6714	10.8426	-0.6043
CHB	NPIP	28007517	6.972	7.6237	-0.2513
CHB	NPIP	28012517	8.051	8.6402	-0.2025
CHB	NPIP	28017517	9.2996	12.3673	-0.7544
CHB	NPIP	28022517	10.3448	12.1979	-0.4617
CHB	NPIP	28027517	7.2241	9.1484	-0.6274
CHB	NPIP	28032517	3.0148	7.1154	-1.684
CHB	NPIP	28037517	5.3023	8.4708	-1.1092
CHB	NPIP	28042517	5.6246	8.4708	-0.9964
CHB	NPIP	28047517	7.2875	9.8261	-0.7745
CHB	NPIP	28052517	10.5215	12.1979	-0.4177
CHB	NPIP	28057517	13.345	11.3508	0.5317
CHB	NPIP	28062517	15.0487	11.1814	1.0457
CHB	NPIP	28067517	13.1074	12.0285	0.2724
CHB	NPIP	28072517	14.1729	13.2144	0.2214
CHB	NPIP	28077517	13.3666	12.3673	0.2457
CHB	NPIP	28082517	15.6952	12.5367	0.7668
CHB	NPIP	28087517	14.467	11.5202	0.7748
CHB	NPIP	28092517	9.2998	11.5202	-0.5838
CHB	NPIP	28097517	10.2994	14.5697	-0.8991
CHB	NPIP	28102517	10.1813	13.5532	-0.7604
CHB	NPIP	28107517	8.498	10.8426	-0.6525
CHB	NPIP	28112517	6.4032	8.979	-0.8545
CHB	NPIP	28117517	8.2844	9.6567	-0.4255
CHB	NPIP	28122517	7.9655	8.8096	-0.285
CHB	NPIP	28127517	5.8471	7.9625	-0.7839
CHB	NPIP	28132517	5.683	8.8096	-1.0556
CHB	NPIP	28137517	6.4241	8.4708	-0.7165
CHB	NPIP	28142517	7.0141	8.4708	-0.51
CHB	NPIP	28147517	9.4074	10.6731	-0.3575
CHB	NPIP	28152517	7.9539	9.9955	-0.6131
CHB	NPIP	28157517	6.4889	8.1319	-0.5972
CHB	NPIP	28162517	7.2202	8.4708	-0.4378
CHB	NPIP	28167517	6.1988	9.4872	-1.0366
CHB	NPIP	28172517	7.2627	9.3178	-0.6587
CHB	NPIP	28177517	7.7457	9.3178	-0.5039
CHB	NPIP	28182517	8.9906	12.0285	-0.7669
CHB	NPIP	28187517	8.7447	13.045	-1.0056
CHB	NPIP	28192517	6.3534	10.1649	-1.1267
CHB	NPIP	28197517	9.0208	10.3343	-0.3823
CHB	NPIP	28202517	7.628	9.4872	-0.5861
CHB	NPIP	28207517	5.6139	8.1319	-0.9152
CHB	NPIP	28212517	6.8962	9.3178	-0.7762
CHB	NPIP	28217517	7.7601	8.979	-0.4043

CHB	NPIP	28222517	9.4401	9.9955	-0.1668
CHB	NPIP	28227517	10.566	10.8426	-0.077
CHB	NPIP	28232517	18.046	15.2474	0.5643
CHB	NPIP	28237517	18.0943	16.4333	0.3118
CHB	NPIP	28242517	14.4101	13.892	0.1141
CHB	NPIP	28247517	15.792	14.7391	0.2193
CHB	NPIP	28252517	13.0942	13.5532	-0.1035
CHB	NPIP	28257517	10.6216	11.3508	-0.1944
CHB	NPIP	28262517	8.4171	10.6731	-0.6372
CHB	NPIP	28267517	6.4481	10.1649	-1.0987
CHB	NPIP	28272517	5.8233	9.4872	-1.1549
CHB	NPIP	28277517	4.7252	9.6567	-1.5292
CHB	NPIP	28282517	5.1591	10.1649	-1.4798
CHB	NPIP	28287517	7.418	10.1649	-0.812
CHB	NPIP	28292517	8.3666	8.979	-0.2032
CHB	NPIP	28297517	5.0699	6.7766	-0.7326
CHB	NPIP	28302517	3.2273	5.0825	-1.03
CHB	NPIP	28307517	4.6172	5.4213	-0.4216
CHB	NPIP	28312517	3.2608	4.913	-0.9451
CHB	NPIP	28317517	2.8142	3.7271	-0.6632
CHB	NPIP	28322517	5.7331	5.5907	0.0727
CHB	NPIP	28327517	5.5258	5.4213	0.0548
CHB	NPIP	28332517	6.0634	6.2684	-0.0944
CHB	NPIP	28337517	9.5191	8.3013	0.4343
CHB	NPIP	28342517	7.5728	7.1154	0.1878
CHB	NPIP	28347517	5.4634	6.7766	-0.5637
CHB	NPIP	28352517	4.8011	5.4213	-0.3252
CHB	NPIP	28357517	2.0259	2.8801	-0.7702
CHB	NPIP	28362517	0.751	1.3553	-0.9794
CHB	NPIP	28367517	0.8837	1.0165	-0.2643
CHB	NPIP	28372517	2.3783	2.7106	-0.3149
CHB	NPIP	28377517	2.4072	2.5412	-0.1338
CHB	NPIP	28382517	3.0228	2.033	1.1803
CHB	NPIP	28387517	3.695	2.8801	0.7347
CHB	NPIP	28392517	1.74	2.3718	-0.6669
CHB	NPIP	28397517	1.1043	1.6942	-0.8096
CHB	NPIP	28402517	1.0335	1.3553	-0.5215
CHB	NPIP	28407517	0.3619	0.6777	-0.8256
CHB	NPIP	28412517	1.2994	1.6942	-0.5418
CHB	NPIP	28417517	2.9656	2.8801	0.0771
CHB	NPIP	28422517	3.3426	3.7271	-0.2793
CHB	NPIP	28427517	1.7537	3.0495	-1.1144
CHB	NPIP	28432517	0.5254	2.033	-1.7978
CHB	NPIP	28437517	0.6417	2.033	-1.6591
CHB	NPIP	28442517	0.6653	1.5247	-1.2768
CHB	NPIP	28447517	0.6307	1.5247	-1.3282
CHB	NPIP	28452517	0.744	1.8636	-1.4285
CHB	NPIP	28457517	0.5656	1.1859	-1.1072
CHB	NPIP	28462517	0.4721	1.6942	-1.6772

CHB	NPIP	28467517	0.4915	1.8636	-1.7506
CHB	NPIP	28472517	0.0388	0.5082	-1.4735
CHB	NPIP	28477517	0.2421	1.0165	-1.5411
CHB	NPIP	28482517	2.0255	4.7436	-1.6033
CHB	NPIP	28487517	4.9848	9.3178	-1.3889
CHB	NPIP	28492517	5.5521	9.4872	-1.2404
CHB	NPIP	28497517	4.5376	8.1319	-1.3064
CHB	NPIP	28502517	3.3956	8.1319	-1.7215
CHB	NPIP	28507517	5.3541	8.6402	-1.1296
CHB	NPIP	28512517	9.0103	9.9955	-0.2958
CHB	NPIP	28517517	8.2829	8.3013	-0.0066
CHB	NPIP	28522517	7.0779	5.7601	0.6547
CHB	NPIP	28527517	7.8664	7.1154	0.3084
CHB	NPIP	28532517	8.1974	8.1319	0.0238
CHB	NPIP	28537517	8.628	9.1484	-0.1697
CHB	NPIP	28542517	7.0885	10.1649	-0.9094
CHB	NPIP	28547517	3.6265	9.3178	-1.8243
CHB	NPIP	28552517	4.5167	9.1484	-1.5101
CHB	NPIP	28557517	7.2433	11.5202	-1.1245
CHB	NPIP	28562517	7.4002	11.6896	-1.1124
CHB	NPIP	28567517	5.3794	9.9955	-1.3861
CHB	NPIP	28572517	4.2686	9.8261	-1.6956
CHB	NPIP	28577517	5.5953	8.6402	-1.0466
CHB	NPIP	28582517	5.8473	9.1484	-1.0763
CHB	NPIP	28587517	5.7687	10.3343	-1.329
CHB	NPIP	28592517	5.2235	9.4872	-1.344
CHB	NPIP	28597517	3.6514	7.4543	-1.4969
CHB	NPIP	28602517	5.972	9.8261	-1.1759
CHB	NPIP	28607517	8.2374	12.1979	-0.9867
CHB	NPIP	28612517	9.3982	11.1814	-0.4822
CHB	NPIP	28617517	22.3821	18.2968	0.6919
CHB	NPIP	28622517	20.7331	18.6357	0.349
CHB	NPIP	28627517	8.9849	12.3673	-0.8318
CHB	NPIP	28632517	5.7888	10.5037	-1.3517
CHB	NPIP	28637517	2.3236	7.9625	-2.0896
CHB	NPIP	28642517	3.4708	9.4872	-1.8965
CHB	NPIP	28647517	6.6288	13.5532	-1.5616
CHB	NPIP	28652517	7.4363	11.3508	-1.0436
CHB	NPIP	28657517	2.7049	3.7271	-0.7426
CHB	NPIP	28662517	0	0	0
CHB	NPIP	28667517	0	0	0
CHB	NPIP	28672517	0.3952	0.3388	0.2262
CHB	NPIP	28677517	0.4523	0.5082	-0.1755
CHB	NPIP	28682517	0.383	0.5082	-0.393
CHB	NPIP	28687517	0.8195	0.6777	0.3707
CHB	NPIP	28692517	0.9092	1.5247	-0.9144
CHB	NPIP	28697517	0.6379	1.6942	-1.4496
CHB	NPIP	28702517	1.7678	1.6942	0.101
CHB	NPIP	28707517	3.8041	3.7271	0.0559

CHB	NPIP	28712517	2.5189	3.5577	-0.7851
CHB	NPIP	28717517	0.2602	1.0165	-1.5051
CHB	NPIP	28722517	0.558	0.5082	0.1563
CHB	NPIP	28727517	0.558	0.5082	0.1563
CHB	NPIP	28732517	0.309	0.8471	-1.2134
CHB	NPIP	28737517	0.6092	2.033	-1.6979
CHB	NPIP	28742517	0.9654	2.3718	-1.4846
CHB	NPIP	28747517	2.1946	3.5577	-1.0302
CHB	NPIP	28752517	5.9762	5.7601	0.1074
CHB	NPIP	28757517	4.8306	4.5742	0.1561
CHB	NPIP	28762517	3.0324	4.066	-0.697
CHB	NPIP	28767517	3.6189	4.066	-0.3015
CHB	NPIP	28772517	0.9703	1.1859	-0.3848
CHB	NPIP	28777517	0.9732	1.0165	-0.0862
CHB	NPIP	28782517	1.0849	1.3553	-0.4382
CHB	NPIP	28787517	0.1117	0.3388	-0.911
CHB	NPIP	28792517	0	0	0
CHB	NPIP	28797517	1.8487	1.3553	0.7996
CHB	NPIP	28802517	2.5563	2.2024	0.3963
CHB	NPIP	28807517	2.577	2.5412	0.0357
CHB	NPIP	28812517	3.1251	4.066	-0.6345
CHB	NPIP	28817517	2.0238	3.8965	-1.3098
CHB	NPIP	28822517	5.9836	7.6237	-0.6325
CHB	NPIP	28827517	7.3413	10.5037	-0.9066
CHB	NPIP	28832517	5.6063	9.4872	-1.2233
CHB	NPIP	28837517	6.5545	11.012	-1.2226
CHB	NPIP	28842517	7.5212	10.8426	-0.9244
CHB	NPIP	28847517	6.6423	9.3178	-0.8576
CHB	NPIP	28852517	4.0678	7.7931	-1.408
CHB	NPIP	28857517	4.5197	7.4543	-1.1551
YRI	NPIP	27847517	13.2087	13.9492	-0.1619
YRI	NPIP	27852517	20.6694	18.3188	0.3963
YRI	NPIP	27857517	20.2097	19.3272	0.1413
YRI	NPIP	27862517	11.8535	15.6298	-0.7411
YRI	NPIP	27867517	10.0384	14.6214	-0.9585
YRI	NPIP	27872517	11.665	14.2853	-0.5602
YRI	NPIP	27877517	10.8117	16.134	-1.0134
YRI	NPIP	27882517	8.8545	14.9575	-1.249
YRI	NPIP	27887517	12.9116	12.6047	0.0739
YRI	NPIP	27892517	20.5615	15.9659	0.8838
YRI	NPIP	27897517	21.7034	19.3272	0.3805
YRI	NPIP	27902517	16.8894	17.8146	-0.1602
YRI	NPIP	27907517	12.1804	17.8146	-0.9756
YRI	NPIP	27912517	12.1448	19.1591	-1.1326
YRI	NPIP	27917517	12.3023	17.8146	-0.9545
YRI	NPIP	27922517	15.4756	18.991	-0.5725
YRI	NPIP	27927517	17.7148	18.1507	-0.0741
YRI	NPIP	27932517	14.4877	16.4701	-0.3701
YRI	NPIP	27937517	14.3304	15.9659	-0.3145

YRI	NPIP	27942517	20.7512	18.1507	0.4423
YRI	NPIP	27947517	23.8115	18.991	0.785
YRI	NPIP	27952517	17.553	14.1172	0.7429
YRI	NPIP	27957517	13.8902	13.1089	0.1812
YRI	NPIP	27962517	13.2781	14.2853	-0.2153
YRI	NPIP	27967517	11.0125	13.613	-0.582
YRI	NPIP	27972517	10.6462	14.1172	-0.7505
YRI	NPIP	27977517	11.3158	13.445	-0.4822
YRI	NPIP	27982517	9.8965	13.2769	-0.7747
YRI	NPIP	27987517	13.8236	16.302	-0.4672
YRI	NPIP	27992517	16.0523	15.9659	0.0166
YRI	NPIP	27997517	14.1882	14.6214	-0.0906
YRI	NPIP	28002517	17.5641	18.6549	-0.1807
YRI	NPIP	28007517	14.6737	14.9575	-0.0581
YRI	NPIP	28012517	11.8735	11.2602	0.1642
YRI	NPIP	28017517	12.1294	15.2937	-0.634
YRI	NPIP	28022517	12.2838	14.9575	-0.5472
YRI	NPIP	28027517	9.397	11.7644	-0.6082
YRI	NPIP	28032517	6.3113	11.7644	-1.4009
YRI	NPIP	28037517	7.2998	12.1005	-1.201
YRI	NPIP	28042517	7.3378	11.7644	-1.1372
YRI	NPIP	28047517	8.4011	13.9492	-1.2134
YRI	NPIP	28052517	9.9328	16.8062	-1.2586
YRI	NPIP	28057517	10.8335	15.4617	-0.9177
YRI	NPIP	28062517	13.1161	14.1172	-0.2165
YRI	NPIP	28067517	15.2124	16.134	-0.1755
YRI	NPIP	28072517	17.2069	15.4617	0.3461
YRI	NPIP	28077517	14.893	13.2769	0.3704
YRI	NPIP	28082517	17.7746	17.6465	0.0224
YRI	NPIP	28087517	16.8097	17.8146	-0.174
YRI	NPIP	28092517	11.87	14.1172	-0.4859
YRI	NPIP	28097517	14.1298	15.4617	-0.2641
YRI	NPIP	28102517	11.7353	15.4617	-0.7389
YRI	NPIP	28107517	8.5066	13.2769	-1.0933
YRI	NPIP	28112517	8.1138	12.4366	-1.0539
YRI	NPIP	28117517	8.863	12.9408	-0.9575
YRI	NPIP	28122517	8.2024	11.4282	-0.8516
YRI	NPIP	28127517	7.465	10.924	-0.9527
YRI	NPIP	28132517	6.8652	9.4115	-0.8059
YRI	NPIP	28137517	7.071	9.2434	-0.6991
YRI	NPIP	28142517	7.7693	12.1005	-1.0836
YRI	NPIP	28147517	10.8139	13.7811	-0.6564
YRI	NPIP	28152517	10.0279	12.4366	-0.5872
YRI	NPIP	28157517	7.1894	12.7727	-1.3273
YRI	NPIP	28162517	7.3499	11.9324	-1.1617
YRI	NPIP	28167517	7.2949	10.5879	-0.9339
YRI	NPIP	28172517	8.0656	12.6047	-1.0927
YRI	NPIP	28177517	8.5859	13.1089	-1.0492
YRI	NPIP	28182517	9.4609	14.1172	-1.0068

YRI	NPIP	28187517	8.1669	12.7727	-1.095
YRI	NPIP	28192517	6.7817	9.7476	-0.9086
YRI	NPIP	28197517	10.3133	10.756	-0.1237
YRI	NPIP	28202517	9.7004	11.0921	-0.3779
YRI	NPIP	28207517	7.5286	10.4199	-0.8323
YRI	NPIP	28212517	8.814	11.0921	-0.6185
YRI	NPIP	28217517	9.2665	12.1005	-0.709
YRI	NPIP	28222517	10.5922	12.7727	-0.5184
YRI	NPIP	28227517	12.7734	13.2769	-0.1154
YRI	NPIP	28232517	16.5963	16.9743	-0.0686
YRI	NPIP	28237517	16.5823	18.991	-0.3923
YRI	NPIP	28242517	14.6608	16.302	-0.3094
YRI	NPIP	28247517	19.1492	15.9659	0.6122
YRI	NPIP	28252517	19.4439	16.8062	0.483
YRI	NPIP	28257517	15.1659	14.9575	0.0427
YRI	NPIP	28262517	15.2272	14.1172	0.24
YRI	NPIP	28267517	14.5017	13.1089	0.3231
YRI	NPIP	28272517	11.3038	12.7727	-0.3492
YRI	NPIP	28277517	9.8031	15.4617	-1.1221
YRI	NPIP	28282517	9.143	16.302	-1.3496
YRI	NPIP	28287517	7.2763	13.9492	-1.4594
YRI	NPIP	28292517	9.202	13.7811	-1.013
YRI	NPIP	28297517	10.2113	12.6047	-0.5762
YRI	NPIP	28302517	6.4665	7.5628	-0.4244
YRI	NPIP	28307517	5.7117	8.067	-0.8595
YRI	NPIP	28312517	4.7154	9.9157	-1.5679
YRI	NPIP	28317517	2.5106	8.235	-2.0497
YRI	NPIP	28322517	5.0579	9.4115	-1.3779
YRI	NPIP	28327517	5.3801	8.4031	-1.0625
YRI	NPIP	28332517	6.8714	8.7392	-0.6331
YRI	NPIP	28337517	11.1725	12.4366	-0.3082
YRI	NPIP	28342517	8.2413	10.0837	-0.5469
YRI	NPIP	28347517	7.2362	10.2518	-0.8814
YRI	NPIP	28352517	6.4524	8.9073	-0.8176
YRI	NPIP	28357517	1.7844	3.3612	-1.2457
YRI	NPIP	28362517	0.3787	1.6806	-1.791
YRI	NPIP	28367517	0.6754	2.1848	-1.6947
YRI	NPIP	28372517	2.316	4.0335	-1.1621
YRI	NPIP	28377517	2.6427	3.5293	-0.6722
YRI	NPIP	28382517	3.7885	5.2099	-0.7694
YRI	NPIP	28387517	4.7017	6.2183	-0.7011
YRI	NPIP	28392517	2.5109	3.5293	-0.7722
YRI	NPIP	28397517	1.7935	2.1848	-0.4393
YRI	NPIP	28402517	1.9932	1.6806	0.43
YRI	NPIP	28407517	1.0482	0.8403	0.4696
YRI	NPIP	28412517	1.2753	1.6806	-0.5576
YRI	NPIP	28417517	2.7204	3.1932	-0.3899
YRI	NPIP	28422517	2.9892	4.3696	-0.8716
YRI	NPIP	28427517	2.226	3.6974	-1.0725

YRI	NPIP	28432517	1.6173	1.6806	-0.0871
YRI	NPIP	28437517	1.1459	1.6806	-0.7356
YRI	NPIP	28442517	1.33	2.0167	-0.8211
YRI	NPIP	28447517	1.0196	1.5126	-0.7341
YRI	NPIP	28452517	0.6965	3.0251	-2.0088
YRI	NPIP	28457517	0.6504	2.5209	-1.8732
YRI	NPIP	28462517	1.6311	2.1848	-0.6217
YRI	NPIP	28467517	1.6678	2.3529	-0.7252
YRI	NPIP	28472517	0.2919	0.8403	-1.2386
YRI	NPIP	28477517	0.4097	1.6806	-1.7484
YRI	NPIP	28482517	2.1082	5.8822	-1.8338
YRI	NPIP	28487517	5.2432	11.4282	-1.6329
YRI	NPIP	28492517	5.9843	11.0921	-1.3868
YRI	NPIP	28497517	6.0274	9.4115	-1.071
YRI	NPIP	28502517	7.552	12.4366	-1.1909
YRI	NPIP	28507517	9.2773	13.445	-0.9439
YRI	NPIP	28512517	9.2489	15.1256	-1.19
YRI	NPIP	28517517	7.6485	14.7895	-1.4773
YRI	NPIP	28522517	7.0999	11.7644	-1.1983
YRI	NPIP	28527517	8.3022	13.613	-1.1887
YRI	NPIP	28532517	9.008	13.7811	-1.056
YRI	NPIP	28537517	9.8347	13.9492	-0.8998
YRI	NPIP	28542517	7.5632	12.4366	-1.1881
YRI	NPIP	28547517	4.1184	8.5712	-1.5367
YRI	NPIP	28552517	5.6844	9.2434	-1.1454
YRI	NPIP	28557517	8.7194	11.9324	-0.8145
YRI	NPIP	28562517	8.5292	12.2685	-0.9234
YRI	NPIP	28567517	5.4573	11.7644	-1.6203
YRI	NPIP	28572517	6.4368	13.445	-1.5872
YRI	NPIP	28577517	7.8864	11.2602	-0.9032
YRI	NPIP	28582517	7.5868	9.4115	-0.5775
YRI	NPIP	28587517	7.8546	11.4282	-0.9434
YRI	NPIP	28592517	7.2311	12.7727	-1.3174
YRI	NPIP	28597517	6.0128	11.4282	-1.4297
YRI	NPIP	28602517	8.1072	15.6298	-1.4764
YRI	NPIP	28607517	12.3442	19.9994	-1.1861
YRI	NPIP	28612517	14.0794	16.302	-0.419
YRI	NPIP	28617517	24.8945	22.5203	0.328
YRI	NPIP	28622517	25.9728	21.512	0.6442
YRI	NPIP	28627517	17.6019	13.613	0.8928
YRI	NPIP	28632517	11.3443	10.5879	0.2145
YRI	NPIP	28637517	4.5695	8.7392	-1.4134
YRI	NPIP	28642517	7.4333	12.9408	-1.2932
YRI	NPIP	28647517	12.7568	16.6382	-0.7176
YRI	NPIP	28652517	12.042	14.9575	-0.5967
YRI	NPIP	28657517	4.3082	6.0502	-0.8254
YRI	NPIP	28662517	0.1281	0.6722	-1.4244
YRI	NPIP	28667517	0.1281	0.6722	-1.4244
YRI	NPIP	28672517	0.4674	0.8403	-0.8422

YRI	NPIP	28677517	0.6851	1.3445	-1.071
YRI	NPIP	28682517	0.3911	0.8403	-1.0146
YRI	NPIP	28687517	0.864	0.8403	0.0535
YRI	NPIP	28692517	1.7146	1.5126	0.3008
YRI	NPIP	28697517	2.0501	1.5126	0.8004
YRI	NPIP	28702517	2.1752	1.6806	0.6804
YRI	NPIP	28707517	4.2877	4.3696	-0.0517
YRI	NPIP	28712517	4.7731	4.2016	0.3733
YRI	NPIP	28717517	1.6346	1.0084	1.2484
YRI	NPIP	28722517	0.5916	0.5042	0.2747
YRI	NPIP	28727517	0.5916	0.5042	0.2747
YRI	NPIP	28732517	0.1335	0.6722	-1.4103
YRI	NPIP	28737517	1.0293	2.689	-1.5773
YRI	NPIP	28742517	2.123	3.0251	-0.7782
YRI	NPIP	28747517	4.2176	6.0502	-0.8683
YRI	NPIP	28752517	6.596	8.4031	-0.6351
YRI	NPIP	28757517	5.6174	5.2099	0.2206
YRI	NPIP	28762517	4.3377	5.2099	-0.4721
YRI	NPIP	28767517	3.6178	4.3696	-0.4747
YRI	NPIP	28772517	1.2921	1.0084	0.5656
YRI	NPIP	28777517	1.5473	1.5126	0.0517
YRI	NPIP	28782517	2.0869	1.8487	0.3047
YRI	NPIP	28787517	0.5395	0.3361	0.8166
YRI	NPIP	28792517	0	0	0
YRI	NPIP	28797517	1.4898	1.1764	0.5605
YRI	NPIP	28802517	2.8223	2.5209	0.3018
YRI	NPIP	28807517	3.6898	3.5293	0.1217
YRI	NPIP	28812517	3.8352	4.5377	-0.4293
YRI	NPIP	28817517	2.2584	4.3696	-1.3331
YRI	NPIP	28822517	6.394	10.2518	-1.1275
YRI	NPIP	28827517	9.3495	12.4366	-0.7526
YRI	NPIP	28832517	7.9701	8.9073	-0.3121
YRI	NPIP	28837517	6.152	9.2434	-0.9949
YRI	NPIP	28842517	6.9977	9.9157	-0.8798
YRI	NPIP	28847517	7.415	9.5795	-0.6739
YRI	NPIP	28852517	6.0629	8.5712	-0.8656
YRI	NPIP	28857517	7.3547	8.4031	-0.3685
CEU	NPIP	27961329	11.4566	8.8692	0.8707
CEU	NPIP	27966329	10.27	9.0398	0.4067
CEU	NPIP	27971329	12.0623	9.2104	0.9267
CEU	NPIP	27976329	11.4073	7.3342	1.632
CEU	NPIP	27981329	10.3286	6.9931	1.3956
CEU	NPIP	27986329	13.6001	10.4043	0.9271
CEU	NPIP	27991329	13.6395	9.8926	1.1393
CEU	NPIP	27996329	12.5065	9.0398	1.1462
CEU	NPIP	28001329	14.3612	10.4043	1.1479
CEU	NPIP	28006329	10.5784	7.8459	1.0294
CEU	NPIP	28011329	8.8058	8.3576	0.1593
CEU	NPIP	28016329	9.8784	10.7454	-0.244

CEU	NPIP	28021329	10.2286	10.0632	0.0495
CEU	NPIP	28026329	6.5119	7.5047	-0.3895
CEU	NPIP	28031329	3.8182	5.458	-0.8572
CEU	NPIP	28036329	4.2096	5.458	-0.6526
CEU	NPIP	28041329	5.0404	5.9697	-0.4485
CEU	NPIP	28046329	7.5535	6.6519	0.3947
CEU	NPIP	28051329	9.2977	6.6519	1.1584
CEU	NPIP	28056329	10.9536	8.0164	1.0849
CEU	NPIP	28061329	13.1161	9.8926	0.9802
CEU	NPIP	28066329	12.4407	9.5515	0.9077
CEU	NPIP	28071329	12.2785	8.3576	1.3938
CEU	NPIP	28076329	13.8907	8.3576	1.9669
CEU	NPIP	28081329	15.2981	9.7221	1.7232
CEU	NPIP	28086329	11.0118	10.0632	0.2839
CEU	NPIP	28091329	5.167	8.3576	-1.1342
CEU	NPIP	28096329	5.892	7.1636	-0.5205
CEU	NPIP	28101329	5.1532	5.9697	-0.3941
CEU	NPIP	28106329	3.2665	4.9463	-0.958
CEU	NPIP	28111329	2.2626	6.1402	-1.8247
CEU	NPIP	28116329	3.6502	7.6753	-1.5471
CEU	NPIP	28121329	5.0608	6.4814	-0.6367
CEU	NPIP	28126329	2.8099	3.9229	-0.7763
CEU	NPIP	28131329	2.0161	4.2641	-1.4593
CEU	NPIP	28136329	2.8949	6.4814	-1.6075
CEU	NPIP	28141329	3.3467	6.1402	-1.3145
CEU	NPIP	28146329	5.3111	5.2874	0.0127
CEU	NPIP	28151329	4.5599	5.1169	-0.3083
CEU	NPIP	28156329	2.2228	4.6052	-1.4465
CEU	NPIP	28161329	2.9584	4.7757	-1.0688
CEU	NPIP	28166329	3.5077	4.4346	-0.5816
CEU	NPIP	28171329	4.3659	5.6286	-0.6423
CEU	NPIP	28176329	4.5986	5.9697	-0.6617
CEU	NPIP	28181329	2.9777	5.9697	-1.444
CEU	NPIP	28186329	2.7827	5.7991	-1.494
CEU	NPIP	28191329	2.9796	5.458	-1.2956
CEU	NPIP	28196329	5.6912	6.9931	-0.5447
CEU	NPIP	28201329	5.3022	6.9931	-0.7075
CEU	NPIP	28206329	4.0546	7.1636	-1.2727
CEU	NPIP	28211329	4.303	6.1402	-0.8645
CEU	NPIP	28216329	4.3873	6.1402	-0.8249
CEU	NPIP	28221329	4.9652	7.1636	-0.8999
CEU	NPIP	28226329	3.8963	5.7991	-0.9424
CEU	NPIP	28231329	6.7877	7.1636	-0.1539
CEU	NPIP	28236329	6.6481	7.8459	-0.4512
CEU	NPIP	28241329	6.0284	8.6987	-0.9148
CEU	NPIP	28246329	11.0812	11.7688	-0.1777
CEU	NPIP	28251329	12.9028	12.1099	0.1995
CEU	NPIP	28256329	11.1273	10.4043	0.2097
CEU	NPIP	28261329	10.2206	8.8692	0.4547

CEU	NPIP	28266329	7.2476	7.3342	-0.0347
CEU	NPIP	28271329	6.0919	6.6519	-0.2452
CEU	NPIP	28276329	6.7398	8.5281	-0.624
CEU	NPIP	28281329	5.0928	8.3576	-1.1605
CEU	NPIP	28286329	6.0567	7.1636	-0.4531
CEU	NPIP	28291329	9.9873	8.8692	0.3762
CEU	NPIP	28296329	8.2506	8.3576	-0.038
CEU	NPIP	28301329	4.114	5.6286	-0.7704
CEU	NPIP	28306329	3.9524	4.9463	-0.5668
CEU	NPIP	28311329	2.8649	4.0935	-0.8261
CEU	NPIP	28316329	2.4371	3.7524	-0.9529
CEU	NPIP	28321329	3.3053	5.458	-1.1254
CEU	NPIP	28326329	4.9227	6.8225	-0.8129
CEU	NPIP	28331329	6.5974	6.6519	-0.0239
CEU	NPIP	28336329	7.4027	6.6519	0.3287
CEU	NPIP	28341329	6.4722	7.5047	-0.4051
CEU	NPIP	28346329	6.659	7.8459	-0.4471
CEU	NPIP	28351329	5.2882	5.7991	-0.253
CEU	NPIP	28356329	1.8126	2.3879	-0.6059
CEU	NPIP	28361329	1.2323	1.0234	0.4151
CEU	NPIP	28366329	1.0142	0.5117	1.576
CEU	NPIP	28371329	2.6269	1.8762	0.9558
CEU	NPIP	28376329	3.1027	2.8996	0.1827
CEU	NPIP	28381329	3.4228	4.2641	-0.5461
CEU	NPIP	28386329	4.0373	5.2874	-0.6722
CEU	NPIP	28391329	1.7013	2.3879	-0.7231
CEU	NPIP	28396329	0.7326	1.1939	-0.822
CEU	NPIP	28401329	1.0346	1.7056	-0.919
CEU	NPIP	28406329	0.4605	0.6822	-0.5789
CEU	NPIP	28411329	0.8157	0.3411	1.9025
CEU	NPIP	28416329	2.2338	2.2173	0.0184
CEU	NPIP	28421329	3.377	4.4346	-0.6636
CEU	NPIP	28426329	2.4391	3.2407	-0.6573
CEU	NPIP	28431329	1.0812	1.3645	-0.4583
CEU	NPIP	28436329	0.6112	0.8528	-0.5441
CEU	NPIP	28441329	0.415	0.8528	-0.9859
CEU	NPIP	28446329	1.0012	2.2173	-1.3586
CEU	NPIP	28451329	1.7864	3.5818	-1.3532
CEU	NPIP	28456329	1.1901	2.0467	-1.0193
CEU	NPIP	28461329	0.7285	1.5351	-1.196
CEU	NPIP	28466329	0.9691	1.8762	-1.1549
CEU	NPIP	28471329	0.309	0.6822	-0.9745
CEU	NPIP	28476329	0.2226	0.8528	-1.4192
CEU	NPIP	28481329	1.2519	2.3879	-1.1963
CEU	NPIP	28486329	4.8233	4.7757	0.028
CEU	NPIP	28491329	6.7716	6.3108	0.2116
CEU	NPIP	28496329	4.7027	6.4814	-0.7972
CEU	NPIP	28501329	3.9068	5.7991	-0.9372
CEU	NPIP	28506329	7.3425	6.9931	0.1462

CEU	NPIP	28511329	10.0092	8.8692	0.3836
CEU	NPIP	28516329	8.55	8.187	0.1315
CEU	NPIP	28521329	5.5428	6.1402	-0.2811
CEU	NPIP	28526329	8.1635	7.6753	0.1877
CEU	NPIP	28531329	11.0443	8.8692	0.7319
CEU	NPIP	28536329	10.7379	8.5281	0.771
CEU	NPIP	28541329	9.0032	7.5047	0.5879
CEU	NPIP	28546329	4.8048	6.4814	-0.7515
CEU	NPIP	28551329	5.7219	7.5047	-0.6995
CEU	NPIP	28556329	7.1375	6.4814	0.2941
CEU	NPIP	28561329	8.726	6.6519	0.9081
CEU	NPIP	28566329	7.591	7.8459	-0.096
CEU	NPIP	28571329	6.2983	8.0164	-0.6346
CEU	NPIP	28576329	7.9074	7.1636	0.3045
CEU	NPIP	28581329	8.408	7.3342	0.4302
CEU	NPIP	28586329	9.719	8.8692	0.286
CEU	NPIP	28591329	9.0237	8.6987	0.1113
CEU	NPIP	28596329	6.2855	6.6519	-0.1604
CEU	NPIP	28601329	6.8571	8.3576	-0.5334
CEU	NPIP	28606329	11.5343	13.645	-0.4743
CEU	NPIP	28611329	10.4014	11.0866	-0.1873
CEU	NPIP	28616329	26.938	16.7151	1.8934
CEU	NPIP	28621329	25.9588	17.7385	1.4383
CEU	NPIP	28626329	8.5797	9.7221	-0.353
CEU	NPIP	28631329	9.8914	9.5515	0.1068
CEU	NPIP	28636329	5.3781	6.3108	-0.4282
CEU	NPIP	28641329	4.2948	6.3108	-0.9256
CEU	NPIP	28646329	7.6417	9.8926	-0.6844
CEU	NPIP	28651329	9.5902	10.5749	-0.2813
CEU	NPIP	28656329	5.3105	5.458	-0.0771
CEU	NPIP	28661329	0.6104	0.8528	-0.5459
CEU	NPIP	28666329	0.0101	0.1706	-0.9534
CEU	NPIP	28671329	0	0	0
CEU	NPIP	28676329	0.4937	0.3411	0.6117
CEU	NPIP	28681329	0.5904	0.5117	0.2468
CEU	NPIP	28686329	0.9625	0.5117	1.4138
CEU	NPIP	28691329	1.1868	0.6822	1.3177
CEU	NPIP	28696329	0.5368	1.1939	-1.171
CEU	NPIP	28701329	1.9175	1.5351	0.567
CEU	NPIP	28706329	3.6819	2.729	0.9007
CEU	NPIP	28711329	2.1676	3.5818	-1.0659
CEU	NPIP	28716329	0.1874	1.5351	-1.9984
CEU	NPIP	28721329	0.503	0.6822	-0.4679
CEU	NPIP	28726329	0.5813	0.8528	-0.6114
CEU	NPIP	28731329	0.2325	0.8528	-1.3969
CEU	NPIP	28736329	1.0284	1.3645	-0.5437
CEU	NPIP	28741329	1.5613	1.5351	0.0388
CEU	NPIP	28746329	2.2841	3.5818	-0.9781
CEU	NPIP	28751329	7.3022	7.1636	0.0567

CEU	NPIP	28756329	5.952	4.7757	0.6918
CEU	NPIP	28761329	2.9101	2.729	0.1712
CEU	NPIP	28766329	3.4954	3.0701	0.3648
CEU	NPIP	28771329	1.395	1.0234	0.7384
CEU	NPIP	28776329	1.5403	1.0234	1.0271
CEU	NPIP	28781329	1.0462	0.8528	0.4355
CEU	NPIP	28786329	0.0689	0.1706	-0.6041
CEU	NPIP	28791329	0	0	0
CEU	NPIP	28796329	1.0254	1.0234	0.004
CEU	NPIP	28801329	2.4133	2.3879	0.0267
CEU	NPIP	28806329	2.7429	2.5584	0.1838
CEU	NPIP	28811329	2.603	3.0701	-0.4007
CEU	NPIP	28816329	2.3317	2.8996	-0.5107
CEU	NPIP	28821329	7.3527	5.1169	1.2376
CEU	NPIP	28826329	8.9938	7.1636	0.7492
CEU	NPIP	28831329	7.2319	6.1402	0.5137
CEU	NPIP	28836329	9.3793	7.3342	0.8194
CEU	NPIP	28841329	9.5988	8.6987	0.3084
CEU	NPIP	28846329	8.5857	7.5047	0.4241
CEU	NPIP	28851329	6.6129	5.458	0.6038
CEU	NPIP	28856329	5.2123	5.1169	0.0528
CEU	NPIP	28861329	12.2488	9.2104	0.9873
CEU	NPIP	28866329	16.7883	10.916	1.6287
CEU	NPIP	28871329	13.9957	8.3576	2.0042
CEU	NPIP	28876329	8.4337	5.2874	1.6918
CEU	NPIP	28881329	5.0212	4.7757	0.1444
CEU	NPIP	28886329	5.8861	5.2874	0.3219
CEU	NPIP	28891329	7.3394	6.8225	0.2212
CEU	NPIP	28896329	7.5636	8.3576	-0.2822
CEU	NPIP	28901329	6.1398	6.3108	-0.0785
CEU	NPIP	28906329	5.6131	5.2874	0.1751
CEU	NPIP	28911329	4.2092	5.1169	-0.5024
CEU	NPIP	28916329	5.1727	5.7991	-0.3103
CEU	NPIP	28921329	5.706	6.8225	-0.4777
CEU	NPIP	28926329	7.5088	7.1636	0.1413
CEU	NPIP	28931329	7.1119	6.3108	0.3678
CEU	NPIP	28936329	4.5733	5.9697	-0.6739
CEU	NPIP	28941329	4.209	6.3108	-0.965
CEU	NPIP	28946329	2.7238	5.2874	-1.3785
CEU	NPIP	28951329	2.9872	4.9463	-1.1173
CEU	NPIP	28956329	4.8654	6.3108	-0.6636
CEU	NPIP	28961329	4.7273	6.1402	-0.6649
CEU	NPIP	28966329	3.9942	6.3108	-1.0636
CHB	NPIP	27961329	9.8449	9.8261	0.0057
CHB	NPIP	27966329	9.9634	9.6567	0.0951
CHB	NPIP	27971329	9.561	10.6731	-0.3141
CHB	NPIP	27976329	10.8098	8.4708	0.8188
CHB	NPIP	27981329	11.7424	8.8096	0.9902
CHB	NPIP	27986329	11.1563	9.8261	0.4058

CHB	NPIP	27991329	9.5233	8.4708	0.3685
CHB	NPIP	27996329	7.9985	9.3178	-0.4229
CHB	NPIP	28001329	8.8926	11.012	-0.5813
CHB	NPIP	28006329	6.7679	7.9625	-0.4427
CHB	NPIP	28011329	6.5641	7.4543	-0.3504
CHB	NPIP	28016329	9.0963	10.8426	-0.486
CHB	NPIP	28021329	10.9524	13.045	-0.4893
CHB	NPIP	28026329	7.3269	9.6567	-0.7224
CHB	NPIP	28031329	4.0994	7.4543	-1.3206
CHB	NPIP	28036329	4.5108	9.1484	-1.512
CHB	NPIP	28041329	5.2439	8.1319	-1.0497
CHB	NPIP	28046329	7.925	8.8096	-0.2987
CHB	NPIP	28051329	10.4638	11.8591	-0.357
CHB	NPIP	28056329	11.7903	12.0285	-0.0601
CHB	NPIP	28061329	13.8138	10.8426	0.8269
CHB	NPIP	28066329	13.2387	11.3508	0.5033
CHB	NPIP	28071329	11.9872	12.0285	-0.0104
CHB	NPIP	28076329	14.4511	13.045	0.3288
CHB	NPIP	28081329	17.7816	13.5532	0.9536
CHB	NPIP	28086329	14.5288	11.8591	0.683
CHB	NPIP	28091329	9.9126	11.6896	-0.4608
CHB	NPIP	28096329	11.1078	13.7226	-0.5828
CHB	NPIP	28101329	10.1739	13.5532	-0.7621
CHB	NPIP	28106329	7.21	11.012	-1.0429
CHB	NPIP	28111329	6.2875	9.6567	-1.0447
CHB	NPIP	28116329	7.8711	9.3178	-0.4637
CHB	NPIP	28121329	8.8547	8.8096	0.0152
CHB	NPIP	28126329	6.7449	8.979	-0.7411
CHB	NPIP	28131329	5.1565	8.4708	-1.1602
CHB	NPIP	28136329	6.355	8.3013	-0.6941
CHB	NPIP	28141329	7.5361	8.4708	-0.3272
CHB	NPIP	28146329	9.1953	9.9955	-0.2403
CHB	NPIP	28151329	7.8125	9.6567	-0.5718
CHB	NPIP	28156329	5.9933	8.6402	-0.9098
CHB	NPIP	28161329	7.7795	9.8261	-0.6244
CHB	NPIP	28166329	6.8063	9.4872	-0.8451
CHB	NPIP	28171329	6.0249	8.1319	-0.7658
CHB	NPIP	28176329	7.2749	8.6402	-0.4693
CHB	NPIP	28181329	8.6735	11.6896	-0.7822
CHB	NPIP	28186329	9.8508	13.5532	-0.835
CHB	NPIP	28191329	7.1089	11.012	-1.0706
CHB	NPIP	28196329	8.3959	9.6567	-0.3909
CHB	NPIP	28201329	7.9446	9.3178	-0.4402
CHB	NPIP	28206329	6.2496	8.979	-0.9054
CHB	NPIP	28211329	6.3078	9.1484	-0.9261
CHB	NPIP	28216329	7.0889	8.8096	-0.581
CHB	NPIP	28221329	8.5191	9.9955	-0.4433
CHB	NPIP	28226329	8.0263	9.8261	-0.5491
CHB	NPIP	28231329	16.2002	13.7226	0.5522

CHB	NPIP	28236329	19.6087	16.0944	0.673
CHB	NPIP	28241329	16.5338	14.9085	0.3348
CHB	NPIP	28246329	15.9763	15.2474	0.147
CHB	NPIP	28251329	13.8527	13.2144	0.1474
CHB	NPIP	28256329	11.9255	12.0285	-0.026
CHB	NPIP	28261329	8.9812	11.3508	-0.6317
CHB	NPIP	28266329	5.9429	10.8426	-1.3636
CHB	NPIP	28271329	5.9977	10.3343	-1.2623
CHB	NPIP	28276329	5.6097	9.4872	-1.2223
CHB	NPIP	28281329	4.7444	9.6567	-1.5232
CHB	NPIP	28286329	6.1259	9.4872	-1.0596
CHB	NPIP	28291329	8.9792	9.6567	-0.2101
CHB	NPIP	28296329	6.2275	7.6237	-0.5384
CHB	NPIP	28301329	2.463	4.5742	-1.2856
CHB	NPIP	28306329	4.3398	4.913	-0.3279
CHB	NPIP	28311329	4.035	5.5907	-0.7938
CHB	NPIP	28316329	3.2429	4.5742	-0.8107
CHB	NPIP	28321329	4.2107	4.7436	-0.3143
CHB	NPIP	28326329	5.8549	5.7601	0.0471
CHB	NPIP	28331329	7.158	6.7766	0.1637
CHB	NPIP	28336329	8.3874	6.946	0.605
CHB	NPIP	28341329	7.4243	6.6072	0.3589
CHB	NPIP	28346329	6.5951	8.1319	-0.5586
CHB	NPIP	28351329	5.0689	6.2684	-0.5524
CHB	NPIP	28356329	2.0259	2.8801	-0.7702
CHB	NPIP	28361329	1.0786	1.5247	-0.6627
CHB	NPIP	28366329	0.7116	0.5082	0.6385
CHB	NPIP	28371329	2.3686	2.5412	-0.1724
CHB	NPIP	28376329	2.5793	3.0495	-0.4044
CHB	NPIP	28381329	2.6419	2.033	0.7261
CHB	NPIP	28386329	3.695	2.8801	0.7347
CHB	NPIP	28391329	1.7559	2.033	-0.3304
CHB	NPIP	28396329	1.1043	1.6942	-0.8096
CHB	NPIP	28401329	1.4081	1.8636	-0.5812
CHB	NPIP	28406329	0.3619	0.6777	-0.8256
CHB	NPIP	28411329	0.5214	0.8471	-0.7345
CHB	NPIP	28416329	1.8897	1.8636	0.0333
CHB	NPIP	28421329	3.5251	3.7271	-0.1467
CHB	NPIP	28426329	2.8296	4.066	-0.8337
CHB	NPIP	28431329	1.1209	2.8801	-1.5861
CHB	NPIP	28436329	0.5936	1.6942	-1.5104
CHB	NPIP	28441329	0.6556	1.3553	-1.134
CHB	NPIP	28446329	0.6789	1.8636	-1.5115
CHB	NPIP	28451329	0.7537	2.033	-1.5256
CHB	NPIP	28456329	0.5656	1.1859	-1.1072
CHB	NPIP	28461329	0.4721	1.6942	-1.6772
CHB	NPIP	28466329	0.4915	1.8636	-1.7506
CHB	NPIP	28471329	0.0291	0.3388	-1.2423
CHB	NPIP	28476329	0.0388	0.5082	-1.4735

CHB	NPIP	28481329	1.335	4.4048	-1.9317
CHB	NPIP	28486329	4.4319	8.8096	-1.478
CHB	NPIP	28491329	5.9623	9.1484	-1.0388
CHB	NPIP	28496329	4.1336	7.6237	-1.3459
CHB	NPIP	28501329	3.3006	7.7931	-1.6979
CHB	NPIP	28506329	6.1591	9.4872	-1.0491
CHB	NPIP	28511329	8.2915	10.3343	-0.5946
CHB	NPIP	28516329	8.2482	8.4708	-0.0779
CHB	NPIP	28521329	6.5096	5.4213	0.5707
CHB	NPIP	28526329	7.3539	6.6072	0.328
CHB	NPIP	28531329	8.9328	8.1319	0.2911
CHB	NPIP	28536329	7.988	8.8096	-0.2774
CHB	NPIP	28541329	7.2916	9.3178	-0.6495
CHB	NPIP	28546329	4.9048	9.8261	-1.5015
CHB	NPIP	28551329	4.9772	10.5037	-1.5844
CHB	NPIP	28556329	6.1072	10.8426	-1.3179
CHB	NPIP	28561329	7.3628	11.3508	-1.0632
CHB	NPIP	28566329	6.08	9.9955	-1.1758
CHB	NPIP	28571329	4.2144	9.6567	-1.6875
CHB	NPIP	28576329	5.5599	9.8261	-1.3016
CHB	NPIP	28581329	5.9022	8.979	-1.0207
CHB	NPIP	28586329	5.6887	9.4872	-1.1974
CHB	NPIP	28591329	5.4752	9.9955	-1.3574
CHB	NPIP	28596329	4.0421	7.6237	-1.3812
CHB	NPIP	28601329	5.1816	9.3178	-1.3258
CHB	NPIP	28606329	8.6423	13.045	-1.0295
CHB	NPIP	28611329	7.7379	10.1649	-0.7175
CHB	NPIP	28616329	21.7847	17.1109	0.8441
CHB	NPIP	28621329	21.5054	18.6357	0.4775
CHB	NPIP	28626329	7.3161	11.1814	-1.0451
CHB	NPIP	28631329	7.2693	11.6896	-1.1463
CHB	NPIP	28636329	4.3678	9.4872	-1.6137
CHB	NPIP	28641329	3.6787	10.1649	-1.9174
CHB	NPIP	28646329	5.8363	12.5367	-1.6267
CHB	NPIP	28651329	7.0948	11.5202	-1.1635
CHB	NPIP	28656329	3.7386	5.7601	-1.0043
CHB	NPIP	28661329	0.3897	0.3388	0.2042
CHB	NPIP	28666329	0	0	0
CHB	NPIP	28671329	0	0	0
CHB	NPIP	28676329	0.4523	0.5082	-0.1755
CHB	NPIP	28681329	0.5344	0.6777	-0.3746
CHB	NPIP	28686329	0.8195	0.6777	0.3707
CHB	NPIP	28691329	1.0251	1.3553	-0.5352
CHB	NPIP	28696329	0.6379	1.6942	-1.4496
CHB	NPIP	28701329	1.7721	1.5247	0.3675
CHB	NPIP	28706329	3.7556	3.0495	0.6072
CHB	NPIP	28711329	2.6425	4.066	-0.9599
CHB	NPIP	28716329	0.3088	1.6942	-1.9013
CHB	NPIP	28721329	0.558	0.5082	0.1563

CHB	NPIP	28726329	0.558	0.5082	0.1563
CHB	NPIP	28731329	0.173	0.6777	-1.3194
CHB	NPIP	28736329	0.5898	1.6942	-1.5157
CHB	NPIP	28741329	1.0916	2.3718	-1.3514
CHB	NPIP	28746329	1.5273	2.7106	-1.1212
CHB	NPIP	28751329	5.8456	5.5907	0.1301
CHB	NPIP	28756329	5.5172	5.7601	-0.1207
CHB	NPIP	28761329	2.6973	4.066	-0.923
CHB	NPIP	28766329	3.1175	3.8965	-0.5448
CHB	NPIP	28771329	1.4457	1.5247	-0.1174
CHB	NPIP	28776329	1.4746	1.1859	0.5153
CHB	NPIP	28781329	1.0849	1.3553	-0.4382
CHB	NPIP	28786329	0.1117	0.3388	-0.911
CHB	NPIP	28791329	0	0	0
CHB	NPIP	28796329	0.9701	1.0165	-0.0923
CHB	NPIP	28801329	2.5563	2.2024	0.3963
CHB	NPIP	28806329	2.6252	2.3718	0.2675
CHB	NPIP	28811329	2.7007	3.3883	-0.5415
CHB	NPIP	28816329	2.8252	4.066	-0.8367
CHB	NPIP	28821329	6.0721	7.7931	-0.6504
CHB	NPIP	28826329	6.775	10.1649	-1.0021
CHB	NPIP	28831329	4.9488	8.4708	-1.233
CHB	NPIP	28836329	6.6861	9.9955	-0.9938
CHB	NPIP	28841329	7.1601	11.3508	-1.1172
CHB	NPIP	28846329	6.7434	10.5037	-1.0781
CHB	NPIP	28851329	4.902	8.3013	-1.2123
CHB	NPIP	28856329	3.9303	6.4378	-1.1275
CHB	NPIP	28861329	9.6269	10.5037	-0.2514
CHB	NPIP	28866329	12.2157	11.3508	0.2306
CHB	NPIP	28871329	10.2168	8.979	0.4106
CHB	NPIP	28876329	6.6343	6.7766	-0.0611
CHB	NPIP	28881329	3.6821	5.4213	-0.912
CHB	NPIP	28886329	5.0147	8.6402	-1.2462
CHB	NPIP	28891329	5.885	8.3013	-0.8618
CHB	NPIP	28896329	5.272	6.7766	-0.6459
CHB	NPIP	28901329	5.2145	8.1319	-1.0604
CHB	NPIP	28906329	5.3564	7.6237	-0.8743
CHB	NPIP	28911329	4.4245	8.1319	-1.3475
CHB	NPIP	28916329	4.4691	8.8096	-1.4655
CHB	NPIP	28921329	4.0929	8.6402	-1.5631
CHB	NPIP	28926329	6.5648	8.8096	-0.7579
CHB	NPIP	28931329	6.617	6.4378	0.0806
CHB	NPIP	28936329	4.3033	6.0989	-0.8476
CHB	NPIP	28941329	4.6707	6.6072	-0.8505
CHB	NPIP	28946329	3.3404	5.7601	-1.2022
CHB	NPIP	28951329	3.831	6.0989	-1.0705
CHB	NPIP	28956329	6.7082	7.4543	-0.2937
CHB	NPIP	28961329	7.114	6.946	0.0705
CHB	NPIP	28966329	6.676	6.7766	-0.0432

YRI	NPIP	27961329	13.1343	13.2769	-0.0327
YRI	NPIP	27966329	10.1647	12.1005	-0.4843
YRI	NPIP	27971329	10.6363	14.1172	-0.7527
YRI	NPIP	27976329	10.9939	14.2853	-0.7037
YRI	NPIP	27981329	11.3486	14.9575	-0.7386
YRI	NPIP	27986329	14.3253	15.9659	-0.3155
YRI	NPIP	27991329	15.2222	15.4617	-0.0475
YRI	NPIP	27996329	14.7308	15.6298	-0.1764
YRI	NPIP	28001329	18.4514	17.8146	0.1103
YRI	NPIP	28006329	15.4368	15.1256	0.063
YRI	NPIP	28011329	10.6374	12.1005	-0.366
YRI	NPIP	28016329	11.2072	13.445	-0.5068
YRI	NPIP	28021329	12.9557	15.1256	-0.4394
YRI	NPIP	28026329	9.3772	13.1089	-0.8656
YRI	NPIP	28031329	7.0912	11.0921	-1.0863
YRI	NPIP	28036329	7.0975	12.2685	-1.277
YRI	NPIP	28041329	7.1353	12.4366	-1.2924
YRI	NPIP	28046329	9.2378	13.1089	-0.898
YRI	NPIP	28051329	9.7353	16.4701	-1.2573
YRI	NPIP	28056329	10.0185	16.302	-1.1846
YRI	NPIP	28061329	11.9832	12.7727	-0.1877
YRI	NPIP	28066329	14.6825	15.7978	-0.2167
YRI	NPIP	28071329	14.4525	15.9659	-0.291
YRI	NPIP	28076329	15.338	13.613	0.3861
YRI	NPIP	28081329	19.6835	17.8146	0.3236
YRI	NPIP	28086329	17.7411	18.4868	-0.1246
YRI	NPIP	28091329	13.4131	15.1256	-0.3468
YRI	NPIP	28096329	13.9456	14.6214	-0.1413
YRI	NPIP	28101329	11.7996	14.9575	-0.6463
YRI	NPIP	28106329	8.2535	12.4366	-1.0198
YRI	NPIP	28111329	7.3253	12.7727	-1.295
YRI	NPIP	28116329	8.7166	13.613	-1.0959
YRI	NPIP	28121329	9.1732	11.7644	-0.6657
YRI	NPIP	28126329	7.1569	11.0921	-1.0684
YRI	NPIP	28131329	6.4305	9.5795	-0.9804
YRI	NPIP	28136329	7.7316	9.5795	-0.5753
YRI	NPIP	28141329	8.851	12.1005	-0.813
YRI	NPIP	28146329	10.1811	13.613	-0.7681
YRI	NPIP	28151329	8.8191	11.5963	-0.7232
YRI	NPIP	28156329	7.6297	12.1005	-1.1185
YRI	NPIP	28161329	8.7456	13.1089	-1.0121
YRI	NPIP	28166329	7.3533	11.4282	-1.0758
YRI	NPIP	28171329	7.1961	11.7644	-1.1736
YRI	NPIP	28176329	7.8106	11.9324	-1.0449
YRI	NPIP	28181329	9.4131	13.9492	-0.992
YRI	NPIP	28186329	9.4795	13.9492	-0.9775
YRI	NPIP	28191329	6.9442	10.4199	-1.0005
YRI	NPIP	28196329	9.7967	10.5879	-0.2244
YRI	NPIP	28201329	9.3133	10.756	-0.4032

YRI	NPIP	28206329	8.1767	10.924	-0.7567
YRI	NPIP	28211329	8.3888	10.924	-0.6982
YRI	NPIP	28216329	8.6919	11.7644	-0.7893
YRI	NPIP	28221329	10.5228	12.6047	-0.5012
YRI	NPIP	28226329	10.382	11.9324	-0.393
YRI	NPIP	28231329	15.999	15.9659	0.0064
YRI	NPIP	28236329	16.8088	18.3188	-0.2546
YRI	NPIP	28241329	15.0601	17.1423	-0.3741
YRI	NPIP	28246329	18.9214	16.9743	0.3531
YRI	NPIP	28251329	19.6425	15.9659	0.7071
YRI	NPIP	28256329	17.7985	15.9659	0.3524
YRI	NPIP	28261329	15.9733	15.4617	0.1014
YRI	NPIP	28266329	13.3386	12.7727	0.1345
YRI	NPIP	28271329	11.3213	12.4366	-0.2719
YRI	NPIP	28276329	10.3448	14.6214	-0.8944
YRI	NPIP	28281329	9.069	15.9659	-1.3264
YRI	NPIP	28286329	7.5681	14.1172	-1.4161
YRI	NPIP	28291329	9.8345	14.4534	-0.9766
YRI	NPIP	28296329	10.2456	13.7811	-0.7822
YRI	NPIP	28301329	5.8551	8.067	-0.8071
YRI	NPIP	28306329	5.5138	7.8989	-0.8873
YRI	NPIP	28311329	5.4798	9.7476	-1.3074
YRI	NPIP	28316329	3.3213	8.235	-1.7594
YRI	NPIP	28321329	4.3388	9.2434	-1.5784
YRI	NPIP	28326329	5.4061	8.9073	-1.1661
YRI	NPIP	28331329	7.1756	9.0754	-0.6219
YRI	NPIP	28336329	10.0948	10.756	-0.1848
YRI	NPIP	28341329	8.3371	10.0837	-0.5184
YRI	NPIP	28346329	8.5478	12.4366	-0.9481
YRI	NPIP	28351329	6.8818	9.5795	-0.8399
YRI	NPIP	28356329	1.7751	3.1932	-1.1695
YRI	NPIP	28361329	0.4661	1.6806	-1.6708
YRI	NPIP	28366329	0.5184	1.6806	-1.5988
YRI	NPIP	28371329	2.2381	4.0335	-1.2148
YRI	NPIP	28376329	2.8089	4.2016	-0.9098
YRI	NPIP	28381329	3.7574	4.7057	-0.5614
YRI	NPIP	28386329	4.711	6.3864	-0.7562
YRI	NPIP	28391329	2.1724	3.8654	-1.1881
YRI	NPIP	28396329	1.7935	2.1848	-0.4393
YRI	NPIP	28401329	2.4592	2.0167	0.5291
YRI	NPIP	28406329	1.0482	0.8403	0.4696
YRI	NPIP	28411329	0.5309	0.6722	-0.3699
YRI	NPIP	28416329	1.6981	1.8487	-0.1926
YRI	NPIP	28421329	3.5808	4.8738	-0.7422
YRI	NPIP	28426329	3.2484	5.0419	-0.9993
YRI	NPIP	28431329	1.7701	2.1848	-0.4656
YRI	NPIP	28436329	1.1274	1.3445	-0.3526
YRI	NPIP	28441329	1.3208	1.8487	-0.6752
YRI	NPIP	28446329	1.0198	1.6806	-0.9091

YRI	NPIP	28451329	0.7058	3.1932	-2.0514
YRI	NPIP	28456329	0.6687	2.689	-1.92
YRI	NPIP	28461329	1.6311	2.1848	-0.6217
YRI	NPIP	28466329	1.6678	2.3529	-0.7252
YRI	NPIP	28471329	0.2919	0.8403	-1.2386
YRI	NPIP	28476329	0.3097	0.8403	-1.1984
YRI	NPIP	28481329	1.3359	4.8738	-2.0309
YRI	NPIP	28486329	5.0235	11.0921	-1.6477
YRI	NPIP	28491329	6.5777	10.924	-1.1971
YRI	NPIP	28496329	4.4643	9.0754	-1.5094
YRI	NPIP	28501329	5.8289	10.756	-1.3769
YRI	NPIP	28506329	11.0227	13.7811	-0.6102
YRI	NPIP	28511329	10.0504	15.7978	-1.1165
YRI	NPIP	28516329	7.5052	14.7895	-1.5069
YRI	NPIP	28521329	6.5964	12.6047	-1.4464
YRI	NPIP	28526329	7.366	12.7727	-1.2854
YRI	NPIP	28531329	9.3833	13.1089	-0.8642
YRI	NPIP	28536329	9.6643	13.7811	-0.9108
YRI	NPIP	28541329	8.3572	13.1089	-1.1022
YRI	NPIP	28546329	4.9685	9.4115	-1.4062
YRI	NPIP	28551329	5.5575	9.7476	-1.2836
YRI	NPIP	28556329	7.6358	12.2685	-1.144
YRI	NPIP	28561329	8.7811	11.7644	-0.7664
YRI	NPIP	28566329	6.7216	10.924	-1.1574
YRI	NPIP	28571329	6.3712	13.7811	-1.6393
YRI	NPIP	28576329	7.503	12.6047	-1.2281
YRI	NPIP	28581329	7.4148	9.7476	-0.7146
YRI	NPIP	28586329	8.1231	10.0837	-0.5819
YRI	NPIP	28591329	7.2188	12.1005	-1.2213
YRI	NPIP	28596329	6.2702	12.6047	-1.5249
YRI	NPIP	28601329	7.4318	14.4534	-1.4847
YRI	NPIP	28606329	12.0901	20.3355	-1.2572
YRI	NPIP	28611329	12.7558	16.302	-0.6685
YRI	NPIP	28616329	24.0146	21.0078	0.4443
YRI	NPIP	28621329	25.1151	21.68	0.4924
YRI	NPIP	28626329	15.3518	13.2769	0.4755
YRI	NPIP	28631329	14.5775	12.2685	0.5702
YRI	NPIP	28636329	7.6694	8.4031	-0.2579
YRI	NPIP	28641329	6.6848	12.1005	-1.3549
YRI	NPIP	28646329	10.5852	16.6382	-1.1191
YRI	NPIP	28651329	12.8823	16.134	-0.6191
YRI	NPIP	28656329	6.978	8.5712	-0.5498
YRI	NPIP	28661329	0.2193	1.0084	-1.5732
YRI	NPIP	28666329	0.1281	0.6722	-1.4244
YRI	NPIP	28671329	0.0276	0.3361	-1.2385
YRI	NPIP	28676329	0.6851	1.3445	-1.071
YRI	NPIP	28681329	0.7944	1.1764	-0.6832
YRI	NPIP	28686329	0.864	0.8403	0.0535
YRI	NPIP	28691329	1.7326	1.5126	0.3276

YRI	NPIP	28696329	2.0501	1.5126	0.8004
YRI	NPIP	28701329	2.1043	1.5126	0.8811
YRI	NPIP	28706329	3.393	3.3612	0.0251
YRI	NPIP	28711329	4.8626	4.5377	0.1985
YRI	NPIP	28716329	2.5292	2.0167	0.6128
YRI	NPIP	28721329	0.5644	0.3361	0.9165
YRI	NPIP	28726329	0.5916	0.5042	0.2747
YRI	NPIP	28731329	0.055	0.6722	-1.6158
YRI	NPIP	28736329	0.7476	2.0167	-1.5174
YRI	NPIP	28741329	2.1235	3.0251	-0.7778
YRI	NPIP	28746329	3.4372	5.378	-1.0214
YRI	NPIP	28751329	6.6827	8.235	-0.5558
YRI	NPIP	28756329	6.4666	6.3864	0.0362
YRI	NPIP	28761329	3.9355	5.0419	-0.6165
YRI	NPIP	28766329	3.1284	4.2016	-0.701
YRI	NPIP	28771329	1.7127	1.5126	0.298
YRI	NPIP	28776329	2.2497	1.8487	0.5129
YRI	NPIP	28781329	2.0869	1.8487	0.3047
YRI	NPIP	28786329	0.5395	0.3361	0.8166
YRI	NPIP	28791329	0	0	0
YRI	NPIP	28796329	0.9656	0.8403	0.283
YRI	NPIP	28801329	2.7855	2.1848	0.6745
YRI	NPIP	28806329	2.9513	3.0251	-0.0637
YRI	NPIP	28811329	3.4735	4.2016	-0.4756
YRI	NPIP	28816329	3.3931	4.2016	-0.5281
YRI	NPIP	28821329	6.2353	9.2434	-0.9681
YRI	NPIP	28826329	8.1216	11.9324	-0.966
YRI	NPIP	28831329	7.1936	9.0754	-0.616
YRI	NPIP	28836329	7.1056	9.9157	-0.8472
YRI	NPIP	28841329	6.8538	10.0837	-0.9587
YRI	NPIP	28846329	7.1564	9.4115	-0.7137
YRI	NPIP	28851329	6.8142	9.0754	-0.7402
YRI	NPIP	28856329	7.3215	8.4031	-0.3801
YRI	NPIP	28861329	11.4001	10.756	0.18
YRI	NPIP	28866329	11.875	12.9408	-0.2503
YRI	NPIP	28871329	10.3635	11.2602	-0.24
YRI	NPIP	28876329	7.4776	7.7309	-0.0961
YRI	NPIP	28881329	4.0252	6.0502	-0.9595
YRI	NPIP	28886329	5.5179	6.5544	-0.457
YRI	NPIP	28891329	7.0942	8.4031	-0.46
YRI	NPIP	28896329	7.0644	8.9073	-0.6138
YRI	NPIP	28901329	5.9285	7.7309	-0.6839
YRI	NPIP	28906329	6.4117	9.4115	-0.9494
YRI	NPIP	28911329	5.182	9.5795	-1.3691
YRI	NPIP	28916329	4.4482	8.7392	-1.4545
YRI	NPIP	28921329	4.3671	8.7392	-1.482
YRI	NPIP	28926329	7.4733	9.4115	-0.6134
YRI	NPIP	28931329	8.2585	8.4031	-0.0508
YRI	NPIP	28936329	5.5429	7.2267	-0.6794

YRI	NPIP	28941329	5.1962	8.067	-1.0476
YRI	NPIP	28946329	3.5608	8.235	-1.6736
YRI	NPIP	28951329	3.7705	7.2267	-1.3946
YRI	NPIP	28956329	7.8964	10.2518	-0.6884
YRI	NPIP	28961329	8.3101	11.7644	-0.8874
YRI	NPIP	28966329	7.5849	10.5879	-0.8516
CEU	NPIP	28142891	2.9841	5.2874	-1.2385
CEU	NPIP	28147891	5.4622	5.7991	-0.1669
CEU	NPIP	28152891	4.6275	5.7991	-0.5803
CEU	NPIP	28157891	1.7017	3.9229	-1.5492
CEU	NPIP	28162891	2.5203	3.7524	-0.8926
CEU	NPIP	28167891	3.9755	4.9463	-0.5536
CEU	NPIP	28172891	4.8477	6.3108	-0.6717
CEU	NPIP	28177891	4.7807	6.6519	-0.8192
CEU	NPIP	28182891	2.8268	5.7991	-1.4722
CEU	NPIP	28187891	2.6395	5.7991	-1.5649
CEU	NPIP	28192891	2.9796	5.2874	-1.2409
CEU	NPIP	28197891	5.4253	6.4814	-0.4734
CEU	NPIP	28202891	5.4277	7.6753	-0.8639
CEU	NPIP	28207891	3.8736	6.8225	-1.2618
CEU	NPIP	28212891	5.7852	7.1636	-0.5642
CEU	NPIP	28217891	4.7281	6.4814	-0.7859
CEU	NPIP	28222891	4.1946	5.7991	-0.7947
CEU	NPIP	28227891	5.4736	6.9931	-0.6358
CEU	NPIP	28232891	6.3412	7.6753	-0.5128
CEU	NPIP	28237891	6.245	7.6753	-0.5498
CEU	NPIP	28242891	8.6155	9.2104	-0.1933
CEU	NPIP	28247891	11.589	12.1099	-0.131
CEU	NPIP	28252891	10.3308	10.916	-0.1623
CEU	NPIP	28257891	9.7721	8.6987	0.3677
CEU	NPIP	28262891	10.1802	9.2104	0.3151
CEU	NPIP	28267891	8.3311	7.8459	0.1828
CEU	NPIP	28272891	5.7889	6.8225	-0.4423
CEU	NPIP	28277891	5.0643	8.187	-1.1313
CEU	NPIP	28282891	5.3069	8.3576	-1.0844
CEU	NPIP	28287891	7.4088	8.3576	-0.3373
CEU	NPIP	28292891	9.3606	7.8459	0.5706
CEU	NPIP	28297891	7.7049	7.3342	0.1485
CEU	NPIP	28302891	4.604	6.4814	-0.8415
CEU	NPIP	28307891	3.3581	4.9463	-0.9057
CEU	NPIP	28312891	2.3751	3.4112	-0.8138
CEU	NPIP	28317891	2.1605	3.9229	-1.2292
CEU	NPIP	28322891	4.6118	6.1402	-0.7192
CEU	NPIP	28327891	4.8724	6.3108	-0.6604
CEU	NPIP	28332891	6.3598	6.6519	-0.1279
CEU	NPIP	28337891	8.3891	8.3576	0.0112
CEU	NPIP	28342891	6.4708	7.6753	-0.463
CEU	NPIP	28347891	5.6524	5.7991	-0.0727
CEU	NPIP	28352891	4.5059	4.7757	-0.1587

CEU	NPIP	28357891	2.3643	2.5584	-0.1934
CEU	NPIP	28362891	0.9068	0.8528	0.1216
CEU	NPIP	28367891	0.6289	0.6822	-0.1392
CEU	NPIP	28372891	2.6761	2.2173	0.5126
CEU	NPIP	28377891	2.9464	2.729	0.2055
CEU	NPIP	28382891	3.4137	4.2641	-0.552
CEU	NPIP	28387891	4.0171	4.9463	-0.5299
CEU	NPIP	28392891	1.7386	2.2173	-0.5348
CEU	NPIP	28397891	0.7326	1.1939	-0.822
CEU	NPIP	28402891	0.9571	1.5351	-0.8571
CEU	NPIP	28407891	0.4605	0.6822	-0.5789
CEU	NPIP	28412891	1.7753	2.0467	-0.3229
CEU	NPIP	28417891	3.2507	2.8996	0.3158
CEU	NPIP	28422891	2.8874	3.2407	-0.2897
CEU	NPIP	28427891	1.4222	2.5584	-1.1318
CEU	NPIP	28432891	0.6112	0.8528	-0.5441
CEU	NPIP	28437891	0.6213	1.0234	-0.799
CEU	NPIP	28442891	0.4251	1.0234	-1.1889
CEU	NPIP	28447891	1.0113	2.3879	-1.4497
CEU	NPIP	28452891	1.7763	3.4112	-1.2841
CEU	NPIP	28457891	1.1699	1.7056	-0.7337
CEU	NPIP	28462891	0.7285	1.5351	-1.196
CEU	NPIP	28467891	0.9691	1.8762	-1.1549
CEU	NPIP	28472891	0.3292	0.8528	-1.1791
CEU	NPIP	28477891	0.2529	1.3645	-1.7983
CEU	NPIP	28482891	2.6421	2.8996	-0.2316
CEU	NPIP	28487891	5.8046	5.2874	0.2781
CEU	NPIP	28492891	5.8398	6.1402	-0.1414
CEU	NPIP	28497891	4.7934	6.4814	-0.7566
CEU	NPIP	28502891	4.6254	6.1402	-0.7128
CEU	NPIP	28507891	7.7385	6.8225	0.392
CEU	NPIP	28512891	10.2495	9.3809	0.2775
CEU	NPIP	28517891	7.4675	8.0164	-0.2027
CEU	NPIP	28522891	6.0771	6.3108	-0.1073
CEU	NPIP	28527891	8.7957	8.3576	0.1557
CEU	NPIP	28532891	10.3539	8.8692	0.4996
CEU	NPIP	28537891	11.2128	8.5281	0.9367
CEU	NPIP	28542891	8.0316	6.9931	0.4345
CEU	NPIP	28547891	3.9433	5.9697	-0.978
CEU	NPIP	28552891	5.8724	6.8225	-0.4065
CEU	NPIP	28557891	8.5922	7.6753	0.3524
CEU	NPIP	28562891	8.845	7.8459	0.3764
CEU	NPIP	28567891	6.0928	6.6519	-0.2448
CEU	NPIP	28572891	5.9759	7.1636	-0.4862
CEU	NPIP	28577891	8.7419	8.0164	0.268
CEU	NPIP	28582891	9.268	8.5281	0.2582
CEU	NPIP	28587891	8.6444	8.0164	0.232
CEU	NPIP	28592891	8.1635	7.3342	0.3323
CEU	NPIP	28597891	5.9973	6.3108	-0.1439

CEU	NPIP	28602891	9.4587	10.4043	-0.2743
CEU	NPIP	28607891	11.4548	13.4744	-0.4593
CEU	NPIP	28612891	14.9307	12.2805	0.658
CEU	NPIP	28617891	27.6819	18.4207	1.5627
CEU	NPIP	28622891	21.0076	15.8623	1.0019
CEU	NPIP	28627891	11.3117	10.2337	0.3176
CEU	NPIP	28632891	7.6625	8.5281	-0.302
CEU	NPIP	28637891	2.0006	4.2641	-1.4693
CEU	NPIP	28642891	4.6568	6.9931	-0.9775
CEU	NPIP	28647891	9.6368	11.2571	-0.4366
CEU	NPIP	28652891	9.6106	9.5515	0.0186
CEU	NPIP	28657891	3.2068	3.5818	-0.2826
CEU	NPIP	28662891	0.0202	0.3411	-1.2864
CEU	NPIP	28667891	0.0101	0.1706	-0.9534
CEU	NPIP	28672891	0.3795	0.1706	1.2409
CEU	NPIP	28677891	0.4937	0.3411	0.6117
CEU	NPIP	28682891	0.6155	0.5117	0.3255
CEU	NPIP	28687891	0.9625	0.5117	1.4138
CEU	NPIP	28692891	0.8023	0.6822	0.3136
CEU	NPIP	28697891	0.5368	1.1939	-1.171
CEU	NPIP	28702891	1.9577	1.8762	0.1038
CEU	NPIP	28707891	3.7324	3.5818	0.1135
CEU	NPIP	28712891	2.1072	3.0701	-0.826
CEU	NPIP	28717891	0.1369	0.6822	-1.4239
CEU	NPIP	28722891	0.5813	0.8528	-0.6114
CEU	NPIP	28727891	0.5813	0.8528	-0.6114
CEU	NPIP	28732891	0.49	0.8528	-0.817
CEU	NPIP	28737891	1.1806	1.8762	-0.8856
CEU	NPIP	28742891	1.2558	1.8762	-0.7899
CEU	NPIP	28747891	3.683	4.9463	-0.7205
CEU	NPIP	28752891	7.282	6.8225	0.1966
CEU	NPIP	28757891	4.4212	3.2407	0.968
CEU	NPIP	28762891	3.2839	2.8996	0.3456
CEU	NPIP	28767891	4.0381	3.0701	0.8303
CEU	NPIP	28772891	1.0414	1.0234	0.0358
CEU	NPIP	28777891	0.9773	0.6822	0.7706
CEU	NPIP	28782891	1.0159	0.5117	1.5813
CEU	NPIP	28787891	0.0689	0.1706	-0.6041
CEU	NPIP	28792891	0	0	0
CEU	NPIP	28797891	1.7811	1.5351	0.3648
CEU	NPIP	28802891	2.8027	2.8996	-0.0871
CEU	NPIP	28807891	2.7199	2.5584	0.1609
CEU	NPIP	28812891	2.8147	3.0701	-0.2191
CEU	NPIP	28817891	1.6984	3.0701	-1.1766
CEU	NPIP	28822891	7.2533	5.2874	1.0571
CEU	NPIP	28827891	9.9048	6.9931	1.2183
CEU	NPIP	28832891	8.1303	6.3108	0.8354
CEU	NPIP	28837891	9.3527	8.3576	0.3537
CEU	NPIP	28842891	10.1142	9.2104	0.2937

CEU	NPIP	28847891	8.43	7.1636	0.5184
CEU	NPIP	28852891	5.5929	4.9463	0.3688
CEU	NPIP	28857891	6.3329	4.9463	0.7908
CEU	NPIP	28862891	14.2977	9.8926	1.3395
CEU	NPIP	28867891	18.4587	11.9394	1.6621
CEU	NPIP	28872891	11.0614	6.9931	1.7022
CEU	NPIP	28877891	5.939	4.6052	0.8098
CEU	NPIP	28882891	5.0313	4.9463	0.0485
CEU	NPIP	28887891	5.2737	4.7757	0.2929
CEU	NPIP	28892891	8.2776	7.6753	0.2315
CEU	NPIP	28897891	7.0719	7.8459	-0.2916
CEU	NPIP	28902891	5.2956	5.2874	0.0044
CEU	NPIP	28907891	5.663	5.458	0.1072
CEU	NPIP	28912891	5.0711	5.9697	-0.4337
CEU	NPIP	28917891	6.6061	6.4814	0.0559
CEU	NPIP	28922891	8.8409	7.6753	0.448
CEU	NPIP	28927891	6.744	6.9931	-0.1042
CEU	NPIP	28932891	3.9784	5.9697	-0.961
CEU	NPIP	28937891	4.4059	5.9697	-0.7547
CEU	NPIP	28942891	3.2822	5.6286	-1.1935
CEU	NPIP	28947891	2.9847	4.9463	-1.1187
CEU	NPIP	28952891	4.343	5.458	-0.5829
CEU	NPIP	28957891	4.5807	6.6519	-0.9068
CEU	NPIP	28962891	3.5677	5.9697	-1.1592
CEU	NPIP	28967891	3.4467	5.2874	-0.9898
CEU	NPIP	28972891	4.1377	4.9463	-0.4611
CEU	NPIP	28977891	6.4084	6.6519	-0.1066
CEU	NPIP	28982891	6.0752	7.8459	-0.667
CEU	NPIP	28987891	4.9258	6.3108	-0.6359
CEU	NPIP	28992891	4.6261	5.7991	-0.581
CEU	NPIP	28997891	4.4616	6.4814	-0.9053
CEU	NPIP	29002891	4.8468	6.8225	-0.8454
CEU	NPIP	29007891	6.3919	9.3809	-0.9549
CEU	NPIP	29012891	6.6603	9.3809	-0.8691
CEU	NPIP	29017891	5.9619	6.6519	-0.3021
CEU	NPIP	29022891	5.9996	6.6519	-0.2856
CEU	NPIP	29027891	5.5762	6.6519	-0.471
CEU	NPIP	29032891	5.2583	6.9931	-0.7259
CEU	NPIP	29037891	4.7728	7.3342	-1.0263
CEU	NPIP	29042891	4.8691	5.6286	-0.3863
CEU	NPIP	29047891	3.1161	2.8996	0.1947
CEU	NPIP	29052891	2.2389	2.3879	-0.1569
CEU	NPIP	29057891	3.6638	3.5818	0.0618
CEU	NPIP	29062891	5.0258	4.6052	0.2554
CEU	NPIP	29067891	4.7448	4.2641	0.312
CEU	NPIP	29072891	4.008	2.8996	0.9968
CEU	NPIP	29077891	4.155	3.4112	0.5842
CEU	NPIP	29082891	4.9101	5.458	-0.2864
CEU	NPIP	29087891	5.7487	6.1402	-0.1842

CEU	NPIP	29092891	7.6423	6.6519	0.4336
CEU	NPIP	29097891	7.6434	7.5047	0.0544
CEU	NPIP	29102891	7.0695	8.187	-0.4048
CEU	NPIP	29107891	8.9773	7.8459	0.4262
CEU	NPIP	29112891	12.2356	8.187	1.4667
CEU	NPIP	29117891	13.5277	10.2337	0.9704
CEU	NPIP	29122891	10.5768	8.8692	0.5746
CEU	NPIP	29127891	15.7075	10.4043	1.5384
CEU	NPIP	29132891	20.765	12.7922	1.9046
CEU	NPIP	29137891	22.3002	13.1333	2.136
CEU	NPIP	29142891	19.6422	12.1099	1.8949
CEU	NPIP	29147891	18.0202	13.3039	1.0856
CEU	NPIP	29152891	26.9505	17.0562	1.7975
CHB	NPIP	28142891	8.5662	9.9955	-0.4292
CHB	NPIP	28147891	9.5159	10.6731	-0.3268
CHB	NPIP	28152891	6.3922	8.3013	-0.6809
CHB	NPIP	28157891	6.7548	8.3013	-0.5516
CHB	NPIP	28162891	7.2493	8.979	-0.5738
CHB	NPIP	28167891	6.1551	9.1484	-0.9759
CHB	NPIP	28172891	7.2817	9.1484	-0.6086
CHB	NPIP	28177891	7.7748	9.8261	-0.6259
CHB	NPIP	28182891	9.7747	12.3673	-0.6376
CHB	NPIP	28187891	9.1443	12.7061	-0.8539
CHB	NPIP	28192891	6.2797	10.3343	-1.1802
CHB	NPIP	28197891	8.242	9.9955	-0.5265
CHB	NPIP	28202891	6.9551	9.1484	-0.7151
CHB	NPIP	28207891	5.6236	8.3013	-0.955
CHB	NPIP	28212891	8.0767	9.8261	-0.5337
CHB	NPIP	28217891	8.1296	9.4872	-0.4279
CHB	NPIP	28222891	8.9568	9.3178	-0.1157
CHB	NPIP	28227891	11.1251	11.012	0.031
CHB	NPIP	28232891	18.135	15.7556	0.465
CHB	NPIP	28237891	17.2044	16.0944	0.2126
CHB	NPIP	28242891	15.4405	14.0615	0.3003
CHB	NPIP	28247891	15.7631	14.4003	0.2901
CHB	NPIP	28252891	11.192	12.5367	-0.3265
CHB	NPIP	28257891	10.7492	11.5202	-0.2027
CHB	NPIP	28262891	8.8959	11.5202	-0.69
CHB	NPIP	28267891	6.9941	10.5037	-1.0062
CHB	NPIP	28272891	5.3543	8.8096	-1.1666
CHB	NPIP	28277891	4.042	9.1484	-1.6649
CHB	NPIP	28282891	5.1688	10.1649	-1.4769
CHB	NPIP	28287891	7.4083	10.1649	-0.8149
CHB	NPIP	28292891	8.3569	8.979	-0.2064
CHB	NPIP	28297891	5.471	6.946	-0.6191
CHB	NPIP	28302891	3.2273	5.0825	-1.03
CHB	NPIP	28307891	4.2161	5.0825	-0.481
CHB	NPIP	28312891	3.2511	4.7436	-0.8804
CHB	NPIP	28317891	2.8894	3.8965	-0.7044

CHB	NPIP	28322891	5.7331	5.5907	0.0727
CHB	NPIP	28327891	5.47	5.5907	-0.0616
CHB	NPIP	28332891	6.5545	6.6072	-0.0231
CHB	NPIP	28337891	9.6471	8.4708	0.4118
CHB	NPIP	28342891	7.608	7.6237	-0.0061
CHB	NPIP	28347891	5.316	6.2684	-0.4386
CHB	NPIP	28352891	4.2747	4.5742	-0.1824
CHB	NPIP	28357891	2.284	3.0495	-0.6583
CHB	NPIP	28362891	0.751	1.3553	-0.9794
CHB	NPIP	28367891	0.8812	1.3553	-0.7684
CHB	NPIP	28372891	2.3783	2.7106	-0.3149
CHB	NPIP	28377891	2.1515	2.033	0.1413
CHB	NPIP	28382891	3.0421	2.2024	0.9402
CHB	NPIP	28387891	3.7047	3.0495	0.5635
CHB	NPIP	28392891	1.7207	2.2024	-0.5394
CHB	NPIP	28397891	1.0946	1.5247	-0.639
CHB	NPIP	28402891	1.0335	1.3553	-0.5215
CHB	NPIP	28407891	0.3619	0.6777	-0.8256
CHB	NPIP	28412891	1.5053	1.8636	-0.4571
CHB	NPIP	28417891	2.9656	2.8801	0.0771
CHB	NPIP	28422891	3.1367	3.5577	-0.3182
CHB	NPIP	28427891	1.7537	3.0495	-1.1144
CHB	NPIP	28432891	0.5254	2.033	-1.7978
CHB	NPIP	28437891	0.6417	2.033	-1.6591
CHB	NPIP	28442891	0.6653	1.5247	-1.2768
CHB	NPIP	28447891	0.7579	1.6942	-1.285
CHB	NPIP	28452891	0.744	1.8636	-1.4285
CHB	NPIP	28457891	0.4384	1.0165	-1.1505
CHB	NPIP	28462891	0.4721	1.6942	-1.6772
CHB	NPIP	28467891	0.4915	1.8636	-1.7506
CHB	NPIP	28472891	0.0388	0.5082	-1.4735
CHB	NPIP	28477891	0.4337	1.5247	-1.6208
CHB	NPIP	28482891	2.3727	5.2519	-1.5528
CHB	NPIP	28487891	4.8317	8.979	-1.3758
CHB	NPIP	28492891	5.2241	9.1484	-1.2795
CHB	NPIP	28497891	4.4991	7.9625	-1.2834
CHB	NPIP	28502891	3.6547	8.1319	-1.6273
CHB	NPIP	28507891	6.1038	9.4872	-1.0665
CHB	NPIP	28512891	9.2046	9.9955	-0.2375
CHB	NPIP	28517891	7.5332	7.4543	0.0311
CHB	NPIP	28522891	6.6052	5.5907	0.5176
CHB	NPIP	28527891	7.8664	7.1154	0.3084
CHB	NPIP	28532891	8.1974	8.1319	0.0238
CHB	NPIP	28537891	8.6377	9.3178	-0.218
CHB	NPIP	28542891	7.0885	10.1649	-0.9094
CHB	NPIP	28547891	3.8192	9.4872	-1.7867
CHB	NPIP	28552891	4.5361	9.4872	-1.5607
CHB	NPIP	28557891	7.0699	11.5202	-1.1701
CHB	NPIP	28562891	7.3904	11.5202	-1.0858

CHB	NPIP	28567891	5.3505	9.6567	-1.3353
CHB	NPIP	28572891	4.2589	9.6567	-1.6737
CHB	NPIP	28577891	6.0046	9.4872	-1.0978
CHB	NPIP	28582891	6.082	9.6567	-1.1084
CHB	NPIP	28587891	5.5102	9.9955	-1.3469
CHB	NPIP	28592891	4.9888	8.979	-1.3237
CHB	NPIP	28597891	3.5201	7.2848	-1.5133
CHB	NPIP	28602891	6.6973	10.3343	-1.0587
CHB	NPIP	28607891	8.2277	12.0285	-0.9595
CHB	NPIP	28612891	11.3543	12.0285	-0.1702
CHB	NPIP	28617891	22.5363	19.8216	0.4257
CHB	NPIP	28622891	18.0615	17.4498	0.1084
CHB	NPIP	28627891	8.8307	10.8426	-0.5599
CHB	NPIP	28632891	5.8449	10.5037	-1.3357
CHB	NPIP	28637891	2.3139	7.7931	-2.0708
CHB	NPIP	28642891	3.7405	10.1649	-1.8991
CHB	NPIP	28647891	6.9884	14.0615	-1.5404
CHB	NPIP	28652891	7.1007	10.5037	-0.9756
CHB	NPIP	28657891	2.3453	3.2189	-0.7182
CHB	NPIP	28662891	0	0	0
CHB	NPIP	28667891	0	0	0
CHB	NPIP	28672891	0.3952	0.3388	0.2262
CHB	NPIP	28677891	0.4523	0.5082	-0.1755
CHB	NPIP	28682891	0.383	0.5082	-0.393
CHB	NPIP	28687891	0.8195	0.6777	0.3707
CHB	NPIP	28692891	0.9092	1.5247	-0.9144
CHB	NPIP	28697891	0.6379	1.6942	-1.4496
CHB	NPIP	28702891	1.8968	2.033	-0.1624
CHB	NPIP	28707891	3.8235	3.8965	-0.0511
CHB	NPIP	28712891	2.3899	3.2189	-0.6816
CHB	NPIP	28717891	0.2408	0.8471	-1.3672
CHB	NPIP	28722891	0.558	0.5082	0.1563
CHB	NPIP	28727891	0.558	0.5082	0.1563
CHB	NPIP	28732891	0.309	0.8471	-1.2134
CHB	NPIP	28737891	0.6092	2.033	-1.6979
CHB	NPIP	28742891	0.9751	2.5412	-1.5639
CHB	NPIP	28747891	3.0868	4.066	-0.6603
CHB	NPIP	28752891	5.9762	5.7601	0.1074
CHB	NPIP	28757891	3.9383	4.066	-0.0861
CHB	NPIP	28762891	3.1527	4.2354	-0.7048
CHB	NPIP	28767891	3.6189	4.066	-0.3015
CHB	NPIP	28772891	0.8403	0.8471	-0.0153
CHB	NPIP	28777891	0.9732	1.0165	-0.0862
CHB	NPIP	28782891	1.0849	1.3553	-0.4382
CHB	NPIP	28787891	0.1117	0.3388	-0.911
CHB	NPIP	28792891	0	0	0
CHB	NPIP	28797891	1.8487	1.3553	0.7996
CHB	NPIP	28802891	2.6225	2.3718	0.2646
CHB	NPIP	28807891	2.746	2.7106	0.0335

CHB	NPIP	28812891	3.3236	4.066	-0.5006
CHB	NPIP	28817891	1.874	3.8965	-1.4146
CHB	NPIP	28822891	5.7285	7.6237	-0.7308
CHB	NPIP	28827891	7.3318	10.5037	-0.9094
CHB	NPIP	28832891	5.6063	9.4872	-1.2233
CHB	NPIP	28837891	6.8291	11.1814	-1.1768
CHB	NPIP	28842891	7.5212	10.8426	-0.9244
CHB	NPIP	28847891	6.387	9.3178	-0.9394
CHB	NPIP	28852891	4.4774	8.1319	-1.3283
CHB	NPIP	28857891	5.1192	7.4543	-0.9192
CHB	NPIP	28862891	10.6402	10.6731	-0.0093
CHB	NPIP	28867891	13.8007	11.8591	0.4967
CHB	NPIP	28872891	8.2736	7.9625	0.1153
CHB	NPIP	28877891	4.2994	5.5907	-0.6588
CHB	NPIP	28882891	3.7403	6.2684	-1.1644
CHB	NPIP	28887891	4.4818	7.9625	-1.2898
CHB	NPIP	28892891	6.4184	7.9625	-0.5722
CHB	NPIP	28897891	5.0615	7.6237	-0.9881
CHB	NPIP	28902891	4.6712	8.1319	-1.2579
CHB	NPIP	28907891	5.7255	7.4543	-0.6805
CHB	NPIP	28912891	5.0925	8.8096	-1.255
CHB	NPIP	28917891	4.8983	9.3178	-1.4166
CHB	NPIP	28922891	7.2511	9.3178	-0.6625
CHB	NPIP	28927891	6.3154	8.3013	-0.7083
CHB	NPIP	28932891	3.6433	5.5907	-0.9936
CHB	NPIP	28937891	4.6495	6.4378	-0.8041
CHB	NPIP	28942891	3.7891	5.7601	-0.9792
CHB	NPIP	28947891	3.8686	5.5907	-0.8787
CHB	NPIP	28952891	5.3948	6.946	-0.6511
CHB	NPIP	28957891	6.0076	7.1154	-0.4549
CHB	NPIP	28962891	6.5064	7.1154	-0.2501
CHB	NPIP	28967891	5.9044	6.2684	-0.1676
CHB	NPIP	28972891	5.0503	7.6237	-0.9924
CHB	NPIP	28977891	6.1323	8.8096	-0.9039
CHB	NPIP	28982891	7.0297	8.4708	-0.5045
CHB	NPIP	28987891	6.3023	8.979	-0.8879
CHB	NPIP	28992891	5.0407	7.4543	-0.9501
CHB	NPIP	28997891	5.1786	6.2684	-0.5019
CHB	NPIP	29002891	6.4062	8.6402	-0.7679
CHB	NPIP	29007891	8.3733	10.6731	-0.6496
CHB	NPIP	29012891	7.7409	9.4872	-0.5505
CHB	NPIP	29017891	6.6726	7.9625	-0.478
CHB	NPIP	29022891	7.7537	8.4708	-0.251
CHB	NPIP	29027891	8.3703	8.979	-0.2019
CHB	NPIP	29032891	8.2254	8.8096	-0.1972
CHB	NPIP	29037891	5.6789	7.7931	-0.7991
CHB	NPIP	29042891	3.9206	5.4213	-0.7869
CHB	NPIP	29047891	2.5279	2.7106	-0.1731
CHB	NPIP	29052891	2.0839	2.3718	-0.3039

CHB	NPIP	29057891	3.8712	3.7271	0.1047
CHB	NPIP	29062891	4.4637	4.5742	-0.0673
CHB	NPIP	29067891	4.4875	4.4048	0.052
CHB	NPIP	29072891	4.3598	3.5577	0.6062
CHB	NPIP	29077891	3.7727	3.0495	0.622
CHB	NPIP	29082891	4.3811	4.5742	-0.1176
CHB	NPIP	29087891	4.4158	5.4213	-0.5273
CHB	NPIP	29092891	5.1239	6.7766	-0.7094
CHB	NPIP	29097891	5.5627	8.979	-1.1333
CHB	NPIP	29102891	5.9248	9.1484	-1.051
CHB	NPIP	29107891	7.0897	8.6402	-0.533
CHB	NPIP	29112891	8.5682	8.979	-0.1363
CHB	NPIP	29117891	9.2572	10.1649	-0.2683
CHB	NPIP	29122891	7.7731	8.8096	-0.35
CHB	NPIP	29127891	13.8704	10.5037	0.9652
CHB	NPIP	29132891	21.4428	13.7226	1.7207
CHB	NPIP	29137891	24.9225	15.5862	1.8434
CHB	NPIP	29142891	19.8426	14.7391	1.0628
CHB	NPIP	29147891	17.2932	14.2309	0.6594
CHB	NPIP	29152891	25.0737	17.4498	1.3512
YRI	NPIP	28142891	8.5881	13.2769	-1.0746
YRI	NPIP	28147891	10.6745	13.613	-0.6577
YRI	NPIP	28152891	9.1912	11.2602	-0.5539
YRI	NPIP	28157891	7.2839	12.7727	-1.3049
YRI	NPIP	28162891	7.3134	11.7644	-1.1435
YRI	NPIP	28167891	7.5706	10.756	-0.8902
YRI	NPIP	28172891	8.4823	12.7727	-1.02
YRI	NPIP	28177891	8.8249	13.445	-1.0463
YRI	NPIP	28182891	9.5903	14.4534	-1.0283
YRI	NPIP	28187891	8.179	12.4366	-1.038
YRI	NPIP	28192891	6.6327	9.9157	-0.9898
YRI	NPIP	28197891	9.6105	10.4199	-0.233
YRI	NPIP	28202891	9.4201	10.924	-0.4142
YRI	NPIP	28207891	7.7025	10.4199	-0.7823
YRI	NPIP	28212891	9.9066	11.4282	-0.4017
YRI	NPIP	28217891	10.1064	12.9408	-0.6656
YRI	NPIP	28222891	10.2213	12.6047	-0.5738
YRI	NPIP	28227891	13.3084	13.1089	0.0463
YRI	NPIP	28232891	16.5851	17.1423	-0.1001
YRI	NPIP	28237891	15.2285	18.4868	-0.5445
YRI	NPIP	28242891	16.2373	16.302	-0.0122
YRI	NPIP	28247891	18.9542	15.7978	0.6132
YRI	NPIP	28252891	17.0403	15.9659	0.2066
YRI	NPIP	28257891	15.6424	14.7895	0.1764
YRI	NPIP	28262891	16.3829	15.1256	0.2546
YRI	NPIP	28267891	14.695	13.9492	0.1631
YRI	NPIP	28272891	10.2308	12.4366	-0.5378
YRI	NPIP	28277891	9.1509	14.6214	-1.1441
YRI	NPIP	28282891	9.1414	15.9659	-1.3124

YRI	NPIP	28287891	7.4642	14.2853	-1.4584
YRI	NPIP	28292891	9.1392	13.613	-1.0013
YRI	NPIP	28297891	10.4599	12.2685	-0.4466
YRI	NPIP	28302891	6.5111	7.5628	-0.4071
YRI	NPIP	28307891	5.2392	8.067	-1.0319
YRI	NPIP	28312891	4.6617	9.9157	-1.5841
YRI	NPIP	28317891	2.5014	8.067	-2.0309
YRI	NPIP	28322891	5.1205	9.4115	-1.3581
YRI	NPIP	28327891	5.6906	8.7392	-1.0334
YRI	NPIP	28332891	7.5016	9.5795	-0.6469
YRI	NPIP	28337891	11.0612	12.6047	-0.3716
YRI	NPIP	28342891	8.3647	10.924	-0.7049
YRI	NPIP	28347891	7.037	9.7476	-0.8304
YRI	NPIP	28352891	5.627	7.0586	-0.5902
YRI	NPIP	28357891	1.8728	3.5293	-1.256
YRI	NPIP	28362891	0.3787	1.6806	-1.791
YRI	NPIP	28367891	0.6331	2.5209	-1.8906
YRI	NPIP	28372891	2.316	4.0335	-1.1621
YRI	NPIP	28377891	2.5966	3.0251	-0.3696
YRI	NPIP	28382891	3.8341	5.378	-0.8125
YRI	NPIP	28387891	4.7566	6.5544	-0.7926
YRI	NPIP	28392891	2.4653	3.3612	-0.7078
YRI	NPIP	28397891	1.7387	1.8487	-0.1407
YRI	NPIP	28402891	1.9932	1.6806	0.43
YRI	NPIP	28407891	1.0482	0.8403	0.4696
YRI	NPIP	28412891	1.5057	1.8487	-0.4387
YRI	NPIP	28417891	2.7204	3.1932	-0.3899
YRI	NPIP	28422891	2.7587	4.2016	-0.9425
YRI	NPIP	28427891	2.226	3.6974	-1.0725
YRI	NPIP	28432891	1.6173	1.6806	-0.0871
YRI	NPIP	28437891	1.1459	1.6806	-0.7356
YRI	NPIP	28442891	1.33	2.0167	-0.8211
YRI	NPIP	28447891	1.2867	2.3529	-1.1286
YRI	NPIP	28452891	0.6965	3.0251	-2.0088
YRI	NPIP	28457891	0.3834	1.6806	-1.7846
YRI	NPIP	28462891	1.6311	2.1848	-0.6217
YRI	NPIP	28467891	1.6678	2.3529	-0.7252
YRI	NPIP	28472891	0.2919	0.8403	-1.2386
YRI	NPIP	28477891	0.428	1.8487	-1.8172
YRI	NPIP	28482891	2.3573	6.2183	-1.7849
YRI	NPIP	28487891	5.3985	11.4282	-1.5919
YRI	NPIP	28492891	5.8655	10.924	-1.3932
YRI	NPIP	28497891	5.9436	9.7476	-1.1653
YRI	NPIP	28502891	7.8969	12.4366	-1.1068
YRI	NPIP	28507891	9.7946	14.4534	-0.9851
YRI	NPIP	28512891	9.1972	15.1256	-1.2004
YRI	NPIP	28517891	7.1304	13.445	-1.4301
YRI	NPIP	28522891	6.7308	11.7644	-1.2932
YRI	NPIP	28527891	8.2585	13.7811	-1.2218

YRI	NPIP	28532891	8.9535	13.613	-1.0429
YRI	NPIP	28537891	9.7987	13.7811	-0.881
YRI	NPIP	28542891	7.5632	12.4366	-1.1881
YRI	NPIP	28547891	4.1184	8.5712	-1.5367
YRI	NPIP	28552891	5.9795	9.7476	-1.1543
YRI	NPIP	28557891	8.7286	12.1005	-0.8436
YRI	NPIP	28562891	8.3894	12.1005	-0.9284
YRI	NPIP	28567891	5.6023	11.7644	-1.5831
YRI	NPIP	28572891	6.3361	13.2769	-1.5907
YRI	NPIP	28577891	8.2182	11.0921	-0.7803
YRI	NPIP	28582891	8.1247	9.9157	-0.54
YRI	NPIP	28587891	7.3779	11.5963	-1.0985
YRI	NPIP	28592891	6.648	12.2685	-1.388
YRI	NPIP	28597891	6.1482	11.7644	-1.4428
YRI	NPIP	28602891	8.9616	16.9743	-1.4533
YRI	NPIP	28607891	12.1902	19.3272	-1.1428
YRI	NPIP	28612891	15.7403	16.6382	-0.166
YRI	NPIP	28617891	26.2472	24.7051	0.1948
YRI	NPIP	28622891	23.6773	20.1675	0.5394
YRI	NPIP	28627891	16.3924	11.7644	1.189
YRI	NPIP	28632891	11.1245	10.2518	0.2551
YRI	NPIP	28637891	4.4449	8.7392	-1.4557
YRI	NPIP	28642891	8.1691	13.2769	-1.1706
YRI	NPIP	28647891	13.4279	16.9743	-0.6432
YRI	NPIP	28652891	11.2969	14.4534	-0.6674
YRI	NPIP	28657891	3.6186	5.378	-0.9259
YRI	NPIP	28662891	0.1281	0.6722	-1.4244
YRI	NPIP	28667891	0.1464	0.8403	-1.5673
YRI	NPIP	28672891	0.4859	1.1764	-1.235
YRI	NPIP	28677891	0.6668	1.1764	-0.9114
YRI	NPIP	28682891	0.3726	0.5042	-0.4136
YRI	NPIP	28687891	0.864	0.8403	0.0535
YRI	NPIP	28692891	1.7146	1.5126	0.3008
YRI	NPIP	28697891	2.0684	1.6806	0.5335
YRI	NPIP	28702891	2.2391	2.0167	0.2659
YRI	NPIP	28707891	4.718	4.3696	0.22
YRI	NPIP	28712891	4.7092	3.8654	0.5921
YRI	NPIP	28717891	1.186	0.8403	0.7808
YRI	NPIP	28722891	0.5916	0.5042	0.2747
YRI	NPIP	28727891	0.5916	0.5042	0.2747
YRI	NPIP	28732891	0.1335	0.6722	-1.4103
YRI	NPIP	28737891	1.0293	2.689	-1.5773
YRI	NPIP	28742891	2.2375	3.3612	-0.8878
YRI	NPIP	28747891	5.2555	6.7225	-0.6321
YRI	NPIP	28752891	6.579	8.235	-0.5929
YRI	NPIP	28757891	4.5795	4.5377	0.0255
YRI	NPIP	28762891	4.5504	5.378	-0.4355
YRI	NPIP	28767891	3.6178	4.3696	-0.4747
YRI	NPIP	28772891	1.0003	1.0084	-0.0161

YRI	NPIP	28777891	1.5473	1.5126	0.0517
YRI	NPIP	28782891	2.0684	1.5126	0.8276
YRI	NPIP	28787891	0.5395	0.3361	0.8166
YRI	NPIP	28792891	0	0	0
YRI	NPIP	28797891	1.4898	1.1764	0.5605
YRI	NPIP	28802891	2.9019	2.689	0.2023
YRI	NPIP	28807891	3.6898	3.5293	0.1217
YRI	NPIP	28812891	3.8699	4.5377	-0.4081
YRI	NPIP	28817891	2.5997	4.8738	-1.3054
YRI	NPIP	28822891	6.2799	10.0837	-1.129
YRI	NPIP	28827891	9.0082	11.9324	-0.7413
YRI	NPIP	28832891	7.9794	9.0754	-0.3588
YRI	NPIP	28837891	6.198	9.5795	-1.0528
YRI	NPIP	28842891	6.9884	9.7476	-0.8452
YRI	NPIP	28847891	7.7548	9.7476	-0.6105
YRI	NPIP	28852891	6.722	9.2434	-0.8115
YRI	NPIP	28857891	7.7336	8.4031	-0.2353
YRI	NPIP	28862891	11.7196	11.2602	0.123
YRI	NPIP	28867891	13.3955	14.2853	-0.1902
YRI	NPIP	28872891	8.7497	9.7476	-0.3057
YRI	NPIP	28877891	5.9661	6.2183	-0.1166
YRI	NPIP	28882891	3.9619	5.8822	-0.9331
YRI	NPIP	28887891	4.813	6.0502	-0.5862
YRI	NPIP	28892891	7.7611	9.2434	-0.477
YRI	NPIP	28897891	6.8156	9.2434	-0.7813
YRI	NPIP	28902891	5.7025	7.8989	-0.8171
YRI	NPIP	28907891	6.2821	9.2434	-0.953
YRI	NPIP	28912891	5.7862	9.4115	-1.1474
YRI	NPIP	28917891	4.4374	8.5712	-1.4266
YRI	NPIP	28922891	7.8634	9.9157	-0.6188
YRI	NPIP	28927891	7.9307	9.2434	-0.4225
YRI	NPIP	28932891	4.9728	7.2267	-0.9095
YRI	NPIP	28937891	5.5793	7.5628	-0.7679
YRI	NPIP	28942891	3.9775	7.7309	-1.4241
YRI	NPIP	28947891	3.6881	8.7392	-1.7122
YRI	NPIP	28952891	5.8485	9.0754	-1.0563
YRI	NPIP	28957891	7.4836	9.7476	-0.6935
YRI	NPIP	28962891	7.7133	11.4282	-0.9807
YRI	NPIP	28967891	7.6355	10.5879	-0.8373
YRI	NPIP	28972891	6.616	10.0837	-1.0293
YRI	NPIP	28977891	6.554	10.756	-1.1742
YRI	NPIP	28982891	9.2082	12.1005	-0.7236
YRI	NPIP	28987891	9.7946	13.2769	-0.7981
YRI	NPIP	28992891	6.7176	9.7476	-0.9282
YRI	NPIP	28997891	6.6893	8.7392	-0.6949
YRI	NPIP	29002891	11.8201	12.4366	-0.1503
YRI	NPIP	29007891	13.6747	15.2937	-0.3244
YRI	NPIP	29012891	8.5786	12.6047	-0.9692
YRI	NPIP	29017891	7.6687	9.9157	-0.6775

YRI	NPIP	29022891	9.157	11.7644	-0.6699
YRI	NPIP	29027891	9.4412	11.9324	-0.6315
YRI	NPIP	29032891	9.6899	10.0837	-0.1169
YRI	NPIP	29037891	7.2518	9.0754	-0.5969
YRI	NPIP	29042891	5.0397	9.7476	-1.4422
YRI	NPIP	29047891	2.9647	6.3864	-1.5443
YRI	NPIP	29052891	2.819	2.8571	-0.0345
YRI	NPIP	29057891	3.6316	3.6974	-0.048
YRI	NPIP	29062891	5.2485	6.0502	-0.3799
YRI	NPIP	29067891	5.894	6.8906	-0.4199
YRI	NPIP	29072891	3.9804	5.2099	-0.6655
YRI	NPIP	29077891	3.7676	4.8738	-0.635
YRI	NPIP	29082891	4.3959	7.2267	-1.1423
YRI	NPIP	29087891	5.3744	8.235	-1.0243
YRI	NPIP	29092891	8.3152	9.9157	-0.4826
YRI	NPIP	29097891	7.8795	12.6047	-1.1375
YRI	NPIP	29102891	6.7998	13.1089	-1.4635
YRI	NPIP	29107891	9.3746	10.756	-0.386
YRI	NPIP	29112891	12.0948	11.5963	0.1298
YRI	NPIP	29117891	11.9801	14.9575	-0.6093
YRI	NPIP	29122891	10.0824	14.4534	-0.9242
YRI	NPIP	29127891	14.684	15.9659	-0.2465
YRI	NPIP	29132891	18.8037	16.4701	0.4356
YRI	NPIP	29137891	20.9585	19.8313	0.1761
YRI	NPIP	29142891	19.3874	21.3439	-0.2847
YRI	NPIP	29147891	16.1946	19.8313	-0.5681
YRI	NPIP	29152891	16.9939	22.0162	-0.7092
CEU	NPIP	28886310	5.8861	5.2874	0.3219
CEU	NPIP	28891310	7.3394	6.8225	0.2212
CEU	NPIP	28896310	7.5636	8.3576	-0.2822
CEU	NPIP	28901310	6.1398	6.3108	-0.0785
CEU	NPIP	28906310	5.6131	5.2874	0.1751
CEU	NPIP	28911310	4.2092	5.1169	-0.5024
CEU	NPIP	28916310	5.1727	5.7991	-0.3103
CEU	NPIP	28921310	5.706	6.8225	-0.4777
CEU	NPIP	28926310	7.5088	7.1636	0.1413
CEU	NPIP	28931310	7.1119	6.3108	0.3678
CEU	NPIP	28936310	4.5733	5.9697	-0.6739
CEU	NPIP	28941310	4.209	6.3108	-0.965
CEU	NPIP	28946310	2.7238	5.2874	-1.3785
CEU	NPIP	28951310	2.9872	4.9463	-1.1173
CEU	NPIP	28956310	4.8654	6.3108	-0.6636
CEU	NPIP	28961310	4.7273	6.1402	-0.6649
CEU	NPIP	28966310	3.9942	6.3108	-1.0636
CEU	NPIP	28971310	3.5102	5.6286	-1.0775
CEU	NPIP	28976310	5.5477	5.6286	-0.0412
CEU	NPIP	28981310	6.2377	6.9931	-0.3161
CEU	NPIP	28986310	4.8273	6.6519	-0.7988
CEU	NPIP	28991310	5.2185	6.4814	-0.5661

CEU	NPIP	28996310	4.4599	6.3108	-0.8498
CEU	NPIP	29001310	3.858	6.1402	-1.0739
CEU	NPIP	29006310	4.9532	8.6987	-1.2832
CEU	NPIP	29011310	7.214	9.3809	-0.6922
CEU	NPIP	29016310	6.4234	6.6519	-0.1
CEU	NPIP	29021310	5.3836	6.8225	-0.6157
CEU	NPIP	29026310	7.464	7.6753	-0.0812
CEU	NPIP	29031310	4.8633	6.8225	-0.8383
CEU	NPIP	29036310	4.6209	6.6519	-0.8892
CEU	NPIP	29041310	5.4991	6.4814	-0.4403
CEU	NPIP	29046310	3.3181	4.0935	-0.5214
CEU	NPIP	29051310	1.7159	1.1939	0.9302
CEU	NPIP	29056310	3.222	3.4112	-0.1486
CEU	NPIP	29061310	6.0526	5.9697	0.04
CEU	NPIP	29066310	4.2533	3.7524	0.3629
CEU	NPIP	29071310	3.5079	2.5584	0.9459
CEU	NPIP	29076310	4.0546	3.2407	0.6674
CEU	NPIP	29081310	5.7248	5.6286	0.0489
CEU	NPIP	29086310	6.2707	6.4814	-0.0944
CEU	NPIP	29091310	7.1478	5.9697	0.5686
CEU	NPIP	29096310	6.2769	6.3108	-0.0156
CEU	NPIP	29101310	7.1482	8.0164	-0.3207
CEU	NPIP	29106310	8.8903	8.5281	0.1264
CEU	NPIP	29111310	10.1383	7.6753	0.9467
CEU	NPIP	29116310	13.9299	9.5515	1.3755
CEU	NPIP	29121310	11.2779	9.3809	0.606
CEU	NPIP	29126310	13.8885	9.7221	1.2875
CEU	NPIP	29131310	19.1746	12.1099	1.7773
CEU	NPIP	29136310	21.2723	12.7922	2.0258
CEU	NPIP	29141310	21.5794	13.4744	1.8433
CEU	NPIP	29146310	18.1994	13.1333	1.1805
CEU	NPIP	29151310	25.1717	15.6917	1.8651
CEU	NPIP	29156310	27.2637	16.0329	2.1647
CEU	NPIP	29161310	17.8433	12.1099	1.4424
CEU	NPIP	29166310	12.8169	10.7454	0.5831
CEU	NPIP	29171310	7.8002	8.0164	-0.0799
CEU	NPIP	29176310	4.758	6.3108	-0.7129
CEU	NPIP	29181310	8.2678	7.8459	0.1589
CEU	NPIP	29186310	19.038	11.7688	1.8786
CEU	NPIP	29191310	22.4858	12.2805	2.5337
CEU	NPIP	29196310	13.5308	9.8926	1.1063
CEU	NPIP	29201310	9.6586	9.7221	-0.0196
CEU	NPIP	29206310	9.6693	10.2337	-0.1663
CEU	NPIP	29211310	12.9773	11.7688	0.3123
CEU	NPIP	29216310	10.8728	8.187	0.973
CEU	NPIP	29221310	7.4776	6.6519	0.3615
CEU	NPIP	29226310	10.5397	9.0398	0.4959
CEU	NPIP	29231310	14.4051	10.0632	1.2994
CEU	NPIP	29236310	17.5708	13.3039	0.9822

CEU	NPIP	29241310	17.9126	14.3272	0.7693
CEU	NPIP	29246310	16.4523	12.1099	1.0924
CEU	NPIP	29251310	13.7963	10.5749	0.9204
CEU	NPIP	29256310	14.5512	10.7454	1.0712
CEU	NPIP	29261310	17.6632	12.4511	1.2773
CEU	NPIP	29266310	20.1328	14.8389	1.0985
CEU	NPIP	29271310	33.0559	18.9324	2.3212
CEU	NPIP	29276310	44.04	19.7852	3.8208
CEU	NPIP	29281310	38.7143	17.0562	3.9346
CEU	NPIP	29286310	34.2791	16.0329	3.5169
CEU	NPIP	29291310	22.1113	11.2571	2.9248
CEU	NPIP	29296310	16.087	9.3809	2.1423
CEU	NPIP	29301310	20.7963	13.8156	1.5504
CEU	NPIP	29306310	14.8303	11.9394	0.7371
CEU	NPIP	29311310	10.6914	10.0632	0.188
CEU	NPIP	29316310	14.5966	12.9627	0.3855
CEU	NPIP	29321310	10.7572	14.6684	-0.8206
CEU	NPIP	29326310	6.1424	12.9627	-1.609
CEU	NPIP	29331310	5.805	10.2337	-1.3047
CEU	NPIP	29336310	5.0173	9.3809	-1.394
CEU	NPIP	29341310	8.4568	9.8926	-0.4366
CEU	NPIP	29346310	8.7409	10.0632	-0.3957
CEU	NPIP	29351310	6.5481	8.0164	-0.5423
CEU	NPIP	29356310	6.1022	6.3108	-0.0958
CEU	NPIP	29361310	4.511	4.9463	-0.2483
CEU	NPIP	29366310	3.5539	4.7757	-0.7186
CEU	NPIP	29371310	2.2859	3.7524	-1.0624
CEU	NPIP	29376310	0.1806	1.8762	-2.1588
CEU	NPIP	29381310	1.1387	1.7056	-0.7764
CEU	NPIP	29386310	3.1515	2.5584	0.5908
CEU	NPIP	29391310	5.4539	5.2874	0.0895
CEU	NPIP	29396310	4.5065	4.7757	-0.1583
CEU	NPIP	29401310	3.0482	4.4346	-0.8699
CEU	NPIP	29406310	2.9225	5.458	-1.3255
CEU	NPIP	29411310	5.1328	7.3342	-0.882
CEU	NPIP	29416310	6.8157	10.0632	-0.9719
CEU	NPIP	29421310	5.0643	8.6987	-1.2451
CEU	NPIP	29426310	6.1453	8.0164	-0.6911
CEU	NPIP	29431310	4.9278	7.3342	-0.9642
CEU	NPIP	29436310	7.8825	10.4043	-0.7316
CEU	NPIP	29441310	7.8681	8.187	-0.1155
CEU	NPIP	29446310	1.4183	2.3879	-1.0211
CEU	NPIP	29451310	0.2888	1.3645	-1.7403
CEU	NPIP	29456310	0.0402	0.3411	-1.2062
CEU	NPIP	29461310	0.0402	0.3411	-1.2062
CEU	NPIP	29466310	0.0585	0.1706	-0.6659
CEU	NPIP	29471310	0.5955	0.6822	-0.2264
CEU	NPIP	29476310	0.6065	0.8528	-0.5547
CEU	NPIP	29481310	0.1192	0.5117	-1.231

CEU	NPIP	29486310	0.0497	0.1706	-0.7182
CEU	NPIP	29491310	1.0458	0.5117	1.6751
CEU	NPIP	29496310	1.0559	0.6822	0.9758
CEU	NPIP	29501310	0.0693	0.3411	-1.0896
CEU	NPIP	29506310	0.1097	0.6822	-1.495
CEU	NPIP	29511310	5.127	2.3879	2.8846
CEU	NPIP	29516310	5.3344	2.0467	3.912
CEU	NPIP	29521310	0.2781	0.5117	-0.7326
CEU	NPIP	29526310	0.0202	0.3411	-1.2864
CEU	NPIP	29531310	0.0303	0.3411	-1.2459
CEU	NPIP	29536310	0.0505	0.6822	-1.6496
CEU	NPIP	29541310	0.0303	0.5117	-1.5098
CEU	NPIP	29546310	0.9911	0.6822	0.8066
CEU	NPIP	29551310	0.981	0.5117	1.4718
CEU	NPIP	29556310	1.2344	0.8528	0.8593
CEU	NPIP	29561310	1.6228	2.0467	-0.5044
CEU	NPIP	29566310	1.0921	2.3879	-1.3646
CEU	NPIP	29571310	1.8563	2.8996	-0.9383
CEU	NPIP	29576310	3.2	3.4112	-0.1659
CEU	NPIP	29581310	4.7372	3.2407	1.2271
CEU	NPIP	29586310	3.9291	3.2407	0.5645
CEU	NPIP	29591310	3.0478	5.458	-1.26
CEU	NPIP	29596310	2.9874	6.4814	-1.5661
CEU	NPIP	29601310	4.4851	5.6286	-0.5817
CEU	NPIP	29606310	6.6302	6.8225	-0.0823
CEU	NPIP	29611310	6.63	8.0164	-0.5121
CEU	NPIP	29616310	4.6652	6.6519	-0.8698
CEU	NPIP	29621310	2.3843	5.1169	-1.5125
CEU	NPIP	29626310	2.867	5.2874	-1.3015
CEU	NPIP	29631310	2.7217	5.1169	-1.3258
CEU	NPIP	29636310	3.5619	4.9463	-0.7895
CEU	NPIP	29641310	10.021	6.1402	1.8262
CEU	NPIP	29646310	9.4197	5.6286	1.9284
CEU	NPIP	29651310	6.0175	5.7991	0.1082
CEU	NPIP	29656310	7.6155	6.8225	0.3393
CEU	NPIP	29661310	5.879	5.6286	0.1274
CEU	NPIP	29666310	7.141	7.3342	-0.0774
CEU	NPIP	29671310	7.8227	8.187	-0.132
CEU	NPIP	29676310	7.5776	7.5047	0.0286
CEU	NPIP	29681310	9.6219	8.3576	0.4494
CEU	NPIP	29686310	7.7077	6.8225	0.3788
CEU	NPIP	29691310	8.2313	5.9697	1.0915
CEU	NPIP	29696310	8.2758	6.3108	0.9022
CEU	NPIP	29701310	5.0653	6.1402	-0.5058
CEU	NPIP	29706310	3.4341	5.6286	-1.1163
CEU	NPIP	29711310	4.3335	6.3108	-0.9078
CEU	NPIP	29716310	5.5261	6.1402	-0.289
CEU	NPIP	29721310	5.7689	6.4814	-0.3194
CEU	NPIP	29726310	5.1655	7.6753	-0.9647

CEU	NPIP	29731310	5.2096	8.187	-1.0786
CEU	NPIP	29736310	5.6996	7.5047	-0.7082
CEU	NPIP	29741310	5.2517	6.4814	-0.5512
CEU	NPIP	29746310	7.5102	7.8459	-0.1265
CEU	NPIP	29751310	7.1472	7.6753	-0.203
CEU	NPIP	29756310	3.7333	7.6753	-1.5152
CEU	NPIP	29761310	5.1235	9.2104	-1.328
CEU	NPIP	29766310	4.262	7.3342	-1.2309
CEU	NPIP	29771310	0.8283	6.3108	-2.5171
CEU	NPIP	29776310	1.457	6.9931	-2.3164
CEU	NPIP	29781310	1.6939	5.2874	-1.9322
CEU	NPIP	29786310	2.1177	5.9697	-1.859
CEU	NPIP	29791310	2.0499	6.9931	-2.0683
CEU	NPIP	29796310	1.458	5.458	-2.0911
CEU	NPIP	29801310	1.0412	4.6052	-2.1639
CEU	NPIP	29806310	0.9163	4.2641	-2.1732
CEU	NPIP	29811310	0.6733	3.0701	-2.0559
CEU	NPIP	29816310	0.677	4.6052	-2.385
CEU	NPIP	29821310	0.9136	6.4814	-2.4956
CEU	NPIP	29826310	2.5412	6.9931	-1.8628
CEU	NPIP	29831310	5.0879	8.0164	-1.0817
CEU	NPIP	29836310	4.7231	6.3108	-0.7289
CEU	NPIP	29841310	3.8844	4.2641	-0.2465
CEU	NPIP	29846310	3.6693	4.6052	-0.5682
CEU	NPIP	29851310	2.3185	4.9463	-1.4986
CEU	NPIP	29856310	2.6759	5.2874	-1.4042
CEU	NPIP	29861310	3.147	4.7757	-0.9579
CEU	NPIP	29866310	2.8242	3.9229	-0.7663
CEU	NPIP	29871310	3.3875	4.0935	-0.4747
CEU	NPIP	29876310	4.8631	5.9697	-0.5341
CEU	NPIP	29881310	5.0672	5.458	-0.2043
CEU	NPIP	29886310	2.7394	4.2641	-0.9897
CEU	NPIP	29891310	2.6961	5.9697	-1.5799
CEU	NPIP	29896310	4.0373	6.8225	-1.1918
CEU	NPIP	29901310	3.899	6.1402	-1.0546
CHB	NPIP	28886310	5.0147	8.6402	-1.2462
CHB	NPIP	28891310	5.885	8.3013	-0.8618
CHB	NPIP	28896310	5.272	6.7766	-0.6459
CHB	NPIP	28901310	5.2145	8.1319	-1.0604
CHB	NPIP	28906310	5.3564	7.6237	-0.8743
CHB	NPIP	28911310	4.4245	8.1319	-1.3475
CHB	NPIP	28916310	4.4691	8.8096	-1.4655
CHB	NPIP	28921310	4.0929	8.6402	-1.5631
CHB	NPIP	28926310	6.5648	8.8096	-0.7579
CHB	NPIP	28931310	6.617	6.4378	0.0806
CHB	NPIP	28936310	4.3033	6.0989	-0.8476
CHB	NPIP	28941310	4.6707	6.6072	-0.8505
CHB	NPIP	28946310	3.3404	5.7601	-1.2022
CHB	NPIP	28951310	3.831	6.0989	-1.0705

CHB	NPIP	28956310	6.7082	7.4543	-0.2937
CHB	NPIP	28961310	7.114	6.946	0.0705
CHB	NPIP	28966310	6.676	6.7766	-0.0432
CHB	NPIP	28971310	5.4809	7.9625	-0.9196
CHB	NPIP	28976310	5.288	8.1319	-1.0337
CHB	NPIP	28981310	6.4527	8.3013	-0.6593
CHB	NPIP	28986310	6.2507	8.1319	-0.6838
CHB	NPIP	28991310	5.6768	7.4543	-0.6997
CHB	NPIP	28996310	5.1694	7.4543	-0.8994
CHB	NPIP	29001310	6.135	8.979	-0.9434
CHB	NPIP	29006310	6.7544	9.9955	-0.9732
CHB	NPIP	29011310	7.4108	9.3178	-0.6113
CHB	NPIP	29016310	7.0072	7.9625	-0.354
CHB	NPIP	29021310	7.2861	7.6237	-0.1302
CHB	NPIP	29026310	9.8995	9.3178	0.1865
CHB	NPIP	29031310	8.0874	9.1484	-0.3459
CHB	NPIP	29036310	6.1957	8.3013	-0.751
CHB	NPIP	29041310	4.8839	6.4378	-0.6987
CHB	NPIP	29046310	2.7097	3.3883	-0.5344
CHB	NPIP	29051310	1.2559	1.1859	0.1249
CHB	NPIP	29056310	3.5413	3.5577	-0.0124
CHB	NPIP	29061310	5.5143	5.7601	-0.1221
CHB	NPIP	29066310	3.5267	3.2189	0.2531
CHB	NPIP	29071310	3.8629	3.2189	0.5295
CHB	NPIP	29076310	4.2145	3.8965	0.2224
CHB	NPIP	29081310	5.2798	4.5742	0.4297
CHB	NPIP	29086310	4.7259	5.5907	-0.4412
CHB	NPIP	29091310	4.9497	6.4378	-0.6691
CHB	NPIP	29096310	5.2482	7.6237	-0.9161
CHB	NPIP	29101310	6.1462	9.6567	-1.0885
CHB	NPIP	29106310	6.7874	9.3178	-0.8111
CHB	NPIP	29111310	7.3242	8.4708	-0.4014
CHB	NPIP	29116310	9.3185	9.8261	-0.1549
CHB	NPIP	29121310	7.3859	7.7931	-0.1539
CHB	NPIP	29126310	11.6792	10.1649	0.4476
CHB	NPIP	29131310	18.7213	13.7226	1.1141
CHB	NPIP	29136310	23.8951	14.7391	1.9067
CHB	NPIP	29141310	23.5254	15.4168	1.6178
CHB	NPIP	29146310	18.5753	14.2309	0.9354
CHB	NPIP	29151310	21.6958	16.9415	0.8668
CHB	NPIP	29156310	24.5791	17.4498	1.2635
CHB	NPIP	29161310	18.666	14.7391	0.8178
CHB	NPIP	29166310	10.0898	13.892	-0.8376
CHB	NPIP	29171310	5.1196	10.1649	-1.4915
CHB	NPIP	29176310	4.4377	8.979	-1.5065
CHB	NPIP	29181310	5.293	14.2309	-1.9245
CHB	NPIP	29186310	14.1133	17.4498	-0.5913
CHB	NPIP	29191310	19.2677	16.6027	0.4954
CHB	NPIP	29196310	11.463	12.8755	-0.3344

CHB	NPIP	29201310	7.6817	10.1649	-0.7341
CHB	NPIP	29206310	8.1993	10.6731	-0.6987
CHB	NPIP	29211310	11.3408	16.6027	-0.9781
CHB	NPIP	29216310	9.8976	15.7556	-1.1448
CHB	NPIP	29221310	7.3164	9.3178	-0.6415
CHB	NPIP	29226310	9.1527	8.8096	0.1158
CHB	NPIP	29231310	13.0765	10.6731	0.6788
CHB	NPIP	29236310	17.2859	16.7721	0.0946
CHB	NPIP	29241310	13.6916	16.6027	-0.5411
CHB	NPIP	29246310	12.1953	11.6896	0.1311
CHB	NPIP	29251310	13.8738	10.3343	1.0303
CHB	NPIP	29256310	15.0788	10.6731	1.2443
CHB	NPIP	29261310	17.2895	13.045	0.9925
CHB	NPIP	29266310	18.7836	17.1109	0.3021
CHB	NPIP	29271310	29.8641	22.1934	1.0785
CHB	NPIP	29276310	41.2884	25.0734	2.0257
CHB	NPIP	29281310	39.7476	24.7346	1.9004
CHB	NPIP	29286310	35.2127	20.8381	2.1479
CHB	NPIP	29291310	22.1327	14.4003	1.6463
CHB	NPIP	29296310	18.6873	15.2474	0.6936
CHB	NPIP	29301310	27.6779	23.8875	0.4963
CHB	NPIP	29306310	20.8633	20.4992	0.0553
CHB	NPIP	29311310	12.2692	12.0285	0.0608
CHB	NPIP	29316310	16.9231	14.0615	0.6232
CHB	NPIP	29321310	19.1848	16.9415	0.409
CHB	NPIP	29326310	13.3516	15.0779	-0.3518
CHB	NPIP	29331310	7.4375	11.012	-0.9805
CHB	NPIP	29336310	5.8039	8.4708	-0.9336
CHB	NPIP	29341310	11.5003	9.3178	0.6996
CHB	NPIP	29346310	12.838	9.4872	1.0562
CHB	NPIP	29351310	9.0727	7.7931	0.4836
CHB	NPIP	29356310	6.7196	7.2848	-0.2272
CHB	NPIP	29361310	4.1976	5.2519	-0.5686
CHB	NPIP	29366310	4.5631	5.4213	-0.45
CHB	NPIP	29371310	2.9425	3.8965	-0.6672
CHB	NPIP	29376310	0.5909	1.6942	-1.5142
CHB	NPIP	29381310	1.8054	2.8801	-0.969
CHB	NPIP	29386310	3.3585	3.7271	-0.2678
CHB	NPIP	29391310	5.5155	6.2684	-0.3468
CHB	NPIP	29396310	5.1338	4.913	0.1263
CHB	NPIP	29401310	4.2656	4.913	-0.3703
CHB	NPIP	29406310	4.3575	6.0989	-0.822
CHB	NPIP	29411310	7.5134	8.6402	-0.3873
CHB	NPIP	29416310	8.4148	11.1814	-0.7481
CHB	NPIP	29421310	4.8462	7.9625	-1.1548
CHB	NPIP	29426310	5.4409	6.7766	-0.5734
CHB	NPIP	29431310	4.4722	6.4378	-0.8838
CHB	NPIP	29436310	6.6635	9.3178	-0.8508
CHB	NPIP	29441310	6.8241	7.7931	-0.3662

CHB	NPIP	29446310	1.36	2.033	-0.8026
CHB	NPIP	29451310	0.0678	0.6777	-1.5945
CHB	NPIP	29456310	0.0192	0.1694	-0.8925
CHB	NPIP	29461310	0.1055	0.6777	-1.4959
CHB	NPIP	29466310	0.0862	0.5082	-1.3247
CHB	NPIP	29471310	0.5408	0.6777	-0.3579
CHB	NPIP	29476310	0.6156	1.1859	-1.018
CHB	NPIP	29481310	0.2454	0.8471	-1.3568
CHB	NPIP	29486310	0.19	0.5082	-0.9989
CHB	NPIP	29491310	0.0485	0.6777	-1.6449
CHB	NPIP	29496310	0.3564	1.1859	-1.4806
CHB	NPIP	29501310	0.6851	1.1859	-0.8939
CHB	NPIP	29506310	0.7002	1.1859	-0.867
CHB	NPIP	29511310	4.999	2.5412	2.4543
CHB	NPIP	29516310	4.8787	2.033	3.3935
CHB	NPIP	29521310	0.2701	0.6777	-1.0656
CHB	NPIP	29526310	0.0482	0.5082	-1.444
CHB	NPIP	29531310	0.0967	0.6777	-1.5189
CHB	NPIP	29536310	0.0967	0.6777	-1.5189
CHB	NPIP	29541310	0.0097	0.1694	-0.949
CHB	NPIP	29546310	0.8397	0.5082	1.0406
CHB	NPIP	29551310	0.83	0.3388	1.9704
CHB	NPIP	29556310	0.7752	1.5247	-1.1135
CHB	NPIP	29561310	1.7653	2.8801	-1.0051
CHB	NPIP	29566310	2.6269	3.0495	-0.3634
CHB	NPIP	29571310	2.9149	2.8801	0.0314
CHB	NPIP	29576310	2.5505	1.8636	0.8764
CHB	NPIP	29581310	2.7398	3.2189	-0.3939
CHB	NPIP	29586310	2.6273	4.7436	-1.2484
CHB	NPIP	29591310	3.2671	5.9295	-1.2889
CHB	NPIP	29596310	3.5294	7.1154	-1.4727
CHB	NPIP	29601310	4.5601	6.946	-1.0015
CHB	NPIP	29606310	5.3006	6.6072	-0.5739
CHB	NPIP	29611310	4.0815	5.7601	-0.834
CHB	NPIP	29616310	2.6314	6.4378	-1.7115
CHB	NPIP	29621310	1.9819	5.7601	-1.8771
CHB	NPIP	29626310	2.1921	4.5742	-1.4505
CHB	NPIP	29631310	1.6632	5.0825	-1.8983
CHB	NPIP	29636310	2.4003	6.4378	-1.8154
CHB	NPIP	29641310	4.4458	7.4543	-1.1842
CHB	NPIP	29646310	3.7165	6.7766	-1.3136
CHB	NPIP	29651310	6.7942	7.7931	-0.3775
CHB	NPIP	29656310	10.5073	7.9625	0.943
CHB	NPIP	29661310	7.198	7.9625	-0.2833
CHB	NPIP	29666310	8.7514	8.8096	-0.0196
CHB	NPIP	29671310	11.0356	8.1319	1.0554
CHB	NPIP	29676310	8.8587	7.2848	0.6327
CHB	NPIP	29681310	8.0329	7.4543	0.2278
CHB	NPIP	29686310	6.9162	6.2684	0.2984

CHB	NPIP	29691310	8.3929	7.1154	0.5246
CHB	NPIP	29696310	7.06	7.4543	-0.1552
CHB	NPIP	29701310	2.5248	5.7601	-1.6074
CHB	NPIP	29706310	1.6088	6.4378	-2.1713
CHB	NPIP	29711310	2.4517	6.946	-1.8865
CHB	NPIP	29716310	3.6286	9.8261	-1.8909
CHB	NPIP	29721310	3.9541	9.6567	-1.7683
CHB	NPIP	29726310	3.1531	7.6237	-1.724
CHB	NPIP	29731310	2.6482	9.9955	-2.2063
CHB	NPIP	29736310	2.5663	9.1484	-2.146
CHB	NPIP	29741310	2.7925	6.946	-1.7435
CHB	NPIP	29746310	5.6238	9.1484	-1.1492
CHB	NPIP	29751310	5.8599	10.5037	-1.3314
CHB	NPIP	29756310	3.1784	9.3178	-1.9679
CHB	NPIP	29761310	4.4034	9.8261	-1.6545
CHB	NPIP	29766310	4.4647	10.3343	-1.7086
CHB	NPIP	29771310	2.3906	10.3343	-2.3123
CHB	NPIP	29776310	2.339	10.5037	-2.3408
CHB	NPIP	29781310	2.0782	7.1154	-2.0686
CHB	NPIP	29786310	3.1643	6.6072	-1.5122
CHB	NPIP	29791310	3.5117	9.1484	-1.8378
CHB	NPIP	29796310	2.5243	8.3013	-2.0603
CHB	NPIP	29801310	1.6716	6.946	-2.214
CHB	NPIP	29806310	0.9895	5.4213	-2.3239
CHB	NPIP	29811310	0.9834	3.5577	-1.9455
CHB	NPIP	29816310	1.0375	5.4213	-2.2987
CHB	NPIP	29821310	1.086	7.1154	-2.4761
CHB	NPIP	29826310	2.1498	7.7931	-2.1329
CHB	NPIP	29831310	3.4982	7.4543	-1.5572
CHB	NPIP	29836310	3.1222	6.2684	-1.449
CHB	NPIP	29841310	2.3611	6.4378	-1.833
CHB	NPIP	29846310	1.9994	5.7601	-1.8684
CHB	NPIP	29851310	1.4091	5.0825	-2.0394
CHB	NPIP	29856310	1.4626	6.0989	-2.1885
CHB	NPIP	29861310	2.1106	7.2848	-2.0799
CHB	NPIP	29866310	2.3845	6.6072	-1.8547
CHB	NPIP	29871310	2.0489	6.0989	-1.9118
CHB	NPIP	29876310	2.3482	7.1154	-1.9577
CHB	NPIP	29881310	2.8945	6.2684	-1.5539
CHB	NPIP	29886310	3.9259	5.0825	-0.6421
CHB	NPIP	29891310	7.0539	5.5907	0.7466
CHB	NPIP	29896310	8.1005	6.0989	0.9448
CHB	NPIP	29901310	6.2964	5.9295	0.1776
YRI	NPIP	28886310	5.5179	6.5544	-0.457
YRI	NPIP	28891310	7.0942	8.4031	-0.46
YRI	NPIP	28896310	7.0551	8.7392	-0.5709
YRI	NPIP	28901310	5.9285	7.7309	-0.6839
YRI	NPIP	28906310	6.421	9.5795	-0.9833
YRI	NPIP	28911310	5.182	9.5795	-1.3691

YRI	NPIP	28916310	4.4482	8.7392	-1.4545
YRI	NPIP	28921310	4.3671	8.7392	-1.482
YRI	NPIP	28926310	7.4733	9.4115	-0.6134
YRI	NPIP	28931310	8.2585	8.4031	-0.0508
YRI	NPIP	28936310	5.5429	7.2267	-0.6794
YRI	NPIP	28941310	5.1962	8.067	-1.0476
YRI	NPIP	28946310	3.5608	8.235	-1.6736
YRI	NPIP	28951310	3.7705	7.2267	-1.3946
YRI	NPIP	28956310	7.8964	10.2518	-0.6884
YRI	NPIP	28961310	8.3101	11.7644	-0.8874
YRI	NPIP	28966310	7.5849	10.5879	-0.8516
YRI	NPIP	28971310	7.7285	11.0921	-0.9132
YRI	NPIP	28976310	5.9232	10.2518	-1.2651
YRI	NPIP	28981310	8.0838	11.5963	-0.9147
YRI	NPIP	28986310	9.2782	12.4366	-0.77
YRI	NPIP	28991310	7.2739	10.2518	-0.8704
YRI	NPIP	28996310	7.192	9.4115	-0.7025
YRI	NPIP	29001310	12.1322	12.2685	-0.0337
YRI	NPIP	29006310	11.8245	14.1172	-0.4957
YRI	NPIP	29011310	8.5886	12.2685	-0.9087
YRI	NPIP	29016310	8.3099	10.756	-0.6836
YRI	NPIP	29021310	7.6804	10.924	-0.8933
YRI	NPIP	29026310	10.7549	12.4366	-0.41
YRI	NPIP	29031310	10.3391	11.4282	-0.2875
YRI	NPIP	29036310	8.0819	9.5795	-0.4663
YRI	NPIP	29041310	5.5019	9.2434	-1.2041
YRI	NPIP	29046310	3.288	7.2267	-1.5893
YRI	NPIP	29051310	1.9407	3.0251	-0.9355
YRI	NPIP	29056310	3.47	3.1932	0.2283
YRI	NPIP	29061310	6.2329	6.7225	-0.211
YRI	NPIP	29066310	5.0142	5.5461	-0.2724
YRI	NPIP	29071310	4.2291	5.5461	-0.6744
YRI	NPIP	29076310	3.8089	5.5461	-0.8896
YRI	NPIP	29081310	4.7043	6.2183	-0.6999
YRI	NPIP	29086310	5.6134	8.067	-0.8953
YRI	NPIP	29091310	7.7709	9.5795	-0.5631
YRI	NPIP	29096310	7.2949	11.9324	-1.1756
YRI	NPIP	29101310	7.4419	13.7811	-1.4024
YRI	NPIP	29106310	8.6104	10.756	-0.5996
YRI	NPIP	29111310	10.1907	10.4199	-0.066
YRI	NPIP	29116310	12.8508	15.1256	-0.4606
YRI	NPIP	29121310	9.8069	13.1089	-0.766
YRI	NPIP	29126310	13.0879	14.7895	-0.352
YRI	NPIP	29131310	17.9881	17.6465	0.0597
YRI	NPIP	29136310	19.6822	18.3188	0.2299
YRI	NPIP	29141310	20.9755	21.68	-0.101
YRI	NPIP	29146310	18.0105	21.1758	-0.4641
YRI	NPIP	29151310	16.8313	21.1758	-0.637
YRI	NPIP	29156310	15.922	20.8397	-0.7323

YRI	NPIP	29161310	16.3454	20.1675	-0.5874
YRI	NPIP	29166310	16.3672	23.6968	-0.9639
YRI	NPIP	29171310	11.0185	20.3355	-1.4205
YRI	NPIP	29176310	10.7641	17.1423	-1.146
YRI	NPIP	29181310	13.3835	17.6465	-0.7449
YRI	NPIP	29186310	18.2726	17.8146	0.0793
YRI	NPIP	29191310	18.8903	19.1591	-0.0434
YRI	NPIP	29196310	12.4907	15.9659	-0.6683
YRI	NPIP	29201310	11.8028	12.6047	-0.193
YRI	NPIP	29206310	13.5604	14.1172	-0.1204
YRI	NPIP	29211310	20.9876	21.1758	-0.0276
YRI	NPIP	29216310	24.0368	21.0078	0.4476
YRI	NPIP	29221310	20.6418	17.9827	0.4563
YRI	NPIP	29226310	15.0679	17.1423	-0.3727
YRI	NPIP	29231310	13.7489	15.4617	-0.3396
YRI	NPIP	29236310	19.0837	22.1842	-0.4346
YRI	NPIP	29241310	20.0293	24.8732	-0.6078
YRI	NPIP	29246310	19.1736	19.3272	-0.0246
YRI	NPIP	29251310	15.5995	15.9659	-0.0705
YRI	NPIP	29256310	15.6729	16.134	-0.0878
YRI	NPIP	29261310	20.0832	19.6633	0.0661
YRI	NPIP	29266310	25.0996	23.6968	0.1845
YRI	NPIP	29271310	39.4055	27.8983	1.2915
YRI	NPIP	29276310	47.8366	30.0831	1.8515
YRI	NPIP	29281310	43.9107	28.0664	1.7679
YRI	NPIP	29286310	41.1824	26.7219	1.6924
YRI	NPIP	29291310	26.2036	19.9994	0.9613
YRI	NPIP	29296310	21.4087	18.991	0.3937
YRI	NPIP	29301310	37.5919	30.5873	0.7187
YRI	NPIP	29306310	33.1126	29.9151	0.3353
YRI	NPIP	29311310	17.5237	18.991	-0.2389
YRI	NPIP	29316310	16.7531	17.8146	-0.1838
YRI	NPIP	29321310	21.9753	22.5203	-0.0753
YRI	NPIP	29326310	18.709	19.4952	-0.1248
YRI	NPIP	29331310	9.3391	14.6214	-1.1047
YRI	NPIP	29336310	7.0773	14.9575	-1.6127
YRI	NPIP	29341310	11.2246	15.9659	-0.9118
YRI	NPIP	29346310	12.2167	16.8062	-0.8404
YRI	NPIP	29351310	9.8683	15.1256	-1.0646
YRI	NPIP	29356310	7.3512	10.2518	-0.8478
YRI	NPIP	29361310	6.6068	8.5712	-0.6779
YRI	NPIP	29366310	6.5305	9.5795	-0.9493
YRI	NPIP	29371310	3.2532	6.2183	-1.3707
YRI	NPIP	29376310	1.2484	3.3612	-1.6692
YRI	NPIP	29381310	1.8355	3.1932	-1.1197
YRI	NPIP	29386310	2.9611	3.6974	-0.5367
YRI	NPIP	29391310	5.8766	7.3947	-0.5999
YRI	NPIP	29396310	5.6225	5.8822	-0.1262
YRI	NPIP	29401310	3.8338	8.235	-1.5759

YRI	NPIP	29406310	4.1073	10.756	-1.858
YRI	NPIP	29411310	6.9562	10.756	-1.0618
YRI	NPIP	29416310	8.0971	14.4534	-1.344
YRI	NPIP	29421310	4.5192	12.1005	-1.8967
YRI	NPIP	29426310	4.0431	10.5879	-1.8561
YRI	NPIP	29431310	4.36	11.2602	-1.8472
YRI	NPIP	29436310	9.3995	16.302	-1.3013
YRI	NPIP	29441310	8.0466	12.6047	-1.0973
YRI	NPIP	29446310	0.5133	2.689	-2.0677
YRI	NPIP	29451310	0.0919	0.8403	-1.6904
YRI	NPIP	29456310	0.0278	0.3361	-1.2377
YRI	NPIP	29461310	0.0278	0.3361	-1.2377
YRI	NPIP	29466310	0.0093	0.1681	-0.944
YRI	NPIP	29471310	0.6211	0.8403	-0.4951
YRI	NPIP	29476310	1.0384	1.1764	-0.2468
YRI	NPIP	29481310	0.523	0.6722	-0.3906
YRI	NPIP	29486310	0.1771	0.3361	-0.6383
YRI	NPIP	29491310	0.1906	0.6722	-1.2608
YRI	NPIP	29496310	0.1831	1.3445	-1.8863
YRI	NPIP	29501310	0.3182	1.5126	-1.7785
YRI	NPIP	29506310	0.346	1.5126	-1.7371
YRI	NPIP	29511310	4.6882	2.8571	1.6559
YRI	NPIP	29516310	4.715	2.5209	2.1973
YRI	NPIP	29521310	0.1737	1.0084	-1.6641
YRI	NPIP	29526310	0.0461	0.5042	-1.4397
YRI	NPIP	29531310	0.0093	0.1681	-0.944
YRI	NPIP	29536310	0.0554	0.6722	-1.6147
YRI	NPIP	29541310	0.2394	0.8403	-1.3572
YRI	NPIP	29546310	0.8872	1.1764	-0.5172
YRI	NPIP	29551310	0.6939	0.8403	-0.3307
YRI	NPIP	29556310	1.033	3.3612	-1.8394
YRI	NPIP	29561310	1.8821	5.7141	-1.9108
YRI	NPIP	29566310	1.5662	4.3696	-1.7701
YRI	NPIP	29571310	2.0042	4.3696	-1.4936
YRI	NPIP	29576310	2.3842	3.6974	-0.9572
YRI	NPIP	29581310	3.8501	5.2099	-0.736
YRI	NPIP	29586310	3.9214	7.5628	-1.4097
YRI	NPIP	29591310	4.0059	8.4031	-1.5454
YRI	NPIP	29596310	4.5688	9.4115	-1.5327
YRI	NPIP	29601310	5.9725	10.4199	-1.2803
YRI	NPIP	29606310	6.6227	10.2518	-1.0607
YRI	NPIP	29611310	4.8445	8.9073	-1.3531
YRI	NPIP	29616310	3.9154	8.5712	-1.6067
YRI	NPIP	29621310	2.6934	8.5712	-2.0284
YRI	NPIP	29626310	2.6477	8.5712	-2.0442
YRI	NPIP	29631310	2.7786	8.067	-1.9298
YRI	NPIP	29636310	5.0566	9.0754	-1.3155
YRI	NPIP	29641310	9.7496	9.7476	0.0006
YRI	NPIP	29646310	9.6476	10.5879	-0.2667

YRI	NPIP	29651310	9.1162	11.7644	-0.6803
YRI	NPIP	29656310	11.4429	11.4282	0.0039
YRI	NPIP	29661310	8.7636	10.2518	-0.435
YRI	NPIP	29666310	10.4169	11.7644	-0.3462
YRI	NPIP	29671310	13.5709	13.2769	0.0674
YRI	NPIP	29676310	11.9749	13.9492	-0.4318
YRI	NPIP	29681310	11.2984	14.6214	-0.695
YRI	NPIP	29686310	9.248	13.1089	-0.8956
YRI	NPIP	29691310	9.6076	12.7727	-0.7525
YRI	NPIP	29696310	8.1388	12.1005	-0.9911
YRI	NPIP	29701310	5.1554	10.756	-1.5651
YRI	NPIP	29706310	3.974	8.9073	-1.6431
YRI	NPIP	29711310	4.7278	10.5879	-1.6619
YRI	NPIP	29716310	7.7653	13.613	-1.3088
YRI	NPIP	29721310	8.3732	11.5963	-0.8393
YRI	NPIP	29726310	7.1051	10.5879	-0.9877
YRI	NPIP	29731310	9.3413	14.2853	-1.0571
YRI	NPIP	29736310	9.3729	13.1089	-0.8666
YRI	NPIP	29741310	7.1759	9.0754	-0.6218
YRI	NPIP	29746310	11.8086	10.756	0.2941
YRI	NPIP	29751310	18.8583	14.6214	0.8861
YRI	NPIP	29756310	16.999	14.9575	0.4178
YRI	NPIP	29761310	14.4818	13.9492	0.1165
YRI	NPIP	29766310	15.0695	14.9575	0.0229
YRI	NPIP	29771310	15.5405	17.1423	-0.2878
YRI	NPIP	29776310	15.4938	16.302	-0.1524
YRI	NPIP	29781310	11.2854	12.4366	-0.2807
YRI	NPIP	29786310	11.6634	11.0921	0.1551
YRI	NPIP	29791310	13.0852	10.924	0.5952
YRI	NPIP	29796310	9.501	9.5795	-0.0244
YRI	NPIP	29801310	4.5154	7.5628	-1.1797
YRI	NPIP	29806310	3.7356	8.4031	-1.6404
YRI	NPIP	29811310	4.1861	10.0837	-1.7505
YRI	NPIP	29816310	3.6876	10.2518	-1.9185
YRI	NPIP	29821310	3.5481	10.5879	-1.9965
YRI	NPIP	29826310	6.0722	12.1005	-1.5082
YRI	NPIP	29831310	9.3104	12.6047	-0.793
YRI	NPIP	29836310	8.1238	11.9324	-0.9655
YRI	NPIP	29841310	6.3075	9.5795	-1.0187
YRI	NPIP	29846310	5.3765	6.5544	-0.5193
YRI	NPIP	29851310	3.8924	7.3947	-1.3839
YRI	NPIP	29856310	5.1622	9.9157	-1.4332
YRI	NPIP	29861310	6.3475	11.0921	-1.2882
YRI	NPIP	29866310	6.5487	10.924	-1.205
YRI	NPIP	29871310	5.9026	9.4115	-1.1105
YRI	NPIP	29876310	6.0983	11.4282	-1.4071
YRI	NPIP	29881310	7.0627	11.5963	-1.1806
YRI	NPIP	29886310	6.7456	9.7476	-0.9196
YRI	NPIP	29891310	8.0345	10.756	-0.7605

YRI	NPIP	29896310	8.7574	9.7476	-0.3033
YRI	NPIP	29901310	6.9485	8.235	-0.4606
CEU	NPIP	29727897	4.6599	8.187	-1.2778
CEU	NPIP	29732897	5.2919	7.5047	-0.8682
CEU	NPIP	29737897	6.2804	6.8225	-0.232
CEU	NPIP	29742897	5.2657	6.8225	-0.6662
CEU	NPIP	29747897	7.7555	8.0164	-0.0964
CEU	NPIP	29752897	6.3849	8.0164	-0.6026
CEU	NPIP	29757897	3.0435	8.187	-1.8634
CEU	NPIP	29762897	5.2092	8.6987	-1.1955
CEU	NPIP	29767897	4.0851	6.4814	-1.0741
CEU	NPIP	29772897	1.0287	6.8225	-2.4792
CEU	NPIP	29777897	1.3012	6.8225	-2.3626
CEU	NPIP	29782897	1.9058	4.9463	-1.734
CEU	NPIP	29787897	2.0746	6.9931	-2.058
CEU	NPIP	29792897	2.1288	6.8225	-2.0084
CEU	NPIP	29797897	1.4539	4.7757	-1.9537
CEU	NPIP	29802897	0.502	4.4346	-2.4674
CEU	NPIP	29807897	0.8291	3.4112	-2.028
CEU	NPIP	29812897	0.6052	3.0701	-2.1144
CEU	NPIP	29817897	0.7275	5.6286	-2.493
CEU	NPIP	29822897	1.8817	7.3342	-2.1847
CEU	NPIP	29827897	3.0626	6.9931	-1.6446
CEU	NPIP	29832897	4.4035	7.3342	-1.1742
CEU	NPIP	29837897	5.236	6.1402	-0.4255
CEU	NPIP	29842897	3.8571	4.2641	-0.2642
CEU	NPIP	29847897	2.8516	4.2641	-0.9169
CEU	NPIP	29852897	2.3286	5.1169	-1.5434
CEU	NPIP	29857897	3.1387	5.1169	-1.095
CEU	NPIP	29862897	3.5411	4.7757	-0.7261
CEU	NPIP	29867897	2.798	4.0935	-0.8711
CEU	NPIP	29872897	3.2581	4.6052	-0.8179
CEU	NPIP	29877897	5.0136	5.6286	-0.3128
CEU	NPIP	29882897	5.242	5.2874	-0.0244
CEU	NPIP	29887897	2.4708	5.1169	-1.4647
CEU	NPIP	29892897	2.9332	5.6286	-1.371
CEU	NPIP	29897897	4.5725	6.3108	-0.7981
CEU	NPIP	29902897	4.4877	6.3108	-0.837
CEU	NPIP	29907897	6.6564	5.2874	0.7361
CEU	NPIP	29912897	6.6832	4.6052	1.2616
CEU	NPIP	29917897	4.8701	5.2874	-0.2244
CEU	NPIP	29922897	6.0023	5.9697	0.0157
CEU	NPIP	29927897	6.4224	5.7991	0.3087
CEU	NPIP	29932897	8.1767	6.6519	0.6676
CEU	NPIP	29937897	7.4966	6.6519	0.3698
CEU	NPIP	29942897	6.0602	5.6286	0.2195
CEU	NPIP	29947897	5.6741	5.2874	0.2079
CEU	NPIP	29952897	3.3416	4.4346	-0.6858
CEU	NPIP	29957897	4.6933	5.2874	-0.3195

CEU	NPIP	29962897	5.7857	5.1169	0.3702
CEU	NPIP	29967897	6.7925	4.7757	1.1862
CEU	NPIP	29972897	6.0373	4.0935	1.3071
CEU	NPIP	29977897	5.8081	4.2641	1.0023
CEU	NPIP	29982897	8.2482	6.4814	0.7919
CEU	NPIP	29987897	5.2839	4.4346	0.5329
CEU	NPIP	29992897	5.2352	3.9229	0.9153
CEU	NPIP	29997897	6.0082	5.1169	0.4934
CEU	NPIP	30002897	3.3638	4.9463	-0.9025
CEU	NPIP	30007897	3.9064	5.9697	-0.9958
CEU	NPIP	30012897	7.9262	8.6987	-0.2647
CEU	NPIP	30017897	8.8071	8.0164	0.292
CEU	NPIP	30022897	7.3191	4.9463	1.3532
CEU	NPIP	30027897	7.3529	5.2874	1.1106
CEU	NPIP	30032897	7.5857	6.9931	0.248
CEU	NPIP	30037897	7.4529	6.9931	0.1924
CEU	NPIP	30042897	6.887	4.9463	1.1068
CEU	NPIP	30047897	9.3781	5.7991	1.7727
CEU	NPIP	30052897	8.476	6.1402	1.0992
CEU	NPIP	30057897	5.5962	5.2874	0.166
CEU	NPIP	30062897	5.608	6.1402	-0.2504
CEU	NPIP	30067897	4.4895	5.7991	-0.6486
CEU	NPIP	30072897	2.1697	4.2641	-1.3596
CEU	NPIP	30077897	2.2727	4.2641	-1.2927
CEU	NPIP	30082897	2.8771	4.7757	-1.1166
CEU	NPIP	30087897	3.0146	5.1169	-1.1637
CEU	NPIP	30092897	5.1789	6.6519	-0.6449
CEU	NPIP	30097897	5.0773	7.1636	-0.854
CEU	NPIP	30102897	3.7365	6.1402	-1.1311
CEU	NPIP	30107897	2.7654	4.9463	-1.2438
CEU	NPIP	30112897	2.3989	4.7757	-1.3979
CEU	NPIP	30117897	6.4869	7.8459	-0.512
CEU	NPIP	30122897	6.9615	7.3342	-0.1493
CEU	NPIP	30127897	3.3944	4.6052	-0.7351
CEU	NPIP	30132897	3.348	4.7757	-0.8397
CEU	NPIP	30137897	6.1276	6.4814	-0.1586
CEU	NPIP	30142897	6.1072	6.8225	-0.3061
CEU	NPIP	30147897	3.0388	5.1169	-1.1503
CEU	NPIP	30152897	5.5537	6.9931	-0.6023
CEU	NPIP	30157897	8.0239	7.5047	0.2037
CEU	NPIP	30162897	10.9984	7.6753	1.2773
CEU	NPIP	30167897	11.0763	8.0164	1.1302
CEU	NPIP	30172897	6.1082	5.7991	0.1531
CEU	NPIP	30177897	5.7947	5.2874	0.2728
CEU	NPIP	30182897	6.173	5.7991	0.1852
CEU	NPIP	30187897	3.5114	5.1169	-0.8887
CEU	NPIP	30192897	2.0614	5.458	-1.7757
CEU	NPIP	30197897	2.4461	4.9463	-1.4259
CEU	NPIP	30202897	1.1575	1.7056	-0.7507

CEU	NPIP	30207897	0.0499	0.5117	-1.4483
CEU	NPIP	30212897	0.1655	0.8528	-1.5478
CEU	NPIP	30217897	0.1156	0.3411	-0.904
CEU	NPIP	30222897	0.4937	0.5117	-0.0565
CEU	NPIP	30227897	0.5038	0.6822	-0.4659
CEU	NPIP	30232897	0.0303	0.3411	-1.2459
CEU	NPIP	30237897	0.0202	0.1706	-0.8934
CEU	NPIP	30242897	0	0	0
CEU	NPIP	30247897	0	0	0
CEU	NPIP	30252897	0	0	0
CEU	NPIP	30257897	0	0	0
CEU	NPIP	30262897	0.0499	0.3411	-1.1673
CEU	NPIP	30267897	0.0802	0.8528	-1.7399
CEU	NPIP	30272897	0.0404	0.6822	-1.6759
CEU	NPIP	30277897	0.0101	0.1706	-0.9534
CEU	NPIP	30282897	0.6151	0.5117	0.3243
CEU	NPIP	30287897	1.1631	0.8528	0.6988
CEU	NPIP	30292897	1.563	1.3645	0.3211
CEU	NPIP	30297897	1.0251	1.3645	-0.5491
CEU	NPIP	30302897	0.5187	0.6822	-0.4269
CEU	NPIP	30307897	1.0796	1.0234	0.1117
CEU	NPIP	30312897	1.0705	1.0234	0.0936
CEU	NPIP	30317897	1.5401	1.7056	-0.2267
CEU	NPIP	30322897	3.9025	3.4112	0.3859
CEU	NPIP	30327897	6.5723	4.7757	1.0567
CEU	NPIP	30332897	7.122	6.4814	0.2871
CEU	NPIP	30337897	6.0561	6.3108	-0.1169
CEU	NPIP	30342897	5.9314	6.1402	-0.0983
CEU	NPIP	30347897	5.6988	6.9931	-0.5416
CEU	NPIP	30352897	3.8633	5.9697	-1.0166
CEU	NPIP	30357897	4.4385	5.458	-0.533
CEU	NPIP	30362897	4.5203	5.9697	-0.6995
CEU	NPIP	30367897	2.4444	4.4346	-1.2487
CEU	NPIP	30372897	3.5529	4.6052	-0.6389
CEU	NPIP	30377897	4.3474	6.4814	-0.9565
CEU	NPIP	30382897	3.1472	6.1402	-1.4084
CEU	NPIP	30387897	3.9013	5.1169	-0.6729
CEU	NPIP	30392897	5.5545	5.9697	-0.2004
CEU	NPIP	30397897	5.1973	7.3342	-0.8562
CEU	NPIP	30402897	4.5143	6.9931	-1.0372
CEU	NPIP	30407897	4.3781	5.6286	-0.6361
CEU	NPIP	30412897	3.5121	3.5818	-0.0525
CEU	NPIP	30417897	2.9031	2.5584	0.3434
CEU	NPIP	30422897	2.8409	2.5584	0.2814
CEU	NPIP	30427897	3.6755	3.0701	0.5193
CEU	NPIP	30432897	4.556	4.6052	-0.0299
CEU	NPIP	30437897	3.9004	4.7757	-0.5148
CEU	NPIP	30442897	3.7846	4.6052	-0.4982
CEU	NPIP	30447897	4.5951	5.9697	-0.6634

CEU	NPIP	30452897	3.5253	5.6286	-1.0699
CEU	NPIP	30457897	4.6809	6.3108	-0.7483
CEU	NPIP	30462897	5.7695	5.9697	-0.0966
CEU	NPIP	30467897	6.4787	6.1402	0.1593
CEU	NPIP	30472897	6.4395	7.5047	-0.4179
CEU	NPIP	30477897	5.1342	6.6519	-0.6645
CEU	NPIP	30482897	4.4842	5.1169	-0.3502
CEU	NPIP	30487897	4.8275	4.6052	0.135
CEU	NPIP	30492897	7.0959	5.2874	0.9724
CEU	NPIP	30497897	7.697	5.6286	1.0521
CEU	NPIP	30502897	7.4463	6.6519	0.3478
CEU	NPIP	30507897	8.2977	6.8225	0.6312
CEU	NPIP	30512897	11.859	8.3576	1.2446
CEU	NPIP	30517897	10.2007	8.5281	0.5836
CEU	NPIP	30522897	4.2126	4.9463	-0.4184
CEU	NPIP	30527897	2.7324	4.6052	-1.1371
CEU	NPIP	30532897	3.1635	5.2874	-1.142
CEU	NPIP	30537897	2.9326	3.2407	-0.2526
CEU	NPIP	30542897	2.2358	3.2407	-0.824
CEU	NPIP	30547897	3.4952	4.9463	-0.8276
CEU	NPIP	30552897	4.9254	6.8225	-0.8118
CEU	NPIP	30557897	4.0078	6.6519	-1.1576
CEU	NPIP	30562897	2.1338	5.6286	-1.7777
CEU	NPIP	30567897	1.6891	6.6519	-2.1728
CEU	NPIP	30572897	1.798	5.458	-1.9134
CEU	NPIP	30577897	1.4939	3.5818	-1.5736
CEU	NPIP	30582897	2.0144	3.5818	-1.1813
CEU	NPIP	30587897	3.0219	4.0935	-0.7206
CEU	NPIP	30592897	2.616	4.6052	-1.2077
CEU	NPIP	30597897	1.2773	4.0935	-1.8937
CEU	NPIP	30602897	1.8159	3.4112	-1.253
CEU	NPIP	30607897	2.0913	3.5818	-1.1234
CEU	NPIP	30612897	2.4234	3.5818	-0.8731
CEU	NPIP	30617897	1.9623	3.0701	-0.9503
CEU	NPIP	30622897	1.9027	3.0701	-1.0014
CEU	NPIP	30627897	2.3682	3.5818	-0.9147
CEU	NPIP	30632897	4.6343	4.0935	0.3636
CEU	NPIP	30637897	6.1929	4.9463	0.7109
CEU	NPIP	30642897	8.9202	6.6519	0.9931
CEU	NPIP	30647897	10.5813	7.5047	1.2071
CEU	NPIP	30652897	5.6867	4.6052	0.6566
CEU	NPIP	30657897	2.3331	2.2173	0.1294
CEU	NPIP	30662897	1.6976	2.729	-0.9749
CEU	NPIP	30667897	2.1855	2.8996	-0.6422
CEU	NPIP	30672897	1.3896	3.0701	-1.4415
CEU	NPIP	30677897	0.804	4.2641	-2.2461
CEU	NPIP	30682897	1.1897	4.0935	-1.9526
CEU	NPIP	30687897	2.0674	4.6052	-1.5408
CEU	NPIP	30692897	3.0161	5.458	-1.2766

CEU	NPIP	30697897	2.2008	3.9229	-1.2011
CEU	NPIP	30702897	2.5611	3.2407	-0.5573
CEU	NPIP	30707897	2.1064	4.2641	-1.4007
CEU	NPIP	30712897	2.0897	4.6052	-1.5273
CEU	NPIP	30717897	2.5296	4.0935	-1.0516
CEU	NPIP	30722897	2.1381	5.1169	-1.6488
CEU	NPIP	30727897	2.3142	5.6286	-1.6859
CEU	NPIP	30732897	1.0555	3.9229	-1.9999
CEU	NPIP	30737897	1.0567	3.4112	-1.8493
CEU	NPIP	30742897	2.4943	4.0935	-1.0753
CHB	NPIP	29727897	2.8978	7.7931	-1.8502
CHB	NPIP	29732897	2.5757	9.8261	-2.2121
CHB	NPIP	29737897	2.9922	8.979	-1.986
CHB	NPIP	29742897	2.9183	6.4378	-1.5825
CHB	NPIP	29747897	5.9539	10.5037	-1.3044
CHB	NPIP	29752897	5.5024	10.8426	-1.4862
CHB	NPIP	29757897	2.4234	7.9625	-2.0526
CHB	NPIP	29762897	4.806	10.3343	-1.6092
CHB	NPIP	29767897	4.5374	10.3343	-1.6874
CHB	NPIP	29772897	2.5315	10.5037	-2.2856
CHB	NPIP	29777897	2.2644	10.3343	-2.349
CHB	NPIP	29782897	2.0792	5.9295	-1.864
CHB	NPIP	29787897	3.6156	7.4543	-1.511
CHB	NPIP	29792897	3.558	9.4872	-1.869
CHB	NPIP	29797897	1.8597	7.6237	-2.2228
CHB	NPIP	29802897	1.1159	6.4378	-2.3929
CHB	NPIP	29807897	0.9122	4.2354	-2.1634
CHB	NPIP	29812897	1.0445	4.7436	-2.182
CHB	NPIP	29817897	1.2545	6.7766	-2.3704
CHB	NPIP	29822897	1.5635	6.946	-2.2594
CHB	NPIP	29827897	2.3322	7.2848	-1.9908
CHB	NPIP	29832897	3.0423	6.6072	-1.5657
CHB	NPIP	29837897	3.1942	6.4378	-1.4584
CHB	NPIP	29842897	2.268	6.2684	-1.8424
CHB	NPIP	29847897	1.7148	5.2519	-1.9077
CHB	NPIP	29852897	1.4582	5.9295	-2.1646
CHB	NPIP	29857897	1.7952	6.946	-2.1621
CHB	NPIP	29862897	2.687	7.6237	-1.9037
CHB	NPIP	29867897	2.1047	6.2684	-1.9177
CHB	NPIP	29872897	1.9429	6.0989	-1.9618
CHB	NPIP	29877897	2.7341	6.7766	-1.7353
CHB	NPIP	29882897	4.3257	5.9295	-0.7764
CHB	NPIP	29887897	4.2983	5.2519	-0.5143
CHB	NPIP	29892897	5.3962	5.4213	-0.0132
CHB	NPIP	29897897	7.8932	5.9295	0.9507
CHB	NPIP	29902897	7.3587	5.9295	0.6919
CHB	NPIP	29907897	8.0162	6.6072	0.6188
CHB	NPIP	29912897	7.1553	6.0989	0.4987
CHB	NPIP	29917897	5.3476	5.7601	-0.2049

CHB	NPIP	29922897	5.9558	6.7766	-0.3523
CHB	NPIP	29927897	6.4498	6.7766	-0.1403
CHB	NPIP	29932897	7.7044	7.9625	-0.0956
CHB	NPIP	29937897	6.7487	6.7766	-0.012
CHB	NPIP	29942897	5.8843	5.2519	0.3411
CHB	NPIP	29947897	5.288	6.0989	-0.3828
CHB	NPIP	29952897	2.9143	5.9295	-1.4597
CHB	NPIP	29957897	3.9006	6.6072	-1.1888
CHB	NPIP	29962897	5.1532	5.7601	-0.3015
CHB	NPIP	29967897	6.331	5.2519	0.582
CHB	NPIP	29972897	5.553	4.913	0.3661
CHB	NPIP	29977897	6.186	4.913	0.7282
CHB	NPIP	29982897	8.134	5.5907	1.2976
CHB	NPIP	29987897	4.6351	4.4048	0.1449
CHB	NPIP	29992897	4.7245	5.5907	-0.442
CHB	NPIP	29997897	5.2324	6.0989	-0.409
CHB	NPIP	30002897	2.8214	4.913	-1.1964
CHB	NPIP	30007897	3.2121	5.4213	-1.1584
CHB	NPIP	30012897	6.1015	7.9625	-0.6896
CHB	NPIP	30017897	6.9086	8.8096	-0.6418
CHB	NPIP	30022897	6.3337	6.4378	-0.0468
CHB	NPIP	30027897	6.3082	4.913	0.7981
CHB	NPIP	30032897	6.1108	5.4213	0.3616
CHB	NPIP	30037897	7.321	7.1154	0.0844
CHB	NPIP	30042897	7.5901	6.4378	0.5181
CHB	NPIP	30047897	8.1536	6.7766	0.5911
CHB	NPIP	30052897	6.6482	8.1319	-0.5393
CHB	NPIP	30057897	4.1614	6.4378	-1.0235
CHB	NPIP	30062897	4.7149	6.4378	-0.7747
CHB	NPIP	30067897	4.73	7.4543	-1.0724
CHB	NPIP	30072897	2.9309	6.0989	-1.4954
CHB	NPIP	30077897	3.0095	7.4543	-1.7496
CHB	NPIP	30082897	2.7927	7.4543	-1.8349
CHB	NPIP	30087897	2.5243	5.5907	-1.5645
CHB	NPIP	30092897	5.1536	8.1319	-1.0825
CHB	NPIP	30097897	5.0177	7.2848	-0.9113
CHB	NPIP	30102897	3.744	4.2354	-0.3199
CHB	NPIP	30107897	3.1296	5.4213	-1.2017
CHB	NPIP	30112897	2.0714	5.7601	-1.8326
CHB	NPIP	30117897	5.2968	7.9625	-0.9878
CHB	NPIP	30122897	5.7278	8.4708	-0.9602
CHB	NPIP	30127897	2.7849	5.0825	-1.2756
CHB	NPIP	30132897	3.7552	4.5742	-0.4987
CHB	NPIP	30137897	6.8468	6.2684	0.2664
CHB	NPIP	30142897	6.1685	6.946	-0.3264
CHB	NPIP	30147897	2.5604	5.0825	-1.4002
CHB	NPIP	30152897	3.6164	6.0989	-1.1718
CHB	NPIP	30157897	5.4712	7.4543	-0.7806
CHB	NPIP	30162897	8.478	7.4543	0.403

CHB	NPIP	30167897	8.1498	6.946	0.5053
CHB	NPIP	30172897	4.4786	6.2684	-0.8243
CHB	NPIP	30177897	4.8951	7.1154	-0.9118
CHB	NPIP	30182897	5.0044	7.1154	-0.8669
CHB	NPIP	30187897	3.0038	6.946	-1.6548
CHB	NPIP	30192897	1.218	5.5907	-2.231
CHB	NPIP	30197897	1.2254	3.7271	-1.8174
CHB	NPIP	30202897	0.8251	1.6942	-1.1927
CHB	NPIP	30207897	0	0	0
CHB	NPIP	30212897	0.0386	0.3388	-1.2042
CHB	NPIP	30217897	0.0484	0.5082	-1.4434
CHB	NPIP	30222897	0.1542	0.3388	-0.7405
CHB	NPIP	30227897	0.1445	0.1694	-0.148
CHB	NPIP	30232897	0.0192	0.1694	-0.8925
CHB	NPIP	30237897	0.0192	0.1694	-0.8925
CHB	NPIP	30242897	0	0	0
CHB	NPIP	30247897	0	0	0
CHB	NPIP	30252897	0	0	0
CHB	NPIP	30257897	0	0	0
CHB	NPIP	30262897	0.1277	0.1694	-0.2478
CHB	NPIP	30267897	0.1472	0.5082	-1.1332
CHB	NPIP	30272897	0.0194	0.3388	-1.2812
CHB	NPIP	30277897	0	0	0
CHB	NPIP	30282897	0.5635	0.6777	-0.2986
CHB	NPIP	30287897	0.8327	1.3553	-0.847
CHB	NPIP	30292897	0.7049	2.2024	-1.6768
CHB	NPIP	30297897	0.4455	1.8636	-1.8093
CHB	NPIP	30302897	0.1776	1.1859	-1.7998
CHB	NPIP	30307897	0.3954	2.5412	-2.1427
CHB	NPIP	30312897	0.412	2.2024	-2.0048
CHB	NPIP	30317897	0.4287	1.5247	-1.6283
CHB	NPIP	30322897	1.6276	5.2519	-1.9547
CHB	NPIP	30327897	2.9103	7.6237	-1.8176
CHB	NPIP	30332897	2.6257	6.7766	-1.7818
CHB	NPIP	30337897	2.0346	6.946	-2.0616
CHB	NPIP	30342897	2.3362	6.0989	-1.7762
CHB	NPIP	30347897	2.107	5.9295	-1.8505
CHB	NPIP	30352897	2.0278	5.2519	-1.7389
CHB	NPIP	30357897	2.4826	5.0825	-1.4434
CHB	NPIP	30362897	1.6722	6.946	-2.2137
CHB	NPIP	30367897	0.739	5.9295	-2.5128
CHB	NPIP	30372897	1.1123	5.4213	-2.2595
CHB	NPIP	30377897	1.7704	6.2684	-2.0716
CHB	NPIP	30382897	1.4734	5.2519	-2.0379
CHB	NPIP	30387897	1.5252	5.4213	-2.043
CHB	NPIP	30392897	1.8525	6.7766	-2.1137
CHB	NPIP	30397897	1.7729	6.946	-2.1715
CHB	NPIP	30402897	1.4795	5.5907	-2.0976
CHB	NPIP	30407897	1.3122	4.066	-1.857

CHB	NPIP	30412897	1.2675	3.3883	-1.6702
CHB	NPIP	30417897	1.0729	4.066	-2.0183
CHB	NPIP	30422897	1.395	4.7436	-1.9753
CHB	NPIP	30427897	1.8624	4.5742	-1.6513
CHB	NPIP	30432897	2.506	4.913	-1.3768
CHB	NPIP	30437897	2.4727	5.5907	-1.5909
CHB	NPIP	30442897	1.6749	7.2848	-2.255
CHB	NPIP	30447897	2.2596	8.979	-2.229
CHB	NPIP	30452897	2.4265	7.9625	-2.0515
CHB	NPIP	30457897	2.3137	6.7766	-1.9158
CHB	NPIP	30462897	2.6204	6.946	-1.8157
CHB	NPIP	30467897	2.2846	7.4543	-2.0349
CHB	NPIP	30472897	1.3552	6.2684	-2.2628
CHB	NPIP	30477897	1.2168	4.066	-1.9213
CHB	NPIP	30482897	1.3301	4.066	-1.8449
CHB	NPIP	30487897	2.8028	6.4378	-1.6344
CHB	NPIP	30492897	4.1907	9.3178	-1.6434
CHB	NPIP	30497897	4.657	8.8096	-1.402
CHB	NPIP	30502897	4.6292	7.4543	-1.112
CHB	NPIP	30507897	4.7312	6.6072	-0.824
CHB	NPIP	30512897	7.2381	8.4708	-0.4315
CHB	NPIP	30517897	5.3001	8.4708	-1.11
CHB	NPIP	30522897	2.1032	4.7436	-1.5575
CHB	NPIP	30527897	1.5399	4.066	-1.7034
CHB	NPIP	30532897	0.8532	4.4048	-2.2348
CHB	NPIP	30537897	0.7874	4.066	-2.2109
CHB	NPIP	30542897	0.5378	3.7271	-2.317
CHB	NPIP	30547897	0.8627	5.4213	-2.3904
CHB	NPIP	30552897	1.7537	6.0989	-2.0511
CHB	NPIP	30557897	1.4194	5.7601	-2.1566
CHB	NPIP	30562897	0.7472	5.7601	-2.4905
CHB	NPIP	30567897	0.8348	5.7601	-2.447
CHB	NPIP	30572897	0.6063	4.5742	-2.4161
CHB	NPIP	30577897	0.861	5.2519	-2.3682
CHB	NPIP	30582897	1.2104	6.2684	-2.3295
CHB	NPIP	30587897	1.47	5.5907	-2.1025
CHB	NPIP	30592897	1.1401	5.2519	-2.2176
CHB	NPIP	30597897	0.5673	3.8965	-2.3285
CHB	NPIP	30602897	0.7773	4.4048	-2.2826
CHB	NPIP	30607897	0.7188	4.066	-2.2571
CHB	NPIP	30612897	1.4436	5.5907	-2.1159
CHB	NPIP	30617897	1.3459	5.7601	-2.1931
CHB	NPIP	30622897	0.9065	5.2519	-2.3436
CHB	NPIP	30627897	1.3588	6.2684	-2.2612
CHB	NPIP	30632897	2.4283	6.6072	-1.8354
CHB	NPIP	30637897	3.012	6.6072	-1.5791
CHB	NPIP	30642897	3.7733	5.9295	-1.0438
CHB	NPIP	30647897	5.0596	6.6072	-0.6797
CHB	NPIP	30652897	3.1034	5.0825	-1.0988

CHB	NPIP	30657897	0.8782	4.2354	-2.1855
CHB	NPIP	30662897	1.181	5.2519	-2.1956
CHB	NPIP	30667897	1.4963	6.0989	-2.1726
CHB	NPIP	30672897	0.8713	5.7601	-2.4289
CHB	NPIP	30677897	0.7727	4.2354	-2.2542
CHB	NPIP	30682897	0.7533	3.8965	-2.1984
CHB	NPIP	30687897	1.0583	5.0825	-2.2342
CHB	NPIP	30692897	1.9779	6.0989	-1.9453
CHB	NPIP	30697897	1.6499	6.6072	-2.1773
CHB	NPIP	30702897	1.3663	5.7601	-2.1829
CHB	NPIP	30707897	1.2465	5.0825	-2.1297
CHB	NPIP	30712897	1.4346	6.0989	-2.2018
CHB	NPIP	30717897	1.1136	4.7436	-2.1412
CHB	NPIP	30722897	1.2018	4.913	-2.1228
CHB	NPIP	30727897	1.6889	5.9295	-2.0529
CHB	NPIP	30732897	1.0335	5.2519	-2.2751
CHB	NPIP	30737897	0.7674	5.7601	-2.4805
CHB	NPIP	30742897	0.9556	5.5907	-2.3649
YRI	NPIP	29727897	7.8907	12.1005	-1.0532
YRI	NPIP	29732897	9.6737	14.1172	-0.9608
YRI	NPIP	29737897	8.3473	11.4282	-0.8134
YRI	NPIP	29742897	7.5939	8.7392	-0.3882
YRI	NPIP	29747897	17.2857	14.1172	0.6851
YRI	NPIP	29752897	19.4985	15.9659	0.6794
YRI	NPIP	29757897	13.089	13.1089	-0.0046
YRI	NPIP	29762897	15.2446	13.9492	0.2833
YRI	NPIP	29767897	14.3692	15.4617	-0.2166
YRI	NPIP	29772897	15.5751	16.4701	-0.1671
YRI	NPIP	29777897	15.6639	15.2937	0.0742
YRI	NPIP	29782897	9.2042	11.9324	-0.6916
YRI	NPIP	29787897	13.4678	11.5963	0.4873
YRI	NPIP	29792897	13.4475	10.5879	0.811
YRI	NPIP	29797897	6.3555	7.8989	-0.5742
YRI	NPIP	29802897	3.9357	7.8989	-1.4744
YRI	NPIP	29807897	3.4297	8.4031	-1.748
YRI	NPIP	29812897	3.7304	9.5795	-1.821
YRI	NPIP	29817897	4.4151	11.7644	-1.8881
YRI	NPIP	29822897	5.1187	12.1005	-1.7467
YRI	NPIP	29827897	6.4279	10.756	-1.2095
YRI	NPIP	29832897	8.6172	12.7727	-0.9879
YRI	NPIP	29837897	8.8255	12.9408	-0.9663
YRI	NPIP	29842897	5.5608	7.5628	-0.775
YRI	NPIP	29847897	4.3567	6.2183	-0.8606
YRI	NPIP	29852897	4.3194	8.4031	-1.4353
YRI	NPIP	29857897	5.3703	10.0837	-1.399
YRI	NPIP	29862897	7.7352	12.1005	-1.0921
YRI	NPIP	29867897	6.2497	10.2518	-1.1697
YRI	NPIP	29872897	5.0374	10.4199	-1.5495
YRI	NPIP	29877897	6.8374	12.2685	-1.3412

YRI	NPIP	29882897	7.7757	9.9157	-0.6452
YRI	NPIP	29887897	6.1782	9.2434	-0.9865
YRI	NPIP	29892897	7.5456	9.9157	-0.7146
YRI	NPIP	29897897	8.4594	9.7476	-0.3946
YRI	NPIP	29902897	7.6085	9.5795	-0.6136
YRI	NPIP	29907897	9.7092	10.4199	-0.2046
YRI	NPIP	29912897	7.4686	8.4031	-0.3284
YRI	NPIP	29917897	5.3455	7.2267	-0.7591
YRI	NPIP	29922897	6.8697	8.5712	-0.5872
YRI	NPIP	29927897	8.0026	9.4115	-0.4459
YRI	NPIP	29932897	9.5601	11.0921	-0.4159
YRI	NPIP	29937897	8.5884	8.7392	-0.0511
YRI	NPIP	29942897	8.506	7.3947	0.4391
YRI	NPIP	29947897	7.9013	8.9073	-0.3351
YRI	NPIP	29952897	5.4524	7.7309	-0.8645
YRI	NPIP	29957897	5.9645	8.235	-0.813
YRI	NPIP	29962897	5.782	8.235	-0.8783
YRI	NPIP	29967897	7.2169	8.7392	-0.516
YRI	NPIP	29972897	7.2754	9.0754	-0.5892
YRI	NPIP	29977897	6.1289	7.8989	-0.6585
YRI	NPIP	29982897	7.7577	9.2434	-0.4781
YRI	NPIP	29987897	5.6788	7.8989	-0.8259
YRI	NPIP	29992897	5.3935	7.2267	-0.7397
YRI	NPIP	29997897	5.7915	7.8989	-0.784
YRI	NPIP	30002897	4.1623	7.3947	-1.2773
YRI	NPIP	30007897	6.5087	8.5712	-0.7118
YRI	NPIP	30012897	10.7363	10.756	-0.0055
YRI	NPIP	30017897	9.2354	9.2434	-0.0026
YRI	NPIP	30022897	6.1231	6.8906	-0.3234
YRI	NPIP	30027897	6.4209	8.235	-0.6495
YRI	NPIP	30032897	7.2058	9.5795	-0.739
YRI	NPIP	30037897	7.9888	9.2434	-0.4038
YRI	NPIP	30042897	8.1101	8.9073	-0.2655
YRI	NPIP	30047897	9.5965	10.0837	-0.1446
YRI	NPIP	30052897	9.8808	11.9324	-0.5201
YRI	NPIP	30057897	8.4659	12.9408	-1.0508
YRI	NPIP	30062897	7.6134	12.4366	-1.1759
YRI	NPIP	30067897	6.068	10.2518	-1.2228
YRI	NPIP	30072897	3.1724	8.4031	-1.8384
YRI	NPIP	30077897	3.9363	10.0837	-1.8246
YRI	NPIP	30082897	5.2799	9.9157	-1.3977
YRI	NPIP	30087897	5.3808	9.5795	-1.3072
YRI	NPIP	30092897	7.2394	11.5963	-1.1346
YRI	NPIP	30097897	5.5376	9.9157	-1.32
YRI	NPIP	30102897	3.4406	7.0586	-1.4915
YRI	NPIP	30107897	3.4719	8.235	-1.7055
YRI	NPIP	30112897	3.9093	8.9073	-1.6646
YRI	NPIP	30117897	7.9843	10.0837	-0.6231
YRI	NPIP	30122897	8.4192	10.2518	-0.5356

YRI	NPIP	30127897	5.1009	9.2434	-1.3332
YRI	NPIP	30132897	4.6229	8.235	-1.2933
YRI	NPIP	30137897	7.6366	9.7476	-0.6467
YRI	NPIP	30142897	7.6856	9.5795	-0.5896
YRI	NPIP	30147897	2.9103	4.7057	-1.0628
YRI	NPIP	30152897	5.1925	6.8906	-0.7155
YRI	NPIP	30157897	8.7219	8.5712	0.052
YRI	NPIP	30162897	12.4587	8.7392	1.2608
YRI	NPIP	30167897	12.8405	9.0754	1.2325
YRI	NPIP	30172897	8.1155	6.7225	0.6002
YRI	NPIP	30177897	8.4006	6.7225	0.7231
YRI	NPIP	30182897	9.116	7.5628	0.6013
YRI	NPIP	30187897	6.6339	6.3864	0.1117
YRI	NPIP	30192897	3.5185	6.5544	-1.3385
YRI	NPIP	30197897	2.5482	5.8822	-1.62
YRI	NPIP	30202897	1.4488	2.0167	-0.679
YRI	NPIP	30207897	0.1515	0.5042	-1.1084
YRI	NPIP	30212897	0.2158	1.1764	-1.7181
YRI	NPIP	30217897	0.0643	0.6722	-1.5914
YRI	NPIP	30222897	0.5038	0.1681	1.9956
YRI	NPIP	30227897	0.5038	0.1681	1.9956
YRI	NPIP	30232897	0.0369	0.5042	-1.4686
YRI	NPIP	30237897	0.0369	0.5042	-1.4686
YRI	NPIP	30242897	0.0093	0.1681	-0.944
YRI	NPIP	30247897	0.0093	0.1681	-0.944
YRI	NPIP	30252897	0	0	0
YRI	NPIP	30257897	0	0	0
YRI	NPIP	30262897	0.1966	0.8403	-1.4539
YRI	NPIP	30267897	0.2059	1.0084	-1.5999
YRI	NPIP	30272897	0.0093	0.1681	-0.944
YRI	NPIP	30277897	0.0891	0.1681	-0.4696
YRI	NPIP	30282897	1.1364	1.1764	-0.0715
YRI	NPIP	30287897	2.3186	2.3529	-0.0363
YRI	NPIP	30292897	4.2576	3.1932	0.8778
YRI	NPIP	30297897	3.5639	3.1932	0.3057
YRI	NPIP	30302897	1.1959	2.3529	-1.2247
YRI	NPIP	30307897	1.4368	3.1932	-1.4485
YRI	NPIP	30312897	1.3584	2.689	-1.2646
YRI	NPIP	30317897	1.4136	3.1932	-1.4677
YRI	NPIP	30322897	3.4273	7.8989	-1.6635
YRI	NPIP	30327897	6.2004	9.2434	-0.9793
YRI	NPIP	30332897	7.4818	8.235	-0.2697
YRI	NPIP	30337897	6.2683	6.8906	-0.2622
YRI	NPIP	30342897	5.5157	6.3864	-0.393
YRI	NPIP	30347897	6.265	9.0754	-0.92
YRI	NPIP	30352897	4.1088	7.5628	-1.3371
YRI	NPIP	30357897	3.5727	6.8906	-1.3981
YRI	NPIP	30362897	4.4933	9.0754	-1.4999
YRI	NPIP	30367897	3.1575	7.2267	-1.642

YRI	NPIP	30372897	3.7059	6.8906	-1.3419
YRI	NPIP	30377897	4.7209	8.5712	-1.3288
YRI	NPIP	30382897	4.6663	6.7225	-0.886
YRI	NPIP	30387897	4.5275	5.378	-0.4476
YRI	NPIP	30392897	6.178	6.5544	-0.1659
YRI	NPIP	30397897	6.8902	8.235	-0.4815
YRI	NPIP	30402897	5.1051	9.7476	-1.4222
YRI	NPIP	30407897	4.8265	8.9073	-1.3591
YRI	NPIP	30412897	5.5518	7.8989	-0.8732
YRI	NPIP	30417897	5.0927	6.3864	-0.5839
YRI	NPIP	30422897	4.2249	5.0419	-0.4552
YRI	NPIP	30427897	3.585	6.0502	-1.168
YRI	NPIP	30432897	4.3761	7.3947	-1.1928
YRI	NPIP	30437897	4.416	7.2267	-1.1342
YRI	NPIP	30442897	4.9058	8.067	-1.1535
YRI	NPIP	30447897	6.26	10.4199	-1.1975
YRI	NPIP	30452897	4.9334	10.0837	-1.5287
YRI	NPIP	30457897	5.7299	9.5795	-1.1985
YRI	NPIP	30462897	5.4928	8.235	-0.9819
YRI	NPIP	30467897	6.8626	10.0837	-0.9561
YRI	NPIP	30472897	6.8964	11.0921	-1.1392
YRI	NPIP	30477897	4.1288	7.7309	-1.3667
YRI	NPIP	30482897	3.946	7.2267	-1.3238
YRI	NPIP	30487897	6.4597	9.2434	-0.8959
YRI	NPIP	30492897	9.7429	11.2602	-0.4062
YRI	NPIP	30497897	8.2114	10.924	-0.7471
YRI	NPIP	30502897	7.2494	10.2518	-0.8775
YRI	NPIP	30507897	9.1323	10.0837	-0.2824
YRI	NPIP	30512897	12.5506	12.9408	-0.0916
YRI	NPIP	30517897	10.8798	13.1089	-0.5171
YRI	NPIP	30522897	5.1796	9.0754	-1.2753
YRI	NPIP	30527897	3.6892	7.2267	-1.4275
YRI	NPIP	30532897	4.2996	8.4031	-1.4422
YRI	NPIP	30537897	5.0028	11.5963	-1.717
YRI	NPIP	30542897	4.0479	9.0754	-1.6457
YRI	NPIP	30547897	3.7779	9.0754	-1.7341
YRI	NPIP	30552897	4.1035	9.5795	-1.7049
YRI	NPIP	30557897	4.0824	9.0754	-1.6344
YRI	NPIP	30562897	3.5981	10.0837	-1.925
YRI	NPIP	30567897	4.0997	10.2518	-1.7981
YRI	NPIP	30572897	4.1331	9.2434	-1.6446
YRI	NPIP	30577897	2.8454	5.8822	-1.4756
YRI	NPIP	30582897	2.8675	6.5544	-1.6255
YRI	NPIP	30587897	5.2471	8.4031	-1.1092
YRI	NPIP	30592897	6.5814	9.5795	-0.9334
YRI	NPIP	30597897	4.9406	8.4031	-1.2169
YRI	NPIP	30602897	5.74	6.8906	-0.4848
YRI	NPIP	30607897	5.3662	5.7141	-0.1735
YRI	NPIP	30612897	6.0514	7.0586	-0.4152

YRI	NPIP	30617897	5.8293	8.235	-0.8614
YRI	NPIP	30622897	4.3506	7.5628	-1.2435
YRI	NPIP	30627897	4.9232	9.2434	-1.3904
YRI	NPIP	30632897	6.2677	11.0921	-1.3099
YRI	NPIP	30637897	6.8328	9.4115	-0.8161
YRI	NPIP	30642897	7.4871	8.4031	-0.3219
YRI	NPIP	30647897	9.6902	10.924	-0.3398
YRI	NPIP	30652897	6.8228	9.0754	-0.7374
YRI	NPIP	30657897	3.0119	6.0502	-1.4396
YRI	NPIP	30662897	3.3568	6.7225	-1.4503
YRI	NPIP	30667897	4.9513	6.8906	-0.8172
YRI	NPIP	30672897	3.9353	6.8906	-1.2453
YRI	NPIP	30677897	2.6348	9.2434	-2.1268
YRI	NPIP	30682897	2.9336	9.4115	-2.0502
YRI	NPIP	30687897	4.9929	10.0837	-1.511
YRI	NPIP	30692897	7.1529	10.0837	-0.8699
YRI	NPIP	30697897	6.9295	9.4115	-0.7855
YRI	NPIP	30702897	6.0468	10.5879	-1.2878
YRI	NPIP	30707897	5.1221	10.756	-1.5744
YRI	NPIP	30712897	6.5488	11.4282	-1.2882
YRI	NPIP	30717897	4.6732	9.2434	-1.4708
YRI	NPIP	30722897	4.0694	8.4031	-1.5231
YRI	NPIP	30727897	5.7906	8.5712	-0.9596
YRI	NPIP	30732897	3.084	7.3947	-1.7034
YRI	NPIP	30737897	2.7543	7.3947	-1.8337
YRI	NPIP	30742897	3.6001	7.7309	-1.5673
CEU	NPIP	73879986	4.1416	5.6286	-0.7564
CEU	NPIP	73884986	4.8371	5.6286	-0.4026
CEU	NPIP	73889986	6.4032	6.8225	-0.1794
CEU	NPIP	73894986	5.4129	7.1636	-0.7166
CEU	NPIP	73899986	3.8703	5.9697	-1.0132
CEU	NPIP	73904986	4.0363	5.7991	-0.8731
CEU	NPIP	73909986	5.0893	6.6519	-0.6841
CEU	NPIP	73914986	7.0651	7.3342	-0.1078
CEU	NPIP	73919986	6.7258	8.3576	-0.5801
CEU	NPIP	73924986	3.5137	6.1402	-1.236
CEU	NPIP	73929986	3.3948	4.9463	-0.8848
CEU	NPIP	73934986	3.0266	5.6286	-1.3235
CEU	NPIP	73939986	1.5628	4.2641	-1.7535
CEU	NPIP	73944986	1.9258	2.8996	-0.8758
CEU	NPIP	73949986	3.5413	4.2641	-0.4692
CEU	NPIP	73954986	5.2884	7.3342	-0.8197
CEU	NPIP	73959986	4.6256	6.8225	-0.94
CEU	NPIP	73964986	4.739	5.2874	-0.2949
CEU	NPIP	73969986	5.214	5.458	-0.1276
CEU	NPIP	73974986	5.569	5.1169	0.2502
CEU	NPIP	73979986	5.2321	6.4814	-0.56
CEU	NPIP	73984986	4.1066	6.4814	-1.0644
CEU	NPIP	73989986	3.3125	5.458	-1.1216

CEU	NPIP	73994986	4.0268	6.3108	-1.0486
CEU	NPIP	73999986	3.9097	5.6286	-0.8743
CEU	NPIP	74004986	3.8396	5.1169	-0.707
CEU	NPIP	74009986	7.6428	6.1402	0.7071
CEU	NPIP	74014986	7.3049	5.9697	0.6444
CEU	NPIP	74019986	5.43	5.2874	0.0767
CEU	NPIP	74024986	5.4739	6.4814	-0.4516
CEU	NPIP	74029986	4.865	6.8225	-0.8376
CEU	NPIP	74034986	5.4372	5.6286	-0.0974
CEU	NPIP	74039986	5.8472	5.2874	0.301
CEU	NPIP	74044986	4.9307	5.458	-0.2757
CEU	NPIP	74049986	3.6436	4.9463	-0.7429
CEU	NPIP	74054986	7.7209	6.3108	0.6474
CEU	NPIP	74059986	11.464	8.3576	1.1042
CEU	NPIP	74064986	7.1643	6.6519	0.2243
CEU	NPIP	74069986	5.5854	6.6519	-0.4669
CEU	NPIP	74074986	10.1431	10.7454	-0.1695
CEU	NPIP	74079986	10.5191	10.0632	0.1364
CEU	NPIP	74084986	9.0495	8.5281	0.1819
CEU	NPIP	74089986	7.2931	8.0164	-0.2672
CEU	NPIP	74094986	5.1979	6.4814	-0.5753
CEU	NPIP	74099986	5.9074	7.8459	-0.7303
CEU	NPIP	74104986	8.5277	9.5515	-0.3216
CEU	NPIP	74109986	7.1604	7.5047	-0.1351
CEU	NPIP	74114986	5.6576	5.6286	0.0148
CEU	NPIP	74119986	10.5702	8.0164	0.9433
CEU	NPIP	74124986	13.5403	10.916	0.7278
CEU	NPIP	74129986	10.5786	11.5982	-0.2671
CEU	NPIP	74134986	6.5866	9.3809	-0.8927
CEU	NPIP	74139986	8.922	12.4511	-0.8649
CEU	NPIP	74144986	10.2266	13.8156	-0.7971
CEU	NPIP	74149986	8.5106	10.2337	-0.5076
CEU	NPIP	74154986	6.9549	9.2104	-0.7329
CEU	NPIP	74159986	4.9918	8.3576	-1.1964
CEU	NPIP	74164986	7.1066	7.8459	-0.2785
CEU	NPIP	74169986	11.341	8.5281	0.9815
CEU	NPIP	74174986	12.3643	10.916	0.4017
CEU	NPIP	74179986	10.7322	10.0632	0.2002
CEU	NPIP	74184986	9.5354	7.5047	0.7967
CEU	NPIP	74189986	8.2979	6.8225	0.6313
CEU	NPIP	74194986	10.8677	9.2104	0.5385
CEU	NPIP	74199986	23.2072	14.3272	1.9052
CEU	NPIP	74204986	31.4649	18.4207	2.2011
CEU	NPIP	74209986	19.7629	13.8156	1.3209
CEU	NPIP	74214986	9.4968	9.7221	-0.0696
CEU	NPIP	74219986	7.1486	8.5281	-0.4813
CEU	NPIP	74224986	7.9431	6.9931	0.3975
CEU	NPIP	74229986	9.8077	7.5047	0.9036
CEU	NPIP	74234986	8.7646	7.1636	0.6554

CEU	NPIP	74239986	10.0132	8.3576	0.5885
CEU	NPIP	74244986	10.1985	7.8459	0.8863
CEU	NPIP	74249986	9.4637	7.6753	0.6874
CEU	NPIP	74254986	8.6152	7.5047	0.4357
CEU	NPIP	74259986	10.2259	7.8459	0.8966
CEU	NPIP	74264986	9.6813	8.8692	0.2733
CEU	NPIP	74269986	6.7576	8.6987	-0.665
CEU	NPIP	74274986	5.4704	7.6753	-0.8475
CEU	NPIP	74279986	5.1721	7.6753	-0.9622
CEU	NPIP	74284986	5.0517	7.1636	-0.8645
CEU	NPIP	74289986	3.1977	4.6052	-0.8546
CEU	NPIP	74294986	2.39	6.4814	-1.8338
CEU	NPIP	74299986	4.3939	8.8692	-1.5059
CEU	NPIP	74304986	4.0175	6.9931	-1.245
CEU	NPIP	74309986	4.2845	6.9931	-1.1333
CEU	NPIP	74314986	5.5073	6.9931	-0.6217
CEU	NPIP	74319986	7.9781	8.3576	-0.1349
CEU	NPIP	74324986	8.2569	10.7454	-0.7004
CEU	NPIP	74329986	4.2259	8.5281	-1.5011
CEU	NPIP	74334986	3.0503	5.7991	-1.3615
CEU	NPIP	74339986	2.9509	4.9463	-1.138
CEU	NPIP	74344986	3.1826	6.3108	-1.4362
CEU	NPIP	74349986	3.7858	6.8225	-1.2994
CEU	NPIP	74354986	4.7858	6.1402	-0.6373
CEU	NPIP	74359986	7.8033	8.0164	-0.0787
CEU	NPIP	74364986	7.0676	8.6987	-0.5588
CEU	NPIP	74369986	4.5014	8.0164	-1.2983
CEU	NPIP	74374986	5.0626	6.9931	-0.8078
CEU	NPIP	74379986	5.312	7.6753	-0.9084
CEU	NPIP	74384986	6.8226	10.2337	-1.0049
CEU	NPIP	74389986	9.1003	10.916	-0.5036
CEU	NPIP	74394986	7.8826	8.8692	-0.332
CEU	NPIP	74399986	4.4776	5.458	-0.5125
CEU	NPIP	74404986	4.7316	6.1402	-0.6628
CEU	NPIP	74409986	4.3739	6.1402	-0.8312
CEU	NPIP	74414986	2.9493	4.4346	-0.9319
CEU	NPIP	74419986	3.1251	3.9229	-0.5564
CEU	NPIP	74424986	9.6392	7.5047	0.8375
CEU	NPIP	74429986	12.4162	11.2571	0.3123
CEU	NPIP	74434986	7.06	7.8459	-0.2961
CEU	NPIP	74439986	5.7749	5.2874	0.2621
CEU	NPIP	74444986	4.0171	3.7524	0.1918
CEU	NPIP	74449986	3.006	3.4112	-0.3183
CEU	NPIP	74454986	7.5933	7.6753	-0.0315
CEU	NPIP	74459986	11.8864	11.4277	0.1219
CEU	NPIP	74464986	13.8522	12.2805	0.3902
CEU	NPIP	74469986	17.4937	14.3272	0.6794
CEU	NPIP	74474986	18.4455	15.0095	0.7053
CEU	NPIP	74479986	13.5232	11.4277	0.5567

CEU	NPIP	74484986	8.8959	8.8692	0.009
CEU	NPIP	74489986	7.598	8.6987	-0.3771
CEU	NPIP	74494986	8.8866	9.0398	-0.0507
CEU	NPIP	74499986	13.3537	11.4277	0.5117
CEU	NPIP	74504986	10.6592	9.8926	0.2331
CEU	NPIP	74509986	6.8736	8.6987	-0.6253
CEU	NPIP	74514986	7.2387	9.5515	-0.7266
CEU	NPIP	74519986	6.1417	8.0164	-0.6924
CEU	NPIP	74524986	8.6938	9.8926	-0.3645
CEU	NPIP	74529986	8.9654	11.2571	-0.6175
CEU	NPIP	74534986	7.3022	11.4277	-1.0961
CEU	NPIP	74539986	5.7928	10.2337	-1.3083
CEU	NPIP	74544986	5.847	10.0632	-1.2618
CEU	NPIP	74549986	7.9309	11.5982	-0.9609
CEU	NPIP	74554986	5.8423	9.0398	-1.0572
CEU	NPIP	74559986	6.9784	9.5515	-0.8084
CEU	NPIP	74564986	9.894	10.916	-0.2835
CEU	NPIP	74569986	8.8767	8.6987	0.061
CEU	NPIP	74574986	8.9215	9.7221	-0.2474
CEU	NPIP	74579986	13.1214	10.916	0.6117
CEU	NPIP	74584986	13.7823	9.2104	1.4856
CEU	NPIP	74589986	12.765	8.8692	1.3109
CEU	NPIP	74594986	13.7831	9.3809	1.4063
CEU	NPIP	74599986	10.2894	7.8459	0.9205
CEU	NPIP	74604986	8.3937	8.5281	-0.0469
CEU	NPIP	74609986	9.9608	10.2337	-0.0804
CEU	NPIP	74614986	10.6696	10.5749	0.0271
CEU	NPIP	74619986	12.8376	11.4277	0.3746
CEU	NPIP	74624986	14.3616	12.7922	0.3749
CEU	NPIP	74629986	13.3923	12.7922	0.1434
CEU	NPIP	74634986	13.0932	10.0632	0.9068
CEU	NPIP	74639986	11.9254	9.5515	0.7458
CEU	NPIP	74644986	12.4065	10.916	0.4134
CEU	NPIP	74649986	14.4411	10.916	0.9777
CEU	NPIP	74654986	15.2773	11.9394	0.851
CEU	NPIP	74659986	12.9021	11.7688	0.2929
CEU	NPIP	74664986	11.8174	10.0632	0.525
CEU	NPIP	74669986	12.5683	10.4043	0.6278
CEU	NPIP	74674986	12.3521	10.2337	0.6241
CEU	NPIP	74679986	11.0971	9.3809	0.5483
CEU	NPIP	74684986	9.9186	9.7221	0.0607
CEU	NPIP	74689986	11.4574	10.7454	0.2004
CEU	NPIP	74694986	12.4694	11.0866	0.378
CEU	NPIP	74699986	14.9192	11.9394	0.7597
CEU	NPIP	74704986	14.7761	11.9394	0.7232
CEU	NPIP	74709986	11.7813	10.4043	0.3995
CEU	NPIP	74714986	8.015	9.3809	-0.4364
CEU	NPIP	74719986	6.8938	10.0632	-0.9485
CEU	NPIP	74724986	5.3941	9.3809	-1.2736

CEU	NPIP	74729986	4.0526	8.0164	-1.464
CEU	NPIP	74734986	18.0029	12.7922	1.2448
CEU	NPIP	74739986	24.9212	15.1801	1.9781
CEU	NPIP	74744986	16.2728	13.1333	0.7315
CEU	NPIP	74749986	11.248	12.9627	-0.4045
CEU	NPIP	74754986	10.6933	11.0866	-0.1075
CEU	NPIP	74759986	7.9328	8.3576	-0.151
CEU	NPIP	74764986	4.9011	7.6753	-1.0663
CEU	NPIP	74769986	6.898	9.5515	-0.8336
CEU	NPIP	74774986	7.4743	10.916	-0.9546
CEU	NPIP	74779986	6.6428	8.8692	-0.7492
CEU	NPIP	74784986	11.0934	11.5982	-0.1323
CEU	NPIP	74789986	12.1049	12.6216	-0.125
CEU	NPIP	74794986	13.8685	13.1333	0.1713
CEU	NPIP	74799986	12.8388	13.4744	-0.1445
CEU	NPIP	74804986	9.9454	10.7454	-0.2252
CEU	NPIP	74809986	12.9326	12.1099	0.207
CEU	NPIP	74814986	10.8703	10.916	-0.0127
CEU	NPIP	74819986	12.5572	11.2571	0.3503
CEU	NPIP	74824986	15.685	13.1333	0.5946
CEU	NPIP	74829986	14.4799	12.1099	0.5962
CEU	NPIP	74834986	12.1482	10.2337	0.564
CEU	NPIP	74839986	10.3723	8.8692	0.5058
CEU	NPIP	74844986	8.8262	8.6987	0.0437
CEU	NPIP	74849986	7.6162	8.5281	-0.3182
CEU	NPIP	74854986	8.679	12.7922	-0.9826
CEU	NPIP	74859986	10.3509	17.2268	-1.2373
CEU	NPIP	74864986	13.1408	15.8623	-0.5299
CEU	NPIP	74869986	14.2441	15.0095	-0.1571
CEU	NPIP	74874986	31.8969	23.0259	1.207
CEU	NPIP	74879986	35.8504	22.8554	1.7809
CEU	NPIP	74884986	23.7292	16.8857	1.2553
CHB	NPIP	73879986	3.0036	11.5202	-2.2391
CHB	NPIP	73884986	2.92	10.1649	-2.1417
CHB	NPIP	73889986	4.7733	9.8261	-1.5416
CHB	NPIP	73894986	4.7251	7.4543	-1.0743
CHB	NPIP	73899986	4.435	8.8096	-1.477
CHB	NPIP	73904986	3.6859	8.979	-1.7559
CHB	NPIP	73909986	3.3394	8.3013	-1.7696
CHB	NPIP	73914986	4.661	10.3343	-1.6514
CHB	NPIP	73919986	4.2447	10.5037	-1.7944
CHB	NPIP	73924986	2.7469	8.4708	-2.0038
CHB	NPIP	73929986	3.3718	7.9625	-1.7012
CHB	NPIP	73934986	3.2994	8.6402	-1.8358
CHB	NPIP	73939986	2.1466	7.9625	-2.1552
CHB	NPIP	73944986	2.2621	5.9295	-1.7754
CHB	NPIP	73949986	3.941	5.5907	-0.8417
CHB	NPIP	73954986	4.9903	7.4543	-0.9699
CHB	NPIP	73959986	4.1557	6.7766	-1.1251

CHB	NPIP	73964986	5.2823	7.1154	-0.7528
CHB	NPIP	73969986	5.6534	8.3013	-0.9444
CHB	NPIP	73974986	5.6362	8.1319	-0.9071
CHB	NPIP	73979986	5.2896	7.7931	-0.9462
CHB	NPIP	73984986	4.34	6.6072	-0.9958
CHB	NPIP	73989986	3.3965	6.2684	-1.3227
CHB	NPIP	73994986	4.2796	7.2848	-1.208
CHB	NPIP	73999986	4.2974	7.6237	-1.2827
CHB	NPIP	74004986	4.0268	8.3013	-1.5245
CHB	NPIP	74009986	7.165	9.3178	-0.6901
CHB	NPIP	74014986	6.1306	6.946	-0.3423
CHB	NPIP	74019986	5.0666	7.1154	-0.8414
CHB	NPIP	74024986	6.0312	9.1484	-1.0163
CHB	NPIP	74029986	5.7455	7.6237	-0.7243
CHB	NPIP	74034986	6.3318	7.6237	-0.4982
CHB	NPIP	74039986	7.5245	8.3013	-0.277
CHB	NPIP	74044986	7.3463	8.3013	-0.3406
CHB	NPIP	74049986	5.2275	7.4543	-0.8765
CHB	NPIP	74054986	8.1972	8.6402	-0.1523
CHB	NPIP	74059986	11.1609	11.5202	-0.0945
CHB	NPIP	74064986	6.9878	8.979	-0.6605
CHB	NPIP	74069986	6.1232	9.4872	-1.0604
CHB	NPIP	74074986	9.5707	11.012	-0.3953
CHB	NPIP	74079986	9.7213	8.8096	0.3078
CHB	NPIP	74084986	8.7129	8.4708	0.0848
CHB	NPIP	74089986	7.2974	9.4872	-0.6903
CHB	NPIP	74094986	6.0615	9.9955	-1.1813
CHB	NPIP	74099986	6.1034	10.6731	-1.2907
CHB	NPIP	74104986	8.2279	12.0285	-0.9595
CHB	NPIP	74109986	8.3836	10.3343	-0.5678
CHB	NPIP	74114986	5.8688	9.9955	-1.2392
CHB	NPIP	74119986	8.3194	13.3838	-1.1558
CHB	NPIP	74124986	10.1748	14.2309	-0.8733
CHB	NPIP	74129986	6.3739	13.2144	-1.5801
CHB	NPIP	74134986	4.7483	10.5037	-1.6501
CHB	NPIP	74139986	8.8127	10.6731	-0.5254
CHB	NPIP	74144986	9.9848	11.012	-0.2818
CHB	NPIP	74149986	8.3952	10.5037	-0.6045
CHB	NPIP	74154986	5.7177	10.6731	-1.3996
CHB	NPIP	74159986	3.1449	9.1484	-1.9574
CHB	NPIP	74164986	7.7632	9.8261	-0.6294
CHB	NPIP	74169986	13.6109	9.8261	1.1547
CHB	NPIP	74174986	13.4407	10.3343	0.9042
CHB	NPIP	74179986	11.3379	10.6731	0.1878
CHB	NPIP	74184986	10.0763	8.4708	0.562
CHB	NPIP	74189986	10.3196	7.9625	0.8735
CHB	NPIP	74194986	11.815	9.1484	0.8694
CHB	NPIP	74199986	21.2658	13.3838	1.7989
CHB	NPIP	74204986	30.3965	16.2638	2.6793

CHB	NPIP	74209986	19.3969	11.012	2.2999
CHB	NPIP	74214986	8.3758	8.1319	0.0887
CHB	NPIP	74219986	5.5456	7.7931	-0.8494
CHB	NPIP	74224986	7.4849	8.4708	-0.3451
CHB	NPIP	74229986	10.0131	9.3178	0.2229
CHB	NPIP	74234986	8.0619	8.8096	-0.2524
CHB	NPIP	74239986	9.237	10.6731	-0.4056
CHB	NPIP	74244986	9.5254	9.4872	0.012
CHB	NPIP	74249986	8.8925	7.2848	0.6462
CHB	NPIP	74254986	7.7088	8.3013	-0.2113
CHB	NPIP	74259986	8.0786	8.6402	-0.193
CHB	NPIP	74264986	7.5475	7.4543	0.0367
CHB	NPIP	74269986	5.0091	8.8096	-1.2832
CHB	NPIP	74274986	4.0388	9.9955	-1.7887
CHB	NPIP	74279986	4.1259	10.8426	-1.8693
CHB	NPIP	74284986	4.1568	11.5202	-1.936
CHB	NPIP	74289986	2.8456	10.5037	-2.1956
CHB	NPIP	74294986	3.5844	8.4708	-1.7106
CHB	NPIP	74299986	5.8492	7.2848	-0.5771
CHB	NPIP	74304986	4.6398	6.7766	-0.9173
CHB	NPIP	74309986	5.2281	8.1319	-1.0555
CHB	NPIP	74314986	6.335	9.4872	-0.9936
CHB	NPIP	74319986	6.9744	10.3343	-0.978
CHB	NPIP	74324986	6.4384	8.979	-0.8428
CHB	NPIP	74329986	3.4666	6.7766	-1.4209
CHB	NPIP	74334986	2.9263	6.2684	-1.5393
CHB	NPIP	74339986	3.0961	5.2519	-1.1627
CHB	NPIP	74344986	3.8763	7.6237	-1.4451
CHB	NPIP	74349986	4.2669	8.6402	-1.5033
CHB	NPIP	74354986	4.3828	8.8096	-1.4946
CHB	NPIP	74359986	6.2041	9.9955	-1.1385
CHB	NPIP	74364986	5.1591	9.1484	-1.3007
CHB	NPIP	74369986	3.3326	11.012	-2.1064
CHB	NPIP	74374986	3.8361	11.6896	-2.0367
CHB	NPIP	74379986	3.9537	11.1814	-1.9543
CHB	NPIP	74384986	5.3463	13.892	-1.8826
CHB	NPIP	74389986	7.0643	13.3838	-1.4423
CHB	NPIP	74394986	6.3111	9.8261	-1.0724
CHB	NPIP	74399986	4.2351	7.7931	-1.3447
CHB	NPIP	74404986	4.7599	8.8096	-1.3673
CHB	NPIP	74409986	4.5349	7.7931	-1.2314
CHB	NPIP	74414986	2.5332	4.913	-1.3612
CHB	NPIP	74419986	2.723	3.3883	-0.5239
CHB	NPIP	74424986	9.3269	6.2684	1.4086
CHB	NPIP	74429986	12.5313	8.979	1.1784
CHB	NPIP	74434986	7.1527	6.4378	0.3214
CHB	NPIP	74439986	5.0828	4.5742	0.3097
CHB	NPIP	74444986	3.8755	4.066	-0.1285
CHB	NPIP	74449986	2.7788	5.7601	-1.4812

CHB	NPIP	74454986	6.4649	9.1484	-0.8749
CHB	NPIP	74459986	11.7179	9.4872	0.7032
CHB	NPIP	74464986	15.2001	11.8591	0.8548
CHB	NPIP	74469986	18.0902	16.4333	0.311
CHB	NPIP	74474986	18.3906	16.4333	0.3674
CHB	NPIP	74479986	12.0563	11.8591	0.0505
CHB	NPIP	74484986	6.7039	9.3178	-0.8379
CHB	NPIP	74489986	5.8836	10.6731	-1.3527
CHB	NPIP	74494986	6.3119	11.6896	-1.3946
CHB	NPIP	74499986	9.0962	12.7061	-0.8654
CHB	NPIP	74504986	7.7146	11.1814	-0.9374
CHB	NPIP	74509986	4.5271	9.8261	-1.6167
CHB	NPIP	74514986	3.9928	9.8261	-1.7797
CHB	NPIP	74519986	4.091	9.4872	-1.701
CHB	NPIP	74524986	6.5682	9.3178	-0.8814
CHB	NPIP	74529986	6.5198	8.8096	-0.7731
CHB	NPIP	74534986	4.6007	10.1649	-1.6448
CHB	NPIP	74539986	4.4285	10.8426	-1.7851
CHB	NPIP	74544986	5.9695	9.8261	-1.1767
CHB	NPIP	74549986	6.1947	9.6567	-1.0735
CHB	NPIP	74554986	3.4441	9.1484	-1.8598
CHB	NPIP	74559986	4.1565	9.4872	-1.6804
CHB	NPIP	74564986	6.8932	10.5037	-1.0351
CHB	NPIP	74569986	6.7026	9.1484	-0.7974
CHB	NPIP	74574986	5.5958	9.1484	-1.1583
CHB	NPIP	74579986	8.0798	12.7061	-1.109
CHB	NPIP	74584986	9.0918	12.1979	-0.7739
CHB	NPIP	74589986	8.3903	8.8096	-0.1416
CHB	NPIP	74594986	8.8226	9.4872	-0.2095
CHB	NPIP	74599986	7.0721	10.8426	-1.0493
CHB	NPIP	74604986	5.6107	9.6567	-1.2546
CHB	NPIP	74609986	6.4584	10.5037	-1.1598
CHB	NPIP	74614986	6.8346	11.1814	-1.1753
CHB	NPIP	74619986	7.2526	11.3508	-1.0926
CHB	NPIP	74624986	8.8456	12.8755	-0.954
CHB	NPIP	74629986	9.9772	11.3508	-0.3662
CHB	NPIP	74634986	10.3014	11.012	-0.1949
CHB	NPIP	74639986	8.6235	11.5202	-0.7616
CHB	NPIP	74644986	8.2998	10.8426	-0.7077
CHB	NPIP	74649986	10.1072	11.012	-0.2482
CHB	NPIP	74654986	10.49	12.1979	-0.4255
CHB	NPIP	74659986	8.4788	11.012	-0.6948
CHB	NPIP	74664986	8.2012	10.8426	-0.7351
CHB	NPIP	74669986	8.399	10.8426	-0.6801
CHB	NPIP	74674986	8.4308	9.9955	-0.4699
CHB	NPIP	74679986	8.3284	11.3508	-0.8058
CHB	NPIP	74684986	7.0019	9.1484	-0.6998
CHB	NPIP	74689986	7.7826	9.1484	-0.4453
CHB	NPIP	74694986	7.9395	12.1979	-1.0609

CHB	NPIP	74699986	9.5928	14.9085	-1.095
CHB	NPIP	74704986	9.5307	14.9085	-1.1078
CHB	NPIP	74709986	7.8601	12.7061	-1.1617
CHB	NPIP	74714986	7.2037	10.5037	-0.9461
CHB	NPIP	74719986	8.1695	8.8096	-0.2161
CHB	NPIP	74724986	6.2794	7.7931	-0.5721
CHB	NPIP	74729986	4.0122	8.6402	-1.5908
CHB	NPIP	74734986	12.9046	13.892	-0.2175
CHB	NPIP	74739986	17.0735	16.4333	0.1202
CHB	NPIP	74744986	12.2678	15.2474	-0.6008
CHB	NPIP	74749986	8.4672	16.0944	-1.4605
CHB	NPIP	74754986	8.2172	14.9085	-1.3784
CHB	NPIP	74759986	8.4642	11.012	-0.6988
CHB	NPIP	74764986	7.8002	10.8426	-0.8467
CHB	NPIP	74769986	8.3253	12.0285	-0.9349
CHB	NPIP	74774986	7.4002	10.5037	-0.8898
CHB	NPIP	74779986	7.3172	8.4708	-0.4038
CHB	NPIP	74784986	11.0055	10.5037	0.1439
CHB	NPIP	74789986	11.3425	13.045	-0.3981
CHB	NPIP	74794986	11.1847	14.5697	-0.7127
CHB	NPIP	74799986	10.0468	13.2144	-0.7317
CHB	NPIP	74804986	7.4495	11.5202	-1.0703
CHB	NPIP	74809986	8.3665	13.892	-1.2173
CHB	NPIP	74814986	5.9926	11.3508	-1.4285
CHB	NPIP	74819986	8.1395	9.4872	-0.4248
CHB	NPIP	74824986	11.0535	12.7061	-0.3962
CHB	NPIP	74829986	8.0944	12.3673	-1.0508
CHB	NPIP	74834986	5.4653	9.3178	-1.2349
CHB	NPIP	74839986	6.1681	9.1484	-0.9717
CHB	NPIP	74844986	9.2263	12.0285	-0.7074
CHB	NPIP	74849986	8.0819	11.5202	-0.904
CHB	NPIP	74854986	5.3246	10.6731	-1.5106
CHB	NPIP	74859986	6.969	12.7061	-1.3753
CHB	NPIP	74864986	10.8222	12.1979	-0.3427
CHB	NPIP	74869986	11.5662	14.5697	-0.6324
CHB	NPIP	74874986	28.8804	25.4123	0.4276
CHB	NPIP	74879986	31.7365	23.2099	1.148
CHB	NPIP	74884986	16.3427	19.4827	-0.5006
YRI	NPIP	73879986	8.8721	15.9659	-1.3642
YRI	NPIP	73884986	8.5363	15.9659	-1.4288
YRI	NPIP	73889986	8.6313	14.6214	-1.2527
YRI	NPIP	73894986	6.7976	13.1089	-1.464
YRI	NPIP	73899986	8.4859	16.6382	-1.5072
YRI	NPIP	73904986	8.9481	17.4785	-1.5044
YRI	NPIP	73909986	8.0043	16.4701	-1.5804
YRI	NPIP	73914986	9.4707	16.134	-1.2687
YRI	NPIP	73919986	9.2455	14.6214	-1.1243
YRI	NPIP	73924986	6.9357	14.4534	-1.5896
YRI	NPIP	73929986	7.5219	14.2853	-1.4461

YRI	NPIP	73934986	8.0903	13.2769	-1.1887
YRI	NPIP	73939986	6.9387	12.9408	-1.4094
YRI	NPIP	73944986	7.1467	13.9492	-1.4877
YRI	NPIP	73949986	7.4129	14.6214	-1.5075
YRI	NPIP	73954986	6.5777	13.2769	-1.5354
YRI	NPIP	73959986	6.5841	13.1089	-1.5135
YRI	NPIP	73964986	6.6918	14.1172	-1.6056
YRI	NPIP	73969986	6.4374	11.7644	-1.3685
YRI	NPIP	73974986	7.6023	11.0921	-0.9475
YRI	NPIP	73979986	6.9526	15.1256	-1.655
YRI	NPIP	73984986	5.7046	14.4534	-1.8499
YRI	NPIP	73989986	6.0986	10.756	-1.3015
YRI	NPIP	73994986	7.9886	12.4366	-1.0844
YRI	NPIP	73999986	6.3304	13.1089	-1.5724
YRI	NPIP	74004986	6.4437	12.6047	-1.4831
YRI	NPIP	74009986	10.9992	14.2853	-0.7026
YRI	NPIP	74014986	8.8756	12.7727	-0.9265
YRI	NPIP	74019986	6.925	10.924	-1.1014
YRI	NPIP	74024986	8.6128	12.9408	-1.0163
YRI	NPIP	74029986	7.342	12.4366	-1.2421
YRI	NPIP	74034986	7.3877	11.5963	-1.0959
YRI	NPIP	74039986	8.4487	12.6047	-1.0005
YRI	NPIP	74044986	8.8706	12.4366	-0.8694
YRI	NPIP	74049986	9.2028	12.6047	-0.8189
YRI	NPIP	74054986	11.6536	15.1256	-0.703
YRI	NPIP	74059986	13.5636	18.1507	-0.7802
YRI	NPIP	74064986	8.6502	14.4534	-1.2271
YRI	NPIP	74069986	10.2209	14.2853	-0.869
YRI	NPIP	74074986	14.09	16.8062	-0.4974
YRI	NPIP	74079986	11.6874	16.302	-0.87
YRI	NPIP	74084986	11.9307	15.9659	-0.776
YRI	NPIP	74089986	10.5672	15.2937	-0.947
YRI	NPIP	74094986	8.3881	13.1089	-1.0951
YRI	NPIP	74099986	10.8069	13.2769	-0.5661
YRI	NPIP	74104986	14.4027	16.302	-0.3581
YRI	NPIP	74109986	11.7611	14.6214	-0.5982
YRI	NPIP	74114986	9.8371	12.6047	-0.6662
YRI	NPIP	74119986	17.0792	16.134	0.18
YRI	NPIP	74124986	20.0194	20.5036	-0.0732
YRI	NPIP	74129986	15.4059	18.991	-0.5838
YRI	NPIP	74134986	9.9855	14.1172	-0.8934
YRI	NPIP	74139986	14.5104	15.4617	-0.1886
YRI	NPIP	74144986	16.3926	14.9575	0.2937
YRI	NPIP	74149986	11.4811	13.9492	-0.5398
YRI	NPIP	74154986	9.9161	14.9575	-1.0318
YRI	NPIP	74159986	7.3193	11.9324	-1.1694
YRI	NPIP	74164986	9.3731	12.9408	-0.8377
YRI	NPIP	74169986	11.4873	15.7978	-0.8374
YRI	NPIP	74174986	9.3562	17.4785	-1.4324

YRI	NPIP	74179986	8.1477	15.6298	-1.4684
YRI	NPIP	74184986	8.66	13.613	-1.1086
YRI	NPIP	74189986	8.8067	14.4534	-1.194
YRI	NPIP	74194986	11.6428	12.7727	-0.2686
YRI	NPIP	74199986	23.8635	15.7978	1.5669
YRI	NPIP	74204986	32.7138	20.1675	1.9283
YRI	NPIP	74209986	20.7838	14.7895	1.24
YRI	NPIP	74214986	9.9124	10.756	-0.2357
YRI	NPIP	74219986	7.8507	9.2434	-0.4482
YRI	NPIP	74224986	9.0356	11.0921	-0.5584
YRI	NPIP	74229986	10.9134	13.445	-0.5733
YRI	NPIP	74234986	9.3244	10.924	-0.4406
YRI	NPIP	74239986	10.7292	11.2602	-0.1421
YRI	NPIP	74244986	10.8778	10.0837	0.2357
YRI	NPIP	74249986	9.5138	9.5795	-0.0205
YRI	NPIP	74254986	9.2379	10.5879	-0.3829
YRI	NPIP	74259986	10.4359	9.5795	0.2666
YRI	NPIP	74264986	9.44	9.4115	0.009
YRI	NPIP	74269986	7.6636	11.4282	-0.9939
YRI	NPIP	74274986	7.2164	12.7727	-1.3209
YRI	NPIP	74279986	8.143	13.9492	-1.2698
YRI	NPIP	74284986	8.7433	14.1172	-1.162
YRI	NPIP	74289986	6.2473	11.4282	-1.3678
YRI	NPIP	74294986	4.3789	9.7476	-1.6446
YRI	NPIP	74299986	4.5339	10.4199	-1.6944
YRI	NPIP	74304986	4.4858	11.2602	-1.8135
YRI	NPIP	74309986	5.4155	12.4366	-1.7117
YRI	NPIP	74314986	5.9623	12.7727	-1.6191
YRI	NPIP	74319986	8.4508	11.2602	-0.7521
YRI	NPIP	74324986	8.8534	11.9324	-0.7805
YRI	NPIP	74329986	5.2839	11.0921	-1.577
YRI	NPIP	74334986	4.0028	8.067	-1.483
YRI	NPIP	74339986	4.1998	8.7392	-1.5387
YRI	NPIP	74344986	6.2622	13.1089	-1.5882
YRI	NPIP	74349986	6.4351	14.4534	-1.6954
YRI	NPIP	74354986	6.7397	13.613	-1.5384
YRI	NPIP	74359986	9.5812	15.7978	-1.2077
YRI	NPIP	74364986	8.2671	14.6214	-1.3289
YRI	NPIP	74369986	7.5252	15.1256	-1.539
YRI	NPIP	74374986	8.6109	15.4617	-1.3585
YRI	NPIP	74379986	7.2692	12.6047	-1.2844
YRI	NPIP	74384986	8.7914	15.1256	-1.2826
YRI	NPIP	74389986	11.0284	16.134	-0.9721
YRI	NPIP	74394986	9.3453	12.7727	-0.8148
YRI	NPIP	74399986	6.0806	10.2518	-1.2191
YRI	NPIP	74404986	6.0718	12.6047	-1.5727
YRI	NPIP	74409986	5.4688	10.756	-1.4775
YRI	NPIP	74414986	3.6084	5.0419	-0.7987
YRI	NPIP	74419986	3.1539	4.3696	-0.7676

YRI	NPIP	74424986	8.6277	11.5963	-0.773
YRI	NPIP	74429986	11.3303	16.302	-0.9373
YRI	NPIP	74434986	7.1507	10.756	-1.0075
YRI	NPIP	74439986	5.9532	6.8906	-0.395
YRI	NPIP	74444986	4.9507	5.8822	-0.4526
YRI	NPIP	74449986	3.6526	6.0502	-1.136
YRI	NPIP	74454986	7.1761	9.9157	-0.826
YRI	NPIP	74459986	11.5415	13.1089	-0.3636
YRI	NPIP	74464986	13.2789	14.6214	-0.2808
YRI	NPIP	74469986	18.3345	17.8146	0.09
YRI	NPIP	74474986	18.7492	18.3188	0.0726
YRI	NPIP	74479986	12.0133	14.2853	-0.4858
YRI	NPIP	74484986	8.4984	12.2685	-0.931
YRI	NPIP	74489986	8.3316	10.924	-0.714
YRI	NPIP	74494986	9.4262	12.2685	-0.7019
YRI	NPIP	74499986	10.9058	15.4617	-0.9034
YRI	NPIP	74504986	8.9771	13.613	-1.0376
YRI	NPIP	74509986	7.4965	12.4366	-1.2044
YRI	NPIP	74514986	6.8872	10.4199	-1.017
YRI	NPIP	74519986	5.6817	9.9157	-1.2766
YRI	NPIP	74524986	7.1121	12.2685	-1.2734
YRI	NPIP	74529986	8.3965	13.7811	-1.1912
YRI	NPIP	74534986	8.7194	15.7978	-1.3751
YRI	NPIP	74539986	7.1878	13.613	-1.4381
YRI	NPIP	74544986	7.07	12.9408	-1.3786
YRI	NPIP	74549986	7.6927	14.1172	-1.3892
YRI	NPIP	74554986	4.6951	10.2518	-1.6241
YRI	NPIP	74559986	5.5769	9.4115	-1.2136
YRI	NPIP	74564986	8.6935	12.6047	-0.9415
YRI	NPIP	74569986	8.3979	11.5963	-0.8329
YRI	NPIP	74574986	8.0856	11.4282	-0.8825
YRI	NPIP	74579986	10.4971	13.1089	-0.6058
YRI	NPIP	74584986	11.1215	12.2685	-0.2832
YRI	NPIP	74589986	10.1556	11.5963	-0.3752
YRI	NPIP	74594986	10.0219	11.4282	-0.3713
YRI	NPIP	74599986	6.8461	11.5963	-1.237
YRI	NPIP	74604986	4.9837	10.756	-1.6131
YRI	NPIP	74609986	6.247	10.756	-1.26
YRI	NPIP	74614986	6.7904	11.2602	-1.1966
YRI	NPIP	74619986	8.1403	13.613	-1.2249
YRI	NPIP	74624986	8.8885	14.6214	-1.1989
YRI	NPIP	74629986	8.9264	14.2853	-1.1458
YRI	NPIP	74634986	8.5197	12.6047	-0.9834
YRI	NPIP	74639986	7.229	10.924	-1.0177
YRI	NPIP	74644986	7.8766	13.445	-1.2611
YRI	NPIP	74649986	9.8425	14.9575	-1.0468
YRI	NPIP	74654986	11.3255	14.1172	-0.6036
YRI	NPIP	74659986	9.1568	10.756	-0.4469
YRI	NPIP	74664986	6.8107	10.756	-1.1025

YRI	NPIP	74669986	7.1025	13.445	-1.4364
YRI	NPIP	74674986	7.7164	12.6047	-1.1768
YRI	NPIP	74679986	6.8639	10.924	-1.1182
YRI	NPIP	74684986	5.4142	11.7644	-1.6314
YRI	NPIP	74689986	6.5511	14.1172	-1.636
YRI	NPIP	74694986	8.3519	15.1256	-1.3716
YRI	NPIP	74699986	10.8227	18.1507	-1.2464
YRI	NPIP	74704986	10.8472	16.8062	-1.0911
YRI	NPIP	74709986	10.493	13.2769	-0.638
YRI	NPIP	74714986	8.8185	11.4282	-0.689
YRI	NPIP	74719986	7.9432	12.2685	-1.0681
YRI	NPIP	74724986	7.503	12.9408	-1.2769
YRI	NPIP	74729986	7.5971	11.5963	-1.0414
YRI	NPIP	74734986	19.0463	17.6465	0.2446
YRI	NPIP	74739986	24.3557	21.3439	0.4383
YRI	NPIP	74744986	19.9905	18.6549	0.2213
YRI	NPIP	74749986	18.3056	18.991	-0.1116
YRI	NPIP	74754986	13.1455	18.4868	-0.8926
YRI	NPIP	74759986	7.616	15.1256	-1.5206
YRI	NPIP	74764986	7.5702	14.2853	-1.4358
YRI	NPIP	74769986	9.3012	14.9575	-1.1576
YRI	NPIP	74774986	8.8191	14.1172	-1.1456
YRI	NPIP	74779986	7.4521	11.4282	-1.0497
YRI	NPIP	74784986	9.9455	13.445	-0.7925
YRI	NPIP	74789986	13.8983	17.4785	-0.6314
YRI	NPIP	74794986	17.954	19.8313	-0.2932
YRI	NPIP	74799986	15.0325	17.9827	-0.5063
YRI	NPIP	74804986	11.7236	16.302	-0.8631
YRI	NPIP	74809986	13.115	17.1423	-0.7236
YRI	NPIP	74814986	10.4495	15.2937	-0.9706
YRI	NPIP	74819986	11.0604	16.302	-0.9882
YRI	NPIP	74824986	15.4677	17.1423	-0.3009
YRI	NPIP	74829986	15.5056	16.134	-0.1196
YRI	NPIP	74834986	11.7488	14.7895	-0.629
YRI	NPIP	74839986	10.8005	13.613	-0.6295
YRI	NPIP	74844986	11.3584	13.9492	-0.5666
YRI	NPIP	74849986	9.6951	15.2937	-1.1218
YRI	NPIP	74854986	9.8226	18.6549	-1.4632
YRI	NPIP	74859986	12.0717	21.8481	-1.3909
YRI	NPIP	74864986	13.2109	22.6884	-1.3
YRI	NPIP	74869986	17.8602	24.5371	-0.8489
YRI	NPIP	74874986	37.7794	31.9318	0.5754
YRI	NPIP	74879986	40.3051	31.4276	0.8871
YRI	NPIP	74884986	33.4465	28.0664	0.6003
CEU	PGA3	60708515	8.6547	6.9931	0.6952
CEU	PGA3	60713515	7.3137	8.8692	-0.5234
CEU	PGA3	60718515	6.4123	9.2104	-0.9092
CEU	PGA3	60723515	6.2296	8.0164	-0.66
CEU	PGA3	60728515	6.2204	8.8692	-0.8913

CEU	PGA3	60733515	5.61	8.187	-0.9336
CEU	PGA3	60738515	3.8901	6.4814	-1.1615
CEU	PGA3	60743515	6.2233	8.8692	-0.8904
CEU	PGA3	60748515	9.5696	11.0866	-0.4147
CEU	PGA3	60753515	13.3457	11.7688	0.4075
CEU	PGA3	60758515	13.1787	11.4277	0.4652
CEU	PGA3	60763515	12.0542	11.0866	0.2645
CEU	PGA3	60768515	14.7895	12.9627	0.431
CEU	PGA3	60773515	14.4477	13.1333	0.3063
CEU	PGA3	60778515	13.0278	13.9861	-0.2104
CEU	PGA3	60783515	12.1119	12.9627	-0.2007
CEU	PGA3	60788515	12.0969	10.5749	0.4349
CEU	PGA3	60793515	11.1121	10.916	0.0544
CEU	PGA3	60798515	6.4714	8.3576	-0.6705
CEU	PGA3	60803515	4.8141	6.1402	-0.624
CEU	PGA3	60808515	7.2031	7.3342	-0.0525
CEU	PGA3	60813515	6.6496	7.3342	-0.2743
CEU	PGA3	60818515	6.4492	9.0398	-0.8565
CEU	PGA3	60823515	10.8167	11.7688	-0.2461
CEU	PGA3	60828515	13.2952	11.5982	0.4446
CEU	PGA3	60833515	14.4234	12.6216	0.4359
CEU	PGA3	60838515	9.3072	10.0632	-0.2262
CEU	PGA3	60843515	2.9691	4.7757	-1.0625
CEU	PGA3	60848515	2.6984	3.7524	-0.7636
CEU	PGA3	60853515	5.2736	6.3108	-0.4762
CEU	PGA3	60858515	13.5477	10.7454	0.7888
CEU	PGA3	60863515	15.8369	11.5982	1.1106
CEU	PGA3	60868515	12.711	10.7454	0.5533
CEU	PGA3	60873515	9.1985	8.6987	0.1712
CEU	PGA3	60878515	5.7572	6.4814	-0.3246
CEU	PGA3	60883515	4.7625	7.1636	-0.9829
CEU	PGA3	60888515	7.3964	8.5281	-0.3949
CEU	PGA3	60893515	11.1995	10.5749	0.1785
CEU	PGA3	60898515	8.9097	9.0398	-0.043
CEU	PGA3	60903515	8.6582	7.5047	0.4526
CEU	PGA3	60908515	19.2614	12.1099	1.7991
CEU	PGA3	60913515	21.6197	13.1333	1.9774
CEU	PGA3	60918515	17.0921	10.0632	2.1035
CEU	PGA3	60923515	17.3045	10.0632	2.167
CEU	PGA3	60928515	11.1031	9.0398	0.6822
CEU	PGA3	60933515	6.2853	6.3108	-0.0117
CEU	PGA3	60938515	6.3754	4.9463	0.815
CEU	PGA3	60943515	6.2614	4.9463	0.75
CEU	PGA3	60948515	8.1093	5.7991	1.1442
CEU	PGA3	60953515	8.9355	6.6519	0.9998
CEU	PGA3	60958515	6.9621	6.4814	0.2155
CEU	PGA3	60963515	6.8273	5.7991	0.5093
CEU	PGA3	60968515	12.1507	7.8459	1.6217
CEU	PGA3	60973515	8.8182	5.458	1.7566

CEU	PGA3	60978515	1.4174	1.3645	0.0856
CEU	PGA3	60983515	0.9099	1.0234	-0.2255
CEU	PGA3	60988515	0	0	0
CEU	PGA3	60993515	0.2035	0.5117	-0.9666
CEU	PGA3	60998515	0.7062	0.8528	-0.3301
CEU	PGA3	61003515	0.5634	1.0234	-0.914
CEU	PGA3	61008515	0.1099	0.8528	-1.673
CEU	PGA3	61013515	2.8598	7.1636	-1.7618
CEU	PGA3	61018515	3.604	10.0632	-1.933
CEU	PGA3	61023515	1.169	6.1402	-2.3393
CEU	PGA3	61028515	0.556	4.9463	-2.5038
CEU	PGA3	61033515	0.5993	4.0935	-2.3496
CEU	PGA3	61038515	0.7596	6.1402	-2.532
CEU	PGA3	61043515	1.1497	7.1636	-2.4618
CEU	PGA3	61048515	1.3603	5.6286	-2.1711
CEU	PGA3	61053515	0.8532	6.1402	-2.4879
CEU	PGA3	61058515	0.913	6.3108	-2.4782
CEU	PGA3	61063515	0.907	4.0935	-2.1427
CEU	PGA3	61068515	0.6419	2.8996	-2.0304
CEU	PGA3	61073515	0.8118	3.9229	-2.1699
CEU	PGA3	61078515	1.0542	4.7757	-2.1888
CEU	PGA3	61083515	1.2686	5.1169	-2.1301
CEU	PGA3	61088515	1.4222	6.1402	-2.2202
CEU	PGA3	61093515	1.0717	6.3108	-2.4054
CEU	PGA3	61098515	0.7565	6.3108	-2.5501
CEU	PGA3	61103515	2.1647	7.8459	-2.1402
CEU	PGA3	61108515	2.3956	7.5047	-2.0046
CEU	PGA3	61113515	1.0278	6.8225	-2.4795
CEU	PGA3	61118515	0.5799	5.9697	-2.6012
CEU	PGA3	61123515	0.5479	4.4346	-2.4386
CEU	PGA3	61128515	0.6836	4.4346	-2.3535
CEU	PGA3	61133515	0.832	4.2641	-2.2279
CEU	PGA3	61138515	0.7745	5.6286	-2.4691
CEU	PGA3	61143515	1.2907	6.8225	-2.367
CEU	PGA3	61148515	1.5083	6.1402	-2.1796
CEU	PGA3	61153515	1.3651	5.1169	-2.0767
CEU	PGA3	61158515	2.357	5.1169	-1.5277
CEU	PGA3	61163515	3.2581	6.6519	-1.4859
CEU	PGA3	61168515	2.5624	5.6286	-1.5597
CEU	PGA3	61173515	1.576	3.7524	-1.5768
CEU	PGA3	61178515	2.5925	5.2874	-1.4491
CEU	PGA3	61183515	2.4865	5.458	-1.5534
CEU	PGA3	61188515	1.6329	4.2641	-1.708
CEU	PGA3	61193515	3.242	6.9931	-1.5695
CEU	PGA3	61198515	4.5675	8.6987	-1.4153
CEU	PGA3	61203515	4.3364	6.1402	-0.8488
CEU	PGA3	61208515	3.8656	6.4814	-1.1724
CEU	PGA3	61213515	3.4649	7.5047	-1.585
CEU	PGA3	61218515	3.6817	7.1636	-1.4253

CEU	PGA3	61223515	3.424	6.1402	-1.2782
CEU	PGA3	61228515	3.1227	5.7991	-1.3256
CEU	PGA3	61233515	2.9538	6.3108	-1.5413
CEU	PGA3	61238515	2.254	6.4814	-1.8948
CEU	PGA3	61243515	2.3296	5.9697	-1.7568
CEU	PGA3	61248515	2.6559	4.9463	-1.3062
CEU	PGA3	61253515	3.8266	7.8459	-1.5141
CEU	PGA3	61258515	6.665	11.2571	-1.2374
CEU	PGA3	61263515	9.4203	15.0095	-1.1473
CEU	PGA3	61268515	9.0771	14.1567	-1.1023
CEU	PGA3	61273515	7.3562	10.5749	-0.9196
CEU	PGA3	61278515	8.6852	9.7221	-0.3204
CEU	PGA3	61283515	5.867	7.8459	-0.7455
CEU	PGA3	61288515	1.6858	6.9931	-2.2207
CEU	PGA3	61293515	1.2925	4.9463	-2.0838
CEU	PGA3	61298515	0.9134	4.2641	-2.1751
CEU	PGA3	61303515	1.692	5.6286	-2.0024
CEU	PGA3	61308515	1.1754	3.7524	-1.867
CEU	PGA3	61313515	1.021	3.5818	-1.9301
CEU	PGA3	61318515	1.7862	6.8225	-2.155
CEU	PGA3	61323515	1.7314	7.3342	-2.2449
CEU	PGA3	61328515	1.4152	6.4814	-2.2707
CEU	PGA3	61333515	1.2255	5.9697	-2.2896
CEU	PGA3	61338515	1.236	5.7991	-2.2601
CEU	PGA3	61343515	0.9046	4.6052	-2.2468
CEU	PGA3	61348515	1.4537	4.7757	-1.9538
CEU	PGA3	61353515	1.9528	5.6286	-1.8697
CEU	PGA3	61358515	1.5615	4.9463	-1.9303
CEU	PGA3	61363515	1.1422	3.7524	-1.891
CEU	PGA3	61368515	1.3593	4.9463	-2.0457
CEU	PGA3	61373515	2.032	5.7991	-1.8658
CEU	PGA3	61378515	3.4587	4.2641	-0.5228
CEU	PGA3	61383515	3.9992	5.6286	-0.8288
CEU	PGA3	61388515	4.2892	7.1636	-1.1766
CEU	PGA3	61393515	4.535	7.5047	-1.1652
CEU	PGA3	61398515	5.9294	8.0164	-0.7708
CEU	PGA3	61403515	8.4595	8.5281	-0.0239
CEU	PGA3	61408515	9.7262	8.6987	0.352
CEU	PGA3	61413515	8.0625	6.9931	0.4475
CEU	PGA3	61418515	6.0625	5.458	0.316
CEU	PGA3	61423515	6.522	6.8225	-0.1286
CEU	PGA3	61428515	7.2863	7.5047	-0.0857
CEU	PGA3	61433515	7.9285	7.1636	0.3131
CEU	PGA3	61438515	10.8501	8.0164	1.0466
CEU	PGA3	61443515	10.3649	8.6987	0.5708
CEU	PGA3	61448515	6.3542	7.6753	-0.5078
CEU	PGA3	61453515	5.2826	7.1636	-0.77
CEU	PGA3	61458515	5.76	6.8225	-0.4546
CEU	PGA3	61463515	6.8235	6.8225	0.0004

CEU	PGA3	61468515	5.3364	7.8459	-0.9454
CEU	PGA3	61473515	3.388	5.6286	-1.1397
CEU	PGA3	61478515	6.1437	5.2874	0.4604
CEU	PGA3	61483515	7.3148	6.3108	0.461
CEU	PGA3	61488515	5.9615	5.6286	0.1693
CEU	PGA3	61493515	5.7186	5.7991	-0.0399
CEU	PGA3	61498515	5.3906	6.1402	-0.3527
CEU	PGA3	61503515	8.2377	7.3342	0.362
CEU	PGA3	61508515	8.3818	6.8225	0.6672
CEU	PGA3	61513515	8.1435	7.5047	0.2506
CEU	PGA3	61518515	9.892	7.5047	0.9367
CEU	PGA3	61523515	10.9336	6.4814	1.9955
CEU	PGA3	61528515	9.6467	8.5281	0.3903
CEU	PGA3	61533515	8.4603	8.0164	0.164
CEU	PGA3	61538515	7.1482	5.7991	0.6682
CEU	PGA3	61543515	4.2711	4.9463	-0.3851
CEU	PGA3	61548515	5.3931	5.1169	0.1529
CEU	PGA3	61553515	5.1159	4.9463	0.0967
CEU	PGA3	61558515	2.5898	4.0935	-1.0111
CEU	PGA3	61563515	2.4137	3.5818	-0.8804
CEU	PGA3	61568515	4.1901	3.4112	0.6118
CEU	PGA3	61573515	4.1608	3.9229	0.1659
CEU	PGA3	61578515	5.4762	5.6286	-0.0775
CEU	PGA3	61583515	5.7345	5.6286	0.0539
CEU	PGA3	61588515	3.8971	4.4346	-0.3372
CEU	PGA3	61593515	5.5349	4.9463	0.3357
CEU	PGA3	61598515	7.4354	6.3108	0.5163
CEU	PGA3	61603515	7.7285	6.9931	0.3077
CEU	PGA3	61608515	6.3852	7.1636	-0.3186
CEU	PGA3	61613515	6.823	8.0164	-0.4408
CEU	PGA3	61618515	9.6236	9.0398	0.193
CEU	PGA3	61623515	13.9765	10.0632	1.1711
CEU	PGA3	61628515	14.7194	10.2337	1.3215
CEU	PGA3	61633515	12.2156	11.0866	0.3086
CEU	PGA3	61638515	9.6966	10.0632	-0.1097
CEU	PGA3	61643515	5.7504	6.1402	-0.1834
CEU	PGA3	61648515	2.8864	3.9229	-0.7229
CEU	PGA3	61653515	7.2492	6.3108	0.4308
CEU	PGA3	61658515	8.999	8.187	0.2942
CEU	PGA3	61663515	4.7411	5.6286	-0.4514
CEU	PGA3	61668515	5.9406	5.458	0.2523
CEU	PGA3	61673515	5.9427	6.6519	-0.3105
CEU	PGA3	61678515	4.2292	5.2874	-0.569
CEU	PGA3	61683515	6.6526	7.5047	-0.3343
CEU	PGA3	61688515	10.0967	10.2337	-0.0404
CEU	PGA3	61693515	12.384	10.5749	0.5169
CEU	PGA3	61698515	12.4712	10.0632	0.7206
CEU	PGA3	61703515	11.0429	8.5281	0.8774
CHB	PGA3	60708515	7.3103	10.3343	-0.8802

CHB	PGA3	60713515	5.514	9.6567	-1.2846
CHB	PGA3	60718515	4.8034	8.8096	-1.3526
CHB	PGA3	60723515	4.6998	9.9955	-1.5902
CHB	PGA3	60728515	2.7569	9.1484	-2.0839
CHB	PGA3	60733515	2.003	7.1154	-2.0995
CHB	PGA3	60738515	1.6309	7.1154	-2.2523
CHB	PGA3	60743515	2.3384	9.1484	-2.2203
CHB	PGA3	60748515	4.1775	10.6731	-1.8346
CHB	PGA3	60753515	7.2671	11.1814	-1.0584
CHB	PGA3	60758515	8.6551	11.012	-0.6465
CHB	PGA3	60763515	9.9743	12.0285	-0.5186
CHB	PGA3	60768515	11.8722	12.7061	-0.1999
CHB	PGA3	60773515	9.8946	10.6731	-0.2199
CHB	PGA3	60778515	8.8633	11.3508	-0.6632
CHB	PGA3	60783515	7.9376	10.6731	-0.7726
CHB	PGA3	60788515	6.9623	7.1154	-0.0629
CHB	PGA3	60793515	10.1144	9.9955	0.0357
CHB	PGA3	60798515	8.4424	10.1649	-0.5092
CHB	PGA3	60803515	4.8184	7.1154	-0.9433
CHB	PGA3	60808515	5.807	7.6237	-0.7006
CHB	PGA3	60813515	4.8513	7.6237	-1.0691
CHB	PGA3	60818515	3.7639	6.946	-1.3357
CHB	PGA3	60823515	7.8637	8.979	-0.37
CHB	PGA3	60828515	9.687	11.012	-0.3634
CHB	PGA3	60833515	9.4394	12.5367	-0.752
CHB	PGA3	60838515	7.434	11.012	-0.9814
CHB	PGA3	60843515	3.9397	7.6237	-1.4207
CHB	PGA3	60848515	2.9461	7.2848	-1.744
CHB	PGA3	60853515	3.6895	8.979	-1.7547
CHB	PGA3	60858515	5.997	10.8426	-1.3486
CHB	PGA3	60863515	7.0544	10.1649	-0.9195
CHB	PGA3	60868515	7.2998	11.1814	-1.0495
CHB	PGA3	60873515	4.5188	9.6567	-1.5932
CHB	PGA3	60878515	2.225	6.7766	-1.9538
CHB	PGA3	60883515	2.1464	6.7766	-1.9876
CHB	PGA3	60888515	1.6503	8.979	-2.4312
CHB	PGA3	60893515	1.5458	10.5037	-2.5682
CHB	PGA3	60898515	1.1915	8.4708	-2.5483
CHB	PGA3	60903515	1.3192	7.4543	-2.4149
CHB	PGA3	60908515	2.4097	12.7061	-2.4683
CHB	PGA3	60913515	2.4877	14.2309	-2.5285
CHB	PGA3	60918515	1.7729	11.012	-2.5342
CHB	PGA3	60923515	1.6134	11.5202	-2.6047
CHB	PGA3	60928515	1.0464	7.6237	-2.5364
CHB	PGA3	60933515	0.8416	5.4213	-2.4015
CHB	PGA3	60938515	1.0272	6.946	-2.4845
CHB	PGA3	60943515	1.4408	7.4543	-2.3671
CHB	PGA3	60948515	1.757	7.9625	-2.2996
CHB	PGA3	60953515	1.4771	8.3013	-2.4338

CHB	PGA3	60958515	1.4186	8.3013	-2.4547
CHB	PGA3	60963515	1.2132	8.6402	-2.5529
CHB	PGA3	60968515	3.5473	10.5037	-1.9944
CHB	PGA3	60973515	3.2199	5.9295	-1.3118
CHB	PGA3	60978515	0.2699	1.0165	-1.4858
CHB	PGA3	60983515	0.1057	1.1859	-1.9281
CHB	PGA3	60988515	0.0194	0.3388	-1.2812
CHB	PGA3	60993515	0.261	1.0165	-1.5035
CHB	PGA3	60998515	0.7651	1.1859	-0.7511
CHB	PGA3	61003515	0.533	0.5082	0.0779
CHB	PGA3	61008515	0.2829	0.6777	-1.0321
CHB	PGA3	61013515	3.0699	10.5037	-2.1312
CHB	PGA3	61018515	3.9265	13.5532	-2.171
CHB	PGA3	61023515	1.4437	5.5907	-2.1159
CHB	PGA3	61028515	0.909	4.913	-2.2903
CHB	PGA3	61033515	1.0815	5.4213	-2.2757
CHB	PGA3	61038515	1.1692	6.6072	-2.3884
CHB	PGA3	61043515	1.5262	8.4708	-2.4311
CHB	PGA3	61048515	1.1298	6.6072	-2.4057
CHB	PGA3	61053515	0.8454	6.0989	-2.4799
CHB	PGA3	61058515	1.2309	7.6237	-2.4652
CHB	PGA3	61063515	1.4639	6.4378	-2.2364
CHB	PGA3	61068515	1.2096	5.2519	-2.1801
CHB	PGA3	61073515	0.9924	5.4213	-2.3224
CHB	PGA3	61078515	1.2865	7.1154	-2.3938
CHB	PGA3	61083515	1.8528	8.979	-2.364
CHB	PGA3	61088515	1.8814	7.4543	-2.1936
CHB	PGA3	61093515	1.7196	7.6237	-2.2768
CHB	PGA3	61098515	1.4799	7.6237	-2.3692
CHB	PGA3	61103515	1.6794	7.6237	-2.2923
CHB	PGA3	61108515	2.0761	7.6237	-2.1393
CHB	PGA3	61113515	1.732	5.2519	-1.8984
CHB	PGA3	61118515	1.5517	6.4378	-2.197
CHB	PGA3	61123515	1.0603	6.946	-2.4706
CHB	PGA3	61128515	0.6223	5.7601	-2.5526
CHB	PGA3	61133515	0.5681	6.2684	-2.6254
CHB	PGA3	61138515	1.2109	7.1154	-2.4248
CHB	PGA3	61143515	2.0668	5.9295	-1.87
CHB	PGA3	61148515	1.5351	5.5907	-2.0693
CHB	PGA3	61153515	0.9725	5.4213	-2.3328
CHB	PGA3	61158515	1.3108	5.2519	-2.1256
CHB	PGA3	61163515	1.5989	7.4543	-2.3048
CHB	PGA3	61168515	1.3215	7.6237	-2.4303
CHB	PGA3	61173515	0.9869	5.5907	-2.349
CHB	PGA3	61178515	1.2732	4.913	-2.082
CHB	PGA3	61183515	1.0769	4.7436	-2.1629
CHB	PGA3	61188515	0.9985	4.913	-2.2391
CHB	PGA3	61193515	1.8051	6.7766	-2.1341
CHB	PGA3	61198515	2.1839	9.1484	-2.2707

CHB	PGA3	61203515	2.0751	8.6402	-2.2567
CHB	PGA3	61208515	1.9161	6.946	-2.1114
CHB	PGA3	61213515	1.6457	7.1154	-2.2462
CHB	PGA3	61218515	1.596	7.9625	-2.3592
CHB	PGA3	61223515	2.3415	7.7931	-2.0604
CHB	PGA3	61228515	2.4422	7.1154	-1.9191
CHB	PGA3	61233515	1.3775	6.2684	-2.2526
CHB	PGA3	61238515	1.1213	6.946	-2.445
CHB	PGA3	61243515	1.1392	7.1154	-2.4542
CHB	PGA3	61248515	1.2128	8.1319	-2.5149
CHB	PGA3	61253515	1.5989	10.8426	-2.5726
CHB	PGA3	61258515	2.5311	12.1979	-2.4084
CHB	PGA3	61263515	3.4384	14.2309	-2.3238
CHB	PGA3	61268515	4.7657	12.1979	-1.8517
CHB	PGA3	61273515	6.21	9.3178	-0.9962
CHB	PGA3	61278515	9.0872	9.3178	-0.0739
CHB	PGA3	61283515	6.7093	7.7931	-0.4096
CHB	PGA3	61288515	1.7805	8.1319	-2.3086
CHB	PGA3	61293515	1.3946	7.4543	-2.3853
CHB	PGA3	61298515	0.7967	6.4378	-2.5364
CHB	PGA3	61303515	2.325	7.6237	-2.0433
CHB	PGA3	61308515	2.1513	6.2684	-1.8962
CHB	PGA3	61313515	1.3181	5.0825	-2.0899
CHB	PGA3	61318515	1.3953	5.7601	-2.1685
CHB	PGA3	61323515	2.0366	6.7766	-2.0347
CHB	PGA3	61328515	2.2545	6.2684	-1.8487
CHB	PGA3	61333515	0.9487	6.6072	-2.4853
CHB	PGA3	61338515	1.2363	8.6402	-2.545
CHB	PGA3	61343515	1.1133	7.2848	-2.4807
CHB	PGA3	61348515	1.1275	6.4378	-2.3877
CHB	PGA3	61353515	1.822	7.1154	-2.1738
CHB	PGA3	61358515	1.4843	6.6072	-2.25
CHB	PGA3	61363515	0.7215	5.4213	-2.4644
CHB	PGA3	61368515	1.2024	5.2519	-2.184
CHB	PGA3	61373515	1.4232	7.1154	-2.3376
CHB	PGA3	61378515	1.853	7.4543	-2.2048
CHB	PGA3	61383515	2.3059	5.7601	-1.7161
CHB	PGA3	61388515	3.9191	8.3013	-1.5629
CHB	PGA3	61393515	4.6806	10.3343	-1.6457
CHB	PGA3	61398515	3.9756	9.3178	-1.7124
CHB	PGA3	61403515	3.5926	8.979	-1.7868
CHB	PGA3	61408515	3.301	8.6402	-1.8353
CHB	PGA3	61413515	2.6918	7.1154	-1.8166
CHB	PGA3	61418515	1.7984	5.4213	-1.8997
CHB	PGA3	61423515	1.8896	5.7601	-1.923
CHB	PGA3	61428515	2.5191	7.1154	-1.8876
CHB	PGA3	61433515	4.3781	8.1319	-1.3644
CHB	PGA3	61438515	8.8715	8.6402	0.0795
CHB	PGA3	61443515	9.5123	8.8096	0.2373

CHB	PGA3	61448515	6.9341	6.946	-0.005
CHB	PGA3	61453515	6.7331	6.0989	0.2994
CHB	PGA3	61458515	5.8496	6.4378	-0.2645
CHB	PGA3	61463515	5.7687	6.0989	-0.1559
CHB	PGA3	61468515	4.6958	6.946	-0.9445
CHB	PGA3	61473515	3.3455	6.6072	-1.4326
CHB	PGA3	61478515	5.6497	6.7766	-0.4837
CHB	PGA3	61483515	6.5997	7.4543	-0.3364
CHB	PGA3	61488515	5.9271	7.1154	-0.488
CHB	PGA3	61493515	6.044	6.6072	-0.2474
CHB	PGA3	61498515	5.2859	6.2684	-0.4525
CHB	PGA3	61503515	6.616	7.6237	-0.3886
CHB	PGA3	61508515	6.7099	7.6237	-0.3524
CHB	PGA3	61513515	6.5089	7.6237	-0.4299
CHB	PGA3	61518515	8.5035	7.4543	0.413
CHB	PGA3	61523515	10.3998	8.4708	0.6753
CHB	PGA3	61528515	8.676	8.8096	-0.0451
CHB	PGA3	61533515	4.2526	6.2684	-0.9284
CHB	PGA3	61538515	3.0628	6.2684	-1.4764
CHB	PGA3	61543515	3.1719	5.9295	-1.335
CHB	PGA3	61548515	4.2833	4.5742	-0.1771
CHB	PGA3	61553515	3.8422	3.8965	-0.038
CHB	PGA3	61558515	1.6659	3.0495	-1.1899
CHB	PGA3	61563515	2.114	3.3883	-1.0035
CHB	PGA3	61568515	4.3246	4.066	0.1744
CHB	PGA3	61573515	4.2193	4.4048	-0.1167
CHB	PGA3	61578515	6.0255	4.7436	0.7562
CHB	PGA3	61583515	6.071	4.2354	1.195
CHB	PGA3	61588515	3.9751	4.2354	-0.1695
CHB	PGA3	61593515	5.7658	4.913	0.4878
CHB	PGA3	61598515	7.612	5.7601	0.9201
CHB	PGA3	61603515	7.8348	6.6072	0.5392
CHB	PGA3	61608515	5.7228	6.946	-0.5135
CHB	PGA3	61613515	5.2126	9.3178	-1.3159
CHB	PGA3	61618515	7.7093	10.5037	-0.8011
CHB	PGA3	61623515	10.2444	9.9955	0.0747
CHB	PGA3	61628515	10.9282	13.5532	-0.592
CHB	PGA3	61633515	8.1841	14.5697	-1.3445
CHB	PGA3	61638515	5.4186	11.012	-1.5342
CHB	PGA3	61643515	4.514	7.9625	-1.2779
CHB	PGA3	61648515	3.0308	6.6072	-1.5708
CHB	PGA3	61653515	8.3548	8.979	-0.2071
CHB	PGA3	61658515	9.9079	10.1649	-0.076
CHB	PGA3	61663515	4.3986	8.3013	-1.3919
CHB	PGA3	61668515	4.186	8.4708	-1.5
CHB	PGA3	61673515	2.8264	7.4543	-1.8217
CHB	PGA3	61678515	2.1639	5.5907	-1.7484
CHB	PGA3	61683515	4.3179	7.9625	-1.3506
CHB	PGA3	61688515	5.9472	10.8426	-1.3624

CHB	PGA3	61693515	7.3791	11.1814	-1.0281
CHB	PGA3	61698515	8.7251	9.6567	-0.2889
CHB	PGA3	61703515	9.9732	9.9955	-0.0067
YRI	PGA3	60708515	11.756	12.1005	-0.0862
YRI	PGA3	60713515	10.522	13.1089	-0.6001
YRI	PGA3	60718515	10.3185	14.6214	-0.8999
YRI	PGA3	60723515	10.1462	13.1089	-0.6872
YRI	PGA3	60728515	9.47	10.924	-0.4005
YRI	PGA3	60733515	7.8566	9.5795	-0.5364
YRI	PGA3	60738515	5.9684	10.4199	-1.2814
YRI	PGA3	60743515	7.037	12.7727	-1.3636
YRI	PGA3	60748515	10.6345	14.4534	-0.8075
YRI	PGA3	60753515	13.4532	15.2937	-0.3688
YRI	PGA3	60758515	11.993	16.302	-0.8123
YRI	PGA3	60763515	11.2693	14.4534	-0.6733
YRI	PGA3	60768515	13.9426	13.9492	-0.0014
YRI	PGA3	60773515	11.5141	14.4534	-0.6215
YRI	PGA3	60778515	10.7075	14.1172	-0.7373
YRI	PGA3	60783515	13.151	14.2853	-0.2425
YRI	PGA3	60788515	11.5345	11.7644	-0.0591
YRI	PGA3	60793515	14.1742	14.1172	0.0123
YRI	PGA3	60798515	11.1631	13.7811	-0.5792
YRI	PGA3	60803515	6.8372	11.4282	-1.212
YRI	PGA3	60808515	9.3601	13.1089	-0.8696
YRI	PGA3	60813515	8.2607	12.9408	-1.099
YRI	PGA3	60818515	6.8087	12.7727	-1.4179
YRI	PGA3	60823515	11.3249	17.1423	-1.0452
YRI	PGA3	60828515	16.3203	19.4952	-0.5042
YRI	PGA3	60833515	17.5343	17.9827	-0.077
YRI	PGA3	60838515	14.5307	15.2937	-0.1529
YRI	PGA3	60843515	10.8137	13.1089	-0.5324
YRI	PGA3	60848515	9.3525	11.7644	-0.6196
YRI	PGA3	60853515	12.0952	12.1005	-0.0013
YRI	PGA3	60858515	17.0274	14.7895	0.463
YRI	PGA3	60863515	17.6577	15.9659	0.3254
YRI	PGA3	60868515	17.5191	17.1423	0.0677
YRI	PGA3	60873515	13.9897	14.2853	-0.0632
YRI	PGA3	60878515	9.931	10.924	-0.2735
YRI	PGA3	60883515	10.0185	10.756	-0.2061
YRI	PGA3	60888515	12.544	13.445	-0.2041
YRI	PGA3	60893515	13.2059	15.2937	-0.4183
YRI	PGA3	60898515	9.3751	12.4366	-0.7464
YRI	PGA3	60903515	9.7182	12.6047	-0.6949
YRI	PGA3	60908515	16.4966	17.3104	-0.1449
YRI	PGA3	60913515	14.121	16.8062	-0.4917
YRI	PGA3	60918515	9.5646	13.445	-0.8788
YRI	PGA3	60923515	11.1499	13.1089	-0.4544
YRI	PGA3	60928515	7.6058	12.9408	-1.2527
YRI	PGA3	60933515	5.7045	11.5963	-1.5343

YRI	PGA3	60938515	5.4938	9.4115	-1.2399
YRI	PGA3	60943515	7.5241	9.7476	-0.6811
YRI	PGA3	60948515	10.0532	10.0837	-0.0091
YRI	PGA3	60953515	10.7983	9.9157	0.2661
YRI	PGA3	60958515	10.776	11.9324	-0.2931
YRI	PGA3	60963515	10.061	15.2937	-1.0485
YRI	PGA3	60968515	13.2417	18.6549	-0.8968
YRI	PGA3	60973515	8.1695	10.4199	-0.6478
YRI	PGA3	60978515	1.7296	1.5126	0.3231
YRI	PGA3	60983515	1.3012	1.5126	-0.3148
YRI	PGA3	60988515	0.0639	0.3361	-1.0928
YRI	PGA3	60993515	2.0659	1.1764	1.5909
YRI	PGA3	60998515	2.5691	1.3445	1.989
YRI	PGA3	61003515	0.5677	0.8403	-0.6157
YRI	PGA3	61008515	0.8133	1.0084	-0.389
YRI	PGA3	61013515	9.2065	12.4366	-0.7875
YRI	PGA3	61018515	12.769	18.991	-1.0132
YRI	PGA3	61023515	5.8698	10.4199	-1.3098
YRI	PGA3	61028515	6.1365	9.9157	-1.1394
YRI	PGA3	61033515	6.9167	11.7644	-1.2454
YRI	PGA3	61038515	5.8344	11.5963	-1.5004
YRI	PGA3	61043515	8.1128	12.1005	-0.9976
YRI	PGA3	61048515	6.1597	8.7392	-0.8744
YRI	PGA3	61053515	4.9339	7.7309	-1.0613
YRI	PGA3	61058515	6.5263	10.5879	-1.1519
YRI	PGA3	61063515	5.678	9.5795	-1.2147
YRI	PGA3	61068515	5.3421	7.8989	-0.9512
YRI	PGA3	61073515	6.8772	10.0837	-0.9517
YRI	PGA3	61078515	7.5029	12.4366	-1.2028
YRI	PGA3	61083515	9.8426	12.4366	-0.6324
YRI	PGA3	61088515	9.3849	10.756	-0.3832
YRI	PGA3	61093515	6.5687	10.5879	-1.1398
YRI	PGA3	61098515	6.5975	9.7476	-0.965
YRI	PGA3	61103515	7.9911	10.756	-0.7726
YRI	PGA3	61108515	9.1105	11.4282	-0.6119
YRI	PGA3	61113515	7.4235	9.9157	-0.7514
YRI	PGA3	61118515	7.3043	10.924	-0.9969
YRI	PGA3	61123515	6.2565	8.9073	-0.8829
YRI	PGA3	61128515	4.617	7.5628	-1.1404
YRI	PGA3	61133515	5.0039	10.4199	-1.5591
YRI	PGA3	61138515	7.393	12.2685	-1.204
YRI	PGA3	61143515	11.0485	11.2602	-0.0567
YRI	PGA3	61148515	9.6031	9.0754	0.1727
YRI	PGA3	61153515	6.5897	8.9073	-0.7719
YRI	PGA3	61158515	6.0326	9.4115	-1.0694
YRI	PGA3	61163515	7.3422	8.5712	-0.4241
YRI	PGA3	61168515	6.7653	8.4031	-0.5756
YRI	PGA3	61173515	5.4255	8.235	-1.006
YRI	PGA3	61178515	6.2511	8.9073	-0.8847

YRI	PGA3	61183515	6.0109	8.235	-0.7964
YRI	PGA3	61188515	5.9105	7.5628	-0.6397
YRI	PGA3	61193515	7.3626	10.5879	-0.9147
YRI	PGA3	61198515	10.3133	11.5963	-0.3341
YRI	PGA3	61203515	10.2362	10.5879	-0.0997
YRI	PGA3	61208515	8.2354	10.756	-0.7044
YRI	PGA3	61213515	8.8319	11.4282	-0.6854
YRI	PGA3	61218515	9.7689	10.4199	-0.1874
YRI	PGA3	61223515	8.0787	9.0754	-0.3263
YRI	PGA3	61228515	8.9822	9.9157	-0.2815
YRI	PGA3	61233515	9.1779	10.2518	-0.3139
YRI	PGA3	61238515	7.8481	10.0837	-0.6636
YRI	PGA3	61243515	7.3564	10.0837	-0.8095
YRI	PGA3	61248515	9.5588	11.7644	-0.5666
YRI	PGA3	61253515	14.0233	16.134	-0.4019
YRI	PGA3	61258515	12.8411	18.4868	-0.9435
YRI	PGA3	61263515	12.4446	20.5036	-1.219
YRI	PGA3	61268515	11.5417	18.3188	-1.1425
YRI	PGA3	61273515	7.3987	11.9324	-1.1493
YRI	PGA3	61278515	9.1636	12.1005	-0.7348
YRI	PGA3	61283515	11.2828	13.7811	-0.5527
YRI	PGA3	61288515	8.5085	11.5963	-0.8041
YRI	PGA3	61293515	5.1452	9.5795	-1.3805
YRI	PGA3	61298515	5.2749	11.2602	-1.6023
YRI	PGA3	61303515	6.4287	10.756	-1.2093
YRI	PGA3	61308515	4.6368	7.5628	-1.1327
YRI	PGA3	61313515	3.9888	7.7309	-1.4198
YRI	PGA3	61318515	4.5682	11.2602	-1.7915
YRI	PGA3	61323515	7.2876	12.7727	-1.304
YRI	PGA3	61328515	7.9432	11.4282	-0.9201
YRI	PGA3	61333515	6.4016	9.0754	-0.8753
YRI	PGA3	61338515	8.2848	10.5879	-0.6531
YRI	PGA3	61343515	8.3519	11.9324	-0.9077
YRI	PGA3	61348515	8.6195	10.756	-0.597
YRI	PGA3	61353515	9.6734	11.9324	-0.5727
YRI	PGA3	61358515	6.7796	11.2602	-1.1995
YRI	PGA3	61363515	4.9195	11.0921	-1.6759
YRI	PGA3	61368515	4.7769	12.1005	-1.8322
YRI	PGA3	61373515	6.0251	12.2685	-1.5418
YRI	PGA3	61378515	6.8782	10.756	-1.0836
YRI	PGA3	61383515	5.8118	9.4115	-1.1393
YRI	PGA3	61388515	10.153	13.1089	-0.6857
YRI	PGA3	61393515	14.1278	17.3104	-0.5665
YRI	PGA3	61398515	13.511	15.7978	-0.4442
YRI	PGA3	61403515	11.5395	14.7895	-0.6723
YRI	PGA3	61408515	11.698	16.134	-0.8446
YRI	PGA3	61413515	11.2791	14.1172	-0.6137
YRI	PGA3	61418515	7.0138	11.2602	-1.1368
YRI	PGA3	61423515	5.7427	11.7644	-1.547

YRI	PGA3	61428515	7.4425	11.9324	-1.1382
YRI	PGA3	61433515	8.3024	11.2602	-0.7918
YRI	PGA3	61438515	8.1181	11.0921	-0.8075
YRI	PGA3	61443515	7.2222	9.5795	-0.7339
YRI	PGA3	61448515	5.2002	8.235	-1.0866
YRI	PGA3	61453515	5.0511	9.4115	-1.38
YRI	PGA3	61458515	6.5818	10.924	-1.1959
YRI	PGA3	61463515	7.1822	11.9324	-1.2042
YRI	PGA3	61468515	7.0191	11.4282	-1.164
YRI	PGA3	61473515	5.3137	8.9073	-1.1969
YRI	PGA3	61478515	7.8894	10.756	-0.8011
YRI	PGA3	61483515	8.9631	11.0921	-0.578
YRI	PGA3	61488515	6.1009	9.5795	-1.083
YRI	PGA3	61493515	5.8962	9.9157	-1.2119
YRI	PGA3	61498515	5.8081	8.9073	-1.0322
YRI	PGA3	61503515	6.8211	9.9157	-0.933
YRI	PGA3	61508515	7.0061	12.6047	-1.3477
YRI	PGA3	61513515	6.5666	12.1005	-1.3845
YRI	PGA3	61518515	7.9504	9.5795	-0.5072
YRI	PGA3	61523515	11.4907	11.2602	0.0617
YRI	PGA3	61528515	10.6404	13.445	-0.6352
YRI	PGA3	61533515	7.8242	11.7644	-1.0123
YRI	PGA3	61538515	6.6895	9.9157	-0.9727
YRI	PGA3	61543515	3.459	7.7309	-1.6209
YRI	PGA3	61548515	2.2758	5.7141	-1.7145
YRI	PGA3	61553515	2.5057	5.7141	-1.5999
YRI	PGA3	61558515	1.5621	5.7141	-2.0704
YRI	PGA3	61563515	1.6504	7.0586	-2.2295
YRI	PGA3	61568515	2.1968	6.8906	-1.9778
YRI	PGA3	61573515	2.0197	5.5461	-1.8058
YRI	PGA3	61578515	2.2922	7.3947	-2.0163
YRI	PGA3	61583515	2.4464	8.5712	-2.1137
YRI	PGA3	61588515	3.0484	8.7392	-1.929
YRI	PGA3	61593515	3.9476	9.2434	-1.7043
YRI	PGA3	61598515	5.3895	11.4282	-1.5942
YRI	PGA3	61603515	8.4283	13.445	-1.1361
YRI	PGA3	61608515	8.4499	13.7811	-1.1794
YRI	PGA3	61613515	11.293	14.4534	-0.6683
YRI	PGA3	61618515	16.6238	15.4617	0.2304
YRI	PGA3	61623515	17.8977	18.823	-0.152
YRI	PGA3	61628515	18.1067	21.8481	-0.5323
YRI	PGA3	61633515	15.0877	22.8565	-1.0581
YRI	PGA3	61638515	13.0686	19.4952	-1.0205
YRI	PGA3	61643515	11.5995	14.7895	-0.6599
YRI	PGA3	61648515	8.499	13.613	-1.1446
YRI	PGA3	61653515	11.2342	13.445	-0.5007
YRI	PGA3	61658515	12.6212	15.9659	-0.6432
YRI	PGA3	61663515	8.6626	15.2937	-1.3287
YRI	PGA3	61668515	7.8969	13.1089	-1.209

YRI	PGA3	61673515	6.6765	11.5963	-1.2811
YRI	PGA3	61678515	5.216	9.5795	-1.3585
YRI	PGA3	61683515	7.3688	12.1005	-1.1838
YRI	PGA3	61688515	10.1889	14.7895	-0.9517
YRI	PGA3	61693515	12.271	15.2937	-0.6057
YRI	PGA3	61698515	12.7569	15.6298	-0.5638
YRI	PGA3	61703515	11.1682	13.7811	-0.5781
CEU	PRAMEF14	12846892	9.7893	11.5982	-0.4739
CEU	PRAMEF14	12851892	16.7456	18.7619	-0.3343
CEU	PRAMEF14	12856892	21.6483	19.6147	0.323
CEU	PRAMEF14	12861892	20.839	28.3134	-0.8321
CEU	PRAMEF14	12866892	29.6277	59.1852	-1.5961
CEU	PRAMEF14	12871892	26.4409	46.9047	-1.3897
CEU	PRAMEF14	12876892	14.3539	14.3272	0.0057
CEU	PRAMEF14	12881892	17.9588	13.8156	0.9202
CEU	PRAMEF14	12886892	22.727	16.374	1.2001
CEU	PRAMEF14	12891892	19.4835	15.6917	0.746
CEU	PRAMEF14	12896892	21.6038	24.7316	-0.3971
CEU	PRAMEF14	12901892	16.9388	22.6848	-0.7932
CEU	PRAMEF14	12906892	9.1203	15.0095	-1.2088
CEU	PRAMEF14	12911892	12.5656	19.9558	-1.1546
CEU	PRAMEF14	12916892	19.9852	27.6311	-0.8716
CEU	PRAMEF14	12921892	18.9324	24.7316	-0.7363
CEU	PRAMEF14	12926892	7.295	10.2337	-0.8658
CEU	PRAMEF14	12931892	6.7001	7.8459	-0.4316
CEU	PRAMEF14	12936892	11.7546	12.9627	-0.285
CEU	PRAMEF14	12941892	16.0483	17.9091	-0.3226
CEU	PRAMEF14	12946892	11.6223	12.4511	-0.2031
CEU	PRAMEF14	12951892	5.6698	9.8926	-1.284
CEU	PRAMEF14	12956892	4.4224	8.0164	-1.3275
CEU	PRAMEF14	12961892	2.9054	2.8996	0.0052
CEU	PRAMEF14	12966892	1.0027	1.1939	-0.3407
CEU	PRAMEF14	12971892	0.2263	0.5117	-0.8951
CEU	PRAMEF14	12976892	0.5964	1.7056	-1.5192
CEU	PRAMEF14	12981892	2.5364	6.1402	-1.6958
CEU	PRAMEF14	12986892	4.0317	6.3108	-1.0464
CEU	PRAMEF14	12991892	3.3587	4.4346	-0.6751
CEU	PRAMEF14	12996892	3.4875	6.3108	-1.2962
CEU	PRAMEF14	13001892	2.3078	3.9229	-1.1265
CEU	PRAMEF14	13006892	1.3344	2.2173	-0.9864
CEU	PRAMEF14	13011892	4.8369	6.1402	-0.6133
CEU	PRAMEF14	13016892	3.7982	5.6286	-0.9311
CEU	PRAMEF14	13021892	0.3577	1.7056	-1.8461
CEU	PRAMEF14	13026892	0.1796	0.6822	-1.3124
CEU	PRAMEF14	13031892	0.3756	0.6822	-0.8006
CEU	PRAMEF14	13036892	3.7852	2.3879	1.4715
CEU	PRAMEF14	13041892	3.4197	1.8762	1.9651
CEU	PRAMEF14	13046892	4.7734	7.3342	-1.026
CEU	PRAMEF14	13051892	5.8868	8.187	-0.8333

CEU	PRAMEF14	13056892	1.1134	0.8528	0.5869
CEU	PRAMEF14	13061892	0	0	0
CEU	PRAMEF14	13066892	0	0	0
CEU	PRAMEF14	13071892	0	0	0
CEU	PRAMEF14	13076892	0	0	0
CEU	PRAMEF14	13081892	0	0	0
CEU	PRAMEF14	13086892	0	0	0
CEU	PRAMEF14	13091892	0	0	0
CEU	PRAMEF14	13096892	0	0	0
CEU	PRAMEF14	13101892	0	0	0
CEU	PRAMEF14	13106892	1.242	1.1939	0.0857
CEU	PRAMEF14	13111892	1.242	1.1939	0.0857
CEU	PRAMEF14	13116892	0.9544	2.3879	-1.5096
CEU	PRAMEF14	13121892	7.2367	10.916	-1.0205
CEU	PRAMEF14	13126892	13.6203	16.8857	-0.599
CEU	PRAMEF14	13131892	11.41	11.0866	0.0884
CEU	PRAMEF14	13136892	8.1909	5.1169	1.7015
CEU	PRAMEF14	13141892	5.6034	4.7757	0.4868
CEU	PRAMEF14	13146892	2.4653	4.6052	-1.2992
CEU	PRAMEF14	13151892	1.5003	2.5584	-1.054
CEU	PRAMEF14	13156892	1.3117	2.2173	-1.0117
CEU	PRAMEF14	13161892	1.6731	2.8996	-1.103
CEU	PRAMEF14	13166892	1.9946	2.0467	-0.062
CEU	PRAMEF14	13171892	7.0289	7.8459	-0.3078
CEU	PRAMEF14	13176892	14.4067	19.103	-0.7652
CEU	PRAMEF14	13181892	15.94	19.103	-0.5154
CEU	PRAMEF14	13186892	13.5123	12.1099	0.3528
CEU	PRAMEF14	13191892	6.678	5.9697	0.3418
CEU	PRAMEF14	13196892	0.9194	2.3879	-1.5465
CEU	PRAMEF14	13201892	2.1886	3.2407	-0.8627
CEU	PRAMEF14	13206892	2.1008	1.8762	0.286
CEU	PRAMEF14	13211892	1.3088	2.0467	-0.878
CEU	PRAMEF14	13216892	1.6945	2.8996	-1.0838
CEU	PRAMEF14	13221892	0.5931	1.0234	-0.855
CEU	PRAMEF14	13226892	0	0	0
CEU	PRAMEF14	13231892	0	0	0
CEU	PRAMEF14	13236892	0	0	0
CEU	PRAMEF14	13241892	0	0	0
CEU	PRAMEF14	13246892	0	0	0
CEU	PRAMEF14	13251892	0	0	0
CEU	PRAMEF14	13256892	0	0	0
CEU	PRAMEF14	13261892	0	0	0
CEU	PRAMEF14	13266892	0	0	0
CEU	PRAMEF14	13271892	0	0	0
CEU	PRAMEF14	13276892	0	0	0
CEU	PRAMEF14	13281892	0	0	0
CEU	PRAMEF14	13286892	0	0	0
CEU	PRAMEF14	13291892	0	0	0
CEU	PRAMEF14	13296892	0	0	0

CEU	PRAMEF14	13301892	0	0	0
CEU	PRAMEF14	13306892	0	0	0
CEU	PRAMEF14	13311892	0	0	0
CEU	PRAMEF14	13316892	0	0	0
CEU	PRAMEF14	13321892	0	0	0
CEU	PRAMEF14	13326892	0.6654	2.0467	-1.6436
CEU	PRAMEF14	13331892	0.6654	2.5584	-1.8857
CEU	PRAMEF14	13336892	0.0101	0.6822	-1.7551
CEU	PRAMEF14	13341892	0.7172	1.0234	-0.6084
CEU	PRAMEF14	13346892	1.1989	1.7056	-0.694
CEU	PRAMEF14	13351892	4.0612	7.3342	-1.3114
CEU	PRAMEF14	13356892	3.7149	6.8225	-1.3297
CEU	PRAMEF14	13361892	1.4242	1.1939	0.4104
CEU	PRAMEF14	13366892	1.5366	1.0234	1.0198
CEU	PRAMEF14	13371892	3.1179	1.3645	2.8366
CEU	PRAMEF14	13376892	10.3927	5.7991	2.2752
CEU	PRAMEF14	13381892	14.0944	9.8926	1.2776
CEU	PRAMEF14	13386892	6.5617	5.2874	0.6852
CEU	PRAMEF14	13391892	0	0	0
CEU	PRAMEF14	13396892	0	0	0
CEU	PRAMEF14	13401892	0	0	0
CEU	PRAMEF14	13406892	0	0	0
CEU	PRAMEF14	13411892	2.965	1.8762	1.3862
CEU	PRAMEF14	13416892	2.965	1.8762	1.3862
CEU	PRAMEF14	13421892	0.4877	0.3411	0.5877
CEU	PRAMEF14	13426892	0.5659	0.5117	0.17
CEU	PRAMEF14	13431892	0.0781	0.1706	-0.5495
CEU	PRAMEF14	13436892	0	0	0
CEU	PRAMEF14	13441892	0	0	0
CEU	PRAMEF14	13446892	1.3636	1.1939	0.3024
CEU	PRAMEF14	13451892	1.9771	1.8762	0.1285
CEU	PRAMEF14	13456892	0.6135	0.6822	-0.1794
CEU	PRAMEF14	13461892	1.3686	1.1939	0.3113
CEU	PRAMEF14	13466892	1.9272	1.5351	0.5814
CEU	PRAMEF14	13471892	1.0845	1.7056	-0.8507
CEU	PRAMEF14	13476892	3.569	3.0701	0.4279
CEU	PRAMEF14	13481892	5.5236	4.4346	0.6833
CEU	PRAMEF14	13486892	5.455	5.6286	-0.0883
CEU	PRAMEF14	13491892	3.283	3.2407	0.0347
CEU	PRAMEF14	13496892	2.5698	2.5584	0.0114
CEU	PRAMEF14	13501892	2.9342	3.0701	-0.1166
CEU	PRAMEF14	13506892	0.7594	1.0234	-0.5246
CEU	PRAMEF14	13511892	2.0959	1.1939	1.6074
CEU	PRAMEF14	13516892	2.8738	3.9229	-0.7317
CEU	PRAMEF14	13521892	0.8644	2.8996	-1.8303
CEU	PRAMEF14	13526892	0	0	0
CEU	PRAMEF14	13531892	1.6549	1.3645	0.4698
CEU	PRAMEF14	13536892	1.6549	1.3645	0.4698
CEU	PRAMEF14	13541892	0.3004	0.1706	0.771

CEU	PRAMEF14	13546892	0.851	0.5117	1.0641
CEU	PRAMEF14	13551892	1.517	1.0234	0.9808
CEU	PRAMEF14	13556892	0.9664	0.6822	0.7421
CEU	PRAMEF14	13561892	0	0	0
CEU	PRAMEF14	13566892	0	0	0
CEU	PRAMEF14	13571892	0	0	0
CEU	PRAMEF14	13576892	0	0	0
CEU	PRAMEF14	13581892	0	0	0
CEU	PRAMEF14	13586892	0	0	0
CEU	PRAMEF14	13591892	0	0	0
CEU	PRAMEF14	13596892	0	0	0
CEU	PRAMEF14	13601892	0	0	0
CEU	PRAMEF14	13606892	0	0	0
CEU	PRAMEF14	13611892	0	0	0
CEU	PRAMEF14	13616892	0	0	0
CEU	PRAMEF14	13621892	0	0	0
CEU	PRAMEF14	13626892	0	0	0
CEU	PRAMEF14	13631892	1.4345	3.0701	-1.403
CEU	PRAMEF14	13636892	1.6079	3.4112	-1.4163
CEU	PRAMEF14	13641892	0.1934	0.5117	-0.9983
CEU	PRAMEF14	13646892	0.02	0.1706	-0.8946
CEU	PRAMEF14	13651892	0.0101	0.1706	-0.9534
CEU	PRAMEF14	13656892	0.0202	0.3411	-1.2864
CEU	PRAMEF14	13661892	0.0101	0.1706	-0.9534
CEU	PRAMEF14	13666892	1.5242	0.6822	2.1987
CEU	PRAMEF14	13671892	1.6236	1.0234	1.1926
CEU	PRAMEF14	13676892	0.0994	0.3411	-0.9689
CEU	PRAMEF14	13681892	0	0	0
CEU	PRAMEF14	13686892	0.1769	0.6822	-1.3195
CEU	PRAMEF14	13691892	1.0369	2.8996	-1.6752
CEU	PRAMEF14	13696892	0.86	2.2173	-1.5164
CEU	PRAMEF14	13701892	2.6236	3.2407	-0.506
CEU	PRAMEF14	13706892	3.7291	4.6052	-0.5319
CEU	PRAMEF14	13711892	1.9375	1.8762	0.078
CEU	PRAMEF14	13716892	3.1725	3.9229	-0.5234
CEU	PRAMEF14	13721892	2.7396	4.2641	-0.9896
CEU	PRAMEF14	13726892	0.3991	0.8528	-1.0217
CEU	PRAMEF14	13731892	2.6024	1.7056	1.2283
CEU	PRAMEF14	13736892	3.3599	2.2173	1.2765
CEU	PRAMEF14	13741892	0.7879	0.8528	-0.1462
CEU	PRAMEF14	13746892	0.0303	0.3411	-1.2459
CEU	PRAMEF14	13751892	0	0	0
CEU	PRAMEF14	13756892	0	0	0
CEU	PRAMEF14	13761892	0	0	0
CEU	PRAMEF14	13766892	0.4455	0.8528	-0.9172
CEU	PRAMEF14	13771892	2.9093	4.2641	-0.8795
CEU	PRAMEF14	13776892	4.5331	5.1169	-0.3231
CEU	PRAMEF14	13781892	15.7471	10.4043	1.5499
CEU	PRAMEF14	13786892	20.4943	14.4978	1.2722

CEU	PRAMEF14	13791892	17.2501	12.9627	1.0115
CEU	PRAMEF14	13796892	16.1666	12.6216	0.8577
CEU	PRAMEF14	13801892	12.0546	11.7688	0.0739
CEU	PRAMEF14	13806892	13.484	13.4744	0.0022
CEU	PRAMEF14	13811892	13.4484	11.9394	0.3847
CEU	PRAMEF14	13816892	12.9093	11.0866	0.4982
CEU	PRAMEF14	13821892	16.4959	13.4744	0.6872
CEU	PRAMEF14	13826892	14.7538	14.1567	0.1296
CEU	PRAMEF14	13831892	11.9268	13.9861	-0.4521
CEU	PRAMEF14	13836892	12.9052	11.7688	0.2937
CEU	PRAMEF14	13841892	11.8134	10.0632	0.5238
CHB	PRAMEF14	12846892	10.8094	12.3673	-0.3831
CHB	PRAMEF14	12851892	17.6309	19.3133	-0.2705
CHB	PRAMEF14	12856892	22.2981	21.0075	0.1913
CHB	PRAMEF14	12861892	18.8898	28.6311	-1.0696
CHB	PRAMEF14	12866892	23.5326	58.4482	-1.9035
CHB	PRAMEF14	12871892	21.4057	45.7421	-1.6892
CHB	PRAMEF14	12876892	10.9457	15.2474	-0.8674
CHB	PRAMEF14	12881892	13.1258	16.9415	-0.6957
CHB	PRAMEF14	12886892	18.11	18.6357	-0.0875
CHB	PRAMEF14	12891892	15.924	17.1109	-0.2143
CHB	PRAMEF14	12896892	31.9281	26.5982	0.6287
CHB	PRAMEF14	12901892	31.7391	23.5487	1.0874
CHB	PRAMEF14	12906892	23.6997	17.6192	1.0677
CHB	PRAMEF14	12911892	32.9104	22.7016	1.4043
CHB	PRAMEF14	12916892	32.4942	27.4452	0.5777
CHB	PRAMEF14	12921892	23.6166	23.3793	0.0317
CHB	PRAMEF14	12926892	9.3099	9.3178	-0.0025
CHB	PRAMEF14	12931892	7.3733	7.2848	0.0356
CHB	PRAMEF14	12936892	10.2543	14.2309	-0.8562
CHB	PRAMEF14	12941892	13.7144	20.4992	-1.03
CHB	PRAMEF14	12946892	9.3486	14.7391	-1.1226
CHB	PRAMEF14	12951892	4.2182	11.5202	-1.9198
CHB	PRAMEF14	12956892	4.4575	8.979	-1.4999
CHB	PRAMEF14	12961892	2.9271	3.5577	-0.4766
CHB	PRAMEF14	12966892	0.5839	2.5412	-1.9545
CHB	PRAMEF14	12971892	0.1355	1.8636	-2.2048
CHB	PRAMEF14	12976892	0.305	2.033	-2.0607
CHB	PRAMEF14	12981892	2.6423	5.7601	-1.549
CHB	PRAMEF14	12986892	3.8192	5.7601	-0.9643
CHB	PRAMEF14	12991892	2.6811	4.066	-0.9339
CHB	PRAMEF14	12996892	2.8214	5.5907	-1.413
CHB	PRAMEF14	13001892	1.6318	3.7271	-1.5222
CHB	PRAMEF14	13006892	0.9482	1.5247	-0.8565
CHB	PRAMEF14	13011892	3.2102	4.2354	-0.6674
CHB	PRAMEF14	13016892	2.4816	4.066	-1.0684
CHB	PRAMEF14	13021892	0.2654	1.1859	-1.6431
CHB	PRAMEF14	13026892	0.0748	0.3388	-1.059
CHB	PRAMEF14	13031892	0.4582	0.5082	-0.157

CHB	PRAMEF14	13036892	4.1017	2.7106	1.3181
CHB	PRAMEF14	13041892	3.6532	2.3718	1.3526
CHB	PRAMEF14	13046892	11.3802	8.979	0.7966
CHB	PRAMEF14	13051892	12.6577	10.1649	0.7369
CHB	PRAMEF14	13056892	1.2873	1.3553	-0.1102
CHB	PRAMEF14	13061892	0	0	0
CHB	PRAMEF14	13066892	0	0	0
CHB	PRAMEF14	13071892	0	0	0
CHB	PRAMEF14	13076892	0	0	0
CHB	PRAMEF14	13081892	0	0	0
CHB	PRAMEF14	13086892	0	0	0
CHB	PRAMEF14	13091892	0	0	0
CHB	PRAMEF14	13096892	0	0	0
CHB	PRAMEF14	13101892	0.0097	0.1694	-0.949
CHB	PRAMEF14	13106892	1.2136	0.6777	1.401
CHB	PRAMEF14	13111892	1.2039	0.5082	2.1839
CHB	PRAMEF14	13116892	2.2545	1.5247	1.0842
CHB	PRAMEF14	13121892	5.9431	8.8096	-0.9678
CHB	PRAMEF14	13126892	8.9218	15.2474	-1.2754
CHB	PRAMEF14	13131892	8.8064	11.5202	-0.7135
CHB	PRAMEF14	13136892	7.1949	5.7601	0.7128
CHB	PRAMEF14	13141892	6.608	4.5742	1.2384
CHB	PRAMEF14	13146892	6.9898	5.2519	0.9373
CHB	PRAMEF14	13151892	4.4987	3.2189	1.0522
CHB	PRAMEF14	13156892	1.1464	1.5247	-0.562
CHB	PRAMEF14	13161892	1.3649	2.7106	-1.2751
CHB	PRAMEF14	13166892	1.9798	2.8801	-0.8117
CHB	PRAMEF14	13171892	9.4338	8.979	0.1509
CHB	PRAMEF14	13176892	28.5302	21.3463	1.0488
CHB	PRAMEF14	13181892	29.2343	22.5322	0.9286
CHB	PRAMEF14	13186892	15.6969	14.0615	0.3562
CHB	PRAMEF14	13191892	7.3741	6.2684	0.5092
CHB	PRAMEF14	13196892	1.5797	3.0495	-1.264
CHB	PRAMEF14	13201892	2.6101	4.2354	-1.0581
CHB	PRAMEF14	13206892	1.656	2.8801	-1.1037
CHB	PRAMEF14	13211892	1.7323	2.2024	-0.5264
CHB	PRAMEF14	13216892	2.6886	3.7271	-0.7544
CHB	PRAMEF14	13221892	1.023	2.033	-1.2044
CHB	PRAMEF14	13226892	0	0	0
CHB	PRAMEF14	13231892	0	0	0
CHB	PRAMEF14	13236892	0	0	0
CHB	PRAMEF14	13241892	0	0	0
CHB	PRAMEF14	13246892	0	0	0
CHB	PRAMEF14	13251892	0	0	0
CHB	PRAMEF14	13256892	0	0	0
CHB	PRAMEF14	13261892	0	0	0
CHB	PRAMEF14	13266892	0	0	0
CHB	PRAMEF14	13271892	0	0	0
CHB	PRAMEF14	13276892	0	0	0

CHB	PRAMEF14	13281892	0	0	0
CHB	PRAMEF14	13286892	0	0	0
CHB	PRAMEF14	13291892	0	0	0
CHB	PRAMEF14	13296892	0	0	0
CHB	PRAMEF14	13301892	0	0	0
CHB	PRAMEF14	13306892	0	0	0
CHB	PRAMEF14	13311892	0	0	0
CHB	PRAMEF14	13316892	0	0	0
CHB	PRAMEF14	13321892	0	0	0
CHB	PRAMEF14	13326892	2.462	2.2024	0.2907
CHB	PRAMEF14	13331892	2.4717	2.8801	-0.3682
CHB	PRAMEF14	13336892	0.0097	0.6777	-1.7463
CHB	PRAMEF14	13341892	0.7369	0.8471	-0.2485
CHB	PRAMEF14	13346892	1.0364	2.033	-1.1885
CHB	PRAMEF14	13351892	3.0643	6.7766	-1.5936
CHB	PRAMEF14	13356892	2.7938	5.9295	-1.518
CHB	PRAMEF14	13361892	1.2142	1.0165	0.3934
CHB	PRAMEF14	13366892	1.4748	0.8471	1.4155
CHB	PRAMEF14	13371892	3.0057	1.5247	2.2002
CHB	PRAMEF14	13376892	8.8923	5.7601	1.5561
CHB	PRAMEF14	13381892	12.7403	9.6567	0.9562
CHB	PRAMEF14	13386892	6.5642	5.2519	0.7078
CHB	PRAMEF14	13391892	0	0	0
CHB	PRAMEF14	13396892	0	0	0
CHB	PRAMEF14	13401892	0	0	0
CHB	PRAMEF14	13406892	0	0	0
CHB	PRAMEF14	13411892	3.276	2.5412	0.7338
CHB	PRAMEF14	13416892	3.276	2.5412	0.7338
CHB	PRAMEF14	13421892	0.8094	0.3388	1.8877
CHB	PRAMEF14	13426892	1.2378	0.5082	2.2903
CHB	PRAMEF14	13431892	0.4283	0.1694	1.5384
CHB	PRAMEF14	13436892	0	0	0
CHB	PRAMEF14	13441892	0	0	0
CHB	PRAMEF14	13446892	1.0632	1.1859	-0.219
CHB	PRAMEF14	13451892	2.0701	1.8636	0.2635
CHB	PRAMEF14	13456892	1.0069	0.6777	0.8606
CHB	PRAMEF14	13461892	1.7419	1.1859	0.9925
CHB	PRAMEF14	13466892	2.1256	1.5247	0.8927
CHB	PRAMEF14	13471892	2.6895	1.6942	1.3659
CHB	PRAMEF14	13476892	5.6227	2.8801	2.4728
CHB	PRAMEF14	13481892	7.7952	4.7436	1.8001
CHB	PRAMEF14	13486892	8.3621	5.9295	1.1777
CHB	PRAMEF14	13491892	4.1348	3.2189	0.753
CHB	PRAMEF14	13496892	2.8235	2.5412	0.2819
CHB	PRAMEF14	13501892	3.4649	2.7106	0.7147
CHB	PRAMEF14	13506892	0.8923	0.6777	0.561
CHB	PRAMEF14	13511892	1.6196	1.0165	1.2002
CHB	PRAMEF14	13516892	2.2886	3.2189	-0.7648
CHB	PRAMEF14	13521892	0.6787	2.3718	-1.7872

CHB	PRAMEF14	13526892	0.0097	0.1694	-0.949
CHB	PRAMEF14	13531892	1.6981	1.1859	0.9143
CHB	PRAMEF14	13536892	1.6981	1.1859	0.9143
CHB	PRAMEF14	13541892	0.3762	0.3388	0.15
CHB	PRAMEF14	13546892	0.6155	0.8471	-0.5223
CHB	PRAMEF14	13551892	0.9235	1.5247	-0.8932
CHB	PRAMEF14	13556892	0.6842	1.0165	-0.6613
CHB	PRAMEF14	13561892	0	0	0
CHB	PRAMEF14	13566892	0	0	0
CHB	PRAMEF14	13571892	0	0	0
CHB	PRAMEF14	13576892	0	0	0
CHB	PRAMEF14	13581892	0	0	0
CHB	PRAMEF14	13586892	0	0	0
CHB	PRAMEF14	13591892	0	0	0
CHB	PRAMEF14	13596892	0	0	0
CHB	PRAMEF14	13601892	0	0	0
CHB	PRAMEF14	13606892	0	0	0
CHB	PRAMEF14	13611892	0	0	0
CHB	PRAMEF14	13616892	0	0	0
CHB	PRAMEF14	13621892	0	0	0
CHB	PRAMEF14	13626892	0	0	0
CHB	PRAMEF14	13631892	0.9098	2.5412	-1.6291
CHB	PRAMEF14	13636892	1.6926	2.8801	-1.0707
CHB	PRAMEF14	13641892	0.9018	0.5082	1.2356
CHB	PRAMEF14	13646892	0.119	0.1694	-0.2995
CHB	PRAMEF14	13651892	0.0097	0.1694	-0.949
CHB	PRAMEF14	13656892	0.0097	0.1694	-0.949
CHB	PRAMEF14	13661892	0	0	0
CHB	PRAMEF14	13666892	1.3333	1.1859	0.2631
CHB	PRAMEF14	13671892	1.4099	1.5247	-0.1706
CHB	PRAMEF14	13676892	0.0765	0.3388	-1.0522
CHB	PRAMEF14	13681892	0	0	0
CHB	PRAMEF14	13686892	0.189	0.6777	-1.2776
CHB	PRAMEF14	13691892	0.7036	2.8801	-1.9624
CHB	PRAMEF14	13696892	0.5146	2.2024	-1.8899
CHB	PRAMEF14	13701892	2.9412	2.8801	0.0551
CHB	PRAMEF14	13706892	4.0272	3.8965	0.0914
CHB	PRAMEF14	13711892	2.201	2.7106	-0.4829
CHB	PRAMEF14	13716892	3.2077	4.913	-0.9754
CHB	PRAMEF14	13721892	2.5334	4.2354	-1.108
CHB	PRAMEF14	13726892	0.4504	1.1859	-1.3129
CHB	PRAMEF14	13731892	2.1409	1.6942	0.613
CHB	PRAMEF14	13736892	2.8816	2.2024	0.7605
CHB	PRAMEF14	13741892	0.7893	1.3553	-0.9173
CHB	PRAMEF14	13746892	0.0388	0.6777	-1.6703
CHB	PRAMEF14	13751892	0	0	0
CHB	PRAMEF14	13756892	0	0	0
CHB	PRAMEF14	13761892	0	0	0
CHB	PRAMEF14	13766892	0.5654	0.5082	0.1796

CHB	PRAMEF14	13771892	4.8905	3.2189	1.3743
CHB	PRAMEF14	13776892	7.0356	4.2354	1.8229
CHB	PRAMEF14	13781892	17.9402	10.6731	2.0525
CHB	PRAMEF14	13786892	23.6048	16.0944	1.4382
CHB	PRAMEF14	13791892	20.7575	16.0944	0.8929
CHB	PRAMEF14	13796892	17.9111	13.7226	0.9335
CHB	PRAMEF14	13801892	12.9589	9.9955	0.8899
CHB	PRAMEF14	13806892	15.0343	12.8755	0.5111
CHB	PRAMEF14	13811892	14.8547	15.4168	-0.1121
CHB	PRAMEF14	13816892	16.0746	14.9085	0.2402
CHB	PRAMEF14	13821892	19.9111	15.4168	0.8967
CHB	PRAMEF14	13826892	19.2397	17.1109	0.3844
CHB	PRAMEF14	13831892	14.3697	17.2803	-0.5207
CHB	PRAMEF14	13836892	12.2214	13.7226	-0.3346
CHB	PRAMEF14	13841892	12.9587	12.5367	0.1025
YRI	PRAMEF14	12846892	12.67	16.6382	-0.7336
YRI	PRAMEF14	12851892	16.4846	19.9994	-0.5446
YRI	PRAMEF14	12856892	18.9731	20.8397	-0.278
YRI	PRAMEF14	12861892	15.899	16.6382	-0.1367
YRI	PRAMEF14	12866892	11.4674	13.7811	-0.5119
YRI	PRAMEF14	12871892	13.0186	15.4617	-0.4844
YRI	PRAMEF14	12876892	16.7236	17.8146	-0.1889
YRI	PRAMEF14	12881892	22.5697	23.3606	-0.1055
YRI	PRAMEF14	12886892	26.0323	30.9234	-0.4966
YRI	PRAMEF14	12891892	22.287	27.8983	-0.6298
YRI	PRAMEF14	12896892	24.2507	35.125	-0.9748
YRI	PRAMEF14	12901892	19.8177	32.9402	-1.2525
YRI	PRAMEF14	12906892	14.2363	23.6968	-1.2442
YRI	PRAMEF14	12911892	16.2253	28.4025	-1.3431
YRI	PRAMEF14	12916892	23.5587	29.747	-0.6525
YRI	PRAMEF14	12921892	23.6077	25.0413	-0.1787
YRI	PRAMEF14	12926892	10.8535	14.2853	-0.7338
YRI	PRAMEF14	12931892	9.4636	13.613	-0.9287
YRI	PRAMEF14	12936892	15.8817	22.8565	-0.9499
YRI	PRAMEF14	12941892	22.9952	30.9234	-0.8049
YRI	PRAMEF14	12946892	17.5382	26.2177	-1.0348
YRI	PRAMEF14	12951892	7.7876	16.6382	-1.6363
YRI	PRAMEF14	12956892	5.8312	11.5963	-1.5013
YRI	PRAMEF14	12961892	3.545	6.3864	-1.2824
YRI	PRAMEF14	12966892	2.0617	4.0335	-1.3341
YRI	PRAMEF14	12971892	1.6494	3.5293	-1.4254
YRI	PRAMEF14	12976892	1.387	2.1848	-0.8958
YRI	PRAMEF14	12981892	4.0235	6.3864	-1.0664
YRI	PRAMEF14	12986892	4.875	8.067	-1.1648
YRI	PRAMEF14	12991892	6.2555	5.7141	0.27
YRI	PRAMEF14	12996892	8.5124	7.0586	0.5993
YRI	PRAMEF14	13001892	4.295	4.7057	-0.2431
YRI	PRAMEF14	13006892	2.0357	2.5209	-0.4859
YRI	PRAMEF14	13011892	6.6443	6.5544	0.0396

YRI	PRAMEF14	13016892	5.6171	4.8738	0.4267
YRI	PRAMEF14	13021892	1.075	0.8403	0.5301
YRI	PRAMEF14	13026892	0.3242	0.8403	-1.1657
YRI	PRAMEF14	13031892	0.6207	1.3445	-1.1756
YRI	PRAMEF14	13036892	3.0844	4.0335	-0.6422
YRI	PRAMEF14	13041892	2.9458	4.3696	-0.899
YRI	PRAMEF14	13046892	5.4225	13.7811	-1.8492
YRI	PRAMEF14	13051892	6.3683	14.1172	-1.6755
YRI	PRAMEF14	13056892	1.2878	1.5126	-0.3347
YRI	PRAMEF14	13061892	0	0	0
YRI	PRAMEF14	13066892	0	0	0
YRI	PRAMEF14	13071892	0	0	0
YRI	PRAMEF14	13076892	0	0	0
YRI	PRAMEF14	13081892	0	0	0
YRI	PRAMEF14	13086892	0	0	0
YRI	PRAMEF14	13091892	0	0	0
YRI	PRAMEF14	13096892	0	0	0
YRI	PRAMEF14	13101892	0	0	0
YRI	PRAMEF14	13106892	1.6795	1.8487	-0.2164
YRI	PRAMEF14	13111892	1.9676	2.3529	-0.4079
YRI	PRAMEF14	13116892	1.1242	2.5209	-1.3987
YRI	PRAMEF14	13121892	7.2873	9.2434	-0.6295
YRI	PRAMEF14	13126892	15.7143	15.6298	0.0166
YRI	PRAMEF14	13131892	15.1641	13.2769	0.4325
YRI	PRAMEF14	13136892	10.075	7.5628	0.9725
YRI	PRAMEF14	13141892	6.4688	5.8822	0.285
YRI	PRAMEF14	13146892	3.402	5.378	-1.0399
YRI	PRAMEF14	13151892	1.4259	2.689	-1.2004
YRI	PRAMEF14	13156892	1.3265	2.3529	-1.0865
YRI	PRAMEF14	13161892	1.9727	3.3612	-1.097
YRI	PRAMEF14	13166892	2.3498	2.5209	-0.1713
YRI	PRAMEF14	13171892	7.885	9.4115	-0.4831
YRI	PRAMEF14	13176892	15.5751	24.201	-1.1115
YRI	PRAMEF14	13181892	17.3213	27.2261	-1.1384
YRI	PRAMEF14	13186892	14.6301	17.9827	-0.5753
YRI	PRAMEF14	13191892	7.9383	7.8989	0.0147
YRI	PRAMEF14	13196892	2.1996	3.8654	-1.169
YRI	PRAMEF14	13201892	3.9267	5.8822	-0.9502
YRI	PRAMEF14	13206892	4.0888	5.0419	-0.531
YRI	PRAMEF14	13211892	1.6918	4.0335	-1.5844
YRI	PRAMEF14	13216892	1.7767	4.2016	-1.584
YRI	PRAMEF14	13221892	0.7876	1.6806	-1.2285
YRI	PRAMEF14	13226892	0	0	0
YRI	PRAMEF14	13231892	0	0	0
YRI	PRAMEF14	13236892	0	0	0
YRI	PRAMEF14	13241892	0	0	0
YRI	PRAMEF14	13246892	0	0	0
YRI	PRAMEF14	13251892	0	0	0
YRI	PRAMEF14	13256892	0	0	0

YRI	PRAMEF14	13261892	0	0	0
YRI	PRAMEF14	13266892	0	0	0
YRI	PRAMEF14	13271892	0	0	0
YRI	PRAMEF14	13276892	0	0	0
YRI	PRAMEF14	13281892	0	0	0
YRI	PRAMEF14	13286892	0	0	0
YRI	PRAMEF14	13291892	0	0	0
YRI	PRAMEF14	13296892	0	0	0
YRI	PRAMEF14	13301892	0	0	0
YRI	PRAMEF14	13306892	0	0	0
YRI	PRAMEF14	13311892	0	0	0
YRI	PRAMEF14	13316892	0	0	0
YRI	PRAMEF14	13321892	0.0093	0.1681	-0.944
YRI	PRAMEF14	13326892	1.4065	2.5209	-1.116
YRI	PRAMEF14	13331892	1.425	2.8571	-1.2951
YRI	PRAMEF14	13336892	0.0278	0.5042	-1.4972
YRI	PRAMEF14	13341892	0.5495	0.8403	-0.6568
YRI	PRAMEF14	13346892	1.9984	2.689	-0.6563
YRI	PRAMEF14	13351892	5.2291	9.0754	-1.2591
YRI	PRAMEF14	13356892	3.8624	7.5628	-1.4325
YRI	PRAMEF14	13361892	1.4682	1.3445	0.2009
YRI	PRAMEF14	13366892	1.702	1.5126	0.282
YRI	PRAMEF14	13371892	2.7487	4.0335	-0.8693
YRI	PRAMEF14	13376892	8.3256	9.4115	-0.3437
YRI	PRAMEF14	13381892	12.5596	12.1005	0.1149
YRI	PRAMEF14	13386892	6.6668	6.2183	0.2073
YRI	PRAMEF14	13391892	0	0	0
YRI	PRAMEF14	13396892	0	0	0
YRI	PRAMEF14	13401892	0	0	0
YRI	PRAMEF14	13406892	0	0	0
YRI	PRAMEF14	13411892	3.1168	2.5209	0.5968
YRI	PRAMEF14	13416892	3.1168	2.5209	0.5968
YRI	PRAMEF14	13421892	0.0996	0.3361	-0.9494
YRI	PRAMEF14	13426892	0.1449	0.5042	-1.1292
YRI	PRAMEF14	13431892	0.0453	0.1681	-0.73
YRI	PRAMEF14	13436892	0	0	0
YRI	PRAMEF14	13441892	0	0	0
YRI	PRAMEF14	13446892	2.6596	1.3445	2.136
YRI	PRAMEF14	13451892	3.4117	2.0167	1.6679
YRI	PRAMEF14	13456892	0.7522	0.6722	0.2094
YRI	PRAMEF14	13461892	1.4245	1.1764	0.4437
YRI	PRAMEF14	13466892	1.7736	1.8487	-0.0961
YRI	PRAMEF14	13471892	0.7586	2.3529	-1.6876
YRI	PRAMEF14	13476892	1.9987	3.0251	-0.8854
YRI	PRAMEF14	13481892	4.1096	5.2099	-0.5956
YRI	PRAMEF14	13486892	4.8068	6.5544	-0.7705
YRI	PRAMEF14	13491892	2.6964	3.3612	-0.5252
YRI	PRAMEF14	13496892	1.2334	3.3612	-1.6811
YRI	PRAMEF14	13501892	1.0151	3.6974	-1.9551

YRI	PRAMEF14	13506892	0.1917	1.0084	-1.6282
YRI	PRAMEF14	13511892	1.7858	2.0167	-0.2761
YRI	PRAMEF14	13516892	2.667	4.7057	-1.2068
YRI	PRAMEF14	13521892	1.1633	3.3612	-1.7364
YRI	PRAMEF14	13526892	0.2821	0.6722	-1.0213
YRI	PRAMEF14	13531892	2.5774	1.5126	1.5855
YRI	PRAMEF14	13536892	2.5774	1.5126	1.5855
YRI	PRAMEF14	13541892	0.5035	0.1681	1.9939
YRI	PRAMEF14	13546892	1.0657	0.8403	0.5091
YRI	PRAMEF14	13551892	1.3837	1.8487	-0.5948
YRI	PRAMEF14	13556892	0.8216	1.1764	-0.6346
YRI	PRAMEF14	13561892	0	0	0
YRI	PRAMEF14	13566892	0	0	0
YRI	PRAMEF14	13571892	0	0	0
YRI	PRAMEF14	13576892	0	0	0
YRI	PRAMEF14	13581892	0	0	0
YRI	PRAMEF14	13586892	0	0	0
YRI	PRAMEF14	13591892	0	0	0
YRI	PRAMEF14	13596892	0	0	0
YRI	PRAMEF14	13601892	0	0	0
YRI	PRAMEF14	13606892	0	0	0
YRI	PRAMEF14	13611892	0	0	0
YRI	PRAMEF14	13616892	0	0	0
YRI	PRAMEF14	13621892	0	0	0
YRI	PRAMEF14	13626892	0	0	0
YRI	PRAMEF14	13631892	1.1751	2.1848	-1.1337
YRI	PRAMEF14	13636892	1.3451	2.8571	-1.3674
YRI	PRAMEF14	13641892	0.3345	1.1764	-1.5058
YRI	PRAMEF14	13646892	0.1645	0.5042	-1.0676
YRI	PRAMEF14	13651892	0.0276	0.1681	-0.8352
YRI	PRAMEF14	13656892	0.0276	0.1681	-0.8352
YRI	PRAMEF14	13661892	0	0	0
YRI	PRAMEF14	13666892	1.6047	2.3529	-0.792
YRI	PRAMEF14	13671892	1.7055	3.1932	-1.2269
YRI	PRAMEF14	13676892	0.1008	0.8403	-1.6703
YRI	PRAMEF14	13681892	0	0	0
YRI	PRAMEF14	13686892	1.1727	0.8403	0.7508
YRI	PRAMEF14	13691892	1.5428	3.0251	-1.2787
YRI	PRAMEF14	13696892	0.3701	2.1848	-2.0375
YRI	PRAMEF14	13701892	2.8191	3.6974	-0.6402
YRI	PRAMEF14	13706892	4.099	5.7141	-0.8054
YRI	PRAMEF14	13711892	2.4804	4.3696	-1.1929
YRI	PRAMEF14	13716892	3.3979	5.0419	-0.916
YRI	PRAMEF14	13721892	2.721	4.0335	-0.8881
YRI	PRAMEF14	13726892	0.5874	1.6806	-1.5039
YRI	PRAMEF14	13731892	3.3082	3.3612	-0.0419
YRI	PRAMEF14	13736892	4.2812	3.5293	0.5701
YRI	PRAMEF14	13741892	1.165	1.3445	-0.2915
YRI	PRAMEF14	13746892	0.1282	0.8403	-1.6084

YRI	PRAMEF14	13751892	0	0	0
YRI	PRAMEF14	13756892	0	0	0
YRI	PRAMEF14	13761892	0	0	0
YRI	PRAMEF14	13766892	1.413	1.0084	0.8066
YRI	PRAMEF14	13771892	4.5626	6.3864	-0.8231
YRI	PRAMEF14	13776892	6.3097	9.4115	-0.9817
YRI	PRAMEF14	13781892	17.456	15.1256	0.4719
YRI	PRAMEF14	13786892	22.3996	19.4952	0.4612
YRI	PRAMEF14	13791892	21.2839	19.8313	0.2269
YRI	PRAMEF14	13796892	20.562	20.6717	-0.0165
YRI	PRAMEF14	13801892	12.6932	16.4701	-0.7051
YRI	PRAMEF14	13806892	11.8301	20.1675	-1.2814
YRI	PRAMEF14	13811892	18.0805	23.5287	-0.7215
YRI	PRAMEF14	13816892	20.8904	19.8313	0.1654
YRI	PRAMEF14	13821892	21.9111	20.3355	0.2402
YRI	PRAMEF14	13826892	23.1641	22.6884	0.0653
YRI	PRAMEF14	13831892	17.9637	20.3355	-0.3616
YRI	PRAMEF14	13836892	13.4068	16.6382	-0.5974
YRI	PRAMEF14	13841892	12.999	16.302	-0.6227
CEU	PRAMEF20	12915450	18.0081	25.2432	-0.9005
CEU	PRAMEF20	12920450	20.2049	26.7783	-0.7725
CEU	PRAMEF20	12925450	10.8439	15.3506	-0.9055
CEU	PRAMEF20	12930450	6.7374	7.5047	-0.3011
CEU	PRAMEF20	12935450	7.7077	9.0398	-0.4404
CEU	PRAMEF20	12940450	14.8041	16.8857	-0.3818
CEU	PRAMEF20	12945450	14.9411	16.0329	-0.2104
CEU	PRAMEF20	12950450	7.1377	11.4277	-1.1398
CEU	PRAMEF20	12955450	3.7916	7.8459	-1.5273
CEU	PRAMEF20	12960450	3.0066	3.0701	-0.0545
CEU	PRAMEF20	12965450	2.9645	2.5584	0.4045
CEU	PRAMEF20	12970450	0.4477	0.5117	-0.2007
CEU	PRAMEF20	12975450	0.4193	0.6822	-0.6865
CEU	PRAMEF20	12980450	2.3067	4.6052	-1.3955
CEU	PRAMEF20	12985450	3.6627	6.6519	-1.3087
CEU	PRAMEF20	12990450	3.409	6.1402	-1.2852
CEU	PRAMEF20	12995450	2.8194	5.458	-1.3794
CEU	PRAMEF20	13000450	2.5073	4.0935	-1.0666
CEU	PRAMEF20	13005450	2.5485	3.7524	-0.8722
CEU	PRAMEF20	13010450	4.5261	5.1169	-0.327
CEU	PRAMEF20	13015450	3.7485	5.2874	-0.8275
CEU	PRAMEF20	13020450	0.6685	2.729	-1.9476
CEU	PRAMEF20	13025450	0.2292	1.0234	-1.5781
CEU	PRAMEF20	13030450	0.0101	0.1706	-0.9534
CEU	PRAMEF20	13035450	3.1293	1.8762	1.5954
CEU	PRAMEF20	13040450	3.7852	2.3879	1.4715
CEU	PRAMEF20	13045450	4.1692	5.458	-0.6738
CEU	PRAMEF20	13050450	5.8868	8.187	-0.8333
CEU	PRAMEF20	13055450	2.3735	3.2407	-0.7111
CEU	PRAMEF20	13060450	0	0	0

CEU	PRAMEF20	13065450	0	0	0
CEU	PRAMEF20	13070450	0	0	0
CEU	PRAMEF20	13075450	0	0	0
CEU	PRAMEF20	13080450	0	0	0
CEU	PRAMEF20	13085450	0	0	0
CEU	PRAMEF20	13090450	0	0	0
CEU	PRAMEF20	13095450	0	0	0
CEU	PRAMEF20	13100450	0	0	0
CEU	PRAMEF20	13105450	0.5543	0.6822	-0.334
CEU	PRAMEF20	13110450	1.242	1.1939	0.0857
CEU	PRAMEF20	13115450	1.2542	2.0467	-0.943
CEU	PRAMEF20	13120450	4.9577	6.9931	-0.8516
CEU	PRAMEF20	13125450	12.5364	16.2034	-0.6997
CEU	PRAMEF20	13130450	13.6489	14.6684	-0.2139
CEU	PRAMEF20	13135450	8.2678	5.6286	1.3425
CEU	PRAMEF20	13140450	5.4212	4.4346	0.619
CEU	PRAMEF20	13145450	3.6652	4.9463	-0.7306
CEU	PRAMEF20	13150450	1.223	3.0701	-1.5844
CEU	PRAMEF20	13155450	1.4665	2.5584	-1.0877
CEU	PRAMEF20	13160450	1.4881	2.5584	-1.0662
CEU	PRAMEF20	13165450	1.3123	1.7056	-0.5387
CEU	PRAMEF20	13170450	5.3946	6.4814	-0.4871
CEU	PRAMEF20	13175450	11.8029	15.5212	-0.7392
CEU	PRAMEF20	13180450	16.269	19.7852	-0.5539
CEU	PRAMEF20	13185450	16.8386	16.374	0.0878
CEU	PRAMEF20	13190450	8.283	7.1636	0.4582
CEU	PRAMEF20	13195450	0.8992	2.0467	-1.3654
CEU	PRAMEF20	13200450	2.5242	3.2407	-0.5875
CEU	PRAMEF20	13205450	2.121	2.2173	-0.1076
CEU	PRAMEF20	13210450	1.2703	1.8762	-0.7714
CEU	PRAMEF20	13215450	1.6945	2.8996	-1.0838
CEU	PRAMEF20	13220450	0.6908	1.3645	-1.0899
CEU	PRAMEF20	13225450	0	0	0
CEU	PRAMEF20	13230450	0	0	0
CEU	PRAMEF20	13235450	0	0	0
CEU	PRAMEF20	13240450	0	0	0
CEU	PRAMEF20	13245450	0	0	0
CEU	PRAMEF20	13250450	0	0	0
CEU	PRAMEF20	13255450	0	0	0
CEU	PRAMEF20	13260450	0	0	0
CEU	PRAMEF20	13265450	0	0	0
CEU	PRAMEF20	13270450	0	0	0
CEU	PRAMEF20	13275450	0	0	0
CEU	PRAMEF20	13280450	0	0	0
CEU	PRAMEF20	13285450	0	0	0
CEU	PRAMEF20	13290450	0	0	0
CEU	PRAMEF20	13295450	0	0	0
CEU	PRAMEF20	13300450	0	0	0
CEU	PRAMEF20	13305450	0	0	0

CEU	PRAMEF20	13310450	0	0	0
CEU	PRAMEF20	13315450	0	0	0
CEU	PRAMEF20	13320450	0	0	0
CEU	PRAMEF20	13325450	0.5428	1.8762	-1.6976
CEU	PRAMEF20	13330450	0.6654	2.0467	-1.6436
CEU	PRAMEF20	13335450	0.1328	0.8528	-1.6214
CEU	PRAMEF20	13340450	0.0101	0.6822	-1.7551
CEU	PRAMEF20	13345450	1.032	1.5351	-0.746
CEU	PRAMEF20	13350450	4.6599	7.8459	-1.2002
CEU	PRAMEF20	13355450	3.8819	6.9931	-1.3018
CEU	PRAMEF20	13360450	0.254	0.6822	-1.1182
CEU	PRAMEF20	13365450	1.5366	1.0234	1.0198
CEU	PRAMEF20	13370450	2.9761	1.7056	1.7401
CEU	PRAMEF20	13375450	6.8322	3.2407	2.945
CEU	PRAMEF20	13380450	14.8582	9.3809	1.7498
CEU	PRAMEF20	13385450	10.1222	7.8459	0.8575
CEU	PRAMEF20	13390450	0.6568	1.0234	-0.7285
CEU	PRAMEF20	13395450	0	0	0
CEU	PRAMEF20	13400450	0	0	0
CEU	PRAMEF20	13405450	0	0	0
CEU	PRAMEF20	13410450	2.965	1.8762	1.3862
CEU	PRAMEF20	13415450	2.965	1.8762	1.3862
CEU	PRAMEF20	13420450	0.4877	0.3411	0.5877
CEU	PRAMEF20	13425450	0.5659	0.5117	0.17
CEU	PRAMEF20	13430450	0.0781	0.1706	-0.5495
CEU	PRAMEF20	13435450	0	0	0
CEU	PRAMEF20	13440450	0	0	0
CEU	PRAMEF20	13445450	1.3636	1.1939	0.3024
CEU	PRAMEF20	13450450	1.3636	1.1939	0.3024
CEU	PRAMEF20	13455450	0.6135	0.6822	-0.1794
CEU	PRAMEF20	13460450	0.6135	0.6822	-0.1794
CEU	PRAMEF20	13465450	1.9272	1.5351	0.5814
CEU	PRAMEF20	13470450	2.4531	2.8996	-0.4016
CEU	PRAMEF20	13475450	0.5259	1.3645	-1.3567
CEU	PRAMEF20	13480450	4.786	3.7524	0.7488
CEU	PRAMEF20	13485450	8.498	7.3342	0.4663
CEU	PRAMEF20	13490450	3.9423	3.7524	0.1376
CEU	PRAMEF20	13495450	2.1754	2.2173	-0.0468
CEU	PRAMEF20	13500450	2.8409	2.8996	-0.0528
CEU	PRAMEF20	13505450	1.1538	1.3645	-0.3409
CEU	PRAMEF20	13510450	1.2997	1.1939	0.1885
CEU	PRAMEF20	13515450	2.8637	3.7524	-0.6438
CEU	PRAMEF20	13520450	1.8322	3.2407	-1.1549
CEU	PRAMEF20	13525450	0.0101	0.1706	-0.9534
CEU	PRAMEF20	13530450	0	0	0
CEU	PRAMEF20	13535450	1.6549	1.3645	0.4698
CEU	PRAMEF20	13540450	1.9553	1.5351	0.6231
CEU	PRAMEF20	13545450	0.5931	0.3411	1.0102
CEU	PRAMEF20	13550450	1.3418	0.8528	1.1012

CEU	PRAMEF20	13555450	1.2243	0.8528	0.8366
CEU	PRAMEF20	13560450	0.1752	0.1706	0.0273
CEU	PRAMEF20	13565450	0	0	0
CEU	PRAMEF20	13570450	0	0	0
CEU	PRAMEF20	13575450	0	0	0
CEU	PRAMEF20	13580450	0	0	0
CEU	PRAMEF20	13585450	0	0	0
CEU	PRAMEF20	13590450	0	0	0
CEU	PRAMEF20	13595450	0	0	0
CEU	PRAMEF20	13600450	0	0	0
CEU	PRAMEF20	13605450	0	0	0
CEU	PRAMEF20	13610450	0	0	0
CEU	PRAMEF20	13615450	0	0	0
CEU	PRAMEF20	13620450	0	0	0
CEU	PRAMEF20	13625450	0	0	0
CEU	PRAMEF20	13630450	1.4345	3.0701	-1.403
CEU	PRAMEF20	13635450	1.4345	3.0701	-1.403
CEU	PRAMEF20	13640450	0.1734	0.3411	-0.6723
CEU	PRAMEF20	13645450	0.1934	0.5117	-0.9983
CEU	PRAMEF20	13650450	0.0301	0.3411	-1.2467
CEU	PRAMEF20	13655450	0.0202	0.3411	-1.2864
CEU	PRAMEF20	13660450	0.0101	0.1706	-0.9534
CEU	PRAMEF20	13665450	1.5242	0.6822	2.1987
CEU	PRAMEF20	13670450	1.5242	0.6822	2.1987
CEU	PRAMEF20	13675450	0.0994	0.3411	-0.9689
CEU	PRAMEF20	13680450	0.0994	0.3411	-0.9689
CEU	PRAMEF20	13685450	0.1769	0.6822	-1.3195
CEU	PRAMEF20	13690450	0.1769	0.6822	-1.3195
CEU	PRAMEF20	13695450	0.86	2.2173	-1.5164
CEU	PRAMEF20	13700450	2.9287	4.7757	-1.0863
CEU	PRAMEF20	13705450	3.7291	4.6052	-0.5319
CEU	PRAMEF20	13710450	2.142	2.2173	-0.0841
CEU	PRAMEF20	13715450	2.2375	3.4112	-0.9218
CEU	PRAMEF20	13720450	3.08	4.4346	-0.8499
CEU	PRAMEF20	13725450	1.3342	1.3645	-0.049
CEU	PRAMEF20	13730450	1.9002	1.3645	0.8666
CEU	PRAMEF20	13735450	2.6024	1.7056	1.2283
CEU	PRAMEF20	13740450	1.5001	1.3645	0.2194
CEU	PRAMEF20	13745450	0.7879	0.8528	-0.1462
CEU	PRAMEF20	13750450	0	0	0
CEU	PRAMEF20	13755450	0	0	0
CEU	PRAMEF20	13760450	0	0	0
CEU	PRAMEF20	13765450	0.4354	0.6822	-0.6445
CEU	PRAMEF20	13770450	1.6399	2.8996	-1.1329
CEU	PRAMEF20	13775450	3.3484	4.2641	-0.5944
CEU	PRAMEF20	13780450	14.6836	10.2337	1.311
CEU	PRAMEF20	13785450	18.9701	13.8156	1.1448
CEU	PRAMEF20	13790450	18.1604	13.3039	1.1179
CEU	PRAMEF20	13795450	18.4061	13.645	1.0699

CEU	PRAMEF20	13800450	11.5168	10.2337	0.378
CEU	PRAMEF20	13805450	13.5541	13.1333	0.0981
CEU	PRAMEF20	13810450	12.578	13.1333	-0.1294
CEU	PRAMEF20	13815450	10.7813	9.2104	0.5105
CEU	PRAMEF20	13820450	17.9949	14.1567	0.8329
CEU	PRAMEF20	13825450	15.6058	14.8389	0.1591
CEU	PRAMEF20	13830450	11.8132	13.3039	-0.3431
CEU	PRAMEF20	13835450	11.8687	11.4277	0.1172
CEU	PRAMEF20	13840450	12.3539	10.5749	0.5083
CEU	PRAMEF20	13845450	12.9188	11.4277	0.3962
CEU	PRAMEF20	13850450	15.4937	12.1099	0.8513
CEU	PRAMEF20	13855450	18.134	15.0095	0.6413
CEU	PRAMEF20	13860450	12.1909	13.8156	-0.3608
CEU	PRAMEF20	13865450	11.3948	11.7688	-0.0967
CEU	PRAMEF20	13870450	19.9942	15.8623	0.8046
CEU	PRAMEF20	13875450	15.6349	14.4978	0.2412
CEU	PRAMEF20	13880450	8.9546	9.7221	-0.2372
CEU	PRAMEF20	13885450	14.8477	12.4511	0.5873
CEU	PRAMEF20	13890450	15.7747	14.6684	0.2321
CEU	PRAMEF20	13895450	13.0703	13.645	-0.1292
CEU	PRAMEF20	13900450	13.3591	11.5982	0.4614
CEU	PRAMEF20	13905450	15.8625	10.7454	1.4403
CEU	PRAMEF20	13910450	12.67	9.3809	1.0507
CEU	PRAMEF20	13915450	10.8114	9.5515	0.3958
CHB	PRAMEF20	12915450	34.1526	26.0899	0.9691
CHB	PRAMEF20	12920450	26.6366	24.904	0.2179
CHB	PRAMEF20	12925450	13.7356	14.2309	-0.1066
CHB	PRAMEF20	12930450	7.2873	7.4543	-0.0657
CHB	PRAMEF20	12935450	7.4755	8.6402	-0.4003
CHB	PRAMEF20	12940450	13.2703	19.991	-1.0452
CHB	PRAMEF20	12945450	11.6212	18.9745	-1.2025
CHB	PRAMEF20	12950450	5.0126	12.7061	-1.8443
CHB	PRAMEF20	12955450	3.6006	9.1484	-1.8088
CHB	PRAMEF20	12960450	3.1957	3.5577	-0.2736
CHB	PRAMEF20	12965450	2.5248	3.5577	-0.7806
CHB	PRAMEF20	12970450	0.1645	2.2024	-2.2819
CHB	PRAMEF20	12975450	0.2477	1.8636	-2.0617
CHB	PRAMEF20	12980450	2.4896	5.4213	-1.5373
CHB	PRAMEF20	12985450	3.8765	5.9295	-0.9939
CHB	PRAMEF20	12990450	2.786	4.2354	-0.9436
CHB	PRAMEF20	12995450	1.7493	4.4048	-1.671
CHB	PRAMEF20	13000450	1.6893	4.066	-1.6027
CHB	PRAMEF20	13005450	2.0495	3.2189	-0.9614
CHB	PRAMEF20	13010450	3.1342	3.8965	-0.5332
CHB	PRAMEF20	13015450	2.4245	3.7271	-0.9463
CHB	PRAMEF20	13020450	0.3413	1.5247	-1.7581
CHB	PRAMEF20	13025450	0.1319	0.6777	-1.4269
CHB	PRAMEF20	13030450	0	0	0
CHB	PRAMEF20	13035450	3.1711	2.3718	0.8437

CHB	PRAMEF20	13040450	4.1017	2.7106	1.3181
CHB	PRAMEF20	13045450	7.9115	6.0989	0.8556
CHB	PRAMEF20	13050450	12.6674	10.3343	0.6791
CHB	PRAMEF20	13055450	5.6865	4.5742	0.6773
CHB	PRAMEF20	13060450	0	0	0
CHB	PRAMEF20	13065450	0	0	0
CHB	PRAMEF20	13070450	0	0	0
CHB	PRAMEF20	13075450	0	0	0
CHB	PRAMEF20	13080450	0	0	0
CHB	PRAMEF20	13085450	0	0	0
CHB	PRAMEF20	13090450	0	0	0
CHB	PRAMEF20	13095450	0	0	0
CHB	PRAMEF20	13100450	0	0	0
CHB	PRAMEF20	13105450	0.4826	0.3388	0.5768
CHB	PRAMEF20	13110450	1.2136	0.6777	1.401
CHB	PRAMEF20	13115450	1.5555	0.6777	2.2948
CHB	PRAMEF20	13120450	5.131	6.6072	-0.6484
CHB	PRAMEF20	13125450	9.0504	15.4168	-1.2702
CHB	PRAMEF20	13130450	9.5894	13.2144	-0.8374
CHB	PRAMEF20	13135450	7.264	6.0989	0.55
CHB	PRAMEF20	13140450	5.7594	4.4048	0.8524
CHB	PRAMEF20	13145450	7.5243	5.5907	0.9866
CHB	PRAMEF20	13150450	4.8813	3.5577	1.0003
CHB	PRAMEF20	13155450	1.8442	1.8636	-0.0248
CHB	PRAMEF20	13160450	1.3562	2.7106	-1.2834
CHB	PRAMEF20	13165450	1.0992	1.8636	-0.9753
CHB	PRAMEF20	13170450	6.1732	6.2684	-0.0438
CHB	PRAMEF20	13175450	21.3191	17.7886	0.6143
CHB	PRAMEF20	13180450	31.2311	23.8875	0.9615
CHB	PRAMEF20	13185450	23.7887	18.6357	0.8574
CHB	PRAMEF20	13190450	8.7367	7.7931	0.3566
CHB	PRAMEF20	13195450	1.5603	2.8801	-1.1899
CHB	PRAMEF20	13200450	2.9598	4.2354	-0.8304
CHB	PRAMEF20	13205450	1.6657	2.8801	-1.0949
CHB	PRAMEF20	13210450	1.3444	2.2024	-0.9607
CHB	PRAMEF20	13215450	2.6983	3.8965	-0.838
CHB	PRAMEF20	13220450	1.4761	2.3718	-0.9455
CHB	PRAMEF20	13225450	0	0	0
CHB	PRAMEF20	13230450	0	0	0
CHB	PRAMEF20	13235450	0	0	0
CHB	PRAMEF20	13240450	0	0	0
CHB	PRAMEF20	13245450	0	0	0
CHB	PRAMEF20	13250450	0	0	0
CHB	PRAMEF20	13255450	0	0	0
CHB	PRAMEF20	13260450	0	0	0
CHB	PRAMEF20	13265450	0	0	0
CHB	PRAMEF20	13270450	0	0	0
CHB	PRAMEF20	13275450	0	0	0
CHB	PRAMEF20	13280450	0	0	0

CHB	PRAMEF20	13285450	0	0	0
CHB	PRAMEF20	13290450	0	0	0
CHB	PRAMEF20	13295450	0	0	0
CHB	PRAMEF20	13300450	0	0	0
CHB	PRAMEF20	13305450	0	0	0
CHB	PRAMEF20	13310450	0	0	0
CHB	PRAMEF20	13315450	0	0	0
CHB	PRAMEF20	13320450	0	0	0
CHB	PRAMEF20	13325450	2.1837	2.033	0.1797
CHB	PRAMEF20	13330450	2.4717	2.3718	0.1055
CHB	PRAMEF20	13335450	0.288	0.8471	-1.2608
CHB	PRAMEF20	13340450	0	0.5082	-1.5953
CHB	PRAMEF20	13345450	1.0364	2.033	-1.1885
CHB	PRAMEF20	13350450	3.7723	7.4543	-1.4493
CHB	PRAMEF20	13355450	2.7938	5.9295	-1.518
CHB	PRAMEF20	13360450	0.0579	0.5082	-1.4136
CHB	PRAMEF20	13365450	1.4748	0.8471	1.4155
CHB	PRAMEF20	13370450	2.9151	1.5247	2.0656
CHB	PRAMEF20	13375450	5.1681	3.2189	1.6025
CHB	PRAMEF20	13380450	13.3261	9.3178	1.2848
CHB	PRAMEF20	13385450	10.2884	7.7931	0.9431
CHB	PRAMEF20	13390450	0.6901	1.0165	-0.6496
CHB	PRAMEF20	13395450	0	0	0
CHB	PRAMEF20	13400450	0	0	0
CHB	PRAMEF20	13405450	0	0	0
CHB	PRAMEF20	13410450	3.276	2.5412	0.7338
CHB	PRAMEF20	13415450	3.276	2.5412	0.7338
CHB	PRAMEF20	13420450	0.8094	0.3388	1.8877
CHB	PRAMEF20	13425450	1.2378	0.5082	2.2903
CHB	PRAMEF20	13430450	0.4283	0.1694	1.5384
CHB	PRAMEF20	13435450	0	0	0
CHB	PRAMEF20	13440450	0	0	0
CHB	PRAMEF20	13445450	1.0632	1.1859	-0.219
CHB	PRAMEF20	13450450	1.0632	1.1859	-0.219
CHB	PRAMEF20	13455450	1.0069	0.6777	0.8606
CHB	PRAMEF20	13460450	1.0069	0.6777	0.8606
CHB	PRAMEF20	13465450	2.1256	1.5247	0.8927
CHB	PRAMEF20	13470450	4.4314	2.8801	1.3987
CHB	PRAMEF20	13475450	2.3057	1.3553	1.5403
CHB	PRAMEF20	13480450	6.6155	3.8965	1.9017
CHB	PRAMEF20	13485450	11.679	7.4543	1.663
CHB	PRAMEF20	13490450	5.1296	3.8965	0.8625
CHB	PRAMEF20	13495450	2.4137	2.2024	0.2366
CHB	PRAMEF20	13500450	3.4026	2.5412	0.8602
CHB	PRAMEF20	13505450	1.3021	1.0165	0.5684
CHB	PRAMEF20	13510450	0.8666	0.6777	0.4938
CHB	PRAMEF20	13515450	2.2886	3.2189	-0.7648
CHB	PRAMEF20	13520450	1.6789	3.0495	-1.1787
CHB	PRAMEF20	13525450	0.0097	0.1694	-0.949

CHB	PRAMEF20	13530450	0	0	0
CHB	PRAMEF20	13535450	1.6981	1.1859	0.9143
CHB	PRAMEF20	13540450	2.0742	1.5247	0.8164
CHB	PRAMEF20	13545450	0.5041	0.5082	-0.0129
CHB	PRAMEF20	13550450	0.786	1.1859	-0.7138
CHB	PRAMEF20	13555450	0.7955	1.3553	-0.9073
CHB	PRAMEF20	13560450	0.1374	0.3388	-0.8079
CHB	PRAMEF20	13565450	0	0	0
CHB	PRAMEF20	13570450	0	0	0
CHB	PRAMEF20	13575450	0	0	0
CHB	PRAMEF20	13580450	0	0	0
CHB	PRAMEF20	13585450	0	0	0
CHB	PRAMEF20	13590450	0	0	0
CHB	PRAMEF20	13595450	0	0	0
CHB	PRAMEF20	13600450	0	0	0
CHB	PRAMEF20	13605450	0	0	0
CHB	PRAMEF20	13610450	0	0	0
CHB	PRAMEF20	13615450	0	0	0
CHB	PRAMEF20	13620450	0	0	0
CHB	PRAMEF20	13625450	0	0	0
CHB	PRAMEF20	13630450	0.9098	2.5412	-1.6291
CHB	PRAMEF20	13635450	0.9098	2.5412	-1.6291
CHB	PRAMEF20	13640450	0.7828	0.3388	1.781
CHB	PRAMEF20	13645450	0.9018	0.5082	1.2356
CHB	PRAMEF20	13650450	0.1287	0.3388	-0.8428
CHB	PRAMEF20	13655450	0.0097	0.1694	-0.949
CHB	PRAMEF20	13660450	0	0	0
CHB	PRAMEF20	13665450	1.3333	1.1859	0.2631
CHB	PRAMEF20	13670450	1.3333	1.1859	0.2631
CHB	PRAMEF20	13675450	0.0765	0.3388	-1.0522
CHB	PRAMEF20	13680450	0.0765	0.3388	-1.0522
CHB	PRAMEF20	13685450	0.189	0.6777	-1.2776
CHB	PRAMEF20	13690450	0.189	0.6777	-1.2776
CHB	PRAMEF20	13695450	0.5146	2.2024	-1.8899
CHB	PRAMEF20	13700450	2.9682	4.913	-1.1124
CHB	PRAMEF20	13705450	4.0272	3.8965	0.0914
CHB	PRAMEF20	13710450	1.9964	2.2024	-0.2307
CHB	PRAMEF20	13715450	2.5243	4.2354	-1.1139
CHB	PRAMEF20	13720450	3.2159	4.7436	-0.9012
CHB	PRAMEF20	13725450	1.1338	1.8636	-0.9311
CHB	PRAMEF20	13730450	1.5962	1.5247	0.1062
CHB	PRAMEF20	13735450	2.1601	1.8636	0.3783
CHB	PRAMEF20	13740450	1.3436	1.6942	-0.4812
CHB	PRAMEF20	13745450	0.7603	1.0165	-0.5099
CHB	PRAMEF20	13750450	0	0	0
CHB	PRAMEF20	13755450	0	0	0
CHB	PRAMEF20	13760450	0	0	0
CHB	PRAMEF20	13765450	0.5654	0.5082	0.1796
CHB	PRAMEF20	13770450	3.0624	1.8636	1.5295

CHB	PRAMEF20	13775450	5.5637	3.3883	1.7132
CHB	PRAMEF20	13780450	17.3158	10.1649	2.1139
CHB	PRAMEF20	13785450	21.925	14.9085	1.4454
CHB	PRAMEF20	13790450	21.1612	16.4333	0.8875
CHB	PRAMEF20	13795450	20.5125	15.0779	1.1075
CHB	PRAMEF20	13800450	12.5721	9.6567	0.904
CHB	PRAMEF20	13805450	15.0598	12.5367	0.6126
CHB	PRAMEF20	13810450	14.301	15.2474	-0.1908
CHB	PRAMEF20	13815450	13.0074	13.5532	-0.1231
CHB	PRAMEF20	13820450	21.3617	15.7556	1.0956
CHB	PRAMEF20	13825450	19.5024	16.7721	0.5026
CHB	PRAMEF20	13830450	14.8367	16.2638	-0.2706
CHB	PRAMEF20	13835450	12.1643	14.0615	-0.4132
CHB	PRAMEF20	13840450	13.0546	13.7226	-0.1489
CHB	PRAMEF20	13845450	14.3154	13.892	0.0933
CHB	PRAMEF20	13850450	16.513	13.892	0.5774
CHB	PRAMEF20	13855450	18.9298	17.1109	0.3285
CHB	PRAMEF20	13860450	13.7053	15.925	-0.4294
CHB	PRAMEF20	13865450	13.8246	14.2309	-0.0875
CHB	PRAMEF20	13870450	23.538	16.0944	1.4254
CHB	PRAMEF20	13875450	19.2858	12.8755	1.5176
CHB	PRAMEF20	13880450	9.9345	10.5037	-0.1632
CHB	PRAMEF20	13885450	15.9143	14.2309	0.3625
CHB	PRAMEF20	13890450	19.237	16.0944	0.6018
CHB	PRAMEF20	13895450	14.715	16.7721	-0.3787
CHB	PRAMEF20	13900450	11.6954	14.4003	-0.5759
CHB	PRAMEF20	13905450	13.6665	12.5367	0.2743
CHB	PRAMEF20	13910450	11.5719	12.8755	-0.3086
CHB	PRAMEF20	13915450	11.6259	13.045	-0.3318
YRI	PRAMEF20	12915450	22.2868	29.5789	-0.7731
YRI	PRAMEF20	12920450	24.3389	27.7303	-0.3829
YRI	PRAMEF20	12925450	14.5076	17.4785	-0.5239
YRI	PRAMEF20	12930450	10.1536	13.613	-0.7743
YRI	PRAMEF20	12935450	9.8166	17.1423	-1.3162
YRI	PRAMEF20	12940450	21.4788	27.7303	-0.7058
YRI	PRAMEF20	12945450	23.3365	30.5873	-0.744
YRI	PRAMEF20	12950450	9.84	19.9994	-1.5741
YRI	PRAMEF20	12955450	5.2669	12.1005	-1.7096
YRI	PRAMEF20	12960450	3.8831	7.2267	-1.3492
YRI	PRAMEF20	12965450	3.1973	5.0419	-1.0278
YRI	PRAMEF20	12970450	1.7494	3.8654	-1.4849
YRI	PRAMEF20	12975450	0.9488	2.3529	-1.4863
YRI	PRAMEF20	12980450	3.562	5.378	-0.9557
YRI	PRAMEF20	12985450	5.6115	8.4031	-0.9811
YRI	PRAMEF20	12990450	6.5475	6.7225	-0.0754
YRI	PRAMEF20	12995450	6.5251	5.8822	0.3124
YRI	PRAMEF20	13000450	4.483	5.0419	-0.3114
YRI	PRAMEF20	13005450	4.1658	4.0335	0.0895
YRI	PRAMEF20	13010450	6.1719	6.3864	-0.0968

YRI	PRAMEF20	13015450	5.6356	5.0419	0.3308
YRI	PRAMEF20	13020450	1.5474	1.0084	1.0746
YRI	PRAMEF20	13025450	0.3242	0.8403	-1.1657
YRI	PRAMEF20	13030450	0.1585	0.6722	-1.3448
YRI	PRAMEF20	13035450	2.7773	3.6974	-0.6706
YRI	PRAMEF20	13040450	3.066	3.8654	-0.561
YRI	PRAMEF20	13045450	4.2304	10.0837	-1.7373
YRI	PRAMEF20	13050450	6.7103	15.2937	-1.7199
YRI	PRAMEF20	13055450	2.7869	5.5461	-1.413
YRI	PRAMEF20	13060450	0	0	0
YRI	PRAMEF20	13065450	0	0	0
YRI	PRAMEF20	13070450	0	0	0
YRI	PRAMEF20	13075450	0	0	0
YRI	PRAMEF20	13080450	0	0	0
YRI	PRAMEF20	13085450	0	0	0
YRI	PRAMEF20	13090450	0	0	0
YRI	PRAMEF20	13095450	0	0	0
YRI	PRAMEF20	13100450	0	0	0
YRI	PRAMEF20	13105450	0.4735	0.1681	1.8155
YRI	PRAMEF20	13110450	1.9676	2.3529	-0.4079
YRI	PRAMEF20	13115450	2.1301	3.3612	-0.9726
YRI	PRAMEF20	13120450	4.7338	6.7225	-0.8569
YRI	PRAMEF20	13125450	14.053	14.6214	-0.1189
YRI	PRAMEF20	13130450	17.1439	14.9575	0.4475
YRI	PRAMEF20	13135450	10.6314	8.4031	0.7832
YRI	PRAMEF20	13140450	6.8968	6.0502	0.4011
YRI	PRAMEF20	13145450	4.5564	6.0502	-0.7078
YRI	PRAMEF20	13150450	1.2621	3.0251	-1.5209
YRI	PRAMEF20	13155450	1.4677	2.5209	-1.0547
YRI	PRAMEF20	13160450	1.4446	3.0251	-1.3634
YRI	PRAMEF20	13165450	1.4656	2.0167	-0.6589
YRI	PRAMEF20	13170450	6.5165	6.5544	-0.0167
YRI	PRAMEF20	13175450	13.0526	19.8313	-1.0588
YRI	PRAMEF20	13180450	17.242	28.5706	-1.2423
YRI	PRAMEF20	13185450	18.046	23.0245	-0.6732
YRI	PRAMEF20	13190450	9.6745	9.5795	0.0296
YRI	PRAMEF20	13195450	2.0897	3.0251	-0.8069
YRI	PRAMEF20	13200450	4.2989	6.2183	-0.8873
YRI	PRAMEF20	13205450	4.0888	5.2099	-0.6068
YRI	PRAMEF20	13210450	1.8995	3.8654	-1.3796
YRI	PRAMEF20	13215450	1.8866	4.8738	-1.7147
YRI	PRAMEF20	13220450	0.8611	2.1848	-1.4862
YRI	PRAMEF20	13225450	0	0	0
YRI	PRAMEF20	13230450	0	0	0
YRI	PRAMEF20	13235450	0	0	0
YRI	PRAMEF20	13240450	0	0	0
YRI	PRAMEF20	13245450	0	0	0
YRI	PRAMEF20	13250450	0	0	0
YRI	PRAMEF20	13255450	0	0	0

YRI	PRAMEF20	13260450	0	0	0
YRI	PRAMEF20	13265450	0	0	0
YRI	PRAMEF20	13270450	0	0	0
YRI	PRAMEF20	13275450	0	0	0
YRI	PRAMEF20	13280450	0	0	0
YRI	PRAMEF20	13285450	0	0	0
YRI	PRAMEF20	13290450	0	0	0
YRI	PRAMEF20	13295450	0	0	0
YRI	PRAMEF20	13300450	0	0	0
YRI	PRAMEF20	13305450	0	0	0
YRI	PRAMEF20	13310450	0	0	0
YRI	PRAMEF20	13315450	0	0	0
YRI	PRAMEF20	13320450	0	0	0
YRI	PRAMEF20	13325450	1.3521	2.3529	-1.0594
YRI	PRAMEF20	13330450	1.4065	2.5209	-1.116
YRI	PRAMEF20	13335450	0.0821	0.6722	-1.5448
YRI	PRAMEF20	13340450	0.0278	0.5042	-1.4972
YRI	PRAMEF20	13345450	1.24	2.0167	-0.9287
YRI	PRAMEF20	13350450	5.4184	9.5795	-1.2955
YRI	PRAMEF20	13355450	4.6207	8.235	-1.2941
YRI	PRAMEF20	13360450	0.4424	0.6722	-0.6016
YRI	PRAMEF20	13365450	1.5663	1.3445	0.3602
YRI	PRAMEF20	13370450	3.1085	3.8654	-0.5312
YRI	PRAMEF20	13375450	5.3819	6.8906	-0.6357
YRI	PRAMEF20	13380450	11.875	11.5963	0.0726
YRI	PRAMEF20	13385450	9.7462	8.9073	0.2794
YRI	PRAMEF20	13390450	1.7108	1.6806	0.0415
YRI	PRAMEF20	13395450	0	0	0
YRI	PRAMEF20	13400450	0	0	0
YRI	PRAMEF20	13405450	0	0	0
YRI	PRAMEF20	13410450	3.1168	2.5209	0.5968
YRI	PRAMEF20	13415450	3.1168	2.5209	0.5968
YRI	PRAMEF20	13420450	0.0996	0.3361	-0.9494
YRI	PRAMEF20	13425450	0.1449	0.5042	-1.1292
YRI	PRAMEF20	13430450	0.0453	0.1681	-0.73
YRI	PRAMEF20	13435450	0	0	0
YRI	PRAMEF20	13440450	0	0	0
YRI	PRAMEF20	13445450	2.6596	1.3445	2.136
YRI	PRAMEF20	13450450	2.6596	1.3445	2.136
YRI	PRAMEF20	13455450	0.7522	0.6722	0.2094
YRI	PRAMEF20	13460450	0.7522	0.6722	0.2094
YRI	PRAMEF20	13465450	1.7736	1.8487	-0.0961
YRI	PRAMEF20	13470450	2.1831	3.5293	-1.0207
YRI	PRAMEF20	13475450	0.4095	1.6806	-1.7487
YRI	PRAMEF20	13480450	3.4941	4.5377	-0.6377
YRI	PRAMEF20	13485450	6.396	7.8989	-0.5591
YRI	PRAMEF20	13490450	3.2663	3.8654	-0.4204
YRI	PRAMEF20	13495450	1.1096	3.0251	-1.6524
YRI	PRAMEF20	13500450	0.9785	3.3612	-1.8824

YRI	PRAMEF20	13505450	0.3154	1.3445	-1.6714
YRI	PRAMEF20	13510450	0.8311	1.6806	-1.1687
YRI	PRAMEF20	13515450	2.667	4.7057	-1.2068
YRI	PRAMEF20	13520450	2.2002	4.2016	-1.3074
YRI	PRAMEF20	13525450	0.2821	0.6722	-1.0213
YRI	PRAMEF20	13530450	0	0	0
YRI	PRAMEF20	13535450	2.5774	1.5126	1.5855
YRI	PRAMEF20	13540450	3.0809	1.6806	1.9264
YRI	PRAMEF20	13545450	0.7955	0.3361	1.8443
YRI	PRAMEF20	13550450	1.2133	1.3445	-0.2131
YRI	PRAMEF20	13555450	1.0917	1.6806	-0.8102
YRI	PRAMEF20	13560450	0.1704	0.5042	-1.049
YRI	PRAMEF20	13565450	0	0	0
YRI	PRAMEF20	13570450	0	0	0
YRI	PRAMEF20	13575450	0	0	0
YRI	PRAMEF20	13580450	0	0	0
YRI	PRAMEF20	13585450	0	0	0
YRI	PRAMEF20	13590450	0	0	0
YRI	PRAMEF20	13595450	0	0	0
YRI	PRAMEF20	13600450	0	0	0
YRI	PRAMEF20	13605450	0	0	0
YRI	PRAMEF20	13610450	0	0	0
YRI	PRAMEF20	13615450	0	0	0
YRI	PRAMEF20	13620450	0	0	0
YRI	PRAMEF20	13625450	0	0	0
YRI	PRAMEF20	13630450	1.1751	2.1848	-1.1337
YRI	PRAMEF20	13635450	1.1751	2.1848	-1.1337
YRI	PRAMEF20	13640450	0.17	0.6722	-1.3147
YRI	PRAMEF20	13645450	0.3345	1.1764	-1.5058
YRI	PRAMEF20	13650450	0.1921	0.6722	-1.2569
YRI	PRAMEF20	13655450	0.0276	0.1681	-0.8352
YRI	PRAMEF20	13660450	0	0	0
YRI	PRAMEF20	13665450	1.6047	2.3529	-0.792
YRI	PRAMEF20	13670450	1.6687	2.8571	-1.0747
YRI	PRAMEF20	13675450	0.1008	0.8403	-1.6703
YRI	PRAMEF20	13680450	0.0369	0.3361	-1.2012
YRI	PRAMEF20	13685450	1.1727	0.8403	0.7508
YRI	PRAMEF20	13690450	1.1727	0.8403	0.7508
YRI	PRAMEF20	13695450	0.3701	2.1848	-2.0375
YRI	PRAMEF20	13700450	2.6762	5.5461	-1.4697
YRI	PRAMEF20	13705450	4.099	5.7141	-0.8054
YRI	PRAMEF20	13710450	2.5944	3.3612	-0.6058
YRI	PRAMEF20	13715450	2.416	4.2016	-1.1664
YRI	PRAMEF20	13720450	3.0744	5.2099	-1.1559
YRI	PRAMEF20	13725450	1.5693	2.5209	-0.953
YRI	PRAMEF20	13730450	2.2505	2.3529	-0.1084
YRI	PRAMEF20	13735450	3.2987	3.1932	0.087
YRI	PRAMEF20	13740450	2.2683	2.5209	-0.253
YRI	PRAMEF20	13745450	1.1107	1.1764	-0.1175

YRI	PRAMEF20	13750450	0	0	0
YRI	PRAMEF20	13755450	0	0	0
YRI	PRAMEF20	13760450	0	0	0
YRI	PRAMEF20	13765450	1.413	1.0084	0.8066
YRI	PRAMEF20	13770450	2.9158	4.2016	-0.8399
YRI	PRAMEF20	13775450	4.7006	7.8989	-1.1898
YRI	PRAMEF20	13780450	16.7818	15.2937	0.2982
YRI	PRAMEF20	13785450	21.2155	18.4868	0.456
YRI	PRAMEF20	13790450	21.9561	19.9994	0.3032
YRI	PRAMEF20	13795450	22.4139	21.512	0.1302
YRI	PRAMEF20	13800450	12.8449	15.9659	-0.6002
YRI	PRAMEF20	13805450	11.2248	18.3188	-1.196
YRI	PRAMEF20	13810450	15.7958	23.1926	-0.9933
YRI	PRAMEF20	13815450	19.3262	20.3355	-0.1539
YRI	PRAMEF20	13820450	23.1432	20.3355	0.4281
YRI	PRAMEF20	13825450	23.0372	22.8565	0.0246
YRI	PRAMEF20	13830450	18.8022	20.6717	-0.2806
YRI	PRAMEF20	13835450	14.032	16.9743	-0.5337
YRI	PRAMEF20	13840450	13.6729	16.9743	-0.5988
YRI	PRAMEF20	13845450	13.8905	17.8146	-0.6795
YRI	PRAMEF20	13850450	20.155	20.6717	-0.0775
YRI	PRAMEF20	13855450	24.8172	24.7051	0.0142
YRI	PRAMEF20	13860450	18.3915	24.0329	-0.7319
YRI	PRAMEF20	13865450	18.6435	23.1926	-0.6109
YRI	PRAMEF20	13870450	24.1625	22.1842	0.2773
YRI	PRAMEF20	13875450	17.7233	18.6549	-0.1543
YRI	PRAMEF20	13880450	10.4661	18.4868	-1.3404
YRI	PRAMEF20	13885450	18.3508	24.5371	-0.7866
YRI	PRAMEF20	13890450	25.7638	27.2261	-0.1681
YRI	PRAMEF20	13895450	22.4097	21.512	0.1296
YRI	PRAMEF20	13900450	17.4954	17.4785	0.003
YRI	PRAMEF20	13905450	18.1173	16.6382	0.2735
YRI	PRAMEF20	13910450	15.4406	14.1172	0.2862
YRI	PRAMEF20	13915450	15.0003	15.2937	-0.0588
CEU	PRAMEF5	12759198	3.8518	5.6286	-0.9038
CEU	PRAMEF5	12764198	3.8633	5.2874	-0.7657
CEU	PRAMEF5	12769198	4.5731	7.3342	-1.1063
CEU	PRAMEF5	12774198	6.9132	9.7221	-0.868
CEU	PRAMEF5	12779198	9.1231	10.4043	-0.3717
CEU	PRAMEF5	12784198	12.3599	11.9394	0.1072
CEU	PRAMEF5	12789198	11.6999	10.7454	0.2687
CEU	PRAMEF5	12794198	11.4958	9.7221	0.5481
CEU	PRAMEF5	12799198	14.7477	10.2337	1.3299
CEU	PRAMEF5	12804198	11.0761	8.3576	0.9663
CEU	PRAMEF5	12809198	5.2342	7.6753	-0.9383
CEU	PRAMEF5	12814198	7.8747	10.0632	-0.6549
CEU	PRAMEF5	12819198	10.8176	10.7454	0.0203
CEU	PRAMEF5	12824198	10.0606	9.0398	0.3375
CEU	PRAMEF5	12829198	10.5442	9.0398	0.4974

CEU	PRAMEF5	12834198	8.0476	11.4277	-0.898
CEU	PRAMEF5	12839198	6.3352	9.7221	-1.0467
CEU	PRAMEF5	12844198	7.8114	7.8459	-0.013
CEU	PRAMEF5	12849198	13.6108	14.1567	-0.1185
CEU	PRAMEF5	12854198	19.8458	19.9558	-0.0172
CEU	PRAMEF5	12859198	19.9963	22.6848	-0.3711
CEU	PRAMEF5	12864198	26.0478	45.3696	-1.3558
CEU	PRAMEF5	12869198	29.376	57.8207	-1.5718
CEU	PRAMEF5	12874198	18.3591	28.8251	-1.1449
CEU	PRAMEF5	12879198	15.1548	11.5982	0.9319
CEU	PRAMEF5	12884198	21.9848	17.9091	0.7066
CEU	PRAMEF5	12889198	22.6156	16.5446	1.1355
CEU	PRAMEF5	12894198	24.202	22.5142	0.2347
CEU	PRAMEF5	12899198	18.6019	24.0493	-0.7106
CEU	PRAMEF5	12904198	7.5904	12.7922	-1.2426
CEU	PRAMEF5	12909198	10.2524	17.3974	-1.2736
CEU	PRAMEF5	12914198	17.0657	25.2432	-1.0178
CEU	PRAMEF5	12919198	20.2385	26.7783	-0.7686
CEU	PRAMEF5	12924198	13.5032	18.4207	-0.8298
CEU	PRAMEF5	12929198	7.8928	8.6987	-0.2761
CEU	PRAMEF5	12934198	6.5306	7.6753	-0.44
CEU	PRAMEF5	12939198	13.0795	15.6917	-0.5139
CEU	PRAMEF5	12944198	17.016	17.7385	-0.1264
CEU	PRAMEF5	12949198	8.5397	10.916	-0.6591
CEU	PRAMEF5	12954198	3.8623	8.5281	-1.628
CEU	PRAMEF5	12959198	3.3591	4.9463	-0.9052
CEU	PRAMEF5	12964198	2.7584	2.3879	0.3902
CEU	PRAMEF5	12969198	0.4578	0.6822	-0.586
CEU	PRAMEF5	12974198	0.2263	0.5117	-0.8951
CEU	PRAMEF5	12979198	0.9592	3.2407	-1.8708
CEU	PRAMEF5	12984198	4.0618	6.9931	-1.2265
CEU	PRAMEF5	12989198	4.5529	6.8225	-0.9712
CEU	PRAMEF5	12994198	2.7992	5.2874	-1.3379
CEU	PRAMEF5	12999198	2.711	4.7757	-1.2143
CEU	PRAMEF5	13004198	1.8771	2.8996	-0.9196
CEU	PRAMEF5	13009198	4.2331	4.6052	-0.2259
CEU	PRAMEF5	13014198	4.4401	6.3108	-0.8589
CEU	PRAMEF5	13019198	0.9615	3.2407	-1.8689
CEU	PRAMEF5	13024198	0.2191	0.8528	-1.4271
CEU	PRAMEF5	13029198	0.0101	0.1706	-0.9534
CEU	PRAMEF5	13034198	0.3958	0.8528	-1.0291
CEU	PRAMEF5	13039198	3.7852	2.3879	1.4715
CEU	PRAMEF5	13044198	5.8015	4.4346	0.8576
CEU	PRAMEF5	13049198	5.8868	8.187	-0.8333
CEU	PRAMEF5	13054198	3.4848	5.458	-1.0315
CEU	PRAMEF5	13059198	0	0	0
CEU	PRAMEF5	13064198	0	0	0
CEU	PRAMEF5	13069198	0	0	0
CEU	PRAMEF5	13074198	0	0	0

CEU	PRAMEF5	13079198	0	0	0
CEU	PRAMEF5	13084198	0	0	0
CEU	PRAMEF5	13089198	0	0	0
CEU	PRAMEF5	13094198	0	0	0
CEU	PRAMEF5	13099198	0	0	0
CEU	PRAMEF5	13104198	0.5442	0.5117	0.1019
CEU	PRAMEF5	13109198	1.242	1.1939	0.0857
CEU	PRAMEF5	13114198	0.7382	1.3645	-1.0132
CEU	PRAMEF5	13119198	3.3053	4.6052	-0.7892
CEU	PRAMEF5	13124198	9.9388	14.3272	-0.9415
CEU	PRAMEF5	13129198	15.1763	16.7151	-0.285
CEU	PRAMEF5	13134198	9.2705	7.3342	0.7758
CEU	PRAMEF5	13139198	5.4587	4.6052	0.5182
CEU	PRAMEF5	13144198	5.616	5.6286	-0.0064
CEU	PRAMEF5	13149198	1.3107	3.2407	-1.5826
CEU	PRAMEF5	13154198	1.6067	2.729	-1.0608
CEU	PRAMEF5	13159198	1.3793	2.2173	-0.9362
CEU	PRAMEF5	13164198	1.3424	1.8762	-0.6796
CEU	PRAMEF5	13169198	4.1466	4.7757	-0.37
CEU	PRAMEF5	13174198	9.7456	11.9394	-0.5593
CEU	PRAMEF5	13179198	15.8407	20.2969	-0.6849
CEU	PRAMEF5	13184198	17.5286	18.9324	-0.2307
CEU	PRAMEF5	13189198	10.0581	8.5281	0.5338
CEU	PRAMEF5	13194198	2.2564	2.8996	-0.5784
CEU	PRAMEF5	13199198	1.8573	2.729	-0.8239
CEU	PRAMEF5	13204198	1.9237	2.2173	-0.328
CEU	PRAMEF5	13209198	1.3554	2.2173	-0.9629
CEU	PRAMEF5	13214198	1.8615	2.5584	-0.6942
CEU	PRAMEF5	13219198	1.2826	1.7056	-0.5793
CEU	PRAMEF5	13224198	0.0404	0.5117	-1.4781
CEU	PRAMEF5	13229198	0	0	0
CEU	PRAMEF5	13234198	0	0	0
CEU	PRAMEF5	13239198	0	0	0
CEU	PRAMEF5	13244198	0	0	0
CEU	PRAMEF5	13249198	0	0	0
CEU	PRAMEF5	13254198	0	0	0
CEU	PRAMEF5	13259198	0	0	0
CEU	PRAMEF5	13264198	0	0	0
CEU	PRAMEF5	13269198	0	0	0
CEU	PRAMEF5	13274198	0	0	0
CEU	PRAMEF5	13279198	0	0	0
CEU	PRAMEF5	13284198	0	0	0
CEU	PRAMEF5	13289198	0	0	0
CEU	PRAMEF5	13294198	0	0	0
CEU	PRAMEF5	13299198	0	0	0
CEU	PRAMEF5	13304198	0	0	0
CEU	PRAMEF5	13309198	0	0	0
CEU	PRAMEF5	13314198	0	0	0
CEU	PRAMEF5	13319198	0	0	0

CEU	PRAMEF5	13324198	0.5024	1.5351	-1.5313
CEU	PRAMEF5	13329198	0.6654	2.0467	-1.6436
CEU	PRAMEF5	13334198	0.1631	1.0234	-1.7095
CEU	PRAMEF5	13339198	0.0101	0.6822	-1.7551
CEU	PRAMEF5	13344198	1.0421	1.7056	-0.9087
CEU	PRAMEF5	13349198	4.6599	7.8459	-1.2002
CEU	PRAMEF5	13354198	3.8819	6.9931	-1.3018
CEU	PRAMEF5	13359198	0.254	0.6822	-1.1182
CEU	PRAMEF5	13364198	1.5366	1.0234	1.0198
CEU	PRAMEF5	13369198	1.5366	1.0234	1.0198
CEU	PRAMEF5	13374198	5.0736	2.3879	2.8283
CEU	PRAMEF5	13379198	13.7794	9.0398	1.567
CEU	PRAMEF5	13384198	11.8808	8.6987	1.0902
CEU	PRAMEF5	13389198	3.175	2.0467	1.3426
CEU	PRAMEF5	13394198	0	0	0
CEU	PRAMEF5	13399198	0	0	0
CEU	PRAMEF5	13404198	0	0	0
CEU	PRAMEF5	13409198	2.965	1.8762	1.3862
CEU	PRAMEF5	13414198	2.965	1.8762	1.3862
CEU	PRAMEF5	13419198	0.4096	0.1706	1.4197
CEU	PRAMEF5	13424198	0.4877	0.3411	0.5877
CEU	PRAMEF5	13429198	0.1563	0.3411	-0.7408
CEU	PRAMEF5	13434198	0.0781	0.1706	-0.5495
CEU	PRAMEF5	13439198	0	0	0
CEU	PRAMEF5	13444198	1.3636	1.1939	0.3024
CEU	PRAMEF5	13449198	1.3636	1.1939	0.3024
CEU	PRAMEF5	13454198	0.6135	0.6822	-0.1794
CEU	PRAMEF5	13459198	0.6135	0.6822	-0.1794
CEU	PRAMEF5	13464198	1.9272	1.5351	0.5814
CEU	PRAMEF5	13469198	1.9272	1.5351	0.5814
CEU	PRAMEF5	13474198	0.5259	1.3645	-1.3567
CEU	PRAMEF5	13479198	4.5984	4.6052	-0.0041
CEU	PRAMEF5	13484198	8.498	7.3342	0.4663
CEU	PRAMEF5	13489198	4.6557	4.2641	0.2542
CEU	PRAMEF5	13494198	2.1754	2.2173	-0.0468
CEU	PRAMEF5	13499198	2.8409	2.8996	-0.0528
CEU	PRAMEF5	13504198	1.1538	1.3645	-0.3409
CEU	PRAMEF5	13509198	1.2997	1.1939	0.1885
CEU	PRAMEF5	13514198	2.8637	3.7524	-0.6438
CEU	PRAMEF5	13519198	1.8322	3.2407	-1.1549
CEU	PRAMEF5	13524198	0.0101	0.1706	-0.9534
CEU	PRAMEF5	13529198	0	0	0
CEU	PRAMEF5	13534198	1.6549	1.3645	0.4698
CEU	PRAMEF5	13539198	1.9553	1.5351	0.6231
CEU	PRAMEF5	13544198	0.3004	0.1706	0.771
CEU	PRAMEF5	13549198	1.3418	0.8528	1.1012
CEU	PRAMEF5	13554198	1.517	1.0234	0.9808
CEU	PRAMEF5	13559198	0.1752	0.1706	0.0273
CEU	PRAMEF5	13564198	0	0	0

CEU	PRAMEF5	13569198	0	0	0
CEU	PRAMEF5	13574198	0	0	0
CEU	PRAMEF5	13579198	0	0	0
CEU	PRAMEF5	13584198	0	0	0
CEU	PRAMEF5	13589198	0	0	0
CEU	PRAMEF5	13594198	0	0	0
CEU	PRAMEF5	13599198	0	0	0
CEU	PRAMEF5	13604198	0	0	0
CEU	PRAMEF5	13609198	0	0	0
CEU	PRAMEF5	13614198	0	0	0
CEU	PRAMEF5	13619198	0	0	0
CEU	PRAMEF5	13624198	0	0	0
CEU	PRAMEF5	13629198	0	0	0
CEU	PRAMEF5	13634198	1.4345	3.0701	-1.403
CEU	PRAMEF5	13639198	1.6079	3.4112	-1.4163
CEU	PRAMEF5	13644198	0.1934	0.5117	-0.9983
CEU	PRAMEF5	13649198	0.0301	0.3411	-1.2467
CEU	PRAMEF5	13654198	0.0101	0.1706	-0.9534
CEU	PRAMEF5	13659198	0.0101	0.1706	-0.9534
CEU	PRAMEF5	13664198	0.0884	0.3411	-1.013
CEU	PRAMEF5	13669198	1.5242	0.6822	2.1987
CEU	PRAMEF5	13674198	1.5452	0.8528	1.5593
CEU	PRAMEF5	13679198	0.0994	0.3411	-0.9689
CEU	PRAMEF5	13684198	0.1769	0.6822	-1.3195
CEU	PRAMEF5	13689198	0.1769	0.6822	-1.3195
CEU	PRAMEF5	13694198	0.86	2.2173	-1.5164
CEU	PRAMEF5	13699198	2.9186	4.6052	-1.024
CEU	PRAMEF5	13704198	2.724	3.9229	-0.8362
CEU	PRAMEF5	13709198	1.6706	2.2173	-0.6108
CEU	PRAMEF5	13714198	3.2426	4.0935	-0.5722
CEU	PRAMEF5	13719198	3.5615	4.6052	-0.6337
CEU	PRAMEF5	13724198	1.3342	1.3645	-0.049
CEU	PRAMEF5	13729198	1.5325	1.1939	0.6034
CEU	PRAMEF5	13734198	2.6024	1.7056	1.2283
CEU	PRAMEF5	13739198	1.8578	1.3645	0.7981
CEU	PRAMEF5	13744198	0.7879	0.8528	-0.1462
CEU	PRAMEF5	13749198	0.0101	0.1706	-0.9534
CEU	PRAMEF5	13754198	0	0	0
CHB	PRAMEF5	12759198	8.9355	9.1484	-0.0694
CHB	PRAMEF5	12764198	7.8287	9.1484	-0.4303
CHB	PRAMEF5	12769198	9.3625	9.6567	-0.0912
CHB	PRAMEF5	12774198	12.3111	12.0285	0.0713
CHB	PRAMEF5	12779198	11.5275	12.7061	-0.2825
CHB	PRAMEF5	12784198	11.9623	13.2144	-0.2892
CHB	PRAMEF5	12789198	10.8064	12.1979	-0.3467
CHB	PRAMEF5	12794198	10.2214	10.8426	-0.1729
CHB	PRAMEF5	12799198	13.2633	12.5367	0.1764
CHB	PRAMEF5	12804198	9.9747	12.0285	-0.5185
CHB	PRAMEF5	12809198	4.5228	9.9955	-1.6433

CHB	PRAMEF5	12814198	7.0865	10.3343	-0.9454
CHB	PRAMEF5	12819198	10.1972	11.012	-0.2235
CHB	PRAMEF5	12824198	10.0219	11.3508	-0.3543
CHB	PRAMEF5	12829198	11.2223	12.8755	-0.3914
CHB	PRAMEF5	12834198	8.4507	15.0779	-1.3506
CHB	PRAMEF5	12839198	6.4954	12.7061	-1.4889
CHB	PRAMEF5	12844198	8.7344	10.5037	-0.5073
CHB	PRAMEF5	12849198	14.4055	15.2474	-0.1698
CHB	PRAMEF5	12854198	20.2016	20.3298	-0.0196
CHB	PRAMEF5	12859198	19.4961	22.5322	-0.4207
CHB	PRAMEF5	12864198	23.0859	44.7256	-1.5356
CHB	PRAMEF5	12869198	23.9692	56.2458	-1.8276
CHB	PRAMEF5	12874198	13.6208	28.97	-1.6662
CHB	PRAMEF5	12879198	10.7322	15.0779	-0.8856
CHB	PRAMEF5	12884198	16.2793	19.8216	-0.5554
CHB	PRAMEF5	12889198	18.3526	18.2968	0.0094
CHB	PRAMEF5	12894198	30.365	24.7346	0.7127
CHB	PRAMEF5	12899198	31.5904	24.3958	0.923
CHB	PRAMEF5	12904198	16.4377	14.7391	0.3537
CHB	PRAMEF5	12909198	28.4412	20.4992	1.2056
CHB	PRAMEF5	12914198	37.4242	26.937	1.222
CHB	PRAMEF5	12919198	28.4645	26.0899	0.2854
CHB	PRAMEF5	12924198	17.1207	16.7721	0.0642
CHB	PRAMEF5	12929198	8.4555	7.7931	0.2504
CHB	PRAMEF5	12934198	6.3073	7.6237	-0.5076
CHB	PRAMEF5	12939198	10.9457	17.4498	-1.1527
CHB	PRAMEF5	12944198	13.5829	20.4992	-1.0499
CHB	PRAMEF5	12949198	6.9596	13.2144	-1.4448
CHB	PRAMEF5	12954198	3.7339	9.9955	-1.8802
CHB	PRAMEF5	12959198	3.4972	5.4213	-1.0089
CHB	PRAMEF5	12964198	2.4959	3.2189	-0.5944
CHB	PRAMEF5	12969198	0.2031	2.5412	-2.3348
CHB	PRAMEF5	12974198	0.1729	1.6942	-2.0878
CHB	PRAMEF5	12979198	1.0289	3.3883	-1.8581
CHB	PRAMEF5	12984198	3.9802	6.4378	-1.105
CHB	PRAMEF5	12989198	4.0624	5.7601	-0.8435
CHB	PRAMEF5	12994198	1.7207	4.2354	-1.6371
CHB	PRAMEF5	12999198	1.8736	4.5742	-1.6445
CHB	PRAMEF5	13004198	1.6701	2.7106	-0.9859
CHB	PRAMEF5	13009198	2.7401	3.3883	-0.5105
CHB	PRAMEF5	13014198	2.8325	4.4048	-0.9894
CHB	PRAMEF5	13019198	0.7354	2.033	-1.5474
CHB	PRAMEF5	13024198	0.1319	0.6777	-1.4269
CHB	PRAMEF5	13029198	0	0	0
CHB	PRAMEF5	13034198	0.5347	1.0165	-0.9588
CHB	PRAMEF5	13039198	4.1017	2.7106	1.3181
CHB	PRAMEF5	13044198	7.9547	5.2519	1.4577
CHB	PRAMEF5	13049198	12.6674	10.3343	0.6791
CHB	PRAMEF5	13054198	8.2796	6.7766	0.6452

CHB	PRAMEF5	13059198	0	0	0
CHB	PRAMEF5	13064198	0	0	0
CHB	PRAMEF5	13069198	0	0	0
CHB	PRAMEF5	13074198	0	0	0
CHB	PRAMEF5	13079198	0	0	0
CHB	PRAMEF5	13084198	0	0	0
CHB	PRAMEF5	13089198	0	0	0
CHB	PRAMEF5	13094198	0	0	0
CHB	PRAMEF5	13099198	0	0	0
CHB	PRAMEF5	13104198	0.4826	0.3388	0.5768
CHB	PRAMEF5	13109198	1.2136	0.6777	1.401
CHB	PRAMEF5	13114198	0.731	0.3388	1.5732
CHB	PRAMEF5	13119198	4.1167	3.8965	0.154
CHB	PRAMEF5	13124198	7.5738	12.8755	-1.2551
CHB	PRAMEF5	13129198	10.5648	15.2474	-0.9442
CHB	PRAMEF5	13134198	7.7598	7.7931	-0.0126
CHB	PRAMEF5	13139198	5.372	4.7436	0.3707
CHB	PRAMEF5	13144198	8.5722	5.9295	1.2794
CHB	PRAMEF5	13149198	5.3075	3.8965	0.9869
CHB	PRAMEF5	13154198	2.564	2.5412	0.0228
CHB	PRAMEF5	13159198	1.2699	2.2024	-1.0441
CHB	PRAMEF5	13164198	1.1369	2.033	-1.0686
CHB	PRAMEF5	13169198	4.736	4.7436	-0.0045
CHB	PRAMEF5	13174198	14.2328	13.892	0.0751
CHB	PRAMEF5	13179198	30.8412	24.0569	0.8822
CHB	PRAMEF5	13184198	29.8528	21.6851	1.1744
CHB	PRAMEF5	13189198	10.6115	9.3178	0.4147
CHB	PRAMEF5	13194198	2.5629	3.3883	-0.65
CHB	PRAMEF5	13199198	2.2937	3.5577	-0.9553
CHB	PRAMEF5	13204198	1.6377	3.0495	-1.2142
CHB	PRAMEF5	13209198	1.1411	2.8801	-1.5679
CHB	PRAMEF5	13214198	2.6781	3.5577	-0.6648
CHB	PRAMEF5	13219198	2.3842	2.7106	-0.3093
CHB	PRAMEF5	13224198	0.0676	0.5082	-1.3831
CHB	PRAMEF5	13229198	0	0	0
CHB	PRAMEF5	13234198	0	0	0
CHB	PRAMEF5	13239198	0	0	0
CHB	PRAMEF5	13244198	0	0	0
CHB	PRAMEF5	13249198	0	0	0
CHB	PRAMEF5	13254198	0	0	0
CHB	PRAMEF5	13259198	0	0	0
CHB	PRAMEF5	13264198	0	0	0
CHB	PRAMEF5	13269198	0	0	0
CHB	PRAMEF5	13274198	0	0	0
CHB	PRAMEF5	13279198	0	0	0
CHB	PRAMEF5	13284198	0	0	0
CHB	PRAMEF5	13289198	0	0	0
CHB	PRAMEF5	13294198	0	0	0
CHB	PRAMEF5	13299198	0	0	0

CHB	PRAMEF5	13304198	0	0	0
CHB	PRAMEF5	13309198	0	0	0
CHB	PRAMEF5	13314198	0	0	0
CHB	PRAMEF5	13319198	0	0	0
CHB	PRAMEF5	13324198	2.1072	1.6942	0.5668
CHB	PRAMEF5	13329198	2.4717	2.3718	0.1055
CHB	PRAMEF5	13334198	0.3646	1.1859	-1.466
CHB	PRAMEF5	13339198	0	0.5082	-1.5953
CHB	PRAMEF5	13344198	1.0364	2.033	-1.1885
CHB	PRAMEF5	13349198	3.7723	7.4543	-1.4493
CHB	PRAMEF5	13354198	2.7938	5.9295	-1.518
CHB	PRAMEF5	13359198	0.0579	0.5082	-1.4136
CHB	PRAMEF5	13364198	1.4748	0.8471	1.4155
CHB	PRAMEF5	13369198	1.4748	0.8471	1.4155
CHB	PRAMEF5	13374198	4.2501	2.3718	1.9827
CHB	PRAMEF5	13379198	12.7144	8.8096	1.3184
CHB	PRAMEF5	13384198	11.2064	8.6402	0.8821
CHB	PRAMEF5	13389198	2.7421	2.2024	0.6043
CHB	PRAMEF5	13394198	0	0	0
CHB	PRAMEF5	13399198	0	0	0
CHB	PRAMEF5	13404198	0	0	0
CHB	PRAMEF5	13409198	3.276	2.5412	0.7338
CHB	PRAMEF5	13414198	3.276	2.5412	0.7338
CHB	PRAMEF5	13419198	0.3811	0.1694	1.258
CHB	PRAMEF5	13424198	0.8094	0.3388	1.8877
CHB	PRAMEF5	13429198	0.8567	0.3388	2.0775
CHB	PRAMEF5	13434198	0.4283	0.1694	1.5384
CHB	PRAMEF5	13439198	0	0	0
CHB	PRAMEF5	13444198	1.0632	1.1859	-0.219
CHB	PRAMEF5	13449198	1.0632	1.1859	-0.219
CHB	PRAMEF5	13454198	1.0069	0.6777	0.8606
CHB	PRAMEF5	13459198	1.0069	0.6777	0.8606
CHB	PRAMEF5	13464198	2.1256	1.5247	0.8927
CHB	PRAMEF5	13469198	2.1256	1.5247	0.8927
CHB	PRAMEF5	13474198	2.3057	1.3553	1.5403
CHB	PRAMEF5	13479198	8.0261	4.913	1.7807
CHB	PRAMEF5	13484198	11.679	7.4543	1.663
CHB	PRAMEF5	13489198	6.0247	4.2354	1.1648
CHB	PRAMEF5	13494198	2.4137	2.2024	0.2366
CHB	PRAMEF5	13499198	3.4026	2.5412	0.8602
CHB	PRAMEF5	13504198	1.3021	1.0165	0.5684
CHB	PRAMEF5	13509198	0.8666	0.6777	0.4938
CHB	PRAMEF5	13514198	2.2886	3.2189	-0.7648
CHB	PRAMEF5	13519198	1.6789	3.0495	-1.1787
CHB	PRAMEF5	13524198	0.0097	0.1694	-0.949
CHB	PRAMEF5	13529198	0	0	0
CHB	PRAMEF5	13534198	1.6981	1.1859	0.9143
CHB	PRAMEF5	13539198	2.0742	1.5247	0.8164
CHB	PRAMEF5	13544198	0.3762	0.3388	0.15

CHB	PRAMEF5	13549198	0.786	1.1859	-0.7138
CHB	PRAMEF5	13554198	0.9235	1.5247	-0.8932
CHB	PRAMEF5	13559198	0.1374	0.3388	-0.8079
CHB	PRAMEF5	13564198	0	0	0
CHB	PRAMEF5	13569198	0	0	0
CHB	PRAMEF5	13574198	0	0	0
CHB	PRAMEF5	13579198	0	0	0
CHB	PRAMEF5	13584198	0	0	0
CHB	PRAMEF5	13589198	0	0	0
CHB	PRAMEF5	13594198	0	0	0
CHB	PRAMEF5	13599198	0	0	0
CHB	PRAMEF5	13604198	0	0	0
CHB	PRAMEF5	13609198	0	0	0
CHB	PRAMEF5	13614198	0	0	0
CHB	PRAMEF5	13619198	0	0	0
CHB	PRAMEF5	13624198	0	0	0
CHB	PRAMEF5	13629198	0	0	0
CHB	PRAMEF5	13634198	0.9098	2.5412	-1.6291
CHB	PRAMEF5	13639198	1.6926	2.8801	-1.0707
CHB	PRAMEF5	13644198	0.9018	0.5082	1.2356
CHB	PRAMEF5	13649198	0.1287	0.3388	-0.8428
CHB	PRAMEF5	13654198	0.0097	0.1694	-0.949
CHB	PRAMEF5	13659198	0	0	0
CHB	PRAMEF5	13664198	0.0952	0.5082	-1.2965
CHB	PRAMEF5	13669198	1.3333	1.1859	0.2631
CHB	PRAMEF5	13674198	1.3147	1.0165	0.5934
CHB	PRAMEF5	13679198	0.0765	0.3388	-1.0522
CHB	PRAMEF5	13684198	0.189	0.6777	-1.2776
CHB	PRAMEF5	13689198	0.189	0.6777	-1.2776
CHB	PRAMEF5	13694198	0.5146	2.2024	-1.8899
CHB	PRAMEF5	13699198	2.9682	4.913	-1.1124
CHB	PRAMEF5	13704198	3.0088	3.3883	-0.2989
CHB	PRAMEF5	13709198	1.6217	1.6942	-0.0995
CHB	PRAMEF5	13714198	3.5427	4.7436	-0.7084
CHB	PRAMEF5	13719198	3.5905	5.2519	-0.896
CHB	PRAMEF5	13724198	1.1338	1.8636	-0.9311
CHB	PRAMEF5	13729198	1.2646	1.1859	0.1405
CHB	PRAMEF5	13734198	2.1504	1.6942	0.6261
CHB	PRAMEF5	13739198	1.6655	1.8636	-0.2527
CHB	PRAMEF5	13744198	0.77	1.1859	-0.7424
CHB	PRAMEF5	13749198	0.0097	0.1694	-0.949
CHB	PRAMEF5	13754198	0	0	0
YRI	PRAMEF5	12759198	10.0804	14.6214	-0.9497
YRI	PRAMEF5	12764198	9.9656	14.4534	-0.9489
YRI	PRAMEF5	12769198	12.3696	16.302	-0.7413
YRI	PRAMEF5	12774198	15.2815	18.823	-0.5817
YRI	PRAMEF5	12779198	15.4029	18.3188	-0.4916
YRI	PRAMEF5	12784198	17.2346	17.3104	-0.0135
YRI	PRAMEF5	12789198	17.3991	17.1423	0.0461

YRI	PRAMEF5	12794198	16.2377	15.4617	0.1539
YRI	PRAMEF5	12799198	19.5625	15.9659	0.6917
YRI	PRAMEF5	12804198	19.294	17.3104	0.3531
YRI	PRAMEF5	12809198	16.3719	16.4701	-0.0183
YRI	PRAMEF5	12814198	15.1837	14.4534	0.1544
YRI	PRAMEF5	12819198	13.1998	14.7895	-0.3289
YRI	PRAMEF5	12824198	11.8048	15.4617	-0.7251
YRI	PRAMEF5	12829198	13.6263	14.6214	-0.2081
YRI	PRAMEF5	12834198	11.8817	14.2853	-0.5139
YRI	PRAMEF5	12839198	8.0802	12.1005	-1.0058
YRI	PRAMEF5	12844198	9.9354	13.1089	-0.7361
YRI	PRAMEF5	12849198	15.5728	18.4868	-0.487
YRI	PRAMEF5	12854198	18.7308	21.8481	-0.4435
YRI	PRAMEF5	12859198	17.4108	20.3355	-0.4459
YRI	PRAMEF5	12864198	13.2579	14.7895	-0.3168
YRI	PRAMEF5	12869198	11.6557	13.1089	-0.3371
YRI	PRAMEF5	12874198	13.7236	15.2937	-0.3146
YRI	PRAMEF5	12879198	19.3686	20.3355	-0.1474
YRI	PRAMEF5	12884198	26.3679	29.5789	-0.3404
YRI	PRAMEF5	12889198	25.1264	30.5873	-0.5603
YRI	PRAMEF5	12894198	26.0582	33.2763	-0.6822
YRI	PRAMEF5	12899198	22.0019	34.1166	-1.1173
YRI	PRAMEF5	12904198	11.5667	23.5287	-1.584
YRI	PRAMEF5	12909198	13.8898	24.8732	-1.3782
YRI	PRAMEF5	12914198	21.4352	31.0915	-0.9752
YRI	PRAMEF5	12919198	25.0015	29.0748	-0.4391
YRI	PRAMEF5	12924198	17.529	19.8313	-0.3596
YRI	PRAMEF5	12929198	10.666	13.9492	-0.718
YRI	PRAMEF5	12934198	8.9148	14.9575	-1.2367
YRI	PRAMEF5	12939198	17.6498	23.8648	-0.8118
YRI	PRAMEF5	12944198	25.1968	32.9402	-0.7391
YRI	PRAMEF5	12949198	12.8835	23.0245	-1.3714
YRI	PRAMEF5	12954198	5.5903	13.445	-1.7789
YRI	PRAMEF5	12959198	4.9301	9.2434	-1.3881
YRI	PRAMEF5	12964198	2.971	4.5377	-0.9573
YRI	PRAMEF5	12969198	1.9928	3.6974	-1.2424
YRI	PRAMEF5	12974198	1.0132	2.5209	-1.5099
YRI	PRAMEF5	12979198	1.5041	4.3696	-1.8093
YRI	PRAMEF5	12984198	5.7366	8.5712	-0.9782
YRI	PRAMEF5	12989198	7.7049	7.3947	0.1226
YRI	PRAMEF5	12994198	6.2908	6.0502	0.114
YRI	PRAMEF5	12999198	5.4384	5.7141	-0.1375
YRI	PRAMEF5	13004198	3.0587	2.689	0.3513
YRI	PRAMEF5	13009198	5.616	5.5461	0.0358
YRI	PRAMEF5	13014198	7.0231	6.5544	0.2066
YRI	PRAMEF5	13019198	2.1032	1.8487	0.3255
YRI	PRAMEF5	13024198	0.1841	0.3361	-0.6102
YRI	PRAMEF5	13029198	0.1585	0.6722	-1.3448
YRI	PRAMEF5	13034198	0.7626	2.0167	-1.4995

YRI	PRAMEF5	13039198	3.066	3.8654	-0.561
YRI	PRAMEF5	13044198	5.2072	7.8989	-1.0014
YRI	PRAMEF5	13049198	6.7103	15.2937	-1.7199
YRI	PRAMEF5	13054198	3.965	9.9157	-1.7941
YRI	PRAMEF5	13059198	0	0	0
YRI	PRAMEF5	13064198	0	0	0
YRI	PRAMEF5	13069198	0	0	0
YRI	PRAMEF5	13074198	0	0	0
YRI	PRAMEF5	13079198	0	0	0
YRI	PRAMEF5	13084198	0	0	0
YRI	PRAMEF5	13089198	0	0	0
YRI	PRAMEF5	13094198	0	0	0
YRI	PRAMEF5	13099198	0	0	0
YRI	PRAMEF5	13104198	0.4735	0.1681	1.8155
YRI	PRAMEF5	13109198	1.9676	2.3529	-0.4079
YRI	PRAMEF5	13114198	1.5219	2.689	-1.1092
YRI	PRAMEF5	13119198	2.2505	4.0335	-1.2064
YRI	PRAMEF5	13124198	10.9001	12.2685	-0.3379
YRI	PRAMEF5	13129198	18.82	16.8062	0.3687
YRI	PRAMEF5	13134198	12.1218	10.2518	0.5466
YRI	PRAMEF5	13139198	7.2847	6.2183	0.493
YRI	PRAMEF5	13144198	6.2958	6.7225	-0.1839
YRI	PRAMEF5	13149198	1.6814	3.6974	-1.4694
YRI	PRAMEF5	13154198	1.8608	2.8571	-0.901
YRI	PRAMEF5	13159198	1.4263	2.8571	-1.2939
YRI	PRAMEF5	13164198	1.6038	2.1848	-0.6523
YRI	PRAMEF5	13169198	5.1231	4.7057	0.2471
YRI	PRAMEF5	13174198	11.54	14.9575	-0.6994
YRI	PRAMEF5	13179198	17.2254	28.7386	-1.2554
YRI	PRAMEF5	13184198	17.8907	26.2177	-0.9928
YRI	PRAMEF5	13189198	10.8943	11.0921	-0.0537
YRI	PRAMEF5	13194198	3.6936	4.2016	-0.3318
YRI	PRAMEF5	13199198	2.8495	5.0419	-1.2215
YRI	PRAMEF5	13204198	3.6062	5.378	-0.9325
YRI	PRAMEF5	13209198	3.175	4.8738	-0.9752
YRI	PRAMEF5	13214198	2.1314	4.7057	-1.5239
YRI	PRAMEF5	13219198	1.2436	2.689	-1.3737
YRI	PRAMEF5	13224198	0.3019	0.5042	-0.6358
YRI	PRAMEF5	13229198	0	0	0
YRI	PRAMEF5	13234198	0	0	0
YRI	PRAMEF5	13239198	0	0	0
YRI	PRAMEF5	13244198	0	0	0
YRI	PRAMEF5	13249198	0	0	0
YRI	PRAMEF5	13254198	0	0	0
YRI	PRAMEF5	13259198	0	0	0
YRI	PRAMEF5	13264198	0	0	0
YRI	PRAMEF5	13269198	0	0	0
YRI	PRAMEF5	13274198	0	0	0
YRI	PRAMEF5	13279198	0	0	0

YRI	PRAMEF5	13284198	0	0	0
YRI	PRAMEF5	13289198	0	0	0
YRI	PRAMEF5	13294198	0	0	0
YRI	PRAMEF5	13299198	0	0	0
YRI	PRAMEF5	13304198	0	0	0
YRI	PRAMEF5	13309198	0	0	0
YRI	PRAMEF5	13314198	0	0	0
YRI	PRAMEF5	13319198	0	0	0
YRI	PRAMEF5	13324198	1.1077	2.0167	-1.0868
YRI	PRAMEF5	13329198	1.4065	2.5209	-1.116
YRI	PRAMEF5	13334198	0.3265	1.0084	-1.3595
YRI	PRAMEF5	13339198	0.0278	0.5042	-1.4972
YRI	PRAMEF5	13344198	1.24	2.0167	-0.9287
YRI	PRAMEF5	13349198	5.4184	9.5795	-1.2955
YRI	PRAMEF5	13354198	4.6207	8.235	-1.2941
YRI	PRAMEF5	13359198	0.4424	0.6722	-0.6016
YRI	PRAMEF5	13364198	1.5663	1.3445	0.3602
YRI	PRAMEF5	13369198	1.702	1.5126	0.282
YRI	PRAMEF5	13374198	3.7004	5.7141	-1.0041
YRI	PRAMEF5	13379198	10.8171	11.9324	-0.2827
YRI	PRAMEF5	13384198	11.4277	10.0837	0.3989
YRI	PRAMEF5	13389198	4.1753	3.6974	0.3483
YRI	PRAMEF5	13394198	0	0	0
YRI	PRAMEF5	13399198	0	0	0
YRI	PRAMEF5	13404198	0	0	0
YRI	PRAMEF5	13409198	3.1076	2.3529	0.7989
YRI	PRAMEF5	13414198	3.1168	2.5209	0.5968
YRI	PRAMEF5	13419198	0.0636	0.3361	-1.094
YRI	PRAMEF5	13424198	0.0996	0.3361	-0.9494
YRI	PRAMEF5	13429198	0.0905	0.3361	-0.986
YRI	PRAMEF5	13434198	0.0453	0.1681	-0.73
YRI	PRAMEF5	13439198	0	0	0
YRI	PRAMEF5	13444198	2.6596	1.3445	2.136
YRI	PRAMEF5	13449198	2.6596	1.3445	2.136
YRI	PRAMEF5	13454198	0.7522	0.6722	0.2094
YRI	PRAMEF5	13459198	0.7522	0.6722	0.2094
YRI	PRAMEF5	13464198	1.7736	1.8487	-0.0961
YRI	PRAMEF5	13469198	1.7736	1.8487	-0.0961
YRI	PRAMEF5	13474198	0.4095	1.6806	-1.7487
YRI	PRAMEF5	13479198	3.4819	5.5461	-1.0571
YRI	PRAMEF5	13484198	6.396	7.8989	-0.5591
YRI	PRAMEF5	13489198	3.688	4.5377	-0.5192
YRI	PRAMEF5	13494198	1.1096	3.0251	-1.6524
YRI	PRAMEF5	13499198	0.9418	3.1932	-1.8568
YRI	PRAMEF5	13504198	0.3154	1.3445	-1.6714
YRI	PRAMEF5	13509198	0.8045	1.6806	-1.2053
YRI	PRAMEF5	13514198	2.667	4.7057	-1.2068
YRI	PRAMEF5	13519198	2.2634	4.3696	-1.3299
YRI	PRAMEF5	13524198	0.2821	0.6722	-1.0213

YRI	PRAMEF5	13529198	0	0	0
YRI	PRAMEF5	13534198	2.5774	1.5126	1.5855
YRI	PRAMEF5	13539198	3.0809	1.6806	1.9264
YRI	PRAMEF5	13544198	0.5035	0.1681	1.9939
YRI	PRAMEF5	13549198	1.2133	1.3445	-0.2131
YRI	PRAMEF5	13554198	1.3837	1.8487	-0.5948
YRI	PRAMEF5	13559198	0.1704	0.5042	-1.049
YRI	PRAMEF5	13564198	0	0	0
YRI	PRAMEF5	13569198	0	0	0
YRI	PRAMEF5	13574198	0	0	0
YRI	PRAMEF5	13579198	0	0	0
YRI	PRAMEF5	13584198	0	0	0
YRI	PRAMEF5	13589198	0	0	0
YRI	PRAMEF5	13594198	0	0	0
YRI	PRAMEF5	13599198	0	0	0
YRI	PRAMEF5	13604198	0	0	0
YRI	PRAMEF5	13609198	0	0	0
YRI	PRAMEF5	13614198	0	0	0
YRI	PRAMEF5	13619198	0	0	0
YRI	PRAMEF5	13624198	0	0	0
YRI	PRAMEF5	13629198	0	0	0
YRI	PRAMEF5	13634198	1.1751	2.1848	-1.1337
YRI	PRAMEF5	13639198	1.3451	2.8571	-1.3674
YRI	PRAMEF5	13644198	0.3345	1.1764	-1.5058
YRI	PRAMEF5	13649198	0.1645	0.5042	-1.0676
YRI	PRAMEF5	13654198	0.0276	0.1681	-0.8352
YRI	PRAMEF5	13659198	0.0276	0.1681	-0.8352
YRI	PRAMEF5	13664198	0.2092	1.3445	-1.8439
YRI	PRAMEF5	13669198	1.6047	2.3529	-0.792
YRI	PRAMEF5	13674198	1.4964	1.8487	-0.4506
YRI	PRAMEF5	13679198	0.1008	0.8403	-1.6703
YRI	PRAMEF5	13684198	1.1727	0.8403	0.7508
YRI	PRAMEF5	13689198	1.1727	0.8403	0.7508
YRI	PRAMEF5	13694198	0.3701	2.1848	-2.0375
YRI	PRAMEF5	13699198	2.6762	5.5461	-1.4697
YRI	PRAMEF5	13704198	2.946	4.2016	-0.8202
YRI	PRAMEF5	13709198	2.0771	3.0251	-0.8178
YRI	PRAMEF5	13714198	3.569	5.7141	-1.0697
YRI	PRAMEF5	13719198	3.5917	5.5461	-1.0008
YRI	PRAMEF5	13724198	1.5693	2.5209	-0.953
YRI	PRAMEF5	13729198	1.5987	1.3445	0.4129
YRI	PRAMEF5	13734198	3.2444	3.0251	0.1892
YRI	PRAMEF5	13739198	2.8924	3.3612	-0.3704
YRI	PRAMEF5	13744198	1.165	1.3445	-0.2915
YRI	PRAMEF5	13749198	0.0276	0.1681	-0.8352
YRI	PRAMEF5	13754198	0	0	0
CEU	PRAMEF8	12786223	13.5298	11.7688	0.4551
CEU	PRAMEF8	12791223	10.2587	9.0398	0.403
CEU	PRAMEF8	12796223	10.502	9.2104	0.4197

CEU	PRAMEF8	12801223	15.9798	11.0866	1.3375
CEU	PRAMEF8	12806223	10.0171	8.6987	0.4517
CEU	PRAMEF8	12811223	3.8244	7.8459	-1.515
CEU	PRAMEF8	12816223	8.4465	9.3809	-0.2985
CEU	PRAMEF8	12821223	12.7267	10.7454	0.5577
CEU	PRAMEF8	12826223	11.0618	9.7221	0.414
CEU	PRAMEF8	12831223	8.565	9.0398	-0.157
CEU	PRAMEF8	12836223	6.3395	10.7454	-1.2401
CEU	PRAMEF8	12841223	5.8899	9.0398	-1.0414
CEU	PRAMEF8	12846223	8.9794	10.7454	-0.4971
CEU	PRAMEF8	12851223	16.8073	17.2268	-0.0755
CEU	PRAMEF8	12856223	21.0907	19.7852	0.2057
CEU	PRAMEF8	12861223	20.3438	26.4372	-0.7251
CEU	PRAMEF8	12866223	29.5318	57.4795	-1.5534
CEU	PRAMEF8	12871223	26.6815	50.8276	-1.5151
CEU	PRAMEF8	12876223	16.544	16.7151	-0.0317
CEU	PRAMEF8	12881223	16.8204	12.6216	1.0158
CEU	PRAMEF8	12886223	21.0916	15.8623	1.0183
CEU	PRAMEF8	12891223	21.7843	16.7151	0.9389
CEU	PRAMEF8	12896223	23.3548	25.4138	-0.2546
CEU	PRAMEF8	12901223	16.7864	22.8554	-0.8317
CEU	PRAMEF8	12906223	8.1771	14.1567	-1.2976
CEU	PRAMEF8	12911223	12.5561	20.1264	-1.1731
CEU	PRAMEF8	12916223	19.9242	26.6077	-0.7904
CEU	PRAMEF8	12921223	19.2975	24.9021	-0.7068
CEU	PRAMEF8	12926223	8.726	12.4511	-0.9129
CEU	PRAMEF8	12931223	6.516	7.5047	-0.3879
CEU	PRAMEF8	12936223	9.3837	10.4043	-0.2961
CEU	PRAMEF8	12941223	14.8498	16.8857	-0.3734
CEU	PRAMEF8	12946223	13.9641	15.0095	-0.2146
CEU	PRAMEF8	12951223	7.0724	11.4277	-1.1571
CEU	PRAMEF8	12956223	3.893	7.6753	-1.4538
CEU	PRAMEF8	12961223	2.6638	2.5584	0.105
CEU	PRAMEF8	12966223	1.611	1.8762	-0.3376
CEU	PRAMEF8	12971223	0.4578	0.6822	-0.586
CEU	PRAMEF8	12976223	0.516	0.8528	-0.7585
CEU	PRAMEF8	12981223	2.3861	5.1169	-1.5115
CEU	PRAMEF8	12986223	3.8543	6.9931	-1.3133
CEU	PRAMEF8	12991223	3.3195	5.458	-1.118
CEU	PRAMEF8	12996223	2.5904	5.1169	-1.3985
CEU	PRAMEF8	13001223	2.5073	4.0935	-1.0666
[truncated: 461,082 more chars]
